# Expression profiles of microRNAs in skeletal muscle of sheep by deep sequencing

**DOI:** 10.5713/ajas.18.0473

**Published:** 2018-11-27

**Authors:** Zhijin Liu, Cunyuan Li, Xiaoyue Li, Yang Yao, Wei Ni, Xiangyu Zhang, Yang Cao, Wureli Hazi, Dawei Wang, Renzhe Quan, Shuting Yu, Yuyu Wu, Songmin Niu, Yulong Cui, Yaseen Khan, Shengwei Hu

**Affiliations:** 1 College of Life Sciences, Shihezi University, Shihezi, Xinjiang 832003, China; 2 College of Animal Science and Technology, Shihezi University, Shihezi, Xinjiang, 832003, China

**Keywords:** Sheep, Skeletal Muscle, MicroRNAs, High-throughput Sequencing

## Abstract

**Objective:**

MicroRNAs are a class of endogenous small regulatory RNAs that regulate cell proliferation, differentiation and apoptosis. Recent studies on miRNAs are mainly focused on mice, human and pig. However, the studies on miRNAs in skeletal muscle of sheep are not comprehensive.

**Methods:**

RNA-seq technology was used to perform genomic analysis of miRNAs in prenatal and postnatal skeletal muscle of sheep. Targeted genes were predicted using miRanda software and miRNA-mRNA interactions were verified by quantitative real-time polymerase chain reaction. To further investigate the function of miRNAs, candidate targeted genes were enriched for analysis using gene ontology (GO) and Kyoto encyclopedia of genes and genomes (KEGG) enrichment.

**Results:**

The results showed total of 1,086 known miRNAs and 40 new candidate miRNAs were detected in prenatal and postnatal skeletal muscle of sheep. In addition, 345 miRNAs (151 up-regulated, 94 down-regulated) were differentially expressed. Moreover, miRanda software was performed to predict targeted genes of miRNAs, resulting in a total of 2,833 predicted targets, especially miR-381 which targeted multiple muscle-related mRNAs. Furthermore, GO and KEGG pathway analysis confirmed that targeted genes of miRNAs were involved in development of skeletal muscles.

**Conclusion:**

This study supplements the miRNA database of sheep, which provides valuable information for further study of the biological function of miRNAs in sheep skeletal muscle.

## INTRODUCTION

MicroRNAs (miRNAs) are a class of endogenous regulatory non-coding RNAs that are ~22nt in length. Most of the miRNAs are highly conserved, timing and tissue-specific in eukaryotes. As one of the negative regulatory factors, miRNA not only inhibit the expression of target mRNA at post-transcription level by incomplete complementary pairing, but also affect the stability of the mRNA by binding to its 3′untranslated region (3′-UTR) [1]. So far, a series of studies have shown that miRNAs play an important role in proliferation, differentiation and apoptosis of cells, nervous system development and even tumorigenesis [2].

*Ovis aries*, an important agricultural animal, has received considerable attention, because of the rich nutritional value of mutton. Skeletal muscle, which is an important source of mutton, is affected by the number and size of muscle fibers. Fetal muscle fibers determine the amount of meat produced in adult sheep [3]. So far, a majority of studies have shown that miRNAs play an important role in regulation of proliferation and differentiation of myoblasts which were closely related to the growth and development of animal skeletal muscle. Thus, muscle-specific miRNAs regulated the expression of myogenic genes affecting the quantitative traits of muscle [4]. miR-206 can promote muscle differentiation by inhibiting myogenic transcription factors (Id1-3 and MyoR) during muscle growth and development [5]. miR-1 and miR-133 are transcribed in a tissue-specific manner together during development, however, both have different functions. miR-1 promotes myogenesis by targeting HDAC4, while miR-133 can regulate cell proliferation by inhibiting the expression of myogenic genes serum response factor [6].

In this study, prenatal and postnatal sheep were selected as research objects, and differential expression profiles of miRNAs were evaluated using deep sequencing approach, which not only provided abundant information for sheep genomic research, but also provided a basis for further study on the function of the candidate genes and the expression regulation of the genes related to mutton quality traits. In addition, this study can also provide useful DNA markers for molecular selection of sheep.

## MATERIALS AND METHODS

### Animal care

The experimental procedures were approved by the Institutional Animal Care and Use Committee at Shihezi University.

### Sample collection and total RNA isolation

We collected the longissimus dorsi of three 1-year-old healthy female Kazak sheep were from a slaughterhouse. Surgery was carried out in a sterile environment to obtain the longissimus dorsi of three prenatal sheep. Tissue samples were immediately frozen in liquid nitrogen and stored at −80°C, respectively. The total RNA was subsequently extracted from the pooled samples using Trizol reagent (Invitrogen, Carlsbad, CA, USA) according to the manufacturer’s instructions.

### Sequencing and data analysis of small RNAs

The cDNA was synthesized by reverse transcriptase polymerase chain reaction (PCR) and the cDNA library was sequenced using Hi Seq 2000 (Illumina, San Diego, CA, USA). To improve the quality of the sequencing, it was necessary to strictly filter reads that were contaminated with primer or adapter sequence or was of low quality. The small RNAs were mapped to the reference genome sequences of sheep by Bowtie software and their expression and distribution on the reference sequences were analyzed. Integrated with miRBase database, the sRNAs aligned to the reference sequences were found and separated as much as possible from other small RNA fragments.

### Prediction of novel miRNA

Hairpin structure of the mRNA precursor can be used to predict new miRNAs. We integrated miREvo [7] and mirdeep2 software [8] to predict novel miRNA, according to the signature hairpin structure of the miRNA precursor.

### Differential expression of miRNA

To compare the differential expression of miRNAs in two libraries, we firstly normalized expression levels of miRNAs by using transcript per million (TPM) [9]. For biologically repeated samples, the analysis was performed using DESeq2 based on the negative binomial distribution [10]. For non-biologically repeated samples, TPM was used to standardize the read count data, followed by differential expression analysis with DEGseq [11]. The screening criteria for differentially expressed miRNAs were usually set by default to: p-value <0.05.

### Prediction of targeted genes and enrichment analysis

In this paper, the miRanda [12] was performed to predict targeted genes of differentially expressed miRNAs in skeletal muscle of prenatal and postnatal sheep. GOseq based on Wallenius non-central hyper-geometric distribution that eliminates gene length preference was used for gene ontology (GO) enrichment analysis [13]. And we performed Kyoto encyclopedia of genes and genomes (KEGG) pathway enrichment analysis of candidate targeted genes using KOBAS [14] software.

### Quantitative real-time polymerase chain reaction

Five miRNAs (miR-181a, mir-199, mir-127, mir-133, and mir-1) were selected from differentially expressed miRNAs that were negatively correlated with their targeted genes and their targeted genes (calsequestrin 1 [*CASQ1*], muscleblind-like splicing regulator 1 [*MBNL1*], carbonic anhydrase 4 [*CA4*], insulin-like growth factor 2 [*IGF2*], and myelin expression factor 2 [*MYEF2*]) were characterized and quantified by quantitative PCR (qPCR). The cDNA was synthesized by reverse transcription for further experiments. We performed qPCR with each sample repeated three times. Quantitative real-time (RT)-PCR was performed as follows: 1 ng of cDNA was added to reaction mixture containing 5 μL 2×UltraSYBR Mixture (CWBIO, Beijing, China), 0.6 μL primers and water added to final volume of 10 μL. The miRNA detection program was 95°C for 10 min, followed by 45 cycles of 95°C for 25 s, 58°C for 10 s, 72°C for 10 s. The β-actin gene was used as reference gene. Genes expression levels were evaluated as 2^−ΔΔct^. The primers used in this experiment were shown in Supplementary Table S1.

## RESULTS

### Summary of the raw sequence reads

**Figure 1:**
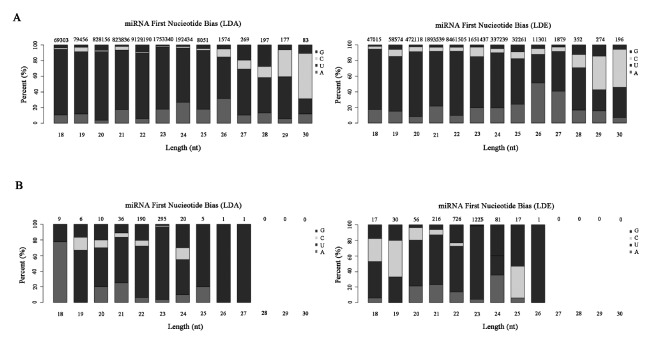
First Nucleotide Bias of miRNAs. (A) The nucleotide distributions of known miRNAs first bases of different lengths in the prenatal stage (LDE) and postnatal stage (LDA). (B) The nucleotide distributions of new miRNAs first bases of different lengths in LDA and LDE. The X-axis is the length of miRNAs, and the Y-axis is the percentage of A/U/C/G in the first base of length of the miRNA. The number above the column is the total number of miRNAs of that length. LDE, longissimus dorsi_embryo (prenatal stage); LDA, longissimus dorsi_adult (postnatal stage).

To detect miRNAs expression profile in sheep skeletal muscle, we constructed two sRNA libraries of the longissimus dorsi of sheep by high-throughput sequencing. We obtained about 16 and 15 million raw sequence reads per sample from prenatal library (Longissimus dorsi embryo [LDE, prenatal stage]) and postnatal library (Longissimus dorsi adult [LDA, pastnatal stage]), respectively (Table 1). After removing an adaptor and low quality reads, each library finally obtained 15.9 and 14.9 million clean reads with lengths between 18 to 35nt and length distribution peak at 22nt (Supplementary Figure S1), of which 88.81% and 94.24% were mapped to the sheep reference sequence, respectively. Among these sRNAs, most of them were aligned with non-miRNAs databases, such as rRNA, tRNA, snRNA (Supplementary Figure S2). Integrated with miRBase database, 1,086 of miRNAs were aligned to mature known miRNAs, which were first base biased U+G (Figure 1A). The remaining candidate sequences were used to predict potentially new miRNAs.

### Prediction of novel miRNAs

**Figure 2:**
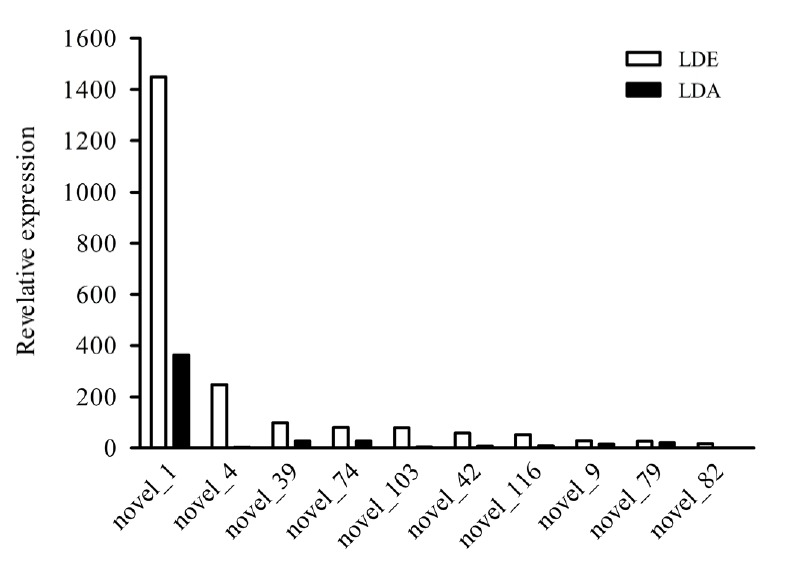
Comparison of expression of the top ten miRNAs between LDA and LDE. The X-axis is different new miRNAs, the Y-axis is the expression levels of miRNAs. The white and black icons are the expression levels of new miRNAs in LDE and LDA, respectively. LDE, longissimus dorsi_embryo (prenatal stage); LDA, longissimus dorsi_adult (postnatal stage).

To further validate the miRNA sequences, new miRNAs were predicted using miREvo and mirdeep2 software. In result, a total of 40 novel RNAs were found in LDE and LDA (Supplementary Table S2). It was worth noting that the expression of Novel_1 was the highest in two different periods (Figure 2). Interestingly, through statistical analysis of the first base preference at different lengths of miRNAs, the data indicated that the first base was predominantly preferred for U, followed by G and C at two developmental stages (Figure 1B).

### miRNA expression and difference analysis

**Figure 3:**
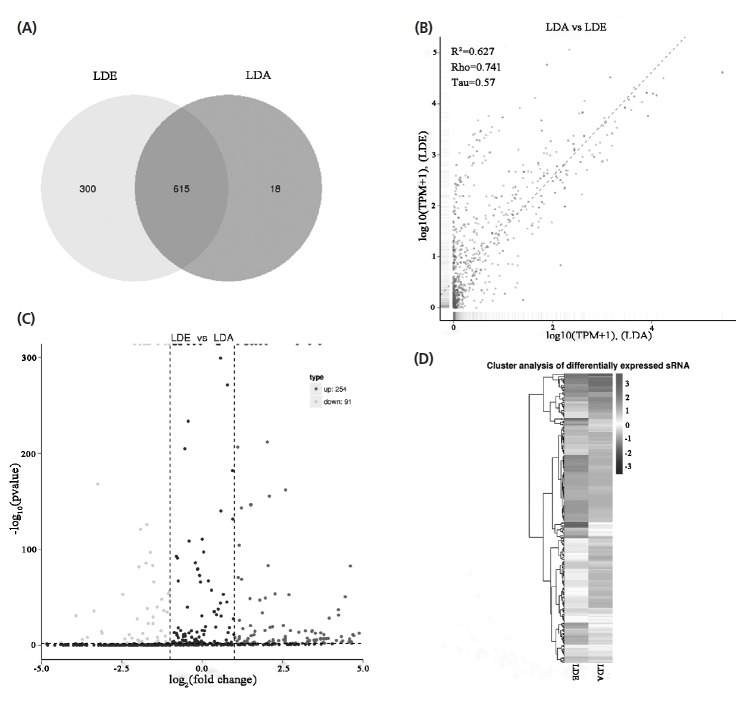
The expression of miRNAs during skeletal muscle development. (A) Venn diagram of miRNAs discovered in LDA and LDE using Illumina sequencing. (B) The scatter plot shows the correlation of miRNAs between LDA and LDE via Illumina sequencing. The X and Y-axis are the log10 (TPM+1) of the LDA and LDE, respectively (R^2^, square of the correlation coefficient of pearson; Rho, the correlation coefficient of spearman; Tau, the correlation coefficient of kendall-tau). (C) Volcano plot of differential expression miRNAs via Illumina sequencing. The X-axis represents fold change of miRNA in LDA and LDE groups, and the Y-axis represents the expression changes of miRNA statistically significant degree. The scatter in the figure represents the miRNAs, the black dots indicate the miRNAs with no significant differences, the dark gray dots indicating a significant up-regulation of the miRNA, and the light gray dots represent a significant down-regulation of the miRNA. (D) Heat map of miRNAs in two development stages via Illumina sequencing. The miRNAs were clustered with log10 (TPM+1) values. Dark gray is high expression of miRNA, and black is low expression miRNA. LDE, longissimus dorsi_embryo (prenatal stage); LDA, longissimus dorsi_adult (postnatal stage).

**Figure 4:**
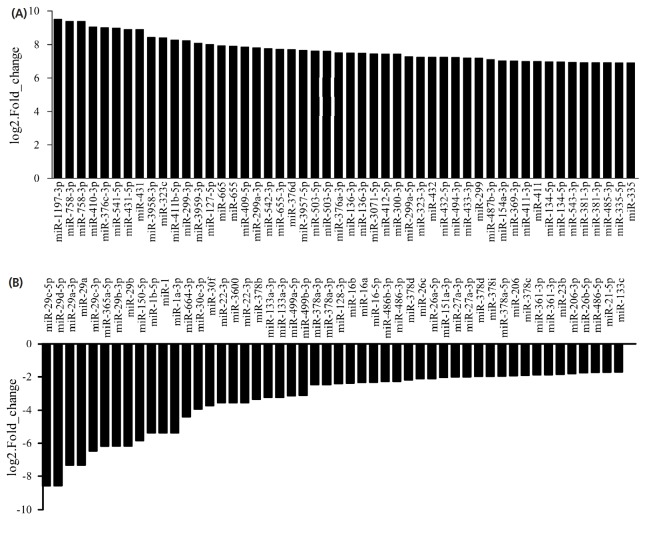
Expression levels of the top of 50 miRNAs. (A) The up-regulated top of 50 miRNAs with the most significant differentially expressed in prenatal and postnatal stages of skeletal muscle of sheep. (B) The down-regulated top of 50 miRNAs with the most significant differentially expressed in prenatal and postnatal stages of skeletal muscle of sheep.

Statistical analysis of the expression levels of known and new miRNAs in each sample was performed and normalized with TPM. Among these 1,000 miRNAs, 615 were co-expressed, while 300 and 18 were expressed in LDE and LDA, respectively (Figure 3A). Correlations of miRNAs between LDE and LDA were lower (R^2^ = 0.627, Figure 3B). To generate miRNAs with differential expressions during sheep muscle development, we detected the level of miRNAs expressions at two development stages and identified 254 up-regulated, 91 down-regulated (Figure 3C), and the top of 50 miRNA, significantly differentially expressed are shown in Figure 4. Meanwhile, hierarchical clustering of differentially expressed genes was performed, resulting in 16 subgroups. Among those subgroups, the largest subclass contained 50 differentially expressed genes (Figure 3D).

### miRNA targeted genes prediction and enrichment analysis

**Figure 5:**
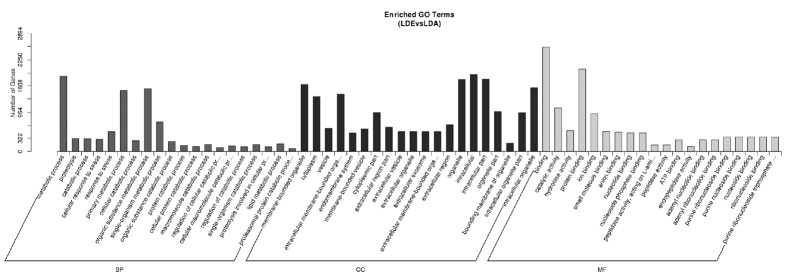
GO annotation analysis for miRNA target genes. The X-axis is the GO term, and the Y-axis is the number of candidate target genes under the one term. BP (dark gray), CC (black), and MF (light gray) are short for the biological processes, cell components and molecular function, respectively. GO, gene ontology.

Targeted genes prediction of miRNAs was performed by the miRanda software, resulting in a predicted total of 2,833 targeted genes (Supplementary Table S1). We found that different miRNAs target the same gene, conversely, the same miRNA target different genes (Table 2). The distribution of differential expressed genes in GO can be used to study the physiological functions and the biological processes in different stages of sheep (Figure 5). In the skeletal muscle of sheep, the analysis for differentially expressed genes through GO enrichment analysis showed that 184 terms were preferentially enriched in cell components, including: endoplasmic reticulum, nuclear lumen, Golgi apparatus etc. while 204 terms were significantly enriched in molecular functions including: protein binding, nucleoside phosphate binding, adenyl ribonucleotide binding etc. And 982 terms were mostly enriched in biological processes, such as metabolic process, negative regulation of signaling, positive regulation of cellular catabolic process, enzyme linked receptor protein signaling pathway etc. These results suggested that miRNAs may be involved in the regulation of muscle development by participating in protein metabolic signaling pathways.

KEGG pathway analysis of miRNAs targets showed that 273 terms were enriched. Among these pathways, the main signaling transduction pathways associated with muscle development were: Wnt signaling pathway, transforming growth factor beta signaling pathway, Hippo signaling pathway, Thyroid hormone signaling pathway, mitogen-activated protein kinase (MAPK) signaling pathway etc. (Supplementary Table S3). These results indicated that miRNAs may be involved in regulation of the development and growth of skeletal muscle.

### Regulatory network analysis of miRNA-mRNA

**Figure 6:**
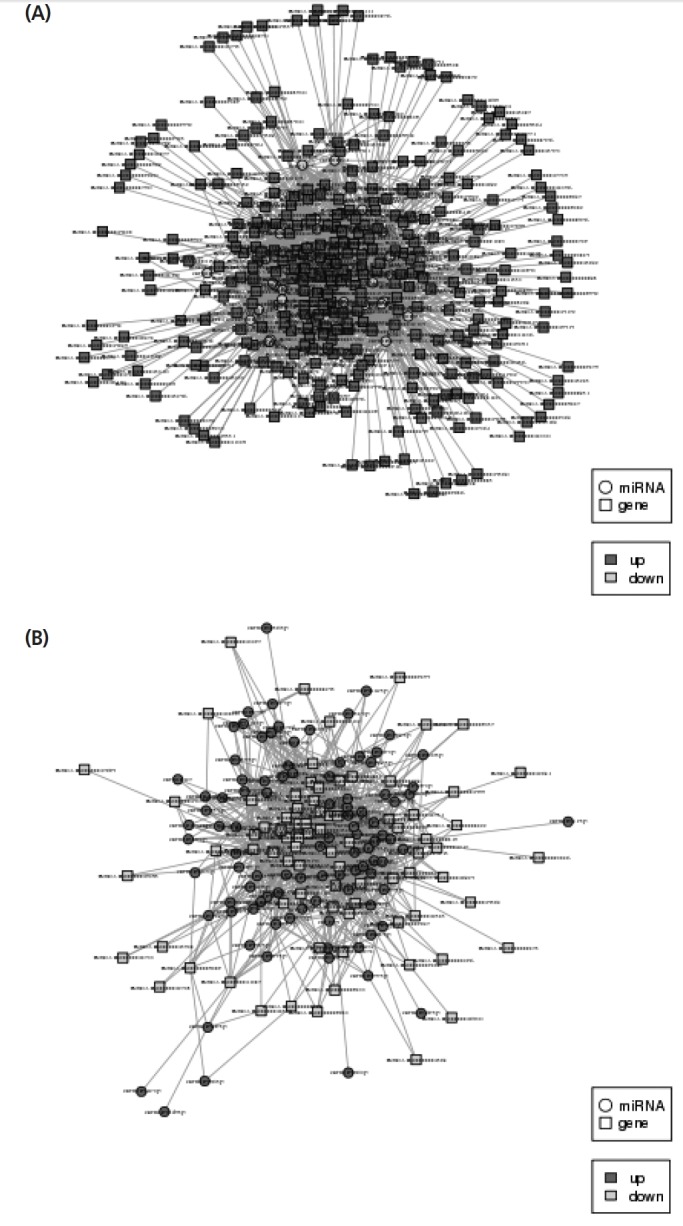
Interaction network of miRNA-mRNA for regulating skeletal muscle growth and development. (A) Down-regulated miRNA versus up-regulated mRNA. (B) Up-regulated miRNA versus down-regulated mRNA in skeletal muscle. Round is miRNA, square is gene, dark gray is the up-regulated gene, light gray is the down-regulated gene.

miRNAs, as regulatory factors, negatively regulate targeted gene expression at the post-transcriptional level by translational repression or by silencing its targeted genes. Therefore, we obtained some significant regulatory networks by analyzing down-regulation of miRNA versus up-regulated genes, and up-regulation of miRNA versus down-regulated genes, respectively. Through regulatory network analysis for down-regulated miRNA versus up-regulated mRNA, the results showed that 29 down-regulated miRNAs were correlated with 290 targeted genes (Figure 6A). Among them, 81% targeted genes may be regulated by miR-143, -miR-23a, miR-29b, miR-30c, miR-23b, miR-26a, miR-221, miR-150, miR-30a-3p, miR-191, miR-22-3p, miR-21, and miR-133. Among these targeted genes, oar-miR-133 might be regulating gene expression of insulin-like growth factor 2 (*IGF2*), intraflagellar transport 122, thyroid hormone receptor interactor 13, fat mass and obesity-associated protein (*FTO*), and ETS proto-oncogene 1 etc. In addition, novel_175 was correlated with insulin like growth factor 2 mRNA binding protein 1, fibroblast growth factor 14 (*FGF14*), v-myb avian myeloblastosis viral oncogene homolog-like 2, formin 2, meis homeobox 3 etc. Similarly, the network of up-regulated miRNA versus down-regulated mRNA in skeletal muscle is shown in Figure 6B. The result showed that 95 up-regulated miRNAs were associated with 62 targeted genes. MiR-106b may be regulate genes expression of paraoxonase 3, thioredoxin interacting protein, pyruvate dehydrogenase kinase 4, interferon related developmental regulator 1, muscular LMNA interacting protein, and tubulin alpha-8 chain. Novel_140 regulated carnitine O-acetyltransferase, solute carrier family 27 member 6, glycogenin 1, LIM and cysteine rich domains 1, solute carrier family 25 member 33, chloride voltage-gated channel 1, NADH:ubiquinone oxidoreductase core subunit S7, and myosin light chain 3.

### Verification of differentially expressed miRNA by qRT-PCR

**Figure 7:**
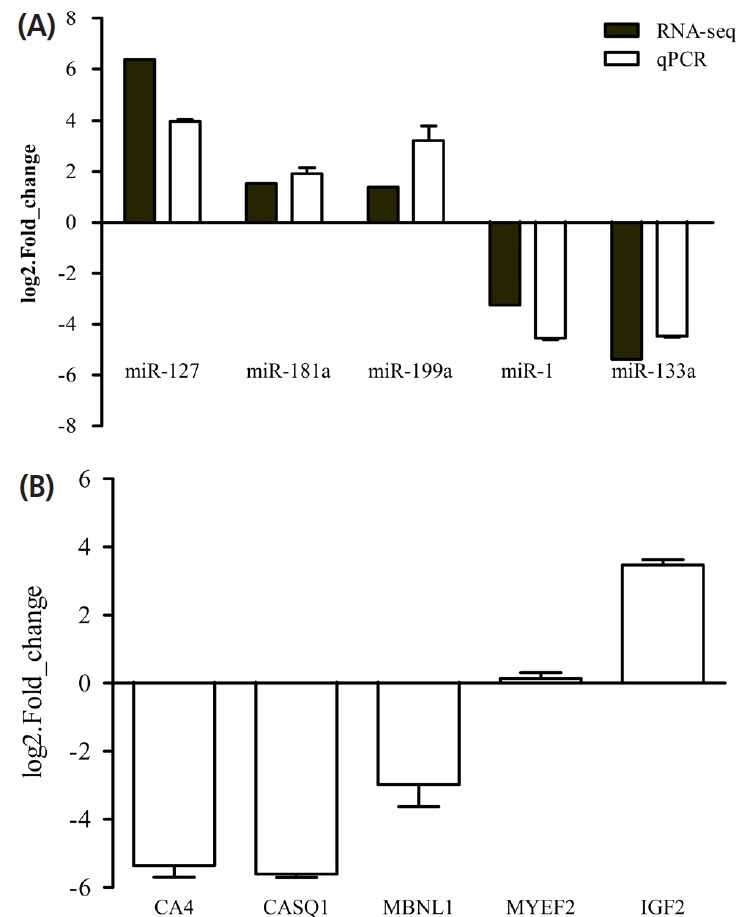
Verification of miRNAs and their target genes. (A) Verification of 5 miRNAs by qRT-PCR. The expression levels of 3 up-regulated (miR-127, miR-181a, miR-191a) and 2 down-regulated (miR-1 and miR-133a) miRNAs were consistent with the sequencing data. Black is the RNA-seq data of 5 differential miRNAs (miR-127, miR-181a, miR-191a, miR-1 and miR-133a) that were negatively correlated with their target genes; white is the relative expression level of 5 differential miRNAs detected by qPCR. (B) The relative expression level of target genes (*CA4*, *CASQ1*, *MBNL1*, *MYEF2*, and *IGF2*) detected by qPCR. There were 3 target genes (*MBNL1*, *CASQ1*, and *CA4*) that were down-regulated, and 2 target genes (*MYEF2* and *IGF2*) that were up-regulated, which were negatively correlated with their corresponding miRNA. qRT-PCR, quantitative real-time polymerase chain reaction; *CA4*, Carbonic anhydrase 4 ; *CASQ1*, calsequestrin 1; *MBNL1*, muscleblind-like splicing regulator 1; *MYEF2*, myelin expression factor 2; *IGF2*, insulin-like growth factor 2. The data are expressed as the mean±standard deviation (n = 3).

To verify the reliability of the sequencing data, we performed qRT-PCR to detect the relative expression levels of five miRNAs selected from differentially expressed miRNAs that were negatively correlated with their targeted genes. The results showed that 3 miRNAs (miR-199a, miR-181a, and miR-127) were up-regulated, 2 miRNAs (miR-1 and miR-133a) were down-regulated, which were correlated with the RNA-seq analysis (Figure 7A). Moreover, we detected the expression of mRNA of these 5 miRNAs targeted genes by qPCR. There were 3 targeted genes (*MBNL1*, *CASQ1*, and *CA4*) that were down-regulated, and 2 targeted genes (*MYEF2* and *IGF2*) that were up-regulated, which were negatively correlated with their corresponding miRNA (Figure 7B). Further, it has been proved from the results that the Illumina HiSeqTM2500 sequencing data was reliable.

## DISCUSSION

With the rapid development of high throughput sequencing technology, a growing number of miRNAs can be found, meanwhile, increasing evidence indicates the important functions of miRNAs in skeletal muscle development. So far, a great number of miRNAs have been detected in sheep muscle using RNA-seq. In this paper, we have obtained 1,126 miRNAs in LDE and LDA using RNA-seq and bioinformatics analysis.

Hairpin structure of precursor miRNA plays a major role in the annotation of miRNA. Thus, the hairpin structure can be used in computer scan prediction of high throughput sequencing [15]. In this study, we analyzed 40 new genes by analyzing the distribution of sequencing reads in the sheep genome. Liu et al [16] screened 22 new genes from Cashmere goat skin. Jinming Huang et al identified 258 new genes from Holstein Cattle [17]. Despite the screening of many new genes, the function and regulatory mechanisms of these new genes still require further experimental validation.

We found that subtypes of miRNAs were differentially ex pressed in skeletal muscle at different stages of sheep, indicating that miRNAs in sheep skeletal muscle were time-specific. Previous studies have shown that the number of muscle fibers were determined at the prenatal stage, which may affect muscle traits [3]. Our study showed that the expression level of miR-127 in the prenatal stage was higher than that in the postnatal stage, indicating that miR-127 may play an important role in embryonic myogenesis. miR-181a, up-regulated miRNA, was well known in control of myoblast differentiation of mammalian [18]. However, the expression of miR-1 and miR-133 were decreased, suggesting that muscle-specific miRNAs in adult skeletal muscle may play a role in responding to overload the function [19].

miRNAs as regulatory factors can regulate gene expres sion by binding to 3′ UTR of its target mRNAs. Here, a total of 2,833 targeted genes were predicted using miRanda software. Among these targeted genes, Sirt1, a NAD+-dependent histone deacetylase enzyme, is an important protein for muscle precursor cell proliferation [20], myoblast survival and muscle differentiation [21]. MEF2C, myocyte-specific enhancer factor 2C, promotes skeletal muscle differentiation [22]. IGF2, insulin like growth factor 2, is an important growth regulator that promotes skeletal muscle differentiation [23]. FGF8, a fibroblast growth factor, promotes muscle terminal differentiation [24].

The growth and development of skeletal muscle are regu lated by multiple signaling pathways. For example, the essential pathway for mammalian skeletal muscle formation is the Wnt signaling pathway, which facilitates the regeneration of skeletal muscle cells and induces myogenesis by activating transcription factors Myf5 and MyoD [25,26]. MAPK signal transduction pathway is another pathway targeted by miRNA in skeletal muscle, which is associated with regulation of muscle differentiation by affecting the activities of myogenic transcription factors [27]. There are additional pathways involved in skeletal muscle growth and development. Essential amino acids can stimulate human muscle protein synthesis. Through the regulation of miRNA expression, taking of essential amino acids can increase the expression of muscle protein to promote muscle growth by activating muscle transcription factors and inhibiting myostatin and MEF2C expression [28]. Gap junction connexins was involved in the cell cycle control by promoting extensive nuclear expression of cyclin-dependent kinase inhibitors to complete the differentiation of skeletal muscle [29]. Moreover, thyroid hormones regulate muscle differentiation by affecting the myogenic transcription factor activities [30]. Collectively, miRNAs in all these pathways play an essential role in the growth and development of skeletal muscle.

In conclusion, 40 potentially novel miRNAs have been identified in sheep skeletal muscle using high-throughput sequencing, which may be sheep-specific. Meantime, we screened differentially expressed miRNAs in prenatal and postnatal skeletal muscle of sheep involved in skeletal muscle development, potential muscle disease. Our results clearly demonstrated that miRNAs play an important role in regulating growth and development of sheep skeletal muscle. This study enriches the miRNA database of sheep and provides valuable references for miRNAs functions.

## Supplemental Information



## Figures and Tables

**Table 1:** The summary of sequencing data

Sample	Total reads	Clean reads	Known miRNA	Novel miRNA
Longissimus dorsi_embryo (prenatal stage)	16,245,409	15,935,490	1,042	39
Longissimus dorsi_adult (postnatal stage)	15,088,402	14,894,902	836	27

**Table 2:** Potential target genes of the partial miRNAs in sheep skeletal muscle

Target gene	Description	miRNAs
*MEF2C*	Myocyte enhancer factor 2C	miR-133c, miR-181b, miR-455, miR-135, miR-21, miR-494, miR-381
*MYEF2*	Myelin expression factor 2	miR-199a, miR-27b, miR-26a, miR-23b, miR-214, miR-499b, miR-26a, miR-125b
*IGF2*	Insulin-like growth factor 2	miR-133a, miR-214, miR-34a, miR-381
*CASQ1*	Calsequestrin 1 fast-twitch, skeletal muscle	miR-26a, miR-615, miR-181a, miR-125a, miR-1b, miR-23a, miR-214
*CDK4*	Cyclin-dependent kinase 4	miR-26a, miR-133a, miR-615, miR-23a, miR-181a, miR-214, miR-208b
*MYBL2*	V-myb avian myeloblastosis viral oncogene homolog-like 2	miR-125b, miR-1a, miR-214, miR-199b, miR-26c
*MBNL1*	Muscleblind-like splicing regulator 1	miR-23b, miR-221, miR-181a, miR-133c, miR-200a, miR-381
*Sirt1*	NAD+-dependent histone deacetylase enzyme	miR-199a, miR-26a, miR-181a, miR-499a, miR-125b
*FGF8*	Fibroblast growth factor	miR-181a, miR-615, miR-1b, miR-221, miR-133a, miR-222, miR-221
