# Supplementary material for: Expression profiles of microRNAs in skeletal muscle of sheep by deep sequencing
Source: Asian-Australas J Anim Sci. 2018 Nov 27;32(6):757–66. doi: 10.5713/ajas.18.0473 (PMC6498074; doi:10.5713/ajas.18.0473)
Supplement: Supplementary file 1 [file ajas-18-0473-suppl.pdf]

Asian-Australasian Journal of Animal Science

Supplemental Information

Expression profile of microRNAs in skeletal muscle of sheep by deep sequencing

Zhijin *Liu*[1], Cunyuan *Li*[1], Xiaoyue *Li*[1], Yang *Yao*[1], Wei *Ni*[1,*], Xiangyu *Zhang*[1], Yang *Cao*[1], Wureli *Hazi*[2], Dawei *Wang*[1], Renzhe *Quan*[1], Shuting *Yu*[1], Yuyu *Wu*[1], Songmin *Niu*[1], Yulong *Cui*[1], Yaseen *Khan*[1], and Shengwei *Hu*[1,*]

[*]**Corresponding Author: Shengwei Hu**

Tel: 0993-2058002, Fax: 0993-2058002, Email: hushengwei@163.com

Wei Ni

Tel: 0993-2058002, Fax: 0993-2058002, Email: niweiwonderful@sina.com

[1]College of Life Sciences, Shihezi University, Shihezi, Xinjiang, 832003, China

[2]College of Animal Science and Technology, Shihezi University, Shihezi, Xinjiang, 832003, China

Table S1. Primer sequences for reverse transcription PCR and qRT-PCR

	primer sequences	Tm value	Product size
miR-199a RT	CTCAACTGGTGTCGTGGAGTCGGCAATTCAGTTGAGAACA	37℃	40bp
miR-181a RT	GTCGTATCCAGTGCAGGGTCCGAGGTATTCGCACTGGATACGACACTC	37℃	48bp
miR-127 RT	GTCGTATCCAGTGCAGGGTCCGAGGTATTCGCACTGGATACGACAGCC	37℃	48bp
miR-1 RT	CTCAACTGGTGTCGTGGAGTCGGCAATTCAGTTGAGTACA	37℃	40bp
miR-133a RT	CTCAACTGGTGTCGTGGAGTCGGCAATTCAGTTGAGCAGCTG	37℃	42bp
miR-199a-F	CCCAGTGTTCAGACTACC	59℃	58bp
miR-199a-R	TGGTGTCGTGGAGTCG		
miR-181a-F	AACATTCAACGCTGTCGGT	59℃	67bp
miR-181a-R	CAGTGCAGGGTCCGAGGTAT		
miR-127-F	TCGGATCCGTCTGAGCTT	59℃	66bp
miR-127-R	CAGTGCAGGGTCCGAGGTAT		
miR-1-F	TGGAATGTAAAGAAGTA	46℃	57bp
miR-1-R	TGGTGTCGTGGAGTCG		
miR-133a-F	TTTGGTCCCCTTCAAC	53℃	58bp
miR-133a-R	TGGTGTCGTGGAGTCG		
β-actin-F	CCAACCGTGAGAAGATGACC	59℃	97bp
β-actin-R	CCAGAGGCGTACAGGGACAG		

MYEF2-F	TGTCCCTCATGAAGACTACCG	58℃	115bp
MYEF2-R	CTGGCTGGCACTAATAGGTTG		
IGF2-F	TGGCATCGTGGAAGAGTGT	58℃	134bp
IGF2-R	GGTATGCTGTGAAGTCGTCC		
MBNL1-F	CGGACAGACAGACTTGAGGTAT	58℃	170bp
MBNL1-R	CACTTTTCCCGAGAGCATC		
CASQ1-F	GATTGAGGGTGAACGAGAGC	58℃	138bp
CASQ1-R	GGGATGTAGGGGTGGAACT		
CA4-F	TGACATCCCCAGACCCAATA	58℃	107bp
CA4-R	AGCCCAGGTAGCGGAAGTA		

Table S2. The expression level of novel mi RNAs

mi RNA	LDE	LDA	mi RNA	LDE	LDA
novel_1	1452	362	novel_23	6	2
novel_4	248	3	novel_63	6	2
novel_39	99	28	novel_83	5	3
novel_74	81	28	novel_27	4	2
novel_103	80	5	novel_68	4	0
novel_42	59	9	novel_124	3	0

novel_116	53	10	novel_32	3	0
novel_9	29	16	novel_73	3	10
novel_79	27	22	novel_87	3	1
novel_82	18	1	novel_91	3	0
novel_48	15	0	novel_111	2	7
novel_69	14	2	novel_120	2	0
novel_44	9	8	novel_121	2	0
novel_96	9	5	novel_60	2	0
novel_101	8	3	novel_127	1	0
novel_25	8	4	novel_133	1	0
novel_78	8	9	novel_51	1	0
novel_17	7	5	novel_94	1	7
novel_115	6	0	novel_99	1	0
novel_19	6	1	novel_107	0	1

Table S3. KEGG pathways enriched for target gene functions of mi RNAs related to skeletal muscle in sheep

KEGG ID	Term	Gene number	Gene symbol
oas04310	Wnt signaling pathway	28	PRKCG, DKK1, PLCB4, RUVBL1, LRP6, DAAM1,

			CUL1, TBL1X, TBL1XR1, FZD3, CAMK2G, CSNK1A1, GSK3B, ROCK2, CTNNB1, WNT5A, PRKACA, NFATC3, PPP3CA, AXIN1, APC, DVL2, FBXW11, FZD4, SENP2, CAMK2A, LOC101102299
oas04010	MAPK signaling pathway	38	PRKCG, TRAF6, GADD45B, IL1B, RPS6KA1, MAP2K2, MAPK8IP3, FGF7, LOC101103463, MAP3K2, ELK4, ARRB1, MAP4K4, MAP3K5, NF1, STK4, FGF19, TAB1, FAS, RASGRP1, ECSIT, PLA2G4D, CASP3, PDGFRA, CRK, DUSP10, MEF2C, MECOM, NFATC3, LOC101114987, PAK2, HSPA8, MAPKAPK5, PRKACA, RASGRP2, PPP3CA, RASGRF1, MAPK3
oas04390	Hippo signaling pathway	28	LLGL2, CSNK1D, LLGL1, AFP, PARD3, YWHAB, BMP4, PPP1CA, SMAD2, PATJ, FZD3, FZD4, ID2, GSK3B, CTNNB1, WNT5A, APC, DVL2, ACTG1, FBXW11, TP73, YAP1, RASSF1, BMPR1B, AXIN1, WWTR1, CDKL3, LOC101102299
oas04540	Gap junction	27	TUBA8, PRKCG, CSNK1D, PLCB4, MAPK3,

			MAP3K2, PDGFD, PDGFRA, CDK1, TUBB, LOC101115640, LOC101102968, GJD2, TUBAL3, ITPR2, MAP2K2, PRKACA, LOC101108550, ADCY9, DRD2, LOC105606030, LOC105606031
oas04020	Calcium signaling pathway	26	PRKCG, ATP2A1, PLCB4, P2RX4, PTK2B, ADRA1A, LOC105605952, PDGFRA, TACR3, PDE1A, LOC101115640, PPP3CA, LOC101104306, ITPR2, LOC101108550, EDNRB, CAMK2G, CALM3, ADCY9, ORAI3, LOC101111432, PRKACA, VDAC2, CAMK2A, LOC101106702, LOC105606070
oas04919	Thyroid hormone signaling pathway	24	PRKCG, MAP2K2, PLCB4, HDAC3, HDAC1, ITGAV, ATP1B1, NCOA3, PIK3R1, SLC9A1, TSC2, GATA4, ATP1B3, GSK3B, ATP1A1, STAT4, CTNNB1, MED16, PRKACA, RXRB, MAPK3, ACTG1, KAT2A, MED13L
oas04912	GnRH signaling pathway	17	MAPK3, MAP3K2, LOC105606031, CAMK2, CAMK2G, LOC101115640, MMP14, HBEGF, PLD2, MAP2K2, PLA2G4D, ITPR2, PLCB4,

			ADCY9, LOC105606030, PTK2B, PRKACA
oas04340	Hedgehog signaling pathway	10	BMP4, FBXW11, WNT5A, PRKACA, LRP2, CSNK1A1, CSNK1D, CSNK1G3, GSK3B LOC101102299
oas04350	TGF-beta signaling pathway	14	MAPK3, CDKL3, BMP4, IFNG, CHRD, DCN, ID2, CUL1, ID3, SMAD9, PITX2, BMPR1B, SMAD2, ZFYVE9
oas04012	ErbB signaling pathway	10	MAPK3, PRKCG, PAK2, PIK3R1, CRK, MAP2K2, HBEGF, CAMK2G, CAMK2A, GSK3B
oas04210	Apoptosis	19	PRKAR1A, LOC101103784, BPHL, CAPN2, PPP3CA, IRAK3, CASP7, DFFA, NFKBIA, FAS, CAPN1, PIK3R1, CASP3, PRKACA, IL1B LOC101103463, CASP6, CASP8, LOC101103049
oas00400	Phenylalanine, tyrosine and tryptophan biosynthesis	3	LOC101103614, TAT, GOT2

Target gene	geneID	Description	sRNA
FIBP	ENSOARG0(	fibrobla	miR-3184-5p,miR-539-3p,miR-423-5p,miR-2478,miR-432-3p,miR-6525,miR-329a-5p,miR-1343-5p,novel_96,miR-543-5p,miR-491-5p,miR-324-3p,miR-3141,miR-301a-5p,miR-339a,novel_121,miR-487a-3p,miR-95-3p,miR-339-5p,novel_79,miR-485-3p,miR-26b-3p,miR-329-5p,miR-16-1-3p,miR-3963,miR-16-1-3p,miR-339b,miR-1343-5p,miR-2408,miR-3141,miR-301b-5p
PIGS	ENSOARG0(	phosphat	miR-454-3p,miR-874-5p,miR-17-3p,miR-664a,miR-155-5p,miR-324-3p,miR-664a-5p,miR-3604,miR-665,miR-122-5p,miR-6134,miR-10b-5p,miR-425-5p,miR-27b-5p,novel_32,miR-22-3p,miR-155-5p,miR-301a-3p,-miR-10a,miR-216b-3p,miR-30c-1-3p,miR-30b-3p,miR-299-3p,miR-324-3p,miR-196a-3p,novel_17,miR-486-3p,miR-143-3p,miR-664b-3p,miR-181b-1-3p,miR-377-5p,miR-140-3p,miR-5703,novel_133,miR-2300b-3p,miR-193b-5p,miR-532-3p,novel_83,miR-383-5p,miR-7859,miR-23b-3p,miR-23c,miR-148b-3p,miR-340-5p,miR-548e-3p,miR-4726-5p,miR-382-3p,miR-1285-5p,miR-361-3p,novel_111,miR-668-5p,miR-378j,miR-25,miR-3596,miR-3064-5p,miR-10b,miR-3085-3p,miR-130b-3p,miR-1306-5p,miR-4532,miR-8485,miR-30c-1-3p,miR-23a-3p,miR-1388-3p,miR-1388-5p,miR-30b-3p,miR-296-5p,miR-10b,miR-145b,miR-181b-2-3p,novel_78,miR-486b-3p,miR-6740-5p,miR-34c-3p,miR-1260b,miR-193a-5p,miR-1248,miR-329b,miR-23b,miR-488-3p,miR-2285aa,miR-370-3p,miR-152-3p,miR-17-3p,miR-30c-2-3p,miR-1260a,miR-3064-5p,miR-4286,miR-155-5p,miR-10a-5p,miR-1290,miR-299a-3p,miR-6517,miR-22-5p,miR-6119-3p,miR-33a-3p,miR-425-5p,miR-301b-3p,miR-2428,miR-323c,miR-371b-3p,miR-24-3p,miR-17-3p,miR-1306,miR-21c,miR-2387,miR-148a-3p,miR-677,miR-181b-3p,miR-299b-3p,miR-10a-5p,novel_51,miR-455-5p,miR-7-5p,miR-296-3p,miR-296-3p,miR-455-5p,miR-30b-3p,miR-21-5p,miR-1260b,-miR-197-3p,miR-22-3p,miR-145a-5p,miR-432,miR-7b-5p,miR-194-3p,miR-432-5p,miR-130a-3p,miR-377-5p,miR-26b-3p
ARHGAP28	ENSOARG0(	Rho GTPa	miR-374c-5p,miR-877-3p,miR-323c,miR-539-5p,miR-3184-3p,miR-145a-3p,miR-203b-5p,miR-1468-5p,miR-147-5p,miR-130b-5p,novel_74,miR-655-3p,miR-665,miR-22-3p,miR-331-5p,miR-3082-5p,miR-21-3p,miR-221-5p,miR-10b-3p,miR-107,miR-3591-3p,miR-655,miR-376a-5p,miR-2483-5p,miR-130b-5p,miR-221-5p,miR-1277-5p,miR-107,miR-1468,miR-124-5p,miR-200c-3p,miR-2285af,miR-200c,miR-2284z,miR-216a-3p,miR-103,miR-200b,miR-429-3p,miR-103a-3p,miR-2284aa,miR-200b-3p,miR-361-5p,miR-378d,miR-493-3p,miR-33b-3p,miR-22-3p,miR-1434-5p,miR-2300b-3p,miR-1434-3p

DCLK3	ENSOARG0(	doubleco	[illegible] 3p,miR-495-3p,miR-17-3p,miR-92a-3p,miR-147-5p,miR-203b-5p,miR-133a-5p,miR-20a-5p,miR-502-5p,miR-29a-3p,miR-670-3p,miR-146b-3p,miR-10a-5p,miR-6395,miR-6516-3p,miR-2285ab,miR-376b-3p,miR-21c,miR-654-3p,miR-345-5p,miR-130b-5p,miR-101-5p,miR-17-5p,miR-106a-5p,miR-330-3p,miR-2284d,miR-2285g,miR-544b,miR-20b,miR-187-3p,miR-377-5p,miR-29b,miR-21-5p,miR-380-3p,miR-2284ab,miR-181a-5p,miR-1434-3p,miR-145a-5p,miR-93,miR-323-3p,miR-1306-5p,miR-34c-3p,miR-29b-3p,miR-3969,miR-154b-5p,miR-199a-5p,miR-671-5p,miR-190a-3p,miR-7144-5p,miR-488-3p,miR-381-3p,miR-3154,miR-17-5p,miR-320a,miR-199a-5p,miR-3065-3p,miR-181b-5p,miR-17-3p,miR-92a-3p,miR-376b-3p,miR-217,miR-30c-2-3p,miR-483-3p,miR-1271-5p,miR-6516-5p,miR-2411-5p,miR-300,miR-10a-5p,miR-134,miR-345-3p,miR-29d-3p,miR-7134-5p,miR-212-3p,miR-181d-5p,miR-2285f,miR-2285y,miR-371a-5p,miR-489,miR-376d,miR-452-3p,miR-20a-3p,miR-7977,miR-199b-5p,miR-30c-1-3p,miR-106a-5p,miR-1839-3p,miR-494-3p,miR-326,miR-1306-5p,miR-342,miR-320b,miR-2320-3p,miR-2285e,miR-9788-3p,miR-216a-3p,miR-151a-3p,novel_4,miR-409b,miR-101a-5p,miR-450b-5p,miR-544a,miR-2284aa,miR-10b,miR-484,miR-6529a,miR-206-3p,miR-10b-5p,miR-338-3p,miR-4510,miR-539-5p,miR-3074-2-3p,miR-425-5p,miR-1271,miR-6516-3p,miR-17-3p,miR-450b-3p,miR-320e,miR-324-3p,miR-6130,miR-6516,miR-544-3p,novel_32,novel_25,miR-204-3p,miR-545-3p,miR-3065-3p,miR-2312,miR-582-5p,novel_99,miR-29b-1-5p,miR-2411,miR-20a,miR-760-3p,miR-106a,miR-320d,miR-2284m,miR-532-3p,novel_83,miR-4429,miR-378d,miR-2300b-3p,miR-5703,miR-1193,miR-2904,miR-199b-5p,miR-2285m,miR-29c-3p,miR-1839-3p,miR-206,miR-29a,miR-6119-3p,novel_87,miR-
BCLAF1	ENSOARG0(	BCL2-ass	miR-2957,miR-200a-5p,miR-26b-3p,miR-148b-5p,miR-669,miR-148b-5p,miR-148a-5p
PBX2	ENSOARG0(	pre-B-ce	miR-147a,let-7g-5p,miR-149-5p,miR-2300a-5p,miR-409b,miR-31-5p,miR-187-3p,miR-450b-5p,miR-1839-5p,miR-542-3p,miR-2284r,miR-22-3p,miR-432-3p,miR-8485,miR-296-3p,let-7a-5p,miR-376b,let-7e,miR-466f-3p,miR-1343-5p,miR-125a-5p,miR-296-3p,miR-320b,miR-182-5p,miR-197-5p,miR-2483-3p,miR-6535,miR-320c,let-7d-5p,novel_68,miR-541-5p,miR-1291,miR-877-3p,miR-505-3p,miR-6395,let-7e-5p,miR-379-5p,miR-18a-3p,miR-877-3p,miR-423-5p,miR-8095,miR-3059-5p,miR-2285t,miR-218-5p,let-7i,miR-505-5p,miR-328-3p,miR-18a-3p,miR-505-3p,let-7d,miR-4429,miR-378d,miR-1961,miR-1290,miR-1197-5p,miR-1193,miR-1343-5p,miR-490-3p,let-7g,miR-2424,miR-486-3p,miR-4286,let-7f-5p,miR-466i-5p,miR-98-5p,miR-320d,miR-450a-2-3p,let-7k,miR-543-5p,novel_127,miR-6119-5p,novel_17,miR-125b,miR-92a-1-5p,let-7c-5p,miR-324-3p,miR-6130,let-7i-5p,miR-22-3p,miR-874-3p,miR-545-3p,miR-296-3p,miR-182-5p,miR-125a,miR-23a-5p,novel_48,miR-320a,miR-129b-3p,miR-1197-3p,miR-6525,miR-6134,miR-3184-5p,let-7b-5p,miR-4510,miR-539-5p,let-7b,miR-486b-3p,miR-487a-5p,miR-4492,miR-2332,let-7f,miR-2433,miR-3969,miR-125b-5p

ZNF282	ENSOARG0(	zinc fin	[illegible]1248,miR-7144-5p,miR-874-3p,miR-574-5p,miR-215-5p,miR-141-3p,miR-615-5p,miR-2285aa,miR-598-3p,miR-542-5p,miR-2355-5p,miR-154b-5p,miR-376c-3p,miR-324-5p,miR-199a-5p,miR-5100,miR-1-5p,miR-29b-3p,miR-1306-5p,novel_42,miR-28a-3p,miR-1260b,miR-1197-3p,miR-500b-5p,miR-3578,miR-330-5p,miR-29d-3p,miR-1343-5p,miR-300,miR-6517,miR-2285ad,miR-431,miR-1224-5p,miR-412,miR-129-1-3p,novel_121,miR-214-5p,miR-545-5p,miR-582-3p,miR-450a-1-3p,miR-125a-3p,miR-1260a,miR-3071-3p,miR-181b-5p,miR-1a-2-5p,miR-127-5p,miR-17-3p,miR-28-3p,miR-543-5p,novel_96,miR-200a-3p,miR-152-3p,miR-107,miR-181a-2-3p,miR-3064-5p,miR-2478,miR-2285b,miR-490-3p,miR-199a-5p,miR-148a-3p,miR-2387,miR-1306,miR-107,miR-1291,miR-505-3p,miR-3120-3p,miR-7-1-3p,miR-670-3p,miR-411b-3p,miR-29a-3p,miR-2404,miR-576-3p,miR-6529b,miR-485-3p,novel_1,miR-29a,miR-128-1-5p,miR-6119-3p,miR-502b,miR-656-5p,miR-1a-1-5p,miR-219b-3p,miR-135a-2-3p,miR-29c-3p,miR-17-3p,miR-3957-3p,miR-24-3p,miR-2428,miR-199b-5p,miR-326-3p,novel_27,miR-1434-3p,miR-7b-5p,miR-1843b-5p,miR-181a-5p,miR-708-3p,miR-194-5p,miR-450c-3p,miR-1260b,miR-29b,miR-188-5p,miR-27b-3p,miR-2889,miR-7862,miR-103a-3p,miR-133b-5p,miR-31-5p,miR-340-3p,miR-377-5p,miR-665,miR-185-5p,miR-134-3p,miR-544b,miR-103,miR-194-3p,miR-377-3p,miR-7-5p,novel_51,miR-194a,miR-330-3p,miR-190a,miR-378a-5p,miR-1343-5p,miR-6240,miR-130b-5p,miR-363-5p,miR-2483-5p,miR-432-3p,miR-654-3p,miR-432-3p,miR-2285r,miR-450a-1-3p,miR-21a-3p,miR-129b-3p,miR-598-3p,novel_99,miR-186-5p,miR-1895,miR-543-3p,miR-2331-3p,miR-504-5p,miR-552-3p,miR-139-5p,miR-216b-5p,miR-6130,miR-544-3p,miR-132-5p,miR-376e-3p,miR-192-
EDNRB	ENSOARG0(	endothel	miR-7705,miR-211,miR-382-5p,miR-19b-1-5p,miR-2440,miR-204-5p,miR-6123,novel_1,novel_74,miR-211-5p,miR-452-3p,miR-548o-3p,miR-574-5p,miR-223-5p,miR-2483-3p,miR-6516,miR-18a-3p,novel_99,miR-1814c,miR-186-5p,miR-1248,miR-493-5p,miR-19a-3p,miR-668-5p,miR-2318,miR-493-5p,miR-6240,miR-758-3p,miR-2310,miR-29a-5p,miR-1957a,miR-8485,miR-2284l,miR-148b-5p,miR-330-3p,novel_17,novel_127,miR-196b-5p,miR-330-3p,miR-18a-3p,miR-148b-5p,miR-6516-5p,miR-3431,miR-758-3p,miR-2113,miR-196a-5p,miR-19b-2-5p,miR-148a-5p,miR-19b-3p,miR-450b-5p

			[illegible] 5p,miR-214-3p,miR-381-5p,miR-199b-5p,miR-1298-3p,miR-411b-5p,miR-2459,miR-541,miR-3956-3p,miR-190b-5p,novel_111,miR-877-3p,miR-361-3p,miR-423-5p,miR-2440,novel_82,miR-378g,miR-3596,miR-409-5p,miR-504,miR-3963,miR-377-5p,miR-376a-5p,miR-486-3p,miR-422a,miR-92a-1-5p,miR-324-3p,miR-98-5p,let-7k,miR-106a,miR-18a-3p,miR-328-3p,miR-1961,miR-378i,miR-217-5p,miR-378a-3p,miR-33b-3p,miR-493-3p,miR-34a-5p,miR-664-5p,miR-3074-5p,let-7b,miR-214,miR-769-5p,miR-122-5p,miR-3184-5p,miR-2285j,let-7f,miR-665,miR-190a-5p,miR-2331-3p,miR-504-5p,miR-30c-1-3p,miR-296-3p,miR-216b-3p,miR-216b-5p,miR-10a,let-7i-5p,miR-2285c,miR-433-3p,miR-339b,miR-186-5p,miR-885-3p,miR-582,miR-296-3p,miR-105-5p,miR-378a-5p,miR-103a-3p,miR-31-5p,miR-339-5p,miR-134-3p,miR-3431,miR-194-3p,miR-149-5p,miR-542-3p,miR-22-3p,miR-381-5p,miR-199b-5p,miR-412-5p,miR-6123,miR-3074-5p,miR-181b-3p,miR-320c,miR-677,miR-216a-3p,miR-150-5p,miR-3970,let-7e-5p,miR-378f,miR-3064-5p,miR-2285b,let-7g,novel_39,let-7f-5p,novel_96,novel_127,miR-6517,miR-412,novel_79,miR-1224-5p,miR-486-5p,miR-330-5p,miR-23b-5p,miR-1343-5p,miR-885-3p,miR-1260b,miR-125b-5p,miR-3068-3p,miR-125a,miR-1271,miR-2355-5p,miR-2285aa,miR-300-3p,miR-20b-5p,miR-106a-5p,miR-2330-5p,let-7a-5p,miR-30c-1-3p,miR-4532,miR-326,novel_103,miR-5126,novel_124,miR-3085-3p,let-7e,miR-105-1,miR-2300a-5p,miR-2285e,miR-9788-3p,miR-216a-3p,miR-500-5p,miR-10b,miR-378b,miR-484,miR-296-5p,miR-30b-3p,miR-412-3p,miR-7857,miR-450b-5p,miR-378c,miR-541-3p,novel_60,miR-1296-5p,miR-2285f,miR-362-5p,miR-107-5p,miR-769-5p,miR-1973,miR-7134-3p,miR-10b,miR-452-
GML	ENSOARG0(	glycosyl	3p,miR-3064-5p,miR-499b-5p,miR-489,miR-378e,let-7d-

ZNF836	ENSOARG0(	zinc fin	[illegible]106a-5p,let-7a-5p,miR-378c,miR-412-3p,miR-143-5p,miR-378b,miR-500-5p,miR-142-3p,miR-27a-3p,miR-222-5p,miR-5010-3p,miR-2284q,miR-371a-5p,miR-30b-5p,miR-219a-2-3p,miR-8095,miR-362-5p,miR-181d-5p,miR-452-5p,miR-2285f,miR-3059-5p,miR-129b-5p,miR-27a-3p,let-7d-5p,miR-3082-5p,miR-7857-3p,miR-378e,miR-499b-5p,miR-541-5p,miR-664b,miR-34b-5p,miR-221-5p,miR-760-3p,miR-466i-5p,miR-106a,miR-216a-5p,miR-2411,miR-146a-5p,miR-125b-2-3p,miR-130a-5p,miR-20a,miR-19b-3p,miR-188-3p,miR-1197-5p,miR-2904,miR-376a-5p,miR-1246,novel_133,miR-199a-3p,miR-532-3p,miR-222-5p,miR-3600,miR-548e-3p,miR-450b-3p,miR-29b-2-5p,miR-19b-1-5p,miR-338-3p,let-7b-5p,miR-3074-2-3p,miR-4510,miR-145a-3p,miR-378c,miR-539-5p,miR-30f,miR-21a-3p,novel_32,miR-6130,miR-532-5p,miR-21-3p,miR-221-5p,miR-140-5p,miR-378a-3p,miR-1343-5p,miR-106a-5p,miR-17-5p,miR-190a,miR-345-5p,miR-654-3p,miR-1260b,miR-138,miR-181a-5p,miR-1434-3p,miR-20b,miR-6238,let-7g-5p,miR-665,miR-133b-5p,miR-199c,miR-656-5p,let-7i,miR-34c,miR-20a-5p,miR-502-5p,miR-449a,miR-326-3p,miR-24-3p,miR-211,novel_101,miR-495-3p,miR-135a-2-3p,miR-19a-3p,miR-450b-3p,miR-2285ab,miR-34b,miR-378b,miR-181b-5p,let-7c-5p,miR-30c-2-3p,miR-1260a,miR-4286,miR-30a-3p,miR-29b-2-5p,let-7a-2-3p,miR-496,miR-582-3p,let-7d,miR-411-3p,miR-545-5p,miR-378h,miR-2411-5p,miR-204-5p,miR-138-5p,miR-331-3p,miR-26c,miR-5100,miR-365a-5p,miR-378c,miR-145-3p,miR-93,miR-500b-5p,miR-17-5p,miR-362-5p,miR-503-3p,miR-34c-5p,miR-598-3p,miR-214-3p,miR-146b,miR-1185-5p,miR-106b-5p,miR-199a-3p,miR-1b-5p,miR-378d,miR-744-3p,miR-23a-3p,miR-101b-3p,miR-2459,miR-1185-3p,miR-421,miR-455-3p,miR-147a,miR-548w,miR-199b-3p,miR-490-
ACTR5	ENSOARG0(	ARP5 act	miR-378b,miR-361-3p,miR-7975,miR-6395,miR-499b-5p,miR-26b-3p,miR-7977,miR-2285ad,miR-328-3p,miR-331-3p,miR-874-5p,miR-543-5p,miR-345-3p,miR-382-3p,miR-423-5p,novel_27,miR-3184-5p

SKI	ENSOARG0(	SKI prot	miR-34c,miR-20a-5p,miR-6123,miR-128-1-5p,miR-92a-3p,miR-29a,miR-3957-3p,miR-29c-3p,miR-326-3p,miR-449a,miR-2285m,miR-2428,miR-211,miR-1307-3p,miR-1306,miR-21c,miR-376b-5p,miR-2387,miR-9851-3p,miR-450b-3p,miR-10a-5p,miR-363,miR-329-5p,miR-669,miR-299b-3p,miR-34b,miR-29a-3p,miR-2285v,miR-32,miR-106a-5p,miR-17-5p,miR-452-5p,miR-2285r,miR-450c-3p,miR-22-3p,novel_23,miR-1434-3p,miR-2889,miR-29b,miR-21-5p,miR-665,novel_116,miR-194-3p,miR-149-5p,miR-20b,miR-100-3p,miR-2433,novel_63,miR-2284k,miR-29b-3p,miR-2332,miR-204-5p,miR-486b-3p,miR-1306-5p,miR-93,miR-93-5p,miR-376c-5p,miR-3154,miR-17-5p,miR-30e-5p,miR-424-3p,miR-376c-5p,miR-23a-5p,miR-20b-5p,miR-329b,miR-370-3p,miR-34c-5p,miR-365a-3p,miR-25-3p,miR-543-5p,miR-92a-3p,miR-376c-5p,miR-376b-5p,miR-3065-3p,miR-92b-3p,miR-29d-3p,miR-330-5p,miR-30a-5p,miR-155-5p,miR-10a-5p,miR-299a-3p,miR-450a-1-3p,miR-505-5p,miR-6516-5p,miR-2285n,miR-4726-5p,miR-2447,miR-423-5p,novel_94,miR-504,miR-2285u,miR-211-5p,miR-25,miR-106b-5p,miR-1306-5p,miR-326,let-7c-3p,miR-33a-5p,miR-124a,miR-106a-5p,miR-10b,miR-143-5p,miR-450b-5p,miR-548w,miR-216a-3p,miR-2459,miR-129-5p,miR-4492,miR-450b-3p,miR-2330-3p,miR-3184-3p,miR-3184-5p,miR-10b-5p,miR-6525,miR-30d-5p,miR-450a-1-3p,miR-625-5p,miR-582-5p,let-7g-3p,miR-30f,miR-3065-3p,miR-32-5p,miR-504-5p,miR-2331-3p,miR-204-3p,miR-22-3p,miR-155-5p,miR-582-5p,miR-10a,miR-2284s,miR-6516,miR-185-3p,miR-208b-3p,miR-34b-5p,miR-30d,miR-299-3p,miR-106a,miR-20a,miR-2285w,miR-486-3p,miR-421-5p,miR-124-3p,miR-5703,miR-30c-5p,novel_133,miR-2408,miR-193b-5p,miR-2300b-3p,miR-34a-5p,miR-378d,miR-184-3p,miR-548e-3p,miR-106a,novel_83,miR-2284h-5p,miR-7859

CHRNA1	ENSOARG0(	choliner	[illegible] 3p,miR-155-5p,miR-219b-3p,miR-324-3p,miR-665,miR-664a-5p,miR-2285j,miR-6134,miR-181c-5p,miR-769-5p,miR-6525,miR-224-5p,miR-3958-5p,miR-2330-3p,miR-214,miR-129-2-3p,miR-625-5p,miR-1827,let-7g-3p,miR-30d-5p,miR-21a-3p,miR-3613-5p,miR-204-3p,miR-155-5p,miR-376e-3p,miR-216b-5p,miR-296-3p,miR-552-3p,miR-182-5p,miR-30c-1-3p,miR-140-5p,miR-221-5p,miR-548o-3p,miR-221-5p,miR-30d,miR-299-3p,miR-21-3p,miR-760-3p,miR-92a-1-5p,miR-185-3p,miR-30b,novel_17,miR-486-3p,miR-2411,miR-143-3p,miR-376c-3p,miR-181b-1-3p,miR-125b-2-3p,miR-130a-5p,miR-329a-5p,miR-664-5p,miR-361-5p,miR-493-3p,miR-33b-3p,miR-2904,miR-5703,miR-1197-5p,miR-188-3p,miR-30c-5p,miR-362-3p,miR-2300b-3p,miR-193b-5p,miR-532-3p,miR-487a-3p,miR-7859,miR-3600,miR-548e-3p,miR-2284q,miR-450a-5p,miR-769-5p,miR-371a-5p,miR-30b-5p,miR-2440,miR-219a-2-3p,miR-2285f,miR-452-5p,miR-181d-5p,miR-3059-5p,miR-2447,miR-335-3p,miR-376a-3p,miR-181a-3p,miR-136-5p,miR-376d,miR-1185-2-3p,miR-4443,miR-3082-5p,miR-211-5p,miR-24-1-5p,miR-4792,miR-214-3p,miR-3955-3p,miR-505,miR-362-3p,miR-2957,miR-182-5p,novel_103,miR-1306-5p,miR-326,miR-3187-3p,miR-30c-1-3p,miR-101b-3p,miR-382-3p,miR-362-3p,miR-101a-5p,miR-125b-2-3p,miR-2284r,miR-329-3p,miR-1185-3p,miR-2459,miR-376b-3p,miR-181b-2-3p,miR-222-5p,miR-145b,miR-105-1,miR-409b,novel_78,miR-2300a-5p,miR-330-3p,miR-592,miR-486b-3p,miR-204-5p,miR-331-5p,novel_9,miR-144,miR-551b-3p,miR-29b-3p,miR-2332,miR-5100,miR-181c-3p,miR-1260b,miR-876-3p,miR-329a,miR-30e-5p,miR-331-5p,miR-376c-3p,miR-374c-3p,miR-3959-5p,miR-769,miR-370-3p,miR-2285aa,miR-17-3p,miR-2898,miR-127-5p,miR-181b-5p,miR-6536,miR-30c-2-3p,miR-30f,miR-181c-miR-377-3p,miR-224-5p,miR-342,novel_51,miR-103a-2-5p,miR-153-3p,miR-1343-5p,let-7f-2-3p,let-7f-2-3p,miR-758-3p,miR-484,miR-758-3p,novel_23,miR-374b-3p,miR-2426,miR-21-5p,miR-1839-5p,miR-1343-3p,miR-31-5p,miR-134-3p,miR-153,miR-2459,miR-1185-3p,miR-216a-3p,miR-148b-5p,miR-142b,miR-2285n,miR-107-5p,miR-2285y,miR-374a-3p,miR-656-5p,miR-22-5p,miR-203b-5p,miR-135a-2-3p,miR-342-3p,miR-2447,miR-219a-5p,miR-2284w,miR-361-3p,miR-21c,miR-136-5p,miR-412-3p,miR-1983,miR-20a-3p,miR-7977,miR-3120-3p,miR-378g,miR-1185-2-3p,miR-200c,miR-217,miR-324-3p,miR-200c-3p,miR-148b-5p,novel_133,miR-1193,miR-217-5p,miR-1343-5p,miR-148a-5p,miR-378d,miR-200b-3p,miR-429-3p,miR-2285ad,miR-200b,miR-383-5p,miR-532-3p,miR-582-3p,miR-135a-1-3p,miR-551b-3p,miR-320e,miR-100-3p,miR-374b-3p,miR-208b-5p,miR-103b,miR-374a-3p,miR-224-5p,miR-
C1orf68	ENSOARG0(	chromoso	338-3p,miR-23a-5p,miR-1271,miR-3965,miR-377-3p,miR-204-3p

SZT2	ENSOARG0(	seizure	[illegible] 424-5p,miR-664a,miR-6516-3p,miR-204-3p,novel_32,miR-6130,miR-323-5p,miR-216c-5p,miR-183-5p,miR-2432,miR-146a-5p,miR-29b-1-5p,miR-185-3p,miR-196a-3p,miR-34b-5p,miR-21-3p,miR-466i-5p,miR-760-3p,miR-1b-3p,miR-505-3p,novel_83,miR-383-5p,miR-15a-5p,miR-2904,miR-140-3p,miR-1193,miR-5703,miR-2300b-3p,miR-2408,miR-378d,miR-181d-5p,miR-541-3p,miR-107-5p,miR-2285t,miR-7977,let-7d-5p,miR-361-3p,novel_94,miR-877-3p,let-7a-5p,miR-424-5p,miR-382-3p,miR-2330-5p,miR-4532,miR-30c-1-3p,novel_103,miR-326,miR-2285o,let-7e,miR-222-5p,novel_4,miR-216a-3p,miR-9788-3p,miR-2285e,miR-125b-2-3p,miR-296-5p,miR-143-5p,miR-3120-5p,miR-365a-5p,miR-29b-3p,miR-410-5p,miR-1-5p,miR-486b-3p,miR-138-5p,miR-370-3p,miR-34c-5p,miR-3154,miR-488-3p,miR-671-5p,miR-485-5p,let-7g-3p,miR-29b-2-5p,miR-1957a,miR-30c-2-3p,let-7c-5p,miR-339a,miR-107,miR-543-5p,miR-450a-2-3p,miR-191-3p,miR-181b-5p,miR-214-5p,miR-29d-3p,miR-218-2-3p,miR-134,miR-128-3p,let-7d,miR-135a-2-3p,miR-449a,miR-326-3p,miR-2428,miR-371b-3p,miR-24-3p,miR-16a,miR-34c,let-7i,miR-15a,miR-6529b,miR-6402,miR-199c,miR-128-1-5p,miR-146b-3p,miR-323a-5p,miR-29a-3p,miR-34b,miR-18a-3p,miR-505-3p,miR-195a-5p,miR-6516-3p,miR-1291,miR-450b-3p,miR-107,miR-130b-5p,miR-503-5p,miR-452-5p,miR-124-5p,miR-133b-5p,miR-103,miR-2285g,miR-503-5p,let-7g-5p,miR-185-5p,miR-873a-5p,miR-653,miR-1285,miR-181a-5p,miR-2889,miR-138,miR-29b,miR-665-5p,miR-214,miR-433-5p,let-7b,miR-3074-5p,miR-3184-5p,miR-122-5p,miR-181c-5p,miR-6525,novel_44,let-7f,miR-4492,miR-129-5p,miR-874-5p,miR-130b-5p,miR-30c-1-3p,let-7i-5p,miR-15b-5p,miR-2285c,miR-885-3p,miR-625-5p,miR-1827,miR-186-5p,miR-339b,miR-329a-
RASGEF1B	ENSOARG0(	RasGEF d	miR-16b,miR-365b-5p,miR-452-5p,miR-424-5p,miR-30a-3p,miR-143-3p,miR-8485,miR-450b-5p,miR-16b,miR-497-5p,miR-3960,miR-30d-3p,miR-2320-3p,miR-15a-5p,miR-322-5p,novel_83,miR-30e-3p,miR-15b-3p,miR-15a,miR-665,miR-16a,miR-30a-3p,miR-2285t,miR-424-5p,miR-4510,miR-16-5p,miR-365a-5p,miR-433-3p,miR-195a-5p,miR-452-3p,miR-6130,miR-15b-5p

INSR	ENSOARG0(	insulin	miR-7641,miR-1224-5p,miR-3600,miR-2285ad,miR-532-3p,miR-582-3p,miR-545-5p,miR-2284h-5p,novel_121,miR-322-5p,miR-505-5p,miR-15a-5p,miR-378h,miR-378a-3p,miR-1434-5p,novel_133,miR-378i,miR-362-3p,miR-493-3p,miR-2887,miR-4286,miR-216c-5p,miR-2285w,novel_39,miR-200a,miR-1271-3p,miR-28b,miR-29b-1-5p,miR-148b-5p,miR-142a-3p,miR-125b-2-3p,miR-500a-5p,miR-422a,miR-125b,miR-205-5p,miR-1260a,miR-543-5p,miR-21-3p,miR-370-5p,miR-532-5p,miR-125a,miR-140-5p,miR-378a-3p,miR-874-3p,miR-15b-5p,miR-2284s,miR-129b-3p,miR-3154,miR-625-5p,miR-485-5p,miR-329a,miR-16-5p,miR-378c,miR-378c,miR-1260b,miR-125b-5p,miR-665,miR-370-5p,miR-1468-5p,miR-424-5p,miR-1843b-3p,miR-27a-3p,novel_78,novel_4,miR-149-5p,miR-142-3p,miR-185-5p,miR-134-3p,miR-1298-3p,miR-197-3p,miR-374b-3p,miR-125b-2-3p,miR-873a-5p,miR-484,miR-378b,miR-2284r,miR-329-3p,miR-497-5p,miR-27b-3p,miR-16b,miR-362-3p,miR-378c,miR-1260b,miR-127-3p,miR-424-5p,miR-2330-5p,miR-8485,miR-1b-5p,miR-378d,miR-2285r,miR-29b-1-5p,miR-362-3p,miR-3141,miR-16b,novel_51,miR-125a-5p,miR-1468,miR-25-5p,miR-378b,miR-452-3p,miR-27a-3p,miR-454-5p,miR-4443,miR-378g,miR-3596,miR-378e,miR-2483-3p,miR-378b,miR-361-3p,miR-3141,miR-378f,miR-195a-5p,miR-136-5p,miR-1193,miR-24-3p,miR-16a,miR-2285y,miR-15a,miR-4324,miR-28c
KPRP	ENSOARG0(	keratino	miR-502b,miR-382-5p,miR-502-5p,miR-4324,miR-576-3p,miR-326-3p,miR-211,miR-24-3p,miR-1839-3p,miR-1193,miR-7975,miR-146b-5p,miR-2319a,miR-379-5p,miR-1291,miR-19a-3p,miR-150-5p,miR-376b-5p,miR-670-3p,miR-3120-3p,miR-3074-5p,miR-125a-5p,miR-660,miR-466f-3p,miR-2331-5p,novel_51,miR-7-5p,miR-146a,miR-345-5p,miR-432-3p,miR-101-5p,miR-188-5p,miR-219a-1-3p,miR-22-3p,miR-542-3p,miR-7b-5p,novel_116,miR-3431,miR-26b-3p,miR-3432a,miR-204-5p,miR-28-5p,miR-125b-5p,miR-26c,miR-409-3p,miR-370-5p,miR-500b-5p,miR-193a-5p,miR-144-5p,miR-376c-5p,miR-23a-5p,miR-3074-1-3p,miR-190a-3p,miR-485-5p,miR-671-5p,miR-10b-3p,miR-345-5p,miR-329a,miR-362-5p,miR-376c-5p,miR-125a,miR-215-5p,miR-574-5p,miR-543-5p,miR-450a-2-3p,miR-30c-2-3p,miR-205-5p,miR-664-3p,miR-125a-3p,miR-3956-5p,miR-2478,miR-29b-2-5p,miR-330-5p,miR-1290,novel_121,miR-2285ad,miR-2285t,miR-4726-5p,miR-301a-5p,novel_82,miR-7705,miR-362-5p,miR-452-5p,miR-3059-5p,miR-2447,miR-335-3p,miR-378j,miR-361-3p,miR-144-5p,miR-211-5p,miR-7977,miR-664b,miR-146b,miR-3955-3p,miR-362-3p,miR-326,miR-8485,miR-193a,miR-30c-1-3p,miR-376b,miR-1839-3p,miR-362-3p,miR-296-5p,miR-329-3p,miR-301b-5p,miR-455-3p,miR-500-5p,miR-147a,miR-1298-3p,novel_78,miR-708-5p,miR-487a-5p,miR-29b-2-5p,miR-665,miR-2285j,miR-338-3p,miR-145a-3p,miR-3074-5p,miR-1827,miR-345-5p,miR-129b-3p,miR-22-3p,miR-204-3p,miR-877-5p,miR-192-5p,miR-370-5p,miR-30c-1-3p,miR-221-5p,miR-660-5p,miR-1277-5p,miR-221-5p,miR-324-3p,miR-500a-5p,miR-125b,miR-146a-5p,miR-376a-5p,miR-345-3p,miR-361-5p,miR-378d,miR-19b-3p,miR-1197-5p,miR-1193,miR-5703,novel_133,miR-215-5p,miR-1246,miR-362-3p,miR-212-5p

FGB	ENSOARG0(	fibrinog	[illegible] 5p,miR-154b-5p,miR-488-3p,miR-301,miR-331-5p,miR-93-5p,miR-17-5p,miR-3154,miR-329a,miR-345-5p,novel_48,miR-20b-5p,miR-671-5p,miR-6128,miR-193a-5p,miR-103b,miR-93,miR-365a-5p,miR-181c-3p,miR-494-5p,miR-331-5p,miR-134-5p,miR-191-5p,miR-486b-3p,miR-2285ad,miR-429-3p,miR-7641,novel_79,miR-431,miR-302a-5p,miR-758-5p,miR-2411-5p,miR-582-3p,miR-134-5p,novel_121,miR-1290,miR-134,miR-3578,miR-2427,miR-330-5p,miR-23b-5p,miR-411-3p,miR-105-2,miR-200b-3p,miR-3529-3p,miR-30a-3p,miR-181a-2-3p,miR-2284f,miR-450b-5p,miR-148b-5p,miR-200a,miR-29a-5p,miR-490-3p,miR-205-5p,miR-2285af,miR-200c,miR-30c-2-3p,miR-3071-3p,miR-339a,miR-17-3p,miR-450a-2-3p,miR-200c-3p,miR-127-5p,miR-200a-3p,miR-320b,miR-3120-3p,novel_69,miR-34b,miR-3970,miR-21c,miR-3065-5p,miR-410-5p,miR-2319a,miR-146b-5p,miR-342-3p,miR-135a-2-3p,novel_101,miR-495-3p,miR-17-3p,miR-2285x,novel_27,miR-374c-5p,miR-449a,miR-326-3p,miR-4324,miR-576-3p,miR-20a-5p,miR-34c,miR-2284o,novel_87,miR-379-3p,novel_1,miR-31-5p,miR-133b-5p,miR-665,miR-339-5p,miR-377-5p,miR-3535,miR-3431,miR-149-5p,miR-2355-3p,miR-20b,miR-873a-5p,miR-1285,miR-197-3p,miR-3071-5p,miR-219a-1-3p,miR-7862,miR-130b-5p,miR-493-5p,miR-432-3p,miR-432-3p,miR-296-3p,miR-345-5p,miR-224-5p,miR-377-3p,miR-296-3p,miR-146a,miR-365b-5p,miR-190a,miR-105-5p,novel_51,miR-106a-5p,miR-17-5p,miR-30c-1-3p,miR-140-5p,miR-221-5p,miR-2331-3p,miR-216b-3p,miR-377-3p,miR-216b-5p,miR-21a-3p,miR-27b-5p,miR-500a-3p,miR-487b-5p,miR-411,miR-339b,miR-345-5p,novel_99,miR-186-5p,miR-539-5p,miR-145a-3p,miR-3958-5p,miR-425-5p,novel_44,miR-224-5p,miR-338-3p,miR-30e-3p,miR-30a-3p,miR-338-5p,novel_74,miR-450b-3p,miR-487b-
LOX	ENSOARG0(	lysyl ox	miR-199c,miR-218-5p,novel_111,miR-423-5p,miR-2284w,miR-212-3p,miR-2284j,miR-493-5p,miR-378j,miR-3065-5p,miR-3591-3p,miR-25,miR-411b-3p,miR-2483-3p,miR-203-3p,miR-677,miR-320c,miR-181b-3p,miR-7-1-3p,miR-320b,miR-365b-5p,novel_51,miR-2284l,miR-193a,miR-2310,miR-130b-5p,miR-493-5p,miR-668-3p,miR-101a-5p,miR-21b,miR-2284x,miR-1434-3p,miR-125b-2-3p,novel_23,miR-145a-5p,miR-455-3p,miR-2355-3p,miR-199b-3p,miR-181b-2-3p,miR-145b,miR-26b-3p,miR-708-5p,miR-28-5p,miR-130b-5p,miR-2284k,miR-135a-1-3p,miR-365a-5p,miR-3184-5p,miR-3184-3p,miR-2318,miR-1814c,miR-582-5p,miR-301,miR-320a,miR-582-5p,miR-21-3p,miR-2355-5p,miR-141-3p,miR-2284m,miR-202-5p,miR-320d,miR-200a-3p,miR-200a,miR-2285b,miR-181a-2-3p,miR-582-3p,miR-4429

[illegible]
5p,miR-877-3p,novel_60,miR-32-3p,miR-212-3p,miR-
2285f,miR-452-5p,miR-181d-5p,miR-502-3p,miR-9-3p,miR-
154a,miR-371a-5p,miR-2284q,miR-105-1,miR-9788-3p,miR-
484,miR-30b-3p,miR-544a,miR-33a-5p,miR-326,miR-1306-
5p,miR-3085-3p,miR-3955-3p,miR-542-5p,miR-660-5p,miR-32-
3p,miR-21-3p,miR-544-3p,miR-139-5p,miR-6130,miR-22-
3p,miR-204-3p,miR-3613-5p,let-7f-1-3p,miR-27b-5p,let-7g-
3p,miR-2312,novel_99,miR-4510,miR-206-3p,miR-338-3p,miR-
6134,miR-19b-1-5p,miR-450b-3p,miR-17-3p,miR-708-5p,miR-
548e-3p,miR-200b,miR-2904,miR-1193,miR-1197-5p,miR-
5703,let-7a-3p,miR-130a-5p,miR-146a-5p,miR-2411,miR-542-
5p,miR-216a-5p,miR-760-3p,miR-2404,miR-2285ab,miR-
21c,miR-18a-3p,miR-6395,miR-542-5p,miR-17-3p,miR-211,miR-
24-3p,miR-323c,miR-326-3p,novel_27,miR-3613,miR-382-
5p,miR-92a-3p,novel_1,miR-656-5p,miR-147-5p,miR-26b-
3p,miR-185-5p,miR-544b,miR-653,miR-181a-5p,miR-30b-
3p,miR-2284ab,miR-21-5p,miR-455-5p,miR-744-5p,miR-130b-
5p,miR-2483-5p,miR-654-3p,miR-296-3p,miR-345-5p,miR-455-
5p,miR-124-5p,miR-660,miR-141-3p,miR-154b-5p,miR-
3154,miR-10b-3p,miR-7144-5p,miR-23a-5p,miR-671-5p,miR-
1306-5p,miR-3607-3p,miR-6128,miR-103b,miR-374a-3p,miR-
410-5p,miR-551b-3p,miR-26c,miR-3969,miR-767-5p,miR-374b-
3p,miR-204-5p,miR-28-5p,miR-483-3p,miR-2411-5p,miR-345-
3p,miR-615-3p,miR-134,miR-218-2-3p,miR-501-3p,miR-105-
2,miR-200b-3p,miR-1,miR-181a-2-3p,miR-2284a,miR-200c,miR-
17-3p,miR-200c-3p,miR-181b-5p,miR-92a-3p,miR-139-5p,miR-
2284g,miR-211-5p,miR-25,miR-500-3p,miR-1185-2-3p,miR-
299b-5p,miR-181a-3p,miR-493-5p,miR-9-3p,miR-2447,miR-363-
3p,miR-3956-3p,miR-7705,miR-218-5p,miR-615,miR-490-
LAMA1 ENSOARG0(laminin, 5p,miR-1343-3p,miR-455-3p,miR-148b-5p,miR-421,miR-
miR-148b-5p,miR-146a-5p,miR-192-3p,miR-216c-5p,miR-
2285w,miR-3529-3p,miR-4286,miR-2284m,miR-450a-2-3p,miR-
3653-3p,novel_96,miR-2285p,miR-185-3p,miR-2284h-5p,miR-
222,miR-411-3p,miR-582-3p,miR-155-5p,miR-148a-5p,miR-
3578,miR-2300b-3p,miR-29d-3p,miR-1434-5p,miR-140-3p,miR-
500b-5p,miR-6525,miR-144-5p,miR-138-5p,novel_74,miR-155-
5p,miR-664a,miR-487a-5p,miR-2284k,miR-222-3p,miR-34c-
3p,miR-29b-3p,miR-19b-1-5p,miR-409-3p,miR-664a-5p,miR-
135a-5p,miR-499b-3p,miR-139-5p,novel_32,miR-204-3p,miR-
155-5p,miR-2284n,miR-21-3p,miR-2318,miR-485-5p,miR-362-
5p,miR-3154,miR-2284l,miR-376b,miR-2284b,miR-491-5p,miR-
124-5p,miR-146b,miR-146a,miR-539-5p,miR-221-3p,miR-500-
5p,miR-148b-5p,miR-26b-3p,miR-138,miR-29b,miR-19b-2-
5p,miR-708-3p,miR-301b-5p,miR-541,miR-197-3p,miR-3071-
5p,miR-362-5p,novel_27,miR-2284w,miR-2428,miR-135a-2-
3p,miR-3059-5p,miR-2447,miR-541-3p,miR-29c-3p,miR-
2284j,miR-628-3p,miR-3968,miR-29a,miR-502b,miR-2285n,miR-
2440,miR-301a-5p,miR-499a-3p,miR-29a-3p,miR-144-5p,miR-
669,miR-139-5p,miR-378b,miR-146b-5p,miR-135b-5p,miR-409-
SRFBP1 ENSOARG0(serum re 5p,miR-221,miR-3065-5p,miR-3591-3p

MAP3K5	ENSOARG0(	mitogen-	miR-199a-3p,miR-582-3p,miR-222-5p,miR-6516-5p,miR-299a-3p,miR-106a,miR-128-3p,miR-105-2,miR-300,miR-30a-5p,miR-2300b-3p,miR-146a-5p,miR-2432,miR-376a-5p,miR-125b-2-3p,miR-20a,miR-369-3p,miR-181a-2-3p,miR-30d,miR-299-3p,novel_96,miR-2284m,miR-106a,miR-466i-5p,miR-30f,miR-374a-5p,miR-664-3p,novel_17,miR-210-5p,miR-133c,miR-2284n,miR-6516,miR-544-3p,miR-300-3p,miR-499b-3p,miR-216b-3p,miR-2355-5p,miR-552-3p,miR-20b-5p,novel_99,miR-1814c,miR-10b-3p,miR-93-5p,miR-655,miR-17-5p,miR-433-3p,miR-30d-5p,miR-381-3p,miR-30e-5p,miR-93,miR-1197-3p,miR-193a-5p,miR-665-5p,miR-425-5p,miR-374b,miR-129-5p,miR-708-5p,miR-28-5p,miR-6740-5p,miR-655-3p,miR-2285j,miR-2284k,miR-544b,miR-3535,miR-27a-3p,miR-222-5p,miR-26b-3p,miR-105-1,novel_78,miR-27b-3p,miR-2284ab,miR-544a,miR-450b-5p,miR-4791,miR-2310,miR-2284l,miR-382,miR-363-5p,miR-23a-3p,miR-1388-3p,miR-106a-5p,miR-146b,miR-103a-2-5p,miR-491-5p,miR-105-5p,miR-2957,miR-106b-5p,miR-17-5p,miR-106a-5p,miR-199a-3p,miR-146a,miR-1306-5p,miR-144-3p,miR-203-3p,miR-27a-3p,miR-499a-3p,miR-3082-5p,miR-299b-3p,miR-499b-5p,miR-374b-5p,miR-664b,miR-146b-5p,miR-877-3p,miR-1306,miR-378j,miR-216a-3p,miR-374c-5p,miR-495-3p,miR-3059-5p,miR-885-5p,miR-2284q,miR-147-5p,miR-6119-3p,miR-382-5p,miR-107-5p,miR-142b,miR-20a-5p

			novel_12,miR-[illegible]-5p,miR-1260b,miR-[illegible],miR-[illegible]-5p,miR-125b-5p,miR-2433,miR-486b-3p,miR-138-5p,miR-1271,miR-125a,miR-615-5p,miR-370-3p,miR-542-5p,miR-2355-5p,miR-874-3p,miR-154b-5p,miR-376c-5p,miR-485-5p,miR-7144-5p,miR-376c-5p,miR-4286,miR-1271-3p,miR-148b-5p,miR-30c-2-3p,miR-1260a,miR-543-5p,novel_127,novel_96,miR-17-3p,miR-1224-5p,miR-6517,miR-129-1-3p,miR-1271-5p,miR-2411-5p,miR-330-5p,miR-345-3p,miR-615-3p,miR-885-3p,miR-17-3p,miR-193b-3p,miR-326-3p,miR-24-3p,miR-6529b,miR-147-5p,miR-320b,miR-3074-5p,miR-18a-3p,miR-1306,miR-376b-5p,miR-2285ab,miR-6395,miR-1291,miR-628-5p,miR-296-3p,miR-432-3p,miR-2411-3p,novel_51,miR-296-3p,miR-193a-3p,miR-125a-5p,miR-187-3p,miR-411-5p,miR-194-3p,miR-3431,miR-2285g,miR-22-3p,miR-744-5p,miR-7862,miR-1260b,miR-138,miR-665-5p,miR-214,miR-425-5p,miR-1271,miR-129-2-3p,miR-3074-5p,miR-4510,miR-338-3p,miR-3184-5p,miR-665,miR-4492,miR-874-5p,miR-664a,miR-17-3p,miR-552-3p,miR-504-5p,miR-221-5p,miR-30c-1-3p,miR-151b,miR-22-3p,miR-193b-3p,miR-6130,miR-129b-3p,miR-2285c,miR-186-5p,miR-1827,miR-1895,miR-885-3p,miR-2312,miR-345-3p,miR-216c-5p,miR-486-3p,miR-2411,miR-125b,miR-185-3p,miR-345-3p,miR-324-3p,miR-210-5p,miR-21-3p,miR-221-5p,miR-760-3p,miR-212-5p,miR-3960,miR-18a-3p,miR-328-3p,miR-151-5p,miR-1197-5p,miR-5703,miR-188-3p,miR-2904,novel_133,miR-148a-5p,miR-941,miR-2285f,miR-3956-3p,novel_60,miR-7705,miR-423-5p,miR-615,novel_82,miR-2285n,miR-4726-5p,miR-3968,miR-7977,miR-452-3p,miR-4443,miR-3596,miR-136-5p,miR-504,miR-299b-5p,miR-96-5p,miR-4532,miR-30c-1-3p,novel_19,miR-1306-5p,miR-326,miR-214-3p,miR-491-5p,novel_78,miR-2300a-5p,miR-2285e,miR-148b-5p,miR-9788-3p,miR-1298-5p,miR-296-5p,miR-
RNPEP	ENSOARG0(	arginyl	

PLXDC2	ENSOARG0(	plexin d	[illegible] 378f,miR-195a-5p,miR-2319a,miR-9851-3p,miR-146b-5p,miR-410-5p,miR-378b,miR-3591-3p,miR-150-5p,miR-376b-5p,miR-2284w,miR-326-3p,miR-211,miR-495-3p,miR-29c-3p,miR-2285x,miR-1a-1-5p,miR-335-5p,novel_120,miR-625-3p,novel_1,miR-29a,miR-92a-3p,miR-16a,miR-28c,miR-576-3p,miR-20a-5p,miR-15a,miR-20b,miR-134-3p,miR-2284d,miR-412-5p,miR-3535,miR-26b-3p,miR-187-3p,miR-3955-5p,miR-31-5p,miR-188-5p,miR-27b-3p,miR-2284x,miR-7862,miR-497-5p,miR-219a-1-3p,miR-16b,miR-29b,miR-30b-3p,miR-197-3p,miR-223-3p,miR-1843b-5p,miR-653,miR-654-3p,miR-628-5p,miR-101-5p,miR-668-3p,miR-363-5p,miR-6240,miR-660,novel_51,miR-17-5p,miR-106a-5p,miR-32,miR-105-5p,miR-330-3p,miR-146a,miR-2284n,miR-25-3p,miR-2285aa,miR-671-5p,miR-190a-3p,miR-376c-5p,miR-20b-5p,let-7g-3p,miR-767,miR-376c-5p,miR-17-5p,miR-93-5p,miR-488-3p,miR-378c,miR-93,miR-16-5p,miR-1306-5p,miR-204-5p,miR-28-5p,miR-410-3p,miR-767-5p,miR-6740-5p,novel_63,miR-29b-3p,miR-1-5p,miR-2284k,miR-582-3p,miR-1271-5p,miR-378h,miR-322-5p,miR-299a-3p,miR-105-2,miR-411-3p,miR-330-5p,miR-218-2-3p,miR-2427,miR-29d-3p,miR-490-3p,miR-2285b,miR-2284a,miR-200a,miR-29a-5p,miR-1957a,miR-2284y,miR-28b,miR-450b-5p,miR-92b-3p,miR-92a-3p,miR-1a-2-5p,miR-2898,miR-30c-2-3p,miR-3071-3p,miR-6119-5p,miR-2285af,miR-27a-3p,miR-4443,miR-203-3p,miR-378e,miR-378g,miR-211-5p,miR-541-5p,miR-2319b,miR-2284g,miR-96-5p,miR-129b-5p,miR-154a,miR-1285-5p,miR-8095,miR-2284j,miR-3059-5p,miR-2447,miR-2285t,miR-335,miR-3968,miR-107-5p,miR-101-3p,miR-147a,miR-539-5p,miR-1298-3p,miR-27a-3p,miR-5010-3p,miR-330-3p,miR-105-1,miR-409b,miR-3120-5p,miR-450b-5p,miR-101a-5p,miR-378c,miR-

GRAMD1B	ENSOARG0(	GRAM dom	[illegible] 5p,miR-7641,novel_79,miR-412,miR-18b-5p,miR-2285ad,miR-6517,let-7a-2-3p,miR-1343-5p,miR-330-5p,miR-501-3p,miR-23b-5p,miR-3591-5p,miR-200a,miR-2284y,miR-2284a,miR-2478,miR-28b,miR-92b-3p,miR-2403,novel_127,miR-543-5p,novel_96,miR-92a-3p,miR-152-3p,miR-181b-5p,miR-127-5p,miR-3071-3p,miR-339a,miR-1260a,miR-205-5p,miR-664-3p,miR-154b-5p,miR-769,miR-365a-3p,miR-25-3p,miR-2285aa,miR-3956,miR-874-3p,miR-7144-5p,miR-23a-5p,miR-376c-5p,miR-1248,miR-2318,miR-376c-5p,miR-18a,miR-3154,miR-488-3p,miR-301,miR-145-3p,miR-1260b,miR-193a-5p,miR-2284v,miR-28-5p,miR-486b-3p,miR-26c,miR-100-3p,miR-2284k,novel_63,miR-194-3p,miR-2355-3p,miR-544b,miR-2284d,miR-412-5p,miR-339-5p,miR-26b-3p,miR-133b-5p,miR-744-5p,miR-7862,miR-188-5p,miR-2284x,miR-2284ab,miR-1260b,miR-374b-3p,miR-7b-5p,miR-181a-5p,miR-1843b-5p,let-7a-2-3p,miR-654-3p,miR-382,miR-668-3p,miR-130b-5p,miR-493-5p,miR-1343-5p,miR-660,miR-483-3p,miR-32,miR-3141,novel_51,miR-7-5p,miR-6535,miR-670-3p,miR-374c-3p,miR-320b,miR-3120-3p,miR-3074-5p,miR-6395,miR-1291,miR-412-3p,miR-1306,miR-378b,miR-3591-3p,miR-18a-3p,miR-376b-5p,miR-148a-3p,miR-3141,miR-18b,miR-326-3p,miR-374c-5p,miR-1307-3p,miR-135a-2-3p,miR-203b-5p,miR-147-5p,miR-6119-3p,miR-92a-3p,novel_87,miR-502-5p,miR-4324,miR-28c,miR-6402,miR-2284h-5p,miR-532-3p,miR-328-3p,miR-148b-3p,miR-18a-3p,miR-2284u,miR-378d,miR-148a-5p,miR-493-3p,miR-2904,miR-1434-5p,miR-1197-5p,miR-2411,miR-486-3p,miR-2432,miR-376a-5p,miR-125b-2-3p,miR-130a-5p,miR-345-3p,miR-216c-5p,miR-2285w,miR-1277-5p,miR-30b-3p,miR-21-3p,miR-2284m,miR-760-3p,miR-466i-5p,miR-219b-5p,miR-204-3p,miR-877-5p,miR-544-3p,miR-216b-5p,miR-

LLGL1	ENSOARG0(	lethal g	miR-135a-2-3p,miR-326-3p,miR-449a,miR-34c,miR-4324,miR-20a-5p,miR-203b-5p,miR-92a-3p,miR-206,miR-7-1-3p,miR-669,novel_69,miR-34b,miR-3065-5p,miR-3591-3p,miR-3141,miR-146b-5p,miR-450b-3p,miR-1291,miR-412-3p,miR-363-5p,miR-345-5p,miR-3141,miR-32,miR-2331-5p,novel_51,miR-106a-5p,miR-17-5p,miR-146a,miR-377-3p,miR-1343-5p,miR-125a-5p,miR-483-3p,miR-194-3p,miR-20b,miR-708-3p,miR-4508,miR-26a-2-3p,miR-744-5p,miR-138,miR-219a-1-3p,miR-93,miR-125b-5p,miR-31-3p,miR-138-5p,miR-2355-5p,miR-615-5p,miR-125a,miR-34c-5p,miR-300-3p,miR-25-3p,miR-3959-5p,miR-93-5p,miR-17-5p,miR-3154,miR-20b-5p,miR-345-5p,miR-3064-5p,novel_39,miR-92b-3p,novel_96,miR-92a-3p,miR-127-5p,miR-6517,miR-483-3p,miR-1343-5p,miR-330-5p,miR-2447,miR-4454,miR-363-3p,miR-541-3p,miR-877-3p,miR-1973,miR-3064-5p,miR-3082-5p,miR-361-3p,miR-140-5p,miR-1983,miR-877-3p,miR-504,miR-1388-3p,miR-106a-5p,miR-8485,miR-106b-5p,miR-326,miR-146b,miR-214-3p,miR-491-5p,miR-3085-3p,miR-1343-3p,miR-9788-3p,miR-216a-3p,miR-147a,miR-541,miR-143-5p,miR-214,miR-145a-3p,miR-6134,miR-206-3p,miR-665,miR-4492,miR-21-3p,miR-216b-3p,miR-552-3p,miR-504-5p,miR-32-5p,miR-2331-3p,miR-204-3p,miR-377-3p,miR-27a-5p,miR-345-5p,miR-20a,miR-146a-5p,miR-125b,miR-185-3p,miR-34b-5p,miR-2484,miR-106a,miR-212-5p,miR-548e-3p,miR-106a,miR-532-3p,miR-7859,miR-5703,miR-188-3p,miR-1197-5p,novel_133,miR-2366,miR-2300b-3p,miR-34a-5p

LELP1	ENSOARG0(	late cor	[illegible] 335-3p,miR-9-3p,miR-129b-5p,miR-361-3p,miR-652-3p,miR-7857-3p,miR-489,miR-211-5p,miR-7977,miR-214-3p,miR-103a-2-5p,miR-3955-3p,miR-106b-5p,miR-320b,miR-431-5p,novel_115,miR-326,miR-3187-3p,miR-30c-1-3p,miR-1388-3p,miR-106a-5p,miR-1388-5p,miR-2426,miR-412-3p,miR-30b-3p,miR-484,miR-455-3p,miR-9788-3p,miR-2459,miR-181b-2-3p,novel_4,miR-1343-3p,miR-874-5p,miR-129-5p,miR-130b-5p,miR-17-3p,miR-6516-3p,miR-665,miR-320e,miR-224-5p,miR-214,miR-590-3p,miR-145a-3p,miR-3074-5p,miR-2331-3p,miR-140-5p,miR-30c-1-3p,miR-21-3p,miR-106a,miR-320d,miR-324-3p,miR-345-3p,miR-618,miR-421-5p,miR-130a-5p,miR-181b-1-3p,miR-20a,miR-216c-5p,miR-378d,miR-4429,miR-33b-3p,miR-5703,miR-1246,miR-193b-5p,miR-2300b-3p,miR-1247-5p,miR-340-5p,miR-3600,miR-212-5p,miR-18a-3p,miR-106a,miR-625-3p,miR-6123,miR-128-1-5p,miR-4324,miR-502-5p,miR-20a-5p,miR-326-3p,miR-2284w,miR-2428,miR-211,miR-17-3p,novel_101,miR-2319a,miR-6516-3p,miR-216a-3p,miR-18a-3p,miR-677,miR-320c,miR-669,miR-181b-3p,miR-670-3p,miR-320b,miR-3074-5p,miR-378a-5p,miR-17-5p,miR-106a-5p,miR-2411-3p,miR-7-5p,miR-224-5p,miR-654-3p,miR-432-3p,miR-432-3p,miR-363-5p,miR-628-5p,miR-130b-5p,miR-2889,miR-7862,miR-30b-3p,miR-219a-1-3p,miR-1260b,miR-7b-5p,miR-223-3p,miR-1434-3p,miR-194-3p,miR-149-5p,miR-20b,miR-3431,miR-31-5p,miR-133b-5p,miR-204-5p,miR-331-3p,miR-3969,miR-410-5p,miR-93,miR-323-3p,miR-1260b,miR-193a-5p,miR-28a-3p,miR-20b-5p,miR-7144-5p,miR-671-5p,miR-93-5p,miR-17-5p,miR-320a,miR-3154,miR-301,miR-324-5p,miR-574-5p,miR-874-3p,novel_127,miR-543-5p,miR-2898,miR-17-3p,miR-28-3p,miR-30f,miR-30c-2-3p,miR-1260a,novel_39,miR-200a,miR-450b-5p,miR-128-3p,miR-7134-5p,miR-23b-5p,miR-

			[illegible] 5p,miR-139-5p,miR-4443,miR-499a-3p,miR-378g,miR-140-5p,novel_94,miR-299b-5p,miR-504,miR-181d-5p,miR-2284j,miR-219a-5p,miR-3059-5p,miR-423-5p,miR-877-3p,miR-371a-5p,miR-301a-5p,novel_82,miR-2285t,miR-4726-5p,miR-3968,miR-1343-3p,novel_78,novel_4,miR-216a-3p,miR-9788-3p,miR-455-3p,miR-1298-3p,miR-539-5p,miR-1a-3p,miR-7857,miR-30b-3p,miR-301b-5p,miR-10b,miR-2284aa,miR-299a-5p,miR-2426,miR-1388-3p,miR-424-5p,miR-4532,miR-193a,miR-30c-1-3p,miR-29b-1-5p,miR-16b,novel_115,miR-146b,miR-214-3p,miR-3085-3p,miR-491-5p,miR-25-5p,miR-3065-3p,miR-216b-3p,miR-296-3p,miR-30c-1-3p,miR-504-5p,miR-545-3p,miR-1277-3p,miR-22-3p,miR-15b-5p,miR-499b-3p,miR-10a,miR-132-5p,miR-139-5p,miR-6130,miR-129b-3p,miR-1827,miR-345-5p,miR-339b,miR-3074-2-3p,miR-214,miR-539-5p,miR-3074-5p,miR-4510,miR-3184-3p,miR-3184-5p,miR-338-3p,miR-181c-5p,miR-10b-5p,miR-6525,miR-206-3p,miR-30a-3p,miR-664a-5p,miR-665,miR-320e,miR-30e-3p,miR-424-5p,miR-708-5p,miR-17-3p,miR-30d-3p,miR-15a-5p,miR-2904,miR-5703,miR-1197-5p,miR-140-3p,novel_133,miR-133a-3p,miR-34a-5p,miR-33b-3p,miR-142-3p,miR-5100,miR-345-3p,miR-503-5p,miR-486-3p,miR-146a-5p,miR-29b-1-5p,miR-130a-5p,miR-324-3p,miR-185-3p,miR-345-3p,miR-196a-3p,miR-210-5p,miR-34b-5p,miR-3653-3p,miR-2284m,miR-760-3p,miR-320b,miR-10a-5p,novel_68,miR-378b,miR-3074-5p,miR-146b-3p,miR-29a-3p,miR-34b,miR-378b,miR-3065-5p,miR-376b-5p,miR-2387,miR-3141,miR-146b-5p,miR-9851-3p,miR-195a-5p,miR-17-3p,miR-29c-3p,miR-135a-2-3p,miR-374c-5p,miR-449a,miR-2428,miR-211,miR-16a,miR-34c,miR-4324,miR-15a,miR-28c,miR-656-5p,miR-147-5p,miR-29a,miR-339-5p,miR-187-3p,miR-149-5p,novel_116,miR-412-5p,miR-2284d,miR-134-3p,miR-
SLC22A6	ENSOARGO(	solute c	

[illegible]
224-5p,miR-124-5p,miR-187-3p,miR-20b,miR-2355-3p,miR-
6238,miR-185-5p,miR-432-5p,miR-145a-5p,miR-181a-5p,miR-
7862,miR-2889,miR-30b-3p,miR-138,miR-380-
3p,novel_101,miR-495-3p,miR-17-3p,miR-3957-3p,miR-136-
3p,miR-342-3p,miR-449a,miR-16a,miR-34c,miR-20a-5p,miR-
6402,miR-15a,miR-147-5p,miR-656-5p,miR-22-5p,novel_1,miR-
128-1-5p,miR-10a-5p,miR-670-3p,miR-34b,miR-146b-3p,miR-
323a-5p,miR-18a-3p,miR-2387,miR-195a-5p,miR-379-5p,miR-
376a-2-5p,miR-19a-3p,miR-181a-2-3p,miR-29b-2-5p,miR-3529-
3p,miR-1271-3p,miR-1957a,miR-199a-5p,miR-28b,miR-380-
5p,miR-200c,miR-200c-3p,miR-181b-5p,miR-17-3p,miR-450a-2-
3p,miR-2898,miR-376a-5p,miR-378h,miR-2411-5p,miR-134,miR-
10a-5p,miR-105-2,miR-1306-5p,miR-378c,miR-93,miR-
3969,miR-1306-3p,miR-138-5p,miR-215-5p,miR-598-3p,miR-
370-3p,miR-154b-5p,miR-199a-5p,miR-365a-3p,miR-769,miR-
34c-5p,miR-374c-3p,miR-17-5p,miR-3154,miR-2448-3p,miR-
485-5p,miR-671-5p,miR-1839-3p,miR-424-5p,miR-494-3p,miR-
106a-5p,miR-758-3p,miR-4532,miR-1306-5p,miR-5126,miR-
342,miR-1468,miR-105-1,miR-2300a-5p,miR-30b-3p,miR-
10b,miR-484,miR-378b,miR-3120-5p,miR-299a-5p,miR-
378c,miR-452-5p,miR-181d-5p,miR-541-3p,novel_60,miR-219a-
2-3p,miR-107-5p,miR-1973,miR-769-5p,miR-2285t,miR-
335,miR-4726-5p,miR-378e,miR-199b-5p,novel_94,miR-
20a,miR-216c-5p,miR-2411,miR-130a-5p,miR-196a-3p,miR-30b-
3p,miR-34b-5p,miR-466i-5p,miR-760-3p,miR-106a,miR-199a-
3p,novel_83,miR-532-3p,miR-15a-5p,miR-1197-5p,miR-
1193,miR-140-3p,miR-2904,miR-2300b-3p,miR-215-5p,miR-
2408,novel_133,miR-19b-3p,miR-3074-2-3p,miR-4510,miR-
378c,miR-338-3p,miR-10b-5p,miR-29b-2-5p,miR-324-3p,miR-
FAM217A ENSOARG0(family w 424-5p,miR-17-3p,miR-378a-3p,miR-140-5p,novel_32,miR-22-

PPP1R16B	ENSOARG0(	protein	miR-[illegible],miR-[illegible],miR-[illegible]-3p,miR-[illegible],novel_[illegible],miR-2459,miR-1306-5p,miR-326,miR-320b,miR-214-3p,let-7e,miR-3085-3p,miR-33a-5p,miR-23a-3p,miR-1388-3p,let-7a-5p,miR-30c-1-3p,miR-378d,miR-129b-5p,miR-1306-3p,miR-9-3p,miR-877-3p,miR-211-5p,miR-20a-3p,miR-3064-5p,let-7d-5p,miR-378g,miR-378e,miR-499a-3p,miR-2285y,miR-335,miR-3968,miR-4726-5p,miR-190b-5p,miR-181d-5p,miR-3059-5p,miR-7705,miR-423-5p,miR-361-3p,miR-188-3p,miR-378a-3p,miR-1961,miR-2408,miR-378i,miR-378d,miR-34a-5p,novel_73,miR-4429,miR-30d-3p,miR-548e-3p,miR-532-3p,miR-200b,miR-7859,miR-23b-3p,miR-422a,novel_17,miR-196a-3p,miR-21-3p,let-7k,miR-34b-5p,miR-99a-3p,miR-760-3p,miR-466i-5p,miR-98-5p,miR-2284m,miR-320d,miR-329a-5p,miR-143-3p,miR-486-3p,miR-376c-3p,miR-376a-5p,miR-433-3p,miR-1827,miR-543-3p,miR-3065-3p,miR-216b-3p,miR-30c-1-3p,miR-2331-3p,miR-378a-3p,let-7i-5p,miR-376e-3p,miR-499b-3p,miR-320e,miR-30a-3p,miR-30e-3p,let-7f,miR-708-5p,miR-190a-5p,miR-129-5p,miR-4492,miR-664a,miR-214,miR-129-2-3p,miR-378c,let-7b,miR-181c-5p,miR-338-3p,miR-6134,let-7b-5p,miR-3184-5p,miR-6525,novel_23,miR-374b-3p,miR-181a-5p,miR-1285,miR-7b-5p,miR-708-3p,miR-1260b,miR-29b,miR-665,miR-31-5p,miR-3955-5p,miR-149-5p,miR-134-3p,let-7g-5p,miR-3431,miR-2331-5p,miR-190a,miR-7-5p,miR-196b-5p,miR-382,miR-654-3p,miR-1306,miR-150-5p,miR-2387,miR-378f,let-7e-5p,miR-329-5p,novel_68,miR-320c,miR-669,miR-34b,miR-29a-3p,let-7i,miR-382-5p,miR-34c,miR-28c,miR-6123,miR-147-5p,miR-502b,miR-656-5p,miR-29a,miR-29c-3p,miR-335-5p,miR-135a-2-3p,miR-2428,miR-326-3p,miR-449a,miR-211,miR-330-5p,miR-29d-3p,miR-196a-5p,miR-200b-3p,let-7d,miR-429-3p,miR-6517,miR-2285ad,miR-129-1-3p,miR-378h,let-7c-5p,miR-30f,miR-30c-2-3p,miR-200c,miR-
HYI	ENSOARG0(	hydroxyp	miR-214,miR-2428,miR-877-5p,miR-214-3p,miR-9788-3p,miR-3431
PHC3	ENSOARG0(	polyhome	miR-409-3p,miR-218-5p,miR-2285j,miR-2332,miR-487a-5p,miR-628-3p,novel_1,miR-452-5p,miR-539-5p,miR-378c,novel_27,miR-378c,miR-411,miR-378f,let-7g-3p,miR-378a-3p,miR-499b-3p,miR-499a-3p,miR-378e,miR-197-5p,miR-30f,miR-422a,let-7c-3p,miR-376b,miR-378d,miR-378a-3p,miR-378i,miR-378b,miR-16-1-3p,miR-378c,miR-16-1-3p,miR-582-3p,miR-9788-3p,miR-378h

COPS5	ENSOARG0(	COP9 sig	miR-103b,miR-1197-3p,miR-224-5p,miR-10b-5p,miR-409-3p,miR-338-5p,miR-410-5p,miR-2284k,novel_63,miR-130b-5p,miR-592,miR-664a,miR-2285aa,miR-503-3p,miR-496-3p,miR-204-3p,miR-323-5p,miR-10a,miR-374c-3p,miR-2284s,miR-433-3p,novel_99,miR-1827,miR-485-5p,miR-671-5p,miR-7144-5p,miR-345-5p,miR-345-5p,miR-3529-3p,miR-329a-5p,miR-2424,miR-29b-1-5p,miR-500a-5p,miR-200c,miR-21-3p,miR-200c-3p,miR-760-3p,miR-3600,miR-429-3p,miR-582-3p,miR-532-3p,miR-545-5p,miR-200b,miR-7859,miR-1197-5p,miR-188-3p,novel_133,miR-2284u,miR-148a-5p,miR-10a-5p,miR-496,miR-200b-3p,miR-2284j,miR-2285f,miR-2447,miR-154a,miR-107-5p,miR-218-5p,miR-656-5p,miR-7977,miR-10a-5p,miR-3120-3p,miR-655-5p,miR-10b,miR-329-5p,miR-2319b,miR-20a-3p,novel_68,miR-2284g,miR-1185-2-3p,miR-203-3p,miR-670-3p,miR-323a-5p,miR-2285ab,miR-544-5p,novel_94,miR-2319a,miR-136-5p,miR-33a-5p,let-7f-2-3p,let-7f-2-3p,miR-2284b,miR-130b-5p,miR-1b-5p,miR-345-5p,miR-2285r,miR-29b-1-5p,miR-224-5p,miR-103a-2-5p,miR-452-5p,miR-145b,miR-3955-5p,miR-31-5p,miR-2459,miR-1185-3p,miR-455-3p,miR-149-5p,miR-148b-5p,miR-1298-3p,miR-3431,miR-496-5p,miR-30b-3p,miR-145a-5p,miR-10b,miR-6529a,miR-484,miR-2284x,miR-30b-3p
SLC13A1	ENSOARG0(	solute c	miR-2284x,miR-16-1-3p,miR-2426,miR-380-3p,miR-2284ab,novel_23,miR-301b-5p,miR-484,miR-2459,miR-216a-3p,miR-6238,miR-130a-3p,miR-665,miR-130b-3p,miR-483-3p,miR-224-5p,miR-199a-3p,miR-654-3p,miR-2330-5p,miR-668-5p,miR-19a-3p,miR-3065-5p,miR-2387,novel_69,miR-411b-3p,miR-499a-3p,miR-7-1-3p,miR-3120-3p,miR-329-5p,miR-3964,miR-379-3p,miR-301a-5p,miR-576-3p,miR-502-5p,miR-154a,miR-301b-3p,miR-9-5p,miR-1a-1-5p,miR-219b-3p,miR-664-5p,miR-16-1-3p,miR-19b-3p,miR-411-3p,miR-5703,miR-199a-3p,miR-483-3p,miR-340-5p,miR-6516-5p,miR-9-5p,miR-7859,miR-548e-3p,miR-1a-2-5p,miR-760-3p,miR-2284m,miR-6119-5p,miR-2285af,miR-380-3p,miR-29a-5p,miR-3065-3p,miR-1957a,miR-345-3p,miR-369-3p,miR-3529-3p,miR-329a-5p,novel_99,miR-1827,miR-2318,miR-329b,miR-876-3p,let-7g-3p,miR-655,miR-503-3p,miR-21a-3p,miR-216b-5p,miR-6130,miR-499b-3p,miR-6516,miR-532-5p,miR-3065-3p,miR-2285aa,miR-548o-3p,miR-140-5p,miR-454-3p,miR-6516-3p,miR-655-3p,miR-100-3p,miR-1-5p,miR-224-5p,miR-3074-2-3p,miR-4510,miR-590-3p,miR-539-5p

SNX31	ENSOARG0(	sorting	miR-345-3p,miR-361-5p,miR-142-3p,miR-184-3p,miR-129-1-3p,miR-2284z,miR-582-3p,miR-9-5p,miR-23b-3p,miR-345-3p,miR-1260a,miR-30b-3p,miR-3653-3p,miR-181b-5p,miR-29b-2-5p,miR-195a-3p,miR-142a-3p,miR-24-2-5p,miR-148b-5p,miR-450b-5p,miR-433-3p,let-7f-1-3p,miR-671-5p,miR-3074-1-3p,miR-10b-3p,miR-216b-3p,miR-2285aa,miR-133c,miR-29b-2-5p,miR-5100,let-7b-3p,miR-129-2-3p,miR-181c-5p,miR-145-3p,miR-10b-3p,miR-1260b,miR-181a-5p,miR-653,miR-2284aa,miR-1260b,miR-105-1,miR-6238,miR-142-3p,miR-1298-3p,miR-153,miR-2411-3p,miR-105-5p,miR-153-3p,miR-23a-3p,miR-101-5p,miR-382-3p,miR-381-3p,miR-9-3p,miR-7689-3p,miR-24-1-5p,miR-3082-5p,miR-382-3p,miR-6123,miR-3958-3p,miR-495-3p,miR-181d-5p,miR-9-5p,miR-3059-5p,miR-9-3p,miR-24-3p
CTTN	ENSOARG0(	cortacti	miR-323-5p,miR-2284n,miR-370-5p,miR-370-3p,miR-182-5p,miR-329a,miR-30f,miR-625-5p,miR-7144-5p,miR-30e-5p,miR-381-3p,miR-30d-5p,miR-23b,miR-362-5p,miR-500b-5p,miR-6134,miR-2284v,miR-708-5p,miR-130b-5p,miR-28-5p,miR-2285j,miR-2433,miR-370-5p,miR-331-3p,miR-665,miR-664a-5p,miR-23b-3p,miR-23c,miR-1247-5p,miR-2284z,miR-2284h-5p,miR-2285ad,miR-493-3p,miR-300,miR-19b-3p,miR-105-2,miR-30c,miR-148a-5p,miR-30c-5p,miR-362-3p,miR-30a-5p,miR-1197-5p,miR-148b-5p,miR-2432,miR-450c-5p,miR-490-3p,miR-2285w,miR-503-5p,miR-4286,miR-2284m,miR-107,miR-30d,miR-21-3p,miR-30b,miR-30f,miR-500a-5p,miR-323a-5p,miR-3596,miR-670-3p,miR-677,miR-181b-3p,miR-541-5p,miR-320b,miR-3120-3p,miR-7977,miR-19a-3p,miR-107,miR-361-3p,miR-150-5p,miR-378b,miR-362-5p,miR-877-3p,miR-361-3p,miR-8095,miR-2284w,miR-885-5p,miR-335-5p,miR-1193,miR-450a-5p,miR-335,miR-30b-5p,miR-2285n,miR-6402,miR-2285y,miR-503-5p,miR-9788-3p,miR-500-5p,miR-103,miR-3955-5p,miR-105-1,miR-103a-3p,miR-181b-2-3p,miR-2284ab,miR-362-3p,miR-381-5p,miR-2284aa,miR-744-5p,miR-484,miR-329-3p,miR-7b-5p,miR-2285r,miR-6412,miR-4532,miR-8485,miR-130b-5p,miR-23a-3p,miR-628-5p,novel_124,miR-2285l,miR-381-5p,miR-503-5p,miR-7-5p,miR-105-5p,miR-362-3p,miR-2957,miR-182-5p
NUMB	ENSOARG0(	numb hom	miR-10a-5p,miR-497-5p,miR-664-5p,miR-16b,miR-450c-3p,miR-10b,novel_121,miR-450a-1-3p,miR-545-5p,miR-15a-5p,miR-322-5p,miR-26b-3p,miR-31-5p,miR-146b,miR-378a-5p,miR-450a-2-3p,miR-16b,miR-185-3p,miR-92a-1-5p,miR-182-5p,miR-146a,miR-146a-5p,miR-2424,let-7c-3p,miR-424-5p,miR-195a-5p,miR-146b-5p,miR-10b-3p,miR-1291,miR-129b-5p,miR-378b,miR-450a-1-3p,miR-488-3p,miR-15b-5p,novel_69,miR-6130,miR-146b-3p,miR-10a,miR-7977,miR-10a-5p,miR-182-5p,miR-370-3p,miR-2355-5p,miR-424-5p,miR-31-3p,miR-450b-3p,miR-3604,miR-16a,miR-133a-5p,miR-15a,miR-145-3p,miR-10b-5p,miR-16-5p,miR-4510,novel_42

ADGRG5	ENSOARG0(	adhesion	miR-27a-3p,novel_4,miR-216a-3p,miR-9788-3p,miR-30b-3p,miR-296-5p,miR-3120-5p,miR-2426,miR-544a,miR-376b,miR-494-3p,miR-424-5p,let-7a-5p,miR-30c-1-3p,miR-1306-5p,novel_115,miR-5126,miR-326,miR-342,miR-320b,let-7e,miR-3085-3p,novel_124,miR-7977,miR-3064-5p,miR-27a-3p,let-7d-5p,miR-7857-3p,miR-361-3p,novel_94,miR-1296-5p,miR-2285f,miR-541-3p,novel_60,miR-769-5p,miR-1973,miR-30b-5p,miR-2284q,miR-335,miR-1b-3p,miR-548e-3p,miR-532-3p,miR-200b,miR-383-5p,miR-188-3p,miR-1197-5p,miR-5703,miR-140-3p,miR-2904,miR-2300b-3p,miR-378d,miR-4429,miR-19b-3p,miR-146a-5p,miR-2411,miR-29b-1-5p,miR-125b,miR-185-3p,miR-196a-3p,miR-21-3p,miR-299-3p,miR-30b-3p,miR-30d,miR-320d,miR-341-3p,miR-532-5p,miR-370-5p,miR-3065-3p,miR-22-3p,miR-204-3p,miR-193b-3p,miR-544-3p,miR-21a-3p,miR-30f,miR-18a-5p,miR-2312,miR-145a-3p,miR-539-5p,miR-6134,let-7b-5p,miR-487a-5p,miR-708-5p,miR-424-5p,miR-664a,miR-133b-5p,miR-103,miR-503-5p,miR-185-5p,let-7g-5p,miR-432-5p,miR-145a-5p,miR-1285,miR-1434-3p,miR-455-5p,miR-219a-1-3p,miR-138,miR-29b,miR-30b-3p,miR-455-5p,miR-330-3p,miR-503-5p,miR-224-5p,miR-1343-5p,miR-452-5p,miR-125a-5p,miR-124-5p,miR-29a-3p,miR-146b-3p,miR-2404,miR-378b,miR-18a-3p,miR-18b,miR-2285ab,miR-195a-5p,miR-6395,miR-9851-3p,miR-19a-3p,miR-107,miR-1291,miR-450b-3p,miR-495-3p,novel_101,miR-1193,miR-3957-3p,miR-342-3p,miR-2428,miR-326-3p,miR-24-3p,let-7i,miR-16a,miR-15a,miR-203b-5p,miR-2411-5p,miR-483-3p,miR-30a-5p,miR-29d-3p,miR-345-3p,miR-200b-3p,let-7d,miR-1271-3p,miR-3065-3p,miR-1957a,miR-28b,miR-339a,let-7c-5p,miR-30c-2-3p,miR-200c,miR-2285af,miR-107,miR-200c-3p,miR-191-3p,miR-450a-2-3p,miR-2898,miR-370-3p,miR-3957-5p,miR-154b-5p,miR-3957,miR-365a-3p,miR-769,miR-18a,miR-
TTC17	ENSOARG0(	tetratri	miR-378j,miR-1193,miR-197-5p,miR-379-5p,miR-1248,novel_111,miR-432-3p,miR-3596

MOCS1	ENSOARG0(	molybden	miR-423-5p,miR-361-3p,miR-2285f,miR-17-3p,miR-219a-5p,miR-2284j,miR-3059-5p,miR-541-3p,miR-4726-5p,miR-2285n,miR-2440,miR-6535,miR-320c,miR-3596,miR-499a-3p,miR-670-3p,miR-320b,miR-10a-5p,miR-7977,miR-2284g,miR-10b,miR-1291,miR-1306,miR-378b,miR-2285ab,miR-361-3p,miR-2387,miR-2285r,miR-23a-3p,let-7c-3p,miR-130b-5p,miR-2330-5p,miR-483-3p,miR-491-5p,miR-29b-1-5p,miR-320b,miR-7-5p,miR-1306-5p,miR-9788-3p,miR-2284d,miR-4508,miR-30b-3p,miR-1260b,miR-30b-3p,miR-7b-5p,miR-10b,miR-541,miR-1843b-5p,miR-3184-5p,miR-338-3p,miR-10b-5p,miR-1260b,miR-129-2-3p,miR-2284v,miR-500,miR-708-5p,miR-486b-3p,miR-130b-5p,miR-28-5p,miR-17-3p,miR-29b-2-5p,miR-665,miR-320e,miR-2284k,miR-2433,miR-2285j,miR-2284n,miR-10a,miR-499b-3p,miR-370-3p,miR-7144-5p,miR-23a-5p,miR-671-5p,miR-500a-3p,miR-320a,miR-23b,miR-200a,miR-486-3p,novel_39,miR-2284y,miR-2285b,miR-2478,miR-29b-1-5p,miR-29b-2-5p,miR-543-5p,novel_96,miR-320d,miR-17-3p,miR-185-3p,miR-217,miR-1260a,miR-582-3p,miR-129-1-3p,miR-23b-3p,miR-23c,miR-483-3p,miR-1b-3p,miR-2285ad,miR-423-3p,miR-2284u,miR-10a-5p,miR-493-3p,miR-582-3p,miR-4429,miR-2904,miR-217-5p,miR-1197-5p,miR-23b-5p,miR-5703,novel_133,miR-2300b-3p,miR-2366
TXLNA	ENSOARG0(	taxilin	miR-1271-3p,miR-490-3p,miR-199a-5p,let-7g,miR-3065-3p,miR-1,miR-3064-5p,miR-127-5p,let-7f-5p,novel_96,miR-543-5p,miR-200a-3p,miR-664-3p,miR-125a-3p,miR-2285af,miR-205-5p,miR-376b-3p,let-7c-5p,miR-30c-2-3p,miR-3071-3p,miR-758-5p,miR-2285ad,miR-18b-5p,let-7d,miR-411-3p,miR-10a-5p,miR-1343-5p,miR-29d-3p,miR-93,miR-133a-3p,miR-138-5p,miR-486b-3p,miR-592,miR-28-5p,miR-126b-3p,miR-99b-3p,miR-2332,miR-29b-3p,novel_63,miR-135a-5p,miR-26c,miR-331-3p,miR-769,miR-300-3p,miR-199a-5p,miR-141-3p,miR-99a-3p,miR-329a,novel_91,miR-20b-5p,miR-18a,miR-17-5p,miR-93-5p,miR-432-3p,miR-2285r,miR-432-3p,miR-452-5p,miR-1343-5p,miR-377-3p,miR-194a,novel_51,miR-17-5p,miR-106a-5p,let-7g-5p,miR-134-3p,miR-185-5p,miR-2355-3p,miR-20b,miR-138,miR-29b,miR-26a-2-3p,miR-2113,miR-19b-2-5p,miR-873a-5p,miR-194-5p,miR-22-3p,miR-24-3p,miR-199b-5p,novel_27,miR-342-3p,miR-495-3p,novel_101,miR-29c-3p,miR-3957-3p,miR-206,miR-29a,miR-379-3p,miR-142b,miR-20a-5p,miR-208a-3p,let-7i,miR-670-3p,miR-29a-3p,miR-323a-5p,miR-216b-3p,miR-10a-5p,miR-3120-3p,miR-412-3p,miR-135b-5p,let-7e-5p,miR-9851-3p,miR-18b,miR-376b-5p,miR-376b-3p,miR-29b-1-5p,miR-664b-3p,miR-486-3p,miR-216c-5p,miR-345-3p,miR-20a,miR-760-3p,miR-98-5p,miR-99a-3p,miR-106a,miR-21-3p,let-7k,miR-221-5p,miR-208b-3p,miR-210-5p,miR-324-3p,miR-500a-5p,miR-487a-3p,miR-7859,miR-95-3p,miR-106a,miR-548e-3p,miR-3960,miR-485-3p,miR-1b-3p,miR-33b-3p,miR-378d,miR-664-5p,miR-133a-3p,miR-362-3p,miR-2300b-3p,miR-1961,miR-1193,miR-1197-5p,miR-2904,miR-206-3p,miR-346,miR-769-5p,miR-6525,miR-10b-5p,miR-338-3p,miR-3184-5p,let-7b-5p,miR-4510,miR-539-5p,let-7b,miR-214,miR-3074-2-3p,miR-664a,miR-708-5p,let-7f,miR-664a-5p,miR-324-

SRRM2	ENSOARG0(	serine/a	miR-122-3p,miR-151a-3p,miR-134-3p,miR-147a,miR-3431,miR-432-5p,miR-496-5p,miR-2459,miR-9788-3p,miR-149-5p,miR-7b-5p,miR-329-3p,miR-432,miR-708-3p,miR-194-5p,miR-1260b,miR-362-3p,miR-744-5p,miR-432-3p,miR-432-3p,miR-1306-5p,novel_103,miR-431-5p,miR-7-5p,miR-342,miR-194a,miR-362-3p,miR-3074-5p,miR-541-5p,miR-320b,miR-411b-3p,miR-3082-5p,novel_69,miR-323a-5p,miR-378g,miR-4443,miR-652-3p,miR-18b,miR-18a-3p,miR-3970,miR-1306,miR-147-3p,miR-877-3p,miR-3059-5p,miR-342-3p,miR-194b-5p,miR-24-3p,miR-3959-3p,miR-877-3p,miR-2428,miR-423-5p,miR-576-3p,miR-133a-5p,novel_82,miR-128-1-5p,miR-3968,miR-502b,miR-6517,miR-18a-3p,miR-431,miR-18b-5p,miR-1224-5p,miR-3600,miR-486-5p,miR-151-5p,miR-532-3p,miR-2408,miR-362-3p,miR-2300b-3p,miR-2366,miR-5703,miR-2904,miR-4286,miR-345-3p,miR-148b-5p,novel_39,miR-486-3p,miR-2285p,miR-1260a,miR-185-3p,miR-466i-5p,miR-450a-2-3p,novel_127,miR-574-5p,miR-3957-5p,miR-296-3p,miR-2355-5p,miR-370-3p,miR-323-5p,miR-3957,miR-154b-5p,miR-151b,miR-21a-3p,miR-18a,miR-329a,miR-18a-5p,miR-1827,miR-671-5p,miR-485-5p,miR-3074-5p,miR-665-5p,miR-2330-3p,miR-1260b,miR-6134,miR-338-3p,miR-3184-5p,miR-2433,miR-551b-3p,miR-664a-5p,miR-151-3p,miR-331-3p,miR-664a,miR-486b-3p,miR-28-5p,miR-708-5p,miR-874-5p,miR-4492
NLRP12	ENSOARG0(	NLR fami	miR-2957,miR-362-3p,miR-16b,miR-106b-5p,miR-320b,miR-1306-5p,miR-146b,miR-3085-3p,novel_124,miR-25-5p,miR-3955-3p,miR-744-3p,miR-376b,miR-106a-5p,miR-424-5p,miR-3187-3p,miR-758-3p,miR-2310,miR-30c-1-3p,miR-378d,miR-758-3p,miR-125b-2-3p,miR-6529a,miR-484,miR-329-3p,miR-378b,miR-301b-5p,miR-1388-5p,miR-378c,miR-362-3p,miR-2426,novel_4,miR-105-1,novel_78,miR-2300a-5p,miR-548w,miR-1343-3p,miR-455-3p,miR-425-3p,miR-1298-3p,miR-371a-5p,miR-2285y,miR-301a-5p,miR-218-5p,miR-2285t,miR-3968,miR-2285f,miR-2447,miR-212-3p,miR-423-5p,miR-1285-5p,miR-877-3p,miR-1983,miR-877-3p,miR-299b-5p,miR-136-5p,miR-504,miR-499b-5p,miR-3064-5p,miR-139-5p,miR-4792,miR-452-3p,miR-2319b,miR-378e,miR-216a-5p,miR-500a-5p,miR-185-3p,miR-92a-1-5p,miR-422a,miR-345-3p,miR-30b-3p,miR-21-3p,miR-299-3p,miR-320d,miR-106a,miR-20a,miR-345-3p,miR-329a-5p,miR-216c-5p,miR-183-5p,miR-2285w,miR-146a-5p,miR-2432,miR-664b-3p,miR-377-5p,miR-2904,miR-1193,miR-378a-3p,miR-378i,novel_133,miR-2300b-3p,miR-362-3p,miR-193b-5p,miR-493-3p,miR-4429,miR-18a-3p,miR-106a,miR-2284h-5p,miR-15a-5p,miR-19b-1-5p,miR-3604,miR-664a-5p,miR-320e,miR-15b-3p,miR-487a-5p,miR-4492,miR-708-5p,miR-424-5p,miR-450b-3p,miR-2330-3p,miR-378c,miR-3184-3p,miR-129-2-3p,miR-3184-5p,miR-6525,miR-346,miR-27b-5p,miR-450a-1-3p,miR-625-5p,miR-186-5p,miR-133b-3p,miR-2312,miR-345-5p,miR-370-5p,miR-3065-3p,miR-552-3p,miR-296-3p,miR-30c-1-3p,miR-504-5p,miR-378a-3p,miR-204-3p,miR-15b-5p,miR-6516,miR-139-5p,miR-105-5p,miR-2331-5p,miR-106a-5p,miR-17-5p,miR-2411-3p,miR-146a,miR-124-5p,miR-378a-5p,miR-363-5p,miR-345-5p,miR-654-3p,miR-450c-3p,miR-19b-2-5p,miR-497-5p,miR-7862,miR-16b,miR-29b,miR-26b-3p,miR-377-5p,miR-31-5p,miR-194-3p,miR-20b,miR-2355-

KDM4A	ENSOARG0(	lysine (	miR-[illegible]-5p,miR-[illegible]-5p,miR-[illegible]-5p,let-7g-5p,miR-[illegible] 3p,miR-149-5p,novel_116,miR-1285,miR-708-3p,miR-873a-5p,miR-3432b,miR-1260b,miR-16b,miR-138,miR-29b,miR-2889,miR-497-5p,miR-6240,miR-380-5p,miR-2285r,miR-377-3p,novel_51,miR-365b-5p,miR-330-3p,miR-483-3p,miR-378a-5p,miR-660,miR-125a-5p,novel_68,miR-26b-5p,novel_69,miR-29a-3p,miR-299b-3p,miR-146b-3p,miR-6535,miR-148a-3p,miR-2285ab,miR-18a-3p,miR-150-5p,miR-1306,miR-1291,miR-195a-5p,miR-6395,let-7e-5p,miR-335-5p,miR-342-3p,miR-495-3p,miR-29c-3p,miR-211,miR-2284w,miR-28c,miR-576-3p,miR-15a,miR-502-5p,let-7i,miR-16a,miR-29a,miR-203b-5p,miR-656-5p,miR-429-3p,miR-299a-3p,miR-2411-5p,miR-483-3p,miR-322-5p,miR-9-5p,miR-214-5p,miR-2284z,miR-501-3p,miR-29d-3p,miR-582-3p,let-7d,miR-200b-3p,miR-3064-5p,miR-380-5p,miR-28b,miR-1271-3p,miR-2285b,let-7g,miR-3065-3p,miR-200c,miR-125a-3p,miR-2285af,miR-1260a,let-7c-5p,let-7f-5p,miR-200c-3p,miR-543-5p,miR-152-3p,novel_127,miR-503-3p,miR-874-3p,miR-1271,miR-125a,miR-615-5p,miR-2355-5p,miR-370-3p,miR-133c,miR-2448-3p,miR-331-5p,miR-23b,miR-362-5p,miR-3154,miR-137-3p,miR-329a,miR-485-5p,miR-1248,miR-7144-5p,miR-181a-2-3p,miR-16-5p,miR-1197-3p,miR-1260b,miR-500b-5p,miR-2903,miR-365a-5p,miR-29b-3p,miR-551b-3p,miR-331-3p,miR-125b-5p,miR-331-5p,miR-138-5p,miR-204-5p,miR-486b-3p,miR-1343-3p,miR-330-3p,miR-2300a-5p,miR-548w,miR-126a-5p,miR-500-5p,miR-455-3p,miR-541,miR-329-3p,miR-484,miR-296-5p,miR-501-3p,miR-2426,miR-362-3p,miR-2284aa,miR-424-5p,let-7a-5p,miR-744-3p,miR-23a-3p,miR-8485,miR-1306-5p,novel_103,miR-342,miR-16b,miR-362-3p,miR-3085-3p,let-7e,miR-3064-5p,miR-139-5p,miR-7977,miR-211-5p,miR-126b-5p,miR-3082-5p,miR-25,miR-500-3p,let-7d-5p,miR-361-3p,miR-504,miR-335-

ITGA9	ENSOARG0(	integrin	miR-3064-5p,miR-2284f,miR-148b-5p,miR-2284y,miR-200a,miR-29a-5p,miR-450c-5p,miR-2285b,miR-192-3p,miR-205-5p,miR-664-3p,miR-127-5p,novel_127,miR-200a-3p,miR-2285ad,miR-429-3p,miR-6517,miR-1224-5p,miR-505-5p,miR-129-1-3p,miR-1290,miR-1343-5p,miR-1260b,miR-1197-3p,miR-125b-5p,miR-125a,miR-1271,miR-331-5p,miR-223-5p,miR-345-5p,miR-329b,miR-628-5p,let-7f-2-3p,let-7f-2-3p,miR-432-3p,miR-2284l,miR-296-3p,miR-146a,miR-105-5p,novel_51,miR-2411-3p,miR-483-3p,miR-193a-3p,miR-3955-5p,miR-134-3p,miR-149-5p,miR-197-3p,novel_23,miR-27b-3p,miR-335-5p,miR-193b-3p,miR-758-5p,miR-199b-5p,miR-142b,miR-2284o,miR-6119-3p,miR-379-3p,miR-6123,miR-3074-5p,miR-329-5p,miR-3120-3p,miR-677,miR-320c,miR-181b-3p,miR-216a-3p,miR-3065-5p,miR-146b-5p,miR-329a-5p,miR-503-5p,miR-376a-5p,miR-181b-1-3p,miR-2424,miR-210-5p,miR-500a-5p,miR-324-3p,miR-345-3p,miR-92a-1-5p,miR-2484,miR-18a-3p,miR-380-5p,miR-2284h-5p,miR-217-5p,miR-33b-3p,miR-2284u,miR-361-5p,miR-3074-5p,miR-3184-3p,miR-129-2-3p,miR-6525,miR-769-5p,miR-346,miR-224-5p,miR-15b-3p,miR-2285j,miR-665,novel_74,miR-487b-5p,miR-4492,miR-504-5p,miR-2331-3p,miR-548o-3p,miR-216b-3p,miR-552-3p,miR-296-3p,miR-499b-3p,miR-433-3p,miR-345-5p,miR-1895,miR-1827,miR-1388-3p,miR-2284b,miR-2310,miR-505,miR-29b-1-5p,miR-153-3p,miR-146b,miR-199b-5p,novel_78,miR-181b-2-3p,miR-153,miR-455-3p,miR-1185-3p,miR-421,miR-2459,miR-1298-5p,miR-301b-5p,miR-1388-5p,miR-101c,miR-2447,let-7i-3p,miR-361-3p,miR-301a-5p,miR-3968,miR-490-5p,miR-2284g,miR-24-1-5p,miR-499a-3p,miR-378g,miR-3596,miR-203-3p,miR-1185-2-3p,miR-140-5p,miR-221,miR-378j,miR-299b-5p,miR-504,miR-3529-3p,miR-29b-2-5p,miR-181a-2-3p,miR-24-2-5p,miR-199a-5p,miR-3065-3p,miR-490-3p,miR-2284a,miR-1260a,miR-200c,miR-217,miR-

PTPRM	ENSOARG0(	protein	miR-2285c,miR-345-5p,miR-1827,miR-625-5p,miR-885-3p,miR-504-5p,miR-2331-3p,miR-30c-1-3p,miR-296-3p,novel_107,miR-154a-3p,miR-301a-3p,miR-377-3p,let-7i-5p,miR-15b-3p,let-7f,miR-665,miR-151-3p,miR-130b-5p,miR-454-3p,miR-4492,miR-129-2-3p,let-7b,novel_44,miR-769-5p,miR-122-5p,miR-1961,miR-2366,novel_73,miR-361-5p,miR-34a-5p,miR-133a-3p,miR-7859,miR-92a-1-5p,miR-324-3p,miR-98-5p,let-7k,miR-487a,miR-376a-5p,miR-143-3p,miR-486-3p,miR-221,miR-378j,miR-504,miR-4792,miR-7689-3p,miR-2483-3p,miR-3596,miR-615,miR-101-3p,miR-3968,miR-154-3p,miR-1843a-3p,miR-3956-3p,miR-2447,miR-758-3p,miR-1388-5p,miR-1839-5p,miR-1343-3p,miR-330-3p,novel_78,miR-153,miR-2459,miR-130b-3p,miR-153-3p,miR-8485,miR-345-5p,miR-874-3p,miR-574-5p,miR-2285aa,miR-6740-5p,miR-193a-5p,miR-1260b,miR-1197-3p,miR-1290,miR-330-5p,miR-23b-5p,miR-2427,miR-885-3p,miR-429-3p,miR-6517,miR-129-1-3p,miR-125a-3p,miR-205-5p,let-7f-5p,miR-152-3p,miR-3064-5p,miR-29a-5p,let-7g,miR-148a-3p,miR-216a-3p,miR-2284e,let-7e-5p,novel_69,miR-374c-3p,miR-669,miR-485-3p,miR-193b-3p,miR-2285m,miR-301b-3p,miR-223-3p,miR-542-3p,miR-432,miR-22-3p,miR-4508,miR-31-5p,miR-3955-5p,miR-3431,miR-194-3p,miR-296-3p,miR-377-3p,miR-105-5p,miR-365b-5p,miR-483-3p,miR-378a-5p,miR-193a-3p,miR-6240,miR-668-3p,miR-21a-3p,miR-193b-3p,miR-132-5p,miR-6516,miR-877-5p,miR-22-3p,novel_32,miR-204-3p,miR-222-3p,miR-29b-2-5p,miR-132-5p,miR-664a,miR-208b-5p,miR-145a-3p,miR-3074-2-3p,miR-425-5p,miR-6134,let-7b-5p,miR-5703,miR-188-3p,miR-140-3p,miR-378d,miR-548e-3p,miR-485-3p,miR-200b,miR-148b-3p,miR-487a-3p,miR-383-5p,miR-532-3p,novel_83,miR-542-5p,miR-185-3p,miR-466i-5p,miR-21-3p,miR-34b-5p,miR-2411,miR-361-3p,miR-147-
PRPF4B	ENSOARG0(	pre-mRNA	miR-371a-5p,miR-495-3p,miR-329b
CDCA7	ENSOARG0(	cell div	let-7e,miR-196b-5p,miR-330-3p,miR-2957,miR-106b-5p,miR-16b,miR-182-5p,miR-17-5p,miR-106a-5p,miR-7-5p,miR-758-3p,miR-432-3p,miR-363-5p,miR-628-5p,let-7a-5p,miR-124a,miR-424-5p,miR-6240,miR-106a-5p,miR-497-5p,miR-16b,miR-101a-5p,miR-758-3p,miR-145a-5p,miR-2113,miR-7b-5p,miR-541,miR-9788-3p,miR-2459,miR-20b,let-7g-5p,miR-185-5p,miR-145b,miR-330-3p,miR-490-5p,miR-4726-5p,miR-16a,let-7i,miR-15a,miR-20a-5p,miR-8095,miR-758-5p,miR-541-3p,miR-195a-5p,novel_94,miR-135b-5p,let-7e-5p,miR-450b-3p,miR-668-5p,miR-2387,let-7d-5p,miR-499a-3p,miR-541-5p,miR-24-1-5p,miR-452-3p,let-7k,miR-106a,miR-2898,miR-98-5p,let-7f-5p,miR-6536,let-7c-5p,miR-2285af,miR-205-5p,let-7g,miR-490-3p,miR-24-2-5p,miR-20a,miR-361-5p,miR-142-3p,let-7d,miR-124-3p,miR-196a-5p,miR-1246,miR-1961,miR-2408,miR-322-5p,miR-7859,miR-15a-5p,miR-380-5p,miR-548e-3p,miR-106a,miR-424-5p,miR-100-3p,miR-135a-5p,let-7f,let-7b-5p,miR-6134,miR-93,miR-16-5p,let-7b,miR-3184-3p,miR-20b-5p,miR-3074-1-3p,miR-93-5p,miR-17-5p,miR-15b-5p,let-7i-5p,miR-499b-3p,miR-216b-5p,miR-2285aa,miR-182-5p,miR-548o-3p

HNRNPD	ENSOARG0(	heteroge	miR-6525,miR-10b-5p,novel_82,miR-339a,miR-450a-2-3p,miR-107,miR-103a-3p,miR-10a-5p,novel_79,miR-339-5p,miR-10a,miR-103,miR-10b,miR-7134-5p,miR-107,miR-339b,miR-10a-5p
LRRC31	ENSOARG0(	leucine	miR-190a-3p,miR-412-3p,miR-30f,miR-129b-3p,miR-320e,miR-2432,miR-411b-3p,miR-412
IDH3A	ENSOARG0(	isocitra	miR-3065-5p,miR-6516-3p,miR-195a-5p,miR-379-5p,miR-2319a,miR-136-5p,miR-7-1-3p,miR-3120-3p,miR-2319b,miR-500-3p,miR-454-5p,miR-203-3p,miR-320c,miR-670-3p,miR-16a,miR-6402,miR-15a,miR-502b,miR-6123,miR-421-5p,miR-206,miR-9-5p,novel_101,miR-452-5p,novel_27,miR-502-3p,miR-371b-3p,miR-758-3p,miR-501-3p,miR-450c-3p,miR-1a-3p,miR-7857,miR-101c,miR-497-5p,miR-450b-5p,miR-16b,miR-409b,miR-16b,miR-197-5p,miR-105-5p,let-7d-3p,miR-320b,miR-214-3p,miR-452-5p,miR-487b-5p,miR-424-5p,miR-130b-5p,miR-503-3p,miR-758-3p,miR-8485,miR-433-3p,miR-320a,miR-503-3p,miR-450a-1-3p,miR-488-3p,miR-21a-3p,miR-1827,miR-7144-5p,miR-543-3p,miR-767,miR-874-3p,miR-503-3p,miR-574-5p,miR-15b-5p,miR-6130,miR-6516,miR-665,miR-6740-5p,miR-34c-3p,miR-130b-5p,miR-134-5p,miR-424-5p,miR-767-5p,miR-450b-3p,miR-214,miR-6128,miR-16-5p,miR-4510,miR-122-5p,miR-206-3p,miR-6525,miR-323-3p,miR-501-3p,miR-23b-5p,miR-5703,miR-2366,miR-3578,miR-134,miR-4429,novel_73,miR-493-3p,miR-1224-5p,miR-6517,miR-134-5p,novel_83,miR-2284z,miR-582-3p,miR-450a-1-3p,miR-545-5p,miR-15a-5p,miR-2411-5p,miR-6516-5p,miR-322-5p,miR-9-5p,miR-1277-5p,miR-450a-2-3p,miR-320d,miR-1,miR-2385-3p,miR-1271-3p,miR-2411
PLXNB1	ENSOARG0(	plexin B	miR-4508,miR-4532,miR-4492,miR-3653-3p,novel_94,miR-3957-3p,miR-185-3p

LDB3	ENSOARG0(	LIM doma	miR-146b-5p,miR-195a-5p,let-7e-5p,miR-107,miR-19a-3p,miR-2387,miR-3120-3p,novel_68,miR-16a,let-7i,miR-15a,miR-28c,miR-211,miR-24-3p,miR-17-3p,novel_101,miR-2285x,miR-342-3p,miR-1a-1-5p,miR-497-5p,miR-26a-2-3p,miR-30b-3p,miR-16b,miR-1260b,miR-653,miR-181a-5p,miR-103,miR-134-3p,let-7g-5p,miR-339-5p,miR-103a-3p,miR-466f-3p,miR-330-3p,miR-2411-3p,miR-146a,miR-377-3p,let-7a-2-3p,miR-654-3p,miR-130b-5p,miR-6240,miR-3074-1-3p,miR-7144-5p,miR-671-5p,miR-1248,miR-485-5p,miR-1814c,miR-10b-3p,let-7g-3p,miR-3154,miR-223-5p,miR-2285aa,miR-141-3p,miR-874-3p,miR-204-5p,miR-28-5p,miR-6740-5p,miR-331-3p,miR-1-5p,miR-1260b,miR-16-5p,let-7a-2-3p,let-7d,miR-2427,miR-345-3p,miR-129-1-3p,miR-322-5p,miR-302a-5p,miR-2411-5p,novel_79,miR-2285ad,miR-107,miR-200a-3p,miR-450a-2-3p,miR-17-3p,miR-127-5p,miR-1a-2-5p,miR-181b-5p,let-7f-5p,miR-339a,let-7c-5p,miR-2285af,miR-1260a,let-7g,miR-200a,miR-2285b,miR-28b,miR-544-5p,miR-96-5p,let-7d-5p,miR-378g,miR-2285u,miR-7689-3p,miR-211-5p,miR-452-3p,miR-2284q,miR-371a-5p,miR-1973,miR-2285y,miR-107-5p,miR-2285n,miR-2440,miR-361-3p,let-7i-3p,miR-452-5p,miR-181d-5p,miR-212-3p,miR-1839-5p,miR-3120-5p,miR-30b-3p,miR-125b-2-3p,miR-9788-3p,miR-2300a-5p,miR-330-3p,miR-146b,miR-214-3p,let-7e,miR-2285o,miR-103a-2-5p,miR-16b,miR-342,miR-8485,miR-2310,let-7a-5p,miR-424-5p,miR-494-3p,novel_99,let-7g-3p,miR-339b,miR-877-5p,miR-15b-5p,let-7i-5p,miR-377-3p,miR-2284s,miR-216b-3p,miR-2331-3p,miR-1277-3p,miR-548o-3p,miR-424-5p,miR-708-5p,miR-130b-5p,miR-664a,miR-17-3p,let-7f,let-7b-5p,miR-6134,miR-181c-5p,miR-6525,novel_44,miR-1271,miR-214,miR-3074-2-3p,let-7b,miR-433-5p,miR-129-2-3p,miR-361-5p,miR-19b-3p,miR-1961,novel_83,miR-2284b-5p,miR-616-3p,miR-15a-

ADGRG3	ENSOARG0(	adhesion	let-7f-5p,miR-200a-3p,novel_96,novel_127,miR-125a-3p,miR-200a,let-7g,miR-2285b,miR-3064-5p,miR-155-5p,miR-1343-5p,miR-330-5p,miR-505-5p,miR-450a-1-3p,miR-486-5p,miR-2285ad,miR-6517,miR-412,miR-1224-5p,miR-592,miR-125b-5p,miR-1260b,novel_42,miR-193a-5p,miR-345-5p,miR-200a-5p,miR-23b,miR-2355-5p,miR-542-5p,miR-125a,miR-106b-3p,miR-193a-3p,miR-146a,miR-3141,miR-365b-5p,novel_51,miR-6240,miR-493-5p,miR-628-5p,miR-26a-2-3p,miR-27b-3p,miR-432,miR-1843b-5p,miR-542-3p,miR-223-3p,miR-450c-3p,miR-197-3p,miR-22-3p,miR-412-5p,miR-134-3p,miR-194-3p,miR-149-5p,miR-31-5p,miR-29a,miR-4324,miR-1247-3p,miR-193b-3p,miR-29c-3p,miR-410-5p,miR-412-3p,miR-146b-5p,miR-2319a,let-7e-5p,miR-3141,miR-1306,miR-3970,miR-150-5p,miR-216a-3p,miR-3065-5p,novel_69,miR-374c-3p,miR-6535,miR-677,miR-181b-3p,miR-329-5p,miR-202-5p,miR-98-5p,let-7k,novel_17,miR-618,miR-210-5p,miR-92a-1-5p,miR-376a-5p,miR-181b-1-3p,miR-486-3p,miR-329a-5p,miR-345-3p,miR-133a-3p,miR-218-1-3p,miR-34a-5p,miR-1961,miR-217-5p,miR-23b-3p,miR-7859,miR-23c,miR-18a-3p,novel_74,miR-129-5p,miR-4492,miR-874-5p,let-7f,miR-665,miR-769-5p,miR-224-5p,miR-3184-5p,let-7b,miR-3958-5p,miR-665-5p,miR-214,miR-345-5p,miR-1827,miR-186-5p,miR-450a-1-3p,miR-129b-3p,miR-2284s,let-7i-5p,miR-2331-3p,miR-504-5p,miR-548o-3p,miR-552-3p,miR-296-3p,miR-491-5p,miR-146b,miR-214-3p,miR-1388-3p,miR-23a-3p,miR-744-3p,miR-2284r,miR-301b-5p,miR-541,miR-501-3p,miR-455-3p,miR-330-3p,miR-181b-2-3p,miR-499a-5p,miR-2440,miR-301a-5p,miR-361-3p,novel_111,miR-423-5p,miR-3956-3p,miR-504,miR-493-5p,miR-1983,miR-140-5p,miR-378g,miR-25,miR-500-3p,miR-543-5p,miR-1260a,let-7c-5p,miR-217,miR-3065-3p,miR-1957a,miR-490-3p,miR-29b-2-5p,miR-2385-3p,miR-
IQSEC1	ENSOARG0(	IQ motif	miR-329a,miR-3074-1-3p,miR-671-5p,miR-30c-1-3p,miR-574-5p,miR-377-3p,miR-544-3p,miR-193b-3p,miR-133c,miR-204-3p,miR-15b-5p,miR-2433,miR-144,miR-665,miR-664a,miR-424-5p,miR-374b,miR-3184-3p,miR-1271,miR-16-5p,miR-346,miR-3184-5p,miR-362-3p,miR-2300b-3p,miR-217-5p,miR-200b-3p,miR-218-1-3p,miR-3960,miR-299a-3p,miR-429-3p,miR-3600,miR-322-5p,miR-15a-5p,miR-1271-5p,miR-200b,miR-545-5p,miR-200c,miR-374a-5p,miR-3071-3p,miR-30c-2-3p,miR-217,miR-200c-3p,miR-760-3p,miR-299-3p,miR-329a-5p,miR-369-3p,miR-29b-1-5p,miR-148b-5p,miR-2285i,miR-143-3p,miR-381-3p,miR-96-5p,miR-2285k,miR-195a-5p,miR-20a-3p,miR-320b,miR-374b-5p,miR-299b-3p,miR-144-3p,miR-27a-3p,miR-15a,miR-6402,miR-16a,miR-490-5p,miR-342-3p,miR-2447,miR-212-3p,miR-193b-3p,miR-2285x,miR-423-5p,miR-2428,miR-329-3p,miR-143-5p,miR-362-3p,miR-16b,miR-544a,miR-101a-5p,miR-497-5p,miR-27b-3p,novel_78,miR-1343-3p,miR-330-3p,miR-5010-3p,miR-27a-3p,miR-544b,miR-342,miR-377-3p,miR-330-3p,miR-29b-1-5p,miR-362-3p,miR-16b,miR-193a-3p,miR-6240,miR-424-5p,miR-101b-3p,miR-494-3p,let-7f-2-3p,let-7f-2-3p,miR-30c-1-3p,miR-8485,miR-193a

KCNU1	ENSOARG0(	potassiu	miR-200b,miR-124-3p,miR-2300b-3p,miR-1961,miR-215-5p,miR-30c-5p,novel_133,miR-218-1-3p,novel_73,miR-487a,miR-143-3p,miR-345-3p,miR-26a-5p,miR-30b,let-7k,miR-30d,miR-98-5p,miR-154a-3p,let-7i-5p,miR-204-3p,miR-192-5p,miR-487b-5p,miR-30d-5p,miR-186-5p,miR-339b,miR-30f,miR-345-5p,miR-154b-3p,miR-665-5p,miR-3074-5p,miR-145a-3p,let-7b,let-7b-5p,miR-224-5p,miR-6525,miR-665,let-7f,miR-130b-5p,miR-4492,miR-487b-5p,miR-145b,miR-9788-3p,miR-376b-3p,miR-758-3p,miR-412-3p,miR-484,miR-3120-5p,miR-2426,miR-33a-5p,miR-2330-5p,let-7a-5p,miR-124a,miR-2310,miR-758-3p,miR-326,let-7e,miR-1185-5p,miR-499b-5p,miR-2319b,miR-20a-3p,miR-10b,miR-664b,let-7d-5p,miR-144-5p,miR-3082-5p,miR-2447,miR-3059-5p,miR-7705,miR-877-3p,miR-2285y,miR-30b-5p,miR-154-3p,miR-2285t,miR-412,novel_79,miR-429-3p,novel_121,miR-330-5p,miR-23b-5p,miR-30a-5p,miR-218-2-3p,miR-3591-5p,miR-345-3p,miR-196a-5p,miR-200b-3p,miR-30c,let-7d,let-7g,miR-339a,let-7c-5p,miR-664-3p,miR-200c,miR-376b-3p,miR-200c-3p,let-7f-5p,miR-215-5p,miR-487a-3p,miR-30e-5p,miR-345-5p,miR-144-5p,miR-193a-5p,miR-331-3p,miR-494-5p,miR-2332,miR-31-3p,miR-339-5p,miR-31-5p,miR-194-3p,let-7g-5p,miR-145a-5p,miR-542-3p,miR-2113,miR-1843b-5p,miR-130b-5p,miR-345-5p,miR-654-3p,miR-2411-3p,miR-224-5p,miR-196b-5p,miR-26b-5p,miR-3074-5p,miR-216b-3p,let-7e-5p,miR-7975,miR-450b-3p,miR-2284w,miR-326-3p,let-7i

			miR-665,miR-450b-3p,miR-17-3p,miR-664a,miR-487a-5p,miR-424-5p,miR-129-2-3p,miR-10b-5p,miR-6525,miR-346,miR-206-3p,miR-450a-1-3p,miR-2312,novel_99,miR-186-5p,miR-660-5p,miR-21-3p,miR-296-3p,miR-377-3p,miR-10a,miR-193b-3p,miR-15b-5p,miR-210-5p,miR-92a-1-5p,miR-466i-5p,miR-299-3p,miR-216c-5p,miR-146a-5p,miR-2366,miR-362-3p,miR-2904,miR-5703,miR-1434-5p,miR-140-3p,miR-33b-3p,miR-16-1-3p,miR-378d,miR-18a-3p,miR-15a-5p,miR-148b-3p,miR-107-5p,miR-3968,miR-335,miR-450a-5p,miR-490-5p,miR-2285t,miR-885-5p,miR-541-3p,miR-2285f,miR-452-5p,miR-219a-5p,miR-199b-5p,miR-136-5p,miR-2319b,miR-3082-5p,miR-1306-5p,miR-362-3p,miR-16b,miR-491-5p,miR-146b,miR-199b-5p,miR-103a-2-5p,miR-424-5p,miR-33a-5p,miR-376b,miR-2284r,miR-484,miR-329-3p,miR-541,miR-10b,miR-1a-3p,miR-30b-3p,miR-362-3p,miR-1388-5p,miR-101a-5p,miR-2284aa,miR-101c,miR-3120-5p,novel_4,miR-409b,miR-548w,miR-539-5p,miR-455-3p,miR-5100,miR-331-3p,miR-409-3p,miR-592,miR-16-5p,miR-137-3p,miR-10b-3p,miR-329a,miR-7144-5p,miR-671-5p,miR-300-3p,miR-374c-3p,miR-199a-5p,miR-17-3p,miR-6536,novel_127,miR-543-5p,novel_96,miR-152-3p,miR-2385-3p,miR-1,miR-4286,miR-199a-5p,miR-200a,miR-1957a,miR-450c-5p,miR-490-3p,miR-218-2-3p,miR-582-3p,miR-10a-5p,miR-299a-3p,miR-505-5p,miR-322-5p,miR-450a-1-3p,miR-2284z,miR-129-1-3p,miR-15a,miR-576-3p,miR-485-3p,miR-16a,miR-206,miR-147-5p,miR-219b-3p,miR-335-5p,miR-193b-3p,miR-17-3p,miR-199b-5p,miR-2285ab,miR-148a-3p,miR-150-5p,miR-1306,miR-3591-3p,miR-18a-3p,miR-146b-5p,miR-2319a,miR-195a-5p,miR-10a-5p,miR-374c-3p,miR-299b-3p,miR-377-3p,miR-146a,miR-455-5p,miR-660,miR-193a-3p,miR-432-3p,miR-542-3p,miR-450c-3p,novel_23,miR-3071-5p,miR-374b-3p,miR-30b-3p,miR-16b,miR-16-1-3p,miR-497-5p,miR-455-
ESX1	ENSOARG0(	ESX home	
			novel_115,miR-2411-3p,miR-323b-3p,miR-29b-1-5p,miR-124-5p,miR-33a-5p,miR-432-3p,miR-432-3p,miR-6529a,miR-3071-5p,miR-30b-3p,miR-323b,miR-30b-3p,miR-138,miR-134-3p,miR-147a,miR-455-3p,miR-9788-3p,miR-216a-3p,miR-4324,novel_1,miR-335,miR-3958-3p,miR-147-5p,miR-203b-5p,miR-3956-3p,miR-335-5p,novel_60,miR-2447,miR-17-3p,miR-3065-5p,miR-504,miR-505-3p,miR-452-3p,miR-378g,miR-345-3p,miR-127-5p,miR-17-3p,miR-200a-3p,miR-376c-3p,miR-29b-1-5p,miR-664b-3p,miR-2285b,miR-2366,miR-345-3p,miR-5703,novel_73,miR-505-3p,miR-1b-3p,miR-340-5p,miR-758-5p,miR-320e,miR-665,miR-3969,miR-6740-5p,miR-17-3p,miR-138-5p,miR-1306-5p,miR-539-5p,miR-425-5p,miR-6128,miR-6525,miR-323-3p,miR-488-3p,miR-3154,miR-27b-5p,miR-504-5p,miR-545-3p,miR-141-3p,miR-2285aa,miR-376e-3p,miR-376c-3p,miR-204-3p
IL1RAPL2	ENSOARG0(	interleu	

ATP6V0B	ENSOARG0(	ATPase,	miR-497-5p,miR-1260b,miR-29b,miR-16b,miR-30b-3p,miR-30b-3p,miR-7b-5p,miR-147a,miR-377-5p,novel_4,miR-2300a-5p,miR-125a-5p,miR-16b,miR-2285v,miR-1306-5p,miR-7-5p,miR-4532,miR-432-3p,miR-424-5p,miR-195a-5p,miR-450b-3p,miR-3970,miR-1306,miR-361-3p,novel_69,miR-29a-3p,miR-29a,miR-2284o,miR-16a,miR-615,miR-15a,miR-423-5p,miR-29c-3p,miR-3957-3p,miR-2447,miR-188-3p,miR-29d-3p,novel_133,miR-615-3p,miR-532-3p,miR-15a-5p,miR-322-5p,miR-505-5p,miR-383-5p,miR-200a-3p,miR-30b-3p,miR-466i-5p,miR-2898,miR-125b,miR-92a-1-5p,miR-185-3p,miR-324-3p,miR-1260a,miR-3065-3p,miR-377-5p,miR-2284f,miR-485-5p,miR-671-5p,miR-1248,miR-23a-5p,miR-15b-5p,miR-204-3p,miR-296-3p,miR-141-3p,miR-552-3p,miR-125a,miR-2355-5p,miR-3065-3p,miR-28-5p,miR-708-5p,miR-874-5p,miR-424-5p,miR-664a,miR-665,miR-331-3p,miR-29d-5p,miR-125b-5p,miR-29b-3p,miR-6134,miR-3184-5p,miR-29c-5p,miR-1260b,miR-665-5p,miR-16-5p,miR-6128,miR-3184-3p,miR-539-5p
SCN3B	ENSOARG0(	sodium c	miR-222-5p,miR-148b-3p,miR-200b,miR-23b-3p,miR-1247-5p,miR-616-3p,miR-1b-3p,miR-3600,miR-33b-3p,miR-34a-5p,miR-2284u,miR-133a-3p,miR-193b-5p,novel_133,miR-5703,miR-125b-2-3p,miR-664b-3p,miR-376a-5p,miR-143-3p,miR-2424,miR-369-3p,miR-466i-5p,miR-30b-3p,miR-34b-5p,miR-185-3p,miR-92a-1-5p,miR-216a-5p,miR-193b-3p,miR-6130,miR-499b-3p,miR-6516,miR-27a-5p,miR-877-5p,miR-204-3p,miR-30c-1-3p,miR-296-3p,miR-182-5p,miR-3065-3p,miR-345-5p,miR-18a-5p,novel_99,miR-625-5p,miR-21a-3p,miR-433-3p,miR-129b-3p,miR-142a-5p,miR-6134,miR-3184-5p,miR-3184-3p,miR-129-2-3p,miR-3074-5p,miR-4510,miR-539-5p,miR-425-5p,miR-17-3p,miR-664a,miR-130b-5p,miR-708-5p,miR-190a-5p,miR-2285j,miR-665,miR-539-5p,miR-142-5p,miR-1298-3p,miR-147a,miR-425-3p,miR-9788-3p,novel_78,miR-2300a-5p,miR-145b,miR-27a-3p,miR-450b-5p,miR-2426,miR-544a,miR-4791,miR-143-5p,miR-484,miR-7857,miR-323b,miR-501-3p,miR-412-3p,miR-30c-1-3p,miR-8485,miR-494-3p,miR-1388-3p,miR-33a-5p,miR-2284b,miR-23a-3p,miR-3085-3p,miR-103a-2-5p,miR-1306-5p,miR-182-5p,miR-505,miR-323b-3p,miR-3082-5p,miR-489,miR-3596,miR-499a-3p,miR-500-3p,miR-27a-3p,miR-20a-3p,miR-452-3p,miR-664b,miR-2284g,miR-3064-5p,miR-499b-5p,miR-541-5p,miR-1983,novel_94,miR-335-3p,miR-361-3p,miR-129b-5p,miR-378j,novel_111,miR-361-3p,miR-423-5p,miR-1285-5p,miR-502-3p,miR-2447,miR-190b-5p,miR-181d-5p,miR-2440,novel_82,miR-218-5p,miR-107-5p,miR-6516-5p,miR-129-1-3p,novel_121,miR-545-5p,miR-429-3p,miR-18b-5p,miR-412,miR-200b-3p,miR-3578,miR-501-3p,miR-23b-5p,miR-2427,miR-29d-3p,miR-148b-5p,miR-28b,miR-2478,miR-2285b,miR-2284a,miR-1957a,miR-200a,miR-376c-5p,miR-3065-3p,miR-376b-5p,miR-2284y,miR-2403,miR-3064-5p,miR-181b-5p,miR-200c-3p,miR-17-3p,miR-152-3p,miR-

PTP4A2	ENSOARG0(	protein	miR-301a-5p,miR-769-5p,miR-2285n,miR-2285t,miR-335,miR-499a-5p,miR-628-3p,miR-2284j,miR-541-3p,miR-212-3p,miR-885-5p,miR-3059-5p,miR-140-5p,miR-493-5p,miR-136-5p,miR-211-5p,miR-2284g,miR-2483-3p,miR-106b-5p,miR-505,miR-29b-1-5p,miR-326,novel_103,miR-342,miR-153-3p,miR-130b-3p,miR-487b-5p,miR-2284b,miR-23a-3p,miR-106a-5p,miR-1388-3p,miR-2310,miR-1b-5p,miR-8485,miR-1a-3p,miR-7857,miR-301b-5p,miR-10b,miR-541,miR-6529a,miR-2284aa,miR-544a,miR-145b,novel_78,miR-421,miR-148b-5p,miR-153,miR-338-5p,miR-664a-5p,miR-324-3p,miR-2285j,miR-454-3p,miR-155-5p,miR-425-5p,miR-145a-3p,miR-3184-3p,miR-338-3p,miR-206-3p,miR-769-5p,miR-10b-5p,miR-27b-5p,miR-21a-3p,miR-186-5p,novel_99,let-7g-3p,miR-660-5p,miR-221-5p,miR-155-5p,miR-193b-3p,miR-377-3p,miR-10a,miR-301a-3p,miR-27a-5p,miR-345-3p,miR-500a-5p,novel_17,miR-221-5p,miR-1277-5p,miR-760-3p,miR-106a,miR-195a-3p,miR-20a,miR-487a,miR-216c-5p,miR-143-3p,miR-125b-2-3p,miR-29b-1-5p,miR-376a-5p,miR-188-3p,miR-1197-5p,miR-191,novel_133,miR-148a-5p,miR-378d,miR-2284u,miR-218-1-3p,miR-505-3p,miR-3600,miR-106a,miR-23c,miR-23b-3p,miR-487a-3p,novel_87,miR-6402,miR-20a-5p,miR-6123,miR-206,miR-2285x,miR-495-3p,miR-193b-3p,miR-335-5p,miR-342-3p,miR-301b-3p,miR-326-3p,miR-3959-3p,miR-323c,miR-371b-3p,miR-211,miR-3065-5p,miR-505-3p,miR-10a-5p,miR-3120-3p,miR-411b-3p,miR-17-5p,novel_51,miR-106a-5p,miR-455-5p,miR-2411-3p,miR-377-3p,miR-193a-3p,miR-660,miR-483-3p,miR-668-3p,miR-493-5p,miR-380-5p,let-7j,miR-539-3p,miR-145a-5p,miR-432,miR-455-5p,miR-380-3p,miR-2284ab,miR-130a-3p,miR-133b-5p,miR-20b,miR-544b,novel_116,miR-3431,miR-432-5p,miR-2284d,miR-135a-1-3p,miR-34c-3p,miR-2284k,miR-191-5p,miR-204-5p,miR-2284y,miR-1306-5p,miR-93,miR-

B4GALT2	ENSOARG0(	UDP-Gal:	miR-2285aa,miR-2355-5p,miR-125a,miR-1271,miR-574-5p,miR-874-3p,miR-25-3p,miR-1248,miR-876-3p,miR-2318,miR-329a,miR-345-5p,miR-16-5p,miR-1260b,miR-1197-3p,miR-125b-5p,miR-2433,miR-412,novel_79,miR-18b-5p,miR-299a-3p,miR-2285ad,miR-6517,novel_121,miR-129-1-3p,miR-505-5p,miR-322-5p,miR-885-3p,miR-3064-5p,miR-29a-5p,novel_39,miR-92b-3p,miR-148b-5p,miR-3071-3p,miR-205-5p,miR-125a-3p,novel_127,miR-152-3p,miR-3074-5p,miR-677,miR-181b-3p,miR-299b-3p,miR-1306,miR-148a-3p,miR-410-5p,miR-412-3p,miR-1839-3p,miR-193b-3p,miR-758-5p,miR-2284w,miR-485-3p,miR-28c,miR-6119-3p,miR-339-5p,miR-103a-3p,miR-3431,miR-134-3p,miR-197-3p,miR-22-3p,miR-432,miR-2113,miR-223-3p,miR-542-3p,miR-497-5p,miR-369-5p,miR-105-5p,miR-32,novel_51,miR-2411-3p,miR-377-3p,miR-296-3p,miR-193a-3p,miR-216b-3p,miR-296-3p,miR-2331-3p,miR-32-5p,miR-15b-5p,miR-377-3p,miR-499b-3p,miR-129b-3p,miR-625-5p,miR-885-3p,miR-1895,miR-1827,miR-345-5p,miR-339b,miR-3958-5p,miR-214,miR-129-2-3p,miR-3074-5p,miR-3184-5p,miR-122-5p,miR-6525,miR-346,miR-665,let-7b-3p,miR-874-5p,miR-4492,miR-380-5p,miR-212-5p,miR-18a-3p,miR-1247-5p,miR-193b-5p,miR-664-5p,miR-133a-3p,miR-34a-5p,miR-503-5p,miR-486-3p,miR-664b-3p,miR-181b-1-3p,miR-500a-5p,miR-345-3p,miR-210-5p,novel_17,miR-3653-3p,miR-4792,miR-24-1-5p,miR-378g,miR-499a-3p,miR-2483-3p,miR-381-3p,miR-1983,miR-96-5p,miR-136-5p,miR-2447,miR-363-3p,miR-423-5p,miR-877-3p,miR-361-3p,miR-301a-5p,miR-218-5p,miR-1843a-3p,miR-181b-2-3p,miR-1343-3p,miR-147a,miR-323b,miR-2284r,miR-329-3p,miR-301b-5p,miR-362-3p,miR-574-3p,miR-8485,miR-323b-3p,miR-199a-3p,miR-214-3p,miR-34c-5p,miR-18a,miR-362-5p,miR-3154,miR-381-3p,miR-23a-5p,miR-6128,miR-1306-5p,miR-500b-5p,miR-331-3p,miR-409-3p,miR-

TNNT3	ENSOARG0(	troponin	miR-214,miR-665-5p,miR-433-5p,miR-6525,miR-10b-5p,novel_44,miR-324-3p,miR-19b-1-5p,miR-151-3p,miR-29b-2-5p,miR-4492,miR-874-5p,miR-129-5p,novel_74,miR-451a,miR-664a,miR-370-5p,miR-3065-3p,miR-2331-3p,miR-140-5p,miR-30c-1-3p,miR-221-5p,miR-548o-3p,miR-204-3p,novel_25,miR-22-3p,miR-10a,miR-487b-5p,miR-543-3p,miR-625-5p,miR-1895,miR-186-5p,novel_99,miR-18a-5p,miR-345-5p,miR-195a-3p,miR-2424,miR-181b-1-3p,miR-219b-5p,miR-185-3p,miR-125b,miR-221-5p,miR-21-3p,miR-202-5p,miR-466i-5p,miR-380-5p,miR-212-5p,miR-199a-3p,miR-2904,miR-1197-5p,miR-140-3p,novel_133,miR-2408,miR-361-5p,miR-493-3p,miR-6527,let-7i-3p,miR-9-5p,miR-452-5p,miR-3059-5p,miR-2447,miR-541-3p,miR-877-3p,novel_111,miR-107-5p,miR-218-5p,miR-2285t,miR-4726-5p,miR-3958-3p,miR-499b-5p,miR-7977,miR-3064-5p,miR-452-3p,miR-20a-3p,miR-2319b,miR-203-3p,miR-376d,miR-7857-3p,miR-378g,miR-489,miR-378j,miR-361-3p,miR-140-5p,miR-376a-3p,miR-544-5p,novel_94,miR-877-3p,miR-33a-5p,miR-744-3p,miR-574-3p,miR-2330-5p,miR-4532,miR-193a,miR-30c-1-3p,miR-2957,miR-199a-3p,miR-342,miR-431-5p,miR-326,miR-1306-5p,miR-2285o,miR-214-3p,miR-103a-2-5p,miR-491-5p,miR-3085-3p,miR-3955-3p,miR-5010-3p,miR-181b-2-3p,miR-199b-3p,miR-151a-3p,miR-1298-3p,miR-376b-3p,miR-2284r,miR-6529a,miR-484,miR-10b,miR-541,miR-1388-5p,miR-101a-5p,novel_42,miR-145-3p,miR-1260b,miR-331-3p,miR-125b-5p,miR-100-3p,miR-6740-5p,miR-135a-5p,miR-370-5p,miR-494-5p,miR-5100,miR-2355-5p,miR-370-3p,miR-125a,miR-1271,miR-141-3p,miR-874-3p,miR-365a-3p,miR-137-3p,miR-18a,miR-488-3p,miR-424-3p,miR-7144-5p,miR-671-5p,miR-2474,miR-345-5p,novel_91,miR-3064-5p,miR-29b-2-5p,miR-4286,miR-181a-2-3p,miR-3065-3p,miR-200a,miR-490-3p,miR-28b,miR-30c-2-3p,miR-376b-3p,miR-

ERLIN2	ENSOARG0(	ER lipid	miR-101c,miR-1839-5p,miR-544a,miR-125b-2-3p,miR-541,miR-421,miR-216a-3p,miR-5010-3p,miR-29b-1-5p,miR-320b,miR-2284b,miR-494-3p,miR-2330-5p,miR-101b-3p,miR-1388-3p,novel_94,miR-3963,miR-4443,miR-144-5p,miR-452-3p,miR-139-5p,miR-2284g,miR-335,miR-628-3p,miR-3958-3p,miR-101-3p,miR-615,novel_82,miR-181d-5p,miR-541-3p,miR-2284u,miR-361-5p,miR-148a-5p,miR-133a-3p,miR-33b-3p,miR-4429,miR-184-3p,miR-140-3p,miR-328-3p,miR-532-3p,miR-505-3p,miR-18a-3p,miR-760-3p,miR-320d,miR-92a-1-5p,novel_17,miR-486-3p,miR-125b-2-3p,miR-377-5p,miR-29b-1-5p,miR-421-5p,miR-345-3p,miR-582-5p,miR-543-3p,miR-345-5p,miR-27b-5p,miR-22-3p,miR-139-5p,miR-377-3p,miR-544-3p,miR-582-5p,miR-296-3p,miR-2331-3p,miR-140-5p,miR-708-5p,miR-129-5p,miR-1468-5p,novel_74,miR-665,miR-3604,miR-320e,miR-181c-5p,miR-224-5p,miR-145a-3p,miR-7862,miR-29b,miR-22-3p,miR-197-3p,miR-542-3p,miR-1843b-5p,miR-181a-5p,miR-2113,miR-2355-3p,miR-194-3p,miR-412-5p,miR-377-5p,miR-378a-5p,miR-483-3p,miR-196b-5p,miR-377-3p,miR-224-5p,miR-345-5p,miR-503-3p,miR-2285r,miR-6395,miR-505-3p,miR-2319a,miR-450b-3p,miR-216a-3p,miR-18a-3p,miR-3970,miR-181b-3p,miR-320c,miR-677,miR-670-3p,miR-29a-3p,miR-7-1-3p,miR-6123,miR-147-5p,miR-421-5p,miR-22-5p,miR-29a,miR-33a-3p,miR-28c,miR-495-3p,novel_101,miR-29c-3p,miR-335-5p,miR-128-3p,miR-23b-5p,miR-29d-3p,miR-196a-5p,miR-615-3p,miR-582-3p,miR-483-3p,miR-24-2-5p,miR-7641,novel_127,miR-181b-5p,miR-1271-3p,miR-2284a,miR-2284y,miR-2478,miR-28b,miR-345-5p,miR-876-3p,miR-3154,miR-320a,miR-137-3p,miR-324-5p,miR-28-5p,miR-486b-3p,miR-29b-3p,miR-2433,miR-144-5p
TGDS	ENSOARG0(	TDP-gluc	miR-219a-2-3p,miR-21-3p,miR-214-3p,miR-494-3p,miR-219-3p,miR-214,miR-219b-3p
TRAV17	ENSOARG0(	T cell r	miR-2284k,miR-2332,miR-338-5p,novel_74,miR-374b,miR-539-5p,miR-2284v,miR-129-2-3p,miR-6128,miR-193a-5p,miR-1197-3p,miR-206-3p,miR-10b-3p,miR-876-3p,miR-154b-3p,miR-7144-5p,novel_99,miR-1248,miR-140-5p,miR-487a-3p,miR-548o-3p,miR-2284n,miR-2285af,miR-200c,miR-500a-5p,miR-185-3p,miR-2284m,miR-200c-3p,novel_127,miR-299-3p,miR-487a,miR-1,miR-195a-3p,miR-130a-5p,miR-2424,miR-140-3p,novel_73,miR-200b-3p,miR-299a-3p,miR-18a-3p,miR-429-3p,miR-1224-5p,miR-30d-3p,miR-487a-3p,miR-222-5p,miR-200b,miR-483-3p,miR-129-1-3p,miR-133a-5p,miR-206,miR-154-3p,miR-212-3p,miR-2284j,miR-452-5p,miR-150-5p,miR-18a-3p,miR-450b-3p,miR-374b-5p,miR-299b-3p,miR-1185-2-3p,miR-323b-3p,miR-483-3p,let-7f-2-3p,let-7f-2-3p,miR-654-3p,miR-539-3p,miR-873a-5p,miR-1a-3p,miR-323b,miR-2284ab,miR-2284x,miR-3955-5p,miR-5010-3p,miR-222-5p,miR-2284d,miR-1185-3p

RAB12	ENSOARG0(	RAB12, m	miR-20b,miR-221-3p,miR-503-5p,miR-26b-3p,miR-339-5p,miR-330-3p,novel_78,miR-29b,miR-125b-2-3p,miR-412-3p,miR-323b,miR-2113,miR-1843b-5p,miR-143-5p,miR-301b-5p,let-7a-2-3p,miR-8485,miR-30c-1-3p,miR-6240,miR-199b-5p,miR-106b-5p,miR-106a-5p,miR-17-5p,miR-330-3p,miR-505,miR-503-5p,miR-4443,miR-29a-3p,miR-3082-5p,miR-25,miR-329-5p,miR-664b,miR-378b,miR-199b-5p,miR-221,miR-148a-3p,miR-7705,miR-199b-5p,miR-1285-5p,novel_27,miR-29c-3p,miR-495-3p,miR-6527,miR-147-5p,miR-374a-3p,miR-22-5p,miR-29a,miR-301a-5p,miR-33a-3p,miR-382-5p,miR-20a-5p,novel_83,miR-148b-3p,novel_79,miR-412,miR-6517,miR-222,miR-361-5p,miR-378d,let-7a-2-3p,miR-188-3p,miR-29d-3p,miR-3591-5p,miR-1271-3p,miR-200a,miR-199a-5p,miR-2432,miR-148b-5p,miR-20a,miR-503-5p,miR-329a-5p,miR-21-3p,miR-200a-3p,miR-152-3p,miR-30b-3p,miR-339a,miR-30c-2-3p,miR-664-3p,miR-2397-5p,novel_32,miR-199a-5p,miR-3965,miR-3956,miR-141-3p,miR-2355-5p,miR-30c-1-3p,miR-20b-5p,miR-339b,let-7g-3p,miR-329b,miR-17-5p,miR-93-5p,miR-338-3p,miR-374a-3p,miR-93,miR-539-5p,miR-374b-3p,miR-29b-3p
ITIH4	ENSOARG0(	inter-al	miR-370-3p,miR-324-5p,miR-199a-5p,miR-34c-5p,miR-3154,miR-488-3p,miR-671-5p,miR-10b-3p,miR-1306-5p,miR-378c,miR-10b-3p,miR-409-3p,miR-1306-3p,miR-370-5p,miR-486b-3p,miR-28-5p,miR-7641,miR-222,miR-378h,miR-483-3p,miR-6516-5p,miR-345-3p,miR-134,miR-10a-5p,miR-582-3p,miR-495-5p,miR-1,miR-30a-3p,miR-2403,miR-181a-2-3p,miR-3065-3p,miR-199a-5p,miR-28b,miR-24-2-5p,miR-1260a,miR-2898,miR-450a-2-3p,miR-10a-5p,miR-146b-3p,miR-34b,miR-2285ab,miR-2387,miR-9851-3p,miR-6395,miR-195a-5p,miR-1291,miR-450b-3p,miR-19a-3p,miR-1193,miR-342-3p,miR-326-3p,miR-449a,miR-24-3p,miR-16a,miR-34c,miR-502-5p,miR-15a,miR-6529b,miR-22-5p,miR-502b,novel_1,miR-26b-3p,miR-377-5p,miR-133b-5p,miR-544b,miR-2355-3p,miR-432-5p,miR-145a-5p,miR-1285,miR-6239,miR-7862,miR-30b-3p,miR-219a-1-3p,miR-1260b,miR-101-5p,miR-363-5p,miR-330-3p,miR-452-5p,miR-125a-5p,miR-3065-3p,miR-370-5p,miR-378a-3p,miR-221-5p,miR-204-3p,novel_25,miR-27a-5p,miR-6516,miR-193b-3p,miR-21a-3p,miR-2312,miR-2330-3p,miR-378c,miR-145a-3p,miR-6134,miR-338-3p,miR-10b-5p,miR-206-3p,miR-222-3p,miR-424-5p,miR-708-5p,miR-208b-5p,miR-450b-3p,miR-3600,miR-3960,miR-532-3p,miR-148b-3p,miR-15a-5p,miR-188-3p,miR-1197-5p,miR-5703,novel_133,miR-378d,miR-19b-3p,miR-29b-1-5p,miR-130a-5p,miR-125b,miR-185-3p,miR-34b-5p,miR-221-5p,miR-30b-3p,miR-760-3p,miR-7977,miR-3064-5p,miR-10b,miR-7857-3p,miR-378e,miR-489,miR-3082-5p,miR-361-3p,miR-199b-5p,novel_94,miR-2285f,miR-452-5p,miR-541-3p,miR-2285y,miR-107-5p,miR-4726-5p,miR-335,novel_4,miR-2300a-5p,miR-221-3p,miR-9788-3p,miR-216a-3p,miR-412-3p,miR-30b-3p,miR-378b,miR-484,miR-296-5p,miR-10b,miR-143-5p,miR-378c,miR-101a-5p,miR-2426,miR-544a,miR-652-5p,miR-494-3p,miR-2330-
ADH4	ENSOARG0(	alcohol	miR-582-5p,miR-190a-5p,miR-466i-5p,miR-101a-5p,miR-3604,miR-190a,miR-26a-5p,miR-582-5p,miR-450b-5p,miR-26b-5p,miR-190b-5p,miR-101-5p

CSPP1	ENSOARG0(	centroso	miR-493-5p,miR-668-5p,miR-96-5p,miR-361-3p,miR-27a-3p,miR-1185-2-3p,miR-203-3p,miR-499a-3p,miR-211-5p,miR-541-5p,miR-20a-3p,miR-3064-5p,miR-2284q,miR-335,miR-218-5p,miR-8095,miR-362-5p,miR-101c,miR-1185-3p,miR-500-5p,miR-27a-3p,miR-181b-2-3p,miR-2300a-5p,miR-3085-3p,miR-16b,miR-199a-3p,miR-193a,miR-3187-3p,miR-4532,miR-8485,miR-23a-3p,miR-424-5p,miR-124a,miR-1388-3p,miR-1827,miR-129b-3p,let-7f-1-3p,miR-21a-3p,miR-877-5p,miR-15b-5p,miR-499b-3p,miR-370-5p,miR-424-5p,miR-874-5p,novel_74,miR-665,let-7b-3p,miR-98-3p,miR-1271,let-7a-3p,miR-218-1-3p,miR-19b-3p,miR-140-3p,miR-217-5p,miR-98-3p,miR-124-3p,miR-328-3p,miR-199a-3p,miR-2284h-5p,miR-222-5p,miR-15a-5p,miR-340-5p,miR-23c,miR-23b-3p,miR-18a-3p,miR-21-3p,miR-466i-5p,miR-181b-1-3p,miR-2285w,miR-6395,miR-195a-5p,miR-135b-5p,miR-19a-3p,miR-1291,miR-18a-3p,miR-216a-3p,miR-181b-3p,miR-677,miR-2404,miR-421-5p,miR-382-5p,miR-16a,miR-133a-5p,miR-576-3p,miR-15a,miR-502-5p,novel_27,miR-211,miR-1193,miR-335-5p,miR-27b-3p,miR-497-5p,miR-219a-1-3p,miR-16b,miR-197-3p,miR-2113,miR-1434-3p,miR-412-5p,miR-2285g,miR-3955-5p,let-7a-2-3p,miR-503-3p,miR-2285r,miR-493-5p,novel_48,let-7g-3p,miR-362-5p,miR-23b,miR-200a-5p,miR-615-5p,miR-3068-3p,miR-204-5p,miR-135a-5p,miR-370-5p,miR-34c-3p,miR-500b-5p,miR-16-5p,let-7a-2-3p,miR-128-3p,miR-214-5p,miR-758-5p,miR-1271-5p,miR-322-5p,miR-6517,novel_127,miR-181c-3p,miR-217,miR-125a-3p,miR-1957a,miR-3064-5p,miR-2385-3p

KLF17	ENSOARG0(	Kruppel-	[illegible] 3p,miR-618,miR-195a-3p,miR-2285w,miR-2424,miR-181b-1-3p,miR-98-3p,miR-217-5p,miR-193b-5p,miR-2366,miR-361-5p,miR-142-3p,miR-380-5p,miR-212-5p,miR-2284h-5p,miR-874-5p,miR-129-5p,miR-130b-5p,miR-3074-5p,miR-98-3p,miR-224-5p,miR-129b-3p,miR-433-3p,miR-543-3p,miR-186-5p,miR-1827,miR-345-5p,miR-30c-1-3p,miR-32-5p,miR-582-5p,miR-323b-3p,miR-505,miR-182-5p,miR-491-5p,miR-124a,miR-323b,miR-758-3p,miR-2284r,miR-1839-5p,miR-181b-2-3p,miR-1343-3p,miR-455-3p,miR-2459,miR-1298-3p,miR-539-5p,novel_82,miR-3964,miR-628-3p,miR-499a-5p,miR-190b-5p,miR-363-3p,miR-3956-3p,miR-885-5p,miR-877-3p,miR-361-3p,miR-378j,miR-493-5p,miR-96-5p,miR-2285u,miR-2319b,miR-144-5p,miR-203-3p,miR-378g,miR-3071-3p,miR-205-5p,novel_96,miR-152-3p,miR-28-3p,miR-3064-5p,miR-29a-5p,miR-92b-3p,miR-1343-5p,miR-330-5p,miR-23b-5p,miR-3591-5p,miR-1290,miR-412,miR-1224-5p,miR-2285ad,miR-429-3p,miR-6517,novel_9,miR-144-5p,miR-28a-3p,miR-1260b,miR-301,miR-345-5p,miR-329b,miR-874-3p,miR-223-5p,miR-25-3p,miR-365b-5p,miR-32,miR-2411-3p,miR-483-3p,miR-628-5p,miR-380-5p,miR-493-5p,miR-503-3p,miR-2285r,miR-26a-2-3p,miR-3955-5p,miR-31-5p,miR-103a-3p,miR-149-5p,miR-194-3p,miR-3535,miR-134-3p,miR-485-3p,miR-576-3p,miR-208a-3p,miR-142b,miR-758-5p,miR-3970,miR-150-5p,miR-216a-3p,miR-376b-5p,miR-148a-3p,miR-2319a,miR-3120-3p,novel_68,miR-3074-5p,miR-677,miR-181b-3p,novel_69,miR-221-5p,miR-30b-3p,miR-760-3p,miR-2411,miR-2432,miR-125b-2-3p,miR-124-3p,miR-1197-5p,miR-5703,miR-2300b-3p,let-7a-3p,miR-3600,miR-148b-3p,miR-324-3p,miR-320e,miR-208b-5p,miR-1271,miR-539-5p,miR-21a-3p,let-7f-1-3p,novel_99,miR-582-5p,miR-2312,let-7g-3p,miR-370-5p,miR-

ZNF410	ENSOARG0(	zinc fin	miR-6240,miR-654-3p,miR-330-3p,miR-2411-3p,miR-31-5p,miR-133b-5p,let-7g-5p,miR-3431,miR-194-3p,miR-181a-5p,miR-145a-5p,miR-138,novel_101,miR-326-3p,miR-142b,let-7i,miR-147-5p,miR-6123,miR-10a-5p,miR-26b-5p,miR-3120-3p,miR-2404,miR-411b-3p,miR-320c,miR-144-3p,miR-3591-3p,let-7e-5p,miR-181a-2-3p,miR-2478,miR-200a,let-7g,miR-3065-3p,miR-30c-2-3p,let-7c-5p,let-7f-5p,miR-127-5p,miR-181b-5p,miR-543-5p,miR-505-5p,miR-758-5p,miR-345-3p,miR-330-5p,let-7d,miR-10a-5p,miR-496,miR-145-3p,miR-144,miR-138-5p,miR-487a-3p,miR-503-3p,miR-2285aa,miR-615-5p,miR-300-3p,miR-320a,miR-10b-3p,miR-1814c,let-7a-5p,miR-101b-3p,miR-30c-1-3p,miR-2310,miR-320b,miR-326,miR-323b-3p,miR-25-5p,let-7e,novel_4,miR-409b,miR-330-3p,miR-2300a-5p,miR-145b,miR-142-5p,miR-455-3p,miR-421,miR-143-5p,miR-10b,miR-323b,miR-450b-5p,miR-16-2-3p,miR-212-3p,miR-452-5p,miR-181d-5p,miR-10b,miR-2319b,miR-2285u,miR-489,let-7d-5p,miR-487a,miR-195a-3p,miR-2432,miR-26a-5p,miR-219b-5p,miR-345-3p,miR-185-3p,miR-320d,miR-98-5p,miR-30b-3p,let-7k,miR-548e-3p,miR-3600,miR-7859,miR-383-5p,miR-1961,miR-2904,miR-5703,miR-1193,miR-4429,let-7b,miR-4510,miR-6525,miR-142a-5p,miR-10b-5p,let-7b-5p,miR-181c-5p,miR-6134,miR-2285j,miR-15b-3p,let-7f,miR-665,miR-3604,miR-6516-3p,miR-4492,miR-129-5p,miR-30c-1-3p,miR-496-3p,miR-548o-3p,miR-154a-3p,miR-3065-3p,miR-21-3p,miR-10a,miR-6130,let-7i-5p,miR-433-3p,miR-154b-3p
GLUD1	ENSOARG0(	Ovis ari	miR-29a-5p,miR-2310,miR-6240,miR-34b-5p,miR-103a-2-5p,miR-124-5p,miR-487b-5p,miR-330-3p,miR-134-5p,miR-330-3p,miR-34a-5p,miR-21-5p,novel_73,miR-134,miR-3184-5p,miR-449a,miR-423-5p,miR-1296-5p,miR-4510,miR-134-5p,miR-592,novel_1,miR-487b-5p,miR-34c,miR-107-5p,miR-2440,miR-34c-5p,miR-6130,miR-34b,miR-365a-3p,miR-598-3p,miR-1827,miR-1814c,miR-598-3p,miR-21c

EIF3A	ENSOARG0(	eukaryot	miR-3120-3p,miR-26b-5p,miR-216b-3p,miR-144-3p,miR-411b-3p,miR-378f,miR-146b-5p,miR-335-5p,miR-2284w,miR-28c,miR-142b,miR-339-5p,miR-487b-3p,miR-103a-3p,miR-31-5p,miR-134-3p,miR-22-3p,miR-7b-5p,miR-19b-2-5p,miR-497-5p,miR-16-1-3p,miR-16b,miR-628-5p,let-7f-2-3p,let-7f-2-3p,miR-493-5p,miR-380-5p,let-7j,miR-2285r,novel_51,miR-194a,miR-2411-3p,miR-105-5p,miR-377-3p,miR-146a,miR-7-5p,miR-125a,miR-2285aa,miR-874-3p,miR-503-3p,miR-223-5p,miR-300-3p,miR-93-5p,miR-30e-5p,miR-1248,miR-20b-5p,miR-329a,miR-345-5p,miR-767,miR-16-5p,miR-1260b,miR-125b-5p,miR-6740-5p,novel_63,miR-2433,miR-134-5p,miR-592,novel_9,miR-18b-5p,novel_79,miR-6517,novel_121,miR-450a-1-3p,miR-134-5p,miR-322-5p,miR-330-5p,miR-1343-5p,miR-450b-5p,miR-148b-5p,miR-8117,miR-664-3p,miR-125a-3p,miR-205-5p,novel_127,miR-126b-5p,miR-374b-5p,miR-7689-3p,miR-1185-2-3p,miR-4443,miR-203-3p,miR-499a-3p,miR-3596,miR-378g,miR-544-5p,miR-376a-3p,miR-493-5p,miR-668-5p,miR-299b-5p,miR-7705,miR-877-3p,miR-615,miR-3968,miR-628-3p,miR-145b,novel_78,miR-199b-3p,miR-1185-3p,miR-421,miR-126a-5p,miR-148b-5p,miR-1298-3p,miR-376b-3p,miR-329-3p,miR-1839-5p,miR-1388-5p,miR-362-3p,miR-744-3p,miR-101b-3p,miR-127-3p,miR-2310,miR-8485,miR-378d,miR-106b-5p,miR-16b,miR-182-5p,miR-505,miR-199a-3p,miR-146b,miR-552-3p,miR-30c-1-3p,miR-15b-5p,miR-499b-3p,miR-10a,miR-377-3p,miR-30d-5p,miR-299,miR-186-5p,miR-543-3p,miR-345-5p,miR-339b,miR-433-5p,miR-122-5p,novel_44,miR-665,miR-106a,miR-18a-3p,miR-7859,miR-378a-3p,miR-217-5p,miR-2366,miR-378i,miR-148a-5p,miR-16-1-3p,miR-218-1-3p,novel_73,miR-33b-3p,miR-2424,miR-422a,miR-345-3p,miR-324-3p,miR-500a-5p,miR-26a-5p,miR-618,miR-210-5p,miR-2484,miR-3653-3p,miR-10a-5p,miR-7-1-3p,miR-320b,miR-376b-

HEBP2	ENSOARG0(	heme bin	miR-548w,miR-153,miR-376b-3p,miR-147a,miR-421,miR-541,miR-323b,miR-758-3p,miR-101c,miR-1839-5p,miR-124a,miR-1388-3p,miR-127-3p,miR-744-3p,miR-378d,miR-8485,miR-199a-3p,miR-323b-3p,miR-182-5p,miR-491-5p,miR-487b-5p,miR-153-3p,miR-139-5p,miR-4792,miR-211-5p,miR-3596,miR-378g,miR-203-3p,miR-140-5p,miR-381-3p,miR-378j,miR-668-5p,miR-376a-3p,miR-9-3p,miR-2447,miR-3956-3p,miR-877-3p,novel_111,miR-18a-3p,miR-380-5p,miR-7859,miR-1247-5p,miR-378i,miR-378a-3p,miR-493-3p,miR-664-5p,miR-16-1-3p,miR-218-1-3p,miR-34a-5p,miR-142a-3p,miR-486-3p,miR-143-3p,miR-210-5p,novel_17,miR-324-3p,miR-345-3p,miR-422a,miR-2484,miR-10a,miR-433-3p,miR-339b,miR-345-5p,miR-543-3p,miR-1827,miR-186-5p,miR-3958-5p,miR-6525,miR-15b-3p,miR-3604,miR-665,miR-874-5p,miR-4492,miR-130b-5p,miR-3955-5p,miR-31-5p,miR-339-5p,miR-3431,miR-134-3p,miR-149-5p,miR-194-3p,miR-411-5p,miR-19b-2-5p,novel_23,miR-16-1-3p,miR-2285r,miR-296-3p,novel_51,miR-2411-3p,miR-194a,miR-3120-3p,miR-150-5p,miR-1306,miR-216a-3p,miR-3065-5p,miR-410-5p,miR-378f,miR-758-5p,miR-4324,miR-142b,miR-625-3p,miR-429-3p,novel_79,miR-1224-5p,miR-2284z,miR-486-5p,novel_121,miR-1290,novel_39,miR-30f,novel_127,novel_96,miR-874-3p,miR-2285aa,miR-125a,miR-3959-5p,miR-345-5p,miR-28a-3p,miR-2284k,miR-125b-5p,miR-142-3p,miR-216a-3p,miR-484,miR-378b,miR-296-5p,miR-10b,miR-378c,miR-544a,miR-2284aa,miR-3120-5p,let-7c-3p,miR-33a-5p,miR-758-3p,miR-1306-5p,miR-3955-3p,miR-10b,miR-20a-3p,miR-499b-5p,miR-378e,miR-7857-3p,miR-454-5p,miR-376d,novel_94,novel_60,miR-541-3p,miR-194b-5p,miR-548e-3p,miR-222-5p,miR-200b,miR-616-3p,novel_83,miR-199a-3p,miR-532-3p,miR-2904,miR-124-3p,miR-188-3p,miR-

FAM161B	ENSOARG00	family w	miR-125b-5p,miR-3969,miR-1-5p,miR-6128,miR-365a-5p,miR-1197-3p,miR-200a-5p,miR-137-3p,miR-503-3p,miR-190a-3p,miR-329a,miR-2285aa,miR-370-3p,miR-141-3p,miR-125a,miR-3068-3p,miR-376c-3p,miR-34c-5p,miR-30f,miR-3071-3p,miR-376b-3p,miR-219-3p,miR-200a-3p,miR-1a-2-5p,miR-2285b,miR-192-3p,miR-2284a,miR-196a-5p,miR-3578,miR-128-3p,miR-10a-5p,miR-7641,miR-582-3p,novel_121,miR-486-5p,miR-34c,miR-147-5p,novel_1,novel_101,miR-1a-1-5p,miR-449a,miR-3959-3p,miR-3591-3p,miR-376b-3p,miR-216a-3p,miR-378b,miR-2285ab,miR-410-5p,miR-412-3p,miR-10a-5p,miR-3120-3p,miR-7-1-3p,miR-34b,miR-330-3p,miR-365b-5p,miR-7-5p,miR-125a-5p,miR-452-5p,miR-660,miR-378a-5p,miR-196b-5p,miR-2483-5p,miR-382,miR-628-5p,miR-493-5p,miR-6240,miR-654-3p,miR-22841,miR-197-3p,novel_23,miR-432,miR-873a-5p,miR-19b-2-5p,miR-708-3p,miR-223-3p,miR-7b-5p,miR-1434-3p,miR-188-5p,miR-30b-3p,miR-16-1-3p,miR-133b-5p,miR-6238,miR-432-5p,miR-19b-1-5p,miR-219b-3p,miR-214,miR-539-5p,miR-3184-3p,miR-6134,miR-10b-5p,miR-129b-3p,miR-299,miR-543-3p,miR-186-5p,miR-2312,let-7g-3p,miR-21-3p,miR-140-5p,miR-221-5p,miR-660-5p,miR-877-5p,miR-376e-3p,miR-582-5p,miR-10a,miR-2284s,miR-139-5p,miR-125b,miR-221-5p,miR-34b-5p,miR-21-3p,miR-216c-5p,miR-2424,miR-143-3p,miR-664b-3p,miR-376c-3p,miR-130a-5p,miR-1246,miR-376a-5p,miR-362-3p,miR-2300b-3p,miR-148a-5p,miR-34a-5p,miR-212-5p,miR-548e-3p,novel_83,miR-7859,miR-2285n,miR-2440,miR-101-3p,miR-2285f,miR-219a-2-3p,miR-7705,miR-381-3p,miR-409-5p,miR-376a-3p,miR-493-5p,miR-1983,miR-299b-5p,miR-147-3p,miR-136-5p,miR-2285u,miR-139-5p,miR-10b,miR-452-3p,miR-203-3p,miR-376d,miR-362-3p,novel_103,miR-214-3p,miR-2284b,miR-652-5p,miR-193a,miR-8485,miR-3187-3p,miR-30b-3p,miR-329-3p,miR-
CALY	ENSOARG00	calcyon	miR-4532,miR-193b-3p,miR-193b-3p,miR-126b-3p,miR-193a-3p,miR-25-5p,novel_19,miR-3141,miR-3141,miR-5126
ERI3	ENSOARG00	ERI1 exo	miR-4726-5p,miR-128-1-5p,miR-129b-3p,miR-433-3p,miR-182-5p,miR-6516,miR-6516-5p,miR-182-5p,miR-1343-3p
SERPINA7	ENSOARG00	serpin p	miR-217,miR-3613,miR-218-5p,miR-205-5p,miR-30b-3p,miR-199b-5p,miR-130b-5p,miR-130b-5p,novel_27,miR-199a-5p,miR-199b-5p,miR-148b-5p,miR-377-5p,miR-129b-3p,miR-217-5p,miR-432,miR-199b-5p,miR-3578,miR-3613-5p,miR-1224-5p,miR-3600,miR-377-5p,miR-31-5p,miR-2331-3p,miR-20a-3p,miR-545-5p,miR-199a-5p,miR-432-5p,miR-539-5p
TPO	ENSOARG00	thyroid	novel_19,miR-4726-5p,novel_9,miR-2432

HDAC3	ENSOARG0(	histone	miR-1-5p,miR-144,miR-100-3p,miR-1197-3p,miR-1260b,miR-500b-5p,miR-378c,miR-3607-3p,miR-345-5p,miR-10b-3p,miR-3074-1-3p,miR-7144-5p,miR-503-3p,miR-362-5p,miR-3154,miR-320a,miR-223-5p,miR-503-3p,miR-574-5p,miR-615-5p,miR-1a-2-5p,miR-17-3p,miR-543-5p,miR-6119-5p,miR-205-5p,miR-2285af,miR-1260a,miR-217,miR-30c-2-3p,miR-450b-5p,miR-1271-3p,miR-2285b,miR-1957a,miR-345-3p,miR-196a-5p,miR-330-5p,miR-483-3p,miR-2411-5p,miR-758-5p,miR-378h,miR-505-5p,novel_121,miR-412,miR-6123,miR-625-3p,miR-576-3p,miR-371b-3p,miR-326-3p,miR-1a-1-5p,miR-342-3p,miR-135a-2-3p,miR-17-3p,miR-193b-3p,miR-412-3p,miR-378f,miR-2285ab,miR-150-5p,miR-2404,miR-144-3p,miR-320c,miR-378b,miR-3120-3p,miR-124-5p,miR-196b-5p,miR-483-3p,miR-193a-3p,miR-296-3p,miR-503-5p,novel_51,miR-455-5p,miR-2411-3p,miR-330-3p,miR-2284l,miR-432-3p,miR-432-3p,miR-345-5p,miR-296-3p,miR-6240,miR-1260b,miR-455-5p,miR-26a-2-3p,miR-542-3p,novel_23,miR-503-5p,miR-2285g,miR-412-5p,miR-149-5p,miR-665,miR-17-3p,novel_74,miR-487a-5p,miR-208b-5p,miR-2285j,miR-320e,miR-665,miR-346,miR-142a-5p,miR-338-3p,miR-145a-3p,miR-378c,miR-345-5p,let-7g-3p,miR-2312,novel_99,miR-2285c,miR-129b-3p,miR-216b-5p,miR-193b-3p,novel_32,novel_25,miR-204-3p,miR-548o-3p,miR-30c-1-3p,miR-504-5p,miR-221-5p,miR-378a-3p,miR-296-3p,miR-216b-3p,miR-760-3p,miR-320d,miR-221-5p,miR-618,miR-210-5p,miR-422a,miR-345-3p,miR-500a-5p,miR-324-3p,miR-130a-5p,miR-2411,miR-503-5p,miR-2285w,miR-4429,miR-378d,miR-378i,miR-1197-5p,miR-217-5p,miR-378a-3p,miR-5703,miR-7859,miR-532-3p,miR-2284h-5p,miR-3600,miR-628-3p,miR-4726-5p,miR-2284q,miR-3964,miR-382-3p,miR-362-5p,miR-361-3p,miR-8095,miR-3956-3p,miR-2447,novel_60,miR-3059-5p,miR-2285f,miR-504,miR-1983,miR-

PRKCG	ENSOARGO(	protein	miR-6395,miR-379-5p,miR-195a-5p,let-7e-5p,miR-1291,miR-410-5p,miR-1306,miR-320c,miR-669,miR-323a-5p,miR-216b-3p,let-7i,miR-425-5p,miR-16a,miR-28c,miR-6529b,miR-4324,miR-15a,miR-2428,miR-326-3p,miR-24-3p,novel_101,miR-1193,miR-744-5p,miR-497-5p,miR-4508,miR-1260b,miR-16b,miR-1285,miR-873a-5p,miR-503-5p,miR-134-3p,miR-185-5p,miR-3431,let-7g-5p,miR-3955-5p,miR-1343-5p,miR-125a-5p,miR-2285l,novel_51,miR-197-5p,miR-503-5p,let-7a-2-3p,miR-628-5p,miR-485-5p,miR-23a-5p,let-7g-3p,miR-320a,miR-324-5p,miR-615-5p,miR-125a,miR-486b-3p,miR-125b-5p,miR-100-3p,miR-370-5p,miR-2433,miR-1260b,miR-133a-3p,miR-16-5p,novel_42,let-7a-2-3p,let-7d,miR-330-5p,miR-1343-5p,miR-218-2-3p,miR-214-5p,miR-322-5p,miR-9-5p,miR-505-5p,miR-6517,miR-423-3p,novel_96,miR-127-5p,let-7f-5p,let-7c-5p,miR-205-5p,miR-1260a,miR-1271-3p,let-7g,miR-28b,miR-2403,miR-877-3p,miR-504,miR-147-3p,miR-361-3p,let-7d-5p,miR-3082-5p,miR-378g,miR-499a-5p,miR-3968,miR-2285n,novel_82,miR-423-5p,miR-361-3p,miR-877-3p,miR-9-5p,miR-2447,miR-21b,miR-1839-5p,miR-1388-5p,miR-484,miR-6529a,miR-421,miR-9788-3p,miR-425-3p,miR-1343-3p,miR-151a-3p,miR-214-3p,let-7e,miR-25-5p,miR-491-5p,miR-16b,miR-1306-5p,miR-5126,miR-326,miR-320b,miR-4532,miR-652-5p,miR-127-3p,miR-424-5p,miR-574-3p,let-7a-5p,miR-1827,miR-1895,miR-133b-3p,miR-21a-3p,miR-877-5p,miR-15b-5p,miR-151b,let-7i-5p,miR-204-3p,miR-6130,miR-323-5p,miR-296-3p,miR-370-5p,miR-504-5p,miR-2331-3p,miR-424-5p,miR-665,miR-151-3p,let-7f,miR-6134,let-7b-5p,miR-3184-5p,miR-214,miR-665-5p,miR-3958-5p,miR-4510,let-7b,miR-4429,miR-1193,miR-5703,miR-2904,miR-2300b-3p,miR-1961,miR-328-3p,miR-532-3p,miR-151-5p,miR-15a-5p,miR-210-3p,let-7k,miR-21-3p,miR-30b-3p,miR-466i-5p,miR-760-
XAF1	ENSOARGO(	XIAP ass	miR-676-3p,miR-1388-3p,miR-130b-5p,miR-127-3p,miR-2285o,miR-29b-1-5p,novel_51,miR-320b,miR-582,miR-326,miR-2355-3p,miR-425-3p,miR-134-3p,miR-339-5p,miR-340-3p,miR-31-5p,miR-1343-3p,miR-544a,miR-22-3p,novel_27,miR-326-3p,miR-9-3p,miR-877-3p,miR-2285f,miR-17-3p,miR-2285x,miR-1193,miR-2447,miR-320c,miR-669,miR-378g,miR-320b,miR-7977,miR-9-3p,miR-6395,miR-1291,miR-2285k,miR-378j,miR-18a-3p,miR-140-5p,miR-2285ab,miR-2424,miR-29b-1-5p,miR-2285i,miR-130a-5p,miR-2403,miR-216c-5p,novel_127,miR-320d,miR-2898,miR-17-3p,miR-324-3p,miR-92a-1-5p,miR-339a,miR-185-3p,miR-671-3p,miR-328-3p,miR-383-5p,novel_79,miR-18a-3p,miR-582-3p,miR-4429,miR-330-5p,miR-23b-5p,miR-3591-5p,miR-130b-5p,novel_74,miR-664a,miR-1843b-3p,miR-17-3p,miR-331-3p,miR-320e,miR-2332,miR-370-5p,miR-204-3p,miR-22-3p,miR-544-3p,miR-370-5p,miR-542-5p,miR-296-3p,miR-2331-3p,miR-874-3p,miR-7144-5p,miR-671-5p,miR-2312,miR-339b,miR-320a

CUEDC2	ENSOARG0(	CUE doma	miR-7705,miR-423-5p,miR-541-3p,miR-3059-5p,miR-194b-5p,miR-335,miR-218-5p,miR-371a-5p,miR-378e,miR-452-3p,miR-664b,miR-3064-5p,miR-4792,miR-7977,miR-499b-5p,miR-378d,miR-30c-1-3p,miR-4532,miR-382-3p,miR-424-5p,miR-106a-5p,miR-1388-3p,miR-25-5p,miR-3085-3p,miR-214-3p,miR-381-5p,miR-146b,miR-326,novel_103,miR-320b,miR-342,miR-106b-5p,miR-16b,miR-29b-1-5p,novel_19,miR-1298-3p,miR-147a,miR-496-5p,miR-2459,miR-2285e,miR-151a-3p,miR-1343-3p,miR-222-5p,miR-2426,miR-378c,miR-21b,miR-2284aa,miR-1839-5p,miR-541,miR-6529a,miR-484,miR-378b,miR-30b-3p,novel_44,miR-6525,miR-6134,miR-3184-5p,miR-122-5p,miR-145a-3p,miR-378c,miR-214,miR-665-5p,miR-3074-2-3p,miR-450b-3p,miR-208b-5p,miR-500,miR-874-5p,miR-708-5p,miR-424-5p,miR-4492,miR-320e,miR-151-3p,miR-193b-3p,miR-15b-5p,miR-877-5p,novel_25,novel_32,miR-378a-3p,miR-30c-1-3p,novel_107,miR-133b-3p,miR-1827,miR-625-5p,miR-1895,miR-2285c,miR-450a-1-3p,miR-500a-3p,miR-377-5p,miR-29b-1-5p,miR-2432,miR-146a-5p,miR-345-3p,miR-195a-3p,miR-20a,miR-320d,miR-106a,miR-21-3p,miR-210-5p,miR-125b,miR-422a,miR-185-3p,miR-122-3p,miR-345-3p,miR-324-3p,miR-219b-5p,miR-500a-5p,miR-15a-5p,miR-2284h-5p,miR-1247-5p,miR-106a,miR-18a-3p,miR-212-5p,novel_73,miR-4429,miR-16-1-3p,miR-2300b-3p,miR-378i,miR-1197-5p,miR-378a-3p,miR-2904,miR-371b-3p,miR-24-3p,miR-1307-3p,miR-2428,miR-326-3p,miR-335-5p,miR-342-3p,novel_101,miR-193b-3p,miR-128-1-5p,miR-6123,miR-502b,miR-22-5p,miR-656-5p,miR-6402,miR-133a-5p,miR-28c,miR-6529b,miR-20a-5p,miR-502-5p,miR-15a,miR-4324,miR-16a,miR-374c-3p,miR-146b-3p,miR-144-3p,miR-320c,miR-6535,miR-363,miR-655-5p,miR-107,miR-1291,miR-378f,miR-195a-5p,miR-146b-5p,miR-
CCDC176	ENSOARG0(	coiled-c	miR-92a-1-5p,miR-455-5p,miR-210-5p,miR-30b-3p,miR-2284b,miR-382-3p,miR-380-5p,miR-192-3p,miR-2284a,miR-2284y,miR-380-5p,miR-376a-5p,miR-148b-5p,miR-412-3p,miR-197-3p,miR-2300b-3p,miR-873a-5p,miR-2284r,miR-30c-5p,miR-2284u,miR-455-5p,miR-33b-3p,miR-544a,miR-2284ab,miR-380-5p,miR-412,miR-3600,miR-544b,novel_121,miR-9788-3p,miR-2284d,miR-505-5p,miR-2284k,miR-218-5p,miR-147-5p,miR-758-5p,miR-2284j,miR-2284v,miR-3059-5p,novel_120,miR-154a,novel_27,miR-8095,miR-2448-3p,miR-140-5p,miR-30e-5p,miR-541-5p,miR-655-5p,miR-2284g,miR-2284n,miR-670-3p,miR-544-3p,miR-582-5p
MECOM	ENSOARG0(	MDS1 and	miR-378b,miR-133a-3p,miR-2483-5p,miR-499b-5p,miR-2355-5p,miR-133b-3p,miR-1468,miR-133a-3p,miR-1468-5p,miR-382-3p,miR-1434-5p
HM13	ENSOARG0(	histocom	miR-199a-5p,miR-328-3p,miR-411b-5p,miR-3064-5p,miR-6517,miR-370-3p,miR-2285u,miR-1271,miR-147-3p,miR-6395,miR-671-5p,miR-199b-5p,miR-330-5p,miR-326-3p,miR-199a-5p,miR-4532,miR-199b-5p,miR-3064-5p,miR-3085-3p,miR-199b-5p,miR-412-5p,miR-2285j,miR-326,miR-412-5p,miR-324-3p

ATF6B	ENSOARG0(	activati	[illegible] 200c-3p,miR-2898,miR-200a-3p,novel_127,miR-1,miR-3064-5p,miR-28b,miR-1271-3p,miR-3065-3p,novel_39,miR-345-3p,miR-23b-5p,miR-1343-5p,miR-300,miR-10a-5p,miR-128-3p,miR-200b-3p,miR-429-3p,novel_79,miR-2411-5p,miR-505-5p,miR-2433,miR-331-3p,miR-767-5p,miR-486b-3p,miR-204-5p,miR-28-5p,miR-1260b,miR-1197-3p,miR-500b-5p,miR-381-3p,miR-488-3p,miR-320a,miR-362-5p,miR-3154,miR-345-5p,miR-329a,miR-767,miR-671-5p,miR-485-5p,miR-141-3p,miR-598-3p,miR-370-3p,miR-154b-5p,miR-223-5p,miR-2411-3p,miR-194a,miR-197-5p,miR-124-5p,miR-106b-3p,miR-1343-5p,miR-382,miR-345-5p,miR-542-3p,miR-1843b-5p,miR-19b-2-5p,miR-873a-5p,miR-194-5p,miR-1260b,miR-30b-3p,miR-188-5p,miR-7862,miR-339-5p,miR-185-5p,miR-134-3p,miR-544b,miR-6529b,miR-28c,miR-502-5p,miR-382-5p,miR-206,miR-502b,miR-22-5p,miR-656-5p,novel_101,miR-542-5p,miR-24-3p,miR-211,miR-2428,miR-2387,miR-18a-3p,miR-216a-3p,miR-1306,miR-1291,miR-505-3p,miR-9851-3p,miR-3074-5p,miR-363,miR-10a-5p,miR-320b,miR-2404,miR-146b-3p,miR-6535,miR-320c,miR-542-5p,miR-2285p,miR-345-3p,miR-500a-5p,miR-466i-5p,miR-320d,miR-21-3p,miR-183-5p,miR-345-3p,miR-2411,miR-486-3p,miR-2424,miR-2300b-3p,miR-362-3p,miR-1197-5p,miR-5703,miR-140-3p,miR-2904,miR-4429,miR-18a-3p,miR-505-3p,miR-200b,miR-210-3p,miR-532-3p,miR-132-5p,miR-324-3p,miR-19b-1-5p,miR-664a,miR-4492,miR-708-5p,miR-4510,miR-145a-3p,miR-3074-5p,miR-206-3p,miR-10b-5p,miR-6525,miR-6134,miR-598-3p,miR-129b-3p,miR-339b,miR-345-5p,miR-1827,miR-625-5p,miR-548o-3p,miR-296-3p,miR-3065-3p,miR-6130,miR-544-3p,miR-132-5p,miR-10a,miR-1306-5p,novel_103,miR-320b,miR-362-3p,miR-3955-3p,miR-25-5p,miR-3085-3p,miR-542-5p,miR-2330-5p,miR-382-3p,miR-
ALDH6A1	ENSOARG0(	aldehyde	miR-432-5p,miR-216a-3p,miR-20b,miR-27a-3p,miR-16b,miR-1388-5p,miR-497-5p,miR-27b-3p,miR-432,miR-873a-5p,miR-301b-5p,miR-1434-3p,miR-501-3p,miR-30c-1-3p,miR-22841,miR-8485,let-7j,miR-2310,miR-424-5p,miR-106a-5p,miR-487b-5p,miR-214-3p,miR-342,miR-29b-1-5p,miR-17-5p,miR-16b,miR-106b-5p,miR-106a-5p,miR-299b-3p,miR-27a-3p,miR-500-3p,miR-329-5p,miR-499b-5p,miR-96-5p,miR-195a-5p,miR-140-5p,miR-2285ab,miR-3970,miR-3065-5p,miR-877-3p,novel_111,miR-502-3p,novel_120,miR-342-3p,miR-335-5p,miR-2285f,novel_101,miR-335,miR-2285t,miR-15a,miR-4324,miR-20a-5p,miR-6402,miR-16a,miR-301a-5p,miR-33a-3p,miR-322-5p,miR-1271-5p,miR-758-5p,miR-15a-5p,novel_83,miR-299a-3p,miR-106a,miR-215-5p,miR-193b-5p,miR-501-3p,miR-29b-1-5p,miR-29a-5p,miR-329a-5p,miR-216c-5p,miR-3529-3p,miR-20a,miR-29b-2-5p,miR-345-3p,miR-106a,miR-221-5p,miR-299-3p,miR-2285af,miR-30c-2-3p,miR-192-5p,novel_25,miR-15b-5p,miR-140-5p,miR-30c-1-3p,miR-221-5p,miR-3068-3p,miR-370-3p,miR-598-3p,miR-216b-3p,miR-215-5p,miR-876-3p,miR-2312,miR-20b-5p,miR-1814c,miR-598-3p,miR-93-5p,miR-17-5p,miR-93,miR-2284v,miR-1271,miR-16-5p,miR-3074-2-3p,miR-214,novel_74,miR-424-5p,miR-34c-3p,miR-29b-2-5p,miR-665

SEC23B	ENSOARGO(	Sec23 ho	miR-2889,miR-378c,miR-1388-5p,miR-323b,miR-1a-3p,miR-378b,miR-223-3p,miR-216a-3p,miR-1185-3p,miR-421,miR-539-5p,miR-503-5p,miR-3431,miR-339-5p,miR-31-5p,miR-1343-3p,novel_4,miR-193a-3p,miR-214-3p,miR-483-3p,novel_124,miR-378a-5p,miR-197-5p,miR-323b-3p,miR-29b-1-5p,miR-2331-5p,miR-503-5p,miR-326,miR-1b-5p,miR-345-5p,miR-378d,let-7f-2-3p,let-7f-2-3p,miR-1388-3p,miR-2330-5p,miR-378f,miR-6516-3p,miR-299b-5p,miR-129b-5p,miR-3065-5p,miR-1185-2-3p,miR-378e,miR-3596,miR-2404,miR-3082-5p,miR-489,miR-7977,miR-126b-5p,miR-10b,miR-656-5p,miR-625-3p,miR-22-5p,miR-2284q,miR-206,miR-450a-5p,miR-33a-3p,miR-2440,miR-133a-5p,miR-6529b,miR-326-3p,miR-193b-3p,novel_101,miR-17-3p,miR-2447,novel_73,miR-2427,miR-330-5p,miR-378a-3p,miR-378i,miR-3578,miR-376a-5p,miR-193b-5p,miR-545-5p,miR-486-5p,miR-378h,miR-483-3p,miR-3600,novel_79,miR-2285ad,miR-6517,miR-21-3p,miR-17-3p,miR-339a,miR-422a,miR-6119-5p,miR-125a-3p,miR-29a-5p,miR-450c-5p,miR-376a-5p,miR-29b-1-5p,miR-130a-5p,miR-1,miR-503-5p,miR-7144-5p,miR-671-5p,miR-345-5p,miR-339b,miR-345-5p,miR-301,miR-204-3p,miR-376c-3p,miR-376e-3p,miR-6130,miR-193b-3p,miR-532-5p,miR-378a-3p,miR-2331-3p,miR-129-5p,miR-4492,miR-17-3p,miR-378c,miR-338-3p,miR-206-3p,miR-193a-5p,miR-3074-2-3p,miR-214,miR-425-5p,miR-378c,miR-590-3p,miR-4510
NMNAT2	ENSOARGO(	nicotina	miR-582-5p,miR-10a,miR-377-3p,miR-139-5p,miR-204-3p,miR-30c-1-3p,miR-548o-3p,miR-339b,miR-582-5p,miR-1827,miR-186-5p,miR-129b-3p,miR-10b-5p,miR-769-5p,miR-6525,miR-206-3p,miR-129-2-3p,miR-3184-3p,miR-3958-5p,miR-214,miR-3074-2-3p,miR-4492,miR-15b-3p,miR-95-3p,miR-7859,miR-493-3p,miR-142-3p,miR-378d,miR-2411,miR-216c-5p,miR-760-3p,miR-466i-5p,miR-500a-5p,miR-3082-5p,miR-203-3p,miR-139-5p,miR-3064-5p,miR-20a-3p,miR-2285u,miR-499b-5p,miR-7977,miR-378j,miR-2447,novel_60,miR-3956-3p,miR-212-3p,miR-452-5p,miR-194b-5p,miR-335,miR-2285n,miR-615,miR-769-5p,miR-2285y,miR-9788-3p,miR-2459,miR-409b,miR-296-5p,miR-10b,miR-323b,miR-1a-3p,miR-30c-1-3p,miR-652-5p,miR-491-5p,miR-3085-3p,miR-25-5p,miR-214-3p,miR-342,miR-2957,miR-323b-3p,miR-769,miR-154b-5p,miR-2285aa,miR-2355-5p,miR-323-3p,miR-1260b,miR-103b,miR-2433,miR-34c-3p,miR-29b-3p,miR-5100,miR-6740-5p,miR-2411-5p,miR-129-1-3p,miR-2285ad,miR-6517,novel_79,miR-10a-5p,miR-615-3p,miR-200a,miR-1,miR-3064-5p,miR-450a-2-3p,novel_96,miR-1260a,miR-30c-2-3p,miR-339a,miR-374c-3p,miR-29a-3p,miR-670-3p,miR-669,novel_68,miR-10a-5p,miR-410-5p,miR-7975,miR-2387,miR-3141,miR-3065-5p,miR-2284w,miR-2285m,miR-342-3p,miR-335-5p,miR-29c-3p,miR-206,miR-29a,novel_87,miR-3431,miR-185-5p,miR-3955-5p,miR-339-5p,miR-26b-3p,miR-29b,miR-1260b,miR-708-3p,miR-1434-3p,miR-194-5p,miR-654-3p,miR-377-3p,miR-3141,miR-197-5p,miR-194a

BRF2	ENSOARG0C	BRF2, RN	miR-4510,miR-129-2-3p,miR-6525,miR-93,miR-338-3p,miR-145-3p,miR-99b-3p,miR-125b-5p,miR-592,miR-208b-5p,miR-129-5p,miR-4492,miR-532-5p,miR-125a,miR-141-3p,miR-99a-3p,miR-365a-3p,miR-6130,miR-204-3p,miR-17-5p,miR-411,miR-129b-3p,miR-93-5p,let-7g-3p,miR-20b-5p,miR-3064-5p,miR-20a,miR-125b-2-3p,miR-450b-5p,miR-200a,novel_17,miR-125b,miR-324-3p,miR-99a-3p,miR-106a,miR-200a-3p,miR-2484,miR-30b-3p,miR-106a,miR-423-3p,miR-6517,miR-2285ad,miR-412,miR-129-1-3p,miR-330-5p,miR-1434-5p,miR-184-3p,miR-3059-5p,miR-2447,miR-495-3p,miR-326-3p,miR-133a-5p,miR-101-3p,miR-20a-5p,miR-382-3p,miR-4324,miR-2285n,miR-33a-3p,miR-2285y,miR-1843a-3p,miR-452-3p,miR-3064-5p,miR-129b-5p,miR-2330-5p,miR-101b-3p,miR-106a-5p,miR-296-3p,miR-296-3p,miR-326,miR-17-5p,miR-106b-5p,miR-106a-5p,miR-3085-3p,miR-125a-5p,miR-412-5p,miR-20b,miR-412-3p,miR-2284x
PLA2G5	ENSOARG0C	phosphol	miR-34c-5p,miR-2355-5p,miR-141-3p,miR-125a,miR-329b,let-7g-3p,miR-2318,miR-329a,miR-23a-5p,miR-671-5p,miR-1248,miR-320a,miR-145-3p,miR-181c-3p,miR-16-5p,miR-138-5p,miR-28-5p,miR-134-5p,miR-551b-3p,miR-410-5p,miR-29b-3p,miR-2332,miR-370-5p,miR-100-3p,miR-125b-5p,miR-6740-5p,miR-3969,miR-322-5p,miR-134-5p,miR-214-5p,novel_121,miR-129-1-3p,miR-222,novel_79,miR-196a-2-3p,miR-128-3p,let-7a-2-3p,miR-134,miR-29d-3p,miR-1343-5p,miR-330-5p,miR-23b-5p,miR-28b,novel_39,miR-3065-3p,miR-1271-3p,miR-3956-5p,miR-3529-3p,miR-17-3p,miR-107,miR-200a-3p,novel_127,miR-205-5p,miR-181c-3p,miR-339a,miR-146b-3p,miR-29a-3p,miR-34b,miR-669,miR-320c,novel_68,miR-1291,miR-107,miR-2319a,miR-195a-5p,miR-150-5p,miR-3065-5p,miR-18a-3p,miR-216a-3p,miR-371b-3p,miR-326-3p,miR-449a,miR-3957-3p,miR-1839-3p,miR-29c-3p,miR-1193,miR-17-3p,miR-495-3p,novel_1,miR-29a,miR-147-5p,miR-502b,miR-15a,miR-28c,miR-34c,miR-16a,miR-432-5p,miR-503-5p,miR-194-3p,novel_116,miR-149-5p,miR-103,miR-103a-3p,miR-339-5p,miR-377-5p,miR-29b,miR-138,miR-16b,miR-16-1-3p,miR-497-5p,miR-7862,miR-19b-2-5p,miR-432,let-7a-2-3p,miR-125a-5p,miR-1343-5p,miR-503-5p,novel_51,miR-136-3p,miR-499b-3p,miR-6130,miR-204-3p,miR-151b,miR-15b-5p,miR-504-5p,miR-3065-3p,miR-216b-3p,miR-370-5p,miR-339b,miR-625-5p,miR-129b-3p,miR-6525,miR-145a-3p,miR-4510,miR-129-2-3p,novel_74,miR-6516-3p,miR-487b-5p,miR-17-3p,miR-487a-5p,miR-708-5p,miR-424-5p,miR-222-3p,miR-19b-1-5p,miR-320e,miR-665,miR-7859,miR-15a-5p,miR-151-5p,miR-532-3p,miR-548e-3p,miR-18a-3p,miR-493-3p,miR-33b-3p,miR-4429,miR-16-1-3p,miR-34a-5p,miR-378d,miR-2300b-3p,miR-362-3p,miR-124-3p,miR-188-3p,miR-5703,miR-140-3p,miR-377-5p,miR-503-5p,miR-320d,miR-34b-

WASL	ENSOARG0(	Wiskott-	miR-1185-2-3p,novel_69,miR-2483-3p,miR-331-5p,miR-300-3p,miR-3120-3p,miR-26b-5p,miR-329-5p,miR-452-3p,miR-3068-3p,miR-32-5p,miR-146b-5p,miR-543-3p,let-7g-3p,miR-3154,miR-137-3p,miR-338-3p,miR-181c-5p,miR-181d-5p,miR-2330-3p,miR-885-5p,miR-363-3p,miR-539-5p,novel_120,miR-28a-3p,miR-2284q,miR-129-5p,novel_74,novel_63,miR-1185-3p,miR-3431,miR-142-3p,miR-3955-5p,miR-450b-5p,miR-1434-5p,miR-3071-5p,miR-181a-5p,let-7j,miR-146a-5p,miR-142a-3p,let-7c-3p,miR-195a-3p,miR-329a-5p,miR-1277-5p,miR-146b,miR-181b-5p,miR-28-3p,miR-32,miR-146a,novel_103,miR-2285af,miR-196a-3p
PSMA4	ENSOARG0(	proteaso	miR-322-5p,miR-2284z,miR-450a-1-3p,miR-2285ad,miR-412,miR-18b-5p,miR-450b-5p,miR-192-3p,miR-3064-5p,miR-6536,miR-205-5p,miR-125a-3p,miR-3071-3p,miR-300-3p,miR-3959-5p,miR-133c,miR-2285aa,miR-1271,miR-329a,miR-345-5p,miR-1248,miR-30e-5p,miR-331-5p,miR-301,miR-137-3p,miR-23b,miR-16-5p,miR-331-5p,novel_9,miR-3432a,miR-100-3p,miR-412-5p,miR-3431,miR-134-3p,miR-194-3p,miR-149-5p,miR-31-5p,miR-16b,miR-3432b,miR-16-1-3p,miR-497-5p,miR-432,miR-19b-2-5p,miR-2113,miR-7b-5p,miR-542-3p,miR-450c-3p,novel_23,miR-2285r,miR-6240,miR-7-5p,miR-146a,novel_69,miR-3074-5p,miR-410-5p,miR-412-3p,miR-146b-5p,miR-3970,miR-2284w,miR-1839-3p,miR-29c-3p,miR-29a,miR-421-5p,miR-4324,miR-142b,miR-7859,miR-23b-3p,miR-23c,miR-30d-3p,miR-184-3p,miR-664-5p,miR-16-1-3p,miR-218-1-3p,miR-361-5p,miR-34a-5p,miR-30c-5p,miR-193b-5p,miR-2285i,miR-142a-3p,miR-377-5p,miR-345-3p,miR-30b,miR-618,miR-219b-5p,miR-122-3p,miR-92a-1-5p,miR-499b-3p,miR-15b-5p,miR-30c-1-3p,miR-2331-3p,miR-216b-3p,miR-296-3p,miR-345-5p,miR-2285c,miR-450a-1-3p,miR-30d-5p,miR-769-5p,miR-3184-5p,miR-3074-5p,miR-3958-5p,miR-1468-5p,miR-874-5p,miR-129-5p,miR-2285j,miR-30e-3p,miR-151-3p,miR-30a-3p,miR-3604,miR-664a-5p,miR-153,miR-539-5p,miR-455-3p,miR-421,miR-126a-5p,miR-330-3p,miR-362-3p,miR-1839-5p,miR-329-3p,miR-323b,miR-758-3p,miR-8485,miR-124a,miR-23a-3p,miR-744-3p,miR-146b,miR-153-3p,miR-323b-3p,miR-182-5p,miR-16b,miR-499a-3p,miR-378g,miR-203-3p,miR-1983,miR-361-3p,novel_111,miR-423-5p,miR-2447,miR-3968,miR-218-5p,miR-582-3p,miR-545-5p,miR-582-3p,miR-29d-3p,miR-30a-5p,miR-1957a,miR-1271-3p,miR-490-3p,miR-30a-3p,miR-191-3p,miR-30c-2-3p,miR-34c-5p,miR-769,miR-365a-3p,miR-370-3p,miR-615-5p,miR-3074-1-3p,miR-488-3p,miR-

EXOC6	ENSOARG0(	exocyst	miR-28c,miR-6402,miR-502-5p,miR-1247-3p,miR-4324,miR-15a,miR-16a,novel_1,miR-128-1-5p,miR-22-5p,miR-203b-5p,novel_101,miR-3959-3p,miR-1307-3p,miR-301b-3p,miR-376b-3p,miR-1306,miR-19a-3p,miR-412-3p,miR-107,miR-195a-5p,miR-7975,miR-329-5p,miR-3120-3p,miR-7-1-3p,miR-320b,miR-411b-3p,miR-181b-3p,miR-677,miR-144-3p,miR-296-3p,miR-7-5p,novel_51,miR-483-3p,miR-493-5p,miR-668-3p,miR-432-3p,miR-296-3p,miR-7b-5p,miR-19b-2-5p,miR-1260b,miR-16b,miR-138,miR-744-5p,miR-4508,miR-497-5p,miR-103a-3p,miR-26b-3p,miR-130a-3p,miR-2285g,miR-544b,miR-103,miR-409-3p,miR-144,miR-138-5p,miR-486b-3p,miR-28-5p,novel_42,miR-1306-5p,miR-16-5p,miR-1260b,miR-424-3p,miR-23b,miR-874-3p,miR-215-5p,miR-370-3p,miR-154b-5p,miR-324-5p,miR-2397-5p,miR-1260a,miR-205-5p,miR-376b-3p,miR-181c-3p,miR-191-3p,miR-450a-2-3p,novel_96,miR-543-5p,miR-107,miR-3529-3p,miR-4286,miR-28b,miR-23b-5p,miR-885-3p,miR-222,miR-483-3p,miR-322-5p,miR-2440,miR-107-5p,miR-628-3p,miR-2284q,miR-3956-3p,miR-541-3p,miR-32-3p,novel_60,miR-2447,miR-423-5p,miR-221,miR-378j,miR-9-3p,miR-1983,miR-376a-3p,miR-493-5p,miR-541-5p,miR-3082-5p,miR-376d,miR-4443,miR-1306-5p,miR-5126,miR-16b,miR-25-5p,miR-130b-3p,miR-491-5p,miR-214-3p,miR-2285o,miR-103a-2-5p,miR-2330-5p,miR-424-5p,miR-127-3p,miR-124a,miR-676-3p,miR-23a-3p,miR-4532,miR-541,miR-296-5p,miR-6529a,miR-125b-2-3p,miR-544a,novel_78,miR-548w,miR-2300a-5p,miR-181b-2-3p,miR-376b-3p,miR-2285e,miR-148b-5p,miR-221-3p,miR-30e-3p,miR-222-3p,miR-2285j,miR-30a-3p,miR-19b-1-5p,miR-708-5p,miR-4492,miR-454-3p,miR-4510,miR-214,miR-3184-5p,miR-2285c,miR-21a-3p,miR-885-3p,miR-32-3p,miR-6130,miR-192-5p,miR-544-3p,miR-301a-3p,miR-582-5p,miR-15b-5p,miR-204-3p,novel_17,miR-196a-3p,miR-185-3p,miR-
SLC13A5	ENSOARG0(	solute c	miR-22-3p,miR-670-3p,miR-2284s,miR-378g,miR-615-5p,miR-320b,miR-2355-5p,miR-504-5p,miR-2284g,miR-9851-3p,miR-504,miR-767,miR-18a-3p,miR-362-5p,miR-150-5p,miR-3970,miR-1306,miR-129b-3p,miR-361-3p,novel_27,miR-362-5p,miR-500b-5p,miR-361-3p,miR-214,miR-665-5p,miR-135a-2-3p,miR-486b-3p,miR-203b-5p,miR-767-5p,miR-4726-5p,miR-128-1-5p,miR-320e,miR-665,miR-324-3p,miR-485-3p,miR-2284o,miR-500-5p,miR-134-3p,miR-340-3p,miR-18a-3p,miR-2284u,miR-133a-3p,miR-30b-3p,miR-30b-3p,miR-2904,miR-22-3p,miR-223-3p,miR-2366,miR-2284a,miR-486-3p,miR-2284y,miR-125b-2-3p,miR-2284b,miR-2284f,novel_127,miR-214-3p,miR-127-5p,miR-2957,miR-1306-5p

FCHSD1	ENSOARG0(	FCH and	miR-670-3p,miR-323a-5p,miR-7-1-3p,miR-6395,miR-195a-5p,miR-107,miR-1291,miR-18a-3p,miR-2285ab,miR-2428,novel_27,miR-326-3p,miR-24-3p,miR-211,miR-17-3p,novel_101,miR-2285x,miR-135a-2-3p,miR-147-5p,miR-199c,miR-128-1-5p,miR-16a,miR-502-5p,miR-15a,miR-103,miR-185-5p,miR-2285g,miR-377-5p,miR-26b-3p,miR-455-5p,miR-30b-3p,miR-873a-5p,miR-345-5p,miR-363-5p,miR-130b-5p,miR-125a-5p,miR-1343-5p,miR-455-5p,miR-199a-5p,miR-615-5p,miR-215-5p,miR-23a-5p,miR-362-5p,miR-3154,miR-378c,miR-500b-5p,miR-204-5p,miR-486b-3p,miR-28-5p,miR-26c,miR-331-3p,miR-2332,miR-5100,miR-378h,miR-1271-5p,miR-2411-5p,miR-128-3p,miR-490-3p,miR-3065-3p,miR-199a-5p,miR-28b,miR-181a-2-3p,miR-4286,miR-2385-3p,miR-107,miR-2898,miR-17-3p,miR-339a,miR-378e,miR-452-3p,miR-877-3p,miR-199b-5p,miR-219a-2-3p,miR-362-5p,miR-2285f,miR-541-3p,miR-212-3p,novel_60,miR-3059-5p,miR-4726-5p,miR-2285e,miR-500-5p,miR-142-3p,miR-2300a-5p,novel_4,miR-378c,miR-30b-3p,miR-412-3p,miR-484,miR-296-5p,miR-378b,miR-1839-3p,miR-424-5p,miR-494-3p,miR-2285o,miR-1306-5p,miR-326,miR-155-5p,miR-323-5p,miR-6130,miR-132-5p,miR-192-5p,miR-3065-3p,miR-378a-3p,miR-2312,let-7g-3p,miR-6134,miR-338-3p,miR-1271,miR-145a-3p,miR-4510,miR-378c,miR-424-5p,miR-708-5p,miR-664a,miR-17-3p,miR-324-3p,miR-532-3p,miR-148b-3p,miR-15a-5p,miR-383-5p,miR-1197-5p,miR-140-3p,miR-5703,miR-2904,miR-124-3p,miR-215-5p,miR-2300b-3p,novel_133,miR-2411,miR-130a-5p,miR-125b-2-3p,miR-21-3p,miR-760-3p,miR-466i-5p,miR-125b,miR-185-3p,miR-196a-3p,miR-669,novel_69,miR-3074-5p,novel_68,miR-378f,miR-216a-3p,miR-150-5p,miR-1306,miR-148a-3p,miR-199b-5p,miR-1839-3p,miR-6123,miR-28c,miR-576-3p,miR-4324,miR-149-5p,miR-194-3p,miR-134-3p,miR-
GCFC2	ENSOARG0(	GC-rich	miR-6238,miR-376c-5p,miR-376b-5p,miR-376b-5p,miR-376c-5p,miR-376c-5p,miR-16-2-3p,novel_48

GOT1L1	ENSOARG0(	glutamic	miR-500a-5p,miR-125b,miR-92a-1-5p,miR-210-5p,miR-1277-5p,miR-92a-3p,miR-2898,miR-450a-2-3p,miR-3064-5p,miR-4286,miR-376b-5p,miR-3065-3p,miR-376c-5p,miR-490-3p,miR-143-3p,miR-376a-5p,miR-92b-3p,miR-377-5p,miR-2427,miR-29d-3p,miR-2904,miR-188-3p,miR-330-5p,miR-2408,miR-193b-5p,novel_73,miR-1224-5p,miR-199a-3p,miR-532-3p,novel_121,miR-129-1-3p,miR-6740-5p,miR-125b-5p,miR-665,miR-29b-3p,miR-551b-3p,novel_74,miR-433-5p,miR-129-2-3p,miR-500b-5p,miR-224-5p,miR-129b-3p,miR-362-5p,miR-488-3p,miR-2285c,miR-7144-5p,miR-543-3p,miR-671-5p,miR-1248,miR-10b-3p,miR-2355-5p,miR-3065-3p,miR-532-5p,miR-125a,miR-32-5p,miR-3068-3p,miR-377-3p,miR-25-3p,miR-3965,miR-216b-5p,miR-32,miR-224-5p,miR-199a-3p,novel_103,miR-377-3p,miR-326,miR-452-5p,miR-125a-5p,miR-3085-3p,miR-363-5p,miR-744-3p,miR-193a,miR-145a-5p,miR-542-3p,miR-16-2-3p,miR-7862,miR-21-5p,miR-29b,miR-450b-5p,miR-665,miR-377-5p,miR-145b,miR-133b-5p,miR-500-5p,novel_116,miR-2285e,miR-2285g,miR-3431,miR-185-5p,miR-4324,miR-101-3p,miR-92a-3p,miR-29a,miR-29c-3p,miR-495-3p,novel_60,miR-3059-5p,miR-3956-3p,miR-363-3p,miR-326-3p,miR-362-5p,miR-24-3p,miR-21c,miR-3065-5p,miR-9-3p,miR-136-5p,miR-499b-5p,miR-7977,miR-7-1-3p,miR-3064-5p,miR-452-3p,miR-378b,miR-216b-3p,miR-29a-3p
SYNDIG1L	ENSOARG0(	synapse	miR-300-3p,miR-154b-5p,miR-133c,miR-324-5p,miR-199a-5p,miR-1271,miR-615-5p,miR-215-5p,miR-2355-5p,miR-1248,miR-7144-5p,miR-331-5p,miR-1260b,miR-378c,miR-16-5p,miR-331-5p,miR-204-5p,miR-134-5p,miR-486b-3p,miR-5100,miR-378h,miR-6516-5p,miR-9-5p,miR-322-5p,miR-214-5p,miR-134-5p,miR-6517,miR-299a-3p,miR-222,miR-431,miR-7641,let-7d,miR-105-2,miR-134,miR-23b-5p,miR-1343-5p,miR-490-3p,let-7g,novel_39,miR-199a-5p,miR-3065-3p,miR-4286,miR-30a-3p,miR-3064-5p,miR-2385-3p,miR-29b-2-5p,let-7f-5p,miR-127-5p,miR-450a-2-3p,miR-2898,miR-543-5p,novel_96,novel_127,miR-107,miR-6119-5p,miR-1260a,let-7c-5p,novel_69,miR-299b-3p,miR-146b-3p,miR-2404,miR-181b-3p,miR-677,miR-669,miR-3120-3p,miR-655-5p,miR-107,miR-378f,miR-6395,miR-195a-5p,let-7e-5p,miR-9851-3p,miR-18a-3p,miR-150-5p,miR-1306,miR-211,miR-199b-5p,miR-3957-3p,miR-502-5p,miR-15a,miR-33a-3p,let-7i,miR-16a,let-7g-5p,miR-134-3p,miR-103,miR-411-5p,miR-103a-3p,miR-219a-1-3p,miR-1260b,miR-30b-3p,miR-16b,miR-27b-3p,miR-188-5p,miR-497-5p,miR-1843b-5p,miR-374b-3p,miR-22-3p,miR-431-3p,miR-432-3p,miR-432-3p,miR-2285r,miR-503-3p,miR-6412,miR-378a-5p,miR-1343-5p,miR-2285v,miR-105-5p,miR-6516,miR-582-5p,miR-192-5p,miR-15b-5p,let-7i-5p,miR-22-3p,novel_32,miR-548o-3p,miR-221-5p,miR-378a-3p,miR-182-5p,miR-296-3p,miR-3065-3p,miR-1827,novel_99,miR-582-5p,miR-129b-3p,miR-346,miR-6525,miR-338-3p,miR-6134,let-7b-5p,miR-145a-3p,let-7b,miR-378c,novel_74,miR-424-5p,miR-874-5p,miR-30e-3p,miR-222-3p,let-7f,miR-29b-2-5p,miR-665,miR-30a-3p,miR-151-3p,miR-15a-5p,miR-328-3p,miR-18a-3p,miR-30d-3p,miR-941,miR-493-3p,miR-215-5p,miR-1961,miR-378i,miR-378a-3p,miR-5703,miR-1193,miR-2904,miR-664b-3p,miR-486-3p,miR-105-3p,miR-183-5p,miR-98-

NOX1	ENSOARG0(	NADPH ox	miR-15a,miR-6402,miR-16a,miR-6119-3p,miR-379-3p,miR-656-5p,miR-147-5p,miR-3059-5p,novel_120,miR-194b-5p,miR-190b-5p,miR-2285x,miR-8095,miR-3065-5p,miR-3963,miR-493-5p,miR-195a-5p,miR-6516-3p,miR-139-5p,miR-655-5p,miR-541-5p,miR-190a,miR-16b,miR-194a,miR-214-3p,miR-424-5p,miR-432-3p,miR-2310,miR-4791,miR-1298-5p,miR-194-5p,miR-2284ab,miR-16b,miR-26a-2-3p,miR-497-5p,miR-548w,miR-3535,miR-148b-5p,miR-421,miR-30e-3p,miR-135a-1-3p,miR-30a-3p,miR-655-3p,miR-331-5p,miR-6516-3p,miR-424-5p,miR-190a-5p,miR-590-3p,miR-16-5p,miR-214,miR-145-3p,miR-21a-3p,miR-331-5p,miR-655,miR-543-3p,miR-186-5p,miR-1814c,miR-1271,miR-27a-5p,miR-139-5p,miR-15b-5p,miR-205-5p,miR-2285p,miR-3071-3p,miR-500a-5p,miR-217,miR-2284m,miR-127-5p,miR-1a-2-5p,miR-21-3p,miR-216c-5p,miR-30a-3p,miR-450b-5p,miR-2478,miR-125b-2-3p,miR-1197-5p,miR-1434-5p,miR-217-5p,miR-411-3p,miR-148a-5p,miR-30d-3p,miR-322-5p,miR-505-5p,miR-758-5p,miR-15a-5p,novel_121
RALBP1	ENSOARG0(	ralA bin	miR-181a-2-3p,miR-340-3p,miR-4443,novel_39
MUC5B	ENSOARG0(	mucin 5B	miR-1260b,miR-361-3p,miR-4510,miR-342-3p,miR-193a-5p,miR-3957-3p,miR-128-1-5p,miR-576-3p,miR-6529b,miR-665,miR-320e,miR-6740-5p,miR-331-3p,novel_69,miR-670-3p,miR-6130,miR-146b-3p,miR-377-3p,miR-4443,miR-874-3p,miR-552-3p,miR-329a,miR-450b-3p,novel_94,miR-1827,miR-625-5p,miR-361-3p,miR-378j,miR-3154,miR-29b-1-5p,miR-143-3p,novel_39,miR-191-3p,miR-25-5p,miR-1343-5p,miR-377-3p,miR-342,miR-1260a,novel_51,miR-29b-1-5p,miR-362-3p,miR-185-5p,miR-1343-3p,miR-145b,miR-3600,miR-1260b,miR-362-3p,miR-1285,miR-2366,miR-362-3p,miR-2408,miR-329-3p,miR-296-5p,miR-145a-5p,miR-1343-5p

INTS3	ENSOARG0(	integrat	miR-214,miR-425-5p,miR-3074-2-3p,miR-378c,miR-6134,miR-3184-5p,miR-6525,miR-665,miR-708-5p,miR-874-5p,miR-4492,miR-424-5p,miR-664a,miR-17-3p,miR-3065-3p,miR-378a-3p,miR-30c-1-3p,miR-15b-5p,miR-323-5p,miR-499b-3p,miR-411,miR-1827,miR-183-5p,miR-329a-5p,miR-486-3p,miR-29b-1-5p,miR-345-3p,miR-422a,miR-185-3p,miR-299-3p,miR-21-3p,miR-2484,miR-30b-3p,miR-760-3p,miR-212-5p,miR-3600,miR-328-3p,miR-15a-5p,miR-378a-3p,miR-378i,novel_133,miR-378d,miR-3059-5p,miR-423-5p,miR-2284q,miR-211-5p,miR-499b-5p,miR-3064-5p,miR-4792,miR-499a-3p,miR-378e,miR-378j,miR-361-3p,miR-424-5p,miR-1388-3p,miR-3187-3p,miR-193a,miR-4532,miR-30c-1-3p,miR-378d,miR-16b,miR-29b-1-5p,miR-326,miR-214-3p,miR-3085-3p,miR-222-5p,miR-1343-3p,miR-455-3p,miR-7857,miR-378b,miR-2284r,miR-3120-5p,miR-378c,miR-16-5p,novel_42,miR-145-3p,miR-378c,miR-2433,miR-486b-3p,miR-28-5p,miR-204-5p,miR-31-3p,miR-2355-5p,miR-3957-5p,miR-3957,miR-3154,miR-301,miR-671-5p,miR-485-5p,miR-23a-5p,let-7g-3p,miR-10b-3p,miR-3064-5p,miR-490-3p,miR-192-3p,miR-3065-3p,miR-28b,miR-148b-5p,miR-30c-2-3p,miR-2285af,miR-543-5p,miR-17-3p,miR-450a-2-3p,miR-6517,miR-299a-3p,novel_121,miR-483-3p,miR-378h,miR-505-5p,miR-322-5p,miR-23b-5p,miR-330-5p,miR-1343-5p,miR-345-3p,let-7a-2-3p,miR-411-3p,miR-17-3p,miR-3957-3p,miR-2428,novel_27,miR-326-3p,miR-211,miR-16a,miR-28c,miR-133a-5p,miR-15a,miR-147-5p,miR-379-3p,miR-128-1-5p,miR-329-5p,miR-378b,miR-6535,miR-299b-3p,miR-323a-5p,miR-378f,miR-6395,miR-195a-5p,miR-363-5p,miR-2483-5p,let-7a-2-3p,miR-432-3p,miR-2285r,miR-2411-3p,novel_51,miR-466f-3p,miR-1343-5p,miR-483-3p,miR-194-3p,miR-3071-5p,miR-197-3p,miR-1285,miR-4508,miR-497-5p,miR-16b

KIF2C	ENSOARG0(	kinesin	miR-29c-3p,miR-2285x,miR-17-3p,miR-1a-1-5p,miR-335-5p,novel_27,miR-326-3p,miR-449a,miR-2428,miR-301b-3p,miR-2284w,miR-34c,miR-502-5p,miR-1247-3p,miR-576-3p,miR-625-3p,miR-22-5p,miR-199c,miR-29a,miR-379-3p,miR-320b,miR-655-5p,miR-10a-5p,miR-378b,miR-677,miR-181b-3p,miR-323a-5p,miR-146b-3p,miR-29a-3p,miR-411b-3p,miR-299b-3p,miR-670-3p,miR-34b,miR-18a-3p,miR-376b-3p,miR-148a-3p,miR-2387,miR-376a-2-5p,miR-135b-5p,miR-668-3p,miR-345-5p,miR-654-3p,miR-432-3p,miR-432-3p,miR-377-3p,miR-660,miR-466f-3p,miR-130a-3p,miR-6238,miR-2355-3p,miR-432-5p,miR-2284d,miR-134-3p,miR-185-5p,miR-22-3p,miR-374b-3p,miR-3071-5p,miR-432,miR-653,miR-1285,miR-2284x,miR-29b,miR-30b-3p,miR-16-1-3p,miR-2284v,miR-500b-5p,miR-331-3p,miR-409-3p,miR-135a-5p,miR-29b-3p,miR-1-5p,miR-2284k,miR-494-5p,miR-5100,miR-134-5p,miR-28-5p,novel_9,miR-767-5p,miR-2355-5p,miR-487a-3p,miR-3068-3p,miR-874-3p,miR-133c,miR-34c-5p,miR-365a-3p,miR-362-5p,miR-320a,miR-485-5p,miR-876-3p,miR-767,miR-345-5p,miR-2318,miR-329a,miR-3064-5p,miR-200a,novel_39,miR-2284y,miR-3065-3p,miR-29a-5p,miR-2284a,miR-490-3p,miR-450b-5p,miR-30c-2-3p,miR-376b-3p,miR-664-3p,miR-543-5p,miR-152-3p,novel_127,miR-17-3p,miR-6536,miR-1a-2-5p,miR-412,miR-376a-5p,miR-299a-3p,miR-2285ad,miR-134-5p,miR-214-5p,miR-24-2-5p,miR-6516-5p,miR-29d-3p,miR-23b-5p,miR-330-5p,miR-134,miR-10a-5p,miR-411-3p,miR-582-3p,miR-2284j,miR-2447,miR-3059-5p,miR-541-3p,miR-362-5p,miR-361-3p,miR-2285y,miR-301a-5p,miR-218-5p,miR-2440,miR-490-5p,miR-154-3p,miR-3968,miR-3958-3p,miR-335,miR-2285u,miR-7977,miR-664b,miR-139-5p,miR-2284g,miR-3064-5p,miR-20a-3p,miR-203-3p,miR-376d,miR-7857-3p,miR-378g,miR-361-3p,miR-376a-3p,miR-877-3p,miR-2284b,miR-382-3p,miR-193a,miR-30c-1-3p,miR-362-

HMGN5	ENSOARG0(high mob	miR-493-5p,miR-101-5p,miR-2284l,miR-345-5p,miR-503-3p,miR-146a,miR-124-5p,miR-378a-5p,miR-466f-3p,miR-133b-5p,miR-130a-3p,miR-2285g,miR-3431,miR-2113,miR-1434-3p,miR-450c-3p,novel_23,miR-16b,miR-497-5p,miR-495-3p,miR-301b-3p,miR-15a,miR-142b,miR-16a,miR-425-5p,miR-6119-3p,miR-656-5p,miR-421-5p,miR-411b-3p,miR-144-3p,miR-320c,miR-677,miR-181b-3p,miR-148a-3p,miR-18b,miR-3065-5p,miR-19a-3p,miR-412-3p,miR-146b-5p,miR-195a-5p,miR-181a-2-3p,miR-380-5p,miR-450b-5p,miR-148b-5p,miR-192-3p,miR-2285af,miR-200c,miR-30f,miR-217,miR-200c-3p,novel_96,miR-152-3p,miR-429-3p,miR-18b-5p,miR-322-5p,miR-6516-5p,miR-545-5p,miR-582-3p,miR-450a-1-3p,novel_121,miR-345-3p,miR-30a-5p,miR-300,miR-155-5p,miR-1306-5p,miR-16-5p,miR-410-5p,miR-144,miR-409-3p,miR-374b-3p,miR-2285aa,miR-598-3p,miR-769,miR-376c-3p,miR-381-3p,miR-30e-5p,miR-301,miR-2448-3p,miR-320a,miR-18a,miR-23b,miR-10b-3p,miR-345-5p,miR-671-5p,miR-424-5p,miR-23a-3p,miR-33a-5p,miR-758-3p,miR-320b,miR-182-5p,miR-16b,miR-491-5p,miR-3955-3p,miR-130b-3p,miR-146b,miR-181b-2-3p,miR-539-5p,miR-142-3p,miR-2285e,miR-6529a,miR-296-5p,miR-484,miR-758-3p,miR-1388-5p,miR-2426,miR-1839-5p,miR-21b,miR-3120-5p,miR-885-5p,miR-8095,miR-769-5p,miR-490-5p,miR-2285t,miR-20a-3p,miR-541-5p,miR-454-5p,miR-140-5p,miR-493-5p,miR-1983,miR-195a-3p,miR-421-5p,miR-376a-5p,miR-142a-3p,miR-376c-3p,miR-181b-1-3p,miR-146a-5p,miR-2432,miR-2285p,miR-500a-5p,miR-345-3p,miR-320d,miR-1277-5p,miR-30b-3p,miR-2484,miR-3600,miR-485-3p,miR-23b-3p,miR-487a-3p,miR-7859,miR-15a-5p,miR-23c,miR-148b-3p,novel_133,miR-1246,miR-1434-5p,miR-217-5p,miR-140-3p,miR-4429,miR-142-3p,miR-19b-3p,miR-218-1-3p,miR-361-5p,miR-769-5p,miR-
CHRNA3	ENSOARG0(choliner	miR-21c,miR-2319a,miR-671-5p,miR-10a-5p,miR-10a,miR-670-3p,miR-203-3p,miR-665,miR-628-3p,miR-2284q,miR-6128,miR-214,miR-361-3p,miR-10b-5p,miR-10b,miR-21-5p,novel_73,miR-2889,miR-10a-5p,miR-665,miR-26b-3p,miR-142-3p,miR-2284h-5p,miR-199a-3p,miR-199a-3p,miR-374a-5p,miR-125a-3p,miR-2397-5p,miR-191-3p,miR-214-3p,miR-2285w,miR-142a-3p

GIMAP8	ENSOARG0(	GTPase,	miR-296-3p,miR-3065-3p,miR-504-5p,miR-221-5p,miR-2331-3p,miR-204-3p,miR-139-5p,miR-376e-3p,miR-450a-1-3p,miR-1827,miR-186-5p,miR-345-5p,miR-133b-3p,miR-214,miR-145a-3p,miR-6134,miR-346,miR-338-5p,miR-320e,miR-665,miR-30a-3p,miR-874-5p,miR-664a,miR-450b-3p,novel_74,miR-485-3p,miR-30d-3p,miR-3600,miR-18a-3p,miR-328-3p,miR-2284h-5p,miR-532-3p,miR-487a-3p,miR-5703,miR-2904,miR-376a-5p,miR-2284u,miR-148a-5p,miR-16-1-3p,miR-4429,novel_73,miR-345-3p,miR-195a-3p,miR-2285w,miR-216c-5p,miR-376c-3p,miR-376a-5p,miR-421-5p,miR-29b-1-5p,miR-125b,miR-345-3p,miR-92a-1-5p,novel_17,miR-221-5p,miR-1277-5p,miR-320d,miR-2284m,miR-7977,miR-211-5p,miR-126b-5p,miR-20a-3p,miR-2284g,miR-139-5p,miR-129b-5p,miR-504,miR-136-5p,miR-2284j,miR-541-3p,miR-2447,miR-7705,miR-107-5p,miR-490-5p,miR-3968,miR-2300a-5p,miR-1343-3p,miR-126a-5p,miR-455-3p,miR-148b-5p,miR-425-3p,miR-323b,miR-541,miR-2284r,miR-544a,miR-2284b,miR-33a-5p,miR-494-3p,miR-2310,miR-4532,miR-323b-3p,miR-29b-1-5p,novel_115,miR-320b,miR-214-3p,miR-103a-2-5p,miR-1185-5p,miR-125a,miR-542-5p,miR-3068-3p,miR-874-3p,miR-2284n,miR-331-5p,miR-374c-3p,miR-320a,miR-200a-5p,miR-2448-3p,miR-301,miR-381-3p,miR-485-5p,miR-671-5p,miR-1814c,miR-345-5p,miR-767,miR-10b-3p,miR-133a-3p,miR-2284v,miR-145-3p,miR-1260b,miR-3969,miR-331-3p,miR-125b-5p,miR-551b-3p,miR-2284k,miR-29b-3p,miR-204-5p,miR-767-5p,miR-138-5p,miR-545-5p,miR-450a-1-3p,miR-758-5p,miR-29d-3p,miR-345-3p,miR-3591-5p,miR-300,miR-2403,miR-181a-2-3p,miR-30a-3p,miR-1271-3p,miR-2284a,miR-192-3p,miR-3065-3p,miR-2284y,miR-380-5p,miR-205-5p,miR-1260a,novel_127,miR-107,miR-127-5p,miR-3120-3p,miR-216b-3p,miR-320c,miR-374c-3p,miR-29a-3p,miR-18a-3p,miR-3065-

NXPE4	ENSOARG0(	neurexop	miR-450a-2-3p, miR-2898, miR-200c-3p, novel_127, miR-205-5p, miR-1260a, miR-200c, miR-125a-3p, miR-219-3p, miR-199a-5p, miR-200a, novel_39, miR-1957a, miR-376c-5p, miR-376b-5p, miR-3065-3p, miR-450c-5p, miR-2285b, miR-29b-2-5p, miR-2385-3p, miR-4286, miR-181a-2-3p, miR-411-3p, miR-200b-3p, let-7a-2-3p, miR-345-3p, miR-1290, miR-218-2-3p, miR-378h, miR-1271-5p, miR-450a-1-3p, miR-486-5p, novel_121, miR-214-5p, miR-429-3p, miR-204-5p, miR-551b-3p, miR-3969, miR-323-3p, miR-500b-5p, miR-93, miR-1260b, miR-378c, miR-365a-5p, miR-374b, miR-1306-5p, miR-133a-3p, miR-876-3p, miR-10b-3p, miR-345-5p, let-7g-3p, miR-7144-5p, miR-20b-5p, miR-671-5p, miR-485-5p, miR-1248, miR-1814c, miR-488-3p, miR-93-5p, miR-17-5p, miR-362-5p, miR-3154, miR-34c-5p, miR-223-5p, miR-199a-5p, miR-574-5p, miR-3068-3p, miR-874-3p, miR-542-5p, miR-2355-5p, miR-3956, miR-378a-5p, miR-224-5p, miR-377-3p, miR-365b-5p, miR-106a-5p, novel_51, miR-2411-3p, miR-17-5p, miR-2285r, let-7a-2-3p, miR-345-5p, miR-130b-5p, miR-668-3p, let-7f-2-3p, let-7f-2-3p, miR-1260b, miR-2284x, miR-188-5p, miR-873a-5p, miR-1434-3p, miR-1285, miR-3431, miR-185-5p, miR-20b, miR-2355-3p, miR-31-5p, miR-379-3p, miR-203b-5p, miR-656-5p, miR-6123, miR-502-5p, miR-20a-5p, miR-34c, miR-382-5p, miR-211, miR-323c, miR-449a, miR-199b-5p, miR-219b-3p, miR-342-3p, miR-2285x, miR-1291, miR-2319a, miR-6395, miR-378f, miR-2387, miR-378b, miR-18a-3p, miR-2404, miR-34b, miR-677, miR-669, miR-3074-5p, miR-106a, miR-202-5p, miR-466i-5p, miR-221-5p, miR-34b-5p, miR-2484, miR-21-3p, novel_17, miR-345-3p, miR-422a, miR-664b-3p, miR-376a-5p, miR-125b-2-3p, miR-20a, miR-369-3p, miR-345-3p, miR-218-1-3p, miR-34a-5p, miR-378d, miR-361-5p, miR-378i, miR-1246, miR-376a-5p, miR-2366, miR-1197-5p, miR-140-3p, miR-378a-3p, miR-5703, miR-222-5p, miR-200b, novel_83, miR-328-3p, miR-18a-3p, miR-106a, miR-
TUBB2A	ENSOARG0(	tubulin,	novel_111, miR-145-3p, let-7b-5p, miR-338-3p, let-7b, miR-136-3p, miR-103b, let-7f, miR-132-5p, let-7i, miR-377-3p, miR-411b-3p, miR-2483-3p, let-7d-5p, let-7i-5p, miR-4792, miR-320b, miR-542-5p, miR-1291, let-7e-5p, miR-2387, miR-148a-3p, miR-216a-3p, miR-432-3p, miR-432-3p, miR-8485, let-7g, let-7a-5p, miR-2898, miR-450a-2-3p, miR-98-5p, let-7f-5p, miR-30b-3p, let-7e, miR-152-3p, let-7k, miR-205-5p, miR-7-5p, miR-377-3p, let-7c-5p, miR-148b-3p, let-7g-5p, miR-26b-3p, let-7d, miR-128-3p, miR-2366, miR-7b-5p, miR-1961, miR-431-3p

			[illegible] 142-5p,miR-1a-3p,miR-329-3p,miR-362-3p,miR-8485,miR-16b,miR-199a-3p,miR-214-3p,miR-103a-2-5p,miR-199b-5p,miR-203-3p,miR-144-5p,miR-25,miR-499a-3p,miR-1983,miR-335-3p,miR-493-5p,miR-504,miR-9-5p,miR-6527,miR-363-3p,miR-1285-5p,miR-2440,novel_82,miR-628-3p,miR-499a-5p,miR-3968,miR-380-5p,miR-18a-3p,miR-2284h-5p,miR-7859,miR-218-1-3p,miR-142-3p,miR-33b-3p,miR-329a-5p,miR-376c-3p,miR-181b-1-3p,miR-664b-3p,miR-376a-5p,miR-122-3p,miR-500a-5p,miR-618,miR-2285p,miR-210-5p,miR-30c-1-3p,miR-504-5p,miR-2331-3p,miR-151b,miR-15b-5p,miR-10a,miR-376e-3p,miR-655,miR-2285c,miR-1827,miR-339b,miR-214,miR-3184-3p,miR-142a-5p,miR-3604,miR-665,miR-664a-5p,miR-151-3p,miR-219b-3p,miR-2285j,miR-130b-5p,miR-129-5p,miR-339-5p,miR-103a-3p,miR-194-3p,miR-149-5p,miR-412-5p,miR-3535,miR-22-3p,miR-2113,miR-7b-5p,miR-542-3p,miR-223-3p,miR-188-5p,miR-2284x,miR-27b-3p,miR-497-5p,miR-26a-2-3p,miR-16b,miR-493-5p,miR-380-5p,miR-2284l,miR-2285r,miR-105-5p,miR-296-3p,miR-7-5p,miR-193a-3p,miR-106b-3p,miR-3120-3p,miR-216b-3p,miR-329-5p,novel_68,miR-181b-3p,miR-144-3p,miR-669,miR-677,miR-374c-3p,miR-411b-3p,miR-3970,miR-148a-3p,miR-2319a,miR-412-3p,miR-758-5p,miR-193b-3p,miR-199b-5p,miR-374c-5p,novel_87,miR-142b,miR-4324,miR-625-3p,miR-206,novel_79,miR-412,miR-1224-5p,miR-6517,miR-486-5p,miR-134-5p,miR-2284z,miR-322-5p,miR-9-5p,miR-192-3p,miR-200a,miR-92b-3p,miR-219-3p,miR-152-3p,novel_127,miR-200a-3p,miR-127-5p,miR-125a,miR-3956,miR-574-5p,miR-376c-3p,miR-25-3p,miR-300-3p,miR-331-5p,miR-424-3p,miR-1248,miR-329b,miR-329a,miR-144-5p,miR-16-5p,miR-1197-3p,miR-125b-5p,novel_63,miR-134-5p,miR-
ARFGEF1	ENSOARG0(	ADP-ribo	27a-3p,miR-2300a-5p,miR-409b,miR-151a-3p,miR-2285e,miR-

ITGA6	ENSOARG0(	integrin	miR-758-3p,miR-2310,miR-8485,miR-378d,miR-676-3p,miR-23a-3p,miR-127-3p,miR-101b-3p,miR-381-5p,miR-487b-5p,miR-29b-1-5p,miR-320b,miR-199a-3p,miR-2285e,miR-27a-3p,miR-222-5p,novel_4,miR-105-1,miR-330-3p,miR-2300a-5p,miR-3120-5p,miR-450b-5p,miR-378c,miR-1388-5p,miR-758-3p,miR-125b-2-3p,miR-143-5p,miR-378b,miR-154a,miR-1285-5p,miR-452-5p,miR-181d-5p,miR-2285f,miR-3956-3p,novel_60,miR-3958-3p,miR-4726-5p,miR-3968,miR-2440,miR-27a-3p,miR-454-5p,miR-2483-3p,miR-378e,miR-499a-3p,miR-7977,miR-211-5p,miR-374b-5p,miR-10b,miR-2319b,miR-1983,novel_94,miR-335-3p,miR-381-3p,miR-409-5p,miR-361-3p,miR-143-3p,miR-2432,miR-125b-2-3p,miR-377-5p,miR-29b-1-5p,miR-195a-3p,miR-329a-5p,miR-216c-5p,miR-3653-3p,miR-320d,miR-202-5p,miR-92a-1-5p,miR-422a,miR-324-3p,miR-210-5p,miR-196a-3p,miR-199a-3p,miR-616-3p,miR-1247-5p,miR-148b-3p,miR-23c,miR-200b,miR-222-5p,miR-23b-3p,miR-7859,miR-548e-3p,miR-378d,miR-142-3p,miR-184-3p,miR-19b-3p,miR-4429,miR-140-3p,miR-378a-3p,miR-2904,miR-378i,miR-30c-5p,miR-6134,miR-6525,miR-145a-3p,miR-4510,miR-3074-5p,miR-378c,miR-590-3p,miR-208b-5p,miR-487b-5p,miR-665,miR-664a-5p,miR-6130,miR-216b-5p,miR-193b-3p,miR-376e-3p,miR-6516,miR-377-3p,miR-499b-3p,miR-296-3p,miR-532-5p,miR-552-3p,miR-216b-3p,miR-21-3p,miR-370-5p,miR-545-3p,miR-32-5p,miR-378a-3p,miR-1827,miR-625-5p,miR-411,miR-487b-5p,miR-30d-5p,miR-27b-5p,miR-2285c,miR-296-3p,miR-432-3p,miR-2285r,miR-6240,miR-193a-3p,miR-136-3p,novel_51,miR-32,miR-105-5p,miR-330-3p,miR-296-3p,miR-377-3p,miR-149-5p,miR-2285g,miR-377-5p,miR-487b-3p,miR-188-5p,miR-27b-3p,miR-1260b,miR-381-5p,novel_23,miR-2113,miR-2285m,miR-211,novel_101,miR-495-3p,miR-1193,miR-193b-3p,miR-136-3p,miR-147-5p,miR-6119-3p,novel_87,miR-

KIAA1841	ENSOARG0(	KIAA1841	miR-3529-3p,miR-30a-3p,miR-380-5p,miR-1957a,miR-200c,miR-217,miR-200c-3p,miR-181b-5p,miR-7641,miR-483-3p,miR-378h,miR-582-3p,miR-545-5p,miR-345-3p,miR-134,miR-200b-3p,miR-105-2,let-7a-2-3p,miR-1306-5p,miR-6128,miR-103b,miR-500b-5p,miR-374a-3p,miR-145-3p,miR-378c,miR-5100,miR-331-3p,miR-3969,miR-31-3p,miR-410-3p,miR-374b-3p,miR-141-3p,miR-769,miR-2448-3p,miR-362-5p,miR-320a,miR-10b-3p,let-7g-3p,novel_48,miR-130b-5p,miR-101-5p,miR-539-3p,miR-345-5p,miR-224-5p,miR-330-3p,miR-125a-5p,miR-133b-5p,miR-665,miR-26b-3p,miR-2284d,miR-2355-3p,miR-653,miR-181a-5p,miR-2284ab,miR-219b-3p,novel_101,miR-495-3p,miR-323c,miR-3959-3p,miR-24-3p,miR-502-5p,miR-6402,miR-656-5p,miR-147-5p,miR-7-1-3p,miR-1291,miR-6516-3p,miR-216c-5p,miR-146a-5p,miR-196a-3p,miR-125b,miR-320d,miR-299-3p,miR-548e-3p,miR-3600,miR-485-3p,miR-487a-3p,miR-383-5p,miR-200b,miR-340-5p,miR-148b-3p,miR-151-5p,miR-199a-3p,miR-532-3p,miR-1246,miR-1197-5p,miR-5703,miR-188-3p,miR-4429,miR-378c,miR-539-5p,miR-2330-3p,miR-6516-3p,miR-487a-5p,miR-378a-3p,miR-21-3p,miR-182-5p,miR-532-5p,miR-27a-5p,miR-544-3p,miR-204-3p,novel_25,miR-27b-5p,let-7g-3p,miR-382-3p,miR-1839-3p,let-7c-3p,miR-376b,miR-758-3p,miR-320b,miR-3955-3p,miR-105-1,novel_4,miR-500-5p,miR-9788-3p,miR-216a-3p,miR-296-5p,miR-378b,miR-484,miR-125b-2-3p,miR-378c,miR-101a-5p,miR-544a,miR-2284aa,miR-3059-5p,novel_60,miR-181d-5p,miR-362-5p,miR-382-3p,miR-769-5p,miR-335,miR-450a-5p,miR-2285t,miR-374a-3p,miR-664b,miR-378e,miR-129b-5p,novel_94,miR-450b-5p,miR-148b-5p,miR-29a-5p,miR-2284y,novel_39,miR-450c-5p,miR-664-3p,miR-30f,miR-28-3p,miR-127-5p,miR-152-3p,miR-200a-3p,miR-299a-3p,miR-429-3p,miR-758-5p,miR-2284z,miR-134-
TXLNB	ENSOARG0(	taxilin	novel_51,miR-330-3p,miR-377-3p,miR-214-3p,let-7e,miR-153-3p,miR-130b-3p,miR-196b-5p,miR-3085-3p,miR-491-5p,miR-376b,miR-668-3p,miR-6240,miR-1839-3p,miR-101b-3p,miR-130b-5p,let-7a-5p,miR-2310,let-7a-2-3p,miR-22-3p,miR-197-3p,miR-10b,miR-296-5p,miR-16-1-3p,miR-380-3p,miR-130a-3p,miR-5010-3p,miR-330-3p,miR-455-3p,let-7g-5p,miR-1298-3p,miR-153,novel_87,let-7i,miR-769-5p,miR-2440,miR-2284q,miR-6119-3p,miR-1296-5p,miR-1839-3p,miR-2447,miR-877-3p,miR-211,miR-1306-3p,miR-3591-3p,miR-361-3p,miR-379-5p,let-7e-5p,miR-504,miR-10a-5p,miR-211-5p,miR-4792,miR-3064-5p,let-7d-5p,let-7c-5p,novel_17,miR-8117,miR-6119-5p,miR-2285af,miR-21-3p,novel_96,let-7k,miR-98-5p,let-7f-5p,miR-3064-5p,miR-380-3p,miR-143-3p,let-7g,miR-1957a,miR-130a-5p,miR-5703,miR-1434-5p,miR-1961,miR-215-5p,miR-196a-5p,miR-10a-5p,let-7a-2-3p,miR-16-1-3p,miR-142-3p,let-7d,miR-6517,miR-129-1-3p,miR-6516-5p,miR-383-5p,miR-3604,miR-1306-3p,let-7f,miR-204-5p,miR-130b-5p,miR-487a-5p,miR-208b-5p,miR-31-3p,novel_9,miR-214,miR-3074-2-3p,miR-129-2-3p,let-7b,miR-539-5p,let-7b-5p,miR-10b-5p,miR-769-5p,miR-6525,miR-200a-5p,miR-1814c,let-7g-3p,let-7g-3p,miR-215-5p,miR-21-3p,miR-504-5p,let-7i-5p,miR-204-3p,miR-22-3p,miR-769,miR-192-5p,miR-10a,miR-6516,miR-377-3p

SERPINI1	ENSOARG0(	serpin p	miR-365a-5p,miR-2284w,miR-3184-3p,miR-103b,miR-9-5p,miR-495-3p,miR-452-5p,miR-208b-5p,novel_82,miR-371a-5p,miR-382-5p,miR-544-3p,miR-154b-5p,miR-2355-5p,miR-296-3p,miR-625-5p,miR-544-5p,miR-3065-5p,miR-200a,miR-146a-5p,let-7j,miR-2424,miR-4286,miR-450a-2-3p,miR-200c-3p,miR-452-5p,miR-2484,miR-200c,miR-146a,miR-365b-5p,miR-505,miR-505-5p,miR-9-5p,miR-429-3p,miR-145b,miR-544a,novel_73,miR-21b,miR-16-2-3p,miR-873a-5p,miR-125b-2-3p,miR-145a-5p
TNXB	ENSOARG0(	tenascin	miR-371b-3p,miR-2428,miR-3607-3p,miR-29c-3p,miR-138-5p,miR-29a,miR-29b-3p,miR-331-3p,miR-1973,miR-29a-3p,miR-4443,novel_68,miR-30c-1-3p,miR-4792,miR-10b,miR-19a-3p,miR-30e-5p,miR-137-3p,miR-30d-5p,miR-30c-1-3p,miR-432-3p,novel_39,miR-127-3p,miR-363-5p,miR-2483-5p,miR-491-5p,miR-2898,miR-30d,novel_96,miR-30f,miR-30c-2-3p,miR-2331-5p,miR-149-5p,miR-3960,miR-138,miR-29b,miR-19b-3p,miR-218-1-3p,miR-29d-3p,miR-5703,miR-30a-5p,miR-1193
STAR	ENSOARG0(	steroido	miR-181b-1-3p,miR-664b-3p,miR-376a-5p,miR-380-3p,miR-2424,miR-486-3p,miR-2432,miR-2285w,miR-466i-5p,miR-202-5p,miR-34b-5p,miR-221-5p,miR-208b-3p,miR-2285p,miR-92a-1-5p,miR-487a-3p,miR-2284h-5p,miR-3600,miR-1b-3p,novel_73,miR-142-3p,miR-378d,miR-34a-5p,miR-218-1-3p,miR-362-3p,miR-1246,miR-140-3p,miR-1197-5p,miR-346,miR-10b-5p,miR-338-3p,miR-539-5p,miR-3074-2-3p,miR-214,miR-17-3p,miR-664a,miR-320e,miR-10a,miR-582-5p,miR-877-5p,novel_32,miR-660-5p,miR-140-5p,miR-221-5p,miR-32-5p,miR-2331-3p,miR-345-5p,miR-339b,let-7g-3p,miR-1895,miR-2285c,miR-3613-5p,miR-129b-3p,miR-2310,miR-3955-3p,miR-214-3p,miR-362-3p,miR-147a,miR-2285e,miR-500-5p,miR-409b,miR-199b-3p,miR-181b-2-3p,miR-222-5p,miR-362-3p,miR-3120-5p,miR-1839-5p,miR-10b,miR-1298-5p,miR-329-3p,miR-7857,miR-362-5p,miR-194b-5p,miR-9-5p,miR-490-5p,miR-2440,novel_82,miR-371a-5p,miR-2285y,miR-25,miR-4443,miR-454-5p,miR-203-3p,miR-452-3p,miR-211-5p,miR-499b-5p,miR-493-5p,miR-361-3p,miR-140-5p,miR-129b-5p,miR-148b-5p,miR-92b-3p,miR-29a-5p,novel_39,miR-1957a,miR-2284f,miR-127-5p,miR-17-3p,miR-2898,miR-92a-3p,miR-125a-3p,miR-2285af,miR-1260a,miR-339a,miR-3071-3p,miR-9-5p,miR-582-3p,miR-2285ad,novel_79,miR-411-3p,miR-10a-5p,let-7a-2-3p,miR-1290,miR-29d-3p,miR-1260b,miR-500b-5p,miR-193a-5p,miR-6128,miR-486b-3p,miR-204-5p,miR-5100,miR-29b-3p,miR-2284k,miR-135a-5p,miR-100-3p,miR-331-3p,miR-25-3p,miR-365a-3p,miR-34c-5p,miR-331-5p,miR-2284n,miR-223-5p,miR-2355-5p,miR-345-5p,miR-329a,let-7g-3p,miR-876-3p,miR-1814c,miR-7144-5p,miR-362-5p,miR-200a-5p,miR-345-5p,let-7a-2-3p,miR-6412,miR-296-3p,miR-493-5p,miR-378a-5p,miR-660,miR-452-5p,miR-296-3p,miR-455-5p,miR-194a,novel_51,miR-32,miR-2285g,miR-432-

ACCSL	ENSOARG0(	1-aminoc	novel_69,miR-320c,miR-144-3p,miR-18a-3p,miR-150-5p,miR-19a-3p,miR-6395,miR-146b-5p,novel_120,miR-1839-3p,miR-1307-3p,miR-211,miR-2285m,miR-6402,miR-142b,miR-133a-5p,miR-576-3p,novel_1,miR-147-5p,miR-22-5p,miR-656-5p,miR-31-5p,miR-187-3p,miR-26b-3p,miR-185-5p,miR-432-5p,miR-2284d,miR-1285,miR-542-3p,miR-7b-5p,miR-1434-3p,miR-432,miR-708-3p,miR-19b-2-5p,miR-653,novel_23,miR-22-3p,miR-197-3p,miR-1260b,miR-2284ab,miR-4508,miR-101-5p,let-7j,miR-146a,miR-7-5p,miR-466f-3p,miR-574-5p,miR-1271,miR-370-3p,miR-3965,miR-2284n,miR-320a,miR-671-5p,miR-485-5p,miR-7144-5p,miR-3074-1-3p,miR-190a-3p,miR-2284v,miR-1260b,miR-2284k,miR-2433,miR-34c-3p,miR-144,miR-204-5p,miR-134-5p,miR-410-3p,miR-429-3p,miR-2285ad,novel_121,miR-134-5p,miR-2284z,miR-134,miR-200b-3p,miR-3529-3p,miR-4286,miR-192-3p,miR-3065-3p,miR-200c,miR-1260a,miR-205-5p,miR-30f,miR-200c-3p,miR-450a-2-3p,novel_127,miR-20a-3p,miR-211-5p,miR-499b-5p,miR-2483-3p,novel_94,miR-541-3p,miR-4454,miR-3059-5p,miR-2284j,miR-2285n,miR-2285y,miR-628-3p,miR-151a-3p,miR-1343-3p,miR-541,miR-484,miR-758-3p,miR-7857,miR-450b-5p,miR-2284aa,miR-2330-5p,miR-1839-3p,miR-494-3p,miR-101b-3p,miR-652-5p,miR-33a-5p,miR-23a-3p,miR-758-3p,miR-8485,miR-320b,novel_124,miR-146b,miR-3065-3p,miR-22-3p,novel_25,miR-21a-3p,miR-539-5p,miR-6525,miR-338-5p,miR-151-3p,miR-19b-1-5p,miR-6516-3p,miR-208b-5p,miR-548e-3p,miR-18a-3p,miR-200b,miR-23c,miR-23b-3p,miR-7859,miR-328-3p,miR-2284h-5p,miR-2366,novel_133,miR-1197-5p,miR-19b-3p,miR-4429,miR-361-5p,miR-378d,miR-2285w,miR-216c-5p,miR-143-3p,miR-146a-5p,miR-2432,miR-2285p,miR-92a-1-5p,miR-500a-5p,miR-2284m,miR-320d,miR-202-5p,miR-30b-3p,miR-3653-3p,miR-1277-5p
NMBR	ENSOARG0(	neuromed	miR-3959-3p,miR-363-3p,miR-539-5p,miR-487b-5p,miR-92a-3p,miR-4324,miR-33a-3p,novel_87,miR-655-3p,miR-125b-5p,miR-19b-1-5p,miR-371a-5p,miR-365a-3p,miR-25-3p,miR-146b-3p,novel_25,miR-32-5p,novel_68,miR-125a,miR-6395,miR-2448-3p,miR-381-3p,miR-488-3p,miR-378b,miR-655,miR-150-5p,miR-380-5p,miR-92b-3p,miR-376a-5p,miR-148b-5p,miR-143-3p,miR-3187-3p,miR-539-3p,miR-380-5p,miR-369-3p,miR-195a-3p,miR-196b-5p,miR-92a-3p,miR-466f-3p,miR-125a-5p,miR-374a-5p,miR-125b,miR-32,miR-500a-5p,miR-758-5p,miR-421,miR-328-3p,miR-532-3p,miR-485-3p,miR-26b-3p,miR-3600,miR-219a-1-3p,miR-16-1-3p,miR-16-1-3p,miR-196a-5p,miR-19b-2-5p,miR-1193,miR-188-3p,miR-1434-5p,miR-218-2-3p

PEX13	ENSOARGO(	peroxiso	miR-376a-2-5p,miR-195a-5p,miR-2319a,miR-2483-3p,miR-2319b,miR-139-5p,novel_1,miR-2285t,miR-15a,miR-382-3p,miR-382-5p,miR-33a-3p,miR-485-3p,miR-16a,miR-2285y,miR-2428,miR-2284w,miR-1193,miR-380-3p,miR-16b,miR-497-5p,miR-1434-3p,miR-653,miR-323b,miR-412-5p,novel_116,miR-26b-3p,miR-491-5p,miR-199a-3p,miR-16b,miR-323b-3p,miR-1b-5p,miR-424-5p,miR-130b-5p,let-7c-3p,miR-582-5p,miR-200a-5p,miR-139-5p,miR-582-5p,miR-15b-5p,miR-370-5p,miR-664a,miR-130b-5p,miR-424-5p,miR-370-5p,miR-338-5p,miR-6525,miR-590-3p,miR-16-5p,miR-200b-3p,miR-15a-5p,miR-200b,miR-222-5p,miR-322-5p,miR-2284z,miR-199a-3p,miR-429-3p,miR-548e-3p,miR-2285ad,miR-376a-5p,miR-485-3p,miR-200c-3p,novel_96,miR-200c,miR-125b-2-3p,miR-380-3p,miR-2385-3p
GJE1	ENSOARGO(	gap junc	miR-767-5p,miR-2285t,miR-2284q,miR-34c-3p,miR-3969,miR-378c,miR-1285-5p,miR-7705,miR-378c,miR-767,miR-329b,miR-107,miR-9851-3p,miR-378f,miR-216a-3p,miR-378e,miR-377-3p,miR-365a-3p,miR-27a-3p,miR-454-5p,miR-378a-3p,miR-378b,miR-329-5p,miR-216b-3p,miR-20a-3p,miR-107,miR-2285af,miR-377-3p,miR-422a,miR-378d,miR-329a-5p,miR-378c,miR-128-3p,miR-1839-5p,miR-27b-3p,miR-378i,miR-873a-5p,miR-378b,novel_23,miR-3071-5p,miR-378a-3p,miR-378h,miR-545-5p,miR-103,miR-103a-3p,miR-27a-3p
GPIHBP1	ENSOARGO(	glycosyl	miR-1343-5p,miR-450a-2-3p,miR-2285v,miR-182-5p,miR-582,miR-3187-3p,novel_39,miR-486-3p,miR-490-3p,miR-432-3p,miR-4286,miR-582-3p,miR-1343-5p,miR-145a-5p,miR-2300b-3p,miR-1843b-5p,miR-541,miR-151-5p,miR-147a,miR-145b,miR-31-5p,miR-486b-3p,novel_82,miR-2440,miR-6527,miR-541-3p,miR-329b,miR-129b-5p,miR-151b,miR-3082-5p,miR-3956,miR-182-5p

LY6H	ENSOARG0(	lymphocy	miR-4492,miR-190a-5p,miR-665,miR-2285j,miR-122-5p,miR-3958-5p,miR-1895,miR-339b,miR-129b-3p,miR-151b,miR-15b-5p,miR-10a,miR-216b-3p,miR-30c-1-3p,miR-504-5p,miR-3653-3p,miR-99a-3p,miR-219b-5p,miR-324-3p,miR-422a,novel_17,miR-210-5p,miR-486-3p,miR-377-5p,miR-2285w,miR-493-3p,miR-184-3p,miR-378a-3p,miR-378i,miR-2284h-5p,miR-328-3p,miR-499a-5p,miR-3968,novel_82,miR-361-3p,miR-6527,miR-190b-5p,miR-544-5p,miR-1983,miR-504,miR-2285u,miR-211-5p,miR-4792,miR-146b,miR-199b-5p,miR-491-5p,miR-505,miR-16b,miR-8485,miR-378d,miR-1388-3p,miR-101c,miR-1839-5p,miR-362-3p,miR-1388-5p,miR-329-3p,miR-541,miR-2459,novel_78,miR-331-5p,miR-100-3p,miR-125b-5p,miR-6740-5p,miR-1260b,miR-16-5p,miR-329b,miR-329a,miR-331-5p,miR-99a-3p,miR-125a,miR-574-5p,miR-874-3p,novel_96,miR-127-5p,miR-664-3p,novel_39,miR-148b-5p,miR-450b-5p,miR-3064-5p,miR-1343-5p,miR-23b-5p,miR-505-5p,miR-322-5p,novel_79,miR-299a-3p,miR-429-3p,miR-6123,miR-199b-5p,miR-193b-3p,miR-146b-5p,miR-378f,miR-410-5p,miR-412-3p,miR-1306,miR-3970,miR-150-5p,miR-216a-3p,miR-374c-3p,miR-299b-3p,miR-193a-3p,miR-378a-5p,miR-105-5p,novel_51,miR-146a,miR-2285r,miR-668-3p,miR-497-5p,miR-16b,miR-432,miR-542-3p,miR-1843b-5p,miR-194-3p,miR-149-5p,miR-3535,miR-3431,miR-339-5p,miR-340-3p,miR-31-5p,miR-708-5p,miR-424-5p,miR-664a,miR-324-3p,miR-132-5p,miR-320e,miR-10b-5p,miR-2330-3p,miR-378c,novel_99,miR-2312,miR-323-5p,miR-193b-3p,miR-3065-3p,miR-370-5p,miR-532-5p,miR-221-5p,miR-378a-3p,miR-221-5p,miR-299-3p,miR-466i-5p,miR-125b,miR-2411,miR-146a-5p,miR-216c-5p,miR-378d,miR-2904,miR-5703,novel_133,miR-2300b-3p,miR-362-3p,miR-532-3p,novel_83,miR-151-5p,miR-15a-5p,miR-200b,miR-1b-3p,miR-4726-5p,miR-382-3p,miR-
NDEL1	ENSOARG0(	nudE neu	miR-5126,miR-432,miR-25-5p,miR-491-5p,miR-486b-3p,novel_96,miR-432-5p,miR-4443,miR-423-5p,novel_39,miR-486-3p,miR-3184-5p,miR-669

ATP6AP2	ENSOARG0(	ATPase,	miR-3184-3p,miR-425-5p,miR-133a-3p,miR-3074-2-3p,miR-103b,miR-374a-3p,miR-10b-5p,miR-145-3p,miR-135a-1-3p,miR-5100,miR-15b-3p,novel_63,miR-3604,miR-219b-3p,miR-487b-5p,miR-138-5p,miR-130b-5p,miR-374b-3p,miR-410-3p,miR-216b-3p,miR-10a,miR-376e-3p,miR-377-3p,miR-6516,miR-499b-3p,miR-376c-3p,miR-299,miR-376c-5p,miR-3154,miR-345-5p,miR-345-5p,miR-133b-3p,novel_99,miR-1248,miR-625-5p,miR-376c-5p,miR-23a-5p,miR-376c-3p,miR-125b-2-3p,miR-2284y,miR-219-3p,miR-210-5p,miR-205-5p,miR-217,miR-219b-5p,miR-216a-5p,miR-127-5p,miR-181b-5p,miR-152-3p,miR-107,miR-2285ad,miR-148b-3p,miR-6516-5p,novel_121,miR-2284z,miR-1290,miR-376a-5p,miR-126-3p,miR-217-5p,miR-218-2-3p,miR-582-3p,miR-2284u,miR-10a-5p,miR-136-3p,miR-342-3p,miR-135a-2-3p,miR-181d-5p,miR-219a-2-3p,miR-2428,miR-6402,novel_87,miR-33a-3p,miR-371a-5p,miR-2285y,novel_1,miR-6119-3p,miR-147-5p,miR-374a-3p,miR-2284g,miR-10a-5p,miR-670-3p,miR-411b-3p,miR-499a-3p,miR-6535,miR-148a-3p,miR-376b-5p,miR-107,miR-299b-5p,miR-1291,novel_94,miR-1388-3p,miR-130b-5p,miR-668-3p,miR-101-5p,miR-345-5p,miR-377-3p,miR-342,miR-136-3p,miR-153-3p,miR-548w,miR-103a-3p,miR-153,miR-126a-5p,miR-103,miR-194-3p,miR-10b,miR-181a-5p,miR-143-5p,miR-125b-2-3p,novel_23,miR-101a-5p,miR-299a-5p,miR-2426,miR-138,miR-21b,miR-2284aa

CYP26C1	ENSOARG0(	cytochro	miR-5010-3p,miR-27a-3p,miR-145b,novel_4,miR-330-3p,miR-1343-3p,miR-500-5p,miR-9788-3p,miR-7857,miR-2284r,miR-296-5p,miR-541,miR-10b,miR-1298-5p,miR-301b-5p,miR-21b,miR-544a,miR-127-3p,miR-2330-5p,miR-505,miR-2957,miR-1306-5p,miR-431-5p,miR-214-3p,miR-381-5p,miR-491-5p,miR-3085-3p,miR-7689-3p,miR-7977,miR-4792,miR-139-5p,miR-3064-5p,miR-2319b,miR-452-3p,miR-652-3p,miR-27a-3p,miR-4443,miR-3596,miR-378g,miR-3082-5p,miR-489,miR-361-3p,novel_94,miR-1983,miR-2285f,miR-2447,miR-541-3p,miR-219a-2-3p,miR-362-5p,miR-2285y,miR-301a-5p,novel_82,miR-30b-5p,miR-3968,miR-335,miR-1b-3p,miR-18a-3p,miR-548e-3p,miR-2284h-5p,miR-7859,miR-1197-5p,miR-30c-5p,miR-2366,miR-193b-5p,miR-664-5p,miR-133a-3p,miR-941,miR-142-3p,miR-345-3p,miR-125b-2-3p,miR-377-5p,miR-324-3p,miR-125b,miR-345-3p,miR-92a-1-5p,miR-30b,miR-210-5p,miR-30d,miR-202-5p,miR-760-3p,miR-296-3p,miR-2331-3p,miR-22-3p,novel_32,miR-204-3p,miR-544-3p,miR-10a,miR-2284s,miR-139-5p,miR-6130,miR-129b-3p,miR-30d-5p,miR-450a-1-3p,miR-625-5p,miR-2312,miR-339b,miR-30f,miR-345-5p,miR-3074-2-3p,miR-214,miR-4510,miR-10b-5p,miR-224-5p,miR-219b-3p,miR-324-3p,miR-664a-5p,miR-665,miR-874-5p,miR-130b-5p,miR-450b-3p,miR-664a,miR-339-5p,miR-665,miR-377-5p,miR-149-5p,miR-544b,miR-432-5p,miR-185-5p,miR-134-3p,miR-3431,miR-450c-3p,miR-22-3p,miR-374b-3p,novel_23,miR-145a-5p,miR-432,miR-873a-5p,miR-1843b-5p,miR-1285,miR-542-3p,miR-223-3p,miR-1434-3p,miR-4508,miR-26a-2-3p,miR-744-5p,miR-2889,miR-27b-3p,miR-138,miR-1260b,miR-381-5p,miR-363-5p,miR-130b-5p,miR-6240,miR-296-3p,miR-345-5p,miR-2285r,miR-432-3p,miR-330-3p,miR-2285v,novel_51,miR-2411-3p,miR-224-5p,miR-296-3p,miR-125a-5p,miR-483-3p,miR-196b-5p,miR-378a-5p,miR-

MDM1	ENSOARG0(	Mdm1 nuc	miR-101b-3p,miR-382-3p,miR-1388-3p,miR-652-5p,miR-33a-5p,miR-23a-3p,miR-326,miR-1306-5p,miR-199a-3p,miR-505,miR-3955-3p,miR-130b-3p,miR-214-3p,miR-199b-3p,novel_78,miR-151a-3p,miR-1343-3p,miR-222-5p,miR-181b-2-3p,miR-27a-3p,miR-1298-3p,miR-142-3p,miR-500-5p,miR-301b-5p,miR-484,miR-296-5p,miR-30b-3p,miR-3962,miR-1a-3p,miR-1388-5p,miR-21b,miR-2284aa,miR-32-3p,novel_111,miR-361-3p,miR-362-5p,miR-218-5p,miR-301a-5p,miR-2285t,miR-20a-3p,miR-139-5p,miR-664b,miR-7977,miR-489,miR-3082-5p,miR-25,miR-7857-3p,miR-27a-3p,miR-203-3p,miR-129b-5p,miR-381-3p,miR-136-5p,miR-345-3p,miR-195a-3p,miR-181b-1-3p,miR-376c-3p,miR-142a-3p,miR-130a-5p,miR-376a-5p,miR-380-3p,miR-143-3p,miR-2424,miR-125b,miR-122-3p,miR-500a-5p,miR-760-3p,miR-2284m,miR-34b-5p,miR-30d-3p,miR-3600,miR-222-5p,miR-148b-3p,miR-23b-3p,miR-532-3p,miR-199a-3p,miR-1246,miR-19b-3p,miR-34a-5p,miR-539-5p,miR-590-3p,miR-3074-2-3p,miR-214,miR-206-3p,miR-346,miR-6525,miR-338-3p,miR-30e-3p,miR-2285j,miR-29b-2-5p,miR-151-3p,miR-19b-1-5p,miR-30a-3p,miR-6516-3p,miR-17-3p,miR-664a,miR-450b-3p,miR-130b-5p,miR-454-3p,miR-500,miR-874-5p,miR-1468-5p,miR-32-3p,miR-2331-3p,miR-139-5p,miR-27a-5p,miR-301a-3p,miR-582-5p,miR-450a-1-3p,miR-21a-3p,miR-433-3p,miR-129b-3p,miR-500a-3p,miR-345-5p,miR-582-5p,miR-186-5p,miR-380-5p,miR-130b-5p,miR-345-5p,miR-466f-3p,miR-125a-5p,miR-452-5p,miR-122-3p,miR-31-5p,miR-26b-3p,miR-665,miR-130a-3p,miR-185-5p,miR-2284d,miR-411-5p,novel_116,miR-19b-2-5p,miR-374b-3p,miR-3071-5p,miR-450c-3p,miR-30b-3p,miR-380-3p,miR-2284ab,miR-7862,miR-27b-3p,novel_120,miR-2285x,miR-17-3p,miR-495-3p,miR-24-3p,miR-301b-3p,miR-2428,miR-2284w,miR-449a,miR-326-3p,miR-4324,miR-382-5p,miR-34c,miR-206,miR-502b,miR-

CHI3L1	ENSOARG0(	chitinas	miR-7977,miR-452-3p,miR-3064-5p,miR-489,miR-361-3p,novel_94,miR-181d-5p,miR-107-5p,miR-371a-5p,miR-769-5p,miR-30b-5p,miR-2284q,miR-2285t,miR-5010-3p,novel_4,miR-2300a-5p,miR-9788-3p,miR-216a-3p,miR-3962,miR-30b-3p,miR-6529a,miR-484,miR-3120-5p,miR-2284aa,miR-450b-5p,miR-2426,miR-2330-5p,miR-382-3p,miR-2957,miR-326,miR-3955-3p,miR-3085-3p,novel_124,miR-3065-3p,miR-545-3p,miR-155-5p,miR-22-3p,novel_32,novel_25,miR-193b-3p,miR-323-5p,miR-6130,novel_99,miR-30f,miR-3074-2-3p,miR-145a-3p,miR-4510,miR-6134,miR-320e,miR-132-5p,miR-708-5p,miR-6516-3p,miR-155-5p,miR-664a,miR-450b-3p,novel_83,miR-532-3p,miR-616-3p,miR-1197-5p,miR-140-3p,miR-188-3p,miR-2904,miR-2300b-3p,miR-378d,miR-2887,miR-216c-5p,miR-183-5p,miR-125b-2-3p,miR-185-3p,miR-125b,miR-21-3p,miR-30b-3p,miR-30d,miR-34b-5p,miR-760-3p,miR-320b,miR-670-3p,miR-34b,miR-29a-3p,miR-323a-5p,miR-2404,miR-18a-3p,miR-2387,miR-6395,miR-7975,miR-450b-3p,novel_101,miR-2428,miR-326-3p,miR-449a,miR-24-3p,miR-34c,miR-6529b,miR-6402,miR-133b-5p,miR-187-3p,miR-503-5p,miR-185-5p,miR-432-5p,miR-1434-3p,miR-181a-5p,miR-873a-5p,miR-744-5p,miR-2889,miR-6239,miR-7862,miR-125b-1-3p,miR-29b,miR-30b-3p,miR-363-5p,miR-345-5p,miR-224-5p,miR-503-5p,miR-125a-5p,miR-452-5p,miR-1343-5p,miR-370-3p,miR-154b-5p,miR-365a-3p,miR-769,miR-34c-5p,miR-3154,miR-381-3p,miR-485-5p,miR-7144-5p,miR-10b-3p,miR-331-3p,miR-29b-3p,miR-410-5p,miR-28-5p,miR-214-5p,miR-582-3p,miR-30a-5p,miR-29d-3p,miR-3578,miR-128-3p,miR-300,miR-4286,miR-30a-3p,miR-490-3p,miR-1271-3p,miR-3065-3p,miR-6119-5p,miR-543-5p,miR-181b-5p,miR-2898,miR-4792,miR-4443,miR-203-3p,miR-378g,miR-25,miR-378j,miR-409-5p,miR-504,miR-6527,miR-7705,novel_111,miR-361-
PSMG4	ENSOARG0(	proteaso	miR-4510,miR-129-2-3p,miR-6130,novel_39,miR-129-1-3p,miR-873a-5p,miR-542-3p,miR-2889
NAPG	ENSOARG0(	N-ethylm	miR-615,miR-615-3p,miR-1197-3p

TINAGL1	ENSOARG0(	tubuloin	novel_17,miR-185-3p,miR-125b,miR-122-3p,miR-92a-1-5p,miR-422a,miR-324-3p,miR-760-3p,miR-466i-5p,miR-21-3p,miR-221-5p,miR-30b-3p,miR-10a-3p,miR-345-3p,miR-377-5p,miR-181b-1-3p,miR-2432,miR-193b-5p,miR-1246,miR-2366,novel_133,miR-378i,miR-378a-3p,miR-5703,miR-188-3p,miR-2904,miR-19b-3p,miR-361-5p,miR-378d,miR-133a-3p,miR-18a-3p,miR-212-5p,miR-132-5p,miR-6516-3p,miR-17-3p,miR-487b-5p,miR-450b-3p,miR-874-5p,miR-4492,miR-708-5p,miR-378c,miR-6525,miR-769-5p,miR-338-3p,miR-6134,miR-3184-5p,miR-450a-1-3p,miR-21a-3p,miR-345-5p,novel_99,miR-186-5p,miR-1827,miR-1895,miR-885-3p,miR-504-5p,miR-140-5p,miR-221-5p,miR-378a-3p,miR-296-3p,miR-3065-3p,miR-370-5p,miR-216b-3p,miR-544-3p,miR-2284s,miR-22-3p,novel_25,miR-204-3p,miR-1306-5p,miR-5126,miR-326,miR-323b-3p,miR-487b-5p,miR-25-5p,miR-3085-3p,miR-199b-5p,miR-33a-5p,miR-676-3p,miR-378d,miR-484,miR-378b,miR-30b-3p,miR-7857,miR-323b,miR-544a,miR-378c,miR-3120-5p,miR-16-2-3p,miR-105-1,miR-409b,novel_78,miR-181b-2-3p,miR-455-3p,miR-9788-3p,miR-769-5p,miR-490-5p,miR-2447,miR-423-5p,miR-199b-5p,miR-140-5p,miR-361-3p,miR-378j,miR-504,miR-9-3p,miR-452-3p,miR-3064-5p,miR-211-5p,miR-7689-3p,miR-25,miR-378e,miR-378g,miR-4443,miR-125a-3p,miR-1260a,miR-450a-2-3p,miR-17-3p,miR-2898,novel_127,miR-3064-5p,miR-192-3p,miR-3065-3p,miR-199a-5p,miR-134,miR-330-5p,miR-1343-5p,miR-885-3p,miR-105-2,miR-6517,miR-2285ad,miR-378h,miR-505-5p,miR-450a-1-3p,miR-134-5p,miR-370-5p,miR-2433,miR-3969,miR-331-3p,miR-125b-5p,miR-6740-5p,miR-138-5p,miR-204-5p,miR-134-5p,miR-28-5p,miR-31-3p,novel_42,miR-103b,miR-1260b,miR-1197-3p,miR-378c,miR-488-3p,miR-345-5p,miR-876-3p,miR-10a-3p,miR-671-5p,miR-7144-5p,miR-3074-1-3p,miR-615-

ACVR2B	ENSOARG0(	activin	miR-365a-3p,miR-199a-5p,miR-574-5p,miR-1271,miR-3956,miR-370-3p,miR-2285aa,miR-671-5p,miR-331-5p,miR-301,miR-320a,miR-374b,miR-16-5p,miR-331-5p,miR-486b-3p,miR-592,miR-5100,miR-409-3p,miR-144,miR-322-5p,novel_121,miR-2284z,miR-545-5p,miR-222,miR-411-3p,miR-128-3p,miR-345-3p,miR-330-5p,miR-501-3p,miR-23b-5p,miR-7134-5p,miR-450c-5p,miR-199a-5p,miR-29a-5p,miR-3065-3p,miR-181b-5p,miR-107,miR-8117,miR-2285af,miR-670-3p,miR-323a-5p,miR-181b-3p,miR-144-3p,miR-320c,miR-6535,miR-677,miR-107,miR-195a-5p,miR-216a-3p,miR-199b-5p,miR-2284w,miR-326-3p,novel_1,miR-379-3p,miR-199c,miR-133a-5p,miR-15a,miR-4324,miR-16a,miR-103,miR-103a-3p,miR-125b-1-3p,miR-16b,miR-497-5p,miR-181a-5p,miR-194-5p,miR-145a-5p,miR-197-3p,let-7j,miR-6240,miR-628-5p,miR-2483-5p,miR-452-5p,miR-194a,novel_51,miR-2411-3p,miR-330-3p,miR-197-5p,miR-323-5p,miR-15b-5p,miR-532-5p,miR-3065-3p,miR-543-3p,miR-625-5p,miR-1895,miR-21a-3p,miR-411,miR-500a-3p,miR-129b-3p,miR-181c-5p,miR-6134,miR-3184-5p,miR-665-5p,miR-424-5p,miR-500,miR-222-3p,miR-665,miR-15a-5p,miR-7859,miR-548e-3p,miR-212-5p,miR-4429,miR-181b-1-3p,miR-29b-1-5p,miR-486-3p,miR-345-3p,miR-466i-5p,miR-320d,miR-21-3p,miR-1277-5p,miR-3653-3p,miR-374a-5p,miR-345-3p,miR-3596,miR-500-3p,miR-652-3p,miR-20a-3p,miR-374b-5p,miR-499b-5p,miR-199b-5p,miR-409-5p,miR-221,miR-877-3p,miR-361-3p,miR-502-3p,miR-423-5p,miR-212-3p,miR-3059-5p,miR-181d-5p,miR-194b-5p,miR-450a-5p,miR-4726-5p,miR-221-3p,miR-455-3p,miR-216a-3p,miR-199b-3p,miR-1343-3p,miR-2300a-5p,miR-330-3p,miR-145b,miR-181b-2-3p,miR-5010-3p,miR-450b-5p,miR-2284aa,miR-1839-5p,miR-6529a,miR-125b-2-3p,miR-501-3p,miR-2310,miR-3187-3p,miR-424-5p,miR-101b-3p,miR-382-3p,miR-3955-3p,novel_124,miR-199b-5p,miR-
YLPM1	ENSOARG0(	YLP moti	miR-146b,miR-146b-5p,miR-136-5p,miR-132-5p,miR-5100,miR-146a,miR-877-5p,miR-8485,miR-7859,miR-548e-3p,miR-1306-5p

HECTD3	ENSOARG0(	HECT dom	miR-6134,miR-145-3p,miR-16-5p,miR-4510,miR-28-5p,miR-486b-3p,miR-708-5p,miR-4492,miR-424-5p,miR-331-3p,miR-2332,miR-15b-5p,miR-199a-5p,miR-6130,miR-374c-3p,miR-874-3p,miR-221-5p,novel_99,miR-329b,miR-320a,miR-3154,miR-2285b,miR-490-3p,miR-486-3p,miR-199a-5p,miR-28b,miR-4286,miR-183-5p,novel_96,novel_127,miR-21-3p,miR-152-3p,miR-221-5p,miR-450a-2-3p,miR-345-3p,miR-185-3p,miR-324-3p,miR-26a-5p,miR-532-3p,miR-199a-3p,miR-15a-5p,miR-148b-3p,miR-505-5p,miR-322-5p,miR-302a-5p,miR-18a-3p,miR-378d,miR-4429,miR-5703,miR-1343-5p,miR-2904,miR-2366,miR-345-3p,miR-7705,miR-199b-5p,miR-2428,miR-3957-3p,miR-541-3p,miR-3059-5p,miR-342-3p,miR-490-5p,miR-3968,miR-16a,miR-28c,miR-15a,miR-6535,miR-374c-3p,miR-3082-5p,miR-2404,miR-378g,miR-26b-5p,miR-2319b,miR-9-3p,miR-195a-5p,miR-18a-3p,miR-148a-3p,miR-199b-5p,miR-3187-3p,miR-432-3p,miR-2284l,miR-432-3p,miR-33a-5p,miR-2483-5p,miR-382,miR-2330-5p,miR-424-5p,miR-2285o,miR-199b-5p,miR-1343-5p,miR-2285l,miR-3955-3p,miR-491-5p,miR-124-5p,miR-2411-3p,miR-16b,novel_115,miR-320b,miR-199a-3p,miR-342,miR-9788-3p,miR-216a-3p,miR-455-3p,miR-134-3p,miR-432-5p,miR-31-5p,miR-497-5p,miR-219a-1-3p,miR-30b-3p,miR-16b,miR-374b-3p,miR-30b-3p,miR-541,miR-1285,miR-143-5p,miR-223-3p,miR-432
REL	ENSOARG0(	v-rel av	miR-22-5p,miR-147-5p,miR-877-3p,miR-6128,novel_60,miR-3059-5p,miR-3956-3p,miR-7144-5p,miR-1248,novel_94,miR-582-5p,miR-1306,miR-378b,miR-23b,miR-2387,miR-203-3p,miR-223-5p,miR-582-5p,miR-3965,miR-541-5p,novel_68,miR-20a-3p,miR-543-5p,miR-181c-3p,miR-1306-5p,miR-210-5p,miR-2424,miR-2432,miR-654-3p,miR-23a-3p,let-7f-2-3p,let-7f-2-3p,miR-33a-5p,miR-26a-2-3p,miR-1193,miR-149-5p,miR-23b-3p,miR-7859,miR-1298-3p,miR-3431,miR-23c,miR-3600,miR-7641,miR-548e-3p
FAM45A	ENSOARG0(	family w	miR-30a-3p,miR-3074-2-3p,miR-1260b,miR-1260a,miR-30a-3p,miR-1260b,miR-142-3p,miR-16-1-3p,miR-487b-5p,miR-410-3p,miR-16-1-3p
KIAA0141	ENSOARG0(	KIAA0141	miR-1343-3p,miR-2300a-5p,miR-340-3p,miR-31-5p,miR-3431,miR-432-5p,miR-194-3p,miR-484,miR-432,miR-30b-3p,miR-3071-5p,miR-7857,miR-1260b,miR-30b-3p,miR-2284aa,miR-2330-5p,miR-146a,miR-2285v,miR-1185-5p,miR-124-5p,miR-146b,miR-139-5p,miR-3120-3p,miR-320b,miR-323a-5p,miR-203-3p,miR-2387,miR-18a-3p,miR-19a-3p,novel_94,miR-146b-5p,novel_120,miR-6527,miR-877-3p,miR-2284w,miR-2440,miR-502-5p,miR-382-5p,miR-4726-5p,miR-147-5p,miR-18a-3p,miR-2285ad,miR-6516-5p,miR-505-5p,miR-2284z,miR-5703,miR-2904,miR-19b-3p,miR-378d,miR-125b-2-3p,miR-2432,miR-146a-5p,miR-376c-5p,miR-376b-5p,miR-210-5p,novel_17,miR-1260a,novel_127,miR-2484,miR-548o-3p,miR-139-5p,miR-323-5p,miR-6516,miR-582-5p,miR-877-5p,miR-133b-3p,miR-1827,miR-582-5p,miR-671-5p,miR-7144-5p,miR-539-5p,miR-665-5p,miR-133a-3p,miR-193a-5p,miR-6128,novel_44,miR-1260b,miR-5100,miR-409-3p,miR-664a,miR-208b-5p,miR-31-3p

			[illegible] 3431,novel_23,miR-450c-3p,miR-22-3p,miR-7b-5p,miR-432,miR-27b-3p,miR-2284x,miR-4508,miR-497-5p,miR-3432b,miR-381-5p,miR-16b,miR-628-5p,miR-6240,miR-432-3p,miR-2285r,miR-432-3p,miR-2411-3p,novel_51,miR-3141,miR-2285v,miR-146a,miR-7-5p,miR-378a-5p,miR-483-3p,miR-3074-5p,novel_68,miR-320c,novel_69,miR-216a-3p,miR-3065-5p,miR-150-5p,miR-3141,miR-148a-3p,miR-2319a,miR-146b-5p,miR-199b-5p,miR-2284o,miR-28c,novel_79,miR-6517,miR-2285ad,miR-450a-1-3p,miR-322-5p,miR-9-5p,miR-330-5p,miR-2427,miR-885-3p,miR-2284f,miR-3064-5p,miR-3956-5p,miR-450c-5p,miR-2285b,miR-2284y,miR-200a,miR-148b-5p,miR-152-3p,novel_96,miR-200a-3p,miR-125a,miR-3956,miR-542-5p,miR-2355-5p,miR-331-5p,miR-345-5p,miR-2474,miR-16-5p,miR-2284v,novel_42,miR-1260b,miR-6740-5p,miR-125b-5p,miR-2284k,miR-2433,miR-331-5p,miR-330-3p,miR-1343-3p,miR-2459,miR-148b-5p,miR-1298-3p,miR-539-5p,miR-501-3p,miR-2284b,miR-127-3p,miR-16b,miR-199a-3p,miR-214-3p,miR-381-5p,miR-103a-2-5p,miR-146b,miR-199b-5p,miR-491-5p,miR-1185-5p,miR-2284g,miR-500-3p,miR-25,miR-378j,miR-335-3p,miR-136-5p,miR-96-5p,let-7i-3p,miR-9-5p,miR-2284j,miR-423-5p,miR-615,miR-101-3p,miR-3968,miR-499a-5p,miR-217-5p,miR-2366,miR-148a-5p,miR-2284u,miR-664-5p,miR-33b-3p,miR-184-3p,miR-503-5p,miR-486-3p,miR-377-5p,miR-92a-1-5p,miR-345-3p,miR-500a-5p,miR-324-3p,miR-2484,miR-202-5p,miR-296-3p,miR-30c-1-3p,miR-2331-3p,miR-15b-5p,miR-216b-5p,miR-500a-3p,miR-129b-3p,miR-450a-1-3p,miR-885-3p,miR-625-5p,miR-345-5p,miR-339b,miR-665-5p,miR-214,miR-3074-5p,miR-433-5p,miR-3184-5p,miR-122-5p,miR-3604,miR-665,miR-130b-
METAP1D	ENSOARG0(	methiony	5p,miR-500,miR-377-5p,miR-26b-3p,miR-503-5p,miR-185-

GATAD2B	ENSOARG0(	GATA zin	miR-216b-3p,miR-3065-3p,miR-30c-1-3p,miR-221-5p,miR-32-5p,miR-504-5p,miR-545-3p,novel_32,miR-27a-5p,miR-21a-3p,miR-2285c,miR-625-5p,miR-1827,novel_99,miR-339b,miR-214,miR-6134,miR-338-3p,miR-6525,miR-219b-3p,miR-2285j,miR-130b-5p,miR-669a-3p,miR-664a,miR-212-5p,miR-505-3p,miR-106a,miR-532-3p,miR-1247-5p,miR-200b,miR-1197-5p,miR-217-5p,miR-191,miR-2366,miR-2300b-3p,miR-664-5p,miR-361-5p,miR-34a-5p,miR-33b-3p,miR-20a,miR-183-5p,miR-486-3p,miR-2432,miR-216a-5p,miR-345-3p,miR-125b,miR-92a-1-5p,novel_17,miR-2285p,miR-34b-5p,miR-221-5p,miR-30b-3p,miR-1277-5p,miR-106a,miR-466i-5p,miR-760-3p,miR-541-5p,miR-7689-3p,miR-211-5p,miR-454-5p,miR-361-3p,miR-140-5p,miR-199b-5p,miR-3963,miR-668-5p,miR-504,miR-1296-5p,novel_60,miR-3059-5p,miR-363-3p,miR-3956-3p,miR-8095,miR-219a-2-3p,miR-361-3p,miR-877-3p,miR-371a-5p,miR-301a-5p,miR-4726-5p,novel_78,miR-2300a-5p,novel_4,miR-330-3p,miR-216a-3p,miR-9788-3p,miR-2285e,miR-30b-3p,miR-6529a,miR-484,miR-143-5p,miR-301b-5p,miR-1298-5p,miR-1839-5p,miR-676-3p,miR-106a-5p,miR-8485,miR-2310,miR-30c-1-3p,miR-106b-5p,miR-1306-5p,miR-199b-5p,miR-214-3p,miR-491-5p,miR-487b-5p,miR-2355-5p,miR-125a,miR-199a-5p,miR-34c-5p,miR-25-3p,miR-93-5p,miR-200a-5p,miR-17-5p,miR-424-3p,miR-23a-5p,miR-7144-5p,miR-1248,miR-1814c,miR-6128,miR-1306-5p,miR-145-3p,miR-93,miR-1197-3p,miR-1260b,miR-125b-5p,miR-34c-3p,miR-2433,miR-2332,miR-191-5p,miR-486b-3p,miR-204-5p,novel_79,miR-429-3p,miR-582-3p,miR-483-3p,miR-1343-5p,miR-345-3p,miR-200b-3p,miR-128-3p,miR-411-3p,miR-181a-2-3p,novel_39,miR-3065-3p,miR-199a-5p,miR-490-3p,miR-92b-3p,miR-30c-2-3p,miR-217,miR-339a,miR-1260a,miR-664-3p,miR-219-3p,miR-200c,novel_96,miR-92a-3p,miR-450a-2-
TPST2	ENSOARG0(	tyrosylp	miR-486b-3p,miR-345-3p,miR-2411-3p,miR-2428,miR-486-3p,novel_39,miR-432-3p,miR-432-3p,miR-101c,miR-219a-1-3p,miR-30b-3p,miR-5703,miR-30b-3p,miR-3154,miR-2366,miR-345-3p,miR-9788-3p

TES	ENSOARG0(	testin L	novel_121,miR-129-1-3p,miR-545-5p,miR-2284z,miR-200b,miR-212-5p,miR-429-3p,miR-105-2,miR-200b-3p,let-7d,miR-300,miR-142-3p,miR-19b-3p,miR-218-2-3p,miR-1961,let-7g,miR-29b-1-5p,miR-29b-2-5p,miR-369-3p,miR-195a-3p,miR-200a-3p,let-7k,let-7f-5p,miR-200c-3p,miR-98-5p,let-7c-5p,miR-324-3p,miR-200c,miR-2285p,miR-2285af,let-7i-5p,miR-301a-3p,miR-374c-3p,miR-141-3p,miR-370-3p,miR-186-5p,novel_99,miR-671-5p,miR-7144-5p,miR-3154,miR-362-5p,miR-655,miR-381-3p,let-7b-5p,miR-500b-5p,miR-425-5p,miR-129-2-3p,miR-145a-3p,let-7b,miR-454-3p,novel_74,miR-29b-2-5p,miR-655-3p,miR-324-3p,let-7f,miR-34c-3p,miR-1185-3p,miR-500-5p,let-7g-5p,miR-153,miR-26b-3p,miR-130a-3p,miR-199b-3p,miR-105-1,miR-7862,miR-2284x,miR-1839-5p,miR-2284aa,miR-2426,novel_23,miR-1843b-5p,miR-101b-3p,let-7a-5p,miR-103a-2-5p,let-7e,miR-153-3p,miR-130b-3p,miR-105-5p,miR-29b-1-5p,miR-1185-2-3p,miR-203-3p,let-7d-5p,miR-3596,novel_68,let-7e-5p,miR-19a-3p,miR-129b-5p,miR-381-3p,miR-154a,miR-301b-3p,novel_111,miR-371b-3p,miR-362-5p,miR-147-5p,miR-199c,miR-2284q,miR-22-5p,let-7i,miR-107-5p,miR-371a-5p,miR-1973
ADCK3	ENSOARG0(	aarF dom	miR-2285u,miR-182-5p,miR-669,miR-23b,miR-2285ab,miR-2285c,miR-543-3p,miR-1814c,miR-6395,miR-96-5p,miR-2312,miR-1271,miR-2285f,miR-190b-5p,miR-181d-5p,miR-541-3p,novel_27,miR-181c-5p,miR-2285n,miR-4324,miR-190a-5p,miR-3600,miR-2285e,miR-328-3p,miR-23b-3p,miR-432-5p,miR-2285g,miR-23c,miR-1271-5p,miR-432,miR-181a-5p,miR-541,miR-23a-3p,miR-382-3p,miR-8485,miR-143-3p,miR-2310,miR-2285r,miR-29b-1-5p,miR-190a,miR-29b-1-5p,miR-216a-5p,miR-182-5p,miR-2285af,miR-125a-3p,novel_17,miR-181b-5p
HIVEP2	ENSOARG0(	human im	novel_1,miR-3184-3p

STAT3	ENSOARG0(	signal t	miR-2284z,miR-505-5p,miR-582-3p,miR-29d-3p,miR-1290,miR-2285b,miR-181b-5p,miR-664-3p,miR-219-3p,miR-324-5p,miR-3965,miR-3959-5p,miR-2285aa,miR-125a,miR-215-5p,miR-20b-5p,miR-345-5p,miR-93-5p,miR-17-5p,miR-320a,miR-93,miR-144-5p,miR-31-3p,miR-138-5p,miR-331-3p,miR-125b-5p,miR-551b-3p,miR-34c-3p,miR-2433,miR-29b-3p,miR-20b,miR-432-5p,miR-134-3p,miR-185-5p,miR-665,miR-133b-5p,miR-744-5p,miR-27b-3p,miR-29b,miR-138,miR-21-5p,miR-219a-1-3p,novel_23,miR-432,miR-708-3p,miR-296-3p,miR-345-5p,miR-2285r,miR-628-5p,miR-130b-5p,miR-493-5p,miR-125a-5p,miR-17-5p,miR-106a-5p,miR-296-3p,miR-377-3p,miR-320c,miR-29a-3p,miR-9851-3p,miR-2319a,miR-1291,miR-3970,miR-21c,miR-378b,miR-371b-3p,miR-24-3p,miR-3957-3p,miR-29c-3p,miR-2285x,miR-342-3p,miR-128-1-5p,miR-29a,miR-3613,miR-20a-5p,miR-106a,miR-361-5p,miR-493-3p,miR-4429,miR-1197-5p,miR-5703,miR-188-3p,novel_133,miR-2300b-3p,miR-215-5p,miR-2424,miR-143-3p,miR-20a,miR-221-5p,miR-2484,miR-106a,miR-320d,miR-125b,miR-196a-3p,novel_32,miR-377-3p,miR-192-5p,miR-216b-5p,miR-552-3p,miR-296-3p,miR-221-5p,miR-1895,let-7g-3p,miR-345-5p,miR-27b-5p,miR-129b-3p,miR-3613-5p,miR-3184-5p,miR-6134,miR-214,miR-665-5p,miR-4492,miR-130b-5p,miR-219b-3p,miR-132-5p,miR-320e,miR-665,miR-9788-3p,miR-1298-3p,miR-27a-3p,miR-2284aa,miR-1388-5p,miR-6529a,miR-301b-5p,miR-2330-5p,miR-106a-5p,miR-103a-2-5p,miR-214-3p,miR-106b-5p,miR-342,miR-320b,miR-144-5p,miR-27a-3p,miR-378g,miR-664b,miR-10b,miR-20a-3p,miR-493-5p,miR-361-3p,miR-423-5p,miR-219a-2-3p,miR-181d-5p,miR-3059-5p,miR-212-3p,miR-2284q,miR-107-5p,miR-301a-5p,miR-101-3p
TMEM150B	ENSOARG0(	transmem	miR-187-3p,miR-29a-5p,miR-216b-5p,miR-185-3p,miR-1343-5p,miR-324-3p,miR-6529b,miR-193b-5p,miR-378d,miR-1343-5p,miR-4492
ZNF616	ENSOARG0(	zinc fin	miR-1-5p,miR-21a-3p,miR-23b,miR-29b-2-5p,miR-1a-2-5p,miR-1a-1-5p,miR-3120-3p,miR-29b-2-5p,miR-23a-3p,miR-499a-3p,miR-23b-3p,miR-1b-5p
AIG1	ENSOARG0(	androgen	miR-326,miR-142b,miR-107-5p,miR-1197-5p,miR-330-5p,miR-664a,miR-127-5p,miR-136-5p,miR-486b-3p,miR-485-5p,miR-103a-2-5p,miR-3059-5p,miR-326-3p,miR-486-3p

SCAPER	ENSOARG0(	S-phase	miR-1895,novel_48,miR-21a-3p,miR-199a-5p,miR-204-3p,miR-6516,miR-139-5p,miR-221-5p,miR-5100,miR-1260b,miR-224-5p,novel_42,miR-145a-3p,miR-105-2,miR-615-3p,miR-7859,miR-6516-5p,miR-2411-5p,miR-483-3p,miR-505-3p,miR-548e-3p,miR-221-5p,miR-2898,miR-324-3p,miR-376b-3p,miR-1260a,miR-2285af,miR-6119-5p,miR-2411,miR-2424,miR-199a-5p,miR-2285b,miR-376a-3p,miR-505-3p,miR-376b-3p,miR-129b-5p,miR-140-5p,miR-199b-5p,miR-376d,miR-2404,miR-374c-3p,miR-3082-5p,miR-2483-3p,miR-499b-5p,miR-139-5p,miR-20a-3p,miR-128-1-5p,novel_1,miR-371a-5p,miR-33a-3p,miR-615,miR-199b-5p,miR-361-3p,miR-3957-3p,miR-2447,miR-2284x,miR-30b-3p,miR-1260b,miR-101a-5p,miR-197-3p,miR-30b-3p,miR-758-3p,miR-7b-5p,miR-376b-3p,miR-105-1,novel_78,miR-199b-5p,miR-466f-3p,miR-483-3p,miR-105-5p,miR-7-5p,miR-224-5p,miR-5126,miR-4532,miR-758-3p,miR-668-3p,miR-33a-5p
TMEM165	ENSOARG0(	transmem	novel_79,miR-412,miR-6517,miR-450a-1-3p,miR-2284z,miR-134-5p,miR-129-1-3p,miR-486-5p,novel_121,miR-9-5p,miR-322-5p,miR-2427,miR-330-5p,miR-3591-5p,miR-1290,miR-155-5p,miR-30c,miR-200a,miR-29a-5p,miR-192-3p,miR-30f,miR-205-5p,miR-219-3p,miR-664-3p,miR-8117,miR-200a-3p,miR-1a-2-5p,miR-2285aa,miR-3068-3p,miR-503-3p,miR-223-5p,miR-23b,miR-424-3p,miR-329a,miR-329b,miR-345-5p,miR-16-5p,miR-193a-5p,miR-494-5p,miR-134-5p,miR-339-5p,miR-103a-3p,miR-149-5p,novel_116,miR-134-3p,miR-197-3p,miR-450c-3p,miR-22-3p,novel_23,miR-19b-2-5p,miR-1843b-5p,miR-497-5p,miR-27b-3p,miR-16b,miR-16-1-3p,miR-493-5p,miR-432-3p,miR-365b-5p,miR-296-3p,miR-377-3p,miR-193a-3p,novel_68,miR-144-3p,miR-193b-3p,miR-1a-1-5p,miR-374c-5p,miR-485-3p,miR-4324,miR-6123,miR-30d-3p,miR-7859,miR-23b-3p,miR-23c,miR-30c-5p,miR-16-1-3p,miR-493-3p,miR-142-3p,miR-369-3p,miR-345-3p,miR-122-3p,miR-30b,miR-216b-3p,miR-296-3p,miR-30c-1-3p,miR-15b-5p,miR-377-3p,miR-10a,miR-582-5p,miR-129b-3p,miR-30d-5p,miR-655,miR-433-3p,miR-487b-5p,miR-2285c,miR-450a-1-3p,miR-1827,miR-339b,miR-345-5p,miR-129-2-3p,miR-181c-5p,miR-6525,miR-30a-3p,miR-219b-3p,miR-3604,miR-665,miR-2285j,miR-30e-3p,miR-454-3p,miR-129-5p,miR-190a-5p,miR-4492,miR-130b-5p,miR-487b-5p,miR-145b,novel_78,miR-548w,miR-1343-3p,miR-539-5p,miR-147a,miR-329-3p,miR-362-3p,miR-23a-3p,miR-101b-3p,miR-8485,miR-2310,miR-16b,miR-1185-5p,miR-130b-3p,miR-374b-5p,miR-203-3p,miR-378g,miR-3596,miR-2483-3p,miR-378j,miR-381-3p,miR-140-5p,miR-493-5p,miR-136-5p,miR-6527,miR-9-5p,miR-190b-5p,miR-361-3p,miR-222,miR-582-3p,miR-545-5p,miR-214-5p,miR-302a-5p,miR-6516-5p,miR-134,miR-496,miR-10a-5p,miR-411-3p,miR-30a-3p,miR-3065-3p,miR-30c-2-3p,miR-339a,miR-2285af,miR-2397-

ITIH1	ENSOARG0(	inter-al	miR-450b-3p,miR-9851-3p,miR-6395,miR-2387,miR-148a-3p,miR-3065-5p,miR-3591-3p,miR-320c,miR-3120-3p,miR-206,miR-656-5p,miR-502-5p,miR-4324,miR-2428,miR-495-3p,miR-2285x,miR-2284x,miR-450c-3p,novel_23,miR-3071-5p,miR-31-5p,miR-665,miR-124-5p,miR-125a-5p,miR-296-3p,miR-2411-3p,miR-432-3p,miR-432-3p,miR-2284l,miR-296-3p,miR-345-5p,miR-363-5p,miR-345-5p,miR-671-5p,miR-301,miR-3154,miR-320a,miR-300-3p,miR-376c-3p,miR-2284n,miR-125a,miR-486b-3p,miR-134-5p,miR-2284k,miR-125b-5p,miR-100-3p,miR-3969,miR-323-3p,miR-1306-5p,miR-2284v,miR-6128,miR-103b,miR-345-3p,miR-134,miR-9-5p,miR-302a-5p,miR-2411-5p,miR-134-5p,miR-450a-1-3p,miR-429-3p,miR-2898,miR-152-3p,miR-30c-2-3p,miR-2285b,miR-192-3p,miR-1,miR-504,miR-9-3p,miR-361-3p,miR-140-5p,miR-2483-3p,miR-4443,miR-499b-5p,miR-7977,miR-4726-5p,miR-101-3p,miR-361-3p,miR-9-3p,miR-423-5p,miR-2447,novel_60,miR-3956-3p,miR-212-3p,miR-9-5p,miR-2284j,miR-1388-5p,miR-450b-5p,miR-6529a,miR-484,miR-1a-3p,miR-147a,miR-539-5p,miR-9788-3p,miR-455-3p,miR-2459,novel_4,miR-548w,miR-1185-5p,miR-487b-5p,miR-214-3p,miR-199a-3p,miR-320b,miR-29b-1-5p,miR-30c-1-3p,miR-3187-3p,miR-124a,miR-1388-3p,miR-2330-5p,miR-345-5p,miR-625-5p,miR-186-5p,miR-1827,let-7f-1-3p,miR-376e-3p,novel_25,miR-204-3p,miR-504-5p,miR-221-5p,miR-30c-1-3p,miR-21-3p,let-7b-3p,miR-3604,miR-320e,miR-206-3p,miR-98-3p,miR-3184-5p,miR-539-5p,miR-214,miR-4429,miR-133a-3p,miR-378d,let-7a-3p,miR-124-3p,miR-98-3p,miR-5703,miR-148b-3p,miR-340-5p,miR-2284h-5p,miR-199a-3p,miR-532-3p,miR-328-3p,miR-212-5p,miR-2284m,miR-320d,miR-221-5p,miR-324-3p,miR-125b,miR-345-3p,miR-376a-5p,miR-664b-3p,miR-29b-1-5p,miR-376c-3p,miR-125b-2-3p,miR-2411,miR-486-3p,miR-2285w

PIEZO2	ENSOARG0(	piezo-ty	novel_94,miR-361-3p,miR-129b-5p,miR-378e,miR-7857-3p,miR-27a-3p,miR-3064-5p,miR-7977,miR-450a-5p,miR-2285n,miR-769-5p,miR-154a,miR-212-3p,miR-452-5p,miR-219a-5p,miR-450b-5p,miR-378c,miR-3120-5p,miR-296-5p,miR-378b,miR-30b-3p,miR-221-3p,novel_4,miR-409b,miR-151a-3p,miR-27a-3p,miR-3085-3p,miR-1306-5p,miR-5126,miR-326,miR-320b,miR-193a,miR-494-3p,miR-106a-5p,miR-154b-3p,novel_99,miR-582-5p,miR-21a-3p,miR-193b-3p,miR-6130,miR-139-5p,miR-132-5p,miR-378a-3p,miR-182-5p,miR-664a,miR-208b-5p,miR-338-5p,miR-320e,miR-29b-2-5p,miR-19b-1-5p,miR-4510,miR-145a-3p,miR-378c,miR-3074-2-3p,miR-4429,miR-1197-5p,miR-140-3p,miR-487a-3p,miR-616-3p,miR-485-3p,miR-3600,miR-466i-5p,miR-106a,miR-320d,miR-21-3p,miR-196a-3p,miR-125b,miR-185-3p,miR-20a,miR-9851-3p,miR-2387,miR-29a-3p,miR-670-3p,miR-2404,miR-378b,miR-320b,miR-147-5p,miR-6402,miR-20a-5p,miR-24-3p,miR-323c,miR-2428,miR-326-3p,miR-29b,miR-138,miR-30b-3p,miR-1285,miR-873a-5p,miR-653,miR-503-5p,miR-20b,miR-187-3p,miR-2285l,miR-1343-5p,miR-125a-5p,miR-224-5p,miR-503-5p,miR-17-5p,miR-106a-5p,miR-190a,miR-345-5p,miR-539-3p,miR-130b-5p,miR-10a-3p,miR-485-5p,miR-671-5p,miR-7144-5p,miR-488-3p,miR-17-5p,miR-320a,miR-769,miR-3965,miR-154b-5p,miR-487a-3p,miR-141-3p,miR-615-5p,miR-598-3p,miR-370-3p,miR-138-5p,miR-486b-3p,miR-2332,miR-135a-1-3p,miR-29b-3p,miR-410-5p,miR-144,miR-331-3p,miR-93,miR-323-3p,miR-378c,miR-365a-5p,miR-128-3p,miR-345-3p,miR-29d-3p,miR-378h,miR-196a-2-3p,miR-200c-3p,miR-450a-2-3p,miR-543-5p,miR-200c,miR-28b,miR-1271-3p,miR-4286,miR-181a-2-3p,miR-29b-2-5p,miR-544-5p,miR-335-3p,miR-493-5p,miR-221,miR-378g,miR-203-3p,miR-139-5p,miR-2285u,miR-154-3p,miR-3964,miR-218-5p,novel_82,miR-361-3p,miR-877-3p,miR-885-5p,miR-190b-
PEX3	ENSOARG0(	peroxiso	miR-301a-3p,miR-323-5p,miR-199a-5p,miR-2331-3p,miR-296-3p,miR-1248,miR-1827,miR-21a-3p,miR-323-3p,miR-145a-3p,miR-3074-2-3p,novel_74,miR-17-3p,miR-454-3p,miR-130b-5p,miR-2285j,miR-135a-5p,miR-7859,miR-199a-3p,miR-2284z,miR-548e-3p,miR-7641,miR-3600,miR-582-3p,miR-16-1-3p,miR-148a-5p,miR-378d,miR-2284u,miR-2300b-3p,miR-376a-5p,miR-1343-5p,miR-23b-5p,miR-2284y,miR-199a-5p,miR-146a-5p,miR-29a-5p,miR-143-3p,miR-2284a,miR-490-3p,miR-503-5p,miR-2284f,miR-202-5p,miR-17-3p,miR-30b-3p,miR-1277-5p,miR-543-5p,miR-2285af,miR-664-3p,miR-92a-1-5p,miR-323a-5p,miR-2404,miR-25,miR-2483-3p,miR-489,miR-652-3p,miR-6535,miR-2284g,miR-664b,miR-541-5p,miR-3120-3p,miR-7-1-3p,miR-2284e,miR-7975,miR-146b-5p,miR-544-5p,miR-135b-5p,miR-199b-5p,miR-301b-3p,miR-199b-5p,novel_60,miR-3059-5p,miR-3956-3p,miR-17-3p,miR-495-3p,miR-6119-3p,miR-199c,miR-218-5p,miR-6402,miR-2284o,miR-3535,miR-412-5p,miR-503-5p,miR-148b-5p,miR-9788-3p,miR-3955-5p,miR-548w,miR-199b-3p,novel_78,miR-130a-3p,miR-145b,miR-16-1-3p,miR-26a-2-3p,miR-2284aa,miR-2889,miR-455-5p,miR-2284x,miR-4791,miR-143-5p,miR-145a-5p,miR-130b-5p,miR-382,miR-628-5p,miR-130b-3p,miR-1343-5p,miR-146b,miR-199b-5p,miR-199a-3p,miR-503-5p,novel_115,miR-146a,miR-455-5p

RIPK1	ENSOARG0(	receptor	miR-1291,miR-2319a,miR-6395,miR-361-3p,miR-2285ab,miR-409-5p,miR-148a-3p,miR-18a-3p,miR-3065-5p,miR-378g,miR-146b-3p,miR-34b,novel_69,miR-29a-3p,miR-2319b,miR-4726-5p,miR-29a,miR-502b,novel_82,miR-2285n,miR-615,miR-28c,miR-576-3p,miR-34c,miR-107-5p,miR-361-3p,miR-877-3p,miR-449a,miR-2284w,miR-3059-5p,miR-2447,miR-2285f,miR-6527,miR-452-5p,miR-29c-3p,miR-29b,miR-4508,miR-7862,miR-432,miR-143-5p,miR-2285g,miR-432-5p,miR-134-3p,miR-503-5p,miR-2285e,novel_4,miR-222-5p,miR-2285l,miR-487b-5p,miR-125a-5p,miR-214-3p,miR-103a-2-5p,miR-503-5p,miR-29b-1-5p,miR-505,miR-2957,novel_51,miR-136-3p,miR-2285r,miR-432-3p,miR-432-3p,miR-124a,miR-2330-5p,miR-2312,miR-876-3p,miR-625-5p,miR-2285c,miR-3154,miR-34c-5p,novel_32,miR-204-3p,miR-324-5p,miR-140-5p,miR-2331-3p,miR-221-5p,miR-874-3p,miR-99a-3p,miR-370-3p,miR-2355-5p,miR-125a,miR-1271,miR-874-5p,miR-208b-5p,miR-29b-3p,miR-99b-3p,miR-331-3p,miR-125b-5p,miR-665,miR-6525,miR-338-3p,miR-3074-2-3p,miR-214,miR-33b-3p,miR-664-5p,miR-34a-5p,miR-615-3p,miR-3578,miR-2300b-3p,miR-29d-3p,miR-124-3p,miR-1193,miR-95-3p,miR-148b-3p,miR-1247-5p,novel_83,miR-545-5p,miR-328-3p,miR-18a-3p,miR-99a-3p,miR-760-3p,miR-221-5p,miR-34b-5p,miR-21-3p,novel_127,miR-152-3p,miR-205-5p,miR-324-3p,miR-125b,miR-185-3p,miR-28b,miR-29b-1-5p,miR-490-3p,miR-503-5p,miR-105-3p,miR-5100,miR-4286

NAA10	ENSOARG0(	N(alpha)	miR-15a,miR-16a,miR-128-1-5p,miR-342-3p,novel_120,miR-135a-2-3p,miR-3957-3p,miR-17-3p,miR-24-3p,miR-1307-3p,miR-18b,miR-18a-3p,miR-378b,miR-1291,miR-2285k,miR-19a-3p,miR-107,miR-195a-5p,miR-379-5p,miR-10a-5p,miR-455-5p,miR-452-5p,miR-1343-5p,miR-363-5p,miR-374b-3p,miR-3071-5p,miR-30b-3p,miR-455-5p,miR-2889,miR-187-3p,miR-103,miR-544b,miR-486b-3p,miR-28-5p,miR-103b,miR-133a-3p,miR-378c,miR-365a-5p,miR-488-3p,miR-503-3p,miR-3154,miR-18a,miR-7144-5p,miR-485-5p,miR-615-5p,miR-374c-3p,miR-199a-5p,miR-339a,miR-2898,miR-450a-2-3p,miR-17-3p,miR-107,miR-543-5p,miR-30a-3p,miR-28b,miR-199a-5p,miR-345-3p,miR-582-3p,miR-10a-5p,miR-378h,miR-2285n,miR-335,miR-2284q,miR-3059-5p,miR-541-3p,miR-361-3p,miR-199b-5p,miR-3064-5p,miR-10b,miR-499b-5p,miR-7977,miR-378e,miR-3082-5p,miR-27a-3p,miR-342,novel_103,miR-5126,novel_124,miR-3085-3p,miR-25-5p,miR-2330-5p,miR-424-5p,miR-33a-5p,miR-3187-3p,miR-193a,miR-4532,miR-378b,miR-484,miR-10b,miR-143-5p,miR-30b-3p,miR-378c,miR-2426,miR-544a,novel_4,miR-2300a-5p,miR-27a-3p,miR-320e,miR-17-3p,miR-708-5p,miR-424-5p,miR-539-5p,miR-378c,miR-145a-3p,miR-4510,miR-3074-2-3p,miR-10b-5p,miR-6134,miR-338-3p,miR-133b-3p,miR-18a-5p,miR-378a-3p,miR-21-3p,miR-182-5p,miR-544-3p,miR-6130,miR-193b-3p,miR-22-3p,novel_32,miR-196a-3p,miR-185-3p,miR-466i-5p,miR-760-3p,miR-30b-3p,miR-2904,miR-188-3p,miR-140-3p,miR-5703,miR-19b-3p,miR-378d,miR-3960,miR-3600,miR-1b-3p,miR-15a-5p,miR-532-3p,miR-28c,miR-625-3p,miR-6123,miR-335-5p,miR-193b-3p,miR-199b-5p,miR-2285m,miR-3970,miR-150-5p,miR-3591-3p,miR-378f,miR-3074-5p,miR-3120-3p,novel_69,miR-374c-3p,miR-669,miR-7-5p,miR-582,miR-365b-5p,miR-2411-3p,novel_51,miR-378a-5p,miR-196b-5p,miR-
RABL6	ENSOARG0(	RAB, mem	miR-378g,miR-134-3p,miR-664a,miR-486-3p,miR-486b-3p,miR-1343-3p
ARHGAP22	ENSOARG0(	Rho GTPa	novel_68,miR-3064-5p,miR-7689-3p,miR-10a-5p,miR-378g,miR-6535,miR-136-5p,miR-9851-3p,novel_94,miR-541-3p,miR-2428,novel_82,miR-142b,miR-576-3p,miR-2440,miR-1973,miR-2285t,miR-1343-3p,miR-26b-3p,miR-185-5p,miR-134-3p,miR-149-5p,miR-2284r,miR-541,miR-10b,miR-30b-3p,miR-30b-3p,miR-1260b,miR-455-5p,miR-3187-3p,miR-345-5p,miR-2285v,miR-455-5p,miR-3085-3p,miR-1343-5p,miR-214-3p,miR-574-5p,miR-2331-3p,miR-370-3p,miR-141-3p,miR-10a,miR-345-5p,miR-345-5p,miR-485-5p,miR-214,miR-10b-5p,miR-1260b,miR-6134,miR-5100,miR-324-3p,miR-664a,novel_83,miR-214-5p,novel_133,miR-1343-5p,miR-2904,miR-493-3p,miR-133a-3p,miR-10a-5p,miR-3064-5p,miR-181a-2-3p,miR-1271-3p,miR-1260a,miR-210-5p,novel_17,miR-324-3p,miR-2898,miR-760-3p,miR-21-3p,miR-200a-3p
CNOT1	ENSOARG0(	CCR4-NOT	miR-376e-3p,miR-194-3p,miR-216a-3p,miR-486-3p,miR-376c-3p,miR-488-3p,miR-576-3p,miR-486b-3p

FUCA2	ENSOARG0(	fucosida	miR-3068-3p,miR-2355-5p,miR-544-3p,miR-6516,miR-377-3p,miR-204-3p,miR-2285c,miR-301,miR-129b-3p,miR-362-5p,miR-2312,miR-345-5p,miR-345-5p,miR-3074-1-3p,miR-671-5p,miR-129-2-3p,miR-103b,miR-500b-5p,miR-1260b,miR-338-3p,miR-410-5p,miR-34c-3p,miR-29b-2-5p,novel_74,miR-410-3p,miR-299a-3p,miR-429-3p,miR-3600,miR-412,miR-212-5p,miR-6516-5p,miR-148b-3p,miR-200b,miR-532-3p,miR-129-1-3p,novel_133,miR-1290,miR-217-5p,miR-188-3p,miR-200b-3p,miR-29b-2-5p,miR-181a-2-3p,miR-450b-5p,miR-148b-5p,miR-130a-5p,miR-2285af,miR-1260a,miR-200c,miR-542-5p,miR-125a-3p,miR-500a-5p,miR-217,miR-200c-3p,miR-152-3p,miR-299-3p,miR-21-3p,miR-20a-3p,miR-2319b,miR-2285u,miR-7977,miR-299b-3p,miR-27a-3p,miR-1185-2-3p,miR-361-3p,miR-2285ab,miR-148a-3p,miR-2447,miR-3059-5p,miR-2285f,miR-542-5p,miR-877-3p,miR-362-5p,miR-218-5p,miR-2285n,miR-382-5p,miR-499a-5p,miR-656-5p,miR-147-5p,miR-31-5p,miR-145b,miR-27a-3p,miR-2285g,miR-3431,miR-500-5p,miR-544b,miR-2285e,miR-484,miR-197-3p,miR-412-3p,miR-145a-5p,miR-1260b,miR-544a,miR-27b-3p,miR-33a-5p,let-7f-2-3p,let-7f-2-3p,miR-2285r,miR-4532,miR-345-5p,miR-377-3p,miR-2331-5p,miR-3955-3p,miR-542-5p
PCDH12	ENSOARG0(	protocad	miR-218-2-3p,miR-29d-3p,miR-501-3p,let-7d,miR-128-3p,miR-2411-5p,miR-214-5p,miR-376b-3p,miR-2285af,miR-339a,let-7c-5p,miR-2898,miR-450a-2-3p,miR-191-3p,miR-107,miR-543-5p,miR-30a-3p,miR-2385-3p,miR-28b,miR-2284a,miR-1271-3p,miR-10b-3p,miR-3074-1-3p,miR-485-5p,miR-370-3p,miR-215-5p,miR-34c-5p,miR-374c-3p,miR-29b-3p,miR-370-5p,miR-99b-3p,miR-331-3p,miR-3969,miR-204-5p,miR-486b-3p,miR-3607-3p,miR-873a-5p,miR-1285,miR-3071-5p,miR-145a-5p,miR-380-3p,miR-29b,miR-2889,miR-133b-5p,miR-432-5p,let-7g-5p,miR-6238,miR-103,miR-2355-3p,miR-544b,miR-1343-5p,miR-376b-3p,miR-18a-3p,miR-19a-3p,miR-107,miR-6516-3p,miR-655-5p,miR-29a-3p,miR-34b,miR-34c,let-7i,miR-656-5p,miR-203b-5p,novel_101,miR-1193,miR-211,miR-3959-3p,novel_27,miR-449a,miR-326-3p,novel_133,miR-215-5p,miR-1197-5p,miR-1193,miR-19b-3p,miR-3600,miR-671-3p,miR-760-3p,miR-30b-3p,miR-34b-5p,miR-130a-5p,miR-2411,let-7g-3p,miR-545-3p,miR-370-5p,miR-182-5p,miR-544-3p,miR-192-5p,miR-193b-3p,miR-6130,miR-204-3p,miR-22-3p,miR-19b-1-5p,miR-450b-3p,miR-145a-3p,miR-4510,miR-425-5p,let-7b-5p,miR-338-3p,miR-544a,miR-5010-3p,miR-27a-3p,miR-9788-3p,miR-216a-3p,novel_115,miR-326,miR-3955-3p,let-7e,let-7a-5p,miR-382-3p,miR-652-5p,miR-361-3p,miR-877-3p,miR-452-3p,miR-20a-3p,miR-541-5p,miR-489,let-7d-5p,miR-27a-3p,miR-376d,miR-4726-5p,miR-335,novel_60,miR-3059-5p,miR-1296-5p,miR-452-5p,miR-502-3p,miR-154a,miR-1343-5p,miR-330-5p,miR-23b-5p,miR-6517,novel_79,miR-431,miR-9-5p,miR-450a-1-3p,miR-129-1-3p,miR-486-5p,let-7f-5p,novel_127,novel_96,miR-148b-5p,miR-450b-5p,let-7g,miR-2284y,miR-200a,miR-2285b,miR-329b,miR-874-3p,miR-99a-3p,miR-1271,miR-6740-5p,novel_42,miR-19b-2-5p,miR-708-3p,miR-432,miR-542-3p,miR-1843b-5p,miR-450c-3p,miR-22-

HSPBP1	ENSOARG0(	HSPA (he	miR-6123,miR-128-1-5p,miR-34c,miR-16a,miR-425-5p,novel_87,miR-382-5p,miR-502-5p,miR-15a,miR-4324,miR-6529b,miR-449a,miR-199b-5p,miR-211,miR-17-3p,miR-342-3p,miR-9851-3p,miR-195a-5p,miR-1291,miR-450b-3p,miR-3141,miR-2387,miR-6535,miR-34b,novel_69,miR-670-3p,miR-329-5p,miR-2285l,miR-3141,miR-330-3p,novel_51,miR-224-5p,miR-503-5p,miR-377-3p,miR-654-3p,miR-382,miR-6240,miR-497-5p,miR-26a-2-3p,miR-27b-3p,miR-30b-3p,miR-16b,miR-1260b,miR-16-1-3p,miR-381-5p,miR-450c-3p,novel_23,miR-432,miR-1843b-5p,miR-2113,novel_116,miR-2355-3p,miR-432-5p,miR-2285g,miR-412-5p,miR-134-3p,miR-503-5p,miR-185-5p,miR-339-5p,miR-377-5p,miR-187-3p,miR-204-5p,miR-486b-3p,miR-28-5p,miR-331-3p,miR-409-3p,miR-2433,miR-500b-5p,miR-1260b,miR-16-5p,miR-193a-5p,novel_42,miR-23a-5p,miR-485-5p,miR-1814c,miR-1248,novel_91,miR-329a,miR-329b,miR-200a-5p,miR-362-5p,miR-3154,miR-488-3p,miR-381-3p,miR-503-3p,miR-199a-5p,miR-324-5p,miR-154b-5p,miR-3957,miR-34c-5p,miR-370-3p,miR-1271,miR-3957-5p,miR-17-3p,miR-2898,miR-30c-2-3p,miR-217,miR-339a,miR-1260a,miR-199a-5p,novel_39,miR-29a-5p,miR-490-3p,miR-2478,miR-30a-3p,miR-4286,miR-885-3p,miR-300,miR-2427,miR-23b-5p,miR-615-3p,miR-545-5p,miR-450a-1-3p,novel_121,miR-505-5p,miR-9-5p,miR-322-5p,miR-2411-5p,miR-6516-5p,miR-412,novel_79,miR-2285ad,miR-6517,miR-4726-5p,miR-107-5p,miR-218-5p,novel_82,miR-2440,miR-615,miR-423-5p,miR-362-5p,miR-9-5p,miR-2447,miR-335-3p,miR-544-5p,miR-877-3p,miR-136-5p,miR-668-5p,miR-378j,miR-361-3p,miR-199b-5p,miR-1185-2-3p,miR-27a-3p,miR-378g,miR-489,miR-211-5p,miR-452-3p,miR-2319b,miR-199b-5p,miR-103a-2-5p,miR-381-5p,novel_124,miR-25-5p,miR-505,miR-362-3p,miR-16b,miR-342,miR-5126,novel_103,miR-3187-3p,miR-193a,miR-

TMEM185A	ENSOARG0(	transmem	miR-378a-5p,miR-196b-5p,miR-377-3p,miR-146a,novel_51,miR-3141,miR-365b-5p,miR-432-3p,miR-432-3p,miR-503-3p,let-7a-2-3p,miR-493-5p,let-7f-2-3p,let-7f-2-3p,miR-16b,miR-27b-3p,miR-497-5p,miR-2113,miR-22-3p,miR-134-3p,miR-194-3p,miR-411-5p,miR-103a-3p,miR-31-5p,miR-339-5p,miR-4324,miR-425-5p,miR-199b-5p,miR-146b-5p,miR-148a-3p,miR-3141,miR-3065-5p,miR-216a-3p,miR-3591-3p,miR-181b-3p,miR-677,miR-669,novel_68,miR-3120-3p,miR-127-5p,miR-152-3p,novel_127,miR-8117,miR-450c-5p,novel_39,miR-3064-5p,miR-196a-5p,miR-330-5p,miR-23b-5p,miR-322-5p,miR-134-5p,miR-429-3p,miR-2285ad,novel_79,miR-1224-5p,miR-134-5p,miR-125b-5p,miR-100-3p,miR-1197-3p,miR-1260b,novel_42,miR-16-5p,miR-345-5p,miR-876-3p,miR-300-3p,miR-376c-3p,miR-874-3p,miR-1271,miR-125a,miR-2285aa,miR-2355-5p,miR-487b-5p,miR-130b-3p,miR-214-3p,miR-199b-5p,miR-146b,miR-16b,miR-29b-1-5p,miR-8485,miR-574-3p,miR-124a,miR-1388-3p,miR-21b,miR-301b-5p,miR-541,miR-2284r,miR-147a,miR-1185-3p,miR-2459,miR-455-3p,miR-548w,miR-1343-3p,miR-3968,miR-615,miR-218-5p,miR-301a-5p,novel_111,miR-361-3p,miR-877-3p,miR-423-5p,miR-3956-3p,miR-136-5p,miR-504,miR-96-5p,miR-493-5p,miR-221,miR-3596,miR-378g,miR-4443,miR-1185-2-3p,miR-4792,miR-211-5p,miR-2285u,miR-2484,novel_17,miR-210-5p,miR-324-3p,miR-181b-1-3p,miR-377-5p,miR-376c-3p,miR-376a-5p,miR-345-3p,miR-142-3p,miR-493-3p,miR-34a-5p,miR-133a-3p,miR-2366,miR-328-3p,miR-18a-3p,miR-212-5p,miR-487b-5p,novel_74,miR-130b-5p,miR-874-5p,miR-4492,miR-454-3p,miR-2285j,miR-664a-5p,miR-665,miR-151-3p,miR-346,miR-6525,miR-3184-5p,miR-3184-3p,miR-214,miR-339b,miR-345-5p,miR-1827,miR-299,miR-376e-3p,miR-10a,miR-2284s,miR-377-3p,miR-15b-5p,miR-2331-3p,miR-504-5p,miR-30c-1-

MYH10	ENSOARG0(	myosin,	miR-32-5p,miR-141-3p,miR-552-3p,miR-2355-5p,miR-370-3p,miR-25-3p,miR-3959-5p,miR-323-5p,miR-6130,miR-6516,miR-151b,miR-154b-5p,novel_32,miR-381-3p,miR-411,miR-376c-5p,miR-1827,miR-671-5p,miR-543-3p,miR-885-3p,miR-1895,miR-376c-5p,miR-625-5p,miR-3184-3p,miR-3074-5p,miR-4510,miR-224-5p,miR-6134,miR-181c-5p,miR-365a-5p,miR-3184-5p,miR-2433,miR-2285j,miR-664a-5p,miR-3604,miR-331-3p,miR-6516-3p,miR-208b-5p,miR-487a-5p,miR-874-5p,miR-2285ad,miR-505-3p,miR-7641,miR-6516-5p,miR-151-5p,miR-2284h-5p,miR-671-3p,miR-2408,miR-330-5p,miR-1434-5p,miR-1343-5p,miR-124-3p,miR-300,miR-885-3p,miR-503-5p,miR-3064-5p,miR-142a-3p,miR-181b-1-3p,miR-92b-3p,novel_39,miR-200a,miR-376c-5p,miR-210-5p,miR-374a-5p,miR-324-3p,miR-466i-5p,miR-181b-5p,miR-200a-3p,miR-543-5p,novel_96,miR-92a-3p,miR-3074-5p,miR-3064-5p,miR-499b-5p,miR-541-5p,miR-323a-5p,miR-181b-3p,miR-27a-3p,miR-677,miR-6535,miR-376b-5p,miR-3970,miR-412-3p,miR-9-3p,miR-505-3p,miR-1983,miR-363-3p,miR-181d-5p,miR-2285x,miR-1285-5p,miR-326-3p,miR-423-5p,miR-9-3p,miR-2285y,miR-3958-3p,miR-92a-3p,miR-3964,miR-490-5p,miR-330-3p,miR-2300a-5p,miR-181b-2-3p,miR-27a-3p,miR-503-5p,miR-142-3p,miR-421,miR-143-5p,miR-181a-5p,miR-223-3p,miR-450b-5p,miR-27b-3p,miR-382-3p,miR-6240,miR-1388-3p,miR-124a,miR-376b,miR-326,novel_115,miR-224-5p,miR-503-5p,miR-32,miR-330-3p,miR-365b-5p,miR-3085-3p,miR-491-5p,miR-1343-5p,miR-106b-3p

BTK	ENSOARG0(	Bruton a	miR-335,miR-2285t,miR-615,miR-769-5p,miR-361-3p,miR-3059-5p,miR-190b-5p,miR-181d-5p,miR-668-5p,miR-504,miR-3963,miR-877-3p,miR-1983,miR-335-3p,miR-361-3p,miR-381-3p,miR-3596,miR-27a-3p,miR-203-3p,miR-4792,miR-139-5p,miR-2284g,miR-7977,miR-2285u,miR-7689-3p,miR-3955-3p,miR-214-3p,miR-381-5p,miR-326,miR-106b-5p,miR-29b-1-5p,miR-2957,miR-106a-5p,miR-1388-3p,miR-2284b,miR-23a-3p,miR-2426,miR-1388-5p,miR-3120-5p,miR-2284aa,miR-21b,miR-296-5p,miR-484,miR-412-3p,miR-147a,miR-2320-3p,miR-496-5p,miR-148b-5p,miR-9788-3p,miR-1343-3p,miR-2300a-5p,miR-27a-3p,miR-181b-2-3p,miR-5010-3p,miR-190a-5p,let-7b-3p,miR-2285j,miR-324-3p,miR-132-5p,miR-98-3p,miR-769-5p,miR-181c-5p,miR-3074-5p,miR-145a-3p,miR-425-5p,miR-214,miR-186-5p,miR-625-5p,let-7f-1-3p,miR-139-5p,miR-132-5p,miR-2284s,miR-151b,miR-877-5p,novel_32,novel_25,miR-504-5p,miR-2331-3p,novel_107,miR-106a,novel_17,miR-618,miR-125b,miR-2285i,miR-376a-5p,miR-29b-1-5p,miR-2432,miR-486-3p,miR-216c-5p,miR-2887,miR-20a,novel_73,miR-33b-3p,let-7a-3p,miR-378d,miR-2284u,miR-148a-5p,miR-16-1-3p,miR-126-3p,miR-5703,miR-2904,miR-98-3p,miR-23c,miR-23b-3p,miR-328-3p,miR-151-5p,miR-106a,miR-3960,miR-212-5p,novel_1,miR-6402,miR-20a-5p,miR-4324,miR-1307-5p,miR-371b-3p,miR-3959-3p,miR-323c,miR-24-3p,miR-326-3p,miR-335-5p,miR-412-3p,miR-2285k,miR-379-5p,miR-6395,miR-7975,miR-21c,miR-3065-5p,miR-374c-3p,novel_69,miR-670-3p,miR-144-3p,miR-677,miR-3074-5p,novel_68,miR-3120-3p,miR-483-3p,miR-466f-3p,miR-125a-5p,miR-7-5p,miR-17-5p,novel_51,miR-106a-5p,miR-190a,miR-2285v,miR-432-3p,miR-2285r,miR-363-5p,miR-16-1-3p,miR-381-5p,miR-219a-1-3p,miR-138,miR-7862,miR-27b-3p,miR-181a-5p,miR-7b-
ZNF263	ENSOARG0(	zinc fin	miR-3154,miR-199b-5p,miR-1983,miR-412-3p,miR-141-3p,miR-216b-3p,miR-874-3p,miR-199a-5p,miR-29b-2-5p,miR-2284k,miR-130b-5p,miR-208b-5p,miR-6516-3p,miR-138-5p,miR-2284j,miR-3059-5p,miR-199b-5p,miR-2284w,miR-1260b,miR-323-3p,miR-188-3p,miR-125b-2-3p,miR-181a-5p,miR-542-3p,miR-1843b-5p,miR-2284x,miR-2284aa,miR-1260b,miR-138,miR-377-5p,novel_78,novel_116,miR-2284z,miR-147a,miR-210-3p,miR-2397-5p,novel_17,miR-1260a,miR-543-5p,miR-200a-3p,miR-199b-5p,miR-466i-5p,miR-127-5p,miR-181b-5p,miR-2284m,miR-29b-2-5p,miR-5100,miR-130b-5p,miR-503-3p,miR-1271-3p,miR-199a-5p,miR-8485,miR-2432,miR-125b-2-3p,miR-377-5p
FMOD	ENSOARG0(	fibromod	miR-365b-5p,miR-105-5p,miR-365a-5p,miR-345-5p,miR-105-2,miR-1291,miR-299a-5p,miR-345-5p,miR-345-5p,miR-542-5p,miR-548e-3p,miR-2300a-5p,miR-105-1,novel_116,miR-214-5p,miR-24-2-5p

CPA6	ENSOARG0(	carboxyp	miR-654-3p, let-7j, let-7a-2-3p, miR-130b-5p, miR-493-5p, miR-628-5p, miR-378a-5p, miR-196b-5p, miR-125a-5p, miR-224-5p, miR-377-3p, miR-190a, miR-2411-3p, miR-194a, miR-432-5p, miR-134-3p, miR-149-5p, miR-194-3p, miR-31-5p, miR-130a-3p, miR-339-5p, miR-26b-3p, miR-1260b, miR-2284x, miR-19b-2-5p, miR-432, miR-1843b-5p, miR-181a-5p, miR-450c-3p, miR-194-5p, miR-3071-5p, miR-211, miR-326-3p, miR-301b-3p, miR-1a-1-5p, miR-17-3p, miR-6119-3p, novel_1, miR-502b, miR-199c, miR-502-5p, miR-6402, miR-485-3p, novel_87, miR-299b-3p, miR-670-3p, miR-669, novel_68, miR-3074-5p, miR-320b, miR-6516-3p, miR-505-3p, miR-2285ab, miR-1306, miR-18a-3p, miR-2285b, miR-490-3p, miR-192-3p, miR-29b-2-5p, miR-17-3p, miR-181b-5p, miR-1a-2-5p, novel_127, miR-543-5p, miR-1260a, miR-181c-3p, miR-339a, miR-302a-5p, miR-9-5p, miR-450a-1-3p, miR-486-5p, novel_121, miR-214-5p, miR-222, miR-299a-3p, miR-2285ad, novel_79, miR-7641, let-7a-2-3p, miR-345-3p, miR-196a-5p, miR-3578, miR-218-2-3p, miR-23b-5p, miR-330-5p, miR-1260b, miR-193a-5p, miR-144-5p, miR-331-5p, miR-410-3p, miR-204-5p, miR-1-5p, miR-5100, miR-135a-1-3p, miR-125b-5p, miR-26c, miR-409-3p, miR-223-5p, miR-154b-5p, miR-874-3p, miR-125a, miR-10b-3p, let-7g-3p, miR-7144-5p, miR-190a-3p, miR-671-5p, miR-1814c, miR-331-5p, miR-23b, miR-1b-5p, miR-758-3p, miR-2310, miR-23a-3p, miR-33a-5p, miR-130b-3p, miR-3955-3p, miR-103a-2-5p, miR-199a-3p, miR-1306-5p, miR-326, miR-182-5p, miR-147a, miR-9788-3p, miR-221-3p, miR-199b-3p, novel_4, novel_78, miR-1388-5p, miR-101a-5p, miR-16-2-3p, miR-3120-5p, miR-484, miR-758-3p, miR-3962, miR-877-3p, novel_111, miR-8095, miR-7705, miR-3059-5p, novel_60, miR-3956-3p, miR-2285f, miR-194b-5p, miR-9-5p, miR-190b-5p, miR-181d-5p, miR-3968, miR-499a-5p, miR-2285t, miR-218-5p, miR-107-5p, miR-378g, miR-7857-3p, miR-2483-3p, miR-203-3p, miR-
RGS22	ENSOARG0(	regulato	miR-320d, let-7g-3p, miR-4429, let-7a-2-3p, miR-320b, miR-218-5p, miR-320a, miR-320c, let-7a-2-3p

TSPAN3	ENSOARG0(	tetraspa	miR-582-5p,miR-7144-5p,let-7g-3p,miR-2318,miR-200a-5p,miR-137-3p,miR-3613-5p,miR-488-3p,miR-15b-5p,novel_25,miR-582-5p,miR-2355-5p,miR-154a-3p,miR-487a-3p,miR-378a-3p,miR-424-5p,miR-29b-3p,miR-338-3p,miR-378c,miR-3184-5p,miR-1197-3p,miR-214,miR-3607-3p,miR-16-5p,miR-6128,miR-378c,miR-378d,let-7a-2-3p,novel_73,miR-378a-3p,miR-330-5p,miR-29d-3p,miR-378i,miR-2284z,miR-483-3p,miR-15a-5p,miR-340-5p,miR-378h,miR-487a-3p,miR-322-5p,miR-24-2-5p,miR-21-3p,miR-422a,miR-29a-5p,novel_39,miR-146a-5p,miR-4286,miR-487a,miR-379-5p,miR-195a-5p,miR-378f,miR-146b-5p,miR-3963,miR-361-3p,miR-3082-5p,miR-29a-3p,miR-378e,miR-378g,miR-2404,miR-3596,miR-147-5p,miR-154-3p,miR-625-3p,miR-29a,miR-6119-3p,miR-301a-5p,miR-16a,miR-3613,miR-218-5p,miR-15a,miR-2284w,miR-326-3p,miR-423-5p,miR-1285-5p,miR-29c-3p,miR-3956-3p,novel_60,miR-342-3p,miR-1839-5p,miR-497-5p,miR-2284aa,miR-29b,miR-16b,miR-378c,miR-1843b-5p,miR-7b-5p,miR-542-3p,miR-301b-5p,miR-378b,miR-432,miR-134-3p,miR-432-5p,miR-412-5p,miR-214-3p,miR-146b,miR-483-3p,miR-16b,miR-146a,miR-326,miR-7-5p,miR-342,let-7a-2-3p,miR-1b-5p,miR-539-3p,let-7j,miR-378d,miR-2483-5p,miR-668-3p,miR-2330-5p,miR-424-5p
NCF2	ENSOARG0(	neutroph	miR-199b-5p,miR-106b-3p,miR-16b,miR-136-3p,miR-503-5p,miR-326,let-7a-2-3p,miR-2310,miR-424-5p,miR-497-5p,miR-3120-5p,miR-2889,miR-16b,miR-16-1-3p,miR-544a,miR-1a-3p,miR-7857,miR-125b-2-3p,miR-2284r,miR-223-3p,miR-1434-3p,miR-148b-5p,miR-544b,miR-539-5p,miR-503-5p,miR-3431,miR-340-3p,miR-7134-3p,miR-206,miR-379-3p,novel_1,miR-16a,miR-382-5p,miR-15a,miR-218-5p,miR-326-3p,miR-199b-5p,miR-877-3p,miR-211,miR-371b-3p,miR-136-3p,miR-335-3p,miR-1983,miR-877-3p,miR-195a-5p,miR-147-3p,miR-412-3p,miR-18a-3p,miR-199b-5p,miR-670-3p,miR-320b,miR-211-5p,miR-26b-5p,miR-7-1-3p,miR-3074-5p,miR-20a-3p,miR-2484,novel_127,miR-21-3p,miR-760-3p,miR-466i-5p,miR-219b-5p,miR-618,miR-26a-5p,miR-29a-5p,miR-199a-5p,miR-1957a,miR-125b-2-3p,miR-1,miR-345-3p,miR-503-5p,miR-16-1-3p,miR-218-1-3p,miR-148a-5p,let-7a-2-3p,miR-1193,miR-330-5p,novel_133,miR-582-3p,miR-545-5p,miR-322-5p,miR-15a-5p,miR-18a-3p,miR-424-5p,miR-204-5p,miR-206-3p,miR-16-5p,miR-1306-5p,miR-3074-5p,miR-543-3p,miR-1814c,miR-671-5p,let-7g-3p,miR-199a-5p,miR-15b-5p,miR-544-3p,miR-216b-3p,miR-370-3p,miR-2285aa,miR-615-5p,miR-3068-3p,miR-874-3p

CD19	ENSOARG0(	CD19 mol	miR-2330-3p,let-7b,let-7b-5p,miR-224-5p,miR-206-3p,miR-320e,miR-665,miR-135a-1-3p,let-7f,miR-874-5p,miR-450b-3p,miR-141-3p,let-7i-5p,miR-204-3p,miR-499b-3p,miR-3154,miR-137-3p,miR-2285c,miR-186-5p,miR-7144-5p,miR-625-5p,let-7g-3p,miR-345-3p,let-7g,miR-376a-5p,let-7c-5p,miR-345-3p,miR-200c,miR-200a-3p,let-7k,miR-107,let-7f-5p,miR-200c-3p,miR-98-5p,miR-1224-5p,miR-429-3p,miR-450a-1-3p,miR-545-5p,miR-200b,miR-7859,miR-505-5p,miR-1197-5p,miR-1961,miR-345-3p,let-7a-2-3p,miR-200b-3p,miR-582-3p,let-7d,novel_120,miR-2447,novel_111,miR-371b-3p,let-7i,miR-576-3p,miR-199c,miR-625-3p,miR-206,miR-6119-3p,miR-20a-3p,let-7d-5p,miR-670-3p,miR-374c-3p,miR-489,miR-499a-3p,miR-1306,miR-2285ab,miR-361-3p,let-7e-5p,miR-107,miR-299b-5p,miR-450b-3p,miR-33a-5p,miR-101b-3p,let-7a-5p,let-7a-2-3p,miR-6412,miR-2285r,novel_51,miR-2411-3p,miR-1306-5p,miR-224-5p,let-7e,miR-26b-3p,miR-222-5p,miR-199b-3p,miR-103a-3p,miR-31-5p,miR-103,miR-2285e,miR-455-3p,let-7g-5p,miR-3431,miR-2285g,miR-374b-3p,novel_23,miR-450c-3p,miR-223-3p,miR-484,miR-2284x
GOT2	ENSOARG0(	glutamic	miR-664a,miR-502b,miR-22-5p,miR-2440,miR-2285y,miR-877-3p,miR-181c-5p,miR-326-3p,miR-181d-5p,miR-1271,miR-3958-5p,miR-96-5p,miR-410-5p,novel_99,miR-485-5p,miR-582-5p,miR-1248,novel_48,miR-335-3p,miR-299,miR-21a-3p,miR-3970,miR-139-5p,miR-378g,miR-582-5p,miR-140-5p,miR-139-5p,miR-552-3p,miR-181b-5p,miR-21-3p,miR-30b-3p,miR-326,novel_51,miR-503-3p,miR-2285w,miR-369-3p,miR-299a-5p,miR-142-3p,miR-181a-5p,miR-330-5p,miR-197-3p,miR-134-3p,miR-3431,miR-1298-3p,miR-1271-5p,novel_121,miR-216a-3p,novel_116,miR-545-5p,miR-532-3p,miR-2284h-5p,novel_78,miR-2285ad,miR-3955-5p

ZNF174	ENSOARG0(	zinc fin	miR-1343-3p,novel_78,novel_4,miR-2300a-5p,miR-5010-3p,miR-181b-2-3p,miR-9788-3p,miR-500-5p,miR-421,miR-2459,miR-6529a,miR-301b-5p,miR-323b,miR-758-3p,let-7a-5p,miR-424-5p,miR-127-3p,miR-33a-5p,miR-376b,miR-758-3p,miR-1b-5p,miR-342,miR-320b,miR-1306-5p,miR-29b-1-5p,miR-323b-3p,miR-1185-5p,miR-3955-3p,miR-214-3p,miR-103a-2-5p,let-7e,miR-20a-3p,miR-211-5p,miR-378g,let-7d-5p,miR-454-5p,miR-4443,miR-668-5p,miR-335-3p,miR-877-3p,miR-9-3p,novel_60,miR-3956-3p,miR-219a-5p,miR-362-5p,novel_82,miR-30b-5p,miR-382-3p,miR-2285n,miR-218-5p,miR-2285y,miR-107-5p,miR-301a-5p,miR-335,miR-490-5p,miR-3600,miR-340-5p,miR-15a-5p,miR-2284h-5p,novel_83,miR-532-3p,miR-30c-5p,miR-2366,miR-1961,miR-2300b-3p,miR-193b-5p,miR-2904,miR-1197-5p,miR-188-3p,miR-142-3p,miR-33b-3p,miR-4429,miR-664-5p,miR-378d,miR-345-3p,miR-29b-1-5p,miR-181b-1-3p,miR-2432,miR-30b,miR-210-5p,miR-125b,miR-345-3p,miR-320d,miR-760-3p,miR-466i-5p,miR-98-5p,miR-30d,let-7k,miR-2331-3p,miR-216b-3p,miR-21-3p,miR-552-3p,miR-582-5p,miR-377-3p,miR-193b-3p,novel_25,let-7i-5p,miR-877-5p,miR-15b-5p,miR-3613-5p,miR-500a-3p,miR-27b-5p,miR-30d-5p,miR-411,miR-345-5p,miR-30f,miR-339b,let-7b,miR-145a-3p,miR-214,miR-2330-3p,miR-346,miR-29c-5p,miR-224-5p,let-7b-5p,let-7f,miR-29d-5p,miR-324-3p,miR-450b-3p,miR-17-3p,miR-487a-5p,miR-500,miR-103a-3p,miR-133b-5p,miR-339-5p,miR-26b-3p,miR-3431,miR-134-3p,let-7g-5p,miR-194-3p,miR-103,miR-2355-3p,miR-223-3p,miR-1843b-5p,miR-450c-3p,miR-197-3p,miR-374b-3p,novel_23,miR-138,miR-2284ab,miR-497-5p,miR-654-3p,miR-2284l,miR-345-5p,miR-224-5p,miR-377-3p,miR-105-5p,novel_51,miR-2411-3p,miR-378a-5p,miR-1343-5p,miR-125a-5p,miR-193a-3p,novel_68,miR-3120-3p,miR-670-3p,miR-374c-
ADH6	ENSOARG0(	alcohol	miR-6740-5p,miR-1973,novel_82,miR-147-5p,miR-487a-5p,miR-2285t,miR-208b-5p,novel_1,miR-1839-3p,miR-4510,miR-129b-3p,novel_99,miR-1827,miR-339b,miR-876-3p,miR-10b-3p,miR-3120-3p,miR-216b-3p,miR-139-5p,miR-6130,miR-139-5p,miR-374c-3p,miR-2404,miR-339a,miR-205-5p,miR-30b-3p,miR-153-3p,novel_124,miR-376b,miR-1839-3p,miR-216c-5p,miR-188-3p,novel_23,miR-5703,miR-197-3p,miR-2300b-3p,miR-708-3p,miR-3120-5p,novel_79,miR-339-5p,novel_78,miR-6517,miR-133b-5p,novel_121,miR-411-5p,miR-153

ZC2HC1B	ENSOARG0(	zinc fin	miR-107,miR-195a-5p,miR-135b-5p,miR-2285ab,miR-2387,miR-378b,miR-21c,miR-146b-3p,miR-7-1-3p,miR-203b-5p,miR-502b,miR-15a,miR-133a-5p,miR-16a,miR-33a-3p,miR-24-3p,miR-326-3p,miR-2428,novel_120,miR-2285x,miR-30b-3p,miR-138,miR-21-5p,miR-181a-5p,miR-1434-3p,miR-3071-5p,miR-145a-5p,miR-432-5p,miR-103,miR-133b-5p,miR-124-5p,miR-224-5p,miR-330-3p,miR-197-5p,miR-539-3p,miR-296-3p,miR-101-5p,let-7g-3p,novel_48,miR-1814c,miR-485-5p,miR-488-3p,miR-381-3p,miR-769,miR-487a-3p,miR-138-5p,miR-28-5p,miR-486b-3p,miR-1-5p,miR-135a-1-3p,miR-331-3p,miR-135a-5p,miR-10b-3p,miR-1306-5p,miR-6128,miR-3607-3p,miR-133a-3p,miR-103b,miR-582-3p,miR-300,let-7a-2-3p,miR-3578,miR-218-2-3p,miR-30a-5p,miR-2411-5p,miR-6516-5p,miR-222,miR-2898,miR-200c-3p,miR-107,miR-2285af,miR-200c,miR-30c-2-3p,miR-217,miR-1957a,miR-2284a,miR-3529-3p,miR-4286,novel_94,miR-7857-3p,miR-489,miR-541-5p,miR-7977,miR-4726-5p,miR-335,miR-30b-5p,miR-371a-5p,miR-769-5p,miR-8095,miR-154a,novel_60,miR-2285f,miR-101a-5p,miR-450b-5p,miR-3120-5p,miR-484,miR-30b-3p,miR-125b-2-3p,miR-142-3p,miR-216a-3p,miR-221-3p,miR-151a-3p,miR-3955-3p,novel_103,miR-1306-5p,miR-326,miR-362-3p,miR-30c-1-3p,miR-3187-3p,miR-424-5p,miR-2330-5p,let-7g-3p,miR-133b-3p,miR-2312,miR-154b-3p,miR-30f,novel_99,miR-27b-5p,miR-6516,miR-6130,miR-139-5p,miR-22-3p,miR-877-5p,miR-532-5p,miR-6516-3p,miR-664a,miR-208b-5p,miR-708-5p,miR-424-5p,miR-222-3p,miR-369-5p,miR-324-3p,miR-145a-3p,miR-4510,miR-362-3p,miR-2300b-3p,miR-2904,miR-1193,miR-487a-3p,miR-15a-5p,novel_83,miR-3600,miR-485-3p,miR-30d,miR-30b-3p,miR-2411,miR-2432,miR-216c-5p,miR-2319a,miR-1306,miR-150-5p,miR-6535,miR-329-5p,miR-379-3p,miR-142b,novel_87,miR-1a-1-5p,miR-335-5p,miR-16b,miR-16-1-
MDFIC	ENSOARG0(	MyoD fam	miR-2285p,miR-145a-5p,miR-145b

TMEM176A	ENSOARG0(	transmem	miR-4792,novel_68,miR-20a-3p,miR-541-5p,miR-211-5p,miR-378g,miR-146b-3p,miR-374c-3p,miR-3082-5p,miR-203-3p,miR-144-5p,miR-669,miR-129b-5p,miR-6516-3p,miR-195a-5p,miR-135b-5p,miR-541-3p,miR-3957-3p,novel_101,miR-452-5p,miR-211,miR-24-3p,miR-323c,miR-423-5p,novel_27,miR-8095,miR-2428,miR-219a-2-3p,miR-15a,miR-28c,miR-16a,miR-3968,miR-31-5p,novel_4,miR-26b-3p,miR-134-3p,miR-194-3p,miR-149-5p,miR-455-3p,miR-484,miR-708-3p,miR-653,miR-541,miR-374b-3p,miR-30b-3p,miR-1388-5p,miR-30b-3p,miR-380-3p,miR-16b,miR-497-5p,miR-424-5p,miR-363-5p,miR-33a-5p,miR-654-3p,miR-3187-3p,miR-503-3p,miR-377-3p,miR-29b-1-5p,miR-16b,miR-3955-3p,miR-660,miR-660-5p,miR-296-3p,miR-377-3p,miR-331-5p,miR-374c-3p,miR-6130,miR-15b-5p,miR-10b-3p,miR-7144-5p,miR-885-3p,miR-23a-5p,miR-671-5p,miR-1827,miR-539-5p,miR-4510,miR-145a-3p,miR-16-5p,miR-6128,miR-144-5p,miR-3184-5p,miR-219b-3p,miR-135a-5p,miR-320e,miR-29b-2-5p,miR-6516-3p,miR-664a,miR-708-5p,miR-424-5p,miR-204-5p,miR-486b-3p,miR-28-5p,miR-3600,miR-322-5p,miR-15a-5p,miR-532-3p,novel_83,miR-2904,miR-1197-5p,miR-5703,miR-885-3p,miR-184-3p,miR-218-1-3p,miR-378d,miR-29b-2-5p,miR-2385-3p,miR-376a-5p,miR-29b-1-5p,miR-450b-5p,miR-28b,miR-486-3p,miR-200a,miR-2432,miR-219-3p,miR-2285p,miR-92a-1-5p,miR-2898
SLURP1	ENSOARG0(	secreted	miR-433-3p,let-7g-3p,miR-1827,miR-885-3p,miR-1895,miR-504-5p,miR-2331-3p,miR-552-3p,miR-3065-3p,miR-193b-3p,miR-323-5p,miR-132-5p,miR-151b,miR-22-3p,miR-320e,miR-132-5p,miR-17-3p,miR-874-5p,miR-145a-3p,novel_44,miR-3184-5p,miR-2366,miR-362-3p,miR-34a-5p,miR-378d,miR-664-5p,miR-133a-3p,miR-151-5p,novel_17,miR-125b,miR-216a-5p,miR-760-3p,miR-34b-5p,miR-345-3p,miR-2887,miR-486-3p,miR-361-3p,miR-378j,miR-504,novel_94,miR-3064-5p,miR-7977,miR-3082-5p,miR-378g,miR-4443,miR-2440,miR-4726-5p,miR-541-3p,miR-6527,miR-877-3p,miR-361-3p,miR-362-5p,miR-423-5p,miR-541,miR-329-3p,miR-758-3p,miR-30b-3p,miR-362-3p,miR-3120-5p,miR-1343-3p,miR-222-5p,miR-147a,miR-500-5p,miR-455-3p,novel_19,miR-362-3p,miR-3085-3p,miR-2330-5p,miR-758-3p,miR-8485,miR-4532,miR-362-5p,miR-329a,miR-1248,miR-485-5p,miR-23a-5p,miR-141-3p,miR-125a,miR-370-3p,miR-2355-5p,miR-34c-5p,miR-135a-5p,miR-125b-5p,novel_9,miR-138-5p,miR-134-5p,miR-486b-3p,miR-1197-3p,miR-1260b,miR-134,miR-885-3p,miR-6517,miR-214-5p,miR-134-5p,miR-1260a,miR-205-5p,miR-17-3p,miR-2898,miR-200a-3p,miR-543-5p,miR-4286,miR-181a-2-3p,miR-3064-5p,miR-1271-3p,novel_39,miR-3065-3p,miR-2387,miR-135b-5p,novel_68,miR-3120-3p,miR-670-3p,miR-34b,miR-323a-5p,miR-6535,miR-34c,miR-203b-5p,miR-17-3p,miR-193b-3p,miR-371b-3p,miR-24-3p,miR-449a,miR-7b-5p,miR-22-3p,miR-1260b,miR-30b-3p,miR-138,miR-380-3p,miR-744-5p,miR-6239,miR-133b-5p,miR-665,miR-134-3p,novel_116,miR-194-3p,miR-7-5p,miR-193a-3p,miR-125a-5p
AKR1A1	ENSOARG0(	aldo-ket	miR-432-3p,miR-185-5p,miR-490-5p,miR-143-3p,miR-671-5p

NKAIN1	ENSOARG0(	Na+/K+ t	miR-423-5p,miR-326-3p,miR-2428,miR-877-3p,miR-24-3p,miR-2285f,miR-17-3p,miR-541-3p,miR-136-5p,miR-18a-3p,miR-2285ab,miR-361-3p,miR-3141,miR-146b-3p,miR-3082-5p,miR-411b-3p,miR-124-5p,miR-483-3p,miR-3141,miR-2331-5p,miR-326,miR-377-3p,miR-432-3p,miR-2284l,miR-432-3p,miR-574-3p,miR-30b-3p,miR-138,miR-1260b,miR-30b-3p,miR-541,miR-149-5p,miR-2459,miR-340-3p,novel_4,miR-4492,miR-486b-3p,miR-138-5p,miR-17-3p,miR-664a,miR-1843b-3p,miR-2433,miR-3184-5p,miR-1260b,miR-665-5p,novel_42,miR-4510,miR-129-2-3p,miR-885-3p,miR-625-5p,miR-7144-5p,miR-485-5p,miR-2312,miR-129b-3p,miR-137-3p,miR-3154,miR-301,miR-377-3p,miR-6130,miR-365a-3p,miR-2285aa,miR-2355-5p,miR-615-5p,miR-532-5p,miR-3653-3p,novel_127,miR-17-3p,miR-760-3p,miR-466i-5p,miR-324-3p,miR-1260a,miR-210-5p,miR-486-3p,miR-2285w,miR-664-5p,miR-378d,miR-885-3p,miR-2904,miR-1197-5p,miR-5703,miR-330-5p,miR-1193,novel_133,miR-2284h-5p,miR-1247-5p,miR-129-1-3p,novel_121,miR-505-5p,miR-483-3p,miR-18a-3p,miR-6517
CATHL3	ENSOARG0(	cathelic	miR-4324,miR-576-3p,miR-382-5p,miR-342-3p,novel_101,miR-214,novel_44,miR-1307-3p,miR-1285-5p,miR-329a,miR-625-5p,miR-485-5p,miR-30c-1-3p,miR-499b-5p,miR-377-3p,miR-299b-3p,miR-411b-3p,miR-342,miR-377-3p,miR-3071-3p,miR-362-3p,miR-500a-5p,miR-30c-2-3p,miR-124-5p,miR-153-3p,miR-214-3p,miR-299-3p,miR-466f-3p,miR-30c-1-3p,miR-2285r,miR-125b-2-3p,miR-2284l,miR-200a,miR-2411,miR-8485,novel_39,miR-2432,miR-329-3p,miR-362-3p,miR-412-3p,miR-362-3p,miR-299a-3p,miR-7641,miR-412,miR-153,miR-2411-5p

THEM6	ENSOARG0(	thioeste	miR-2285n,miR-301a-5p,miR-361-3p,miR-3956-3p,novel_60,miR-668-5p,miR-1983,miR-361-3p,miR-378g,miR-20a-3p,miR-25-5p,miR-214-3p,miR-146b,miR-431-5p,miR-1306-5p,miR-342,miR-16b,miR-3187-3p,miR-4532,miR-2330-5p,miR-424-5p,miR-1388-3p,miR-574-3p,miR-23a-3p,miR-3120-5p,miR-10b,miR-301b-5p,miR-296-5p,miR-30b-3p,miR-147a,miR-2459,miR-421,miR-2300a-5p,miR-1343-3p,miR-330-3p,miR-664a,miR-424-5p,miR-874-5p,miR-664a-5p,miR-665,miR-324-3p,miR-6525,miR-10b-5p,miR-214,miR-1827,miR-129b-3p,miR-323-5p,miR-10a,miR-15b-5p,novel_32,miR-22-3p,miR-221-5p,miR-296-3p,miR-2484,miR-221-5p,miR-210-5p,miR-125b,miR-324-3p,miR-125b-2-3p,miR-664b-3p,miR-486-3p,miR-146a-5p,miR-345-3p,miR-19b-3p,miR-2366,miR-126-3p,novel_133,miR-140-3p,miR-188-3p,miR-2904,miR-15a-5p,miR-23c,miR-148b-3p,miR-23b-3p,novel_83,miR-18a-3p,miR-505-3p,miR-128-1-5p,miR-625-3p,miR-15a,miR-16a,miR-24-3p,miR-371b-3p,miR-342-3p,miR-412-3p,miR-19a-3p,miR-1291,miR-195a-5p,miR-505-3p,miR-146b-5p,miR-9851-3p,miR-2387,miR-148a-3p,miR-18a-3p,miR-150-5p,miR-1306,miR-411b-3p,novel_69,miR-2404,miR-323a-5p,miR-7-1-3p,miR-10a-5p,miR-378a-5p,miR-125a-5p,miR-146a,novel_51,miR-330-3p,miR-6240,miR-363-5p,miR-30b-3p,miR-138,miR-16b,miR-2889,miR-497-5p,miR-873a-5p,miR-22-3p,miR-197-3p,miR-134-3p,miR-2355-3p,miR-194-3p,miR-149-5p,miR-767-5p,miR-138-5p,miR-486b-3p,miR-2433,miR-331-3p,miR-125b-5p,miR-1306-5p,miR-193a-5p,miR-16-5p,miR-767,miR-23b,miR-125a,miR-141-3p,miR-2355-5p,miR-542-5p,novel_127,miR-200a-3p,miR-152-3p,miR-200a,miR-181a-2-3p,miR-10a-5p,miR-322-5p,miR-505-5p,miR-486-5p,miR-214-5p,miR-6517,miR-431

LARP7	ENSOARG0(	La ribon	miR-181b-2-3p,miR-1343-3p,miR-330-3p,miR-1185-3p,miR-500-5p,miR-148b-5p,miR-539-5p,miR-376b-3p,miR-484,miR-2284r,miR-21b,miR-101c,miR-101a-5p,miR-2426,miR-1388-5p,miR-652-5p,miR-33a-5p,miR-106a-5p,miR-8485,miR-30c-1-3p,miR-182-5p,miR-106b-5p,miR-505,miR-2957,miR-320b,miR-199b-5p,miR-3955-3p,miR-7977,miR-20a-3p,miR-139-5p,miR-376d,miR-1185-2-3p,miR-7857-3p,miR-199b-5p,miR-140-5p,miR-493-5p,miR-376a-3p,miR-96-5p,miR-190b-5p,miR-181d-5p,miR-154a,novel_111,miR-362-5p,miR-2440,miR-3968,miR-30d-3p,miR-505-3p,miR-380-5p,miR-3600,miR-106a,miR-532-3p,miR-23c,miR-23b-3p,miR-5703,miR-188-3p,miR-30c-5p,miR-34a-5p,miR-148a-5p,miR-4429,miR-33b-3p,miR-20a,miR-329a-5p,miR-2424,miR-2411,miR-181b-1-3p,miR-377-5p,miR-376a-5p,miR-92a-1-5p,miR-125b,miR-500a-5p,miR-216a-5p,miR-210-5p,miR-299-3p,miR-21-3p,miR-30d,miR-34b-5p,miR-30b-3p,miR-99a-3p,miR-320d,miR-106a,miR-182-5p,miR-545-3p,miR-30c-1-3p,novel_25,miR-216b-5p,miR-139-5p,miR-6130,miR-6516,miR-377-3p,miR-582-5p,miR-30d-5p,miR-21a-3p,miR-582-5p,miR-30f,miR-18a-5p,miR-1271,miR-4510,miR-539-5p,miR-338-3p,miR-181c-5p,miR-6525,miR-30a-3p,miR-30e-3p,miR-130b-5p,miR-190a-5p,miR-664a,miR-17-3p,miR-377-5p,miR-665,miR-103a-3p,miR-31-5p,miR-103,miR-20b,miR-185-5p,miR-181a-5p,miR-873a-5p,miR-29b,miR-21-5p,let-7f-2-3p,let-7f-2-3p,miR-382,miR-6240,miR-380-5p,miR-130b-5p,let-7j,miR-17-5p,miR-106a-5p,miR-105-5p,miR-190a,miR-330-3p,miR-377-3p,miR-466f-3p,miR-125a-5p,miR-3120-3p,miR-329-5p,miR-181b-3p,miR-677,miR-320c,miR-299b-3p,miR-670-3p,miR-29a-3p,miR-34b,miR-146b-3p,miR-21c,miR-376b-3p,miR-3065-5p,miR-150-5p,miR-2387,miR-18b,miR-505-3p,miR-6516-3p,miR-107,miR-758-5p,miR-29c-3p,miR-17-3p,miR-495-3p,miR-2285x,miR-199b-5p,miR-449a,miR-24-

			[illegible]126b-5p,miR-3596,miR-489,miR-2483-3p,miR-500-3p,miR-27a-3p,miR-4443,miR-361-3p,miR-199b-5p,miR-378j,miR-493-5p,miR-1983,miR-2447,miR-885-5p,miR-541-3p,miR-6527,miR-181d-5p,miR-361-3p,miR-1285-5p,miR-8095,miR-502-3p,miR-423-5p,miR-2285n,miR-628-3p,miR-199b-3p,miR-1343-3p,miR-151a-3p,miR-27a-3p,miR-222-5p,miR-147a,miR-1298-3p,miR-421,miR-126a-5p,miR-541,miR-143-5p,miR-501-3p,miR-2426,miR-21b,miR-124a,miR-382-3p,miR-101b-3p,miR-106a-5p,miR-23a-3p,miR-33a-5p,miR-744-3p,miR-30c-1-3p,miR-8485,miR-2310,miR-320b,miR-106b-5p,miR-199b-5p,miR-214-3p,miR-30c-1-3p,miR-221-5p,miR-548o-3p,miR-216b-5p,miR-139-5p,miR-204-3p,miR-22-3p,miR-151b,miR-155-5p,miR-30d-5p,let-7g-3p,miR-543-3p,miR-186-5p,miR-1827,miR-145a-3p,miR-3074-5p,miR-214,miR-122-5p,miR-3184-5p,miR-181c-5p,miR-338-3p,miR-151-3p,miR-320e,miR-3604,miR-665,novel_74,miR-155-5p,miR-17-3p,miR-208b-5p,miR-548e-3p,miR-106a,miR-7859,miR-23b-3p,miR-148b-3p,miR-222-5p,miR-23c,miR-2284h-5p,miR-151-5p,novel_133,miR-376a-5p,miR-124-3p,miR-2904,miR-1434-5p,miR-140-3p,miR-4429,miR-19b-3p,miR-183-5p,miR-2285w,miR-20a,miR-345-3p,miR-664b-3p,miR-125b-2-3p,miR-320d,miR-106a,miR-221-5p,miR-21-3p,miR-3074-5p,miR-670-3p,miR-677,miR-320c,miR-148a-3p,miR-2387,miR-3970,miR-216a-3p,miR-1291,miR-412-3p,miR-19a-3p,miR-376a-2-5p,miR-136-3p,miR-17-3p,miR-199b-5p,miR-576-3p,miR-6402,miR-20a-5p,novel_1,miR-203b-5p,miR-147-5p,miR-199c,miR-31-5p,miR-133b-5p,miR-412-5p,miR-185-5p,miR-20b,miR-223-3p,miR-181a-5p,miR-22-3p,novel_23,miR-1260b,miR-27b-3p,miR-2889,miR-2284x,miR-493-5p,miR-363-5p,miR-628-5p,let-7a-2-3p,miR-197-5p,miR-
PIP4K2A	ENSOARG0(	phosphat	106a-5p,miR-17-5p,miR-124-5p,miR-452-5p,miR-466f-3p,miR-

CDH5	ENSOARG0(	cadherin	miR-2428,novel_27,miR-326-3p,miR-135a-2-3p,miR-17-3p,miR-2285x,miR-1193,novel_1,miR-502b,miR-147-5p,miR-199c,miR-203b-5p,miR-6402,miR-670-3p,miR-323a-5p,miR-378b,miR-320b,miR-1291,miR-450b-3p,miR-6395,miR-9851-3p,miR-18b,miR-2387,miR-654-3p,miR-466f-3p,miR-125a-5p,miR-503-5p,miR-455-5p,miR-503-5p,miR-185-5p,miR-544b,miR-6238,miR-187-3p,miR-30b-3p,miR-138,miR-455-5p,miR-181a-5p,miR-1434-3p,miR-873a-5p,miR-194-5p,miR-3071-5p,miR-374b-3p,miR-2903,miR-145-3p,miR-378c,miR-133a-3p,miR-138-5p,miR-410-3p,miR-370-5p,miR-410-5p,miR-409-3p,miR-144,miR-769,miR-374c-3p,miR-199a-5p,miR-370-3p,miR-485-5p,miR-3074-1-3p,miR-7144-5p,miR-23a-5p,miR-18a,miR-3154,miR-2284a,miR-199a-5p,miR-2403,miR-4286,miR-181b-5p,miR-17-3p,miR-450a-2-3p,miR-2285af,miR-30c-2-3p,miR-378h,miR-128-3p,miR-105-2,miR-134,miR-345-3p,miR-501-3p,miR-30a-5p,miR-218-2-3p,miR-502-3p,miR-212-3p,miR-3059-5p,miR-194b-5p,miR-181d-5p,miR-4726-5p,miR-7134-3p,miR-30b-5p,miR-382-3p,miR-769-5p,miR-378e,miR-27a-3p,miR-7977,miR-199b-5p,miR-30c-1-3p,miR-491-3p,miR-193a,miR-1306-5p,novel_115,novel_103,miR-326,miR-362-3p,miR-142-3p,miR-216a-3p,miR-9788-3p,miR-105-1,miR-409b,miR-222-5p,miR-27a-3p,miR-101a-5p,miR-2426,miR-544a,miR-378c,miR-143-5p,miR-378b,miR-30b-3p,miR-7857,miR-6134,miR-338-3p,miR-4510,miR-145a-3p,miR-378c,miR-3074-2-3p,miR-6516-3p,miR-664a,miR-17-3p,miR-155-5p,miR-139-5p,miR-323-5p,miR-6130,miR-544-3p,miR-155-5p,miR-204-3p,novel_32,miR-22-3p,miR-378a-3p,miR-221-5p,miR-370-5p,miR-30f,miR-18a-5p,miR-133b-3p,miR-582-5p,miR-466i-5p,miR-760-3p,miR-21-3p,miR-30d,miR-221-5p,miR-30b-3p,miR-125b,miR-148b-3p,miR-362-3p,miR-2300b-3p,novel_133,miR-140-3p,miR-5703,miR-2904,miR-124-3p,miR-
GNB1	ENSOARG0(	guanine	miR-431,novel_83,miR-148b-5p,miR-194-3p,novel_133,miR-19b-2-5p,miR-2284r,miR-541,miR-133a-3p,miR-218-1-3p,miR-148a-5p,miR-493-3p,miR-2385-3p,miR-29b-2-5p,miR-494-3p,miR-3065-3p,miR-2432,miR-654-3p,miR-376a-5p,miR-148b-5p,miR-197-5p,miR-3071-3p,miR-2957,miR-219-3p,miR-431-5p,miR-370-3p,miR-3065-3p,miR-3120-3p,miR-615-5p,miR-129b-3p,miR-361-3p,miR-1983,miR-485-5p,miR-1248,miR-410-5p,miR-299b-5p,miR-412-3p,miR-3958-5p,miR-6128,miR-495-3p,miR-3074-2-3p,miR-541-3p,miR-3184-5p,miR-423-5p,miR-219a-2-3p,miR-2428,miR-2285m,miR-361-3p,miR-19b-1-5p,miR-219b-3p,miR-425-5p,miR-665,miR-29b-2-5p,miR-410-5p,novel_82,miR-15b-3p,miR-203b-5p,miR-2285t,miR-628-3p,miR-664a
PITPNM3	ENSOARG0(	PITPNM f	miR-33a-5p,miR-2330-5p,miR-424-5p,miR-503-5p,miR-130b-5p,miR-1343-5p,miR-16b,novel_51,miR-217,novel_115,miR-503-5p,miR-503-5p,miR-15a-5p,miR-322-5p,miR-6517,miR-497-5p,miR-16b,miR-217-5p,miR-1343-5p,miR-2904,miR-541,miR-1285-5p,miR-346,miR-16-5p,miR-541-3p,novel_42,miR-130b-5p,miR-147-5p,miR-502b,miR-424-5p,miR-331-3p,miR-16a,miR-15a,miR-15b-5p,miR-20a-3p,miR-195a-5p,miR-7144-5p

VSTM4	ENSOARG0(	V-set an	miR-532-3p,novel_83,miR-2355-3p,novel_69,miR-30c-1-3p,miR-548w,miR-1343-3p,miR-1260b,miR-124-3p,miR-2904,miR-193b-5p,miR-193a,miR-30c-1-3p,miR-1260b,miR-24-3p,miR-124a,miR-1973,miR-30c-2-3p,miR-4324,miR-1260a
CLUAP1	ENSOARG0(	clusteri	miR-3068-3p,miR-874-3p,miR-204-3p,miR-17-5p,miR-320a,miR-93-5p,miR-2285c,miR-20b-5p,miR-2312,miR-2330-3p,miR-3074-2-3p,miR-1271,miR-206-3p,miR-93,miR-320e,miR-219b-3p,miR-34c-3p,miR-2285j,miR-129-5p,miR-4492,miR-212-5p,miR-106a,miR-18a-3p,novel_83,miR-1271-5p,miR-330-5p,miR-1197-5p,miR-4429,miR-493-3p,miR-181a-2-3p,miR-1,miR-20a,miR-3529-3p,miR-2285b,miR-29b-1-5p,miR-185-3p,miR-219-3p,novel_127,miR-543-5p,miR-466i-5p,miR-106a,miR-320d,miR-7-1-3p,miR-320b,miR-320c,miR-3082-5p,miR-18a-3p,miR-3065-5p,miR-2285ab,miR-361-3p,miR-96-5p,miR-2285f,miR-2447,miR-219a-2-3p,miR-326-3p,miR-20a-5p,miR-147-5p,miR-206,novel_1,miR-499a-5p,miR-145b,miR-105-1,miR-20b,miR-2285e,miR-2285g,miR-145a-5p,miR-1a-3p,miR-7b-5p,miR-744-3p,miR-668-3p,miR-106a-5p,miR-2285r,novel_51,miR-106b-5p,miR-17-5p,miR-106a-5p,miR-505,miR-105-5p,miR-29b-1-5p,miR-326,miR-7-5p,miR-320b,miR-378a-5p
PSMG2	ENSOARG0(	proteaso	miR-374b-3p,miR-21b,miR-1839-5p,miR-380-3p,miR-2426,miR-421,miR-412-5p,miR-147a,miR-197-5p,miR-146a,miR-146b,miR-452-5p,miR-196b-5p,miR-628-5p,miR-382-3p,let-7a-2-3p,miR-361-3p,miR-2387,miR-146b-5p,miR-335-3p,miR-1983,miR-379-5p,miR-412-3p,miR-541-5p,miR-669,miR-146b-3p,miR-133a-5p,miR-28c,miR-374a-3p,miR-335,miR-1193,miR-3059-5p,miR-335-5p,miR-423-5p,miR-8095,miR-7134-5p,miR-23b-5p,novel_133,miR-196a-5p,miR-664-5p,miR-361-5p,let-7a-2-3p,miR-184-3p,miR-423-3p,miR-545-5p,miR-582-3p,miR-7859,miR-185-3p,miR-6119-5p,miR-3529-3p,miR-2432,miR-146a-5p,miR-28b,miR-376a-5p,miR-3154,miR-485-5p,miR-10b-3p,let-7g-3p,let-7g-3p,miR-2285aa,miR-370-5p,miR-2355-5p,miR-296-3p,miR-6130,miR-370-5p,miR-708-5p,miR-31-3p,miR-374b-3p,miR-28-5p,miR-6128,miR-3607-3p,miR-4510,miR-3184-5p,miR-6134,miR-374a-3p,miR-1197-3p

CCDC42	ENSOARG0(	coiled-c	miR-2285c,miR-2312,miR-186-5p,miR-378a-3p,miR-370-5p,miR-21-3p,miR-544-3p,miR-139-5p,miR-6130,miR-22-3p,let-7i-5p,let-7f,miR-19b-1-5p,let-7b,miR-378c,miR-3074-5p,miR-4510,let-7b-5p,miR-338-3p,miR-378i,novel_133,miR-2366,miR-1961,miR-2300b-3p,miR-2904,miR-1197-5p,miR-378a-3p,miR-5703,miR-4429,miR-148a-5p,miR-2284u,miR-125b,miR-422a,miR-345-3p,miR-320d,miR-98-5p,miR-30b-3p,miR-299-3p,let-7k,miR-216c-5p,miR-329a-5p,miR-29b-1-5p,miR-486-3p,miR-146a-5p,miR-1983,miR-9-3p,miR-2284g,miR-139-5p,miR-211-5p,miR-378g,miR-378e,miR-203-3p,let-7d-5p,miR-4443,miR-2285y,miR-301a-5p,miR-2285t,novel_60,miR-3956-3p,miR-2285f,miR-9-3p,miR-378b,miR-301b-5p,miR-30b-3p,miR-378c,miR-544a,miR-101c,miR-9788-3p,miR-148b-5p,miR-2285e,miR-320b,novel_115,miR-1306-5p,miR-29b-1-5p,miR-491-5p,miR-3955-3p,miR-146b,let-7e,let-7a-5p,miR-382-3p,miR-744-3p,miR-378d,miR-3154,miR-320a,miR-1248,miR-125a,miR-1271,miR-615-5p,miR-370-5p,miR-331-3p,miR-125b-5p,miR-204-5p,miR-486b-3p,miR-378c,miR-345-3p,let-7d,miR-2285ad,miR-299a-3p,miR-24-2-5p,miR-378h,miR-2285af,let-7c-5p,miR-127-5p,let-7f-5p,novel_96,miR-148b-5p,let-7g,novel_39,miR-2284y,miR-2284a,miR-2285ab,miR-1306,miR-412-3p,miR-146b-5p,miR-378f,let-7e-5p,miR-329-5p,miR-3074-5p,miR-299b-3p,novel_69,miR-320c,miR-669,miR-6535,miR-576-3p,let-7i,miR-22-5p,miR-147-5p,miR-1193,miR-211,miR-24-3p,miR-2428,miR-19b-2-5p,miR-22-3p,miR-30b-3p,miR-219a-1-3p,miR-188-5p,miR-412-5p,miR-2285g,let-7g-5p,miR-134-3p,miR-194-3p,miR-544b,miR-146a,miR-2411-3p,novel_51,miR-125a-5p,miR-432-3p,miR-432-3p
ORC5	ENSOARG0(	origin r	miR-27b-3p,miR-3120-5p,miR-135b-5p,miR-128-3p,miR-127-5p,miR-135a-5p,miR-216a-3p,miR-27a-3p,miR-616-3p,miR-505-5p,miR-27a-3p

ARHGAP26	ENSOARG0(	Rho GTPa	miR-2285n,miR-30b-5p,miR-2285y,miR-107-5p,novel_60,miR-3059-5p,miR-181d-5p,miR-9-3p,miR-129b-5p,miR-20a-3p,miR-10b,miR-7977,miR-7857-3p,miR-3082-5p,miR-342,novel_103,miR-326,miR-542-5p,miR-424-5p,miR-33a-5p,miR-193a,miR-10b,miR-2300a-5p,miR-5010-3p,miR-222-5p,miR-142-3p,miR-216a-3p,miR-2285e,miR-324-3p,miR-6516-3p,miR-664a,miR-424-5p,miR-4510,miR-10b-5p,miR-338-3p,miR-6134,miR-30f,novel_99,miR-660-5p,miR-21-3p,miR-323-5p,miR-6130,novel_32,miR-204-3p,miR-877-5p,miR-542-5p,miR-216a-5p,miR-125b,miR-185-3p,miR-760-3p,miR-30d,miR-34b-5p,miR-2411,miR-2904,miR-548e-3p,miR-3600,miR-200b,miR-340-5p,miR-15a-5p,miR-15a,miR-6402,miR-34c,miR-16a,miR-33a-3p,miR-203b-5p,miR-199c,miR-147-5p,miR-342-3p,miR-2285x,miR-542-5p,novel_101,miR-495-3p,miR-211,miR-3959-3p,novel_27,miR-449a,miR-326-3p,miR-18a-3p,miR-1291,miR-107,miR-9851-3p,miR-195a-5p,miR-655-5p,miR-320b,miR-10a-5p,miR-323a-5p,miR-34b,miR-670-3p,miR-330-3p,miR-455-5p,miR-1343-5p,miR-125a-5p,miR-660,miR-466f-3p,miR-130b-5p,miR-382,miR-654-3p,miR-345-5p,miR-181a-5p,miR-1434-3p,miR-145a-5p,miR-380-3p,miR-1260b,miR-219a-1-3p,miR-455-5p,miR-744-5p,miR-133b-5p,miR-2285g,miR-103,miR-31-3p,miR-204-5p,miR-486b-3p,miR-6128,miR-3607-3p,miR-381-3p,miR-488-3p,miR-7144-5p,miR-671-5p,miR-485-5p,miR-141-3p,miR-34c-5p,miR-1260a,miR-200c,miR-339a,miR-217,miR-450a-2-3p,miR-181b-5p,miR-200c-3p,miR-107,miR-3529-3p,miR-30a-3p,miR-2478,miR-1957a,miR-2284a,miR-30a-5p,miR-300,miR-128-3p,miR-200b-3p,miR-10a-5p,miR-2411-5p,novel_82,miR-301a-5p,miR-490-5p,miR-2447,miR-3956-3p,miR-2284j,novel_111,miR-423-5p,miR-335-3p,miR-9-3p,miR-544-5p,miR-1983,miR-2284g,miR-211-5p,miR-203-3p,miR-1185-2-

IFNG	ENSOARG0(	interfer	novel_121,miR-134-5p,miR-302a-5p,miR-330-5p,miR-29d-3p,miR-134,miR-345-3p,miR-29b-2-5p,miR-30a-3p,miR-3065-3p,novel_39,miR-450b-5p,miR-3071-3p,miR-664-3p,miR-1260a,miR-2285af,miR-200a-3p,miR-181b-5p,miR-127-5p,miR-17-3p,miR-141-3p,miR-125a,miR-320a,miR-3154,miR-1814c,miR-671-5p,miR-345-5p,miR-10b-3p,miR-374a-3p,miR-1260b,miR-323-3p,miR-3969,miR-100-3p,miR-125b-5p,miR-6740-5p,miR-29b-3p,novel_63,miR-134-5p,miR-374b-3p,miR-2355-3p,miR-6238,miR-134-3p,miR-3071-5p,miR-22-3p,miR-181a-5p,miR-653,miR-19b-2-5p,miR-27b-3p,miR-26a-2-3p,miR-219a-1-3p,miR-1260b,miR-29b,miR-101-5p,miR-363-5p,miR-6240,miR-345-5p,miR-432-3p,miR-432-3p,miR-2411-3p,miR-330-3p,miR-224-5p,miR-660,miR-125a-5p,miR-124-5p,miR-216b-3p,miR-181b-3p,miR-320c,miR-677,miR-29a-3p,miR-3065-5p,miR-2285ab,miR-6516-3p,miR-505-3p,miR-29c-3p,miR-495-3p,miR-17-3p,miR-2285x,miR-2284w,miR-326-3p,miR-24-3p,miR-33a-3p,miR-4324,miR-147-5p,miR-6123,miR-656-5p,miR-203b-5p,miR-29a,miR-30d-3p,miR-505-3p,miR-340-5p,miR-5703,miR-124-3p,miR-1246,novel_133,miR-361-5p,miR-4429,miR-195a-3p,miR-143-3p,miR-181b-1-3p,miR-125b-2-3p,miR-376a-5p,miR-125b,miR-345-3p,miR-216a-5p,novel_17,miR-221-5p,miR-1277-5p,miR-320d,miR-3065-3p,miR-660-5p,miR-504-5p,miR-221-5p,miR-545-3p,miR-22-3p,miR-204-3p,miR-216b-5p,miR-6130,miR-656-3p,miR-433-3p,miR-21a-3p,miR-543-3p,miR-345-5p,miR-2312,miR-3184-3p,miR-4510,miR-181c-5p,miR-224-5p,miR-29b-2-5p,miR-30a-3p,miR-19b-1-5p,miR-151-3p,miR-30e-3p,miR-6516-3p,miR-17-3p,miR-27a-3p,miR-181b-2-3p,miR-330-3p,miR-1343-3p,miR-151a-3p,miR-421,miR-455-3p,miR-9788-3p,miR-216a-3p,miR-125b-2-3p,miR-323b,miR-484,miR-3120-5p,miR-33a-5p,miR-124a,miR-2310,miR-323b-3p,miR-326,novel_115,miR-320b,miR-

MFAP5	ENSOARG0(	microfib	miR-425-5p,miR-3958-5p,miR-3074-5p,miR-129-2-3p,miR-4510,miR-378c,miR-539-5p,miR-6134,miR-181c-5p,miR-6525,miR-769-5p,miR-320e,miR-665,miR-664a-5p,miR-29b-2-5p,miR-424-5p,miR-6516-3p,miR-17-3p,miR-450b-3p,miR-30c-1-3p,miR-221-5p,miR-378a-3p,miR-15b-5p,miR-204-3p,novel_25,miR-139-5p,miR-6130,miR-27b-5p,miR-450a-1-3p,miR-186-5p,miR-345-5p,miR-20a,miR-2411,miR-486-3p,miR-181b-1-3p,miR-376c-3p,miR-345-3p,miR-422a,miR-500a-5p,novel_17,miR-221-5p,miR-320d,miR-106a,miR-2284m,miR-106a,miR-548e-3p,miR-532-3p,miR-340-5p,miR-23c,miR-15a-5p,miR-222-5p,miR-23b-3p,miR-7859,miR-1197-5p,miR-5703,miR-378a-3p,miR-378i,miR-218-1-3p,miR-133a-3p,miR-142-3p,novel_73,miR-4429,miR-19b-3p,miR-181d-5p,miR-2284j,miR-885-5p,miR-3956-3p,miR-541-3p,novel_60,miR-3059-5p,miR-2447,miR-4454,miR-8095,miR-361-3p,miR-877-3p,miR-107-5p,miR-371a-5p,miR-769-5p,miR-218-5p,miR-374a-3p,miR-499a-5p,miR-499b-5p,miR-541-5p,miR-139-5p,miR-378e,miR-129b-5p,miR-140-5p,miR-9-3p,miR-493-5p,miR-299b-5p,miR-101b-3p,miR-106a-5p,miR-424-5p,miR-2310,miR-30c-1-3p,miR-378d,miR-106b-5p,miR-16b,miR-505,novel_115,miR-326,miR-431-5p,miR-320b,miR-103a-2-5p,miR-1185-5p,miR-181b-2-3p,miR-2300a-5p,miR-455-3p,miR-9788-3p,miR-216a-3p,miR-147a,miR-30b-3p,miR-541,miR-143-5p,miR-378b,miR-3120-5p,miR-101c,miR-21b,miR-1388-5p,miR-378c,miR-16-5p,miR-6128,miR-1306-5p,miR-378c,miR-1260b,miR-374a-3p,miR-93,miR-323-3p,miR-3969,miR-144,miR-2332,miR-2284k,miR-410-5p,miR-134-5p,miR-374b-3p,miR-486b-3p,novel_9,miR-1271,miR-2285aa,miR-503-3p,miR-3068-3p,miR-2284n,miR-376c-3p,miR-769,miR-17-5p,miR-3154,miR-320a,miR-93-5p,miR-1814c,miR-1248,miR-23a-5p,miR-20b-5p,miR-345-5p,miR-10b-3p,miR-29b-2-5p,miR-28b,miR-30c-2-

BAC5	ENSOARG0(	cathelic	miR-2284d,miR-2285g,miR-503-5p,miR-134-3p,miR-31-5p,miR-497-5p,miR-30b-3p,miR-29b,miR-2284ab,miR-16b,miR-1260b,miR-873a-5p,miR-1434-3p,miR-345-5p,miR-654-3p,miR-2284l,miR-382,miR-668-3p,miR-628-5p,miR-380-5p,miR-125a-5p,miR-193a-3p,miR-124-5p,miR-483-3p,miR-378a-5p,miR-2411-3p,miR-503-5p,miR-320c,miR-29a-3p,miR-655-5p,miR-195a-5p,miR-6395,miR-1291,miR-376b-3p,miR-2284w,miR-1839-3p,miR-193b-3p,novel_101,miR-17-3p,miR-29c-3p,miR-6123,miR-29a,miR-16a,miR-382-5p,miR-4324,miR-15a,miR-28c,miR-142b,miR-2284z,miR-322-5p,miR-505-5p,miR-483-3p,miR-2411-5p,miR-758-5p,miR-412,miR-582-3p,miR-29d-3p,miR-23b-5p,miR-345-3p,miR-380-5p,miR-28b,miR-2403,miR-543-5p,novel_96,miR-17-3p,miR-30c-2-3p,miR-1260a,miR-376b-3p,miR-2285af,miR-125a-3p,miR-154b-5p,miR-2284n,miR-3965,miR-215-5p,miR-125a,miR-345-5p,miR-320a,miR-3154,miR-488-3p,miR-2448-3p,miR-503-3p,miR-1260b,miR-374a-3p,miR-16-5p,miR-2284v,miR-374b-3p,miR-592,miR-486b-3p,miR-125b-5p,miR-29b-3p,miR-2284k,miR-1306-3p,miR-2285e,miR-425-3p,miR-142-3p,miR-376b-3p,miR-1298-3p,miR-5010-3p,miR-1343-3p,miR-2300a-5p,miR-2284aa,miR-1388-5p,miR-2426,miR-412-3p,miR-30b-3p,miR-4532,miR-8485,miR-3187-3p,miR-30c-1-3p,miR-676-3p,miR-424-5p,miR-1839-3p,miR-214-3p,miR-1185-5p,miR-491-5p,miR-182-5p,miR-16b,miR-320b,miR-376d,miR-378g,miR-541-5p,miR-376a-3p,miR-129b-5p,miR-1306-3p,miR-423-5p,miR-7705,miR-154a,miR-2284j,miR-219a-5p,miR-3059-5p,novel_60,miR-3956-3p,miR-374a-3p,miR-2284q,novel_82,novel_83,miR-328-3p,miR-15a-5p,miR-4429,miR-140-3p,miR-2300b-3p,miR-215-5p,miR-2411,miR-2432,miR-486-3p,miR-143-3p,miR-142a-3p,miR-503-5p,miR-21-3p,miR-2284m,miR-320d,miR-324-3p,miR-125b,miR-345-3p,miR-185-3p,miR-196a-3p,miR-877-

KPNA4	ENSOARG0(	karyophe	miR-1197-5p,miR-188-3p,miR-19b-3p,miR-548e-3p,miR-532-3p,miR-23b-3p,miR-15a-5p,miR-23c,miR-345-3p,miR-3653-3p,miR-345-3p,miR-2424,miR-376a-5p,miR-130a-5p,miR-543-3p,miR-3065-3p,miR-545-3p,miR-32-3p,novel_25,miR-15b-5p,miR-377-3p,miR-2285j,miR-424-5p,miR-208b-5p,miR-129-2-3p,miR-181c-5p,novel_44,miR-101c,miR-181b-2-3p,miR-105-1,miR-376b-3p,miR-505,miR-16b,miR-342,miR-199b-5p,miR-23a-3p,miR-33a-5p,miR-1388-3p,miR-424-5p,miR-101b-3p,miR-491-3p,miR-199b-5p,miR-376a-3p,miR-493-5p,miR-335-3p,miR-2285u,miR-664b,miR-20a-3p,miR-376d,miR-500-3p,miR-378g,miR-371a-5p,miR-101-3p,miR-374a-3p,miR-181d-5p,miR-32-3p,miR-502-3p,miR-1285-5p,miR-877-3p,miR-29d-3p,miR-501-3p,miR-345-3p,miR-3591-5p,miR-105-2,miR-7641,miR-376a-5p,miR-129-1-3p,miR-322-5p,miR-24-2-5p,miR-376b-3p,miR-664-3p,miR-200a-3p,miR-181b-5p,miR-3065-3p,miR-200a,miR-199a-5p,miR-490-3p,miR-2478,miR-331-5p,miR-7144-5p,let-7g-3p,miR-141-3p,miR-199a-5p,miR-300-3p,miR-100-3p,miR-3969,miR-135a-5p,miR-29b-3p,novel_63,miR-331-5p,miR-16-5p,miR-3071-5p,miR-181a-5p,miR-26a-2-3p,miR-497-5p,miR-29b,miR-16b,miR-3955-5p,miR-149-5p,miR-134-3p,miR-105-5p,miR-2411-3p,miR-377-3p,miR-124-5p,miR-493-5p,let-7j,let-7a-2-3p,miR-654-3p,miR-2285r,miR-432-3p,miR-376b-3p,miR-3065-5p,miR-135b-5p,miR-195a-5p,miR-376a-2-5p,miR-450b-3p,miR-19a-3p,novel_68,miR-677,miR-181b-3p,miR-29a-3p,miR-411b-3p,miR-16a,miR-15a,miR-6123,miR-29a,novel_1,miR-29c-3p,miR-2285x,miR-342-3p,miR-199b-5p

AICDA	ENSOARG0(	activati	[illegible] 5p,miR-378c,miR-539-5p,miR-590-3p,miR-129-2-3p,miR-129-5p,miR-487a-5p,novel_74,miR-487b-5p,miR-155-5p,miR-151-3p,miR-155-5p,miR-6516,miR-376e-3p,miR-140-5p,miR-2331-3p,miR-30c-1-3p,miR-378a-3p,miR-543-3p,miR-129b-3p,miR-299,miR-21a-3p,miR-2424,miR-2432,miR-376a-5p,miR-664b-3p,miR-376c-3p,miR-130a-5p,miR-5100,miR-487a,miR-21-3p,miR-466i-5p,miR-219b-5p,miR-422a,miR-125b,miR-2285p,novel_17,miR-487a-3p,miR-7859,miR-200b,miR-380-5p,miR-3600,miR-485-3p,miR-548e-3p,miR-133a-3p,miR-361-5p,miR-378a-3p,miR-217-5p,miR-378i,miR-2408,miR-2366,miR-1246,novel_111,novel_60,miR-3059-5p,miR-3956-3p,miR-374a-3p,miR-628-3p,miR-371a-5p,miR-218-5p,miR-27a-3p,miR-378e,miR-2483-3p,miR-3082-5p,miR-499b-5p,miR-7977,miR-664b,miR-452-3p,miR-20a-3p,miR-335-3p,miR-877-3p,miR-378j,miR-140-5p,miR-193a,miR-2310,miR-30c-1-3p,miR-378d,miR-33a-5p,miR-376b,miR-1388-3p,miR-101b-3p,miR-214-3p,miR-455-3p,miR-27a-3p,miR-151a-3p,miR-2300a-5p,miR-21b,miR-2284aa,miR-378c,miR-299a-5p,miR-378b,miR-484,miR-378c,miR-323-3p,miR-374a-3p,miR-374b-3p,miR-592,miR-331-5p,miR-26c,miR-144,miR-125b-5p,miR-2433,miR-376c-3p,miR-331-5p,miR-370-3p,miR-125a,miR-141-3p,miR-487a-3p,miR-376c-5p,miR-23a-5p,miR-1814c,miR-671-5p,novel_91,miR-2318,miR-3154,miR-376c-5p,miR-331-5p,miR-2448-3p,miR-1957a,miR-3956-5p,miR-148b-5p,miR-380-5p,miR-200a-3p,miR-181b-5p,miR-200c-3p,miR-30c-2-3p,miR-30f,miR-217,miR-2285af,miR-200c,miR-664-3p,miR-2284z,miR-129-1-3p,novel_121,miR-505-5p,miR-6516-5p,miR-378h,miR-6517,miR-429-3p,miR-200b-3p,miR-155-5p,miR-218-2-3p,novel_27,miR-2285m,miR-2285x,miR-758-5p,miR-656-5p,miR-421-5p,miR-203b-5p,miR-485-3p,miR-502-5p,miR-

DRGX	ENSOARG0(	dorsal r	miR-543-3p,miR-1827,miR-186-5p,miR-345-5p,miR-129b-3p,miR-500a-3p,miR-655,miR-10a,miR-216b-3p,miR-32-5p,miR-2331-3p,miR-30c-1-3p,miR-500,miR-130b-5p,miR-30a-3p,miR-665,miR-30e-3p,miR-3184-5p,miR-769-5p,miR-6525,miR-224-5p,miR-214,miR-3074-5p,miR-664-5p,novel_73,miR-191,miR-193b-5p,miR-2284h-5p,miR-328-3p,miR-30d-3p,miR-3653-3p,miR-324-3p,miR-122-3p,miR-92a-1-5p,miR-210-5p,miR-486-3p,miR-329a-5p,miR-2285w,miR-493-5p,miR-1983,miR-136-5p,miR-203-3p,miR-1185-2-3p,miR-7689-3p,miR-139-5p,miR-2284g,miR-2319b,miR-490-5p,miR-3968,miR-499a-5p,miR-101-3p,miR-423-5p,miR-877-3p,miR-361-3p,miR-2284j,miR-2447,miR-363-3p,miR-1839-5p,miR-362-3p,miR-329-3p,miR-1185-3p,miR-421,miR-2459,miR-145b,miR-1343-3p,miR-330-3p,miR-199b-5p,miR-214-3p,miR-182-5p,miR-199a-3p,miR-431-5p,miR-124a,miR-101b-3p,miR-1248,miR-345-5p,miR-329a,miR-329b,miR-223-5p,miR-300-3p,miR-25-3p,miR-2285aa,miR-125a,miR-503-3p,miR-874-3p,miR-592,miR-191-5p,miR-125b-5p,miR-100-3p,miR-2284k,miR-1197-3p,miR-1260b,miR-193a-5p,novel_42,miR-330-5p,miR-3591-5p,novel_121,miR-505-5p,miR-1224-5p,miR-431,miR-6517,miR-200a-3p,miR-2284y,miR-2285b,miR-92b-3p,miR-2319a,miR-412-3p,miR-1306,miR-3591-3p,miR-6535,miR-374c-3p,miR-3074-5p,miR-329-5p,miR-6123,miR-199b-5p,miR-193b-3p,miR-335-5p,miR-197-3p,miR-19b-2-5p,miR-432,miR-7b-5p,miR-542-3p,miR-31-5p,miR-193a-3p,miR-32,novel_51,miR-7-5p,miR-2285r,miR-432-3p,let-7f-2-3p,let-7f-2-3p,miR-6240,miR-493-5p,novel_99,miR-2312,miR-27b-5p,miR-204-3p,novel_32,miR-132-5p,miR-544-3p,miR-139-5p,miR-6130,miR-193b-3p,miR-3065-3p,miR-21-3p,miR-182-5p,miR-496-3p,miR-708-5p,miR-6516-3p,miR-664a,miR-19b-1-5p,miR-655-3p,miR-320e,miR-6134,miR-338-3p,miR-10b-5p,miR-145a-3p,miR-4510,miR-

GPR83	ENSOARG0(	G protei	miR-20a,miR-216c-5p,miR-130a-5p,miR-29b-1-5p,miR-185-3p,miR-125b,miR-21-3p,miR-760-3p,miR-106a,miR-532-3p,novel_83,miR-148b-3p,miR-15a-5p,miR-200b,miR-5703,miR-2904,miR-4510,miR-145a-3p,miR-539-5p,miR-487a-5p,miR-424-5p,miR-708-5p,miR-208b-5p,miR-17-3p,miR-664a,miR-370-5p,novel_25,miR-6130,miR-193b-3p,miR-139-5p,miR-376b,miR-494-3p,miR-106a-5p,miR-424-5p,miR-30c-1-3p,novel_103,miR-326,miR-342,miR-3085-3p,miR-27a-3p,miR-105-1,miR-409b,miR-216a-3p,miR-9788-3p,miR-30b-3p,miR-125b-2-3p,miR-6529a,miR-484,miR-3120-5p,miR-2284aa,miR-181d-5p,miR-541-3p,miR-3059-5p,miR-219a-2-3p,miR-2285n,miR-2285t,miR-335,miR-4726-5p,miR-3064-5p,miR-454-5p,miR-27a-3p,miR-129b-5p,miR-361-3p,miR-877-3p,miR-4286,miR-30c-2-3p,miR-200c,miR-2285af,miR-376b-3p,miR-1260a,miR-107,miR-200c-3p,miR-181b-5p,miR-17-3p,miR-214-5p,miR-105-2,miR-128-3p,miR-200b-3p,miR-6128,miR-365a-5p,miR-93,miR-135a-5p,miR-370-5p,miR-410-5p,miR-204-5p,miR-28-5p,miR-31-3p,miR-138-5p,miR-141-3p,miR-615-5p,miR-2284n,miR-17-5p,miR-3154,miR-485-5p,novel_48,miR-345-5p,miR-296-3p,miR-106a-5p,miR-17-5p,miR-330-3p,miR-224-5p,miR-1343-5p,miR-125a-5p,miR-26b-3p,miR-665,miR-133b-5p,miR-103,miR-20b,miR-185-5p,miR-432-5p,miR-2284d,miR-145a-5p,miR-181a-5p,miR-873a-5p,miR-1260b,miR-2284ab,miR-138,miR-30b-3p,miR-1193,novel_101,miR-17-3p,miR-3957-3p,miR-342-3p,novel_120,miR-2428,miR-326-3p,miR-371b-3p,miR-24-3p,miR-211,miR-382-5p,miR-16a,miR-20a-5p,miR-6402,miR-15a,miR-203b-5p,novel_1,miR-146b-3p,miR-195a-5p,miR-135b-5p,miR-107,miR-329a-5p,miR-2424,novel_17,miR-106a,miR-7859,miR-148a-5p,miR-16-1-3p,miR-493-3p,miR-214,miR-3074-5p,miR-181c-5p,miR-122-5p,miR-3184-5p,miR-224-5p,miR-219b-3p,miR-2285i,miR-4492,miR-2331-3p,miR-

XAB2	ENSOARG0(	XPA bind	novel_68,miR-411b-3p,miR-323a-5p,miR-18a-3p,miR-1306,miR-150-5p,miR-135b-5p,miR-195a-5p,miR-412-3p,miR-1291,miR-193b-3p,miR-2428,miR-326-3p,miR-24-3p,novel_87,miR-16a,miR-28c,miR-15a,miR-6123,miR-377-5p,novel_116,miR-149-5p,miR-3431,miR-22-3p,miR-497-5p,miR-30b-3p,miR-16b,miR-363-5p,miR-2331-5p,miR-2411-3p,miR-193a-3p,miR-615-5p,miR-3956,miR-370-3p,miR-2355-5p,miR-874-3p,miR-324-5p,miR-3154,miR-485-5p,miR-1248,miR-16-5p,miR-374a-3p,miR-135a-5p,miR-100-3p,miR-2433,miR-374b-3p,miR-28-5p,miR-486b-3p,miR-412,miR-6517,miR-486-5p,miR-322-5p,miR-330-5p,miR-7134-5p,miR-345-3p,miR-4286,miR-3064-5p,miR-2285b,miR-1271-3p,miR-3065-3p,miR-28b,miR-217,novel_127,novel_96,miR-2898,miR-7977,miR-7689-3p,miR-10b,miR-3064-5p,miR-361-3p,miR-1983,miR-877-3p,miR-374a-3p,miR-4726-5p,miR-2300a-5p,miR-1343-3p,miR-151a-3p,miR-30b-3p,miR-412-3p,miR-2284r,miR-296-5p,miR-3120-5p,miR-1839-5p,let-7c-3p,miR-424-5p,miR-182-5p,miR-16b,miR-1306-5p,miR-326,miR-214-3p,miR-25-5p,miR-491-5p,miR-3085-3p,miR-532-5p,miR-182-5p,miR-3065-3p,miR-15b-5p,miR-22-3p,miR-193b-3p,miR-323-5p,miR-500a-3p,miR-129b-3p,miR-27b-5p,miR-1827,miR-1895,let-7g-3p,miR-665-5p,miR-214,miR-145a-3p,miR-6134,miR-346,miR-6525,miR-664a-5p,miR-151-3p,miR-132-5p,miR-424-5p,miR-500,miR-4492,miR-708-5p,miR-664a,miR-3600,miR-18a-3p,miR-532-3p,miR-1247-5p,miR-15a-5p,miR-217-5p,miR-188-3p,miR-2366,novel_133,miR-378d,miR-345-3p,miR-105-3p,miR-486-3p,miR-377-5p,miR-345-3p,miR-185-3p,miR-324-3p,miR-500a-5p,miR-2484,miR-21-3p,miR-760-3p

TMEM69	ENSOARG0(	transmem	miR-370-5p,miR-29b-3p,miR-3969,miR-767-5p,miR-6128,miR-3607-3p,miR-500b-5p,miR-323-3p,miR-365a-5p,miR-2448-3p,miR-503-3p,miR-488-3p,miR-320a,miR-362-5p,miR-3154,miR-7144-5p,miR-190a-3p,miR-487a-3p,miR-769,miR-200c,let-7c-5p,miR-200c-3p,miR-181b-5p,miR-2898,miR-2385-3p,miR-1,miR-30a-3p,miR-2284a,miR-1957a,miR-345-3p,miR-30a-5p,miR-29d-3p,let-7d,miR-10a-5p,miR-200b-3p,miR-128-3p,miR-7641,miR-6516-5p,miR-582-3p,let-7i,miR-147-5p,miR-656-5p,miR-22-5p,miR-203b-5p,miR-1193,miR-378b,miR-505-3p,miR-6516-3p,miR-7975,miR-10a-5p,miR-670-3p,miR-29a-3p,miR-466f-3p,miR-345-5p,miR-539-3p,miR-181a-5p,miR-653,miR-3071-5p,miR-145a-5p,miR-219a-1-3p,miR-29b,miR-30b-3p,miR-2284ab,miR-185-5p,let-7g-5p,miR-432-5p,miR-2284d,miR-544b,miR-338-5p,miR-6516-3p,miR-208b-5p,miR-145a-3p,miR-539-5p,miR-2330-3p,miR-425-5p,miR-206-3p,miR-10b-5p,miR-338-3p,let-7b-5p,miR-30f,miR-154b-3p,novel_99,miR-221-5p,miR-182-5p,miR-21-3p,miR-370-5p,miR-544-3p,miR-6516,novel_25,miR-204-3p,miR-185-3p,miR-216a-5p,miR-466i-5p,miR-2284m,miR-320d,miR-221-5p,miR-216c-5p,miR-2432,miR-2300b-3p,miR-5703,miR-4429,miR-485-3p,miR-505-3p,miR-3600,miR-148b-3p,miR-340-5p,miR-200b,miR-222-5p,miR-487a-3p,miR-383-5p,miR-199a-3p,miR-769-5p,miR-2285t,miR-3059-5p,miR-452-5p,miR-181d-5p,miR-362-5p,miR-154a,miR-877-3p,novel_94,miR-7977,miR-499b-5p,let-7d-5p,novel_115,miR-320b,miR-2957,miR-3955-3p,let-7e,let-7a-5p,miR-758-3p,miR-10b,miR-6529a,miR-125b-2-3p,miR-30b-3p,miR-544a,miR-16-2-3p,miR-2284aa,miR-142-3p,miR-9788-3p,miR-500-5p,miR-216a-3p,miR-2284k,miR-6740-5p,miR-2284v,miR-28a-3p,miR-193a-5p,miR-301,miR-30e-5p,miR-23b,miR-200a-5p,miR-345-5p,miR-2318,miR-767,miR-1248,let-7f-5p,miR-127-5p,miR-28-3p,miR-152-3p,miR-450b-
NADK	ENSOARG0(	NAD kina	miR-1306-5p,miR-206-3p,miR-132-5p,miR-151-3p,miR-2284k,miR-370-3p,miR-1271,novel_25,miR-2284n,miR-216b-5p,miR-424-3p,miR-1827,let-7g-3p,miR-3064-5p,miR-216c-5p,miR-2432,novel_39,miR-2285b,miR-1271-3p,miR-412-5p,miR-216a-5p,novel_96,miR-2284m,miR-127-5p,miR-466i-5p,miR-760-3p,miR-1343-5p,miR-1197-5p,novel_133,miR-133a-3p,miR-128-3p,miR-3059-5p,miR-1285-5p,miR-361-3p,miR-301a-5p,miR-412-5p,miR-206,miR-3064-5p,miR-669,miR-27a-3p,miR-378g,miR-216a-3p,miR-493-5p,miR-628-5p,miR-493-5p,miR-1343-5p,miR-3085-3p,miR-491-5p,miR-378a-5p,miR-27a-3p,miR-151a-3p,miR-149-5p,miR-9788-3p,miR-411-5p,miR-411b-5p,miR-2284d,miR-147a,miR-134-3p,miR-1a-3p,miR-301b-5p,miR-744-5p,miR-2284x,miR-27b-3p,miR-2284ab

CYBRD1	ENSOARG0(	cytochro	miR-93-5p,miR-23b,miR-17-5p,miR-30e-5p,novel_48,miR-20b-5p,miR-671-5p,miR-329b,miR-2285aa,miR-324-5p,miR-2284n,miR-34c-5p,miR-769,miR-2284k,miR-5100,miR-204-5p,miR-374b-3p,miR-486b-3p,miR-6128,miR-133a-3p,miR-2284v,miR-93,miR-374a-3p,miR-1260b,miR-2427,miR-30a-5p,miR-3591-5p,miR-3578,miR-105-2,miR-2285ad,miR-222,miR-2284z,novel_121,miR-9-5p,miR-24-2-5p,miR-505-5p,miR-217,miR-2285af,miR-1260a,miR-4286,miR-3529-3p,miR-2285b,miR-2478,miR-24-2-5p,miR-3591-3p,miR-9851-3p,miR-378b,miR-329-5p,miR-677,miR-323a-5p,miR-34b,miR-34c,novel_87,miR-6402,miR-20a-5p,miR-421-5p,miR-502b,novel_1,miR-136-3p,miR-449a,miR-211,miR-24-3p,miR-371b-3p,miR-22-3p,miR-1285,miR-2113,miR-380-3p,miR-2284ab,miR-1260b,miR-20b,miR-3431,miR-134-3p,miR-105-5p,miR-17-5p,miR-106a-5p,miR-668-3p,miR-363-5p,miR-130b-5p,miR-500a-3p,miR-433-3p,miR-30d-5p,novel_99,miR-186-5p,let-7g-3p,miR-133b-3p,miR-21-3p,miR-296-3p,miR-182-5p,miR-552-3p,miR-548o-3p,miR-22-3p,miR-216b-5p,miR-323-5p,miR-6130,miR-15b-3p,miR-222-3p,miR-208b-5p,miR-500,miR-4492,miR-130b-5p,novel_74,miR-214,miR-3074-2-3p,miR-145a-3p,miR-4510,miR-142a-5p,miR-769-5p,miR-124-3p,miR-5703,miR-1434-5p,miR-217-5p,miR-378d,miR-34a-5p,miR-361-5p,novel_73,miR-106a,miR-23b-3p,miR-23c,miR-30b,novel_17,miR-618,miR-374a-5p,miR-34b-5p,miR-30d,miR-106a,miR-2284m,miR-20a,miR-345-3p,miR-329a-5p,miR-216c-5p,miR-486-3p,miR-2432,miR-143-3p,miR-380-3p,miR-376a-5p,miR-664b-3p,miR-378j,miR-129b-5p,miR-221,miR-211-5p,miR-24-1-5p,miR-2319b,miR-203-3p,miR-378g,miR-7857-3p,miR-769-5p,miR-371a-5p,miR-2285y,miR-30b-5p,miR-2285t,miR-374a-3p,miR-2284q,miR-4726-5p,miR-9-5p,miR-452-5p,novel_60,miR-3956-3p,novel_111,miR-323b,miR-6529a,miR-

NR3C1	ENSOARG0(	nuclear	miR-299a-5p,miR-2426,miR-544a,miR-378c,miR-3120-5p,miR-16-2-3p,miR-1839-5p,miR-2284aa,miR-378b,miR-758-3p,miR-30b-3p,miR-1a-3p,miR-147a,miR-142-3p,miR-421,miR-1185-3p,miR-455-3p,miR-148b-5p,miR-222-5p,miR-27a-3p,miR-181b-2-3p,miR-214-3p,miR-103a-2-5p,miR-146b,miR-320b,miR-342,miR-106b-5p,miR-378d,miR-758-3p,miR-8485,miR-124a,miR-744-3p,miR-652-5p,let-7c-3p,miR-136-5p,miR-299b-5p,miR-3963,miR-361-3p,miR-381-3p,miR-378e,miR-27a-3p,miR-1185-2-3p,miR-452-3p,miR-211-5p,miR-499b-5p,miR-4726-5p,miR-218-5p,miR-30b-5p,miR-382-3p,miR-107-5p,miR-361-3p,miR-7705,miR-154a,miR-3059-5p,miR-452-5p,miR-181d-5p,miR-2285f,miR-184-3p,miR-142-3p,miR-4429,miR-361-5p,let-7a-3p,miR-148a-5p,miR-30c-5p,miR-378i,miR-1193,miR-378a-3p,miR-5703,miR-98-3p,miR-124-3p,miR-2904,miR-222-5p,miR-200b,miR-7859,miR-328-3p,novel_83,miR-30d-3p,miR-3600,miR-466i-5p,miR-202-5p,miR-320d,miR-2484,miR-1277-5p,miR-30d,miR-30b-3p,miR-30b,miR-422a,miR-92a-1-5p,miR-216a-5p,miR-142a-3p,miR-181b-1-3p,miR-376a-5p,miR-146a-5p,miR-2432,miR-183-5p,miR-20a,miR-30f,let-7g-3p,miR-2312,miR-582-5p,miR-1827,miR-543-3p,let-7f-1-3p,miR-299,miR-433-3p,miR-487b-5p,miR-30d-5p,miR-129b-3p,miR-323-5p,miR-582-5p,miR-544-3p,miR-377-3p,miR-22-3p,miR-660-5p,miR-140-5p,miR-378a-3p,miR-370-5p,miR-3065-3p,miR-21-3p,miR-17-3p,miR-487b-5p,miR-30e-3p,miR-2285j,let-7b-3p,miR-29b-2-5p,miR-665,miR-206-3p,miR-98-3p,miR-129-2-3p,miR-378c,miR-539-5p,miR-3074-2-3p,miR-214,miR-30b-3p,miR-29b,miR-21-5p,miR-7862,miR-27b-3p,miR-2284x,miR-181a-5p,miR-3071-5p,miR-22-3p,miR-544b,miR-103,miR-20b,miR-103a-3p,miR-660,miR-106b-3p,miR-146a,miR-377-3p,miR-106a-5p,miR-17-5p,miR-432-3p,miR-2284l,miR-380-5p,let-7f-2-3p,let-7f-2-
USP9X	ENSOARG0(	ubiquiti	miR-3578,miR-7857,miR-544a,miR-1260b,miR-219a-1-3p,miR-1388-5p,miR-2284aa,novel_4,miR-3955-5p,miR-548e-3p,miR-431,miR-505-5p,miR-7859,miR-2284z,miR-431-5p,miR-196a-3p,miR-1260a,miR-3071-3p,miR-505,miR-29b-1-5p,miR-760-3p,miR-200a-3p,miR-221-5p,miR-329a-5p,miR-29b-1-5p,miR-432-3p,miR-2478,miR-490-3p,miR-345-5p,miR-409-5p,miR-345-5p,miR-345-5p,miR-186-5p,miR-329-5p,miR-221-5p,miR-126b-5p,miR-141-3p,miR-3120-3p,miR-216b-3p,miR-3959-5p,miR-670-3p,miR-544-3p,miR-499b-3p,miR-499a-3p,miR-502b,miR-490-5p,miR-885-5p,miR-212-3p,miR-1260b

SMC4	ENSOARG0(	structur	miR-378d,miR-140-3p,miR-2408,miR-362-3p,novel_83,miR-616-3p,miR-199a-3p,miR-487a-3p,miR-383-5p,miR-148b-3p,miR-15a-5p,miR-3600,miR-485-3p,miR-221-5p,miR-21-3p,miR-299-3p,miR-2284m,miR-125b,miR-196a-3p,miR-146a-5p,miR-2432,miR-29b-1-5p,miR-582-5p,miR-133b-3p,let-7g-3p,miR-18a-5p,miR-27b-5p,miR-21a-3p,let-7f-1-3p,miR-22-3p,miR-877-5p,miR-6516,miR-139-5p,miR-21-3p,miR-182-5p,miR-221-5p,miR-708-5p,miR-424-5p,miR-450b-3p,miR-17-3p,miR-29b-2-5p,miR-338-5p,miR-6134,miR-3074-2-3p,miR-145a-3p,miR-2284aa,miR-2426,miR-125b-2-3p,miR-484,miR-500-5p,miR-142-3p,miR-3085-3p,miR-362-3p,miR-33a-5p,miR-424-5p,miR-877-3p,novel_94,miR-129b-5p,miR-7857-3p,miR-499b-5p,miR-541-5p,miR-3064-5p,miR-3958-3p,miR-8095,miR-219a-2-3p,miR-362-5p,miR-181d-5p,miR-212-3p,let-7a-2-3p,miR-582-3p,miR-7134-5p,miR-29d-3p,miR-302a-5p,miR-6516-5p,miR-17-3p,miR-181b-5p,miR-181c-3p,miR-217,miR-339a,miR-1260a,miR-1271-3p,miR-2478,miR-30a-3p,miR-29b-2-5p,miR-7144-5p,miR-10b-3p,let-7g-3p,miR-362-5p,miR-18a,miR-503-3p,miR-28-5p,miR-204-5p,miR-410-5p,miR-34c-3p,miR-29b-3p,miR-500b-5p,miR-133a-3p,miR-29b,miR-2284ab,miR-21-5p,miR-1260b,miR-3071-5p,miR-653,miR-1285,miR-1434-3p,miR-181a-5p,miR-2284d,miR-665,miR-26b-3p,miR-125a-5p,miR-190a,miR-330-3p,miR-539-3p,miR-382,miR-379-5p,miR-6395,miR-195a-5p,miR-21c,miR-378b,miR-18a-3p,miR-18b,miR-2404,miR-670-3p,miR-29a-3p,miR-656-5p,miR-147-5p,novel_1,miR-16a,miR-382-5p,miR-502-5p,miR-15a,novel_27,miR-211,miR-3959-3p,novel_101,miR-17-3p,miR-2285x,novel_120,miR-16-1-3p,miR-361-5p,miR-33b-3p,miR-217-5p,miR-1434-5p,miR-328-3p,miR-7859,miR-380-5p,miR-30d-3p,miR-18a-3p,miR-324-3p,miR-500a-5p,miR-219b-5p,miR-2285p,miR-26a-5p,novel_17,miR-2424,miR-376a-5p,miR-142a-3p,miR-329a-

IPP	ENSOARG0(	intracis	miR-6529a,miR-1a-3p,miR-2426,miR-450b-5p,miR-544a,miR-3120-5p,miR-16-2-3p,miR-2284aa,miR-21b,miR-330-3p,miR-27a-3p,miR-181b-2-3p,miR-142-5p,miR-153,miR-2459,miR-1185-3p,miR-500-5p,novel_103,miR-3955-3p,miR-153-3p,miR-146b,miR-124a,miR-33a-5p,miR-193a,miR-3187-3p,miR-361-3p,miR-129b-5p,novel_94,miR-335-3p,miR-20a-3p,miR-499a-3p,miR-1185-2-3p,miR-27a-3p,miR-203-3p,miR-335,miR-3968,miR-212-3p,miR-32-3p,miR-3059-5p,miR-219a-5p,miR-194b-5p,miR-2284j,miR-362-5p,miR-877-3p,miR-2300b-3p,miR-5703,miR-124-3p,miR-19b-3p,miR-378d,miR-222-5p,miR-383-5p,miR-210-5p,miR-345-3p,miR-185-3p,miR-216a-5p,miR-500a-5p,miR-2284m,miR-329a-5p,miR-10a-3p,miR-146a-5p,miR-21a-3p,miR-129b-3p,miR-18a-5p,novel_99,miR-32-3p,miR-545-3p,miR-216b-5p,miR-499b-3p,miR-544-3p,miR-377-3p,miR-155-5p,miR-665,miR-19b-1-5p,miR-155-5p,miR-664a,miR-208b-5p,miR-708-5p,miR-129-5p,miR-4492,miR-539-5p,miR-2330-3p,miR-206-3p,miR-142a-5p,miR-338-3p,miR-1285,miR-1434-3p,miR-708-3p,miR-19b-2-5p,miR-194-5p,miR-3071-5p,miR-2284ab,miR-27b-3p,miR-188-5p,miR-133b-5p,miR-31-5p,miR-2284d,miR-544b,miR-146a,miR-377-3p,miR-194a,miR-2411-3p,miR-330-3p,miR-483-3p,miR-106b-3p,miR-6240,let-7f-2-3p,let-7f-2-3p,miR-382,miR-668-3p,miR-18b,miR-2387,miR-378b,miR-19a-3p,miR-6516-3p,miR-9851-3p,miR-146b-5p,miR-329-5p,miR-181b-3p,miR-677,miR-142b,miR-6402,miR-502-5p,novel_87,miR-382-5p,miR-485-3p,miR-206,miR-625-3p,miR-203b-5p,miR-1a-1-5p,miR-335-5p,miR-323c,miR-2428,miR-2284w,novel_27,miR-134,miR-345-3p,miR-300,miR-155-5p,miR-18b-5p,miR-483-3p,miR-302a-5p,novel_121,miR-2284z,miR-134-5p,miR-30f,miR-1a-2-5p,miR-543-5p,miR-200a-3p,miR-181a-2-3p,miR-1,miR-2285b,miR-490-3p,miR-331-5p,miR-381-3p,miR-362-5p,miR-18a,miR-10a-3p,miR-7144-
SRRM4	ENSOARG0(	serine/a	miR-361-3p,miR-488-3p,miR-1291,miR-485-5p,miR-221-5p,miR-542-5p,miR-2355-5p,miR-323a-5p,miR-34c-5p,novel_69,miR-323-5p,miR-34b,miR-4443,miR-34c,miR-665,miR-2284q,miR-3059-5p,miR-145a-3p,miR-214,miR-2330-3p,miR-877-3p,miR-449a,miR-196a-5p,miR-296-5p,miR-5703,miR-374b-3p,miR-34a-5p,miR-3120-5p,miR-665,miR-222-5p,miR-2284h-5p,miR-194-3p,novel_17,miR-196b-5p,miR-2898,miR-221-5p,miR-34b-5p,miR-214-3p

SHPRH	ENSOARG0(	SNF2 his	miR-181c-5p,miR-122-5p,miR-378c,miR-3074-5p,miR-378c,miR-592,miR-222-3p,miR-144,miR-132-5p,miR-378a-3p,miR-30f, let-7g-3p,miR-424-3p,miR-2448-3p,miR-30e-5p,miR-30d-5p,miR-130a-5p,miR-380-5p,miR-450c-5p,miR-466i-5p,miR-200c-3p,miR-181b-5p,miR-2484,miR-30d,miR-664-3p,miR-200c,miR-2285af,miR-30b,miR-422a,miR-378h,miR-200b,miR-24-2-5p,miR-429-3p,miR-2285ad,miR-222,miR-142-3p,miR-30c,miR-200b-3p,novel_133,miR-30c-5p,miR-378i,miR-30a-5p,miR-378a-3p,miR-1343-5p,miR-323c,miR-9-3p,miR-181d-5p,miR-450a-5p,miR-3958-3p,miR-147-5p,miR-656-5p,miR-30b-5p,miR-670-3p,miR-489,miR-378e,miR-144-3p,miR-3074-5p,miR-20a-3p,miR-664b,miR-412-3p,miR-9-3p,miR-1983,miR-378f,miR-2319a,miR-221,miR-378j,miR-378d,miR-380-5p,miR-33a-5p, let-7c-3p,miR-1343-5p,miR-221-3p,miR-31-5p,miR-378c,miR-1388-5p,miR-181a-5p,miR-143-5p,miR-1434-3p,miR-378b,miR-708-3p,miR-484,miR-7857
APOOL	ENSOARG0(	apolipop	miR-493-5p,miR-411,miR-377-3p,miR-154b-5p,miR-493-5p
GDF3	ENSOARG0(	growth d	miR-1197-5p,miR-196a-5p,miR-10a-5p,miR-155-5p,miR-33b-3p,miR-212-5p,miR-582-3p,miR-210-3p,miR-30f,miR-125a-3p,miR-221-5p,miR-466i-5p,miR-181b-5p,miR-127-5p,miR-345-3p,miR-490-3p,miR-1271-3p,miR-376b-5p,miR-29a-5p,miR-376c-5p,miR-200a-5p,miR-1895,miR-376c-5p,miR-2312,miR-10b-3p,miR-552-3p,miR-3956,miR-221-5p,miR-504-5p,miR-155-5p,miR-10a,miR-151-3p,miR-5100,miR-155-5p,novel_74,miR-3184-3p,miR-181c-5p,miR-365a-5p,miR-145-3p,miR-10b-5p,miR-145a-5p,miR-10b,miR-181a-5p,miR-708-3p,miR-484,miR-188-5p,miR-145b,miR-222-5p,miR-151a-3p,miR-199b-3p,miR-539-5p,miR-365b-5p,miR-103a-2-5p,miR-196b-5p,miR-1b-5p,miR-2284l,miR-378j,miR-150-5p,miR-376b-5p,miR-2285ab,miR-877-3p,miR-504,miR-10a-5p,miR-203-3p,miR-107-5p,miR-2440,miR-199c,miR-490-5p,miR-181d-5p,novel_101,miR-2285f,miR-3059-5p,miR-877-3p
GRK5	ENSOARG0(	G protei	miR-133a-3p,novel_133,miR-615-3p,miR-501-3p,miR-9-5p,miR-23b-3p,miR-532-3p,miR-2285ad,miR-18a-3p,miR-3600,miR-760-3p,novel_127,miR-3653-3p,miR-125a-3p,miR-1260a,miR-185-3p,miR-500a-5p,miR-28b,miR-664b-3p,miR-490-3p,miR-1271-3p,miR-192-3p,miR-199a-5p,novel_39,miR-3064-5p,miR-23a-5p,miR-3154,miR-23b,miR-216b-5p,miR-300-3p,miR-132-5p,miR-199a-5p,miR-664a,miR-138-5p,miR-4492,miR-2332,miR-3604,miR-1260b,miR-433-5p,miR-214,miR-1260b,miR-30b-3p,miR-138,miR-21-5p,miR-455-5p,miR-2889,miR-1285,miR-541,miR-296-5p,miR-432,miR-2284r,miR-30b-3p,miR-3071-5p,miR-758-3p,miR-501-3p,miR-134-3p,miR-432-5p,miR-149-5p,miR-216a-3p,miR-1343-3p,miR-3955-5p,miR-3085-3p,miR-103a-2-5p,miR-214-3p,miR-199b-5p,novel_103,novel_51,miR-455-5p,miR-432-3p,miR-432-3p,miR-758-3p,miR-1b-5p,miR-3187-3p,miR-1388-3p,miR-23a-3p,miR-412-3p,miR-1291,miR-1983,miR-335-3p,miR-199b-5p,miR-2387,miR-381-3p,miR-21c,miR-18a-3p,miR-411b-3p,miR-378g,miR-4443,miR-500-3p,miR-378b,miR-3064-5p,miR-615,miR-28c,miR-107-5p,miR-382-5p,miR-2285y,miR-877-3p,miR-2428,miR-199b-5p,novel_27,miR-502-3p,miR-541-3p,miR-3059-5p,miR-9-5p

LAPTM5	ENSOARG0(	lysosoma	miR-182-5p,miR-2411-3p,miR-105-5p,miR-330-3p,miR-296-3p,miR-193a-3p,miR-214-3p,miR-660,miR-130b-3p,miR-491-5p,miR-376b,miR-6240,miR-296-3p,miR-6239,miR-29b,miR-181b-2-3p,miR-130a-3p,miR-330-3p,miR-105-1,miR-133b-5p,miR-1843a-3p,miR-29a,miR-29c-3p,miR-193b-3p,miR-301b-3p,miR-378b,miR-148a-3p,miR-6516-3p,miR-7977,miR-320b,miR-20a-3p,miR-181b-3p,miR-6535,miR-677,miR-29a-3p,miR-3596,miR-185-3p,miR-345-3p,miR-217,miR-543-5p,miR-152-3p,novel_96,miR-21-3p,miR-28-3p,miR-143-3p,miR-29a-5p,miR-3065-3p,miR-181b-1-3p,miR-140-3p,miR-217-5p,miR-29d-3p,miR-134,miR-193b-5p,miR-345-3p,miR-105-2,miR-582-3p,miR-1224-5p,miR-134-5p,miR-148b-3p,miR-7859,miR-6740-5p,miR-100-3p,miR-29b-3p,miR-34c-3p,miR-134-5p,miR-4492,miR-487a-5p,miR-454-3p,miR-214,miR-28a-3p,miR-1197-3p,miR-301,novel_99,miR-1827,miR-876-3p,miR-182-5p,miR-296-3p,miR-2285aa,miR-3065-3p,miR-660-5p,miR-3068-3p,novel_32,miR-193b-3p,miR-301a-3p
DZIP1	ENSOARG0(	DAZ inte	miR-504,miR-493-5p,miR-544-5p,miR-221,miR-129b-5p,miR-7857-3p,miR-203-3p,miR-1185-2-3p,miR-27a-3p,miR-139-5p,miR-3064-5p,miR-452-3p,miR-7689-3p,miR-126b-5p,miR-450a-5p,miR-335,miR-7134-3p,miR-218-5p,miR-101-3p,miR-301a-5p,miR-362-5p,novel_111,miR-154a,miR-2285f,miR-181d-5p,miR-450b-5p,miR-21b,miR-101c,miR-3120-5p,miR-2284r,miR-301b-5p,miR-125b-2-3p,miR-147a,miR-500-5p,miR-216a-3p,miR-221-3p,miR-148b-5p,miR-1185-3p,miR-330-3p,novel_78,miR-2300a-5p,miR-27a-3p,miR-181b-2-3p,miR-491-5p,miR-3085-3p,miR-542-5p,miR-320b,miR-199a-3p,miR-29b-1-5p,miR-16b,miR-182-5p,miR-193a,miR-1b-5p,miR-1388-3p,miR-494-3p,miR-424-5p,miR-23a-3p,miR-2312,miR-345-5p,miR-625-5p,novel_99,miR-1827,miR-433-3p,miR-6516,miR-2284s,miR-139-5p,miR-6130,miR-15b-5p,miR-504-5p,miR-21-3p,miR-532-5p,miR-182-5p,miR-664a,miR-424-5p,miR-708-5p,miR-208b-5p,miR-2285j,miR-6525,miR-346,miR-181c-5p,miR-129-2-3p,miR-4510,miR-2330-3p,miR-184-3p,novel_73,miR-148a-5p,miR-34a-5p,miR-2300b-3p,miR-7859,miR-23b-3p,miR-95-3p,miR-15a-5p,miR-200b,miR-23c,miR-199a-3p,miR-380-5p,miR-320d,miR-466i-5p,miR-3653-3p,miR-34b-5p,miR-299-3p,miR-542-5p,miR-2285p,miR-618,miR-500a-5p,miR-345-3p,miR-29b-1-5p,miR-130a-5p,miR-486-3p,miR-143-3p,miR-345-3p,miR-107,miR-195a-5p,miR-2285ab,miR-3591-3p,miR-216a-3p,miR-299b-3p,miR-411b-3p,miR-670-3p,miR-34b,miR-669,miR-320c,miR-677,miR-181b-3p,miR-320b,miR-3120-3p,novel_1,miR-502-5p,miR-15a,miR-28c,miR-34c,miR-485-3p,miR-16a,miR-382-5p,miR-323c,miR-449a,miR-335-5p,miR-3957-3p,miR-2285x,miR-542-5p,miR-758-5p,miR-16b,miR-219a-1-3p,miR-497-5p,miR-27b-3p,miR-7862,miR-432,miR-181a-5p,miR-1843b-5p,miR-197-3p,miR-432-5p,miR-185-5p,miR-3431,novel_116,miR-103,miR-103a-3p,miR-

SF3B2	ENSOARG0(	splicing	miR-18a-3p,miR-1224-5p,miR-322-5p,miR-378h,miR-758-5p,miR-15a-5p,miR-2284z,miR-450a-1-3p,miR-214-5p,miR-378i,miR-1197-5p,miR-378a-3p,miR-378d,miR-34a-5p,miR-24-2-5p,miR-380-5p,miR-1957a,miR-199a-5p,miR-2285af,miR-2397-5p,miR-125a-3p,miR-219b-5p,miR-422a,miR-34b-5p,novel_127,miR-543-5p,miR-21-3p,miR-378a-3p,miR-3068-3p,miR-21-3p,miR-2355-5p,miR-182-5p,miR-34c-5p,miR-6130,miR-199a-5p,miR-15b-5p,miR-154b-5p,miR-877-5p,miR-2448-3p,miR-450a-1-3p,miR-129b-3p,miR-671-5p,miR-378c,novel_42,miR-4510,miR-3184-3p,miR-16-5p,miR-1271,miR-1197-3p,miR-122-5p,miR-378c,miR-338-3p,miR-2433,miR-664a-5p,miR-3969,miR-665,miR-320e,miR-450b-3p,miR-424-5p,miR-487a-5p,miR-130b-5p,miR-2300a-5p,miR-455-3p,miR-2459,miR-378b,miR-1843b-5p,miR-450c-3p,miR-16b,miR-378c,miR-2284aa,miR-497-5p,miR-380-5p,miR-130b-5p,miR-424-5p,miR-376b,miR-378d,miR-4532,miR-5126,novel_19,miR-16b,miR-182-5p,novel_51,novel_124,miR-487b-5p,miR-199b-5p,miR-103a-2-5p,miR-24-1-5p,miR-216b-3p,miR-452-3p,miR-7689-3p,miR-378e,miR-3596,miR-670-3p,miR-34b,miR-2483-3p,miR-140-5p,miR-2387,miR-199b-5p,miR-18a-3p,miR-96-5p,miR-378f,miR-195a-5p,novel_60,miR-3956-3p,miR-452-5p,novel_101,miR-361-3p,miR-24-3p,miR-449a,miR-199b-5p,miR-15a,miR-6529b,miR-34c,miR-16a,miR-107-5p,miR-4726-5p,miR-628-3p,novel_1,miR-203b-5p,miR-22-5p,miR-147-5p

CAND1	ENSOARG0(	cullin-a	miR-371a-5p,novel_82,miR-3964,miR-3958-3p,let-7i-3p,miR-181d-5p,miR-363-3p,miR-8095,miR-877-3p,novel_111,miR-378j,miR-140-5p,miR-361-3p,miR-221,miR-335-3p,miR-1983,miR-541-5p,miR-7689-3p,miR-139-5p,miR-452-3p,miR-144-5p,miR-203-3p,miR-27a-3p,miR-454-5p,miR-499a-3p,miR-25,miR-2483-3p,miR-505,miR-106b-5p,miR-431-5p,novel_103,miR-153-3p,miR-214-3p,miR-130b-3p,miR-744-3p,miR-101b-3p,miR-382-3p,miR-106a-5p,miR-8485,miR-1b-5p,miR-491-3p,miR-1a-3p,miR-4791,miR-143-5p,miR-1298-5p,miR-2284aa,miR-3120-5p,miR-101a-5p,miR-181b-2-3p,miR-27a-3p,miR-221-3p,miR-153,miR-664a-5p,miR-222-3p,miR-2285j,miR-208b-5p,miR-708-5p,miR-454-3p,miR-214,miR-3184-3p,miR-346,miR-206-3p,miR-411,miR-21a-3p,miR-625-5p,miR-133b-3p,miR-3065-3p,miR-532-5p,miR-140-5p,miR-32-5p,miR-499b-3p,miR-301a-3p,miR-377-3p,miR-582-5p,miR-139-5p,miR-500a-5p,novel_17,miR-1277-5p,miR-21-3p,miR-106a,miR-20a,miR-2411,miR-130a-5p,miR-181b-1-3p,miR-133a-3p,miR-218-1-3p,miR-361-5p,miR-19b-3p,miR-505-3p,miR-548e-3p,miR-106a,novel_83,miR-7859,miR-148b-3p,miR-133a-5p,miR-28c,miR-20a-5p,miR-92a-3p,miR-6119-3p,miR-379-3p,miR-206,miR-29a,novel_1,novel_101,miR-2285x,miR-29c-3p,miR-1a-1-5p,miR-301b-3p,miR-2284w,miR-2285m,miR-323c,miR-148a-3p,miR-505-3p,miR-412-3p,miR-19a-3p,miR-3120-3p,miR-216b-3p,miR-677,miR-144-3p,miR-181b-3p,miR-29a-3p,miR-32,miR-106a-5p,miR-17-5p,miR-377-3p,miR-452-5p,miR-668-3p,miR-197-3p,miR-653,miR-181a-5p,miR-542-3p,miR-1434-3p,miR-27b-3p,miR-29b,miR-16-1-3p,miR-130a-3p,miR-20b,miR-3535,miR-144,miR-26c,miR-409-3p,miR-1-5p,miR-29b-3p,miR-2332,miR-28-5p,miR-134-5p,miR-6128,miR-133a-3p,miR-144-5p,miR-323-3p,miR-93,miR-93-5p,miR-17-5p,miR-488-3p,miR-20b-5p,miR-3074-1-3p,miR-2285aa,miR-
ATP2A1	ENSOARG0(	ATPase,	miR-148b-5p,novel_39,miR-503-5p,miR-3064-5p,miR-450a-2-3p,miR-191-3p,miR-543-5p,novel_96,novel_127,miR-200a-3p,miR-210-5p,miR-8117,miR-324-3p,miR-339a,miR-345-3p,miR-185-3p,miR-92a-1-5p,miR-483-3p,miR-129-1-3p,miR-18a-3p,miR-1224-5p,novel_79,miR-212-5p,miR-431,miR-941,miR-615-3p,miR-345-3p,miR-2300b-3p,miR-2904,miR-5703,miR-769-5p,miR-4510,miR-129-2-3p,miR-425-5p,miR-874-5p,miR-3432a,miR-4492,miR-135a-1-3p,miR-331-3p,miR-323-5p,miR-769,miR-6130,miR-2331-3p,miR-874-3p,miR-2355-5p,miR-370-3p,miR-296-3p,miR-141-3p,miR-1271,miR-339b,miR-23a-5p,miR-625-5p,miR-1248,miR-129b-3p,miR-296-3p,miR-1388-3p,miR-1839-3p,miR-3085-3p,miR-491-5p,miR-483-3p,miR-25-5p,miR-3955-3p,miR-503-5p,miR-431-5p,miR-296-3p,miR-3141,novel_51,miR-2411-3p,miR-503-5p,miR-9788-3p,miR-2355-3p,miR-31-5p,miR-1343-3p,miR-339-5p,miR-30b-3p,miR-3120-5p,miR-30b-3p,miR-361-3p,novel_111,miR-3059-5p,miR-1839-3p,miR-576-3p,miR-615,miR-769-5p,miR-425-5p,miR-323a-5p,miR-6535,miR-3064-5p,miR-499b-5p,miR-1291,miR-361-3p,miR-3141,miR-18a-3p

CRNKL1	ENSOARG0(	crooked	miR-452-5p,miR-1285-5p,miR-154a,miR-2285n,novel_82,miR-2440,miR-154-3p,miR-335,miR-452-3p,miR-20a-3p,miR-203-3p,miR-652-3p,miR-27a-3p,miR-7857-3p,miR-2483-3p,miR-129b-5p,miR-140-5p,miR-335-3p,miR-1388-3p,miR-494-3p,miR-29b-1-5p,miR-320b,novel_103,miR-146b,miR-222-5p,miR-27a-3p,miR-199b-3p,miR-105-1,miR-330-3p,miR-148b-5p,miR-1298-3p,miR-1a-3p,miR-7857,miR-544a,miR-3074-2-3p,miR-3074-5p,miR-206-3p,novel_44,miR-29b-2-5p,miR-208b-5p,miR-669a-3p,miR-450b-3p,miR-6516-3p,miR-17-3p,miR-216b-3p,miR-552-3p,miR-2331-3p,miR-544-3p,miR-377-3p,miR-582-5p,miR-21a-3p,miR-450a-1-3p,miR-543-3p,miR-582-5p,miR-154b-3p,miR-146a-5p,miR-2432,miR-380-3p,miR-29b-1-5p,miR-125b-2-3p,miR-216a-5p,miR-125b,miR-2285p,miR-34b-5p,miR-21-3p,miR-320d,miR-1b-3p,miR-212-5p,novel_83,miR-328-3p,miR-200b,miR-188-3p,miR-218-1-3p,miR-148a-5p,miR-34a-5p,novel_73,miR-4429,miR-184-3p,miR-17-3p,novel_101,miR-335-5p,miR-449a,miR-374c-5p,miR-323c,miR-34c,miR-4324,miR-576-3p,miR-22-5p,miR-147-5p,miR-199c,miR-6119-3p,novel_1,miR-206,miR-216b-3p,miR-3074-5p,miR-320c,miR-2404,miR-34b,miR-670-3p,miR-150-5p,miR-3065-5p,miR-216a-3p,miR-146b-5p,miR-6516-3p,miR-6395,miR-1291,miR-382,miR-6240,miR-330-3p,miR-105-5p,miR-455-5p,miR-146a,miR-377-3p,miR-125a-5p,miR-26b-3p,miR-3955-5p,novel_116,miR-544b,miR-432-5p,miR-185-5p,miR-450c-3p,miR-431-3p,miR-374b-3p,miR-432,miR-542-3p,miR-1285,miR-2113,miR-27b-3p,miR-7862,miR-455-5p,miR-380-3p,miR-2284ab,miR-1260b,miR-3607-3p,miR-2284v,miR-1260b,miR-125b-5p,miR-6740-5p,miR-34c-3p,miR-494-5p,miR-592,miR-125a,miR-34c-5p,miR-331-5p,miR-374c-3p,miR-320a,miR-488-3p,miR-2448-3p,miR-1,miR-29b-2-5p,miR-3529-3p,miR-1271-3p,miR-2285af,miR-205-5p,miR-1260a,miR-200c,miR-125a-3p,miR-17-
YIPF5	ENSOARG0(	Yip1 dom	miR-2404,miR-25,miR-2284s,miR-2284r,miR-129b-5p
RAB32	ENSOARG0(	RAB32, m	miR-193a,miR-1388-3p,miR-3184-3p,miR-502b,miR-21-3p,miR-196a-3p,miR-664-3p,miR-505-5p,miR-185-5p,miR-145b,miR-664b,miR-6517,miR-668-5p,miR-145a-5p,miR-484,miR-21a-3p
KDELR2	ENSOARG0(	KDEL (Ly	miR-31-5p,miR-1224-5p,miR-125a,miR-323a-5p,miR-323-5p,miR-330-5p,miR-96-5p,miR-138,miR-7144-5p,miR-493-5p,miR-493-5p,miR-181a-2-3p,miR-2285r,miR-326-3p,novel_63,miR-326,miR-210-5p,miR-125b-5p,miR-125b,miR-655-3p,miR-138-5p,miR-127-5p,miR-125a-5p

METTL8	ENSOARG0(	methyltr	miR-29b-1-5p,miR-214-3p,miR-103a-2-5p,miR-1388-3p,miR-1839-3p,miR-382-3p,miR-23a-3p,miR-33a-5p,miR-193a,miR-8485,miR-758-3p,miR-1b-5p,miR-301b-5p,miR-758-3p,miR-101c,novel_4,miR-1343-3p,miR-151a-3p,miR-5010-3p,miR-27a-3p,miR-9788-3p,miR-148b-5p,miR-455-3p,miR-126a-5p,miR-218-5p,miR-101-3p,miR-769-5p,miR-301a-5p,miR-107-5p,miR-3958-3p,miR-2285t,novel_60,miR-3956-3p,miR-212-3p,miR-9-5p,miR-219a-5p,miR-129b-5p,miR-668-5p,miR-136-5p,novel_94,miR-4792,miR-24-1-5p,miR-20a-3p,miR-7689-3p,miR-126b-5p,miR-25,miR-203-3p,miR-454-5p,miR-27a-3p,novel_17,miR-219b-5p,miR-185-3p,miR-21-3p,miR-299-3p,miR-2285w,miR-376a-5p,miR-29b-1-5p,miR-130a-5p,miR-125b-2-3p,miR-380-3p,miR-191,miR-217-5p,miR-188-3p,miR-142-3p,miR-218-1-3p,miR-148a-5p,miR-212-5p,miR-23b-3p,miR-23c,miR-2284h-5p,miR-532-3p,miR-151-3p,miR-320e,miR-29b-2-5p,miR-665,miR-3604,miR-17-3p,miR-4492,miR-590-3p,miR-145a-3p,miR-3074-2-3p,miR-214,miR-769-5p,miR-224-5p,miR-21a-3p,miR-433-3p,miR-186-5p,novel_99,miR-2331-3p,miR-216b-3p,miR-377-3p,miR-656-3p,miR-224-5p,miR-377-3p,miR-365b-5p,miR-466f-3p,miR-654-3p,miR-2285r,miR-3071-5p,miR-380-3p,miR-27b-3p,miR-122-3p,miR-103a-3p,miR-185-5p,miR-3431,miR-103,miR-502-5p,miR-485-3p,miR-6119-3p,novel_1,miR-421-5p,miR-147-5p,miR-1a-1-5p,miR-1839-3p,miR-495-3p,miR-17-3p,miR-2285x,miR-371b-3p,miR-216a-3p,miR-107,miR-7-1-3p,miR-2404,miR-299b-3p,miR-217,miR-450a-2-3p,miR-17-3p,miR-1a-2-5p,miR-107,miR-2385-3p,miR-29b-2-5p,miR-24-2-5p,miR-1271-3p,miR-128-3p,miR-299a-3p,miR-9-5p,novel_121,miR-1-5p,novel_63,miR-6740-5p,miR-100-3p,miR-409-3p,miR-191-5p,miR-592,miR-1306-5p,miR-193a-5p,miR-365a-5p,miR-301,miR-23b,miR-10b-3p,miR-23a-5p,miR-3074-1-3p,miR-

RNMT	ENSOARG0(	RNA (gua	miR-598-3p,miR-30d-5p,miR-186-5p,miR-30c-1-3p,miR-2331-3p,miR-548o-3p,miR-301a-3p,miR-499b-3p,miR-15b-5p,let-7i-5p,let-7f,miR-151-3p,miR-3604,miR-664a-5p,miR-454-3p,let-7b,miR-214,miR-142a-5p,miR-181c-5p,miR-30c-5p,miR-1961,miR-1434-5p,miR-361-5p,miR-2284u,miR-106a,miR-7859,miR-2284h-5p,miR-30b,miR-210-5p,novel_17,miR-500a-5p,miR-219b-5p,miR-345-3p,miR-202-5p,miR-98-5p,let-7k,miR-2285w,miR-345-3p,miR-664b-3p,miR-486-3p,miR-335-3p,miR-2284g,miR-499a-3p,miR-3596,miR-203-3p,miR-4443,novel_82,miR-101-3p,miR-301a-5p,miR-9-5p,novel_111,miR-2284r,miR-329-3p,miR-301b-5p,miR-362-3p,miR-101c,miR-1839-5p,miR-199b-3p,miR-145b,miR-425-3p,miR-147a,miR-142-5p,miR-455-3p,miR-421,miR-16b,miR-106b-5p,miR-1185-5p,miR-130b-3p,miR-214-3p,miR-127-3p,miR-2284b,miR-8485,miR-1b-5p,miR-30e-5p,miR-93-5p,miR-329a,miR-20b-5p,miR-1248,miR-3068-3p,miR-2285aa,miR-300-3p,miR-2284k,novel_9,miR-592,miR-28a-3p,miR-16-5p,miR-1260b,miR-1197-3p,miR-196a-5p,miR-330-5p,miR-155-5p,miR-30c,miR-322-5p,miR-9-5p,novel_121,miR-28-3p,let-7f-5p,miR-200a-3p,miR-3064-5p,let-7g,miR-2284y,novel_39,miR-2285b,miR-3141,miR-150-5p,miR-1306,miR-3591-3p,miR-2319a,let-7e-5p,miR-3120-3p,miR-576-3p,miR-142b,novel_87,miR-421-5p,miR-193b-3p,miR-301b-3p,miR-708-3p,miR-542-3p,miR-223-3p,miR-16b,miR-497-5p,miR-27b-3p,miR-340-3p,miR-103a-3p,miR-487b-3p,miR-194-3p,miR-105-5p,miR-3141,miR-2411-3p,miR-196b-5p,miR-193a-3p,miR-432-3p,miR-432-3p,miR-21a-3p,miR-30f,miR-3065-3p,miR-21-3p,miR-193b-3p,miR-155-5p,miR-324-3p,miR-132-5p,miR-155-5p,miR-208b-5p,miR-424-5p,let-7b-5p,novel_133,miR-362-3p,miR-376a-5p,miR-5703,miR-19b-3p,miR-378d,miR-3600,miR-222-5p,miR-15a-5p,miR-532-3p,miR-196a-3p,miR-106a,miR-
MPRIP	ENSOARG0(	myosin p	miR-452-3p,miR-140-3p,miR-1197-5p,novel_101,miR-185-3p,miR-21-3p,miR-128-1-5p
PRELID2	ENSOARG0(	PRELI do	miR-582-3p,miR-184-3p,miR-330-5p,miR-22-3p,miR-1290,miR-302a-5p,miR-2300a-5p,miR-18a-3p,miR-2285ad,miR-214-3p,novel_127,miR-3955-3p,miR-3085-3p,miR-185-3p,miR-219b-5p,novel_17,miR-326,miR-345-5p,miR-2432,miR-376a-5p,miR-3064-5p,miR-216c-5p,miR-1388-3p,miR-135b-5p,miR-345-5p,miR-345-5p,miR-3591-3p,miR-18a-3p,miR-2285ab,miR-22-3p,miR-223-5p,miR-499a-3p,miR-499b-3p,miR-21-3p,miR-874-3p,miR-3064-5p,miR-490-5p,miR-335,miR-135a-5p,miR-2332,miR-326-3p,miR-877-3p,miR-214,miR-3074-2-3p,miR-2285f,miR-335-5p,miR-145a-3p,miR-3184-3p

TLK1	ENSOARG0(	tousled-	miR-195a-5p,miR-493-5p,miR-3065-5p,miR-3591-3p,miR-489,miR-211-5p,miR-7-1-3p,miR-2285t,miR-490-5p,miR-371a-5p,miR-16a,miR-133a-5p,miR-15a,novel_27,miR-211,miR-9-5p,miR-193b-3p,miR-497-5p,miR-1260b,miR-16b,miR-3071-5p,miR-653,miR-503-5p,miR-153,miR-105-1,miR-193a-3p,miR-153-3p,miR-16b,miR-105-5p,miR-503-5p,let-7a-2-3p,miR-539-3p,let-7c-3p,miR-668-3p,miR-493-5p,miR-424-5p,novel_48,let-7g-3p,let-7g-3p,miR-3154,miR-500a-3p,miR-15b-5p,miR-154b-5p,miR-193b-3p,miR-532-5p,miR-1271,miR-141-3p,miR-21-3p,miR-204-5p,miR-500,miR-424-5p,miR-487b-5p,miR-338-5p,miR-2285j,miR-1260b,miR-16-5p,miR-3607-3p,miR-378d,let-7a-2-3p,miR-218-1-3p,miR-188-3p,miR-2427,miR-532-3p,miR-15a-5p,miR-487a-3p,miR-9-5p,miR-322-5p,miR-485-3p,miR-3600,miR-7641,miR-6517,miR-200a-3p,miR-1260a,miR-29a-5p,miR-376a-5p,miR-3529-3p,miR-503-5p
AIPL1	ENSOARG0(	aryl hyd	miR-330-5p,miR-23b-5p,miR-218-2-3p,miR-10a-5p,miR-128-3p,miR-18b-5p,novel_79,miR-6517,miR-129-1-3p,miR-545-5p,miR-2284z,miR-483-3p,miR-2411-5p,miR-24-2-5p,miR-302a-5p,miR-217,miR-339a,miR-30c-2-3p,miR-1260a,novel_96,miR-152-3p,miR-127-5p,miR-2898,miR-3064-5p,miR-490-3p,miR-1957a,miR-199a-5p,miR-29a-5p,miR-380-5p,miR-18a,miR-3154,miR-2448-3p,miR-671-5p,miR-1248,miR-485-5p,miR-3074-1-3p,miR-125a,miR-615-5p,miR-2285aa,miR-370-3p,miR-2355-5p,miR-3068-3p,miR-199a-5p,miR-125b-5p,miR-6740-5p,miR-592,miR-1197-3p,miR-1260b,miR-194-5p,novel_23,miR-22-3p,miR-223-3p,miR-432,miR-188-5p,miR-1260b,miR-30b-3p,miR-377-5p,miR-26b-3p,miR-130a-3p,miR-339-5p,miR-149-5p,miR-194-3p,miR-134-3p,miR-432-5p,miR-194a,novel_51,miR-330-3p,miR-125a-5p,miR-483-3p,miR-378a-5p,miR-2483-5p,miR-382,miR-6240,miR-380-5p,miR-432-3p,miR-654-3p,miR-216a-3p,miR-3970,miR-1306,miR-148a-3p,miR-18b,miR-19a-3p,miR-10a-5p,miR-26b-5p,miR-378b,novel_68,novel_69,miR-33a-3p,novel_87,miR-4324,miR-502b,miR-6123,miR-203b-5p,miR-128-1-5p,miR-758-5p,miR-335-5p,miR-219b-3p,miR-199b-5p,miR-301b-3p,miR-2428,miR-326-3p,miR-3959-3p,miR-1197-5p,miR-140-3p,miR-217-5p,miR-2300b-3p,novel_133,miR-378d,miR-664-5p,miR-184-3p,miR-19b-3p,miR-493-3p,miR-3600,miR-380-5p,miR-548e-3p,novel_83,miR-2284h-5p,miR-148b-3p,miR-7859,miR-125b,miR-219b-5p,miR-26a-5p,miR-210-5p,miR-21-3p,miR-221-5p,miR-202-5p,miR-345-3p,miR-2285w,miR-2411,miR-376c-3p,miR-377-5p,miR-29b-1-5p,miR-411,novel_99,miR-339b,miR-18a-5p,miR-216b-3p,miR-2331-3p,miR-30c-1-3p,miR-221-5p,miR-504-5p,miR-22-3p,miR-204-3p,miR-6130,miR-301a-3p,miR-10a,miR-320e,miR-2285j,miR-669a-3p,miR-454-3p,miR-4492,miR-4510,miR-129-2-3p,miR-10b-5p,miR-6525,miR-30b-
HMSD	ENSOARG0(	histocom	miR-17-3p,novel_44,miR-346,miR-206-3p,miR-6134,miR-449a,miR-2440,miR-34c,miR-206,miR-17-3p,miR-17-3p,miR-34b-5p,miR-625-3p,miR-3120-3p,miR-34b,miR-34c-5p,miR-2284r,miR-21a-3p,miR-34a-5p

MYOF	ENSOARGO(	myoferli	miR-320b,miR-326,miR-29b-1-5p,miR-182-5p,miR-153-3p,miR-2330-5p,let-7c-3p,miR-744-3p,miR-376b,miR-758-3p,miR-2310,miR-2284r,miR-301b-5p,miR-758-3p,miR-101a-5p,miR-544a,miR-2284aa,miR-199b-3p,novel_78,miR-151a-3p,miR-409b,miR-548w,miR-27a-3p,miR-153,miR-539-5p,miR-142-3p,miR-455-3p,miR-1185-3p,miR-2440,miR-301a-5p,miR-2447,miR-3059-5p,miR-877-3p,miR-129b-5p,miR-136-5p,miR-493-5p,miR-452-3p,miR-499b-5p,miR-211-5p,miR-2483-3p,miR-489,miR-203-3p,miR-144-5p,miR-1185-2-3p,miR-27a-3p,miR-219b-5p,miR-125b,miR-202-5p,miR-320d,miR-466i-5p,miR-3653-3p,miR-216c-5p,miR-2887,miR-29b-1-5p,miR-377-5p,miR-142a-3p,miR-2432,miR-376a-5p,miR-1434-5p,miR-4429,miR-19b-3p,miR-380-5p,miR-148b-3p,miR-340-5p,miR-151-3p,miR-29b-2-5p,novel_74,miR-17-3p,miR-487a-5p,miR-130b-5p,miR-669a-3p,miR-539-5p,miR-129-2-3p,miR-6134,miR-338-3p,let-7g-3p,miR-345-5p,novel_99,miR-186-5p,miR-660-5p,miR-182-5p,miR-6516,miR-544-3p,miR-204-3p,miR-125a-5p,miR-660,miR-130b-5p,miR-493-5p,miR-345-5p,let-7a-2-3p,miR-653,miR-26a-2-3p,miR-27b-3p,miR-31-5p,miR-377-5p,miR-6238,miR-544b,miR-502-5p,miR-33a-3p,novel_1,miR-199c,miR-17-3p,miR-758-5p,miR-211,novel_27,miR-326-3p,miR-2284w,miR-148a-3p,miR-3970,miR-3065-5p,miR-378b,miR-19a-3p,miR-320b,miR-3120-3p,miR-7-1-3p,miR-320c,miR-17-3p,miR-28-3p,miR-152-3p,miR-3529-3p,miR-29b-2-5p,miR-181a-2-3p,miR-218-2-3p,miR-330-5p,let-7a-2-3p,miR-1224-5p,miR-6516-5p,miR-758-5p,miR-2284z,miR-129-1-3p,novel_121,novel_63,miR-125b-5p,miR-409-3p,miR-204-5p,miR-28a-3p,miR-103b,miR-144-5p,miR-1197-3p,miR-301,miR-320a,let-7g-3p,miR-2318,miR-345-5p,miR-23a-5p,miR-1814c,miR-1248,miR-125a,miR-331-5p,miR-3959-5p
OGDHL	ENSOARGO(	oxogluta	miR-3596,miR-185-5p,miR-1298-3p,novel_83,miR-582-3p,miR-149-5p,miR-134-5p,miR-2285aa,miR-2355-5p,miR-138,miR-1291,miR-455-5p,miR-361-3p,miR-6529a,miR-134,miR-2903,miR-423-5p,miR-3184-5p,miR-486-3p,miR-138-5p,miR-378a-5p,miR-664a,miR-4492,miR-134-5p,miR-486b-3p,miR-331-3p,miR-1973,miR-185-3p,miR-455-5p
SCHIP1	ENSOARGO(	schwanno	miR-2355-3p,miR-432-3p,miR-142-5p,miR-432-3p,miR-142a-5p,miR-141-3p,miR-877-3p,miR-200a-3p,miR-135b-5p,miR-3958-3p,miR-338-5p,miR-135a-5p,miR-140-3p,miR-21a-3p

STXBP2	ENSOARG0(	syntaxin	miR-3074-2-3p,miR-214,miR-3184-5p,miR-6525,miR-142a-5p,miR-224-5p,miR-338-5p,miR-2285j,miR-708-5p,miR-487a-5p,miR-874-5p,miR-4492,miR-130b-5p,miR-17-3p,miR-21-3p,miR-370-5p,miR-552-3p,miR-296-3p,miR-30c-1-3p,miR-2331-3p,miR-504-5p,miR-6516,miR-544-3p,miR-323-5p,miR-139-5p,miR-433-3p,miR-1827,novel_99,miR-133b-3p,miR-18a-5p,miR-486-3p,miR-143-3p,miR-376a-5p,miR-29b-1-5p,miR-130a-5p,miR-142a-3p,miR-324-3p,miR-92a-1-5p,miR-345-3p,miR-30b-3p,miR-21-3p,miR-466i-5p,miR-3600,miR-532-3p,miR-199a-3p,miR-23b-3p,miR-222-5p,miR-340-5p,miR-23c,miR-200b,miR-2904,miR-140-3p,miR-188-3p,novel_133,miR-2300b-3p,miR-133a-3p,miR-142-3p,miR-452-5p,novel_60,miR-3956-3p,miR-423-5p,miR-7705,miR-361-3p,miR-371a-5p,novel_82,miR-218-5p,miR-2440,miR-2284q,miR-499a-5p,miR-335,miR-450a-5p,miR-499b-5p,miR-7977,miR-139-5p,miR-3064-5p,miR-10b,miR-2319b,miR-20a-3p,miR-1185-2-3p,miR-2483-3p,miR-140-5p,miR-361-3p,miR-199b-5p,miR-335-3p,novel_94,miR-1983,miR-147-3p,miR-668-5p,miR-504,miR-23a-3p,miR-376b,miR-33a-5p,miR-1388-3p,miR-193a,miR-2310,miR-1b-5p,miR-30c-1-3p,miR-29b-1-5p,miR-199a-3p,miR-326,miR-153-3p,miR-199b-5p,miR-214-3p,miR-3085-3p,miR-487b-5p,miR-145b,novel_78,miR-2300a-5p,miR-9788-3p,miR-455-3p,miR-1185-3p,miR-153,miR-496-5p,miR-142-5p,miR-142-3p,miR-484,miR-2284aa,miR-3120-5p,miR-1388-5p,miR-544a,miR-3607-3p,miR-193a-5p,miR-133a-3p,miR-103b,miR-28a-3p,miR-323-3p,miR-1260b,miR-1197-3p,miR-409-3p,miR-2332,miR-370-5p,miR-3432a,miR-134-5p,miR-486b-3p,miR-28-5p,miR-2355-5p,miR-542-5p,miR-370-3p,miR-2285aa,miR-1271,miR-615-5p,miR-574-5p,miR-199a-5p,miR-3965,miR-3959-5p,miR-365a-3p,miR-3154,miR-18a,miR-23b,miR-488-3p,miR-301,miR-503-3p,miR-7144-5p,miR-671-

IDO1	ENSOARG0(	indoleam	miR-541,miR-329-3p,miR-30b-3p,miR-362-3p,miR-1388-5p,miR-101c,miR-409b,miR-330-3p,miR-181b-2-3p,miR-376b-3p,miR-455-3p,miR-320b,miR-16b,miR-362-3p,miR-3955-3p,miR-424-5p,miR-33a-5p,miR-30c-1-3p,miR-136-5p,miR-9-3p,miR-335-3p,miR-376a-3p,miR-2483-3p,miR-3082-5p,miR-7857-3p,miR-3596,miR-376d,miR-3968,miR-154-3p,miR-2284q,miR-541-3p,miR-3059-5p,miR-9-5p,novel_111,miR-154a,miR-2366,miR-193b-5p,miR-362-3p,miR-5703,miR-2904,miR-19b-3p,miR-142-3p,miR-4429,miR-184-3p,miR-485-3p,miR-15a-5p,miR-487a-3p,miR-345-3p,miR-216a-5p,miR-202-5p,miR-320d,miR-221-5p,miR-503-5p,miR-487a,miR-345-3p,miR-142a-3p,miR-181b-1-3p,miR-130a-5p,miR-376c-3p,miR-486-3p,miR-450a-1-3p,miR-345-5p,miR-154b-3p,novel_99,miR-221-5p,miR-30c-1-3p,miR-532-5p,miR-370-5p,miR-154a-3p,miR-2284s,miR-376e-3p,miR-377-3p,miR-15b-5p,miR-877-5p,miR-15b-3p,miR-6516-3p,miR-17-3p,miR-450b-3p,miR-424-5p,miR-3074-2-3p,miR-3958-5p,miR-374b-3p,miR-450c-3p,miR-197-3p,miR-219a-1-3p,miR-1260b,miR-16b,miR-30b-3p,miR-497-5p,miR-503-5p,miR-3431,miR-377-3p,miR-503-5p,miR-2411-3p,miR-330-3p,miR-483-3p,miR-1343-5p,miR-6240,miR-380-5p,miR-432-3p,miR-432-3p,miR-345-5p,miR-539-3p,miR-378b,miR-376b-3p,miR-216a-3p,miR-3970,miR-19a-3p,miR-410-5p,miR-195a-5p,miR-6516-3p,miR-378b,miR-655-5p,miR-374c-3p,miR-181b-3p,miR-320c,miR-677,miR-15a,novel_87,miR-16a,novel_1,miR-1193,novel_101,miR-17-3p,miR-2428,miR-345-3p,miR-1343-5p,miR-128-3p,miR-483-3p,miR-758-5p,miR-9-5p,miR-322-5p,novel_121,miR-450a-1-3p,miR-376b-3p,miR-2285af,miR-1260a,miR-30f,miR-30c-2-3p,miR-17-3p,miR-543-5p,miR-380-5p,novel_39,miR-2448-3p,miR-3154,miR-320a,miR-329a,miR-345-5p,miR-1248,miR-487a-3p,miR-2355-5p,miR-331-5p,miR-374c-3p,miR-376c-3p,miR-5100,miR-370-5p,miR-551b-3p,miR-
RAD54L	ENSOARG0(	RAD54-li	miR-23a-5p,miR-7144-5p,miR-1248,miR-329b,miR-329a,miR-361-3p,miR-677,miR-181b-3p,miR-378g,miR-499a-3p,miR-27a-5p,miR-499b-3p,miR-211-5p,miR-3074-5p,miR-203b-5p,miR-204-5p,miR-130b-5p,miR-4726-5p,miR-30a-3p,miR-665,miR-30e-3p,miR-211,miR-371b-3p,miR-3074-2-3p,miR-3059-5p,miR-129-2-3p,miR-3074-5p,miR-885-5p,miR-362-3p,miR-2904,miR-23b-5p,miR-188-3p,novel_133,miR-329-3p,miR-362-3p,miR-1285,miR-1247-5p,miR-2284h-5p,miR-421,miR-129-1-3p,miR-3431,miR-134-3p,miR-1224-5p,miR-181b-2-3p,miR-324-3p,miR-362-3p,miR-181b-1-3p,miR-30a-3p,miR-130b-5p,miR-2285w

GRXCR2	ENSOARGO(	glutared	miR-625-5p,let-7f-1-3p,miR-129b-3p,miR-500a-3p,miR-6130,miR-15b-5p,miR-877-5p,miR-30c-1-3p,miR-548o-3p,miR-216b-3p,miR-154a-3p,miR-487a-5p,miR-500,miR-424-5p,miR-30e-3p,miR-30a-3p,miR-29b-2-5p,miR-338-5p,miR-3184-5p,miR-539-5p,miR-4510,miR-2284u,miR-361-5p,let-7a-3p,miR-1246,miR-140-3p,miR-5703,miR-487a-3p,miR-7859,miR-15a-5p,miR-199a-3p,miR-548e-3p,miR-30d-3p,miR-212-5p,miR-485-3p,miR-3653-3p,miR-21-3p,miR-185-3p,miR-345-3p,miR-376a-5p,miR-487a,miR-195a-3p,miR-499a-3p,miR-203-3p,miR-500-3p,miR-1185-2-3p,miR-2284g,miR-154-3p,miR-107-5p,miR-877-3p,miR-362-5p,miR-502-3p,miR-423-5p,miR-3059-5p,miR-6527,miR-2284j,miR-2426,miR-21b,miR-3120-5p,miR-6529a,miR-501-3p,miR-1298-3p,miR-500-5p,miR-9788-3p,miR-1185-3p,miR-548w,miR-2300a-5p,miR-103a-2-5p,miR-199a-3p,miR-431-5p,miR-16b,miR-30c-1-3p,miR-8485,miR-1839-3p,miR-424-5p,miR-376b,miR-2284b,miR-10b-3p,miR-2318,novel_48,miR-7144-5p,miR-190a-3p,miR-362-5p,miR-487a-3p,miR-370-3p,miR-592,miR-2284k,miR-29b-3p,miR-135a-5p,miR-500b-5p,miR-2284v,miR-16-5p,miR-345-3p,miR-218-2-3p,miR-29d-3p,miR-501-3p,miR-322-5p,novel_121,miR-214-5p,miR-431,miR-6119-5p,miR-30f,miR-30c-2-3p,miR-2478,miR-450b-5p,miR-2284y,miR-2284a,miR-29b-2-5p,miR-30a-3p,miR-4286,miR-195a-5p,miR-135b-5p,miR-150-5p,miR-376b-3p,miR-378b,miR-29a-3p,miR-6535,novel_1,miR-29a,miR-147-5p,miR-4324,miR-15a,miR-6402,miR-16a,miR-1839-3p,miR-29c-3p,miR-16b,miR-29b,miR-497-5p,miR-223-3p,miR-2284d,miR-2355-3p,miR-122-3p,miR-665,miR-466f-3p,miR-2411-3p,miR-2285r,miR-539-3p,miR-668-3p,let-7f-2-3p,let-7f-2-3p
TRAV8-7	ENSOARGO(	T cell r	miR-411b-3p,miR-769,miR-204-3p,miR-140-5p,miR-504-5p,miR-2355-5p,miR-125a,miR-296-3p,miR-876-3p,miR-504,novel_48,novel_94,miR-361-3p,miR-450a-1-3p,miR-23b,miR-769-5p,miR-24-3p,miR-6134,miR-2447,miR-3607-3p,novel_74,miR-450b-3p,novel_1,miR-6123,miR-2285j,miR-5100,miR-2440,miR-125b-5p,miR-769-5p,miR-153,miR-23b-3p,miR-383-5p,miR-23c,miR-450a-1-3p,miR-545-5p,miR-30b-3p,miR-16-1-3p,miR-16-1-3p,miR-3591-5p,miR-1843b-5p,miR-7b-5p,miR-2904,miR-450c-3p,miR-30b-3p,miR-5703,miR-143-3p,miR-3529-3p,miR-23a-3p,miR-125a-5p,miR-153-3p,miR-7-5p,miR-618,miR-216a-5p,miR-92a-1-5p,miR-125b

SETBP1	ENSOARG0(	SET bind	miR-582-5p,miR-500a-3p,miR-21a-3p,miR-2285c,miR-15b-5p,miR-377-3p,miR-582-5p,miR-370-5p,miR-660-5p,miR-874-5p,miR-500,miR-129-5p,miR-424-5p,miR-30a-3p,miR-320e,miR-3604,miR-2285j,miR-6525,miR-206-3p,miR-3074-2-3p,miR-539-5p,miR-4429,novel_73,miR-19b-3p,miR-217-5p,miR-1197-5p,miR-1246,miR-362-3p,miR-193b-5p,miR-1247-5p,miR-15a-5p,miR-505-3p,miR-212-5p,miR-3960,miR-3653-3p,miR-320d,miR-324-3p,miR-92a-1-5p,miR-130a-5p,miR-377-5p,miR-369-3p,miR-877-3p,miR-1983,miR-129b-5p,miR-140-5p,miR-361-3p,miR-203-3p,miR-489,miR-2483-3p,miR-2285u,miR-211-5p,miR-4792,miR-20a-3p,miR-2319b,miR-3964,miR-335,miR-2440,miR-1285-5p,miR-877-3p,miR-3059-5p,miR-212-3p,miR-362-3p,miR-101a-5p,miR-1a-3p,miR-329-3p,miR-455-3p,miR-2285e,miR-147a,novel_78,miR-25-5p,miR-2957,miR-505,miR-362-3p,miR-16b,miR-320b,miR-8485,miR-424-5p,miR-329a,miR-3154,miR-320a,miR-488-3p,miR-424-3p,miR-301,miR-133c,miR-154b-5p,miR-3965,miR-141-3p,miR-204-5p,miR-138-5p,miR-26c,miR-135a-5p,miR-29b-3p,miR-410-5p,novel_63,miR-34c-3p,miR-370-5p,miR-6128,miR-3607-3p,miR-16-5p,miR-29d-3p,miR-214-5p,novel_121,miR-322-5p,miR-200a-3p,miR-450a-2-3p,miR-30f,miR-217,miR-192-3p,miR-1,miR-2319a,miR-195a-5p,miR-505-3p,miR-135b-5p,miR-450b-3p,miR-19a-3p,miR-412-3p,miR-21c,miR-3065-5p,miR-6535,miR-320c,miR-29a-3p,miR-655-5p,miR-378b,miR-656-5p,miR-147-5p,miR-206,novel_1,miR-29a,miR-16a,miR-33a-3p,miR-15a,novel_27,miR-374c-5p,miR-2285m,miR-211,miR-1193,miR-29c-3p,miR-335-5p,miR-497-5p,miR-7862,miR-188-5p,miR-138,miR-21-5p,miR-29b,miR-16b,miR-223-3p,miR-1434-3p,miR-2285g,miR-377-5p,miR-660,miR-452-5p,miR-377-3p,miR-654-3p,miR-2285r,miR-2483-5p,miR-101-5p

KMT2E	ENSOARG0(	lysine (	miR-664a,miR-155-5p,miR-450b-3p,miR-424-5p,miR-4492,miR-874-5p,miR-665,miR-6134,miR-3184-5p,miR-214,miR-30f,miR-133b-3p,miR-582-5p,miR-450a-1-3p,miR-21a-3p,miR-30d-5p,miR-582-5p,miR-155-5p,miR-15b-5p,miR-22-3p,miR-504-5p,miR-106a,miR-21-3p,miR-30d,miR-208b-3p,miR-185-3p,miR-29b-1-5p,miR-143-3p,miR-486-3p,miR-345-3p,miR-20a,miR-1193,miR-200b,miR-15a-5p,miR-328-3p,miR-106a,miR-4726-5p,miR-2285t,miR-218-5p,miR-423-5p,miR-452-5p,miR-219a-5p,miR-504,miR-147-3p,miR-381-3p,miR-7857-3p,miR-1185-2-3p,miR-4443,miR-203-3p,miR-499b-5p,miR-25-5p,miR-214-3p,miR-106b-5p,miR-16b,miR-29b-1-5p,miR-106a-5p,miR-2330-5p,miR-424-5p,miR-450b-5p,miR-2426,miR-3120-5p,miR-21b,miR-30b-3p,miR-1185-3p,miR-5010-3p,miR-138-5p,miR-486b-3p,miR-134-5p,miR-409-3p,miR-100-3p,miR-133a-3p,miR-16-5p,miR-20b-5p,miR-30e-5p,miR-17-5p,miR-3154,miR-200a-5p,miR-93-5p,miR-874-3p,miR-3068-3p,miR-574-5p,miR-141-3p,miR-1271,miR-615-5p,miR-370-3p,miR-200c-3p,miR-2898,miR-200a-3p,miR-543-5p,miR-200c,miR-200b-3p,miR-155-5p,miR-134,miR-30a-5p,miR-322-5p,miR-134-5p,miR-450a-1-3p,miR-582-3p,miR-2284z,miR-429-3p,miR-1224-5p,miR-20a-5p,miR-133a-5p,miR-208a-3p,miR-15a,miR-16a,miR-371b-3p,miR-2428,miR-1291,miR-6395,miR-195a-5p,miR-6535,miR-320b,miR-452-5p,miR-106a-5p,miR-17-5p,miR-432-3p,let-7f-2-3p,let-7f-2-3p,miR-363-5p,miR-138,miR-30b-3p,miR-16b,miR-497-5p,miR-1843b-5p,miR-1285,miR-432,miR-22-3p,miR-450c-3p,miR-197-3p,miR-432-5p,miR-20b,miR-2355-3p
GRID2IP	ENSOARG0(	glutamat	miR-2903,miR-874-5p,miR-744-5p,miR-340-3p,miR-4286
TFEB	ENSOARG0(	transcri	miR-3074-5p,miR-2428,miR-326-3p,miR-449a,miR-331-3p,miR-34c,miR-128-1-5p,miR-6123,miR-28-5p,miR-486b-3p,miR-4492,miR-708-5p,miR-3074-5p,miR-34b,miR-378g,miR-34c-5p,miR-377-3p,miR-4443,miR-22-3p,miR-433-3p,miR-2411,novel_39,miR-486-3p,miR-8485,miR-377-3p,miR-326,miR-324-3p,miR-34b-5p,miR-134-3p,miR-2411-5p,miR-412-5p,miR-330-5p,miR-125b-2-3p,miR-30b-3p,miR-22-3p,miR-30b-3p,miR-34a-5p

ATG9B	ENSOARG0(	autophag	miR-3141,miR-150-5p,miR-1306,miR-3970,miR-216a-3p,miR-410-5p,miR-2284e,miR-378f,novel_68,novel_69,miR-411b-3p,miR-6535,miR-677,miR-181b-3p,miR-4324,miR-335-5p,miR-1839-3p,miR-193b-3p,miR-199b-5p,miR-7b-5p,miR-197-3p,miR-16b,miR-16-1-3p,miR-497-5p,miR-27b-3p,miR-31-5p,miR-340-3p,miR-339-5p,miR-134-3p,miR-194-3p,miR-7-5p,miR-377-3p,miR-3141,novel_51,miR-193a-3p,miR-668-3p,miR-2285r,miR-424-3p,miR-345-5p,miR-874-3p,miR-3068-3p,miR-2285aa,miR-2355-5p,miR-125a,miR-2433,miR-100-3p,miR-125b-5p,miR-592,miR-193a-5p,miR-16-5p,miR-1197-3p,miR-1260b,miR-1290,miR-330-5p,miR-429-3p,novel_79,miR-1224-5p,miR-18b-5p,miR-431,miR-9-5p,miR-505-5p,miR-322-5p,miR-486-5p,miR-129-1-3p,miR-127-5p,novel_96,novel_127,miR-3064-5p,novel_39,miR-29a-5p,miR-504,miR-139-5p,miR-378g,miR-2483-3p,miR-500-3p,miR-4443,novel_82,miR-301a-5p,miR-490-5p,miR-2447,miR-9-5p,miR-423-5p,miR-541,miR-301b-5p,miR-501-3p,miR-758-3p,miR-1388-5p,miR-21b,novel_78,miR-181b-2-3p,miR-2459,miR-431-5p,miR-29b-1-5p,novel_19,miR-16b,miR-491-5p,miR-199b-5p,miR-214-3p,miR-1388-3p,miR-378d,miR-2285c,miR-598-3p,miR-345-5p,miR-339b,miR-504-5p,miR-2331-3p,miR-30c-1-3p,miR-216b-3p,miR-2284s,miR-377-3p,miR-582-5p,miR-15b-5p,miR-151b,miR-665,miR-4492,miR-130b-5p,miR-129-2-3p,miR-3958-5p,miR-665-5p,miR-214,miR-769-5p,miR-346,miR-224-5p,miR-3184-5p,miR-378i,miR-193b-5p,miR-378a-3p,miR-142-3p,miR-16-1-3p,miR-133a-3p,miR-18a-3p,miR-212-5p,miR-1247-5p,miR-2284h-5p,miR-328-3p,novel_17,miR-422a,miR-202-5p,miR-377-5p,miR-181b-1-3p,miR-486-3p,miR-143-3p,miR-2285ab,miR-18b,miR-2387,miR-18a-3p,miR-378b,miR-450b-3p,miR-1291,miR-6395,miR-195a-5p,miR-320b,miR-15a,miR-16a,miR-128-1-5p,miR-22-5p,miR-135a-2-3p,miR-1193,miR-

TNMD	ENSOARG0(	tenomodu	miR-548e-3p,miR-106a,miR-322-5p,miR-7859,miR-505-5p,miR-15a-5p,miR-545-5p,miR-214-5p,miR-3591-5p,miR-193b-5p,miR-2904,miR-411-3p,miR-105-2,miR-128-3p,miR-378d,miR-361-5p,miR-496,miR-183-5p,miR-20a,miR-29b-2-5p,miR-29b-1-5p,miR-199a-5p,miR-1957a,miR-29a-5p,miR-2285b,miR-192-3p,miR-219b-5p,miR-106a,miR-107,miR-200a-3p,miR-140-5p,miR-503-3p,miR-496-3p,miR-2285aa,miR-2355-5p,miR-370-3p,miR-141-3p,miR-1271,miR-300-3p,novel_32,miR-199a-5p,miR-15b-5p,miR-488-3p,miR-21a-3p,miR-503-3p,miR-2285c,miR-93-5p,miR-17-5p,miR-7144-5p,miR-20b-5p,miR-625-5p,miR-1827,miR-16-5p,miR-425-5p,miR-93,miR-2433,miR-410-5p,miR-5100,miR-331-3p,miR-29b-2-5p,miR-665,miR-6516-3p,miR-129-5p,miR-424-5p,miR-103a-3p,miR-105-1,novel_4,miR-2300a-5p,miR-3535,miR-2285g,miR-539-5p,miR-149-5p,miR-455-3p,miR-2459,miR-103,miR-2285e,miR-20b,miR-653,miR-708-3p,miR-1285,miR-1434-3p,novel_23,miR-16b,miR-101a-5p,miR-497-5p,miR-424-5p,miR-106a-5p,miR-33a-5p,miR-8485,miR-29b-1-5p,miR-105-5p,miR-106a-5p,miR-16b,miR-106b-5p,miR-17-5p,miR-124-5p,miR-199b-5p,miR-216b-3p,miR-499b-5p,miR-374c-3p,miR-670-3p,miR-203-3p,miR-2285ab,miR-361-3p,miR-199b-5p,miR-216a-3p,miR-299b-5p,miR-136-5p,miR-107,miR-335-3p,miR-195a-5p,novel_120,miR-2285f,novel_27,miR-199b-5p,miR-218-5p,novel_82,miR-15a,miR-20a-5p,miR-2440,miR-16a,miR-379-3p,novel_1

SLC35C1	ENSOARG0(	solute c	miR-1185-3p,miR-147a,miR-1298-3p,miR-181b-2-3p,miR-199b-3p,miR-1343-3p,novel_78,miR-1839-5p,miR-541,miR-8485,miR-1388-3p,miR-124a,miR-103a-2-5p,miR-491-5p,miR-487b-5p,miR-182-5p,miR-431-5p,miR-652-3p,miR-4443,miR-1185-2-3p,miR-378g,miR-3596,miR-2319b,miR-1983,miR-504,miR-668-5p,miR-409-5p,miR-423-5p,miR-190b-5p,miR-9-5p,miR-2447,miR-628-3p,novel_82,miR-218-5p,miR-1247-5p,miR-2284h-5p,miR-328-3p,miR-30d-3p,miR-18a-3p,miR-218-1-3p,miR-34a-5p,miR-493-3p,miR-184-3p,miR-217-5p,miR-2366,miR-1961,miR-486-3p,miR-181b-1-3p,miR-2285w,miR-3653-3p,miR-2484,let-7k,miR-202-5p,miR-98-5p,miR-324-3p,miR-92a-1-5p,miR-26a-5p,let-7i-5p,miR-216b-3p,miR-296-3p,miR-2331-3p,miR-504-5p,miR-543-3p,miR-1827,miR-339b,miR-345-5p,miR-3184-5p,miR-6525,let-7b,miR-3184-3p,miR-129-2-3p,miR-190a-5p,miR-130b-5p,miR-30a-3p,miR-665,let-7f,miR-30e-3p,miR-149-5p,miR-134-3p,miR-3431,miR-339-5p,miR-31-5p,miR-2284x,miR-197-3p,miR-22-3p,miR-431-3p,miR-432,miR-1843b-5p,miR-2113,miR-432-3p,miR-668-3p,let-7f-2-3p,miR-628-5p,let-7f-2-3p,novel_51,miR-296-3p,miR-677,miR-669,miR-320c,miR-181b-3p,miR-374c-3p,miR-299b-3p,miR-26b-5p,novel_68,let-7e-5p,miR-412-3p,miR-1306,miR-3970,miR-148a-3p,miR-576-3p,miR-28c,miR-129-1-3p,miR-505-5p,miR-9-5p,novel_79,miR-431,miR-299a-3p,miR-429-3p,miR-330-5p,miR-1290,let-7g,miR-450c-5p,miR-192-3p,miR-450b-5p,miR-3064-5p,miR-152-3p,novel_96,novel_127,miR-200a-3p,let-7f-5p,miR-205-5p,miR-2355-5p,miR-125a,miR-1271,miR-3068-3p,miR-874-3p,miR-876-3p,miR-345-5p,miR-329b,miR-1260b,novel_9,miR-6740-5p,miR-125b-5p,miR-216a-3p,novel_4,miR-2300a-5p,miR-3120-5p,miR-30b-3p,miR-484,miR-4532,miR-652-5p,let-7a-5p,miR-494-3p,let-7e,miR-3085-3p,miR-3955-3p,miR-
ZMAT4	ENSOARG0(	zinc fin	miR-204-5p,miR-6516-3p,miR-138-5p,miR-3969,miR-151-3p,miR-6134,miR-338-3p,miR-378c,miR-378c,miR-1895,miR-376c-5p,miR-376c-5p,miR-200a-5p,miR-22-3p,novel_25,miR-377-3p,miR-496-3p,miR-503-3p,miR-2331-3p,miR-378a-3p,miR-221-5p,miR-221-5p,miR-345-3p,miR-217,miR-422a,miR-664-3p,novel_17,miR-2284a,miR-376c-5p,miR-376b-5p,miR-2284y,miR-142a-3p,miR-2284u,miR-496,miR-142-3p,miR-378a-3p,miR-217-5p,miR-501-3p,miR-2427,miR-378i,miR-3591-5p,miR-345-3p,miR-378h,miR-33a-3p,miR-502-3p,miR-211,miR-3059-5p,miR-342-3p,miR-378f,miR-376b-5p,miR-500-3p,miR-669,miR-378e,miR-211-5p,miR-664b,miR-2411-3p,miR-377-3p,miR-342,miR-2285r,miR-378d,miR-2284b,miR-138,miR-378c,miR-145a-5p,miR-501-3p,miR-22-3p,miR-7857,miR-378b,miR-194-3p,miR-142-3p,miR-145b,miR-151a-3p

TUFM	ENSOARG0(	Tu trans	miR-365a-5p,miR-93,miR-1197-3p,miR-16-5p,miR-133a-3p,miR-28a-3p,miR-486b-3p,miR-28-5p,miR-138-5p,miR-29b-3p,miR-769,miR-25-3p,miR-370-3p,miR-215-5p,miR-874-3p,miR-7144-5p,miR-23a-5p,miR-20b-5p,miR-329b,miR-93-5p,miR-3154,miR-18a,miR-17-5p,novel_39,miR-1271-3p,miR-92b-3p,miR-2385-3p,miR-30a-3p,miR-181a-2-3p,miR-152-3p,novel_96,novel_127,miR-92a-3p,miR-17-3p,miR-450a-2-3p,miR-28-3p,miR-127-5p,miR-217,miR-376b-3p,miR-664-3p,miR-2284z,miR-582-3p,miR-322-5p,miR-758-5p,miR-18b-5p,miR-6517,miR-29d-3p,miR-1343-5p,miR-23b-5p,miR-345-3p,novel_27,miR-2428,miR-24-3p,miR-193b-3p,miR-1839-3p,miR-17-3p,miR-29c-3p,novel_120,miR-335-5p,miR-6123,miR-92a-3p,miR-29a,miR-16a,miR-15a,miR-4324,miR-20a-5p,miR-669,miR-670-3p,novel_69,miR-29a-3p,miR-320b,miR-3120-3p,novel_68,miR-3074-5p,miR-378b,miR-6395,miR-6516-3p,miR-195a-5p,miR-410-5p,miR-412-3p,miR-3970,miR-150-5p,miR-378b,miR-376b-3p,miR-18a-3p,miR-18b,miR-148a-3p,miR-654-3p,miR-432-3p,miR-432-3p,miR-6240,miR-1343-5p,miR-193a-3p,miR-32,miR-365b-5p,miR-330-3p,miR-17-5p,miR-106a-5p,miR-455-5p,miR-2411-3p,miR-7-5p,miR-20b,miR-2355-3p,miR-134-3p,miR-185-5p,miR-26b-3p,miR-133b-5p,miR-497-5p,miR-455-5p,miR-29b,miR-138,miR-30b-3p,miR-16b,miR-145a-5p,miR-542-3p,miR-7b-5p,miR-769-5p,miR-6525,miR-3958-5p,miR-425-5p,miR-214,miR-145a-3p,miR-3074-5p,miR-4492,miR-708-5p,miR-424-5p,miR-17-3p,miR-6516-3p,miR-664a,miR-451a,miR-30a-3p,miR-665,miR-30e-3p,miR-15b-5p,miR-877-5p,miR-192-5p,miR-582-5p,miR-2284s,miR-216b-5p,miR-139-5p,miR-193b-3p,miR-296-3p,miR-532-5p,miR-552-3p,miR-2331-3p,miR-140-5p,miR-32-5p,miR-1827,miR-582-5p,miR-186-5p,miR-18a-5p,miR-486-3p,miR-2424,miR-143-3p,miR-664b-3p,miR-195a-3p,miR-20a,miR-345-
ZDHHC4	ENSOARG0(	zinc fin	miR-2285k,miR-96-5p,miR-1291,miR-195a-5p,miR-9851-3p,miR-299b-3p,miR-677,miR-664b,miR-541-5p,miR-3968,miR-6119-3p,miR-576-3p,miR-218-5p,miR-502-5p,miR-15a,miR-16a,miR-371b-3p,miR-544a,miR-16b,miR-30b-3p,miR-497-5p,miR-296-5p,miR-374b-3p,miR-30b-3p,miR-412-5p,miR-544b,novel_116,miR-2300a-5p,miR-31-5p,miR-1185-5p,miR-1343-5p,miR-582,miR-224-5p,miR-16b,novel_19,miR-382-3p,miR-424-5p,miR-1388-3p,novel_99,miR-7144-5p,miR-27a-5p,miR-544-3p,miR-15b-5p,miR-574-5p,miR-221-5p,miR-2355-5p,miR-3065-3p,miR-424-5p,miR-3604,miR-224-5p,miR-2330-3p,miR-1271,miR-3607-3p,miR-16-5p,miR-378d,miR-193b-5p,miR-1343-5p,miR-1271-5p,miR-15a-5p,miR-505-5p,miR-322-5p,miR-6517,miR-299a-3p,miR-3960,miR-1b-3p,miR-21-3p,miR-299-3p,miR-221-5p,miR-664-3p,miR-210-5p,miR-2285i,miR-29a-5p,miR-3065-3p,miR-181a-2-3p

HNRNPDL	ENSOARG0(	heteroge	miR-205-5p,miR-664-3p,miR-3071-3p,miR-181b-5p,miR-200c-3p,miR-1,miR-29b-2-5p,miR-30a-3p,miR-3064-5p,miR-4286,miR-181a-2-3p,miR-29a-5p,novel_39,miR-345-3p,miR-134,miR-411-3p,miR-200b-3p,miR-299a-3p,miR-429-3p,miR-1224-5p,miR-1271-5p,miR-6516-5p,miR-2411-5p,miR-134-5p,miR-450a-1-3p,miR-2284k,novel_63,miR-125b-5p,miR-135a-5p,miR-592,miR-134-5p,miR-486b-3p,miR-376c-5p,miR-320a,miR-3154,miR-10b-3p,let-7g-3p,miR-376c-5p,miR-574-5p,miR-2355-5p,miR-125a,miR-1271,miR-769,miR-223-5p,miR-146a,miR-190a,miR-2411-3p,miR-455-5p,miR-124-5p,miR-125a-5p,miR-493-5p,miR-2285r,miR-432-3p,miR-432-3p,miR-223-3p,miR-181a-5p,miR-450c-3p,miR-30b-3p,miR-219a-1-3p,miR-16-1-3p,miR-455-5p,miR-2284x,miR-3955-5p,miR-26b-3p,miR-134-3p,novel_1,miR-206,miR-203b-5p,novel_101,miR-2428,miR-376b-5p,miR-412-3p,miR-146b-5p,miR-6516-3p,miR-505-3p,miR-135b-5p,miR-379-5p,miR-329-5p,novel_69,miR-299b-3p,miR-670-3p,miR-669,miR-320c,miR-677,miR-181b-3p,miR-2285p,novel_17,miR-324-3p,miR-125b,miR-345-3p,miR-320d,miR-2284m,miR-466i-5p,miR-221-5p,miR-3653-3p,miR-299-3p,miR-21-3p,miR-29b-1-5p,miR-130a-5p,miR-181b-1-3p,miR-2411,miR-146a-5p,miR-486-3p,miR-2366,miR-1193,miR-5703,miR-4429,miR-16-1-3p,miR-3600,miR-505-3p,miR-200b,miR-30a-3p,miR-665,miR-29b-2-5p,miR-450b-3p,miR-6516-3p,miR-487b-5p,miR-129-5p,miR-190a-5p,miR-1271,miR-2330-3p,miR-3074-2-3p,miR-769-5p,miR-206-3p,miR-181c-5p,miR-450a-1-3p,miR-221-5p,miR-140-5p,miR-182-5p,miR-6516,miR-320b,novel_115,miR-29b-1-5p,miR-182-5p,miR-3085-3p,miR-146b,miR-6529a,miR-143-5p,miR-1a-3p,miR-30b-3p,miR-1388-5p,miR-450b-5p,miR-101c,miR-5010-3p,miR-181b-2-3p,miR-455-3p,miR-9788-3p,miR-371a-5p,miR-769-5p,miR-885-5p,miR-181d-5p,miR-2284i,miR-190b-5p,miR-7705,miR-96-
KISS1	ENSOARG0(	KiSS-1 m	miR-450b-5p,miR-107,miR-221-5p,novel_127,miR-17-3p,miR-466i-5p,miR-412-5p,miR-92a-1-5p,miR-185-3p,miR-125a-3p,novel_121,miR-210-3p,miR-322-5p,miR-483-3p,miR-15a-5p,miR-412,miR-431,miR-18a-3p,miR-128-3p,miR-193b-5p,miR-16-5p,miR-193a-5p,miR-424-5p,miR-874-5p,miR-4492,miR-17-3p,miR-320e,miR-5100,miR-370-5p,miR-22-3p,miR-15b-5p,miR-154b-5p,miR-370-3p,miR-370-5p,miR-3956,miR-296-3p,miR-221-5p,miR-874-3p,miR-137-3p,miR-628-5p,miR-424-5p,miR-6240,miR-452-5p,miR-103a-2-5p,miR-483-3p,novel_19,miR-330-3p,miR-16b,miR-431-5p,miR-216a-3p,miR-194-3p,miR-411b-5p,miR-103,miR-134-3p,miR-147a,miR-103a-3p,miR-330-3p,miR-497-5p,miR-4508,miR-16b,miR-197-3p,miR-412-3p,miR-22-3p,miR-2284r,miR-1843b-5p,miR-143-5p,miR-2428,miR-1307-3p,miR-24-3p,miR-3957-3p,miR-17-3p,miR-1193,novel_60,miR-3956-3p,miR-6123,miR-1307-5p,miR-16a,miR-107-5p,miR-502-5p,miR-15a,miR-412-5p,miR-2440,miR-669,miR-378g,miR-369-5p,miR-195a-5p,miR-107,miR-18a-3p,miR-129b-5p,miR-216a-3p,miR-361-3p,miR-2387

IFRD1	ENSOARG0(	interfer	miR-545-5p,miR-24-2-5p,miR-7641,let-7a-2-3p,miR-105-2,miR-200b-3p,miR-411-3p,miR-30a-5p,miR-29d-3p,miR-3578,miR-345-3p,miR-1957a,miR-28b,miR-4286,miR-181a-2-3p,miR-1,miR-3529-3p,miR-200c-3p,miR-17-3p,miR-450a-2-3p,miR-217,miR-30c-2-3p,miR-6119-5p,miR-200c,miR-376b-3p,miR-2284n,miR-374c-3p,miR-615-5p,miR-1814c,miR-671-5p,let-7g-3p,miR-320a,miR-17-5p,miR-362-5p,miR-93,miR-500b-5p,miR-133a-3p,miR-3607-3p,miR-6128,miR-374b,miR-410-3p,miR-138-5p,miR-3969,miR-370-5p,miR-29b-3p,miR-410-5p,miR-20b,miR-185-5p,miR-2285g,miR-2284d,miR-2889,miR-455-5p,miR-138,miR-2284ab,miR-29b,miR-30b-3p,miR-380-3p,miR-3071-5p,miR-194-5p,miR-374b-3p,miR-1434-3p,miR-873a-5p,miR-345-5p,miR-539-3p,miR-1343-5p,miR-106a-5p,miR-455-5p,miR-17-5p,miR-330-3p,miR-190a,miR-224-5p,miR-29a-3p,miR-670-3p,miR-2404,miR-655-5p,miR-6516-3p,miR-195a-5p,miR-7975,miR-376b-3p,miR-18a-3p,miR-2428,miR-24-3p,miR-1193,miR-17-3p,miR-342-3p,novel_120,miR-502b,miR-203b-5p,novel_1,miR-16a,miR-20a-5p,miR-15a,miR-532-3p,miR-15a-5p,miR-200b,miR-487a-3p,miR-485-3p,miR-548e-3p,miR-4429,miR-5703,miR-188-3p,miR-2904,miR-124-3p,miR-20a,miR-216c-5p,miR-21-3p,miR-30d,miR-1277-5p,miR-30b-3p,miR-466i-5p,miR-320d,miR-106a,miR-2284m,miR-374a-5p,miR-196a-3p,miR-22-3p,novel_25,miR-204-3p,miR-139-5p,miR-532-5p,miR-370-5p,miR-30f,miR-154b-3p,let-7g-3p,miR-133b-3p,let-7f-1-3p,miR-21a-3p,miR-206-3p,miR-145a-3p,miR-424-5p,miR-664a,miR-6516-3p,miR-17-3p,miR-324-3p,miR-2285e,miR-500-5p,miR-105-1,miR-151a-3p,miR-2284aa,miR-2426,miR-30b-3p,miR-4791,miR-484,miR-758-3p,miR-491-3p,miR-30c-1-3p,miR-33a-5p,let-7c-3p,miR-424-5p,miR-106a-5p,miR-320b,miR-342,miR-376d,miR-7977,miR-541-5p,miR-8095,miR-

PPP2R5C	ENSOARG0(protein	miR-212-3p,miR-769-5p,miR-2285y,miR-1973,miR-382-3p,miR-2285n,miR-2285t,miR-2284q,miR-2285u,miR-211-5p,miR-2284g,miR-3064-5p,miR-1185-2-3p,miR-129b-5p,miR-378j,miR-877-3p,miR-504,miR-1388-3p,miR-424-5p,miR-3187-3p,miR-16b,miR-214-3p,miR-3085-3p,miR-9788-3p,miR-1185-3p,miR-425-3p,miR-21b,miR-2426,miR-214,miR-769-5p,miR-665,miR-424-5p,miR-450b-3p,miR-296-3p,miR-504-5p,miR-15b-5p,miR-193b-3p,miR-27b-5p,miR-299,miR-450a-1-3p,let-7g-3p,miR-18a-5p,miR-2887,miR-329a-5p,miR-183-5p,miR-486-3p,miR-299-3p,miR-202-5p,miR-3960,miR-15a-5p,miR-200b,miR-188-3p,miR-5703,miR-2366,miR-2284u,miR-378d,miR-184-3p,miR-3957-3p,miR-193b-3p,miR-2428,miR-2285m,miR-211,miR-16a,miR-425-5p,miR-15a,miR-329-5p,miR-299b-3p,miR-3141,miR-18b,miR-7975,miR-135b-5p,miR-195a-5p,miR-107,let-7f-2-3p,miR-380-5p,let-7a-2-3p,miR-654-3p,miR-3141,miR-1343-5p,miR-193a-3p,miR-378a-5p,miR-31-5p,miR-187-3p,miR-103a-3p,miR-103,miR-185-5p,miR-450c-3p,miR-374b-3p,miR-3071-5p,miR-708-3p,miR-542-3p,miR-497-5p,miR-16b,miR-16-5p,miR-1306-5p,miR-10b-3p,miR-135a-5p,miR-134-5p,miR-204-5p,miR-486b-3p,miR-767-5p,miR-542-5p,miR-615-5p,miR-574-5p,miR-300-3p,miR-769,miR-200a-5p,miR-3154,miR-18a,miR-2448-3p,miR-7144-5p,miR-671-5p,miR-767,let-7g-3p,miR-329b,miR-3064-5p,miR-2403,miR-2284y,miR-1271-3p,miR-450b-5p,miR-380-5p,miR-2285af,miR-200c,miR-107,miR-450a-2-3p,miR-200c-3p,miR-18b-5p,miR-299a-3p,miR-2285ad,miR-6517,miR-429-3p,miR-134-5p,miR-450a-1-3p,miR-214-5p,miR-24-2-5p,miR-322-5p,miR-1343-5p,miR-345-3p,miR-134,miR-1290,miR-200b-3p,let-7a-2-3p

SDF2	ENSOARG0(	stromal	miR-127-5p,miR-1a-2-5p,miR-17-3p,miR-200a-3p,novel_96,miR-1260a,miR-2285af,miR-2478,miR-1271-3p,miR-199a-5p,miR-181a-2-3p,miR-4286,miR-29b-2-5p,miR-3064-5p,miR-2385-3p,miR-300,miR-128-3p,miR-1290,miR-2427,miR-29d-3p,miR-1343-5p,miR-378h,miR-483-3p,miR-322-5p,novel_121,miR-582-3p,miR-545-5p,miR-6517,miR-222,miR-767-5p,miR-204-5p,miR-29b-3p,miR-1-5p,miR-3969,miR-1197-3p,miR-1260b,miR-500b-5p,miR-378c,miR-145-3p,miR-374b,miR-193a-5p,miR-3607-3p,miR-16-5p,miR-767,miR-381-3p,miR-362-5p,miR-34c-5p,miR-374c-3p,miR-199a-5p,miR-1271,miR-141-3p,miR-2355-5p,miR-370-3p,miR-378a-5p,miR-483-3p,miR-193a-3p,miR-1343-5p,miR-377-3p,miR-330-3p,miR-432-3p,miR-6240,miR-1260b,miR-29b,miR-16b,miR-744-5p,miR-188-5p,miR-497-5p,miR-1434-3p,miR-432,miR-374b-3p,miR-185-5p,miR-134-3p,miR-432-5p,miR-412-5p,miR-544b,miR-194-3p,miR-340-3p,miR-26b-3p,miR-29a,novel_1,miR-128-1-5p,miR-502b,miR-6529b,miR-15a,miR-16a,miR-34c,miR-211,miR-199b-5p,miR-449a,miR-335-5p,miR-1a-1-5p,miR-17-3p,miR-29c-3p,miR-193b-3p,miR-412-3p,miR-195a-5p,miR-378f,miR-216a-3p,miR-150-5p,miR-374c-3p,miR-670-3p,miR-29a-3p,miR-34b,miR-26b-5p,miR-320b,miR-34b-5p,miR-210-5p,miR-374a-5p,miR-26a-5p,miR-2285p,miR-422a,miR-500a-5p,miR-29b-1-5p,miR-183-5p,miR-34a-5p,miR-378d,miR-2366,miR-193b-5p,miR-378i,miR-5703,miR-378a-3p,miR-124-3p,miR-2904,miR-15a-5p,miR-30d-3p,miR-17-3p,miR-6516-3p,miR-424-5p,miR-487a-5p,miR-222-3p,miR-664a-5p,miR-29b-2-5p,miR-132-5p,miR-3184-5p,miR-145a-3p,miR-378c,novel_99,miR-582-5p,miR-193b-3p,miR-582-5p,miR-544-3p,miR-2284s,miR-377-3p,miR-15b-5p,miR-548o-3p,miR-378a-3p,miR-140-5p,miR-182-5p,miR-3955-3p,miR-491-5p,miR-3085-3p,miR-199b-5p,miR-16b,miR-182-5p,miR-29b-1-5p,miR-

DDX3X	ENSOARG0(	DEAD (As	miR-135a-5p,miR-6740-5p,novel_63,miR-29b-3p,miR-374b-3p,miR-767-5p,miR-16-5p,miR-6128,miR-2284v,miR-1306-5p,miR-145-3p,miR-1197-3p,miR-7144-5p,miR-767,miR-1271,miR-215-5p,miR-141-3p,miR-370-3p,miR-2284n,miR-199a-5p,miR-25-3p,miR-217,miR-200c,miR-2285af,miR-92a-3p,miR-200a-3p,miR-127-5p,miR-6536,miR-181b-5p,miR-200c-3p,miR-30a-3p,miR-29b-2-5p,miR-2285b,miR-192-3p,miR-199a-5p,miR-92b-3p,miR-29d-3p,miR-2427,miR-200b-3p,miR-582-3p,miR-7641,miR-429-3p,miR-545-5p,miR-2284z,miR-322-5p,miR-505-5p,miR-382-5p,miR-16a,miR-208a-3p,miR-133a-5p,miR-15a,miR-29a,miR-6119-3p,miR-92a-3p,miR-495-3p,miR-29c-3p,miR-1839-3p,miR-342-3p,miR-219b-3p,miR-199b-5p,miR-2284w,miR-323c,miR-135b-5p,miR-195a-5p,miR-19a-3p,miR-7-1-3p,miR-29a-3p,miR-2404,miR-330-3p,miR-32,miR-466f-3p,miR-493-5p,miR-6240,miR-2284l,miR-2285r,miR-181a-5p,miR-1285,miR-497-5p,miR-29b,miR-30b-3p,miR-16b,miR-2284ab,miR-133b-5p,miR-3431,miR-2284d,miR-664a-5p,miR-29b-2-5p,miR-30a-3p,miR-324-3p,miR-30e-3p,miR-500,miR-424-5p,miR-4492,miR-129-5p,miR-6516-3p,miR-3074-2-3p,miR-433-5p,miR-181c-5p,miR-500a-3p,miR-582-5p,novel_99,miR-186-5p,miR-543-3p,miR-296-3p,miR-140-5p,miR-32-5p,miR-15b-5p,miR-192-5p,miR-499b-3p,miR-92a-1-5p,miR-208b-3p,miR-210-5p,miR-1277-5p,miR-30b-3p,miR-202-5p,miR-216c-5p,miR-2432,miR-217-5p,miR-1197-5p,miR-124-3p,miR-215-5p,miR-361-5p,miR-133a-3p,miR-19b-3p,miR-30d-3p,miR-212-5p,miR-485-3p,miR-548e-3p,miR-616-3p,miR-200b,miR-15a-5p,miR-7859,miR-487a-3p,miR-101-3p,miR-374a-3p,miR-181d-5p,miR-199b-5p,miR-493-5p,miR-20a-3p,miR-10b,miR-454-5p,miR-2483-3p,miR-3082-5p,miR-25,miR-499a-3p,miR-16b,miR-505,miR-2957,miR-342,miR-199b-5p,miR-652-5p,let-7e-3p,miR-1839-3p,miR-424-5p,miR-382-3p,miR-124a,miR-
DIAPH2	ENSOARG0(	diaphano	miR-21a-3p,miR-335-3p,miR-3074-1-3p,miR-135b-5p,novel_99,miR-574-5p,miR-221-5p,miR-329-5p,miR-370-5p,miR-3120-3p,miR-532-5p,miR-7-1-3p,miR-7857-3p,miR-377-3p,miR-3082-5p,miR-203-3p,miR-370-5p,miR-371a-5p,miR-107-5p,miR-135a-5p,miR-22-5p,miR-3059-5p,miR-1193,miR-376a-5p,miR-1197-5p,miR-30b-3p,miR-30b-3p,miR-33b-3p,miR-450b-5p,miR-188-5p,miR-330-3p,miR-222-5p,miR-411-5p,miR-377-3p,miR-618,miR-330-3p,miR-466i-5p,miR-30b-3p,miR-221-5p,miR-466f-3p,miR-103a-2-5p,miR-329a-5p,miR-6240,miR-369-3p,miR-495-5p,miR-376a-5p,miR-2424,miR-8485
CHEK2	ENSOARG0(	checkpoi	novel_1,miR-219-3p,miR-219b-3p,miR-219a-2-3p,miR-199b-5p,miR-199a-5p,let-7c-3p,miR-23a-3p,let-7g-3p,miR-199b-5p,miR-140-5p,miR-23b,miR-23c,miR-23b-3p,miR-199a-5p,miR-409b

GNL3	ENSOARG0(	guanine	miR-193b-3p,miR-17-3p,miR-495-3p,novel_120,miR-1a-1-5p,novel_27,miR-301b-3p,miR-2428,miR-199b-5p,miR-211,miR-323c,miR-16a,miR-382-5p,miR-15a,miR-142b,miR-502b,miR-379-3p,miR-320b,novel_68,miR-378b,miR-216b-3p,miR-6535,miR-411b-3p,miR-3065-5p,miR-216a-3p,miR-21c,miR-378b,miR-376b-5p,miR-148a-3p,miR-146b-5p,miR-7975,miR-195a-5p,miR-107,miR-19a-3p,miR-628-5p,miR-539-3p,miR-2284l,miR-7-5p,miR-146a,miR-193a-3p,miR-378a-5p,miR-130a-3p,miR-26b-3p,miR-103a-3p,miR-103,miR-3535,miR-3071-5p,miR-145a-5p,miR-7b-5p,miR-497-5p,miR-27b-3p,miR-21-5p,miR-2284ab,miR-16b,miR-16-1-3p,miR-16-5p,miR-2284v,miR-181c-3p,miR-500b-5p,miR-410-5p,miR-1-5p,novel_63,miR-494-5p,miR-410-3p,miR-204-5p,miR-370-3p,miR-2285aa,miR-3956,miR-141-3p,miR-874-3p,miR-3068-3p,miR-199a-5p,miR-331-5p,miR-769,miR-376c-5p,miR-362-5p,miR-30e-5p,miR-376c-5p,miR-3064-5p,miR-181a-2-3p,miR-199a-5p,miR-3065-3p,miR-29a-5p,miR-3956-5p,miR-192-3p,miR-1271-3p,miR-148b-5p,miR-450b-5p,miR-2478,miR-30f,miR-181c-3p,miR-125a-3p,miR-107,miR-152-3p,miR-200a-3p,miR-17-3p,miR-1a-2-5p,miR-222,miR-582-3p,novel_121,miR-214-5p,miR-129-1-3p,miR-486-5p,miR-322-5p,miR-758-5p,miR-23b-5p,miR-3578,miR-30c,miR-128-3p,miR-411-3p,miR-6527,miR-2447,miR-1285-5p,miR-362-5p,miR-769-5p,miR-30b-5p,miR-1843a-3p,miR-490-5p,miR-628-3p,miR-7689-3p,miR-211-5p,miR-3064-5p,miR-20a-3p,miR-10b,miR-27a-3p,miR-499a-3p,miR-7857-3p,miR-199b-5p,miR-221,miR-181a-3p,miR-136-5p,let-7c-3p,miR-1388-3p,miR-424-5p,miR-758-3p,miR-1b-5p,miR-505,miR-16b,miR-146b,miR-199b-5p,miR-3085-3p,miR-1185-5p,miR-130b-3p,miR-145b,miR-27a-3p,miR-1343-3p,miR-151a-3p,miR-548w,miR-500-5p,miR-221-3p,miR-148b-5p,miR-142-3p,miR-142-5p,miR-147a,miR-
PREX2	ENSOARG0(	phosphat	miR-136-3p,miR-342-3p,miR-22-5p,miR-490-5p,miR-6402,miR-144-5p,miR-203-3p,miR-6535,miR-499a-3p,miR-10b,miR-2319b,miR-2284g,novel_94,miR-146b-5p,miR-129b-5p,miR-1306,miR-654-3p,miR-432-3p,miR-2284b,miR-363-5p,miR-23a-3p,miR-6240,miR-1388-3p,miR-146b,miR-1343-5p,miR-182-5p,miR-330-3p,miR-1306-5p,miR-146a,miR-342,miR-185-5p,miR-3431,miR-26b-3p,miR-330-3p,miR-31-5p,miR-340-3p,miR-450b-5p,miR-1388-5p,miR-30b-3p,miR-380-3p,miR-3071-5p,miR-374b-3p,miR-30b-3p,miR-484,miR-144-5p,miR-133a-3p,miR-2330-3p,miR-3607-3p,miR-155-5p,miR-155-5p,miR-365a-3p,miR-499b-3p,miR-182-5p,miR-140-5p,miR-671-5p,miR-625-5p,miR-876-3p,miR-10b-3p,miR-133b-3p,miR-23b,miR-129b-3p,miR-137-3p,miR-380-3p,miR-2284a,miR-376c-5p,miR-376b-5p,miR-2284y,miR-146a-5p,miR-376a-5p,miR-200c-3p,miR-202-5p,miR-200c,miR-2285p,novel_121,miR-23c,miR-200b,miR-23b-3p,miR-302a-5p,miR-7859,miR-505-5p,miR-3600,miR-429-3p,miR-548e-3p,miR-2284u,miR-133a-3p,miR-155-5p,miR-200b-3p,miR-140-3p,miR-1343-5p

MLF1	ENSOARG0(	myeloid	miR-2284w,miR-323c,miR-211,miR-190b-5p,miR-29c-3p,miR-495-3p,miR-335-5p,miR-885-5p,miR-135a-2-3p,miR-29a,miR-335,novel_87,miR-371a-5p,miR-20a-5p,miR-6402,miR-101-3p,miR-3613,miR-376d,miR-1185-2-3p,miR-411b-3p,miR-29a-3p,miR-2483-3p,miR-2404,miR-7857-3p,miR-146b-3p,miR-499a-3p,miR-211-5p,miR-7689-3p,miR-329-5p,miR-139-5p,miR-3064-5p,miR-1983,miR-335-3p,miR-376a-3p,miR-412-3p,miR-216a-3p,miR-376b-3p,miR-221,miR-8485,miR-432-3p,miR-106a-5p,miR-452-5p,miR-3085-3p,miR-106a-5p,novel_51,miR-106b-5p,miR-17-5p,miR-105-5p,miR-1185-3p,miR-20b,miR-221-3p,miR-376b-3p,novel_4,miR-105-1,miR-101c,miR-16-2-3p,miR-450b-5p,miR-380-3p,miR-29b,novel_23,miR-1843b-5p,miR-223-3p,miR-1434-3p,miR-93,miR-425-5p,miR-590-3p,miR-204-5p,miR-767-5p,miR-3604,miR-3969,miR-338-5p,miR-320e,miR-664a-5p,miR-222-3p,miR-34c-3p,miR-29b-3p,miR-877-5p,miR-139-5p,miR-216b-5p,miR-499b-3p,miR-141-3p,miR-532-5p,miR-3065-3p,miR-2285aa,miR-485-5p,miR-20b-5p,miR-767,miR-433-3p,miR-17-5p,miR-93-5p,miR-3613-5p,miR-488-3p,miR-380-3p,miR-143-3p,miR-3065-3p,miR-421-5p,miR-3064-5p,miR-20a,miR-329a-5p,miR-200a-3p,miR-106a,miR-2285p,miR-376b-3p,miR-106a,miR-222,miR-128-3p,miR-142-3p,miR-1193,miR-29d-3p
PGC	ENSOARG0(	progastr	miR-3955-3p,miR-125a-5p,let-7e,miR-1306-5p,miR-190a,miR-365b-5p,miR-29b-1-5p,novel_51,miR-2284l,let-7a-5p,miR-2483-5p,miR-744-3p,miR-1260b,miR-3432b,miR-2426,miR-3120-5p,miR-744-5p,miR-143-5p,miR-10b,miR-197-3p,miR-147a,let-7g-5p,miR-2355-3p,miR-665,miR-181b-2-3p,miR-421-5p,novel_82,miR-34c,let-7i,miR-24-3p,miR-423-5p,miR-449a,miR-2447,miR-190b-5p,miR-219a-5p,miR-136-5p,miR-335-3p,let-7e-5p,miR-1306,miR-18a-3p,miR-499a-3p,miR-34b,miR-411b-3p,let-7d-5p,miR-677,miR-181b-3p,miR-10a-5p,miR-760-3p,miR-98-5p,let-7f-5p,miR-34b-5p,miR-543-5p,let-7k,novel_127,miR-1260a,miR-2285p,miR-125a-3p,miR-216a-5p,let-7c-5p,miR-125b,miR-450b-5p,miR-29b-1-5p,miR-181b-1-3p,miR-130a-5p,miR-2424,let-7g,miR-29b-2-5p,let-7d,miR-34a-5p,miR-10a-5p,miR-134,miR-1961,miR-2366,miR-5703,miR-140-3p,miR-134-5p,novel_121,miR-18a-3p,miR-1b-3p,miR-190a-5p,miR-592,miR-134-5p,let-7f,miR-125b-5p,miR-29b-2-5p,miR-3969,miR-10b-5p,miR-346,miR-1260b,miR-3184-5p,miR-365a-5p,let-7b-5p,let-7b,miR-145a-3p,miR-1248,miR-10a,miR-499b-3p,miR-34c-5p,miR-365a-3p,miR-3959-5p,miR-204-3p,let-7i-5p,miR-874-3p,miR-370-3p,miR-125a,miR-1271

EPB41	ENSOARG0(	erythroc	miR-2285r,miR-432-3p,miR-2284l,miR-668-3p,miR-483-3p,miR-378a-5p,miR-146a,miR-365b-5p,miR-2285v,miR-194a,novel_51,miR-3535,miR-134-3p,miR-3431,miR-149-5p,miR-339-5p,miR-16b,miR-497-5p,miR-2284x,miR-432,miR-1843b-5p,miR-542-3p,miR-2113,miR-22-3p,miR-2285m,miR-625-3p,miR-4324,miR-28c,miR-2284o,miR-144-3p,miR-669,miR-320c,novel_68,miR-329-5p,miR-146b-5p,miR-148a-3p,miR-3970,miR-148b-5p,novel_39,miR-2284y,miR-192-3p,miR-2284f,miR-127-5p,miR-152-3p,novel_127,novel_96,miR-205-5p,miR-322-5p,miR-129-1-3p,novel_79,miR-1343-5p,miR-330-5p,miR-1260b,miR-1197-3p,miR-16-5p,novel_9,novel_63,miR-2284k,miR-6740-5p,miR-3959-5p,miR-874-3p,miR-542-5p,miR-1271,miR-876-3p,miR-329b,miR-20b-5p,miR-93-5p,miR-2284b,miR-491-5p,miR-146b,miR-103a-2-5p,miR-29b-1-5p,novel_19,miR-106b-5p,miR-16b,miR-1298-3p,miR-455-3p,miR-145b,miR-2284r,miR-541,miR-877-3p,miR-361-3p,miR-423-5p,miR-2447,miR-3956-3p,miR-190b-5p,miR-2284j,miR-615,miR-499a-3p,miR-378g,miR-24-1-5p,miR-2284g,miR-126b-5p,miR-504,miR-136-5p,miR-409-5p,miR-378j,miR-2285i,miR-2285w,miR-345-3p,miR-210-5p,miR-500a-5p,miR-1247-5p,miR-2284h-5p,miR-18a-3p,miR-106a,miR-30d-3p,miR-212-5p,miR-33b-3p,miR-2284u,miR-193b-5p,miR-769-5p,miR-224-5p,miR-3184-5p,miR-181c-5p,miR-129-2-3p,miR-190a-5p,miR-4492,miR-874-5p,miR-130b-5p,miR-30e-3p,miR-30a-3p,miR-10a,miR-499b-3p,miR-2284s,miR-15b-5p,miR-2331-3p,miR-504-5p,miR-30c-1-3p,miR-552-3p,miR-339b,miR-1895,miR-543-3p,miR-654-3p,miR-130b-5p,miR-382,miR-1343-5p,miR-224-5p,miR-190a,miR-106a-5p,miR-455-5p,miR-17-5p,miR-432-5p,miR-544b,miR-20b,miR-2355-3p,miR-138,miR-30b-3p,miR-2284ab,miR-219a-1-3p,miR-1260b,miR-455-5p,miR-653,miR-1285,miR-181a-5p,miR-1434-3p,miR-145a-5p,miR-194-5p,miR-

SLC22A18	ENSOARG0(	solute c	miR-665-5p,miR-214,miR-145a-3p,miR-4510,miR-6134,miR-3184-5p,miR-346,novel_44,miR-6525,miR-10b-5p,miR-664a-5p,miR-320e,miR-324-3p,miR-130b-5p,miR-4492,miR-874-5p,miR-664a,miR-182-5p,miR-21-3p,miR-3065-3p,miR-30c-1-3p,miR-504-5p,miR-22-3p,miR-6130,miR-323-5p,miR-139-5p,miR-10a,miR-377-3p,miR-625-5p,miR-345-5p,miR-339b,miR-345-3p,miR-486-3p,miR-2411,miR-181b-1-3p,miR-130a-5p,miR-185-3p,miR-500a-5p,miR-324-3p,novel_17,miR-299-3p,miR-30b-3p,miR-34b-5p,miR-760-3p,miR-466i-5p,miR-320d,miR-3960,miR-18a-3p,miR-328-3p,miR-1247-5p,miR-5703,miR-2904,miR-2300b-3p,miR-2366,miR-34a-5p,miR-378d,miR-148a-5p,miR-133a-3p,miR-16-1-3p,miR-4429,miR-3956-3p,miR-541-3p,novel_60,miR-423-5p,novel_111,miR-301a-5p,miR-2440,miR-218-5p,miR-490-5p,miR-3968,miR-664b,miR-4792,miR-3064-5p,miR-139-5p,miR-4443,miR-499a-3p,miR-378g,miR-361-3p,miR-1983,miR-877-3p,miR-504,miR-193a,miR-4532,miR-8485,miR-30c-1-3p,miR-182-5p,miR-1306-5p,miR-5126,miR-320b,miR-214-3p,miR-491-5p,miR-3085-3p,miR-181b-2-3p,miR-2300a-5p,novel_4,miR-455-3p,miR-9788-3p,miR-148b-5p,miR-147a,miR-30b-3p,miR-10b,miR-541,miR-301b-5p,miR-2284r,miR-296-5p,miR-21b,novel_42,miR-331-3p,miR-100-3p,miR-6740-5p,miR-2433,miR-486b-3p,miR-370-3p,miR-874-3p,miR-574-5p,miR-34c-5p,miR-320a,miR-3154,miR-671-5p,miR-485-5p,miR-7144-5p,miR-345-5p,novel_91,miR-4286,miR-3064-5p,miR-3529-3p,miR-1271-3p,miR-3065-3p,novel_39,miR-148b-5p,miR-181c-3p,miR-339a,miR-30c-2-3p,miR-664-3p,miR-125a-3p,novel_127,miR-543-5p,novel_96,novel_79,miR-299a-3p,miR-2411-5p,miR-505-5p,miR-10a-5p,miR-2428,miR-449a,miR-24-3p,miR-33a-3p,miR-34c,miR-576-3p,miR-147-5p,miR-656-5p,miR-10a-5p,miR-320b,miR-378b,novel_68,miR-181b-3p,miR-669,miR-320c,miR-
ZNF277	ENSOARG0(	zinc fin	miR-181b-5p,miR-2284m,miR-21b,miR-181a-5p,miR-205-5p,miR-30c-2-3p,miR-500a-5p,miR-30c-1-3p,miR-2284n,miR-181c-5p,miR-30c-1-3p,miR-187-3p,miR-181d-5p

CFAP61	ENSOARG0(	cilia an	miR-490-3p,miR-199a-5p,miR-2478,miR-4286,miR-29b-2-5p,miR-2385-3p,novel_127,miR-181b-5p,miR-200c-3p,miR-17-3p,miR-450a-2-3p,miR-339a,miR-30f,miR-664-3p,miR-200c,miR-2285af,miR-214-5p,miR-758-5p,miR-2411-5p,miR-1271-5p,miR-505-5p,miR-322-5p,miR-302a-5p,novel_79,miR-429-3p,miR-496,miR-200b-3p,miR-30a-5p,miR-218-2-3p,miR-1343-5p,miR-1290,miR-345-3p,miR-365a-5p,miR-323-3p,miR-3607-3p,miR-16-5p,miR-28-5p,miR-204-5p,miR-410-5p,miR-2433,miR-199a-5p,miR-3959-5p,miR-374c-3p,miR-1271,miR-598-3p,miR-2285aa,miR-503-3p,miR-485-5p,miR-1248,miR-345-5p,miR-329a,miR-10b-3p,miR-23b,miR-301,miR-424-3p,miR-30e-5p,miR-345-5p,miR-101-5p,miR-106b-3p,miR-1343-5p,miR-455-5p,miR-2411-3p,miR-365b-5p,miR-149-5p,miR-339-5p,miR-133b-5p,miR-455-5p,miR-497-5p,miR-26a-2-3p,miR-16b,miR-380-3p,miR-374b-3p,miR-145a-5p,miR-22-3p,miR-2113,miR-181a-5p,miR-19b-2-5p,miR-873a-5p,miR-199b-5p,miR-2428,miR-2284w,miR-211,miR-17-3p,novel_101,miR-2285x,miR-3957-3p,miR-6123,miR-656-5p,miR-425-5p,miR-485-3p,miR-16a,miR-6402,miR-6529b,miR-502-5p,miR-15a,miR-6535,miR-374c-3p,novel_68,miR-195a-5p,miR-1291,miR-18a-3p,miR-2387,miR-380-3p,miR-2411,miR-29b-1-5p,miR-345-3p,miR-345-3p,miR-185-3p,miR-500a-5p,novel_17,miR-210-5p,miR-532-3p,miR-15a-5p,miR-200b,miR-23c,miR-7859,miR-23b-3p,miR-210-3p,miR-212-5p,miR-3600,miR-548e-3p,miR-18a-3p,miR-218-1-3p,miR-941,miR-184-3p,miR-1197-5p,miR-188-3p,miR-2904,miR-2408,miR-362-3p,miR-2366,miR-6134,miR-181c-5p,miR-3184-5p,miR-6525,miR-1271,miR-4510,miR-874-5p,miR-424-5p,miR-708-5p,miR-17-3p,miR-664a,miR-665,miR-29b-2-5p,miR-19b-1-5p,miR-15b-5p,novel_25,miR-22-3p,miR-6130,miR-216b-5p,miR-2284s,novel_107,miR-548o-3p,miR-496-3p,miR-543-3p,miR-625-5p,miR-339b,miR-345-5p,miR-30f,miR-

AHSA1	ENSOARG0(	AHA1, ac	miR-224-5p,miR-197-5p,miR-660,miR-363-5p,miR-345-5p,miR-296-3p,miR-1434-3p,miR-653,miR-194-5p,miR-1260b,miR-138,miR-30b-3p,miR-744-5p,miR-133b-5p,miR-130a-3p,miR-432-5p,miR-203b-5p,miR-656-5p,novel_120,miR-342-3p,miR-17-3p,miR-24-3p,miR-2428,novel_27,miR-18a-3p,miR-9851-3p,miR-378b,miR-2404,miR-200c,miR-1260a,miR-200c-3p,miR-17-3p,miR-4286,miR-1,miR-2284a,miR-1957a,miR-3065-3p,miR-345-3p,miR-582-3p,miR-300,miR-496,let-7a-2-3p,miR-200b-3p,miR-128-3p,miR-7641,miR-378h,miR-24-2-5p,miR-214-5p,miR-582-3p,miR-135a-1-3p,miR-410-5p,miR-409-3p,miR-331-3p,miR-138-5p,miR-6128,miR-500b-5p,miR-378c,miR-503-3p,miR-381-3p,miR-488-3p,miR-362-5p,miR-3154,let-7g-3p,miR-671-5p,miR-141-3p,novel_103,miR-1306-5p,miR-342,miR-2330-5p,miR-33a-5p,miR-652-5p,miR-296-5p,miR-484,miR-378b,miR-30b-3p,miR-3962,miR-7857,miR-378c,miR-3120-5p,miR-409b,novel_4,miR-27a-3p,miR-500-5p,miR-9788-3p,miR-216a-3p,miR-107-5p,miR-371a-5p,miR-2285y,miR-3059-5p,miR-452-5p,miR-194b-5p,miR-362-5p,miR-8095,miR-361-3p,miR-129b-5p,novel_94,miR-452-3p,miR-10b,miR-20a-3p,miR-7977,miR-541-5p,miR-3082-5p,miR-378e,miR-27a-3p,miR-185-3p,miR-760-3p,miR-221-5p,miR-30b-3p,miR-2300b-3p,novel_133,miR-1193,miR-188-3p,miR-140-3p,miR-2904,miR-548e-3p,miR-1b-3p,miR-200b,miR-532-3p,miR-17-3p,miR-155-5p,miR-539-5p,miR-378c,miR-3074-2-3p,miR-206-3p,miR-21a-3p,let-7g-3p,miR-660-5p,miR-496-3p,miR-378a-3p,miR-140-5p,miR-221-5p,miR-3065-3p,miR-193b-3p,miR-139-5p,miR-155-5p,miR-204-3p,miR-296-3p,miR-7-5p,miR-2411-3p,novel_51,miR-194a,miR-196b-5p,miR-378a-5p,miR-193a-3p,miR-106b-3p,miR-628-5p,miR-2284l,let-7a-2-3p,miR-503-3p,miR-7b-5p,miR-432,miR-3432b,miR-16-1-3p,miR-27b-3p,miR-31-5p,miR-194-3p,miR-149-5p,miR-411-5p,miR-576-

FRS3	ENSOARG0(	fibrobla	miR-2320-3p,miR-216a-3p,miR-9788-3p,miR-105-1,novel_4,miR-409b,miR-27a-3p,miR-378c,miR-544a,miR-3120-5p,miR-484,miR-378b,miR-6529a,miR-296-5p,miR-412-3p,miR-30b-3p,miR-30c-1-3p,miR-3187-3p,miR-2330-5p,miR-424-5p,miR-106a-5p,miR-25-5p,miR-5126,miR-326,miR-378e,miR-489,miR-3082-5p,miR-27a-3p,miR-541-5p,novel_94,miR-361-3p,miR-8095,novel_60,miR-541-3p,miR-1296-5p,miR-181d-5p,miR-2284q,miR-2285y,miR-15a-5p,miR-532-3p,miR-3960,miR-3600,miR-378d,novel_133,miR-1246,miR-2904,miR-5703,miR-125b-2-3p,miR-20a,miR-106a,miR-760-3p,miR-221-5p,miR-34b-5p,miR-30b-3p,miR-299-3p,miR-185-3p,miR-6130,miR-193b-3p,novel_25,miR-204-3p,miR-22-3p,miR-877-5p,miR-221-5p,miR-140-5p,miR-378a-3p,miR-3065-3p,miR-206-3p,miR-6134,miR-378c,miR-145a-3p,miR-4510,miR-17-3p,miR-664a,miR-424-5p,miR-185-5p,miR-544b,miR-20b,miR-30b-3p,miR-138,miR-1260b,miR-455-5p,miR-181a-5p,miR-345-5p,miR-124-5p,miR-1343-5p,miR-224-5p,miR-455-5p,miR-106a-5p,miR-17-5p,miR-146b-3p,miR-34b,miR-1291,miR-195a-5p,miR-18a-3p,miR-371b-3p,miR-24-3p,miR-449a,novel_27,miR-326-3p,novel_101,miR-17-3p,miR-502b,miR-15a,miR-20a-5p,miR-6529b,miR-34c,miR-16a,miR-33a-3p,miR-483-3p,miR-378h,miR-545-5p,miR-105-2,miR-615-3p,miR-380-5p,miR-3065-3p,miR-2284a,miR-1,miR-30a-3p,miR-181a-2-3p,miR-2898,miR-450a-2-3p,miR-17-3p,miR-181b-5p,miR-1260a,miR-2285af,miR-30c-2-3p,miR-34c-5p,miR-365a-3p,miR-485-5p,miR-1814c,miR-2448-3p,miR-3154,miR-17-5p,miR-93,miR-145-3p,miR-378c,miR-6128,miR-103b,miR-138-5p,miR-486b-3p,miR-2332,miR-126a-5p,miR-421,miR-1343-3p,miR-2284r,miR-541,miR-1a-3p,miR-378d,miR-2310,miR-127-3p,miR-106b-5p,miR-16b,miR-3596,miR-378g,miR-203-3p,miR-652-3p,miR-4443,miR-2284g,miR-4792,miR-2319b,miR-126b-

CLEC4G	ENSOARG0(	C-type l	miR-203-3p,miR-454-5p,miR-7857-3p,miR-25,miR-378g,miR-378e,miR-499b-5p,miR-7689-3p,miR-7977,miR-664b,miR-4792,miR-452-3p,miR-1983,novel_94,miR-504,miR-136-5p,miR-361-3p,miR-140-5p,miR-219a-2-3p,novel_111,miR-2285f,miR-2447,miR-4454,miR-541-3p,miR-212-3p,miR-2285t,miR-154-3p,miR-2284q,miR-4726-5p,novel_82,miR-615,miR-421,miR-425-3p,novel_4,miR-1343-3p,miR-409b,miR-3120-5p,miR-378c,miR-323b,miR-30b-3p,miR-125b-2-3p,miR-758-3p,miR-378b,miR-2284r,miR-541,miR-143-5p,miR-193a,miR-758-3p,miR-30c-1-3p,miR-378d,miR-23a-3p,miR-376b,miR-1388-3p,miR-424-5p,miR-381-5p,miR-491-5p,miR-487b-5p,miR-323b-3p,miR-29b-1-5p,miR-16b,miR-326,miR-204-3p,miR-22-3p,miR-155-5p,miR-877-5p,miR-15b-5p,miR-377-3p,miR-2284s,miR-6130,miR-216b-3p,miR-154a-3p,miR-30c-1-3p,miR-378a-3p,miR-504-5p,miR-660-5p,miR-548o-3p,miR-1827,miR-2312,miR-345-5p,miR-154b-3p,miR-299,miR-122-5p,miR-6525,miR-378c,miR-145a-3p,miR-4510,miR-3184-3p,miR-129-2-3p,miR-487a-5p,miR-4492,miR-129-5p,miR-424-5p,miR-155-5p,miR-487b-5p,miR-451a,miR-219b-3p,miR-338-5p,miR-2285j,miR-487a-3p,miR-23b-3p,miR-383-5p,miR-15a-5p,miR-23c,miR-485-3p,miR-16-1-3p,miR-218-1-3p,miR-33b-3p,miR-2904,miR-378a-3p,miR-1197-5p,novel_133,miR-378i,miR-193b-5p,miR-2366,miR-2300b-3p,miR-486-3p,miR-143-3p,miR-29b-1-5p,miR-376a-5p,miR-377-5p,miR-345-3p,miR-216c-5p,miR-487a,miR-21-3p,miR-216a-5p,miR-324-3p,miR-125b,miR-185-3p,miR-422a,miR-92a-1-5p,miR-345-3p,miR-669,miR-2404,miR-670-3p,miR-378f,miR-195a-5p,miR-107,miR-412-3p,miR-2285ab,miR-326-3p,miR-24-3p,novel_101,miR-135a-2-3p,miR-22-5p,miR-16a,miR-15a,miR-194-3p,miR-2355-3p,miR-103,miR-134-3p,miR-26b-3p,miR-377-5p,miR-103a-3p,miR-133b-5p,miR-497-5p,miR-30b-3p,miR-138,miR-16b,miR-16-1-3p,miR-381-
EPG5	ENSOARG0(	ectopic	miR-376c-5p,miR-30d-5p,miR-30e-5p,miR-21a-3p,miR-376c-5p,miR-1248,miR-329a,miR-30f,miR-32-3p,novel_25,miR-6130,miR-665-5p,miR-1306-5p,miR-4510,miR-365a-5p,miR-6525,miR-7134-5p,miR-30a-5p,miR-345-3p,miR-30c-5p,miR-362-3p,miR-2300b-3p,miR-24-2-5p,miR-345-3p,miR-30b,miR-30d,novel_96,miR-490-3p,miR-143-3p,miR-3065-5p,miR-376b-5p,miR-2285ab,miR-140-5p,miR-299b-5p,miR-3120-3p,miR-411b-3p,miR-30b-5p,miR-203b-5p,miR-147-5p,miR-4726-5p,miR-2285f,novel_101,miR-32-3p,miR-2428,miR-197-3p,miR-708-3p,miR-329-3p,miR-7862,miR-362-3p,miR-299a-5p,miR-219a-1-3p,miR-665,miR-487b-3p,miR-330-3p,miR-9788-3p,miR-185-5p,miR-365b-5p,miR-330-3p,miR-362-3p,miR-2411-3p,miR-466f-3p,miR-491-5p,miR-574-3p,miR-6240,miR-8485,miR-193a,miR-432-3p
SPTLC2	ENSOARG0(	serine p	miR-203-3p,miR-29a-3p,miR-431,miR-1343-3p,miR-3074-5p,miR-3064-5p,miR-4792,miR-363,miR-1843b-5p,miR-6134,miR-29c-3p,miR-3064-5p,miR-3074-5p,miR-29a,miR-3085-3p,miR-6402,miR-431-5p

MYO3B	ENSOARG0(	myosin I	miR-16a,miR-15a,miR-424-5p,miR-487b-5p,miR-16-5p,miR-3059-5p,miR-7705,miR-326-3p,miR-1827,miR-195a-5p,miR-107,let-7g-3p,miR-552-3p,miR-499b-5p,miR-15b-5p,novel_32,miR-670-3p,miR-489,miR-378g,miR-499a-3p,miR-499b-3p,miR-16b,miR-125a-3p,miR-326,miR-381-5p,miR-107,miR-378a-5p,miR-363-5p,let-7c-3p,miR-2330-5p,miR-424-5p,miR-30b-3p,miR-330-5p,miR-1285,miR-542-3p,miR-497-5p,miR-450b-5p,miR-381-5p,miR-16b,miR-30b-3p,miR-103a-3p,miR-1343-3p,miR-421,miR-103,miR-582-3p,miR-134-3p,miR-3431,miR-15a-5p,miR-322-5p
ATRN	ENSOARG0(	attracti	miR-30f,miR-30e-5p,miR-2285c,miR-411,miR-30d-5p,miR-2284n,miR-30c-1-3p,miR-221-5p,miR-141-3p,miR-1271,miR-532-5p,miR-615-5p,miR-129-5p,miR-874-5p,miR-486b-3p,miR-2284k,miR-222-3p,miR-3184-5p,miR-6134,miR-181c-5p,miR-1306-5p,miR-2284v,miR-145a-3p,miR-411-3p,miR-30c,miR-3591-5p,miR-30c-5p,miR-1246,miR-30a-5p,miR-302a-5p,miR-483-3p,miR-2284z,novel_121,miR-222,miR-2284m,miR-760-3p,miR-466i-5p,miR-181b-5p,miR-221-5p,miR-30d,miR-200a-3p,miR-30b,miR-2285p,novel_17,miR-6119-5p,miR-324-3p,miR-30c-2-3p,miR-185-3p,miR-130a-5p,miR-486-3p,miR-192-3p,miR-1271-3p,miR-3064-5p,miR-221,miR-129b-5p,miR-27a-3p,miR-3064-5p,miR-499a-5p,miR-379-3p,miR-502-5p,miR-30b-5p,miR-4324,miR-33a-3p,miR-423-5p,novel_60,miR-3059-5p,miR-3956-3p,miR-3957-3p,novel_101,miR-495-3p,miR-2284j,miR-181d-5p,miR-2284ab,miR-101a-5p,miR-2284aa,miR-2284x,miR-27b-3p,miR-708-3p,miR-296-5p,miR-143-5p,miR-181a-5p,miR-125b-2-3p,miR-2285g,miR-185-5p,novel_116,miR-221-3p,miR-2285e,miR-1343-3p,miR-26b-3p,miR-27a-3p,miR-3085-3p,miR-483-3p,novel_103,let-7d-3p,novel_51,miR-30c-1-3p,miR-382-3p,miR-628-5p
PIK3C2B	ENSOARG0(	phosphat	miR-4443,miR-532-3p,miR-377-3p,miR-145b,miR-615-5p,miR-105-1,miR-2889,miR-877-3p,miR-105-2,miR-4508,miR-329a,miR-1260b,miR-362-3p,miR-145a-5p,miR-362-3p,miR-329-3p,novel_39,miR-1260b,miR-342-3p,miR-21-3p,miR-760-3p,miR-362-3p,miR-105-5p,miR-371a-5p,miR-377-3p,miR-1260a,miR-342
MAP4	ENSOARG0(	microtub	miR-194-5p,miR-7b-5p,miR-2300b-3p,miR-3141,miR-184-3p,miR-1291,miR-425-5p,miR-3141,miR-2440,miR-5126,miR-7-5p,miR-17-3p,miR-128-1-5p,miR-17-3p,miR-17-3p,miR-194b-5p

CES4A	ENSOARG0(	carboxyl	miR-2300a-5p,miR-330-3p,miR-181b-2-3p,miR-145b,miR-27a-3p,miR-147a,miR-1298-3p,miR-153,miR-2459,miR-216a-3p,miR-9788-3p,miR-455-3p,miR-4791,miR-484,miR-378b,miR-30b-3p,miR-412-3p,miR-2426,miR-450b-5p,miR-544a,miR-378c,miR-1388-5p,miR-1839-5p,miR-1839-3p,miR-382-3p,let-7a-5p,miR-1388-3p,miR-376b,miR-30c-1-3p,miR-378d,miR-3187-3p,miR-193a,miR-1306-5p,miR-326,miR-199a-3p,miR-3085-3p,novel_124,miR-491-5p,let-7e,miR-153-3p,miR-2319b,miR-452-3p,miR-3064-5p,miR-7977,miR-211-5p,miR-489,miR-7857-3p,miR-378e,miR-27a-3p,let-7d-5p,miR-1983,miR-493-5p,miR-3956-3p,novel_60,miR-2447,miR-190b-5p,miR-452-5p,miR-2285f,novel_111,miR-423-5p,miR-2440,miR-4726-5p,miR-2284q,miR-2285t,miR-3960,miR-200b,miR-328-3p,miR-199a-3p,miR-2284h-5p,miR-1961,miR-2300b-3p,miR-378i,miR-378a-3p,miR-5703,miR-2904,miR-34a-5p,miR-664-5p,miR-2285w,miR-503-5p,miR-183-5p,miR-345-3p,miR-181b-1-3p,miR-125b-2-3p,miR-486-3p,miR-422a,miR-185-3p,miR-219b-5p,miR-98-5p,miR-760-3p,miR-466i-5p,let-7k,miR-34b-5p,miR-3653-3p,miR-30b-3p,miR-378a-3p,miR-30c-1-3p,miR-3065-3p,miR-6130,miR-6516,miR-544-3p,miR-377-3p,let-7i-5p,miR-204-3p,miR-129b-3p,miR-18a-5p,miR-2312,miR-1827,miR-4510,miR-378c,let-7b,miR-2330-3p,miR-665-5p,miR-6525,miR-6134,miR-338-3p,miR-122-5p,miR-3184-5p,let-7b-5p,miR-30e-3p,let-7f,miR-30a-3p,miR-487a-5p,miR-190a-5p,miR-874-5p,miR-187-3p,miR-3955-5p,miR-665,let-7g-5p,miR-503-5p,miR-3431,miR-3535,miR-544b,miR-149-5p,novel_116,miR-1434-3p,miR-145a-5p,miR-30b-3p,miR-29b,miR-455-5p,miR-27b-3p,miR-188-5p,miR-2889,miR-493-5p,miR-6240,miR-668-3p,miR-377-3p,miR-503-5p,novel_51,miR-455-5p,miR-190a,miR-330-3p,miR-365b-5p,miR-483-3p,miR-452-5p,miR-378b,novel_68,miR-411b-3p,novel_69,miR-29a-3p,miR-

PPP1R9A	ENSOARG0(	protein	[illegible] 5p,miR-196a-5p,miR-1290,miR-134-5p,miR-450a-1-3p,miR-486-5p,miR-129-1-3p,novel_121,novel_79,miR-2285ad,miR-429-3p,miR-6517,miR-592,miR-134-5p,miR-6740-5p,miR-1260b,let-7e-3p,miR-30e-5p,miR-223-5p,miR-133c,miR-2355-5p,miR-2285aa,miR-1271,miR-483-3p,miR-196b-5p,miR-105-5p,miR-194a,miR-377-3p,let-7a-2-3p,miR-2285r,miR-380-5p,miR-6240,miR-27b-3p,miR-3432b,miR-450c-3p,novel_23,miR-339-5p,miR-29a,novel_87,miR-4324,miR-142b,miR-199b-5p,miR-29c-3p,miR-378f,miR-3065-5p,miR-216a-3p,miR-148a-3p,miR-6535,miR-3120-3p,miR-26b-5p,miR-329-5p,miR-216b-3p,miR-500a-5p,miR-3601,miR-324-3p,miR-422a,miR-30b,novel_17,miR-618,miR-26a-5p,miR-380-3p,miR-664b-3p,miR-345-3p,miR-329a-5p,miR-487a,miR-664-5p,miR-218-1-3p,miR-33b-3p,miR-378a-3p,miR-217-5p,miR-378i,miR-30c-5p,miR-2366,miR-7859,miR-30d-3p,miR-4492,miR-500,miR-129-5p,miR-151-3p,miR-30a-3p,miR-3604,miR-665,miR-2285j,miR-15b-3p,miR-30e-3p,miR-346,miR-129-2-3p,miR-625-5p,miR-1827,miR-339b,miR-500a-3p,miR-30d-5p,miR-2285c,miR-450a-1-3p,miR-377-3p,miR-499b-3p,miR-216b-3p,miR-154a-3p,miR-30c-1-3p,miR-504-5p,miR-199b-5p,miR-29b-1-5p,miR-8485,miR-378d,miR-1388-3p,miR-574-3p,miR-1839-5p,miR-455-3p,miR-425-3p,miR-147a,miR-539-5p,novel_78,miR-548w,miR-330-3p,miR-490-5p,miR-154-3p,miR-499a-5p,novel_82,miR-493-5p,miR-299b-5p,miR-136-5p,miR-504,miR-140-5p,miR-221,miR-499a-3p,miR-2285u,miR-211-5p,miR-2319b,miR-17-3p,miR-200c-3p,miR-181b-5p,miR-30c-2-3p,miR-217,miR-339a,miR-1260a,miR-200c,miR-199a-5p,miR-490-3p,miR-30a-3p,miR-29b-2-5p,miR-2403,miR-4286,miR-181a-2-3p,miR-200b-3p,miR-105-2,miR-128-3p,let-7a-2-3p,miR-582-3p,miR-29d-3p,miR-30a-5p,miR-134,miR-582-3p,miR-545-5p,miR-214-

C16orf70	ENSOARG0(	chromoso	let-7a-5p,miR-574-3p,miR-494-3p,miR-4532,miR-378d,miR-320b,novel_103,miR-1306-5p,miR-326,let-7e,miR-214-3p,miR-103a-2-5p,miR-3085-3p,miR-130b-3p,miR-145b,miR-1343-3p,novel_4,miR-330-3p,miR-216a-3p,miR-1185-3p,miR-147a,miR-1298-3p,miR-7857,miR-30b-3p,miR-125b-2-3p,miR-2284r,miR-296-5p,miR-378b,miR-484,miR-541,miR-10b,miR-378c,miR-450b-5p,miR-101a-5p,miR-2285f,miR-181d-5p,miR-541-3p,miR-423-5p,miR-877-3p,miR-2285y,miR-107-5p,miR-2285n,miR-218-5p,miR-615,miR-4726-5p,miR-335,miR-541-5p,miR-2285u,miR-7977,miR-3064-5p,miR-24-1-5p,miR-452-3p,let-7d-5p,miR-1185-2-3p,miR-4443,miR-378e,miR-489,miR-493-5p,miR-181a-3p,miR-96-5p,miR-219b-5p,miR-324-3p,miR-185-3p,miR-422a,miR-26a-5p,miR-30b-3p,miR-221-5p,let-7k,miR-320d,miR-98-5p,miR-466i-5p,miR-212-5p,miR-3960,miR-532-3p,novel_83,miR-328-3p,miR-148b-3p,miR-378a-3p,novel_133,miR-378i,miR-2300b-3p,miR-1961,miR-133a-3p,miR-378d,miR-4429,miR-941,miR-19b-3p,miR-33b-3p,miR-1271,miR-214,miR-2330-3p,miR-665-5p,miR-378c,let-7b,miR-129-2-3p,miR-145a-3p,miR-3184-3p,miR-3074-5p,miR-4510,miR-3184-5p,let-7b-5p,miR-338-3p,miR-6134,miR-10b-5p,miR-346,miR-324-3p,miR-19b-1-5p,miR-320e,let-7f,miR-4492,miR-208b-5p,miR-454-3p,novel_74,miR-21-3p,miR-552-3p,miR-378a-3p,miR-221-5p,let-7i-5p,miR-10a,miR-377-3p,miR-301a-3p,miR-6516,miR-6130,miR-129b-3p,miR-133b-3p,let-7g-3p,miR-2312,miR-339b,miR-101-5p,miR-363-5p,let-7f-2-3p,let-7f-2-3p,miR-628-5p,miR-6240,miR-493-5p,miR-2285r,miR-330-3p,miR-105-5p,miR-2285v,miR-7-5p,miR-377-3p,miR-339-5p,miR-130a-3p,miR-31-5p,miR-3955-5p,miR-149-5p,miR-194-3p,miR-3431,let-7g-5p,miR-145a-5p,miR-19b-2-5p,miR-2113,miR-7b-5p,miR-4508,miR-2284x,miR-30b-3p,miR-125b-1-3p,miR-3957-3p,novel_120,miR-335-5p,miR-326-
VEPH1	ENSOARG0(	ventricu	miR-877-3p,miR-154a,miR-105-3p,miR-539-5p,miR-130b-5p,let-7f-2-3p,let-7f-2-3p,miR-483-3p,miR-103a-2-5p,miR-130b-5p,miR-153-3p,miR-221-5p,miR-218-5p,miR-182-5p,miR-107-5p,miR-323-5p,miR-483-3p,miR-153,miR-323a-5p,miR-1185-3p,miR-1185-2-3p,miR-221-5p,miR-655-5p,novel_99,miR-1827,novel_94,miR-2319a,miR-433-3p

CBX2	ENSOARG0(	chromobo	miR-182-5p,miR-16b,miR-323b-3p,miR-199b-5p,miR-127-3p,miR-124a,miR-1b-5p,miR-8485,miR-378d,miR-758-3p,miR-501-3p,miR-323b,miR-301b-5p,miR-2284r,miR-329-3p,miR-362-3p,miR-181b-2-3p,miR-145b,miR-1343-3p,miR-199b-3p,novel_78,miR-330-3p,miR-2459,miR-148b-5p,miR-1298-3p,miR-496-5p,miR-301a-5p,miR-218-5p,miR-2284j,miR-2447,miR-361-3p,miR-211-5p,miR-2285u,miR-7689-3p,miR-24-1-5p,miR-4443,miR-500-3p,miR-25,miR-3596,miR-499a-3p,miR-378g,miR-345-3p,miR-422a,miR-92a-1-5p,miR-324-3p,miR-210-5p,miR-30b,let-7k,miR-98-5p,miR-345-3p,miR-503-5p,miR-10a-3p,miR-486-3p,miR-181b-1-3p,miR-421-5p,miR-376a-5p,miR-378a-3p,miR-1434-5p,miR-193b-5p,miR-1961,miR-378i,miR-30c-5p,miR-148a-5p,miR-34a-5p,miR-133a-3p,miR-218-1-3p,miR-212-5p,miR-18a-3p,miR-665,let-7f,miR-130b-5p,miR-500,miR-4492,miR-129-2-3p,let-7b,miR-346,miR-224-5p,miR-6525,miR-30d-5p,miR-500a-3p,miR-450a-1-3p,miR-1827,miR-625-5p,miR-885-3p,miR-1895,miR-296-3p,miR-548o-3p,miR-30c-1-3p,miR-2331-3p,miR-15b-5p,let-7i-5p,miR-216b-5p,miR-377-3p,miR-2284s,miR-499b-3p,miR-194a,miR-2411-3p,novel_51,miR-582,miR-377-3p,miR-193a-3p,miR-483-3p,miR-6240,miR-2285r,novel_23,miR-450c-3p,miR-708-3p,miR-2284x,miR-27b-3p,miR-497-5p,miR-4508,miR-16b,miR-340-3p,miR-149-5p,miR-134-3p,miR-576-3p,miR-6123,miR-421-5p,miR-29a,miR-6119-3p,miR-29c-3p,miR-193b-3p,miR-199b-5p,miR-3065-5p,miR-148a-3p,let-7e-5p,miR-378f,miR-3120-3p,novel_68,miR-181b-3p,miR-677,miR-299b-3p,miR-30f,miR-152-3p,novel_127,let-7f-5p,miR-28-3p,miR-3064-5p,novel_39,miR-200a,let-7g,miR-2284y,miR-148b-5p,miR-330-5p,miR-23b-5p,miR-1343-5p,miR-30c,miR-885-3p,miR-412,miR-299a-3p,miR-2285ad,miR-129-1-3p,miR-2284z,miR-450a-1-3p,miR-322-5p,miR-505-5p,miR-100-3p,miR-125b-

PANK3	ENSOARG0(	pantothe	miR-29b-1-5p,miR-130a-5p,miR-329a-5p,miR-30d,miR-299-3p,miR-30b,miR-196a-3p,miR-26a-5p,miR-2285p,miR-7859,miR-23b-3p,miR-23c,miR-200b,miR-222-5p,miR-212-5p,miR-548e-3p,miR-16-1-3p,miR-218-1-3p,let-7a-3p,miR-148a-5p,miR-5703,miR-188-3p,miR-1193,miR-217-5p,miR-30c-5p,miR-142a-5p,miR-539-5p,miR-190a-5p,miR-708-5p,novel_74,miR-6516-3p,miR-151-3p,miR-664a-5p,miR-15b-3p,novel_25,miR-544-3p,miR-27a-5p,miR-2331-3p,miR-548o-3p,miR-582-5p,miR-2312,let-7g-3p,miR-30f,miR-30d-5p,miR-2285c,miR-8485,miR-23a-3p,let-7c-3p,miR-652-5p,miR-2284b,miR-101b-3p,miR-494-3p,miR-487b-5p,miR-29b-1-5p,miR-326,miR-148b-5p,miR-2459,miR-1185-3p,miR-2285e,miR-421,miR-142-5p,miR-409b,miR-105-1,miR-1343-3p,miR-151a-3p,miR-101c,miR-2426,miR-544a,miR-8095,miR-2285f,miR-9-5p,miR-190b-5p,miR-3059-5p,miR-212-3p,miR-30b-5p,miR-101-3p,miR-203-3p,miR-454-5p,miR-1185-2-3p,miR-3596,miR-7857-3p,miR-2483-3p,miR-499b-5p,miR-541-5p,miR-126b-5p,miR-2284g,miR-452-3p,miR-20a-3p,miR-3963,miR-129b-5p,miR-200a,miR-2284y,miR-2284a,miR-28b,miR-181a-2-3p,miR-200a-3p,miR-200c-3p,miR-30f,miR-217,miR-200c,novel_121,miR-9-5p,miR-376a-5p,miR-222,miR-299a-3p,miR-6517,miR-429-3p,miR-105-2,miR-30c,miR-200b-3p,miR-582-3p,miR-330-5p,miR-30a-5p,miR-3607-3p,miR-28-5p,miR-135a-5p,miR-34c-3p,miR-133c,miR-365a-3p,miR-2285aa,miR-141-3p,miR-3068-3p,miR-7144-5p,novel_48,miR-485-5p,miR-10b-3p,miR-23b,miR-30e-5p,miR-628-5p,miR-466f-3p,miR-105-5p,miR-190a,miR-197-5p,miR-6238,miR-544b,miR-2285g,miR-133b-5p,miR-4508,miR-16-1-3p,miR-374b-3p,miR-326-3p,miR-495-3p,miR-147-5p,miR-28c,miR-576-3p,miR-299b-3p,miR-655-5p,miR-26b-5p,miR-329-5p,miR-376a-2-5p,miR-135b-5p,miR-2285ab
STX8	ENSOARG0(	syntaxin	miR-424-5p,miR-30a-3p,miR-29b-2-5p,miR-29b-1-5p,miR-377-3p,miR-16b,miR-182-5p,miR-29b-1-5p,novel_127,miR-466f-3p,miR-1343-5p,miR-18a-3p,miR-30d-3p,miR-26b-3p,miR-15a-5p,miR-322-5p,miR-545-5p,miR-1343-5p,miR-16b,miR-188-5p,miR-133a-3p,miR-497-5p,miR-16-5p,miR-6527,miR-30e-3p,miR-15a,miR-29b-2-5p,miR-16a,miR-30a-3p,miR-424-5p,miR-378b,miR-182-5p,miR-323-5p,miR-323a-5p,miR-377-3p,miR-454-5p,miR-15b-5p,miR-18a-3p,miR-10b-3p,miR-195a-5p

ZNF619	ENSOARG0(	zinc fin	miR-148b-5p,miR-216a-3p,miR-455-3p,miR-147a,miR-27a-3p,novel_78,miR-105-1,miR-548w,miR-101c,miR-1388-5p,miR-450b-5p,miR-412-3p,miR-193a,miR-3187-3p,miR-30c-1-3p,miR-23a-3p,miR-424-5p,miR-491-5p,miR-3085-3p,miR-3955-3p,miR-16b,miR-326,miR-4443,miR-27a-3p,miR-378g,miR-7689-3p,miR-499b-5p,miR-211-5p,miR-3064-5p,miR-24-1-5p,miR-2319b,miR-452-3p,miR-20a-3p,miR-361-3p,miR-361-3p,miR-877-3p,miR-2285f,miR-3059-5p,miR-2447,miR-885-5p,miR-2285t,miR-490-5p,miR-3968,miR-450a-5p,novel_82,miR-671-3p,miR-487a-3p,miR-23b-3p,miR-23c,miR-15a-5p,miR-485-3p,miR-30d-3p,miR-505-3p,miR-133a-3p,miR-148a-5p,miR-33b-3p,miR-1197-5p,miR-140-3p,miR-376a-5p,miR-215-5p,miR-2411,miR-2424,miR-2432,miR-376a-5p,miR-345-3p,miR-216c-5p,miR-221-5p,miR-466i-5p,miR-760-3p,miR-185-3p,miR-204-3p,miR-15b-5p,miR-6516,miR-192-5p,miR-323-5p,miR-545-3p,miR-30c-1-3p,miR-221-5p,miR-496-3p,miR-885-3p,miR-345-5p,miR-598-3p,miR-6134,miR-6525,miR-224-5p,miR-3074-2-3p,miR-539-5p,miR-129-2-3p,miR-424-5p,miR-4492,miR-30a-3p,miR-324-3p,miR-29b-2-5p,miR-665,miR-30e-3p,miR-149-5p,miR-194-3p,miR-432-5p,miR-3431,miR-134-3p,miR-31-5p,miR-497-5p,miR-27b-3p,miR-455-5p,miR-188-5p,miR-16b,miR-1260b,miR-197-3p,miR-708-3p,miR-432,miR-1285,miR-1434-3p,miR-2113,miR-345-5p,miR-2285r,miR-654-3p,miR-483-3p,miR-378a-5p,miR-2285l,miR-105-5p,miR-455-5p,miR-224-5p,miR-669,miR-6535,miR-2404,miR-323a-5p,miR-320b,miR-378b,miR-505-3p,miR-195a-5p,miR-379-5p,miR-135b-5p,miR-1291,miR-450b-3p,miR-3065-5p,miR-2285ab,miR-326-3p,miR-2428,miR-211,miR-371b-3p,miR-1839-3p,novel_101,miR-495-3p,novel_120,miR-656-5p,miR-625-3p,miR-147-5p,miR-128-1-5p,miR-16a,miR-1247-3p,miR-15a,miR-6402,novel_121,miR-129-1-3p,miR-505-5p,miR-322-5p,miR-483-3p,miR-6516-

EDEM3	ENSOARGO(	ER degra	miR-6119-5p,miR-664-3p,miR-200c,miR-2285af,miR-200c-3p,miR-1a-2-5p,miR-127-5p,miR-107,miR-450b-5p,miR-3065-3p,miR-29d-3p,miR-496,miR-200b-3p,miR-429-3p,novel_121,miR-2332,miR-370-5p,miR-34c-3p,miR-1-5p,miR-29b-3p,miR-135a-5p,miR-138-5p,miR-6128,miR-1197-3p,miR-323-3p,miR-145-3p,miR-376c-5p,miR-320a,miR-329a,miR-345-5p,miR-876-3p,miR-10b-3p,miR-1248,miR-376c-5p,miR-503-3p,miR-1271,miR-365a-3p,miR-190a,miR-330-3p,miR-452-5p,miR-493-5p,miR-6240,let-7f-2-3p,let-7f-2-3p,miR-345-5p,miR-1434-3p,miR-542-3p,miR-223-3p,miR-653,miR-138,miR-29b,miR-103a-3p,miR-31-5p,miR-3955-5p,miR-3535,miR-103,miR-425-5p,novel_1,miR-29a,miR-656-5p,miR-1a-1-5p,miR-29c-3p,miR-1193,miR-2285x,miR-323c,miR-3959-3p,miR-2387,miR-376b-5p,miR-1306,miR-107,miR-412-3p,miR-379-5p,miR-135b-5p,miR-26b-5p,miR-29a-3p,miR-181b-3p,miR-677,miR-320c,miR-2285p,miR-26a-5p,miR-196a-3p,miR-202-5p,miR-320d,miR-30b-3p,miR-181b-1-3p,miR-125b-2-3p,miR-362-3p,novel_133,miR-140-3p,miR-1434-5p,miR-1193,miR-4429,miR-142-3p,miR-148a-5p,miR-133a-3p,miR-3600,miR-200b,miR-2284h-5p,miR-2285j,miR-3604,miR-320e,miR-324-3p,miR-487a-5p,miR-190a-5p,miR-3184-3p,miR-145a-3p,miR-214,miR-425-5p,miR-345-5p,let-7g-3p,miR-582-5p,miR-496-3p,miR-140-5p,miR-370-5p,miR-3065-3p,miR-582-5p,miR-2284s,novel_25,novel_103,miR-1306-5p,miR-320b,miR-362-3p,miR-2957,miR-214-3p,miR-376b,let-7c-3p,miR-8485,miR-4791,miR-484,miR-2284r,miR-329-3p,miR-1388-5p,miR-362-3p,miR-1839-5p,miR-330-3p,miR-181b-2-3p,miR-421,miR-1185-3p,miR-148b-5p,miR-499a-5p,miR-190b-5p,miR-452-5p,miR-409-5p,miR-378j,miR-136-5p,miR-299b-5p,miR-1983,miR-493-5p,miR-664b,miR-126b-5p,miR-2483-3p,miR-1185-2-3p

EXOC3L1	ENSOARG0(	exocyst	miR-1839-3p,miR-335-5p,novel_27,miR-326-3p,miR-371b-3p,miR-24-3p,miR-6529b,miR-656-5p,miR-502b,novel_1,miR-144-3p,miR-146b-3p,miR-670-3p,miR-150-5p,miR-1306,miR-18a-3p,miR-3141,miR-135b-5p,miR-382,miR-668-3p,miR-3141,miR-2411-3p,novel_51,miR-125a-5p,miR-124-5p,miR-339-5p,miR-340-3p,miR-31-5p,novel_116,miR-2355-3p,miR-3535,miR-450c-3p,miR-223-3p,miR-744-5p,miR-30b-3p,miR-16-1-3p,miR-6128,miR-28a-3p,miR-145-3p,miR-1197-3p,miR-144,miR-331-3p,miR-125b-5p,miR-135a-5p,miR-410-3p,miR-592,novel_9,miR-2355-5p,miR-615-5p,miR-215-5p,miR-125a,miR-365a-3p,miR-7144-5p,miR-23a-5p,miR-3074-1-3p,miR-671-5p,miR-1248,miR-10b-3p,miR-329a,miR-200a,miR-148b-5p,miR-450b-5p,miR-339a,miR-200c,novel_127,miR-28-3p,miR-200c-3p,miR-7641,novel_79,miR-412,miR-450a-1-3p,miR-545-5p,miR-129-1-3p,miR-505-5p,miR-758-5p,miR-7134-5p,miR-330-5p,miR-345-3p,miR-1290,miR-2447,miR-423-5p,miR-877-3p,novel_111,miR-371a-5p,miR-107-5p,miR-382-3p,miR-4726-5p,miR-335,miR-7977,miR-10b,miR-20a-3p,miR-378j,miR-335-3p,miR-544-5p,miR-136-5p,let-7c-3p,miR-33a-5p,miR-652-5p,miR-574-3p,miR-1388-3p,miR-1839-3p,miR-382-3p,miR-101b-3p,miR-362-3p,novel_103,miR-1306-5p,miR-326,miR-103a-2-5p,miR-214-3p,miR-3955-3p,miR-25-5p,miR-412-3p,miR-30b-3p,miR-329-3p,miR-484,miR-4791,miR-1839-5p,miR-3120-5p,miR-362-3p,miR-214,miR-129-2-3p,miR-3184-5p,miR-338-5p,miR-15b-3p,miR-4492,miR-450b-3p,miR-341-3p,miR-2331-3p,miR-204-3p,miR-151b,miR-192-5p,miR-216b-5p,miR-411,miR-21a-3p,miR-450a-1-3p,let-7g-3p,miR-339b,miR-195a-3p,miR-345-3p,miR-664b-3p,miR-376a-5p,miR-125b,miR-345-3p,miR-185-3p,novel_17,miR-21-3p,miR-202-5p,miR-3600,miR-18a-3p,miR-151-5p,miR-210-3p,miR-2366,miR-215-5p,miR-362-3p,miR-16-1-3p,miR-361-5p
ATP6V0A2	ENSOARG0(	ATPase,	miR-7859,miR-532-3p,miR-2284z,miR-544b,miR-548e-3p,novel_78,miR-300,miR-544a,miR-21b,miR-2284aa,miR-873a-5p,miR-542-3p,miR-1434-3p,miR-1290,miR-2478,let-7j,miR-2285b,let-7c-3p,miR-376b,miR-2284m,miR-224-5p,novel_17,miR-125a-3p,miR-3071-3p,miR-544-3p,miR-374c-3p,miR-133c,miR-548o-3p,let-7g-3p,miR-1248,miR-381-3p,miR-200a-5p,miR-381-3p,miR-129b-5p,miR-378j,miR-224-5p,miR-24-3p,miR-499a-5p,miR-6119-3p,miR-487a-5p,miR-669a-3p,miR-15b-3p,miR-142b

IRAK3	ENSOARG0(	interleu	miR-26b-5p,miR-3074-5p,miR-216b-3p,miR-329-5p,miR-320c,miR-374c-3p,miR-299b-3p,miR-216a-3p,miR-21c,miR-18a-3p,miR-505-3p,miR-378f,miR-495-3p,miR-2285x,miR-1a-1-5p,miR-2428,miR-301b-3p,miR-2284w,miR-133a-5p,miR-6402,miR-421-5p,miR-147-5p,miR-199c,miR-379-3p,miR-130a-3p,miR-26b-3p,miR-3955-5p,miR-6238,miR-194-3p,miR-2285g,miR-185-5p,miR-181a-5p,miR-7b-5p,miR-542-3p,miR-1434-3p,miR-380-3p,miR-21-5p,miR-16-1-3p,let-7a-2-3p,miR-190a,miR-105-5p,miR-365b-5p,miR-2411-3p,miR-2331-5p,miR-7-5p,miR-2285aa,miR-3957-5p,miR-3957,miR-374c-3p,miR-320a,novel_48,miR-1248,miR-10b-3p,miR-2318,miR-329b,let-7g-3p,miR-3607-3p,miR-374b,miR-378c,miR-365a-5p,miR-331-3p,miR-100-3p,miR-6740-5p,miR-1-5p,miR-592,miR-299a-3p,miR-582-3p,novel_121,miR-378h,miR-345-3p,miR-128-3p,let-7a-2-3p,miR-411-3p,miR-3529-3p,miR-200a,miR-29a-5p,miR-30c-2-3p,miR-3071-3p,miR-2285af,novel_127,miR-450a-2-3p,miR-127-5p,miR-1a-2-5p,miR-6536,miR-181b-5p,miR-374b-5p,miR-499b-5p,miR-7977,miR-139-5p,miR-4792,miR-452-3p,miR-454-5p,miR-378e,miR-3082-5p,miR-378j,miR-504,miR-190b-5p,miR-181d-5p,novel_60,miR-3956-3p,miR-154a,novel_82,miR-490-5p,miR-2285t,miR-2284q,miR-628-3p,miR-3968,miR-199b-3p,miR-105-1,miR-409b,miR-455-3p,miR-216a-3p,miR-421,miR-2285e,miR-6529a,miR-378b,miR-143-5p,miR-21b,miR-378c,miR-494-3p,miR-193a,miR-1b-5p,miR-378d,miR-30c-1-3p,miR-320b,miR-199a-3p,miR-130b-3p,miR-552-3p,miR-30c-1-3p,miR-504-5p,miR-221-5p,miR-378a-3p,miR-545-3p,novel_25,miR-301a-3p,miR-2284s,miR-139-5p,miR-6130,miR-655,miR-2285c,miR-1895,miR-186-5p,let-7g-3p,miR-378c,miR-539-5p,miR-3074-5p,miR-4510,miR-145a-3p,miR-181c-5p,miR-6525,miR-346,miR-664a-5p,miR-454-3p,miR-190a-5p,miR-3600,miR-505-3p,miR-18a-
UBR3	ENSOARG0(	ubiquiti	miR-105-5p,miR-103a-2-5p,let-7a-2-3p,miR-30c-1-3p,miR-374b-3p,miR-197-3p,miR-541,miR-21-5p,miR-105-1,miR-2300a-5p,miR-148b-5p,novel_87,miR-107-5p,miR-4324,miR-218-5p,miR-2285x,miR-1193,miR-541-3p,miR-219b-3p,miR-323c,miR-211,miR-544-5p,miR-505-3p,miR-136-5p,miR-3120-3p,miR-211-5p,miR-26b-5p,miR-329-5p,miR-378b,miR-203-3p,miR-6535,miR-670-3p,miR-3082-5p,miR-3596,miR-2404,miR-216a-5p,miR-30c-2-3p,miR-3071-3p,miR-26a-5p,miR-2285p,miR-21-3p,miR-181a-2-3p,miR-329a-5p,miR-2285b,miR-148a-5p,let-7a-3p,let-7a-2-3p,miR-105-2,miR-493-3p,miR-505-3p,miR-1224-5p,miR-2332,miR-370-5p,miR-204-5p,miR-410-3p,miR-590-3p,miR-145-3p,miR-301,miR-21a-3p,miR-625-5p,let-7g-3p,miR-10b-3p,miR-370-5p,miR-548o-3p,miR-30c-1-3p,miR-545-3p,miR-365a-3p,miR-300-3p

HCK	ENSOARG0(	HCK prot	miR-30a-5p,miR-345-3p,miR-200b-3p,miR-300,miR-429-3p,miR-2285ad,miR-505-5p,miR-219-3p,miR-200c,miR-125a-3p,miR-1260a,miR-200c-3p,miR-181a-2-3p,miR-4286,miR-1271-3p,novel_39,miR-199a-5p,miR-450b-5p,miR-3154,miR-30e-5p,miR-381-3p,miR-671-5p,miR-1814c,miR-7144-5p,miR-23a-5p,miR-345-5p,miR-2285aa,miR-2284n,miR-199a-5p,miR-3965,miR-3959-5p,miR-34c-5p,miR-135a-5p,miR-26c,miR-331-3p,miR-2332,novel_63,miR-486b-3p,miR-410-3p,miR-6128,miR-365a-5p,miR-1260b,miR-194-5p,miR-145a-5p,miR-374b-3p,miR-542-3p,miR-708-3p,miR-432,miR-873a-5p,miR-19b-2-5p,miR-653,miR-188-5p,miR-2284x,miR-1260b,miR-219a-1-3p,miR-21-5p,miR-30b-3p,miR-26b-3p,miR-31-5p,miR-194-3p,miR-3431,miR-134-3p,miR-432-5p,miR-3535,miR-2411-3p,miR-194a,miR-365b-5p,miR-197-5p,let-7f-2-3p,let-7f-2-3p,miR-668-3p,miR-130b-5p,miR-345-5p,miR-2284l,miR-432-3p,miR-432-3p,miR-21c,miR-1306,miR-2387,miR-135b-5p,miR-412-3p,miR-2285k,miR-320b,miR-181b-3p,miR-669,miR-677,miR-374c-3p,miR-34b,miR-146b-3p,miR-34c,miR-576-3p,miR-208a-3p,miR-4324,miR-342-3p,miR-219b-3p,miR-199b-5p,miR-2428,miR-374c-5p,miR-449a,miR-5703,miR-2904,miR-1246,miR-2300b-3p,novel_133,miR-34a-5p,miR-664-5p,miR-218-1-3p,miR-33b-3p,miR-30d-3p,miR-1b-3p,miR-2284h-5p,miR-200b,miR-92a-1-5p,miR-345-3p,miR-216a-5p,miR-208b-3p,miR-34b-5p,miR-221-5p,miR-2284m,miR-202-5p,miR-2285w,miR-486-3p,miR-2424,miR-181b-1-3p,miR-2285i,miR-30d-5p,miR-129b-3p,miR-186-5p,miR-345-5p,miR-552-3p,miR-221-5p,miR-504-5p,novel_25,miR-6130,miR-320e,miR-19b-1-5p,miR-219b-3p,miR-324-3p,miR-30a-3p,miR-30e-3p,miR-130b-5p,miR-874-5p,miR-487a-5p,miR-3074-2-3p,miR-4510,miR-145a-3p,miR-590-3p,miR-539-5p,miR-758-3p,miR-30b-3p,miR-301b-5p,miR-4791,miR-541,miR-101c,miR-21b,miR-101a-5p,miR-181b-2-

PSTPIP2	ENSOARG0(	proline-	miR-299-3p,miR-221-5p,miR-30b-3p,miR-106a,miR-320d,miR-2284m,miR-125b,miR-542-5p,miR-196a-3p,miR-2411,miR-125b-2-3p,miR-20a,miR-19b-3p,miR-4429,miR-1197-5p,miR-188-3p,miR-5703,miR-362-3p,miR-376a-5p,novel_83,miR-15a-5p,miR-3600,miR-548e-3p,miR-424-5p,miR-708-5p,miR-155-5p,miR-6516-3p,miR-664a,miR-29b-2-5p,miR-145a-3p,novel_99,miR-18a-5p,miR-2312,miR-3613-5p,miR-877-5p,miR-155-5p,miR-22-3p,miR-204-3p,miR-139-5p,miR-532-5p,miR-3065-3p,miR-21-3p,miR-221-5p,miR-542-5p,miR-3955-3p,miR-362-3p,novel_115,miR-326,miR-320b,miR-342,miR-491-3p,miR-3187-3p,miR-30c-1-3p,miR-2330-5p,miR-424-5p,miR-106a-5p,miR-3120-5p,miR-2426,miR-412-3p,miR-2285e,miR-9788-3p,miR-5010-3p,miR-2284q,miR-107-5p,miR-769-5p,miR-9-3p,miR-452-5p,miR-2285f,miR-3059-5p,miR-199b-5p,miR-7977,miR-499b-5p,miR-452-3p,miR-450a-2-3p,miR-339a,miR-30c-2-3p,miR-2285af,miR-490-3p,miR-3065-3p,miR-199a-5p,miR-2478,miR-4286,miR-181a-2-3p,miR-29b-2-5p,miR-134,miR-345-3p,miR-2411-5p,miR-302a-5p,miR-486b-3p,miR-204-5p,miR-28-5p,miR-31-3p,miR-551b-3p,miR-1-5p,miR-93,miR-323-3p,miR-103b,miR-671-5p,miR-23a-5p,miR-17-5p,miR-18a,miR-320a,miR-199a-5p,miR-769,miR-125a-5p,miR-124-5p,miR-17-5p,miR-106a-5p,miR-7862,miR-219a-1-3p,miR-1285,miR-2355-3p,miR-20b,miR-185-5p,miR-2285g,miR-203b-5p,miR-16a,miR-20a-5p,miR-133a-5p,miR-3613,miR-15a,miR-2428,miR-326-3p,miR-24-3p,miR-211,miR-542-5p,novel_120,miR-135a-2-3p,miR-342-3p,miR-219b-3p,miR-6395,miR-6516-3p,miR-195a-5p,miR-19a-3p,miR-18a-3p,miR-18b,miR-2285ab,miR-670-3p,miR-2404,miR-2484,miR-345-3p,miR-324-3p,miR-500a-5p,novel_17,miR-618,miR-486-3p,miR-376a-5p,miR-345-3p,miR-361-5p,miR-142-3p,miR-2366,miR-328-3p,miR-7859,miR-106a,miR-18a-3p,miR-874-

IGHM	ENSOARG0(	immunogl	miR-3064-5p,miR-1271-3p,miR-199a-5p,miR-28b,miR-30c-2-3p,novel_96,miR-107,miR-18b-5p,miR-1224-5p,miR-423-3p,miR-6517,miR-299a-3p,miR-134-5p,miR-378h,miR-505-5p,miR-330-5p,miR-134,miR-128-3p,miR-133a-3p,miR-378c,miR-331-3p,miR-125b-5p,miR-6740-5p,miR-134-5p,miR-592,miR-486b-3p,miR-28-5p,miR-615-5p,miR-1271,miR-125a,miR-370-3p,miR-2355-5p,miR-574-5p,miR-324-5p,miR-199a-5p,miR-34c-5p,miR-18a,miR-485-5p,miR-2318,miR-345-5p,miR-329a,miR-876-3p,miR-345-5p,miR-654-3p,miR-455-5p,miR-146a,miR-377-3p,miR-125a-5p,miR-378a-5p,miR-103a-3p,miR-103,miR-2355-3p,miR-432-5p,miR-22-3p,miR-432,miR-2889,miR-455-5p,miR-4508,miR-342-3p,miR-199b-5p,miR-326-3p,miR-449a,miR-24-3p,miR-371b-3p,miR-34c,miR-28c,miR-6123,miR-181b-3p,miR-677,miR-669,novel_69,miR-34b,miR-299b-3p,miR-216a-3p,miR-18b,miR-6395,miR-378f,miR-146b-5p,miR-107,miR-412-3p,miR-1291,miR-2424,miR-486-3p,miR-146a-5p,miR-181b-1-3p,miR-185-3p,miR-125b,miR-422a,miR-299-3p,miR-34b-5p,miR-760-3p,miR-466i-5p,miR-328-3p,miR-210-3p,miR-378a-3p,miR-5703,miR-2408,miR-362-3p,miR-378i,novel_133,miR-378d,miR-34a-5p,miR-133a-3p,miR-33b-3p,miR-214,miR-665-5p,miR-378c,miR-338-3p,miR-3184-5p,miR-132-5p,miR-4492,miR-500,miR-708-5p,miR-6516-3p,miR-664a,miR-296-3p,novel_107,miR-140-5p,miR-378a-3p,miR-504-5p,miR-2331-3p,miR-30c-1-3p,miR-22-3p,miR-2284s,miR-377-3p,miR-500a-3p,miR-1827,miR-1895,miR-18a-5p,miR-345-5p,miR-133b-3p,miR-4532,miR-8485,miR-378d,miR-30c-1-3p,miR-362-3p,miR-326,miR-342,miR-214-3p,miR-199b-5p,miR-146b,miR-3085-3p,miR-491-5p,miR-181b-2-3p,miR-9788-3p,miR-216a-3p,miR-541,miR-301b-5p,miR-2284r,miR-329-3p,miR-378b,miR-362-3p,miR-378c,miR-541-3p,miR-1285-5p,miR-423-5p,miR-361-3p,miR-301a-5p,miR-

CYP4B1	ENSOARG0(	cytochro	miR-127-5p,novel_127,miR-543-5p,miR-1260a,miR-125a-3p,miR-30c-2-3p,miR-24-2-5p,miR-148b-5p,miR-199a-5p,miR-200a,miR-2284y,miR-2284a,miR-1271-3p,miR-885-3p,miR-3591-5p,miR-615-3p,miR-29d-3p,miR-23b-5p,miR-9-5p,miR-24-2-5p,miR-322-5p,miR-2411-5p,miR-450a-1-3p,miR-214-5p,miR-412,miR-1224-5p,miR-767-5p,miR-592,miR-29b-3p,miR-370-5p,miR-331-3p,miR-135a-5p,miR-1260b,miR-16-5p,miR-2474,miR-767,miR-10b-3p,miR-671-5p,miR-1248,miR-3154,miR-34c-5p,miR-769,miR-199a-5p,miR-324-5p,miR-376c-3p,miR-2355-5p,miR-378a-5p,miR-377-3p,novel_51,miR-194a,miR-455-5p,miR-432-3p,miR-654-3p,miR-130b-5p,miR-493-5p,miR-16b,miR-29b,miR-1260b,miR-497-5p,miR-26a-2-3p,miR-455-5p,miR-873a-5p,miR-432,miR-450c-3p,miR-145a-5p,miR-194-5p,miR-432-5p,miR-412-5p,miR-194-3p,miR-544b,miR-133b-5p,miR-377-5p,miR-29a,miR-15a,miR-6402,miR-576-3p,miR-16a,miR-34c,miR-371b-3p,miR-449a,miR-199b-5p,novel_120,miR-342-3p,miR-335-5p,novel_101,miR-29c-3p,miR-1193,miR-2285x,miR-9851-3p,miR-195a-5p,miR-135b-5p,miR-379-5p,miR-2387,miR-150-5p,miR-18a-3p,miR-323a-5p,miR-411b-3p,miR-29a-3p,miR-374c-3p,miR-670-3p,miR-34b,miR-669,miR-320b,miR-3653-3p,miR-34b-5p,miR-221-5p,miR-196a-3p,novel_17,miR-92a-1-5p,miR-376c-3p,miR-377-5p,miR-2411,miR-2432,miR-216c-5p,miR-2285w,miR-345-3p,miR-33b-3p,miR-133a-3p,miR-148a-5p,miR-2284u,miR-34a-5p,miR-2300b-3p,miR-124-3p,miR-1197-5p,miR-1193,miR-383-5p,miR-15a-5p,miR-2284h-5p,novel_83,miR-532-3p,miR-18a-3p,miR-450b-3p,novel_74,miR-6516-3p,miR-424-5p,miR-874-5p,miR-130b-5p,miR-665,miR-338-5p,miR-769-5p,miR-142a-5p,miR-539-5p,miR-145a-3p,miR-425-5p,miR-214,let-7g-3p,miR-885-3p,miR-1827,miR-450a-1-3p,miR-129b-3p,miR-544-3p,miR-27a-5p,miR-376e-3p,miR-2284s,miR-377-3p,miR-323-
PRR36	ENSOARG0(	proline	miR-3154,miR-199b-5p,miR-671-5p,miR-1827,miR-485-5p,miR-1895,miR-7689-3p,miR-574-5p,miR-2331-3p,miR-6535,miR-199a-5p,miR-6130,miR-193b-3p,miR-615,miR-2433,miR-486b-3p,miR-28-5p,miR-708-5p,miR-664a,miR-128-1-5p,miR-214,miR-665-5p,miR-193a-5p,miR-193b-3p,miR-4510,miR-376a-3p,miR-199b-5p,miR-2428,miR-6134,miR-423-5p,miR-3184-5p,miR-5703,miR-2904,miR-7857,miR-2300b-3p,miR-615-3p,miR-484,miR-378d,miR-1839-5p,miR-431,miR-214-5p,miR-9788-3p,miR-505-5p,miR-383-5p,novel_51,miR-324-3p,miR-431-5p,miR-543-5p,miR-193a-3p,miR-214-3p,miR-199b-5p,miR-760-3p,miR-2898,miR-127-3p,miR-143-3p,miR-199a-5p,miR-486-3p

BYSL	ENSOARG0(	bystin-l	miR-185-3p,miR-324-3p,miR-299-3p,miR-21-3p,miR-221-5p,miR-34b-5p,miR-760-3p,miR-99a-3p,miR-105-3p,miR-216c-5p,miR-2424,miR-146a-5p,miR-486-3p,miR-1197-5p,miR-188-3p,miR-217-5p,miR-124-3p,miR-191,miR-34a-5p,miR-493-3p,miR-1b-3p,miR-548e-3p,miR-328-3p,miR-532-3p,novel_83,miR-148b-3p,miR-200b,miR-7859,miR-210-3p,miR-3604,miR-665,miR-655-3p,miR-151-3p,miR-15b-3p,miR-708-5p,miR-4492,miR-129-5p,miR-664a,miR-214,miR-4510,miR-338-3p,miR-3184-5p,miR-769-5p,miR-655,miR-2285c,miR-1827,miR-625-5p,miR-339b,miR-548o-3p,miR-660-5p,miR-504-5p,miR-221-5p,miR-2331-3p,miR-877-5p,miR-6130,miR-326,novel_103,miR-1306-5p,miR-2285o,miR-103a-2-5p,miR-214-3p,miR-146b,miR-3085-3p,miR-652-5p,miR-494-3p,miR-124a,miR-758-3p,miR-758-3p,miR-7857,miR-412-3p,miR-2426,miR-1388-5p,miR-5010-3p,miR-151a-3p,miR-2285e,miR-2459,miR-107-5p,miR-769-5p,miR-615,miR-2440,miR-2284q,miR-4726-5p,miR-194b-5p,miR-6527,miR-3956-3p,miR-3059-5p,novel_60,miR-423-5p,miR-361-3p,miR-1306-3p,miR-361-3p,miR-504,miR-96-5p,miR-7977,miR-211-5p,miR-20a-3p,miR-4792,miR-3064-5p,miR-4443,miR-378g,miR-217,miR-339a,miR-200c,miR-8117,miR-1260a,miR-200a-3p,miR-152-3p,miR-200c-3p,miR-3064-5p,miR-3956-5p,novel_39,miR-29a-5p,miR-380-5p,miR-330-5p,miR-3578,miR-615-3p,miR-200b-3p,novel_79,miR-1224-5p,miR-412,miR-6517,miR-429-3p,miR-299a-3p,miR-582-3p,miR-1271-5p,miR-135a-5p,miR-331-3p,miR-1306-3p,miR-99b-3p,miR-2433,miR-204-5p,miR-486b-3p,miR-28-5p,miR-191-5p,miR-592,miR-31-3p,miR-138-5p,miR-193a-5p,miR-1197-3p,miR-1260b,miR-2448-3p,miR-876-3p,miR-141-3p,miR-99a-3p,miR-769,miR-34c-5p,novel_51,miR-194a,miR-146a,miR-466f-3p,miR-660,miR-628-5p,miR-382,miR-380-5p,miR-654-3p,miR-432-3p,miR-194-5p,miR-873a-5p,miR-

OAS1	ENSOARG0(	2'-5'-ol	miR-18a-3p,miR-3600,miR-485-3p,miR-95-3p,miR-487a-3p,miR-340-5p,miR-200b,miR-15a-5p,miR-616-3p,miR-2284h-5p,miR-191,miR-30c-5p,miR-376a-5p,miR-1193,miR-5703,miR-184-3p,miR-33b-3p,miR-16-1-3p,miR-133a-3p,miR-378d,miR-329a-5p,miR-2285w,miR-487a,miR-345-3p,miR-29b-1-5p,miR-181b-1-3p,miR-130a-5p,miR-30b,miR-216a-5p,miR-324-3p,miR-345-3p,miR-125b,miR-185-3p,miR-202-5p,miR-21-3p,miR-30c-1-3p,miR-154a-3p,miR-216b-3p,miR-544-3p,miR-582-5p,miR-377-3p,miR-15b-5p,miR-598-3p,miR-30d-5p,let-7g-3p,miR-30f,miR-154b-3p,miR-339b,miR-345-5p,miR-582-5p,novel_99,miR-3074-2-3p,miR-6525,miR-6134,miR-181c-5p,miR-2285j,miR-665,miR-17-3p,miR-664a,miR-874-5p,miR-424-5p,miR-4492,miR-199b-3p,miR-105-1,miR-5010-3p,miR-181b-2-3p,miR-376b-3p,miR-216a-3p,miR-455-3p,miR-1185-3p,miR-2284r,miR-6529a,miR-484,miR-3962,miR-450b-5p,miR-544a,miR-2426,miR-101c,miR-1839-5p,miR-2284aa,miR-1388-3p,miR-2330-5p,miR-424-5p,miR-744-3p,miR-30c-1-3p,miR-8485,miR-2310,miR-342,miR-326,miR-29b-1-5p,miR-16b,miR-491-5p,miR-664b,miR-4792,miR-541-5p,miR-7977,miR-3082-5p,miR-1185-2-3p,miR-376d,miR-361-3p,miR-129b-5p,miR-378j,miR-493-5p,miR-376a-3p,miR-1983,miR-877-3p,miR-3059-5p,miR-452-5p,miR-181d-5p,miR-877-3p,miR-361-3p,miR-30b-5p,miR-490-5p,miR-6517,miR-429-3p,miR-7641,novel_79,miR-376a-5p,miR-322-5p,miR-2284z,novel_121,miR-196a-5p,miR-345-3p,miR-3578,miR-1343-5p,miR-30a-5p,miR-330-5p,miR-105-2,miR-200b-3p,miR-128-3p,miR-2385-3p,miR-181a-2-3p,miR-380-5p,miR-148b-5p,miR-376b-5p,miR-376c-5p,miR-1271-3p,miR-2285af,miR-376b-3p,miR-8117,miR-664-3p,miR-200c,miR-30c-2-3p,miR-339a,miR-17-3p,miR-200c-3p,miR-127-5p,miR-181b-5p,novel_127,novel_96,miR-487a-3p,miR-2355-5p,miR-598-3p,miR-1271,miR-125a,miR-374c-3p,miR-154b-
CTNNB1	ENSOARG0(	catenin	miR-148b-5p,miR-532-3p,miR-320c,miR-300-3p,miR-340-5p,miR-2285aa,miR-3120-3p,miR-496-3p,miR-503-3p,miR-543-3p,miR-148a-5p,miR-496,miR-4429,miR-1260b,miR-320a,miR-21a-3p,miR-181a-5p,miR-2285b,miR-181c-5p,miR-148b-5p,miR-1260b,miR-181d-5p,miR-1306-5p,miR-320d,miR-181b-5p,miR-2957,miR-320b,miR-1260a,miR-618
SPDL1	ENSOARG0(	spindle	miR-141-3p,miR-26b-5p,miR-221-5p,novel_68,miR-548o-3p,miR-27a-3p,miR-377-3p,miR-23b,miR-6516-3p,miR-145a-3p,miR-331-3p,miR-33a-3p,miR-29b-2-5p,miR-22-5p,miR-3600,miR-27a-3p,miR-548e-3p,miR-330-3p,miR-7859,miR-23b-3p,miR-23c,miR-27b-3p,novel_73,miR-29b-2-5p,miR-23a-3p,miR-6240,miR-143-3p,miR-29b-1-5p,miR-130a-5p,miR-330-3p,miR-29b-1-5p,miR-26a-5p,miR-377-3p,miR-221-5p,miR-466f-3p,miR-200a-3p,miR-21-3p,miR-760-3p
DHX30	ENSOARG0(	DEAH (As	miR-204-3p,miR-2447,miR-320b,miR-4286,miR-128-1-5p,miR-450b-3p,miR-4492,miR-324-3p

MPI	ENSOARG0(	mannose	miR-2285v,miR-380-5p,miR-2285r,miR-654-3p,miR-345-5p,miR-197-3p,miR-374b-3p,miR-16b,miR-1260b,miR-497-5p,miR-188-5p,miR-339-5p,miR-26b-3p,miR-2285g,miR-134-3p,miR-185-5p,miR-3431,miR-149-5p,miR-2355-3p,miR-15a,miR-16a,miR-22-5p,miR-199c,miR-3957-3p,novel_101,miR-758-5p,miR-17-3p,miR-199b-5p,miR-2285ab,miR-148a-3p,miR-2387,miR-18a-3p,miR-216a-3p,miR-410-5p,miR-195a-5p,miR-3074-5p,miR-320b,miR-2404,miR-374c-3p,miR-1260a,miR-200c,miR-339a,miR-17-3p,miR-200c-3p,miR-152-3p,novel_127,miR-181a-2-3p,miR-4286,miR-380-5p,miR-199a-5p,miR-134,miR-200b-3p,miR-128-3p,miR-429-3p,novel_79,miR-322-5p,miR-24-2-5p,miR-505-5p,miR-134-5p,miR-129-1-3p,miR-551b-3p,miR-26c,miR-3969,miR-31-3p,miR-28-5p,miR-134-5p,miR-1306-5p,miR-16-5p,miR-1260b,miR-2448-3p,miR-329a,miR-345-5p,miR-23a-5p,miR-7144-5p,miR-671-5p,miR-485-5p,miR-1248,miR-2285aa,miR-1271,miR-300-3p,miR-374c-3p,miR-199a-5p,miR-362-3p,miR-16b,miR-25-5p,miR-199b-5p,miR-424-5p,miR-2284r,miR-329-3p,miR-143-5p,miR-125b-2-3p,miR-362-3p,miR-1839-5p,miR-3120-5p,miR-199b-3p,miR-5010-3p,miR-222-5p,miR-1298-3p,miR-216a-3p,miR-9788-3p,miR-2285e,miR-421,miR-2440,miR-371a-5p,miR-7134-3p,miR-2285f,miR-219a-5p,miR-361-3p,miR-1285-5p,miR-140-5p,miR-199b-5p,miR-139-5p,miR-20a-3p,miR-2319b,miR-378g,miR-3082-5p,miR-203-3p,miR-210-5p,miR-30b-3p,miR-216c-5p,miR-376a-5p,miR-2424,miR-376a-5p,miR-362-3p,miR-98-3p,miR-140-3p,miR-188-3p,miR-664-5p,let-7a-3p,miR-548e-3p,miR-18a-3p,miR-380-5p,miR-7859,miR-148b-3p,miR-15a-5p,miR-200b,miR-532-3p,let-7b-3p,miR-664a,miR-17-3p,miR-4492,miR-708-5p,miR-424-5p,miR-129-2-3p,miR-3074-5p,miR-3958-5p,miR-665-5p,miR-346,miR-98-3p,miR-122-5p,miR-2285c,let-7f-1-3p,miR-129b-3p,miR-2312,miR-345-5p,miR-339b,miR-139-
TIPARP	ENSOARG0(	TCDD-ind	miR-138,miR-16b,miR-497-5p,miR-2113,miR-223-3p,miR-3962,miR-330-3p,miR-16b,miR-330-3p,miR-101b-3p,miR-6240,miR-424-5p,miR-1388-3p,miR-652-5p,miR-363-5p,miR-23a-3p,miR-136-5p,miR-19a-3p,novel_94,miR-505-3p,miR-195a-5p,miR-2285ab,miR-3065-5p,miR-18a-3p,miR-378j,miR-3082-5p,miR-2319b,miR-499a-5p,miR-6119-3p,miR-203b-5p,miR-142b,miR-15a,miR-16a,miR-2285f,miR-19b-3p,miR-885-3p,miR-140-3p,miR-2427,miR-218-2-3p,miR-23c,miR-15a-5p,miR-23b-3p,miR-7859,miR-322-5p,miR-616-3p,miR-548e-3p,miR-18a-3p,miR-505-3p,miR-30d-3p,miR-21-3p,novel_127,miR-2285p,miR-30a-3p,let-7g-3p,miR-2312,novel_99,miR-885-3p,miR-23b,miR-15b-5p,miR-545-3p,miR-552-3p,miR-615-5p,miR-2285aa,miR-138-5p,miR-424-5p,miR-30e-3p,miR-3604,miR-30a-3p,miR-16-5p

DOCK4	ENSOARG0(	dedicato	miR-429-3p,miR-106a,miR-200b,miR-24-2-5p,miR-582-3p,miR-545-5p,miR-3591-5p,miR-30c-5p,miR-1434-5p,miR-30a-5p,miR-33b-3p,miR-200b-3p,miR-20a,miR-24-2-5p,miR-380-5p,miR-2285b,miR-664-3p,miR-26a-5p,miR-200c,miR-3071-3p,miR-500a-5p,miR-30f,miR-200c-3p,miR-106a,miR-92a-3p,miR-21-3p,miR-30d,miR-32-5p,miR-1271,miR-2448-3p,miR-2285c,miR-30e-5p,miR-299,miR-30d-5p,miR-17-5p,miR-655,miR-362-5p,miR-93-5p,miR-500a-3p,miR-186-5p,miR-20b-5p,miR-665-5p,miR-193a-5p,miR-93,miR-500b-5p,miR-145-3p,miR-34c-3p,miR-655-3p,miR-338-5p,miR-324-3p,miR-500,miR-199b-3p,miR-1343-3p,miR-2285g,miR-2285e,miR-20b,miR-500-5p,miR-2113,novel_23,miR-299a-5p,miR-106a-5p,miR-101b-3p,miR-380-5p,let-7c-3p,miR-106b-5p,miR-17-5p,miR-106a-5p,miR-32,miR-24-1-5p,miR-664b,miR-26b-5p,miR-2404,miR-299b-5p,miR-362-5p,miR-1285-5p,miR-20a-5p,miR-502-5p,miR-92a-3p,miR-199c,miR-3964
ADCY9	ENSOARG0(	adenylat	miR-378d,miR-8485,miR-124a,miR-1839-3p,miR-3085-3p,miR-7-5p,miR-326,novel_51,miR-194-3p,miR-1185-3p,miR-409b,novel_4,miR-378c,miR-4508,miR-744-5p,miR-7862,miR-296-5p,miR-378b,miR-7b-5p,miR-541,miR-211,miR-326-3p,miR-541-3p,miR-1839-3p,miR-2285x,miR-17-3p,miR-1193,miR-4726-5p,miR-2440,miR-7857-3p,miR-378e,miR-3596,miR-669,miR-1185-2-3p,miR-3064-5p,miR-3074-5p,miR-320b,miR-2285u,miR-211-5p,miR-378f,miR-18b,miR-376c-3p,miR-2285b,miR-105-3p,miR-3064-5p,miR-4286,miR-345-3p,miR-17-3p,miR-466i-5p,miR-3601,miR-422a,miR-210-3p,miR-378h,miR-7641,miR-18b-5p,miR-378i,miR-215-5p,miR-124-3p,miR-378a-3p,miR-23b-5p,miR-330-5p,miR-140-3p,miR-378c,miR-378c,miR-3074-5p,miR-2330-3p,miR-17-3p,miR-874-5p,miR-4492,miR-208b-5p,miR-204-5p,miR-551b-3p,miR-370-5p,miR-3969,miR-376e-3p,miR-192-5p,miR-378a-3p,miR-370-5p,miR-615-5p,miR-215-5p,miR-18a-5p,miR-625-5p,miR-1895,miR-671-5p,miR-18a

MGAT4B	ENSOARG0(	mannosyl	miR-2300a-5p,miR-330-3p,miR-1343-3p,novel_4,miR-9788-3p,miR-216a-3p,miR-455-3p,miR-376b-3p,miR-142-5p,miR-496-5p,miR-501-3p,miR-2284r,miR-1388-3p,miR-193a,miR-4532,novel_115,miR-5126,miR-199b-5p,miR-25-5p,novel_124,miR-126b-5p,miR-4443,miR-376d,miR-500-3p,miR-199b-5p,miR-409-5p,miR-361-3p,miR-376a-3p,miR-194b-5p,miR-2447,miR-502-3p,miR-877-3p,miR-30b-5p,miR-4726-5p,miR-3968,miR-3960,miR-328-3p,miR-2904,miR-2300b-3p,miR-2366,miR-191,miR-30c-5p,miR-218-1-3p,miR-664-5p,miR-493-3p,miR-486-3p,miR-421-5p,miR-125b,miR-30b,miR-2484,miR-30d,miR-760-3p,miR-202-5p,miR-552-3p,miR-3065-3p,miR-204-3p,novel_25,miR-6130,miR-2284s,miR-30d-5p,miR-500a-3p,miR-598-3p,miR-21a-3p,miR-1827,miR-186-5p,miR-30f,miR-145a-3p,miR-4510,miR-338-3p,miR-6134,miR-142a-5p,miR-4492,miR-500,miR-194-3p,miR-194-5p,miR-431-3p,miR-7862,miR-744-5p,miR-26a-2-3p,miR-29b,miR-6240,miR-380-5p,miR-194a,miR-365b-5p,miR-330-3p,miR-1343-5p,miR-125a-5p,miR-3120-3p,miR-378b,miR-6535,miR-669,miR-29a-3p,miR-374c-3p,miR-216a-3p,miR-376b-3p,miR-6395,miR-1291,miR-29c-3p,miR-3957-3p,miR-199b-5p,miR-28c,miR-29a,miR-128-1-5p,novel_121,miR-214-5p,miR-30a-5p,miR-501-3p,miR-29d-3p,miR-1343-5p,miR-3578,miR-30c,miR-128-3p,miR-1271-3p,miR-199a-5p,miR-1957a,miR-3065-3p,miR-380-5p,miR-28b,miR-30f,miR-376b-3p,miR-205-5p,miR-125a,miR-598-3p,miR-370-3p,miR-199a-5p,miR-2448-3p,miR-30e-5p,miR-23a-5p,miR-10b-3p,novel_42,miR-365a-5p,miR-331-3p,miR-125b-5p,miR-29b-3p,miR-2433,miR-191-5p,miR-486b-3p
PSMD10	ENSOARG0(	proteaso	miR-499a-5p,miR-450b-3p,miR-487b-5p,miR-382-5p,miR-382-3p,miR-133a-3p,miR-133b-3p,miR-30f,miR-2318,miR-30d-5p,miR-30e-5p,miR-450a-1-3p,miR-6535,miR-324-5p,miR-7857-3p,miR-30c-1-3p,miR-487b-5p,miR-30c-2-3p,miR-2285p,miR-143-3p,miR-30c-1-3p,miR-494-3p,miR-30c,miR-101a-5p,miR-142-3p,miR-450c-3p,miR-30a-5p,miR-873a-5p,miR-30c-5p,miR-450a-1-3p,miR-421,miR-505-3p
FSCN1	ENSOARG0(	fascin a	miR-145b,miR-133b-5p,miR-1343-3p,miR-31-5p,miR-185-5p,miR-425-3p,miR-30b-3p,miR-145a-5p,miR-541,miR-744-5p,miR-29b,miR-30b-3p,miR-4532,miR-326,miR-214-3p,miR-125a-5p,miR-3085-3p,miR-483-3p,miR-7977,miR-3064-5p,miR-29a-3p,novel_69,miR-150-5p,miR-2387,miR-361-3p,miR-29c-3p,miR-541-3p,miR-2428,miR-423-5p,miR-326-3p,miR-24-3p,miR-361-3p,miR-1307-5p,novel_82,miR-4324,miR-29a,miR-4726-5p,miR-212-5p,miR-429-3p,miR-6517,miR-151-5p,miR-483-3p,miR-200b,miR-505-5p,miR-330-5p,miR-5703,miR-2427,miR-29d-3p,miR-200b-3p,miR-582-3p,miR-2403,miR-4286,miR-3064-5p,miR-3065-3p,miR-29a-5p,miR-486-3p,miR-185-3p,miR-125b,miR-125a-3p,miR-200c,miR-21-3p,miR-200c-3p,miR-450a-2-3p,miR-615-5p,miR-125a,miR-3065-3p,miR-151b,miR-300-3p,miR-129b-3p,miR-488-3p,miR-214,miR-3184-5p,miR-6525,miR-665,miR-125b-5p,miR-2433,miR-29b-3p,miR-486b-3p,miR-4492

STON2	ENSOARG0(	stonin 2	miR-5703,miR-1193,miR-140-3p,miR-191,miR-2300b-3p,miR-4429,miR-142-3p,miR-485-3p,miR-30d-3p,miR-548e-3p,miR-671-3p,miR-7859,miR-487a-3p,miR-23b-3p,miR-23c,miR-324-3p,miR-345-3p,novel_17,miR-3653-3p,miR-30b-3p,miR-320d,miR-216c-5p,miR-146a-5p,miR-2432,miR-421-5p,miR-181b-1-3p,miR-543-3p,miR-625-5p,miR-345-5p,miR-532-5p,miR-2331-3p,miR-22-3p,miR-877-5p,miR-10a,miR-30a-3p,miR-30e-3p,miR-487a-5p,miR-129-5p,miR-708-5p,novel_74,miR-3074-2-3p,miR-665-5p,miR-6134,miR-10b-5p,miR-758-3p,miR-10b,miR-450b-5p,miR-181b-2-3p,miR-145b,miR-105-1,miR-421,miR-153,miR-505,miR-320b,miR-146b,miR-153-3p,miR-23a-3p,miR-1388-3p,miR-193a,miR-8485,miR-758-3p,miR-378j,miR-129b-5p,miR-3963,miR-211-5p,miR-2483-3p,miR-7134-3p,miR-452-5p,miR-3059-5p,miR-7705,novel_111,miR-345-3p,miR-105-2,miR-10a-5p,novel_121,miR-24-2-5p,miR-505-5p,miR-2898,miR-127-5p,miR-1a-2-5p,miR-30a-3p,miR-4286,miR-2285b,miR-450b-5p,miR-320a,miR-23b,miR-488-3p,miR-331-5p,miR-7144-5p,miR-345-5p,miR-365a-3p,miR-100-3p,miR-135a-5p,miR-1-5p,miR-410-5p,miR-5100,miR-204-5p,miR-28-5p,miR-191-5p,miR-331-5p,miR-193a-5p,let-7e-3p,miR-22-3p,miR-145a-5p,novel_23,miR-432,miR-653,miR-873a-5p,miR-1843b-5p,miR-7b-5p,miR-1434-3p,miR-188-5p,miR-26b-3p,novel_116,miR-432-5p,miR-185-5p,miR-105-5p,miR-2411-3p,miR-7-5p,miR-146a,miR-378a-5p,let-7j,miR-539-3p,miR-345-5p,miR-150-5p,miR-146b-5p,miR-135b-5p,miR-10a-5p,miR-216b-3p,miR-320c,miR-677,miR-181b-3p,miR-4324,miR-576-3p,miR-22-5p,miR-421-5p,miR-128-1-5p,novel_101,miR-1a-1-5p,novel_27,miR-211
SSR3	ENSOARG0(	signal s	miR-1814c,miR-1827,miR-2319a,miR-320a,miR-6130,miR-499b-3p,miR-499a-3p,miR-320c,miR-664b,miR-211-5p,miR-499b-5p,miR-6119-3p,miR-204-5p,miR-147-5p,miR-4492,miR-218-5p,miR-382-5p,novel_87,miR-301a-5p,miR-100-3p,miR-371a-5p,miR-6525,miR-211,miR-361-3p,miR-3184-5p,miR-423-5p,miR-4510,miR-101a-5p,miR-4429,miR-301b-5p,miR-3431,miR-496-5p,miR-2284z,miR-455-3p,miR-450a-2-3p,miR-491-5p,miR-320d,novel_96,miR-664-3p,miR-320b,miR-185-3p,miR-505,miR-2310,miR-503-3p,miR-8485

CFAP52	ENSOARG0(	cilia an	miR-3068-3p,miR-874-3p,miR-769,miR-34c-5p,miR-30e-5p,miR-100-5p,miR-376c-5p,miR-1248,miR-7144-5p,miR-1306-5p,miR-16-5p,miR-378c,miR-29b-3p,miR-551b-3p,miR-331-3p,miR-592,miR-2285ad,miR-431,miR-483-3p,miR-378h,miR-322-5p,miR-450a-1-3p,miR-23b-5p,miR-30a-5p,miR-29d-3p,miR-1343-5p,miR-99a-5p,miR-3956-5p,miR-192-3p,miR-99a,miR-217,miR-2898,novel_96,novel_68,miR-3120-3p,miR-655-5p,miR-29a-3p,miR-34b,miR-2404,miR-376b-5p,miR-3065-5p,miR-1306,miR-412-3p,miR-2285k,miR-378f,miR-195a-5p,miR-29c-3p,miR-3957-3p,miR-371b-3p,miR-24-3p,miR-2284w,miR-449a,miR-4324,miR-15a,miR-1307-5p,miR-16a,miR-34c,miR-29a,miR-122-3p,miR-3955-5p,miR-503-5p,miR-412-5p,miR-3535,miR-149-5p,miR-450c-3p,miR-16-1-3p,miR-16b,miR-29b,miR-30b-3p,miR-497-5p,miR-6240,miR-654-3p,miR-503-5p,miR-99a-5p,miR-330-3p,miR-483-3p,miR-1343-5p,miR-452-5p,miR-140-5p,miR-545-3p,miR-378a-3p,miR-296-3p,miR-532-5p,miR-877-5p,miR-15b-5p,miR-204-3p,miR-450a-1-3p,miR-21a-3p,miR-30d-5p,miR-186-5p,novel_99,miR-1827,miR-590-3p,miR-378c,miR-214,miR-2330-3p,miR-346,miR-769-5p,miR-665,miR-450b-3p,miR-424-5p,miR-340-5p,miR-15a-5p,miR-193b-5p,miR-378i,miR-217-5p,miR-378a-3p,miR-34a-5p,miR-16-1-3p,miR-503-5p,miR-216c-5p,miR-345-3p,miR-195a-3p,miR-130a-5p,miR-2285i,miR-2285p,miR-92a-1-5p,miR-422a,miR-122-3p,miR-324-3p,miR-466i-5p,miR-34b-5p,miR-30d,miR-20a-3p,miR-7977,miR-2285u,miR-3082-5p,miR-378e,miR-203-3p,miR-409-5p,miR-361-3p,miR-381-3p,miR-378j,miR-99b-5p,miR-147-3p,miR-877-3p,miR-1983,miR-212-3p,miR-2447,miR-30b-5p,miR-2285n,miR-107-5p,miR-371a-5p,miR-769-5p,miR-3968,miR-330-3p,novel_4,miR-548w,miR-4791,miR-6529a,miR-378b,miR-484,miR-30b-3p,miR-2426,miR-378c,miR-21b,miR-16-2-3p,miR-424-5p,miR-2330-5p,miR-1388-

GZMB	ENSOARG0(	granzyme	miR-544a,miR-2426,miR-1388-5p,miR-16-2-3p,miR-21b,miR-2284r,miR-484,miR-1298-3p,miR-455-3p,miR-409b,novel_78,miR-1343-3p,miR-548w,miR-27a-3p,miR-5010-3p,miR-3085-3p,miR-491-5p,novel_115,miR-431-5p,novel_103,miR-1306-5p,miR-326,miR-16b,miR-182-5p,miR-505,miR-30c-1-3p,miR-2310,miR-8485,miR-424-5p,miR-1388-3p,let-7c-3p,miR-136-5p,miR-181a-3p,miR-96-5p,miR-9-3p,miR-361-3p,miR-378j,miR-129b-5p,miR-378g,miR-27a-3p,miR-2319b,miR-3064-5p,miR-139-5p,miR-7977,miR-211-5p,miR-2285u,miR-335,miR-3958-3p,miR-4726-5p,miR-490-5p,miR-101-3p,novel_82,novel_111,miR-361-3p,miR-1285-5p,miR-423-5p,miR-9-3p,miR-885-5p,miR-184-3p,miR-378d,miR-140-3p,miR-1434-5p,miR-5703,miR-2904,miR-15a-5p,miR-616-3p,miR-18a-3p,miR-212-5p,miR-2484,miR-21-3p,miR-221-5p,miR-2285p,miR-125b,miR-185-3p,miR-92a-1-5p,miR-324-3p,miR-376a-5p,miR-380-3p,miR-2424,miR-216c-5p,miR-195a-3p,miR-582-5p,miR-1895,miR-450a-1-3p,miR-21a-3p,miR-139-5p,miR-544-3p,miR-582-5p,miR-6516,miR-15b-5p,miR-22-3p,miR-548o-3p,miR-30c-1-3p,miR-221-5p,miR-296-3p,miR-552-3p,miR-182-5p,miR-370-5p,miR-3065-3p,miR-17-3p,miR-450b-3p,miR-130b-5p,miR-708-5p,miR-424-5p,miR-208b-5p,miR-4492,miR-2285j,miR-3604,miR-6525,miR-338-3p,miR-6134,miR-3184-5p,miR-3074-5p,miR-129-2-3p,miR-3074-2-3p,miR-665-5p,miR-1271,miR-3958-5p,miR-29b,miR-380-3p,miR-16b,miR-7862,miR-27b-3p,miR-188-5p,miR-455-5p,miR-497-5p,miR-7b-5p,miR-542-3p,miR-1434-3p,miR-223-3p,miR-3071-5p,miR-450c-3p,miR-22-3p,miR-3431,miR-134-3p,miR-544b,miR-194-3p,miR-149-5p,miR-187-3p,miR-3955-5p,miR-665,miR-483-3p,miR-452-5p,miR-125a-5p,miR-7-5p,miR-455-5p,novel_51,miR-432-3p,miR-432-3p,miR-654-3p,let-7i,miR-130b-5p,miR-450b-3p,miR-410-5p,miR-

DDX52	ENSOARG0(	DEAD (As	miR-125b-2-3p,miR-301b-5p,miR-143-5p,miR-378b,miR-101c,miR-2284aa,miR-544a,miR-378c,miR-27a-3p,miR-145b,miR-105-1,miR-1185-3p,miR-421,miR-455-3p,miR-9788-3p,miR-182-5p,miR-505,miR-199a-3p,miR-103a-2-5p,miR-3955-3p,miR-130b-3p,miR-652-5p,miR-744-3p,let-7c-3p,miR-23a-3p,miR-1388-3p,miR-2310,miR-3187-3p,miR-378d,miR-381-3p,miR-378j,miR-361-3p,novel_94,miR-877-3p,miR-181a-3p,miR-3963,miR-211-5p,miR-7977,miR-7689-3p,miR-499b-5p,miR-2319b,miR-27a-3p,miR-1185-2-3p,miR-2483-3p,miR-25,miR-378e,miR-107-5p,miR-301a-5p,miR-371a-5p,miR-218-5p,miR-2285n,miR-154-3p,miR-9-5p,miR-181d-5p,miR-3956-3p,miR-212-3p,miR-3059-5p,novel_60,miR-154a,miR-7705,novel_111,miR-1197-5p,miR-378a-3p,miR-188-3p,miR-5703,miR-140-3p,miR-1434-5p,miR-98-3p,miR-378i,let-7a-3p,miR-218-1-3p,miR-212-5p,miR-3600,miR-380-5p,miR-548e-3p,miR-532-3p,miR-199a-3p,miR-23c,miR-487a-3p,miR-23b-3p,miR-7859,miR-422a,miR-345-3p,miR-500a-5p,miR-216a-5p,miR-208b-3p,miR-2285p,miR-2484,miR-221-5p,miR-466i-5p,miR-105-3p,miR-487a,miR-216c-5p,miR-143-3p,miR-376c-3p,miR-433-3p,let-7f-1-3p,miR-582-5p,miR-186-5p,miR-543-3p,miR-625-5p,miR-154b-3p,miR-339b,miR-296-3p,miR-552-3p,miR-182-5p,miR-21-3p,miR-548o-3p,miR-378a-3p,miR-221-5p,novel_25,miR-323-5p,miR-544-3p,miR-376e-3p,miR-301a-3p,miR-6516,miR-582-5p,miR-338-5p,let-7b-3p,miR-2285j,miR-454-3p,miR-6516-3p,miR-425-5p,miR-378c,miR-338-3p,miR-6134,miR-181c-5p,miR-98-3p,novel_23,miR-145a-5p,miR-197-3p,miR-7b-5p,miR-542-3p,miR-181a-5p,miR-223-3p,miR-432,miR-27b-3p,miR-7862,miR-26b-3p,miR-339-5p,miR-130a-3p,miR-103a-3p,miR-103,miR-2355-3p,miR-544b,miR-185-5p,miR-432-5p,miR-2411-3p,miR-105-5p,miR-7-5p,miR-378a-5p,miR-483-3p,let-7f-2-3p,let-7f-2-3p,miR-380-5p,miR-539-

MND1	ENSOARG0(	meiotic	miR-8095,miR-3059-5p,novel_60,miR-32-3p,miR-194b-5p,miR-452-5p,miR-4726-5p,miR-450a-5p,miR-382-3p,miR-2285n,miR-2285y,miR-1973,miR-107-5p,miR-376d,miR-10b,miR-452-3p,miR-541-5p,miR-499b-5p,miR-877-3p,miR-361-3p,miR-199b-5p,miR-129b-5p,miR-30c-1-3p,miR-3187-3p,miR-758-3p,miR-3955-3p,miR-320b,miR-1306-5p,miR-326,miR-216a-3p,miR-9788-3p,miR-221-3p,miR-105-1,miR-2300a-5p,miR-222-5p,miR-2426,miR-2284aa,miR-6529a,miR-484,miR-10b,miR-412-3p,miR-10b-5p,miR-338-3p,miR-539-5p,miR-145a-3p,miR-17-3p,miR-6516-3p,miR-222-3p,miR-324-3p,miR-19b-1-5p,miR-320e,miR-27a-5p,miR-193b-3p,miR-22-3p,miR-204-3p,miR-877-5p,miR-221-5p,miR-545-3p,miR-32-3p,miR-532-5p,miR-154b-3p,miR-21a-3p,miR-2432,miR-146a-5p,miR-2284m,miR-320d,miR-30b-3p,miR-221-5p,miR-21-3p,miR-196a-3p,miR-374a-5p,miR-487a-3p,miR-200b,miR-548e-3p,miR-3600,miR-4429,miR-378d,novel_133,miR-1197-5p,miR-140-3p,miR-1193,miR-211,miR-323c,miR-326-3p,miR-495-3p,miR-17-3p,miR-2285x,miR-92a-3p,miR-656-5p,miR-199c,miR-147-5p,miR-133a-5p,miR-33a-3p,miR-382-5p,miR-10a-5p,miR-9851-3p,miR-135b-5p,miR-21c,miR-376b-3p,miR-539-3p,miR-345-5p,miR-1343-5p,miR-466f-3p,miR-2284d,miR-2355-3p,miR-125b-1-3p,miR-7862,miR-653,miR-194-5p,miR-145a-5p,miR-374b-3p,miR-145-3p,miR-1306-5p,miR-374b,miR-3607-3p,miR-204-5p,miR-34c-3p,miR-1306-3p,miR-144,miR-331-3p,miR-135a-5p,miR-199a-5p,miR-154b-5p,miR-487a-3p,miR-10b-3p,miR-671-5p,miR-488-3p,miR-320a,miR-199a-5p,miR-1957a,miR-376c-5p,miR-376b-5p,miR-17-3p,miR-200c-3p,miR-543-5p,miR-92a-3p,miR-376b-3p,miR-2285af,miR-200c,miR-2397-5p,miR-30c-2-3p,miR-222,miR-582-3p,miR-200b-3p,miR-105-2,miR-10a-5p,miR-345-3p,miR-134,miR-7134-5p,miR-361-3p,miR-7705,miR-2447,miR-363-3p,miR-3956-3p,miR-2284i,miR-154-3p,miR-499a-3p,miR-
FAM71F1	ENSOARG0(	family w	miR-544a,miR-4429,miR-3963,miR-1814c,miR-155-5p,miR-1197-5p,miR-320a,miR-188-3p,miR-150-5p,miR-147a,miR-544-3p,miR-7857-3p,miR-155-5p,miR-544b,miR-320c,miR-182-5p,miR-155-5p,miR-320d,miR-130b-5p,miR-208b-5p,miR-31-3p,miR-142b,novel_17,miR-320b,miR-182-5p,miR-130a-5p,miR-2478,miR-877-3p,miR-2310,miR-130b-5p,miR-3059-5p

NME2	ENSOARG0(	NME/NM23	miR-1224-5p,miR-412,miR-429-3p,miR-2285ad,miR-486-5p,miR-450a-1-3p,miR-758-5p,miR-1290,miR-155-5p,miR-192-3p,miR-2285b,let-7g,novel_39,miR-450b-5p,miR-30f,miR-3071-3p,miR-205-5p,let-7f-5p,miR-127-5p,miR-2285aa,miR-200a-5p,miR-93-5p,miR-20b-5p,miR-345-5p,miR-2318,miR-2284v,miR-1260b,miR-494-5p,miR-2284k,miR-31-5p,miR-3955-5p,miR-134-3p,miR-450c-3p,miR-223-3p,miR-2113,miR-1843b-5p,miR-542-3p,miR-19b-2-5p,miR-432,miR-2284x,miR-628-5p,miR-380-5p,let-7a-2-3p,miR-2284l,miR-432-3p,miR-432-3p,novel_51,miR-2411-3p,miR-146a,miR-378a-5p,miR-329-5p,miR-181b-3p,miR-320c,miR-677,miR-216a-3p,miR-3065-5p,let-7e-5p,miR-146b-5p,novel_87,miR-421-5p,miR-379-3p,miR-30d-3p,miR-106a,miR-328-3p,miR-95-3p,miR-217-5p,miR-1434-5p,miR-193b-5p,miR-1961,miR-133a-3p,miR-33b-3p,novel_73,miR-493-3p,miR-195a-3p,miR-329a-5p,miR-181b-1-3p,miR-664b-3p,miR-345-3p,miR-2285p,let-7k,miR-3653-3p,miR-98-5p,miR-504-5p,miR-2331-3p,let-7i-5p,miR-487b-5p,miR-450a-1-3p,miR-186-5p,miR-625-5p,miR-345-5p,miR-214,let-7b,miR-346,miR-224-5p,miR-6525,miR-3604,miR-30a-3p,miR-30e-3p,let-7f,miR-874-5p,miR-487b-5p,miR-145b,miR-181b-2-3p,miR-548w,miR-2459,miR-455-3p,miR-147a,miR-323b,miR-301b-5p,miR-106b-5p,miR-323b-3p,miR-199a-3p,miR-214-3p,miR-146b,miR-487b-5p,miR-7689-3p,miR-139-5p,miR-2483-3p,miR-378g,miR-3596,miR-381-3p,miR-378j,miR-140-5p,miR-9-3p,miR-504,miR-2284j,miR-3956-3p,miR-1285-5p,novel_111,miR-877-3p,miR-301a-5p,miR-3964,miR-490-5p,miR-3968,miR-545-5p,miR-6516-5p,miR-2411-5p,miR-345-3p,let-7a-2-3p,miR-128-3p,let-7d,miR-582-3p,miR-411-3p,miR-30a-3p,miR-1271-3p,miR-380-5p,miR-2478,let-7c-5p,miR-217,miR-200c,miR-2285af,miR-1260a,miR-200c-3p,miR-17-3p,miR-370-3p,miR-17-5p,miR-320a,miR-3154,miR-2448-
DOCK2	ENSOARG0(	dedicato	miR-3956,miR-132-5p,miR-193b-3p,miR-299,miR-543-3p,miR-877-3p,miR-671-5p,miR-107,miR-193b-3p,miR-214,miR-17-3p,novel_42,miR-1306-5p,novel_27,miR-132-5p,miR-369-5p,miR-371a-5p,miR-29b-2-5p,miR-301a-5p,miR-551b-3p,miR-28c,novel_74,miR-17-3p,miR-6517,miR-103a-3p,miR-2355-3p,miR-103,miR-214-5p,miR-496-5p,miR-301b-5p,miR-369-5p,miR-7862,miR-3120-5p,miR-125b-1-3p,miR-495-5p,miR-29b-2-5p,miR-1388-3p,miR-494-3p,miR-143-3p,miR-28b,miR-29b-1-5p,miR-29b-1-5p,miR-107,miR-106b-3p,miR-193a-3p,miR-214-3p,miR-21-3p,miR-17-3p
BAG3	ENSOARG0(	BCL2-ass	miR-2330-3p,miR-345-3p,miR-129-2-3p,miR-345-5p,miR-143-3p,miR-29a-5p,miR-361-3p,miR-217,miR-3969,miR-19b-1-5p,miR-196a-3p,miR-2484,miR-452-3p,miR-129-1-3p,miR-203-3p,miR-217-5p,miR-3591-5p,miR-345-5p,miR-345-5p

PON3	ENSOARG0(	paraoxon	miR-100-3p,miR-144-5p,miR-16-5p,miR-1248,miR-20b-5p,miR-329a,miR-345-5p,miR-23b,miR-200a-5p,miR-93-5p,miR-30e-5p,miR-133c,miR-3959-5p,miR-331-5p,novel_127,miR-152-3p,miR-3071-3p,miR-30f,miR-125a-3p,miR-664-3p,miR-8117,miR-205-5p,miR-2285b,miR-2284y,miR-29a-5p,miR-450b-5p,miR-30c,miR-330-5p,miR-1343-5p,novel_121,miR-2284z,miR-758-5p,miR-322-5p,miR-9-5p,novel_79,miR-429-3p,miR-299a-3p,miR-6123,miR-6119-3p,miR-4324,miR-199b-5p,miR-301b-3p,miR-1839-3p,miR-378f,miR-2319a,miR-216a-3p,miR-3591-3p,miR-150-5p,miR-148a-3p,miR-181b-3p,miR-144-3p,miR-677,miR-669,miR-299b-3p,miR-374c-3p,miR-3120-3p,miR-26b-5p,miR-3074-5p,miR-378a-5p,miR-2411-3p,miR-2285v,miR-582,miR-7-5p,let-7j,let-7f-2-3p,let-7f-2-3p,miR-380-5p,miR-497-5p,miR-16-1-3p,miR-16b,novel_23,miR-7b-5p,miR-223-3p,miR-3431,miR-412-5p,miR-339-5p,miR-103a-3p,miR-31-5p,miR-130b-5p,miR-454-3p,miR-190a-5p,miR-151-3p,miR-2285j,miR-3184-5p,miR-346,miR-3074-5p,miR-186-5p,miR-339b,miR-345-5p,miR-30d-5p,miR-129b-3p,miR-2285c,miR-15b-5p,miR-10a,miR-301a-3p,miR-345-3p,miR-122-3p,miR-422a,miR-324-3p,miR-500a-5p,novel_17,miR-26a-5p,miR-30b,miR-181b-1-3p,miR-376a-5p,miR-148a-5p,miR-2284u,miR-16-1-3p,novel_73,miR-378a-3p,miR-30c-5p,miR-378i,miR-23c,miR-210-3p,miR-7859,miR-23b-3p,miR-106a,miR-18a-3p,miR-3968,novel_82,miR-423-5p,miR-877-3p,miR-9-5p,miR-3956-3p,miR-668-5p,miR-3963,miR-96-5p,miR-378j,miR-1185-2-3p,miR-144-5p,miR-203-3p,miR-2285u,miR-2319b,miR-2284g,miR-139-5p,miR-103a-2-5p,miR-199b-5p,miR-130b-3p,miR-16b,miR-106b-5p,miR-199a-3p,miR-2310,miR-8485,miR-378d,miR-23a-3p,miR-101b-3p,miR-124a,miR-1388-3p,miR-1839-5p,miR-1388-5p,miR-362-3p,miR-541,miR-329-3p,miR-1185-3p,miR-2459,miR-148b-5p,miR-455-

LEMD3	ENSOARG0(	LEM doma	miR-204-5p,miR-134-5p,miR-592,miR-191-5p,miR-125b-5p,miR-135a-5p,miR-370-5p,miR-323-3p,miR-193a-5p,miR-3607-3p,miR-103b,novel_48,miR-7144-5p,miR-3074-1-3p,miR-1248,miR-671-5p,miR-137-3p,miR-331-5p,miR-300-3p,miR-3965,miR-370-3p,miR-125a,miR-215-5p,miR-487a-3p,miR-874-3p,miR-107,miR-181b-5p,miR-6536,miR-30f,miR-339a,miR-2285af,miR-664-3p,miR-29a-5p,miR-2284y,miR-192-3p,miR-2284a,miR-1271-3p,miR-2385-3p,miR-30a-3p,miR-29b-2-5p,miR-4286,miR-411-3p,miR-330-5p,miR-345-3p,miR-3591-5p,miR-134,miR-1290,miR-134-5p,miR-450a-1-3p,novel_121,miR-505-5p,miR-6516-5p,miR-483-3p,novel_79,miR-412,miR-431,miR-222,miR-6517,miR-199c,miR-6119-3p,novel_1,miR-379-3p,miR-4324,miR-6529b,miR-326-3p,miR-211,miR-2285x,miR-1193,novel_120,miR-135b-5p,miR-107,miR-19a-3p,miR-3970,miR-3065-5p,miR-21c,miR-378b,miR-411b-3p,miR-7-1-3p,miR-329-5p,miR-216b-3p,miR-125a-5p,miR-466f-3p,miR-483-3p,miR-2285l,miR-194a,miR-455-5p,miR-2411-3p,miR-377-3p,miR-2285r,miR-130b-5p,miR-455-5p,miR-380-3p,miR-21-5p,miR-2284ab,miR-219a-1-3p,miR-450c-3p,miR-194-5p,miR-653,miR-181a-5p,miR-103,miR-339-5p,miR-26b-3p,miR-103a-3p,miR-208b-5p,miR-130b-5p,miR-487b-5p,miR-30a-3p,miR-665,miR-3604,miR-222-3p,miR-30e-3p,miR-181c-5p,miR-338-3p,miR-142a-5p,miR-3074-2-3p,miR-186-5p,novel_99,miR-154b-3p,miR-339b,miR-411,miR-6516,miR-377-3p,miR-192-5p,miR-216b-5p,miR-139-5p,miR-154a-3p,miR-370-5p,miR-532-5p,miR-545-3p,miR-466i-5p,miR-324-3p,miR-345-3p,miR-125b,miR-2285p,miR-2424,miR-29b-1-5p,miR-664b-3p,miR-216c-5p,miR-329a-5p,miR-2285w,miR-487a,miR-218-1-3p,miR-2284u,miR-33b-3p,miR-19b-3p,miR-124-3p,miR-140-3p,miR-191,miR-215-5p,miR-1246,miR-2300b-3p,miR-2366,miR-2284b-
AGT	ENSOARG0(	angioten	miR-2408,miR-199b-5p,miR-210-3p,miR-199a-5p,miR-199b-5p,miR-199a-5p,miR-199b-5p
SRL	ENSOARG0(	sarcalum	miR-1197-3p,miR-28b,miR-28c,miR-382-3p
NDUFA3	ENSOARG0(	NADH deh	miR-2447,miR-326-3p,miR-361-3p,miR-320e,miR-2433,miR-4492,miR-486b-3p,miR-4726-5p,miR-664a,miR-320b,miR-204-3p,miR-6535,miR-377-3p,miR-18a-3p,miR-378b,miR-485-5p,miR-339b,miR-363-5p,miR-4286,miR-8485,miR-486-3p,miR-29b-1-5p,miR-29b-1-5p,miR-339a,novel_51,miR-7-5p,miR-326,miR-377-3p,miR-1343-5p,novel_127,miR-2898,miR-339-5p,novel_79,miR-212-5p,miR-18a-3p,novel_78,miR-9788-3p,miR-95-3p,miR-185-5p,miR-7857,miR-1343-5p,miR-330-5p,miR-5703,miR-484,miR-7b-5p,miR-378d,miR-744-5p
AP3M2	ENSOARG0(	adaptor-	novel_23,miR-329a-5p,miR-1434-3p,miR-329-5p,miR-2284z,miR-2284aa,miR-450b-5p

RTN3	ENSOARG0(	reticulo	miR-432-3p,miR-432-3p,let-7a-2-3p,miR-668-3p,miR-378a-5p,miR-483-3p,miR-377-3p,miR-105-5p,miR-365b-5p,novel_51,miR-412-5p,miR-3431,miR-134-3p,miR-16b,miR-16-1-3p,miR-497-5p,miR-27b-3p,miR-2284x,miR-432,miR-19b-2-5p,miR-1843b-5p,miR-223-3p,miR-450c-3p,novel_23,miR-301b-3p,miR-29c-3p,miR-29a,miR-1247-3p,miR-144-3p,miR-6535,miR-148a-3p,miR-1306,miR-148b-5p,miR-200a,miR-29a-5p,miR-6536,novel_96,miR-152-3p,miR-205-5p,miR-125a-3p,miR-322-5p,miR-9-5p,miR-450a-1-3p,miR-134-5p,miR-129-1-3p,novel_121,miR-486-5p,miR-155-5p,miR-1290,miR-23b-5p,novel_42,miR-16-5p,miR-144-5p,miR-134-5p,miR-2284k,miR-133c,miR-3068-3p,miR-2355-5p,miR-2285aa,miR-1271,miR-876-3p,miR-329a,miR-301,miR-2310,miR-491-5p,miR-130b-3p,miR-103a-2-5p,miR-29b-1-5p,miR-16b,miR-147a,miR-148b-5p,miR-421,miR-1343-3p,miR-362-3p,miR-101c,miR-329-3p,miR-501-3p,miR-758-3p,novel_111,miR-423-5p,miR-9-5p,miR-2284j,miR-3968,miR-628-3p,miR-218-5p,miR-2440,miR-3596,miR-378g,miR-25,miR-203-3p,miR-144-5p,miR-4443,miR-500-3p,miR-504,miR-668-5p,miR-136-5p,miR-335-3p,miR-9-3p,miR-378j,miR-486-3p,miR-143-3p,miR-202-5p,miR-324-3p,miR-92a-1-5p,miR-1247-5p,miR-328-3p,miR-493-3p,miR-33b-3p,miR-16-1-3p,miR-361-5p,miR-148a-5p,miR-142a-5p,miR-224-5p,miR-3184-5p,miR-129-2-3p,novel_74,miR-487b-5p,miR-500,miR-4492,miR-454-3p,miR-129-5p,miR-874-5p,miR-130b-5p,miR-301a-3p,miR-377-3p,miR-15b-5p,miR-151b,miR-504-5p,miR-552-3p,miR-625-5p,miR-186-5p,miR-450a-1-3p,miR-500a-3p,miR-129b-3p,miR-487b-5p,miR-130b-5p,miR-363-5p,miR-382,miR-452-5p,miR-660,miR-224-5p,miR-197-5p,miR-432-5p,miR-185-5p,miR-6238,miR-544b,miR-133b-5p,miR-130a-3p,miR-29b,miR-30b-3p,miR-219a-1-3p,miR-7862,miR-873a-5p,miR-374b-3p,miR-3071-
C8orf34	ENSOARG0(	chromoso	miR-96-5p,novel_48,miR-135b-5p,miR-331-5p,miR-3613-5p,miR-331-5p,miR-34c-5p,miR-769,novel_69,miR-3965,miR-34b,miR-216b-3p,miR-541-5p,miR-532-5p,miR-1271,miR-331-5p,miR-669a-3p,miR-486b-3p,miR-135a-1-3p,miR-769-5p,miR-34c,miR-331-3p,miR-2285y,miR-135a-5p,miR-301a-5p,miR-142a-5p,miR-769-5p,miR-1197-3p,miR-8095,miR-449a,miR-1271,miR-425-5p,miR-214,miR-34a-5p,miR-2408,miR-301b-5p,miR-487a-3p,miR-1271-5p,miR-142-5p,miR-2284h-5p,novel_121,miR-2285ad,miR-409b,miR-548w,miR-485-3p,miR-2898,miR-34b-5p,miR-214-3p,miR-125a-3p,miR-500a-5p,miR-125b-2-3p,miR-486-3p,miR-539-3p,miR-376c-5p,miR-2432,miR-376b-5p,miR-2285w,miR-382-3p,miR-652-5p

RASAL3	ENSOARG0(	RAS prot	miR-660-5p,miR-378a-3p,miR-2331-3p,miR-182-5p,miR-370-5p,miR-21-3p,miR-377-3p,miR-301a-3p,miR-15b-5p,novel_32,miR-2285c,miR-655,miR-433-3p,miR-1827,novel_99,miR-378c,miR-214,miR-1271,miR-2285j,miR-655-3p,miR-219b-3p,miR-17-3p,miR-424-5p,miR-454-3p,miR-485-3p,miR-15a-5p,miR-487a-3p,miR-532-3p,miR-362-3p,miR-378i,miR-5703,miR-378a-3p,miR-2904,miR-19b-3p,miR-378d,miR-34a-5p,miR-345-3p,miR-181b-1-3p,miR-143-3p,miR-486-3p,miR-422a,miR-500a-5p,miR-760-3p,miR-34b-5p,miR-664b,miR-7977,miR-3596,miR-378e,miR-27a-3p,miR-199b-5p,miR-378j,miR-136-5p,miR-96-5p,miR-181a-3p,miR-877-3p,miR-493-5p,miR-885-5p,miR-541-3p,miR-452-5p,miR-219a-2-3p,miR-3968,miR-105-1,miR-2300a-5p,novel_4,miR-330-3p,miR-27a-3p,miR-181b-2-3p,miR-2285e,miR-9788-3p,miR-541,miR-484,miR-296-5p,miR-329-3p,miR-378b,miR-758-3p,miR-323b,miR-378c,miR-362-3p,miR-3120-5p,miR-101c,miR-424-5p,miR-382-3p,miR-1388-3p,miR-33a-5p,miR-378d,miR-758-3p,miR-8485,miR-1306-5p,miR-16b,miR-182-5p,miR-505,miR-362-3p,miR-323b-3p,miR-130b-3p,miR-3955-3p,miR-491-5p,miR-214-3p,miR-199b-5p,miR-374c-3p,miR-34c-5p,miR-154b-5p,miR-199a-5p,miR-3154,miR-2318,miR-329a,miR-1248,miR-1306-5p,miR-16-5p,miR-1260b,miR-378c,miR-181c-3p,miR-370-5p,miR-135a-1-3p,miR-29b-3p,miR-138-5p,miR-486b-3p,miR-483-3p,miR-378h,miR-1271-5p,miR-505-5p,miR-322-5p,miR-1290,miR-2427,miR-29d-3p,miR-1343-5p,miR-105-2,miR-4286,miR-495-5p,miR-199a-5p,miR-219-3p,miR-664-3p,miR-1260a,miR-181c-3p,miR-17-3p,novel_96,miR-378b,novel_68,miR-7-1-3p,miR-34b,miR-374c-3p,miR-29a-3p,miR-181b-3p,miR-677,miR-3591-3p,miR-3065-5p,miR-150-5p,miR-1306,miR-19a-3p,miR-195a-5p,miR-378f,miR-29c-3p,miR-1193,miR-17-3p,miR-371b-3p,miR-301b-3p,miR-199b-
TIMM44	ENSOARG0(	transloc	miR-486-3p,miR-1271-3p,miR-3064-5p,miR-4286,miR-221-5p,miR-324-3p,miR-125b,miR-1260a,miR-210-5p,miR-532-3p,miR-486-5p,miR-412,miR-431,miR-6517,miR-128-3p,miR-378d,miR-33b-3p,miR-29d-3p,miR-188-3p,miR-615-3p,miR-1260b,miR-193a-5p,miR-103b,novel_42,miR-4510,miR-708-5p,miR-874-5p,miR-4492,miR-28-5p,miR-486b-3p,miR-125b-5p,miR-664a-5p,miR-29b-3p,miR-370-5p,miR-2332,miR-22-3p,miR-154b-5p,miR-6130,miR-193b-3p,miR-2355-5p,miR-370-5p,miR-125a,miR-2331-3p,miR-221-5p,miR-548o-3p,miR-1895,miR-382,miR-125a-5p,miR-103a-2-5p,miR-193a-3p,miR-3085-3p,miR-431-5p,miR-1306-5p,miR-194-3p,miR-1185-3p,miR-3431,miR-134-3p,novel_4,miR-1343-3p,miR-2300a-5p,miR-29b,miR-30b-3p,miR-1260b,miR-412-3p,miR-22-3p,miR-197-3p,miR-7857,miR-30b-3p,miR-296-5p,miR-2284r,miR-542-3p,miR-541,miR-877-3p,miR-371b-3p,miR-193b-3p,miR-1193,miR-29c-3p,miR-541-3p,miR-128-1-5p,miR-29a,miR-107-5p,miR-382-5p,miR-615,miR-1185-2-3p,miR-25,miR-7857-3p,miR-378g,miR-29a-3p,miR-7977,miR-3064-5p,miR-877-3p,miR-1306,miR-216a-3p,miR-361-3p

GALNT2	ENSOARG0(	polypept	miR-499b-5p,miR-2284g,miR-154b-5p,miR-4443,miR-151b,miR-132-5p,miR-485-5p,miR-1827,miR-3957-3p,miR-2284j,miR-665-5p,miR-2284v,miR-3184-5p,miR-423-5p,miR-2428,novel_111,miR-1260b,miR-1307-5p,miR-132-5p,miR-2284k,miR-133a-5p,miR-147-5p,miR-664a,miR-429-3p,miR-151-5p,miR-2284d,miR-1298-3p,miR-200b,miR-30b-3p,miR-3591-5p,miR-200b-3p,miR-2284x,miR-7862,miR-2284u,miR-30b-3p,miR-1260b,miR-363-5p,miR-2284b,let-7j,miR-2284y,miR-4532,miR-2284a,miR-654-3p,miR-1260a,miR-200c,miR-106b-3p,miR-2898,miR-2284m,miR-760-3p,miR-200c-3p,miR-191-3p
PGBD5	ENSOARG0(	piggyBac	miR-431-3p,miR-505,miR-381-5p,miR-381-5p,miR-1b-3p,novel_42,miR-2411,miR-2411-5p
ADAMTSL2	ENSOARG0(	ADAMTS-l	miR-1291,miR-412-3p,miR-1895,miR-1983,miR-378f,miR-361-3p,miR-378e,novel_69,miR-154b-5p,miR-378a-3p,miR-378b,miR-2355-5p,miR-7977,miR-1271,miR-2433,miR-5100,miR-615,miR-28c,miR-665,novel_111,miR-1260b,miR-1197-3p,miR-1285-5p,miR-378c,miR-378c,novel_60,novel_42,miR-145a-3p,miR-3956-3p,miR-378c,miR-1260b,miR-33b-3p,miR-615-3p,miR-378b,miR-484,miR-378i,miR-378a-3p,miR-505-5p,miR-185-5p,miR-378h,miR-194-3p,miR-149-5p,miR-2459,novel_78,novel_4,miR-1343-3p,miR-760-3p,miR-1260a,miR-324-3p,miR-422a,miR-28b,miR-378d,miR-376c-5p,novel_39,miR-2432,miR-376b-5p,miR-1271-3p,miR-574-3p
PLAT	ENSOARG0(	plasmino	novel_82,miR-140-5p,miR-101-3p,miR-30c-2-3p,miR-5703,miR-147-5p,miR-135a-2-3p,miR-140-5p,miR-30c-1-3p,miR-129-2-3p,miR-345-3p,miR-665-5p,miR-30c-1-3p,miR-129-1-3p,miR-6134

USP43	ENSOARG0(	ubiquiti	miR-378d,miR-5703,miR-362-3p,novel_133,novel_83,miR-199a-3p,miR-505-3p,miR-3600,miR-548e-3p,miR-21-3p,miR-30b-3p,miR-760-3p,miR-185-3p,miR-216a-5p,miR-2411,miR-216c-5p,let-7g-3p,miR-27b-5p,miR-21a-3p,miR-155-5p,miR-22-3p,miR-6130,miR-323-5p,miR-6516,miR-27a-5p,miR-3065-3p,miR-487a-5p,miR-155-5p,miR-338-5p,miR-324-3p,let-7b-5p,miR-4510,miR-3120-5p,miR-450b-5p,miR-2426,miR-484,miR-500-5p,miR-9788-3p,miR-222-5p,miR-27a-3p,let-7e,miR-3955-3p,miR-3085-3p,miR-362-3p,novel_103,miR-193a,miR-30c-1-3p,miR-376b,miR-494-3p,let-7a-5p,miR-877-3p,miR-27a-3p,miR-454-5p,let-7d-5p,miR-7977,miR-541-5p,miR-499b-5p,miR-10b,miR-664b,miR-3064-5p,miR-2284q,miR-2285t,miR-335,miR-769-5p,miR-8095,miR-362-5p,miR-541-3p,miR-3059-5p,let-7a-2-3p,miR-300,let-7d,miR-582-3p,miR-30a-5p,miR-29d-3p,miR-214-5p,miR-545-5p,miR-2411-5p,miR-6516-5p,miR-7641,miR-423-3p,miR-92a-3p,let-7c-5p,miR-30c-2-3p,miR-490-3p,miR-3065-3p,miR-28b,miR-30a-3p,miR-495-5p,miR-2385-3p,miR-7144-5p,novel_91,let-7g-3p,miR-362-5p,miR-503-3p,miR-381-3p,miR-769,miR-141-3p,miR-370-3p,miR-486b-3p,miR-204-5p,miR-29b-3p,miR-410-5p,miR-145-3p,miR-500b-5p,miR-103b,miR-6128,miR-7862,miR-29b,miR-374b-3p,let-7g-5p,miR-665,miR-124-5p,miR-197-5p,miR-296-3p,miR-505-3p,miR-1291,miR-18a-3p,miR-29a-3p,miR-323a-5p,miR-2404,miR-147-5p,miR-199c,miR-656-5p,novel_1,miR-92a-3p,let-7i,miR-6402,miR-24-3p,miR-211,miR-2285x,miR-148a-5p,miR-16-1-3p,miR-493-3p,miR-1961,miR-2366,miR-7859,miR-30d-3p,miR-380-5p,miR-18a-3p,let-7k,miR-98-5p,miR-202-5p,miR-324-3p,miR-2285p,novel_17,miR-486-3p,miR-376c-3p,miR-181b-1-3p,miR-345-3p,miR-195a-3p,miR-186-5p,miR-543-3p,miR-625-5p,miR-433-3p,miR-30d-5p,miR-500a-3p,let-7i-5p,miR-2284s,miR-376e-3p,miR-552-3p,miR-2331-3p,miR-

MPP1	ENSOARG0(	membrane	miR-199a-5p,novel_39,miR-3065-3p,miR-192-3p,miR-3064-5p,miR-29b-2-5p,miR-4286,miR-2898,novel_96,miR-543-5p,miR-92a-3p,novel_127,miR-2285af,miR-8117,miR-30c-2-3p,miR-486-5p,miR-129-1-3p,miR-1224-5p,miR-23b-5p,miR-1306-5p,novel_42,miR-204-5p,miR-100-3p,miR-25-3p,miR-3959-5p,miR-199a-5p,miR-370-3p,miR-615-5p,miR-329a,miR-345-5p,miR-7144-5p,miR-23a-5p,miR-301,miR-137-3p,miR-345-5p,miR-130b-5p,miR-6240,miR-493-5p,miR-363-5p,miR-124-5p,miR-466f-3p,miR-7-5p,miR-330-3p,miR-32,miR-197-5p,miR-105-5p,miR-134-3p,miR-3431,novel_116,miR-2355-3p,miR-4508,miR-7b-5p,miR-431-3p,miR-22-3p,miR-145a-5p,miR-211,miR-24-3p,miR-371b-3p,miR-199b-5p,miR-92a-3p,miR-147-5p,miR-4324,miR-133a-5p,miR-146b-3p,miR-378b,miR-3120-3p,miR-450b-3p,miR-412-3p,miR-7975,miR-6395,miR-379-5p,miR-1306,miR-3970,miR-3065-5p,miR-18a-3p,miR-376a-5p,miR-2424,miR-760-3p,miR-324-3p,miR-185-3p,miR-328-3p,miR-18a-3p,miR-212-5p,novel_133,miR-362-3p,miR-124-3p,miR-5703,miR-3184-5p,miR-6134,miR-129-2-3p,novel_74,miR-664a,miR-500,miR-129-5p,miR-4492,miR-130b-5p,miR-324-3p,miR-29b-2-5p,miR-664a-5p,miR-665,novel_25,miR-22-3p,miR-204-3p,miR-2331-3p,miR-30c-1-3p,miR-32-5p,miR-504-5p,miR-3065-3p,let-7g-3p,miR-345-5p,miR-21a-3p,miR-500a-3p,miR-30c-1-3p,miR-4532,miR-124a,miR-2330-5p,miR-3085-3p,novel_124,miR-491-5p,miR-199b-5p,miR-1306-5p,miR-362-3p,miR-1298-3p,miR-9788-3p,novel_78,miR-105-1,miR-1343-3p,miR-330-3p,miR-145b,miR-362-3p,miR-1839-5p,miR-6529a,miR-329-3p,miR-361-3p,miR-423-5p,miR-3059-5p,novel_60,miR-2447,miR-363-3p,miR-3956-3p,miR-1296-5p,miR-4726-5p,miR-2440,miR-3596,miR-378g,miR-2483-3p,miR-203-3p,miR-4443,miR-3064-5p,miR-211-5p,miR-504,miR-493-5p,miR-1983,miR-361-3p,miR-

FOXI1	ENSOARG0(	forkhead	miR-211,miR-371b-3p,miR-24-3p,miR-449a,miR-326-3p,miR-2428,miR-17-3p,novel_101,miR-29c-3p,miR-128-1-5p,miR-29a,miR-4324,miR-502-5p,miR-34c,miR-382-5p,miR-2404,miR-323a-5p,miR-29a-3p,miR-34b,miR-677,miR-669,novel_68,miR-3074-5p,miR-329-5p,miR-655-5p,miR-2284e,miR-376a-2-5p,miR-2387,miR-1306,miR-378b,miR-18a-3p,miR-539-3p,miR-345-5p,miR-130b-5p,miR-668-3p,miR-363-5p,miR-628-5p,miR-378a-5p,miR-483-3p,miR-466f-3p,miR-7-5p,miR-503-5p,miR-377-3p,miR-2285v,miR-197-5p,novel_51,miR-503-5p,miR-134-3p,miR-185-5p,miR-3431,miR-149-5p,miR-194-3p,miR-3955-5p,miR-187-3p,miR-29b,miR-21-5p,miR-1260b,miR-188-5p,miR-873a-5p,miR-7b-5p,miR-450c-3p,miR-22-3p,miR-1260b,novel_9,miR-486b-3p,miR-28-5p,miR-204-5p,miR-592,miR-410-5p,miR-29b-3p,miR-5100,miR-6740-5p,miR-331-3p,miR-34c-5p,miR-300-3p,miR-154b-5p,miR-487a-3p,miR-874-3p,miR-2355-5p,miR-876-3p,miR-345-5p,miR-329b,miR-7144-5p,miR-671-5p,miR-485-5p,miR-1814c,miR-3154,miR-3065-3p,novel_39,miR-3064-5p,miR-17-3p,miR-2898,novel_127,miR-1260a,miR-125a-3p,miR-3071-3p,miR-483-3p,miR-450a-1-3p,miR-545-5p,miR-6517,miR-1224-5p,miR-376a-5p,miR-3591-5p,miR-29d-3p,miR-330-5p,miR-877-3p,miR-8095,miR-2447,let-7i-3p,miR-2285t,miR-154-3p,miR-218-5p,miR-301a-5p,miR-378g,miR-7857-3p,miR-3082-5p,miR-4792,miR-139-5p,miR-3064-5p,miR-452-3p,miR-499b-5p,miR-7689-3p,miR-541-5p,miR-211-5p,miR-3963,miR-668-5p,miR-504,miR-877-3p,miR-361-3p,miR-378j,miR-2310,miR-376b,miR-3085-3p,miR-487b-5p,miR-153-3p,miR-1468,miR-214-3p,novel_103,miR-326,miR-1306-5p,miR-29b-1-5p,miR-153,miR-1298-3p,miR-147a,miR-9788-3p,miR-455-3p,miR-421,novel_78,miR-1343-3p,miR-2300a-5p,miR-5010-3p,miR-181b-2-3p,miR-1388-5p,miR-2426,miR-2284r,miR-301b-5p,miR-122-5p,miR-539-
LMAN1L	ENSOARG0(	lectin,	miR-503-3p,miR-491-3p,miR-432-3p,miR-628-5p,miR-363-5p,miR-382,miR-6240,miR-3085-3p,miR-491-5p,miR-330-3p,miR-3141,miR-2355-3p,miR-134-3p,miR-185-5p,miR-432-5p,miR-330-3p,miR-122-3p,miR-4508,miR-1260b,miR-30b-3p,miR-30b-3p,miR-501-3p,miR-197-3p,miR-412-3p,miR-432,miR-484,miR-502-3p,miR-361-3p,miR-3956-3p,novel_60,miR-6123,miR-656-5p,miR-22-5p,miR-3968,miR-6402,miR-218-5p,miR-4443,miR-500-3p,miR-378g,miR-3064-5p,miR-18a-3p,miR-409-5p,miR-3141,miR-361-3p,miR-1271-3p,miR-486-3p,miR-3064-5p,novel_127,novel_96,miR-191-3p,miR-450a-2-3p,miR-185-3p,miR-122-3p,miR-219b-5p,miR-1260a,miR-1247-5p,miR-532-3p,miR-196a-2-3p,miR-412,miR-18a-3p,miR-378d,miR-501-3p,miR-1197-5p,miR-1260b,miR-6525,miR-4510,miR-486b-3p,miR-331-5p,miR-324-3p,miR-331-3p,miR-2433,miR-6130,miR-1271,miR-2355-5p,miR-370-3p,miR-874-3p,miR-3068-3p,miR-2474,miR-331-5p
PUS7	ENSOARG0(	pseudour	miR-371b-3p,miR-181d-5p,miR-33a-5p,miR-668-3p,novel_1,miR-181b-5p,miR-320d,miR-320b,miR-345-3p,miR-3604,miR-2411-3p,miR-340-5p,miR-320c,novel_116,novel_78,miR-548e-3p,miR-4429,miR-671-5p,miR-7975,miR-3578,miR-345-3p,miR-708-3p,miR-125b-2-3p,miR-320a

FMN2	ENSOARG0(	formin 2	miR-330-3p,miR-2411-3p,miR-224-5p,miR-377-3p,miR-1343-5p,miR-125a-5p,miR-363-5p,miR-668-3p,miR-130b-5p,miR-6240,miR-539-3p,miR-3071-5p,miR-708-3p,miR-1434-3p,miR-1285,miR-542-3p,miR-1843b-5p,miR-26a-2-3p,miR-497-5p,miR-188-5p,miR-380-3p,miR-16b,miR-16-1-3p,miR-130a-3p,miR-339-5p,miR-103a-3p,miR-2355-3p,miR-103,miR-3535,miR-2285g,miR-3431,miR-16a,miR-382-5p,miR-15a,miR-28c,miR-142b,miR-576-3p,novel_1,miR-495-3p,novel_101,miR-17-3p,miR-135a-2-3p,miR-342-3p,novel_27,miR-301b-3p,miR-211,miR-323c,miR-18a-3p,miR-376b-3p,miR-376b-5p,miR-18b,miR-195a-5p,miR-505-3p,miR-6516-3p,miR-1291,miR-107,miR-10a-5p,miR-3120-3p,miR-7-1-3p,miR-144-3p,miR-411b-3p,miR-3071-3p,miR-339a,miR-376b-3p,miR-219-3p,miR-200c,miR-6119-5p,miR-107,miR-200a-3p,novel_127,miR-450a-2-3p,miR-17-3p,miR-6536,miR-200c-3p,miR-30a-3p,miR-200a,miR-28b,miR-1343-5p,miR-345-3p,miR-200b-3p,miR-496,miR-10a-5p,novel_79,miR-412,miR-18b-5p,miR-222,miR-429-3p,miR-545-5p,miR-322-5p,miR-2411-5p,miR-144,miR-6740-5p,miR-125b-5p,miR-409-3p,miR-3969,miR-204-5p,miR-767-5p,novel_9,miR-16-5p,miR-320a,miR-18a,miR-376c-5p,miR-671-5p,miR-10b-3p,miR-767,miR-2318,miR-2285aa,miR-125a,miR-141-3p,miR-874-3p,miR-503-3p,miR-154b-5p,miR-365a-3p,miR-16b,miR-342,miR-320b,miR-214-3p,miR-130b-3p,miR-101b-3p,miR-424-5p,miR-3187-3p,miR-412-3p,miR-125b-2-3p,miR-484,miR-4791,miR-10b,miR-101c,miR-3120-5p,miR-1388-5p,miR-330-3p,novel_78,miR-221-3p,miR-2285e,miR-421,miR-2459,miR-147a,miR-376b-3p,miR-1298-3p,miR-218-5p,miR-2440,miR-101-3p,miR-490-5p,miR-3968,miR-452-5p,miR-2447,miR-885-5p,miR-8095,miR-9-3p,miR-219a-2-3p,miR-221,miR-376a-3p,miR-9-3p,miR-499b-5p,miR-541-5p,miR-211-5p,miR-4792,miR-452-3p,miR-
C1orf198	ENSOARG0(	chromoso	miR-130b-5p,miR-6134,miR-2284w,miR-338-3p,miR-2285x,miR-625-5p,miR-2448-3p,miR-18a-3p,miR-3596,miR-6535,novel_25,miR-491-5p,miR-450a-2-3p,miR-2898,novel_96,novel_127,miR-2285af,miR-196a-3p,miR-380-5p,miR-758-3p,miR-2285b,miR-380-5p,miR-130b-5p,miR-363-5p,miR-758-3p,miR-197-3p,miR-412-3p,miR-758-5p,miR-421,miR-2459,miR-18a-3p,miR-412

CANX	ENSOARG0(	calnexin	miR-486-5p,miR-450a-1-3p,miR-545-5p,miR-483-3p,miR-1271-5p,miR-302a-5p,miR-431,miR-6517,miR-429-3p,miR-222,miR-200b-3p,miR-128-3p,miR-300,miR-411-3p,miR-1343-5p,miR-2284a,miR-192-3p,miR-2284y,novel_39,miR-28b,miR-450b-5p,miR-148b-5p,miR-24-2-5p,miR-30a-3p,miR-29b-2-5p,miR-152-3p,novel_127,miR-181b-5p,miR-191-3p,miR-205-5p,miR-1260a,miR-324-5p,miR-223-5p,miR-215-5p,miR-370-3p,miR-574-5p,miR-485-5p,miR-23b,miR-381-3p,miR-1260b,miR-193a-5p,miR-486b-3p,miR-144,miR-370-5p,miR-194-3p,miR-149-5p,miR-134-3p,miR-185-5p,miR-3535,miR-26b-3p,miR-487b-3p,miR-130a-3p,miR-31-5p,miR-744-5p,miR-27b-3p,miR-1260b,miR-380-3p,miR-194-5p,miR-374b-3p,miR-450c-3p,miR-181a-5p,miR-708-3p,miR-19b-2-5p,miR-2285r,miR-1343-5p,miR-483-3p,miR-124-5p,miR-194a,miR-136-3p,miR-377-3p,miR-224-5p,miR-144-3p,novel_69,miR-411b-3p,miR-3120-3p,miR-216b-3p,miR-329-5p,novel_68,miR-6516-3p,miR-505-3p,miR-9851-3p,miR-2319a,miR-450b-3p,miR-3591-3p,miR-18a-3p,miR-216a-3p,miR-148a-3p,miR-301b-3p,miR-1307-3p,miR-3959-3p,miR-495-3p,miR-1193,miR-2285x,miR-342-3p,miR-199c,miR-379-3p,novel_1,miR-6119-3p,miR-128-1-5p,novel_87,miR-33a-3p,miR-576-3p,miR-28c,miR-142b,miR-2284h-5p,miR-148b-3p,miR-200b,miR-23c,miR-7859,miR-23b-3p,miR-505-3p,miR-18a-3p,let-7a-3p,miR-378d,miR-148a-5p,miR-33b-3p,miR-140-3p,miR-188-3p,miR-2904,miR-98-3p,miR-215-5p,miR-2300b-3p,miR-380-3p,miR-143-3p,miR-486-3p,miR-2285w,miR-329a-5p,miR-216c-5p,miR-760-3p,miR-122-3p,miR-6130,miR-656-3p,miR-192-5p,miR-544-3p,miR-301a-3p,miR-377-3p,miR-370-5p,miR-216b-3p,miR-21-3p,miR-2331-3p,miR-504-5p,miR-625-5p,miR-2312,miR-411,miR-500a-3p,miR-27b-5p,let-7f-1-3p,miR-450a-1-3p,miR-21a-3p,miR-338-3p,miR-181c-5p,miR-3184-5p,miR-122-5p,miR-98-3p,miR-

INPP5F	ENSOARG0(	inositol	miR-324-5p, miR-365a-3p, miR-34c-5p, miR-331-5p, miR-874-3p, miR-1248, miR-301, miR-488-3p, miR-381-3p, miR-1197-3p, miR-2284v, miR-1306-5p, miR-592, miR-6740-5p, miR-331-3p, miR-100-3p, miR-494-5p, novel_63, miR-2284k, miR-214-5p, novel_121, miR-582-3p, miR-450a-1-3p, miR-376a-5p, miR-429-3p, miR-6517, miR-155-5p, miR-200b-3p, miR-300, miR-330-5p, miR-2284a, miR-192-3p, miR-2284y, miR-4286, miR-152-3p, miR-200c-3p, miR-1a-2-5p, miR-127-5p, miR-450a-2-3p, miR-200c, miR-34b, miR-26b-5p, miR-655-5p, miR-379-5p, miR-376a-2-5p, miR-2319a, miR-412-3p, miR-1291, miR-450b-3p, miR-148a-3p, miR-2284w, novel_27, miR-326-3p, miR-449a, miR-24-3p, miR-135a-2-3p, miR-203b-5p, novel_1, novel_87, miR-33a-3p, miR-34c, miR-6402, miR-142b, miR-133a-5p, miR-576-3p, miR-412-5p, miR-2355-3p, miR-194-3p, miR-134-3p, miR-432-5p, miR-2284d, miR-2284x, miR-26a-2-3p, miR-3071-5p, miR-450c-3p, miR-432, miR-6240, miR-466f-3p, miR-330-3p, miR-377-3p, miR-224-5p, miR-155-5p, miR-204-3p, miR-216b-5p, miR-377-3p, miR-27a-5p, miR-499b-3p, miR-532-5p, novel_99, miR-1895, miR-487b-5p, miR-500a-3p, miR-450a-1-3p, let-7f-1-3p, miR-98-3p, miR-224-5p, miR-6525, miR-3074-2-3p, miR-500, miR-664a, miR-155-5p, miR-450b-3p, miR-3604, let-7b-3p, miR-2284h-5p, miR-200b, miR-148b-3p, miR-23c, miR-487a-3p, miR-23b-3p, miR-7859, miR-485-3p, miR-34a-5p, let-7a-3p, miR-98-3p, miR-143-3p, miR-2432, miR-142a-3p, miR-130a-5p, miR-29b-1-5p, miR-2285w, miR-21-3p, miR-34b-5p, miR-2284m, miR-500a-5p, miR-412-5p, miR-26a-5p, miR-3082-5p, miR-499a-3p, miR-378g, miR-2285u, miR-499b-5p, miR-452-3p, miR-2319b, miR-1983, miR-381-3p, miR-378j, miR-140-5p, miR-154a, miR-2284j, miR-452-5p, miR-212-3p, miR-3956-3p, miR-2447, novel_60, miR-3964, novel_82, miR-2285n, miR-411b-5p, miR-2459, miR-421, miR-216a-3p, miR-147a, miR-142-3p, miR-330-3p, novel_78, miR-
CHORDC1	ENSOARG0(	cysteine	miR-412, miR-504-5p, miR-15b-5p, miR-203-3p, miR-15a-5p, miR-322-5p, miR-412-3p, miR-195a-5p, miR-497-5p, miR-7144-5p, miR-504, miR-16b, miR-10b-3p, miR-345-3p, miR-16-5p, miR-424-5p, miR-16b, miR-16a, miR-15a, miR-21-3p, miR-424-5p

C3orf33	ENSOARG0(	chromoso	novel_1,miR-6402,miR-15a,miR-16a,miR-3959-3p,miR-24-3p,miR-211,miR-2428,novel_27,miR-374c-5p,miR-135a-2-3p,miR-1193,miR-17-3p,miR-19a-3p,miR-195a-5p,miR-2319a,miR-2285ab,miR-3065-5p,miR-21c,miR-1306,miR-670-3p,miR-411b-3p,novel_69,miR-181b-3p,miR-677,miR-320c,miR-6535,miR-3074-5p,novel_68,miR-10a-5p,miR-7-1-3p,miR-106b-3p,miR-136-3p,miR-2411-3p,miR-2284l,miR-432-3p,miR-432-3p,miR-345-5p,miR-16-1-3p,miR-219a-1-3p,miR-21-5p,miR-138,miR-30b-3p,miR-16b,miR-188-5p,miR-497-5p,miR-432,miR-708-3p,miR-374b-3p,miR-22-3p,miR-450c-3p,miR-134-3p,miR-432-5p,miR-2285g,miR-31-5p,miR-138-5p,miR-486b-3p,miR-204-5p,miR-28-5p,miR-370-5p,miR-494-5p,miR-1306-3p,miR-3969,miR-500b-5p,miR-145-3p,miR-28a-3p,miR-16-5p,miR-3607-3p,miR-345-5p,miR-10b-3p,miR-1814c,miR-671-5p,miR-1248,miR-7144-5p,miR-30e-5p,miR-320a,miR-362-5p,miR-3154,miR-769,miR-874-3p,miR-503-3p,miR-1271,miR-2285aa,miR-200c-3p,miR-28-3p,miR-17-3p,novel_96,miR-181a-2-3p,miR-3064-5p,miR-582-3p,miR-10a-5p,miR-30c,miR-200b-3p,miR-345-3p,miR-9-5p,miR-322-5p,miR-450a-1-3p,miR-545-5p,miR-6517,miR-429-3p,miR-412,miR-2285t,miR-101-3p,novel_82,miR-30b-5p,miR-218-5p,miR-301a-5p,miR-769-5p,miR-371a-5p,miR-362-5p,miR-154a,miR-9-5p,miR-2285f,miR-504,novel_94,miR-9-3p,miR-1306-3p,miR-129b-5p,miR-2483-3p,miR-489,miR-499a-3p,miR-7857-3p,miR-378g,miR-4443,miR-10b,miR-3064-5p,miR-126b-5p,miR-211-5p,miR-3955-3p,miR-3085-3p,miR-491-5p,miR-214-3p,miR-1306-5p,novel_115,miR-199a-3p,miR-320b,miR-182-5p,miR-16b,miR-2957,miR-2310,miR-8485,miR-424-5p,miR-1388-3p,miR-2426,miR-101c,miR-301b-5p,miR-10b,miR-2284r,miR-484,miR-125b-2-3p,miR-30b-3p,miR-412-3p,miR-1298-3p,miR-2459,miR-455-3p,miR-500-5p,miR-1343-3p,miR-2300a-5p,miR-

GNPAT	ENSOARG0(	glyceron	miR-6119-5p,miR-8117,miR-1260a,novel_127,miR-3064-5p,miR-28b,miR-450c-5p,miR-200a,miR-3065-3p,miR-345-3p,miR-330-5p,miR-29d-3p,miR-300,miR-411-3p,miR-128-3p,miR-105-2,miR-6517,miR-1224-5p,miR-758-5p,miR-2411-5p,miR-378h,miR-302a-5p,miR-1306-3p,miR-5100,miR-29b-3p,miR-135a-5p,miR-28-5p,miR-486b-3p,miR-133a-3p,miR-3607-3p,miR-193a-5p,miR-1260b,miR-365a-5p,miR-378c,miR-381-3p,miR-200a-5p,miR-345-5p,miR-671-5p,miR-485-5p,miR-1248,miR-7144-5p,miR-1271,miR-2285aa,miR-370-3p,miR-365a-3p,miR-300-3p,miR-324-5p,miR-377-3p,miR-296-3p,miR-224-5p,miR-194a,miR-2411-3p,miR-365b-5p,miR-330-3p,miR-2285v,miR-105-5p,miR-193a-3p,miR-6240,miR-2285r,miR-345-5p,miR-296-3p,miR-1285,miR-145a-5p,novel_23,miR-194-5p,miR-22-3p,miR-219a-1-3p,miR-1260b,miR-30b-3p,miR-29b,miR-27b-3p,miR-3955-5p,miR-26b-3p,miR-185-5p,miR-134-3p,miR-3431,miR-2285g,miR-149-5p,miR-28c,miR-1247-3p,miR-379-3p,miR-29a,miR-6119-3p,miR-22-5p,miR-29c-3p,miR-193b-3p,miR-3957-3p,miR-326-3p,novel_27,miR-216a-3p,miR-18a-3p,miR-1306,miR-3970,miR-410-5p,miR-135b-5p,miR-378f,miR-7975,miR-3074-5p,novel_68,novel_69,miR-29a-3p,miR-323a-5p,miR-2404,miR-677,miR-345-3p,miR-422a,miR-324-3p,miR-216a-5p,miR-466i-5p,miR-760-3p,miR-21-3p,miR-3653-3p,miR-105-3p,miR-29b-1-5p,miR-486-3p,miR-2411,miR-2432,miR-2408,miR-378i,miR-1434-5p,miR-378a-3p,miR-140-3p,miR-124-3p,miR-133a-3p,miR-18a-3p,miR-3600,miR-340-5p,miR-2284h-5p,miR-2285j,miR-3604,miR-665,miR-151-3p,miR-664a,miR-874-5p,miR-708-5p,miR-3184-3p,miR-3074-5p,miR-378c,miR-3958-5p,miR-224-5p,miR-6525,miR-3184-5p,miR-129b-3p,miR-345-5p,miR-133b-3p,novel_99,miR-1895,miR-140-5p,miR-378a-3p,miR-182-5p,miR-296-3p,miR-3065-3p,miR-193b-3p,miR-323-5p,miR-377-3p,miR-22-3p,miR-1306-5p,miR-326,miR-182-
SLC29A4	ENSOARG0(	solute c	miR-221-5p,miR-3085-3p,miR-760-3p,miR-324-3p,novel_51,miR-455-5p,miR-185-3p,miR-339a,miR-1306-5p,miR-486-3p,miR-3065-3p,miR-1271-3p,miR-3064-5p,miR-4286,miR-2889,miR-3120-5p,miR-455-5p,miR-29b,miR-33b-3p,miR-7134-5p,miR-29d-3p,miR-188-3p,miR-296-5p,miR-215-5p,miR-149-5p,miR-532-3p,novel_79,miR-339-5p,miR-340-3p,miR-3960,novel_4,miR-1343-3p,miR-874-5p,miR-486b-3p,miR-29a,miR-664a,miR-2433,miR-29b-3p,miR-576-3p,miR-2428,miR-2903,miR-24-3p,miR-346,miR-665-5p,miR-29c-3p,novel_60,novel_42,miR-3956-3p,miR-1895,miR-1291,miR-339b,miR-1306,miR-3154,miR-361-3p,miR-192-5p,miR-29a-3p,miR-3065-3p,miR-341-3p,miR-615-5p,miR-215-5p,novel_68,miR-221-5p,miR-3064-5p

CACFD1	ENSOARG0(	calcium	miR-10a-5p,miR-4792,miR-2319b,miR-677,miR-181b-3p,miR-378e,miR-299b-3p,miR-3591-3p,miR-361-3p,miR-378f,miR-1291,miR-504,miR-218-5p,miR-576-3p,miR-339-5p,miR-181b-2-3p,novel_78,miR-2459,miR-296-5p,miR-378b,miR-10b,miR-378c,miR-219a-1-3p,miR-668-3p,miR-378d,miR-432-3p,miR-29b-1-5p,miR-483-3p,miR-491-5p,miR-21-3p,miR-370-3p,miR-2355-5p,miR-552-3p,miR-615-5p,miR-504-5p,miR-378a-3p,miR-10a,miR-339b,miR-378c,miR-378c,miR-10b-5p,miR-331-3p,miR-665,miR-29b-2-5p,miR-2433,miR-708-5p,miR-874-5p,miR-28-5p,miR-486b-3p,novel_79,miR-299a-3p,miR-1247-5p,miR-483-3p,miR-378h,miR-378a-3p,miR-378i,miR-10a-5p,miR-29b-2-5p,miR-486-3p,miR-29b-1-5p,miR-324-3p,miR-339a,miR-422a,miR-125a-3p,miR-299-3p,novel_96
INA	ENSOARG0(	internex	miR-148a-5p,miR-27b-3p,miR-34a-5p,miR-4429,miR-2426,miR-1388-5p,miR-5703,miR-1246,novel_121,miR-221-3p,miR-148b-5p,miR-376b-3p,miR-487a-3p,miR-505-3p,miR-27a-3p,miR-485-3p,miR-222,miR-660,miR-34b-5p,miR-320d,novel_103,miR-205-5p,miR-376b-3p,miR-320b,miR-539-3p,miR-125b-2-3p,miR-505-3p,miR-376a-3p,miR-376b-3p,miR-320a,miR-221,miR-27a-3p,miR-376d,miR-320c,miR-203-3p,miR-34b,miR-769,miR-34c-5p,miR-3120-3p,miR-320b,miR-548o-3p,miR-660-5p,miR-329-5p,miR-20a-3p,miR-769-5p,miR-324-3p,miR-34c,miR-6740-5p,miR-2440,miR-15b-3p,miR-218-5p,miR-222-3p,miR-449a,miR-877-3p,miR-769-5p,miR-495-3p,miR-3059-5p,miR-219b-3p

HK3	ENSOARG0(	hexokina	let-7e-5p,miR-6395,miR-379-5p,miR-1291,miR-412-3p,miR-1306,miR-216a-3p,miR-18a-3p,miR-6535,miR-669,miR-670-3p,miR-299b-3p,miR-320b,miR-216b-3p,miR-128-1-5p,miR-382-5p,let-7i,miR-425-5p,miR-28c,miR-326-3p,miR-2428,miR-24-3p,miR-3959-3p,miR-193b-3p,miR-17-3p,miR-542-5p,miR-135a-2-3p,miR-26a-2-3p,miR-4508,miR-27b-3p,miR-1260b,miR-381-5p,miR-194-5p,miR-873a-5p,miR-2113,miR-3535,let-7g-5p,miR-339-5p,miR-122-3p,miR-1343-5p,miR-466f-3p,miR-193a-3p,miR-197-5p,miR-194a,novel_51,miR-377-3p,miR-345-5p,miR-654-3p,miR-363-5p,miR-485-5p,miR-345-5p,miR-2355-5p,miR-370-3p,miR-1271,miR-615-5p,miR-874-3p,miR-486b-3p,miR-331-3p,miR-2433,miR-1260b,miR-1197-3p,novel_42,miR-128-3p,let-7d,miR-1343-5p,miR-330-5p,miR-1290,miR-3578,miR-582-3p,miR-505-5p,miR-196a-2-3p,novel_79,miR-299a-3p,miR-6517,novel_127,miR-543-5p,miR-450a-2-3p,miR-17-3p,miR-2898,let-7f-5p,miR-30c-2-3p,miR-339a,let-7c-5p,miR-1260a,miR-125a-3p,novel_39,let-7g,miR-490-3p,miR-1271-3p,miR-28b,miR-3064-5p,miR-4286,miR-1983,miR-504,miR-668-5p,let-7d-5p,miR-454-5p,miR-27a-3p,miR-3596,miR-499a-3p,miR-7977,miR-3064-5p,miR-2319b,miR-20a-3p,miR-4726-5p,miR-423-5p,miR-194b-5p,miR-541-3p,miR-1839-5p,miR-7857,miR-2284r,miR-541,miR-4791,miR-1298-5p,miR-9788-3p,miR-2459,miR-496-5p,miR-27a-3p,miR-2300a-5p,miR-1343-3p,miR-381-5p,miR-214-3p,miR-542-5p,let-7e,miR-3085-3p,miR-3955-3p,miR-1306-5p,miR-326,miR-8485,miR-491-3p,miR-30c-1-3p,let-7a-5p,miR-1388-3p,miR-2330-5p,miR-625-5p,miR-1827,miR-345-5p,miR-339b,miR-129b-3p,miR-411,miR-877-5p,let-7i-5p,miR-151b,miR-499b-3p,miR-377-3p,miR-6130,miR-193b-3p,miR-552-3p,miR-2331-3p,miR-30c-1-3p,miR-504-5p,miR-4492,miR-874-5p,miR-17-3p,miR-664a,miR-132-5p,let-7f,miR-3184-5p,let-

FAM129A	ENSOARG0(	family w	miR-433-3p,novel_25,miR-22-3p,miR-204-3p,miR-155-5p,miR-182-5p,miR-2331-3p,miR-208b-5p,miR-130b-5p,miR-664a,miR-338-5p,miR-2285j,miR-3184-5p,miR-6134,miR-224-5p,miR-214,miR-145a-3p,miR-3074-5p,miR-148a-5p,miR-378d,miR-2284u,miR-33b-3p,miR-140-3p,miR-1197-5p,miR-2408,miR-1247-5p,miR-21-3p,miR-760-3p,miR-324-3p,novel_17,miR-2411,miR-376a-5p,miR-130a-5p,miR-195a-3p,miR-20a,miR-335-3p,miR-378g,miR-2285u,miR-7977,miR-211-5p,miR-2284g,miR-664b,miR-2319b,miR-1843a-3p,miR-423-5p,miR-361-3p,miR-452-5p,miR-2447,miR-3059-5p,miR-885-5p,miR-1388-5p,miR-101a-5p,miR-2426,miR-450b-5p,miR-30b-3p,miR-296-5p,miR-484,miR-4791,miR-148b-5p,miR-105-1,miR-1343-3p,miR-2300a-5p,miR-381-5p,miR-214-3p,miR-182-5p,miR-106b-5p,miR-431-5p,miR-193a,miR-2310,miR-744-3p,miR-652-5p,miR-2284b,miR-1388-3p,miR-20b-5p,miR-1814c,miR-10b-3p,miR-30e-5p,miR-300-3p,miR-141-3p,miR-204-5p,miR-144,miR-331-3p,miR-1260b,miR-6128,miR-1306-5p,miR-155-5p,miR-23b-5p,miR-302a-5p,miR-2411-5p,miR-1224-5p,miR-431,miR-429-3p,miR-107,miR-200a-3p,miR-2898,miR-127-5p,miR-200c-3p,miR-30f,miR-1260a,miR-2285af,miR-664-3p,miR-200c,miR-2284y,miR-1271-3p,miR-2284a,miR-28b,miR-107,miR-2387,miR-144-3p,miR-3074-5p,miR-656-5p,miR-128-1-5p,miR-382-5p,miR-502-5p,miR-4324,miR-20a-5p,miR-28c,miR-211,novel_120,miR-30b-3p,miR-381-5p,miR-1260b,miR-197-3p,miR-22-3p,miR-103,miR-3535,miR-134-3p,miR-185-5p,miR-31-5p,miR-103a-3p,miR-122-3p,miR-378a-5p,miR-105-5p,miR-106a-5p,miR-224-5p,miR-130b-5p
EGLN1	ENSOARG0(	egl-9 fa	miR-30a-3p,miR-664a-5p,miR-34c-3p,miR-30e-3p,miR-487a-5p,miR-424-5p,miR-129-5p,miR-450b-3p,miR-16-5p,miR-129-2-3p,miR-2284v,miR-93,novel_44,miR-93-5p,miR-30d-5p,miR-23b,miR-30e-5p,miR-21a-3p,miR-450a-1-3p,miR-20b-5p,let-7g-3p,miR-329b,miR-370-3p,miR-21-3p,miR-182-5p,miR-223-5p,novel_25,miR-15b-5p,miR-196a-3p,miR-376b-3p,miR-200c,miR-125a-3p,miR-30b-3p,miR-30d,miR-21-3p,miR-106a,miR-466i-5p,miR-200c-3p,miR-20a,miR-3064-5p,miR-30a-3p,miR-29a-5p,miR-143-3p,miR-29b-1-5p,miR-1343-5p,miR-330-5p,miR-30a-5p,miR-3591-5p,miR-429-3p,miR-106a,miR-450a-1-3p,miR-129-1-3p,miR-23b-3p,miR-322-5p,miR-302a-5p,miR-15a-5p,miR-16a,miR-382-5p,miR-15a,miR-576-3p,miR-20a-5p,miR-326-3p,miR-8095,miR-376b-3p,miR-3591-3p,miR-378b,miR-376a-3p,miR-195a-5p,miR-450b-3p,miR-541-5p,miR-320b,miR-3120-3p,miR-3064-5p,miR-452-3p,miR-203-3p,miR-677,miR-4443,miR-376d,miR-1185-2-3p,miR-181b-3p,miR-378g,miR-2957,miR-29b-1-5p,miR-16b,miR-182-5p,miR-106b-5p,miR-326,miR-1343-5p,miR-3085-3p,miR-382,miR-23a-3p,miR-101-5p,let-7f-2-3p,miR-376b,let-7f-2-3p,miR-424-5p,miR-106a-5p,miR-503-3p,miR-2284l,miR-450c-3p,miR-30b-3p,miR-3962,miR-873a-5p,miR-1285,miR-497-5p,miR-16b,miR-30b-3p,miR-2284ab,miR-181b-2-3p,miR-548w,miR-20b,miR-1185-3p,miR-2459,miR-2285e,miR-2285g,miR-376b-3p,miR-185-5p,miR-134-3p

NIPAL2	ENSOARGO(	NIPA-lik	miR-151-3p,miR-2284k,novel_63,miR-486b-3p,miR-664a,miR-138-5p,miR-450b-3p,miR-3607-3p,miR-2284v,miR-4510,miR-3074-5p,miR-1306-5p,miR-122-5p,miR-6525,miR-3154,miR-450a-1-3p,miR-186-5p,novel_48,miR-215-5p,miR-2285aa,miR-487a-3p,miR-376c-3p,miR-2284n,miR-204-3p,miR-6130,miR-376e-3p,miR-499b-3p,miR-345-3p,miR-92a-1-5p,miR-3071-3p,miR-2397-5p,miR-2285p,miR-26a-5p,novel_96,miR-30b-3p,miR-2284m,miR-3064-5p,miR-487a,miR-216c-5p,miR-2284a,miR-490-3p,miR-2285b,miR-2284y,miR-2411,miR-486-3p,miR-377-5p,miR-376c-3p,miR-5703,miR-23b-5p,miR-124-3p,miR-215-5p,miR-2366,novel_133,miR-345-3p,miR-378d,miR-2284u,miR-19b-3p,miR-485-3p,miR-3600,miR-2285ad,miR-671-3p,miR-2284z,miR-450a-1-3p,miR-2411-5p,miR-9-5p,miR-487a-3p,novel_87,miR-485-3p,miR-6402,miR-2285n,miR-1247-3p,novel_1,miR-2284j,miR-9-5p,miR-3956-3p,miR-2447,novel_60,miR-2428,miR-3970,miR-2387,miR-19a-3p,miR-450b-3p,miR-1291,miR-26b-5p,miR-2285u,miR-655-5p,miR-3074-5p,miR-4792,miR-2284g,miR-3064-5p,miR-3082-5p,miR-499a-3p,miR-2411-3p,novel_103,miR-491-5p,miR-3085-3p,miR-2284b,miR-628-5p,miR-124a,miR-432-3p,miR-654-3p,novel_23,miR-30b-3p,miR-197-3p,miR-450c-3p,miR-4791,miR-223-3p,miR-484,miR-7862,miR-2284x,miR-101a-5p,miR-138,miR-30b-3p,miR-1388-5p,miR-2284ab,miR-377-5p,miR-151a-3p,miR-548w,miR-544b,miR-411-5p,miR-1298-3p,miR-185-5p,miR-3535,miR-2284d

WFIKKN2	ENSOARG0(	WAP, fol	miR-145a-5p,miR-194-5p,miR-374b-3p,miR-1434-3p,miR-1285,miR-29b,miR-1260b,miR-2355-3p,miR-103,miR-2285g,miR-503-5p,miR-197-5p,miR-330-3p,miR-503-5p,miR-125a-5p,miR-124-5p,miR-363-5p,miR-130b-5p,miR-345-5p,miR-2285ab,miR-18b,miR-6395,miR-195a-5p,miR-1291,miR-107,miR-320b,miR-323a-5p,miR-146b-3p,miR-2404,miR-29a-3p,miR-16a,miR-382-5p,miR-502-5p,miR-15a,miR-6529b,miR-6402,miR-203b-5p,miR-1193,novel_120,miR-342-3p,miR-326-3p,miR-2428,miR-3959-3p,miR-24-3p,miR-29d-3p,miR-501-3p,miR-345-3p,miR-214-5p,miR-483-3p,miR-378h,miR-30c-2-3p,miR-1260a,miR-107,miR-30a-3p,miR-4286,miR-181a-2-3p,miR-3065-3p,miR-199a-5p,miR-1271-3p,miR-380-5p,miR-28b,miR-18a,miR-2448-3p,miR-7144-5p,miR-671-5p,miR-10b-3p,novel_91,miR-199a-5p,miR-365a-3p,miR-29b-3p,miR-135a-1-3p,miR-370-5p,miR-1306-3p,miR-486b-3p,miR-6128,miR-378c,miR-378b,miR-143-5p,miR-378c,miR-101a-5p,miR-5010-3p,miR-27a-3p,miR-222-5p,miR-2300a-5p,miR-9788-3p,miR-216a-3p,miR-2285e,miR-2957,miR-342,miR-326,miR-1306-5p,miR-3085-3p,miR-3955-3p,miR-376b,miR-424-5p,miR-3187-3p,miR-30c-1-3p,miR-361-3p,miR-199b-5p,miR-147-3p,miR-664b,miR-3064-5p,miR-27a-3p,miR-378e,miR-3082-5p,miR-489,miR-371a-5p,miR-7134-3p,miR-450a-5p,miR-2285f,miR-194b-5p,novel_60,miR-3059-5p,miR-502-3p,miR-219a-2-3p,miR-2904,miR-1197-5p,miR-188-3p,novel_133,miR-376a-5p,miR-2300b-3p,miR-378d,miR-3600,miR-532-3p,miR-383-5p,miR-15a-5p,miR-216a-5p,miR-125b,miR-185-3p,miR-221-5p,miR-760-3p,miR-466i-5p,miR-183-5p,miR-21a-3p,miR-2312,miR-18a-5p,miR-370-5p,miR-3065-3p,miR-532-5p,miR-221-5p,miR-545-3p,miR-378a-3p,novel_25,miR-204-3p,miR-22-3p,miR-27a-5p,miR-323-5p,miR-6130,miR-193b-3p,miR-324-3p,miR-338-5p,miR-[illegible],miR-424-5p,miR-487a-5p,miR-664a,miR-3074-2-3p,miR-

DPEP3	ENSOARG0(	dipeptid	miR-3074-2-3p,miR-214,miR-1271,let-7b,let-7b-5p,miR-346,miR-206-3p,miR-10b-5p,miR-320e,miR-29b-2-5p,miR-30a-3p,miR-324-3p,miR-30e-3p,let-7f,miR-190a-5p,miR-454-3p,miR-6516-3p,miR-17-3p,miR-155-5p,miR-450b-3p,miR-504-5p,miR-30c-1-3p,miR-2331-3p,miR-155-5p,let-7i-5p,miR-204-3p,miR-139-5p,miR-301a-3p,miR-376e-3p,miR-10a,miR-499b-3p,miR-129b-3p,miR-450a-1-3p,novel_99,miR-625-5p,miR-345-5p,miR-2312,miR-20a,miR-143-3p,miR-486-3p,miR-2424,miR-376c-3p,miR-181b-1-3p,miR-345-3p,miR-2285p,miR-210-5p,miR-299-3p,let-7k,miR-3653-3p,miR-760-3p,miR-98-5p,miR-320d,miR-106a,miR-30d-3p,miR-106a,miR-2284h-5p,miR-1193,miR-5703,miR-1961,novel_133,miR-378d,miR-148a-5p,miR-4429,miR-190b-5p,miR-2285f,miR-541-3p,miR-2447,miR-9-3p,novel_111,miR-361-3p,miR-301a-5p,novel_82,miR-335,miR-10b,miR-139-5p,miR-24-1-5p,miR-203-3p,let-7d-5p,miR-499a-3p,miR-3596,miR-9-3p,miR-335-3p,miR-136-5p,miR-504,miR-96-5p,miR-2284b,miR-106a-5p,let-7a-5p,miR-2310,miR-30c-1-3p,miR-106b-5p,miR-323b-3p,miR-326,novel_115,novel_103,miR-431-5p,miR-320b,miR-214-3p,let-7e,miR-3955-3p,miR-130b-3p,miR-181b-2-3p,novel_4,miR-330-3p,miR-148b-5p,miR-216a-3p,miR-455-3p,miR-125b-2-3p,miR-7857,miR-323b,miR-1a-3p,miR-10b,miR-541,miR-301b-5p,miR-2284r,miR-101c,miR-1839-5p,miR-2284aa,miR-1388-5p,miR-28a-3p,miR-145-3p,miR-1260b,miR-323-3p,miR-93,novel_63,miR-486b-3p,miR-3432a,miR-376c-3p,miR-376c-5p,miR-17-5p,miR-3154,miR-320a,miR-93-5p,miR-671-5p,miR-1814c,miR-7144-5p,miR-20b-5p,miR-376c-5p,miR-23a-5p,novel_91,miR-345-5p,miR-2318,miR-876-3p,miR-10b-3p,miR-181a-2-3p,miR-4286,miR-29b-2-5p,miR-1,miR-30a-3p,miR-1271-3p,miR-490-3p,miR-2284a,let-7g,miR-2284y,novel_39,miR-24-2-5p,let-7c-5p,miR-30c-2-3p,miR-
TRAV10	ENSOARG0(	T cell r	miR-3962,miR-2366,miR-484,miR-378d,miR-1291,miR-10b-3p,miR-330-3p,miR-6517,miR-4792,miR-331-3p,miR-2957,miR-330-3p,miR-125a-3p,miR-6402,miR-378a-5p,miR-450b-3p,miR-1185-5p,miR-2330-3p,miR-3956-3p,miR-6240,novel_60,miR-7705,miR-1271-3p,miR-654-3p

RNF121	ENSOARG0(	ring fin	miR-486-3p,miR-503-5p,miR-345-3p,miR-760-3p,miR-4661-5p,miR-320d,miR-21-3p,miR-299-3p,miR-221-5p,miR-210-5p,miR-125b,miR-185-3p,miR-324-3p,miR-500a-5p,miR-216a-5p,miR-7859,miR-1247-5p,miR-18a-3p,miR-548e-3p,miR-505-3p,miR-30d-3p,miR-212-5p,miR-3600,miR-1b-3p,miR-4429,miR-941,miR-378d,miR-218-1-3p,miR-2366,miR-2300b-3p,miR-140-3p,miR-1197-5p,miR-10b-5p,miR-6134,miR-145a-3p,miR-3074-5p,miR-665-5p,miR-214,miR-664a,novel_74,miR-874-5p,miR-500,miR-708-5p,miR-4492,miR-30e-3p,miR-665,miR-320e,miR-324-3p,miR-132-5p,miR-30a-3p,miR-193b-3p,miR-377-3p,miR-10a,miR-27a-5p,miR-2284s,novel_32,miR-22-3p,miR-204-3p,miR-32-3p,miR-140-5p,miR-30c-1-3p,miR-2331-3p,miR-221-5p,miR-345-5p,miR-500a-3p,miR-129b-3p,miR-30c-1-3p,miR-8485,miR-2330-5p,miR-127-3p,miR-574-3p,miR-1388-3p,miR-3085-3p,miR-1185-5p,miR-491-5p,miR-103a-2-5p,miR-214-3p,miR-199b-5p,miR-1306-5p,miR-326,miR-320b,miR-342,miR-9788-3p,novel_4,miR-2300a-5p,miR-145b,miR-2426,miR-10b,miR-301b-5p,miR-2284r,miR-484,miR-30b-3p,miR-877-3p,miR-1285-5p,miR-3956-3p,novel_60,miR-32-3p,miR-2447,miR-3059-5p,miR-3968,miR-4726-5p,novel_82,miR-2285n,miR-107-5p,miR-301a-5p,miR-378g,miR-203-3p,miR-452-3p,miR-20a-3p,miR-3064-5p,miR-7977,novel_94,miR-199b-5p,miR-361-3p,miR-28b,miR-148b-5p,miR-199a-5p,novel_39,miR-4286,miR-181a-2-3p,miR-3064-5p,miR-30a-3p,novel_96,miR-543-5p,novel_127,miR-6119-5p,miR-125a-3p,miR-1260a,miR-2285af,miR-30c-2-3p,miR-134-5p,miR-6517,miR-299a-3p,miR-1224-5p,miR-411-3p,miR-10a-5p,miR-134,miR-3578,miR-23b-5p,miR-330-5p,miR-29d-3p,miR-1260b,miR-1197-3p,miR-365a-5p,miR-193a-5p,miR-6128,miR-138-5p,miR-28-5p,miR-134-5p,miR-486b-3p,miR-592,miR-29b-3p,miR-409-3p,miR-331-3p,miR-125b-5p,miR-154b-5p,miR-

PDGFD	ENSOARG0(	platelet	miR-7859,miR-487a-3p,miR-222-5p,miR-15a-5p,miR-532-3p,miR-2284h-5p,miR-106a,miR-30d-3p,miR-485-3p,miR-4429,miR-361-5p,miR-378i,miR-2300b-3p,miR-124-3p,miR-378a-3p,miR-1193,miR-217-5p,miR-188-3p,miR-5703,miR-377-5p,miR-2424,miR-183-5p,miR-329a-5p,miR-2285w,miR-20a,miR-106a,miR-320d,miR-30b-3p,miR-1277-5p,miR-2285p,miR-618,miR-500a-5p,miR-125b,miR-422a,miR-6516,miR-301a-3p,novel_25,miR-378a-3p,miR-496-3p,miR-532-5p,miR-552-3p,miR-133b-3p,let-7g-3p,miR-18a-5p,novel_99,miR-27b-5p,miR-500a-3p,miR-6525,miR-206-3p,miR-433-5p,miR-378c,miR-2330-3p,miR-6516-3p,miR-487b-5p,miR-17-3p,miR-454-3p,miR-500,miR-874-5p,miR-208b-5p,miR-130b-5p,miR-15b-3p,miR-2285j,miR-222-3p,miR-30e-3p,miR-30a-3p,miR-148b-5p,miR-500-5p,miR-221-3p,miR-105-1,novel_78,miR-330-3p,miR-378c,miR-1388-5p,miR-2284aa,miR-378b,miR-296-5p,miR-1298-5p,miR-1a-3p,miR-125b-2-3p,miR-30b-3p,miR-378d,miR-124a,miR-106a-5p,miR-424-5p,miR-1185-5p,miR-130b-3p,miR-3955-3p,miR-103a-2-5p,miR-320b,miR-505,miR-106b-5p,miR-16b,miR-378e,miR-3596,miR-378g,miR-3082-5p,miR-203-3p,miR-664b,miR-4792,miR-452-3p,miR-211-5p,miR-136-5p,miR-361-3p,miR-221,miR-378j,miR-361-3p,miR-362-5p,novel_111,miR-1296-5p,miR-450a-5p,miR-335,miR-374a-3p,miR-2285n,miR-2440,miR-2285y,miR-107-5p,miR-322-5p,miR-505-5p,miR-6516-5p,miR-378h,miR-2284z,novel_121,miR-2285ad,miR-222,miR-18b-5p,miR-105-2,miR-496,miR-1290,miR-29d-3p,miR-1957a,miR-200a,miR-450c-5p,miR-2285b,miR-30a-3p,miR-1,miR-2284f,miR-450a-2-3p,miR-17-3p,miR-1a-2-5p,miR-200a-3p,miR-543-5p,miR-6119-5p,miR-664-3p,miR-125a-3p,miR-217,miR-3965,miR-133c,miR-503-3p,miR-370-3p,miR-141-3p,miR-125a,miR-329b,miR-20b-5p,miR-488-3p,miR-93-5p,miR-3154,miR-18a,miR-376c-5p,miR-
DPEP2	ENSOARG0(	dipeptid	miR-210-5p,miR-217,miR-125b,miR-760-3p,miR-214-3p,miR-125a-5p,miR-494-3p,miR-4286,miR-654-3p,miR-664b-3p,miR-2432,miR-2113,miR-217-5p,miR-1197-5p,miR-450c-3p,miR-133a-3p,miR-151a-3p,miR-1343-3p,miR-505-5p,miR-149-5p,miR-411-5p,miR-216a-3p,miR-532-3p,miR-450a-1-3p,miR-194-3p,miR-218-5p,miR-324-3p,miR-151-3p,miR-125b-5p,miR-450b-3p,miR-2285t,miR-874-5p,miR-4492,miR-3074-5p,miR-214,miR-346,miR-145-3p,miR-450a-1-3p,miR-488-3p,miR-379-5p,miR-625-5p,miR-3074-5p,miR-4792,novel_68,miR-125a,miR-370-3p,miR-489,miR-670-3p,miR-154b-5p,miR-6535
EXOC2	ENSOARG0(	exocyst	miR-380-5p,miR-4443,miR-486-3p,miR-133c,miR-330-3p,miR-6240,miR-380-5p,miR-3120-3p,miR-760-3p,miR-486b-3p,miR-543-5p,miR-6529b,miR-2448-3p,miR-21a-3p,miR-330-3p

STX12	ENSOARG0(	syntaxin	miR-17-3p, miR-6536, miR-200c-3p, miR-30b-3p, miR-196a-3p, miR-200c, miR-30c-2-3p, miR-122-3p, miR-142a-3p, miR-486-3p, miR-3529-3p, miR-1, miR-195a-3p, miR-300, miR-200b-3p, miR-105-2, miR-5703, miR-200b, miR-2284z, miR-2284h-5p, miR-2285ad, miR-429-3p, miR-196a-2-3p, miR-664a, miR-17-3p, miR-486b-3p, miR-5100, miR-135a-1-3p, miR-370-5p, miR-409-3p, miR-338-5p, miR-206-3p, miR-539-5p, miR-425-5p, miR-30f, miR-582-5p, miR-299, miR-381-3p, miR-30e-5p, miR-582-5p, miR-365a-3p, miR-30c-1-3p, miR-370-5p, miR-552-3p, miR-330-3p, miR-105-5p, miR-30c-1-3p, miR-6240, let-7f-2-3p, let-7f-2-3p, miR-1a-3p, miR-142-3p, miR-126a-5p, miR-1185-3p, miR-31-5p, miR-105-1, miR-330-3p, miR-628-3p, miR-206, miR-2285t, miR-2284q, miR-382-3p, miR-877-3p, novel_111, novel_27, miR-2428, miR-212-3p, miR-1193, miR-17-3p, miR-7857-3p, miR-1185-2-3p, miR-4443, miR-320b
DHRS7C	ENSOARG0(	dehydrog	[illegible] 2284x, miR-103a-3p, miR-339-5p, miR-149-5p, miR-628-5p, miR-668-3p, miR-432-3p, miR-432-3p, let-7a-2-3p, miR-3065-5p, miR-216a-3p, miR-1306, miR-378f, miR-4324, miR-425-5p, miR-206, miR-335-5p, miR-301b-3p, miR-23b-5p, miR-330-5p, novel_79, miR-505-5p, novel_121, miR-134-5p, miR-2284z, miR-219-3p, miR-30f, miR-6536, novel_127, miR-3064-5p, miR-2285b, novel_39, miR-200a, miR-345-5p, miR-876-3p, miR-874-3p, miR-1271, miR-3956, miR-125a, miR-300-3p, miR-2284k, novel_63, miR-125b-5p, novel_9, miR-134-5p, miR-541, miR-301b-5p, miR-1a-3p, miR-1388-5p, miR-1343-3p, miR-1185-3p, miR-455-3p, miR-148b-5p, miR-199a-3p, miR-214-3p, miR-2284b, miR-378d, miR-8485, miR-504, miR-335-3p, miR-2284g, miR-211-5p, miR-2483-3p, miR-4443, miR-1185-2-3p, miR-218-5p, novel_82, miR-301a-5p, miR-3956-3p, miR-885-5p, miR-2447, miR-2284j, miR-361-3p, miR-423-5p, miR-378i, miR-378a-3p, miR-142-3p, miR-33b-3p, miR-493-3p, miR-148a-5p, miR-18a-3p, miR-422a, miR-503-5p, miR-421-5p, miR-664b-3p, miR-376a-5p, miR-2285c, miR-339b, miR-345-5p, miR-1827, miR-30c-1-3p, miR-504-5p, miR-2331-3p, miR-301a-3p, miR-582-5p, miR-151b, miR-15b-3p, miR-151-3p, miR-219b-3p, miR-130b-5p, miR-4492, miR-454-3p, miR-214, miR-769-5p, miR-3184-5p, miR-653, miR-374b-3p, miR-3071-5p, miR-138, miR-2284ab, miR-2889, miR-503-5p, miR-185-5p, miR-432-5p, miR-2285g, miR-103, miR-503-5p, miR-197-5p, miR-125a-5p, miR-130b-5p, miR-101-5p, miR-345-5p, miR-2387, miR-2285ab, miR-18a-3p, miR-107, miR-19a-3p, miR-505-3p, miR-6516-3p, miR-2404, miR-6402, miR-147-5p, miR-502b, miR-203b-5p, miR-17-3p, miR-3959-3p, miR-211, miR-2428, miR-326-3p, miR-134, miR-3578, let-7a-2-3p, miR-128-3p, miR-378h, miR-2411-5p, miR-339a, miR-30c-2-3p, miR-17-3p, miR-107, miR-3529-3p, miR-1, miR-2284a, miR-490-

ZNF667	ENSOARG0(	zinc fin	miR-452-3p,miR-664b,miR-211-5p,miR-541-5p,miR-7689-3p,miR-3082-5p,miR-2483-3p,miR-7857-3p,miR-27a-3p,miR-4443,miR-199b-5p,miR-299b-5p,miR-544-5p,miR-9-3p,miR-335-3p,miR-493-5p,miR-541-3p,miR-3059-5p,miR-362-5p,miR-154a,miR-628-3p,miR-1343-3p,miR-199b-3p,miR-2300a-5p,miR-330-3p,miR-145b,miR-27a-3p,miR-147a,miR-421,miR-9788-3p,miR-500-5p,miR-455-3p,miR-541,miR-484,miR-6529a,miR-30b-3p,miR-125b-2-3p,miR-101a-5p,miR-450b-5p,miR-101c,miR-382-3p,miR-424-5p,miR-2330-5p,miR-744-3p,miR-23a-3p,miR-2310,miR-4532,novel_115,miR-320b,miR-182-5p,miR-16b,miR-29b-1-5p,miR-491-5p,miR-542-5p,miR-199b-5p,miR-221-5p,miR-296-3p,miR-182-5p,miR-532-5p,miR-21-3p,miR-582-5p,miR-192-5p,miR-15b-5p,miR-3613-5p,miR-339b,miR-582-5p,miR-2330-3p,miR-224-5p,miR-664a,miR-6516-3p,miR-424-5p,miR-485-3p,miR-212-5p,miR-3600,miR-15a-5p,miR-23b-3p,miR-487a-3p,miR-7859,miR-2284h-5p,miR-199a-3p,miR-215-5p,miR-1246,miR-1434-5p,miR-5703,miR-2904,miR-4429,miR-33b-3p,miR-2285w,miR-329a-5p,miR-183-5p,miR-2887,miR-29b-1-5p,miR-2424,miR-486-3p,miR-542-5p,miR-26a-5p,miR-92a-1-5p,miR-345-3p,miR-324-3p,miR-500a-5p,miR-466i-5p,miR-320d,miR-2484,miR-21-3p,miR-221-5p,miR-216b-3p,miR-329-5p,miR-26b-5p,miR-374c-3p,miR-670-3p,miR-144-3p,miR-320c,miR-669,miR-2387,miR-3591-3p,miR-150-5p,miR-195a-5p,miR-542-5p,miR-3957-3p,miR-211,miR-199b-5p,miR-2428,miR-15a,miR-3613,novel_87,miR-16a,novel_1,miR-6119-3p,miR-199c,miR-31-5p,miR-339-5p,miR-3431,novel_116,miR-145a-5p,miR-374b-3p,miR-30b-3p,miR-16b,miR-188-5p,miR-27b-3p,miR-497-5p,miR-26a-2-3p,miR-493-5p,miR-6240,miR-668-3p,miR-432-3p,miR-432-3p,miR-654-3p,miR-503-3p,miR-539-3p,miR-296-3p,miR-296-3p,miR-224-5p,miR-2411-3p,novel_51,miR-330-3p,miR-483-3p,miR-1343-5p,miR-215-
TRIM2	ENSOARG0(	triparti	miR-2284g,miR-145b,miR-2284b,miR-2320-3p,miR-2284y,novel_25,miR-145-3p,miR-2284a,miR-145a-5p,miR-2284x,miR-2284u
LUC7L3	ENSOARG0(	LUC7-lik	miR-490-5p,miR-6402,miR-500b-5p,miR-362-5p,miR-335-3p,miR-362-5p,miR-3591-3p,miR-301,miR-299b-3p,miR-300-3p,miR-3120-3p,miR-21-3p,miR-20a-3p,miR-378b,miR-221-5p,novel_68,miR-299-3p,miR-221-5p,miR-217,miR-455-5p,miR-500a-5p,miR-197-5p,miR-2285af,miR-192-3p,let-7j,miR-652-5p,miR-455-5p,miR-218-1-3p,miR-217-5p,miR-149-5p,miR-500-5p,miR-299a-3p
ZBTB7B	ENSOARG0(	zinc fin	miR-432-3p,miR-30c-1-3p,miR-486-3p,miR-30c-2-3p,miR-324-3p,miR-3141,miR-760-3p,miR-2285l,miR-25-5p,miR-1277-5p,miR-30b-3p,miR-340-3p,miR-412,miR-1298-3p,miR-9-5p,miR-532-3p,miR-5703,miR-1197-5p,miR-412-3p,miR-2904,miR-2889,miR-133a-3p,miR-665-5p,miR-9-5p,miR-133a-3p,miR-103b,miR-6128,miR-361-3p,miR-3184-5p,miR-423-5p,miR-2285n,novel_63,miR-665,miR-100-3p,miR-324-3p,miR-486b-3p,miR-2284q,miR-208b-5p,miR-4492,miR-30c-1-3p,miR-2355-5p,novel_69,miR-6535,miR-3141,miR-361-3p,miR-3154,miR-137-3p,miR-133b-3p,novel_94,miR-7144-5p

NFATC3	ENSOARG0(	nuclear	miR-541-3p,miR-3059-5p,miR-194b-5p,miR-502-3p,miR-30b-5p,miR-382-3p,miR-2285n,miR-107-5p,miR-4726-5p,miR-20a-3p,miR-3064-5p,miR-7977,miR-7857-3p,miR-376d,miR-199b-5p,miR-361-3p,miR-877-3p,miR-1839-3p,miR-382-3p,miR-494-3p,miR-424-5p,miR-2330-5p,miR-376b,miR-193a,miR-3187-3p,miR-326,miR-1306-5p,miR-320b,miR-25-5p,miR-3085-3p,miR-105-1,novel_4,miR-2300a-5p,miR-221-3p,miR-9788-3p,miR-216a-3p,miR-30b-3p,miR-2426,miR-2284aa,miR-4510,miR-145a-3p,miR-3074-2-3p,miR-1271,miR-206-3p,miR-6134,miR-222-3p,miR-320e,miR-664a,miR-17-3p,miR-487a-5p,miR-424-5p,miR-221-5p,miR-545-3p,miR-370-5p,miR-3065-3p,miR-323-5p,miR-6130,miR-192-5p,miR-22-3p,miR-21a-3p,miR-30f,miR-2411,miR-196a-3p,miR-185-3p,miR-125b,miR-216a-5p,miR-320d,miR-21-3p,miR-30d,miR-30b-3p,miR-221-5p,miR-505-3p,miR-340-5p,miR-15a-5p,miR-148b-3p,novel_83,miR-532-3p,miR-215-5p,miR-1197-5p,miR-140-3p,miR-2904,miR-4429,miR-378d,miR-219b-3p,miR-495-3p,miR-17-3p,miR-1193,miR-371b-3p,miR-211,miR-2428,novel_27,miR-326-3p,miR-15a,miR-16a,miR-203b-5p,miR-378b,miR-29a-3p,miR-670-3p,miR-323a-5p,miR-376b-3p,miR-18a-3p,miR-378b,miR-1291,miR-505-3p,miR-195a-5p,miR-379-5p,miR-9851-3p,miR-363-5p,miR-503-5p,miR-330-3p,miR-466f-3p,miR-125a-5p,miR-26b-3p,miR-503-5p,miR-185-5p,miR-432-5p,miR-653,miR-3071-5p,miR-194-5p,miR-1260b,miR-29b,miR-30b-3p,miR-6128,miR-3607-3p,miR-145-3p,miR-365a-5p,miR-2332,miR-370-5p,miR-135a-1-3p,miR-29b-3p,miR-1-5p,miR-3969,miR-486b-3p,miR-204-5p,miR-31-3p,miR-141-3p,miR-215-5p,miR-370-3p,miR-199a-5p,miR-488-3p,miR-381-3p,miR-320a,miR-10b-3p,miR-7144-5p,miR-4286,miR-181a-2-3p,miR-1,miR-30a-3p,miR-28b,miR-24-2-5p,miR-490-3p,miR-1271-3p,miR-199a-5p,miR-1957a,miR-3065-3p,miR-376b-3p,miR-

FNIP2	ENSOARG0(	follicul	miR-3959-3p,miR-2428,miR-2285x,miR-495-3p,miR-92a-3p,miR-133a-5p,miR-6529b,miR-15a,let-7i,miR-16a,miR-670-3p,miR-2404,miR-7-1-3p,miR-320b,miR-19a-3p,miR-195a-5p,miR-7975,miR-2387,miR-376b-3p,miR-539-3p,miR-130b-5p,miR-1343-5p,miR-503-5p,miR-224-5p,miR-330-3p,let-7g-5p,miR-503-5p,miR-133b-5p,miR-377-5p,miR-219a-1-3p,miR-1260b,miR-21-5p,miR-138,miR-181a-5p,miR-653,miR-374b-3p,miR-133a-3p,miR-6128,miR-3607-3p,miR-138-5p,miR-28-5p,miR-486b-3p,miR-99b-3p,miR-3969,miR-365a-3p,miR-2284n,miR-487a-3p,miR-141-3p,miR-215-5p,miR-370-3p,miR-10b-3p,miR-671-5p,miR-488-3p,miR-320a,miR-3154,miR-28b,miR-490-3p,miR-1271-3p,miR-3065-3p,miR-191-3p,miR-181b-5p,miR-450a-2-3p,miR-92a-3p,miR-1260a,miR-376b-3p,let-7c-5p,miR-30c-2-3p,miR-6516-5p,miR-483-3p,miR-214-5p,miR-545-5p,miR-222,miR-196a-2-3p,let-7d,miR-411-3p,miR-496,miR-345-3p,miR-218-2-3p,miR-8095,miR-9-3p,miR-541-3p,miR-212-3p,novel_60,miR-452-5p,miR-181d-5p,miR-4726-5p,miR-2284q,miR-371a-5p,miR-3082-5p,miR-489,miR-376d,let-7d-5p,miR-452-3p,miR-7977,miR-499b-5p,miR-541-5p,miR-361-3p,miR-30c-1-3p,miR-424-5p,let-7a-5p,miR-652-5p,let-7c-3p,miR-3955-3p,let-7e,miR-2285o,novel_103,miR-320b,miR-362-3p,miR-2957,miR-2320-3p,miR-216a-3p,miR-221-3p,miR-2300a-5p,miR-544a,miR-2284aa,miR-296-5p,miR-125b-2-3p,miR-6134,miR-338-3p,let-7b-5p,miR-145a-3p,miR-3074-2-3p,miR-2330-3p,miR-155-5p,miR-664a,miR-450b-3p,miR-424-5p,miR-708-5p,miR-222-3p,miR-320e,miR-324-3p,miR-132-5p,miR-544-3p,miR-192-5p,miR-6516,miR-155-5p,novel_32,miR-22-3p,miR-204-3p,miR-496-3p,miR-545-3p,miR-21-3p,miR-3065-3p,miR-154b-3p,let-7g-3p,miR-133b-3p,novel_99,let-7f-1-3p,miR-27b-5p,miR-125b-2-3p,miR-2284m,miR-320d,miR-21-3p,miR-30b-3p,miR-196a-3p,miR-185-
DCST2	ENSOARG0(	DC-STAMP	miR-1827,let-7g-3p,let-7i-5p,miR-22-3p,miR-6130,miR-486b-3p,miR-3432a,let-7f,let-7b-5p,miR-2330-3p,miR-103b,miR-3074-2-3p,miR-4510,let-7b,miR-378d,miR-33b-3p,let-7d,miR-330-5p,miR-501-3p,miR-2366,miR-1961,miR-2411-5p,miR-1224-5p,novel_96,miR-543-5p,let-7k,let-7f-5p,miR-760-3p,miR-98-5p,let-7c-5p,miR-185-3p,miR-125a-3p,miR-2411,let-7g,miR-486-3p,novel_39,let-7e-5p,miR-493-5p,miR-1291,miR-361-3p,miR-500-3p,let-7d-5p,novel_69,miR-25,miR-378g,miR-320b,let-7i,miR-502-3p,miR-326-3p,miR-361-3p,miR-541-3p,miR-7862,miR-744-5p,miR-4508,miR-501-3p,miR-22-3p,miR-1843b-5p,miR-541,miR-1434-3p,miR-653,miR-9788-3p,miR-134-3p,let-7g-5p,novel_78,let-7e,miR-491-5p,novel_51,miR-197-5p,miR-326,miR-4532,miR-628-5p,miR-493-5p,let-7a-5p
RFC5	ENSOARG0(	replicat	miR-153-3p,miR-30d,miR-30b,miR-7-5p,miR-654-3p,miR-664b-3p,miR-8485,miR-758-3p,let-7a-2-3p,miR-668-3p,miR-885-3p,miR-133a-3p,miR-30c,miR-101c,let-7a-2-3p,miR-19b-2-5p,miR-30c-5p,miR-134,miR-7b-5p,miR-758-3p,miR-30a-5p,miR-7859,miR-153,miR-134-5p,miR-134-5p,miR-4324,miR-30b-5p,miR-101-3p,miR-19b-1-5p,miR-346,miR-338-3p,let-7g-3p,miR-30f,miR-885-3p,miR-30e-5p,miR-30d-5p,miR-2355-5p

HNRNPH1	ENSOARG0(	heteroge	miR-6536,miR-124-5p,miR-4726-5p,miR-320d,miR-502b,miR-2285p,miR-320b,miR-409-3p,miR-2331-5p,miR-100-3p,miR-29a-5p,miR-382,miR-4429,miR-380-3p,miR-200b-3p,miR-188-3p,miR-320a,miR-200b,miR-532-3p,miR-320c,miR-548o-3p,miR-216b-3p,miR-548e-3p,miR-212-5p
MME	ENSOARG0(	membrane	miR-301a-5p,miR-100-3p,miR-301b-5p,miR-1285,novel_82,miR-664b-3p
C11orf84	ENSOARG0(	chromoso	miR-362-5p,miR-154a,miR-8095,miR-212-3p,novel_60,miR-3059-5p,miR-450a-5p,miR-3958-3p,miR-30b-5p,miR-489,miR-378e,miR-27a-3p,miR-452-3p,miR-20a-3p,miR-3064-5p,miR-7977,miR-541-5p,novel_94,miR-361-3p,miR-30c-1-3p,miR-193a,miR-424-5p,let-7c-3p,miR-3955-3p,miR-3085-3p,novel_103,miR-326,miR-320b,miR-2957,miR-362-3p,miR-9788-3p,miR-500-5p,miR-216a-3p,miR-27a-3p,miR-222-5p,miR-544a,miR-101a-5p,miR-378c,miR-3120-5p,miR-484,miR-378b,miR-6529a,miR-296-5p,miR-6134,miR-145a-3p,miR-378c,miR-664a,miR-17-3p,miR-708-5p,miR-208b-5p,miR-424-5p,miR-320e,miR-19b-1-5p,miR-324-3p,miR-193b-3p,miR-139-5p,miR-27a-5p,miR-544-3p,miR-877-5p,miR-22-3p,novel_25,miR-660-5p,miR-378a-3p,miR-182-5p,miR-30f,miR-18a-5p,miR-582-5p,novel_99,miR-125b-2-3p,miR-5100,miR-760-3p,miR-466i-5p,miR-320d,miR-299-3p,miR-21-3p,miR-34b-5p,miR-30d,miR-125b,miR-185-3p,miR-148b-3p,miR-15a-5p,miR-671-3p,miR-532-3p,miR-4429,miR-19b-3p,miR-378d,miR-362-3p,miR-2300b-3p,novel_133,miR-1197-5p,miR-1193,miR-140-3p,miR-24-3p,miR-2428,miR-449a,miR-326-3p,miR-17-3p,miR-199c,miR-203b-5p,miR-6402,miR-6529b,miR-502-5p,miR-15a,miR-34c,miR-16a,miR-670-3p,miR-34b,miR-2404,miR-378b,miR-320b,miR-19a-3p,miR-195a-5p,miR-6395,miR-2387,miR-18b,miR-378b,miR-18a-3p,miR-296-3p,miR-130b-5p,miR-363-5p,miR-124-5p,miR-466f-3p,miR-125a-5p,miR-660,miR-224-5p,miR-330-3p,miR-185-5p,miR-544b,miR-6238,miR-133b-5p,miR-130a-3p,miR-665,miR-1260b,miR-138,miR-7862,miR-2889,miR-500b-5p,miR-145-3p,miR-378c,miR-138-5p,miR-28-5p,miR-486b-3p,miR-135a-1-3p,miR-409-3p,miR-331-3p,miR-365a-3p,miR-34c-5p,miR-324-5p,miR-615-5p,miR-370-3p,miR-485-5p,miR-488-3p,miR-320a,miR-18a,miR-362-5p,miR-3154,miR-28b,miR-1271-3p,miR-
ZIM2	ENSOARG0(	zinc fin	miR-1827,let-7e-5p,miR-137-3p,miR-378b,let-7d-5p,let-7i-5p,miR-155-5p,novel_69,miR-320b,miR-141-3p,miR-3064-5p,miR-130b-5p,miR-147-5p,miR-138-5p,miR-450b-3p,miR-155-5p,miR-301a-5p,let-7i,miR-107-5p,let-7f,miR-133a-5p,let-7b-5p,miR-452-5p,let-7b,miR-1839-5p,miR-155-5p,miR-138,let-7d,miR-142-3p,miR-188-3p,miR-484,miR-301b-5p,miR-1961,miR-450a-1-3p,miR-421,let-7g-5p,miR-106b-3p,miR-200a-3p,miR-103a-2-5p,let-7k,let-7e,miR-3085-3p,let-7f-5p,miR-98-5p,miR-505,let-7c-5p,let-7g,miR-200a,miR-654-3p,miR-432-3p,miR-3064-5p,let-7a-5p,miR-130b-5p

RALGAPA2	ENSOARGO(	Ral GTPa	miR-181a-2-3p,miR-193a-5p,miR-144-5p,miR-374a-3p,miR-145-3p,novel_63,miR-135a-1-3p,miR-370-5p,miR-3969,miR-134-5p,miR-374b-3p,miR-215-5p,miR-3965,miR-23b,miR-3154,miR-3074-1-3p,miR-485-5p,miR-2284f,miR-2284y,miR-2284a,miR-490-3p,miR-664-3p,miR-17-3p,miR-2898,miR-107,miR-2285ad,miR-6517,miR-483-3p,miR-134-5p,novel_121,miR-214-5p,miR-134,miR-1343-5p,miR-330-5p,miR-128-3p,miR-17-3p,miR-1193,miR-24-3p,miR-326-3p,miR-4324,miR-576-3p,miR-2284o,miR-3074-5p,miR-2404,miR-374c-3p,miR-677,miR-6535,miR-181b-3p,miR-21c,miR-216a-3p,miR-450b-3p,miR-107,miR-6240,miR-377-3p,miR-330-3p,miR-2331-5p,miR-483-3p,miR-1343-5p,miR-133b-5p,miR-103a-3p,miR-432-5p,miR-134-3p,miR-185-5p,miR-149-5p,miR-103,miR-432,miR-1843b-5p,miR-542-3p,miR-21-5p,miR-27b-3p,miR-539-5p,miR-4510,miR-3074-5p,miR-665-5p,miR-3074-2-3p,miR-132-5p,miR-665,miR-17-3p,miR-664a,miR-370-5p,miR-192-5p,miR-377-3p,miR-6130,miR-139-5p,miR-204-3p,miR-1895,miR-376a-5p,miR-181b-1-3p,miR-3653-3p,miR-21-3p,miR-23b-3p,miR-383-5p,miR-222-5p,miR-23c,miR-2366,miR-215-5p,miR-140-3p,miR-1434-5p,miR-2284u,miR-378d,miR-2447,miR-212-3p,miR-1296-5p,miR-877-3p,miR-361-3p,novel_82,miR-2285n,miR-2285y,miR-4726-5p,miR-3968,miR-374a-3p,miR-664b,miR-139-5p,miR-4792,miR-2284g,miR-452-3p,miR-2285u,miR-378g,miR-144-5p,miR-27a-3p,miR-4443,miR-361-3p,miR-877-3p,miR-382-3p,miR-23a-3p,miR-2284b,miR-326,miR-330-3p,miR-181b-2-3p,miR-27a-3p,miR-2459,miR-421,miR-6529a,miR-484,miR-125b-2-3p
WASF2	ENSOARGO(	WAS prot	miR-211,miR-8095,miR-212-3p,miR-9-5p,miR-452-5p,miR-4726-5p,novel_1,miR-218-5p,miR-2285y,miR-378g,novel_69,miR-4443,miR-541-5p,miR-211-5p,miR-10a-5p,miR-7977,miR-504,miR-9851-3p,miR-148a-3p,miR-2387,miR-124a,miR-2330-5p,miR-6240,miR-214-3p,miR-7-5p,novel_103,miR-330-3p,novel_51,miR-432-5p,miR-134-3p,miR-3955-5p,miR-330-3p,miR-1343-3p,miR-2300a-5p,miR-30b-3p,miR-1388-5p,miR-2426,miR-432,miR-7b-5p,miR-10b,miR-30b-3p,miR-10b-5p,miR-122-5p,miR-4510,miR-193a-5p,miR-214,miR-665-5p,miR-204-5p,miR-331-5p,miR-10a,miR-6130,novel_32,miR-574-5p,miR-504-5p,miR-2474,miR-625-5p,miR-1827,miR-671-5p,miR-21a-3p,miR-760-3p,miR-543-5p,miR-152-3p,miR-196a-3p,miR-9-5p,miR-148b-3p,miR-1224-5p,miR-196a-2-3p,miR-10a-5p,novel_133,miR-124-3p
STK32A	ENSOARGO(	serine/t	miR-194a,miR-505,miR-219b-5p,miR-6119-5p,miR-200a-3p,miR-378a-5p,miR-124-5p,miR-23a-3p,miR-382-3p,miR-130b-5p,miR-3071-5p,miR-194-5p,miR-30b-3p,miR-30a-5p,miR-1193,miR-873a-5p,miR-296-5p,miR-3120-5p,miR-27b-3p,miR-133a-3p,miR-128-3p,miR-30b-3p,miR-885-3p,miR-27a-3p,miR-3600,miR-1224-5p,miR-23b-3p,miR-320e,miR-370-5p,miR-130b-5p,miR-495-3p,miR-194b-5p,miR-1193,miR-6527,miR-3059-5p,novel_27,miR-216a-3p,miR-23b,miR-30d-5p,miR-30e-5p,miR-885-3p,miR-625-5p,miR-2318,miR-141-3p,miR-370-5p,miR-27a-3p

LAP3	ENSOARG0(	leucine	miR-2355-3p,miR-134-3p,miR-185-5p,miR-3431,miR-3535,miR-2284d,miR-339-5p,miR-27b-3p,miR-188-5p,miR-16-1-3p,miR-2284ab,miR-29b,miR-194-5p,miR-1285,miR-1434-3p,miR-345-5p,miR-432-3p,miR-2284l,miR-2285r,miR-432-3p,miR-493-5p,miR-380-5p,miR-378a-5p,miR-194a,novel_51,miR-32,miR-146a,miR-181b-3p,miR-677,miR-299b-3p,miR-670-3p,miR-29a-3p,miR-2404,miR-3120-3p,miR-3074-5p,miR-329-5p,miR-6516-3p,miR-6395,miR-146b-5p,miR-2285ab,miR-376b-5p,miR-2284w,miR-323c,miR-211,miR-29c-3p,miR-147-5p,miR-29a,miR-379-3p,miR-92a-3p,novel_87,miR-502-5p,miR-758-5p,miR-2411-5p,miR-6516-5p,miR-302a-5p,miR-1224-5p,novel_79,miR-299a-3p,miR-582-3p,miR-411-3p,miR-29d-3p,miR-2285b,miR-3065-3p,miR-92b-3p,miR-380-5p,miR-2478,miR-2385-3p,miR-92a-3p,miR-200c-3p,miR-339a,miR-30c-2-3p,miR-200c,miR-154b-5p,miR-3957,miR-370-3p,miR-3957-5p,miR-376c-5p,miR-345-5p,miR-376c-5p,miR-301,miR-2448-3p,miR-1306-5p,miR-204-5p,miR-3969,miR-135a-1-3p,miR-34c-3p,miR-29b-3p,miR-455-3p,miR-539-5p,miR-181b-2-3p,miR-27a-3p,novel_4,novel_78,miR-3120-5p,miR-21b,miR-101c,miR-101a-5p,miR-758-3p,miR-7857,miR-4791,miR-758-3p,miR-3187-3p,miR-30c-1-3p,miR-124a,miR-146b,miR-4443,miR-27a-3p,miR-489,miR-499a-3p,miR-378g,miR-211-5p,miR-139-5p,miR-493-5p,miR-299b-5p,miR-3963,miR-194b-5p,miR-2285f,miR-363-3p,miR-2284q,miR-490-5p,miR-628-3p,miR-3968,miR-101-3p,miR-2440,miR-328-3p,miR-2284h-5p,miR-548e-3p,miR-16-1-3p,miR-218-1-3p,miR-188-3p,miR-5703,miR-124-3p,miR-2411,miR-146a-5p,miR-181b-1-3p,miR-376a-5p,miR-345-3p,miR-2285w,miR-329a-5p,miR-299-3p,miR-219b-5p,miR-196a-3p,miR-216b-5p,miR-139-5p,miR-499b-3p,miR-6516,miR-27a-5p,miR-532-5p,miR-3065-3p,miR-32-5p,miR-30c-1-3p,miR-1827,miR-345-5p,miR-339b,miR-2312,miR-411,miR-

PDZK1IP1	ENSOARG0(	PDZK1 in	miR-152-3p,novel_127,miR-2898,let-7f-5p,miR-30c-2-3p,let-7c-5p,miR-125a-3p,let-7g,miR-490-3p,miR-28b,miR-3064-5p,miR-1,let-7d,miR-300,miR-322-5p,miR-6516-5p,miR-1224-5p,miR-222,miR-28-5p,miR-486b-3p,miR-331-3p,miR-100-3p,miR-365a-5p,miR-16-5p,miR-7144-5p,miR-671-5p,miR-485-5p,miR-329a,miR-381-3p,miR-365a-3p,miR-2355-5p,miR-370-3p,miR-574-5p,miR-365b-5p,miR-7-5p,miR-224-5p,miR-146a,miR-377-3p,miR-296-3p,miR-539-3p,miR-296-3p,miR-503-3p,miR-654-3p,let-7f-2-3p,let-7f-2-3p,miR-497-5p,miR-30b-3p,miR-16b,miR-22-3p,miR-145a-5p,miR-7b-5p,miR-1843b-5p,miR-542-3p,miR-194-3p,miR-2355-3p,let-7g-5p,miR-134-3p,miR-377-5p,miR-26b-3p,miR-133b-5p,miR-206,miR-16a,let-7i,miR-502-5p,miR-15a,miR-576-3p,miR-28c,miR-133a-5p,miR-2428,miR-2284w,miR-495-3p,miR-146b-5p,let-7e-5p,miR-195a-5p,miR-450b-3p,miR-19a-3p,miR-1306,miR-3065-5p,miR-18a-3p,miR-378b,miR-148a-3p,miR-677,miR-181b-3p,miR-2404,miR-3074-5p,let-7k,miR-98-5p,miR-324-3p,miR-185-3p,miR-196a-3p,miR-146a-5p,miR-486-3p,miR-181b-1-3p,miR-125b-2-3p,miR-377-5p,miR-216c-5p,miR-218-1-3p,miR-142-3p,miR-19b-3p,miR-188-3p,miR-1197-5p,miR-1193,miR-1434-5p,miR-5703,miR-140-3p,novel_133,miR-1961,miR-362-3p,novel_83,miR-383-5p,miR-487a-3p,miR-148b-3p,miR-15a-5p,miR-212-5p,miR-485-3p,miR-18a-3p,miR-708-5p,miR-424-5p,miR-4492,miR-664a,miR-664a-5p,miR-222-3p,let-7f,miR-3184-5p,let-7b-5p,miR-6134,miR-338-3p,miR-224-5p,miR-206-3p,miR-665-5p,let-7b,miR-3074-5p,miR-145a-3p,miR-4510,miR-1827,miR-129b-3p,novel_32,miR-22-3p,miR-204-3p,miR-15b-5p,let-7i-5p,miR-377-3p,miR-6516,miR-132-5p,miR-6130,miR-552-3p,miR-296-3p,miR-30c-1-3p,miR-146b,let-7e,miR-3085-3p,miR-362-3p,miR-16b,miR-1306-5p,novel_103,miR-4532,miR-30c-1-3p,miR-744-3p,let-7a-

PSMC1	ENSOARG0(	proteaso	miR-210-3p,miR-505-5p,miR-483-3p,miR-6516-5p,novel_83,miR-450a-1-3p,miR-151-5p,miR-18a-3p,miR-212-5p,miR-493-3p,miR-941,miR-128-3p,miR-345-3p,novel_133,miR-5703,miR-1197-5p,miR-23b-5p,miR-28b,miR-199a-5p,miR-486-3p,miR-503-5p,miR-191-3p,miR-760-3p,miR-127-5p,miR-543-5p,novel_127,miR-324-3p,miR-345-3p,miR-6516,miR-6130,miR-199a-5p,miR-151b,miR-2331-3p,miR-504-5p,miR-874-3p,miR-370-3p,miR-2355-5p,miR-1271,miR-1895,miR-485-5p,miR-450a-1-3p,miR-3154,miR-346,miR-3184-5p,miR-4510,miR-3184-3p,miR-214,miR-665-5p,miR-450b-3p,miR-138-5p,miR-486b-3p,miR-2433,miR-331-3p,miR-132-5p,miR-412-5p,miR-432-5p,miR-134-3p,miR-503-5p,miR-9788-3p,miR-149-5p,miR-216a-3p,miR-194-3p,miR-421,miR-340-3p,miR-1343-3p,miR-30b-3p,miR-138,miR-744-5p,miR-432,miR-296-5p,miR-484,miR-2284r,miR-450c-3p,miR-30b-3p,miR-654-3p,miR-363-5p,novel_124,miR-483-3p,miR-2285l,miR-199b-5p,miR-106b-3p,miR-214-3p,miR-503-5p,miR-2411-3p,novel_51,miR-2331-5p,miR-2404,miR-378g,novel_69,miR-670-3p,novel_68,miR-4792,miR-20a-3p,miR-7977,miR-450b-3p,miR-504,miR-412-3p,miR-1983,miR-2387,miR-199b-5p,miR-150-5p,miR-18a-3p,miR-216a-3p,miR-361-3p,miR-371b-3p,miR-1307-3p,miR-24-3p,miR-423-5p,miR-199b-5p,novel_60,miR-3956-3p,miR-335-5p,miR-3957-3p,miR-495-3p,novel_101,miR-128-1-5p,miR-335,miR-6123,miR-502-5p,miR-28c

PITX2	ENSOARG0(paired-l	miR-2285e,miR-1298-3p,miR-496-5p,miR-425-3p,miR-5010-3p,miR-548w,miR-1343-3p,miR-1839-5p,miR-2426,miR-450b-5p,miR-362-3p,miR-30b-3p,miR-1a-3p,miR-143-5p,miR-329-3p,miR-1b-5p,miR-2310,miR-8485,miR-30c-1-3p,miR-424-5p,miR-494-3p,miR-214-3p,miR-491-5p,miR-16b,miR-362-3p,miR-29b-1-5p,miR-1306-5p,miR-4443,miR-378g,miR-7977,miR-211-5p,miR-499b-5p,miR-452-3p,miR-544-5p,miR-335-3p,miR-493-5p,miR-140-5p,miR-361-3p,miR-423-5p,miR-361-3p,miR-452-5p,miR-6527,miR-2285f,miR-2447,miR-3059-5p,miR-4726-5p,miR-3968,miR-1973,miR-615,miR-2440,miR-2285n,novel_83,miR-1247-5p,miR-15a-5p,miR-30d-3p,miR-548e-3p,miR-18a-3p,miR-34a-5p,miR-142-3p,miR-184-3p,miR-33b-3p,miR-362-3p,miR-2300b-3p,miR-380-3p,miR-486-3p,miR-125b-2-3p,miR-29b-1-5p,miR-2887,miR-216c-5p,miR-329a-5p,miR-34b-5p,miR-92a-1-5p,miR-324-3p,novel_17,miR-15b-5p,miR-204-3p,miR-6130,miR-216b-5p,miR-296-3p,miR-140-5p,miR-30c-1-3p,miR-186-5p,miR-1827,let-7g-3p,miR-2312,miR-27b-5p,miR-6134,miR-3184-5p,miR-206-3p,miR-224-5p,miR-214,miR-3074-2-3p,miR-4510,miR-3074-5p,miR-3184-3p,miR-874-5p,miR-424-5p,miR-708-5p,miR-6516-3p,miR-487b-5p,miR-665,miR-29b-2-5p,miR-30a-3p,miR-324-3p,miR-30e-3p,miR-194-3p,miR-3431,miR-134-3p,miR-2285g,miR-188-5p,miR-2284x,miR-497-5p,miR-30b-3p,miR-16b,miR-21-5p,miR-380-3p,miR-1843b-5p,miR-542-3p,miR-873a-5p,miR-493-5p,miR-466f-3p,miR-1343-5p,miR-452-5p,miR-2285l,novel_51,miR-224-5p,miR-669,miR-34b,miR-329-5p,miR-3074-5p,miR-195a-5p,miR-3065-5p,miR-18a-3p,miR-1306,miR-3970,miR-150-5p,miR-2285ab,miR-449a,miR-211,miR-2285x,miR-1a-1-5p,miR-502b,miR-206,miR-16a,miR-34c,miR-6402,miR-28c,miR-4324,miR-15a,miR-134-5p,miR-582-3p,miR-505-5p,miR-322-5p,miR-2427,miR-7134-5p,miR-1343-5p,miR-

FUNDC2	ENSOARG0(	FUN14 do	miR-10a-5p,miR-200b-3p,miR-300,miR-2411-5p,miR-1271-5p,miR-24-2-5p,miR-339a,miR-217,miR-200c,miR-1260a,miR-543-5p,miR-181b-5p,miR-200c-3p,miR-2898,miR-181a-2-3p,miR-2284a,miR-1957a,miR-199a-5p,miR-3065-3p,miR-2478,miR-503-3p,miR-381-3p,miR-671-5p,miR-485-5p,miR-7144-5p,miR-10a-3p,miR-215-5p,miR-370-3p,miR-2284n,miR-199a-5p,miR-769,miR-409-3p,miR-3969,miR-99b-3p,miR-5100,miR-34c-3p,miR-28-5p,miR-374b,miR-145a-5p,miR-181a-5p,miR-873a-5p,miR-744-5p,miR-1260b,miR-30b-3p,miR-26b-3p,miR-130a-3p,miR-122-3p,miR-544b,miR-2355-3p,miR-432-5p,miR-2284d,miR-224-5p,miR-1343-5p,miR-125a-5p,miR-363-5p,miR-130b-5p,miR-654-3p,miR-378b,miR-2387,miR-6516-3p,miR-1291,miR-10a-5p,miR-655-5p,miR-2404,miR-323a-5p,miR-133a-5p,miR-199c,miR-147-5p,miR-22-5p,miR-128-1-5p,novel_101,miR-542-5p,miR-136-3p,miR-326-3p,miR-3959-3p,miR-323c,miR-24-3p,miR-140-3p,miR-362-3p,miR-215-5p,miR-378d,miR-3600,miR-532-3p,miR-199a-3p,miR-200b,miR-125b,miR-216a-5p,miR-374a-5p,miR-542-5p,miR-196a-3p,miR-299-3p,miR-21-3p,miR-221-5p,miR-466i-5p,miR-2284m,miR-2411,miR-146a-5p,let-7f-1-3p,miR-21a-3p,novel_99,miR-532-5p,miR-182-5p,miR-21-3p,miR-3065-3p,miR-221-5p,miR-155-5p,miR-22-3p,miR-193b-3p,miR-323-5p,miR-192-5p,miR-544-3p,miR-338-5p,miR-320e,miR-708-5p,miR-6516-3p,miR-155-5p,miR-2330-3p,miR-1271,miR-338-3p,miR-6134,miR-10b-5p,miR-30b-3p,miR-412-3p,miR-7857,miR-143-5p,miR-10b,miR-6529a,miR-2284aa,miR-450b-5p,miR-544a,miR-27a-3p,miR-222-5p,miR-362-3p,miR-2957,miR-326,miR-542-5p,miR-1839-3p,miR-199b-5p,novel_94,miR-499b-5p,miR-27a-3p,miR-107-5p,miR-769-5p,miR-335,miR-450a-5p,miR-181d-5p,novel_60,miR-154a,miR-330-5p,miR-23b-5p,miR-1343-5p,miR-1290,miR-155-5p,miR-412,novel_79,miR-429-3p,miR-
TH	ENSOARG0(	tyrosine	miR-486-3p,miR-486b-3p,miR-138,miR-138-5p,miR-664a,novel_19,miR-361-3p,miR-5126
MCMBP	ENSOARG0(	minichro	miR-129-1-3p,miR-421,miR-374c-3p,miR-3082-5p,miR-3074-1-3p,miR-493-5p,miR-378d,miR-2284x,miR-194-5p,miR-219a-2-3p,miR-23a-3p,miR-194b-5p,miR-493-5p,miR-885-5p,miR-129-2-3p,miR-3964,miR-127-5p,miR-219b-3p,miR-194a,miR-142b,miR-219-3p

PDK4	ENSOARGO(	pyruvate	miR-2310,miR-124a,miR-382-3p,miR-106a-5p,miR-101b-3p,miR-153-3p,miR-29b-1-5p,miR-182-5p,miR-106b-5p,miR-1306-5p,miR-153,miR-181b-2-3p,miR-151a-3p,novel_78,miR-16-2-3p,miR-2426,miR-501-3p,miR-412-3p,miR-502-3p,miR-374a-3p,miR-154-3p,miR-450a-5p,miR-500-3p,miR-7857-3p,miR-489,miR-181a-3p,miR-668-5p,miR-129b-5p,miR-2432,miR-664b-3p,miR-376a-5p,miR-181b-1-3p,miR-20a,miR-329a-5p,miR-2285w,miR-487a,miR-221-5p,miR-1277-5p,miR-106a,miR-345-3p,miR-1247-5p,miR-2284h-5p,miR-487a-3p,miR-148b-3p,miR-212-5p,miR-18a-3p,miR-106a,miR-361-5p,miR-124-3p,miR-1197-5p,miR-2366,miR-376a-5p,miR-122-5p,miR-129-2-3p,miR-500,miR-708-5p,miR-17-3p,miR-151-3p,miR-22-3p,miR-376e-3p,miR-154a-3p,miR-532-5p,miR-182-5p,miR-221-5p,miR-339b,miR-154b-3p,miR-500a-3p,miR-539-3p,miR-363-5p,miR-124-5p,novel_51,miR-17-5p,miR-106a-5p,miR-2411-3p,miR-20b,miR-2355-3p,miR-412-5p,miR-3431,miR-339-5p,miR-188-5p,miR-22-3p,miR-3071-5p,miR-542-3p,novel_27,miR-17-3p,miR-20a-5p,miR-28c,miR-144-3p,miR-677,miR-181b-3p,miR-2404,novel_69,miR-329-5p,miR-1306,miR-18a-3p,miR-148a-3p,miR-29a-5p,miR-450c-5p,miR-28b,miR-152-3p,miR-543-5p,novel_127,miR-17-3p,miR-2898,miR-200c-3p,miR-339a,miR-200c,miR-129-1-3p,novel_79,miR-412,miR-429-3p,miR-7134-5p,miR-501-3p,miR-345-3p,miR-181c-3p,miR-93,miR-374a-3p,miR-374b-3p,miR-28-5p,miR-144,miR-3969,miR-5100,miR-376c-3p,miR-331-5p,miR-487a-3p,miR-874-3p,novel_48,miR-20b-5p,miR-1814c,miR-1248,miR-329b,miR-93-5p,miR-3154,miR-17-5p,miR-30e-5p
CHRNB3	ENSOARGO(	choliner	miR-2285ab,miR-129b-5p,miR-96-5p,miR-107,miR-2284e,miR-9-3p,miR-378f,miR-195a-5p,miR-2319b,miR-25,miR-378e,miR-299b-3p,miR-454-5p,novel_82,miR-15a,miR-769-5p,miR-16a,miR-2285y,miR-2284o,novel_1,miR-656-5p,miR-885-5p,miR-2285f,miR-452-5p,miR-1193,miR-9-3p,miR-484,miR-378b,miR-1434-3p,miR-2113,miR-378c,miR-16b,miR-1388-5p,miR-26a-2-3p,miR-3120-5p,miR-31-5p,miR-103a-3p,miR-185-5p,miR-3431,novel_116,miR-455-3p,miR-6238,miR-103,miR-16b,miR-196b-5p,miR-3955-3p,miR-660,miR-424-5p,miR-654-3p,miR-378d,miR-345-5p,miR-655,miR-2312,miR-345-5p,miR-345-5p,miR-485-5p,miR-378a-3p,miR-660-5p,miR-496-3p,miR-2355-5p,miR-370-5p,miR-141-3p,miR-27a-5p,miR-769,miR-6130,miR-877-5p,miR-15b-5p,novel_63,miR-370-5p,miR-30e-3p,miR-30a-3p,miR-320e,miR-655-3p,novel_74,miR-208b-5p,miR-592,miR-378c,miR-4510,miR-3184-3p,miR-1271,miR-16-5p,miR-769-5p,miR-378c,miR-378i,miR-196a-5p,miR-1290,miR-378a-3p,miR-582-3p,miR-218-1-3p,miR-496,miR-299a-3p,miR-2285ad,miR-548e-3p,miR-1224-5p,miR-7859,miR-322-5p,miR-15a-5p,miR-340-5p,miR-1271-5p,miR-378h,miR-1247-5p,miR-422a,miR-107,miR-200a-3p,miR-299-3p,miR-30a-3p,miR-495-5p,miR-369-3p,miR-345-3p,miR-2284f,miR-130a-5p,miR-29a-5p

XPOT	ENSOARGO(	exportin	miR-1b-5p,miR-8485,miR-101b-3p,miR-214-3p,miR-153-3p,miR-146b,miR-505,miR-323b-3p,miR-199a-3p,miR-126a-5p,miR-1185-3p,miR-455-3p,miR-153,miR-425-3p,miR-145b,novel_78,miR-330-3p,miR-101c,miR-21b,miR-323b,miR-541,miR-2284r,miR-7705,novel_111,miR-361-3p,miR-2284j,miR-3956-3p,miR-2447,miR-490-5p,miR-3968,miR-499a-5p,miR-615,miR-2440,miR-101-3p,miR-1185-2-3p,miR-3596,miR-126b-5p,miR-2285u,miR-4792,miR-139-5p,miR-9-3p,miR-335-3p,miR-378j,miR-1306-3p,miR-381-3p,miR-380-3p,miR-143-3p,miR-486-3p,miR-376c-3p,miR-2285i,miR-376a-5p,miR-195a-3p,miR-500a-5p,miR-26a-5p,miR-30b,miR-16-1-3p,miR-184-3p,novel_73,miR-217-5p,miR-2366,miR-30c-5p,miR-346,miR-214,miR-129-5p,novel_74,miR-3604,miR-665,miR-664a-5p,miR-376e-3p,miR-2284s,miR-548o-3p,miR-2331-3p,miR-30c-1-3p,miR-1827,miR-129b-3p,let-7a-2-3p,miR-2284l,miR-2285r,miR-432-3p,miR-6240,miR-380-5p,miR-146a,miR-7-5p,novel_116,miR-149-5p,miR-31-5p,miR-188-5p,miR-16-1-3p,novel_23,miR-7b-5p,miR-2113,miR-19b-2-5p,miR-2284w,miR-1839-3p,miR-6123,miR-425-5p,miR-28c,miR-320c,miR-669,miR-144-3p,miR-3120-3p,miR-26b-5p,miR-2319a,miR-146b-5p,miR-3591-3p,miR-148a-3p,novel_39,miR-200a-3p,miR-152-3p,miR-125a-3p,novel_121,miR-2284z,miR-505-5p,miR-429-3p,miR-30c,miR-1343-5p,miR-1197-3p,miR-2284v,novel_9,miR-2284k,novel_63,miR-376c-3p,miR-2285aa,miR-3068-3p,miR-503-3p,miR-2318,miR-200a-5p,miR-301,miR-30e-5p,miR-30c-1-3p,miR-376b,miR-2330-5p,miR-1839-3p,miR-2957,novel_103,miR-320b,miR-500-5p,miR-2320-3p,miR-2300a-5p,miR-409b,miR-3120-5p,miR-2284aa,miR-101a-5p,miR-143-5p,miR-6529a,miR-362-5p,miR-541-3p,novel_60,miR-2285t,miR-30b-5p,miR-382-3p,miR-2285n,miR-454-5p,miR-7977,miR-20a-3p,novel_94,miR-129b-

B3GALNT2	ENSOARG0(	beta-1,3	miR-154a,miR-2284j,miR-452-5p,miR-9-5p,miR-32-3p,miR-2284q,miR-490-5p,miR-2440,novel_82,miR-376d,miR-27a-3p,miR-1185-2-3p,miR-489,miR-499a-3p,miR-7977,miR-20a-3p,miR-2284g,miR-1983,miR-369-5p,miR-376a-3p,miR-136-5p,miR-96-5p,miR-199b-5p,miR-140-5p,miR-2310,miR-2284b,miR-424-5p,miR-214-3p,miR-1468,miR-153-3p,miR-146b,miR-199b-5p,miR-3955-3p,miR-487b-5p,miR-491-5p,miR-16b,miR-29b-1-5p,miR-326,miR-1185-3p,miR-455-3p,miR-216a-3p,miR-376b-3p,miR-147a,miR-153,miR-27a-3p,miR-181b-2-3p,miR-330-3p,miR-16-2-3p,miR-2284aa,miR-2426,miR-1388-5p,miR-125b-2-3p,miR-1298-5p,miR-143-5p,miR-2284r,miR-181c-5p,miR-338-3p,miR-214,miR-2330-3p,miR-1271,miR-539-5p,miR-130b-5p,miR-424-5p,miR-1468-5p,miR-664a,novel_74,miR-29b-2-5p,miR-664a-5p,miR-369-5p,miR-15b-5p,miR-155-5p,miR-192-5p,miR-499b-3p,miR-296-3p,miR-21-3p,miR-32-3p,miR-1827,novel_99,miR-345-5p,miR-133b-3p,miR-487b-5p,miR-146a-5p,miR-181b-1-3p,miR-29b-1-5p,miR-345-3p,miR-195a-3p,miR-329a-5p,miR-21-3p,miR-466i-5p,miR-2284m,miR-26a-5p,miR-328-3p,novel_83,miR-2284h-5p,miR-532-3p,miR-148b-3p,miR-200b,miR-15a-5p,miR-383-5p,miR-2284u,miR-16-1-3p,miR-140-3p,miR-1197-5p,miR-188-3p,miR-1246,miR-215-5p,miR-199b-5p,miR-2428,miR-326-3p,miR-128-1-5p,miR-16a,miR-2284o,miR-28c,miR-6529b,miR-15a,miR-181b-3p,miR-677,miR-670-3p,miR-26b-5p,miR-329-5p,miR-195a-5p,miR-6395,miR-146b-5p,miR-412-3p,miR-450b-3p,miR-1291,miR-216a-3p,miR-376b-3p,miR-3591-3p,miR-148a-3p,miR-345-5p,miR-296-3p,miR-654-3p,let-7f-2-3p,let-7f-2-3p,miR-6240,miR-130b-5p,miR-452-5p,miR-1343-5p,miR-124-5p,miR-330-3p,miR-146a,miR-296-3p,miR-2355-3p,miR-3431,miR-412-5p,miR-2284d,miR-27b-3p,miR-744-5p,miR-497-5p,miR-369-5p,miR-16-1-3p,miR-219a-1-3p,miR-2284ab,miR-
SNTB2	ENSOARG0(	syntroph	miR-205-5p,miR-181b-5p,miR-34b-5p,miR-181b-1-3p,miR-29a-5p,miR-2411,miR-2432,miR-3578,miR-124-3p,miR-5703,miR-148a-5p,miR-34a-5p,miR-2285ad,miR-1224-5p,miR-2411-5p,miR-545-5p,miR-331-3p,miR-3969,miR-665,miR-135a-5p,novel_42,miR-4510,miR-6128,miR-181c-5p,miR-27b-5p,miR-129b-3p,miR-10b-3p,miR-1248,miR-582-5p,miR-186-5p,miR-874-3p,miR-2285aa,miR-34c-5p,miR-582-5p,miR-6130,novel_25,miR-487b-5p,miR-153-3p,miR-124a,miR-2330-5p,miR-628-5p,miR-2113,miR-181a-5p,miR-1434-3p,miR-145a-5p,miR-450b-5p,miR-101a-5p,miR-101c,miR-145b,miR-181b-2-3p,miR-26b-3p,miR-153,miR-134-3p,miR-185-5p,miR-9788-3p,miR-148b-5p,miR-149-5p,miR-218-5p,miR-2440,miR-34c,miR-2285y,miR-6119-3p,miR-7134-3p,miR-203b-5p,miR-181d-5p,miR-449a,miR-9-3p,miR-140-5p,miR-409-5p,miR-21c,miR-147-3p,miR-136-5p,miR-9-3p,novel_94,miR-135b-5p,novel_68,miR-378g,miR-34b,miR-489,miR-677,miR-181b-3p

RNF170	ENSOARG0(	ring fin	miR-450a-1-3p,miR-487a-3p,miR-9-5p,miR-758-5p,miR-485-3p,miR-16-1-3p,miR-105-2,miR-2284u,miR-300,miR-2904,miR-5703,miR-23b-5p,miR-188-3p,miR-2366,miR-2284y,miR-2424,miR-2284a,miR-2285b,miR-380-3p,miR-2478,miR-181b-1-3p,miR-195a-3p,miR-216c-5p,miR-200a-3p,miR-17-3p,miR-181b-5p,miR-92a-1-5p,miR-22-3p,miR-324-5p,miR-544-3p,miR-331-5p,miR-6130,miR-139-5p,miR-141-3p,miR-140-5p,novel_99,miR-27b-5p,miR-3154,miR-381-3p,miR-331-5p,miR-450a-1-3p,miR-181c-5p,miR-142a-5p,miR-3607-3p,miR-2330-3p,miR-4510,miR-31-3p,miR-204-5p,miR-450b-3p,miR-17-3p,miR-331-5p,miR-338-5p,miR-2284k,miR-455-3p,miR-9788-3p,miR-544b,miR-1298-3p,miR-134-3p,miR-142-5p,miR-181b-2-3p,miR-27a-3p,miR-105-1,miR-16-2-3p,miR-21b,miR-27b-3p,miR-3120-5p,miR-7862,miR-380-3p,miR-2284ab,miR-16-1-3p,miR-544a,miR-22-3p,miR-450c-3p,miR-194-5p,miR-181a-5p,miR-2284l,miR-2284b,miR-25-5p,miR-105-5p,miR-194a,miR-677,miR-27a-3p,miR-181b-3p,miR-378g,miR-3082-5p,miR-211-5p,miR-139-5p,miR-2284g,miR-7975,miR-1983,miR-6516-3p,miR-412-3p,miR-140-5p,miR-2284w,miR-211,miR-194b-5p,miR-181d-5p,miR-17-3p,miR-9-5p,miR-2285t,miR-3968,miR-485-3p
RAB1B	ENSOARG0(	RAB1B, m	miR-26b-3p,miR-103a-3p,miR-2355-3p,miR-103,miR-134-3p,miR-185-5p,miR-145a-5p,miR-22-3p,miR-2113,miR-873a-5p,miR-7862,miR-744-5p,miR-497-5p,miR-1260b,miR-16b,miR-138,miR-30b-3p,miR-628-5p,miR-668-3p,miR-6240,novel_51,miR-105-5p,miR-330-3p,miR-3141,miR-377-3p,miR-181b-3p,miR-6535,miR-669,miR-677,novel_69,miR-146b-3p,miR-18a-3p,miR-3141,miR-18b,miR-195a-5p,miR-135b-5p,miR-19a-3p,miR-107,miR-1291,miR-335-5p,miR-342-3p,miR-301b-3p,novel_27,miR-326-3p,miR-24-3p,miR-16a,miR-502-5p,miR-15a,miR-147-5p,miR-128-1-5p,miR-18b-5p,miR-6517,miR-214-5p,miR-322-5p,miR-330-5p,miR-615-3p,miR-105-2,miR-300,miR-490-3p,miR-200a,novel_39,miR-1260a,miR-543-5p,novel_96,novel_127,miR-107,miR-127-5p,miR-450a-2-3p,miR-1271,miR-2355-5p,miR-574-5p,miR-154b-5p,miR-365a-3p,miR-18a,miR-381-3p,miR-671-5p,miR-485-5p,miR-7144-5p,miR-329a,miR-10b-3p,miR-193a-5p,miR-16-5p,miR-1197-3p,miR-1260b,miR-135a-5p,miR-331-3p,miR-2433,miR-486b-3p,miR-592,miR-138-5p,miR-181b-2-3p,miR-145b,miR-105-1,miR-1343-3p,miR-2300a-5p,miR-330-3p,miR-147a,miR-30b-3p,miR-541,miR-296-5p,miR-329-3p,miR-484,miR-1839-5p,miR-362-3p,miR-652-5p,miR-424-5p,miR-1388-3p,miR-8485,miR-16b,miR-362-3p,miR-5126,miR-326,miR-342,novel_124,miR-491-5p,miR-2285u,miR-378g,miR-3596,miR-361-3p,miR-541-3p,miR-423-5p,miR-615,miR-2285n,miR-335,miR-4726-5p,miR-3960,miR-18a-3p,miR-15a-5p,miR-5703,miR-140-3p,miR-362-3p,novel_133,miR-19b-3p,miR-184-3p,miR-486-3p,miR-181b-1-3p,miR-185-3p,miR-324-3p,miR-21-3p,miR-466i-5p,miR-552-3p,miR-15b-5p,miR-22-3p,miR-6130,miR-301a-3p,miR-2284s,miR-377-3p,miR-129b-3p,miR-1827,miR-18a-5p,miR-425-5p,miR-4510,miR-3184-5p,miR-6525,miR-665,miR-320e,miR-424-5p,miR-874-5p,miR-454-3p,miR-664a

CD164L2	ENSOARG0(	CD164 si	miR-4726-5p,miR-2898,miR-708-5p,miR-34b-5p,miR-28-5p,miR-2440,miR-28c,miR-324-3p,miR-34c,miR-28b,novel_39,miR-449a,miR-2330-5p,miR-485-5p,miR-34a-5p,novel_133,miR-2300b-3p,miR-378g,miR-34c-5p,miR-34b,miR-134-3p
MYBL2	ENSOARG0(	v-myb av	miR-331-5p,novel_9,miR-134-5p,miR-125b-5p,miR-100-3p,miR-1197-3p,miR-1260b,miR-16-5p,miR-345-5p,miR-331-5p,miR-30e-5p,miR-300-3p,miR-223-5p,miR-574-5p,miR-125a,miR-1271,miR-2285aa,miR-1a-2-5p,novel_127,miR-200a-3p,miR-30f,miR-29a-5p,miR-200a,novel_39,miR-3064-5p,miR-30c,miR-1290,miR-2427,miR-9-5p,miR-322-5p,miR-134-5p,miR-429-3p,miR-299a-3p,miR-2285ad,miR-18b-5p,miR-412,novel_79,miR-29a,miR-6123,miR-1a-1-5p,miR-29c-3p,miR-3141,miR-3591-3p,miR-1306,miR-299b-3p,miR-669,miR-6535,miR-320c,miR-3120-3p,miR-378a-5p,miR-483-3p,miR-3141,miR-628-5p,miR-16b,miR-2284x,miR-27b-3p,miR-497-5p,miR-2113,miR-1843b-5p,miR-542-3p,novel_23,miR-450c-3p,miR-3431,miR-134-3p,miR-412-5p,novel_116,miR-149-5p,miR-194-3p,miR-31-5p,miR-339-5p,miR-487b-5p,miR-130b-5p,miR-874-5p,miR-4492,miR-30e-3p,miR-665,miR-664a-5p,miR-30a-3p,miR-346,miR-6525,miR-769-5p,miR-122-5p,miR-3184-5p,miR-3184-3p,miR-214,miR-665-5p,miR-339b,miR-345-5p,miR-186-5p,miR-543-3p,miR-625-5p,miR-2285c,miR-30d-5p,miR-487b-5p,miR-2284s,miR-15b-5p,miR-2331-3p,miR-2484,miR-210-5p,miR-30b,miR-92a-1-5p,miR-3601,miR-324-3p,miR-376a-5p,miR-143-3p,miR-486-3p,novel_73,miR-34a-5p,miR-193b-5p,miR-30c-5p,miR-7859,miR-210-3p,miR-18a-3p,miR-212-5p,miR-30d-3p,novel_82,novel_111,miR-361-3p,miR-877-3p,miR-423-5p,miR-9-5p,miR-136-5p,miR-96-5p,miR-544-5p,miR-378g,miR-500-3p,miR-4443,miR-2319b,miR-214-3p,miR-16b,miR-29b-1-5p,miR-505,miR-101c,miR-541,miR-2284r,miR-501-3p,miR-1298-3p,miR-421,miR-199b-3p,miR-1343-3p,miR-138-5p,miR-486b-3p,miR-31-3p,miR-370-5p,miR-5100,miR-29b-3p,miR-1-5p,miR-409-3p,miR-26c,miR-1306-5p,miR-103b,miR-3607-3p,miR-6128,miR-18a,miR-320a,miR-769,miR-34c-5p,miR-154b-5p,miR-141-3p,miR-370-3p,miR-
DSTYK	ENSOARG0(	dual ser	miR-450b-3p,miR-378f,miR-2319a,miR-361-3p,miR-376b-5p,miR-670-3p,miR-3082-5p,miR-299b-3p,miR-323a-5p,miR-378e,novel_68,miR-3064-5p,miR-499b-5p,miR-2285u,miR-2284q,miR-101-3p,miR-107-5p,miR-326-3p,miR-1285-5p,miR-9-5p,miR-378c,miR-2284x,miR-1434-3p,miR-4791,miR-378b,miR-450c-3p,miR-1298-3p,miR-3535,miR-2300a-5p,miR-378a-5p,miR-3085-3p,miR-103a-2-5p,miR-326,novel_51,miR-197-5p,miR-654-3p,miR-378d,miR-101b-3p,miR-186-5p,miR-376c-5p,miR-450a-1-3p,miR-3154,miR-376c-5p,miR-500a-3p,miR-323-5p,miR-3068-3p,miR-378a-3p,miR-1271,miR-370-3p,novel_74,miR-450b-3p,miR-500,miR-409-3p,miR-378c,miR-378c,miR-3074-2-3p,miR-103b,miR-2330-3p,miR-378i,miR-1197-5p,miR-330-5p,miR-378a-3p,miR-23b-5p,miR-378h,miR-9-5p,miR-486-5p,miR-450a-1-3p,miR-2284h-5p,miR-299a-3p,miR-3600,miR-1b-3p,miR-760-3p,miR-21-3p,miR-299-3p,miR-6119-5p,miR-422a,miR-216a-5p,miR-148b-5p,miR-29a-5p,miR-3064-5p

TERF2	ENSOARG0(	telomeri	let-7c-3p,miR-363-5p,miR-432-3p,miR-29b-1-5p,miR-490-3p,miR-1271-3p,novel_39,miR-2432,miR-2285af,miR-455-5p,miR-3071-3p,miR-105-5p,miR-29b-1-5p,miR-3955-3p,miR-130b-3p,miR-2484,miR-152-3p,miR-21-3p,miR-105-1,miR-7641,miR-130a-3p,miR-134-3p,miR-148b-3p,miR-24-2-5p,miR-214-5p,miR-450a-1-3p,miR-1843b-5p,miR-5703,miR-21-5p,miR-455-5p,miR-105-2,miR-155-5p,miR-2285f,miR-301b-3p,miR-338-3p,novel_82,miR-33a-3p,miR-155-5p,miR-450b-3p,miR-454-3p,miR-378b,miR-7977,miR-655-5p,miR-411b-3p,miR-378g,miR-301a-3p,miR-155-5p,miR-148a-3p,miR-21a-3p,miR-2285ab,miR-21c,miR-3154,let-7g-3p,miR-2312,miR-186-5p
LYST	ENSOARG0(	lysosoma	miR-409-5p,miR-381-3p,miR-136-5p,miR-299b-5p,miR-664b,miR-499b-5p,miR-25,miR-4443,miR-30b-5p,miR-3059-5p,miR-2447,miR-194b-5p,miR-452-5p,miR-2285f,novel_111,miR-423-5p,miR-4791,miR-296-5p,miR-7857,miR-450b-5p,miR-299a-5p,miR-101a-5p,miR-3120-5p,miR-199b-3p,miR-151a-3p,miR-2300a-5p,miR-330-3p,miR-1185-3p,miR-216a-3p,miR-106b-5p,miR-16b,miR-3955-3p,miR-106a-5p,miR-2330-5p,miR-424-5p,miR-1388-3p,miR-33a-5p,miR-23a-3p,miR-3187-3p,miR-299,miR-30d-5p,miR-411,miR-500a-3p,miR-27b-5p,miR-339b,miR-30f,miR-2312,miR-582-5p,miR-186-5p,miR-1827,miR-32-5p,miR-21-3p,miR-3065-3p,miR-656-3p,miR-2284s,miR-582-5p,miR-15b-5p,miR-204-3p,miR-30e-3p,miR-324-3p,miR-151-3p,miR-30a-3p,miR-19b-1-5p,miR-669a-3p,miR-130b-5p,miR-4492,miR-500,miR-424-5p,miR-590-3p,miR-2330-3p,miR-6525,miR-338-3p,miR-3184-5p,miR-122-5p,miR-30c-5p,miR-1197-5p,miR-142-3p,miR-19b-3p,miR-2284u,miR-148a-5p,miR-34a-5p,miR-106a,miR-212-5p,miR-23c,miR-222-5p,miR-15a-5p,miR-23b-3p,novel_83,miR-374a-5p,miR-210-5p,miR-30b,miR-196a-3p,miR-185-3p,miR-345-3p,miR-219b-5p,miR-500a-5p,miR-324-3p,miR-106a,miR-299-3p,miR-30d,miR-34b-5p,miR-3653-3p,miR-345-3p,miR-20a,miR-380-3p,miR-486-3p,miR-2285ab,miR-19a-3p,miR-450b-3p,miR-195a-5p,miR-34b,miR-299b-3p,miR-2404,miR-20a-5p,miR-576-3p,miR-15a,miR-33a-3p,miR-34c,miR-16a,miR-6119-3p,miR-92a-3p,miR-199c,miR-495-3p,miR-449a,miR-19b-2-5p,miR-194-5p,miR-16b,miR-21-5p,miR-380-3p,miR-497-5p,miR-26b-3p,miR-339-5p,miR-3431,miR-3535,miR-20b,miR-194-3p,novel_116,miR-194a,miR-106a-5p,novel_51,miR-17-5p,miR-2411-3p,miR-330-3p,miR-32,miR-466f-3p,miR-6240,miR-130b-5p,miR-101-5p,miR-432-3p,miR-30e-5p,miR-23b,miR-17-5p,miR-93-5p,miR-20b-5p,miR-141-3p,miR-2285aa,miR-25-
CYB5B	ENSOARG0(	cytochro	miR-1388-5p,miR-29b,miR-544a,miR-128-3p,miR-484,miR-29d-3p,miR-2285g,miR-544b,miR-299a-3p,miR-330-3p,miR-3600,miR-17-3p,miR-760-3p,miR-299-3p,miR-224-5p,miR-330-3p,miR-148b-5p,miR-654-3p,miR-3065-3p,miR-494-3p,miR-6240,miR-23a-5p,miR-150-5p,miR-216a-3p,miR-29a-3p,miR-299b-3p,miR-3065-3p,miR-532-5p,miR-29a,miR-17-3p,miR-147-5p,miR-2285j,miR-29b-3p,miR-502-5p,miR-576-3p,miR-425-5p,miR-224-5p,miR-6134,miR-539-5p,miR-3184-3p,miR-29c-3p,miR-17-3p

AP5Z1	ENSOARG0(	adaptor-	miR-760-3p,miR-4661-5p,miR-34b-5p,miR-30b-3p,miR-221-5p,miR-21-3p,miR-26a-5p,miR-324-3p,miR-345-3p,miR-125b,miR-185-3p,miR-125b-2-3p,miR-486-3p,miR-503-5p,miR-2887,miR-345-3p,miR-142-3p,miR-16-1-3p,miR-34a-5p,miR-191,novel_133,miR-1197-5p,miR-15a-5p,miR-1247-5p,miR-328-3p,miR-18a-3p,miR-4492,miR-500,miR-874-5p,miR-708-5p,miR-424-5p,miR-132-5p,miR-10b-5p,miR-3184-5p,miR-433-5p,miR-145a-3p,miR-214,miR-339b,miR-885-3p,miR-1827,novel_99,miR-21a-3p,miR-500a-3p,miR-132-5p,miR-10a,miR-22-3p,miR-15b-5p,miR-30c-1-3p,miR-2331-3p,miR-221-5p,miR-370-5p,miR-21-3p,miR-552-3p,miR-532-5p,miR-3085-3p,miR-199b-5p,miR-214-3p,miR-342,miR-326,miR-1306-5p,novel_19,miR-16b,miR-30c-1-3p,miR-424-5p,miR-3120-5p,miR-484,miR-296-5p,miR-541,miR-10b,miR-30b-3p,miR-1298-3p,miR-147a,miR-1343-3p,novel_4,miR-330-3p,novel_82,miR-218-5p,miR-2285n,miR-615,miR-2440,miR-371a-5p,miR-2285y,miR-361-3p,miR-877-3p,miR-423-5p,novel_60,miR-541-3p,miR-3956-3p,miR-361-3p,miR-199b-5p,miR-378g,miR-203-3p,miR-3064-5p,miR-452-3p,miR-7689-3p,miR-7977,miR-126b-5p,miR-450a-2-3p,miR-2898,novel_127,miR-543-5p,miR-125a-3p,miR-30c-2-3p,miR-339a,miR-28b,miR-199a-5p,miR-3064-5p,miR-2385-3p,miR-4286,miR-181a-2-3p,miR-885-3p,miR-10a-5p,miR-615-3p,miR-345-3p,miR-134,miR-1343-5p,miR-330-5p,miR-322-5p,miR-134-5p,miR-2285ad,miR-7641,novel_79,miR-138-5p,miR-486b-3p,miR-134-5p,miR-191-5p,miR-28-5p,miR-2433,miR-370-5p,miR-125b-5p,miR-331-3p,miR-1197-3p,novel_42,miR-16-5p,miR-876-3p,miR-329b,miR-485-5p,miR-34c-5p,miR-199a-5p,miR-370-3p,miR-2355-5p,miR-1271,miR-615-5p,miR-125a,miR-125a-5p,miR-1343-5p,miR-503-5p,miR-330-3p,novel_51,miR-6240,miR-363-5p,miR-628-5p,miR-16b,miR-
IGF2	ENSOARG0(	insulin-	miR-9-5p,miR-449a,miR-371b-3p,let-7i,miR-34c,miR-769-5p,miR-1247-3p,miR-218-5p,miR-490-5p,miR-128-1-5p,let-7d-5p,miR-669,miR-6535,miR-34b,miR-299b-3p,miR-7857-3p,let-7e-5p,miR-493-5p,miR-147-3p,miR-668-3p,miR-493-5p,let-7a-5p,miR-296-3p,miR-4532,miR-365b-5p,miR-296-3p,miR-381-5p,let-7e,miR-214-3p,miR-25-5p,miR-2459,let-7g-5p,miR-147a,miR-19b-2-5p,miR-1839-5p,miR-381-5p,miR-214,miR-665-5p,miR-133a-3p,miR-193a-5p,let-7b,let-7b-5p,miR-365a-5p,miR-122-5p,miR-2903,miR-769-5p,miR-19b-1-5p,miR-324-3p,let-7f,miR-2433,miR-486b-3p,miR-296-3p,miR-3956,miR-574-5p,let-7i-5p,miR-769,miR-34c-5p,miR-133b-3p,miR-192-3p,miR-1271-3p,miR-486-3p,let-7g,let-7c-5p,miR-92a-1-5p,miR-185-3p,let-7k,miR-299-3p,miR-3653-3p,miR-34b-5p,miR-98-5p,miR-466i-5p,let-7f-5p,miR-299a-3p,miR-210-3p,miR-9-5p,miR-1197-5p,miR-193b-5p,miR-1961,miR-34a-5p,miR-133a-3p,miR-33b-3p,let-7d,miR-142-3p

CERS4	ENSOARG0(	ceramide	miR-147a,miR-455-3p,miR-1185-3p,miR-1343-3p,miR-548w,novel_78,miR-1388-5p,miR-101c,miR-323b,miR-378d,miR-2310,miR-1388-3p,miR-23a-3p,miR-744-3p,novel_19,miR-323b-3p,miR-378g,miR-2483-3p,miR-1185-2-3p,miR-139-5p,miR-2319b,miR-211-5p,miR-504,miR-335-3p,miR-361-3p,miR-877-3p,miR-423-5p,miR-2447,miR-6527,miR-3968,miR-490-5p,miR-23b-3p,miR-7859,miR-23c,miR-1247-5p,miR-18a-3p,miR-30d-3p,miR-16-1-3p,miR-378i,miR-2366,miR-378a-3p,miR-376a-5p,miR-486-3p,miR-2424,miR-143-3p,novel_17,miR-324-3p,miR-422a,miR-345-3p,miR-216b-5p,miR-2331-3p,miR-504-5p,miR-345-5p,miR-186-5p,miR-299,miR-129b-3p,miR-346,miR-224-5p,miR-3184-5p,miR-3074-5p,miR-129-2-3p,miR-3958-5p,novel_74,miR-4492,miR-129-5p,miR-130b-5p,miR-30e-3p,miR-30a-3p,miR-3431,miR-134-3p,miR-31-5p,miR-340-3p,miR-16-1-3p,miR-4508,miR-27b-3p,miR-188-5p,miR-432,miR-19b-2-5p,miR-223-3p,miR-2113,miR-22-3p,miR-432-3p,miR-432-3p,miR-668-3p,let-7f-2-3p,let-7f-2-3p,miR-106b-3p,miR-2285v,novel_51,miR-2411-3p,novel_69,miR-677,miR-320c,novel_68,miR-3074-5p,miR-3120-3p,miR-410-5p,miR-378f,miR-376b-5p,miR-150-5p,miR-29c-3p,miR-29a,miR-134-5p,miR-129-1-3p,miR-2285ad,miR-6517,miR-1290,miR-1343-5p,miR-330-5p,novel_39,miR-3064-5p,miR-127-5p,novel_127,miR-200a-3p,miR-874-3p,miR-503-3p,miR-2285aa,miR-1271,miR-345-5p,miR-30e-5p,miR-23b,miR-376c-5p,let-7e-3p,miR-193a-5p,novel_9,miR-134-5p,miR-592,miR-9788-3p,novel_4,miR-27a-3p,miR-378c,miR-544a,miR-16-2-3p,miR-3120-5p,miR-484,miR-378b,miR-30b-3p,miR-193a,miR-4532,miR-376b,miR-3085-3p,miR-3955-3p,miR-320b,novel_115,miR-326,miR-378e,miR-489,miR-27a-3p,miR-3064-5p,miR-541-5p,miR-147-3p,novel_94,miR-877-3p,miR-361-3p,miR-129b-5p,miR-8095,miR-154a,miR-2285t,miR-

HOOK3	ENSOARG0(	hook mic	miR-4429,miR-124-3p,miR-30c-5p,miR-2284h-5p,miR-148b-3p,miR-30d-3p,miR-505-3p,miR-106a,miR-3960,miR-30d,miR-30b-3p,miR-320d,miR-106a,miR-345-3p,miR-324-3p,miR-2285p,miR-210-5p,miR-30b,miR-130a-5p,miR-125b-2-3p,miR-29b-1-5p,miR-20a,miR-2285w,miR-30f,miR-345-5p,miR-2312,miR-30d-5p,miR-2285c,miR-21a-3p,miR-544-3p,miR-377-3p,miR-499b-3p,miR-532-5p,miR-21-3p,miR-1277-3p,miR-130b-5p,miR-208b-5p,miR-708-5p,miR-664a,miR-665,miR-338-5p,miR-320e,miR-30a-3p,miR-324-3p,miR-2285j,miR-181c-5p,miR-224-5p,miR-346,miR-6525,miR-769-5p,miR-2330-3p,miR-1271,miR-3074-5p,miR-21b,miR-2284aa,miR-16-2-3p,miR-544a,miR-2426,miR-143-5p,miR-541,miR-2285e,miR-1298-3p,miR-539-5p,miR-199b-5p,miR-3085-3p,miR-106b-5p,miR-29b-1-5p,miR-431-5p,miR-320b,miR-8485,let-7c-3p,miR-106a-5p,miR-382-3p,miR-2330-5p,miR-124a,miR-1388-3p,miR-544-5p,miR-96-5p,miR-378j,miR-1306-3p,miR-199b-5p,miR-499a-3p,miR-211-5p,miR-7977,miR-499b-5p,miR-541-5p,miR-452-3p,miR-2319b,miR-3064-5p,miR-664b,miR-2285t,miR-769-5p,novel_82,miR-30b-5p,miR-2285n,miR-154a,miR-194b-5p,miR-181d-5p,miR-2285f,miR-6527,miR-3956-3p,miR-885-5p,miR-541-3p,miR-212-3p,novel_60,let-7a-2-3p,miR-30c,miR-300,miR-411-3p,miR-30a-5p,miR-218-2-3p,miR-1290,miR-3578,miR-3591-5p,miR-345-3p,miR-545-5p,miR-2284z,miR-1271-5p,miR-505-5p,miR-431,miR-152-3p,miR-181b-5p,miR-450a-2-3p,miR-3071-3p,miR-664-3p,miR-2285b,novel_39,miR-29a-5p,miR-199a-5p,miR-380-5p,miR-2478,miR-450b-5p,miR-3064-5p,miR-30a-3p,miR-485-5p,miR-671-5p,miR-20b-5p,miR-7144-5p,miR-345-5p,let-7g-3p,miR-876-3p,miR-3154,miR-17-5p,miR-320a,miR-93-5p,miR-2448-3p,miR-381-3p,miR-30e-5p,miR-199a-5p,miR-223-5p,miR-769,miR-204-5p,miR-28-5p,miR-1306-3p,miR-93,miR-
C2orf73	ENSOARG0(	chromoso	miR-2318

NFAT5	ENSOARG0(	nuclear	miR-2483-3p,miR-10b,miR-2284g,miR-139-5p,novel_94,miR-9-3p,miR-335-3p,miR-493-5p,miR-504,miR-378j,miR-181d-5p,miR-194b-5p,miR-2284j,miR-32-3p,miR-2284q,miR-374a-3p,miR-1973,miR-218-5p,miR-2285n,miR-2285e,miR-455-3p,miR-216a-3p,miR-147a,miR-27a-3p,novel_78,miR-548w,miR-450b-5p,miR-101a-5p,miR-10b,miR-8485,miR-2284b,miR-33a-5p,miR-652-5p,miR-494-3p,miR-1388-3p,miR-487b-5p,miR-491-5p,miR-182-5p,miR-2957,miR-139-5p,miR-216b-5p,miR-376e-3p,miR-582-5p,miR-10a,miR-296-3p,miR-182-5p,miR-3065-3p,miR-216b-3p,miR-21-3p,miR-32-3p,miR-548o-3p,miR-504-5p,miR-140-5p,miR-625-5p,miR-133b-3p,miR-655,miR-411,miR-30d-5p,miR-2285c, let-7f-1-3p,miR-299,miR-21a-3p,miR-181c-5p,miR-122-5p,miR-98-3p,miR-6525,miR-10b-5p,miR-129-2-3p,miR-130b-5p,miR-669a-3p,novel_74,miR-655-3p,miR-2285j,let-7b-3p,novel_83,miR-2284h-5p,miR-340-5p,miR-7859,miR-548e-3p,miR-148a-5p,miR-361-5p,miR-2284u,miR-218-1-3p,miR-133a-3p,novel_73,miR-33b-3p,miR-5703,miR-98-3p,miR-376a-5p,miR-2366,miR-2432,miR-376c-3p,miR-130a-5p,miR-376a-5p,miR-2285w,miR-3653-3p,miR-2284m,miR-345-3p,miR-92a-1-5p,miR-2285p,novel_17,miR-208b-3p,miR-6535,miR-374c-3p,miR-2404,miR-10a-5p,miR-216b-3p,novel_68,miR-450b-3p,miR-3591-3p,miR-378b,miR-3970,miR-376b-5p,miR-374c-5p,novel_101,miR-495-3p,miR-2285x,novel_120,novel_1,miR-33a-3p,miR-208a-3p,miR-142b,miR-149-5p,miR-3431,miR-2285g,miR-26b-3p,miR-31-5p,miR-27b-3p,miR-2284x,miR-188-5p,miR-374b-3p,miR-194-5p,miR-7b-5p,miR-223-3p,miR-181a-5p,miR-296-3p,miR-432-3p,miR-432-3p,miR-382,miR-363-5p,miR-493-5p,miR-130b-5p,miR-466f-3p,miR-2411-3p,miR-194a,miR-296-3p,miR-7-5p,miR-2284n,miR-376c-3p,miR-324-5p,miR-2285aa,miR-574-5p,miR-376c-5p,novel_48,miR-876-3p,miR-376c-5p,miR-

LRP2	ENSOARG0(	low dens	[illegible] 3p,miR-222-5p,miR-485-3p,miR-548e-3p,miR-378d,miR-19b-3p,miR-4429,miR-1197-5p,miR-362-3p,miR-146a-5p,miR-216c-5p,miR-21-3p,miR-221-5p,miR-34b-5p,miR-320d,miR-155-5p,miR-204-3p,miR-6130,miR-6516,miR-3065-3p,miR-221-5p,novel_99,miR-2312,miR-27b-5p,miR-21a-3p,miR-10b-5p,miR-3074-2-3p,miR-4510,miR-145a-3p,miR-708-5p,miR-208b-5p,miR-424-5p,miR-6516-3p,miR-664a,miR-155-5p,miR-17-3p,miR-450b-3p,miR-320e,miR-222-3p,miR-221-3p,miR-500-5p,miR-142-3p,miR-222-5p,miR-409b,novel_4,miR-151a-3p,miR-450b-5p,miR-7857,miR-4791,miR-10b,miR-484,miR-3187-3p,miR-30c-1-3p,miR-33a-5p,miR-424-5p,miR-494-3p,miR-2330-5p,miR-3085-3p,miR-362-3p,miR-320b,miR-342,miR-20a-3p,miR-664b,miR-3064-5p,novel_94,miR-877-3p,miR-199b-5p,miR-361-3p,miR-362-5p,miR-452-5p,miR-181d-5p,miR-2285f,miR-3059-5p,miR-2284q,miR-4726-5p,miR-769-5p,miR-371a-5p,miR-6516-5p,miR-222,miR-10a-5p,miR-300,miR-134,miR-345-3p,miR-2284a,miR-490-3p,miR-199a-5p,miR-3065-3p,miR-4286,miR-181a-2-3p,miR-30a-3p,miR-29b-2-5p,miR-543-5p,miR-107,miR-181b-5p,miR-17-3p,miR-30c-2-3p,miR-1260a,miR-199a-5p,miR-769,miR-34c-5p,miR-615-5p,miR-485-5p,miR-671-5p,miR-1814c,novel_48,miR-7144-5p,miR-10b-3p,miR-320a,miR-362-5p,miR-381-3p,miR-145-3p,miR-500b-5p,miR-103b,miR-3607-3p,miR-1306-5p,miR-486b-3p,miR-28-5p,miR-204-5p,miR-135a-5p,miR-5100,miR-103,miR-185-5p,miR-130a-3p,miR-133b-5p,miR-219a-1-3p,miR-1260b,miR-380-3p,miR-374b-3p,miR-3071-5p,miR-145a-5p,miR-181a-5p,miR-345-5p,miR-296-3p,miR-654-3p,miR-2483-5p,miR-130b-5p,miR-452-5p,miR-124-5p,miR-670-3p,miR-34b,miR-10a-5p,miR-320b,miR-195a-5p,miR-135b-5p,miR-107,miR-19a-3p,miR-1291,miR-2387,miR-2285ab,novel_27,miR-449a,miR-24-
DPYSL3	ENSOARG0(	dihydrop	miR-186-5p,miR-1248,miR-1827,miR-23a-5p,miR-1895,miR-6130,miR-216b-5p,miR-141-3p,miR-221-5p,miR-204-5p,miR-31-3p,miR-29b-3p,miR-1-5p,miR-3184-5p,miR-1260b,miR-4510,miR-3074-5p,miR-1197-5p,miR-330-5p,miR-2427,miR-29d-3p,miR-1247-5p,miR-200a-3p,miR-221-5p,miR-1a-2-5p,miR-2898,miR-92a-1-5p,miR-324-3p,miR-3071-3p,novel_17,miR-2285af,miR-1260a,miR-4286,miR-195a-3p,miR-216c-5p,miR-9-3p,miR-136-5p,miR-668-5p,miR-361-3p,miR-3082-5p,miR-29a-3p,miR-7-1-3p,miR-211-5p,miR-7977,miR-3074-5p,miR-29a,miR-128-1-5p,miR-326-3p,miR-423-5p,miR-9-3p,miR-323c,miR-211,miR-29c-3p,miR-452-5p,miR-1a-1-5p,miR-1260b,miR-29b,miR-421,miR-330-3p,miR-187-3p,miR-31-5p,miR-330-3p,miR-505,miR-326,miR-1b-5p,miR-494-3p,miR-6240

SKOR2	ENSOARG0(	SKI fami	miR-139-5p,miR-452-3p,miR-3082-5p,novel_69,miR-140-5p,miR-147-3p,miR-410-5p,miR-335-5p,miR-1193,miR-9-5p,miR-326-3p,miR-4324,miR-335,miR-409b,miR-151a-3p,miR-1343-3p,miR-145b,miR-2285g,miR-2285e,miR-2355-3p,miR-145a-5p,miR-1388-5p,miR-1260b,miR-450b-5p,miR-2426,miR-539-3p,miR-1b-5p,miR-326,miR-125a-5p,miR-214-3p,miR-370-5p,miR-125a,miR-6516,miR-139-5p,miR-133c,miR-2285c,miR-200a-5p,miR-500a-3p,miR-129-2-3p,miR-3607-3p,miR-3958-5p,miR-214,miR-1260b,miR-370-5p,miR-151-3p,miR-125b-5p,miR-500,miR-486b-3p,miR-485-3p,miR-9-5p,miR-487a-3p,miR-758-5p,miR-6516-5p,miR-2284h-5p,miR-129-1-3p,miR-1434-5p,miR-330-5p,miR-216c-5p,miR-2285w,miR-195a-3p,miR-4286,miR-29a-5p,miR-486-3p,miR-1260a,novel_17,miR-125b,miR-21-3p
LGALS8	ENSOARG0(	lectin,	miR-23b,miR-345-5p,miR-1248,miR-574-5p,miR-1271,miR-125a,miR-133c,novel_63,miR-125b-5p,miR-6740-5p,miR-1260b,miR-1197-3p,miR-1290,miR-196a-5p,miR-3591-5p,miR-330-5p,miR-429-3p,miR-299a-3p,miR-2285ad,miR-18b-5p,miR-1224-5p,miR-9-5p,miR-129-1-3p,miR-219-3p,miR-127-5p,let-7f-5p,miR-152-3p,miR-3064-5p,miR-148b-5p,miR-3956-5p,let-7g,miR-148a-3p,miR-150-5p,miR-1306,let-7e-5p,miR-2319a,miR-299b-3p,miR-411b-3p,miR-320c,miR-208a-3p,miR-28c,miR-576-3p,miR-485-3p,miR-29a,miR-206,miR-421-5p,miR-29c-3p,miR-199b-5p,miR-2113,miR-542-3p,miR-7b-5p,miR-19b-2-5p,miR-197-3p,miR-431-3p,miR-3955-5p,miR-31-5p,miR-134-3p,miR-149-5p,miR-194-3p,miR-7-5p,miR-365b-5p,miR-378a-5p,miR-196b-5p,miR-493-5p,let-7f-2-3p,miR-628-5p,let-7f-2-3p,miR-668-3p,miR-503-3p,let-7a-2-3p,miR-411,miR-500a-3p,miR-345-5p,miR-186-5p,miR-1827,miR-30c-1-3p,miR-504-5p,miR-552-3p,let-7i-5p,miR-30e-3p,let-7f,miR-151-3p,miR-219b-3p,miR-30a-3p,miR-130b-5p,miR-500,miR-129-5p,miR-129-2-3p,let-7b,miR-769-5p,miR-6525,miR-3184-5p,miR-122-5p,miR-193b-5p,miR-1961,miR-148a-5p,miR-30d-3p,miR-23c,miR-7859,miR-23b-3p,miR-210-5p,miR-208b-3p,miR-500a-5p,miR-3601,miR-98-5p,let-7k,miR-345-3p,miR-195a-3p,miR-664b-3p,miR-421-5p,miR-2424,miR-486-3p,miR-221,miR-378j,miR-504,miR-544-5p,miR-493-5p,miR-2319b,miR-3596,miR-378g,miR-4443,miR-1185-2-3p,miR-101-3p,miR-301a-5p,miR-499a-5p,miR-3956-3p,miR-9-5p,miR-877-3p,miR-423-5p,miR-301b-5p,miR-758-3p,miR-1a-3p,miR-1388-5p,miR-1839-5p,miR-1343-3p,miR-147a,miR-425-3p,miR-421,miR-1185-3p,miR-148b-5p,miR-153-3p,miR-199b-5p,miR-101b-3p,miR-574-3p,miR-23a-3p,miR-2310,miR-488-3p,miR-18a,miR-320a,let-7g-3p,miR-1814c,miR-671-5p,miR-485-5p,miR-7144-5p,miR-769,miR-199a-5p,miR-324-5p,miR-135a-1-3p,miR-

ZNF548	ENSOARG0(	zinc fin	miR-181a-2-3p,miR-2284f,miR-3529-3p,novel_39,miR-200a,miR-3065-3p,miR-2284y,miR-192-3p,miR-450c-5p,miR-2284a,miR-450b-5p,miR-205-5p,miR-200c,miR-200a-3p,novel_127,miR-17-3p,miR-127-5p,miR-181b-5p,miR-200c-3p,miR-376a-5p,miR-2285ad,miR-429-3p,miR-6517,miR-545-5p,miR-134-5p,miR-214-5p,miR-9-5p,miR-24-2-5p,miR-2411-5p,miR-501-3p,miR-330-5p,miR-345-3p,miR-3578,miR-134,miR-1290,miR-200b-3p,miR-582-3p,miR-193a-5p,miR-103b,miR-145-3p,miR-1197-3p,miR-409-3p,miR-2284k,miR-1306-3p,miR-486b-3p,miR-134-5p,miR-138-5p,miR-141-3p,miR-223-5p,miR-3965,miR-3959-5p,miR-3154,miR-320a,miR-7144-5p,miR-671-5p,miR-10b-3p,miR-345-5p,miR-2318,miR-628-5p,let-7f-2-3p,let-7f-2-3p,miR-130b-5p,miR-539-3p,miR-345-5p,miR-432-3p,miR-432-3p,miR-2284l,miR-105-5p,miR-2411-3p,miR-224-5p,miR-466f-3p,miR-378a-5p,miR-544b,miR-3431,miR-197-3p,miR-145a-5p,miR-19b-2-5p,miR-181a-5p,miR-1285,miR-188-5p,miR-30b-3p,miR-138,miR-2284ab,miR-21-5p,miR-219a-1-3p,miR-17-3p,miR-135a-2-3p,miR-326-3p,miR-2428,miR-2284o,miR-502b,novel_1,miR-655-5p,miR-3120-3p,miR-378b,miR-320c,miR-677,miR-323a-5p,miR-21c,miR-3065-5p,miR-18a-3p,miR-2319a,miR-9851-3p,miR-376a-2-5p,miR-2284e,miR-183-5p,miR-216c-5p,miR-486-3p,miR-2411,miR-2424,miR-143-3p,miR-376a-5p,miR-130a-5p,miR-345-3p,miR-21-3p,miR-320d,miR-2284m,miR-1b-3p,miR-3600,miR-485-3p,miR-18a-3p,miR-7859,miR-487a-3p,miR-200b,miR-2904,miR-5703,miR-376a-5p,miR-2284u,miR-361-5p,miR-4429,miR-142-3p,miR-214,miR-665-5p,miR-425-5p,miR-3074-2-3p,miR-4510,miR-181c-5p,miR-338-3p,miR-224-5p,miR-19b-1-5p,miR-30a-3p,miR-655-3p,miR-338-5p,miR-2285j,miR-30e-3p,miR-500,miR-4492,miR-130b-5p,miR-17-3p,miR-3065-3p,miR-552-3p,miR-296-3p,miR-182-5p,miR-548o-3p,novel_32,miR-544-[illegible]

FBXW11	ENSOARG0(	F-box an	miR-708-5p,miR-17-3p,miR-324-3p,let-7b-5p,miR-6134,miR-1271,miR-539-5p,miR-4510,novel_99,miR-582-5p,miR-2312,miR-133b-3p,let-7g-3p,miR-204-3p,miR-6516,miR-544-3p,miR-6130,miR-341-3p,miR-182-5p,miR-32-3p,miR-30b-3p,miR-299-3p,miR-21-3p,miR-106a,miR-320d,miR-125b,miR-125b-2-3p,miR-20a,miR-216c-5p,miR-4429,miR-5703,miR-1197-5p,miR-362-3p,miR-199a-3p,novel_83,miR-532-3p,miR-200b,miR-3600,miR-374a-3p,miR-2284q,miR-2285f,miR-194b-5p,miR-3059-5p,novel_60,miR-32-3p,miR-877-3p,let-7d-5p,miR-27a-3p,miR-3082-5p,miR-499b-5p,miR-20a-3p,miR-2285o,let-7e,miR-362-3p,miR-342,miR-320b,miR-1306-5p,let-7c-3p,let-7a-5p,miR-382-3p,miR-106a-5p,miR-16-2-3p,miR-2426,miR-544a,miR-7857,miR-30b-3p,miR-484,miR-4791,miR-9788-3p,miR-216a-3p,miR-27a-3p,miR-410-3p,miR-374b-3p,miR-28-5p,miR-486b-3p,miR-204-5p,miR-767-5p,miR-331-3p,miR-26c,miR-29b-3p,miR-93,miR-374a-3p,miR-133a-3p,miR-3074-1-3p,miR-7144-5p,miR-320a,miR-17-5p,miR-381-3p,miR-488-3p,miR-503-3p,miR-374c-3p,miR-141-3p,miR-17-3p,miR-200c-3p,let-7c-5p,miR-2285af,miR-200c,miR-490-3p,miR-2403,miR-200b-3p,miR-300,let-7d,miR-29d-3p,miR-3578,miR-6516-5p,miR-1271-5p,miR-7641,miR-502b,miR-147-5p,miR-128-1-5p,novel_1,let-7i,miR-33a-3p,miR-20a-5p,novel_27,miR-211,miR-24-3p,miR-3959-3p,miR-17-3p,miR-342-3p,novel_120,miR-9851-3p,miR-1291,miR-2285ab,miR-2387,miR-146b-3p,miR-29a-3p,miR-655-5p,miR-1343-5p,miR-125a-5p,miR-330-3p,miR-106a-5p,miR-455-5p,miR-17-5p,miR-345-5p,miR-101-5p,miR-744-5p,miR-455-5p,miR-29b,miR-30b-3p,miR-194-5p,miR-873a-5p,miR-1434-3p,miR-20b,miR-544b,miR-432-5p,let-7g-5p,miR-665,miR-130a-3p,miR-133b-5p,miR-874-5p,miR-454-3p,miR-451a,miR-3604,let-7f,miR-214,let-7b,miR-3074-5p,miR-129-2-3p,miR-186-5p,miR-345-

EWSR1	ENSOARG0(	EWS RNA-	miR-92a-1-5p,miR-3601,miR-324-3p,miR-345-3p,miR-377-5p,miR-486-3p,miR-193b-5p,miR-2366,miR-33b-3p,miR-34a-5p,miR-18a-3p,miR-212-5p,miR-328-3p,miR-665,miR-874-5p,miR-4492,miR-3074-5p,miR-214,miR-3958-5p,miR-769-5p,miR-3184-5p,miR-345-5p,miR-339b,miR-1827,miR-885-3p,miR-1895,miR-30c-1-3p,miR-504-5p,miR-2331-3p,miR-296-3p,miR-552-3p,miR-216b-3p,miR-377-3p,miR-499b-3p,miR-582-5p,miR-15b-5p,miR-16b,miR-29b-1-5p,miR-491-5p,miR-214-3p,miR-381-5p,miR-199b-5p,miR-574-3p,miR-541,novel_78,miR-181b-2-3p,miR-147a,miR-2459,miR-455-3p,novel_82,miR-3968,miR-490-5p,miR-3956-3p,miR-2447,miR-877-3p,miR-361-3p,miR-423-5p,miR-504,miR-96-5p,miR-4792,miR-139-5p,miR-24-1-5p,miR-211-5p,miR-499a-3p,miR-378g,miR-8117,novel_96,novel_127,miR-3064-5p,miR-1343-5p,miR-885-3p,novel_79,miR-1224-5p,miR-505-5p,miR-322-5p,miR-134-5p,miR-6740-5p,miR-125b-5p,miR-134-5p,novel_42,miR-16-5p,miR-193a-5p,miR-1197-3p,miR-345-5p,miR-329b,miR-1248,miR-874-3p,miR-574-5p,miR-125a,miR-1271,miR-2355-5p,miR-542-5p,miR-331-5p,miR-377-3p,miR-7-5p,novel_51,miR-378a-5p,miR-193a-3p,let-7j,miR-1843b-5p,miR-7b-5p,miR-432,miR-381-5p,miR-16b,miR-4508,miR-497-5p,miR-3955-5p,miR-340-3p,miR-339-5p,miR-134-3p,miR-149-5p,miR-28c,miR-1247-3p,miR-193b-3p,miR-1839-3p,miR-199b-5p,miR-1306,miR-3970,miR-410-5p,miR-3074-5p,novel_68,miR-3120-3p,novel_69,miR-374c-3p,miR-181b-3p,miR-6535,miR-669,miR-677,miR-542-5p,miR-125b,miR-185-3p,miR-466i-5p,miR-760-3p,miR-34b-5p,miR-30b-3p,miR-221-5p,miR-2887,miR-29b-1-5p,novel_133,miR-5703,miR-1193,miR-2904,miR-378d,miR-1b-3p,miR-15a-5p,miR-532-3p,miR-29b-2-5p,miR-664a,miR-17-3p,miR-424-5p,miR-4510,miR-2330-3p,miR-1271,miR-6134,miR-21a-3p,miR-582-5p,novel_99,miR-
ARFIP1	ENSOARG0(	ADP-ribo	miR-148a-3p,miR-152-3p,miR-410-3p,miR-148b-3p

EFNA1	ENSOARG0(	ephrin-A	miR-135a-2-3p,miR-193b-3p,miR-495-3p,miR-6123,miR-147-5p,miR-15a,miR-576-3p,miR-6529b,miR-16a,novel_69,miR-677,miR-181b-3p,novel_68,miR-320b,miR-450b-3p,miR-1291,miR-195a-5p,miR-150-5p,miR-18a-3p,miR-378b,miR-654-3p,miR-296-3p,miR-2483-5p,miR-483-3p,miR-193a-3p,miR-296-3p,novel_51,miR-149-5p,miR-138,miR-30b-3p,miR-16b,miR-497-5p,miR-19b-2-5p,miR-873a-5p,miR-145a-5p,miR-16-5p,miR-138-5p,miR-486b-3p,miR-6740-5p,miR-365a-3p,miR-370-3p,miR-141-3p,miR-7144-5p,miR-485-5p,miR-488-3p,miR-301,miR-3154,miR-200a,novel_39,novel_127,miR-543-5p,novel_96,miR-200a-3p,miR-2285af,miR-30c-2-3p,miR-322-5p,miR-483-3p,miR-1271-5p,miR-2411-5p,miR-129-1-3p,miR-885-3p,miR-3578,miR-423-5p,miR-2447,novel_60,miR-3956-3p,miR-301a-5p,miR-203-3p,miR-7977,miR-96-5p,novel_94,miR-140-5p,miR-361-3p,miR-30c-1-3p,miR-8485,miR-758-3p,miR-424-5p,miR-652-5p,miR-491-5p,miR-214-3p,miR-505,miR-2957,miR-16b,miR-455-3p,miR-2300a-5p,miR-1343-3p,miR-181b-2-3p,miR-145b,miR-1388-5p,miR-484,miR-301b-5p,miR-758-3p,miR-30b-3p,miR-6525,miR-3184-5p,miR-4510,miR-129-2-3p,miR-145a-3p,miR-1271,miR-2330-3p,miR-214,miR-424-5p,miR-4492,miR-874-5p,miR-19b-1-5p,miR-324-3p,miR-582-5p,miR-6130,miR-193b-3p,miR-204-3p,miR-15b-5p,miR-30c-1-3p,miR-2331-3p,miR-221-5p,miR-885-3p,miR-129b-3p,miR-181b-1-3p,miR-2411,miR-486-3p,miR-183-5p,miR-5100,miR-345-3p,miR-221-5p,miR-219b-5p,miR-500a-5p,miR-185-3p,miR-15a-5p,miR-532-3p,novel_83,miR-18a-3p,miR-188-3p
CCNL2	ENSOARG0(	cyclin L	miR-1291,miR-107,miR-1827,miR-877-3p,miR-129b-5p,miR-132-5p,miR-216b-3p,miR-552-3p,miR-26b-5p,miR-128-1-5p,miR-129-5p,miR-203b-5p,miR-2433,miR-6525,novel_111,novel_101,miR-941,miR-142-3p,miR-664-5p,miR-196a-5p,miR-126-3p,miR-2904,miR-412-3p,miR-3962,miR-188-3p,miR-2411-5p,miR-532-3p,miR-411-5p,miR-149-5p,miR-103,miR-214-5p,miR-103a-3p,miR-412,miR-491-5p,miR-196b-5p,miR-2898,miR-107,miR-30b-3p,novel_96,novel_17,miR-26a-5p,miR-2411,miR-363-5p,miR-4286
SMAD2	ENSOARG0(	SMAD fam	miR-3604,miR-769-5p,miR-365b-5p,miR-30b,miR-30b-5p,miR-30d,miR-127-5p,miR-212-3p,miR-365a-5p,miR-8485,miR-769-5p,miR-18a,miR-30d-5p,miR-30a-5p,miR-18b,miR-30e-5p,miR-30c-5p,miR-30c,miR-18a-5p,miR-30f,let-7g-3p,miR-18b-5p,novel_78,miR-3955-5p,miR-455-3p,miR-769,miR-3596

ZNF550	ENSOARG0(	zinc fin	miR-378e,miR-20a-3p,miR-211-5p,miR-668-5p,miR-9-3p,miR-335-3p,miR-199b-5p,miR-361-3p,miR-129b-5p,miR-154a,miR-3059-5p,miR-2447,miR-181d-5p,miR-2284j,miR-2285t,miR-218-5p,miR-2285y,miR-1298-3p,miR-9788-3p,miR-216a-3p,miR-409b,miR-1388-5p,miR-378c,miR-2284aa,miR-101c,miR-378b,miR-125b-2-3p,miR-30b-3p,miR-378d,miR-3187-3p,miR-2330-5p,miR-424-5p,miR-1388-3p,miR-130b-3p,miR-199b-5p,miR-1306-5p,miR-320b,miR-16b,miR-301a-3p,miR-155-5p,miR-15b-5p,miR-204-3p,miR-378a-3p,miR-532-5p,miR-370-5p,miR-345-5p,novel_99,miR-186-5p,miR-543-3p,miR-21a-3p,miR-487b-5p,miR-129b-3p,miR-6134,miR-378c,miR-539-5p,miR-425-5p,miR-2330-3p,miR-6516-3p,miR-155-5p,miR-454-3p,miR-424-5p,miR-665,miR-15a-5p,miR-200b,miR-212-5p,miR-4429,miR-33b-3p,miR-19b-3p,miR-16-1-3p,miR-378i,miR-378a-3p,miR-5703,miR-217-5p,miR-377-5p,miR-486-3p,miR-503-5p,miR-216c-5p,miR-345-3p,miR-466i-5p,miR-320d,miR-21-3p,novel_17,miR-26a-5p,miR-422a,miR-125b,miR-345-3p,miR-185-3p,miR-324-3p,miR-670-3p,miR-320c,novel_68,miR-26b-5p,miR-19a-3p,miR-1291,miR-195a-5p,miR-378f,miR-6516-3p,miR-2319a,miR-1306,miR-24-3p,miR-211,miR-199b-5p,miR-301b-3p,miR-2428,miR-1193,miR-203b-5p,miR-502-5p,miR-15a,miR-16a,miR-503-5p,miR-2284d,miR-377-5p,miR-130a-3p,miR-219a-1-3p,miR-16-1-3p,miR-30b-3p,miR-16b,miR-2284ab,miR-497-5p,miR-2113,miR-7b-5p,miR-181a-5p,miR-432-3p,miR-2284l,miR-432-3p,miR-345-5p,miR-628-5p,miR-125a-5p,miR-7-5p,miR-503-5p,miR-2411-3p,miR-154b-5p,miR-199a-5p,miR-324-5p,miR-125a,miR-345-5p,miR-876-3p,miR-1248,miR-671-5p,miR-320a,miR-3154,miR-378c,miR-2284v,miR-16-5p,miR-204-5p,miR-134-5p,miR-486b-3p,miR-370-5p,miR-494-5p,miR-2284k,miR-125b-5p,miR-331-3p,miR-378h,miR-322-5p,miR-505-5p,miR-2284z,miR-134-5p,miR-429-3p,miR-
WDTC1	ENSOARG0(	WD and t	miR-107,miR-195a-5p,miR-6395,miR-378f,miR-1306,miR-378e,miR-154b-5p,miR-15b-5p,miR-378b,miR-378a-3p,miR-424-5p,miR-15a,miR-16a,miR-331-3p,miR-378c,miR-378c,miR-16-5p,miR-30b-3p,miR-16b,miR-378c,miR-497-5p,miR-4791,miR-378b,novel_133,miR-378i,miR-378a-3p,miR-30b-3p,miR-15a-5p,miR-378h,miR-322-5p,miR-3535,miR-328-3p,miR-103,miR-149-5p,miR-103a-3p,novel_124,miR-107,miR-1306-5p,miR-16b,miR-422a,miR-378d,miR-424-5p,miR-4286
MED22	ENSOARG0(	mediator	miR-378g,miR-134-3p,miR-199a-5p,novel_4,miR-30b-3p,miR-199b-5p,miR-30b-3p,miR-330-5p,miR-3184-5p,miR-199a-5p,miR-423-5p,miR-326-3p,miR-199b-5p,miR-128-1-5p,miR-760-3p,miR-199b-5p,miR-326,miR-185-3p
BECN1	ENSOARG0(	beclin 1	miR-30d,miR-544a,miR-30a-5p,miR-30d-5p,miR-30f,miR-30e-5p,miR-544b,miR-544-3p

MBNL1	ENSOARG0(	musclebl	miR-183-5p,miR-130a-5p,miR-2411,miR-2424,miR-2432,miR-196a-3p,miR-30b,miR-2285p,novel_17,miR-500a-5p,miR-345-3p,miR-2284m,miR-760-3p,miR-466i-5p,miR-221-5p,miR-30d,miR-548e-3p,miR-23b-3p,miR-7859,miR-23c,miR-30c-5p,miR-193b-5p,miR-218-1-3p,miR-2284u,miR-148a-5p,miR-590-3p,miR-425-5p,miR-346,miR-181c-5p,miR-2285j,miR-19b-1-5p,miR-338-5p,miR-664a,miR-500,miR-454-3p,miR-669a-3p,miR-221-5p,miR-140-5p,miR-545-3p,miR-496-3p,miR-548o-3p,miR-6516,miR-301a-3p,miR-2284s,let-7f-1-3p,miR-500a-3p,miR-30d-5p,miR-18a-5p,miR-30f,miR-186-5p,novel_99,miR-101b-3p,miR-23a-3p,miR-2284b,miR-3187-3p,miR-323b-3p,miR-3955-3p,miR-130b-3p,miR-2300a-5p,miR-199b-3p,miR-330-3p,miR-539-5p,miR-147a,miR-148b-5p,miR-1185-3p,miR-484,miR-2284r,miR-143-5p,miR-323b,miR-3059-5p,miR-885-5p,miR-181d-5p,miR-2284j,miR-9-5p,miR-30b-5p,miR-101-3p,miR-371a-5p,miR-2284g,miR-2319b,miR-211-5p,miR-1185-2-3p,miR-381-3p,miR-129b-5p,miR-99b-5p,miR-668-5p,miR-1983,miR-29b-2-5p,miR-181a-2-3p,miR-380-5p,miR-29a-5p,miR-200a,miR-2284y,miR-2284a,miR-2285b,miR-99a,miR-3071-3p,miR-181b-5p,miR-107,miR-200a-3p,miR-18b-5p,miR-9-5p,miR-758-5p,miR-2411-5p,miR-6516-5p,miR-545-5p,miR-134-5p,novel_121,miR-345-3p,miR-134,miR-30a-5p,miR-300,miR-99a-5p,let-7a-2-3p,miR-496,miR-2284v,novel_63,miR-410-5p,miR-2284k,miR-144,novel_9,miR-204-5p,miR-134-5p,miR-370-3p,miR-141-3p,miR-374c-3p,miR-133c,miR-2284n,miR-381-3p,miR-2448-3p,miR-200a-5p,miR-18a,miR-23b,miR-100-5p,let-7g-3p,miR-23a-5p,miR-380-5p,miR-6240,miR-2483-5p,let-7f-2-3p,let-7f-2-3p,miR-432-3p,miR-296-3p,let-7a-2-3p,miR-296-3p,miR-330-3p,miR-99a-5p,miR-2411-3p,miR-452-5p,miR-187-3p,miR-103a-3p,miR-130a-3p,miR-412-5p,miR-2284d,miR-3431,miR-194-3p,miR-103,miR-2355-3p,miR-19b-2-
ACTN2	ENSOARG0(	actinin,	miR-2397-5p,miR-2484,miR-30b-3p,miR-221-5p,miR-216c-5p,miR-490-3p,miR-654-3p,miR-2285r,miR-2478,miR-140-3p,novel_23,miR-542-3p,miR-450b-5p,miR-411-3p,miR-26b-3p,miR-5010-3p,miR-548w,miR-3960,miR-455-3p,miR-6516-5p,miR-338-5p,miR-2285j,miR-208b-5p,miR-379-3p,miR-495-3p,miR-2285f,miR-6128,miR-539-5p,miR-8095,miR-142a-5p,miR-361-3p,miR-3591-3p,miR-3065-5p,miR-137-3p,miR-200a-5p,miR-2285ab,novel_48,miR-668-5p,miR-2312,miR-3963,miR-7-1-3p,miR-21-3p,miR-541-5p,miR-2285u,miR-221-5p,miR-6516

MAPK3	ENSOARG0(	mitogen-	miR-769-5p,miR-4726-5p,miR-2285t,miR-212-3p,miR-541-3p,miR-452-5p,miR-181d-5p,miR-362-5p,miR-502-3p,miR-361-3p,miR-129b-5p,novel_94,miR-3064-5p,miR-452-3p,miR-10b,miR-7977,miR-3082-5p,miR-27a-3p,miR-454-5p,miR-342,novel_103,miR-326,miR-1306-5p,miR-2957,miR-3085-3p,miR-542-5p,miR-424-5p,miR-652-5p,miR-30c-1-3p,miR-4532,miR-296-5p,miR-484,miR-10b,miR-7857,miR-30b-3p,miR-2426,miR-3120-5p,novel_4,miR-27a-3p,miR-9788-3p,miR-500-5p,miR-17-3p,miR-664a,miR-424-5p,miR-708-5p,miR-208b-5p,miR-145a-3p,miR-4510,miR-10b-5p,miR-206-3p,miR-6134,miR-21a-3p,miR-133b-3p,miR-221-5p,miR-3065-3p,miR-182-5p,miR-532-5p,miR-6130,miR-22-3p,miR-542-5p,miR-125b,miR-185-3p,miR-466i-5p,miR-34b-5p,miR-30b-3p,miR-221-5p,miR-21-3p,miR-183-5p,miR-125b-2-3p,miR-2904,miR-5703,miR-19b-3p,miR-378d,miR-1b-3p,miR-383-5p,miR-148b-3p,miR-15a-5p,novel_83,miR-15a,miR-6529b,miR-34c,miR-16a,miR-199c,miR-147-5p,miR-342-3p,miR-3957-3p,miR-17-3p,miR-542-5p,novel_101,miR-211,miR-24-3p,miR-326-3p,miR-449a,miR-2428,miR-2387,miR-18a-3p,miR-1291,miR-19a-3p,miR-107,miR-195a-5p,miR-320b,miR-10a-5p,miR-2404,miR-146b-3p,miR-34b,miR-670-3p,miR-224-5p,miR-197-5p,miR-455-5p,miR-1343-5p,miR-125a-5p,miR-363-5p,miR-2483-5p,miR-345-5p,miR-1285,miR-181a-5p,miR-30b-3p,miR-219a-1-3p,miR-455-5p,miR-665,miR-377-5p,miR-2355-3p,miR-103,miR-551b-3p,miR-5100,miR-99b-3p,miR-331-3p,miR-26c,miR-767-5p,miR-486b-3p,miR-28-5p,miR-204-5p,miR-1306-5p,miR-6128,miR-133a-3p,miR-500b-5p,miR-145-3p,miR-362-5p,miR-23a-5p,miR-190a-3p,miR-485-5p,miR-370-3p,miR-34c-5p,miR-769,miR-6119-5p,miR-30c-2-3p,miR-2898,miR-17-3p,miR-181b-5p,miR-107,miR-543-5p,miR-3529-3p,miR-1,miR-4286,miR-28b,miR-2478,miR-3065-3p,miR-2284a,miR-1271-3p,miR-134,miR-501-

STK10	ENSOARG0(	serine/t	miR-1839-5p, miR-378c, miR-2426, miR-296-5p, miR-378b, miR-6529a, miR-484, miR-145b, miR-27a-3p, miR-105-1, miR-146b, miR-542-5p, miR-103a-2-5p, miR-214-3p, miR-3085-3p, miR-3955-3p, miR-29b-1-5p, miR-16b, miR-182-5p, miR-342, miR-1306-5p, miR-193a, miR-2310, miR-378d, miR-382-3p, miR-424-5p, miR-9-3p, miR-361-3p, miR-27a-3p, miR-499a-3p, miR-378e, miR-3064-5p, miR-490-5p, miR-107-5p, miR-1285-5p, miR-142-3p, miR-2904, miR-378a-3p, miR-378i, novel_133, miR-193b-5p, miR-2366, miR-7859, miR-340-5p, miR-15a-5p, miR-148b-3p, miR-3600, miR-548e-3p, miR-18a-3p, miR-21-3p, miR-422a, miR-92a-1-5p, miR-542-5p, novel_17, miR-146a-5p, miR-143-3p, miR-29b-1-5p, miR-183-5p, miR-885-3p, miR-625-5p, miR-1827, miR-133b-3p, miR-450a-1-3p, miR-15b-5p, miR-499b-3p, miR-377-3p, miR-370-5p, miR-182-5p, miR-378a-3p, miR-548o-3p, miR-4492, miR-424-5p, miR-450b-3p, miR-324-3p, miR-665, miR-664a-5p, miR-338-3p, miR-214, miR-539-5p, miR-378c, miR-497-5p, miR-27b-3p, miR-21-5p, miR-16b, miR-3432b, miR-1260b, miR-450c-3p, miR-3071-5p, miR-145a-5p, miR-2113, miR-185-5p, miR-133b-5p, miR-483-3p, miR-378a-5p, miR-105-5p, miR-365b-5p, miR-377-3p, miR-146a, miR-101-5p, miR-363-5p, miR-146b-5p, miR-195a-5p, miR-378f, miR-1291, miR-450b-3p, miR-1306, miR-18a-3p, miR-21c, miR-148a-3p, miR-6535, miR-670-3p, miR-3120-3p, miR-16a, miR-15a, miR-576-3p, novel_27, miR-2284w, miR-24-3p, miR-323c, miR-1193, miR-542-5p, miR-342-3p, miR-885-3p, miR-23b-5p, miR-450a-1-3p, miR-322-5p, miR-483-3p, miR-378h, miR-7641, miR-152-3p, novel_127, miR-1260a, miR-490-3p, miR-3064-5p, miR-1814c, miR-542-5p, miR-370-3p, miR-2355-5p, miR-1271, miR-592, miR-370-5p, miR-145-3p, miR-378c, miR-365a-5p, miR-1260b, miR-16-5p, miR-133a-3p

SLC50A1	ENSOARG0(	solute c	miR-137-3p,miR-424-3p,miR-301,miR-329a,miR-345-5p,miR-1271,miR-125a,miR-874-3p,miR-3068-3p,miR-133c,miR-125b-5p,miR-16-5p,miR-193a-5p,miR-1260b,miR-330-5p,miR-1343-5p,miR-431,novel_79,miR-2285ad,miR-9-5p,miR-322-5p,miR-125a-3p,miR-664-3p,miR-200a-3p,miR-152-3p,miR-200a,miR-216a-3p,miR-3970,miR-148a-3p,miR-3141,miR-378f,miR-146b-5p,miR-2319a,miR-410-5p,miR-3074-5p,miR-216b-3p,miR-144-3p,miR-669,novel_69,miR-485-3p,miR-421-5p,miR-29a,miR-29c-3p,miR-301b-3p,miR-22-3p,miR-7b-5p,miR-223-3p,miR-2284x,miR-26a-2-3p,miR-497-5p,miR-16-1-3p,miR-16b,miR-339-5p,miR-103a-3p,miR-3955-5p,miR-194-3p,miR-149-5p,miR-134-3p,miR-2411-3p,miR-3141,miR-365b-5p,miR-146a,miR-7-5p,miR-378a-5p,miR-493-5p,miR-6240,miR-2285c,miR-345-5p,miR-339b,miR-15b-5p,miR-301a-3p,miR-582-5p,miR-10a,miR-664a-5p,miR-30a-3p,miR-30e-3p,miR-130b-5p,miR-129-5p,miR-4492,miR-454-3p,novel_74,miR-665-5p,miR-214,miR-3958-5p,miR-3074-5p,miR-122-5p,miR-346,miR-378a-3p,miR-378i,miR-361-5p,miR-16-1-3p,miR-33b-3p,miR-422a,miR-345-3p,miR-324-3p,novel_17,miR-210-5p,miR-345-3p,miR-2285i,miR-409-5p,miR-140-5p,miR-493-5p,miR-136-5p,miR-211-5p,miR-2319b,miR-24-1-5p,miR-4443,miR-378g,miR-218-5p,miR-9-5p,miR-3956-3p,miR-2447,miR-877-3p,miR-541,miR-329-3p,miR-362-3p,miR-145b,miR-1343-3p,miR-2459,miR-147a,miR-539-5p,miR-16b,miR-29b-1-5p,miR-431-5p,miR-103a-2-5p,miR-214-3p,miR-146b,miR-130b-3p,miR-1388-3p,miR-124a,miR-574-3p,miR-378d,miR-3154,novel_48,miR-23a-5p,miR-141-3p,miR-370-3p,miR-331-3p,miR-144,miR-5100,miR-29b-3p,miR-374b-3p,miR-204-5p,miR-31-3p,miR-138-5p,miR-145-3p,miR-378c,miR-365a-5p,miR-374a-3p,miR-29d-3p,miR-345-3p,miR-10a-5p,miR-128-3p,miR-582-3p,miR-378b,miR-339a,miR-1260a,miR-543-5p,miR-

SUCNR1	ENSOARG0(	succinat	miR-219-3p,miR-664-3p,miR-30c-2-3p,let-7c-5p,miR-217,miR-450a-2-3p,miR-181b-5p,miR-6536,let-7f-5p,miR-3529-3p,miR-1,miR-2478,miR-29a-5p,let-7g,let-7d,miR-582-3p,miR-155-5p,miR-299a-3p,miR-6517,miR-376a-5p,miR-545-5p,novel_121,miR-410-5p,miR-125b-5p,miR-331-3p,miR-410-3p,miR-374b-3p,miR-1306-5p,miR-3607-3p,miR-6128,miR-193a-5p,miR-500b-5p,miR-323-3p,miR-374a-3p,miR-2448-3p,miR-301,miR-503-3p,miR-137-3p,miR-3154,miR-23b,miR-362-5p,miR-10b-3p,miR-345-5p,miR-2318,miR-329a,miR-3074-1-3p,miR-1814c,miR-503-3p,miR-2355-5p,miR-125a,miR-331-5p,miR-300-3p,miR-769,miR-223-5p,miR-146a,miR-377-3p,miR-190a,miR-197-5p,miR-105-5p,miR-106b-3p,miR-452-5p,miR-125a-5p,miR-466f-3p,miR-493-5p,miR-101-5p,miR-654-3p,miR-2285r,miR-345-5p,miR-181a-5p,miR-2113,miR-3071-5p,miR-21-5p,miR-26a-2-3p,miR-665,miR-185-5p,let-7g-5p,miR-2355-3p,miR-4324,miR-3613,miR-33a-3p,let-7i,miR-206,novel_1,miR-335-5p,novel_27,miR-2285ab,miR-2387,miR-21c,miR-450b-3p,miR-412-3p,miR-146b-5p,miR-6516-3p,let-7e-5p,miR-376a-2-5p,miR-378b,miR-320b,miR-146b-3p,miR-2404,miR-299b-3p,miR-210-5p,novel_17,miR-618,miR-500a-5p,miR-125b,miR-98-5p,miR-466i-5p,miR-299-3p,let-7k,miR-216c-5p,miR-376a-5p,miR-664b-3p,miR-29b-1-5p,miR-146a-5p,novel_133,miR-1961,miR-362-3p,miR-2300b-3p,miR-193b-5p,miR-376a-5p,miR-1197-5p,miR-5703,miR-217-5p,novel_73,miR-148a-5p,miR-548e-3p,miR-23b-3p,miR-7859,miR-340-5p,miR-23c,let-7f,miR-2285j,miR-219b-3p,miR-320e,miR-3604,miR-664a-5p,miR-155-5p,miR-6516-3p,miR-190a-5p,miR-208b-5p,miR-129-5p,miR-669a-3p,miR-590-3p,let-7b,miR-145a-3p,miR-3184-3p,miR-3074-2-3p,miR-769-5p,miR-206-3p,let-7b-5p,miR-338-3p,miR-181c-5p,miR-21a-3p,miR-3613-5p,miR-27b-5p,miR-129b-3p,let-7g-3p,miR-345-
COPG1	ENSOARG0(	coatomer	miR-221-5p,miR-664-3p,miR-412-5p,miR-185-3p,miR-664b-3p,miR-450b-5p,miR-142a-3p,miR-329a-5p,miR-503-5p,miR-3529-3p,novel_133,miR-193b-5p,miR-2366,miR-708-5p,miR-28-5p,miR-2285j,miR-2332,miR-6740-5p,miR-320e,miR-142a-5p,miR-145-3p,miR-3184-5p,miR-876-3p,miR-129b-3p,miR-300-3p,miR-365a-3p,novel_32,miR-221-5p,miR-216b-3p,miR-542-5p,miR-615-5p,miR-182-5p,miR-341-3p,miR-503-5p,miR-342,miR-5126,novel_19,miR-182-5p,miR-4508,miR-744-5p,miR-503-5p,miR-142-3p,miR-142-5p,miR-194-3p,miR-411b-5p,novel_4,miR-2300a-5p,miR-409b,miR-128-1-5p,miR-7134-3p,miR-412-5p,miR-1247-3p,miR-6529b,miR-361-3p,miR-423-5p,miR-342-3p,miR-135a-2-3p,miR-4454,miR-2387,miR-3065-5p,miR-25,miR-3596,novel_68,miR-664b

IST1	ENSOARG0(	increase	miR-424-5p,miR-664a,miR-664a-5p,let-7f,let-7b-5p,miR-6134,miR-3958-5p,miR-2330-3p,let-7b,miR-4510,miR-1827,miR-204-3p,let-7i-5p,miR-877-5p,miR-15b-5p,miR-6130,miR-182-5p,miR-504-5p,miR-3653-3p,miR-2484,let-7k,miR-106a,miR-98-5p,miR-125b,miR-196a-3p,miR-29b-1-5p,miR-20a,miR-2904,miR-1961,miR-1247-5p,miR-616-3p,miR-15a-5p,miR-148b-3p,miR-212-5p,miR-505-3p,miR-106a,miR-499a-5p,miR-1973,miR-107-5p,miR-382-3p,miR-2285n,miR-877-3p,miR-9-5p,novel_60,miR-3059-5p,miR-2447,miR-3956-3p,miR-504,miR-361-3p,miR-199b-5p,miR-221,let-7d-5p,miR-4443,miR-27a-3p,miR-378g,miR-2483-3p,miR-199b-5p,let-7e,miR-103a-2-5p,miR-29b-1-5p,miR-16b,miR-106b-5p,miR-182-5p,miR-431-5p,miR-676-3p,let-7a-5p,miR-106a-5p,miR-382-3p,miR-424-5p,miR-7857,miR-125b-2-3p,miR-6529a,miR-143-5p,miR-221-3p,miR-216a-3p,miR-421,miR-147a,miR-27a-3p,novel_78,miR-125b-5p,miR-410-5p,miR-365a-5p,miR-93,miR-16-5p,miR-6128,miR-20b-5p,miR-671-5p,miR-485-5p,miR-10b-3p,miR-93-5p,miR-17-5p,miR-488-3p,miR-199a-5p,miR-154b-5p,miR-370-3p,miR-1271,miR-125a,miR-152-3p,miR-2898,let-7f-5p,miR-3071-3p,let-7c-5p,miR-205-5p,miR-2397-5p,let-7g,miR-29a-5p,miR-199a-5p,miR-128-3p,let-7d,miR-2427,miR-196a-5p,miR-322-5p,miR-9-5p,miR-431,miR-16a,let-7i,miR-4324,miR-15a,miR-20a-5p,miR-133a-5p,miR-199b-5p,miR-323c,novel_101,novel_120,miR-7975,miR-505-3p,miR-195a-5p,let-7e-5p,miR-410-5p,miR-216a-3p,miR-2387,miR-148a-3p,miR-125a-5p,miR-196b-5p,miR-378a-5p,miR-365b-5p,miR-106a-5p,miR-17-5p,miR-296-3p,miR-296-3p,miR-654-3p,miR-628-5p,miR-497-5p,miR-27b-3p,miR-16b,miR-219a-1-3p,miR-432,miR-708-3p,miR-873a-5p,miR-1843b-5p,miR-2355-3p,miR-20b,miR-432-5p,miR-412-5p,miR-185-5p,miR-134-3p,let-7g-5p,miR-3955-5p,miR-133b-5p,miR-122-3p

MATN2	ENSOARG0(	matrilin	novel_9,miR-331-5p,miR-592,novel_63,miR-125b-5p,miR-1260b,miR-16-5p,miR-876-3p,miR-329b,miR-331-5p,miR-301,miR-874-3p,miR-2285aa,miR-2355-5p,miR-125a,miR-127-5p,novel_96,miR-152-3p,miR-200a-3p,miR-29a-5p,miR-200a,miR-3956-5p,miR-885-3p,miR-155-5p,miR-3591-5p,miR-1343-5p,miR-330-5p,miR-9-5p,miR-505-5p,miR-322-5p,miR-450a-1-3p,novel_121,miR-299a-3p,miR-2285ad,miR-429-3p,miR-1224-5p,miR-412,miR-18b-5p,miR-625-3p,miR-6123,miR-4324,miR-576-3p,miR-28c,miR-199b-5p,miR-193b-3p,miR-410-5p,miR-146b-5p,miR-2319a,miR-148a-3p,miR-3970,miR-299b-3p,miR-144-3p,miR-6535,novel_68,miR-329-5p,miR-193a-3p,miR-377-3p,miR-582,miR-146a,miR-2285v,miR-105-5p,miR-2411-3p,miR-432-3p,miR-2284l,miR-493-5p,miR-6240,miR-16b,miR-16-1-3p,miR-26a-2-3p,miR-542-3p,miR-1843b-5p,miR-197-3p,miR-450c-3p,miR-103a-3p,miR-129-5p,miR-874-5p,miR-4492,miR-130b-5p,miR-664a-5p,miR-6525,miR-3184-5p,miR-122-5p,miR-181c-5p,miR-3958-5p,miR-543-3p,miR-885-3p,miR-186-5p,miR-450a-1-3p,miR-411,miR-377-3p,miR-582-5p,miR-15b-5p,miR-2331-3p,miR-1277-3p,miR-216b-3p,miR-552-3p,miR-618,miR-219b-5p,miR-324-3p,miR-500a-5p,miR-345-3p,miR-376a-5p,miR-664b-3p,miR-377-5p,miR-486-3p,miR-143-3p,miR-329a-5p,miR-142-3p,miR-16-1-3p,miR-2366,miR-7859,miR-210-3p,miR-328-3p,miR-212-5p,miR-499a-5p,miR-628-3p,miR-490-5p,miR-101-3p,miR-2440,miR-361-3p,miR-877-3p,miR-423-5p,miR-7705,miR-2447,miR-9-5p,miR-99b-5p,miR-668-5p,miR-493-5p,miR-381-3p,miR-378j,miR-203-3p,miR-139-5p,miR-7689-3p,miR-211-5p,miR-491-5p,miR-146b,miR-153-3p,miR-199b-5p,miR-505,miR-16b,miR-2310,miR-124a,miR-1388-3p,miR-744-3p,miR-1839-5p,miR-758-3p,miR-153,miR-147a,miR-455-3p,miR-126a-5p,miR-330-3p,miR-1343-3p,miR-145b,miR-486b-3p,miR-204-
AADAC	ENSOARG0(	arylacet	miR-216c-5p,miR-424-5p,miR-16-5p,miR-1193,miR-143-3p,miR-15a,miR-370-5p,miR-16a,miR-16b,miR-127-5p,miR-487b-5p,miR-424-5p,miR-2285t,miR-21-3p,miR-370-5p,miR-322-5p,miR-15a-5p,miR-6238,miR-216a-3p,miR-2459,miR-15b-5p,miR-21a-3p,miR-140-5p,miR-16b,miR-329b,miR-497-5p,miR-2319a,miR-195a-5p
IRF4	ENSOARG0(	interfer	miR-377-3p,miR-324-5p,novel_32,miR-199a-5p,miR-21-3p,miR-125a,miR-2312,miR-339b,miR-23b,miR-3184-5p,miR-6134,miR-664a,miR-125b-5p,miR-665,miR-23b-3p,miR-23c,miR-328-3p,miR-6517,novel_79,miR-493-3p,miR-2300b-3p,miR-2366,miR-330-5p,miR-140-3p,miR-125b-2-3p,miR-199a-5p,miR-490-3p,novel_96,miR-125a-3p,miR-542-5p,miR-324-3p,miR-216a-5p,miR-339a,miR-125b,miR-320b,miR-3120-3p,miR-1291,miR-9851-3p,miR-6395,miR-2285ab,miR-361-3p,miR-199b-5p,miR-3591-3p,miR-361-3p,miR-24-3p,miR-326-3p,miR-423-5p,miR-2428,miR-199b-5p,miR-342-3p,miR-541-3p,miR-2285f,miR-542-5p,novel_101,miR-4324,novel_78,miR-339-5p,miR-4508,miR-541,miR-432-3p,miR-4532,miR-23a-3p,miR-491-5p,miR-125a-5p,miR-199b-5p,miR-542-5p,miR-342,miR-326,miR-377-3p,novel_51

AP1G1	ENSOARG0(	adaptor-	miR-1a-3p,miR-323b,miR-301b-5p,miR-541,miR-484,miR-3120-5p,miR-16-2-3p,miR-2426,miR-145b,miR-2459,miR-421,miR-1185-3p,miR-9788-3p,miR-455-3p,miR-216a-3p,miR-142-5p,miR-323b-3p,miR-199b-5p,miR-3085-3p,miR-376b,miR-101b-3p,miR-124a,miR-2310,miR-199b-5p,miR-877-3p,miR-335-3p,miR-3064-5p,miR-139-5p,miR-1185-2-3p,miR-203-3p,miR-3082-5p,miR-301a-5p,miR-371a-5p,miR-154-3p,miR-7134-3p,miR-490-5p,miR-541-3p,miR-3059-5p,miR-423-5p,miR-877-3p,miR-5703,miR-217-5p,miR-124-3p,miR-2904,miR-191,miR-378d,miR-19b-3p,miR-30d-3p,miR-485-3p,miR-3600,miR-487a-3p,miR-125b,miR-216a-5p,miR-3653-3p,miR-466i-5p,miR-195a-3p,miR-487a,miR-625-5p,miR-154b-3p,miR-18a-5p,miR-216b-3p,miR-154a-3p,miR-21-3p,miR-660-5p,miR-139-5p,miR-301a-3p,miR-665,miR-30a-3p,miR-30e-3p,miR-130b-5p,miR-454-3p,miR-487a-5p,miR-3184-5p,miR-206-3p,miR-142a-5p,miR-145a-5p,miR-1285,miR-1434-3p,miR-29b,miR-26b-3p,miR-130a-3p,miR-665,miR-185-5p,miR-365b-5p,miR-1343-5p,miR-125a-5p,miR-660,miR-628-5p,miR-363-5p,miR-130b-5p,miR-539-3p,miR-2285r,miR-3591-3p,miR-378b,miR-18b,miR-3120-3p,miR-216b-3p,novel_69,miR-29a-3p,miR-670-3p,miR-382-5p,miR-6402,miR-142b,miR-4324,miR-147-5p,miR-502b,miR-29a,miR-206,miR-2285x,miR-29c-3p,miR-495-3p,miR-199b-5p,miR-301b-3p,miR-24-3p,miR-1343-5p,miR-29d-3p,miR-3578,miR-18b-5p,miR-545-5p,miR-217,miR-205-5p,miR-2898,miR-1,miR-3064-5p,miR-30a-3p,miR-199a-5p,miR-18a,miR-3154,miR-1814c,miR-329b,miR-767,miR-125a,miR-487a-3p,miR-199a-5p,miR-409-3p,miR-125b-5p,miR-29b-3p,miR-191-5p,miR-767-5p,miR-365a-5p
SLC9A1	ENSOARG0(	solute c	miR-135a-2-3p,miR-4510,miR-486-3p,miR-148b-5p,miR-92a-1-5p,miR-486b-3p,miR-760-3p,miR-3600,novel_68,novel_116,miR-149-5p,miR-455-3p,miR-6130,miR-185-5p,miR-30b-3p,miR-2387,miR-30b-3p,miR-1291
ZP4	ENSOARG0(	zona pel	miR-9788-3p,miR-203-3p,novel_25,miR-539-5p,miR-301a-3p,miR-130a-3p,miR-504-5p,miR-504,miR-1193,miR-124-3p,miR-1434-3p,miR-143-3p,novel_39,miR-29b-1-5p,miR-654-3p,miR-17-3p,miR-382,miR-124a,miR-28-5p,novel_96,miR-708-5p,miR-454-3p,miR-22-5p,miR-130b-3p,miR-17-3p,miR-17-3p,miR-491-5p,miR-29b-1-5p,miR-4324
NOX4	ENSOARG0(	NADPH ox	miR-7b-5p,miR-450c-3p,miR-380-3p,miR-2426,miR-3120-5p,miR-2284x,miR-187-3p,miR-130a-3p,miR-421,miR-7-5p,miR-199a-3p,miR-99a-5p,miR-136-3p,miR-130b-3p,let-7j,miR-2310,miR-2387,miR-99b-5p,miR-96-5p,miR-136-5p,miR-9-3p,miR-20a-3p,miR-211-5p,miR-26b-5p,miR-25,miR-3596,miR-670-3p,miR-142b,miR-2440,miR-92a-3p,miR-421-5p,miR-3059-5p,miR-363-3p,miR-136-3p,miR-452-5p,miR-9-5p,miR-211,miR-323c,miR-3959-3p,miR-9-3p,miR-301b-3p,miR-99a-5p,miR-9-5p,miR-222-5p,miR-1271-5p,miR-450a-1-3p,miR-199a-3p,miR-99a,miR-26a-5p,miR-92a-3p,miR-29b-2-5p,miR-421-5p,miR-92b-3p,miR-450a-1-3p,miR-500a-3p,miR-100-5p,miR-3074-1-3p,miR-1248,miR-1814c,miR-216b-3p,miR-301a-3p,miR-25-3p,miR-133c,novel_63,miR-29b-2-5p,miR-3604,miR-3969,miR-450b-3p,miR-500,miR-454-3p,miR-204-5p,miR-1271

RPS5	ENSOARG0(	ribosoma	miR-128-3p,miR-615-3p,miR-378i,miR-2366,miR-2300b-3p,miR-1343-5p,miR-5703,miR-378a-3p,miR-24-2-5p,miR-148b-3p,miR-378h,miR-532-3p,miR-3960,miR-380-5p,miR-450a-2-3p,miR-221-5p,miR-30b-3p,miR-152-3p,miR-1260a,miR-422a,miR-105-3p,miR-3064-5p,miR-29b-2-5p,miR-4286,miR-1895,miR-485-5p,miR-500a-3p,miR-3154,miR-769,miR-154b-5p,miR-221-5p,miR-140-5p,miR-504-5p,miR-378a-3p,miR-2355-5p,miR-500,miR-665,miR-29b-2-5p,miR-769-5p,miR-1260b,miR-122-5p,miR-378c,miR-365a-5p,miR-378c,novel_42,miR-3074-5p,miR-30b-3p,miR-378c,miR-1260b,miR-3120-5p,miR-484,miR-378b,miR-541,miR-3071-5p,miR-30b-3p,miR-185-5p,miR-31-5p,novel_78,novel_4,miR-1343-3p,miR-2300a-5p,miR-3085-3p,miR-1343-5p,miR-1306-5p,miR-3141,miR-365b-5p,novel_51,miR-378d,miR-4532,miR-1388-3p,miR-1291,miR-147-3p,miR-504,miR-378f,miR-361-3p,miR-3141,miR-148a-3p,miR-2387,miR-150-5p,miR-1306,miR-216a-3p,miR-2404,miR-378e,novel_69,miR-3064-5p,miR-3074-5p,miR-499b-5p,miR-615,miR-769-5p,miR-877-3p,miR-361-3p,miR-371b-3p,miR-24-3p,novel_27,novel_60,miR-3956-3p,miR-541-3p,miR-758-5p
RCOR2	ENSOARG0(	REST cor	miR-664a,miR-138-5p,miR-4726-5p,miR-147-5p,miR-6123,miR-2285t,miR-874-5p,miR-2433,miR-6740-5p,novel_111,miR-361-3p,miR-3074-5p,miR-541-3p,miR-2330-3p,miR-1291,miR-485-5p,miR-6395,miR-146b-5p,miR-1895,miR-3154,miR-137-3p,miR-1306,miR-6535,miR-324-5p,miR-22-3p,miR-3074-5p,miR-7977,miR-499b-5p,miR-320b,miR-146b,miR-210-5p,miR-146a,miR-1306-5p,miR-654-3p,miR-146a-5p,miR-2285w,miR-363-5p,miR-941,miR-138,miR-30b-3p,miR-541,miR-1285,miR-2284r,miR-1197-5p,miR-30b-3p,miR-5703,miR-22-3p,miR-2427,miR-1298-3p,miR-328-3p,miR-2284h-5p,novel_83,novel_4,miR-2300a-5p,miR-31-5p
AADACL2	ENSOARG0(	arylacet	miR-200a-3p,miR-450a-2-3p,miR-28-3p,miR-200c-3p,miR-127-5p,miR-216a-5p,miR-2285af,miR-200c,miR-29a-5p,miR-664b-3p,miR-29b-1-5p,miR-3529-3p,miR-487a,miR-200b-3p,miR-1290,miR-3578,miR-23b-3p,miR-487a-3p,miR-200b,miR-23c,miR-429-3p,miR-592,miR-144,miR-6740-5p,novel_63,miR-365a-5p,miR-144-5p,miR-103b,miR-133a-3p,miR-28a-3p,miR-133b-3p,miR-154b-3p,miR-27b-5p,miR-23b,miR-488-3p,miR-499b-3p,miR-582-5p,miR-141-3p,miR-574-5p,miR-487a-3p,miR-548o-3p,miR-1185-5p,miR-365b-5p,miR-29b-1-5p,miR-539-3p,miR-654-3p,miR-23a-3p,miR-668-3p,miR-744-3p,miR-219a-1-3p,miR-708-3p,miR-216a-3p,miR-1185-3p,miR-8095,novel_111,miR-452-5p,miR-3059-5p,novel_120,miR-885-5p,miR-877-3p,miR-3065-5p,miR-378j,miR-144-3p,miR-144-5p,miR-1185-2-3p,miR-499a-3p,miR-25,miR-670-3p,miR-2483-3p,miR-541-5p,miR-7-1-3p
DUSP22	ENSOARG0(	dual spe	miR-744-5p,novel_19,miR-324-3p,novel_78

DYM	ENSOARG0(	dymeclin	miR-34b-5p,miR-543-5p,miR-219b-5p,miR-30f,miR-24-2-5p,miR-148b-5p,miR-195a-3p,miR-668-3p,miR-30a-3p,miR-181a-2-3p,miR-34a-5p,miR-1388-5p,miR-219a-1-3p,miR-1298-5p,miR-214-5p,miR-147a,miR-665,miR-3600,miR-181b-2-3p,miR-30a-3p,miR-34c,miR-320e,miR-382-3p,miR-30e-3p,miR-449a,miR-877-3p,novel_101,miR-3059-5p,miR-625-5p,miR-582-5p,miR-136-5p,miR-677,novel_25,miR-203-3p,miR-877-5p,miR-181b-3p,miR-34c-5p,miR-582-5p,miR-34b,miR-216b-5p,miR-3120-3p,miR-24-1-5p
SCAP	ENSOARG0(	SREBF ch	miR-26b-3p,miR-340-3p,novel_78,miR-2300a-5p,miR-1343-3p,miR-432-5p,miR-503-5p,miR-30b-3p,miR-484,miR-432,miR-2284r,miR-2889,miR-138,miR-30b-3p,miR-574-3p,novel_19,miR-505,novel_51,miR-503-5p,miR-7-5p,miR-326,miR-125a-5p,miR-199b-5p,miR-3085-3p,miR-3064-5p,miR-3074-5p,novel_69,miR-18a-3p,miR-2285ab,miR-199b-5p,miR-9851-3p,miR-1291,miR-2285f,miR-495-3p,novel_27,miR-326-3p,miR-423-5p,miR-199b-5p,miR-361-3p,novel_82,miR-128-1-5p,miR-18a-3p,miR-330-5p,miR-378d,miR-582-3p,miR-3064-5p,miR-503-5p,miR-199a-5p,miR-486-3p,novel_39,miR-185-3p,miR-125b,novel_17,miR-543-5p,novel_127,miR-370-3p,miR-125a,miR-545-3p,miR-874-3p,miR-199a-5p,miR-1827,miR-2312,miR-193a-5p,miR-3074-5p,miR-3184-5p,miR-346,miR-331-3p,miR-125b-5p,miR-2433,miR-486b-3p,miR-138-5p
ZNF248	ENSOARG0(	zinc fin	miR-335-3p,miR-877-3p,miR-136-5p,miR-320c,miR-3596,miR-541-5p,miR-664b,miR-20a-3p,miR-329-5p,miR-421-5p,miR-301a-5p,miR-33a-3p,miR-576-3p,miR-8095,miR-219a-2-3p,miR-877-3p,miR-219a-5p,miR-181d-5p,novel_60,miR-885-5p,miR-3956-3p,miR-2284ab,miR-197-3p,miR-758-3p,miR-653,miR-301b-5p,miR-181a-5p,novel_116,miR-2284d,miR-133b-5p,miR-2411-3p,miR-320b,miR-377-3p,miR-758-3p,let-7a-2-3p,miR-382-3p,miR-582-5p,let-7g-3p,miR-329b,miR-320a,miR-582-5p,miR-377-3p,miR-140-5p,miR-134-5p,miR-664a,miR-219b-3p,miR-29b-2-5p,miR-2284k,miR-181c-5p,miR-6525,let-7a-2-3p,miR-4429,miR-188-3p,miR-345-3p,miR-134,miR-134-5p,miR-758-5p,miR-543-5p,miR-320d,miR-466i-5p,miR-181b-5p,miR-219b-5p,miR-219-3p,miR-664-3p,miR-29a-5p,miR-29b-2-5p,miR-329a-5p

TYR	ENSOARGO(	tyrosina	miR-493-5p,miR-9-3p,miR-6516-3p,miR-504,miR-668-5p,miR-320c,miR-1185-2-3p,novel_69,miR-3082-5p,miR-320b,miR-329-5p,miR-2284q,miR-206,miR-335,miR-33a-3p,miR-20a-5p,miR-2428,miR-219a-2-3p,novel_111,novel_101,miR-17-3p,miR-335-5p,miR-212-3p,miR-16-2-3p,miR-101c,miR-2284aa,miR-380-3p,miR-219a-1-3p,miR-450b-5p,miR-1a-3p,miR-3071-5p,miR-708-3p,miR-455-3p,miR-9788-3p,miR-20b,miR-1185-3p,miR-3955-5p,miR-330-3p,miR-125a-5p,miR-3955-3p,miR-365b-5p,miR-330-3p,miR-197-5p,miR-106a-5p,miR-106b-5p,miR-17-5p,miR-2411-3p,miR-224-5p,miR-320b,miR-4532,miR-3187-3p,miR-432-3p,miR-432-3p,miR-23a-3p,miR-382,let-7f-2-3p,let-7f-2-3p,miR-6240,miR-493-5p,miR-106a-5p,miR-20b-5p,let-7g-3p,miR-329b,miR-93-5p,miR-17-5p,miR-23b,miR-3154,miR-320a,miR-488-3p,miR-216b-5p,miR-125a,miR-552-3p,miR-532-5p,miR-504-5p,miR-129-5p,miR-486b-3p,novel_74,miR-17-3p,miR-664a,miR-125b-5p,miR-219b-3p,miR-665,miR-410-5p,miR-365a-5p,miR-6134,miR-93,miR-224-5p,miR-206-3p,miR-193a-5p,miR-200b-3p,miR-493-3p,miR-4429,miR-5703,miR-345-3p,miR-3591-5p,miR-2366,miR-2284z,novel_121,miR-23b-3p,miR-200b,miR-23c,miR-3600,miR-1b-3p,miR-1224-5p,miR-548e-3p,miR-429-3p,miR-106a,miR-106a,miR-320d,miR-17-3p,miR-200c-3p,miR-125b,miR-345-3p,miR-2285af,miR-200c,miR-219-3p,miR-618,miR-486-3p,miR-380-3p,miR-664b-3p,miR-148b-5p,miR-125b-2-3p,miR-1,miR-20a,miR-216c-5p,miR-329a-5p
TAT	ENSOARGO(	tyrosine	miR-486b-3p,miR-24-3p,miR-6525,miR-423-5p,miR-3184-5p,miR-145-3p,miR-145a-3p,miR-214,miR-147-3p,miR-7144-5p,miR-18a-3p,miR-216a-3p,miR-574-5p,miR-7977,miR-2355-5p,miR-370-3p,miR-466i-5p,miR-214-3p,novel_127,miR-143-3p,miR-486-3p,miR-128-3p,miR-1285,miR-1246,miR-193b-5p,miR-145a-5p,miR-5703,miR-3431,miR-1298-3p,miR-455-3p,miR-1343-3p,miR-18a-3p,miR-145b

ZNF19	ENSOARG0(	zinc fin	miR-125a-3p,miR-301f,novel_127,miR-152-3p,miR-29a-5p,novel_39,miR-330-5p,miR-299a-3p,miR-412,miR-9-5p,miR-322-5p,miR-2284z,novel_121,miR-2284k,miR-125b-5p,miR-6740-5p,miR-592,miR-16-5p,miR-1197-3p,miR-767,miR-345-5p,miR-574-5p,miR-874-3p,miR-125a,miR-7-5p,miR-377-3p,miR-365b-5p,miR-105-5p,miR-2411-3p,miR-432-3p,miR-2284l,miR-432-3p,miR-1843b-5p,miR-542-3p,miR-7b-5p,miR-22-3p,novel_23,miR-16b,miR-497-5p,miR-2284x,miR-31-5p,miR-103a-3p,miR-412-5p,miR-149-5p,miR-194-3p,miR-28c,miR-576-3p,miR-206,miR-29a,miR-625-3p,miR-335-5p,miR-29c-3p,miR-376b-5p,miR-148a-3p,miR-1306,miR-3591-3p,miR-2319a,miR-378f,miR-3074-5p,miR-3120-3p,miR-299b-3p,miR-320c,miR-144-3p,miR-500a-5p,miR-345-3p,miR-422a,miR-345-3p,miR-376a-5p,miR-486-3p,miR-143-3p,miR-378i,miR-2366,miR-378a-3p,miR-18a-3p,miR-7859,miR-95-3p,miR-328-3p,miR-3604,miR-664a-5p,miR-500,miR-3074-5p,miR-181c-5p,miR-500a-3p,miR-129b-3p,miR-433-3p,miR-345-5p,miR-625-5p,miR-543-3p,miR-186-5p,miR-548o-3p,miR-377-3p,miR-15b-5p,miR-16b,miR-182-5p,miR-153-3p,miR-574-3p,miR-101b-3p,miR-378d,miR-2310,miR-2284r,miR-301b-5p,miR-501-3p,miR-1a-3p,miR-758-3p,miR-101c,miR-1343-3p,novel_78,miR-153,miR-148b-5p,miR-2459,miR-101-3p,miR-301a-5p,miR-490-5p,miR-2447,miR-9-5p,miR-877-3p,miR-9-3p,miR-24-1-5p,miR-2319b,miR-500-3p,miR-6119-5p,miR-2898,miR-17-3p,miR-181b-5p,miR-107,miR-29b-2-5p,miR-1,miR-24-2-5p,miR-28b,miR-2478,miR-3065-3p,miR-345-3p,miR-29d-3p,miR-501-3p,miR-105-2,miR-378h,miR-29b-3p,miR-410-5p,miR-5100,miR-370-5p,miR-135a-1-3p,miR-144,miR-138-5p,miR-767-5p,miR-486b-3p,miR-500b-5p,miR-378c,miR-365a-5p,miR-488-3p,miR-3154,miR-362-5p,miR-320a,miR-10b-3p,novel_48,miR-376c-5p,miR-1814c,miR-485-
HSP90AA1	ENSOARG0(	heat sho	miR-3529-3p,miR-216c-5p,miR-1b-5p,miR-491-3p,miR-8485,miR-2478,miR-144,miR-362-3p,miR-2285p,miR-1247-3p,miR-221-5p,miR-27a-3p,miR-216b-3p,miR-655-5p,miR-21-3p,miR-221-5p,miR-27a-3p,miR-144-3p,miR-3591-3p,miR-542-3p,miR-2300b-3p,miR-362-3p,miR-1434-3p,miR-329-3p,miR-27b-3p,miR-186-5p,miR-885-3p,miR-329a,miR-885-3p,miR-362-3p

ZNF23	ENSOARG0(	zinc fin	miR-877-3p,miR-362-5p,miR-499a-5p,miR-450a-5p,miR-2285t,miR-490-5p,miR-101-3p,miR-107-5p,miR-378e,miR-203-3p,miR-4792,miR-211-5p,miR-136-5p,miR-1983,miR-140-5p,miR-378d,miR-1388-3p,miR-106a-5p,miR-491-5p,miR-381-5p,miR-103a-2-5p,miR-320b,miR-29b-1-5p,miR-2957,miR-106b-5p,miR-147a,miR-216a-3p,miR-500-5p,miR-151a-3p,miR-145b,miR-181b-2-3p,miR-378c,miR-101a-5p,miR-2426,miR-101c,miR-378b,miR-484,miR-412-3p,miR-346,miR-378c,miR-433-5p,miR-2330-3p,miR-450b-3p,miR-17-3p,miR-151-3p,miR-320e,miR-29b-2-5p,miR-27a-5p,miR-377-3p,miR-140-5p,miR-378a-3p,miR-2331-3p,miR-552-3p,miR-133b-3p,miR-450a-1-3p,miR-29b-1-5p,miR-181b-1-3p,miR-2432,miR-486-3p,miR-329a-5p,miR-20a,miR-106a,miR-202-5p,miR-320d,miR-3653-3p,miR-34b-5p,miR-299-3p,miR-21-3p,miR-422a,miR-328-3p,miR-106a,miR-30d-3p,miR-4429,miR-142-3p,miR-34a-5p,miR-378i,novel_133,miR-215-5p,miR-378a-3p,miR-1197-5p,miR-211,miR-449a,miR-2284w,novel_101,miR-17-3p,miR-203b-5p,miR-20a-5p,miR-34c,miR-146b-3p,miR-34b,miR-299b-3p,miR-677,miR-320c,miR-181b-3p,novel_68,miR-329-5p,miR-216b-3p,miR-320b,miR-412-3p,miR-378f,miR-6395,miR-2387,miR-216a-3p,miR-101-5p,miR-628-5p,miR-124-5p,miR-378a-5p,miR-377-3p,miR-106a-5p,miR-17-5p,novel_116,miR-20b,miR-381-5p,miR-188-5p,miR-2284x,miR-450c-3p,miR-145a-5p,miR-93,miR-500b-5p,miR-1197-3p,miR-378c,miR-133a-3p,miR-486b-3p,miR-374b-3p,miR-204-5p,miR-34c-5p,miR-154b-5p,miR-215-5p,miR-329b,miR-20b-5p,miR-503-3p,miR-93-5p,miR-17-5p,miR-362-5p,miR-320a,miR-450c-5p,miR-29b-2-5p,miR-2385-3p,miR-17-3p,novel_96,miR-205-5p,miR-505-5p,miR-378h,miR-450a-1-3p,miR-299a-3p,miR-6517,miR-1224-5p,miR-412,miR-128-3p,miR-23b-5p
SMARCD3	ENSOARG0(	SWI/SNF	miR-18a-3p,miR-1306,miR-21a-3p,miR-1827,miR-6395,miR-1291,miR-3065-3p,miR-30c-1-3p,miR-6130,miR-3596,miR-146b-3p,miR-324-3p,miR-6529b,miR-374b-3p,miR-4492,novel_1,miR-138-5p,miR-4726-5p,miR-452-5p,miR-4510,miR-3184-5p,miR-423-5p,miR-374a-3p,miR-24-3p,novel_23,miR-1343-5p,miR-542-3p,miR-148a-5p,miR-138,miR-2300a-5p,miR-18a-3p,miR-31-5p,miR-328-3p,novel_83,miR-148b-5p,miR-505-5p,miR-185-3p,miR-30c-2-3p,miR-618,miR-1306-5p,novel_127,novel_96,miR-1343-5p,miR-491-5p,miR-192-3p,miR-490-3p,miR-3187-3p,miR-8485,miR-3065-3p,miR-30c-1-3p

HNF4A	ENSOARG0(	hepatocy	miR-2300a-5p,miR-1343-3p,miR-1298-3p,miR-2285e,miR-216a-3p,miR-9788-3p,miR-301b-5p,miR-378b,miR-296-5p,miR-2284r,miR-30b-3p,miR-2426,miR-378c,miR-1388-5p,miR-424-5p,miR-378d,miR-193a,miR-326,miR-1306-5p,miR-320b,miR-16b,miR-3085-3p,miR-214-3p,miR-3064-5p,miR-7977,miR-378e,miR-361-3p,miR-140-5p,miR-1983,miR-3956-3p,novel_60,miR-361-3p,miR-101-3p,miR-2440,miR-301a-5p,miR-148b-3p,miR-15a-5p,miR-2366,miR-378i,novel_133,miR-1197-5p,miR-1193,miR-188-3p,miR-378a-3p,miR-4429,miR-33b-3p,miR-34a-5p,miR-125b-2-3p,miR-2432,miR-486-3p,novel_17,miR-422a,miR-324-3p,miR-99a-3p,miR-320d,miR-299-3p,miR-34b-5p,miR-30b-3p,miR-140-5p,miR-378a-3p,miR-370-5p,miR-193b-3p,miR-6130,miR-6516,miR-15b-5p,miR-877-5p,novel_32,miR-2285c,miR-129-2-3p,miR-3074-5p,miR-4510,miR-378c,miR-214,miR-665-5p,miR-3074-2-3p,miR-425-5p,miR-346,miR-224-5p,miR-6134,miR-29b-2-5p,miR-665,miR-17-3p,miR-424-5p,miR-2285g,miR-149-5p,miR-30b-3p,miR-16b,miR-497-5p,miR-432-3p,miR-224-5p,miR-193a-3p,miR-452-5p,miR-3074-5p,miR-378b,miR-216b-3p,miR-299b-3p,miR-670-3p,miR-34b,miR-6535,miR-320c,miR-148a-3p,miR-2387,miR-150-5p,miR-1306,miR-412-3p,miR-378f,miR-195a-5p,miR-1193,miR-17-3p,miR-193b-3p,miR-326-3p,miR-449a,miR-15a,novel_87,miR-16a,miR-34c,miR-299a-3p,miR-6516-5p,miR-378h,miR-322-5p,miR-129-1-3p,miR-1290,miR-330-5p,miR-181a-2-3p,miR-29b-2-5p,miR-3064-5p,miR-2285b,miR-17-3p,miR-152-3p,miR-99a-3p,miR-2355-5p,miR-2285aa,miR-34c-5p,miR-324-5p,miR-488-3p,miR-3154,miR-320a,miR-1248,miR-7144-5p,miR-16-5p,miR-323-3p,miR-378c,miR-370-5p,miR-99b-3p,miR-2433,miR-486b-3p

VIP	ENSOARG0(	vasoacti	miR-9-3p,miR-499a-3p,miR-27a-3p,miR-2284g,miR-4792,miR-126b-5p,miR-211-5p,miR-30b-5p,miR-9-3p,miR-2447,miR-32-3p,miR-2284j,miR-181d-5p,miR-544a,miR-450b-5p,miR-101a-5p,miR-362-3p,miR-329-3p,miR-1a-3p,miR-153,miR-105-1,novel_4,miR-27a-3p,miR-181b-2-3p,miR-3955-3p,miR-153-3p,novel_103,miR-320b,miR-182-5p,miR-362-3p,miR-1b-5p,miR-8485,miR-382-3p,miR-494-3p,miR-652-5p,miR-339b,miR-30f,miR-186-5p,novel_99,miR-543-3p,miR-411,miR-30d-5p,miR-192-5p,miR-499b-3p,miR-544-3p,miR-582-5p,miR-877-5p,miR-204-3p,miR-32-3p,miR-496-3p,miR-182-5p,miR-2285j,miR-3604,miR-206-3p,miR-181c-5p,miR-145a-3p,miR-4429,miR-2284u,miR-361-5p,miR-378d,miR-1246,miR-193b-5p,miR-215-5p,miR-362-3p,miR-30c-5p,miR-200b,miR-320d,miR-2284m,miR-30d,miR-30b,miR-216a-5p,miR-181b-1-3p,miR-130a-5p,miR-216c-5p,miR-181b-3p,miR-677,miR-144-3p,miR-320c,miR-7-1-3p,miR-206,novel_1,miR-147-5p,miR-576-3p,miR-382-5p,miR-3959-3p,miR-211,miR-2428,miR-2284w,novel_27,miR-1a-1-5p,miR-27b-3p,miR-2284x,miR-181a-5p,miR-3431,miR-2284d,miR-544b,miR-187-3p,miR-31-5p,miR-339-5p,miR-105-5p,miR-329a,miR-3074-1-3p,miR-30e-5p,miR-3154,miR-320a,miR-2284n,miR-215-5p,miR-370-3p,miR-134-5p,miR-204-5p,miR-2284k,miR-1-5p,miR-144,miR-2284v,miR-496,miR-105-2,miR-30c,miR-200b-3p,miR-134,miR-1290,miR-30a-5p,miR-134-5p,miR-429-3p,novel_79,miR-200c-3p,miR-1a-2-5p,miR-181b-5p,miR-200c,miR-2285af,miR-339a,miR-30f,miR-450b-5p,miR-2284a,miR-2284y,miR-1

NUCB2	ENSOARG0(	nucleobi	miR-320a,miR-3154,miR-301,miR-503-3p,miR-7144-5p,miR-876-3p,miR-2318,miR-345-5p,miR-329a,miR-141-3p,miR-125a,miR-133c,miR-2284n,miR-376c-3p,miR-34c-5p,miR-769,miR-6740-5p,miR-125b-5p,miR-135a-5p,miR-3969,miR-2284k,miR-410-5p,miR-370-5p,miR-486b-3p,miR-16-5p,miR-1260b,miR-330-5p,miR-1290,miR-105-2,miR-10a-5p,miR-411-3p,miR-1224-5p,miR-2285ad,miR-582-3p,miR-486-5p,miR-9-5p,miR-322-5p,miR-2285af,miR-1260a,miR-2397-5p,miR-200a-3p,miR-17-3p,miR-29b-2-5p,miR-29a-5p,miR-490-3p,miR-2285b,miR-2478,miR-148b-5p,miR-150-5p,miR-2285ab,miR-146b-5p,miR-135b-5p,miR-195a-5p,miR-450b-3p,miR-10a-5p,miR-216b-3p,miR-378b,miR-320c,miR-669,miR-34b,miR-34c,miR-16a,miR-1247-3p,miR-15a,miR-133a-5p,miR-576-3p,miR-656-5p,novel_1,miR-206,miR-379-3p,miR-1839-3p,miR-17-3p,miR-1193,miR-326-3p,miR-449a,miR-24-3p,miR-323c,novel_23,miR-3071-5p,miR-19b-2-5p,miR-708-3p,miR-1285,miR-2113,miR-497-5p,miR-2284ab,miR-16b,miR-1260b,miR-194-3p,miR-2285g,miR-185-5p,miR-105-5p,miR-296-3p,miR-146a,miR-452-5p,miR-125a-5p,let-7f-2-3p,let-7f-2-3p,let-7j,miR-296-3p,miR-345-5p,miR-129b-3p,miR-2285c,miR-582-5p,miR-186-5p,miR-2312,miR-345-5p,miR-370-5p,miR-140-5p,miR-204-3p,miR-15b-5p,miR-151b,miR-376e-3p,miR-582-5p,miR-10a,miR-139-5p,miR-6130,miR-19b-1-5p,miR-29b-2-5p,miR-2285j,miR-424-5p,miR-17-3p,miR-425-5p,miR-214,miR-3074-2-3p,miR-145a-3p,miR-4510,miR-338-3p,miR-6525,miR-769-5p,miR-10b-5p,miR-206-3p,miR-1197-5p,miR-5703,miR-188-3p,miR-1193,miR-1246,miR-362-3p,miR-148a-5p,miR-378d,miR-34a-5p,miR-4429,miR-548e-3p,miR-151-5p,miR-2284h-5p,miR-532-3p,miR-7859,miR-15a-5p,miR-500a-5p,miR-125b,miR-34b-5p,miR-21-3p,miR-2284m,miR-320d,miR-345-3p,miR-216c-5p,miR-2285w,miR-486-3p,miR-146a-5p,miR-2424,miR-130a-
GPATCH3	ENSOARG0(	G patch	miR-664a,miR-4492,miR-130b-5p,let-7f,miR-135a-1-3p,miR-370-5p,miR-19b-1-5p,miR-125b-5p,let-7b-5p,miR-539-5p,let-7b,miR-3958-5p,miR-485-5p,miR-129b-3p,miR-2284s,let-7i-5p,miR-874-3p,miR-370-5p,miR-125a,let-7f-5p,miR-760-3p,miR-98-5p,let-7k,miR-196a-3p,miR-210-5p,let-7c-5p,miR-125b,miR-185-3p,miR-148b-5p,let-7g,miR-329a-5p,miR-2403,miR-941,let-7d,miR-184-3p,miR-148a-5p,miR-378d,miR-193b-5p,miR-1961,miR-7134-5p,miR-124-3p,miR-383-5p,novel_83,miR-128-1-5p,miR-2285n,miR-1973,let-7i,miR-877-3p,miR-1193,miR-410-5p,miR-379-5p,let-7e-5p,miR-3970,miR-129b-5p,miR-378g,novel_69,miR-6535,let-7d-5p,miR-329-5p,miR-7977,miR-2285l,miR-125a-5p,let-7e,miR-197-5p,novel_19,miR-330-3p,miR-2957,miR-432-3p,let-7a-5p,miR-130b-5p,miR-124a,miR-6240,miR-101a-5p,miR-2426,miR-1839-5p,miR-2284r,miR-484,miR-19b-2-5p,miR-143-5p,miR-425-3p,miR-134-3p,miR-3431,let-7g-5p,miR-455-3p,miR-148b-5p,novel_78,miR-330-3p
CDK18	ENSOARG0(	cyclin-d	miR-21-3p,miR-486b-3p,miR-490-5p,miR-184-3p,miR-2904,miR-2300b-3p,miR-455-3p,miR-486-3p,miR-323-5p,miR-323a-5p,miR-2355-5p,miR-105-3p

HNRNPM	ENSOARG0(	heteroge	miR-133b-3p,miR-876-3p,miR-505-3p,miR-133a-3p,miR-409-5p,miR-873a-5p,miR-2483-3p,miR-9-5p,novel_121,miR-154b-5p,miR-548w,miR-505-3p,miR-487b-5p,miR-2285af,miR-216a-5p,miR-8485,miR-212-3p,miR-133a-3p,miR-9-5p
SEC23IP	ENSOARG0(	SEC23 in	miR-452-5p,miR-1343-5p,miR-7-5p,miR-224-5p,miR-2411-3p,miR-32,miR-105-5p,miR-330-3p,miR-22841,miR-539-3p,miR-6240,miR-363-5p,miR-2284ab,miR-181a-5p,miR-7b-5p,miR-432,miR-873a-5p,miR-374b-3p,miR-3431,miR-2285g,miR-432-5p,miR-2355-3p,novel_116,miR-339-5p,miR-206,miR-92a-3p,miR-625-3p,miR-502-5p,miR-4324,miR-199b-5p,miR-374c-5p,miR-335-5p,miR-1a-1-5p,miR-17-3p,miR-495-3p,miR-2285x,miR-1839-3p,miR-3957-3p,miR-410-5p,miR-505-3p,miR-148a-3p,miR-2387,miR-3065-5p,miR-18a-3p,miR-3591-3p,miR-3970,miR-374c-3p,miR-2404,miR-323a-5p,novel_68,miR-7-1-3p,miR-127-5p,miR-1a-2-5p,miR-181b-5p,miR-17-3p,miR-450a-2-3p,novel_127,miR-92a-3p,miR-152-3p,miR-2285af,miR-339a,miR-217,miR-30c-2-3p,miR-92b-3p,miR-199a-5p,miR-29a-5p,miR-3529-3p,miR-4286,miR-181a-2-3p,miR-495-5p,miR-300,miR-30c,miR-105-2,miR-1290,miR-3591-5p,miR-345-3p,miR-30a-5p,miR-1343-5p,miR-6516-5p,miR-9-5p,novel_121,miR-129-1-3p,miR-2285ad,novel_79,miR-196a-2-3p,miR-412,novel_9,miR-374b-3p,miR-191-5p,miR-28-5p,miR-34c-3p,miR-1-5p,miR-100-3p,miR-374a-3p,miR-144-5p,miR-6128,miR-876-3p,miR-1248,miR-7144-5p,miR-503-3p,miR-301,miR-381-3p,miR-23b,miR-25-3p,miR-300-3p,miR-374c-3p,miR-199a-5p,miR-133c,miR-503-3p,miR-874-3p,miR-141-3p,miR-25-5p,miR-199b-5p,miR-323b-3p,miR-505,miR-29b-1-5p,miR-30c-1-3p,miR-758-3p,miR-8485,miR-494-3p,miR-1839-3p,let-7c-3p,miR-23a-3p,miR-2426,miR-450b-5p,miR-1388-5p,miR-3120-5p,miR-541,miR-758-3p,miR-412-3p,miR-323b,miR-142-5p,miR-421,miR-2285e,miR-2459,miR-455-3p,novel_78,miR-105-1,novel_4,miR-330-3p,miR-2300a-5p,miR-222-5p,miR-335,miR-3968,miR-2284q,miR-374a-3p,miR-30b-5p,miR-2285n,miR-2285y,miR-371a-5p,miR-877-3p,miR-1285-5p,miR-541-3p,miR-212-3p,miR-363-3p,miR-3059-5p,miR-

CTSC	ENSOARG0(	cathepsi	miR-664b-3p,miR-329a-5p,miR-345-3p,miR-760-3p,miR-2284m,miR-21-3p,miR-34b-5p,miR-26a-5p,miR-210-5p,miR-2285p,miR-196a-3p,miR-200b,miR-328-3p,miR-616-3p,miR-505-3p,miR-3600,miR-34a-5p,miR-664-5p,miR-2300b-3p,miR-217-5p,miR-124-3p,miR-346,miR-142a-5p,miR-181c-5p,miR-3184-3p,miR-3074-5p,miR-214,miR-500,miR-190a-5p,miR-30e-3p,miR-15b-3p,miR-665,miR-3604,miR-30a-3p,miR-323-5p,miR-6516,miR-548o-3p,miR-140-5p,miR-30c-1-3p,miR-345-5p,miR-500a-3p,miR-30c-1-3p,miR-3187-3p,miR-382-3p,miR-124a,miR-3085-3p,miR-214-3p,miR-323b-3p,miR-142-5p,miR-421,novel_78,miR-409b,miR-1388-5p,miR-3120-5p,miR-16-2-3p,miR-484,miR-501-3p,miR-323b,miR-877-3p,miR-502-3p,miR-885-5p,miR-3059-5p,miR-181d-5p,miR-190b-5p,miR-382-3p,miR-2483-3p,miR-500-3p,miR-203-3p,miR-452-3p,miR-3064-5p,miR-499b-5p,miR-129b-5p,miR-450b-5p,miR-200a,miR-181a-2-3p,miR-30a-3p,miR-3064-5p,miR-200c-3p,miR-181b-5p,miR-28-3p,miR-200c,miR-217,miR-30c-2-3p,miR-6516-5p,miR-505-5p,miR-582-3p,miR-429-3p,miR-7641,miR-200b-3p,miR-501-3p,miR-29d-3p,miR-145-3p,miR-28a-3p,miR-6128,miR-3607-3p,miR-2332,miR-29b-3p,miR-2284k,miR-135a-5p,miR-34c-5p,miR-331-5p,miR-300-3p,miR-2284n,miR-154b-5p,miR-3068-3p,miR-2355-5p,miR-345-5p,miR-329b,miR-10b-3p,miR-654-3p,miR-345-5p,miR-455-5p,miR-190a,miR-432-5p,miR-149-5p,miR-6238,miR-194-3p,miR-133b-5p,miR-31-5p,miR-2284ab,miR-29b,miR-455-5p,miR-188-5p,miR-181a-5p,miR-1843b-5p,miR-223-3p,miR-432,miR-449a,miR-29c-3p,novel_101,miR-2285x,novel_1,miR-29a,miR-147-5p,miR-142b,miR-576-3p,miR-34c,miR-670-3p,miR-29a-3p,miR-34b,miR-323a-5p,miR-2404,miR-329-5p,miR-3074-5p,miR-26b-5p,miR-6395,miR-135b-5p,miR-505-3p

LRTOMT	ENSOARG0(	leucine	miR-345-5p,miR-329b,miR-331-5p,miR-154b-5p,miR-300-3p,miR-615-5p,miR-125a,miR-2355-5p,miR-874-3p,miR-191-5p,miR-134-5p,miR-486b-3p,miR-331-5p,miR-331-3p,miR-125b-5p,miR-100-3p,miR-5100,miR-2332,miR-378c,miR-365a-5p,miR-133a-3p,miR-193a-5p,miR-16-5p,miR-10a-5p,miR-200b-3p,let-7d,miR-330-5p,miR-134,miR-1290,miR-3591-5p,miR-196a-5p,miR-214-5p,miR-134-5p,miR-378h,miR-483-3p,miR-322-5p,miR-505-5p,novel_79,miR-429-3p,miR-299a-3p,miR-2285ad,novel_127,miR-107,let-7f-5p,miR-200c-3p,miR-2898,miR-450a-2-3p,miR-217,miR-339a,let-7c-5p,miR-664-3p,miR-125a-3p,miR-200c,miR-490-3p,miR-1271-3p,miR-29a-5p,miR-200a,let-7g,novel_39,miR-28b,miR-3064-5p,miR-3529-3p,let-7e-5p,miR-6395,miR-195a-5p,miR-379-5p,miR-378f,miR-2319a,miR-7975,miR-412-3p,miR-107,miR-1291,miR-18a-3p,miR-2387,miR-6535,miR-299b-3p,miR-670-3p,miR-323a-5p,miR-10a-5p,miR-655-5p,miR-320b,miR-329-5p,miR-3074-5p,miR-6119-3p,let-7i,miR-425-5p,miR-16a,miR-28c,miR-4324,miR-1247-3p,miR-15a,miR-2284w,miR-326-3p,miR-24-3p,miR-369-5p,miR-497-5p,miR-30b-3p,miR-16b,miR-2355-3p,miR-103,novel_116,miR-3431,let-7g-5p,miR-412-5p,miR-26b-3p,miR-339-5p,miR-103a-3p,miR-125a-5p,miR-196b-5p,miR-483-3p,novel_51,miR-365b-5p,miR-377-3p,miR-345-5p,miR-2483-5p,miR-668-3p,miR-1827,miR-345-5p,miR-339b,miR-133b-3p,miR-129b-3p,miR-500a-3p,miR-21a-3p,let-7i-5p,miR-877-5p,miR-15b-5p,miR-323-5p,miR-10a,miR-377-3p,miR-296-3p,miR-532-5p,miR-182-5p,miR-216b-3p,miR-140-5p,miR-378a-3p,miR-2331-3p,miR-504-5p,miR-4492,miR-500,miR-874-5p,miR-424-5p,miR-664a,miR-151-3p,miR-369-5p,miR-324-3p,let-7f,miR-2285j,miR-338-3p,miR-6134,let-7b-5p,miR-346,miR-10b-5p,miR-6525,miR-214,miR-665-5p,miR-3074-5p,miR-378e,let-7b,miR-378d,miR-133a-3p,miR-184-
RAB7A	ENSOARG0(	RAB7A, m	miR-378g,miR-134-3p,miR-3600,miR-9851-3p,miR-30c,miR-3120-5p,miR-744-5p,miR-147-3p,miR-30b-3p,miR-30f,miR-500a-3p,miR-30d-5p,miR-30a-5p,miR-30b-3p,miR-30e-5p,miR-488-3p,miR-30c-5p,miR-2387,miR-423-5p,miR-3184-5p,miR-143-3p,miR-3607-3p,miR-2285x,miR-500,miR-30b,miR-30b-5p,novel_82,miR-5126,novel_17

ERLEC1	ENSOARG0(	endoplas	miR-152-3p,miR-543-5p,novel_127,miR-127-5p,miR-200c-3p,let-7f-5p,miR-181b-5p,miR-17-3p,miR-217,let-7c-5p,miR-30c-2-3p,miR-200c,miR-490-3p,miR-2285b,miR-2284a,novel_39,let-7g,miR-199a-5p,miR-29a-5p,miR-2284y,miR-148b-5p,miR-181a-2-3p,miR-30a-3p,miR-128-3p,miR-200b-3p,let-7d,miR-23b-5p,miR-3578,miR-134,miR-345-3p,miR-545-5p,miR-134-5p,miR-450a-1-3p,miR-758-5p,miR-7641,miR-429-3p,miR-299a-3p,miR-134-5p,novel_9,miR-331-3p,miR-26c,miR-125b-5p,miR-5100,miR-2332,miR-2284k,miR-145-3p,miR-323-3p,miR-2284v,miR-1306-5p,miR-1248,miR-23a-5p,miR-345-5p,miR-3154,miR-137-3p,miR-200a-5p,miR-424-3p,miR-2448-3p,miR-199a-5p,miR-769,miR-125a,miR-125a-5p,miR-136-3p,miR-194a,miR-2411-3p,miR-377-3p,miR-503-3p,miR-345-5p,let-7j,miR-432-3p,miR-2284l,miR-654-3p,miR-432-3p,miR-382,miR-27b-3p,miR-2284ab,miR-145a-5p,miR-3071-5p,miR-194-5p,miR-450c-3p,miR-1843b-5p,miR-1434-3p,miR-181a-5p,miR-134-3p,let-7g-5p,miR-2284d,miR-412-5p,miR-665,miR-31-5p,miR-502b,miR-382-5p,let-7i,miR-133a-5p,miR-6402,miR-2428,miR-199b-5p,novel_27,miR-24-3p,miR-17-3p,novel_101,miR-136-3p,let-7e-5p,miR-412-3p,miR-450b-3p,miR-3065-5p,miR-216a-3p,miR-18a-3p,miR-378b,miR-2387,miR-148a-3p,miR-299b-3p,miR-3120-3p,miR-320b,miR-3074-5p,miR-378b,miR-216b-3p,miR-2484,miR-299-3p,let-7k,miR-221-5p,miR-760-3p,miR-98-5p,miR-466i-5p,miR-202-5p,miR-125b,miR-185-3p,miR-92a-1-5p,miR-345-3p,miR-500a-5p,miR-130a-5p,miR-29b-1-5p,miR-345-3p,miR-195a-3p,miR-216c-5p,miR-148a-5p,miR-664-5p,novel_73,miR-33b-3p,miR-1197-5p,miR-217-5p,miR-5703,miR-1434-5p,miR-2300b-3p,miR-1961,novel_133,miR-199a-3p,miR-148b-3p,miR-200b,miR-222-5p,miR-7859,miR-30d-3p,miR-18a-3p,miR-548e-3p,miR-129-5p,miR-4492,miR-1468-
LIPG	ENSOARG0(	lipase,	miR-7977,novel_68,miR-664b,miR-181b-3p,miR-677,miR-669,miR-144-5p,miR-374c-3p,miR-670-3p,miR-18a-3p,miR-1306,miR-409-5p,miR-361-3p,miR-1983,novel_94,miR-6516-3p,miR-7975,miR-146b-5p,miR-412-3p,miR-1291,miR-190b-5p,miR-2285x,miR-2284w,miR-7705,miR-371b-3p,miR-382-5p,novel_87,miR-2285y,novel_82,miR-6123,miR-490-5p,miR-4726-5p,miR-181b-2-3p,novel_78,miR-133b-5p,miR-2355-3p,miR-544b,miR-2285e,miR-9788-3p,miR-149-5p,novel_116,miR-503-5p,miR-496-5p,miR-153,miR-2285g,miR-22-3p,miR-1434-3p,miR-484,miR-744-5p,miR-1839-5p,miR-101c,miR-2426,miR-544a,miR-138,miR-668-3p,miR-2284l,novel_51,miR-29b-1-5p,miR-146a,miR-1306-5p,miR-503-5p,miR-214-3p,miR-146b,miR-153-3p,miR-1343-5p,miR-378a-5p,miR-2285aa,miR-874-3p,miR-2331-3p,miR-376c-3p,miR-22-3p,miR-6130,miR-376e-3p,miR-544-3p,miR-129b-3p,miR-500a-3p,miR-301,miR-2285c,miR-1827,miR-23a-5p,miR-885-3p,miR-214,miR-425-5p,miR-144-5p,miR-4510,miR-29b-2-5p,miR-132-5p,miR-500,miR-410-3p,miR-874-5p,miR-6516-3p,novel_74,miR-138-5p,miR-6517,miR-18a-3p,miR-582-3p,miR-23b-5p,miR-1343-5p,novel_133,miR-184-3p,miR-885-3p,miR-4286,miR-29b-2-5p,miR-495-5p,miR-503-5p,miR-146a-5p,miR-376c-3p,miR-29b-1-5p,miR-324-3p,miR-125a-3p,miR-664-3p,novel_127,miR-760-3p,miR-450a-2-3p,miR-2898

TTPAL	ENSOARG0(	tocopher	miR-2285p,miR-8117,miR-92a-1-5p,miR-17-3p,miR-106a,miR-543-5p,miR-299-3p,miR-221-5p,miR-2403,miR-20a,miR-1,miR-29b-2-5p,miR-5100,miR-380-5p,miR-29b-1-5p,miR-2284a,miR-143-3p,miR-192-3p,novel_39,miR-29a-5p,miR-2284y,miR-193b-5p,miR-140-3p,miR-10a-5p,miR-148a-5p,miR-106a,miR-299a-3p,miR-18b-5p,miR-1b-3p,miR-210-3p,miR-450a-1-3p,miR-29b-2-5p,miR-3604,miR-331-3p,miR-664a,miR-17-3p,miR-450b-3p,miR-130b-5p,miR-1306-5p,miR-133a-3p,miR-6128,miR-206-3p,miR-10b-5p,miR-93,miR-365a-5p,miR-450a-1-3p,miR-2448-3p,miR-488-3p,miR-17-5p,miR-18a,miR-93-5p,miR-200a-5p,miR-137-3p,miR-18a-5p,miR-133b-3p,miR-20b-5p,miR-221-5p,miR-296-3p,miR-21-3p,miR-10a,miR-154b-5p,miR-324-5p,novel_51,miR-106b-5p,miR-17-5p,miR-106a-5p,miR-365b-5p,miR-29b-1-5p,miR-103a-2-5p,miR-452-5p,miR-106a-5p,miR-380-5p,miR-130b-5p,miR-503-3p,miR-193a,miR-10b,miR-181a-5p,miR-296-5p,miR-3071-5p,miR-450c-3p,miR-1a-3p,novel_4,miR-425-3p,miR-496-5p,miR-20b,miR-411-5p,miR-148b-5p,miR-20a-5p,novel_82,novel_87,miR-107-5p,miR-206,miR-3968,miR-2284q,miR-219b-3p,miR-181d-5p,miR-17-3p,miR-24-3p,miR-2428,miR-1285-5p,miR-18b,miR-2387,miR-3591-3p,miR-3970,miR-877-3p,miR-10a-5p,miR-499b-5p,miR-299b-3p,miR-489,miR-4443
TRAPPC9	ENSOARG0(	traffick	miR-216a-5p,miR-185-3p,miR-125b,miR-30d,miR-21-3p,miR-760-3p,miR-216c-5p,miR-183-5p,miR-29b-1-5p,miR-124-3p,miR-140-3p,miR-188-3p,miR-5703,miR-1193,novel_133,miR-2300b-3p,miR-215-5p,miR-376a-5p,miR-505-3p,miR-548e-3p,miR-532-3p,miR-148b-3p,miR-200b,miR-19b-1-5p,miR-324-3p,miR-655-3p,miR-708-5p,miR-6516-3p,miR-1271,miR-425-5p,miR-2330-3p,miR-6134,let-7g-3p,miR-30f,miR-21-3p,miR-182-5p,miR-545-3p,miR-22-3p,novel_25,miR-544-3p,miR-192-5p,novel_103,miR-1306-5p,miR-326,miR-3085-3p,miR-3955-3p,miR-2330-5p,miR-30b-3p,miR-484,miR-3120-5p,miR-544a,miR-216a-3p,miR-2320-3p,miR-30b-5p,miR-7134-3p,miR-4726-5p,miR-335,miR-452-5p,miR-194b-5p,novel_60,miR-3059-5p,miR-212-3p,miR-541-3p,miR-129b-5p,miR-361-3p,miR-499b-5p,miR-7977,miR-3064-5p,miR-7857-3p,miR-3082-5p,miR-489,miR-1260a,miR-200c,miR-543-5p,miR-200c-3p,miR-490-3p,miR-1271-3p,miR-2284a,miR-218-2-3p,miR-30a-5p,miR-3578,miR-200b-3p,miR-128-3p,let-7a-2-3p,miR-7641,miR-376a-5p,miR-423-3p,miR-545-5p,miR-302a-5p,miR-1271-5p,miR-331-3p,miR-1306-3p,miR-5100,miR-28-5p,miR-486b-3p,miR-138-5p,let-7g-3p,miR-615-5p,miR-141-3p,miR-215-5p,miR-154b-5p,miR-365a-3p,miR-224-5p,miR-125a-5p,miR-124-5p,miR-2483-5p,miR-345-5p,miR-3071-5p,miR-194-5p,miR-1434-3p,miR-1285,miR-744-5p,miR-2889,miR-138,miR-30b-3p,miR-1260b,miR-130a-3p,miR-665,miR-133b-5p,miR-544b,miR-432-5p,miR-147-5p,miR-502b,novel_1,miR-2285x,novel_120,novel_27,miR-326-3p,miR-2428,miR-24-3p,miR-1307-3p,miR-18a-3p,miR-2387,miR-6516-3p,miR-505-3p,miR-376a-2-5p,miR-1291,miR-320b,miR-363,miR-324-3p,miR-30b,miR-210-5p,miR-208b-3p,novel_17,miR-369-3p,miR-486-3p,miR-143-3p,miR-664b-3p,miR-30c-5p,miR-218-1-3p,miR-2284u,miR-493-3p,miR-33b-3p,miR-142-3p,miR-212-

TNPO3	ENSOARG0(	transpor	miR-2428,miR-3968,miR-101-3p,miR-378e,miR-664b,miR-378b,miR-6395,miR-378f,miR-1291,miR-216a-3p,miR-2387,miR-2310,miR-378d,miR-378a-5p,miR-29b-1-5p,miR-2957,miR-9788-3p,miR-216a-3p,miR-185-5p,miR-339-5p,miR-1343-3p,miR-3120-5p,miR-378c,miR-1260b,miR-125b-2-3p,miR-873a-5p,miR-378b,miR-484,miR-542-3p,miR-1843b-5p,miR-378c,miR-122-5p,miR-1260b,miR-3074-2-3p,miR-539-5p,miR-378c,miR-145a-3p,miR-3432a,miR-664a,miR-6516-3p,miR-665,miR-151b,miR-532-5p,miR-574-5p,miR-378a-3p,miR-221-5p,miR-1814c,miR-339b,miR-329b,miR-143-3p,miR-29b-1-5p,miR-664b-3p,miR-221-5p,miR-466i-5p,miR-339a,miR-422a,miR-1260a,miR-664-3p,miR-532-3p,miR-151-5p,miR-328-3p,miR-378h,novel_79,miR-128-3p,miR-378a-3p,miR-378i
ETFDH	ENSOARG0(	electron	miR-769-5p,miR-2440,novel_82,miR-3958-3p,miR-452-5p,miR-541-3p,miR-877-3p,miR-362-5p,miR-199b-5p,miR-493-5p,miR-20a-3p,miR-378e,miR-378g,miR-16b,miR-362-3p,miR-326,miR-320b,miR-199b-5p,miR-424-5p,miR-124a,miR-491-3p,miR-8485,miR-378d,miR-3962,miR-4791,miR-541,miR-484,miR-296-5p,miR-378b,miR-329-3p,miR-3120-5p,miR-544a,miR-378c,miR-362-3p,miR-2300a-5p,miR-500-5p,miR-455-3p,miR-665,miR-19b-1-5p,miR-4492,miR-155-5p,miR-17-3p,miR-378c,miR-769-5p,miR-1827,miR-216b-3p,miR-378a-3p,miR-155-5p,miR-15b-5p,miR-544-3p,miR-422a,miR-210-5p,miR-760-3p,miR-466i-5p,miR-320d,miR-5703,miR-378a-3p,miR-124-3p,miR-362-3p,miR-378i,miR-4429,miR-142-3p,miR-493-3p,miR-3600,miR-18a-3p,miR-200b,miR-15a-5p,miR-16a,miR-28c,miR-133a-5p,miR-15a,miR-17-3p,miR-219b-3p,miR-2428,miR-199b-5p,miR-326-3p,miR-24-3p,miR-21c,miR-18a-3p,miR-378f,miR-195a-5p,miR-107,miR-1291,miR-363,miR-320c,miR-365b-5p,miR-7-5p,miR-466f-3p,miR-196b-5p,miR-2483-5p,miR-493-5p,miR-7b-5p,miR-19b-2-5p,miR-432,miR-653,miR-4508,miR-497-5p,miR-16b,miR-21-5p,miR-103a-3p,miR-103,miR-544b,miR-134-3p,miR-3535,miR-432-5p,miR-6740-5p,miR-135a-1-3p,miR-16-5p,miR-365a-5p,miR-378c,miR-500b-5p,miR-323-3p,miR-362-5p,miR-320a,miR-7144-5p,miR-329b,miR-329a,miR-2285aa,miR-199a-5p,miR-223-5p,miR-769,novel_127,miR-107,miR-17-3p,miR-181a-2-3p,miR-199a-5p,miR-28b,miR-148b-5p,miR-330-5p,miR-196a-5p,miR-155-5p,miR-200b-3p,miR-429-3p,novel_121,miR-378h,miR-322-5p

ACAA2	ENSOARG0(	acetyl-C	miR-133b-5p,miR-31-5p,miR-130a-3p,miR-134-3p,miR-1843b-5p,miR-1434-3p,miR-450c-3p,miR-29b,miR-188-5p,miR-382,miR-432-3p,novel_51,miR-29a-3p,miR-299b-3p,miR-2285ab,miR-1306,miR-412-3p,miR-29c-3p,miR-1193,miR-211,miR-199b-5p,miR-301b-3p,miR-2284w,miR-382-5p,miR-29a,miR-203b-5p,miR-656-5p,miR-299a-3p,miR-412,miR-450a-1-3p,miR-30a-5p,miR-29d-3p,miR-300,miR-30c,miR-181a-2-3p,miR-4286,miR-30a-3p,miR-3064-5p,miR-2478,miR-1271-3p,miR-490-3p,miR-199a-5p,miR-125a-3p,miR-30c-2-3p,miR-1271,miR-2355-5p,miR-769,miR-199a-5p,miR-30e-5p,miR-2318,miR-876-3p,miR-10b-3p,miR-193a-5p,miR-370-5p,miR-29b-3p,miR-204-5p,novel_4,miR-455-3p,miR-541,miR-484,miR-412-3p,miR-299a-5p,miR-744-3p,miR-676-3p,miR-30c-1-3p,miR-1306-5p,miR-182-5p,miR-29b-1-5p,miR-130b-3p,miR-3085-3p,miR-199b-5p,miR-3064-5p,miR-139-5p,miR-211-5p,miR-499b-5p,miR-541-5p,miR-7857-3p,miR-378g,miR-199b-5p,miR-361-3p,miR-129b-5p,miR-668-5p,miR-1983,miR-541-3p,miR-2285f,miR-8095,miR-218-5p,miR-30b-5p,miR-1973,miR-769-5p,miR-3968,miR-499a-5p,miR-2285t,miR-30d-3p,miR-3600,miR-1247-5p,novel_83,miR-30c-5p,novel_133,miR-1193,miR-188-3p,miR-2904,miR-142-3p,miR-493-3p,miR-216c-5p,miR-125b-2-3p,miR-376a-5p,miR-29b-1-5p,miR-210-5p,miR-30b,miR-92a-1-5p,miR-299-3p,miR-30c-1-3p,miR-2331-3p,miR-296-3p,miR-182-5p,miR-370-5p,miR-139-5p,miR-6130,miR-2284s,miR-301a-3p,miR-450a-1-3p,miR-299,miR-30f,miR-2312,miR-186-5p,miR-4510,miR-145a-3p,miR-3074-2-3p,miR-769-5p,miR-338-3p,miR-30e-3p,miR-30a-3p,miR-450b-3p,miR-129-5p,miR-454-3p,miR-208b-5p
MFSD4	ENSOARG0(	major fa	miR-664a,miR-2428,miR-129-1-3p,miR-485-5p,miR-103a-2-5p,miR-129-2-3p,miR-107-5p

SERINC3	ENSOARG0(	serine i	miR-941,miR-4429,miR-19b-3p,miR-128-3p,miR-134,miR-188-3p,miR-29d-3p,miR-15a-5p,miR-1271-5p,miR-322-5p,miR-7859,miR-129-1-3p,miR-214-5p,miR-2284z,miR-134-5p,miR-532-3p,miR-431,miR-212-5p,miR-320d,miR-17-3p,miR-107,miR-1277-5p,novel_17,miR-2285af,miR-324-3p,miR-3071-3p,miR-376c-3p,miR-29b-1-5p,miR-490-3p,miR-1271-3p,miR-3065-3p,miR-181a-2-3p,miR-30a-3p,miR-671-5p,miR-7144-5p,miR-376c-5p,miR-424-3p,miR-488-3p,miR-299,miR-21a-3p,miR-320a,miR-376c-5p,miR-769,miR-376e-3p,miR-377-3p,miR-301a-3p,miR-376c-3p,miR-15b-5p,miR-2331-3p,miR-140-5p,miR-3065-3p,miR-370-3p,miR-17-3p,miR-138-5p,miR-134-5p,miR-424-5p,miR-454-3p,miR-30e-3p,miR-5100,miR-29b-3p,miR-30a-3p,miR-769-5p,miR-129-2-3p,miR-144-5p,miR-1271,miR-16-5p,miR-299a-5p,miR-138,miR-29b,miR-16b,miR-26a-2-3p,miR-497-5p,miR-2284aa,miR-1298-5p,novel_23,miR-103,miR-6238,novel_78,miR-330-3p,miR-133b-5p,miR-103a-3p,miR-31-5p,miR-130a-3p,miR-377-3p,miR-431-5p,miR-320b,miR-16b,miR-29b-1-5p,miR-330-3p,miR-2284l,miR-1b-5p,miR-424-5p,miR-6240,miR-1388-3p,miR-19a-3p,miR-107,miR-96-5p,miR-195a-5p,miR-9-3p,miR-2285ab,miR-361-3p,miR-216a-3p,miR-150-5p,miR-3970,miR-29a-3p,miR-320c,miR-2285u,miR-29a,miR-128-1-5p,miR-2440,miR-15a,novel_82,miR-382-5p,miR-769-5p,miR-16a,miR-877-3p,miR-301b-3p,miR-9-3p,novel_27,miR-3059-5p,miR-17-3p,miR-29c-3p,miR-2285f

EPN3	ENSOARG0(	epsin 3	miR-148a-3p,miR-3141,miR-2319a,miR-412-3p,miR-329-5p,miR-6535,miR-669,miR-299b-3p,miR-374c-3p,miR-485-3p,miR-1247-3p,miR-576-3p,miR-28c,miR-193b-3p,miR-1a-1-5p,miR-450c-3p,miR-2113,miR-4508,miR-27b-3p,miR-188-5p,miR-339-5p,miR-31-5p,miR-194-3p,miR-134-3p,miR-2285v,miR-3141,miR-105-5p,miR-365b-5p,miR-194a,novel_51,miR-2411-3p,miR-193a-3p,miR-483-3p,let-7f-2-3p,let-7f-2-3p,miR-6240,miR-2285r,miR-23b,miR-331-5p,miR-2474,miR-2285aa,miR-125a,miR-376c-3p,miR-331-5p,miR-300-3p,miR-125b-5p,miR-2433,novel_63,miR-331-5p,novel_9,novel_42,miR-1197-3p,miR-2427,miR-1343-5p,miR-330-5p,miR-885-3p,novel_79,miR-1224-5p,miR-18b-5p,miR-299a-3p,miR-429-3p,miR-2284z,miR-450a-1-3p,novel_121,miR-505-5p,miR-152-3p,novel_96,miR-1a-2-5p,miR-376a-3p,miR-1983,miR-504,miR-139-5p,miR-2319b,miR-203-3p,miR-4443,miR-1185-2-3p,miR-378g,miR-25,miR-3596,miR-218-5p,miR-2440,miR-3964,miR-3968,miR-361-3p,miR-1839-5p,miR-199b-3p,miR-330-3p,miR-2459,miR-1185-3p,miR-1298-3p,miR-376b-3p,novel_19,miR-199a-3p,miR-214-3p,miR-491-5p,miR-23a-3p,miR-1388-3p,miR-101b-3p,miR-450a-1-3p,miR-885-3p,miR-1827,miR-339b,miR-30c-1-3p,miR-504-5p,miR-376e-3p,miR-10a,miR-665,miR-2285j,miR-4492,miR-129-5p,miR-665-5p,miR-214,miR-122-5p,miR-6525,miR-346,novel_44,miR-218-1-3p,miR-493-3p,miR-33b-3p,miR-2284h-5p,miR-328-3p,miR-23b-3p,miR-7859,miR-23c,miR-324-3p,miR-92a-1-5p,miR-345-3p,miR-369-3p,miR-345-3p,miR-2285w,miR-143-3p,miR-376c-3p,miR-376b-3p,miR-2285ab,miR-18b,miR-379-5p,miR-6395,miR-505-3p,miR-450b-3p,miR-1291,miR-19a-3p,miR-10a-5p,miR-146b-3p,miR-670-3p,miR-199c,miR-147-5p,miR-128-1-5p,novel_101,miR-136-3p,novel_27,miR-326-3p,miR-2428,miR-24-3p,miR-371b-3p,miR-194-5p,miR-3071-5p,miR-744-5p,miR-

STRA6	ENSOARGO(	stimulat	miR-147a,miR-1298-3p,miR-2300a-5p,miR-199b-3p,novel_78,miR-1343-3p,miR-181b-2-3p,miR-1388-5p,miR-378c,miR-544a,miR-450b-5p,miR-21b,miR-2284r,miR-484,miR-378b,miR-301b-5p,miR-10b,miR-7857,miR-125b-2-3p,miR-378d,miR-8485,miR-193a,miR-33a-5p,miR-3085-3p,miR-199b-5p,miR-103a-2-5p,miR-214-3p,miR-199a-3p,miR-320b,miR-1306-5p,miR-378g,miR-378e,miR-489,miR-4443,miR-3064-5p,miR-2319b,miR-452-3p,miR-20a-3p,miR-211-5p,miR-126b-5p,miR-504,miR-1983,miR-199b-5p,miR-378j,miR-877-3p,miR-361-3p,novel_111,miR-423-5p,miR-3059-5p,miR-452-5p,novel_82,miR-107-5p,miR-301a-5p,miR-95-3p,miR-199a-3p,miR-532-3p,miR-328-3p,miR-18a-3p,miR-4429,miR-664-5p,miR-378d,miR-378i,miR-2300b-3p,miR-378a-3p,miR-1197-5p,miR-5703,miR-1193,miR-376a-5p,miR-181b-1-3p,miR-486-3p,miR-320d,miR-760-3p,miR-99a-3p,miR-466i-5p,miR-21-3p,miR-299-3p,miR-324-3p,miR-500a-5p,miR-345-3p,miR-125b,miR-422a,miR-10a,miR-544-3p,miR-6516,miR-377-3p,miR-323-5p,miR-6130,miR-216b-5p,novel_32,miR-378a-3p,miR-140-5p,miR-504-5p,miR-216b-3p,let-7g-3p,miR-339b,miR-345-5p,miR-625-5p,miR-21a-3p,miR-450a-1-3p,miR-6525,miR-10b-5p,miR-206-3p,miR-224-5p,miR-346,miR-3184-5p,miR-338-3p,miR-378c,miR-539-5p,miR-145a-3p,miR-4510,miR-3074-5p,miR-129-2-3p,miR-214,miR-665-5p,miR-450b-3p,miR-664a,miR-208b-5p,miR-874-5p,miR-324-3p,miR-320e,miR-432-5p,miR-134-3p,miR-3431,miR-194-3p,miR-149-5p,miR-544b,miR-339-5p,miR-138,miR-455-5p,miR-7862,miR-873a-5p,miR-432,miR-450c-3p,let-7a-2-3p,miR-345-5p,miR-380-5p,miR-668-3p,miR-378a-5p,miR-125a-5p,miR-224-5p,miR-377-3p,novel_51,miR-455-5p,miR-2411-3p,miR-323a-5p,miR-299b-3p,miR-670-3p,miR-677,miR-320c,miR-6535,miR-181b-3p,novel_68,miR-378h,miR-3074-5p,miR-10a-5p,miR-1291,miR-

NUB1	ENSOARG0(	negative	novel_73,miR-142-3p,miR-33b-3p,miR-34a-5p,miR-664-5p,miR-193b-5p,miR-1434-5p,miR-217-5p,miR-95-3p,miR-18a-3p,miR-30d-3p,miR-212-5p,miR-210-5p,miR-92a-1-5p,miR-324-3p,miR-3601,miR-143-3p,miR-486-3p,miR-503-5p,miR-345-5p,miR-1827,miR-885-3p,miR-2285c,miR-450a-1-3p,miR-216b-5p,miR-15b-5p,miR-504-5p,novel_74,miR-130b-5p,miR-1468-5p,miR-874-5p,miR-664a-5p,miR-224-5p,miR-346,miR-6525,miR-181c-5p,miR-3184-5p,miR-122-5p,miR-1388-5p,miR-101c,miR-301b-5p,miR-541,miR-1a-3p,miR-501-3p,miR-2459,miR-455-3p,miR-330-3p,novel_78,miR-181b-2-3p,miR-381-5p,miR-199a-3p,miR-182-5p,miR-16b,miR-2310,miR-1388-3p,miR-504,miR-335-3p,miR-500-3p,miR-4443,miR-2319b,miR-139-5p,miR-24-1-5p,miR-211-5p,miR-499a-5p,miR-3968,novel_82,miR-301a-5p,miR-877-3p,miR-423-5p,miR-2447,miR-9-5p,miR-885-3p,miR-1290,miR-330-5p,miR-9-5p,miR-322-5p,miR-505-5p,miR-486-5p,novel_121,miR-134-5p,miR-450a-1-3p,miR-429-3p,miR-412,miR-200a-3p,novel_127,miR-152-3p,miR-8117,miR-125a-3p,miR-450b-5p,miR-29a-5p,miR-2284f,miR-3064-5p,miR-345-5p,miR-2474,miR-1248,miR-574-5p,miR-1271,miR-125a,miR-2355-5p,miR-134-5p,miR-125b-5p,miR-6740-5p,miR-1260b,miR-16-5p,miR-193a-5p,miR-381-5p,miR-16b,miR-188-5p,miR-497-5p,miR-542-3p,miR-7b-5p,miR-450c-3p,miR-3431,miR-194-3p,miR-149-5p,miR-31-5p,miR-106b-3p,miR-582,miR-7-5p,miR-3141,miR-105-5p,miR-6240,miR-2284e,miR-148a-3p,miR-3141,miR-1306,miR-150-5p,miR-3970,miR-181b-3p,miR-669,miR-677,miR-206,miR-28c,miR-4324,miR-2284o,miR-1246,miR-2300b-3p,miR-1197-5p,miR-188-3p,miR-140-3p,miR-5703,miR-200b,miR-148b-3p,miR-15a-5p,miR-487a-3p,miR-383-5p,miR-199a-3p,novel_83,miR-466i-5p,miR-760-3p,miR-2284m,miR-1277-5p,miR-34b-5p,miR-125b,miR-185-3p,miR-216a-5p,let-7g-3p,miR-21a-3p,miR-139-5p,miR-

PTPN13	ENSOARGO(	protein	miR-376a-5p,miR-1961,miR-140-3p,miR-33b-3p,miR-34a-5p,miR-378d,miR-3600,miR-200b,miR-148b-3p,miR-23c,miR-23b-3p,miR-383-5p,miR-210-3p,miR-26a-5p,miR-345-3p,miR-500a-5p,miR-98-5p,miR-202-5p,let-7k,miR-2484,miR-34b-5p,miR-30b-3p,miR-345-3p,miR-195a-3p,miR-377-5p,miR-376a-5p,miR-143-3p,miR-2432,miR-2285c,miR-21a-3p,miR-433-3p,miR-30d-5p,miR-129b-3p,miR-345-5p,novel_99,miR-1827,miR-504-5p,miR-545-3p,miR-216b-3p,miR-656-3p,miR-216b-5p,miR-6130,miR-10a,miR-301a-3p,miR-6516,miR-877-5p,let-7i-5p,miR-204-3p,miR-2285j,let-7f,miR-320e,miR-19b-1-5p,miR-132-5p,miR-130b-5p,miR-190a-5p,miR-454-3p,miR-4510,miR-3074-5p,let-7b,miR-224-5p,miR-6525,miR-10b-5p,let-7b-5p,miR-10b,miR-4791,miR-1298-5p,miR-6529a,miR-484,miR-758-3p,miR-2426,miR-1388-5p,miR-1839-5p,miR-16-2-3p,miR-2284aa,miR-101c,novel_78,miR-105-1,miR-2300a-5p,miR-145b,miR-539-5p,miR-2459,miR-2285e,miR-148b-5p,miR-9788-3p,miR-1306-5p,miR-326,miR-3955-3p,miR-130b-3p,miR-3085-3p,let-7e,miR-103a-2-5p,miR-2285o,miR-494-3p,miR-1388-3p,let-7a-5p,miR-33a-5p,miR-652-5p,miR-23a-3p,miR-758-3p,miR-193a,miR-504,miR-3963,miR-9-3p,miR-877-3p,miR-452-3p,miR-20a-3p,miR-2319b,miR-3064-5p,miR-7977,miR-2483-3p,miR-378g,miR-25,miR-4443,miR-203-3p,let-7d-5p,miR-101-3p,miR-382-3p,miR-218-5p,miR-107-5p,miR-371a-5p,miR-3964,miR-3956-3p,novel_60,miR-2447,miR-190b-5p,miR-2285f,miR-7705,miR-1290,miR-134,miR-345-3p,miR-3591-5p,miR-23b-5p,miR-330-5p,miR-30a-5p,let-7d,miR-10a-5p,miR-105-2,miR-200b-3p,miR-429-3p,miR-6517,miR-196a-2-3p,miR-6516-5p,miR-505-5p,miR-134-5p,miR-2284z,miR-664-3p,miR-200c,let-7c-5p,miR-200c-3p,miR-127-5p,let-7f-5p,miR-2898,miR-152-3p,miR-543-5p,miR-200a-3p,miR-3529-3p,miR-3064-5p,miR-

LACTB2	ENSOARG0(	lactamas	miR-339-5p,miR-377-5p,miR-31-5p,miR-133b-5p,miR-544b,miR-2284d,miR-185-5p,miR-450c-3p,novel_23,miR-653,miR-873a-5p,miR-7b-5p,miR-1434-3p,miR-29b,miR-2284ab,miR-30b-3p,miR-219a-1-3p,miR-16-1-3p,miR-363-5p,let-7f-2-3p,let-7f-2-3p,miR-380-5p,miR-130b-5p,miR-345-5p,miR-2285r,miR-32,miR-105-5p,novel_51,miR-224-5p,miR-7-5p,miR-146a,miR-660,miR-452-5p,miR-125a-5p,miR-378a-5p,miR-7-1-3p,miR-3120-3p,miR-6535,miR-299b-3p,novel_69,miR-29a-3p,miR-670-3p,miR-1306,miR-3970,miR-3591-3p,miR-216a-3p,miR-18b,miR-146b-5p,miR-135b-5p,miR-410-5p,miR-3957-3p,miR-758-5p,miR-29c-3p,miR-17-3p,miR-335-5p,novel_27,miR-2284w,miR-211,miR-24-3p,miR-4324,miR-1247-3p,miR-28c,miR-421-5p,miR-502b,miR-92a-3p,novel_1,miR-29a,miR-7641,novel_79,miR-18b-5p,miR-431,miR-2285ad,miR-299a-3p,miR-2284z,miR-450a-1-3p,miR-129-1-3p,miR-9-5p,miR-218-2-3p,miR-29d-3p,miR-30a-5p,miR-128-3p,miR-105-2,miR-155-5p,miR-29b-2-5p,miR-30a-3p,miR-181a-2-3p,miR-4286,miR-2403,miR-490-3p,miR-380-5p,miR-28b,miR-92b-3p,miR-30c-2-3p,miR-3071-3p,miR-339a,miR-92a-3p,miR-200a-3p,miR-17-3p,miR-28-3p,miR-125a,miR-141-3p,miR-2284n,miR-25-3p,miR-365a-3p,miR-769,miR-18a,miR-3154,miR-30e-5p,miR-301,miR-2448-3p,miR-7144-5p,miR-1248,miR-876-3p,miR-345-5p,miR-329a,miR-3607-3p,miR-103b,miR-28a-3p,miR-100-3p,miR-125b-5p,miR-135a-5p,miR-29b-3p,novel_63,miR-135a-1-3p,miR-486b-3p,miR-204-5p,miR-592,miR-28-5p,miR-5010-3p,miR-105-1,miR-1185-3p,miR-425-3p,miR-147a,miR-30b-3p,miR-329-3p,miR-2284aa,miR-362-3p,miR-1388-5p,miR-450b-5p,miR-544a,miR-33a-5p,miR-494-3p,miR-3187-3p,miR-30c-1-3p,miR-2957,miR-29b-1-5p,miR-362-3p,novel_103,miR-431-5p,miR-1306-5p,miR-146b,miR-214-3p,miR-1185-5p,miR-211-5p,miR-203-3p,miR-1185-2-3p,miR-3596,miR-25,miR-3082-5p,miR-
ZNF536	ENSOARG0(	zinc fin	miR-151-5p,miR-199a-3p,miR-214-5p,miR-1224-5p,miR-7641,miR-431,miR-30c,miR-378d,miR-885-3p,miR-493-3p,miR-33b-3p,miR-4429,miR-1343-5p,miR-2904,miR-5703,miR-30a-5p,miR-30c-5p,miR-615-3p,miR-486-3p,miR-143-3p,miR-450b-5p,miR-142a-3p,miR-495-5p,miR-29b-2-5p,miR-30d,miR-320d,miR-760-3p,miR-181b-5p,miR-219b-5p,miR-125b,miR-205-5p,miR-30b,miR-542-5p,miR-210-5p,novel_32,miR-151b,miR-877-5p,miR-3957,miR-193b-3p,miR-6130,miR-370-3p,miR-542-5p,miR-125a,miR-615-5p,miR-3957-5p,miR-140-5p,miR-885-3p,miR-1895,miR-1248,miR-1827,miR-671-5p,miR-876-3p,miR-30f,miR-27b-5p,miR-320a,miR-30d-5p,miR-30e-5p,miR-181c-5p,miR-193a-5p,miR-214,miR-665-5p,novel_42,miR-4510,miR-145a-3p,miR-708-5p,miR-4492,miR-486b-3p,miR-28-5p,miR-592,miR-125b-5p,miR-29b-2-5p,miR-2459,miR-2355-3p,miR-432-5p,miR-142-3p,miR-1343-3p,miR-7862,miR-125b-2-3p,miR-432,miR-181a-5p,miR-654-3p,miR-2330-5p,miR-125a-5p,miR-1343-5p,miR-193a-3p,miR-542-5p,miR-214-3p,novel_124,miR-320b,miR-199a-3p,miR-431-5p,miR-1306-5p,miR-320c,miR-669,miR-670-3p,miR-499b-5p,miR-1291,miR-1306,miR-2428,miR-361-3p,miR-877-3p,miR-193b-3p,miR-542-5p,miR-219a-5p,miR-181d-5p,miR-30b-5p,miR-502-5p,miR-615

OXSM	ENSOARG0(	3-oxoacy	miR-876-3p,miR-345-5p,miR-329a,miR-20b-5p,miR-93-5p,miR-133c,miR-2355-5p,novel_9,novel_63,miR-1197-3p,miR-1260b,novel_42,miR-28a-3p,miR-129-1-3p,miR-429-3p,miR-1224-5p,miR-28-3p,let-7f-5p,miR-1a-2-5p,novel_96,miR-148b-5p,let-7g,novel_39,miR-29a-5p,miR-2285b,miR-3064-5p,miR-410-5p,let-7e-5p,miR-150-5p,miR-3065-5p,miR-3591-3p,novel_69,novel_68,miR-3074-5p,miR-3120-3p,miR-26b-5p,miR-6119-3p,miR-199b-5p,miR-1a-1-5p,miR-193b-3p,miR-708-3p,miR-542-3p,miR-1843b-5p,miR-7b-5p,miR-22-3p,miR-194-3p,miR-149-5p,miR-103a-3p,miR-487b-3p,miR-378a-5p,miR-193a-3p,miR-7-5p,miR-2285v,miR-365b-5p,let-7j,let-7a-2-3p,miR-628-5p,miR-345-5p,miR-543-3p,miR-1827,miR-129b-3p,miR-500a-3p,miR-411,miR-216b-5p,let-7i-5p,miR-504-5p,miR-2331-3p,miR-129-5p,miR-500,miR-4492,miR-2285j,let-7f,miR-665,miR-6525,miR-769-5p,miR-98-3p,miR-181c-5p,let-7b,miR-129-2-3p,miR-3074-5p,miR-3958-5p,miR-214,novel_73,miR-33b-3p,miR-34a-5p,miR-2366,miR-1961,miR-98-3p,miR-217-5p,miR-328-3p,miR-106a,miR-98-5p,miR-3653-3p,miR-2484,let-7k,miR-26a-5p,miR-324-3p,miR-3601,miR-376a-5p,miR-143-3p,miR-504,miR-9-3p,miR-381-3p,miR-3596,miR-2483-3p,miR-139-5p,miR-2319b,miR-211-5p,miR-1843a-3p,miR-2440,miR-301a-5p,miR-877-3p,miR-361-3p,miR-1285-5p,miR-2447,miR-1388-5p,miR-362-3p,miR-329-3p,miR-301b-5p,miR-323b,miR-758-3p,miR-455-3p,miR-491-5p,miR-199b-5p,miR-214-3p,miR-103a-2-5p,miR-323b-3p,miR-505,miR-106b-5p,miR-182-5p,miR-1388-3p,miR-10b-3p,let-7g-3p,miR-7144-5p,miR-671-5p,miR-3154,miR-17-5p,miR-34c-5p,miR-365a-3p,miR-769,miR-324-5p,miR-199a-5p,miR-370-3p,miR-138-5p,miR-204-5p,miR-1-5p,miR-551b-3p,miR-331-3p,miR-26c,miR-135a-5p,miR-93,miR-365a-5p,miR-3607-3p,miR-6128,let-7d,miR-200b-3p,let-7a-2-3p,miR-3578,miR-218-2-

DDRGK1	ENSOARG0(	DDRGK do	miR-200a,novel_127,miR-152-3p,miR-322-5p,miR-505-5p,miR-1224-5p,miR-6517,miR-30c,miR-1290,miR-196a-5p,miR-16-5p,miR-494-5p,miR-300-3p,miR-2355-5p,miR-542-5p,miR-874-3p,miR-1248,miR-20b-5p,miR-876-3p,miR-93-5p,miR-301,miR-30e-5p,miR-432-3p,miR-432-3p,let-7f-2-3p,let-7f-2-3p,miR-380-5p,miR-193a-3p,miR-196b-5p,miR-483-3p,miR-378a-5p,novel_51,miR-2411-3p,miR-146a,miR-411-5p,miR-194-3p,miR-3431,miR-134-3p,miR-497-5p,miR-16b,miR-22-3p,miR-199b-5p,miR-758-5p,miR-193b-3p,miR-421-5p,miR-379-3p,miR-576-3p,miR-669,miR-320c,novel_69,miR-26b-5p,miR-216b-3p,novel_68,miR-378f,miR-146b-5p,miR-2319a,miR-1306,miR-148a-3p,miR-421-5p,miR-422a,miR-345-3p,miR-324-3p,miR-26a-5p,miR-618,miR-210-5p,novel_17,miR-30b,miR-328-3p,miR-7859,miR-380-5p,miR-106a,miR-18a-3p,miR-34a-5p,miR-142-3p,miR-217-5p,miR-378a-3p,miR-378i,miR-30c-5p,miR-6525,miR-214,miR-130b-5p,miR-874-5p,miR-500,miR-4492,miR-487b-5p,miR-451a,miR-151-3p,miR-15b-5p,miR-216b-5p,miR-2284s,miR-1277-3p,miR-548o-3p,miR-30c-1-3p,miR-186-5p,miR-1827,miR-433-3p,miR-30d-5p,miR-487b-5p,miR-500a-3p,miR-8485,miR-378d,miR-214-3p,miR-103a-2-5p,miR-146b,miR-153-3p,miR-199b-5p,miR-487b-5p,miR-1185-5p,miR-16b,miR-106b-5p,miR-2459,miR-1185-3p,miR-148b-5p,miR-455-3p,miR-153,miR-21b,miR-501-3p,miR-301b-5p,miR-2284r,miR-1285-5p,miR-877-3p,miR-361-3p,miR-3956-3p,miR-3964,miR-301a-5p,miR-2440,miR-218-5p,miR-1185-2-3p,miR-500-3p,miR-203-3p,miR-378g,miR-140-5p,miR-490-3p,miR-199a-5p,miR-380-5p,miR-181a-2-3p,miR-543-5p,miR-450a-2-3p,miR-2898,miR-17-3p,miR-217,miR-30c-2-3p,miR-582-3p,miR-378h,miR-483-3p,miR-411-3p,miR-501-3p,miR-30a-5p,miR-218-2-3p,miR-3578,miR-345-3p,miR-378c,miR-93,miR-409-3p,miR-135a-5p,miR-199a-5p,miR-34c-5p,miR-671-5p,miR-17-5p,miR-

PHYHIPL	ENSOARG0(	phytanoy	miR-3059-5p,miR-2447,miR-452-5p,miR-194b-5p,miR-877-3p,novel_82,miR-628-3p,miR-374a-3p,miR-3964,miR-2284q,miR-2284g,miR-10b,miR-541-5p,miR-2483-3p,miR-3082-5p,miR-199b-5p,miR-129b-5p,miR-1983,miR-101b-3p,miR-23a-3p,miR-2284b,miR-30c-1-3p,miR-3187-3p,miR-2310,miR-199a-3p,miR-29b-1-5p,miR-199b-5p,miR-199b-3p,miR-1298-3p,miR-216a-3p,miR-2459,miR-10b,miR-299a-5p,miR-544a,miR-450b-5p,miR-16-2-3p,miR-101c,miR-4510,miR-3074-2-3p,miR-10b-5p,miR-19b-1-5p,miR-29b-2-5p,miR-450b-3p,miR-6516-3p,miR-30c-1-3p,miR-221-5p,miR-660-5p,miR-32-3p,miR-548o-3p,miR-3065-3p,miR-21-3p,miR-544-3p,miR-10a,miR-6130,miR-204-3p,miR-877-5p,miR-450a-1-3p,miR-411,miR-195a-3p,miR-421-5p,miR-29b-1-5p,miR-130a-5p,miR-376c-3p,miR-618,novel_17,miR-92a-1-5p,miR-345-3p,miR-221-5p,miR-548e-3p,miR-7859,miR-23b-3p,miR-222-5p,miR-199a-3p,novel_73,miR-19b-3p,miR-2284u,miR-135a-2-3p,miR-2285x,miR-2284w,miR-199b-5p,miR-6402,miR-133a-5p,miR-576-3p,miR-33a-3p,novel_1,miR-199c,miR-147-5p,miR-10a-5p,miR-7-1-3p,miR-2404,miR-2387,miR-3065-5p,miR-3591-3p,miR-412-3p,miR-107,miR-19a-3p,miR-363-5p,miR-382,miR-101-5p,miR-628-5p,miR-432-3p,miR-2285r,miR-432-3p,miR-2411-3p,miR-194a,miR-660,miR-31-5p,miR-103a-3p,miR-26b-3p,miR-412-5p,miR-103,miR-544b,miR-2355-3p,miR-19b-2-5p,miR-653,miR-542-3p,miR-450c-3p,novel_23,miR-194-5p,miR-3071-5p,miR-34c-3p,miR-592,miR-503-3p,miR-2355-5p,miR-2285aa,miR-141-3p,miR-331-5p,miR-199a-5p,miR-488-3p,miR-331-5p,miR-23b,miR-3154,miR-329b,miR-1814c,miR-3529-3p,miR-29b-2-5p,miR-2478,miR-450b-5p,miR-200a,miR-199a-5p,miR-2284y,miR-3065-3p,miR-2285b,miR-2284a,miR-30c-2-3p,miR-107,miR-200a-3p,miR-376a-5p,miR-450a-1-3p,miR-486-5p,novel_121,miR-345-3p,miR-10a-5p

ALDOA	ENSOARG0(	aldolase	miR-34b,miR-411b-3p,miR-374c-3p,miR-299b-3p,miR-669,novel_68,miR-216b-3p,miR-655-5p,miR-7-1-3p,miR-9851-3p,miR-195a-5p,miR-148a-3p,miR-18b,miR-2387,miR-1306,miR-3065-5p,miR-18a-3p,miR-3591-3p,miR-378b,miR-3959-3p,miR-449a,miR-326-3p,miR-2428,miR-199b-5p,novel_120,miR-335-5p,miR-3957-3p,miR-17-3p,miR-206,miR-22-5p,miR-199c,miR-147-5p,miR-15a,miR-485-3p,miR-34c,miR-16a,miR-3535,miR-412-5p,miR-2285g,miR-503-5p,miR-134-3p,miR-194-3p,miR-149-5p,miR-31-5p,miR-30b-3p,miR-16b,miR-16-1-3p,miR-497-5p,miR-27b-3p,miR-188-5p,miR-1434-3p,miR-7b-5p,miR-374b-3p,miR-2284l,miR-539-3p,miR-130b-5p,miR-124-5p,miR-1343-5p,miR-7-5p,miR-503-5p,miR-105-5p,novel_51,miR-374c-3p,miR-34c-5p,miR-769,miR-199a-5p,miR-3957,miR-574-5p,miR-3957-5p,miR-874-3p,miR-2285aa,miR-10b-3p,miR-7144-5p,miR-23a-5p,miR-485-5p,miR-1814c,miR-331-5p,miR-3154,miR-18a,miR-374a-3p,miR-28a-3p,miR-3607-3p,miR-16-5p,miR-133a-3p,miR-331-5p,miR-486b-3p,miR-374b-3p,miR-134-5p,miR-410-5p,miR-135a-1-3p,miR-1306-3p,miR-331-3p,miR-100-3p,miR-322-5p,miR-1271-5p,miR-134-5p,miR-129-1-3p,miR-299a-3p,miR-423-3p,miR-412,miR-18b-5p,miR-615-3p,miR-134,miR-1343-5p,miR-330-5p,miR-148b-5p,miR-199a-5p,miR-2285b,miR-490-3p,miR-1,miR-2385-3p,miR-3064-5p,miR-4286,miR-28-3p,miR-17-3p,novel_127,miR-152-3p,miR-378g,miR-3082-5p,miR-27a-3p,miR-3064-5p,miR-139-5p,miR-20a-3p,miR-7977,miR-96-5p,miR-299b-5p,miR-668-5p,miR-877-3p,miR-361-3p,miR-199b-5p,miR-1306-3p,miR-129b-5p,miR-361-3p,miR-3059-5p,miR-2447,miR-541-3p,miR-885-5p,miR-452-5p,miR-3968,miR-335,miR-2285t,miR-374a-3p,miR-2284q,novel_82,miR-615,miR-769-5p,miR-371a-5p,miR-153,miR-216a-3p,miR-148b-5p,miR-455-3p,miR-2285e,miR-2459,novel_78,miR-199b-3p,miR-27a-3p,miR-2426,miR-
TMEM135	ENSOARG0(	transmem	miR-4510,miR-494-3p,miR-376b,miR-877-3p,miR-376a-5p,miR-99b-3p,miR-505,miR-664a-5p,miR-124-5p,miR-99a-3p,miR-487a-5p,miR-200a-3p,miR-139-5p,miR-99a-3p,miR-141-3p,miR-139-5p,miR-6130,miR-324-5p,miR-27b-5p
PMPCA	ENSOARG0(	peptidas	miR-320a,miR-10b-3p,miR-671-5p,miR-1827,miR-874-3p,miR-2331-3p,miR-370-3p,miR-21-3p,miR-151b,miR-29b-2-5p,miR-664a,miR-138-5p,miR-4492,miR-3432a,miR-3184-3p,miR-145a-3p,miR-214,miR-3074-2-3p,miR-1260b,miR-346,miR-338-3p,miR-145-3p,miR-2366,miR-2300b-3p,miR-140-3p,miR-5703,miR-2904,miR-4429,miR-493-3p,miR-378d,miR-6517,miR-18a-3p,miR-483-3p,miR-214-5p,miR-328-3p,miR-532-3p,miR-151-5p,miR-1260a,miR-320d,novel_127,miR-2887,miR-29b-2-5p,miR-29b-1-5p,miR-3591-3p,miR-18a-3p,miR-147-3p,miR-1291,miR-6395,miR-4792,miR-378g,miR-320c,miR-4324,miR-128-1-5p,miR-194b-5p,miR-3957-3p,miR-361-3p,miR-2428,miR-1285,miR-194-5p,miR-197-3p,miR-1260b,miR-2426,miR-138,novel_78,miR-1343-3p,novel_4,miR-134-3p,miR-2459,miR-9788-3p,miR-320b,novel_51,miR-194a,miR-2331-5p,miR-29b-1-5p,miR-483-3p,miR-214-3p,miR-494-3p,miR-432-3p,miR-432-3p,miR-8485

SWT1	ENSOARG0(	SWT1 RNA	miR-1388-3p,let-7a-5p,miR-2284b,miR-23a-3p,miR-3187-3p,miR-7-5p,miR-99a-5p,miR-2411-3p,miR-197-5p,miR-105-5p,let-7e,miR-133b-5p,miR-105-1,miR-26b-3p,let-7g-5p,miR-2284d,miR-148b-5p,miR-216a-3p,miR-7b-5p,miR-6529a,novel_23,miR-412-3p,miR-2284x,miR-135a-2-3p,miR-219a-5p,miR-24-3p,miR-142b,miR-208a-3p,let-7i,miR-22-5p,miR-2319b,miR-2284g,novel_68,miR-320b,miR-670-3p,let-7d-5p,miR-216a-3p,miR-99b-5p,miR-96-5p,let-7e-5p,miR-376a-2-5p,miR-2319a,miR-2385-3p,miR-2284y,let-7g,miR-210-5p,miR-99a,miR-208b-3p,miR-6119-5p,miR-2285af,let-7c-5p,miR-345-3p,miR-98-5p,let-7f-5p,miR-2898,miR-543-5p,let-7k,miR-548e-3p,miR-376a-5p,miR-412,miR-1271-5p,miR-23c,miR-383-5p,miR-23b-3p,miR-3578,miR-1961,miR-345-3p,miR-1197-5p,let-7d,miR-378d,miR-2284u,miR-148a-5p,miR-155-5p,miR-105-2,miR-128-3p,miR-99a-5p,miR-4510,let-7b,miR-1271,let-7b-5p,let-7f,miR-324-3p,miR-155-5p,miR-6130,miR-155-5p,let-7i-5p,miR-100-5p,miR-23b,miR-329b,miR-625-5p
NGLY1	ENSOARG0(	N-glycan	miR-211,miR-183-5p,miR-211-5p,miR-329b,miR-9851-3p,miR-208b-5p,miR-1343-5p,miR-204-5p,novel_99,miR-1343-5p,miR-1434-5p
FZD4	ENSOARG0(	frizzled	miR-224-5p,novel_44,miR-346,miR-433-5p,let-7b,miR-129-2-3p,miR-3184-3p,novel_74,miR-874-5p,miR-454-3p,let-7f,miR-665,miR-664a-5p,miR-3604,miR-582-5p,miR-301a-3p,let-7i-5p,miR-151b,miR-15b-5p,miR-2331-3p,miR-30c-1-3p,miR-1277-3p,miR-548o-3p,miR-296-3p,miR-552-3p,miR-1895,miR-885-3p,miR-543-3p,miR-625-5p,miR-186-5p,miR-411,miR-433-3p,miR-30d-5p,miR-376a-5p,miR-181b-1-3p,miR-143-3p,miR-503-5p,miR-345-3p,miR-98-5p,miR-2484,let-7k,miR-30b,miR-324-3p,miR-500a-5p,miR-422a,miR-7859,miR-328-3p,miR-380-5p,miR-142-3p,miR-133a-3p,miR-218-1-3p,miR-2284u,miR-378i,miR-30c-5p,miR-1961,miR-193b-5p,miR-217-5p,miR-378a-3p,miR-877-3p,novel_82,miR-615,miR-101-3p,miR-500-3p,miR-4443,miR-2284g,miR-211-5p,miR-136-5p,miR-1983,miR-378j,miR-378d,miR-8485,miR-1388-3p,miR-124a,miR-101b-3p,miR-491-5p,miR-130b-3p,miR-153-3p,miR-199b-5p,miR-505,miR-323b-3p,miR-153,miR-2459,miR-199b-3p,miR-1343-3p,miR-101c,miR-541,miR-323b,miR-501-3p,miR-1260b,let-7e-3p,miR-16-5p,miR-3432a,miR-6740-5p,miR-125b-5p,miR-300-3p,miR-542-5p,miR-1271,miR-125a,miR-767,miR-329b,miR-1248,miR-30e-5p,miR-450b-5p,let-7g,miR-2284y,miR-192-3p,miR-3064-5p,miR-127-5p,let-7f-5p,novel_96,miR-200a-3p,miR-152-3p,miR-30f,miR-322-5p,miR-2284z,novel_121,miR-129-1-3p,miR-2285ad,miR-429-3p,miR-18b-5p,miR-885-3p,miR-30c,miR-1290,miR-1343-5p,miR-330-5p,miR-23b-5p,miR-374c-5p,miR-2284w,miR-199b-5p,miR-301b-3p,miR-376a-3p,miR-193b-3p,miR-29c-3p,miR-758-5p,miR-29a,miR-421-5p,miR-28c,novel_87,miR-374c-3p,miR-6535,miR-677,miR-181b-3p,miR-3120-3p,miR-412-3p,miR-378f,let-7e-5p,miR-148a-3p,miR-150-5p,miR-216a-3p,miR-2284l,miR-668-3p,let-7f-2-3p,let-7f-2-3p,miR-193a-3p,novel_51,miR-194a,miR-412-5p,miR-3535,miR-411-5p,miR-194-3p,miR-31-5p,miR-497-

OTUB1	ENSOARG0(	OTU deub	miR-885-3p,let-7d,miR-1343-5p,miR-29d-3p,miR-196a-5p,miR-505-5p,novel_79,miR-2285ad,novel_127,miR-17-3p,let-7f-5p,miR-339a,let-7c-5p,miR-125a-3p,miR-2397-5p,miR-3065-3p,let-7g,novel_39,miR-28b,miR-3064-5p,miR-4286,miR-7144-5p,miR-485-5p,miR-671-5p,miR-876-3p,miR-329b,miR-3154,miR-488-3p,miR-2355-5p,miR-125a,miR-574-5p,miR-874-3p,miR-486b-3p,miR-331-3p,miR-125b-5p,miR-6740-5p,miR-29b-3p,miR-2433,miR-1306-5p,miR-26a-2-3p,miR-455-5p,miR-744-5p,miR-30b-3p,miR-29b,miR-197-3p,miR-1843b-5p,miR-194-3p,miR-503-5p,let-7g-5p,miR-339-5p,miR-377-5p,miR-1343-5p,miR-125a-5p,miR-196b-5p,miR-330-3p,miR-455-5p,miR-503-5p,miR-432-3p,miR-363-5p,miR-628-5p,miR-6240,miR-6395,let-7e-5p,miR-1291,miR-150-5p,miR-1306,miR-18a-3p,miR-6535,miR-29a-3p,miR-128-1-5p,miR-29a,let-7i,miR-4324,miR-133a-5p,miR-28c,miR-2428,miR-24-3p,miR-3957-3p,miR-29c-3p,miR-758-5p,miR-17-3p,miR-133a-3p,miR-19b-3p,miR-2904,miR-5703,miR-1961,novel_83,miR-328-3p,miR-380-5p,miR-3960,miR-18a-3p,miR-21-3p,let-7k,miR-98-5p,miR-324-3p,miR-185-3p,miR-125b,miR-2285p,miR-210-5p,miR-486-3p,miR-377-5p,miR-130a-5p,miR-503-5p,miR-885-3p,miR-1827,miR-339b,novel_32,let-7i-5p,miR-499b-3p,miR-6130,miR-3065-3p,miR-552-3p,miR-548o-3p,miR-4492,miR-17-3p,miR-664a,miR-665,let-7f,let-7b-5p,miR-122-5p,miR-3184-5p,miR-6134,miR-214,let-7b,miR-145a-3p,miR-3184-3p,miR-4510,miR-30b-3p,miR-296-5p,miR-484,miR-301b-5p,miR-541,miR-147a,miR-5010-3p,miR-330-3p,novel_78,novel_4,miR-1343-3p,miR-214-3p,let-7e,miR-3085-3p,miR-25-5p,miR-487b-5p,miR-1306-5p,miR-5126,let-7a-5p,miR-877-3p,miR-361-3p,let-7d-5p,miR-499a-3p,miR-2483-3p,miR-7977,miR-3064-5p,miR-301a-5p,miR-423-5p,novel_60,miR-3956-3p,miR-541-3p

ELK4	ENSOARG0(	ELK4, ET	miR-6395,miR-195a-5p,miR-135b-5p,miR-6516-3p,miR-107,miR-1291,miR-378b,miR-2285ab,miR-34b,miR-2404,miR-7-1-3p,miR-378b,miR-199c,miR-203b-5p,miR-92a-3p,let-7i,miR-382-5p,miR-34c,miR-16a,miR-15a,miR-449a,miR-323c,miR-3959-3p,novel_101,miR-495-3p,miR-17-3p,miR-219a-1-3p,miR-145a-5p,miR-1434-3p,miR-544b,miR-103,let-7g-5p,miR-133b-5p,miR-466f-3p,miR-125a-5p,miR-124-5p,miR-330-3p,miR-224-5p,miR-539-3p,miR-654-3p,miR-382,miR-101-5p,miR-130b-5p,miR-7144-5p,miR-320a,miR-362-5p,miR-199a-5p,miR-769,miR-34c-5p,miR-141-3p,miR-487a-3p,miR-374b-3p,miR-486b-3p,miR-135a-5p,miR-26c,miR-5100,miR-2332,miR-1-5p,miR-378c,miR-374a-3p,miR-323-3p,miR-500b-5p,miR-3607-3p,let-7d,miR-582-3p,miR-545-5p,miR-378h,miR-2411-5p,miR-24-2-5p,miR-7641,miR-92a-3p,miR-107,miR-17-3p,miR-2898,let-7c-5p,miR-217,miR-490-3p,miR-199a-5p,miR-1957a,novel_94,miR-129b-5p,miR-199b-5p,miR-361-3p,let-7d-5p,miR-378e,miR-7977,miR-541-5p,miR-3064-5p,miR-2284q,miR-374a-3p,miR-2285t,miR-769-5p,miR-8095,miR-362-5p,miR-452-5p,miR-2285f,miR-212-3p,miR-2284aa,miR-2426,miR-544a,miR-378c,miR-125b-2-3p,miR-484,miR-378b,miR-6529a,miR-500-5p,miR-2300a-5p,let-7e,miR-3085-3p,miR-362-3p,miR-1306-5p,novel_103,miR-320b,miR-491-3p,miR-193a,miR-3187-3p,miR-1839-3p,miR-424-5p,let-7a-5p,miR-154b-3p,miR-2312,miR-21a-3p,miR-204-3p,miR-6130,miR-544-3p,miR-27a-5p,miR-21-3p,miR-221-5p,miR-545-3p,miR-378a-3p,miR-424-5p,miR-6516-3p,miR-17-3p,miR-338-5p,miR-655-3p,miR-324-3p,miR-338-3p,miR-6134,let-7b-5p,miR-2330-3p,miR-3074-2-3p,miR-4510,miR-378c,miR-539-5p,miR-378d,miR-4429,miR-140-3p,miR-362-3p,miR-15a-5p,miR-148b-3p,miR-487a-3p,miR-383-5p,miR-485-3p,miR-21-3p,miR-299-3p,miR-34b-5p,miR-221-5p,miR-466i-5p,miR-

			[illegible]
			125b,miR-542-5p,miR-30b,miR-486-3p,miR-329a-5p,miR-218-1-
			3p,miR-30c-5p,novel_133,miR-1247-5p,miR-616-3p,miR-199a-
			3p,miR-210-3p,miR-30d-3p,miR-3600,miR-18a-3p,miR-874-
			5p,miR-190a-5p,miR-29b-2-5p,miR-30a-3p,miR-30e-3p,miR-
			6134,miR-214,miR-3184-3p,miR-30f,miR-339b,miR-345-5p,miR-
			30d-5p,miR-129b-3p,miR-2285c,novel_32,miR-193b-3p,miR-
			323-5p,miR-544-3p,miR-216b-3p,miR-30c-1-3p,miR-504-
			5p,miR-221-5p,miR-542-5p,miR-214-3p,miR-199b-5p,miR-3955-
			3p,miR-491-5p,novel_124,miR-3085-3p,miR-2957,miR-326,miR-
			199a-3p,miR-2310,miR-4532,miR-30c-1-3p,miR-1388-3p,miR-
			3120-5p,miR-2426,miR-544a,miR-1388-5p,miR-484,miR-
			2285e,miR-2459,miR-147a,miR-145b,miR-1343-3p,miR-
			2285y,miR-30b-5p,novel_111,miR-877-3p,miR-190b-5p,miR-
			6527,miR-3956-3p,miR-3059-5p,novel_60,miR-1983,miR-
			504,miR-3963,miR-378j,miR-199b-5p,miR-3082-5p,miR-2483-
			3p,miR-378g,miR-7977,miR-7689-3p,miR-3064-
			5p,novel_127,novel_96,miR-200a-3p,miR-127-5p,miR-
			339a,miR-30f,miR-30c-2-3p,miR-1260a,miR-29a-5p,miR-199a-
			5p,miR-4286,miR-30a-3p,miR-3064-5p,miR-29b-2-5p,miR-
			30c,miR-30a-5p,miR-330-5p,miR-1343-5p,miR-1290,miR-
			134,miR-134-5p,novel_79,miR-299a-3p,miR-2285ad,miR-
			592,miR-134-5p,miR-486b-3p,miR-410-3p,miR-138-5p,miR-
			125b-5p,miR-410-5p,miR-1260b,miR-3607-3p,miR-6128,miR-
			1814c,miR-1248,miR-7144-5p,miR-23a-5p,miR-345-5p,miR-
			329b,miR-876-3p,miR-3154,miR-30e-5p,miR-488-3p,miR-154b-
			5p,miR-133c,miR-199a-5p,miR-374c-3p,miR-141-3p,miR-
			125a,miR-2285aa,miR-2355-5p,miR-874-3p,miR-574-5p,miR-
			193a-3p,miR-1343-5p,miR-125a-5p,miR-455-5p,miR-190a,miR-
			345-5p,miR-628-5p,miR-455-5p,miR-4508,miR-1260b,miR-
WDR86	ENSOARG0(	WD repea	138,miR-145a-5p,miR-3071-5p,miR-1285,miR-542-3p,miR-
			novel_42,miR-214,miR-3184-5p,miR-2285j,miR-29b-3p,miR-
			6740-5p,miR-450b-3p,miR-138-5p,miR-221-5p,miR-504-5p,miR-
			30c-1-3p,miR-3065-3p,miR-450a-1-3p,miR-3154,miR-671-
			5p,miR-3064-5p,miR-181b-1-3p,miR-3065-3p,miR-2432,miR-
			490-3p,novel_17,miR-30c-2-3p,miR-92a-1-5p,miR-760-3p,miR-
			466i-5p,miR-30b-3p,miR-221-5p,miR-412,miR-210-3p,miR-
			450a-1-3p,miR-1247-5p,miR-29d-3p,miR-124-3p,miR-135a-2-
			3p,miR-3957-3p,miR-29c-3p,miR-423-5p,miR-2284w,miR-371a-
MYO3A	ENSOARG0(	myosin I	5p,miR-128-1-5p,miR-4726-5p,miR-29a,miR-502b,miR-3064-
			5p,miR-7689-3p,miR-29a-3p,miR-677,miR-181b-3p,miR-361-
			3p,miR-1306,miR-1291,miR-504,miR-124a,miR-6240,miR-30c-1-
			3p,miR-4532,miR-758-3p,miR-7-5p,miR-1306-5p,miR-330-
			3p,novel_51,miR-3085-3p,miR-214-3p,miR-340-3p,miR-2300a-
			5p,novel_78,novel_4,miR-330-3p,miR-5010-3p,miR-181b-2-
			3p,miR-185-5p,miR-873a-5p,miR-7b-5p,miR-412-3p,miR-450c-
			3p,miR-758-3p,miR-29b,miR-138,miR-4508

PPM1H	ENSOARG0(protein	miR-7859,miR-23b-3p,miR-23c,miR-2284h-5p,miR-328-3p,miR-212-5p,miR-33b-3p,miR-361-5p,miR-34a-5p,miR-98-3p,miR-664b-3p,miR-376a-5p,miR-421-5p,miR-377-5p,miR-142a-3p,miR-143-3p,miR-2285w,miR-345-3p,miR-3653-3p,miR-26a-5p,miR-618,miR-3601,miR-324-3p,miR-92a-1-5p,miR-345-3p,miR-499b-3p,miR-151b,miR-15b-5p,miR-30c-1-3p,miR-32-5p,miR-216b-3p,miR-296-3p,miR-552-3p,miR-345-5p,miR-625-5p,miR-1895,miR-186-5p,miR-500a-3p,miR-30d-5p,miR-6525,miR-98-3p,miR-665-5p,miR-214,miR-129-5p,miR-500,miR-4492,miR-130b-5p,miR-2285j,miR-3604,miR-153,miR-1298-3p,miR-2459,miR-199b-3p,novel_78,miR-330-3p,miR-145b,miR-541,miR-1a-3p,miR-8485,miR-1388-3p,miR-23a-3p,miR-744-3p,miR-491-5p,miR-199b-5p,miR-146b,miR-153-3p,miR-214-3p,miR-431-5p,miR-505,miR-16b,miR-182-5p,miR-25,miR-499a-3p,miR-378g,miR-3596,miR-2483-3p,miR-203-3p,miR-4443,miR-139-5p,miR-2319b,miR-2285u,miR-126b-5p,miR-211-5p,miR-99b-5p,miR-335-3p,miR-9-3p,miR-221,miR-2447,miR-885-5p,miR-3956-3p,miR-499a-5p,novel_82,miR-615,miR-322-5p,miR-134-5p,novel_121,miR-299a-3p,miR-2285ad,miR-1224-5p,miR-412,miR-431,miR-1290,miR-2427,miR-1343-5p,miR-92b-3p,miR-29a-5p,miR-200a,novel_39,miR-450c-5p,miR-3956-5p,miR-127-5p,miR-200a-3p,novel_96,miR-125a-3p,miR-25-3p,miR-3068-3p,miR-2285aa,miR-2355-5p,miR-876-3p,miR-345-5p,miR-30e-5p,miR-23b,miR-1197-3p,miR-1260b,miR-193a-5p,miR-16-5p,miR-134-5p,miR-2433,miR-134-3p,miR-194-3p,miR-31-5p,miR-16b,miR-497-5p,miR-27b-3p,miR-19b-2-5p,miR-542-3p,miR-223-3p,miR-432-3p,miR-2284l,miR-6240,miR-378a-5p,miR-146a,miR-105-5p,miR-32,novel_51,miR-2411-3p,miR-299b-3p,miR-320c,miR-3120-3p,miR-26b-5p,miR-146b-5p,miR-3591-3p,miR-199b-5p,miR-1839-3p,miR-6119-3p,miR-206,miR-421-5p,miR-142b,miR-576-3p,novel_87,miR-

CCDC6	ENSOARG0(	coiled-c	miR-378h,miR-545-5p,novel_121,miR-299a-3p,miR-376a-5p,miR-105-2,miR-128-3p,miR-3591-5p,miR-345-3p,miR-218-2-3p,miR-330-5p,miR-92b-3p,miR-3064-5p,miR-127-5p,miR-6536,miR-92a-3p,miR-200a-3p,miR-152-3p,miR-125a-3p,miR-25-3p,miR-574-5p,miR-141-3p,miR-1271,miR-329b,miR-671-5p,miR-30e-5p,miR-374a-3p,miR-378c,miR-374b-3p,miR-5100,miR-6740-5p,miR-130a-3p,miR-219a-1-3p,miR-16-1-3p,miR-188-5p,miR-27b-3p,miR-1843b-5p,miR-1285,miR-2113,novel_23,miR-130b-5p,miR-363-5p,miR-124-5p,miR-146a,miR-32,miR-105-5p,miR-2411-3p,miR-299b-3p,miR-670-3p,miR-144-3p,miR-669,miR-3074-5p,miR-26b-5p,miR-19a-3p,miR-146b-5p,miR-378f,miR-148a-3p,miR-3591-3p,miR-216a-3p,miR-211,novel_27,miR-326-3p,miR-301b-3p,miR-2285x,novel_101,miR-92a-3p,novel_1,miR-6123,miR-576-3p,miR-148b-3p,miR-3600,miR-19b-3p,miR-218-1-3p,miR-16-1-3p,miR-148a-5p,miR-378i,miR-376a-5p,miR-378a-3p,miR-130a-5p,miR-146a-5p,miR-195a-3p,miR-30d,miR-2484,miR-299-3p,miR-196a-3p,miR-26a-5p,miR-422a,miR-345-3p,miR-301a-3p,miR-544-3p,miR-378a-3p,miR-32-5p,miR-21-3p,miR-552-3p,let-7g-3p,miR-30f,let-7f-1-3p,miR-500a-3p,miR-30d-5p,miR-122-5p,miR-6134,miR-378c,miR-3074-5p,miR-665-5p,miR-6516-3p,miR-500,miR-454-3p,miR-130b-5p,let-7b-3p,miR-324-3p,miR-665,miR-148b-5p,miR-105-1,miR-27a-3p,miR-378c,miR-544a,miR-6529a,miR-378b,miR-323b,miR-125b-2-3p,miR-378d,miR-8485,let-7c-3p,miR-3085-3p,miR-130b-3p,miR-146b,miR-326,miR-2957,miR-505,miR-323b-3p,miR-378e,miR-2483-3p,miR-27a-3p,miR-3064-5p,miR-126b-5p,miR-211-5p,miR-7977,miR-7705,miR-4454,miR-363-3p,miR-628-3p,miR-374a-3p,miR-2284q
PML	ENSOARG0(	promyelo	novel_17,miR-541,miR-146a,miR-5703,miR-133b-3p,miR-146b-5p,miR-146b,miR-133a-3p,miR-541-3p,miR-1388-3p,miR-133a-3p,miR-2355-5p,miR-6134,miR-146a-5p
SLC45A3	ENSOARG0(	solute c	miR-485-5p,miR-378f,miR-1827,miR-34a-5p,miR-378c,miR-378a-3p,miR-378i,miR-361-3p,miR-378b,miR-378e,miR-34c-5p,miR-34b,miR-503-5p,miR-378h,miR-31-5p,miR-378a-3p,miR-874-5p,miR-34b-5p,miR-34c,miR-422a,miR-665,miR-503-5p,miR-378c,miR-449a,miR-1271-3p,miR-378d,miR-378c,miR-503-5p

CPEB4	ENSOARG0(	cytoplas	miR-450b-5p,miR-2426,miR-362-3p,miR-329-3p,miR-125b-2-3p,miR-3962,miR-421,miR-500-5p,miR-105-1,miR-199b-3p,miR-1343-3p,miR-548w,miR-222-5p,miR-145b,miR-27a-3p,miR-130b-3p,miR-491-5p,miR-214-3p,miR-362-3p,miR-2310,miR-491-3p,miR-382-3p,miR-376b,miR-23a-3p,miR-136-5p,miR-668-5p,miR-96-5p,miR-544-5p,miR-381-3p,miR-378j,miR-27a-3p,miR-203-3p,miR-10b,miR-20a-3p,miR-2319b,miR-139-5p,miR-664b,miR-24-1-5p,miR-211-5p,miR-541-5p,miR-374b-5p,miR-2285u,miR-335,miR-30b-5p,miR-2285n,miR-371a-5p,miR-2285y,miR-362-5p,miR-8095,miR-181d-5p,miR-9-5p,miR-2285f,miR-19b-3p,miR-362-3p,miR-30c-5p,miR-5703,miR-23c,miR-222-5p,miR-148b-3p,miR-487a-3p,miR-23b-3p,miR-30d-3p,miR-30d,miR-3653-3p,novel_17,miR-374a-5p,miR-2285p,miR-30b,miR-185-3p,miR-500a-5p,miR-216a-5p,miR-376c-3p,miR-377-5p,miR-421-5p,miR-380-3p,miR-486-3p,miR-345-3p,miR-30f,miR-2312,miR-582-5p,novel_99,miR-186-5p,miR-543-3p,miR-21a-3p,miR-30d-5p,miR-656-3p,miR-139-5p,miR-377-3p,miR-376e-3p,miR-301a-3p,miR-140-5p,miR-32-5p,miR-21-3p,miR-155-5p,miR-487b-5p,miR-130b-5p,miR-487a-5p,miR-454-3p,miR-30e-3p,miR-29b-2-5p,miR-19b-1-5p,miR-30a-3p,miR-6525,miR-181c-5p,miR-145a-3p,miR-214,miR-1271,miR-1260b,miR-138,miR-380-3p,miR-2284ab,miR-27b-3p,miR-181a-5p,miR-708-3p,miR-19b-2-5p,novel_23,miR-145a-5p,miR-185-5p,miR-544b,miR-377-5p,miR-130a-3p,miR-466f-3p,miR-377-3p,miR-32,miR-105-5p,miR-654-3p,let-7j,miR-130b-5p,let-7f-2-3p,let-7f-2-3p,miR-19a-3p,miR-2319a,miR-148a-3p,miR-2285ab,miR-150-5p,miR-3120-3p,novel_1,miR-92a-3p,miR-199c,miR-33a-3p,miR-211,miR-301b-3p,miR-335-5p,miR-2285x,miR-582-3p,miR-105-2,miR-30c,miR-134,miR-30a-5p,miR-23b-5p,miR-2427,miR-1271-5p,miR-24-2-5p,miR-9-5p,novel_121,miR-134-5p,miR-545-

GRM6	ENSOARG0(	glutamat	miR-154b-5p,miR-3959-5p,miR-1271,miR-376c-5p,miR-671-5p,miR-876-3p,miR-376c-5p,miR-3154,miR-301,miR-2448-3p,miR-378c,novel_63,miR-450a-1-3p,novel_121,miR-129-1-3p,miR-378h,miR-412,miR-299a-3p,miR-10a-5p,miR-1343-5p,miR-3578,miR-2284y,miR-1271-3p,miR-2284a,miR-380-5p,miR-4286,miR-181a-2-3p,novel_96,miR-2898,miR-6536,miR-30c-2-3p,miR-217,miR-205-5p,miR-6535,miR-669,miR-299b-3p,miR-7-1-3p,miR-10a-5p,miR-378f,miR-450b-3p,miR-19a-3p,miR-376b-5p,miR-2284w,miR-24-3p,novel_101,miR-342-3p,miR-147-5p,miR-6123,novel_1,miR-3431,miR-185-5p,miR-26b-3p,miR-31-5p,miR-30b-3p,miR-450c-3p,miR-197-3p,miR-653,miR-1434-3p,miR-2483-5p,miR-101-5p,miR-380-5p,miR-493-5p,miR-106b-3p,miR-1343-5p,miR-224-5p,miR-877-5p,miR-10a,miR-216b-5p,miR-532-5p,miR-182-5p,miR-378a-3p,miR-2331-3p,miR-30c-1-3p,miR-625-5p,miR-186-5p,let-7g-3p,miR-129b-3p,miR-450a-1-3p,miR-10b-5p,miR-6525,miR-224-5p,miR-214,miR-378c,miR-3184-3p,miR-129-2-3p,miR-450b-3p,miR-665,miR-2285j,miR-671-3p,miR-532-3p,miR-7859,miR-548e-3p,miR-218-1-3p,miR-378d,miR-2284u,miR-19b-3p,miR-5703,miR-188-3p,miR-217-5p,miR-378a-3p,miR-378i,novel_133,miR-1246,miR-299-3p,miR-422a,novel_17,miR-7857-3p,miR-378e,miR-3596,miR-489,miR-10b,miR-493-5p,miR-361-3p,miR-361-3p,miR-877-3p,miR-6527,miR-2285t,miR-3958-3p,miR-301a-5p,miR-2285n,miR-455-3p,miR-9788-3p,miR-421,novel_4,miR-409b,miR-21b,miR-16-2-3p,miR-378c,miR-450b-5p,miR-412-3p,miR-125b-2-3p,miR-30b-3p,miR-6529a,miR-378b,miR-10b,miR-301b-5p,miR-8485,miR-491-3p,miR-30c-1-3p,miR-378d,miR-2284b,miR-214-3p,miR-491-5p,miR-182-5p,miR-342

ILVBL	ENSOARG0(	ilvB (ba	miR-2319b,miR-211-5p,miR-504,novel_94,miR-361-3p,miR-423-5p,miR-2447,miR-3059-5p,miR-9-5p,miR-2284q,miR-382-3p,miR-371a-5p,miR-301a-5p,miR-539-5p,miR-1298-3p,miR-455-3p,miR-1343-3p,novel_4,miR-450b-5p,miR-101c,miR-484,miR-301b-5p,miR-7857,miR-30c-1-3p,miR-193a,miR-1388-3p,novel_124,miR-5126,miR-326,miR-29b-1-5p,miR-2284s,miR-27a-5p,miR-6516,miR-22-3p,miR-204-3p,miR-30c-1-3p,miR-504-5p,miR-221-5p,miR-21-3p,miR-296-3p,miR-345-5p,miR-339b,miR-885-3p,miR-1895,miR-625-5p,miR-1827,miR-6525,miR-3184-5p,miR-3074-5p,miR-3958-5p,miR-3074-2-3p,miR-664a,miR-17-3p,miR-874-5p,miR-4492,miR-130b-5p,miR-15b-3p,miR-30e-3p,miR-30a-3p,miR-19b-1-5p,miR-2284h-5p,miR-328-3p,miR-18a-3p,miR-3600,miR-16-1-3p,miR-2366,miR-2408,miR-2904,miR-5703,miR-140-3p,miR-29b-1-5p,miR-130a-5p,miR-2285w,miR-345-3p,miR-466i-5p,miR-30b-3p,miR-221-5p,miR-299-3p,miR-185-3p,miR-125b,miR-299b-3p,miR-3074-5p,miR-410-5p,miR-135b-5p,miR-6395,miR-3970,miR-18a-3p,miR-3591-3p,miR-211,miR-371b-3p,miR-326-3p,miR-2284w,miR-2428,miR-219b-3p,miR-17-3p,miR-147-5p,miR-4324,miR-502-5p,miR-185-5p,miR-194-3p,miR-339-5p,miR-16-1-3p,miR-4508,miR-744-5p,miR-19b-2-5p,miR-1434-3p,miR-7b-5p,miR-22-3p,miR-296-3p,miR-345-5p,miR-130b-5p,miR-452-5p,miR-1343-5p,miR-125a-5p,miR-7-5p,miR-296-3p,miR-365b-5p,novel_51,miR-374c-3p,miR-874-3p,miR-2355-5p,miR-125a,miR-345-5p,miR-7144-5p,miR-671-5p,miR-485-5p,miR-1197-3p,miR-365a-5p,miR-134-5p,miR-204-5p,miR-125b-5p,miR-135a-5p,miR-9-5p,miR-6516-5p,miR-134-5p,miR-582-3p,miR-299a-3p,miR-6517,novel_79,miR-885-3p,miR-134,miR-1343-5p,miR-330-5p,miR-148b-5p,miR-1271-3p,miR-2385-3p,miR-30a-3p,miR-17-3p,novel_127,miR-543-5p,miR-30c-2-3p,miR-339a

CALHM3	ENSOARG0(	calcium	miR-193a-5p,miR-6128,miR-133a-3p,miR-145-3p,miR-1260b,miR-331-3p,miR-6740-5p,miR-125b-5p,miR-409-3p,miR-29b-3p,miR-28-5p,novel_9,miR-370-3p,miR-2355-5p,miR-125a,miR-141-3p,miR-615-5p,miR-487a-3p,miR-874-3p,miR-34c-5p,miR-18a,miR-381-3p,miR-7144-5p,miR-485-5p,miR-1814c,miR-329a,miR-345-5p,miR-329b,miR-3064-5p,miR-29b-2-5p,miR-4286,miR-181a-2-3p,miR-3065-3p,miR-376c-5p,miR-376b-5p,miR-1271-3p,miR-28b,miR-1260a,miR-125a-3p,miR-664-3p,miR-450a-2-3p,miR-17-3p,miR-2898,miR-1224-5p,miR-7641,miR-18b-5p,miR-2285ad,miR-299a-3p,miR-545-5p,miR-486-5p,miR-129-1-3p,miR-505-5p,miR-9-5p,miR-483-3p,miR-6516-5p,miR-1343-5p,miR-29d-3p,miR-1290,miR-128-3p,miR-300,miR-29c-3p,miR-17-3p,miR-2285x,novel_120,miR-449a,miR-2284w,miR-2428,miR-24-3p,miR-34c,miR-33a-3p,miR-502-5p,miR-4324,miR-28c,miR-421-5p,miR-22-5p,miR-656-5p,miR-29a,miR-3120-3p,miR-3074-5p,miR-329-5p,miR-216b-3p,miR-146b-3p,miR-299b-3p,miR-374c-3p,miR-34b,miR-29a-3p,miR-216a-3p,miR-18b,miR-6395,miR-1291,miR-412-3p,miR-628-5p,miR-539-3p,miR-345-5p,miR-197-5p,novel_51,miR-224-5p,miR-1343-5p,miR-125a-5p,miR-483-3p,miR-187-3p,miR-340-3p,miR-31-5p,miR-133b-5p,miR-194-3p,miR-149-5p,miR-432-5p,miR-3535,miR-134-3p,miR-185-5p,miR-3431,miR-432,miR-1843b-5p,miR-7862,miR-29b,miR-30b-3p,miR-219a-1-3p,miR-1260b,miR-2330-3p,miR-129-2-3p,miR-3074-5p,miR-4510,miR-6525,miR-224-5p,miR-346,miR-324-3p,miR-151-3p,miR-665,miR-29b-2-5p,miR-500,miR-708-5p,miR-4492,novel_74,miR-17-3p,miR-664a,miR-154a-3p,miR-216b-3p,miR-3065-3p,miR-552-3p,miR-182-5p,miR-532-5p,miR-221-5p,miR-2331-3p,novel_25,miR-877-5p,miR-6516,miR-216b-5p,miR-6130,miR-129b-3p,miR-500a-3p,miR-1895,miR-1827,miR-133b-3p,miR-345-5p,miR-154b-3p,miR-18a-5p,miR-

TOP2B	ENSOARG0(	topoisom	miR-383-5p,miR-222-5p,novel_83,miR-671-3p,miR-199a-3p,miR-1b-3p,novel_133,miR-362-3p,miR-2904,miR-188-3p,miR-29b-1-5p,miR-130a-5p,miR-146a-5p,miR-760-3p,miR-30d,miR-30b-3p,miR-34b-5p,miR-221-5p,miR-21-3p,miR-542-5p,miR-125b,miR-185-3p,miR-544-3p,miR-6516,miR-193b-3p,miR-6130,novel_32,miR-140-5p,miR-221-5p,miR-370-5p,let-7g-3p,miR-30f,miR-18a-5p,novel_99,let-7b-5p,miR-6134,miR-4510,miR-145a-3p,miR-3074-2-3p,miR-17-3p,miR-708-5p,miR-208b-5p,miR-324-3p,miR-216a-3p,novel_4,miR-5010-3p,miR-299a-5p,miR-544a,miR-101a-5p,miR-2426,miR-484,miR-125b-2-3p,miR-30b-3p,miR-30c-1-3p,miR-4532,miR-758-3p,let-7a-5p,miR-382-3p,miR-33a-5p,novel_124,miR-3085-3p,miR-25-5p,miR-3955-3p,miR-542-5p,let-7e,miR-342,novel_103,miR-362-3p,miR-489,let-7d-5p,miR-3064-5p,miR-20a-3p,novel_60,miR-541-3p,miR-452-5p,miR-30b-5p,miR-371a-5p,miR-6516-5p,miR-483-3p,miR-545-5p,miR-423-3p,let-7d,miR-615-3p,miR-134,miR-30a-5p,miR-4286,miR-181a-2-3p,miR-17-3p,miR-2898,miR-191-3p,miR-107,miR-92a-3p,miR-2397-5p,miR-30c-2-3p,miR-181c-3p,let-7c-5p,miR-34c-5p,miR-370-3p,miR-141-3p,miR-10b-3p,miR-1814c,miR-503-3p,miR-18a,miR-3154,miR-6128,miR-138-5p,miR-28-5p,miR-486b-3p,miR-370-5p,let-7g-5p,miR-544b,miR-103,miR-2355-3p,miR-133b-5p,miR-122-3p,miR-26b-3p,miR-138,miR-30b-3p,miR-145a-5p,miR-3071-5p,miR-374b-3p,miR-130b-5p,miR-101-5p,miR-382,miR-124-5p,miR-452-5p,miR-125a-5p,miR-34b,miR-363,miR-107,miR-6395,miR-18b,novel_27,miR-449a,miR-219b-3p,miR-342-3p,miR-542-5p,miR-17-3p,miR-1193,miR-92a-3p,miR-656-5p,miR-502-5p,miR-6529b,miR-34c,let-7i,miR-23b-3p,miR-210-3p,miR-23c,miR-328-3p,miR-142-3p,miR-34a-5p,miR-30c-5p,miR-1961,miR-193b-5p,miR-376a-5p,miR-486-3p,miR-345-3p,miR-98-5p,let-7k,miR-

SNX9	ENSOARG0(	sorting	miR-30f,miR-125a-3p,miR-200a-3p,miR-200a,miR-450c-5p,miR-450b-5p,miR-148b-5p,miR-1343-5p,miR-155-5p,miR-30c,miR-1224-5p,miR-6517,miR-2284z,miR-505-5p,miR-9-5p,novel_63,novel_9,miR-193a-5p,miR-1197-3p,miR-93-5p,miR-30e-5p,miR-20b-5p,miR-329a,miR-345-5p,miR-2355-5p,miR-1271,miR-574-5p,miR-133c,miR-3141,novel_51,miR-377-3p,miR-493-5p,let-7a-2-3p,miR-22-3p,miR-450c-3p,novel_23,miR-2113,miR-223-3p,miR-27b-3p,miR-16-1-3p,miR-381-5p,miR-31-5p,novel_116,miR-412-5p,miR-1247-3p,miR-142b,miR-576-3p,miR-28c,miR-421-5p,miR-6123,miR-29a,miR-29c-3p,miR-3065-5p,miR-3591-3p,miR-3141,miR-2319a,miR-412-3p,miR-329-5p,miR-669,miR-320c,miR-677,miR-181b-3p,miR-374c-3p,novel_69,miR-500a-5p,miR-30b,miR-202-5p,miR-143-3p,miR-664b-3p,miR-181b-1-3p,miR-1434-5p,miR-217-5p,miR-30c-5p,miR-2366,miR-193b-5p,miR-16-1-3p,miR-148a-5p,miR-184-3p,miR-106a,miR-210-3p,miR-665,miR-15b-3p,miR-874-5p,miR-4492,miR-665-5p,miR-3184-5p,miR-769-5p,miR-6525,miR-30d-5p,miR-433-3p,miR-625-5p,miR-345-5p,miR-30c-1-3p,miR-504-5p,miR-548o-3p,miR-10a,miR-2284s,miR-377-3p,miR-106b-5p,miR-381-5p,miR-1388-3p,miR-8485,miR-329-3p,miR-2284r,miR-541,miR-1298-5p,miR-362-3p,miR-181b-2-3p,miR-1343-3p,miR-148b-5p,miR-2459,miR-126a-5p,miR-218-5p,miR-3968,miR-9-5p,miR-2447,miR-423-5p,miR-877-3p,miR-361-3p,novel_111,miR-378j,miR-493-5p,miR-1983,miR-9-3p,miR-299b-5p,miR-96-5p,miR-504,miR-2285u,miR-211-5p,miR-2319b,miR-4443,miR-2483-3p,miR-30c-2-3p,miR-217,miR-450a-2-3p,miR-29b-2-5p,miR-3529-3p,miR-1957a,miR-3065-3p,miR-28b,miR-218-2-3p,miR-29d-3p,miR-30a-5p,let-7a-2-3p,miR-10a-5p,miR-423-3p,miR-1271-5p,miR-26c,miR-29b-3p,miR-2332,miR-204-5p,miR-1306-5p,miR-93,miR-320a,miR-17-5p,miR-3074-1-3p,miR-7144-5p,miR-23a-
STK39	ENSOARG0(	serine t	miR-885-5p,miR-212-3p,novel_120,miR-17-3p,miR-3959-3p,miR-877-3p,miR-2428,miR-4324,novel_87,miR-7857-3p,miR-27a-3p,miR-1306,miR-7975,miR-124a,miR-744-3p,miR-1306-5p,novel_51,miR-29b-1-5p,miR-2285l,miR-2285o,miR-27a-3p,miR-425-3p,miR-223-3p,miR-374b-3p,miR-30b-3p,miR-125b-2-3p,miR-16-1-3p,miR-2426,miR-30b-3p,miR-27b-3p,miR-145a-3p,miR-4510,miR-539-5p,miR-1306-5p,miR-3074-2-3p,miR-6134,miR-145-3p,miR-410-5p,miR-551b-3p,miR-665,miR-664a,miR-17-3p,miR-548o-3p,miR-6130,miR-877-5p,miR-503-3p,miR-10b-3p,miR-671-5p,novel_99,miR-1827,miR-543-3p,miR-2285w,miR-3529-3p,miR-2403,miR-130a-5p,miR-376a-5p,miR-29b-1-5p,miR-2478,miR-192-3p,miR-29a-5p,miR-2285p,miR-30f,miR-17-3p,miR-1b-3p,miR-2284h-5p,miR-582-3p,miR-2300b-3p,miR-1197-5p,miR-124-3p,miR-142-3p

CEP89	ENSOARG0(	centroso	miR-218-1-3p,miR-34a-5p,miR-215-5p,miR-1193,miR-188-3p,miR-1434-5p,miR-140-3p,miR-487a-3p,miR-23b-3p,miR-15a-5p,miR-23c,miR-18a-3p,miR-1b-3p,miR-485-3p,miR-212-5p,miR-30b-3p,miR-34b-5p,miR-21-3p,miR-324-3p,miR-125b,miR-185-3p,miR-345-3p,miR-181b-1-3p,miR-486-3p,miR-143-3p,miR-503-5p,miR-345-3p,miR-2312,miR-345-5p,miR-339b,miR-1827,miR-27b-5p,miR-192-5p,miR-544-3p,miR-6130,miR-204-3p,miR-15b-5p,miR-877-5p,miR-30c-1-3p,miR-370-5p,miR-552-3p,miR-6516-3p,miR-664a,miR-4492,miR-874-5p,miR-129-5p,miR-424-5p,miR-324-3p,miR-665,miR-664a-5p,miR-3604,miR-769-5p,miR-4510,miR-544a,miR-323b,miR-30b-3p,miR-496-5p,miR-216a-3p,miR-1185-3p,miR-1343-3p,miR-2300a-5p,miR-181b-2-3p,miR-1185-5p,miR-25-5p,novel_103,miR-505,miR-323b-3p,miR-16b,miR-30c-1-3p,miR-4532,miR-8485,miR-424-5p,miR-101b-3p,miR-23a-3p,let-7c-3p,miR-877-3p,miR-1983,miR-361-3p,miR-3596,miR-3082-5p,miR-4443,miR-1185-2-3p,miR-10b,miR-7689-3p,novel_82,miR-101-3p,miR-371a-5p,miR-769-5p,miR-361-3p,miR-7705,miR-2447,miR-2285f,miR-452-5p,let-7a-2-3p,miR-345-3p,miR-3578,miR-29d-3p,miR-322-5p,miR-758-5p,miR-214-5p,novel_79,miR-196a-2-3p,miR-200a-3p,novel_127,miR-30c-2-3p,miR-339a,novel_39,miR-200a,miR-490-3p,miR-1271-3p,miR-345-5p,let-7g-3p,miR-23a-5p,miR-485-5p,miR-23b,miR-34c-5p,miR-769,miR-542-5p,miR-141-3p,miR-1271,miR-125a,miR-215-5p,miR-486b-3p,miR-2433,miR-29b-3p,miR-370-5p,miR-331-3p,miR-125b-5p,miR-26c,miR-16-5p,miR-29b,miR-16b,miR-30b-3p,miR-497-5p,miR-744-5p,miR-6239,miR-542-3p,miR-503-5p,miR-194-3p,miR-544b,miR-339-5p,miR-125a-5p,miR-466f-3p,miR-503-5p,novel_51,miR-432-3p,miR-539-3p,let-7a-2-3p,miR-345-5p,miR-363-5p,miR-1291,miR-450b-3p,miR-412-3p,miR-195a-5p,miR-2285ab,miR-

PLXNA3	ENSOARG0(	plexin A	miR-502b,miR-203b-5p,miR-34c,miR-28c,miR-142b,miR-4324,miR-2428,miR-199b-5p,novel_27,miR-449a,miR-371b-3p,miR-24-3p,miR-17-3p,miR-193b-3p,miR-379-5p,miR-6395,miR-1291,miR-216a-3p,miR-18a-3p,miR-148a-3p,miR-669,miR-6535,novel_69,miR-34b,miR-26b-5p,miR-3074-5p,miR-193a-3p,miR-125a-5p,miR-2331-5p,miR-197-5p,miR-330-3p,miR-503-3p,miR-432-3p,miR-363-5p,miR-6240,miR-27b-3p,miR-7862,miR-16-1-3p,miR-1260b,miR-138,miR-30b-3p,miR-1843b-5p,miR-1434-3p,novel_116,miR-194-3p,miR-149-5p,miR-134-3p,miR-31-5p,miR-592,miR-486b-3p,miR-28-5p,miR-31-3p,miR-138-5p,miR-331-3p,miR-125b-5p,miR-100-3p,miR-5100,miR-2433,miR-145-3p,miR-1260b,miR-500b-5p,miR-133a-3p,novel_42,miR-1306-5p,miR-671-5p,miR-485-5p,miR-7144-5p,miR-2474,miR-362-5p,miR-3154,miR-199a-5p,miR-365a-3p,miR-34c-5p,miR-615-5p,miR-215-5p,miR-125a,miR-503-3p,miR-874-3p,miR-152-3p,novel_96,novel_127,miR-127-5p,miR-2898,miR-17-3p,miR-125a-3p,miR-1260a,novel_39,miR-3065-3p,miR-199a-5p,miR-1957a,miR-28b,miR-450b-5p,miR-30a-3p,miR-3064-5p,miR-29b-2-5p,miR-128-3p,miR-885-3p,miR-23b-5p,miR-3578,miR-3591-5p,novel_121,miR-450a-1-3p,miR-545-5p,miR-505-5p,miR-490-5p,miR-4726-5p,miR-3968,miR-2440,miR-423-5p,miR-362-5p,miR-361-3p,miR-136-5p,miR-378j,miR-199b-5p,miR-361-3p,miR-27a-3p,miR-4443,miR-3082-5p,miR-378g,miR-452-3p,miR-2319b,miR-3064-5p,miR-214-3p,miR-199b-5p,miR-1185-5p,miR-3085-3p,miR-491-5p,novel_19,miR-29b-1-5p,miR-3187-3p,miR-376b,miR-33a-5p,miR-2330-5p,miR-124a,miR-21b,miR-1839-5p,miR-30b-3p,miR-296-5p,miR-500-5p,miR-9788-3p,miR-539-5p,miR-27a-3p,miR-2300a-5p,miR-330-3p,miR-151a-3p,miR-487a-5p,miR-4492,miR-874-5p,miR-708-5p,miR-17-3p,miR-450b-3p,miR-320e,miR-29b-2-5p,miR-151-3p,miR-324-3p,miR-30a-3p,miR-

SRP72	ENSOARG0(	signal r	miR-345-5p,miR-548o-3p,miR-32-3p,miR-532-5p,miR-21-3p,miR-3065-3p,miR-6130,miR-377-3p,miR-10a,miR-15b-5p,miR-30e-3p,miR-665,miR-30a-3p,miR-130b-5p,miR-424-5p,miR-208b-5p,miR-4510,miR-3074-2-3p,miR-346,miR-10b-5p,miR-2366,miR-1434-5p,miR-140-3p,miR-5703,miR-1193,miR-2904,miR-664-5p,miR-30d-3p,miR-3600,miR-15a-5p,miR-200b,miR-7859,miR-2284h-5p,miR-2285p,novel_17,miR-196a-3p,miR-760-3p,miR-21-3p,miR-3653-3p,miR-2285w,miR-664b-3p,miR-146a-5p,miR-199b-5p,miR-376a-3p,miR-452-3p,miR-211-5p,miR-2483-3p,miR-3082-5p,miR-7857-3p,miR-376d,miR-144-5p,novel_82,miR-2285n,miR-218-5p,miR-382-3p,miR-107-5p,miR-3968,miR-490-5p,miR-3956-3p,novel_60,miR-32-3p,miR-10b,miR-30b-3p,miR-323b,miR-2426,miR-105-1,miR-1343-3p,miR-145b,miR-376b-3p,miR-539-5p,miR-153,miR-9788-3p,miR-455-3p,miR-342,miR-16b,miR-323b-3p,miR-491-5p,miR-1185-5p,miR-103a-2-5p,miR-153-3p,miR-146b,miR-199b-5p,miR-494-3p,miR-1839-3p,miR-424-5p,miR-744-3p,miR-1b-5p,miR-8485,miR-3154,miR-345-5p,miR-329b,let-7g-3p,miR-1248,miR-3074-1-3p,miR-365a-3p,miR-374c-3p,miR-199a-5p,miR-5100,miR-2332,miR-1-5p,miR-29b-3p,miR-3969,miR-134-5p,miR-204-5p,miR-592,let-7e-3p,miR-144-5p,miR-16-5p,miR-365a-5p,miR-134,miR-29d-3p,miR-10a-5p,miR-200b-3p,miR-105-2,miR-128-3p,miR-429-3p,miR-322-5p,miR-134-5p,miR-200c,miR-6119-5p,miR-376b-3p,miR-205-5p,miR-1a-2-5p,miR-127-5p,miR-200c-3p,novel_96,miR-107,miR-3529-3p,miR-30a-3p,miR-192-3p,miR-3065-3p,novel_39,miR-199a-5p,miR-376b-5p,miR-3065-5p,miR-376b-3p,miR-216a-3p,miR-3591-3p,miR-107,miR-195a-5p,miR-6516-3p,miR-2319a,miR-146b-5p,miR-10a-5p,novel_69,miR-670-3p,miR-374c-3p,miR-29a-3p,miR-677,miR-133a-5p,miR-6402,miR-15a,miR-16a,novel_1,miR-29a,miR-128-1-5p,miR-147-5p,miR-1a-1-
MTDH	ENSOARG0(	metadher	miR-30d,miR-500a-5p,miR-30b,miR-26a-5p,miR-450b-5p,miR-24-2-5p,miR-487a,miR-30c,miR-300,miR-30a-5p,miR-30c-5p,miR-1290,miR-582-3p,miR-2284z,miR-7859,miR-487a-3p,miR-24-2-5p,miR-485-3p,miR-2285j,miR-2332,miR-3607-3p,miR-1814c,miR-10b-3p,miR-876-3p,let-7g-3p,miR-154b-3p,miR-30f,miR-30d-5p,miR-30e-5p,miR-299,miR-21a-3p,miR-381-3p,miR-22-3p,miR-2285aa,miR-154a-3p,miR-487a-3p,miR-452-5p,miR-466f-3p,miR-330-3p,miR-539-3p,miR-2310,miR-628-5p,miR-493-5p,miR-6240,miR-2284aa,miR-27b-3p,miR-22-3p,miR-6238,miR-27a-3p,miR-330-3p,miR-548w,miR-154-3p,miR-335,miR-1973,miR-33a-3p,novel_87,miR-30b-5p,miR-374c-5p,novel_60,miR-335-5p,miR-3956-3p,miR-493-5p,miR-544-5p,miR-3065-5p,miR-27a-3p,miR-25,miR-7-1-3p,miR-26b-5p,miR-24-1-5p,miR-2319b

NEURL1	ENSOARG0(	neuraliz	miR-151-3p,miR-30a-3p,miR-665,miR-3604,miR-874-5p,miR-4492,miR-669a-3p,novel_74,miR-433-5p,miR-3184-3p,miR-6525,miR-769-5p,miR-224-5p,novel_44,miR-411,miR-30d-5p,miR-450a-1-3p,miR-1895,miR-625-5p,miR-543-3p,miR-186-5p,miR-216b-3p,miR-2331-3p,miR-2284s,miR-324-3p,miR-422a,miR-345-3p,miR-30b,novel_17,miR-329a-5p,miR-486-3p,miR-143-3p,miR-664b-3p,miR-377-5p,miR-378a-3p,miR-30c-5p,miR-378i,miR-193b-5p,miR-34a-5p,miR-142-3p,miR-33b-3p,miR-30d-3p,miR-212-5p,miR-18a-3p,miR-1247-5p,miR-328-3p,miR-95-3p,miR-23b-3p,miR-23c,miR-218-5p,novel_82,miR-615,miR-3968,miR-2447,miR-1285-5p,miR-361-3p,miR-877-3p,miR-136-5p,miR-211-5p,miR-3596,miR-378g,miR-505,miR-29b-1-5p,miR-491-5p,miR-23a-3p,miR-744-3p,miR-8485,miR-2310,miR-378d,miR-329-3p,miR-2284r,miR-21b,miR-362-3p,miR-181b-2-3p,miR-330-3p,miR-1343-3p,miR-125b-5p,miR-1197-3p,miR-1260b,miR-23b,miR-30e-5p,miR-1248,miR-2318,miR-329a,miR-2285aa,miR-2355-5p,miR-125a,miR-874-3p,miR-8117,novel_96,miR-152-3p,novel_127,miR-3064-5p,miR-29a-5p,miR-2285b,miR-148b-5p,miR-1343-5p,miR-330-5p,miR-30c,miR-1224-5p,miR-18b-5p,miR-299a-3p,miR-2285ad,miR-6517,miR-450a-1-3p,miR-505-5p,miR-4324,miR-28c,miR-193b-3p,miR-1839-3p,miR-335-5p,miR-1306,miR-150-5p,miR-216a-3p,miR-3065-5p,miR-148a-3p,miR-378f,miR-329-5p,miR-677,novel_69,miR-299b-3p,miR-2411-3p,miR-194a,novel_51,miR-7-5p,miR-193a-3p,miR-628-5p,miR-6240,miR-2285r,miR-432-3p,miR-432-3p,miR-450c-3p,miR-19b-2-5p,miR-432,miR-7b-5p,miR-1843b-5p,miR-223-3p,miR-27b-3p,miR-188-5p,miR-31-5p,novel_116,miR-194-3p,miR-134-3p,miR-19b-1-5p,miR-320e,miR-487a-5p,miR-450b-3p,miR-664a,miR-17-3p,miR-3074-2-3p,miR-378c,miR-4510,miR-6134,miR-338-3p,miR-3613-5p,miR-30f,miR-18a-5p,miR-221-

KIF1C	ENSOARG0(	kinesin	miR-200b-3p,miR-300,miR-330-5p,miR-30a-5p,miR-615-3p,miR-3578,miR-545-5p,miR-486-5p,miR-9-5p,miR-431,miR-299a-3p,miR-222,miR-2285ad,miR-429-3p,miR-200a-3p,miR-17-3p,miR-28-3p,miR-200c-3p,miR-181b-5p,miR-30f,miR-30c-2-3p,miR-200c,miR-664-3p,novel_39,miR-1957a,miR-376c-5p,miR-376b-5p,miR-490-3p,miR-148b-5p,miR-4286,miR-181a-2-3p,miR-7144-5p,miR-362-5p,miR-23b,miR-30e-5p,miR-381-3p,miR-488-3p,miR-133c,miR-374c-3p,miR-769,miR-125a,miR-141-3p,miR-191-5p,miR-125b-5p,miR-331-3p,miR-135a-5p,miR-3969,miR-135a-1-3p,miR-500b-5p,miR-6128,miR-28a-3p,miR-744-5p,miR-16-1-3p,miR-1285,miR-2355-3p,miR-2285g,miR-503-5p,miR-134-3p,miR-185-5p,miR-377-5p,miR-3955-5p,miR-125a-5p,novel_51,miR-503-5p,miR-146a,miR-2285r,miR-363-5p,miR-146b-5p,miR-505-3p,miR-135b-5p,miR-1306,miR-150-5p,miR-378b,miR-677,miR-6535,miR-181b-3p,miR-299b-3p,miR-670-3p,miR-320b,miR-625-3p,miR-656-5p,miR-147-5p,miR-502b,miR-4324,miR-133a-5p,miR-326-3p,miR-2428,miR-24-3p,miR-17-3p,miR-16-1-3p,miR-218-1-3p,miR-378d,miR-148a-5p,miR-1197-5p,miR-188-3p,miR-140-3p,miR-191,miR-2366,miR-2300b-3p,miR-532-3p,miR-7859,miR-23b-3p,miR-23c,miR-200b,miR-505-3p,miR-548e-3p,miR-30d,miR-221-5p,miR-299-3p,miR-324-3p,miR-125b,novel_17,miR-146a-5p,miR-377-5p,miR-130a-5p,miR-181b-1-3p,miR-345-3p,miR-216c-5p,miR-503-5p,miR-1895,miR-186-5p,miR-1827,miR-500a-3p,miR-30d-5p,miR-2285c,miR-499b-3p,miR-140-5p,miR-2331-3p,miR-30c-1-3p,miR-221-5p,miR-504-5p,miR-548o-3p,miR-500,miR-874-5p,miR-6516-3p,miR-17-3p,miR-664a,miR-2285j,miR-222-3p,miR-3184-5p,miR-338-3p,miR-6525,miR-769-5p,miR-7857,miR-301b-5p,miR-143-5p,miR-148b-5p,miR-500-5p,miR-221-3p,miR-2285e,miR-421,miR-126a-5p,miR-181b-2-3p,novel_78,miR-1343-3p,miR-2300a-5p,miR-146b,miR-103a-2-

PIK3C2A	ENSOARG0(	phosphat	miR-2285af,miR-376b-3p,miR-200c,miR-30f,miR-28-3p,miR-200c-3p,miR-1a-2-5p,miR-127-5p,miR-6536,miR-181b-5p,novel_127,miR-152-3p,miR-200a-3p,miR-29b-2-5p,miR-30a-3p,miR-3064-5p,miR-181a-2-3p,miR-450b-5p,miR-200a,novel_39,miR-2284a,miR-345-3p,miR-3591-5p,miR-1290,miR-134,miR-582-3p,miR-200b-3p,miR-128-3p,miR-2285ad,miR-429-3p,miR-412,miR-18b-5p,miR-9-5p,miR-483-3p,miR-134-5p,miR-214-5p,miR-2284k,miR-1-5p,miR-494-5p,miR-6740-5p,miR-100-3p,miR-767-5p,novel_9,miR-374b-3p,miR-134-5p,miR-28a-3p,miR-133a-3p,miR-323-3p,miR-145-3p,miR-301,miR-18a,miR-767,miR-345-5p,miR-23a-5p,miR-1814c,miR-671-5p,miR-487a-3p,miR-3068-3p,miR-2285aa,miR-141-3p,miR-331-5p,miR-133c,miR-2284n,miR-190a,miR-194a,miR-2411-3p,miR-483-3p,miR-130b-5p,miR-382,miR-101-5p,miR-363-5p,miR-2483-5p,let-7f-2-3p,let-7f-2-3p,miR-654-3p,miR-432-3p,miR-432-3p,miR-539-3p,let-7j,miR-345-5p,miR-708-3p,miR-181a-5p,miR-1285,miR-1843b-5p,miR-223-3p,miR-2113,miR-194-5p,novel_23,miR-2284ab,miR-26a-2-3p,miR-7862,miR-2284x,miR-130a-3p,miR-26b-3p,miR-2284d,miR-3431,miR-149-5p,miR-6238,miR-2355-3p,miR-576-3p,novel_1,miR-421-5p,miR-625-3p,miR-22-5p,miR-203b-5p,miR-1a-1-5p,miR-495-3p,miR-323c,miR-3959-3p,miR-374c-5p,miR-301b-3p,miR-2285ab,miR-18b,miR-148a-3p,miR-216a-3p,miR-376b-3p,miR-3065-5p,miR-18a-3p,miR-19a-3p,miR-2284e,miR-9851-3p,novel_68,miR-7-1-3p,miR-2404,miR-670-3p,miR-216a-5p,miR-345-3p,miR-2284m,miR-466i-5p,miR-2484,miR-216c-5p,miR-487a,miR-2285w,miR-105-3p,miR-376a-5p,miR-421-5p,miR-130a-5p,miR-125b-2-3p,miR-2432,miR-143-3p,miR-376a-5p,miR-124-3p,miR-98-3p,miR-1197-5p,miR-188-3p,miR-1434-5p,miR-19b-3p,miR-142-3p,miR-664-5p,let-7a-3p,miR-361-5p,miR-18a-3p,miR-485-3p,miR-30d-3p,miR-23b-

RPS6KA1	ENSOARG0(	ribosoma	miR-3064-5p,novel_39,miR-1271-3p,miR-148b-5p,miR-380-5p,miR-30c-2-3p,miR-6119-5p,miR-200c,miR-543-5p,miR-200c-3p,miR-429-3p,novel_121,miR-214-5p,miR-505-5p,miR-1343-5p,miR-330-5p,miR-345-3p,miR-200b-3p,miR-133a-3p,miR-93,miR-331-3p,miR-125b-5p,miR-6740-5p,miR-3969,miR-410-5p,miR-486b-3p,miR-138-5p,miR-125a,miR-1271,miR-615-5p,miR-3068-3p,miR-93-5p,miR-320a,miR-17-5p,miR-3154,miR-2448-3p,miR-20b-5p,miR-876-3p,miR-363-5p,miR-380-5p,miR-432-3p,miR-432-3p,novel_51,miR-106a-5p,miR-17-5p,miR-2411-3p,miR-125a-5p,miR-1343-5p,miR-193a-3p,miR-133b-5p,miR-149-5p,miR-194-3p,miR-2355-3p,miR-20b,miR-1285,miR-1434-3p,miR-4508,miR-138,miR-30b-3p,miR-219a-1-3p,miR-193b-3p,miR-342-3p,miR-326-3p,miR-2284w,miR-2428,miR-20a-5p,miR-320c,miR-669,miR-6395,miR-1291,miR-20a,miR-486-3p,miR-345-3p,miR-125b,miR-221-5p,miR-21-3p,miR-320d,miR-106a,miR-760-3p,miR-106a,miR-532-3p,novel_83,miR-328-3p,miR-200b,miR-124-3p,miR-5703,miR-148a-5p,miR-4429,miR-665-5p,miR-6134,miR-6525,miR-4492,miR-30c-1-3p,miR-504-5p,miR-221-5p,miR-2284s,miR-193b-3p,miR-21a-3p,miR-133b-3p,miR-124a,miR-106a-5p,miR-30c-1-3p,miR-2957,miR-323b-3p,miR-106b-5p,miR-342,miR-320b,miR-326,novel_115,miR-103a-2-5p,miR-3085-3p,miR-1343-3p,miR-9788-3p,miR-148b-5p,miR-323b,miR-30b-3p,miR-2284r,miR-541,miR-3059-5p,miR-541-3p,miR-107-5p,miR-3968,miR-3064-5p,miR-2319b,miR-3082-5p,miR-140-5p,miR-877-3p,miR-504
C10orf107	ENSOARG0(	chromoso	miR-30d-5p,miR-30a-5p,miR-1843b-5p,miR-30e-5p,miR-30c-5p,miR-1248,miR-30c,miR-450b-5p,miR-133b-3p,miR-3065-3p,novel_68,miR-3064-5p,miR-877-5p,miR-3431,miR-7857-3p,miR-107-5p,miR-409-3p,miR-30f,miR-30b,miR-30b-5p,miR-103a-2-5p,miR-208b-5p,miR-30d,miR-3085-3p,miR-2898,miR-133a-3p,miR-3064-5p,miR-6134,miR-145-3p,miR-2432,miR-3065-3p
DYNLT1	ENSOARG0(	dynein,	miR-330-3p,miR-29b-1-5p,miR-214-3p,miR-382-3p,miR-6240,miR-494-3p,miR-345-5p,let-7a-2-3p,miR-8485,miR-30b-3p,miR-181a-5p,miR-301b-5p,miR-484,miR-744-5p,miR-4508,miR-30b-3p,miR-138,miR-330-3p,novel_116,miR-455-3p,miR-216a-3p,miR-185-5p,miR-412-5p,miR-301a-5p,novel_87,miR-2440,novel_82,miR-335,miR-1193,miR-181d-5p,miR-335-5p,miR-3059-5p,miR-361-3p,miR-1983,miR-6395,miR-412-3p,miR-1291,miR-499b-5p,miR-3074-5p,miR-185-3p,miR-324-3p,miR-125a-3p,miR-181b-5p,miR-127-5p,miR-345-3p,miR-200a,miR-486-3p,miR-130a-5p,miR-29b-1-5p,miR-134,miR-3591-5p,let-7a-2-3p,miR-328-3p,miR-134-5p,miR-6740-5p,miR-370-5p,miR-2433,miR-486b-3p,miR-134-5p,miR-410-3p,miR-138-5p,novel_74,miR-214,miR-3074-5p,miR-6134,miR-181c-5p,miR-1197-3p,miR-582-5p,miR-543-3p,miR-345-5p,let-7g-3p,miR-345-5p,miR-876-3p,miR-216b-3p,miR-370-5p,miR-574-5p,miR-223-5p,miR-582-5p

ARID5B	ENSOARG0(	AT rich	miR-299b-5p,miR-376a-3p,miR-335-3p,miR-544-5p,miR-877-3p,miR-361-3p,miR-25,miR-378e,miR-203-3p,miR-1185-2-3p,miR-376d,miR-4792,miR-20a-3p,miR-2319b,miR-7977,miR-3968,miR-3964,miR-30b-5p,novel_82,miR-301a-5p,miR-877-3p,novel_111,miR-1285-5p,miR-3059-5p,miR-2447,miR-452-5p,miR-378c,miR-450b-5p,miR-378b,miR-2284r,miR-301b-5p,miR-7857,miR-323b,miR-1a-3p,miR-758-3p,miR-376b-3p,miR-148b-5p,miR-1185-3p,miR-2285e,miR-330-3p,miR-105-1,miR-491-5p,miR-130b-3p,miR-320b,miR-342,miR-323b-3p,miR-378d,miR-8485,miR-758-3p,miR-2310,miR-382-3p,miR-2330-5p,miR-33a-5p,miR-30f,miR-345-5p,miR-299,miR-2285c,miR-30d-5p,miR-6516,novel_32,miR-204-3p,miR-140-5p,miR-378a-3p,miR-545-3p,miR-216b-3p,miR-21-3p,miR-296-3p,miR-454-3p,miR-129-5p,miR-130b-5p,miR-19b-1-5p,miR-665,miR-320e,miR-6525,miR-338-3p,miR-378c,miR-4429,novel_73,miR-19b-3p,miR-664-5p,miR-218-1-3p,miR-148a-5p,miR-30c-5p,novel_133,miR-378i,miR-378a-3p,miR-1197-5p,miR-1193,miR-95-3p,miR-200b,miR-1247-5p,miR-212-5p,miR-320d,miR-466i-5p,miR-30b-3p,miR-1277-5p,miR-30b,miR-500a-5p,miR-422a,miR-664b-3p,miR-2411,miR-486-3p,miR-2424,miR-143-3p,miR-183-5p,miR-19a-3p,miR-135b-5p,miR-378f,miR-376b-5p,miR-376b-3p,miR-3591-3p,miR-2404,miR-670-3p,miR-320c,miR-329-5p,miR-3120-3p,miR-7-1-3p,novel_1,miR-22-5p,miR-502-5p,miR-6529b,miR-6402,miR-576-3p,miR-301b-3p,miR-2284w,miR-342-3p,novel_101,miR-495-3p,miR-188-5p,miR-6239,miR-432,miR-19b-2-5p,miR-1434-3p,miR-223-3p,miR-450c-3p,miR-374b-3p,miR-432-5p,miR-2285g,miR-194-3p,miR-2355-3p,miR-31-5p,miR-124-5p,miR-378a-5p,miR-466f-3p,miR-296-3p,miR-330-3p,miR-105-5p,miR-296-3p,miR-345-5p,miR-130b-5p,miR-6240,let-7f-2-3p,let-7f-2-3p,miR-876-3p,miR-345-5p,miR-329b,miR-7144-5p,miR-

SLC7A10	ENSOARG0(	solute c	miR-30f,miR-18a-5p,miR-885-3p,miR-500a-3p,miR-30d-5p,miR-2284s,miR-139-5p,miR-6130,miR-204-3p,miR-22-3p,miR-151b,miR-378a-3p,miR-3065-3p,miR-552-3p,miR-296-3p,miR-874-5p,miR-500,miR-129-5p,miR-30e-3p,miR-219b-3p,miR-324-3p,miR-30a-3p,miR-665,miR-320e,miR-206-3p,miR-122-5p,miR-3184-5p,miR-433-5p,miR-378c,miR-4510,miR-145a-3p,miR-129-2-3p,miR-425-5p,miR-214,miR-34a-5p,novel_133,miR-30c-5p,miR-378i,miR-362-3p,miR-2904,miR-378a-3p,miR-5703,miR-148b-3p,miR-151-5p,miR-1247-5p,miR-2284h-5p,miR-328-3p,miR-3960,miR-18a-3p,miR-3600,miR-1b-3p,miR-30d-3p,miR-34b-5p,miR-30d,miR-21-3p,miR-30b,miR-210-5p,miR-500a-5p,miR-324-3p,miR-422a,miR-122-3p,miR-29b-1-5p,miR-377-5p,miR-130a-5p,miR-125b-2-3p,miR-2424,miR-486-3p,miR-2285w,miR-147-3p,miR-136-5p,miR-361-3p,miR-199b-5p,miR-378e,miR-378g,miR-4443,miR-500-3p,miR-27a-3p,miR-4792,miR-139-5p,miR-4726-5p,miR-30b-5p,miR-615,miR-2285y,miR-361-3p,miR-502-3p,miR-423-5p,miR-219a-2-3p,miR-2447,miR-541-3p,miR-378c,miR-362-3p,miR-16-2-3p,miR-3120-5p,miR-329-3p,miR-378b,miR-484,miR-541,miR-1a-3p,miR-501-3p,miR-9788-3p,miR-455-3p,miR-2459,novel_4,miR-27a-3p,miR-199b-5p,miR-214-3p,miR-5126,miR-1306-5p,miR-326,miR-362-3p,miR-2957,miR-29b-1-5p,miR-378d,miR-3187-3p,miR-193a,miR-4532,miR-491-3p,miR-676-3p,miR-329a,miR-23a-5p,miR-7144-5p,miR-1248,miR-485-5p,miR-30e-5p,miR-18a,miR-34c-5p,miR-199a-5p,miR-324-5p,miR-154b-5p,miR-874-3p,miR-370-3p,miR-2355-5p,miR-615-5p,miR-138-5p,miR-486b-3p,miR-592,miR-331-3p,miR-378c,miR-181a-2-3p,miR-6128,miR-3607-3p,miR-885-3p,miR-582-3p,miR-30c,miR-615-3p,miR-23b-5p,miR-330-5p,miR-30a-5p,miR-501-3p,miR-24-2-5p,miR-505-5p,miR-378h,miR-129-1-3p,miR-2285ad,miR-6517,miR-18b-5p,miR-2898,miR-543-5p,novel_127,miR-152-

ERICH6	ENSOARG0(	glutamat	miR-21a-3p,miR-378a-3p,miR-21-3p,miR-323-5p,miR-544-3p,miR-338-5p,miR-29b-2-5p,miR-324-3p,miR-664a,miR-487a-5p,miR-378c,miR-338-3p,miR-6134,miR-362-3p,miR-1246,miR-376a-5p,miR-188-3p,miR-1197-5p,miR-5703,miR-19b-3p,miR-4429,miR-222-5p,miR-199a-3p,novel_83,miR-616-3p,miR-532-3p,miR-196a-3p,miR-106a,miR-320d,miR-299-3p,miR-21-3p,miR-30b-3p,miR-20a,miR-29b-1-5p,miR-361-3p,miR-129b-5p,novel_94,miR-452-3p,miR-20a-3p,miR-7977,miR-541-5p,miR-3082-5p,miR-378e,miR-769-5p,miR-3958-3p,miR-2285t,miR-3059-5p,miR-362-5p,miR-8095,miR-484,miR-378b,miR-30b-3p,miR-412-3p,miR-2426,miR-544a,miR-378c,miR-3120-5p,miR-16-2-3p,miR-2300a-5p,miR-105-1,miR-222-5p,miR-9788-3p,miR-500-5p,miR-216a-3p,miR-326,miR-320b,miR-342,miR-362-3p,miR-3955-3p,miR-1839-3p,miR-382-3p,miR-106a-5p,miR-376b,miR-33a-5p,miR-30c-1-3p,miR-758-3p,miR-3187-3p,miR-381-3p,miR-362-5p,miR-17-5p,miR-3154,miR-320a,let-7g-3p,miR-10b-3p,miR-485-5p,miR-1814c,miR-7144-5p,miR-141-3p,miR-769,miR-29b-3p,miR-144,miR-767-5p,miR-204-5p,miR-1306-5p,miR-374b,miR-6128,miR-500b-5p,miR-93,miR-378c,miR-145-3p,miR-134,miR-345-3p,miR-29d-3p,miR-218-2-3p,miR-300,let-7a-2-3p,miR-105-2,miR-128-3p,miR-483-3p,miR-378h,miR-1260a,miR-181c-3p,miR-30c-2-3p,miR-495-5p,miR-2385-3p,miR-29b-2-5p,miR-2478,miR-490-3p,miR-2285ab,miR-19a-3p,miR-6516-3p,miR-7975,miR-7-1-3p,miR-320b,miR-670-3p,miR-29a-3p,miR-2404,miR-323a-5p,miR-146b-3p,miR-20a-5p,miR-203b-5p,miR-656-5p,miR-342-3p,novel_101,miR-24-3p,miR-211,miR-2428,miR-326-3p,miR-3071-5p,miR-374b-3p,miR-1260b,miR-29b,miR-30b-3p,miR-187-3p,miR-26b-3p,miR-377-5p,miR-665,miR-130a-3p,miR-544b,miR-20b,miR-224-5p,miR-106a-5p,miR-17-5p,miR-190a,miR-654-3p,miR-30c-1-3p,miR-2331-
TCAIM	ENSOARG0(	T cell a	miR-185-3p,let-7c-5p,miR-200c,miR-2397-5p,miR-2285p,miR-1277-5p,let-7k,let-7f-5p,miR-200c-3p,miR-98-5p,let-7g,miR-199a-5p,miR-192-3p,miR-142a-3p,miR-1961,miR-1290,let-7d,miR-485-3p,miR-429-3p,miR-616-3p,miR-487a-3p,miR-322-5p,miR-15a-5p,miR-144,miR-135a-5p,miR-15b-3p,let-7f,miR-424-5p,miR-4492,miR-487b-5p,miR-16-5p,miR-3074-2-3p,let-7b,let-7b-5p,miR-769-5p,miR-3154,miR-2285aa,miR-133c,miR-199a-5p,let-7i-5p,miR-15b-5p,miR-331-5p,miR-769,miR-197-5p,miR-330-3p,miR-16b,miR-7-5p,miR-199b-5p,let-7e,miR-124-5p,miR-628-5p,let-7a-5p,miR-424-5p,miR-6240,miR-7b-5p,miR-1843b-5p,miR-542-3p,miR-2113,miR-497-5p,miR-1839-5p,miR-16b,miR-330-3p,miR-142-3p,let-7g-5p,miR-1298-3p,miR-3431,miR-485-3p,miR-769-5p,miR-16a,let-7i,miR-15a,miR-218-5p,miR-203b-5p,miR-6119-3p,miR-495-3p,novel_120,miR-219b-3p,miR-8095,miR-199b-5p,miR-199b-5p,miR-2319a,miR-1983,miR-195a-5p,miR-135b-5p,let-7e-5p,miR-136-5p,miR-412-3p,miR-541-5p,miR-320b,miR-7-1-3p,miR-2319b,miR-144-3p,let-7d-5p,miR-411b-3p,miR-2483-3p

SYTL3	ENSOARG0(	synaptot	miR-221,miR-148a-3p,miR-216a-3p,miR-1306,miR-1291,miR-378f,miR-493-5p,miR-3120-3p,miR-7977,miR-2285u,miR-378e,miR-2404,miR-6535,novel_82,miR-4324,miR-502-5p,miR-301a-5p,miR-33a-3p,miR-107-5p,miR-206,miR-2284q,miR-2285t,miR-3956-3p,miR-541-3p,miR-342-3p,novel_60,miR-2447,miR-3059-5p,miR-758-5p,miR-3957-3p,miR-2428,miR-301b-5p,miR-2113,miR-541,miR-378b,miR-296-5p,miR-329-3p,miR-1a-3p,miR-362-3p,miR-378c,miR-3120-5p,novel_4,miR-2300a-5p,miR-133b-5p,miR-330-3p,miR-1298-3p,miR-2320-3p,miR-3431,miR-2285g,miR-2285e,miR-221-3p,miR-1306-5p,novel_103,miR-342,miR-362-3p,miR-330-3p,miR-365b-5p,miR-466f-3p,miR-103a-2-5p,miR-214-3p,miR-493-5p,miR-6240,miR-380-5p,miR-378d,miR-654-3p,miR-2448-3p,miR-2285c,miR-21a-3p,miR-329a,miR-485-5p,novel_99,miR-2331-3p,miR-221-5p,miR-378a-3p,miR-1271,miR-204-3p,miR-222-3p,miR-664a-5p,miR-664a,miR-4492,miR-1306-5p,miR-378c,miR-214,miR-206-3p,miR-378c,miR-365a-5p,miR-362-3p,miR-3591-5p,miR-378i,miR-378a-3p,miR-1197-5p,miR-133a-3p,miR-128-3p,miR-222,miR-380-5p,miR-3600,miR-148b-3p,miR-378h,miR-210-3p,miR-671-3p,miR-210-5p,miR-422a,miR-185-3p,miR-92a-1-5p,miR-466i-5p,miR-152-3p,miR-221-5p,miR-345-3p,miR-1,miR-380-5p
DLX6	ENSOARG0(	distal-l	miR-1185-2-3p,miR-27a-3p,miR-4443,miR-2483-3p,miR-211-5p,miR-10b,miR-452-3p,miR-2319b,miR-20a-3p,miR-3064-5p,miR-335-3p,miR-504,miR-96-5p,miR-378j,miR-140-5p,miR-877-3p,miR-3956-3p,miR-2447,novel_60,miR-1843a-3p,miR-628-3p,miR-107-5p,miR-769-5p,miR-615,miR-218-5p,miR-1185-3p,miR-9788-3p,miR-145b,miR-27a-3p,miR-5010-3p,miR-199b-3p,miR-105-1,miR-2300a-5p,novel_4,miR-1343-3p,miR-330-3p,miR-2284aa,miR-2426,miR-450b-5p,miR-544a,miR-758-3p,miR-30b-3p,miR-412-3p,miR-10b,miR-143-5p,miR-2310,miR-758-3p,miR-193a,miR-30c-1-3p,miR-127-3p,miR-494-3p,miR-103a-2-5p,miR-491-5p,miR-3085-3p,miR-326,miR-320b,miR-342,miR-204-3p,novel_32,miR-6130,miR-377-3p,miR-544-3p,miR-10a,miR-552-3p,miR-504-5p,miR-140-5p,miR-30c-1-3p,miR-625-5p,miR-411,miR-129b-3p,miR-769-5p,miR-10b-5p,miR-6525,miR-3074-2-3p,miR-1271,miR-4510,miR-145a-3p,miR-3074-5p,miR-539-5p,miR-130b-5p,miR-129-5p,miR-874-5p,miR-664a,miR-338-5p,miR-320e,miR-29b-2-5p,miR-2285j,miR-222-3p,miR-383-5p,miR-3600,miR-18a-3p,miR-378d,miR-218-1-3p,miR-4429,miR-19b-3p,miR-1197-5p,miR-5703,miR-1434-5p,miR-2904,novel_133,miR-191,miR-486-3p,miR-421-5p,miR-376a-5p,miR-183-5p,miR-21-3p,miR-30b-3p,miR-1277-5p,miR-3653-3p,miR-320d,miR-125b,novel_17,miR-6535,miR-320c,miR-146b-3p,miR-10a-5p,miR-655-5p,miR-3074-5p,miR-363,miR-6516-3p,miR-135b-5p,miR-19a-3p,miR-107,miR-3065-5p,miR-18a-3p,miR-150-5p,miR-2428,miR-2284w,miR-326-3p,miR-24-3p,miR-211,novel_101,miR-2285x,miR-342-3p,miR-199c,miR-421-5p,miR-379-3p,novel_1,miR-2284o,miR-6402,miR-502-5p,miR-4324,miR-544b,miR-103,novel_116,miR-185-5p,miR-412-5p,miR-26b-3p,miR-103a-3p,miR-3955-5p,miR-31-5p,miR-27b-3p,miR-188-5p,miR-26a-2-3p,miR-30b-3p,miR-380-3p,miR-

GNA12	ENSOARG0(	guanine	miR-28b,miR-450b-5p,miR-199a-5p,miR-200a,miR-2403,miR-29b-2-5p,miR-30a-3p,miR-2898,miR-450a-2-3p,miR-543-5p,novel_127,miR-2285af,miR-30c-2-3p,miR-1271-5p,miR-24-2-5p,miR-505-5p,miR-134-5p,miR-582-3p,miR-885-3p,miR-128-3p,miR-134,miR-345-3p,miR-615-3p,miR-1343-5p,novel_42,miR-138-5p,miR-134-5p,miR-204-5p,miR-486b-3p,miR-5100,miR-410-5p,miR-2433,miR-331-3p,miR-365a-3p,miR-3965,miR-199a-5p,miR-874-3p,miR-574-5p,miR-2355-5p,miR-345-5p,miR-876-3p,miR-671-5p,miR-23a-5p,miR-320a,miR-345-5p,miR-193a-3p,miR-1343-5p,miR-2331-5p,novel_51,miR-2411-3p,miR-134-3p,miR-2285g,miR-187-3p,miR-340-3p,miR-219a-1-3p,miR-138,miR-21-5p,miR-27b-3p,miR-4508,miR-369-5p,miR-3071-5p,miR-24-3p,miR-211,miR-2285m,miR-199b-5p,miR-193b-3p,miR-128-1-5p,miR-421-5p,miR-28c,miR-6529b,miR-146b-3p,miR-181b-3p,miR-677,miR-320c,miR-669,miR-320b,miR-450b-3p,miR-1291,miR-6395,miR-9851-3p,miR-2387,miR-216a-3p,miR-21c,miR-18a-3p,miR-1306,miR-181b-1-3p,miR-29b-1-5p,miR-421-5p,miR-486-3p,miR-2432,miR-105-3p,miR-216c-5p,miR-466i-5p,miR-760-3p,miR-320d,miR-2484,miR-185-3p,miR-345-3p,miR-324-3p,miR-219b-5p,miR-222-5p,miR-328-3p,miR-1247-5p,novel_83,miR-151-5p,miR-199a-3p,miR-18a-3p,miR-30d-3p,miR-4429,miR-378d,miR-218-1-3p,miR-2408,novel_133,miR-124-3p,miR-2904,miR-346,miR-6134,miR-338-3p,miR-3184-5p,miR-214,miR-665-5p,miR-1271,miR-664a,miR-129-5p,miR-4492,miR-30e-3p,miR-29b-2-5p,miR-369-5p,miR-30a-3p,miR-132-5p,miR-193b-3p,miR-151b,miR-30c-1-3p,miR-140-5p,miR-552-3p,novel_107,miR-345-5p,miR-1827,miR-885-3p,miR-1895,miR-2285c,miR-30c-1-3p,miR-8485,miR-3187-3p,miR-2330-5p,miR-124a,miR-1388-3p,miR-214-3p,miR-199b-5p,novel_103,miR-1306-5p,miR-320b,miR-199a-3p,miR-29b-1-
PCCA	ENSOARG0(	propiony	miR-1285-5p,miR-374c-5p,miR-371b-3p,miR-24-3p,miR-452-5p,novel_60,miR-2447,miR-3956-3p,miR-541-3p,miR-22-5p,miR-4324,miR-142b,miR-320c,miR-203-3p,miR-378g,miR-7857-3p,miR-489,miR-24-1-5p,miR-363,miR-2319b,miR-335-3p,miR-7975,novel_94,miR-6395,miR-450b-3p,miR-1291,miR-2285k,miR-1306,miR-21c,miR-18b,miR-193a,miR-130b-5p,miR-3955-3p,miR-105-5p,miR-320b,miR-1306-5p,miR-582,novel_103,miR-411-5p,miR-142-5p,miR-134-3p,miR-105-1,novel_4,miR-151a-3p,miR-7862,miR-3120-5p,miR-21-5p,miR-30b-3p,miR-380-3p,miR-3071-5p,miR-30b-3p,miR-541,miR-542-3p,miR-142a-5p,miR-129-2-3p,miR-129-5p,miR-130b-5p,novel_74,miR-331-3p,miR-30a-3p,miR-151-3p,miR-100-3p,miR-409-3p,miR-655-3p,miR-30e-3p,miR-204-3p,miR-324-5p,miR-216b-3p,miR-370-3p,miR-574-5p,miR-140-5p,miR-874-3p,miR-18a-5p,miR-655,miR-18a,miR-320a,miR-3154,miR-488-3p,miR-503-3p,miR-29a-5p,miR-3956-5p,miR-143-3p,miR-2285b,miR-380-3p,miR-2285i,miR-24-2-5p,miR-130a-5p,miR-369-3p,miR-30a-3p,miR-1277-5p,miR-21-3p,miR-320d,miR-760-3p,miR-216a-5p,miR-324-3p,miR-217,miR-2284h-5p,miR-129-1-3p,miR-328-3p,miR-18b-5p,miR-30d-3p,miR-105-2,miR-4429,miR-217-5p,miR-2366
XRN2	ENSOARG0(	5'-3' ex	miR-1434-3p,miR-488-3p,miR-2447,miR-204-3p,miR-1298-3p

RNF11	ENSOARG0(	ring fin	miR-2113,miR-2285af,miR-2285j,miR-218-2-3p,miR-504,miR-504-5p,novel_68,miR-24-3p,miR-2483-3p,miR-149-5p
ERAL1	ENSOARG0(	Era-like	miR-1343-5p,miR-330-5p,miR-200b-3p,miR-429-3p,novel_79,miR-505-5p,miR-214-5p,miR-200c,miR-6119-5p,miR-30c-2-3p,miR-339a,miR-17-3p,miR-200c-3p,miR-200a-3p,novel_96,miR-4286,miR-28b,miR-148b-5p,novel_39,miR-488-3p,miR-301,miR-3154,miR-23b,miR-329b,miR-671-5p,miR-1248,miR-2285aa,miR-2355-5p,miR-141-3p,miR-376c-3p,miR-144,miR-3969,miR-1306-5p,miR-181a-2-3p,miR-6128,miR-144-5p,miR-1197-3p,miR-542-3p,miR-22-3p,miR-30b-3p,miR-455-5p,miR-7862,miR-31-5p,miR-339-5p,miR-377-5p,miR-26b-3p,miR-3431,miR-149-5p,miR-194-3p,miR-544b,novel_51,miR-455-5p,miR-1343-5p,miR-660,miR-130b-5p,miR-432-3p,miR-2387,miR-1306,miR-1291,miR-450b-3p,miR-6395,novel_68,miR-216b-3p,miR-329-5p,miR-320b,miR-146b-3p,novel_69,miR-144-3p,miR-28c,miR-576-3p,miR-142b,miR-425-5p,novel_1,miR-656-5p,miR-625-3p,miR-17-3p,miR-542-5p,miR-326-3p,miR-2284w,novel_133,miR-188-3p,novel_73,miR-148a-5p,miR-378d,miR-3600,miR-7859,miR-383-5p,miR-23b-3p,miR-200b,novel_83,miR-532-3p,miR-2284h-5p,miR-328-3p,miR-542-5p,miR-324-3p,miR-760-3p,miR-329a-5p,miR-2285w,miR-377-5p,miR-376c-3p,miR-2424,miR-129b-3p,miR-339b,miR-1827,miR-30c-1-3p,miR-660-5p,miR-182-5p,miR-544-3p,miR-376e-3p,miR-6130,miR-139-5p,miR-22-3p,miR-204-3p,miR-3604,miR-665,miR-17-3p,miR-451a,miR-664a,miR-874-5p,miR-130b-5p,miR-4510,miR-425-5p,miR-214,miR-3074-2-3p,miR-6525,miR-3184-5p,miR-484,miR-296-5p,miR-323b,miR-30b-3p,miR-544a,miR-3120-5p,miR-409b,miR-2320-3p,miR-455-3p,miR-148b-5p,miR-421,miR-2459,miR-1306-5p,miR-326,miR-323b-3p,miR-182-5p,miR-491-5p,miR-542-5p,miR-214-3p,miR-127-3p,miR-494-3p,miR-2330-5p,miR-101b-3p,miR-23a-3p,miR-33a-5p,miR-30c-1-3p,miR-361-3p,miR-139-5p,miR-20a-3p,miR-7689-3p,miR-7977,miR-144-5p,miR-4726-5p,miR-2447,miR-423-
KCTD15	ENSOARG0(	potassiu	miR-6119-5p,miR-200c,miR-125a-3p,miR-760-3p,miR-200c-3p,novel_127,miR-183-5p,miR-181a-2-3p,miR-2411,miR-134,miR-2366,miR-29d-3p,miR-188-3p,miR-142-3p,miR-133a-3p,miR-200b-3p,miR-18a-3p,miR-429-3p,miR-412,miR-2411-5p,miR-200b,novel_83,miR-134-5p,miR-29b-3p,miR-324-3p,miR-331-3p,miR-665,miR-664a,miR-874-5p,miR-134-5p,miR-214,miR-3074-2-3p,miR-346,miR-338-3p,miR-1827,miR-504-5p,miR-2355-5p,miR-615-5p,miR-544-3p,miR-132-5p,miR-324-5p,miR-3141,miR-214-3p,miR-574-3p,miR-628-5p,miR-33a-5p,miR-484,miR-873a-5p,miR-301b-5p,miR-541,miR-412-3p,miR-30b-3p,miR-30b-3p,miR-29b,miR-544a,miR-26b-3p,miR-1298-3p,miR-3431,miR-301a-5p,miR-128-1-5p,miR-29a,miR-2285t,miR-541-3p,miR-1296-5p,miR-29c-3p,novel_101,miR-452-5p,miR-361-3p,miR-3141,miR-18a-3p,miR-504,miR-877-3p,miR-452-3p,miR-7977,miR-146b-3p,miR-29a-3p,novel_69,miR-669

TTC39A	ENSOARG0(	tetratri	miR-144-5p,miR-20b-5p,miR-2318,miR-876-3p,miR-23b,miR-93-5p,miR-300-3p,miR-874-3p,novel_127,novel_96,miR-200a-3p,miR-127-5p,miR-125a-3p,novel_39,miR-200a,miR-3064-5p,miR-330-5p,miR-1290,miR-196a-5p,miR-3591-5p,miR-129-1-3p,novel_121,miR-758-5p,novel_79,miR-412,miR-6517,miR-299a-3p,miR-2285ad,miR-29a,miR-576-3p,miR-28c,miR-199b-5p,miR-29c-3p,miR-193b-3p,miR-335-5p,miR-412-3p,miR-410-5p,miR-216a-3p,miR-3065-5p,miR-181b-3p,miR-6535,miR-677,miR-669,miR-320c,miR-299b-3p,miR-3120-3p,miR-193a-3p,miR-196b-5p,miR-483-3p,miR-2411-3p,novel_51,miR-296-3p,miR-7-5p,miR-432-3p,miR-432-3p,miR-2285r,miR-6240,miR-27b-3p,miR-2284x,miR-26a-2-3p,miR-16-1-3p,novel_23,miR-22-3p,miR-197-3p,miR-542-3p,miR-7b-5p,miR-432,miR-411-5p,miR-134-3p,miR-412-5p,miR-339-5p,miR-3955-5p,miR-4492,miR-190a-5p,miR-451a,novel_74,miR-3604,miR-665,miR-151-3p,miR-30a-3p,miR-30e-3p,miR-224-5p,miR-769-5p,miR-6525,miR-214,miR-3958-5p,miR-129-2-3p,miR-3184-3p,miR-590-3p,miR-1827,miR-339b,miR-433-3p,miR-129b-3p,miR-216b-5p,miR-499b-3p,miR-10a,miR-2284s,miR-582-5p,miR-552-3p,miR-296-3p,miR-30c-1-3p,miR-504-5p,miR-3653-3p,miR-345-3p,miR-324-3p,miR-2285p,novel_17,miR-618,miR-486-3p,miR-2424,miR-181b-1-3p,miR-2285w,miR-16-1-3p,miR-493-3p,miR-217-5p,miR-2366,miR-2284h-5p,miR-23c,miR-23b-3p,miR-212-5p,miR-30d-3p,miR-106a,miR-18a-3p,miR-3964,miR-615,novel_82,miR-1285-5p,novel_111,miR-361-3p,miR-877-3p,miR-190b-5p,miR-2447,miR-1983,miR-9-3p,miR-376a-3p,miR-504,miR-96-5p,miR-99b-5p,miR-378j,miR-140-5p,miR-4443,miR-144-5p,miR-203-3p,miR-378g,miR-499a-3p,miR-214-3p,miR-103a-2-5p,miR-199b-5p,miR-491-5p,miR-106b-5p,miR-323b-3p,miR-199a-3p,miR-744-3p,miR-23a-3p,miR-124a,miR-101c,miR-1839-5p,miR-1388-5p,miR-

YWHAB	ENSOARG0(	tyrosine	novel_60,miR-3059-5p,miR-541-3p,miR-212-3p,miR-452-5p,miR-362-5p,miR-2285n,miR-769-5p,miR-335,miR-374a-3p,miR-3064-5p,miR-20a-3p,miR-7977,miR-378e,miR-3082-5p,miR-27a-3p,miR-199b-5p,miR-129b-5p,miR-106a-5p,let-7c-3p,miR-33a-5p,miR-30c-1-3p,miR-3187-3p,miR-3085-3p,miR-27a-3p,miR-500-5p,miR-484,miR-6529a,miR-378b,miR-143-5p,miR-10b,miR-412-3p,miR-125b-2-3p,miR-378c,miR-450b-5p,miR-101a-5p,miR-2284aa,miR-378c,miR-10b-5p,miR-206-3p,miR-132-5p,miR-664a,miR-378a-3p,miR-192-5p,miR-27a-5p,miR-323-5p,miR-877-5p,let-7g-3p,miR-133b-3p,miR-20a,miR-125b-2-3p,miR-130a-5p,miR-146a-5p,miR-196a-3p,miR-185-3p,miR-2284m,miR-106a,miR-760-3p,miR-30b-3p,miR-299-3p,miR-3600,miR-383-5p,miR-200b,miR-148b-3p,novel_133,miR-215-5p,miR-135a-2-3p,novel_101,miR-323c,miR-2428,miR-20a-5p,miR-22-5p,miR-10a-5p,miR-2404,miR-323a-5p,miR-379-5p,miR-101-5p,miR-296-3p,miR-345-5p,miR-17-5p,miR-106a-5p,miR-124-5p,miR-452-5p,miR-466f-3p,miR-432-5p,miR-185-5p,miR-2355-3p,miR-20b,miR-2284ab,miR-138,miR-1260b,miR-2889,miR-133a-3p,miR-93,miR-500b-5p,miR-374a-3p,miR-378c,miR-145-3p,miR-5100,miR-26c,miR-138-5p,miR-410-3p,miR-486b-3p,miR-374b-3p,miR-141-3p,miR-215-5p,miR-365a-3p,miR-769,miR-199a-5p,miR-2284n,miR-488-3p,miR-17-5p,miR-362-5p,miR-10b-3p,let-7g-3p,miR-376c-5p,miR-3074-1-3p,miR-671-5p,miR-485-5p,miR-1814c,miR-1,miR-30a-3p,miR-181a-2-3p,miR-199a-5p,miR-1260a,miR-2285af,miR-200c,miR-30c-2-3p,miR-339a,miR-217,miR-2898,miR-191-3p,miR-200c-3p,miR-543-5p,miR-378h,miR-545-5p,miR-134,miR-30a-5p,miR-582-3p,miR-128-3p,miR-200b-3p,miR-10a-5p,let-7a-2-3p,miR-3956-3p,miR-6527,miR-2284j,miR-9-5p,miR-218-5p,miR-2440,miR-490-5p,miR-203-3p,miR-1185-2-3p,miR-4443,miR-335-3p,miR-

EGR2	ENSOARG0(early gr	novel_83,miR-383-5p,miR-340-5p,miR-148b-3p,miR-200b,miR-3600,miR-548e-3p,let-7a-3p,miR-4429,miR-124-3p,miR-2904,miR-1197-5p,miR-5703,miR-215-5p,miR-2411,miR-29b-1-5p,miR-125b-2-3p,miR-20a,miR-216c-5p,miR-34b-5p,miR-1277-5p,miR-30b-3p,miR-221-5p,miR-320d,miR-106a,miR-2284m,miR-760-3p,miR-125b,miR-196a-3p,miR-374a-5p,miR-6516,miR-544-3p,miR-192-5p,miR-139-5p,miR-656-3p,miR-370-5p,miR-182-5p,miR-221-5p,miR-140-5p,miR-496-3p,miR-660-5p,novel_99,let-7g-3p,miR-10b-5p,miR-425-5p,miR-539-5p,miR-664a,miR-320e,miR-216a-3p,miR-2285e,miR-142-3p,miR-27a-3p,miR-2300a-5p,novel_4,miR-2284aa,miR-544a,miR-450b-5p,miR-101a-5p,miR-484,miR-6529a,miR-10b,miR-33a-5p,miR-106a-5p,miR-320b,miR-27a-3p,miR-7857-3p,miR-664b,miR-20a-3p,miR-10b,novel_94,miR-129b-5p,miR-154a,miR-194b-5p,novel_60,miR-541-3p,miR-2285t,miR-374a-3p,miR-2284q,miR-769-5p,miR-371a-5p,miR-2285y,miR-2285n,miR-545-5p,miR-6516-5p,miR-2411-5p,miR-200b-3p,miR-496,let-7a-2-3p,miR-10a-5p,miR-345-3p,miR-2284a,miR-4286,miR-107,miR-92a-3p,miR-543-5p,miR-200c-3p,miR-217,miR-2285af,miR-200c,miR-324-5p,miR-2284n,miR-34c-5p,miR-769,miR-215-5p,novel_48,let-7g-3p,miR-320a,miR-17-5p,miR-381-3p,miR-503-3p,miR-93,miR-374a-3p,miR-6128,miR-374b,miR-486b-3p,miR-138-5p,miR-331-3p,miR-135a-5p,miR-34c-3p,miR-370-5p,miR-544b,miR-103,miR-20b,miR-432-5p,miR-2284d,miR-2285g,miR-185-5p,miR-26b-3p,miR-133b-5p,miR-7862,miR-2284ab,miR-138,miR-145a-5p,miR-194-5p,miR-1434-3p,miR-296-3p,miR-345-5p,miR-654-3p,miR-125a-5p,miR-660,miR-330-3p,miR-106a-5p,miR-17-5p,miR-224-5p,miR-34b,miR-320b,miR-10a-5p,miR-378b,miR-9851-3p,miR-7975,miR-135b-5p,miR-107,miR-378b,miR-449a,miR-24-3p,novel_101,miR-1193,miR-2285x,miR-92a-3p,miR-34c,miR-

HELLS	ENSOARG0(	helicase	miR-181b-5p,miR-200c-3p,miR-28-3p,miR-107,miR-200c,miR-2285af,miR-30c-2-3p,miR-30f,miR-2285b,miR-199a-5p,miR-3064-5p,miR-200b-3p,miR-30c,miR-1290,miR-30a-5p,miR-486-5p,novel_121,miR-582-3p,miR-429-3p,miR-6517,miR-2285ad,miR-1224-5p,novel_9,miR-374b-3p,miR-370-5p,miR-410-5p,miR-34c-3p,miR-3969,miR-144,miR-374a-3p,miR-145-3p,miR-28a-3p,let-7e-3p,miR-329b,miR-485-5p,miR-7144-5p,novel_48,miR-30e-5p,miR-320a,miR-34c-5p,miR-199a-5p,miR-2285aa,miR-466f-3p,miR-660,miR-7-5p,novel_51,miR-194a,miR-30b-3p,miR-181a-5p,miR-7b-5p,miR-19b-2-5p,miR-145a-5p,miR-194-5p,miR-197-3p,miR-3431,miR-412-5p,miR-2285g,miR-103,novel_116,miR-103a-3p,miR-31-5p,miR-26b-3p,miR-206,miR-656-5p,miR-576-3p,miR-6402,miR-142b,miR-133a-5p,miR-34c,miR-199b-5p,miR-2428,miR-449a,miR-1193,miR-2285x,miR-107,miR-412-3p,miR-2284e,miR-3591-3p,miR-150-5p,miR-34b,miR-181b-3p,miR-320c,miR-144-3p,miR-669,miR-677,novel_68,miR-3120-3p,miR-26b-5p,miR-320d,miR-30d,miR-34b-5p,miR-2285p,miR-26a-5p,miR-30b,miR-216a-5p,miR-125b-2-3p,miR-181b-1-3p,miR-376a-5p,miR-29b-1-5p,miR-2424,miR-183-5p,miR-369-3p,miR-4429,miR-148a-5p,miR-34a-5p,miR-361-5p,miR-1246,miR-30c-5p,miR-188-3p,miR-200b,miR-487a-3p,miR-532-3p,miR-212-5p,miR-664a,miR-2285j,miR-320e,miR-655-3p,miR-19b-1-5p,miR-206-3p,miR-181c-5p,miR-433-5p,miR-30f,novel_99,miR-625-5p,miR-2285c,miR-655,miR-30d-5p,miR-6130,miR-660-5p,miR-548o-3p,miR-30c-1-3p,miR-182-5p,miR-21-3p,miR-370-5p,miR-487b-5p,miR-3085-3p,miR-199b-5p,novel_103,miR-320b,miR-182-5p,miR-2957,miR-323b-3p,miR-29b-1-5p,miR-30c-1-3p,miR-33a-5p,miR-101a-5p,miR-2426,miR-1388-5p,miR-21b,miR-484,miR-30b-3p,miR-323b,miR-2285e,miR-148b-5p,novel_78,miR-145b,miR-181b-2-3p,miR-499a-5p,miR-
DHDDS	ENSOARG0(	dehydrod	miR-340-3p,novel_4,miR-10a-5p,miR-10a,miR-10b-5p,miR-484,miR-10b,miR-2904,miR-874-5p,miR-10a-5p,miR-543-5p

DLX5	ENSOARG0(	distal-l	miR-874-3p,miR-503-3p,miR-574-5p,miR-487a-3p,miR-615-5p,miR-2355-5p,miR-2285aa,miR-769,miR-154b-5p,miR-301,miR-30e-5p,miR-362-5p,miR-3154,miR-767,miR-10b-3p,miR-1814c,miR-485-5p,miR-7144-5p,novel_48,miR-23a-5p,miR-2284v,miR-1306-5p,miR-16-5p,miR-3607-3p,miR-500b-5p,miR-2903,novel_63,miR-2284k,miR-767-5p,miR-204-5p,miR-486b-3p,miR-592,miR-3432a,miR-196a-2-3p,miR-1271-5p,miR-322-5p,miR-505-5p,novel_121,miR-2284z,miR-615-3p,miR-23b-5p,miR-30a-5p,miR-501-3p,miR-1343-5p,miR-7134-5p,miR-496,miR-4286,miR-2385-3p,miR-24-2-5p,miR-28b,miR-148b-5p,miR-2285b,miR-1957a,miR-29a-5p,miR-376b-3p,miR-2285af,miR-450a-2-3p,novel_127,miR-3120-3p,miR-655-5p,miR-411b-3p,novel_69,miR-323a-5p,miR-669,miR-6535,miR-2387,miR-18a-3p,miR-376b-3p,miR-3065-5p,miR-1306,miR-2285k,miR-1291,miR-505-3p,miR-195a-5p,miR-9851-3p,miR-342-3p,miR-24-3p,miR-211,miR-28c,miR-15a,miR-4324,miR-16a,miR-485-3p,novel_1,miR-147-5p,miR-199c,miR-31-5p,miR-377-5p,miR-134-3p,miR-432-5p,miR-2284d,miR-1843b-5p,miR-432,miR-197-3p,miR-30b-3p,miR-2284ab,miR-16b,miR-2284x,miR-497-5p,miR-26a-2-3p,miR-6240,let-7f-2-3p,let-7f-2-3p,miR-628-5p,miR-363-5p,miR-668-3p,miR-2284l,miR-296-3p,miR-539-3p,miR-296-3p,miR-377-3p,novel_51,miR-330-3p,miR-2285l,miR-1343-5p,miR-496-3p,miR-221-5p,miR-2331-3p,miR-504-5p,miR-154a-3p,miR-323-5p,miR-216b-5p,miR-139-5p,miR-656-3p,miR-6130,miR-582-5p,miR-377-3p,miR-499b-3p,miR-151b,miR-15b-5p,miR-21a-3p,miR-30d-5p,miR-27b-5p,miR-129b-3p,miR-154b-3p,miR-582-5p,miR-625-5p,miR-4510,miR-145a-3p,miR-539-5p,miR-214,miR-1271,miR-769-5p,miR-142a-5p,miR-6134,miR-3184-5p,miR-122-5p,miR-487b-5p,miR-129-5p,miR-1468-5p,miR-424-5p,miR-4492,miR-18a-3p,miR-505-3p,miR-485-3p,miR-15a-5p,miR-487a-3p,miR-151-

PCSK7	ENSOARG0(	proprote	miR-3085-3p,miR-491-5p,miR-29b-1-5p,novel_19,miR-1306-5p,miR-320b,miR-342,miR-3187-3p,miR-8485,miR-744-3p,miR-1388-3p,miR-3120-5p,miR-2426,miR-544a,miR-30b-3p,miR-7857,miR-541,miR-143-5p,miR-2284r,miR-376b-3p,miR-181b-2-3p,miR-1343-3p,miR-2300a-5p,miR-2284q,miR-490-5p,miR-769-5p,miR-2440,miR-423-5p,miR-877-3p,miR-9-5p,miR-541-3p,novel_94,miR-376a-3p,miR-504,miR-376d,miR-4443,miR-378g,miR-211-5p,miR-7977,miR-2319b,miR-2284g,miR-3064-5p,miR-34b-5p,miR-760-3p,miR-320d,miR-125b,miR-185-3p,miR-486-3p,miR-181b-1-3p,miR-29b-1-5p,miR-378d,miR-2284u,miR-34a-5p,miR-664-5p,miR-133a-3p,miR-218-1-3p,miR-4429,miR-5703,miR-2408,miR-2300b-3p,miR-328-3p,miR-2284h-5p,novel_83,miR-3600,miR-18a-3p,miR-130b-5p,miR-129-5p,miR-4492,miR-17-3p,miR-664a,miR-665,miR-132-5p,miR-2285j,miR-6134,miR-3184-5p,miR-6525,miR-769-5p,miR-4510,miR-129b-3p,miR-6130,miR-544-3p,miR-132-5p,miR-377-3p,miR-545-3p,miR-504-5p,miR-2331-3p,miR-125a-5p,miR-378a-5p,miR-483-3p,novel_51,miR-377-3p,miR-432-3p,miR-628-5p,miR-130b-5p,miR-744-5p,miR-30b-3p,miR-1285,miR-542-3p,miR-2355-3p,miR-544b,miR-149-5p,miR-134-3p,miR-26b-3p,miR-133b-5p,miR-340-3p,miR-6123,miR-128-1-5p,miR-34c,miR-449a,miR-211,miR-17-3p,miR-342-3p,miR-6395,miR-2319a,miR-1291,miR-376b-3p,miR-18a-3p,miR-3065-5p,miR-216a-3p,miR-378b,miR-1306,miR-2387,miR-181b-3p,miR-677,miR-320c,miR-34b,novel_69,novel_68,miR-543-5p,novel_96,miR-200a-3p,novel_127,miR-450a-2-3p,miR-2898,miR-17-3p,miR-3071-3p,miR-376b-3p,miR-29a-5p,miR-200a,novel_39,miR-2284y,miR-148b-5p,miR-2385-3p,miR-3064-5p,miR-128-3p,miR-23b-5p,miR-2427,miR-134,miR-134-5p,miR-582-3p,miR-483-3p,miR-9-5p,miR-204-5p,miR-134-5p,miR-486b-3p,miR-409-3p,miR-331-3p,miR-125b-5p,miR-100-3p,miR-
PRKAG2	ENSOARG0(	protein	miR-2331-3p,miR-545-3p,miR-499b-3p,miR-300-3p,miR-204-3p,miR-381-3p,miR-320a,miR-433-3p,miR-329a,miR-885-3p,miR-539-5p,miR-1197-3p,miR-338-3p,miR-2285j,miR-3604,miR-100-3p,miR-191-5p,miR-208b-5p,miR-2285ad,miR-18a-3p,miR-505-3p,miR-485-3p,miR-148b-3p,miR-487a-3p,miR-302a-5p,miR-545-5p,miR-362-3p,miR-191,miR-217-5p,miR-188-3p,miR-124-3p,novel_73,miR-300,miR-4429,miR-885-3p,miR-148a-5p,miR-128-3p,miR-130a-5p,miR-2285b,miR-29a-5p,miR-200a,miR-2285af,miR-181c-3p,miR-217,miR-320d,miR-152-3p,novel_127,miR-216b-3p,novel_68,miR-2285u,miR-499a-3p,miR-203-3p,miR-320c,miR-148a-3p,miR-216a-3p,miR-3065-5p,miR-18a-3p,miR-299b-5p,miR-9-3p,miR-505-3p,miR-212-3p,miR-342-3p,miR-2447,miR-495-3p,miR-2285m,miR-576-3p,miR-2285n,miR-485-3p,miR-2285y,miR-147-5p,novel_78,miR-2300a-5p,novel_4,miR-26b-3p,miR-148b-5p,miR-216a-3p,miR-329-3p,miR-450b-5p,miR-362-3p,miR-494-3p,miR-124a,miR-574-3p,miR-539-3p,miR-320b,miR-342,miR-362-3p,miR-2957,miR-124-5p,miR-452-5p

EPS15	ENSOARG0(	epiderma	miR-2285ad,miR-429-3p,miR-505-5p,miR-2284z,novel_121,miR-129-1-3p,miR-3578,miR-29d-3p,miR-218-2-3p,miR-200b-3p,miR-181a-2-3p,miR-4286,miR-24-2-5p,miR-380-5p,miR-2285b,miR-192-3p,miR-205-5p,miR-200c,miR-664-3p,miR-17-3p,miR-127-5p,miR-1a-2-5p,miR-181b-5p,miR-200c-3p,miR-152-3p,miR-2285aa,miR-374c-3p,miR-300-3p,miR-3965,miR-2284n,miR-2448-3p,miR-23b,miR-376c-5p,miR-767,miR-2318,miR-329b,novel_48,miR-7144-5p,miR-376c-5p,miR-1306-5p,miR-6128,miR-133a-3p,novel_63,miR-29b-3p,miR-2284k,miR-1-5p,miR-144,miR-3969,miR-767-5p,miR-130a-3p,miR-2284d,miR-185-5p,miR-194-3p,miR-19b-2-5p,miR-181a-5p,miR-1843b-5p,miR-223-3p,miR-3071-5p,miR-145a-5p,miR-29b,miR-2284ab,miR-7862,miR-27b-3p,miR-380-5p,miR-6240,miR-668-3p,miR-2284l,let-7j,miR-503-3p,miR-193a-3p,miR-26b-5p,miR-3120-3p,miR-411b-3p,miR-29a-3p,miR-6535,miR-144-3p,miR-376b-5p,miR-148a-3p,miR-3970,miR-150-5p,miR-378b,miR-2319a,miR-342-3p,miR-1a-1-5p,miR-193b-3p,miR-29c-3p,miR-758-5p,miR-17-3p,miR-301b-3p,miR-29a,miR-3600,miR-380-5p,miR-23b-3p,miR-23c,miR-200b,miR-148b-3p,miR-1246,miR-98-3p,miR-140-3p,novel_73,miR-216c-5p,miR-125b-2-3p,miR-196a-3p,novel_17,miR-26a-5p,miR-216a-5p,miR-466i-5p,miR-1277-5p,miR-545-3p,miR-548o-3p,miR-582-5p,miR-301a-3p,miR-193b-3p,miR-139-5p,let-7f-1-3p,miR-655,miR-133b-3p,miR-543-3p,miR-625-5p,miR-129-2-3p,miR-98-3p,miR-181c-5p,let-7b-3p,miR-2285j,miR-19b-1-5p,miR-655-3p,miR-664a-5p,miR-17-3p,miR-487a-5p,miR-454-3p,miR-669a-3p,miR-145b,miR-27a-3p,miR-147a,miR-1298-3p,miR-216a-3p,miR-455-3p,miR-126a-5p,miR-421,miR-6529a,miR-21b,miR-2284aa,miR-1839-5p,miR-1388-3p,miR-494-3p,miR-23a-3p,miR-1b-5p,miR-342,miR-505,miR-130b-3p,miR-139-5p,miR-24-1-5p,miR-664b,miR-2285u,miR-7857-3p,miR-
EIF2A	ENSOARG0(	eukaryot	miR-299-3p,miR-452-5p,miR-490-5p,miR-190a-5p,miR-100-3p,miR-190a,miR-2440,miR-133a-5p,miR-190b-5p,miR-376c-5p,miR-200b-3p,miR-450b-5p,miR-376c-5p,miR-376b-5p,miR-200b,miR-299b-3p,miR-6516-5p,miR-6516,miR-429-3p,miR-299a-3p

C1R	ENSOARG0(	compleme	miR-1,miR-2478,miR-29a-5p,miR-2285af,miR-1260a,miR-200c,miR-219-3p,miR-125a-3p,miR-339a,miR-450a-2-3p,miR-17-3p,miR-200c-3p,miR-6536,novel_127,miR-200a-3p,miR-299a-3p,miR-429-3p,miR-6517,miR-7641,novel_79,miR-18b-5p,miR-431,miR-9-5p,miR-378h,miR-483-3p,miR-450a-1-3p,miR-134-5p,miR-2284z,novel_121,miR-1290,miR-134,miR-23b-5p,miR-200b-3p,let-7a-2-3p,miR-193a-5p,miR-6128,miR-133a-3p,miR-1260b,miR-378c,miR-410-5p,miR-370-5p,miR-409-3p,miR-134-5p,miR-874-3p,miR-2285aa,miR-141-3p,miR-215-5p,miR-488-3p,miR-503-3p,miR-301,miR-18a,miR-345-5p,let-7g-3p,miR-7144-5p,miR-671-5p,miR-130b-5p,miR-493-5p,miR-382,miR-668-3p,miR-101-5p,miR-432-3p,miR-345-5p,let-7a-2-3p,miR-7-5p,miR-483-3p,miR-378a-5p,miR-452-5p,miR-339-5p,miR-2285g,miR-432-5p,miR-412-5p,miR-6238,miR-544b,miR-2355-3p,miR-653,miR-432,miR-873a-5p,miR-2113,miR-7b-5p,miR-223-3p,miR-197-3p,miR-450c-3p,miR-22-3p,novel_23,miR-30b-3p,miR-1260b,miR-188-5p,miR-7862,miR-17-3p,miR-2285x,miR-1193,miR-24-3p,miR-371b-3p,novel_27,miR-374c-5p,miR-2428,miR-2284w,miR-502-5p,miR-133a-5p,miR-142b,miR-33a-3p,miR-206,miR-421-5p,miR-625-3p,miR-22-5p,miR-378b,miR-216b-3p,miR-655-5p,miR-320b,miR-26b-5p,miR-299b-3p,miR-2285ab,miR-18b,miR-3970,miR-150-5p,miR-18a-3p,miR-410-5p,miR-450b-3p,miR-6516-3p,miR-378f,miR-216c-5p,miR-2285w,miR-29b-1-5p,miR-142a-3p,miR-130a-5p,miR-26a-5p,miR-2285p,miR-219b-5p,miR-422a,miR-202-5p,miR-21-3p,miR-299-3p,miR-18a-3p,miR-548e-3p,miR-3600,miR-1b-3p,miR-7859,miR-200b,miR-222-5p,miR-199a-3p,miR-616-3p,miR-2284h-5p,miR-378i,miR-1246,miR-215-5p,miR-376a-5p,miR-124-3p,miR-1197-5p,miR-378a-3p,miR-188-3p,miR-1434-5p,miR-140-3p,miR-493-3p,miR-142-3p,miR-378e,miR-3958-5p,miR-214,miR-3074-2-3p,miR-
AFF1	ENSOARG0(	AF4/FMR2	miR-378d,miR-654-3p,miR-3187-3p,miR-1388-3p,miR-5100,miR-422a,miR-378h,miR-544b,miR-31-5p,miR-30d-3p,miR-378c,miR-1388-5p,miR-544a,miR-19b-3p,miR-378i,novel_133,miR-378b,miR-3578,miR-1197-5p,miR-378a-3p,miR-378c,miR-3059-5p,miR-378c,miR-3074-2-3p,miR-452-5p,miR-502b,miR-30e-3p,miR-30a-3p,miR-544-3p,miR-378e,miR-378a-3p,miR-19a-3p,miR-378f

CINP	ENSOARG0(	cyclin-d	miR-181d-5p,miR-2447,miR-361-3p,miR-154-3p,miR-1843a-3p,miR-450a-5p,miR-211-5p,miR-664b,miR-376d,miR-378g,miR-3596,miR-199b-5p,miR-1983,miR-376a-3p,miR-668-5p,miR-494-3p,miR-424-5p,miR-127-3p,miR-382-3p,miR-8485,miR-16b,miR-29b-1-5p,miR-214-3p,miR-199b-5p,miR-130b-3p,miR-3955-3p,novel_78,miR-2459,miR-148b-5p,miR-376b-3p,miR-1a-3p,miR-412-3p,miR-484,miR-3120-5p,miR-1388-5p,miR-3074-2-3p,miR-214,miR-145a-3p,miR-590-3p,miR-181c-5p,miR-206-3p,miR-2285j,miR-424-5p,miR-454-3p,miR-874-5p,miR-4492,miR-664a,miR-552-3p,miR-154a-3p,miR-370-5p,miR-3065-3p,miR-15b-5p,novel_32,novel_25,miR-204-3p,miR-129b-3p,miR-1895,miR-154b-3p,miR-345-3p,miR-487a,miR-2432,miR-2424,miR-130a-5p,miR-29b-1-5p,miR-92a-1-5p,miR-21-3p,miR-485-3p,miR-3600,miR-548e-3p,novel_83,miR-15a-5p,miR-487a-3p,miR-7859,miR-1197-5p,miR-140-3p,novel_133,miR-148a-5p,miR-493-3p,novel_101,miR-1193,miR-301b-3p,miR-199b-5p,miR-374c-5p,miR-24-3p,miR-323c,miR-211,miR-33a-3p,miR-16a,miR-6402,miR-15a,miR-656-5p,miR-22-5p,miR-206,miR-7-1-3p,miR-144-3p,miR-378b,miR-376b-3p,miR-3065-5p,miR-216a-3p,miR-195a-5p,miR-412-3p,miR-1291,miR-539-3p,miR-2331-5p,miR-7-5p,miR-466f-3p,miR-452-5p,miR-1343-5p,miR-130a-3p,miR-149-5p,miR-3431,miR-134-3p,miR-412-5p,miR-3071-5p,miR-7b-5p,miR-1434-3p,miR-181a-5p,miR-744-5p,miR-497-5p,miR-138,miR-16b,miR-16-5p,miR-1197-3p,miR-144,miR-370-5p,miR-2433,miR-204-5p,miR-138-5p,miR-1271,miR-615-5p,miR-370-3p,miR-487a-3p,miR-199a-5p,miR-485-5p,miR-671-5p,miR-181a-2-3p,miR-1,miR-490-3p,miR-450c-5p,miR-199a-5p,miR-3065-3p,miR-664-3p,miR-376b-3p,miR-543-5p,miR-181b-5p,miR-412,novel_121,miR-214-5p,miR-322-5p,miR-1343-5p,miR-1290,miR-128-3p

FAM50A	ENSOARG0(	family w	novel_87,miR-625-3p,miR-199b-5p,miR-150-5p,miR-3591-3p,miR-216a-3p,miR-3065-5p,miR-3141,miR-146b-5p,miR-26b-5p,miR-3120-3p,miR-669,miR-365b-5p,miR-3141,novel_51,miR-7-5p,miR-146a,miR-296-3p,let-7a-2-3p,miR-450c-3p,miR-22-3p,miR-197-3p,novel_23,miR-7b-5p,miR-542-3p,miR-497-5p,miR-4508,miR-2284x,miR-16b,miR-16-1-3p,miR-31-5p,miR-3955-5p,miR-103a-3p,miR-149-5p,miR-3535,miR-592,miR-16-5p,miR-23b,miR-345-5p,miR-2355-5p,miR-3956,miR-3068-3p,miR-223-5p,miR-205-5p,miR-664-3p,novel_127,novel_96,miR-127-5p,miR-3064-5p,miR-192-3p,miR-148b-5p,miR-1343-5p,miR-330-5p,miR-1290,miR-18b-5p,miR-429-3p,miR-450a-1-3p,miR-322-5p,miR-505-5p,miR-218-5p,miR-101-3p,miR-499a-5p,miR-2447,miR-423-5p,miR-7705,miR-361-3p,miR-877-3p,novel_111,miR-140-5p,miR-409-5p,miR-335-3p,miR-544-5p,miR-96-5p,miR-668-5p,miR-2285u,miR-7689-3p,miR-139-5p,miR-4792,miR-2319b,miR-203-3p,miR-16b,miR-146b,miR-199b-5p,miR-103a-2-5p,miR-491-5p,miR-487b-5p,miR-23a-3p,miR-1388-3p,miR-8485,miR-2310,miR-541,miR-101c,miR-21b,miR-1388-5p,miR-145b,miR-455-3p,miR-2459,miR-147a,miR-142-5p,miR-665,miR-664a-5p,miR-4492,miR-874-5p,miR-669a-3p,miR-130b-5p,novel_74,miR-487b-5p,miR-665-5p,miR-3184-5p,miR-181c-5p,miR-6525,miR-769-5p,miR-142a-5p,miR-433-3p,miR-450a-1-3p,miR-625-5p,miR-186-5p,miR-1827,miR-345-5p,miR-296-3p,miR-30c-1-3p,miR-548o-3p,miR-151b,miR-15b-5p,miR-500a-5p,miR-324-3p,miR-92a-1-5p,miR-26a-5p,novel_17,miR-210-5p,miR-2484,miR-664b-3p,miR-2366,miR-16-1-3p,miR-34a-5p,miR-142-3p,miR-18a-3p,miR-328-3p,miR-7859,miR-23b-3p,miR-23c,miR-34c,miR-16a,miR-15a,miR-502-5p,miR-502b,novel_101,miR-2285x,miR-1193,miR-135a-2-3p,novel_27,miR-449a,miR-326-3p,miR-3959-3p,miR-323c,miR-

CCDC105	ENSOARG0(	coiled-c	[illegible] 5p,miR-30c-1-3p,miR-8485,miR-124a,miR-744-3p,let-7c-3p,miR-2426,miR-1388-5p,miR-101c,miR-541,miR-6529a,miR-125b-2-3p,miR-30b-3p,miR-147a,miR-153,miR-1185-3p,miR-455-3p,miR-216a-3p,miR-148b-5p,novel_4,miR-1343-3p,miR-2300a-5p,miR-330-3p,miR-27a-3p,miR-145b,miR-3968,miR-2284q,miR-490-5p,miR-218-5p,miR-769-5p,miR-154a,miR-363-3p,miR-541-3p,miR-2447,miR-4454,miR-504,miR-136-5p,miR-544-5p,miR-493-5p,miR-361-3p,miR-25,miR-378g,miR-3596,miR-27a-3p,miR-1185-2-3p,miR-20a-3p,miR-10b,miR-24-1-5p,miR-139-5p,miR-4792,miR-7977,miR-211-5p,miR-21-3p,miR-2484,miR-221-5p,miR-210-5p,novel_17,miR-196a-3p,miR-345-3p,miR-125b,miR-219b-5p,miR-29b-1-5p,miR-486-3p,miR-2285w,miR-345-3p,miR-19b-3p,miR-148a-5p,miR-16-1-3p,miR-218-1-3p,miR-2300b-3p,miR-2366,miR-1197-5p,miR-5703,miR-124-3p,miR-2284h-5p,miR-3600,miR-17-3p,miR-6516-3p,miR-155-5p,miR-664a,miR-450b-3p,miR-500,let-7b-3p,miR-338-5p,miR-664a-5p,miR-665,miR-206-3p,miR-6525,miR-769-5p,miR-4510,miR-3074-5p,miR-433-5p,miR-425-5p,miR-665-5p,let-7g-3p,miR-133b-3p,miR-582-5p,novel_99,let-7f-1-3p,miR-450a-1-3p,miR-21a-3p,miR-487b-5p,miR-411,miR-500a-3p,miR-6130,miR-139-5p,miR-582-5p,miR-27a-5p,miR-155-5p,miR-204-3p,miR-496-3p,miR-30c-1-3p,miR-504-5p,miR-221-5p,miR-32-5p,miR-532-5p,miR-1343-5p,miR-125a-5p,miR-7-5p,miR-2411-3p,novel_51,miR-330-3p,miR-32,miR-654-3p,miR-6240,miR-493-5p,let-7f-2-3p,let-7f-2-3p,miR-1260b,miR-16-1-3p,miR-30b-3p,miR-27b-3p,miR-7b-5p,miR-1285,miR-3071-5p,miR-145a-5p,miR-450c-3p,miR-197-3p,miR-185-5p,miR-134-3p,miR-412-5p,miR-2355-3p,miR-149-5p,miR-26b-3p,miR-206,miR-6119-3p,miR-92a-3p,miR-22-5p,miR-133a-5p,miR-1247-3p,novel_87,miR-24-3p,miR-211,miR-2428,miR-17-
TMTC4	ENSOARG0(	transmem	miR-1468-5p,miR-129-5p,miR-138-5p,miR-33a-3p,miR-219b-3p,miR-2332,miR-2285j,let-7b-3p,miR-219a-2-3p,miR-2285m,miR-98-3p,miR-2285x,miR-3059-5p,miR-129b-5p,let-7f-1-3p,miR-30e-5p,miR-1185-2-3p,miR-27a-3p,miR-1468,miR-219-3p,miR-2285af,miR-8485,miR-193a,let-7a-3p,miR-27b-3p,miR-450b-5p,miR-138,miR-30a-5p,miR-98-3p,miR-1185-3p,miR-2284h-5p,miR-27a-3p
ZADH2	ENSOARG0(	zinc bin	miR-329a-5p,miR-30c-1-3p,miR-2285b,miR-29a-5p,novel_17,miR-182-5p,miR-3071-3p,miR-505,miR-30c-2-3p,miR-216a-5p,miR-383-5p,miR-134-5p,miR-134,miR-215-5p,miR-188-3p,miR-3071-5p,miR-5703,miR-885-5p,miR-539-5p,miR-452-5p,miR-6128,miR-769-5p,miR-2284w,miR-338-3p,novel_27,miR-133a-5p,miR-5100,miR-4324,miR-769-5p,miR-3968,miR-134-5p,miR-329-5p,miR-30c-1-3p,miR-215-5p,miR-182-5p,miR-656-3p,miR-769,miR-3082-5p,miR-192-5p,miR-154b-5p,miR-3154,miR-136-5p

NKIRAS1	ENSOARG0(	NFKB inh	miR-339-5p,miR-665,miR-133b-5p,miR-103a-3p,miR-31-5p,miR-20b,miR-103,miR-194-3p,miR-3431,miR-412-5p,miR-374b-3p,miR-197-3p,miR-1285,miR-223-3p,miR-873a-5p,miR-7862,miR-27b-3p,miR-497-5p,miR-30b-3p,miR-16b,miR-628-5p,miR-382,miR-6240,miR-130b-5p,miR-539-3p,miR-17-5p,miR-106a-5p,miR-136-3p,miR-2411-3p,miR-330-3p,miR-105-5p,miR-193a-3p,miR-125a-5p,miR-106b-3p,miR-26b-5p,miR-10a-5p,miR-3074-5p,novel_68,miR-669,miR-677,miR-670-3p,miR-2404,miR-3065-5p,miR-18a-3p,miR-216a-3p,miR-3970,miR-195a-5p,miR-9851-3p,miR-412-3p,miR-19a-3p,miR-107,miR-2284e,miR-1291,miR-495-3p,miR-17-3p,miR-193b-3p,miR-326-3p,miR-1307-3p,novel_87,miR-425-5p,miR-2284o,miR-16a,miR-20a-5p,miR-133a-5p,miR-15a,novel_1,novel_79,miR-7641,miR-429-3p,miR-2285ad,miR-6516-5p,miR-1271-5p,miR-322-5p,miR-23b-5p,miR-330-5p,miR-1290,miR-345-3p,miR-10a-5p,miR-128-3p,miR-200b-3p,miR-300,miR-2284f,miR-2385-3p,miR-1271-3p,miR-200a,miR-380-5p,miR-339a,miR-664-3p,miR-200c,miR-2285af,novel_127,miR-107,miR-200c-3p,miR-17-3p,miR-125a,miR-2285aa,miR-487a-3p,miR-374c-3p,miR-23b,miR-17-5p,miR-93-5p,miR-2448-3p,miR-381-3p,miR-1814c,miR-7144-5p,miR-20b-5p,miR-329a,miR-329b,miR-133a-3p,miR-16-5p,miR-1306-5p,miR-93,miR-323-3p,miR-3969,miR-125b-5p,miR-410-5p,miR-28-5p,miR-27a-3p,novel_78,novel_4,miR-330-3p,miR-2459,miR-216a-3p,miR-142-5p,miR-1298-3p,miR-147a,miR-30b-3p,miR-758-3p,miR-323b,miR-301b-5p,miR-10b,miR-329-3p,miR-296-5p,miR-2284r,miR-484,miR-3120-5p,miR-101c,miR-2426,miR-450b-5p,miR-362-3p,miR-744-3p,miR-23a-3p,miR-106a-5p,miR-424-5p,miR-124a,miR-574-3p,miR-758-3p,miR-2310,miR-8485,miR-16b,miR-106b-5p,miR-505,miR-323b-3p,miR-362-3p,miR-326,miR-199a-3p,miR-214-3p,miR-7689-3p,miR-452-3p,miR-10b,miR-664b,miR-27a-
UBE2E1	ENSOARG0(	ubiquiti	miR-3604,miR-486b-3p,miR-204-5p,miR-500,miR-410-3p,miR-1260b,miR-142a-5p,miR-3154,miR-320a,miR-500a-3p,miR-129b-3p,miR-876-3p,miR-141-3p,miR-376e-3p,miR-544-3p,miR-376c-3p,novel_17,miR-1260a,miR-345-3p,miR-320d,miR-200a-3p,miR-3529-3p,miR-329a-5p,miR-181a-2-3p,miR-376c-3p,miR-143-3p,novel_39,miR-486-3p,miR-3578,miR-345-3p,miR-5703,miR-140-3p,miR-124-3p,miR-582-3p,miR-4429,miR-105-2,miR-16-1-3p,miR-222-5p,miR-486-5p,miR-199a-3p,miR-2284z,miR-532-3p,novel_1,miR-499a-5p,miR-211,miR-2428,miR-3065-5p,miR-877-3p,miR-329-5p,miR-452-3p,miR-20a-3p,miR-211-5p,miR-499b-5p,miR-320b,miR-320c,novel_115,miR-320b,miR-199a-3p,miR-2411-3p,miR-105-5p,miR-3955-3p,miR-124-5p,miR-124a,miR-652-5p,miR-628-5p,miR-432-3p,miR-432-3p,miR-193a,miR-542-3p,novel_23,miR-3071-5p,miR-30b-3p,miR-101a-5p,miR-16-1-3p,miR-544a,miR-1260b,miR-219a-1-3p,miR-30b-3p,miR-1388-5p,miR-101c,miR-2284aa,miR-2300a-5p,miR-105-1,miR-142-5p,miR-544b,miR-455-3p,miR-9788-3p

INPPL1	ENSOARG0(	inositol	miR-125a,miR-1271,miR-2355-5p,miR-370-3p,miR-2285aa,miR-3957-5p,miR-3957,miR-17-5p,miR-93-5p,miR-671-5p,miR-1814c,miR-20b-5p,miR-23a-5p,miR-193a-5p,novel_42,miR-93,miR-331-3p,miR-125b-5p,miR-2433,miR-29b-3p,miR-412,miR-2285ad,novel_121,miR-129-1-3p,miR-483-3p,miR-302a-5p,miR-29d-3p,miR-4286,miR-3064-5p,novel_39,miR-3065-3p,miR-30c-2-3p,miR-205-5p,miR-2898,miR-320b,novel_68,miR-29a-3p,miR-3141,miR-450b-3p,miR-1291,miR-29c-3p,miR-335-5p,miR-135a-2-3p,miR-2428,miR-371b-3p,miR-20a-5p,miR-4324,miR-29a,miR-26b-3p,miR-133b-5p,miR-20b,miR-149-5p,miR-22-3p,miR-1285,miR-873a-5p,miR-2284x,miR-29b,miR-363-5p,miR-130b-5p,novel_51,miR-17-5p,miR-106a-5p,miR-3141,miR-125a-5p,miR-483-3p,miR-296-3p,miR-3065-3p,miR-548o-3p,miR-30c-1-3p,miR-22-3p,novel_32,miR-216b-5p,miR-132-5p,miR-499b-3p,novel_99,miR-214,miR-129-2-3p,miR-433-5p,miR-6134,miR-346,miR-665,miR-19b-1-5p,miR-130b-5p,miR-874-5p,miR-4492,miR-1468-5p,miR-664a,miR-1b-3p,miR-106a,miR-532-3p,miR-2300b-3p,miR-378d,miR-184-3p,miR-345-3p,miR-20a,miR-143-3p,miR-376a-5p,miR-125b,miR-92a-1-5p,miR-185-3p,miR-219b-5p,miR-324-3p,miR-2285p,miR-760-3p,miR-106a,miR-7977,miR-2285u,miR-3064-5p,miR-489,miR-499a-3p,miR-140-5p,miR-3956-3p,novel_60,miR-3059-5p,miR-361-3p,miR-2285y,miR-335,miR-3968,miR-2300a-5p,novel_4,miR-412-3p,miR-2284r,miR-106a-5p,miR-2310,miR-30c-1-3p,miR-106b-5p,novel_19,miR-1468,miR-214-3p,miR-3085-3p

DAP3	ENSOARG0(	death as	miR-371a-5p, miR-107-5p, miR-4726-5p, miR-450a-5p, miR-2285f, miR-181d-5p, novel_60, miR-212-3p, miR-361-3p, miR-199b-5p, miR-877-3p, miR-499b-5p, miR-541-5p, miR-7977, miR-452-3p, miR-20a-3p, miR-378e, miR-489, miR-3082-5p, miR-320b, miR-326, miR-1306-5p, miR-1468, miR-33a-5p, miR-494-3p, miR-3187-3p, miR-758-3p, miR-30c-1-3p, miR-412-3p, miR-30b-3p, miR-378b, miR-484, miR-143-5p, miR-10b, miR-3120-5p, miR-378c, miR-450b-5p, miR-2426, novel_4, miR-2300a-5p, miR-409b, miR-151a-3p, miR-221-3p, miR-2285e, miR-19b-1-5p, miR-222-3p, miR-17-3p, miR-6516-3p, miR-664a, miR-539-5p, miR-378c, miR-145a-3p, miR-4510, miR-6134, miR-338-3p, miR-10b-5p, miR-206-3p, miR-27b-5p, novel_99, miR-2312, miR-18a-5p, miR-182-5p, miR-140-5p, miR-378a-3p, miR-660-5p, miR-22-3p, novel_32, miR-204-3p, miR-6130, miR-193b-3p, miR-216a-5p, miR-185-3p, miR-34b-5p, miR-299-3p, miR-2284m, miR-320d, miR-466i-5p, miR-125b-2-3p, miR-124-3p, miR-1197-5p, miR-5703, novel_133, miR-376a-5p, miR-2300b-3p, miR-4429, miR-3600, miR-199a-3p, miR-222-5p, miR-34c, miR-133a-5p, miR-656-5p, miR-203b-5p, novel_101, miR-17-3p, miR-135a-2-3p, novel_27, miR-326-3p, miR-449a, miR-2428, miR-211, miR-24-3p, miR-2285ab, miR-18b, miR-7975, miR-9851-3p, miR-6395, miR-376a-2-5p, miR-7-1-3p, miR-10a-5p, miR-670-3p, miR-34b, miR-224-5p, miR-660, miR-130b-5p, miR-345-5p, miR-374b-3p, miR-3071-5p, miR-145a-5p, miR-181a-5p, miR-6239, miR-30b-3p, miR-2284ab, miR-377-5p, miR-2355-3p, miR-432-5p, miR-2285g, miR-185-5p, miR-331-3p, miR-3969, miR-5100, miR-204-5p, miR-486b-3p, miR-3607-3p, miR-378c, miR-145-3p, miR-18a, miR-320a, miR-3154, miR-7144-5p, miR-3074-1-3p, miR-23a-5p, miR-1814c, miR-671-5p, miR-10b-3p, miR-370-3p, miR-3957-5p, miR-199a-5p, miR-3957, miR-2284n, miR-34c-5p, miR-30c-2-3p, miR-543-5p, miR-450a-2-3p, miR-17-3p, miR-181b-5p, miR-199a-5p, miR-490-

EEFSEC	ENSOARG0(	eukaryot	miR-378e,miR-1185-2-3p,miR-452-3p,miR-3064-5p,miR-499b-5p,miR-136-5p,miR-504,miR-3963,miR-361-3p,miR-3956-3p,novel_60,miR-6527,miR-4726-5p,miR-615,miR-769-5p,miR-1185-3p,miR-216a-3p,novel_78,miR-1343-3p,novel_4,miR-181b-2-3p,miR-378c,miR-362-3p,miR-378b,miR-2284r,miR-329-3p,miR-296-5p,miR-484,miR-378d,miR-8485,miR-4532,miR-127-3p,miR-1388-3p,miR-124a,miR-3085-3p,miR-491-5p,miR-326,miR-5126,miR-362-3p,miR-193b-3p,miR-2284s,miR-2331-3p,miR-504-5p,miR-378a-3p,miR-532-5p,miR-216b-3p,miR-3065-3p,miR-345-5p,miR-1827,miR-1895,miR-224-5p,miR-6525,miR-769-5p,miR-338-3p,miR-378c,miR-425-5p,miR-665,miR-324-3p,miR-328-3p,miR-1247-5p,miR-532-3p,miR-361-5p,miR-34a-5p,miR-378d,miR-362-3p,miR-378i,miR-378a-3p,miR-2904,miR-124-3p,miR-181b-1-3p,miR-125b-2-3p,miR-503-5p,miR-760-3p,miR-34b-5p,miR-422a,miR-125b,miR-185-3p,miR-92a-1-5p,novel_69,miR-34b,miR-181b-3p,miR-669,miR-677,novel_68,miR-1291,miR-378f,miR-6395,miR-9851-3p,miR-2387,miR-371b-3p,miR-449a,miR-326-3p,miR-193b-3p,miR-379-3p,miR-128-1-5p,miR-6123,miR-34c,miR-185-5p,miR-503-5p,miR-149-5p,miR-194-3p,miR-122-3p,miR-744-5p,miR-2889,miR-4508,miR-1285,miR-7b-5p,miR-345-5p,let-7f-2-3p,let-7f-2-3p,miR-363-5p,miR-483-3p,miR-193a-3p,miR-125a-5p,miR-106b-3p,miR-503-5p,miR-7-5p,miR-224-5p,miR-769,miR-34c-5p,miR-3957,miR-3957-5p,miR-574-5p,miR-125a,miR-1271,miR-2355-5p,miR-329a,miR-345-5p,miR-876-3p,miR-1197-3p,miR-378c,novel_42,miR-2433,miR-331-3p,miR-125b-5p,miR-378h,miR-483-3p,miR-505-5p,miR-6517,miR-2285ad,miR-7641,miR-196a-2-3p,miR-1224-5p,miR-411-3p,miR-615-3p,miR-330-5p,miR-3065-3p,miR-3064-5p,miR-127-5p,miR-191-3p,miR-2898,novel_96

COL22A1	ENSOARGO(	collagen	miR-130b-5p,miR-296-3p,let-7j,miR-345-5p,miR-194a,novel_51,miR-2411-3p,miR-503-5p,miR-377-3p,miR-296-3p,miR-125a-5p,miR-466f-3p,miR-483-3p,miR-378a-5p,miR-187-3p,miR-122-3p,miR-103a-3p,miR-149-5p,miR-103,miR-2355-3p,miR-432-5p,miR-503-5p,miR-134-3p,miR-450c-3p,miR-22-3p,miR-374b-3p,miR-194-5p,miR-432,miR-1843b-5p,miR-497-5p,miR-16b,miR-138,miR-29b,miR-1260b,miR-3957-3p,novel_101,miR-17-3p,miR-29c-3p,miR-342-3p,miR-326-3p,miR-2284w,miR-199b-5p,miR-2428,miR-24-3p,miR-371b-3p,miR-1307-5p,miR-16a,novel_87,miR-15a,miR-576-3p,miR-6529b,miR-203b-5p,miR-502b,miR-6119-3p,miR-128-1-5p,miR-29a,novel_1,miR-216b-3p,miR-3074-5p,miR-320c,miR-669,miR-677,miR-181b-3p,miR-323a-5p,miR-670-3p,miR-374c-3p,miR-29a-3p,miR-1306,miR-18a-3p,miR-216a-3p,miR-195a-5p,miR-107,miR-412-3p,miR-3064-5p,novel_39,miR-199a-5p,miR-376c-5p,miR-376b-5p,miR-3065-3p,miR-490-3p,miR-205-5p,miR-1260a,miR-125a-3p,miR-6119-5p,miR-107,novel_127,novel_96,miR-17-3p,miR-191-3p,miR-127-5p,miR-6517,miR-450a-1-3p,miR-214-5p,miR-322-5p,miR-483-3p,miR-29d-3p,miR-23b-5p,miR-330-5p,miR-345-3p,miR-3578,miR-128-3p,miR-6128,miR-16-5p,miR-181a-2-3p,miR-1260b,miR-26c,miR-331-3p,miR-125b-5p,miR-29b-3p,miR-486b-3p,miR-592,miR-138-5p,miR-615-5p,miR-125a,miR-874-3p,miR-199a-5p,miR-154b-5p,miR-374c-3p,miR-200a-5p,miR-3154,miR-320a,miR-671-5p,miR-485-5p,miR-1248,miR-345-5p,miR-744-3p,miR-33a-5p,miR-424-5p,miR-382-3p,miR-8485,miR-182-5p,miR-16b,miR-342,miR-320b,miR-1306-5p,miR-326,miR-199b-5p,miR-214-3p,miR-491-5p,miR-3085-3p,miR-181b-2-3p,miR-1343-3p,miR-148b-5p,miR-216a-3p,miR-1298-3p,miR-484,miR-2426,miR-190b-5p,miR-194b-5p,miR-2447,miR-3059-5p,miR-212-3p,miR-423-5p,miR-877-3p,miR-361-3p,miR-382-3p,miR-

SLC1A6	ENSOARG0(	solute c	miR-7-5p,miR-194a,miR-196b-5p,miR-124-5p,miR-493-5p,miR-380-5p,miR-668-3p,miR-7b-5p,miR-1434-3p,miR-653,miR-873a-5p,novel_23,miR-194-5p,miR-219a-1-3p,miR-16-1-3p,miR-7862,miR-3955-5p,miR-339-5p,miR-665,miR-185-5p,miR-412-5p,miR-6238,miR-149-5p,miR-502-5p,novel_87,novel_1,miR-22-5p,miR-342-3p,miR-3957-3p,miR-2428,miR-326-3p,miR-148a-3p,miR-376b-5p,miR-412-3p,miR-6395,miR-505-3p,miR-3074-5p,miR-10a-5p,miR-323a-5p,miR-339a,miR-3071-3p,miR-127-5p,miR-152-3p,miR-200a-3p,miR-3529-3p,miR-4286,miR-30a-3p,miR-380-5p,miR-148b-5p,miR-2478,miR-2285b,miR-2284a,miR-29a-5p,miR-196a-5p,miR-330-5p,miR-10a-5p,novel_79,miR-24-2-5p,miR-486-5p,miR-2332,miR-331-3p,miR-2448-3p,miR-3154,miR-1814c,miR-1248,miR-7144-5p,miR-376c-5p,miR-141-3p,miR-2355-5p,miR-365a-3p,miR-223-5p,miR-326,miR-342,miR-199a-3p,miR-505,miR-2957,miR-1185-5p,miR-214-3p,miR-494-3p,miR-2284b,miR-2310,miR-541,miR-10b,miR-16-2-3p,miR-151a-3p,miR-2300a-5p,miR-148b-5p,miR-216a-3p,miR-218-5p,miR-490-5p,miR-541-3p,miR-2447,miR-194b-5p,miR-9-3p,miR-361-3p,miR-504,miR-3963,miR-299b-5p,miR-1983,miR-9-3p,miR-493-5p,miR-7977,miR-3082-5p,miR-203-3p,miR-105-3p,miR-345-3p,miR-195a-3p,novel_133,miR-5703,miR-2904,miR-493-3p,miR-2284u,miR-148a-5p,miR-16-1-3p,miR-505-3p,miR-30d-3p,miR-148b-3p,miR-328-3p,miR-199a-3p,miR-30e-3p,miR-151-3p,miR-30a-3p,miR-208b-5p,miR-4492,miR-3074-5p,miR-214,miR-10b-5p,miR-339b,let-7g-3p,miR-186-5p,miR-545-3p,miR-504-5p,miR-216b-5p,miR-323-5p,miR-10a,miR-204-3p
GDI1	ENSOARG0(	GDP diss	miR-134-3p,miR-340-3p,miR-199b-3p,miR-6517,miR-30b-3p,miR-296-5p,miR-542-3p,miR-541,miR-2113,miR-30b-3p,miR-296-3p,miR-8485,miR-486-3p,miR-101b-3p,miR-363-5p,miR-1185-5p,miR-466f-3p,miR-342,novel_17,miR-377-3p,miR-296-3p,miR-185-3p,miR-92a-1-5p,miR-378g,miR-377-3p,miR-139-5p,novel_32,miR-669,miR-139-5p,miR-541-5p,miR-2355-5p,miR-625-5p,miR-9851-3p,miR-1827,miR-1248,miR-361-3p,miR-150-5p,miR-206-3p,miR-8095,miR-342-3p,miR-541-3p,miR-206,miR-4492,miR-656-5p,miR-22-5p,miR-199c,miR-486b-3p,miR-147-5p,miR-34c-3p

PM20D1	ENSOARG0(peptidas	miR-450c-5p,miR-3071-3p,miR-664-3p,novel_96,novel_127,miR-127-5p,novel_79,miR-431,miR-2285ad,miR-23b-5p,miR-3591-5p,miR-1290,miR-2284v,miR-1260b,miR-2284k,miR-592,miR-331-5p,miR-300-3p,miR-331-5p,miR-3959-5p,miR-331-5p,miR-767,miR-329a,miR-345-5p,miR-668-3p,let-7f-2-3p,let-7f-2-3p,miR-380-5p,miR-2411-3p,novel_51,miR-296-3p,miR-378a-5p,miR-339-5p,miR-149-5p,novel_116,miR-3431,miR-22-3p,novel_23,miR-432,miR-542-3p,miR-26a-2-3p,miR-188-5p,miR-2284x,miR-29c-3p,miR-485-3p,novel_87,miR-142b,miR-576-3p,miR-6123,miR-29a,miR-3074-5p,miR-6535,miR-677,miR-181b-3p,miR-2319a,miR-412-3p,miR-486-3p,miR-143-3p,miR-181b-1-3p,miR-324-3p,miR-500a-5p,miR-345-3p,miR-3653-3p,miR-18a-3p,miR-1434-5p,miR-193b-5p,miR-133a-3p,novel_73,miR-3184-3p,miR-3074-5p,miR-181c-5p,miR-6525,miR-142a-5p,miR-665,let-7b-3p,miR-874-5p,miR-4492,miR-130b-5p,novel_74,miR-30c-1-3p,miR-582-5p,miR-129b-3p,miR-433-3p,miR-625-5p,miR-1827,miR-339b,miR-345-5p,miR-1388-3p,miR-323b-3p,miR-29b-1-5p,miR-431-5p,miR-153-3p,miR-103a-2-5p,miR-491-5p,miR-181b-2-3p,novel_78,miR-148b-5p,miR-455-3p,miR-2459,miR-1185-3p,miR-153,miR-425-3p,miR-142-5p,miR-323b,miR-758-3p,miR-329-3p,miR-301b-5p,miR-1388-5p,miR-362-3p,miR-2284j,miR-877-3p,miR-361-3p,miR-301a-5p,novel_82,miR-218-5p,miR-211-5p,miR-2319b,miR-203-3p,miR-4443,miR-1185-2-3p,miR-3596,miR-381-3p,miR-9-3p,miR-544-5p,miR-1983,miR-96-5p,miR-29b-2-5p,miR-4286,miR-2403,miR-1957a,miR-3065-3p,miR-490-3p,miR-380-5p,miR-30c-2-3p,miR-339a,miR-1260a,miR-450a-2-3p,miR-2898,miR-181b-5p,miR-7641,miR-302a-5p,miR-6516-5p,miR-1271-5p,miR-29d-3p,miR-345-3p,miR-6128,miR-145-3p,miR-500b-5p,miR-409-3p,miR-29b-3p,miR-135a-1-3p,miR-486b-3p,miR-204-5p,miR-767-5p,miR-

ZNF385D	ENSOARG0(	zinc fin	miR-299a-5p,miR-101a-5p,miR-101c,miR-2284aa,miR-16-2-3p,miR-301b-5p,miR-4791,miR-541,miR-1a-3p,miR-142-3p,miR-1298-3p,miR-1185-3p,miR-2285e,novel_78,miR-3955-3p,miR-1468,miR-8485,miR-494-3p,miR-1388-3p,miR-33a-5p,miR-1983,novel_94,miR-335-3p,miR-2483-3p,miR-7857-3p,miR-499a-3p,miR-1185-2-3p,miR-10b,miR-335,miR-101-3p,novel_82,miR-301a-5p,miR-154a,miR-541-3p,miR-194b-5p,miR-2285f,miR-378d,miR-2300b-3p,novel_133,miR-188-3p,miR-5703,miR-148b-3p,miR-340-5p,miR-210-3p,miR-532-3p,miR-1247-5p,novel_83,miR-466i-5p,miR-21-3p,miR-1277-5p,miR-210-5p,miR-92a-1-5p,miR-142a-3p,miR-130a-5p,miR-664b-3p,miR-2411,let-7g-3p,miR-2312,miR-582-5p,miR-450a-1-3p,miR-2285c,miR-21a-3p,miR-433-3p,miR-129b-3p,miR-499b-3p,miR-582-5p,miR-155-5p,miR-660-5p,miR-296-3p,miR-155-5p,miR-450b-3p,miR-1468-5p,miR-320e,miR-665,miR-206-3p,miR-338-3p,miR-3184-3p,miR-539-5p,miR-590-3p,miR-665-5p,miR-219a-1-3p,miR-138,miR-188-5p,miR-653,miR-432,miR-873a-5p,miR-194-5p,novel_23,miR-450c-3p,miR-3535,miR-432-5p,miR-412-5p,miR-2285g,miR-26b-3p,miR-2285l,miR-124-5p,miR-466f-3p,miR-660,miR-194a,miR-197-5p,miR-432-3p,miR-654-3p,let-7a-2-3p,let-7f-2-3p,let-7f-2-3p,miR-412-3p,miR-2319a,miR-148a-3p,miR-2285ab,miR-376b-5p,miR-670-3p,miR-378b,miR-7-1-3p,miR-26b-5p,miR-206,novel_1,miR-147-5p,miR-142b,miR-335-5p,miR-219b-3p,miR-495-3p,novel_101,let-7a-2-3p,miR-155-5p,miR-2411-5p,miR-450a-1-3p,miR-2284z,miR-6517,miR-152-3p,miR-2285b,miR-1,miR-329b,let-7g-3p,miR-671-5p,miR-1248,miR-190a-3p,miR-7144-5p,miR-3154,miR-154b-5p,miR-3068-3p,miR-138-5p,miR-135a-1-3p,miR-26c,miR-331-3p,let-7e-3p,miR-193a-5p
PICALM	ENSOARG0(	phosphat	miR-30d-5p,miR-30e-5p,miR-1248,miR-329b,miR-30f,let-7g-3p,miR-10b-3p,miR-216b-3p,miR-660-5p,miR-155-5p,miR-2284n,miR-377-3p,miR-2284k,miR-669a-3p,miR-155-5p,miR-206-3p,miR-1197-3p,miR-30a-5p,miR-1434-5p,miR-217-5p,miR-1290,miR-3578,miR-30c-5p,let-7a-2-3p,miR-2284u,miR-218-1-3p,miR-155-5p,miR-1224-5p,novel_121,miR-148b-3p,miR-302a-5p,miR-217,miR-664-3p,miR-2285af,miR-21-3p,miR-152-3p,miR-181b-5p,miR-2284m,miR-195a-3p,miR-3529-3p,miR-2284y,miR-129b-5p,miR-3065-5p,miR-1306,miR-148a-3p,miR-6516-3p,miR-7975,miR-493-5p,miR-541-5p,miR-378b,miR-664b,miR-2284g,miR-670-3p,miR-6402,miR-6123,miR-502b,miR-2285t,miR-206,miR-495-3p,miR-181d-5p,miR-2284j,miR-219b-3p,miR-2284w,miR-8095,miR-3959-3p,miR-3071-5p,miR-181a-5p,miR-2284x,miR-2284ab,miR-330-3p,miR-421,miR-2284d,miR-330-3p,miR-1306-5p,miR-377-3p,miR-660,miR-6240,let-7a-2-3p,miR-503-3p
KIF26B	ENSOARG0(	kinesin	miR-106a,miR-30a-3p,miR-17-5p,miR-106b-5p,miR-106a-5p,miR-20a-5p,miR-30e-3p,miR-2284y,miR-2284a,miR-93,miR-20a,miR-30a-3p,miR-2284b,miR-574-3p,miR-106a-5p,miR-20b-5p,miR-2284u,miR-93-5p,miR-17-5p,miR-1298-5p,miR-20b,miR-670-3p,miR-499b-5p,miR-30d-3p,miR-187-3p,miR-2284g,miR-452-3p,miR-106a

C1S	ENSOARG0(	compleme	miR-6395,miR-135b-5p,miR-6516-3p,miR-107,miR-1291,miR-18a-3p,miR-21c,miR-3591-3p,miR-148a-3p,miR-376b-5p,miR-3120-3p,miR-655-5p,miR-378b,miR-656-5p,miR-203b-5p,miR-22-5p,novel_1,miR-502-5p,miR-199b-5p,miR-326-3p,miR-24-3p,miR-211,miR-17-3p,novel_101,miR-2284x,miR-16-1-3p,miR-138,miR-30b-3p,miR-21-5p,miR-3071-5p,miR-374b-3p,miR-145a-5p,miR-197-3p,miR-181a-5p,miR-1434-3p,miR-2113,miR-544b,miR-103,miR-3431,miR-412-5p,miR-3535,miR-339-5p,miR-103a-3p,miR-466f-3p,miR-125a-5p,miR-378a-5p,miR-2411-3p,novel_51,miR-105-5p,miR-197-5p,let-7a-2-3p,miR-345-5p,miR-2284l,miR-654-3p,let-7f-2-3p,let-7f-2-3p,miR-130b-5p,miR-1248,miR-485-5p,miR-7144-5p,miR-376c-5p,let-7g-3p,miR-345-5p,miR-10b-3p,miR-376c-5p,miR-3154,miR-137-3p,miR-301,miR-503-3p,miR-154b-5p,miR-199a-5p,miR-125a,miR-874-3p,miR-134-5p,miR-204-5p,miR-138-5p,miR-3969,miR-409-3p,miR-135a-5p,miR-125b-5p,miR-6740-5p,miR-135a-1-3p,miR-410-5p,miR-2284k,miR-145-3p,miR-1197-3p,miR-193a-5p,miR-2284v,let-7a-2-3p,miR-200b-3p,miR-105-2,miR-330-5p,miR-134,miR-345-3p,miR-486-5p,miR-134-5p,miR-2284z,miR-6516-5p,miR-302a-5p,novel_79,miR-196a-2-3p,miR-412,miR-6517,miR-429-3p,novel_127,miR-152-3p,miR-107,miR-127-5p,miR-200c-3p,miR-181b-5p,miR-17-3p,miR-339a,miR-3071-3p,miR-200c,miR-2285af,miR-205-5p,miR-192-3p,miR-200a,miR-199a-5p,miR-29a-5p,miR-148b-5p,miR-181a-2-3p,miR-877-3p,miR-136-5p,miR-199b-5p,miR-1185-2-3p,miR-211-5p,miR-499b-5p,miR-20a-3p,miR-452-3p,miR-2440,miR-7705,miR-154a,miR-181d-5p,miR-2284j,miR-452-5p,miR-2447,miR-3120-5p,miR-2284aa,miR-2426,miR-544a,miR-1388-5p,miR-30b-3p,miR-758-3p,miR-412-3p,miR-323b,miR-4791,miR-296-5p,miR-6529a,miR-484,miR-1185-3p,miR-9788-3p,miR-455-3p,miR-1298-3p,miR-145b,miR-105-1,novel_4,miR-

PHF7	ENSOARG0(	PHD fing	miR-2285e,miR-216a-3p,miR-9788-3p,miR-30b-3p,miR-484,miR-296-5p,miR-3120-5p,miR-2284aa,miR-16-2-3p,miR-450b-5p,miR-2426,miR-424-5p,miR-106a-5p,miR-2330-5p,miR-758-3p,miR-362-3p,miR-1306-5p,novel_115,miR-326,miR-320b,miR-3085-3p,miR-10b,miR-3064-5p,novel_94,miR-452-5p,miR-181d-5p,miR-2285f,miR-541-3p,miR-30b-5p,miR-2285n,miR-2285t,novel_83,miR-199a-3p,miR-532-3p,miR-148b-3p,miR-15a-5p,miR-200b,miR-222-5p,miR-383-5p,miR-1197-5p,miR-5703,miR-2904,miR-2300b-3p,miR-362-3p,miR-126-3p,miR-378d,miR-4429,miR-941,miR-20a,miR-105-3p,miR-146a-5p,miR-2411,miR-299-3p,miR-21-3p,miR-466i-5p,miR-2284m,miR-106a,miR-320d,miR-182-5p,miR-3065-3p,novel_32,miR-22-3p,miR-6516,miR-21a-3p,novel_99,miR-18a-5p,miR-30f,let-7g-3p,miR-2312,miR-424-5p,miR-17-3p,miR-20b,miR-185-5p,miR-2285g,miR-2284d,miR-374b-3p,miR-1434-3p,miR-181a-5p,miR-219a-1-3p,miR-30b-3p,miR-2284ab,miR-363-5p,miR-130b-5p,miR-345-5p,miR-654-3p,miR-106a-5p,miR-17-5p,miR-466f-3p,miR-1343-5p,miR-2285l,miR-146b-3p,miR-378b,miR-18b,miR-2285ab,miR-195a-5p,miR-6395,miR-9851-3p,miR-2285k,miR-17-3p,miR-495-3p,miR-135a-2-3p,miR-2428,miR-326-3p,miR-211,miR-16a,miR-6402,miR-20a-5p,miR-15a,novel_1,miR-214-5p,miR-582-3p,miR-6516-5p,miR-2411-5p,miR-134,miR-345-3p,miR-200b-3p,miR-4286,miR-2385-3p,miR-30a-3p,miR-3529-3p,miR-1957a,miR-3065-3p,miR-200c,miR-2397-5p,miR-200c-3p,miR-181b-5p,miR-17-3p,miR-2284n,miR-374c-3p,miR-18a,miR-320a,miR-17-5p,miR-3154,let-7g-3p,miR-103b,miR-93,miR-204-5p,miR-486b-3p,miR-410-3p,miR-181b-2-3p,miR-1343-3p,miR-2459,miR-455-3p,miR-758-3p,miR-541,miR-329-3p,miR-101c,miR-362-3p,miR-182-5p,miR-106b-5p,miR-16b,miR-199a-3p,miR-146b,miR-491-5p,miR-211-5p,miR-378g,miR-9-3p,miR-136-5p,miR-504,miR-

C18orf63	ENSOARG0(	chromoso	miR-199a-5p,miR-1957a,miR-376b-5p,miR-376c-5p,miR-29a-5p,miR-380-5p,miR-2478,miR-28b,miR-450b-5p,miR-29b-2-5p,miR-1,miR-30a-3p,miR-3529-3p,miR-107,miR-200a-3p,miR-543-5p,miR-17-3p,miR-6536,miR-200c-3p,miR-2285af,miR-6119-5p,miR-200c,miR-450a-1-3p,miR-545-5p,miR-2284z,novel_121,miR-302a-5p,miR-322-5p,miR-429-3p,miR-200b-3p,miR-496,miR-411-3p,miR-3578,miR-1290,miR-16-5p,miR-103b,miR-486b-3p,miR-28-5p,miR-2284k,miR-135a-1-3p,miR-494-5p,miR-199a-5p,miR-133c,miR-2285aa,miR-141-3p,miR-574-5p,miR-3068-3p,miR-376c-5p,miR-1248,miR-1814c,miR-10b-3p,miR-329a,miR-200a-5p,miR-376c-5p,miR-2448-3p,miR-2284l,miR-380-5p,miR-130b-5p,miR-452-5p,miR-124-5p,miR-224-5p,miR-7-5p,miR-149-5p,novel_116,miR-103,miR-544b,miR-103a-3p,miR-497-5p,miR-2284x,miR-16b,miR-380-3p,miR-22-3p,miR-450c-3p,novel_23,miR-873a-5p,miR-1434-3p,miR-7b-5p,miR-199b-5p,miR-323c,miR-24-3p,miR-17-3p,novel_101,miR-22-5p,miR-502b,miR-206,miR-379-3p,miR-16a,miR-15a,miR-28c,miR-677,miR-181b-3p,miR-7-1-3p,miR-3120-3p,novel_68,miR-329-5p,miR-195a-5p,miR-379-5p,miR-450b-3p,miR-19a-3p,miR-107,miR-3065-5p,miR-376b-5p,miR-2387,miR-2424,miR-486-3p,miR-380-3p,miR-29b-1-5p,miR-181b-1-3p,miR-329a-5p,miR-216c-5p,miR-21-3p,miR-2284m,miR-202-5p,miR-466i-5p,miR-2285p,miR-532-3p,miR-7859,miR-200b,miR-15a-5p,miR-19b-3p,miR-2904,miR-188-3p,miR-140-3p,miR-2300b-3p,miR-362-3p,miR-122-5p,miR-3184-5p,miR-224-5p,miR-206-3p,miR-3074-2-3p,miR-708-5p,miR-424-5p,miR-130b-5p,miR-450b-3p,miR-487b-5p,miR-17-3p,miR-151-3p,miR-30a-3p,miR-29b-2-5p,miR-664a-5p,miR-30e-3p,miR-22-3p,miR-204-3p,miR-15b-5p,miR-544-3p,miR-582-5p,miR-2331-3p,miR-496-3p,miR-186-5p,miR-582-5p,miR-154b-3p,miR-129b-3p,miR-411,miR-487b-5p,miR-21a-3p,miR-

WDR11	ENSOARG0(	WD repea	miR-2387,miR-1306,miR-18a-3p,miR-3065-5p,miR-3591-3p,miR-1291,miR-450b-3p,miR-146b-5p,miR-2319a,miR-195a-5p,miR-655-5p,miR-26b-5p,miR-411b-3p,miR-320c,miR-669,miR-15a,miR-6402,miR-20a-5p,miR-16a,novel_87,miR-335-5p,miR-3957-3p,miR-193b-3p,miR-17-3p,miR-211,miR-24-3p,miR-371b-3p,miR-326-3p,miR-199b-5p,miR-873a-5p,miR-181a-5p,miR-1843b-5p,miR-2113,miR-1434-3p,miR-16b,miR-30b-3p,miR-497-5p,miR-2284x,miR-744-5p,miR-3955-5p,miR-340-3p,miR-31-5p,miR-133b-5p,miR-503-5p,miR-134-3p,miR-149-5p,miR-194-3p,miR-20b,miR-503-5p,miR-224-5p,miR-146a,miR-365b-5p,miR-330-3p,novel_51,miR-17-5p,miR-106a-5p,miR-378a-5p,miR-125a-5p,miR-466f-3p,miR-193a-3p,miR-380-5p,miR-6240,miR-2448-3p,miR-93-5p,miR-320a,miR-17-5p,miR-20b-5p,miR-485-5p,miR-874-3p,miR-125a,miR-374c-3p,miR-300-3p,miR-199a-5p,miR-125b-5p,miR-26c,miR-3969,miR-28-5p,miR-134-5p,miR-204-5p,miR-6128,miR-16-5p,miR-193a-5p,miR-93,miR-1197-3p,miR-365a-5p,miR-134,miR-330-5p,miR-322-5p,miR-758-5p,miR-134-5p,miR-214-5p,miR-30c-2-3p,miR-217,miR-17-3p,miR-181b-5p,novel_127,miR-543-5p,miR-3064-5p,miR-2385-3p,miR-380-5p,miR-199a-5p,miR-3065-3p,miR-1271-3p,miR-199b-5p,miR-378j,miR-9-3p,miR-877-3p,miR-4792,miR-3064-5p,miR-452-3p,miR-20a-3p,miR-2319b,miR-2285u,miR-211-5p,miR-499a-3p,miR-378g,miR-3082-5p,miR-218-5p,miR-101-3p,miR-107-5p,miR-499a-5p,miR-335,miR-3059-5p,miR-2447,miR-541-3p,miR-181d-5p,miR-452-5p,novel_111,miR-296-5p,miR-541,miR-30b-3p,miR-101c,miR-1343-3p,novel_4,miR-330-3p,miR-147a,miR-455-3p,miR-9788-3p,miR-320b,miR-326,miR-1306-5p,miR-106b-5p,miR-16b,miR-3085-3p,miR-146b,miR-199b-5p,miR-103a-2-5p,miR-214-3p,miR-574-3p,miR-424-5p,miR-382-3p,miR-106a-5p,miR-30c-1-3p,miR-8485,miR-4532,miR-411,novel_99,miR-30c-1-3p,miR-

REEP3	ENSOARG0(	receptor	miR-7-5p,miR-106a-5p,miR-17-5p,miR-197-5p,miR-539-3p,miR-668-3p,miR-101-5p,miR-16-1-3p,miR-1260b,miR-7b-5p,miR-1434-3p,miR-1843b-5p,miR-3071-5p,miR-450c-3p,miR-197-3p,miR-3431,miR-412-5p,miR-103,miR-20b,miR-149-5p,miR-103a-3p,miR-31-5p,miR-3955-5p,miR-26b-3p,miR-130a-3p,novel_1,miR-6119-3p,miR-6123,miR-656-5p,miR-133a-5p,miR-20a-5p,miR-33a-3p,miR-34c,miR-323c,miR-371b-3p,miR-301b-3p,miR-2284w,miR-449a,miR-495-3p,miR-107,miR-19a-3p,miR-376b-3p,miR-18a-3p,miR-378b,miR-411b-3p,miR-374c-3p,miR-34b,miR-299b-3p,miR-144-3p,miR-320c,miR-26b-5p,miR-7-1-3p,miR-200c-3p,miR-543-5p,miR-200a-3p,novel_127,miR-107,miR-219-3p,miR-200c,miR-6119-5p,miR-376b-3p,miR-1260a,miR-2478,miR-2285b,miR-450c-5p,miR-200a,miR-30a-3p,miR-3064-5p,miR-300,miR-200b-3p,miR-30c,miR-3578,miR-1290,miR-30a-5p,miR-9-5p,novel_121,miR-582-3p,miR-450a-1-3p,miR-429-3p,miR-299a-3p,miR-2332,miR-3969,miR-100-3p,miR-1260b,miR-93,miR-329a,miR-2318,miR-671-5p,miR-20b-5p,miR-3074-1-3p,miR-381-3p,miR-488-3p,miR-320a,miR-17-5p,miR-200a-5p,miR-93-5p,miR-365a-3p,miR-374c-3p,miR-34c-5p,miR-487a-3p,miR-141-3p,miR-2285aa,miR-130b-3p,miR-3955-3p,miR-3085-3p,miR-153-3p,miR-320b,miR-106b-5p,miR-29b-1-5p,miR-505,miR-362-3p,miR-106a-5p,miR-124a,miR-652-5p,miR-33a-5p,miR-362-3p,miR-329-3p,miR-484,miR-147a,miR-539-5p,miR-376b-3p,miR-153,miR-2459,miR-421,miR-455-3p,novel_78,miR-409b,miR-548w,miR-151a-3p,miR-450a-5p,miR-154-3p,miR-2285t,miR-490-5p,miR-30b-5p,novel_111,miR-154a,miR-219a-2-3p,miR-1285-5p,miR-212-3p,miR-32-3p,miR-9-5p,miR-190b-5p,miR-299b-5p,miR-376a-3p,miR-7857-3p,miR-376d,miR-454-5p,miR-203-3p,miR-20a-3p,miR-3064-5p,miR-466i-5p,miR-106a,miR-320d,miR-299-3p,miR-34b-5p,miR-

NRF1	ENSOARG0(	nuclear	miR-182-5p,miR-21-3p,miR-32-5p,miR-504-5p,miR-151b,novel_32,miR-22-3p,miR-6130,miR-377-3p,miR-500a-3p,miR-1827,miR-339b,miR-214,miR-145a-3p,miR-129-2-3p,miR-4510,miR-539-5p,miR-224-5p,miR-6525,miR-665,miR-324-3p,miR-2285j,miR-874-5p,miR-500,miR-485-3p,miR-3600,miR-151-5p,miR-200b,miR-222-5p,miR-7859,miR-487a-3p,miR-1197-5p,miR-1434-5p,miR-362-3p,miR-2887,miR-329a-5p,miR-216c-5p,miR-377-5p,miR-664b-3p,miR-92a-1-5p,novel_17,miR-210-5p,miR-760-3p,miR-10b,miR-500-3p,miR-668-5p,miR-504,miR-2284j,miR-3956-3p,miR-212-3p,miR-363-3p,miR-885-5p,novel_60,miR-502-3p,miR-371a-5p,miR-2285n,miR-2285t,miR-4726-5p,miR-222-5p,miR-1343-3p,novel_4,miR-2300a-5p,miR-421,miR-9788-3p,miR-455-3p,miR-2320-3p,miR-1298-3p,miR-30b-3p,miR-501-3p,miR-329-3p,miR-3120-5p,miR-2426,miR-362-3p,miR-1388-5p,miR-744-3p,miR-2330-5p,miR-1388-3p,miR-8485,miR-193a,miR-182-5p,miR-505,miR-362-3p,miR-342,miR-214-3p,miR-491-5p,miR-141-3p,miR-3068-3p,miR-2284n,miR-25-3p,miR-381-3p,miR-485-5p,miR-7144-5p,novel_48,miR-329b,novel_91,miR-329a,miR-28a-3p,miR-1197-3p,miR-331-3p,miR-2284k,miR-410-5p,novel_63,miR-1224-5p,novel_79,miR-7641,miR-429-3p,miR-214-5p,miR-129-1-3p,miR-483-3p,miR-501-3p,miR-3578,miR-200b-3p,miR-300,miR-4286,miR-181a-2-3p,novel_39,miR-29a-5p,miR-92b-3p,miR-148b-5p,miR-339a,miR-125a-3p,miR-200c,miR-92a-3p,miR-543-5p,novel_96,miR-200a-3p,miR-200c-3p,miR-450a-2-3p,miR-28-3p,miR-329-5p,miR-3591-3p,miR-342-3p,miR-576-3p,novel_1,miR-92a-3p,miR-377-5p,miR-339-5p,miR-411-5p,miR-3431,miR-2284d,miR-197-3p,miR-22-3p,miR-7b-5p,miR-223-3p,miR-873a-5p,miR-708-3p,miR-125b-1-3p,miR-30b-3p,miR-503-3p,miR-539-3p,miR-2284l,miR-377-3p,miR-224-5p,miR-7-5p,miR-466f-3p,miR-483-3p
FAM187B	ENSOARG0(	family w	miR-129-2-3p,miR-4510,miR-214,miR-665-5p,miR-133a-3p,miR-6134,miR-874-5p,miR-30c-1-3p,miR-574-5p,miR-6130,miR-204-3p,miR-2448-3p,miR-129b-3p,miR-133b-3p,miR-329a,miR-1827,miR-216c-5p,miR-181a-2-3p,miR-380-5p,miR-28b,novel_39,miR-490-3p,miR-196a-3p,miR-210-5p,miR-30c-2-3p,miR-760-3p,miR-2484,miR-543-5p,miR-152-3p,novel_127,miR-18a-3p,miR-758-5p,miR-148b-3p,miR-582-3p,novel_83,miR-129-1-3p,miR-214-5p,novel_133,miR-362-3p,miR-124-3p,miR-1343-5p,miR-330-5p,miR-1197-5p,miR-2447,miR-326-3p,novel_82,miR-28c,miR-4726-5p,miR-7977,miR-670-3p,miR-6535,miR-4443,miR-148a-3p,miR-18a-3p,miR-3065-5p,miR-450b-3p,miR-412-3p,miR-1983,miR-380-5p,miR-124a,miR-30c-1-3p,miR-654-3p,miR-326,miR-362-3p,miR-1343-5p,miR-214-3p,miR-455-3p,miR-2355-3p,miR-329-3p,miR-7857,miR-362-3p,miR-2426,miR-744-5p

STK4	ENSOARG0(	serine/t	miR-149-5p,miR-2459,miR-2355-3p,miR-134-3p,miR-185-5p,novel_4,miR-1343-3p,miR-138,miR-450b-5p,miR-653,miR-296-5p,miR-1843b-5p,miR-8485,let-7a-2-3p,miR-30c-1-3p,miR-668-3p,miR-628-5p,miR-380-5p,miR-124a,miR-146b,miR-466f-3p,miR-491-5p,miR-5126,miR-146a,miR-326,miR-3596,miR-499a-3p,miR-378g,miR-411b-3p,novel_69,miR-34b,miR-7977,novel_68,miR-664b,miR-146b-5p,miR-877-3p,miR-18a-3p,miR-326-3p,miR-449a,miR-423-5p,miR-7705,miR-452-5p,novel_101,miR-2447,miR-6123,miR-128-1-5p,miR-34c,miR-151-5p,novel_121,miR-486-5p,miR-2411-5p,miR-212-5p,miR-548e-3p,miR-18a-3p,miR-218-1-3p,let-7a-2-3p,miR-34a-5p,miR-124-3p,miR-23b-5p,miR-330-5p,miR-5703,miR-126-3p,miR-2411,miR-146a-5p,miR-143-3p,miR-664b-3p,miR-380-5p,miR-4286,miR-181a-2-3p,miR-34b-5p,novel_127,novel_96,miR-760-3p,miR-99a-3p,miR-30c-2-3p,miR-185-3p,miR-8117,miR-664-3p,miR-204-3p,miR-151b,miR-34c-5p,miR-499b-3p,miR-6130,miR-99a-3p,miR-30c-1-3p,miR-7144-5p,miR-1248,miR-671-5p,let-7g-3p,let-7g-3p,miR-500a-3p,miR-200a-5p,miR-3154,miR-433-3p,miR-2448-3p,miR-3184-5p,miR-6134,miR-6525,miR-4510,miR-874-5p,miR-500,miR-138-5p,miR-34c-3p,miR-2433,miR-99b-3p
CPQ	ENSOARG0(	carboxyp	miR-93-5p,miR-320a,miR-20b-5p,miR-9-3p,miR-320c,miR-154b-5p,miR-299b-3p,miR-365a-3p,miR-338-5p,miR-3969,miR-382-5p,miR-218-5p,miR-421-5p,miR-708-5p,miR-28-5p,novel_120,miR-3059-5p,miR-145a-3p,miR-9-3p,miR-145-3p,miR-93,miR-361-3p,miR-371b-3p,miR-1434-3p,miR-16-1-3p,miR-4429,miR-16-1-3p,miR-299a-3p,miR-106a,miR-194-3p,miR-20b,miR-539-5p,miR-320b,novel_17,miR-299-3p,miR-320d,miR-106a,miR-3955-3p,miR-106a-5p,miR-2432,miR-1957a,miR-421-5p,miR-22841
ATP6AP1	ENSOARG0(	ATPase,	miR-664a-5p,miR-1260b,novel_42,miR-539-5p,miR-181a-2-3p,miR-3074-5p,miR-4510,miR-214,miR-133a-3p,miR-133b-3p,miR-1248,miR-21a-3p,miR-129b-3p,miR-3154,miR-499b-3p,miR-6130,miR-204-3p,miR-199a-5p,miR-30c-1-3p,miR-370-3p,miR-552-3p,miR-2898,miR-1277-5p,novel_96,miR-1260a,novel_17,miR-30c-2-3p,miR-199a-5p,miR-490-3p,miR-503-5p,miR-3064-5p,miR-133a-3p,miR-1290,miR-1343-5p,miR-1197-5p,miR-5703,miR-505-5p,miR-576-3p,miR-1307-5p,miR-1307-3p,miR-199b-5p,miR-2447,miR-361-3p,miR-199b-5p,miR-3141,miR-499a-3p,miR-6535,miR-3064-5p,miR-3074-5p,miR-7689-3p,miR-3120-3p,miR-491-5p,miR-3085-3p,miR-199b-5p,miR-1343-5p,miR-214-3p,miR-503-5p,miR-7-5p,miR-296-3p,miR-3141,novel_51,miR-30c-1-3p,miR-296-3p,miR-4532,miR-363-5p,miR-1260b,miR-188-5p,miR-744-5p,miR-873a-5p,miR-7b-5p,miR-503-5p,miR-185-5p,miR-194-3p,miR-421,miR-2300a-5p

LHX3	ENSOARGO(	LIM home	miR-3082-5p,miR-378g,miR-378e,miR-454-5p,miR-4792,miR-24-1-5p,miR-1983,miR-361-3p,miR-361-3p,miR-423-5p,miR-541-3p,miR-2447,miR-301a-5p,miR-1298-3p,miR-409b,miR-1343-3p,miR-151a-3p,miR-181b-2-3p,miR-2426,miR-450b-5p,miR-378c,miR-301b-5p,miR-541,miR-6529a,miR-484,miR-378b,miR-30b-3p,miR-30c-1-3p,miR-378d,miR-4532,miR-424-5p,miR-2330-5p,miR-1388-3p,miR-491-5p,novel_103,miR-326,miR-182-5p,miR-16b,miR-6130,miR-323-5p,miR-15b-5p,novel_32,miR-204-3p,miR-22-3p,miR-378a-3p,miR-30c-1-3p,miR-221-5p,miR-296-3p,miR-182-5p,miR-370-5p,novel_99,miR-21a-3p,miR-6525,miR-3184-5p,miR-4510,miR-3184-3p,miR-145a-3p,miR-378c,miR-2330-3p,miR-424-5p,miR-874-5p,miR-151-3p,miR-19b-1-5p,miR-15a-5p,miR-378i,novel_133,miR-378a-3p,miR-1197-5p,miR-181b-1-3p,miR-486-3p,miR-345-3p,miR-221-5p,miR-196a-3p,miR-125b,miR-422a,novel_69,miR-323a-5p,miR-181b-3p,miR-677,miR-6535,miR-3120-3p,miR-412-3p,miR-1291,miR-195a-5p,miR-378f,miR-2387,miR-326-3p,novel_120,miR-1193,miR-2285x,miR-6402,miR-28c,miR-502-5p,miR-1247-3p,miR-15a,miR-16a,miR-134-3p,miR-185-5p,miR-31-5p,miR-26b-3p,miR-138,miR-30b-3p,miR-16b,miR-4508,miR-497-5p,miR-542-3p,miR-7b-5p,miR-19b-2-5p,miR-374b-3p,miR-22-3p,miR-125a-5p,miR-452-5p,miR-1343-5p,miR-7-5p,miR-1271,miR-615-5p,miR-125a,miR-542-5p,miR-370-3p,miR-23a-5p,miR-378c,miR-6128,miR-16-5p,miR-193a-5p,novel_9,miR-138-5p,miR-486b-3p,miR-370-5p,miR-2433,miR-331-3p,miR-125b-5p,miR-758-5p,miR-378h,miR-322-5p,miR-7641,miR-3578,miR-330-5p,miR-1343-5p,miR-28b,miR-24-2-5p,miR-490-3p,miR-2285b,novel_39,miR-543-5p,novel_96,miR-30c-2-3p

THEGL	ENSOARG0(	theg spe	miR-885-5p,miR-363-3p,miR-2447,miR-190b-5p,miR-194b-5p,miR-3968,miR-490-5p,miR-2319b,miR-24-1-5p,miR-7977,miR-4443,miR-504,miR-136-5p,miR-877-3p,miR-493-5p,miR-424-5p,miR-16b,miR-29b-1-5p,miR-105-1,miR-548w,miR-330-3p,novel_78,novel_4,miR-145b,miR-539-5p,miR-142-5p,miR-484,miR-412-3p,miR-3120-5p,miR-21b,miR-224-5p,miR-142a-5p,miR-30e-3p,miR-2285j,miR-655-3p,miR-324-3p,miR-30a-3p,miR-664a,miR-17-3p,miR-424-5p,miR-4492,miR-190a-5p,miR-660-5p,miR-2331-3p,miR-504-5p,miR-323-5p,miR-376e-3p,miR-15b-5p,miR-204-3p,miR-21a-3p,miR-655,miR-433-3p,miR-129b-3p,miR-345-5p,miR-1827,miR-2285w,miR-329a-5p,miR-130a-5p,miR-376c-3p,miR-29b-1-5p,miR-2432,miR-486-3p,miR-202-5p,miR-21-3p,miR-30d-3p,miR-148b-3p,miR-15a-5p,miR-1247-5p,miR-2284h-5p,miR-193b-5p,miR-1434-5p,miR-17-3p,miR-15a,miR-16a,miR-147-5p,miR-656-5p,miR-378b,miR-329-5p,miR-3120-3p,miR-323a-5p,miR-2404,miR-148a-3p,miR-195a-5p,miR-493-5p,miR-6240,miR-668-3p,miR-363-5p,miR-345-5p,miR-224-5p,miR-194a,miR-190a,miR-330-3p,miR-105-5p,miR-660,miR-31-5p,miR-185-5p,miR-3431,miR-194-3p,miR-1843b-5p,miR-145a-5p,miR-194-5p,miR-138,miR-16b,miR-2284x,miR-2889,miR-497-5p,miR-16-5p,miR-138-5p,miR-486b-3p,miR-141-3p,miR-370-3p,miR-2355-5p,miR-376c-3p,miR-301,miR-345-5p,miR-329b,miR-671-5p,miR-485-5p,miR-30a-3p,miR-24-2-5p,miR-125a-3p,miR-17-3p,miR-2898,miR-200a-3p,miR-152-3p,miR-412,miR-322-5p,miR-24-2-5p,novel_121,miR-218-2-3p,miR-582-3p,miR-105-2
FOXN2	ENSOARG0(	forkhead	miR-15a,miR-2284k,miR-135a-5p,novel_87,miR-16a,miR-628-3p,miR-424-5p,miR-2284v,miR-378c,miR-16-5p,miR-378c,novel_27,miR-503-3p,miR-378j,miR-195a-5p,miR-378f,miR-135b-5p,miR-378b,miR-496-3p,miR-503-3p,miR-378a-3p,miR-30c-1-3p,miR-3120-3p,miR-378e,miR-15b-5p,miR-422a,miR-16b,miR-30c-2-3p,miR-216a-5p,miR-760-3p,miR-2284m,miR-424-5p,miR-2284b,miR-30c-1-3p,miR-378d,miR-2284a,miR-193a,miR-378i,miR-378b,miR-378a-3p,miR-378c,miR-2284ab,miR-16b,miR-2284u,miR-188-5p,miR-496,miR-497-5p,miR-2284aa,miR-3600,miR-196a-2-3p,miR-15a-5p,miR-378h,miR-322-5p,miR-2284d,miR-2284z

IL34	ENSOARG0(	interleu	miR-3074-5p,novel_68,miR-669,miR-411b-3p,miR-34b,novel_69,miR-2404,miR-323a-5p,miR-146b-3p,miR-3591-3p,miR-1306,miR-3141,miR-379-5p,miR-1291,miR-1193,miR-2428,miR-449a,miR-382-5p,miR-34c,miR-208a-3p,miR-576-3p,miR-22-5p,miR-128-1-5p,miR-31-5p,miR-340-3p,miR-149-5p,miR-3431,miR-432-5p,miR-197-3p,miR-22-3p,miR-542-3p,miR-432,miR-2889,miR-744-5p,miR-16-1-3p,miR-30b-3p,miR-493-5p,miR-6240,miR-345-5p,miR-432-3p,novel_51,miR-330-3p,miR-365b-5p,miR-3141,miR-197-5p,miR-105-5p,miR-1343-5p,miR-141-3p,miR-1271,miR-615-5p,miR-370-3p,miR-542-5p,miR-2285aa,miR-2355-5p,miR-874-3p,miR-34c-5p,miR-671-5p,miR-345-5p,miR-365a-5p,miR-145-3p,miR-1197-3p,miR-6740-5p,miR-331-3p,miR-370-5p,miR-5100,miR-2433,miR-486b-3p,miR-134-5p,miR-28-5p,novel_9,miR-6517,miR-134-5p,miR-1271-5p,miR-9-5p,miR-1343-5p,miR-134,miR-615-3p,miR-490-3p,miR-1271-3p,miR-3065-3p,miR-200a,novel_39,miR-30c-2-3p,novel_96,miR-200a-3p,miR-450a-2-3p,miR-7977,miR-2319b,miR-4443,miR-3596,miR-129b-5p,miR-361-3p,miR-493-5p,miR-504,miR-96-5p,miR-9-5p,miR-541-3p,miR-3059-5p,miR-4454,miR-2447,miR-7705,miR-423-5p,novel_111,miR-361-3p,miR-615,novel_82,miR-4726-5p,miR-1343-3p,miR-330-3p,miR-2300a-5p,miR-151a-3p,miR-2459,miR-9788-3p,miR-496-5p,miR-30b-3p,miR-541,miR-484,miR-296-5p,miR-3120-5p,miR-1839-5p,miR-2330-5p,miR-1388-3p,miR-193a,miR-30c-1-3p,miR-505,miR-29b-1-5p,miR-1306-5p,miR-491-5p,miR-3065-3p,miR-21-3p,miR-370-5p,miR-504-5p,miR-30c-1-3p,miR-151b,miR-204-3p,miR-22-3p,miR-6130,miR-323-5p,miR-1895,miR-345-5p,let-7g-3p,miR-3074-2-3p,miR-1271,miR-3074-5p,miR-145a-3p,miR-3184-3p,miR-4510,miR-6134,miR-122-5p,miR-3184-5p,miR-6525,miR-665,miR-324-3p,miR-151-3p,miR-708-5p,miR-
NACC2	ENSOARG0(	NACC fam	miR-129-2-3p,miR-3074-5p,miR-590-3p,novel_42,miR-214,miR-665-5p,miR-2433,miR-144,miR-132-5p,miR-331-3p,miR-664a,miR-486b-3p,miR-4492,miR-874-5p,miR-129-5p,miR-874-3p,miR-221-5p,miR-2355-5p,miR-377-3p,miR-300-3p,novel_32,miR-21a-3p,miR-23b,miR-329a,miR-485-5p,miR-1895,miR-625-5p,miR-3064-5p,miR-130a-5p,miR-486-3p,miR-205-5p,miR-185-3p,novel_127,miR-221-5p,miR-3960,miR-18a-3p,miR-2285ad,miR-212-5p,miR-1224-5p,miR-23c,miR-222-5p,miR-23b-3p,miR-505-5p,miR-129-1-3p,miR-532-3p,miR-362-3p,miR-2366,novel_133,miR-1197-5p,miR-1343-5p,miR-300,miR-128-3p,miR-541-3p,miR-342-3p,miR-3059-5p,miR-371b-3p,miR-361-3p,miR-6402,miR-6529b,miR-4324,miR-371a-5p,miR-2285y,miR-128-1-5p,miR-199c,miR-6123,miR-3074-5p,miR-3064-5p,miR-3120-3p,miR-7977,miR-320b,miR-27a-3p,miR-6535,miR-144-3p,miR-361-3p,miR-3065-5p,miR-216a-3p,miR-18a-3p,miR-1291,miR-101b-3p,miR-574-3p,miR-1388-3p,miR-23a-3p,miR-432-3p,miR-4532,miR-8485,miR-377-3p,miR-5126,novel_103,miR-342,novel_51,miR-455-5p,miR-2331-5p,miR-362-3p,miR-3085-3p,miR-214-3p,miR-466f-3p,miR-1343-5p,miR-199b-3p,miR-222-5p,miR-27a-3p,miR-432-5p,miR-541,miR-296-5p,miR-432,miR-329-3p,miR-362-3p,miR-744-5p,miR-27b-3p,miR-2889,miR-455-5p,miR-1839-5p
MBD2	ENSOARG0(	methyl-C	miR-341-3p,miR-542-5p

UBAC1	ENSOARGO(	UBA doma	miR-145-3p,miR-16-5p,miR-134-5p,miR-551b-3p,miR-2433,miR-331-3p,miR-6740-5p,miR-125b-5p,miR-769,miR-874-3p,miR-125a,miR-615-5p,miR-2355-5p,miR-329b,miR-671-5p,miR-3154,miR-1271-3p,miR-3065-3p,miR-181a-2-3p,miR-3064-5p,miR-2898,novel_127,miR-543-5p,novel_96,miR-219-3p,miR-205-5p,miR-339a,miR-30c-2-3p,miR-322-5p,miR-214-5p,miR-486-5p,miR-134-5p,miR-6517,miR-299a-3p,novel_79,miR-134,miR-345-3p,miR-330-5p,miR-2428,miR-326-3p,miR-542-5p,miR-15a,miR-16a,miR-299b-3p,miR-669,miR-378b,novel_68,miR-1291,miR-6395,miR-195a-5p,miR-2387,miR-18a-3p,miR-654-3p,miR-363-5p,miR-378a-5p,miR-125a-5p,miR-377-3p,miR-224-5p,miR-2411-3p,miR-432-5p,miR-2355-3p,miR-149-5p,novel_116,miR-339-5p,miR-16b,miR-2889,miR-497-5p,miR-432,miR-346,miR-224-5p,miR-769-5p,miR-145a-3p,miR-665-5p,miR-214,novel_74,miR-4492,miR-424-5p,miR-665,miR-219b-3p,miR-377-3p,miR-15b-5p,miR-221-5p,miR-2331-3p,miR-30c-1-3p,miR-3065-3p,miR-339b,miR-466i-5p,miR-760-3p,miR-299-3p,miR-221-5p,miR-542-5p,miR-125b,miR-185-3p,miR-324-3p,miR-3601,miR-15a-5p,miR-328-3p,miR-18a-3p,miR-493-3p,miR-378d,miR-133a-3p,miR-218-1-3p,miR-193b-5p,miR-2904,miR-877-3p,miR-361-3p,miR-219a-2-3p,miR-541-3p,miR-4726-5p,miR-2284q,miR-218-5p,miR-301a-5p,miR-769-5p,miR-371a-5p,miR-2483-3p,miR-452-3p,miR-20a-3p,miR-3064-5p,miR-7977,novel_94,miR-544-5p,miR-361-3p,miR-378j,miR-30c-1-3p,miR-2330-5p,miR-424-5p,miR-127-3p,miR-33a-5p,miR-3085-3p,miR-491-5p,miR-214-3p,miR-542-5p,miR-326,miR-16b,novel_4,miR-2426,miR-3120-5p,miR-301b-5p,miR-541,miR-125b-2-3p
MMP13	ENSOARGO(	matrix m	miR-2284z,miR-134-5p,miR-1185-3p,miR-2284d,miR-9-5p,miR-30d-3p,miR-3955-5p,miR-105-1,miR-105-2,miR-16-1-3p,miR-2284aa,miR-362-3p,miR-2284ab,miR-16-1-3p,miR-124-3p,miR-218-2-3p,miR-3071-5p,miR-3591-5p,miR-653,miR-329-3p,miR-134,miR-362-3p,miR-2432,miR-491-3p,miR-654-3p,miR-30a-3p,let-7f-2-3p,let-7f-2-3p,miR-124a,miR-105-3p,miR-466f-3p,miR-2284m,miR-127-5p,miR-99a-3p,miR-105-5p,miR-362-3p,miR-2284n,miR-1185-2-3p,novel_69,miR-99a-3p,miR-2285aa,miR-211-5p,miR-7144-5p,miR-135b-5p,miR-329a,miR-21c,miR-488-3p,miR-211,miR-3957-3p,miR-2284j,miR-9-5p,miR-2284v,miR-22-5p,miR-134-5p,miR-204-5p,novel_9,miR-30a-3p,miR-135a-5p,miR-409-3p,miR-30e-3p,miR-99b-3p
WWTR1	ENSOARGO(	WW domai	miR-615,miR-370-5p,miR-142b,let-7f,novel_87,let-7i,miR-324-3p,miR-451a,miR-486b-3p,miR-134-5p,miR-130b-5p,miR-145a-3p,miR-4510,miR-1193,miR-193b-3p,miR-3607-3p,miR-224-5p,miR-21a-3p,miR-412-3p,miR-1827,miR-1983,miR-2319b,miR-370-5p,miR-2285aa,miR-6130,miR-193b-3p,miR-544-3p,miR-4443,let-7i-5p,novel_103,miR-224-5p,miR-3071-3p,let-7f-5p,miR-98-5p,miR-193a-3p,miR-543-5p,miR-21-3p,miR-130b-5p,miR-192-3p,miR-486-3p,miR-200a,let-7g,miR-134,miR-143-5p,miR-615-3p,miR-544a,miR-5010-3p,let-7g-5p,miR-126a-5p,miR-544b,miR-134-5p,miR-545-5p,miR-582-3p

SF3B3	ENSOARG0(	splicing	miR-206,miR-154-3p,miR-2284q,miR-22-5p,miR-885-5p,miR-181d-5p,miR-452-5p,miR-2387,miR-3141,miR-181a-3p,miR-2319a,miR-2319b,miR-670-3p,miR-2404,miR-25,novel_103,miR-455-5p,miR-3141,miR-330-3p,miR-378a-5p,miR-483-3p,miR-6240,miR-382-3p,miR-574-3p,miR-668-3p,miR-432-3p,miR-539-3p,let-7j,miR-8485,miR-1298-5p,miR-1843b-5p,miR-181a-5p,miR-873a-5p,miR-653,miR-484,miR-1a-3p,miR-450c-3p,miR-197-3p,miR-455-5p,miR-330-3p,miR-2300a-5p,miR-187-3p,miR-665,miR-142-5p,miR-411-5p,miR-338-5p,miR-6740-5p,miR-145a-3p,miR-3184-3p,miR-6128,miR-206-3p,miR-346,miR-142a-5p,miR-181c-5p,miR-181c-3p,miR-301,miR-3154,miR-154b-3p,miR-1827,miR-186-5p,miR-548o-3p,miR-487a-3p,miR-300-3p,miR-154b-5p,miR-216a-5p,miR-500a-5p,miR-181b-5p,miR-2484,miR-30b-3p,miR-487a,miR-1,miR-376a-5p,miR-192-3p,miR-2285b,miR-490-3p,miR-143-3p,novel_39,miR-200a,miR-1246,miR-2366,miR-1197-5p,miR-188-3p,miR-5703,miR-133a-3p,miR-485-3p,miR-483-3p,miR-487a-3p,novel_121,miR-450a-1-3p
SIRT1	ENSOARG0(	sirtuin	miR-29a-3p,miR-135b-5p,miR-216a-3p,miR-3591-3p,miR-199b-5p,novel_27,miR-211,miR-758-5p,miR-29c-3p,miR-135a-2-3p,miR-147-5p,miR-29a,novel_1,miR-382-5p,miR-502-5p,miR-544b,miR-2284d,miR-26a-2-3p,miR-138,miR-29b,miR-2284ab,novel_23,miR-22-3p,miR-542-3p,miR-1285,miR-181a-5p,miR-653,miR-539-3p,miR-2284l,miR-382,miR-6240,miR-380-5p,miR-660,miR-2284n,miR-199a-5p,miR-300-3p,miR-2355-5p,miR-487a-3p,novel_48,miR-23b,miR-2448-3p,miR-301,miR-30e-5p,miR-488-3p,miR-323-3p,miR-2284v,miR-204-5p,miR-138-5p,miR-409-3p,miR-135a-5p,miR-29b-3p,miR-2284k,miR-2284z,miR-758-5p,miR-9-5p,miR-128-3p,miR-155-5p,miR-30a-5p,miR-29d-3p,miR-3591-5p,miR-199a-5p,miR-380-5p,miR-29b-2-5p,miR-181b-5p,miR-450a-2-3p,miR-30f,miR-203-3p,miR-2483-3p,miR-7857-3p,miR-499a-3p,miR-211-5p,miR-20a-3p,miR-544-5p,miR-129b-5p,miR-199b-5p,miR-9-5p,miR-181d-5p,miR-2284j,miR-154-3p,miR-126a-5p,miR-2284aa,miR-16-2-3p,miR-544a,miR-2426,miR-450b-5p,miR-125b-2-3p,miR-143-5p,miR-3187-3p,miR-23a-3p,miR-494-3p,miR-199b-5p,miR-29b-1-5p,miR-155-5p,miR-22-3p,miR-656-3p,miR-544-3p,miR-499b-3p,miR-552-3p,miR-21-3p,miR-154a-3p,miR-548o-3p,miR-660-5p,miR-625-5p,miR-543-3p,miR-154b-3p,miR-181c-5p,miR-6525,miR-665-5p,miR-155-5p,miR-29b-2-5p,miR-532-3p,miR-340-5p,miR-23c,miR-487a-3p,miR-23b-3p,miR-485-3p,miR-380-5p,miR-664-5p,miR-142-3p,miR-140-3p,miR-188-3p,miR-29b-1-5p,miR-421-5p,miR-487a,miR-216c-5p,miR-3653-3p,miR-2284m,miR-196a-3p

STARD6	ENSOARG0(	StAR-rel	miR-217-5p,miR-193b-5p,miR-16-1-3p,miR-184-3p,miR-30d-3p,miR-106a,miR-7859,miR-210-5p,miR-26a-5p,miR-202-5p,miR-503-5p,miR-486-3p,miR-376a-5p,miR-411,miR-433-3p,miR-1827,miR-543-3p,miR-339b,miR-552-3p,miR-548o-3p,miR-15b-5p,miR-582-5p,miR-377-3p,miR-10a,miR-665,miR-30a-3p,miR-30e-3p,miR-2285j,miR-130b-5p,miR-129-5p,miR-451a,miR-3074-5p,miR-3184-3p,miR-769-5p,miR-758-3p,miR-541,miR-329-3p,miR-362-3p,miR-1343-3p,miR-330-3p,miR-1185-3p,miR-455-3p,miR-147a,miR-153,miR-16b,miR-103a-2-5p,miR-153-3p,miR-146b,miR-101b-3p,miR-124a,miR-1b-5p,miR-378j,miR-221,miR-544-5p,miR-136-5p,miR-211-5p,miR-4792,miR-139-5p,miR-1185-2-3p,miR-4443,miR-203-3p,miR-25,miR-218-5p,miR-3968,miR-3956-3p,miR-2447,miR-877-3p,miR-1343-5p,miR-155-5p,novel_79,miR-429-3p,miR-2285ad,miR-322-5p,miR-3071-3p,miR-30f,miR-205-5p,miR-200a-3p,miR-3064-5p,miR-29a-5p,miR-148b-5p,miR-1248,miR-20b-5p,miR-329a,miR-2318,novel_9,miR-16-5p,novel_42,miR-2113,miR-27b-3p,miR-497-5p,miR-16-1-3p,miR-16b,miR-339-5p,miR-31-5p,miR-194a,miR-146a,miR-377-3p,miR-378a-5p,miR-628-5p,let-7f-2-3p,let-7f-2-3p,miR-668-3p,miR-6240,miR-146b-5p,miR-2319a,miR-26b-5p,miR-3074-5p,miR-144-3p,miR-374c-3p,miR-576-3p,miR-28c,miR-140-3p,miR-1197-5p,miR-5703,miR-124-3p,miR-2904,miR-362-3p,miR-2300b-3p,miR-3600,miR-548e-3p,novel_83,miR-15a-5p,miR-222-5p,miR-383-5p,miR-185-3p,miR-374a-5p,miR-466i-5p,miR-106a,miR-20a,miR-183-5p,miR-146a-5p,novel_99,miR-582-5p,miR-370-5p,miR-140-5p,miR-155-5p,miR-204-3p,miR-323-5p,miR-139-5p,miR-544-3p,miR-320e,miR-338-5p,miR-324-3p,miR-222-3p,miR-208b-5p,miR-708-5p,miR-424-5p,miR-17-3p,miR-6516-3p,miR-155-5p,miR-539-5p,miR-10b-5p,miR-30b-3p,miR-10b,miR-484,miR-101a-5p,miR-450b-5p,miR-544a,miR-
CTDSP2	ENSOARG0(	CTD (car	miR-432-3p,miR-664b-3p,miR-155-5p,miR-2319b,miR-124a,miR-214,miR-155-5p,miR-214-3p,miR-128-3p,miR-30b-3p,miR-155-5p,miR-3071-5p,miR-216a-3p,miR-132-5p,miR-124-3p
SYT11	ENSOARG0(	synaptot	miR-7144-5p,miR-7975,miR-877-3p,miR-33b-3p,miR-329b,miR-5703,miR-345-3p,miR-154b-5p,miR-300-3p,miR-483-3p,miR-142-3p,miR-541-5p,miR-329-5p,miR-490-5p,miR-3958-3p,miR-483-3p,miR-664a,miR-2411-3p,miR-345-3p,novel_17,miR-8095,miR-2285b,miR-2428,miR-130a-5p,miR-142a-3p,miR-2285x,miR-329a-5p

TTYH3	ENSOARG0(	tweety f	miR-24-3p,novel_27,miR-449a,miR-423-5p,miR-541-3p,let-7i-3p,miR-3957-3p,miR-4726-5p,miR-2440,miR-28c,miR-576-3p,miR-769-5p,miR-34c,miR-34b,miR-4443,miR-320b,miR-504,miR-96-5p,miR-1291,miR-6395,miR-335-3p,miR-9851-3p,miR-3141,miR-150-5p,miR-1306,miR-668-3p,miR-25-5p,miR-124-5p,miR-214-3p,miR-125a-5p,miR-1343-5p,miR-1306-5p,miR-5126,miR-3141,novel_19,miR-496-5p,miR-2459,miR-194-3p,miR-744-5p,miR-541,miR-296-5p,miR-1197-3p,miR-769-5p,miR-3184-5p,miR-4510,miR-129-2-3p,miR-214,miR-1271,miR-28-5p,miR-486b-3p,miR-708-5p,miR-665,miR-331-3p,miR-125b-5p,miR-132-5p,miR-6130,miR-769,miR-132-5p,miR-34c-5p,miR-27a-5p,miR-504-5p,miR-2331-3p,miR-1271,miR-125a,miR-671-5p,miR-885-3p,miR-129b-3p,miR-28b,miR-2411,miR-486-3p,novel_39,miR-466i-5p,miR-127-5p,miR-191-3p,miR-34b-5p,miR-125b,miR-122-3p,miR-324-3p,miR-2411-5p,miR-1271-5p,miR-302a-5p,miR-328-3p,miR-129-1-3p,miR-532-3p,miR-6517,miR-1224-5p,miR-184-3p,miR-885-3p,miR-34a-5p,miR-5703,miR-188-3p,miR-140-3p,miR-1343-5p,miR-2904
ANKRA2	ENSOARG0(	ankyrin	miR-338-3p,miR-3958-5p,miR-214,miR-4510,miR-130b-5p,miR-450b-3p,miR-664a,miR-487b-5p,miR-17-3p,miR-665,miR-29b-2-5p,miR-2285j,miR-376e-3p,miR-6516,miR-323-5p,miR-193b-3p,miR-216b-5p,miR-6130,miR-139-5p,miR-154a-3p,miR-296-3p,miR-552-3p,miR-221-5p,miR-140-5p,miR-545-3p,miR-504-5p,novel_99,miR-1827,miR-154b-3p,miR-30f,miR-30d-5p,miR-21a-3p,miR-2285c,miR-450a-1-3p,miR-2424,miR-377-5p,miR-376c-3p,miR-345-3p,miR-487a,miR-34b-5p,miR-30d,miR-221-5p,miR-299-3p,miR-760-3p,miR-92a-1-5p,miR-30b,novel_83,miR-616-3p,miR-328-3p,miR-487a-3p,miR-340-5p,miR-30d-3p,miR-485-3p,miR-218-1-3p,miR-34a-5p,miR-30c-5p,miR-1246,miR-8095,miR-452-5p,miR-212-3p,miR-490-5p,miR-154-3p,miR-2285y,miR-30b-5p,miR-2440,miR-4443,miR-3596,miR-378g,miR-2285u,miR-541-5p,miR-7977,miR-664b,miR-139-5p,miR-20a-3p,miR-2319b,miR-544-5p,miR-504,miR-378j,miR-140-5p,miR-8485,miR-3187-3p,miR-193a,miR-744-3p,miR-652-5p,miR-2330-5p,miR-214-3p,miR-487b-5p,miR-3955-3p,miR-505,miR-216a-3p,miR-2459,miR-2285e,miR-3120-5p,miR-1388-5p,miR-450b-5p,miR-145-3p,miR-1260b,miR-374a-3p,miR-6128,miR-134-5p,miR-374b-3p,miR-135a-5p,miR-135a-1-3p,miR-5100,miR-154b-5p,miR-376c-3p,miR-34c-5p,miR-300-3p,miR-331-5p,miR-487a-3p,miR-874-3p,miR-7144-5p,miR-329b,miR-3154,miR-381-3p,miR-30e-5p,miR-2448-3p,miR-29a-5p,miR-192-3p,miR-2284a,miR-1271-3p,miR-2285b,miR-380-5p,miR-29b-2-5p,miR-17-3p,miR-191-3p,miR-1260a,miR-664-3p,miR-6119-5p,miR-545-5p,miR-134-5p,miR-450a-1-3p,novel_121,miR-486-5p,miR-6516-5p,miR-2285ad,miR-299a-3p,miR-30c,miR-300,miR-2427,miR-218-2-3p,miR-30a-5p,miR-134,miR-449a,miR-371b-3p,miR-193b-3p,miR-17-3p,miR-495-3p,miR-2285x,miR-34c,miR-2404,miR-323a-5p,miR-299b-3p,novel_69,miR-34b,miR-3120-3p,novel_68,miR-135b-5p,miR-

NRD1	ENSOARG0(	nardilys	miR-196a-5p,miR-2284r,miR-148a-3p,miR-378j,miR-3962,miR-129b-5p,miR-216a-3p,miR-19a-3p,miR-19b-3p,miR-128-3p,miR-671-5p,miR-130a-3p,miR-301a-3p,miR-3431,miR-142-3p,miR-148b-3p,miR-133c,miR-199a-3p,miR-199a-3p,miR-100-3p,miR-485-3p,miR-17-3p,miR-196b-5p,miR-130b-3p,miR-17-3p,miR-454-3p,miR-208b-5p,miR-152-3p,miR-17-3p,miR-142a-3p,miR-301b-3p
TM4SF18	ENSOARG0(	transmem	miR-382,miR-3529-3p,miR-4510,miR-3187-3p,miR-2284y,miR-664b-3p,miR-382-5p,miR-219b-5p,miR-500a-5p,miR-142b,miR-421-5p,miR-452-5p,miR-378a-5p,miR-21-3p,miR-1224-5p,miR-3600,miR-2355-3p,miR-203-3p,miR-323-5p,miR-6130,miR-582-5p,miR-323a-5p,miR-3065-5p,miR-5703,miR-3591-3p,miR-877-3p,miR-186-5p,miR-582-5p,miR-2284u,miR-543-3p
FGF14	ENSOARG0(	fibrobla	novel_9,novel_1,miR-2285j,miR-135a-5p,miR-1307-3p,miR-145-3p,let-7g-3p,miR-10b-3p,let-7g-3p,miR-136-5p,miR-2319a,miR-1814c,miR-376a-2-5p,miR-1248,miR-135b-5p,miR-598-3p,miR-433-3p,miR-25,miR-133c,miR-2319b,miR-598-3p,miR-760-3p,miR-2285af,miR-29b-1-5p,miR-197-5p,miR-29b-1-5p,miR-376a-5p,let-7a-2-3p,miR-2310,miR-2887,let-7a-2-3p,miR-484,miR-218-2-3p,novel_78
ETV2	ENSOARG0(	ets vari	miR-199b-5p,miR-29c-3p,miR-6119-3p,miR-379-3p,miR-29a,miR-320c,miR-411b-3p,novel_68,miR-3074-5p,miR-146b-5p,miR-378f,miR-150-5p,miR-1306,miR-3065-5p,miR-3591-3p,miR-503-3p,miR-2285r,miR-432-3p,miR-2284l,miR-432-3p,miR-668-3p,let-7f-2-3p,let-7f-2-3p,miR-6240,miR-378a-5p,novel_51,miR-2411-3p,miR-146a,miR-194-3p,miR-149-5p,miR-411-5p,miR-134-3p,miR-3431,miR-339-5p,miR-3955-5p,miR-103a-3p,miR-497-5p,miR-2284x,miR-16b,miR-16-1-3p,miR-450c-3p,miR-22-3p,miR-197-3p,novel_23,miR-432,miR-223-3p,miR-193a-5p,miR-16-5p,miR-2284v,miR-134-5p,miR-100-3p,miR-2284k,miR-2433,miR-133c,miR-25-3p,miR-2355-5p,miR-2285aa,miR-1248,miR-345-5p,miR-424-3p,miR-301,novel_39,miR-2285b,miR-450b-5p,miR-148b-5p,miR-92b-3p,novel_127,miR-219-3p,miR-450a-1-3p,miR-2284z,miR-134-5p,miR-322-5p,miR-758-5p,novel_79,miR-18b-5p,miR-2285ad,miR-330-5p,miR-1285-5p,miR-423-5p,miR-877-3p,miR-2284j,miR-2447,miR-3956-3p,miR-363-3p,miR-3968,novel_82,miR-2440,miR-1185-2-3p,miR-378g,miR-25,miR-139-5p,miR-9-3p,miR-504,miR-378j,miR-1306-3p,miR-1b-5p,miR-378d,miR-744-3p,miR-124a,miR-146b,miR-199b-5p,miR-214-3p,miR-103a-2-5p,miR-29b-1-5p,miR-323b-3p,miR-16b,miR-199a-3p,miR-455-3p,miR-1185-3p,miR-539-5p,miR-147a,miR-199b-3p,novel_78,miR-330-3p,miR-1388-5p,miR-323b,miR-2284r,miR-3184-5p,miR-224-5p,miR-214,miR-665-5p,miR-3074-5p,miR-3184-3p,miR-4492,miR-129-5p,miR-130b-5p,miR-219b-3p,miR-15b-5p,miR-10a,miR-582-5p,miR-296-3p,miR-2331-3p,miR-504-5p,miR-186-5p,miR-339b,miR-345-5p,miR-129b-3p,miR-411,miR-450a-1-3p,miR-664b-3p,miR-2484,miR-202-5p,miR-324-3p,miR-422a,miR-345-3p,miR-92a-1-5p,novel_17,miR-210-3p,miR-23b-3p,miR-23c,miR-212-5p,miR-18a-3p,miR-16-1-3p,miR-664-5p,miR-133a-3p,miR-378a-

COG4	ENSOARG0(	componen	miR-328-3p,miR-505-5p,miR-378g,miR-134-3p,miR-6395,miR-1260b,miR-2300b-3p,miR-2387,miR-200a,miR-1260b,miR-432-3p,miR-1839-3p,miR-214,miR-1839-3p,miR-214-3p,miR-664a,miR-2285v,miR-3969,miR-1260a,miR-4324,novel_17
BTF3L4	ENSOARG0(	basic tr	miR-2285ad,miR-429-3p,miR-431,miR-24-2-5p,miR-758-5p,miR-378h,miR-582-3p,miR-2284z,miR-345-3p,miR-330-5p,let-7d,miR-200b-3p,miR-2385-3p,miR-29b-2-5p,miR-24-2-5p,miR-380-5p,let-7g,miR-29a-5p,miR-2285af,miR-2397-5p,miR-200c,let-7c-5p,miR-28-3p,let-7f-5p,miR-200c-3p,miR-370-3p,miR-2285aa,miR-769,miR-2284n,miR-488-3p,miR-2448-3p,miR-331-5p,miR-93-5p,miR-200a-5p,miR-17-5p,miR-329a,miR-20b-5p,miR-3074-1-3p,miR-28a-3p,let-7e-3p,miR-2284v,miR-3607-3p,miR-93,miR-378c,miR-2284k,miR-100-3p,miR-138-5p,miR-331-5p,miR-410-3p,miR-204-5p,miR-187-3p,miR-130a-3p,let-7g-5p,miR-185-5p,novel_116,miR-20b,miR-873a-5p,miR-2113,miR-1434-3p,miR-194-5p,miR-3071-5p,miR-145a-5p,miR-30b-3p,miR-138,miR-2284ab,miR-380-5p,miR-6240,miR-382,miR-432-3p,miR-2284l,miR-503-3p,miR-190a,miR-105-5p,miR-330-3p,miR-194a,miR-17-5p,miR-106a-5p,miR-2411-3p,miR-466f-3p,novel_68,miR-19a-3p,miR-505-3p,let-7e-5p,miR-378f,miR-335-5p,novel_101,miR-211,novel_27,miR-326-3p,miR-301b-3p,miR-20a-5p,let-7i,miR-382-5p,novel_1,miR-548e-3p,miR-106a,miR-505-3p,miR-7859,miR-200b,miR-2284h-5p,miR-378i,miR-362-3p,miR-1961,miR-5703,miR-378a-3p,miR-19b-3p,miR-361-5p,miR-216c-5p,miR-2285w,miR-20a,miR-29b-1-5p,miR-422a,miR-345-3p,miR-106a,miR-2284m,miR-98-5p,let-7k,miR-378a-3p,miR-301a-3p,miR-877-5p,let-7i-5p,miR-21a-3p,miR-129b-3p,miR-500a-3p,miR-186-5p,miR-378c,let-7b,miR-769-5p,miR-6525,let-7b-5p,let-7f,miR-2285j,miR-29b-2-5p,miR-665,miR-664a,miR-208b-5p,miR-500,miR-190a-5p,miR-454-3p,miR-129-5p,miR-669a-3p,miR-548w,miR-105-1,miR-1343-3p,miR-330-3p,miR-145b,miR-9788-3p,miR-329-3p,miR-6529a,miR-378b,miR-30b-3p,miR-378c,miR-362-3p,miR-2426,miR-101c,miR-2284aa,let-7a-5p,miR-494-3p,miR-106a-
KLHDC10	ENSOARG0(	kelch do	miR-141-3p,miR-3120-3p,miR-7857-3p,miR-185-5p,miR-6130,miR-455-3p,miR-21a-3p,miR-378j,miR-3154,miR-7144-5p,miR-1248,miR-212-3p,miR-4510,miR-2432,novel_27,miR-486-3p,miR-142b,miR-324-3p,miR-3604,miR-486b-3p,miR-200a-3p
TRPT1	ENSOARG0(	tRNA pho	miR-504,miR-1271,miR-125a,miR-3065-3p,miR-320b,miR-3074-5p,miR-660-5p,miR-504-5p,miR-3064-5p,miR-301a-5p,miR-125b-5p,miR-324-3p,miR-3074-5p,miR-541-3p,miR-1306-5p,miR-3071-5p,miR-197-3p,miR-7b-5p,miR-541,miR-301b-5p,miR-5010-3p,novel_4,miR-6517,miR-660,miR-125a-5p,miR-3085-3p,miR-125b,miR-29b-1-5p,miR-324-3p,miR-7-5p,novel_39,miR-3065-3p,miR-664b-3p,miR-29b-1-5p,miR-3064-5p,miR-668-3p,miR-382-3p
RAB7B	ENSOARG0(	RAB7B, m	miR-185-3p,miR-2904,miR-5126,miR-378d,miR-744-5p,miR-4508,miR-4492,miR-3085-3p,miR-615-5p,miR-3064-5p,miR-3064-5p,miR-1307-3p

TMEM171	ENSOARG0(	transmem	miR-2285ad,miR-429-3p,miR-758-5p,miR-2284z,miR-450a-1-3p,novel_121,miR-3591-5p,miR-885-3p,miR-29a-5p,novel_39,miR-2284y,miR-2285b,miR-30f,miR-127-5p,novel_96,miR-503-3p,miR-2285aa,miR-300-3p,miR-3959-5p,miR-133c,miR-200a-5p,miR-23b,miR-329a,miR-1248,miR-2284v,miR-1260b,miR-1197-3p,miR-2284k,miR-6740-5p,novel_9,miR-103a-3p,miR-411-5p,miR-194-3p,miR-1843b-5p,miR-450c-3p,miR-380-5p,miR-493-5p,miR-432-3p,miR-432-3p,miR-146a,miR-194a,miR-2411-3p,miR-483-3p,miR-378a-5p,miR-411b-3p,miR-144-3p,miR-320c,miR-150-5p,miR-1306,miR-3591-3p,miR-146b-5p,miR-199b-5p,miR-301b-3p,miR-142b,miR-576-3p,miR-6123,miR-30d-3p,miR-23b-3p,miR-23c,miR-2284h-5p,miR-193b-5p,miR-98-3p,miR-1434-5p,miR-493-3p,miR-2284u,miR-2285w,miR-664b-3p,miR-486-3p,miR-2285p,miR-210-5p,miR-618,miR-324-3p,miR-219b-5p,miR-345-3p,miR-202-5p,miR-504-5p,miR-2331-3p,miR-30c-1-3p,miR-301a-3p,miR-10a,miR-450a-1-3p,miR-2285c,miR-655,miR-885-3p,miR-625-5p,miR-186-5p,miR-98-3p,miR-3184-5p,let-7b-3p,miR-30a-3p,novel_74,miR-454-3p,miR-4492,miR-548w,miR-455-3p,miR-2459,miR-329-3p,miR-362-3p,miR-1388-5p,miR-101c,miR-124a,miR-101b-3p,miR-23a-3p,miR-2284b,miR-1b-5p,miR-29b-1-5p,miR-323b-3p,miR-1185-5p,miR-491-5p,miR-130b-3p,miR-199b-5p,miR-146b,miR-139-5p,miR-2284g,miR-4792,miR-211-5p,miR-2483-3p,miR-504,miR-493-5p,miR-3956-3p,miR-2284j,miR-423-5p,miR-7705,miR-222,miR-376a-5p,miR-483-3p,miR-345-3p,miR-3578,miR-200b-3p,miR-496,miR-10a-5p,miR-30a-3p,miR-29b-2-5p,miR-181a-2-3p,miR-380-5p,miR-199a-5p,miR-2284a,miR-2285af,miR-1260a,miR-200c,miR-6119-5p,miR-30c-2-3p,miR-200c-3p,miR-107,miR-370-3p,miR-215-5p,miR-199a-5p,miR-2284n,miR-2448-3p,miR-3154,miR-320a,novel_48,miR-135a-1-3p,miR-144,miR-138-5p,miR-204-
RUVBL1	ENSOARG0(	RuvB-lik	miR-340-3p,novel_78,miR-330-3p,miR-2300a-5p,miR-1343-3p,novel_116,miR-503-5p,miR-134-3p,miR-30b-3p,miR-329-3p,miR-296-5p,miR-7862,miR-362-3p,miR-30b-3p,miR-1388-3p,miR-6240,miR-4532,let-7a-2-3p,miR-330-3p,novel_19,miR-362-3p,novel_51,miR-503-5p,miR-320b,miR-5126,miR-125a-5p,miR-3085-3p,miR-2285u,miR-3120-3p,miR-3064-5p,miR-4792,miR-320c,miR-323a-5p,miR-378g,miR-411b-3p,miR-150-5p,miR-3591-3p,miR-18b,miR-148a-3p,miR-7975,miR-504,miR-423-5p,miR-2428,miR-24-3p,miR-2285y,miR-4324,miR-2285n,miR-1247-3p,miR-128-1-5p,miR-18b-5p,miR-2285ad,miR-3960,miR-2284h-5p,miR-505-5p,miR-148b-3p,miR-2904,miR-7134-5p,novel_133,miR-362-3p,let-7a-2-3p,miR-941,miR-4429,miR-3064-5p,miR-2887,miR-4286,miR-2285w,miR-503-5p,miR-486-3p,miR-3065-3p,miR-2432,miR-1271-3p,miR-125b,miR-185-3p,miR-30b-3p,miR-152-3p,miR-320d,miR-3065-3p,miR-598-3p,miR-21-3p,miR-125a,miR-504-5p,miR-27a-5p,miR-323-5p,miR-598-3p,miR-320a,miR-18a,miR-21a-3p,miR-488-3p,miR-671-5p,let-7g-3p,miR-329a,miR-18a-5p,miR-433-5p,miR-3184-3p,miR-3184-5p,miR-6525,miR-346,miR-1197-3p,miR-125b-5p,miR-665,miR-2433,miR-4492,miR-486b-3p

NF2	ENSOARG0(	neurofib	miR-30d-5p,miR-3141,miR-671-5p,miR-30f,miR-211-5p,miR-370-5p,miR-30c-1-3p,miR-3064-5p,miR-4792,miR-669,miR-331-3p,miR-151-3p,miR-142b,miR-370-5p,miR-30b-5p,miR-204-5p,miR-4492,miR-1193,miR-3607-3p,miR-24-3p,miR-211,miR-142a-5p,miR-877-3p,miR-217-5p,miR-7b-5p,miR-1285,miR-542-3p,miR-30c-5p,miR-548w,miR-151a-3p,miR-194-3p,miR-142-5p,miR-217,miR-3141,miR-219b-5p,miR-30c-2-3p,miR-210-5p,miR-7-5p,miR-205-5p,miR-30d,miR-3085-3p,miR-3064-5p,miR-1388-3p,miR-29a-5p,miR-30c-1-3p
KMT2B	ENSOARG0(	lysine (	miR-484,miR-296-5p,miR-30b-3p,miR-450b-5p,miR-544a,miR-2426,miR-2300a-5p,miR-216a-3p,miR-9788-3p,miR-1306-5p,miR-5126,miR-326,miR-362-3p,miR-3085-3p,miR-25-5p,miR-2330-5p,miR-106a-5p,let-7c-3p,miR-30c-1-3p,miR-361-3p,miR-199b-5p,novel_94,miR-877-3p,miR-3064-5p,miR-20a-3p,miR-499b-5p,miR-489,miR-454-5p,miR-2285n,miR-769-5p,miR-2285y,miR-107-5p,miR-4726-5p,miR-2285t,miR-2284q,miR-541-3p,miR-502-3p,miR-154a,miR-126-3p,novel_133,miR-362-3p,miR-376a-5p,miR-124-3p,miR-1197-5p,miR-19b-3p,miR-378d,miR-3960,miR-148b-3p,miR-532-3p,miR-199a-3p,miR-185-3p,miR-125b,miR-106a,miR-760-3p,miR-466i-5p,miR-34b-5p,miR-30b-3p,miR-221-5p,miR-21-3p,miR-20a,miR-29b-1-5p,miR-146a-5p,miR-21a-3p,miR-18a-5p,miR-140-5p,miR-221-5p,miR-660-5p,miR-370-5p,miR-544-3p,miR-6130,miR-193b-3p,novel_25,miR-19b-1-5p,miR-324-3p,miR-29b-2-5p,miR-338-5p,miR-6516-3p,miR-664a,miR-708-5p,miR-145a-3p,miR-4510,miR-1271,miR-3074-2-3p,miR-206-3p,miR-6134,miR-1434-3p,miR-1285,miR-145a-5p,miR-30b-3p,miR-1260b,miR-744-5p,miR-130a-3p,miR-26b-3p,miR-503-5p,miR-544b,miR-20b,miR-224-5p,miR-503-5p,miR-197-5p,miR-106a-5p,miR-17-5p,miR-124-5p,miR-452-5p,miR-125a-5p,miR-660,miR-1343-5p,miR-363-5p,miR-296-3p,miR-345-5p,miR-18b,miR-18a-3p,miR-1291,miR-19a-3p,miR-7975,miR-9851-3p,miR-378b,miR-320b,miR-2404,miR-670-3p,miR-34b,miR-20a-5p,miR-34c,miR-33a-3p,miR-502b,miR-199c,miR-135a-2-3p,novel_101,miR-1193,miR-24-3p,novel_27,miR-449a,miR-326-3p,miR-2428,miR-345-3p,miR-3578,miR-501-3p,miR-411-3p,miR-7641,miR-1271-5p,miR-483-3p,miR-214-5p,miR-1260a,miR-30c-2-3p,miR-2898,miR-543-5p,miR-29b-2-5p,miR-1,miR-495-5p,miR-181a-2-3p,miR-380-5p,miR-28b,miR-24-2-5p,miR-2478,miR-199a-5p,miR-490-3p,miR-1271-3p,miR-488-

CCDC68	ENSOARG0(	coiled-c	miR-[illegible],miR-[illegible],miR-[illegible],miR-20a,miR-[illegible]5p,miR-2285w,miR-1277-5p,miR-221-5p,miR-106a,miR-320d,miR-466i-5p,miR-219b-5p,miR-92a-1-5p,miR-345-3p,miR-2285p,miR-2284h-5p,miR-148b-3p,miR-106a,miR-16-1-3p,miR-361-5p,miR-4429,miR-2904,miR-5703,miR-362-3p,miR-215-5p,miR-338-3p,miR-224-5p,miR-425-5p,miR-665-5p,miR-539-5p,miR-145a-3p,miR-3074-5p,miR-708-5p,miR-454-3p,miR-190a-5p,novel_74,miR-450b-3p,miR-6516-3p,miR-151-3p,miR-219b-3p,miR-324-3p,miR-301a-3p,miR-376e-3p,miR-192-5p,miR-377-3p,miR-139-5p,miR-216b-3p,miR-532-5p,miR-182-5p,miR-221-5p,miR-30c-1-3p,miR-625-5p,let-7g-3p,miR-433-3p,miR-21a-3p,miR-450a-1-3p,miR-8485,miR-30c-1-3p,miR-106a-5p,miR-494-3p,miR-101b-3p,miR-130b-3p,miR-362-3p,miR-2957,miR-106b-5p,miR-182-5p,miR-320b,miR-342,novel_115,miR-9788-3p,miR-148b-5p,miR-455-3p,miR-27a-3p,miR-151a-3p,miR-105-1,miR-101c,miR-1839-5p,miR-16-2-3p,miR-362-3p,miR-30b-3p,miR-329-3p,miR-6529a,miR-9-3p,miR-8095,miR-219a-2-3p,miR-361-3p,miR-452-5p,miR-190b-5p,miR-490-5p,miR-2285t,miR-628-3p,miR-3968,miR-218-5p,miR-101-3p,miR-27a-3p,miR-378g,miR-2483-3p,miR-541-5p,miR-139-5p,miR-2319b,miR-452-3p,miR-493-5p,miR-9-3p,novel_94,miR-136-5p,novel_39,miR-200a,miR-3529-3p,miR-200a-3p,miR-152-3p,miR-127-5p,miR-200c-3p,miR-3071-3p,miR-30c-2-3p,miR-2285af,miR-200c,miR-125a-3p,miR-219-3p,miR-450a-1-3p,miR-134-5p,novel_121,miR-486-5p,miR-758-5p,miR-429-3p,miR-105-2,miR-29d-3p,miR-345-3p,miR-134,miR-93,miR-103b,miR-410-3p,miR-134-5p,miR-28-5p,miR-144,miR-331-3p,miR-29b-3p,miR-2332,miR-5100,miR-376c-3p,miR-141-3p,miR-215-5p,miR-574-5p,miR-20b-5p,miR-7144-5p,miR-10b-3p,miR-329a,miR-93-5p,miR-200a-5p,miR-320a,miR-17-5p,miR-3154,miR-488-3p,let-7j,miR-296-3p,miR-

NPAS4	ENSOARG0(	neuronal	miR-3074-5p,miR-378b,miR-10a-5p,miR-655-5p,miR-299b-3p,miR-374c-3p,miR-29a-3p,miR-146b-3p,miR-669,miR-320c,miR-3141,miR-378b,miR-18a-3p,miR-1306,miR-378f,miR-146b-5p,miR-2319a,miR-335-5p,miR-219b-3p,novel_101,miR-542-5p,miR-29c-3p,miR-193b-3p,miR-24-3p,miR-2284w,miR-326-3p,miR-374c-5p,miR-206,novel_1,miR-29a,miR-203b-5p,miR-340-3p,miR-412-5p,miR-432-5p,miR-2355-3p,novel_116,miR-1434-3p,miR-1843b-5p,miR-432,novel_23,miR-1260b,miR-29b,miR-30b-3p,miR-380-3p,miR-744-5p,miR-6240,miR-628-5p,miR-363-5p,miR-2284l,miR-2285r,miR-345-5p,miR-146a,miR-224-5p,novel_51,miR-3141,miR-330-3p,miR-124-5p,miR-378a-5p,miR-193a-3p,miR-452-5p,miR-542-5p,miR-2285aa,miR-223-5p,miR-301,miR-362-5p,miR-3154,miR-320a,miR-345-5p,miR-329b,miR-1248,miR-7144-5p,miR-193a-5p,miR-6128,miR-1260b,miR-500b-5p,miR-378c,miR-5100,miR-29b-3p,novel_63,miR-331-3p,miR-100-3p,miR-6740-5p,miR-486b-3p,miR-6517,miR-299a-3p,miR-6516-5p,miR-378h,novel_121,miR-486-5p,miR-2284z,miR-330-5p,miR-29d-3p,miR-885-3p,miR-10a-5p,miR-4286,miR-1,miR-30a-3p,miR-3956-5p,miR-1271-3p,miR-3065-3p,novel_39,miR-29a-5p,miR-2285af,miR-1260a,miR-127-5p,miR-1a-2-5p,miR-2898,miR-543-5p,novel_127,miR-10b,miR-2319b,miR-139-5p,miR-7977,miR-499b-5p,miR-3082-5p,miR-378e,miR-3596,miR-409-5p,miR-361-3p,miR-504,miR-136-5p,miR-877-3p,miR-335-3p,miR-1296-5p,miR-361-3p,miR-362-5p,miR-371a-5p,miR-335,miR-4726-5p,miR-3968,miR-2285t,miR-548w,novel_4,miR-330-3p,miR-1298-3p,miR-496-5p,miR-500-5p,miR-10b,miR-2284r,miR-378b,miR-30b-3p,miR-758-3p,miR-323b,miR-1a-3p,miR-450b-5p,miR-378c,miR-2284aa,miR-2330-5p,miR-1388-3p,miR-23a-3p,miR-378d,miR-758-3p,miR-193a,miR-4532,miR-326,miR-1306-5p,miR-320b,miR-182-
TTC21B	ENSOARG0(	tetratri	miR-496-3p,miR-503-3p,miR-376a-5p,miR-10b-3p,miR-302a-5p,miR-3082-5p,miR-496

NBR1	ENSOARG0(	neighbor	miR-16a,miR-2284o,miR-382-5p,miR-15a,miR-4324,miR-576-3p,miR-6402,miR-142b,miR-2428,miR-2284w,miR-323c,miR-371b-3p,miR-193b-3p,miR-2285x,miR-1193,miR-342-3p,miR-135a-2-3p,miR-2319a,miR-195a-5p,miR-412-3p,miR-2284e,miR-3591-3p,miR-18a-3p,miR-2387,miR-148a-3p,miR-677,miR-181b-3p,miR-655-5p,miR-320b,miR-1343-5p,miR-660,miR-193a-3p,miR-124-5p,miR-330-3p,miR-365b-5p,miR-2411-3p,miR-224-5p,miR-296-3p,miR-296-3p,miR-503-3p,let-7a-2-3p,miR-2284l,miR-363-5p,miR-668-3p,miR-130b-5p,miR-6240,miR-497-5p,miR-2284x,miR-2284ab,miR-138,miR-16b,miR-450c-3p,miR-197-3p,miR-145a-5p,miR-374b-3p,miR-873a-5p,miR-1843b-5p,miR-181a-5p,miR-194-3p,miR-544b,miR-2284d,miR-412-5p,miR-134-3p,miR-339-5p,miR-486b-3p,miR-134-5p,miR-28-5p,miR-138-5p,miR-331-3p,miR-409-3p,miR-2284k,miR-5100,miR-370-5p,miR-135a-1-3p,miR-365a-5p,miR-193a-5p,miR-16-5p,miR-103b,novel_42,miR-2284v,miR-7144-5p,miR-485-5p,miR-1814c,miR-1248,miR-876-3p,let-7g-3p,miR-154b-5p,miR-2284n,miR-2285aa,miR-874-3p,miR-152-3p,novel_96,novel_127,miR-2898,miR-181b-5p,miR-127-5p,miR-200c-3p,miR-30c-2-3p,miR-339a,miR-205-5p,miR-2285af,miR-200c,miR-664-3p,miR-2284y,miR-200a,miR-3065-3p,miR-29a-5p,miR-1271-3p,miR-490-3p,miR-2284a,miR-3064-5p,miR-4286,miR-2284f,miR-200b-3p,let-7a-2-3p,miR-1343-5p,miR-345-3p,miR-1290,miR-134,miR-450a-1-3p,miR-134-5p,miR-2284z,miR-129-1-3p,miR-486-5p,miR-322-5p,miR-6516-5p,miR-758-5p,miR-1224-5p,novel_79,miR-429-3p,miR-423-3p,miR-4726-5p,miR-301a-5p,miR-423-5p,miR-154a,miR-877-3p,novel_111,miR-2284j,miR-181d-5p,novel_60,miR-3956-3p,miR-1983,miR-7857-3p,miR-378g,miR-3082-5p,miR-7977,miR-664b,miR-2284g,miR-3064-5p,miR-20a-3p,miR-214-3p,miR-3085-3p,miR-491-5p,miR-29b-1-5p,miR-16b,miR-

STXBP4	ENSOARG0(	syntaxin	novel_103,miR-16b,miR-106b-5p,miR-199b-5p,miR-1388-3p,miR-424-5p,miR-106a-5p,miR-652-5p,miR-378d,miR-378b,miR-143-5p,miR-378c,miR-544a,miR-3120-5p,miR-1343-3p,miR-2300a-5p,miR-548w,miR-27a-3p,miR-147a,miR-421,miR-2285e,miR-2285n,miR-382-3p,miR-371a-5p,miR-2284q,miR-2447,miR-885-5p,miR-2285f,miR-194b-5p,miR-361-3p,novel_111,miR-423-5p,miR-8095,miR-140-5p,miR-199b-5p,miR-378j,miR-136-5p,novel_94,miR-2319b,miR-541-5p,miR-7977,miR-378g,miR-378e,miR-203-3p,miR-4443,miR-27a-3p,miR-196a-3p,miR-324-3p,miR-125b,miR-185-3p,miR-422a,miR-106a,miR-202-5p,miR-466i-5p,miR-3653-3p,miR-2285w,miR-20a,miR-376a-5p,miR-421-5p,miR-125b-2-3p,miR-378i,miR-126-3p,miR-378a-3p,miR-5703,miR-142-3p,miR-33b-3p,miR-18a-3p,miR-106a,miR-505-3p,miR-383-5p,miR-340-5p,miR-15a-5p,miR-2284h-5p,miR-15b-3p,miR-320e,miR-665,miR-6516-3p,miR-487b-5p,miR-424-5p,miR-669a-3p,miR-378c,miR-129-2-3p,miR-145a-3p,miR-2330-3p,miR-6525,miR-3184-5p,miR-122-5p,miR-6134,miR-21a-3p,miR-299,miR-2285c,miR-411,miR-2312,miR-625-5p,miR-543-3p,miR-582-5p,novel_99,miR-2331-3p,miR-378a-3p,miR-660-5p,miR-370-5p,miR-544-3p,miR-582-5p,miR-204-3p,miR-22-3p,miR-15b-5p,miR-7-5p,miR-194a,miR-455-5p,miR-106a-5p,miR-17-5p,miR-124-5p,miR-125a-5p,miR-660,miR-101-5p,miR-628-5p,miR-2285r,miR-653,miR-7b-5p,miR-1285,miR-22-3p,miR-194-5p,miR-3071-5p,miR-16b,miR-497-5p,miR-27b-3p,miR-455-5p,miR-2285g,miR-185-5p,miR-134-3p,miR-544b,miR-20b,miR-502-5p,miR-4324,miR-15a,miR-28c,miR-142b,miR-20a-5p,miR-16a,novel_1,miR-1193,miR-2285m,miR-199b-5p,miR-2285ab,miR-150-5p,miR-3065-5p,miR-18a-3p,miR-1291,miR-2319a,miR-505-3p,miR-195a-5p,miR-378f,miR-7-1-3p,novel_69,miR-3071-3p,miR-127-5p,novel_127,miR-3529-

PHF12	ENSOARG0(	PHD fing	miR-1306-5p,miR-5126,miR-326,miR-16b,miR-25-5p,miR-3955-3p,miR-130b-3p,novel_124,miR-491-5p,miR-214-3p,miR-424-5p,miR-2330-5p,miR-33a-5p,miR-30c-1-3p,miR-758-3p,miR-4532,miR-2284r,miR-758-3p,miR-30b-3p,miR-412-3p,miR-2426,miR-101c,miR-105-1,miR-199b-3p,miR-2300a-5p,miR-222-5p,miR-5010-3p,miR-147a,miR-1298-3p,miR-1185-3p,miR-9788-3p,miR-2440,novel_82,miR-2285n,miR-2284q,miR-490-5p,miR-2285t,miR-32-3p,miR-6527,novel_111,miR-378j,miR-136-5p,miR-96-5p,miR-877-3p,miR-493-5p,miR-20a-3p,miR-664b,miR-541-5p,miR-2285u,miR-4443,miR-1185-2-3p,miR-203-3p,miR-2285p,miR-185-3p,miR-125b,miR-466i-5p,miR-30b-3p,miR-221-5p,miR-503-5p,miR-369-3p,miR-125b-2-3p,miR-376a-5p,miR-664b-3p,miR-2411,miR-2432,miR-376a-5p,miR-2300b-3p,miR-140-3p,miR-19b-3p,miR-18a-3p,miR-148b-3p,miR-15a-5p,miR-200b,miR-210-3p,miR-151-5p,miR-2285j,miR-665,miR-320e,miR-655-3p,miR-324-3p,miR-664a,miR-17-3p,miR-129-5p,miR-208b-5p,miR-454-3p,miR-424-5p,miR-4510,miR-539-5p,miR-214,miR-1271,novel_44,miR-224-5p,miR-346,miR-6525,miR-6134,miR-655,miR-27b-5p,miR-18a-5p,let-7g-3p,miR-186-5p,miR-1827,miR-625-5p,miR-32-3p,miR-30c-1-3p,miR-221-5p,miR-552-3p,miR-216b-3p,miR-6130,miR-301a-3p,miR-6516,miR-27a-5p,miR-151b,miR-15b-5p,miR-7-5p,miR-224-5p,miR-503-5p,miR-455-5p,novel_51,miR-105-5p,miR-2285l,miR-378a-5p,miR-126a-3p,miR-125a-5p,miR-493-5p,miR-668-3p,miR-363-5p,miR-1843b-5p,miR-7b-5p,miR-432,miR-3071-5p,miR-374b-3p,miR-30b-3p,miR-138,miR-16b,miR-455-5p,miR-744-5p,miR-497-5p,miR-31-5p,miR-26b-3p,miR-130a-3p,miR-503-5p,miR-185-5p,miR-432-5p,novel_116,miR-411-5p,miR-4324,miR-15a,miR-1247-3p,miR-33a-3p,miR-16a,miR-6119-3p,miR-199c,miR-22-5p,miR-219b-3p,miR-495-3p,novel_101,miR-17-3p,miR-371b-

PTDSS1	ENSOARG0(	phosphat	miR-324-5p,miR-874-3p,miR-329a,miR-320a,miR-3154,miR-488-3p,miR-365a-5p,miR-2903,miR-193a-5p,miR-3969,miR-370-5p,miR-5100,novel_63,miR-410-5p,novel_121,miR-2284z,novel_79,miR-6517,miR-299a-3p,miR-411-3p,miR-1343-5p,miR-1271-3p,miR-490-3p,miR-3065-3p,novel_39,miR-24-2-5p,miR-4286,miR-3064-5p,miR-29b-2-5p,miR-3529-3p,novel_96,miR-339a,miR-30c-2-3p,miR-219-3p,miR-664-3p,miR-320c,miR-299b-3p,miR-450b-3p,miR-3591-3p,miR-1306,novel_27,miR-1193,miR-2285x,miR-147-5p,miR-203b-5p,miR-379-3p,miR-128-1-5p,miR-1247-3p,miR-544b,miR-185-5p,miR-377-5p,miR-339-5p,miR-133b-5p,miR-3955-5p,miR-340-3p,miR-31-5p,miR-188-5p,miR-744-5p,miR-2284x,miR-6412,miR-2285r,miR-363-5p,miR-130b-5p,miR-466f-3p,miR-1343-5p,novel_51,miR-365b-5p,miR-204-3p,miR-27a-5p,miR-544-3p,miR-21-3p,miR-370-5p,miR-3065-3p,miR-140-5p,miR-30c-1-3p,miR-339b,miR-411,miR-6134,miR-346,miR-3074-2-3p,miR-145a-3p,miR-130b-5p,miR-29b-2-5p,miR-324-3p,miR-219b-3p,miR-2285j,miR-1247-5p,miR-3600,miR-4429,miR-1197-5p,miR-1434-5p,miR-124-3p,miR-2300b-3p,miR-362-3p,novel_133,miR-130a-5p,miR-377-5p,miR-29b-1-5p,miR-183-5p,miR-299-3p,miR-30b-3p,miR-320d,miR-202-5p,miR-324-3p,miR-664b,miR-24-1-5p,miR-3064-5p,miR-9-3p,miR-219a-2-3p,miR-9-3p,miR-541-3p,miR-3059-5p,miR-2447,miR-1298-3p,novel_4,miR-2284aa,miR-544a,miR-450b-5p,miR-2426,miR-362-3p,miR-125b-2-3p,miR-541,miR-329-3p,miR-8485,miR-193a,miR-3187-3p,miR-30c-1-3p,miR-124a,miR-25-5p,miR-491-5p,miR-3085-3p,miR-362-3p,miR-29b-1-5p,miR-1306-5p,miR-320b
TNPO1	ENSOARG0(	transpor	miR-27a-3p,miR-299b-3p,miR-2285u,miR-296-3p,miR-10b,miR-329-5p,miR-452-3p,miR-299b-5p,miR-1306,miR-655,miR-299,miR-148a-3p,miR-424-3p,miR-452-5p,miR-22-5p,miR-107-5p,miR-655-3p,miR-2285n,novel_83,miR-2459,miR-148b-3p,miR-196a-2-3p,miR-665,miR-5010-3p,miR-27a-3p,miR-299a-3p,miR-27b-3p,miR-299a-5p,miR-218-2-3p,miR-2300b-3p,miR-29a-5p,miR-296-3p,miR-4286,miR-103a-2-5p,miR-299-3p,miR-152-3p,miR-6536,miR-92a-1-5p,novel_51,miR-196a-3p,novel_103,miR-296-3p,miR-1306-5p

DSEL	ENSOARG0(	dermatan	miR-411-3p,miR-582-3p,miR-10a-5p,miR-9-5p,miR-2285ad,miR-6517,miR-412,miR-450a-2-3p,miR-1a-2-5p,miR-2285af,miR-664-3p,miR-219-3p,miR-217,miR-2478,miR-3529-3p,miR-29b-2-5p,miR-1,miR-7144-5p,miR-376c-5p,miR-1248,miR-93-5p,miR-3154,miR-376c-5p,miR-23b,miR-362-5p,miR-34c-5p,miR-769,miR-133c,miR-2285aa,miR-125a,miR-215-5p,miR-204-5p,miR-191-5p,miR-410-5p,miR-1-5p,miR-100-3p,miR-125b-5p,miR-3969,miR-135a-5p,miR-500b-5p,miR-93,miR-6128,miR-103b,miR-27b-3p,miR-145a-5p,miR-412-5p,miR-3535,miR-134-3p,miR-185-5p,miR-411-5p,miR-149-5p,miR-125a-5p,miR-296-3p,miR-377-3p,miR-296-3p,miR-135b-5p,miR-376b-5p,miR-376b-3p,miR-3065-5p,miR-670-3p,miR-34b,miR-677,miR-181b-3p,novel_68,miR-329-5p,miR-3074-5p,miR-10a-5p,miR-6119-3p,miR-379-3p,miR-206,novel_1,miR-203b-5p,miR-656-5p,miR-502-5p,miR-4324,miR-20a-5p,miR-576-3p,miR-34c,miR-211,miR-24-3p,miR-374c-5p,miR-449a,miR-342-3p,miR-135a-2-3p,miR-1a-1-5p,miR-495-3p,miR-142-3p,miR-941,miR-16-1-3p,miR-34a-5p,miR-191,miR-215-5p,miR-2366,miR-2904,miR-1434-5p,miR-5703,miR-217-5p,miR-383-5p,miR-23b-3p,miR-23c,miR-616-3p,miR-3600,miR-466i-5p,miR-30b-3p,miR-34b-5p,miR-2484,novel_17,miR-125b,miR-29b-1-5p,miR-181b-1-3p,miR-125b-2-3p,miR-130a-5p,miR-329a-5p,miR-20a,miR-543-3p,miR-582-5p,novel_99,miR-411,miR-192-5p,miR-10a,miR-377-3p,miR-582-5p,miR-2284s,miR-656-3p,miR-139-5p,miR-204-3p,miR-2331-3p,miR-216b-3p,miR-532-5p,miR-669a-3p,miR-324-3p,miR-219b-3p,miR-665,miR-29b-2-5p,miR-6525,miR-769-5p,miR-10b-5p,miR-206-3p,miR-6134,miR-433-5p,miR-3074-5p,miR-2426,miR-4791,miR-10b,miR-301b-5p,miR-1a-3p,miR-412-3p,miR-147a,miR-500-5p,miR-455-3p,miR-421,miR-548w,miR-5010-3p,miR-181b-2-3p,miR-27a-3p,miR-145b,miR-342,novel_103,miR-29b-1-5p,miR-106b-5p,miR-1b-
ZNF333	ENSOARG0(	zinc fin	miR-6134,miR-8095,miR-6527,miR-193b-3p,miR-193a-5p,miR-4510,miR-3059-5p,miR-7134-3p,miR-107-5p,miR-331-3p,miR-502-5p,miR-193b-3p,novel_69,miR-323-5p,miR-6130,miR-25,miR-323a-5p,miR-2404,miR-320b,miR-541-5p,miR-329-5p,miR-3064-5p,miR-329b,miR-107,miR-216a-3p,miR-2387,miR-140-5p,miR-432-3p,miR-3064-5p,miR-363-5p,miR-1388-3p,miR-329a-5p,miR-193a-3p,miR-543-5p,miR-103a-2-5p,miR-106b-3p,miR-107,miR-483-3p,miR-3085-3p,miR-2898,miR-378a-5p,miR-3601,novel_17,miR-103,miR-216a-3p,miR-222-5p,miR-483-3p,miR-210-3p,miR-103a-3p,miR-2889,miR-7862,miR-128-3p,miR-142-3p,miR-493-3p,miR-23b-5p,miR-5703,miR-188-3p,miR-1290
ZFYVE9	ENSOARG0(	zinc fin	miR-106a,miR-130a-3p,miR-3600,miR-145b,miR-20b,miR-93-5p,miR-17-5p,miR-145a-5p,miR-20b-5p,miR-877-3p,miR-106a-5p,miR-20a,miR-93,miR-301b-3p,miR-20a-5p,miR-106a-5p,miR-106b-5p,miR-17-5p,miR-106a,miR-130b-3p,miR-454-3p

SETD2	ENSOARG0(	SET doma	miR-3065-5p,miR-1306,miR-378f,miR-146b-5p,miR-412-3p,novel_68,miR-669,miR-576-3p,miR-28c,miR-4324,miR-625-3p,miR-193b-3p,miR-197-3p,miR-27b-3p,miR-497-5p,miR-16-1-3p,miR-16b,miR-3955-5p,miR-3431,miR-134-3p,novel_51,miR-146a,miR-377-3p,miR-296-3p,miR-193a-3p,miR-6240,miR-2285r,miR-424-3p,miR-329a,miR-1271,miR-125a,miR-2355-5p,miR-574-5p,miR-125b-5p,miR-2284k,novel_63,miR-134-5p,miR-16-5p,miR-2284v,novel_42,miR-1260b,miR-23b-5p,miR-330-5p,miR-155-5p,miR-431,miR-1224-5p,miR-6517,miR-2285ad,miR-134-5p,miR-505-5p,miR-9-5p,miR-322-5p,miR-205-5p,novel_96,miR-3064-5p,miR-2284y,miR-148b-5p,miR-378j,miR-221,miR-140-5p,miR-1983,miR-544-5p,miR-668-5p,miR-299b-5p,miR-99b-5p,miR-211-5p,miR-2319b,miR-378g,miR-499a-3p,miR-2440,miR-218-5p,miR-9-5p,miR-2284j,miR-2447,miR-423-5p,miR-877-3p,miR-329-3p,miR-362-3p,miR-1388-5p,miR-199b-3p,miR-1343-3p,miR-330-3p,miR-182-5p,miR-16b,miR-505,miR-431-5p,miR-103a-2-5p,miR-214-3p,miR-146b,miR-491-5p,miR-2284b,miR-124a,miR-1388-3p,miR-378d,miR-655,miR-129b-3p,miR-1827,miR-30c-1-3p,miR-15b-5p,miR-377-3p,miR-499b-3p,miR-10a,miR-665,miR-30a-3p,miR-151-3p,miR-30e-3p,miR-2285j,miR-4492,miR-874-5p,novel_74,miR-214,miR-3184-5p,miR-224-5p,miR-6525,miR-1434-5p,miR-378a-3p,miR-193b-5p,miR-378i,miR-34a-5p,miR-16-1-3p,miR-664-5p,miR-33b-3p,miR-493-3p,miR-212-5p,miR-30d-3p,miR-328-3p,miR-1247-5p,miR-422a,miR-324-3p,miR-210-5p,miR-345-3p,miR-369-3p,miR-143-3p,miR-2387,miR-2285ab,miR-6395,miR-195a-5p,miR-450b-3p,miR-1291,miR-7-1-3p,miR-10a-5p,miR-34b,miR-670-3p,miR-34c,miR-16a,miR-502-5p,miR-15a,miR-199c,miR-203b-5p,miR-2428,miR-326-3p,miR-449a,miR-371b-3p,miR-211,miR-1434-3p,miR-1285,miR-7862,miR-1260b,miR-2284ab,miR-133b-5p,miR-185-5p,miR-

HBP1	ENSOARG0(	HMG-box	miR-1b-5p,miR-106a-5p,miR-199b-5p,miR-130b-3p,miR-106b-5p,miR-2957,miR-421,miR-9788-3p,miR-148b-5p,miR-1298-3p,miR-142-5p,miR-222-5p,miR-412-3p,miR-541,miR-877-3p,miR-452-5p,miR-885-5p,miR-212-3p,miR-541-3p,miR-144-5p,miR-3082-5p,miR-378g,miR-499b-5p,miR-2284g,novel_94,miR-136-5p,miR-299b-5p,miR-96-5p,miR-381-3p,miR-199b-5p,miR-130a-5p,miR-20a,miR-21-3p,miR-221-5p,miR-106a,miR-185-3p,miR-196a-3p,miR-212-5p,miR-106a,miR-2284u,miR-148a-5p,miR-19b-3p,miR-217-5p,miR-140-3p,miR-188-3p,miR-376a-5p,miR-338-3p,miR-142a-5p,miR-1271,miR-4492,miR-208b-5p,miR-454-3p,miR-155-5p,miR-664a,miR-6516-3p,miR-155-5p,miR-193b-3p,miR-301a-3p,miR-3065-3p,miR-21-3p,miR-221-5p,miR-27b-5p,miR-21a-3p,miR-668-3p,miR-193a-3p,miR-17-5p,miR-106a-5p,miR-20b,miR-134-3p,miR-130a-3p,miR-665,miR-29b,miR-21-5p,miR-197-3p,miR-2113,miR-301b-3p,miR-199b-5p,miR-29c-3p,miR-193b-3p,miR-502b,miR-6123,miR-29a,miR-33a-3p,miR-20a-5p,miR-29a-3p,miR-320b,miR-2319a,miR-9851-3p,miR-19a-3p,miR-1291,miR-21c,miR-3591-3p,miR-2387,miR-2284a,miR-3956-5p,miR-2284y,miR-199a-5p,miR-3065-3p,miR-2478,miR-181a-2-3p,miR-495-5p,miR-127-5p,miR-6536,miR-217,miR-205-5p,miR-2285af,miR-1271-5p,miR-505-5p,miR-412,miR-155-5p,miR-300,miR-29d-3p,miR-145-3p,miR-1197-3p,miR-93,miR-144-5p,miR-1306-5p,miR-767-5p,miR-29b-3p,miR-199a-5p,miR-2355-5p,miR-1248,miR-7144-5p,miR-20b-5p,miR-767,miR-17-5p,miR-93-5p,miR-200a-5p,miR-488-3p,miR-381-3p
PHF10	ENSOARG0(	PHD fing	miR-16a,novel_82,miR-15a,miR-20a-5p,miR-208a-3p,miR-6529b,miR-656-5p,miR-2284q,miR-3957-3p,miR-3970,miR-18a-3p,miR-129b-5p,miR-140-5p,miR-2387,miR-9851-3p,miR-195a-5p,novel_94,miR-96-5p,miR-2285k,miR-329-5p,miR-144-3p,miR-16b,miR-106b-5p,miR-106a-5p,miR-17-5p,miR-224-5p,miR-106a-5p,miR-424-5p,miR-484,miR-497-5p,miR-16b,miR-1388-5p,miR-450b-5p,miR-339-5p,miR-31-5p,novel_78,miR-20b,miR-3535,miR-185-5p,miR-3431,miR-26c,miR-144,miR-29b-2-5p,miR-665,miR-129-5p,miR-424-5p,miR-1271,miR-16-5p,miR-539-5p,miR-28a-3p,miR-145a-3p,miR-93,miR-224-5p,miR-93-5p,miR-17-5p,miR-20b-5p,miR-339b,miR-216b-3p,miR-370-3p,miR-1271,miR-140-5p,miR-874-3p,miR-15b-5p,miR-6516,miR-339a,miR-2285af,miR-200c,miR-125a-3p,miR-208b-3p,miR-21-3p,novel_127,miR-28-3p,miR-106a,miR-200c-3p,miR-29b-2-5p,miR-20a,miR-181a-2-3p,miR-4286,miR-329a-5p,miR-2432,miR-2285i,miR-218-2-3p,miR-188-3p,miR-3591-5p,miR-200b-3p,novel_79,miR-18a-3p,miR-106a,miR-429-3p,miR-532-3p,miR-322-5p,miR-15a-5p,miR-200b,miR-1271-5p,miR-6516-5p,miR-758-5p

PRODH2	ENSOARG0(	proline	miR-545-5p,miR-378h,miR-2411-5p,miR-200b-3p,miR-496,miR-300,miR-30a-5p,miR-134,miR-1957a,miR-199a-5p,miR-1271-3p,miR-380-5p,miR-30a-3p,miR-181a-2-3p,miR-2403,miR-17-3p,miR-2898,miR-200c-3p,miR-30c-2-3p,miR-339a,miR-217,miR-200c,miR-199a-5p,miR-154b-5p,miR-374c-3p,miR-365a-3p,miR-615-5p,miR-7144-5p,miR-671-5p,miR-485-5p,miR-10b-3p,miR-362-5p,miR-3154,miR-381-3p,miR-503-3p,miR-2448-3p,miR-378c,miR-500b-5p,miR-3607-3p,miR-133a-3p,miR-103b,miR-486b-3p,miR-331-3p,miR-3969,miR-551b-3p,miR-432-5p,miR-185-5p,miR-122-3p,miR-744-5p,miR-30b-3p,miR-219a-1-3p,miR-1285,miR-345-5p,miR-125a-5p,miR-22851,miR-197-5p,miR-330-3p,miR-224-5p,miR-146b-3p,miR-2404,miR-378b,miR-1291,miR-18a-3p,miR-2387,miR-326-3p,miR-2428,miR-3957-3p,novel_101,miR-17-3p,miR-656-5p,miR-147-5p,miR-199c,novel_1,miR-382-5p,novel_83,miR-151-5p,miR-200b,miR-1b-3p,miR-548e-3p,miR-378d,miR-2904,miR-1197-5p,miR-188-3p,miR-5703,miR-140-3p,novel_133,miR-1246,miR-2408,miR-2432,miR-2411,miR-221-5p,miR-21-3p,miR-760-3p,miR-125b,miR-204-3p,miR-22-3p,miR-877-5p,miR-6130,miR-140-5p,miR-221-5p,miR-378a-3p,miR-32-3p,miR-496-3p,miR-133b-3p,miR-21a-3p,miR-6134,miR-3074-2-3p,miR-2330-3p,miR-378c,miR-539-5p,miR-4510,miR-450b-3p,miR-17-3p,miR-664a,miR-324-3p,miR-500-5p,miR-222-5p,miR-409b,novel_4,miR-151a-3p,miR-378c,miR-450b-5p,miR-7857,miR-30b-3p,miR-484,miR-378b,miR-143-5p,miR-3187-3p,miR-30c-1-3p,miR-494-3p,miR-2330-5p,miR-3085-3p,miR-326,miR-1306-5p,miR-378e,miR-3082-5p,miR-541-5p,miR-499b-5p,miR-3064-5p,miR-452-3p,miR-20a-3p,miR-877-3p,miR-361-3p,miR-199b-5p,miR-362-5p,miR-32-3p,miR-2285t,miR-1973,miR-2285n,miR-134-5p,miR-450a-1-3p,novel_121,miR-9-5p,miR-505-5p,miR-1224-5p,novel_79,miR-431,miR-6517,miR-
MMP10	ENSOARG0(	matrix m	miR-452-3p,miR-10b,miR-363,miR-10a-5p,miR-7977,miR-211-5p,miR-1185-2-3p,miR-18a-3p,miR-136-5p,miR-504,miR-342-3p,miR-190b-5p,miR-495-3p,miR-9-5p,miR-2284j,miR-211,miR-20a-5p,novel_82,miR-548w,miR-105-1,miR-31-5p,miR-145b,miR-3431,miR-1185-3p,miR-455-3p,miR-10b,miR-1285,miR-873a-5p,miR-329-3p,miR-145a-5p,miR-30b-3p,miR-362-3p,miR-30b-3p,miR-2284x,miR-188-5p,miR-106a-5p,let-7f-2-3p,let-7f-2-3p,miR-101-5p,miR-8485,miR-342,miR-106a-5p,miR-17-5p,miR-105-5p,miR-190a,miR-362-3p,miR-874-3p,miR-504-5p,miR-365a-3p,miR-10a,miR-324-5p,miR-93-5p,miR-329a,miR-10b-3p,miR-20b-5p,novel_48,miR-2330-3p,miR-93,miR-10b-5p,miR-2284k,miR-665,miR-30a-3p,miR-592,miR-204-5p,miR-190a-5p,miR-18a-3p,miR-3600,miR-9-5p,miR-362-3p,novel_133,miR-10a-5p,miR-664-5p,miR-105-2,miR-30a-3p,miR-376a-5p,miR-148b-5p,miR-2424,miR-2284m,novel_127

GLT6D1	ENSOARG0(	glycosyl	miR-532-5p,miR-496-3p,miR-221-5p,miR-545-3p,miR-22-3p,miR-193b-3p,miR-6130,miR-27a-5p,miR-192-5p,novel_99,miR-2312,miR-3074-2-3p,miR-4510,miR-539-5p,miR-6134,let-7b-5p,miR-338-5p,miR-29b-2-5p,miR-222-3p,miR-208b-5p,miR-708-5p,miR-424-5p,miR-1843b-3p,miR-6516-3p,miR-17-3p,miR-548e-3p,miR-200b,miR-148b-3p,miR-15a-5p,miR-5703,miR-1197-5p,miR-188-3p,miR-2904,miR-124-3p,miR-2300b-3p,miR-215-5p,miR-1246,novel_133,let-7a-3p,miR-378d,miR-19b-3p,miR-5100,miR-183-5p,miR-2432,miR-125b-2-3p,miR-29b-1-5p,miR-185-3p,miR-299-3p,miR-21-3p,miR-221-5p,miR-466i-5p,miR-664b,let-7d-5p,miR-3082-5p,miR-7857-3p,miR-129b-5p,miR-199b-5p,novel_94,miR-877-3p,miR-2285f,miR-541-3p,miR-3059-5p,miR-154a,miR-371a-5p,miR-335,miR-222-5p,miR-221-3p,miR-125b-2-3p,miR-412-3p,miR-6529a,miR-484,miR-2426,miR-299a-5p,miR-33a-5p,miR-424-5p,let-7a-5p,miR-4532,miR-3187-3p,miR-30c-1-3p,miR-1306-5p,miR-326,miR-342,let-7e,miR-3955-3p,novel_124,miR-215-5p,miR-3957-5p,miR-3957,miR-199a-5p,miR-3965,miR-365a-3p,miR-374c-3p,miR-2448-3p,miR-671-5p,miR-7144-5p,miR-3607-3p,miR-331-3p,miR-2332,miR-29b-3p,miR-410-5p,miR-204-5p,miR-28-5p,miR-767-5p,miR-222,miR-545-5p,miR-29d-3p,miR-218-2-3p,miR-345-3p,miR-615-3p,miR-496,miR-200b-3p,let-7d,miR-4286,miR-2385-3p,miR-30a-3p,miR-29b-2-5p,miR-490-3p,miR-199a-5p,miR-380-5p,miR-28b,miR-181c-3p,let-7c-5p,miR-30c-2-3p,miR-200c,miR-543-5p,miR-200c-3p,miR-191-3p,miR-2898,miR-17-3p,miR-7-1-3p,miR-29a-3p,miR-2404,miR-378b,miR-18a-3p,miR-2285ab,miR-195a-5p,miR-9851-3p,miR-19a-3p,miR-17-3p,miR-2285x,miR-3957-3p,miR-136-3p,miR-342-3p,miR-2428,miR-326-3p,miR-323c,miR-371b-3p,miR-211,let-7i,miR-16a,miR-15a,miR-203b-5p,novel_1,miR-665,miR-2355-3p,let-7g-

DFFA	ENSOARG0(	DNA frag	miR-219a-2-3p,miR-7705,miR-154a,miR-1285-5p,novel_111,miR-2284j,miR-3059-5p,miR-2447,miR-2284q,miR-2285t,miR-107-5p,miR-769-5p,miR-2285y,miR-2285n,miR-218-5p,miR-27a-3p,miR-4443,let-7d-5p,miR-378g,miR-3596,miR-2319b,miR-3064-5p,miR-96-5p,miR-381-3p,miR-199b-5p,miR-140-5p,miR-2310,miR-758-3p,miR-3187-3p,miR-33a-5p,miR-101b-3p,let-7a-5p,miR-1468,miR-542-5p,let-7e,miR-103a-2-5p,miR-146b,miR-199b-5p,miR-153-3p,miR-25-5p,miR-487b-5p,miR-491-5p,miR-3085-3p,miR-505,miR-199a-3p,miR-320b,miR-216a-3p,miR-1298-3p,miR-153,miR-145b,miR-27a-3p,miR-181b-2-3p,miR-330-3p,novel_78,miR-409b,miR-544a,miR-30b-3p,miR-758-3p,miR-6529a,miR-296-5p,miR-6134,let-7b-5p,miR-224-5p,miR-769-5p,miR-1271,miR-145a-3p,miR-4510,miR-3184-3p,miR-3074-5p,let-7b,miR-669a-3p,miR-708-5p,miR-4492,miR-129-5p,miR-1468-5p,miR-17-3p,miR-665,miR-3604,miR-320e,miR-219b-3p,miR-324-3p,let-7f,miR-2285j,miR-877-5p,let-7i-5p,miR-204-3p,miR-6130,miR-544-3p,miR-21-3p,miR-660-5p,novel_99,miR-543-3p,miR-487b-5p,let-7f-1-3p,miR-380-3p,miR-146a-5p,miR-2432,miR-181b-1-3p,miR-130a-5p,miR-125b-2-3p,miR-2285w,miR-216c-5p,miR-329a-5p,let-7k,miR-34b-5p,miR-760-3p,miR-98-5p,miR-2284m,miR-320d,miR-185-3p,miR-324-3p,miR-542-5p,miR-328-3p,miR-199a-3p,miR-532-3p,miR-2284h-5p,miR-200b,miR-222-5p,miR-7859,miR-548e-3p,miR-34a-5p,miR-4429,miR-493-3p,miR-5703,miR-188-3p,miR-1961,miR-199b-5p,miR-449a,miR-323c,miR-3959-3p,miR-17-3p,miR-542-5p,miR-6119-3p,let-7i,miR-34c,miR-576-3p,miR-4324,miR-181b-3p,miR-320c,miR-144-3p,miR-677,miR-411b-3p,miR-34b,miR-320b,miR-3074-5p,miR-329-5p,miR-135b-5p,miR-6395,miR-6516-3p,let-7e-5p,miR-376a-2-5p,miR-146b-5p,miR-450b-3p,miR-216a-3p,miR-3591-3p,miR-
ARHGEF2	ENSOARG0(	Rho/Rac	miR-363-5p,miR-1247-5p,miR-378g,miR-134-3p,novel_51,novel_17,miR-2300b-3p,miR-5126,miR-2898,miR-184-3p

CDH7	ENSOARG0(	cadherin	miR-140-5p,miR-378j,miR-96-5p,miR-544-5p,miR-9-3p,miR-335-3p,miR-493-5p,miR-2319b,miR-126b-5p,miR-211-5p,miR-1185-2-3p,miR-2440,miR-3956-3p,miR-2447,miR-2284j,novel_111,miR-877-3p,miR-541,miR-758-3p,miR-1839-5p,miR-548w,novel_78,miR-199b-3p,miR-181b-2-3p,miR-142-5p,miR-126a-5p,miR-148b-5p,miR-455-3p,miR-199a-3p,miR-16b,miR-199b-5p,miR-146b,miR-744-3p,miR-23a-3p,miR-1b-5p,miR-299,miR-30d-5p,miR-598-3p,miR-339b,miR-345-5p,miR-377-3p,miR-15b-5p,let-7b-3p,miR-15b-3p,miR-665,miR-451a,miR-130b-5p,miR-129-5p,miR-3074-5p,miR-3184-3p,miR-98-3p,miR-142a-5p,miR-181c-5p,miR-30c-5p,miR-1434-5p,miR-98-3p,miR-493-3p,miR-148a-5p,miR-23c,miR-618,novel_17,miR-30b,miR-500a-5p,miR-181b-1-3p,miR-148a-3p,miR-3591-3p,miR-2319a,miR-146b-5p,miR-3074-5p,miR-3120-3p,miR-181b-3p,miR-677,miR-144-3p,miR-576-3p,miR-28c,miR-1a-1-5p,miR-1839-3p,miR-2284w,miR-199b-5p,miR-708-3p,miR-16b,miR-369-5p,miR-497-5p,miR-103a-3p,miR-339-5p,miR-146a,miR-377-3p,novel_51,miR-105-5p,miR-196b-5p,miR-493-5p,miR-628-5p,let-7f-2-3p,let-7f-2-3p,miR-432-3p,miR-2284l,miR-2285r,miR-432-3p,miR-301,miR-30e-5p,miR-137-3p,miR-200a-5p,miR-345-5p,miR-767,miR-2355-5p,miR-2285aa,miR-300-3p,miR-331-5p,miR-223-5p,miR-2284k,miR-100-3p,miR-134-5p,miR-2284v,miR-16-5p,miR-196a-5p,miR-30c,miR-429-3p,miR-1224-5p,miR-412,novel_79,miR-322-5p,miR-486-5p,novel_121,miR-134-5p,miR-664-3p,miR-205-5p,miR-127-5p,miR-1a-2-5p,miR-152-3p,miR-200a-3p,miR-450b-5p,miR-200a,miR-29a-5p,miR-199b-5p,miR-129b-5p,miR-664b,miR-7977,miR-541-5p,miR-499b-5p,miR-3082-5p,miR-489,miR-7857-3p,miR-30b-5p,miR-382-3p,miR-371a-5p,miR-212-3p,miR-541-3p,novel_60,miR-3059-5p,miR-452-5p,miR-8095,miR-143-5p,miR-412-3p,miR-450b-5p,miR-544a,miR-105-
HPS3	ENSOARG0(	Hermansk	miR-664b-3p,miR-378d,miR-199a-5p,miR-6119-5p,miR-2285p,miR-455-5p,miR-422a,miR-2898,miR-200a-3p,miR-30b-3p,miR-199b-5p,miR-151a-3p,miR-199b-3p,miR-1343-3p,miR-378h,miR-6516-5p,novel_121,miR-545-5p,miR-411-5p,miR-541,miR-378i,miR-378b,miR-378a-3p,miR-5703,miR-7857,miR-2904,miR-450b-5p,miR-378c,miR-455-5p,miR-16-2-3p,miR-145a-3p,miR-541-3p,miR-378c,miR-2285x,miR-452-5p,miR-3957-3p,miR-199b-5p,miR-378c,miR-151-3p,miR-3968,novel_74,miR-147-5p,miR-199c,miR-208b-5p,miR-216b-3p,miR-545-3p,miR-378a-3p,miR-7-1-3p,miR-141-3p,miR-216b-3p,miR-3082-5p,miR-378e,miR-6516,miR-199a-5p,miR-199b-5p,miR-3065-5p,miR-378f

AFAP1L1	ENSOARG0(	actin fi	miR-142-3p,miR-1298-3p,miR-221-3p,miR-199b-3p,miR-548w,miR-105-1,miR-222-5p,miR-181b-2-3p,miR-544a,miR-362-3p,miR-101c,miR-143-5p,miR-484,miR-329-3p,miR-30c-1-3p,miR-8485,miR-101b-3p,miR-124a,miR-3955-3p,miR-130b-3p,miR-491-5p,miR-1185-5p,miR-542-5p,miR-199b-5p,miR-326,novel_103,miR-320b,miR-199a-3p,miR-362-3p,miR-499a-3p,miR-378g,miR-203-3p,miR-20a-3p,miR-2319b,miR-139-5p,miR-668-5p,miR-96-5p,miR-9-3p,miR-1983,miR-221,miR-199b-5p,miR-378j,novel_111,miR-877-3p,miR-361-3p,miR-7705,miR-154a,miR-1285-5p,miR-2447,miR-9-5p,miR-2285f,miR-3968,miR-2285t,miR-218-5p,miR-222-5p,miR-148b-3p,miR-7859,miR-328-3p,miR-616-3p,miR-199a-3p,miR-4429,miR-33b-3p,miR-34a-5p,miR-361-5p,miR-362-3p,novel_133,miR-1197-5p,miR-124-3p,miR-181b-1-3p,miR-142a-3p,miR-377-5p,miR-130a-5p,miR-329a-5p,miR-760-3p,miR-202-5p,miR-320d,miR-34b-5p,miR-30b-3p,miR-542-5p,miR-345-3p,miR-216b-5p,miR-139-5p,miR-6130,miR-499b-3p,miR-544-3p,miR-301a-3p,miR-204-3p,miR-545-3p,miR-140-5p,miR-30c-1-3p,miR-552-3p,miR-532-5p,miR-582-5p,miR-543-3p,miR-433-3p,miR-27b-5p,miR-6525,miR-6134,miR-181c-5p,miR-4510,miR-129-2-3p,miR-664a,miR-6516-3p,miR-17-3p,miR-454-3p,miR-222-3p,miR-2285j,miR-320e,miR-19b-1-5p,miR-134-3p,miR-544b,miR-149-5p,novel_116,miR-133b-5p,miR-31-5p,miR-377-5p,miR-130a-3p,miR-665,miR-21-5p,miR-29b,miR-2284x,miR-181a-5p,miR-19b-2-5p,novel_23,miR-432-3p,miR-432-3p,miR-2285r,miR-296-3p,miR-363-5p,miR-483-3p,miR-296-3p,novel_51,miR-2411-3p,miR-365b-5p,miR-105-5p,miR-34b,miR-670-3p,miR-29a-3p,miR-181b-3p,miR-677,miR-320c,miR-378b,miR-329-5p,miR-655-5p,miR-412-3p,miR-6395,miR-135b-5p,miR-148a-3p,miR-2387,miR-2285ah,miR-3065-5p,miR-21c,miR-371b-3p,miR-3959-

TRPA1	ENSOARG0(	transien	miR-23b-3p,miR-15a-5p,miR-148b-3p,miR-328-3p,miR-18a-3p,miR-3600,miR-30d-3p,miR-493-3p,miR-142-3p,miR-16-1-3p,miR-2300b-3p,miR-362-3p,miR-376a-5p,miR-1197-5p,miR-140-3p,miR-376a-5p,miR-421-5p,miR-181b-1-3p,miR-143-3p,miR-216c-5p,miR-329a-5p,miR-487a,miR-466i-5p,novel_17,miR-500a-5p,miR-216a-5p,miR-125b,miR-345-3p,miR-301a-3p,miR-204-3p,miR-877-5p,miR-15b-5p,miR-545-3p,miR-3065-3p,miR-21-3p,miR-1827,miR-186-5p,miR-2285c,miR-433-3p,miR-655,miR-224-5p,miR-206-3p,miR-3184-5p,miR-181c-5p,miR-539-5p,miR-145a-3p,miR-3074-5p,miR-3184-3p,miR-3958-5p,miR-665-5p,miR-3074-2-3p,miR-2330-3p,miR-664a,miR-6516-3p,miR-454-3p,miR-708-5p,miR-424-5p,miR-222-3p,miR-30e-3p,miR-30a-3p,miR-19b-1-5p,miR-29b-2-5p,miR-655-3p,miR-500-5p,miR-9788-3p,miR-1185-3p,miR-2285e,miR-1343-3p,novel_78,miR-5010-3p,miR-181b-2-3p,miR-145b,miR-362-3p,miR-2426,miR-450b-5p,miR-3120-5p,miR-329-3p,miR-7857,miR-1a-3p,miR-125b-2-3p,miR-193a,miR-1388-3p,miR-424-5p,miR-23a-3p,miR-33a-5p,miR-130b-3p,miR-103a-2-5p,miR-326,miR-362-3p,miR-16b,miR-489,miR-3082-5p,miR-1185-2-3p,miR-10b,miR-335-3p,miR-877-3p,miR-1983,miR-362-5p,miR-1285-5p,miR-423-5p,novel_60,miR-2447,miR-3956-3p,miR-6527,miR-181d-5p,miR-9-5p,miR-628-3p,miR-374a-3p,miR-218-5p,miR-107-5p,miR-322-5p,miR-9-5p,miR-134-5p,miR-486-5p,miR-222,miR-6517,miR-429-3p,miR-7641,miR-300,miR-128-3p,miR-345-3p,miR-3578,miR-134,miR-29d-3p,miR-330-5p,miR-450b-5p,miR-148b-5p,miR-3065-3p,miR-200a,miR-1957a,miR-3956-5p,miR-3529-3p,miR-1,miR-30a-3p,miR-29b-2-5p,miR-2898,miR-200c-3p,miR-1a-2-5p,miR-181b-5p,novel_127,miR-152-3p,miR-200a-3p,miR-1260a,miR-6119-5p,miR-200c,miR-3959-5p,miR-487a-3p,miR-370-3p,miR-141-3p,miR-125a,miR-767,miR-329a,miR-2318,miR-23a-5p,miR-

PSMB1	ENSOARG0(	proteaso	miR-877-5p,novel_32,miR-204-3p,miR-3065-3p,miR-370-5p,miR-660-5p,miR-221-5p,miR-545-3p,miR-582-5p,miR-18a-5p,miR-338-3p,miR-6134,let-7b-5p,miR-29c-5p,miR-145a-3p,miR-664a,miR-29d-5p,miR-19b-3p,miR-1197-5p,miR-2904,miR-124-3p,novel_133,miR-146a-5p,miR-29b-1-5p,miR-21-3p,miR-34b-5p,miR-30d,miR-221-5p,miR-2284m,miR-216a-5p,miR-27a-3p,let-7d-5p,miR-3082-5p,miR-7857-3p,miR-7977,miR-3064-5p,novel_94,miR-361-3p,miR-452-5p,miR-541-3p,miR-3059-5p,novel_60,miR-4726-5p,miR-371a-5p,miR-216a-3p,miR-27a-3p,novel_4,miR-30b-3p,miR-7857,miR-296-5p,miR-484,miR-491-3p,miR-30c-1-3p,miR-382-3p,let-7a-5p,let-7e,miR-3085-3p,novel_115,miR-1306-5p,miR-342,miR-2284n,miR-365a-3p,miR-374c-3p,miR-34c-5p,miR-141-3p,miR-370-3p,miR-485-5p,miR-7144-5p,miR-23a-5p,novel_48,miR-18a,miR-3154,miR-2448-3p,miR-488-3p,miR-365a-5p,miR-374a-3p,miR-323-3p,miR-6128,miR-3607-3p,miR-204-5p,miR-374b-3p,miR-331-3p,miR-370-5p,miR-29b-3p,miR-545-5p,let-7d,miR-30a-5p,miR-29d-3p,miR-3065-3p,miR-380-5p,miR-28b,miR-181a-2-3p,miR-30a-3p,miR-543-5p,miR-191-3p,miR-450a-2-3p,miR-2898,miR-339a,let-7c-5p,miR-30c-2-3p,miR-1260a,miR-29a-3p,miR-34b,miR-146b-3p,miR-320b,miR-363,miR-505-3p,miR-7975,miR-19a-3p,miR-18a-3p,miR-18b,miR-2428,miR-449a,miR-3959-3p,miR-24-3p,miR-211,miR-1193,miR-2285x,miR-342-3p,miR-656-5p,let-7i,miR-34c,miR-502-5p,miR-2355-3p,miR-185-5p,let-7g-5p,miR-432-5p,miR-187-3p,miR-219a-1-3p,miR-1260b,miR-29b,miR-30b-3p,miR-380-3p,miR-2284ab,miR-345-5p,miR-654-3p,miR-363-5p,miR-466f-3p,miR-660,let-7i-5p,miR-216b-5p,miR-582-5p,miR-30c-1-3p,miR-625-5p,miR-345-5p,miR-339b,miR-487b-5p,miR-30d-5p,miR-500a-3p,miR-665-5p,miR-214,miR-3184-3p,miR-3074-5p,miR-590-3p,let-7b,miR-500,miR-664a-5p,miR-665,miR-30a-
TBX20	ENSOARG0(	T-box 20	miR-206-3p,miR-424-5p,miR-16-5p,miR-1,miR-206,miR-21-3p,miR-424-5p,miR-107,miR-135a-1-3p,miR-15a,miR-16b,miR-16a,miR-15a-5p,miR-487a-3p,miR-322-5p,miR-15b-5p,miR-103,miR-652-3p,miR-103a-3p,miR-504-5p,miR-485-3p,miR-370-3p,miR-107,miR-504,miR-16b,miR-195a-5p,miR-497-5p,miR-1a-3p
FBXW8	ENSOARG0(	F-box an	miR-216b-3p,miR-139-5p,miR-552-3p,miR-139-5p,miR-142-3p,miR-142a-3p,miR-377-3p,miR-361-3p,miR-669,miR-2366,miR-377-3p,miR-6529b,miR-216a-3p,miR-128-3p
SLK	ENSOARG0(	STE20-li	miR-2285y,miR-140-3p,miR-2285ad
HLTF	ENSOARG0(	helicase	miR-9-5p,miR-144-5p,miR-376c-3p,miR-2284l,miR-877-3p,miR-30c-1-3p,miR-1b-5p,miR-2285p,miR-320b,miR-2285af,miR-30c-2-3p,miR-320d,miR-592,miR-548w,miR-30c-1-3p,miR-185-5p,miR-376e-3p,miR-9-5p,miR-376c-3p,miR-320c,novel_32,miR-144-5p,miR-320a,miR-188-3p,miR-137-3p,miR-27b-5p,miR-4429,miR-1388-5p,miR-2284x,miR-505-3p

ALKBH6	ENSOARG0(	alkB hom	miR-410-5p,miR-450b-3p,miR-1306,miR-146b-3p,miR-670-3p,miR-299b-3p,miR-6535,miR-329-5p,miR-128-1-5p,miR-656-5p,miR-142b,miR-28c,miR-133a-5p,miR-323c,miR-342-3p,miR-335-5p,miR-1193,miR-30b-3p,miR-125b-1-3p,miR-1260b,miR-7862,miR-374b-3p,novel_116,miR-31-5p,miR-665,miR-339-5p,miR-26b-3p,miR-1343-5p,novel_51,miR-345-5p,miR-130b-5p,miR-345-5p,miR-1814c,miR-365a-3p,miR-324-5p,miR-154b-5p,miR-2355-5p,miR-141-3p,miR-486b-3p,miR-134-5p,miR-370-5p,miR-331-3p,miR-1260b,miR-582-3p,miR-200b-3p,miR-1290,miR-134,miR-1343-5p,miR-505-5p,miR-134-5p,miR-129-1-3p,miR-299a-3p,miR-429-3p,miR-1224-5p,novel_79,miR-2898,miR-200c-3p,miR-200a-3p,miR-543-5p,miR-1260a,miR-125a-3p,miR-200c,miR-339a,miR-450b-5p,miR-28b,miR-148b-5p,miR-490-3p,miR-29b-2-5p,miR-495-5p,miR-4286,miR-2403,miR-877-3p,miR-361-3p,miR-409-5p,miR-2483-3p,miR-454-5p,miR-4792,miR-452-3p,miR-2319b,miR-20a-3p,miR-499a-5p,miR-335,miR-2284q,miR-877-3p,miR-361-3p,novel_60,miR-2447,miR-3956-3p,miR-1296-5p,miR-1388-5p,miR-2284r,miR-296-5p,miR-6529a,miR-484,miR-30b-3p,miR-425-3p,miR-148b-5p,miR-2459,miR-1343-3p,novel_78,miR-222-5p,miR-3955-3p,miR-2285o,miR-214-3p,miR-342,miR-1306-5p,miR-2957,miR-505,miR-2310,miR-345-5p,miR-339b,miR-1827,miR-129b-3p,miR-204-3p,novel_32,miR-140-5p,miR-370-5p,miR-6516-3p,miR-4492,miR-130b-5p,miR-15b-3p,miR-2285j,miR-29b-2-5p,miR-665,miR-6134,miR-129-2-3p,miR-3958-5p,miR-214,novel_73,miR-148a-5p,novel_133,miR-2300b-3p,miR-2904,miR-222-5p,miR-200b,miR-1247-5p,miR-2284h-5p,miR-212-5p,miR-760-3p,miR-299-3p,miR-21-3p,novel_17,miR-324-3p,miR-185-3p,miR-486-3p,miR-329a-5p,miR-2285w
CARTPT	ENSOARG0(	CART pre	miR-133a-3p,miR-148a-5p,miR-300,miR-1197-5p,novel_121,miR-487a-3p,miR-485-3p,miR-18a-3p,miR-21-3p,novel_96,novel_127,miR-2284m,miR-1260a,miR-210-5p,miR-200a,miR-3065-3p,miR-216c-5p,miR-625-5p,miR-598-3p,miR-433-3p,miR-21a-3p,miR-381-3p,miR-223-5p,miR-27a-5p,miR-377-3p,miR-21-3p,miR-598-3p,miR-3065-3p,miR-1271,miR-487a-5p,miR-204-5p,miR-331-3p,miR-3604,miR-2284k,miR-365a-5p,miR-181c-3p,miR-6525,miR-1260b,miR-214,miR-539-5p,miR-2284ab,miR-219a-1-3p,miR-1260b,miR-432,miR-2113,miR-148b-5p,miR-432-5p,miR-2284d,novel_78,miR-452-5p,miR-466f-3p,miR-103a-2-5p,miR-214-3p,miR-491-5p,miR-365b-5p,miR-342,miR-377-3p,miR-8485,miR-193a,miR-491-3p,miR-668-3p,miR-376b,miR-494-3p,miR-493-5p,miR-335-3p,miR-493-5p,miR-181a-3p,miR-3970,miR-18a-3p,miR-3591-3p,miR-669,miR-7857-3p,miR-2285u,miR-211-5p,miR-22-5p,miR-147-5p,miR-485-3p,miR-107-5p,miR-2285n,miR-211,miR-2284j,miR-452-5p,miR-219a-5p,miR-342-3p,miR-3059-5p

GYG1	ENSOARG0(	glycogen	miR-223-5p,miR-376c-3p,miR-3957,miR-34c-5p,miR-331-5p,miR-769,miR-2285aa,miR-615-5p,miR-125a,miR-3957-5p,miR-3074-1-3p,miR-1248,miR-345-5p,miR-2318,miR-329b,miR-23b,miR-3154,miR-320a,miR-488-3p,miR-331-5p,miR-301,miR-378c,miR-365a-5p,miR-193a-5p,miR-6128,miR-486b-3p,miR-191-5p,miR-204-5p,miR-331-5p,miR-26c,miR-331-3p,miR-125b-5p,miR-135a-1-3p,miR-582-3p,novel_121,miR-9-5p,miR-302a-5p,miR-758-5p,miR-1271-5p,miR-378h,miR-412,miR-431,miR-376a-5p,miR-423-3p,miR-2427,miR-330-5p,miR-345-3p,novel_39,miR-29a-5p,miR-1957a,miR-450c-5p,miR-2285b,miR-490-3p,miR-92b-3p,miR-495-5p,miR-30a-3p,miR-181a-2-3p,miR-92a-3p,miR-450a-2-3p,miR-181b-5p,miR-320c,miR-677,miR-181b-3p,miR-2404,miR-323a-5p,miR-146b-3p,miR-670-3p,miR-34b,miR-3120-3p,miR-26b-5p,miR-378b,miR-329-5p,miR-378f,miR-376a-2-5p,miR-412-3p,miR-21c,miR-326-3p,novel_27,miR-449a,miR-2428,miR-211,miR-24-3p,miR-2285x,miR-219b-3p,miR-656-5p,miR-147-5p,miR-92a-3p,miR-34c,miR-425-5p,novel_87,miR-133a-5p,miR-142b,miR-194-3p,miR-412-5p,miR-3431,miR-31-5p,miR-26a-2-3p,miR-30b-3p,miR-21-5p,miR-219a-1-3p,miR-194-5p,miR-19b-2-5p,miR-542-3p,miR-181a-5p,miR-1843b-5p,miR-539-3p,miR-345-5p,miR-432-3p,miR-432-3p,miR-363-5p,miR-125a-5p,miR-378a-5p,miR-365b-5p,miR-190a,miR-194a,miR-2411-3p,novel_25,miR-499b-3p,miR-323-5p,miR-216b-5p,miR-296-3p,miR-545-3p,miR-378a-3p,let-7g-3p,miR-345-5p,miR-129b-3p,miR-411,miR-3184-5p,miR-338-3p,miR-6134,miR-769-5p,miR-1271,miR-2330-3p,miR-665-5p,miR-378c,miR-4492,miR-190a-5p,miR-664a,miR-30a-3p,miR-19b-1-5p,miR-338-5p,miR-30e-3p,miR-7859,miR-487a-3p,miR-23b-3p,miR-3600,miR-30d-3p,miR-485-3p,miR-133a-3p,miR-34a-5p,miR-361-5p,miR-33b-3p,miR-4429,miR-5703,miR-378a-3p,miR-378i,miR-191,miR-

SEC61A1	ENSOARG0(	Sec61 al	miR-194-3p,miR-544b,miR-412-5p,miR-503-5p,miR-22-3p,miR-873a-5p,miR-1285,miR-2113,miR-542-3p,miR-744-5p,miR-138,miR-2284ab,miR-363-5p,miR-654-3p,miR-2284l,miR-503-5p,miR-1343-5p,miR-125a-5p,miR-660,miR-196b-5p,miR-483-3p,miR-329-5p,miR-669,miR-6535,miR-374c-3p,miR-411b-3p,novel_69,miR-670-3p,miR-34b,miR-1306,miR-216a-3p,miR-18a-3p,miR-2387,miR-18b,miR-6395,miR-378f,miR-3957-3p,novel_101,miR-1193,miR-342-3p,miR-335-5p,novel_27,miR-449a,miR-34c,miR-502-5p,miR-6123,miR-128-1-5p,novel_1,miR-196a-2-3p,miR-1224-5p,miR-18b-5p,miR-6517,miR-134-5p,miR-545-5p,miR-2284z,miR-505-5p,miR-483-3p,miR-2411-5p,miR-378h,miR-1343-5p,miR-615-3p,miR-196a-5p,miR-134,miR-128-3p,miR-495-5p,miR-4286,novel_39,miR-3065-3p,miR-1271-3p,miR-490-3p,novel_127,novel_96,miR-200a-3p,miR-2898,miR-370-3p,miR-2285aa,miR-1271,miR-141-3p,miR-125a,miR-615-5p,miR-3956,miR-874-3p,miR-324-5p,miR-34c-5p,miR-374c-3p,miR-3154,miR-18a,miR-23a-5p,miR-485-5p,miR-329b,miR-193a-5p,miR-133a-3p,novel_42,miR-378c,miR-181c-3p,miR-1197-3p,miR-125b-5p,miR-331-3p,miR-3969,miR-5100,miR-370-5p,miR-28-5p,miR-134-5p,miR-486b-3p,miR-138-5p,miR-2300a-5p,miR-1343-3p,miR-216a-3p,miR-496-5p,miR-142-3p,miR-147a,miR-378b,miR-2284r,miR-301b-5p,miR-2284aa,miR-1388-5p,miR-378c,miR-101a-5p,miR-544a,miR-382-3p,miR-101b-3p,miR-4532,miR-378d,miR-505,novel_19,miR-2957,miR-342,miR-5126,miR-1306-5p,miR-491-5p,novel_124,miR-4792,miR-4443,miR-378e,miR-361-3p,novel_94,miR-181a-3p,miR-219a-5p,miR-423-5p,miR-1973,miR-371a-5p,miR-301a-5p,miR-218-5p,miR-615,miR-4726-5p,miR-335,miR-18a-3p,miR-1247-5p,novel_83,miR-532-3p,miR-328-3p,miR-383-5p,miR-378a-3p,miR-1197-5p,miR-188-3p,novel_133,miR-378i,miR-218-1-

ENO2	ENSOARG0(	enolase	miR-1260b,miR-1197-3p,novel_42,let-7e-3p,miR-193a-5p,miR-331-5p,miR-6740-5p,miR-3959-5p,miR-874-3p,miR-2355-5p,miR-2285aa,miR-329a,miR-345-5p,miR-329b,miR-331-5p,novel_39,miR-3064-5p,novel_96,miR-152-3p,miR-205-5p,miR-505-5p,miR-9-5p,miR-486-5p,novel_121,miR-450a-1-3p,miR-6517,miR-429-3p,miR-299a-3p,miR-2285ad,miR-1224-5p,novel_79,miR-196a-5p,miR-23b-5p,miR-1343-5p,miR-2284w,miR-193b-3p,miR-6123,miR-28c,miR-576-3p,novel_87,miR-299b-3p,miR-181b-3p,miR-6535,miR-669,miR-677,miR-320c,miR-3074-5p,miR-3120-3p,miR-148a-3p,miR-3065-5p,miR-216a-3p,miR-432-3p,miR-432-3p,miR-6240,miR-378a-5p,miR-196b-5p,miR-193a-3p,miR-377-3p,miR-7-5p,novel_51,miR-2285v,miR-134-3p,miR-3431,novel_116,miR-31-5p,miR-339-5p,miR-16-1-3p,miR-3432b,miR-542-3p,miR-7b-5p,miR-708-3p,novel_23,miR-450c-3p,miR-6525,miR-3074-5p,miR-433-5p,miR-214,miR-130b-5p,miR-4492,miR-874-5p,miR-665,miR-377-3p,miR-2284s,miR-30c-1-3p,miR-2331-3p,miR-296-3p,miR-345-5p,miR-339b,miR-1827,miR-1895,miR-450a-1-3p,miR-2285c,miR-129b-3p,miR-377-5p,miR-376a-5p,miR-486-3p,novel_17,miR-92a-1-5p,miR-324-3p,miR-219b-5p,miR-328-3p,miR-212-5p,miR-184-3p,miR-34a-5p,miR-16-1-3p,miR-664-5p,miR-218-1-3p,miR-877-3p,miR-361-3p,miR-3956-3p,miR-2447,miR-9-5p,miR-6527,miR-3968,miR-499a-5p,miR-378g,miR-139-5p,miR-7689-3p,miR-136-5p,miR-378j,miR-8485,miR-1388-3p,miR-491-5p,miR-214-3p,miR-103a-2-5p,miR-199a-3p,miR-147a,miR-455-3p,miR-1343-3p,novel_78,miR-330-3p,miR-145b,miR-362-3p,miR-1388-5p,miR-2284r,miR-329-3p,miR-138-5p,miR-486b-3p,miR-28-5p,miR-3969,miR-331-3p,miR-34c-5p,miR-3957,miR-3957-5p,miR-615-5p,miR-10b-3p,miR-485-5p,miR-671-5p,miR-23a-5p,miR-3154,miR-320a,miR-28b,miR-3529-3p,miR-181a-2-

MCCC2	ENSOARG0(	methylcr	miR-143-3p,miR-29b-1-5p,miR-142a-3p,miR-369-3p,miR-20a,miR-2887,miR-105-3p,miR-1277-5p,miR-30b-3p,miR-3653-3p,miR-21-3p,miR-106a,miR-320d,miR-99a-3p,miR-122-3p,miR-125b,miR-196a-3p,miR-210-5p,novel_17,miR-532-3p,novel_83,miR-23b-3p,miR-23c,miR-505-3p,miR-30d-3p,miR-548e-3p,miR-106a,miR-218-1-3p,miR-33b-3p,novel_73,miR-4429,miR-140-3p,miR-1197-5p,miR-5703,miR-1193,miR-193b-5p,miR-181c-5p,miR-1271,miR-433-5p,novel_74,miR-6516-3p,miR-30a-3p,miR-219b-3p,miR-29b-2-5p,miR-665,miR-320e,miR-30e-3p,miR-204-3p,miR-155-5p,miR-499b-3p,miR-27a-5p,miR-139-5p,miR-216b-3p,miR-182-5p,miR-552-3p,miR-504-5p,miR-543-3p,miR-1827,let-7g-3p,miR-27b-5p,miR-433-3p,miR-3613-5p,miR-193a,miR-8485,miR-3187-3p,miR-23a-3p,miR-676-3p,miR-106a-5p,miR-382-3p,miR-25-5p,miR-29b-1-5p,miR-106b-5p,miR-182-5p,miR-320b,novel_103,miR-9788-3p,miR-2459,miR-1185-3p,miR-425-3p,miR-496-5p,miR-539-5p,miR-142-3p,miR-1298-3p,miR-27a-3p,miR-181b-2-3p,miR-330-3p,novel_4,miR-1343-3p,miR-548w,miR-2284aa,miR-1839-5p,miR-3120-5p,miR-2426,miR-30b-3p,miR-541,miR-219a-2-3p,miR-877-3p,miR-2285f,miR-6527,miR-219a-5p,miR-194b-5p,miR-181d-5p,miR-2447,miR-541-3p,miR-499a-5p,miR-335,miR-218-5p,miR-101-3p,miR-203-3p,miR-27a-3p,miR-1185-2-3p,miR-499a-3p,miR-139-5p,miR-452-3p,miR-877-3p,miR-9-3p,miR-96-5p,miR-504,miR-361-3p,miR-29a-5p,novel_39,miR-3956-5p,miR-28b,miR-2478,miR-30a-3p,miR-29b-2-5p,miR-2403,miR-3529-3p,miR-450a-2-3p,miR-1a-2-5p,miR-181b-5p,miR-1260a,miR-219-3p,miR-2284z,miR-505-5p,miR-1271-5p,miR-196a-2-3p,miR-155-5p,let-7a-2-3p,miR-582-3p,miR-10b-3p,miR-93,miR-1260b,miR-6128,miR-126b-3p,miR-100-3p,miR-125b-5p,miR-3969,miR-1-5p,miR-99b-3p,miR-5100,miR-2332,miR-223-5p,miR-154b-5p,miR-331-5p,miR-3965,miR-365a-

G2E3	ENSOARG0(	G2/M-pha	miR-130a-5p,miR-29b-1-5p,miR-376a-5p,miR-380-3p,miR-2285w,miR-329a-5p,miR-21-3p,miR-374a-5p,miR-500a-5p,miR-324-3p,miR-7859,miR-2284h-5p,miR-376a-5p,miR-188-3p,miR-206-3p,miR-142a-5p,miR-3074-2-3p,miR-6516-3p,miR-155-5p,miR-208b-5p,miR-2285j,miR-655-3p,miR-324-3p,miR-544-3p,miR-877-5p,miR-155-5p,novel_32,miR-660-5p,miR-496-3p,miR-140-5p,miR-532-5p,miR-21-3p,miR-339b,miR-2312,let-7g-3p,novel_99,miR-21a-3p,miR-299,miR-655,miR-1b-5p,miR-1185-5p,miR-29b-1-5p,miR-142-5p,miR-1185-3p,miR-421,miR-548w,miR-330-3p,miR-409b,miR-145b,miR-544a,miR-1388-5p,miR-16-2-3p,miR-21b,miR-6529a,miR-125b-2-3p,miR-1a-3p,novel_111,miR-194b-5p,miR-2285f,miR-499a-5p,miR-2284q,miR-374a-3p,miR-382-3p,miR-2285n,miR-218-5p,miR-3596,miR-1185-2-3p,miR-2319b,miR-211-5p,miR-374b-5p,miR-2285u,miR-299b-5p,miR-129b-5p,miR-2478,miR-29a-5p,miR-200a,miR-1,miR-200c-3p,miR-200c,miR-339a,miR-302a-5p,miR-545-5p,miR-429-3p,miR-2285ad,novel_79,miR-411-3p,let-7a-2-3p,miR-496,miR-155-5p,miR-218-2-3p,miR-374a-3p,miR-374b,miR-331-5p,miR-204-5p,miR-374b-3p,miR-410-3p,miR-2332,miR-135a-5p,miR-100-3p,miR-331-5p,miR-324-5p,miR-3068-3p,miR-503-3p,miR-141-3p,let-7g-3p,miR-329b,miR-331-5p,miR-424-3p,let-7a-2-3p,miR-6240,miR-660,miR-194a,novel_51,miR-330-3p,miR-2284d,miR-544b,miR-339-5p,miR-16-1-3p,miR-380-3p,miR-188-5p,miR-2113,miR-194-5p,miR-145a-5p,miR-323c,miR-211,miR-2284w,miR-374c-5p,miR-206,miR-33a-3p,miR-329-5p,miR-1291,miR-135b-5p,miR-2285ab,miR-3591-3p
CDC42BPB	ENSOARG0(	CDC42 bi	miR-4532,miR-363-5p,miR-146b,miR-214-3p,miR-199a-3p,miR-146a,miR-505,miR-29b-1-5p,novel_19,novel_51,miR-149-5p,miR-31-5p,miR-199b-3p,miR-548w,novel_4,miR-1343-3p,miR-665,miR-27a-3p,miR-30b-3p,miR-744-5p,miR-27b-3p,miR-296-5p,miR-301b-5p,miR-541,miR-22-3p,miR-7857,miR-30b-3p,miR-361-3p,miR-24-3p,miR-371b-3p,miR-423-5p,miR-2428,novel_120,miR-2447,miR-541-3p,miR-335-5p,miR-128-1-5p,miR-335,miR-490-5p,miR-199c,novel_82,miR-301a-5p,miR-669,miR-27a-3p,miR-4443,miR-499b-5p,miR-2285u,miR-320b,miR-1291,miR-19a-3p,miR-504,miR-9851-3p,miR-146b-5p,miR-6395,miR-361-3p,miR-148a-3p,miR-29b-1-5p,novel_39,miR-146a-5p,miR-486-3p,miR-29b-2-5p,miR-2898,miR-152-3p,miR-200a-3p,miR-205-5p,miR-185-3p,miR-24-2-5p,miR-7859,miR-148b-3p,miR-199a-3p,miR-134-5p,miR-328-3p,miR-548e-3p,miR-19b-3p,miR-134,miR-218-2-3p,miR-1193,miR-122-5p,miR-3184-5p,miR-433-5p,miR-214,miR-3074-2-3p,miR-664a,miR-4492,miR-486b-3p,miR-134-5p,miR-15b-3p,miR-2433,miR-2285j,miR-331-3p,miR-29b-2-5p,miR-204-3p,novel_25,miR-22-3p,miR-574-5p,miR-504-5p,miR-2355-5p,miR-141-3p,miR-552-3p,miR-485-5p,miR-1827,miR-129b-3p

AARS	ENSOARG0(	alanyl-t	miR-365a-5p,miR-378c,novel_42,miR-193a-5p,miR-6128,miR-767-5p,miR-134-5p,miR-29b-3p,miR-25-3p,miR-376c-3p,miR-542-5p,miR-370-3p,miR-1271,miR-767,miR-503-3p,miR-3154,miR-92b-3p,miR-2284y,miR-3065-3p,miR-2284a,miR-490-3p,miR-1271-3p,miR-3064-5p,miR-1,miR-127-5p,novel_127,miR-92a-3p,miR-2285af,miR-125a-3p,miR-9-5p,miR-483-3p,miR-378h,miR-134-5p,miR-486-5p,miR-412,miR-411-3p,miR-582-3p,miR-134,miR-1343-5p,miR-29d-3p,miR-2428,miR-342-3p,miR-29c-3p,miR-92a-3p,miR-128-1-5p,miR-206,miR-379-3p,miR-29a,novel_1,miR-22-5p,miR-576-3p,miR-6529b,miR-323a-5p,miR-29a-3p,miR-6535,novel_68,miR-320b,miR-412-3p,miR-9851-3p,miR-378f,miR-2285ab,miR-18a-3p,miR-2285r,miR-493-5p,miR-101-5p,miR-363-5p,miR-483-3p,miR-1343-5p,miR-365b-5p,miR-32,miR-149-5p,miR-29b,miR-30b-3p,miR-26a-2-3p,miR-206-3p,miR-378c,miR-4510,miR-214,miR-665-5p,miR-664a,miR-129-5p,miR-4492,miR-664a-5p,miR-376e-3p,miR-6130,miR-323-5p,miR-221-5p,miR-32-5p,miR-378a-3p,miR-3065-3p,miR-2312,let-7g-3p,miR-129b-3p,miR-411,miR-125b-2-3p,miR-376c-3p,miR-216c-5p,miR-221-5p,miR-26a-5p,miR-185-3p,miR-422a,miR-383-5p,miR-532-3p,miR-18a-3p,miR-218-1-3p,miR-2284u,novel_133,miR-378i,miR-1197-5p,miR-188-3p,miR-378a-3p,miR-140-3p,miR-361-3p,miR-877-3p,novel_60,miR-3059-5p,miR-3956-3p,miR-363-3p,miR-2285f,miR-9-5p,miR-452-5p,miR-4726-5p,miR-301a-5p,miR-378e,miR-3064-5p,miR-2284g,miR-452-3p,miR-3963,miR-493-5p,miR-1983,miR-361-3p,miR-378d,miR-4532,miR-758-3p,miR-1388-3p,miR-2284b,miR-3085-3p,miR-214-3p,miR-342,miR-1343-3p,miR-2300a-5p,miR-1388-5p,miR-378c,miR-450b-5p,miR-296-5p,miR-378b,miR-301b-5p,miR-1a-3p,miR-412-3p,miR-125b-2-3p,miR-30b-3p,miR-3962,miR-758-3p

KIAA1328	ENSOARG0(	KIAA1328	miR-125a,miR-370-3p,miR-874-3p,miR-376c-3p,miR-324-5p,miR-300-3p,miR-3154,miR-30e-5p,miR-485-5p,miR-671-5p,miR-7144-5p,miR-345-5p,miR-876-3p,miR-10a-3p,miR-10b-3p,miR-16-5p,miR-1197-3p,miR-331-3p,miR-125b-5p,novel_79,miR-222,miR-582-3p,miR-322-5p,miR-24-2-5p,miR-505-5p,miR-330-5p,miR-30a-5p,miR-10a-5p,miR-30c,miR-181a-2-3p,miR-3064-5p,miR-30a-3p,miR-3529-3p,novel_39,miR-1957a,miR-24-2-5p,miR-339a,miR-30f,miR-30c-2-3p,miR-6119-5p,novel_127,novel_96,miR-107,miR-2898,miR-10a-5p,miR-320b,miR-329-5p,novel_68,novel_69,miR-374c-3p,miR-323a-5p,miR-18a-3p,miR-2387,miR-195a-5p,miR-6395,miR-9851-3p,miR-107,miR-335-5p,miR-2428,miR-326-3p,miR-371b-3p,miR-16a,miR-6402,miR-15a,miR-502b,miR-421-5p,miR-26b-3p,miR-339-5p,miR-103a-3p,miR-187-3p,miR-2355-3p,miR-103,miR-194-3p,miR-149-5p,miR-185-5p,miR-134-3p,miR-503-5p,miR-3071-5p,novel_23,miR-22-3p,miR-1843b-5p,miR-873a-5p,miR-7862,miR-497-5p,miR-3432b,miR-30b-3p,miR-16b,miR-363-5p,miR-345-5p,miR-432-3p,miR-654-3p,novel_51,miR-503-5p,miR-125a-5p,miR-378a-5p,miR-504-5p,miR-30c-1-3p,miR-15b-5p,miR-22-3p,miR-216b-5p,miR-323-5p,miR-6130,miR-499b-3p,miR-376e-3p,miR-10a,miR-30d-5p,miR-129b-3p,let-7f-1-3p,miR-625-5p,miR-30f,miR-345-5p,miR-339b,miR-425-5p,miR-2330-3p,miR-4510,miR-6134,miR-6525,miR-10b-5p,miR-320e,miR-665,miR-30a-3p,miR-30e-3p,miR-222-3p,miR-424-5p,miR-4492,miR-664a,miR-30d-3p,miR-212-5p,miR-18a-3p,miR-328-3p,miR-616-3p,miR-532-3p,miR-15a-5p,miR-383-5p,miR-1197-5p,miR-30c-5p,miR-33b-3p,miR-503-5p,miR-10a-3p,miR-329a-5p,miR-183-5p,miR-130a-5p,miR-376c-3p,miR-421-5p,miR-185-3p,miR-125b,miR-324-3p,miR-210-5p,miR-30b,miR-30b-3p,miR-30d,miR-760-3p,miR-202-5p,miR-7689-

MMP1	ENSOARG0(	matrix m	miR-2411,miR-376a-5p,miR-20a,miR-345-3p,miR-216c-5p,miR-329a-5p,miR-30d,miR-106a,miR-500a-5p,miR-125b,miR-92a-1-5p,miR-345-3p,novel_17,miR-199a-3p,miR-1247-5p,miR-7859,miR-106a,miR-16-1-3p,miR-4429,novel_73,miR-2904,miR-1193,miR-5703,miR-2300b-3p,miR-338-3p,miR-4510,miR-130b-5p,miR-669a-3p,miR-450b-3p,miR-17-3p,miR-219b-3p,miR-19b-1-5p,miR-3604,miR-2285j,miR-204-3p,miR-544-3p,miR-2284s,miR-499b-3p,miR-377-3p,miR-193b-3p,miR-6130,miR-139-5p,miR-296-3p,miR-182-5p,miR-625-5p,miR-543-3p,miR-186-5p,miR-2312,miR-30d-5p,miR-450a-1-3p,miR-2285c,miR-193a,miR-106a-5p,miR-494-3p,miR-101b-3p,miR-103a-2-5p,miR-106b-5p,miR-182-5p,miR-199a-3p,miR-320b,miR-326,miR-455-3p,miR-216a-3p,miR-2285e,miR-409b,miR-199b-3p,novel_78,miR-2284aa,miR-544a,miR-3962,miR-484,miR-2284r,miR-541,miR-219a-2-3p,miR-2285f,miR-3059-5p,novel_60,miR-2447,miR-541-3p,miR-3956-3p,miR-7134-3p,miR-3968,miR-371a-5p,miR-107-5p,miR-218-5p,miR-499a-3p,miR-7977,miR-4792,miR-139-5p,miR-335-3p,miR-136-5p,miR-1306-3p,miR-140-5p,miR-450b-5p,miR-17-3p,miR-127-5p,miR-1260a,miR-219-3p,miR-450a-1-3p,miR-134-5p,miR-2284z,miR-2411-5p,miR-330-5p,miR-345-3p,miR-134,miR-93,miR-1260b,miR-6128,miR-134-5p,miR-125b-5p,miR-135a-5p,miR-410-5p,miR-1306-3p,miR-2355-5p,miR-2285aa,miR-125a,miR-20b-5p,miR-7144-5p,miR-2318,miR-329b,miR-93-5p,miR-320a,miR-17-5p,let-7j,miR-296-3p,miR-432-3p,miR-668-3p,miR-130b-5p,miR-125a-5p,miR-452-5p,miR-193a-3p,miR-17-5p,miR-106a-5p,miR-2411-3p,miR-296-3p,novel_116,miR-544b,miR-20b,miR-2285g,miR-185-5p,miR-6239,miR-1260b,miR-16-1-3p,miR-450c-3p,miR-3071-5p,miR-19b-2-5p,miR-708-3p,miR-1434-3p,miR-542-3p,novel_27,miR-326-3p,miR-2284w,miR-24-3p,miR-193b-3p,miR-17-3p,miR-495-

COG5	ENSOARG0(	componen	miR-362-5p,miR-9-3p,miR-452-5p,miR-2285n,miR-30b-5p,miR-7857-3p,miR-378e,miR-376d,let-7d-5p,miR-20a-3p,miR-3064-5p,miR-499b-5p,miR-541-5p,miR-129b-5p,miR-30c-1-3p,miR-193a,let-7a-5p,miR-33a-5p,miR-3955-3p,miR-3085-3p,miR-2285o,let-7e,novel_103,miR-320b,miR-2957,miR-500-5p,miR-9788-3p,novel_4,miR-2300a-5p,miR-151a-3p,miR-5010-3p,miR-101a-5p,miR-450b-5p,miR-378c,miR-16-2-3p,miR-4791,miR-10b,miR-378b,miR-206-3p,miR-10b-5p,let-7b-5p,miR-378c,miR-2330-3p,miR-155-5p,miR-655-3p,miR-155-5p,miR-378a-3p,miR-27b-5p,miR-29b-1-5p,miR-2432,miR-21-3p,miR-30d,miR-216a-5p,miR-616-3p,miR-548e-3p,miR-4429,miR-378d,miR-1197-5p,miR-1193,miR-5703,miR-3959-3p,miR-24-3p,novel_27,miR-136-3p,novel_101,novel_1,miR-147-5p,miR-199c,miR-656-5p,miR-22-5p,let-7i,miR-382-5p,miR-10a-5p,miR-2285k,miR-135b-5p,miR-6516-3p,miR-7975,miR-2387,miR-376b-3p,miR-378b,miR-654-3p,miR-2285l,miR-503-5p,let-7g-5p,miR-503-5p,miR-185-5p,miR-2355-3p,miR-6238,miR-187-3p,miR-377-5p,miR-1260b,miR-1434-3p,miR-653,miR-323-3p,miR-500b-5p,miR-378c,miR-365a-5p,miR-767-5p,miR-1-5p,miR-135a-5p,miR-409-3p,miR-331-3p,miR-141-3p,novel_91,miR-10b-3p,novel_48,miR-503-3p,miR-381-3p,miR-362-5p,miR-320a,miR-1,miR-30a-3p,miR-2898,miR-543-5p,miR-6119-5p,miR-376b-3p,miR-1260a,let-7c-5p,miR-30c-2-3p,miR-378h,miR-582-3p,miR-545-5p,let-7d,miR-300,miR-411-3p,miR-10a-5p,miR-134,miR-345-3p,miR-30a-5p,novel_111,miR-885-5p,miR-499a-5p,miR-628-3p,miR-3964,miR-2440,miR-301a-5p,miR-25,miR-203-3p,miR-2319b,miR-7689-3p,miR-2285u,miR-136-5p,miR-9-3p,miR-376a-3p,miR-378d,miR-8485,miR-101b-3p,miR-1388-3p,miR-744-3p,miR-491-5p,miR-214-3p,miR-29b-1-5p,miR-505,miR-142-5p,miR-376b-3p,miR-421,miR-199b-3p,miR-1839-5p,miR-301b-

CDK4	ENSOARG0(	cyclin-d	miR-335-5p,miR-576-3p,miR-3074-5p,miR-329-5p,miR-320c,miR-1306,miR-3141,miR-376b-5p,miR-378f,miR-2284e,miR-2284l,miR-432-3p,novel_51,miR-365b-5p,miR-3141,miR-378a-5p,miR-339-5p,miR-103a-3p,miR-194-3p,novel_116,miR-3431,miR-134-3p,miR-3535,miR-22-3p,miR-197-3p,miR-1843b-5p,miR-19b-2-5p,miR-2284x,miR-188-5p,miR-497-5p,miR-16b,miR-16-5p,miR-2284v,miR-1197-3p,miR-6740-5p,miR-2284k,miR-1271,miR-2285aa,miR-376c-5p,miR-1248,miR-329b,miR-876-3p,miR-3064-5p,miR-200a-3p,novel_96,miR-127-5p,miR-18b-5p,miR-1224-5p,novel_79,miR-412,novel_121,miR-129-1-3p,miR-322-5p,miR-2427,miR-2284j,miR-190b-5p,miR-3956-3p,miR-423-5p,novel_111,miR-877-3p,miR-361-3p,miR-2440,novel_82,miR-211-5p,miR-2285u,miR-378g,miR-544-5p,miR-504,miR-299b-5p,miR-676-3p,miR-2310,miR-378d,miR-16b,miR-29b-1-5p,miR-491-5p,miR-145b,miR-1343-3p,miR-148b-5p,miR-147a,miR-665-5p,miR-3074-5p,miR-129-2-3p,miR-3184-5p,miR-769-5p,miR-664a-5p,miR-2285j,miR-500,miR-190a-5p,miR-1468-5p,miR-4492,miR-129-5p,miR-874-5p,novel_74,miR-296-3p,miR-548o-3p,miR-504-5p,miR-15b-5p,miR-216b-5p,miR-10a,miR-500a-3p,miR-543-3p,miR-339b,miR-345-3p,miR-329a-5p,miR-377-5p,miR-422a,miR-92a-1-5p,miR-324-3p,miR-26a-5p,miR-2285p,miR-618,novel_17,miR-212-5p,miR-328-3p,miR-7859,miR-378a-3p,miR-1434-5p,miR-193b-5p,miR-378i,miR-148a-5p,miR-133a-3p,miR-33b-3p,miR-17-3p,miR-136-3p,miR-2428,miR-323c,miR-371b-3p,miR-211,miR-16a,miR-6529b,miR-15a,miR-656-5p,miR-22-5p,miR-10a-5p,miR-2404,miR-378b,miR-18b,miR-6395,miR-195a-5p,miR-107,miR-2483-5p,miR-654-3p,miR-136-3p,miR-190a,miR-377-5p,miR-103,miR-544b,miR-2355-3p,miR-374b-3p,miR-145a-5p,miR-873a-5p,miR-2284ab,miR-30b-3p,miR-1306-5p,miR-378c,miR-365a-5p,miR-

DPY19L2	ENSOARG0(	dpy-19-l	miR-23a-3p,miR-199a-3p,miR-16b,miR-182-5p,miR-103a-2-5p,miR-146b,miR-330-3p,novel_78,miR-548w,miR-181b-2-3p,miR-425-3p,miR-455-3p,miR-329-3p,miR-1388-5p,miR-362-3p,miR-101c,miR-885-5p,miR-2447,miR-1285-5p,miR-628-3p,miR-1843a-3p,miR-2319b,miR-2285u,miR-374b-5p,miR-2483-3p,miR-25,miR-378g,miR-203-3p,miR-221,miR-381-3p,miR-9-3p,miR-369-3p,miR-181b-1-3p,miR-377-5p,miR-486-3p,miR-2424,miR-26a-5p,miR-2285p,miR-345-3p,miR-202-5p,miR-23c,miR-7859,miR-23b-3p,miR-30c-5p,miR-1434-5p,miR-217-5p,miR-33b-3p,novel_73,miR-361-5p,miR-16-1-3p,miR-218-1-3p,miR-3074-5p,miR-433-5p,miR-224-5p,miR-142a-5p,miR-181c-5p,miR-665,miR-219b-3p,miR-4492,miR-500,miR-30c-1-3p,miR-15b-5p,miR-30d-5p,miR-411,miR-655,miR-129b-3p,miR-500a-3p,miR-186-5p,miR-6240,miR-628-5p,miR-668-3p,miR-2284l,miR-432-3p,miR-432-3p,miR-146a,miR-2411-3p,miR-3955-5p,miR-3431,miR-134-3p,miR-2113,miR-542-3p,miR-16-1-3p,miR-16b,miR-2284x,miR-497-5p,miR-2284w,miR-374c-5p,miR-379-3p,miR-3074-5p,miR-26b-5p,miR-374c-3p,miR-181b-3p,miR-320c,miR-677,miR-3065-5p,miR-3970,miR-146b-5p,miR-3064-5p,miR-2285b,novel_39,miR-29a-5p,miR-219-3p,miR-30f,miR-127-5p,miR-200a-3p,miR-429-3p,miR-2285ad,miR-758-5p,miR-322-5p,miR-505-5p,miR-486-5p,miR-2284z,miR-134-5p,miR-1290,miR-330-5p,miR-2284v,miR-16-5p,miR-2284k,miR-134-5p,miR-574-5p,miR-2285aa,miR-30e-5p,miR-23b,miR-329a,miR-767,miR-424-5p,miR-33a-5p,miR-30c-1-3p,novel_115,miR-326,miR-320b,miR-362-3p,miR-3955-3p,miR-3085-3p,novel_4,miR-5010-3p,miR-221-3p,miR-9788-3p,miR-30b-3p,miR-2284aa,miR-181d-5p,miR-452-5p,miR-219a-2-3p,miR-2285n,miR-30b-5p,miR-107-5p,miR-371a-5p,miR-2284q,miR-374a-3p,miR-20a-3p,miR-3064-5p,miR-499b-5p,miR-7857-3p,miR-129b-5p,novel_94,miR-183-5p,miR-130a-5p,miR-

HEPH	ENSOARG0(	hephaest	miR-1197-3p,miR-1260b,miR-16-5p,miR-134-5p,miR-125b-5p,novel_63,miR-3959-5p,miR-125a,miR-1271,miR-2285aa,miR-874-3p,miR-1248,miR-2318,miR-345-5p,miR-876-3p,miR-200a-5p,miR-137-3p,miR-301,miR-29a-5p,novel_39,miR-148b-5p,miR-152-3p,novel_127,miR-205-5p,miR-129-1-3p,miR-450a-1-3p,miR-134-5p,miR-322-5p,miR-18b-5p,miR-1224-5p,miR-429-3p,miR-330-5p,miR-1290,miR-301b-3p,miR-199b-5p,miR-29c-3p,miR-29a,miR-576-3p,miR-4324,miR-181b-3p,miR-677,miR-374c-3p,miR-411b-3p,miR-3120-3p,miR-329-5p,novel_68,miR-410-5p,miR-3591-3p,miR-150-5p,miR-3970,miR-148a-3p,miR-432-3p,miR-432-3p,miR-668-3p,miR-380-5p,miR-194a,miR-2411-3p,novel_51,miR-105-5p,miR-296-3p,novel_116,miR-411-5p,miR-149-5p,miR-3431,miR-188-5p,miR-27b-3p,miR-497-5p,miR-16-1-3p,miR-16b,miR-197-3p,miR-450c-3p,miR-223-3p,miR-142a-5p,miR-214,miR-3958-5p,miR-129-2-3p,miR-454-3p,novel_74,miR-30a-3p,miR-30e-3p,miR-15b-5p,miR-301a-3p,miR-10a,miR-154a-3p,miR-2331-3p,miR-543-3p,miR-345-5p,miR-450a-1-3p,miR-143-3p,miR-2424,miR-486-3p,miR-181b-1-3p,miR-377-5p,miR-376a-5p,miR-195a-3p,miR-487a,miR-2285w,miR-329a-5p,miR-345-3p,miR-500a-5p,novel_17,miR-210-5p,miR-618,miR-328-3p,miR-2284h-5p,miR-30d-3p,miR-18a-3p,miR-16-1-3p,novel_73,miR-361-3p,miR-2447,miR-154-3p,miR-2440,novel_82,miR-218-5p,miR-2483-3p,miR-3596,miR-24-1-5p,miR-544-5p,miR-3963,miR-96-5p,miR-8485,miR-103a-2-5p,miR-214-3p,miR-199b-5p,miR-130b-3p,miR-1185-5p,miR-16b,miR-29b-1-5p,miR-2459,miR-455-3p,miR-148b-5p,miR-539-5p,miR-1298-3p,miR-142-5p,miR-181b-2-3p,miR-548w,novel_78,miR-1343-3p,miR-758-3p,miR-541,miR-145-3p,miR-374a-3p,miR-103b,miR-6128,miR-486b-3p,miR-374b-3p,miR-204-5p,miR-31-3p,miR-138-5p,miR-331-3p,miR-29b-3p,miR-410-5p,miR-154b-5p,miR-199a-5p,miR-
LRRC23	ENSOARG0(	leucine	miR-324-3p,miR-362-3p,miR-185-3p,miR-194a,miR-342,miR-5126,miR-30b-3p,novel_96,miR-491-5p,novel_124,miR-25-5p,miR-4532,novel_39,miR-3065-3p,miR-2478,miR-194-5p,miR-30b-3p,miR-484,miR-329-3p,miR-362-3p,miR-4508,miR-362-3p,miR-30b-3p,miR-2285ad,miR-548w,miR-149-5p,miR-9788-3p,miR-2459,miR-6740-5p,miR-2285y,miR-26c,novel_82,miR-2285n,miR-4492,miR-4726-5p,miR-1296-5p,miR-193a-5p,miR-194b-5p,miR-665-5p,miR-342-3p,miR-3184-5p,miR-423-5p,miR-24-3p,novel_48,miR-3963,miR-329a,miR-3065-3p,miR-10b,miR-4443,miR-877-5p,miR-670-3p
TSPAN31	ENSOARG0(	tetraspa	miR-3059-5p,miR-452-5p,miR-214,miR-376a-5p,miR-29a-5p,miR-338-3p,miR-380-3p,miR-2284w,miR-6134,miR-502-5p,miR-576-3p,miR-6740-5p,miR-107-5p,miR-135a-5p,miR-21-3p,miR-214-3p,miR-6517,miR-323a-5p,miR-432-5p,miR-323-5p,miR-185-5p,miR-432,miR-193b-5p,miR-5703,miR-380-3p,miR-10b-3p,miR-3432b,miR-218-1-3p,miR-6516-3p,miR-135b-5p

SCFD1	ENSOARG0(	sec1 fam	miR-21-3p,miR-320d,miR-4661-5p,miR-219b-5p,miR-500a-5p,miR-125b,miR-345-3p,miR-196a-3p,miR-146a-5p,miR-2411,miR-2432,miR-376a-5p,miR-130a-5p,miR-148a-5p,miR-4429,miR-33b-3p,miR-2904,miR-5703,miR-376a-5p,miR-2300b-3p,novel_83,miR-1247-5p,miR-148b-3p,miR-3600,miR-487a-5p,miR-130b-5p,miR-450b-3p,miR-155-5p,miR-6516-3p,miR-664a,miR-219b-3p,miR-665,miR-655-3p,miR-6134,miR-338-3p,miR-2330-3p,miR-539-5p,miR-4510,novel_99,miR-582-5p,miR-345-5p,miR-655,miR-450a-1-3p,miR-155-5p,miR-582-5p,miR-139-5p,miR-6130,miR-370-5p,miR-552-3p,miR-2331-3p,miR-32-5p,miR-504-5p,miR-146b,miR-1185-5p,miR-3955-3p,miR-2957,miR-320b,novel_115,miR-3187-3p,miR-1b-5p,miR-376b,miR-101c,miR-21b,miR-2284aa,miR-101a-5p,miR-30b-3p,miR-484,miR-9788-3p,miR-455-3p,miR-148b-5p,miR-2459,miR-539-5p,miR-105-1,miR-374a-3p,miR-335,miR-218-5p,miR-1285-5p,miR-219a-2-3p,miR-9-5p,miR-3059-5p,miR-363-3p,miR-335-3p,miR-9-3p,miR-504,miR-136-5p,miR-129b-5p,miR-361-3p,miR-7857-3p,miR-2483-3p,miR-3082-5p,miR-211-5p,miR-126b-5p,miR-7977,miR-139-5p,miR-2319b,miR-20a-3p,miR-200a-3p,miR-92a-3p,miR-152-3p,miR-450a-2-3p,miR-2898,miR-28-3p,miR-6536,miR-1a-2-5p,miR-1260a,miR-6119-5p,miR-219-3p,miR-2397-5p,novel_39,miR-200a,miR-450b-5p,miR-92b-3p,miR-495-5p,miR-105-2,miR-155-5p,let-7a-2-3p,miR-300,miR-2427,miR-345-3p,miR-582-3p,miR-450a-1-3p,miR-2284z,miR-302a-5p,miR-9-5p,miR-2411-5p,miR-204-5p,miR-374b-3p,miR-138-5p,miR-767-5p,miR-125b-5p,miR-1-5p,miR-410-5p,miR-370-5p,miR-145-3p,miR-374a-3p,miR-1260b,miR-193a-5p,miR-28a-3p,miR-7144-5p,miR-23a-5p,miR-485-5p,miR-1248,miR-767,miR-10b-3p,miR-345-5p,let-7g-3p,miR-320a,miR-3154,miR-25-3p,miR-370-3p,miR-141-3p,miR-125a,miR-3068-3p,miR-125a-5p,miR-105-5p,miR-32,miR-2411-
SUN5	ENSOARG0(	Sad1 and	novel_68,miR-2319b,miR-3074-5p,miR-20a-3p,miR-541-5p,miR-299b-3p,miR-203-3p,miR-378b,miR-129b-5p,miR-216a-3p,miR-452-5p,miR-8095,miR-7705,miR-485-3p,miR-206,novel_1,miR-421-5p,miR-656-5p,miR-374a-3p,miR-2284q,miR-147a,miR-216a-3p,miR-223-3p,miR-1a-3p,novel_23,miR-758-3p,miR-1260b,miR-2284aa,miR-130b-5p,let-7c-3p,miR-33a-5p,miR-296-3p,miR-758-3p,let-7a-2-3p,miR-431-5p,miR-296-3p,miR-2957,miR-29b-1-5p,novel_51,miR-2331-3p,miR-370-3p,miR-552-3p,miR-532-5p,miR-27a-5p,miR-216b-5p,novel_25,miR-155-5p,miR-488-3p,miR-2448-3p,let-7g-3p,miR-485-5p,miR-3074-5p,miR-3607-3p,miR-193a-5p,miR-6525,miR-206-3p,miR-374a-3p,miR-1260b,miR-15b-3p,miR-155-5p,miR-129-5p,miR-130b-5p,miR-374b-3p,miR-134-5p,miR-548e-3p,miR-299a-3p,miR-6517,miR-431,miR-7859,miR-2411-5p,miR-2284h-5p,miR-134-5p,miR-2284z,miR-532-3p,miR-134,miR-140-3p,miR-128-3p,miR-155-5p,let-7a-2-3p,miR-183-5p,miR-2285w,miR-1,miR-29b-1-5p,miR-450b-5p,miR-130a-5p,miR-125b-2-3p,miR-2411,miR-1271-3p,miR-1260a,miR-2285p,miR-374a-5p,miR-760-3p,miR-466i-5p,miR-127-5p,miR-299-3p,miR-543-5p,miR-2484

SLC10A2	ENSOARG0(	solute c	miR-380-5p,miR-323-3p,miR-362-5p,miR-500b-5p,miR-380-5p,miR-133a-3p,miR-466i-5p,miR-2285j,miR-3604,miR-323b-3p,miR-500a-5p,miR-500-5p,miR-219a-1-3p,miR-133b-3p,novel_99,miR-2448-3p,miR-503-3p,miR-362-5p,miR-129b-5p,miR-323b
ST3GAL1	ENSOARG0(	ST3 beta	miR-221-5p,novel_68,miR-125a,miR-7977,miR-6395,miR-485-5p,miR-885-3p,miR-3141,miR-450a-1-3p,miR-433-3p,miR-361-3p,miR-6525,miR-6134,miR-3184-5p,miR-423-5p,miR-145a-3p,miR-129-2-3p,novel_42,miR-664a,miR-128-1-5p,miR-450b-3p,miR-874-5p,miR-30e-3p,miR-218-5p,miR-320e,miR-409-3p,miR-125b-5p,miR-30a-3p,miR-129-1-3p,miR-328-3p,miR-214-5p,miR-450a-1-3p,novel_4,miR-2300a-5p,miR-1343-3p,miR-30d-3p,miR-412,miR-885-3p,miR-218-1-3p,miR-1843b-5p,miR-6529a,miR-140-3p,miR-5703,miR-450c-3p,miR-412-3p,miR-124-3p,miR-1343-5p,miR-432-3p,miR-2424,miR-105-3p,miR-124a,miR-216c-5p,miR-30a-3p,miR-495-5p,miR-125a-5p,miR-1343-5p,miR-221-5p,miR-125b,miR-3141
DUSP8	ENSOARG0(	dual spe	miR-2433,miR-1247-3p,miR-1343-5p,miR-2411-5p,miR-2411,miR-1343-5p,miR-625-5p
MRPL2	ENSOARG0(	mitochon	miR-3960,miR-2355-5p,miR-2285u,miR-133a-3p,miR-133b-3p,miR-874-5p,miR-133a-3p,miR-2285j,miR-324-3p,miR-2427
NEDD4L	ENSOARG0(	neural p	miR-454-3p,miR-2285j,miR-15b-3p,miR-30e-3p,miR-30a-3p,miR-142a-5p,miR-3184-5p,miR-181c-5p,miR-1271,miR-665-5p,let-7g-3p,miR-339b,miR-1827,miR-433-3p,miR-301a-3p,miR-6516,miR-27a-5p,miR-499b-3p,miR-204-3p,novel_32,miR-151b,miR-216b-3p,miR-106a,miR-760-3p,miR-3653-3p,miR-34b-5p,miR-618,miR-125b,miR-377-5p,miR-181b-1-3p,miR-125b-2-3p,miR-486-3p,miR-146a-5p,miR-143-3p,miR-380-3p,miR-216c-5p,miR-105-3p,miR-5100,miR-20a,miR-345-3p,miR-33b-3p,miR-34a-5p,miR-140-3p,miR-188-3p,miR-1434-5p,miR-7859,miR-23b-3p,miR-23c,novel_83,miR-151-5p,miR-106a,miR-3600,miR-505-3p,miR-3968,miR-628-3p,miR-2284q,miR-2285n,miR-371a-5p,miR-2285y,miR-877-3p,miR-8095,miR-423-5p,miR-2447,miR-541-3p,miR-2285f,miR-181d-5p,miR-96-5p,miR-499a-3p,miR-2483-3p,miR-203-3p,miR-4443,miR-541-5p,miR-7977,miR-130b-3p,miR-146b,miR-2957,miR-106b-5p,miR-2330-5p,miR-382-3p,miR-106a-5p,miR-23a-3p,miR-450b-5p,miR-101c,miR-2284aa,miR-484,miR-143-5p,miR-541,miR-425-3p,miR-496-5p,miR-142-5p,miR-9788-3p,miR-2300a-5p,miR-181b-2-3p,miR-145b,miR-486b-3p,miR-125b-5p,miR-93,miR-1260b,miR-145-3p,miR-10b-3p,let-7g-3p,miR-20b-5p,miR-671-5p,miR-301,miR-93-5p,miR-23b,miR-3154,miR-17-5p,miR-34c-5p,miR-503-3p,miR-1271,miR-125a,miR-181b-5p,miR-1260a,miR-6119-5p,miR-339a,miR-148b-5p,novel_39,miR-2285b,miR-490-3p,miR-30a-3p,miR-2403,let-7a-2-3p,miR-1290,miR-1271-5p,miR-6516-5p,miR-582-3p,miR-2284z,miR-2285ad,miR-6517,miR-1224-5p,novel_79,miR-6119-3p,miR-203b-5p,miR-22-5p,miR-20a-5p,miR-34c,novel_87,miR-24-3p,miR-449a,miR-301b-3p,novel_120,miR-3957-3p,miR-495-3p,miR-1291,miR-146b-5p,miR-505-3p,miR-2285ab,miR-34b,miR-677,miR-181b-3p,miR-124-5p,miR-2285l,miR-125a-5p,miR-146a,miR-106a-5p,miR-17-5p,let-7a-2-3p,miR-668-

ZNF420	ENSOARG0(	zinc fin	miR-23b,miR-320a,miR-137-3p,miR-488-3p,miR-485-5p,miR-7144-5p,miR-3074-1-3p,miR-329b,miR-10b-3p,miR-374c-3p,miR-16-5p,miR-3591-5p,miR-345-3p,miR-196a-5p,miR-322-5p,miR-30f,miR-664-3p,miR-191-3p,miR-30a-3p,miR-1,miR-29b-2-5p,miR-490-3p,miR-21c,miR-150-5p,miR-2387,miR-195a-5p,miR-19a-3p,miR-3120-3p,miR-7-1-3p,miR-3074-5p,miR-378b,miR-329-5p,miR-181b-3p,miR-677,miR-320c,miR-16a,miR-142b,miR-20a-5p,miR-4324,miR-15a,miR-199c,miR-421-5p,miR-206,novel_27,miR-371b-3p,miR-3071-5p,miR-22-3p,miR-1434-3p,miR-653,miR-497-5p,miR-21-5p,miR-16b,miR-130a-3p,miR-330-3p,miR-196b-5p,miR-6240,miR-539-3p,miR-22841,miR-432-3p,miR-21a-3p,novel_99,miR-625-5p,miR-21-3p,miR-545-3p,miR-221-5p,miR-15b-5p,miR-22-3p,miR-656-3p,miR-216b-5p,miR-499b-3p,miR-301a-3p,miR-29b-2-5p,miR-30a-3p,miR-487a-5p,miR-424-5p,miR-454-3p,miR-3074-5p,miR-590-3p,miR-539-5p,miR-206-3p,miR-188-3p,miR-124-3p,miR-1246,miR-378d,miR-218-1-3p,miR-4429,miR-19b-3p,miR-485-3p,miR-3600,miR-106a,novel_83,miR-15a-5p,miR-23b-3p,miR-487a-3p,miR-345-3p,miR-324-3p,novel_17,miR-2484,miR-221-5p,miR-3653-3p,miR-106a,miR-320d,miR-20a,miR-195a-3p,miR-380-3p,miR-181b-1-3p,miR-421-5p,miR-378j,miR-668-5p,miR-452-3p,miR-664b,miR-499a-3p,miR-301a-5p,miR-490-5p,miR-3968,miR-3956-3p,miR-3059-5p,novel_60,miR-125b-2-3p,miR-1a-3p,miR-323b,miR-301b-5p,miR-181b-2-3p,miR-222-5p,miR-330-3p,miR-199b-3p,miR-539-5p,miR-16b,miR-323b-3p,novel_103,miR-320b,miR-130b-3p,miR-376b,miR-23a-3p,miR-424-5p,miR-106a-5p,miR-124a,miR-2310,miR-1b-5p,miR-3187-3p,miR-8485,miR-193a
NDRG1	ENSOARG0(	N-myc do	miR-2483-5p,let-7c-3p,miR-2330-5p,miR-2285r,miR-29b-1-5p,miR-2957,miR-377-3p,miR-452-5p,miR-1343-3p,miR-105-1,miR-2459,miR-1298-3p,miR-134-3p,novel_23,miR-369-5p,miR-188-5p,miR-2426,miR-9-5p,miR-2447,miR-1285-5p,miR-211,miR-2285t,miR-3968,miR-211-5p,miR-2319b,miR-329-5p,miR-6535,miR-2404,miR-499a-3p,miR-378g,miR-378j,miR-2319a,miR-450b-3p,miR-136-5p,miR-29b-2-5p,miR-329a-5p,miR-2432,miR-29b-1-5p,miR-450b-5p,miR-324-3p,miR-216a-5p,miR-2285af,novel_17,miR-548e-3p,miR-7859,miR-9-5p,miR-2904,miR-2300b-3p,miR-664-5p,miR-105-2,miR-133a-3p,miR-145a-3p,miR-145-3p,miR-6740-5p,miR-369-5p,miR-29b-2-5p,miR-4492,miR-204-5p,novel_9,miR-2355-5p,miR-2285aa,miR-204-3p,miR-499b-3p,miR-377-3p,miR-7144-5p,miR-1827,miR-133b-3p,miR-10b-3p

CSNK1A1	ENSOARG0(	casein k	miR-2285g,novel_116,miR-6238,miR-103,miR-20b,miR-103a-3p,miR-16-1-3p,miR-22-3p,miR-194-5p,miR-3071-5p,miR-493-5p,miR-6240,miR-2483-5p,miR-363-5p,miR-224-5p,miR-105-5p,miR-330-3p,miR-194a,miR-106a-5p,miR-17-5p,miR-677,miR-181b-3p,miR-410-5p,miR-107,miR-2285ab,miR-24-3p,miR-323c,novel_27,miR-17-3p,miR-495-3p,miR-2285x,novel_1,miR-22-5p,miR-147-5p,miR-6402,miR-20a-5p,miR-133a-5p,miR-9-5p,miR-24-2-5p,miR-758-5p,miR-545-5p,miR-2284z,miR-222,miR-105-2,miR-30c,miR-30a-5p,miR-2284y,miR-376c-5p,miR-29a-5p,miR-17-3p,miR-450a-2-3p,miR-127-5p,miR-107,miR-200a-3p,miR-664-3p,miR-331-5p,miR-769,miR-133c,miR-154b-5p,miR-141-3p,miR-3074-1-3p,miR-20b-5p,miR-30e-5p,miR-331-5p,miR-93-5p,miR-137-3p,miR-17-5p,miR-3154,miR-93,miR-1197-3p,miR-6128,miR-331-5p,miR-592,miR-2284k,miR-331-3p,miR-153,miR-455-3p,miR-148b-5p,miR-221-3p,miR-2285e,miR-105-1,miR-330-3p,miR-409b,miR-5010-3p,miR-181b-2-3p,miR-21b,miR-2284aa,miR-382-3p,miR-106a-5p,miR-23a-3p,miR-652-5p,miR-153-3p,miR-103a-2-5p,miR-106b-5p,miR-203-3p,miR-664b,miR-2284g,miR-126b-5p,miR-668-5p,miR-493-5p,miR-361-3p,miR-221,miR-2285f,miR-194b-5p,miR-9-5p,miR-2284j,miR-499a-5p,miR-30b-5p,miR-769-5p,miR-371a-5p,miR-107-5p,miR-7859,miR-23c,miR-548e-3p,miR-106a,novel_73,miR-16-1-3p,miR-2284u,miR-148a-5p,miR-30c-5p,miR-2904,miR-5703,miR-125b-2-3p,miR-181b-1-3p,miR-2432,miR-216c-5p,miR-20a,miR-106a,miR-2284m,miR-466i-5p,miR-221-5p,miR-30d,miR-21-3p,miR-30b,miR-210-5p,miR-185-3p,miR-6130,miR-216b-5p,miR-22-3p,miR-221-5p,miR-532-5p,miR-296-3p,miR-2312,miR-30f,miR-186-5p,miR-21a-3p,miR-2285c,miR-30d-5p,miR-769-5p,miR-224-5p,miR-4510,miR-3958-5p,miR-17-3p,miR-208b-5p,miR-4492,miR-222-3p,miR-338-5p,miR-664a-5p

DCDC2C	ENSOARG0(	doubleco	miR-134-5p,miR-545-5p,miR-582-3p,miR-758-5p,miR-483-3p,miR-376a-5p,novel_79,miR-429-3p,miR-299a-3p,miR-10a-5p,miR-105-2,miR-200b-3p,miR-23b-5p,miR-134,miR-1290,miR-345-3p,miR-2285b,miR-200a,miR-3065-3p,miR-29b-2-5p,miR-200a-3p,miR-200c-3p,miR-450a-2-3p,miR-339a,miR-200c,miR-615-5p,miR-141-3p,miR-125a,miR-671-5p,let-7g-3p,miR-2318,miR-329a,miR-362-5p,miR-200a-5p,miR-145-3p,miR-500b-5p,miR-6128,novel_42,miR-28-5p,miR-134-5p,miR-144,miR-125b-5p,miR-6740-5p,miR-370-5p,miR-2433,miR-544b,miR-2355-3p,novel_116,miR-149-5p,miR-26b-3p,miR-339-5p,miR-487b-3p,miR-340-3p,miR-187-3p,miR-31-5p,miR-27b-3p,miR-2889,miR-16-1-3p,miR-3071-5p,miR-22-3p,miR-197-3p,miR-1285,miR-873a-5p,let-7a-2-3p,miR-432-3p,miR-654-3p,miR-125a-5p,miR-483-3p,miR-2411-3p,miR-105-5p,miR-144-3p,miR-6535,miR-299b-3p,miR-2404,miR-10a-5p,miR-3074-5p,miR-378b,miR-376a-2-5p,miR-9851-3p,miR-2319a,miR-410-5p,miR-2284w,novel_27,miR-1193,miR-656-5p,novel_87,miR-485-3p,miR-133a-5p,miR-3613,miR-502-5p,novel_83,miR-532-3p,miR-671-3p,miR-616-3p,miR-200b,miR-340-5p,miR-3600,miR-16-1-3p,miR-1197-5p,miR-188-3p,miR-1193,miR-362-3p,novel_133,miR-2424,miR-29b-1-5p,miR-664b-3p,miR-345-3p,miR-299-3p,miR-30b-3p,miR-125b,miR-345-3p,miR-500a-5p,miR-324-3p,miR-216a-5p,miR-22-3p,novel_25,miR-204-3p,miR-544-3p,miR-10a,miR-552-3p,miR-3065-3p,miR-216b-3p,miR-370-5p,miR-504-5p,miR-625-5p,miR-339b,miR-411,miR-3613-5p,miR-10b-5p,miR-2330-3p,miR-3958-5p,miR-3074-5p,miR-145a-3p,miR-708-5p,miR-874-5p,miR-6516-3p,novel_74,miR-29b-2-5p,miR-500-5p,miR-1298-3p,miR-153,miR-27a-3p,miR-1343-3p,miR-105-1,novel_78,miR-3120-5p,miR-544a,miR-362-3p,miR-758-3p,miR-10b,miR-541,miR-484,miR-329-3p,miR-758-3p,miR-8485,miR-744-3p,miR-2330-
DNAJB1	ENSOARG0(	DnaJ (Hs	miR-125b,miR-500a-5p,miR-324-3p,miR-181b-5p,miR-450a-2-3p,miR-34b-5p,miR-3064-5p,miR-125b-2-3p,miR-376a-5p,miR-192-3p,miR-486-3p,miR-2366,miR-2300b-3p,miR-193b-5p,miR-2904,miR-29d-3p,miR-19b-3p,let-7a-2-3p,miR-34a-5p,miR-128-3p,miR-155-5p,miR-6517,miR-15a-5p,miR-322-5p,miR-9-5p,miR-383-5p,miR-302a-5p,miR-328-3p,miR-5100,miR-29b-3p,miR-665,miR-331-3p,miR-324-3p,miR-125b-5p,miR-155-5p,miR-138-5p,miR-486b-3p,miR-424-5p,miR-4510,miR-1306-5p,miR-133a-3p,miR-16-5p,miR-346,miR-224-5p,miR-500b-5p,miR-6525,miR-181c-5p,miR-122-5p,miR-362-5p,miR-329b,let-7g-3p,miR-133b-3p,miR-1827,miR-543-3p,miR-1271,miR-125a,miR-370-3p,miR-6130,miR-377-3p,miR-34c-5p,miR-155-5p,miR-15b-5p,miR-377-3p,miR-224-5p,miR-16b,novel_51,miR-3085-3p,miR-125a-5p,miR-424-5p,miR-654-3p,let-7a-2-3p,let-7j,miR-181a-5p,miR-125b-2-3p,miR-219a-1-3p,miR-138,miR-16b,miR-29b,miR-3120-5p,miR-497-5p,novel_78,miR-133b-5p,miR-31-5p,miR-194-3p,miR-500-5p,miR-142b,miR-15a,miR-34c,miR-16a,miR-29a,miR-3956-3p,novel_60,miR-495-3p,novel_101,miR-29c-3p,miR-9-5p,miR-181d-5p,miR-362-5p,miR-361-3p,miR-2285m,miR-449a,miR-361-3p,miR-216a-3p,miR-19a-3p,miR-6395,miR-195a-5p,miR-3064-5p,miR-3120-3p,miR-3082-5p,miR-29a-3p,miR-34b,miR-2404,miR-6535

			miR-[illegible],miR-[illegible],miR-[illegible],miR-[illegible],miR-199c,novel_1,miR-17-3p,miR-24-3p,miR-371b-3p,miR-211,miR-2387,miR-195a-5p,miR-412-3p,miR-107,miR-2285k,miR-7-1-3p,miR-378b,miR-216b-3p,miR-320c,miR-669,miR-299b-3p,novel_69,miR-670-3p,miR-2404,miR-323a-5p,miR-2411-3p,novel_51,miR-582,miR-125a-5p,miR-378a-5p,let-7a-2-3p,miR-539-3p,novel_23,miR-374b-3p,miR-3071-5p,miR-450c-3p,miR-223-3p,miR-873a-5p,miR-708-3p,miR-7862,miR-497-5p,miR-381-5p,miR-1260b,miR-30b-3p,miR-16b,miR-665,miR-133b-5p,miR-103a-3p,miR-103,miR-125b-5p,miR-331-3p,miR-494-5p,miR-34c-3p,miR-204-5p,miR-486b-3p,miR-16-5p,miR-1306-5p,miR-1260b,miR-320a,miR-3154,miR-381-3p,miR-485-5p,miR-2318,miR-329a,let-7g-3p,miR-10a-3p,miR-141-3p,miR-125a,miR-3071-3p,miR-200c,miR-6119-5p,miR-1260a,miR-205-5p,miR-200a-3p,miR-107,miR-127-5p,miR-200c-3p,miR-17-3p,miR-181a-2-3p,miR-192-3p,miR-200a,miR-148b-5p,miR-218-2-3p,miR-3578,miR-1290,miR-345-3p,miR-200b-3p,miR-300,miR-429-3p,miR-6517,miR-299a-3p,miR-582-3p,miR-450a-1-3p,miR-545-5p,miR-1271-5p,miR-322-5p,miR-218-5p,miR-2285t,miR-499a-5p,miR-3968,miR-3059-5p,miR-2447,miR-154a,miR-8095,miR-877-3p,miR-378j,miR-381-3p,miR-1983,novel_94,miR-96-5p,miR-126b-5p,miR-211-5p,miR-541-5p,miR-452-3p,miR-203-3p,miR-3082-5p,miR-3596,miR-16b,miR-362-3p,miR-323b-3p,miR-29b-1-5p,miR-199a-3p,miR-320b,miR-1468,miR-381-5p,miR-424-5p,miR-8485,miR-30b-3p,miR-323b,miR-2284r,miR-484,miR-329-3p,miR-1388-5p,miR-362-3p,miR-151a-3p,miR-199b-3p,miR-2300a-5p,miR-455-3p,miR-147a,miR-1298-3p,miR-324-3p,miR-151-3p,miR-424-5p,miR-17-3p,miR-664a,miR-450b-3p,miR-425-5p,miR-1271,miR-539-5p,miR-346,miR-487b-5p,miR-450a-1-3p,miR-
ELP2	ENSOARG0(	elongato	21a-3p,miR-1895,miR-552-3p,miR-216b-3p,miR-15b-

CRELD1	ENSOARG0(	cysteine	miR-370-5p,miR-331-3p,miR-132-5p,miR-100-3p,miR-320e,miR-708-5p,miR-486b-3p,miR-28-5p,miR-145a-3p,miR-1271,miR-193a-5p,miR-93,miR-6525,miR-346,miR-3184-5p,miR-93-5p,miR-23b,miR-17-5p,let-7g-3p,miR-10b-3p,miR-20b-5p,miR-1895,miR-1814c,miR-2331-3p,miR-2355-5p,miR-370-5p,miR-377-3p,miR-27a-5p,miR-22-3p,miR-151b,miR-877-5p,miR-324-3p,miR-185-3p,miR-2898,miR-106a,miR-127-5p,miR-466i-5p,miR-107,miR-152-3p,novel_127,miR-21-3p,miR-20a,miR-4286,miR-376a-5p,miR-486-3p,miR-2432,miR-143-3p,miR-2300b-3p,miR-1290,miR-1343-5p,miR-124-3p,miR-330-5p,miR-33b-3p,miR-128-3p,miR-18a-3p,miR-106a,miR-6517,miR-412,miR-1b-3p,miR-431,miR-23b-3p,miR-148b-3p,miR-1271-5p,miR-222-5p,miR-23c,miR-199a-3p,novel_83,miR-151-5p,novel_82,miR-502-5p,miR-4324,miR-20a-5p,miR-33a-3p,miR-628-3p,miR-7134-3p,miR-1193,miR-452-5p,miR-361-3p,miR-371b-3p,miR-423-5p,miR-326-3p,miR-361-3p,miR-148a-3p,miR-216a-3p,miR-18a-3p,miR-96-5p,miR-107,miR-136-5p,miR-877-3p,miR-378g,miR-2404,miR-7-5p,miR-199a-3p,miR-431-5p,miR-377-3p,miR-326,miR-106b-5p,miR-106a-5p,miR-17-5p,miR-1343-5p,miR-124a,miR-106a-5p,miR-494-3p,miR-23a-3p,miR-363-5p,miR-2310,miR-503-3p,miR-296-5p,miR-2284r,miR-1285,miR-7b-5p,miR-542-3p,miR-1434-3p,miR-412-3p,miR-22-3p,miR-103a-3p,miR-1343-3p,miR-134-3p,miR-147a,miR-9788-3p,novel_116,miR-216a-3p,miR-20b,miR-103,miR-2459

TRIM63	ENSOARG0(	triparti	miR-487a,miR-216c-5p,miR-329a-5p,miR-345-3p,miR-181b-1-3p,miR-29b-1-5p,miR-618,novel_17,miR-30b,miR-345-3p,miR-125b,miR-92a-1-5p,miR-299-3p,miR-30b-3p,miR-18a-3p,miR-485-3p,miR-23c,miR-487a-3p,miR-23b-3p,miR-532-3p,miR-2366,miR-362-3p,miR-30c-5p,miR-124-3p,miR-378d,miR-16-1-3p,miR-145a-3p,miR-3074-5p,miR-206-3p,miR-769-5p,miR-665,miR-17-3p,miR-450b-3p,miR-130b-5p,miR-208b-5p,miR-708-5p,miR-129-5p,miR-1277-3p,miR-548o-3p,miR-30c-1-3p,miR-296-3p,miR-154a-3p,miR-377-3p,miR-204-3p,miR-22-3p,miR-450a-1-3p,miR-30d-5p,miR-154b-3p,miR-30f,miR-339b,novel_99,miR-186-5p,miR-101b-3p,miR-124a,miR-2284b,miR-23a-3p,miR-30c-1-3p,miR-1306-5p,miR-326,miR-505,miR-362-3p,miR-29b-1-5p,miR-3085-3p,miR-199b-5p,miR-409b,miR-1343-3p,miR-2300a-5p,miR-181b-2-3p,miR-329-3p,miR-30b-3p,miR-1a-3p,miR-2426,miR-1388-5p,miR-362-3p,miR-885-5p,miR-3059-5p,miR-2447,miR-101-3p,miR-30b-5p,novel_82,miR-769-5p,miR-4726-5p,miR-154-3p,miR-3064-5p,miR-2285u,miR-2483-3p,miR-4443,miR-199b-5p,miR-381-3p,miR-4286,miR-181a-2-3p,miR-1,miR-3064-5p,miR-28b,miR-490-3p,miR-199a-5p,miR-1260a,miR-339a,miR-3071-3p,miR-30c-2-3p,miR-127-5p,miR-17-3p,novel_127,miR-6517,miR-299a-3p,novel_79,miR-214-5p,miR-450a-1-3p,miR-345-3p,miR-330-5p,miR-30a-5p,miR-29d-3p,miR-300,miR-411-3p,miR-2284v,miR-1306-5p,miR-1260b,miR-145-3p,novel_63,miR-29b-3p,miR-125b-5p,miR-767-5p,miR-28-5p,miR-410-3p,miR-874-3p,miR-487a-3p,miR-615-5p,miR-125a,miR-769,miR-199a-5p,miR-381-3p,miR-30e-5p,miR-23b,miR-329a,miR-767,miR-1248,miR-485-5p,miR-7144-5p,miR-130b-5p,miR-2285r,miR-539-3p,miR-377-3p,miR-2411-3p,novel_51,miR-125a-5p,miR-339-5p,miR-450c-3p,miR-22-3p,miR-197-3p,miR-16-1-3p,miR-1260b,miR-30b-3p,miR-29b,miR-2889,miR-29c-3p,miR-17-

ZIC4	ENSOARGO(	Zic fami	miR-21a-3p,miR-1827,miR-504-5p,miR-140-5p,miR-221-5p,miR-378a-3p,miR-545-3p,miR-216b-3p,miR-132-5p,miR-301a-3p,miR-499b-3p,miR-27a-5p,miR-6130,novel_32,miR-204-3p,miR-30e-3p,miR-30a-3p,miR-132-5p,miR-320e,miR-6516-3p,miR-17-3p,miR-664a,miR-874-5p,miR-454-3p,miR-129-5p,miR-378c,miR-3074-5p,miR-145a-3p,miR-4510,miR-1271,miR-3074-2-3p,miR-6525,miR-206-3p,miR-6134,miR-378i,miR-2904,miR-124-3p,miR-378a-3p,miR-188-3p,miR-140-3p,miR-493-3p,miR-4429,miR-218-1-3p,miR-16-1-3p,miR-18a-3p,miR-30d-3p,miR-212-5p,miR-383-5p,miR-23b-3p,miR-23c,miR-15a-5p,novel_83,miR-532-3p,novel_17,miR-422a,miR-185-3p,miR-345-3p,miR-320d,miR-760-3p,miR-466i-5p,miR-1277-5p,miR-221-5p,miR-21-3p,miR-299-3p,miR-664b-3p,miR-142a-3p,miR-130a-5p,miR-2432,miR-486-3p,miR-361-3p,miR-409-5p,miR-129b-5p,miR-96-5p,miR-299b-5p,miR-504,miR-20a-3p,miR-541-5p,miR-211-5p,miR-499a-3p,miR-378g,miR-378e,miR-2483-3p,miR-203-3p,miR-454-5p,miR-500-3p,miR-4443,miR-218-5p,miR-2285n,miR-2440,miR-2285y,miR-3968,miR-4726-5p,miR-335,miR-450a-5p,miR-7134-3p,miR-2285t,miR-2447,miR-181d-5p,miR-9-5p,miR-452-5p,miR-877-3p,miR-502-3p,miR-8095,miR-378b,miR-484,miR-1a-3p,miR-501-3p,miR-1388-5p,miR-378c,miR-2426,miR-3120-5p,miR-105-1,novel_78,miR-1343-3p,novel_4,miR-409b,miR-425-3p,miR-539-5p,miR-142-3p,miR-9788-3p,miR-320b,miR-342,novel_103,miR-1306-5p,miR-491-5p,novel_124,miR-130b-3p,miR-3955-3p,miR-25-5p,miR-2285o,miR-1388-3p,miR-124a,miR-2330-5p,miR-424-5p,miR-23a-3p,miR-378d,miR-4532,miR-2310,miR-320a,miR-23b,miR-3154,miR-876-3p,miR-7144-5p,miR-23a-5p,miR-485-5p,miR-671-5p,miR-1814c,miR-542-5p,miR-2355-5p,miR-615-5p,miR-374c-3p,miR-34c-3p,miR-2433,miR-135a-1-3p,miR-144,miR-6740-5p,miR-3969,miR-204-
USP7	ENSOARGO(	ubiquiti	miR-148a-3p,miR-3970,miR-381-3p,miR-3065-5p,miR-3591-3p,miR-299b-5p,miR-107,miR-412-3p,miR-505-3p,miR-1827,miR-1983,miR-195a-5p,miR-221-5p,miR-21-3p,novel_69,miR-15b-5p,miR-15a,miR-16a,miR-100-3p,miR-138-5p,miR-4492,miR-424-5p,miR-16-5p,miR-1197-3p,novel_27,miR-2427,novel_23,miR-16b,miR-138,miR-299a-5p,miR-497-5p,miR-103a-3p,miR-133b-5p,miR-505-3p,miR-322-5p,miR-412-5p,miR-15a-5p,miR-148b-3p,miR-616-3p,miR-194-3p,miR-103,miR-421,miR-205-5p,miR-196a-3p,miR-16b,miR-185-3p,miR-221-5p,miR-107,miR-152-3p,miR-424-5p,miR-4532,miR-2310

KLC4	ENSOARGO(	kinesin	miR-615-5p,miR-370-3p,miR-154b-5p,miR-1248,miR-7144-5p,miR-1306-5p,miR-3607-3p,miR-16-5p,miR-1260b,miR-145-3p,miR-365a-5p,miR-34c-3p,miR-100-3p,miR-28-5p,miR-126b-3p,miR-6517,miR-222,miR-758-5p,miR-322-5p,let-7d,miR-28b,novel_39,let-7g,miR-8117,miR-1260a,let-7c-5p,let-7f-5p,novel_96,novel_127,miR-3074-5p,novel_68,miR-18a-3p,miR-1291,let-7e-5p,miR-195a-5p,miR-24-3p,miR-2428,miR-28c,miR-15a,miR-1247-3p,let-7i,miR-16a,miR-128-1-5p,miR-665,let-7g-5p,miR-185-5p,miR-2285g,miR-149-5p,miR-873a-5p,miR-381-5p,miR-219a-1-3p,miR-1260b,miR-16b,miR-30b-3p,miR-2284x,miR-497-5p,miR-432-3p,novel_51,miR-365b-5p,miR-2331-3p,miR-532-5p,miR-132-5p,let-7i-5p,miR-15b-5p,miR-877-5p,miR-151b,miR-2285c,miR-1827,miR-543-3p,miR-3074-5p,miR-3184-3p,let-7b,miR-214,miR-665-5p,miR-6525,miR-3184-5p,let-7b-5p,miR-222-3p,let-7f,miR-665,miR-874-5p,miR-4492,miR-424-5p,miR-708-5p,miR-18a-3p,miR-15a-5p,miR-151-5p,miR-199a-3p,miR-1961,miR-140-3p,miR-1197-5p,miR-33b-3p,miR-345-3p,miR-29b-1-5p,miR-185-3p,miR-324-3p,miR-98-5p,miR-760-3p,let-7k,let-7d-5p,miR-409-5p,miR-221,miR-361-3p,miR-140-5p,miR-668-5p,miR-541-3p,miR-361-3p,miR-423-5p,miR-107-5p,miR-301a-5p,miR-1343-3p,miR-2459,miR-2285e,miR-221-3p,miR-9788-3p,miR-455-3p,miR-301b-5p,miR-541,miR-296-5p,miR-30b-3p,miR-450b-5p,miR-1388-5p,miR-1839-5p,miR-424-5p,let-7a-5p,miR-193a,miR-4532,miR-199a-3p,miR-16b,miR-29b-1-5p,miR-487b-5p,miR-1185-5p,miR-491-5p,miR-381-5p,miR-103a-2-5p,let-7e,miR-214-3p
GIPC1	ENSOARGO(	GIPC PDZ	miR-2284r,miR-2387,miR-1306,miR-30b-3p,miR-30b-3p,miR-2474,miR-1260b,novel_4,miR-615-5p,miR-2284s,miR-532-3p,miR-1260a,miR-1306-5p,miR-185-3p,miR-4726-5p,miR-4492,miR-486b-3p,miR-125b-2-3p,miR-1260b,miR-423-5p,miR-486-3p,miR-3184-5p
FAM155A	ENSOARGO(	family w	miR-29b-1-5p,miR-224-5p,miR-3957-3p,miR-320d,novel_124,miR-466i-5p,miR-29b-1-5p,miR-320b,miR-224-5p,miR-320c,miR-147a,miR-128-3p,miR-4429,miR-216a-3p,miR-320a,miR-125b-2-3p

SLC30A2	ENSOARG0(	solute c	miR-93-5p,miR-3154,miR-17-5p,miR-20b-5p,miR-7144-5p,miR-485-5p,miR-671-5p,miR-2474,miR-370-3p,miR-2355-5p,miR-2285aa,miR-125a,miR-1271,miR-331-3p,miR-125b-5p,miR-6740-5p,miR-2433,miR-6128,miR-193a-5p,miR-365a-5p,miR-2903,miR-93,miR-1197-3p,miR-1260b,miR-1343-5p,miR-23b-5p,miR-330-5p,miR-615-3p,miR-345-3p,miR-10a-5p,miR-582-3p,miR-222,miR-2285ad,miR-423-3p,miR-429-3p,miR-6517,miR-582-3p,miR-505-5p,miR-1271-5p,miR-483-3p,miR-2285af,miR-1260a,miR-200c,miR-6119-5p,miR-125a-3p,miR-543-5p,novel_96,miR-200c-3p,miR-191-3p,miR-3064-5p,novel_39,miR-29a-5p,miR-3065-3p,miR-1271-3p,miR-28b,miR-148b-5p,miR-1306,miR-3141,miR-6395,miR-450b-3p,miR-1291,miR-320b,miR-3120-3p,miR-10a-5p,miR-677,miR-6535,miR-669,miR-181b-3p,miR-2404,miR-146b-3p,novel_69,miR-502-5p,miR-28c,miR-6529b,miR-20a-5p,miR-128-1-5p,miR-542-5p,miR-342-3p,miR-326-3p,miR-24-3p,miR-145a-5p,miR-432,miR-1843b-5p,miR-7b-5p,miR-30b-3p,miR-1260b,miR-377-5p,miR-3955-5p,miR-194-3p,miR-149-5p,miR-20b,miR-432-5p,miR-134-3p,miR-3431,miR-503-5p,miR-365b-5p,miR-3141,miR-2411-3p,novel_51,miR-106a-5p,miR-17-5p,miR-7-5p,miR-503-5p,miR-377-3p,miR-125a-5p,miR-1343-5p,miR-483-3p,miR-363-5p,miR-130b-5p,miR-432-3p,miR-1827,miR-216b-3p,miR-3065-3p,miR-532-5p,miR-296-3p,miR-182-5p,miR-504-5p,miR-2284s,miR-10a,miR-377-3p,miR-6130,miR-324-3p,miR-132-5p,miR-665,miR-222-3p,miR-4492,miR-130b-5p,novel_74,miR-664a,miR-214,miR-2330-3p,miR-665-5p,miR-3074-2-3p,miR-4510,miR-145a-3p,miR-3184-3p,miR-6134,miR-10b-5p,miR-6525,miR-124-3p,miR-5703,miR-1193,novel_133,miR-376a-5p,miR-2300b-3p,miR-218-1-3p,miR-148a-5p,miR-378d,miR-212-5p,miR-106a,miR-532-3p,novel_83,miR-199a-3p,miR-671-3p,miR-2284b-5p,miR-328-

HK1	ENSOARG0(	hexokina	miR-2285aa,miR-2355-5p,miR-125a,miR-1271,miR-3068-3p,miR-874-3p,miR-25-3p,miR-23b,miR-876-3p,miR-345-5p,miR-329a,miR-193a-5p,miR-1197-3p,miR-125b-5p,miR-100-3p,miR-134-5p,miR-412,miR-2285ad,miR-299a-3p,miR-429-3p,miR-134-5p,miR-129-1-3p,novel_121,miR-330-5p,miR-23b-5p,miR-155-5p,miR-3064-5p,miR-29a-5p,miR-192-3p,miR-3956-5p,miR-2285b,miR-92b-3p,miR-450b-5p,miR-125a-3p,novel_96,novel_127,novel_68,miR-3074-5p,miR-669,miR-299b-3p,miR-1306,miR-3591-3p,miR-412-3p,miR-29c-3p,miR-374c-5p,miR-2284w,miR-199b-5p,miR-1307-5p,miR-485-3p,novel_87,miR-142b,miR-6123,miR-29a,miR-31-5p,miR-194-3p,novel_116,miR-149-5p,miR-412-5p,miR-432,miR-7b-5p,miR-3432b,miR-628-5p,miR-6240,miR-105-5p,novel_51,miR-7-5p,miR-552-3p,miR-2331-3p,miR-504-5p,miR-151b,miR-129b-3p,miR-1827,miR-345-5p,miR-665-5p,miR-214,miR-3074-5p,miR-129-2-3p,miR-122-5p,miR-6525,miR-30a-3p,miR-665,miR-30e-3p,miR-4492,miR-129-5p,miR-130b-5p,novel_74,miR-30d-3p,miR-18a-3p,miR-1247-5p,miR-2284h-5p,miR-23b-3p,miR-23c,miR-193b-5p,miR-2366,miR-218-1-3p,miR-34a-5p,miR-33b-3p,miR-345-3p,miR-2285w,miR-486-3p,miR-376a-5p,miR-324-3p,novel_17,miR-210-5p,miR-4792,miR-378j,miR-335-3p,miR-1983,miR-136-5p,miR-668-5p,miR-504,miR-2447,miR-363-3p,miR-361-3p,novel_111,novel_82,miR-3968,miR-1343-3p,miR-199b-3p,miR-330-3p,miR-421,miR-2459,miR-425-3p,miR-323b,miR-758-3p,miR-329-3p,miR-2284r,miR-101c,miR-1388-5p,miR-362-3p,miR-23a-3p,miR-744-3p,miR-8485,miR-323b-3p,miR-29b-1-5p,miR-199a-3p,miR-199b-5p,miR-214-3p,miR-103a-2-5p,miR-491-5p,miR-487b-5p,miR-370-3p,miR-199a-5p,miR-154b-5p,miR-34c-5p,miR-488-3p,miR-7144-5p,miR-671-5p,miR-485-5p,miR-10b-3p,miR-145-3p,miR-331-3p,miR-551b-3p,miR-29b-

CD70	ENSOARG0(	CD70 mol	miR-154b-3p,miR-2318,miR-329a,miR-2312,miR-767,miR-362-5p,miR-200a-5p,miR-450a-1-3p,miR-376c-3p,miR-2284n,miR-199a-5p,miR-544-3p,miR-377-3p,miR-376e-3p,miR-300-3p,miR-3068-3p,miR-503-3p,miR-487a-3p,miR-204-5p,miR-130b-5p,miR-767-5p,miR-450b-3p,miR-409-3p,miR-664a-5p,miR-30a-3p,miR-30e-3p,miR-181c-3p,miR-6525,miR-500b-5p,miR-2330-3p,miR-3074-5p,miR-590-3p,miR-539-5p,miR-148a-5p,miR-105-2,miR-664-5p,miR-140-3p,miR-1197-5p,miR-362-3p,miR-2284z,miR-450a-1-3p,miR-545-5p,miR-9-5p,miR-487a-3p,miR-485-3p,miR-30d-3p,miR-18a-3p,novel_127,miR-2898,miR-202-5p,miR-450a-2-3p,miR-92a-1-5p,novel_17,miR-6119-5p,miR-490-3p,novel_39,miR-29a-5p,miR-199a-5p,miR-376c-3p,miR-130a-5p,miR-376a-5p,miR-2478,miR-30a-3p,miR-487a,novel_94,miR-877-3p,miR-2319a,miR-136-5p,miR-181a-3p,miR-129b-5p,miR-18a-3p,miR-199b-5p,miR-2285ab,miR-4443,miR-670-3p,miR-2404,miR-378g,miR-211-5p,miR-452-3p,miR-3074-5p,miR-4792,miR-199c,miR-22-5p,novel_1,miR-3958-3p,miR-502-5p,miR-199b-5p,miR-362-5p,miR-211,miR-9-5p,miR-2285f,miR-3059-5p,miR-342-3p,miR-2284aa,miR-101c,miR-26a-2-3p,miR-2426,miR-544a,miR-362-3p,miR-3071-5p,miR-450c-3p,miR-197-3p,miR-329-3p,miR-544b,miR-500-5p,novel_116,miR-411-5p,miR-148b-5p,miR-3431,miR-134-3p,miR-153,miR-5010-3p,miR-199b-3p,miR-105-1,miR-199b-5p,miR-153-3p,miR-105-5p,miR-362-3p,miR-377-3p,miR-342,miR-8485,miR-539-3p,miR-33a-5p,miR-130b-5p,miR-574-3p

TKTL1	ENSOARG0(	transket	miR-181c-5p,miR-10b-5p,miR-206-3p,miR-4510,miR-424-5p,miR-874-5p,miR-4492,novel_74,miR-155-5p,miR-17-3p,miR-664a,miR-320e,miR-3604,miR-155-5p,miR-15b-5p,miR-151b,miR-376e-3p,miR-10a,miR-499b-3p,miR-377-3p,miR-192-5p,miR-6130,miR-3065-3p,miR-21-3p,miR-370-5p,miR-182-5p,miR-30c-1-3p,miR-504-5p,miR-2331-3p,novel_99,miR-30f,miR-30d-5p,miR-21a-3p,miR-2285c,miR-2411,miR-29b-1-5p,miR-30d,miR-3653-3p,miR-21-3p,miR-299-3p,miR-219b-5p,miR-3601,miR-92a-1-5p,miR-185-3p,miR-345-3p,miR-196a-3p,miR-2285p,novel_17,miR-210-5p,novel_83,miR-151-5p,miR-616-3p,miR-487a-3p,miR-23b-3p,miR-148b-3p,miR-23c,miR-15a-5p,miR-222-5p,miR-1b-3p,miR-3600,miR-380-5p,miR-485-3p,miR-16-1-3p,novel_73,miR-124-3p,miR-5703,miR-217-5p,miR-140-3p,miR-30c-5p,miR-191,miR-1246,miR-215-5p,miR-2366,miR-2300b-3p,miR-452-5p,miR-181d-5p,novel_60,miR-3956-3p,miR-541-3p,miR-212-3p,miR-490-5p,miR-3964,miR-2285t,miR-2285y,miR-107-5p,miR-301a-5p,miR-30b-5p,miR-2285n,miR-101-3p,miR-615,miR-1185-2-3p,miR-3596,miR-499a-3p,miR-7977,miR-24-1-5p,miR-10b,miR-877-3p,novel_94,miR-136-5p,miR-504,miR-140-5p,miR-8485,miR-3187-3p,miR-2310,miR-1b-5p,miR-30c-1-3p,miR-23a-3p,miR-124a,miR-2330-5p,miR-382-3p,miR-424-5p,miR-103a-2-5p,miR-491-5p,miR-29b-1-5p,miR-182-5p,miR-16b,novel_115,miR-9788-3p,miR-1185-3p,miR-421,miR-2285e,miR-145b,miR-2300a-5p,miR-548w,miR-330-3p,miR-2284aa,miR-1a-3p,miR-7857,miR-541,miR-10b,miR-301b-5p,miR-323-3p,miR-16-5p,miR-191-5p,miR-138-5p,miR-26c,miR-144,miR-135a-5p,miR-1-5p,miR-370-5p,miR-2332,miR-215-5p,miR-1814c,miR-485-5p,miR-876-3p,miR-2318,miR-23b,miR-30e-5p,miR-381-3p,novel_39,miR-3065-3p,miR-24-2-5p,miR-1,miR-181a-2-3p,miR-107,novel_96,miR-152-3p,miR-450a-2-3p,miR-17-3p,miR-127-

RAB14	ENSOARG0(	RAB14, m	miR-2285ad,miR-3600,miR-505-5p,miR-148b-3p,miR-582-3p,miR-151-5p,miR-545-5p,novel_121,miR-30c-5p,miR-29d-3p,miR-30a-5p,miR-23b-5p,miR-300,miR-19b-3p,let-7a-2-3p,miR-3064-5p,miR-28b,miR-148b-5p,miR-142a-3p,miR-3065-3p,miR-2285af,miR-30b,miR-8117,miR-3071-3p,miR-216a-5p,miR-17-3p,miR-2898,miR-152-3p,miR-200a-3p,miR-21-3p,miR-3065-3p,miR-2285aa,miR-21-3p,miR-370-5p,miR-141-3p,miR-301a-3p,miR-6130,miR-151b,miR-381-3p,miR-30e-5p,miR-21a-3p,miR-503-3p,miR-2285c,miR-376c-5p,miR-30d-5p,let-7g-3p,miR-30f,let-7g-3p,miR-7144-5p,miR-625-5p,miR-376c-5p,miR-1827,miR-671-5p,miR-3184-3p,miR-4510,miR-214,miR-144-5p,miR-338-3p,miR-2285j,miR-29b-3p,miR-370-5p,miR-324-3p,miR-665,miR-17-3p,miR-454-3p,miR-708-5p,miR-28-5p,miR-130b-5p,miR-31-5p,miR-130a-3p,miR-496-5p,miR-2285g,miR-185-5p,miR-142-3p,miR-1185-3p,miR-2285e,miR-301b-5p,miR-1434-3p,miR-374b-3p,miR-29b,miR-188-5p,miR-3120-5p,miR-130b-5p,let-7c-3p,miR-363-5p,let-7f-2-3p,let-7f-2-3p,let-7a-2-3p,miR-3085-3p,miR-130b-3p,miR-214-3p,miR-3064-5p,miR-3120-3p,miR-126b-5p,miR-29a-3p,miR-144-5p,miR-203-3p,miR-1185-2-3p,miR-376b-5p,miR-148a-3p,miR-3591-3p,miR-19a-3p,miR-9-3p,miR-17-3p,miR-29c-3p,miR-452-5p,miR-1193,miR-1285-5p,miR-301b-3p,novel_82,miR-30b-5p,miR-6402,miR-28c,miR-2285y,miR-301a-5p,miR-382-5p,miR-29a,miR-2285t,miR-2284q
MCFD2	ENSOARG0(	multiple	miR-30c-2-3p,miR-664-3p,miR-200c,miR-8117,miR-6119-5p,miR-107,miR-450a-2-3p,miR-17-3p,miR-466i-5p,miR-200c-3p,miR-3064-5p,miR-329a-5p,miR-199a-5p,miR-664-5p,miR-200b-3p,miR-429-3p,miR-199a-3p,miR-129-1-3p,miR-23b-3p,miR-322-5p,miR-7859,miR-200b,miR-15a-5p,miR-23c,miR-151-3p,miR-664a-5p,miR-665,miR-424-5p,miR-592,miR-130b-5p,novel_74,miR-17-3p,miR-16-5p,miR-129-2-3p,miR-1197-3p,miR-23b,miR-3154,novel_99,miR-186-5p,miR-2285aa,miR-30c-1-3p,miR-199a-5p,miR-154b-5p,miR-15b-5p,miR-139-5p,miR-2957,miR-16b,miR-199b-5p,miR-3085-3p,miR-23a-3p,miR-652-5p,miR-130b-5p,miR-424-5p,miR-6240,miR-30c-1-3p,miR-145a-5p,novel_23,miR-101c,miR-2284x,miR-16b,miR-145b,miR-199b-3p,miR-151a-3p,miR-103a-3p,miR-103,miR-16a,miR-15a,miR-218-5p,miR-101-3p,miR-147-5p,miR-199c,miR-335,miR-17-3p,miR-3059-5p,miR-335-5p,miR-199b-5p,miR-361-3p,miR-361-3p,miR-199b-5p,miR-195a-5p,miR-107,miR-541-5p,miR-139-5p,miR-3064-5p,miR-664b
DLGAP2	ENSOARG0(	discs, l	miR-2411-5p,miR-1307-3p,miR-2411,miR-744-5p,miR-183-5p

USP5	ENSOARG0(	ubiquiti	miR-212-3p,miR-335-5p,miR-219b-3p,miR-194b-5p,miR-1839-3p,miR-2285f,miR-2428,miR-423-5p,novel_82,miR-107-5p,miR-335,miR-4726-5p,miR-452-3p,miR-3064-5p,novel_69,miR-670-3p,miR-146b-3p,miR-144-5p,miR-2285ab,miR-361-3p,miR-129b-5p,miR-6395,miR-127-3p,miR-1839-3p,miR-8485,miR-4532,miR-194a,miR-25-5p,miR-3085-3p,miR-103a-2-5p,miR-381-5p,miR-1343-5p,miR-409b,novel_78,novel_4,miR-2300a-5p,miR-185-5p,miR-2285g,miR-2285e,miR-149-5p,miR-1285,miR-484,miR-296-5p,miR-194-5p,miR-381-5p,miR-2889,miR-3074-2-3p,miR-144-5p,miR-1197-3p,miR-346,miR-3184-5p,miR-2433,miR-551b-3p,miR-19b-1-5p,miR-664a,novel_74,miR-486b-3p,miR-874-5p,miR-4492,miR-3432a,miR-129-5p,miR-2331-3p,miR-215-5p,miR-370-3p,miR-2355-5p,miR-192-5p,miR-154b-5p,miR-2285c,miR-2312,miR-2474,miR-485-5p,miR-1827,miR-1895,miR-4286,miR-3064-5p,miR-486-3p,miR-210-5p,miR-185-3p,miR-324-3p,miR-2898,miR-450a-2-3p,miR-3600,miR-24-2-5p,miR-328-3p,miR-215-5p,novel_133,miR-5703,miR-1343-5p,miR-378d
MEST	ENSOARG0(	mesoderm	miR-1814c,novel_48,miR-17-5p,miR-199a-5p,miR-769,miR-365a-3p,miR-615-5p,miR-370-3p,miR-487a-3p,miR-204-5p,miR-374b-3p,miR-767-5p,miR-370-5p,miR-135a-1-3p,miR-374a-3p,miR-93,miR-3607-3p,miR-10a-5p,miR-105-2,miR-411-3p,miR-134,miR-345-3p,miR-545-5p,miR-222,miR-181b-5p,miR-339a,miR-1271-3p,miR-490-3p,miR-2284a,miR-1957a,miR-199a-5p,miR-3065-3p,miR-181a-2-3p,miR-1,miR-2385-3p,miR-29b-2-5p,miR-6395,miR-1291,miR-670-3p,miR-10a-5p,miR-378b,miR-656-5p,miR-20a-5p,miR-326-3p,miR-211,miR-2285x,miR-1193,miR-135a-2-3p,miR-30b-3p,miR-145a-5p,miR-374b-3p,miR-181a-5p,miR-1285,miR-20b,miR-544b,miR-377-5p,miR-125a-5p,miR-1343-5p,miR-106a-5p,miR-17-5p,miR-190a,miR-330-3p,miR-130b-5p,let-7g-3p,miR-21a-3p,miR-544-3p,miR-532-5p,miR-182-5p,miR-370-5p,miR-3065-3p,miR-545-3p,miR-487a-5p,miR-208b-5p,miR-664a,miR-29b-2-5p,miR-19b-1-5p,miR-222-3p,miR-6134,miR-206-3p,miR-10b-5p,miR-378d,miR-362-3p,novel_83,miR-151-5p,miR-383-5p,miR-505-3p,miR-3600,miR-299-3p,miR-106a,miR-125b,miR-146a-5p,miR-2432,miR-130a-5p,miR-29b-1-5p,miR-20a,miR-199b-5p,miR-7857-3p,miR-499b-5p,miR-2285t,miR-374a-3p,miR-4726-5p,miR-371a-5p,miR-769-5p,miR-181d-5p,miR-452-5p,miR-541-3p,miR-212-3p,novel_60,miR-3059-5p,miR-3120-5p,miR-544a,miR-30b-3p,miR-3962,miR-412-3p,miR-10b,miR-484,miR-221-3p,miR-9788-3p,miR-105-1,miR-3955-3p,miR-362-3p,novel_103,miR-1306-5p,miR-326,miR-491-3p,miR-193a,miR-376b,let-7c-3p,miR-106a-5p,miR-20b-5p,miR-329a,miR-767,miR-23b,miR-93-5p,miR-223-5p,miR-1271,miR-125a,miR-2285aa,miR-2355-5p,miR-874-3p,miR-503-3p,miR-134-5p,miR-592,miR-125b-5p,miR-23b-5p,miR-330-5p,miR-1343-5p,miR-129-1-3p,miR-134-5p,miR-505-5p,miR-9-5p,miR-431,novel_79,miR-412,miR-299a-3p,miR-127-5p,miR-6536,miR-

MPHOSPH9	ENSOARG0(	M-phase	miR-338-3p,miR-6134,miR-1271,miR-4510,miR-208b-5p,miR-487a-5p,miR-424-5p,miR-6516-3p,miR-22-3p,miR-139-5p,miR-6130,miR-193b-3p,miR-27a-5p,miR-182-5p,miR-21-3p,novel_99,miR-2312,miR-27b-5p,miR-2411,miR-125b-2-3p,miR-183-5p,miR-21-3p,miR-34b-5p,miR-2284m,miR-532-3p,miR-15a-5p,miR-340-5p,miR-200b,miR-383-5p,miR-548e-3p,miR-19b-3p,miR-188-3p,miR-124-3p,miR-2904,miR-2300b-3p,novel_133,miR-8095,miR-181d-5p,miR-2285f,novel_60,miR-2285t,miR-107-5p,miR-489,miR-499b-5p,miR-541-5p,miR-664b,miR-652-5p,miR-376b,miR-424-5p,miR-2285o,miR-1306-5p,miR-2285e,miR-9788-3p,novel_4,miR-16-2-3p,miR-2284aa,miR-30b-3p,miR-125b-2-3p,miR-7857,miR-143-5p,miR-4791,miR-145-3p,miR-323-3p,miR-3607-3p,miR-1306-5p,miR-135a-5p,miR-144,miR-5100,miR-1-5p,miR-34c-3p,miR-2284n,miR-154b-5p,miR-3965,miR-34c-5p,miR-141-3p,miR-671-5p,miR-23a-5p,novel_48,miR-488-3p,miR-381-3p,miR-2284a,miR-28b,miR-30a-3p,miR-107,miR-200c-3p,miR-181b-5p,miR-2898,miR-339a,miR-200c,miR-2285af,miR-582-3p,miR-545-5p,miR-1271-5p,miR-2411-5p,miR-24-2-5p,miR-200b-3p,miR-300,miR-134,miR-345-3p,miR-449a,miR-3959-3p,miR-211,miR-495-3p,novel_101,miR-2285x,miR-136-3p,novel_120,novel_1,miR-33a-3p,miR-34c,miR-16a,miR-133a-5p,miR-15a,miR-502-5p,miR-34b,miR-195a-5p,miR-135b-5p,miR-7975,miR-19a-3p,miR-107,miR-1291,miR-18a-3p,miR-21c,miR-2387,miR-2285ab,miR-2483-5p,miR-190a,miR-103,miR-185-5p,miR-2285g,miR-2284d,miR-26b-3p,miR-130a-3p,miR-133b-5p,miR-7862,miR-30b-3p,miR-21-5p,miR-145a-5p,miR-3071-5p,miR-181a-5p,miR-653,miR-181c-5p,miR-214,miR-3958-5p,miR-454-3p,miR-129-5p,miR-190a-5p,miR-665,miR-30a-3p,miR-30e-3p,miR-2285j,miR-15b-5p,miR-301a-3p,miR-552-3p,miR-548o-3p,miR-1827,miR-543-3p,miR-625-5p,miR-

ARHGEF37	ENSOARG0(	Rho guan	miR-322-5p,miR-24-2-5p,miR-378h,miR-6517,miR-7641,miR-412,miR-155-5p,miR-28b,miR-148b-5p,miR-3065-3p,miR-2284y,miR-29a-5p,miR-2284a,miR-17-3p,novel_127,novel_96,miR-2285af,miR-205-5p,miR-6119-5p,miR-219-3p,miR-133c,miR-874-3p,miR-329a,miR-23a-5p,miR-1248,miR-485-5p,miR-320a,miR-378c,miR-374b,miR-16-5p,miR-144-5p,miR-486b-3p,miR-28-5p,miR-331-3p,miR-409-3p,miR-134-3p,miR-149-5p,miR-133b-5p,miR-377-5p,miR-26b-3p,miR-16b,miR-30b-3p,miR-2284ab,miR-497-5p,miR-27b-3p,miR-2113,miR-22-3p,miR-654-3p,miR-432-3p,let-7j,miR-6240,miR-124-5p,miR-452-5p,miR-660,miR-193a-3p,miR-377-3p,miR-330-3p,miR-411b-3p,miR-320c,miR-3074-5p,miR-378b,miR-320b,miR-450b-3p,miR-6395,miR-378f,miR-195a-5p,miR-3970,miR-1306,miR-18a-3p,miR-374c-5p,miR-342-3p,miR-193b-3p,miR-17-3p,novel_1,miR-199c,miR-4324,miR-15a,miR-576-3p,miR-28c,miR-16a,miR-425-5p,miR-15a-5p,miR-2284h-5p,miR-532-3p,miR-328-3p,miR-18a-3p,miR-3600,miR-4429,miR-218-1-3p,miR-378i,miR-362-3p,miR-2366,miR-140-3p,miR-378a-3p,miR-377-5p,miR-486-3p,miR-183-5p,miR-2285w,miR-369-3p,miR-320d,miR-2484,miR-21-3p,novel_17,miR-324-3p,miR-92a-1-5p,miR-422a,miR-27a-5p,miR-377-3p,miR-582-5p,miR-193b-3p,miR-139-5p,miR-204-3p,miR-22-3p,miR-15b-5p,miR-155-5p,miR-378a-3p,miR-660-5p,miR-3065-3p,miR-216b-3p,let-7g-3p,miR-582-5p,miR-129b-3p,miR-655,miR-378c,miR-539-5p,miR-3074-5p,miR-155-5p,miR-6516-3p,miR-664a,miR-17-3p,miR-708-5p,miR-424-5p,miR-2285j,miR-324-3p,miR-219b-3p,miR-655-3p,miR-216a-3p,novel_4,miR-199b-3p,miR-330-3p,miR-1343-3p,miR-2300a-5p,miR-27a-3p,miR-378c,miR-362-3p,miR-2426,miR-101a-5p,miR-3120-5p,miR-6529a,miR-378b,miR-329-3p,miR-412-3p,miR-758-3p,miR-30b-3p,miR-378d,miR-3187-3p,miR-758-
COA7	ENSOARG0(	cytochro	miR-181a-2-3p,miR-195a-3p,miR-30a-3p,miR-130a-5p,miR-380-3p,miR-125a-3p,miR-200c,novel_17,miR-1260a,miR-205-5p,miR-217,miR-30f,miR-200c-3p,miR-202-5p,miR-543-5p,miR-429-3p,miR-30d-3p,miR-7641,miR-200b,miR-302a-5p,miR-199a-3p,miR-1197-5p,miR-23b-5p,miR-217-5p,miR-1343-5p,miR-582-3p,miR-200b-3p,miR-664-5p,miR-3074-5p,miR-2330-3p,miR-214,miR-1260b,miR-338-3p,miR-145-3p,miR-3184-5p,miR-30e-3p,miR-135a-5p,miR-30a-3p,miR-130b-5p,miR-660-5p,miR-140-5p,miR-21-3p,miR-499b-3p,miR-377-3p,miR-137-3p,miR-345-5p,miR-345-5p,miR-130b-5p,miR-345-5p,miR-1306-5p,miR-377-3p,miR-199a-3p,miR-2285l,miR-214-3p,miR-452-5p,miR-660,miR-1343-5p,novel_4,miR-31-5p,miR-145b,miR-222-5p,miR-26b-3p,miR-665,miR-432-5p,miR-455-3p,miR-1434-3p,miR-541,miR-6529a,miR-432,miR-873a-5p,miR-145a-5p,miR-1260b,miR-380-3p,miR-3120-5p,miR-188-5p,miR-541-3p,miR-3956-3p,novel_60,miR-3059-5p,miR-2285x,miR-2284w,miR-423-5p,miR-101-3p,miR-3958-3p,miR-452-3p,miR-20a-3p,miR-3074-5p,miR-499a-3p,miR-140-5p,miR-376b-5p,miR-3591-3p,miR-1306,miR-150-5p,miR-136-5p,miR-135b-5p

PKN1	ENSOARG0(	protein	miR-1260a,novel_127,miR-181a-2-3p,miR-3064-5p,miR-28b,miR-3065-3p,novel_39,miR-1343-5p,miR-128-3p,miR-423-3p,miR-299a-3p,miR-483-3p,miR-2411-5p,miR-370-5p,miR-1306-3p,miR-125b-5p,miR-331-3p,miR-138-5p,miR-486b-3p,novel_42,miR-1306-5p,miR-1260b,miR-10b-3p,miR-671-5p,miR-23a-5p,miR-125a,miR-1271,miR-542-5p,miR-370-3p,miR-34c-5p,miR-3141,miR-483-3p,miR-1343-5p,miR-125a-5p,miR-130b-5p,miR-432-3p,miR-197-3p,miR-1260b,miR-30b-3p,miR-138,miR-188-5p,miR-7862,miR-134-3p,miR-149-5p,miR-28c,miR-1247-3p,miR-34c,miR-136-3p,miR-342-3p,miR-1193,novel_101,miR-3957-3p,miR-24-3p,miR-449a,miR-3141,miR-2285ab,miR-216a-3p,miR-18a-3p,miR-1306,miR-1291,miR-450b-3p,miR-6395,miR-9851-3p,miR-3074-5p,miR-320b,miR-299b-3p,miR-34b,miR-2404,miR-125b,miR-324-3p,miR-760-3p,miR-299-3p,miR-34b-5p,miR-2887,miR-130a-5p,miR-376a-5p,miR-2411,miR-486-3p,miR-1197-5p,miR-33b-3p,miR-34a-5p,miR-18a-3p,miR-212-5p,miR-3600,miR-222-5p,miR-328-3p,miR-199a-3p,miR-532-3p,miR-665,miR-132-5p,miR-664a,miR-130b-5p,miR-4492,miR-129-5p,miR-145a-3p,miR-3074-5p,miR-129b-3p,miR-2312,miR-2331-3p,miR-532-5p,miR-370-5p,miR-3065-3p,miR-204-3p,novel_32,miR-5126,miR-1306-5p,miR-199a-3p,miR-342,miR-3085-3p,novel_124,miR-153-3p,miR-127-3p,miR-301b-5p,miR-541,miR-143-5p,miR-484,miR-30b-3p,miR-3120-5p,novel_4,miR-1343-3p,miR-153,miR-421,miR-216a-3p,miR-301a-5p,miR-499a-5p,miR-4726-5p,miR-490-5p,miR-541-3p,miR-2447,miR-3059-5p,let-7i-3p,miR-2285f,miR-6527,miR-877-3p,miR-361-3p,miR-1306-3p,miR-129b-5p,miR-668-5p,novel_94,miR-493-5p,miR-3064-5p,miR-7977,miR-489,miR-378g
DDX41	ENSOARG0(	DEAD (As	miR-9-5p,miR-299a-3p,miR-34a-5p,miR-378d,miR-218-1-3p,miR-1197-5p,miR-5703,miR-2904,miR-1343-5p,miR-2366,miR-362-3p,miR-2300b-3p,novel_133,miR-200a,miR-486-3p,novel_39,miR-377-5p,miR-28b,miR-183-5p,miR-200a-3p,miR-299-3p,miR-21-3p,miR-34b-5p,miR-466i-5p,miR-202-5p,miR-22-3p,novel_32,miR-323-5p,miR-6130,miR-34c-5p,miR-2284s,miR-182-5p,miR-141-3p,miR-370-3p,miR-574-5p,miR-2331-3p,miR-1248,miR-485-5p,miR-329a,miR-3154,miR-21a-3p,miR-122-5p,miR-6525,miR-214,miR-4510,miR-28-5p,miR-486b-3p,miR-592,miR-130b-5p,miR-874-5p,miR-708-5p,miR-664a,novel_74,miR-138-5p,miR-665,miR-6740-5p,miR-410-5p,miR-149-5p,miR-194-3p,miR-412-5p,miR-377-5p,novel_4,miR-3120-5p,miR-101a-5p,miR-362-3p,miR-138,miR-22-3p,miR-1843b-5p,miR-1434-3p,miR-2113,miR-329-3p,miR-2284r,miR-101-5p,miR-382-3p,miR-1388-3p,miR-130b-5p,miR-103a-2-5p,miR-214-3p,miR-1343-5p,novel_51,miR-182-5p,miR-362-3p,miR-3141,miR-4443,miR-669,miR-34b,miR-299b-3p,miR-323a-5p,miR-655-5p,miR-320b,miR-20a-3p,miR-1983,miR-412-3p,miR-3141,miR-361-3p,miR-154a,miR-2428,novel_27,miR-449a,miR-1285-5p,miR-9-5p,miR-452-5p,miR-2285t,miR-107-5p,miR-34c,miR-28c,miR-4324,miR-218-5p

ZNF532	ENSOARG0(	zinc fin	miR-216b-3p,miR-214,novel_68,miR-340-3p,miR-363,miR-149-5p,miR-30b-3p,miR-2331-5p,miR-2113,miR-3432a,miR-744-5p,miR-214-3p,miR-30b-3p
MGLL	ENSOARG0(	monoglyc	miR-216a-3p,miR-18a-3p,miR-3059-5p,miR-370-3p,miR-628-5p,miR-1271,miR-466i-5p,miR-23a-5p,novel_127,miR-1197-5p,miR-23b-5p,miR-18a-3p
MARVELD2	ENSOARG0(	MARVEL d	miR-3074-5p,let-7b,miR-378c,miR-425-5p,miR-10b-5p,let-7b-5p,let-7f,miR-338-5p,miR-29b-2-5p,miR-665,miR-320e,miR-1843b-3p,miR-669a-3p,miR-190a-5p,miR-378a-3p,miR-140-5p,miR-296-3p,miR-3065-3p,miR-216b-3p,miR-139-5p,miR-216b-5p,miR-10a,miR-877-5p,let-7i-5p,miR-204-3p,miR-21a-3p,miR-30f,miR-2285w,miR-125b-2-3p,miR-146a-5p,miR-422a,miR-219b-5p,miR-216a-5p,miR-466i-5p,miR-98-5p,let-7k,miR-30b-3p,miR-23c,miR-200b,miR-23b-3p,miR-7859,miR-2284h-5p,miR-532-3p,miR-2408,miR-1961,novel_133,miR-378i,miR-378a-3p,miR-1197-5p,miR-217-5p,miR-5703,miR-2904,miR-124-3p,novel_73,miR-16-1-3p,miR-218-1-3p,miR-541-3p,miR-2447,miR-9-5p,miR-452-5p,miR-8095,miR-1285-5p,novel_82,miR-2284q,miR-20a-3p,miR-452-3p,miR-3064-5p,miR-139-5p,miR-211-5p,miR-541-5p,miR-489,miR-3082-5p,miR-2483-3p,miR-378g,miR-378e,miR-203-3p,let-7d-5p,miR-140-5p,miR-668-5p,miR-1983,miR-1839-3p,miR-124a,let-7a-5p,miR-33a-5p,miR-23a-3p,miR-378d,miR-8485,miR-326,miR-342,miR-323b-3p,miR-3085-3p,let-7e,miR-146b,miR-330-3p,miR-145b,miR-539-5p,miR-455-3p,miR-216a-3p,miR-10b,miR-143-5p,miR-1298-5p,miR-541,miR-378b,miR-3962,miR-125b-2-3p,miR-323b,miR-450b-5p,miR-378c,miR-21b,miR-2284aa,miR-101c,miR-193a-5p,miR-1197-3p,miR-1260b,miR-323-3p,miR-378c,miR-135a-1-3p,miR-29b-3p,miR-1-5p,miR-3969,miR-100-3p,miR-138-5p,miR-204-5p,miR-410-3p,miR-31-3p,miR-574-5p,miR-370-3p,miR-23b,let-7g-3p,miR-329b,miR-10b-3p,miR-29b-2-5p,miR-3064-5p,miR-2478,let-7g,miR-3065-3p,miR-1957a,miR-6119-5p,miR-200c,miR-205-5p,miR-2285af,miR-1260a,let-7c-5p,miR-217,miR-127-5p,miR-1a-2-5p,miR-200c-3p,let-7f-5p,miR-429-3p,miR-6517,miR-376a-5p,miR-1224-5p,miR-7641,miR-378h,miR-9-5p,miR-2284z,miR-545-5p,miR-3591-5p,miR-196a-5p,miR-23b-5p,miR-
KIF22	ENSOARG0(	kinesin	miR-125a,miR-1b-3p,miR-2411-5p,miR-532-3p,miR-652-3p,miR-541,miR-2284r,miR-129b-5p,miR-1343-5p,miR-450b-5p,miR-125b-1-3p,miR-744-5p,miR-4508,miR-541-3p,miR-216c-5p,miR-676-3p,miR-2411,miR-6402,novel_17,miR-4324,novel_51,miR-125b,miR-125b-5p,miR-191-3p,miR-1343-5p,miR-452-5p,miR-208b-5p,miR-125a-5p
DPP3	ENSOARG0(	dipeptid	novel_4,miR-320b,miR-2459,novel_32

RNF167	ENSOARG0(	ring fin	miR-2904,miR-188-3p,miR-1197-5p,novel_133,miR-2284u,miR-148a-5p,miR-33b-3p,miR-184-3p,miR-19b-3p,miR-505-3p,miR-2284h-5p,miR-328-3p,miR-324-3p,miR-185-3p,miR-92a-1-5p,miR-2285p,miR-618,miR-2284m,miR-466i-5p,miR-369-3p,miR-2285w,miR-2432,miR-146a-5p,miR-486-3p,miR-29b-1-5p,miR-376a-5p,miR-377-5p,miR-130a-5p,miR-655,miR-625-5p,miR-339b,miR-18a-5p,miR-552-3p,miR-296-3p,miR-182-5p,miR-140-5p,miR-30c-1-3p,miR-504-5p,miR-545-3p,miR-660-5p,miR-27a-5p,miR-10a,miR-324-3p,miR-219b-3p,miR-655-3p,miR-665,miR-874-5p,miR-4492,miR-669a-3p,miR-130b-5p,miR-3074-2-3p,miR-539-5p,miR-3184-5p,miR-6134,miR-10b-5p,miR-758-3p,miR-10b,miR-101c,miR-330-3p,miR-455-3p,miR-9788-3p,miR-148b-5p,miR-421,miR-147a,miR-1298-3p,miR-29b-1-5p,miR-182-5p,miR-5126,novel_103,miR-146b,miR-199b-5p,miR-103a-2-5p,miR-491-5p,miR-3085-3p,miR-2284b,miR-4532,miR-8485,miR-2310,miR-758-3p,miR-30c-1-3p,miR-361-3p,miR-199b-5p,miR-1983,miR-99b-5p,miR-504,miR-136-5p,miR-7977,miR-211-5p,miR-24-1-5p,miR-2284g,miR-3064-5p,miR-10b,miR-203-3p,miR-378g,miR-107-5p,miR-490-5p,miR-7134-3p,miR-2284q,miR-4726-5p,miR-335,miR-9-5p,miR-2284j,miR-3059-5p,novel_60,miR-212-3p,miR-3956-3p,miR-885-5p,miR-423-5p,miR-1285-5p,miR-219a-2-3p,miR-361-3p,miR-877-3p,miR-1343-5p,miR-134,miR-99a-5p,miR-10a-5p,novel_79,miR-7641,miR-18b-5p,miR-134-5p,miR-582-3p,novel_121,miR-24-2-5p,miR-9-5p,miR-30c-2-3p,miR-339a,miR-99a,miR-125a-3p,miR-219-3p,miR-6119-5p,miR-543-5p,novel_96,miR-2898,miR-3064-5p,miR-2284f,miR-4286,miR-199a-5p,miR-2284y,miR-376c-5p,miR-2284a,miR-148b-5p,miR-24-2-5p,miR-137-3p,miR-100-5p,miR-18a,miR-488-3p,miR-7144-5p,miR-1814c,miR-370-3p,miR-1271,miR-223-5p,miR-199a-5p,miR-2284p,miR-365a-3p,miR-144,miR-135a-5p,miR-

PSMD8	ENSOARG0(	proteaso	miR-1343-3p,miR-2300a-5p,miR-181b-2-3p,miR-1185-3p,miR-2459,miR-455-3p,miR-143-5p,miR-10b,miR-301b-5p,miR-2284r,miR-296-5p,miR-412-3p,miR-3187-3p,miR-326,miR-29b-1-5p,miR-2957,miR-487b-5p,miR-146b,miR-10b,miR-211-5p,miR-499b-5p,miR-499a-3p,miR-1185-2-3p,miR-652-3p,miR-409-5p,miR-140-5p,miR-361-3p,miR-504,novel_94,miR-544-5p,miR-2447,miR-32-3p,miR-361-3p,miR-154a,miR-615,miR-301a-5p,miR-371a-5p,miR-2285y,miR-548e-3p,miR-212-5p,miR-7859,miR-532-3p,miR-2300b-3p,miR-188-3p,miR-217-5p,miR-2904,miR-33b-3p,miR-378d,miR-133a-3p,miR-329a-5p,miR-345-3p,miR-181b-1-3p,miR-29b-1-5p,miR-143-3p,miR-486-3p,miR-146a-5p,miR-345-3p,miR-324-3p,miR-216a-5p,miR-202-5p,miR-660-5p,miR-32-3p,miR-2331-3p,miR-504-5p,miR-296-3p,miR-6130,miR-499b-3p,miR-10a,novel_25,novel_32,miR-204-3p,miR-22-3p,miR-345-5p,miR-339b,miR-625-5p,miR-885-3p,miR-145a-3p,miR-4510,miR-346,miR-10b-5p,miR-338-3p,miR-6134,miR-3604,miR-320e,miR-664a,miR-487b-5p,miR-1843b-3p,miR-130b-5p,miR-874-5p,miR-4492,miR-133b-5p,miR-187-3p,miR-339-5p,miR-487b-3p,miR-185-5p,miR-2355-3p,miR-149-5p,novel_116,miR-873a-5p,miR-22-3p,miR-29b,miR-744-5p,miR-2889,miR-130b-5p,miR-432-3p,miR-345-5p,miR-296-3p,miR-296-3p,miR-146a,miR-2411-3p,novel_51,miR-378a-5p,miR-660,miR-329-5p,miR-10a-5p,miR-670-3p,miR-29a-3p,miR-2404,miR-181b-3p,miR-677,miR-450b-3p,miR-146b-5p,miR-29c-3p,miR-24-3p,miR-211,miR-2428,miR-326-3p,miR-28c,novel_87,novel_1,miR-29a,miR-6119-3p,miR-6123,miR-22-5p,miR-2285ad,miR-412,novel_79,miR-505-5p,miR-214-5p,miR-582-3p,miR-134-5p,miR-134,miR-345-3p,miR-615-3p,miR-330-5p,miR-29d-3p,miR-885-3p,miR-10a-5p,miR-28b,miR-2285b,miR-1271-3p,miR-6119-5p,miR-125a-3p,miR-217,miR-339a,miR-200a-3p,miR-141-3p,miR-2355-5p,miR-370-3p,miR-

TRAF1	ENSOARG0(	TNF rece	miR-378g,let-7d-5p,miR-4443,miR-1983,miR-361-3p,miR-409-5p,miR-361-3p,miR-877-3p,miR-423-5p,miR-3059-5p,miR-9-5p,miR-2284j,miR-2285n,miR-2285y,miR-107-5p,miR-153,miR-455-3p,miR-421,novel_4,miR-2300a-5p,miR-1343-3p,miR-409b,miR-145b,miR-2284aa,miR-1839-5p,miR-16-2-3p,miR-2284r,miR-8485,let-7a-5p,miR-574-3p,miR-2330-5p,miR-25-5p,miR-487b-5p,miR-153-3p,miR-214-3p,let-7e,miR-103a-2-5p,miR-199a-3p,miR-1306-5p,miR-29b-1-5p,novel_19,let-7i-5p,miR-221-5p,miR-548o-3p,miR-532-5p,miR-625-5p,miR-885-3p,miR-1895,miR-1827,miR-3184-5p,let-7b-5p,let-7b,miR-129-2-3p,miR-214,miR-3074-2-3p,miR-874-5p,miR-4492,let-7f,miR-132-5p,miR-340-5p,miR-532-3p,miR-199a-3p,miR-616-3p,miR-328-3p,miR-3600,miR-212-5p,miR-378d,novel_133,miR-193b-5p,miR-1961,miR-188-3p,miR-1197-5p,miR-5703,miR-29b-1-5p,miR-345-3p,miR-760-3p,miR-98-5p,miR-221-5p,let-7k,miR-210-5p,miR-185-3p,miR-92a-1-5p,miR-670-3p,miR-6535,novel_68,miR-412-3p,let-7e-5p,miR-6395,miR-1306,miR-24-3p,miR-2428,miR-136-3p,miR-128-1-5p,miR-147-5p,miR-28c,miR-576-3p,let-7i,miR-2284d,miR-412-5p,miR-134-3p,miR-3431,let-7g-5p,miR-665,miR-4508,miR-188-5p,miR-708-3p,miR-653,miR-2113,novel_23,miR-145a-5p,miR-432-3p,miR-483-3p,miR-378a-5p,miR-466f-3p,miR-197-5p,miR-136-3p,novel_51,miR-324-5p,miR-133c,miR-874-3p,miR-615-5p,miR-1271,miR-23a-5p,miR-671-5p,miR-1197-3p,miR-145-3p,miR-1306-5p,miR-2284k,miR-26c,miR-9-5p,miR-483-3p,miR-2284z,miR-129-1-3p,miR-2285ad,miR-6517,miR-885-3p,let-7d,miR-23b-5p,miR-28b,miR-148b-5p,miR-2478,let-7g,novel_39,let-7f-5p,miR-205-5p,miR-125a-3p,let-7c-5p
PLSCR4	ENSOARG0(	phosphol	miR-335-5p,miR-335
PHF19	ENSOARG0(	PHD fing	miR-371b-3p,miR-361-3p,miR-2447,novel_101,miR-495-3p,miR-450a-5p,miR-4726-5p,miR-628-3p,miR-15a,miR-769-5p,miR-2285y,miR-16a,novel_69,miR-499a-3p,miR-652-3p,miR-4792,miR-7977,miR-195a-5p,miR-361-3p,miR-18a-3p,miR-150-5p,let-7j,miR-424-5p,miR-25-5p,miR-214-3p,miR-125a-5p,miR-377-3p,miR-16b,miR-185-5p,miR-187-3p,miR-450b-5p,miR-16b,miR-497-5p,miR-873a-5p,miR-484,miR-769-5p,novel_42,miR-214,miR-16-5p,miR-424-5p,miR-665,miR-125b-5p,miR-769,miR-377-3p,miR-499b-3p,miR-15b-5p,miR-204-3p,miR-874-3p,miR-221-5p,miR-125a,miR-552-3p,miR-3065-3p,miR-370-3p,miR-1895,miR-301,miR-130a-5p,miR-143-3p,miR-450c-5p,miR-3065-3p,miR-760-3p,novel_127,miR-221-5p,miR-125a-3p,miR-125b,miR-15a-5p,miR-322-5p,miR-2284h-5p,miR-18a-3p,miR-1224-5p
MIOS	ENSOARG0(	missing	miR-885-5p,miR-424-5p,miR-16-5p,miR-877-3p,miR-28b,let-7j,miR-125a-3p,miR-28c,miR-15a,miR-16b,miR-16a,miR-424-5p,miR-107,miR-103a-3p,miR-18b-5p,miR-15a-5p,miR-7857-3p,miR-322-5p,miR-15b-5p,miR-103,miR-18b,miR-542-3p,miR-18a,miR-18a-5p,miR-107,miR-16b,miR-195a-5p,miR-497-5p
KAT7	ENSOARG0(	K(lysine	miR-335,miR-582,miR-149-5p,miR-6535,miR-145-3p,miR-192-3p,miR-146b-3p,miR-432-3p,miR-335-5p

PSMD5	ENSOARG0(	proteaso	miR-27b-5p,miR-129b-3p,miR-548o-3p,miR-452-3p,miR-1271,miR-299b-3p,miR-133c,miR-669,miR-2285j,miR-7134-3p,miR-2285t,miR-145a-3p,miR-539-5p,miR-346,miR-7705,miR-326-3p,miR-1290,miR-330-5p,miR-30b-3p,miR-30b-3p,miR-548e-3p,miR-299a-3p,miR-7859,miR-505-5p,miR-671-3p,miR-9788-3p,miR-326,miR-2285af,miR-29b-1-5p,miR-491-5p,miR-299-3p,novel_96,miR-494-3p,miR-29b-1-5p
PIDD1	ENSOARG0(	p53-indu	miR-148a-3p,miR-450b-3p,miR-147-3p,novel_99,novel_94,miR-125a,miR-34c-5p,miR-193b-3p,miR-34b,miR-4324,miR-125b-5p,miR-34c,miR-331-3p,miR-4726-5p,miR-7134-3p,miR-486b-3p,novel_42,miR-342-3p,miR-193b-3p,miR-449a,novel_133,miR-2366,miR-2904,miR-450b-5p,miR-2426,miR-34a-5p,miR-6517,miR-148b-3p,miR-2284h-5p,miR-342,novel_51,miR-125b,miR-34b-5p,miR-125a-5p,miR-193a-3p,miR-152-3p,miR-2285w,miR-486-3p,miR-4532
PAQR6	ENSOARG0(	progesti	novel_39,miR-199a-5p,miR-3065-3p,miR-2285b,miR-3064-5p,miR-30a-3p,novel_96,miR-1260a,miR-505-5p,miR-378h,miR-885-3p,miR-29d-3p,miR-3578,miR-378c,miR-374a-3p,miR-1260b,miR-31-3p,miR-374b-3p,miR-486b-3p,miR-331-3p,miR-26c,miR-3969,miR-29b-3p,miR-199a-5p,miR-2285aa,miR-370-3p,miR-1271,miR-574-5p,miR-376c-5p,miR-7144-5p,miR-329a,miR-329b,miR-376c-5p,miR-628-5p,miR-130b-5p,miR-149-5p,miR-3535,miR-134-3p,miR-29b,miR-30b-3p,miR-381-5p,miR-1260b,miR-22-3p,novel_23,miR-199b-5p,miR-371b-3p,miR-24-3p,miR-29c-3p,miR-335-5p,miR-22-5p,miR-502b,miR-29a,miR-669,miR-146b-3p,miR-29a-3p,novel_68,miR-3074-5p,miR-329-5p,miR-378f,miR-1291,miR-1306,miR-376b-5p,miR-2387,miR-486-3p,miR-329a-5p,miR-466i-5p,miR-324-3p,miR-185-3p,miR-422a,miR-532-3p,miR-30d-3p,miR-142-3p,miR-33b-3p,miR-124-3p,miR-378a-3p,miR-378i,miR-362-3p,miR-122-5p,miR-6134,miR-425-5p,miR-378c,miR-4510,miR-3074-5p,miR-4492,miR-130b-5p,miR-324-3p,miR-30a-3p,miR-665,miR-30e-3p,miR-22-3p,miR-6130,miR-3065-3p,miR-2331-3p,miR-378a-3p,miR-885-3p,miR-758-3p,miR-378d,miR-124a,miR-1388-3p,miR-199b-5p,miR-381-5p,miR-491-5p,miR-3085-3p,miR-362-3p,novel_19,miR-1306-5p,miR-496-5p,miR-1343-3p,miR-2300a-5p,miR-362-3p,miR-378c,miR-450b-5p,miR-758-3p,miR-30b-3p,miR-378b,miR-329-3p,miR-296-5p,miR-484,miR-541,miR-4791,miR-361-3p,novel_60,miR-541-3p,miR-3956-3p,miR-374a-3p,miR-335,miR-4443,miR-378e,miR-378g,miR-7977,miR-3064-5p,novel_94,miR-3963,miR-199b-5p

C11orf58	ENSOARG0(	chromoso	miR-363-5p,miR-382,miR-654-3p,miR-345-5p,miR-296-3p,miR-330-3p,miR-197-5p,miR-124-5p,miR-466f-3p,miR-26b-3p,miR-2284d,miR-194-5p,miR-3071-5p,miR-380-3p,miR-1193,miR-17-3p,miR-2285x,miR-24-3p,novel_27,miR-326-3p,miR-6402,miR-502-5p,miR-382-5p,novel_1,miR-147-5p,miR-203b-5p,miR-670-3p,miR-3529-3p,miR-181a-2-3p,miR-29b-2-5p,miR-30a-3p,miR-1271-3p,miR-3065-3p,miR-200c,miR-2285af,miR-339a,miR-217,miR-30c-2-3p,miR-200c-3p,miR-17-3p,miR-222,miR-7641,miR-24-2-5p,miR-545-5p,miR-300,miR-128-3p,miR-105-2,miR-200b-3p,miR-374b,miR-133a-3p,miR-500b-5p,miR-370-5p,miR-5100,miR-2332,miR-1-5p,miR-409-3p,miR-3969,miR-144,miR-28-5p,miR-410-3p,miR-215-5p,miR-141-3p,miR-370-3p,miR-2284n,miR-503-3p,miR-381-3p,miR-362-5p,miR-3154,miR-10b-3p,miR-190a-3p,miR-7144-5p,miR-494-3p,miR-652-5p,let-7c-3p,miR-30c-1-3p,miR-326,miR-3955-3p,miR-3085-3p,miR-105-1,novel_4,miR-27a-3p,miR-221-3p,miR-500-5p,miR-143-5p,miR-4791,miR-484,miR-125b-2-3p,miR-541-3p,miR-452-5p,miR-194b-5p,miR-362-5p,miR-2285n,miR-371a-5p,miR-2285y,miR-2284q,miR-2285t,miR-3064-5p,miR-7977,miR-499b-5p,miR-3082-5p,miR-27a-3p,miR-129b-5p,miR-877-3p,miR-216c-5p,miR-29b-1-5p,miR-374a-5p,miR-185-3p,miR-216a-5p,miR-2284m,miR-21-3p,miR-221-5p,miR-30b-3p,miR-3600,miR-148b-3p,miR-200b,miR-383-5p,miR-215-5p,miR-2300b-3p,novel_133,miR-140-3p,miR-5703,miR-2904,let-7a-3p,miR-145a-3p,miR-539-5p,miR-425-5p,miR-3074-2-3p,miR-206-3p,miR-338-3p,miR-222-3p,miR-655-3p,miR-29b-2-5p,miR-17-3p,miR-450b-3p,miR-708-5p,miR-221-5p,miR-3065-3p,miR-370-5p,miR-139-5p,miR-192-5p,miR-877-5p,let-7f-1-3p,miR-21a-3p,miR-133b-3p,let-7g-3p,novel_99,miR-6240,miR-2284l,miR-2285r,miR-296-3p,miR-194a,miR-105-5p,miR-339-5p,miR-412-5p,miR-3535,miR-223-

CEP164	ENSOARG0(	centroso	miR-22841,miR-503-3p,let-7j,miR-6240,miR-363-5p,miR-1343-5p,miR-125a-5p,miR-377-3p,miR-146a,miR-7-5p,miR-330-3p,miR-3141,miR-103,miR-133b-5p,miR-103a-3p,miR-16b,miR-7862,miR-497-5p,miR-4508,miR-181a-5p,miR-7b-5p,miR-873a-5p,miR-371b-3p,miR-24-3p,miR-326-3p,novel_27,miR-135a-2-3p,novel_101,miR-3957-3p,miR-22-5p,miR-28c,miR-4324,miR-15a,miR-16a,novel_69,miR-299b-3p,miR-677,miR-26b-5p,miR-320b,miR-107,miR-1291,miR-195a-5p,miR-379-5p,miR-135b-5p,miR-146b-5p,miR-3141,miR-1306,miR-450b-5p,miR-28b,miR-181a-2-3p,miR-181b-5p,miR-2898,miR-107,miR-664-3p,miR-2285af,miR-2411-5p,miR-322-5p,novel_121,miR-2284z,miR-134-5p,miR-6517,miR-2285ad,miR-299a-3p,miR-1224-5p,miR-7641,miR-134,miR-330-5p,miR-1343-5p,miR-374a-3p,miR-1197-3p,miR-1306-5p,miR-193a-5p,miR-16-5p,miR-374b-3p,miR-28-5p,miR-134-5p,miR-486b-3p,miR-410-3p,miR-135a-5p,miR-331-3p,miR-125b-5p,miR-769,miR-125a,miR-671-5p,miR-488-3p,miR-3154,miR-758-3p,miR-4532,miR-424-5p,miR-382-3p,miR-1468,miR-103a-2-5p,miR-146b,miR-326,miR-1306-5p,miR-199a-3p,miR-16b,miR-29b-1-5p,miR-147a,miR-330-3p,miR-151a-3p,miR-181b-2-3p,miR-2426,miR-2284aa,miR-484,miR-758-3p,miR-361-3p,miR-2447,miR-219a-5p,miR-181d-5p,miR-4726-5p,miR-2284q,miR-374a-3p,miR-107-5p,miR-2285y,miR-769-5p,miR-3082-5p,miR-452-3p,miR-4792,miR-664b,miR-139-5p,miR-504,miR-361-3p,miR-129b-5p,miR-29b-1-5p,miR-2432,miR-2411,miR-486-3p,miR-146a-5p,miR-466i-5p,miR-760-3p,miR-202-5p,miR-299-3p,miR-30b-3p,miR-2285p,miR-26a-5p,miR-185-3p,miR-125b,miR-324-3p,miR-222-5p,miR-15a-5p,miR-199a-3p,miR-184-3p,miR-378d,miR-133a-3p,miR-2300b-3p,miR-769-5p,miR-181c-5p,miR-3184-3p,miR-145a-3p,miR-665-5p,miR-1468-5p,miR-874-5p,miR-708-5p,miR-2285j,miR-665,miR-151-3p,miR-324-3p,miR-139-5p,miR-377-
CDK7	ENSOARG0(	cyclin-d	miR-155-5p,miR-148a-5p,novel_99,miR-2284ab,miR-145a-5p,miR-148b-5p,novel_83,miR-199a-3p,miR-155-5p,miR-2284d,miR-222-5p,miR-145b,miR-126b-5p,miR-552-3p,miR-532-5p,miR-155-5p,miR-3604,miR-2284k,miR-199a-3p,miR-2285j,miR-2284y,miR-2284a,miR-148b-5p,miR-452-5p,miR-2284v
SLA	ENSOARG0(	Src-like	miR-363,miR-26b-3p,miR-505-3p,miR-21-3p,miR-2355-5p,miR-3600,miR-6516-5p,miR-383-5p,miR-6516,miR-7b-5p,miR-1285,miR-542-3p,miR-361-3p,miR-3591-3p,miR-433-3p,miR-450b-5p,miR-455-5p,miR-505-3p,miR-23a-5p,novel_101,miR-1271-3p,miR-8485,miR-376c-5p,miR-125a-3p,miR-7-5p,miR-665,miR-455-5p

EPAS1	ENSOARG0(	endothel	miR-485-3p,miR-769-5p,miR-576-3p,miR-2284q,miR-502b,miR-2285t,miR-17-3p,miR-452-5p,miR-495-3p,miR-2447,miR-7705,miR-323c,miR-376b-3p,miR-3065-5p,miR-148a-3p,miR-376a-3p,miR-299b-5p,miR-450b-3p,miR-4792,miR-376d,novel_69,miR-3596,miR-2404,miR-455-5p,novel_51,miR-182-5p,miR-105-5p,miR-1468,let-7a-2-3p,miR-8485,novel_23,miR-412-3p,miR-296-5p,miR-2889,miR-455-5p,miR-2284aa,miR-1260b,miR-101a-5p,miR-544a,miR-138,miR-105-1,miR-544b,miR-2459,miR-9788-3p,miR-376b-3p,miR-320e,miR-1468-5p,miR-500,miR-874-5p,miR-4492,miR-664a,miR-17-3p,miR-138-5p,miR-1306-5p,miR-1260b,miR-769-5p,miR-6525,miR-500a-3p,miR-485-5p,miR-582-5p,miR-190a-3p,miR-7144-5p,let-7g-3p,let-7g-3p,miR-182-5p,miR-21-3p,miR-503-3p,miR-496-3p,miR-154b-5p,miR-151b,miR-204-3p,miR-769,miR-544-3p,miR-582-5p,miR-125a-3p,miR-2285af,miR-196a-3p,miR-1260a,miR-376b-3p,miR-152-3p,miR-543-5p,miR-466i-5p,miR-127-5p,miR-17-3p,miR-450a-2-3p,miR-369-3p,miR-2285w,miR-3529-3p,miR-1271-3p,miR-2432,novel_39,miR-148b-5p,miR-5703,miR-3578,miR-496,let-7a-2-3p,miR-3600,miR-412,miR-214-5p,miR-671-3p,miR-151-5p,miR-2284z,miR-2284h-5p,miR-532-3p,miR-148b-3p
LMAN1	ENSOARG0(	lectin, :	miR-2284w,miR-449a,novel_1,miR-199c,miR-22-5p,miR-6402,miR-4324,miR-34c,miR-411b-3p,miR-34b,novel_68,miR-412-3p,miR-135b-5p,miR-376b-5p,miR-2285ab,miR-3591-3p,miR-216a-3p,miR-2284l,miR-2285r,miR-345-5p,miR-6240,miR-130b-5p,miR-628-5p,miR-2483-5p,miR-101-5p,miR-125a-5p,miR-330-3p,miR-2284d,miR-544b,miR-2355-3p,miR-1260b,miR-138,miR-2284ab,miR-27b-3p,miR-188-5p,miR-7862,miR-181a-5p,miR-542-3p,miR-374b-3p,miR-450c-3p,miR-22-3p,miR-197-3p,miR-1260b,miR-2284v,miR-3607-3p,miR-138-5p,miR-2284k,miR-135a-5p,miR-409-3p,miR-125b-5p,miR-34c-5p,miR-503-3p,miR-125a,miR-2355-5p,miR-345-5p,miR-2318,miR-876-3p,miR-1814c,miR-1248,miR-376c-5p,miR-376c-5p,miR-490-3p,miR-2284a,miR-2284y,miR-2385-3p,miR-181b-5p,miR-200c-3p,miR-200c,miR-2397-5p,miR-2285af,miR-1260a,miR-217,miR-302a-5p,miR-9-5p,novel_121,miR-450a-1-3p,miR-2284z,miR-545-5p,miR-429-3p,miR-582-3p,miR-496,miR-128-3p,miR-200b-3p,miR-1290,novel_111,miR-885-5p,miR-541-3p,miR-3059-5p,miR-181d-5p,miR-2284j,miR-9-5p,miR-2285f,miR-101-3p,miR-2440,miR-2285n,miR-2285y,miR-27a-3p,miR-2284g,miR-2285u,miR-1983,novel_94,miR-335-3p,miR-361-3p,miR-140-5p,miR-378j,miR-2310,miR-382-3p,miR-124a,miR-2284b,miR-23a-3p,miR-153-3p,miR-199a-3p,miR-142-3p,miR-153,miR-148b-5p,miR-199b-3p,novel_78,miR-330-3p,miR-27a-3p,miR-2426,miR-544a,miR-3120-5p,miR-2284aa,miR-541,miR-484,miR-181c-5p,miR-3184-3p,miR-450b-3p,miR-130b-5p,miR-208b-5p,miR-544-3p,miR-22-3p,miR-496-3p,miR-221-5p,miR-2331-3p,novel_107,miR-216b-3p,miR-21-3p,miR-345-5p,miR-543-3p,miR-450a-1-3p,miR-129b-3p,miR-130a-5p,miR-142a-3p,miR-664b-3p,miR-216c-5p,miR-369-3p,miR-760-3p,miR-2284m,miR-221-5p,miR-34b-5p,miR-196a-3p,miR-92a-1-5p,miR-125b,miR-200b,miR-

GLG1	ENSOARG0(golgi gl	miR-142-3p,miR-184-3p,miR-378a-3p,miR-378i,miR-328-3p,miR-1247-5p,miR-212-5p,miR-18a-3p,miR-422a,miR-92a-1-5p,miR-210-5p,miR-143-3p,miR-376c-3p,miR-369-3p,miR-329a-5p,miR-1895,miR-625-5p,miR-339b,miR-129b-3p,miR-15b-5p,miR-151b,miR-377-3p,miR-376e-3p,miR-10a,miR-301a-3p,miR-2284s,miR-582-5p,miR-296-3p,miR-454-3p,miR-4492,miR-664a-5p,miR-665,miR-15b-3p,miR-2285j,miR-3184-5p,miR-346,miR-665-5p,miR-3074-5p,miR-362-3p,miR-1a-3p,miR-541,miR-2284r,miR-329-3p,miR-1185-3p,miR-381-5p,miR-146b,miR-130b-3p,miR-491-5p,miR-16b,miR-8485,miR-378d,miR-744-3p,miR-676-3p,miR-574-3p,miR-1983,miR-9-3p,miR-493-5p,miR-378j,miR-4443,miR-1185-2-3p,miR-203-3p,miR-2483-3p,miR-378g,miR-2285u,miR-2319b,miR-24-1-5p,miR-2440,miR-423-5p,miR-1285-5p,novel_111,miR-3956-3p,miR-2447,miR-23b-5p,miR-330-5p,miR-1343-5p,novel_121,miR-134-5p,miR-322-5p,miR-18b-5p,novel_79,miR-2285ad,novel_127,novel_96,miR-200a-3p,miR-28-3p,miR-125a-3p,miR-148b-5p,miR-1248,miR-329b,miR-329a,miR-2318,miR-767,miR-376c-3p,miR-1271,miR-125a,miR-542-5p,miR-874-3p,miR-3068-3p,miR-134-5p,miR-125b-5p,miR-2433,miR-1197-3p,miR-193a-5p,miR-16-5p,miR-28a-3p,miR-188-5p,miR-26a-2-3p,miR-497-5p,miR-381-5p,miR-16b,miR-197-3p,miR-1843b-5p,miR-7b-5p,miR-194-3p,miR-149-5p,miR-134-3p,miR-339-5p,miR-31-5p,miR-193a-3p,novel_51,miR-377-3p,miR-146a,miR-7-5p,let-7a-2-3p,let-7j,miR-432-3p,miR-432-3p,let-7f-2-3p,let-7f-2-3p,miR-493-5p,miR-378f,miR-2319a,miR-146b-5p,miR-412-3p,miR-3591-3p,miR-1306,miR-669,miR-6535,miR-3120-3p,miR-3074-5p,miR-329-5p,novel_68,miR-6123,miR-206,miR-425-5p,novel_87,miR-576-3p,miR-4324,miR-301b-3p,miR-193b-3p,miR-378d,miR-941,miR-1197-5p,miR-188-3p,miR-1193,miR-140-3p,miR-362-

FAM117A	ENSOARG0(	family w	miR-6527,miR-9-5p,miR-2447,miR-423-5p,miR-219a-2-3p,miR-361-3p,miR-2285y,miR-2285n,miR-3964,miR-4726-5p,miR-499a-5p,miR-499b-5p,miR-7977,miR-664b,miR-139-5p,miR-24-1-5p,miR-3064-5p,miR-2319b,miR-20a-3p,miR-7857-3p,miR-499a-3p,miR-378g,miR-3596,miR-2483-3p,miR-381-3p,miR-378j,miR-361-3p,miR-199b-5p,miR-96-5p,miR-23a-3p,miR-744-3p,miR-124a,miR-2330-5p,miR-106a-5p,miR-30c-1-3p,miR-29b-1-5p,miR-106b-5p,miR-199a-3p,miR-320b,miR-326,miR-199b-5p,miR-3085-3p,miR-3955-3p,miR-5010-3p,miR-199b-3p,novel_78,miR-330-3p,miR-1343-3p,miR-216a-3p,miR-1298-3p,miR-412-3p,miR-296-5p,miR-1271,miR-425-5p,miR-2330-3p,miR-129-2-3p,miR-3074-5p,miR-4510,miR-3184-5p,miR-6134,miR-219b-3p,miR-665,miR-3604,miR-4492,miR-874-5p,miR-708-5p,miR-450b-3p,miR-487b-5p,miR-451a,miR-17-3p,miR-664a,miR-3065-3p,miR-21-3p,miR-216b-3p,miR-296-3p,miR-552-3p,miR-30c-1-3p,novel_25,novel_32,miR-22-3p,miR-204-3p,miR-499b-3p,miR-582-5p,miR-6130,miR-139-5p,miR-450a-1-3p,miR-885-3p,miR-345-5p,miR-20a,miR-329a-5p,miR-486-3p,miR-143-3p,miR-664b-3p,miR-29b-1-5p,miR-122-3p,miR-185-3p,miR-2285p,miR-21-3p,miR-320d,miR-106a,miR-212-5p,miR-30d-3p,miR-18a-3p,miR-3960,miR-106a,miR-616-3p,miR-199a-3p,miR-532-3p,miR-7859,miR-23b-3p,miR-210-3p,miR-23c,miR-124-3p,novel_133,miR-4429,miR-17-3p,novel_101,miR-29c-3p,novel_27,miR-326-3p,miR-2284w,miR-199b-5p,miR-1307-5p,miR-425-5p,miR-20a-5p,miR-421-5p,miR-199c,miR-502b,miR-6119-3p,novel_1,miR-29a,miR-379-3p,miR-320b,miR-329-5p,miR-3074-5p,miR-669,miR-320c,miR-6535,miR-2404,miR-670-3p,novel_69,miR-29a-3p,miR-150-5p,miR-3591-3p,miR-18a-3p,miR-378b,miR-3141,miR-9851-3p,miR-19a-3p,miR-363-5p,miR-668-3p,miR-6240,miR-345-5p,miR-654-3p,miR-432-3p,miR-330-3p,miR-
MEOX1	ENSOARG0(	mesenchy	miR-30b-3p,miR-541,miR-873a-5p,miR-432,miR-3120-5p,miR-26a-2-3p,miR-450b-5p,miR-30b-3p,miR-138,miR-222-5p,novel_4,miR-1343-3p,miR-455-3p,miR-1298-3p,miR-147a,miR-3431,miR-432-5p,novel_51,miR-182-5p,miR-3085-3p,miR-491-5p,miR-378a-5p,miR-628-5p,miR-432-3p,miR-432-3p,miR-30c-1-3p,miR-18a-3p,miR-129b-5p,miR-3591-3p,miR-3970,miR-361-3p,miR-504,miR-1291,miR-655-5p,miR-499b-5p,miR-452-3p,miR-3064-5p,miR-669,miR-3082-5p,novel_69,miR-769-5p,miR-371a-5p,miR-2285n,miR-502-5p,miR-490-5p,miR-3956-3p,miR-212-3p,miR-541-3p,novel_60,miR-423-5p,miR-1285-5p,novel_111,miR-3959-3p,miR-24-3p,miR-140-3p,miR-188-3p,miR-5703,miR-2427,miR-193b-5p,miR-155-5p,miR-493-3p,miR-6517,miR-18a-3p,miR-2285ad,miR-486-5p,miR-616-3p,miR-222-5p,miR-92a-1-5p,miR-185-3p,miR-30c-2-3p,novel_17,novel_127,novel_96,miR-21-3p,miR-466i-5p,miR-345-3p,miR-3064-5p,miR-29a-5p,miR-486-3p,miR-21a-3p,miR-485-5p,miR-876-3p,miR-296-3p,miR-182-5p,miR-216b-3p,miR-21-3p,miR-874-3p,miR-30c-1-3p,miR-2331-3p,miR-545-3p,miR-504-5p,miR-155-5p,miR-324-5p,miR-769,miR-6740-5p,miR-135a-1-3p,miR-486b-3p,miR-129-5p,miR-155-5p,miR-138-5p,miR-145a-3p,miR-338-3p,miR-6134,miR-3184-5p,miR-769-5p,miR-6525
TMEM144	ENSOARG0(	transmem	miR-196a-5p,miR-196b-5p,miR-193a

AK8	ENSOARG0(	adenylat	miR-2285e,miR-2459,miR-216a-3p,novel_4,miR-1343-3p,miR-409b,miR-3120-5p,miR-2426,miR-30b-3p,miR-412-3p,miR-541,miR-296-5p,miR-3187-3p,miR-4532,miR-23a-3p,let-7a-5p,let-7e,miR-25-5p,miR-3085-3p,miR-29b-1-5p,novel_103,miR-1306-5p,miR-326,miR-203-3p,let-7d-5p,miR-2483-3p,miR-378g,miR-7977,miR-2319b,miR-10b,miR-3064-5p,miR-504,miR-361-3p,miR-423-5p,miR-361-3p,miR-9-5p,miR-1296-5p,miR-6527,miR-541-3p,miR-2447,miR-4454,miR-490-5p,miR-335,miR-4726-5p,miR-769-5p,novel_82,miR-328-3p,novel_83,miR-23c,miR-487a-3p,miR-23b-3p,miR-212-5p,miR-485-3p,miR-380-5p,miR-18a-3p,miR-33b-3p,miR-5703,miR-2366,miR-1961,miR-2300b-3p,miR-2424,miR-486-3p,miR-125b-2-3p,miR-377-5p,miR-29b-1-5p,miR-183-5p,let-7k,miR-99a-3p,miR-760-3p,miR-98-5p,miR-125b,miR-185-3p,miR-92a-1-5p,miR-210-5p,let-7i-5p,miR-204-3p,miR-22-3p,miR-6130,miR-377-3p,miR-6516,miR-296-3p,miR-532-5p,miR-552-3p,miR-370-5p,miR-660-5p,miR-2331-3p,miR-504-5p,miR-1827,miR-1895,miR-129b-3p,miR-2285c,miR-338-3p,miR-3184-5p,let-7b-5p,miR-122-5p,miR-224-5p,miR-6525,miR-769-5p,miR-4510,let-7b,miR-874-5p,miR-4492,miR-708-5p,miR-664a,miR-29b-2-5p,miR-665,let-7f,miR-15b-3p,miR-194-3p,miR-185-5p,miR-134-3p,let-7g-5p,miR-2285g,miR-377-5p,miR-187-3p,miR-744-5p,miR-2889,miR-219a-1-3p,miR-30b-3p,miR-22-3p,miR-197-3p,miR-1843b-5p,miR-296-3p,miR-363-5p,miR-668-3p,miR-2483-5p,miR-660,miR-125a-5p,miR-378a-5p,miR-2331-5p,miR-3141,miR-377-3p,miR-296-3p,miR-224-5p,miR-669,novel_69,miR-411b-3p,miR-2404,miR-146b-3p,miR-320b,miR-6395,let-7e-5p,miR-1291,miR-18a-3p,miR-1306,miR-2387,miR-3141,miR-2428,miR-326-3p,miR-371b-3p,miR-1193,miR-758-5p,miR-335-5p,miR-135a-2-3p,let-7i,miR-28c,miR-6529b,miR-4324,miR-6516-
ZBTB21	ENSOARG0(	zinc fin	miR-30d-3p,miR-187-3p,miR-669,miR-185-5p,miR-3970,miR-27b-5p,miR-485-5p,miR-345-5p,miR-345-5p,miR-3963,miR-425-5p,miR-30a-3p,miR-345-5p,miR-2478,miR-185-3p,miR-30a-3p,miR-30e-3p,miR-543-5p,miR-25-5p
SLC9A9	ENSOARG0(	solute c	miR-2426,miR-1260b,miR-26a-2-3p,miR-134-3p,miR-146b,miR-1343-5p,miR-146a,miR-2285r,miR-654-3p,miR-539-3p,miR-503-3p,miR-130b-5p,miR-1388-3p,miR-668-3p,miR-504,miR-335-3p,miR-9851-3p,miR-146b-5p,miR-18a-3p,miR-378g,miR-3082-5p,miR-670-3p,miR-6535,miR-3120-3p,miR-7977,novel_82,miR-28c,miR-6529b,novel_87,miR-2284w,miR-452-5p,miR-2904,miR-1343-5p,miR-487a-3p,miR-505-5p,miR-214-5p,miR-18a-3p,miR-485-3p,novel_127,miR-205-5p,miR-1260a,miR-125a-3p,miR-216a-5p,miR-28b,miR-146a-5p,miR-2285b,miR-490-3p,miR-183-5p,miR-4286,miR-767,let-7g-3p,miR-2312,miR-625-5p,miR-424-3p,miR-216b-5p,miR-2331-3p,miR-504-5p,miR-548o-3p,novel_107,miR-2355-5p,miR-615-5p,miR-767-5p,miR-130b-5p,miR-34c-3p,miR-3969,miR-1260b

SMG5	ENSOARG0(	SMG5 non	let-7a-2-3p,miR-7641,miR-214-5p,miR-545-5p,miR-2411-5p,miR-1271-5p,miR-339a,miR-217,miR-30c-2-3p,miR-376b-3p,miR-181a-2-3p,miR-4286,miR-29b-2-5p,miR-1271-3p,miR-490-3p,miR-362-5p,miR-671-5p,miR-7144-5p,miR-23a-5p,let-7g-3p,miR-215-5p,miR-370-3p,miR-154b-5p,miR-2284n,miR-331-3p,miR-2332,miR-486b-3p,miR-204-5p,miR-138-5p,miR-133a-3p,miR-500b-5p,miR-374b-3p,miR-873a-5p,miR-7862,miR-30b-3p,miR-138,miR-2284ab,miR-185-5p,miR-503-5p,miR-2284d,miR-503-5p,miR-224-5p,miR-125a-5p,miR-363-5p,miR-654-3p,miR-18a-3p,miR-376b-3p,miR-2387,miR-2285ab,miR-9851-3p,miR-1291,miR-2404,miR-323a-5p,miR-146b-3p,miR-502-5p,miR-147-5p,miR-203b-5p,miR-128-1-5p,miR-135a-2-3p,miR-342-3p,miR-2428,novel_27,miR-24-3p,miR-371b-3p,miR-211,miR-1197-5p,miR-124-3p,miR-362-3p,miR-2300b-3p,miR-215-5p,miR-548e-3p,miR-383-5p,miR-125b,miR-185-3p,miR-196a-3p,miR-21-3p,miR-221-5p,miR-105-3p,miR-216c-5p,miR-2411,miR-146a-5p,miR-125b-2-3p,miR-29b-1-5p,miR-27b-5p,novel_99,let-7g-3p,miR-133b-3p,miR-182-5p,miR-221-5p,miR-877-5p,novel_32,miR-22-3p,miR-204-3p,miR-323-5p,miR-139-5p,miR-193b-3p,miR-192-5p,miR-29b-2-5p,miR-320e,miR-324-3p,miR-132-5p,miR-664a,miR-1271,miR-6134,miR-30b-3p,miR-125b-2-3p,miR-412-3p,miR-7857,miR-484,miR-296-5p,miR-6529a,miR-2300a-5p,miR-409b,miR-9788-3p,miR-500-5p,miR-362-3p,miR-5126,miR-1306-5p,miR-342,miR-25-5p,miR-3955-3p,miR-3085-3p,miR-33a-5p,miR-1839-3p,miR-30c-1-3p,miR-361-3p,novel_94,miR-147-3p,miR-7977,miR-499b-5p,miR-20a-3p,miR-3064-5p,miR-376d,miR-371a-5p,miR-2284q,miR-2285t,miR-4726-5p,miR-2285f,miR-541-3p,novel_60,miR-362-5p,miR-1290,miR-885-3p,novel_79,miR-412,novel_127,miR-3064-5p,miR-1248,miR-329a,miR-329b,miR-1271,miR-125a,miR-2355-5p,miR-542-
EMP2	ENSOARG0(	epitheli	miR-92a-1-5p,miR-422a,miR-205-5p,miR-1260a,miR-125a-3p,miR-34b-5p,miR-3653-3p,miR-221-5p,miR-152-3p,miR-760-3p,miR-30a-3p,miR-329a-5p,miR-2424,miR-200a,novel_39,miR-1271-3p,miR-3956-5p,miR-148b-5p,miR-376c-3p,miR-124-3p,miR-23b-5p,miR-1197-5p,miR-378a-3p,miR-5703,miR-378i,miR-2408,miR-105-2,miR-34a-5p,miR-148a-5p,miR-3600,miR-7641,miR-30d-3p,miR-486-5p,miR-148b-3p,miR-378h,miR-6740-5p,miR-30a-3p,miR-2285j,miR-494-5p,miR-30e-3p,miR-708-5p,miR-487a-5p,miR-874-5p,miR-28-5p,miR-487b-5p,miR-6516-3p,miR-378c,miR-145a-3p,miR-4510,miR-378c,miR-1260b,miR-487b-5p,miR-3154,miR-1827,miR-2318,miR-329b,miR-370-3p,miR-296-3p,miR-3956,miR-221-5p,miR-378a-3p,miR-376c-3p,miR-376e-3p,miR-34c-5p,miR-300-3p,miR-132-5p,miR-27a-5p,miR-323-5p,miR-6130,miR-105-5p,miR-1306-5p,miR-296-3p,miR-376b,miR-124a,miR-8485,miR-296-3p,miR-193a,miR-378d,miR-378b,miR-143-5p,miR-26a-2-3p,miR-378c,miR-1260b,miR-3955-5p,miR-409b,miR-105-1,miR-1343-3p,miR-455-3p,miR-148b-5p,miR-2459,miR-34c,miR-2440,miR-576-3p,miR-7134-3p,miR-147-5p,miR-6527,miR-135a-2-3p,miR-885-5p,miR-449a,miR-1307-3p,novel_111,miR-1306,miR-361-3p,miR-148a-3p,miR-544-5p,miR-378f,miR-9-3p,miR-2285u,miR-329-5p,miR-378b,miR-4443,miR-378e,miR-323a-5p,miR-3596,miR-34b

MAPRE2	ENSOARG0(	microtub	miR-382,miR-6240,miR-130b-5p,miR-345-5p,miR-296-3p,miR-194a,miR-182-5p,miR-29b-1-5p,miR-330-3p,miR-3141,novel_103,miR-296-3p,miR-224-5p,miR-214-3p,miR-125a-5p,miR-491-5p,miR-330-3p,novel_4,miR-2320-3p,miR-147a,miR-194-5p,miR-22-3p,miR-1434-3p,miR-301b-5p,miR-296-5p,miR-1260b,miR-29b,miR-29c-3p,miR-194b-5p,miR-2447,miR-423-5p,miR-211,miR-382-5p,miR-301a-5p,miR-4324,miR-2285n,miR-29a,miR-211-5p,miR-655-5p,miR-2319b,miR-29a-3p,miR-489,miR-3141,miR-135b-5p,miR-7975,miR-1291,miR-450b-3p,miR-410-5p,miR-345-3p,miR-29b-2-5p,miR-490-3p,miR-192-3p,miR-1271-3p,novel_39,miR-486-3p,miR-125b-2-3p,miR-29b-1-5p,miR-125b,miR-92a-1-5p,miR-216a-5p,miR-1260a,novel_96,miR-200a-3p,miR-21-3p,miR-466i-5p,miR-450a-2-3p,miR-2285ad,novel_83,miR-582-3p,miR-140-3p,miR-5703,miR-29d-3p,miR-885-3p,miR-411-3p,miR-214,miR-3958-5p,miR-4510,miR-539-5p,miR-145-3p,miR-3184-5p,miR-1260b,miR-224-5p,miR-6525,miR-135a-5p,miR-3604,miR-29b-2-5p,miR-125b-5p,miR-29b-3p,miR-486b-3p,miR-130b-5p,miR-204-5p,miR-141-3p,miR-296-3p,miR-3956,miR-182-5p,miR-125a,miR-548o-3p,miR-204-3p,miR-22-3p,miR-6130,miR-411,miR-21a-3p,miR-1827,miR-7144-5p,miR-885-3p,miR-345-5p,miR-345-5p
PPM1M	ENSOARG0(	protein	miR-500b-5p,miR-6525,miR-769-5p,miR-362-5p,novel_44,miR-346,miR-145-3p,miR-4492,miR-486b-3p,miR-769-5p,miR-132-5p,miR-769,novel_32,miR-151b,miR-221-5p,miR-370-3p,novel_94,miR-18a-3p,miR-362-5p,miR-486-3p,miR-2898,miR-30b-3p,miR-221-5p,novel_127,miR-2397-5p,miR-450a-1-3p,miR-500-5p,miR-151-5p,miR-18a-3p,miR-423-3p,miR-212-5p,miR-3120-5p,novel_133,miR-218-2-3p,miR-2904,miR-140-3p

MED13L	ENSOARG0(	mediator	miR-193a-5p,miR-2284v,miR-1260b,miR-1197-3p,miR-6740-5p,miR-2284k,novel_63,miR-331-5p,miR-2355-5p,miR-2285aa,miR-1271,miR-331-5p,miR-23b,miR-376c-5p,miR-331-5p,miR-1248,miR-876-3p,miR-2318,miR-3064-5p,miR-29a-5p,miR-200a,miR-2284y,miR-2285b,miR-148b-5p,miR-205-5p,miR-664-3p,miR-200a-3p,novel_96,novel_127,miR-152-3p,miR-1224-5p,miR-2285ad,miR-2284z,miR-450a-1-3p,novel_121,miR-23b-5p,miR-3591-5p,miR-155-5p,miR-335-5p,miR-199b-5p,miR-576-3p,miR-26b-5p,miR-3120-3p,miR-6535,miR-320c,miR-411b-3p,miR-3591-3p,miR-216a-3p,miR-3065-5p,miR-376b-5p,miR-148a-3p,miR-668-3p,miR-6240,miR-493-5p,let-7j,miR-503-3p,let-7a-2-3p,miR-2285r,miR-432-3p,miR-432-3p,miR-194a,miR-7-5p,miR-377-3p,miR-483-3p,miR-340-3p,miR-194-3p,novel_116,miR-149-5p,miR-3535,miR-450c-3p,miR-432,miR-7b-5p,miR-27b-3p,miR-665-5p,miR-590-3p,miR-3184-3p,miR-181c-5p,miR-142a-5p,miR-769-5p,miR-224-5p,miR-30a-3p,miR-2285j,miR-30e-3p,miR-874-5p,miR-129-5p,miR-4492,miR-190a-5p,miR-2331-3p,miR-377-3p,miR-129b-3p,miR-655,miR-450a-1-3p,miR-625-5p,miR-543-3p,miR-186-5p,miR-195a-3p,miR-486-3p,miR-377-5p,miR-324-3p,miR-210-5p,miR-26a-5p,miR-618,miR-2285p,miR-202-5p,miR-30d-3p,miR-18a-3p,miR-23b-3p,miR-23c,miR-1434-5p,miR-193b-5p,miR-218-1-3p,miR-34a-5p,miR-2284u,miR-148a-5p,miR-493-3p,miR-190b-5p,miR-2284j,miR-2447,novel_82,miR-101-3p,miR-2285u,miR-7689-3p,miR-126b-5p,miR-211-5p,miR-2284g,miR-2319b,miR-4443,miR-3596,miR-381-3p,miR-140-5p,miR-221,miR-335-3p,miR-493-5p,miR-9-3p,miR-668-5p,miR-23a-3p,miR-2284b,miR-8485,miR-29b-1-5p,miR-199b-5p,miR-491-5p,miR-199b-3p,novel_78,miR-330-3p,miR-455-3p,miR-148b-5p,miR-2459,miR-126a-5p,miR-421,miR-142-5p,miR-2284r,miR-1839-5p,miR-1388-5p,miR-
ACTN4	ENSOARG0(	actinin,	miR-378d,miR-30b-3p,miR-1343-5p,miR-1197-5p,miR-30b-3p,miR-330-5p,miR-296-5p,miR-2366,miR-1298-3p,miR-185-5p,miR-147a,miR-1343-3p,miR-30b-3p,miR-1343-5p,miR-214-3p,miR-466i-5p,miR-324-3p,miR-30c-2-3p,miR-3141,miR-185-3p,miR-326,miR-4532,miR-432-3p,miR-30c-1-3p,miR-28b,miR-654-3p,miR-432-3p,miR-183-5p,miR-127-3p,miR-1291,miR-129b-3p,miR-361-3p,miR-3141,miR-6535,miR-323a-5p,miR-323-5p,miR-3082-5p,miR-542-5p,miR-2285aa,miR-2355-5p,miR-574-5p,miR-30c-1-3p,miR-4726-5p,miR-128-1-5p,miR-331-3p,miR-665,miR-2433,miR-28c,miR-326-3p,novel_101,miR-214
MECP2	ENSOARG0(	methyl C	miR-1343-5p,miR-103a-2-5p,miR-214-3p,miR-431-5p,novel_17,miR-1306-5p,miR-326,miR-185-3p,miR-2331-5p,miR-28b,miR-486-3p,miR-490-3p,miR-450b-5p,miR-7862,miR-484,miR-450c-3p,miR-1343-5p,miR-412-3p,miR-197-3p,miR-374b-3p,miR-330-5p,miR-412-5p,miR-134-3p,miR-151-5p,miR-450a-1-3p,miR-340-3p,miR-3955-5p,miR-6517,miR-1343-3p,miR-412,miR-431,miR-212-5p,miR-450b-3p,miR-128-1-5p,miR-874-5p,miR-4492,miR-486b-3p,miR-6123,miR-2433,miR-502-5p,miR-28c,miR-324-3p,miR-24-3p,miR-1307-3p,miR-326-3p,miR-6134,miR-1306-5p,miR-193a-5p,miR-214,miR-450a-1-3p,miR-1306,miR-129b-5p,miR-3596,miR-378g,miR-374c-3p,novel_32,miR-151b,miR-1271

SLC35B1	ENSOARG0(	solute c	miR-193b-3p,miR-17-3p,miR-211,miR-326-3p,miR-2284w,miR-2285m,miR-4324,miR-133a-5p,miR-20a-5p,let-7i,novel_68,miR-320b,novel_69,miR-411b-3p,miR-374c-3p,miR-320c,miR-150-5p,miR-376b-3p,miR-3065-5p,miR-3591-3p,miR-1291,miR-7975,miR-6395,let-7e-5p,miR-130b-5p,miR-668-3p,miR-363-5p,miR-2285r,miR-432-3p,miR-503-5p,miR-197-5p,miR-105-5p,novel_51,miR-106a-5p,miR-17-5p,miR-196b-5p,miR-193a-3p,miR-133b-5p,miR-665,miR-26b-3p,miR-432-5p,miR-2284d,let-7g-5p,miR-134-3p,miR-503-5p,miR-185-5p,miR-149-5p,miR-20b,miR-432,miR-2113,miR-1843b-5p,miR-374b-3p,miR-138,miR-2284ab,miR-30b-3p,miR-188-5p,miR-2284v,miR-133a-3p,miR-93,miR-145-3p,miR-331-3p,miR-138-5p,miR-204-5p,miR-28-5p,miR-2285aa,miR-2355-5p,miR-215-5p,miR-324-5p,miR-488-3p,miR-2448-3p,miR-93-5p,miR-320a,miR-17-5p,miR-876-3p,miR-20b-5p,miR-1248,miR-485-5p,miR-3529-3p,miR-4286,let-7g,miR-1271-3p,miR-376b-3p,miR-125a-3p,let-7c-5p,miR-2898,miR-17-3p,let-7f-5p,miR-543-5p,miR-2411-5p,novel_121,miR-196a-5p,miR-330-5p,let-7d,miR-105-2,miR-2447,miR-877-3p,miR-7705,miR-2285n,miR-2440,miR-107-5p,miR-499a-5p,miR-20a-3p,miR-7689-3p,miR-211-5p,miR-7977,miR-3596,miR-378g,let-7d-5p,miR-376d,miR-361-3p,miR-129b-5p,miR-376a-3p,miR-544-5p,let-7a-5p,miR-106a-5p,miR-652-5p,miR-376b,miR-320b,miR-326,miR-182-5p,miR-106b-5p,miR-1185-5p,miR-103a-2-5p,let-7e,novel_4,miR-105-1,miR-222-5p,miR-147a,miR-376b-3p,miR-9788-3p,miR-2459,miR-421,miR-296-5p,miR-30b-3p,miR-2426,let-7b,miR-4510,miR-145a-3p,miR-6525,let-7b-5p,miR-338-3p,miR-6134,let-7f,miR-324-3p,miR-665,miR-17-3p,miR-664a,miR-129-5p,miR-487a-5p,miR-874-5p,miR-708-5p,miR-4492,miR-130b-5p,miR-545-3p,miR-21-3p,miR-182-5p,miR-192-5p,miR-193b-3p,miR-6130,miR-204-3p,let-7i-
BAG2	ENSOARG0(	BCL2-ass	novel_1,miR-22-5p,miR-147-5p,miR-6123,miR-2285f,miR-495-3p,miR-362-5p,miR-8095,miR-2285ab,miR-3065-5p,miR-7975,miR-544-5p,miR-139-5p,miR-329-5p,miR-378b,miR-541-5p,miR-2285u,miR-7857-3p,miR-677,miR-1185-2-3p,miR-181b-3p,miR-105-5p,miR-196b-5p,miR-466f-3p,miR-494-3p,miR-382,miR-101-5p,miR-33a-5p,miR-539-3p,miR-3962,miR-30b-3p,miR-30b-3p,miR-1388-5p,miR-101a-5p,miR-3120-5p,miR-133b-5p,miR-105-1,miR-181b-2-3p,miR-500-5p,miR-1185-3p,miR-2285j,miR-494-5p,miR-30e-3p,miR-324-3p,miR-30a-3p,miR-665,novel_74,miR-487b-5p,miR-155-5p,miR-4492,miR-665-5p,miR-6525,miR-500b-5p,miR-362-5p,miR-3154,miR-487b-5p,miR-2312,miR-329b,miR-582-5p,miR-548o-3p,miR-2285aa,miR-141-3p,miR-582-5p,miR-139-5p,miR-155-5p,miR-2397-5p,miR-324-3p,miR-450a-2-3p,miR-200a-3p,miR-329a-5p,miR-30a-3p,miR-196a-5p,miR-23b-5p,miR-1434-5p,miR-105-2,miR-155-5p,miR-361-5p,miR-30d-3p,miR-487a-3p,miR-7859,miR-545-5p

XRCC2	ENSOARGO(	X-ray re	miR-215-5p,miR-376a-5p,miR-2366,miR-301b-5p,miR-873a-5p,miR-125b-2-3p,miR-3071-5p,miR-145a-5p,miR-26a-2-3p,miR-1343-3p,miR-2285ad,miR-145b,miR-383-5p,miR-2459,miR-1185-3p,miR-9788-3p,miR-2285p,miR-2957,miR-487b-5p,miR-17-3p,miR-103a-2-5p,miR-494-3p,miR-3529-3p,miR-329a-5p,let-7f-2-3p,miR-3065-5p,miR-136-5p,miR-671-5p,miR-329-5p,miR-215-5p,miR-2483-3p,miR-192-5p,miR-1185-2-3p,miR-502-5p,novel_87,miR-301a-5p,miR-107-5p,miR-2285y,miR-487b-5p,miR-17-3p,miR-6119-3p,miR-669a-3p,miR-3956-3p,miR-212-3p,novel_60,miR-2285x,miR-17-3p,miR-452-5p,miR-495-3p,miR-2428,novel_27
LGALS4	ENSOARGO(	lectin,	miR-16b,miR-296-3p,miR-326,miR-320b,miR-1343-5p,miR-199b-5p,miR-363-5p,miR-424-5p,miR-380-5p,miR-296-3p,miR-193a,miR-432-3p,miR-432-3p,miR-374b-3p,miR-450c-3p,miR-143-5p,miR-101c,miR-497-5p,miR-16b,miR-455-3p,miR-216a-3p,miR-134-3p,miR-185-5p,miR-425-3p,miR-16a,miR-34c,miR-15a,miR-2285t,miR-203b-5p,novel_1,miR-452-5p,miR-3957-3p,miR-219b-3p,miR-199b-5p,miR-2428,miR-154a,miR-326-3p,miR-449a,miR-423-5p,miR-216a-3p,miR-199b-5p,miR-140-5p,miR-361-3p,miR-376b-5p,miR-195a-5p,miR-541-5p,miR-378b,miR-2319b,miR-320c,miR-3082-5p,miR-374c-3p,miR-34b,miR-378g,miR-30b-3p,miR-34b-5p,miR-2898,miR-320d,miR-2403,miR-4286,miR-2385-3p,miR-183-5p,miR-143-3p,miR-2285b,miR-2411,miR-29a-5p,miR-199a-5p,miR-376c-5p,miR-486-3p,miR-380-5p,miR-2478,miR-330-5p,miR-1343-5p,miR-2904,miR-193b-5p,miR-1246,miR-378d,miR-34a-5p,miR-128-3p,miR-142-3p,miR-4429,miR-885-3p,novel_121,miR-450a-1-3p,miR-15a-5p,miR-2411-5p,miR-322-5p,miR-505-5p,miR-665,miR-5100,miR-486b-3p,miR-424-5p,miR-874-5p,miR-450b-3p,novel_74,miR-16-5p,miR-4510,miR-3184-5p,miR-411,miR-376c-5p,miR-433-3p,miR-320a,miR-450a-1-3p,miR-2448-3p,novel_99,miR-885-3p,miR-2318,miR-296-3p,miR-15b-5p,novel_25,miR-199a-5p,miR-6130,miR-34c-5p,miR-27a-5p,miR-374c-3p

AIFM2	ENSOARG0(	apoptosi	miR-3956-3p,novel_60,miR-3059-5p,miR-2447,miR-181d-5p,miR-2284j,miR-877-3p,miR-219a-2-3p,miR-2440,novel_82,miR-30b-5p,miR-335,miR-4726-5p,miR-3964,miR-20a-3p,miR-2284g,miR-211-5p,miR-499b-5p,miR-489,miR-3596,miR-25,miR-378g,miR-203-3p,let-7d-5p,miR-378j,miR-129b-5p,miR-504,miR-99b-5p,miR-96-5p,miR-335-3p,miR-382-3p,let-7a-5p,miR-33a-5p,miR-2284b,miR-199a-3p,let-7e,miR-214-3p,novel_78,miR-105-1,miR-539-5p,miR-147a,miR-1298-3p,miR-2285e,miR-2459,miR-148b-5p,miR-9788-3p,miR-455-3p,miR-3962,miR-3120-5p,miR-2284aa,miR-3074-5p,miR-4510,let-7b,miR-214,miR-1271,miR-181c-5p,miR-338-3p,let-7b-5p,miR-122-5p,let-7f,miR-2285j,miR-324-3p,miR-219b-3p,miR-17-3p,miR-6516-3p,novel_74,miR-4492,miR-504-5p,miR-221-5p,miR-21-3p,miR-216b-3p,miR-6130,miR-6516,let-7i-5p,miR-204-3p,novel_25,miR-3613-5p,miR-2285c,miR-30d-5p,miR-30f,miR-345-5p,miR-1827,miR-186-5p,miR-543-3p,miR-130a-5p,miR-376a-5p,miR-2285i,novel_17,miR-30b,miR-196a-3p,miR-345-3p,miR-324-3p,miR-466i-5p,miR-98-5p,miR-2284m,miR-2484,let-7k,miR-30d,miR-221-5p,miR-340-5p,miR-222-5p,miR-210-3p,miR-199a-3p,novel_83,miR-1961,miR-30c-5p,miR-1197-5p,miR-188-3p,miR-5703,miR-378d,miR-148a-5p,miR-2284u,miR-133a-3p,miR-335-5p,miR-135a-2-3p,novel_101,miR-29c-3p,miR-17-3p,miR-3957-3p,miR-211,miR-2428,novel_27,miR-1247-3p,miR-502-5p,let-7i,miR-29a,miR-147-5p,miR-3074-5p,miR-7-1-3p,miR-29a-3p,novel_69,miR-2404,miR-3065-5p,miR-376b-3p,miR-3591-3p,miR-21c,miR-3970,miR-2285k,miR-6516-3p,miR-135b-5p,let-7e-5p,miR-363-5p,miR-2284l,miR-432-3p,miR-432-3p,miR-345-5p,miR-7-5p,novel_51,miR-2411-3p,miR-99a-5p,miR-105-5p,miR-196b-5p,miR-452-5p,miR-665,miR-185-5p,miR-134-3p,let-7g-5p,miR-2284d,miR-
CASP6	ENSOARG0(	caspase	miR-224-5p,miR-199a-3p,miR-125b,miR-2411-3p,miR-345-3p,miR-105-5p,miR-330-3p,miR-3955-3p,miR-378a-5p,miR-125a-5p,miR-6240,miR-495-5p,let-7a-2-3p,miR-29a-5p,miR-1290,miR-484,miR-345-3p,miR-188-3p,miR-412-3p,miR-300,miR-450b-5p,miR-1388-5p,let-7a-2-3p,miR-105-1,miR-330-3p,miR-409b,miR-2285ad,miR-376a-5p,miR-665,miR-412,miR-1298-3p,miR-3431,miR-129-1-3p,miR-199a-3p,miR-455-3p,miR-148b-5p,miR-6529b,miR-142b,miR-2285j,miR-2433,miR-218-5p,miR-2285n,miR-125b-5p,miR-2285y,miR-499a-5p,miR-129-2-3p,miR-2447,miR-224-5p,miR-381-3p,miR-361-3p,let-7g-3p,miR-450b-3p,let-7g-3p,miR-376a-2-5p,novel_94,miR-2319b,miR-125a,miR-2355-5p,miR-877-5p,miR-204-3p
CCT3	ENSOARG0(	chaperon	miR-6535,miR-144-3p,novel_68,miR-221-5p,miR-320b,novel_94,miR-1827,miR-2285c,miR-216a-3p,miR-24-3p,miR-219a-2-3p,miR-335-5p,miR-335,miR-656-5p,miR-129-5p,miR-669a-3p,miR-502-5p,miR-219b-3p,miR-144,miR-100-3p,miR-665,miR-210-3p,miR-2285g,miR-149-5p,miR-545-5p,novel_121,miR-2285e,miR-377-5p,miR-33b-3p,miR-128-3p,miR-188-5p,miR-1197-5p,miR-450b-5p,miR-377-5p,miR-101b-3p,miR-760-3p,miR-221-5p,miR-219-3p,miR-324-3p,miR-185-3p,miR-136-3p

ECH1	ENSOARG0(	enoyl Co	[illegible] 5p,miR-181a-5p,miR-1285,miR-1434-3p,miR-3071-5p,miR-2285g,miR-2284d,miR-2355-3p,miR-377-5p,miR-660,miR-455-5p,miR-197-5p,miR-130b-5p,miR-2483-5p,miR-363-5p,miR-1291,miR-195a-5p,miR-6395,miR-379-5p,miR-2387,miR-2285ab,miR-18a-3p,miR-2404,miR-6402,miR-15a,miR-16a,miR-24-3p,miR-211,miR-2428,novel_27,miR-326-3p,novel_120,miR-542-5p,miR-2285x,novel_101,miR-300,miR-128-3p,miR-378h,miR-483-3p,miR-2411-5p,miR-24-2-5p,miR-181b-5p,miR-2898,miR-1260a,miR-339a,miR-30c-2-3p,miR-2478,miR-24-2-5p,miR-2284a,miR-490-3p,miR-3065-3p,miR-199a-5p,miR-4286,miR-671-5p,miR-485-5p,miR-7144-5p,miR-381-3p,miR-488-3p,miR-320a,miR-769,miR-2284n,miR-324-5p,miR-199a-5p,miR-615-5p,miR-138-5p,miR-486b-3p,miR-374b-3p,miR-204-5p,miR-34c-3p,miR-3969,miR-331-3p,miR-374a-3p,miR-378c,miR-1306-5p,miR-2426,miR-378c,miR-2284aa,miR-378b,miR-484,miR-30b-3p,miR-2285e,miR-409b,miR-3085-3p,miR-542-5p,miR-326,novel_103,miR-1306-5p,miR-320b,miR-362-3p,miR-30c-1-3p,miR-4532,miR-193a,miR-424-5p,novel_94,miR-877-3p,miR-199b-5p,miR-361-3p,miR-3082-5p,miR-378e,miR-20a-3p,miR-3064-5p,miR-7977,miR-374a-3p,miR-7134-3p,miR-2285n,miR-2285y,miR-769-5p,miR-371a-5p,miR-541-3p,miR-3059-5p,novel_60,miR-181d-5p,miR-2285f,miR-4429,miR-378d,miR-362-3p,novel_133,miR-140-3p,miR-1197-5p,miR-188-3p,miR-15a-5p,miR-3600,miR-760-3p,miR-2284m,miR-320d,miR-30b-3p,miR-221-5p,miR-542-5p,miR-185-3p,miR-2411,miR-5100,miR-2312,miR-132-5p,miR-155-5p,miR-877-5p,novel_32,miR-204-3p,miR-660-5p,miR-221-5p,miR-378a-3p,miR-3065-3p,miR-155-5p,miR-664a,miR-424-5p,miR-320e,miR-324-3p,miR-378c,miR-16b,miR-2284x,miR-497-5p,miR-223-3p,miR-1843b-5p,miR-542-3p,miR-708-3p,miR-
SLC25A22	ENSOARG0(	solute c	miR-4286,miR-185-3p,novel_42,miR-5126,miR-2300a-5p,miR-133a-3p
LRBA	ENSOARG0(	LPS-resp	novel_120,novel_27,miR-107-5p,miR-4492,miR-154-3p,miR-130b-5p,miR-154a-3p,miR-487a-3p,miR-660-5p,miR-320c,miR-320a,miR-1248,miR-544-5p,miR-154b-3p,miR-130b-5p,miR-487a,miR-105-3p,miR-2432,miR-185-3p,miR-320b,miR-2285af,miR-660,miR-320d,miR-485-3p,miR-2284z,miR-487a-3p,miR-432-5p,miR-539-5p,miR-708-3p,miR-432,miR-2284aa,miR-4429,miR-299a-5p
TSGA13	ENSOARG0(	testis s	miR-1271,miR-2285i,miR-1260b,miR-4532,miR-1260a,miR-2433,miR-1306-5p,miR-1307-5p,miR-4726-5p,miR-615-5p,miR-544-3p,miR-1271-5p,miR-3431,novel_116,miR-544b,miR-1306,miR-5703,miR-96-5p,miR-876-3p,miR-1260b,miR-941,miR-544a,miR-2285k,miR-744-5p

BEND6	ENSOARG0(	BEN doma	novel_25,miR-182-5p,miR-154a-3p,miR-216b-3p,miR-548o-3p,miR-660-5p,miR-378a-3p,miR-140-5p,miR-30c-1-3p,novel_99,miR-186-5p,miR-1827,miR-543-3p,miR-154b-3p,miR-30f,miR-345-5p,let-7g-3p,miR-433-3p,miR-30d-5p,miR-2285c,miR-206-3p,miR-3074-2-3p,miR-3958-5p,miR-3074-5p,miR-129-2-3p,miR-590-3p,miR-378c,miR-539-5p,miR-129-5p,miR-451a,miR-219b-3p,miR-30a-3p,miR-324-3p,miR-30e-3p,miR-616-3p,miR-199a-3p,miR-200b,miR-23c,miR-383-5p,miR-23b-3p,miR-7859,miR-30d-3p,miR-212-5p,miR-106a,miR-548e-3p,miR-361-5p,miR-378d,miR-218-1-3p,miR-16-1-3p,miR-142-3p,miR-1197-5p,miR-1193,miR-378a-3p,miR-217-5p,miR-378i,miR-30c-5p,miR-486-3p,miR-130a-5p,miR-181b-1-3p,miR-29b-1-5p,miR-376a-5p,miR-421-5p,miR-195a-3p,miR-20a,miR-487a,miR-3653-3p,miR-760-3p,miR-202-5p,miR-106a,miR-92a-1-5p,miR-422a,miR-345-3p,miR-219b-5p,miR-30b,miR-1185-2-3p,miR-489,miR-3596,miR-378e,miR-499b-5p,miR-541-5p,miR-20a-3p,miR-877-3p,novel_94,miR-335-3p,miR-136-5p,miR-668-5p,miR-129b-5p,miR-378j,miR-361-3p,miR-140-5p,miR-219a-2-3p,miR-8095,miR-361-3p,miR-877-3p,miR-452-5p,miR-2285f,miR-3956-3p,novel_60,miR-154-3p,miR-335,miR-3958-3p,miR-107-5p,miR-2285y,miR-30b-5p,miR-218-5p,miR-1185-3p,miR-2285e,miR-216a-3p,miR-153,miR-222-5p,miR-181b-2-3p,miR-2300a-5p,miR-199b-3p,miR-378c,miR-758-3p,miR-1a-3p,miR-6529a,miR-378b,miR-484,miR-758-3p,miR-2310,miR-491-3p,miR-378d,miR-30c-1-3p,miR-652-5p,let-7c-3p,miR-23a-3p,miR-106a-5p,miR-127-3p,miR-103a-2-5p,miR-153-3p,miR-182-5p,miR-29b-1-5p,miR-199a-3p,miR-2284n,miR-331-5p,miR-1271,miR-487a-3p,miR-1814c,miR-7144-5p,miR-20b-5p,miR-376c-5p,miR-345-5p,miR-10b-3p,miR-376c-5p,miR-17-5p,miR-23b,miR-137-3p,miR-93-5p,miR-503-3p,miR-30e-5p,miR-378c,miR-145-3p,miR-93,miR-

PIK3R1	ENSOARG0(	phosphoi	miR-493-5p,miR-6240,miR-2285r,miR-432-3p,novel_51,miR-483-3p,miR-103a-3p,miR-134-3p,miR-542-3p,novel_23,miR-16b,miR-497-5p,miR-335-5p,miR-29c-3p,miR-576-3p,miR-28c,miR-29a,miR-421-5p,miR-625-3p,miR-3074-5p,miR-329-5p,miR-411b-3p,miR-320c,miR-669,miR-148a-3p,miR-3065-5p,miR-216a-3p,miR-150-5p,miR-378f,miR-2319a,miR-3064-5p,miR-2285b,miR-2284y,miR-219-3p,miR-205-5p,miR-3071-3p,miR-127-5p,miR-28-3p,miR-152-3p,miR-200a-3p,novel_96,miR-6517,miR-2285ad,miR-1224-5p,miR-9-5p,miR-322-5p,novel_121,miR-486-5p,miR-2284z,miR-3591-5p,miR-1343-5p,miR-155-5p,miR-2284v,miR-28a-3p,miR-16-5p,miR-193a-5p,miR-2284k,novel_9,miR-3068-3p,miR-2285aa,miR-2355-5p,miR-542-5p,miR-223-5p,miR-301,miR-23b,miR-137-3p,miR-93-5p,miR-767,miR-876-3p,miR-20b-5p,miR-2284b,miR-23a-3p,miR-378d,miR-8485,miR-199a-3p,miR-16b,miR-106b-5p,miR-323b-3p,miR-487b-5p,miR-491-5p,miR-153-3p,miR-330-3p,novel_78,miR-1343-3p,miR-147a,miR-153,miR-455-3p,miR-2284r,miR-323b,miR-1388-5p,miR-2284j,miR-9-5p,miR-877-3p,miR-7705,miR-2440,miR-218-5p,miR-628-3p,miR-499a-5p,miR-490-5p,miR-2319b,miR-2284g,miR-2285u,miR-2483-3p,miR-378g,miR-203-3p,miR-221,miR-381-3p,miR-668-5p,miR-504,miR-136-5p,miR-9-3p,miR-493-5p,miR-329a-5p,miR-143-3p,miR-486-3p,miR-422a,miR-324-3p,miR-202-5p,miR-2484,miR-106a,miR-23c,miR-7859,miR-23b-3p,miR-328-3p,miR-2284h-5p,miR-378i,miR-378a-3p,miR-142-3p,miR-33b-3p,miR-2284u,miR-3074-5p,miR-6525,miR-15b-3p,miR-3604,miR-219b-3p,novel_74,miR-129-5p,miR-1277-3p,miR-504-5p,miR-2331-3p,miR-30c-1-3p,miR-582-5p,miR-15b-5p,miR-299,miR-411,miR-186-5p,miR-654-3p,miR-17-5p,miR-106a-5p,miR-330-3p,miR-466f-3p,miR-660,miR-1343-5p,miR-133b-5p,miR-665,miR-185-5p,miR-2284d,miR-20b,miR-103,miR-6238,miR-

U2SURP	ENSOARG0(	U2 snRNP·	miR-222-3p,miR-320e,miR-3604,miR-17-3p,miR-451a,miR-208b-5p,miR-224-5p,miR-18a-5p,let-7g-3p,novel_99,miR-625-5p,miR-548o-3p,miR-3065-3p,miR-544-3p,miR-192-5p,miR-106a,miR-320d,miR-34b-5p,miR-2285w,miR-329a-5p,miR-345-3p,miR-20a,miR-2432,miR-215-5p,miR-217-5p,miR-4429,novel_73,miR-34a-5p,miR-106a,miR-548e-3p,miR-7859,miR-2284h-5p,miR-2440,miR-2285n,miR-3968,miR-3956-3p,novel_60,novel_111,miR-199b-5p,miR-221,miR-361-3p,miR-126b-5p,miR-2285u,miR-454-5p,novel_103,miR-320b,miR-342,miR-106b-5p,miR-182-5p,miR-199b-5p,miR-106a-5p,miR-382-3p,let-7c-3p,miR-2310,miR-758-3p,miR-758-3p,miR-7857,miR-2426,miR-544a,miR-199b-3p,novel_4,miR-222-5p,miR-181b-2-3p,miR-221-3p,miR-29b-3p,miR-100-3p,miR-28a-3p,miR-3607-3p,miR-93,miR-365a-5p,miR-18a,miR-376c-5p,miR-3154,miR-17-5p,miR-320a,miR-93-5p,miR-1814c,miR-376c-5p,miR-20b-5p,miR-215-5p,miR-365a-3p,miR-34c-5p,miR-374c-3p,miR-199a-5p,miR-205-5p,miR-217,miR-127-5p,miR-17-3p,miR-28-3p,miR-3529-3p,miR-4286,miR-199a-5p,miR-3065-3p,miR-1290,miR-29d-3p,miR-222,miR-18b-5p,miR-483-3p,miR-20a-5p,novel_87,miR-34c,novel_1,miR-29a,miR-199c,miR-421-5p,novel_120,miR-219b-3p,miR-342-3p,miR-17-3p,miR-29c-3p,miR-323c,miR-199b-5p,miR-449a,novel_27,miR-18b,miR-376b-5p,miR-2319a,miR-329-5p,miR-34b,miR-374c-3p,miR-670-3p,miR-29a-3p,miR-320c,miR-677,miR-7-5p,miR-224-5p,miR-17-5p,miR-106a-5p,miR-365b-5p,miR-483-3p,miR-378a-5p,miR-542-3p,miR-7b-5p,novel_23,miR-374b-3p,miR-29b,miR-31-5p,miR-20b

DDX31	ENSOARG0(	DEAD (As	novel_68,miR-320b,miR-2404,miR-323a-5p,miR-34b,miR-669,miR-144-3p,miR-376b-5p,miR-18a-3p,miR-216a-3p,miR-378b,miR-146b-5p,miR-9851-3p,miR-335-5p,miR-2285x,miR-495-3p,miR-1193,miR-24-3p,miR-449a,novel_27,miR-2428,miR-2284w,miR-576-3p,miR-34c,miR-502b,miR-31-5p,miR-3535,miR-412-5p,miR-185-5p,miR-503-5p,miR-194-3p,miR-2355-3p,miR-2113,miR-450c-3p,miR-197-3p,miR-380-3p,miR-16-1-3p,miR-7862,miR-6240,miR-363-5p,miR-2285r,miR-503-3p,miR-503-5p,miR-146a,miR-105-5p,miR-190a,miR-330-3p,miR-2331-5p,novel_51,miR-2411-3p,miR-1343-5p,miR-466f-3p,miR-874-3p,miR-34c-5p,miR-488-3p,miR-200a-5p,miR-376c-5p,miR-3154,miR-23b,miR-10b-3p,miR-329a,miR-7144-5p,miR-376c-5p,miR-23a-5p,miR-671-5p,miR-1814c,miR-1306-5p,miR-6128,miR-193a-5p,miR-1197-3p,miR-2433,miR-370-5p,miR-6740-5p,miR-486b-3p,miR-412,miR-302a-5p,miR-450a-1-3p,miR-345-3p,miR-615-3p,miR-3578,miR-1343-5p,miR-128-3p,miR-105-2,miR-3064-5p,miR-495-5p,miR-30a-3p,miR-181a-2-3p,miR-3065-3p,novel_39,miR-29a-5p,miR-6119-5p,miR-125a-3p,miR-2898,novel_96,novel_127,miR-3064-5p,miR-10b,miR-452-3p,miR-499b-5p,miR-25,miR-203-3p,miR-4443,miR-409-5p,miR-378j,miR-668-5p,miR-2447,miR-541-3p,miR-190b-5p,miR-361-3p,miR-423-5p,miR-382-3p,novel_82,miR-101-3p,miR-615,miR-2440,miR-301a-5p,miR-4726-5p,miR-628-3p,miR-335,miR-490-5p,miR-105-1,miR-2300a-5p,miR-151a-3p,miR-330-3p,miR-1343-3p,miR-147a,miR-9788-3p,miR-216a-3p,miR-296-5p,miR-329-3p,miR-541,miR-301b-5p,miR-412-3p,miR-362-3p,miR-1388-5p,miR-450b-5p,miR-2426,miR-101c,miR-574-3p,miR-1388-3p,miR-23a-3p,miR-652-5p,miR-4532,miR-2310,miR-362-3p,miR-491-5p,novel_124,miR-3085-3p,miR-3955-3p,miR-146b,miR-214-3p,miR-2285o,miR-2331-3p,miR-3065-3p,miR-370-5p,miR-216b-

LY6G6D	ENSOARG0(	lymphocy	miR-345-5p,miR-1827,miR-625-5p,miR-500a-3p,miR-129b-3p,miR-377-3p,miR-376e-3p,miR-499b-3p,miR-2284s,miR-32-5p,miR-296-3p,miR-130b-5p,miR-1468-5p,miR-500,miR-129-5p,miR-664a-5p,miR-665,miR-151-3p,miR-769-5p,miR-6525,miR-3184-5p,miR-129-2-3p,miR-3074-5p,miR-665-5p,miR-33b-3p,miR-16-1-3p,miR-193b-5p,miR-217-5p,miR-23c,miR-23b-3p,miR-106a,miR-18a-3p,miR-212-5p,miR-202-5p,miR-210-5p,miR-92a-1-5p,miR-345-3p,miR-324-3p,miR-181b-1-3p,miR-376c-3p,miR-376a-5p,miR-143-3p,miR-486-3p,miR-329a-5p,miR-345-3p,miR-136-5p,miR-299b-5p,miR-1983,miR-1306-3p,miR-25,miR-378g,miR-499a-3p,miR-4792,miR-211-5p,miR-7689-3p,miR-490-5p,novel_82,miR-218-5p,miR-361-3p,miR-877-3p,miR-423-5p,miR-363-3p,miR-3956-3p,miR-1388-5p,miR-2284r,miR-758-3p,miR-147a,miR-1298-3p,miR-1343-3p,novel_78,miR-145b,miR-181b-2-3p,miR-491-5p,miR-103a-2-5p,miR-199b-5p,miR-146b,miR-106b-5p,miR-29b-1-5p,novel_19,miR-1388-3p,miR-23a-3p,miR-345-5p,miR-767,miR-20b-5p,miR-23b,miR-93-5p,miR-25-3p,miR-376c-3p,miR-874-3p,miR-1271,miR-2285aa,novel_63,miR-1290,miR-330-5p,miR-505-5p,miR-129-1-3p,novel_121,miR-6517,miR-2285ad,miR-299a-3p,miR-1a-2-5p,novel_96,novel_127,miR-125a-3p,miR-205-5p,miR-92b-3p,miR-148b-5p,novel_39,miR-412-3p,miR-146b-5p,miR-3591-3p,miR-216a-3p,miR-3065-5p,miR-1306,miR-299b-3p,miR-411b-3p,miR-181b-3p,miR-677,miR-329-5p,miR-3074-5p,novel_68,miR-576-3p,miR-4324,novel_87,miR-199b-5p,miR-1a-1-5p,miR-193b-3p,miR-1839-3p,miR-16-1-3p,miR-188-5p,miR-26a-2-3p,miR-542-3p,miR-708-3p,miR-450c-3p,miR-134-3p,miR-3431,miR-412-5p,miR-149-5p,miR-194-3p,miR-483-3p,miR-193a-3p,miR-296-3p,miR-377-3p,miR-146a,novel_51,miR-2411-3p,miR-32,miR-365b-5p,miR-2285r,miR-668-3p,miR-3613-5p,miR-193b-3p,miR-
KHSRP	ENSOARG0(	KH-type	miR-5100,miR-2300a-5p
ATE1	ENSOARG0(	arginylt	miR-30d,miR-107,miR-21-3p,miR-103a-2-5p,miR-324-3p,miR-16b,miR-30b,miR-2285b,miR-380-5p,miR-1388-3p,miR-380-5p,miR-424-5p,miR-497-5p,miR-30c,miR-101c,miR-16b,miR-23b-5p,miR-30a-5p,miR-30c-5p,miR-103,miR-322-5p,miR-15a-5p,miR-103a-3p,miR-424-5p,miR-16a,miR-135a-5p,miR-107-5p,miR-15a,miR-30b-5p,miR-502-5p,miR-1197-3p,miR-16-5p,miR-135b-5p,miR-195a-5p,miR-450b-3p,miR-107,miR-30f,miR-30d-5p,miR-21a-3p,miR-30e-5p,miR-2448-3p,miR-15b-5p,miR-3120-3p
SAR1A	ENSOARG0(	secretio	miR-2284z,miR-378h,miR-128-3p,miR-2284u,miR-885-3p,miR-2904,miR-1343-5p,miR-5703,miR-140-3p,miR-378a-3p,miR-378i,miR-2284y,miR-486-3p,miR-2284a,miR-130a-5p,miR-183-5p,miR-28-3p,miR-2284m,miR-122-3p,miR-422a,novel_17,miR-3957,miR-2284n,miR-370-3p,miR-1271,miR-378a-3p,miR-3957-5p,miR-885-3p,novel_99,miR-378c,miR-28a-3p,miR-378c,miR-2284v,miR-486b-3p,miR-2284k,miR-216a-3p,miR-455-3p,miR-2284d,miR-1298-3p,novel_4,miR-2284aa,miR-2284x,miR-2284ab,miR-378c,miR-197-3p,miR-378b,miR-4532,miR-378d,miR-628-5p,miR-2284b,miR-382-3p,miR-1343-5p,miR-505,miR-1306-5p,miR-378e,miR-411b-3p,miR-2284g,miR-378f,miR-150-5p,miR-1306,miR-216a-3p,miR-409-5p,miR-2284j,novel_60,miR-3956-3p

PFKFB1	ENSOARG0(	6-phosph	miR-1434-5p,miR-5703,miR-217-5p,miR-2300b-3p,miR-34a-5p,novel_73,miR-19b-3p,miR-30d-3p,miR-18a-3p,miR-328-3p,miR-200b,miR-23c,miR-148b-3p,miR-23b-3p,miR-185-3p,miR-196a-3p,miR-299-3p,miR-3653-3p,miR-34b-5p,miR-30b-3p,miR-202-5p,miR-195a-3p,miR-329a-5p,miR-2411,miR-486-3p,miR-146a-5p,miR-130a-5p,miR-181b-1-3p,miR-376a-5p,novel_99,miR-1827,miR-625-5p,miR-3065-3p,miR-140-5p,novel_32,miR-301a-3p,miR-10a,miR-30a-3p,miR-30e-3p,miR-454-3p,miR-208b-5p,miR-451a,miR-425-5p,miR-433-5p,miR-539-5p,miR-181c-5p,miR-346,miR-10b-5p,miR-6525,miR-3962,miR-30b-3p,miR-10b,miR-541,miR-6529a,miR-222-5p,miR-181b-2-3p,miR-5010-3p,miR-455-3p,miR-147a,miR-376b-3p,miR-153,miR-2957,miR-431-5p,miR-146b,miR-153-3p,miR-130b-3p,miR-23a-3p,miR-382-3p,miR-381-3p,miR-129b-5p,miR-140-5p,novel_94,miR-335-3p,miR-376a-3p,miR-493-5p,miR-668-5p,miR-20a-3p,miR-10b,miR-376d,miR-144-5p,miR-7857-3p,miR-2440,miR-218-5p,miR-3958-3p,miR-4726-5p,miR-181d-5p,miR-541-3p,miR-877-3p,miR-23b-5p,miR-29d-3p,miR-1290,miR-196a-5p,miR-10a-5p,miR-200b-3p,miR-300,miR-376a-5p,miR-431,miR-429-3p,miR-299a-3p,novel_121,miR-545-5p,miR-2411-5p,miR-217,miR-3071-3p,miR-125a-3p,miR-200c,miR-376b-3p,novel_127,miR-181b-5p,miR-200c-3p,miR-450a-2-3p,miR-30a-3p,miR-490-3p,miR-3065-3p,miR-23b,miR-3154,miR-381-3p,miR-671-5p,miR-485-5p,miR-2318,miR-876-3p,miR-10b-3p,miR-2285aa,miR-370-3p,miR-34c-5p,miR-3969,miR-29b-3p,miR-486b-3p,miR-138-5p,miR-144-5p,miR-193a-5p,miR-6128,miR-374b-3p,novel_23,miR-3071-5p,miR-181a-5p,miR-7862,miR-29b,miR-138,miR-30b-3p,miR-665,miR-130a-3p,miR-31-5p,miR-340-3p,miR-194-3p,miR-185-5p,miR-146a,miR-196b-5p,miR-493-5p,miR-503-3p,miR-432-3p,miR-2285r,miR-432-3p,miR-376b-3p,miR-18a-3p,miR-148a-3p,miR-

MTMR3	ENSOARG0(	myotubul	miR-2284y,miR-29a-5p,let-7g,miR-92b-3p,miR-3064-5p,let-7f-5p,novel_121,miR-450a-1-3p,miR-2284z,miR-9-5p,miR-505-5p,miR-322-5p,miR-431,miR-2285ad,miR-23b-5p,miR-1343-5p,miR-3591-5p,miR-196a-5p,miR-16-5p,miR-592,miR-3432a,miR-6740-5p,miR-2433,miR-25-3p,miR-2355-5p,miR-574-5p,miR-20b-5p,miR-345-5p,miR-329a,miR-93-5p,let-7a-2-3p,miR-668-3p,miR-6240,miR-493-5p,miR-193a-3p,miR-196b-5p,miR-378a-5p,miR-483-3p,novel_51,miR-194a,miR-32,miR-3141,miR-377-3p,miR-146a,miR-149-5p,miR-31-5p,miR-369-5p,miR-497-5p,miR-16b,miR-197-3p,miR-450c-3p,miR-22-3p,miR-432,miR-193b-3p,miR-335-5p,miR-28c,miR-4324,novel_69,miR-329-5p,miR-378f,let-7e-5p,miR-146b-5p,miR-1306,miR-150-5p,miR-3141,miR-329a-5p,let-7k,miR-98-5p,miR-422a,miR-324-3p,miR-210-5p,miR-328-3p,miR-2284h-5p,miR-212-5p,miR-106a,miR-2284u,miR-218-1-3p,miR-33b-3p,miR-378a-3p,miR-1961,miR-378i,miR-122-5p,novel_44,miR-346,miR-214,let-7b,miR-129-5p,miR-151-3p,let-7f,miR-15b-5p,miR-151b,let-7i-5p,miR-377-3p,miR-2284s,miR-10a,miR-2331-3p,miR-32-5p,miR-504-5p,miR-1827,miR-345-5p,miR-129b-3p,miR-450a-1-3p,miR-8485,miR-378d,miR-2284b,miR-1388-3p,miR-214-3p,miR-146b,miR-106b-5p,miR-16b,miR-431-5p,miR-455-3p,miR-147a,miR-330-3p,novel_78,miR-362-3p,miR-2284r,miR-329-3p,miR-361-3p,miR-363-3p,miR-1843a-3p,miR-2440,miR-218-5p,novel_82,miR-4443,miR-25,miR-3596,miR-2284g,miR-493-5p,miR-504,miR-99b-5p,miR-96-5p,miR-3065-3p,miR-28b,miR-4286,miR-181a-2-3p,miR-92a-3p,miR-543-5p,miR-2898,miR-17-3p,let-7c-5p,miR-99a,miR-214-5p,miR-483-3p,miR-1271-5p,miR-378h,let-7a-2-3p,miR-10a-5p,miR-99a-5p,let-7d,miR-378c,miR-93,miR-103b,miR-28-5p,miR-410-3p,miR-370-5p,miR-410-5p,novel_48,miR-100-5p,miR-17-5p,miR-345-5p,miR-1343-
GET4	ENSOARG0(	golgi to	miR-532-3p,miR-1260b,miR-1260b,miR-877-3p,miR-361-3p,miR-1260a
CDH20	ENSOARG0(	cadherin	miR-130b-5p,novel_39,miR-1306-5p,miR-125b,miR-324-3p,miR-760-3p,miR-491-5p,novel_96,miR-125a-5p,miR-106b-3p,miR-6516-5p,miR-147a,miR-214-5p,miR-149-5p,novel_83,miR-2408,miR-296-5p,miR-378d,miR-361-3p,miR-338-3p,miR-2285j,miR-2433,miR-218-5p,miR-125b-5p,miR-664a,miR-130b-5p,novel_68,miR-7977,miR-125a,miR-6516,miR-1306,miR-329b,miR-543-3p

TRMT61A	ENSOARG0(	tRNA met	miR-664a,miR-138-5p,miR-874-5p,miR-320e,miR-125b-5p,miR-331-3p,miR-1197-3p,miR-6134,miR-3184-5p,miR-3074-5p,miR-129-2-3p,miR-4510,let-7g-3p,miR-18a-5p,let-7g-3p,miR-485-5p,miR-1827,miR-671-5p,miR-301,miR-488-3p,miR-18a,miR-193b-3p,miR-6130,miR-2284s,miR-544-3p,novel_32,miR-2331-3p,miR-125a,miR-2355-5p,novel_96,miR-125b,miR-185-3p,miR-1271-3p,novel_39,miR-345-3p,miR-2887,miR-4286,miR-3064-5p,let-7a-2-3p,miR-2366,miR-615-3p,miR-330-5p,miR-1197-5p,miR-129-1-3p,miR-6517,miR-2285ad,miR-18b-5p,miR-4726-5p,miR-6123,miR-615,miR-2285n,miR-301a-5p,miR-2285y,miR-24-3p,miR-361-3p,miR-326-3p,miR-423-5p,miR-3956-3p,novel_101,miR-193b-3p,novel_94,miR-18b,miR-361-3p,miR-2483-3p,miR-2404,miR-146b-3p,miR-378g,miR-4443,miR-669,miR-3074-5p,miR-3064-5p,miR-2285u,miR-25-5p,novel_124,miR-3085-3p,miR-491-5p,miR-193a-3p,miR-125a-5p,miR-326,miR-5126,let-7a-2-3p,miR-4532,miR-544a,miR-219a-1-3p,miR-138,miR-301b-5p,miR-2284r,miR-185-5p,miR-134-3p,miR-149-5p,miR-9788-3p,miR-194-3p,novel_4,novel_78,miR-1343-3p,miR-133b-5p,miR-2300a-5p
SEPHS1	ENSOARG0(	selenoph	miR-148b-5p,miR-219-3p,miR-125a-3p,miR-200a-3p,novel_127,miR-127-5p,novel_79,miR-1224-5p,miR-412,miR-429-3p,miR-2284z,miR-758-5p,miR-9-5p,miR-1290,miR-3591-5p,miR-155-5p,miR-193a-5p,miR-494-5p,miR-592,novel_9,miR-2355-5p,miR-874-3p,miR-503-3p,miR-376c-5p,miR-93-5p,miR-301,miR-20b-5p,miR-329b,miR-345-5p,miR-876-3p,miR-628-5p,miR-668-3p,miR-493-5p,miR-503-3p,miR-2285r,miR-377-3p,miR-483-3p,miR-339-5p,miR-31-5p,novel_116,miR-3431,miR-134-3p,miR-22-3p,miR-542-3p,miR-2284x,miR-16-1-3p,miR-335-5p,miR-2284o,miR-142b,miR-576-3p,miR-4324,miR-3120-3p,miR-181b-3p,miR-144-3p,miR-6535,miR-677,miR-374c-3p,miR-3591-3p,miR-150-5p,miR-376b-5p,miR-378f,miR-2319a,miR-345-3p,miR-369-3p,miR-195a-3p,miR-376a-5p,miR-92a-1-5p,miR-422a,miR-212-5p,miR-30d-3p,miR-106a,miR-18a-3p,miR-1434-5p,miR-378a-3p,miR-217-5p,miR-378i,miR-148a-5p,miR-16-1-3p,miR-214,miR-665,miR-219b-3p,miR-30a-3p,miR-30e-3p,miR-2285j,miR-4492,novel_74,miR-296-3p,miR-216b-3p,miR-504-5p,miR-377-3p,miR-487b-5p,miR-433-3p,miR-129b-3p,miR-625-5p,miR-339b,miR-345-5p,miR-2310,miR-378d,miR-182-5p,miR-106b-5p,miR-29b-1-5p,miR-214-3p,miR-1185-5p,miR-181b-2-3p,miR-145b,miR-1343-3p,novel_78,miR-2459,miR-421,miR-148b-5p,miR-455-3p,miR-539-5p,miR-147a,miR-758-3p,miR-501-3p,miR-301b-5p,miR-2284r,miR-1839-5p,miR-9-5p,miR-885-5p,miR-2447,miR-7705,miR-1285-5p,miR-877-3p,miR-301a-5p,miR-499a-5p,miR-7689-3p,miR-2319b,miR-24-1-5p,miR-139-5p,miR-500-3p,miR-203-3p,miR-2483-3p,miR-378g,miR-3596,miR-381-3p,miR-378j,miR-544-5p,miR-493-5p,miR-504,miR-136-5p,miR-181a-2-3p,miR-29b-2-5p,miR-30a-3p,miR-1271-3p,miR-376c-5p,miR-376b-5p,miR-24-2-5p,miR-217,miR-339a,miR-200c,miR-191-3p,miR-200c-3p,miR-17-3p,miR-450a-2-3p,miR-483-3p,miR-378h,miR-501-

CCDC178	ENSOARG0(	coiled-c	miR-2411-5p,miR-30a-5p,miR-345-3p,miR-3578,miR-134,let-7d,miR-181a-2-3p,miR-3529-3p,miR-199a-5p,miR-380-5p,miR-28b,miR-181c-3p,miR-217,let-7c-5p,miR-1260a,miR-107,miR-92a-3p,miR-141-3p,miR-3957-5p,miR-487a-3p,miR-199a-5p,miR-154b-5p,miR-3957,miR-34c-5p,miR-17-5p,miR-320a,miR-2448-3p,novel_48,miR-485-5p,miR-10b-3p,miR-3607-3p,miR-365a-5p,miR-93,miR-374a-3p,miR-135a-1-3p,miR-28-5p,miR-374b-3p,miR-486b-3p,miR-544b,miR-103,miR-20b,miR-2355-3p,let-7g-5p,miR-145a-5p,miR-873a-5p,miR-455-5p,miR-30b-3p,miR-1260b,miR-219a-1-3p,miR-382,miR-130b-5p,miR-539-3p,miR-330-3p,miR-190a,miR-106a-5p,miR-455-5p,miR-17-5p,miR-224-5p,miR-452-5p,miR-320b,miR-378b,miR-670-3p,miR-34b,miR-378b,miR-505-3p,miR-195a-5p,miR-450b-3p,miR-107,miR-219b-3p,miR-449a,miR-326-3p,miR-2428,miR-24-3p,miR-16a,miR-34c,let-7i,miR-382-5p,miR-15a,miR-20a-5p,miR-22-5p,miR-199c,miR-147-5p,miR-92a-3p,novel_1,miR-505-3p,miR-485-3p,miR-487a-3p,miR-222-5p,miR-15a-5p,miR-188-3p,miR-5703,novel_133,miR-2300b-3p,miR-1246,miR-376a-5p,miR-4429,miR-20a,miR-216c-5p,miR-2411,miR-130a-5p,miR-216a-5p,miR-30d,miR-34b-5p,miR-21-3p,miR-299-3p,miR-320d,miR-106a,miR-466i-5p,miR-21-3p,miR-32-3p,miR-544-3p,miR-139-5p,miR-21a-3p,novel_99,miR-30f,miR-154b-3p,miR-539-5p,let-7b-5p,miR-324-3p,miR-320e,miR-708-5p,miR-424-5p,miR-664a,miR-409b,novel_4,miR-9788-3p,miR-30b-3p,miR-6529a,miR-143-5p,miR-544a,miR-33a-5p,let-7a-5p,miR-424-5p,miR-106a-5p,miR-193a,miR-758-3p,miR-320b,novel_115,miR-326,miR-1306-5p,let-7e,miR-25-5p,miR-664b,miR-20a-3p,let-7d-5p,miR-7857-3p,miR-489,miR-3082-5p,miR-129b-5p,miR-361-3p,miR-199b-5p,miR-3059-5p,miR-32-3p,miR-212-3p,miR-9-3p,miR-382-3p,miR-374a-3p,miR-2285ad,miR-299a-3p,miR-134-5p,miR-322-5p,miR-330-5p,miR-
MAN1C1	ENSOARG0(	mannosid	miR-206-3p,miR-346,miR-654-3p,miR-541-3p,miR-495-5p,miR-206,miR-2284q,miR-1468,miR-1468-5p,miR-1343-5p,novel_87,miR-489,miR-328-3p,miR-548o-3p,miR-320b,miR-1291,miR-485-5p,miR-6395,miR-541,miR-3578,miR-140-5p,miR-1343-5p
NUBP1	ENSOARG0(	nucleoti	miR-224-5p,miR-211,novel_27,miR-541-3p,miR-3059-5p,miR-9-5p,miR-3607-3p,miR-4726-5p,miR-204-5p,miR-486b-3p,miR-28-5p,miR-708-5p,miR-421-5p,miR-331-3p,miR-139-5p,miR-2404,miR-2284s,miR-133c,miR-139-5p,miR-211-5p,miR-320b,miR-186-5p,miR-1895,miR-3065-5p,miR-129b-3p,miR-377-5p,miR-192-3p,miR-1957a,miR-4532,miR-486-3p,miR-216c-5p,miR-124a,let-7c-3p,miR-760-3p,miR-191-3p,miR-196a-3p,miR-224-5p,miR-185-3p,novel_51,miR-2331-5p,miR-105-5p,miR-7859,miR-9-5p,miR-455-3p,miR-194-3p,miR-105-1,miR-548e-3p,miR-377-5p,miR-16-1-3p,miR-30b-3p,miR-188-5p,miR-105-2,miR-16-1-3p,miR-541,miR-125b-2-3p,novel_23,miR-30b-3p,miR-124-3p,miR-2904
FIGN	ENSOARG0(	fidgetin	miR-181a-5p,miR-493-3p,novel_9,miR-181b-5p,miR-466i-5p,miR-345-5p,miR-345-5p,miR-450a-5p,miR-1248,miR-2355-5p,miR-1298-3p,novel_69,novel_27,miR-450c-5p,miR-345-5p

ARAP1	ENSOARG0(	ArfGAP w	miR-342,miR-1343-5p,miR-543-5p,miR-214-3p,novel_96,miR-491-5p,miR-378a-5p,miR-3600,miR-26b-3p,miR-145b,miR-149-5p,miR-486-5p,miR-1343-5p,miR-145a-5p,miR-23b-5p,novel_133,miR-541,miR-10b,miR-10a-5p,miR-744-5p,miR-885-3p,miR-342-3p,miR-541-3p,miR-145-3p,miR-423-5p,miR-3184-5p,miR-10b-5p,miR-6525,miR-370-3p,miR-10a-5p,miR-504-5p,miR-10b,miR-669,miR-10a,miR-670-3p,novel_69,miR-129b-3p,miR-361-3p,miR-488-3p,miR-2387,miR-23a-5p,miR-885-3p,let-7g-3p,miR-504
DTX3	ENSOARG0(	deltex 3	miR-4492,miR-130b-5p,miR-29b-2-5p,miR-320e,miR-3184-5p,miR-6134,miR-6525,miR-3958-5p,miR-214,miR-2330-3p,miR-4510,miR-3074-5p,miR-3184-3p,miR-625-5p,miR-1895,miR-1827,miR-129b-3p,miR-21a-3p,novel_32,miR-877-5p,miR-6130,miR-139-5p,miR-296-3p,miR-545-3p,miR-2331-3p,miR-21-3p,miR-760-3p,miR-466i-5p,miR-219b-5p,miR-92a-1-5p,miR-185-3p,novel_17,miR-486-3p,miR-29b-1-5p,miR-5100,miR-664-5p,miR-941,novel_73,miR-5703,miR-532-3p,miR-1b-3p,miR-212-5p,miR-3960,miR-4726-5p,miR-107-5p,miR-423-5p,miR-361-3p,miR-877-3p,miR-361-3p,miR-27a-3p,miR-378g,miR-7977,miR-3064-5p,miR-139-5p,miR-103a-2-5p,miR-214-3p,miR-3085-3p,miR-491-5p,miR-29b-1-5p,miR-342,miR-4532,miR-1388-5p,miR-30b-3p,miR-147a,miR-27a-3p,miR-486b-3p,miR-2433,miR-410-5p,miR-1260b,novel_42,miR-7144-5p,miR-1248,miR-876-3p,miR-3154,miR-424-3p,miR-324-5p,miR-154b-5p,miR-2285aa,miR-107,novel_96,miR-2898,miR-1260a,miR-28b,miR-29b-2-5p,miR-3064-5p,miR-4286,miR-3529-3p,miR-1343-5p,miR-3578,miR-24-2-5p,miR-423-3p,miR-128-1-5p,miR-6402,miR-28c,miR-495-3p,miR-342-3p,miR-9851-3p,miR-410-5p,miR-107,miR-3141,miR-6535,miR-146b-3p,miR-2404,novel_69,miR-3074-5p,miR-1343-5p,miR-3141,miR-2331-5p,novel_51,miR-296-3p,miR-296-3p,miR-432-3p,miR-363-5p,miR-130b-5p,miR-4508,miR-744-5p,miR-27b-3p,miR-7862,miR-2889,miR-30b-3p,miR-1260b,miR-1843b-5p,miR-149-5p,miR-194-3p,miR-103,miR-134-3p,miR-185-5p,miR-665,miR-103a-3p,miR-133b-5p

MMP20	ENSOARG0(	matrix m	novel_99,miR-345-5p,miR-433-3p,miR-129b-3p,miR-2285c,miR-299,miR-582-5p,miR-376e-3p,miR-182-5p,miR-548o-3p,miR-140-5p,miR-221-5p,miR-487a-5p,miR-665,miR-320e,miR-2285j,miR-346,miR-665-5p,miR-3958-5p,miR-145a-3p,miR-539-5p,miR-361-5p,miR-19b-3p,miR-188-3p,miR-2300b-3p,miR-532-3p,miR-200b,miR-487a-3p,miR-485-3p,miR-548e-3p,miR-221-5p,miR-466i-5p,miR-125b,miR-345-3p,miR-500a-5p,miR-374a-5p,miR-618,miR-2432,miR-376c-3p,miR-130a-5p,miR-376a-5p,miR-216c-5p,miR-136-5p,miR-378j,miR-140-5p,miR-1185-2-3p,miR-25,miR-2285u,miR-2284q,miR-450a-5p,miR-452-5p,miR-1296-5p,miR-3956-3p,novel_60,miR-1839-5p,miR-2426,miR-299a-5p,miR-1388-5p,miR-30b-3p,miR-125b-2-3p,miR-3962,miR-1298-5p,miR-1185-3p,miR-2285e,miR-539-5p,miR-147a,miR-103a-2-5p,miR-182-5p,miR-505,miR-1306-5p,miR-2310,miR-8485,miR-376b,miR-2330-5p,miR-101b-3p,miR-1814c,miR-671-5p,miR-1248,miR-7144-5p,miR-3074-1-3p,novel_48,miR-345-5p,miR-10b-3p,miR-2448-3p,miR-331-5p,miR-376c-3p,miR-133c,miR-223-5p,miR-365a-3p,miR-125a,miR-370-3p,miR-3068-3p,miR-331-5p,miR-138-5p,miR-409-3p,miR-135a-5p,miR-144,miR-26c,miR-125b-5p,miR-135a-1-3p,miR-5100,miR-1260b,miR-193a-5p,miR-200b-3p,miR-411-3p,miR-2427,miR-3578,miR-345-3p,miR-3591-5p,miR-6516-5p,miR-758-5p,miR-376a-5p,miR-1224-5p,miR-429-3p,miR-127-5p,miR-200c-3p,miR-200c,miR-1260a,miR-450c-5p,miR-1957a,miR-29a-5p,miR-450b-5p,miR-380-5p,miR-376a-2-5p,miR-135b-5p,miR-7975,miR-19a-3p,miR-410-5p,miR-21c,miR-1306,miR-144-3p,miR-2404,miR-625-3p,miR-379-3p,miR-33a-3p,miR-485-3p,miR-133a-5p,miR-2284w,novel_27,miR-3959-3p,novel_101,miR-495-3p,miR-188-5p,miR-1260b,miR-138,miR-21-5p,miR-30b-3p,miR-380-3p,novel_23,miR-2113,miR-6238,miR-3431,miR-2285g,miR-340-

SUN1	ENSOARG0(	Sad1 and	miR-199b-5p,miR-668-5p,miR-335-3p,miR-2285u,miR-3082-5p,miR-2483-3p,miR-1185-2-3p,miR-2285n,miR-769-5p,miR-3968,miR-3964,miR-363-3p,miR-9-5p,miR-452-5p,miR-181d-5p,miR-2285f,miR-544a,miR-450b-5p,miR-1388-5p,miR-1839-5p,miR-153,miR-1185-3p,miR-2285e,novel_103,miR-106b-5p,miR-29b-1-5p,miR-199b-5p,miR-153-3p,miR-146b,miR-106a-5p,miR-494-3p,miR-652-5p,miR-1b-5p,miR-4532,miR-2285c,miR-500a-3p,miR-2312,miR-885-3p,miR-32-5p,miR-3065-3p,miR-6130,miR-544-3p,miR-2285j,miR-29b-2-5p,miR-500,miR-4492,miR-4510,miR-769-5p,miR-338-3p,miR-181c-5p,miR-2408,miR-217-5p,miR-941,miR-33b-3p,miR-106a,miR-3960,miR-340-5p,miR-7859,miR-2284h-5p,miR-2285p,miR-185-3p,miR-500a-5p,miR-106a,miR-1277-5p,miR-30b-3p,miR-345-3p,miR-20a,miR-29b-1-5p,miR-146a-5p,miR-486-3p,miR-2285ab,miR-150-5p,miR-146b-5p,miR-320b,novel_69,miR-29a-3p,miR-20a-5p,miR-4324,novel_87,miR-29a,miR-6119-3p,miR-92a-3p,miR-29c-3p,miR-199b-5p,miR-181a-5p,miR-138,miR-29b,miR-2285g,miR-544b,miR-20b,miR-146a,miR-17-5p,miR-106a-5p,miR-32,miR-106b-3p,let-7f-2-3p,let-7f-2-3p,miR-2483-5p,let-7a-2-3p,miR-17-5p,miR-93-5p,let-7g-3p,miR-767,miR-23a-5p,miR-20b-5p,miR-2355-5p,miR-370-3p,miR-769,miR-25-3p,miR-199a-5p,miR-223-5p,miR-29b-3p,miR-2284k,miR-767-5p,miR-138-5p,miR-486b-3p,miR-93,miR-3591-5p,miR-23b-5p,miR-29d-3p,miR-885-3p,let-7a-2-3p,miR-1224-5p,miR-9-5p,miR-2285af,miR-217,miR-181b-5p,miR-92a-3p,miR-543-5p,miR-29b-2-5p,miR-92b-3p,miR-3065-3p,miR-199a-5p

NCCRP1	ENSOARGO(	non-spec	let-7b,miR-4510,miR-665-5p,miR-10b-5p,miR-224-5p,miR-346,let-7b-5p,miR-3184-5p,let-7f,miR-324-3p,miR-665,miR-454-3p,miR-874-5p,miR-4492,miR-30c-1-3p,miR-221-5p,miR-504-5p,miR-545-3p,miR-216b-3p,miR-21-3p,miR-296-3p,miR-10a,miR-6516,miR-301a-3p,miR-323-5p,miR-216b-5p,miR-6130,let-7i-5p,miR-877-5p,miR-21a-3p,miR-27b-5p,miR-345-5p,miR-186-5p,miR-216c-5p,miR-20a,miR-345-3p,miR-2432,novel_17,miR-210-5p,miR-324-3p,miR-106a,miR-98-5p,miR-221-5p,miR-3653-3p,miR-299-3p,let-7k,miR-18a-3p,miR-106a,miR-505-3p,miR-148b-3p,miR-199a-3p,novel_133,miR-1246,miR-1961,miR-5703,miR-16-1-3p,miR-218-1-3p,miR-378d,miR-148a-5p,miR-361-5p,miR-541-3p,miR-423-5p,miR-3968,miR-2285t,miR-490-5p,miR-664b,miR-3064-5p,miR-10b,miR-203-3p,let-7d-5p,miR-144-5p,miR-1185-2-3p,miR-361-3p,miR-129b-5p,miR-504,miR-1983,miR-544-5p,novel_94,let-7a-5p,miR-1388-3p,miR-106a-5p,miR-30c-1-3p,miR-199a-3p,miR-326,novel_103,miR-106b-5p,miR-491-5p,miR-3085-3p,miR-3955-3p,miR-130b-3p,miR-153-3p,let-7e,miR-409b,miR-153,miR-425-3p,miR-1298-3p,miR-9788-3p,miR-455-3p,miR-148b-5p,miR-1185-3p,miR-2459,miR-6529a,miR-484,miR-541,miR-10b,miR-143-5p,miR-3962,miR-30b-3p,miR-3120-5p,miR-193a-5p,miR-144-5p,miR-93,miR-365a-5p,miR-6740-5p,miR-331-3p,miR-370-3p,miR-141-3p,miR-300-3p,miR-365a-3p,miR-3965,miR-93-5p,miR-17-5p,miR-345-5p,miR-3074-1-3p,miR-20b-5p,miR-3064-5p,miR-495-5p,miR-2403,miR-450b-5p,let-7g,miR-1957a,miR-376b-5p,miR-376c-5p,miR-490-3p,miR-2285b,miR-664-3p,miR-30c-2-3p,let-7c-5p,miR-2898,let-7f-5p,miR-200a-3p,miR-543-5p,novel_96,novel_127,miR-152-3p,miR-299a-3p,miR-6517,miR-6516-5p,miR-196a-5p,miR-330-5p,let-7d,miR-10a-5p,miR-374c-5p,miR-326-3p,miR-301b-3p,miR-1247-3p,miR-
MADD	ENSOARGO(	MAP-kina	miR-874-3p,novel_68,miR-1271,miR-216b-3p,miR-370-3p,miR-320b,miR-193b-3p,miR-411b-3p,miR-4443,miR-21a-3p,miR-18a-3p,miR-877-3p,miR-3059-5p,miR-214,miR-193b-3p,miR-346,miR-2428,miR-2433,miR-665,miR-664a,miR-128-1-5p,miR-2284q,novel_4,miR-18a-3p,miR-505-5p,miR-421,miR-149-5p,miR-2284h-5p,miR-2366,miR-2285w,miR-345-3p,miR-8485,novel_39,miR-210-5p,miR-2285af,miR-760-3p,miR-491-5p,miR-378a-5p,novel_127,novel_96,miR-193a-3p,miR-214-3p,miR-21-3p,miR-106b-3p
IQGAP3	ENSOARGO(	IQ motif	miR-148a-3p,miR-3591-5p,miR-3154,miR-1343-5p,miR-148b-3p,miR-505-5p,miR-7977,miR-664a,miR-450a-2-3p,miR-4726-5p,miR-152-3p,miR-543-5p,miR-1343-5p,miR-6525,miR-148b-5p,miR-2428,miR-2330-5p

TVP23A	ENSOARG0(	trans-go	miR-411-3p,miR-128-3p,miR-1343-5p,miR-330-5p,miR-30a-5p,miR-322-5p,miR-505-5p,miR-2411-5p,miR-545-5p,miR-2285ad,miR-6517,miR-412,miR-2898,novel_96,miR-205-5p,miR-28b,miR-148b-5p,miR-199a-5p,novel_39,miR-2285b,miR-181a-2-3p,miR-10b-3p,miR-329a,miR-329b,miR-485-5p,miR-671-5p,miR-30e-5p,miR-362-5p,miR-3154,miR-199a-5p,miR-574-5p,miR-3068-3p,miR-370-3p,miR-1271,miR-486b-3p,miR-28-5p,miR-2433,miR-331-3p,miR-144,miR-500b-5p,miR-145-3p,miR-16-5p,miR-16b,miR-497-5p,miR-873a-5p,miR-1843b-5p,miR-1434-3p,miR-185-5p,miR-134-3p,miR-26b-3p,miR-1343-5p,miR-432-3p,miR-130b-5p,miR-363-5p,miR-668-3p,miR-1291,miR-195a-5p,miR-150-5p,miR-216a-3p,miR-323a-5p,miR-6535,miR-144-3p,miR-320b,miR-379-3p,miR-502-5p,miR-4324,miR-15a,miR-28c,miR-16a,miR-326-3p,miR-199b-5p,novel_101,miR-378d,novel_133,miR-362-3p,miR-5703,miR-1197-5p,miR-95-3p,miR-222-5p,miR-15a-5p,miR-199a-3p,miR-151-5p,miR-760-3p,miR-30b-3p,miR-30d,miR-2484,miR-210-5p,miR-324-3p,miR-219b-5p,miR-486-3p,miR-2411,miR-105-3p,miR-1895,miR-625-5p,novel_99,miR-1827,miR-582-5p,miR-30d-5p,miR-2284s,miR-582-5p,miR-6130,miR-323-5p,miR-204-3p,miR-15b-5p,miR-151b,miR-216b-3p,miR-664a,miR-6516-3p,miR-874-5p,miR-424-5p,miR-708-5p,miR-130b-5p,miR-665,miR-6525,miR-346,miR-338-3p,miR-6134,miR-539-5p,miR-4510,miR-145a-3p,miR-214,miR-362-3p,miR-1388-5p,miR-2426,miR-101c,miR-329-3p,miR-541,miR-412-3p,miR-500-5p,miR-455-3p,miR-2459,novel_4,miR-2300a-5p,miR-1343-3p,miR-491-5p,miR-199b-5p,miR-214-3p,miR-103a-2-5p,miR-199a-3p,miR-326,novel_103,miR-362-3p,miR-16b,miR-3187-3p,miR-8485,miR-4532,miR-424-5p,miR-2330-5p,miR-744-3p,miR-668-5p,miR-199b-5p,miR-3596,miR-378g,miR-489,miR-203-3p,miR-4792,miR-20a-3p,miR-4726-5p,miR-3964,miR-

GCA	ENSOARG0(	grancalc	miR-548e-3p,miR-7641,miR-9-5p,novel_83,miR-2284h-5p,miR-376a-5p,miR-1434-5p,miR-19b-3p,let-7a-3p,miR-148a-5p,miR-128-3p,miR-155-5p,miR-2285w,miR-664b-3p,miR-92b-3p,miR-380-3p,miR-200a,miR-2285p,miR-3071-3p,miR-181b-5p,miR-202-5p,miR-2284m,miR-92a-3p,miR-200a-3p,miR-1277-5p,miR-3068-3p,miR-32-3p,miR-548o-3p,miR-545-3p,miR-140-5p,miR-32-5p,miR-141-3p,miR-216b-3p,miR-2285aa,miR-25-3p,miR-499b-3p,miR-301a-3p,miR-155-5p,miR-204-3p,miR-2285c,let-7f-1-3p,miR-299,miR-137-3p,miR-2318,miR-7144-5p,novel_48,miR-590-3p,miR-323-3p,miR-494-5p,miR-19b-1-5p,miR-155-5p,miR-130b-5p,miR-204-5p,miR-454-3p,novel_78,miR-145b,miR-130a-3p,miR-1298-3p,miR-2285g,miR-412-5p,miR-2284d,miR-1185-3p,miR-2285e,miR-126a-5p,miR-2355-3p,miR-149-5p,miR-148b-5p,miR-181a-5p,miR-19b-2-5p,miR-2284r,novel_23,miR-145a-5p,miR-125b-2-3p,miR-374b-3p,miR-299a-5p,miR-450b-5p,miR-380-3p,miR-3120-5p,miR-382-3p,miR-493-5p,miR-130b-5p,let-7f-2-3p,let-7f-2-3p,miR-491-3p,let-7j,novel_103,novel_51,miR-32,miR-505,miR-103a-2-5p,miR-216b-3p,miR-3120-3p,miR-211-5p,miR-2483-3p,miR-2404,miR-499a-3p,miR-3596,miR-1185-2-3p,miR-216a-3p,miR-136-5p,miR-19a-3p,miR-450b-3p,miR-376a-2-5p,miR-493-5p,miR-363-3p,miR-3956-3p,novel_60,miR-219b-3p,miR-32-3p,miR-2447,miR-181d-5p,miR-9-5p,miR-2285x,miR-211,miR-301b-3p,miR-154a,novel_27,miR-107-5p,miR-485-3p,miR-92a-3p,miR-2284q,miR-147-5p

SLC26A4	ENSOARG0(	solute c	miR-493-5p,miR-1983,miR-409-5p,miR-199b-5p,miR-129b-5p,miR-499a-3p,miR-2483-3p,let-7d-5p,miR-454-5p,miR-2319b,miR-450a-5p,miR-490-5p,miR-3964,miR-218-5p,miR-382-3p,miR-769-5p,miR-2447,miR-3059-5p,miR-363-3p,miR-541-3p,miR-2285f,miR-181d-5p,miR-9-5p,miR-544a,miR-1839-5p,miR-2284aa,miR-16-2-3p,miR-484,miR-10b,miR-541,miR-153,miR-539-5p,miR-455-3p,miR-2285e,miR-105-1,novel_78,miR-151a-3p,miR-181b-2-3p,miR-491-5p,miR-3955-3p,miR-153-3p,miR-199b-5p,let-7e,miR-16b,miR-1b-5p,let-7a-5p,miR-424-5p,miR-494-3p,miR-23a-3p,miR-744-3p,miR-2312,miR-345-5p,novel_99,miR-2285c,miR-10a,miR-544-3p,miR-499b-3p,miR-204-3p,novel_25,let-7i-5p,miR-15b-5p,miR-32-5p,miR-548o-3p,miR-216b-3p,miR-532-5p,miR-450b-3p,miR-487b-5p,miR-208b-5p,miR-424-5p,miR-130b-5p,miR-2285j,let-7f,miR-151-3p,miR-769-5p,miR-10b-5p,let-7b-5p,miR-181c-5p,miR-539-5p,let-7b,miR-142-3p,miR-19b-3p,miR-218-1-3p,miR-1961,miR-1246,miR-1434-5p,miR-217-5p,miR-23b-3p,miR-7859,miR-383-5p,miR-23c,miR-200b,miR-15a-5p,miR-148b-3p,miR-1247-5p,miR-328-3p,miR-548e-3p,miR-98-5p,miR-3653-3p,let-7k,miR-26a-5p,miR-618,miR-125b-2-3p,miR-181b-1-3p,miR-2285w,miR-195a-3p,miR-412-3p,miR-19a-3p,miR-6395,miR-195a-5p,let-7e-5p,miR-2285ab,miR-148a-3p,miR-144-3p,miR-677,miR-181b-3p,miR-26b-5p,miR-10a-5p,miR-92a-3p,miR-656-5p,miR-147-5p,miR-15a,miR-6402,miR-16a,let-7i,miR-323c,miR-199b-5p,miR-1a-1-5p,miR-16b,miR-497-5p,miR-1285,miR-2113,miR-223-3p,miR-3071-5p,miR-2285g,miR-3431,let-7g-5p,miR-194-3p,miR-544b,miR-31-5p,miR-133b-5p,miR-378a-5p,miR-483-3p,miR-32,miR-197-5p,miR-365b-5p,miR-105-5p,let-7j,miR-345-5p,miR-130b-5p,miR-493-5p,miR-345-5p,miR-190a-3p,miR-488-3p,miR-200a-5p,miR-23b,miR-3154,miR-331-5p,miR-769,miR-

GMFG	ENSOARG0(	glia mat	miR-2285r,miR-296-3p,miR-493-5p,miR-380-5p,miR-660,miR-296-3p,miR-582,miR-224-5p,miR-17-5p,miR-106a-5p,miR-412-5p,miR-20b,miR-411-5p,miR-31-5p,miR-377-5p,miR-665,miR-1260b,miR-6239,miR-1285,miR-199b-5p,miR-326-3p,miR-542-5p,miR-576-3p,miR-20a-5p,miR-320c,miR-2285k,miR-450b-3p,miR-9851-3p,miR-2285ab,miR-3065-5p,miR-1306,miR-380-5p,miR-490-3p,miR-199a-5p,miR-29a-5p,miR-1957a,miR-4286,miR-200a-3p,miR-1260a,miR-2411-5p,miR-758-5p,miR-214-5p,miR-129-1-3p,miR-134-5p,miR-1224-5p,miR-7641,miR-412,miR-496,miR-134,miR-330-5p,miR-1260b,miR-374a-3p,miR-323-3p,miR-93,miR-133a-3p,miR-374b-3p,miR-134-5p,miR-494-5p,miR-3969,miR-6740-5p,miR-199a-5p,miR-503-3p,miR-574-5p,miR-215-5p,miR-1271,miR-141-3p,miR-542-5p,miR-329b,miR-485-5p,miR-671-5p,miR-1248,miR-23a-5p,miR-20b-5p,miR-2448-3p,miR-17-5p,miR-3154,miR-320a,miR-93-5p,miR-3187-3p,miR-106a-5p,miR-652-5p,miR-1468,miR-542-5p,miR-199b-5p,miR-326,miR-1306-5p,miR-320b,miR-106b-5p,miR-323b-3p,miR-9788-3p,miR-148b-5p,miR-2300a-5p,miR-301b-5p,miR-484,miR-2284r,miR-412-3p,miR-323b,miR-361-3p,miR-154a,miR-423-5p,miR-3956-3p,novel_60,miR-2447,miR-2285f,miR-374a-3p,novel_82,miR-301a-5p,miR-4443,miR-2319b,miR-504,miR-9-3p,miR-493-5p,miR-199b-5p,miR-361-3p,miR-377-5p,miR-2285i,miR-2411,miR-2285w,miR-345-3p,miR-20a,miR-106a,miR-320d,miR-2484,miR-542-5p,miR-324-3p,miR-340-5p,miR-2284h-5p,miR-1247-5p,miR-106a,miR-548e-3p,miR-4429,miR-148a-5p,miR-133a-3p,miR-215-5p,miR-140-3p,miR-5703,miR-2904,miR-224-5p,miR-338-3p,miR-3184-5p,miR-4510,miR-129-2-3p,miR-539-5p,miR-6516-3p,miR-874-5p,miR-1468-5p,miR-6130,miR-192-5p,miR-877-5p,miR-204-3p,miR-660-5p,miR-504-5p,miR-133b-3p,miR-2312,miR-1827,miR-129b-3p
AKNA	ENSOARG0(	AT-hook	miR-4532,miR-4443,miR-6130,miR-483-3p,miR-4510,miR-485-5p,miR-483-3p,miR-25-5p,miR-324-3p,miR-3601,miR-2904,miR-1197-5p,miR-488-3p,miR-296-5p

SIX2	ENSOARG0(	SIX home	miR-542-5p,miR-2355-5p,miR-125a,miR-1271,miR-574-5p,miR-3068-3p,miR-503-3p,miR-1248,miR-767,miR-345-5p,miR-329b,miR-1260b,miR-1197-3p,novel_42,novel_9,miR-125b-5p,miR-2433,miR-758-5p,miR-1224-5p,miR-299a-3p,miR-1343-5p,miR-330-5p,novel_39,miR-2285b,miR-3064-5p,miR-200a-3p,miR-152-3p,miR-125a-3p,miR-219-3p,miR-320c,miR-299b-3p,miR-3120-3p,novel_68,miR-146b-5p,miR-378f,miR-412-3p,miR-3970,miR-216a-3p,miR-148a-3p,miR-2284w,miR-301b-3p,miR-193b-3p,miR-335-5p,miR-6119-3p,miR-425-5p,miR-4324,miR-149-5p,miR-194-3p,miR-412-5p,miR-134-3p,miR-31-5p,miR-103a-3p,miR-188-5p,miR-22-3p,miR-197-3p,novel_23,miR-432,miR-2113,miR-6240,miR-193a-3p,miR-483-3p,miR-378a-5p,novel_51,miR-194a,miR-146a,miR-151b,miR-301a-3p,miR-2284s,miR-548o-3p,miR-1895,miR-543-3p,miR-1827,miR-345-5p,miR-2285c,miR-3184-5p,miR-181c-5p,miR-6525,miR-769-5p,miR-214,miR-3184-3p,miR-454-3p,miR-874-5p,miR-4492,miR-130b-5p,miR-219b-3p,miR-30a-3p,miR-664a-5p,miR-665,miR-3604,miR-30e-3p,miR-30d-3p,miR-133a-3p,miR-34a-5p,miR-142-3p,miR-378a-3p,miR-378i,miR-486-3p,miR-2424,miR-143-3p,miR-92a-1-5p,miR-422a,miR-210-5p,miR-1185-2-3p,miR-4443,miR-3596,miR-378g,miR-211-5p,miR-1983,miR-9-3p,miR-423-5p,miR-361-3p,novel_111,miR-2447,miR-3956-3p,miR-218-5p,miR-2440,miR-1185-3p,miR-153,miR-147a,miR-330-3p,miR-1839-5p,miR-8485,miR-378d,miR-676-3p,miR-127-3p,miR-146b,miR-153-3p,miR-214-3p,miR-130b-3p,miR-34c-5p,miR-769,miR-370-3p,miR-141-3p,miR-7144-5p,miR-23a-5p,miR-485-5p,let-7g-3p,miR-320a,miR-362-5p,miR-378c,miR-6128,miR-1306-5p,miR-486b-3p,miR-204-5p,miR-28-5p,miR-138-5p,miR-767-5p,miR-5100,miR-483-3p,miR-378h,miR-128-3p,miR-496,miR-376c-5p,miR-1957a,miR-3065-3p,miR-490-3p,miR-30a-3p,miR-
TNRC6C	ENSOARG0(	trinucle	miR-320c,miR-6517,miR-2300a-5p,miR-542-5p,miR-4429,miR-101c,miR-2284r,miR-320a,miR-2428,novel_42,miR-181a-2-3p,miR-4286,miR-128-1-5p,miR-320d,miR-106b-3p,miR-4492,miR-320b,miR-208a-3p,miR-5126,miR-208b-3p
LRRC20	ENSOARG0(	leucine	miR-330-5p,miR-3120-5p,miR-128-3p,miR-1224-5p,miR-2300a-5p,miR-133b-5p,miR-216a-3p,miR-134-3p,miR-326,miR-214-3p,miR-760-3p,miR-3085-3p,miR-3064-5p,miR-2483-5p,miR-143-3p,miR-490-3p,miR-24-2-5p,miR-216a-3p,miR-2285ab,miR-7144-5p,miR-450b-3p,miR-2312,miR-552-3p,miR-3074-5p,miR-3064-5p,miR-2331-3p,miR-24-1-5p,miR-204-3p,miR-378g,miR-656-5p,miR-203b-5p,miR-214,miR-2285f,miR-6527,miR-3074-5p,miR-2447,miR-326-3p,miR-6525

PIP4K2C	ENSOARG0(	phosphat	miR-15b-5p, miR-22-3p, miR-199a-5p, miR-769, miR-193b-3p, miR-17-5p, miR-93-5p, miR-450a-1-3p, miR-20b-5p, miR-16-5p, miR-1260b, miR-769-5p, miR-93, miR-424-5p, miR-450b-3p, miR-412, miR-106a, miR-328-3p, miR-450a-1-3p, miR-15a-5p, miR-383-5p, miR-322-5p, miR-188-3p, novel_133, miR-20a, miR-199a-5p, miR-142a-3p, miR-2478, miR-1260a, miR-106a, miR-3120-3p, miR-452-3p, miR-3596, miR-146b-3p, miR-199b-5p, miR-6395, miR-195a-5p, novel_101, miR-193b-3p, miR-199b-5p, miR-361-3p, miR-16a, miR-769-5p, miR-20a-5p, miR-15a, miR-499a-5p, miR-421, miR-20b, miR-142-3p, miR-412-3p, miR-450c-3p, miR-22-3p, miR-6529a, miR-497-5p, miR-1260b, miR-16b, miR-101-5p, miR-424-5p, miR-106a-5p, miR-3187-3p, miR-16b, miR-106b-5p, miR-17-5p, miR-106a-5p, miR-505, miR-193a-3p, miR-199b-5p
PLS1	ENSOARG0(	plastin	miR-128-3p, miR-1290, miR-3591-5p, miR-582-3p, miR-758-5p, miR-429-3p, miR-299a-3p, miR-299-3p, miR-21-3p, miR-1277-5p, miR-6536, miR-181b-5p, miR-200c-3p, miR-127-5p, miR-2284m, miR-17-3p, miR-200c, miR-2285af, miR-490-3p, miR-192-3p, miR-2432, miR-29b-1-5p, miR-2312, miR-411, miR-137-3p, miR-200a-5p, miR-204-3p, miR-216b-5p, miR-2285aa, miR-17-3p, miR-19b-1-5p, miR-5100, miR-2284k, miR-181c-5p, novel_44, miR-142a-5p, miR-3607-3p, miR-6128, miR-145a-3p, miR-2284v, miR-27b-3p, miR-2284x, miR-3120-5p, miR-101c, miR-16-1-3p, miR-2284ab, novel_23, miR-323b, miR-181a-5p, miR-1434-3p, miR-873a-5p, miR-708-3p, miR-19b-2-5p, miR-216a-3p, miR-142-5p, miR-27a-3p, miR-548w, miR-3955-3p, miR-323b-3p, miR-29b-1-5p, miR-1b-5p, miR-8485, miR-376b, miR-382, miR-382-3p, miR-6516-3p, miR-3065-5p, miR-216a-3p, miR-378j, miR-2285ab, miR-27a-3p, miR-299b-3p, miR-7-1-3p, miR-541-5p, novel_1, miR-133a-5p, miR-101-3p, miR-8095, miR-2284j, miR-17-3p, miR-181d-5p, miR-2285f, miR-2447

CCDC109B	ENSOARG0(	coiled-c	miR-106a,miR-2284u,miR-193b-5p,miR-3781,miR-378a-3p,miR-181b-1-3p,miR-143-3p,miR-486-3p,miR-2484,novel_17,miR-345-3p,miR-422a,miR-500a-5p,miR-216b-5p,miR-582-5p,miR-15b-5p,miR-30c-1-3p,miR-2331-3p,miR-345-5p,miR-186-5p,miR-411,miR-3074-5p,miR-590-3p,novel_74,miR-190a-5p,miR-2285j,miR-665,miR-151-3p,miR-539-5p,miR-1298-3p,miR-2459,miR-455-3p,miR-1343-3p,miR-145b,miR-181b-2-3p,miR-1388-5p,miR-101c,miR-758-3p,miR-378d,miR-1b-5p,miR-8485,miR-1388-3p,miR-103a-2-5p,miR-16b,miR-106b-5p,miR-29b-1-5p,miR-2319b,miR-139-5p,miR-211-5p,miR-668-5p,miR-544-5p,miR-1983,miR-493-5p,miR-140-5p,miR-877-3p,miR-7705,miR-2447,miR-190b-5p,miR-2284j,miR-499a-5p,miR-322-5p,miR-2284z,miR-299a-3p,miR-330-5p,miR-23b-5p,miR-1343-5p,miR-2285b,novel_39,miR-200a,miR-29a-5p,miR-3064-5p,miR-6536,miR-127-5p,miR-331-5p,miR-2318,miR-345-5p,miR-20b-5p,miR-331-5p,miR-93-5p,miR-16-5p,miR-331-5p,novel_63,miR-2284k,miR-412-5p,miR-411-5p,miR-194-3p,novel_116,miR-3955-5p,miR-16b,miR-188-5p,miR-2284x,miR-497-5p,miR-223-3p,miR-2113,miR-19b-2-5p,miR-432-3p,miR-432-3p,miR-503-3p,let-7a-2-3p,miR-493-5p,miR-380-5p,miR-194a,miR-2411-3p,miR-105-5p,miR-299b-3p,miR-181b-3p,miR-6535,miR-320c,miR-677,miR-3074-5p,miR-3120-3p,miR-412-3p,miR-378f,miR-2319a,miR-3065-5p,miR-3591-3p,miR-150-5p,miR-625-3p,novel_87,miR-340-5p,miR-15a-5p,miR-4429,miR-1246,miR-215-5p,novel_133,miR-1197-5p,miR-5703,miR-2904,miR-130a-5p,miR-29b-1-5p,miR-20a,miR-106a,miR-2284m,miR-320d,miR-299-3p,miR-21-3p,miR-30b-3p,miR-216a-5p,miR-139-5p,miR-544-3p,miR-192-5p,miR-877-5p,miR-204-3p,miR-140-5p,miR-378a-3p,miR-21-3p,miR-154b-3p,let-7g-3p,miR-21a-3p,miR-145a-3p,miR-378c,miR-539-5p,miR-425-5p,miR-17-3p,miR-424-5p,miR-29b-2-5p,miR-
GAREM	ENSOARG0(	GRB2 ass	miR-361-3p,miR-17-3p,miR-214,miR-219b-3p,novel_42,miR-486b-3p,miR-28-5p,miR-708-5p,miR-4492,miR-17-3p,miR-128-1-5p,novel_69,miR-670-3p,miR-499b-5p,miR-3065-3p,miR-1983,miR-412-3p,novel_91,miR-361-3p,miR-486-3p,miR-2432,miR-3065-3p,miR-4532,miR-495-5p,miR-127-3p,miR-214-3p,miR-17-3p,novel_19,miR-5126,miR-412-5p,miR-222-5p,miR-412,miR-2300a-5p,miR-744-5p,miR-2426,miR-412-3p,miR-1290,miR-484
TMEM57	ENSOARG0(	transmem	miR-548e-3p,miR-3600,miR-432-5p,miR-7859,miR-411-5p,miR-148b-5p,miR-1185-3p,miR-432,miR-329-3p,miR-19b-2-5p,miR-10b,miR-542-3p,miR-362-3p,miR-124-3p,miR-188-3p,miR-362-3p,miR-10a-5p,miR-148a-5p,miR-2284u,miR-124a,let-7f-2-3p,let-7f-2-3p,miR-2284b,miR-148b-5p,miR-200a,miR-2284y,miR-2284a,miR-362-3p,miR-466f-3p,miR-214-3p,miR-2484,miR-21-3p,miR-2284g,miR-452-3p,miR-499b-5p,miR-532-5p,miR-10a-5p,miR-10a,miR-6535,miR-154b-5p,miR-1185-2-3p,miR-378b,miR-133b-3p,miR-450b-3p,miR-412-3p,miR-329a,miR-7144-5p,miR-625-5p,miR-335-3p,miR-1983,miR-3059-5p,miR-133a-3p,miR-214,novel_101,miR-10b-5p,novel_82,miR-19b-1-5p,miR-665

ZFP36	ENSOARG0(	ZFP36 ri	miR-29b,miR-1260b,miR-2426,miR-16-2-3p,miR-744-5p,miR-2284x,miR-296-5p,miR-145a-5p,miR-496-5p,miR-134-3p,miR-221-3p,miR-149-5p,miR-145b,miR-491-5p,miR-483-3p,miR-146b,miR-199b-5p,miR-326,miR-146a,miR-2331-5p,novel_51,miR-182-5p,miR-30c-1-3p,miR-6412,miR-1388-3p,miR-494-3p,miR-3963,miR-412-3p,miR-668-5p,miR-146b-5p,miR-135b-5p,miR-1983,miR-361-3p,miR-2285ab,miR-199b-5p,miR-1306-3p,miR-378g,novel_69,miR-2483-3p,miR-29a-3p,novel_68,miR-3074-5p,miR-320b,miR-211-5p,miR-3968,miR-29a,miR-147-5p,miR-615,miR-142b,miR-371a-5p,miR-2285y,miR-211,miR-24-3p,miR-326-3p,miR-1285-5p,miR-199b-5p,miR-136-3p,miR-2285f,novel_101,miR-29c-3p,miR-17-3p,miR-184-3p,miR-300,novel_133,miR-615-3p,miR-29d-3p,miR-1434-5p,miR-330-5p,miR-483-3p,miR-2411-5p,miR-151-5p,miR-532-3p,miR-222,miR-2285ad,miR-7641,miR-1224-5p,miR-17-3p,miR-760-3p,miR-1277-5p,novel_96,miR-1260a,miR-125a-3p,miR-324-3p,miR-30c-2-3p,miR-2411,novel_39,miR-146a-5p,miR-199a-5p,miR-2432,miR-195a-3p,miR-5100,miR-2312,miR-876-3p,miR-485-5p,miR-381-3p,miR-598-3p,miR-200a-5p,miR-27a-5p,miR-2284s,miR-199a-5p,miR-151b,miR-30c-1-3p,miR-2355-5p,miR-598-3p,miR-182-5p,miR-17-3p,miR-708-5p,miR-204-5p,miR-28-5p,miR-29b-3p,miR-2433,miR-551b-3p,miR-1306-3p,miR-338-5p,miR-135a-5p,miR-1260b,novel_42,miR-181a-2-3p,miR-3074-5p,miR-3074-2-3p

SDF4	ENSOARG0(	stromal	miR-2355-5p,miR-125a,miR-874-3p,miR-300-3p,miR-23b,miR-193a-5p,novel_42,miR-1260b,miR-1197-3p,miR-125b-5p,miR-6740-5p,miR-592,novel_79,miR-1224-5p,miR-412,miR-6517,miR-505-5p,miR-1343-5p,miR-330-5p,miR-196a-5p,miR-885-3p,miR-3064-5p,miR-200a,novel_39,miR-3956-5p,miR-2285b,miR-192-3p,miR-148b-5p,miR-125a-3p,miR-200a-3p,novel_127,miR-127-5p,miR-3120-3p,novel_68,miR-669,miR-150-5p,miR-1306,miR-216a-3p,miR-378f,miR-412-3p,miR-193b-3p,miR-29c-3p,miR-199b-5p,miR-208a-3p,miR-28c,miR-29a,miR-487b-3p,miR-339-5p,miR-149-5p,miR-194-3p,miR-412-5p,miR-3431,miR-134-3p,miR-197-3p,miR-7b-5p,miR-628-5p,novel_51,miR-2411-3p,miR-7-5p,miR-193a-3p,miR-378a-5p,miR-483-3p,miR-196b-5p,miR-552-3p,miR-2331-3p,miR-504-5p,miR-10a,miR-2284s,miR-2285c,miR-885-3p,miR-543-3p,miR-1827,miR-339b,miR-665-5p,miR-214,miR-30a-3p,miR-664a-5p,miR-665,miR-30e-3p,miR-874-5p,miR-4492,miR-30d-3p,miR-212-5p,miR-18a-3p,miR-2284h-5p,miR-23b-3p,miR-23c,miR-378a-3p,miR-378i,miR-2366,miR-193b-5p,miR-133a-3p,miR-34a-5p,miR-33b-3p,miR-195a-3p,miR-2285w,miR-486-3p,miR-219b-5p,miR-3601,miR-324-3p,miR-422a,miR-345-3p,novel_17,miR-208b-3p,miR-210-5p,miR-2484,miR-7689-3p,miR-24-1-5p,miR-4792,miR-2319b,miR-4443,miR-378g,miR-1983,miR-504,miR-2447,miR-7705,miR-877-3p,miR-361-3p,miR-218-5p,miR-145b,novel_78,miR-1343-3p,miR-1298-3p,miR-147a,miR-2284r,miR-541,miR-23a-3p,miR-676-3p,miR-744-3p,miR-574-3p,miR-378d,miR-505,miR-199b-5p,miR-214-3p,miR-1185-5p,miR-370-3p,miR-141-3p,miR-615-5p,miR-199a-5p,miR-34c-5p,miR-3154,miR-3074-1-3p,miR-485-5p,miR-671-5p,miR-6128,miR-378c,miR-145-3p,miR-331-3p,miR-3969,miR-551b-3p,miR-29b-3p,miR-370-5p,miR-31-3p,miR-486b-3p,miR-28-5p,miR-378b,miR-483-3p,miR-29d-3p,miR-345-3p,miR-128-

MEP1B	ENSOARG0(	meprin A	miR-2411-3p,miR-130b-5p,miR-345-5p,miR-432-3p,miR-432-3p,miR-2284l,miR-22-3p,miR-1285,miR-181a-5p,miR-1434-3p,miR-188-5p,miR-29b,miR-2284ab,miR-380-3p,miR-411-5p,miR-2284d,miR-134-3p,miR-185-5p,miR-142b,miR-421-5p,miR-147-5p,miR-29a,miR-379-3p,novel_1,miR-29c-3p,miR-1193,miR-219b-3p,miR-199b-5p,miR-2428,miR-3959-3p,miR-18a-3p,miR-2285ab,miR-2285k,miR-3120-3p,miR-320c,miR-669,miR-29a-3p,miR-30c-2-3p,miR-181c-3p,miR-2285af,miR-200a-3p,novel_127,miR-127-5p,miR-181b-5p,miR-3064-5p,miR-181a-2-3p,miR-2403,miR-199a-5p,miR-200a,novel_39,miR-2284a,miR-490-3p,miR-29d-3p,miR-345-3p,miR-134,miR-134-5p,miR-758-5p,miR-3969,miR-29b-3p,miR-410-5p,novel_63,miR-2332,miR-370-5p,miR-134-5p,miR-486b-3p,miR-6128,miR-1306-5p,miR-2284v,miR-323-3p,miR-3154,miR-320a,miR-381-3p,miR-671-5p,miR-345-5p,miR-2355-5p,miR-141-3p,miR-199a-5p,miR-154b-5p,miR-300-3p,miR-320b,novel_115,miR-199b-5p,miR-3085-3p,miR-2284b,miR-8485,miR-30c-1-3p,miR-125b-2-3p,miR-301b-5p,miR-16-2-3p,miR-2426,miR-9788-3p,miR-148b-5p,miR-216a-3p,miR-421,miR-425-3p,miR-301a-5p,novel_82,miR-382-3p,miR-490-5p,miR-2284q,miR-2285f,miR-181d-5p,miR-423-5p,miR-1285-5p,miR-381-3p,miR-361-3p,miR-140-5p,miR-199b-5p,miR-9-3p,novel_94,miR-499b-5p,miR-126b-5p,miR-3064-5p,miR-203-3p,miR-378g,miR-2483-3p,miR-219b-5p,miR-216a-5p,miR-324-3p,miR-345-3p,novel_17,miR-221-5p,miR-1277-5p,miR-3653-3p,miR-2484,miR-320d,miR-195a-3p,miR-183-5p,miR-216c-5p,miR-486-3p,miR-380-3p,miR-664b-3p,miR-2285i,miR-5703,miR-1197-5p,miR-2300b-3p,miR-133a-3p,miR-148a-5p,miR-4429,miR-3600,miR-18a-3p,miR-616-3p,novel_83,miR-320e,miR-2285j,miR-4492,miR-130b-5p,miR-425-5p,miR-539-5p,miR-145a-3p,miR-3184-5p,miR-181c-5p,miR-6525,miR-129b-
COL4A1	ENSOARG0(	collagen	miR-767-5p,miR-29a,miR-424-5p,miR-2433,miR-29b-3p,miR-15a,miR-222-3p,miR-16a,miR-145-3p,miR-4510,miR-16-5p,miR-29c-3p,novel_101,miR-9-5p,miR-767,miR-10b-3p,miR-195a-5p,miR-671-5p,miR-221,miR-377-3p,miR-6130,novel_69,miR-29a-3p,miR-6535,miR-15b-5p,miR-378b,miR-377-3p,miR-324-3p,miR-16b,miR-185-3p,miR-4532,miR-424-5p,miR-363-5p,miR-676-3p,miR-29b,miR-16b,miR-497-5p,miR-29d-3p,miR-9-5p,miR-322-5p,miR-15a-5p,miR-221-3p,miR-421,miR-222

GPATCH4	ENSOARG0(	G patch	miR-1248,miR-329b,miR-345-5p,miR-300-3p,miR-2285aa,miR-874-3p,miR-574-5p,miR-134-5p,miR-2433,novel_42,miR-330-5p,miR-2427,miR-486-5p,miR-129-1-3p,novel_121,miR-2284z,miR-134-5p,miR-450a-1-3p,miR-505-5p,novel_79,novel_96,miR-200a-3p,novel_127,miR-127-5p,let-7f-5p,miR-2285b,let-7g,miR-378f,let-7e-5p,miR-3065-5p,miR-181b-3p,miR-6535,miR-677,miR-144-3p,miR-374c-3p,miR-411b-3p,miR-6123,miR-576-3p,miR-28c,miR-26a-2-3p,miR-381-5p,miR-450c-3p,miR-22-3p,miR-1843b-5p,miR-134-3p,miR-339-5p,miR-103a-3p,miR-106b-3p,miR-483-3p,miR-378a-5p,miR-194a,miR-2411-3p,miR-365b-5p,let-7f-2-3p,let-7f-2-3p,miR-668-3p,miR-6240,miR-339b,miR-345-5p,miR-30d-5p,miR-450a-1-3p,let-7i-5p,miR-552-3p,miR-2331-3p,miR-130b-5p,miR-4492,miR-129-5p,miR-665,let-7f,miR-122-5p,miR-3184-5p,miR-224-5p,miR-129-2-3p,let-7b,miR-361-5p,miR-133a-3p,miR-33b-3p,miR-378a-3p,miR-1961,miR-378i,miR-30c-5p,miR-328-3p,miR-18a-3p,let-7k,miR-98-5p,miR-422a,miR-345-3p,miR-92a-1-5p,miR-219b-5p,miR-324-3p,novel_17,miR-618,miR-143-3p,miR-486-3p,miR-181b-1-3p,miR-377-5p,miR-668-5p,miR-136-5p,miR-3963,miR-1306-3p,miR-140-5p,miR-1185-2-3p,miR-203-3p,miR-378g,miR-25,miR-3968,miR-301a-5p,miR-2440,miR-423-5p,miR-877-3p,miR-6527,miR-885-5p,miR-2447,miR-301b-5p,miR-1185-3p,miR-2459,miR-496-5p,miR-181b-2-3p,miR-1343-3p,miR-330-3p,novel_78,miR-381-5p,miR-103a-2-5p,miR-491-5p,miR-505,novel_19,miR-378d,miR-124a,miR-7144-5p,miR-3074-1-3p,miR-3154,miR-365a-3p,miR-141-3p,miR-28-5p,miR-486b-3p,miR-138-5p,miR-3969,miR-144,miR-331-3p,miR-5100,miR-1306-3p,miR-410-5p,miR-378c,miR-365a-5p,miR-133a-3p,miR-1306-5p,let-7d,miR-30a-5p,miR-134,miR-345-3p,miR-214-5p,miR-378h,miR-483-3p,miR-7641,miR-107,miR-17-3p,miR-
WFDC8	ENSOARG0(	WAP four	novel_78,miR-548w,miR-126b-5p,miR-182-5p,miR-3431,miR-300-3p,miR-505-5p,miR-129-1-3p,miR-455-3p,miR-532-3p,miR-140-3p,miR-7144-5p,miR-129-2-3p,miR-224-5p,miR-450b-5p,miR-224-5p,miR-182-5p

RMND5B	ENSOARG0(	required	miR-345-3p,miR-3064-5p,miR-376c-3p,miR-125b-2-3p,miR-29b-1-5p,miR-2284y,miR-542-5p,miR-92a-1-5p,miR-422a,miR-3071-3p,miR-127-5p,miR-21-3p,miR-152-3p,miR-212-5p,miR-148b-3p,miR-483-3p,miR-378h,miR-129-1-3p,novel_83,miR-378i,miR-1197-5p,miR-140-3p,miR-378a-3p,miR-5703,miR-33b-3p,miR-2284u,miR-129-2-3p,miR-3074-5p,miR-378c,miR-214,miR-378c,miR-2332,miR-410-5p,miR-100-3p,miR-2331-3p,miR-378a-3p,miR-296-3p,miR-1271,miR-2285aa,miR-2355-5p,miR-370-3p,miR-139-5p,miR-376e-3p,miR-376c-3p,miR-22-3p,miR-21a-3p,let-7g-3p,miR-671-5p,miR-485-5p,miR-2330-5p,let-7c-3p,miR-378d,miR-8485,miR-296-3p,miR-296-3p,miR-7-5p,miR-29b-1-5p,miR-3141,miR-483-3p,miR-3085-3p,miR-214-3p,miR-542-5p,miR-548w,miR-2300a-5p,miR-421,novel_116,miR-216a-3p,miR-194-3p,miR-7b-5p,miR-1285,miR-541,miR-223-3p,miR-378b,miR-6529a,miR-30b-3p,miR-22-3p,miR-30b-3p,miR-378c,miR-188-5p,miR-541-3p,novel_101,miR-542-5p,novel_82,miR-6123,miR-3074-5p,miR-2319b,miR-3064-5p,miR-139-5p,miR-2284g,miR-3120-3p,miR-378e,miR-669,miR-203-3p,miR-148a-3p,miR-3141,miR-3065-5p,miR-150-5p,miR-1291,miR-378f
NSMCE4A	ENSOARG0(	NSE4 hom	miR-542-5p,miR-323c,miR-502-5p,miR-15a,miR-16a,miR-206,miR-6119-3p,miR-203b-5p,miR-329-5p,novel_68,miR-3120-3p,miR-320b,miR-374c-3p,miR-323a-5p,miR-181b-3p,miR-320c,miR-677,miR-2387,miR-378b,miR-150-5p,miR-1306,miR-107,miR-505-3p,miR-195a-5p,miR-2319a,miR-493-5p,miR-363-5p,miR-654-3p,let-7a-2-3p,miR-539-3p,miR-296-3p,miR-296-3p,miR-105-5p,miR-378a-5p,miR-1343-5p,miR-103a-3p,miR-432-5p,miR-103,miR-149-5p,miR-432,miR-145a-5p,miR-138,miR-16b,miR-2284x,miR-27b-3p,miR-26a-2-3p,miR-497-5p,miR-16-5p,miR-6128,miR-1197-3p,miR-145-3p,miR-138-5p,miR-134-5p,miR-487a-3p,miR-2285aa,miR-301,miR-3154,miR-320a,miR-200a-5p,let-7g-3p,miR-1,miR-30a-3p,miR-29b-2-5p,miR-3065-3p,miR-6119-5p,miR-664-3p,miR-2285af,miR-2898,miR-107,miR-222,miR-7641,miR-2411-5p,miR-322-5p,miR-134-5p,miR-582-3p,miR-134,miR-1343-5p,let-7a-2-3p,miR-105-2,miR-3956-3p,novel_60,miR-3059-5p,miR-7705,miR-301a-5p,miR-20a-3p,miR-664b,miR-139-5p,miR-489,miR-27a-3p,miR-203-3p,miR-221,miR-129b-5p,miR-381-3p,novel_94,miR-493-5p,miR-424-5p,miR-652-5p,miR-4532,miR-1306-5p,miR-320b,miR-182-5p,miR-16b,miR-29b-1-5p,miR-542-5p,miR-153-3p,miR-105-1,miR-27a-3p,miR-181b-2-3p,miR-145b,miR-1298-3p,miR-153,miR-421,miR-221-3p,miR-9788-3p,miR-301b-5p,miR-1a-3p,miR-2426,miR-206-3p,miR-98-3p,miR-30e-3p,miR-222-3p,let-7b-3p,miR-29b-2-5p,miR-664a-5p,miR-30a-3p,miR-487b-5p,novel_74,miR-424-5p,miR-182-5p,miR-296-3p,miR-3065-3p,miR-139-5p,miR-323-5p,miR-582-5p,miR-15b-5p,let-7f-1-3p,miR-21a-3p,miR-433-3p,miR-129b-3p,miR-1827,miR-582-5p,miR-625-5p,miR-543-3p,miR-487a,miR-105-3p,miR-329a-5p,miR-29b-1-5p,miR-143-3p,miR-2424,miR-2411,miR-2285p,miR-542-5p,miR-210-5p,miR-320d,miR-21-3p,miR-1277-5p,miR-505-3p,miR-30d-3p,miR-

ZNF618	ENSOARG0(	zinc fin	miR-411,miR-216a-3p,miR-7144-5p,miR-1248,miR-677,miR-6535,miR-22-3p,miR-500-3p,miR-181b-3p,miR-2433,miR-5100,miR-1973,miR-425-5p,miR-450a-5p,miR-486b-3p,miR-130b-5p,miR-224-5p,miR-502-3p,miR-2408,miR-7b-5p,miR-22-3p,miR-501-3p,miR-140-3p,miR-501-3p,miR-2426,miR-128-3p,novel_78,miR-181b-2-3p,miR-505-5p,miR-147a,novel_121,miR-224-5p,miR-7-5p,miR-2397-5p,miR-2898,miR-378a-5p,miR-3955-3p,miR-466i-5p,miR-130b-5p,let-7c-3p,miR-363-5p,miR-181b-1-3p,miR-130a-5p,miR-125b-2-3p,miR-8485,miR-486-3p,miR-450c-5p
BBS9	ENSOARG0(	Bardet-B	miR-431,miR-2285ad,miR-582-3p,miR-322-5p,miR-758-5p,miR-30a-5p,miR-1290,miR-3064-5p,miR-181a-2-3p,miR-4286,miR-200a,miR-490-3p,miR-450b-5p,miR-380-5p,miR-217,miR-2285af,miR-107,miR-543-5p,miR-17-3p,miR-127-5p,miR-2355-5p,miR-2285aa,miR-370-3p,miR-574-5p,miR-487a-3p,miR-137-3p,miR-2448-3p,miR-1814c,miR-345-5p,miR-16-5p,miR-144-5p,novel_63,miR-204-5p,miR-31-5p,miR-103a-3p,miR-103,miR-432-5p,miR-22-3p,miR-432,miR-497-5p,miR-26a-2-3p,miR-188-5p,miR-16b,miR-380-5p,miR-539-3p,let-7j,miR-345-5p,miR-654-3p,miR-377-3p,miR-660,miR-3120-3p,miR-670-3p,miR-2387,miR-195a-5p,miR-107,novel_101,miR-17-3p,miR-374c-5p,miR-211,miR-16a,miR-15a,miR-133a-5p,miR-656-5p,miR-502b,miR-485-3p,miR-548e-3p,miR-7859,miR-487a-3p,miR-15a-5p,miR-2904,miR-217-5p,novel_133,miR-664-5p,miR-133a-3p,miR-361-5p,miR-369-3p,miR-487a,miR-618,miR-221-5p,miR-216b-3p,miR-154a-3p,miR-140-5p,miR-221-5p,miR-660-5p,miR-548o-3p,novel_25,miR-22-3p,miR-15b-5p,miR-377-3p,miR-2284s,miR-129b-3p,miR-345-5p,miR-154b-3p,miR-145a-3p,miR-320e,miR-424-5p,miR-129-5p,miR-17-3p,miR-758-3p,miR-2284r,miR-101a-5p,miR-33a-5p,miR-424-5p,miR-758-3p,miR-2310,miR-16b,miR-431-5p,miR-103a-2-5p,miR-3085-3p,miR-3955-3p,miR-211-5p,miR-7977,miR-3064-5p,miR-452-3p,miR-20a-3p,miR-144-5p,miR-7857-3p,miR-2483-3p,miR-378j,miR-219a-5p,miR-3059-5p,miR-2285y,miR-107-5p,miR-218-5p,miR-2285n,miR-154-3p,miR-3968,miR-3958-3p,miR-4726-5p

SUPT5H	ENSOARG0(	suppress	miR-2478,miR-199a-5p,novel_39,miR-3065-3p,miR-490-3p,miR-450c-5p,miR-181b-5p,miR-6536,miR-107,novel_96,miR-6119-5p,miR-125a-3p,miR-30f,miR-339a,miR-505-5p,miR-378h,miR-214-5p,miR-129-1-3p,miR-6517,miR-412,novel_79,miR-7641,miR-411-3p,miR-196a-5p,miR-330-5p,miR-30a-5p,miR-1197-3p,miR-378c,novel_42,miR-193a-5p,miR-6128,miR-767-5p,miR-592,miR-486b-3p,miR-331-3p,miR-199a-5p,miR-154b-5p,miR-2355-5p,miR-2285aa,miR-615-5p,miR-876-3p,miR-767,miR-329a,miR-3074-1-3p,miR-1248,miR-30e-5p,miR-503-3p,miR-296-3p,miR-503-3p,miR-363-5p,miR-196b-5p,miR-378a-5p,miR-296-3p,miR-377-3p,novel_51,miR-412-5p,miR-3431,novel_116,miR-2355-3p,miR-103,miR-103a-3p,miR-339-5p,miR-377-5p,miR-26b-3p,miR-219a-1-3p,miR-16-1-3p,miR-2284x,miR-7862,miR-2889,miR-873a-5p,miR-1434-3p,miR-181a-5p,miR-197-3p,miR-24-3p,miR-326-3p,miR-199b-5p,miR-1839-3p,novel_101,miR-128-1-5p,miR-379-3p,miR-22-5p,miR-502-5p,miR-6529b,miR-670-3p,miR-669,miR-216b-3p,miR-655-5p,miR-3120-3p,miR-1291,miR-107,miR-2319a,miR-378f,miR-377-5p,miR-486-3p,miR-216c-5p,miR-195a-3p,miR-30b,novel_17,miR-422a,miR-487a-3p,miR-222-5p,miR-199a-3p,miR-485-3p,miR-664-5p,miR-16-1-3p,miR-378i,miR-30c-5p,miR-362-3p,miR-2904,miR-1197-5p,miR-378a-3p,miR-5703,miR-6525,miR-6134,miR-338-3p,miR-181c-5p,miR-378c,miR-145a-3p,miR-4510,miR-129-2-3p,miR-665-5p,miR-4492,miR-324-3p,miR-320e,miR-377-3p,miR-139-5p,miR-216b-5p,miR-6130,miR-378a-3p,miR-216b-3p,miR-3065-3p,miR-339b,miR-30f,miR-186-5p,miR-21a-3p,miR-30d-5p,miR-378d,miR-4532,miR-193a,miR-1839-3p,miR-491-5p,miR-487b-5p,miR-199b-5p,miR-199a-3p,miR-5126,miR-326,miR-362-3p,miR-455-3p,miR-1343-3p,novel_78,miR-378c,miR-362-3p,miR-329-3p,miR-378b,miR-412-3p,miR-125b-2-3p,miR-
PREPL	ENSOARG0(	prolyl e	miR-3187-3p,miR-432-3p,miR-378d,miR-424-5p,miR-1185-5p,miR-455-5p,miR-16b,miR-326,miR-103,miR-2459,miR-145b,novel_4,miR-103a-3p,miR-1343-3p,miR-497-5p,miR-455-5p,miR-378c,miR-16b,miR-450b-5p,miR-145a-5p,miR-378b,miR-326-3p,miR-2285f,miR-203b-5p,miR-147-5p,novel_1,miR-16a,miR-218-5p,miR-15a,miR-142b,miR-6535,miR-378e,novel_69,miR-378b,miR-7975,miR-195a-5p,miR-378f,miR-107,miR-129b-5p,miR-2285ab,miR-107,miR-21-3p,miR-2898,miR-422a,miR-129-1-3p,miR-383-5p,miR-322-5p,miR-15a-5p,miR-378h,miR-6516-5p,miR-582-3p,miR-142-3p,miR-33b-3p,miR-330-5p,miR-378a-3p,miR-378i,miR-2300b-3p,miR-378c,miR-16-5p,miR-378c,miR-129-2-3p,miR-145a-3p,miR-424-5p,miR-767-5p,miR-15b-5p,miR-6516,miR-2285aa,miR-378a-3p,miR-1248,novel_99,miR-2312,miR-767,miR-3154

RASL10B	ENSOARG00	RAS-like	miR-188-5p,miR-22-3p,miR-542-3p,miR-7b-5p,miR-1843b-5p,novel_116,miR-3431,miR-134-3p,miR-339-5p,miR-31-5p,miR-193a-3p,novel_51,miR-105-5p,miR-377-3p,miR-7-5p,miR-432-3p,miR-628-5p,miR-380-5p,miR-378f,let-7e-5p,miR-412-3p,miR-3970,miR-1306,miR-150-5p,miR-6535,miR-669,miR-374c-3p,miR-216b-3p,novel_68,miR-29a,miR-4324,miR-29c-3p,miR-758-5p,miR-1839-3p,miR-193b-3p,miR-335-5p,miR-330-5p,miR-1343-5p,miR-129-1-3p,miR-758-5p,miR-505-5p,miR-9-5p,novel_79,miR-412,miR-6517,miR-2285ad,novel_127,novel_96,miR-127-5p,let-7f-5p,miR-125a-3p,let-7g,miR-200a,miR-29a-5p,miR-1248,miR-876-3p,miR-767,miR-301,miR-376c-3p,miR-125a,miR-2285aa,miR-2355-5p,miR-874-3p,miR-125b-5p,miR-2433,miR-1260b,miR-193a-5p,miR-1839-5p,miR-1388-5p,miR-301b-5p,miR-455-3p,miR-147a,miR-1298-3p,miR-145b,miR-1343-3p,miR-214-3p,miR-491-5p,miR-8485,miR-378d,miR-127-3p,miR-1983,miR-504,miR-203-3p,miR-3596,miR-378g,miR-211-5p,miR-4792,miR-3968,miR-301a-5p,novel_82,miR-423-5p,novel_111,miR-877-3p,miR-361-3p,miR-9-5p,miR-2284j,miR-184-3p,miR-493-3p,miR-378a-3p,miR-1961,miR-378i,miR-7859,miR-212-5p,miR-380-5p,miR-18a-3p,let-7k,miR-98-5p,miR-202-5p,miR-422a,miR-92a-1-5p,miR-324-3p,novel_17,miR-618,miR-486-3p,miR-376c-3p,miR-377-5p,miR-664b-3p,miR-345-3p,miR-1827,miR-186-5p,miR-339b,miR-2285c,let-7i-5p,miR-376e-3p,miR-377-3p,miR-10a,miR-216b-3p,miR-548o-3p,miR-504-5p,miR-874-5p,miR-4492,miR-665,let-7f,miR-2285j,miR-3184-5p,miR-346,miR-224-5p,miR-6525,miR-769-5p,miR-665-5p,miR-214,miR-129-2-3p,let-7b,miR-455-5p,miR-1260b,miR-138,miR-30b-3p,miR-29b,miR-2284ab,miR-145a-5p,miR-873a-5p,miR-185-5p,let-7g-5p,miR-2285g,miR-2284d,miR-26b-3p,miR-377-5p,miR-133b-5p,miR-125a-5p,miR-1343-5p,miR-455-5p,miR-
GRIA2	ENSOARG00	glutamat	miR-483-3p,miR-2404,miR-483-3p,miR-101c
ISG20L2	ENSOARG00	interfer	novel_82,miR-218-5p,miR-107-5p,miR-29a,miR-628-3p,miR-29c-3p,miR-194b-5p,miR-1193,miR-211,miR-18b,miR-3591-3p,miR-135b-5p,miR-146b-5p,miR-10a-5p,miR-211-5p,miR-29a-3p,miR-3596,miR-181b-3p,miR-4443,miR-677,miR-146a,miR-224-5p,novel_51,miR-194a,miR-2331-5p,miR-330-3p,miR-103a-2-5p,miR-146b,miR-6240,miR-33a-5p,miR-652-5p,miR-101-5p,miR-8485,miR-10b,miR-194-5p,miR-22-3p,miR-450c-3p,miR-29b,miR-138,miR-330-3p,miR-1343-3p,miR-181b-2-3p,miR-370-5p,miR-29b-3p,miR-135a-5p,miR-6740-5p,miR-132-5p,miR-664a,miR-767-5p,miR-138-5p,miR-450b-3p,miR-204-5p,miR-103b,miR-224-5p,miR-10b-5p,miR-450a-1-3p,miR-488-3p,miR-18a,miR-18a-5p,miR-767,miR-7144-5p,miR-552-3p,miR-370-5p,miR-21-3p,miR-10a,miR-22-3p,miR-125a-3p,novel_17,miR-6119-5p,miR-196a-3p,miR-324-3p,miR-2898,miR-181b-1-3p,miR-125b-2-3p,miR-146a-5p,miR-193b-5p,miR-29d-3p,miR-10a-5p,miR-6517,miR-18b-5p,miR-24-2-5p,miR-450a-1-3p,miR-532-3p,miR-1247-5p

CD300LG	ENSOARG0(	CD300 mo	miR-216a-5p,miR-125b,miR-320d,miR-760-3p,miR-4661-5p,miR-221-5p,miR-34b-5p,miR-21-3p,miR-299-3p,miR-2887,miR-29b-1-5p,miR-130a-5p,miR-146a-5p,miR-362-3p,miR-1246,miR-2904,miR-188-3p,miR-1197-5p,miR-140-3p,miR-5703,miR-4429,miR-548e-3p,miR-148b-3p,miR-532-3p,miR-616-3p,miR-19b-1-5p,miR-320e,miR-29b-2-5p,miR-450b-3p,miR-17-3p,miR-664a,miR-708-5p,miR-487a-5p,miR-378c,miR-539-5p,miR-145a-3p,miR-3074-2-3p,miR-10b-5p,miR-338-3p,miR-21a-3p,miR-30f,novel_99,miR-140-5p,miR-221-5p,miR-378a-3p,miR-21-3p,miR-3065-3p,miR-532-5p,miR-182-5p,miR-6516,miR-656-3p,novel_32,miR-22-3p,miR-320b,miR-1306-5p,miR-326,miR-362-3p,miR-3085-3p,miR-3955-3p,miR-2330-5p,miR-376b,miR-30c-1-3p,miR-193a,miR-3187-3p,miR-491-3p,miR-378b,miR-484,miR-10b,miR-378c,miR-101a-5p,miR-2284aa,miR-16-2-3p,miR-3120-5p,miR-105-1,miR-2300a-5p,novel_4,miR-5010-3p,miR-222-5p,miR-9788-3p,miR-30b-5p,miR-769-5p,miR-371a-5p,miR-3059-5p,novel_60,miR-541-3p,miR-199b-5p,miR-3064-5p,miR-7977,miR-378e,miR-7857-3p,miR-1260a,miR-30c-2-3p,miR-339a,miR-2898,miR-17-3p,miR-191-3p,miR-3529-3p,miR-29b-2-5p,miR-28b,miR-3065-3p,miR-199a-5p,miR-490-3p,miR-134,miR-29d-3p,miR-30a-5p,miR-105-2,miR-10a-5p,miR-483-3p,miR-378h,miR-6516-5p,miR-545-5p,miR-29b-3p,miR-410-5p,miR-331-3p,miR-409-3p,miR-138-5p,miR-28-5p,miR-204-5p,miR-6128,miR-365a-5p,miR-378c,miR-3154,miR-320a,miR-10b-3p,miR-7144-5p,miR-671-5p,miR-1814c,miR-141-3p,miR-34c-5p,miR-769,miR-199a-5p,miR-154b-5p,miR-190a,miR-330-3p,miR-452-5p,miR-125a-5p,miR-1343-5p,miR-130b-5p,miR-363-5p,miR-1434-3p,miR-1285,miR-138,miR-29b,miR-1260b,miR-219a-1-3p,miR-122-3p,miR-432-5p,miR-185-5p,miR-502-5p,miR-34c,novel_1,miR-199c,miR-147-5p,novel_101,miR-17-3p,miR-211,miR-371b-3p,miR-449a,miR-

TDRD3	ENSOARG0(	tudor do	miR-544-5p,miR-1983,miR-136-5p,miR-181d-5p,miR-194b-5p,miR-363-3p,miR-3059-5p,miR-107-5p,miR-2285n,miR-154-3p,miR-335,miR-181b-2-3p,miR-1343-3p,miR-151a-3p,miR-421,miR-2459,miR-216a-3p,miR-4791,miR-33a-5p,miR-494-3p,miR-1b-5p,miR-2310,miR-182-5p,miR-29b-1-5p,novel_103,miR-103a-2-5p,miR-182-5p,miR-532-5p,miR-21-3p,miR-154a-3p,miR-548o-3p,miR-2331-3p,miR-32-5p,miR-376e-3p,miR-377-3p,miR-27b-5p,novel_99,miR-543-3p,miR-154b-3p,miR-539-5p,miR-181c-5p,miR-224-5p,miR-664a-5p,miR-3604,miR-151-3p,miR-130b-5p,miR-485-3p,miR-505-3p,miR-487a-3p,miR-1197-5p,miR-1193,miR-217-5p,miR-191,miR-218-1-3p,miR-487a,miR-216c-5p,miR-143-3p,miR-2424,miR-181b-1-3p,miR-376c-3p,miR-376a-5p,miR-29b-1-5p,miR-125b,miR-2285p,miR-196a-3p,miR-299-3p,miR-320b,miR-181b-3p,miR-677,miR-411b-3p,miR-299b-3p,miR-374c-3p,miR-2404,miR-3591-3p,miR-3970,miR-505-3p,miR-412-3p,miR-1291,miR-3957-3p,miR-335-5p,miR-24-3p,miR-371b-3p,miR-133a-5p,miR-4324,miR-502b,miR-203b-5p,miR-625-3p,novel_1,miR-6119-3p,miR-92a-3p,miR-3955-5p,miR-2355-3p,miR-6238,miR-185-5p,miR-3535,miR-432-5p,miR-194-5p,miR-374b-3p,miR-181a-5p,miR-223-3p,miR-432,miR-2284x,miR-6240,miR-130b-5p,miR-539-3p,miR-654-3p,novel_51,miR-194a,miR-32,miR-330-3p,miR-377-3p,miR-224-5p,miR-125a-5p,miR-125a,miR-487a-3p,miR-154b-5p,miR-376c-3p,miR-365a-3p,miR-25-3p,miR-200a-5p,miR-1814c,miR-190a-3p,miR-125b-5p,miR-135a-1-3p,miR-34c-3p,miR-191-5p,miR-299a-3p,miR-486-5p,miR-3591-5p,miR-4286,miR-2285b,miR-192-3p,miR-92b-3p,miR-217,miR-205-5p,miR-92a-3p,miR-127-5p,miR-181b-5p

SYNDIG1	ENSOARG0(	synapse	miR-342-3p,miR-193b-3p,miR-17-3p,miR-449a,miR-326-3p,miR-15a,miR-142b,miR-28c,miR-576-3p,miR-16a,miR-34c,miR-128-1-5p,miR-656-5p,miR-502b,novel_68,miR-3074-5p,miR-146b-3p,miR-34b,miR-374c-3p,miR-299b-3p,miR-669,miR-2285ab,miR-18b,miR-2387,miR-216a-3p,miR-1291,miR-9851-3p,miR-2319a,miR-195a-5p,miR-6395,miR-668-3p,miR-363-5p,miR-654-3p,miR-432-3p,let-7a-2-3p,miR-345-5p,miR-503-5p,miR-377-3p,miR-365b-5p,miR-105-5p,miR-194a,miR-378a-5p,miR-125a-5p,miR-106b-3p,miR-193a-3p,miR-665,miR-3535,miR-2285g,miR-134-3p,miR-185-5p,miR-503-5p,novel_116,miR-653,miR-1843b-5p,miR-22-3p,miR-374b-3p,miR-194-5p,miR-30b-3p,miR-16b,miR-1260b,miR-26a-2-3p,miR-497-5p,miR-188-5p,novel_42,miR-1306-5p,miR-16-5p,miR-193a-5p,miR-133a-3p,miR-1260b,miR-145-3p,miR-365a-5p,miR-34c-3p,miR-2433,miR-125b-5p,miR-331-3p,miR-767-5p,miR-134-5p,miR-486b-3p,miR-28-5p,miR-574-5p,miR-2355-5p,miR-125a,miR-615-5p,miR-34c-5p,miR-374c-3p,miR-488-3p,miR-18a,miR-767,miR-2474,miR-10b-3p,miR-345-5p,let-7g-3p,miR-485-5p,miR-3529-3p,miR-3064-5p,miR-4286,miR-28b,miR-200a,miR-3065-3p,novel_39,miR-490-3p,miR-1271-3p,miR-2285af,miR-1260a,miR-664-3p,miR-125a-3p,miR-217,miR-17-3p,miR-450a-2-3p,novel_96,miR-2285ad,miR-299a-3p,miR-18b-5p,miR-322-5p,miR-2411-5p,miR-134-5p,miR-134,miR-3578,miR-7134-5p,miR-330-5p,miR-582-3p,miR-105-2,miR-155-5p,miR-128-3p,let-7a-2-3p,miR-2447,miR-541-3p,miR-2285f,miR-194b-5p,miR-361-3p,miR-877-3p,novel_111,miR-2285n,novel_82,miR-2440,miR-107-5p,miR-301a-5p,miR-664b,miR-3064-5p,miR-2319b,miR-541-5p,miR-2285u,miR-378g,miR-25,miR-2483-3p,miR-203-3p,miR-361-3p,miR-378j,miR-668-5p,miR-136-5p,miR-1388-3p,miR-124a,miR-127-3p,miR-424-5p,miR-676-3p,miR-376b,miR-

TBX3	ENSOARG0(	T-box 3	miR-147-5p,miR-625-3p,miR-20a-5p,miR-24-3p,miR-211,miR-3957-3p,miR-410-5p,miR-378f,miR-9851-3p,miR-146b-5p,miR-2285ab,miR-18a-3p,miR-3065-5p,miR-1306,novel_69,miR-2404,miR-296-3p,miR-146a,miR-106a-5p,novel_51,miR-17-5p,miR-455-5p,miR-365b-5p,miR-296-3p,miR-668-3p,miR-16-1-3p,miR-2284x,miR-188-5p,miR-455-5p,miR-4508,miR-1434-3p,miR-19b-2-5p,miR-3071-5p,novel_23,miR-2285g,miR-20b,miR-194-3p,miR-31-5p,miR-204-5p,novel_63,miR-2433,miR-93,miR-378c,miR-365a-5p,miR-1306-5p,miR-6128,miR-329a,miR-10b-3p,miR-1814c,miR-485-5p,miR-20b-5p,miR-7144-5p,miR-301,miR-424-3p,miR-17-5p,miR-3154,miR-93-5p,miR-769,miR-300-3p,miR-376c-3p,miR-874-3p,miR-141-3p,miR-2355-5p,miR-127-5p,miR-6536,novel_96,miR-200a-3p,novel_127,miR-664-3p,miR-205-5p,miR-3065-3p,novel_39,miR-378h,miR-2285ad,miR-1224-5p,miR-2285n,novel_82,miR-769-5p,miR-2285y,miR-361-3p,miR-3956-3p,novel_60,miR-2447,miR-2285f,miR-544-5p,miR-378j,miR-489,miR-499a-3p,miR-378e,miR-1185-2-3p,miR-664b,miR-211-5p,miR-491-5p,miR-146b,miR-5126,miR-1306-5p,miR-106b-5p,miR-362-3p,miR-505,miR-378d,miR-2310,miR-193a,miR-106a-5p,miR-2426,miR-450b-5p,miR-378c,miR-362-3p,miR-378b,miR-329-3p,miR-2285e,miR-1185-3p,novel_4,miR-409b,miR-548w,miR-1343-3p,miR-664a,miR-129-5p,miR-2285j,miR-664a-5p,miR-665,miR-324-3p,miR-19b-1-5p,miR-769-5p,miR-378c,miR-3958-5p,miR-2312,miR-625-5p,miR-2285c,miR-27b-5p,miR-376e-3p,miR-2284s,miR-499b-3p,miR-204-3p,miR-378a-3p,miR-3065-3p,miR-106a,miR-1277-5p,miR-30b-3p,miR-2285p,miR-422a,miR-216a-5p,miR-376c-3p,miR-376a-5p,miR-146a-5p,miR-105-3p,miR-20a,miR-16-1-3p,miR-362-3p,miR-378i,miR-1193,miR-1197-5p,miR-140-3p,miR-378a-3p,miR-188-3p,miR-7859,novel_83,miR-106a,miR-18a-3p
CD109	ENSOARG0(	CD109 mo	miR-3968,miR-301a-5p,miR-502-5p,miR-374c-5p,miR-2428,miR-362-5p,miR-2285x,miR-541-3p,miR-150-5p,miR-1306,miR-669,miR-6535,miR-2483-3p,miR-374c-3p,miR-320b,miR-216b-3p,miR-452-3p,miR-491-5p,miR-182-5p,miR-1306-5p,novel_103,miR-345-5p,miR-124a,miR-2330-5p,miR-3120-5p,miR-16-1-3p,miR-197-3p,miR-484,miR-301b-5p,miR-541,miR-500-5p,novel_4,miR-4492,miR-6740-5p,miR-3969,miR-665,miR-320e,miR-655-3p,miR-6525,miR-500b-5p,miR-346,miR-1306-5p,miR-625-5p,miR-345-5p,miR-345-5p,miR-129b-3p,miR-655,miR-3154,miR-362-5p,miR-381-3p,miR-374c-3p,miR-300-3p,miR-182-5p,miR-296-3p,miR-21-3p,novel_96,miR-500a-5p,miR-185-3p,miR-2285af,miR-6119-5p,miR-192-3p,miR-2887,miR-4286,miR-216c-5p,miR-16-1-3p,miR-142-3p,miR-582-3p,miR-300,miR-2904,miR-124-3p,miR-2408,miR-193b-5p,miR-2300b-3p,miR-1b-3p

GUK1	ENSOARG0(	guanylat	miR-486b-3p,miR-4492,miR-874-5p,miR-665,miR-3969,miR-132-5p,miR-331-3p,miR-551b-3p,miR-2433,miR-6134,miR-122-5p,miR-224-5p,miR-346,miR-1260b,miR-1197-3p,miR-6525,miR-214,miR-665-5p,miR-6128,miR-3074-5p,miR-1827,miR-485-5p,miR-671-5p,miR-23a-5p,miR-329a,miR-129b-3p,miR-200a-5p,miR-22-3p,miR-3065-3p,miR-2355-5p,miR-370-3p,miR-874-3p,miR-574-5p,miR-140-5p,miR-299-3p,novel_127,novel_96,miR-21-3p,miR-760-3p,miR-466i-5p,miR-185-3p,miR-1260a,miR-143-3p,miR-486-3p,miR-3065-3p,miR-130a-5p,miR-125b-2-3p,miR-4286,miR-2887,miR-3064-5p,miR-378d,miR-1197-5p,miR-330-5p,miR-362-3p,miR-193b-5p,miR-486-5p,miR-1247-5p,miR-431,miR-18a-3p,miR-299a-3p,miR-6123,miR-147-5p,miR-128-1-5p,miR-3968,miR-4726-5p,miR-4324,miR-326-3p,novel_111,miR-361-3p,miR-219a-5p,miR-541-3p,miR-136-5p,miR-18a-3p,miR-140-5p,miR-361-3p,miR-489,novel_69,miR-299b-3p,miR-670-3p,miR-7977,miR-3074-5p,miR-3064-5p,novel_68,miR-214-3p,miR-3085-3p,miR-491-5p,miR-455-5p,miR-362-3p,miR-5126,novel_103,miR-326,miR-431-5p,miR-224-5p,miR-432-3p,miR-744-5p,miR-455-5p,miR-3120-5p,miR-4508,miR-1260b,miR-362-3p,miR-125b-2-3p,miR-22-3p,miR-541,miR-1285,miR-873a-5p,miR-329-3p,miR-484,miR-296-5p,miR-149-5p,miR-455-3p,miR-147a,novel_78,miR-1343-3p
AGPAT5	ENSOARG0(	1-acylgl	miR-335,novel_1,miR-379-3p,miR-628-3p,miR-2285t,miR-34c,novel_111,miR-211,miR-449a,novel_27,miR-374c-5p,miR-335-5p,miR-17-3p,miR-194b-5p,miR-19a-3p,miR-299b-5p,miR-378f,miR-2387,miR-3591-3p,miR-381-3p,miR-216a-3p,miR-34b,miR-378e,miR-320c,miR-329-5p,miR-2319b,miR-211-5p,miR-3120-3p,miR-26b-5p,miR-320b,miR-2331-5p,miR-194a,miR-2957,miR-505,miR-2284l,miR-378d,miR-124a,miR-544a,miR-1260b,miR-378c,miR-432,miR-378b,miR-2284r,miR-194-5p,miR-432-5p,miR-544b,miR-149-5p,miR-148b-5p,miR-194-3p,miR-17-3p,miR-28-5p,miR-204-5p,miR-191-5p,miR-874-5p,miR-708-5p,miR-4492,miR-30e-3p,let-7b-3p,miR-30a-3p,miR-1260b,miR-1197-3p,miR-378c,miR-129-2-3p,miR-1306-5p,miR-378c,miR-539-5p,miR-3074-2-3p,miR-1248,let-7f-1-3p,miR-320a,miR-27b-5p,miR-544-3p,miR-34c-5p,miR-324-5p,miR-548o-3p,miR-545-3p,miR-378a-3p,miR-2355-5p,miR-3065-3p,miR-21-3p,miR-127-5p,miR-320d,miR-17-3p,miR-34b-5p,miR-26a-5p,miR-1260a,miR-122-3p,miR-422a,miR-148b-5p,miR-3065-3p,miR-105-3p,miR-183-5p,miR-329a-5p,miR-30a-3p,miR-19b-3p,miR-4429,miR-411-3p,let-7a-3p,miR-148a-5p,miR-34a-5p,miR-128-3p,miR-2300b-3p,miR-191,novel_133,miR-378i,miR-378a-3p,miR-7134-5p,miR-124-3p,miR-378h,miR-302a-5p,miR-129-1-3p,miR-532-3p,miR-545-5p,miR-30d-3p

KDSR	ENSOARG0(	3-ketodi	miR-329-3p,miR-1434-3p,miR-125b-2-3p,miR-374b-3p,miR-362-3p,miR-16b,miR-219a-1-3p,miR-4508,miR-497-5p,miR-744-5p,miR-340-3p,miR-339-5p,miR-153,miR-134-3p,miR-221-3p,miR-2459,miR-362-3p,miR-3141,miR-16b,miR-199b-5p,miR-153-3p,miR-660,miR-214-3p,miR-1388-3p,miR-124a,miR-2330-5p,miR-494-3p,miR-424-5p,miR-676-3p,miR-491-3p,miR-221,miR-199b-5p,miR-3141,miR-335-3p,miR-9851-3p,miR-877-3p,miR-376a-2-5p,miR-195a-5p,miR-4792,miR-452-3p,miR-20a-3p,miR-329-5p,miR-2285u,miR-320b,miR-3596,miR-378g,miR-374c-3p,miR-299b-3p,miR-34b,miR-6535,miR-2285n,miR-15a,miR-34c,miR-1307-5p,miR-16a,miR-3059-5p,miR-24-3p,miR-449a,miR-1285-5p,miR-199b-5p,miR-126-3p,miR-362-3p,miR-124-3p,miR-2904,miR-582-3p,miR-34a-5p,miR-222,miR-2285ad,miR-299a-3p,miR-548e-3p,novel_79,miR-30d-3p,miR-376a-5p,miR-212-5p,miR-7859,miR-322-5p,miR-15a-5p,miR-582-3p,novel_17,miR-210-5p,miR-216a-5p,miR-185-3p,miR-339a,miR-202-5p,miR-34b-5p,miR-21-3p,miR-299-3p,miR-200a-3p,miR-329a-5p,miR-30a-3p,miR-664b-3p,miR-199a-5p,miR-2424,miR-129b-3p,miR-2474,miR-329a,miR-339b,miR-485-5p,miR-3957-5p,miR-2331-3p,miR-660-5p,miR-548o-3p,miR-2285aa,miR-141-3p,miR-34c-5p,miR-374c-3p,miR-656-3p,miR-199a-5p,miR-324-5p,miR-15b-5p,miR-3957,miR-222-3p,miR-30e-3p,miR-30a-3p,miR-3969,miR-320e,miR-6516-3p,miR-129-5p,miR-4492,miR-424-5p,miR-181a-2-3p,miR-16-5p,miR-6128,miR-214,miR-6134,miR-338-3p

CD4	ENSOARG0(	T-cell s	miR-2433,miR-125b-5p,miR-331-3p,miR-486b-3p,miR-1306-5p,miR-193a-5p,miR-500b-5p,miR-381-3p,miR-30e-5p,miR-362-5p,miR-3154,miR-10b-3p,miR-2318,miR-485-5p,miR-671-5p,miR-874-3p,miR-2355-5p,miR-2285aa,miR-1271,miR-125a,miR-769,miR-154b-5p,miR-200c,miR-125a-3p,let-7c-5p,miR-17-3p,miR-200c-3p,miR-127-5p,let-7f-5p,miR-191-3p,miR-181b-5p,novel_127,novel_96,miR-495-5p,miR-3064-5p,miR-2403,miR-450b-5p,let-7g,novel_39,miR-1271-3p,miR-615-3p,miR-1290,miR-3578,miR-30a-5p,miR-330-5p,miR-501-3p,miR-582-3p,let-7d,miR-30c,miR-128-3p,miR-200b-3p,miR-222,miR-6517,miR-429-3p,miR-1271-5p,miR-2411-5p,miR-582-3p,miR-129-1-3p,miR-4324,miR-133a-5p,let-7i,miR-22-5p,miR-656-5p,miR-135a-2-3p,miR-342-3p,miR-542-5p,miR-17-3p,novel_101,miR-326-3p,miR-2428,miR-3141,miR-1306,miR-18a-3p,miR-216a-3p,miR-378b,miR-412-3p,miR-146b-5p,let-7e-5p,miR-3074-5p,miR-329-5p,miR-320b,miR-669,miR-377-3p,miR-296-3p,miR-146a,miR-3141,miR-2285v,miR-194a,miR-125a-5p,miR-668-3p,miR-654-3p,miR-296-3p,miR-181a-5p,miR-1285,miR-542-3p,miR-223-3p,miR-2113,miR-194-5p,miR-16-1-3p,miR-27b-3p,miR-31-5p,miR-133b-5p,miR-377-5p,miR-134-3p,miR-185-5p,let-7g-5p,miR-149-5p,miR-6238,miR-544b,let-7f,miR-222-3p,miR-664a,miR-17-3p,miR-874-5p,miR-500,miR-4492,let-7b,miR-129-2-3p,miR-3184-3p,miR-3074-5p,miR-4510,miR-1271,miR-214,miR-769-5p,miR-3184-5p,let-7b-5p,miR-6134,miR-181c-5p,miR-500a-3p,miR-30d-5p,miR-30f,miR-1827,miR-2331-3p,miR-216b-3p,miR-296-3p,miR-182-5p,miR-544-3p,miR-499b-3p,miR-377-3p,miR-2284s,miR-139-5p,miR-6130,novel_25,let-7i-5p,miR-30b,novel_17,miR-2285p,miR-542-5p,miR-324-3p,miR-125b,miR-92a-1-5p,miR-185-3p,miR-98-5p,miR-30d,let-7k,miR-329a-5p,miR-2887,miR-376a-5p,miR-29b-1-5p,miR-377-5p,miR-146a-5p,miR-2411,miR-
VPS4B	ENSOARG0(	vacuolar	novel_107,novel_82,miR-4792,miR-548o-3p
DCTN2	ENSOARG0(	dynactin	miR-147-5p,novel_82,miR-2285n,miR-301a-5p,miR-877-3p,miR-211,miR-361-3p,miR-3959-3p,miR-24-3p,miR-326-3p,miR-135a-2-3p,miR-3059-5p,miR-2285f,miR-504,miR-6395,miR-2285ab,miR-361-3p,miR-18a-3p,miR-129b-5p,miR-411b-3p,miR-3082-5p,miR-677,miR-181b-3p,novel_68,miR-2319b,miR-10a-5p,miR-211-5p,miR-214-3p,miR-466f-3p,novel_115,miR-326,miR-105-5p,miR-330-3p,novel_51,miR-30c-1-3p,miR-345-5p,miR-491-3p,miR-758-3p,let-7a-2-3p,miR-130b-5p,miR-574-3p,miR-101b-3p,miR-6240,miR-668-3p,miR-16-1-3p,miR-3120-5p,miR-484,miR-4791,miR-10b,miR-301b-5p,miR-7857,miR-758-3p,miR-3071-5p,miR-3535,miR-149-5p,miR-105-1,miR-330-3p,novel_78,miR-151a-3p,miR-339-5p,miR-500,miR-708-5p,miR-129-5p,miR-130b-5p,miR-204-5p,miR-28-5p,miR-30e-3p,miR-30a-3p,miR-324-3p,miR-151-3p,miR-10b-5p,miR-338-3p,miR-103b,miR-214,miR-345-5p,miR-339b,miR-345-5p,let-7g-3p,miR-21a-3p,miR-503-3p,miR-500a-3p,miR-10a,novel_32,miR-504-5p,miR-30c-1-3p,miR-874-3p,miR-2355-5p,miR-760-3p,novel_127,miR-543-5p,miR-21-3p,miR-324-3p,miR-30c-2-3p,miR-339a,miR-185-3p,miR-181b-1-3p,miR-30a-3p,miR-4286,miR-582-3p,miR-16-1-3p,miR-105-2,miR-218-1-3p,let-7a-2-3p,miR-10a-5p,miR-2300b-3p,miR-330-5p,miR-328-3p,miR-18a-3p,novel_79,miR-30d-3p

CST7	ENSOARG0(	cystatin	miR-345-3p,miR-105-3p,miR-486-3p,miR-92a-1-5p,miR-219b-5p,miR-210-5p,miR-196a-3p,miR-21-3p,miR-221-5p,miR-34b-5p,miR-30b-3p,miR-466i-5p,miR-3600,miR-3960,miR-532-3p,novel_83,miR-1247-5p,miR-148b-3p,miR-210-3p,miR-188-3p,miR-1246,miR-2408,miR-34a-5p,miR-378d,miR-19b-3p,miR-214,miR-425-5p,miR-3958-5p,miR-3074-5p,miR-4510,miR-145a-3p,miR-6525,miR-769-5p,miR-29b-2-5p,miR-665,miR-324-3p,miR-487a-5p,miR-4492,miR-874-5p,miR-664a,miR-17-3p,miR-487b-5p,novel_74,miR-296-3p,miR-221-5p,miR-504-5p,miR-2331-3p,miR-22-3p,miR-6130,miR-2285c,miR-345-5p,miR-2312,miR-376b,miR-744-3p,miR-1839-3p,miR-193a,miR-4532,miR-326,miR-214-3p,miR-199b-5p,miR-25-5p,miR-487b-5p,miR-491-5p,miR-3085-3p,miR-27a-3p,miR-145b,novel_4,novel_78,miR-1343-3p,miR-2285e,miR-455-3p,miR-147a,miR-496-5p,miR-30b-3p,miR-7857,miR-1298-5p,miR-541,miR-296-5p,miR-101c,miR-2285f,miR-212-3p,miR-541-3p,miR-361-3p,miR-769-5p,miR-2440,miR-1843a-3p,miR-4726-5p,miR-3968,miR-7977,miR-211-5p,miR-452-3p,miR-3064-5p,miR-4443,miR-27a-3p,miR-378g,miR-199b-5p,miR-504,miR-3064-5p,miR-29b-2-5p,miR-2385-3p,miR-490-3p,novel_39,miR-200a,miR-199a-5p,miR-125a-3p,novel_96,miR-543-5p,miR-200a-3p,miR-152-3p,miR-107,miR-191-3p,miR-17-3p,miR-2898,novel_121,miR-23b-5p,miR-330-5p,miR-29d-3p,miR-1343-5p,let-7a-2-3p,miR-300,miR-411-3p,miR-365a-5p,miR-29b-3p,miR-592,miR-486b-3p,miR-204-5p,miR-141-3p,miR-1271,miR-2355-5p,miR-370-3p,miR-874-3p,miR-3068-3p,miR-574-5p,miR-154b-5p,miR-199a-5p,miR-769,miR-34c-5p,miR-381-3p,miR-485-5p,miR-1248,miR-23a-5p,let-7g-3p,miR-345-5p,miR-2318,miR-876-3p,miR-628-5p,let-7a-2-3p,miR-345-5p,miR-654-3p,miR-2285r,novel_51,miR-365b-5p,miR-106b-3p,miR-1343-5p,miR-124-5p,miR-378a-5p,miR-103a-3p,miR-
XRN1	ENSOARG0(	5'-3' ex	miR-1271-3p,miR-1b-5p,let-7j,miR-93,miR-20a,miR-1a-1-5p,miR-493-5p,miR-106a-5p,miR-1a-2-5p,miR-106a,miR-106b-5p,miR-17-5p,miR-106a-5p,miR-20a-5p,miR-1-5p,miR-20b,miR-106a,miR-2284x,miR-20b-5p,miR-493-5p,miR-33b-3p,miR-30b-3p,miR-17-5p,miR-30b-3p,miR-93-5p,miR-1434-3p,miR-1246
ACSBG2	ENSOARG0(	acyl-CoA	miR-107-5p,miR-1291,miR-206,miR-103a-2-5p,miR-3059-5p,miR-10b,miR-193a-5p,miR-665-5p,miR-206-3p
POC1A	ENSOARG0(	POC1 cen	miR-504,miR-1843b-5p,miR-2459,miR-7857-3p,miR-211-5p,miR-7977,miR-2355-5p,miR-3074-5p,miR-504-5p,miR-3064-5p,miR-204-5p,miR-760-3p,miR-3085-3p,miR-502-5p,miR-1271-3p,miR-211,miR-345-3p,miR-3064-5p,miR-3074-5p
DMRTB1	ENSOARG0(	DMRT-lik	miR-374c-3p,miR-3965,miR-3596,miR-181b-3p,miR-203-3p,miR-677,miR-21-5p,miR-378d,miR-874-5p,miR-34c-3p,novel_51,miR-21c

GCG	ENSOARG0(	glucagon	miR-374a-3p,miR-539-5p,miR-425-5p,novel_9,novel_74,miR-374b-3p,miR-130b-5p,miR-454-3p,miR-31-3p,miR-29b-3p,miR-409-3p,miR-135a-5p,miR-193b-3p,miR-301a-3p,miR-2284s,miR-300-3p,miR-503-3p,miR-182-5p,miR-2285aa,miR-1248,miR-7144-5p,novel_48,miR-381-3p,miR-320a,miR-137-3p,miR-143-3p,miR-329a-5p,miR-200c-3p,miR-466i-5p,miR-320d,miR-125a-3p,miR-200c,miR-30f,miR-216a-5p,miR-582-3p,miR-429-3p,miR-300,miR-4429,miR-218-1-3p,miR-3591-5p,miR-188-3p,miR-29d-3p,miR-301b-3p,miR-2284w,miR-1285-5p,novel_101,miR-29c-3p,miR-193b-3p,miR-29a,miR-3958-3p,miR-374a-3p,miR-142b,miR-2483-3p,miR-29a-3p,miR-1185-2-3p,miR-320c,miR-329-5p,miR-3120-3p,miR-135b-5p,miR-6516-3p,miR-140-5p,miR-654-3p,miR-758-3p,miR-2310,miR-494-3p,miR-130b-5p,let-7f-2-3p,miR-487b-5p,miR-130b-3p,miR-193a-3p,miR-320b,miR-182-5p,miR-2320-3p,miR-1185-3p,miR-130a-3p,miR-219a-1-3p,miR-29b,miR-21b,miR-1434-3p,miR-2284r,miR-758-3p
ASF1B	ENSOARG0(	anti-sil	miR-1296-5p,miR-3059-5p,miR-2285n,miR-2285t,miR-4726-5p,miR-7977,miR-3064-5p,miR-20a-3p,let-7d-5p,miR-378e,miR-3082-5p,miR-129b-5p,miR-33a-5p,let-7a-5p,miR-424-5p,miR-758-3p,miR-326,let-7e,novel_124,miR-3085-3p,miR-5010-3p,miR-2300a-5p,miR-9788-3p,miR-412-3p,miR-30b-3p,miR-378b,miR-296-5p,miR-484,miR-378c,miR-2426,miR-378c,miR-145a-3p,let-7b-5p,miR-206-3p,miR-324-3p,miR-424-5p,miR-708-5p,miR-450b-3p,miR-155-5p,miR-221-5p,miR-378a-3p,novel_32,miR-877-5p,miR-155-5p,miR-193b-3p,miR-21a-3p,let-7g-3p,miR-216c-5p,miR-216a-5p,miR-221-5p,miR-34b-5p,miR-30b-3p,miR-299-3p,miR-21-3p,miR-2284m,miR-760-3p,miR-466i-5p,miR-3600,novel_83,miR-148b-3p,miR-200b,miR-15a-5p,miR-222-5p,miR-2904,miR-188-3p,miR-1197-5p,novel_133,miR-2300b-3p,miR-378d,miR-19b-3p,miR-326-3p,miR-449a,miR-24-3p,miR-16a,miR-34c,let-7i,miR-15a,miR-656-5p,miR-128-1-5p,miR-320b,miR-2404,miR-34b,miR-195a-5p,miR-450b-3p,miR-19a-3p,miR-2483-5p,miR-296-3p,miR-190a,miR-455-5p,miR-503-5p,miR-466f-3p,miR-124-5p,miR-130a-3p,miR-665,miR-503-5p,miR-185-5p,let-7g-5p,miR-145a-5p,miR-455-5p,miR-744-5p,miR-30b-3p,miR-2284ab,miR-1260b,miR-6128,miR-1306-5p,miR-378c,miR-323-3p,miR-331-3p,miR-34c-3p,miR-5100,miR-486b-3p,miR-28-5p,miR-370-3p,miR-154b-5p,miR-2284n,miR-34c-5p,miR-2448-3p,miR-485-5p,miR-1,miR-181a-2-3p,miR-4286,miR-380-5p,miR-24-2-5p,let-7c-5p,miR-1260a,miR-200c,miR-450a-2-3p,miR-200c-3p,miR-24-2-5p,miR-378h,miR-483-3p,miR-200b-3p,let-7d,miR-2284j,miR-190b-5p,miR-423-5p,miR-877-3p,novel_82,miR-2285u,miR-7689-3p,miR-24-1-5p,miR-3596,miR-378g,miR-493-5p,miR-1983,miR-544-5p,miR-3963,miR-504,miR-744-3p,miR-378d,miR-16b,miR-214-3p,miR-130b-3p,miR-145b,miR-1343-3p,miR-2459,miR-1298-3p,miR-1a-

CLEC16A	ENSOARG0(	C-type l	miR-615-3p,miR-1193,miR-1197-5p,miR-29d-3p,miR-1306,miR-1260b,miR-1291,miR-29b,miR-1248,miR-125a,miR-216b-3p,miR-3065-3p,miR-29a-3p,miR-216b-5p,miR-615,miR-1306-5p,miR-1260a,miR-29b-3p,miR-125b,miR-107-5p,miR-92a-1-5p,miR-125b-5p,miR-29a,miR-466f-3p,miR-103a-2-5p,miR-125a-5p,miR-145a-3p,miR-29c-3p,miR-1260b,miR-877-3p,miR-3065-3p
FKBP15	ENSOARG0(	FK506 bi	miR-132-5p,miR-324-3p,miR-664a,miR-708-5p,miR-539-5p,miR-3074-5p,miR-145a-3p,miR-129-2-3p,miR-3074-2-3p,miR-142a-5p,miR-769-5p,miR-224-5p,miR-6134,miR-1827,miR-582-5p,miR-30c-1-3p,miR-3065-3p,miR-532-5p,miR-296-3p,miR-27a-5p,miR-377-3p,miR-582-5p,novel_32,miR-125b,miR-320d,miR-106a,miR-34b-5p,miR-30b-3p,miR-2484,miR-2285w,miR-20a,miR-125b-2-3p,miR-146a-5p,miR-2432,miR-143-3p,novel_133,miR-1246,miR-2904,miR-188-3p,miR-140-3p,miR-184-3p,miR-19b-3p,miR-4429,miR-361-5p,miR-378d,miR-34a-5p,miR-18a-3p,miR-106a,miR-1b-3p,miR-3600,miR-23b-3p,miR-95-3p,miR-23c,miR-2284h-5p,miR-2285y,miR-769-5p,miR-3968,miR-2285t,miR-212-3p,miR-541-3p,miR-452-5p,miR-361-3p,miR-361-3p,miR-1983,miR-664b,miR-3064-5p,miR-452-3p,miR-2319b,miR-20a-3p,miR-374b-5p,miR-378g,miR-27a-3p,miR-4443,miR-320b,miR-505,miR-106b-5p,miR-3085-3p,miR-146b,miR-1388-3p,miR-106a-5p,miR-23a-3p,miR-30c-1-3p,miR-484,miR-541,miR-412-3p,miR-101c,miR-3120-5p,miR-105-1,miR-199b-3p,miR-2300a-5p,miR-1343-3p,miR-27a-3p,miR-147a,miR-142-5p,miR-1298-3p,miR-216a-3p,miR-2284k,miR-125b-5p,miR-28-5p,miR-374b,miR-93,miR-93-5p,miR-17-5p,miR-23b,miR-320a,miR-3074-1-3p,novel_48,miR-20b-5p,miR-23a-5p,miR-671-5p,miR-485-5p,miR-503-3p,miR-874-3p,miR-370-3p,miR-125a,miR-141-3p,miR-34c-5p,miR-769,miR-324-5p,miR-223-5p,miR-664-3p,miR-125a-3p,miR-30c-2-3p,novel_127,miR-200a-3p,miR-3529-3p,miR-2385-3p,miR-3064-5p,miR-148b-5p,miR-3065-3p,miR-411-3p,miR-128-3p,miR-105-2,miR-2285ad,miR-6517,miR-412,miR-582-3p,miR-129-1-3p,novel_121,miR-214-5p,miR-4324,miR-133a-5p,miR-20a-5p,miR-34c,miR-379-3p,miR-203b-5p,novel_101,miR-371b-3p,miR-24-3p,miR-449a,miR-2284w,miR-18a-3p,miR-216a-3p,miR-412-3p,miR-19a-3p,miR-146b-5p,miR-3074-5p,miR-

ALDH1L1	ENSOARG0(	aldehyde	miR-504,miR-299b-5p,miR-1983,miR-544-5p,miR-493-5p,miR-3596,miR-378g,miR-1185-2-3p,miR-2319b,miR-4792,miR-211-5p,miR-2285u,miR-3968,miR-615,miR-218-5p,miR-301a-5p,miR-361-3p,miR-423-5p,miR-2447,miR-9-5p,miR-1388-5p,miR-301b-5p,miR-541,miR-1298-3p,miR-142-5p,miR-1185-3p,miR-148b-5p,miR-1343-3p,miR-330-3p,miR-181b-2-3p,miR-1185-5p,miR-431-5p,miR-199a-3p,novel_19,miR-378d,miR-1388-3p,miR-124a,miR-339b,miR-1827,miR-625-5p,miR-1895,miR-2285c,miR-10a,miR-151b,miR-548o-3p,miR-504-5p,miR-2331-3p,miR-216b-3p,miR-129-5p,miR-4492,miR-874-5p,miR-30e-3p,miR-665,miR-30a-3p,miR-142a-5p,miR-3184-5p,miR-665-5p,miR-142-3p,miR-493-3p,miR-148a-5p,miR-34a-5p,miR-2366,miR-193b-5p,miR-378i,miR-378a-3p,miR-1247-5p,miR-30d-3p,miR-208b-3p,miR-210-5p,miR-422a,miR-92a-1-5p,miR-324-3p,miR-181b-1-3p,miR-377-5p,miR-142a-3p,miR-486-3p,miR-345-3p,miR-412-3p,miR-378f,miR-148a-3p,miR-3591-3p,miR-1306,miR-411b-3p,miR-181b-3p,miR-677,miR-6535,miR-669,miR-208a-3p,miR-28c,miR-4324,miR-425-5p,miR-2284w,miR-1843b-5p,miR-7b-5p,miR-542-3p,miR-223-3p,novel_23,miR-197-3p,miR-22-3p,miR-134-3p,miR-149-5p,miR-339-5p,miR-7-5p,novel_51,miR-194a,miR-105-5p,miR-2284l,miR-493-5p,miR-6240,miR-876-3p,miR-767,miR-1248,miR-301,miR-200a-5p,miR-874-3p,miR-125a,miR-1271,miR-542-5p,miR-2355-5p,miR-3432a,miR-494-5p,miR-2433,miR-125b-5p,miR-6740-5p,miR-1260b,miR-330-5p,miR-23b-5p,miR-1343-5p,miR-505-5p,miR-9-5p,miR-6517,miR-431,novel_79,miR-152-3p,miR-125a-3p,miR-205-5p,miR-148b-5p,miR-2285b,novel_39,miR-2284y,miR-3064-5p,novel_94,miR-361-3p,miR-3082-5p,miR-7857-3p,miR-378e,miR-3064-5p,miR-7977,miR-4726-5p,miR-541-3p,miR-194b-5p,miR-101a-5p,miR-378c,miR-10b,miR-378b,miR-484,miR-296-5p,miR-30b-3p,miR-142-3p,miR-2285o,miR-9788-

GPR137	ENSOARG0(	G protei	miR-378d,miR-2330-5p,miR-424-5p,let-7a-5p,miR-491-5p,let-7e,miR-103a-2-5p,miR-199b-5p,novel_115,miR-326,miR-16b,miR-199b-3p,miR-409b,miR-2300a-5p,miR-151a-3p,miR-27a-3p,miR-378c,miR-3120-5p,miR-378b,miR-296-5p,miR-484,miR-30b-3p,miR-877-3p,miR-423-5p,miR-3956-3p,novel_60,miR-452-5p,miR-2285f,miR-4726-5p,miR-107-5p,miR-2285y,miR-378e,miR-27a-3p,let-7d-5p,miR-2319b,miR-211-5p,miR-7977,miR-504,miR-3963,miR-96-5p,miR-199b-5p,miR-361-3p,miR-378j,miR-377-5p,miR-143-3p,miR-486-3p,miR-345-3p,miR-760-3p,miR-98-5p,let-7k,miR-221-5p,miR-92a-1-5p,miR-345-3p,miR-185-3p,miR-216a-5p,miR-15a-5p,miR-383-5p,miR-328-3p,miR-1247-5p,miR-18a-3p,miR-1961,miR-378i,miR-378a-3p,miR-217-5p,miR-6525,miR-338-3p,miR-3184-5p,let-7b-5p,miR-4510,let-7b,miR-539-5p,miR-378c,miR-2330-3p,miR-665-5p,miR-1271,miR-130b-5p,miR-874-5p,miR-708-5p,miR-424-5p,miR-4492,let-7f,miR-320e,miR-151-3p,miR-6130,let-7i-5p,miR-15b-5p,miR-140-5p,miR-504-5p,miR-221-5p,miR-296-3p,miR-1827,miR-885-3p,miR-1895,miR-2285r,miR-296-3p,miR-130b-5p,miR-483-3p,miR-296-3p,miR-2411-3p,miR-185-5p,let-7g-5p,miR-149-5p,novel_116,miR-31-5p,miR-377-5p,miR-30b-3p,miR-16b,miR-27b-3p,miR-497-5p,miR-1843b-5p,miR-1285,miR-873a-5p,miR-371b-3p,miR-24-3p,miR-211,miR-199b-5p,miR-2285m,miR-326-3p,miR-135a-2-3p,novel_101,miR-128-1-5p,miR-199c,miR-656-5p,miR-15a,let-7i,miR-16a,novel_69,miR-374c-3p,miR-6535,miR-216b-3p,miR-378b,miR-320b,miR-1291,miR-195a-5p,miR-6395,miR-378f,let-7e-5p,miR-9851-3p,miR-2285ab,miR-216a-3p,miR-18a-3p,miR-490-3p,miR-199a-5p,let-7g,miR-4286,miR-181a-2-3p,let-7f-5p,miR-543-5p,novel_127,novel_96,miR-6119-5p,miR-125a-3p,miR-217,let-7c-5p,miR-483-3p,miR-1271-5p,miR-322-5p,miR-

COL12A1	ENSOARG0(	collagen	miR-1a-2-5p,miR-17-3p,novel_127,miR-30b-3p,miR-34b-5p,miR-208b-3p,novel_17,miR-125a-3p,miR-181b-1-3p,miR-130a-5p,miR-2285b,miR-2284a,miR-2284y,miR-2411,miR-885-3p,miR-2284u,miR-34a-5p,miR-361-5p,miR-1193,miR-330-5p,miR-124-3p,miR-15a-5p,miR-2411-5p,miR-302a-5p,miR-9-5p,miR-322-5p,miR-199a-3p,miR-222,miR-18a-3p,miR-30d-3p,miR-431,miR-1224-5p,miR-412,miR-17-3p,miR-424-5p,miR-2332,novel_63,miR-1-5p,let-7b-3p,miR-2284k,miR-222-3p,miR-34c-3p,miR-135a-5p,miR-100-3p,miR-3074-5p,miR-2284v,miR-3074-2-3p,miR-665-5p,miR-16-5p,miR-3607-3p,miR-2318,miR-329b,novel_99,miR-485-5p,miR-885-3p,miR-23a-5p,miR-411,miR-34c-5p,miR-15b-5p,miR-32-3p,miR-140-5p,miR-552-3p,miR-1271,miR-370-3p,miR-103a-2-5p,miR-431-5p,miR-326,miR-1306-5p,miR-199a-3p,miR-16b,miR-432-3p,miR-424-5p,miR-1388-3p,miR-124a,miR-2284b,miR-2426,miR-16b,miR-497-5p,miR-4791,miR-873a-5p,miR-145a-5p,miR-412-3p,miR-539-5p,miR-2284d,miR-3535,miR-221-3p,miR-187-3p,miR-145b,miR-181b-2-3p,miR-208a-3p,miR-2440,miR-15a,miR-107-5p,miR-16a,miR-34c,miR-323c,miR-2284w,miR-326-3p,miR-449a,miR-212-3p,miR-3956-3p,miR-1a-1-5p,miR-219b-3p,novel_60,miR-32-3p,miR-17-3p,let-7i-3p,miR-9-5p,miR-2284j,miR-668-5p,miR-135b-5p,miR-544-5p,miR-2319a,miR-221,miR-381-3p,miR-129b-5p,miR-18a-3p,miR-1306,miR-34b,miR-181b-3p,miR-677,miR-3074-5p,miR-2319b,miR-2284g
MED27	ENSOARG0(	mediator	miR-181a-2-3p,miR-665-5p,miR-2447,miR-342-3p,miR-2330-5p,miR-1343-3p,miR-204-3p,miR-134-5p,miR-95-3p,miR-197-3p,miR-342,miR-134,miR-6529b,miR-134-5p,miR-7862
HMGCLL1	ENSOARG0(	3-hydrox	miR-361-3p,miR-365a-3p,miR-224-5p,miR-99a-5p,novel_87,miR-100-5p,miR-224-5p,miR-361-3p,miR-3578,miR-99a,miR-99a-5p,miR-99b-5p,miR-450b-5p

PLXNA4	ENSOARG0(	plexin A	miR-505-5p,miR-214-5p,miR-6517,miR-299a-3p,miR-1224-5p,let-7d,miR-148b-5p,miR-490-3p,miR-1957a,let-7g,miR-4286,miR-3064-5p,let-7f-5p,miR-2898,novel_96,miR-125a-3p,miR-2285af,let-7c-5p,miR-34c-5p,miR-154b-5p,miR-542-5p,miR-2355-5p,miR-370-3p,miR-485-5p,miR-3154,miR-103b,miR-1306-3p,miR-134-3p,let-7g-5p,miR-185-5p,miR-2285g,miR-194-3p,miR-26a-2-3p,miR-1843b-5p,miR-194-5p,miR-432-3p,miR-654-3p,miR-432-3p,miR-2285r,miR-130b-5p,miR-628-5p,miR-363-5p,miR-194a,novel_51,novel_69,miR-299b-3p,miR-34b,miR-2404,miR-6535,miR-1291,let-7e-5p,miR-2387,miR-2285ab,miR-1306,miR-3959-3p,miR-2285m,miR-449a,miR-542-5p,miR-208a-3p,let-7i,miR-34c,miR-151-5p,miR-212-5p,miR-3600,miR-142-3p,miR-34a-5p,miR-133a-3p,miR-2300b-3p,miR-1961,miR-140-3p,miR-1197-5p,miR-188-3p,miR-2904,miR-183-5p,miR-98-5p,let-7k,miR-299-3p,miR-34b-5p,miR-542-5p,miR-208b-3p,miR-6130,let-7i-5p,miR-151b,novel_32,miR-204-3p,miR-504-5p,miR-140-5p,miR-2331-3p,miR-1827,miR-2285c,miR-6134,let-7b-5p,miR-3184-5p,miR-4510,let-7b,novel_74,miR-130b-5p,miR-874-5p,let-7f,miR-664a-5p,miR-151-3p,miR-324-3p,miR-147a,miR-496-5p,miR-153,miR-2285e,miR-455-3p,miR-9788-3p,miR-216a-3p,miR-151a-3p,miR-1839-5p,miR-301b-5p,miR-296-5p,miR-8485,let-7a-5p,miR-574-3p,novel_124,miR-491-5p,miR-3085-3p,let-7e,miR-542-5p,miR-153-3p,miR-1306-5p,miR-2957,miR-489,miR-25,miR-378g,let-7d-5p,miR-3064-5p,miR-504,miR-299b-5p,novel_94,miR-1306-3p,miR-877-3p,miR-154a,miR-423-5p,miR-212-3p,miR-2447,miR-3059-5p,miR-4454,miR-194b-5p,miR-4726-5p,miR-218-5p,miR-301a-5p

			[illegible] 3074-1-3p,miR-190a-3p,miR-2318,miR-18a,miR-320a,miR-331-5p,miR-381-3p,miR-365a-5p,miR-6128,miR-16-5p,miR-331-5p,miR-144,miR-135a-1-3p,miR-2332,novel_63,miR-410-5p,novel_121,miR-486-5p,miR-450a-1-3p,miR-545-5p,miR-6516-5p,miR-322-5p,miR-18b-5p,miR-429-3p,miR-200b-3p,miR-300,miR-582-3p,miR-1290,miR-345-3p,miR-196a-5p,miR-3591-5p,miR-450b-5p,miR-148b-5p,miR-24-2-5p,miR-29b-2-5p,miR-107,miR-200c-3p,miR-127-5p,miR-200c,miR-677,miR-144-3p,miR-320c,miR-411b-3p,miR-7-1-3p,miR-3120-3p,miR-195a-5p,miR-505-3p,miR-19a-3p,miR-107,miR-3065-5p,miR-18b,miR-2285ab,miR-2428,miR-323c,miR-495-3p,novel_1,miR-6119-3p,novel_87,miR-33a-3p,miR-16a,miR-142b,miR-15a,miR-103,miR-3431,miR-134-3p,miR-2284d,miR-3535,miR-665,miR-103a-3p,miR-2284x,miR-497-5p,miR-16b,miR-194-5p,miR-145a-5p,miR-542-3p,miR-1434-3p,miR-708-3p,miR-2284l,miR-2285r,miR-6240,miR-493-5p,miR-196b-5p,miR-2411-3p,miR-194a,miR-330-3p,miR-190a,miR-365b-5p,miR-15b-5p,novel_25,miR-6130,miR-6516,miR-582-5p,miR-532-5p,miR-182-5p,miR-216b-3p,miR-32-3p,miR-18a-5p,miR-2312,let-7g-3p,miR-21a-3p,miR-6525,miR-4510,miR-190a-5p,miR-424-5p,miR-664a,miR-29b-2-5p,miR-2285j,miR-2284h-5p,novel_83,miR-15a-5p,miR-340-5p,miR-200b,miR-505-3p,miR-212-5p,miR-3600,miR-19b-3p,miR-4429,miR-1434-5p,miR-2904,miR-1246,miR-2424,miR-130a-5p,miR-345-3p,miR-2285w,miR-21-3p,miR-2284m,miR-320d,miR-345-3p,miR-500a-5p,miR-216a-5p,miR-196a-3p,miR-2483-3p,miR-378g,miR-24-1-5p,miR-9-3p,miR-493-5p,miR-335-3p,miR-129b-5p,miR-194b-5p,miR-190b-5p,miR-2285f,miR-32-3p,miR-3964,miR-628-3p,miR-107-5p,miR-126a-5p,miR-539-5p,miR-153,miR-222-
ARHGAP5	ENSOARG0(	Rho GTPa	5p,miR-145b,miR-181b-2-3p,miR-330-3p,miR-3120-5p,miR-

SERPINB12	ENSOARG0(	serpin p	miR-2319b,miR-139-5p,miR-126b-5p,miR-378g,miR-144-5p,miR-652-3p,miR-504,miR-3963,miR-96-5p,miR-2447,miR-423-5p,miR-154-3p,miR-330-3p,miR-1343-3p,miR-181b-2-3p,miR-153,miR-126a-5p,miR-455-3p,miR-8485,miR-182-5p,miR-29b-1-5p,miR-130b-3p,miR-491-5p,miR-103a-2-5p,miR-146b,miR-153-3p,miR-504-5p,miR-30c-1-3p,miR-10a,miR-299,miR-30d-5p,miR-345-5p,miR-1827,miR-3184-3p,miR-224-5p,miR-769-5p,miR-3184-5p,miR-30e-3p,miR-30a-3p,miR-151-3p,novel_74,miR-4492,miR-454-3p,miR-874-5p,miR-129-5p,miR-30d-3p,miR-380-5p,miR-7859,miR-1247-5p,miR-2366,miR-30c-5p,miR-16-1-3p,miR-487a,miR-195a-3p,miR-181b-1-3p,miR-2285i,miR-421-5p,miR-500a-5p,novel_68,miR-3120-3p,miR-299b-3p,miR-374c-3p,miR-411b-3p,miR-181b-3p,miR-677,miR-669,miR-320c,miR-3591-3p,miR-1306,miR-150-5p,miR-146b-5p,miR-2319a,miR-335-5p,miR-29c-3p,miR-758-5p,miR-193b-3p,miR-142b,miR-29a,miR-31-5p,miR-134-3p,miR-194-3p,novel_116,miR-149-5p,miR-223-3p,miR-7b-5p,miR-432,miR-16-1-3p,miR-6240,miR-628-5p,miR-668-3p,miR-432-3p,miR-432-3p,miR-146a,miR-7-5p,miR-105-5p,miR-196b-5p,miR-193a-3p,miR-3956,miR-542-5p,miR-30e-5p,miR-345-5p,miR-329b,miR-144-5p,miR-1260b,miR-1197-3p,miR-6740-5p,miR-429-3p,miR-299a-3p,miR-758-5p,miR-505-5p,miR-2284z,miR-3591-5p,miR-196a-5p,miR-330-5p,miR-1343-5p,miR-3064-5p,miR-148b-5p,miR-2285b,miR-200a,novel_39,miR-664-3p,miR-127-5p,miR-200a-3p,novel_96,miR-10b,miR-664b,miR-3064-5p,miR-452-5p,miR-2285f,miR-107-5p,miR-769-5p,miR-335,miR-4726-5p,miR-151a-3p,miR-105-1,miR-9788-3p,miR-10b,miR-6529a,miR-484,miR-30b-3p,miR-2284aa,miR-16-2-3p,miR-652-5p,miR-30c-1-3p,miR-3187-3p,novel_103,miR-1306-5p,miR-326,miR-320b,miR-2957,miR-3085-3p,novel_124,miR-140-5p,miR-221-5p,miR-182-5p,miR-532-

IL19	ENSOARG0(	interleu	miR-211-5p,miR-499b-5p,miR-3064-5p,miR-4792,miR-500-3p,miR-203-3p,miR-2483-3p,miR-3082-5p,miR-7857-3p,miR-499a-3p,miR-199b-5p,miR-877-3p,miR-668-5p,miR-136-5p,miR-1296-5p,miR-3059-5p,miR-502-3p,miR-1285-5p,miR-361-3p,miR-301a-5p,miR-2285y,miR-2440,miR-3958-3p,miR-181b-2-3p,miR-2300a-5p,miR-1343-3p,miR-330-3p,miR-151a-3p,miR-421,miR-216a-3p,miR-142-5p,miR-501-3p,miR-301b-5p,miR-6529a,miR-21b,miR-1839-5p,miR-101c,miR-1388-3p,miR-124a,miR-2310,miR-431-5p,miR-320b,miR-199b-5p,miR-3085-3p,miR-552-3p,miR-32-3p,miR-140-5p,miR-221-5p,miR-151b,miR-155-5p,miR-216b-5p,miR-193b-3p,miR-377-3p,miR-499b-3p,miR-433-3p,miR-27b-5p,miR-129b-3p,novel_99,miR-145a-3p,miR-6134,miR-224-5p,miR-142a-5p,miR-6525,miR-665,miR-320e,miR-151-3p,miR-2285j,miR-130b-5p,miR-208b-5p,miR-874-5p,miR-155-5p,novel_74,miR-548e-3p,miR-328-3p,novel_83,miR-151-5p,miR-7859,miR-383-5p,miR-217-5p,miR-1197-5p,miR-140-3p,miR-1193,miR-5703,miR-124-3p,novel_133,miR-378d,miR-4429,miR-2424,miR-2411,miR-181b-1-3p,miR-125b-2-3p,miR-664b-3p,miR-92a-1-5p,miR-185-3p,miR-299-3p,miR-221-5p,miR-320d,miR-3120-3p,miR-655-5p,miR-320b,miR-181b-3p,miR-677,miR-320c,miR-299b-3p,miR-374c-3p,miR-378b,miR-216a-3p,miR-150-5p,miR-6395,miR-9851-3p,miR-1291,miR-495-3p,miR-193b-3p,miR-1a-1-5p,miR-199b-5p,miR-211,miR-576-3p,miR-147-5p,miR-22-5p,miR-26b-3p,miR-487b-3p,miR-665,miR-31-5p,miR-340-3p,miR-411-5p,miR-149-5p,miR-194-3p,miR-432-5p,miR-374b-3p,miR-3071-5p,miR-197-3p,miR-1843b-5p,miR-1285,miR-7b-5p,miR-1434-3p,miR-873a-5p,miR-432,miR-188-5p,miR-744-5p,miR-363-5p,miR-6240,miR-130b-5p,let-7j,miR-432-3p,miR-2285r,miR-330-3p,miR-377-3p,miR-7-5p,miR-224-5p,miR-193a-3p,miR-615-5p,miR-2355-5p,miR-3068-3p,miR-199a-5p,miR-320a,miR-
CDHR5	ENSOARG0(	cadherin	miR-200c,miR-5126,miR-431-5p,miR-326,miR-330-3p,novel_51,miR-185-3p,miR-491-5p,miR-200c-3p,miR-214-3p,novel_96,novel_127,miR-216c-5p,miR-6240,miR-29b-2-5p,miR-4532,miR-486-3p,novel_133,miR-542-3p,miR-330-5p,miR-30b-3p,miR-188-3p,miR-30b-3p,miR-200b-3p,miR-18a-3p,miR-2300a-5p,miR-429-3p,miR-330-3p,miR-431,miR-3431,miR-532-3p,miR-455-3p,miR-1306-3p,miR-331-3p,miR-665,miR-29b-2-5p,miR-874-5p,miR-486b-3p,miR-2447,miR-214,miR-103b,miR-361-3p,miR-2903,miR-371b-3p,miR-326-3p,miR-3184-5p,miR-423-5p,miR-122-5p,miR-18a-3p,miR-1306-3p,miR-876-3p,miR-23a-5p,miR-9851-3p,miR-1895,miR-671-5p,miR-574-5p,miR-140-5p,miR-615-5p,miR-670-3p,miR-669,miR-204-3p
DPP4	ENSOARG0(	dipeptid	miR-143-5p,miR-148a-3p,miR-100-3p,miR-148b-3p,miR-378a-5p,miR-152-3p,miR-208b-5p

CPNE9	ENSOARG0(	copine f	miR-10b-3p,miR-329b,miR-3074-1-3p,miR-485-5p,miR-488-3p,miR-301,miR-3154,miR-320a,miR-3965,miR-769,miR-370-3p,miR-134-5p,miR-486b-3p,miR-204-5p,miR-2433,miR-135a-1-3p,miR-331-3p,miR-365a-5p,miR-3607-3p,miR-134,miR-1343-5p,miR-330-5p,miR-9-5p,miR-505-5p,miR-24-2-5p,miR-134-5p,miR-214-5p,miR-450a-2-3p,miR-127-5p,novel_127,miR-30f,miR-2385-3p,miR-19a-3p,miR-146b-5p,miR-2387,miR-3141,miR-150-5p,miR-18a-3p,novel_69,miR-670-3p,miR-320c,miR-669,miR-3120-3p,miR-128-1-5p,miR-203b-5p,miR-502-5p,miR-576-3p,miR-211,miR-24-3p,miR-326-3p,miR-2428,miR-135a-2-3p,miR-3432b,miR-219a-1-3p,miR-4508,miR-188-5p,miR-744-5p,miR-432,miR-708-3p,miR-1285,miR-223-3p,miR-542-3p,miR-22-3p,miR-145a-5p,miR-432-5p,miR-3535,miR-134-3p,miR-31-5p,miR-1343-5p,miR-146a,miR-365b-5p,miR-190a,miR-3141,novel_51,miR-432-3p,let-7f-2-3p,let-7f-2-3p,miR-21a-3p,let-7f-1-3p,miR-433-3p,miR-139-5p,miR-22-3p,miR-204-3p,miR-504-5p,miR-182-5p,miR-4492,miR-874-5p,miR-190a-5p,miR-15b-3p,let-7b-3p,miR-19b-1-5p,miR-3604,miR-665,miR-769-5p,miR-98-3p,miR-346,miR-3184-5p,miR-6134,miR-433-5p,miR-19b-3p,miR-184-3p,miR-4429,let-7a-3p,miR-2366,miR-193b-5p,miR-2300b-3p,miR-98-3p,miR-2904,miR-5703,miR-18a-3p,miR-320d,miR-618,miR-2285p,miR-185-3p,miR-92a-1-5p,miR-376a-5p,miR-2432,miR-486-3p,miR-146a-5p,miR-216c-5p,miR-504,miR-335-3p,miR-9-3p,miR-381-3p,miR-378g,miR-2483-3p,miR-1185-2-3p,miR-4792,miR-139-5p,miR-7977,miR-211-5p,miR-4726-5p,miR-3968,miR-2285t,miR-101-3p,miR-769-5p,miR-1285-5p,miR-9-3p,miR-423-5p,miR-2447,miR-541-3p,miR-9-5p,miR-190b-5p,miR-101c,miR-296-5p,miR-541,miR-4791,miR-455-3p,miR-1185-3p,miR-145b,miR-1185-5p,miR-146b,miR-320b,miR-326,miR-182-5p,miR-2330-5p
DSG3	ENSOARG0(	desmogle	miR-345-3p,miR-217,miR-2411-3p,miR-105-5p,miR-219b-5p,miR-374a-5p,miR-26a-5p,miR-466f-3p,novel_96,miR-199b-5p,miR-181b-5p,miR-202-5p,miR-491-5p,miR-181a-2-3p,miR-382-3p,miR-380-3p,miR-2432,miR-199a-5p,miR-130a-5p,miR-145a-5p,miR-217-5p,miR-3578,miR-181a-5p,miR-345-3p,miR-708-3p,miR-1839-5p,miR-2426,miR-380-3p,miR-145b,miR-151a-3p,miR-105-1,miR-548e-3p,miR-3604,miR-151-3p,miR-181d-5p,miR-665-5p,miR-193a-5p,miR-32-3p,miR-374b,miR-135a-2-3p,miR-181c-5p,miR-199b-5p,miR-423-5p,miR-3184-5p,miR-129b-5p,miR-150-5p,miR-199b-5p,novel_94,miR-668-5p,miR-21-3p,miR-374b-5p,miR-32-3p,miR-20a-3p,miR-548o-3p,miR-133c,miR-199a-5p,miR-146b-3p,miR-3596,miR-27a-5p

GLRB	ENSOARG0(	glycine	miR-4510,miR-539-5p,miR-664a-5p,miR-29b-2-5p,miR-222-3p,miR-487a-5p,novel_74,miR-216b-3p,miR-323-5p,miR-6130,miR-6516,miR-433-3p,miR-411,miR-30d-5p,miR-30f,miR-195a-3p,miR-380-3p,miR-125b-2-3p,miR-377-5p,miR-130a-5p,miR-210-5p,miR-30b,miR-30d,miR-760-3p,miR-1b-3p,miR-18a-3p,miR-200b,miR-340-5p,miR-23b-3p,miR-383-5p,miR-5703,miR-140-3p,miR-2904,miR-124-3p,miR-30c-5p,miR-361-5p,miR-194b-5p,miR-6527,miR-212-3p,miR-101-3p,miR-30b-5p,miR-374a-3p,miR-628-3p,miR-211-5p,miR-499b-5p,miR-20a-3p,miR-2319b,miR-664b,miR-27a-3p,miR-203-3p,miR-489,miR-3082-5p,miR-381-3p,miR-378j,miR-129b-5p,miR-221,miR-376b,miR-23a-3p,miR-2330-5p,miR-382-3p,miR-124a,miR-2310,miR-505,miR-3955-3p,miR-27a-3p,miR-5010-3p,miR-1343-3p,miR-409b,miR-221-3p,miR-3962,miR-450b-5p,miR-2426,miR-374a-3p,miR-135a-5p,miR-6740-5p,miR-374b-3p,miR-204-5p,miR-3956,miR-1271,miR-141-3p,miR-370-3p,miR-874-3p,miR-3965,miR-23b,miR-30e-5p,miR-381-3p,miR-1814c,miR-485-5p,novel_48,miR-181a-2-3p,miR-29b-2-5p,miR-3956-5p,miR-29a-5p,miR-450b-5p,miR-2478,miR-200c,miR-664-3p,miR-205-5p,novel_127,miR-200a-3p,miR-200c-3p,miR-429-3p,miR-222,novel_121,miR-6516-5p,miR-30a-5p,miR-1290,miR-200b-3p,miR-30c,miR-300,miR-2284w,miR-211,novel_1,miR-378b,miR-329-5p,novel_68,miR-323a-5p,miR-3065-5p,miR-18a-3p,miR-135b-5p,miR-7975,miR-2319a,miR-6240,miR-432-3p,miR-194a,miR-330-3p,miR-452-5p,miR-377-5p,miR-185-5p,miR-194-5p,miR-3071-5p,miR-873a-5p,miR-708-3p,miR-27b-3p,miR-380-3p

PRKACA	ENSOARG0(	cAMP-dep	miR-300-3p,miR-769,miR-874-3p,miR-615-5p,miR-125a,miR-10a-3p,miR-485-5p,miR-3154,miR-1260b,miR-378c,miR-138-5p,miR-486b-3p,miR-2433,miR-331-3p,miR-125b-5p,miR-6740-5p,miR-3969,miR-378h,miR-483-3p,miR-214-5p,miR-299a-3p,miR-2285ad,novel_79,miR-10a-5p,miR-1343-5p,miR-330-5p,miR-200a,miR-1271-3p,miR-495-5p,miR-3064-5p,miR-181a-2-3p,miR-450a-2-3p,miR-2898,novel_127,novel_96,miR-2285af,miR-1260a,miR-664-3p,miR-30c-2-3p,miR-339a,novel_69,miR-299b-3p,miR-6535,miR-669,miR-378b,miR-3074-5p,miR-320b,miR-10a-5p,miR-3120-3p,miR-378f,miR-2387,miR-3141,miR-18a-3p,miR-326-3p,miR-2428,miR-4324,miR-339-5p,miR-138,miR-30b-3p,miR-1260b,miR-125b-1-3p,miR-4508,miR-873a-5p,miR-19b-2-5p,miR-130b-5p,miR-363-5p,miR-483-3p,miR-125a-5p,miR-1343-5p,miR-582,miR-2285v,miR-3141,miR-10a,miR-2284s,miR-216b-5p,miR-6130,miR-504-5p,miR-378a-3p,miR-221-5p,miR-30c-1-3p,miR-339b,miR-625-5p,miR-1827,miR-21a-3p,miR-10b-5p,miR-769-5p,miR-346,miR-3184-5p,miR-539-5p,miR-378c,miR-4510,miR-3074-5p,miR-664a,miR-4492,miR-130b-5p,miR-19b-1-5p,miR-532-3p,miR-18a-3p,miR-378d,miR-378i,miR-5703,miR-378a-3p,miR-1197-5p,miR-486-3p,miR-2432,miR-10a-3p,miR-345-3p,miR-30b-3p,miR-221-5p,miR-299-3p,miR-422a,miR-125b,miR-185-3p,miR-378e,miR-3082-5p,miR-664b,miR-3064-5p,miR-499b-5p,miR-2285u,miR-504,miR-335-3p,miR-361-3p,miR-423-5p,miR-2285n,miR-769-5p,miR-1343-3p,miR-378c,miR-2426,miR-378b,miR-2284r,miR-10b,miR-30b-3p,miR-30c-1-3p,miR-378d,miR-4532,miR-382-3p,miR-491-5p,miR-3085-3p,miR-25-5p,miR-326

MARS	ENSOARGO(	methiony	miR-2426,miR-544a,miR-1388-5p,miR-362-3p,miR-125b-2-3p,miR-30b-3p,miR-412-3p,miR-329-3p,miR-484,miR-9788-3p,miR-425-3p,miR-181b-2-3p,miR-222-5p,novel_4,miR-151a-3p,miR-214-3p,miR-103a-2-5p,miR-199b-5p,miR-106b-5p,miR-362-3p,novel_19,novel_103,miR-1306-5p,miR-320b,miR-1839-3p,miR-106a-5p,miR-504,miR-181a-3p,miR-199b-5p,miR-361-3p,miR-4443,miR-3082-5p,miR-499a-3p,miR-3596,miR-211-5p,miR-2285u,miR-664b,miR-2285t,miR-335,miR-3958-3p,miR-628-3p,miR-4726-5p,miR-107-5p,miR-769-5p,miR-371a-5p,novel_82,miR-219a-2-3p,miR-452-5p,miR-378d,miR-664-5p,miR-142-3p,miR-4429,miR-140-3p,miR-188-3p,miR-1193,miR-362-3p,miR-193b-5p,miR-376a-5p,miR-191,miR-616-3p,miR-1247-5p,miR-222-5p,miR-487a-3p,miR-485-3p,miR-106a,miR-18a-3p,miR-221-5p,miR-760-3p,miR-320d,miR-106a,miR-92a-1-5p,miR-324-3p,novel_17,miR-196a-3p,miR-143-3p,miR-380-3p,miR-486-3p,miR-2432,miR-2424,miR-376a-5p,miR-20a,miR-329a-5p,miR-216c-5p,miR-27b-5p,miR-22-3p,miR-323-5p,miR-216b-5p,miR-499b-3p,miR-544-3p,miR-582-5p,miR-296-3p,miR-552-3p,miR-370-5p,miR-221-5p,miR-504-5p,miR-2331-3p,miR-4492,miR-874-5p,miR-129-5p,miR-17-3p,miR-6516-3p,miR-665,miR-320e,miR-219b-3p,miR-19b-1-5p,miR-151-3p,miR-30a-3p,miR-30e-3p,miR-338-3p,miR-769-5p,miR-214,miR-665-5p,miR-425-5p,miR-744-5p,miR-2889,miR-455-5p,miR-26a-2-3p,miR-30b-3p,miR-380-3p,miR-197-3p,miR-22-3p,miR-1285,miR-1843b-5p,miR-7b-5p,miR-19b-2-5p,miR-708-3p,miR-20b,miR-544b,miR-2355-3p,novel_116,miR-194-3p,miR-149-5p,miR-185-5p,miR-665,miR-31-5p,miR-106a-5p,miR-455-5p,miR-17-5p,miR-197-5p,miR-7-5p,miR-539-3p,miR-654-3p,miR-628-5p,miR-382,miR-379-5p,miR-216a-3p,miR-18a-3p,miR-1306,miR-150-5p,miR-2387,miR-677,miR-320c,miR-323a-5p,miR-146b-3p,miR-655-5p,miR-329-

KIAA1958	ENSOARG0(	KIAA1958	miR-23a-5p,miR-381-3p,miR-320a,miR-154b-5p,miR-141-3p,miR-138-5p,miR-1-5p,miR-323-3p,miR-378c,miR-1306-5p,miR-3607-3p,let-7d,miR-582-3p,miR-300,miR-105-2,miR-128-3p,miR-345-3p,miR-3578,miR-501-3p,miR-24-2-5p,miR-302a-5p,miR-483-3p,miR-1271-5p,miR-378h,miR-214-5p,miR-450a-2-3p,miR-181b-5p,miR-92a-3p,miR-6119-5p,let-7c-5p,miR-2478,miR-24-2-5p,miR-1271-3p,miR-490-3p,miR-181a-2-3p,miR-4286,miR-1291,miR-195a-5p,miR-505-3p,miR-135b-5p,miR-6516-3p,miR-2387,miR-18a-3p,miR-378b,miR-320b,miR-92a-3p,novel_1,miR-147-5p,miR-502b,miR-199c,miR-15a,miR-16a,let-7i,miR-24-3p,miR-326-3p,miR-2428,miR-495-3p,miR-138,miR-653,miR-181a-5p,miR-145a-5p,miR-2285g,miR-503-5p,miR-185-5p,let-7g-5p,miR-122-3p,miR-665,miR-377-5p,miR-503-5p,miR-539-3p,miR-345-5p,miR-130b-5p,let-7g-3p,miR-21a-3p,miR-27b-5p,miR-193b-3p,miR-204-3p,novel_32,miR-545-3p,miR-378a-3p,miR-532-5p,miR-182-5p,miR-664a,miR-208b-5p,miR-424-5p,miR-324-3p,miR-320e,let-7b-5p,miR-338-3p,miR-378c,miR-145a-3p,miR-1271,miR-4429,miR-941,miR-378d,novel_133,miR-362-3p,miR-2408,miR-188-3p,miR-5703,miR-140-3p,miR-1193,miR-487a-3p,miR-15a-5p,miR-148b-3p,miR-151-5p,novel_83,miR-532-3p,miR-3960,miR-3600,miR-485-3p,miR-505-3p,miR-320d,miR-760-3p,miR-466i-5p,miR-21-3p,miR-185-3p,miR-125b-2-3p,novel_94,miR-378e,let-7d-5p,miR-664b,miR-3064-5p,miR-10b,miR-20a-3p,miR-7977,miR-4726-5p,miR-2285n,miR-1973,miR-2285y,miR-107-5p,miR-502-3p,novel_60,miR-212-3p,miR-541-3p,miR-2285f,miR-181d-5p,miR-378c,miR-450b-5p,miR-6529a,miR-378b,miR-9788-3p,miR-216a-3p,miR-2285e,miR-105-1,miR-151a-3p,miR-2300a-5p,miR-409b,miR-5010-3p,miR-222-5p,miR-3085-3p,novel_124,miR-1468,let-7e,miR-320b,novel_115,miR-326,miR-362-3p,let-7a-5p,miR-
TMEM30A	ENSOARG0(	transmem	miR-193a-3p,miR-378d,miR-545-5p,miR-193b-3p,miR-193b-3p

SSR4	ENSOARG0(	signal s	miR-374c-3p,miR-677,miR-669,miR-181b-3p,novel_68,miR-329-5p,miR-3120-3p,miR-3141,miR-3591-3p,miR-758-5p,miR-6123,miR-4324,miR-1307-5p,miR-134-3p,miR-194-3p,miR-149-5p,miR-3955-5p,miR-31-5p,miR-339-5p,miR-16b,miR-16-1-3p,miR-497-5p,miR-27b-3p,miR-197-3p,miR-22-3p,miR-432-3p,miR-628-5p,miR-365b-5p,miR-3141,miR-105-5p,novel_51,miR-194a,miR-874-3p,miR-2285aa,miR-2355-5p,miR-125a,miR-2474,miR-23b,novel_42,miR-16-5p,miR-592,miR-2433,miR-125b-5p,miR-100-3p,miR-6740-5p,miR-322-5p,miR-505-5p,miR-129-1-3p,miR-2285ad,novel_79,miR-18b-5p,miR-1343-5p,miR-330-5p,miR-29a-5p,miR-3956-5p,miR-2285b,miR-192-3p,miR-3064-5p,novel_96,novel_127,miR-664-3p,miR-125a-3p,miR-378g,miR-499a-3p,miR-500-3p,miR-4443,miR-211-5p,miR-504,miR-668-5p,miR-544-5p,miR-140-5p,miR-361-3p,miR-7705,miR-2447,miR-3956-3p,miR-190b-5p,miR-3968,miR-218-5p,miR-2440,miR-425-3p,miR-1298-3p,miR-147a,miR-421,miR-199b-3p,novel_78,miR-181b-2-3p,miR-2284r,miR-501-3p,miR-8485,miR-2310,miR-1388-3p,miR-23a-3p,miR-491-5p,miR-103a-2-5p,miR-214-3p,miR-29b-1-5p,miR-16b,miR-2284s,miR-499b-3p,miR-216b-5p,miR-15b-5p,miR-151b,miR-504-5p,miR-216b-3p,novel_107,miR-552-3p,miR-339b,miR-1895,miR-625-5p,miR-1827,miR-129b-3p,miR-500a-3p,miR-433-3p,miR-6525,miR-769-5p,miR-129-2-3p,miR-665-5p,miR-214,miR-500,miR-4492,miR-190a-5p,miR-874-5p,miR-130b-5p,miR-665,miR-664a-5p,miR-7859,miR-23b-3p,miR-23c,miR-2284h-5p,miR-18a-3p,miR-380-5p,miR-493-3p,miR-184-3p,miR-33b-3p,miR-16-1-3p,miR-34a-5p,miR-2366,miR-2285i,miR-181b-1-3p,miR-486-3p,miR-143-3p,miR-329a-5p,miR-2285w,miR-503-5p,miR-345-3p,novel_17,miR-323a-5p,miR-146b-3p,miR-34b,miR-320b,miR-1291,miR-2285k,miR-9851-3p,miR-195a-5p,miR-2387,miR-18b,miR-18a-

ATP1B3	ENSOARG0(	ATPase,	novel_79,miR-1224-5p,miR-483-3p,miR-9-5p,miR-330-5p,miR-218-2-3p,miR-345-3p,miR-10a-5p,miR-4286,miR-181a-2-3p,miR-2285b,novel_39,miR-199a-5p,miR-2478,miR-28b,miR-339a,miR-125a-3p,miR-152-3p,miR-127-5p,miR-2355-5p,miR-370-3p,miR-874-3p,miR-487a-3p,miR-324-5p,miR-199a-5p,miR-133c,miR-223-5p,miR-374c-3p,miR-23b,miR-3154,miR-6128,miR-365a-5p,miR-6740-5p,miR-34c-3p,miR-486b-3p,miR-28-5p,miR-339-5p,novel_116,miR-197-3p,miR-708-3p,miR-27b-3p,miR-188-5p,miR-455-5p,miR-219a-1-3p,miR-30b-3p,miR-493-5p,miR-130b-5p,miR-296-3p,miR-432-3p,miR-432-3p,miR-455-5p,miR-2411-3p,miR-365b-5p,miR-296-3p,miR-224-5p,miR-452-5p,miR-124-5p,miR-483-3p,miR-10a-5p,miR-3120-3p,miR-320b,miR-329-5p,miR-150-5p,miR-148a-3p,miR-6516-3p,miR-7975,miR-2319a,miR-3957-3p,miR-2428,miR-199b-5p,miR-326-3p,miR-28c,miR-203b-5p,miR-128-1-5p,miR-148b-3p,miR-23c,miR-23b-3p,miR-95-3p,miR-5703,miR-140-3p,miR-188-3p,novel_133,miR-148a-5p,miR-142-3p,miR-487a,miR-216c-5p,miR-329a-5p,miR-486-3p,miR-345-3p,miR-185-3p,miR-210-5p,miR-196a-3p,miR-182-5p,miR-154a-3p,miR-216b-3p,miR-32-3p,miR-2331-3p,miR-10a,miR-299,miR-186-5p,miR-154b-3p,miR-339b,miR-425-5p,miR-145a-3p,miR-224-5p,miR-10b-5p,miR-130b-5p,miR-708-5p,miR-27a-3p,miR-548w,novel_78,miR-9788-3p,miR-30b-3p,miR-10b,miR-2426,miR-23a-3p,miR-182-5p,novel_115,novel_103,miR-326,miR-199b-5p,miR-7689-3p,miR-2319b,miR-27a-3p,miR-454-5p,miR-25,miR-199b-5p,miR-493-5p,miR-9-5p,miR-452-5p,miR-32-3p,novel_82,miR-154-3p

IDH3G	ENSOARG0(	isocitra	miR-424-5p,miR-652-5p,miR-30c-1-3p,miR-4532,miR-320b,miR-1306-5p,miR-5126,miR-3085-3p,novel_124,miR-3955-3p,novel_4,miR-2300a-5p,miR-5010-3p,miR-2285e,miR-296-5p,miR-484,miR-10b,miR-30b-3p,miR-450b-5p,novel_60,miR-541-3p,miR-212-3p,miR-2285f,miR-769-5p,miR-107-5p,miR-4726-5p,miR-3064-5p,miR-499b-5p,miR-7977,miR-3082-5p,miR-361-3p,miR-877-3p,miR-130a-5p,miR-196a-3p,miR-185-3p,miR-320d,miR-760-3p,miR-466i-5p,miR-221-5p,miR-34b-5p,miR-30b-3p,miR-299-3p,miR-21-3p,miR-15a-5p,miR-532-3p,novel_133,miR-2408,miR-2300b-3p,miR-1197-5p,miR-1193,miR-140-3p,miR-5703,miR-4429,miR-378d,miR-425-5p,miR-10b-5p,miR-6134,miR-324-3p,miR-338-5p,miR-320e,miR-17-3p,miR-664a,miR-424-5p,miR-221-5p,miR-370-5p,miR-3065-3p,miR-182-5p,miR-132-5p,miR-193b-3p,miR-139-5p,miR-22-3p,novel_32,miR-21a-3p,miR-2312,miR-130b-5p,miR-363-5p,miR-654-3p,miR-197-5p,miR-1343-5p,miR-130a-3p,miR-26b-3p,miR-377-5p,miR-2285g,miR-1285,miR-138,miR-30b-3p,miR-1260b,miR-744-5p,miR-7862,miR-2889,miR-1193,miR-17-3p,miR-211,miR-24-3p,miR-3959-3p,miR-371b-3p,miR-449a,miR-15a,miR-6402,miR-6529b,miR-34c,miR-16a,miR-128-1-5p,miR-656-5p,miR-22-5p,miR-147-5p,miR-363,miR-378b,miR-320b,miR-10a-5p,miR-146b-3p,miR-34b,miR-2285ab,miR-18a-3p,miR-450b-3p,miR-1291,miR-9851-3p,miR-195a-5p,miR-379-5p,miR-181a-2-3p,miR-3065-3p,miR-490-3p,miR-1271-3p,miR-1260a,miR-30c-2-3p,miR-339a,miR-17-3p,miR-2898,miR-543-5p,miR-24-2-5p,miR-483-3p,miR-214-5p,miR-615-3p,miR-582-3p,miR-10a-5p,miR-1306-5p,miR-410-5p,miR-370-5p,miR-331-3p,miR-138-5p,miR-486b-3p,miR-204-5p,miR-370-3p,miR-615-5p,miR-34c-5p,miR-769,miR-154b-5p,miR-320a,miR-3074-1-3p,miR-485-5p,miR-1388-3p,miR-505,miR-182-5p,miR-16b,miR-130b-3p,miR-193a-2-5p,miR-381-
IGF2BP1	ENSOARG0(	insulin-	miR-125b-5p,miR-6740-5p,miR-664a,novel_9,miR-181a-2-3p,miR-129-2-3p,miR-6128,miR-665-5p,miR-6525,miR-365a-5p,miR-2285c,miR-2312,miR-339b,miR-23a-5p,miR-671-5p,miR-3956,miR-125a,miR-34c-5p,miR-2284s,miR-199a-5p,miR-204-3p,miR-412-5p,miR-125b,miR-339a,miR-185-3p,miR-92a-1-5p,miR-34b-5p,novel_96,miR-125b-2-3p,miR-199a-5p,miR-2411,miR-146a-5p,miR-2285b,miR-23b-5p,miR-5703,miR-128-3p,miR-34a-5p,novel_79,miR-2411-5p,miR-483-3p,miR-129-1-3p,miR-412-5p,miR-4324,miR-101-3p,miR-34c,novel_120,miR-2447,miR-541-3p,miR-6527,miR-2285f,miR-449a,miR-154a,miR-199b-5p,miR-2428,miR-2285ab,miR-199b-5p,miR-3141,miR-216a-3p,miR-450b-3p,miR-146b-5p,miR-655-5p,miR-126b-5p,miR-34b,miR-670-3p,miR-146a,miR-330-3p,miR-365b-5p,miR-3141,miR-483-3p,miR-491-5p,miR-146b,miR-199b-5p,miR-125a-5p,miR-1388-3p,miR-6240,miR-33a-5p,let-7j,miR-193a,miR-6529a,miR-1285,miR-541,miR-330-3p,novel_4,miR-665,miR-339-5p,miR-222-5p,miR-2285g,miR-9788-3p,miR-216a-3p,miR-2355-3p,miR-411b-5p,miR-2285e

INTS8	ENSOARG0(	integrat	miR-499a-5p,miR-101-3p,miR-218-5p,miR-30b-5p,miR-9-3p,miR-212-3p,miR-32-3p,miR-3059-5p,miR-194b-5p,miR-96-5p,miR-9-3p,miR-493-5p,miR-221,miR-199b-5p,miR-140-5p,miR-7857-3p,miR-499a-3p,miR-27a-3p,miR-4443,miR-203-3p,miR-3064-5p,miR-2284g,miR-664b,miR-2285u,miR-487b-5p,miR-130b-3p,miR-3085-3p,miR-199b-5p,miR-323b-3p,miR-29b-1-5p,miR-2310,miR-4532,miR-101b-3p,miR-124a,miR-376b,miR-23a-3p,miR-2426,miR-3120-5p,miR-101c,miR-4791,miR-323b,miR-221-3p,miR-216a-3p,miR-151a-3p,miR-27a-3p,miR-17-3p,miR-6516-3p,miR-454-3p,miR-487a-5p,miR-30e-3p,miR-222-3p,miR-29b-2-5p,miR-664a-5p,miR-151-3p,miR-324-3p,miR-30a-3p,miR-224-5p,miR-338-3p,miR-3074-2-3p,miR-425-5p,miR-1271,miR-30f,miR-345-5p,novel_99,miR-21a-3p,miR-30d-5p,miR-411,miR-216b-5p,miR-499b-3p,miR-548o-3p,miR-32-3p,miR-660-5p,miR-2331-3p,miR-21-3p,miR-30d,miR-2285p,miR-208b-3p,miR-196a-3p,miR-30b,miR-122-3p,miR-92a-1-5p,miR-376a-5p,miR-29b-1-5p,miR-486-3p,novel_73,miR-2284u,miR-218-1-3p,miR-16-1-3p,miR-376a-5p,miR-215-5p,miR-30c-5p,miR-1434-5p,miR-1193,miR-188-3p,miR-124-3p,miR-200b,miR-23c,miR-23b-3p,miR-30d-3p,miR-6402,miR-2428,miR-301b-3p,miR-199b-5p,miR-17-3p,miR-376a-2-5p,miR-2319a,miR-3065-5p,miR-374c-3p,miR-196b-5p,miR-660,miR-224-5p,miR-7-5p,novel_51,miR-194a,miR-432-3p,miR-432-3p,miR-345-5p,miR-493-5p,miR-16-1-3p,miR-2284ab,miR-27b-3p,miR-7b-5p,miR-1434-3p,miR-708-3p,miR-653,miR-194-5p,miR-3535,miR-194-3p,miR-133b-5p,miR-130a-3p,miR-486b-3p,miR-134-5p,miR-410-3p,miR-410-5p,novel_63,miR-3969,miR-1197-3p,miR-28a-3p,miR-345-5p,miR-1814c,miR-301,miR-381-3p,miR-30e-5p,miR-23b,miR-374c-3p,miR-199a-5p,miR-133c,miR-223-5p,miR-6536,miR-200c-3p,miR-17-3p,miR-28-3p,miR-200c,miR-664-3p,miR-450b-5p,miR-192-
GPM6A	ENSOARG0(	glycopro	miR-210-5p,miR-224-5p,miR-32,miR-6536,miR-487b-5p,miR-92a-3p,miR-181a-2-3p,miR-432-3p,miR-432-3p,let-7a-2-3p,miR-2432,miR-1290,miR-1843b-5p,miR-6529a,miR-708-3p,miR-142-3p,let-7a-2-3p,miR-133a-3p,miR-3955-5p,miR-758-5p,miR-328-3p,miR-216a-3p,novel_87,miR-425-5p,miR-664a,miR-92a-3p,miR-204-5p,miR-28-5p,miR-500,miR-708-5p,miR-203b-5p,miR-539-5p,miR-452-5p,miR-144-5p,miR-224-5p,miR-211,miR-6134,miR-2428,miR-409-5p,miR-3591-3p,miR-433-3p,miR-3065-5p,miR-500a-3p,miR-200a-5p,let-7g-3p,miR-876-3p,miR-6395,miR-20a-3p,miR-32-5p,miR-7-1-3p,miR-211-5p,miR-21-3p,miR-25-3p,miR-216b-5p,miR-144-5p,miR-223-5p

HSDL2	ENSOARG0(	hydroxys	miR-129-1-3p,miR-134-5p,miR-1224-5p,miR-6517,miR-2285ad,let-7d,miR-330-5p,miR-2427,miR-1290,miR-134,miR-196a-5p,miR-345-3p,miR-192-3p,miR-1271-3p,let-7g,miR-200a,novel_39,miR-30a-3p,miR-29b-2-5p,miR-3529-3p,miR-200a-3p,miR-181b-5p,let-7f-5p,miR-2898,miR-17-3p,let-7c-5p,miR-6119-5p,miR-125a-3p,miR-1260a,miR-141-3p,miR-2285aa,miR-671-5p,miR-1814c,miR-3074-1-3p,miR-345-5p,miR-10b-3p,miR-362-5p,miR-3154,miR-145-3p,miR-1260b,miR-133a-3p,miR-134-5p,miR-592,miR-486b-3p,miR-144,miR-100-3p,miR-6740-5p,novel_63,miR-149-5p,let-7g-5p,miR-3535,miR-130a-3p,miR-381-5p,miR-16-1-3p,miR-1260b,miR-219a-1-3p,miR-30b-3p,miR-3071-5p,miR-374b-3p,miR-1843b-5p,miR-181a-5p,miR-873a-5p,miR-345-5p,miR-432-3p,miR-432-3p,miR-6240,miR-196b-5p,miR-2411-3p,novel_51,miR-330-3p,miR-377-3p,miR-181b-3p,miR-144-3p,miR-677,miR-374c-3p,miR-6395,miR-505-3p,let-7e-5p,miR-1291,miR-3065-5p,miR-1306,miR-2428,miR-301b-3p,miR-326-3p,miR-17-3p,novel_1,miR-33a-3p,let-7i,novel_87,miR-328-3p,miR-199a-3p,miR-532-3p,miR-222-5p,miR-212-5p,miR-505-3p,miR-30d-3p,let-7a-3p,miR-361-5p,miR-33b-3p,miR-1197-5p,miR-188-3p,miR-5703,miR-1961,miR-2366,miR-1246,miR-143-3p,miR-2424,miR-486-3p,miR-181b-1-3p,miR-376a-5p,miR-29b-1-5p,miR-345-3p,let-7k,miR-21-3p,miR-98-5p,miR-92a-1-5p,miR-345-3p,novel_17,miR-618,let-7i-5p,miR-204-3p,miR-656-3p,miR-377-3p,miR-301a-3p,miR-543-3p,miR-345-5p,miR-133b-3p,miR-27b-5p,let-7f-1-3p,miR-181c-5p,let-7b-5p,miR-224-5p,miR-142a-5p,miR-3074-2-3p,miR-129-2-3p,miR-145a-3p,miR-590-3p,let-7b,miR-454-3p,miR-17-3p,miR-29b-2-5p,miR-30a-3p,let-7f,let-7b-3p,miR-455-3p,miR-9788-3p,miR-500-5p,miR-142-5p,miR-181b-2-3p,miR-330-3p,miR-409b,miR-1343-3p,miR-30b-3p,miR-143-5p,miR-4791,miR-23a-

LAG3	ENSOARG0(	lymphocy	miR-199b-5p,miR-29c-3p,miR-206,miR-29a,miR-6123,miR-425-5p,novel_69,miR-6535,miR-669,miR-3074-5p,miR-3120-3p,miR-412-3p,miR-146b-5p,miR-2319a,miR-148a-3p,miR-3141,miR-3065-5p,miR-216a-3p,miR-1306,miR-432-3p,miR-432-3p,miR-2285r,miR-628-5p,miR-668-3p,miR-483-3p,miR-378a-5p,miR-146a,miR-296-3p,novel_51,miR-3141,miR-134-3p,miR-412-5p,miR-149-5p,miR-411-5p,miR-194-3p,miR-31-5p,miR-339-5p,miR-16b,miR-497-5p,miR-542-3p,miR-2113,miR-708-3p,miR-197-3p,miR-1197-3p,miR-181a-2-3p,novel_42,miR-193a-5p,miR-16-5p,miR-592,miR-125b-5p,miR-100-3p,miR-3068-3p,miR-874-3p,miR-574-5p,miR-1271,miR-125a,miR-542-5p,miR-1248,miR-450b-5p,novel_39,miR-200a,miR-3064-5p,miR-152-3p,novel_96,miR-125a-3p,miR-322-5p,novel_121,miR-6517,novel_79,miR-330-5p,novel_111,miR-361-3p,miR-423-5p,miR-3956-3p,miR-2447,miR-499a-5p,novel_82,miR-301a-5p,miR-378g,miR-4443,miR-4792,miR-211-5p,miR-3963,miR-1983,miR-376a-3p,miR-8485,miR-574-3p,miR-491-5p,miR-214-3p,miR-199b-5p,miR-146b,miR-16b,miR-323b-3p,miR-376b-3p,miR-1298-3p,miR-425-3p,novel_78,miR-1388-5p,miR-301b-5p,miR-758-3p,miR-323b,miR-1a-3p,miR-6525,miR-769-5p,miR-3184-5p,miR-3074-5p,miR-3184-3p,miR-214,miR-130b-5p,miR-874-5p,miR-4492,miR-3604,miR-15b-5p,miR-2331-3p,miR-552-3p,miR-296-3p,miR-216b-3p,miR-339b,miR-1827,miR-625-5p,miR-655,miR-129b-3p,miR-377-5p,miR-2285i,miR-376a-5p,miR-143-3p,miR-486-3p,miR-345-3p,miR-92a-1-5p,miR-324-3p,miR-210-3p,miR-328-3p,miR-142-3p,miR-184-3p,miR-34a-5p,miR-2366,miR-211,miR-326-3p,miR-449a,miR-135a-2-3p,miR-342-3p,miR-1193,novel_1,miR-656-5p,miR-15a,miR-16a,miR-34c,miR-29a-3p,miR-670-3p,miR-34b,miR-323a-5p,miR-146b-3p,miR-320b,miR-2285k,miR-450b-3p,miR-1291,miR-195a-5p,miR-
ABHD14B	ENSOARG0(	abhydrol	miR-130a-3p,miR-412-5p,miR-1298-3p,miR-185-5p,miR-147a,miR-9788-3p,miR-2284r,miR-143-5p,miR-450c-3p,miR-4508,miR-744-5p,miR-455-5p,miR-127-3p,miR-4532,miR-320b,miR-1306-5p,miR-197-5p,miR-455-5p,miR-2331-5p,miR-491-5p,miR-130b-3p,miR-660,miR-1343-5p,miR-214-3p,miR-4792,miR-299b-3p,miR-6535,miR-320c,miR-361-3p,miR-148a-3p,miR-1306,miR-450b-3p,miR-96-5p,miR-3059-5p,novel_60,miR-335-5p,miR-3956-3p,miR-361-3p,miR-877-3p,miR-24-3p,miR-423-5p,miR-301b-3p,miR-4324,miR-28c,novel_87,miR-128-1-5p,miR-4726-5p,miR-335,miR-490-5p,miR-299a-3p,miR-6517,miR-210-3p,miR-1271-5p,miR-148b-3p,miR-450a-1-3p,miR-214-5p,novel_133,miR-2366,miR-1343-5p,miR-1434-5p,miR-5703,miR-4429,miR-582-3p,miR-133a-3p,miR-2385-3p,miR-181a-2-3p,miR-28b,miR-142a-3p,miR-1271-3p,miR-30f,miR-185-3p,miR-320d,miR-2898,miR-466i-5p,miR-543-5p,miR-299-3p,novel_96,miR-152-3p,miR-2331-3p,miR-660-5p,miR-301a-3p,miR-324-5p,miR-450a-1-3p,miR-320a,let-7g-3p,miR-7144-5p,miR-485-5p,miR-1271,miR-214,novel_44,miR-145-3p,miR-3184-5p,miR-6134,miR-551b-3p,miR-331-3p,miR-450b-3p,miR-664a,miR-454-3p

ALDH5A1	ENSOARG0(	aldehyde	miR-128-3p,miR-411-3p,miR-214-5p,miR-6516-5p,miR-483-3p,miR-378h,miR-543-5p,miR-107,miR-450a-2-3p,miR-30c-2-3p,miR-2285af,miR-490-3p,miR-2284a,miR-1271-3p,miR-376b-5p,miR-376c-5p,miR-24-2-5p,miR-2478,miR-30a-3p,miR-3529-3p,miR-7144-5p,miR-320a,miR-17-5p,miR-503-3p,miR-204-5p,miR-144,miR-331-3p,miR-2332,miR-378c,miR-93,miR-133a-3p,miR-103b,miR-30b-3p,miR-1434-3p,miR-103,miR-20b,miR-2284d,miR-432-5p,miR-377-5p,miR-130a-3p,miR-133b-5p,miR-466f-3p,miR-1343-5p,miR-125a-5p,miR-106a-5p,miR-17-5p,miR-197-5p,miR-330-3p,miR-654-3p,miR-101-5p,miR-195a-5p,miR-6395,miR-379-5p,miR-19a-3p,miR-107,miR-2285k,miR-670-3p,miR-7-1-3p,miR-320b,miR-378b,miR-33a-3p,miR-16a,miR-6402,miR-20a-5p,miR-15a,miR-326-3p,miR-24-3p,miR-211,miR-19b-3p,miR-4429,miR-188-3p,miR-5703,miR-532-3p,novel_83,miR-616-3p,miR-15a-5p,miR-383-5p,miR-3600,miR-106a,miR-320d,miR-125b,miR-185-3p,miR-20a,novel_99,miR-133b-3p,miR-21a-3p,miR-139-5p,miR-193b-3p,miR-182-5p,miR-378a-3p,miR-487a-5p,miR-208b-5p,miR-424-5p,miR-6516-3p,miR-450b-3p,miR-324-3p,miR-378c,miR-3120-5p,miR-2284aa,miR-101a-5p,miR-544a,miR-378c,miR-30b-3p,miR-378b,miR-216a-3p,miR-27a-3p,miR-3955-3p,novel_103,miR-326,miR-1306-5p,miR-320b,miR-3187-3p,miR-30c-1-3p,miR-376b,miR-33a-5p,miR-494-3p,miR-382-3p,miR-106a-5p,miR-424-5p,novel_94,miR-129b-5p,miR-454-5p,miR-27a-3p,miR-3082-5p,miR-378e,miR-20a-3p,miR-3958-3p,miR-219a-2-3p,miR-452-5p,miR-330-5p,miR-1343-5p,miR-2284z,miR-450a-1-3p,miR-322-5p,miR-505-5p,miR-6517,miR-2285ad,novel_96,miR-219-3p,miR-125a-3p,miR-2284y,miR-148b-5p,miR-20b-5p,miR-329b,miR-93-5p,miR-137-3p,miR-125a,miR-3956,miR-592,miR-125b-5p,miR-2284k,miR-16-5p,miR-193a-5p,miR-2284y,miR-27b-3p,miR-26a-2-3p,miR-497-

SUSD1	ENSOARG0(	sushi do	miR-146b-5p,miR-378f,miR-412-3p,miR-150-5p,miR-216a-3p,miR-376b-5p,miR-6535,miR-677,miR-144-3p,miR-181b-3p,miR-3120-3p,miR-3074-5p,miR-206,miR-2284o,novel_87,miR-425-5p,miR-4324,miR-576-3p,miR-335-5p,miR-26a-2-3p,miR-497-5p,miR-16b,miR-16-1-3p,miR-22-3p,miR-450c-3p,novel_23,miR-432,miR-7b-5p,novel_116,miR-3535,miR-412-5p,miR-3431,miR-31-5p,miR-378a-5p,miR-483-3p,miR-7-5p,miR-377-3p,miR-146a,miR-6240,miR-1248,miR-2318,miR-345-5p,miR-376c-5p,miR-30e-5p,miR-3959-5p,miR-2285aa,miR-3068-3p,miR-503-3p,miR-592,miR-100-3p,miR-1197-3p,miR-16-5p,miR-144-5p,miR-28a-3p,miR-30c,miR-2427,miR-450a-1-3p,novel_121,miR-9-5p,miR-322-5p,miR-505-5p,miR-1224-5p,miR-2285ad,miR-429-3p,miR-200a-3p,miR-28-3p,miR-30f,miR-205-5p,miR-664-3p,miR-200a,miR-29a-5p,miR-2284y,miR-450b-5p,miR-148b-5p,miR-2284f,miR-376a-3p,miR-1983,miR-9-3p,miR-136-5p,miR-144-5p,miR-4443,miR-25,miR-3596,miR-2483-3p,miR-211-5p,miR-139-5p,miR-2284g,miR-301a-5p,novel_82,miR-2440,miR-1285-5p,miR-9-5p,let-7i-3p,miR-190b-5p,miR-3956-3p,miR-1388-5p,miR-1a-3p,miR-301b-5p,miR-148b-5p,miR-2459,miR-1298-3p,miR-376b-3p,miR-147a,miR-142-5p,miR-181b-2-3p,miR-330-3p,miR-548w,miR-146b,miR-103a-2-5p,miR-1185-5p,miR-29b-1-5p,miR-16b,miR-8485,miR-2310,miR-378d,miR-2284b,miR-1388-3p,miR-124a,miR-625-5p,miR-543-3p,miR-1827,miR-345-5p,miR-129b-3p,miR-500a-3p,miR-30d-5p,miR-433-3p,miR-2285c,miR-450a-1-3p,miR-15b-5p,miR-10a,miR-2284s,miR-377-3p,miR-582-5p,miR-552-3p,miR-548o-3p,miR-190a-5p,miR-500,miR-874-5p,miR-4492,miR-130b-5p,miR-181c-5p,miR-142a-5p,miR-769-5p,miR-6525,miR-3074-5p,miR-16-1-3p,miR-664-5p,miR-2284u,miR-148a-5p,miR-493-3p,miR-378a-3p,miR-30c-5p,miR-378i,miR-2366,miR-2284b-5p,miR-328-3p,miR-7859,miR-500a-
TEX40	ENSOARG0(	testis e	miR-125b-2-3p,miR-376a-5p,miR-490-3p,miR-2403,miR-4286,miR-181a-2-3p,miR-760-3p,miR-2898,miR-320d,miR-450a-2-3p,miR-2484,miR-30b-3p,miR-34b-5p,miR-210-5p,miR-185-3p,miR-324-3p,miR-30c-2-3p,miR-483-3p,miR-487a-3p,miR-6517,miR-3960,miR-4429,miR-493-3p,miR-496,miR-34a-5p,miR-215-5p,miR-3578,miR-330-5p,miR-5703,miR-769-5p,miR-142a-5p,miR-6134,miR-539-5p,miR-665-5p,miR-6128,miR-138-5p,miR-4492,miR-31-3p,miR-5100,miR-370-5p,miR-2433,miR-769,miR-365a-3p,miR-192-5p,miR-34c-5p,novel_32,miR-204-3p,miR-496-3p,miR-503-3p,miR-30c-1-3p,miR-140-5p,miR-215-5p,miR-370-5p,let-7g-3p,miR-10b-3p,miR-876-3p,miR-1814c,miR-1827,miR-320a,miR-3154,miR-30c-1-3p,let-7a-2-3p,miR-2310,miR-2330-5p,miR-6240,miR-574-3p,miR-744-3p,miR-25-5p,miR-483-3p,miR-378a-5p,miR-2285o,miR-326,miR-320b,miR-3141,miR-330-3p,miR-142-5p,miR-1298-3p,miR-496-5p,miR-425-3p,miR-6238,miR-330-3p,miR-1343-3p,miR-31-5p,miR-145b,miR-138,miR-30b-3p,miR-7862,miR-744-5p,miR-4508,miR-296-5p,miR-30b-3p,miR-145a-5p,novel_111,miR-361-3p,miR-2428,miR-449a,miR-326-3p,miR-3956-3p,miR-2447,novel_60,miR-1193,miR-1296-5p,miR-2285t,miR-2440,miR-34c,miR-769-5p,novel_69,miR-34b,miR-2404,miR-203-3p,miR-320c,miR-669,miR-2319b,miR-2387,miR-3141,miR-361-3p,miR-378j

RGS7BP	ENSOARG0(	regulato	miR-6516-3p,miR-3065-5p,miR-129b-5p,miR-378j,miR-181b-3p,miR-320c,miR-677,miR-2404,miR-490-5p,miR-335,miR-499a-5p,miR-4726-5p,miR-7705,miR-495-3p,miR-335-5p,miR-3956-3p,miR-2447,novel_60,miR-3120-5p,miR-450b-5p,miR-7b-5p,miR-1843b-5p,miR-653,miR-19b-2-5p,miR-539-5p,miR-153,miR-187-3p,miR-153-3p,miR-2331-5p,miR-296-3p,miR-320b,miR-7-5p,miR-296-3p,miR-33a-5p,miR-382,miR-1248,miR-10a-3p,miR-320a,miR-30d-5p,miR-503-3p,miR-30e-5p,miR-204-3p,miR-376e-3p,miR-6516,miR-296-3p,miR-503-3p,miR-140-5p,miR-410-3p,miR-6516-3p,miR-655-3p,miR-19b-1-5p,miR-100-3p,miR-338-3p,miR-6134,miR-539-5p,miR-4429,miR-1434-5p,miR-30a-5p,miR-1290,novel_83,miR-6516-5p,miR-21-3p,miR-6536,miR-127-5p,miR-320d,miR-500a-5p,miR-2285p,novel_17,miR-376c-5p,miR-2432,miR-2424,miR-10a-3p
RCAN3	ENSOARG0(	RCAN fam	miR-30c-1-3p,miR-552-3p,miR-370-5p,miR-544-3p,miR-10a,miR-15b-5p,miR-299,miR-30d-5p,miR-30f,novel_99,miR-425-5p,miR-665-5p,miR-10b-5p,miR-6516-3p,miR-424-5p,miR-190a-5p,miR-106a,miR-548e-3p,miR-18a-3p,miR-212-5p,miR-15a-5p,miR-7859,miR-2284h-5p,miR-30c-5p,miR-140-3p,miR-34a-5p,miR-2285w,miR-20a,miR-29b-1-5p,miR-486-3p,miR-30b,miR-500a-5p,miR-466i-5p,miR-106a,miR-34b-5p,miR-30d,miR-3653-3p,miR-20a-3p,miR-2483-3p,miR-27a-3p,miR-199b-5p,miR-381-3p,miR-3956-3p,miR-541-3p,miR-212-3p,novel_60,miR-190b-5p,miR-218-5p,miR-30b-5p,miR-301a-5p,miR-371a-5p,miR-499a-5p,miR-374a-3p,miR-330-3p,novel_78,miR-27a-3p,miR-10b,miR-541,miR-301b-5p,miR-125b-2-3p,miR-544a,miR-101c,miR-424-5p,miR-106a-5p,miR-30c-1-3p,miR-1b-5p,miR-8485,miR-106b-5p,miR-16b,miR-29b-1-5p,miR-199b-5p,miR-3068-3p,miR-874-3p,miR-2355-5p,miR-3959-5p,miR-34c-5p,miR-199a-5p,miR-301,miR-30e-5p,miR-376c-5p,miR-17-5p,miR-93-5p,miR-671-5p,miR-7144-5p,miR-20b-5p,miR-376c-5p,miR-28a-3p,miR-16-5p,miR-374a-3p,miR-93,miR-370-5p,novel_63,miR-374b-3p,miR-592,miR-486b-3p,miR-376a-5p,miR-322-5p,miR-505-5p,novel_121,miR-30a-5p,miR-10a-5p,miR-30c,miR-450b-5p,miR-199a-5p,miR-376c-5p,miR-125a-3p,miR-2285af,miR-30f,miR-30c-2-3p,miR-3071-3p,miR-28-3p,novel_127,miR-3120-3p,miR-10a-5p,miR-34b,miR-669,miR-376b-5p,miR-18a-3p,miR-195a-5p,miR-376a-2-5p,miR-1193,miR-199b-5p,miR-449a,miR-20a-5p,miR-4324,miR-502-5p,miR-15a,miR-33a-3p,miR-16a,miR-34c,miR-147-5p,miR-203b-5p,miR-20b,miR-544b,miR-6238,miR-1285,miR-708-3p,miR-873a-5p,miR-219a-1-3p,miR-16b,miR-27b-3p,miR-497-5p,miR-6240,miR-654-3p,miR-17-5p,miR-106a-5p,miR-330-3p,miR-190a

UGCG	ENSOARG0C	UDP-gluc	let-7a-2-3p,let-7g,miR-130a-5p,miR-450b-5p,miR-3529-3p,let-7a-5p,let-7e,let-7k,miR-1468,miR-181b-5p,miR-98-5p,let-7f-5p,miR-2284m,miR-217,let-7c-5p,miR-320b,miR-126a-5p,let-7g-5p,miR-2284d,miR-376a-5p,let-7a-2-3p,miR-1839-5p,let-7d,miR-4429,miR-2284ab,miR-217-5p,miR-23b-5p,miR-1961,let-7b-5p,miR-2284j,miR-181d-5p,miR-136-3p,let-7b,miR-129-5p,miR-410-3p,miR-1468-5p,miR-3604,let-7i,let-7f,miR-2284k,miR-2284n,let-7i-5p,let-7d-5p,miR-300-3p,miR-2285u,let-7e-5p,novel_99,miR-376a-2-5p,let-7g-3p,miR-3065-5p,miR-320a
PHF14	ENSOARG0C	PHD fing	miR-217-5p,miR-30d-3p,miR-200b,miR-125b,miR-345-3p,miR-466i-5p,miR-195a-3p,miR-380-3p,miR-143-3p,miR-146a-5p,miR-181b-1-3p,miR-377-5p,miR-29b-1-5p,miR-376a-5p,miR-433-3p,novel_99,miR-582-5p,miR-625-5p,miR-345-5p,miR-182-5p,miR-532-5p,miR-496-3p,miR-504-5p,miR-6516,miR-376e-3p,miR-582-5p,miR-338-5p,miR-132-5p,miR-19b-1-5p,miR-15b-3p,miR-425-5p,miR-125b-2-3p,miR-323b,miR-4791,miR-301b-5p,miR-484,miR-181b-2-3p,miR-27a-3p,miR-105-1,miR-548w,miR-9788-3p,miR-455-3p,miR-182-5p,miR-29b-1-5p,miR-323b-3p,miR-326,novel_115,miR-342,miR-103a-2-5p,miR-146b,miR-3085-3p,miR-652-5p,miR-382-3p,miR-2330-5p,miR-1388-3p,miR-381-3p,novel_94,miR-136-5p,miR-504,miR-126b-5p,miR-499b-5p,miR-452-3p,miR-3064-5p,miR-24-1-5p,miR-27a-3p,miR-107-5p,miR-301a-5p,miR-371a-5p,miR-101-3p,miR-2284q,miR-452-5p,miR-9-5p,miR-1296-5p,miR-212-3p,miR-3059-5p,miR-154a,miR-877-3p,miR-330-5p,miR-23b-5p,miR-2427,miR-134,miR-345-3p,miR-496,miR-105-2,miR-200b-3p,miR-376a-5p,miR-429-3p,miR-134-5p,miR-6516-5p,miR-758-5p,miR-9-5p,miR-24-2-5p,miR-217,miR-2285af,miR-200c-3p,miR-2898,miR-495-5p,miR-3064-5p,miR-1271-3p,miR-1957a,miR-24-2-5p,miR-137-3p,miR-503-3p,miR-671-5p,miR-345-5p,miR-125a,miR-370-3p,miR-503-3p,miR-3068-3p,miR-300-3p,miR-331-5p,miR-3969,miR-125b-5p,miR-134-5p,miR-3071-5p,miR-197-3p,miR-19b-2-5p,miR-27b-3p,miR-455-5p,miR-2284x,miR-380-3p,miR-377-5p,miR-31-5p,miR-3431,miR-3535,miR-2285g,miR-455-5p,miR-2411-3p,miR-105-5p,miR-146a,miR-466f-3p,miR-125a-5p,miR-452-5p,miR-124-5p,miR-382,miR-345-5p,let-7j,miR-432-3p,miR-654-3p,miR-432-3p,miR-6516-3p,miR-376a-2-5p,miR-146b-5p,miR-655-5p,miR-181b-3p,miR-677,miR-6402,miR-4324,miR-656-5p,miR-2285x,miR-342-3p,novel_120,miR-135a-2-3p,miR-

NIPAL3	ENSOARG0(	NIPA-lik	miR-532-3p,miR-322-5p,miR-483-3p,miR-15a-5p,miR-1b-3p,miR-18a-3p,miR-345-3p,miR-126-3p,miR-486-3p,novel_127,miR-28-3p,miR-92a-1-5p,miR-345-3p,miR-185-3p,miR-1260a,miR-125a-3p,miR-15b-5p,miR-877-5p,miR-6130,miR-370-5p,miR-21-3p,miR-552-3p,miR-874-3p,miR-485-5p,miR-329b,miR-487b-5p,miR-6134,miR-346,miR-1260b,miR-16-5p,miR-665-5p,miR-2330-3p,miR-28a-3p,miR-4510,miR-3074-5p,miR-4492,miR-424-5p,miR-486b-3p,miR-130b-5p,miR-100-3p,miR-324-3p,miR-370-5p,miR-494-5p,miR-9788-3p,miR-149-5p,miR-1185-3p,novel_4,novel_78,miR-497-5p,miR-3120-5p,miR-16b,miR-1260b,miR-2426,miR-7b-5p,miR-541,miR-542-3p,miR-654-3p,miR-363-5p,let-7f-2-3p,let-7f-2-3p,miR-130b-5p,miR-424-5p,miR-483-3p,miR-3955-3p,miR-197-5p,miR-2411-3p,miR-16b,miR-7-5p,miR-1306-5p,miR-1185-2-3p,miR-146b-3p,miR-541-5p,novel_68,miR-4792,miR-3074-5p,miR-378b,miR-195a-5p,novel_94,miR-1306,miR-3591-3p,miR-18a-3p,miR-8095,miR-154a,miR-1193,miR-541-3p,miR-16a,miR-576-3p
BCAR1	ENSOARG0(	breast c	miR-30b-3p,miR-1260b,miR-7862,miR-374b-3p,miR-412-5p,miR-185-5p,let-7g-5p,miR-3431,miR-6238,novel_116,miR-149-5p,miR-194-3p,miR-31-5p,miR-133b-5p,miR-339-5p,miR-378a-5p,miR-125a-5p,miR-466f-3p,novel_51,miR-432-3p,miR-363-5p,miR-1291,let-7e-5p,miR-6395,miR-378f,miR-150-5p,miR-1306,miR-18a-3p,miR-378b,miR-299b-3p,miR-374c-3p,miR-677,miR-669,miR-181b-3p,miR-3120-3p,miR-6529b,miR-576-3p,let-7i,miR-211,miR-326-3p,miR-2284w,miR-335-5p,miR-1839-3p,miR-17-3p,novel_101,miR-495-3p,let-7d,miR-1290,miR-330-5p,miR-2411-5p,miR-378h,miR-2285ad,miR-299a-3p,novel_79,miR-412,miR-17-3p,miR-127-5p,miR-191-3p,let-7f-5p,miR-543-5p,novel_127,miR-205-5p,miR-1260a,miR-125a-3p,miR-339a,let-7c-5p,miR-148b-5p,miR-450b-5p,novel_39,let-7g,miR-3065-3p,miR-1271-3p,miR-490-3p,miR-3064-5p,miR-495-5p,miR-4286,miR-2474,miR-7144-5p,miR-23a-5p,miR-671-5p,miR-1248,miR-485-5p,miR-3154,miR-374c-3p,miR-769,miR-324-5p,miR-874-3p,miR-2355-5p,miR-370-3p,miR-1271,miR-615-5p,miR-125a,miR-28-5p,miR-486b-3p,miR-204-5p,miR-331-3p,miR-125b-5p,miR-1260b,miR-378c,novel_42,miR-103b,miR-378c,miR-1839-5p,miR-484,miR-296-5p,miR-378b,miR-2284r,miR-301b-5p,miR-541,miR-412-3p,miR-125b-2-3p,miR-30b-3p,miR-539-5p,miR-147a,miR-455-3p,miR-148b-5p,novel_4,miR-1343-3p,miR-181b-2-3p,miR-3085-3p,miR-3955-3p,miR-1468,let-7e,miR-214-3p,miR-326,miR-1306-5p,miR-2957,miR-378d,miR-8485,let-7a-5p,miR-1839-3p,miR-33a-5p,miR-147-3p,miR-504,miR-335-3p,miR-877-3p,miR-361-3p,miR-378j,miR-378e,let-7d-5p,miR-4443,miR-3064-5p,miR-20a-3p,miR-211-5p,miR-4726-5p,miR-3968,miR-335,miR-2285n,miR-218-5p,miR-769-5p,miR-2285y,miR-301a-5p,miR-361-3p,miR-3059-5p,miR-541-3p,let-7i-3p,miR-219a-5p,miR-184-3p,miR-133a-3p,miR-148a-5p,miR-361-5p,miR-

STPG1	ENSOARG0(	sperm-ta	miR-486-3p,miR-345-5p,miR-1271-3p,miR-432-3p,miR-668-3p,miR-628-5p,miR-21-3p,miR-466f-3p,miR-214-3p,miR-760-3p,miR-339a,miR-326,miR-339-5p,novel_79,miR-3960,miR-30b-3p,miR-3432b,miR-1197-5p,miR-330-5p,miR-30b-3p,miR-296-5p,miR-541,miR-1843b-5p,miR-326-3p,miR-24-3p,miR-214,miR-665-5p,miR-145a-3p,miR-541-3p,miR-486b-3p,miR-6123,miR-4726-5p,miR-100-3p,miR-370-3p,miR-339b,miR-345-5p,miR-345-5p,miR-21a-3p
MLF2	ENSOARG0(	myeloid	miR-296-5p,miR-541,miR-300,miR-219a-1-3p,miR-34a-5p,miR-2284x,miR-2285ad,miR-199b-3p,miR-6517,miR-2284d,miR-151-5p,miR-125b,miR-185-3p,miR-2284m,miR-125a-5p,miR-34b-5p,miR-1388-3p,miR-101b-3p,miR-101-5p,miR-125b-2-3p,miR-486-3p,miR-8485,miR-361-3p,miR-488-3p,miR-381-3p,miR-139-5p,miR-125a,miR-34c-5p,miR-139-5p,novel_69,miR-489,miR-34b,miR-151b,miR-2284n,miR-2284k,miR-4324,miR-2440,miR-34c,miR-125b-5p,miR-2285y,miR-4726-5p,miR-4492,miR-199c,miR-486b-3p,miR-4454,miR-2284v,miR-541-3p,miR-2285x,novel_101,miR-2284j,miR-361-3p,miR-423-5p,miR-449a,miR-3184-5p,miR-6134
RNF180	ENSOARG0(	ring fin	miR-382-5p,miR-4324,novel_101,miR-17-3p,miR-326-3p,miR-24-3p,miR-3065-5p,miR-21c,miR-505-3p,miR-378f,miR-6516-3p,miR-146b-5p,miR-107,miR-26b-5p,miR-3120-3p,miR-378b,miR-181b-3p,miR-320c,miR-677,miR-194a,miR-190a,miR-330-3p,miR-105-5p,miR-365b-5p,miR-146a,miR-660,miR-124-5p,miR-363-5p,miR-668-3p,miR-6240,miR-493-5p,miR-194-5p,novel_23,miR-450c-3p,miR-181a-5p,miR-2113,miR-223-3p,miR-432,miR-744-5p,miR-27b-3p,miR-26a-2-3p,miR-16-1-3p,miR-21-5p,miR-339-5p,miR-103a-3p,miR-3955-5p,miR-103,miR-3431,miR-432-5p,miR-409-3p,miR-100-3p,miR-331-3p,miR-5100,miR-34c-3p,miR-2433,miR-486b-3p,miR-410-3p,novel_9,miR-1306-5p,miR-365a-5p,miR-181c-3p,miR-378c,miR-1197-3p,miR-320a,miR-137-3p,miR-503-3p,miR-1814c,miR-190a-3p,miR-7144-5p,miR-215-5p,miR-300-3p,miR-339a,miR-125a-3p,miR-200c,novel_96,miR-543-5p,miR-107,miR-200c-3p,miR-181b-5p,miR-17-3p,miR-2898,miR-2385-3p,miR-450c-5p,miR-29a-5p,miR-24-2-5p,miR-450b-5p,miR-330-5p,miR-105-2,miR-200b-3p,miR-431,miR-7641,novel_79,miR-429-3p,miR-450a-1-3p,miR-378h,miR-9-5p,miR-24-2-5p,miR-218-5p,miR-3964,miR-450a-5p,miR-194b-5p,miR-190b-5p,miR-181d-5p,miR-9-5p,miR-452-5p,miR-3956-3p,miR-885-5p,miR-2447,novel_60,miR-7705,miR-1285-5p,miR-493-5p,miR-668-5p,miR-181a-3p,miR-7977,miR-2285u,miR-24-1-5p,miR-139-5p,miR-27a-3p,miR-7857-3p,miR-378e,miR-326,miR-431-5p,miR-320b,miR-146b,miR-3955-3p,miR-491-5p,miR-33a-5p,miR-744-3p,miR-494-3p,miR-101b-3p,miR-2310,miR-193a,miR-378d,miR-378b,miR-6529a,miR-101c,miR-378c,miR-181b-2-3p,miR-27a-3p,miR-151a-3p,miR-105-1,novel_78,miR-330-3p,miR-548w,miR-338-5p,miR-665,miR-655-3p,miR-151-3p,miR-190a-5p,miR-6516-3p,miR-17-3p,miR-450b-3p,miR-145a-3p,miR-378c,miR-539-5p,miR-338-3p,miR-

UBE2Z	ENSOARG0(	ubiquiti	miR-450a-1-3p,miR-433-3p,miR-655,miR-129b-3p,miR-339b,miR-345-5p,miR-1827,miR-625-5p,miR-543-3p,miR-32-5p,miR-30c-1-3p,miR-552-3p,miR-216b-3p,miR-377-3p,miR-15b-5p,let-7i-5p,miR-30e-3p,miR-15b-3p,let-7f,miR-664a-5p,miR-665,miR-219b-3p,miR-30a-3p,novel_74,miR-130b-5p,miR-129-5p,miR-874-5p,miR-4492,let-7b,miR-3958-5p,miR-1961,miR-33b-3p,miR-142-3p,miR-361-5p,miR-106a,miR-30d-3p,miR-212-5p,miR-23c,miR-7859,miR-23b-3p,miR-328-3p,miR-2284h-5p,miR-2285p,miR-210-5p,miR-98-5p,let-7k,miR-195a-3p,miR-378j,miR-381-3p,miR-136-5p,miR-3963,miR-211-5p,miR-2285u,miR-7689-3p,miR-3596,miR-378g,miR-1185-2-3p,miR-3956-3p,miR-2447,miR-9-5p,novel_111,miR-361-3p,miR-329-3p,miR-2284r,miR-362-3p,miR-1839-5p,miR-330-3p,novel_78,miR-145b,miR-181b-2-3p,miR-147a,miR-153,miR-2459,miR-1185-3p,miR-106b-5p,miR-16b,miR-505,miR-491-5p,miR-1185-5p,miR-103a-2-5p,miR-153-3p,miR-199b-5p,miR-124a,miR-23a-3p,miR-331-5p,miR-23b,miR-93-5p,miR-329a,miR-345-5p,miR-876-3p,miR-20b-5p,miR-574-5p,miR-125a,miR-2355-5p,miR-300-3p,miR-331-5p,miR-2284k,novel_63,miR-125b-5p,miR-6740-5p,miR-331-5p,miR-134-5p,miR-592,novel_42,miR-16-5p,miR-1260b,miR-330-5p,miR-1343-5p,miR-6517,miR-2285ad,miR-18b-5p,novel_79,miR-9-5p,miR-322-5p,novel_121,miR-486-5p,miR-134-5p,miR-450a-1-3p,miR-664-3p,miR-125a-3p,miR-219-3p,miR-30f,miR-1a-2-5p,let-7f-5p,miR-200a-3p,novel_96,miR-3064-5p,miR-92b-3p,miR-2285b,let-7g,novel_39,miR-29a-5p,miR-216a-3p,miR-150-5p,miR-410-5p,let-7e-5p,novel_68,novel_69,miR-181b-3p,miR-677,miR-142b,miR-576-3p,miR-4324,miR-1247-3p,miR-6123,miR-335-5p,miR-1a-1-5p,miR-199b-5p,miR-2113,miR-542-3p,miR-1843b-5p,miR-450c-3p,miR-16b,miR-2284x,miR-497-5p,miR-340-

PSMC3	ENSOARG0(	proteaso	miR-185-3p,miR-34b-5p,miR-299-3p,miR-760-3p,miR-4661-5p,miR-2887,miR-183-5p,miR-2285w,miR-2424,miR-2411,miR-486-3p,miR-124-3p,miR-1197-5p,miR-5703,miR-193b-5p,miR-2300b-3p,miR-378d,miR-34a-5p,miR-3600,miR-212-5p,miR-18a-3p,miR-2284h-5p,miR-532-3p,miR-328-3p,miR-148b-3p,miR-15a-5p,miR-19b-1-5p,miR-324-3p,miR-132-5p,miR-29d-5p,miR-664a-5p,miR-665,miR-487a-5p,miR-874-5p,miR-708-5p,miR-424-5p,miR-4492,miR-1468-5p,miR-664a,miR-214,miR-3074-2-3p,miR-665-5p,miR-2330-3p,miR-129-2-3p,miR-145a-3p,miR-4510,miR-122-5p,miR-3184-5p,miR-6134,miR-338-3p,novel_44,miR-206-3p,miR-29c-5p,miR-543-3p,miR-1895,miR-1827,miR-2312,miR-339b,miR-345-5p,miR-3065-3p,miR-552-3p,miR-30c-1-3p,miR-2331-3p,miR-22-3p,novel_32,miR-15b-5p,miR-544-3p,miR-6130,miR-193b-3p,novel_19,miR-16b,miR-5126,miR-1306-5p,miR-214-3p,miR-1468,miR-103a-2-5p,miR-491-5p,miR-3085-3p,miR-376b,miR-652-5p,miR-124a,miR-424-5p,miR-491-3p,miR-30c-1-3p,miR-412-3p,miR-30b-3p,miR-296-5p,miR-2284r,miR-143-5p,miR-541,miR-301b-5p,miR-544a,novel_78,miR-199b-3p,novel_4,miR-330-3p,miR-2300a-5p,miR-1343-3p,miR-455-3p,miR-9788-3p,miR-147a,miR-107-5p,miR-301a-5p,miR-218-5p,miR-615,miR-1843a-3p,miR-4726-5p,miR-2285f,miR-6527,miR-3059-5p,miR-541-3p,miR-423-5p,miR-361-3p,miR-877-3p,miR-1306-3p,miR-361-3p,miR-147-3p,miR-7977,miR-4792,miR-3064-5p,miR-2319b,miR-4443,miR-3596,miR-7857-3p,miR-25,miR-30c-2-3p,miR-30f,miR-339a,miR-1260a,miR-125a-3p,miR-8117,miR-152-3p,novel_96,novel_127,miR-191-3p,miR-127-5p,miR-3064-5p,miR-4286,novel_39,miR-3065-3p,miR-2478,miR-28b,miR-29d-3p,miR-1343-5p,miR-615-3p,miR-134,novel_79,miR-412,miR-299a-3p,miR-6517,miR-582-3p,miR-134-5p,miR-214-5p,miR-129-1-3p,miR-322-5p,miR-505-

SLFN14	ENSOARG0(	schlafen	miR-758-3p,miR-33a-5p,miR-382-3p,miR-3085-3p,miR-3955-3p,miR-362-3p,miR-320b,novel_103,novel_115,miR-326,miR-1306-5p,miR-2285e,miR-2300a-5p,miR-2284aa,miR-378c,miR-544a,miR-2426,miR-378b,miR-8095,miR-1296-5p,miR-2285f,miR-452-5p,miR-181d-5p,novel_60,miR-541-3p,miR-2284q,miR-769-5p,miR-371a-5p,miR-7857-3p,miR-378e,miR-541-5p,miR-3064-5p,miR-20a-3p,novel_94,miR-129b-5p,miR-2411,miR-2432,miR-29b-1-5p,miR-125b-2-3p,miR-5100,miR-216c-5p,miR-34b-5p,miR-299-3p,miR-320d,miR-2284m,miR-216a-5p,miR-196a-3p,novel_83,miR-199a-3p,miR-532-3p,miR-487a-3p,miR-222-5p,miR-3600,miR-485-3p,miR-4429,miR-124-3p,miR-1197-5p,miR-188-3p,miR-140-3p,miR-362-3p,miR-376a-5p,miR-2408,miR-338-3p,miR-3074-2-3p,miR-378c,miR-450b-3p,miR-155-5p,miR-17-3p,miR-320e,miR-655-3p,miR-22-3p,miR-204-3p,miR-155-5p,miR-544-3p,miR-139-5p,miR-182-5p,miR-378a-3p,novel_99,let-7g-3p,miR-2312,miR-539-3p,miR-363-5p,miR-1343-5p,miR-197-5p,miR-544b,miR-2285g,miR-432-5p,miR-665,miR-380-3p,miR-138,miR-2284ab,miR-145a-5p,miR-374b-3p,miR-873a-5p,miR-181a-5p,novel_27,miR-326-3p,miR-449a,miR-2428,miR-24-3p,miR-495-3p,miR-17-3p,miR-22-5p,miR-502b,novel_1,miR-34c,miR-6402,miR-34b,miR-378b,miR-376a-2-5p,miR-450b-3p,miR-2285ab,miR-490-3p,miR-450a-2-3p,miR-17-3p,miR-181b-5p,miR-2285af,miR-378h,miR-2411-5p,miR-196a-2-3p,miR-376a-5p,miR-300,miR-615-3p,miR-345-3p,miR-134,miR-378c,miR-365a-5p,miR-1306-5p,miR-138-5p,miR-331-3p,miR-2284n,miR-3957,miR-34c-5p,miR-769,miR-370-3p,miR-141-3p,miR-3957-5p,miR-1814c,miR-671-5p,miR-320a,miR-488-3p,miR-381-3p,miR-8485,miR-1b-5p,miR-2310,miR-378d,miR-124a,miR-1388-3p,miR-153-3p,miR-491-5p,miR-29b-1-5p,miR-182-5p,miR-199a-3p,miR-153,miR-1298-3p,miR-145b,miR-1343-
YAP1	ENSOARG0(	yorkie h	miR-16b,miR-2284ab,miR-497-5p,miR-1285,miR-484,miR-7857,miR-15a-5p,miR-2284d,miR-322-5p,miR-199a-3p,miR-199b-3p,miR-2284m,miR-193a-3p,miR-21-3p,miR-199a-3p,miR-16b,miR-324-3p,miR-424-5p,miR-216c-5p,miR-186-5p,miR-195a-5p,miR-7144-5p,miR-21a-3p,miR-2483-3p,miR-193b-3p,miR-15b-5p,miR-503-3p,miR-370-3p,miR-7689-3p,miR-2355-5p,miR-199c,miR-424-5p,miR-15a,miR-16a,miR-331-3p,miR-145-3p,miR-2284v,miR-495-3p,miR-2330-3p,miR-193b-3p,miR-16-5p
UCK1	ENSOARG0(	uridine-	miR-371b-3p,miR-30c-1-3p,miR-3064-5p,miR-3085-3p,miR-101-3p,novel_87,miR-30c-2-3p,miR-328-3p,miR-3064-5p,miR-30c-1-3p,miR-1271,miR-18b-5p,miR-212-5p,miR-18a-5p,miR-6395,miR-378d,miR-1839-5p,miR-101c,miR-18b,miR-18a
TDRD9	ENSOARG0(	tudor do	miR-450a-2-3p,miR-16-1-3p,miR-16-1-3p,miR-7-5p,miR-7b-5p,miR-331-3p,miR-330-3p,miR-2904,miR-182-5p,miR-6525,miR-6240,miR-330-3p,miR-196a-2-3p,miR-182-5p

ESRRA	ENSOARG0(	estrogen	miR-15b-5p,miR-204-3p,miR-216b-5p,miR-193b-3p,miR-132-5p,miR-10a,miR-296-3p,miR-3065-3p,miR-30c-1-3p,miR-2331-3p,miR-1827,let-7g-3p,miR-3184-5p,miR-10b-5p,miR-214,miR-145a-3p,miR-129-2-3p,miR-424-5p,miR-4492,miR-324-3p,miR-132-5p,miR-2285j,miR-328-3p,miR-2284h-5p,miR-15a-5p,miR-3600,miR-18a-3p,miR-34a-5p,miR-133a-3p,miR-142-3p,miR-2904,miR-2408,miR-143-3p,miR-486-3p,miR-421-5p,miR-2285w,miR-34b-5p,miR-760-3p,miR-202-5p,miR-92a-1-5p,miR-125b,miR-185-3p,miR-324-3p,miR-4443,miR-500-3p,miR-7977,miR-3064-5p,miR-147-3p,miR-361-3p,miR-423-5p,miR-502-3p,miR-877-3p,miR-1296-5p,miR-2447,miR-1973,miR-9788-3p,miR-496-5p,novel_4,miR-16-2-3p,miR-450b-5p,miR-2426,miR-501-3p,miR-10b,miR-4791,miR-4532,miR-8485,miR-30c-1-3p,miR-744-3p,miR-424-5p,miR-214-3p,miR-3085-3p,novel_124,miR-16b,miR-326,miR-1306-5p,miR-154b-5p,miR-365a-3p,miR-34c-5p,miR-615-5p,miR-1271,miR-125a,miR-2355-5p,miR-874-3p,miR-671-5p,miR-485-5p,let-7g-3p,miR-137-3p,miR-16-5p,miR-486b-3p,miR-3969,miR-125b-5p,miR-2433,miR-129-1-3p,miR-302a-5p,miR-322-5p,miR-1224-5p,miR-2285ad,miR-10a-5p,let-7a-2-3p,miR-128-3p,miR-501-3p,miR-330-5p,miR-3065-3p,miR-148b-5p,miR-495-5p,miR-3064-5p,novel_127,miR-543-5p,miR-2898,miR-30c-2-3p,miR-669,miR-34b,miR-10a-5p,miR-195a-5p,miR-6395,miR-1291,miR-216a-3p,miR-18a-3p,miR-1306,miR-2428,novel_27,miR-326-3p,miR-449a,miR-193b-3p,miR-421-5p,miR-425-5p,miR-16a,miR-34c,miR-6529b,miR-502-5p,miR-4324,miR-15a,miR-2355-3p,miR-432-5p,miR-3535,miR-340-3p,miR-497-5p,miR-16b,miR-1843b-5p,miR-432,let-7a-2-3p,miR-363-5p,miR-193a-3p,miR-125a-5p,novel_51

EVC	ENSOARG0(	Ellis va	miR-497-5p,miR-16b,miR-450c-3p,miR-22-3p,miR-1843b-5p,miR-7b-5p,miR-194-3p,miR-149-5p,miR-3431,miR-134-3p,miR-412-5p,miR-31-5p,miR-378a-5p,miR-2411-3p,miR-2285v,miR-3141,miR-7-5p,let-7j,miR-432-3p,miR-628-5p,miR-668-3p,miR-216a-3p,miR-3591-3p,miR-3065-5p,miR-1306,miR-150-5p,miR-3141,miR-376b-5p,miR-669,miR-320c,miR-6123,miR-206,miR-6119-3p,novel_87,miR-1307-5p,miR-576-3p,miR-28c,miR-4324,miR-758-5p,miR-330-5p,miR-1290,miR-129-1-3p,miR-134-5p,miR-450a-1-3p,miR-758-5p,miR-322-5p,miR-18b-5p,miR-412,miR-1224-5p,miR-429-3p,novel_96,miR-200a-3p,miR-664-3p,miR-125a-3p,miR-205-5p,miR-450c-5p,miR-2285b,miR-200a,miR-29a-5p,miR-450b-5p,miR-3064-5p,miR-1248,miR-345-5p,miR-2318,miR-376c-5p,miR-300-3p,miR-125a,miR-2355-5p,miR-134-5p,miR-125b-5p,miR-1260b,miR-16-5p,miR-758-3p,miR-1a-3p,miR-301b-5p,miR-541,miR-1298-3p,miR-199b-3p,miR-1343-3p,novel_78,miR-487b-5p,miR-491-5p,miR-16b,miR-505,miR-1b-5p,miR-8485,miR-544-5p,miR-136-5p,miR-504,miR-140-5p,miR-2483-3p,miR-378g,miR-4792,miR-3964,miR-490-5p,miR-3968,miR-301a-5p,novel_82,novel_111,miR-361-3p,miR-2447,miR-34a-5p,miR-218-1-3p,miR-142-3p,miR-493-3p,miR-2366,miR-212-5p,miR-380-5p,miR-345-3p,miR-210-5p,novel_17,miR-2285p,miR-143-3p,miR-377-5p,miR-186-5p,miR-625-5p,miR-345-5p,miR-655,miR-129b-3p,miR-450a-1-3p,miR-2285c,miR-15b-5p,miR-2331-3p,miR-504-5p,miR-30c-1-3p,miR-130b-5p,miR-874-5p,miR-769-5p,miR-129-2-3p,miR-3184-3p,miR-455-5p,miR-1260b,miR-30b-3p,miR-1434-3p,miR-1285,miR-2285g,miR-377-5p,miR-466f-3p,miR-125a-5p,miR-124-5p,miR-455-5p,miR-197-5p,miR-345-5p,miR-654-3p,miR-130b-5p,miR-195a-5p,miR-9851-3p,miR-18b,miR-2387,miR-2285ab,miR-34b,miR-323a-5p,miR-655-5p,miR-320b,miR-

CDX1	ENSOARG0(	caudal t	novel_60,miR-3059-5p,miR-219a-5p,miR-2285f,miR-30b-5p,miR-382-3p,miR-371a-5p,miR-769-5p,miR-2285t,miR-452-3p,miR-7977,miR-499b-5p,miR-378e,miR-7857-3p,miR-199b-5p,miR-361-3p,novel_94,miR-877-3p,miR-424-5p,miR-33a-5p,miR-758-3p,miR-3187-3p,miR-193a,miR-326,novel_115,miR-1306-5p,novel_124,novel_4,miR-222-5p,miR-2320-3p,miR-2285e,miR-9788-3p,miR-4791,miR-484,miR-378b,miR-296-5p,miR-378c,miR-2284aa,miR-145a-3p,miR-4510,miR-378c,miR-2330-3p,miR-206-3p,miR-338-5p,miR-320e,miR-655-3p,miR-664a,miR-708-5p,miR-424-5p,miR-378a-3p,miR-532-5p,miR-182-5p,miR-3065-3p,miR-21-3p,miR-193b-3p,miR-6130,miR-877-5p,novel_25,miR-204-3p,miR-21a-3p,miR-30f,miR-2312,novel_99,miR-105-3p,miR-29b-1-5p,miR-2411,miR-196a-3p,miR-125b,miR-760-3p,miR-2284m,miR-21-3p,miR-299-3p,miR-485-3p,miR-15a-5p,miR-487a-3p,miR-383-5p,miR-532-3p,miR-188-3p,miR-1193,miR-140-3p,miR-124-3p,novel_101,miR-495-3p,miR-24-3p,miR-211,miR-2428,miR-326-3p,novel_27,miR-6402,miR-15a,miR-382-5p,miR-16a,novel_1,miR-378b,miR-29a-3p,miR-146b-3p,miR-2387,miR-2285ab,miR-18a-3p,miR-378b,miR-450b-3p,miR-1291,miR-6395,miR-135b-5p,miR-195a-5p,miR-379-5p,miR-382,miR-539-3p,miR-296-3p,miR-224-5p,miR-455-5p,miR-330-3p,miR-197-5p,miR-1343-5p,miR-125a-5p,miR-665,miR-185-5p,miR-2285g,miR-1260b,miR-29b,miR-2284ab,miR-455-5p,miR-6128,miR-378c,miR-365a-5p,miR-5100,miR-29b-3p,miR-3969,miR-135a-5p,miR-331-3p,miR-28-5p,miR-486b-3p,miR-204-5p,miR-141-3p,miR-3965,miR-769,miR-2284n,miR-154b-5p,miR-324-5p,miR-199a-5p,miR-488-3p,miR-3154,miR-10a-3p,miR-671-5p,miR-485-5p,miR-7144-5p,novel_48,miR-3529-3p,miR-181a-2-3p,miR-4286,miR-1,miR-2385-3p,miR-28b,miR-3065-3p,miR-199a-5p,miR-1260a,miR-543-5p,miR-378b,miR-

RRP9	ENSOARG0(	ribosoma	miR-30a-3p,miR-3064-5p,miR-181a-2-3p,miR-3065-3p,novel_39,miR-2478,miR-92b-3p,miR-339a,miR-1260a,miR-92a-3p,novel_127,miR-17-3p,miR-7641,novel_79,miR-6517,miR-505-5p,miR-2411-5p,miR-3591-5p,miR-193a-5p,miR-1197-3p,miR-1260b,miR-331-3p,miR-135a-5p,miR-370-5p,miR-135a-1-3p,miR-28-5p,miR-486b-3p,miR-370-3p,miR-1271,miR-874-3p,miR-34c-5p,miR-25-3p,miR-488-3p,miR-7144-5p,miR-1814c,miR-671-5p,miR-1248,miR-32,miR-2331-5p,miR-503-5p,miR-146a,miR-339-5p,miR-340-3p,miR-149-5p,miR-194-3p,miR-2285g,miR-503-5p,miR-197-3p,miR-3071-5p,miR-7862,miR-30b-3p,miR-1260b,miR-1193,miR-17-3p,miR-342-3p,miR-449a,novel_27,miR-2285m,miR-2428,miR-24-3p,miR-34c,miR-6402,miR-625-3p,miR-6123,miR-128-1-5p,miR-92a-3p,miR-206,novel_68,miR-3074-5p,miR-6535,miR-146b-3p,miR-2404,novel_69,miR-411b-3p,miR-670-3p,miR-34b,miR-1306,miR-18a-3p,miR-146b-5p,miR-135b-5p,miR-412-3p,miR-503-5p,miR-2411,miR-486-3p,miR-2432,miR-146a-5p,miR-185-3p,miR-196a-3p,miR-221-5p,miR-34b-5p,miR-21-3p,miR-30d-3p,miR-18a-3p,miR-140-3p,miR-5703,novel_133,miR-2300b-3p,miR-193b-5p,miR-34a-5p,miR-378d,miR-3184-3p,miR-4510,miR-145a-3p,miR-3074-5p,miR-3184-5p,miR-206-3p,miR-324-3p,miR-30a-3p,miR-665,miR-320e,miR-15b-3p,miR-30e-3p,miR-874-5p,miR-129-5p,miR-708-5p,miR-664a,miR-17-3p,miR-3065-3p,miR-216b-3p,miR-370-5p,miR-545-3p,miR-504-5p,miR-32-5p,miR-2331-3p,miR-221-5p,miR-204-3p,miR-877-5p,miR-6130,miR-433-3p,miR-2285c,miR-625-5p,miR-543-3p,miR-1827,miR-339b,miR-744-3p,miR-8485,miR-2310,miR-505,miR-342,miR-1306-5p,miR-146b,miR-103a-2-5p,miR-3085-3p,miR-3955-3p,miR-1343-3p,novel_78,miR-9788-3p,miR-2285e,miR-421,miR-147a,miR-30b-3p,miR-296-5p,miR-484,miR-301b-5p,miR-2426,miR-2447,miR-363-3p,miR-423-5p,miR-107-

PSMD14	ENSOARG0(	proteaso	miR-5010-3p,miR-105-1,novel_4,novel_78,miR-2300a-5p,miR-2459,miR-1a-3p,miR-501-3p,miR-30b-3p,miR-758-3p,miR-125b-2-3p,miR-329-3p,miR-541,miR-2284aa,miR-362-3p,miR-23a-3p,miR-1388-3p,let-7a-5p,miR-494-3p,miR-106a-5p,miR-8485,miR-758-3p,miR-30c-1-3p,miR-362-3p,miR-106b-5p,miR-1306-5p,novel_103,miR-5126,let-7e,miR-3955-3p,miR-487b-5p,miR-25-5p,miR-2285u,miR-211-5p,miR-4792,miR-24-1-5p,miR-20a-3p,let-7d-5p,miR-500-3p,miR-4443,miR-378g,miR-378j,miR-335-3p,miR-493-5p,miR-136-5p,miR-504,miR-4454,miR-3059-5p,miR-363-3p,miR-885-5p,miR-541-3p,miR-502-3p,miR-877-3p,novel_111,miR-769-5p,miR-2285y,miR-2285n,miR-30b-5p,miR-2285t,miR-374a-3p,miR-2284q,miR-505-3p,miR-532-3p,miR-23b-3p,miR-7859,miR-200b,miR-222-5p,miR-98-3p,miR-217-5p,miR-1197-5p,miR-140-3p,miR-30c-5p,miR-2300b-3p,miR-362-3p,miR-1961,miR-195a-3p,miR-20a,miR-2887,miR-345-3p,miR-329a-5p,miR-2432,miR-421-5p,miR-216a-5p,miR-219b-5p,miR-125b,miR-30b,miR-26a-5p,miR-30d,let-7k,miR-98-5p,miR-216b-3p,miR-532-5p,miR-30c-1-3p,miR-32-5p,miR-504-5p,miR-22-3p,let-7i-5p,miR-27a-5p,miR-132-5p,miR-6130,miR-656-3p,miR-500a-3p,miR-30d-5p,miR-450a-1-3p,miR-625-5p,miR-30f,miR-154b-3p,let-7b,miR-3184-3p,miR-129-2-3p,miR-4510,let-7b-5p,miR-6134,miR-338-3p,miR-769-5p,miR-98-3p,miR-206-3p,miR-224-5p,miR-132-5p,miR-338-5p,let-7f,miR-500,miR-708-5p,miR-4492,miR-208b-5p,miR-129-5p,miR-130b-5p,miR-450b-3p,miR-664a,miR-6516-3p,miR-26b-3p,miR-3955-5p,miR-194-3p,miR-20b,miR-3431,miR-134-3p,miR-185-5p,let-7g-5p,miR-22-3p,miR-450c-3p,miR-3071-5p,miR-873a-5p,miR-2284x,miR-7862,miR-2284ab,miR-30b-3p,miR-130b-5p,miR-296-3p,miR-32,miR-105-5p,novel_51,miR-224-5p,miR-296-3p,miR-125a-5p,miR-124-5p,miR-26b-5p,miR-329-5p,miR-144-3p,miR-

PLD2	ENSOARG0(	phosphol	miR-7857-3p,miR-20a-3p,miR-7977,miR-877-3p,novel_94,miR-361-3p,miR-199b-5p,miR-362-5p,novel_60,miR-4726-5p,miR-335,miR-2285t,miR-371a-5p,miR-769-5p,miR-2320-3p,miR-9788-3p,miR-500-5p,novel_4,miR-2300a-5p,miR-409b,miR-2426,miR-101a-5p,miR-2284aa,miR-16-2-3p,miR-4791,miR-412-3p,miR-30b-3p,miR-3962,miR-193a,miR-758-3p,miR-33a-5p,novel_103,miR-1306-5p,miR-326,miR-132-5p,miR-6130,miR-204-3p,miR-155-5p,miR-140-5p,miR-221-5p,miR-660-5p,miR-496-3p,miR-3065-3p,miR-18a-5p,novel_99,miR-539-5p,miR-4510,miR-2330-3p,miR-450b-3p,miR-664a,miR-6516-3p,miR-155-5p,miR-17-3p,miR-708-5p,miR-320e,miR-148b-3p,novel_83,miR-151-5p,miR-532-3p,miR-3600,miR-505-3p,miR-485-3p,miR-378d,novel_133,miR-5703,miR-1197-5p,miR-188-3p,miR-130a-5p,miR-125b-2-3p,miR-216c-5p,miR-2887,miR-2284m,miR-466i-5p,miR-221-5p,miR-34b-5p,miR-299-3p,miR-196a-3p,miR-146b-3p,miR-29a-3p,miR-34b,miR-135b-5p,miR-505-3p,miR-18b,miR-18a-3p,miR-24-3p,miR-326-3p,novel_27,miR-449a,miR-135a-2-3p,novel_101,miR-17-3p,miR-133a-5p,miR-34c,miR-432-5p,miR-2284d,miR-185-5p,miR-133b-5p,miR-138,miR-30b-3p,miR-29b,miR-2284ab,miR-380-3p,miR-1260b,miR-7862,miR-455-5p,miR-653,miR-3071-5p,miR-145a-5p,miR-374b-3p,miR-539-3p,miR-660,miR-466f-3p,miR-330-3p,miR-455-5p,miR-34c-5p,miR-769,miR-324-5p,miR-199a-5p,miR-2284n,miR-23a-5p,miR-485-5p,miR-671-5p,miR-381-3p,miR-503-3p,miR-18a,miR-362-5p,miR-3154,miR-500b-5p,miR-365a-5p,miR-6128,miR-138-5p,miR-486b-3p,miR-28-5p,miR-29b-3p,miR-410-5p,miR-135a-1-3p,miR-5100,miR-331-3p,miR-3969,miR-409-3p,miR-135a-5p,miR-545-5p,miR-196a-2-3p,miR-300,miR-496,miR-134,miR-29d-3p,miR-2478,miR-28b,miR-3065-3p,miR-199a-5p,miR-30a-3p,miR-2385-3p,miR-181a-2-3p,miR-450a-2-3p,miR-17-3p,miR-2898,miR-
OLFM4	ENSOARG0(	olfactom	miR-5703,miR-150-5p,miR-6535,miR-545-5p,miR-365a-3p,miR-7977,miR-125a,miR-30d-3p,miR-874-3p,miR-125a-5p,miR-125b,miR-324-3p,miR-125b-5p,miR-30a-3p,miR-4286,miR-30a-3p
TXNDC11	ENSOARG0(	thioredo	miR-1197-3p,miR-133a-3p,miR-486b-3p,miR-4492,miR-125b-5p,miR-544-3p,miR-615-5p,miR-125a,miR-141-3p,miR-2285aa,miR-548o-3p,miR-485-5p,novel_99,miR-1827,let-7g-3p,miR-133b-3p,miR-320a,novel_39,miR-486-3p,miR-2432,miR-421-5p,miR-181a-2-3p,miR-2285w,miR-200a-3p,miR-1277-5p,miR-320d,miR-185-3p,miR-125b,miR-210-5p,miR-2284h-5p,miR-9-5p,miR-7859,let-7a-2-3p,miR-128-3p,miR-133a-3p,miR-4429,miR-493-3p,miR-7705,miR-2428,novel_27,miR-24-3p,miR-452-5p,miR-181d-5p,miR-9-5p,miR-2284q,miR-421-5p,miR-107-5p,miR-1973,miR-218-5p,miR-320c,miR-6535,miR-411b-3p,miR-489,miR-374c-3p,miR-378g,miR-3596,miR-216a-3p,miR-378b,let-7a-2-3p,miR-432-3p,miR-103a-2-5p,miR-125a-5p,miR-378a-5p,miR-505,miR-320b,miR-544b,miR-194-3p,miR-216a-3p,miR-134-3p,miR-26b-3p,miR-3120-5p,miR-544a,miR-1388-5p,miR-374b-3p,miR-143-5p,miR-296-5p

PI15	ENSOARG0(	peptidas	miR-409b,miR-151a-3p,miR-27a-3p,miR-455-3p,miR-221-3p,miR-2285e,miR-2459,miR-541,miR-101c,miR-2284aa,miR-3120-5p,miR-101b-3p,miR-652-5p,miR-2284b,miR-8485,novel_115,miR-431-5p,miR-1306-5p,miR-2957,miR-146b,miR-103a-2-5p,miR-139-5p,miR-2284g,miR-20a-3p,miR-126b-5p,miR-3596,miR-144-5p,miR-27a-3p,miR-221,miR-129b-5p,miR-493-5p,miR-541-3p,miR-2285f,miR-2284j,miR-101-3p,miR-769-5p,miR-107-5p,miR-3958-3p,miR-2284q,miR-7859,miR-383-5p,miR-2300b-3p,miR-188-3p,miR-1434-5p,miR-5703,miR-19b-3p,miR-142-3p,miR-33b-3p,miR-16-1-3p,miR-2284u,miR-216c-5p,miR-369-3p,miR-146a-5p,miR-380-3p,miR-219b-5p,miR-345-3p,miR-2284m,miR-466i-5p,miR-3653-3p,miR-1277-5p,novel_107,miR-139-5p,novel_32,miR-2285c,miR-450a-1-3p,miR-655,miR-2312,miR-345-5p,miR-186-5p,miR-590-3p,miR-539-5p,miR-145a-3p,miR-769-5p,miR-2285j,miR-222-3p,miR-151-3p,miR-320e,miR-655-3p,miR-664a,miR-708-5p,miR-103a-3p,miR-26b-3p,miR-412-5p,miR-2284d,miR-2285g,miR-6238,miR-103,miR-653,miR-223-3p,miR-1434-3p,miR-3071-5p,miR-138,miR-380-3p,miR-2284ab,miR-16-1-3p,miR-27b-3p,miR-493-5p,miR-382,miR-2284l,miR-296-3p,miR-345-5p,miR-146a,miR-296-3p,miR-2411-3p,miR-124-5p,miR-452-5p,miR-466f-3p,miR-655-5p,miR-3120-3p,miR-670-3p,miR-374c-3p,miR-2285ab,miR-1306,miR-216a-3p,miR-107,miR-19a-3p,miR-2284e,miR-146b-5p,miR-2319a,miR-135a-2-3p,miR-495-3p,miR-2285x,miR-374c-5p,miR-4324,miR-28c,miR-2284o,miR-22-5p,miR-502b,miR-222,miR-431,miR-2284z,novel_121,miR-214-5p,miR-345-3p,miR-3578,miR-300,miR-128-3p,miR-2284f,miR-28b,miR-1957a,miR-2284y,miR-2285b,miR-192-3p,miR-2284a,miR-2285af,miR-181c-3p,miR-107,miR-300-3p,miR-374c-3p,miR-769,miR-223-5p,miR-2284[illegible],miR-381-3p,miR-345-5p,miR-485-5p,miR-2284[illegible],miR-144-
CHCHD3	ENSOARG0(	coiled-c	miR-2426,miR-2898,miR-1248,miR-200a-3p,miR-193b-5p,miR-1197-5p,miR-2284l,miR-141-3p,miR-212-5p,miR-6128
ZNF318	ENSOARG0(	zinc fin	miR-1983,miR-412-3p,miR-125b-2-3p,novel_133,miR-2366,miR-2432,miR-29a-5p,miR-1343-3p

ZNF689	ENSOARG0(	zinc fin	miR-145a-3p,miR-3074-5p,miR-3184-3p,miR-129-2-3p,miR-4510,miR-378c,let-7b,miR-214,miR-2330-3p,novel_44,miR-338-3p,let-7b-5p,miR-30e-3p,let-7f,miR-3604,miR-324-3p,miR-30a-3p,miR-664a,miR-130b-5p,miR-500,miR-660-5p,miR-30c-1-3p,miR-504-5p,miR-2331-3p,miR-32-5p,miR-545-3p,miR-378a-3p,miR-139-5p,miR-6130,miR-582-5p,miR-192-5p,miR-27a-5p,let-7i-5p,miR-204-3p,novel_25,miR-500a-3p,miR-129b-3p,miR-345-5p,miR-133b-3p,novel_99,miR-582-5p,miR-2285w,miR-329a-5p,miR-130a-5p,miR-181b-1-3p,miR-210-5p,miR-542-5p,miR-422a,miR-324-3p,miR-216a-5p,miR-98-5p,let-7k,miR-299-3p,miR-2484,miR-21-3p,miR-34b-5p,miR-18a-3p,miR-30d-3p,miR-3600,miR-2284h-5p,miR-215-5p,miR-2366,miR-1961,miR-378i,miR-378a-3p,miR-5703,miR-1193,miR-2904,miR-33b-3p,miR-34a-5p,miR-133a-3p,miR-885-5p,miR-3956-3p,novel_60,miR-3059-5p,miR-2447,miR-452-5p,miR-190b-5p,miR-362-5p,miR-1285-5p,miR-2440,miR-371a-5p,miR-7134-3p,miR-20a-3p,miR-139-5p,miR-3064-5p,miR-499b-5p,miR-378e,let-7d-5p,miR-504,miR-136-5p,miR-299b-5p,miR-544-5p,miR-877-3p,miR-2330-5p,let-7a-5p,miR-1388-3p,miR-33a-5p,miR-652-5p,miR-30c-1-3p,miR-378d,miR-4532,miR-193a,miR-326,miR-431-5p,miR-323b-3p,miR-3955-3p,miR-3085-3p,miR-491-5p,miR-542-5p,let-7e,miR-214-3p,miR-330-3p,miR-2300a-5p,miR-181b-2-3p,miR-539-5p,miR-425-3p,miR-455-3p,miR-500-5p,miR-484,miR-378b,miR-323b,miR-2426,miR-378c,miR-1388-5p,miR-2284aa,miR-1839-5p,miR-101c,miR-133a-3p,miR-1260b,miR-500b-5p,miR-378c,miR-365a-5p,miR-409-3p,miR-331-3p,miR-100-3p,miR-141-3p,miR-215-5p,miR-370-3p,miR-25-3p,miR-34c-5p,miR-154b-5p,miR-2448-3p,miR-488-3p,miR-362-5p,miR-3154,miR-345-5p,miR-671-5p,miR-3529-3p,miR-4286,miR-181a-2-3p,miR-2403,miR-30a-3p,miR-3064-5p,miR-380-5p,miR-148b-5p,miR-92b-3p,miR-200a,let-

IRAK1BP1	ENSOARG0(	interleu	miR-378a-5p,miR-377-3p,miR-582,miR-7-5p,novel_51,miR-365b-5p,miR-22841,miR-2285r,miR-628-5p,let-7f-2-3p,let-7f-2-3p,miR-668-3p,miR-7b-5p,miR-432,miR-708-3p,novel_23,miR-3431,miR-3535,miR-31-5p,miR-339-5p,miR-2285m,miR-301b-3p,miR-758-5p,miR-2319a,miR-376b-5p,miR-1306,miR-299b-3p,miR-320c,miR-216b-3p,novel_68,miR-28-3p,miR-200a-3p,novel_127,novel_96,miR-219-3p,miR-3071-3p,miR-29a-5p,miR-2284y,miR-200a,miR-1290,miR-3591-5p,miR-23b-5p,miR-2284z,miR-429-3p,miR-6517,miR-299a-3p,novel_79,miR-592,miR-2433,miR-1260b,miR-181c-3p,miR-28a-3p,miR-193a-5p,miR-2318,miR-345-5p,miR-1248,miR-301,miR-93-5p,miR-331-5p,miR-3068-3p,miR-503-3p,miR-2355-5p,miR-130b-3p,miR-491-5p,miR-199a-3p,miR-106b-5p,miR-1b-5p,miR-2284b,miR-101c,miR-758-3p,miR-147a,miR-142-5p,miR-425-3p,miR-2459,miR-1185-3p,miR-455-3p,novel_78,miR-548w,miR-145b,miR-3968,miR-490-5p,miR-3964,miR-101-3p,miR-2440,novel_82,miR-877-3p,miR-3956-3p,miR-885-5p,miR-181a-3p,miR-140-5p,miR-381-3p,miR-2483-3p,miR-1185-2-3p,miR-203-3p,miR-2284g,miR-4792,miR-139-5p,miR-210-5p,miR-500a-5p,miR-380-3p,miR-2424,miR-503-5p,miR-345-3p,miR-142-3p,miR-184-3p,miR-361-5p,miR-148a-5p,miR-2284u,miR-218-1-3p,miR-664-5p,miR-98-3p,miR-7859,miR-328-3p,miR-106a,miR-18a-3p,miR-380-5p,novel_74,miR-669a-3p,miR-130b-5p,miR-454-3p,miR-129-5p,miR-2285j,let-7b-3p,miR-219b-3p,miR-98-3p,miR-346,miR-6525,miR-142a-5p,miR-769-5p,miR-339b,miR-345-5p,miR-186-5p,miR-433-3p,miR-129b-3p,miR-377-3p,miR-301a-3p,miR-216b-3p,miR-466f-3p,miR-660,miR-503-5p,miR-654-3p,miR-345-5p,miR-130b-5p,miR-1260b,miR-380-3p,miR-30b-3p,miR-2284ab,miR-3071-5p,miR-145a-5p,miR-503-5p,miR-432-5p,miR-130a-3p,miR-22-5p,miR-656-5p,miR-20a-5p,miR-24-3p,miR-17-
MLIP	ENSOARG0(	muscular	miR-20a,miR-1271-3p,miR-192-3p,miR-125a-3p,miR-200c,miR-2285p,miR-30b,miR-376b-3p,miR-30f,miR-500a-5p,miR-200c-3p,miR-106a,miR-30d,miR-106a,miR-200b,miR-340-5p,miR-222-5p,novel_121,miR-214-5p,miR-199a-3p,novel_133,miR-30c-5p,miR-30a-5p,miR-30c,miR-200b-3p,miR-590-3p,miR-3074-2-3p,miR-374a-3p,miR-500b-5p,miR-93,miR-370-5p,miR-135a-1-3p,miR-374b-3p,miR-487a-5p,miR-190a-5p,miR-532-5p,miR-296-3p,miR-552-3p,miR-370-5p,miR-323-5p,miR-2284s,novel_25,miR-30e-5p,miR-17-5p,miR-30d-5p,miR-362-5p,miR-93-5p,miR-6240,miR-106a-5p,miR-1388-3p,miR-2285r,miR-758-3p,miR-199a-3p,miR-17-5p,novel_51,miR-106b-5p,miR-330-3p,miR-190a,miR-409b,miR-1343-3p,miR-2300a-5p,miR-500-5p,miR-1285,miR-1434-3p,miR-2284r,miR-758-3p,miR-1193,miR-362-5p,miR-20a-5p,miR-30b-5p,miR-502-5p,miR-382-5p,miR-499a-5p,miR-374a-3p,miR-2483-3p,miR-3082-5p,miR-670-3p,miR-323a-5p,miR-150-5p,miR-877-3p

LECT1	ENSOARG0(	leukocyt	miR-296-3p,miR-552-3p,miR-548o-3p,miR-15b-5p,miR-216b-5p,miR-376e-3p,miR-500a-3p,miR-129b-3p,miR-450a-1-3p,miR-1827,miR-345-5p,miR-339b,miR-3958-5p,miR-3074-5p,miR-129-2-3p,miR-3184-5p,miR-346,miR-30a-3p,miR-30e-3p,miR-130b-5p,miR-129-5p,miR-500,miR-30d-3p,miR-380-5p,miR-378a-3p,miR-2366,miR-378i,miR-148a-5p,miR-2284u,miR-361-5p,miR-16-1-3p,miR-133a-3p,miR-345-3p,miR-329a-5p,miR-2424,miR-486-3p,miR-181b-1-3p,miR-422a,miR-345-3p,miR-92a-1-5p,miR-324-3p,novel_17,miR-26a-5p,miR-211-5p,miR-139-5p,miR-2284g,miR-500-3p,miR-378g,miR-221,miR-493-5p,miR-376a-3p,miR-96-5p,miR-9-5p,miR-2284j,let-7i-3p,miR-6527,miR-2447,miR-423-5p,miR-361-3p,miR-490-5p,miR-3968,miR-181b-2-3p,miR-421,miR-455-3p,miR-148b-5p,miR-376b-3p,miR-1298-3p,miR-153,miR-758-3p,miR-501-3p,miR-541,miR-1839-5p,miR-101c,miR-1388-5p,miR-2310,miR-378d,miR-16b,miR-29b-1-5p,miR-103a-2-5p,miR-153-3p,miR-487b-5p,miR-2285aa,miR-2355-5p,miR-503-3p,miR-376c-3p,miR-331-5p,miR-137-3p,miR-2318,miR-329b,miR-345-5p,miR-876-3p,miR-16-5p,miR-28a-3p,miR-100-3p,novel_63,miR-2284k,miR-592,novel_79,miR-1224-5p,miR-2285ad,miR-129-1-3p,novel_121,miR-450a-1-3p,miR-9-5p,miR-505-5p,miR-322-5p,miR-2427,miR-1290,miR-3591-5p,miR-155-5p,miR-3956-5p,miR-2285b,miR-148b-5p,miR-450b-5p,miR-125a-3p,miR-205-5p,miR-28-3p,miR-3120-3p,miR-26b-5p,miR-329-5p,miR-3074-5p,miR-181b-3p,miR-677,novel_69,miR-216a-3p,miR-150-5p,miR-378f,miR-410-5p,miR-758-5p,miR-425-5p,miR-4324,miR-339-5p,miR-103a-3p,miR-31-5p,miR-3955-5p,miR-194-3p,miR-411-5p,miR-134-3p,miR-3431,novel_23,miR-450c-3p,miR-197-3p,miR-22-3p,miR-7b-5p,miR-708-3p,miR-2284x,miR-188-5p,miR-497-5p,miR-16-1-3p,miR-16b,miR-628-5p,miR-493-5p,miR-380-5p,let-7a-2-
ACOT8	ENSOARG0(	acyl-CoA	miR-493-3p,miR-378i,miR-378a-3p,miR-330-5p,miR-1193,miR-5703,miR-378h,miR-3960,miR-6517,miR-30b-3p,miR-221-5p,miR-543-5p,miR-210-5p,miR-3071-3p,miR-30c-2-3p,miR-422a,miR-185-3p,miR-122-3p,novel_39,miR-3064-5p,miR-1827,miR-3154,miR-6130,miR-204-3p,miR-324-5p,miR-140-5p,miR-221-5p,miR-2331-3p,miR-378a-3p,miR-30c-1-3p,miR-4492,miR-708-5p,miR-28-5p,miR-331-3p,miR-320e,miR-665,miR-1197-3p,miR-378c,miR-378c,novel_42,miR-3074-5p,miR-4510,miR-378c,miR-21-5p,miR-1388-5p,miR-219a-1-3p,miR-450b-5p,miR-4508,miR-744-5p,miR-378b,miR-296-5p,miR-2284r,miR-541,miR-758-3p,miR-3955-5p,novel_78,miR-3085-3p,miR-326,miR-1306-5p,miR-378d,miR-30c-1-3p,miR-432-3p,miR-432-3p,miR-758-3p,miR-1291,miR-450b-3p,miR-412-3p,miR-1983,miR-378f,miR-361-3p,miR-1306,miR-21c,miR-378e,miR-652-3p,miR-4443,miR-3064-5p,miR-3074-5p,miR-128-1-5p,miR-4726-5p,miR-326-3p,miR-2428,miR-2447,miR-541-3p,novel_101

PROP1	ENSOARG0(	PROP pai	miR-432,miR-708-3p,miR-145a-5p,novel_23,miR-374b-3p,miR-30b-3p,miR-16b,miR-2284ab,miR-16-1-3p,miR-497-5p,miR-2284x,miR-31-5p,miR-665,miR-432-5p,miR-2284d,miR-3431,miR-194-3p,miR-411-5p,miR-146a,miR-105-5p,miR-2483-5p,miR-654-3p,miR-2387,miR-1306,miR-18a-3p,miR-410-5p,miR-412-3p,miR-146b-5p,miR-195a-5p,miR-329-5p,miR-411b-3p,miR-299b-3p,miR-15a,miR-16a,miR-33a-3p,miR-379-3p,novel_1,miR-147-5p,novel_120,miR-335-5p,miR-1839-3p,miR-199b-5p,miR-2284w,miR-1290,miR-23b-5p,miR-30a-5p,miR-411-3p,miR-105-2,miR-200b-3p,miR-30c,miR-299a-3p,miR-429-3p,miR-7641,miR-412,miR-322-5p,miR-6516-5p,miR-545-5p,novel_121,miR-200c,miR-30c-2-3p,miR-30f,miR-200c-3p,miR-1a-2-5p,miR-181b-5p,miR-200a-3p,miR-543-5p,novel_127,miR-450b-5p,miR-199a-5p,miR-30e-5p,miR-329b,miR-141-3p,miR-331-5p,miR-199a-5p,miR-376c-3p,miR-2284n,miR-2284k,miR-2332,miR-6740-5p,novel_9,miR-28-5p,miR-374b-3p,miR-592,miR-1306-5p,miR-16-5p,miR-6128,miR-144-5p,miR-374a-3p,miR-2284r,miR-484,miR-412-3p,miR-30b-3p,miR-1388-5p,miR-101c,miR-105-1,miR-548w,miR-539-5p,miR-199a-3p,miR-1306-5p,miR-16b,miR-199b-5p,miR-146b,miR-124a,miR-1388-3p,miR-1839-3p,miR-424-5p,miR-494-3p,miR-30c-1-3p,miR-8485,miR-361-3p,miR-199b-5p,miR-381-3p,miR-129b-5p,miR-1983,miR-499b-5p,miR-3596,miR-7857-3p,miR-499a-3p,miR-144-5p,miR-30b-5p,novel_82,miR-2285y,miR-4726-5p,miR-499a-5p,miR-335,miR-3964,miR-374a-3p,miR-2447,novel_60,miR-3956-3p,miR-2284j,miR-181d-5p,miR-30c-5p,miR-2366,miR-124-3p,novel_73,miR-16-1-3p,miR-361-5p,miR-18a-3p,miR-200b,miR-15a-5p,miR-199a-3p,miR-30b,miR-2285p,miR-216a-5p,miR-500a-5p,miR-2284m,miR-30d,miR-1277-5p,miR-2484,miR-299-3p,miR-329a-5p,miR-376a-5p,miR-376c-3p,miR-

SERPINB11	ENSOARG0(	serpin p	miR-362-3p,miR-329-3p,miR-455-3p,miR-539-5p,miR-330-3p,miR-199b-3p,miR-103a-2-5p,miR-491-5p,miR-29b-1-5p,miR-2310,miR-8485,miR-378d,miR-101b-3p,miR-504,miR-299b-5p,miR-140-5p,miR-4443,miR-211-5p,miR-7689-3p,miR-139-5p,miR-2440,miR-7705,novel_111,miR-34a-5p,miR-361-5p,miR-133a-3p,miR-16-1-3p,miR-184-3p,miR-378a-3p,miR-378i,miR-95-3p,miR-30d-3p,miR-380-5p,miR-2484,miR-122-3p,miR-92a-1-5p,miR-422a,miR-219b-5p,miR-377-5p,miR-376a-5p,miR-186-5p,miR-1827,miR-625-5p,miR-339b,miR-433-3p,miR-151b,miR-377-3p,miR-2284s,miR-552-3p,miR-216b-3p,miR-504-5p,miR-129-5p,miR-874-5p,miR-30a-3p,miR-30e-3p,novel_44,miR-6525,miR-665-5p,miR-3184-3p,miR-129-2-3p,miR-2284x,miR-27b-3p,miR-16-1-3p,miR-542-3p,miR-2113,miR-19b-2-5p,novel_116,miR-3431,miR-3535,miR-339-5p,miR-340-3p,miR-378a-5p,miR-377-3p,let-7a-2-3p,let-7j,miR-432-3p,miR-432-3p,miR-6240,miR-378f,miR-216a-3p,miR-3591-3p,miR-148a-3p,miR-6535,miR-374c-3p,miR-411b-3p,miR-3120-3p,miR-216b-3p,miR-28c,miR-576-3p,miR-4324,miR-2284w,miR-758-5p,miR-335-5p,miR-330-5p,miR-1290,miR-486-5p,miR-129-1-3p,miR-134-5p,miR-758-5p,miR-505-5p,novel_79,miR-412,miR-429-3p,miR-152-3p,novel_96,novel_39,miR-148b-5p,miR-3064-5p,miR-329a,miR-876-3p,miR-125a,miR-3068-3p,miR-574-5p,miR-134-5p,miR-126b-3p,miR-125b-5p,miR-1260b,miR-193a-5p,miR-299a-5p,miR-450b-5p,miR-378c,miR-125b-2-3p,miR-412-3p,miR-4791,miR-378b,miR-296-5p,miR-216a-3p,miR-9788-3p,miR-27a-3p,novel_4,miR-409b,miR-2300a-5p,miR-3955-3p,miR-3085-3p,miR-362-3p,miR-326,novel_103,miR-376b,miR-652-5p,miR-33a-5p,miR-877-3p,novel_94,miR-129b-5p,miR-27a-3p,miR-378e,miR-3064-5p,miR-335,miR-107-5p,miR-1197-5p,miR-1193,miR-140-3p,miR-5703,miR-2904,miR-362-3p,miR-2408,miR-215-5p,miR-2300b-3p,novel_133,novel_83,miR-671-

CFL2	ENSOARG0(	cofilin	miR-429-3p, miR-7641, miR-23b-3p, miR-23c, miR-222-5p, miR-199a-3p, novel_121, miR-30c-5p, miR-3591-5p, miR-3578, miR-140-3p, miR-30a-5p, miR-1434-5p, miR-5703, novel_73, miR-19b-3p, miR-30c, miR-16-1-3p, miR-183-5p, miR-20a, miR-450b-5p, miR-130a-5p, miR-142a-3p, miR-2284y, miR-199a-5p, miR-146a-5p, miR-376c-5p, miR-2284a, miR-205-5p, miR-30b, miR-125a-3p, miR-200c, miR-30f, miR-200c-3p, miR-30d, novel_96, miR-200a-3p, miR-545-3p, miR-503-3p, miR-2285aa, miR-141-3p, miR-300-3p, miR-301a-3p, miR-199a-5p, miR-30e-5p, miR-21a-3p, miR-3613-5p, miR-93-5p, miR-17-5p, miR-23b, miR-30d-5p, miR-2318, miR-30f, miR-20b-5p, miR-625-5p, miR-539-5p, miR-2330-3p, miR-425-5p, miR-93, miR-551b-3p, miR-19b-1-5p, miR-409-3p, miR-664a-5p, novel_74, miR-6516-3p, miR-451a, miR-454-3p, miR-133b-5p, miR-130a-3p, miR-145b, miR-153, miR-142-3p, miR-194-3p, miR-2459, miR-1185-3p, miR-20b, miR-19b-2-5p, miR-1434-3p, miR-145a-5p, novel_23, miR-194-5p, miR-16-1-3p, miR-382-3p, miR-382, miR-23a-3p, let-7f-2-3p, let-7f-2-3p, miR-3187-3p, miR-342, miR-199a-3p, miR-146a, miR-505, miR-194a, miR-106b-5p, miR-106a-5p, miR-17-5p, miR-491-5p, miR-130b-3p, miR-153-3p, miR-146b, miR-199b-5p, miR-216b-3p, miR-3120-3p, miR-411b-3p, miR-1185-2-3p, miR-199b-5p, miR-2387, miR-3065-5p, miR-378b, miR-21c, miR-299b-5p, miR-19a-3p, miR-335-3p, miR-146b-5p, miR-9-3p, miR-342-3p, miR-212-3p, miR-495-3p, miR-194b-5p, miR-1285-5p, miR-7705, miR-199b-5p, miR-301b-3p, miR-3613, miR-30b-5p, miR-20a-5p

DIMT1	ENSOARG0(	DIM1 dim	miR-320b,miR-323b-3p,miR-130b-3p,miR-487b-5p,miR-1468,miR-199b-5p,miR-146b,miR-2310,miR-3962,miR-1a-3p,miR-501-3p,miR-450b-5p,miR-2284aa,miR-199b-3p,miR-548w,miR-1343-3p,miR-222-5p,miR-181b-2-3p,miR-27a-3p,miR-539-5p,miR-216a-3p,miR-101-3p,miR-2440,miR-2285n,miR-371a-5p,miR-499a-5p,miR-154-3p,miR-3956-3p,miR-885-5p,novel_60,miR-2447,miR-190b-5p,novel_111,miR-502-3p,miR-199b-5p,miR-493-5p,miR-499b-5p,miR-489,miR-27a-3p,miR-500-3p,miR-92a-1-5p,miR-466i-5p,miR-320d,miR-2484,miR-1277-5p,miR-34b-5p,miR-487a,miR-329a-5p,miR-369-3p,miR-181b-1-3p,miR-2432,miR-2424,miR-146a-5p,miR-215-5p,miR-5703,miR-4429,miR-493-3p,miR-34a-5p,miR-548e-3p,miR-210-3p,miR-487a-3p,miR-7859,miR-2285j,miR-324-3p,miR-487b-5p,miR-454-3p,miR-3184-3p,miR-206-3p,miR-6525,miR-21a-3p,miR-154b-3p,let-7g-3p,miR-186-5p,miR-660-5p,miR-296-3p,miR-154a-3p,miR-21-3p,miR-193b-3p,miR-301a-3p,miR-204-3p,miR-146a,miR-455-5p,miR-196b-5p,miR-193a-3p,miR-660,miR-493-5p,miR-668-3p,miR-2285r,miR-1434-3p,miR-542-3p,miR-3071-5p,miR-197-3p,miR-219a-1-3p,miR-455-5p,miR-27b-3p,miR-130a-3p,miR-33a-3p,miR-382-5p,miR-34c,miR-206,miR-199c,miR-495-3p,miR-2285x,miR-193b-3p,miR-301b-3p,miR-199b-5p,miR-2284w,miR-374c-5p,miR-449a,miR-3591-3p,miR-216a-3p,miR-450b-3p,miR-376a-2-5p,miR-146b-5p,miR-329-5p,miR-3120-3p,miR-374c-3p,miR-411b-3p,miR-34b,miR-181b-3p,miR-320c,miR-30f,miR-543-5p,miR-1,miR-2285b,miR-200a,miR-199a-5p,miR-196a-5p,miR-501-3p,miR-128-3p,miR-2284z,miR-135a-1-3p,miR-2332,novel_63,miR-410-5p,miR-3969,miR-410-3p,miR-6128,miR-3607-3p,miR-145-3p,miR-320a,miR-2318,miR-10b-3p,miR-1814c,novel_48,miR-3068-3p,miR-487a-3p,miR-215-5p,miR-2285aa,miR-331-5p,miR-34c-5p,miR-199a-5p

BAZ1A	ENSOARG0(	bromodom	miR-582-3p,miR-134,miR-23b-5p,miR-302a-5p,miR-483-3p,miR-758-5p,miR-378h,miR-134-5p,miR-7641,miR-17-3p,miR-28-3p,miR-376b-3p,miR-664-3p,miR-219-3p,miR-200a,miR-29a-5p,miR-3956-5p,miR-30a-3p,miR-3064-5p,miR-181a-2-3p,miR-4286,miR-10b-3p,miR-485-5p,miR-488-3p,miR-137-3p,miR-200a-5p,miR-154b-5p,miR-487a-3p,miR-542-5p,miR-615-5p,miR-138-5p,miR-134-5p,miR-374b-3p,miR-6740-5p,miR-409-3p,miR-374a-3p,miR-378c,miR-28a-3p,miR-3607-3p,miR-138,miR-30b-3p,miR-653,miR-194-3p,novel_116,miR-149-5p,miR-544b,miR-483-3p,miR-197-5p,miR-654-3p,let-7j,miR-130b-5p,miR-493-5p,miR-2483-5p,let-7f-2-3p,let-7f-2-3p,miR-7975,miR-2319a,miR-6395,miR-378f,miR-378b,miR-323a-5p,miR-669,miR-378b,miR-320b,miR-655-5p,miR-7-1-3p,miR-421-5p,miR-502b,miR-382-5p,miR-2428,miR-542-5p,miR-17-3p,miR-142-3p,miR-378d,novel_133,miR-378i,miR-193b-5p,miR-378a-3p,miR-7859,miR-222-5p,miR-328-3p,miR-548e-3p,miR-1b-3p,miR-30d-3p,miR-202-5p,miR-542-5p,miR-92a-1-5p,miR-185-3p,miR-422a,miR-421-5p,miR-29b-1-5p,miR-142a-3p,miR-216c-5p,miR-487a,miR-345-3p,miR-154b-3p,novel_99,miR-582-5p,miR-3613-5p,miR-129b-3p,miR-433-3p,miR-582-5p,miR-544-3p,miR-323-5p,miR-378a-3p,miR-548o-3p,miR-216b-3p,miR-154a-3p,miR-552-3p,miR-296-3p,miR-17-3p,miR-487b-5p,miR-129-5p,miR-874-5p,miR-130b-5p,miR-15b-3p,miR-30e-3p,miR-30a-3p,miR-219b-3p,miR-338-5p,miR-665,miR-320e,miR-378c,miR-145a-3p,miR-378c,miR-544a,miR-2426,miR-101c,miR-378b,miR-758-3p,miR-30b-3p,miR-142-3p,miR-2459,miR-1185-3p,miR-548w,novel_78,miR-222-5p,miR-3085-3p,miR-542-5p,miR-29b-1-5p,miR-378d,miR-758-3p,let-7c-3p,miR-744-3p,miR-493-5p,novel_94,miR-3596,miR-378e,miR-1185-2-3p,miR-3064-5p,miR-664b,miR-3968,miR-3958-3p,miR-374a-3p,miR-154-3p,miR-2285p,miR-877-3p,miR-

SERPINB7	ENSOARG0(	serpin p	miR-362-5p,miR-3154,miR-301,miR-30e-5p,miR-329b,miR-876-3p,miR-141-3p,miR-370-3p,miR-154b-5p,miR-199a-5p,miR-223-5p,miR-2284k,miR-3607-3p,miR-6128,miR-365a-5p,miR-500b-5p,miR-323-3p,miR-23b-5p,miR-30a-5p,miR-2427,miR-218-2-3p,miR-3578,miR-196a-5p,miR-411-3p,miR-450a-1-3p,miR-6516-5p,miR-2285af,miR-200a-3p,miR-6536,miR-1,miR-495-5p,miR-2284a,miR-199a-5p,miR-376c-5p,miR-2284y,miR-323a-5p,miR-146b-3p,miR-502b,miR-656-5p,miR-206,miR-495-3p,miR-335-5p,miR-199b-5p,miR-2284w,miR-323c,miR-145a-5p,miR-374b-3p,miR-450c-3p,miR-708-3p,miR-2284x,miR-380-3p,miR-26b-3p,miR-31-5p,miR-3431,miR-365b-5p,miR-224-5p,miR-452-5p,miR-196b-5p,miR-101-5p,miR-30d-5p,miR-450a-1-3p,miR-1827,miR-30f,miR-139-5p,miR-216b-5p,miR-323-5p,miR-6516,miR-324-3p,miR-2285j,miR-487a-5p,miR-450b-3p,miR-539-5p,miR-433-5p,miR-6134,miR-122-5p,miR-224-5p,miR-206-3p,miR-188-3p,miR-124-3p,miR-30c-5p,miR-142-3p,miR-1247-5p,miR-210-5p,novel_17,miR-30b,miR-2484,miR-30d,miR-466i-5p,miR-2284m,miR-195a-3p,miR-143-3p,miR-380-3p,miR-2424,miR-125b-2-3p,miR-199b-5p,miR-2285u,miR-2319b,miR-452-3p,miR-4792,miR-139-5p,miR-4443,miR-203-3p,miR-107-5p,miR-2440,miR-30b-5p,miR-2284q,miR-335,miR-499a-5p,miR-628-3p,miR-4726-5p,let-7i-3p,miR-2284j,miR-541-3p,miR-885-5p,miR-362-5p,miR-3962,miR-1a-3p,miR-541,miR-484,miR-1839-5p,miR-2426,miR-450b-5p,miR-145b,miR-5010-3p,miR-548w,miR-2459,miR-9788-3p,miR-500-5p,miR-147a,miR-103a-2-5p,miR-199b-5p,miR-3955-3p,miR-376b,miR-124a

PHIP	ENSOARG0(	pleckstr	let-7c-3p,miR-33a-5p,miR-124a,miR-424-5p,miR-106a-5p,miR-8485,miR-1b-5p,miR-758-3p,miR-362-3p,miR-106b-5p,miR-16b,miR-199b-5p,miR-145b,miR-105-1,miR-548w,miR-2285e,miR-758-3p,miR-329-3p,miR-4791,miR-1839-5p,miR-16-2-3p,miR-2284aa,miR-362-3p,miR-1388-5p,miR-544a,miR-450b-5p,miR-299a-5p,miR-2285f,miR-190b-5p,miR-212-3p,miR-7705,miR-877-3p,miR-371a-5p,miR-30b-5p,miR-3964,miR-499a-5p,miR-7689-3p,miR-7977,miR-126b-5p,miR-20a-3p,miR-203-3p,miR-2483-3p,miR-381-3p,miR-199b-5p,miR-493-5p,novel_94,miR-96-5p,miR-195a-3p,miR-20a,miR-369-3p,miR-216c-5p,miR-376a-5p,miR-421-5p,miR-125b-2-3p,miR-130a-5p,miR-500a-5p,miR-30b,miR-26a-5p,miR-30d,miR-1277-5p,miR-106a,miR-466i-5p,miR-548e-3p,miR-106a,novel_83,miR-148b-3p,miR-15a-5p,miR-98-3p,miR-124-3p,miR-5703,miR-217-5p,miR-30c-5p,miR-362-3p,miR-215-5p,miR-376a-5p,let-7a-3p,novel_73,miR-142-3p,miR-19b-3p,miR-1271,miR-2330-3p,miR-3074-2-3p,miR-3074-5p,miR-129-2-3p,miR-338-3p,miR-98-3p,miR-655-3p,miR-3604,let-7b-3p,miR-129-5p,miR-190a-5p,miR-669a-3p,miR-155-5p,miR-6516-3p,miR-660-5p,miR-155-5p,miR-877-5p,miR-15b-5p,miR-544-3p,miR-192-5p,miR-6516,miR-129b-3p,miR-411,miR-655,miR-30d-5p,miR-299,let-7f-1-3p,miR-2285c,miR-543-3p,miR-186-5p,novel_99,miR-133b-3p,let-7g-3p,miR-2312,miR-345-5p,miR-30f,miR-101-5p,miR-493-5p,let-7j,miR-503-3p,miR-345-5p,miR-654-3p,miR-190a,miR-105-5p,miR-17-5p,miR-106a-5p,miR-660,miR-466f-3p,miR-378a-5p,miR-31-5p,miR-6238,miR-544b,miR-20b,miR-3535,miR-2285g,miR-185-5p,miR-3431,miR-145a-5p,novel_23,miR-653,miR-497-5p,miR-380-3p,miR-16b,miR-374c-5p,miR-199b-5p,miR-2284w,miR-3959-3p,miR-323c,miR-16a,miR-33a-3p,novel_87,miR-15a,miR-4324,miR-20a-5p,miR-142b,miR-203b-5p,miR-147-5p,miR-6119-
CDH2	ENSOARG0(	cadherin	miR-380-5p,miR-23a-3p,miR-668-3p,miR-2310,miR-758-3p,miR-342,miR-1306-5p,miR-377-3p,miR-326,novel_51,miR-3085-3p,miR-26b-3p,miR-145b,miR-142-3p,miR-3431,miR-539-5p,miR-544b,miR-126a-5p,miR-19b-2-5p,miR-223-3p,miR-145a-5p,miR-758-3p,miR-125b-2-3p,miR-544a,miR-1260b,miR-21b,miR-188-5p,miR-2889,miR-342-3p,miR-17-3p,miR-326-3p,miR-154a,miR-6402,miR-371a-5p,miR-425-5p,miR-6119-3p,miR-3968,miR-421-5p,miR-203b-5p,miR-502b,miR-3064-5p,miR-20a-3p,miR-3074-5p,miR-329-5p,miR-2319b,miR-499a-3p,miR-411b-3p,miR-489,miR-3082-5p,miR-148a-3p,miR-1306,miR-150-5p,miR-183-5p,miR-329a-5p,miR-2285w,miR-195a-3p,miR-3064-5p,miR-380-5p,miR-142a-3p,miR-130a-5p,miR-2432,miR-1260a,miR-210-5p,novel_17,miR-17-3p,miR-6536,miR-21-3p,miR-152-3p,miR-548e-3p,miR-23b-3p,miR-7859,miR-758-5p,miR-23c,miR-148b-3p,novel_83,miR-2284h-5p,miR-532-3p,miR-140-3p,miR-330-5p,miR-155-5p,miR-590-3p,miR-3074-5p,miR-103b,miR-425-5p,miR-323-3p,miR-1260b,miR-494-5p,miR-19b-1-5p,miR-338-5p,miR-17-3p,miR-155-5p,miR-767-5p,miR-592,miR-532-5p,miR-544-3p,miR-377-3p,miR-155-5p,miR-2448-3p,miR-301,miR-23b,miR-487b-5p,miR-767,miR-1814c
RSL1D1	ENSOARG0(	ribosoma	miR-331-5p,miR-432-3p,miR-214,miR-214-3p,miR-331-5p,novel_103,miR-2448-3p,miR-331-5p

CELF1	ENSOARG0(	CUGBP, E	miR-505-3p,miR-409b,miR-105-1,miR-544b,miR-148b-5p,miR-1343-5p,miR-2904,miR-362-3p,miR-329-3p,miR-2889,miR-105-2,miR-544a,miR-362-3p,miR-3064-5p,miR-329a-5p,miR-185-3p,miR-105-5p,miR-362-3p,miR-1343-5p,miR-487b-5p,miR-124-5p,miR-3085-3p,miR-21-3p,miR-329-5p,miR-452-3p,miR-3064-5p,miR-489,miR-323-5p,miR-300-3p,miR-323a-5p,miR-487b-5p,miR-3591-3p,miR-140-5p,miR-186-5p,miR-505-3p,miR-329a,miR-495-3p,miR-3604,miR-494-5p,miR-2433,miR-487b-5p
KARS	ENSOARG0(	lysyl-tR	miR-184-3p,miR-148a-5p,miR-378d,miR-378i,miR-362-3p,miR-378a-3p,miR-1197-5p,miR-210-3p,miR-383-5p,miR-15a-5p,miR-199a-3p,miR-532-3p,miR-18a-3p,miR-3600,miR-212-5p,miR-505-3p,novel_17,miR-422a,miR-345-3p,miR-29b-1-5p,miR-181b-1-3p,miR-125b-2-3p,miR-130a-5p,miR-2312,miR-543-3p,miR-625-5p,novel_99,miR-2285c,miR-433-3p,miR-15b-5p,miR-378a-3p,miR-30c-1-3p,miR-2331-3p,miR-548o-3p,miR-3065-3p,novel_107,miR-296-3p,miR-424-5p,miR-222-3p,miR-2285j,miR-19b-1-5p,miR-224-5p,miR-181c-5p,miR-378c,miR-129-2-3p,miR-3074-2-3p,miR-1388-5p,miR-378c,miR-362-3p,miR-16-2-3p,miR-3120-5p,miR-484,miR-6529a,miR-329-3p,miR-378b,miR-412-3p,miR-425-3p,miR-539-5p,miR-148b-5p,miR-221-3p,miR-2285e,miR-421,miR-548w,novel_78,miR-145b,miR-27a-3p,miR-181b-2-3p,novel_124,miR-487b-5p,miR-25-5p,miR-103a-2-5p,miR-199a-3p,miR-326,miR-29b-1-5p,miR-362-3p,miR-16b,miR-30c-1-3p,miR-378d,miR-193a,miR-8485,miR-1b-5p,miR-424-5p,miR-652-5p,miR-335-3p,novel_94,miR-221,miR-378e,miR-3596,miR-2483-3p,miR-203-3p,miR-27a-3p,miR-24-1-5p,miR-2319b,miR-541-5p,miR-628-3p,miR-3958-3p,miR-4726-5p,miR-335,miR-218-5p,miR-2285n,miR-101-3p,miR-371a-5p,miR-1973,miR-107-5p,miR-877-3p,novel_111,miR-8095,miR-4454,miR-3059-5p,miR-212-3p,miR-1296-5p,miR-2285f,miR-181d-5p,miR-219a-5p,miR-452-5p,miR-9-5p,miR-411-3p,miR-345-3p,miR-3578,miR-2427,miR-330-5p,miR-505-5p,miR-9-5p,miR-322-5p,miR-302a-5p,miR-378h,miR-582-3p,miR-129-1-3p,miR-222,miR-7641,miR-412,miR-181b-5p,novel_127,miR-200a-3p,miR-1260a,miR-125a-3p,miR-30c-2-3p,miR-24-2-5p,miR-148b-5p,miR-1957a,novel_39,miR-3065-3p,miR-1271-3p,miR-495-5p,miR-2403,miR-876-3p,miR-10b-3p,miR-329a,miR-7144-5p,miR-1248,miR-671-5p,miR-365a-3p,miR-141-3p,novel_9,miR-

PLEKHH2	ENSOARG0(	pleckstr	miR-8485,miR-30c-1-3p,miR-376b,miR-424-5p,miR-103a-2-5p,miR-146b,miR-182-5p,miR-29b-1-5p,miR-505,miR-320b,miR-126a-5p,miR-455-3p,miR-376b-3p,miR-147a,miR-27a-3p,miR-330-3p,miR-1a-3p,miR-412-3p,miR-154a,miR-9-5p,miR-2447,miR-2284q,miR-628-3p,miR-107-5p,miR-2440,miR-101-3p,miR-27a-3p,miR-376d,miR-203-3p,miR-211-5p,miR-126b-5p,miR-7977,miR-2319b,miR-376a-3p,miR-3963,miR-409-5p,miR-361-3p,miR-143-3p,miR-2432,miR-2424,miR-146a-5p,miR-130a-5p,miR-29b-1-5p,miR-195a-3p,miR-2285w,miR-329a-5p,miR-2484,miR-299-3p,miR-34b-5p,miR-466i-5p,miR-219b-5p,miR-500a-5p,miR-2284h-5p,miR-15a-5p,miR-210-3p,miR-487a-3p,miR-485-3p,miR-34a-5p,miR-4429,miR-188-3p,miR-5703,miR-2904,miR-2300b-3p,miR-206-3p,miR-6525,miR-3074-5p,miR-130b-5p,miR-129-5p,miR-487a-5p,miR-708-5p,miR-155-5p,miR-29b-2-5p,miR-665,miR-3604,miR-15b-5p,miR-155-5p,miR-204-3p,miR-27a-5p,miR-182-5p,miR-3065-3p,miR-21-3p,miR-216b-3p,miR-548o-3p,miR-140-5p,miR-30c-1-3p,miR-2331-3p,novel_99,miR-625-5p,miR-18a-5p,miR-133b-3p,miR-433-3p,miR-27b-5p,miR-598-3p,miR-539-3p,miR-6240,miR-130b-5p,miR-330-3p,miR-377-3p,miR-146a,miR-149-5p,miR-3431,miR-412-5p,miR-187-3p,miR-3955-5p,miR-27b-3p,miR-219a-1-3p,miR-16-1-3p,miR-29b,miR-21-5p,miR-542-3p,miR-1285,miR-1843b-5p,miR-2284w,miR-449a,miR-211,miR-29c-3p,miR-6123,miR-29a,miR-206,novel_1,miR-379-3p,miR-33a-3p,miR-34c,miR-133a-5p,miR-6535,miR-669,miR-670-3p,miR-299b-3p,miR-34b,miR-29a-3p,miR-7-1-3p,miR-3074-5p,miR-329-5p,miR-195a-5p,miR-146b-5p,miR-2319a,miR-450b-3p,miR-216a-3p,miR-376b-3p,miR-3591-3p,miR-18b,miR-2285b,miR-1271-3p,miR-29a-5p,miR-3065-3p,miR-2478,miR-1,miR-29b-2-5p,miR-2385-3p,miR-6536,miR-450a-2-3p,miR-30f,miR-30c-2-3p,miR-6119-5p,miR-376b-3p,novel_121,miR-

C9orf152	ENSOARG0(	chromoso	miR-345-5p,miR-767,miR-671-5p,miR-485-5p,miR-190a-3p,miR-3154,miR-199a-5p,miR-133c,miR-503-3p,miR-2355-5p,miR-767-5p,miR-28-5p,miR-2332,miR-29b-3p,miR-3969,miR-6128,let-7d,miR-10a-5p,miR-496,miR-128-3p,miR-615-3p,miR-330-5p,miR-29d-3p,miR-758-5p,miR-483-3p,miR-9-5p,miR-302a-5p,miR-2284z,miR-2285ad,let-7f-5p,miR-17-3p,miR-2898,miR-152-3p,miR-543-5p,miR-125a-3p,let-7c-5p,miR-28b,let-7g,miR-199a-5p,novel_39,miR-1957a,miR-4286,let-7e-5p,miR-148a-3p,miR-216a-3p,miR-150-5p,miR-29a-3p,miR-411b-3p,miR-10a-5p,novel_1,miR-29a,miR-199c,miR-656-5p,miR-28c,miR-6402,miR-382-5p,let-7i,miR-199b-5p,miR-2428,miR-326-3p,miR-342-3p,miR-17-3p,miR-758-5p,miR-29c-3p,miR-30b-3p,miR-29b,miR-27b-3p,miR-4508,miR-1285,miR-7b-5p,miR-873a-5p,miR-194-5p,miR-134-3p,let-7g-5p,miR-185-5p,miR-31-5p,miR-483-3p,miR-377-3p,miR-7-5p,miR-194a,miR-2284l,miR-345-5p,miR-130b-5p,miR-382,miR-345-5p,miR-6130,miR-10a,miR-377-3p,let-7i-5p,miR-140-5p,miR-504-5p,miR-17-3p,miR-664a,miR-130b-5p,miR-708-5p,miR-4492,let-7f,miR-324-3p,miR-151-3p,miR-6525,miR-10b-5p,let-7b-5p,miR-4510,let-7b,miR-433-5p,miR-3074-2-3p,miR-425-5p,novel_73,miR-378d,miR-218-1-3p,miR-1961,miR-2300b-3p,miR-140-3p,miR-5703,miR-124-3p,miR-148b-3p,miR-3600,miR-380-5p,miR-98-5p,let-7k,novel_17,miR-185-3p,miR-219b-5p,miR-421-5p,miR-143-3p,miR-2887,miR-504,miR-199b-5p,miR-140-5p,miR-361-3p,miR-378g,miR-27a-3p,let-7d-5p,miR-203-3p,miR-452-3p,miR-7977,miR-3968,miR-615,miR-2285n,miR-2285y,miR-361-3p,miR-877-3p,miR-452-5p,miR-194b-5p,miR-9-5p,miR-2284aa,miR-10b,miR-6529a,miR-3962,miR-758-3p,miR-30b-3p,miR-9788-3p,miR-199b-3p,miR-151a-3p,miR-548w,miR-27a-3p,let-7e,miR-199b-5p,miR-326,miR-342,miR-2310,miR-758-

RSF1	ENSOARG0(	remodeli	miR-758-3p,miR-101c,miR-1839-5p,miR-181b-2-3p,miR-548w,miR-455-3p,miR-148b-5p,miR-1185-3p,miR-1298-3p,miR-539-5p,miR-505,miR-29b-1-5p,miR-16b,miR-103a-2-5p,miR-487b-5p,miR-23a-3p,miR-2284b,miR-8485,miR-2310,miR-1983,miR-96-5p,miR-2285u,miR-126b-5p,miR-2284g,miR-2319b,miR-1185-2-3p,miR-3596,miR-2483-3p,novel_82,miR-9-5p,miR-190b-5p,miR-2284j,miR-2447,miR-217-5p,miR-1961,miR-193b-5p,miR-2284u,miR-30d-3p,miR-7859,miR-23b-3p,miR-23c,miR-500a-5p,miR-618,let-7k,miR-98-5p,miR-99a-3p,miR-2285w,miR-486-3p,miR-181b-1-3p,miR-598-3p,miR-487b-5p,miR-548o-3p,let-7i-5p,miR-15b-5p,miR-377-3p,miR-30a-3p,miR-219b-3p,let-7f,miR-30e-3p,miR-190a-5p,novel_74,miR-487b-5p,miR-3958-5p,let-7b,miR-181c-5p,miR-6525,novel_44,miR-197-3p,miR-497-5p,miR-2284x,miR-16b,miR-31-5p,novel_116,miR-412-5p,miR-3431,miR-105-5p,miR-377-3p,miR-193a-3p,miR-6240,let-7j,let-7a-2-3p,miR-2284l,miR-150-5p,miR-3591-3p,miR-376b-5p,let-7e-5p,miR-410-5p,miR-412-3p,novel_68,miR-216b-3p,miR-677,miR-320c,miR-181b-3p,miR-374c-3p,miR-576-3p,miR-193b-3p,miR-1a-1-5p,miR-374c-5p,miR-2427,miR-3591-5p,miR-1290,miR-412,miR-2285ad,miR-6517,miR-429-3p,novel_121,miR-486-5p,miR-9-5p,miR-322-5p,miR-219-3p,miR-8117,miR-200a-3p,let-7f-5p,miR-1a-2-5p,miR-2284y,let-7g,miR-376c-5p,miR-23b,miR-1248,miR-876-3p,miR-2318,miR-99a-3p,miR-2285aa,miR-503-3p,miR-300-3p,miR-2284k,miR-494-5p,miR-592,miR-16-5p,miR-1260b,miR-7857,miR-412-3p,miR-125b-2-3p,miR-3120-5p,miR-2426,miR-105-1,novel_4,miR-320b,let-7e,let-7c-3p,let-7a-5p,miR-424-5p,miR-758-3p,let-7d-5p,miR-2285y,miR-107-5p,miR-2285n,miR-2285t,miR-2284q,miR-3958-3p,miR-4726-5p,miR-181d-5p,miR-212-3p,miR-219a-2-3p,miR-2904,miR-126-3p,miR-

ZNF354B	ENSOARG0(	zinc fin	miR-148a-5p,miR-16-1-3p,miR-19b-3p,miR-4429,miR-33b-3p,miR-5703,miR-98-3p,miR-1961,miR-199a-3p,miR-148b-3p,miR-505-3p,miR-3600,let-7k,miR-221-5p,miR-98-5p,miR-466i-5p,miR-2284m,miR-320d,miR-345-3p,miR-486-3p,miR-130a-5p,miR-181b-1-3p,miR-125b-2-3p,miR-664b-3p,novel_99,miR-582-5p,miR-345-5p,miR-133b-3p,let-7g-3p,let-7f-1-3p,let-7i-5p,miR-204-3p,miR-582-5p,miR-301a-3p,miR-6516,miR-377-3p,miR-216b-3p,miR-3065-3p,miR-496-3p,miR-504-5p,miR-221-5p,miR-545-3p,miR-208b-5p,miR-454-3p,miR-129-5p,miR-17-3p,miR-6516-3p,miR-451a,let-7f,miR-15b-3p,miR-2285j,let-7b-3p,let-7b-5p,miR-98-3p,miR-6525,miR-2330-3p,miR-129-2-3p,let-7b,miR-539-5p,miR-101c,miR-450b-5p,miR-101a-5p,miR-1388-5p,miR-125b-2-3p,miR-30b-3p,miR-501-3p,miR-323b,miR-1185-3p,miR-148b-5p,miR-9788-3p,miR-455-3p,miR-147a,miR-1298-3p,miR-145b,miR-181b-2-3p,miR-105-1,miR-548w,let-7e,miR-199b-5p,miR-3955-3p,miR-130b-3p,miR-491-5p,miR-323b-3p,miR-505,miR-2957,miR-326,novel_115,novel_103,miR-199a-3p,miR-342,miR-320b,miR-2310,miR-491-3p,let-7a-5p,miR-9-3p,miR-504,miR-129b-5p,miR-381-3p,miR-199b-5p,miR-500-3p,miR-1185-2-3p,miR-144-5p,let-7d-5p,miR-2483-3p,miR-3082-5p,miR-7857-3p,miR-126b-5p,miR-211-5p,miR-2285u,miR-499b-5p,miR-452-3p,miR-2284q,miR-628-3p,miR-499a-5p,miR-502-3p,miR-1285-5p,miR-9-5p,miR-190b-5p,miR-885-5p,miR-2447,miR-3059-5p,miR-4454,miR-496,miR-105-2,let-7d,miR-582-3p,miR-411-3p,miR-501-3p,miR-330-5p,miR-134,miR-345-3p,novel_121,miR-129-1-3p,miR-214-5p,miR-134-5p,miR-483-3p,miR-758-5p,miR-6516-5p,miR-9-5p,miR-302a-5p,novel_96,miR-200a-3p,miR-152-3p,miR-127-5p,miR-6536,let-7f-5p,miR-17-3p,let-7c-5p,miR-3071-3p,miR-664-3p,miR-2285b,novel_39,miR-200a,let-7g,miR-3065-3p,miR-
TENM3	ENSOARG0(	teneurin	miR-4492,miR-625-5p,miR-185-3p,novel_51,miR-1306,novel_19,miR-1306-5p,miR-6134,miR-2428,miR-4532,miR-423-5p,miR-3184-5p,miR-423-3p,miR-2331-3p
CAMK2A	ENSOARG0(	calcium/	miR-1306-5p,miR-214-3p,miR-125a-5p,miR-3085-3p,miR-378a-5p,miR-376b,miR-363-5p,miR-3187-3p,miR-432-3p,miR-30c-1-3p,miR-30b-3p,miR-4791,miR-542-3p,miR-484,miR-1260b,miR-30b-3p,miR-377-5p,miR-26b-3p,miR-2300a-5p,miR-31-5p,miR-3535,miR-576-3p,miR-6529b,miR-30b-5p,miR-4324,miR-450a-5p,miR-4726-5p,miR-877-3p,miR-18a-3p,miR-1306,miR-135b-5p,miR-504,miR-216b-3p,miR-329-5p,miR-3064-5p,miR-7857-3p,miR-125b,miR-185-3p,miR-30f,miR-324-3p,miR-30c-2-3p,miR-1260a,miR-30b,novel_127,miR-221-5p,miR-30d,miR-30b-3p,miR-760-3p,miR-127-5p,miR-2898,miR-3064-5p,miR-495-5p,miR-329a-5p,miR-450c-5p,miR-3065-3p,miR-377-5p,miR-130a-5p,miR-376a-5p,miR-1197-5p,miR-30a-5p,miR-5703,miR-3578,novel_133,miR-30c-5p,miR-30c,miR-184-3p,miR-212-5p,miR-18a-3p,miR-758-5p,miR-320e,miR-135a-5p,miR-331-3p,miR-125b-5p,miR-487a-5p,miR-4492,miR-214,miR-6128,miR-4510,miR-1260b,miR-30d-5p,miR-30e-5p,miR-1827,miR-671-5p,miR-625-5p,miR-10b-3p,miR-552-3p,miR-125a,miR-2355-5p,miR-3065-3p,miR-30c-1-3p,miR-504-5p,miR-221-5p,miR-154b-5p,miR-6130

PSAP	ENSOARG0(	prosapos	miR-1193,miR-19b-2-5p,miR-450b-5p,miR-184-3p,miR-380-5p,miR-505-3p,miR-548e-3p,miR-2300a-5p,miR-421,miR-185-5p,miR-503-5p,miR-6516-5p,miR-503-5p,miR-542-5p,miR-199b-5p,miR-214-3p,miR-542-5p,miR-503-5p,miR-3065-3p,miR-199a-5p,miR-129b-3p,miR-199b-5p,miR-505-3p,miR-3065-3p,miR-2355-5p,miR-615-5p,miR-3120-3p,miR-199a-5p,miR-6516,miR-6130,miR-19b-1-5p,miR-421-5p,miR-542-5p,miR-214,miR-758-5p,miR-1306-5p,miR-4510,miR-199b-5p,miR-877-3p,miR-361-3p,miR-3959-3p
BPIFB1	ENSOARG0(	BPI fold	miR-128-3p,miR-10a-5p,miR-1343-5p,miR-1197-5p,miR-2300b-3p,miR-215-5p,miR-2408,miR-383-5p,miR-505-5p,miR-148b-3p,miR-6517,miR-221-5p,miR-152-3p,miR-127-5p,miR-30c-2-3p,miR-185-3p,miR-125b,miR-181c-3p,miR-125a-3p,miR-2432,miR-181b-1-3p,miR-3064-5p,miR-1,miR-7144-5p,miR-3154,novel_32,miR-301a-3p,miR-10a,miR-192-5p,miR-499b-3p,miR-323-5p,miR-370-3p,miR-125a,miR-215-5p,miR-615-5p,miR-30c-1-3p,miR-504-5p,miR-221-5p,miR-454-3p,miR-6516-3p,miR-664a,miR-125b-5p,miR-135a-5p,miR-551b-3p,miR-181c-3p,miR-365a-5p,miR-6134,miR-10b-5p,miR-206-3p,miR-3074-5p,miR-3184-3p,miR-455-5p,miR-1388-5p,miR-1a-3p,miR-873a-5p,miR-10b,miR-1843b-5p,miR-1185-3p,miR-185-5p,miR-147a,miR-2320-3p,miR-130a-3p,miR-181b-2-3p,miR-31-5p,miR-340-3p,miR-330-3p,novel_78,novel_4,miR-125a-5p,miR-1343-5p,miR-124-5p,miR-3085-3p,miR-130b-3p,miR-330-3p,miR-3141,miR-365b-5p,miR-455-5p,miR-30c-1-3p,miR-628-5p,miR-6240,miR-135b-5p,miR-181a-3p,miR-504,miR-216a-3p,miR-148a-3p,miR-3141,miR-2387,miR-677,miR-203-3p,miR-1185-2-3p,miR-181b-3p,miR-499a-3p,miR-323a-5p,miR-146b-3p,miR-489,miR-655-5p,miR-10a-5p,miR-3064-5p,miR-3074-5p,miR-490-5p,miR-206,novel_27,miR-301b-3p,miR-2428,miR-24-3p,miR-1296-5p,miR-135a-2-3p,miR-219b-3p
FCRL1	ENSOARG0(	Fc recep	miR-22-5p,miR-186-5p,miR-669a-3p,miR-1248,miR-2355-5p,miR-361-3p,miR-101-3p

TGM6	ENSOARG0(	transglu	miR-3653-3p,miR-202-5p,miR-320d,miR-26a-5p,miR-183-5p,miR-503-5p,miR-184-3p,miR-4429,miR-2904,miR-1193,miR-140-3p,miR-1197-5p,miR-5703,miR-215-5p,miR-2366,miR-15a-5p,miR-505-3p,miR-487a-5p,miR-424-5p,miR-129-5p,miR-874-5p,miR-487b-5p,miR-219b-3p,miR-665,miR-15b-3p,miR-6134,miR-206-3p,miR-129-2-3p,novel_99,miR-345-5p,miR-129b-3p,miR-487b-5p,miR-15b-5p,miR-499b-3p,miR-544-3p,miR-377-3p,miR-192-5p,miR-370-5p,miR-30c-1-3p,miR-199b-5p,miR-103a-2-5p,miR-3085-3p,miR-2957,miR-505,miR-16b,miR-320b,miR-2310,miR-30c-1-3p,miR-376b,miR-424-5p,miR-544a,miR-1a-3p,miR-541,miR-2320-3p,miR-628-3p,miR-3968,miR-107-5p,miR-219a-2-3p,novel_111,miR-9-5p,miR-452-5p,miR-3059-5p,miR-541-3p,miR-493-5p,miR-877-3p,miR-199b-5p,miR-7857-3p,miR-499a-3p,miR-2483-3p,miR-3082-5p,miR-7977,miR-3064-5p,miR-4792,miR-452-3p,miR-2898,miR-30c-2-3p,miR-205-5p,miR-219-3p,miR-199a-5p,miR-200a,miR-450b-5p,miR-3064-5p,miR-1,miR-2385-3p,miR-4286,miR-1343-5p,miR-3591-5p,miR-129-1-3p,miR-322-5p,miR-9-5p,miR-1224-5p,miR-135a-1-3p,miR-370-5p,miR-365a-5p,miR-16-5p,miR-6128,miR-1306-5p,miR-1814c,miR-345-5p,miR-320a,miR-199a-5p,miR-374c-3p,miR-615-5p,miR-215-5p,miR-1343-5p,miR-378a-5p,miR-365b-5p,novel_51,miR-503-5p,miR-296-3p,miR-377-3p,miR-296-3p,miR-345-5p,miR-654-3p,miR-432-3p,miR-493-5p,miR-497-5p,miR-16b,miR-374b-3p,miR-3071-5p,miR-873a-5p,miR-1843b-5p,miR-544b,miR-503-5p,miR-26b-3p,miR-133b-5p,miR-656-5p,miR-206,miR-16a,miR-15a,miR-199b-5p,miR-1193,miR-7975,miR-195a-5p,miR-505-3p,miR-320c,miR-677,miR-374c-3p,miR-320b,miR-26b-5p

SERPINB1(	ENSOARG0(	serpin p	miR-541-3p,miR-2447,miR-423-5p,miR-2285y,novel_82,miR-154-3p,miR-2285t,miR-450a-5p,miR-7977,miR-452-3p,miR-139-5p,miR-4443,miR-27a-3p,miR-203-3p,miR-3082-5p,miR-25,miR-7857-3p,miR-378g,miR-129b-5p,miR-378j,miR-1983,miR-544-5p,novel_94,miR-33a-5p,miR-23a-3p,miR-494-3p,miR-2310,miR-758-3p,miR-193a,miR-8485,miR-30c-1-3p,miR-182-5p,miR-29b-1-5p,miR-1306-5p,novel_103,miR-214-3p,miR-25-5p,miR-3955-3p,miR-145b,miR-27a-3p,novel_78,miR-2300a-5p,novel_4,miR-409b,miR-548w,miR-455-3p,miR-147a,miR-1298-3p,miR-2320-3p,miR-425-3p,miR-758-3p,miR-30b-3p,miR-10b,miR-541,miR-484,miR-2284aa,miR-21b,miR-101c,miR-101a-5p,miR-450b-5p,miR-1388-5p,miR-214,miR-665-5p,miR-3074-2-3p,miR-3074-5p,miR-4510,miR-6134,miR-3184-5p,novel_44,miR-10b-5p,miR-665,miR-29b-2-5p,miR-4492,miR-17-3p,miR-182-5p,miR-3065-3p,miR-154a-3p,miR-21-3p,miR-30c-1-3p,miR-2331-3p,miR-204-3p,miR-6130,miR-139-5p,miR-10a,miR-132-5p,miR-582-5p,miR-2284s,miR-6516,miR-433-3p,miR-582-5p,novel_99,miR-1827,miR-186-5p,miR-625-5p,miR-885-3p,miR-339b,miR-154b-3p,miR-183-5p,miR-216c-5p,miR-2424,miR-486-3p,miR-29b-1-5p,miR-376a-5p,miR-125b,miR-324-3p,miR-216a-5p,miR-299-3p,miR-485-3p,miR-3600,miR-3960,miR-1247-5p,miR-23c,miR-487a-3p,miR-23b-3p,miR-1193,miR-140-3p,miR-1434-5p,miR-5703,miR-2904,miR-2300b-3p,novel_133,miR-361-5p,miR-16-1-3p,miR-184-3p,miR-17-3p,miR-3957-3p,novel_87,miR-142b,miR-576-3p,miR-6529b,miR-4324,miR-502-5p,miR-147-5p,miR-6123,miR-22-5p,miR-379-3p,novel_1,miR-128-1-5p,miR-10a-5p,miR-3074-5p,miR-6535,miR-411b-3p,miR-299b-3p,miR-3591-3p,miR-216a-3p,miR-378b,miR-1306,miR-150-5p,miR-376a-2-5p,miR-412-3p,miR-432-3p,novel_51,miR-1343-5p,miR-452-5p,miR-125a-5p,miR-483-3p,miR-339-5p,miR-133b-5p,miR-31-

AQP4	ENSOARGO(	aquapori	miR-216a-3p,miR-3065-5p,miR-2285ab,let-7e-5p,miR-2319a,miR-7-1-3p,miR-3120-3p,miR-329-5p,miR-144-3p,miR-2404,let-7i,miR-33a-3p,miR-425-5p,miR-203b-5p,miR-421-5p,miR-495-3p,miR-199b-5p,miR-2284w,miR-323c,miR-1434-3p,miR-19b-2-5p,miR-2284x,miR-2355-3p,let-7g-5p,miR-134-3p,miR-503-5p,miR-3535,miR-365b-5p,miR-190a,miR-105-5p,miR-503-5p,miR-196b-5p,miR-363-5p,miR-503-3p,miR-539-3p,miR-2318,miR-141-3p,miR-1271,miR-487a-3p,miR-199a-5p,miR-409-3p,miR-6740-5p,miR-331-3p,miR-365a-5p,miR-374a-3p,miR-1290,miR-196a-5p,miR-128-3p,miR-105-2,let-7d,miR-1224-5p,miR-412,miR-129-1-3p,miR-545-5p,miR-2284z,miR-6516-5p,let-7c-5p,miR-30c-2-3p,miR-200a-3p,let-7f-5p,miR-6536,miR-30a-3p,miR-3529-3p,miR-2285b,let-7g,miR-199a-5p,miR-199b-5p,miR-140-5p,miR-2285u,miR-2319b,let-7d-5p,miR-378g,miR-2440,miR-2285n,miR-154-3p,miR-374a-3p,miR-190b-5p,miR-2285f,miR-154a,miR-1285-5p,miR-361-3p,miR-412-3p,miR-4791,miR-2284aa,miR-450b-5p,miR-151a-3p,miR-105-1,miR-147a,miR-1298-3p,let-7e,miR-199b-5p,miR-487b-5p,miR-3955-3p,miR-1185-5p,miR-101b-3p,let-7a-5p,miR-491-3p,miR-8485,miR-30c-1-3p,miR-433-3p,miR-27b-5p,miR-582-5p,miR-154b-3p,miR-2312,miR-154a-3p,miR-545-3p,miR-30c-1-3p,let-7i-5p,miR-6516,miR-582-5p,miR-30a-3p,miR-151-3p,miR-19b-1-5p,miR-30e-3p,let-7f,miR-4492,miR-129-5p,miR-190a-5p,novel_74,miR-129-2-3p,miR-590-3p,let-7b,let-7b-5p,miR-5703,miR-1961,miR-193b-5p,miR-664-5p,novel_73,miR-30d-3p,miR-485-3p,miR-3600,miR-487a-3p,miR-185-3p,let-7k,miR-98-5p,miR-466i-5p,miR-503-5p,miR-487a,miR-130a-5p,miR-664b-3p,miR-421-5p

RASSF7	ENSOARG0(	Ras asso	miR-193a-5p,miR-1260b,miR-10b-3p,miR-1306-3p,miR-2433,miR-331-3p,miR-138-5p,miR-486b-3p,miR-874-3p,miR-574-5p,miR-615-5p,miR-1271,miR-370-3p,miR-2355-5p,miR-2285aa,miR-300-3p,miR-34c-5p,miR-199a-5p,miR-3154,let-7g-3p,miR-329b,miR-10b-3p,miR-485-5p,miR-1814c,miR-7144-5p,miR-4286,miR-181a-2-3p,miR-1271-3p,novel_39,miR-376b-5p,miR-199a-5p,miR-376c-5p,miR-125a-3p,miR-1260a,miR-30c-2-3p,miR-181b-5p,miR-191-3p,miR-17-3p,miR-543-5p,miR-152-3p,novel_96,novel_127,miR-6517,miR-299a-3p,miR-7641,miR-758-5p,miR-505-5p,miR-214-5p,miR-486-5p,miR-345-3p,miR-23b-5p,miR-330-5p,miR-1343-5p,miR-885-3p,let-7a-2-3p,miR-10a-5p,miR-135a-2-3p,miR-342-3p,miR-17-3p,novel_101,miR-193b-3p,miR-24-3p,miR-199b-5p,miR-449a,miR-326-3p,miR-34c,miR-22-5p,miR-329-5p,miR-378b,miR-10a-5p,miR-26b-5p,miR-34b,miR-299b-3p,miR-670-3p,miR-323a-5p,miR-669,miR-3141,miR-148a-3p,miR-2387,miR-2285ab,miR-3591-3p,miR-3065-5p,miR-18a-3p,miR-1306,miR-150-5p,miR-363-5p,miR-432-3p,miR-432-3p,miR-2285r,let-7a-2-3p,miR-296-3p,miR-296-3p,miR-7-5p,miR-2411-3p,miR-3141,miR-193a-3p,miR-1343-5p,miR-340-3p,miR-31-5p,miR-185-5p,miR-432-5p,miR-2285g,miR-3535,miR-544b,miR-194-3p,miR-181a-5p,miR-1285,miR-7b-5p,miR-542-3p,miR-873a-5p,miR-432,miR-1260b,miR-30b-3p,miR-138,miR-6239,miR-744-5p,miR-145a-3p,miR-4510,miR-214,miR-346,miR-10b-5p,miR-6525,miR-6134,miR-181c-5p,miR-3184-5p,miR-122-5p,miR-665,miR-320e,miR-664a,miR-17-3p,miR-500,miR-4492,miR-1468-5p,miR-221-5p,miR-30c-1-3p,miR-2331-3p,miR-504-5p,miR-296-3p,miR-21-3p,miR-6130,miR-323-5p,miR-193b-3p,miR-544-3p,miR-582-5p,miR-10a,miR-2284s,miR-151b,novel_32,miR-2285c,miR-129b-3p,miR-500a-3p,let-7g-3p,miR-2312,miR-582-5p,miR-186-5p,miR-1827,miR-1895,miR-885-3p,miR-625-

MTRF1	ENSOARG0(	mitochon	miR-10a,miR-139-5p,miR-296-3p,miR-30c-1-3p,miR-545-3p,miR-378a-3p,miR-32-3p,miR-129b-3p,miR-21a-3p,miR-10b-5p,miR-590-3p,miR-378c,miR-487b-5p,miR-30a-3p,miR-30e-3p,miR-383-5p,miR-3600,miR-30d-3p,miR-16-1-3p,miR-2904,miR-5703,miR-378a-3p,miR-378i,miR-142a-3p,miR-329a-5p,miR-422a,miR-345-3p,miR-618,miR-2285p,miR-203-3p,miR-27a-3p,miR-1185-2-3p,miR-378e,miR-3082-5p,miR-374b-5p,miR-139-5p,miR-664b,miR-20a-3p,novel_94,miR-378j,miR-140-5p,miR-9-5p,miR-32-3p,miR-3964,miR-499a-5p,miR-3968,miR-107-5p,miR-101-3p,miR-455-3p,miR-1185-3p,miR-496-5p,miR-425-3p,miR-2320-3p,miR-142-3p,miR-27a-3p,miR-21b,miR-1388-5p,miR-378c,miR-2426,miR-484,miR-2284r,miR-378b,miR-10b,miR-378d,miR-30c-1-3p,miR-2330-5p,miR-2285o,miR-103a-2-5p,miR-3955-3p,miR-487b-5p,miR-342,novel_115,miR-223-5p,miR-133c,miR-365a-3p,miR-3068-3p,miR-7144-5p,miR-671-5p,miR-2318,miR-378c,miR-6128,miR-374b,miR-28a-3p,miR-331-3p,miR-24-2-5p,miR-9-5p,miR-483-3p,miR-378h,miR-10a-5p,miR-345-3p,miR-3591-5p,novel_39,miR-192-3p,miR-450b-5p,miR-30a-3p,miR-2403,miR-543-5p,miR-28-3p,miR-200c-3p,miR-30c-2-3p,miR-664-3p,miR-200c,miR-125a-3p,miR-411b-3p,miR-7-1-3p,miR-10a-5p,miR-2319a,miR-378f,miR-24-3p,miR-342-3p,miR-136-3p,miR-656-5p,miR-502-5p,miR-432-5p,miR-3431,miR-26a-2-3p,miR-27b-3p,miR-380-3p,miR-16-1-3p,miR-432,miR-873a-5p,miR-1285,miR-223-3p,miR-296-3p,miR-2285r,miR-452-5p,miR-483-3p,miR-2285l,miR-2411-3p,miR-296-3p

MYL3	ENSOARG0(	myosin,	miR-200b-3p,miR-496,miR-10a-5p,miR-885-3p,miR-330-5p,miR-501-3p,miR-345-3p,miR-322-5p,miR-1271-5p,miR-429-3p,novel_127,novel_96,miR-2898,miR-200c-3p,miR-30c-2-3p,miR-1260a,miR-219-3p,miR-200c,novel_39,miR-29b-2-5p,miR-3064-5p,miR-4286,miR-671-5p,miR-1248,miR-767,miR-876-3p,miR-329a,miR-2318,miR-320a,miR-362-5p,miR-488-3p,miR-503-3p,miR-154b-5p,miR-300-3p,miR-34c-5p,miR-3965,miR-2355-5p,miR-503-3p,miR-874-3p,miR-486b-3p,miR-767-5p,miR-135a-5p,miR-3969,miR-1-5p,miR-365a-5p,miR-500b-5p,miR-323-3p,miR-1260b,miR-16-5p,miR-497-5p,miR-7862,miR-16b,miR-30b-3p,miR-219a-1-3p,miR-16-1-3p,miR-1260b,miR-3071-5p,miR-1434-3p,novel_116,miR-149-5p,miR-134-3p,miR-3431,miR-185-5p,miR-665,miR-340-3p,miR-133b-5p,miR-193a-3p,miR-466f-3p,miR-124-5p,miR-365b-5p,miR-3141,novel_51,miR-2411-3p,miR-377-3p,miR-296-3p,miR-296-3p,miR-432-3p,miR-654-3p,miR-432-3p,miR-2483-5p,miR-135b-5p,miR-6395,miR-195a-5p,miR-410-5p,miR-1306,miR-3065-5p,miR-18a-3p,miR-3141,miR-6535,miR-320c,miR-146b-3p,miR-34b,novel_69,miR-10a-5p,novel_68,miR-3074-5p,miR-199c,miR-6119-3p,miR-34c,miR-16a,novel_87,miR-1247-3p,miR-15a,miR-326-3p,novel_27,miR-449a,miR-2428,miR-323c,miR-371b-3p,miR-3957-3p,miR-193b-3p,miR-342-3p,miR-1a-1-5p,miR-136-3p,miR-16-1-3p,miR-34a-5p,miR-378d,miR-4429,miR-5703,miR-2408,miR-362-3p,miR-2366,novel_83,miR-199a-3p,miR-328-3p,miR-200b,miR-15a-5p,miR-18a-3p,miR-34b-5p,miR-221-5p,miR-21-3p,miR-2484,miR-320d,miR-760-3p,miR-219b-5p,miR-324-3p,miR-500a-5p,miR-216a-5p,miR-345-3p,miR-185-3p,miR-486-3p,miR-183-5p,miR-885-3p,miR-1827,miR-129b-3p,novel_32,miR-15b-5p,miR-10a,miR-377-3p,miR-2284s,miR-193b-3p,miR-216b-3p,miR-140-5p,miR-30c-1-3p,miR-221-5p,miR-2331-3p,miR-496-3p,miR-487a-5p,miR-

LRRC1	ENSOARG0(	leucine	miR-142-3p,miR-421,miR-9788-3p,miR-1343-3p,miR-151a-3p,miR-362-3p,miR-143-5p,miR-6529a,miR-329-3p,miR-3962,miR-125b-2-3p,miR-30b-3p,miR-1b-5p,miR-2310,miR-8485,miR-424-5p,miR-124a,miR-3085-3p,miR-491-5p,miR-214-3p,miR-320b,miR-199a-3p,miR-16b,miR-29b-1-5p,miR-505,miR-362-3p,miR-3082-5p,miR-25,miR-2319b,miR-3064-5p,miR-4792,miR-7977,miR-499b-5p,miR-129b-5p,miR-154a,miR-9-5p,miR-3968,miR-2284q,miR-154-3p,miR-2440,miR-218-5p,miR-15a-5p,miR-222-5p,miR-487a-3p,miR-199a-3p,miR-151-5p,novel_73,miR-4429,miR-2300b-3p,miR-362-3p,miR-126-3p,miR-188-3p,miR-1197-5p,miR-5703,miR-124-3p,miR-130a-5p,miR-142a-3p,miR-29b-1-5p,miR-486-3p,miR-487a,miR-503-5p,miR-320d,miR-2285p,novel_17,miR-196a-3p,miR-92a-1-5p,miR-500a-5p,miR-6130,miR-193b-3p,miR-151b,miR-15b-5p,miR-22-3p,miR-660-5p,miR-32-5p,miR-296-3p,miR-154a-3p,miR-1827,miR-21a-3p,miR-598-3p,miR-338-3p,miR-4510,miR-3074-5p,miR-145a-3p,miR-2330-3p,miR-214,miR-664a,miR-130b-5p,miR-424-5p,miR-874-5p,miR-2285j,miR-665,miR-320e,miR-151-3p,miR-503-5p,miR-103,novel_116,miR-194-3p,miR-103a-3p,miR-3955-5p,miR-30b-3p,miR-16b,miR-497-5p,miR-2113,miR-374b-3p,miR-22-3p,miR-539-3p,miR-130b-5p,miR-380-5p,miR-668-3p,miR-483-3p,miR-378a-5p,miR-466f-3p,miR-193a-3p,miR-660,miR-582,miR-503-5p,miR-2285v,miR-32,miR-411b-3p,miR-670-3p,miR-6535,miR-320c,miR-3074-5p,miR-320b,miR-107,miR-195a-5p,miR-1a-1-5p,novel_120,novel_101,miR-495-3p,miR-193b-3p,miR-92a-3p,miR-147-5p,miR-421-5p,miR-22-5p,miR-28c,miR-208a-3p,miR-33a-3p,miR-16a,miR-483-3p,miR-9-5p,miR-322-5p,novel_121,miR-134-5p,miR-582-3p,miR-6517,miR-134,miR-380-5p,miR-92b-3p,miR-28b,miR-181a-2-3p,miR-3064-5p,miR-1a-2-5p,miR-450a-2-3p,miR-200a-3p,novel_96,miR-92a-
KCTD1	ENSOARG0(	potassiu	miR-153-3p,miR-376c-5p,novel_103,miR-1434-3p,miR-653,miR-130a-5p,miR-23b-3p,miR-153,miR-211,miR-211-5p,miR-23a-3p,miR-329-5p,miR-2331-3p,miR-329a-5p
TRIM25	ENSOARG0(	triparti	miR-188-5p,miR-34a-5p,miR-2889,miR-30b-3p,miR-1260b,miR-2426,miR-188-3p,miR-5703,miR-758-3p,miR-30b-3p,miR-532-3p,miR-328-3p,miR-20b,miR-339-5p,novel_79,miR-106a,miR-34b-5p,miR-214-3p,miR-106a,miR-378a-5p,miR-17-5p,miR-106b-5p,miR-106a-5p,miR-339a,miR-1260a,miR-3065-3p,novel_39,miR-4532,miR-758-3p,miR-20a,miR-106a-5p,miR-20b-5p,miR-9851-3p,miR-1827,miR-6395,miR-1291,miR-339b,miR-93-5p,miR-17-5p,miR-677,miR-204-3p,miR-4443,miR-3957,miR-34c-5p,miR-34b,miR-3065-3p,miR-211-5p,miR-615-5p,miR-3957-5p,miR-204-5p,miR-34c,miR-132-5p,miR-20a-5p,miR-449a,miR-7705,miR-2428,miR-211,miR-93,miR-1260b,miR-214,miR-2447

ESRP1	ENSOARG0(	epitheli	novel_83,miR-2284h-5p,miR-149-5p,novel_121,miR-23b-3p,miR-200b,miR-6516-5p,miR-23c,miR-429-3p,miR-200b-3p,miR-496,miR-3962,miR-5703,miR-2300b-3p,miR-2310,miR-654-3p,miR-369-3p,miR-23a-3p,miR-181a-2-3p,miR-452-5p,miR-2898,miR-200c-3p,miR-365b-5p,miR-200c,miR-2285p,miR-204-3p,miR-6516,miR-582-5p,miR-126b-5p,miR-496-3p,miR-503-3p,miR-1814c,miR-450b-3p,miR-23b,miR-129b-5p,miR-655,miR-301,miR-365a-5p,miR-1296-5p,miR-3074-2-3p,miR-2447,miR-145a-3p,miR-147-5p,miR-655-3p,miR-382-5p,miR-2440
CEP126	ENSOARG0(	centroso	miR-1248,miR-154b-3p,miR-136-5p,miR-200a-5p,miR-450a-1-3p,miR-148a-3p,miR-2483-3p,miR-154a-3p,miR-487a-3p,miR-154-3p,miR-628-3p,miR-450b-3p,novel_27,miR-142a-5p,miR-3059-5p,miR-590-3p,miR-1388-5p,miR-145a-5p,miR-23b-5p,miR-450c-3p,miR-143-5p,miR-6529a,miR-345-3p,miR-2459,miR-9788-3p,miR-450a-1-3p,miR-3431,miR-148b-3p,miR-142-5p,miR-487a-3p,miR-383-5p,miR-145b,miR-222-5p,miR-665,miR-31-5p,miR-3955-5p,miR-152-3p,miR-2411-3p,miR-345-3p,miR-6119-5p,let-7j,miR-539-3p,miR-376a-5p,miR-664b-3p,miR-421-5p,miR-487a,miR-216c-5p
NUP214	ENSOARG0(	nucleopo	miR-30b-3p,miR-125b-2-3p,miR-6529a,miR-484,miR-378b,miR-296-5p,miR-143-5p,miR-2284aa,miR-3120-5p,miR-378c,miR-544a,miR-2300a-5p,miR-409b,miR-9788-3p,miR-2285e,miR-320b,novel_115,miR-1306-5p,novel_103,miR-326,miR-542-5p,miR-3085-3p,miR-3955-3p,miR-2330-5p,miR-494-3p,miR-424-5p,miR-106a-5p,miR-758-3p,miR-30c-1-3p,novel_94,miR-664b,miR-3064-5p,miR-10b,miR-378e,miR-489,miR-769-5p,miR-2285y,miR-107-5p,miR-4726-5p,miR-2285f,miR-3059-5p,novel_60,miR-2904,miR-1197-5p,miR-5703,miR-378d,miR-4429,miR-548e-3p,miR-199a-3p,miR-148b-3p,miR-222-5p,miR-15a-5p,miR-125b,miR-185-3p,miR-542-5p,miR-34b-5p,miR-221-5p,miR-30b-3p,miR-106a,miR-320d,miR-2284m,miR-760-3p,miR-20a,miR-2887,miR-125b-2-3p,miR-21a-3p,novel_99,miR-2312,miR-3065-3p,miR-532-5p,miR-378a-3p,miR-221-5p,miR-22-3p,miR-544-3p,miR-193b-3p,miR-6130,miR-324-3p,miR-29b-2-5p,miR-208b-5p,miR-424-5p,miR-450b-3p,miR-17-3p,miR-1271,miR-3074-2-3p,miR-539-5p,miR-378c,miR-4510,miR-338-3p,miR-206-3p,miR-145a-5p,miR-1434-3p,miR-7862,miR-29b,miR-30b-3p,miR-219a-1-3p,miR-544b,miR-2285g,miR-185-5p,miR-503-5p,miR-17-5p,miR-224-5p,miR-503-5p,miR-1343-5p,miR-125a-5p,miR-466f-3p,miR-363-5p,miR-382,miR-2483-5p,miR-130b-5p,miR-345-5p,miR-2285ab,miR-2387,miR-195a-5p,miR-1291,miR-320b,miR-378b,miR-2404,miR-670-3p,miR-34b,miR-29a-3p,miR-34c,miR-16a,miR-502-5p,miR-15a,miR-6529b,miR-20a-5p,miR-203b-5p,miR-147-5p,miR-128-1-5p,miR-17-3p,miR-542-5p,miR-449a,miR-326-3p,miR-2428,miR-211,miR-24-3p,miR-29d-3p,miR-345-3p,miR-134,miR-582-3p,miR-545-5p,miR-1271-5p,miR-378h,miR-30c-2-3p,miR-2285af,miR-543-5p,miR-2898,miR-17-3p,miR-191-3p,miR-29b-2-5p,miR-1,miR-4286,miR-376c-5p,miR-376b-5p,miR-3065-3p,miR-490-3p,miR-3154,miR-17-5p,miR-320a,miR-488-3p,miR-
LMNTD2	ENSOARG0(	lamin ta	miR-128-1-5p,miR-151-5p,miR-744-5p,miR-151b,miR-502-5p,miR-7977

LMBR1	ENSOARG0(	limb dev	miR-371a-5p,novel_19,miR-1a-3p,miR-2904,miR-191-3p,miR-206,miR-574-5p,miR-1,miR-6527,miR-2284s,miR-361-3p,miR-206-3p,miR-9788-3p,miR-2428
GCLC	ENSOARG0(	glutamat	novel_63,miR-100-3p,miR-125b-5p,novel_9,miR-126b-3p,novel_42,miR-16-5p,miR-1260b,miR-30e-5p,miR-345-5p,miR-874-3p,miR-542-5p,miR-2355-5p,miR-125a,miR-1271,miR-125a-3p,miR-30f,miR-127-5p,novel_127,miR-148b-5p,miR-192-3p,miR-1290,miR-30c,miR-299a-3p,miR-6517,miR-9-5p,miR-322-5p,miR-2284z,novel_121,miR-486-5p,miR-4324,miR-576-3p,miR-206,miR-2284w,miR-3065-5p,miR-412-3p,novel_68,miR-3074-5p,miR-3120-3p,miR-299b-3p,miR-374c-3p,miR-377-3p,miR-493-5p,miR-2285r,miR-432,miR-542-3p,miR-22-3p,miR-197-3p,miR-16b,miR-497-5p,miR-4508,miR-2284x,miR-340-3p,miR-3955-5p,miR-134-3p,miR-411-5p,miR-194-3p,miR-30e-3p,miR-30a-3p,miR-665,novel_74,miR-129-5p,miR-874-5p,miR-433-5p,miR-3074-5p,miR-214,miR-665-5p,miR-181c-5p,miR-2285c,miR-433-3p,miR-30d-5p,miR-345-5p,miR-543-3p,miR-30c-1-3p,miR-504-5p,miR-548o-3p,miR-216b-3p,miR-377-3p,miR-582-5p,miR-15b-5p,miR-30b,miR-210-5p,miR-2285p,miR-618,miR-503-5p,miR-345-3p,miR-380-3p,miR-143-3p,miR-30c-5p,miR-184-3p,miR-218-1-3p,miR-34a-5p,miR-18a-3p,miR-7859,miR-218-5p,miR-101-3p,miR-2440,miR-3968,miR-9-5p,miR-877-3p,novel_111,miR-1285-5p,miR-409-5p,miR-378j,miR-99b-5p,miR-504,miR-493-5p,miR-1983,miR-544-5p,miR-2285u,miR-378g,miR-25,miR-199a-3p,miR-16b,miR-214-3p,miR-103a-2-5p,miR-124a,miR-101b-3p,miR-2310,miR-1a-3p,miR-101c,miR-548w,miR-199b-3p,miR-1343-3p,miR-145b,miR-425-3p,miR-496-5p,miR-147a,miR-2459,miR-34c-3p,miR-370-5p,miR-135a-5p,miR-409-3p,miR-138-5p,miR-6128,miR-103b,miR-381-3p,miR-3154,miR-100-5p,miR-7144-5p,miR-1814c,miR-671-5p,miR-3957-5p,miR-370-3p,miR-34c-5p,miR-374c-3p,miR-3965,miR-3957,miR-1260a,miR-99a,miR-30c-2-3p,miR-17-3p,miR-2898,miR-181b-5p,miR-543-5p,miR-30a-3p,miR-1,miR-181a-2-3p,miR-

THADA	ENSOARG0(	thyroid	miR-505-3p,miR-383-5p,miR-15a-5p,novel_133,miR-215-5p,miR-2904,miR-1197-5p,miR-188-3p,miR-5703,miR-4429,miR-19b-3p,miR-130a-5p,miR-125b-2-3p,miR-2411,miR-216a-5p,miR-320d,miR-760-3p,miR-34b-5p,miR-221-5p,miR-140-5p,miR-378a-3p,miR-221-5p,miR-3065-3p,miR-370-5p,miR-192-5p,miR-139-5p,miR-204-3p,miR-22-3p,miR-155-5p,miR-3613-5p,miR-18a-5p,miR-378c,miR-2330-3p,miR-3074-2-3p,miR-320e,miR-29b-2-5p,miR-17-3p,miR-155-5p,miR-664a,miR-708-5p,miR-424-5p,miR-222-5p,miR-500-5p,miR-216a-3p,miR-378b,miR-30b-3p,miR-378c,miR-2426,miR-2330-5p,miR-424-5p,miR-320b,miR-326,miR-5126,miR-2957,miR-3085-3p,miR-3064-5p,miR-452-3p,miR-20a-3p,miR-378e,miR-3082-5p,miR-361-3p,miR-154a,miR-30b-5p,miR-371a-5p,miR-2284q,miR-376a-5p,miR-2411-5p,miR-378h,miR-545-5p,miR-582-3p,miR-214-5p,miR-345-3p,miR-134,miR-30a-3p,miR-29b-2-5p,miR-3065-3p,miR-2285af,miR-17-3p,miR-370-3p,miR-215-5p,miR-615-5p,miR-34c-5p,miR-374c-3p,miR-488-3p,miR-3154,miR-18a,miR-320a,miR-10a-3p,miR-7144-5p,miR-1814c,miR-485-5p,miR-1306-5p,miR-378c,miR-551b-3p,miR-410-5p,miR-34c-3p,miR-370-5p,miR-331-3p,miR-28-5p,miR-130a-3p,miR-432-5p,miR-653,miR-1285,miR-1434-3p,miR-374b-3p,miR-30b-3p,miR-744-5p,miR-345-5p,miR-330-3p,miR-1343-5p,miR-655-5p,miR-320b,miR-34b,miR-18b,miR-450b-3p,miR-19a-3p,miR-9851-3p,miR-135b-5p,miR-505-3p,miR-195a-5p,miR-376a-2-5p,miR-219b-3p,novel_101,miR-1193,miR-17-3p,miR-371b-3p,miR-326-3p,miR-449a,miR-2428,miR-15a,miR-3613,miR-16a,miR-34c,novel_1,miR-199c,miR-212-5p,miR-7859,miR-1247-5p,miR-378i,miR-378a-3p,miR-493-3p,miR-16-1-3p,miR-664-5p,miR-34a-5p,miR-10a-3p,miR-195a-3p,miR-181b-1-3p,miR-30b,novel_17,miR-422a,miR-345-3p,miR-2484,miR-548o-3p,miR-216b-3p,miR-301a-3p,miR-499b-3p,miR-15b-
GSPT1	ENSOARG0(	G1 to S	miR-548w,miR-1343-3p,miR-2319b,miR-421,miR-539-5p,miR-433-3p,miR-1285,miR-7975,miR-2319a,miR-539-5p,miR-1a-1-5p,novel_27,miR-503-3p,miR-6525,miR-1-5p,miR-1a-2-5p,miR-6536
FAM206A	ENSOARG0(	family w	miR-3431,miR-885-3p,miR-1827,novel_99,miR-22-5p,miR-885-3p
ARHGEF7	ENSOARG0(	Rho guan	miR-452-3p,miR-504-5p,miR-29a-3p,miR-378g,miR-301a-3p,miR-361-3p,miR-504,miR-19a-3p,miR-671-5p,miR-369-5p,miR-214,miR-29c-3p,miR-24-3p,miR-1197-3p,miR-361-3p,miR-301b-3p,miR-28c,miR-2440,miR-29b-3p,miR-551b-3p,miR-1307-5p,miR-29a,miR-3432a,miR-874-5p,miR-454-3p,miR-2300a-5p,miR-130a-3p,miR-3431,miR-134-3p,miR-210-3p,miR-2904,miR-29d-3p,miR-19b-3p,miR-142-3p,miR-1388-5p,miR-29b,miR-744-5p,miR-4508,miR-28b,miR-5126,miR-582,miR-210-5p,miR-185-3p,miR-2285v,novel_19,miR-130b-3p,miR-543-5p,miR-214-3p,miR-21-3p

ELOVL7	ENSOARG0(	ELOVL fa	miR-31-5p,miR-122-3p,miR-103a-3p,miR-130a-3p,miR-377-5p,miR-26b-3p,miR-412-5p,novel_116,miR-6238,miR-103,miR-7b-5p,miR-1843b-5p,miR-1434-3p,miR-450c-3p,miR-194-5p,novel_23,miR-30b-3p,miR-380-3p,miR-16b,miR-2284x,miR-6240,miR-7-5p,miR-582,miR-330-3p,miR-194a,miR-124-5p,miR-466f-3p,miR-329-5p,miR-26b-5p,miR-7-1-3p,miR-2404,miR-299b-3p,miR-148a-3p,miR-3970,miR-3065-5p,miR-376b-3p,miR-3591-3p,miR-450b-3p,miR-107,miR-2285k,miR-6516-3p,miR-195a-5p,miR-335-5p,miR-24-3p,miR-326-3p,miR-374c-5p,miR-301b-3p,miR-2428,miR-133a-5p,miR-16a,miR-6119-3p,miR-379-3p,miR-656-5p,miR-299a-3p,miR-429-3p,miR-322-5p,miR-24-2-5p,miR-6516-5p,miR-450a-1-3p,miR-545-5p,miR-30a-5p,miR-330-5p,miR-200b-3p,miR-2385-3p,miR-30a-3p,miR-3064-5p,miR-376b-3p,miR-205-5p,miR-200c,miR-217,miR-200c-3p,miR-107,miR-152-3p,miR-574-5p,miR-2285aa,miR-769,miR-30e-5p,miR-381-3p,miR-301,let-7g-3p,miR-23a-5p,miR-1248,miR-374b,miR-16-5p,miR-193a-5p,miR-144-5p,miR-323-3p,miR-374a-3p,miR-34c-3p,miR-2332,miR-31-3p,miR-374b-3p,miR-1343-3p,miR-330-3p,miR-376b-3p,miR-455-3p,miR-126a-5p,miR-301b-5p,miR-30b-3p,miR-2426,miR-101c,miR-124a,miR-424-5p,miR-101b-3p,miR-33a-5p,miR-8485,miR-199a-3p,miR-326,miR-16b,miR-3085-3p,miR-130b-3p,miR-3064-5p,miR-452-3p,miR-10b,miR-374b-5p,miR-2285u,miR-203-3p,miR-376d,miR-454-5p,miR-381-3p,miR-378j,miR-299b-5p,miR-504,miR-376a-3p,miR-194b-5p,miR-219a-5p,miR-877-3p,miR-154a,miR-218-5p,miR-101-3p,miR-2440,miR-769-5p,miR-301a-5p,miR-499a-5p,miR-335,miR-374a-3p,miR-490-5p,miR-548e-3p,miR-1b-3p,miR-30d-3p,miR-7859,miR-487a-3p,miR-148b-3p,miR-15a-5p,miR-222-5p,miR-200b,miR-199a-3p,miR-2366,miR-1246,miR-2300b-3p,miR-2904,miR-124-3p,miR-188-3p,miR-1197-5p,miR-217-5p,miR-

MRPS22	ENSOARG0(	mitochon	miR-134-5p,miR-450a-1-3p,novel_121,miR-758-5p,miR-29d-3p,miR-345-3p,miR-134,miR-1290,let-7a-2-3p,novel_39,miR-1271-3p,miR-490-3p,miR-217,miR-543-5p,miR-6536,miR-331-5p,miR-374c-3p,miR-3154,miR-320a,miR-2448-3p,miR-331-5p,miR-7144-5p,miR-1814c,miR-671-5p,miR-144-5p,miR-103b,miR-29b-3p,miR-2284k,miR-494-5p,miR-2332,miR-204-5p,miR-134-5p,miR-486b-3p,miR-28-5p,miR-331-5p,miR-134-3p,miR-3431,miR-450c-3p,miR-873a-5p,miR-1434-3p,miR-188-5p,miR-2284x,miR-29b,miR-30b-3p,miR-219a-1-3p,let-7a-2-3p,miR-2285r,miR-432-3p,miR-432-3p,miR-2411-3p,miR-452-5p,miR-660,miR-329-5p,miR-320c,miR-29a-3p,miR-2285ab,miR-376a-2-5p,miR-6516-3p,novel_101,miR-29c-3p,miR-2284w,miR-2428,miR-211,miR-323c,miR-33a-3p,miR-421-5p,miR-625-3p,miR-147-5p,miR-29a,miR-532-3p,miR-616-3p,miR-1197-5p,miR-217-5p,miR-5703,miR-2408,miR-4429,miR-33b-3p,miR-329a-5p,miR-486-3p,miR-2432,miR-421-5p,miR-500a-5p,miR-345-3p,novel_17,miR-2284m,miR-320d,miR-532-5p,miR-660-5p,miR-548o-3p,miR-487b-5p,miR-450a-1-3p,miR-186-5p,miR-2312,miR-2330-3p,miR-214,miR-3074-2-3p,miR-433-5p,miR-539-5p,miR-6525,miR-665,miR-320e,miR-2285j,miR-708-5p,miR-450b-3p,miR-487b-5p,miR-6516-3p,miR-455-3p,miR-216a-3p,miR-126a-5p,miR-539-5p,miR-30b-3p,miR-3962,miR-2284r,miR-301b-5p,miR-101c,miR-1388-5p,miR-382-3p,miR-2310,miR-320b,miR-214-3p,miR-3955-3p,miR-211-5p,miR-20a-3p,miR-203-3p,miR-144-5p,miR-378g,miR-378j,miR-335-3p,miR-9-3p,miR-2285f,miR-2284j,miR-3059-5p,novel_60,miR-3956-3p,miR-877-3p,miR-301a-5p,miR-101-3p,miR-1843a-3p,miR-2284q,miR-3968,miR-3958-3p

IKBKAP	ENSOARG0(	inhibito	miR-182-5p,miR-370-5p,miR-21-3p,miR-30c-1-3p,miR-504-5p,miR-221-5p,miR-151b,miR-155-5p,miR-15b-5p,miR-204-3p,miR-6130,miR-377-3p,miR-544-3p,miR-487b-5p,miR-129b-3p,miR-625-5p,miR-543-3p,miR-345-5p,miR-2312,miR-3074-2-3p,miR-129-2-3p,miR-4510,miR-539-5p,miR-181c-5p,miR-224-5p,miR-346,miR-142a-5p,miR-132-5p,miR-151-3p,miR-15b-3p,miR-129-5p,miR-208b-5p,miR-424-5p,miR-4492,miR-155-5p,miR-17-3p,miR-487b-5p,miR-3600,miR-548e-3p,miR-151-5p,novel_83,miR-532-3p,miR-200b,miR-15a-5p,miR-23c,miR-7859,miR-23b-3p,miR-140-3p,miR-188-3p,miR-2366,miR-1246,miR-2408,miR-2300b-3p,novel_133,miR-133a-3p,miR-4429,novel_73,miR-345-3p,miR-195a-3p,miR-216c-5p,miR-2411,miR-181b-1-3p,miR-125b-2-3p,miR-345-3p,miR-216a-5p,miR-324-3p,miR-210-5p,miR-299-3p,miR-221-5p,miR-466i-5p,miR-320d,miR-452-3p,miR-1185-2-3p,miR-4443,miR-203-3p,miR-129b-5p,miR-1983,miR-335-3p,miR-504,miR-452-5p,miR-181d-5p,miR-2285f,miR-2447,miR-361-3p,miR-371a-5p,miR-2285y,miR-2440,miR-490-5p,miR-3964,miR-2285t,miR-3968,miR-145b,miR-181b-2-3p,miR-5010-3p,miR-548w,miR-330-3p,miR-151a-3p,miR-421,miR-1185-3p,miR-2459,miR-455-3p,miR-142-5p,miR-539-5p,miR-125b-2-3p,miR-323b,miR-1839-5p,miR-16-2-3p,miR-544a,miR-33a-5p,miR-23a-3p,let-7c-3p,miR-424-5p,miR-382-3p,miR-8485,miR-30c-1-3p,miR-16b,miR-182-5p,miR-323b-3p,miR-431-5p,miR-326,miR-320b,miR-487b-5p,miR-370-3p,miR-2285aa,miR-3068-3p,miR-133c,miR-324-5p,miR-331-5p,miR-320a,miR-23b,miR-137-3p,miR-301,miR-2448-3p,miR-381-3p,miR-671-5p,miR-3074-1-3p,miR-7144-5p,miR-345-5p,let-7g-3p,miR-2318,miR-767,miR-16-5p,miR-365a-5p,miR-374a-3p,miR-1260b,miR-3969,miR-409-3p,miR-26c,miR-370-5p,miR-494-5p,miR-5100,miR-29b-3p,miR-134-5p,miR-374b-3p,miR-767-5p,novel_9,miR-431,miR-429-
CLSTN1	ENSOARG0(	calsynte	miR-193b-3p,miR-1839-3p,miR-541-3p,miR-4510,miR-4492,miR-138-5p,miR-3968,miR-541-5p,miR-504-5p,miR-6130,miR-193b-3p,miR-885-3p,miR-493-5p,miR-504,miR-329a,miR-4286,miR-493-5p,miR-1839-3p,miR-574-3p,miR-2411,miR-185-3p,miR-362-3p,miR-7-5p,miR-193a-3p,novel_96,miR-491-5p,miR-532-3p,miR-2284z,miR-2411-5p,miR-2904,miR-362-3p,miR-7b-5p,miR-541,miR-223-3p,miR-329-3p,miR-21b,miR-219a-1-3p,miR-138,miR-885-3p,miR-362-3p

CD302	ENSOARG0(	CD302 mo	miR-6516-5p,novel_121,miR-582-3p,miR-1290,miR-3591-5p,miR-1343-5p,miR-582-3p,let-7a-2-3p,miR-105-2,miR-155-5p,miR-29b-2-5p,miR-30a-3p,miR-199a-5p,miR-200a,miR-1957a,miR-3065-3p,miR-29a-5p,miR-6119-5p,miR-2285af,miR-217,miR-30f,miR-181b-5p,miR-1a-2-5p,miR-28-3p,miR-450a-2-3p,miR-17-3p,miR-200a-3p,novel_96,miR-3068-3p,miR-141-3p,miR-215-5p,miR-2285aa,miR-374c-3p,miR-223-5p,miR-199a-5p,miR-30e-5p,miR-488-3p,miR-362-5p,miR-17-5p,miR-137-3p,miR-200a-5p,let-7g-3p,miR-876-3p,miR-3074-1-3p,miR-20b-5p,miR-28a-3p,miR-6128,miR-374a-3p,miR-500b-5p,miR-323-3p,miR-135a-1-3p,miR-494-5p,novel_63,miR-1-5p,miR-34c-3p,miR-409-3p,miR-144,novel_9,miR-374b-3p,miR-26b-3p,miR-3535,miR-2284d,miR-411-5p,miR-181a-5p,miR-19b-2-5p,miR-653,miR-145a-5p,novel_23,miR-374b-3p,miR-16-1-3p,miR-380-3p,miR-30b-3p,miR-7862,miR-6240,miR-493-5p,miR-101-5p,miR-22841,miR-654-3p,let-7a-2-3p,miR-224-5p,miR-17-5p,miR-105-5p,miR-330-3p,miR-197-5p,miR-378a-5p,miR-1343-5p,miR-329-5p,miR-374c-3p,miR-323a-5p,miR-181b-3p,miR-6535,miR-144-3p,miR-677,miR-1a-1-5p,miR-219b-3p,miR-17-3p,miR-2285x,miR-323c,miR-199b-5p,miR-2284w,miR-6402,miR-20a-5p,miR-502-5p,miR-33a-3p,novel_1,miR-147-5p,miR-199c,miR-106a,miR-30d-3p,miR-3600,miR-7859,miR-616-3p,miR-199a-3p,miR-215-5p,miR-376a-5p,miR-1197-5p,miR-1193,miR-217-5p,miR-361-5p,miR-16-1-3p,miR-218-1-3p,miR-183-5p,miR-216c-5p,miR-195a-3p,miR-20a,miR-125b-2-3p,miR-664b-3p,miR-376a-5p,miR-29b-1-5p,miR-143-3p,miR-380-3p,miR-210-5p,miR-216a-5p,miR-500a-5p,miR-760-3p,miR-106a,miR-1277-5p,miR-140-5p,miR-532-5p,miR-552-3p,miR-3065-3p,miR-656-3p,miR-323-5p,miR-139-5p,miR-6516,miR-582-5p,miR-155-5p,miR-877-5p,miR-487b-5p,miR-433-3p,miR-655,let-7g-3p,novel_99,miR-582-5p,miR-

TCOF1	ENSOARG0(	Treacher	miR-29b,miR-30b-3p,miR-2284ab,miR-544b,miR-185-5p,miR-2284d,miR-26b-3p,miR-339-5p,miR-1343-5p,miR-196b-5p,miR-377-3p,miR-345-5p,miR-539-3p,miR-668-3p,miR-493-5p,miR-378f,miR-450b-3p,miR-677,miR-144-3p,miR-29a-3p,miR-411b-3p,miR-3074-5p,miR-378b,miR-6123,miR-29a,novel_1,miR-502-5p,miR-199b-5p,miR-326-3p,miR-1193,miR-29c-3p,miR-17-3p,miR-330-5p,miR-29d-3p,miR-1343-5p,miR-196a-5p,miR-2284z,miR-378h,miR-505-5p,novel_79,miR-543-5p,miR-17-3p,miR-339a,miR-205-5p,miR-199a-5p,miR-2478,miR-345-5p,miR-3154,miR-23b,miR-2284n,miR-154b-5p,miR-199a-5p,miR-615-5p,miR-598-3p,miR-2355-5p,miR-487a-3p,miR-486b-3p,miR-592,miR-144,miR-100-3p,miR-370-5p,miR-29b-3p,miR-378c,miR-2284v,miR-21b,miR-2284aa,miR-544a,miR-378c,miR-30b-3p,miR-378b,miR-296-5p,miR-484,miR-181b-2-3p,novel_4,novel_78,miR-2300a-5p,miR-103a-2-5p,miR-199b-5p,miR-29b-1-5p,novel_103,miR-326,miR-3187-3p,miR-193a,miR-8485,miR-378d,miR-744-3p,miR-23a-3p,miR-2330-5p,miR-493-5p,miR-136-5p,miR-3963,miR-199b-5p,miR-361-3p,miR-378e,miR-154-3p,miR-107-5p,miR-101-3p,novel_82,miR-423-5p,miR-361-3p,miR-2447,miR-33b-3p,miR-378a-3p,miR-378i,miR-151-5p,miR-23c,miR-487a-3p,miR-7859,miR-23b-3p,miR-485-3p,miR-1b-3p,miR-1277-5p,miR-2284m,miR-422a,miR-185-3p,miR-196a-3p,miR-486-3p,miR-29b-1-5p,miR-487a,miR-216c-5p,miR-582-5p,miR-1827,miR-154b-3p,miR-339b,miR-345-5p,let-7g-3p,miR-598-3p,miR-500a-3p,miR-151b,miR-877-5p,miR-204-3p,miR-216b-5p,miR-6130,miR-377-3p,miR-544-3p,miR-582-5p,miR-216b-3p,miR-154a-3p,miR-370-5p,miR-378a-3p,miR-500,miR-17-3p,miR-665,miR-6134,miR-338-3p,miR-3184-5p,miR-4510,miR-3074-5p,miR-378c

HOXB1	ENSOARG0(	homeobox	miR-9-3p,miR-361-3p,miR-877-3p,novel_111,miR-9-5p,miR-452-5p,miR-3059-5p,novel_60,miR-2447,miR-3956-3p,miR-2284q,miR-4726-5p,miR-2285y,miR-301a-5p,miR-2285n,let-7d-5p,miR-203-3p,miR-4443,miR-378e,miR-378g,miR-489,miR-2285u,miR-499b-5p,miR-7977,miR-2319b,miR-9-3p,miR-299b-5p,miR-504,miR-361-3p,miR-378d,miR-23a-3p,miR-1388-3p,miR-124a,let-7a-5p,let-7e,novel_124,miR-130b-3p,miR-2957,miR-320b,miR-5126,novel_103,miR-326,miR-9788-3p,miR-1298-3p,novel_4,miR-105-1,miR-1343-3p,miR-330-3p,miR-378c,miR-1388-5p,miR-299a-5p,miR-450b-5p,miR-7857,miR-30b-3p,miR-296-5p,miR-378b,miR-2284r,miR-301b-5p,miR-143-5p,miR-4791,let-7b-5p,miR-6525,miR-539-5p,let-7b,miR-378c,miR-145a-3p,miR-3074-5p,miR-129-2-3p,miR-3184-3p,miR-500,miR-4492,miR-874-5p,miR-454-3p,miR-665,miR-3604,let-7f,miR-204-3p,let-7i-5p,miR-2284s,miR-301a-3p,miR-3065-3p,miR-378a-3p,miR-2331-3p,miR-504-5p,miR-543-3p,miR-625-5p,miR-1827,miR-133b-3p,miR-345-5p,miR-339b,miR-500a-3p,miR-21a-3p,miR-486-3p,miR-143-3p,miR-130a-5p,miR-377-5p,let-7k,miR-320d,miR-98-5p,miR-92a-1-5p,miR-185-3p,miR-422a,miR-345-3p,miR-208b-3p,novel_17,miR-671-3p,miR-532-3p,miR-23b-3p,miR-23c,miR-378d,miR-493-3p,miR-4429,miR-124-3p,miR-188-3p,miR-1197-5p,miR-378a-3p,miR-5703,novel_133,miR-378i,miR-193b-5p,miR-1961,miR-2300b-3p,miR-326-3p,miR-301b-3p,miR-2428,miR-24-3p,miR-1307-3p,let-7i,miR-1247-3p,miR-208a-3p,miR-28c,miR-6535,miR-320c,miR-146b-3p,miR-670-3p,miR-655-5p,miR-3074-5p,miR-378f,let-7e-5p,miR-150-5p,miR-3141,miR-345-5p,miR-2285r,miR-363-5p,miR-6240,miR-452-5p,miR-330-3p,miR-365b-5p,miR-3141,miR-197-5p,miR-105-5p,novel_51,miR-455-5p,miR-2411-3p,novel_116,miR-149-5p,miR-432-5p,miR-3535,let-7g-5p,miR-134-3p,miR-185-
TTK	ENSOARG0(	TTK prot	miR-411,miR-500a-3p,miR-488-3p,miR-1827,miR-582-5p,miR-2312,miR-141-3p,miR-552-3p,miR-21-3p,miR-3065-3p,miR-32-3p,miR-548o-3p,miR-216b-5p,miR-139-5p,miR-656-3p,miR-582-5p,miR-34c-5p,miR-377-3p,miR-144,miR-135a-1-3p,miR-1-5p,miR-592,miR-191-5p,miR-500,miR-425-5p,miR-338-3p,miR-6525,miR-191,miR-3591-5p,miR-34a-5p,miR-105-2,miR-582-3p,miR-411-3p,miR-493-3p,miR-485-3p,miR-3600,miR-548e-3p,miR-2284z,miR-24-2-5p,miR-487a-3p,miR-7859,miR-125a-3p,miR-200a-3p,miR-543-5p,miR-34b-5p,miR-1a-2-5p,miR-127-5p,miR-2898,miR-450a-2-3p,miR-329a-5p,miR-216c-5p,miR-143-3p,miR-3065-3p,miR-200a,miR-125b-2-3p,miR-421-5p,miR-24-2-5p,miR-3591-3p,miR-381-3p,miR-140-5p,miR-2285ab,miR-329-5p,miR-378b,miR-24-1-5p,miR-139-5p,miR-677,miR-144-3p,miR-34b,miR-3596,miR-371a-5p,miR-485-3p,miR-34c,miR-101-3p,miR-2440,miR-656-5p,miR-22-5p,miR-379-3p,novel_1,miR-3968,miR-2285f,miR-1a-1-5p,miR-212-3p,miR-32-3p,miR-2284w,novel_27,miR-449a,miR-361-3p,miR-374b-3p,miR-1434-3p,miR-4791,miR-2284aa,miR-330-3p,miR-105-1,miR-6238,miR-185-5p,miR-3535,miR-105-5p,miR-330-3p,miR-377-3p,miR-744-3p,miR-628-5p,miR-363-5p,miR-382-3p,miR-6240,miR-101b-3p,miR-1b-5p,miR-539-3p,miR-193a,miR-2285r

SNX29	ENSOARG0(	sorting	miR-30c-2-3p,miR-16b,miR-194a,novel_124,miR-25-5p,miR-21-3p,miR-424-5p,miR-30c-1-3p,miR-200a,miR-3065-3p,miR-3187-3p,miR-484,miR-194-5p,miR-16b,miR-544a,miR-497-5p,miR-378d,miR-744-5p,miR-26b-3p,miR-322-5p,miR-1298-3p,miR-15a-5p,miR-544b,miR-15a,miR-370-5p,miR-16a,miR-132-5p,miR-135a-5p,miR-424-5p,miR-3964,miR-16-5p,miR-194b-5p,miR-1193,miR-1307-3p,miR-412-3p,miR-1827,miR-135b-5p,miR-195a-5p,miR-1983,miR-30c-1-3p,miR-3065-3p,miR-370-5p,miR-615-5p,miR-544-3p,miR-15b-5p
SRP54	ENSOARG0(	signal r	miR-31-5p,miR-3431,miR-544b,miR-181a-5p,novel_23,miR-380-3p,miR-16-1-3p,miR-2284x,miR-380-5p,miR-493-5p,let-7f-2-3p,let-7f-2-3p,let-7a-2-3p,miR-146a,miR-125a-5p,miR-193a-3p,novel_68,miR-329-5p,miR-378b,miR-3120-3p,miR-144-3p,miR-6535,miR-677,miR-181b-3p,miR-148a-3p,miR-3065-5p,miR-7975,miR-146b-5p,miR-378f,miR-6516-3p,miR-335-5p,miR-193b-3p,miR-323c,miR-24-3p,miR-142b,novel_87,miR-379-3p,novel_1,miR-203b-5p,miR-656-5p,miR-22-5p,miR-147-5p,miR-429-3p,miR-6517,miR-758-5p,miR-378h,miR-2411-5p,miR-582-3p,novel_121,miR-3591-5p,miR-1290,miR-411-3p,miR-200b-3p,let-7a-2-3p,miR-3529-3p,miR-495-5p,miR-30a-3p,miR-380-5p,miR-450c-5p,miR-2285af,miR-125a-3p,miR-200c,miR-6119-5p,miR-217,miR-6536,miR-127-5p,miR-181b-5p,miR-200c-3p,miR-200a-3p,novel_96,miR-152-3p,miR-598-3p,miR-141-3p,miR-125a,miR-331-5p,miR-365a-3p,miR-154b-5p,miR-2448-3p,miR-3154,miR-23b,miR-10b-3p,let-7g-3p,miR-3074-1-3p,miR-374b,miR-378c,miR-135a-1-3p,miR-125b-5p,novel_9,miR-548w,miR-1343-3p,miR-181b-2-3p,miR-142-5p,miR-421,miR-126a-5p,miR-484,miR-378b,miR-1388-5p,miR-378c,miR-544a,miR-101c,miR-3120-5p,miR-494-3p,miR-382-3p,miR-23a-3p,miR-744-3p,miR-33a-5p,miR-378d,miR-193a,miR-491-5p,miR-1185-5p,miR-3955-3p,miR-146b,miR-541-5p,miR-374b-5p,miR-378e,miR-7857-3p,miR-489,miR-1185-2-3p,miR-493-5p,miR-212-3p,miR-181d-5p,miR-452-5p,miR-8095,miR-628-3p,miR-335,miR-450a-5p,miR-548e-3p,miR-3600,miR-7859,miR-23b-3p,miR-23c,miR-222-5p,miR-200b,miR-148b-3p,miR-378i,miR-2300b-3p,miR-1197-5p,miR-1193,miR-378a-3p,miR-217-5p,miR-16-1-3p,miR-133a-3p,miR-361-5p,miR-378d,miR-329a-5p,miR-216c-5p,miR-369-3p,miR-125b-2-3p,miR-181b-1-3p,miR-146a-5p,miR-2411,miR-2424,miR-2285p,miR-210-5p,miR-324-3p,miR-125b,miR-422a,miR-466i-
VWA8	ENSOARG0(	von Will	miR-199b-5p,miR-877-3p,miR-24-3p,miR-1306-5p,miR-147-5p,miR-371a-5p,novel_87,novel_25,miR-199a-5p,miR-377-3p,miR-3082-5p,miR-3120-3p,miR-3064-5p,miR-20a-3p,miR-299b-5p,miR-21a-3p,miR-2387,miR-199b-5p,miR-199a-5p,miR-101-5p,miR-3064-5p,miR-452-5p,miR-153-3p,miR-199b-5p,miR-3085-3p,miR-205-5p,miR-196a-3p,miR-377-3p,novel_116,miR-616-3p,miR-153,miR-133b-5p,miR-378d,miR-2427,miR-1197-5p,miR-1285
PYGB	ENSOARG0(	phosphor	miR-145a-3p,miR-378g,miR-361-3p,miR-134-3p,miR-320c,miR-320b,miR-320a,miR-1197-5p,miR-320e,miR-320d,novel_1,miR-4429,miR-133a-3p,miR-147-5p

RNF215	ENSOARG0(	ring fin	miR-10b-3p,miR-876-3p,miR-615-5p,miR-125a,miR-2355-5p,miR-874-3p,miR-331-3p,miR-125b-5p,miR-2433,miR-486b-3p,miR-103b,miR-16-5p,miR-193a-5p,miR-330-5p,miR-345-3p,miR-615-3p,miR-582-3p,miR-6517,miR-299a-3p,novel_121,miR-322-5p,miR-29b-2-5p,miR-450c-5p,miR-490-3p,novel_39,miR-28b,miR-1306,miR-3141,miR-195a-5p,miR-2319a,miR-450b-3p,miR-1291,miR-26b-5p,miR-320b,miR-3074-5p,miR-669,miR-299b-3p,miR-2404,miR-323a-5p,miR-16a,miR-28c,miR-6402,miR-15a,miR-502-5p,novel_1,miR-128-1-5p,miR-326-3p,miR-371b-3p,miR-145a-5p,miR-22-3p,miR-1843b-5p,miR-744-5p,miR-497-5p,miR-16b,miR-133b-5p,miR-411-5p,miR-2285g,miR-2411-3p,novel_51,miR-3141,miR-377-3p,miR-125a-5p,miR-378a-5p,miR-124-5p,miR-432-3p,miR-2285c,miR-1827,miR-625-5p,miR-1895,miR-2331-3p,miR-504-5p,miR-15b-5p,miR-204-3p,miR-22-3p,novel_32,miR-323-5p,miR-377-3p,miR-132-5p,miR-2284s,miR-29b-2-5p,miR-324-3p,miR-1468-5p,miR-874-5p,miR-424-5p,miR-664a,miR-214,miR-3074-5p,miR-6134,miR-188-3p,miR-1193,miR-2904,miR-2366,miR-133a-3p,miR-664-5p,miR-532-3p,miR-15a-5p,miR-345-3p,miR-92a-1-5p,miR-125b,miR-185-3p,miR-324-3p,miR-210-5p,miR-26a-5p,miR-299-3p,miR-486-3p,miR-361-3p,miR-504,miR-7977,miR-20a-3p,miR-2319b,miR-4792,miR-652-3p,miR-107-5p,miR-1973,miR-615,miR-6527,miR-3059-5p,miR-2447,miR-877-3p,miR-296-5p,miR-484,miR-145b,novel_4,miR-2300a-5p,miR-1343-3p,miR-2285e,miR-2459,miR-147a,miR-16b,miR-326,miR-1306-5p,novel_103,miR-103a-2-5p,miR-1468,miR-214-3p,miR-424-5p,miR-1388-3p,miR-4532
SHMT2	ENSOARG0(	serine h	miR-2300a-5p,miR-429-3p,miR-3431,miR-200b,miR-24-2-5p,miR-1197-5p,miR-194-5p,miR-218-2-3p,miR-2300b-3p,miR-200b-3p,miR-4429,miR-30c-1-3p,miR-2285r,novel_51,miR-194a,miR-30c-2-3p,miR-200c,miR-374a-5p,miR-320b,miR-200c-3p,miR-320d,miR-655-5p,miR-2355-5p,miR-30c-1-3p,miR-320c,miR-6130,miR-320a,miR-3970,miR-361-3p,miR-410-5p,miR-194b-5p,miR-665-5p,miR-3958-5p,miR-4510,novel_42,miR-3059-5p,miR-361-3p,miR-320e,miR-6402
RWDD4	ENSOARG0(	RWD doma	miR-205-5p,miR-493-5p,miR-487a,miR-494-3p,miR-1388-3p,miR-2284f,miR-2284b,miR-2284a,miR-1b-5p,miR-2284y,novel_73,miR-2284u,miR-134-3p,miR-142-5p,miR-135a-1-3p,miR-6402,miR-2332,miR-142b,miR-655-3p,miR-19b-1-5p,miR-2284o,miR-6516-3p,miR-331-5p,miR-154-3p,miR-490-5p,miR-136-3p,novel_120,miR-6128,miR-142a-5p,miR-374c-5p,miR-331-5p,miR-655,miR-2284e,miR-136-5p,miR-10b-3p,novel_48,miR-493-5p,miR-140-5p,miR-2284g,miR-487a-3p,miR-545-3p,miR-139-5p,miR-7689-3p,miR-139-5p,miR-331-5p,miR-378g
BCKDHB	ENSOARG0(	branched	miR-7641,miR-3959-5p,miR-1247-3p,miR-329-3p,miR-362-3p,miR-362-3p,miR-2904,miR-3601,miR-362-3p,miR-451a,miR-329a,miR-708-5p,miR-30b-3p,miR-147-5p,miR-28-5p

RAD23B	ENSOARG0(	RAD23 ho	miR-30b,miR-320d,miR-106a,miR-2284m,miR-181b-5p,novel_127,miR-20a,miR-376a-5p,miR-125b-2-3p,miR-199a-5p,miR-200a,miR-376c-5p,miR-29a-5p,miR-376b-5p,miR-30c-5p,miR-3591-5p,miR-376a-5p,miR-30a-5p,miR-4429,miR-30c,miR-18a-3p,miR-106a,miR-376a-5p,miR-322-5p,miR-23b-3p,miR-340-5p,miR-23c,miR-15a-5p,miR-2284k,miR-135a-1-3p,miR-3604,novel_74,miR-331-5p,miR-424-5p,miR-874-5p,miR-2284v,miR-16-5p,miR-93,miR-181c-5p,miR-30e-5p,miR-503-3p,miR-331-5p,miR-93-5p,miR-23b,miR-376c-5p,miR-320a,miR-17-5p,miR-3154,miR-30f,miR-376c-5p,miR-20b-5p,miR-186-5p,miR-140-5p,miR-132-5p,miR-199a-5p,miR-15b-5p,miR-320b,miR-377-3p,miR-16b,miR-106b-5p,novel_51,miR-17-5p,miR-106a-5p,miR-199b-5p,miR-106a-5p,miR-424-5p,miR-101-5p,miR-23a-3p,miR-744-3p,miR-2285r,miR-181a-5p,miR-1434-3p,miR-4791,miR-1388-5p,miR-16b,miR-497-5p,miR-3535,miR-2284d,miR-147a,miR-20b,miR-15a,miR-30b-5p,miR-20a-5p,miR-6402,miR-142b,miR-16a,miR-6119-3p,novel_1,miR-2284j,miR-181d-5p,novel_111,miR-199b-5p,miR-376b-5p,miR-199b-5p,miR-18a-3p,miR-195a-5p,novel_94,miR-376a-2-5p,miR-2404,miR-320c
KIF26A	ENSOARG0(	kinesin	miR-485-5p,miR-361-3p,miR-24-3p,miR-92a-1-5p,miR-140-3p,novel_42
SS18	ENSOARG0(	synovial	miR-144,miR-409-3p,miR-2284k,miR-5100,miR-6128,miR-144-5p,miR-28a-3p,miR-1814c,miR-137-3p,miR-23b,miR-488-3p,miR-424-3p,miR-3965,miR-2285aa,miR-503-3p,miR-28-3p,miR-30f,miR-205-5p,miR-148b-5p,miR-30a-3p,miR-2385-3p,miR-4286,miR-181a-2-3p,miR-128-3p,miR-345-3p,miR-1271-5p,miR-376a-5p,miR-2285ad,miR-2285m,miR-371b-3p,miR-3959-3p,miR-376a-2-5p,miR-3065-5p,miR-3591-3p,miR-216a-3p,miR-144-3p,miR-26b-5p,miR-329-5p,miR-3074-5p,miR-466f-3p,miR-2411-3p,miR-194a,miR-539-3p,miR-2284l,miR-130b-5p,miR-493-5p,miR-6239,miR-2284x,miR-2284ab,miR-219a-1-3p,miR-197-3p,miR-194-5p,novel_23,miR-145a-5p,miR-374b-3p,miR-708-3p,miR-411-5p,miR-2284d,miR-1468-5p,miR-130b-5p,novel_74,miR-487b-5p,miR-30a-3p,miR-3604,miR-664a-5p,miR-338-5p,miR-30e-3p,miR-142a-5p,miR-1271,miR-665-5p,miR-3184-3p,miR-3074-5p,miR-582-5p,miR-411,let-7f-1-3p,miR-21-3p,miR-548o-3p,miR-21-3p,miR-2284m,miR-219b-5p,miR-345-3p,miR-26a-5p,miR-2424,miR-376a-5p,miR-142a-3p,miR-345-3p,miR-216c-5p,miR-183-5p,miR-329a-5p,miR-1434-5p,miR-188-3p,miR-376a-5p,miR-2300b-3p,miR-487a-3p,miR-7859,miR-383-5p,miR-23b-3p,miR-23c,miR-340-5p,miR-485-3p,miR-30d-3p,miR-548e-3p,miR-2285y,miR-371a-5p,miR-107-5p,miR-2285n,miR-101-3p,miR-877-3p,miR-194b-5p,miR-2284j,miR-212-3p,miR-493-5p,miR-877-3p,miR-96-5p,miR-381-3p,miR-144-5p,miR-203-3p,miR-2483-3p,miR-3082-5p,miR-2285u,miR-452-3p,miR-103a-2-5p,miR-1468,miR-487b-5p,novel_103,miR-2310,miR-23a-3p,miR-744-3p,miR-101b-3p,miR-101a-5p,miR-6529a,miR-216a-3p,miR-142-5p,miR-142-3p,miR-145b,miR-548w

FAM177A1	ENSOARG0(	family w	miR-330-3p,miR-2285ad,miR-145b,miR-1b-3p,novel_121,miR-194-3p,miR-216a-3p,miR-545-5p,miR-1193,miR-3071-5p,miR-145a-5p,miR-128-3p,miR-6240,miR-574-3p,miR-183-5p,miR-195a-3p,miR-2483-5p,miR-664b-3p,miR-2478,miR-2285b,miR-376b-5p,miR-376c-5p,miR-2432,miR-205-5p,miR-330-3p,miR-487b-5p,miR-660,miR-660-5p,miR-211-5p,miR-126b-5p,miR-2284s,miR-331-5p,miR-154b-5p,miR-203-3p,miR-331-5p,miR-361-3p,miR-140-5p,miR-216a-3p,miR-27b-5p,miR-876-3p,miR-543-3p,miR-7975,miR-3074-1-3p,miR-145a-3p,miR-361-3p,miR-211,miR-2285j,miR-34c-3p,miR-338-5p,miR-665,miR-100-3p,miR-331-5p,miR-204-5p
PRSS45	ENSOARG0(	protease	miR-424-5p,miR-382-3p,miR-378d,miR-8485,miR-193a,miR-320b,miR-326,novel_103,miR-2957,miR-505,miR-16b,miR-483-3p,miR-125a-5p,miR-1343-5p,miR-340-3p,miR-3955-5p,miR-103a-3p,miR-133b-5p,miR-5010-3p,miR-3431,miR-103,miR-653,miR-873a-5p,miR-378b,miR-181a-5p,miR-125b-2-3p,miR-30b-3p,miR-30b-3p,miR-378c,miR-16b,miR-101a-5p,miR-497-5p,miR-2447,miR-181d-5p,miR-2285x,miR-326-3p,miR-154a,miR-4324,miR-15a,miR-16a,miR-33a-3p,miR-502b,miR-2284q,miR-378e,miR-3082-5p,miR-374c-3p,miR-320c,miR-6535,miR-361-3p,miR-21c,miR-3065-5p,miR-378b,miR-1291,miR-107,miR-195a-5p,miR-378f,miR-3529-3p,miR-4286,novel_39,miR-490-3p,miR-196a-3p,miR-422a,miR-125b,miR-320d,miR-466i-5p,miR-181b-5p,miR-107,miR-200a-3p,miR-548e-3p,miR-1224-5p,miR-322-5p,miR-7859,miR-483-3p,miR-15a-5p,miR-378h,novel_83,miR-129-1-3p,miR-378i,miR-1343-5p,miR-1197-5p,miR-378a-3p,miR-5703,miR-330-5p,miR-4429,miR-142-3p,miR-378d,miR-378c,miR-129-2-3p,miR-16-5p,miR-1197-3p,miR-378c,miR-181c-5p,miR-410-5p,miR-125b-5p,miR-665,miR-331-5p,miR-378a-3p,miR-548o-3p,miR-216b-3p,miR-2285aa,miR-2355-5p,miR-141-3p,miR-125a,miR-552-3p,miR-2284s,miR-331-5p,miR-582-5p,miR-204-3p,miR-15b-5p,miR-331-5p,miR-320a,miR-3154,miR-625-5p,miR-7144-5p,miR-582-5p,miR-671-5p
TJAP1	ENSOARG0(	tight ju	miR-424-5p,novel_51,miR-16b,miR-3141,miR-146a,miR-326,miR-214-3p,miR-1343-5p,miR-146b,miR-25-5p,miR-1343-3p,miR-216a-3p,miR-194-3p,miR-143-5p,miR-484,miR-744-5p,miR-497-5p,miR-4508,miR-138,miR-16b,miR-2447,miR-449a,miR-326-3p,miR-24-3p,miR-371b-3p,miR-1307-3p,miR-361-3p,miR-16a,miR-34c,miR-502-5p,miR-1247-3p,miR-15a,miR-669,miR-34b,miR-670-3p,miR-18a-3p,miR-3141,miR-195a-5p,miR-146b-5p,novel_39,miR-486-3p,miR-146a-5p,miR-92a-1-5p,miR-185-3p,novel_127,miR-34b-5p,miR-760-3p,miR-191-3p,miR-6517,miR-18a-3p,miR-15a-5p,miR-322-5p,miR-330-5p,miR-1343-5p,miR-34a-5p,miR-214,miR-16-5p,miR-338-3p,miR-324-3p,miR-2433,miR-486b-3p,miR-4492,miR-424-5p,miR-138-5p,miR-296-3p,miR-874-3p,miR-15b-5p,miR-204-3p,miR-34c-5p,miR-1827,miR-10b-3p,miR-2474
7-Mar	ENSOARG0(	membrane	miR-193a,novel_83,miR-668-5p,miR-33b-3p

MICU1	ENSOARG0(	mitochon	miR-670-3p,miR-2404,miR-10a-5p,miR-655-5p,miR-320b,miR-195a-5p,miR-6516-3p,miR-135b-5p,miR-1291,miR-2428,miR-326-3p,miR-371b-3p,miR-24-3p,miR-17-3p,miR-1193,miR-147-5p,novel_1,miR-16a,miR-6402,miR-15a,miR-185-5p,miR-377-5p,miR-130a-3p,miR-30b-3p,miR-145a-5p,miR-374b-3p,miR-1285,miR-653,miR-130b-5p,miR-365a-3p,miR-374c-3p,miR-141-3p,miR-370-3p,miR-671-5p,miR-485-5p,miR-1814c,miR-10b-3p,miR-362-5p,miR-3154,miR-2448-3p,miR-365a-5p,miR-500b-5p,miR-6128,miR-1306-5p,miR-28-5p,miR-486b-3p,miR-409-3p,miR-135a-5p,miR-370-5p,miR-10a-5p,miR-3578,miR-345-3p,miR-1271-3p,miR-1957a,miR-380-5p,miR-24-2-5p,miR-4286,miR-1,miR-17-3p,miR-450a-2-3p,miR-339a,miR-27a-3p,miR-454-5p,miR-489,miR-7977,miR-20a-3p,miR-452-3p,novel_94,miR-877-3p,miR-129b-5p,miR-154a,miR-362-5p,miR-1296-5p,miR-541-3p,miR-212-3p,novel_60,miR-3059-5p,miR-107-5p,miR-382-3p,miR-500-5p,miR-9788-3p,miR-222-5p,miR-27a-3p,miR-2300a-5p,novel_4,miR-30b-3p,miR-125b-2-3p,miR-10b,miR-296-5p,miR-484,miR-3187-3p,miR-652-5p,miR-424-5p,miR-3955-3p,novel_124,miR-362-3p,miR-326,novel_103,novel_25,miR-204-3p,miR-6130,miR-21-3p,miR-370-5p,miR-221-5p,miR-545-3p,novel_99,miR-6134,miR-338-3p,miR-206-3p,miR-10b-5p,miR-4510,miR-145a-3p,miR-708-5p,miR-424-5p,miR-6516-3p,miR-664a,miR-17-3p,miR-338-5p,miR-15a-5p,miR-383-5p,miR-3600,miR-5703,miR-2904,miR-362-3p,miR-2432,miR-146a-5p,miR-29b-1-5p,miR-183-5p,miR-21-3p,miR-299-3p,miR-221-5p,miR-760-3p,miR-466i-5p,miR-185-3p,miR-181b-3p,miR-669,miR-677,miR-299b-3p,miR-374c-3p,miR-3120-3p,miR-3074-5p,novel_68,miR-2319a,miR-146b-5p,miR-412-3p,miR-3065-5p,miR-301b-3p,miR-758-5p,miR-206,miR-6119-3p,miR-4324,miR-1247-3p,miR-411-5p,miR-149-5p,miR-339-5p,miR-31-5p,miR-3955-5p,miR-27b-3p,miR-497-
EFR3A	ENSOARG0(	EFR3 hom	miR-505-5p,miR-302a-5p,miR-328-3p,miR-545-5p,miR-2285ad,miR-485-3p,miR-30d-3p,miR-3600,miR-148a-5p,miR-16-1-3p,miR-218-1-3p,miR-501-3p,miR-130a-5p,miR-148b-5p,miR-92b-3p,miR-490-3p,miR-380-3p,miR-2424,miR-345-3p,miR-2385-3p,miR-92a-3p,miR-26a-5p,miR-125a-3p,miR-25-3p,miR-139-5p,miR-582-5p,miR-154b-5p,miR-32-5p,miR-552-3p,miR-216b-3p,miR-767,miR-2312,miR-424-3p,miR-2285c,miR-488-3p,miR-200a-5p,miR-224-5p,miR-590-3p,miR-539-5p,miR-767-5p,miR-129-5p,miR-3969,miR-100-3p,miR-539-5p,miR-2285g,miR-544b,miR-2285e,miR-148b-5p,miR-16-1-3p,miR-21-5p,miR-380-3p,miR-2284x,miR-1839-5p,miR-16-2-3p,miR-26a-2-3p,miR-708-3p,miR-873a-5p,miR-3071-5p,miR-501-3p,miR-2284l,miR-8485,miR-539-3p,miR-493-5p,miR-382,miR-676-3p,miR-224-5p,miR-32,miR-500-3p,miR-144-3p,miR-139-5p,miR-26b-5p,miR-126b-5p,miR-3120-3p,miR-136-5p,miR-9-3p,miR-6395,miR-493-5p,miR-2387,miR-2285ab,miR-361-3p,miR-21c,miR-7705,miR-502-3p,miR-9-3p,miR-363-3p,miR-452-5p,miR-2285f,miR-499a-5p,miR-92a-3p,miR-6123,miR-6402,miR-382-5p,miR-2285y

GPBP1	ENSOARG0(	GC-rich	miR-4429,miR-345-3p,miR-181a-5p,miR-22-3p,novel_23,miR-3071-5p,miR-217-5p,miR-145a-5p,miR-188-3p,miR-23b-3p,miR-539-5p,miR-23c,miR-221-3p,miR-2355-3p,miR-222,miR-3600,miR-145b,miR-320d,miR-17-3p,miR-181b-5p,miR-320b,miR-1306-5p,miR-217,miR-345-3p,miR-2411-3p,miR-664b-3p,miR-130a-5p,miR-503-3p,miR-23a-3p,miR-625-5p,miR-543-3p,novel_94,miR-9-3p,miR-582-5p,miR-424-3p,miR-221,miR-2387,miR-1306,miR-320a,miR-23b,miR-582-5p,miR-6535,miR-320c,miR-22-3p,miR-378b,miR-664a,miR-17-3p,miR-22-5p,miR-222-3p,miR-2440,miR-409-3p,miR-338-5p,miR-323-3p,novel_27,miR-181c-5p,miR-539-5p,miR-6527,miR-17-3p,miR-181d-5p,miR-2330-3p
MCU	ENSOARG0(	mitochon	miR-744-3p,miR-424-5p,miR-124a,miR-2310,miR-30c-1-3p,miR-16b,miR-3085-3p,miR-409b,miR-421,miR-2285e,miR-147a,miR-125b-2-3p,miR-30b-3p,miR-2426,miR-450b-5p,miR-452-5p,miR-363-3p,miR-212-3p,miR-3059-5p,miR-8095,miR-361-3p,miR-2285n,miR-7977,miR-2285u,miR-541-5p,miR-2319b,miR-3064-5p,miR-4443,miR-203-3p,miR-25,miR-504,miR-329a-5p,miR-185-3p,miR-324-3p,miR-202-5p,miR-485-3p,novel_83,miR-532-3p,miR-15a-5p,miR-487a-3p,miR-188-3p,miR-124-3p,novel_133,miR-378d,miR-218-1-3p,miR-16-1-3p,miR-184-3p,miR-142-3p,miR-129-2-3p,miR-4510,miR-145a-3p,miR-6134,miR-338-3p,miR-19b-1-5p,miR-424-5p,miR-17-3p,miR-552-3p,miR-2331-3p,miR-504-5p,miR-30c-1-3p,miR-32-5p,miR-15b-5p,miR-323-5p,miR-6130,miR-27b-5p,miR-2285c,miR-21a-3p,novel_99,miR-543-3p,miR-628-5p,miR-668-3p,miR-539-3p,miR-2285r,novel_51,miR-32,miR-466f-3p,miR-483-3p,miR-6238,miR-185-5p,miR-2285g,novel_23,miR-1843b-5p,miR-19b-2-5p,miR-497-5p,miR-16-1-3p,miR-219a-1-3p,miR-16b,miR-138,miR-30b-3p,miR-17-3p,miR-24-3p,miR-16a,miR-485-3p,miR-133a-5p,miR-15a,miR-92a-3p,miR-3120-3p,miR-329-5p,miR-669,miR-323a-5p,miR-2404,miR-150-5p,miR-195a-5p,miR-3064-5p,miR-192-3p,miR-92b-3p,miR-30f,miR-30c-2-3p,miR-2285af,miR-92a-3p,miR-17-3p,miR-2898,miR-129-1-3p,miR-483-3p,miR-322-5p,miR-3591-5p,miR-300,miR-16-5p,miR-6128,miR-145-3p,miR-2332,novel_63,miR-2433,miR-138-5p,miR-25-3p,miR-300-3p,miR-381-3p,miR-488-3p,miR-1814c,miR-329b,miR-10b-3p

			miR-101a-5p,miR-362-3p,miR-378c,miR-30b-3p,miR-541,miR-378b,miR-329-3p,miR-2285e,miR-455-3p,miR-9788-3p,miR-1298-3p,miR-425-3p,miR-222-5p,miR-199b-3p,miR-548w,miR-330-3p,miR-199b-5p,miR-1185-5p,miR-16b,miR-182-5p,miR-362-3p,miR-431-5p,novel_103,miR-2310,miR-193a,miR-378d,miR-652-5p,miR-2284b,miR-23a-3p,miR-424-5p,miR-494-3p,miR-129b-5p,miR-199b-5p,miR-361-3p,miR-203-3p,miR-3082-5p,miR-378e,miR-499b-5p,miR-2284g,miR-139-5p,miR-2284q,miR-1843a-3p,miR-2285t,miR-3968,miR-1285-5p,miR-2284j,miR-541-3p,miR-2284u,miR-133a-3p,miR-19b-3p,miR-1197-5p,miR-378a-3p,miR-2904,miR-362-3p,miR-378i,miR-15a-5p,miR-23c,miR-7859,miR-23b-3p,miR-21-3p,miR-221-5p,miR-1277-5p,miR-466i-5p,miR-2284m,miR-422a,miR-125b,miR-125b-2-3p,miR-216c-5p,miR-186-5p,miR-2285c,miR-15b-5p,miR-139-5p,miR-6516,miR-132-5p,miR-182-5p,miR-532-5p,miR-378a-3p,miR-221-5p,miR-424-5p,novel_74,miR-324-3p,miR-346,miR-378c,miR-7862,miR-188-5p,miR-2284x,miR-497-5p,miR-30b-3p,miR-16b,miR-374b-3p,miR-149-5p,miR-194-3p,miR-2284d,miR-412-5p,miR-2285g,miR-26b-3p,miR-466f-3p,miR-125a-5p,miR-330-3p,miR-365b-5p,miR-2284l,miR-6240,miR-378f,miR-195a-5p,miR-7975,miR-19a-3p,miR-150-5p,miR-374c-3p,miR-670-3p,miR-146b-3p,miR-3120-3p,miR-147-5p,miR-199c,novel_1,miR-382-5p,miR-16a,miR-4324,miR-15a,miR-199b-5p,miR-3957-3p,miR-1a-1-5p,miR-135a-2-3p,miR-3578,novel_121,miR-545-5p,miR-378h,miR-6516-5p,miR-322-5p,miR-431,miR-1a-2-5p,miR-6119-5p,miR-2397-5p,miR-192-3p,miR-1271-3p,miR-2284a,miR-199a-5p,miR-1957a,miR-2284y,miR-2403,miR-1814c,miR-7144-5p,miR-329a,let-7g-3p,miR-23b,miR-488-3p,miR-199a-5p,miR-331-5p,miR-374c-3p,miR-615-5p,miR-125a,miR-2355-5p,miR-3068-3p,miR-125b-5p,miR-1-5p,miR-2284k,miR-365a-5p,miR-378c,miR-16-5p,miR-
CYP2B6	ENSOARG0(	cytochro	
ANGPTL5	ENSOARG0(	angiopoi	miR-369-3p,miR-590-3p,miR-329a-5p,miR-146a-5p,miR-500a-5p,miR-182-5p,miR-218-5p,miR-146a,miR-146b,miR-532-5p,miR-182-5p,miR-2331-3p,miR-329-5p,novel_78,miR-545-5p,miR-656-3p,miR-539-5p,miR-376a-5p,miR-1839-5p,miR-146b-5p,miR-379-5p

GNB2L1	ENSOARG0(	guanine	miR-152-3p,novel_96,novel_127,let-7f-5p,let-7g,miR-148b-5p,miR-1343-5p,miR-330-5p,miR-30c,miR-299a-3p,miR-134-5p,miR-2284z,miR-322-5p,miR-9-5p,miR-6740-5p,miR-2284k,miR-2433,miR-134-5p,miR-16-5p,miR-193a-5p,miR-181a-2-3p,miR-1260b,miR-30e-5p,miR-1248,miR-574-5p,novel_51,miR-193a-3p,miR-378a-5p,miR-668-3p,miR-628-5p,miR-6240,let-7j,miR-432-3p,miR-2284l,miR-22-3p,miR-197-3p,miR-223-3p,miR-497-5p,miR-4508,miR-16b,miR-3955-5p,miR-31-5p,miR-103a-3p,miR-194-3p,miR-149-5p,miR-134-3p,miR-425-5p,miR-412-5p,miR-4324,miR-576-3p,miR-193b-3p,miR-301b-3p,miR-150-5p,miR-3970,miR-148a-3p,let-7e-5p,miR-378f,miR-412-3p,miR-6535,miR-669,miR-299b-3p,miR-324-3p,miR-422a,let-7k,miR-98-5p,miR-486-3p,miR-377-5p,miR-378a-3p,miR-30c-5p,miR-378i,miR-1961,miR-133a-3p,miR-34a-5p,miR-18a-3p,miR-328-3p,miR-210-3p,miR-95-3p,miR-151-3p,let-7f,miR-2285j,miR-454-3p,miR-4492,miR-214,miR-590-3p,let-7b,miR-769-5p,miR-6525,miR-346,miR-30d-5p,miR-625-5p,miR-1827,miR-296-3p,miR-30c-1-3p,miR-504-5p,miR-15b-5p,let-7i-5p,miR-301a-3p,miR-16b,miR-199a-3p,miR-214-3p,miR-491-5p,miR-130b-3p,miR-487b-5p,miR-8485,miR-2310,miR-378d,miR-2284r,miR-541,miR-330-3p,miR-1343-3p,miR-1185-3p,miR-2459,miR-421,miR-411b-5p,miR-1298-3p,miR-147a,miR-218-5p,miR-3968,miR-628-3p,miR-2284j,miR-9-5p,miR-2447,miR-361-3p,miR-140-5p,miR-1983,miR-544-5p,miR-504,miR-136-5p,miR-211-5p,miR-4792,miR-2319b,miR-1185-2-3p,miR-378g,miR-30c-2-3p,let-7c-5p,miR-1260a,miR-107,miR-1957a,miR-30a-5p,miR-134,let-7d,miR-582-3p,miR-378h,miR-26c,miR-410-5p,miR-370-5p,miR-204-5p,miR-374b-3p,miR-486b-3p,miR-3607-3p,miR-6128,miR-103b,miR-378c,miR-374a-3p,miR-7144-5p,miR-485-5p,miR-1814c,miR-615-5p,miR-154b-5p,miR-2284n,miR-34c-5p,miR-
PPP2R3C	ENSOARG0(	protein	miR-412-3p,miR-133b-3p,miR-411-3p,miR-107,miR-133a-3p,novel_48,miR-151a-3p,miR-103a-3p,miR-541-5p,miR-216b-3p,miR-412,miR-203-3p,miR-103,novel_121,miR-151-3p,miR-379-3p,miR-107,miR-592,miR-452-5p,miR-133a-3p,miR-8095

HRH4	ENSOARG0(	histamin	miR-143-3p,miR-486-3p,miR-2285p,miR-196a-3p,miR-345-3p,miR-324-3p,miR-760-3p,miR-2284m,miR-2484,miR-148b-3p,miR-222-5p,miR-210-3p,novel_83,miR-2366,miR-193b-5p,miR-188-3p,miR-217-5p,miR-5703,novel_73,miR-142-3p,miR-148a-5p,miR-2284u,miR-664-5p,miR-3184-3p,miR-10b-5p,miR-2285j,miR-338-5p,miR-17-3p,miR-669a-3p,miR-130b-5p,miR-32-3p,miR-660-5p,miR-182-5p,miR-296-3p,miR-10a,miR-204-3p,miR-339b,miR-494-3p,miR-652-5p,miR-23a-3p,miR-193a,miR-326,miR-1306-5p,novel_103,miR-182-5p,miR-323b-3p,miR-505,miR-1185-5p,miR-153-3p,miR-1343-3p,miR-27a-3p,miR-539-5p,miR-376b-3p,miR-153,miR-455-3p,miR-148b-5p,miR-10b,miR-125b-2-3p,miR-323b,miR-2426,miR-101a-5p,miR-2284aa,miR-885-5p,miR-3956-3p,miR-32-3p,novel_60,miR-2447,miR-2284j,miR-361-3p,miR-2440,miR-371a-5p,miR-335,miR-499a-5p,miR-2284g,miR-211-5p,miR-2483-3p,miR-27a-3p,miR-376d,miR-4443,miR-203-3p,miR-361-3p,miR-129b-5p,miR-668-5p,miR-136-5p,miR-335-3p,miR-376a-3p,miR-181a-2-3p,miR-148b-5p,miR-2284a,novel_39,miR-29a-5p,miR-2284y,miR-8117,miR-376b-3p,miR-339a,miR-217,miR-17-3p,miR-200a-3p,miR-152-3p,miR-107,novel_79,novel_121,miR-134-5p,miR-2284z,miR-134,miR-345-3p,miR-330-5p,miR-10a-5p,miR-2284v,miR-2332,novel_63,miR-2284k,miR-6740-5p,novel_9,miR-134-5p,miR-486b-3p,miR-204-5p,miR-141-3p,miR-331-5p,miR-2284n,miR-424-3p,miR-200a-5p,miR-485-5p,miR-7144-5p,miR-130b-5p,miR-2411-3p,miR-124-5p,miR-660,miR-103a-3p,miR-26b-3p,miR-339-5p,miR-2284d,miR-432-5p,miR-103,miR-149-5p,novel_116,miR-223-3p,miR-1434-3p,miR-432,miR-3071-5p,miR-2284ab,miR-27b-3p,miR-335-5p,miR-17-3p,miR-495-3p,miR-323c,miR-3959-3p,miR-211,miR-326-3p,miR-133a-5p,novel_1,miR-378b,novel_68,miR-10a-5p,miR-411b-3p,miR-
UBE3D	ENSOARG0(	ubiquiti	novel_74,miR-21-3p,miR-34b-5p,miR-320e,miR-34c,miR-449a,miR-4510,miR-186-5p,miR-34a-5p,miR-1827,novel_133,miR-21a-3p,miR-188-3p,miR-3970,miR-34b,miR-6130,miR-487a-3p,miR-34c-5p,miR-421,miR-485-3p

CD1E	ENSOARG0(	CD1e mol	miR-204-5p,miR-331-3p,miR-378c,miR-103b,miR-7144-5p,miR-3074-1-3p,miR-488-3p,miR-301,miR-223-5p,miR-374c-3p,miR-365a-3p,miR-503-3p,novel_96,let-7f-5p,miR-3071-3p,miR-30c-2-3p,let-7c-5p,miR-2285af,let-7g,miR-2285b,miR-1,miR-30a-3p,miR-128-3p,miR-496,let-7d,miR-196a-5p,miR-1290,miR-450a-1-3p,miR-9-5p,miR-378h,miR-7641,miR-412,miR-299a-3p,miR-222,novel_1,miR-206,miR-379-3p,let-7i,miR-502-5p,miR-142b,miR-6402,miR-576-3p,miR-2428,miR-211,miR-323c,miR-2285x,miR-146b-5p,miR-378f,let-7e-5p,miR-3591-3p,miR-216a-3p,miR-2387,miR-3141,miR-299b-3p,miR-196b-5p,miR-3141,miR-7-5p,miR-146a,let-7j,miR-628-5p,miR-27b-3p,miR-21-5p,miR-30b-3p,miR-16-1-3p,miR-22-3p,miR-450c-3p,miR-7b-5p,miR-544b,miR-412-5p,miR-3535,let-7g-5p,miR-3431,miR-665,miR-450b-3p,miR-664a,miR-30a-3p,miR-655-3p,let-7f,miR-222-3p,miR-30e-3p,let-7b-5p,miR-6525,miR-206-3p,miR-378c,let-7b,miR-543-3p,miR-27b-5p,miR-655,miR-450a-1-3p,novel_25,miR-22-3p,let-7i-5p,miR-544-3p,miR-21-3p,miR-532-5p,miR-378a-3p,miR-30c-1-3p,miR-496-3p,miR-3653-3p,let-7k,miR-299-3p,miR-98-5p,miR-216a-5p,miR-422a,miR-196a-3p,novel_17,miR-146a-5p,miR-2432,miR-143-3p,miR-345-3p,miR-2285w,miR-33b-3p,miR-378a-3p,miR-378i,miR-2300b-3p,miR-1961,miR-2284h-5p,miR-30d-3p,miR-499a-5p,miR-1285-5p,miR-9-5p,miR-3059-5p,novel_60,miR-212-3p,miR-3956-3p,miR-3963,miR-221,miR-409-5p,let-7d-5p,miR-27a-3p,miR-378e,miR-211-5p,miR-20a-3p,miR-452-3p,miR-146b,let-7e,miR-491-5p,miR-3955-3p,miR-378d,miR-30c-1-3p,miR-652-5p,let-7a-5p,miR-1388-3p,miR-382-3p,miR-378c,miR-544a,miR-412-3p,miR-7857,miR-1a-3p,miR-3962,miR-125b-2-3p,miR-30b-3p,miR-378b,miR-4791,miR-221-3p,miR-455-3p,miR-148b-5p,miR-2459,miR-27a-3p

ID3	ENSOARG0(	inhibito	miR-1290,miR-134-5p,miR-9-5p,miR-322-5p,novel_79,miR-431,miR-429-3p,miR-6517,miR-200a-3p,miR-125a-3p,novel_39,miR-2284y,miR-767,miR-876-3p,miR-345-5p,miR-300-3p,miR-3959-5p,miR-503-3p,miR-134-5p,miR-6740-5p,miR-2284k,miR-1260b,miR-1197-3p,miR-193a-5p,miR-16-5p,novel_42,miR-2284v,miR-26a-2-3p,miR-497-5p,miR-27b-3p,miR-188-5p,miR-16b,novel_23,miR-432,miR-149-5p,miR-3431,miR-339-5p,miR-340-3p,miR-193a-3p,miR-378a-5p,miR-377-3p,miR-146a,miR-668-3p,miR-380-5p,miR-146b-5p,miR-410-5p,miR-1306,miR-3591-3p,novel_68,miR-3074-5p,miR-29a,miR-193b-3p,miR-29c-3p,miR-335-5p,miR-2284u,miR-33b-3p,novel_73,miR-193b-5p,miR-328-3p,miR-210-3p,miR-212-5p,miR-30d-3p,miR-500a-5p,novel_17,miR-2285p,miR-143-3p,miR-376a-5p,miR-377-5p,miR-1827,miR-339b,miR-345-5p,miR-500a-3p,miR-129b-3p,miR-299,miR-15b-5p,miR-377-3p,miR-552-3p,miR-1468-5p,miR-874-5p,miR-500,miR-130b-5p,miR-451a,miR-30a-3p,miR-30e-3p,miR-6525,miR-769-5p,miR-224-5p,miR-3958-5p,miR-433-5p,miR-3074-5p,miR-1388-5p,miR-758-3p,miR-541,miR-147a,miR-1298-3p,miR-1343-3p,miR-146b,miR-29b-1-5p,miR-16b,miR-431-5p,miR-2284b,miR-136-5p,miR-140-5p,miR-4443,miR-7689-3p,miR-211-5p,miR-4792,miR-139-5p,miR-2284g,miR-628-3p,miR-499a-5p,miR-7705,miR-877-3p,miR-6527,miR-2284j,miR-9-5p,miR-2447,miR-3956-3p,miR-200b-3p,miR-496,miR-29d-3p,miR-134,miR-582-3p,miR-6516-5p,miR-2898,miR-450a-2-3p,miR-200c-3p,miR-339a,miR-1260a,miR-200c,miR-2397-5p,miR-3065-3p,miR-490-3p,miR-1271-3p,miR-2284a,miR-380-5p,miR-29b-2-5p,miR-30a-3p,miR-181a-2-3p,miR-7144-5p,miR-485-5p,miR-671-5p,miR-10b-3p,miR-362-5p,miR-488-3p,miR-2448-3p,miR-2284n,miR-769,miR-141-3p,miR-215-5p,miR-28-5p,miR-204-5p,miR-138-5p,miR-767-5p,miR-29b-3p,miR-370-5p,miR-

MTCH2	ENSOARG0(	mitochon	miR-885-3p,miR-1343-5p,miR-330-5p,miR-322-5p,miR-9-5p,miR-429-3p,miR-412,miR-6536,miR-200a-3p,miR-664-3p,miR-450b-5p,miR-29a-5p,miR-2284y,miR-200a,miR-3064-5p,miR-876-3p,miR-767,miR-345-5p,miR-2318,miR-301,miR-331-5p,miR-300-3p,miR-133c,miR-223-5p,miR-503-3p,miR-2355-5p,miR-125a,miR-331-5p,miR-100-3p,miR-125b-5p,miR-16-5p,miR-16b,miR-16-1-3p,miR-26a-2-3p,miR-497-5p,miR-223-3p,miR-7b-5p,miR-412-5p,miR-3431,miR-149-5p,miR-103a-3p,miR-7-5p,miR-105-5p,miR-194a,let-7j,miR-6240,miR-668-3p,miR-378f,miR-1306,miR-411b-3p,miR-6535,miR-144-3p,miR-669,miR-677,novel_68,miR-329-5p,miR-26b-5p,miR-3120-3p,miR-206,miR-625-3p,miR-374c-5p,miR-199b-5p,miR-218-1-3p,miR-378i,miR-2366,miR-378a-3p,miR-1434-5p,miR-7859,miR-210-3p,miR-23c,miR-2284h-5p,miR-212-5p,miR-26a-5p,miR-2285p,miR-210-5p,miR-324-3p,miR-92a-1-5p,miR-422a,miR-664b-3p,miR-2285i,miR-376a-5p,miR-329a-5p,miR-2285w,miR-345-5p,miR-625-5p,miR-885-3p,miR-543-3p,miR-1827,miR-655,miR-15b-5p,miR-504-5p,miR-548o-3p,miR-216b-3p,novel_74,miR-487b-5p,miR-130b-5p,miR-2285j,miR-664a-5p,miR-6525,miR-346,miR-3184-5p,miR-214,miR-1388-5p,miR-1a-3p,miR-147a,miR-455-3p,miR-421,miR-330-3p,miR-181b-2-3p,miR-487b-5p,miR-199b-5p,miR-214-3p,miR-182-5p,miR-16b,miR-378d,miR-1b-5p,miR-23a-3p,miR-504,miR-136-5p,miR-221,miR-139-5p,miR-2285u,miR-2440,miR-423-5p,miR-1285-5p,miR-2447,miR-9-5p,miR-300,miR-105-2,miR-200b-3p,miR-496,miR-3578,miR-302a-5p,miR-6516-5p,miR-378h,miR-222,miR-450a-2-3p,miR-107,miR-1957a,miR-199a-5p,miR-2284a,miR-1,miR-181a-2-3p,miR-7144-5p,miR-485-5p,miR-381-3p,miR-362-5p,miR-374c-3p,miR-199a-5p,miR-141-3p,miR-767-5p,miR-5100,miR-370-5p,miR-135a-1-3p,miR-331-3p,miR-135a-5p,miR-323-3p,miR-500b-5p,miR-378c,miR-1306-5p,miR-30b-
AKAP11	ENSOARG0(	A kinase	miR-148a-5p,miR-493-3p,miR-1290,miR-2284h-5p,miR-487a-3p,miR-7859,miR-322-5p,miR-15a-5p,miR-148b-3p,miR-3600,miR-485-3p,miR-548e-3p,miR-152-3p,miR-450a-2-3p,miR-500a-5p,miR-122-3p,novel_17,miR-125a-3p,miR-29b-1-5p,miR-216c-5p,miR-2285w,miR-186-5p,miR-500a-3p,miR-362-5p,miR-488-3p,miR-15b-5p,miR-300-3p,miR-301a-3p,miR-552-3p,miR-296-3p,miR-874-3p,miR-500,miR-454-3p,miR-424-5p,miR-592,miR-451a,miR-664a,miR-19b-1-5p,miR-3604,miR-2285j,miR-500b-5p,miR-6525,miR-16-5p,miR-193a-5p,miR-425-5p,miR-497-5p,miR-2889,miR-16b,miR-3071-5p,miR-19b-2-5p,miR-2113,miR-223-3p,miR-1434-3p,miR-148b-5p,miR-500-5p,miR-216a-3p,miR-194-3p,miR-539-5p,miR-130a-3p,miR-122-3p,miR-466f-3p,miR-130b-3p,miR-29b-1-5p,miR-16b,miR-539-3p,miR-8485,miR-193a,miR-2285r,miR-1388-3p,miR-424-5p,miR-544-5p,miR-877-3p,miR-195a-5p,miR-150-5p,miR-3065-5p,miR-148a-3p,miR-670-3p,miR-499b-5p,miR-541-5p,miR-147-5p,miR-6119-3p,miR-16a,novel_87,miR-502-5p,miR-15a,miR-101-3p,miR-8095,miR-301b-3p,miR-362-5p,miR-371b-3p,miR-2285x,miR-452-5p,miR-3059-5p

DOPEY1	ENSOARG0(	dopey fa	miR-330-3p,miR-3600,miR-6516-5p,miR-2285g,miR-412-5p,miR-2285e,novel_121,miR-134-5p,miR-143-5p,miR-301b-5p,miR-134,miR-5703,miR-197-3p,miR-98-3p,miR-450b-5p,miR-101a-5p,miR-1388-5p,let-7a-3p,miR-6240,miR-3064-5p,miR-363-5p,miR-28b,miR-30c-1-3p,miR-26a-5p,miR-2285af,miR-30c-2-3p,miR-330-3p,miR-2898,miR-3085-3p,miR-3064-5p,miR-30c-1-3p,miR-26b-5p,miR-2285aa,miR-6516,miR-300-3p,miR-223-5p,miR-2285c,let-7f-1-3p,miR-381-3p,miR-412-3p,miR-767,miR-181a-3p,miR-9-3p,miR-485-5p,miR-1983,miR-9851-3p,novel_48,miR-452-5p,miR-98-3p,miR-323-3p,miR-6134,miR-181c-3p,miR-6402,miR-28c,novel_82,let-7b-3p,miR-301a-5p,miR-767-5p,miR-4726-5p,miR-134-5p,miR-625-3p
SDS	ENSOARG0(	serine d	miR-138-5p,miR-494-5p,miR-2332,miR-331-3p,miR-125b-5p,miR-1260b,miR-145-3p,miR-378c,miR-181a-2-3p,miR-16-5p,miR-103b,miR-876-3p,let-7g-3p,miR-23a-5p,miR-485-5p,miR-301,miR-137-3p,miR-200a-5p,miR-374c-3p,miR-199a-5p,miR-154b-5p,miR-141-3p,miR-125a,miR-615-5p,miR-17-3p,miR-181b-5p,miR-107,miR-200a-3p,novel_127,miR-1260a,miR-125a-3p,miR-664-3p,miR-339a,miR-1957a,novel_39,miR-199a-5p,miR-490-3p,miR-192-3p,miR-1271-3p,miR-4286,miR-2284f,let-7a-2-3p,miR-3591-5p,miR-1343-5p,miR-23b-5p,miR-322-5p,miR-378h,miR-483-3p,miR-299a-3p,miR-423-3p,novel_79,novel_1,miR-502b,miR-4324,miR-15a,miR-576-3p,miR-1307-5p,miR-16a,miR-2284o,miR-323c,miR-24-3p,miR-199b-5p,miR-2428,miR-135a-2-3p,miR-17-3p,novel_101,miR-412-3p,miR-107,miR-7975,miR-195a-5p,miR-378f,miR-2285ab,miR-3141,miR-18a-3p,miR-323a-5p,miR-374c-3p,novel_69,miR-299b-3p,miR-320b,miR-483-3p,miR-452-5p,miR-1343-5p,miR-125a-5p,miR-7-5p,miR-3141,miR-455-5p,miR-136-3p,let-7a-2-3p,miR-503-3p,miR-668-3p,miR-363-5p,miR-138,miR-16b,miR-1260b,miR-26a-2-3p,miR-497-5p,miR-455-5p,miR-7862,miR-873a-5p,miR-432,miR-181a-5p,miR-7b-5p,miR-542-3p,miR-432-5p,miR-134-3p,miR-149-5p,novel_116,miR-103,miR-340-3p,miR-133b-5p,miR-103a-3p,miR-665,miR-339-5p,miR-487b-5p,miR-17-3p,miR-208b-5p,miR-424-5p,miR-2285j,miR-151-3p,miR-320e,miR-664a-5p,novel_44,miR-181c-5p,miR-378c,miR-4510,miR-214,let-7g-3p,miR-339b,miR-543-3p,miR-625-5p,miR-27b-5p,miR-129b-3p,miR-487b-5p,miR-27a-5p,miR-6130,miR-323-5p,miR-216b-5p,miR-204-3p,miR-15b-5p,miR-378a-3p,miR-221-5p,miR-296-3p,miR-466i-5p,miR-760-3p,miR-221-5p,miR-30b-3p,miR-299-3p,miR-21-3p,miR-185-3p,miR-125b,miR-422a,miR-29b-1-5p,miR-5100,miR-378i,miR-2300b-

NDST1	ENSOARG0(	N-deacet	miR-432-5p,miR-2355-3p,miR-130a-3p,miR-30b-3p,miR-7862,miR-873a-5p,miR-145a-5p,miR-654-3p,miR-130b-5p,miR-363-5p,miR-1343-5p,miR-224-5p,miR-34b,miR-670-3p,miR-323a-5p,miR-2404,miR-146b-3p,miR-450b-3p,miR-6516-3p,miR-2285ab,miR-378b,miR-376b-3p,miR-18a-3p,miR-211,miR-326-3p,miR-449a,novel_1,miR-22-5p,miR-6402,miR-502-5p,miR-34c,miR-483-3p,miR-582-3p,miR-7641,let-7a-2-3p,miR-218-2-3p,miR-28b,miR-1271-3p,miR-2284a,miR-4286,miR-181a-2-3p,miR-200c-3p,miR-2898,miR-543-5p,miR-200c,miR-376b-3p,miR-339a,miR-3965,miR-34c-5p,miR-215-5p,miR-615-5p,miR-370-3p,miR-598-3p,let-7g-3p,miR-485-5p,miR-671-5p,miR-7144-5p,miR-362-5p,miR-3154,miR-500b-5p,miR-103b,miR-6128,miR-28-5p,miR-486b-3p,miR-204-5p,miR-135a-1-3p,miR-331-3p,miR-9788-3p,miR-500-5p,miR-216a-3p,miR-2300a-5p,miR-222-5p,miR-2426,miR-3120-5p,miR-143-5p,miR-30b-3p,let-7c-3p,novel_124,miR-3085-3p,miR-5126,novel_103,miR-1306-5p,miR-326,miR-376d,miR-20a-3p,miR-3064-5p,miR-664b,miR-7977,novel_94,miR-361-3p,miR-362-5p,novel_60,miR-3059-5p,miR-452-5p,miR-2285f,miR-335,miR-2285t,miR-7134-3p,miR-2285n,miR-382-3p,miR-371a-5p,miR-222-5p,miR-383-5p,miR-532-3p,novel_83,miR-199a-3p,miR-548e-3p,miR-3600,miR-1b-3p,miR-215-5p,miR-5703,miR-1197-5p,miR-124-3p,miR-29b-1-5p,miR-146a-5p,miR-2432,miR-2887,miR-760-3p,miR-221-5p,miR-34b-5p,miR-6130,miR-323-5p,miR-192-5p,miR-132-5p,miR-877-5p,miR-204-3p,miR-22-3p,miR-140-5p,miR-221-5p,miR-182-5p,miR-2312,novel_99,miR-582-5p,miR-206-3p,miR-6134,miR-338-3p,miR-145a-3p,miR-4510,miR-1271,miR-6516-3p,miR-664a,miR-708-5p,miR-324-3p,miR-134-3p,miR-3431,miR-194-3p,miR-149-5p,novel_116,miR-339-5p,miR-542-3p,miR-708-3p,miR-432,novel_23,miR-22-3p,miR-2285r,let-7a-2-3p,miR-
OSBPL1A	ENSOARG0(	oxystero	miR-2332,miR-6402,miR-660-5p,miR-4286,miR-499a-5p,miR-660,novel_116

C4BPA	ENSOARG0(	compleme	miR-193b-3p,miR-29c-3p,novel_27,miR-301b-3p,miR-2428,miR-211,miR-323c,let-7i,miR-4324,miR-576-3p,miR-625-3p,miR-147-5p,novel_1,miR-29a,miR-10a-5p,miR-3120-3p,novel_68,miR-144-3p,miR-6535,miR-670-3p,miR-29a-3p,miR-3970,miR-150-5p,miR-1306,miR-216a-3p,miR-376b-3p,miR-376b-5p,miR-148a-3p,miR-2387,miR-18b,miR-135b-5p,let-7e-5p,miR-6516-3p,miR-412-3p,miR-382,miR-130b-5p,miR-493-5p,miR-2331-5p,novel_51,miR-125a-5p,miR-193a-3p,miR-130a-3p,miR-377-5p,miR-187-3p,miR-133b-5p,miR-2355-3p,miR-412-5p,miR-3535,miR-185-5p,miR-134-3p,let-7g-5p,miR-431-3p,miR-3071-5p,miR-708-3p,miR-1285,miR-1434-3p,miR-29b,miR-219a-1-3p,miR-1260b,miR-193a-5p,miR-133a-3p,miR-1260b,miR-144,miR-125b-5p,miR-135a-5p,miR-29b-3p,miR-486b-3p,miR-134-5p,miR-204-5p,miR-2355-5p,miR-215-5p,miR-1271,miR-125a,miR-376c-3p,miR-300-3p,miR-769,miR-200a-5p,miR-18a,miR-488-3p,miR-7144-5p,miR-23a-5p,miR-329b,miR-2385-3p,miR-4286,miR-181a-2-3p,let-7g,miR-2284y,miR-1957a,miR-1271-3p,miR-192-3p,miR-2478,let-7c-5p,miR-376b-3p,miR-1260a,miR-2285af,miR-125a-3p,miR-200c,miR-219-3p,miR-543-5p,miR-152-3p,miR-2898,miR-200c-3p,let-7f-5p,miR-18b-5p,miR-2285ad,miR-6517,miR-429-3p,miR-134-5p,miR-545-5p,novel_121,miR-29d-3p,miR-23b-5p,miR-134,miR-200b-3p,miR-128-3p,miR-10a-5p,let-7d,miR-582-3p,miR-452-5p,miR-2447,miR-219a-2-3p,miR-371a-5p,miR-769-5p,miR-107-5p,novel_82,miR-3968,miR-499a-5p,miR-541-5p,miR-7977,miR-211-5p,miR-2284g,miR-139-5p,let-7d-5p,miR-376d,miR-378g,miR-3082-5p,miR-361-3p,miR-140-5p,miR-376a-3p,miR-493-5p,miR-1983,miR-2284b,miR-1388-3p,let-7a-5p,miR-124a,miR-2330-5p,miR-29b-1-5p,miR-199a-3p,miR-1306-5p,miR-103a-2-5p,let-7e,miR-130b-3p,miR-5010-3p,miR-9788-3p,miR-148b-5p,miR-455-3p,miR-421,miR-376b-

ATXN7L3	ENSOARG0(	ataxin 7	miR-760-3p,miR-34b-5p,miR-221-5p,miR-30b-3p,miR-299-3p,miR-216a-5p,miR-125b,miR-2432,miR-2411,miR-378d,novel_133,miR-362-3p,miR-2300b-3p,miR-1246,miR-2904,miR-1197-5p,miR-5703,miR-140-3p,miR-487a-3p,miR-15a-5p,miR-532-3p,miR-3600,miR-485-3p,miR-450b-3p,miR-17-3p,miR-664a,miR-424-5p,miR-708-5p,miR-10b-5p,miR-338-3p,miR-378c,miR-145a-3p,miR-4510,miR-425-5p,let-7g-3p,miR-154b-3p,miR-544-3p,miR-27a-5p,miR-6130,novel_32,miR-204-3p,miR-221-5p,miR-378a-3p,miR-660-5p,miR-3065-3p,miR-3085-3p,miR-326,miR-1306-5p,miR-362-3p,miR-4532,miR-382-3p,miR-2330-5p,miR-424-5p,miR-378c,miR-544a,miR-484,miR-378b,miR-10b,miR-125b-2-3p,miR-30b-3p,miR-9788-3p,novel_4,miR-2300a-5p,miR-5010-3p,miR-2284q,miR-2285y,miR-541-3p,miR-1296-5p,miR-361-3p,miR-199b-5p,miR-378e,miR-3064-5p,miR-452-3p,miR-10b,miR-7977,miR-17-3p,miR-1260a,miR-6119-5p,miR-28b,miR-199a-5p,miR-3065-3p,miR-490-3p,miR-1271-3p,miR-2284a,miR-3529-3p,miR-4286,miR-181a-2-3p,miR-128-3p,miR-10a-5p,let-7a-2-3p,miR-134,miR-218-2-3p,miR-378h,miR-483-3p,miR-2411-5p,miR-545-5p,miR-423-3p,miR-138-5p,miR-204-5p,miR-486b-3p,miR-28-5p,miR-331-3p,miR-323-3p,miR-378c,let-7g-3p,miR-7144-5p,miR-23a-5p,miR-485-5p,miR-488-3p,miR-3154,miR-34c-5p,miR-199a-5p,miR-487a-3p,miR-370-3p,miR-125a-5p,miR-660,miR-197-5p,miR-330-3p,miR-654-3p,miR-539-3p,miR-130b-5p,miR-363-5p,miR-138,miR-30b-3p,miR-1260b,miR-7862,miR-374b-3p,miR-145a-5p,miR-185-5p,miR-544b,miR-133b-5p,miR-377-5p,novel_1,miR-15a,miR-502-5p,miR-6402,miR-34c,miR-16a,miR-211,miR-371b-3p,miR-323c,miR-1307-3p,miR-24-3p,miR-326-3p,novel_27,miR-449a,miR-2428,miR-135a-2-3p,miR-2285x,novel_101,miR-17-3p,miR-1291,miR-450b-3p,miR-7975,miR-6395,miR-195a-

FAM216B	ENSOARG0(	family w	novel_83,miR-148b-3p,miR-340-5p,miR-15a-5p,miR-3600,miR-548e-3p,miR-148a-5p,miR-34a-5p,miR-16-1-3p,miR-1197-5p,miR-5703,miR-2904,miR-2300b-3p,miR-376a-5p,novel_133,miR-143-3p,miR-2432,miR-181b-1-3p,miR-345-3p,miR-183-5p,miR-329a-5p,miR-34b-5p,miR-466i-5p,miR-202-5p,miR-345-3p,miR-185-3p,miR-500a-5p,miR-2285p,miR-196a-3p,miR-15b-5p,miR-6130,miR-6516,miR-301a-3p,miR-532-5p,miR-3065-3p,miR-216b-3p,miR-30c-1-3p,miR-140-5p,miR-1827,miR-345-5p,miR-18a-5p,miR-2312,miR-433-3p,miR-2285c,miR-450a-1-3p,miR-21a-3p,miR-6134,miR-3184-5p,miR-224-5p,miR-129-2-3p,miR-4510,miR-539-5p,miR-454-3p,miR-4492,miR-424-5p,miR-17-3p,miR-664a,miR-450b-3p,miR-29b-2-5p,miR-665,miR-320e,miR-222-3p,miR-2285e,miR-421,miR-9788-3p,miR-148b-5p,miR-221-3p,miR-455-3p,miR-147a,miR-376b-3p,miR-181b-2-3p,miR-145b,miR-2300a-5p,novel_4,miR-409b,miR-330-3p,miR-2284aa,miR-1388-5p,miR-758-3p,miR-30b-3p,miR-501-3p,miR-2284r,miR-484,miR-491-3p,miR-758-3p,miR-1b-5p,miR-2310,miR-30c-1-3p,miR-424-5p,miR-130b-3p,miR-16b,miR-2957,miR-326,miR-376d,miR-500-3p,miR-489,miR-499a-3p,miR-7857-3p,miR-126b-5p,miR-499b-5p,miR-1983,miR-376a-3p,miR-221,miR-140-5p,miR-361-3p,miR-502-3p,miR-423-5p,novel_111,miR-9-5p,miR-219a-5p,miR-452-5p,miR-2285f,miR-212-3p,miR-374a-3p,miR-335,miR-4726-5p,miR-2440,miR-129-1-3p,miR-450a-1-3p,miR-582-3p,miR-2284z,miR-545-5p,miR-6516-5p,miR-322-5p,miR-9-5p,miR-505-5p,miR-18b-5p,miR-6517,miR-222,miR-128-3p,miR-330-5p,miR-501-3p,miR-29d-3p,miR-345-3p,miR-3065-3p,novel_39,miR-148b-5p,miR-29b-2-5p,miR-152-3p,miR-107,miR-127-5p,miR-17-3p,miR-30c-2-3p,miR-2397-5p,miR-6119-5p,miR-376b-3p,miR-133c,miR-34c-5p,miR-2285aa,miR-1814c,miR-7144-5p,miR-345-5p,miR-767,miR-876-3p,miR-18a,miR-301,miR-145-

YIPF3	ENSOARG0(	Yip1 dom	miR-17-3p,novel_101,miR-449a,novel_27,miR-2428,miR-34c,miR-382-5p,miR-425-5p,miR-576-3p,miR-128-1-5p,miR-320b,novel_68,miR-146b-3p,miR-2404,miR-299b-3p,novel_69,miR-34b,miR-18b,miR-148a-3p,miR-378f,miR-363-5p,miR-668-3p,miR-628-5p,miR-539-3p,miR-432-3p,miR-2284l,miR-197-5p,miR-6238,miR-149-5p,miR-544b,miR-432-5p,miR-185-5p,miR-134-3p,miR-3071-5p,miR-145a-5p,miR-432,miR-19b-2-5p,miR-138,miR-219a-1-3p,miR-133a-3p,miR-378c,miR-145-3p,miR-6740-5p,miR-135a-1-3p,miR-410-3p,miR-31-3p,miR-3432a,miR-28-5p,miR-138-5p,miR-598-3p,miR-615-5p,miR-3956,miR-487a-3p,miR-874-3p,miR-324-5p,miR-300-3p,miR-34c-5p,miR-137-3p,miR-18a,miR-488-3p,miR-7144-5p,miR-10b-3p,miR-876-3p,miR-30a-3p,miR-3064-5p,miR-181a-2-3p,miR-4286,novel_39,miR-30c-2-3p,miR-125a-3p,novel_96,miR-152-3p,miR-17-3p,miR-450a-2-3p,miR-412,miR-18b-5p,miR-299a-3p,miR-378h,miR-3591-5p,miR-6527,miR-1296-5p,miR-4454,miR-3059-5p,miR-361-3p,miR-2284q,miR-154-3p,miR-499b-5p,miR-3064-5p,miR-4792,miR-378e,miR-378g,miR-140-5p,miR-544-5p,miR-877-3p,miR-652-5p,miR-376b,miR-4532,miR-30c-1-3p,miR-378d,novel_19,miR-3085-3p,novel_124,miR-491-5p,miR-487b-5p,miR-3955-3p,miR-145b,miR-412-3p,miR-378b,miR-296-5p,miR-101c,miR-3120-5p,miR-378c,miR-544a,miR-2330-3p,miR-378c,miR-539-5p,miR-145a-3p,miR-346,miR-30a-3p,miR-19b-1-5p,miR-665,miR-320e,miR-30e-3p,miR-874-5p,miR-487a-5p,miR-708-5p,miR-17-3p,miR-487b-5p,miR-154a-3p,miR-140-5p,miR-30c-1-3p,miR-2331-3p,miR-378a-3p,novel_32,miR-151b,miR-544-3p,miR-598-3p,miR-625-5p,miR-133b-3p,miR-18a-5p,miR-154b-3p,miR-487a,miR-324-3p,miR-216a-5p,miR-422a,miR-196a-3p,novel_17,miR-210-5p,miR-34b-5p,miR-299-3p,miR-485-3p,miR-30d-3p,novel_83,miR-151-5p,miR-1247-5p,miR-487a-

FAM58A	ENSOARG0(	family w	miR-378h,miR-1271-5p,miR-134-5p,miR-2284z,miR-2285ad,miR-6517,miR-412,miR-7641,miR-18b-5p,miR-128-3p,miR-615-3p,miR-345-3p,miR-134,miR-330-5p,miR-2478,miR-199a-5p,novel_39,miR-2284y,miR-29a-5p,miR-2285b,miR-2284a,miR-450a-2-3p,miR-2898,miR-1a-2-5p,novel_127,miR-543-5p,novel_96,miR-1260a,miR-199a-5p,miR-2284n,miR-2355-5p,miR-370-3p,miR-485-5p,miR-671-5p,miR-18a,miR-1260b,miR-378c,novel_42,miR-2284v,miR-592,miR-134-5p,miR-2284k,miR-1-5p,miR-370-5p,miR-409-3p,miR-412-5p,miR-2284d,miR-134-3p,miR-194-3p,miR-31-5p,miR-2284ab,miR-1260b,miR-708-3p,miR-653,miR-542-3p,miR-432-3p,miR-6240,miR-193a-3p,miR-3141,miR-330-3p,miR-2411-3p,miR-411b-3p,miR-669,miR-3074-5p,miR-1291,miR-9851-3p,miR-505-3p,miR-378f,miR-18b,miR-3141,miR-216a-3p,miR-3591-3p,miR-18a-3p,miR-326-3p,novel_27,miR-199b-5p,miR-1a-1-5p,miR-193b-3p,miR-2285x,miR-1193,miR-421-5p,miR-6123,miR-576-3p,miR-382-5p,miR-95-3p,miR-23c,miR-532-3p,miR-2284h-5p,miR-18a-3p,miR-505-3p,miR-212-5p,miR-493-3p,miR-378d,miR-2284u,novel_133,miR-378i,miR-2904,miR-378a-3p,miR-140-3p,miR-1193,miR-188-3p,miR-2285w,miR-2284m,miR-30b-3p,miR-500a-5p,miR-422a,miR-345-3p,miR-185-3p,miR-193b-3p,miR-6130,miR-2331-3p,miR-378a-3p,miR-21-3p,miR-370-5p,miR-18a-5p,miR-543-3p,miR-346,miR-338-3p,miR-378c,miR-539-5p,miR-4510,miR-3074-5p,miR-1271,miR-2330-3p,miR-4492,miR-874-5p,miR-216a-3p,miR-9788-3p,miR-330-3p,miR-378c,miR-2284aa,miR-378b,miR-143-5p,miR-412-3p,miR-323b,miR-758-3p,miR-125b-2-3p,miR-378d,miR-8485,miR-3187-3p,miR-758-3p,miR-2330-5p,miR-382-3p,miR-2284b,miR-491-5p,miR-199b-5p,miR-103a-2-5p,miR-326,novel_115,miR-323b-3p,miR-378e,miR-378g,miR-489,miR-203-3p,miR-2284g,miR-541-5p,miR-7977,miR-96-5p,miR-

PSMA6	ENSOARG0(	proteaso	miR-106a-5p,miR-17-5p,miR-1343-5p,let-7f-2-3p,let-7f-2-3p,miR-382,miR-2284l,miR-654-3p,miR-432-3p,miR-223-3p,miR-1434-3p,miR-145a-5p,miR-16-1-3p,miR-219a-1-3p,miR-380-3p,miR-29b,miR-16b,miR-497-5p,miR-31-5p,miR-339-5p,miR-3431,miR-20b,novel_116,miR-20a-5p,miR-6402,miR-15a,miR-16a,miR-206,miR-29a,miR-22-5p,miR-656-5p,miR-625-3p,miR-203b-5p,miR-335-5p,miR-1a-1-5p,miR-29c-3p,miR-1193,miR-24-3p,miR-211,miR-2284w,miR-326-3p,miR-148a-3p,miR-3065-5p,miR-412-3p,miR-195a-5p,miR-2319a,miR-3074-5p,miR-26b-5p,miR-10a-5p,miR-29a-3p,miR-374c-3p,miR-6119-5p,miR-219-3p,miR-2285af,miR-217,miR-339a,miR-30c-2-3p,miR-127-5p,miR-1a-2-5p,miR-152-3p,miR-1,miR-30a-3p,miR-495-5p,miR-29b-2-5p,miR-3065-3p,miR-1957a,miR-1290,miR-30a-5p,miR-330-5p,miR-1343-5p,miR-29d-3p,miR-10a-5p,miR-155-5p,miR-30c,novel_79,miR-322-5p,miR-214-5p,miR-370-5p,miR-29b-3p,miR-409-3p,miR-331-3p,novel_9,miR-204-5p,miR-16-5p,miR-3607-3p,miR-1197-3p,miR-93,miR-30e-5p,miR-23b,miR-17-5p,miR-93-5p,miR-20b-5p,miR-365a-3p,miR-3959-5p,novel_103,miR-326,miR-106b-5p,miR-16b,miR-2957,miR-424-5p,miR-106a-5p,miR-376b,miR-23a-3p,miR-30c-1-3p,miR-1b-5p,miR-8485,miR-10b,miR-484,miR-125b-2-3p,miR-1a-3p,miR-16-2-3p,miR-151a-3p,miR-145b,miR-5010-3p,miR-421,miR-1185-3p,miR-101-3p,miR-30b-5p,miR-335,miR-499a-5p,miR-2447,novel_111,miR-219a-2-3p,miR-423-5p,miR-504,miR-1983,novel_94,miR-2319b,miR-139-5p,miR-211-5p,miR-7689-3p,miR-3082-5p,miR-1185-2-3p,miR-618,miR-2285p,miR-26a-5p,miR-30b,miR-106a,miR-221-5p,miR-30d,miR-20a,miR-195a-3p,miR-130a-5p,miR-380-3p,miR-30c-5p,miR-5703,miR-217-5p,miR-16-1-3p,miR-106a,miR-30d-3p,miR-3600,miR-148b-3p,miR-23c,miR-15a-5p,miR-23b-3p,miR-30e-3p,miR-2285j,miR-29b-2-5p,miR-664a-
PGM3	ENSOARG0(	phosphog	miR-212-3p,miR-29b-2-5p,miR-3607-3p,miR-142a-3p,miR-29b-1-5p,miR-200a,miR-5100,miR-29b-2-5p,miR-29b-1-5p,miR-30d,miR-142-3p,miR-216a-3p,miR-455-3p,miR-199a-3p,miR-30e-5p,miR-30d-5p,miR-582-3p

HLA-DOB	ENSOARG0(	major hi	miR-219b-5p,miR-500a-5p,miR-345-3p,miR-122-3p,miR-210-5p,miR-2424,miR-329a-5p,miR-503-5p,miR-2285w,miR-361-5p,miR-2284u,miR-148a-5p,miR-2284h-5p,miR-23b-3p,miR-7859,miR-23c,miR-4492,novel_74,miR-665,miR-3604,miR-2285j,miR-181c-5p,miR-665-5p,miR-625-5p,miR-885-3p,miR-1895,miR-1827,miR-186-5p,miR-339b,miR-345-5p,miR-129b-3p,miR-450a-1-3p,miR-499b-3p,miR-2284s,miR-377-3p,miR-10a,miR-582-5p,miR-30c-1-3p,miR-1277-3p,miR-29b-1-5p,miR-323b-3p,miR-182-5p,miR-23a-3p,miR-744-3p,miR-2284b,miR-101b-3p,miR-21b,miR-362-3p,miR-758-3p,miR-2284r,miR-329-3p,miR-455-3p,miR-148b-5p,miR-1185-3p,miR-147a,miR-3964,miR-628-3p,novel_82,miR-2440,miR-101-3p,miR-877-3p,miR-2284j,miR-493-5p,miR-1983,miR-381-3p,miR-203-3p,miR-1185-2-3p,miR-499a-3p,miR-211-5p,miR-139-5p,miR-2284g,miR-2319b,miR-200a-3p,miR-30f,miR-200a,miR-2284y,miR-450c-5p,miR-3064-5p,miR-885-3p,miR-196a-5p,miR-1290,miR-450a-1-3p,miR-134-5p,miR-758-5p,novel_79,miR-1224-5p,miR-299a-3p,miR-134-5p,miR-2284k,miR-193a-5p,miR-2284v,miR-345-5p,miR-329b,miR-329a,miR-23b,miR-2285aa,miR-503-3p,miR-874-3p,miR-196b-5p,miR-483-3p,miR-365b-5p,novel_51,miR-2411-3p,miR-7-5p,miR-377-3p,let-7j,miR-432-3p,miR-2285r,miR-432-3p,miR-493-5p,miR-4508,miR-2284x,miR-27b-3p,miR-450c-3p,miR-7b-5p,novel_116,miR-3431,miR-339-5p,miR-3955-5p,miR-31-5p,miR-6119-3p,miR-29a,miR-576-3p,miR-28c,miR-29c-3p,miR-412-3p,miR-150-5p,miR-3591-3p,miR-320c,miR-144-3p,miR-374c-3p,miR-299b-3p,miR-3120-3p,miR-329-5p,miR-30b-3p,miR-299-3p,miR-21-3p,miR-2284m,miR-320d,miR-466i-5p,miR-760-3p,miR-216a-5p,miR-185-3p,miR-29b-1-5p,miR-130a-5p,miR-19b-3p,miR-4429,miR-5703,miR-140-3p,miR-1193,novel_133,miR-362-3p,miR-2300b-3p,miR-215-5p,miR-
DNAJB6	ENSOARG0(	DnaJ (Hs	miR-214,miR-141-3p,miR-221-5p,miR-429-3p,miR-200b-3p,miR-221-5p,miR-214-3p,miR-21-3p,miR-200a-3p,miR-138-5p,miR-138,miR-200c-3p,miR-200c

DCAF15	ENSOARG0(	DDB1 and	miR-320b,let-7d-5p,miR-6535,novel_69,miR-378g,miR-129b-5p,miR-1306,miR-2387,miR-3141,miR-361-3p,miR-140-5p,let-7e-5p,miR-1983,miR-195a-5p,miR-412-3p,miR-668-5p,miR-9-5p,miR-541-3p,miR-3956-3p,novel_60,miR-423-5p,miR-326-3p,miR-371b-3p,miR-361-3p,let-7i,miR-16a,novel_82,miR-15a,miR-4726-5p,miR-5010-3p,miR-1343-3p,miR-2285e,miR-134-3p,let-7g-5p,miR-147a,miR-496-5p,miR-2285g,novel_23,miR-542-3p,miR-541,miR-7862,miR-744-5p,miR-497-5p,miR-4508,miR-16b,miR-628-5p,miR-424-5p,let-7a-5p,miR-1388-3p,novel_51,miR-16b,miR-3141,miR-29b-1-5p,novel_103,miR-1306-5p,miR-5126,miR-326,let-7e,miR-125a-5p,miR-483-3p,miR-196b-5p,miR-1271,miR-215-5p,miR-125a,miR-370-3p,miR-140-5p,miR-15b-5p,let-7i-5p,novel_32,miR-192-5p,miR-2285c,miR-1827,miR-885-3p,miR-1895,miR-665-5p,miR-16-5p,let-7b,let-7b-5p,miR-3184-5p,miR-665,miR-125b-5p,miR-331-3p,let-7f,miR-486b-3p,miR-424-5p,miR-874-5p,miR-4492,miR-214-5p,miR-582-3p,miR-532-3p,miR-483-3p,miR-15a-5p,miR-9-5p,miR-322-5p,miR-330-5p,miR-2904,miR-1961,miR-215-5p,miR-2408,miR-196a-5p,novel_133,miR-378d,miR-582-3p,let-7d,miR-885-3p,miR-4286,miR-486-3p,let-7g,miR-29b-1-5p,miR-185-3p,miR-125b,let-7c-5p,miR-324-3p,let-7k,miR-466i-5p,miR-98-5p,let-7f-5p
PLXNA1	ENSOARG0(	plexin A	miR-2284r,miR-2904,miR-191-3p,miR-504,miR-2285k,miR-3085-3p,miR-4492,miR-874-3p,miR-504-5p,miR-3064-5p,miR-3064-5p,miR-2285i,miR-1271-3p,miR-151b,miR-151-5p

CASP3	ENSOARG0(	caspase	miR-382-5p,let-7i,miR-4324,miR-199c,miR-22-5p,miR-17-3p,miR-342-3p,miR-3959-3p,miR-24-3p,miR-371b-3p,miR-1306,miR-2387,let-7e-5p,miR-146b-5p,miR-9851-3p,miR-412-3p,miR-450b-3p,miR-3120-3p,miR-26b-5p,miR-329-5p,novel_68,let-7d-3p,miR-146a,miR-7-5p,miR-101-5p,miR-2285r,miR-181a-5p,miR-7b-5p,miR-432,miR-2889,miR-2284x,miR-138,miR-544b,miR-194-3p,miR-3431,let-7g-5p,miR-432-5p,novel_63,miR-2284k,miR-134-5p,miR-374b-3p,miR-138-5p,miR-374a-3p,miR-137-3p,miR-381-3p,miR-30e-5p,miR-329a,miR-329b,miR-1271,miR-874-3p,miR-769,let-7c-5p,miR-30f,miR-125a-3p,miR-664-3p,let-7f-5p,miR-181b-5p,miR-17-3p,miR-2284a,let-7g,miR-3065-3p,miR-2284y,miR-30a-5p,miR-2427,miR-134,miR-3591-5p,miR-30c,miR-300,let-7d,miR-6517,miR-129-1-3p,novel_121,miR-134-5p,miR-2411-5p,miR-107-5p,miR-301a-5p,miR-769-5p,miR-371a-5p,miR-30b-5p,miR-374a-3p,miR-181d-5p,miR-452-5p,miR-1983,miR-544-5p,miR-299b-5p,miR-20a-3p,miR-452-3p,miR-664b,let-7d-5p,miR-3596,miR-7857-3p,miR-499a-3p,miR-362-3p,miR-1306-5p,miR-342,miR-199a-3p,miR-103a-2-5p,let-7e,miR-146b,miR-2284b,miR-382-3p,let-7a-5p,miR-574-3p,miR-491-3p,miR-8485,miR-301b-5p,miR-484,miR-329-3p,miR-544a,miR-362-3p,miR-199b-3p,miR-1343-3p,novel_78,miR-216a-3p,let-7f,miR-2285j,miR-17-3p,miR-129-2-3p,let-7b,miR-181c-5p,let-7b-5p,miR-769-5p,miR-30d-5p,miR-21a-3p,miR-552-3p,miR-3065-3p,let-7i-5p,miR-499b-3p,miR-544-3p,miR-26a-5p,miR-30b,let-7k,miR-30d,miR-3653-3p,miR-760-3p,miR-98-5p,miR-2284m,miR-329a-5p,miR-216c-5p,miR-146a-5p,miR-2411,miR-188-3p,miR-362-3p,miR-1961,miR-30c-5p,novel_133,miR-378d,miR-548e-3p,miR-199a-3p,miR-7859
CCDC122	ENSOARG0(	coiled-c	miR-454-3p,miR-204-5p,miR-191-5p,novel_63,miR-29b-3p,miR-30e-3p,miR-324-3p,miR-30a-3p,miR-100-3p,miR-10b-5p,miR-93,miR-1197-3p,miR-224-5p,miR-144-5p,miR-18a-5p,miR-20b-5p,miR-625-5p,miR-671-5p,novel_99,miR-488-3p,miR-93-5p,miR-18a,miR-17-5p,miR-433-3p,miR-10a,miR-301a-3p,miR-216b-5p,novel_25,miR-204-3p,miR-3065-3p,miR-141-3p,miR-532-5p,miR-2898,miR-106a,miR-200c-3p,miR-21-3p,miR-200a-3p,miR-152-3p,miR-200c,novel_17,miR-148b-5p,miR-200a,miR-3065-3p,miR-192-3p,miR-20a,miR-30a-3p,miR-200b-3p,miR-148a-5p,miR-10a-5p,miR-615-3p,miR-191,miR-1246,miR-29d-3p,miR-23b-5p,miR-505-5p,miR-302a-5p,miR-200b,miR-148b-3p,miR-1247-5p,novel_121,miR-106a,miR-429-3p,miR-6517,miR-30d-3p,miR-18b-5p,miR-335,miR-29a,miR-2285t,miR-615,miR-20a-5p,miR-301a-5p,miR-211,miR-301b-3p,miR-2447,miR-342-3p,miR-3059-5p,miR-885-5p,miR-335-5p,miR-29c-3p,novel_94,miR-148a-3p,miR-18b,miR-29a-3p,miR-144-5p,miR-2319b,miR-10b,miR-320b,miR-499b-5p,miR-10a-5p,miR-211-5p,miR-130b-3p,miR-342,miR-296-3p,miR-106b-5p,miR-106a-5p,miR-17-5p,miR-2331-5p,miR-296-3p,miR-503-3p,miR-106a-5p,miR-676-3p,miR-101-5p,miR-668-3p,miR-363-5p,miR-29b,miR-101c,miR-10b,miR-301b-5p,miR-374b-3p,miR-3431,miR-148b-5p,novel_116,miR-20b,miR-421,miR-1343-3p,miR-130a-3p

SPTA1	ENSOARG0(	spectrin	miR-320a,miR-23b,miR-136-5p,miR-10b-3p,miR-6516-3p,miR-1248,miR-543-3p,miR-3074-1-3p,miR-7975,miR-20a-3p,miR-299b-3p,miR-376e-3p,miR-376c-3p,miR-877-5p,miR-22-3p,miR-144-3p,miR-301a-5p,miR-144,miR-26c,miR-2285y,miR-155-5p,novel_1,novel_74,miR-31-3p,miR-6525,miR-301b-5p,miR-7b-5p,miR-1290,miR-873a-5p,miR-188-3p,miR-22-3p,miR-323b,miR-4429,miR-155-5p,miR-299a-3p,miR-2285ad,miR-23c,miR-23b-3p,miR-2355-3p,novel_116,miR-205-5p,miR-7-5p,miR-320b,miR-323b-3p,miR-330-3p,miR-3955-3p,miR-491-5p,miR-2484,miR-299-3p,novel_96,miR-6240,miR-101b-3p,miR-33a-5p,miR-181a-2-3p,miR-23a-3p,miR-376a-5p,miR-143-3p,miR-8485
NFKBIA	ENSOARG0(	NF-kappa	miR-145b,miR-181b-2-3p,novel_4,miR-31-5p,miR-421,miR-544b,miR-9788-3p,miR-125b-2-3p,miR-145a-5p,miR-30b-3p,miR-541,miR-329-3p,miR-4508,miR-544a,miR-362-3p,miR-138,miR-30b-3p,miR-668-3p,miR-130b-5p,miR-1b-5p,miR-8485,miR-539-3p,miR-432-3p,novel_51,miR-362-3p,miR-1306-5p,miR-320b,miR-103a-2-5p,miR-106b-3p,miR-196b-5p,miR-211-5p,miR-499b-5p,miR-2319b,miR-378b,miR-329-5p,miR-20a-3p,miR-181b-3p,miR-677,miR-320c,miR-34b,miR-411b-3p,miR-670-3p,miR-381-3p,miR-1306,miR-18b,miR-505-3p,miR-2285x,miR-541-3p,miR-2284w,miR-449a,miR-877-3p,miR-211,miR-107-5p,miR-34c,miR-769-5p,miR-1307-5p,miR-101-3p,miR-4324,miR-147-5p,novel_1,miR-505-3p,miR-18b-5p,miR-485-3p,miR-532-3p,miR-487a-3p,miR-1193,miR-188-3p,miR-362-3p,miR-196a-5p,let-7a-3p,miR-34a-5p,miR-300,miR-4429,miR-345-3p,miR-329a-5p,miR-1271-3p,miR-29a-5p,miR-181b-1-3p,miR-130a-5p,miR-185-3p,miR-3071-3p,miR-21-3p,miR-34b-5p,miR-221-5p,miR-320d,miR-221-5p,miR-769,miR-544-3p,miR-2284s,miR-34c-5p,miR-320a,miR-18a,miR-129b-3p,miR-381-3p,miR-488-3p,miR-485-5p,miR-23a-5p,miR-329b,miR-329a,miR-18a-5p,miR-181a-2-3p,miR-1306-5p,miR-6134,miR-769-5p,miR-3969,miR-3604,miR-132-5p,miR-2332,miR-130b-5p,miR-204-5p,miR-129-5p,miR-664a,miR-138-5p
POLR1C	ENSOARG0(	polymera	miR-455-3p,miR-328-3p,novel_78,miR-1343-3p,miR-133b-5p,miR-30d-3p,miR-1260b,miR-4429,miR-128-3p,miR-22-3p,miR-432-3p,miR-432-3p,miR-486-3p,miR-3187-3p,miR-668-3p,miR-5100,miR-30a-3p,miR-181a-2-3p,miR-320d,miR-124-5p,miR-127-5p,miR-221-5p,miR-320b,miR-1260a,miR-210-5p,miR-216a-5p,miR-499a-3p,miR-499b-3p,miR-411b-3p,miR-320c,miR-22-3p,miR-454-5p,miR-221-5p,miR-452-3p,miR-2355-5p,miR-1271,miR-532-5p,miR-9-3p,miR-6395,miR-361-3p,miR-301,miR-216a-3p,miR-320a,miR-877-3p,miR-361-3p,miR-24-3p,miR-374a-3p,miR-1260b,miR-2428,miR-335-5p,miR-145a-3p,miR-2285x,novel_74,miR-664a,miR-335,miR-374a-3p,miR-874-5p,miR-486b-3p,miR-374b-3p,miR-101-3p,miR-30e-3p,miR-30a-3p,miR-320e

IL6ST	ENSOARG0(	interleu	miR-301a-3p,miR-877-5p,miR-21a-3p,miR-433-3p,miR-411,miR-129b-3p,miR-590-3p,miR-665-5p,miR-346,miR-320e,miR-6516-3p,miR-487b-5p,miR-454-3p,miR-4492,miR-129-5p,miR-708-5p,miR-106a,miR-148b-3p,miR-23c,miR-23b-3p,miR-199a-3p,miR-1434-5p,miR-4429,miR-33b-3p,miR-19b-3p,miR-493-3p,miR-361-5p,miR-218-1-3p,miR-345-3p,miR-20a,miR-421-5p,miR-29b-1-5p,miR-219b-5p,miR-500a-5p,miR-106a,miR-320d,miR-299-3p,miR-30b-3p,miR-452-3p,miR-24-1-5p,miR-664b,miR-499b-5p,miR-541-5p,miR-3082-5p,miR-7857-3p,miR-378g,miR-1185-2-3p,miR-4443,miR-668-5p,miR-544-5p,novel_94,miR-493-5p,miR-219a-5p,miR-452-5p,miR-194b-5p,novel_111,miR-8095,miR-2440,miR-3968,miR-421,miR-1185-3p,miR-106a-5p,miR-382-3p,miR-23a-3p,miR-2310,miR-320b,miR-199a-3p,miR-106b-5p,miR-29b-1-5p,miR-130b-3p,miR-1185-5p,miR-874-3p,miR-503-3p,miR-574-5p,miR-320a,miR-17-5p,miR-93-5p,miR-200a-5p,miR-2318,miR-329b,miR-767,miR-1814c,miR-7144-5p,novel_48,miR-20b-5p,miR-3074-1-3p,miR-3607-3p,miR-93,miR-2332,miR-5100,novel_9,miR-767-5p,miR-28-5p,miR-429-3p,miR-299a-3p,miR-411-3p,miR-24-2-5p,miR-2285b,miR-200c,miR-664-3p,miR-200c-3p,miR-152-3p,miR-3120-3p,miR-670-3p,miR-299b-3p,miR-320c,miR-148a-3p,miR-3065-5p,miR-19a-3p,miR-6516-3p,miR-495-3p,miR-301b-3p,novel_27,miR-20a-5p,miR-379-3p,miR-421-5p,miR-22-5p,miR-3955-5p,miR-31-5p,miR-130a-3p,miR-134-3p,miR-2355-3p,miR-20b,miR-223-3p,miR-194-5p,miR-493-5p,miR-628-5p,miR-2284l,miR-194a,miR-17-5p,miR-106a-5p,miR-197-5p

TMEM61	ENSOARG0(	transmem	let-7f-5p,novel_96,miR-152-3p,novel_127,miR-3064-5p,miR-29a-5p,let-7g,novel_39,miR-1343-5p,miR-330-5p,miR-155-5p,miR-299a-3p,miR-6517,novel_79,miR-505-5p,miR-2284z,miR-134-5p,novel_121,miR-2284k,miR-134-5p,miR-30e-5p,miR-93-5p,miR-767,miR-329b,miR-20b-5p,miR-1271,miR-146a,miR-105-5p,miR-365b-5p,novel_51,miR-380-5p,miR-668-3p,miR-2285r,miR-432-3p,let-7j,miR-432,miR-1843b-5p,miR-2113,miR-16-1-3p,miR-27b-3p,miR-188-5p,miR-339-5p,miR-134-3p,miR-194-3p,miR-149-5p,miR-576-3p,miR-28c,miR-142b,miR-425-5p,miR-6119-3p,miR-29a,miR-625-3p,miR-421-5p,miR-758-5p,miR-29c-3p,miR-301b-3p,miR-148a-3p,miR-1306,miR-3591-3p,miR-412-3p,miR-146b-5p,miR-2319a,miR-378f,let-7e-5p,novel_68,miR-216b-3p,miR-3120-3p,miR-411b-3p,novel_69,miR-299b-3p,miR-6535,miR-2285p,miR-324-3p,miR-422a,miR-98-5p,let-7k,miR-421-5p,miR-486-3p,miR-143-3p,miR-30c-5p,miR-378i,miR-1961,miR-217-5p,miR-378a-3p,miR-33b-3p,miR-184-3p,miR-16-1-3p,miR-34a-5p,miR-361-5p,miR-18a-3p,miR-106a,miR-380-5p,miR-30d-3p,miR-210-3p,miR-328-3p,let-7f,miR-30e-3p,miR-30a-3p,miR-665,miR-500,miR-874-5p,miR-4492,miR-454-3p,let-7b,miR-665-5p,miR-6525,miR-224-5p,miR-3184-5p,miR-500a-3p,miR-30d-5p,miR-339b,miR-625-5p,miR-1895,miR-1827,miR-504-5p,miR-548o-3p,miR-216b-3p,miR-552-3p,miR-296-3p,miR-301a-3p,miR-499b-3p,let-7i-5p,miR-106b-5p,miR-182-5p,miR-491-5p,miR-130b-3p,miR-146b,miR-124a,miR-1388-3p,miR-378d,miR-8485,miR-2310,miR-199b-3p,miR-1343-3p,novel_78,miR-2459,miR-3968,miR-2447,miR-2284j,miR-877-3p,miR-361-3p,miR-1285-5p,miR-423-5p,miR-221,miR-378j,miR-504,miR-335-3p,miR-1983,miR-4792,miR-2319b,miR-378g,miR-499a-3p,miR-217,let-7c-5p,miR-339a,miR-17-3p,miR-30a-3p,miR-181a-2-3p,miR-4286,miR-380-5p,miR-28b,miR-3065-3p,miR-
CDK5RAP3	ENSOARG0(	CDK5 reg	miR-296-5p,miR-873a-5p

PLEKHN1	ENSOARG0(	pleckstr	miR-15a-5p,miR-328-3p,miR-18a-3p,miR-212-5p,miR-34a-5p,miR-133a-3p,novel_133,miR-378i,miR-378a-3p,miR-1197-5p,miR-29b-1-5p,miR-2285i,miR-486-3p,miR-202-5p,miR-34b-5p,miR-2285p,miR-125b,miR-185-3p,miR-422a,miR-324-3p,miR-216a-5p,miR-323-5p,miR-6130,miR-6516,miR-377-3p,miR-15b-5p,miR-504-5p,miR-140-5p,miR-378a-3p,miR-552-3p,miR-3065-3p,miR-345-5p,miR-133b-3p,miR-1827,novel_44,miR-3184-5p,miR-4510,miR-3074-5p,miR-378c,miR-1271,miR-664a,miR-487b-5p,miR-17-3p,miR-130b-5p,miR-708-5p,miR-874-5p,miR-424-5p,miR-4492,miR-320e,miR-29b-2-5p,miR-324-3p,miR-147a,miR-2459,miR-409b,miR-378c,miR-541,miR-301b-5p,miR-378b,miR-296-5p,miR-378d,miR-1b-5p,miR-8485,miR-1839-3p,miR-424-5p,miR-487b-5p,miR-3085-3p,miR-326,miR-342,miR-16b,miR-29b-1-5p,miR-378g,miR-378e,miR-4443,miR-3064-5p,miR-7977,miR-504,miR-96-5p,miR-423-5p,miR-3956-3p,miR-541-3p,novel_60,miR-452-5p,miR-4726-5p,miR-218-5p,miR-301a-5p,miR-6516-5p,miR-1271-5p,miR-378h,miR-322-5p,miR-6517,miR-1224-5p,miR-23b-5p,miR-330-5p,miR-29d-3p,novel_39,miR-29a-5p,miR-3065-3p,miR-181a-2-3p,miR-29b-2-5p,miR-3064-5p,miR-17-3p,miR-2898,miR-543-5p,novel_127,miR-125a-3p,miR-34c-5p,miR-154b-5p,miR-615-5p,miR-125a,miR-2355-5p,miR-345-5p,miR-485-5p,miR-378c,miR-133a-3p,miR-6128,miR-16-5p,miR-138-5p,miR-486b-3p,miR-28-5p,miR-29b-3p,miR-2433,miR-331-3p,miR-125b-5p,miR-134-3p,miR-412-5p,miR-544b,miR-2355-3p,miR-149-5p,miR-133b-5p,miR-340-3p,miR-16b,miR-138,miR-29b,miR-7862,miR-497-5p,miR-432-3p,miR-345-5p,miR-130b-5p,miR-363-5p,miR-378a-5p,miR-125a-5p,miR-377-3p,novel_51,miR-29a-3p,miR-34b,miR-323a-5p,miR-146b-3p,miR-669,miR-3074-5p,miR-216b-3p,miR-2285k,miR-6395,miR-195a-5p,miR-378f,miR-2319a,miR-2387,miR-18a-3p,miR-326-3p,miR-

ADCYAP1R1	ENSOARG0(	adenylat	miR-708-5p,miR-874-5p,miR-664a,miR-320e,miR-665,miR-655-3p,miR-324-3p,miR-2285j,miR-6134,miR-206-3p,miR-346,miR-6525,miR-665-5p,miR-3184-3p,miR-145a-3p,miR-129-2-3p,miR-378c,miR-1827,miR-543-3p,miR-625-5p,miR-345-5p,miR-18a-5p,miR-2312,miR-411,miR-655,miR-129b-3p,miR-22-3p,miR-27a-5p,miR-376e-3p,miR-552-3p,miR-182-5p,miR-21-3p,miR-496-3p,miR-504-5p,miR-378a-3p,miR-299-3p,miR-30b-3p,miR-760-3p,miR-320d,miR-125b,miR-422a,miR-92a-1-5p,miR-500a-5p,miR-324-3p,miR-486-3p,miR-2432,miR-376c-3p,miR-345-3p,miR-183-5p,miR-378d,miR-664-5p,miR-4429,miR-378a-3p,miR-5703,miR-188-3p,miR-140-3p,miR-2904,miR-193b-5p,miR-376a-5p,miR-378i,novel_133,miR-328-3p,miR-532-3p,miR-212-5p,miR-3958-3p,miR-4726-5p,miR-628-3p,miR-371a-5p,miR-2285y,novel_82,miR-1285-5p,novel_111,miR-877-3p,miR-2285f,miR-541-3p,novel_94,miR-504,miR-1185-2-3p,miR-378e,miR-4792,miR-153-3p,novel_124,miR-491-5p,miR-182-5p,novel_19,miR-505,novel_103,miR-1306-5p,miR-320b,miR-2310,miR-3187-3p,miR-8485,miR-378d,miR-744-3p,miR-1388-3p,miR-3120-5p,miR-378c,miR-143-5p,miR-541,miR-296-5p,miR-378b,miR-2284r,miR-6529a,miR-1185-3p,miR-2459,miR-9788-3p,miR-147a,miR-1298-3p,miR-496-5p,miR-153,miR-330-3p,miR-105-1,novel_78,novel_4,miR-134-5p,miR-28-5p,miR-486b-3p,miR-138-5p,miR-135a-5p,miR-125b-5p,miR-331-3p,miR-5100,miR-378c,miR-145-3p,miR-1197-3p,miR-1260b,miR-181a-2-3p,miR-485-5p,miR-1248,miR-1814c,miR-7144-5p,miR-23a-5p,miR-345-5p,miR-18a,miR-320a,miR-154b-5p,miR-376c-3p,miR-300-3p,miR-331-5p,miR-141-3p,miR-125a,miR-3956,miR-370-3p,miR-2355-5p,miR-503-3p,miR-200a-3p,novel_96,miR-107,miR-450a-2-3p,miR-125a-3p,miR-2285af,miR-205-5p,miR-1260a,miR-490-3p,miR-200a,novel_39,miR-148b-5p,miR-2478,miR-4286,miR-3529-
PTPRN2	ENSOARG0(	protein	miR-204-5p,miR-211,miR-2404,novel_51,miR-211-5p,miR-3960,miR-1434-3p
CENPU	ENSOARG0(	centrome	miR-2284u,miR-199b-5p,miR-376b-5p,miR-411-5p,miR-199a-5p,miR-222-5p,miR-487a-3p,miR-7859,miR-485-3p,miR-329-5p,miR-3074-5p,miR-151a-3p,miR-548e-3p,miR-592,miR-199b-5p,miR-485-3p,miR-151-3p,miR-330-3p,miR-2285p,miR-4324,miR-2284a,miR-199b-5p,miR-199a-5p,miR-2284y,miR-2284b,miR-2330-3p,miR-195a-3p,miR-3074-5p,miR-6240,miR-329a-5p,miR-590-3p

LPAR2	ENSOARG0(	lysophos	miR-342-3p,miR-326-3p,miR-449a,miR-24-3p,miR-34c,miR-16a,miR-15a,miR-6529b,miR-203b-5p,miR-7-1-3p,miR-378b,miR-670-3p,miR-34b,miR-2285ab,miR-6516-3p,miR-195a-5p,miR-330-3p,miR-197-5p,miR-2331-5p,miR-125a-5p,miR-377-5p,miR-133b-5p,miR-2355-3p,miR-2285g,miR-432-5p,miR-185-5p,miR-873a-5p,miR-653,miR-1285,miR-30b-3p,miR-219a-1-3p,miR-1260b,miR-378c,miR-500b-5p,miR-3969,miR-135a-1-3p,miR-5100,miR-28-5p,miR-486b-3p,miR-370-3p,miR-199a-5p,miR-324-5p,miR-2284n,miR-34c-5p,miR-769,miR-362-5p,novel_48,miR-7144-5p,miR-671-5p,miR-1814c,miR-485-5p,miR-199a-5p,miR-1271-3p,miR-490-3p,miR-28b,miR-30c-2-3p,miR-1260a,miR-6516-5p,miR-483-3p,miR-378h,miR-1271-5p,miR-128-3p,miR-1296-5p,miR-2285f,miR-32-3p,miR-219a-2-3p,miR-362-5p,miR-769-5p,miR-2285y,miR-541-5p,miR-7977,miR-664b,miR-7857-3p,miR-378e,miR-199b-5p,novel_94,miR-1839-3p,miR-424-5p,miR-758-3p,miR-30c-1-3p,miR-362-3p,miR-342,novel_103,miR-1306-5p,miR-326,novel_4,miR-151a-3p,miR-409b,miR-2300a-5p,miR-216a-3p,miR-9788-3p,miR-500-5p,miR-2285e,miR-30b-3p,miR-296-5p,miR-378b,miR-484,miR-3120-5p,miR-378c,miR-1271,miR-378c,miR-324-3p,miR-708-5p,miR-424-5p,miR-450b-3p,miR-21-3p,miR-221-5p,miR-378a-3p,miR-32-3p,miR-204-3p,novel_32,miR-6516,miR-21a-3p,miR-2312,miR-183-5p,miR-216c-5p,miR-130a-5p,miR-216a-5p,miR-125b,miR-34b-5p,miR-221-5p,miR-21-3p,miR-2284m,miR-3600,miR-1b-3p,miR-548e-3p,miR-151-5p,miR-532-3p,miR-383-5p,miR-15a-5p,miR-2904,miR-126-3p,novel_133,miR-362-3p,miR-2300b-3p,miR-1839-3p,miR-2284w,miR-199b-5p,miR-4324,miR-1247-3p,miR-28c,miR-6535,miR-1306,miR-216a-3p,miR-3591-3p,miR-378f,miR-412-3p,miR-6240,miR-432-3p,miR-432-3p,novel_51,miR-483-3p,miR-3955-5p,miR-31-5p,miR-149-

GAS2	ENSOARG0(	growth a	miR-181a-2-3p,miR-29b-2-5p,miR-2385-3p,miR-2478,miR-376c-5p,miR-199a-5p,miR-376b-5p,miR-200c,miR-2397-5p,miR-6119-5p,miR-376b-3p,miR-1260a,miR-339a,miR-217,miR-200c-3p,miR-450a-2-3p,miR-107,miR-302a-5p,miR-24-2-5p,miR-582-3p,miR-545-5p,miR-30a-5p,miR-300,let-7a-2-3p,miR-200b-3p,miR-1306-5p,miR-133a-3p,miR-6128,miR-374a-3p,miR-145-3p,miR-410-5p,miR-331-3p,miR-26c,miR-138-5p,miR-374b-3p,miR-487a-3p,miR-141-3p,miR-598-3p,miR-365a-3p,miR-374c-3p,miR-199a-5p,miR-381-3p,let-7g-3p,miR-10b-3p,miR-671-5p,miR-376c-5p,miR-2483-5p,miR-654-3p,miR-539-3p,miR-190a,miR-22851,miR-544b,miR-103,miR-653,miR-194-5p,miR-374b-3p,miR-219a-1-3p,miR-1260b,miR-138,miR-21-5p,miR-219b-3p,miR-135a-2-3p,miR-2285x,miR-3959-3p,miR-6402,miR-15a,miR-382-5p,miR-16a,novel_1,miR-656-5p,miR-670-3p,miR-376b-3p,miR-21c,miR-107,miR-195a-5p,miR-29b-1-5p,miR-374a-5p,miR-196a-3p,miR-185-3p,miR-221-5p,miR-30d,miR-485-3p,miR-1b-3p,miR-200b,miR-15a-5p,miR-487a-3p,miR-362-3p,miR-2408,miR-1197-5p,miR-188-3p,miR-2904,miR-145a-3p,miR-539-5p,miR-3074-2-3p,miR-338-3p,miR-29b-2-5p,miR-132-5p,miR-155-5p,miR-208b-5p,miR-487a-5p,miR-221-5p,miR-182-5p,miR-139-5p,miR-544-3p,miR-132-5p,miR-155-5p,miR-877-5p,novel_25,miR-21a-3p,miR-154b-3p,miR-30f,miR-133b-3p,let-7g-3p,novel_99,miR-582-5p,miR-424-5p,miR-33a-5p,miR-376b,miR-362-3p,miR-151a-3p,miR-216a-3p,miR-4791,miR-484,miR-544a,miR-3120-5p,miR-16-2-3p,miR-2284aa,miR-452-5p,miR-194b-5p,miR-219a-2-3p,miR-8095,miR-30b-5p,miR-382-3p,miR-2285t,miR-20a-3p,miR-541-5p,miR-499b-5p,miR-3082-5p,miR-376d,miR-199b-5p,novel_94,miR-219-3p,miR-30f,miR-200a-3p,miR-429-3p,miR-2285ad,novel_79,miR-1224-5p,miR-9-5p,miR-322-5p,miR-486-5p,miR-2284z,miR-196a-5p,miR-3591-5p,miR-2427,miR-
PARN	ENSOARG0(	poly(A)-	miR-365b-5p,miR-130b-5p,miR-106b-3p,miR-138-5p,miR-130b-5p,miR-7705,miR-365a-5p,novel_111,miR-1248,miR-3120-5p,miR-544a,miR-138,miR-542-5p,miR-3957-5p,miR-3957,miR-544b,miR-544-3p
PLEKHA1	ENSOARG0(	pleckstr	miR-29a-3p,miR-374c-3p,miR-27a-3p,miR-154b-5p,miR-181b-3p,miR-677,miR-548o-3p,miR-216b-3p,miR-598-3p,miR-3065-3p,miR-1827,miR-1814c,miR-2387,miR-21c,miR-487b-5p,miR-598-3p,miR-224-5p,miR-1197-3p,miR-145a-3p,miR-342-3p,miR-29c-3p,miR-29a,miR-487b-5p,miR-199c,miR-494-5p,miR-29b-3p,miR-410-5p,miR-2285n,miR-2285y,miR-340-5p,miR-7859,miR-199b-3p,miR-2285ad,miR-548e-3p,miR-27a-3p,miR-222-5p,miR-181b-2-3p,miR-2426,miR-29b,miR-21-5p,miR-27b-3p,miR-2366,miR-484,miR-140-3p,novel_23,miR-374b-3p,miR-29d-3p,miR-181b-1-3p,miR-2310,miR-3065-3p,miR-200a,miR-8485,miR-377-3p,miR-224-5p,miR-342

PTGS2	ENSOARG0(	prostagl	miR-7705,miR-212-3p,miR-2447,novel_1,miR-2284q,miR-208b-5p,miR-101-3p,miR-576-3p,miR-2285j,miR-485-3p,miR-1185-2-3p,miR-204-3p,miR-216b-3p,miR-2284g,miR-26b-5p,miR-21-3p,let-7g-3p,miR-107,miR-450b-3p,novel_99,miR-7144-5p,miR-301,miR-21a-3p,miR-129b-5p,miR-3591-3p,miR-148b-5p,miR-2284y,miR-8485,miR-101b-3p,miR-1388-3p,let-7f-2-3p,let-7f-2-3p,miR-2284b,miR-543-5p,miR-107,miR-3653-3p,miR-26a-5p,miR-2285af,miR-7859,miR-1185-3p,miR-103,miR-411-5p,miR-148b-5p,miR-103a-3p,novel_78,miR-548e-3p,miR-148a-5p,miR-2284u,miR-218-1-3p,miR-2300b-3p,miR-3071-5p,miR-125b-2-3p
C6orf58	ENSOARG0(	chromoso	miR-7641,miR-6516-5p,miR-378h,miR-322-5p,miR-9-5p,miR-134-5p,miR-134,miR-345-3p,miR-330-5p,miR-23b-5p,miR-582-3p,miR-496,miR-2403,miR-2385-3p,miR-148b-5p,novel_39,miR-199a-5p,miR-205-5p,miR-181c-3p,miR-127-5p,miR-543-5p,miR-223-5p,miR-199a-5p,miR-3154,miR-23b,miR-345-5p,miR-671-5p,miR-7144-5p,miR-23a-5p,miR-6128,miR-3607-3p,miR-16-5p,miR-193a-5p,miR-378c,miR-135a-1-3p,miR-2332,miR-1306-3p,miR-3969,novel_9,miR-486b-3p,miR-134-5p,miR-204-5p,miR-26b-3p,miR-130a-3p,miR-432-5p,miR-149-5p,miR-542-3p,miR-223-3p,miR-432,miR-3071-5p,miR-197-3p,miR-16-1-3p,miR-30b-3p,miR-16b,miR-188-5p,miR-27b-3p,miR-497-5p,miR-130b-5p,miR-432-3p,miR-432-3p,miR-654-3p,miR-2285r,miR-345-5p,miR-224-5p,miR-2411-3p,novel_51,miR-3074-5p,miR-378b,novel_68,miR-2404,miR-323a-5p,miR-181b-3p,miR-677,miR-2285ab,miR-378b,miR-6516-3p,miR-195a-5p,miR-378f,miR-371b-3p,miR-3959-3p,miR-211,miR-2428,miR-301b-3p,miR-199b-5p,novel_27,miR-326-3p,miR-15a,miR-502-5p,miR-16a,miR-421-5p,miR-548e-3p,miR-15a-5p,miR-222-5p,miR-23c,miR-23b-3p,miR-7859,miR-210-3p,miR-616-3p,miR-199a-3p,miR-2284h-5p,miR-378i,miR-378a-3p,miR-140-3p,miR-5703,miR-493-3p,miR-148a-5p,miR-2285w,miR-130a-5p,miR-181b-1-3p,miR-29b-1-5p,miR-143-3p,miR-486-3p,miR-210-5p,miR-2285p,miR-345-3p,miR-422a,miR-221-5p,miR-496-3p,miR-221-5p,miR-140-5p,miR-378a-3p,miR-182-5p,miR-323-5p,miR-301a-3p,miR-6516,miR-15b-5p,miR-433-3p,miR-345-5p,miR-2312,miR-1827,miR-145a-3p,miR-3074-5p,miR-378c,miR-425-5p,miR-224-5p,miR-6525,miR-6516-3p,miR-130b-5p,miR-424-5p,miR-454-3p,miR-2300a-5p,miR-222-5p,miR-181b-2-3p,miR-27a-3p,miR-1298-3p,miR-425-3p,miR-148b-5p,miR-9788-3p,miR-1298-5p,miR-143-5p,miR-378b,miR-484,miR-125b-2-3p,miR-30b-3p,miR-7857,miR-378[illegible],miR-
MCF2L	ENSOARG0(	MCF.2 ce	miR-4429,miR-362-3p,miR-330-5p,miR-2366,miR-362-3p,miR-2284r,miR-329-3p,miR-328-3p,miR-1247-5p,miR-320d,novel_51,miR-324-3p,miR-362-3p,miR-377-3p,miR-1306-5p,miR-326,miR-320b,miR-342,miR-3065-3p,miR-4532,miR-6395,miR-1895,miR-9851-3p,miR-329a,miR-1291,miR-450b-3p,miR-320a,miR-1306,miR-361-3p,miR-320c,miR-377-3p,miR-132-5p,miR-615-5p,miR-1271,miR-370-3p,miR-3065-3p,miR-4726-5p,miR-320e,miR-132-5p,miR-326-3p,miR-361-3p,miR-342-3p

DAPL1	ENSOARG0(	death as	miR-6525,miR-3958-5p,let-7b,miR-129-5p,miR-454-3p,miR-1468-5p,miR-487b-5p,miR-151-3p,miR-30a-3p,miR-30e-3p,let-7f,let-7i-5p,miR-301a-3p,miR-296-3p,miR-2331-3p,miR-1827,miR-186-5p,miR-625-5p,miR-345-5p,miR-30d-5p,miR-299,miR-486-3p,miR-377-5p,miR-181b-1-3p,let-7k,miR-98-5p,miR-345-3p,miR-219b-5p,miR-2285p,miR-30b,miR-30d-3p,miR-16-1-3p,novel_73,miR-1434-5p,miR-1961,miR-193b-5p,miR-30c-5p,miR-361-3p,miR-877-3p,miR-2284j,miR-885-5p,miR-490-5p,novel_82,miR-4443,miR-203-3p,miR-2483-3p,miR-25,miR-3596,miR-211-5p,miR-9-3p,miR-140-5p,miR-2310,miR-8485,miR-103a-2-5p,miR-146b,miR-130b-3p,miR-487b-5p,miR-323b-3p,miR-29b-1-5p,miR-505,miR-421,miR-455-3p,miR-147a,miR-496-5p,miR-181b-2-3p,novel_78,miR-21b,miR-101c,miR-1839-5p,miR-323b,miR-2284v,novel_9,miR-125b-5p,miR-2284k,miR-125a,miR-2355-5p,miR-874-3p,miR-1248,miR-345-5p,miR-876-3p,miR-424-3p,miR-30e-5p,miR-450c-5p,let-7g,novel_39,miR-3064-5p,miR-152-3p,let-7f-5p,miR-127-5p,miR-30f,miR-486-5p,miR-505-5p,miR-412,miR-6517,miR-299a-3p,miR-155-5p,miR-30c,miR-23b-5p,miR-1343-5p,miR-301b-3p,miR-335-5p,novel_87,miR-4324,miR-181b-3p,miR-669,miR-677,miR-320c,miR-299b-3p,miR-374c-3p,let-7e-5p,miR-146b-5p,miR-410-5p,miR-3065-5p,miR-1306,miR-148a-3p,let-7j,miR-2284l,miR-432-3p,miR-628-5p,miR-380-5p,miR-2411-3p,novel_51,miR-146a,miR-194-3p,miR-412-5p,miR-31-5p,miR-2284x,miR-16-1-3p,miR-197-3p,miR-432,miR-338-3p,let-7b-5p,miR-4510,miR-539-5p,miR-487a-5p,miR-155-5p,miR-29b-2-5p,miR-324-3p,miR-155-5p,novel_25,miR-6130,miR-532-5p,miR-660-5p,miR-140-5p,miR-545-3p,novel_99,miR-27b-5p,miR-146a-5p,miR-2432,miR-29b-1-5p,miR-216c-5p,miR-299-3p,miR-21-3p,miR-30d,miR-30b-3p,miR-466i-5p,miR-320d,miR-2284m,miR-
GTF2F2	ENSOARG0(	general	miR-539-5p,miR-541-5p,miR-377-3p,miR-148b-5p,miR-8095,miR-377-3p,miR-3120-5p,novel_48

PSMB8	ENSOARG0(	proteaso	miR-1260b,miR-1197-3p,novel_42,miR-2284v,miR-193a-5p,miR-144-5p,miR-134-5p,miR-2433,miR-2284k,miR-125b-5p,miR-100-3p,miR-574-5p,miR-2285aa,miR-542-5p,miR-125a,miR-2318,miR-329a,miR-345-5p,miR-30e-5p,miR-450b-5p,miR-2284y,miR-29a-5p,novel_39,miR-2285b,miR-6536,miR-200a-3p,miR-125a-3p,miR-9-5p,miR-758-5p,miR-134-5p,miR-412,miR-431,miR-30c,miR-1290,miR-330-5p,miR-335-5p,miR-412-5p,miR-142b,miR-677,miR-320c,miR-181b-3p,miR-3120-3p,miR-2319a,miR-3065-5p,miR-432-3p,miR-432-3p,miR-380-5p,miR-483-3p,miR-2411-3p,miR-3955-5p,miR-4508,miR-2284x,miR-2113,miR-197-3p,miR-346,miR-3184-5p,miR-122-5p,miR-451a,miR-4492,miR-130b-5p,miR-30e-3p,miR-30a-3p,miR-665,miR-2284s,miR-499b-3p,miR-10a,miR-151b,miR-504-5p,miR-2331-3p,miR-548o-3p,miR-552-3p,miR-345-5p,miR-1895,miR-625-5p,miR-543-3p,miR-30d-5p,miR-376a-5p,miR-377-5p,miR-181b-1-3p,miR-2285w,miR-2484,miR-30b,novel_17,miR-210-5p,miR-345-3p,miR-92a-1-5p,miR-2284h-5p,miR-142-3p,miR-2284u,miR-34a-5p,miR-30c-5p,miR-2366,miR-193b-5p,miR-877-3p,miR-361-3p,miR-423-5p,miR-1285-5p,miR-2447,miR-2284j,miR-9-5p,miR-499a-5p,miR-218-5p,miR-301a-5p,miR-499a-3p,miR-2483-3p,miR-203-3p,miR-144-5p,miR-4792,miR-2319b,miR-211-5p,miR-96-5p,miR-504,miR-136-5p,miR-378j,miR-1388-3p,miR-2284b,miR-431-5p,miR-29b-1-5p,miR-323b-3p,miR-147a,miR-455-3p,miR-411b-5p,miR-1343-3p,novel_78,miR-181b-2-3p,miR-145b,miR-362-3p,miR-2284r,miR-329-3p,miR-541,miR-301b-5p,miR-323b,miR-323-3p,miR-6128,miR-3607-3p,miR-204-5p,miR-34c-5p,miR-2284n,miR-3957,miR-3957-5p,miR-141-3p,miR-10b-3p,novel_91,miR-7144-5p,miR-671-5p,miR-2448-3p,miR-320a,miR-3154,miR-380-5p,miR-490-3p,miR-2284a,miR-29b-2-5p,miR-30a-3p,miR-17-3p,miR-543-5p,miR-1260a,miR-6119-
LUZP1	ENSOARG0(	leucine	miR-1185-5p,miR-542-5p,miR-193a-3p,miR-16b,miR-3141,miR-494-3p,miR-424-5p,miR-138,miR-16b,miR-497-5p,miR-1285,miR-4791,miR-2284r,miR-3071-5p,miR-185-5p,miR-147a,miR-3535,miR-149-5p,miR-9788-3p,miR-665,miR-128-1-5p,miR-4726-5p,miR-15a,miR-16a,miR-2447,miR-542-5p,miR-3957-3p,miR-193b-3p,miR-195a-5p,miR-3141,miR-18a-3p,novel_69,miR-670-3p,miR-3596,miR-6535,miR-669,miR-452-3p,miR-4792,miR-320b,novel_127,miR-542-5p,novel_17,miR-185-3p,miR-324-3p,miR-125b-2-3p,miR-664b-3p,miR-143-3p,novel_39,miR-486-3p,miR-378d,miR-15a-5p,miR-322-5p,miR-18a-3p,miR-138-5p,miR-486b-3p,miR-28-5p,miR-4492,miR-708-5p,miR-424-5p,miR-16-5p,miR-3154,miR-193b-3p,miR-154b-5p,miR-15b-5p,novel_32,miR-204-3p,miR-296-3p

LMCD1	ENSOARG0(	LIM and	miR-328-3p,miR-1224-5p,miR-18a-3p,miR-6517,miR-2904,miR-345-3p,miR-1290,miR-2432,miR-199a-5p,miR-3064-5p,miR-345-3p,miR-181a-2-3p,miR-3529-3p,miR-221-5p,novel_127,miR-543-5p,miR-450a-2-3p,miR-345-3p,miR-125b,miR-196a-3p,miR-219-3p,miR-204-3p,miR-223-5p,miR-199a-5p,miR-193b-3p,miR-139-5p,miR-532-5p,miR-182-5p,miR-615-5p,miR-125a,miR-504-5p,miR-221-5p,miR-874-3p,miR-3068-3p,miR-23a-5p,miR-1827,miR-485-5p,miR-1814c,miR-27b-5p,miR-21a-3p,miR-3184-5p,miR-193a-5p,miR-138-5p,miR-125b-5p,miR-219b-3p,miR-135a-5p,miR-665,miR-1185-3p,novel_78,novel_4,miR-2300a-5p,miR-138,miR-197-3p,miR-3071-5p,miR-2113,miR-2310,miR-432-3p,let-7c-3p,miR-382-3p,miR-1839-3p,miR-125a-5p,miR-199b-5p,miR-193a-3p,miR-3085-3p,miR-3955-3p,miR-182-5p,miR-2411-3p,miR-203-3p,miR-1185-2-3p,miR-2404,miR-670-3p,miR-2483-3p,miR-499b-5p,miR-7689-3p,miR-139-5p,miR-3064-5p,miR-335-3p,miR-135b-5p,miR-6395,miR-1983,miR-1291,miR-412-3p,miR-504,miR-18a-3p,miR-199b-5p,miR-423-5p,novel_27,miR-219a-2-3p,miR-199b-5p,miR-371b-3p,miR-193b-3p,miR-1839-3p,miR-6527,miR-2447,miR-147-5p,miR-499a-5p,miR-371a-5p
BGN	ENSOARG0(	biglycan	miR-210-5p,miR-1973,miR-128-1-5p,miR-4726-5p,miR-486b-3p,miR-2330-5p,miR-541-3p,miR-380-5p,miR-665-5p,miR-1197-3p,miR-361-3p,miR-380-5p,miR-486-3p,miR-541,miR-2448-3p,miR-361-3p,miR-2904,miR-22-3p,novel_69,miR-505-5p,miR-27a-5p,miR-22-3p

KIAA0408	ENSOARG0(	KIAA0408	miR-296-3p,miR-105-5p,miR-32,miR-432-3p,miR-2285r,let-7a-2-3p,miR-380-5p,let-7f-2-3p,let-7f-2-3p,miR-2113,miR-708-3p,miR-450c-3p,miR-412-5p,miR-149-5p,miR-31-5p,miR-340-3p,miR-576-3p,miR-4324,novel_87,miR-199b-5p,miR-376b-5p,miR-3970,miR-374c-3p,miR-411b-3p,novel_68,miR-26b-5p,miR-127-5p,miR-28-3p,miR-200a-3p,miR-2285b,miR-192-3p,miR-450c-5p,miR-200a,miR-29a-5p,miR-3064-5p,miR-30c,miR-1290,miR-758-5p,miR-129-1-3p,miR-450a-1-3p,miR-2284z,miR-429-3p,miR-18b-5p,miR-1224-5p,miR-412,miR-125b-5p,miR-6740-5p,miR-28a-3p,miR-1248,miR-30e-5p,miR-376c-5p,miR-200a-5p,miR-25-3p,miR-331-5p,miR-503-3p,miR-3068-3p,miR-125a,miR-2355-5p,miR-487b-5p,miR-214-3p,miR-103a-2-5p,miR-199b-5p,miR-182-5p,miR-505,miR-2310,miR-1b-5p,miR-8485,miR-23a-3p,miR-1388-5p,miR-541,miR-758-3p,miR-501-3p,miR-376b-3p,miR-2459,miR-1185-3p,miR-455-3p,miR-148b-5p,miR-145b,miR-3964,miR-1285-5p,miR-363-3p,miR-2447,miR-2284j,miR-6527,miR-3963,miR-376a-3p,miR-140-5p,miR-499a-3p,miR-500-3p,miR-1185-2-3p,miR-2319b,miR-139-5p,miR-211-5p,miR-202-5p,miR-2484,miR-210-5p,miR-26a-5p,miR-30b,miR-92a-1-5p,miR-329a-5p,miR-369-3p,miR-195a-3p,miR-142-3p,miR-148a-5p,miR-30c-5p,miR-1434-5p,miR-23c,novel_74,miR-130b-5p,miR-500,miR-15b-3p,let-7b-3p,miR-664a-5p,miR-224-5p,miR-346,miR-181c-5p,miR-129-2-3p,miR-214,miR-186-5p,miR-1827,miR-543-3p,miR-625-5p,miR-450a-1-3p,miR-30d-5p,miR-500a-3p,miR-499b-3p,miR-151b,miR-548o-3p,miR-32-5p,miR-2331-3p,miR-296-3p,miR-466f-3p,miR-125a-5p,miR-452-5p,miR-224-5p,miR-296-3p,miR-130b-5p,miR-2284ab,miR-2889,miR-1285,miR-181a-5p,miR-1434-3p,miR-3071-5p,miR-145a-5p,miR-185-5p,miR-544b,novel_1,miR-22-5p,miR-382-5p,miR-3959-3p,miR-323c,miR-24-3p,miR-211,novel_27,novel_120,novel_101,miR-

USP24	ENSOARG0(	ubiquiti	miR-409b,miR-330-3p,miR-376b-3p,miR-2285e,miR-9788-3p,miR-455-3p,miR-301b-5p,miR-143-5p,miR-101c,miR-21b,miR-106a-5p,miR-23a-3p,miR-4532,miR-326,miR-106b-5p,miR-139-5p,miR-499a-3p,miR-378g,miR-376d,miR-361-3p,miR-381-3p,miR-147-3p,miR-877-3p,novel_94,miR-376a-3p,miR-212-3p,miR-885-5p,miR-219a-2-3p,miR-2440,miR-301a-5p,miR-499a-5p,miR-374a-3p,miR-106a,miR-30d-3p,miR-23c,miR-210-3p,miR-23b-3p,miR-328-3p,miR-2300b-3p,miR-98-3p,miR-34a-5p,let-7a-3p,miR-361-5p,miR-218-1-3p,miR-20a,miR-210-5p,miR-196a-3p,miR-185-3p,miR-345-3p,miR-216a-5p,miR-760-3p,miR-106a,miR-34b-5p,miR-1277-5p,miR-221-5p,miR-496-3p,miR-221-5p,miR-370-5p,miR-139-5p,miR-193b-3p,miR-582-5p,miR-499b-3p,miR-2285c,miR-450a-1-3p,miR-411,miR-500a-3p,miR-27b-5p,miR-18a-5p,miR-345-5p,let-7g-3p,miR-582-5p,miR-186-5p,miR-3184-3p,miR-539-5p,miR-98-3p,miR-346,let-7b-3p,miR-2285j,miR-30a-3p,miR-219b-3p,miR-324-3p,miR-664a,miR-450b-3p,miR-500,miR-4492,miR-134-3p,miR-2285g,miR-20b,novel_116,miR-1285,miR-450c-3p,miR-197-3p,miR-138,miR-4508,miR-6240,miR-345-5p,let-7a-2-3p,miR-503-3p,miR-106a-5p,miR-17-5p,miR-2411-3p,miR-330-3p,miR-483-3p,miR-193a-3p,miR-106b-3p,miR-34b,miR-18b,miR-376b-3p,miR-450b-3p,miR-6516-3p,miR-6395,miR-1193,miR-193b-3p,miR-2428,miR-449a,miR-326-3p,miR-20a-5p,miR-34c,miR-379-3p,miR-18b-5p,miR-1224-5p,miR-483-3p,miR-450a-1-3p,miR-3578,miR-345-3p,miR-330-5p,miR-411-3p,let-7a-2-3p,miR-496,miR-181a-2-3p,miR-2285b,miR-29a-5p,miR-219-3p,miR-376b-3p,miR-503-3p,miR-574-5p,miR-34c-5p,miR-324-5p,miR-23b,miR-18a,miR-17-5p,miR-93-5p,let-7g-3p,miR-345-5p,miR-20b-5p,miR-374a-3p,miR-93,miR-135a-1-3p,miR-370-5p,miR-138-5p,miR-374b-3p
UBALD2	ENSOARG0(	UBA-like	miR-125b-2-3p,miR-22-3p,miR-450c-3p,miR-541,miR-2284r,miR-2426,miR-29b,novel_4,miR-149-5p,miR-216a-3p,miR-194-3p,miR-3141,miR-30c-1-3p,miR-216a-3p,miR-18a-3p,miR-378j,miR-3141,miR-2387,miR-1291,miR-450b-3p,miR-139-5p,miR-34b,miR-29a-3p,miR-34c,miR-2440,miR-29a,miR-29c-3p,miR-541-3p,miR-449a,miR-24-3p,miR-188-3p,miR-29d-3p,novel_133,miR-378d,miR-34a-5p,miR-128-3p,miR-885-3p,miR-18a-3p,miR-450a-1-3p,miR-532-3p,miR-92a-1-5p,miR-185-3p,miR-30c-2-3p,miR-2285p,novel_127,miR-34b-5p,miR-4286,miR-143-3p,miR-3065-3p,miR-29a-5p,miR-450a-1-3p,miR-488-3p,miR-1827,miR-885-3p,miR-296-3p,miR-2355-5p,miR-3065-3p,miR-874-3p,miR-30c-1-3p,miR-2331-3p,miR-22-3p,miR-139-5p,miR-34c-5p,miR-331-3p,miR-29b-3p,miR-4492,miR-450b-3p,miR-1306-5p

SPIN1	ENSOARG0(	spindlin	miR-625-5p,miR-154b-3p,miR-30f,miR-154a-3p,miR-2285aa,miR-3065-3p,miR-1277-3p,miR-487a-3p,miR-544-3p,miR-6516,miR-582-5p,miR-29b-2-5p,miR-19b-1-5p,miR-2285j,miR-29b-3p,miR-6128,miR-145-3p,miR-142a-5p,miR-29d-3p,miR-2366,miR-3578,miR-376a-5p,miR-30c-5p,miR-345-3p,miR-105-2,miR-16-1-3p,miR-376a-5p,miR-485-3p,miR-3600,miR-2285ad,miR-299a-3p,miR-2284h-5p,miR-340-5p,miR-148b-3p,miR-6516-5p,miR-487a-3p,miR-345-3p,miR-125a-3p,miR-21-3p,miR-299-3p,miR-152-3p,miR-466i-5p,miR-127-5p,miR-181a-2-3p,miR-29b-2-5p,miR-487a,miR-3529-3p,miR-2285w,miR-192-3p,miR-376b-5p,miR-1957a,miR-146a-5p,miR-3065-3p,miR-376c-5p,miR-148b-5p,miR-29b-1-5p,miR-378j,miR-148a-3p,miR-376a-2-5p,miR-146b-5p,miR-7977,miR-499b-5p,miR-203-3p,miR-652-3p,miR-6535,miR-29a-3p,miR-299b-3p,miR-378g,miR-2285y,miR-576-3p,miR-2440,miR-2285n,miR-154-3p,miR-22-5p,miR-29a,miR-29c-3p,novel_111,miR-125b-2-3p,miR-873a-5p,miR-1839-5p,miR-544a,miR-450b-5p,miR-16-1-3p,miR-29b,miR-5010-3p,miR-105-1,miR-126a-5p,miR-544b,miR-134-3p,miR-142-5p,miR-2411-3p,miR-29b-1-5p,miR-105-5p,miR-146a,miR-466f-3p,miR-146b,miR-124-5p,miR-33a-5p,miR-744-3p,miR-8485,miR-539-3p
CADM3	ENSOARG0(	cell adh	miR-370-3p,miR-615-5p,miR-3154,miR-488-3p,miR-1814c,miR-10b-3p,let-7g-3p,miR-345-5p,miR-329a,miR-16-5p,miR-331-3p,miR-6740-5p,miR-2433,miR-370-5p,miR-486b-3p,miR-138-5p,miR-6517,miR-322-5p,miR-1271-5p,miR-483-3p,miR-1343-5p,miR-2427,miR-345-3p,let-7a-2-3p,miR-3064-5p,miR-4286,miR-3065-3p,novel_39,miR-181c-3p,miR-125a-3p,miR-152-3p,novel_96,novel_127,miR-450a-2-3p,miR-127-5p,miR-363,miR-216b-3p,miR-3074-5p,novel_69,miR-1306,miR-18a-3p,miR-3141,miR-148a-3p,miR-195a-5p,miR-1291,miR-1193,miR-2285m,miR-16a,miR-15a,miR-656-5p,miR-147-5p,miR-377-5p,miR-185-5p,miR-134-3p,miR-22-3p,miR-19b-2-5p,miR-542-3p,miR-7b-5p,miR-497-5p,miR-744-5p,miR-138,miR-16b,miR-6240,let-7a-2-3p,miR-345-5p,miR-432-3p,miR-3141,miR-330-3p,novel_51,miR-2411-3p,miR-7-5p,miR-1343-5p,miR-466f-3p,miR-483-3p,miR-370-5p,novel_107,miR-3065-3p,miR-140-5p,miR-204-3p,miR-22-3p,miR-15b-5p,miR-6130,miR-433-3p,miR-1827,novel_99,let-7g-3p,miR-345-5p,miR-1271,miR-3074-2-3p,miR-214,miR-539-5p,miR-3074-5p,miR-4510,miR-3184-5p,miR-6525,miR-346,miR-132-5p,miR-19b-1-5p,miR-665,miR-874-5p,miR-424-5p,miR-129-5p,miR-4492,miR-18a-3p,novel_83,miR-532-3p,miR-148b-3p,miR-15a-5p,miR-1197-5p,miR-188-3p,miR-2300b-3p,miR-362-3p,miR-493-3p,novel_73,miR-33b-3p,miR-142-3p,miR-486-3p,miR-143-3p,miR-376a-5p,miR-377-5p,miR-324-3p,miR-185-3p,miR-345-3p,miR-21-3p,miR-466i-5p,miR-3064-5p,miR-378g,miR-7857-3p,miR-140-5p,miR-361-3p,miR-96-5p,miR-2447,miR-541-3p,miR-212-3p,miR-423-5p,miR-361-3p,miR-1973,miR-2440,miR-1343-3p,miR-330-3p,miR-455-3p,miR-153,miR-147a,miR-329-3p,miR-296-5p,miR-541,miR-143-5p,miR-3120-5p,miR-362-3p,miR-1388-5p,let-7c-3p,miR-382-3p,miR-2330-5p,miR-424-5p,miR-8485,miR-2310,miR-362-3p,miR-16b,miR-

CXCL16	ENSOARG0(	chemokin	miR-6240,miR-668-3p,miR-432-3p,miR-22841,miR-432-3p,let-7a-2-3p,miR-7-5p,miR-146a,miR-105-5p,miR-2411-3p,miR-106b-3p,miR-31-5p,miR-103a-3p,miR-3431,novel_116,miR-194-3p,miR-149-5p,miR-19b-2-5p,miR-432,miR-7b-5p,miR-450c-3p,miR-3432b,miR-4508,miR-27b-3p,miR-335-5p,miR-29c-3p,miR-142b,miR-29a,miR-6123,miR-329-5p,miR-411b-3p,miR-299b-3p,miR-669,miR-320c,miR-677,miR-6535,miR-144-3p,miR-181b-3p,miR-412-3p,miR-146b-5p,miR-378f,miR-2284y,miR-192-3p,miR-2285b,miR-125a-3p,miR-127-5p,novel_127,miR-200a-3p,miR-299a-3p,miR-429-3p,miR-1224-5p,miR-412,miR-18b-5p,miR-505-5p,miR-758-5p,miR-2284z,miR-450a-1-3p,miR-134-5p,miR-486-5p,miR-885-3p,miR-2284v,miR-1260b,novel_63,miR-331-5p,miR-134-5p,miR-592,miR-3068-3p,miR-874-3p,miR-2355-5p,miR-2285aa,miR-331-5p,miR-767,miR-345-5p,miR-329b,miR-1388-3p,miR-574-3p,miR-2284b,miR-378d,miR-29b-1-5p,miR-505,miR-323b-3p,miR-146b,miR-214-3p,miR-1343-3p,novel_78,miR-181b-2-3p,miR-145b,miR-147a,miR-455-3p,miR-323b,miR-1388-5p,miR-101c,miR-2447,miR-3956-3p,miR-6527,miR-361-3p,miR-423-5p,miR-1285-5p,novel_82,miR-2284g,miR-2285u,miR-211-5p,miR-3596,miR-4443,miR-381-3p,miR-3963,miR-136-5p,miR-504,miR-1983,miR-329a-5p,miR-345-3p,miR-2285i,miR-376a-5p,miR-181b-1-3p,miR-2424,miR-500a-5p,miR-345-3p,miR-422a,miR-92a-1-5p,miR-18a-3p,miR-328-3p,miR-378i,miR-378a-3p,miR-493-3p,miR-133a-3p,miR-361-5p,miR-214,miR-665-5p,miR-3184-5p,miR-181c-5p,miR-2285j,miR-151-3p,miR-451a,miR-4492,miR-504-5p,miR-30c-1-3p,miR-552-3p,miR-296-3p,miR-10a,miR-450a-1-3p,miR-129b-3p,miR-345-5p,miR-543-3p,miR-885-3p,miR-625-5p,miR-363-5p,miR-654-3p,miR-345-5p,miR-197-5p,miR-330-3p,miR-455-5p,miR-2285l,miR-133b-5p,miR-2284d,miR-432-5p,miR-544b,miR-103,miR-1285,miR-
LIPE	ENSOARG0(	lipase,	miR-2355-5p,miR-3957-5p,miR-3957,novel_32,miR-193b-3p,miR-3065-5p,miR-129b-3p,miR-2300b-3p,miR-2387,miR-1827,miR-378d,miR-625-5p,miR-941,miR-193b-3p,miR-361-3p,novel_17,miR-193a-3p,miR-25-5p

ACKR1	ENSOARG0(	atypical	miR-4324,novel_120,miR-758-5p,miR-24-3p,miR-326-3p,miR-199b-5p,miR-2428,miR-2285ab,miR-378b,miR-107,miR-2319a,miR-135b-5p,miR-3120-3p,miR-299b-3p,miR-670-3p,miR-6535,miR-677,miR-181b-3p,miR-7-5p,miR-503-5p,miR-190a,miR-483-3p,miR-452-5p,miR-125a-5p,miR-466f-3p,miR-628-5p,miR-2285r,miR-708-3p,miR-7b-5p,miR-2113,miR-450c-3p,miR-197-3p,miR-3071-5p,miR-16-1-3p,miR-27b-3p,miR-31-5p,miR-103a-3p,miR-503-5p,miR-134-3p,miR-185-5p,miR-194-3p,miR-103,miR-551b-3p,miR-125b-5p,miR-135a-5p,miR-10b-3p,miR-485-5p,miR-671-5p,miR-874-3p,miR-3068-3p,miR-2355-5p,miR-125a,miR-300-3p,miR-769,miR-199a-5p,miR-200c,miR-2898,miR-200c-3p,miR-107,novel_96,miR-3529-3p,miR-29b-2-5p,miR-450b-5p,miR-199a-5p,novel_39,miR-192-3p,miR-330-5p,miR-200b-3p,miR-299a-3p,miR-429-3p,miR-7641,miR-2411-5p,miR-483-3p,miR-545-5p,miR-450a-1-3p,miR-382-3p,miR-769-5p,miR-3968,miR-2285t,miR-2447,miR-541-3p,miR-2285f,miR-190b-5p,miR-423-5p,miR-361-3p,miR-199b-5p,miR-136-5p,miR-139-5p,miR-7689-3p,miR-126b-5p,miR-378g,miR-2483-3p,miR-203-3p,miR-27a-3p,miR-326,miR-491-5p,miR-199b-5p,miR-382-3p,miR-744-3p,miR-8485,miR-2284r,miR-484,miR-541,miR-101a-5p,miR-409b,miR-1343-3p,miR-181b-2-3p,miR-27a-3p,miR-147a,miR-455-3p,miR-216a-3p,miR-29b-2-5p,miR-450b-3p,miR-4492,miR-190a-5p,miR-4510,miR-3184-3p,miR-665-5p,miR-6525,miR-769-5p,miR-346,miR-3184-5p,miR-6134,miR-450a-1-3p,miR-129b-3p,miR-2312,miR-186-5p,miR-582-5p,miR-2331-3p,miR-552-3p,miR-296-3p,miR-27a-5p,miR-6130,miR-139-5p,miR-204-3p,novel_17,miR-92a-1-5p,miR-125b,miR-299-3p,miR-216c-5p,miR-105-3p,miR-503-5p,miR-376a-5p,miR-181b-1-3p,miR-125b-2-3p,miR-2411,miR-2904,miR-188-3p,miR-1197-5p,miR-133a-3p,miR-16-1-3p,miR-380-5p,miR-1b-3p,miR-200b

APCS	ENSOARG0(	amyloid	miR-2285r,miR-503-3p,miR-296-3p,miR-582,miR-377-3p,miR-3141,miR-2285v,miR-365b-5p,miR-2411-3p,miR-378a-5p,miR-483-3p,miR-31-5p,miR-103a-3p,miR-339-5p,miR-3535,miR-134-3p,novel_116,miR-432,miR-450c-3p,miR-431-3p,novel_23,miR-16b,miR-497-5p,miR-369-5p,miR-2284x,miR-199b-5p,miR-2284w,miR-4324,miR-142b,miR-2284o,miR-425-5p,novel_68,miR-6535,miR-320c,miR-3141,miR-3065-5p,miR-3591-3p,miR-3064-5p,miR-2284f,miR-450b-5p,miR-148b-5p,miR-200a,miR-29a-5p,miR-3956-5p,miR-205-5p,miR-664-3p,miR-30f,miR-127-5p,novel_96,miR-200a-3p,miR-2285ad,novel_79,miR-431,miR-9-5p,miR-322-5p,miR-758-5p,miR-450a-1-3p,miR-2427,miR-16-5p,miR-193a-5p,miR-6740-5p,miR-125b-5p,miR-3432a,miR-574-5p,miR-874-3p,miR-2285aa,miR-2355-5p,miR-125a,miR-3956,miR-300-3p,miR-424-3p,miR-301,miR-93-5p,miR-23b,miR-329a,miR-20b-5p,miR-23a-3p,miR-8485,miR-2310,miR-431-5p,miR-323b-3p,miR-505,miR-29b-1-5p,miR-16b,miR-106b-5p,miR-182-5p,miR-491-5p,miR-199b-5p,miR-548w,novel_78,miR-539-5p,miR-147a,miR-455-3p,miR-2459,miR-329-3p,miR-2284r,miR-323b,miR-758-3p,miR-362-3p,miR-2447,miR-6527,miR-9-5p,miR-361-3p,miR-628-3p,miR-3968,miR-25,miR-3596,miR-378g,miR-2483-3p,miR-409-5p,miR-136-5p,miR-335-3p,miR-544-5p,miR-2285p,miR-500a-5p,miR-345-3p,miR-92a-1-5p,miR-106a,miR-212-5p,miR-23b-3p,miR-23c,miR-193b-5p,miR-1434-5p,miR-184-3p,miR-33b-3p,miR-665-5p,miR-769-5p,miR-6525,miR-2285j,miR-30e-3p,miR-151-3p,miR-30a-3p,miR-129-5p,miR-30c-1-3p,miR-377-3p,miR-15b-5p,miR-450a-1-3p,miR-2285c,miR-129b-3p,miR-411,miR-339b,miR-543-3p,miR-654-3p,miR-296-3p,let-7d-3p,miR-106a-5p,miR-17-5p,miR-124-5p,miR-125a-5p,miR-452-5p,miR-133b-5p,miR-665,miR-2285g,miR-432-5p,miR-103,miR-20b,miR-2355-3p,miR-873a-5p,miR-653,miR-1434-3p,miR-
KDM1A	ENSOARG0(	lysine (	miR-3958-5p,miR-129-2-3p,miR-4510,miR-224-5p,miR-665,miR-331-3p,miR-125b-5p,miR-34c-3p,miR-2285j,miR-592,miR-6516-3p,novel_74,miR-182-5p,miR-125a,miR-552-3p,miR-154a-3p,miR-221-5p,miR-376c-3p,miR-877-5p,miR-6130,miR-365a-3p,miR-376e-3p,miR-3154,miR-200a-5p,miR-3074-1-3p,miR-154b-3p,miR-29a-5p,miR-376c-3p,miR-125b,novel_17,miR-664-3p,miR-6119-5p,miR-208b-3p,miR-618,miR-221-5p,miR-1224-5p,miR-214-5p,novel_121,miR-129-1-3p,miR-483-3p,miR-302a-5p,miR-140-3p,miR-128-3p,miR-133a-3p,miR-218-1-3p,miR-33b-3p,miR-19b-3p,novel_101,miR-194b-5p,miR-6527,miR-212-3p,miR-541-3p,miR-107-5p,miR-147-5p,miR-154-3p,miR-490-5p,novel_1,miR-2285u,miR-664b,novel_69,miR-216a-3p,miR-3970,miR-19a-3p,miR-410-5p,miR-491-3p,miR-2284l,miR-194a,miR-182-5p,novel_51,miR-224-5p,miR-103a-2-5p,miR-125a-5p,miR-483-3p,miR-5010-3p,miR-409b,miR-2355-3p,miR-2459,miR-3431,miR-185-5p,miR-1298-3p,miR-432-5p,miR-3071-5p,miR-194-5p,miR-30b-3p,miR-143-5p,miR-541,miR-432,miR-101c,miR-30b-3p
SECISBP2	ENSOARG0(	SECIS bi	miR-3074-1-3p,miR-876-3p,miR-6516,miR-196b-5p,miR-6516-5p,miR-196a-5p

OTOP1	ENSOARG0(	otopetri	miR-7859,miR-758-5p,novel_121,miR-222,miR-548e-3p,miR-3600,miR-142-3p,miR-5703,miR-380-5p,miR-2424,miR-216c-5p,miR-195a-3p,miR-181b-5p,miR-21-3p,miR-219-3p,miR-2285p,novel_17,miR-125a-3p,miR-30f,miR-548o-3p,miR-21-3p,miR-190a-3p,miR-186-5p,miR-21a-3p,let-7f-1-3p,miR-2448-3p,miR-3154,miR-411,miR-6525,miR-181c-5p,let-7b-3p,miR-222-3p,miR-219b-3p,miR-1298-3p,miR-221-3p,miR-2459,miR-340-3p,novel_78,miR-27a-3p,miR-450b-5p,miR-101c,miR-455-5p,miR-27b-3p,miR-873a-5p,miR-181a-5p,novel_23,miR-3071-5p,miR-380-5p,miR-574-3p,miR-101b-3p,miR-382-3p,miR-382,miR-668-3p,miR-124-5p,miR-452-5p,miR-455-5p,novel_69,miR-411b-3p,miR-27a-3p,miR-544-5p,miR-221,miR-3591-3p,miR-381-3p,miR-3959-3p,miR-219a-2-3p,miR-2284w,novel_60,miR-3956-3p,miR-181d-5p,miR-495-3p,miR-628-3p,novel_1,miR-147-5p,miR-101-3p,miR-382-5p
EDEM1	ENSOARG0(	ER degra	let-7i-5p,novel_25,miR-204-3p,miR-769,miR-132-5p,miR-10a,miR-2284s,miR-3956,miR-615-5p,miR-548o-3p,miR-30c-1-3p,miR-504-5p,miR-339b,miR-362-5p,let-7b-5p,miR-10b-5p,miR-500b-5p,miR-769-5p,miR-3074-2-3p,miR-1271,miR-433-5p,let-7b,miR-134-5p,miR-204-5p,miR-17-3p,miR-6740-5p,let-7f,miR-134-5p,miR-1271-5p,novel_79,miR-548e-3p,miR-10a-5p,let-7d,miR-1343-5p,miR-124-3p,miR-1961,miR-134,miR-1290,miR-196a-5p,novel_133,let-7g,miR-376a-5p,miR-345-3p,let-7k,let-7f-5p,miR-98-5p,miR-2898,miR-17-3p,let-7c-5p,miR-339a,miR-181c-3p,miR-92a-1-5p,miR-30c-2-3p,miR-500a-5p,miR-664-3p,miR-27a-3p,let-7d-5p,miR-3596,miR-211-5p,miR-10a-5p,miR-664b,let-7e-5p,miR-504,miR-96-5p,miR-378j,miR-362-5p,miR-211,miR-17-3p,miR-1839-3p,miR-2447,miR-147-5p,miR-499a-5p,let-7i,miR-769-5p,miR-576-3p,miR-2459,miR-500-5p,miR-9788-3p,miR-185-5p,let-7g-5p,miR-27a-3p,miR-339-5p,miR-409b,miR-27b-3p,miR-2426,novel_23,miR-7b-5p,miR-10b,miR-484,miR-2284r,miR-4532,miR-432-3p,miR-432-3p,miR-30c-1-3p,miR-668-3p,miR-382-3p,miR-1839-3p,miR-1388-3p,let-7a-5p,miR-124a,let-7e,miR-1343-5p,miR-196b-5p,miR-1185-5p,miR-7-5p

SKIV2L2	ENSOARG0(	superkil	miR-6516-5p,miR-378h,miR-2411-5p,miR-322-5p,miR-24-2-5p,novel_121,miR-582-3p,miR-450a-1-3p,miR-6517,miR-222,miR-299a-3p,miR-10a-5p,miR-155-5p,miR-345-3p,miR-3591-5p,miR-330-5p,miR-501-3p,miR-24-2-5p,miR-28b,miR-450b-5p,miR-1271-3p,miR-2285b,miR-181a-2-3p,miR-3064-5p,miR-127-5p,miR-6536,miR-450a-2-3p,miR-28-3p,miR-17-3p,miR-2898,miR-200a-3p,miR-219-3p,miR-376b-3p,miR-365a-3p,miR-3959-5p,miR-331-5p,miR-34c-5p,miR-3068-3p,miR-874-3p,miR-141-3p,miR-1271,miR-10b-3p,miR-671-5p,miR-1814c,miR-1248,miR-488-3p,miR-200a-5p,miR-378c,miR-1306-5p,miR-28a-3p,miR-16-5p,miR-193a-5p,miR-28-5p,miR-494-5p,miR-34c-3p,miR-3969,miR-6740-5p,miR-3431,miR-412-5p,miR-194-3p,miR-31-5p,miR-26b-3p,miR-380-3p,miR-16b,miR-497-5p,miR-1285,miR-708-3p,miR-450c-3p,miR-654-3p,miR-6240,miR-378a-5p,miR-124-5p,miR-2411-3p,miR-330-3p,miR-105-5p,miR-34b,miR-299b-3p,miR-677,miR-378b,miR-26b-5p,miR-10a-5p,miR-3120-3p,miR-195a-5p,miR-378f,miR-2319a,miR-3591-3p,miR-376b-3p,miR-1306,miR-371b-3p,miR-326-3p,miR-449a,miR-335-5p,miR-17-3p,miR-495-3p,miR-2285x,miR-199c,miR-203b-5p,miR-28c,miR-15a,miR-34c,miR-16a,miR-15a-5p,miR-487a-3p,miR-7859,miR-95-3p,miR-2284h-5p,miR-548e-3p,miR-485-3p,miR-1b-3p,miR-3600,miR-34a-5p,miR-378d,miR-361-5p,miR-664-5p,miR-378i,miR-1434-5p,miR-1197-5p,miR-378a-3p,miR-130a-5p,miR-421-5p,miR-376a-5p,miR-143-3p,miR-380-3p,miR-2411,miR-2285w,miR-195a-3p,miR-369-3p,miR-466i-5p,miR-21-3p,miR-299-3p,miR-221-5p,miR-34b-5p,miR-26a-5p,miR-210-5p,miR-2285p,miR-374a-5p,miR-422a,miR-345-3p,miR-324-3p,miR-6516,miR-544-3p,miR-10a,miR-15b-5p,miR-877-5p,miR-221-5p,miR-378a-3p,miR-552-3p,miR-21-3p,novel_99,miR-450a-1-3p,miR-21a-3p,miR-487b-5p,miR-129b-3p,miR-10b-5p,miR-6525,miR-

MYO1A	ENSOARG0(	myosin I	miR-125b,miR-196a-3p,miR-542-5p,miR-30d,miR-30b-3p,miR-34b-5p,miR-21-3p,miR-2284m,miR-466i-5p,miR-760-3p,miR-532-3p,miR-487a-3p,miR-148b-3p,miR-15a-5p,miR-124-3p,miR-188-3p,miR-1197-5p,miR-5703,novel_133,miR-215-5p,miR-2300b-3p,miR-19b-3p,miR-425-5p,miR-378c,miR-4510,let-7b-5p,miR-338-3p,miR-6134,miR-19b-1-5p,miR-320e,miR-338-5p,miR-708-5p,miR-424-5p,miR-664a,miR-3065-3p,miR-182-5p,miR-532-5p,miR-378a-3p,miR-545-3p,miR-22-3p,miR-204-3p,miR-877-5p,miR-192-5p,miR-6130,miR-193b-3p,miR-133b-3p,miR-30f,let-7a-5p,miR-424-5p,miR-4532,miR-193a,miR-30c-1-3p,miR-326,miR-1306-5p,let-7e,miR-542-5p,miR-3085-3p,miR-25-5p,miR-3955-3p,novel_4,miR-9788-3p,miR-30b-3p,miR-484,miR-6529a,miR-378b,miR-378c,miR-2426,miR-2285f,novel_60,miR-541-3p,miR-371a-5p,miR-107-5p,miR-4726-5p,miR-7977,miR-3064-5p,miR-20a-3p,miR-452-3p,let-7d-5p,miR-454-5p,miR-378e,miR-489,miR-3082-5p,miR-361-3p,miR-877-3p,miR-4286,miR-3065-3p,miR-2284a,miR-30c-2-3p,miR-339a,let-7c-5p,miR-1260a,miR-2898,miR-7641,miR-378h,miR-218-2-3p,miR-29d-3p,miR-30a-5p,miR-615-3p,miR-345-3p,miR-134,miR-128-3p,let-7d,miR-133a-3p,miR-365a-5p,miR-378c,miR-145-3p,miR-323-3p,miR-26c,miR-3969,miR-410-5p,miR-29b-3p,miR-135a-1-3p,miR-28-5p,miR-486b-3p,miR-138-5p,miR-767-5p,miR-370-3p,miR-615-5p,miR-215-5p,miR-2284n,miR-34c-5p,miR-3154,miR-7144-5p,miR-485-5p,miR-363-5p,miR-296-3p,miR-345-5p,miR-125a-5p,miR-1343-5p,miR-665,miR-130a-3p,miR-133b-5p,miR-6238,miR-2284d,miR-185-5p,let-7g-5p,miR-873a-5p,miR-1285,miR-2889,miR-7862,miR-29b,miR-138,miR-30b-3p,miR-380-3p,miR-2284ab,miR-1260b,miR-3957-3p,novel_101,miR-542-5p,miR-135a-2-3p,miR-449a,miR-326-3p,miR-2428,miR-3959-3p,miR-371b-3p,miR-323c,miR-34c,miR-16a,let-7i,miR-33a-3p,miR-

TMPRSS11I	ENSOARG0(	transmem	miR-6516-3p,miR-450b-3p,miR-338-5p,miR-6134,miR-4510,miR-378c,miR-582-5p,novel_99,let-7f-1-3p,miR-323-5p,miR-6130,miR-544-3p,miR-204-3p,miR-660-5p,miR-378a-3p,miR-221-5p,miR-182-5p,miR-21-3p,miR-221-5p,miR-30b-3p,miR-125b,miR-185-3p,miR-216a-5p,miR-125b-2-3p,miR-146a-5p,miR-2411,miR-216c-5p,let-7a-3p,miR-378d,novel_133,miR-5703,miR-2904,miR-148b-3p,miR-200b,miR-222-5p,miR-199a-3p,miR-3600,miR-371a-5p,miR-2285y,miR-154a,miR-541-3p,novel_60,miR-3059-5p,miR-452-5p,miR-194b-5p,miR-877-3p,miR-199b-5p,miR-129b-5p,miR-3082-5p,miR-378e,miR-27a-3p,miR-452-3p,miR-20a-3p,miR-3955-3p,miR-2957,miR-30c-1-3p,miR-193a,miR-33a-5p,miR-2426,miR-450b-5p,miR-544a,miR-378c,miR-2284aa,miR-4791,miR-378b,miR-30b-3p,miR-7857,miR-2320-3p,miR-9788-3p,miR-105-1,miR-409b,miR-27a-3p,miR-138-5p,miR-486b-3p,miR-204-5p,miR-410-3p,miR-2332,miR-410-5p,miR-409-3p,miR-135a-5p,miR-331-3p,miR-365a-5p,miR-378c,miR-3607-3p,miR-10b-3p,miR-488-3p,miR-3154,miR-154b-5p,miR-199a-5p,miR-200c-3p,miR-2898,miR-543-5p,miR-200c,miR-217,miR-30c-2-3p,miR-490-3p,miR-1271-3p,miR-376c-5p,miR-199a-5p,miR-376b-5p,miR-3529-3p,miR-4286,miR-300,miR-411-3p,miR-200b-3p,miR-105-2,miR-128-3p,miR-134,miR-3578,miR-378h,miR-2411-5p,miR-502b,miR-203b-5p,miR-24-3p,miR-211,miR-495-3p,miR-3957-3p,miR-450b-3p,miR-6516-3p,miR-6395,miR-135b-5p,miR-379-5p,miR-670-3p,miR-146b-3p,miR-323a-5p,miR-378b,miR-655-5p,miR-124-5p,miR-1343-5p,miR-452-5p,miR-125a-5p,miR-660,miR-654-3p,miR-345-5p,miR-130b-5p,miR-363-5p,miR-2483-5p,miR-30b-3p,miR-138,miR-1285,miR-3071-5p,miR-194-5p,miR-432-5p,miR-544b,miR-2355-3p,miR-6238,miR-26b-3p,miR-130a-3p,miR-665,novel_74,miR-130b-5p,miR-454-3p,miR-4492,miR-2285i,let-7b-3p,miR-3604,miR-346,miR-98-3p,miR-6525,miR-
SEMA4D	ENSOARG0(	sema dom	miR-376c-5p,miR-376b-5p,miR-148b-5p,miR-28b,miR-185-3p,miR-342,miR-326,miR-107,miR-660,miR-543-5p,miR-2285o,miR-191-3p,miR-133b-5p,novel_4,miR-103a-3p,miR-2300a-5p,miR-103,miR-134-3p,miR-539-5p,miR-2904,miR-330-5p,miR-342-3p,miR-3074-5p,miR-122-5p,miR-326-3p,miR-664a-5p,miR-425-5p,miR-2285j,miR-2332,miR-135a-1-3p,miR-28c,miR-22-5p,miR-574-5p,miR-3074-5p,miR-660-5p,miR-7857-3p,miR-378g,miR-411,miR-107
ANO5	ENSOARG0(	anoctami	miR-135a-5p,miR-149-5p,miR-135b-5p

ZC3H13	ENSOARG0(	zinc fin	miR-15a-5p,miR-200b,miR-378h,miR-758-5p,miR-23c,miR-6516-5p,miR-322-5p,miR-7859,miR-23b-3p,miR-429-3p,miR-3600,miR-582-3p,miR-200b-3p,miR-128-3p,miR-378i,miR-378a-3p,miR-2427,miR-148b-5p,miR-3065-3p,miR-29b-2-5p,miR-466i-5p,miR-760-3p,miR-200c-3p,miR-21-3p,miR-1277-5p,miR-200c,miR-205-5p,miR-422a,miR-216b-5p,miR-6516,miR-15b-5p,miR-378a-3p,miR-545-3p,miR-3065-3p,miR-2312,miR-1827,novel_48,miR-21a-3p,miR-488-3p,miR-23b,miR-346,miR-378c,miR-3074-5p,miR-378c,miR-16-5p,miR-664a,miR-424-5p,miR-129-5p,miR-149-5p,miR-216a-3p,miR-27a-3p,miR-378c,miR-1388-5p,miR-16b,miR-3120-5p,miR-27b-3p,miR-2284x,miR-497-5p,miR-16-2-3p,miR-542-3p,miR-378b,miR-378d,miR-424-5p,miR-23a-3p,miR-16b,miR-182-5p,miR-378e,miR-27a-3p,miR-4443,miR-378b,miR-3074-5p,novel_68,miR-26b-5p,miR-320b,miR-136-5p,miR-378f,miR-195a-5p,miR-2285ab,miR-3065-5p,miR-129b-5p,miR-216a-3p,miR-2428,miR-2285x,miR-2285f,miR-15a,novel_82,miR-16a
VAT1L	ENSOARG0(	vesicle	miR-1306-3p,miR-1306-3p,miR-153-3p,miR-340-3p,miR-193a-5p,miR-665-5p,miR-3956,miR-103b,miR-7857-3p,miR-153,miR-496-5p,miR-877-3p,miR-3187-3p
C1orf234	ENSOARG0(	chromoso	miR-125a-3p,miR-6119-5p,miR-30f,miR-185-3p,miR-3085-3p,miR-196b-5p,miR-181b-5p,miR-106b-3p,miR-30d,novel_127,miR-214-3p,miR-3064-5p,miR-2887,miR-148b-5p,miR-196a-5p,miR-2284r,miR-181a-5p,miR-30a-5p,miR-18a-3p,novel_4,miR-455-3p,miR-2355-3p,miR-218-5p,miR-6529b,miR-5100,miR-29d-5p,miR-664a,miR-1843a-3p,miR-3964,miR-4492,miR-592,miR-2447,miR-181d-5p,miR-214,miR-361-3p,miR-29c-5p,miR-181c-5p,miR-488-3p,miR-30e-5p,miR-30d-5p,miR-3591-3p,miR-18a-3p,miR-876-3p,miR-543-3p,miR-2319a,miR-877-3p,miR-3064-5p,miR-452-3p,miR-874-3p,miR-3068-3p,miR-21-3p,miR-2285aa,miR-552-3p,miR-2284s,miR-204-3p

SYK	ENSOARG0(	spleen t	miR-6517,miR-2285ad,miR-7641,miR-2411-5p,miR-505-5p,miR-582-3p,miR-450a-1-3p,miR-3578,miR-345-3p,miR-885-3p,miR-105-2,miR-181a-2-3p,miR-3064-5p,miR-495-5p,miR-24-2-5p,miR-28b,miR-450b-5p,miR-1957a,novel_39,miR-3065-3p,miR-29a-5p,miR-2284y,miR-664-3p,miR-1260a,miR-2285af,miR-3071-3p,miR-450a-2-3p,miR-17-3p,novel_96,miR-107,miR-487a-3p,miR-370-3p,miR-488-3p,miR-3154,miR-345-5p,miR-2318,miR-485-5p,miR-671-5p,miR-1248,miR-7144-5p,miR-1306-5p,miR-144-5p,miR-133a-3p,miR-193a-5p,miR-1197-3p,miR-1260b,miR-365a-5p,miR-2433,miR-331-3p,miR-28-5p,miR-133b-5p,miR-103a-3p,miR-122-3p,miR-31-5p,miR-3431,miR-134-3p,miR-185-5p,miR-2285g,miR-103,miR-2355-3p,miR-194-3p,miR-6238,miR-1285,miR-3071-5p,miR-450c-3p,miR-16-1-3p,miR-1260b,miR-30b-3p,miR-2284x,miR-6240,miR-130b-5p,miR-628-5p,miR-2483-5p,miR-101-5p,miR-432-3p,miR-432-3p,miR-345-5p,let-7j,miR-2411-3p,novel_51,miR-365b-5p,miR-330-3p,miR-105-5p,miR-2285l,miR-124-5p,miR-193a-3p,miR-452-5p,miR-26b-5p,miR-3120-3p,miR-320b,miR-2404,miR-146b-3p,miR-6535,miR-2285ab,miR-3970,miR-107,miR-410-5p,miR-17-3p,miR-193b-3p,miR-24-3p,miR-2428,miR-28c,miR-4324,miR-128-1-5p,miR-502b,miR-656-5p,miR-203b-5p,miR-22-5p,miR-212-5p,miR-1b-3p,miR-532-3p,miR-2366,miR-1197-5p,miR-188-3p,miR-1193,miR-140-3p,miR-124-3p,miR-2904,novel_73,miR-2284u,miR-16-1-3p,miR-133a-3p,miR-487a,miR-345-3p,miR-143-3p,miR-2432,miR-2411,novel_17,miR-26a-5p,miR-618,miR-345-3p,miR-92a-1-5p,miR-324-3p,miR-219b-5p,miR-760-3p,miR-2484,miR-21-3p,miR-221-5p,miR-504-5p,miR-221-5p,miR-552-3p,miR-216b-3p,miR-154a-3p,miR-3065-3p,miR-193b-3p,miR-139-5p,miR-6130,miR-499b-3p,miR-132-5p,miR-582-5p,miR-877-5p,novel_32,miR-450a-1-3p,miR-2285c,miR-

CALD1	ENSOARG0(	caldesmo	miR-6240,miR-668-3p,miR-2284l,let-7j,let-7a-2-3p,miR-146a,miR-330-3p,miR-17-5p,miR-106a-5p,miR-483-3p,miR-130a-3p,miR-20b,miR-1434-3p,novel_23,miR-2284ab,miR-219a-1-3p,miR-26a-2-3p,miR-2284x,miR-301b-3p,miR-20a-5p,novel_87,miR-147-5p,miR-378b,miR-670-3p,miR-2387,miR-148a-3p,miR-376b-3p,miR-3065-5p,miR-19a-3p,miR-146b-5p,miR-2319a,miR-378f,miR-505-3p,miR-376a-2-5p,miR-3529-3p,miR-148b-5p,miR-29a-5p,miR-2285b,miR-376b-3p,miR-200c,miR-217,miR-450a-2-3p,miR-200c-3p,miR-152-3p,miR-6517,miR-429-3p,miR-376a-5p,miR-9-5p,miR-483-3p,miR-6516-5p,miR-378h,miR-3591-5p,miR-3578,miR-2427,miR-200b-3p,let-7a-2-3p,miR-93,miR-378c,miR-34c-3p,miR-494-5p,miR-2332,miR-5100,miR-100-3p,miR-3969,miR-3068-3p,miR-93-5p,miR-17-5p,let-7g-3p,miR-20b-5p,miR-1814c,miR-106a-5p,miR-494-3p,miR-378d,miR-2310,miR-106b-5p,miR-182-5p,miR-130b-3p,miR-146b,miR-330-3p,miR-548w,miR-151a-3p,miR-376b-3p,miR-148b-5p,miR-484,miR-378b,miR-2284r,miR-378c,miR-299a-5p,miR-101c,miR-1839-5p,miR-32-3p,miR-9-5p,miR-877-3p,novel_111,miR-154a,miR-7705,miR-2440,miR-2319b,miR-499b-5p,miR-378e,miR-2483-3p,miR-3082-5p,miR-195a-3p,miR-20a,miR-146a-5p,miR-618,miR-422a,miR-106a,miR-466i-5p,miR-106a,miR-505-3p,miR-200b,miR-222-5p,miR-148b-3p,novel_83,miR-378i,miR-5703,miR-1434-5p,miR-217-5p,miR-378a-3p,miR-19b-3p,miR-148a-5p,miR-378c,miR-3184-3p,miR-6525,miR-338-3p,miR-151-3p,miR-454-3p,miR-140-5p,miR-378a-3p,miR-545-3p,miR-32-3p,miR-301a-3p,miR-6516,miR-582-5p,miR-877-5p,miR-299,miR-487b-5p,let-7g-3p,novel_99,miR-582-5p

F10	ENSOARG0(	coagulat	miR-1197-5p,miR-2408,miR-2300b-3p,miR-133a-3p,miR-142-3p,miR-548e-3p,miR-1247-5p,miR-7859,miR-92a-1-5p,miR-125b,miR-185-3p,miR-345-3p,miR-324-3p,miR-30b-3p,miR-760-3p,miR-345-3p,miR-2887,miR-183-5p,miR-29b-1-5p,miR-500a-3p,miR-450a-1-3p,miR-296-3p,miR-3065-3p,miR-548o-3p,miR-2331-3p,miR-504-5p,miR-204-3p,miR-6130,miR-6516,miR-4492,miR-500,miR-664a,miR-450b-3p,miR-214,miR-4510,miR-3074-5p,miR-145a-3p,miR-3184-5p,miR-30b-3p,miR-541,miR-296-5p,miR-3120-5p,miR-145b,novel_78,miR-1343-3p,miR-9788-3p,miR-29b-1-5p,novel_19,miR-5126,miR-326,miR-214-3p,miR-491-5p,miR-3085-3p,miR-2330-5p,miR-574-3p,miR-1388-3p,miR-4532,miR-361-3p,miR-504,miR-147-3p,miR-211-5p,miR-7977,miR-452-3p,miR-3064-5p,miR-4443,miR-3596,miR-615,novel_82,miR-3968,miR-452-5p,miR-541-3p,miR-3059-5p,miR-2447,miR-423-5p,miR-361-3p,miR-877-3p,miR-330-5p,miR-1343-5p,miR-345-3p,miR-615-3p,miR-1224-5p,miR-486-5p,miR-214-5p,miR-450a-1-3p,miR-6516-5p,miR-505-5p,miR-543-5p,novel_96,miR-191-3p,miR-450a-2-3p,miR-181a-2-3p,miR-3064-5p,miR-3065-3p,novel_39,miR-29a-5p,miR-671-5p,miR-485-5p,miR-23a-5p,miR-615-5p,miR-125a,miR-542-5p,miR-370-3p,miR-324-5p,miR-300-3p,miR-331-3p,miR-125b-5p,miR-6740-5p,miR-204-5p,miR-138-5p,miR-193a-5p,miR-181a-2-3p,novel_42,miR-365a-5p,miR-145a-5p,miR-3071-5p,miR-450c-3p,miR-744-5p,miR-4508,miR-125b-1-3p,miR-30b-3p,miR-138,miR-340-3p,miR-187-3p,miR-149-5p,miR-432-5p,novel_51,miR-2411-3p,miR-365b-5p,miR-3141,miR-1343-5p,miR-125a-5p,miR-22851,miR-378a-5p,miR-432-3p,miR-432-3p,miR-3141,miR-1291,miR-3120-3p,miR-320b,miR-3074-5p,novel_68,miR-669,novel_69,miR-6529b,miR-133a-5p,miR-4324,miR-147-5p,miR-128-1-5p,miR-3957-3p,miR-2285m,novel_27,miR-326-3p,miR-24-3p,miR-211
PPAP2B	ENSOARG0(	phosphat	miR-424-5p,miR-34c-3p,miR-370-5p,miR-378c,miR-6134,miR-6128,miR-16-5p,miR-378c,miR-7144-5p,let-7g-3p,miR-339b,miR-204-3p,miR-15b-5p,miR-370-5p,miR-378a-3p,miR-574-5p,miR-504-5p,miR-548o-3p,novel_127,miR-324-3p,miR-339a,miR-217,miR-422a,miR-2285af,miR-2432,miR-130a-5p,miR-128-3p,miR-378d,miR-378a-3p,miR-217-5p,miR-23b-5p,miR-1193,miR-378i,miR-3591-5p,miR-2284z,miR-302a-5p,miR-322-5p,miR-378h,miR-15a-5p,miR-7641,novel_79,miR-18a-3p,miR-2285t,miR-16a,novel_87,miR-15a,miR-877-3p,miR-361-3p,miR-371b-3p,novel_111,miR-495-3p,miR-1193,miR-2447,miR-541-3p,miR-335-3p,miR-2319a,miR-195a-5p,miR-378f,miR-504,miR-18a-3p,miR-378j,miR-216a-3p,miR-2387,miR-669,miR-27a-3p,miR-378e,miR-670-3p,miR-124-5p,miR-16b,miR-296-3p,miR-296-3p,miR-378d,miR-424-5p,miR-497-5p,miR-3120-5p,miR-27b-3p,miR-1388-5p,miR-378c,miR-16b,miR-2426,miR-378b,miR-541,miR-149-5p,novel_116,miR-216a-3p,miR-496-5p,miR-339-5p,miR-27a-3p,miR-222-5p

ANKRD29	ENSOARG0(	ankyrin	miR-217-5p,miR-1246,miR-376a-5p,miR-493-3p,miR-30d-3p,miR-548e-3p,miR-328-3p,miR-23c,miR-200b,miR-23b-3p,miR-7859,miR-125b,miR-210-5p,miR-618,miR-1277-5p,miR-760-3p,miR-466i-5p,miR-2424,miR-2432,miR-376c-3p,miR-433-3p,miR-21a-3p,miR-345-5p,miR-660-5p,miR-216b-5p,miR-376e-3p,miR-30a-3p,miR-30e-3p,miR-17-3p,miR-412-3p,miR-541,miR-6529a,miR-484,miR-3120-5p,miR-2426,miR-101a-5p,miR-5010-3p,miR-1298-3p,miR-505,miR-1306-5p,miR-33a-5p,miR-23a-3p,let-7c-3p,miR-129b-5p,novel_94,miR-136-5p,miR-7977,miR-499b-5p,miR-20a-3p,miR-203-3p,miR-3596,miR-2440,miR-628-3p,miR-3968,miR-194b-5p,miR-541-3p,miR-885-5p,miR-212-3p,miR-3956-3p,novel_60,miR-3059-5p,miR-7705,novel_111,miR-877-3p,miR-200b-3p,miR-128-3p,miR-411-3p,miR-376a-5p,miR-1224-5p,miR-412,miR-217,miR-200c,miR-2397-5p,miR-200a-3p,miR-543-5p,miR-200c-3p,miR-17-3p,miR-181a-2-3p,miR-30a-3p,miR-200a,miR-29a-5p,miR-23b,miR-3074-1-3p,miR-7144-5p,miR-190a-3p,miR-345-5p,let-7g-3p,miR-125a,miR-141-3p,miR-2355-5p,miR-2285aa,miR-376c-3p,miR-223-5p,miR-331-3p,miR-125b-5p,miR-2332,miR-6128,miR-3071-5p,miR-194-5p,miR-26b-3p,novel_116,miR-194a,miR-2285v,miR-466f-3p,miR-125a-5p,miR-660,miR-363-5p,miR-668-3p,miR-345-5p,miR-654-3p,miR-378b,miR-216a-3p,miR-3970,miR-1306,miR-2387,miR-6395,miR-6516-3p,miR-376a-2-5p,miR-3120-3p,miR-146b-3p,miR-576-3p,miR-502-5p,miR-421-5p,miR-379-3p,miR-17-3p,miR-136-3p,miR-2284w,novel_27

AUH	ENSOARG0(	AU RNA b	miR-204-5p,miR-6740-5p,miR-5100,miR-370-5p,miR-378c,miR-133a-3p,miR-16-5p,miR-1248,miR-329a,miR-10b-3p,miR-23b,miR-488-3p,miR-503-3p,miR-574-5p,miR-450a-2-3p,miR-17-3p,miR-339a,miR-490-3p,miR-450c-5p,miR-2285b,miR-450b-5p,miR-30a-3p,miR-1290,miR-3578,miR-345-3p,miR-378h,miR-758-5p,miR-322-5p,miR-9-5p,miR-505-5p,miR-412,novel_79,miR-656-5p,miR-22-5p,miR-16a,miR-15a,miR-2284w,miR-2285m,miR-211,novel_101,miR-17-3p,miR-1193,novel_120,miR-378f,miR-195a-5p,miR-412-3p,miR-378b,miR-3591-3p,miR-3970,miR-669,miR-146b-3p,miR-124-5p,miR-2411-3p,novel_51,miR-296-3p,miR-224-5p,miR-539-3p,let-7j,miR-296-3p,miR-654-3p,miR-101-5p,miR-27b-3p,miR-188-5p,miR-7862,miR-497-5p,miR-4508,miR-16b,miR-145a-5p,novel_23,miR-542-3p,miR-185-5p,miR-3431,miR-412-5p,miR-377-5p,miR-339-5p,miR-133b-5p,miR-129-5p,miR-424-5p,miR-17-3p,miR-6516-3p,novel_74,miR-664a-5p,miR-665,miR-30a-3p,miR-30e-3p,miR-122-5p,miR-224-5p,miR-378c,novel_99,miR-339b,miR-133b-3p,let-7f-1-3p,miR-15b-5p,miR-216b-5p,miR-552-3p,miR-532-5p,miR-370-5p,miR-21-3p,miR-378a-3p,miR-1277-5p,miR-422a,miR-185-3p,miR-345-3p,miR-324-3p,novel_17,miR-210-5p,miR-125b-2-3p,miR-377-5p,let-7a-3p,miR-133a-3p,miR-188-3p,miR-378a-3p,miR-124-3p,miR-2300b-3p,miR-362-3p,miR-378i,miR-15a-5p,miR-23c,miR-487a-3p,miR-95-3p,miR-23b-3p,miR-485-3p,miR-30d-3p,miR-450a-5p,miR-301a-5p,miR-371a-5p,miR-2440,miR-101-3p,miR-8095,miR-361-3p,miR-9-5p,miR-6527,miR-544-5p,miR-1983,miR-129b-5p,miR-378j,miR-361-3p,miR-27a-3p,miR-378e,miR-211-5p,miR-541-5p,miR-153-3p,miR-16b,miR-362-3p,miR-378d,miR-23a-3p,miR-424-5p,miR-124a,miR-2426,miR-362-3p,miR-378c,miR-412-3p,miR-301b-5p,miR-378b,miR-329-3p,miR-2320-3p,miR-153,miR-145b,miR-27a-3p,novel_78
TMEM179	ENSOARG0(	transmem	miR-877-3p,miR-425-3p,miR-361-3p,miR-2403,miR-8117,miR-2433,miR-361-3p,miR-2904

TRIM58	ENSOARG0(	triparti	miR-3064-5p,miR-30a-3p,miR-1271-3p,miR-490-3p,miR-205-5p,miR-376b-3p,miR-2285af,miR-30c-2-3p,miR-3071-3p,miR-339a,miR-2898,miR-2285ad,novel_79,miR-505-5p,miR-2284z,miR-450a-1-3p,novel_121,miR-345-3p,miR-218-2-3p,miR-2427,miR-330-5p,miR-30a-5p,miR-300,miR-155-5p,miR-10a-5p,miR-374b,miR-93,miR-410-5p,miR-135a-1-3p,miR-5100,miR-191-5p,miR-204-5p,miR-370-3p,miR-215-5p,miR-300-3p,miR-133c,miR-223-5p,miR-30e-5p,miR-381-3p,miR-301,miR-93-5p,miR-17-5p,miR-7144-5p,miR-3074-1-3p,miR-20b-5p,miR-1814c,miR-130b-5p,miR-668-3p,miR-363-5p,miR-628-5p,let-7j,miR-7-5p,let-7d-3p,miR-17-5p,miR-106a-5p,miR-2411-3p,miR-339-5p,miR-3535,miR-185-5p,miR-411-5p,miR-2355-3p,miR-20b,miR-708-3p,miR-7b-5p,miR-2113,miR-1843b-5p,miR-1434-3p,miR-450c-3p,miR-16-1-3p,miR-188-5p,miR-219b-3p,novel_120,novel_101,miR-211,miR-24-3p,miR-326-3p,miR-2284w,miR-20a-5p,miR-382-5p,novel_1,miR-199c,miR-216b-3p,miR-655-5p,miR-10a-5p,miR-411b-3p,novel_69,miR-2387,miR-3970,miR-376b-3p,miR-3065-5p,miR-216c-5p,miR-195a-3p,miR-20a,miR-345-3p,miR-130a-5p,miR-2432,miR-618,miR-374a-5p,miR-500a-5p,miR-345-3p,miR-106a,miR-1277-5p,miR-3653-3p,miR-106a,miR-30d-3p,miR-210-3p,miR-340-5p,miR-191,miR-215-5p,miR-2300b-3p,miR-2366,miR-124-3p,miR-1193,miR-1434-5p,miR-188-3p,miR-5703,miR-33b-3p,novel_73,miR-16-1-3p,miR-218-1-3p,miR-539-5p,miR-4510,miR-145a-3p,miR-3074-2-3p,miR-10b-5p,miR-122-5p,miR-15b-3p,miR-30e-3p,miR-30a-3p,miR-151-3p,miR-3604,miR-338-5p,miR-655-3p,miR-450b-3p,novel_74,miR-155-5p,miR-874-5p,miR-130b-5p,miR-504-5p,miR-30c-1-3p,miR-545-3p,miR-552-3p,miR-10a,miR-192-5p,miR-6130,miR-299,miR-450a-1-3p,miR-655,miR-339b,miR-1827,miR-124a,miR-494-3p,miR-106a-5p,miR-30c-1-3p,miR-2310,miR-326,miR-106b-5p,miR-

PRKAA2	ENSOARG0(	5'-AMP-a	miR-2284aa,miR-3120-5p,miR-378c,miR-101a-5p,miR-450b-5p,miR-2426,miR-378b,miR-216a-3p,miR-2285e,miR-27a-3p,novel_4,miR-2300a-5p,miR-3955-3p,novel_103,miR-758-3p,miR-106a-5p,miR-382-3p,miR-494-3p,miR-424-5p,miR-129b-5p,miR-361-3p,miR-199b-5p,miR-27a-3p,miR-378e,miR-3082-5p,miR-541-5p,miR-499b-5p,miR-2285t,miR-374a-3p,miR-2284q,miR-4726-5p,miR-335,miR-382-3p,miR-30b-5p,miR-8095,miR-9-3p,miR-154a,miR-2285f,miR-1296-5p,miR-194b-5p,miR-452-5p,miR-181d-5p,let-7a-3p,miR-1197-5p,miR-140-3p,miR-199a-3p,miR-616-3p,miR-532-3p,miR-200b,miR-340-5p,miR-148b-3p,miR-15a-5p,miR-30b-3p,miR-106a,miR-2284m,miR-216a-5p,miR-125b,miR-2432,miR-146a-5p,miR-29b-1-5p,miR-20a,miR-216c-5p,miR-2312,miR-30f,miR-27b-5p,miR-21a-3p,let-7f-1-3p,miR-6130,miR-656-3p,miR-370-5p,miR-21-3p,miR-182-5p,miR-378a-3p,miR-660-5p,miR-424-5p,miR-29b-2-5p,miR-6134,miR-3074-2-3p,miR-378c,miR-539-5p,miR-4510,miR-380-3p,miR-2284ab,miR-1260b,miR-194-5p,miR-3071-5p,miR-653,miR-181a-5p,miR-1285,miR-1434-3p,miR-20b,miR-2284d,miR-2285g,miR-185-5p,miR-665,miR-130a-3p,miR-26b-3p,miR-133b-5p,miR-1343-5p,miR-125a-5p,miR-660,miR-124-5p,miR-330-3p,miR-17-5p,miR-106a-5p,miR-224-5p,miR-345-5p,miR-101-5p,miR-130b-5p,miR-6395,miR-195a-5p,miR-376a-2-5p,miR-1291,miR-18a-3p,miR-2285ab,miR-655-5p,miR-7-1-3p,miR-92a-3p,novel_1,miR-16a,miR-382-5p,miR-15a,miR-133a-5p,miR-20a-5p,novel_27,miR-1193,miR-495-3p,miR-128-3p,miR-200b-3p,miR-411-3p,miR-3578,miR-545-5p,miR-378h,miR-376a-5p,miR-423-3p,miR-92a-3p,miR-2898,miR-181b-5p,miR-200c-3p,miR-1260a,miR-2285af,miR-200c,miR-199a-5p,miR-490-3p,miR-2284a,miR-29b-2-5p,miR-30a-3p,miR-7144-5p,miR-1814c,miR-10b-3p,miR-17-5p,miR-199a-5p,miR-2284n,miR-154b-5p,miR-3965,miR-370-3p,miR-141-3p,miR-

BRMS1L	ENSOARG0(	breast c	miR-2284ab,miR-219a-1-3p,miR-1260b,miR-3071-5p,miR-653,miR-181a-5p,miR-20b,miR-377-5p,miR-187-3p,miR-660,miR-452-5p,miR-17-5p,miR-106a-5p,miR-345-5p,miR-654-3p,miR-135b-5p,miR-376a-2-5p,miR-18a-3p,miR-670-3p,miR-34b,miR-7-1-3p,miR-363,miR-378b,miR-203b-5p,miR-147-5p,miR-92a-3p,miR-34c,miR-3613,miR-6529b,miR-20a-5p,miR-449a,miR-2428,miR-24-3p,miR-323c,miR-495-3p,miR-17-3p,miR-1193,miR-105-2,miR-200b-3p,let-7a-2-3p,miR-30a-5p,miR-345-3p,miR-378h,miR-6516-5p,miR-7641,miR-376a-5p,miR-222,miR-92a-3p,miR-450a-2-3p,miR-17-3p,miR-181b-5p,miR-200c-3p,miR-1260a,miR-200c,miR-6119-5p,miR-490-3p,miR-2385-3p,miR-30a-3p,miR-29b-2-5p,miR-3529-3p,miR-3074-1-3p,miR-7144-5p,miR-485-5p,miR-10b-3p,let-7g-3p,miR-320a,miR-17-5p,miR-3154,miR-34c-5p,miR-374c-3p,miR-487a-3p,miR-374b-3p,miR-486b-3p,miR-409-3p,miR-135a-5p,miR-3969,miR-1-5p,miR-370-5p,miR-378c,miR-93,miR-374a-3p,miR-378c,miR-101a-5p,miR-450b-5p,miR-299a-5p,miR-125b-2-3p,miR-378b,miR-4791,miR-9788-3p,miR-221-3p,miR-27a-3p,miR-105-1,miR-3955-3p,miR-320b,let-7c-3p,miR-652-5p,miR-494-3p,miR-106a-5p,novel_94,miR-27a-3p,miR-378e,miR-664b,miR-452-3p,miR-4726-5p,miR-371a-5p,miR-30b-5p,miR-2285n,miR-181d-5p,miR-4429,miR-5703,miR-1246,miR-616-3p,miR-383-5p,miR-200b,miR-340-5p,miR-3600,miR-548e-3p,miR-30d,miR-1277-5p,miR-34b-5p,miR-221-5p,miR-21-3p,miR-320d,miR-106a,miR-29b-1-5p,miR-125b-2-3p,miR-20a,miR-183-5p,miR-154b-3p,miR-30f,miR-3613-5p,miR-204-3p,miR-6516,miR-370-5p,miR-21-3p,miR-182-5p,miR-221-5p,miR-545-3p,miR-378a-3p,miR-660-5p,miR-17-3p,miR-338-5p,miR-29b-2-5p,miR-222-3p,miR-338-3p,miR-425-5p,miR-3074-2-3p,miR-378c,miR-2284x,miR-27b-3p,miR-197-3p,miR-450c-3p,novel_23,miR-223-3p,miR-542-3p,miR-411-5p,miR-
PSMB9	ENSOARG0(	proteaso	let-7a-5p,let-7b,miR-2447,miR-541-3p,let-7g,let-7b-5p,miR-326-3p,let-7f,miR-326,miR-6740-5p,let-7i,let-7c-5p,miR-98-5p,let-7f-5p,let-7k,let-7e,miR-412,miR-412-5p,let-7g-5p,miR-204-3p,let-7d-5p,let-7i-5p,miR-541,miR-1961,miR-412-3p,miR-330-5p,let-7d,novel_73,let-7e-5p
GATAD2A	ENSOARG0(	GATA zin	miR-4510,miR-26b-3p,miR-6130,miR-544b,miR-2904,miR-1343-5p,miR-30b-3p,miR-4726-5p,miR-450a-2-3p,miR-30b-3p,miR-544a,miR-381-5p,miR-1343-5p,miR-1248,miR-381-5p
ZNF300	ENSOARG0(	zinc fin	miR-4286,novel_42,miR-380-3p,miR-154a,miR-2432,miR-200a,miR-100-3p,miR-485-3p,miR-2957,miR-2285y,miR-494-5p,miR-135a-1-3p,miR-2440,miR-2285n,miR-21-3p,miR-374b-3p,miR-6516-3p,miR-487b-5p,novel_1,miR-485-3p,miR-2285ad,miR-3596,miR-7857-3p,miR-487b-5p,miR-150-5p,miR-197-3p,miR-2300b-3p,miR-2284x,miR-544-5p,miR-543-3p,miR-380-3p
CEP170B	ENSOARG0(	centroso	miR-132-5p,miR-3065-3p,novel_42,miR-423-3p,miR-3065-3p,miR-2887,miR-138-5p,miR-138,miR-30b-3p,miR-4492,novel_17,miR-2366,miR-1306-5p,miR-1306,novel_51
GAPDH	ENSOARG0(	glyceral	miR-1843b-5p,miR-670-3p,miR-211-5p,miR-615-5p,miR-2355-5p,miR-370-3p,miR-2898,miR-486b-3p,miR-204-5p,miR-4492,novel_17,miR-185-3p,miR-376a-5p,miR-211,miR-6134,miR-486-3p,miR-3059-5p

LRRC63	ENSOARG0(	leucine	miR-6240,miR-363-5p,let-7j,let-7a-2-3p,miR-503-3p,miR-377-3p,miR-330-3p,miR-32,miR-365b-5p,novel_51,miR-483-3p,miR-196b-5p,miR-125a-5p,miR-133b-5p,miR-103a-3p,miR-377-5p,miR-432-5p,miR-412-5p,miR-6238,miR-544b,miR-103,miR-432,miR-542-3p,miR-1843b-5p,miR-197-3p,miR-22-3p,miR-374b-3p,miR-138,miR-30b-3p,miR-380-3p,miR-16-1-3p,miR-125b-1-3p,miR-4508,miR-2284x,miR-7862,miR-342-3p,miR-2285x,novel_101,miR-24-3p,miR-326-3p,miR-2428,miR-4324,miR-142b,miR-92a-3p,miR-502b,novel_68,miR-323a-5p,miR-2404,miR-299b-3p,miR-374c-3p,miR-677,miR-144-3p,miR-181b-3p,miR-148a-3p,miR-18a-3p,miR-3065-5p,miR-216a-3p,miR-450b-3p,miR-410-5p,miR-107,miR-3529-3p,miR-3064-5p,miR-2403,miR-92b-3p,miR-2284y,miR-1957a,miR-2284a,miR-490-3p,miR-192-3p,miR-2285af,miR-219-3p,miR-30f,miR-450a-2-3p,miR-2898,miR-127-5p,miR-107,miR-152-3p,novel_127,miR-92a-3p,miR-2285ad,miR-299a-3p,miR-1224-5p,miR-505-5p,miR-483-3p,miR-1271-5p,miR-6516-5p,miR-582-3p,miR-545-5p,miR-486-5p,miR-3591-5p,miR-345-3p,miR-196a-5p,miR-1290,miR-3578,miR-330-5p,miR-582-3p,miR-128-3p,let-7a-2-3p,miR-1306-5p,miR-6128,miR-1197-3p,miR-365a-5p,miR-410-5p,novel_63,miR-2284k,miR-125b-5p,miR-144,miR-331-3p,miR-6740-5p,miR-138-5p,miR-28-5p,miR-592,miR-3068-3p,miR-370-3p,miR-125a,miR-365a-3p,miR-25-3p,miR-488-3p,miR-503-3p,miR-200a-5p,miR-3154,miR-876-3p,let-7g-3p,miR-7144-5p,miR-485-5p,miR-1814c,miR-382-3p,miR-2284b,miR-8485,miR-2310,miR-758-3p,miR-342,novel_103,miR-326,miR-2957,miR-3085-3p,miR-487b-5p,miR-3955-3p,miR-153-3p,miR-214-3p,miR-548w,novel_4,miR-330-3p,miR-181b-2-3p,miR-153,miR-425-3p,miR-1298-3p,miR-142-5p,miR-455-3p,miR-9788-3p,miR-296-5p,miR-6529a,miR-484,miR-301b-5p,miR-541,miR-30b-3p,miR-758-3p,miR-1388-5p,miR-

TMPRSS4	ENSOARG0(	transmem	novel_115,miR-320b,miR-758-3p,miR-2330-5p,miR-424-5p,miR-652-5p,miR-544a,miR-3120-5p,miR-16-2-3p,miR-484,miR-142-3p,miR-500-5p,miR-216a-3p,miR-5010-3p,miR-335,miR-3958-3p,miR-769-5p,miR-362-5p,miR-502-3p,miR-541-3p,miR-212-3p,miR-3059-5p,miR-181d-5p,miR-3082-5p,miR-7977,miR-541-5p,miR-320d,miR-2284m,miR-21-3p,miR-299-3p,miR-30b-3p,miR-34b-5p,miR-125b,miR-130a-5p,miR-29b-1-5p,miR-4429,miR-378d,novel_133,miR-15a-5p,miR-532-3p,miR-3600,miR-664a,miR-17-3p,miR-208b-5p,miR-424-5p,miR-29b-2-5p,miR-320e,miR-206-3p,miR-145a-3p,miR-539-5p,miR-3074-2-3p,miR-21a-3p,miR-139-5p,miR-27a-5p,miR-544-3p,miR-22-3p,miR-204-3p,miR-496-3p,miR-140-5p,miR-532-5p,miR-1343-5p,miR-452-5p,miR-125a-5p,miR-1260b,miR-138,miR-2284ab,miR-7862,miR-1285,miR-873a-5p,miR-145a-5p,miR-2284d,miR-432-5p,miR-2355-3p,miR-544b,miR-6402,miR-15a,miR-34c,miR-16a,miR-24-3p,miR-211,miR-449a,miR-17-3p,miR-450b-3p,miR-195a-5p,miR-2387,miR-18a-3p,miR-378b,miR-34b,miR-146b-3p,miR-450a-2-3p,miR-17-3p,miR-1260a,miR-2284a,miR-1271-3p,miR-181a-2-3p,miR-1,miR-29b-2-5p,miR-30a-3p,miR-496,miR-345-3p,miR-501-3p,miR-7641,miR-138-5p,miR-204-5p,miR-486b-3p,miR-5100,miR-135a-5p,miR-409-3p,miR-331-3p,miR-500b-5p,miR-145-3p,miR-6128,miR-1814c,miR-485-5p,miR-7144-5p,miR-3074-1-3p,miR-362-5p,miR-320a,miR-3965,miR-769,miR-34c-5p,miR-2284n,miR-154b-5p,miR-141-3p,miR-130b-3p,miR-487b-5p,miR-214-3p,miR-153-3p,miR-431-5p,miR-16b,miR-29b-1-5p,miR-2310,miR-101b-3p,miR-574-3p,miR-2284b,miR-744-3p,miR-101c,miR-541,miR-301b-5p,miR-2284r,miR-758-3p,miR-1a-3p,miR-501-3p,miR-539-5p,miR-1298-3p,miR-147a,miR-153,miR-148b-5p,miR-1343-3p,miR-145b,miR-3968,miR-499a-5p,miR-101-3p,miR-218-5p,novel_82,miR-301a-5p,miR-877-
NOC2L	ENSOARG0(	NOC2-lik	miR-1260b,miR-181c-5p,miR-338-3p,miR-16-5p,miR-138-5p,miR-486b-3p,miR-134-5p,miR-28-5p,miR-424-5p,miR-874-5p,miR-708-5p,miR-665,miR-320e,miR-3965,miR-132-5p,miR-34c-5p,miR-15b-5p,miR-30c-1-3p,miR-2331-3p,miR-504-5p,miR-1271,miR-552-3p,miR-370-3p,miR-2355-5p,miR-339b,miR-1814c,miR-488-3p,miR-28b,miR-2478,miR-486-3p,novel_39,miR-329a-5p,miR-181b-5p,miR-760-3p,miR-127-5p,novel_127,miR-543-5p,miR-21-3p,miR-34b-5p,miR-125a-3p,miR-210-5p,miR-1260a,miR-185-3p,miR-339a,miR-30c-2-3p,miR-324-3p,miR-15a-5p,miR-505-5p,miR-322-5p,novel_83,miR-134-5p,miR-6517,miR-18a-3p,novel_79,miR-34a-5p,miR-16-1-3p,miR-134,miR-5703,miR-330-5p,miR-1343-5p,miR-24-3p,miR-361-3p,miR-326-3p,miR-449a,miR-335-5p,miR-541-3p,miR-181d-5p,miR-335,miR-203b-5p,miR-28c,miR-15a,miR-34c,miR-16a,miR-34b,novel_69,miR-378g,miR-329-5p,novel_68,miR-4792,miR-7977,miR-504,miR-3963,miR-1291,novel_94,miR-195a-5p,miR-3141,miR-361-3p,miR-21c,miR-18a-3p,miR-129b-5p,miR-1306,miR-30c-1-3p,miR-2310,miR-4532,miR-8485,miR-424-5p,miR-127-3p,miR-628-5p,miR-1343-5p,miR-1306-5p,miR-326,miR-16b,miR-3141,miR-134-3p,miR-421,novel_78,miR-339-5p,miR-1260b,miR-16-1-3p,miR-21-5p,miR-138,miR-16b,miR-497-5p,miR-541,miR-873a-5p

NELL1	ENSOARG0(	neural E	miR-16-5p,miR-214,miR-424-5p,miR-16a,miR-16b,miR-15a,miR-424-5p,miR-214-3p,miR-31-5p,miR-15b-5p,miR-374c-3p,miR-322-5p,miR-15a-5p,miR-374c-3p,miR-3082-5p,miR-374b-3p,miR-497-5p,miR-195a-5p,miR-16b
KIAA0226I	ENSOARG0(	KIAA0226	miR-301b-3p,miR-3059-5p,miR-1271,miR-6516-3p,miR-454-3p,miR-130b-5p,miR-2285j,miR-323a-5p,miR-301a-3p,miR-323-5p,miR-2483-3p,miR-545-3p,miR-452-3p,miR-182-5p,miR-147-3p,miR-96-5p,miR-9851-3p,miR-186-5p,miR-6516-3p,miR-1827,miR-301,miR-143-3p,miR-216c-5p,miR-130b-5p,miR-130b-3p,miR-618,miR-182-5p,miR-432-5p,miR-1271-5p,miR-545-5p,miR-455-3p,miR-421,miR-7641,miR-130a-3p,miR-432,miR-2300b-3p,miR-218-2-3p,miR-23b-5p
ZNF169	ENSOARG0(	zinc fin	miR-625-5p,miR-345-5p,miR-339b,miR-10a,miR-301a-3p,miR-151-3p,miR-30a-3p,miR-665,miR-30e-3p,miR-1468-5p,miR-454-3p,miR-4492,miR-190a-5p,miR-669a-3p,miR-130b-5p,miR-3958-5p,miR-214,miR-6525,miR-142a-5p,miR-346,miR-217-5p,miR-378a-3p,miR-378i,miR-2366,miR-16-1-3p,miR-142-3p,novel_73,miR-30d-3p,miR-500a-5p,miR-422a,miR-345-3p,miR-618,novel_17,miR-486-3p,miR-143-3p,miR-181b-1-3p,miR-142a-3p,miR-378j,miR-335-3p,miR-544-5p,miR-9-3p,miR-96-5p,miR-136-5p,miR-139-5p,miR-203-3p,miR-3596,miR-499a-5p,miR-190b-5p,miR-2447,miR-3956-3p,miR-877-3p,miR-323b,miR-1a-3p,miR-758-3p,miR-541,miR-101c,miR-1839-5p,miR-1388-5p,miR-181b-2-3p,miR-142-5p,miR-323b-3p,miR-29b-1-5p,miR-199b-5p,miR-214-3p,miR-130b-3p,miR-1388-3p,miR-101b-3p,miR-2310,miR-378d,miR-137-3p,miR-376c-5p,miR-345-5p,miR-329b,miR-2318,miR-3068-3p,miR-133c,miR-331-5p,miR-592,miR-134-5p,novel_9,miR-1260b,miR-1343-5p,miR-330-5p,miR-3591-5p,miR-412,novel_79,miR-2284z,miR-134-5p,miR-505-5p,miR-758-5p,miR-664-3p,miR-152-3p,miR-127-5p,miR-3064-5p,novel_39,miR-192-3p,miR-3970,miR-3591-3p,miR-216a-3p,miR-376b-5p,miR-148a-3p,miR-2319a,miR-378f,miR-410-5p,miR-320c,miR-677,miR-181b-3p,miR-374c-3p,miR-421-5p,miR-6123,miR-206,miR-193b-3p,miR-199b-5p,miR-301b-3p,novel_23,miR-708-3p,miR-432,miR-223-3p,miR-1843b-5p,miR-26a-2-3p,miR-27b-3p,miR-188-5p,miR-2284x,miR-16-1-3p,miR-339-5p,miR-194-3p,miR-3535,miR-2411-3p,miR-193a-3p,miR-668-3p,let-7a-2-3p,miR-432-3p,miR-432-3p,miR-370-5p,miR-21-3p,miR-221-5p,miR-378a-3p,miR-204-3p,miR-27a-5p,miR-193b-3p,miR-139-5p,miR-29b-2-5p,miR-320e,miR-208b-5p,miR-6516-3p,miR-664a,miR-1271,miR-2330-3p,miR-3074-2-3p,miR-378c,miR-145a-3p,miR-10b-5p,miR-206-3p,miR-

PCID2	ENSOARG0(	PCI doma	miR-22-3p,novel_23,miR-223-3p,miR-4508,miR-2889,miR-2284ab,miR-1260b,miR-130a-3p,miR-340-3p,miR-103a-3p,miR-133b-5p,miR-149-5p,miR-20b,miR-103,miR-2284d,miR-134-3p,miR-3141,miR-106a-5p,miR-17-5p,miR-377-3p,miR-296-3p,miR-1343-5p,miR-125a-5p,miR-126a-3p,miR-668-3p,miR-628-5p,miR-296-3p,miR-150-5p,miR-3591-3p,miR-3065-5p,miR-148a-3p,miR-3141,miR-2387,miR-505-3p,miR-376a-2-5p,miR-378f,miR-1291,miR-107,miR-655-5p,miR-3120-3p,miR-6535,miR-502-5p,miR-4324,miR-6402,miR-576-3p,miR-20a-5p,miR-22-5p,miR-502b,miR-147-5p,miR-6119-3p,miR-128-1-5p,miR-3957-3p,miR-17-3p,miR-1a-1-5p,miR-335-5p,miR-301b-3p,miR-2428,miR-24-3p,miR-1343-5p,miR-30a-5p,miR-885-3p,miR-300,miR-376a-5p,miR-431,miR-6517,miR-2284z,miR-545-5p,miR-378h,miR-30c-2-3p,miR-205-5p,miR-2285af,miR-1260a,miR-219-3p,miR-125a-3p,miR-107,miR-152-3p,miR-17-3p,miR-1a-2-5p,miR-3064-5p,miR-30a-3p,miR-181a-2-3p,novel_39,miR-1271-3p,miR-2285b,miR-148b-5p,miR-450b-5p,miR-93-5p,miR-362-5p,miR-17-5p,miR-30e-5p,miR-381-3p,miR-20b-5p,miR-485-5p,miR-671-5p,miR-598-3p,miR-2355-5p,miR-542-5p,miR-615-5p,miR-125a,miR-324-5p,miR-2284n,miR-125b-5p,miR-551b-3p,miR-2433,miR-2284k,miR-1-5p,miR-126b-3p,novel_42,miR-2284v,miR-378c,miR-500b-5p,miR-93,miR-1197-3p,miR-1260b,miR-323b,miR-758-3p,miR-378b,miR-1839-5p,miR-2284aa,miR-3120-5p,miR-378c,miR-1343-3p,miR-500-5p,miR-455-3p,miR-147a,novel_19,miR-323b-3p,miR-106b-5p,novel_115,miR-5126,miR-431-5p,miR-214-3p,miR-103a-2-5p,miR-3085-3p,miR-25-5p,miR-130b-3p,miR-652-5p,miR-574-3p,miR-101b-3p,miR-106a-5p,miR-494-3p,miR-4532,miR-758-3p,miR-378d,miR-30c-1-3p,miR-361-3p,miR-877-3p,novel_94,miR-504,miR-139-5p,miR-3064-5p,miR-4443,miR-378e,miR-378g,miR-107-5p,miR-101-3p,miR-2284g,miR-4726-
TPD52L1	ENSOARG0(	tumor pr	miR-147a,miR-758-5p

AKAP17A	ENSOARG0(	A kinase	miR-362-3p,miR-501-3p,miR-7857,miR-30b-3p,miR-484,miR-329-3p,miR-296-5p,miR-541,miR-9788-3p,miR-216a-3p,miR-2459,novel_4,miR-330-3p,miR-2300a-5p,miR-199b-5p,miR-214-3p,miR-25-5p,miR-362-3p,novel_19,miR-326,miR-5126,miR-4532,miR-8485,miR-2330-5p,novel_94,miR-1983,miR-877-3p,miR-504,miR-361-3p,miR-199b-5p,miR-500-3p,miR-3596,miR-378g,miR-499b-5p,novel_82,miR-502-3p,miR-423-5p,miR-3059-5p,miR-2447,miR-541-3p,miR-2904,miR-217-5p,miR-140-3p,novel_133,miR-2366,miR-362-3p,miR-21-3p,miR-324-3p,miR-125b,miR-345-3p,miR-185-3p,miR-2285p,novel_17,miR-486-3p,miR-2887,miR-503-5p,miR-1895,miR-1827,miR-345-5p,miR-21a-3p,miR-204-3p,miR-22-3p,miR-193b-3p,miR-6130,miR-370-5p,miR-3065-3p,miR-504-5p,miR-2331-3p,miR-548o-3p,miR-874-5p,miR-664a,miR-664a-5p,miR-320e,miR-665,miR-3184-5p,miR-6134,miR-214,miR-2330-3p,miR-4510,miR-4508,miR-744-5p,miR-2889,miR-138,miR-30b-3p,miR-29b,miR-22-3p,miR-374b-3p,miR-873a-5p,miR-1843b-5p,miR-7b-5p,miR-194-3p,miR-503-5p,miR-134-3p,miR-185-5p,miR-3431,miR-133b-5p,miR-1343-5p,miR-125a-5p,miR-193a-3p,miR-378a-5p,miR-330-3p,miR-365b-5p,miR-3141,miR-2411-3p,miR-2331-5p,miR-503-5p,miR-7-5p,miR-345-5p,miR-6240,miR-9851-3p,miR-1291,miR-412-3p,miR-216a-3p,miR-3141,miR-29a-3p,miR-670-3p,miR-421-5p,miR-29a,miR-4324,miR-6529b,miR-326-3p,miR-199b-5p,miR-371b-3p,miR-323c,miR-24-3p,miR-3957-3p,miR-193b-3p,miR-29c-3p,miR-1193,miR-495-3p,miR-128-3p,miR-7134-5p,miR-29d-3p,miR-1343-5p,miR-330-5p,miR-501-3p,miR-345-3p,miR-543-5p,miR-2898,miR-217,novel_39,miR-199a-5p,miR-3065-3p,miR-23a-5p,miR-7144-5p,miR-671-5p,miR-767,miR-329a,miR-345-5p,miR-3154,miR-199a-5p,miR-324-5p,miR-542-5p,miR-125a,miR-874-3p,miR-486b-3p,miR-138-5p,miR-767-5p,miR-

CNTN5	ENSOARG0(	contacti	miR-2285c,miR-30d-5p,miR-154b-3p,miR-2312,miR-543-3p,miR-154a-3p,miR-370-5p,miR-377-3p,miR-6516,miR-30e-3p,miR-30a-3p,miR-3958-5p,miR-6525,miR-181c-5p,miR-2408,miR-30c-5p,miR-140-3p,miR-493-3p,let-7a-3p,miR-106a,miR-30d-3p,miR-212-5p,miR-3600,miR-200b,miR-487a-3p,miR-2285p,miR-618,miR-374a-5p,miR-30b,miR-2284m,miR-106a,miR-30d,miR-487a,miR-183-5p,miR-20a,miR-181b-1-3p,miR-664b-3p,miR-380-3p,miR-381-3p,miR-136-5p,miR-668-5p,miR-877-3p,miR-9-3p,miR-335-3p,miR-452-3p,miR-10b,miR-664b,miR-3064-5p,miR-378g,miR-3596,miR-1185-2-3p,miR-101-3p,miR-30b-5p,miR-2285n,miR-2285y,miR-499a-5p,miR-154-3p,miR-194b-5p,miR-181d-5p,miR-2285f,miR-7705,miR-9-3p,miR-450b-5p,miR-199b-3p,novel_4,novel_78,miR-548w,miR-181b-2-3p,miR-145b,miR-5010-3p,miR-539-5p,miR-2285e,miR-1185-3p,miR-216a-3p,miR-106b-5p,miR-487b-5p,miR-491-5p,miR-3085-3p,miR-106a-5p,miR-574-3p,miR-744-3p,miR-8485,miR-30e-5p,miR-17-5p,miR-93-5p,miR-3074-1-3p,miR-20b-5p,miR-190a-3p,miR-487a-3p,miR-615-5p,miR-1271,miR-370-5p,novel_63,miR-29b-3p,miR-1-5p,miR-135a-5p,miR-93,miR-145-3p,miR-30a-5p,miR-29d-3p,miR-30c,miR-200b-3p,miR-429-3p,miR-6517,miR-2285ad,miR-6516-5p,miR-486-5p,novel_121,miR-664-3p,miR-200c,miR-205-5p,miR-30f,miR-181b-5p,miR-200c-3p,novel_96,miR-3064-5p,miR-30a-3p,miR-450b-5p,miR-1957a,miR-2285ab,miR-3970,miR-410-5p,miR-135b-5p,miR-655-5p,miR-29a-3p,miR-181b-3p,miR-677,miR-20a-5p,miR-382-5p,miR-29a,novel_1,miR-199c,miR-203b-5p,miR-1a-1-5p,miR-29c-3p,miR-1193,novel_27,miR-1434-3p,miR-181a-5p,miR-3071-5p,miR-145a-5p,miR-194-5p,miR-29b,miR-2284ab,miR-380-3p,miR-7862,miR-26b-3p,miR-134-3p,miR-185-5p,miR-2285g,miR-20b,miR-2355-3p,miR-411-5p,miR-377-3p,miR-194a,miR-106a-5p,miR-17-5p,let-7f-2-
C19orf57	ENSOARG0(	chromoso	miR-361-3p,miR-652-5p,miR-4286,miR-452-5p,miR-128-1-5p,miR-466f-3p,miR-362-3p,miR-146b-3p,miR-4792,miR-6517,miR-362-3p,miR-493-3p,miR-329a,miR-329-3p,miR-361-3p,miR-362-3p

C1QA	ENSOARG0(	compleme	miR-378c,miR-365a-5p,miR-133a-3p,miR-6128,miR-3607-3p,miR-486b-3p,miR-28-5p,miR-410-5p,miR-331-3p,miR-26c,miR-769,miR-365a-3p,miR-141-3p,miR-370-3p,miR-671-5p,miR-485-5p,miR-1814c,miR-1271-3p,miR-3065-3p,miR-4286,miR-2385-3p,miR-1,miR-200c-3p,miR-2898,miR-17-3p,miR-543-5p,miR-107,miR-6119-5p,miR-200c,miR-339a,miR-378h,miR-2411-5p,miR-214-5p,miR-7641,miR-10a-5p,miR-128-3p,miR-200b-3p,miR-345-3p,miR-501-3p,miR-24-3p,miR-326-3p,novel_101,miR-17-3p,miR-3957-3p,miR-199c,miR-656-5p,miR-6529b,miR-15a,miR-16a,miR-670-3p,miR-10a-5p,miR-320b,miR-107,miR-19a-3p,miR-1291,miR-195a-5p,miR-6395,miR-2387,miR-654-3p,miR-345-5p,miR-296-3p,miR-2483-5p,miR-363-5p,miR-1343-5p,miR-224-5p,miR-330-3p,miR-197-5p,miR-432-5p,miR-103,miR-130a-3p,miR-30b-3p,miR-3071-5p,miR-374b-3p,miR-206-3p,miR-10b-5p,miR-6134,miR-539-5p,miR-378c,miR-2330-3p,miR-6516-3p,miR-664a,miR-17-3p,miR-708-5p,miR-424-5p,miR-19b-1-5p,miR-204-3p,novel_32,miR-140-5p,miR-378a-3p,miR-221-5p,miR-545-3p,miR-3065-3p,miR-133b-3p,miR-125b-2-3p,miR-2411,miR-2432,miR-183-5p,miR-466i-5p,miR-760-3p,miR-21-3p,miR-221-5p,miR-148b-3p,miR-15a-5p,miR-200b,miR-532-3p,miR-1b-3p,miR-19b-3p,miR-2408,miR-376a-5p,novel_133,miR-1197-5p,miR-188-3p,miR-5703,miR-502-3p,miR-541-3p,miR-3059-5p,miR-335,miR-450a-5p,miR-4726-5p,miR-107-5p,miR-769-5p,miR-378e,miR-147-3p,miR-877-3p,novel_94,miR-361-3p,miR-193a,miR-424-5p,miR-2330-5p,miR-33a-5p,miR-326,miR-1306-5p,miR-142-3p,miR-216a-3p,novel_4,miR-2300a-5p,miR-450b-5p,miR-2426,miR-378c,miR-3120-5p,miR-10b,miR-378b,miR-484,miR-296-5p,miR-125b-2-3p,miR-30b-3p,miR-1197-3p,novel_42,miR-16-5p,novel_9,miR-592,miR-2433,miR-6740-5p,miR-300-3p,miR-874-3p,miR-574-5p,miR-1271,miR-

MRPS23	ENSOARG0(	mitochon	miR-329a-5p,miR-2285i,miR-421-5p,miR-219b-5p,miR-324-3p,novel_17,miR-26a-5p,let-7k,miR-98-5p,miR-30d-3p,miR-212-5p,miR-18a-3p,miR-217-5p,miR-1961,miR-16-1-3p,miR-34a-5p,novel_73,miR-214,let-7b,miR-433-5p,miR-129-2-3p,miR-769-5p,miR-346,miR-224-5p,miR-30a-3p,miR-3604,miR-665,let-7f,miR-2285j,miR-30e-3p,miR-4492,miR-669a-3p,miR-216b-3p,miR-552-3p,miR-15b-5p,let-7i-5p,miR-377-3p,miR-543-3p,miR-1895,miR-186-5p,miR-1827,miR-2284b,miR-124a,miR-101b-3p,miR-505,miR-182-5p,miR-16b,miR-199a-3p,miR-199b-5p,miR-214-3p,miR-491-5p,miR-330-3p,miR-1185-3p,miR-758-3p,miR-1298-5p,miR-3956-3p,miR-361-3p,miR-877-3p,novel_111,miR-2440,miR-101-3p,miR-3964,miR-2285u,miR-211-5p,miR-24-1-5p,miR-2319b,miR-221,miR-335-3p,miR-1983,miR-96-5p,let-7g,novel_39,miR-29a-5p,miR-148b-5p,miR-125a-3p,miR-664-3p,novel_127,novel_96,miR-152-3p,let-7f-5p,miR-412,miR-299a-3p,miR-129-1-3p,miR-322-5p,miR-1343-5p,miR-16-5p,miR-1260b,miR-100-3p,miR-125b-5p,miR-2355-5p,miR-125a,miR-503-3p,let-7f-2-3p,let-7f-2-3p,miR-380-5p,miR-6240,miR-432-3p,miR-432-3p,miR-365b-5p,novel_51,miR-7-5p,miR-377-3p,miR-149-5p,miR-194-3p,miR-708-3p,miR-7b-5p,miR-542-3p,miR-497-5p,miR-26a-2-3p,miR-16b,miR-16-1-3p,miR-374c-5p,miR-199b-5p,novel_87,miR-4324,miR-28c,miR-576-3p,miR-421-5p,miR-26b-5p,novel_68,miR-329-5p,miR-669,miR-144-3p,miR-320c,miR-299b-3p,novel_69,miR-150-5p,miR-3591-3p,miR-3065-5p,miR-148a-3p,let-7e-5p,miR-412-3p,miR-125b-2-3p,miR-125b,miR-185-3p,miR-221-5p,miR-34b-5p,miR-30b-3p,miR-299-3p,miR-21-3p,miR-320d,miR-760-3p,miR-3600,miR-532-3p,miR-199a-3p,miR-148b-3p,miR-15a-5p,miR-124-3p,miR-1193,miR-188-3p,novel_133,miR-2300b-3p,miR-378d,miR-4429,miR-1271,let-7b-5p,miR-324-3p,miR-320e,miR-222-

GALNT13	ENSOARG0(	polypept	miR-1271,miR-125a,miR-141-3p,miR-3068-3p,miR-2284n,miR-365a-3p,miR-374c-3p,miR-3154,miR-376c-5p,miR-23b,miR-2448-3p,miR-30e-5p,miR-488-3p,miR-1814c,miR-671-5p,miR-376c-5p,miR-7144-5p,miR-345-5p,miR-876-3p,miR-767,miR-144-5p,miR-193a-5p,miR-3607-3p,miR-409-3p,miR-135a-5p,miR-125b-5p,miR-144,miR-5100,miR-370-5p,miR-486b-3p,miR-134-5p,miR-191-5p,miR-767-5p,miR-376a-5p,miR-412,miR-429-3p,miR-450a-1-3p,miR-134-5p,miR-545-5p,miR-483-3p,miR-30a-5p,miR-7134-5p,miR-3578,miR-134,miR-196a-5p,miR-345-3p,miR-128-3p,miR-30c,miR-200b-3p,miR-582-3p,miR-411-3p,miR-2403,miR-29b-2-5p,miR-30a-3p,miR-376c-5p,novel_39,miR-29a-5p,miR-376b-5p,miR-148b-5p,miR-380-5p,miR-200c,miR-376b-3p,miR-200a-3p,miR-200c-3p,miR-127-5p,miR-7-1-3p,miR-26b-5p,miR-3120-3p,miR-378b,miR-144-3p,miR-670-3p,miR-374c-3p,miR-2404,miR-376b-3p,miR-216a-3p,miR-376b-5p,miR-2285ab,miR-376a-2-5p,miR-135b-5p,miR-6516-3p,miR-9851-3p,miR-19a-3p,miR-1193,miR-135a-2-3p,novel_120,miR-2428,miR-2284w,miR-3959-3p,miR-24-3p,miR-323c,miR-6402,miR-133a-5p,miR-576-3p,miR-502b,miR-199c,miR-22-5p,miR-379-3p,novel_1,miR-665,miR-31-5p,miR-6238,miR-149-5p,miR-2285g,miR-412-5p,miR-3535,novel_23,miR-197-3p,miR-450c-3p,miR-1434-3p,miR-455-5p,miR-27b-3p,miR-16-1-3p,miR-219a-1-3p,miR-2284ab,miR-30b-3p,miR-628-5p,miR-2483-5p,miR-380-5p,miR-503-3p,miR-345-5p,miR-432-3p,miR-432-3p,miR-2411-3p,miR-455-5p,miR-224-5p,miR-125a-5p,miR-196b-5p,miR-483-3p,miR-296-3p,miR-370-5p,miR-140-5p,miR-504-5p,miR-151b,miR-877-5p,novel_25,miR-139-5p,miR-30d-5p,miR-2285c,miR-450a-1-3p,miR-21a-3p,novel_99,miR-186-5p,miR-345-5p,miR-30f,miR-2312,miR-3074-2-3p,miR-539-5p,miR-224-5p,miR-6525,miR-29b-2-5p,miR-30a-3p,miR-30e-3p,miR-208b-5p,miR-6516-
PNPLA8	ENSOARG0(	patatin-	miR-3969,miR-320e,novel_17,miR-200a-3p,miR-466f-3p,miR-335,miR-2355-5p,miR-141-3p,miR-26b-5p,miR-545-3p,miR-101b-3p,miR-335-5p,miR-200a,miR-2432,miR-145-3p
LRCH1	ENSOARG0(	leucine-	miR-301b-5p,miR-301a-5p,miR-450a-2-3p,miR-485-5p,miR-22-5p,miR-6517,miR-4792,miR-552-3p,miR-26b-3p,miR-185-5p,miR-6525,miR-6535,miR-669

BRD2	ENSOARG0(	bromodom	miR-2284b,miR-23a-3p,miR-16b,miR-182-5p,miR-323b-3p,miR-214-3p,miR-103a-2-5p,miR-130b-3p,miR-181b-2-3p,novel_78,miR-2459,miR-1185-3p,miR-421,miR-455-3p,miR-147a,miR-323b,miR-541,miR-101c,miR-1388-5p,miR-2284j,miR-3956-3p,miR-2447,miR-7705,miR-423-5p,novel_111,miR-2440,miR-3964,miR-490-5p,miR-3968,miR-499a-5p,miR-211-5p,miR-7689-3p,miR-374b-5p,miR-2319b,miR-24-1-5p,miR-2284g,miR-139-5p,miR-1185-2-3p,miR-144-5p,miR-2483-3p,miR-378j,miR-544-5p,miR-136-5p,miR-369-3p,miR-143-3p,miR-486-3p,miR-181b-1-3p,miR-376a-5p,miR-345-3p,miR-92a-1-5p,miR-324-3p,miR-219b-5p,novel_17,miR-210-5p,miR-2484,miR-30d-3p,miR-328-3p,miR-23c,miR-7859,miR-23b-3p,miR-361-5p,miR-2284u,miR-16-1-3p,miR-214,miR-129-2-3p,miR-3184-5p,miR-769-5p,miR-6525,miR-665,miR-30a-3p,miR-151-3p,miR-30e-3p,miR-15b-3p,miR-2285j,miR-4492,miR-1468-5p,miR-454-3p,miR-874-5p,novel_74,miR-296-3p,miR-2331-3p,miR-15b-5p,miR-301a-3p,miR-433-3p,miR-450a-1-3p,miR-625-5p,miR-885-3p,miR-543-3p,miR-345-5p,let-7a-2-3p,miR-2285r,miR-432-3p,novel_51,miR-2411-3p,miR-3141,miR-3431,miR-450c-3p,miR-197-3p,miR-542-3p,miR-432,miR-19b-2-5p,miR-188-5p,miR-497-5p,miR-16-1-3p,miR-16b,miR-301b-3p,miR-142b,miR-28c,miR-625-3p,miR-6119-3p,miR-3120-3p,novel_68,miR-181b-3p,miR-677,miR-374c-3p,miR-148a-3p,miR-3141,miR-376b-5p,miR-2319a,miR-2285b,miR-2284y,miR-29a-5p,miR-450b-5p,miR-152-3p,miR-6536,miR-127-5p,miR-129-1-3p,miR-134-5p,miR-450a-1-3p,miR-322-5p,miR-23b-5p,miR-2427,miR-885-3p,miR-144-5p,miR-16-5p,miR-2284v,miR-1260b,miR-2284k,miR-2433,novel_63,miR-134-5p,novel_9,miR-376c-5p,miR-23b,miR-137-3p,miR-1248,miR-345-5p,miR-424-5p,miR-5126,miR-1468,novel_4,miR-151a-3p,miR-409b,miR-216a-3p,miR-125b-2-

TRDN	ENSOARG0(	triadin	miR-576-3p,miR-218-5p,miR-22-5p,miR-374a-3p,miR-2285t,miR-628-3p,miR-9-5p,novel_101,miR-1a-1-5p,miR-3059-5p,miR-2447,miR-9-3p,novel_27,miR-326-3p,miR-362-5p,miR-877-3p,miR-211,miR-378b,miR-140-5p,miR-9-3p,miR-335-3p,miR-493-5p,miR-299b-5p,miR-96-5p,miR-211-5p,miR-329-5p,miR-489,miR-3082-5p,miR-378g,miR-29b-1-5p,miR-326,miR-224-5p,miR-487b-5p,miR-378a-5p,miR-483-3p,miR-744-3p,miR-23a-3p,miR-493-5p,miR-2330-5p,miR-345-5p,miR-2310,miR-758-3p,miR-503-3p,miR-758-3p,miR-873a-5p,miR-16-2-3p,miR-450b-5p,miR-3432b,miR-299a-5p,miR-26b-3p,miR-500-5p,miR-134-3p,miR-29b-2-5p,miR-338-5p,miR-3604,miR-320e,miR-30a-3p,miR-19b-1-5p,miR-6740-5p,miR-30e-3p,miR-1-5p,miR-204-5p,miR-129-5p,miR-487b-5p,miR-3074-2-3p,miR-6128,miR-1271,miR-3184-3p,miR-145a-3p,miR-539-5p,miR-6134,miR-122-5p,miR-1197-3p,miR-224-5p,miR-500b-5p,miR-142a-5p,miR-23b,miR-362-5p,miR-299,novel_99,miR-1827,miR-1814c,miR-1248,miR-625-5p,miR-345-5p,miR-345-5p,miR-1271,miR-532-5p,miR-2285aa,miR-140-5p,miR-204-3p,novel_25,miR-223-5p,miR-331-5p,miR-6516,miR-618,miR-543-5p,miR-127-5p,miR-760-3p,miR-29b-2-5p,miR-30a-3p,miR-195a-3p,miR-329a-5p,miR-200a,miR-130a-5p,miR-376a-5p,miR-29b-1-5p,miR-330-5p,miR-1290,miR-1246,miR-361-5p,miR-218-1-3p,miR-142-3p,miR-212-5p,miR-30d-3p,miR-23c,miR-483-3p,miR-340-5p,miR-6516-5p,miR-23b-3p,miR-9-5p
CDYL2	ENSOARG0(	chromodo	miR-17-3p,miR-25-5p,miR-1291,miR-17-3p,miR-17-3p,novel_4,miR-2331-3p,miR-134-3p,miR-378g
CUEDC1	ENSOARG0(	CUE doma	miR-767,miR-1827,miR-1814c,miR-378f,miR-3074-5p,miR-378a-3p,miR-1271,miR-6130,miR-34b,miR-29a-3p,miR-378g,miR-34c-5p,miR-378e,miR-29b-3p,miR-34c,miR-767-5p,miR-29a,miR-4492,miR-3074-5p,miR-4510,miR-378c,miR-29c-3p,miR-1260b,miR-2428,miR-449a,miR-378c,miR-378b,miR-378i,miR-378a-3p,miR-1197-5p,miR-29d-3p,miR-1260b,miR-1388-5p,miR-29b,miR-378c,miR-34a-5p,miR-148a-5p,miR-378h,miR-134-3p,miR-148b-5p,miR-1260a,novel_51,miR-422a,miR-760-3p,miR-34b-5p,miR-30b-3p,miR-654-3p,miR-378d,miR-148b-5p,miR-503-3p,miR-2310
CLVS2	ENSOARG0(	clavesin	miR-6395,miR-544-5p,miR-1291,miR-96-5p,miR-145a-5p,miR-218-2-3p,miR-221,miR-328-3p,miR-221-3p,miR-1271-5p,miR-582-5p,miR-532-5p,miR-145b,miR-216b-3p,miR-222,miR-1468,miR-1468-5p,miR-197-5p,miR-133a-5p,miR-222-3p,miR-1271

TDGF1	ENSOARG0(	teratoca	miR-361-5p,miR-148a-5p,miR-133a-3p,miR-218-1-3p,miR-5703,miR-1197-5p,miR-140-3p,miR-1193,miR-2904,miR-362-3p,miR-199a-3p,miR-532-3p,miR-23c,miR-487a-3p,miR-23b-3p,miR-7859,miR-485-3p,miR-212-5p,miR-548e-3p,miR-299-3p,miR-21-3p,miR-185-3p,miR-345-3p,miR-500a-5p,miR-324-3p,novel_17,miR-618,miR-376c-3p,miR-181b-1-3p,miR-664b-3p,miR-421-5p,miR-345-3p,miR-2285w,novel_99,miR-345-5p,let-7f-1-3p,novel_25,miR-204-3p,miR-323-5p,miR-216b-5p,miR-139-5p,miR-6130,miR-544-3p,miR-376e-3p,miR-377-3p,miR-132-5p,miR-2284s,miR-499b-3p,miR-552-3p,miR-21-3p,miR-548o-3p,miR-140-5p,miR-545-3p,miR-708-5p,miR-208b-5p,miR-6516-3p,miR-338-5p,miR-664a-5p,miR-29b-2-5p,miR-30a-3p,miR-30e-3p,miR-206-3p,miR-6525,miR-3074-2-3p,miR-129-2-3p,miR-4510,miR-539-5p,miR-2284aa,miR-1839-5p,miR-101c,miR-544a,miR-362-3p,miR-1388-5p,miR-758-3p,miR-1a-3p,miR-143-5p,miR-2284r,miR-329-3p,miR-148b-5p,miR-455-3p,miR-539-5p,miR-153,miR-425-3p,miR-145b,miR-199b-3p,miR-105-1,novel_78,miR-548w,novel_4,miR-1343-3p,miR-153-3p,miR-3955-3p,miR-491-5p,miR-362-3p,miR-1306-5p,miR-199a-3p,miR-1b-5p,miR-758-3p,miR-8485,miR-33a-5p,miR-23a-3p,miR-101b-3p,miR-1983,novel_94,miR-136-5p,miR-129b-5p,miR-203-3p,miR-378g,miR-499a-3p,miR-7977,miR-20a-3p,miR-10b,miR-664b,miR-139-5p,miR-2284q,miR-7134-3p,miR-2285t,miR-4726-5p,miR-3968,miR-1973,miR-181d-5p,miR-194b-5p,miR-2447,miR-2427,miR-1290,miR-345-3p,novel_121,miR-129-1-3p,miR-214-5p,miR-2284z,miR-758-5p,miR-299a-3p,novel_96,miR-1a-2-5p,miR-127-5p,miR-181b-5p,miR-450a-2-3p,miR-30f,miR-125a-3p,miR-664-3p,miR-2285b,miR-29a-5p,miR-376c-5p,miR-376b-5p,miR-2478,miR-28b,miR-148b-5p,miR-4286,miR-2403,miR-30a-3p,miR-1,miR-495-5p,miR-29b-2-5p,miR-2385-3p,miR-1248,miR-3074-1-3p,miR-376c-5p,miR-
CCDC62	ENSOARG0(	coiled-c	miR-490-5p,miR-200a-3p,miR-129b-5p,miR-9-5p,miR-331-5p,miR-654-3p,miR-8095,miR-539-5p,miR-3074-5p,miR-3074-5p,miR-541-5p,miR-655-5p,miR-141-3p,miR-3074-2-3p,miR-9-5p
LYPD8	ENSOARG0(	LY6/PLAU	miR-432,miR-542-3p,miR-301b-5p,miR-383-5p,miR-432-5p,miR-216a-3p,miR-7641,miR-18b-5p,miR-181b-2-3p,miR-760-3p,miR-125a-5p,miR-21-3p,miR-125b,miR-432-3p,miR-181b-1-3p,miR-432-3p,miR-8485,miR-668-3p,miR-376b,miR-18a-5p,miR-671-5p,miR-18b,miR-129b-3p,miR-18a,miR-411b-3p,miR-204-3p,miR-677,miR-181b-3p,miR-574-5p,miR-2285aa,miR-2355-5p,miR-125a,miR-708-5p,miR-487a-5p,miR-28-5p,miR-125b-5p,miR-301a-5p,miR-3604,miR-323-3p,miR-122-5p,miR-2447
NSDHL	ENSOARG0(	NAD(P) d	miR-374a-3p,miR-769-5p,miR-101-5p,miR-363-5p,miR-3064-5p,novel_127,miR-4492,miR-3653-3p,miR-374a-3p,miR-4726-5p,miR-3085-3p,miR-769-5p,miR-3071-3p,miR-576-3p,miR-769,miR-185-5p,miR-2285aa,miR-874-3p,miR-18a-3p,miR-3064-5p,miR-877-3p,miR-18a-3p,miR-1434-3p

ORAI3	ENSOARG0(	ORAI cal	miR-130b-5p,miR-708-5p,miR-4492,miR-208b-5p,miR-664a,miR-17-3p,miR-451a,miR-665,miR-324-3p,miR-30a-3p,miR-30e-3p,miR-338-3p,miR-181c-5p,miR-3184-5p,miR-224-5p,miR-145a-3p,miR-3074-5p,miR-433-5p,miR-539-5p,miR-378c,novel_99,miR-625-5p,miR-339b,miR-2312,miR-2285c,novel_32,miR-22-3p,miR-204-3p,miR-6516,miR-192-5p,miR-3065-3p,miR-21-3p,miR-30c-1-3p,miR-504-5p,miR-378a-3p,miR-34b-5p,miR-466i-5p,miR-125b,miR-185-3p,miR-422a,miR-216a-5p,miR-500a-5p,miR-324-3p,miR-196a-3p,miR-2424,miR-486-3p,miR-125b-2-3p,miR-376a-5p,miR-345-3p,miR-2285w,miR-503-5p,miR-183-5p,miR-10a-3p,miR-378d,miR-34a-5p,miR-133a-3p,miR-378a-3p,miR-1197-5p,miR-376a-5p,miR-215-5p,miR-378i,miR-2284h-5p,miR-30d-3p,miR-548e-3p,miR-18a-3p,miR-2285t,miR-3968,miR-301a-5p,miR-2285y,miR-2440,miR-2285n,miR-423-5p,miR-8095,miR-362-5p,miR-361-3p,miR-452-5p,miR-181d-5p,miR-2285f,miR-541-3p,miR-2447,novel_94,miR-136-5p,miR-504,miR-129b-5p,miR-454-5p,miR-1185-2-3p,miR-203-3p,miR-378e,miR-7977,miR-499b-5p,miR-541-5p,miR-452-3p,miR-25-5p,miR-1185-5p,miR-5126,miR-1306-5p,miR-378d,miR-30c-1-3p,miR-744-3p,miR-382-3p,miR-1388-3p,miR-3120-5p,miR-101a-5p,miR-378c,miR-30b-3p,miR-3962,miR-412-3p,miR-301b-5p,miR-541,miR-378b,miR-2285e,miR-1185-3p,miR-500-5p,miR-147a,miR-105-1,miR-2300a-5p,miR-548w,miR-28-5p,miR-486b-3p,miR-409-3p,miR-331-3p,miR-125b-5p,miR-135a-1-3p,miR-410-5p,miR-29b-3p,miR-2433,miR-10b-3p,miR-378c,miR-2903,miR-500b-5p,miR-193a-5p,miR-1306-5p,novel_42,miR-1248,miR-485-5p,miR-7144-5p,miR-2318,miR-876-3p,miR-10b-3p,miR-10a-3p,miR-3154,miR-362-5p,miR-488-3p,miR-324-5p,miR-34c-5p,miR-300-3p,miR-374c-3p,miR-215-5p,miR-125a,miR-370-3p,novel_127,miR-127-5p,miR-181b-5p,miR-2898,miR-17-
PRPF40A	ENSOARG0(	PRP40 pr	miR-371b-3p,miR-199a-5p,miR-199b-5p,miR-3059-5p,miR-487a,miR-3607-3p,miR-17-3p,miR-17-3p,miR-17-3p,miR-199b-5p,miR-30d,miR-154-3p,miR-2285n,novel_82,miR-199a-5p,miR-2285ad,miR-487a-3p,miR-154a-3p,miR-2284ab,miR-154b-3p,miR-101a-5p,miR-30e-5p,miR-2366,miR-199b-5p,miR-30a-5p,miR-30d-5p
TRIB1	ENSOARG0(	tribbles	miR-130a-3p,miR-122-3p,miR-409b,novel_121,miR-214-5p,miR-9788-3p,miR-185-5p,miR-3431,miR-3071-5p,miR-218-2-3p,miR-10b,miR-2284r,miR-3120-5p,miR-10a-5p,miR-345-3p,miR-143-3p,miR-3065-3p,miR-181b-1-3p,miR-2957,miR-1306-5p,miR-664-3p,miR-130b-3p,miR-450a-2-3p,miR-10a-5p,miR-211-5p,miR-3065-3p,miR-2319b,miR-452-3p,miR-664b,miR-181b-3p,miR-677,novel_69,miR-10a,miR-301a-3p,miR-3065-5p,miR-3154,miR-1306,novel_99,miR-1983,miR-19a-3p,miR-412-3p,miR-329b,miR-665-5p,miR-193a-5p,miR-212-3p,miR-3059-5p,miR-301b-3p,miR-6134,miR-1197-3p,miR-10b-5p,miR-211,miR-320e,miR-664a-5p,miR-100-3p,miR-6740-5p,miR-204-5p,miR-454-3p,novel_1

CYP4V2	ENSOARG0(	cytochro	miR-29b-1-5p,miR-181b-1-3p,miR-377-5p,miR-130a-5p,miR-216c-5p,miR-2285w,miR-195a-3p,miR-345-3p,miR-320d,miR-760-3p,miR-3653-3p,miR-2484,miR-21-3p,miR-210-5p,miR-208b-3p,miR-618,miR-324-3p,miR-125b,miR-345-3p,miR-383-5p,miR-7859,miR-148b-3p,miR-340-5p,miR-2284h-5p,novel_83,miR-1247-5p,miR-548e-3p,miR-18a-3p,miR-505-3p,miR-4429,miR-33b-3p,miR-16-1-3p,miR-361-5p,miR-215-5p,miR-362-3p,miR-1246,miR-2366,miR-1197-5p,miR-217-5p,miR-1434-5p,miR-140-3p,miR-5703,miR-6525,miR-142a-5p,miR-224-5p,miR-4510,miR-129-2-3p,miR-3958-5p,miR-425-5p,miR-2330-3p,miR-17-3p,miR-129-5p,miR-487a-5p,miR-130b-5p,miR-324-3p,miR-664a-5p,miR-665,miR-192-5p,miR-323-5p,miR-6130,miR-216b-5p,novel_32,novel_25,miR-30c-1-3p,miR-140-5p,miR-504-5p,miR-548o-3p,miR-370-5p,miR-532-5p,miR-552-3p,let-7g-3p,miR-18a-5p,miR-339b,miR-1827,miR-186-5p,miR-30c-1-3p,miR-8485,miR-1b-5p,miR-1839-3p,miR-494-3p,miR-382-3p,miR-652-5p,miR-491-5p,miR-3085-3p,miR-3955-3p,miR-320b,novel_103,miR-362-3p,miR-29b-1-5p,miR-142-5p,miR-9788-3p,miR-455-3p,miR-216a-3p,novel_4,miR-181b-2-3p,miR-145b,miR-362-3p,miR-101a-5p,miR-16-2-3p,miR-329-3p,miR-301b-5p,miR-143-5p,novel_60,miR-3956-3p,miR-452-5p,miR-194b-5p,miR-9-5p,miR-490-5p,miR-374a-3p,miR-218-5p,miR-301a-5p,miR-2483-3p,miR-489,miR-3064-5p,miR-541-5p,miR-504,miR-668-5p,miR-335-3p,miR-493-5p,miR-140-5p,miR-129b-5p,miR-28b,miR-380-5p,miR-2285b,miR-1271-3p,miR-3529-3p,miR-3064-5p,miR-181a-2-3p,miR-17-3p,miR-450a-2-3p,miR-543-5p,miR-152-3p,novel_96,novel_127,miR-30c-2-3p,miR-217,miR-339a,miR-9-5p,miR-302a-5p,miR-483-3p,miR-758-5p,miR-134-5p,miR-545-5p,miR-129-1-3p,miR-6517,novel_79,miR-18b-5p,miR-582-3p,miR-345-3p,miR-134,miR-1343-5p,miR-2427,miR-374a-

CENPN	ENSOARG0(	centrome	miR-1298-5p,miR-299a-5p,miR-101c,miR-1343-3p,miR-548w,miR-27a-3p,miR-425-3p,miR-148b-5p,miR-9788-3p,miR-5126,miR-326,miR-16b,miR-25-5p,miR-3085-3p,miR-103a-2-5p,miR-424-5p,miR-33a-5p,miR-30c-1-3p,miR-381-3p,miR-299b-5p,miR-544-5p,miR-335-3p,miR-2319b,miR-20a-3p,miR-3064-5p,miR-211-5p,miR-541-5p,miR-3082-5p,miR-499a-3p,miR-4443,miR-27a-3p,miR-107-5p,miR-335,miR-2284q,miR-7134-3p,miR-2284j,miR-194b-5p,miR-9-5p,miR-452-5p,miR-877-3p,miR-8095,miR-2408,miR-1197-5p,miR-3960,miR-3600,miR-15a-5p,miR-200b,miR-532-3p,miR-26a-5p,miR-500a-5p,miR-2284m,miR-216c-5p,miR-125b-2-3p,miR-664b-3p,miR-2411,miR-129b-3p,novel_99,miR-186-5p,miR-30c-1-3p,miR-21-3p,miR-2284s,miR-499b-3p,miR-15b-5p,miR-17-3p,miR-424-5p,miR-590-3p,miR-1843b-5p,miR-223-3p,miR-873a-5p,miR-374b-3p,miR-3071-5p,miR-194-5p,miR-16b,miR-188-5p,miR-27b-3p,miR-2284x,miR-497-5p,miR-26b-3p,miR-185-5p,miR-194a,miR-197-5p,miR-378a-5p,miR-124-5p,miR-2284l,miR-2285r,miR-3591-3p,miR-150-5p,miR-195a-5p,miR-376a-2-5p,miR-329-5p,miR-26b-5p,miR-670-3p,miR-677,miR-15a,miR-1247-3p,miR-16a,novel_1,miR-22-5p,miR-656-5p,miR-335-5p,novel_120,novel_101,miR-17-3p,miR-211,novel_27,miR-326-3p,miR-134,miR-330-5p,miR-300,miR-200b-3p,miR-429-3p,miR-2285ad,miR-376a-5p,miR-1224-5p,miR-2411-5p,miR-322-5p,miR-9-5p,miR-134-5p,miR-582-3p,miR-200c,miR-125a-3p,miR-2285af,miR-205-5p,miR-30c-2-3p,miR-200c-3p,miR-17-3p,miR-200a-3p,miR-181a-2-3p,miR-2403,miR-2385-3p,miR-3064-5p,miR-148b-5p,miR-2285b,miR-29a-5p,novel_39,miR-381-3p,miR-3154,miR-200a-5p,miR-671-5p,novel_48,miR-23a-5p,miR-615-5p,miR-141-3p,miR-542-5p,miR-300-3p,miR-5100,miR-2284k,miR-2433,miR-134-5p,miR-204-5p,miR-410-3p,miR-31-3p,miR-16-5p

FNBP1	ENSOARG0(	formin b	miR-301b-3p,miR-335-5p,miR-758-5p,miR-193b-3p,miR-6123,miR-28c,miR-1307-5p,miR-374c-3p,miR-299b-3p,miR-320c,novel_68,miR-146b-5p,miR-3141,miR-376b-5p,miR-216a-3p,miR-3591-3p,miR-3065-5p,miR-6240,miR-668-3p,miR-193a-3p,miR-146a,miR-2285v,miR-3141,miR-3535,miR-194-3p,miR-149-5p,novel_116,miR-340-3p,miR-3955-5p,miR-27b-3p,miR-2113,novel_23,miR-22-3p,miR-1260b,miR-1197-3p,miR-2433,miR-125b-5p,miR-133c,miR-503-3p,miR-125a,miR-1271,miR-2285aa,miR-2355-5p,miR-2474,miR-424-3p,miR-301,miR-376c-5p,novel_39,miR-3064-5p,novel_96,miR-200a-3p,miR-8117,miR-9-5p,miR-129-1-3p,miR-6517,miR-299a-3p,miR-2285ad,miR-18b-5p,miR-330-5p,miR-1343-5p,novel_111,miR-361-3p,miR-877-3p,miR-423-5p,miR-2447,miR-9-5p,miR-1843a-3p,miR-301a-5p,miR-652-3p,miR-203-3p,miR-139-5p,miR-136-5p,miR-8485,miR-124a,miR-574-3p,miR-130b-3p,miR-491-5p,miR-146b,miR-182-5p,miR-505,miR-29b-1-5p,novel_19,miR-455-3p,miR-199b-3p,miR-1343-3p,novel_78,miR-330-3p,miR-145b,miR-301b-5p,miR-541,miR-1298-5p,miR-2284r,miR-6525,miR-3184-5p,miR-129-2-3p,novel_74,miR-454-3p,miR-4492,miR-665,miR-2284s,miR-301a-3p,miR-151b,miR-2331-3p,miR-552-3p,miR-216b-3p,miR-1827,miR-543-3p,miR-1895,miR-2285c,miR-299,miR-433-3p,miR-129b-3p,miR-486-3p,miR-2285w,miR-345-3p,miR-210-5p,miR-3601,miR-7859,miR-2284h-5p,miR-212-5p,miR-380-5p,miR-142-3p,miR-34a-5p,miR-2366,novel_27,miR-449a,miR-326-3p,miR-342-3p,miR-1193,miR-128-1-5p,miR-199c,miR-6529b,miR-34c,miR-670-3p,miR-34b,miR-2404,miR-378b,miR-363,miR-320b,miR-2387,miR-18b,miR-378b,miR-363-5p,miR-2483-5p,miR-2285l,miR-452-5p,miR-125a-5p,miR-1343-5p,miR-330-3p,miR-185-5p,miR-2285g,miR-544b,miR-187-3p,miR-130a-3p,miR-1260b,miR-138,miR-2889,miR-744-5p,miR-1285,miR-

ITGA2	ENSOARG0(	integrin	miR-19b-3p,miR-148a-5p,miR-1434-5p,miR-23b-3p,miR-23c,miR-15a-5p,novel_83,miR-2284h-5p,miR-616-3p,miR-106a,miR-3600,miR-505-3p,miR-106a,miR-760-3p,miR-1277-5p,miR-21-3p,miR-299-3p,novel_17,miR-500a-5p,miR-324-3p,miR-181b-1-3p,miR-380-3p,miR-143-3p,miR-2285w,miR-20a,miR-345-3p,miR-339b,miR-625-5p,miR-1827,miR-21a-3p,miR-598-3p,miR-6516,miR-27a-5p,novel_32,miR-204-3p,novel_25,miR-155-5p,miR-877-5p,miR-15b-5p,miR-2331-3p,miR-140-5p,miR-30c-1-3p,miR-32-3p,miR-216b-3p,miR-17-3p,miR-155-5p,miR-424-5p,miR-129-5p,miR-151-3p,miR-665,miR-338-5p,miR-29b-2-5p,miR-338-3p,miR-3074-5p,miR-214,miR-3074-2-3p,miR-450b-5p,miR-541,miR-143-5p,miR-30b-3p,miR-9788-3p,miR-148b-5p,miR-221-3p,miR-1185-3p,miR-126a-5p,miR-330-3p,miR-151a-3p,miR-5010-3p,miR-145b,miR-181b-2-3p,miR-27a-3p,miR-3085-3p,miR-199b-5p,miR-103a-2-5p,miR-214-3p,miR-342,novel_115,miR-326,miR-1306-5p,miR-106b-5p,miR-16b,miR-30c-1-3p,miR-8485,miR-2310,miR-424-5p,miR-2330-5p,miR-106a-5p,miR-101b-3p,miR-23a-3p,miR-299b-5p,miR-136-5p,miR-493-5p,miR-544-5p,miR-140-5p,miR-199b-5p,miR-221,miR-381-3p,miR-489,miR-203-3p,miR-1185-2-3p,miR-27a-3p,miR-4792,miR-3064-5p,miR-452-3p,miR-541-5p,miR-374b-5p,miR-211-5p,miR-7977,miR-3968,miR-2285y,miR-107-5p,miR-8095,novel_60,miR-32-3p,miR-2447,miR-3059-5p,miR-541-3p,miR-3956-3p,miR-212-3p,miR-219a-5p,miR-194b-5p,miR-452-5p,miR-300,miR-155-5p,miR-2427,miR-330-5p,miR-322-5p,miR-6516-5p,novel_121,miR-299a-3p,miR-222,miR-2285ad,novel_79,miR-17-3p,miR-127-5p,miR-6536,miR-543-5p,miR-205-5p,miR-3071-3p,miR-30f,miR-30c-2-3p,miR-339a,miR-148b-5p,miR-199a-5p,miR-3064-5p,miR-29b-2-5p,miR-876-3p,miR-2318,miR-329b,miR-23a-5p,miR-20b-5p,miR-1248,miR-1814c,miR-381-3p,miR-93-

PES1	ENSOARG0(	pescadil	miR-345-3p,miR-183-5p,miR-486-3p,miR-376c-3p,miR-185-3p,miR-324-3p,miR-299-3p,miR-21-3p,miR-34b-5p,miR-760-3p,miR-2284m,miR-1b-3p,miR-18a-3p,miR-328-3p,miR-200b,miR-148b-3p,miR-5703,miR-2904,miR-2300b-3p,novel_133,miR-34a-5p,miR-148a-5p,miR-2284u,miR-19b-3p,miR-214,miR-129-2-3p,miR-6134,miR-206-3p,miR-6525,miR-29b-2-5p,miR-665,miR-324-3p,miR-2285j,miR-874-5p,miR-4492,miR-454-3p,miR-129-5p,miR-664a,miR-21-3p,miR-504-5p,miR-140-5p,miR-2331-3p,miR-204-3p,miR-22-3p,miR-301a-3p,miR-376e-3p,miR-129b-3p,miR-345-5p,miR-18a-5p,miR-339b,let-7g-3p,miR-2284b,miR-2330-5p,miR-574-3p,miR-326,miR-342,miR-214-3p,miR-130b-3p,miR-25-5p,novel_124,miR-181b-2-3p,novel_78,novel_4,miR-409b,miR-2300a-5p,miR-2459,miR-9788-3p,miR-148b-5p,miR-1298-3p,miR-30b-3p,miR-1a-3p,miR-484,miR-2284r,miR-3120-5p,miR-2284aa,miR-2426,miR-1388-5p,miR-2284j,miR-9-5p,miR-885-5p,miR-2447,miR-154a,miR-361-3p,miR-1973,miR-2440,miR-211-5p,miR-126b-5p,miR-7689-3p,miR-499b-5p,miR-24-1-5p,miR-2284g,miR-203-3p,miR-3082-5p,miR-3596,miR-129b-5p,miR-361-3p,novel_94,miR-504,miR-4286,miR-29b-2-5p,miR-1,miR-1271-3p,miR-2284a,miR-2284y,miR-24-2-5p,miR-148b-5p,miR-339a,miR-200c,miR-1260a,miR-2285af,miR-200a-3p,novel_127,miR-152-3p,miR-200c-3p,miR-18b-5p,novel_79,miR-429-3p,miR-299a-3p,miR-129-1-3p,miR-2284z,miR-9-5p,miR-24-2-5p,miR-505-5p,miR-330-5p,miR-128-3p,miR-200b-3p,miR-6128,miR-2284v,miR-1260b,miR-2284k,miR-486b-3p,miR-204-5p,miR-141-3p,miR-615-5p,miR-2355-5p,miR-2285aa,miR-874-3p,miR-2284n,miR-376c-3p,miR-133c,miR-34c-5p,miR-18a,miR-488-3p,miR-345-5p,miR-2474,miR-876-3p,miR-668-3p,miR-345-5p,miR-432-3p,miR-378a-5p,miR-339-5p,miR-130a-3p,miR-122-3p,miR-31-5p,miR-

NME8	ENSOARG0(	NME/NM23	miR-485-5p,miR-331-5p,miR-2448-3p,miR-23b,miR-331-5p,miR-3965,miR-125a,miR-1271,miR-331-5p,miR-374b-3p,miR-34c-3p,miR-125b-5p,miR-374a-3p,miR-378c,miR-1306-5p,miR-200b-3p,miR-378h,miR-2285ad,miR-429-3p,miR-200c-3p,miR-181b-5p,novel_96,miR-664-3p,miR-1,miR-30a-3p,miR-378f,miR-21c,miR-669,miR-677,miR-181b-3p,miR-206,miR-656-5p,miR-203b-5p,miR-4324,miR-33a-3p,miR-323c,miR-374c-5p,miR-2285m,miR-335-5p,miR-21-5p,miR-19b-2-5p,miR-542-3p,miR-181a-5p,miR-197-3p,novel_23,miR-145a-5p,miR-6238,miR-31-5p,miR-660,miR-125a-5p,miR-582,miR-377-3p,miR-296-3p,miR-2285v,miR-654-3p,miR-296-3p,miR-493-5p,miR-101-5p,let-7f-2-3p,let-7f-2-3p,novel_99,miR-3613-5p,let-7f-1-3p,miR-377-3p,miR-378a-3p,miR-140-5p,miR-2331-3p,miR-660-5p,let-7b-3p,miR-30e-3p,miR-30a-3p,miR-19b-1-5p,miR-206-3p,miR-98-3p,miR-181c-5p,miR-378c,miR-539-5p,novel_73,miR-33b-3p,miR-378i,miR-98-3p,miR-378a-3p,miR-23b-3p,miR-200b,miR-23c,miR-2284h-5p,miR-30d-3p,miR-466i-5p,miR-92a-1-5p,miR-422a,miR-125b,miR-125b-2-3p,miR-181b-1-3p,miR-216c-5p,miR-2285w,miR-345-3p,miR-136-5p,miR-493-5p,miR-378e,miR-1185-2-3p,miR-4443,miR-664b,miR-2319b,miR-335,miR-218-5p,miR-107-5p,novel_111,miR-3059-5p,miR-885-5p,miR-181d-5p,miR-1388-5p,miR-378c,miR-101a-5p,miR-101c,miR-484,miR-378b,miR-6529a,miR-1a-3p,miR-7857,miR-147a,miR-1185-3p,miR-145b,miR-181b-2-3p,miR-491-5p,miR-3955-3p,miR-103a-2-5p,miR-378d,miR-101b-3p,miR-494-3p,miR-23a-3p,miR-33a-5p

XG	ENSOARG0(	Xg blood	miR-3959-3p,miR-24-3p,novel_27,miR-22-5p,miR-656-5p,miR-203b-5p,miR-4324,miR-502-5p,miR-15a,miR-16a,miR-323a-5p,miR-669,miR-677,miR-6535,miR-181b-3p,miR-216b-3p,miR-410-5p,miR-379-5p,miR-195a-5p,miR-18a-3p,miR-376b-3p,miR-296-3p,miR-130b-5p,miR-363-5p,let-7f-2-3p,let-7f-2-3p,miR-1343-5p,miR-296-3p,miR-197-5p,miR-105-5p,miR-432-5p,miR-3431,miR-149-5p,miR-2355-3p,miR-544b,miR-187-3p,miR-339-5p,miR-26b-3p,miR-16b,miR-369-5p,miR-497-5p,miR-2284x,miR-432,miR-1843b-5p,miR-181a-5p,miR-1197-3p,miR-145-3p,miR-16-5p,miR-486b-3p,miR-99b-3p,miR-331-3p,miR-3969,miR-365a-3p,miR-133c,miR-99a-3p,miR-2355-5p,miR-876-3p,miR-7144-5p,miR-1814c,miR-1248,miR-488-3p,miR-3065-3p,miR-2385-3p,miR-181a-2-3p,miR-191-3p,miR-181b-5p,novel_127,miR-2285af,miR-376b-3p,miR-125a-3p,miR-339a,miR-322-5p,miR-9-5p,miR-582-3p,miR-2284z,miR-2285ad,novel_79,miR-885-3p,miR-105-2,miR-3578,miR-1290,miR-1343-5p,miR-30a-5p,miR-361-3p,novel_111,miR-3059-5p,miR-4454,miR-541-3p,miR-181d-5p,miR-9-5p,miR-3968,miR-2284q,miR-2285n,miR-2440,miR-371a-5p,miR-2285y,miR-1973,miR-301a-5p,miR-1185-2-3p,miR-376d,miR-139-5p,miR-20a-3p,miR-7977,miR-126b-5p,miR-376a-3p,miR-877-3p,miR-140-5p,miR-378j,miR-2310,miR-1388-3p,miR-101b-3p,miR-424-5p,miR-652-5p,miR-214-3p,miR-2957,miR-16b,miR-376b-3p,miR-455-3p,miR-216a-3p,miR-1185-3p,miR-105-1,miR-181b-2-3p,miR-101a-5p,miR-2426,miR-544a,miR-2284aa,miR-101c,miR-541,miR-301b-5p,miR-143-5p,miR-6525,miR-122-5p,miR-181c-5p,miR-6134,miR-145a-3p,miR-3958-5p,miR-214,novel_74,miR-424-5p,miR-130b-5p,miR-369-5p,miR-664a-5p,miR-132-5p,miR-544-3p,miR-323-5p,miR-216b-5p,miR-139-5p,miR-15b-5p,miR-140-5p,miR-3065-3p,novel_107,miR-339b,miR-885-3p,miR-299,miR-30d-5p,miR-664b-3p,miR-181b-
LRRC2	ENSOARG0(	leucine	miR-140-3p,miR-1197-5p,miR-193b-5p,miR-2284u,miR-148a-5p,miR-16-1-3p,miR-33b-3p,miR-411-3p,miR-2285ad,miR-129-1-3p,miR-545-5p,miR-15a-5p,miR-322-5p,miR-9-5p,novel_17,miR-30b-3p,miR-29b-2-5p,miR-2284a,miR-1271-3p,novel_39,miR-2284y,miR-130a-5p,miR-29b-1-5p,miR-411,miR-129b-3p,miR-671-5p,miR-1248,novel_48,miR-345-5p,miR-345-5p,miR-767,miR-1271,miR-660-5p,miR-140-5p,miR-15b-5p,miR-223-5p,miR-3969,miR-665,miR-29b-2-5p,miR-130b-5p,miR-424-5p,miR-874-5p,miR-129-5p,miR-767-5p,miR-144-5p,miR-16-5p,miR-129-2-3p,novel_23,miR-432,miR-16-2-3p,miR-497-5p,miR-101a-5p,miR-16-1-3p,miR-16b,miR-2284ab,miR-1343-3p,miR-216a-3p,miR-148b-5p,miR-455-3p,miR-3431,miR-432-5p,miR-16b,miR-2957,miR-29b-1-5p,miR-660,miR-376b,miR-2284b,miR-363-5p,miR-382-3p,miR-424-5p,miR-130b-5p,miR-1b-5p,miR-345-5p,miR-432-3p,miR-432-3p,miR-140-5p,miR-361-3p,miR-195a-5p,miR-335-3p,miR-9851-3p,miR-7689-3p,miR-2285u,miR-452-3p,miR-2284g,miR-4443,miR-144-5p,miR-7857-3p,miR-16a,miR-2285y,miR-382-3p,miR-2285n,miR-15a,miR-147-5p,miR-22-5p,miR-335,miR-379-3p,miR-9-5p,miR-335-5p,miR-3956-3p,miR-212-3p,miR-3059-5p,novel_60,miR-2284w

RGS19	ENSOARG0(	regulato	miR-488-3p,miR-500a-3p,miR-188-3p,miR-504,miR-504-5p,miR-370-3p,miR-141-3p,miR-1298-3p,novel_69,miR-669,novel_17,miR-331-3p,miR-491-5p,miR-127-5p,miR-500,miR-200a-3p,novel_96,miR-214-3p,miR-214,miR-6525,miR-1271-3p
SCN2B	ENSOARG0(	sodium c	miR-9851-3p,miR-18b,miR-2387,miR-18a-3p,miR-34b,miR-670-3p,miR-29a-3p,miR-146b-3p,miR-10a-5p,miR-320b,novel_1,miR-502b,miR-22-5p,miR-6402,miR-6529b,let-7i,miR-34c,miR-211,miR-2428,miR-449a,miR-2285x,novel_101,miR-3957-3p,miR-219a-1-3p,miR-1260b,miR-138,miR-29b,miR-455-5p,miR-181a-5p,miR-873a-5p,let-7g-5p,miR-2285g,miR-432-5p,miR-1343-5p,miR-224-5p,miR-455-5p,miR-654-3p,miR-345-5p,miR-296-3p,miR-363-5p,miR-10b-3p,miR-485-5p,miR-23a-5p,miR-362-5p,miR-18a,miR-34c-5p,miR-199a-5p,miR-215-5p,miR-370-3p,miR-138-5p,miR-204-5p,miR-486b-3p,miR-28-5p,miR-2332,miR-29b-3p,miR-331-3p,miR-500b-5p,miR-378c,miR-145-3p,miR-1306-5p,miR-133a-3p,miR-6128,let-7d,miR-10a-5p,miR-105-2,miR-501-3p,miR-29d-3p,miR-6516-5p,miR-378h,miR-2411-5p,miR-214-5p,miR-545-5p,miR-7641,miR-181b-5p,miR-6119-5p,miR-2285af,miR-1260a,let-7c-5p,miR-217,miR-339a,miR-30c-2-3p,miR-28b,miR-1271-3p,miR-3065-3p,miR-199a-5p,miR-4286,miR-30a-3p,miR-1,miR-29b-2-5p,novel_94,miR-199b-5p,miR-361-3p,miR-489,miR-3082-5p,miR-378e,miR-7857-3p,miR-27a-3p,let-7d-5p,miR-452-3p,miR-3064-5p,miR-7977,miR-335,miR-4726-5p,miR-2284q,miR-362-5p,miR-502-3p,novel_60,miR-181d-5p,miR-378c,miR-2284aa,miR-10b,miR-143-5p,miR-6529a,miR-378b,miR-296-5p,miR-7857,miR-2285e,miR-500-5p,miR-105-1,miR-2300a-5p,miR-27a-3p,miR-222-5p,miR-5010-3p,miR-3085-3p,let-7e,miR-5126,miR-30c-1-3p,miR-4532,miR-494-3p,miR-2330-5p,let-7a-5p,let-7c-3p,miR-18a-5p,miR-133b-3p,miR-6130,miR-192-5p,miR-6516,miR-27a-5p,novel_32,miR-204-3p,miR-378a-3p,miR-140-5p,miR-221-5p,miR-3065-3p,miR-664a,miR-450b-3p,miR-708-5p,miR-29b-2-5p,miR-324-3p,miR-206-3p,miR-10b-5p,miR-338-3p,miR-6134,let-7b-5p,miR-4510,miR-539-5p,miR-378c,miR-378d,miR-

PAAF1	ENSOARG0(	proteaso	novel_27,miR-326-3p,miR-495-3p,miR-1193,miR-6119-3p,miR-656-5p,miR-20a-5p,miR-3613,miR-502-5p,miR-4324,miR-33a-3p,miR-144-3p,miR-3074-5p,miR-10a-5p,miR-412-3p,miR-450b-3p,miR-378f,miR-7975,miR-2319a,miR-376b-3p,miR-3065-5p,miR-3970,miR-654-3p,miR-345-5p,miR-6240,miR-2483-5p,miR-125a-5p,miR-17-5p,miR-106a-5p,miR-330-3p,miR-105-5p,miR-3431,miR-134-3p,miR-20b,miR-340-3p,miR-31-5p,miR-339-5p,miR-2284x,miR-26a-2-3p,novel_23,miR-3071-5p,miR-374a-3p,miR-93,miR-378c,miR-28a-3p,miR-6128,miR-134-5p,miR-374b-3p,miR-370-5p,miR-34c-3p,miR-3969,miR-144,miR-6740-5p,miR-125b-5p,miR-3959-5p,miR-769,miR-154b-5p,miR-3957,miR-133c,miR-3957-5p,miR-141-3p,miR-125a,miR-2285aa,miR-345-5p,miR-2318,miR-876-3p,miR-1814c,miR-20b-5p,miR-3074-1-3p,miR-301,miR-488-3p,miR-17-5p,miR-93-5p,miR-3529-3p,miR-495-5p,miR-127-5p,miR-28-3p,miR-200a-3p,miR-376b-3p,miR-339a,miR-217,miR-378h,miR-129-1-3p,miR-134-5p,miR-2284z,miR-2285ad,novel_79,miR-10a-5p,miR-134,miR-1290,miR-330-5p,novel_111,miR-361-3p,miR-877-3p,miR-3968,miR-2285n,miR-382-3p,miR-107-5p,miR-2285y,miR-769-5p,miR-378e,miR-3596,miR-378g,miR-454-5p,miR-376d,miR-2319b,miR-504,miR-1983,miR-376a-3p,miR-335-3p,miR-378j,miR-378d,miR-2310,miR-101b-3p,miR-124a,miR-487b-5p,miR-103a-2-5p,miR-326,miR-106b-5p,miR-323b-3p,miR-2957,miR-376b-3p,miR-330-3p,miR-1343-3p,miR-2426,miR-378c,miR-2284aa,miR-10b,miR-484,miR-378b,miR-323b,miR-7857,miR-769-5p,miR-10b-5p,miR-129-2-3p,miR-3074-5p,miR-145a-3p,miR-378c,miR-2330-3p,novel_74,miR-874-5p,miR-10a,novel_32,miR-221-5p,miR-504-5p,miR-378a-3p,miR-370-5p,miR-216b-3p,miR-339b,miR-345-5p,miR-1827,miR-3613-5p,miR-2424,miR-216c-5p,miR-345-3p,miR-20a,miR-760-3p,miR-221-5p,miR-30b-3p,miR-1277-5p,miR-

AMOTL2	ENSOARG0(	angiomot	novel_127,miR-152-3p,novel_96,miR-127-5p,novel_39,miR-2284y,miR-200a,miR-450b-5p,miR-3064-5p,miR-30c,miR-885-3p,miR-2427,miR-330-5p,miR-3591-5p,miR-134-5p,miR-450a-1-3p,novel_121,miR-505-5p,miR-322-5p,novel_79,miR-2285ad,miR-299a-3p,miR-429-3p,miR-6517,miR-134-5p,miR-100-3p,miR-193a-5p,miR-16-5p,miR-30e-5p,miR-3959-5p,miR-3956,miR-106b-3p,miR-193a-3p,miR-105-5p,novel_51,miR-2411-3p,miR-146a,miR-432-3p,miR-432-3p,miR-4508,miR-497-5p,miR-16b,miR-450c-3p,miR-134-3p,miR-339-5p,miR-103a-3p,miR-6119-3p,miR-28c,miR-199b-5p,miR-193b-3p,miR-2319a,miR-146b-5p,miR-1306,miR-150-5p,miR-216a-3p,miR-3065-5p,miR-148a-3p,miR-6535,miR-320c,miR-669,miR-299b-3p,miR-3120-3p,miR-2484,miR-324-3p,miR-345-3p,miR-30b,novel_17,miR-486-3p,miR-376a-5p,miR-218-1-3p,miR-133a-3p,miR-34a-5p,miR-2284u,miR-33b-3p,miR-30c-5p,miR-328-3p,miR-212-5p,miR-18a-3p,miR-4492,miR-669a-3p,miR-30a-3p,miR-665,miR-2285j,miR-30e-3p,miR-6525,miR-224-5p,miR-214,miR-885-3p,miR-625-5p,miR-1827,miR-339b,miR-129b-3p,miR-30d-5p,miR-450a-1-3p,miR-15b-5p,miR-10a,miR-30c-1-3p,miR-199b-5p,miR-146b,miR-153-3p,miR-103a-2-5p,miR-214-3p,miR-491-5p,miR-323b-3p,miR-16b,miR-2284b,miR-574-3p,miR-323b,miR-455-3p,miR-2459,miR-153,miR-147a,miR-145b,miR-1343-3p,novel_82,miR-2440,miR-101-3p,miR-877-3p,miR-2447,miR-885-5p,miR-136-5p,miR-378g,miR-2285u,miR-24-1-5p,miR-2284g,miR-2319b,miR-107,miR-17-3p,miR-200c-3p,miR-181b-5p,miR-30c-2-3p,miR-339a,miR-376b-3p,miR-2285af,miR-200c,miR-1957a,miR-199a-5p,miR-3065-3p,miR-24-2-5p,miR-28b,miR-30a-3p,miR-105-2,miR-200b-3p,miR-128-3p,miR-10a-5p,miR-300,miR-218-2-3p,miR-30a-5p,miR-345-3p,miR-134,miR-582-3p,miR-214-5p,miR-302a-5p,miR-423-3p,miR-28-5p,miR-486b-3p,miR-138-

NCOA5	ENSOARG0(	nuclear	miR-365a-5p,miR-378c,miR-16-5p,miR-767-5p,miR-138-5p,miR-134-5p,miR-410-3p,miR-29b-3p,miR-6740-5p,miR-34c-5p,miR-503-3p,miR-1271,miR-615-5p,miR-141-3p,miR-598-3p,miR-2355-5p,miR-370-3p,miR-345-5p,miR-329b,miR-10b-3p,miR-767,miR-485-5p,miR-190a-3p,miR-488-3p,miR-18a,miR-28b,miR-148b-5p,novel_39,miR-29a-5p,miR-3529-3p,miR-181a-2-3p,miR-30a-3p,miR-450a-2-3p,miR-200a-3p,novel_96,miR-2285af,miR-378h,miR-24-2-5p,miR-322-5p,miR-134-5p,miR-450a-1-3p,miR-222,miR-18b-5p,miR-412,miR-1224-5p,miR-134,miR-330-5p,miR-29d-3p,miR-1343-5p,miR-371b-3p,miR-24-3p,miR-2285m,miR-301b-3p,miR-2284w,miR-326-3p,miR-449a,miR-335-5p,miR-342-3p,miR-29c-3p,novel_101,miR-29a,miR-502b,miR-28c,miR-502-5p,miR-15a,miR-33a-3p,miR-34c,miR-16a,miR-29a-3p,miR-34b,miR-146b-3p,miR-323a-5p,miR-329-5p,novel_68,miR-378f,miR-195a-5p,miR-6516-3p,miR-9851-3p,miR-2319a,miR-18b,miR-3065-5p,miR-1306,miR-503-3p,miR-345-5p,miR-296-3p,miR-124-5p,miR-106b-3p,miR-1343-5p,miR-296-3p,miR-224-5p,novel_51,miR-365b-5p,miR-3431,miR-194-3p,miR-149-5p,miR-122-3p,miR-377-5p,miR-130a-3p,miR-16-1-3p,miR-29b,miR-138,miR-16b,miR-2284x,miR-27b-3p,miR-497-5p,miR-653,miR-450c-3p,miR-197-3p,miR-22-3p,miR-224-5p,miR-346,miR-378c,miR-214,miR-3074-2-3p,miR-664a,miR-450b-3p,miR-454-3p,miR-424-5p,miR-30e-3p,miR-2285j,miR-222-3p,miR-320e,miR-3604,miR-30a-3p,miR-324-3p,miR-323-5p,miR-301a-3p,miR-15b-5p,miR-22-3p,miR-2331-3p,miR-378a-3p,miR-296-3p,miR-18a-5p,miR-345-5p,novel_99,miR-625-5p,miR-450a-1-3p,miR-129b-3p,miR-598-3p,miR-377-5p,miR-2285w,miR-329a-5p,miR-34b-5p,novel_17,miR-196a-3p,miR-92a-1-5p,miR-422a,miR-15a-5p,miR-2284h-5p,miR-30d-3p,miR-34a-5p,miR-16-1-3p,miR-133a-3p,miR-378i,novel_133,miR-378a-3p,miR-
C14orf79	ENSOARG0(	chromoso	miR-491-5p,miR-874-5p,miR-708-5p,miR-130b-5p,miR-28-5p,novel_96,miR-503-5p,miR-125a-3p,miR-135a-2-3p,miR-130b-5p,miR-503-5p,miR-22-3p,miR-378g,miR-503-5p,miR-134-3p,miR-22-3p,miR-370-3p,miR-1271
TPD52	ENSOARG0(	tumor pr	miR-2285aa,miR-2355-5p,miR-216b-3p,miR-548o-3p,miR-545-3p,miR-1185-2-3p,miR-203-3p,novel_25,miR-34b,miR-411b-3p,novel_69,miR-34c-5p,miR-3065-5p,miR-361-3p,miR-9-3p,miR-186-5p,miR-493-5p,miR-107,let-7g-3p,miR-425-5p,miR-28a-3p,miR-449a,miR-409-3p,miR-34c,miR-2440,miR-576-3p,miR-382-3p,miR-218-5p,miR-22-5p,miR-5010-3p,miR-103a-3p,miR-103,miR-1185-3p,miR-582-3p,miR-412-5p,miR-34a-5p,miR-142-3p,miR-582-3p,miR-668-3p,miR-493-5p,miR-2285r,miR-185-3p,miR-6119-5p,miR-34b-5p,miR-107,miR-28-3p

NOL8	ENSOARG0(	nucleola	miR-155-5p,miR-345-3p,miR-134,miR-3578,miR-2284z,miR-134-5p,novel_121,miR-505-5p,miR-758-5p,miR-6516-5p,miR-378h,miR-2411-5p,miR-18b-5p,miR-2285ad,miR-299a-3p,miR-152-3p,miR-543-5p,novel_96,miR-2898,miR-450a-2-3p,miR-181b-5p,miR-127-5p,miR-30f,miR-205-5p,miR-376b-3p,miR-1260a,miR-2285af,miR-6119-5p,novel_39,miR-29a-5p,miR-1271-3p,miR-450b-5p,miR-148b-5p,miR-1,miR-3064-5p,miR-20b-5p,miR-10b-3p,miR-345-5p,let-7g-3p,miR-93-5p,miR-23b,miR-18a,miR-17-5p,miR-320a,miR-3154,miR-154b-5p,miR-370-3p,miR-2355-5p,miR-2285aa,miR-1271,miR-3068-3p,miR-874-3p,miR-31-3p,miR-134-5p,miR-100-3p,miR-409-3p,miR-135a-5p,miR-370-5p,miR-378c,miR-93,miR-1260b,miR-1197-3p,miR-6128,miR-1306-5p,miR-2284x,miR-21-5p,miR-1260b,miR-16-1-3p,miR-194-5p,miR-19b-2-5p,miR-432,miR-1843b-5p,miR-1285,miR-1434-3p,miR-181a-5p,miR-6238,novel_116,miR-20b,miR-432-5p,miR-134-3p,miR-665,miR-31-5p,miR-133b-5p,miR-106b-3p,miR-190a,miR-194a,miR-106a-5p,miR-17-5p,miR-2411-3p,miR-146a,miR-345-5p,miR-654-3p,miR-432-3p,miR-432-3p,miR-2284l,miR-146b-5p,miR-135b-5p,miR-505-3p,miR-378f,miR-376b-3p,miR-3591-3p,miR-21c,miR-148a-3p,miR-18b,miR-669,miR-320c,miR-677,miR-181b-3p,miR-670-3p,miR-299b-3p,miR-378b,miR-3074-5p,miR-203b-5p,miR-147-5p,novel_1,miR-206,miR-4324,miR-20a-5p,miR-2428,miR-1839-3p,miR-1193,miR-16-1-3p,miR-4429,novel_73,miR-33b-3p,miR-142-3p,miR-1434-5p,miR-378a-3p,miR-140-3p,miR-5703,novel_133,miR-378i,miR-2300b-3p,miR-532-3p,miR-199a-3p,miR-23b-3p,miR-7859,miR-23c,miR-340-5p,miR-148b-3p,miR-3600,miR-505-3p,miR-30d-3p,miR-106a,miR-221-5p,miR-299-3p,miR-106a,miR-320d,miR-219b-5p,miR-345-3p,miR-422a,miR-618,miR-210-5p,miR-2411,miR-146a-5p,miR-143-3p,miR-376a-5p,miR-181b-1-

ATP5B	ENSOARG0(	ATP synt	let-7i,miR-502-5p,miR-6402,novel_101,miR-326-3p,miR-24-3p,miR-150-5p,miR-21c,miR-18a-3p,miR-9851-3p,miR-505-3p,let-7e-5p,miR-135b-5p,miR-450b-3p,miR-3074-5p,miR-6535,miR-677,miR-181b-3p,novel_69,miR-365b-5p,miR-197-5p,miR-455-5p,miR-2411-3p,miR-660,miR-196b-5p,miR-363-5p,miR-493-5p,miR-539-3p,miR-2285r,miR-542-3p,miR-2113,miR-4508,miR-26a-2-3p,miR-455-5p,miR-744-5p,miR-138,miR-30b-3p,miR-26b-3p,let-7g-5p,miR-331-3p,miR-135a-5p,miR-409-3p,novel_63,miR-138-5p,miR-103b,miR-1306-5p,miR-365a-5p,miR-500b-5p,miR-362-5p,miR-30e-5p,miR-381-3p,miR-2355-5p,miR-2285aa,miR-487a-3p,miR-874-3p,miR-300-3p,miR-30c-2-3p,miR-217,let-7c-5p,miR-2285af,miR-205-5p,novel_96,novel_127,miR-2898,let-7f-5p,miR-3064-5p,miR-1957a,let-7g,miR-24-2-5p,miR-30a-5p,miR-330-5p,miR-345-3p,miR-196a-5p,miR-300,let-7d,miR-582-3p,miR-1224-5p,miR-431,miR-129-1-3p,miR-6516-5p,miR-107-5p,miR-2285n,miR-490-5p,miR-154-3p,miR-2284q,miR-499a-5p,miR-219a-5p,miR-2447,miR-212-3p,miR-423-5p,miR-361-3p,miR-362-5p,novel_111,miR-129b-5p,miR-378j,miR-361-3p,miR-493-5p,miR-544-5p,miR-7977,miR-24-1-5p,miR-3064-5p,miR-20a-3p,let-7d-5p,miR-25,miR-2483-3p,miR-29b-1-5p,miR-326,miR-431-5p,let-7e,miR-103a-2-5p,miR-491-5p,miR-3085-3p,miR-33a-5p,miR-376b,miR-124a,let-7a-5p,miR-4532,miR-30c-1-3p,miR-30b-3p,miR-3962,miR-484,miR-21b,miR-101c,miR-181b-2-3p,miR-216a-3p,miR-500-5p,miR-421,miR-142-3p,miR-665,let-7f,miR-4492,miR-487a-5p,miR-664a,miR-2330-3p,let-7b,miR-539-5p,miR-129-2-3p,miR-3074-5p,miR-3184-3p,miR-4510,let-7b-5p,miR-3184-5p,miR-129b-3p,miR-27b-5p,miR-30d-5p,miR-21a-3p,miR-625-5p,miR-1827,miR-154b-3p,miR-154a-3p,miR-221-5p,miR-30c-1-3p,miR-660-5p,miR-204-3p,let-7i-5p,miR-877-5p,miR-6516,miR-6130,miR-500a-

TM9SF3	ENSOARG0(	transmem	miR-323a-5p,miR-2404,miR-411b-3p,miR-144-3p,miR-10a-5p,miR-146b-5p,miR-2319a,miR-6516-3p,miR-150-5p,miR-371b-3p,novel_27,miR-199b-5p,miR-376a-3p,miR-2285x,novel_1,miR-379-3p,miR-142b,miR-33a-3p,miR-2355-3p,miR-31-5p,miR-487b-3p,miR-380-3p,miR-2284ab,miR-708-3p,miR-181a-5p,miR-1434-3p,miR-450c-3p,miR-432-3p,let-7a-2-3p,miR-668-3p,miR-377-3p,miR-146a,miR-105-5p,miR-199a-5p,miR-503-3p,miR-329a,let-7g-3p,novel_48,miR-376c-5p,miR-23b,miR-376c-5p,miR-144-5p,miR-133a-3p,miR-134-5p,miR-486b-3p,miR-2433,novel_63,miR-144,miR-24-2-5p,miR-134-5p,miR-582-3p,miR-450a-1-3p,miR-545-5p,miR-2284z,miR-2285ad,miR-222,miR-429-3p,miR-411-3p,miR-105-2,miR-155-5p,miR-200b-3p,let-7a-2-3p,miR-10a-5p,miR-1290,miR-134,miR-218-2-3p,miR-2427,miR-24-2-5p,miR-199a-5p,miR-1957a,miR-2284y,miR-2284a,miR-3529-3p,miR-30a-3p,miR-2385-3p,miR-4286,miR-450a-2-3p,miR-200c-3p,miR-181b-5p,miR-2285af,miR-205-5p,miR-200c,miR-125a-3p,miR-217,miR-144-5p,miR-1185-2-3p,miR-2284g,miR-24-1-5p,miR-10b,miR-20a-3p,miR-2319b,miR-2285u,miR-374b-5p,miR-541-5p,miR-136-5p,miR-668-5p,miR-493-5p,miR-877-3p,miR-221,miR-199b-5p,miR-877-3p,novel_60,miR-3956-3p,miR-181d-5p,miR-2285n,novel_82,miR-153,miR-221-3p,miR-1185-3p,miR-105-1,miR-362-3p,miR-2284aa,miR-101c,miR-21b,miR-16-2-3p,miR-329-3p,miR-10b,miR-758-3p,miR-8485,miR-193a,miR-758-3p,miR-101b-3p,miR-23a-3p,miR-2284b,miR-3955-3p,miR-199b-5p,miR-146b,miR-153-3p,novel_103,miR-2957,miR-362-3p,miR-10a,miR-323-5p,miR-155-5p,miR-2331-3p,miR-548o-3p,miR-216b-3p,miR-133b-3p,let-7g-3p,miR-543-3p,miR-450a-1-3p,miR-129b-3p,miR-10b-5p,miR-181c-5p,miR-539-5p,miR-425-5p,miR-450b-3p,miR-155-5p,miR-6516-3p,miR-208b-5p,miR-129-5p,miR-222-3p,miR-30e-3p,miR-30a-3p,miR-

STX4	ENSOARG0(	syntaxin	miR-2285g,miR-134-3p,miR-185-5p,miR-503-5p,miR-149-5p,miR-194-3p,miR-103,miR-187-3p,miR-103a-3p,miR-339-5p,miR-16b,miR-30b-3p,miR-497-5p,miR-26a-2-3p,miR-744-5p,miR-27b-3p,miR-7862,miR-653,miR-708-3p,miR-22-3p,miR-374b-3p,miR-130b-5p,miR-382,miR-668-3p,miR-125a-5p,miR-660,miR-1343-5p,miR-503-5p,miR-2411-3p,novel_51,miR-374c-3p,miR-677,novel_68,miR-3074-5p,miR-320b,miR-26b-5p,miR-1291,miR-412-3p,miR-107,miR-195a-5p,miR-378f,miR-216a-3p,miR-24-3p,miR-2428,miR-199b-5p,miR-3957-3p,miR-17-3p,miR-128-1-5p,miR-147-5p,miR-15a,miR-4324,miR-16a,miR-322-5p,miR-378h,miR-222,miR-196a-2-3p,novel_79,miR-1224-5p,miR-128-3p,miR-345-3p,miR-3591-5p,miR-1343-5p,miR-200a,miR-199a-5p,miR-3065-3p,miR-29a-5p,miR-192-3p,miR-1271-3p,miR-3529-3p,miR-3064-5p,miR-17-3p,miR-2898,miR-107,miR-200a-3p,miR-125a-3p,miR-30c-2-3p,miR-217,miR-339a,miR-374c-3p,miR-365a-3p,miR-199a-5p,miR-874-3p,miR-2355-5p,miR-141-3p,miR-125a,miR-767,miR-7144-5p,miR-485-5p,miR-1814c,miR-1197-3p,miR-378c,miR-16-5p,miR-3607-3p,miR-767-5p,miR-125b-5p,miR-153,miR-496-5p,miR-455-3p,miR-221-3p,miR-2285e,miR-2300a-5p,miR-409b,miR-27a-3p,miR-181b-2-3p,miR-378c,miR-101c,miR-3120-5p,miR-378b,miR-143-5p,miR-541,miR-30b-3p,miR-378d,miR-30c-1-3p,miR-8485,miR-2310,miR-424-5p,miR-2330-5p,miR-3085-3p,miR-199b-5p,miR-153-3p,novel_103,miR-16b,miR-378g,miR-378e,miR-3082-5p,miR-27a-3p,miR-4443,miR-3064-5p,miR-4792,miR-10b,miR-20a-3p,miR-7977,miR-504,miR-1983,miR-361-3p,miR-199b-5p,miR-221,miR-877-3p,miR-361-3p,miR-7705,miR-541-3p,miR-452-5p,miR-3968,miR-490-5p,miR-2285t,miR-101-3p,miR-15a-5p,miR-151-5p,miR-33b-3p,miR-378d,miR-378i,miR-2408,miR-193b-5p,miR-2904,miR-5703,miR-1197-5p,miR-378a-3p,miR-217-5p,miR-188-3p,miR-216c-
VEGFA	ENSOARG0(	vascular	miR-378j,miR-1197-5p,miR-6130,miR-142-3p,miR-7857-3p,novel_25,miR-3960,miR-125a,miR-592,miR-125a-5p,miR-205-5p,novel_51,miR-125b,miR-125b-5p,novel_111,miR-142a-3p,miR-877-3p,miR-6412,miR-2432,miR-4510,miR-145a-3p

CAMK2G	ENSOARG0(	calcium/	miR-483-3p,miR-125a-5p,miR-1343-5p,miR-377-3p,miR-330-3p,miR-105-5p,miR-3141,miR-6240,miR-628-5p,miR-382,miR-668-3p,miR-138,miR-29b,miR-30b-3p,miR-7862,miR-744-5p,miR-181a-5p,miR-2113,miR-22-3p,miR-3431,miR-544b,miR-149-5p,miR-194-3p,miR-31-5p,miR-29a,miR-147-5p,miR-28c,miR-502-5p,miR-2428,miR-495-3p,miR-29c-3p,miR-17-3p,miR-1291,miR-3141,miR-2285ab,miR-1306,miR-150-5p,miR-29a-3p,miR-669,miR-6535,novel_68,miR-3120-3p,miR-10a-5p,miR-127-5p,miR-181b-5p,miR-17-3p,miR-543-5p,miR-125a-3p,miR-28b,novel_39,miR-3065-3p,miR-3064-5p,miR-582-3p,miR-10a-5p,miR-105-2,miR-1343-5p,miR-29d-3p,miR-483-3p,novel_121,miR-545-5p,miR-196a-2-3p,miR-138-5p,miR-486b-3p,miR-29b-3p,miR-2433,miR-125b-5p,miR-6740-5p,miR-6128,miR-193a-5p,miR-485-5p,miR-1248,miR-3154,miR-769,miR-125a,miR-25-5p,miR-3085-3p,miR-1185-5p,miR-153-3p,miR-1306-5p,miR-5126,novel_19,miR-4532,miR-3187-3p,miR-33a-5p,miR-544a,miR-2426,miR-1388-5p,miR-301b-5p,miR-10b,miR-30b-3p,miR-153,miR-2459,miR-455-3p,miR-105-1,miR-330-3p,novel_78,novel_82,miR-301a-5p,miR-371a-5p,miR-769-5p,miR-877-3p,miR-361-3p,miR-423-5p,miR-219a-5p,miR-181d-5p,miR-452-5p,miR-2285f,miR-136-5p,miR-499a-3p,miR-4443,miR-452-3p,miR-4792,miR-3064-5p,miR-7977,miR-760-3p,miR-2484,miR-21-3p,miR-3653-3p,novel_17,miR-210-5p,miR-185-3p,miR-125b,miR-125b-2-3p,miR-486-3p,miR-133a-3p,miR-188-3p,miR-2904,miR-383-5p,miR-532-3p,novel_83,miR-2284h-5p,miR-3960,miR-212-5p,miR-1b-3p,miR-17-3p,miR-664a,miR-4492,miR-2285j,miR-664a-5p,miR-10b-5p,miR-769-5p,miR-181c-5p,miR-3184-5p,miR-665-5p,miR-2330-3p,miR-2312,miR-1827,miR-625-5p,miR-543-3p,miR-10a,miR-544-3p,miR-499b-3p,miR-377-3p,miR-877-5p,miR-22-3p,miR-532-5p,miR-3065-3p
LDLRAD2	ENSOARG0(	low dens	miR-3065-3p,novel_39,miR-424-5p,miR-16b,miR-194a,miR-15a-5p,miR-322-5p,miR-149-5p,miR-151-5p,novel_78,miR-16b,miR-29b,miR-497-5p,miR-664-5p,miR-296-5p,miR-194-5p,miR-29d-3p,miR-1197-3p,miR-361-3p,novel_42,miR-29c-3p,miR-194b-5p,miR-16-5p,miR-29a,miR-664a,miR-4726-5p,miR-424-5p,miR-410-5p,miR-29b-3p,miR-15a,miR-665,miR-1973,miR-16a,miR-29a-3p,miR-15b-5p,miR-151b,miR-2331-3p,miR-3065-3p,miR-195a-5p,miR-2387,miR-361-3p

TACSTD2	ENSOARG0(	tumor-as	miR-2411,miR-2424,miR-2432,miR-486-3p,miR-143-3p,miR-421-5p,miR-376a-5p,miR-376c-3p,miR-195a-3p,let-7k,miR-466i-5p,miR-760-3p,miR-98-5p,miR-219b-5p,miR-185-3p,miR-210-5p,miR-7859,miR-383-5p,miR-15a-5p,miR-3600,miR-18a-3p,miR-548e-3p,miR-218-1-3p,miR-361-5p,miR-184-3p,miR-2904,novel_133,miR-2300b-3p,miR-1961,let-7b-5p,miR-3184-5p,miR-6134,miR-6525,miR-224-5p,miR-214,let-7b,miR-539-5p,miR-145a-3p,miR-424-5p,miR-669a-3p,miR-130b-5p,novel_74,miR-17-3p,miR-324-3p,miR-664a-5p,let-7f,miR-204-3p,let-7i-5p,miR-15b-5p,miR-582-5p,miR-376e-3p,miR-656-3p,miR-216b-3p,miR-504-5p,miR-30c-1-3p,miR-625-5p,miR-1827,miR-582-5p,miR-2312,let-7g-3p,miR-345-5p,miR-129b-3p,miR-433-3p,let-7f-1-3p,miR-3613-5p,miR-8485,miR-758-3p,miR-30c-1-3p,miR-33a-5p,let-7a-5p,miR-382-3p,miR-424-5p,miR-199b-5p,let-7e,miR-214-3p,miR-3085-3p,miR-16b,miR-342,miR-1306-5p,miR-431-5p,miR-9788-3p,miR-1185-3p,miR-1298-3p,miR-1343-3p,miR-199b-3p,miR-548w,miR-16-2-3p,miR-2284aa,miR-450b-5p,miR-758-3p,miR-125b-2-3p,miR-4791,miR-8095,miR-1285-5p,miR-423-5p,miR-7705,miR-877-3p,miR-361-3p,miR-2285f,miR-9-5p,miR-2447,novel_60,miR-3956-3p,miR-490-5p,miR-2285t,miR-4726-5p,miR-371a-5p,novel_82,miR-2440,let-7d-5p,miR-4443,miR-1185-2-3p,miR-3082-5p,miR-489,miR-499b-5p,miR-541-5p,miR-7977,miR-3064-5p,miR-20a-3p,miR-1983,miR-3963,miR-504,miR-136-5p,miR-129b-5p,miR-381-3p,miR-140-5p,miR-361-3p,miR-199b-5p,miR-200a,miR-199a-5p,let-7g,miR-490-3p,miR-2478,miR-3064-5p,miR-181a-2-3p,novel_127,miR-543-5p,miR-200a-3p,miR-17-3p,miR-450a-2-3p,let-7f-5p,miR-30c-2-3p,let-7c-5p,miR-1260a,miR-2284z,novel_121,miR-9-5p,miR-322-5p,miR-2411-5p,miR-1224-5p,miR-431,miR-429-3p,let-7a-2-3p,let-7d,miR-196a-5p,miR-365a-5p,miR-
CD79A	ENSOARG0(	CD79a mo	miR-1343-3p,miR-339-5p,miR-147a,miR-496-5p,miR-544b,miR-542-3p,miR-301b-5p,miR-450c-3p,miR-544a,miR-1260b,miR-744-5p,miR-2889,miR-1388-3p,miR-376b,miR-668-3p,miR-432-3p,miR-345-5p,miR-4532,miR-1306-5p,novel_51,miR-491-5p,miR-2319b,miR-7977,miR-541-5p,miR-320b,miR-34b,miR-669,miR-2387,miR-378j,miR-129b-5p,miR-1306,miR-1291,novel_111,miR-449a,miR-2440,miR-301a-5p,miR-34c,miR-421-5p,miR-22-5p,miR-30d-3p,novel_79,miR-1224-5p,miR-215-5p,miR-885-3p,miR-493-3p,miR-34a-5p,miR-133a-3p,miR-29b-2-5p,miR-30a-3p,miR-200a,miR-2432,miR-2424,miR-1260a,miR-92a-1-5p,miR-339a,miR-185-3p,miR-324-3p,miR-466i-5p,novel_96,miR-221-5p,miR-34b-5p,miR-874-3p,miR-221-5p,miR-552-3p,miR-296-3p,miR-215-5p,miR-370-3p,miR-544-3p,miR-34c-5p,miR-192-5p,miR-154b-5p,miR-345-5p,miR-345-5p,miR-339b,miR-485-5p,miR-1248,miR-1827,miR-671-5p,miR-7144-5p,miR-885-3p,miR-625-5p,miR-145a-3p,miR-3074-2-3p,miR-1260b,miR-346,miR-30e-3p,miR-5100,miR-34c-3p,miR-664a-5p,miR-29b-2-5p,miR-30a-3p,miR-664a,miR-450b-3p,miR-874-5p,miR-487a-5p,miR-410-3p,miR-4492,miR-129-5p
ARSD	ENSOARG0(	arylsulf	miR-2428,miR-361-3p,miR-1271,miR-214,miR-2887,miR-214-3p,miR-1343-5p,miR-136-5p,miR-450a-2-3p,novel_19,miR-1343-5p

DYNLL2	ENSOARG0(	dynein,	miR-19b-3p,miR-1197-5p,miR-15a-5p,miR-148b-3p,miR-322-5p,miR-328-3p,miR-17-3p,miR-200a-3p,miR-152-3p,miR-107,novel_17,miR-210-5p,miR-2285af,miR-30c-2-3p,miR-200a,miR-181a-2-3p,miR-1827,miR-1814c,miR-1248,miR-301a-3p,miR-15b-5p,miR-30c-1-3p,miR-141-3p,miR-1271,miR-2355-5p,miR-17-3p,miR-424-5p,miR-454-3p,miR-874-5p,miR-135a-1-3p,miR-665,miR-3969,miR-145a-3p,miR-16-5p,miR-16b,miR-497-5p,miR-185-5p,miR-103,miR-455-3p,miR-103a-3p,miR-222-5p,miR-130a-3p,miR-130b-3p,miR-466f-3p,miR-1306-5p,miR-16b,miR-30c-1-3p,miR-2285r,miR-2310,miR-424-5p,let-7f-2-3p,let-7f-2-3p,miR-19a-3p,miR-107,miR-6395,miR-195a-5p,miR-7975,miR-148a-3p,miR-361-3p,miR-1306,miR-1185-2-3p,miR-7977,miR-502b,miR-15a,miR-16a,miR-301b-3p,miR-212-3p,miR-17-3p
CD40	ENSOARG0(	CD40 mol	miR-324-3p,miR-422a,miR-92a-1-5p,miR-2284m,miR-4661-5p,miR-98-5p,miR-30b-3p,miR-21-3p,let-7k,miR-29b-1-5p,miR-376a-5p,miR-130a-5p,miR-2411,miR-378i,novel_133,miR-1961,miR-215-5p,miR-2366,miR-193b-5p,miR-378a-3p,miR-493-3p,miR-148a-5p,miR-18a-3p,miR-3960,miR-210-3p,miR-200b,miR-328-3p,let-7f,miR-19b-1-5p,miR-132-5p,miR-665,miR-664a-5p,miR-450b-3p,miR-6516-3p,miR-708-5p,miR-190a-5p,miR-4492,miR-130b-5p,miR-590-3p,let-7b,miR-378c,miR-145a-3p,miR-1271,miR-214,miR-6525,let-7b-5p,miR-450a-1-3p,miR-18a-5p,miR-625-5p,miR-1895,miR-1827,miR-30c-1-3p,miR-378a-3p,miR-1277-3p,miR-192-5p,miR-544-3p,miR-656-3p,miR-22-3p,miR-204-3p,let-7i-5p,miR-5126,miR-2957,miR-29b-1-5p,novel_124,miR-25-5p,let-7e,miR-214-3p,let-7a-5p,miR-574-3p,miR-1839-3p,miR-33a-5p,miR-378d,miR-30c-1-3p,miR-193a,miR-4532,miR-2310,miR-378b,miR-30b-3p,miR-378c,miR-1388-5p,miR-544a,miR-1343-3p,miR-330-3p,miR-145b,miR-148b-5p,miR-2459,miR-2447,miR-2285f,miR-190b-5p,miR-452-5p,miR-96-5p,miR-136-5p,miR-1983,novel_94,miR-877-3p,miR-20a-3p,miR-2319b,miR-499b-5p,miR-7977,miR-378g,miR-378e,miR-489,let-7d-5p,miR-200c,miR-30c-2-3p,let-7c-5p,miR-181c-3p,let-7f-5p,miR-200c-3p,miR-107,novel_127,miR-200a-3p,miR-181a-2-3p,miR-148b-5p,miR-28b,miR-450b-5p,let-7g,miR-376c-5p,miR-376b-5p,miR-218-2-3p,miR-582-3p,let-7d,miR-200b-3p,miR-6517,miR-429-3p,miR-18b-5p,miR-2411-5p,miR-378h,miR-1271-5p,miR-450a-1-3p,novel_63,miR-331-3p,miR-6740-5p,miR-767-5p,miR-410-3p,miR-28-5p,miR-378c,miR-365a-5p,miR-488-3p,miR-18a,miR-376c-5p,miR-10b-3p,miR-767,miR-876-3p,miR-376c-5p,miR-1248,miR-1814c,miR-3068-3p,miR-2355-5p,miR-215-5p,miR-141-3p,miR-324-5p,miR-190a,miR-330-3p,miR-365b-5p,miR-

GPATCH8	ENSOARG0(	G patch	miR-345-3p,miR-218-2-3p,miR-29d-3p,miR-23b-5p,miR-6517,miR-7641,novel_79,miR-196a-2-3p,miR-322-5p,miR-1260a,miR-30c-2-3p,miR-339a,miR-107,miR-543-5p,miR-152-3p,miR-181a-2-3p,miR-4286,miR-380-5p,miR-3065-3p,miR-29a-5p,miR-2448-3p,miR-200a-5p,miR-3154,miR-7144-5p,miR-23a-5p,miR-1248,miR-487a-3p,miR-125a,miR-1271,miR-769,miR-3959-5p,miR-223-5p,miR-376c-3p,miR-29b-3p,miR-410-5p,miR-494-5p,miR-5100,miR-125b-5p,miR-3969,miR-31-3p,miR-204-5p,miR-16-5p,miR-6128,miR-1260b,miR-365a-5p,miR-145-3p,miR-432,miR-708-3p,miR-7b-5p,miR-3071-5p,miR-29b,miR-16b,miR-1260b,miR-497-5p,miR-188-5p,miR-2284x,miR-133b-5p,miR-103a-3p,miR-130a-3p,miR-339-5p,miR-432-5p,miR-185-5p,miR-149-5p,miR-103,miR-7-5p,miR-365b-5p,miR-2411-3p,novel_51,miR-660,miR-125a-5p,miR-380-5p,miR-654-3p,miR-148a-3p,miR-1306,miR-378b,miR-107,miR-6516-3p,miR-195a-5p,novel_68,miR-320b,miR-670-3p,miR-411b-3p,miR-29a-3p,miR-15a,miR-4324,miR-576-3p,miR-16a,miR-29a,miR-342-3p,miR-29c-3p,miR-211,miR-301b-3p,miR-2300b-3p,miR-193b-5p,miR-376a-5p,miR-188-3p,miR-1197-5p,miR-5703,miR-361-5p,miR-548e-3p,miR-212-5p,miR-7859,miR-487a-3p,miR-148b-3p,miR-15a-5p,miR-199a-3p,miR-532-3p,miR-210-5p,novel_17,miR-324-3p,miR-125b,miR-345-3p,miR-760-3p,miR-221-5p,miR-487a,miR-345-3p,miR-376c-3p,miR-143-3p,miR-129b-3p,miR-487b-5p,let-7g-3p,miR-154b-3p,miR-339b,miR-543-3p,miR-1827,miR-221-5p,miR-30c-1-3p,miR-660-5p,miR-154a-3p,miR-216b-3p,miR-3065-3p,miR-182-5p,miR-301a-3p,miR-376e-3p,miR-499b-3p,miR-27a-5p,miR-2284s,miR-132-5p,miR-216b-5p,novel_25,novel_32,miR-15b-5p,miR-324-3p,miR-665,novel_74,miR-487b-5p,miR-424-5p,miR-129-5p,miR-454-3p,miR-214,miR-769-5p,miR-6529a,miR-2284r,miR-143-5p,miR-7857,miR-3120-5p,miR-2300a-5p,miR-147a,miR-
DBX1	ENSOARG0(	developi	let-7a-2-3p,miR-491-3p,miR-8485,miR-539-3p,miR-4532,miR-6240,miR-494-3p,miR-124a,miR-214-3p,miR-199b-5p,miR-330-3p,miR-320b,miR-412-5p,miR-222-5p,miR-665,miR-330-3p,novel_4,miR-1260b,miR-223-3p,miR-199b-5p,miR-2284w,miR-877-3p,miR-2447,miR-3968,miR-4726-5p,miR-628-3p,miR-502-5p,miR-382-3p,miR-320c,novel_69,miR-670-3p,miR-3120-3p,miR-3074-5p,miR-147-3p,miR-378b,miR-3970,miR-199b-5p,miR-2411,miR-199a-5p,miR-5100,miR-200a-3p,miR-221-5p,miR-320d,miR-1260a,miR-2411-5p,miR-487a-3p,miR-485-3p,miR-3600,miR-4429,miR-885-3p,miR-124-3p,miR-376a-5p,miR-191,novel_133,miR-1260b,miR-1197-3p,miR-214,miR-665-5p,miR-193a-5p,miR-4510,miR-3074-5p,miR-191-5p,miR-592,miR-664a,miR-6740-5p,miR-2433,miR-154b-5p,miR-204-3p,miR-199a-5p,miR-6130,miR-141-3p,miR-2355-5p,miR-221-5p,miR-885-3p,let-7g-3p,miR-320a

TCN2	ENSOARG0(	transcob	miR-330-5p,miR-299a-3p,miR-24-2-5p,miR-302a-5p,miR-505-5p,miR-129-1-3p,miR-200c,miR-125a-3p,miR-450a-2-3p,miR-2898,miR-200c-3p,novel_127,miR-3064-5p,miR-495-5p,miR-30a-3p,miR-4286,miR-24-2-5p,novel_39,miR-29a-5p,miR-490-3p,miR-1271-3p,miR-488-3p,miR-320a,miR-876-3p,miR-345-5p,miR-329a,miR-190a-3p,miR-485-5p,miR-874-3p,miR-2355-5p,miR-99a-3p,miR-615-5p,miR-125a,miR-410-5p,miR-551b-3p,miR-99b-3p,miR-5100,miR-125b-5p,miR-331-3p,miR-100-3p,miR-6740-5p,miR-135a-5p,miR-204-5p,novel_42,miR-103b,miR-323-3p,miR-1843b-5p,miR-22-3p,miR-197-3p,novel_23,miR-30b-3p,miR-744-5p,miR-188-5p,miR-2285g,miR-134-3p,miR-224-5p,miR-3141,miR-2331-5p,miR-378a-5p,miR-660,miR-125a-5p,miR-668-3p,miR-345-5p,miR-2285ab,miR-3141,miR-18a-3p,miR-1291,miR-412-3p,miR-2319a,miR-135b-5p,miR-505-3p,novel_68,miR-3074-5p,miR-2404,miR-670-3p,miR-299b-3p,miR-320c,miR-6535,miR-6402,miR-6123,miR-211,miR-326-3p,miR-362-3p,miR-2366,miR-1197-5p,miR-4429,miR-133a-3p,miR-148a-5p,miR-378d,miR-18a-3p,miR-3960,miR-3600,miR-212-5p,miR-30d-3p,miR-505-3p,miR-151-5p,novel_83,miR-210-5p,miR-324-3p,miR-125b,miR-92a-1-5p,miR-185-3p,miR-320d,miR-760-3p,miR-99a-3p,miR-299-3p,miR-216c-5p,miR-345-3p,miR-664b-3p,miR-29b-1-5p,miR-125b-2-3p,miR-299,miR-2285c,miR-2312,miR-345-5p,miR-625-5p,miR-1827,novel_99,miR-504-5p,miR-140-5p,miR-660-5p,miR-296-3p,miR-6130,miR-22-3p,miR-204-3p,miR-151b,miR-324-3p,miR-30a-3p,miR-320e,miR-664a-5p,miR-4492,miR-874-5p,miR-4510,miR-3074-5p,miR-129-2-3p,miR-214,miR-224-5p,miR-3184-5p,miR-6134,miR-296-5p,miR-329-3p,miR-541,miR-143-5p,miR-30b-3p,miR-362-3p,miR-1839-5p,miR-3120-5p,miR-2300a-5p,novel_78,miR-1343-3p,miR-409b,miR-147a,miR-9788-3p,miR-148b-5p,miR-
THEG	ENSOARG0(	theg spe	miR-541,miR-450b-5p,miR-1388-5p,miR-2284x,miR-2889,miR-2300a-5p,miR-147a,miR-142-3p,novel_115,miR-365b-5p,miR-491-5p,miR-214-3p,miR-103a-2-5p,miR-125a-5p,miR-1343-5p,miR-2330-5p,miR-8485,miR-193a,miR-361-3p,miR-504,miR-450b-3p,miR-7975,miR-2319b,miR-4792,miR-25,miR-6535,miR-669,novel_82,miR-30b-5p,miR-4324,miR-1247-3p,miR-107-5p,miR-425-5p,miR-3968,miR-4726-5p,miR-2285t,miR-541-3p,novel_120,miR-2447,miR-24-3p,miR-2428,miR-423-5p,miR-376a-5p,miR-30c-5p,miR-30a-5p,miR-2904,miR-1343-5p,miR-133a-3p,miR-200b-3p,miR-30c,miR-6517,miR-429-3p,miR-200b,miR-505-5p,miR-532-3p,miR-200c,miR-210-5p,miR-30b,miR-185-3p,miR-125b,miR-30f,miR-200c-3p,novel_96,miR-30d,miR-142a-3p,miR-130a-5p,miR-376a-5p,miR-2432,miR-29a-5p,miR-30e-5p,miR-30d-5p,miR-3154,miR-500a-3p,miR-30f,miR-133b-3p,miR-1827,miR-504-5p,miR-2331-3p,miR-615-5p,miR-125a,miR-2355-5p,miR-6130,miR-204-3p,miR-15b-3p,miR-34c-3p,miR-320e,miR-125b-5p,miR-664a,miR-500,miR-4492,miR-4510,miR-133a-3p,miR-214,miR-193a-5p,miR-3607-3p,miR-6128,miR-365a-5p,miR-3184-5p

CASQ1	ENSOARG0(	calseque	miR-211,miR-371b-3p,novel_27,miR-326-3p,miR-2284w,miR-15a,miR-6529b,miR-28c,miR-382-5p,miR-128-1-5p,miR-22-5p,miR-147-5p,miR-502b,novel_68,miR-216b-3p,miR-3074-5p,miR-3120-3p,miR-10a-5p,miR-146b-3p,miR-299b-3p,miR-6535,miR-2285ab,miR-2387,miR-1306,miR-216a-3p,miR-3591-3p,miR-378b,miR-450b-3p,miR-412-3p,miR-378f,miR-195a-5p,miR-654-3p,miR-432-3p,miR-345-5p,miR-365b-5p,novel_51,miR-194a,miR-124-5p,miR-378a-5p,miR-660,miR-1343-5p,miR-339-5p,miR-26b-3p,miR-3535,miR-412-5p,miR-185-5p,miR-149-5p,miR-194-3p,miR-2355-3p,miR-542-3p,miR-22-3p,miR-197-3p,miR-194-5p,miR-3071-5p,miR-16b,miR-138,miR-497-5p,miR-26a-2-3p,miR-4508,miR-6239,miR-27b-3p,miR-16-5p,miR-1197-3p,miR-365a-5p,miR-378c,novel_63,miR-100-3p,miR-409-3p,miR-3969,miR-138-5p,miR-134-5p,miR-204-5p,miR-592,miR-28-5p,miR-574-5p,miR-874-3p,miR-370-3p,miR-2355-5p,miR-1271,miR-141-3p,miR-615-5p,miR-769,miR-365a-3p,miR-154b-5p,miR-3154,miR-10b-3p,miR-876-3p,miR-345-5p,miR-329a,miR-23a-5p,miR-7144-5p,miR-671-5p,miR-1248,miR-1814c,miR-3064-5p,miR-2385-3p,miR-29b-2-5p,miR-181a-2-3p,miR-28b,miR-24-2-5p,miR-3065-3p,miR-2284y,miR-125a-3p,miR-339a,miR-200a-3p,miR-299a-3p,novel_79,miR-412,miR-322-5p,miR-378h,miR-2411-5p,miR-134-5p,miR-214-5p,miR-3591-5p,miR-134,miR-1343-5p,miR-330-5p,miR-582-3p,miR-128-3p,miR-10a-5p,miR-3059-5p,novel_60,miR-2447,miR-3956-3p,miR-885-5p,miR-212-3p,miR-2285f,miR-1296-5p,miR-194b-5p,miR-361-3p,novel_111,miR-154a,miR-218-5p,novel_82,miR-101-3p,miR-769-5p,miR-490-5p,miR-2284q,miR-2284g,miR-3064-5p,miR-24-1-5p,miR-4792,miR-10b,miR-211-5p,miR-7857-3p,miR-378e,miR-2483-3p,miR-203-3p,miR-27a-3p,miR-361-3p,miR-378i,miR-504,miR-136-5p,miR-1983,miR-1388-3p,miR-2330-

SLC10A7	ENSOARG0(	solute c	miR-376b-3p,miR-27a-3p,miR-199b-3p,miR-105-1,miR-2300a-5p,miR-1839-5p,miR-544a,miR-125b-2-3p,miR-7857,miR-412-3p,miR-1a-3p,miR-484,miR-744-3p,miR-23a-3p,miR-494-3p,let-7a-5p,miR-124a,let-7e,miR-199b-5p,miR-130b-3p,miR-27a-3p,miR-376d,miR-203-3p,let-7d-5p,miR-2285u,miR-20a-3p,miR-452-3p,miR-4792,miR-493-5p,miR-376a-3p,miR-378j,miR-199b-5p,miR-7705,novel_111,miR-194b-5p,miR-2447,miR-7134-3p,miR-3958-3p,miR-371a-5p,miR-2285y,miR-2284h-5p,miR-200b,miR-340-5p,miR-148b-3p,miR-23b-3p,miR-16-1-3p,miR-1193,miR-140-3p,miR-124-3p,miR-1961,miR-421-5p,miR-2285w,miR-329a-5p,miR-216c-5p,let-7k,miR-221-5p,miR-98-5p,miR-500a-5p,miR-216a-5p,miR-2285p,let-7i-5p,miR-204-3p,miR-216b-5p,miR-544-3p,miR-301a-3p,miR-532-5p,miR-21-3p,miR-660-5p,miR-221-5p,miR-625-5p,miR-30f,let-7g-3p,miR-411,miR-21a-3p,let-7b-5p,miR-206-3p,miR-433-5p,let-7b,miR-454-3p,miR-155-5p,miR-664a-5p,miR-665,miR-2285j,let-7f,miR-2355-3p,miR-544b,let-7g-5p,miR-185-5p,miR-130a-3p,miR-27b-3p,miR-16-1-3p,miR-219a-1-3p,miR-3071-5p,novel_23,miR-194-5p,miR-7b-5p,miR-2113,miR-1434-3p,miR-363-5p,miR-493-5p,miR-660,miR-194a,miR-105-5p,miR-7-5p,miR-144-3p,miR-6535,miR-411b-3p,miR-3120-3p,miR-329-5p,miR-135b-5p,let-7e-5p,miR-6516-3p,miR-450b-3p,miR-376b-3p,miR-3065-5p,miR-3591-3p,miR-148a-3p,miR-199b-5p,miR-301b-3p,novel_27,miR-2285x,miR-147-5p,miR-421-5p,miR-625-3p,miR-206,miR-379-3p,let-7i,miR-6529b,miR-486-5p,miR-412,miR-6517,miR-429-3p,miR-2285ad,miR-105-2,miR-200b-3p,miR-300,let-7d,miR-411-3p,miR-1290,miR-3578,let-7g,miR-199a-5p,miR-2478,miR-1,miR-3529-3p,miR-152-3p,let-7f-5p,miR-200c-3p,let-7c-5p,miR-30f,miR-3071-3p,miR-200c,miR-376b-3p,miR-199a-5p,miR-141-3p,miR-370-3p,miR-329b,miR-2318,miR-424-
MAU2	ENSOARG0(	MAU2 sis	miR-338-3p,miR-103b,miR-214,miR-145a-3p,miR-767-5p,miR-29b-3p,miR-204-3p,miR-324-5p,miR-22-3p,miR-132-5p,miR-2355-5p,miR-21-3p,miR-221-5p,miR-1277-3p,miR-23a-5p,miR-2474,miR-767,miR-18a-5p,miR-329a,miR-18a,miR-3064-5p,miR-221-5p,miR-760-3p,novel_83,miR-1247-5p,miR-532-3p,miR-18b-5p,miR-29d-3p,miR-5703,miR-2300b-3p,miR-362-3p,novel_101,miR-29c-3p,miR-2447,miR-147-5p,miR-29a,miR-1973,miR-301a-5p,miR-378g,miR-29a-3p,miR-7689-3p,miR-3120-3p,novel_68,miR-3064-5p,miR-9851-3p,novel_94,miR-3591-3p,miR-361-3p,miR-18b,miR-8485,miR-432-3p,miR-363-5p,miR-214-3p,miR-466f-3p,miR-3085-3p,miR-2285v,miR-2957,miR-362-3p,miR-432-5p,miR-1298-3p,miR-134-3p,miR-31-5p,novel_4,miR-1343-3p,miR-122-3p,miR-29b,miR-362-3p,miR-30b-3p,miR-22-3p,miR-30b-3p,miR-329-3p,miR-432,miR-296-5p,miR-2284r,miR-301b-5p
AAED1	ENSOARG0(	AhpC/TSA	miR-3074-2-3p,miR-4791,miR-2285t,miR-489,miR-3535,miR-1185-5p

LYPD4	ENSOARG0(	LY6/PLAU	miR-130b-5p,miR-424-5p,miR-668-3p,miR-33a-5p,miR-654-3p,miR-296-3p,miR-3187-3p,miR-345-5p,miR-296-3p,miR-2957,miR-16b,miR-3085-3p,miR-199b-5p,miR-1343-5p,miR-145b,miR-222-5p,miR-3431,miR-147a,miR-194-3p,miR-455-3p,miR-22-3p,miR-374b-3p,miR-145a-5p,miR-16b,miR-2426,miR-497-5p,miR-877-3p,miR-24-3p,miR-219a-2-3p,miR-199b-5p,miR-15a,miR-502-5p,miR-28c,miR-6529b,miR-16a,miR-3968,miR-2285t,miR-3064-5p,novel_68,miR-20a-3p,miR-2319b,miR-299b-3p,miR-374c-3p,miR-4443,miR-199b-5p,miR-150-5p,miR-216a-3p,miR-1291,novel_94,miR-135b-5p,miR-195a-5p,miR-216c-5p,miR-3064-5p,miR-181a-2-3p,miR-376a-5p,miR-28b,novel_39,miR-199a-5p,miR-1957a,miR-192-3p,miR-205-5p,miR-200c,miR-210-5p,miR-219-3p,miR-324-3p,miR-760-3p,miR-200c-3p,miR-299-3p,miR-543-5p,miR-299a-3p,miR-429-3p,miR-6517,miR-196a-2-3p,miR-322-5p,miR-200b,miR-15a-5p,miR-222-5p,novel_121,miR-1246,miR-2300b-3p,miR-1343-5p,miR-5703,miR-23b-5p,miR-188-3p,miR-493-3p,miR-155-5p,miR-200b-3p,miR-128-3p,miR-378d,miR-145a-3p,miR-193a-5p,miR-16-5p,miR-6525,miR-338-3p,miR-219b-3p,miR-6740-5p,miR-331-3p,miR-26c,miR-665,miR-135a-5p,miR-155-5p,miR-424-5p,miR-130b-5p,miR-140-5p,miR-2331-3p,miR-374c-3p,miR-199a-5p,miR-22-3p,miR-155-5p,miR-15b-5p,miR-345-5p,miR-345-5p,miR-23a-5p,miR-3074-1-3p,miR-186-5p
GM2A	ENSOARG0(	GM2 gang	miR-432-3p,miR-382,miR-106a-5p,miR-2330-5p,miR-193a-3p,miR-214-3p,miR-130b-3p,miR-106b-5p,novel_51,miR-17-5p,miR-106a-5p,miR-505,miR-342,miR-20b,miR-544b,miR-134-3p,miR-130a-3p,miR-665,miR-1343-3p,miR-31-5p,miR-450b-5p,miR-544a,miR-22-3p,miR-1285,miR-143-5p,miR-484,miR-2284r,miR-296-5p,miR-301b-3p,novel_111,miR-877-3p,miR-361-3p,miR-17-3p,miR-1193,miR-2285x,miR-1296-5p,miR-193b-3p,miR-342-3p,miR-147-5p,miR-2285t,miR-490-5p,novel_1,miR-6529b,miR-20a-5p,miR-299b-3p,miR-670-3p,miR-7857-3p,miR-378g,miR-3120-3p,miR-2319b,miR-452-3p,miR-6395,miR-1291,miR-378j,miR-381-3p,miR-486-3p,novel_39,miR-345-3p,miR-4286,miR-181a-2-3p,miR-20a,miR-299-3p,miR-543-5p,miR-191-3p,miR-17-3p,miR-106a,miR-324-3p,miR-328-3p,miR-532-3p,miR-7859,miR-7641,miR-106a,miR-299a-3p,miR-548e-3p,miR-378d,miR-300,miR-1197-5p,miR-188-3p,miR-2904,miR-1246,miR-338-3p,miR-93,miR-214,miR-4510,miR-486b-3p,miR-31-3p,miR-874-5p,miR-454-3p,miR-155-5p,miR-17-3p,miR-370-5p,miR-2285j,miR-22-3p,novel_32,miR-193b-3p,miR-6130,miR-544-3p,miR-301a-3p,miR-532-5p,miR-370-5p,miR-2285aa,miR-874-3p,miR-485-5p,miR-20b-5p,miR-17-5p,miR-93-5p,miR-381-3p

CAB39L	ENSOARG0(	calcium	miR-129b-3p,miR-433-3p,miR-362-5p,miR-21a-3p,miR-7144-5p,miR-574-5p,miR-140-5p,novel_25,miR-300-3p,miR-30a-3p,novel_63,miR-4492,miR-191-5p,miR-134-5p,miR-17-3p,miR-425-5p,miR-539-5p,miR-145-3p,miR-500b-5p,miR-1260b,miR-206-3p,miR-124-3p,miR-191,miR-134,miR-33b-3p,miR-1224-5p,miR-30d-3p,miR-18a-3p,miR-299a-3p,miR-134-5p,novel_83,miR-383-5p,miR-3071-3p,miR-181c-3p,miR-185-3p,miR-1260a,miR-3653-3p,miR-543-5p,miR-299-3p,novel_127,miR-202-5p,miR-17-3p,miR-760-3p,miR-466i-5p,miR-30a-3p,miR-1,miR-3064-5p,miR-345-3p,miR-216c-5p,miR-29a-5p,miR-664b-3p,miR-2478,miR-1306,miR-150-5p,miR-18a-3p,miR-2387,miR-2319a,miR-7975,miR-544-5p,miR-7977,novel_68,miR-3064-5p,miR-489,miR-299b-3p,novel_82,miR-576-3p,miR-203b-5p,miR-2285t,miR-502b,miR-147-5p,miR-206,miR-495-3p,miR-17-3p,miR-2428,miR-362-5p,miR-1a-3p,miR-323b,miR-197-3p,novel_23,miR-1285,miR-1260b,miR-5010-3p,miR-133b-5p,miR-9788-3p,miR-194-3p,miR-500-5p,miR-539-5p,miR-323b-3p,miR-1306-5p,miR-124-5p,miR-3085-3p,miR-1185-5p,miR-652-5p,miR-124a,miR-8485,miR-654-3p,miR-2285r,miR-2284l
KPNB1	ENSOARG0(	karyophe	miR-483-3p,miR-222-5p,miR-194-3p,miR-2284h-5p,miR-340-3p,miR-1343-3p,miR-330-3p,miR-222-5p,miR-29b,miR-484,miR-541,miR-181a-5p,miR-29d-3p,miR-194-5p,miR-3071-5p,miR-140-3p,miR-2432,miR-1388-3p,miR-2285w,miR-6240,miR-2330-5p,miR-483-3p,miR-181b-5p,novel_17,miR-330-3p,miR-374c-3p,miR-29a-3p,miR-3120-3p,miR-543-3p,novel_99,miR-21a-3p,miR-150-5p,miR-487b-5p,novel_27,miR-154a,miR-181c-5p,miR-541-3p,miR-194b-5p,miR-181d-5p,miR-665-5p,miR-29c-3p,miR-29a,miR-2284q,miR-29b-3p,miR-494-5p,miR-1973

PLAU	ENSOARG0(	urokinas	miR-3064-5p,miR-664b,miR-452-3p,miR-541-5p,miR-7977,miR-7857-3p,miR-3082-5p,let-7d-5p,miR-27a-3p,miR-361-3p,miR-199b-5p,novel_94,novel_60,miR-32-3p,miR-541-3p,miR-2285f,miR-452-5p,miR-181d-5p,miR-362-5p,miR-8095,miR-154a,miR-2285n,miR-769-5p,miR-371a-5p,miR-107-5p,miR-4726-5p,miR-2284q,miR-27a-3p,miR-500-5p,miR-484,miR-30b-3p,miR-125b-2-3p,miR-450b-5p,let-7a-5p,miR-494-3p,miR-33a-5p,miR-652-5p,miR-326,miR-362-3p,miR-3085-3p,let-7e,miR-221-5p,miR-32-3p,miR-3065-3p,miR-21-3p,miR-182-5p,miR-6516,miR-323-5p,miR-193b-3p,miR-139-5p,miR-204-3p,miR-22-3p,novel_99,miR-3074-2-3p,miR-206-3p,let-7b-5p,miR-338-3p,miR-324-3p,miR-320e,miR-450b-3p,miR-664a,miR-17-3p,miR-208b-5p,miR-548e-3p,miR-3600,miR-671-3p,novel_133,miR-362-3p,miR-2904,miR-5703,miR-19b-3p,miR-183-5p,miR-29b-1-5p,miR-125b-2-3p,miR-2411,miR-2284m,miR-221-5p,miR-30b-3p,miR-299-3p,miR-320b,miR-655-5p,miR-323a-5p,miR-2404,miR-670-3p,miR-29a-3p,miR-2285ab,miR-18a-3p,miR-1291,miR-107,miR-19a-3p,miR-7975,miR-9851-3p,miR-6395,miR-135a-2-3p,novel_101,miR-17-3p,miR-323c,miR-24-3p,miR-326-3p,miR-502-5p,let-7i,miR-203b-5p,miR-22-5p,miR-187-3p,miR-2284d,let-7g-5p,miR-103,miR-181a-5p,miR-1285,miR-374b-3p,miR-138,miR-29b,miR-30b-3p,miR-2284ab,miR-7862,miR-130b-5p,miR-2483-5p,miR-382,miR-363-5p,miR-654-3p,miR-296-3p,miR-345-5p,miR-466f-3p,miR-370-3p,miR-615-5p,miR-141-3p,miR-374c-3p,miR-769,miR-324-5p,miR-199a-5p,miR-381-3p,miR-2448-3p,miR-503-3p,miR-3154,miR-362-5p,let-7g-3p,miR-7144-5p,miR-3074-1-3p,miR-485-5p,miR-1306-5p,miR-500b-5p,miR-365a-5p,miR-29b-3p,miR-1-5p,miR-331-3p,miR-3969,miR-138-5p,miR-767-5p,miR-7641,miR-6516-5p,miR-2411-5p,miR-582-3p,miR-545-5p,miR-345-3p,miR-29d-3p,miR-411-3p,miR-
FAM184A	ENSOARG0(	family w	miR-6402,novel_103,miR-1-5p,miR-1a-2-5p,miR-466i-5p,miR-212-3p,miR-1a-1-5p,miR-6240,miR-1b-5p,miR-216a-3p,miR-301,miR-27b-3p,miR-128-3p,miR-2426,miR-27a-3p,miR-27a-3p,miR-455-3p,miR-185-5p

SPEN	ENSOARG0(	spen fam	miR-1343-5p,miR-466f-3p,miR-124-5p,miR-330-3p,miR-224-5p,miR-363-5p,miR-29b,miR-30b-3p,miR-544b,miR-2285g,miR-185-5p,miR-665,miR-130a-3p,miR-26b-3p,miR-133b-5p,miR-128-1-5p,miR-34c,miR-16a,miR-382-5p,miR-502-5p,miR-15a,miR-6402,miR-449a,miR-326-3p,miR-2428,miR-211,miR-323c,miR-3959-3p,miR-542-5p,miR-1193,novel_101,miR-2285x,miR-6516-3p,miR-6395,miR-195a-5p,miR-135b-5p,miR-1291,miR-450b-3p,miR-19a-3p,miR-18a-3p,miR-2387,miR-323a-5p,miR-29a-3p,miR-670-3p,miR-34b,miR-320b,miR-450a-2-3p,miR-191-3p,miR-30c-2-3p,miR-2285af,miR-199a-5p,miR-3065-3p,miR-28b,miR-4286,miR-3529-3p,miR-128-3p,miR-300,miR-29d-3p,miR-30a-5p,miR-134,miR-214-5p,miR-2411-5p,miR-28-5p,miR-486b-3p,miR-204-5p,miR-331-3p,miR-135a-5p,miR-29b-3p,miR-370-5p,miR-10b-3p,miR-145-3p,miR-500b-5p,miR-323-3p,miR-190a-3p,miR-671-5p,miR-485-5p,miR-10b-3p,novel_91,miR-320a,miR-362-5p,miR-3154,miR-381-3p,miR-199a-5p,miR-34c-5p,miR-598-3p,miR-1468,miR-542-5p,miR-3085-3p,miR-25-5p,miR-2957,miR-320b,miR-5126,novel_103,miR-326,miR-758-3p,miR-30c-1-3p,miR-2330-5p,miR-424-5p,miR-544a,miR-450b-5p,miR-2426,miR-125b-2-3p,miR-30b-3p,miR-296-5p,miR-9788-3p,miR-216a-3p,miR-500-5p,miR-2285e,miR-5010-3p,novel_4,miR-4726-5p,miR-2285y,miR-107-5p,miR-30b-5p,miR-362-5p,miR-541-3p,miR-877-3p,miR-129b-5p,miR-361-3p,miR-199b-5p,miR-489,miR-499b-5p,miR-7977,miR-3064-5p,miR-10b,miR-34b-5p,miR-30d,miR-30b-3p,miR-221-5p,miR-21-3p,miR-299-3p,miR-320d,miR-466i-5p,miR-185-3p,miR-542-5p,miR-2411,miR-29b-1-5p,miR-130a-5p,miR-125b-2-3p,miR-183-5p,miR-19b-3p,miR-4429,miR-2904,miR-1197-5p,miR-140-3p,miR-2300b-3p,miR-15a-5p,miR-148b-3p,miR-3600,miR-424-5p,miR-708-5p,miR-450b-3p,miR-664a,miR-155-5p,miR-324-3p,miR-320e,miR-
SUMF1	ENSOARG0(	sulfatas	miR-191,miR-541,miR-412-3p,miR-16b,miR-497-5p,miR-412,miR-425-3p,miR-322-5p,miR-15a-5p,novel_83,miR-411-5p,miR-16b,miR-183-5p,miR-424-5p,miR-2403,miR-130a-5p,miR-2387,miR-3970,miR-3154,miR-129b-5p,miR-10b-3p,miR-7144-5p,miR-671-5p,miR-195a-5p,novel_68,miR-323a-5p,miR-323-5p,miR-15b-5p,miR-15a,miR-16a,miR-3968,miR-424-5p,miR-502b,miR-191-5p,miR-541-3p,miR-16-5p,miR-10b-3p
PEA15	ENSOARG0(	phosphop	miR-18a-3p,miR-1197-5p,miR-331-3p,miR-197-5p,miR-125b-1-3p,miR-493-3p,novel_127,miR-18a-3p,miR-3957-3p,miR-877-3p,miR-8485

TRIML1	ENSOARG0(	triparti	miR-8485,miR-1b-5p,miR-758-3p,miR-1388-3p,miR-124a,miR-494-3p,miR-33a-5p,miR-491-5p,miR-3955-3p,miR-199b-5p,miR-214-3p,miR-103a-2-5p,miR-431-5p,miR-323b-3p,miR-29b-1-5p,miR-182-5p,miR-539-5p,miR-1298-3p,miR-9788-3p,miR-2285e,miR-151a-3p,miR-1343-3p,miR-1388-5p,miR-299a-5p,miR-450b-5p,miR-2426,miR-484,miR-143-5p,miR-412-3p,miR-323b,miR-758-3p,miR-154a,novel_60,miR-3956-3p,miR-2285f,miR-219a-5p,miR-499a-5p,miR-2285t,miR-374a-3p,miR-30b-5p,novel_82,miR-107-5p,miR-3082-5p,miR-489,miR-139-5p,miR-664b,miR-2319b,miR-20a-3p,miR-211-5p,miR-335-3p,novel_94,miR-1983,miR-199b-5p,miR-409-5p,miR-129b-5p,miR-29b-1-5p,miR-376a-5p,miR-125b-2-3p,miR-376c-3p,miR-2432,miR-486-3p,miR-143-3p,miR-216c-5p,miR-105-3p,miR-99a-3p,miR-221-5p,miR-30d,miR-21-3p,miR-30b,miR-196a-3p,miR-219b-5p,miR-200b,miR-532-3p,miR-380-5p,miR-212-5p,miR-33b-3p,miR-16-1-3p,miR-361-5p,miR-378d,miR-30c-5p,miR-2366,miR-124-3p,miR-2904,miR-1197-5p,miR-217-5p,miR-6525,miR-224-5p,novel_44,miR-346,miR-539-5p,miR-214,miR-664a,miR-17-3p,miR-487a-5p,miR-874-5p,miR-208b-5p,miR-30e-3p,miR-30a-3p,miR-151-3p,miR-29b-2-5p,miR-376e-3p,miR-6516,miR-193b-3p,miR-139-5p,miR-656-3p,miR-140-5p,miR-221-5p,miR-2331-3p,miR-660-5p,miR-182-5p,miR-532-5p,miR-2312,miR-30f,miR-18a-5p,miR-186-5p,miR-299,miR-2285c,miR-129b-3p,miR-30d-5p,miR-487b-5p,miR-2285r,miR-654-3p,miR-432-3p,miR-296-3p,miR-660,miR-466f-3p,miR-193a-3p,miR-224-5p,miR-296-3p,miR-412-5p,miR-2285g,miR-3431,miR-194-3p,miR-31-5p,miR-1260b,miR-16-1-3p,miR-653,miR-211,miR-199b-5p,miR-193b-3p,miR-758-5p,miR-17-3p,miR-495-3p,miR-2285x,novel_1,miR-625-3p,miR-656-5p,miR-502-5p,miR-412-3p,miR-9851-3p,miR-2319a,miR-2285ab,miR-18b,miR-378b,miR-199a-5p,miR-490-3p,miR-3956-
ALPL	ENSOARG0(	alkaline	miR-128-3p,miR-582-3p,miR-1343-5p,miR-330-5p,miR-129-1-3p,miR-505-5p,miR-1271-5p,miR-212-5p,novel_17,miR-148b-5p,miR-2403,miR-1827,miR-2312,miR-345-5p,miR-345-5p,miR-362-5p,miR-3154,miR-204-3p,miR-2285aa,miR-370-5p,miR-182-5p,miR-4492,miR-370-5p,miR-5100,miR-1271,miR-6128,miR-214,miR-129-2-3p,miR-26a-2-3p,miR-3120-5p,miR-455-5p,miR-2284x,miR-30b-3p,miR-30b-3p,miR-500-5p,miR-149-5p,miR-216a-3p,miR-425-3p,miR-134-3p,miR-1343-5p,miR-214-3p,miR-455-5p,miR-182-5p,miR-326,miR-8485,miR-345-5p,miR-9851-3p,miR-96-5p,miR-216a-3p,miR-2285ab,miR-361-3p,miR-2387,miR-3596,miR-7857-3p,miR-378g,miR-7977,miR-20a-3p,miR-2319b,miR-203b-5p,miR-7134-3p,novel_1,miR-1973,novel_87,miR-326-3p,miR-361-3p,miR-877-3p,miR-362-5p,miR-24-3p,miR-371b-3p,miR-2285f,miR-1193,miR-2447

ZBTB10	ENSOARG0(	zinc fin	miR-345-3p,miR-181b-1-3p,miR-380-3p,miR-374a-5p,miR-618,miR-320d,miR-30d-3p,miR-3600,miR-15a-5p,miR-383-5p,miR-7859,miR-4429,miR-19b-3p,miR-361-5p,miR-539-5p,miR-214,miR-142a-5p,miR-338-3p,miR-15b-3p,miR-2285j,miR-655-3p,miR-3604,miR-129-5p,miR-208b-5p,miR-548o-3p,miR-32-3p,miR-545-3p,miR-140-5p,miR-532-5p,miR-139-5p,miR-582-5p,miR-15b-5p,miR-2285c,miR-655,miR-598-3p,let-7g-3p,miR-2312,miR-582-5p,miR-625-5p,miR-424-5p,miR-101b-3p,miR-320b,miR-16b,miR-1185-5p,miR-214-3p,miR-548w,miR-105-1,miR-330-3p,miR-145b,miR-181b-2-3p,miR-142-5p,miR-1185-3p,miR-2285e,miR-500-5p,miR-450b-5p,miR-2426,miR-32-3p,miR-194b-5p,miR-2285f,miR-362-5p,miR-8095,miR-1285-5p,miR-2285n,miR-2285y,miR-499a-5p,miR-374a-3p,miR-139-5p,miR-541-5p,miR-2285u,miR-3082-5p,miR-1185-2-3p,miR-203-3p,miR-140-5p,miR-450b-5p,miR-2285b,miR-30f,miR-200a-3p,miR-107,miR-2285ad,miR-322-5p,miR-214-5p,miR-29d-3p,miR-411-3p,let-7a-2-3p,miR-105-2,miR-16-5p,miR-374a-3p,miR-2332,miR-29b-3p,miR-144,miR-374b-3p,miR-141-3p,miR-2285aa,miR-598-3p,miR-374c-3p,miR-320a,miR-362-5p,let-7g-3p,miR-6240,let-7f-2-3p,let-7f-2-3p,miR-2483-5p,miR-668-3p,miR-654-3p,let-7a-2-3p,let-7j,miR-194a,miR-105-5p,miR-330-3p,miR-103a-3p,miR-3955-5p,miR-2285g,miR-103,miR-223-3p,miR-194-5p,novel_23,miR-145a-5p,miR-29b,miR-380-3p,miR-16b,miR-2284x,miR-188-5p,miR-497-5p,miR-29c-3p,novel_101,miR-576-3p,miR-15a,miR-16a,miR-29a,miR-379-3p,miR-6119-3p,miR-3120-3p,miR-374c-3p,miR-29a-3p,miR-181b-3p,miR-144-3p,miR-677,miR-320c,miR-2285ab,miR-3970,miR-19a-3p,miR-107,miR-195a-5p
CD177	ENSOARG0(	CD177 mo	miR-3154,miR-320a,miR-3074-1-3p,miR-329b,miR-345-5p,miR-345-5p,miR-10b-3p,miR-125a,miR-1271,miR-552-3p,miR-21-3p,miR-874-3p,miR-574-5p,miR-376c-3p,miR-22-3p,miR-193b-3p,miR-6130,miR-377-3p,miR-376e-3p,miR-320e,miR-125b-5p,miR-4492,miR-874-5p,novel_74,miR-214,miR-4510,miR-145-3p,miR-1197-3p,miR-6525,miR-5703,miR-330-5p,miR-1343-5p,miR-1290,miR-4429,miR-18a-3p,miR-2285ad,miR-185-3p,miR-125b,miR-219b-5p,miR-664-3p,novel_17,miR-543-5p,novel_127,novel_96,miR-466i-5p,miR-2898,miR-320d,miR-329a-5p,miR-376c-3p,miR-376a-5p,miR-3591-3p,miR-18a-3p,novel_94,miR-7975,miR-147-3p,miR-450b-3p,miR-7977,miR-320b,miR-2285u,miR-329-5p,miR-10b,miR-20a-3p,miR-664b,miR-203-3p,miR-320c,miR-670-3p,miR-2285y,miR-4324,miR-2285n,miR-656-5p,miR-193b-3p,miR-3059-5p,miR-326-3p,novel_27,miR-24-3p,miR-323b,miR-22-3p,miR-197-3p,miR-7b-5p,miR-2889,miR-188-5p,miR-450b-5p,miR-1388-5p,miR-26b-3p,miR-5010-3p,miR-31-5p,miR-187-3p,miR-147a,miR-185-5p,miR-3431,novel_51,miR-323b-3p,miR-377-3p,miR-326,miR-7-5p,miR-320b,miR-193a-3p,miR-214-3p,miR-1343-5p,miR-125a-5p,miR-491-5p,miR-124-5p,miR-33a-5p,miR-363-5p,miR-1388-3p,miR-345-5p,miR-3187-3p

MRPS30	ENSOARG0(	mitochon	miR-6240,miR-345-5p,miR-1957a,novel_39,miR-432-3p,miR-432-3p,miR-664b-3p,miR-2411-3p,miR-345-3p,miR-197-5p,miR-193a-3p,miR-124-5p,miR-17-3p,miR-1343-3p,miR-214-5p,miR-9788-3p,miR-455-3p,miR-758-5p,miR-3535,miR-5703,miR-3071-5p,miR-4791,miR-345-3p,miR-378d,miR-2330-3p,miR-17-3p,miR-193b-3p,miR-3184-3p,miR-1306-5p,miR-376a-3p,miR-2447,novel_27,miR-3969,miR-409-3p,miR-6740-5p,miR-2440,miR-6402,miR-142b,miR-382-3p,miR-502-5p,miR-421-5p,miR-490-5p,miR-6516-3p,miR-17-3p,miR-21-3p,miR-216b-3p,miR-504-5p,miR-204-3p,miR-193b-3p,miR-216b-5p,miR-3154,miR-150-5p,miR-488-3p,miR-379-5p,miR-186-5p,miR-504,miR-345-5p,miR-345-5p
PIK3AP1	ENSOARG0(	phosphoi	miR-320b,miR-182-5p,miR-2331-5p,miR-105-5p,miR-483-3p,miR-378a-5p,miR-214-3p,let-7e,miR-493-5p,let-7a-5p,miR-30c-1-3p,let-7a-2-3p,miR-3187-3p,miR-2113,miR-1434-3p,miR-484,miR-544a,miR-1839-5p,miR-2300a-5p,miR-105-1,miR-181b-2-3p,let-7g-5p,miR-185-5p,miR-1298-3p,miR-3535,miR-544b,miR-148b-5p,miR-142b,let-7i,miR-6123,miR-203b-5p,miR-3059-5p,miR-877-3p,miR-668-5p,let-7e-5p,miR-376a-2-5p,miR-493-5p,miR-489,miR-181b-3p,miR-454-5p,let-7d-5p,miR-320c,miR-677,let-7c-5p,miR-500a-5p,miR-30c-2-3p,miR-127-5p,miR-200c-3p,let-7f-5p,miR-98-5p,miR-320d,let-7k,miR-221-5p,miR-216c-5p,miR-2385-3p,miR-181b-1-3p,miR-376a-5p,miR-490-3p,let-7g,miR-1961,miR-140-3p,miR-4429,let-7d,miR-493-3p,miR-148a-5p,let-7a-2-3p,miR-105-2,miR-376a-5p,miR-212-5p,miR-483-3p,miR-7859,miR-671-3p,let-7f,miR-331-3p,miR-100-3p,novel_74,miR-4510,miR-3184-3p,miR-590-3p,let-7b,miR-214,miR-6128,miR-3607-3p,miR-193a-5p,miR-6134,let-7b-5p,miR-320a,miR-129b-3p,let-7g-3p,let-7g-3p,miR-10b-3p,miR-1248,miR-3074-1-3p,miR-30c-1-3p,miR-221-5p,miR-182-5p,miR-365a-3p,miR-6130,miR-544-3p,let-7i-5p

UHRF1	ENSOARG0(	ubiquiti	let-7b,miR-3074-5p,miR-769-5p,miR-6525,miR-30a-3p,miR-665,let-7f,miR-2285j,miR-30e-3p,miR-874-5p,miR-4492,novel_74,miR-216b-3p,miR-2331-3p,let-7i-5p,miR-2284s,miR-129b-3p,miR-655,miR-2285c,miR-625-5p,miR-1827,miR-2285w,miR-2424,miR-143-3p,miR-377-5p,miR-210-5p,let-7k,miR-98-5p,miR-30d-3p,miR-2284h-5p,miR-7859,miR-95-3p,miR-1434-5p,miR-1961,miR-193b-5p,miR-2366,miR-16-1-3p,miR-218-1-3p,miR-34a-5p,miR-493-3p,miR-142-3p,miR-9-5p,miR-2447,miR-361-3p,novel_111,miR-7689-3p,miR-139-5p,miR-1185-2-3p,miR-25,miR-3596,miR-221,miR-1983,miR-744-3p,miR-124a,miR-101b-3p,miR-323b-3p,miR-29b-1-5p,miR-153-3p,miR-491-5p,miR-145b,miR-1343-3p,miR-199b-3p,miR-1185-3p,miR-153,miR-147a,miR-323b,miR-2284r,miR-193a-5p,miR-28a-3p,novel_42,miR-1197-3p,miR-2355-5p,miR-2285aa,miR-503-3p,miR-137-3p,miR-301,miR-1248,let-7g,miR-664-3p,novel_96,miR-200a-3p,miR-28-3p,let-7f-5p,miR-412,miR-2285ad,miR-6517,miR-9-5p,miR-758-5p,miR-330-5p,miR-1839-3p,miR-29c-3p,miR-374c-5p,miR-6123,miR-29a,miR-3074-5p,miR-669,miR-374c-3p,miR-1306,miR-3591-3p,let-7e-5p,miR-412-3p,let-7f-2-3p,let-7f-2-3p,miR-628-5p,let-7j,miR-432-3p,miR-105-5p,miR-365b-5p,miR-7-5p,miR-3955-5p,miR-194-3p,novel_116,miR-412-5p,miR-197-3p,miR-432,miR-708-3p,miR-1843b-5p,miR-542-3p,miR-7b-5p,miR-16-1-3p,miR-3074-2-3p,miR-4510,let-7b-5p,miR-338-3p,miR-324-3p,miR-132-5p,miR-655-3p,miR-320e,miR-222-3p,miR-708-5p,miR-17-3p,miR-21-3p,miR-496-3p,miR-204-3p,novel_32,miR-544-3p,miR-6130,miR-139-5p,miR-2312,miR-216c-5p,miR-2432,miR-29b-1-5p,miR-216a-5p,miR-185-3p,miR-542-5p,miR-34b-5p,miR-466i-5p,miR-532-3p,miR-124-3p,miR-5703,miR-1197-5p,miR-140-3p,novel_133,miR-19b-3p,miR-2285f,miR-219a-5p,miR-8095,miR-2285y,miR-769-5p,miR-371a-

MCM9	ENSOARG0(	minichro	miR-7641,miR-2285ad,miR-134-5p,miR-2284z,miR-330-5p,miR-1343-5p,miR-1290,miR-134,miR-345-3p,let-7a-2-3p,miR-128-3p,let-7d,miR-885-3p,miR-30a-3p,miR-3064-5p,novel_39,let-7g,miR-2478,let-7c-5p,miR-30f,miR-219-3p,miR-200a-3p,let-7f-5p,miR-141-3p,miR-598-3p,miR-2355-5p,miR-487a-3p,miR-331-5p,miR-3154,miR-23b,miR-362-5p,let-7g-3p,miR-3607-3p,miR-365a-5p,miR-374a-3p,miR-500b-5p,miR-144,miR-6740-5p,miR-2332,novel_63,miR-486b-3p,miR-374b-3p,miR-134-5p,miR-187-3p,miR-31-5p,miR-2355-3p,let-7g-5p,miR-2285g,miR-432-5p,novel_23,miR-22-3p,miR-542-3p,miR-1434-3p,miR-653,miR-432,miR-27b-3p,miR-2284x,miR-16-1-3p,miR-219a-1-3p,miR-30b-3p,miR-363-5p,miR-493-5p,let-7a-2-3p,miR-432-3p,miR-432-3p,miR-2411-3p,miR-365b-5p,miR-377-3p,miR-1343-5p,miR-144-3p,miR-374c-3p,miR-670-3p,miR-216a-3p,miR-2285ab,let-7e-5p,miR-505-3p,miR-495-3p,miR-2285x,miR-2428,miR-326-3p,miR-3959-3p,miR-382-5p,let-7i,miR-502-5p,miR-656-5p,miR-203b-5p,miR-6119-3p,miR-30d-3p,miR-505-3p,miR-616-3p,miR-1247-5p,miR-23c,miR-340-5p,miR-23b-3p,miR-383-5p,miR-5703,miR-140-3p,miR-124-3p,miR-1961,miR-16-1-3p,novel_73,miR-493-3p,miR-345-3p,miR-487a,miR-216c-5p,miR-486-3p,miR-2424,miR-2432,miR-345-3p,miR-185-3p,miR-500a-5p,miR-21-3p,let-7k,miR-98-5p,miR-154a-3p,let-7i-5p,miR-22-3p,miR-377-3p,miR-598-3p,miR-2285c,miR-885-3p,miR-154b-3p,miR-2312,let-7g-3p,miR-214,miR-665-5p,miR-425-5p,let-7b,let-7b-5p,miR-3184-5p,miR-30a-3p,miR-219b-3p,miR-30e-3p,miR-15b-3p,let-7f,miR-208b-5p,miR-4492,novel_74,miR-27a-3p,miR-409b,miR-2285e,miR-9788-3p,miR-500-5p,miR-539-5p,miR-30b-3p,miR-484,miR-2284aa,miR-2426,miR-23a-3p,miR-1388-3p,let-7a-5p,miR-124a,miR-326,let-7e,miR-214-3p,miR-3955-3p,miR-3085-3p,miR-1185-5p,miR-2319b,miR-3064-5p,miR-27a-3p,let-

ACOX1	ENSOARG0(	acyl-CoA	miR-429-3p,miR-7641,miR-1224-5p,miR-6516-5p,miR-483-3p,miR-378h,miR-322-5p,miR-3591-5p,miR-330-5p,miR-2427,miR-300,miR-128-3p,miR-200b-3p,miR-99a-5p,miR-181a-2-3p,miR-495-5p,miR-30a-3p,miR-2385-3p,miR-92b-3p,miR-2285b,miR-199a-5p,miR-1957a,miR-664-3p,miR-99a,miR-200c,miR-1260a,miR-217,miR-3071-3p,miR-200c-3p,miR-200a-3p,miR-92a-3p,miR-141-3p,miR-25-3p,miR-154b-5p,miR-199a-5p,miR-381-3p,miR-488-3p,miR-3154,miR-100-5p,miR-345-5p,miR-1814c,miR-1248,miR-7144-5p,miR-16-5p,miR-1260b,miR-1197-3p,miR-378c,novel_63,miR-135a-5p,miR-409-3p,miR-138-5p,miR-592,miR-486b-3p,miR-1285,miR-1434-3p,miR-542-3p,miR-16-1-3p,miR-1260b,miR-16b,miR-138,miR-497-5p,miR-130b-5p,miR-2285r,miR-345-5p,miR-146a,miR-99a-5p,novel_51,miR-32,miR-2285l,miR-483-3p,miR-106b-3p,novel_68,miR-3120-3p,miR-655-5p,miR-320b,miR-2404,miR-181b-3p,miR-677,miR-669,miR-2387,miR-216a-3p,miR-150-5p,miR-505-3p,miR-378f,miR-195a-5p,miR-135b-5p,miR-146b-5p,miR-219b-3p,miR-2285x,miR-199b-5p,novel_27,miR-326-3p,miR-576-3p,miR-15a,miR-33a-3p,miR-382-5p,miR-16a,novel_1,miR-92a-3p,miR-147-5p,miR-421-5p,miR-30d-3p,miR-505-3p,miR-200b,miR-15a-5p,miR-2284h-5p,miR-532-3p,miR-378i,miR-378a-3p,miR-1197-5p,miR-140-3p,miR-217-5p,miR-378d,miR-218-1-3p,miR-16-1-3p,miR-2285w,miR-183-5p,miR-181b-1-3p,miR-125b-2-3p,miR-29b-1-5p,miR-143-3p,miR-486-3p,miR-146a-5p,miR-2285p,miR-618,miR-422a,miR-760-3p,miR-221-5p,miR-3653-3p,miR-32-3p,miR-221-5p,miR-504-5p,miR-32-5p,miR-378a-3p,miR-2331-3p,miR-296-3p,miR-552-3p,miR-6130,miR-582-5p,miR-6516,miR-15b-5p,miR-877-5p,novel_25,miR-21a-3p,miR-345-5p,miR-582-5p,miR-543-3p,miR-145a-3p,miR-4510,miR-433-5p,miR-378c,miR-30e-3p,miR-2285i,miR-15b-3p,miR-320e,miR-30a-3p,miR-130b-
ERCC6L2	ENSOARG0(	excision	let-7i,miR-301a-5p,miR-142b,novel_82,miR-199c,miR-656-5p,miR-206,miR-190b-5p,miR-181d-5p,miR-301b-3p,novel_27,miR-18a-3p,miR-140-5p,miR-135b-5p,let-7e-5p,miR-136-5p,miR-329-5p,miR-664b,miR-181b-3p,miR-27a-3p,miR-203-3p,miR-677,miR-144-3p,let-7d-5p,miR-499a-3p,miR-2404,miR-2411-3p,miR-190a,miR-199a-3p,let-7e,miR-130b-3p,miR-2483-5p,miR-2330-5p,let-7a-5p,miR-380-5p,miR-1a-3p,miR-301b-5p,miR-181a-5p,miR-3120-5p,miR-27b-3p,miR-21b,miR-450b-5p,miR-219a-1-3p,miR-1388-5p,miR-181b-2-3p,miR-27a-3p,miR-199b-3p,miR-149-5p,miR-216a-3p,miR-455-3p,let-7g-5p,miR-142-5p,miR-29b-2-5p,miR-135a-5p,miR-30a-3p,miR-144,miR-30e-3p,let-7f,miR-190a-5p,miR-454-3p,miR-3074-2-3p,miR-6128,let-7b,miR-181c-5p,let-7b-5p,miR-206-3p,miR-142a-5p,miR-2448-3p,novel_99,miR-582-5p,miR-485-5p,miR-625-5p,miR-2318,miR-140-5p,let-7i-5p,miR-301a-3p,miR-499b-3p,miR-582-5p,miR-345-3p,miR-217,let-7c-5p,miR-664-3p,miR-618,novel_127,miR-543-5p,let-7k,miR-181b-5p,miR-98-5p,let-7f-5p,miR-181a-2-3p,miR-29b-2-5p,miR-1,miR-30a-3p,miR-329a-5p,miR-1271-3p,let-7g,miR-181b-1-3p,miR-380-5p,miR-217-5p,miR-188-3p,miR-140-3p,miR-1961,miR-1246,miR-345-3p,let-7d,miR-30d-3p,miR-212-5p,miR-18a-3p,miR-548e-3p,miR-199a-3p,miR-616-3p,miR-545-5p,miR-758-5p,miR-7859

E2F1	ENSOARG0(	E2F tran	miR-370-5p,miR-331-3p,miR-378c,miR-6128,miR-193a-5p,miR-10b-3p,miR-329a,miR-424-3p,miR-199a-5p,miR-542-5p,miR-1271,miR-2898,miR-127-5p,miR-200c-3p,let-7f-5p,novel_127,miR-200c,let-7c-5p,miR-148b-5p,let-7g,miR-199a-5p,miR-2284a,let-7d,miR-200b-3p,miR-3591-5p,miR-483-3p,miR-758-5p,miR-378h,miR-2411-5p,miR-429-3p,miR-6517,miR-412,miR-128-1-5p,miR-656-5p,let-7i,miR-24-3p,miR-199b-5p,miR-1193,miR-1291,miR-7975,miR-378f,let-7e-5p,miR-1306,miR-18a-3p,novel_69,miR-655-5p,miR-320b,miR-378a-5p,miR-483-3p,miR-22851,miR-296-3p,miR-432-3p,miR-296-3p,miR-130b-5p,miR-432,miR-1843b-5p,miR-22-3p,miR-432-5p,let-7g-5p,miR-31-5p,miR-4492,miR-129-5p,miR-130b-5p,let-7b-3p,let-7f,miR-132-5p,miR-98-3p,let-7b-5p,miR-3184-5p,let-7b,miR-378c,miR-4510,miR-665-5p,miR-1827,let-7f-1-3p,miR-2284s,miR-216b-5p,miR-6130,miR-22-3p,let-7i-5p,miR-151b,miR-140-5p,miR-221-5p,miR-378a-3p,miR-370-5p,miR-296-3p,miR-466i-5p,miR-98-5p,miR-221-5p,let-7k,miR-618,miR-324-3p,miR-92a-1-5p,miR-422a,miR-185-3p,miR-376a-5p,miR-29b-1-5p,miR-130a-5p,miR-2424,miR-2411,miR-493-3p,miR-941,let-7a-3p,miR-148a-5p,miR-378i,miR-362-3p,miR-1961,miR-98-3p,miR-378a-3p,miR-188-3p,miR-200b,miR-532-3p,miR-151-5p,miR-18a-3p,novel_111,miR-423-5p,miR-877-3p,miR-199b-5p,miR-381-3p,miR-378e,miR-7857-3p,let-7d-5p,miR-126b-5p,miR-199b-5p,let-7e,miR-1306-5p,miR-29b-1-5p,miR-362-3p,miR-378d,miR-4532,miR-1388-3p,let-7a-5p,miR-2284b,miR-362-3p,miR-378c,miR-101a-5p,miR-329-3p,miR-296-5p,miR-378b,miR-412-3p,miR-147a,miR-455-3p,miR-148b-5p,novel_4

CFH	ENSOARG0(	compleme	miR-2284w,novel_27,miR-29c-3p,miR-17-3p,miR-1193,miR-502b,miR-22-5p,miR-29a,miR-33a-3p,miR-142b,miR-181b-3p,miR-320c,miR-677,miR-29a-3p,miR-3120-3p,miR-655-5p,novel_68,miR-107,miR-378b,miR-21c,miR-1306,miR-148a-3p,miR-2387,miR-345-5p,let-7a-2-3p,miR-2284l,miR-654-3p,miR-432-3p,miR-668-3p,miR-493-5p,miR-452-5p,novel_51,miR-2411-3p,miR-103,miR-149-5p,miR-3431,miR-2284d,miR-103a-3p,miR-31-5p,miR-26a-2-3p,miR-1260b,miR-219a-1-3p,miR-21-5p,miR-29b,miR-30b-3p,miR-2284ab,novel_23,miR-3071-5p,miR-2113,miR-19b-2-5p,miR-145-3p,miR-1260b,miR-1197-3p,miR-144-5p,miR-6128,miR-3607-3p,miR-2284v,miR-1306-5p,miR-592,miR-3969,miR-5100,miR-370-5p,miR-2284k,miR-29b-3p,miR-2284n,miR-133c,miR-223-5p,miR-3068-3p,miR-1248,miR-7144-5p,miR-345-5p,let-7g-3p,miR-23b,miR-320a,miR-3154,miR-192-3p,miR-2285b,miR-2284a,novel_39,miR-200a,miR-29a-5p,miR-2284y,miR-3529-3p,miR-152-3p,miR-543-5p,miR-107,miR-127-5p,miR-200c-3p,miR-17-3p,miR-217,miR-664-3p,miR-200c,miR-1260a,miR-2285af,miR-486-5p,novel_121,miR-2284z,miR-758-5p,miR-7641,miR-429-3p,miR-222,let-7a-2-3p,miR-200b-3p,miR-29d-3p,miR-3578,miR-345-3p,miR-1285-5p,miR-8095,miR-877-3p,miR-2284j,miR-3956-3p,miR-3059-5p,novel_60,miR-2447,miR-154-3p,miR-107-5p,miR-203-3p,miR-144-5p,miR-3082-5p,miR-2483-3p,miR-541-5p,miR-2319b,miR-20a-3p,miR-2284g,miR-664b,novel_94,miR-335-3p,miR-493-5p,miR-136-5p,miR-129b-5p,miR-221,miR-140-5p,miR-491-3p,miR-3187-3p,miR-33a-5p,miR-23a-3p,let-7c-3p,miR-103a-2-5p,miR-1306-5p,miR-320b,miR-199a-3p,miR-126a-5p,miR-221-3p,miR-9788-3p,miR-455-3p,miR-181b-2-3p,miR-5010-3p,miR-1343-3p,miR-548w,miR-2284aa,miR-16-2-3p,miR-450b-5p,miR-30b-3p,miR-143-5p,miR-3074-2-3p,miR-

SLC25A33	ENSOARG0(	solute c	miR-146a,miR-7-5p,miR-2411-3p,miR-493-5p,miR-2285r,miR-503-3p,let-7a-2-3p,miR-7b-5p,miR-542-3p,miR-19b-2-5p,miR-432,miR-197-3p,miR-16-1-3p,miR-16b,miR-369-5p,miR-497-5p,miR-3955-5p,miR-31-5p,miR-339-5p,miR-134-3p,miR-3431,novel_116,miR-142b,miR-29a,miR-29c-3p,miR-376b-5p,miR-3591-3p,miR-3970,miR-410-5p,miR-378f,miR-146b-5p,miR-2319a,miR-3074-5p,miR-669,miR-6536,miR-3064-5p,miR-29a-5p,miR-3591-5p,miR-23b-5p,miR-429-3p,miR-6517,novel_79,miR-412,miR-322-5p,miR-9-5p,miR-193a-5p,miR-16-5p,miR-1197-3p,miR-181c-3p,miR-301,miR-376c-5p,miR-200a-5p,miR-767,miR-1248,miR-874-3p,miR-331-5p,miR-300-3p,miR-376c-3p,miR-182-5p,miR-16b,miR-29b-1-5p,novel_19,miR-103a-2-5p,miR-146b,miR-378d,miR-1b-5p,miR-501-3p,miR-1839-5p,miR-21b,miR-1343-3p,miR-548w,miR-421,miR-455-3p,miR-2440,miR-218-5p,miR-499a-5p,miR-2447,miR-9-5p,miR-2284j,miR-877-3p,miR-140-5p,miR-136-5p,miR-96-5p,miR-299b-5p,miR-181a-3p,miR-493-5p,miR-2319b,miR-139-5p,miR-2483-3p,miR-378g,miR-500-3p,miR-422a,miR-345-3p,miR-324-3p,miR-202-5p,miR-2285w,miR-376a-5p,miR-378i,miR-378a-3p,miR-33b-3p,miR-34a-5p,miR-16-1-3p,miR-30d-3p,miR-212-5p,miR-2284h-5p,miR-665,miR-30a-3p,miR-130b-5p,miR-3074-5p,miR-3184-3p,miR-665-5p,miR-3958-5p,novel_44,miR-346,miR-6525,miR-339b,miR-1827,miR-543-3p,miR-216b-5p,miR-582-5p,miR-15b-5p,miR-136-3p,miR-2285l,miR-130b-5p,miR-363-5p,miR-654-3p,miR-374b-3p,miR-29b,miR-21-5p,miR-30b-3p,miR-7862,miR-133b-5p,miR-26b-3p,miR-432-5p,miR-2355-3p,miR-544b,miR-6402,miR-133a-5p,miR-6529b,miR-502-5p,miR-15a,miR-382-5p,miR-34c,miR-16a,novel_1,miR-656-5p,miR-203b-5p,miR-22-5p,novel_101,miR-495-3p,miR-17-3p,miR-24-3p,miR-449a,novel_27,miR-378h,miR-21c,miR-505-3p,miR-195a-
LYPD3	ENSOARG0(	LY6/PLAU	miR-381-3p,miR-21c,miR-18a-3p,miR-148a-3p,miR-1291,miR-27a-3p,miR-320c,miR-323a-5p,miR-301a-5p,miR-33a-3p,miR-371a-5p,miR-28c,miR-2284q,miR-374a-3p,miR-4726-5p,miR-499a-5p,miR-2428,novel_111,miR-301b-5p,miR-708-3p,miR-296-5p,miR-27b-3p,miR-455-5p,miR-2426,miR-21-5p,miR-27a-3p,miR-31-5p,miR-503-5p,miR-185-5p,miR-455-5p,miR-320b,miR-503-5p,miR-124-5p,miR-320a,miR-27b-5p,miR-216b-3p,miR-3965,miR-323-5p,miR-320e,miR-2433,miR-486b-3p,miR-374b-3p,miR-28-5p,miR-708-5p,miR-4492,novel_74,miR-28a-3p,miR-6134,miR-374a-3p,novel_44,miR-98-3p,miR-1197-3p,miR-188-3p,miR-5703,miR-98-3p,miR-376a-5p,miR-4429,miR-18a-3p,miR-148b-3p,miR-185-3p,miR-219b-5p,miR-2285af,novel_127,miR-152-3p,miR-2898,miR-320d,miR-28-3p,miR-503-5p,miR-486-3p,miR-28b

RASGRP2	ENSOARG0(	RAS guan	miR-2300a-5p,miR-2285e,miR-30b-3p,miR-7857,miR-143-5p,miR-484,miR-6529a,miR-378b,miR-378c,let-7c-3p,miR-2330-5p,miR-424-5p,let-7a-5p,miR-3187-3p,miR-30c-1-3p,miR-362-3p,novel_103,miR-1306-5p,let-7e,miR-25-5p,miR-3085-3p,miR-7977,miR-499b-5p,miR-452-3p,miR-3064-5p,let-7d-5p,miR-3082-5p,miR-378e,miR-361-3p,miR-2285f,miR-541-3p,novel_60,miR-371a-5p,miR-3958-3p,miR-4726-5p,miR-3600,miR-532-3p,miR-15a-5p,miR-1197-5p,miR-2904,miR-362-3p,novel_133,miR-2411,miR-125b,miR-185-3p,miR-34b-5p,miR-221-5p,miR-466i-5p,miR-370-5p,miR-3065-3p,miR-221-5p,miR-378a-3p,miR-22-3p,novel_32,miR-204-3p,miR-6130,miR-27a-5p,miR-18a-5p,miR-2312,let-7g-3p,miR-3074-2-3p,miR-145a-3p,miR-4510,miR-378c,let-7b-5p,miR-320e,miR-324-3p,miR-708-5p,miR-17-3p,miR-665,let-7g-5p,miR-2285g,miR-432-5p,miR-1285,miR-744-5p,miR-2889,miR-29b,miR-30b-3p,miR-6412,miR-197-5p,miR-224-5p,miR-466f-3p,miR-125a-5p,miR-320b,miR-34b,miR-670-3p,miR-29a-3p,miR-146b-3p,miR-18a-3p,miR-18b,miR-2285ab,miR-195a-5p,miR-6395,miR-9851-3p,miR-450b-3p,miR-1291,miR-17-3p,miR-1193,miR-3957-3p,miR-2428,miR-449a,miR-24-3p,let-7i,miR-34c,miR-16a,miR-15a,miR-147-5p,miR-203b-5p,miR-656-5p,miR-128-1-5p,miR-2411-5p,miR-378h,miR-29d-3p,let-7d,miR-582-3p,miR-181a-2-3p,miR-4286,miR-1271-3p,miR-1957a,miR-3065-3p,miR-28b,let-7c-5p,miR-339a,miR-30c-2-3p,miR-543-5p,miR-17-3p,miR-2898,miR-615-5p,miR-370-3p,miR-34c-5p,miR-3154,miR-18a,miR-485-5p,miR-7144-5p,miR-378c,miR-99b-3p,miR-370-5p,miR-29b-3p,miR-486b-3p,miR-28-5p,miR-410-3p,miR-1343-3p,miR-455-3p,miR-147a,miR-301b-5p,miR-541,miR-329-3p,miR-362-3p,miR-1388-3p,miR-8485,miR-378d,miR-16b,miR-214-3p,miR-381-5p,miR-2319b,miR-4443,miR-1983,miR-504,miR-3956-3p,miR-2447,miR-423-

CNGA2	ENSOARGO(	cyclic n	miR-466i-5p,miR-299-3p,miR-21-3p,miR-2484,miR-26a-5p,miR-345-3p,miR-185-3p,miR-92a-1-5p,miR-216a-5p,miR-377-5p,miR-376a-5p,miR-2411,novel_133,miR-140-3p,miR-616-3p,miR-532-3p,miR-30d-3p,miR-664a,miR-130b-5p,miR-30e-3p,miR-665,miR-30a-3p,miR-324-3p,miR-206-3p,miR-4510,miR-3184-3p,miR-129-2-3p,miR-539-5p,miR-3074-2-3p,miR-214,miR-665-5p,miR-3958-5p,let-7g-3p,miR-129b-3p,miR-6130,miR-27a-5p,miR-548o-3p,miR-30c-1-3p,miR-296-3p,miR-216b-3p,miR-491-5p,miR-214-3p,miR-153-3p,miR-199b-5p,miR-326,miR-1306-5p,miR-342,miR-30c-1-3p,miR-1839-3p,miR-4791,miR-30b-3p,miR-1a-3p,miR-153,miR-2459,miR-216a-3p,novel_4,miR-450a-5p,miR-615,miR-2440,miR-101-3p,miR-371a-5p,novel_111,miR-1983,miR-199b-5p,miR-378g,miR-4443,miR-7689-3p,miR-127-5p,novel_96,miR-6119-5p,miR-2285af,miR-30f,miR-30c-2-3p,miR-450c-5p,miR-200a,miR-376b-5p,novel_39,miR-199a-5p,miR-376c-5p,miR-3529-3p,miR-1,miR-128-3p,miR-615-3p,miR-345-3p,miR-330-5p,miR-2411-5p,miR-758-5p,novel_121,miR-486-5p,miR-129-1-3p,miR-6517,miR-299a-3p,miR-331-5p,miR-138-5p,miR-144,miR-331-3p,miR-1197-3p,miR-193a-5p,novel_91,miR-10b-3p,miR-671-5p,miR-7144-5p,miR-331-5p,miR-3965,miR-300-3p,miR-331-5p,miR-199a-5p,miR-874-3p,miR-1271,miR-370-3p,miR-2355-5p,miR-378a-5p,miR-2411-3p,novel_51,miR-105-5p,miR-130b-5p,miR-363-5p,miR-30b-3p,miR-138,miR-542-3p,miR-2113,miR-432,miR-873a-5p,miR-197-3p,miR-134-3p,miR-3535,miR-432-5p,miR-377-5p,miR-665,miR-206,miR-502b,miR-6123,miR-22-5p,miR-4324,miR-2428,miR-199b-5p,miR-326-3p,miR-219b-3p,miR-342-3p,miR-1839-3p,miR-412-3p,miR-1291,miR-410-5p,miR-216a-3p,miR-3970,miR-1306,miR-150-5p,miR-299b-3p,miR-670-3p,miR-144-3p,miR-3120-3p,miR-26b-5p

TOPBP1	ENSOARG0(	topoisom	miR-1814c,miR-671-5p,miR-376c-5p,miR-10b-3p,miR-18a,miR-320a,miR-154b-5p,miR-34c-5p,miR-374c-3p,miR-28-5p,miR-138-5p,miR-331-3p,miR-370-5p,miR-365a-5p,miR-374a-3p,miR-200b-3p,miR-105-2,miR-501-3p,miR-218-2-3p,miR-345-3p,miR-302a-5p,miR-222,miR-200c-3p,miR-17-3p,miR-339a,miR-30c-2-3p,miR-200c,miR-6119-5p,miR-376b-3p,miR-2284a,miR-181a-2-3p,miR-29b-2-5p,miR-30a-3p,miR-3529-3p,miR-505-3p,miR-6395,miR-195a-5p,miR-376b-3p,miR-18b,miR-670-3p,miR-34b,miR-323a-5p,miR-2404,miR-22-5p,novel_1,miR-34c,miR-16a,miR-133a-5p,miR-15a,miR-449a,miR-17-3p,novel_101,miR-1193,miR-495-3p,miR-2285x,miR-3957-3p,miR-219a-1-3p,miR-30b-3p,miR-138,miR-374b-3p,miR-653,miR-2355-3p,miR-2284d,miR-466f-3p,miR-1343-5p,miR-330-3p,miR-224-5p,miR-296-3p,miR-582-5p,novel_99,miR-18a-5p,miR-27b-5p,miR-21a-3p,novel_25,miR-22-3p,miR-204-3p,miR-656-3p,miR-323-5p,miR-182-5p,miR-532-5p,miR-370-5p,miR-221-5p,miR-545-3p,miR-424-5p,miR-708-5p,miR-17-3p,miR-29b-2-5p,miR-320e,miR-324-3p,miR-19b-1-5p,miR-222-3p,miR-338-3p,miR-2330-3p,miR-3074-2-3p,miR-145a-3p,miR-539-5p,miR-4429,miR-5703,miR-2904,miR-15a-5p,miR-200b,miR-505-3p,miR-3600,miR-34b-5p,miR-30b-3p,miR-221-5p,miR-320d,miR-196a-3p,miR-146a-5p,miR-130a-5p,miR-216c-5p,miR-376d,miR-27a-3p,miR-3082-5p,miR-7857-3p,miR-541-5p,miR-499b-5p,miR-20a-3p,miR-3064-5p,miR-2285t,miR-374a-3p,miR-450a-5p,miR-2285y,miR-2285n,miR-502-3p,miR-452-5p,miR-219a-5p,miR-541-3p,miR-3059-5p,miR-2284aa,miR-101a-5p,miR-30b-3p,miR-7857,miR-4791,miR-484,miR-296-5p,miR-221-3p,miR-142-3p,miR-27a-3p,miR-105-1,miR-3085-3p,novel_103,miR-320b,miR-193a,miR-3187-3p,miR-30c-1-3p,miR-33a-5p,miR-424-5p,miR-494-3p,miR-329b,miR-23b,miR-376c-5p,miR-2285aa,novel_9,miR-2284k,miR-
KIAA0430	ENSOARG0(	KIAA0430	miR-25-5p,miR-125a-5p,miR-660,miR-503-5p,novel_103,miR-182-5p,miR-345-5p,miR-1388-3p,miR-130b-5p,miR-2483-5p,miR-1260b,miR-219a-1-3p,miR-16-2-3p,miR-1843b-5p,miR-22-3p,miR-496-5p,miR-503-5p,miR-149-5p,miR-2355-3p,miR-31-5p,miR-133b-5p,miR-628-3p,miR-128-1-5p,miR-335,novel_1,miR-502-5p,miR-4324,miR-211,miR-335-5p,miR-17-3p,miR-877-3p,miR-18b,miR-378b,miR-2483-3p,miR-454-5p,miR-4443,novel_68,miR-7689-3p,miR-211-5p,miR-3120-3p,miR-17-3p,miR-760-3p,miR-21-3p,miR-1260a,novel_17,miR-3071-3p,miR-125b,miR-148b-5p,miR-2432,miR-503-5p,miR-195a-3p,miR-134,miR-5703,miR-505-5p,miR-134-5p,miR-582-3p,miR-129-1-3p,miR-3600,miR-18b-5p,miR-17-3p,miR-874-5p,miR-134-5p,miR-204-5p,miR-130b-5p,miR-2433,miR-125b-5p,miR-1260b,miR-539-5p,miR-129-2-3p,miR-3607-3p,miR-6128,miR-345-5p,miR-18a-5p,miR-345-5p,miR-7144-5p,miR-485-5p,miR-21a-3p,miR-18a,miR-3959-5p,miR-22-3p,miR-154b-5p,miR-660-5p,miR-182-5p,miR-125a
CRBN	ENSOARG0(	cereblon	miR-3065-3p,miR-877-3p,miR-29c-3p,miR-6240,miR-3074-5p,miR-29a,miR-409-3p,miR-3969,miR-151-3p,miR-330-3p,miR-29b-3p,miR-29a-3p,miR-331-5p,miR-3065-3p,miR-3074-5p,miR-330-3p,miR-151a-3p,miR-877-3p,miR-29b,miR-380-3p,miR-29d-3p,miR-21a-3p

AP3M1	ENSOARG0(	adaptor-	miR-143-5p,miR-125b-2-3p,miR-3962,miR-30b-3p,miR-7857,miR-544a,miR-1388-5p,miR-2284aa,miR-199b-3p,miR-105-1,miR-142-3p,miR-216a-3p,miR-320b,miR-199a-3p,miR-182-5p,miR-16b,miR-3955-3p,miR-3085-3p,miR-214-3p,miR-424-5p,miR-30c-1-3p,miR-491-3p,miR-2310,miR-378j,miR-452-3p,miR-20a-3p,miR-2319b,miR-3064-5p,miR-139-5p,miR-211-5p,miR-2483-3p,miR-499a-3p,miR-378g,miR-218-5p,miR-769-5p,miR-490-5p,miR-212-3p,miR-3059-5p,miR-154a,miR-5703,miR-1197-5p,miR-1193,miR-4429,miR-378d,miR-2284u,miR-548e-3p,miR-15a-5p,miR-222-5p,miR-7859,miR-199a-3p,miR-671-3p,miR-2285p,miR-345-3p,miR-92a-1-5p,miR-216a-5p,miR-320d,miR-202-5p,miR-299-3p,miR-216c-5p,miR-183-5p,miR-345-3p,miR-130a-5p,miR-142a-3p,miR-2424,miR-450a-1-3p,miR-487b-5p,miR-345-5p,miR-1827,miR-186-5p,miR-548o-3p,miR-30c-1-3p,miR-2331-3p,miR-296-3p,miR-182-5p,miR-3065-3p,miR-6130,miR-139-5p,miR-499b-3p,miR-544-3p,miR-15b-5p,novel_32,miR-22-3p,miR-450b-3p,miR-708-5p,miR-208b-5p,miR-424-5p,miR-4492,miR-129-2-3p,miR-3074-5p,miR-4510,miR-590-3p,miR-214,miR-3074-2-3p,miR-3958-5p,miR-769-5p,miR-1285,miR-7b-5p,miR-542-3p,miR-708-3p,miR-374b-3p,miR-22-3p,miR-450c-3p,miR-16-1-3p,miR-1260b,miR-30b-3p,miR-138,miR-29b,miR-16b,miR-497-5p,miR-26a-2-3p,miR-134-3p,miR-412-5p,miR-544b,miR-2355-3p,miR-194-3p,miR-7-5p,miR-2411-3p,miR-105-5p,miR-124-5p,miR-380-5p,miR-628-5p,miR-432-3p,miR-432-3p,miR-345-5p,let-7j,miR-2387,miR-378b,miR-410-5p,miR-195a-5p,miR-3074-5p,miR-320b,miR-655-5p,miR-299b-3p,miR-29a-3p,miR-144-3p,miR-320c,miR-28c,miR-15a,novel_87,miR-16a,miR-29a,novel_1,miR-6119-3p,miR-147-5p,miR-502b,miR-6123,miR-199c,miR-625-3p,miR-135a-2-3p,novel_120,miR-29c-3p,miR-323c,miR-24-3p,miR-211,miR-2428,novel_27,miR-3578,miR-

PHLDB3	ENSOARG0(	pleckstr	miR-3120-3p,novel_68,miR-181b-3p,miR-6535,miR-677,novel_69,miR-299b-3p,miR-374c-3p,miR-18a-3p,miR-18b,miR-148a-3p,miR-2387,let-7e-5p,miR-9851-3p,miR-450b-3p,miR-1193,miR-301b-3p,miR-326-3p,miR-24-3p,let-7i,miR-6529b,miR-28c,miR-576-3p,miR-4324,miR-147-5p,miR-128-1-5p,miR-130a-3p,miR-194-3p,miR-149-5p,let-7g-5p,miR-432-5p,miR-197-3p,miR-1843b-5p,miR-432,miR-708-3p,miR-873a-5p,miR-2284x,miR-744-5p,miR-455-5p,miR-4508,miR-1260b,miR-2483-5p,miR-363-5p,miR-345-5p,novel_51,miR-455-5p,miR-365b-5p,miR-105-5p,miR-452-5p,miR-125a-5p,miR-660,miR-1343-5p,miR-615-5p,miR-1271,miR-125a,miR-215-5p,miR-2355-5p,miR-370-3p,miR-874-3p,miR-574-5p,miR-374c-3p,miR-18a,miR-362-5p,miR-3154,miR-301,miR-671-5p,miR-1248,miR-7144-5p,miR-23a-5p,novel_48,miR-345-5p,miR-193a-5p,miR-28a-3p,miR-365a-5p,miR-1197-3p,miR-1260b,miR-500b-5p,miR-409-3p,miR-331-3p,miR-125b-5p,miR-6740-5p,miR-2332,miR-370-5p,miR-2433,miR-28-5p,miR-126b-3p,miR-18b-5p,miR-6517,miR-2285ad,miR-222,miR-299a-3p,miR-330-5p,miR-1343-5p,miR-615-3p,miR-105-2,let-7d,miR-4286,miR-29b-2-5p,miR-192-3p,miR-3065-3p,let-7g,miR-28b,miR-148b-5p,let-7c-5p,miR-30c-2-3p,miR-1260a,miR-152-3p,miR-543-5p,novel_96,novel_127,let-7f-5p,miR-28-3p,miR-7977,miR-499b-5p,miR-2319b,miR-10b,miR-4792,miR-4443,let-7d-5p,miR-203-3p,miR-489,miR-221,novel_94,miR-136-5p,miR-504,miR-452-5p,miR-212-3p,miR-2447,miR-154a,miR-423-5p,miR-361-3p,miR-362-5p,miR-2285y,miR-615,novel_82,miR-4726-5p,miR-181b-2-3p,miR-105-1,novel_78,miR-1343-3p,miR-2300a-5p,miR-2459,miR-500-5p,miR-148b-5p,miR-216a-3p,miR-455-3p,miR-221-3p,miR-147a,miR-1298-3p,miR-758-3p,miR-3120-5p,miR-1839-5p,miR-21b,miR-450b-5p,miR-1388-5p,miR-2330-5p,miR-124a,let-7a-5p,miR-758-3p,miR-193a,miR-30c-1-
KPNA3	ENSOARG0(	karyophe	miR-212-5p,miR-299a-3p,miR-2285ad,miR-450a-1-3p,miR-134-5p,miR-2411-5p,miR-383-5p,miR-302a-5p,miR-30a-5p,miR-1197-5p,miR-2904,miR-134,miR-30c-5p,miR-30c,novel_73,miR-300,miR-4429,miR-2285w,miR-376c-5p,miR-2411,miR-29b-1-5p,miR-30f,miR-30b,miR-21-3p,miR-299-3p,miR-466i-5p,miR-320d,miR-182-5p,miR-2285aa,miR-548o-3p,novel_25,miR-331-5p,miR-30d-5p,miR-362-5p,miR-320a,miR-500a-3p,miR-450a-1-3p,miR-30e-5p,miR-21a-3p,miR-381-3p,miR-1827,miR-1814c,miR-186-5p,miR-625-5p,miR-30f,miR-767,miR-425-5p,miR-214,miR-500b-5p,miR-5100,miR-374b-3p,miR-592,miR-134-5p,miR-500,miR-767-5p,miR-6516-3p,miR-664a,miR-450b-3p,miR-27a-3p,miR-26b-3p,miR-500-5p,miR-539-5p,miR-194-5p,miR-323b,miR-450c-3p,miR-542-3p,miR-301b-5p,miR-223-3p,miR-1434-3p,miR-873a-5p,miR-188-5p,miR-27b-3p,miR-744-3p,miR-101b-3p,miR-2330-5p,miR-2310,miR-194a,miR-182-5p,miR-29b-1-5p,miR-323b-3p,miR-320b,miR-214-3p,miR-27a-3p,miR-4443,miR-320c,miR-299b-3p,miR-129b-5p,miR-3065-5p,miR-9851-3p,miR-194b-5p,miR-3059-5p,miR-2428,miR-24-3p,miR-362-5p,miR-301a-5p,miR-485-3p,miR-2440,novel_82,miR-30b-5p,miR-1247-3p,miR-203b-5p,miR-499a-5p

PNN	ENSOARG0(	pinin, d	miR-6525,miR-1296-5p,miR-3074-2-3p,miR-2330-3p,miR-495-3p,miR-212-3p,novel_74,novel_1,miR-320e,miR-2284k,novel_63,miR-133a-5p,miR-320c,miR-1185-2-3p,miR-377-3p,miR-499b-5p,miR-7-1-3p,miR-545-3p,miR-543-3p,miR-505-3p,miR-186-5p,miR-10b-3p,miR-2318,miR-107,miR-3154,miR-381-3p,miR-320a,miR-381-3p,miR-503-3p,miR-376a-5p,miR-2285r,miR-654-3p,let-7f-2-3p,let-7f-2-3p,miR-107,miR-320d,miR-3071-3p,miR-500a-5p,miR-105-5p,miR-320b,miR-2285af,miR-377-3p,miR-1185-3p,miR-103,miR-302a-5p,miR-505-3p,miR-105-1,miR-103a-3p,miR-105-2,miR-2284ab,miR-4429,miR-300,miR-30a-5p,miR-2300b-3p,miR-1434-3p
LTF	ENSOARG0(	lactotra	miR-148a-3p,miR-216a-3p,miR-450b-3p,miR-410-5p,miR-195a-5p,miR-135b-5p,miR-216b-3p,miR-655-5p,miR-374c-3p,miR-320c,miR-15a,miR-16a,miR-206,miR-379-3p,miR-147-5p,miR-625-3p,miR-17-3p,miR-2285x,miR-495-3p,miR-301b-3p,miR-1434-3p,miR-22-3p,miR-450c-3p,miR-16-1-3p,miR-30b-3p,miR-16b,miR-2284x,miR-497-5p,miR-4508,miR-130a-3p,miR-2331-5p,miR-378a-5p,miR-493-5p,miR-380-5p,miR-101-5p,miR-2448-3p,miR-320a,miR-3154,miR-329a,miR-1248,miR-3957-5p,miR-374c-3p,miR-300-3p,miR-3957,miR-135a-1-3p,novel_63,miR-135a-5p,miR-331-3p,novel_9,miR-28-5p,miR-592,miR-16-5p,miR-1197-3p,miR-145-3p,miR-2427,miR-218-2-3p,miR-411-3p,miR-128-3p,miR-200b-3p,miR-6517,miR-429-3p,miR-2411-5p,miR-1271-5p,miR-322-5p,miR-214-5p,miR-545-5p,miR-450a-1-3p,miR-219-3p,miR-200c,miR-2285af,miR-200c-3p,miR-17-3p,miR-152-3p,miR-3529-3p,miR-181a-2-3p,miR-1,miR-30a-3p,miR-380-5p,miR-2285b,miR-2284y,miR-361-3p,miR-381-3p,miR-96-5p,miR-877-3p,miR-493-5p,miR-2284g,miR-7689-3p,miR-3082-5p,miR-2440,miR-218-5p,miR-301a-5p,miR-107-5p,miR-371a-5p,miR-499a-5p,miR-3956-3p,miR-2447,novel_60,miR-7705,miR-219a-2-3p,miR-301b-5p,miR-329-3p,miR-296-5p,miR-30b-3p,miR-1a-3p,miR-101a-5p,miR-362-3p,miR-16-2-3p,miR-1343-3p,novel_4,miR-539-5p,miR-1298-3p,miR-421,miR-148b-5p,miR-320b,miR-182-5p,miR-16b,miR-362-3p,miR-3955-3p,miR-130b-3p,miR-103a-2-5p,miR-214-3p,miR-2330-5p,miR-424-5p,miR-124a,miR-652-5p,miR-491-3p,miR-1b-5p,miR-8485,miR-4532,miR-450a-1-3p,let-7f-1-3p,miR-21a-3p,miR-129b-3p,novel_99,miR-1827,miR-186-5p,miR-625-5p,miR-543-3p,miR-548o-3p,miR-296-3p,miR-182-5p,miR-2284s,miR-301a-3p,miR-877-5p,miR-15b-5p,miR-22-3p,miR-204-3p,miR-30e-3p,let-7b-3p,miR-2285j,miR-665,miR-219b-3p,miR-30a-3p,miR-6516-3p,miR-17-3p,novel_74,miR-

MADCAM1	ENSOARG0(	mucosal	miR-9788-3p,miR-216a-3p,miR-455-3p,miR-194-3p,miR-412-5p,miR-147a,miR-339-5p,miR-31-5p,novel_78,miR-497-5p,miR-101c,miR-455-5p,miR-30b-3p,miR-16b,miR-219a-1-3p,miR-412-3p,novel_23,miR-374b-3p,miR-30b-3p,miR-484,miR-6412,miR-432-3p,miR-432-3p,miR-33a-5p,miR-424-5p,miR-103a-2-5p,miR-3955-3p,miR-2285l,miR-16b,miR-455-5p,miR-2411-3p,miR-224-5p,miR-326,novel_115,miR-374c-3p,miR-7977,miR-139-5p,miR-452-3p,miR-20a-3p,miR-7975,miR-195a-5p,miR-1983,miR-147-3p,miR-412-3p,miR-361-3p,miR-326-3p,miR-423-5p,miR-2428,miR-219a-2-3p,miR-361-3p,novel_111,miR-17-3p,miR-135a-2-3p,miR-656-5p,miR-16a,miR-107-5p,miR-15a,miR-502-5p,miR-6402,miR-129-1-3p,miR-486-5p,miR-322-5p,miR-6516-5p,miR-758-5p,miR-15a-5p,novel_79,miR-412,miR-493-3p,miR-184-3p,miR-2904,miR-330-5p,miR-1197-5p,miR-1193,miR-188-3p,miR-5703,miR-345-3p,miR-486-3p,novel_39,miR-3956-5p,miR-376a-5p,miR-28-3p,miR-17-3p,miR-127-5p,miR-345-3p,miR-339a,miR-219-3p,miR-15b-5p,miR-374c-3p,miR-132-5p,miR-6516,miR-139-5p,miR-370-3p,miR-2355-5p,miR-1271,miR-1277-3p,miR-23a-5p,miR-10b-3p,miR-339b,miR-200a-5p,miR-3184-5p,miR-145-3p,miR-224-5p,miR-16-5p,let-7e-3p,miR-129-2-3p,miR-3184-3p,miR-424-5p,miR-874-5p,miR-486b-3p,miR-17-3p,miR-132-5p,miR-551b-3p
TRNT1	ENSOARG0(	tRNA nuc	miR-1271-3p,miR-183-5p,miR-2285w,miR-369-3p,miR-20a,miR-106a,miR-107,miR-21-3p,miR-299-3p,miR-205-5p,miR-216a-5p,miR-181c-3p,miR-7859,miR-2284h-5p,miR-548e-3p,miR-299a-3p,miR-106a,miR-33b-3p,miR-93,miR-4510,miR-410-3p,miR-204-5p,miR-2285j,miR-331-3p,miR-100-3p,miR-409-3p,miR-664a-5p,miR-3604,miR-320e,miR-300-3p,miR-582-5p,miR-374c-3p,miR-6130,miR-154b-5p,miR-2331-3p,miR-216b-3p,miR-2312,miR-345-5p,miR-345-5p,miR-20b-5p,miR-582-5p,miR-2285c,miR-301,miR-3613-5p,miR-93-5p,miR-17-5p,miR-345-5p,miR-491-3p,miR-106a-5p,miR-323b-3p,miR-106a-5p,miR-106b-5p,miR-17-5p,miR-412-5p,miR-2285g,miR-1298-3p,miR-194-3p,miR-411-5p,miR-103,miR-20b,miR-2285e,novel_78,miR-103a-3p,miR-1343-3p,miR-5010-3p,miR-101a-5p,miR-2426,miR-16-2-3p,miR-301b-5p,miR-2113,miR-323b,miR-125b-2-3p,miR-211,miR-1285-5p,miR-7705,novel_60,miR-3059-5p,miR-3956-3p,miR-2285f,miR-452-5p,novel_101,miR-3613,miR-20a-5p,miR-301a-5p,miR-299b-3p,miR-374c-3p,miR-211-5p,miR-450b-3p,miR-107,miR-9851-3p,miR-2285ab,miR-378b

TPGS1	ENSOARGO(	tubulin	miR-218-5p,miR-2440,miR-2284j,miR-2447,miR-3956-3p,miR-1285-5p,miR-361-3p,miR-877-3p,novel_111,miR-140-5p,miR-96-5p,miR-299b-5p,miR-136-5p,miR-7689-3p,miR-4792,miR-4443,miR-378g,miR-499a-3p,miR-29b-1-5p,novel_19,miR-199a-3p,miR-146b,miR-199b-5p,miR-214-3p,miR-491-5p,miR-676-3p,miR-8485,miR-2310,miR-2284r,miR-1839-5p,miR-181b-2-3p,miR-1343-3p,novel_78,miR-2459,miR-147a,miR-142-5p,miR-1298-3p,let-7f,miR-500,miR-874-5p,miR-4492,miR-130b-5p,novel_74,miR-665-5p,miR-214,let-7b,miR-3074-5p,miR-122-5p,miR-142a-5p,miR-6525,miR-500a-3p,miR-129b-3p,miR-299,miR-885-3p,miR-186-5p,miR-1827,miR-345-5p,miR-296-3p,miR-2331-3p,miR-30c-1-3p,let-7i-5p,miR-376e-3p,miR-2284s,miR-10a,miR-377-3p,miR-499b-3p,miR-219b-5p,miR-324-3p,miR-92a-1-5p,miR-618,let-7k,miR-98-5p,miR-345-3p,miR-486-3p,miR-376a-5p,miR-376c-3p,miR-181b-1-3p,miR-193b-5p,miR-1961,miR-34a-5p,miR-142-3p,miR-184-3p,miR-212-5p,miR-1247-5p,miR-328-3p,novel_87,miR-142b,miR-576-3p,miR-6123,miR-29a,miR-206,miR-193b-3p,miR-29c-3p,miR-199b-5p,miR-1306,miR-3141,miR-146b-5p,let-7e-5p,miR-3120-3p,novel_68,miR-3074-5p,miR-669,miR-6535,miR-677,miR-320c,miR-181b-3p,miR-299b-3p,miR-3141,miR-194a,miR-146a,miR-377-3p,miR-193a-3p,miR-378a-5p,miR-432-3p,miR-2284l,miR-22-3p,miR-197-3p,miR-542-3p,miR-1843b-5p,miR-223-3p,miR-2113,miR-2284x,miR-31-5p,miR-194-3p,miR-134-3p,miR-125b-5p,novel_63,miR-2284k,miR-134-5p,novel_42,miR-2284v,miR-1260b,miR-767,miR-876-3p,miR-345-5p,miR-2355-5p,miR-2285aa,miR-125a,miR-574-5p,miR-874-3p,miR-3068-3p,miR-376c-3p,miR-3959-5p,miR-125a-3p,miR-664-3p,novel_96,miR-127-5p,let-7f-5p,miR-3064-5p,let-7g,novel_39,miR-330-5p,miR-155-5p,miR-885-3p,miR-1224-5p,miR-299a-3p,miR-134-5p,miR-2284z,miR-2285t,miR-

PASD1	ENSOARG0(	PAS doma	miR-371a-5p,novel_82,miR-218-5p,miR-101-3p,miR-2284q,miR-1296-5p,miR-181d-5p,miR-190b-5p,miR-2284j,miR-212-3p,miR-502-3p,miR-1285-5p,miR-7705,miR-361-3p,miR-140-5p,miR-9-3p,miR-1983,miR-139-5p,miR-452-3p,miR-144-5p,miR-500-3p,miR-499a-3p,miR-3082-5p,miR-362-3p,miR-16b,miR-342,miR-320b,novel_103,miR-1185-5p,miR-491-5p,miR-487b-5p,miR-652-5p,miR-376b,miR-33a-5p,miR-494-3p,miR-424-5p,miR-30c-1-3p,miR-1a-3p,miR-501-3p,miR-30b-3p,miR-296-5p,miR-6529a,miR-329-3p,miR-484,miR-2284aa,miR-101c,miR-362-3p,miR-450b-5p,miR-1343-3p,novel_4,miR-330-3p,miR-455-3p,miR-9788-3p,miR-216a-3p,miR-338-5p,miR-2285j,miR-487a-5p,miR-190a-5p,miR-208b-5p,miR-424-5p,miR-669a-3p,miR-450b-3p,miR-487b-5p,miR-155-5p,miR-425-5p,miR-145a-3p,miR-129-2-3p,miR-181c-5p,miR-338-3p,miR-6525,miR-206-3p,miR-450a-1-3p,miR-543-3p,miR-186-5p,miR-370-5p,miR-296-3p,miR-552-3p,miR-30c-1-3p,miR-221-5p,miR-2331-3p,miR-545-3p,miR-15b-5p,miR-155-5p,miR-499b-3p,miR-377-3p,miR-139-5p,miR-500a-5p,miR-345-3p,miR-92a-1-5p,miR-26a-5p,miR-221-5p,miR-1277-5p,miR-3653-3p,miR-21-3p,miR-2284m,miR-320d,miR-345-3p,miR-183-5p,miR-2904,miR-5703,miR-126-3p,novel_133,miR-362-3p,miR-16-1-3p,miR-361-5p,miR-4429,novel_73,miR-33b-3p,miR-505-3p,novel_83,miR-383-5p,miR-15a-5p,miR-200b,miR-16a,miR-15a,miR-576-3p,miR-142b,miR-421-5p,miR-656-5p,miR-147-5p,miR-379-3p,miR-206,miR-495-3p,miR-1193,novel_120,miR-135a-2-3p,miR-342-3p,novel_27,miR-24-3p,miR-376b-3p,miR-3065-5p,miR-376b-5p,miR-195a-5p,miR-505-3p,miR-412-3p,miR-107,miR-7-1-3p,miR-26b-5p,miR-3120-3p,miR-320c,miR-144-3p,miR-190a,miR-330-3p,miR-455-5p,miR-2411-3p,miR-377-3p,miR-483-3p,miR-363-5p,miR-628-5p,let-7j,miR-432-3p,miR-432-3p,miR-2284l,miR-450c-3p,miR-708-3p,miR-873a-

DHX57	ENSOARG0(	DEAH (As	miR-4429,miR-133a-3p,miR-2284u,miR-34a-5p,miR-361-5p,miR-376a-5p,miR-217-5p,miR-23c,miR-222-5p,miR-2284h-5p,miR-532-3p,miR-328-3p,miR-18a-3p,miR-212-5p,miR-30d-3p,miR-320d,miR-466i-5p,miR-34b-5p,miR-21-3p,miR-92a-1-5p,miR-185-3p,miR-2285i,miR-130a-5p,miR-146a-5p,miR-543-3p,miR-1827,miR-21a-3p,miR-2285c,miR-129b-3p,miR-6130,miR-323-5p,miR-204-3p,miR-32-3p,miR-532-5p,miR-190a-5p,miR-15b-3p,miR-2285j,miR-30e-3p,miR-324-3p,miR-30a-3p,miR-665,miR-346,miR-181c-5p,miR-539-5p,miR-145a-3p,miR-4510,miR-214,miR-101a-5p,miR-296-5p,miR-376b-3p,miR-147a,miR-2285e,novel_78,miR-145b,miR-3955-3p,miR-146b,miR-214-3p,miR-320b,miR-342,miR-505,miR-2310,miR-1388-3p,miR-494-3p,miR-101b-3p,miR-127-3p,miR-2284b,miR-376a-3p,miR-335-3p,miR-361-3p,miR-378g,miR-3082-5p,miR-144-5p,miR-203-3p,miR-376d,miR-664b,miR-2284g,miR-20a-3p,miR-7689-3p,miR-499b-5p,miR-7977,miR-3958-3p,miR-374a-3p,miR-2284q,miR-2285n,novel_82,miR-2285y,miR-1285-5p,miR-32-3p,miR-2447,miR-212-3p,miR-190b-5p,miR-181d-5p,miR-300,let-7a-2-3p,miR-3591-5p,miR-134,miR-302a-5p,miR-24-2-5p,miR-483-3p,miR-134-5p,miR-582-3p,miR-545-5p,novel_121,miR-2285ad,miR-450a-2-3p,miR-28-3p,miR-181b-5p,novel_127,miR-2285af,miR-376b-3p,miR-1260a,miR-664-3p,miR-217,miR-29a-5p,miR-2284y,miR-2284a,miR-30a-3p,miR-181a-2-3p,let-7g-3p,miR-329b,miR-7144-5p,miR-1814c,miR-488-3p,miR-381-3p,miR-331-5p,miR-320a,miR-34c-5p,miR-874-3p,miR-2285aa,miR-2355-5p,miR-331-5p,miR-374b-3p,miR-134-5p,miR-26c,miR-6740-5p,miR-3969,miR-1260b,miR-374a-3p,miR-145-3p,miR-28a-3p,miR-6128,miR-3607-3p,miR-144-5p,miR-1260b,miR-653,miR-708-3p,miR-181a-5p,miR-1285,miR-145a-5p,miR-3071-5p,miR-2285g,miR-412-5p,miR-134-3p,miR-3431,miR-6238,miR-26b-3p,miR-483-3p,miR-146a,miR-

CCL28	ENSOARG0(	chemokin	miR-345-5p,miR-503-3p,miR-874-3p,miR-574-5p,miR-134-5p,miR-1197-3p,miR-3591-5p,miR-330-5p,miR-758-5p,novel_121,miR-2284z,miR-450a-1-3p,miR-134-5p,novel_79,miR-1224-5p,miR-127-5p,novel_127,miR-2285b,novel_39,miR-378f,miR-2319a,miR-3065-5p,miR-150-5p,miR-1306,miR-181b-3p,miR-677,miR-320c,miR-3074-5p,miR-216b-3p,miR-28c,novel_87,miR-16-1-3p,miR-27b-3p,miR-4508,miR-26a-2-3p,miR-432,miR-19b-2-5p,miR-450c-3p,miR-3431,miR-194-3p,miR-31-5p,miR-339-5p,miR-377-3p,miR-2411-3p,miR-194a,miR-2284l,miR-432-3p,miR-2285r,miR-432-3p,miR-628-5p,miR-668-3p,miR-345-5p,miR-339b,miR-1827,miR-186-5p,miR-450a-1-3p,miR-2285c,miR-411,miR-433-3p,miR-129b-3p,miR-598-3p,miR-10a,miR-377-3p,miR-30c-1-3p,miR-2331-3p,miR-296-3p,miR-216b-3p,novel_74,miR-130b-5p,miR-129-5p,miR-2285j,miR-3604,miR-665,miR-769-5p,miR-6525,miR-3074-5p,miR-16-1-3p,miR-2366,miR-378i,miR-378a-3p,miR-7859,miR-328-3p,miR-2284h-5p,miR-18a-3p,miR-618,miR-2285p,miR-345-3p,miR-422a,miR-92a-1-5p,miR-181b-1-3p,miR-142a-3p,miR-486-3p,miR-2424,miR-2285w,miR-9-3p,miR-335-3p,miR-25,miR-2319b,miR-7689-3p,miR-3968,miR-101-3p,miR-218-5p,miR-1285-5p,miR-885-5p,miR-2447,miR-1388-5p,miR-1298-3p,miR-539-5p,miR-455-3p,miR-199b-3p,miR-181b-2-3p,miR-1185-5p,miR-199a-3p,miR-29b-1-5p,miR-378d,miR-2310,miR-10b-3p,miR-1814c,miR-671-5p,miR-3074-1-3p,miR-7144-5p,miR-503-3p,miR-488-3p,miR-362-5p,miR-320a,miR-3154,miR-769,miR-3965,miR-324-5p,miR-598-3p,miR-486b-3p,miR-409-3p,miR-500b-5p,miR-323-3p,miR-378c,miR-145-3p,miR-411-3p,miR-496,miR-10a-5p,miR-134,miR-345-3p,miR-7134-5p,miR-218-2-3p,miR-378h,miR-2411-5p,miR-545-5p,miR-181b-5p,miR-17-3p,miR-450a-2-3p,miR-543-5p,miR-2285af,miR-339a,miR-30c-2-3p,miR-
CDV3	ENSOARG0(	CDV3 hom	let-7f-1-3p,miR-2448-3p,miR-7144-5p,miR-1248,miR-2331-3p,miR-32-3p,miR-503-3p,miR-370-5p,miR-3065-3p,miR-133c,miR-877-5p,let-7i-5p,miR-15b-5p,let-7b-3p,let-7f,miR-1-5p,miR-370-5p,miR-29b-2-5p,miR-424-5p,let-7b,miR-16-5p,miR-98-3p,let-7b-5p,miR-196a-5p,miR-1961,miR-98-3p,let-7d,let-7a-3p,miR-18a-3p,miR-3600,miR-322-5p,miR-383-5p,miR-15a-5p,let-7c-5p,miR-1a-2-5p,miR-98-5p,let-7f-5p,miR-21-3p,novel_127,let-7k,miR-216c-5p,miR-29b-2-5p,miR-4286,miR-29b-1-5p,miR-376a-5p,miR-2432,let-7g,miR-3065-3p,miR-2285b,miR-409-5p,miR-18a-3p,miR-195a-5p,let-7e-5p,miR-20a-3p,miR-7689-3p,let-7d-5p,miR-15a,miR-16a,let-7i,miR-382-5p,miR-32-3p,miR-1a-1-5p,miR-1193,miR-16b,miR-2426,miR-497-5p,miR-665,let-7g-5p,miR-544b,miR-2459,miR-29b-1-5p,miR-16b,miR-196b-5p,miR-487b-5p,let-7e,let-7a-5p,miR-424-5p,miR-2483-5p

NUS1	ENSOARG0(	NUS1 deh	miR-29b-2-5p,miR-2285j,miR-487a-5p,miR-454-3p,miR-208b-5p,miR-17-3p,miR-450b-3p,novel_74,miR-1271,miR-3184-3p,miR-224-5p,miR-769-5p,miR-30d-5p,miR-450a-1-3p,miR-30f,miR-21-3p,miR-32-3p,miR-548o-3p,novel_25,miR-22-3p,miR-216b-5p,miR-377-3p,miR-544-3p,miR-301a-3p,miR-216a-5p,miR-500a-5p,miR-26a-5p,miR-2285p,miR-30b,miR-30d,miR-2284m,miR-369-3p,miR-2285w,miR-105-3p,miR-143-3p,miR-2432,miR-2424,miR-146a-5p,miR-125b-2-3p,miR-376a-5p,miR-29b-1-5p,miR-376a-5p,miR-362-3p,miR-30c-5p,miR-218-1-3p,miR-184-3p,miR-19b-3p,miR-485-3p,miR-3600,miR-548e-3p,miR-328-3p,miR-2284h-5p,miR-340-5p,miR-222-5p,miR-23c,miR-23b-3p,miR-487a-3p,miR-7859,miR-371a-5p,miR-769-5p,miR-101-3p,miR-382-3p,miR-30b-5p,miR-2284q,miR-3964,miR-490-5p,miR-2285t,miR-499a-5p,miR-181d-5p,miR-2285f,miR-212-3p,miR-3059-5p,miR-32-3p,miR-7705,miR-8095,miR-1285-5p,novel_111,miR-199b-5p,miR-96-5p,miR-126b-5p,miR-541-5p,miR-4792,miR-2483-3p,miR-378g,miR-499a-3p,miR-362-3p,miR-505,miR-29b-1-5p,miR-326,miR-342,miR-146b,miR-199b-5p,miR-130b-3p,miR-744-3p,miR-376b,miR-23a-3p,miR-676-3p,miR-382-3p,miR-1b-5p,miR-2310,miR-8485,miR-329-3p,miR-16-2-3p,miR-544a,miR-362-3p,miR-105-1,miR-1343-3p,miR-455-3p,miR-100-3p,novel_63,miR-2284k,miR-6128,miR-2284v,miR-145-3p,miR-23b,miR-2448-3p,miR-503-3p,miR-30e-5p,miR-1814c,let-7g-3p,miR-329a,miR-2355-5p,miR-2285aa,miR-199a-5p,miR-223-5p,miR-769,miR-30f,miR-2285af,miR-17-3p,miR-29b-2-5p,miR-3529-3p,miR-2285b,miR-199a-5p,miR-380-5p,miR-30a-5p,miR-330-5p,let-7a-2-3p,miR-128-3p,miR-105-2,miR-30c,novel_121,miR-450a-1-3p,miR-545-5p,miR-758-5p,miR-1271-5p,novel_87,miR-133a-5p,miR-421-5p,miR-22-5p,novel_1,miR-6119-3p,miR-17-3p,miR-2285x,miR-342-

FBP2	ENSOARG0(	fructose	miR-4443,miR-500-3p,miR-27a-3p,miR-378e,miR-25,miR-3082-5p,miR-211-5p,miR-9-3p,miR-299b-5p,miR-147-3p,miR-504,miR-129b-5p,miR-199b-5p,miR-502-3p,miR-9-3p,miR-362-5p,novel_111,miR-190b-5p,novel_60,miR-3956-3p,miR-363-3p,miR-107-5p,miR-9788-3p,miR-455-3p,miR-500-5p,miR-421,miR-5010-3p,miR-181b-2-3p,miR-27a-3p,miR-330-3p,novel_78,miR-548w,miR-101c,miR-21b,miR-3120-5p,miR-378c,miR-299a-5p,miR-544a,miR-501-3p,miR-1a-3p,miR-30b-3p,miR-378b,miR-4791,miR-378d,let-7c-3p,miR-124a,miR-382-3p,miR-424-5p,miR-2330-5p,miR-199b-5p,miR-103a-2-5p,miR-3955-3p,miR-505,miR-29b-1-5p,miR-16b,miR-320b,novel_115,miR-15b-5p,miR-6516,miR-544-3p,miR-193b-3p,miR-21-3p,miR-532-5p,miR-552-3p,miR-378a-3p,miR-32-5p,miR-504-5p,novel_99,miR-133b-3p,let-7g-3p,miR-18a-5p,miR-345-5p,miR-433-3p,miR-338-3p,miR-206-3p,novel_44,miR-224-5p,miR-378c,miR-129-2-3p,miR-190a-5p,miR-129-5p,miR-424-5p,miR-130b-5p,miR-29b-2-5p,miR-15b-3p,miR-15a-5p,miR-505-3p,miR-133a-3p,miR-4429,miR-124-3p,miR-5703,miR-378a-3p,miR-378i,novel_133,miR-1246,miR-2424,miR-486-3p,miR-143-3p,miR-380-3p,miR-29b-1-5p,miR-181b-1-3p,miR-329a-5p,miR-1277-5p,miR-3653-3p,miR-320d,miR-345-3p,miR-125b,miR-422a,miR-2285p,miR-320c,miR-677,miR-181b-3p,miR-2404,miR-670-3p,miR-374c-3p,miR-329-5p,miR-378b,miR-9851-3p,miR-195a-5p,miR-505-3p,miR-378f,miR-135b-5p,miR-378b,miR-3591-3p,miR-376b-5p,miR-18b,miR-199b-5p,miR-2428,miR-211,miR-193b-3p,miR-135a-2-3p,miR-6123,miR-502b,miR-92a-3p,miR-6119-3p,miR-206,novel_1,miR-16a,novel_87,miR-15a,miR-194-3p,miR-2355-3p,miR-544b,miR-3535,miR-185-5p,miR-497-5p,miR-2284x,miR-27b-3p,miR-380-3p,miR-30b-3p,miR-16b,miR-219a-1-3p,miR-125b-1-3p,miR-374b-3p,miR-345-5p,miR-432-3p,miR-432-
NPEPPS	ENSOARG0(	aminopep	miR-377-3p,miR-330-3p,miR-3085-3p,miR-6240,miR-376b,miR-491-3p,miR-8485,miR-181a-5p,miR-873a-5p,miR-2426,miR-27b-3p,miR-330-3p,miR-27a-3p,miR-421,miR-500-5p,miR-2285n,miR-2285y,miR-135a-2-3p,miR-2285x,miR-181d-5p,miR-362-5p,miR-154a,miR-2387,miR-378j,miR-1291,miR-544-5p,miR-505-3p,miR-3064-5p,miR-664b,miR-7977,miR-2285u,miR-2483-3p,miR-27a-3p,miR-664-3p,miR-92a-1-5p,miR-500a-5p,miR-181b-5p,miR-30b-3p,miR-3653-3p,miR-4286,miR-181a-2-3p,miR-3064-5p,miR-421-5p,miR-29a-5p,miR-2432,miR-200a,miR-2366,miR-5703,novel_73,miR-2285ad,miR-505-3p,miR-214-5p,miR-665,miR-487a-5p,miR-129-5p,miR-1197-3p,miR-500b-5p,miR-181c-5p,miR-122-5p,miR-3154,miR-362-5p,miR-574-5p,miR-2331-3p,miR-296-3p,miR-656-3p,miR-377-3p
GAN	ENSOARG0(	gigaxoni	miR-335,miR-381-5p,miR-381-5p,novel_39,miR-335-5p,miR-2285j,miR-129b-3p

RNASEH2B	ENSOARG0(	ribonucl	miR-1,miR-2385-3p,miR-29b-2-5p,miR-181a-2-3p,miR-2284y,novel_39,miR-490-3p,miR-192-3p,miR-2285b,miR-2284a,miR-450b-5p,miR-2478,miR-3071-3p,miR-2285af,miR-125a-3p,miR-543-5p,miR-17-3p,miR-127-5p,miR-181b-5p,miR-412,miR-7641,miR-299a-3p,miR-6517,miR-429-3p,miR-545-5p,miR-134-5p,novel_121,miR-1271-5p,miR-758-5p,miR-330-5p,miR-345-3p,miR-134,miR-1290,miR-3578,miR-128-3p,miR-200b-3p,miR-496,miR-6128,miR-193a-5p,miR-1306-5p,miR-2284v,miR-6740-5p,miR-331-3p,miR-409-3p,miR-3969,miR-135a-5p,miR-34c-3p,miR-2284k,miR-592,miR-134-5p,novel_9,miR-2285aa,miR-503-3p,miR-223-5p,miR-133c,miR-154b-5p,miR-374c-3p,miR-3154,miR-301,miR-503-3p,miR-424-3p,miR-7144-5p,miR-1814c,miR-485-5p,miR-345-5p,miR-2318,miR-382,miR-130b-5p,miR-493-5p,miR-6240,miR-345-5p,miR-432-3p,miR-432-3p,miR-22841,miR-330-3p,let-7d-3p,miR-2411-3p,miR-224-5p,miR-377-3p,miR-146a,miR-193a-3p,miR-124-5p,miR-130a-3p,miR-26b-3p,miR-3955-5p,miR-187-3p,novel_116,miR-544b,miR-2284d,miR-2285g,miR-185-5p,miR-3431,miR-197-3p,novel_23,miR-3071-5p,miR-374b-3p,miR-708-3p,miR-873a-5p,miR-653,miR-181a-5p,miR-223-3p,miR-1434-3p,miR-7862,miR-2284x,miR-16-1-3p,miR-193b-3p,miR-17-3p,miR-326-3p,miR-2284w,miR-323c,miR-147-5p,miR-199c,miR-206,novel_1,miR-320b,miR-26b-5p,miR-669,novel_69,miR-374c-3p,miR-299b-3p,miR-3970,miR-3065-5p,miR-378b,miR-3591-3p,miR-216a-3p,miR-2319a,miR-146b-5p,miR-6516-3p,miR-135b-5p,miR-19a-3p,miR-146a-5p,miR-29b-1-5p,miR-219b-5p,miR-216a-5p,miR-500a-5p,miR-345-3p,miR-221-5p,miR-299-3p,miR-2284m,miR-548e-3p,miR-7859,miR-383-5p,miR-200b,miR-340-5p,miR-2904,miR-1434-5p,miR-5703,novel_133,miR-2366,miR-16-1-3p,miR-378d,miR-19b-3p,miR-1271,miR-2330-3p,miR-181c-5p,miR-224-5p,miR-206-
ASAH1	ENSOARG0(	N-acylsp	miR-499a-5p,novel_1,miR-656-5p,miR-147-5p,novel_82,miR-4324,miR-502-5p,miR-371a-5p,novel_111,miR-342-3p,miR-17-3p,miR-376a-3p,miR-493-5p,miR-6516-3p,miR-361-3p,miR-1306,miR-3970,miR-18a-3p,miR-376b-3p,miR-670-3p,miR-203-3p,miR-677,miR-27a-3p,miR-4443,miR-181b-3p,miR-376d,miR-1185-2-3p,miR-139-5p,miR-216b-3p,miR-20a-3p,miR-7689-3p,miR-125a-5p,miR-466f-3p,miR-214-3p,miR-342,miR-431-5p,novel_103,miR-1306-5p,miR-365b-5p,miR-2957,novel_51,miR-758-3p,miR-345-5p,miR-493-5p,miR-628-5p,miR-652-5p,miR-33a-5p,miR-1388-5p,miR-1260b,miR-27b-3p,miR-484,miR-22-3p,miR-431-3p,miR-758-3p,miR-376b-3p,miR-148b-5p,miR-1185-3p,novel_4,novel_78,miR-409b,miR-27a-3p,miR-17-3p,miR-125b-5p,miR-26c,miR-665,miR-1260b,miR-365a-5p,miR-3184-3p,miR-6128,miR-214,miR-345-5p,miR-345-5p,miR-625-5p,miR-376e-3p,miR-139-5p,miR-223-5p,miR-22-3p,miR-376c-3p,miR-1277-3p,miR-874-3p,miR-125a,miR-17-3p,miR-466i-5p,miR-200c-3p,novel_127,miR-21-3p,miR-376b-3p,miR-1260a,miR-200c,miR-6119-5p,miR-219b-5p,miR-30f,miR-125b,miR-185-3p,miR-376c-3p,miR-181b-1-3p,miR-2424,miR-490-3p,miR-181a-2-3p,miR-200b-3p,miR-148a-5p,miR-1434-5p,miR-140-3p,miR-200b,miR-545-5p,miR-2284h-5p,miR-214-5p,miR-18a-3p,miR-429-3p,miR-431

CPEB2	ENSOARG0(	cytoplas	miR-503-3p, miR-543-3p, miR-32-5p, miR-15b-5p, miR-300-3p, miR-544-3p, miR-499b-3p, miR-19b-1-5p, miR-208b-5p, miR-424-5p, miR-669a-3p, miR-486b-3p, novel_74, miR-6516-3p, miR-16-5p, miR-590-3p, miR-224-5p, miR-501-3p, miR-2284u, miR-412, miR-2285ad, novel_121, miR-9-5p, miR-322-5p, miR-15a-5p, miR-543-5p, miR-3529-3p, miR-486-3p, miR-2284a, miR-450b-5p, miR-125b-2-3p, miR-142a-3p, miR-544-5p, miR-195a-5p, miR-136-5p, miR-668-5p, miR-2285u, miR-20a-3p, miR-500-3p, miR-1185-2-3p, miR-499a-3p, miR-7857-3p, novel_69, miR-16a, miR-2285n, miR-4324, miR-15a, miR-3964, miR-452-5p, miR-9-5p, miR-363-3p, miR-502-3p, miR-412-3p, miR-501-3p, miR-374b-3p, miR-432, miR-653, miR-19b-2-5p, miR-21b, miR-497-5p, miR-16b, miR-544a, novel_116, miR-544b, miR-432-5p, miR-142-3p, miR-32, miR-16b, miR-224-5p, miR-652-5p, miR-2284b, miR-424-5p, miR-3187-3p, let-7j, miR-491-3p
DUPD1	ENSOARG0(	dual spe	miR-139-5p, miR-361-3p, miR-2387, miR-1306, miR-107, miR-877-3p, miR-195a-5p, miR-505-3p, miR-9-5p, novel_111, miR-24-3p, miR-15a, miR-16a, miR-33a-3p, miR-2285t, miR-656-5p, miR-103a-3p, miR-425-3p, miR-149-5p, miR-455-3p, miR-421, miR-103, miR-16b, miR-2426, miR-497-5p, miR-424-5p, miR-432-3p, miR-4532, miR-224-5p, miR-1306-5p, miR-29b-1-5p, miR-16b, novel_51, miR-452-5p, miR-125a-5p, miR-99a-3p, miR-125a, miR-615-5p, miR-139-5p, miR-365a-3p, miR-133c, miR-15b-5p, miR-500a-3p, miR-625-5p, miR-186-5p, miR-671-5p, miR-3184-3p, miR-145a-3p, miR-16-5p, miR-224-5p, miR-338-3p, miR-99b-3p, miR-331-3p, miR-125b-5p, miR-100-3p, miR-29b-2-5p, miR-665, miR-500, miR-424-5p, miR-486b-3p, miR-6517, miR-505-3p, miR-322-5p, miR-9-5p, miR-15a-5p, miR-2904, miR-1193, miR-216c-5p, miR-29b-2-5p, miR-4286, miR-2403, miR-29b-1-5p, miR-130a-5p, miR-486-3p, miR-8117, miR-125b, miR-99a-3p, miR-107, miR-543-5p
LCOR	ENSOARG0(	ligand d	miR-196a-5p, miR-330-5p, miR-501-3p, let-7a-2-3p, miR-1271-5p, miR-6516-5p, miR-199a-3p, miR-545-5p, miR-6119-5p, miR-466i-5p, miR-181b-5p, miR-29b-2-5p, miR-142a-3p, miR-181b-1-3p, miR-28b, miR-3154, miR-500a-3p, let-7g-3p, let-7g-3p, miR-876-3p, miR-186-5p, miR-543-3p, miR-140-5p, miR-552-3p, miR-216b-5p, miR-139-5p, miR-6516, miR-135a-5p, miR-29b-2-5p, miR-338-5p, miR-6516-3p, miR-500, miR-1271, miR-181c-5p, miR-181a-5p, miR-484, miR-501-3p, miR-219a-1-3p, miR-450b-5p, miR-27b-3p, miR-27a-3p, miR-26b-3p, miR-539-5p, miR-142-3p, miR-2355-3p, miR-326, miR-1306-5p, miR-199a-3p, miR-196b-5p, miR-101b-3p, miR-494-3p, let-7a-2-3p, miR-8485, miR-3065-5p, miR-150-5p, miR-1306, miR-96-5p, miR-135b-5p, miR-9-3p, miR-4792, miR-139-5p, miR-3120-3p, miR-320b, miR-27a-3p, miR-500-3p, miR-181b-3p, miR-677, miR-28c, miR-2440, novel_87, miR-181d-5p, miR-2285m, miR-326-3p, miR-9-3p, miR-502-3p

SCRN1	ENSOARG0(	secernin	miR-6517,miR-299a-3p,miR-2411-5p,miR-24-2-5p,miR-505-5p,miR-214-5p,miR-3578,miR-1343-5p,miR-128-3p,miR-4286,miR-450b-5p,miR-1271-3p,miR-6119-5p,miR-1260a,miR-205-5p,novel_127,miR-152-3p,miR-331-5p,miR-2448-3p,miR-23b,miR-3154,miR-329a,miR-329b,miR-10b-3p,miR-671-5p,miR-7144-5p,miR-1306-5p,miR-6128,miR-1260b,miR-2433,miR-331-3p,miR-6740-5p,miR-331-5p,miR-28-5p,miR-592,miR-31-5p,miR-185-5p,miR-432-5p,miR-1843b-5p,miR-1285,miR-1434-3p,miR-19b-2-5p,miR-432,miR-3071-5p,miR-194-5p,miR-22-3p,miR-1260b,miR-30b-3p,miR-27b-3p,miR-363-5p,miR-654-3p,miR-296-3p,miR-377-3p,miR-296-3p,miR-224-5p,miR-194a,novel_51,miR-3141,miR-193a-3p,miR-660,miR-1343-5p,miR-3074-5p,novel_68,miR-299b-3p,miR-181b-3p,miR-677,miR-3141,miR-148a-3p,miR-216a-3p,miR-18a-3p,miR-342-3p,miR-542-5p,miR-193b-3p,miR-1307-3p,miR-2428,novel_27,miR-133a-5p,miR-502-5p,miR-1247-3p,miR-485-3p,miR-6119-3p,miR-147-5p,miR-18a-3p,miR-3960,miR-23c,miR-148b-3p,miR-383-5p,miR-23b-3p,miR-2284h-5p,miR-532-3p,miR-362-3p,miR-1197-5p,miR-2904,miR-378d,miR-218-1-3p,miR-2285w,miR-345-3p,miR-181b-1-3p,miR-2411,miR-542-5p,miR-2285p,miR-92a-1-5p,miR-324-3p,miR-760-3p,miR-299-3p,miR-660-5p,miR-32-3p,miR-140-5p,miR-296-3p,miR-193b-3p,miR-377-3p,miR-2284s,novel_25,novel_32,miR-22-3p,miR-2285c,miR-411,miR-129b-3p,miR-3184-3p,miR-3074-5p,miR-539-5p,miR-214,miR-665-5p,miR-224-5p,miR-19b-1-5p,miR-664a,miR-708-5p,miR-129-5p,novel_78,miR-27a-3p,miR-181b-2-3p,miR-147a,miR-421,miR-2285e,miR-2459,miR-9788-3p,miR-541,miR-329-3p,miR-30b-3p,miR-1388-5p,miR-362-3p,miR-23a-3p,miR-3187-3p,miR-8485,miR-342,miR-362-3p,miR-1185-5p,miR-542-5p,miR-214-3p,miR-27a-3p,miR-203-3p,miR-140-5p,novel_94,miR-541-3p,miR-32-3p,miR-3059-5p,miR-194b-

GPR50	ENSOARG0(	melatoni	miR-2285f,miR-382-3p,miR-335,miR-10b,miR-2319b,miR-4792,miR-211-5p,miR-7977,miR-2483-3p,miR-3596,miR-378g,miR-27a-3p,miR-129b-5p,miR-668-5p,miR-136-5p,miR-493-5p,miR-101b-3p,miR-2330-5p,miR-382-3p,let-7c-3p,miR-30c-1-3p,novel_103,miR-182-5p,miR-362-3p,miR-2957,miR-25-5p,miR-491-5p,miR-542-5p,miR-409b,miR-27a-3p,miR-329-3p,miR-758-3p,miR-1a-3p,miR-101a-5p,miR-362-3p,miR-3120-5p,miR-21b,miR-3184-3p,miR-206-3p,miR-6525,miR-665,miR-664a-5p,miR-655-3p,miR-155-5p,miR-129-5p,miR-874-5p,miR-221-5p,miR-30c-1-3p,miR-182-5p,miR-3065-3p,miR-216b-3p,miR-216b-5p,miR-377-3p,miR-6516,miR-155-5p,miR-151b,miR-655,miR-2312,miR-133b-3p,let-7g-3p,miR-195a-3p,miR-421-5p,miR-376a-5p,miR-26a-5p,miR-542-5p,miR-125b,miR-324-3p,miR-21-3p,miR-221-5p,miR-548e-3p,miR-7859,miR-151-5p,miR-362-3p,miR-1197-5p,miR-5703,miR-140-3p,miR-218-1-3p,miR-133a-3p,miR-335-5p,miR-542-5p,miR-2285x,novel_101,miR-29c-3p,miR-24-3p,miR-211,miR-29a,miR-206,miR-6119-3p,miR-421-5p,miR-22-5p,miR-26b-5p,miR-29a-3p,miR-669,miR-6535,miR-2387,miR-2285ab,miR-493-5p,miR-432-3p,miR-654-3p,miR-377-3p,miR-125a-5p,miR-185-5p,miR-134-3p,miR-197-3p,miR-29b,miR-27b-3p,miR-103b,miR-133a-3p,miR-1197-3p,miR-29b-3p,miR-409-3p,miR-3969,miR-125b-5p,novel_9,miR-204-5p,miR-3068-3p,miR-125a,miR-331-5p,miR-329a,miR-10b-3p,miR-4286,miR-181a-2-3p,miR-1,miR-1271-3p,miR-490-3p,miR-3065-3p,miR-30c-2-3p,miR-127-5p,novel_96,miR-6517,miR-7641,miR-6516-5p,miR-302a-5p,miR-29d-3p,miR-155-5p
HEMK1	ENSOARG0(	HemK met	miR-2404,miR-25,miR-29a-3p,miR-324-5p,miR-204-3p,miR-452-3p,miR-3065-3p,miR-2355-5p,miR-7977,miR-3120-3p,miR-767,miR-450b-3p,miR-361-3p,miR-3154,miR-361-3p,miR-1260b,miR-1197-3p,miR-2447,miR-541-3p,miR-145a-3p,miR-1839-3p,miR-29c-3p,miR-767-5p,miR-29a,miR-6123,miR-29b-3p,miR-410-5p,miR-615,miR-324-3p,miR-3969,miR-425-5p,miR-505-5p,miR-1298-3p,miR-532-3p,miR-2285ad,novel_78,miR-29b,miR-1260b,miR-615-3p,miR-541,miR-29d-3p,miR-3065-3p,miR-3187-3p,miR-1839-3p,miR-181a-2-3p,miR-1260a,miR-210-5p,miR-324-3p,miR-185-3p,novel_51
VDAC2	ENSOARG0(	voltage-	miR-370-3p,miR-1271,miR-660-5p,miR-133b-5p,miR-134-5p,miR-1343-5p,miR-217-5p,miR-708-3p,miR-134,miR-503-3p,miR-625-5p,miR-671-5p,miR-329b,miR-16-1-3p,miR-17-3p,miR-217,miR-3604,miR-660,miR-1343-5p,novel_96,miR-134-5p,miR-17-3p,miR-491-5p,miR-17-3p

RXRB	ENSOARG0(	retinoid	miR-2428,miR-361-3p,miR-3956-3p,novel_60,miR-301a-5p,miR-16a,miR-101-3p,miR-6529b,miR-15a,miR-4443,miR-320b,miR-195a-5p,miR-1291,miR-18a-3p,miR-1306,miR-2387,miR-361-3p,miR-4532,miR-424-5p,miR-101b-3p,miR-130b-5p,miR-491-5p,miR-16b,miR-1306-5p,miR-9788-3p,miR-455-3p,miR-3431,miR-185-5p,miR-2300a-5p,miR-409b,miR-31-5p,miR-16-2-3p,miR-101c,miR-497-5p,miR-4508,miR-1260b,miR-16b,miR-197-3p,miR-412-3p,miR-542-3p,miR-301b-5p,miR-2113,miR-484,miR-6134,miR-1260b,miR-6525,miR-16-5p,miR-3184-3p,novel_42,miR-590-3p,miR-1306-5p,miR-130b-5p,miR-424-5p,miR-664a,miR-665,miR-6740-5p,miR-331-3p,miR-877-5p,miR-15b-5p,miR-3957,novel_32,miR-3065-3p,miR-874-3p,miR-221-5p,miR-3957-5p,miR-7144-5p,miR-23a-5p,miR-21a-3p,miR-490-3p,miR-3065-3p,miR-148b-5p,miR-376a-5p,novel_127,novel_96,miR-221-5p,miR-324-3p,miR-1260a,miR-532-3p,miR-15a-5p,miR-322-5p,miR-412,miR-3600,miR-6517,miR-18a-3p,miR-2300b-3p
BSG	ENSOARG0(	basigin	miR-376e-3p,miR-376c-3p,miR-874-3p,miR-361-3p,miR-18a-3p,miR-1197-3p,miR-6134,miR-214,miR-486b-3p,miR-6529b,novel_82,miR-2285n,miR-185-5p,miR-149-5p,miR-194-3p,miR-18a-3p,miR-26b-3p,miR-412,miR-7862,miR-193b-5p,miR-323b,miR-412-3p,miR-2904,miR-376c-3p,miR-486-3p,miR-652-5p,let-7c-3p,miR-191-3p,miR-214-3p,novel_127,miR-30b-3p,miR-185-3p,miR-323b-3p
GOLM1	ENSOARG0(	golgi me	let-7g-3p,miR-329a,miR-3074-1-3p,miR-671-5p,miR-485-5p,miR-320a,miR-34c-5p,miR-300-3p,miR-3959-5p,miR-769,miR-574-5p,miR-370-3p,miR-592,miR-28-5p,miR-370-5p,miR-323-3p,miR-582-3p,miR-128-3p,let-7a-2-3p,miR-3578,miR-302a-5p,miR-483-3p,miR-152-3p,novel_127,miR-2285af,miR-205-5p,miR-217,miR-28b,miR-29a-5p,miR-3064-5p,miR-29b-2-5p,miR-181a-2-3p,miR-1291,miR-9851-3p,miR-2285ab,miR-148a-3p,miR-1306,miR-3065-5p,miR-18a-3p,miR-216a-3p,miR-411b-3p,miR-34b,miR-7-1-3p,miR-3120-3p,miR-502b,miR-6402,miR-6529b,miR-28c,miR-34c,miR-33a-3p,miR-449a,novel_27,miR-2428,miR-2284w,miR-135a-2-3p,miR-342-3p,miR-495-3p,novel_101,miR-1193,miR-542-3p,miR-197-3p,miR-3071-5p,novel_23,miR-145a-5p,miR-2285g,miR-6238,miR-483-3p,miR-377-3p,let-7a-2-3p,miR-382,miR-668-3p,miR-363-5p,miR-101-5p,let-7g-3p,miR-2312,miR-1827,miR-21a-3p,miR-2285c,miR-377-3p,miR-6130,miR-877-5p,miR-2331-3p,miR-545-3p,miR-370-5p,miR-664a,miR-4492,miR-708-5p,miR-665,miR-29b-2-5p,miR-338-5p,miR-769-5p,miR-4510,miR-214,miR-4429,miR-34a-5p,miR-362-3p,miR-2904,miR-5703,miR-140-3p,miR-217-5p,miR-7859,miR-148b-3p,miR-2284h-5p,miR-18a-3p,miR-548e-3p,miR-34b-5p,miR-21-3p,miR-210-5p,miR-216a-5p,miR-3601,miR-500a-5p,miR-29b-1-5p,miR-125b-2-3p,miR-2432,miR-143-3p,miR-2285w,miR-9-3p,miR-3082-5p,miR-3064-5p,miR-20a-3p,miR-452-3p,miR-126b-5p,miR-499a-5p,novel_82,miR-2440,miR-769-5p,miR-371a-5p,miR-2285f,miR-219a-5p,miR-362-3p,miR-101a-5p,miR-329-3p,miR-216a-3p,miR-2285e,miR-421,miR-145b,miR-3085-3p,miR-214-3p,miR-342,miR-320b,miR-1306-5p,novel_103,miR-362-3p,miR-29b-1-5p,miR-652-5p,miR-744-3p

MPZL2	ENSOARG0(	myelin p	miR-124a,miR-382-3p,miR-2284b,miR-652-5p,miR-193a,miR-2310,miR-320b,miR-491-5p,miR-130b-3p,miR-487b-5p,miR-103a-2-5p,miR-330-3p,miR-27a-3p,miR-145b,miR-425-3p,miR-539-5p,miR-216a-3p,miR-148b-5p,miR-455-3p,miR-296-5p,miR-301b-5p,miR-544a,miR-16-2-3p,miR-3059-5p,miR-2447,miR-212-3p,miR-194b-5p,miR-877-3p,miR-2285n,miR-107-5p,miR-301a-5p,miR-2284g,miR-139-5p,miR-2319b,miR-126b-5p,miR-3596,miR-27a-3p,miR-335-3p,miR-664b-3p,miR-130a-5p,miR-3601,miR-500a-5p,miR-185-3p,miR-320d,miR-466i-5p,miR-30b-3p,miR-548e-3p,miR-3600,miR-485-3p,miR-487a-3p,miR-200b,miR-2904,miR-124-3p,miR-1197-5p,miR-5703,miR-1193,miR-4429,novel_73,miR-16-1-3p,miR-2284u,miR-590-3p,miR-2330-3p,miR-6525,miR-122-5p,miR-6134,miR-19b-1-5p,miR-320e,novel_74,miR-155-5p,miR-487b-5p,miR-17-3p,miR-708-5p,miR-454-3p,miR-140-5p,miR-548o-3p,miR-370-5p,miR-544-3p,miR-301a-3p,miR-139-5p,miR-204-3p,miR-155-5p,miR-877-5p,miR-655,miR-30d-5p,miR-345-5p,miR-625-5p,novel_99,miR-6240,miR-628-5p,miR-539-3p,miR-503-3p,miR-345-5p,miR-330-3p,miR-194a,miR-665,miR-130a-3p,miR-2284d,miR-149-5p,miR-6238,miR-194-3p,miR-544b,miR-873a-5p,miR-19b-2-5p,miR-1434-3p,miR-197-3p,novel_23,miR-194-5p,miR-3071-5p,miR-145a-5p,miR-21-5p,miR-2284ab,miR-16-1-3p,miR-26a-2-3p,miR-27b-3p,miR-17-3p,miR-495-3p,miR-1193,miR-2285x,miR-323c,novel_27,miR-301b-3p,miR-2428,miR-1247-3p,miR-576-3p,miR-379-3p,miR-147-5p,miR-378b,miR-146b-3p,miR-320c,miR-2387,miR-21c,miR-216a-3p,miR-2319a,miR-2403,miR-181a-2-3p,novel_39,miR-29a-5p,miR-2284y,miR-2284a,miR-2285b,miR-125a-3p,miR-200c,miR-6119-5p,miR-30f,miR-17-3p,miR-6536,miR-200c-3p,novel_96,miR-429-3p,miR-30a-5p,miR-411-3p,miR-200b-3p,miR-155-5p,miR-128-3p,miR-2284v,miR-3607-3p,miR-

SLC25A34	ENSOARG0(	solute c	miR-128-1-5p,miR-199c,miR-28c,miR-133a-5p,miR-326-3p,miR-2284w,miR-135a-2-3p,miR-335-5p,miR-758-5p,miR-1291,miR-19a-3p,miR-107,miR-6395,miR-216a-3p,miR-378b,miR-670-3p,miR-669,novel_68,miR-320b,miR-196b-5p,miR-1343-5p,miR-7-5p,miR-105-5p,miR-330-3p,novel_51,miR-6240,miR-363-5p,miR-138,miR-4508,miR-7862,miR-432,miR-7b-5p,miR-22-3p,miR-450c-3p,novel_23,miR-145a-5p,miR-432-5p,miR-412-5p,miR-134-3p,miR-3431,miR-185-5p,miR-411-5p,miR-149-5p,miR-103,miR-103a-3p,miR-138-5p,miR-28-5p,miR-134-5p,miR-486b-3p,miR-410-5p,miR-2433,miR-331-3p,miR-1197-3p,miR-10b-3p,miR-193a-5p,miR-2474,miR-7144-5p,miR-23a-5p,miR-485-5p,miR-3154,miR-365a-3p,miR-154b-5p,miR-574-5p,miR-2355-5p,miR-615-5p,miR-2898,miR-107,novel_96,miR-30c-2-3p,miR-28b,novel_39,miR-3956-5p,miR-490-3p,miR-105-2,miR-128-3p,miR-196a-5p,miR-134,miR-2427,miR-1343-5p,miR-330-5p,miR-505-5p,miR-9-5p,miR-302a-5p,miR-450a-1-3p,miR-134-5p,miR-582-3p,miR-545-5p,miR-412,miR-335,miR-2285t,miR-3964,miR-2440,miR-301a-5p,miR-877-3p,miR-423-5p,miR-541-3p,miR-1296-5p,miR-9-5p,miR-504,miR-361-3p,miR-378g,miR-3596,miR-3082-5p,miR-4443,miR-7977,miR-491-5p,miR-1185-5p,miR-25-5p,miR-326,miR-30c-1-3p,miR-376b,miR-2426,miR-450b-5p,miR-101c,miR-16-2-3p,miR-3120-5p,miR-296-5p,miR-301b-5p,miR-541,miR-143-5p,miR-412-3p,miR-758-3p,miR-147a,miR-148b-5p,miR-199b-3p,miR-105-1,novel_78,miR-2300a-5p,miR-1343-3p,miR-330-3p,miR-145b,miR-450b-3p,miR-664a,miR-487a-5p,miR-708-5p,miR-874-5p,miR-4492,miR-664a-5p,miR-665,miR-3184-5p,miR-122-5p,miR-6134,miR-145a-3p,miR-4510,miR-665-5p,miR-2312,novel_99,miR-450a-1-3p,miR-129b-3p,miR-6130,miR-22-3p,miR-2331-3p,miR-504-5p,miR-30c-1-3p,novel_107,miR-296-3p,miR-552-3p,miR-760-3p,miR-30b-3p,miR-2285p,miR-92a-1-
CRAT	ENSOARG0(	carnitin	miR-124-3p,miR-378a-3p,miR-378b,miR-378i,miR-378c,miR-219a-1-3p,novel_78,novel_4,miR-2300a-5p,miR-194-3p,miR-214-5p,miR-134-3p,miR-378h,miR-503-5p,miR-185-3p,miR-422a,miR-503-5p,miR-196a-3p,miR-210-5p,miR-124a,miR-503-5p,novel_39,miR-378d,miR-488-3p,miR-7144-5p,miR-378f,miR-1291,miR-370-3p,miR-378a-3p,miR-669,miR-378g,miR-378e,miR-6130,miR-1973,miR-320e,miR-665,miR-378c,miR-4510,miR-378c

NCSTN	ENSOARG0(	nicastri	miR-339-5p,miR-194-3p,miR-149-5p,miR-19b-2-5p,miR-7b-5p,miR-1843b-5p,miR-542-3p,miR-450c-3p,miR-22-3p,miR-197-3p,miR-27b-3p,miR-2285r,miR-432-3p,miR-432-3p,miR-7-5p,miR-377-3p,miR-296-3p,miR-105-5p,novel_51,miR-2411-3p,miR-483-3p,novel_68,miR-6535,miR-669,miR-320c,miR-3591-3p,miR-576-3p,miR-485-3p,novel_87,miR-412,miR-1224-5p,novel_79,miR-18b-5p,miR-9-5p,miR-450a-1-3p,miR-129-1-3p,miR-3591-5p,miR-2427,miR-330-5p,novel_39,miR-2285b,miR-205-5p,miR-200a-3p,miR-99a-3p,miR-542-5p,miR-125a,miR-876-3p,miR-1248,novel_42,miR-1260b,miR-181c-3p,miR-125b-5p,miR-100-3p,miR-548w,miR-145b,miR-147a,miR-2459,miR-758-3p,miR-1839-5p,miR-574-3p,miR-744-3p,miR-8485,miR-214-3p,miR-24-1-5p,miR-139-5p,miR-4443,miR-140-5p,miR-181a-3p,miR-504,miR-136-5p,miR-2447,miR-3956-3p,miR-885-5p,miR-9-5p,miR-361-3p,novel_111,novel_82,miR-218-5p,miR-2440,miR-328-3p,miR-193b-5p,miR-33b-3p,miR-184-3p,miR-133a-3p,miR-34a-5p,miR-503-5p,miR-345-3p,miR-2285i,miR-664b-3p,miR-486-3p,novel_17,miR-3601,miR-324-3p,miR-92a-1-5p,miR-345-3p,miR-99a-3p,miR-2484,miR-504-5p,miR-30c-1-3p,miR-296-3p,miR-377-3p,miR-299,miR-450a-1-3p,miR-339b,miR-625-5p,miR-1895,miR-1827,miR-129-2-3p,miR-214,miR-6525,miR-769-5p,novel_44,miR-224-5p,novel_74,miR-487b-5p,miR-4492,miR-130b-5p,miR-133b-5p,miR-185-5p,miR-503-5p,miR-2355-3p,miR-1434-3p,miR-145a-5p,miR-138,miR-1260b,miR-455-5p,miR-130b-5p,miR-363-5p,miR-654-3p,miR-296-3p,miR-503-5p,miR-224-5p,miR-455-5p,miR-125a-5p,miR-466f-3p,miR-363,miR-323a-5p,miR-34b,miR-2387,miR-18b,miR-1291,miR-2285k,miR-6395,miR-342-3p,miR-3957-3p,novel_101,miR-1193,miR-2285x,miR-371b-3p,miR-24-3p,novel_27,miR-326-3p,miR-449a,miR-2428,miR-34c,novel_1,miR-24-2-5p,miR-483-3p,miR-345-3p,miR-7134-

SIRT3	ENSOARG0(	sirtuin	miR-378f,miR-146b-5p,miR-150-5p,miR-1306,miR-376b-5p,miR-6535,miR-26b-5p,miR-329-5p,novel_68,miR-4324,miR-199b-5p,miR-193b-3p,miR-497-5p,miR-16b,novel_23,miR-450c-3p,miR-22-3p,miR-1843b-5p,miR-7b-5p,miR-432,novel_116,miR-149-5p,miR-3431,miR-103a-3p,miR-31-5p,miR-193a-3p,novel_51,miR-2285v,miR-582,miR-146a,miR-7-5p,miR-628-5p,miR-493-5p,miR-6240,miR-1248,miR-20b-5p,miR-345-5p,miR-876-3p,miR-376c-5p,miR-93-5p,miR-301,miR-125a,miR-1271,miR-2355-5p,miR-874-3p,miR-3068-3p,miR-574-5p,miR-134-5p,miR-100-3p,miR-6740-5p,miR-125b-5p,miR-1197-3p,miR-1260b,miR-16-5p,miR-885-3p,miR-330-5p,miR-23b-5p,miR-1343-5p,miR-134-5p,miR-450a-1-3p,miR-505-5p,miR-9-5p,miR-322-5p,miR-1224-5p,miR-6517,miR-2285ad,novel_96,novel_127,miR-127-5p,miR-3071-3p,miR-2285b,miR-3956-5p,miR-3064-5p,miR-493-5p,miR-504,miR-500-3p,miR-3596,miR-211-5p,miR-24-1-5p,miR-490-5p,miR-3968,miR-499a-5p,miR-301a-5p,miR-2440,miR-218-5p,miR-423-5p,novel_111,miR-361-3p,miR-9-5p,miR-2447,miR-101c,miR-501-3p,miR-301b-5p,miR-541,miR-2284r,miR-455-3p,miR-148b-5p,miR-1298-3p,miR-548w,miR-199b-3p,miR-330-3p,miR-1343-3p,miR-214-3p,miR-199b-5p,miR-146b,miR-491-5p,miR-16b,miR-106b-5p,miR-199a-3p,miR-378d,miR-2284b,miR-124a,miR-885-3p,miR-625-5p,miR-345-5p,miR-433-3p,miR-598-3p,miR-450a-1-3p,miR-15b-5p,miR-151b,miR-296-3p,miR-216b-3p,miR-2331-3p,miR-504-5p,miR-4492,miR-874-5p,miR-129-5p,miR-665,miR-122-5p,miR-3184-5p,miR-6525,miR-214,miR-34a-5p,miR-148a-5p,miR-133a-3p,miR-33b-3p,miR-142-3p,miR-378a-3p,miR-193b-5p,miR-378i,miR-210-3p,miR-106a,miR-18a-3p,miR-422a,miR-92a-1-5p,miR-3601,miR-500a-5p,miR-324-3p,miR-210-5p,novel_17,miR-26a-5p,miR-486-3p,miR-142a-3p,miR-421-5p,miR-503-5p,miR-379-5p,miR-195a-5p,miR-6516-

CNTN6	ENSOARG0(	contacti	miR-10a-5p,miR-329-5p,miR-144-3p,miR-29a-3p,miR-670-3p,miR-2404,miR-376b-3p,miR-21c,miR-216a-3p,miR-3591-3p,miR-3970,miR-150-5p,miR-1306,miR-18b,miR-148a-3p,miR-2285ab,miR-6516-3p,miR-146b-5p,miR-19a-3p,miR-410-5p,novel_101,miR-17-3p,miR-29c-3p,miR-301b-3p,miR-2284w,novel_27,miR-211,novel_87,miR-382-5p,miR-6402,miR-3613,miR-502-5p,miR-4324,miR-502b,miR-147-5p,miR-421-5p,novel_1,miR-29a,miR-130a-3p,miR-340-3p,miR-2355-3p,miR-544b,novel_116,miR-412-5p,miR-432-5p,miR-3071-5p,novel_23,miR-432,miR-27b-3p,miR-2889,miR-455-5p,miR-2284x,miR-26a-2-3p,miR-125b-1-3p,miR-1260b,miR-21-5p,miR-29b,miR-628-5p,miR-382,miR-2483-5p,miR-493-5p,miR-6240,miR-130b-5p,miR-503-3p,miR-2411-3p,miR-455-5p,miR-330-3p,miR-365b-5p,miR-146a,miR-224-5p,miR-660,miR-452-5p,miR-141-3p,miR-223-5p,miR-374c-3p,miR-18a,miR-503-3p,miR-301,miR-1814c,miR-3074-1-3p,miR-144-5p,miR-28a-3p,miR-365a-5p,miR-1197-3p,miR-1260b,miR-144,miR-5100,miR-29b-3p,miR-34c-3p,miR-204-5p,miR-134-5p,miR-592,miR-18b-5p,miR-582-3p,miR-134-5p,miR-545-5p,miR-758-5p,miR-1271-5p,miR-6516-5p,miR-24-2-5p,miR-9-5p,miR-505-5p,miR-501-3p,miR-29d-3p,miR-3578,miR-134,miR-345-3p,miR-10a-5p,miR-128-3p,miR-582-3p,miR-181a-2-3p,miR-29b-2-5p,miR-30a-3p,miR-3065-3p,miR-200a,miR-450b-5p,miR-217,miR-376b-3p,miR-1260a,miR-200a-3p,miR-152-3p,miR-6536,miR-127-5p,miR-28-3p,miR-17-3p,miR-211-5p,miR-20a-3p,miR-452-3p,miR-139-5p,miR-500-3p,miR-27a-3p,miR-376d,miR-7857-3p,miR-378j,miR-129b-5p,novel_94,miR-376a-3p,miR-335-3p,miR-493-5p,miR-96-5p,miR-9-5p,miR-2285f,miR-885-5p,miR-154a,miR-1285-5p,miR-502-3p,novel_111,miR-877-3p,miR-101-3p,miR-2440,miR-218-5p,miR-2285t,miR-3968,miR-4726-5p,miR-27a-3p,miR-
VANGL2	ENSOARG0(	VANGL pl	miR-138-5p,miR-28-5p,miR-874-5p,miR-708-5p,miR-5100,miR-2433,miR-664a-5p,miR-665,miR-125b-5p,miR-1197-3p,miR-6525,miR-145-3p,miR-365a-5p,miR-3074-5p,miR-4510,miR-539-5p,miR-214,miR-1271,let-7g-3p,let-7g-3p,miR-485-5p,miR-885-3p,miR-3154,miR-6130,miR-193b-3p,miR-125a,miR-3956,miR-615-5p,miR-760-3p,miR-450a-2-3p,miR-21-3p,novel_96,novel_17,miR-542-5p,miR-26a-5p,miR-2285af,miR-125b,miR-185-3p,miR-500a-5p,miR-142a-3p,miR-490-3p,miR-3956-5p,miR-143-3p,miR-2285b,miR-2411,miR-146a-5p,miR-885-3p,let-7a-2-3p,miR-193b-5p,miR-140-3p,miR-1193,miR-5703,miR-1343-5p,miR-2411-5p,miR-1271-5p,miR-6517,miR-6123,miR-361-3p,miR-542-5p,miR-193b-3p,miR-6527,miR-96-5p,miR-146b-5p,miR-361-3p,miR-1306,novel_69,miR-6535,miR-3074-5p,miR-378b,miR-4792,novel_68,miR-26b-5p,miR-124-5p,miR-491-5p,miR-214-3p,miR-193a-3p,miR-542-5p,miR-146b,miR-452-5p,miR-1343-5p,miR-125a-5p,miR-146a,novel_103,miR-1306-5p,miR-365b-5p,miR-432-3p,let-7a-2-3p,miR-138,miR-30b-3p,miR-4508,miR-873a-5p,miR-708-3p,miR-296-5p,miR-30b-3p,miR-3962,miR-142-3p,miR-185-5p,miR-147a,miR-9788-3p,novel_4

C1QTNF7	ENSOARG0(	C1q and	miR-668-3p,miR-2483-5p,miR-345-5p,miR-432-3p,miR-432-3p,novel_51,miR-2411-3p,miR-455-5p,miR-452-5p,miR-466f-3p,miR-124-5p,miR-2285l,miR-187-3p,miR-31-5p,miR-194-3p,miR-2355-3p,miR-185-5p,miR-3431,novel_23,miR-145a-5p,miR-653,miR-542-3p,miR-455-5p,miR-2284x,miR-30b-3p,miR-219a-1-3p,miR-1260b,miR-219b-3p,miR-1a-1-5p,novel_27,miR-2428,miR-199b-5p,miR-24-3p,miR-371b-3p,miR-1247-3p,miR-656-5p,miR-625-3p,miR-199c,novel_1,miR-3120-3p,miR-144-3p,miR-320c,miR-669,miR-374c-3p,miR-299b-3p,miR-18a-3p,miR-6516-3p,miR-29b-2-5p,miR-199a-5p,novel_39,miR-30c-2-3p,miR-1260a,miR-2285af,miR-200c,miR-219-3p,miR-543-5p,novel_127,novel_96,miR-200c-3p,miR-1a-2-5p,miR-299a-3p,miR-429-3p,miR-134-5p,miR-2284z,miR-6516-5p,miR-345-3p,miR-3591-5p,miR-134,miR-1290,miR-200b-3p,miR-496,miR-6128,miR-3607-3p,miR-1306-5p,miR-323-3p,miR-1260b,miR-144,miR-1-5p,miR-2284k,miR-486b-3p,miR-134-5p,miR-331-5p,miR-487a-3p,miR-503-3p,miR-199a-5p,miR-331-5p,miR-200a-5p,miR-3154,miR-320a,miR-331-5p,miR-503-3p,miR-10b-3p,miR-345-5p,miR-1388-3p,miR-101b-3p,miR-8485,miR-193a,miR-3187-3p,miR-30c-1-3p,miR-29b-1-5p,miR-2957,miR-320b,novel_115,miR-199b-5p,miR-153-3p,miR-491-5p,miR-3955-3p,miR-145b,miR-222-5p,miR-199b-3p,miR-2300a-5p,miR-455-3p,miR-9788-3p,miR-216a-3p,miR-126a-5p,miR-153,miR-30b-3p,miR-484,miR-296-5p,miR-1298-5p,miR-101c,miR-2284aa,miR-1388-5p,miR-2426,miR-101a-5p,miR-2284j,miR-2447,miR-885-5p,miR-219a-2-3p,miR-361-3p,novel_111,miR-371a-5p,miR-2285y,miR-2285t,miR-154-3p,miR-2284q,miR-541-5p,miR-139-5p,miR-10b,miR-2319b,miR-452-3p,miR-499a-3p,miR-361-3p,miR-199b-5p,miR-9-3p,miR-183-5p,miR-487a,miR-486-3p,miR-376a-5p,miR-29b-1-5p,miR-130a-5p,miR-

FUS	ENSOARG0(	FUS RNA	miR-329-3p,miR-362-3p,miR-21b,miR-101c,miR-330-3p,miR-145b,miR-199a-3p,miR-16b,miR-29b-1-5p,miR-214-3p,miR-2310,miR-8485,miR-668-5p,miR-299b-5p,miR-96-5p,miR-2319b,miR-2284g,miR-2285u,miR-144-5p,novel_82,miR-3956-3p,miR-2447,miR-2284j,miR-361-3p,miR-877-3p,miR-193b-5p,miR-2366,miR-493-3p,miR-2284u,miR-133a-3p,miR-212-5p,miR-328-3p,miR-2284h-5p,miR-1247-5p,miR-2285p,miR-324-3p,miR-2285w,miR-329a-5p,miR-345-3p,miR-376c-3p,miR-377-5p,miR-143-3p,miR-450a-1-3p,miR-299,miR-655,miR-598-3p,miR-339b,miR-543-3p,miR-548o-3p,miR-30c-1-3p,miR-216b-3p,miR-216b-5p,miR-377-3p,miR-582-5p,miR-10a,miR-376e-3p,miR-15b-5p,miR-2285j,miR-3604,miR-665,novel_74,miR-3074-5p,miR-129-2-3p,miR-214,miR-3958-5p,miR-224-5p,miR-1843b-5p,miR-19b-2-5p,miR-450c-3p,miR-197-3p,miR-16b,miR-188-5p,miR-2284x,miR-497-5p,miR-339-5p,miR-3535,miR-149-5p,miR-377-3p,miR-105-5p,miR-3141,miR-378a-5p,miR-6240,miR-2284l,let-7a-2-3p,miR-3141,miR-376b-5p,miR-3591-3p,miR-3970,miR-150-5p,miR-1306,miR-2284e,miR-410-5p,miR-216b-3p,miR-3074-5p,miR-329-5p,miR-411b-3p,miR-669,miR-320c,miR-6535,miR-142b,novel_87,miR-2284o,miR-379-3p,miR-29a,miR-6119-3p,miR-29c-3p,miR-2284w,miR-1290,miR-330-5p,novel_79,miR-322-5p,miR-129-1-3p,miR-450a-1-3p,miR-127-5p,miR-2284f,miR-450b-5p,miR-376c-5p,miR-329a,miR-503-3p,miR-3956,miR-300-3p,miR-376c-3p,miR-2284k,miR-2284v,miR-144-5p,miR-16-5p,miR-4791,miR-10b,miR-484,miR-30b-3p,miR-2300a-5p,miR-105-1,miR-409b,miR-216a-3p,miR-500-5p,miR-1306-5p,miR-326,miR-320b,miR-362-3p,miR-2957,miR-424-5p,miR-652-5p,miR-33a-5p,miR-30c-1-3p,miR-4532,miR-3187-3p,miR-499b-5p,miR-541-5p,miR-2285n,miR-4726-5p,miR-212-3p,novel_60,miR-3059-5p,miR-362-5p,miR-154a,miR-8095,miR-362-3p,miR-2408,miR-1197-5p,miR-

SH3GLB2	ENSOARG0(	SH3-doma	miR-200c-3p,miR-191-3p,miR-200c,miR-490-3p,miR-29a-5p,miR-2403,miR-3064-5p,miR-200b-3p,miR-615-3p,miR-330-5p,miR-2427,miR-6516-5p,miR-322-5p,miR-450a-1-3p,miR-2284z,miR-429-3p,miR-486b-3p,miR-28-5p,miR-410-5p,miR-125b-5p,miR-1197-3p,miR-365a-5p,miR-16-5p,miR-485-5p,miR-424-3p,miR-3959-5p,miR-324-5p,miR-874-3p,miR-574-5p,miR-125a,miR-125a-5p,miR-660,miR-365b-5p,miR-363-5p,miR-219a-1-3p,miR-16b,miR-30b-3p,miR-744-5p,miR-497-5p,miR-4508,miR-223-3p,miR-708-3p,miR-450c-3p,miR-134-3p,miR-544b,miR-26b-3p,miR-576-3p,miR-15a,miR-16a,miR-24-3p,miR-326-3p,miR-1291,miR-195a-5p,miR-1306,novel_69,miR-329-5p,novel_68,miR-320b,miR-196a-3p,miR-92a-1-5p,miR-185-3p,miR-125b,miR-324-3p,miR-130a-5p,miR-486-3p,miR-329a-5p,miR-2366,miR-1193,miR-1197-5p,miR-200b,miR-15a-5p,miR-616-3p,miR-151-5p,miR-450b-3p,miR-424-5p,miR-4492,miR-708-5p,miR-665,miR-338-3p,miR-4510,miR-582-5p,miR-450a-1-3p,miR-139-5p,miR-6130,miR-582-5p,miR-6516,miR-544-3p,miR-15b-5p,miR-151b,miR-204-3p,miR-660-5p,miR-2331-3p,miR-504-5p,miR-296-3p,miR-3085-3p,miR-326,miR-1306-5p,miR-16b,miR-4532,miR-424-5p,miR-2330-5p,miR-1388-3p,miR-544a,miR-3120-5p,miR-2284aa,miR-296-5p,miR-2284r,miR-484,miR-30b-3p,miR-147a,miR-425-3p,novel_4,novel_78,miR-615,miR-382-3p,novel_82,miR-877-3p,miR-2447,miR-452-5p,miR-136-5p,miR-504,novel_94,miR-3082-5p,miR-378g,miR-2319b,miR-4792,miR-3064-5p,miR-139-5p,miR-499b-5p,miR-7689-3p

SH2D5	ENSOARG0(	SH2 doma	miR-127-5p,novel_96,miR-152-3p,novel_127,miR-148b-5p,miR-450c-5p,miR-155-5p,miR-23b-5p,miR-1343-5p,miR-2427,miR-758-5p,miR-505-5p,miR-134-5p,miR-6517,miR-429-3p,miR-299a-3p,miR-2285ad,novel_79,miR-1224-5p,miR-134-5p,miR-2433,miR-125b-5p,miR-6740-5p,miR-345-5p,miR-2474,miR-300-3p,miR-874-3p,miR-503-3p,miR-125a,miR-2285aa,miR-2355-5p,miR-106b-3p,miR-296-3p,miR-7-5p,novel_51,miR-2411-3p,miR-3141,miR-432-3p,let-7a-2-3p,miR-380-5p,miR-628-5p,let-7f-2-3p,let-7f-2-3p,miR-1843b-5p,miR-7b-5p,miR-3431,miR-134-3p,miR-194-3p,miR-31-5p,miR-339-5p,miR-29a,miR-142b,miR-28c,miR-4324,miR-1247-3p,miR-199b-5p,miR-335-5p,miR-29c-3p,miR-378f,miR-3141,miR-148a-3p,miR-3591-3p,miR-3065-5p,miR-3970,miR-299b-3p,miR-6535,novel_17,miR-345-3p,miR-122-3p,miR-422a,miR-324-3p,miR-377-5p,miR-486-3p,miR-34a-5p,miR-133a-3p,miR-2366,miR-378i,miR-378a-3p,miR-18a-3p,miR-4492,miR-874-5p,miR-500,miR-2285j,miR-665,miR-214,miR-665-5p,miR-345-5p,miR-339b,miR-1827,miR-1895,miR-411,miR-129b-3p,miR-500a-3p,miR-10a,miR-30c-1-3p,miR-2331-3p,miR-504-5p,miR-491-5p,miR-214-3p,miR-199b-5p,miR-378d,miR-124a,miR-676-3p,miR-1388-5p,miR-2284r,miR-758-3p,miR-147a,miR-1185-3p,miR-2459,miR-455-3p,novel_78,miR-499a-5p,miR-2440,miR-877-3p,miR-361-3p,miR-2447,miR-504,miR-409-5p,miR-378g,miR-4443,miR-1185-2-3p,miR-2319b,miR-2285u,miR-200c-3p,miR-17-3p,miR-543-5p,miR-6119-5p,miR-200c,miR-339a,miR-30c-2-3p,miR-380-5p,miR-28b,miR-490-3p,miR-199a-5p,miR-3065-3p,miR-4286,miR-495-5p,miR-496,let-7a-2-3p,miR-10a-5p,miR-134,miR-345-3p,miR-218-2-3p,miR-29d-3p,miR-378h,miR-6516-5p,miR-214-5p,miR-582-3p,miR-7641,miR-138-5p,miR-28-5p,miR-486b-3p,miR-29b-3p,miR-551b-3p,miR-135a-5p,miR-3969,miR-26c,miR-378c,miR-1306-

RING1	ENSOARG0(	ring fin	miR-184-3p,miR-133a-3p,miR-378d,miR-34a-5p,miR-362-3p,miR-2904,miR-1197-5p,miR-140-3p,miR-1193,miR-5703,miR-532-3p,miR-151-5p,miR-760-3p,miR-34b-5p,miR-21-3p,miR-196a-3p,miR-185-3p,miR-376c-3p,miR-377-5p,miR-2411,miR-486-3p,miR-143-3p,miR-216c-5p,miR-5100,miR-2887,let-7g-3p,miR-133b-3p,miR-345-5p,miR-1895,miR-885-3p,miR-625-5p,novel_99,miR-1827,miR-21a-3p,miR-450a-1-3p,miR-129b-3p,miR-376e-3p,miR-544-3p,miR-151b,miR-30c-1-3p,miR-2331-3p,miR-370-5p,miR-552-3p,miR-450b-3p,miR-664a,miR-17-3p,miR-874-5p,miR-1468-5p,miR-208b-5p,miR-4492,miR-2285j,miR-324-3p,miR-338-5p,miR-665,miR-6525,miR-3184-5p,miR-6134,miR-338-3p,miR-3958-5p,miR-214,miR-2330-3p,miR-665-5p,miR-3074-2-3p,miR-362-3p,miR-544a,miR-2426,miR-101c,miR-329-3p,miR-296-5p,miR-6529a,miR-143-5p,miR-541,miR-4791,miR-501-3p,miR-412-3p,miR-7857,miR-125b-2-3p,miR-30b-3p,miR-2459,miR-105-1,miR-2300a-5p,novel_4,miR-1343-3p,miR-5010-3p,miR-3085-3p,novel_124,miR-491-5p,miR-103a-2-5p,miR-214-3p,miR-1468,miR-1306-5p,novel_103,miR-326,miR-362-3p,novel_19,miR-30c-1-3p,miR-4532,miR-491-3p,miR-1388-3p,let-7c-3p,miR-33a-5p,miR-136-5p,novel_94,miR-361-3p,miR-378g,miR-489,miR-4443,miR-500-3p,miR-4792,miR-3064-5p,miR-2319b,miR-452-3p,miR-211-5p,miR-7977,miR-4726-5p,miR-2285t,novel_82,miR-2285n,miR-1973,miR-107-5p,miR-361-3p,miR-423-5p,miR-502-3p,miR-3059-5p,miR-541-3p,miR-9-5p,miR-885-3p,miR-105-2,miR-1290,miR-1343-5p,miR-218-2-3p,miR-330-5p,miR-23b-5p,miR-501-3p,miR-9-5p,miR-505-5p,miR-24-2-5p,miR-758-5p,miR-2411-5p,miR-450a-1-3p,miR-412,miR-17-3p,miR-2898,miR-127-5p,miR-543-5p,novel_96,miR-1260a,miR-30c-2-3p,novel_39,miR-29a-5p,miR-2285b,miR-1271-3p,miR-3064-5p,miR-2385-3p,miR-

MRPL38	ENSOARG0(	mitochon	miR-432,miR-450c-3p,miR-22-3p,miR-1260b,miR-30b-3p,miR-138,miR-16b,miR-7862,miR-2889,miR-497-5p,miR-133b-5p,miR-26b-3p,miR-134-3p,miR-503-5p,miR-3431,miR-432-5p,miR-2355-3p,miR-149-5p,miR-503-5p,miR-2411-3p,miR-330-3p,miR-378a-5p,miR-125a-5p,miR-1343-5p,miR-6240,miR-363-5p,miR-101-5p,miR-432-3p,miR-654-3p,miR-2285r,miR-2387,miR-18a-3p,miR-150-5p,miR-1306,miR-1291,miR-195a-5p,miR-3074-5p,novel_68,miR-320b,miR-34b,miR-669,miR-4324,miR-15a,miR-382-5p,miR-34c,miR-16a,novel_1,miR-128-1-5p,miR-203b-5p,miR-421-5p,miR-335-5p,miR-1307-3p,miR-449a,miR-374c-5p,miR-345-3p,miR-30a-5p,miR-23b-5p,miR-1343-5p,miR-7134-5p,miR-885-3p,miR-6517,miR-431,miR-7641,miR-1224-5p,miR-505-5p,miR-322-5p,miR-24-2-5p,miR-214-5p,miR-450a-1-3p,miR-1260a,miR-30c-2-3p,novel_96,miR-543-5p,miR-200a-3p,novel_127,miR-4286,miR-3064-5p,miR-24-2-5p,miR-148b-5p,miR-1271-3p,miR-3065-3p,novel_39,miR-301,miR-30e-5p,miR-1248,miR-874-3p,miR-1271,miR-141-3p,miR-125a,miR-615-5p,miR-370-3p,miR-2355-5p,miR-365a-3p,miR-769,miR-34c-5p,miR-133c,miR-2332,miR-2433,miR-125b-5p,miR-138-5p,miR-486b-3p,miR-28-5p,miR-31-3p,miR-1306-5p,novel_42,miR-16-5p,miR-193a-5p,miR-1260b,miR-181c-3p,miR-301b-5p,miR-541,miR-296-5p,miR-6529a,miR-30b-3p,novel_78,miR-330-3p,miR-2300a-5p,miR-147a,miR-455-3p,novel_103,miR-1306-5p,miR-5126,miR-431-5p,miR-16b,miR-29b-1-5p,miR-25-5p,miR-491-5p,miR-3085-3p,miR-214-3p,miR-424-5p,miR-652-5p,miR-30c-1-3p,miR-3187-3p,miR-409-5p,miR-361-3p,miR-140-5p,miR-129b-5p,miR-147-3p,miR-181a-3p,miR-24-1-5p,miR-3064-5p,miR-7977,miR-7689-3p,miR-489,miR-378g,miR-4443,miR-652-3p,miR-301a-5p,miR-2285y,miR-769-5p,miR-335,miR-4726-5p,miR-541-3p,miR-361-3p,miR-877-3p,miR-193b-5p,novel_133,miR-1197-5p,miR-33b-
HNRNPLL	ENSOARG0(	heteroge	miR-541-5p,miR-7-1-3p,miR-20a-3p,miR-144-3p,miR-320c,miR-2483-3p,miR-3065-5p,miR-1983,miR-9-3p,miR-412-3p,miR-194b-5p,miR-1a-1-5p,miR-8095,miR-219a-2-3p,miR-371a-5p,miR-133a-5p,miR-576-3p,miR-490-5p,miR-625-3p,miR-330-3p,miR-151a-3p,miR-194-3p,miR-421,miR-412-5p,miR-194-5p,miR-708-3p,let-7c-3p,miR-376b,miR-6240,miR-539-3p,miR-1b-5p,miR-654-3p,miR-30c-1-3p,miR-330-3p,miR-194a,miR-320b,miR-224-5p,miR-466f-3p,miR-2285aa,miR-30c-1-3p,miR-2331-3p,miR-300-3p,miR-200a-5p,miR-129b-3p,miR-320a,miR-23a-5p,novel_99,let-7g-3p,miR-539-5p,miR-224-5p,miR-144,miR-151-3p,miR-3969,miR-1-5p,miR-487a-5p,novel_9,miR-485-3p,novel_121,miR-1246,miR-4429,miR-490-3p,miR-2285b,miR-125b-2-3p,miR-30c-2-3p,miR-216a-5p,miR-196a-3p,miR-2285p,miR-219-3p,miR-320d,miR-1a-2-5p
SLC28A3	ENSOARG0(	solute c	miR-9-3p,miR-142-3p,miR-4429,miR-320a,miR-503-3p,miR-2300b-3p,miR-486-5p,miR-320c,miR-222-5p,miR-411b-3p,miR-670-3p,miR-5010-3p,miR-3065-3p,miR-330-3p,novel_96,miR-147-5p,miR-625-3p,miR-491-5p,miR-330-3p,miR-320b,miR-2428,miR-3065-3p,miR-148b-5p,miR-668-3p,miR-494-3p,miR-6240

OXCT1	ENSOARG0(	3-oxoaci	miR-381-3p,miR-18a-3p,miR-378f,miR-9-3p,miR-379-5p,miR-2319a,miR-504,miR-3120-3p,miR-378b,miR-452-3p,miR-664b,miR-320c,miR-29a-3p,miR-2404,miR-378e,novel_87,miR-22-5p,miR-29a,novel_1,miR-29c-3p,miR-495-3p,miR-452-5p,miR-136-3p,novel_111,miR-323c,miR-378b,miR-708-3p,miR-296-5p,miR-299a-5p,miR-29b,miR-378c,miR-26b-3p,miR-330-3p,miR-9788-3p,miR-185-5p,novel_51,miR-182-5p,miR-330-3p,miR-320b,miR-466f-3p,miR-2285l,miR-491-5p,miR-1185-5p,miR-196b-5p,miR-6240,let-7j,miR-193a,miR-378d,miR-320a,miR-433-3p,miR-3154,miR-129b-3p,miR-299,miR-186-5p,miR-7144-5p,miR-141-3p,miR-182-5p,miR-3065-3p,miR-874-3p,miR-504-5p,miR-378a-3p,miR-6130,miR-216b-5p,miR-331-3p,let-7b-3p,miR-29b-3p,miR-487a-5p,miR-3607-3p,miR-4510,miR-378c,miR-378c,miR-98-3p,miR-6525,miR-378a-3p,miR-188-3p,miR-5703,miR-140-3p,miR-29d-3p,miR-98-3p,miR-3578,miR-196a-5p,miR-378i,miR-378d,miR-200b-3p,miR-4429,miR-493-3p,miR-429-3p,miR-548e-3p,miR-18a-3p,miR-214-5p,miR-532-3p,miR-2284z,miR-2284h-5p,miR-616-3p,miR-200b,miR-378h,miR-7859,miR-422a,miR-92a-1-5p,miR-219b-5p,miR-664-3p,miR-200c,miR-200a-3p,novel_96,novel_127,miR-200c-3p,miR-202-5p,miR-320d,miR-345-3p,miR-195a-3p,miR-3529-3p,miR-2285w,miR-1271-3p,miR-3065-3p,miR-200a,miR-2432
RFX6	ENSOARG0(	regulato	miR-194b-5p,miR-9-5p,miR-452-5p,miR-2285f,miR-212-3p,miR-3059-5p,miR-877-3p,miR-371a-5p,miR-101-3p,miR-2440,miR-30b-5p,miR-490-5p,miR-3958-3p,miR-7977,miR-2285u,miR-499b-5p,miR-27a-3p,miR-1185-2-3p,let-7d-5p,miR-203-3p,miR-335-3p,miR-136-5p,miR-299b-5p,miR-96-5p,miR-33a-5p,miR-382-3p,miR-124a,let-7a-5p,miR-758-3p,miR-2310,miR-182-5p,miR-505,miR-320b,let-7e,miR-153-3p,miR-27a-3p,miR-5010-3p,miR-548w,miR-2459,miR-1185-3p,miR-216a-3p,miR-153,miR-125b-2-3p,miR-758-3p,miR-4791,miR-544a,miR-129-2-3p,miR-539-5p,let-7b,miR-338-3p,let-7b-5p,miR-224-5p,miR-338-5p,miR-30a-3p,miR-30e-3p,miR-2285j,let-7f,miR-130b-5p,miR-208b-5p,miR-129-5p,miR-17-3p,miR-182-5p,let-7i-5p,miR-544-3p,miR-376e-3p,miR-27a-5p,miR-433-3p,miR-30d-5p,miR-21a-3p,novel_99,miR-339b,miR-30f,miR-2312,miR-487a,miR-376c-3p,miR-125b-2-3p,miR-125b,miR-500a-5p,miR-324-3p,miR-210-5p,miR-30b,let-7k,miR-1277-5p,miR-30d,miR-98-5p,miR-320d,miR-30d-3p,miR-222-5p,miR-200b,miR-1434-5p,miR-140-3p,miR-217-5p,miR-124-3p,miR-1961,miR-30c-5p,miR-664-5p,miR-17-3p,miR-29c-3p,novel_27,let-7i,miR-4324,miR-421-5p,miR-29a,miR-379-3p,miR-320c,miR-29a-3p,miR-378b,miR-2285ab,let-7e-5p,miR-410-5p,let-7f-2-3p,let-7f-2-3p,miR-130b-5p,miR-380-5p,miR-2284l,miR-654-3p,miR-194a,novel_51,miR-224-5p,miR-466f-3p,miR-125a-5p,miR-452-5p,miR-339-5p,miR-340-3p,miR-544b,let-7g-5p,miR-3431,miR-3535,miR-194-5p,miR-27b-3p,miR-7862,miR-29b,miR-193a-5p,miR-3607-3p,miR-28a-3p,miR-1197-3p,miR-409-3p,miR-125b-5p,miR-29b-3p,miR-125a,miR-141-3p,miR-487a-3p,miR-376c-3p,miR-320a,miR-137-3p,miR-2448-3p,miR-424-3p,miR-30e-5p,miR-485-5p,miR-1814c,novel_91,miR-30a-3p,miR-2385-3p,miR-192-3p,let-7g,miR-200a,miR-339a,miR-217,let-7c-5p,miR-30f,miR-200c,miR-2285af,miR-205-5p,miR-

TSPAN13	ENSOARG0(	tetraspa	miR-499b-3p,miR-2285aa,miR-30c-1-3p,novel_99,miR-190a-3p,miR-329a,miR-3154,miR-23b,let-7f-1-3p,miR-21a-3p,miR-181c-5p,miR-98-3p,miR-3074-2-3p,miR-665-5p,miR-130b-5p,miR-191-5p,miR-135a-5p,miR-3969,let-7b-3p,miR-1-5p,miR-200b,miR-23c,miR-302a-5p,miR-23b-3p,miR-383-5p,miR-7859,miR-429-3p,let-7a-3p,miR-200b-3p,miR-128-3p,miR-33b-3p,miR-98-3p,miR-218-2-3p,miR-362-3p,miR-3591-5p,miR-191,miR-146a-5p,miR-200a,miR-130a-5p,miR-376a-5p,miR-181a-2-3p,miR-195a-3p,miR-21-3p,miR-1a-2-5p,miR-200c-3p,miR-181b-5p,miR-30c-2-3p,miR-216a-5p,miR-200c,miR-664-3p,miR-1185-2-3p,miR-499a-3p,miR-499b-5p,miR-216b-3p,miR-378b,miR-664b,miR-135b-5p,miR-146b-5p,miR-3065-5p,miR-216a-3p,miR-323c,miR-452-5p,miR-181d-5p,miR-885-5p,miR-1a-1-5p,miR-219b-3p,miR-199c,miR-22-5p,novel_1,miR-371a-5p,miR-576-3p,miR-1185-3p,miR-411-5p,miR-9788-3p,miR-330-3p,miR-199b-3p,miR-362-3p,miR-125b-2-3p,miR-3071-5p,miR-197-3p,miR-181a-5p,miR-542-3p,miR-329-3p,miR-653,miR-503-3p,miR-8485,let-7j,miR-30c-1-3p,let-7f-2-3p,let-7f-2-3p,miR-23a-3p,miR-494-3p,miR-6240,miR-130b-5p,miR-146b,miR-362-3p,miR-330-3p,miR-146a
ZDHHC13	ENSOARG0(	zinc fin	miR-148a-5p,miR-2284ab,miR-1197-5p,miR-140-3p,miR-1a-3p,miR-873a-5p,miR-2285e,miR-455-3p,miR-148b-5p,miR-3431,miR-142-5p,miR-2285g,miR-2284d,miR-146b,miR-216a-5p,miR-3601,miR-146a,miR-2285af,miR-2284a,miR-146a-5p,miR-2284b,miR-1,miR-135b-5p,novel_99,miR-146b-5p,novel_48,miR-2312,miR-1306-3p,miR-200a-5p,miR-424-3p,miR-2285c,miR-2285ab,miR-204-3p,miR-3596,miR-27a-5p,miR-7-1-3p,miR-216b-3p,miR-2284q,miR-206,miR-135a-5p,miR-33a-3p,miR-1306-3p,miR-206-3p,miR-142a-5p,miR-3074-2-3p,miR-2285f,miR-2284v,miR-145a-3p,miR-2447
LPO	ENSOARG0(	lactoper	miR-3154,miR-625-5p,miR-1248,miR-671-5p,miR-485-5p,miR-2355-5p,miR-216b-3p,miR-2285aa,miR-532-5p,miR-615-5p,miR-574-5p,miR-548o-3p,miR-874-3p,miR-204-3p,novel_32,miR-223-5p,miR-22-3p,miR-34c-5p,miR-324-3p,miR-3969,miR-665,miR-874-5p,miR-3607-3p,novel_42,miR-3184-5p,miR-6134,miR-338-3p,miR-6525,miR-1197-3p,miR-1343-5p,miR-330-5p,miR-2366,miR-193b-5p,miR-128-3p,miR-34a-5p,miR-148a-5p,miR-2284u,miR-582-3p,miR-299a-3p,miR-548e-3p,novel_83,miR-7859,miR-324-3p,miR-34b-5p,miR-299-3p,miR-2898,miR-450a-2-3p,miR-760-3p,miR-127-5p,miR-345-3p,miR-4286,miR-2284y,miR-146a-5p,miR-664b-3p,miR-28b,miR-216a-3p,miR-361-3p,miR-146b-5p,miR-2284g,miR-203-3p,miR-677,miR-181b-3p,miR-378g,miR-299b-3p,novel_69,miR-489,miR-34b,miR-34c,miR-218-5p,miR-28c,miR-2440,miR-128-1-5p,miR-135a-2-3p,novel_60,miR-2447,miR-3956-3p,miR-449a,miR-326-3p,miR-423-5p,miR-22-3p,miR-3071-5p,miR-30b-3p,miR-484,miR-2113,miR-30b-3p,miR-181b-2-3p,novel_4,miR-1343-3p,miR-2300a-5p,miR-148b-5p,miR-9788-3p,miR-421,miR-134-3p,miR-185-5p,miR-146a,miR-326,miR-1343-5p,miR-146b,miR-378a-5p,miR-363-5p,miR-2284b,miR-2330-5p,miR-503-3p

TRIM65	ENSOARG0(	triparti	novel_133,miR-1343-5p,miR-188-3p,miR-1197-5p,miR-5703,miR-941,miR-133a-3p,let-7a-2-3p,miR-378d,miR-299a-3p,miR-106a,novel_79,miR-302a-5p,miR-1271-5p,miR-6516-5p,miR-2284h-5p,miR-582-3p,miR-486-5p,miR-2285af,miR-6119-5p,novel_17,miR-2285p,miR-92a-1-5p,miR-339a,miR-106a,miR-760-3p,miR-299-3p,novel_96,miR-183-5p,miR-2285w,miR-20a,miR-1,miR-200a,miR-146a-5p,miR-450c-5p,miR-301,miR-93-5p,miR-17-5p,miR-339b,let-7g-3p,miR-345-5p,miR-345-5p,miR-20b-5p,miR-625-5p,miR-574-5p,miR-874-3p,miR-548o-3p,miR-370-3p,miR-2355-5p,miR-296-3p,miR-6516,miR-300-3p,miR-6130,miR-154b-5p,miR-144,miR-409-3p,miR-708-5p,miR-28-5p,let-7e-3p,miR-145a-3p,miR-4510,miR-3184-3p,miR-6128,miR-1271,miR-665-5p,miR-93,miR-1197-3p,miR-206-3p,miR-122-5p,miR-2284r,miR-1a-3p,miR-145a-5p,miR-187-3p,miR-2300a-5p,miR-339-5p,miR-26b-3p,miR-145b,miR-3431,novel_116,miR-20b,miR-1306-5p,miR-146a,miR-106a-5p,novel_51,miR-106b-5p,miR-17-5p,miR-491-5p,miR-146b,miR-1343-5p,miR-101b-3p,miR-106a-5p,let-7c-3p,miR-744-3p,miR-345-5p,let-7a-2-3p,miR-361-3p,miR-1306,miR-96-5p,miR-9851-3p,miR-146b-5p,miR-2319b,miR-299b-3p,miR-670-3p,miR-144-3p,miR-4443,novel_82,miR-20a-5p,miR-371a-5p,miR-499a-5p,miR-206,novel_1,miR-450a-5p,miR-2284q,miR-877-3p,miR-3959-3p,miR-371b-3p
RALY	ENSOARG0(	RALY het	miR-8485,miR-3187-3p,miR-345-5p,miR-2330-5p,miR-101b-3p,miR-3085-3p,miR-197-5p,miR-505,novel_51,miR-326,miR-500-5p,miR-9788-3p,miR-455-3p,miR-1185-3p,miR-134-3p,miR-3431,miR-133b-5p,miR-2300a-5p,miR-1343-3p,miR-2284x,miR-7862,miR-138,miR-873a-5p,miR-301b-5p,miR-326-3p,miR-877-3p,miR-361-3p,miR-362-5p,novel_111,miR-495-3p,miR-1193,miR-135a-2-3p,miR-3059-5p,miR-2447,miR-335-5p,miR-206,miR-335,miR-301a-5p,miR-2285n,miR-101-3p,miR-576-3p,miR-669,miR-1185-2-3p,miR-378g,miR-670-3p,miR-2285u,miR-499b-5p,miR-3064-5p,miR-3074-5p,miR-9851-3p,miR-1291,miR-18a-3p,miR-18b,miR-148a-3p,miR-2411,novel_39,miR-1271-3p,miR-3064-5p,miR-2887,miR-543-5p,miR-152-3p,novel_127,miR-450a-2-3p,miR-2285af,miR-210-5p,miR-148b-3p,miR-2411-5p,miR-18b-5p,miR-18a-3p,miR-218-1-3p,miR-378d,miR-330-5p,miR-23b-5p,miR-6134,miR-206-3p,miR-4510,miR-3074-5p,miR-874-5p,miR-138-5p,miR-767-5p,miR-324-3p,miR-2433,miR-370-5p,miR-204-3p,miR-6130,miR-216b-5p,miR-370-3p,miR-370-5p,miR-1271,miR-23a-5p,miR-1827,miR-767,miR-18a-5p,miR-345-5p,miR-345-5p,miR-362-5p,miR-18a,miR-3154

MAMLD1	ENSOARG0(	mastermi	miR-345-3p,miR-127-5p,miR-760-3p,miR-200a-3p,novel_96,miR-221-5p,miR-183-5p,miR-3064-5p,miR-377-5p,miR-143-3p,miR-345-3p,miR-330-5p,miR-1343-5p,miR-155-5p,miR-212-5p,miR-1224-5p,miR-483-3p,novel_83,miR-320e,miR-664a-5p,miR-135a-5p,miR-155-5p,miR-665-5p,miR-133a-3p,miR-3958-5p,miR-346,miR-365a-5p,miR-133b-3p,miR-2474,miR-485-5p,miR-3068-3p,miR-2331-3p,miR-221-5p,miR-182-5p,miR-1271,miR-141-3p,miR-370-3p,miR-2284s,miR-155-5p,miR-326,miR-2411-3p,miR-182-5p,miR-365b-5p,miR-3085-3p,miR-491-5p,miR-483-3p,miR-378a-5p,miR-1343-5p,miR-2330-5p,miR-1388-3p,miR-744-3p,miR-432-3p,miR-432-3p,miR-2284r,miR-2426,miR-3120-5p,novel_4,miR-377-5p,miR-134-3p,miR-147-5p,miR-219b-3p,miR-1296-5p,miR-326-3p,miR-3970,miR-1291,miR-410-5p,miR-3963,miR-135b-5p,miR-3064-5p,miR-320b,miR-378g,miR-2404,miR-6535
SLAMF7	ENSOARG0(	SLAM fam	miR-135a-1-3p,miR-2440,miR-5100,miR-382-5p,miR-135a-5p,miR-6740-5p,miR-324-3p,miR-3964,miR-22-5p,miR-2285t,miR-3074-2-3p,miR-133a-3p,miR-8095,miR-326-3p,miR-150-5p,miR-133b-3p,miR-135b-5p,miR-7975,miR-1271,miR-182-5p,miR-541-5p,miR-370-3p,miR-3082-5p,miR-411b-3p,miR-670-3p,miR-181b-3p,miR-677,novel_32,miR-223-5p,miR-326,miR-210-5p,miR-7-5p,miR-182-5p,miR-450a-2-3p,miR-2285w,miR-345-3p,miR-668-3p,miR-181b-1-3p,miR-654-3p,miR-490-3p,miR-7b-5p,novel_133,miR-330-5p,miR-2427,miR-148a-5p,miR-188-5p,miR-2300a-5p,miR-133b-5p,miR-6517,miR-421,miR-148b-5p,novel_116,miR-2284h-5p,miR-545-5p
AGR2	ENSOARG0(	anterior	miR-6516-3p,miR-208b-5p,miR-551b-3p,miR-29d-5p,miR-3604,miR-877-3p,miR-6525,miR-29c-5p,miR-122-5p,miR-2285x,miR-361-3p,miR-200a-5p,miR-411,miR-320a,miR-2284s,miR-323a-5p,miR-323-5p,miR-320c,miR-574-5p,miR-452-3p,miR-660-5p,miR-20a-3p,miR-532-5p,miR-124-5p,miR-320d,miR-450a-2-3p,miR-200c-3p,miR-660,miR-320b,miR-200c,novel_103,miR-92a-1-5p,miR-654-3p,miR-1957a,miR-2424,miR-143-3p,miR-1388-3p,miR-2483-5p,miR-181a-2-3p,miR-33a-5p,miR-4429,miR-3432b,miR-2284aa,miR-200b-3p,miR-200b,miR-222-5p,miR-2284z,miR-31-5p,miR-429-3p,miR-5010-3p
MYH11	ENSOARG0(	myosin,	miR-92a-1-5p,miR-2397-5p,miR-664-3p,miR-34b-5p,miR-450a-2-3p,miR-495-5p,miR-101-5p,miR-345-3p,miR-382-3p,miR-376c-5p,miR-486-3p,miR-376b-5p,miR-2284l,miR-377-5p,miR-1246,miR-218-1-3p,miR-34a-5p,miR-377-5p,novel_4,miR-1247-5p,miR-9-5p,miR-34c,miR-490-5p,miR-129-5p,miR-486b-3p,miR-2285x,miR-9-5p,miR-136-3p,miR-449a,miR-3956,miR-874-3p,miR-133c,miR-34c-5p,miR-216b-5p,miR-34b

TMX4	ENSOARG0(	thioredo	miR-544-3p,miR-370-3p,miR-2285aa,miR-874-3p,miR-186-5p,miR-1895,miR-30f,miR-339b,miR-767,miR-3154,miR-3607-3p,miR-28-5p,miR-874-5p,miR-208b-5p,miR-708-5p,miR-767-5p,miR-135a-5p,miR-100-3p,miR-2332,miR-29b-3p,miR-7859,novel_79,miR-548e-3p,miR-29d-3p,miR-30c-5p,miR-181b-1-3p,miR-376a-5p,miR-2887,miR-183-5p,miR-339a,novel_17,miR-30b,miR-181b-3p,miR-27a-3p,miR-677,miR-29a-3p,miR-541-5p,miR-10b,miR-877-3p,miR-135b-5p,miR-8095,miR-194b-5p,miR-29c-3p,miR-219a-5p,miR-147-5p,miR-29a,miR-3968,miR-371a-5p,miR-30b-5p,miR-544b,miR-181b-2-3p,miR-27a-3p,miR-339-5p,miR-133b-5p,novel_78,miR-31-5p,miR-7862,miR-27b-3p,miR-3120-5p,miR-544a,miR-29b,miR-194-5p,miR-654-3p,miR-378a-5p,miR-194a
LY9	ENSOARG0(	lymphocy	novel_27,miR-122-5p,miR-10b-3p,miR-342-3p,miR-3059-5p,miR-130b-5p,miR-2285t,miR-142b,novel_87,miR-107-5p,miR-3082-5p,miR-670-3p,miR-27a-3p,miR-2331-3p,miR-552-3p,miR-136-5p,miR-186-5p,miR-23b,miR-130a-5p,miR-432-3p,miR-503-3p,miR-382-3p,miR-6240,miR-130b-5p,miR-23a-3p,miR-103a-2-5p,miR-1277-5p,miR-452-5p,miR-618,novel_17,miR-342,miR-330-3p,miR-23c,miR-1298-3p,miR-23b-3p,miR-330-3p,miR-145b,miR-27a-3p,miR-1b-3p,miR-142-3p,miR-27b-3p,miR-2300b-3p,novel_133,miR-145a-5p,miR-2427,miR-218-2-3p
IGHE	ENSOARG0(	immunogl	miR-542-3p,miR-2113,miR-4791,miR-378b,miR-30b-3p,miR-378c,miR-30b-3p,miR-16b,miR-3120-5p,miR-497-5p,miR-2300a-5p,miR-339-5p,miR-147a,miR-3535,miR-149-5p,miR-216a-3p,miR-16b,miR-197-5p,miR-3141,miR-214-3p,miR-193a-3p,miR-424-5p,miR-363-5p,miR-378d,miR-3141,miR-376b-5p,miR-504,miR-147-3p,miR-96-5p,miR-195a-5p,miR-378f,miR-9851-3p,miR-378b,novel_68,miR-211-5p,miR-3082-5p,miR-146b-3p,miR-378e,miR-4443,miR-615,miR-15a,miR-16a,miR-193b-3p,miR-211,miR-361-3p,miR-423-5p,novel_133,miR-378i,miR-615-3p,miR-378a-3p,miR-2904,miR-378d,miR-133a-3p,miR-212-5p,miR-1224-5p,miR-15a-5p,miR-2411-5p,miR-1271-5p,miR-378h,miR-322-5p,miR-185-3p,miR-422a,miR-339a,miR-324-3p,miR-760-3p,miR-466i-5p,miR-2898,miR-543-5p,miR-376b-5p,miR-2411,novel_39,miR-376c-5p,miR-486-3p,miR-376c-5p,miR-339b,miR-485-5p,miR-376c-5p,miR-504-5p,miR-378a-3p,miR-574-5p,miR-193b-3p,miR-3965,miR-6130,miR-27a-5p,miR-15b-5p,miR-29d-5p,miR-204-5p,miR-486b-3p,miR-424-5p,miR-4492,miR-4510,miR-378c,miR-665-5p,miR-214,miR-1271,miR-16-5p,miR-346,miR-29c-5p,miR-378c,miR-3184-5p
INADL	ENSOARG0(	InaD-lik	miR-1843b-5p,miR-34a-5p,miR-142-3p,miR-211-5p,miR-139-5p,miR-30c-1-3p,miR-2459,miR-194-3p,miR-34b,miR-670-3p,miR-139-5p,miR-34c-5p,miR-3604,miR-34c,miR-500a-5p,miR-365b-5p,miR-30c-2-3p,miR-204-5p,miR-34b-5p,miR-2898,miR-499a-5p,miR-183-5p,miR-2285b,miR-449a,miR-365a-5p,miR-323c,miR-30c-1-3p,miR-450b-5p,miR-211

DDOST	ENSOARG0(	dolichyl	miR-3957-3p,miR-17-3p,miR-1193,miR-342-3p,miR-326-3p,miR-449a,miR-2428,miR-211,miR-24-3p,miR-34c,miR-6402,miR-22-5p,miR-147-5p,miR-128-1-5p,novel_1,miR-655-5p,miR-7-1-3p,miR-323a-5p,miR-2404,miR-34b,miR-18a-3p,miR-450b-3p,miR-654-3p,miR-190a,miR-197-5p,miR-99a-5p,miR-503-5p,miR-125a-5p,miR-452-5p,miR-1343-5p,miR-22851,miR-26b-3p,miR-432-5p,miR-2285g,miR-185-5p,miR-503-5p,miR-145a-5p,miR-1285,miR-181a-5p,miR-7862,miR-138,miR-219a-1-3p,miR-1260b,miR-6128,miR-331-3p,miR-3969,miR-409-3p,miR-34c-3p,miR-410-5p,miR-370-5p,miR-204-5p,miR-138-5p,miR-370-3p,miR-487a-3p,miR-199a-5p,miR-34c-5p,miR-374c-3p,miR-3965,miR-320a,miR-3154,miR-100-5p,miR-7144-5p,miR-23a-5p,miR-485-5p,miR-10a-3p,miR-4286,miR-181a-2-3p,miR-199a-5p,miR-490-3p,miR-1271-3p,miR-30c-2-3p,miR-1260a,miR-2285af,miR-6119-5p,miR-99a,miR-17-3p,miR-181b-5p,miR-214-5p,miR-2411-5p,miR-345-3p,miR-134,miR-99a-5p,miR-181d-5p,miR-219a-5p,miR-3059-5p,miR-541-3p,miR-154a,miR-1973,miR-371a-5p,miR-107-5p,miR-4726-5p,miR-499b-5p,miR-3082-5p,miR-361-3p,miR-199b-5p,miR-877-3p,novel_94,miR-30c-1-3p,miR-2957,miR-320b,miR-342,novel_103,miR-326,miR-2285o,miR-9788-3p,miR-2285e,miR-7857,miR-296-5p,miR-484,miR-6529a,miR-143-5p,miR-145a-3p,miR-4510,miR-324-3p,miR-369-5p,miR-19b-1-5p,miR-132-5p,miR-208b-5p,miR-664a,miR-17-3p,miR-370-5p,novel_32,miR-22-3p,miR-193b-3p,miR-323-5p,miR-6130,miR-21a-3p,novel_99,miR-183-5p,miR-146a-5p,miR-2411,miR-2432,miR-125b,miR-1277-5p,miR-34b-5p,miR-320d,miR-466i-5p,miR-760-3p,miR-3600,miR-3960,miR-199a-3p,novel_83,miR-532-3p,miR-487a-3p,miR-148b-3p,miR-340-5p,miR-1197-5p,miR-2408,miR-378d,miR-4429,miR-193b-3p,miR-199b-5p,miR-4324,miR-6123,miR-3120-3p,miR-216b-
SSRP1	ENSOARG0(	structur	miR-378h,miR-15a-5p,miR-322-5p,miR-6517,miR-18a-3p,miR-196a-2-3p,let-7a-2-3p,miR-664-5p,miR-2300b-3p,miR-378i,miR-378a-3p,miR-2904,miR-105-3p,miR-183-5p,miR-17-3p,novel_127,novel_96,miR-21-3p,miR-30b-3p,miR-125a-3p,miR-1260a,miR-422a,miR-185-3p,miR-324-3p,miR-6130,miR-377-3p,miR-15b-5p,miR-324-5p,miR-874-3p,miR-504-5p,miR-378a-3p,miR-1271,miR-2355-5p,let-7g-3p,miR-21a-3p,miR-1260b,miR-338-3p,miR-378c,miR-4510,miR-378c,novel_42,miR-214,miR-16-5p,miR-664a,miR-17-3p,miR-28-5p,miR-204-5p,miR-4492,miR-874-5p,miR-708-5p,miR-424-5p,miR-5100,miR-410-5p,miR-665,miR-331-3p,miR-185-5p,miR-455-3p,miR-1260b,miR-378c,miR-16b,miR-744-5p,miR-497-5p,miR-143-5p,miR-223-3p,miR-2284r,miR-378b,miR-296-5p,miR-374b-3p,miR-3071-5p,miR-378d,miR-1b-5p,let-7a-2-3p,miR-424-5p,miR-1388-3p,miR-491-5p,miR-2285o,miR-214-3p,miR-5126,miR-377-3p,miR-16b,miR-374c-3p,miR-378e,miR-454-5p,miR-6535,miR-7977,miR-211-5p,miR-504,miR-378f,miR-877-3p,miR-195a-5p,miR-2387,miR-361-3p,miR-18a-3p,miR-24-3p,miR-211,miR-2428,novel_27,miR-3059-5p,miR-17-3p,miR-15a,miR-16a

TNFAIP6	ENSOARG0(	tumor ne	miR-665,miR-145b,miR-142-5p,miR-455-3p,miR-1185-3p,miR-2284r,miR-301b-5p,miR-145a-5p,novel_23,miR-450b-5p,miR-2284aa,miR-455-5p,miR-2483-5p,let-7f-2-3p,miR-2284b,let-7f-2-3p,miR-30c-1-3p,miR-345-5p,miR-1b-5p,miR-455-5p,miR-182-5p,miR-199b-5p,miR-2284g,miR-655-5p,miR-7977,miR-323a-5p,miR-670-3p,miR-2483-3p,miR-203-3p,miR-1185-2-3p,miR-454-5p,miR-199b-5p,miR-7975,miR-2319a,novel_94,miR-3059-5p,novel_60,miR-3956-3p,miR-877-3p,novel_27,miR-154a,miR-2284w,miR-199b-5p,miR-142b,miR-301a-5p,novel_1,miR-374a-3p,miR-7641,miR-3600,miR-2284z,miR-188-3p,miR-23b-5p,miR-582-3p,miR-142-3p,miR-155-5p,miR-378d,miR-2284u,miR-130a-5p,miR-2284y,miR-199a-5p,miR-143-3p,miR-2284a,miR-205-5p,miR-30c-2-3p,miR-219b-5p,miR-30c-1-3p,miR-182-5p,miR-582-5p,miR-300-3p,miR-216b-5p,miR-323-5p,miR-199a-5p,miR-155-5p,miR-137-3p,miR-433-3p,miR-345-5p,miR-345-5p,miR-1827,miR-142a-5p,miR-374a-3p,miR-3604,miR-129-5p,miR-374b-3p
SAMD10	ENSOARG0(	sterile	miR-669,miR-329-5p,miR-410-5p,let-7e-5p,miR-3141,miR-216a-3p,miR-1306,miR-3970,miR-2285m,miR-29c-3p,miR-29a,miR-6123,miR-576-3p,miR-134-3p,miR-3431,miR-149-5p,novel_116,miR-31-5p,miR-16b,miR-27b-3p,miR-497-5p,miR-7b-5p,miR-542-3p,miR-450c-3p,miR-432-3p,miR-432-3p,miR-493-5p,miR-196b-5p,miR-7-5p,miR-3141,miR-2285v,miR-331-5p,miR-223-5p,miR-874-3p,miR-125a,miR-329b,miR-329a,miR-30e-5p,miR-1260b,novel_42,miR-193a-5p,miR-16-5p,miR-592,miR-125b-5p,miR-758-5p,miR-322-5p,miR-450a-1-3p,miR-6517,miR-885-3p,miR-196a-5p,miR-330-5p,miR-2427,miR-2285b,let-7g,let-7f-5p,novel_96,novel_127,miR-378g,miR-4443,miR-211-5p,miR-504,miR-493-5p,novel_111,miR-877-3p,miR-423-5p,miR-2447,miR-301a-5p,miR-147a,miR-1298-3p,miR-496-5p,miR-1343-3p,miR-362-3p,miR-21b,miR-301b-5p,miR-541,miR-329-3p,miR-1388-3p,miR-491-5p,miR-214-3p,miR-103a-2-5p,miR-16b,let-7i-5p,miR-15b-5p,miR-30c-1-3p,miR-2331-3p,miR-504-5p,miR-552-3p,miR-1827,miR-885-3p,miR-450a-1-3p,miR-129b-3p,miR-346,miR-6525,miR-3184-5p,let-7b,miR-214,miR-3958-5p,novel_74,miR-4492,miR-874-5p,let-7f,miR-3604,miR-328-3p,miR-18a-3p,miR-212-5p,miR-33b-3p,miR-142-3p,miR-34a-5p,miR-133a-3p,miR-1961,miR-193b-5p,miR-30c-5p,miR-377-5p,miR-664b-3p,miR-143-3p,miR-486-3p,miR-329a-5p,miR-98-5p,let-7k,miR-30b,miR-92a-1-5p,miR-324-3p,miR-219b-5p,miR-29a-3p,miR-670-3p,miR-34b,miR-323a-5p,miR-2404,miR-320b,miR-450b-3p,miR-1291,miR-195a-5p,miR-6395,miR-9851-3p,miR-18a-3p,miR-3959-3p,miR-24-3p,miR-211,miR-326-3p,miR-449a,miR-1193,miR-128-1-5p,miR-6402,miR-15a,miR-3613,let-7i,miR-34c,miR-16a,miR-185-5p,let-7g-5p,miR-6238,miR-133b-5p,miR-26b-3p,miR-377-5p,miR-1260b,miR-29b,miR-30b-3p,miR-2889,miR-7862,miR-1285,miR-2483-5p,miR-382,miR-

IDNK	ENSOARG0(	idnK, gl	miR-8485,miR-2310,miR-744-3p,miR-146b,miR-199b-5p,miR-214-3p,miR-103a-2-5p,miR-491-5p,miR-1185-5p,miR-505,miR-182-5p,miR-2459,miR-126a-5p,miR-147a,miR-181b-2-3p,miR-1343-3p,miR-330-3p,miR-101c,miR-1388-5p,miR-2284r,miR-301b-5p,miR-541,miR-361-3p,miR-2447,miR-3956-3p,miR-3968,miR-499a-5p,miR-301a-5p,miR-2440,miR-144-5p,miR-25,miR-2285u,miR-211-5p,miR-139-5p,miR-335-3p,miR-1983,miR-504,miR-136-5p,miR-221,miR-486-3p,miR-664b-3p,miR-181b-1-3p,miR-345-3p,miR-329a-5p,miR-2285w,miR-3653-3p,miR-202-5p,miR-345-3p,miR-92a-1-5p,miR-122-3p,miR-26a-5p,miR-618,miR-210-5p,miR-2284h-5p,miR-328-3p,miR-7859,miR-380-5p,miR-664-5p,miR-16-1-3p,miR-218-1-3p,miR-361-5p,miR-184-3p,miR-217-5p,miR-2366,miR-193b-5p,miR-6525,miR-769-5p,miR-214,miR-3184-3p,miR-500,miR-129-5p,miR-4492,novel_74,miR-665,miR-2284s,miR-10a,miR-216b-5p,miR-216b-3p,miR-552-3p,miR-30c-1-3p,miR-504-5p,miR-548o-3p,miR-543-3p,miR-625-5p,miR-1895,miR-1827,miR-345-5p,miR-339b,miR-500a-3p,miR-433-3p,miR-299,miR-2285c,miR-432-3p,miR-432-3p,miR-380-5p,miR-6240,miR-483-3p,miR-105-5p,miR-2411-3p,miR-194a,miR-7-5p,miR-146a,miR-149-5p,miR-3431,miR-339-5p,miR-31-5p,miR-27b-3p,miR-188-5p,miR-16-1-3p,miR-2113,miR-7b-5p,miR-199b-5p,miR-758-5p,miR-29c-3p,miR-625-3p,miR-421-5p,miR-29a,miR-4324,miR-28c,miR-6535,miR-677,miR-669,miR-181b-3p,miR-374c-3p,miR-26b-5p,novel_68,miR-329-5p,miR-146b-5p,miR-412-3p,miR-3970,miR-3591-3p,miR-148a-3p,miR-29a-5p,miR-192-3p,miR-148b-5p,novel_96,miR-200a-3p,miR-152-3p,miR-127-5p,miR-205-5p,miR-664-3p,novel_79,miR-2285ad,miR-429-3p,miR-1343-5p,miR-144-5p,miR-191-5p,miR-100-3p,miR-6740-5p,miR-125b-5p,miR-2433,miR-223-5p,miR-133c,miR-300-3p,miR-2355-5p,miR-1271,miR-125a,miR-
SPARC	ENSOARG0(	secreted	miR-1343-5p,miR-130b-3p,miR-3085-3p,miR-196b-5p,novel_51,miR-544b,miR-500-5p,miR-3431,miR-130a-3p,miR-339-5p,miR-31-5p,miR-16-2-3p,miR-544a,miR-1260b,miR-138,miR-1434-3p,miR-653,miR-2284w,miR-301b-3p,miR-449a,miR-211,miR-362-5p,miR-17-3p,miR-502b,miR-2285t,miR-34c,miR-502-5p,miR-669,miR-34b,miR-211-5p,miR-4792,novel_68,miR-3064-5p,miR-18a-3p,miR-148a-3p,miR-361-3p,miR-1271-3p,miR-2285b,miR-192-3p,miR-345-3p,miR-181a-2-3p,miR-3064-5p,miR-3529-3p,novel_127,miR-152-3p,miR-34b-5p,miR-221-5p,miR-760-3p,miR-17-3p,miR-450a-2-3p,miR-339a,miR-1260a,miR-532-3p,miR-148b-3p,miR-3600,novel_79,miR-18a-3p,miR-34a-5p,miR-1343-5p,miR-2408,miR-196a-5p,miR-10b-3p,miR-1260b,miR-6525,miR-500b-5p,miR-204-5p,miR-454-3p,miR-500,miR-17-3p,miR-138-5p,miR-3969,miR-331-3p,novel_25,miR-544-3p,miR-301a-3p,miR-34c-5p,miR-221-5p,miR-2331-3p,miR-339b,miR-362-5p,miR-500a-3p

USP14	ENSOARG0(	ubiquiti	miR-300-3p,miR-2483-3p,miR-2284n,miR-545-3p,miR-140-5p,miR-221-5p,miR-2284g,miR-3074-5p,miR-7-1-3p,miR-876-3p,miR-488-3p,miR-301,miR-2448-3p,miR-18a-3p,miR-326-3p,miR-1306-5p,miR-3074-5p,miR-145a-3p,miR-2284v,miR-2284j,miR-22-5p,miR-203b-5p,miR-147-5p,miR-2284k,miR-2285j,miR-2284d,miR-758-5p,miR-148b-5p,miR-455-3p,miR-18a-3p,miR-148a-5p,miR-2284u,miR-1197-5p,miR-330-5p,miR-380-5p,miR-130a-5p,miR-2284y,miR-8485,miR-380-5p,miR-2284b,miR-221-5p,novel_127,miR-326,miR-30f
SNRPN	ENSOARG0(	small nu	miR-148a-3p,miR-3154,miR-377-3p,miR-27a-3p,miR-20a-3p,miR-26b-5p,miR-101-3p,miR-301a-5p,miR-135a-2-3p,novel_60,miR-3956-3p,miR-2285x,novel_73,miR-21b,miR-27b-3p,miR-301b-5p,miR-124-3p,miR-148b-3p,miR-421,miR-27a-3p,miR-127-5p,miR-452-5p,miR-3653-3p,miR-152-3p,miR-2285af,miR-377-3p,novel_103,miR-26a-5p,miR-3071-3p,miR-2432,miR-124a,let-7c-3p,miR-33a-5p
SESN3	ENSOARG0(	sestrin	miR-27b-5p,miR-433-3p,miR-221,miR-2285u,miR-32-5p,miR-499b-3p,miR-499a-3p,miR-25-3p,miR-411b-3p,miR-2285y,miR-33a-3p,miR-34c-3p,miR-222-3p,miR-2285n,miR-92a-3p,miR-495-3p,miR-363-3p,miR-323-3p,miR-432,miR-6529a,miR-101a-5p,miR-222,miR-2285ad,miR-330-3p,novel_4,miR-221-3p,miR-432-5p,miR-32,miR-330-3p,miR-2285af,miR-466f-3p,miR-92a-3p,miR-202-5p,miR-668-3p,miR-6240,miR-92b-3p,miR-450b-5p
UBE3A	ENSOARG0(	ubiquiti	novel_79,miR-134-5p,miR-486-5p,miR-322-5p,miR-196a-5p,miR-134,miR-105-2,miR-496,miR-300,miR-3064-5p,miR-3529-3p,miR-200a,miR-1957a,miR-29a-5p,miR-2284y,miR-192-3p,miR-2284a,miR-148b-5p,miR-3071-3p,miR-30f,miR-339a,miR-664-3p,miR-125a-3p,miR-200a-3p,miR-17-3p,miR-450a-2-3p,miR-141-3p,miR-503-3p,miR-223-5p,miR-365a-3p,miR-3154,miR-320a,miR-381-3p,novel_48,miR-7144-5p,miR-485-5p,miR-16-5p,miR-374b,miR-1-5p,miR-134-5p,miR-339-5p,miR-133b-5p,miR-412-5p,miR-197-3p,miR-223-3p,miR-7b-5p,miR-497-5p,miR-455-5p,miR-30b-3p,miR-16b,miR-668-3p,miR-130b-5p,miR-493-5p,miR-105-5p,miR-455-5p,miR-7-5p,miR-146a,miR-196b-5p,miR-7-1-3p,miR-3074-5p,miR-411b-3p,miR-3065-5p,miR-146b-5p,miR-195a-5p,miR-412-3p,miR-758-5p,miR-17-3p,miR-1a-1-5p,miR-2428,miR-2284w,miR-16a,miR-15a,miR-576-3p,miR-6119-3p,miR-380-5p,miR-30d-3p,miR-15a-5p,miR-124-3p,novel_133,miR-148a-5p,miR-2284u,miR-493-3p,miR-146a-5p,miR-500a-5p,miR-2285p,miR-3653-3p,miR-21-3p,miR-21-3p,miR-15b-5p,miR-499b-3p,miR-6130,miR-21a-3p,let-7f-1-3p,miR-543-3p,miR-186-5p,let-7g-3p,miR-339b,miR-3184-3p,miR-145a-3p,miR-3074-5p,miR-4510,miR-338-5p,miR-3604,let-7b-3p,miR-129-5p,miR-424-5p,miR-130b-5p,miR-664a,miR-17-3p,miR-105-1,miR-548w,miR-148b-5p,miR-1298-3p,miR-30b-3p,miR-2284r,miR-2284b,miR-124a,miR-424-5p,miR-1b-5p,miR-2957,miR-505,miR-16b,miR-320b,miR-146b,miR-3085-3p,miR-374b-5p,miR-541-5p,miR-2285u,miR-664b,miR-2284g,miR-3064-5p,miR-499a-3p,miR-381-3p,miR-129b-5p,miR-493-5p,miR-1983,miR-668-5p,miR-452-5p,miR-361-3p,miR-2285n,miR-101-3p,miR-2440,miR-628-3p

TUBAL3	ENSOARG0(	tubulin,	miR-147-5p, miR-374a-3p, miR-22-5p, miR-3958-3p, miR-371a-5p, miR-6529b, miR-2440, miR-218-5p, miR-1307-3p, miR-17-3p, novel_94, miR-877-3p, miR-376a-3p, miR-2319a, miR-376b-3p, miR-3591-3p, miR-216a-3p, miR-378b, miR-27a-3p, miR-376d, miR-299b-3p, miR-7977, miR-10b, miR-20a-3p, miR-2284g, miR-466f-3p, miR-1185-5p, novel_51, miR-182-5p, miR-2411-3p, miR-105-5p, miR-377-3p, miR-431-5p, miR-345-5p, miR-654-3p, miR-33a-5p, miR-23a-3p, miR-494-3p, miR-130b-5p, miR-27b-3p, miR-3120-5p, miR-2284x, miR-145a-5p, miR-1285, miR-2355-3p, miR-455-3p, miR-9788-3p, miR-185-5p, miR-376b-3p, miR-26b-3p, miR-145b, miR-27a-3p, miR-339-5p, miR-105-1, novel_78, novel_4, miR-548w, miR-122-3p, miR-130b-5p, miR-374b-3p, miR-17-3p, miR-409-3p, miR-5100, miR-338-3p, miR-374a-3p, miR-665-5p, miR-103b, miR-193a-5p, miR-28a-3p, miR-539-5p, novel_99, miR-1248, miR-7144-5p, miR-339b, miR-345-5p, miR-345-5p, miR-10b-3p, miR-301, miR-365a-3p, miR-377-3p, miR-182-5p, miR-141-3p, miR-21-3p, miR-496-3p, miR-3068-3p, miR-503-3p, miR-2331-3p, miR-221-5p, miR-200a-3p, miR-299-3p, miR-221-5p, miR-28-3p, miR-17-3p, miR-122-3p, miR-345-3p, miR-339a, miR-618, miR-196a-3p, miR-376b-3p, miR-200a, miR-1957a, miR-2432, miR-29a-5p, miR-376a-5p, miR-496, miR-105-2, miR-218-1-3p, miR-128-3p, miR-140-3p, miR-1434-5p, miR-345-3p, miR-23c, miR-23b-3p, miR-431, miR-376a-5p, miR-7641, novel_79, miR-299a-3p, miR-2285ad

C7	ENSOARG0(	compleme	miR-4286,miR-181a-2-3p,miR-490-3p,miR-28b,miR-217,miR-376b-3p,miR-200c,miR-543-5p,miR-191-3p,miR-200c-3p,miR-7641,miR-378h,miR-1271-5p,miR-218-2-3p,miR-30a-5p,miR-3578,miR-200b-3p,miR-128-3p,let-7a-2-3p,miR-300,miR-1306-5p,miR-378c,miR-144,miR-331-3p,miR-551b-3p,miR-486b-3p,miR-767-5p,miR-141-3p,miR-487a-3p,miR-324-5p,miR-365a-3p,miR-381-3p,miR-503-3p,miR-7144-5p,novel_48,let-7g-3p,miR-345-5p,miR-654-3p,miR-330-3p,miR-665,miR-130a-3p,miR-26b-3p,miR-544b,miR-2355-3p,miR-432-5p,miR-3071-5p,miR-873a-5p,miR-7862,miR-2889,miR-30b-3p,miR-2285x,miR-542-5p,miR-135a-2-3p,novel_27,miR-24-3p,miR-16a,miR-15a,miR-6402,miR-133a-5p,miR-656-5p,miR-18a-3p,miR-376b-3p,miR-2387,miR-195a-5p,miR-505-3p,miR-146a-5p,miR-216a-5p,miR-542-5p,miR-30d,miR-30b-3p,miR-21-3p,miR-760-3p,miR-1b-3p,miR-3600,miR-505-3p,miR-485-3p,novel_83,miR-487a-3p,miR-15a-5p,miR-148b-3p,miR-200b,miR-1197-5p,miR-1193,novel_133,miR-1246,miR-2300b-3p,let-7a-3p,miR-1271,miR-2330-3p,miR-3074-2-3p,miR-378c,miR-424-5p,miR-155-5p,miR-378a-3p,miR-545-3p,miR-204-3p,novel_25,miR-155-5p,miR-877-5p,miR-544-3p,miR-21a-3p,let-7f-1-3p,novel_99,miR-582-5p,let-7g-3p,miR-154b-3p,miR-30f,let-7c-3p,miR-33a-5p,miR-382-3p,miR-424-5p,novel_103,miR-542-5p,novel_124,miR-105-1,miR-2300a-5p,miR-9788-3p,miR-412-3p,miR-30b-3p,miR-484,miR-378b,miR-16-2-3p,miR-3120-5p,miR-378c,miR-544a,miR-299a-5p,miR-101a-5p,miR-212-3p,miR-219a-2-3p,miR-371a-5p,miR-30b-5p,miR-2285t,miR-7977,miR-664b,miR-20a-3p,miR-376d,miR-378e,miR-7857-3p,miR-3082-5p,miR-29a-5p,miR-148b-5p,miR-450b-5p,miR-205-5p,miR-125a-3p,miR-664-3p,miR-219-3p,novel_127,miR-152-3p,miR-200a-3p,miR-127-5p,miR-412,miR-431,miR-429-3p,miR-6517,miR-129-1-3p,miR-

IL11RA	ENSOARG0(	interleu	miR-1b-3p,miR-15a-5p,miR-532-3p,miR-362-3p,novel_133,miR-1197-5p,miR-5703,miR-124-3p,miR-378d,miR-216c-5p,miR-2432,miR-466i-5p,miR-21-3p,miR-30b-3p,miR-221-5p,miR-660-5p,miR-378a-3p,miR-221-5p,miR-182-5p,miR-6130,miR-139-5p,miR-204-3p,miR-582-5p,miR-145a-3p,miR-4510,miR-378c,miR-3074-2-3p,miR-206-3p,miR-338-3p,miR-664a,miR-450b-3p,miR-424-5p,miR-222-5p,miR-2285e,miR-500-5p,miR-9788-3p,miR-378b,miR-484,miR-30b-3p,miR-125b-2-3p,miR-412-3p,miR-101a-5p,miR-2426,miR-378c,miR-3120-5p,miR-424-5p,miR-30c-1-3p,miR-758-3p,miR-193a,miR-1306-5p,novel_103,miR-326,miR-362-3p,miR-2285o,miR-7977,miR-499b-5p,miR-489,miR-3082-5p,miR-378e,miR-376d,miR-199b-5p,miR-361-3p,novel_94,novel_60,miR-3059-5p,miR-362-5p,miR-107-5p,miR-335,miR-2284q,miR-2285t,miR-378h,miR-134,miR-615-3p,let-7a-2-3p,miR-128-3p,miR-29b-2-5p,miR-1,miR-28b,miR-490-3p,miR-199a-5p,miR-376b-3p,miR-1260a,miR-339a,miR-217,miR-30c-2-3p,miR-2898,miR-370-3p,miR-374c-3p,miR-324-5p,miR-199a-5p,miR-3154,miR-362-5p,let-7g-3p,miR-671-5p,miR-23a-5p,miR-7144-5p,miR-1306-5p,miR-6128,miR-500b-5p,miR-378c,miR-365a-5p,miR-135a-1-3p,miR-410-5p,miR-3969,miR-133b-5p,miR-377-5p,miR-130a-3p,miR-665,miR-503-5p,miR-185-5p,miR-2285g,miR-653,miR-873a-5p,miR-374b-3p,miR-1260b,miR-30b-3p,miR-363-5p,miR-654-3p,miR-345-5p,miR-296-3p,miR-503-5p,miR-2285l,miR-660,miR-670-3p,miR-146b-3p,miR-376b-3p,miR-18a-3p,miR-450b-3p,miR-1291,miR-6395,miR-195a-5p,miR-379-5p,miR-9851-3p,miR-371b-3p,miR-2428,miR-326-3p,miR-15a,miR-16a,novel_1,miR-502b,miR-18a-3p,miR-328-3p,miR-2284h-5p,miR-193b-5p,miR-378i,miR-378a-3p,miR-217-5p,miR-142-3p,miR-493-3p,miR-133a-3p,miR-664-5p,miR-503-5p,miR-2285w,miR-329a-5p,miR-376c-3p,miR-377-5p,miR-376a-
G3BP1	ENSOARG0(	GTPase a	miR-767-5p,miR-450b-3p,miR-208b-5p,miR-874-5p,miR-4492,miR-3969,miR-219b-3p,miR-19b-1-5p,miR-1197-3p,miR-6134,miR-365a-5p,miR-4510,novel_42,miR-345-5p,miR-345-5p,miR-767,miR-1814c,miR-543-3p,miR-190a-3p,miR-625-5p,miR-450a-1-3p,miR-3154,miR-6130,miR-377-3p,miR-154b-5p,miR-223-5p,miR-3068-3p,miR-545-3p,miR-552-3p,miR-21-3p,miR-2284m,miR-299-3p,miR-125a-3p,miR-219-3p,miR-2285af,miR-345-3p,miR-185-3p,miR-217,miR-216a-5p,miR-181b-1-3p,miR-181a-2-3p,miR-4286,miR-218-1-3p,miR-16-1-3p,miR-345-3p,miR-217-5p,miR-188-3p,miR-5703,miR-140-3p,miR-758-5p,miR-545-5p,miR-2284z,miR-450a-1-3p,miR-299a-3p,miR-505-3p,miR-3600,miR-203b-5p,miR-656-5p,miR-133a-5p,miR-576-3p,miR-219a-2-3p,miR-3956-3p,novel_60,miR-3059-5p,miR-452-5p,miR-495-3p,miR-450b-3p,miR-505-3p,miR-140-5p,miR-3065-5p,miR-3591-3p,miR-411b-3p,miR-299b-3p,miR-181b-3p,miR-677,miR-2319b,miR-1185-5p,miR-452-5p,miR-377-3p,miR-2411-3p,miR-365b-5p,miR-345-5p,miR-2310,let-7j,miR-668-3p,miR-16-1-3p,miR-30b-3p,miR-2284aa,miR-4791,miR-708-3p,miR-19b-2-5p,miR-30b-3p,miR-450c-3p,miR-185-5p,miR-3535,miR-9788-3p,miR-548w,novel_4,miR-1343-3p,miR-409b

EEF1A1	ENSOARG0(	eukaryot	novel_103,miR-664-3p,miR-200c,miR-127-5p,miR-200c-3p,miR-2385-3p,miR-23a-3p,miR-2285w,miR-421-5p,novel_23,miR-542-3p,miR-200b-3p,miR-101c,miR-21-5p,miR-411-3p,miR-582-3p,miR-429-3p,miR-2284h-5p,miR-2285e,miR-23b-3p,miR-2285g,miR-200b,miR-23c,miR-3604,miR-135a-1-3p,miR-708-5p,miR-421-5p,miR-625-3p,miR-2285t,miR-28-5p,miR-2285f,miR-495-3p,novel_60,miR-3956-3p,novel_27,miR-381-3p,miR-23b,miR-21c,miR-2285ab,miR-2285c,miR-335-3p,miR-7975,miR-2312,miR-664b
STAT2	ENSOARG0(	signal t	miR-500b-5p,miR-362-5p,miR-361-3p,miR-23a-3p,miR-214,miR-214-3p,novel_127,miR-21-3p,miR-664-3p,miR-500-5p,miR-486-5p,miR-432-5p,miR-23b-3p,miR-505-5p,miR-656-3p,novel_69,miR-23c,miR-2483-3p,miR-7977,miR-664b,miR-18a-3p,miR-7975,miR-378d,miR-18a-3p,miR-23b,miR-433-3p,miR-362-5p,miR-3154,miR-484,miR-361-3p,miR-432
CCBL1	ENSOARG0(	cysteine	miR-378d,miR-8485,miR-124a,miR-2284b,miR-130b-3p,miR-487b-5p,miR-214-3p,miR-199a-3p,miR-323b-3p,miR-425-3p,miR-539-5p,miR-147a,miR-148b-5p,miR-181b-2-3p,miR-362-3p,miR-2284r,miR-329-3p,miR-541,miR-301b-5p,miR-1298-5p,miR-323b,miR-501-3p,novel_111,miR-423-5p,miR-3956-3p,miR-885-5p,miR-2284j,miR-2440,miR-301a-5p,miR-203-3p,miR-4443,miR-500-3p,miR-2284g,miR-126b-5p,miR-211-5p,miR-299b-5p,miR-140-5p,miR-221,miR-181b-1-3p,miR-2424,miR-380-3p,miR-143-3p,miR-329a-5p,miR-2285w,miR-503-5p,miR-202-5p,miR-30b,miR-345-3p,miR-92a-1-5p,miR-422a,miR-7859,miR-2284h-5p,miR-18a-3p,miR-212-5p,miR-30d-3p,miR-218-1-3p,miR-148a-5p,miR-2284u,miR-30c-5p,miR-378i,miR-193b-5p,miR-378a-3p,miR-769-5p,miR-6525,miR-224-5p,miR-3184-5p,miR-122-5p,miR-3074-5p,miR-129-2-3p,miR-665-5p,miR-214,miR-129-5p,miR-874-5p,miR-454-3p,miR-130b-5p,miR-30a-3p,miR-665,miR-664a-5p,miR-301a-3p,miR-2331-3p,miR-548o-3p,miR-296-3p,miR-543-3p,miR-1895,miR-186-5p,miR-2285c,miR-129b-3p,miR-30d-5p,miR-433-3p,miR-432-3p,miR-432-3p,let-7j,miR-380-5p,miR-483-3p,miR-296-3p,miR-365b-5p,miR-105-5p,miR-2411-3p,novel_51,miR-194-3p,novel_116,miR-149-5p,miR-3955-5p,miR-27b-3p,miR-188-5p,miR-708-3p,miR-22-3p,miR-197-3p,novel_23,miR-301b-3p,miR-29c-3p,miR-29a,miR-28c,miR-299b-3p,miR-411b-3p,miR-677,miR-181b-3p,novel_68,miR-3074-5p,miR-329-5p,miR-378f,miR-148a-3p,miR-1306,miR-148b-5p,miR-2284y,novel_127,miR-152-3p,miR-219-3p,miR-505-5p,miR-758-5p,miR-2284z,miR-129-1-3p,miR-2285ad,miR-299a-3p,miR-412,miR-1224-5p,miR-18b-5p,miR-30c,miR-330-5p,miR-2284v,miR-193a-5p,miR-2284k,miR-6740-5p,miR-574-5p,miR-874-3p,miR-2285aa,miR-1271,miR-767,miR-329b,miR-329a,miR-1248,miR-30e-5p,miR-424-3p,miR-376b,miR-3955-3p,miR-
KCNMA1	ENSOARG0(	potassiu	miR-3965,miR-224-5p,miR-148b-5p,miR-2284x,miR-1343-3p,miR-224-5p

SF1	ENSOARG0(	splicing	novel_17,miR-2285v,miR-760-3p,miR-181b-5p,miR-2898,miR-17-3p,miR-21-3p,miR-1388-3p,miR-363-5p,miR-181a-5p,miR-541,miR-301b-5p,miR-30b-3p,miR-501-3p,miR-501-3p,miR-30b-3p,miR-2889,miR-3120-5p,miR-129-1-3p,miR-214-5p,miR-671-3p,miR-301a-5p,miR-331-3p,miR-17-3p,miR-4726-5p,miR-874-5p,miR-129-2-3p,miR-541-3p,miR-181d-5p,miR-17-3p,miR-346,miR-361-3p,miR-181c-5p,miR-502-3p,novel_27,miR-361-3p,miR-299b-5p,miR-452-3p,miR-2331-3p,miR-7977,miR-365a-3p,miR-489,miR-500-3p
EIF2S2	ENSOARG0(	eukaryot	miR-130a-3p,miR-185-5p,let-7g-5p,miR-2285g,miR-2284d,miR-181a-5p,miR-3071-5p,miR-194-5p,miR-29b,miR-382,miR-345-5p,miR-378b,miR-10a-5p,miR-34b,miR-670-3p,miR-29a-3p,miR-2404,miR-2285ab,miR-450b-3p,miR-505-3p,miR-6516-3p,miR-342-3p,miR-3959-3p,miR-2428,miR-449a,miR-6402,let-7i,miR-34c,novel_1,miR-502b,miR-199c,miR-222,miR-134,miR-345-3p,miR-29d-3p,miR-582-3p,let-7d,miR-10a-5p,miR-128-3p,miR-29b-2-5p,miR-199a-5p,miR-376c-5p,miR-2285af,miR-217,miR-339a,let-7c-5p,miR-30c-2-3p,miR-181b-5p,miR-543-5p,miR-3957-5p,miR-141-3p,miR-769,miR-374c-3p,miR-34c-5p,miR-3957,miR-324-5p,miR-199a-5p,miR-320a,miR-362-5p,miR-485-5p,miR-376c-5p,miR-6128,miR-323-3p,miR-29b-3p,miR-2300a-5p,miR-151a-3p,miR-27a-3p,miR-2285e,miR-500-5p,miR-9788-3p,miR-216a-3p,miR-221-3p,miR-10b,miR-125b-2-3p,miR-3120-5p,miR-1839-3p,let-7a-5p,miR-30c-1-3p,novel_115,miR-320b,miR-342,miR-3955-3p,let-7e,miR-489,miR-7857-3p,miR-27a-3p,let-7d-5p,miR-199b-5p,miR-129b-5p,novel_94,miR-877-3p,miR-541-3p,miR-194b-5p,miR-2285f,miR-362-5p,miR-219a-2-3p,miR-9-3p,miR-769-5p,miR-2284q,miR-548e-3p,miR-505-3p,miR-340-5p,miR-148b-3p,miR-383-5p,miR-151-5p,novel_83,miR-140-3p,miR-5703,miR-124-3p,miR-4429,miR-216c-5p,miR-2432,miR-196a-3p,miR-760-3p,miR-320d,miR-2284m,miR-21-3p,miR-34b-5p,miR-140-5p,miR-21-3p,miR-139-5p,miR-6130,miR-204-3p,novel_25,miR-22-3p,miR-21a-3p,miR-2312,novel_99,miR-4510,miR-10b-5p,miR-338-3p,let-7b-5p,miR-222-3p,miR-29b-2-5p,miR-655-3p,miR-6516-3p,miR-450b-3p,miR-208b-5p,miR-339-5p,miR-134-3p,novel_116,miR-411-5p,miR-708-3p,novel_23,miR-22-3p,miR-450c-3p,miR-188-5p,miR-27b-3p,miR-2284x,miR-432-3p,miR-432-3p,miR-2411-3p,miR-196b-5p,miR-374c-3p,miR-320c,miR-148a-3p,miR-376b-5p,miR-3591-3p,miR-216a-3p,let-

TAX1BP1	ENSOARG0(	Tax1 (hu	novel_79,miR-7641,miR-18b-5p,miR-429-3p,miR-2284z,miR-582-3p,miR-486-5p,miR-302a-5p,miR-9-5p,miR-6516-5p,miR-378h,miR-200b-3p,miR-128-3p,miR-10a-5p,let-7a-2-3p,miR-582-3p,miR-300,miR-3529-3p,miR-29a-5p,miR-1957a,miR-2285b,miR-1271-3p,miR-192-3p,miR-24-2-5p,miR-339a,miR-2285af,miR-205-5p,miR-200c,miR-543-5p,novel_127,miR-181b-5p,miR-200c-3p,miR-370-3p,miR-2285aa,miR-1271,miR-874-3p,miR-324-5p,miR-365a-3p,miR-93-5p,miR-320a,miR-18a,miR-23b,miR-17-5p,miR-488-3p,novel_48,miR-20b-5p,miR-1814c,miR-10b-3p,miR-329b,let-7g-3p,miR-133a-3p,miR-378c,miR-10b-3p,miR-145-3p,miR-93,miR-374a-3p,miR-3969,miR-135a-5p,miR-5100,miR-374b-3p,miR-204-5p,miR-130a-3p,miR-339-5p,miR-26b-3p,miR-20b,miR-3071-5p,miR-145a-5p,miR-19b-2-5p,miR-653,miR-181a-5p,miR-7b-5p,miR-542-3p,miR-1434-3p,miR-380-3p,miR-30b-3p,miR-16-1-3p,let-7f-2-3p,let-7f-2-3p,miR-493-5p,let-7a-2-3p,miR-654-3p,miR-432-3p,miR-17-5p,miR-106a-5p,miR-7-5p,miR-377-3p,miR-660,miR-466f-3p,miR-124-5p,miR-655-5p,miR-320b,miR-7-1-3p,miR-10a-5p,miR-329-5p,miR-216b-3p,miR-320c,miR-323a-5p,miR-670-3p,miR-374c-3p,miR-150-5p,miR-3591-3p,miR-216a-3p,miR-18a-3p,miR-18b,miR-378f,miR-505-3p,miR-135b-5p,miR-19a-3p,miR-374c-5p,novel_27,miR-301b-3p,miR-211,miR-323c,miR-20a-5p,miR-22-5p,miR-147-5p,miR-1b-3p,miR-30d-3p,miR-505-3p,miR-18a-3p,miR-106a,miR-199a-3p,miR-616-3p,miR-7859,miR-23b-3p,miR-23c,miR-340-5p,miR-200b,miR-222-5p,miR-124-3p,miR-378a-3p,miR-140-3p,miR-378i,miR-16-1-3p,miR-19b-3p,miR-33b-3p,miR-4429,miR-20a,miR-329a-5p,miR-380-3p,miR-125b-2-3p,miR-500a-5p,miR-219b-5p,miR-422a,miR-196a-3p,miR-106a,miR-320d,miR-202-5p,miR-21-3p,miR-154a-3p,miR-532-5p,miR-378a-3p,miR-504-5p,miR-660-5p,miR-582-5p,miR-10a,miR-301a-3p,miR-377-

CEACAM19	ENSOARG0(	carcinoe:	miR-708-5p,miR-450b-3p,miR-30a-3p,miR-324-3p,miR-665,miR-30e-3p,miR-3184-5p,miR-346,miR-206-3p,miR-378c,miR-3184-3p,miR-3074-5p,miR-4510,miR-885-3p,miR-625-5p,miR-186-5p,miR-1827,miR-345-5p,miR-411,miR-299,miR-450a-1-3p,miR-877-5p,miR-27a-5p,miR-6130,miR-193b-3p,miR-370-5p,miR-296-3p,miR-2331-3p,miR-140-5p,miR-221-5p,miR-545-3p,miR-378a-3p,miR-221-5p,miR-34b-5p,miR-466i-5p,miR-760-3p,miR-324-3p,miR-185-3p,miR-92a-1-5p,miR-422a,miR-146a-5p,miR-2411,miR-486-3p,miR-143-3p,miR-29b-1-5p,miR-216c-5p,miR-16-1-3p,miR-34a-5p,miR-378d,miR-148a-5p,miR-2284u,miR-378a-3p,miR-1197-5p,miR-217-5p,miR-378i,novel_133,miR-362-3p,miR-2408,miR-2300b-3p,miR-532-3p,novel_83,miR-328-3p,miR-210-3p,miR-23c,miR-222-5p,miR-3600,miR-505-3p,miR-30d-3p,miR-3968,miR-4726-5p,miR-450a-5p,miR-335,miR-1973,miR-371a-5p,miR-107-5p,miR-301a-5p,miR-423-5p,miR-877-3p,miR-1296-5p,miR-194b-5p,novel_60,miR-3059-5p,miR-3956-3p,miR-877-3p,novel_94,miR-1983,miR-181a-3p,miR-378j,miR-27a-3p,miR-3596,miR-378g,miR-378e,miR-499b-5p,miR-2284g,miR-3064-5p,miR-20a-3p,miR-146b,miR-103a-2-5p,miR-491-5p,miR-3085-3p,miR-505,miR-29b-1-5p,miR-362-3p,novel_115,miR-326,miR-1306-5p,miR-8485,miR-378d,miR-23a-3p,miR-33a-5p,miR-744-3p,miR-362-3p,miR-378c,miR-1388-5p,miR-299a-5p,miR-450b-5p,miR-101a-5p,miR-1a-3p,miR-378b,miR-329-3p,miR-143-5p,miR-301b-5p,miR-148b-5p,miR-9788-3p,miR-421,miR-27a-3p,miR-222-5p,miR-145b,miR-1343-3p,miR-409b,miR-28-5p,miR-486b-3p,miR-767-5p,miR-331-3p,miR-6740-5p,miR-2433,miR-370-5p,miR-378c,miR-181c-3p,miR-1260b,miR-193a-5p,miR-6128,novel_42,miR-7144-5p,miR-1248,miR-767,miR-345-5p,miR-329a,miR-488-3p,miR-503-3p,miR-301,miR-324-5p,miR-34c-5p,miR-2285aa,miR-1271,miR-874-3p,novel_96,miR-
ACADSB	ENSOARG0(	acyl-CoA	miR-9851-3p,novel_73,miR-27b-5p,miR-124-3p,miR-296-5p,miR-1843b-5p,miR-5100,miR-2284s,miR-124a

RBM43	ENSOARG0(	RNA bind	miR-374b,miR-1306-5p,miR-204-5p,miR-2332,miR-135a-1-3p,miR-34c-3p,miR-1-5p,miR-135a-5p,miR-331-3p,miR-34c-5p,miR-2284n,miR-141-3p,miR-370-3p,let-7g-3p,miR-7144-5p,miR-23a-5p,miR-488-3p,miR-320a,miR-28b,miR-2478,miR-24-2-5p,miR-2284a,miR-3529-3p,miR-4286,miR-30a-3p,miR-29b-2-5p,miR-181b-5p,miR-200c-3p,miR-2898,miR-450a-2-3p,miR-92a-3p,miR-6119-5p,miR-200c,miR-2285af,miR-1260a,miR-339a,miR-6516-5p,miR-302a-5p,miR-24-2-5p,miR-545-5p,miR-7641,let-7a-2-3p,miR-128-3p,miR-134,miR-323c,miR-3959-3p,miR-371b-3p,miR-211,miR-326-3p,novel_27,miR-449a,miR-2285x,miR-92a-3p,miR-147-5p,miR-22-5p,miR-6402,miR-33a-3p,miR-382-5p,miR-34c,miR-670-3p,miR-34b,miR-2404,miR-7-1-3p,miR-450b-3p,miR-135b-5p,miR-7975,miR-2285ab,miR-345-5p,miR-130b-5p,miR-363-5p,miR-101-5p,miR-452-5p,miR-125a-5p,miR-1343-5p,miR-432-5p,miR-2284d,miR-544b,miR-6238,miR-133b-5p,miR-26b-3p,miR-377-5p,miR-665,miR-1260b,miR-2284ab,miR-30b-3p,miR-7862,miR-1285,miR-1434-3p,miR-181a-5p,miR-873a-5p,miR-653,miR-374b-3p,miR-3071-5p,miR-338-3p,miR-6134,miR-539-5p,miR-2330-3p,miR-3074-2-3p,miR-6516-3p,miR-155-5p,miR-208b-5p,miR-338-5p,miR-29b-2-5p,miR-320e,miR-19b-1-5p,miR-6516,miR-544-3p,miR-877-5p,miR-155-5p,novel_25,miR-204-3p,miR-221-5p,let-7g-3p,miR-2312,novel_99,miR-216c-5p,miR-466i-5p,miR-320d,miR-2284m,miR-21-3p,miR-221-5p,miR-34b-5p,miR-125b,miR-222-5p,miR-532-3p,miR-548e-3p,miR-3600,miR-4429,miR-1246,miR-5703,miR-154a,miR-219a-2-3p,miR-212-3p,miR-181d-5p,miR-2285f,miR-335,miR-450a-5p,miR-2284q,miR-107-5p,miR-371a-5p,miR-2285y,miR-489,miR-7977,miR-129b-5p,miR-758-3p,miR-652-5p,miR-3955-3p,miR-1468,miR-5126,miR-1306-5p,miR-326,miR-320b,miR-216a-3p,miR-151a-3p,miR-222-5p,miR-101a-
MUL1	ENSOARG0(	mitochon	let-7b-5p,miR-378c,let-7b,miR-378c,miR-214,miR-126b-3p,let-7f,miR-29b-3p,miR-365a-3p,let-7i-5p,novel_32,miR-378a-3p,miR-3065-3p,miR-885-3p,miR-3613-5p,miR-3065-3p,let-7g,miR-181a-2-3p,miR-4286,let-7f-5p,miR-98-5p,let-7k,miR-152-3p,miR-185-3p,let-7c-5p,miR-217,miR-422a,miR-148b-3p,miR-378h,miR-212-5p,let-7d,miR-941,miR-885-3p,miR-1961,miR-378i,miR-196a-5p,miR-378a-3p,miR-217-5p,miR-29d-3p,miR-29c-3p,miR-29a,miR-1843a-3p,miR-3613,let-7i,miR-1307-5p,miR-29a-3p,miR-378e,let-7d-5p,miR-378f,let-7e-5p,miR-148a-3p,miR-378d,let-7a-5p,let-7c-3p,miR-196b-5p,let-7e,miR-214-3p,miR-7-5p,let-7g-5p,novel_78,miR-340-3p,miR-29b,miR-378c,miR-7b-5p,miR-378b,miR-3962

NEK3	ENSOARG0(	NIMA-rel	miR-199b-5p,miR-29c-3p,miR-193b-3p,miR-29a,miR-625-3p,miR-421-5p,novel_87,miR-299b-3p,miR-181b-3p,miR-320c,miR-677,miR-329-5p,miR-3074-5p,miR-2284e,miR-3141,miR-3065-5p,miR-432-3p,miR-2284l,miR-432-3p,miR-503-3p,miR-6240,miR-196b-5p,miR-193a-3p,novel_51,miR-2411-3p,miR-3141,miR-134-3p,miR-3535,miR-411-5p,miR-3955-5p,miR-16b,miR-2284x,miR-497-5p,miR-26a-2-3p,miR-223-3p,novel_23,miR-22-3p,miR-2284v,miR-28a-3p,let-7e-3p,novel_42,miR-144-5p,miR-193a-5p,miR-16-5p,miR-134-5p,miR-3432a,miR-2284k,miR-1271,miR-2355-5p,miR-376c-5p,miR-200a-5p,miR-450b-5p,miR-450c-5p,miR-3064-5p,miR-28-3p,novel_127,miR-30f,miR-758-5p,miR-322-5p,miR-9-5p,novel_121,miR-134-5p,miR-2284z,miR-429-3p,miR-299a-3p,miR-2285ad,miR-412,miR-155-5p,miR-1290,miR-196a-5p,miR-23b-5p,novel_111,miR-877-3p,miR-361-3p,miR-423-5p,miR-2447,miR-9-5p,miR-2284j,miR-3964,miR-2440,novel_82,miR-2483-3p,miR-378g,miR-499a-3p,miR-144-5p,miR-2319b,miR-504,miR-3963,miR-335-3p,miR-221,miR-140-5p,miR-214-3p,miR-103a-2-5p,miR-199b-5p,miR-16b,miR-505,miR-29b-1-5p,miR-323b-3p,miR-1298-3p,miR-2459,miR-455-3p,novel_78,miR-1343-3p,miR-330-3p,miR-181b-2-3p,miR-1388-5p,miR-323b,miR-224-5p,miR-346,miR-6525,miR-181c-5p,miR-3184-5p,miR-122-5p,miR-3074-5p,miR-214,miR-4492,miR-874-5p,miR-129-5p,miR-30e-3p,miR-665,miR-30a-3p,miR-499b-3p,miR-15b-5p,miR-504-5p,miR-30c-1-3p,miR-2331-3p,miR-216b-3p,miR-186-5p,miR-1827,miR-2285c,miR-181b-1-3p,miR-2424,miR-329a-5p,miR-202-5p,novel_17,miR-345-3p,miR-324-3p,miR-18a-3p,miR-30d-3p,novel_73,miR-133a-3p,miR-24-3p,miR-371b-3p,miR-2428,novel_101,miR-495-3p,miR-17-3p,novel_1,miR-128-1-5p,miR-147-5p,miR-656-5p,miR-22-5p,miR-203b-5p,miR-15a,miR-16a,miR-29a-3p,miR-
SEMA6B	ENSOARG0(	sema dom	miR-486-3p,miR-541-3p,miR-147-3p,miR-885-3p,miR-486b-3p,miR-885-3p,miR-541,miR-1285,miR-431-3p
CEACAM16	ENSOARG0(	carcinoe	let-7c-5p,let-7k,let-7e,miR-2898,miR-98-5p,let-7f-5p,miR-363-5p,let-7a-5p,let-7g,miR-296-5p,miR-1961,miR-2300b-3p,miR-1843b-5p,miR-128-3p,miR-2889,miR-378d,let-7d,miR-9788-3p,miR-500-5p,miR-328-3p,let-7g-5p,let-7i,let-7f,miR-502-5p,miR-1306-3p,miR-874-5p,miR-1296-5p,miR-133a-3p,let-7b,let-7b-5p,miR-500b-5p,miR-362-5p,miR-362-5p,miR-1306-3p,miR-216a-3p,miR-625-5p,miR-6395,let-7e-5p,miR-671-5p,miR-10b-3p,miR-133b-3p,miR-504,miR-504-5p,novel_68,let-7d-5p,let-7i-5p,miR-154b-5p

DNAI1	ENSOARG0(	dynein,	let-7f-2-3p,let-7f-2-3p,miR-125a-5p,miR-466f-3p,miR-224-5p,miR-365b-5p,miR-197-5p,miR-194a,miR-185-5p,miR-134-3p,miR-149-5p,miR-194-3p,miR-31-5p,miR-339-5p,miR-30b-3p,miR-3432b,miR-1260b,miR-27b-3p,miR-708-3p,miR-2113,miR-1843b-5p,miR-1434-3p,miR-22-3p,miR-194-5p,miR-24-3p,miR-326-3p,miR-2428,miR-199b-5p,miR-3957-3p,miR-22-5p,miR-6123,miR-4324,miR-146b-3p,miR-320c,miR-6535,novel_68,miR-1291,miR-378f,miR-379-5p,miR-2387,miR-199a-5p,novel_39,miR-2284y,miR-2284a,miR-450a-2-3p,miR-191-3p,miR-1260a,miR-339a,miR-378h,miR-2411-5p,novel_121,miR-6517,miR-1224-5p,novel_79,miR-2427,miR-330-5p,miR-1260b,miR-378c,miR-365a-5p,miR-6128,miR-331-5p,miR-486b-3p,miR-331-3p,miR-125b-5p,miR-769,miR-199a-5p,miR-324-5p,miR-154b-5p,miR-874-3p,miR-370-3p,miR-125a,miR-615-5p,miR-329a,miR-485-5p,miR-331-5p,miR-320a,miR-378d,miR-8485,miR-1b-5p,miR-127-3p,novel_124,miR-25-5p,miR-199b-5p,miR-103a-2-5p,miR-320b,miR-326,miR-362-3p,miR-147a,miR-142-3p,miR-1185-3p,novel_4,novel_78,miR-1343-3p,miR-409b,miR-27a-3p,miR-362-3p,miR-378c,miR-1839-5p,miR-378b,miR-484,miR-329-3p,miR-1298-5p,miR-30b-3p,miR-877-3p,miR-423-5p,miR-212-3p,miR-1296-5p,miR-194b-5p,miR-452-5p,miR-499a-5p,miR-4726-5p,miR-382-3p,miR-769-5p,miR-107-5p,miR-378e,miR-378g,miR-3082-5p,miR-27a-3p,miR-1185-2-3p,miR-2284g,miR-452-3p,miR-7977,novel_94,miR-199b-5p,miR-376a-5p,miR-142a-3p,miR-2411,miR-486-3p,miR-5100,miR-345-3p,miR-320d,miR-760-3p,miR-221-5p,miR-324-3p,miR-422a,miR-125b,miR-92a-1-5p,miR-3960,miR-1b-3p,miR-3600,miR-4429,miR-378d,miR-2284u,miR-378i,novel_133,miR-362-3p,miR-2300b-3p,miR-378a-3p,miR-6525,miR-769-5p,miR-224-5p,miR-3184-5p,miR-338-3p,miR-6134,miR-378c,miR-145a-
CDC73	ENSOARG0(	cell div	miR-199b-5p,miR-9-3p,miR-211,miR-181d-5p,miR-2285x,miR-9-5p,miR-885-5p,miR-2284q,miR-379-3p,miR-628-3p,novel_87,miR-4443,miR-320c,miR-299b-3p,miR-3596,miR-211-5p,miR-499b-5p,miR-378b,miR-2319b,miR-329-5p,miR-2284g,miR-664b,miR-135b-5p,novel_94,miR-9-3p,miR-2319a,miR-335-3p,miR-668-5p,miR-378b,miR-150-5p,miR-221,miR-199b-5p,miR-539-3p,miR-8485,miR-193a,miR-30c-1-3p,miR-652-5p,miR-628-5p,miR-101-5p,miR-676-3p,miR-466f-3p,miR-199b-5p,miR-196b-5p,miR-505,novel_103,miR-320b,miR-149-5p,miR-221-3p,miR-539-5p,miR-432-5p,miR-188-5p,miR-2284aa,miR-2284ab,miR-181a-5p,miR-432,miR-181c-5p,miR-3074-2-3p,miR-2284v,miR-539-5p,miR-204-5p,miR-331-5p,miR-3604,miR-135a-5p,miR-15b-3p,miR-222-3p,miR-199a-5p,miR-30c-1-3p,miR-1248,miR-7144-5p,novel_48,miR-10b-3p,miR-3154,miR-320a,miR-331-5p,let-7f-1-3p,miR-199a-5p,miR-2284y,miR-1957a,miR-376a-5p,miR-183-5p,miR-329a-5p,miR-299-3p,miR-181b-5p,miR-2284m,miR-320d,miR-30c-2-3p,miR-3071-3p,miR-664-3p,miR-2285af,miR-196a-3p,miR-2284z,miR-7859,miR-487a-3p,miR-9-5p,miR-299a-3p,miR-222,miR-548e-3p,miR-2284u,miR-664-5p,miR-4429,novel_73,miR-582-3p,miR-411-3p,miR-196a-5p

CHFR	ENSOARG0(	checkpoi	miR-320b,novel_103,miR-326,miR-3085-3p,miR-424-5p,miR-30c-1-3p,miR-758-3p,miR-484,miR-10b,miR-143-5p,miR-412-3p,miR-30b-3p,miR-2426,miR-544a,miR-3120-5p,miR-105-1,novel_4,miR-2300a-5p,miR-27a-3p,miR-221-3p,miR-9788-3p,miR-2285e,miR-2285y,miR-4726-5p,miR-335,miR-450a-5p,miR-2285t,miR-2284q,miR-3059-5p,miR-541-3p,miR-361-3p,miR-199b-5p,miR-877-3p,miR-3064-5p,miR-7857-3p,miR-27a-3p,miR-216a-5p,miR-185-3p,miR-320d,miR-760-3p,miR-34b-5p,miR-21-3p,miR-2887,miR-29b-1-5p,miR-2411,miR-2300b-3p,miR-1246,miR-2904,miR-1197-5p,miR-5703,miR-1193,miR-4429,miR-15a-5p,miR-222-3p,miR-19b-1-5p,miR-17-3p,miR-664a,miR-424-5p,miR-539-5p,miR-145a-3p,miR-425-5p,miR-10b-5p,miR-206-3p,miR-6134,miR-338-3p,miR-27b-5p,miR-18a-5p,miR-370-5p,miR-532-5p,miR-544-3p,miR-193b-3p,miR-323-5p,miR-139-5p,miR-22-3p,miR-204-3p,miR-455-5p,miR-466f-3p,miR-654-3p,miR-138,miR-30b-3p,miR-7862,miR-455-5p,miR-133b-5p,miR-26b-3p,miR-2285g,miR-185-5p,miR-544b,miR-15a,miR-34c,miR-16a,miR-203b-5p,miR-502b,novel_101,miR-1193,miR-17-3p,miR-495-3p,miR-211,miR-371b-3p,miR-24-3p,miR-449a,novel_27,miR-326-3p,miR-2428,miR-18b,miR-2387,miR-1291,miR-9851-3p,miR-6395,miR-195a-5p,miR-320b,miR-10a-5p,miR-2404,miR-323a-5p,miR-146b-3p,miR-670-3p,miR-34b,miR-2285af,miR-30c-2-3p,miR-217,miR-17-3p,miR-543-5p,miR-3529-3p,miR-1,miR-4286,miR-181a-2-3p,miR-380-5p,miR-199a-5p,miR-1271-3p,miR-345-3p,miR-105-2,miR-128-3p,miR-10a-5p,miR-222,miR-483-3p,miR-2411-5p,miR-370-5p,miR-331-3p,miR-138-5p,miR-767-5p,miR-31-3p,miR-204-5p,miR-365a-5p,miR-2448-3p,miR-320a,miR-18a,miR-3074-1-3p,miR-23a-5p,miR-671-5p,miR-485-5p,miR-370-3p,miR-141-3p,miR-34c-5p,miR-365a-3p,miR-199a-5p,miR-154b-5p,miR-431-5p,miR-505,miR-29b-1-5p,miR-
F11R	ENSOARG0(	F11 rece	miR-4726-5p,miR-185-5p
GABRA5	ENSOARG0(	gamma-am	miR-219a-2-3p,miR-371b-3p,miR-17-3p,miR-2447,novel_1,miR-2440,miR-669,miR-677,miR-181b-3p,miR-323a-5p,miR-2483-3p,miR-489,miR-26b-5p,novel_68,miR-3064-5p,miR-452-3p,miR-2319a,miR-493-5p,miR-146b-5p,miR-129b-5p,miR-381-3p,miR-18b,miR-2387,miR-2310,miR-380-5p,miR-494-3p,miR-493-5p,miR-146b,miR-3085-3p,miR-2957,miR-146a,miR-181b-2-3p,miR-409b,miR-219a-1-3p,miR-197-3p,miR-3071-5p,miR-6525,miR-592,miR-17-3p,miR-6740-5p,miR-219b-3p,miR-204-3p,miR-6516,miR-582-5p,miR-365a-3p,miR-323-5p,miR-3068-3p,miR-186-5p,novel_99,miR-582-5p,miR-18a-5p,miR-2318,miR-18a,miR-433-3p,miR-2448-3p,miR-2424,miR-146a-5p,miR-143-3p,miR-380-5p,miR-181b-1-3p,miR-195a-3p,miR-3064-5p,miR-181a-2-3p,miR-216c-5p,miR-450a-2-3p,miR-17-3p,miR-2898,miR-30f,miR-2285af,miR-205-5p,miR-26a-5p,miR-219-3p,miR-532-3p,miR-616-3p,novel_121,miR-6516-5p,miR-222-5p,miR-18b-5p,miR-142-3p,miR-2427

NEK11	ENSOARG0(	NIMA-rel	miR-2284s,miR-499b-3p,miR-6130,miR-532-5p,miR-378a-3p,miR-140-5p,miR-221-5p,miR-548o-3p,miR-133b-3p,miR-2285c,miR-181c-5p,miR-6525,miR-346,miR-3074-2-3p,miR-378c,miR-4510,miR-874-5p,miR-129-5p,miR-664a,miR-324-3p,miR-30a-3p,miR-222-3p,miR-30e-3p,miR-1247-5p,miR-2284h-5p,miR-200b,miR-30d-3p,miR-18a-3p,miR-106a,miR-133a-3p,miR-148a-5p,miR-493-3p,miR-124-3p,miR-378a-3p,miR-378i,miR-193b-5p,miR-376a-5p,miR-20a,miR-2285w,miR-221-5p,miR-106a,miR-324-3p,miR-422a,miR-125b,miR-196a-3p,novel_17,miR-499a-3p,miR-378e,miR-3082-5p,miR-541-5p,miR-4792,miR-24-1-5p,miR-335-3p,miR-544-5p,miR-140-5p,miR-221,miR-8095,miR-877-3p,novel_111,miR-9-5p,miR-194b-5p,miR-3059-5p,novel_60,miR-885-5p,miR-3956-3p,miR-2284q,miR-148b-5p,miR-221-3p,miR-2285e,miR-222-5p,miR-145b,miR-3120-5p,miR-378c,miR-758-3p,miR-378b,miR-758-3p,miR-378d,miR-124a,miR-106a-5p,miR-505,miR-106b-5p,miR-370-3p,miR-141-3p,miR-125a,miR-20b-5p,miR-485-5p,miR-93-5p,miR-17-5p,miR-365a-5p,miR-378c,miR-93,miR-133a-3p,miR-134-5p,miR-125b-5p,miR-6740-5p,miR-134-5p,miR-214-5p,miR-9-5p,miR-378h,miR-222,miR-429-3p,miR-200b-3p,miR-218-2-3p,miR-134,miR-490-3p,miR-1271-3p,miR-24-2-5p,miR-30a-3p,miR-181a-2-3p,miR-200a-3p,novel_127,miR-450a-2-3p,miR-200c-3p,miR-181b-5p,miR-200c,miR-9851-3p,miR-378f,miR-18a-3p,miR-3959-3p,miR-4324,miR-20a-5p,miR-133a-5p,miR-20b,miR-2285g,miR-412-5p,miR-665,miR-31-5p,miR-2889,miR-455-5p,miR-194-5p,novel_23,miR-145a-5p,miR-181a-5p,miR-7b-5p,miR-125a-5p,miR-365b-5p,miR-194a,miR-106a-5p,miR-17-5p,miR-455-5p,miR-7-5p

SLC29A1	ENSOARG0(	solute c	miR-31-5p,miR-103a-3p,miR-411-5p,miR-708-3p,miR-431-3p,miR-4508,miR-668-3p,miR-628-5p,miR-377-3p,miR-365b-5p,novel_51,miR-106b-3p,miR-3074-5p,miR-411b-3p,novel_69,miR-374c-3p,miR-6535,miR-216a-3p,miR-2284e,miR-412-3p,let-7e-5p,miR-378f,miR-2284w,miR-199b-5p,miR-4324,miR-576-3p,miR-2284o,miR-6517,miR-412,miR-1224-5p,miR-505-5p,miR-134-5p,miR-2284z,miR-1343-5p,miR-3064-5p,miR-2284f,let-7g,miR-125a-3p,miR-664-3p,let-7f-5p,novel_96,novel_127,miR-503-3p,miR-874-3p,miR-2355-5p,miR-3956,miR-424-3p,miR-93-5p,miR-345-5p,miR-20b-5p,novel_42,miR-28a-3p,miR-2284v,miR-1197-3p,miR-2433,miR-134-5p,novel_78,miR-145b,miR-147a,miR-1298-3p,miR-126a-5p,miR-2459,miR-301b-5p,miR-758-3p,miR-1388-5p,miR-1388-3p,miR-124a,miR-676-3p,miR-378d,miR-29b-1-5p,miR-106b-5p,miR-491-5p,miR-199b-5p,miR-214-3p,miR-126b-5p,miR-3596,miR-652-3p,miR-203-3p,miR-96-5p,miR-99b-5p,miR-1983,miR-6527,miR-361-3p,miR-877-3p,miR-301a-5p,miR-628-3p,miR-154-3p,miR-18a-3p,miR-106a,miR-23c,miR-378i,miR-1961,miR-378a-3p,miR-664-5p,miR-487a,miR-369-3p,miR-345-3p,miR-486-3p,miR-143-3p,novel_17,miR-210-5p,miR-422a,miR-98-5p,let-7k,miR-2331-3p,miR-154a-3p,miR-377-3p,miR-2284s,let-7i-5p,miR-2285c,miR-129b-3p,miR-433-3p,miR-345-5p,miR-543-3p,miR-625-5p,miR-1827,let-7b,miR-433-5p,miR-3074-5p,miR-665-5p,miR-214,miR-6525,miR-346,miR-224-5p,let-7f,miR-665,miR-4492,miR-874-5p,miR-130b-5p,miR-133b-5p,miR-2284d,miR-2285g,miR-185-5p,let-7g-5p,miR-103,miR-2355-3p,miR-20b,miR-145a-5p,miR-3071-5p,miR-138,miR-30b-3p,miR-2284ab,miR-219a-1-3p,miR-744-5p,miR-455-5p,miR-130b-5p,miR-345-5p,miR-224-5p,miR-197-5p,miR-99a-5p,miR-106a-5p,miR-17-5p,miR-455-5p,miR-1343-5p,miR-378b,miR-323a-5p,miR-2387,miR-18a-3p,miR-378h,miR-

ARAP2	ENSOARGO(	ArfGAP w	miR-181b-1-3p,miR-130a-5p,miR-450b-5p,miR-376b-5p,miR-200a,miR-376c-5p,miR-29b-2-5p,miR-20a,miR-191-3p,miR-106a,miR-200a-3p,miR-299-3p,miR-152-3p,miR-125a-3p,miR-345-3p,miR-185-3p,miR-217,miR-339a,miR-324-3p,miR-216a-5p,miR-758-5p,miR-148b-3p,miR-6516-5p,miR-450a-1-3p,miR-545-5p,miR-106a,miR-299a-3p,miR-30d-3p,miR-212-5p,miR-505-3p,miR-7641,novel_79,miR-300,miR-10a-5p,miR-345-3p,miR-217-5p,miR-1434-5p,miR-93,miR-10b-5p,miR-145-3p,miR-3074-5p,miR-3074-2-3p,miR-450b-3p,miR-454-3p,miR-29b-2-5p,miR-100-3p,miR-144,miR-139-5p,miR-6516,miR-10a,miR-301a-3p,miR-582-5p,miR-133c,miR-548o-3p,miR-141-3p,miR-345-5p,miR-339b,miR-345-5p,let-7g-3p,miR-582-5p,miR-20b-5p,miR-376c-5p,miR-450a-1-3p,miR-2285c,miR-381-3p,miR-376c-5p,miR-17-5p,miR-93-5p,miR-345-5p,miR-8485,miR-3187-3p,miR-106a-5p,let-7c-3p,miR-101-5p,miR-130b-3p,miR-466f-3p,miR-106b-5p,miR-17-5p,miR-106a-5p,miR-2411-3p,miR-105-5p,miR-2285g,miR-421,miR-1185-3p,miR-2459,miR-2285e,miR-20b,miR-216a-3p,miR-133b-5p,miR-548w,miR-122-3p,miR-181b-2-3p,miR-130a-3p,miR-339-5p,miR-101a-5p,miR-2284x,miR-10b,miR-2284r,miR-708-3p,miR-450c-3p,miR-361-3p,miR-301b-3p,miR-3956-3p,novel_60,miR-495-3p,miR-628-3p,miR-20a-5p,novel_82,miR-2285n,novel_69,miR-299b-3p,miR-1185-2-3p,miR-181b-3p,miR-144-3p,miR-677,miR-3074-5p,miR-139-5p,miR-3120-3p,miR-10a-5p,miR-655-5p,miR-450b-3p,miR-877-3p,miR-505-3p,miR-148a-3p,miR-376b-5p,miR-381-3p,miR-150-5p

LYPD6	ENSOARG0(	LY6/PLAU	miR-16-1-3p,miR-133a-3p,let-7a-3p,miR-34a-5p,novel_73,miR-2904,miR-98-3p,miR-1197-5p,miR-188-3p,miR-1193,miR-5703,miR-30c-5p,miR-362-3p,miR-2300b-3p,miR-2366,miR-193b-5p,miR-2284h-5p,miR-616-3p,miR-383-5p,miR-487a-3p,miR-7859,miR-212-5p,miR-548e-3p,miR-18a-3p,miR-106a,miR-34b-5p,miR-30d,miR-1277-5p,miR-106a,miR-500a-5p,miR-216a-5p,miR-345-3p,miR-30b,miR-618,miR-486-3p,miR-181b-1-3p,miR-20a,miR-345-3p,miR-216c-5p,miR-183-5p,miR-543-3p,novel_99,miR-133b-3p,miR-30f,miR-345-5p,miR-129b-3p,miR-30d-5p,let-7f-1-3p,miR-132-5p,miR-27a-5p,miR-6516,miR-3065-3p,miR-296-3p,miR-532-5p,miR-30c-1-3p,miR-140-5p,miR-545-3p,miR-450b-3p,miR-664a,miR-6516-3p,miR-19b-1-5p,miR-132-5p,miR-338-5p,miR-665,miR-15b-3p,let-7b-3p,miR-224-5p,miR-98-3p,miR-129-2-3p,miR-3120-5p,miR-362-3p,miR-450b-5p,miR-2426,miR-2284r,miR-329-3p,miR-153,miR-376b-3p,miR-181b-2-3p,miR-105-1,novel_4,miR-1343-3p,miR-153-3p,miR-103a-2-5p,miR-362-3p,miR-505,miR-106b-5p,miR-326,miR-193a,miR-8485,miR-1b-5p,miR-2310,miR-30c-1-3p,miR-33a-5p,miR-106a-5p,miR-494-3p,miR-376a-3p,miR-381-3p,miR-361-3p,miR-454-5p,miR-25,miR-2483-3p,miR-489,miR-7689-3p,miR-490-5p,miR-2284q,miR-628-3p,miR-107-5p,miR-30b-5p,miR-2440,miR-1285-5p,miR-877-3p,miR-361-3p,miR-3059-5p,miR-30c,miR-29d-3p,miR-2427,miR-30a-5p,miR-330-5p,miR-345-3p,miR-3578,miR-450a-1-3p,miR-545-5p,miR-129-1-3p,miR-758-5p,miR-6516-5p,miR-1224-5p,novel_127,miR-200a-3p,miR-30c-2-3p,miR-205-5p,miR-2285af,miR-376b-3p,novel_39,miR-3065-3p,miR-29a-5p,miR-2478,miR-4286,miR-181a-2-3p,miR-7144-5p,miR-20b-5p,miR-1248,miR-485-5p,miR-1814c,miR-767,miR-329a,miR-345-5p,miR-93-5p,miR-17-5p,miR-30e-5p,miR-488-3p,miR-301,miR-154b-5p,miR-3957,miR-34c-5p,miR-370-
UBAP1	ENSOARG0(	ubiquiti	miR-876-3p,miR-541,miR-182-5p,miR-2285b,miR-2284h-5p,novel_32,miR-452-3p,miR-3956-3p,miR-541-3p,novel_60,miR-552-3p,miR-182-5p

GALNT8	ENSOARG0(	polypept	miR-16-5p,miR-2284v,miR-125b-5p,miR-2433,miR-2284k,miR-125a,miR-2285aa,miR-300-3p,miR-137-3p,miR-93-5p,miR-1248,miR-20b-5p,miR-2318,miR-345-5p,miR-876-3p,novel_39,miR-29a-5p,miR-148b-5p,miR-200a-3p,novel_127,novel_79,miR-2285ad,novel_121,miR-2284z,miR-450a-1-3p,miR-505-5p,miR-322-5p,miR-330-5p,miR-1290,miR-30c,miR-335-5p,miR-28c,miR-181b-3p,miR-677,miR-216a-3p,miR-2319a,miR-6240,miR-493-5p,miR-2284l,miR-432-3p,miR-432-3p,miR-2285r,novel_51,miR-2411-3p,miR-7-5p,miR-339-5p,miR-103a-3p,miR-3955-5p,miR-31-5p,miR-194-3p,miR-149-5p,miR-3431,miR-412-5p,novel_23,miR-450c-3p,miR-197-3p,miR-7b-5p,miR-19b-2-5p,miR-188-5p,miR-4508,miR-497-5p,miR-16-1-3p,miR-16b,miR-214,miR-346,miR-665,miR-664a-5p,miR-30a-3p,miR-30e-3p,miR-500,miR-4492,miR-454-3p,novel_74,miR-296-3p,miR-30c-1-3p,miR-504-5p,miR-15b-5p,miR-216b-5p,miR-10a,miR-30d-5p,miR-598-3p,miR-500a-3p,miR-129b-3p,miR-450a-1-3p,miR-339b,miR-345-5p,miR-2285w,miR-143-3p,miR-486-3p,miR-377-5p,miR-181b-1-3p,miR-345-3p,miR-92a-1-5p,miR-219b-5p,miR-210-5p,miR-30b,miR-212-5p,miR-30d-3p,miR-106a,miR-18a-3p,miR-1247-5p,miR-2284h-5p,miR-7859,miR-2366,miR-30c-5p,miR-16-1-3p,miR-2447,miR-877-3p,miR-2440,miR-3968,miR-2319b,miR-203-3p,miR-499a-3p,miR-335-3p,miR-493-5p,miR-504,miR-1388-3p,miR-8485,miR-106b-5p,miR-16b,miR-214-3p,miR-130b-3p,miR-487b-5p,miR-181b-2-3p,miR-330-3p,miR-455-3p,miR-758-3p,miR-541,miR-1388-5p,miR-1306-5p,miR-374a-3p,miR-93,miR-3969,miR-331-3p,miR-2332,miR-34c-3p,miR-486b-3p,miR-141-3p,miR-370-3p,miR-598-3p,miR-17-5p,miR-3154,miR-488-3p,miR-671-5p,miR-7144-5p,miR-4286,miR-30a-3p,miR-2385-3p,miR-28b,miR-339a,miR-30c-2-3p,miR-543-5p,miR-107,miR-17-3p,miR-214-5p,miR-582-3p,miR-6516-

TMEM86A	ENSOARG0(	transmem	miR-487a,miR-92a-1-5p,miR-125b,miR-422a,miR-345-3p,miR-500a-5p,miR-324-3p,miR-216a-5p,miR-26a-5p,miR-299-3p,miR-21-3p,miR-760-3p,miR-485-3p,miR-616-3p,miR-15a-5p,miR-148b-3p,miR-487a-3p,miR-1197-5p,miR-1193,miR-378a-3p,miR-5703,miR-140-3p,miR-1246,miR-2300b-3p,miR-378i,miR-378d,miR-214,miR-665-5p,miR-378c,miR-338-3p,miR-6134,miR-3184-5p,miR-769-5p,miR-665,miR-324-3p,miR-15b-3p,miR-424-5p,miR-4492,miR-664a,miR-450b-3p,miR-296-3p,miR-552-3p,miR-154a-3p,miR-496-3p,miR-378a-3p,miR-30c-1-3p,miR-877-5p,miR-15b-5p,miR-22-3p,novel_32,miR-204-3p,miR-216b-5p,miR-450a-1-3p,miR-2285c,miR-186-5p,miR-154b-3p,let-7g-3p,miR-33a-5p,let-7c-3p,miR-424-5p,miR-1839-3p,miR-2330-5p,miR-8485,miR-30c-1-3p,miR-378d,miR-16b,miR-505,miR-326,novel_103,miR-214-3p,miR-153-3p,miR-2300a-5p,miR-2285e,miR-9788-3p,miR-500-5p,miR-153,miR-301b-5p,miR-378b,miR-484,miR-296-5p,miR-1388-5p,miR-378c,miR-219a-5p,miR-3059-5p,miR-2447,miR-423-5p,novel_111,miR-877-3p,miR-362-5p,miR-361-3p,miR-301a-5p,miR-769-5p,miR-371a-5p,miR-154-3p,miR-490-5p,miR-2285t,miR-499a-5p,miR-211-5p,miR-7977,miR-10b,miR-664b,miR-454-5p,miR-378e,miR-378g,miR-361-3p,novel_94,miR-30c-2-3p,miR-125a-3p,miR-664-3p,miR-1260a,miR-152-3p,miR-1224-5p,miR-299a-3p,miR-450a-1-3p,miR-378h,miR-24-2-5p,miR-322-5p,miR-330-5p,miR-29d-3p,miR-218-2-3p,miR-345-3p,let-7a-2-3p,miR-496,miR-193a-5p,miR-16-5p,miR-378c,miR-1197-3p,miR-1260b,miR-500b-5p,miR-331-3p,miR-125b-5p,miR-29b-3p,miR-204-5p,miR-125a,miR-370-3p,miR-503-3p,miR-487a-3p,miR-3965,miR-365a-3p,miR-769,miR-362-5p,miR-485-5p,let-7g-3p,miR-876-3p,let-7a-2-3p,miR-539-3p,miR-432-3p,miR-654-3p,novel_51,miR-455-5p,miR-2411-3p,miR-197-5p,miR-125a-5p,miR-149-5p,novel_116,miR-134-3p,miR-2285g,miR-450c-
DKK3	ENSOARG0(	dickkopf	miR-32,novel_23,miR-92a-3p,miR-92a-3p,miR-363,miR-4792,miR-32-5p,miR-2447,miR-363-3p,miR-204-3p,miR-92b-3p,miR-6525,miR-25-3p

UBE2R2	ENSOARG0(	ubiquiti	miR-134,miR-3578,miR-345-3p,miR-330-5p,miR-582-3p,miR-155-5p,miR-200b-3p,miR-429-3p,miR-299a-3p,miR-2285ad,miR-9-5p,miR-24-2-5p,miR-322-5p,miR-505-5p,miR-302a-5p,novel_121,miR-486-5p,miR-214-5p,miR-134-5p,miR-2284z,miR-450a-1-3p,miR-200c,miR-125a-3p,miR-376b-3p,miR-2285af,miR-200c-3p,miR-152-3p,miR-3529-3p,miR-181a-2-3p,miR-3064-5p,miR-29b-2-5p,miR-380-5p,miR-490-3p,miR-200a,novel_39,miR-2448-3p,miR-320a,miR-3154,miR-345-5p,miR-7144-5p,miR-125a,miR-331-5p,miR-34c-5p,miR-374c-3p,miR-133c,miR-2284k,miR-410-5p,miR-409-3p,miR-125b-5p,miR-331-3p,miR-6740-5p,miR-134-5p,miR-486b-3p,miR-204-5p,miR-144-5p,miR-3607-3p,miR-16-5p,miR-1197-3p,miR-323-3p,miR-7b-5p,miR-1285,miR-1434-3p,miR-1843b-5p,miR-653,novel_23,miR-450c-3p,miR-22-3p,miR-16-1-3p,miR-16b,miR-30b-3p,miR-188-5p,miR-455-5p,miR-2284x,miR-497-5p,miR-31-5p,miR-3955-5p,miR-26b-3p,miR-3431,miR-146a,miR-7-5p,miR-2411-3p,miR-455-5p,miR-378a-5p,miR-125a-5p,miR-380-5p,miR-628-5p,miR-382,miR-432-3p,miR-432-3p,miR-345-5p,let-7j,miR-148a-3p,miR-2285ab,miR-3065-5p,miR-376b-3p,miR-6516-3p,miR-195a-5p,miR-146b-5p,miR-216b-3p,miR-3120-3p,miR-374c-3p,miR-299b-3p,miR-670-3p,miR-34b,miR-146b-3p,miR-181b-3p,miR-320c,miR-677,miR-15a,miR-34c,miR-16a,miR-502b,miR-203b-5p,miR-656-5p,miR-335-5p,miR-342-3p,miR-758-5p,miR-495-3p,miR-24-3p,miR-3959-3p,miR-211,miR-2428,miR-326-3p,novel_27,miR-449a,miR-2300b-3p,novel_133,miR-1197-5p,miR-5703,miR-140-3p,miR-4429,miR-378d,miR-34a-5p,miR-16-1-3p,miR-133a-3p,miR-380-5p,miR-200b,miR-148b-3p,miR-15a-5p,miR-199a-3p,miR-374a-5p,miR-2285p,miR-196a-3p,miR-125b,miR-345-3p,miR-185-3p,miR-2284m,miR-320d,miR-21-3p,miR-299-3p,miR-30b-3p,miR-34b-5p,miR-221-5p,miR-216c-5p,miR-181b-

CLUL1	ENSOARG0(	clusteri	miR-151a-3p,miR-548w,miR-9788-3p,miR-221-3p,miR-500-5p,miR-455-3p,miR-376b-3p,miR-125b-2-3p,miR-758-3p,miR-30b-3p,miR-296-5p,miR-4791,miR-541,miR-101c,miR-3120-5p,miR-2426,miR-544a,miR-23a-3p,let-7c-3p,miR-1388-3p,miR-1839-3p,miR-193a,miR-2310,miR-758-3p,miR-29b-1-5p,miR-182-5p,miR-199b-5p,miR-103a-2-5p,miR-211-5p,miR-139-5p,miR-2319b,miR-376d,miR-3596,miR-3082-5p,miR-361-3p,miR-221,miR-199b-5p,miR-376a-3p,miR-504,miR-136-5p,miR-190b-5p,miR-194b-5p,miR-2447,miR-3059-5p,miR-541-3p,miR-362-5p,miR-877-3p,miR-371a-5p,miR-769-5p,miR-107-5p,miR-2440,miR-101-3p,miR-2285t,miR-628-3p,miR-3958-3p,miR-3968,miR-3600,miR-18a-3p,miR-548e-3p,miR-1247-5p,miR-23b-3p,miR-200b,miR-23c,miR-2904,miR-1197-5p,miR-5703,miR-140-3p,miR-34a-5p,miR-142-3p,miR-33b-3p,miR-183-5p,miR-216c-5p,miR-2432,miR-376a-5p,miR-29b-1-5p,miR-377-5p,miR-125b,miR-92a-1-5p,miR-34b-5p,miR-21-3p,miR-202-5p,miR-296-3p,miR-182-5p,miR-504-5p,miR-548o-3p,novel_32,miR-204-3p,novel_25,miR-22-3p,miR-155-5p,miR-544-3p,miR-6130,miR-139-5p,miR-500a-3p,miR-411,miR-450a-1-3p,miR-1827,novel_99,let-7g-3p,miR-345-5p,miR-665-5p,miR-4510,miR-145a-3p,miR-3074-5p,miR-6134,miR-769-5p,miR-151-3p,miR-338-5p,miR-222-3p,miR-2285j,miR-500,miR-190a-5p,miR-130b-5p,miR-450b-3p,miR-155-5p,miR-665,miR-26b-3p,miR-377-5p,miR-31-5p,miR-3955-5p,miR-149-5p,miR-544b,miR-3535,miR-185-5p,miR-450c-3p,miR-22-3p,novel_23,miR-3071-5p,miR-194-5p,miR-188-5p,miR-30b-3p,miR-219a-1-3p,miR-382,miR-380-5p,miR-130b-5p,miR-6412,let-7a-2-3p,miR-345-5p,miR-2285r,miR-654-3p,miR-190a,miR-194a,miR-125a-5p,miR-124-5p,miR-3074-5p,miR-6535,miR-34b,miR-670-3p,miR-150-5p,miR-18a-3p,miR-376b-5p,miR-376a-2-5p,miR-450b-3p,miR-1839-3p,novel_120,miR-
GLRX2	ENSOARG0(	glutared	miR-541-5p,miR-370-3p,miR-7977,miR-378g,miR-769,miR-203-3p,miR-677,miR-22-3p,miR-181b-3p,miR-93-5p,miR-3970,miR-17-5p,miR-410-5p,miR-345-5p,miR-345-5p,miR-20b-5p,miR-6516-3p,miR-539-5p,miR-3958-5p,miR-214,miR-769-5p,miR-93,miR-8095,miR-2284w,miR-2285j,miR-20a-5p,miR-100-3p,miR-769-5p,miR-664a-5p,miR-107-5p,miR-487a-5p,miR-106a,miR-181b-2-3p,miR-222-5p,miR-539-5p,miR-134-3p,miR-1298-3p,miR-199a-3p,miR-671-3p,miR-20b,miR-22-3p,miR-106a-5p,miR-20a,miR-345-3p,miR-376a-5p,miR-181b-1-3p,miR-345-5p,miR-199a-3p,miR-17-5p,miR-106b-5p,miR-106a-5p,miR-106a,miR-103a-2-5p,miR-214-3p

PVRL2	ENSOARG0(	poliovir	miR-181c-5p,miR-6525,miR-769-5p,miR-665-5p,miR-378c,miR-539-5p,miR-708-5p,miR-4492,miR-129-5p,miR-6516-3p,miR-17-3p,miR-320e,miR-665,miR-2285j,novel_32,novel_25,miR-27a-5p,miR-182-5p,miR-370-5p,miR-21-3p,miR-660-5p,miR-140-5p,miR-378a-3p,miR-504-5p,miR-186-5p,miR-345-5p,miR-339b,let-7g-3p,miR-133b-3p,miR-21a-3p,miR-486-3p,miR-181b-1-3p,miR-376a-5p,miR-21-3p,miR-2284m,miR-345-3p,miR-125b,miR-422a,miR-324-3p,miR-199a-3p,miR-222-5p,miR-361-5p,miR-133a-3p,miR-4429,novel_73,miR-5703,miR-378a-3p,miR-1193,miR-124-3p,miR-2300b-3p,miR-378i,miR-194b-5p,miR-181d-5p,miR-2284j,miR-9-5p,miR-3956-3p,novel_60,miR-769-5p,miR-454-5p,miR-378g,miR-378e,miR-452-3p,miR-24-1-5p,miR-9-3p,novel_94,miR-504,miR-129b-5p,miR-140-5p,miR-491-3p,miR-378d,let-7c-3p,miR-124a,miR-3955-3p,miR-182-5p,miR-323b-3p,novel_115,miR-326,miR-320b,miR-199a-3p,miR-421,miR-9788-3p,miR-216a-3p,miR-147a,miR-496-5p,miR-181b-2-3p,novel_4,miR-199b-3p,miR-548w,miR-1388-5p,miR-378c,miR-30b-3p,miR-323b,miR-378b,miR-378c,miR-323-3p,miR-133a-3p,miR-6128,miR-28-5p,miR-134-5p,miR-486b-3p,miR-135a-5p,miR-331-3p,miR-125b-5p,miR-6740-5p,miR-370-5p,miR-5100,miR-135a-1-3p,miR-2284k,miR-769,miR-125a,miR-2355-5p,miR-874-3p,miR-671-5p,miR-345-5p,miR-10b-3p,miR-320a,miR-3154,novel_39,miR-24-2-5p,miR-4286,miR-543-5p,miR-181b-5p,miR-17-3p,miR-339a,miR-486-5p,miR-134-5p,miR-6516-5p,miR-378h,miR-9-5p,novel_79,miR-330-5p,miR-134,miR-345-3p,miR-2428,miR-326-3p,miR-24-3p,miR-17-3p,novel_101,miR-1193,miR-199c,miR-421-5p,miR-485-3p,miR-4324,miR-181b-3p,miR-677,miR-411b-3p,miR-378f,miR-135b-5p,miR-6516-3p,miR-376a-2-5p,miR-3591-3p,miR-345-5p,miR-432-3p,miR-432-3p,miR-628-5p,miR-668-3p,miR-125a-5p,miR-660,miR-124-
ATP1B1	ENSOARG0(	ATPase,	let-7g-3p,miR-885-3p,miR-218-1-3p,miR-885-3p,miR-135b-5p,miR-223-3p,miR-137-3p,miR-500a-3p,miR-142-5p,miR-545-5p,miR-574-5p,miR-199b-3p,miR-329-5p,miR-3120-3p,miR-141-3p,miR-500,miR-200a-3p,miR-199c,miR-15b-3p,novel_63,miR-218-5p,miR-576-3p,miR-135a-5p,miR-142a-5p,miR-329a-5p

FBXL5	ENSOARG0(	F-box an	miR-10a-5p,miR-128-3p,miR-300,miR-345-3p,miR-486-5p,miR-545-5p,miR-378h,miR-7641,miR-1224-5p,miR-423-3p,miR-299a-3p,novel_96,miR-543-5p,miR-6536,miR-205-5p,miR-2285b,miR-200a,miR-29a-5p,miR-148b-5p,miR-30a-3p,miR-485-5p,miR-671-5p,miR-1248,miR-7144-5p,novel_48,miR-20b-5p,miR-200a-5p,miR-93-5p,miR-424-3p,miR-488-3p,miR-381-3p,miR-324-5p,miR-331-5p,miR-1271,miR-99a-3p,miR-5100,miR-99b-3p,miR-378c,miR-365a-5p,miR-93,miR-6128,miR-374b,miR-455-5p,miR-27b-3p,miR-188-5p,miR-223-3p,miR-1434-3p,miR-544b,miR-2355-3p,miR-411-5p,miR-26b-3p,miR-133b-5p,miR-466f-3p,miR-660,miR-124-5p,miR-17-5p,miR-106a-5p,miR-455-5p,miR-2411-3p,miR-365b-5p,miR-224-5p,miR-654-3p,miR-6395,miR-378f,miR-410-5p,miR-216a-3p,miR-3065-5p,miR-3970,miR-150-5p,miR-2387,miR-181b-3p,miR-677,miR-299b-3p,miR-670-3p,miR-10a-5p,miR-26b-5p,miR-3120-3p,miR-655-5p,miR-425-5p,miR-20a-5p,miR-323c,miR-3959-3p,miR-495-3p,miR-148a-5p,miR-140-3p,miR-378a-3p,miR-1193,miR-2366,miR-378i,miR-328-3p,miR-7859,miR-548e-3p,miR-21-3p,miR-299-3p,miR-99a-3p,miR-422a,miR-345-3p,miR-500a-5p,miR-26a-5p,miR-125b-2-3p,miR-181b-1-3p,miR-345-3p,miR-20a,miR-1827,miR-186-5p,let-7g-3p,miR-433-3p,miR-21a-3p,miR-877-5p,miR-216b-5p,miR-10a,miR-544-3p,miR-548o-3p,miR-660-5p,miR-545-3p,miR-504-5p,miR-378a-3p,miR-129-5p,miR-3604,miR-30a-3p,miR-224-5p,miR-10b-5p,miR-665-5p,miR-3958-5p,miR-378c,miR-544a,miR-2426,miR-378c,miR-10b,miR-378b,miR-6529a,miR-148b-5p,miR-216a-3p,miR-153,miR-181b-2-3p,miR-27a-3p,novel_78,miR-548w,miR-153-3p,miR-491-5p,miR-106b-5p,miR-2957,miR-1b-5p,miR-193a,miR-8485,miR-378d,miR-33a-5p,miR-744-3p,miR-652-5p,let-7c-3p,miR-494-3p,miR-106a-5p,miR-101b-3p,miR-877-3p,novel_94,miR-504,miR-129b-5p,miR-27a-3p,miR-378e,miR-
FABP12	ENSOARG0(	fatty ac	miR-5703,miR-140-3p,miR-2366,miR-496,miR-218-1-3p,miR-212-5p,miR-30d-3p,novel_79,miR-2285ad,miR-18a-3p,miR-214-5p,miR-339a,miR-664-3p,novel_127,miR-181b-5p,miR-30a-3p,miR-2385-3p,miR-329a-5p,miR-1271-3p,miR-2285i,miR-450b-5p,miR-3154,miR-200a-5p,miR-500a-3p,miR-424-3p,miR-1827,miR-339b,miR-532-5p,miR-552-3p,miR-2355-5p,miR-496-3p,miR-503-3p,miR-223-5p,miR-30a-3p,miR-30e-3p,miR-204-5p,miR-592,miR-500,miR-129-5p,novel_74,miR-425-5p,miR-3074-5p,miR-181c-5p,miR-194-5p,miR-181a-5p,miR-27b-3p,miR-1388-5p,miR-27a-3p,miR-339-5p,miR-548w,miR-185-5p,miR-194a,miR-493-5p,miR-1b-5p,miR-18a-3p,miR-378j,miR-361-3p,miR-493-5p,miR-335-3p,miR-2285k,miR-211-5p,miR-320b,miR-3074-5p,miR-452-3p,miR-329-5p,miR-664b,miR-27a-3p,miR-33a-3p,miR-2285y,miR-2285x,miR-194b-5p,miR-181d-5p,miR-211

TAPBP	ENSOARG0(	TAP bind	miR-1291,miR-6395,miR-3141,miR-18b,miR-221,miR-361-3p,miR-489,miR-2404,miR-7857-3p,miR-7977,miR-199c,miR-101-3p,miR-615,miR-4324,miR-218-5p,miR-24-3p,miR-2428,miR-326-3p,miR-212-3p,miR-3956-3p,novel_60,miR-30b-3p,miR-6529a,miR-873a-5p,miR-30b-3p,miR-185-5p,miR-425-3p,miR-221-3p,miR-2300a-5p,miR-199b-3p,miR-222-5p,miR-665,miR-125a-5p,miR-326,novel_103,miR-199a-3p,miR-3141,miR-2330-5p,miR-101b-3p,miR-18a-5p,miR-329b,miR-582-5p,novel_99,miR-1248,miR-186-5p,miR-1827,miR-485-5p,miR-18a,miR-6130,miR-132-5p,miR-151b,miR-223-5p,miR-125a,miR-2285aa,miR-2355-5p,miR-134-5p,miR-2433,miR-222-3p,miR-125b-5p,miR-6740-5p,miR-4510,miR-493-3p,miR-2300b-3p,miR-134,miR-1246,miR-2408,miR-615-3p,miR-330-5p,miR-23b-5p,miR-1193,miR-1434-5p,miR-505-5p,miR-328-3p,miR-582-3p,miR-671-3p,miR-199a-3p,miR-134-5p,miR-151-5p,miR-6517,miR-3960,miR-222,miR-18b-5p,miR-127-5p,miR-450a-2-3p,miR-2285af,miR-125b,novel_39,miR-2403
PLA2G2F	ENSOARG0(	phosphol	miR-192-3p,novel_39,miR-3064-5p,miR-760-3p,miR-466i-5p,miR-543-5p,miR-30b-3p,miR-8117,miR-422a,miR-125b,miR-185-3p,miR-92a-1-5p,miR-339a,miR-30c-2-3p,miR-378h,miR-328-3p,novel_79,miR-378d,miR-133a-3p,miR-1246,miR-2366,miR-378i,miR-330-5p,miR-378a-3p,miR-501-3p,miR-124-3p,miR-2904,miR-346,miR-2903,miR-3184-5p,miR-378c,miR-378c,miR-193a-5p,miR-664a,miR-4492,miR-874-5p,miR-551b-3p,miR-320e,miR-665,miR-125b-5p,miR-331-3p,miR-216b-5p,miR-300-3p,miR-378a-3p,miR-30c-1-3p,miR-504-5p,miR-296-3p,miR-125a,miR-1271,miR-216b-3p,miR-345-5p,miR-345-5p,miR-339b,miR-1827,miR-1248,miR-485-5p,miR-23a-5p,miR-378d,miR-30c-1-3p,miR-345-5p,miR-193a,miR-4532,miR-6240,miR-124a,miR-3085-3p,miR-125a-5p,miR-326,miR-1306-5p,novel_51,miR-330-3p,miR-3431,miR-134-3p,miR-1298-3p,miR-2459,miR-149-5p,miR-194-3p,miR-330-3p,miR-187-3p,miR-145b,miR-5010-3p,miR-339-5p,miR-219a-1-3p,miR-378c,miR-301b-5p,miR-296-5p,miR-378b,miR-2284r,miR-145a-5p,miR-7857,miR-501-3p,miR-24-3p,miR-361-3p,miR-877-3p,miR-2428,miR-502-3p,miR-326-3p,miR-423-5p,miR-3956-3p,miR-3059-5p,novel_60,miR-4726-5p,miR-502b,miR-490-5p,novel_82,miR-4324,miR-301a-5p,miR-378e,miR-378g,miR-4443,miR-500-3p,miR-677,miR-378b,miR-3064-5p,miR-7977,miR-320b,miR-504,miR-1291,miR-378f,miR-877-3p,miR-6395,miR-361-3p,miR-1306

ARHGAP30	ENSOARG0(	Rho GTPa	miR-29a,miR-502-5p,miR-211,miR-2284w,miR-199b-5p,miR-1839-3p,miR-29c-3p,miR-1291,miR-2319a,miR-6395,miR-378f,miR-376b-5p,miR-3141,miR-148a-3p,miR-2387,miR-21c,miR-3065-5p,miR-323a-5p,miR-29a-3p,miR-670-3p,miR-378b,miR-10a-5p,miR-3120-3p,miR-7-1-3p,miR-378a-5p,miR-1343-5p,miR-125a-5p,miR-224-5p,miR-296-3p,miR-3141,miR-654-3p,miR-2285r,miR-296-3p,miR-380-5p,miR-29b,miR-21-5p,miR-30b-3p,miR-1260b,miR-219a-1-3p,miR-2284x,miR-708-3p,miR-2113,miR-450c-3p,miR-3071-5p,miR-3535,miR-3431,novel_116,miR-6238,miR-340-3p,miR-31-5p,miR-204-5p,novel_63,miR-551b-3p,miR-29b-3p,miR-125b-5p,miR-331-3p,miR-500b-5p,miR-1197-3p,miR-1260b,miR-10b-3p,miR-378c,miR-28a-3p,miR-1306-5p,miR-181a-2-3p,miR-10b-3p,miR-329b,miR-376c-5p,miR-30e-5p,miR-2448-3p,miR-301,miR-362-5p,miR-376c-5p,miR-223-5p,miR-199a-5p,miR-503-3p,miR-125a,miR-615-5p,miR-450a-2-3p,miR-200c-3p,miR-152-3p,miR-543-5p,miR-205-5p,miR-1260a,miR-200c,miR-30c-2-3p,miR-380-5p,miR-199a-5p,miR-3529-3p,miR-30a-3p,miR-582-3p,miR-200b-3p,miR-10a-5p,miR-615-3p,miR-3591-5p,miR-1343-5p,miR-29d-3p,miR-30a-5p,miR-23b-5p,miR-758-5p,miR-378h,miR-450a-1-3p,miR-429-3p,miR-3958-3p,miR-2284q,miR-30b-5p,miR-615,miR-107-5p,miR-877-3p,miR-362-5p,miR-361-3p,novel_111,miR-423-5p,miR-2447,miR-212-3p,miR-668-5p,miR-504,miR-140-5p,miR-199b-5p,miR-378e,miR-4443,miR-211-5p,miR-199b-5p,miR-103a-2-5p,novel_19,miR-30c-1-3p,miR-378d,miR-2310,miR-758-3p,miR-1839-3p,miR-2330-5p,miR-376b,miR-33a-5p,miR-378c,miR-1388-5p,miR-2426,miR-378b,miR-6529a,miR-10b,miR-4791,miR-30b-3p,miR-758-3p,miR-500-5p,miR-455-3p,miR-2300a-5p,miR-1343-3p,miR-5010-3p,miR-450b-3p,miR-874-5p,miR-487a-5p,miR-30e-3p,miR-30a-3p,miR-320e,miR-10b-5p,miR-224-5p,miR-3184-
AMOTL1	ENSOARG0(	angiomot	miR-421,miR-2424
ATP2C1	ENSOARG0(	ATPase,	miR-2284y,miR-2284a,miR-664b-3p,miR-214,miR-2284b,miR-2284j,miR-2284v,miR-3184-3p,miR-452-5p,miR-214-3p,miR-2284m,novel_87,miR-2284k,novel_83,miR-2284n,miR-2284d,miR-2284g,miR-7975,miR-2284u,miR-148a-5p,miR-876-3p,miR-668-5p,miR-3071-5p
TBCK	ENSOARG0(	TBC1 dom	miR-145a-3p,let-7a-2-3p,miR-301b-3p,miR-145-3p,novel_96,miR-152-3p,miR-454-3p,miR-130b-3p,miR-379-3p,miR-155-5p,miR-491-5p,miR-30d-3p,miR-130a-3p,miR-2319b,miR-155-5p,miR-148b-3p,miR-301a-3p,miR-1434-5p,miR-223-3p,miR-148a-3p,miR-2300b-3p,miR-3120-5p,miR-2319a,miR-155-5p,miR-1388-5p

SRCIN1	ENSOARG0(	SRC kina	miR-1271-5p,miR-322-5p,miR-431,miR-18b-5p,miR-7641,miR-6517,miR-299a-3p,novel_127,novel_96,miR-30c-2-3p,miR-2285af,miR-205-5p,miR-1260a,miR-3064-5p,miR-671-5p,miR-20b-5p,miR-345-5p,miR-876-3p,miR-10b-3p,miR-18a,miR-3154,miR-17-5p,miR-93-5p,miR-301,miR-34c-5p,miR-125a,miR-370-3p,miR-2355-5p,miR-874-3p,miR-574-5p,miR-125b-5p,miR-1260b,miR-93,miR-133a-3p,miR-16-5p,novel_42,miR-4508,miR-497-5p,miR-1260b,miR-16b,miR-145a-5p,miR-2113,miR-873a-5p,miR-20b,miR-185-5p,miR-377-5p,miR-133b-5p,miR-125a-5p,miR-17-5p,novel_51,miR-106a-5p,miR-345-5p,miR-432-3p,miR-195a-5p,miR-18a-3p,miR-378b,miR-150-5p,miR-18b,miR-6535,miR-669,miR-34b,miR-299b-3p,miR-329-5p,novel_87,miR-16a,miR-34c,miR-20a-5p,miR-15a,miR-502-5p,miR-2284w,miR-449a,miR-24-3p,miR-378d,miR-34a-5p,miR-1193,miR-2366,miR-2284h-5p,miR-15a-5p,miR-7859,miR-106a,miR-548e-3p,miR-18a-3p,miR-299-3p,miR-34b-5p,miR-106a,miR-92a-1-5p,miR-125b,miR-185-3p,miR-324-3p,miR-216a-5p,novel_17,miR-125b-2-3p,miR-377-5p,miR-376a-5p,miR-29b-1-5p,miR-345-3p,miR-20a,miR-2285w,miR-329a-5p,miR-345-5p,miR-18a-5p,miR-133b-3p,miR-15b-5p,miR-6130,miR-296-3p,miR-21-3p,miR-548o-3p,miR-30c-1-3p,miR-874-5p,miR-424-5p,miR-664a,miR-132-5p,miR-324-3p,miR-2285j,miR-338-3p,miR-6134,miR-122-5p,miR-2330-3p,miR-1271,miR-4510,miR-3184-3p,miR-3120-5p,miR-2426,miR-301b-5p,miR-541,miR-455-3p,miR-9788-3p,miR-145b,miR-491-5p,miR-3085-3p,miR-106b-5p,miR-16b,miR-29b-1-5p,miR-431-5p,miR-30c-1-3p,miR-106a-5p,miR-424-5p,miR-1388-3p,miR-96-5p,miR-2285u,miR-3064-5p,miR-2284q,miR-4726-5p,miR-301a-5p,miR-382-3p,miR-218-5p,miR-1285-5p,miR-541-3p

PLA2G2D	ENSOARG0(	phosphol	miR-362-3p,miR-329-3p,miR-541,miR-30b-3p,miR-1298-3p,miR-9788-3p,miR-216a-3p,miR-1343-3p,miR-330-3p,novel_103,miR-1306-5p,miR-326,miR-362-3p,miR-29b-1-5p,miR-106b-5p,miR-30c-1-3p,miR-8485,miR-124a,miR-106a-5p,miR-504,miR-1983,miR-877-3p,miR-3596,miR-3958-3p,miR-2284q,novel_82,miR-877-3p,miR-423-5p,miR-541-3p,miR-6527,miR-1296-5p,miR-194b-5p,miR-493-3p,miR-664-5p,miR-378d,novel_133,miR-2300b-3p,miR-193b-5p,miR-362-3p,miR-124-3p,miR-2904,miR-1193,miR-151-5p,miR-106a,miR-106a,miR-221-5p,miR-216a-5p,miR-324-3p,miR-185-3p,miR-29b-1-5p,miR-486-3p,miR-2411,miR-143-3p,miR-20a,miR-339b,miR-1895,miR-1827,miR-433-3p,miR-377-3p,miR-151b,miR-30c-1-3p,miR-221-5p,miR-504-5p,miR-21-3p,miR-29b-2-5p,miR-665,miR-122-5p,miR-3184-5p,miR-129-2-3p,miR-665-5p,miR-3074-2-3p,miR-30b-3p,miR-4508,miR-2284x,miR-7b-5p,miR-194-5p,miR-412-5p,miR-194-3p,miR-20b,miR-339-5p,miR-1343-5p,miR-7-5p,miR-377-3p,miR-330-3p,miR-17-5p,miR-194a,miR-106a-5p,miR-432-3p,miR-6240,miR-412-3p,miR-9851-3p,miR-1306,miR-3591-3p,miR-216a-3p,miR-670-3p,miR-6535,miR-203b-5p,miR-6123,miR-502b,miR-502-5p,miR-20a-5p,miR-28c,miR-371b-3p,miR-326-3p,novel_101,miR-128-3p,miR-3578,miR-1343-5p,miR-330-5p,miR-2411-5p,miR-129-1-3p,novel_79,miR-30c-2-3p,miR-339a,miR-28b,miR-1271-3p,miR-29b-2-5p,miR-329a,miR-20b-5p,miR-301,miR-93-5p,miR-17-5p,miR-486b-3p,miR-551b-3p,miR-331-3p,miR-93,miR-1306-5p,miR-193a-5p
APOC4	ENSOARG0(	apolipop	miR-22-3p,miR-412-3p,miR-197-3p,miR-30b-3p,miR-541,miR-30b-3p,miR-1388-5p,miR-2426,miR-2300a-5p,miR-133b-5p,miR-153,miR-3141,miR-146a,miR-431-5p,miR-153-3p,miR-146b,miR-2285o,miR-491-5p,miR-378a-5p,miR-2285l,miR-668-3p,miR-8485,let-7a-2-3p,miR-3141,miR-146b-5p,miR-877-3p,miR-3963,miR-4792,miR-6535,miR-4443,miR-323a-5p,miR-34b,miR-34c,miR-769-5p,miR-502b,miR-3968,miR-541-3p,miR-449a,miR-2428,miR-7705,miR-3959-3p,miR-5703,miR-193b-5p,miR-218-1-3p,let-7a-2-3p,miR-34a-5p,miR-412,miR-431,miR-212-5p,miR-2411-5p,miR-34b-5p,novel_96,miR-5100,miR-4286,miR-2411,miR-146a-5p,miR-29a-5p,novel_39,miR-2478,let-7g-3p,miR-21-3p,miR-2331-3p,miR-22-3p,novel_32,miR-34c-5p,miR-323-5p,miR-769,miR-665,miR-320e,miR-4492,miR-769-5p,miR-6525

GXYLT2	ENSOARG0(	glucosid	miR-664-5p,miR-33b-3p,miR-2904,miR-188-3p,miR-5703,miR-140-3p,miR-30c-5p,miR-215-5p,miR-2366,miR-376a-5p,miR-532-3p,novel_83,miR-2284h-5p,miR-487a-3p,miR-148b-3p,miR-485-3p,miR-212-5p,miR-221-5p,miR-21-3p,miR-2284m,miR-466i-5p,miR-216a-5p,miR-345-3p,miR-30b,miR-2285p,miR-2424,miR-143-3p,miR-181b-1-3p,miR-183-5p,miR-329a-5p,miR-2285w,miR-105-3p,miR-625-5p,novel_99,let-7g-3p,miR-133b-3p,miR-345-5p,miR-30f,miR-433-3p,miR-21a-3p,miR-450a-1-3p,miR-22-3p,miR-204-3p,miR-544-3p,miR-192-5p,miR-139-5p,miR-656-3p,miR-216b-3p,miR-221-5p,miR-548o-3p,miR-130b-5p,miR-450b-3p,miR-6516-3p,miR-17-3p,miR-324-3p,miR-3604,miR-2285j,miR-15b-3p,miR-206-3p,miR-2330-3p,miR-129-2-3p,miR-101c,miR-3120-5p,miR-544a,miR-412-3p,miR-1a-3p,miR-758-3p,miR-484,miR-6529a,miR-541,miR-4791,miR-301b-5p,miR-455-3p,miR-1185-3p,miR-153,miR-376b-3p,miR-222-5p,miR-181b-2-3p,miR-330-3p,novel_78,miR-2300a-5p,miR-409b,miR-153-3p,miR-2285o,miR-103a-2-5p,miR-3955-3p,miR-2957,miR-505,miR-326,miR-4532,miR-758-3p,miR-491-3p,miR-2310,miR-652-5p,miR-33a-5p,miR-376a-3p,miR-136-5p,miR-668-5p,miR-361-3p,miR-203-3p,miR-1185-2-3p,miR-376d,miR-3596,miR-25,miR-3082-5p,miR-2285u,miR-126b-5p,miR-139-5p,miR-452-3p,miR-499a-5p,miR-3968,miR-2285y,miR-301a-5p,miR-107-5p,miR-2285n,miR-30b-5p,miR-1285-5p,miR-9-5p,miR-452-5p,miR-2447,novel_60,miR-3059-5p,miR-541-3p,miR-3956-3p,miR-30c,miR-29d-3p,miR-1343-5p,miR-23b-5p,miR-330-5p,miR-345-3p,miR-1290,miR-3578,miR-450a-1-3p,miR-129-1-3p,miR-486-5p,miR-9-5p,miR-412,miR-2285ad,miR-152-3p,miR-17-3p,miR-376b-3p,miR-1957a,miR-2285b,miR-490-3p,miR-192-3p,miR-28b,miR-2385-3p,miR-1,miR-181a-2-3p,miR-3529-3p,miR-671-5p,miR-1814c,miR-767,miR-329b,miR-345-5p,miR-137-3p,miR-374c-
HDHD1	ENSOARG0(	haloacid	miR-7144-5p,miR-582-5p,miR-320b,miR-21-3p,miR-7977,miR-544-3p,miR-582-5p,miR-378g,miR-324-5p,miR-154b-5p,miR-101-3p,miR-499a-5p,miR-4492,miR-486b-3p,miR-541-3p,miR-129-2-3p,miR-361-3p,miR-346,miR-2284w,miR-432,miR-541,miR-544a,miR-219a-1-3p,miR-101c,miR-4508,miR-744-5p,miR-432-5p,miR-134-3p,miR-671-3p,miR-544b,miR-129-1-3p,miR-296-3p,novel_19,miR-2898,miR-452-5p,miR-101b-3p,miR-486-3p,miR-296-3p
MPHOSPH6	ENSOARG0(	M-phase	miR-877-5p,miR-2284h-5p,miR-200a,miR-3082-5p,miR-141-3p,miR-125a,miR-493-5p,miR-2285w,miR-200a-3p,miR-493-5p,miR-125a-5p,miR-219a-1-3p,miR-125b,miR-125b-5p,miR-30f

UBE4A	ENSOARG0(	ubiquiti	miR-194b-5p, miR-2447, miR-502-3p, miR-423-5p, miR-371a-5p, miR-30b-5p, miR-374a-3p, miR-3964, miR-628-3p, miR-335, miR-24-1-5p, miR-452-3p, miR-144-5p, miR-27a-3p, miR-500-3p, miR-1185-2-3p, miR-7857-3p, miR-378e, miR-2483-3p, miR-378j, miR-140-5p, miR-1983, miR-744-3p, miR-124a, miR-424-5p, miR-193a, miR-1b-5p, miR-378d, miR-16b, miR-320b, miR-326, miR-27a-3p, miR-181b-2-3p, miR-501-3p, miR-412-3p, miR-296-5p, miR-378b, miR-16-2-3p, miR-378c, miR-3074-2-3p, miR-378c, miR-3074-5p, miR-122-5p, miR-3184-5p, miR-338-5p, miR-3604, miR-29b-2-5p, miR-4492, miR-874-5p, miR-424-5p, miR-370-5p, miR-296-3p, miR-378a-3p, miR-548o-3p, miR-22-3p, miR-204-3p, miR-15b-5p, miR-192-5p, miR-582-5p, miR-129b-3p, miR-30d-5p, miR-3613-5p, novel_99, miR-1827, miR-582-5p, let-7g-3p, miR-154b-3p, miR-30f, miR-329a-5p, miR-181b-1-3p, miR-422a, miR-196a-3p, miR-30b, miR-30d, miR-30b-3p, miR-2484, miR-320d, miR-3600, miR-505-3p, miR-548e-3p, miR-7859, miR-200b, miR-15a-5p, miR-124-3p, miR-378a-3p, miR-1197-5p, miR-5703, miR-217-5p, miR-30c-5p, miR-378i, miR-215-5p, miR-218-1-3p, miR-4429, miR-495-3p, miR-1193, miR-2285x, miR-135a-2-3p, miR-335-5p, miR-326-3p, miR-2284w, miR-3959-3p, miR-16a, miR-15a, miR-3613, miR-28c, miR-6402, miR-502b, miR-320b, miR-7-1-3p, miR-3074-5p, miR-329-5p, miR-320c, miR-181b-3p, miR-670-3p, miR-3970, miR-216a-3p, miR-195a-5p, miR-505-3p, miR-135b-5p, miR-378f, miR-450b-3p, miR-412-3p, miR-107, miR-2483-5p, let-7f-2-3p, let-7f-2-3p, let-7a-2-3p, miR-194a, miR-466f-3p, miR-665, miR-31-5p, miR-103a-3p, miR-6238, miR-103, miR-432-5p, miR-412-5p, miR-22-3p, miR-3071-5p, novel_23, miR-194-5p, miR-432, miR-542-3p, miR-2113, miR-1434-3p, miR-497-5p, miR-26a-2-3p, miR-27b-3p, miR-16b, miR-16-5p, miR-144-5p, miR-378c, miR-145-3p, miR-374a-3p, miR-1197-3p, miR-135a-5p, novel_63, miR-370-5p, miR-31-3p, miR-
KIF5C	ENSOARG0(	kinesin	let-7f-2-3p, let-7f-2-3p, miR-424-5p, miR-503-3p, miR-3956-5p, miR-16b, miR-107, miR-5010-3p, miR-103a-3p, novel_121, miR-103, miR-582-3p, miR-15a-5p, miR-322-5p, novel_23, miR-2284x, miR-497-5p, miR-16b, novel_101, miR-6527, miR-16-5p, miR-193a-5p, miR-4510, miR-371b-3p, miR-665, miR-16a, miR-576-3p, miR-15a, miR-424-5p, novel_1, miR-2285aa, miR-1185-2-3p, miR-15b-5p, miR-6130, miR-2284s, miR-301, miR-361-3p, miR-195a-5p, miR-107, let-7g-3p

BAG1	ENSOARG0(	BCL2-ass	novel_99,miR-21a-3p,miR-204-3p,miR-192-5p,miR-193b-3p,miR-370-5p,miR-221-5p,miR-378a-3p,miR-660-5p,miR-424-5p,miR-450b-3p,miR-17-3p,miR-324-3p,miR-3074-2-3p,miR-378c,miR-145a-3p,miR-378d,miR-2904,miR-1193,miR-188-3p,miR-140-3p,miR-5703,novel_133,miR-362-3p,miR-215-5p,miR-2408,novel_83,miR-15a-5p,miR-222-5p,miR-200b,miR-505-3p,miR-221-5p,miR-21-3p,miR-2284m,miR-216a-5p,miR-185-3p,miR-146a-5p,miR-29b-1-5p,miR-216c-5p,miR-183-5p,miR-199b-5p,miR-378e,miR-3082-5p,miR-664b,miR-452-3p,miR-20a-3p,miR-154a,miR-181d-5p,miR-194b-5p,novel_60,miR-3059-5p,miR-541-3p,miR-2284aa,miR-378c,miR-450b-5p,miR-2426,miR-299a-5p,miR-378b,miR-6529a,miR-5010-3p,novel_4,miR-2300a-5p,miR-3955-3p,miR-362-3p,miR-326,miR-30c-1-3p,miR-382-3p,miR-494-3p,miR-2330-5p,miR-424-5p,miR-485-5p,miR-10b-3p,let-7g-3p,miR-3154,miR-381-3p,miR-199a-5p,miR-2284n,miR-374c-3p,miR-370-3p,miR-215-5p,miR-204-5p,miR-138-5p,miR-331-3p,miR-3969,miR-135a-5p,miR-410-5p,miR-370-5p,miR-378c,miR-3607-3p,miR-200b-3p,let-7a-2-3p,miR-300,miR-345-3p,miR-3578,miR-545-5p,miR-483-3p,miR-378h,miR-107,miR-543-5p,miR-2898,miR-17-3p,miR-200c-3p,miR-181b-5p,miR-30c-2-3p,miR-376b-3p,miR-200c,miR-199a-5p,miR-2385-3p,miR-4286,miR-181a-2-3p,miR-2403,miR-3529-3p,miR-505-3p,miR-195a-5p,miR-135b-5p,miR-450b-3p,miR-107,miR-18a-3p,miR-2387,miR-670-3p,miR-7-1-3p,miR-502b,novel_1,miR-16a,miR-15a,miR-326-3p,miR-211,miR-24-3p,miR-3957-3p,miR-17-3p,miR-1193,miR-495-3p,miR-2285x,miR-138,miR-194-5p,miR-374b-3p,miR-873a-5p,miR-181a-5p,miR-1285,miR-103,miR-2355-3p,miR-2284d,miR-432-5p,miR-26b-3p,miR-133b-5p,miR-1343-5p,miR-452-5p,miR-660,miR-654-3p,miR-543-3p,miR-625-5p,miR-186-5p,miR-1827,miR-433-3p,miR-299,miR-450a-1-
DNAJC16	ENSOARG0(	DnaJ (Hs	miR-17-3p,miR-2285b,miR-10b-3p,miR-200a,miR-99a,miR-2440,novel_87,miR-99a-5p,miR-3601,miR-17-3p,miR-6119-3p,miR-17-3p,miR-21-3p,miR-200a-3p,miR-141-3p,miR-3959-5p,miR-223-5p,miR-100-5p,miR-99b-5p,miR-10b-3p,miR-99a-5p
MAL2	ENSOARG0(	mal, T-c	miR-136-5p,miR-30f,novel_94,miR-381-3p,miR-30e-5p,miR-3082-5p,miR-192-5p,miR-329-5p,miR-215-5p,miR-182-5p,miR-6402,miR-30b-5p,miR-2285n,miR-2285y,miR-326-3p,novel_101,miR-665-5p,miR-33b-3p,miR-300,miR-376a-5p,miR-215-5p,miR-30c-5p,miR-30a-5p,miR-330-5p,miR-194-3p,miR-409b,miR-2285ad,miR-7641,miR-450a-2-3p,miR-125a-3p,miR-326,miR-30b,miR-182-5p,miR-30f,miR-376a-5p,miR-329a-5p

PLCB4	ENSOARG0(	phosphol	miR-3120-5p, miR-455-5p, miR-30b-3p, miR-16-1-3p, miR-2426, miR-197-3p, miR-30b-3p, miR-3071-5p, miR-708-3p, miR-143-5p, miR-301b-5p, miR-9788-3p, miR-421, miR-3955-5p, miR-31-5p, miR-1343-3p, miR-330-3p, miR-214-3p, miR-103a-2-5p, miR-378a-5p, miR-365b-5p, miR-330-3p, miR-455-5p, miR-326, miR-193a, let-7c-3p, miR-2483-5p, miR-380-5p, miR-6240, miR-335-3p, miR-1291, miR-96-5p, miR-3591-3p, miR-148a-3p, miR-2387, miR-652-3p, miR-669, miR-7857-3p, miR-2404, miR-7977, miR-216b-3p, miR-329-5p, miR-2285t, miR-147-5p, miR-371a-5p, miR-301a-5p, miR-107-5p, miR-4324, miR-218-5p, miR-423-5p, miR-326-3p, miR-7705, miR-2284w, miR-361-3p, novel_60, miR-32-3p, novel_120, miR-212-3p, miR-3956-3p, miR-378d, miR-941, miR-330-5p, miR-5703, miR-23b-5p, miR-2300b-3p, miR-134, miR-134-5p, miR-148b-3p, miR-23c, miR-1271-5p, miR-3600, miR-30d-3p, miR-200a-3p, miR-21-3p, miR-152-3p, miR-2898, miR-324-3p, miR-125a-3p, miR-6119-5p, miR-618, miR-200a, miR-1957a, miR-2285b, miR-1271-3p, miR-192-3p, miR-380-5p, miR-29b-2-5p, miR-30a-3p, miR-183-5p, miR-329a-5p, miR-582-5p, let-7g-3p, miR-876-3p, miR-2448-3p, novel_32, miR-582-5p, miR-2355-5p, miR-21-3p, miR-141-3p, miR-140-5p, miR-32-3p, miR-3068-3p, miR-708-5p, miR-28-5p, miR-134-5p, miR-30a-3p, miR-665, miR-320e, miR-29b-2-5p, miR-30e-3p, miR-3184-5p, miR-365a-5p, miR-1197-3p, miR-1271, miR-2330-3p, miR-214

UCKL1	ENSOARG0(	uridine-	miR-18a-3p,miR-378b,miR-150-5p,miR-6395,miR-2319a,miR-146b-5p,miR-1291,miR-10a-5p,miR-3074-5p,miR-669,miR-670-3p,novel_69,miR-29a-3p,miR-146b-3p,miR-33a-3p,miR-6402,miR-1247-3p,miR-502-5p,miR-4324,miR-29a,miR-206,miR-758-5p,miR-29c-3p,miR-2428,miR-326-3p,miR-24-3p,miR-3071-5p,miR-432,miR-4508,miR-1260b,miR-30b-3p,miR-138,miR-29b,miR-26b-3p,miR-544b,miR-2355-3p,miR-149-5p,miR-3431,miR-2285g,miR-432-5p,novel_51,miR-377-3p,miR-146a,miR-125a-5p,miR-628-5p,miR-363-5p,miR-380-5p,miR-345-5p,miR-3154,miR-2448-3p,miR-671-5p,miR-485-5p,miR-7144-5p,miR-23a-5p,miR-345-5p,miR-329a,miR-876-3p,miR-767,miR-125a,miR-1271,miR-215-5p,miR-2355-5p,miR-370-3p,miR-874-3p,miR-154b-5p,miR-3969,miR-125b-5p,miR-331-3p,miR-29b-3p,miR-134-5p,miR-486b-3p,miR-28-5p,miR-767-5p,miR-138-5p,miR-133a-3p,miR-1306-5p,miR-1197-3p,miR-1260b,miR-330-5p,miR-23b-5p,miR-29d-3p,miR-134,miR-3578,miR-10a-5p,miR-2285ad,miR-214-5p,miR-134-5p,miR-2285af,miR-1260a,novel_96,miR-543-5p,novel_127,miR-2898,miR-181a-2-3p,miR-1,miR-3064-5p,miR-29b-2-5p,miR-3065-3p,miR-380-5p,miR-140-5p,miR-877-3p,miR-504,miR-7977,miR-499b-5p,miR-452-3p,miR-3064-5p,miR-4792,miR-2483-3p,miR-301a-5p,miR-2285y,miR-4726-5p,miR-219a-5p,miR-212-3p,miR-2447,miR-423-5p,miR-361-3p,miR-30b-3p,miR-1a-3p,miR-10b,miR-301b-5p,miR-2284r,miR-484,miR-296-5p,miR-329-3p,miR-3120-5p,miR-544a,miR-362-3p,miR-2300a-5p,miR-1343-3p,miR-2285e,miR-1298-3p,miR-147a,miR-362-3p,miR-326,miR-146b,miR-491-5p,miR-3085-3p,let-7c-3p,miR-494-3p,miR-2330-5p,miR-124a,miR-1388-3p,miR-4532,miR-500a-3p,miR-2285c,miR-21a-3p,miR-1827,miR-345-5p,miR-133b-3p,let-7g-3p,miR-3065-3p,miR-140-5p,miR-2331-3p,miR-504-5p,miR-877-5p,novel_32,miR-204-3p,miR-

UNK	ENSOARG0(	unkempt	miR-22-3p,novel_23,miR-1843b-5p,miR-7b-5p,miR-27b-3p,miR-138,miR-29b,miR-2284ab,miR-1260b,miR-16-1-3p,miR-26b-3p,miR-149-5p,miR-194-3p,miR-20b,miR-2284d,miR-3431,miR-134-3p,miR-503-5p,miR-365b-5p,miR-197-5p,miR-106a-5p,novel_51,miR-17-5p,miR-7-5p,miR-503-5p,miR-146a,miR-296-3p,miR-1343-5p,miR-466f-3p,miR-668-3p,miR-296-3p,let-7a-2-3p,miR-654-3p,miR-1306,miR-18a-3p,miR-216a-3p,miR-2319a,miR-146b-5p,miR-450b-3p,miR-1291,miR-320b,miR-7-1-3p,miR-144-3p,miR-6535,novel_69,miR-34b,miR-374c-3p,miR-29a-3p,miR-34c,miR-4324,miR-28c,miR-20a-5p,miR-22-5p,miR-656-5p,miR-128-1-5p,miR-29a,miR-3957-3p,miR-29c-3p,miR-495-3p,miR-17-3p,miR-135a-2-3p,miR-449a,miR-326-3p,miR-24-3p,miR-1307-3p,miR-29d-3p,miR-1343-5p,miR-330-5p,miR-30a-5p,miR-128-3p,miR-30c,let-7a-2-3p,miR-1224-5p,miR-6517,miR-582-3p,miR-2284z,novel_121,miR-129-1-3p,miR-505-5p,miR-30c-2-3p,miR-1260a,miR-6119-5p,novel_127,novel_96,miR-450a-2-3p,miR-17-3p,miR-4286,miR-181a-2-3p,miR-3065-3p,miR-200a,miR-490-3p,miR-28b,miR-93-5p,miR-3154,miR-17-5p,miR-30e-5p,miR-20b-5p,miR-485-5p,miR-1248,let-7g-3p,miR-329a,miR-370-3p,miR-1271,miR-874-3p,miR-34c-5p,miR-769,miR-144,miR-331-3p,miR-29b-3p,miR-3432a,miR-28-5p,miR-486b-3p,miR-138-5p,miR-133a-3p,miR-2284v,miR-365a-5p,miR-93,miR-1260b,miR-125b-2-3p,miR-296-5p,miR-2284r,miR-329-3p,miR-484,miR-16-2-3p,miR-2284aa,miR-3120-5p,miR-362-3p,miR-27a-3p,novel_4,novel_78,miR-409b,miR-2300a-5p,miR-1343-3p,miR-216a-3p,miR-1298-3p,miR-362-3p,miR-106b-5p,miR-1306-5p,miR-5126,miR-326,miR-146b,miR-214-3p,miR-491-5p,novel_124,miR-382-3p,miR-106a-5p,miR-8485,miR-4532,miR-193a,miR-30c-1-3p,miR-361-3p,novel_94,miR-877-3p,miR-147-3p,miR-27a-3p,miR-378g,miR-769-5p,miR-30b-

AKAP3	ENSOARG0(	A kinase	miR-20a,miR-2432,miR-143-3p,miR-125b-2-3p,miR-181b-1-3p,miR-130a-5p,miR-185-3p,miR-92a-1-5p,miR-345-3p,miR-210-5p,miR-34b-5p,miR-21-3p,miR-299-3p,miR-106a,miR-760-3p,miR-1b-3p,miR-3600,miR-380-5p,miR-30d-3p,miR-18a-3p,miR-3960,miR-106a,miR-532-3p,miR-328-3p,miR-2904,miR-5703,novel_133,miR-2300b-3p,miR-133a-3p,miR-34a-5p,miR-378d,miR-493-3p,miR-184-3p,miR-214,miR-145a-3p,miR-3074-5p,miR-3184-3p,miR-4510,miR-3184-5p,miR-338-3p,miR-6525,miR-224-5p,miR-346,miR-30a-3p,miR-665,miR-29b-2-5p,miR-30e-3p,miR-4492,miR-17-3p,miR-487b-5p,miR-3065-3p,miR-552-3p,miR-296-3p,miR-140-5p,miR-504-5p,miR-30c-1-3p,miR-204-3p,miR-6130,miR-193b-3p,miR-139-5p,miR-21a-3p,miR-1895,miR-133b-3p,miR-345-5p,miR-1388-3p,miR-494-3p,miR-106a-5p,miR-101b-3p,miR-30c-1-3p,miR-2957,miR-106b-5p,novel_103,miR-431-5p,miR-5126,miR-326,miR-103a-2-5p,miR-214-3p,miR-491-5p,miR-181b-2-3p,miR-1343-3p,miR-9788-3p,miR-216a-3p,miR-500-5p,miR-455-3p,miR-425-3p,miR-125b-2-3p,miR-484,miR-541,miR-101c,miR-450b-5p,miR-2426,miR-2447,miR-541-3p,miR-8095,miR-423-5p,miR-362-5p,miR-107-5p,miR-101-3p,miR-2440,miR-628-3p,miR-3968,miR-7689-3p,miR-541-5p,miR-211-5p,miR-139-5p,miR-24-1-5p,miR-4443,miR-378g,miR-129b-5p,miR-335-3p,miR-877-3p,novel_94,miR-1983,miR-668-5p,miR-504,miR-30a-3p,miR-29b-2-5p,miR-181a-2-3p,miR-2403,miR-1957a,novel_39,miR-3065-3p,miR-490-3p,miR-28b,miR-380-5p,miR-24-2-5p,miR-148b-5p,miR-30f,miR-30c-2-3p,miR-125a-3p,novel_127,novel_96,miR-17-3p,miR-2898,miR-7641,miR-1224-5p,miR-431,miR-299a-3p,miR-505-5p,miR-24-2-5p,miR-483-3p,miR-758-5p,miR-1343-5p,miR-330-5p,miR-345-3p,miR-133a-3p,miR-1306-5p,miR-500b-5p,miR-93,miR-331-3p,miR-6740-5p,miR-409-3p,miR-2433,miR-551b-3p,miR-204-5p,miR-
PTPN11	ENSOARG0(	protein	miR-130b-3p,miR-2957,miR-2284b,miR-1839-5p,miR-1260b,miR-708-3p,miR-216a-3p,miR-421,miR-376b-3p,miR-130a-3p,miR-133b-5p,miR-203b-5p,miR-2284q,miR-6119-3p,miR-33a-3p,miR-218-5p,miR-301b-3p,miR-371b-3p,miR-3059-5p,miR-376a-3p,miR-6516-3p,miR-505-3p,miR-379-5p,miR-216a-3p,miR-376b-3p,miR-3065-5p,miR-148a-3p,miR-376d,miR-489,miR-670-3p,miR-655-5p,miR-2284g,miR-3653-3p,miR-152-3p,miR-200a-3p,miR-3071-3p,miR-1260a,miR-376b-3p,miR-2284y,miR-200a,miR-2424,miR-2284a,miR-143-3p,miR-192-3p,miR-30a-3p,miR-29b-2-5p,miR-128-3p,miR-2284u,miR-411-3p,miR-98-3p,miR-140-3p,miR-582-3p,miR-7859,miR-148b-3p,miR-1224-5p,miR-505-3p,miR-30d-3p,miR-548e-3p,miR-410-3p,miR-454-3p,miR-487b-5p,miR-30a-3p,miR-29b-2-5p,let-7b-3p,miR-30e-3p,miR-1260b,miR-98-3p,miR-193a-5p,miR-7144-5p,miR-487b-5p,let-7f-1-3p,miR-324-5p,miR-301a-3p,miR-3965,miR-2285aa,miR-532-5p,miR-141-3p
ZMYND10	ENSOARG0(	zinc fin	miR-324-3p,miR-664a,miR-450a-2-3p,miR-491-5p,novel_96,miR-1343-5p,miR-329a-5p,miR-1343-5p,miR-197-3p,miR-329b,miR-2426,miR-876-3p,miR-6395,miR-485-5p,miR-505-3p,miR-329-5p,miR-2300a-5p,miR-1271,miR-505-3p,miR-421,miR-328-3p

SLC26A7	ENSOARG0(	solute c	miR-1814c,miR-199a-5p,miR-2355-5p,miR-2285aa,miR-1271,miR-215-5p,miR-487a-3p,miR-3068-3p,miR-3432a,miR-486b-3p,miR-144,miR-135a-5p,miR-2284k,miR-323-3p,miR-6128,miR-105-2,miR-200b-3p,miR-2427,miR-330-5p,miR-196a-5p,miR-345-3p,miR-582-3p,miR-545-5p,miR-302a-5p,miR-24-2-5p,miR-1224-5p,miR-429-3p,miR-543-5p,novel_96,miR-450a-2-3p,miR-200c-3p,miR-30c-2-3p,miR-200c,novel_39,miR-199a-5p,miR-24-2-5p,miR-30a-3p,miR-3529-3p,miR-2319a,miR-135b-5p,miR-6516-3p,miR-19a-3p,miR-1306,miR-3065-5p,miR-378b,miR-3591-3p,miR-2387,miR-144-3p,miR-677,miR-181b-3p,miR-411b-3p,miR-3120-3p,miR-3074-5p,miR-378b,miR-625-3p,miR-421-5p,miR-203b-5p,miR-502b,miR-199c,novel_1,miR-382-5p,miR-133a-5p,miR-326-3p,novel_27,miR-199b-5p,miR-2428,miR-3959-3p,miR-342-3p,miR-455-5p,miR-2284x,miR-30b-3p,miR-219a-1-3p,miR-223-3p,miR-2113,novel_116,miR-2285g,miR-134-3p,miR-26b-3p,miR-377-5p,miR-3955-5p,miR-452-5p,miR-196b-5p,miR-190a,miR-105-5p,miR-2411-3p,miR-455-5p,miR-224-5p,miR-377-3p,miR-539-3p,miR-503-3p,miR-432-3p,miR-432-3p,miR-668-3p,miR-382,let-7f-2-3p,let-7f-2-3p,miR-130b-5p,miR-543-3p,miR-186-5p,miR-154b-3p,miR-2285c,miR-377-3p,miR-192-5p,miR-216b-5p,miR-139-5p,miR-216b-3p,miR-154a-3p,miR-21-3p,miR-182-5p,miR-30c-1-3p,miR-548o-3p,miR-190a-5p,miR-129-5p,miR-130b-5p,miR-6516-3p,miR-487b-5p,miR-324-3p,miR-30a-3p,miR-3604,miR-338-5p,miR-2285j,miR-30e-3p,miR-6525,miR-224-5p,miR-3184-3p,miR-3074-5p,miR-145a-3p,miR-142-3p,miR-19b-3p,miR-5703,novel_133,miR-215-5p,miR-1246,miR-2284h-5p,miR-7859,miR-487a-3p,miR-200b,miR-30d-3p,miR-2284m,miR-466i-5p,miR-345-3p,miR-196a-3p,miR-618,miR-2285p,miR-486-3p,miR-2424,miR-29b-1-5p,miR-181b-1-3p,miR-125b-2-3p,miR-377-5p,miR-142a-3p,miR-345-3p,miR-183-5p,miR-487a,miR-
FMR1NB	ENSOARG0(	fragile	miR-133a-3p,miR-224-5p,miR-142a-5p,miR-365a-5p,miR-664a,miR-30c-1-3p,miR-216b-5p,miR-6516,miR-374c-3p,miR-2285c,miR-301,miR-488-3p,miR-3154,miR-376c-5p,miR-137-3p,miR-345-5p,miR-345-5p,miR-133b-3p,miR-10b-3p,miR-485-5p,miR-376c-5p,miR-345-3p,miR-376a-5p,novel_39,miR-345-3p,miR-30c-2-3p,miR-466i-5p,miR-450a-2-3p,miR-6516-5p,miR-483-3p,miR-532-3p,miR-345-3p,miR-188-3p,miR-5703,miR-330-5p,miR-1343-5p,miR-218-1-3p,miR-133a-3p,miR-24-3p,miR-2428,miR-326-3p,miR-374c-3p,miR-4443,miR-376b-5p,miR-877-3p,miR-6516-3p,miR-2319a,miR-6240,miR-30c-1-3p,miR-345-5p,miR-8485,miR-326,novel_103,miR-224-5p,miR-2411-3p,miR-330-3p,miR-365b-5p,miR-483-3p,miR-378a-5p,miR-1343-5p,novel_78,miR-330-3p,miR-539-5p,miR-2285g,miR-3535,miR-2285e,miR-4791,miR-125b-2-3p,miR-450b-5p

ANKRD52	ENSOARG0(	ankyrin	miR-1827,miR-885-3p,miR-1895,miR-625-5p,miR-133b-3p,miR-151b,miR-204-3p,miR-22-3p,miR-6130,miR-499b-3p,miR-296-3p,miR-370-5p,miR-2331-3p,miR-140-5p,miR-221-5p,miR-874-5p,miR-665,miR-19b-1-5p,miR-222-3p,miR-6134,miR-338-3p,miR-3184-5p,miR-346,miR-214,miR-129-2-3p,miR-4510,miR-145a-3p,miR-34a-5p,miR-148a-5p,miR-133a-3p,miR-4429,miR-1197-5p,miR-1193,miR-5703,miR-199a-3p,miR-151-5p,miR-2284h-5p,miR-200b,miR-383-5p,miR-7859,miR-106a,miR-548e-3p,miR-18a-3p,miR-34b-5p,miR-30b-3p,miR-221-5p,miR-106a,miR-92a-1-5p,miR-185-3p,miR-219b-5p,miR-324-3p,miR-542-5p,miR-486-3p,miR-2432,miR-125b-2-3p,miR-345-3p,miR-20a,miR-221,miR-361-3p,miR-203-3p,miR-144-5p,miR-499a-3p,miR-378g,miR-499b-5p,miR-452-3p,miR-20a-3p,miR-3064-5p,miR-4792,miR-101-3p,miR-2440,miR-423-5p,miR-361-3p,miR-541-3p,miR-212-3p,miR-2447,miR-30b-3p,miR-541,miR-484,miR-9788-3p,miR-148b-5p,miR-221-3p,miR-539-5p,miR-425-3p,miR-199b-3p,novel_4,miR-1343-3p,miR-2300a-5p,miR-542-5p,miR-214-3p,miR-25-5p,miR-487b-5p,miR-491-5p,miR-3085-3p,miR-106b-5p,miR-2957,miR-320b,miR-199a-3p,miR-342,miR-4532,miR-33a-5p,miR-101b-3p,miR-106a-5p,miR-485-5p,miR-20b-5p,miR-320a,miR-17-5p,miR-3154,miR-93-5p,miR-34c-5p,miR-2355-5p,miR-874-3p,miR-574-5p,miR-486b-3p,miR-134-5p,miR-3969,miR-331-3p,miR-370-5p,miR-5100,miR-2433,miR-93,miR-133a-3p,miR-144-5p,miR-200b-3p,miR-885-3p,miR-134,miR-129-1-3p,miR-214-5p,miR-134-5p,miR-483-3p,miR-429-3p,miR-222,novel_96,novel_127,miR-200c-3p,miR-200c,novel_39,miR-148b-5p,miR-4286,miR-2403,miR-3064-5p,miR-1291,miR-18a-3p,miR-150-5p,miR-3141,miR-2387,miR-669,miR-6535,novel_69,miR-34b,miR-2404,miR-199c,miR-656-5p,miR-128-1-5p,miR-34c,miR-20a-5p,miR-1247-3p,miR-449a,miR-371b-3p,miR-323c,miR-542-5p,miR-1193,miR-342-

PLA2G3	ENSOARG0(	phosphol	miR-214-5p,miR-532-3p,miR-616-3p,miR-148b-3p,miR-212-5p,miR-3600,miR-412,miR-2285ad,miR-218-1-3p,miR-133a-3p,let-7d,miR-1197-5p,miR-330-5p,miR-2904,miR-1343-5p,miR-2427,miR-2300b-3p,miR-1961,miR-193b-5p,miR-486-3p,let-7g,miR-377-5p,miR-130a-5p,miR-450b-5p,miR-4286,novel_96,miR-152-3p,let-7k,miR-21-3p,miR-221-5p,miR-760-3p,let-7f-5p,miR-98-5p,miR-2898,let-7c-5p,miR-125b,miR-324-3p,miR-1260a,let-7i-5p,miR-204-3p,novel_25,miR-323-5p,miR-6130,miR-2284s,miR-544-3p,miR-125a,miR-615-5p,miR-216b-3p,miR-221-5p,miR-186-5p,miR-7144-5p,miR-767,miR-3154,miR-500a-3p,miR-338-3p,let-7b-5p,miR-1260b,miR-214,miR-6128,miR-145a-3p,miR-4510,let-7b,miR-130b-5p,miR-486b-3p,miR-592,miR-500,miR-4492,miR-767-5p,miR-665,miR-100-3p,miR-125b-5p,miR-135a-1-3p,let-7f,miR-544b,let-7g-5p,miR-134-3p,miR-185-5p,miR-377-5p,miR-26b-3p,miR-1343-3p,novel_4,miR-409b,miR-2300a-5p,miR-3120-5p,miR-544a,miR-1260b,miR-412-3p,miR-197-3p,miR-143-5p,miR-541,miR-484,miR-296-5p,miR-4532,miR-652-5p,miR-363-5p,let-7a-5p,miR-130b-5p,miR-214-3p,let-7e,miR-125a-5p,miR-1343-5p,miR-491-5p,miR-326,miR-454-5p,miR-669,let-7d-5p,miR-489,miR-323a-5p,miR-378g,miR-320b,miR-452-3p,miR-2319b,novel_94,let-7e-5p,miR-9851-3p,miR-668-5p,miR-3970,miR-148a-3p,miR-2387,miR-326-3p,miR-24-3p,miR-495-3p,miR-541-3p,miR-2447,let-7i,miR-2285y
LDHA	ENSOARG0(	lactate	miR-29b-1-5p,miR-323b-3p,miR-199a-3p,miR-214-3p,miR-124-5p,miR-101b-3p,miR-493-5p,miR-2284l,miR-432-3p,miR-432-3p,miR-30b-3p,miR-323b,miR-1285,miR-181a-5p,miR-4791,miR-6529a,miR-2284aa,miR-101a-5p,miR-2426,miR-219a-1-3p,miR-380-3p,miR-29b,miR-30b-3p,miR-1388-5p,miR-2284ab,miR-665,miR-133b-5p,miR-1343-3p,miR-103a-3p,miR-103,miR-455-3p,miR-9788-3p,novel_116,miR-216a-3p,miR-3535,miR-2284d,miR-34c,miR-101-3p,miR-30b-5p,miR-502-5p,miR-147-5p,miR-29a,miR-29c-3p,miR-452-5p,miR-181d-5p,miR-219a-2-3p,miR-2428,miR-449a,miR-378j,miR-376b-5p,miR-6516-3p,miR-9-3p,miR-493-5p,miR-668-5p,miR-107,miR-504,miR-7-1-3p,miR-655-5p,miR-216b-3p,miR-144-3p,miR-3082-5p,miR-29a-3p,miR-34b,miR-3596,miR-200c,miR-219-3p,miR-30b,miR-34b-5p,miR-30d,miR-107,miR-181b-5p,miR-127-5p,miR-200c-3p,miR-380-3p,miR-486-3p,novel_39,miR-29b-1-5p,miR-30a-5p,miR-5703,miR-2427,miR-29d-3p,miR-134,miR-30c-5p,miR-34a-5p,miR-30c,miR-218-1-3p,miR-200b-3p,miR-212-5p,miR-429-3p,miR-2284z,miR-199a-3p,miR-134-5p,miR-545-5p,miR-200b,miR-340-5p,miR-383-5p,miR-219b-3p,miR-100-3p,miR-144,miR-29b-3p,miR-34c-3p,miR-2284k,miR-134-5p,miR-486b-3p,miR-874-5p,miR-767-5p,miR-6516-3p,miR-214,miR-2284v,miR-539-5p,miR-181c-5p,miR-323-3p,miR-6525,miR-3154,miR-30d-5p,miR-598-3p,miR-30e-5p,miR-30f,miR-767,miR-598-3p,miR-3068-3p,miR-504-5p,novel_25,miR-27a-5p,miR-34c-5p

NDC80	ENSOARG0(	NDC80 ki	miR-20a,miR-369-3p,miR-329a-5p,miR-2432,miR-2411,miR-142a-3p,miR-130a-5p,miR-376a-5p,miR-29b-1-5p,miR-185-3p,miR-125b,miR-2285p,miR-196a-3p,miR-299-3p,miR-34b-5p,miR-760-3p,miR-106a,miR-202-5p,miR-485-3p,miR-106a,miR-18a-3p,novel_83,miR-23c,miR-23b-3p,miR-487a-3p,miR-1197-5p,miR-217-5p,miR-140-3p,miR-1434-5p,miR-2904,miR-376a-5p,miR-1246,miR-148a-5p,miR-34a-5p,miR-2284u,miR-218-1-3p,miR-214,miR-181c-5p,miR-338-3p,miR-6134,miR-142a-5p,miR-655-3p,miR-2285j,miR-669a-3p,miR-190a-5p,miR-500,miR-129-5p,miR-454-3p,miR-17-3p,miR-450b-3p,miR-182-5p,miR-216b-3p,miR-21-3p,miR-548o-3p,miR-2331-3p,miR-301a-3p,miR-377-3p,miR-655,miR-500a-3p,miR-450a-1-3p,novel_99,miR-543-3p,miR-625-5p,miR-339b,miR-345-5p,miR-133b-3p,let-7g-3p,miR-33a-5p,miR-23a-3p,miR-1839-3p,miR-106a-5p,miR-2330-5p,miR-8485,miR-182-5p,miR-106b-5p,miR-29b-1-5p,miR-326,miR-214-3p,miR-103a-2-5p,miR-199b-5p,miR-130b-3p,miR-3955-3p,miR-199b-3p,novel_4,miR-548w,miR-421,miR-2459,miR-148b-5p,miR-147a,miR-142-3p,miR-501-3p,miR-484,miR-2284aa,miR-190b-5p,miR-194b-5p,miR-2284j,miR-452-5p,miR-181d-5p,miR-212-3p,miR-7705,miR-502-3p,novel_111,miR-107-5p,miR-371a-5p,miR-2440,miR-218-5p,miR-499a-5p,miR-628-3p,miR-211-5p,miR-20a-3p,miR-2284g,miR-500-3p,miR-203-3p,miR-3082-5p,miR-2483-3p,miR-25,miR-3596,miR-378j,miR-129b-5p,miR-199b-5p,miR-361-3p,miR-9-3p,miR-493-5p,miR-335-3p,miR-299b-5p,miR-3529-3p,miR-2284a,miR-2284y,miR-200a,miR-199a-5p,miR-29a-5p,miR-217,miR-339a,miR-3071-3p,miR-543-5p,novel_127,miR-200a-3p,miR-181b-5p,miR-17-3p,miR-28-3p,miR-376a-5p,miR-196a-2-3p,novel_79,miR-6517,miR-2285ad,miR-299a-3p,miR-2284z,miR-582-3p,miR-450a-1-3p,miR-2411-5p,miR-501-3p,miR-330-5p,miR-29d-3p,miR-3591-

FAM114A2	ENSOARG0(	family w	miR-299-3p,miR-320d,miR-2284m,miR-216a-5p,miR-324-3p,miR-185-3p,miR-26a-5p,miR-618,miR-486-3p,miR-380-3p,miR-218-1-3p,miR-4429,miR-2904,miR-217-5p,miR-2366,miR-215-5p,miR-23b-3p,miR-15a-5p,miR-505-3p,miR-4492,miR-424-5p,miR-190a-5p,miR-450b-3p,novel_74,miR-3604,miR-15b-3p,miR-2285j,miR-224-5p,miR-129-2-3p,miR-3184-3p,miR-1827,miR-450a-1-3p,miR-204-3p,miR-15b-5p,miR-377-3p,miR-544-3p,miR-192-5p,miR-27a-5p,miR-323-5p,miR-21-3p,miR-3065-3p,miR-545-3p,miR-16b,miR-320b,miR-23a-3p,miR-1388-3p,miR-424-5p,miR-21b,miR-1839-5p,miR-16-2-3p,miR-544a,miR-412-3p,miR-541,miR-2285t,miR-2440,miR-452-5p,miR-2284j,miR-9-5p,miR-190b-5p,miR-2447,miR-3059-5p,miR-541-3p,miR-212-3p,miR-335-3p,miR-299b-5p,miR-203-3p,miR-144-5p,miR-3082-5p,miR-200c-3p,miR-127-5p,miR-181c-3p,miR-217,miR-2285af,miR-200c,miR-6119-5p,miR-376c-5p,miR-3065-3p,miR-2285b,miR-181a-2-3p,miR-3529-3p,miR-1343-5p,miR-29d-3p,miR-3591-5p,miR-3578,miR-450a-1-3p,miR-129-1-3p,miR-322-5p,miR-9-5p,miR-412,miR-1224-5p,miR-299a-3p,miR-486b-3p,novel_63,miR-34c-3p,miR-29b-3p,miR-1197-3p,miR-16-5p,miR-144-5p,miR-320a,miR-23b,miR-301,miR-2284n,miR-331-5p,miR-374c-3p,miR-215-5p,miR-1343-5p,miR-190a,miR-224-5p,miR-377-3p,miR-497-5p,miR-16b,miR-29b,miR-380-3p,miR-2284ab,miR-450c-3p,miR-374b-3p,miR-411-5p,miR-544b,miR-412-5p,miR-665,miR-26b-3p,novel_1,miR-29a,miR-16a,miR-33a-3p,novel_87,miR-15a,miR-2284w,miR-29c-3p,miR-495-3p,miR-135a-2-3p,miR-219b-3p,miR-195a-5p,miR-505-3p,miR-6516-3p,miR-450b-3p,miR-3591-3p,miR-669,miR-320c,miR-323a-5p,miR-299b-3p,miR-374c-3p,miR-670-3p,miR-29a-3p,miR-26b-5p
KIFC1	ENSOARG0(	kinesin	miR-3529-3p,miR-342-3p,novel_79,miR-339-5p,miR-377-3p,miR-8485,miR-377-3p,miR-342,miR-339a,miR-339b,miR-466f-3p
NHLRC3	ENSOARG0(	NHL repe	miR-433-5p,miR-1271,miR-3074-2-3p,miR-410-5p,miR-2284k,miR-125b-5p,miR-665,miR-29b-2-5p,miR-664a,miR-486b-3p,miR-2331-3p,miR-2355-5p,miR-370-3p,miR-125a,miR-1271,miR-27a-5p,miR-301,miR-200a-5p,miR-3154,miR-345-5p,miR-329a,miR-345-5p,miR-1827,miR-29b-2-5p,miR-181b-1-3p,miR-486-3p,novel_39,miR-143-3p,miR-26a-5p,miR-345-3p,miR-125b,miR-543-5p,miR-1271-5p,miR-345-3p,miR-362-3p,miR-124-3p,miR-5703,miR-1193,miR-582-3p,novel_60,miR-3956-3p,miR-877-3p,miR-7705,miR-2428,miR-655-5p,miR-541-5p,miR-3120-3p,miR-26b-5p,miR-3082-5p,miR-677,miR-181b-3p,miR-129b-5p,miR-96-5p,miR-9851-3p,miR-335-3p,miR-124a,miR-628-5p,miR-432-3p,miR-432-3p,miR-345-5p,miR-7-5p,novel_115,miR-362-3p,miR-2411-3p,miR-125a-5p,miR-381-5p,miR-145b,miR-181b-2-3p,miR-539-5p,miR-9788-3p,miR-329-3p,miR-296-5p,miR-7b-5p,miR-2113,miR-197-3p,miR-145a-5p,miR-30b-3p,miR-362-3p,miR-1388-5p,miR-30b-3p,miR-2284ab,miR-381-5p,miR-219a-1-3p,miR-101c,miR-188-5p

RGS2	ENSOARG0(	regulato	miR-194b-5p,miR-101-3p,miR-30b-5p,miR-15a,miR-16a,miR-6119-3p,miR-452-3p,miR-7977,miR-378g,miR-3596,miR-129b-5p,miR-107,miR-96-5p,miR-195a-5p,miR-424-5p,let-7a-2-3p,miR-194a,miR-362-3p,miR-1468,miR-103a-3p,miR-134-3p,miR-103,miR-9788-3p,miR-542-3p,miR-329-3p,miR-194-5p,miR-22-3p,miR-16b,miR-362-3p,miR-2284x,miR-101c,miR-497-5p,miR-1271,miR-16-5p,miR-338-3p,miR-30e-3p,miR-551b-3p,miR-30a-3p,miR-1468-5p,miR-424-5p,miR-15b-5p,miR-877-5p,miR-22-3p,miR-30e-5p,miR-30d-5p,let-7g-3p,miR-329a,miR-30f,miR-671-5p,miR-7144-5p,miR-30a-3p,miR-125a-3p,novel_17,miR-30b,miR-30f,miR-30d,miR-107,miR-30d-3p,miR-1271-5p,miR-15a-5p,miR-322-5p,miR-582-3p,miR-362-3p,miR-30c-5p,miR-30a-5p,let-7a-2-3p,miR-30c
EPHX2	ENSOARG0(	epoxide	miR-21-3p,miR-154a-3p,miR-370-5p,miR-532-5p,miR-2331-3p,miR-660-5p,miR-15b-5p,miR-6516,miR-544-3p,miR-323-5p,miR-21a-3p,miR-450a-1-3p,miR-1827,miR-345-5p,miR-154b-3p,miR-2330-3p,miR-3074-2-3p,miR-539-5p,miR-6134,miR-338-3p,miR-338-5p,miR-2285j,miR-190a-5p,miR-708-5p,miR-450b-3p,miR-6516-3p,miR-485-3p,miR-487a-3p,miR-15a-5p,miR-140-3p,miR-1197-5p,miR-188-3p,miR-191,miR-2300b-3p,miR-2366,miR-148a-5p,miR-487a,miR-377-5p,miR-345-3p,miR-299-3p,miR-21-3p,miR-760-3p,miR-7977,miR-4792,miR-3064-5p,miR-2284g,miR-27a-3p,miR-1185-2-3p,miR-7857-3p,miR-3082-5p,novel_94,miR-190b-5p,miR-452-5p,miR-154a,miR-1973,miR-107-5p,miR-382-3p,miR-218-5p,miR-3964,miR-154-3p,miR-499a-5p,miR-335,miR-27a-3p,miR-409b,miR-455-3p,miR-148b-5p,miR-1185-3p,miR-3962,miR-484,miR-544a,miR-2284b,miR-1388-3p,miR-494-3p,miR-424-5p,miR-431-5p,miR-103a-2-5p,miR-3085-3p,miR-370-3p,miR-615-5p,miR-1271,miR-487a-3p,miR-488-3p,miR-301,miR-7144-5p,miR-1248,miR-10b-3p,miR-345-5p,miR-16-5p,miR-103b,miR-1306-5p,miR-365a-5p,miR-1260b,miR-370-5p,miR-191-5p,miR-28-5p,miR-138-5p,miR-7641,miR-431,miR-2285ad,miR-299a-3p,miR-450a-1-3p,novel_121,miR-214-5p,miR-322-5p,miR-6516-5p,miR-1343-5p,miR-345-3p,miR-196a-5p,miR-411-3p,miR-3064-5p,miR-200a,miR-28b,miR-3071-3p,miR-1260a,miR-125a-3p,miR-655-5p,miR-323a-5p,miR-299b-3p,miR-150-5p,miR-3065-5p,miR-3591-3p,miR-378b,miR-7975,miR-195a-5p,miR-450b-3p,miR-1193,miR-335-5p,novel_27,miR-374c-5p,miR-2284w,miR-371b-3p,miR-485-3p,miR-16a,miR-502-5p,miR-15a,miR-28c,miR-379-3p,miR-377-5p,miR-544b,miR-185-5p,miR-450c-3p,miR-3071-5p,miR-1285,miR-497-5p,miR-6239,miR-27b-3p,miR-7862,miR-138,miR-1260b,miR-101-5p,let-7f-2-3p,let-7f-2-3p,let-7i,miR-539-3p,miR-345-5p,miR-654-3p,miR-190a,miR-365b-

HOXA10	ENSOARG0(	homeobox	miR-493-5p,miR-27a-3p,miR-218-5p,miR-2285y,miR-3059-5p,miR-2285f,miR-1296-5p,miR-361-3p,miR-296-5p,miR-30b-3p,miR-7857,miR-544a,miR-1343-3p,miR-2300a-5p,miR-548w,miR-27a-3p,miR-9788-3p,miR-320b,miR-16b,miR-182-5p,novel_19,miR-424-5p,miR-30c-1-3p,miR-4532,miR-500a-3p,miR-625-5p,miR-1895,miR-548o-3p,miR-30c-1-3p,miR-2331-3p,miR-182-5p,miR-552-3p,miR-3065-3p,miR-6130,miR-544-3p,miR-15b-5p,miR-22-3p,miR-6516-3p,miR-664a,miR-424-5p,miR-500,miR-4510,miR-129-2-3p,miR-145a-3p,miR-590-3p,miR-191,miR-5703,miR-4429,miR-548e-3p,miR-3960,miR-15a-5p,miR-185-3p,miR-320d,miR-21-3p,miR-503-5p,miR-195a-3p,miR-125b-2-3p,miR-3141,miR-2285ab,miR-107,miR-195a-5p,miR-135b-5p,miR-9851-3p,miR-7-1-3p,miR-29a-3p,miR-320c,miR-144-3p,miR-6529b,miR-502-5p,miR-1247-3p,miR-15a,miR-16a,miR-379-3p,miR-29a,miR-29c-3p,miR-495-3p,miR-3957-3p,miR-371b-3p,miR-1307-3p,miR-22-3p,miR-197-3p,miR-16b,miR-29b,miR-30b-3p,miR-2284x,miR-27b-3p,miR-4508,miR-497-5p,miR-133b-5p,miR-103a-3p,miR-503-5p,miR-185-5p,miR-544b,miR-103,miR-503-5p,miR-3141,miR-493-5p,miR-2285r,miR-331-5p,miR-320a,miR-767,miR-485-5p,miR-574-5p,miR-2285aa,miR-300-3p,novel_63,miR-2433,miR-29b-3p,miR-3969,miR-135a-5p,miR-144,miR-331-5p,miR-767-5p,miR-191-5p,miR-6128,miR-16-5p,miR-2427,miR-29d-3p,miR-411-3p,miR-2285ad,miR-322-5p,miR-24-2-5p,miR-129-1-3p,miR-6119-5p,miR-30c-2-3p,miR-107,miR-3529-3p,miR-490-3p,miR-1957a,miR-3065-3p
CHRNA2	ENSOARG0(	choliner	miR-670-3p,miR-499a-3p,miR-7977,miR-2285u,miR-363,miR-6395,miR-335-3p,miR-148a-3p,miR-3141,miR-361-3p,miR-140-5p,miR-2428,miR-326-3p,miR-361-3p,miR-17-3p,miR-541-3p,miR-3059-5p,miR-4726-5p,miR-2285y,miR-2440,miR-1247-3p,miR-2285n,miR-194-3p,miR-185-5p,miR-330-3p,miR-31-5p,miR-188-5p,miR-744-5p,miR-4508,miR-30b-3p,miR-362-3p,miR-30b-3p,miR-3071-5p,miR-125b-2-3p,miR-197-3p,miR-22-3p,miR-541,miR-1843b-5p,miR-329-3p,miR-4532,miR-654-3p,miR-30c-1-3p,miR-6240,miR-130b-5p,miR-214-3p,miR-125a-5p,miR-491-5p,miR-362-3p,miR-330-3p,miR-3141,miR-2957,miR-326,miR-224-5p,miR-324-5p,miR-22-3p,miR-365a-3p,miR-499b-3p,miR-615-5p,miR-125a,miR-140-5p,miR-30c-1-3p,miR-7144-5p,miR-329a,miR-10b-3p,miR-6134,miR-122-5p,miR-224-5p,miR-214,miR-193a-5p,miR-129-2-3p,miR-134-5p,miR-130b-5p,miR-4492,miR-874-5p,miR-129-5p,miR-17-3p,novel_74,miR-665,miR-125b-5p,miR-324-3p,miR-30a-3p,miR-30e-3p,miR-2433,miR-328-3p,miR-129-1-3p,miR-134-5p,miR-200b,miR-148b-3p,miR-30d-3p,miR-429-3p,miR-2285ad,miR-200b-3p,miR-330-5p,miR-362-3p,miR-134,miR-1271-3p,miR-3956-5p,miR-125b-2-3p,miR-664b-3p,miR-376a-5p,miR-450b-5p,miR-30a-3p,miR-152-3p,novel_96,miR-3653-3p,miR-200c-3p,miR-760-3p,miR-17-3p,miR-125b,miR-185-3p,miR-30f,miR-30c-2-3p,miR-125a-3p,miR-200c,miR-205-5p,miR-2285af

ADAMTS4	ENSOARG0(	ADAM met	miR-3085-3p,miR-214-3p,miR-103a-2-5p,novel_103,miR-1306-5p,miR-326,miR-320b,miR-29b-1-5p,miR-378d,miR-8485,miR-494-3p,miR-101b-3p,miR-124a,miR-744-3p,miR-652-5p,miR-378c,miR-3120-5p,miR-301b-5p,miR-6529a,miR-2284r,miR-378b,miR-216a-3p,miR-500-5p,miR-455-3p,miR-2300a-5p,miR-105-1,miR-27a-3p,miR-181b-2-3p,miR-5010-3p,miR-3968,miR-4726-5p,miR-154-3p,miR-2440,novel_82,miR-218-5p,miR-107-5p,miR-301a-5p,miR-371a-5p,miR-362-5p,miR-877-3p,miR-361-3p,miR-7705,miR-219a-2-3p,miR-212-3p,miR-2447,miR-452-5p,miR-219a-5p,miR-1296-5p,miR-504,miR-544-5p,miR-361-3p,miR-378j,miR-489,miR-378e,miR-3596,miR-378g,miR-454-5p,miR-27a-3p,miR-2319b,miR-452-3p,miR-3064-5p,miR-7977,miR-99a-3p,miR-760-3p,miR-320d,miR-21-3p,miR-374a-5p,miR-92a-1-5p,miR-345-3p,miR-422a,miR-324-3p,miR-500a-5p,miR-130a-5p,miR-29b-1-5p,miR-143-3p,miR-2424,miR-2432,miR-486-3p,miR-2285w,miR-487a,miR-329a-5p,miR-345-3p,miR-4429,miR-378d,miR-664-5p,miR-2300b-3p,miR-2408,miR-193b-5p,miR-378i,miR-378a-3p,miR-1197-5p,miR-188-3p,miR-1193,miR-124-3p,miR-2904,miR-148b-3p,miR-487a-3p,miR-328-3p,miR-532-3p,miR-18a-3p,miR-485-3p,miR-1b-3p,miR-3600,miR-17-3p,miR-6516-3p,miR-708-5p,miR-665,miR-655-3p,miR-320e,miR-29b-2-5p,miR-324-3p,miR-219b-3p,miR-224-5p,miR-3184-3p,miR-4510,miR-3074-5p,miR-378c,miR-214,miR-154b-3p,miR-345-5p,miR-186-5p,novel_99,miR-582-5p,miR-543-3p,miR-655,miR-129b-3p,miR-216b-5p,miR-6130,miR-582-5p,miR-377-3p,miR-2284s,miR-204-3p,novel_32,miR-140-5p,miR-378a-3p,miR-504-5p,miR-2331-3p,miR-552-3p,miR-216b-3p,miR-154a-3p,miR-370-5p,miR-378a-5p,miR-124-5p,miR-377-3p,miR-224-5p,novel_51,miR-2411-3p,miR-105-5p,miR-2285r,miR-345-5p,let-7j,miR-539-3p,miR-382,miR-668-3p,miR-363-5p,miR-1260b,miR-27b-

PTK2B	ENSOARG0(	protein	miR-125b-5p,miR-2433,miR-29b-3p,miR-138-5p,miR-133a-3p,miR-145a-3p,miR-4510,miR-145-3p,miR-320a,miR-186-5p,miR-133b-3p,let-7g-3p,miR-345-5p,miR-345-5p,miR-339b,miR-2355-5p,miR-125a,miR-874-3p,miR-199a-5p,miR-151b,miR-544-3p,miR-6130,miR-125b,miR-345-3p,miR-339a,miR-6119-5p,novel_127,miR-299-3p,miR-320d,miR-2898,miR-466i-5p,miR-181a-2-3p,miR-4286,miR-183-5p,novel_39,miR-200a,miR-199a-5p,miR-181b-1-3p,miR-125b-2-3p,miR-29d-3p,miR-5703,miR-330-5p,miR-345-3p,miR-2300b-3p,miR-493-3p,miR-19b-3p,miR-4429,novel_79,miR-7641,miR-18a-3p,miR-299a-3p,miR-151-5p,miR-23b-3p,miR-23c,miR-340-5p,miR-107-5p,miR-2285n,miR-576-3p,miR-7134-3p,miR-4726-5p,miR-29a,miR-29c-3p,miR-219b-3p,miR-541-3p,miR-326-3p,miR-199b-5p,miR-2428,miR-150-5p,miR-129b-5p,miR-18a-3p,miR-2387,miR-199b-5p,miR-9851-3p,miR-9-3p,miR-1291,miR-19a-3p,miR-320c,miR-677,miR-4443,miR-181b-3p,novel_69,miR-299b-3p,miR-29a-3p,miR-330-3p,miR-2411-3p,novel_51,miR-320b,novel_115,miR-326,miR-125a-5p,miR-199b-5p,miR-103a-2-5p,novel_124,miR-25-5p,miR-363-5p,miR-23a-3p,let-7c-3p,miR-6240,miR-345-5p,miR-432-3p,miR-432-3p,miR-30b-3p,miR-125b-2-3p,miR-541,miR-101c,miR-16-2-3p,miR-29b,miR-138,miR-30b-3p,miR-544a,miR-339-5p,miR-181b-2-3p,miR-1343-3p,miR-330-3p,miR-9788-3p,miR-194-3p,miR-544b,miR-147a
CERS3	ENSOARG0(	ceramide	miR-874-5p,miR-22-5p,miR-103a-2-5p,miR-450a-2-3p,miR-132-5p,miR-107-5p,miR-1271-3p,miR-877-3p,miR-628-5p,miR-26b-3p,miR-3059-5p
DNAJC5	ENSOARG0(	DnaJ (Hs	miR-128-3p,miR-1895,miR-615,miR-296-5p,miR-615-3p,miR-216a-3p,miR-371b-3p,miR-3957-3p
TRIM35	ENSOARG0(	triparti	let-7d,miR-582-3p,miR-378d,miR-1961,miR-134,miR-140-3p,miR-222-5p,miR-505-5p,miR-545-5p,miR-134-5p,miR-106a,miR-2285ad,miR-1b-3p,let-7f-5p,miR-6536,miR-98-5p,miR-106a,miR-21-3p,let-7k,miR-2285af,let-7c-5p,miR-122-3p,miR-130a-5p,miR-2478,miR-664b-3p,miR-192-3p,miR-3065-3p,let-7g,miR-2432,miR-486-3p,miR-3529-3p,miR-345-3p,miR-20a,miR-20b-5p,miR-21a-3p,miR-3154,miR-93-5p,miR-301a-3p,miR-300-3p,let-7i-5p,novel_32,miR-574-5p,miR-2331-3p,miR-3065-3p,miR-21-3p,miR-134-5p,miR-486b-3p,miR-454-3p,let-7f,miR-665,miR-331-3p,miR-93,let-7b-5p,miR-3074-5p,let-7b,miR-1306-5p,miR-3074-2-3p,miR-450b-5p,miR-1839-5p,miR-542-3p,miR-484,miR-125b-2-3p,miR-3071-5p,let-7g-5p,miR-20b,miR-421,miR-149-5p,miR-9788-3p,miR-1343-3p,novel_78,miR-130a-3p,miR-130b-3p,miR-378a-5p,let-7e,novel_103,miR-1306-5p,miR-106b-5p,novel_51,miR-106a-5p,miR-505,miR-2957,miR-106a-5p,let-7a-5p,miR-2483-5p,miR-363-5p,let-7e-5p,miR-3065-5p,miR-3591-3p,miR-150-5p,miR-1306,let-7d-5p,miR-3074-5p,miR-2319b,miR-499b-5p,miR-7689-3p,novel_1,miR-2285t,miR-20a-5p,miR-133a-5p,novel_82,let-7i,miR-2285y,miR-24-3p,miR-301b-3p,miR-3059-5p,miR-495-3p
DYRK4	ENSOARG0(	dual-spe	miR-495-3p,miR-625-5p,miR-362-3p,miR-329a,miR-6536,miR-362-3p,miR-338-5p,miR-329-3p,miR-503-3p,miR-2332,miR-362-3p

SLC43A1	ENSOARG0(	solute c	miR-671-5p,miR-3154,novel_32,novel_25,miR-15b-5p,miR-132-5p,miR-365a-3p,miR-125a,miR-615-5p,miR-874-5p,miR-708-5p,miR-424-5p,miR-204-5p,miR-486b-3p,miR-28-5p,miR-664a,miR-331-3p,miR-6740-5p,miR-125b-5p,miR-29b-2-5p,miR-320e,miR-665,miR-1306-3p,miR-135a-1-3p,miR-3184-5p,miR-1197-3p,miR-16-5p,miR-1271,miR-1306-5p,novel_42,miR-145a-3p,miR-133a-3p,miR-2284u,miR-33b-3p,miR-184-3p,miR-2904,miR-188-3p,miR-23b-5p,miR-5703,miR-2366,miR-2300b-3p,miR-532-3p,novel_83,miR-1247-5p,miR-505-5p,miR-322-5p,miR-1271-5p,miR-15a-5p,miR-1224-5p,miR-212-5p,miR-107,miR-543-5p,miR-450a-2-3p,miR-127-5p,miR-216a-5p,miR-125b,miR-2285af,miR-210-5p,novel_39,miR-486-3p,miR-2284y,miR-2284a,miR-450c-5p,miR-2285b,miR-29b-1-5p,miR-29b-2-5p,miR-1983,miR-195a-5p,miR-96-5p,miR-147-3p,miR-1291,miR-107,miR-668-5p,miR-412-3p,miR-1306-3p,miR-361-3p,miR-2387,miR-6535,miR-378g,miR-3082-5p,miR-211-5p,miR-7977,miR-2284g,miR-128-1-5p,miR-450a-5p,miR-16a,miR-15a,miR-4324,miR-423-5p,miR-361-3p,miR-211,miR-24-3p,miR-371b-3p,miR-3957-3p,novel_101,miR-3059-5p,miR-497-5p,miR-2889,miR-16b,miR-2426,miR-296-5p,miR-7b-5p,miR-1843b-5p,miR-9788-3p,miR-421,miR-103,miR-185-5p,miR-134-3p,novel_78,novel_4,miR-103a-3p,miR-125a-5p,miR-3955-3p,miR-29b-1-5p,miR-16b,novel_51,miR-7-5p,miR-296-3p,miR-296-3p,miR-363-5p,miR-2284b,miR-628-5p,miR-424-5p

PHF1	ENSOARG0(	PHD fing	miR-18b-5p,miR-412,miR-423-3p,miR-299a-3p,miR-2285ad,miR-214-5p,miR-2284z,miR-450a-1-3p,miR-483-3p,miR-758-5p,miR-378h,miR-330-5p,miR-1343-5p,miR-3578,miR-1290,miR-615-3p,miR-99a-5p,miR-30a-3p,miR-29b-2-5p,miR-490-3p,miR-2284a,miR-2284y,miR-99a,miR-125a-3p,miR-1260a,novel_96,miR-543-5p,miR-152-3p,miR-191-3p,miR-17-3p,miR-2898,miR-370-3p,miR-2355-5p,miR-300-3p,miR-18a,miR-17-5p,miR-100-5p,miR-320a,miR-93-5p,miR-485-5p,miR-1814c,miR-20b-5p,miR-7144-5p,miR-345-5p,miR-2474,miR-6128,miR-365a-5p,miR-378c,miR-1260b,miR-93,miR-135a-5p,miR-100-3p,miR-592,miR-377-5p,miR-26b-3p,miR-130a-3p,miR-20b,miR-194-3p,miR-185-5p,miR-134-3p,miR-3431,miR-412-5p,miR-450c-3p,miR-1843b-5p,miR-542-3p,miR-873a-5p,miR-744-5p,miR-2889,miR-7862,miR-1260b,miR-3432b,miR-2483-5p,miR-345-5p,miR-106a-5p,miR-99a-5p,miR-2331-5p,miR-17-5p,miR-365b-5p,miR-197-5p,miR-3141,miR-146a,miR-1343-5p,miR-378a-5p,miR-483-3p,miR-655-5p,miR-3074-5p,miR-6535,miR-320c,miR-669,miR-299b-3p,miR-670-3p,miR-3141,miR-148a-3p,miR-18b,miR-378f,miR-135b-5p,miR-146b-5p,miR-2285k,miR-412-3p,miR-17-3p,miR-301b-3p,miR-326-3p,miR-371b-3p,miR-1307-3p,miR-211,miR-6529b,miR-20a-5p,miR-199c,miR-502b,miR-22-5p,miR-30d-3p,miR-106a,miR-151-5p,miR-532-3p,miR-148b-3p,miR-383-5p,miR-378a-3p,miR-1197-5p,miR-5703,miR-2408,miR-378i,miR-2284u,miR-4429,miR-19b-3p,miR-493-3p,miR-20a,miR-5100,miR-146a-5p,miR-377-5p,miR-2285i,miR-185-3p,miR-92a-1-5p,miR-422a,miR-324-3p,miR-2285p,novel_17,miR-2484,miR-299-3p,miR-3653-3p,miR-466i-5p,miR-320d,miR-106a,miR-296-3p,miR-378a-3p,miR-877-5p,miR-151b,novel_32,miR-301a-3p,miR-450a-1-3p,miR-1827,miR-1895,miR-625-5p,miR-345-5p,miR-18a-5p,miR-665-5p,miR-3074-5p,miR-145a-3p,miR-539-

DLC1	ENSOARG0(	DLC1 Rho	miR-544b,novel_116,miR-6238,miR-411-5p,miR-194-3p,miR-432-5p,miR-2284d,miR-412-5p,miR-377-5p,miR-2284x,miR-1260b,miR-2284ab,miR-29b,miR-30b-3p,novel_23,miR-145a-5p,miR-542-3p,miR-1843b-5p,miR-223-3p,miR-432,miR-653,miR-19b-2-5p,let-7a-2-3p,miR-345-5p,let-7j,miR-2284l,miR-668-3p,miR-493-5p,miR-130b-5p,miR-466f-3p,miR-483-3p,miR-2411-3p,miR-669,miR-299b-3p,novel_69,miR-29a-3p,novel_68,miR-19a-3p,miR-412-3p,miR-410-5p,miR-216a-3p,miR-3065-5p,miR-150-5p,miR-2285ab,miR-2428,miR-2284w,novel_27,miR-374c-5p,miR-24-3p,miR-211,miR-29c-3p,miR-17-3p,miR-495-3p,miR-2285x,miR-335-5p,miR-147-5p,miR-203b-5p,miR-379-3p,miR-29a,novel_1,miR-502-5p,miR-4324,miR-486-5p,miR-2284z,miR-582-3p,miR-483-3p,miR-6516-5p,miR-9-5p,miR-412,miR-429-3p,miR-299a-3p,miR-496,let-7a-2-3p,miR-128-3p,miR-200b-3p,miR-582-3p,miR-411-3p,miR-29d-3p,miR-3578,miR-345-3p,miR-3591-5p,miR-2284a,miR-490-3p,miR-1271-3p,miR-2285b,novel_39,miR-376b-5p,miR-376c-5p,miR-29a-5p,miR-2284y,miR-2478,miR-450b-5p,miR-3064-5p,miR-30a-3p,miR-3529-3p,miR-200a-3p,miR-200c-3p,miR-450a-2-3p,miR-28-3p,miR-17-3p,miR-30c-2-3p,miR-200c,miR-2285af,miR-1260a,miR-2284n,miR-223-5p,miR-769,miR-374c-3p,miR-141-3p,miR-2285aa,miR-503-3p,miR-1814c,miR-1248,miR-23a-5p,miR-345-5p,let-7g-3p,miR-23b,miR-200a-5p,miR-381-3p,miR-1260b,miR-2284v,miR-28a-3p,miR-204-5p,miR-409-3p,novel_63,miR-29b-3p,miR-2284k,miR-216a-3p,miR-148b-5p,miR-145b,miR-151a-3p,miR-2284aa,miR-450b-5p,miR-544a,miR-101a-5p,miR-2426,miR-30b-3p,miR-7857,miR-412-3p,miR-541,miR-484,miR-2310,miR-8485,miR-30c-1-3p,miR-2284b,miR-23a-3p,miR-101b-3p,miR-3085-3p,miR-2957,novel_115,miR-4443,miR-454-5p,miR-3082-5p,miR-3596,miR-211-5p,miR-2285u,miR-3064-5p,miR-1983,miR-493-

ADRA1A	ENSOARG0(	adrenoce	miR-2478,miR-192-3p,miR-1957a,miR-199a-5p,miR-3529-3p,miR-2385-3p,miR-181b-5p,miR-6536,miR-2898,miR-17-3p,miR-8117,miR-125a-3p,miR-6119-5p,miR-378h,miR-1271-5p,miR-322-5p,miR-582-3p,miR-18b-5p,miR-412,miR-885-3p,miR-1290,miR-23b-5p,miR-330-5p,miR-218-2-3p,miR-500b-5p,miR-378c,miR-2284v,miR-16-5p,novel_9,miR-370-5p,miR-5100,miR-2433,novel_63,miR-2284k,miR-331-3p,miR-769,miR-374c-3p,miR-199a-5p,miR-223-5p,miR-2285aa,miR-876-3p,miR-1248,miR-485-5p,miR-3154,miR-18a,miR-362-5p,miR-654-3p,miR-432-3p,miR-539-3p,miR-130b-5p,miR-628-5p,miR-466f-3p,miR-377-3p,miR-503-5p,miR-7-5p,novel_51,miR-134-3p,miR-503-5p,miR-2284d,miR-412-5p,novel_116,miR-31-5p,miR-26b-3p,miR-16-1-3p,miR-30b-3p,miR-16b,miR-2284ab,miR-188-5p,miR-7862,miR-497-5p,miR-7b-5p,miR-181a-5p,miR-542-3p,miR-374b-3p,novel_23,miR-323c,miR-199b-5p,miR-2428,miR-2284w,miR-326-3p,miR-335-5p,miR-219b-3p,miR-17-3p,miR-1193,miR-6119-3p,miR-199c,miR-22-5p,miR-502-5p,miR-15a,miR-4324,novel_87,miR-16a,miR-374c-3p,miR-329-5p,novel_68,miR-19a-3p,miR-378f,miR-195a-5p,miR-18b,miR-125b-2-3p,miR-664b-3p,miR-503-5p,miR-216c-5p,miR-329a-5p,miR-2284m,miR-3653-3p,novel_17,miR-422a,miR-92a-1-5p,miR-500a-5p,miR-15a-5p,miR-7859,miR-487a-3p,miR-548e-3p,miR-485-3p,miR-212-5p,miR-1b-3p,miR-184-3p,miR-19b-3p,miR-493-3p,miR-148a-5p,miR-664-5p,miR-16-1-3p,miR-218-1-3p,miR-1246,miR-2300b-3p,miR-378i,miR-378a-3p,miR-1193,miR-5703,miR-2904,miR-346,miR-769-5p,miR-338-3p,miR-181c-5p,miR-145a-3p,miR-378c,miR-425-5p,miR-2330-3p,miR-1271,miR-17-3p,miR-130b-5p,miR-129-5p,miR-4492,miR-424-5p,miR-2285j,miR-377-3p,miR-15b-5p,miR-204-3p,miR-378a-3p,miR-552-3p,miR-296-3p,miR-370-5p,miR-18a-5p,novel_99,miR-1827,miR-186-5p,miR-543-3p,miR-885-
CCDC181	ENSOARG0(	coiled-c	miR-3591-3p,miR-330-3p,miR-21-3p,miR-129-5p,miR-330-3p,miR-6240,miR-32-3p,miR-32-3p,miR-3074-2-3p,miR-1224-5p,miR-5010-3p,miR-21-3p,miR-323-3p

DPYSL2	ENSOARG0(	dihydrop	miR-378b,miR-299b-3p,miR-669,miR-148a-3p,miR-2319a,miR-135a-2-3p,miR-2285x,miR-326-3p,miR-199b-5p,miR-301b-3p,miR-2428,miR-576-3p,miR-147-5p,miR-3955-5p,miR-133b-5p,miR-130a-3p,miR-185-5p,miR-873a-5p,miR-7b-5p,miR-1285,miR-1434-3p,miR-145a-5p,miR-30b-3p,miR-138,miR-654-3p,miR-345-5p,miR-7-5p,miR-1343-5p,miR-2285aa,miR-769,miR-199a-5p,miR-324-5p,miR-154b-5p,miR-30e-5p,miR-488-3p,miR-424-3p,miR-200a-5p,miR-3154,miR-345-5p,miR-3607-3p,novel_63,miR-138-5p,miR-299a-3p,miR-1224-5p,miR-758-5p,miR-6516-5p,miR-129-1-3p,miR-345-3p,miR-1343-5p,miR-330-5p,miR-30a-5p,miR-3529-3p,miR-3064-5p,miR-199a-5p,miR-3065-3p,miR-2285af,miR-125a-3p,miR-6119-5p,miR-543-5p,miR-152-3p,miR-4792,miR-3064-5p,miR-499b-5p,miR-2483-3p,miR-1185-2-3p,miR-361-3p,miR-199b-5p,novel_94,miR-101-3p,miR-769-5p,miR-301a-5p,miR-1343-3p,miR-145b,miR-147a,miR-9788-3p,miR-1185-3p,miR-484,miR-301b-5p,miR-30b-3p,miR-125b-2-3p,miR-124a,miR-1388-3p,miR-2330-5p,miR-193a,miR-326,miR-3085-3p,miR-130b-3p,miR-199b-5p,miR-2331-3p,miR-21-3p,miR-3065-3p,miR-296-3p,miR-6516,miR-6130,miR-216b-5p,miR-21a-3p,miR-129b-3p,miR-433-3p,miR-30d-5p,miR-30f,miR-345-5p,miR-1895,miR-4510,miR-129-2-3p,miR-3184-3p,miR-769-5p,miR-346,miR-338-3p,miR-6134,miR-2285j,miR-19b-1-5p,miR-3604,miR-665,miR-454-3p,miR-548e-3p,miR-7859,miR-148b-3p,miR-2408,miR-124-3p,miR-140-3p,miR-5703,miR-184-3p,miR-218-1-3p,miR-345-3p,miR-143-3p,miR-324-3p,miR-345-3p,miR-466i-5p,miR-30d,miR-30b-3p,miR-21-3p,miR-299-3p
SH3BGR	ENSOARG0(	SH3 doma	miR-145a-3p,miR-2284v,miR-346,miR-2284k,miR-6740-5p,miR-4492,miR-30c-1-3p,miR-140-5p,miR-2355-5p,miR-2284n,miR-411,miR-1827,miR-148b-5p,miR-2284y,miR-2284a,miR-30c-2-3p,miR-185-3p,miR-2284m,miR-21-3p,novel_96,miR-485-3p,miR-487a-3p,miR-483-3p,miR-758-5p,novel_83,miR-3578,miR-2300b-3p,miR-124-3p,miR-5703,miR-411-3p,miR-128-3p,miR-218-1-3p,miR-2284u,miR-148a-5p,miR-6527,miR-2284j,miR-2428,miR-2440,miR-485-3p,miR-379-3p,miR-22-5p,miR-363,miR-2284g,miR-7857-3p,novel_69,miR-216a-3p,miR-124a,miR-6240,miR-23a-3p,miR-668-3p,miR-2284b,miR-30c-1-3p,miR-503-3p,miR-330-3p,miR-491-5p,miR-483-3p,miR-378a-5p,miR-31-5p,miR-330-3p,novel_78,miR-432-5p,miR-2284d,miR-185-5p,miR-147a,miR-216a-3p,miR-148b-5p,miR-432,miR-1434-3p,miR-2284ab,miR-2284x
SMCHD1	ENSOARG0(	structur	miR-30f,miR-455-5p,miR-224-5p,miR-377-3p,let-7c-3p,miR-2285w,miR-200a,miR-148b-5p,miR-145a-5p,miR-301b-5p,miR-218-1-3p,miR-455-5p,miR-450b-5p,miR-3600,miR-145b,miR-548e-3p,miR-2284h-5p,miR-7859,miR-758-5p,miR-3604,miR-301a-5p,miR-382-3p,miR-218-5p,miR-101-3p,miR-129-5p,miR-3059-5p,miR-4510,miR-122-5p,miR-374c-5p,miR-224-5p,miR-129b-3p,miR-433-3p,miR-1814c,miR-541-5p,miR-363,miR-548o-3p,miR-3068-3p,miR-377-3p,miR-300-3p,miR-6130

NDUFS2	ENSOARG0(	NADH deh	miR-340-3p,miR-548e-3p,miR-2285ad,novel_4,miR-1343-3p,miR-339-5p,novel_79,miR-7859,miR-450a-1-3p,miR-2284h-5p,miR-345-3p,miR-615-3p,miR-450c-3p,miR-22-3p,miR-140-3p,miR-1197-5p,miR-21b,miR-363-5p,miR-376a-5p,miR-8485,miR-200a,miR-143-3p,miR-505,miR-324-3p,miR-2411-3p,miR-185-3p,miR-339a,miR-345-3p,miR-2898,miR-200a-3p,miR-466f-3p,miR-2319b,miR-3074-5p,miR-141-3p,miR-3082-5p,novel_32,miR-22-3p,miR-204-3p,miR-6535,miR-450a-1-3p,miR-378b,miR-339b,miR-625-5p,miR-6516-3p,miR-2447,miR-3074-5p,miR-3957-3p,miR-1296-5p,miR-495-3p,miR-6525,miR-423-5p,miR-3184-5p,miR-615,miR-6740-5p,miR-324-3p,miR-2285y,miR-450b-3p,miR-4726-5p,miR-128-1-5p
LIMK2	ENSOARG0(	LIM doma	miR-34b-5p,miR-214-3p,miR-543-5p,novel_96,miR-3085-3p,miR-491-5p,miR-324-3p,miR-2331-5p,miR-194a,miR-92a-1-5p,miR-326,novel_39,miR-486-3p,miR-432-3p,miR-3064-5p,miR-34a-5p,miR-2889,miR-2904,miR-22-3p,miR-330-5p,miR-188-3p,miR-194-5p,miR-3591-5p,miR-3578,miR-149-5p,miR-532-3p,miR-203b-5p,miR-486b-3p,miR-4726-5p,miR-324-3p,miR-34c,miR-326-3p,miR-449a,miR-361-3p,miR-24-3p,miR-194b-5p,miR-214,miR-2447,miR-1291,miR-2474,miR-204-3p,miR-22-3p,miR-34c-5p,miR-34b,miR-615-5p,miR-296-3p,miR-3064-5p
ADAL	ENSOARG0(	adenosin	miR-3600,miR-616-3p,miR-151-5p,miR-383-5p,miR-23b-3p,miR-23c,miR-340-5p,miR-140-3p,miR-1197-5p,miR-5703,novel_133,miR-218-1-3p,miR-378d,miR-148a-5p,miR-34a-5p,miR-33b-3p,miR-4429,miR-329a-5p,miR-2424,miR-2432,miR-143-3p,miR-181b-1-3p,miR-377-5p,miR-345-3p,miR-185-3p,miR-92a-1-5p,miR-2285p,miR-34b-5p,miR-221-5p,miR-320d,miR-760-3p,miR-21-3p,miR-532-5p,miR-296-3p,miR-2331-3p,miR-30c-1-3p,miR-221-5p,miR-504-5p,miR-204-3p,miR-151b,miR-377-3p,miR-499b-3p,miR-139-5p,miR-6130,miR-433-3p,miR-21a-3p,miR-450a-1-3p,miR-1827,let-7g-3p,miR-18a-5p,miR-4510,miR-129-2-3p,miR-338-3p,miR-769-5p,miR-6525,miR-19b-1-5p,miR-151-3p,miR-338-5p,miR-665,miR-2285j,miR-208b-5p,miR-4492,novel_74,miR-450b-3p,miR-17-3p,miR-5010-3p,miR-181b-2-3p,miR-105-1,novel_4,novel_78,miR-1343-3p,miR-151a-3p,miR-148b-5p,miR-1298-3p,miR-30b-3p,miR-2284r,miR-1839-5p,miR-21b,miR-101c,miR-2426,miR-450b-5p,miR-101a-5p,let-7c-3p,miR-33a-5p,miR-101b-3p,miR-1839-3p,miR-30c-1-3p,miR-2957,miR-320b,miR-326,miR-431-5p,miR-1185-5p,miR-3085-3p,miR-3955-3p,miR-499b-5p,miR-211-5p,miR-3064-5p,miR-139-5p,miR-24-1-5p,miR-20a-3p,miR-452-3p,miR-499a-3p,miR-25,miR-378g,miR-3596,miR-129b-5p,miR-361-3p,miR-335-3p,miR-877-3p,miR-1983,miR-136-5p,miR-504,miR-6527,miR-9-5p,miR-452-5p,miR-3059-5p,miR-2447,miR-877-3p,miR-361-3p,miR-769-5p,miR-101-3p,miR-2440,miR-3964,miR-490-5p,miR-2285t,miR-335,miR-18b-5p,miR-431,miR-6517,miR-134-5p,miR-450a-1-3p,miR-545-5p,miR-129-1-3p,miR-214-5p,miR-505-5p,miR-9-5p,miR-2427,miR-29d-3p,miR-23b-5p,miR-330-5p,miR-345-3p,miR-134,miR-105-2,miR-300,miR-3064-5p,miR-181a-2-3p,miR-376c-5p,novel_39,miR-1957a,miR-376b-5p,miR-1271-3p,miR-28b,miR-24-2-5p,miR-148b-5p,miR-30c-2-3p,miR-

NCOA3	ENSOARG0(	nuclear	miR-29d-3p,miR-345-3p,let-7a-2-3p,miR-10a-5p,miR-105-2,miR-200b-3p,let-7d,miR-411-3p,miR-545-5p,miR-483-3p,let-7c-5p,miR-200c,miR-6119-5p,miR-376b-3p,miR-2285af,miR-200c-3p,miR-181b-5p,miR-2898,miR-17-3p,miR-29b-2-5p,miR-3529-3p,miR-490-3p,miR-199a-5p,miR-17-5p,miR-320a,miR-3154,miR-1814c,let-7g-3p,miR-141-3p,miR-370-3p,miR-487a-3p,miR-199a-5p,miR-374c-3p,miR-135a-5p,miR-409-3p,miR-331-3p,miR-2332,miR-29b-3p,miR-28-5p,miR-486b-3p,miR-767-5p,miR-6128,miR-145-3p,miR-93,miR-1434-3p,miR-181a-5p,miR-653,miR-30b-3p,miR-29b,miR-26b-3p,miR-20b,miR-544b,miR-185-5p,let-7g-5p,miR-432-5p,miR-2285g,miR-17-5p,miR-106a-5p,miR-224-5p,miR-466f-3p,miR-660,miR-382,miR-345-5p,miR-539-3p,miR-376b-3p,miR-378b,miR-135b-5p,miR-505-3p,miR-195a-5p,miR-1291,miR-450b-3p,miR-7-1-3p,miR-10a-5p,miR-320b,miR-29a-3p,miR-670-3p,miR-146b-3p,miR-2404,let-7i,miR-16a,miR-6402,miR-20a-5p,miR-15a,miR-199c,novel_1,miR-17-3p,miR-2285x,miR-3959-3p,miR-188-3p,miR-140-3p,miR-1246,novel_133,miR-378d,miR-4429,miR-505-3p,miR-485-3p,miR-548e-3p,miR-200b,miR-15a-5p,miR-487a-3p,miR-21-3p,miR-30b-3p,miR-1277-5p,miR-320d,miR-106a,miR-20a,miR-216c-5p,miR-2432,miR-125b-2-3p,miR-21a-3p,novel_99,miR-154b-3p,miR-21-3p,miR-660-5p,miR-140-5p,miR-6130,miR-544-3p,miR-29b-2-5p,miR-320e,miR-708-5p,miR-424-5p,miR-17-3p,miR-6516-3p,miR-450b-3p,miR-425-5p,miR-3074-2-3p,miR-2330-3p,miR-145a-3p,miR-4510,miR-6134,let-7b-5p,miR-206-3p,miR-10b-5p,miR-125b-2-3p,miR-30b-3p,miR-412-3p,miR-7857,miR-143-5p,miR-10b,miR-2284aa,miR-101a-5p,miR-544a,miR-2426,miR-27a-3p,miR-151a-3p,miR-105-1,miR-9788-3p,novel_103,miR-1306-5p,miR-320b,let-7e,miR-25-5p,miR-3085-3p,miR-424-5p,miR-106a-5p,let-7a-5p,miR-129b-5p,miR-199b-5p,miR-361-

REV3L	ENSOARG0(	REV3-lik	miR-582-5p,miR-877-5p,miR-548o-3p,miR-32-5p,miR-2331-3p,miR-339b,let-7g-3p,miR-186-5p,miR-582-5p,miR-1827,miR-543-3p,let-7f-1-3p,miR-21a-3p,miR-411,miR-30d-5p,miR-27b-5p,miR-6525,miR-539-5p,miR-487a-5p,miR-208b-5p,miR-30e-3p,miR-29b-2-5p,miR-369-5p,miR-30a-3p,miR-148b-3p,miR-340-5p,miR-23c,miR-23b-3p,miR-4429,miR-1246,novel_133,miR-5703,miR-125b-2-3p,miR-130a-5p,miR-421-5p,miR-329a-5p,miR-760-3p,miR-320d,miR-3653-3p,miR-618,miR-345-3p,miR-1185-2-3p,miR-27a-3p,miR-20a-3p,miR-664b,miR-126b-5p,miR-668-5p,miR-99b-5p,miR-9-3p,miR-544-5p,miR-493-5p,miR-381-3p,miR-877-3p,miR-212-3p,miR-363-3p,miR-452-5p,miR-335,miR-2284q,miR-101-3p,novel_82,miR-107-5p,miR-1185-3p,miR-2459,miR-126a-5p,miR-9788-3p,miR-455-3p,miR-105-1,miR-1343-3p,miR-27a-3p,miR-145b,miR-299a-5p,miR-101a-5p,miR-101c,miR-6529a,miR-30b-3p,miR-412-3p,miR-1b-5p,miR-101b-3p,miR-382-3p,miR-376b,miR-23a-3p,miR-103a-2-5p,miR-326,miR-320b,miR-25-3p,miR-365a-3p,miR-154b-5p,miR-223-5p,miR-141-3p,miR-329b,let-7g-3p,miR-767,miR-1248,miR-503-3p,miR-331-5p,miR-488-3p,miR-30e-5p,miR-23b,miR-320a,miR-100-5p,miR-3154,miR-323-3p,miR-6128,miR-331-5p,miR-767-5p,miR-5100,miR-29b-3p,miR-135a-5p,miR-6740-5p,novel_121,novel_79,miR-412,let-7a-2-3p,miR-105-2,miR-99a-5p,miR-345-3p,miR-330-5p,miR-30a-5p,miR-29d-3p,miR-92b-3p,miR-2285b,miR-1957a,novel_39,miR-29b-2-5p,miR-30a-3p,miR-92a-3p,miR-200a-3p,miR-152-3p,miR-107,miR-664-3p,miR-99a,miR-2285af,miR-339a,miR-30f,miR-29a-3p,miR-670-3p,miR-2404,miR-320c,miR-329-5p,novel_68,miR-3120-3p,miR-655-5p,miR-107,miR-135b-5p,miR-2319a,miR-148a-3p,miR-3065-5p,miR-3970,miR-2284w,miR-2285m,miR-2428,miR-326-3p,miR-335-5p,miR-219b-3p,miR-135a-2-3p,miR-29c-3p,miR-
MITF	ENSOARG0(	micropht	miR-2285r,miR-345-3p,miR-503-5p,miR-493-5p,miR-503-5p,novel_17,novel_121,miR-7859,miR-503-5p,miR-134-3p,miR-6516-5p,miR-212-5p,miR-181b-2-3p,miR-548e-3p,miR-1343-3p,miR-450b-5p,miR-582-3p,miR-1434-5p,miR-145-3p,miR-211,miR-323c,miR-3074-2-3p,miR-500,miR-2284q,miR-204-5p,novel_1,miR-2285n,miR-677,miR-378g,miR-6516,miR-211-5p,miR-216b-3p,miR-493-5p,miR-1827,novel_99,let-7g-3p,miR-2312,miR-500a-3p,miR-381-3p,miR-381-3p

MED16	ENSOARG0(	mediator	miR-484,miR-2284r,miR-296-5p,miR-541,miR-10b,miR-30b-3p,miR-544a,miR-199b-3p,novel_4,novel_78,miR-2300a-5p,miR-330-3p,miR-145b,miR-147a,miR-9788-3p,miR-421,miR-2459,miR-1306-5p,novel_103,miR-505,miR-16b,miR-491-5p,miR-3955-3p,miR-214-3p,miR-574-3p,miR-124a,miR-424-5p,miR-2330-5p,miR-8485,miR-2310,miR-140-5p,miR-361-3p,miR-147-3p,miR-504,miR-668-5p,miR-1983,miR-664b,miR-20a-3p,miR-499b-5p,miR-378g,miR-203-3p,novel_82,miR-2440,miR-2285y,miR-4726-5p,miR-3968,miR-2447,miR-541-3p,miR-877-3p,miR-361-3p,miR-423-5p,miR-2300b-3p,miR-2366,miR-124-3p,miR-1193,miR-493-3p,miR-18a-3p,miR-1b-3p,miR-15a-5p,miR-328-3p,miR-196a-3p,miR-210-5p,miR-324-3p,miR-185-3p,miR-345-3p,miR-760-3p,miR-221-5p,miR-3653-3p,miR-21-3p,miR-299-3p,miR-345-3p,miR-2432,miR-486-3p,miR-380-3p,miR-339b,miR-625-5p,novel_99,miR-1827,miR-221-5p,miR-504-5p,miR-2331-3p,miR-3065-3p,miR-296-3p,miR-552-3p,miR-544-3p,miR-10a,miR-377-3p,miR-193b-3p,miR-204-3p,miR-22-3p,miR-15b-5p,miR-665,miR-17-3p,miR-487b-5p,miR-874-5p,miR-4492,miR-424-5p,miR-214,miR-10b-5p,miR-3184-5p,miR-6134,miR-873a-5p,miR-542-3p,miR-197-3p,miR-22-3p,miR-145a-5p,miR-138,miR-30b-3p,miR-29b,miR-16b,miR-380-3p,miR-1260b,miR-497-5p,miR-7862,miR-455-5p,miR-31-5p,miR-133b-5p,miR-103a-3p,miR-339-5p,miR-26b-3p,miR-134-3p,miR-3431,miR-149-5p,miR-544b,miR-103,miR-377-3p,miR-330-3p,novel_51,miR-455-5p,miR-2411-3p,miR-483-3p,miR-124-5p,miR-1343-5p,miR-193a-3p,miR-6240,miR-628-5p,miR-2285r,miR-2387,miR-1306,miR-150-5p,miR-18a-3p,miR-1291,miR-107,miR-412-3p,miR-9851-3p,miR-195a-5p,miR-6395,miR-135b-5p,miR-379-5p,novel_68,miR-10a-5p,novel_69,miR-29a-3p,miR-299b-3p,miR-6535,miR-15a,miR-16a,miR-128-1-5p,miR-29a,miR-6123,miR-199c,miR-193b-

PIP4K2B	ENSOARG0(	phosphat	miR-744-3p,miR-2330-5p,miR-382-3p,miR-30c-1-3p,miR-2957,novel_19,miR-431-5p,miR-214-3p,miR-103a-2-5p,miR-3085-3p,miR-222-5p,miR-105-1,miR-1343-3p,novel_4,miR-221-3p,miR-148b-5p,miR-2459,miR-30b-3p,miR-6529a,miR-484,miR-1298-5p,miR-1839-5p,miR-2426,miR-450b-5p,miR-3059-5p,novel_60,miR-2447,miR-3956-3p,miR-885-5p,miR-363-3p,miR-877-3p,miR-361-3p,miR-2285y,miR-107-5p,novel_82,miR-2285n,miR-2284q,miR-4726-5p,miR-3064-5p,miR-664b,miR-4443,miR-489,miR-361-3p,miR-221,novel_94,miR-183-5p,miR-143-3p,miR-125b-2-3p,miR-92a-1-5p,novel_17,miR-760-3p,miR-1b-3p,miR-3600,miR-485-3p,miR-487a-3p,miR-210-3p,miR-222-5p,miR-5703,miR-217-5p,miR-2300b-3p,miR-215-5p,miR-218-1-3p,miR-133a-3p,miR-148a-5p,miR-214,miR-3074-5p,miR-145a-3p,miR-6525,miR-224-5p,miR-320e,miR-3604,miR-29b-2-5p,miR-665,miR-222-3p,miR-874-5p,miR-532-5p,miR-30c-1-3p,miR-32-5p,novel_32,miR-204-3p,miR-877-5p,miR-192-5p,miR-411,miR-1895,miR-625-5p,novel_99,miR-101-5p,miR-628-5p,let-7j,miR-296-3p,miR-539-3p,miR-654-3p,miR-432-3p,miR-105-5p,miR-32,miR-3141,novel_51,miR-224-5p,miR-296-3p,miR-466f-3p,miR-483-3p,miR-378a-5p,miR-194-3p,miR-149-5p,miR-432-5p,miR-185-5p,miR-197-3p,miR-708-3p,miR-432,miR-4508,miR-188-5p,miR-30b-3p,miR-219b-3p,miR-24-3p,miR-323c,miR-22-5p,miR-147-5p,miR-128-1-5p,miR-92a-3p,miR-379-3p,novel_68,miR-3074-5p,miR-378b,miR-3141,miR-9851-3p,miR-450b-3p,miR-3064-5p,miR-29b-2-5p,miR-4286,miR-376c-5p,novel_39,miR-2285b,miR-92b-3p,miR-148b-5p,miR-30c-2-3p,miR-217,miR-2285af,miR-664-3p,miR-92a-3p,miR-543-5p,miR-431,miR-2285ad,miR-222,miR-582-3p,novel_121,miR-483-3p,miR-218-2-3p,miR-105-2,miR-411-3p,miR-323-3p,miR-5100,miR-2355-5p,miR-370-3p,miR-215-5p,miR-25-3p,miR-200a-5p,miR-

EXT1	ENSOARG0(	exostosi	miR-3059-5p,miR-181d-5p,miR-877-3p,miR-7705,miR-154a,novel_82,miR-3968,miR-3958-3p,miR-211-5p,miR-378g,miR-499a-3p,miR-489,let-7d-5p,miR-203-3p,miR-1185-2-3p,miR-140-5p,miR-299b-5p,miR-136-5p,miR-504,miR-376a-3p,miR-1983,miR-544-5p,novel_94,miR-124a,let-7a-5p,miR-494-3p,miR-1839-3p,miR-8485,miR-2310,miR-199a-3p,miR-326,miR-1306-5p,miR-29b-1-5p,miR-182-5p,let-7e,miR-214-3p,miR-199b-3p,novel_4,miR-1343-3p,miR-409b,miR-222-5p,miR-181b-2-3p,miR-376b-3p,miR-142-3p,miR-455-3p,miR-1185-3p,miR-2284r,miR-143-5p,miR-3962,miR-125b-2-3p,miR-30b-3p,miR-1388-5p,miR-1839-5p,let-7b,miR-590-3p,miR-129-2-3p,miR-145a-3p,miR-3184-3p,miR-4510,miR-3074-5p,miR-3074-2-3p,miR-214,miR-346,let-7b-5p,miR-122-5p,miR-6134,miR-181c-5p,miR-2285j,let-7f,miR-15b-3p,miR-30a-3p,miR-664a-5p,miR-29b-2-5p,miR-874-5p,miR-208b-5p,miR-129-5p,miR-130b-5p,miR-504-5p,miR-140-5p,miR-660-5p,miR-496-3p,miR-1277-3p,miR-21-3p,miR-216b-3p,miR-182-5p,miR-6516,miR-192-5p,miR-582-5p,miR-6130,novel_25,let-7i-5p,miR-21a-3p,miR-299,miR-433-3p,miR-345-5p,miR-18a-5p,miR-885-3p,miR-582-5p,novel_99,miR-195a-3p,miR-29b-1-5p,miR-376a-5p,miR-130a-5p,miR-181b-1-3p,miR-142a-3p,miR-125b-2-3p,miR-2424,miR-143-3p,novel_17,miR-216a-5p,miR-324-3p,miR-125b,miR-202-5p,miR-98-5p,miR-30b-3p,miR-2484,let-7k,miR-18a-3p,miR-1b-3p,miR-30d-3p,miR-383-5p,miR-200b,miR-532-3p,miR-199a-3p,miR-1961,miR-215-5p,miR-193b-5p,miR-124-3p,miR-1197-5p,miR-5703,miR-140-3p,miR-33b-3p,miR-142-3p,miR-664-5p,miR-361-5p,miR-1a-1-5p,miR-1839-3p,miR-211,miR-24-3p,miR-326-3p,miR-2284w,let-7i,miR-382-5p,miR-33a-3p,miR-22-5p,miR-199c,miR-363,novel_68,miR-329-5p,miR-3074-5p,miR-320b,miR-655-5p,miR-2404,miR-146b-3p,miR-411b-3p,miR-

ERCC2	ENSOARG0(	excision	miR-214-3p,miR-3085-3p,miR-491-5p,novel_19,miR-16b,miR-5126,miR-4532,miR-193a,miR-758-3p,let-7c-3p,miR-1388-3p,miR-424-5p,miR-1839-5p,miR-758-3p,miR-6529a,miR-541,miR-2459,miR-151a-3p,novel_78,miR-2300a-5p,miR-1343-3p,miR-4726-5p,miR-499a-5p,miR-769-5p,miR-361-3p,miR-541-3p,novel_94,miR-877-3p,miR-147-3p,miR-136-5p,miR-504,miR-361-3p,miR-4792,miR-3064-5p,miR-21-3p,miR-324-3p,miR-185-3p,miR-125b,miR-196a-3p,miR-486-3p,miR-2887,miR-105-3p,miR-493-3p,miR-5703,novel_133,miR-2300b-3p,miR-151-5p,novel_83,miR-532-3p,miR-328-3p,miR-15a-5p,miR-3960,miR-18a-3p,miR-874-5p,miR-4492,miR-424-5p,novel_74,miR-664a,miR-324-3p,miR-151-3p,miR-3604,miR-665,miR-6134,miR-769-5p,novel_44,miR-346,miR-214,miR-145a-3p,miR-4510,miR-625-5p,miR-1827,let-7g-3p,miR-339b,miR-345-5p,miR-129b-3p,miR-433-3p,miR-151b,miR-15b-5p,miR-2284s,miR-193b-3p,miR-6130,miR-3065-3p,miR-545-3p,miR-504-5p,miR-125a-5p,miR-1343-5p,miR-193a-3p,miR-483-3p,miR-197-5p,novel_51,miR-7-5p,miR-345-5p,miR-432-3p,miR-668-3p,miR-497-5p,miR-4508,miR-744-5p,miR-7862,miR-29b,miR-16b,miR-197-3p,miR-2113,miR-1285,miR-7b-5p,miR-103,miR-185-5p,miR-339-5p,miR-3955-5p,miR-103a-3p,miR-6123,miR-128-1-5p,miR-29a,miR-16a,miR-2284o,miR-15a,miR-4324,miR-6529b,miR-193b-3p,miR-3957-3p,miR-29c-3p,miR-135a-2-3p,miR-379-5p,miR-6395,miR-195a-5p,miR-1291,miR-107,miR-150-5p,miR-18a-3p,miR-378b,miR-6535,miR-146b-3p,miR-411b-3p,miR-29a-3p,novel_68,miR-107,novel_127,miR-543-5p,novel_96,miR-2898,miR-339a,miR-125a-3p,miR-29a-5p,novel_39,miR-3065-3p,miR-490-3p,miR-1271-3p,miR-3064-5p,miR-2284f,miR-29d-3p,miR-1343-5p,miR-486-5p,miR-214-5p,miR-505-5p,miR-322-5p,miR-483-3p,miR-1224-5p,novel_79,miR-423-3p,miR-3432a,miR-486b-3p,miR-
TBL1Y	ENSOARG0(	transduc	miR-185-3p,miR-219-3p,miR-374a-5p,miR-8117,miR-2285af,miR-543-5p,miR-466i-5p,miR-320d,miR-2432,miR-142a-3p,miR-5703,miR-2904,miR-4429,miR-2285ad,miR-582-3p,miR-2284z,miR-1271-5p,miR-3604,miR-320e,miR-219b-3p,miR-324-3p,miR-2285j,miR-130b-5p,miR-4492,miR-664a,miR-138-5p,miR-3607-3p,miR-1271,miR-374b,miR-6134,miR-3184-5p,miR-224-5p,miR-1197-3p,miR-3154,miR-320a,miR-1248,miR-7144-5p,miR-625-5p,miR-2285aa,miR-660-5p,miR-874-3p,miR-133c,miR-377-3p,miR-194a,miR-136-3p,novel_19,miR-377-3p,miR-224-5p,miR-320b,miR-660,miR-363-5p,miR-130b-5p,miR-4532,miR-194-5p,miR-197-3p,miR-2284aa,miR-4508,miR-138,miR-26b-3p,miR-187-3p,miR-142-3p,miR-2285y,miR-2285n,miR-194b-5p,miR-219a-2-3p,miR-2284w,miR-2428,miR-423-5p,miR-96-5p,miR-3120-3p,miR-2285u,miR-374b-5p,miR-320c,miR-669

ZCCHC24	ENSOARG0(	zinc fin	miR-339b,miR-505-3p,miR-1827,miR-381-3p,miR-378g,miR-377-3p,miR-27a-3p,miR-2319b,miR-32-3p,miR-2331-3p,miR-30c-1-3p,miR-3064-5p,miR-615-5p,miR-141-3p,miR-552-3p,miR-542-5p,miR-2355-5p,miR-3065-3p,miR-450a-5p,miR-6123,miR-486b-3p,miR-331-3p,miR-371b-3p,miR-135a-2-3p,miR-32-3p,miR-300,miR-27b-3p,miR-2300b-3p,miR-484,miR-5703,miR-323b,miR-134-3p,miR-545-5p,miR-9788-3p,miR-27a-3p,miR-505-3p,miR-339-5p,novel_79,miR-3085-3p,miR-200a-3p,miR-377-3p,miR-339a,miR-30c-2-3p,miR-323b-3p,miR-30c-1-3p,miR-450c-5p,miR-486-3p,miR-200a,miR-3065-3p,miR-2432,miR-2330-5p,miR-628-5p,miR-3064-5p
UFM1	ENSOARG0(	ubiquiti	miR-193a-5p,miR-16-5p,miR-2433,miR-3068-3p,miR-2285aa,miR-3959-5p,miR-331-5p,miR-30e-5p,miR-93-5p,miR-200a-5p,miR-329b,miR-345-5p,miR-1248,miR-20b-5p,miR-450c-5p,miR-200a,miR-205-5p,miR-3071-3p,miR-127-5p,novel_127,miR-152-3p,miR-429-3p,miR-299a-3p,miR-2285ad,miR-322-5p,novel_121,miR-450a-1-3p,miR-1290,miR-3591-5p,miR-1839-3p,miR-301b-3p,miR-576-3p,miR-4324,miR-216b-3p,miR-411b-3p,miR-299b-3p,miR-6535,miR-148a-3p,miR-3591-3p,miR-378f,miR-2319a,miR-493-5p,miR-432-3p,miR-432-3p,miR-503-3p,miR-377-3p,miR-194a,miR-105-5p,miR-31-5p,miR-134-3p,miR-411-5p,miR-1843b-5p,miR-223-3p,miR-432,novel_23,miR-450c-3p,miR-16-1-3p,miR-16b,miR-2284x,miR-188-5p,miR-497-5p,miR-590-3p,miR-214,miR-224-5p,miR-346,miR-769-5p,miR-122-5p,miR-3184-5p,miR-30e-3p,miR-3604,miR-665,miR-30a-3p,miR-151-3p,miR-487b-5p,miR-130b-5p,miR-874-5p,miR-500,miR-454-3p,miR-301a-3p,miR-377-3p,miR-15b-5p,miR-450a-1-3p,miR-411,miR-30d-5p,miR-500a-3p,miR-345-5p,miR-186-5p,miR-1827,miR-625-5p,miR-543-3p,miR-345-3p,miR-143-3p,miR-486-3p,miR-618,miR-422a,miR-106a,miR-18a-3p,miR-30d-3p,miR-7859,miR-193b-5p,miR-378i,miR-1434-5p,miR-378a-3p,miR-33b-3p,miR-142-3p,miR-16-1-3p,miR-3956-3p,miR-423-5p,miR-101-3p,miR-301a-5p,miR-211-5p,miR-378g,miR-1185-2-3p,miR-4443,miR-140-5p,miR-136-5p,miR-544-5p,miR-335-3p,miR-493-5p,miR-101b-3p,miR-378d,miR-2310,miR-8485,miR-106b-5p,miR-16b,miR-130b-3p,miR-1185-5p,miR-214-3p,miR-103a-2-5p,miR-153-3p,miR-1298-3p,miR-147a,miR-153,miR-421,miR-2459,miR-1185-3p,miR-301b-5p,miR-1388-5p,miR-1306-5p,miR-103b,miR-3607-3p,miR-93,miR-378c,miR-145-3p,miR-370-5p,miR-2332,miR-551b-3p,miR-34c-3p,miR-204-5p,miR-486b-3p,miR-365a-3p,miR-769,miR-374c-3p,miR-2284p,miR-381-3p,miR-

LAMP5	ENSOARG0(	lysosoma	miR-1343-5p,miR-452-5p,miR-125a-5p,miR-7-5p,miR-2411-3p,miR-130b-5p,miR-29b,miR-4508,miR-2113,miR-7b-5p,miR-432,miR-22-3p,miR-185-5p,miR-3431,miR-432-5p,miR-412-5p,miR-3955-5p,miR-31-5p,miR-26b-3p,miR-339-5p,miR-29a,miR-199c,miR-6123,miR-203b-5p,miR-6402,miR-142b,miR-4324,miR-24-3p,miR-2284w,miR-199b-5p,miR-2428,miR-326-3p,novel_27,miR-335-5p,miR-135a-2-3p,miR-29c-3p,novel_101,miR-412-3p,miR-505-3p,miR-376a-2-5p,miR-2285ab,miR-18a-3p,miR-1306,miR-29a-3p,miR-411b-3p,miR-374c-3p,miR-2404,miR-320c,miR-3074-5p,novel_68,miR-7-1-3p,miR-10a-5p,miR-320b,miR-450a-2-3p,miR-543-5p,novel_96,novel_127,miR-205-5p,miR-339a,miR-2478,miR-199a-5p,miR-29a-5p,miR-3065-3p,miR-3064-5p,miR-10a-5p,miR-345-3p,miR-330-5p,miR-30a-5p,miR-1343-5p,miR-29d-3p,miR-2411-5p,miR-24-2-5p,novel_121,miR-129-1-3p,miR-214-5p,miR-222,miR-431,novel_79,miR-28-5p,miR-126b-3p,miR-135a-1-3p,miR-2332,miR-5100,novel_63,miR-29b-3p,miR-409-3p,miR-125b-5p,miR-6740-5p,miR-193a-5p,miR-10b-3p,miR-876-3p,miR-485-5p,miR-1814c,miR-301,miR-30e-5p,miR-320a,miR-3965,miR-374c-3p,miR-3957,miR-199a-5p,miR-3068-3p,miR-874-3p,miR-574-5p,miR-3957-5p,miR-125a,novel_124,miR-3085-3p,miR-491-5p,miR-1468,miR-199b-5p,miR-153-3p,miR-431-5p,miR-326,miR-1306-5p,miR-320b,miR-199a-3p,miR-182-5p,miR-29b-1-5p,miR-2957,miR-491-3p,miR-2310,miR-382-3p,miR-494-3p,miR-2330-5p,miR-1388-3p,miR-33a-5p,let-7c-3p,miR-3120-5p,miR-10b,miR-6529a,miR-296-5p,miR-484,miR-142-5p,miR-539-5p,miR-153,miR-455-3p,miR-221-3p,miR-199b-3p,novel_78,miR-5010-3p,miR-335,miR-628-3p,miR-3968,miR-2284q,miR-490-5p,miR-2440,miR-30b-5p,miR-218-5p,miR-212-3p,miR-3059-5p,miR-2447,miR-452-5p,miR-2285f,miR-136-5p,miR-

MACC1	ENSOARG0(	metastas	miR-197-5p,miR-105-5p,miR-224-5p,miR-377-3p,miR-146a,miR-193a-3p,miR-466f-3p,miR-483-3p,miR-101-5p,let-7f-2-3p,let-7f-2-3p,miR-628-5p,miR-130b-5p,miR-493-5p,miR-345-5p,miR-654-3p,miR-2284l,miR-197-3p,miR-145a-5p,novel_23,miR-374b-3p,miR-497-5p,miR-188-5p,miR-380-3p,miR-2284ab,miR-16b,miR-31-5p,miR-340-3p,miR-149-5p,miR-412-5p,miR-2285g,miR-2284d,miR-3431,miR-16a,novel_87,miR-15a,miR-656-5p,miR-193b-3p,miR-2285x,novel_101,miR-219b-3p,miR-342-3p,miR-199b-5p,miR-2284w,miR-150-5p,miR-1306,miR-18a-3p,miR-3591-3p,miR-2285ab,miR-148a-3p,miR-2319a,miR-146b-5p,miR-135b-5p,miR-195a-5p,miR-6516-3p,miR-3074-5p,miR-669,miR-6535,miR-3071-3p,miR-30f,miR-205-5p,miR-200c,miR-152-3p,novel_127,miR-200c-3p,miR-3064-5p,miR-199a-5p,miR-490-3p,miR-1271-3p,miR-450b-5p,miR-7134-5p,miR-134,miR-200b-3p,miR-105-2,miR-412,miR-7641,miR-2285ad,miR-429-3p,miR-2284z,miR-134-5p,miR-129-1-3p,miR-322-5p,miR-24-2-5p,miR-483-3p,miR-135a-5p,miR-2284k,miR-2332,miR-5100,miR-31-3p,miR-134-5p,novel_9,miR-16-5p,miR-6128,miR-2284v,miR-323-3p,miR-362-5p,miR-488-3p,miR-424-3p,miR-7144-5p,miR-671-5p,miR-10b-3p,miR-2318,miR-345-5p,miR-329a,miR-2355-5p,miR-199a-5p,miR-223-5p,miR-376c-3p,miR-154b-5p,miR-300-3p,miR-769,miR-29b-1-5p,miR-505,miR-362-3p,miR-16b,miR-342,novel_103,miR-1306-5p,miR-146b,miR-199b-5p,miR-103a-2-5p,miR-3085-3p,miR-3955-3p,miR-33a-5p,miR-424-5p,miR-8485,miR-758-3p,miR-412-3p,miR-758-3p,miR-329-3p,miR-6529a,miR-484,miR-101c,miR-2284aa,miR-362-3p,miR-1388-5p,miR-2426,miR-222-5p,miR-145b,miR-105-1,miR-500-5p,miR-1185-3p,miR-2285e,miR-126a-5p,miR-1298-3p,miR-142-3p,miR-539-5p,miR-769-5p,miR-2285y,miR-371a-5p,miR-107-5p,miR-2285n,miR-2285f,miR-3059-5p,miR-8095,miR-362-
C12orf4	ENSOARG0(	chromoso	miR-33a-3p,miR-125b-5p,miR-142b,miR-130b-5p,miR-206,miR-135a-2-3p,miR-338-3p,miR-206-3p,miR-6525,miR-433-3p,miR-129b-5p,miR-27b-5p,miR-1814c,miR-6395,let-7g-3p,miR-1291,miR-7977,miR-125a,miR-3965,novel_69,miR-365a-3p,miR-185-3p,miR-125b,miR-2957,miR-105-5p,miR-2397-5p,miR-26a-5p,novel_96,miR-125a-5p,miR-760-3p,miR-3955-3p,miR-491-5p,miR-2887,let-7c-3p,miR-1,miR-130b-5p,miR-2310,miR-8485,novel_23,miR-374b-3p,miR-1a-3p,miR-4791,miR-2113,miR-484,miR-105-2,miR-16-1-3p,miR-4508,novel_73,miR-142-3p,miR-16-1-3p,miR-105-1,miR-328-3p,miR-214-5p,miR-2459,novel_116,miR-3535
PSPH	ENSOARG0(	phosphos	miR-6119-3p,miR-200c-3p,miR-144,miR-217,novel_87,miR-200c,miR-369-3p,miR-582-5p,miR-30b-3p,miR-217-5p,miR-30b-3p,miR-2285c,miR-144-3p,miR-2285e,miR-582-5p,miR-2285g,miR-139-5p,miR-139-5p,novel_4,miR-429-3p,miR-548o-3p

SMARCC2	ENSOARG0(	SWI/SNF	miR-6525,miR-3184-5p,miR-433-5p,miR-3074-5p,miR-129-2-3p,miR-3958-5p,miR-665-5p,miR-214,miR-500,miR-874-5p,miR-4492,miR-10a,miR-377-3p,miR-216b-5p,miR-15b-5p,miR-151b,miR-30c-1-3p,miR-548o-3p,miR-296-3p,miR-345-5p,miR-339b,miR-885-3p,miR-543-3p,miR-625-5p,miR-1895,miR-1827,miR-450a-1-3p,miR-598-3p,miR-500a-3p,miR-376a-5p,miR-377-5p,miR-486-3p,miR-143-3p,miR-2285w,novel_17,miR-2285p,miR-219b-5p,miR-324-3p,miR-500a-5p,miR-92a-1-5p,miR-7859,miR-210-3p,miR-2284h-5p,miR-18a-3p,miR-493-3p,novel_73,miR-218-1-3p,miR-34a-5p,miR-423-5p,miR-2447,miR-3968,miR-615,miR-301a-5p,miR-378g,miR-3596,miR-2483-3p,miR-4443,miR-500-3p,miR-4792,miR-2319b,miR-668-5p,miR-1983,miR-140-5p,miR-378j,miR-8485,miR-1b-5p,miR-491-5p,miR-1185-5p,miR-487b-5p,miR-199b-5p,miR-214-3p,miR-199a-3p,miR-505,miR-16b,miR-1298-3p,miR-455-3p,miR-1343-3p,miR-199b-3p,miR-145b,miR-2284r,miR-301b-5p,miR-501-3p,miR-1260b,miR-16-5p,novel_9,miR-134-5p,miR-592,miR-2284k,miR-133c,miR-574-5p,miR-2355-5p,miR-345-5p,miR-329b,miR-148b-5p,novel_39,miR-2285b,novel_127,novel_96,miR-200a-3p,miR-125a-3p,miR-3071-3p,miR-322-5p,miR-505-5p,miR-134-5p,miR-450a-1-3p,miR-129-1-3p,novel_121,miR-2285ad,miR-299a-3p,miR-429-3p,novel_79,miR-1224-5p,miR-412,miR-18b-5p,miR-885-3p,miR-1290,miR-2427,miR-1343-5p,miR-330-5p,miR-23b-5p,miR-199b-5p,miR-1247-3p,miR-142b,novel_69,miR-299b-3p,miR-144-3p,miR-6535,novel_68,miR-3074-5p,miR-3120-3p,miR-410-5p,miR-412-3p,miR-2319a,miR-3970,miR-1306,miR-216a-3p,miR-432-3p,miR-2284l,miR-668-3p,miR-628-5p,miR-483-3p,miR-106b-3p,miR-7-5p,miR-377-3p,miR-2285v,novel_51,miR-134-3p,miR-149-5p,miR-194-3p,miR-339-5p,miR-16b,miR-4508,miR-497-
NEFL	ENSOARG0(	neurofil	miR-6529b,novel_94,miR-7134-3p,miR-582-3p,miR-2319a,miR-29a-5p
R3HDM4	ENSOARG0(	R3H doma	miR-329-5p,miR-1843b-5p,miR-329a-5p,miR-329b,miR-491-5p,miR-128-1-5p,novel_96
CREB3L2	ENSOARG0(	cAMP res	miR-6119-5p,miR-541,miR-21c,miR-876-3p,miR-21-5p,miR-541-3p,miR-4510,miR-140-5p,miR-193a-5p,miR-6130,miR-6134
NEFM	ENSOARG0(	neurofil	miR-450a-5p,miR-127-5p,miR-135b-5p,miR-450c-5p,miR-135a-5p

CDK19	ENSOARG0(	cyclin-d	miR-18a,miR-488-3p,miR-7144-5p,miR-141-3p,miR-487a-3p,miR-324-5p,miR-133c,miR-1-5p,miR-2332,miR-410-3p,miR-138-5p,miR-374a-3p,miR-1260b,miR-1343-5p,miR-330-5p,miR-18b-5p,miR-431,miR-222,miR-545-5p,miR-214-5p,miR-3071-3p,miR-1260a,miR-376b-3p,miR-107,miR-152-3p,miR-200a-3p,miR-450a-2-3p,miR-1a-2-5p,miR-30a-3p,miR-29b-2-5p,miR-376b-3p,miR-148a-3p,miR-18b,miR-2387,miR-450b-3p,miR-19a-3p,miR-107,miR-3120-3p,miR-6535,miR-502-5p,miR-576-3p,miR-22-5p,miR-136-3p,miR-1a-1-5p,miR-326-3p,novel_27,miR-301b-3p,miR-323c,miR-3071-5p,miR-194-5p,miR-145a-5p,miR-653,miR-873a-5p,miR-1434-3p,miR-138,miR-1260b,miR-130a-3p,miR-103a-3p,novel_116,miR-411-5p,miR-103,miR-3431,miR-194a,miR-224-5p,miR-377-3p,miR-1343-5p,miR-124-5p,let-7f-2-3p,let-7f-2-3p,miR-2285r,miR-21a-3p,novel_99,miR-154b-3p,miR-18a-5p,miR-552-3p,miR-548o-3p,miR-204-3p,novel_32,miR-377-3p,miR-301a-3p,miR-139-5p,miR-30a-3p,miR-29b-2-5p,miR-664a-5p,miR-222-3p,miR-30e-3p,miR-454-3p,miR-664a,miR-425-5p,miR-3074-2-3p,miR-539-5p,miR-3184-3p,miR-3184-5p,miR-338-3p,miR-6525,miR-224-5p,miR-1434-5p,miR-126-3p,miR-2366,miR-2300b-3p,miR-361-5p,miR-493-3p,miR-19b-3p,miR-3600,miR-485-3p,miR-487a-3p,miR-383-5p,miR-148b-3p,miR-500a-5p,miR-2285p,novel_17,miR-1277-5p,miR-345-3p,miR-487a,miR-130a-5p,miR-221,miR-376a-3p,miR-668-5p,miR-541-5p,miR-139-5p,miR-376d,miR-1185-2-3p,miR-2483-3p,novel_82,miR-154-3p,miR-499a-5p,miR-452-5p,miR-194b-5p,miR-2447,miR-423-5p,miR-361-3p,miR-296-5p,miR-143-5p,miR-1839-5p,miR-1388-5p,miR-450b-5p,miR-145b,miR-221-3p,miR-1185-3p,miR-2459,miR-376b-3p,miR-326,miR-431-5p,miR-130b-3p,miR-101b-3p,miR-494-3p,miR-1b-5p

MPND	ENSOARG0(	MPN doma	miR-323-5p,miR-139-5p,miR-377-3p,miR-34c-5p,miR-151b,miR-22-3p,novel_32,miR-125a,miR-370-3p,miR-3065-3p,miR-18a-5p,miR-339b,miR-10b-3p,miR-1248,miR-543-3p,miR-21a-3p,miR-18a,miR-23b,miR-1260b,miR-338-3p,miR-181c-5p,miR-3184-5p,miR-214,miR-103b,miR-665-5p,miR-664a,miR-134-5p,miR-486b-3p,miR-130b-5p,miR-4492,miR-125b-5p,miR-331-3p,miR-23c,miR-383-5p,miR-24-2-5p,miR-23b-3p,miR-328-3p,miR-151-5p,miR-134-5p,miR-532-3p,miR-2284z,miR-6517,miR-431,miR-18b-5p,miR-212-5p,novel_79,miR-493-3p,miR-378d,miR-34a-5p,miR-133a-3p,miR-134,miR-188-3p,miR-380-3p,miR-3065-3p,miR-486-3p,miR-345-3p,miR-4286,miR-3064-5p,miR-181b-5p,miR-34b-5p,novel_17,miR-1260a,miR-339a,miR-125b,miR-185-3p,miR-324-3p,miR-411b-3p,miR-34b,novel_69,miR-323a-5p,miR-27a-3p,miR-6535,miR-669,miR-452-3p,miR-3064-5p,miR-139-5p,miR-320b,novel_94,miR-877-3p,miR-6395,miR-9851-3p,miR-18b,miR-1306,miR-150-5p,miR-24-3p,miR-449a,miR-423-5p,miR-342-3p,miR-3059-5p,miR-181d-5p,miR-3968,miR-4324,miR-34c,miR-216a-3p,miR-149-5p,miR-133b-5p,miR-31-5p,miR-27a-3p,miR-339-5p,miR-1260b,miR-380-3p,miR-27b-3p,miR-7862,miR-188-5p,miR-4508,miR-181a-5p,miR-296-5p,miR-22-3p,miR-197-3p,miR-8485,miR-382-3p,miR-130b-5p,miR-23a-3p,miR-668-3p,miR-3085-3p,miR-214-3p,miR-466f-3p,miR-125a-5p,miR-377-3p,miR-1306-5p,miR-431-5p,miR-342,novel_51

ADAM7	ENSOARG0(	ADAM met	let-7g,miR-1957a,miR-29a-5p,let-7f-5p,miR-6536,miR-107,miR-30c-2-3p,miR-3071-3p,let-7c-5p,miR-545-5p,miR-450a-1-3p,novel_121,miR-299a-3p,miR-2285ad,miR-412,let-7d,miR-582-3p,miR-30c,miR-496,let-7a-2-3p,miR-10a-5p,miR-196a-5p,miR-501-3p,miR-30a-5p,miR-93,miR-374a-3p,miR-3607-3p,miR-6128,miR-138-5p,miR-331-5p,miR-374b-3p,miR-34c-3p,miR-135a-1-3p,miR-144,miR-125b-5p,miR-6740-5p,miR-135a-5p,miR-331-5p,miR-300-3p,miR-3068-3p,miR-503-3p,miR-2285aa,miR-125a,miR-2318,novel_91,miR-20b-5p,miR-1248,miR-1814c,miR-30e-5p,miR-503-3p,miR-331-5p,miR-93-5p,miR-320a,miR-17-5p,miR-654-3p,let-7a-2-3p,miR-493-5p,miR-101-5p,miR-196b-5p,miR-125a-5p,miR-146a,miR-190a,miR-17-5p,miR-106a-5p,let-7g-5p,miR-185-5p,miR-20b,miR-103,miR-103a-3p,miR-138,miR-30b-3p,miR-16-1-3p,miR-26a-2-3p,miR-19b-2-5p,miR-873a-5p,miR-1434-3p,miR-450c-3p,miR-3071-5p,miR-219b-3p,novel_120,miR-335-5p,novel_1,miR-147-5p,miR-6402,miR-20a-5p,miR-576-3p,let-7i,miR-146b-3p,miR-299b-3p,miR-144-3p,miR-320c,miR-3074-5p,miR-3120-3p,miR-10a-5p,miR-450b-3p,miR-107,miR-146b-5p,let-7e-5p,miR-505-3p,miR-135b-5p,miR-3970,miR-3591-3p,miR-664b-3p,miR-146a-5p,miR-20a,miR-106a,miR-320d,miR-98-5p,miR-30b-3p,let-7k,miR-299-3p,miR-30b,miR-219b-5p,miR-185-3p,miR-125b,miR-383-5p,miR-340-5p,miR-106a,miR-3600,miR-505-3p,miR-212-5p,miR-30d-3p,miR-493-3p,miR-4429,miR-16-1-3p,miR-664-5p,miR-361-5p,miR-30c-5p,miR-1961,miR-1246,miR-2904,miR-188-3p,miR-142a-5p,miR-10b-5p,let-7b-5p,miR-6134,let-7b,miR-4510,miR-3074-5p,miR-3074-2-3p,miR-450b-3p,miR-6516-3p,miR-190a-5p,miR-487a-5p,miR-4492,let-7f,miR-19b-1-5p,miR-665,miR-664a-5p,miR-10a,miR-6130,let-7i-5p,miR-504-5p,miR-30c-1-3p,miR-496-3p,miR-21-3p,miR-30f,miR-186-5p,miR-21a-
TUBGCP4	ENSOARG0(	tubulin,	miR-185-5p,miR-194-3p,miR-532-3p,miR-133b-5p,miR-665,miR-7-1-3p,miR-222-5p,miR-3956,miR-219a-1-3p,miR-146b-5p,miR-135b-5p,miR-877-3p,miR-873a-5p,miR-541,miR-3065-5p,miR-194-5p,miR-188-3p,miR-1957a,novel_39,miR-146a-5p,miR-486-3p,miR-338-3p,miR-541-3p,miR-194b-5p,miR-146b,miR-208b-5p,miR-486b-3p,miR-146a,miR-101-3p,miR-135a-5p,miR-194a
AKR7A2	ENSOARG0(	aldo-ket	miR-4429,miR-1388-5p,miR-3071-5p,miR-758-3p,miR-5703,miR-323b,miR-345-3p,miR-199a-3p,miR-455-3p,miR-340-5p,miR-3431,miR-496-5p,miR-665,miR-127-5p,miR-320d,miR-17-3p,miR-2411-3p,miR-345-3p,miR-323b-3p,novel_115,miR-199a-3p,miR-320b,miR-758-3p,novel_39,miR-432-3p,miR-432-3p,miR-664b-3p,miR-6516-3p,miR-504,miR-3154,miR-3065-5p,miR-320a,miR-320c,miR-504-5p,miR-140-5p,miR-17-3p,miR-6516-3p,miR-664a,miR-3969,miR-6740-5p,miR-2428,miR-17-3p,novel_101
ADAMDEC1	ENSOARG0(	ADAM-lik	miR-429-3p,miR-26b-3p,miR-181b-2-3p,miR-383-5p,miR-6516-5p,miR-222-5p,miR-2355-3p,miR-125b-2-3p,miR-2426,miR-450b-5p,miR-200b-3p,miR-628-5p,miR-181b-1-3p,miR-125b-2-3p,miR-224-5p,miR-200c,miR-6119-5p,miR-200c-3p,miR-221-5p,miR-221-5p,miR-370-3p,miR-499b-5p,miR-582-5p,miR-6516,miR-677,miR-181b-3p,miR-582-5p,miR-145a-3p,miR-425-5p,miR-224-5p,miR-145-3p,miR-6402

SULT1E1	ENSOARG0(	sulfotra	miR-503-3p,miR-17-5p,miR-10b-3p,miR-671-5p,miR-1814c,miR-485-5p,miR-7144-5p,novel_48,miR-215-5p,miR-34c-5p,miR-2284n,miR-199a-5p,miR-409-3p,miR-331-3p,miR-767-5p,miR-204-5p,miR-93,miR-145-3p,miR-378c,miR-501-3p,miR-218-2-3p,miR-10a-5p,miR-7641,miR-6516-5p,miR-378h,miR-1271-5p,miR-376b-3p,miR-339a,miR-30c-2-3p,miR-181b-5p,miR-450a-2-3p,miR-17-3p,miR-30a-3p,miR-1,miR-29b-2-5p,miR-2478,miR-199a-5p,miR-2387,miR-376b-3p,miR-18a-3p,miR-6395,miR-195a-5p,miR-7975,miR-378b,miR-10a-5p,miR-7-1-3p,miR-34b,miR-20a-5p,miR-502-5p,miR-15a,miR-16a,miR-34c,miR-17-3p,miR-2285x,miR-3959-3p,miR-24-3p,miR-211,novel_27,miR-449a,miR-1434-3p,miR-181a-5p,miR-2284ab,miR-187-3p,miR-2285g,miR-2284d,miR-20b,miR-106a-5p,miR-17-5p,miR-130b-5p,miR-382,miR-654-3p,let-7g-3p,miR-32-3p,miR-378a-3p,miR-182-5p,miR-21-3p,miR-193b-3p,miR-139-5p,miR-192-5p,miR-6516,miR-204-3p,miR-655-3p,miR-29b-2-5p,miR-17-3p,miR-6516-3p,miR-664a,miR-450b-3p,miR-424-5p,miR-145a-3p,miR-378c,miR-3074-2-3p,miR-1271,miR-206-3p,miR-10b-5p,miR-215-5p,miR-1197-5p,miR-140-3p,miR-5703,miR-2904,miR-548e-3p,miR-3600,miR-148b-3p,miR-15a-5p,miR-216a-5p,miR-106a,miR-2284m,miR-34b-5p,miR-216c-5p,miR-20a,miR-199b-5p,miR-877-3p,miR-20a-3p,miR-664b,miR-7977,miR-541-5p,miR-378e,miR-376d,miR-2285n,miR-107-5p,miR-2285t,novel_60,miR-32-3p,miR-181d-5p,miR-8095,miR-502-3p,miR-10b,miR-484,miR-6529a,miR-378b,miR-125b-2-3p,miR-450b-5p,miR-378c,miR-5010-3p,miR-2285e,miR-382-3p,miR-106a-5p,miR-424-5p,miR-33a-5p,let-7c-3p,miR-30c-1-3p,miR-193a,miR-301,miR-331-5p,miR-93-5p,miR-876-3p,miR-767,miR-20b-5p,miR-331-5p,miR-592,miR-16-5p,miR-1290,novel_79,miR-322-5p,miR-9-5p,novel_121,miR-2284z,miR-450a-1-3p,miR-664-3p,miR-205-

RAVER2	ENSOARG0(	ribonucl	miR-345-3p,miR-2424,miR-2411,miR-146a-5p,miR-486-3p,miR-181b-1-3p,miR-376c-3p,miR-125b-2-3p,miR-664b-3p,miR-2285p,miR-210-5p,miR-618,miR-196a-3p,miR-30b,miR-21-3p,miR-1277-5p,miR-30d,miR-485-3p,miR-3600,novel_83,miR-200b,miR-148b-3p,miR-487a-3p,miR-7859,miR-140-3p,miR-217-5p,miR-2904,novel_133,miR-30c-5p,miR-142-3p,novel_73,miR-3074-2-3p,miR-145a-3p,miR-590-3p,miR-3184-5p,miR-142a-5p,miR-320e,miR-19b-1-5p,let-7b-3p,miR-130b-5p,miR-190a-5p,miR-708-5p,miR-487b-5p,miR-450b-3p,miR-532-5p,miR-552-3p,miR-182-5p,miR-216b-3p,miR-21-3p,miR-660-5p,miR-32-5p,miR-204-3p,miR-22-3p,miR-376e-3p,miR-6516,miR-30d-5p,miR-598-3p,let-7f-1-3p,miR-21a-3p,miR-186-5p,miR-885-3p,miR-30f,miR-2312,miR-494-3p,miR-182-5p,miR-323b-3p,novel_103,miR-146b,miR-3955-3p,miR-491-5p,miR-27a-3p,miR-181b-2-3p,miR-330-3p,miR-126a-5p,miR-539-5p,miR-376b-3p,miR-142-5p,miR-3962,miR-323b,miR-4791,miR-3120-5p,miR-450b-5p,miR-190b-5p,miR-452-5p,miR-2285f,miR-2447,miR-423-5p,novel_111,miR-30b-5p,miR-374a-3p,miR-4726-5p,miR-126b-5p,miR-211-5p,miR-541-5p,miR-20a-3p,miR-27a-3p,miR-376d,miR-454-5p,miR-4443,miR-203-3p,miR-25,miR-129b-5p,miR-381-3p,miR-378j,miR-335-3p,miR-376a-3p,miR-493-5p,miR-99b-5p,miR-2285b,miR-490-3p,novel_39,miR-1957a,miR-29a-5p,miR-92b-3p,miR-217,miR-3071-3p,miR-30f,miR-200c,miR-99a,miR-376b-3p,miR-543-5p,miR-152-3p,novel_96,miR-92a-3p,miR-200c-3p,miR-28-3p,miR-2898,miR-429-3p,miR-450a-1-3p,miR-6516-5p,miR-2411-5p,miR-30a-5p,miR-3591-5p,miR-196a-5p,miR-30c,miR-99a-5p,miR-200b-3p,miR-300,miR-582-3p,miR-885-3p,miR-28a-3p,miR-145-3p,miR-374a-3p,miR-323-3p,miR-144,miR-410-5p,miR-34c-3p,miR-486b-3p,miR-374b-3p,miR-204-5p,miR-28-5p,miR-592,miR-1271,miR-370-3p,miR-598-3p,miR-2285aa,miR-
LMOD3	ENSOARG0(	leiomodi	miR-148a-5p,miR-1248,novel_133,miR-296-5p,miR-148b-5p,miR-374c-3p,miR-374c-3p,miR-26b-3p,miR-2331-3p,miR-548o-3p,miR-874-3p,miR-4492,miR-21-3p,miR-2957,miR-409-3p,miR-224-5p,miR-15b-3p,miR-6119-5p,miR-2428,miR-432-3p,miR-224-5p,miR-345-3p,miR-744-3p,miR-1306-5p
POSTN	ENSOARG0(	periosti	miR-188-3p,miR-217-5p,miR-1290,miR-1246,miR-429-3p,miR-2285ad,miR-660,miR-200c-3p,miR-217,miR-200c,miR-219-3p,miR-296-3p,miR-6119-5p,miR-296-3p,miR-130a-5p,miR-376a-5p,miR-2478,miR-19a-3p,miR-10b-3p,miR-541-5p,miR-370-5p,miR-660-5p,miR-20a-3p,miR-545-3p,miR-490-5p,novel_9,miR-499a-5p,miR-2285y,miR-219b-3p,miR-370-5p,miR-219a-2-3p,miR-8095,novel_111,miR-1193

AKR1D1	ENSOARG0(	aldo-ket	miR-193b-5p,miR-33b-3p,miR-2284u,miR-34a-5p,miR-548e-3p,miR-106a,miR-3600,miR-7859,miR-1247-5p,novel_83,novel_17,miR-210-5p,miR-2285p,miR-324-3p,miR-216a-5p,miR-106a,miR-2284m,miR-34b-5p,miR-21-3p,miR-20a,miR-195a-3p,miR-421-5p,miR-125b-2-3p,miR-130a-5p,miR-377-5p,miR-2424,miR-486-3p,miR-2432,miR-380-3p,miR-143-3p,miR-2285c,miR-129b-3p,miR-487b-5p,miR-582-5p,miR-140-5p,miR-1277-3p,miR-370-5p,miR-182-5p,miR-499b-3p,miR-301a-3p,miR-582-5p,miR-323-5p,miR-6130,miR-2285j,miR-324-3p,miR-320e,miR-664a,miR-487b-5p,miR-454-3p,miR-130b-5p,miR-4510,miR-3958-5p,miR-3074-2-3p,miR-769-5p,miR-224-5p,miR-412-3p,miR-125b-2-3p,miR-101a-5p,miR-2284aa,miR-3120-5p,miR-27a-3p,miR-1298-3p,miR-376b-3p,miR-147a,miR-2285e,miR-326,miR-182-5p,miR-106b-5p,miR-130b-3p,miR-3955-3p,miR-494-3p,miR-106a-5p,miR-2284b,miR-140-5p,miR-376a-3p,miR-877-3p,miR-2284g,miR-499b-5p,miR-499a-3p,miR-3082-5p,miR-2483-3p,miR-376d,miR-27a-3p,novel_82,miR-769-5p,miR-3958-3p,miR-2285t,miR-490-5p,novel_60,miR-3059-5p,miR-3956-3p,miR-1296-5p,miR-2284j,miR-877-3p,miR-219a-2-3p,miR-1290,miR-218-2-3p,miR-23b-5p,miR-330-5p,miR-411-3p,miR-128-3p,miR-7641,miR-412,miR-2284z,miR-376b-3p,miR-2285af,miR-8117,miR-219-3p,miR-3071-3p,miR-543-5p,miR-2478,miR-1957a,novel_39,miR-29a-5p,miR-2284y,miR-2284a,miR-93-5p,miR-3154,miR-17-5p,miR-876-3p,miR-329b,miR-20b-5p,miR-874-3p,miR-34c-5p,miR-769,miR-2284n,miR-2284k,miR-494-5p,miR-370-5p,miR-26c,miR-3969,miR-135a-5p,miR-486b-3p,miR-2284v,miR-6128,miR-93,miR-1197-3p,miR-432,miR-653,miR-1285,miR-7b-5p,miR-197-3p,miR-374b-3p,miR-2284ab,miR-380-3p,miR-16-1-3p,miR-27b-3p,miR-455-5p,miR-130a-3p,miR-377-5p,miR-432-5p,miR-412-5p,miR-2284d,miR-2285g,miR-20b,miR-224-5p,miR-7-

LRP1B	ENSOARG0(	low dens	novel_39,miR-380-5p,miR-450b-5p,miR-543-5p,miR-6536,miR-217,miR-450a-1-3p,miR-9-5p,miR-758-5p,miR-299a-3p,miR-155-5p,let-7a-2-3p,miR-411-3p,miR-345-3p,miR-323-3p,miR-3607-3p,miR-331-3p,miR-34c-3p,miR-324-5p,miR-769,miR-2355-5p,miR-487a-3p,miR-7144-5p,miR-671-5p,miR-10b-3p,miR-10a-3p,miR-345-5p,let-7g-3p,miR-3154,miR-320a,miR-2448-3p,let-7a-2-3p,miR-345-5p,miR-432-3p,miR-432-3p,miR-380-5p,miR-466f-3p,miR-2411-3p,miR-185-5p,miR-31-5p,miR-30b-3p,miR-16-1-3p,miR-219a-1-3p,miR-450c-3p,miR-197-3p,miR-653,miR-542-3p,miR-2428,novel_101,miR-342-3p,miR-203b-5p,miR-656-5p,miR-625-3p,miR-147-5p,miR-379-3p,miR-485-3p,miR-133a-5p,miR-320c,miR-299b-3p,miR-7-1-3p,miR-26b-5p,miR-505-3p,miR-6516-3p,miR-3065-5p,miR-2432,miR-376a-5p,miR-329a-5p,miR-10a-3p,miR-487a,miR-299-3p,miR-2484,miR-320d,miR-92a-1-5p,miR-345-3p,miR-26a-5p,miR-618,miR-210-5p,miR-487a-3p,miR-505-3p,miR-485-3p,miR-16-1-3p,miR-133a-3p,miR-378d,miR-4429,miR-5703,miR-217-5p,miR-769-5p,miR-669a-3p,miR-450b-3p,miR-6516-3p,miR-155-5p,miR-665,miR-155-5p,miR-216b-5p,miR-154a-3p,miR-296-3p,miR-552-3p,miR-545-3p,miR-186-5p,miR-154b-3p,miR-345-5p,miR-450a-1-3p,miR-3187-3p,miR-758-3p,miR-33a-5p,miR-494-3p,miR-103a-2-5p,miR-3955-3p,miR-487b-5p,miR-505,miR-320b,miR-342,novel_115,miR-9788-3p,miR-455-3p,miR-1185-3p,miR-409b,miR-548w,miR-101c,miR-1388-5p,miR-30b-3p,miR-758-3p,miR-484,miR-9-3p,miR-877-3p,miR-452-5p,miR-9-5p,miR-212-3p,miR-2284q,miR-154-3p,miR-371a-5p,miR-769-5p,miR-107-5p,miR-1185-2-3p,miR-3596,miR-2483-3p,miR-20a-3p,miR-335-3p,miR-9-3p,miR-129b-5p,miR-361-3p,miR-140-5p

LASP1	ENSOARG0(	LIM and	miR-767-5p,miR-374b-3p,miR-486b-3p,miR-5100,miR-370-5p,miR-29b-3p,miR-135a-5p,miR-3969,miR-331-3p,miR-374a-3p,miR-93,miR-365a-5p,miR-133a-3p,miR-6128,miR-329a,miR-767,miR-20b-5p,miR-7144-5p,miR-23b,miR-3154,miR-17-5p,miR-93-5p,miR-199a-5p,miR-2355-5p,miR-191-3p,miR-125a-3p,miR-30f,miR-2284a,miR-1271-3p,miR-2284y,novel_39,miR-199a-5p,miR-3065-3p,miR-1,miR-128-3p,miR-3578,miR-330-5p,miR-29d-3p,miR-483-3p,miR-9-5p,miR-505-5p,miR-214-5p,miR-6517,miR-299a-3p,miR-1224-5p,miR-206,novel_1,miR-29a,miR-20a-5p,miR-502-5p,miR-33a-3p,miR-382-5p,miR-199b-5p,novel_27,miR-326-3p,miR-342-3p,miR-1193,miR-29c-3p,miR-379-5p,miR-135b-5p,miR-2387,miR-2285ab,miR-216a-3p,miR-150-5p,novel_69,miR-299b-3p,miR-29a-3p,miR-670-3p,miR-146b-3p,miR-323a-5p,miR-2404,miR-6535,miR-3074-5p,miR-483-3p,miR-17-5p,novel_51,miR-106a-5p,miR-365b-5p,miR-330-3p,miR-6240,miR-130b-5p,miR-2483-5p,miR-363-5p,miR-30b-3p,miR-29b,miR-27b-3p,miR-26a-2-3p,miR-542-3p,miR-432,miR-145a-5p,miR-22-3p,miR-185-5p,miR-134-3p,miR-432-5p,miR-3535,miR-544b,miR-20b,miR-194-3p,miR-130b-5p,miR-874-5p,miR-4492,miR-320e,miR-665,miR-324-3p,miR-206-3p,miR-6134,miR-3184-5p,miR-145a-3p,miR-4510,miR-3074-5p,miR-3184-3p,miR-214,miR-2330-3p,miR-2312,miR-133b-3p,miR-625-5p,miR-129b-3p,miR-6130,miR-323-5p,miR-544-3p,miR-22-3p,miR-204-3p,miR-182-5p,miR-3065-3p,miR-370-5p,miR-466i-5p,miR-106a,miR-299-3p,novel_17,miR-185-3p,miR-125b-2-3p,miR-486-3p,miR-20a,miR-33b-3p,miR-148a-5p,miR-2284u,miR-133a-3p,miR-362-3p,miR-2366,miR-188-3p,miR-1197-5p,miR-140-3p,miR-124-3p,miR-23c,miR-23b-3p,miR-532-3p,miR-106a,miR-212-5p,miR-499a-5p,miR-374a-3p,miR-2440,miR-218-5p,miR-301a-5p,miR-1285-5p,miR-8095,miR-423-5p,miR-541-3p,miR-3956-3p,miR-

SULF2	ENSOARG0(	sulfatas	miR-21-3p,miR-552-3p,miR-140-5p,miR-30c-1-3p,miR-1277-3p,miR-22-3p,novel_25,miR-155-5p,miR-6516,miR-544-3p,miR-21a-3p,miR-625-5p,novel_99,miR-1827,miR-345-5p,miR-30f,miR-214,miR-3074-2-3p,miR-539-5p,miR-129-2-3p,miR-3184-5p,miR-122-5p,miR-206-3p,novel_44,miR-224-5p,miR-219b-3p,miR-151-3p,miR-320e,miR-664a-5p,miR-29b-2-5p,miR-2285j,miR-708-5p,miR-4492,miR-6516-3p,miR-487b-5p,miR-3600,miR-30d-3p,miR-2284h-5p,miR-532-3p,miR-616-3p,miR-340-5p,miR-148b-3p,miR-1434-5p,miR-217-5p,miR-188-3p,novel_133,miR-30c-5p,miR-193b-5p,miR-362-3p,miR-2300b-3p,miR-2366,miR-376a-5p,miR-16-1-3p,miR-142-3p,miR-4429,miR-33b-3p,miR-329a-5p,miR-29b-1-5p,miR-130a-5p,miR-125b-2-3p,miR-219b-5p,miR-500a-5p,miR-185-3p,miR-196a-3p,novel_17,miR-299-3p,miR-2484,miR-320d,miR-760-3p,miR-2285u,miR-211-5p,miR-20a-3p,miR-452-3p,miR-203-3p,miR-3082-5p,miR-378j,miR-1306-3p,miR-129b-5p,miR-409-5p,miR-335-3p,miR-99b-5p,miR-147-3p,miR-668-5p,miR-2285f,miR-3059-5p,miR-423-5p,miR-219a-2-3p,miR-877-3p,miR-362-5p,miR-2285y,miR-382-3p,miR-1843a-3p,miR-2285t,miR-499a-5p,miR-145b,miR-105-1,miR-1343-3p,miR-548w,miR-151a-3p,miR-455-3p,miR-500-5p,miR-147a,miR-412-3p,miR-1a-3p,miR-329-3p,miR-362-3p,miR-544a,miR-450b-5p,miR-2426,miR-33a-5p,miR-1388-3p,miR-101b-3p,miR-193a,miR-2310,miR-30c-1-3p,miR-362-3p,miR-29b-1-5p,miR-320b,miR-1306-5p,miR-214-3p,miR-574-5p,miR-874-3p,miR-3068-3p,miR-320a,miR-362-5p,miR-3154,miR-100-5p,miR-7144-5p,miR-1248,miR-1814c,miR-767,miR-876-3p,miR-345-5p,miR-2318,miR-329a,let-7g-3p,miR-103b,miR-1306-5p,miR-500b-5p,miR-1260b,miR-374a-3p,miR-144,miR-3969,miR-409-3p,miR-410-5p,miR-1306-3p,miR-135a-1-3p,miR-28-5p,miR-374b-3p,miR-204-5p,miR-592,miR-138-5p,novel_9,miR-767-5p,miR-

LPIN2	ENSOARG0(	lipin 2	miR-24-3p,miR-323c,miR-449a,miR-326-3p,miR-342-3p,miR-3957-3p,miR-495-3p,miR-17-3p,miR-128-1-5p,miR-15a,miR-6529b,miR-16a,miR-34c,miR-2404,miR-34b,miR-29a-3p,miR-655-5p,miR-450b-3p,miR-9851-3p,miR-195a-5p,miR-18b,miR-2387,miR-654-3p,miR-345-5p,miR-363-5p,miR-125a-5p,miR-126a-3p,miR-330-3p,miR-2331-5p,miR-185-5p,miR-29b,miR-30b-3p,miR-744-5p,miR-500b-5p,miR-378c,miR-133a-3p,miR-486b-3p,miR-29b-3p,miR-34c-5p,miR-324-5p,miR-154b-5p,miR-370-3p,miR-141-3p,let-7g-3p,miR-23a-5p,novel_48,miR-488-3p,miR-362-5p,miR-18a,miR-3154,miR-3065-3p,miR-29b-2-5p,miR-4286,miR-2898,miR-17-3p,miR-543-5p,miR-2285af,miR-339a,miR-483-3p,miR-378h,miR-214-5p,miR-105-2,miR-615-3p,miR-7134-5p,miR-29d-3p,miR-362-5p,novel_60,miR-3059-5p,miR-541-3p,miR-4726-5p,miR-335,miR-2285n,miR-2285y,miR-378e,miR-3064-5p,miR-877-3p,miR-361-3p,miR-3187-3p,miR-424-5p,miR-652-5p,miR-376b,miR-3085-3p,miR-3955-3p,miR-342,miR-326,novel_103,miR-362-3p,miR-9788-3p,miR-500-5p,miR-105-1,miR-409b,miR-378c,miR-2426,miR-484,miR-6529a,miR-378b,miR-143-5p,miR-30b-3p,miR-338-3p,miR-378c,miR-4510,miR-3074-2-3p,miR-425-5p,miR-17-3p,miR-208b-5p,miR-487a-5p,miR-424-5p,miR-19b-1-5p,miR-29b-2-5p,miR-6130,miR-204-3p,miR-140-5p,miR-221-5p,miR-378a-3p,miR-3065-3p,miR-133b-3p,miR-18a-5p,miR-582-5p,miR-29b-1-5p,miR-130a-5p,miR-760-3p,miR-30b-3p,miR-34b-5p,miR-221-5p,miR-21-3p,miR-299-3p,miR-196a-3p,miR-185-3p,miR-125b,miR-383-5p,miR-15a-5p,miR-199a-3p,miR-532-3p,miR-3600,miR-485-3p,miR-941,miR-378d,novel_133,miR-362-3p,miR-1246,miR-2904,miR-188-3p,miR-1197-5p,miR-140-3p,miR-2284w,miR-335-5p,miR-29c-3p,miR-29a,miR-4324,miR-142b,miR-576-3p,miR-208a-3p,miR-299b-3p,novel_69,miR-6535,miR-669,miR-3074-5p,miR-412-

TRAFD1	ENSOARG0(	TRAF-typ	novel_39,miR-3065-3p,miR-2285b,miR-3064-5p,miR-181a-2-3p,miR-2898,miR-450a-2-3p,miR-1a-2-5p,novel_127,novel_96,miR-1260a,miR-339a,miR-505-5p,miR-6516-5p,miR-483-3p,miR-758-5p,miR-6517,novel_79,miR-431,miR-300,miR-105-2,miR-128-3p,miR-345-3p,miR-1290,miR-29d-3p,miR-23b-5p,miR-1197-3p,miR-1260b,miR-193a-5p,miR-103b,miR-767-5p,miR-410-3p,miR-486b-3p,miR-29b-3p,miR-2433,miR-1-5p,miR-5100,miR-144,miR-331-3p,miR-6740-5p,miR-34c-5p,miR-374c-3p,miR-370-3p,miR-2355-5p,miR-2285aa,miR-615-5p,miR-767,miR-345-5p,miR-329a,miR-7144-5p,miR-1248,miR-381-3p,miR-488-3p,miR-503-3p,miR-424-3p,miR-320a,miR-6412,miR-345-5p,miR-130b-5p,miR-382,miR-483-3p,miR-193a-3p,miR-7-5p,miR-224-5p,miR-377-3p,miR-146a,miR-197-5p,miR-105-5p,miR-2411-3p,novel_51,miR-3431,miR-194-3p,miR-2355-3p,miR-187-3p,miR-339-5p,miR-665,miR-29b,miR-1260b,miR-27b-3p,miR-7b-5p,miR-223-3p,miR-542-3p,miR-2113,miR-24-3p,miR-449a,novel_27,miR-2285m,miR-2284w,miR-1a-1-5p,miR-193b-3p,miR-29c-3p,miR-29a,miR-421-5p,miR-6529b,miR-34c,miR-146b-3p,miR-2404,miR-29a-3p,novel_69,miR-670-3p,miR-34b,miR-144-3p,novel_68,miR-3120-3p,miR-412-3p,miR-19a-3p,miR-9851-3p,miR-6395,miR-1306,miR-3591-3p,miR-216a-3p,miR-18a-3p,miR-378b,miR-125b-2-3p,miR-486-3p,miR-146a-5p,miR-2285w,miR-760-3p,miR-34b-5p,miR-21-3p,miR-216a-5p,miR-92a-1-5p,miR-345-3p,miR-2284h-5p,miR-532-3p,miR-328-3p,miR-18a-3p,miR-3600,miR-19b-3p,miR-378d,let-7a-3p,miR-361-5p,miR-34a-5p,miR-362-3p,miR-98-3p,miR-1193,miR-188-3p,miR-140-3p,miR-5703,miR-6525,miR-224-5p,miR-98-3p,miR-338-3p,miR-433-5p,miR-539-5p,miR-3184-3p,miR-3074-2-3p,miR-487b-5p,miR-130b-5p,miR-2285j,let-7b-3p,miR-6516,miR-377-3p,miR-139-5p,miR-193b-3p,novel_25,miR-2331-
PIGU	ENSOARG0(	phosphat	miR-30b-3p,miR-2284r,miR-378b,miR-143-5p,miR-3120-5p,miR-30b-3p,miR-378c,miR-16-1-3p,miR-665,miR-147a,novel_51,miR-199a-3p,miR-193a-3p,miR-491-5p,miR-1388-3p,miR-378d,miR-18a-3p,miR-1306-3p,miR-1983,miR-6395,miR-378f,miR-450b-3p,miR-412-3p,miR-7689-3p,miR-24-1-5p,miR-378b,miR-3074-5p,miR-669,miR-378e,miR-371a-5p,miR-2285y,miR-1247-3p,miR-576-3p,miR-193b-3p,miR-495-3p,novel_60,miR-2447,miR-3956-3p,miR-361-3p,miR-371b-3p,miR-378a-3p,novel_133,miR-378i,miR-16-1-3p,miR-133a-3p,miR-212-5p,miR-2285ad,miR-18a-3p,miR-199a-3p,miR-214-5p,miR-328-3p,miR-24-2-5p,miR-378h,miR-324-3p,miR-422a,novel_96,novel_127,miR-450a-2-3p,miR-29b-2-5p,miR-3065-3p,miR-24-2-5p,miR-3065-3p,miR-140-5p,miR-378a-3p,miR-204-3p,miR-193b-3p,miR-6130,miR-324-3p,miR-3969,miR-29b-2-5p,miR-1306-3p,miR-378c,novel_42,miR-3074-5p,miR-4510,miR-378c,miR-346

ENTPD4	ENSOARG0(	ectonucl	miR-331-3p,miR-125b-5p,miR-135a-5p,miR-2285j,miR-34c-3p,miR-29b-3p,miR-204-5p,miR-664a,miR-425-5p,miR-539-5p,miR-1306-5p,miR-93,miR-93-5p,miR-376c-5p,miR-23b,miR-320a,miR-17-5p,miR-2285c,miR-376c-5p,miR-20b-5p,miR-485-5p,miR-1248,miR-1814c,let-7g-3p,miR-329a,miR-3065-3p,miR-370-3p,miR-125a,miR-1271,miR-532-5p,miR-504-5p,miR-376c-3p,miR-376e-3p,miR-27a-5p,miR-216b-5p,miR-139-5p,miR-125b,novel_17,miR-30b-3p,miR-3653-3p,miR-543-5p,miR-21-3p,miR-320d,miR-466i-5p,miR-345-3p,miR-376c-5p,miR-3065-3p,miR-376b-5p,miR-29b-1-5p,miR-376c-3p,miR-2427,miR-29d-3p,miR-218-2-3p,miR-5703,miR-362-3p,miR-193b-5p,miR-1246,miR-1290,miR-4429,miR-548e-3p,miR-532-3p,miR-328-3p,miR-7859,miR-23b-3p,miR-383-5p,miR-23c,miR-20a-5p,miR-199c,miR-29a,miR-2285f,miR-29c-3p,miR-212-3p,miR-2284w,miR-2428,miR-211,miR-3959-3p,miR-378j,miR-2285ab,miR-376b-5p,miR-135b-5p,miR-6395,miR-1291,miR-299b-5p,miR-504,miR-136-5p,miR-211-5p,miR-363,miR-139-5p,miR-329-5p,miR-203-3p,miR-320c,miR-29a-3p,miR-670-3p,miR-362-3p,miR-29b-1-5p,miR-17-5p,novel_51,miR-106a-5p,miR-320b,novel_103,miR-125a-5p,miR-23a-3p,miR-101-5p,miR-8485,miR-2310,miR-329-3p,miR-873a-5p,miR-1434-3p,miR-1843b-5p,miR-362-3p,miR-29b,miR-219a-1-3p,miR-2426,miR-133b-5p,miR-199b-3p,miR-2300a-5p,miR-9788-3p,miR-216a-3p,miR-411-5p,miR-194-3p,miR-455-3p,miR-20b,miR-2285e,miR-2285g,miR-3431
FEZ2	ENSOARG0(	fascicul	miR-155-5p,miR-219a-2-3p,miR-320c,miR-744-3p,miR-155-5p,miR-4429,miR-320d,miR-9-3p,miR-155-5p,miR-219-3p,miR-320b,miR-320a,miR-2957,miR-219b-3p,miR-7857
JAK1	ENSOARG0(	Janus ki	miR-668-5p,miR-2426,miR-767,miR-29b,miR-410-5p,miR-29d-3p,miR-133c,miR-29a-3p,miR-487a-3p,miR-485-3p,miR-29a,miR-767-5p,miR-92a-1-5p,miR-218-5p,miR-29b-3p,miR-539-3p,miR-29c-3p,miR-668-3p,miR-3958-5p
VWA5A	ENSOARG0(	von Will	miR-708-5p,miR-28-5p,miR-8117,novel_103,miR-6525,miR-425-5p,miR-433-5p,miR-1388-5p,miR-22-3p,miR-2285c,miR-22-3p,miR-2285e,miR-2285g,miR-552-3p,miR-31-5p,novel_78

MYO1D	ENSOARG0(	myosin I	novel_94,miR-147-3p,miR-199b-5p,miR-361-3p,miR-378e,miR-7977,miR-541-5p,miR-335,miR-4726-5p,miR-769-5p,miR-1973,miR-8095,novel_60,miR-2284aa,miR-378c,miR-30b-3p,miR-296-5p,miR-6529a,miR-378b,miR-142-3p,novel_4,miR-2300a-5p,novel_124,miR-5126,miR-326,miR-320b,miR-758-3p,miR-4532,miR-106a-5p,miR-155-5p,miR-877-5p,novel_25,miR-22-3p,miR-323-5p,miR-6516,miR-3065-3p,miR-21-3p,miR-660-5p,miR-140-5p,miR-221-5p,miR-378a-3p,miR-664a,miR-6516-3p,miR-155-5p,miR-320e,miR-324-3p,miR-6134,miR-378c,miR-378d,miR-4429,miR-5703,miR-1193,miR-124-3p,novel_133,miR-616-3p,miR-3960,miR-21-3p,miR-221-5p,miR-760-3p,miR-320d,miR-106a,miR-185-3p,miR-125b,miR-130a-5p,miR-20a,miR-6395,miR-107,miR-1291,miR-18a-3p,miR-2404,miR-323a-5p,miR-320b,miR-147-5p,miR-656-5p,miR-128-1-5p,miR-20a-5p,miR-133a-5p,miR-6529b,miR-2428,miR-326-3p,miR-1307-3p,novel_101,miR-744-5p,miR-2889,miR-30b-3p,miR-3071-5p,miR-145a-5p,miR-1285,miR-103,miR-20b,miR-432-5p,miR-26b-3p,miR-187-3p,miR-660,miR-452-5p,miR-125a-5p,miR-1343-5p,miR-124-5p,miR-17-5p,miR-106a-5p,miR-130b-5p,miR-7144-5p,miR-320a,miR-17-5p,miR-2448-3p,miR-3957,miR-199a-5p,miR-769,miR-615-5p,miR-3957-5p,miR-486b-3p,miR-26c,miR-551b-3p,miR-378c,miR-93,miR-103b,miR-1306-5p,miR-134,miR-6516-5p,miR-378h,miR-107,miR-450a-2-3p,miR-1271-3p,miR-3065-3p,miR-199a-5p,miR-380-5p,miR-28b,miR-4286,miR-30a-3p,miR-1983,miR-335-3p,miR-136-5p,miR-504,miR-140-5p,miR-652-3p,miR-378g,miR-4792,miR-628-3p,miR-301a-5p,miR-218-5p,miR-423-5p,miR-361-3p,miR-9-5p,miR-3956-3p,miR-885-5p,miR-758-3p,miR-301b-5p,miR-147a,miR-496-5p,miR-181b-2-3p,miR-145b,miR-1343-3p,novel_78,miR-214-3p,miR-199b-5p,miR-491-5p,miR-106b-5p,miR-378d,miR-124a,miR-1827,miR-

LOXL2	ENSOARG0(	lysyl ox	miR-541,miR-432,miR-329-3p,miR-873a-5p,miR-30b-3p,miR-16-1-3p,miR-29b,miR-138,miR-30b-3p,miR-362-3p,miR-103a-3p,miR-31-5p,miR-134-3p,miR-432-5p,miR-103,miR-2459,novel_103,miR-29b-1-5p,novel_19,miR-362-3p,miR-3085-3p,miR-1343-5p,miR-744-3p,miR-382,miR-432-3p,miR-432-3p,miR-361-3p,miR-3591-3p,miR-378j,miR-18a-3p,miR-107,miR-1291,miR-877-3p,miR-6395,miR-216b-3p,miR-10b,novel_68,miR-139-5p,miR-4792,miR-3064-5p,miR-26b-5p,miR-7977,miR-3082-5p,miR-29a-3p,miR-378g,miR-203-3p,miR-6535,miR-29a,miR-335,miR-379-3p,novel_1,miR-128-1-5p,miR-147-5p,miR-136-3p,miR-3956-3p,miR-335-5p,miR-541-3p,novel_60,miR-2447,miR-17-3p,miR-29c-3p,miR-371b-3p,miR-2428,miR-134,miR-362-3p,miR-5703,miR-1197-5p,miR-2427,miR-2904,miR-1343-5p,miR-29d-3p,miR-582-3p,miR-411-3p,miR-16-1-3p,miR-133a-3p,miR-6517,miR-18a-3p,miR-1224-5p,miR-383-5p,miR-328-3p,miR-1247-5p,miR-134-5p,miR-26a-5p,miR-185-3p,miR-219b-5p,miR-30f,miR-17-3p,miR-543-5p,novel_127,miR-107,miR-3064-5p,miR-29b-1-5p,miR-2285b,miR-1957a,novel_39,miR-500a-3p,miR-329a,miR-767,miR-1827,miR-485-5p,miR-874-3p,miR-532-5p,miR-552-3p,miR-615-5p,miR-21-3p,miR-139-5p,miR-6130,miR-2284s,miR-204-3p,miR-29b-3p,miR-665,miR-17-3p,miR-767-5p,miR-664a,miR-138-5p,miR-134-5p,miR-874-5p,miR-500,miR-4492,miR-4510,miR-1306-5p,miR-425-5p,miR-6128,miR-6525
CRIM1	ENSOARG0(	cysteine	miR-20b-5p,miR-1814c,miR-876-3p,miR-345-5p,miR-93-5p,miR-18a,miR-17-5p,miR-2448-3p,miR-133c,miR-134-5p,miR-28-5p,miR-1306-3p,miR-378c,miR-181c-3p,miR-93,miR-1260b,miR-16-5p,miR-330-5p,miR-134,miR-582-3p,miR-134-5p,miR-2284z,miR-9-5p,miR-322-5p,miR-378h,miR-376a-5p,miR-18b-5p,miR-222,miR-152-3p,miR-1260a,miR-24-2-5p,miR-3529-3p,miR-378f,miR-195a-5p,miR-3065-5p,miR-148a-3p,miR-18b,miR-374c-3p,miR-3074-5p,miR-378b,miR-16a,miR-15a,miR-20a-5p,miR-326-3p,miR-2284w,miR-2285x,miR-495-3p,miR-342-3p,miR-497-5p,miR-7862,miR-2284x,miR-16b,miR-2284ab,miR-1260b,miR-181a-5p,miR-223-3p,miR-20b,miR-665,miR-466f-3p,miR-17-5p,miR-106a-5p,miR-224-5p,miR-377-3p,miR-345-5p,miR-2285r,miR-432-3p,let-7f-2-3p,let-7f-2-3p,miR-628-5p,miR-493-5p,miR-1895,let-7g-3p,miR-18a-5p,miR-345-5p,miR-15b-5p,miR-377-3p,miR-378a-3p,miR-424-5p,miR-487a-5p,miR-708-5p,miR-665,miR-338-5p,miR-222-3p,miR-181c-5p,miR-224-5p,miR-378c,miR-3074-5p,miR-378a-3p,miR-1197-5p,miR-378i,miR-376a-5p,miR-532-3p,miR-199a-3p,miR-7859,miR-23b-3p,miR-15a-5p,miR-148b-3p,miR-106a,miR-1277-5p,miR-30b-3p,miR-2284m,miR-106a,miR-466i-5p,miR-422a,miR-185-3p,miR-210-5p,miR-125b-2-3p,miR-20a,miR-493-5p,novel_94,miR-181a-3p,miR-1306-3p,miR-221,miR-1185-2-3p,miR-378e,miR-24-1-5p,miR-9-5p,miR-212-3p,miR-2284aa,miR-1839-5p,miR-378c,miR-101a-5p,miR-2426,miR-323b,miR-378b,miR-143-5p,miR-221-3p,miR-1185-3p,miR-421,miR-2300a-5p,miR-323b-3p,miR-106b-5p,miR-16b,miR-342,miR-199a-3p,miR-326,novel_103,miR-8485,miR-2310,miR-378d,miR-23a-3p,miR-376b,miR-424-5p,miR-106a-5p

BACE2	ENSOARG0(	beta-sit	miR-3065-3p,miR-1957a,miR-490-3p,miR-1271-3p,miR-495-5p,miR-181a-2-3p,miR-2898,miR-450a-2-3p,let-7c-5p,miR-1271-5p,miR-222,miR-411-3p,let-7d,miR-134,miR-218-2-3p,miR-500b-5p,miR-145-3p,miR-138-5p,miR-486b-3p,miR-28-5p,miR-410-5p,miR-5100,miR-135a-5p,miR-34c-5p,miR-365a-3p,miR-370-3p,novel_48,miR-485-5p,miR-362-5p,miR-3154,miR-382,miR-330-3p,miR-455-5p,miR-2285g,miR-432-5p,miR-185-5p,let-7g-5p,miR-544b,miR-133b-5p,miR-26b-3p,miR-138,miR-219a-1-3p,miR-455-5p,miR-1434-3p,novel_27,miR-449a,miR-2428,miR-3957-3p,miR-2285x,novel_101,miR-22-5p,miR-15a,miR-133a-5p,miR-34c,miR-16a,miR-382-5p,let-7i,miR-2404,miR-34b,miR-320b,miR-450b-3p,miR-1291,miR-7975,miR-195a-5p,miR-6395,miR-135b-5p,miR-2285ab,miR-2387,miR-34b-5p,miR-221-5p,miR-299-3p,miR-487a-3p,miR-15a-5p,miR-340-5p,miR-548e-3p,miR-3600,miR-485-3p,miR-941,let-7a-3p,novel_133,miR-362-3p,miR-2300b-3p,miR-140-3p,let-7b-5p,miR-145a-3p,miR-1271,miR-425-5p,miR-3074-2-3p,miR-664a,miR-6516-3p,miR-424-5p,miR-708-5p,miR-222-3p,miR-544-3p,novel_32,miR-204-3p,novel_25,miR-877-5p,miR-221-5p,miR-3065-3p,miR-182-5p,miR-2312,let-7g-3p,let-7f-1-3p,miR-491-3p,let-7a-5p,miR-424-5p,miR-494-3p,let-7e,miR-2957,miR-362-3p,miR-142-3p,miR-500-5p,miR-9788-3p,miR-221-3p,miR-2285e,miR-2300a-5p,miR-2426,miR-450b-5p,miR-544a,miR-296-5p,miR-125b-2-3p,miR-362-5p,miR-2285f,miR-3958-3p,miR-2285t,miR-2285n,miR-2285y,miR-489,let-7d-5p,miR-20a-3p,miR-7977,miR-129b-5p,miR-148b-5p,let-7g,miR-2285b,miR-192-3p,let-7f-5p,miR-127-5p,novel_96,miR-205-5p,miR-125a-3p,miR-3071-3p,miR-322-5p,miR-758-5p,miR-134-5p,miR-486-5p,miR-299a-3p,miR-2285ad,miR-431,miR-196a-5p,miR-3591-5p,miR-1197-3p,miR-16-5p,novel_9,miR-134-5p,novel_63,miR-
HNRNPA2B1	ENSOARG0(	heteroge	miR-2285p,miR-618,miR-376b-3p,miR-217,miR-500a-5p,miR-127-5p,miR-2484,miR-107,miR-345-3p,miR-181a-2-3p,miR-3064-5p,miR-450b-5p,miR-1197-5p,miR-330-5p,miR-217-5p,miR-218-2-3p,miR-142-3p,miR-15a-5p,miR-483-3p,miR-322-5p,miR-1306-3p,miR-3969,miR-664a-5p,miR-410-3p,miR-424-5p,miR-190a-5p,miR-214,miR-2330-3p,miR-16-5p,miR-338-3p,miR-2285c,miR-21a-3p,miR-129b-3p,miR-200a-5p,miR-503-3p,miR-21-3p,miR-216b-3p,miR-15b-5p,miR-326,miR-16b,miR-190a,miR-483-3p,miR-3085-3p,miR-103a-2-5p,miR-214-3p,miR-382-3p,miR-424-5p,let-7f-2-3p,miR-33a-5p,let-7f-2-3p,let-7c-3p,miR-16b,miR-188-5p,miR-21b,miR-497-5p,miR-103a-3p,miR-548w,miR-2300a-5p,miR-26b-3p,miR-376b-3p,miR-134-3p,miR-2285g,miR-2285e,miR-1185-3p,miR-103,miR-6238,novel_116,miR-15a,miR-502-5p,miR-107-5p,miR-16a,miR-499a-5p,miR-3956-3p,novel_60,miR-219b-3p,miR-190b-5p,miR-326-3p,miR-376b-3p,miR-1306-3p,miR-3591-3p,miR-107,miR-412-3p,miR-3963,miR-195a-5p,miR-1983,miR-376a-3p,miR-216b-3p,miR-3064-5p,novel_68,miR-411b-3p,miR-378g,miR-376d,miR-1185-2-3p,miR-203-3p,miR-6535

ASGR1	ENSOARG0(	asialogl	miR-16-5p,miR-193a-5p,miR-2284v,miR-1260b,miR-6740-5p,miR-125b-5p,miR-2284k,miR-134-5p,miR-2355-5p,miR-125a,miR-503-3p,miR-874-3p,miR-376c-3p,miR-301,miR-1248,miR-345-5p,miR-2318,novel_39,let-7g,miR-29a-5p,miR-450c-5p,miR-30f,miR-127-5p,let-7f-5p,novel_79,miR-6517,miR-134-5p,miR-2284z,miR-129-1-3p,miR-322-5p,miR-193b-3p,miR-1839-3p,miR-2284w,miR-142b,miR-320c,miR-2319a,miR-378f,let-7e-5p,miR-412-3p,miR-628-5p,miR-432-3p,miR-432-3p,miR-2284l,novel_51,miR-2411-3p,miR-193a-3p,miR-339-5p,miR-103a-3p,miR-3535,miR-3431,miR-19b-2-5p,miR-1843b-5p,miR-497-5p,miR-2284x,miR-16b,miR-16-1-3p,miR-665-5p,let-7b,miR-3184-3p,miR-129-2-3p,miR-181c-5p,miR-6525,miR-346,miR-665,let-7f,miR-874-5p,miR-130b-5p,miR-2331-3p,miR-504-5p,miR-30c-1-3p,miR-548o-3p,miR-15b-5p,let-7i-5p,miR-376e-3p,miR-2284s,miR-216b-5p,miR-299,miR-2285c,miR-186-5p,miR-345-5p,miR-339b,miR-345-3p,miR-2424,miR-486-3p,miR-143-3p,miR-324-3p,miR-422a,miR-345-3p,let-7k,miR-98-5p,miR-378a-3p,miR-217-5p,miR-378i,miR-1961,miR-133a-3p,miR-16-1-3p,novel_73,miR-2284j,miR-2440,miR-2285u,miR-211-5p,miR-2319b,miR-2483-3p,miR-140-5p,miR-409-5p,miR-9-3p,miR-1983,miR-96-5p,miR-504,miR-136-5p,miR-378d,miR-323b-3p,miR-29b-1-5p,miR-16b,miR-145b,miR-548w,novel_78,miR-455-3p,miR-147a,miR-1298-3p,miR-323b,miR-2284r,miR-1839-5p,miR-101c,miR-1388-5p,miR-6128,miR-1306-5p,miR-378c,miR-323-3p,miR-486b-3p,miR-204-5p,miR-370-3p,miR-154b-5p,miR-3154,miR-320a,miR-381-3p,miR-503-3p,miR-7144-5p,miR-4286,miR-30c-2-3p,let-7c-5p,miR-217,miR-339a,miR-1260a,miR-107,miR-543-5p,miR-17-3p,miR-2898,miR-181b-5p,miR-7641,miR-376a-5p,miR-378h,miR-6516-5p,miR-1271-5p,miR-345-3p,miR-134,miR-496,miR-300,let-7d,miR-17-

WDR18	ENSOARG0(	WD repea	miR-30f,miR-205-5p,miR-125a-3p,miR-152-3p,miR-200a-3p,miR-3064-5p,novel_39,miR-192-3p,miR-1343-5p,miR-330-5p,miR-1290,miR-30c,miR-155-5p,miR-412,miR-18b-5p,miR-134-5p,miR-505-5p,miR-322-5p,miR-125b-5p,miR-134-5p,miR-16-5p,miR-1197-3p,miR-1260b,miR-23b,miR-30e-5p,miR-329b,miR-329a,miR-345-5p,miR-2285aa,miR-125a,miR-1271,miR-874-3p,miR-133c,miR-365b-5p,miR-105-5p,miR-2411-3p,miR-194a,novel_51,miR-377-3p,miR-378a-5p,miR-668-3p,miR-432-3p,miR-432-3p,miR-197-3p,miR-1843b-5p,miR-223-3p,miR-497-5p,miR-27b-3p,miR-16b,miR-381-5p,miR-31-5p,miR-103a-3p,miR-149-5p,miR-134-3p,miR-3431,miR-485-3p,miR-425-5p,miR-4324,miR-576-3p,miR-29a,miR-29c-3p,miR-199b-5p,miR-2284w,miR-1306,miR-3970,miR-216a-3p,miR-148a-3p,miR-378f,miR-410-5p,miR-412-3p,novel_68,miR-329-5p,miR-6535,miR-677,miR-181b-3p,novel_69,miR-324-3p,miR-422a,miR-345-3p,miR-92a-1-5p,miR-30b,miR-345-3p,miR-329a-5p,miR-486-3p,miR-143-3p,miR-181b-1-3p,miR-377-5p,miR-1434-5p,miR-378a-3p,miR-30c-5p,miR-378i,miR-133a-3p,miR-218-1-3p,miR-361-5p,miR-34a-5p,miR-493-3p,novel_73,miR-328-3p,miR-23b-3p,miR-23c,miR-665,miR-500,miR-4492,miR-874-5p,miR-454-3p,miR-129-5p,miR-3958-5p,miR-665-5p,miR-214,miR-3184-3p,miR-181c-5p,miR-6525,miR-224-5p,miR-500a-3p,miR-30d-5p,miR-1895,miR-625-5p,miR-345-5p,miR-216b-3p,miR-296-3p,miR-504-5p,miR-2331-3p,miR-30c-1-3p,miR-15b-5p,miR-377-3p,miR-499b-3p,miR-2284s,miR-216b-5p,miR-29b-1-5p,miR-16b,miR-199b-5p,miR-214-3p,miR-381-5p,miR-103a-2-5p,miR-130b-3p,miR-23a-3p,miR-1388-3p,miR-127-3p,miR-8485,miR-378d,miR-329-3p,miR-2284r,miR-541,miR-362-3p,miR-181b-2-3p,miR-199b-3p,novel_78,miR-1343-3p,miR-455-3p,miR-421,miR-2459,miR-1298-3p,miR-147a,novel_82,miR-2440,miR-490-5p,miR-3968,miR-7705,miR-

SUPT20H	ENSOARG0(	suppress	miR-2285n,miR-2285y,miR-107-5p,miR-499a-5p,miR-6527,miR-9-5p,miR-129b-5p,miR-96-5p,miR-544-5p,novel_94,miR-1983,miR-3064-5p,miR-2319b,miR-7689-3p,miR-2285u,miR-211-5p,miR-378e,miR-2483-3p,miR-1185-2-3p,novel_103,miR-323b-3p,miR-29b-1-5p,miR-491-5p,miR-3085-3p,miR-103a-2-5p,miR-1468,miR-1388-3p,miR-376b,miR-652-5p,miR-378d,miR-491-3p,miR-2310,miR-484,miR-378b,miR-4791,miR-323b,miR-1a-3p,miR-30b-3p,miR-378c,miR-450b-5p,miR-2426,miR-101c,miR-21b,miR-1343-3p,miR-145b,miR-9788-3p,miR-455-3p,miR-216a-3p,miR-30e-3p,miR-30a-3p,miR-664a-5p,miR-29b-2-5p,miR-6516-3p,miR-17-3p,miR-129-5p,miR-487a-5p,miR-208b-5p,miR-378c,miR-3184-3p,miR-4510,miR-6525,miR-206-3p,novel_44,miR-338-3p,miR-21a-3p,miR-433-3p,let-7g-3p,miR-345-5p,miR-140-5p,miR-378a-3p,miR-370-5p,miR-552-3p,miR-532-5p,miR-27a-5p,miR-6130,novel_25,miR-2285p,miR-26a-5p,miR-92a-1-5p,miR-422a,miR-2284m,miR-299-3p,miR-329a-5p,miR-5100,miR-376a-5p,miR-664b-3p,miR-29b-1-5p,miR-377-5p,miR-486-3p,miR-2411,miR-378i,miR-193b-5p,miR-1197-5p,miR-378a-3p,miR-5703,miR-142-3p,miR-1b-3p,miR-30d-3p,novel_83,miR-616-3p,miR-328-3p,miR-142b,miR-6402,miR-33a-3p,miR-6119-3p,miR-29a,miR-206,miR-656-5p,miR-502b,miR-147-5p,miR-1a-1-5p,miR-495-3p,miR-29c-3p,miR-17-3p,miR-2285x,miR-1193,miR-211,miR-3959-3p,novel_27,miR-2387,miR-150-5p,miR-3970,miR-216a-3p,miR-378b,miR-450b-3p,miR-412-3p,miR-2319a,miR-6395,miR-378f,miR-6516-3p,miR-329-5p,miR-7-1-3p,miR-26b-5p,miR-3120-3p,miR-2404,miR-374c-3p,miR-670-3p,miR-299b-3p,miR-29a-3p,miR-669,miR-6535,miR-296-3p,miR-365b-5p,miR-483-3p,let-7f-2-3p,miR-654-3p,miR-2285r,miR-2284l,miR-296-3p,miR-345-5p,let-7a-2-3p,miR-503-3p,miR-2113,miR-3071-5p,miR-145a-5p,miR-374b-3p,miR-30b-3p,miR-29b,miR-

TNKS	ENSOARG0(	tankyras	miR-93,miR-323-3p,miR-6740-5p,miR-26c,miR-409-3p,miR-410-3p,miR-138-5p,miR-2285aa,miR-133c,miR-376c-3p,miR-300-3p,miR-93-5p,miR-17-5p,miR-503-3p,miR-2448-3p,miR-23a-5p,miR-7144-5p,miR-20b-5p,miR-671-5p,miR-2474,miR-345-5p,miR-29b-2-5p,miR-3064-5p,miR-3065-3p,novel_39,miR-490-3p,miR-192-3p,miR-2285b,miR-380-5p,miR-28b,miR-24-2-5p,miR-217,miR-2898,miR-17-3p,miR-127-5p,miR-181b-5p,miR-1224-5p,miR-450a-1-3p,miR-9-5p,miR-758-5p,miR-330-5p,miR-3591-5p,miR-196a-5p,miR-155-5p,miR-105-2,miR-411-3p,miR-193b-3p,miR-17-3p,miR-326-3p,miR-371b-3p,novel_87,miR-6402,miR-28c,miR-20a-5p,miR-147-5p,miR-320b,miR-3120-3p,novel_68,miR-144-3p,miR-411b-3p,miR-3065-5p,miR-7975,miR-146b-5p,miR-505-3p,miR-6395,miR-2285k,miR-363-5p,miR-380-5p,miR-6240,miR-345-5p,miR-654-3p,miR-330-3p,miR-105-5p,miR-17-5p,miR-106a-5p,miR-224-5p,miR-146a,miR-377-3p,miR-452-5p,miR-193a-3p,miR-196b-5p,novel_116,miR-194-3p,miR-411-5p,miR-20b,miR-544b,miR-432-5p,miR-134-3p,miR-450c-3p,miR-197-3p,novel_23,miR-432,miR-181a-5p,miR-2113,miR-26a-2-3p,miR-2889,miR-2284x,miR-138,miR-125b-1-3p,miR-145a-3p,miR-3184-5p,miR-181c-5p,miR-6525,miR-224-5p,miR-29b-2-5p,miR-208b-5p,miR-1468-5p,miR-4492,miR-450b-3p,miR-17-3p,miR-451a,miR-216b-3p,novel_107,miR-3065-3p,miR-552-3p,miR-140-5p,miR-32-3p,miR-155-5p,miR-377-3p,miR-544-3p,miR-376e-3p,miR-193b-3p,miR-3613-5p,miR-450a-1-3p,miR-1827,miR-345-5p,miR-20a,miR-146a-5p,miR-2432,miR-2285i,miR-376c-3p,miR-142a-3p,miR-2484,miR-202-5p,miR-106a,miR-3600,miR-505-3p,miR-106a,miR-328-3p,miR-340-5p,miR-217-5p,miR-5703,miR-140-3p,miR-2366,miR-142-3p,miR-181d-5p,miR-9-5p,miR-32-3p,miR-541-3p,miR-212-3p,miR-423-5p,miR-8095,miR-877-3p,novel_111,miR-2440,miR-101-3p,miR-499a-5p,miR-3958-

KLF3	ENSOARG0(	Kruppel-	miR-22-5p,miR-29a,miR-2428,miR-29c-3p,novel_120,miR-335-5p,miR-2319a,miR-150-5p,miR-2404,miR-29a-3p,miR-655-5p,miR-7-1-3p,miR-452-5p,miR-124-5p,miR-190a,miR-194a,miR-2411-3p,miR-432-3p,miR-432-3p,miR-668-3p,miR-493-5p,miR-7862,miR-2284x,miR-29b,miR-21-5p,novel_23,miR-374b-3p,miR-194-5p,miR-1843b-5p,novel_116,miR-149-5p,miR-544b,miR-2284d,miR-3432a,miR-2284k,miR-29b-3p,miR-2332,miR-2284v,miR-1248,miR-10b-3p,miR-23b,miR-3154,miR-30e-5p,miR-324-5p,miR-2355-5p,miR-1271,miR-2898,miR-30f,miR-3064-5p,miR-30a-3p,miR-4286,miR-181a-2-3p,miR-30c,miR-29d-3p,miR-501-3p,miR-30a-5p,miR-345-3p,miR-545-5p,miR-129-1-3p,miR-6516-5p,miR-222,miR-3968,miR-335,miR-371a-5p,miR-30b-5p,miR-218-5p,miR-502-3p,novel_111,miR-194b-5p,miR-190b-5p,miR-2284j,miR-4454,miR-2447,miR-493-5p,novel_94,miR-129b-5p,miR-221,miR-652-3p,miR-500-3p,miR-7857-3p,miR-499a-3p,miR-2483-3p,miR-3064-5p,miR-20a-3p,miR-153-3p,miR-214-3p,miR-1185-5p,miR-3085-3p,miR-5126,miR-23a-3p,miR-450b-5p,miR-544a,miR-7857,miR-501-3p,miR-221-3p,miR-153,miR-1298-3p,miR-548w,novel_78,miR-190a-5p,miR-129-5p,miR-874-5p,novel_74,miR-664a,miR-487b-5p,miR-30a-3p,miR-222-3p,miR-2285j,miR-30e-3p,miR-214,miR-3184-3p,miR-4510,miR-129-2-3p,miR-30d-5p,miR-411,miR-204-3p,miR-877-5p,miR-6516,miR-499b-3p,miR-544-3p,miR-6130,miR-552-3p,miR-140-5p,miR-30b-3p,miR-3653-3p,miR-30d,miR-2284m,miR-202-5p,miR-345-3p,miR-30b,miR-2432,miR-345-3p,miR-218-1-3p,miR-1197-5p,miR-1193,miR-30c-5p,miR-2284h-5p,miR-23b-3p,miR-30d-3p

R3HCC1	ENSOARG0(	R3H doma	miR-363-5p,miR-668-3p,miR-130b-5p,miR-345-5p,miR-432-3p,miR-224-5p,miR-503-5p,miR-377-3p,miR-1343-5p,miR-125a-5p,miR-660,miR-378a-5p,miR-483-3p,miR-339-5p,miR-31-5p,miR-340-3p,miR-194-3p,miR-412-5p,miR-503-5p,miR-134-3p,miR-197-3p,miR-22-3p,miR-708-3p,miR-1285,miR-27b-3p,miR-138,miR-2284ab,miR-30b-3p,miR-1260b,miR-17-3p,miR-1a-1-5p,miR-335-5p,miR-326-3p,miR-199b-5p,miR-24-3p,miR-1307-3p,miR-382-5p,miR-28c,miR-6529b,miR-133a-5p,miR-656-5p,miR-421-5p,miR-6123,miR-320b,miR-10a-5p,miR-216b-3p,miR-146b-3p,miR-299b-3p,miR-411b-3p,miR-670-3p,miR-3970,miR-1306,miR-18a-3p,miR-216a-3p,miR-2285ab,miR-2387,miR-9851-3p,miR-2319a,miR-135b-5p,miR-6395,miR-1291,miR-3064-5p,miR-30a-3p,miR-181a-2-3p,novel_39,miR-3065-3p,miR-199a-5p,miR-490-3p,miR-28b,miR-339a,miR-1260a,miR-6119-5p,miR-200c,novel_96,novel_127,miR-450a-2-3p,miR-17-3p,miR-200c-3p,miR-1a-2-5p,miR-127-5p,novel_79,miR-299a-3p,miR-429-3p,miR-2284z,miR-483-3p,miR-758-5p,miR-1343-5p,miR-330-5p,miR-615-3p,miR-200b-3p,miR-155-5p,miR-128-3p,miR-10a-5p,miR-144-5p,miR-1306-5p,miR-2284v,miR-1260b,miR-125b-5p,miR-135a-5p,miR-3969,miR-1-5p,miR-135a-1-3p,miR-592,miR-138-5p,miR-767-5p,miR-2355-5p,miR-370-3p,miR-2285aa,miR-615-5p,miR-125a,miR-874-3p,miR-199a-5p,miR-376c-3p,miR-424-3p,miR-7144-5p,miR-23a-5p,miR-485-5p,miR-876-3p,miR-767,miR-345-5p,miR-574-3p,miR-2330-5p,miR-8485,miR-1b-5p,miR-505,miR-29b-1-5p,miR-2957,miR-182-5p,miR-1306-5p,miR-326,miR-199b-5p,miR-103a-2-5p,miR-491-5p,miR-3085-3p,miR-25-5p,miR-3955-3p,miR-27a-3p,miR-2300a-5p,miR-1343-3p,miR-148b-5p,miR-216a-3p,miR-1298-3p,miR-30b-3p,miR-484,miR-296-5p,miR-143-5p,miR-10b,miR-2284aa,miR-3120-5p,miR-1388-5p,miR-2285f,miR-1296-5p,miR-32-3p,miR-3059-5p,miR-

RNF144B	ENSOARG0(	ring fin	miR-29a-5p,miR-2285b,miR-29b-2-5p,miR-30a-3p,miR-4286,miR-17-3p,miR-200c-3p,miR-127-5p,miR-200c,miR-2284z,miR-129-1-3p,miR-486-5p,miR-322-5p,miR-302a-5p,miR-758-5p,miR-378h,miR-299a-3p,miR-429-3p,miR-200b-3p,miR-128-3p,miR-582-3p,miR-345-3p,miR-378c,miR-16-5p,miR-144-5p,miR-2284v,miR-6740-5p,miR-144,miR-125b-5p,miR-100-3p,novel_63,miR-410-5p,miR-2284k,miR-5100,miR-133c,miR-2284n,miR-34c-5p,miR-365a-3p,miR-370-3p,miR-125a,miR-3956,miR-487a-3p,miR-574-5p,miR-3068-3p,miR-874-3p,miR-376c-5p,miR-671-5p,miR-329b,miR-376c-5p,miR-488-3p,miR-6412,miR-432-3p,miR-2285r,miR-101-5p,miR-6240,miR-125a-5p,miR-466f-3p,miR-378a-5p,miR-124-5p,miR-330-3p,miR-2411-3p,miR-377-3p,miR-6238,miR-544b,miR-412-5p,miR-2284d,miR-185-5p,miR-26b-3p,miR-497-5p,miR-26a-2-3p,miR-27b-3p,miR-2284ab,miR-16b,miR-16-1-3p,miR-873a-5p,miR-653,miR-1434-3p,miR-449a,miR-371b-3p,miR-17-3p,miR-656-5p,miR-147-5p,miR-6119-3p,novel_1,miR-34c,miR-16a,novel_87,miR-15a,miR-144-3p,miR-670-3p,miR-299b-3p,miR-34b,miR-26b-5p,miR-329-5p,miR-378f,miR-195a-5p,miR-412-3p,miR-150-5p,miR-216a-3p,miR-376b-5p,miR-376a-5p,miR-376c-3p,miR-345-3p,miR-329a-5p,miR-183-5p,miR-487a,miR-34b-5p,miR-299-3p,miR-2284m,miR-466i-5p,miR-125b,miR-422a,miR-345-3p,miR-374a-5p,miR-26a-5p,miR-200b,miR-15a-5p,miR-30d-3p,miR-212-5p,miR-16-1-3p,miR-34a-5p,let-7a-3p,miR-142-3p,miR-98-3p,miR-1193,miR-378a-3p,miR-378i,miR-193b-5p,miR-1246,miR-376a-5p,miR-6525,miR-98-3p,miR-2330-3p,miR-378c,miR-539-5p,miR-129-2-3p,miR-145a-3p,miR-4510,miR-129-5p,miR-424-5p,miR-17-3p,miR-30a-3p,miR-29b-2-5p,miR-320e,let-7b-3p,miR-204-3p,miR-15b-5p,miR-377-3p,miR-544-3p,miR-6130,miR-216b-5p,miR-378a-3p,miR-548o-3p,miR-1827,miR-

MBTPS1	ENSOARG0(	membrane	miR-203b-5p,novel_1,miR-34c,novel_87,miR-449a,miR-2428,miR-301b-3p,miR-495-3p,miR-146b-5p,miR-6395,miR-1291,miR-107,miR-412-3p,miR-18a-3p,miR-18b,miR-320c,miR-146b-3p,miR-34b,miR-320b,miR-3120-3p,miR-216b-3p,miR-660,miR-330-3p,miR-365b-5p,novel_51,miR-146a,miR-539-3p,miR-345-5p,miR-654-3p,miR-363-5p,let-7f-2-3p,let-7f-2-3p,miR-130b-5p,miR-6240,miR-7862,miR-2889,miR-381-5p,miR-22-3p,miR-873a-5p,miR-1843b-5p,miR-2113,miR-194-3p,miR-103,miR-185-5p,miR-3431,miR-130a-3p,miR-31-5p,miR-103a-3p,miR-486b-3p,miR-331-3p,miR-1306-3p,miR-365a-5p,miR-6128,miR-7144-5p,miR-671-5p,miR-485-5p,miR-876-3p,miR-329b,miR-345-5p,miR-320a,miR-18a,miR-154b-5p,miR-34c-5p,miR-2355-5p,miR-615-5p,miR-874-3p,miR-3068-3p,miR-107,novel_127,miR-30c-2-3p,miR-3071-3p,miR-217,miR-2285af,miR-29b-2-5p,miR-181a-2-3p,miR-23b-5p,miR-1290,miR-129-1-3p,miR-9-5p,miR-2411-5p,miR-412,miR-18b-5p,miR-2285ad,miR-4726-5p,miR-1973,miR-2285y,miR-107-5p,miR-2285n,miR-9-5p,miR-541-3p,novel_94,miR-1983,miR-504,miR-1306-3p,miR-361-3p,miR-409-5p,miR-1185-2-3p,miR-3596,miR-20a-3p,miR-146b,miR-381-5p,miR-103a-2-5p,miR-130b-3p,miR-505,miR-320b,miR-193a,miR-30c-1-3p,miR-574-3p,miR-494-3p,miR-412-3p,miR-125b-2-3p,miR-484,miR-296-5p,miR-541,miR-1185-3p,miR-2300a-5p,miR-330-3p,miR-151a-3p,miR-454-3p,miR-874-5p,miR-130b-5p,miR-6516-3p,miR-151-3p,miR-132-5p,miR-29b-2-5p,miR-320e,miR-346,miR-129-2-3p,miR-625-5p,miR-18a-5p,miR-345-5p,miR-433-3p,miR-21a-3p,miR-22-3p,novel_32,miR-301a-3p,novel_107,miR-552-3p,miR-221-5p,miR-30c-1-3p,miR-504-5p,miR-660-5p,miR-221-5p,miR-34b-5p,miR-21-3p,miR-320d,miR-216a-5p,miR-324-3p,novel_17,miR-146a-5p,miR-2411,miR-486-3p,miR-2432,miR-125b-2-3p,miR-34a-5p,miR-4429,miR-5703,miR-1193,miR-188-
SACM1L	ENSOARG0(	SAC1 sup	miR-493-5p,miR-505-3p,miR-3065-5p,miR-3591-3p,miR-21c,miR-320c,miR-203-3p,miR-1185-2-3p,miR-27a-3p,miR-499a-3p,miR-3082-5p,miR-199c,miR-6123,miR-4726-5p,miR-6402,miR-576-3p,miR-2284w,miR-452-5p,miR-3059-5p,miR-2284x,miR-27b-3p,miR-21-5p,miR-544a,miR-145a-5p,miR-708-3p,miR-6529a,miR-653,miR-411-5p,novel_116,miR-148b-5p,miR-1185-3p,miR-544b,miR-665,miR-27a-3p,miR-222-5p,miR-145b,miR-199b-3p,novel_4,miR-548w,miR-214-3p,miR-505,miR-2411-3p,miR-320b,novel_115,miR-491-3p,let-7f-2-3p,let-7f-2-3p,miR-652-5p,miR-130b-5p,miR-493-5p,miR-582-5p,miR-133b-3p,let-7g-3p,miR-320a,miR-301,miR-499b-3p,miR-544-3p,miR-582-5p,miR-3965,miR-6130,miR-365a-3p,miR-21-3p,miR-216b-3p,miR-1271,miR-141-3p,miR-708-5p,miR-130b-5p,miR-28-5p,miR-324-3p,miR-665,miR-409-3p,miR-214,miR-133a-3p,miR-28a-3p,miR-4510,miR-142-3p,miR-4429,miR-23b-5p,miR-188-3p,miR-345-3p,miR-7859,miR-302a-5p,miR-758-5p,miR-3600,miR-505-3p,miR-200a-3p,miR-28-3p,miR-320d,miR-3071-3p,miR-345-3p,miR-196a-3p,miR-29a-5p,miR-2432,miR-148b-5p,miR-130a-5p

GCDH	ENSOARGO(	glutaryl	miR-668-3p,let-7c-3p,miR-124a,let-7j,miR-3187-3p,miR-2284l,miR-432-3p,miR-365b-5p,miR-1306-5p,miR-214-3p,miR-487b-5p,miR-3085-3p,miR-339-5p,novel_78,miR-409b,miR-2300a-5p,miR-421,miR-2459,miR-149-5p,miR-9788-3p,miR-134-3p,miR-1298-3p,miR-412-5p,miR-3071-5p,miR-125b-2-3p,miR-30b-3p,miR-197-3p,miR-301b-5p,miR-2284r,miR-7862,miR-3120-5p,miR-1839-5p,miR-1260b,miR-30b-3p,miR-2284w,miR-8095,miR-361-3p,miR-301a-5p,miR-371a-5p,miR-615,miR-147-5p,miR-6123,miR-3964,miR-499a-5p,miR-128-1-5p,miR-7977,miR-541-5p,miR-3064-5p,miR-669,miR-411b-3p,miR-378g,miR-378j,miR-1306,miR-409-5p,miR-140-5p,miR-6395,miR-1983,miR-19a-3p,miR-412-3p,miR-1291,miR-3064-5p,miR-2285w,miR-3529-3p,miR-3956-5p,miR-143-3p,miR-486-3p,miR-339a,miR-3601,novel_17,miR-210-5p,miR-125a-3p,miR-1260a,miR-221-5p,miR-6536,miR-99a-3p,novel_79,miR-548e-3p,miR-129-1-3p,miR-328-3p,miR-151-5p,miR-1247-5p,miR-2284h-5p,miR-505-5p,miR-7859,miR-188-3p,miR-124-3p,miR-1290,miR-215-5p,miR-193b-5p,novel_133,miR-615-3p,miR-361-5p,miR-19b-3p,miR-3074-2-3p,miR-214,miR-145a-3p,miR-129-2-3p,miR-338-3p,miR-365a-5p,miR-224-5p,miR-1260b,miR-6525,miR-99b-3p,miR-15b-3p,miR-592,miR-486b-3p,miR-28-5p,miR-708-5p,miR-4492,miR-215-5p,miR-99a-3p,miR-2285aa,miR-370-3p,miR-548o-3p,miR-221-5p,miR-151b,miR-216b-5p,miR-132-5p,miR-192-5p,miR-129b-3p,miR-1895,miR-625-5p,miR-339b

NR2F2	ENSOARG0(	nuclear	miR-3578,miR-23b-5p,miR-300,miR-885-3p,let-7a-2-3p,miR-128-3p,miR-423-3p,miR-222,miR-7641,miR-582-3p,miR-1a-2-5p,novel_96,miR-543-5p,miR-3064-5p,miR-24-2-5p,miR-192-3p,miR-2284a,miR-2284y,miR-29a-5p,miR-381-3p,let-7g-3p,miR-329a,miR-876-3p,miR-1248,miR-1814c,miR-3074-1-3p,miR-2355-5p,miR-2285aa,miR-2284n,miR-2433,miR-29b-3p,miR-2284k,miR-1-5p,miR-409-3p,miR-144,miR-331-3p,miR-204-5p,miR-486b-3p,miR-410-3p,miR-2284v,miR-1306-5p,miR-3607-3p,miR-145a-5p,miR-197-3p,miR-16-1-3p,miR-29b,miR-2284ab,miR-27b-3p,miR-503-5p,miR-2284d,novel_116,miR-194-3p,miR-146a,miR-377-3p,miR-503-5p,miR-330-3p,miR-452-5p,miR-6240,miR-101-5p,miR-382,miR-2285r,let-7a-2-3p,miR-6412,miR-2387,miR-3065-5p,miR-216a-3p,miR-150-5p,miR-146b-5p,miR-7-1-3p,miR-320b,miR-29a-3p,miR-181b-3p,miR-677,miR-144-3p,miR-382-5p,miR-29a,miR-6119-3p,miR-1a-1-5p,miR-342-3p,miR-376a-3p,novel_101,miR-29c-3p,miR-495-3p,miR-2285x,miR-323c,miR-211,miR-2428,miR-374c-5p,miR-362-3p,miR-193b-5p,miR-188-3p,miR-140-3p,miR-98-3p,miR-2284u,let-7a-3p,miR-16-1-3p,miR-212-5p,miR-3600,miR-532-3p,miR-618,novel_17,miR-92a-1-5p,miR-324-3p,miR-2284m,miR-202-5p,miR-503-5p,miR-216c-5p,miR-183-5p,miR-369-3p,miR-181b-1-3p,miR-29b-1-5p,miR-2424,miR-486-3p,miR-146a-5p,let-7f-1-3p,miR-655,novel_99,miR-186-5p,miR-1895,miR-885-3p,miR-140-5p,miR-545-3p,miR-296-3p,miR-377-3p,miR-499b-3p,novel_32,miR-222-3p,let-7b-3p,miR-655-3p,miR-664a,miR-6516-3p,miR-208b-5p,miR-346,novel_44,miR-98-3p,miR-3184-5p,miR-1298-5p,miR-2284r,miR-329-3p,miR-299a-5p,miR-101a-5p,miR-362-3p,miR-1388-5p,miR-330-3p,miR-2300a-5p,miR-181b-2-3p,miR-27a-3p,miR-145b,miR-1185-3p,miR-9788-3p,miR-455-3p,miR-216a-3p,miR-221-3p,miR-342,miR-29b-1-5p,miR-362-3p,miR-3085-
RHOBTB2	ENSOARG0(	Rho-rela	miR-3085-3p,miR-491-5p,miR-1185-5p,miR-146b,miR-452-5p,miR-146a,miR-326,miR-505,miR-30c-1-3p,miR-378d,miR-127-3p,miR-380-3p,miR-30b-3p,miR-378c,miR-378b,miR-223-3p,miR-1285,miR-30b-3p,miR-134-3p,miR-147a,miR-185-5p,miR-9788-3p,miR-31-5p,novel_4,miR-1343-3p,miR-181b-2-3p,novel_1,miR-6123,miR-4324,novel_82,miR-218-5p,miR-371b-3p,miR-24-3p,miR-423-5p,miR-326-3p,miR-154a,miR-219b-3p,novel_101,miR-1193,miR-495-3p,miR-146b-5p,miR-378f,miR-361-3p,miR-2387,miR-18a-3p,miR-378e,miR-378g,miR-2483-3p,miR-677,miR-181b-3p,miR-3064-5p,miR-378b,miR-3120-3p,miR-2898,miR-466i-5p,novel_127,novel_96,miR-21-3p,miR-30c-2-3p,miR-422a,miR-450b-5p,miR-125b-2-3p,miR-181b-1-3p,miR-486-3p,novel_39,miR-146a-5p,miR-29a-5p,miR-3064-5p,miR-664-5p,miR-378i,miR-2366,miR-330-5p,miR-378a-3p,miR-378h,miR-582-3p,miR-151-5p,miR-18a-3p,novel_9,miR-4492,miR-874-5p,miR-486b-3p,miR-370-5p,miR-100-3p,miR-6740-5p,miR-3969,miR-320e,miR-6525,miR-2903,miR-3184-5p,miR-378c,miR-1306-5p,miR-378c,miR-539-5p,miR-4510,miR-193a-5p,miR-133a-3p,miR-665-5p,miR-133b-3p,miR-1827,miR-3154,miR-6130,miR-151b,miR-378a-3p,miR-30c-1-3p,miR-874-3p,miR-370-5p

TAPT1	ENSOARG0(	transmem	miR-33b-3p,miR-362-3p,miR-1197-5p,miR-217-5p,miR-188-3p,miR-7859,miR-23b-3p,miR-23c,miR-148b-3p,miR-2284h-5p,miR-106a,miR-106a,miR-221-5p,miR-299-3p,miR-196a-3p,miR-618,miR-324-3p,miR-345-3p,miR-29b-1-5p,miR-181b-1-3p,miR-2424,miR-2285w,miR-20a,miR-2312,miR-339b,miR-345-5p,miR-2285c,miR-377-3p,miR-155-5p,miR-221-5p,miR-504-5p,miR-496-3p,miR-21-3p,miR-182-5p,miR-664a,miR-155-5p,miR-129-5p,miR-208b-5p,miR-130b-5p,miR-2285j,miR-29b-2-5p,miR-6134,miR-1271,miR-362-3p,miR-2426,miR-16-2-3p,miR-296-5p,miR-329-3p,miR-1298-5p,miR-7857,miR-455-3p,miR-2459,miR-2285e,miR-330-3p,miR-2300a-5p,miR-181b-2-3p,miR-3085-3p,miR-1306-5p,miR-326,miR-362-3p,miR-29b-1-5p,miR-106b-5p,miR-182-5p,miR-8485,miR-106a-5p,miR-2330-5p,miR-494-3p,miR-23a-3p,miR-96-5p,miR-668-5p,miR-504,miR-877-3p,miR-361-3p,miR-3596,miR-2483-3p,miR-3082-5p,miR-3064-5p,miR-2319b,miR-499b-5p,miR-2285u,miR-3968,novel_82,miR-2285n,miR-371a-5p,miR-361-3p,miR-2285f,miR-155-5p,miR-496,miR-345-3p,miR-330-5p,miR-1271-5p,miR-2285ad,miR-299a-3p,novel_79,miR-7641,miR-196a-2-3p,miR-2898,miR-200a-3p,miR-152-3p,miR-205-5p,miR-1260a,miR-2285af,miR-217,miR-339a,miR-28b,miR-200a,miR-3064-5p,miR-29b-2-5p,miR-345-5p,miR-329a,miR-3074-1-3p,miR-7144-5p,novel_48,miR-20b-5p,miR-485-5p,miR-488-3p,miR-301,miR-93-5p,miR-17-5p,miR-324-5p,miR-3068-3p,miR-503-3p,miR-2285aa,miR-2355-5p,miR-141-3p,novel_63,miR-144,miR-93,miR-1197-3p,miR-1260b,miR-145-3p,miR-1306-5p,novel_42,miR-1260b,miR-455-5p,miR-744-5p,miR-1434-3p,miR-3071-5p,miR-2285g,novel_116,miR-2355-3p,miR-20b,miR-339-5p,miR-466f-3p,miR-377-3p,miR-330-3p,miR-106a-5p,miR-17-5p,miR-455-5p,miR-2411-3p,miR-345-5p,miR-130b-5p,miR-6240,miR-363-5p,miR-450b-3p,miR-9851-
CTTNBP2	ENSOARG0(	cortacti	miR-204-3p,miR-216b-5p,miR-3431,miR-141-3p,miR-2447,novel_4,miR-129-5p,miR-103a-2-5p,miR-200a-3p,miR-450b-3p,miR-107-5p
TMEM225	ENSOARG0(	transmem	miR-18a-3p,miR-3065-5p,miR-195a-5p,miR-320b,miR-320c,miR-2440,miR-15a,miR-382-5p,miR-16a,miR-92a-3p,miR-199c,miR-2285t,miR-363-3p,miR-2447,miR-194b-5p,miR-1839-3p,novel_111,miR-24-3p,miR-1434-3p,miR-19b-2-5p,miR-329-3p,miR-3962,miR-194-5p,miR-450b-5p,miR-101a-5p,miR-219a-1-3p,miR-16b,miR-362-3p,miR-3120-5p,miR-497-5p,miR-133b-5p,miR-199b-3p,novel_4,miR-2320-3p,miR-142-5p,miR-320b,miR-199a-3p,miR-194a,miR-16b,miR-32,miR-362-3p,miR-196b-5p,miR-1839-3p,miR-494-3p,miR-101b-3p,miR-424-5p,miR-23a-3p,miR-101-5p,miR-2284l,miR-8485,miR-21a-3p,miR-23b,miR-411,miR-320a,miR-329a,miR-3074-1-3p,miR-874-3p,miR-545-3p,miR-32-5p,miR-141-3p,miR-25-3p,miR-6516,miR-300-3p,miR-15b-5p,miR-204-3p,miR-3969,miR-320e,miR-3604,miR-19b-1-5p,miR-100-3p,miR-424-5p,miR-208b-5p,miR-16-5p,miR-142a-5p,miR-2366,miR-362-3p,miR-196a-5p,miR-4429,miR-218-1-3p,miR-18a-3p,miR-548e-3p,miR-15a-5p,miR-6516-5p,miR-23c,miR-23b-3p,miR-322-5p,miR-7859,miR-199a-3p,miR-320d,novel_127,miR-92a-3p,miR-200a-3p,miR-21-3p,miR-3653-3p,miR-3529-3p,miR-216c-5p,miR-92b-3p,miR-490-3p,miR-192-3p,miR-1957a,miR-200a

MYOM1	ENSOARG0(	myomesin	miR-1306-5p,miR-466f-3p
FHAD1	ENSOARG0(	forkhead	miR-2285r,miR-2284l,miR-378a-5p,miR-193a-3p,miR-296-3p,miR-365b-5p,miR-3431,miR-134-3p,miR-194-3p,novel_116,miR-31-5p,miR-103a-3p,miR-339-5p,miR-16b,miR-16-1-3p,miR-497-5p,miR-2284x,miR-432,miR-1843b-5p,miR-542-3p,miR-223-3p,miR-450c-3p,novel_23,miR-376a-3p,miR-193b-3p,miR-28c,miR-374c-3p,miR-299b-3p,miR-144-3p,miR-3074-5p,miR-2319a,miR-148a-3p,miR-216a-3p,miR-450b-5p,miR-200a,miR-3064-5p,miR-200a-3p,novel_96,novel_127,miR-152-3p,miR-9-5p,miR-322-5p,miR-2284z,miR-450a-1-3p,miR-134-5p,miR-299a-3p,miR-2285ad,miR-429-3p,novel_79,miR-431,miR-18b-5p,miR-1290,miR-1343-5p,miR-2427,miR-23b-5p,miR-330-5p,miR-16-5p,novel_9,miR-592,miR-134-5p,novel_63,miR-125b-5p,miR-574-5p,miR-2285aa,miR-2355-5p,miR-125a,miR-345-5p,miR-23b,miR-8485,miR-101b-3p,miR-23a-3p,miR-491-5p,miR-214-3p,miR-103a-2-5p,miR-431-5p,miR-29b-1-5p,miR-323b-3p,miR-16b,miR-182-5p,miR-147a,miR-455-3p,miR-1185-3p,miR-2459,miR-126a-5p,miR-199b-3p,miR-21b,miR-301b-5p,miR-541,miR-323b,miR-423-5p,miR-2447,miR-3956-3p,miR-9-5p,miR-3968,novel_82,miR-301a-5p,miR-378g,miR-3596,miR-1185-2-3p,miR-139-5p,miR-2319b,miR-374b-5p,miR-211-5p,miR-3963,miR-96-5p,miR-504,miR-335-3p,miR-140-5p,miR-409-5p,miR-377-5p,miR-142a-3p,miR-2424,miR-486-3p,miR-195a-3p,miR-345-3p,novel_17,miR-324-3p,miR-92a-1-5p,miR-23b-3p,miR-7859,miR-23c,miR-18a-3p,miR-493-3p,novel_73,miR-33b-3p,miR-218-1-3p,miR-16-1-3p,miR-34a-5p,miR-193b-5p,miR-217-5p,miR-6525,miR-769-5p,miR-224-5p,miR-3184-5p,miR-3184-3p,miR-3074-5p,miR-214,miR-874-5p,miR-1468-5p,miR-151-3p,miR-665,miR-10a,miR-216b-5p,miR-15b-5p,miR-504-5p,miR-216b-3p,miR-552-3p,miR-296-3p,miR-339b,miR-345-5p,miR-186-5p,miR-299,miR-2285c,miR-

AK9	ENSOARG0(	adenylat	miR-1468-5p,miR-424-5p,miR-129-5p,miR-6516-3p,miR-3604,miR-29b-2-5p,miR-6134,miR-145a-3p,miR-186-5p,miR-2285c,miR-15b-5p,miR-2331-3p,miR-660-5p,miR-320d,miR-345-3p,miR-26a-5p,miR-486-3p,miR-664b-3p,miR-376c-3p,miR-195a-3p,miR-369-3p,miR-216c-5p,miR-4429,miR-142-3p,miR-217-5p,miR-1197-5p,miR-140-3p,miR-5703,miR-199a-3p,miR-23b-3p,miR-200b,miR-15a-5p,miR-23c,miR-30d-3p,miR-382-3p,miR-9-3p,miR-8095,miR-3059-5p,miR-335-3p,miR-9-3p,miR-203-3p,miR-499b-5p,miR-541-5p,miR-126b-5p,miR-211-5p,miR-2284g,miR-3064-5p,miR-1468,miR-3085-3p,miR-3955-3p,miR-16b,miR-320b,miR-199a-3p,miR-8485,miR-2310,miR-23a-3p,miR-424-5p,miR-382-3p,miR-16-2-3p,miR-1388-5p,miR-30b-3p,miR-484,miR-4791,miR-9788-3p,miR-455-3p,miR-2285e,miR-376b-3p,miR-222-5p,novel_78,miR-486b-3p,miR-134-5p,miR-135a-5p,miR-409-3p,miR-3969,miR-145-3p,miR-3607-3p,miR-193a-5p,miR-16-5p,miR-3074-1-3p,miR-1814c,miR-137-3p,miR-376c-5p,miR-320a,miR-23b,miR-3154,miR-370-3p,miR-1271,miR-874-3p,miR-127-5p,miR-200c-3p,miR-217,miR-205-5p,miR-376b-3p,miR-200c,novel_39,miR-2284a,miR-490-3p,miR-495-5p,miR-3064-5p,miR-29b-2-5p,miR-2385-3p,miR-181a-2-3p,miR-200b-3p,miR-345-3p,miR-134,miR-134-5p,novel_121,miR-322-5p,miR-758-5p,miR-7641,miR-429-3p,miR-656-5p,miR-22-5p,miR-147-5p,novel_1,miR-16a,miR-15a,miR-2428,miR-211,miR-195a-5p,miR-135b-5p,miR-6516-3p,miR-376b-5p,miR-320c,miR-2404,miR-26b-5p,miR-660,miR-2411-3p,miR-503-3p,miR-497-5p,miR-2284x,miR-188-5p,miR-16b,miR-30b-3p,miR-219a-1-3p,miR-653,miR-412-5p,miR-3535,miR-3431,miR-665,miR-31-5p

PGAM5	ENSOARG0(	phosphog	miR-6395,miR-135b-5p,let-7e-5p,miR-379-5p,miR-450b-3p,miR-1291,miR-21c,miR-3970,miR-1306,miR-18b,miR-148a-3p,miR-181b-3p,miR-144-3p,miR-677,miR-299b-3p,miR-670-3p,miR-2404,miR-26b-5p,miR-3120-3p,miR-3074-5p,miR-147-5p,novel_1,let-7i,miR-576-3p,miR-20a-5p,miR-2284w,miR-326-3p,novel_27,miR-371b-3p,miR-211,miR-3957-3p,miR-135a-2-3p,miR-27b-3p,miR-7862,miR-455-5p,miR-1260b,miR-542-3p,miR-223-3p,miR-181a-5p,miR-2355-3p,miR-20b,miR-411-5p,novel_116,let-7g-5p,miR-134-3p,miR-2284d,miR-26b-3p,miR-665,miR-187-3p,miR-452-5p,miR-483-3p,miR-378a-5p,novel_51,miR-106a-5p,miR-455-5p,miR-2411-3p,miR-17-5p,miR-330-3p,miR-224-5p,miR-432-3p,let-7f-2-3p,let-7f-2-3p,miR-363-5p,miR-6240,miR-671-5p,novel_48,miR-20b-5p,miR-18a,miR-17-5p,miR-93-5p,miR-2284n,miR-324-5p,miR-1271,miR-615-5p,miR-370-3p,miR-874-3p,miR-592,miR-134-5p,miR-204-5p,novel_9,miR-409-3p,miR-135a-5p,miR-144,miR-331-3p,miR-6740-5p,miR-5100,miR-2284k,miR-1260b,miR-93,miR-1306-5p,let-7d,miR-330-5p,miR-134,miR-345-3p,miR-545-5p,miR-134-5p,miR-582-3p,miR-758-5p,miR-483-3p,miR-2411-5p,miR-1271-5p,miR-9-5p,miR-18b-5p,miR-412,miR-2285ad,miR-299a-3p,miR-152-3p,miR-543-5p,let-7f-5p,miR-127-5p,miR-181b-5p,miR-450a-2-3p,let-7c-5p,miR-30c-2-3p,miR-205-5p,miR-1260a,miR-2284a,let-7g,miR-2284y,miR-181a-2-3p,miR-136-5p,miR-96-5p,miR-140-5p,miR-361-3p,miR-27a-3p,miR-1185-2-3p,miR-203-3p,let-7d-5p,miR-25,miR-3596,miR-378g,miR-211-5p,miR-2319b,miR-139-5p,miR-2284g,miR-107-5p,miR-371a-5p,miR-2285y,miR-423-5p,miR-1285-5p,miR-181d-5p,miR-9-5p,miR-2284j,miR-3120-5p,miR-1839-5p,miR-101a-5p,miR-450b-5p,miR-412-3p,miR-484,miR-296-5p,miR-1185-3p,miR-2459,miR-148b-5p,miR-539-5p,miR-1298-3p,miR-181b-2-3p,miR-27a-3p,novel_4,miR-1343-3p,miR-

DYDC2	ENSOARG0(	DPY30 do	let-7a-5p,miR-33a-5p,miR-491-3p,miR-3187-3p,miR-326,novel_115,miR-1306-5p,miR-320b,miR-3955-3p,miR-487b-5p,miR-3085-3p,miR-491-5p,miR-103a-2-5p,let-7e,miR-27a-3p,miR-181b-2-3p,miR-142-3p,miR-455-3p,miR-9788-3p,miR-148b-5p,miR-30b-3p,miR-101a-5p,miR-1388-5p,miR-101c,miR-212-3p,miR-2447,miR-2284j,miR-6527,miR-423-5p,miR-30b-5p,miR-107-5p,miR-2285t,miR-10b,miR-452-3p,miR-20a-3p,miR-139-5p,miR-2284g,miR-3064-5p,miR-7977,miR-499b-5p,miR-7689-3p,miR-7857-3p,miR-378g,miR-27a-3p,miR-4443,let-7d-5p,miR-9-3p,miR-345-3p,miR-195a-3p,miR-142a-3p,miR-181b-1-3p,miR-376a-5p,miR-486-3p,miR-2432,miR-26a-5p,miR-30b,miR-185-3p,miR-98-5p,miR-466i-5p,miR-320d,let-7k,miR-30d,miR-30b-3p,miR-383-5p,miR-95-3p,miR-210-3p,miR-2300b-3p,miR-1961,miR-193b-5p,miR-30c-5p,novel_133,miR-5703,miR-4429,miR-19b-3p,miR-148a-5p,miR-361-5p,miR-218-1-3p,miR-129-2-3p,miR-145a-3p,let-7b,miR-425-5p,miR-338-3p,let-7b-5p,miR-3184-5p,miR-15b-3p,let-7f,miR-320e,miR-17-3p,miR-487b-5p,miR-450b-3p,miR-2331-3p,miR-296-3p,miR-139-5p,miR-582-5p,miR-377-3p,let-7i-5p,miR-204-3p,miR-450a-1-3p,miR-299,miR-30d-5p,miR-487b-5p,miR-30f,miR-582-5p,miR-543-3p,miR-625-5p,miR-382,miR-2483-5p,miR-668-3p,miR-363-5p,miR-432-3p,miR-2284l,miR-432-3p,miR-377-3p,miR-134-3p,let-7g-5p,miR-3431,miR-1843b-5p,miR-873a-5p,miR-374b-3p,miR-450c-3p,miR-138,miR-30b-3p,miR-2284ab,miR-188-5p,miR-27b-3p,miR-17-3p,miR-2428,miR-326-3p,let-7i,miR-33a-3p,miR-425-5p,miR-379-3p,miR-502b,miR-656-5p,miR-203b-5p,miR-216b-3p,miR-26b-5p,miR-181b-3p,miR-320c,miR-677,miR-6535,miR-1306,miR-19a-3p,miR-450b-3p,let-7e-5p,miR-135b-5p,miR-6516-3p,miR-3064-5p,miR-148b-5p,miR-2284a,miR-3956-5p,novel_39,let-7g,miR-376c-5p,miR-376b-5p,let-7c-
SPOPL	ENSOARG0(	speckle-	miR-130b-3p,miR-320d,miR-153-3p,miR-320b,miR-224-5p,miR-217,miR-500a-5p,miR-181b-1-3p,miR-494-3p,miR-124a,miR-345-3p,miR-101-5p,miR-20a,miR-195a-3p,miR-101a-5p,miR-4429,miR-2284r,miR-125b-2-3p,miR-217-5p,miR-124-3p,miR-153,miR-26b-3p,miR-3600,miR-130a-3p,miR-502b,miR-454-3p,miR-6402,miR-370-5p,miR-142b,miR-20a-5p,miR-371a-5p,miR-224-5p,miR-323-3p,miR-301b-3p,miR-1193,miR-103b,miR-6516-3p,miR-9-3p,novel_48,miR-320a,miR-139-5p,miR-301a-3p,miR-499b-3p,miR-300-3p,miR-499a-3p,miR-181b-3p,miR-677,miR-203-3p,miR-223-5p,miR-320c,miR-3068-3p,miR-20a-3p,miR-139-5p,miR-370-5p

SNAP25	ENSOARG0(	synaptos	miR-2411,miR-29b-1-5p,miR-216c-5p,miR-299-3p,miR-221-5p,miR-34b-5p,miR-760-3p,miR-466i-5p,miR-320d,miR-125b,novel_83,miR-532-3p,miR-148b-3p,miR-222-5p,miR-200b,miR-15a-5p,miR-383-5p,miR-4429,miR-19b-3p,miR-140-3p,miR-5703,miR-2904,miR-215-5p,miR-2300b-3p,novel_133,miR-6134,miR-206-3p,miR-10b-5p,miR-3074-2-3p,miR-4510,miR-708-5p,miR-424-5p,miR-6516-3p,miR-17-3p,miR-450b-3p,miR-29b-2-5p,miR-19b-1-5p,miR-132-5p,miR-22-3p,miR-204-3p,miR-323-5p,miR-139-5p,miR-6130,miR-544-3p,miR-132-5p,miR-192-5p,miR-3065-3p,miR-21-3p,miR-221-5p,miR-582-5p,novel_99,miR-18a-5p,miR-2312,miR-133b-3p,miR-21a-3p,miR-193a,miR-3187-3p,miR-1839-3p,miR-424-5p,miR-382-3p,miR-2957,miR-1306-5p,miR-320b,miR-9788-3p,miR-216a-3p,miR-27a-3p,miR-222-5p,miR-3120-5p,miR-544a,miR-7857,miR-143-5p,miR-10b,miR-484,miR-154a,miR-219a-2-3p,miR-452-5p,miR-181d-5p,miR-2285f,miR-541-3p,novel_60,miR-2284q,miR-7134-3p,miR-2285t,miR-2285y,miR-27a-3p,miR-3082-5p,miR-7857-3p,miR-20a-3p,miR-199b-5p,miR-361-3p,miR-199a-5p,miR-3065-3p,miR-380-5p,miR-2403,miR-181a-2-3p,miR-29b-2-5p,miR-1,miR-30a-3p,miR-543-5p,miR-107,miR-181b-5p,miR-200c-3p,miR-2898,miR-17-3p,miR-217,miR-200c,miR-1260a,miR-214-5p,miR-2411-5p, let-7a-2-3p,miR-10a-5p,miR-200b-3p,miR-582-3p,miR-134,miR-345-3p,miR-133a-3p,miR-6128,miR-3607-3p,miR-1306-5p,miR-204-5p,miR-486b-3p,miR-28-5p,miR-138-5p,miR-5100,miR-199a-5p,miR-34c-5p,miR-374c-3p,miR-141-3p,miR-215-5p,miR-1814c,miR-671-5p, let-7g-3p,miR-320a,miR-18a,miR-3154,miR-2448-3p,miR-345-5p,miR-125a-5p,miR-330-3p,miR-190a,miR-503-5p,miR-544b,miR-103,miR-503-5p,miR-185-5p,miR-432-5p,miR-130a-3p,miR-219a-1-3p,miR-1260b,miR-138,miR-380-3p,miR-374b-3p,miR-1434-
CDC42BPG	ENSOARG0(	CDC42 bi	miR-486b-3p,miR-208b-5p,novel_74,miR-2285y,miR-125b-5p,miR-331-3p,miR-324-3p,miR-3184-5p,miR-423-5p,miR-326-3p,miR-361-3p,miR-1271,miR-6395,miR-1291,miR-96-5p,miR-301,miR-454-5p,miR-670-3p,miR-378g,miR-3956,miR-125a,miR-125a-5p,miR-125b,miR-92a-1-5p,novel_51,miR-326,miR-143-3p,miR-486-3p,miR-376b-5p,miR-2330-5p,miR-330-5p,miR-328-3p,miR-134-3p,miR-1271-5p,miR-1343-3p,miR-2285ad
DMPK	ENSOARG0(	dystroph	miR-3141,miR-1306-5p,miR-361-3p,miR-3154,miR-1306,miR-3141,novel_42,miR-361-3p,miR-151b,miR-151-5p
SLC38A3	ENSOARG0(	solute c	miR-2331-3p,miR-615-5p,miR-7977,miR-2355-5p,miR-2285aa,miR-6130,novel_69,miR-193b-3p,miR-378g,miR-151b,miR-488-3p,miR-361-3p,miR-133b-3p,miR-671-5p,miR-146b-5p,miR-4510,miR-28a-3p,miR-495-3p,miR-133a-3p,miR-193b-3p,miR-206-3p,miR-326-3p,miR-6529b,miR-28c,miR-206,miR-4726-5p,miR-134-5p,miR-486b-3p,miR-874-5p,miR-134-3p,miR-2459,miR-148b-5p,miR-9788-3p,novel_83,miR-151-5p,miR-134-5p,miR-194-3p,miR-134,miR-1285,miR-2300b-3p,miR-330-5p,miR-1a-3p,miR-33b-3p,miR-148a-5p,miR-378d,miR-2330-5p,miR-1,miR-148b-5p,miR-28b,miR-143-3p,miR-1271-3p,miR-486-3p,miR-146a-5p,miR-326,miR-146a,miR-196a-3p,miR-2957,miR-1185-5p,miR-28-3p,miR-193a-3p,miR-146b

SMAD9	ENSOARG0(	SMAD fam	miR-181b-2-3p,miR-27a-3p,miR-216a-3p,miR-378b,miR-1a-3p,miR-450b-5p,miR-378c,miR-106a-5p,miR-378d,miR-2310,miR-8485,miR-326,miR-320b,miR-106b-5p,miR-214-3p,miR-499b-5p,miR-378e,miR-7857-3p,miR-27a-3p,miR-203-3p,miR-212-3p,miR-452-5p,miR-2285f,miR-6527,miR-1973,miR-490-5p,miR-2285t,miR-106a,miR-18a-3p,miR-222-5p,miR-210-3p,miR-328-3p,novel_83,miR-532-3p,miR-1246,miR-378i,miR-126-3p,miR-378a-3p,miR-188-3p,miR-4429,miR-329a-5p,miR-345-3p,miR-20a,miR-181b-1-3p,miR-664b-3p,miR-146a-5p,miR-210-5p,miR-374a-5p,miR-422a,miR-320d,miR-106a,miR-21-3p,miR-378a-3p,miR-532-5p,miR-3065-3p,miR-370-5p,miR-193b-3p,miR-6516,miR-22-3p,miR-450a-1-3p,miR-21a-3p,miR-2312,miR-378c,miR-214,miR-206-3p,miR-338-5p,miR-665,miR-324-3p,miR-450b-3p,miR-187-3p,miR-26b-3p,miR-149-5p,miR-708-3p,novel_23,miR-3071-5p,miR-450c-3p,miR-22-3p,miR-27b-3p,miR-455-5p,miR-2285r,miR-17-5p,miR-106a-5p,miR-455-5p,miR-365b-5p,miR-193a-3p,miR-329-5p,novel_68,miR-3120-3p,miR-655-5p,miR-181b-3p,miR-320c,miR-677,miR-669,miR-2387,miR-2285ab,miR-18a-3p,miR-450b-3p,miR-6395,miR-378f,miR-136-3p,miR-1193,miR-193b-3p,novel_27,miR-326-3p,miR-20a-5p,miR-206,miR-6516-5p,miR-378h,miR-302a-5p,miR-450a-1-3p,miR-330-5p,miR-1,miR-490-3p,miR-3956-5p,miR-200a,miR-1957a,miR-29a-5p,miR-3065-3p,novel_127,miR-200a-3p,miR-874-3p,miR-141-3p,miR-17-5p,miR-320a,miR-93-5p,miR-329b,miR-485-5p,miR-1248,miR-1814c,miR-3074-1-3p,miR-20b-5p,miR-1306-5p,miR-93,miR-365a-5p,miR-378c,miR-370-5p,miR-135a-1-3p,miR-100-3p
SPATA32	ENSOARG0(	spermato	miR-4443,miR-154b-5p,miR-2284z,miR-148b-3p,miR-3596,miR-3960,miR-378d,miR-2284aa,miR-1260b,miR-1306,miR-148a-3p,miR-130a-5p,miR-125b-2-3p,miR-1260b,miR-29b-1-5p,miR-28b,miR-28-5p,miR-152-3p,miR-3653-3p,miR-708-5p,miR-4726-5p,miR-665,miR-29b-1-5p,miR-1306-5p,miR-28c,miR-1260a
SARAF	ENSOARG0(	store-op	miR-374b-3p,miR-21c,miR-199b-5p,miR-301,miR-21a-3p,miR-381-3p,miR-300,miR-10b-3p,miR-21-5p,miR-3120-3p,miR-548e-3p,miR-455-3p,miR-199a-5p,miR-539-5p,miR-2483-3p,miR-383-5p,miR-7859,miR-655-3p,miR-2332,miR-205-5p,miR-199b-5p,miR-181a-2-3p,novel_101,miR-369-3p,miR-1306-5p,miR-199b-5p,miR-374c-5p,miR-199a-5p,miR-323c

HSDL1	ENSOARG0(	hydroxys	miR-144-5p,miR-1185-2-3p,miR-3596,miR-499a-3p,miR-2483-3p,miR-374b-5p,miR-7689-3p,miR-211-5p,miR-2284g,miR-139-5p,miR-2319b,miR-3963,miR-221,miR-877-3p,novel_111,miR-2447,miR-154-3p,novel_82,miR-455-3p,miR-1185-3p,miR-496-5p,miR-181b-2-3p,miR-330-3p,miR-199b-3p,miR-1343-3p,miR-323b,miR-1a-3p,miR-8485,miR-1b-5p,miR-23a-3p,miR-101b-3p,miR-146b,miR-491-5p,miR-487b-5p,miR-323b-3p,novel_19,miR-16b,miR-106b-5p,let-7i-5p,miR-15b-5p,miR-377-3p,miR-499b-3p,miR-582-5p,miR-154a-3p,miR-552-3p,miR-296-3p,miR-2331-3p,miR-1277-3p,miR-625-5p,miR-345-5p,miR-655,miR-411,miR-299,miR-2285c,miR-122-5p,miR-6525,miR-224-5p,let-7b,miR-129-2-3p,miR-130b-5p,miR-487b-5p,miR-30a-3p,miR-3604,let-7f,miR-2285j,miR-30e-3p,miR-1247-5p,miR-7859,miR-23b-3p,miR-23c,miR-30d-3p,miR-106a,miR-2284u,miR-493-3p,novel_73,miR-142-3p,miR-217-5p,miR-1961,miR-486-3p,miR-143-3p,miR-181b-1-3p,miR-369-3p,miR-195a-3p,miR-345-3p,miR-487a,let-7k,miR-98-5p,miR-122-3p,miR-92a-1-5p,miR-210-5p,miR-320c,miR-144-3p,miR-677,miR-181b-3p,miR-411b-3p,miR-146b-5p,let-7e-5p,miR-1306,miR-216a-3p,miR-374c-5p,miR-379-3p,miR-206,miR-2284o,miR-4324,miR-142b,miR-28c,miR-103a-3p,miR-497-5p,miR-2284x,miR-16b,novel_23,miR-19b-2-5p,miR-223-3p,miR-1843b-5p,miR-2285r,miR-2284l,miR-668-3p,miR-6240,miR-106b-3p,miR-378a-5p,miR-365b-5p,miR-194a,novel_51,miR-377-3p,miR-146a,miR-331-5p,miR-3068-3p,miR-874-3p,miR-20b-5p,miR-876-3p,miR-345-5p,miR-93-5p,miR-200a-5p,miR-23b,miR-331-5p,miR-1260b,miR-16-5p,miR-144-5p,miR-181a-2-3p,miR-331-5p,miR-2284k,miR-129-1-3p,novel_121,miR-322-5p,miR-2285ad,miR-429-3p,miR-155-5p,miR-3591-5p,miR-29a-5p,let-7g,miR-2284y,miR-192-3p,miR-450b-5p,miR-2284f,novel_96,miR-6536,let-7f-5p,let-

			miR-3600,miR-212-5p,miR-548e-3p,miR-7859,miR-23b-3p,miR-222-5p,miR-23c,miR-124-3p,miR-188-3p,miR-1197-5p,miR-378a-3p,miR-5703,miR-378i,miR-2366,miR-16-1-3p,miR-378d,miR-361-5p,miR-345-3p,miR-216c-5p,miR-2424,miR-377-5p,miR-181b-1-3p,miR-216a-5p,miR-422a,miR-92a-1-5p,miR-30b-3p,miR-221-5p,miR-182-5p,miR-296-3p,miR-545-3p,miR-221-5p,miR-378a-3p,miR-548o-3p,miR-660-5p,miR-204-3p,miR-544-3p,miR-499b-3p,miR-139-5p,miR-129b-3p,miR-433-3p,let-7f-1-3p,miR-450a-1-3p,novel_99,miR-186-5p,miR-2312,miR-345-5p,miR-1271,miR-3074-2-3p,miR-378c,miR-6134,miR-769-5p,miR-224-5p,miR-320e,let-7b-3p,miR-2285j,miR-129-5p,miR-450b-3p,miR-664a,miR-145b,miR-222-5p,miR-181b-2-3p,novel_78,miR-1343-3p,miR-409b,miR-216a-3p,miR-500-5p,miR-455-3p,miR-1185-3p,miR-425-3p,miR-539-5p,miR-296-5p,miR-378b,miR-484,miR-2284aa,miR-1839-5p,miR-378c,miR-1388-5p,miR-544a,miR-23a-3p,miR-33a-5p,miR-124a,miR-8485,miR-378d,miR-182-5p,miR-1306-5p,novel_103,miR-24-1-5p,miR-139-5p,miR-20a-3p,miR-203-3p,miR-1185-2-3p,miR-378e,miR-3596,miR-499a-3p,miR-489,miR-378j,miR-129b-5p,miR-96-5p,miR-299b-5p,miR-2285f,miR-2447,miR-361-3p,miR-362-5p,miR-769-5p,miR-218-5p,miR-2285t,miR-499a-5p,miR-429-3p,miR-2284z,miR-134-5p,miR-450a-1-3p,novel_121,miR-24-2-5p,miR-758-5p,miR-378h,miR-1271-5p,miR-3591-5p,miR-134,miR-128-3p,miR-582-3p,miR-4286,miR-2403,novel_39,miR-1957a,miR-490-3p,miR-2285b,miR-450b-5p,miR-24-2-5p,miR-205-5p,miR-200c,miR-200c-3p,miR-127-5p,miR-154b-5p,miR-769,miR-362-5p,miR-3154,miR-671-5p,miR-1248,miR-10b-3p,miR-767,miR-345-5p,miR-1306-5p,miR-145-3p,miR-378c,miR-500b-5p,miR-331-3p,miR-135a-5p,miR-135a-1-3p,miR-134-5p,miR-767-5p,miR-377-5p,miR-31-5p,novel_116,miR-544b,miR-2355-3p,miR-
C7orf31	ENSOARG0(	chromoso	
RSPH6A	ENSOARG0(	radial s	miR-139-5p,miR-874-3p,miR-105-1,miR-2319b,miR-370-3p,novel_79,miR-339-5p,miR-365a-3p,miR-139-5p,miR-2285ab,miR-2312,miR-339b,miR-105-2,miR-877-3p,miR-3059-5p,miR-2285f,miR-2285x,miR-877-3p,miR-6525,miR-486-3p,miR-105-5p,miR-339a,miR-664a-5p,miR-486b-3p

CIZ1	ENSOARG0(	CDKN1A i	miR-194b-5p,miR-2285f,miR-1296-5p,miR-541-3p,miR-3059-5p,novel_60,miR-4726-5p,miR-2285y,miR-30b-5p,miR-454-5p,miR-27a-3p,miR-3082-5p,miR-7977,miR-452-3p,miR-20a-3p,miR-664b,novel_94,miR-361-3p,miR-3187-3p,miR-33a-5p,miR-652-5p,miR-424-5p,miR-106a-5p,miR-542-5p,miR-25-5p,miR-1306-5p,miR-326,miR-9788-3p,miR-27a-3p,miR-3120-5p,miR-2426,miR-30b-3p,miR-3962,miR-10b,miR-143-5p,miR-4791,miR-484,miR-296-5p,miR-338-3p,miR-10b-5p,miR-424-5p,miR-664a,miR-320e,miR-29b-2-5p,miR-324-3p,miR-877-5p,miR-532-5p,miR-21-3p,miR-221-5p,miR-545-3p,miR-30f,miR-133b-3p,miR-2312,let-7f-1-3p,miR-2432,miR-2411,miR-146a-5p,miR-130a-5p,miR-20a,miR-183-5p,miR-21-3p,miR-30b-3p,miR-34b-5p,miR-221-5p,miR-760-3p,miR-106a,miR-125b,miR-542-5p,miR-15a-5p,miR-1b-3p,miR-3960,miR-378d,let-7a-3p,miR-188-3p,miR-1197-5p,miR-5703,miR-2300b-3p,novel_133,miR-2428,miR-449a,miR-326-3p,miR-24-3p,novel_101,miR-542-5p,miR-203b-5p,miR-128-1-5p,miR-34c,miR-16a,miR-20a-5p,miR-15a,miR-670-3p,miR-34b,miR-29a-3p,miR-146b-3p,miR-10a-5p,miR-320b,miR-195a-5p,miR-1291,miR-378b,miR-2387,miR-2285ab,miR-345-5p,miR-130b-5p,miR-125a-5p,miR-1343-5p,miR-17-5p,miR-106a-5p,miR-20b,miR-185-5p,miR-432-5p,miR-26b-3p,miR-377-5p,miR-138,miR-30b-3p,miR-29b,miR-194-5p,miR-3071-5p,miR-145a-5p,miR-1285,miR-873a-5p,miR-365a-5p,miR-93,miR-133a-3p,miR-103b,miR-1306-5p,miR-486b-3p,miR-138-5p,miR-135a-1-3p,miR-29b-3p,miR-410-5p,miR-324-5p,miR-34c-5p,miR-615-5p,miR-370-3p,miR-485-5p,miR-7144-5p,miR-376c-5p,miR-23a-5p,let-7g-3p,miR-17-5p,miR-3154,miR-2448-3p,miR-381-3p,miR-490-3p,miR-1271-3p,miR-380-5p,miR-2478,miR-4286,miR-29b-2-5p,miR-3529-3p,miR-543-5p,miR-191-3p,miR-2898,miR-450a-2-3p,miR-339a,miR-214-5p,miR-2411-5p,miR-

MKKS	ENSOARG0(	McKusick	miR-181b-1-3p,miR-376c-3p,miR-377-5p,miR-421-5p,miR-664b-3p,miR-376a-5p,miR-143-3p,miR-2424,miR-202-5p,novel_17,miR-92a-1-5p,miR-345-3p,miR-219b-5p,miR-23c,miR-23b-3p,miR-7859,miR-106a,miR-33b-3p,miR-361-5p,miR-664-5p,miR-1434-5p,miR-769-5p,miR-665-5p,miR-487b-5p,miR-2285j,miR-15b-3p,miR-665,miR-377-3p,miR-376e-3p,miR-499b-3p,miR-548o-3p,miR-2331-3p,miR-296-3p,miR-345-5p,miR-186-5p,miR-2285c,miR-450a-1-3p,miR-23a-3p,miR-199b-5p,miR-153-3p,miR-106b-5p,miR-496-5p,miR-153,miR-1185-3p,miR-2459,miR-411b-5p,miR-455-3p,miR-148b-5p,novel_78,miR-181b-2-3p,miR-21b,miR-101c,miR-1839-5p,miR-758-3p,miR-1a-3p,miR-877-3p,miR-7705,miR-1285-5p,miR-3968,miR-101-3p,miR-499a-3p,miR-1185-2-3p,miR-203-3p,miR-7689-3p,miR-136-5p,miR-9-3p,miR-493-5p,miR-335-3p,miR-127-5p,miR-3071-3p,miR-505-5p,novel_121,miR-134-5p,miR-2284z,miR-450a-1-3p,miR-6517,miR-429-3p,miR-196a-5p,miR-1197-3p,miR-134-5p,miR-125b-5p,miR-376c-3p,miR-3068-3p,miR-125a,miR-345-5p,miR-2318,miR-1248,miR-20b-5p,miR-30e-5p,miR-23b,miR-93-5p,miR-493-5p,let-7f-2-3p,let-7f-2-3p,miR-196b-5p,miR-378a-5p,miR-483-3p,miR-377-3p,miR-194a,miR-2411-3p,miR-194-3p,miR-31-5p,miR-16-1-3p,miR-27b-3p,miR-188-5p,miR-19b-2-5p,miR-450c-3p,miR-199b-5p,miR-2284w,miR-1839-3p,miR-379-3p,miR-206,miR-421-5p,miR-625-3p,miR-28c,miR-412-5p,miR-4324,novel_87,miR-181b-3p,miR-669,miR-320c,miR-677,miR-144-3p,miR-329-5p,miR-216b-3p,miR-3065-5p,miR-150-5p,miR-3970,miR-1306,miR-130a-5p,miR-2411,miR-2432,miR-216c-5p,miR-20a,miR-106a,miR-320d,miR-21-3p,miR-196a-3p,miR-125b,miR-216a-5p,miR-412-5p,miR-340-5p,miR-200b,miR-548e-3p,miR-505-3p,miR-4429,miR-215-5p,miR-376a-5p,novel_133,miR-188-3p,miR-2904,miR-206-3p,miR-2330-3p,miR-17-3p,miR-155-

LLGL2	ENSOARG0(	lethal g	miR-193b-3p,miR-29c-3p,miR-2285x,miR-135a-2-3p,miR-449a,miR-2428,miR-371b-3p,miR-34c,novel_87,miR-502-5p,miR-6529b,miR-203b-5p,miR-22-5p,miR-29a,novel_68,miR-3074-5p,miR-6535,miR-323a-5p,miR-411b-3p,novel_69,miR-29a-3p,miR-299b-3p,miR-670-3p,miR-34b,miR-1306,miR-150-5p,miR-376b-3p,miR-18a-3p,miR-6516-3p,miR-505-3p,miR-380-5p,miR-296-3p,miR-432-3p,miR-432-3p,novel_51,miR-2411-3p,miR-7-5p,miR-296-3p,miR-660,miR-193a-3p,miR-124-5p,miR-378a-5p,miR-187-3p,miR-133b-5p,miR-3535,miR-708-3p,miR-7b-5p,miR-1434-3p,miR-29b,miR-30b-3p,miR-219a-1-3p,miR-193a-5p,miR-29b-3p,miR-410-5p,miR-486b-3p,miR-767-5p,miR-2355-5p,miR-34c-5p,miR-769,miR-3154,miR-2448-3p,miR-301,miR-7144-5p,miR-767,miR-181a-2-3p,novel_39,miR-3065-3p,miR-2285b,miR-192-3p,miR-380-5p,miR-376b-3p,miR-6119-5p,novel_127,novel_96,miR-127-5p,miR-196a-2-3p,miR-299a-3p,miR-2285ad,miR-6517,miR-6516-5p,miR-29d-3p,miR-345-3p,miR-877-3p,miR-2285y,miR-769-5p,miR-2285n,novel_82,miR-7977,miR-376d,miR-361-3p,miR-140-5p,miR-376a-3p,miR-877-3p,let-7c-3p,miR-33a-5p,miR-4532,miR-199a-3p,miR-1306-5p,novel_115,miR-214-3p,miR-491-5p,miR-487b-5p,miR-2300a-5p,miR-9788-3p,miR-455-3p,miR-539-5p,miR-142-3p,miR-376b-3p,miR-1298-3p,miR-30b-3p,miR-296-5p,miR-4791,miR-450b-5p,miR-214,miR-665-5p,miR-3074-5p,miR-338-3p,miR-6525,miR-769-5p,miR-665,miR-874-5p,miR-6516-3p,miR-3065-3p,novel_107,miR-532-5p,miR-221-5p,miR-660-5p,miR-548o-3p,miR-6516,miR-193b-3p,miR-216b-5p,miR-323-5p,miR-129b-3p,miR-625-5p,novel_99,let-7g-3p,miR-2887,miR-216c-5p,miR-486-3p,miR-125b-2-3p,miR-142a-3p,miR-345-3p,miR-618,miR-34b-5p,miR-221-5p,miR-299-3p,miR-21-3p,miR-3600,miR-212-5p,miR-505-3p,miR-548o-3p,miR-18a-3p,miR-199a-3p,miR-
DRG1	ENSOARG0(	developm	novel_78,novel_4,miR-216a-3p,miR-544b,miR-432-5p,miR-134-3p,miR-197-3p,miR-432,miR-2284r,miR-6529a,miR-181a-5p,miR-541,miR-29b,miR-544a,miR-23a-3p,miR-493-5p,miR-382-3p,miR-2330-5p,miR-296-3p,miR-432-3p,miR-505,novel_51,miR-320b,miR-296-3p,miR-320b,miR-126b-5p,miR-26b-5p,miR-216b-3p,miR-10b,miR-320c,miR-25,miR-378g,miR-2404,miR-29a-3p,miR-150-5p,miR-493-5p,miR-136-5p,miR-17-3p,miR-29c-3p,miR-181d-5p,miR-541-3p,miR-423-5p,miR-154a,miR-877-3p,miR-2285y,miR-502b,miR-4726-5p,miR-29a,miR-2285ad,miR-214-5p,miR-383-5p,miR-23b-3p,miR-23c,miR-29d-3p,miR-1197-5p,miR-3578,miR-2300b-3p,miR-496,miR-378d,miR-4429,miR-33b-3p,miR-216c-5p,miR-200a,miR-1271-3p,miR-92a-1-5p,miR-26a-5p,miR-200a-3p,miR-320d,miR-17-3p,miR-181b-5p,miR-141-3p,miR-296-3p,miR-503-3p,miR-496-3p,miR-544-3p,miR-320a,miR-23b,miR-3154,miR-488-3p,miR-3074-1-3p,miR-543-3p,let-7g-3p,miR-767,miR-103b,miR-425-5p,miR-3184-5p,miR-181c-5p,miR-6525,miR-665,miR-29b-3p,miR-4492,miR-17-3p,miR-767-5p

SH2D4B	ENSOARG0(	SH2 doma	miR-130b-5p,let-7f-2-3p,miR-668-3p,miR-345-5p,miR-377-3p,miR-146a,novel_51,miR-194a,miR-378a-5p,miR-193a-3p,miR-122-3p,miR-339-5p,miR-665,miR-185-5p,miR-3431,miR-544b,novel_116,miR-542-3p,miR-1434-3p,miR-873a-5p,miR-194-5p,miR-197-3p,miR-1260b,miR-29b,miR-7862,miR-27b-3p,miR-342-3p,miR-2285x,miR-29c-3p,miR-17-3p,miR-495-3p,miR-193b-3p,miR-323c,miR-24-3p,miR-371b-3p,miR-2284w,miR-326-3p,miR-142b,miR-28c,miR-502-5p,novel_1,miR-29a,miR-6119-3p,miR-199c,miR-625-3p,miR-3074-5p,miR-329-5p,novel_68,miR-26b-5p,miR-655-5p,miR-29a-3p,miR-670-3p,miR-2404,miR-6535,miR-320c,miR-148a-3p,miR-3065-5p,miR-1306,miR-450b-3p,miR-135b-5p,miR-2319a,miR-146b-5p,miR-3064-5p,miR-28b,miR-148b-5p,miR-450c-5p,miR-2284a,novel_39,miR-3065-3p,miR-2284y,miR-200c,miR-664-3p,miR-1260a,miR-339a,miR-200c-3p,miR-2898,miR-17-3p,miR-200a-3p,novel_96,miR-152-3p,miR-423-3p,miR-429-3p,miR-6517,miR-2285ad,miR-431,miR-412,novel_79,miR-7641,miR-6516-5p,miR-758-5p,novel_121,miR-129-1-3p,miR-3578,miR-1290,miR-345-3p,miR-330-5p,miR-29d-3p,miR-200b-3p,miR-193a-5p,miR-6128,miR-1260b,miR-145-3p,miR-181c-3p,miR-5100,miR-29b-3p,miR-135a-5p,novel_9,miR-28-5p,miR-141-3p,miR-2355-5p,miR-154b-5p,miR-301,miR-3154,miR-362-5p,miR-320a,miR-329b,miR-345-5p,miR-2318,miR-671-5p,miR-3074-1-3p,miR-101b-3p,miR-494-3p,miR-2284b,miR-33a-5p,miR-1b-5p,miR-758-3p,miR-8485,miR-431-5p,novel_115,miR-1306-5p,miR-326,miR-342,miR-320b,miR-2957,miR-29b-1-5p,miR-1185-5p,miR-3085-3p,miR-491-5p,miR-146b,miR-199b-3p,miR-1343-3p,novel_78,miR-2300a-5p,miR-27a-3p,miR-222-5p,miR-1185-3p,miR-500-5p,miR-455-3p,miR-148b-5p,miR-301b-5p,miR-296-5p,miR-125b-2-3p,miR-758-3p,miR-412-3p,miR-544a,miR-1388-5p,miR-3956-3p,miR-
TMPRSS2	ENSOARG0(	transmem	miR-877-3p,miR-24-3p,miR-449a,miR-6128,miR-665-5p,miR-664a,miR-874-5p,miR-34c,miR-1973,miR-34c-5p,miR-2404,miR-34b,novel_32,novel_68,miR-2331-3p,miR-329-5p,miR-615-5p,miR-10b-3p,miR-1291,miR-2319a,novel_94,miR-1248,miR-485-5p,miR-433-3p,miR-2478,miR-376a-5p,miR-4532,miR-329a-5p,miR-495-5p,miR-491-5p,miR-760-3p,miR-1343-5p,miR-34b-5p,novel_96,miR-3071-3p,miR-92a-1-5p,miR-505-5p,miR-194-3p,miR-149-5p,miR-1247-5p,miR-34a-5p,miR-2889,miR-2300b-3p,miR-143-5p,miR-1343-5p
NSL1	ENSOARG0(	NSL1, MI	miR-320b,miR-2285p,miR-409-3p,novel_74,miR-320d,miR-592,miR-2447,miR-101-5p,miR-190b-5p,miR-130a-5p,miR-4791,miR-320a,miR-33b-3p,miR-4429,miR-335-3p,miR-3955-5p,miR-5010-3p,miR-3535,miR-499b-3p,miR-499a-3p,miR-1298-3p,miR-320c,miR-204-3p,miR-194-3p

CHD2	ENSOARG0(	chromodo	miR-378h,miR-148b-3p,miR-15a-5p,miR-383-5p,miR-322-5p,miR-7641,miR-3600,miR-1224-5p,miR-2285ad,miR-128-3p,miR-4429,miR-19b-3p,miR-378a-3p,miR-1197-5p,miR-140-3p,miR-5703,miR-7134-5p,miR-2408,miR-3591-5p,miR-615-3p,miR-378i,novel_39,miR-28b,miR-2887,novel_96,miR-543-5p,miR-152-3p,miR-221-5p,miR-107,miR-127-5p,miR-99a-3p,miR-181b-5p,miR-320d,miR-422a,miR-185-3p,miR-1260a,miR-205-5p,miR-15b-5p,miR-204-3p,miR-377-3p,miR-296-3p,miR-99a-3p,miR-32-3p,miR-221-5p,miR-2331-3p,miR-378a-3p,miR-625-5p,miR-543-3p,miR-320a,miR-6134,miR-181c-5p,miR-378c,miR-1197-3p,miR-1260b,miR-6525,miR-214,miR-16-5p,miR-378c,miR-4492,miR-424-5p,miR-664a,miR-320e,miR-6740-5p,miR-99b-3p,miR-551b-3p,miR-2285j,miR-103,miR-145b,miR-103a-3p,novel_4,miR-2300a-5p,miR-340-3p,miR-7862,miR-497-5p,miR-1260b,miR-16b,miR-378c,miR-145a-5p,miR-197-3p,miR-181a-5p,miR-378b,miR-2284r,miR-378d,miR-668-3p,miR-382-3p,miR-424-5p,miR-214-3p,miR-491-5p,miR-16b,miR-377-3p,miR-320b,miR-320c,miR-670-3p,miR-378e,miR-3596,miR-7977,miR-195a-5p,miR-378f,miR-107,miR-19a-3p,miR-216a-3p,miR-148a-3p,miR-361-3p,miR-361-3p,miR-181d-5p,miR-32-3p,miR-2447,miR-502b,miR-147-5p,novel_1,miR-128-1-5p,miR-4726-5p,miR-2285y,miR-16a,miR-28c,miR-615,miR-15a
NCOA6	ENSOARG0(	nuclear	[illegible]362-3p,miR-215-5p,miR-1246,miR-376a-5p,miR-2300b-3p,miR-378d,miR-3600,miR-199a-3p,miR-532-3p,miR-148b-3p,miR-216a-5p,miR-196a-3p,miR-30b-3p,miR-34b-5p,miR-299-3p,miR-106a,miR-466i-5p,miR-20a,miR-2432,miR-130a-5p,miR-27b-5p,let-7g-3p,miR-2312,miR-18a-5p,miR-21-3p,miR-532-5p,miR-660-5p,miR-27a-5p,miR-192-5p,miR-320e,miR-664a,miR-539-5p,let-7b-5p,miR-338-3p,miR-10b-5p,miR-296-5p,miR-6529a,miR-10b,miR-16-2-3p,miR-3120-5p,miR-101a-5p,novel_4,miR-2300a-5p,miR-151a-3p,miR-9788-3p,miR-2285e,miR-362-3p,miR-342,miR-1306-5p,let-7e,let-7c-3p,miR-652-5p,let-7a-5p,miR-382-3p,miR-106a-5p,miR-30c-1-3p,miR-129b-5p,novel_94,miR-877-3p,miR-7977,miR-20a-3p,miR-10b,miR-452-3p,let-7d-5p,miR-371a-5p,miR-335,miR-2285f,miR-181d-5p,miR-194b-5p,miR-3059-5p,novel_60,miR-212-3p,miR-218-2-3p,miR-345-3p,miR-128-3p,let-7a-2-3p,miR-10a-5p,let-7d,miR-24-2-5p,miR-483-3p,miR-30c-2-3p,miR-339a,let-7c-5p,miR-1260a,miR-543-5p,miR-92a-3p,miR-181b-5p,miR-181a-2-3p,miR-4286,miR-3529-3p,miR-1271-3p,miR-24-2-5p,miR-380-5p,miR-18a,miR-17-5p,miR-488-3p,miR-2448-3p,miR-7144-5p,miR-671-5p,miR-485-5p,let-7g-3p,miR-615-5p,miR-215-5p,miR-324-5p,miR-34c-5p,miR-374c-3p,miR-331-3p,miR-409-3p,miR-551b-3p,miR-5100,miR-486b-3p,miR-204-5p,miR-138-5p,miR-93,miR-194-5p,miR-374b-3p,miR-145a-5p,miR-1285,miR-138,miR-1260b,miR-130a-3p,miR-665,miR-26b-3p,miR-133b-5p,miR-20b,miR-2285g,miR-185-5p,let-7g-5p,miR-106a-5p,miR-17-5p,miR-224-5p,miR-660,miR-130b-5p,miR-345-5p,miR-654-3p,miR-18a-3p,miR-2285ab,miR-18b,miR-2387,miR-655-5p,miR-320b,miR-7-1-3p,miR-10a-5p,miR-146b-3p,miR-34b,miR-34c,let-7i,miR-6402,miR-20a-5p,miR-92a-3p,miR-2285x,miR-135a-2-3p,miR-

PARP11	ENSOARG0(	poly (AD	miR-1343-5p,miR-378a-5p,miR-2411-3p,novel_51,miR-330-3p,miR-224-5p,miR-345-5p,miR-432-3p,miR-432-3p,let-7f-2-3p,let-7f-2-3p,miR-668-3p,miR-6240,miR-130b-5p,miR-16-1-3p,miR-219a-1-3p,miR-431-3p,miR-181a-5p,miR-223-3p,miR-873a-5p,miR-3431,miR-339-5p,miR-31-5p,miR-187-3p,miR-147-5p,miR-656-5p,miR-206,miR-379-3p,miR-28c,miR-2428,miR-24-3p,novel_101,miR-17-3p,miR-495-3p,miR-1839-3p,miR-150-5p,miR-1306,miR-181b-3p,miR-677,miR-6535,miR-10a-5p,miR-3074-5p,miR-378b,miR-329-5p,miR-216b-3p,miR-200a-3p,miR-200c-3p,miR-127-5p,miR-181b-5p,miR-17-3p,miR-450a-2-3p,miR-339a,miR-30c-2-3p,miR-3071-3p,miR-200c,miR-219-3p,miR-192-3p,miR-1271-3p,novel_39,miR-29a-5p,miR-28b,miR-450b-5p,miR-1,miR-10a-5p,miR-200b-3p,miR-30c,miR-411-3p,miR-30a-5p,miR-1343-5p,miR-134,miR-345-3p,miR-134-5p,miR-6516-5p,miR-2411-5p,novel_79,miR-429-3p,miR-6517,miR-134-5p,miR-191-5p,miR-486b-3p,miR-2332,miR-410-5p,miR-181c-3p,miR-1306-5p,miR-671-5p,miR-7144-5p,miR-2318,miR-345-5p,miR-10b-3p,miR-3154,miR-137-3p,miR-503-3p,miR-30e-5p,miR-769,miR-215-5p,miR-1271,miR-141-3p,miR-370-3p,miR-2285aa,miR-323b-3p,miR-1306-5p,miR-30c-1-3p,miR-33a-5p,miR-1839-3p,miR-1388-3p,miR-3120-5p,miR-2426,miR-450b-5p,miR-544a,miR-101a-5p,miR-1388-5p,miR-323b,miR-1a-3p,miR-10b,miR-143-5p,miR-6529a,miR-1185-3p,miR-148b-5p,miR-455-3p,miR-181b-2-3p,miR-5010-3p,novel_78,novel_4,miR-330-3p,miR-769-5p,miR-2440,miR-30b-5p,miR-219a-2-3p,miR-181d-5p,miR-3059-5p,miR-504,miR-147-3p,miR-181a-3p,miR-1185-2-3p,miR-2483-3p,miR-3082-5p,miR-20a-3p,miR-4792,miR-21-3p,miR-30d,miR-466i-5p,miR-345-3p,miR-30b,miR-486-3p,miR-2411,miR-2424,miR-125b-2-3p,miR-181b-1-3p,miR-376a-5p,miR-345-3p,miR-329a-5p,miR-378d,miR-16-1-3p,novel_73,miR-140-3p,miR-1197-5p,miR-188-
CRCP	ENSOARG0(	CGRP rec	miR-455-3p,miR-769,miR-7857-3p,miR-2355-5p,miR-874-3p,miR-140-5p,miR-18a-3p,let-7a-2-3p,let-7g-3p,miR-299a-5p,miR-299b-5p,miR-129b-5p,miR-188-3p,miR-18a-3p,miR-197-3p,miR-299,let-7a-2-3p,miR-769-5p,miR-181a-2-3p,novel_127,miR-769-5p,novel_17
PHYHIP	ENSOARG0(	phytanoy	miR-199b-5p,miR-3154,miR-132-5p,miR-6130,miR-199a-5p,miR-6535,miR-542-5p,miR-296-3p,miR-17-3p,miR-4492,miR-361-3p,miR-3184-5p,miR-423-5p,miR-199b-5p,miR-4510,miR-17-3p,miR-542-5p,miR-653,miR-296-5p,miR-1343-5p,miR-5703,miR-133b-5p,miR-377-5p,miR-17-3p,miR-2898,miR-199b-5p,miR-1343-5p,miR-542-5p,miR-542-5p,miR-92a-1-5p,miR-185-3p,miR-377-5p,miR-199a-5p,miR-363-5p
MYL12B	ENSOARG0(	myosin,	miR-2284l,miR-3074-2-3p,miR-320d,miR-147-5p,miR-320b,miR-2285af,miR-92a-1-5p,miR-6516-5p,miR-6516,miR-320c,miR-296-3p,miR-2285aa,miR-450b-5p,miR-4429,miR-300,miR-493-3p,miR-625-5p,miR-381-3p,miR-320a,miR-381-3p

PANX3	ENSOARG0(	pannexin	miR-29b-1-5p, miR-505, miR-326, miR-1306-5p, miR-103a-2-5p, let-7e, let-7a-5p, miR-8485, miR-3187-3p, miR-2310, miR-30b-3p, miR-1839-5p, miR-3120-5p, miR-1388-5p, miR-450b-5p, miR-27a-3p, miR-145b, novel_78, miR-330-3p, miR-151a-3p, miR-216a-3p, miR-500-5p, miR-2285e, miR-539-5p, miR-142-3p, miR-107-5p, miR-218-5p, miR-450a-5p, miR-2285f, miR-3059-5p, miR-219a-2-3p, miR-154a, miR-361-3p, miR-362-5p, miR-378j, miR-409-5p, miR-335-3p, miR-877-3p, miR-2285u, miR-499b-5p, miR-211-5p, miR-664b, miR-2319b, let-7d-5p, miR-27a-3p, miR-7857-3p, miR-196a-3p, miR-26a-5p, miR-1277-5p, let-7k, miR-202-5p, miR-98-5p, miR-2285w, miR-380-3p, miR-29b-1-5p, miR-142a-3p, miR-130a-5p, miR-5703, miR-188-3p, miR-1246, miR-1961, miR-16-1-3p, miR-378d, miR-142-3p, miR-33b-3p, miR-3600, miR-485-3p, miR-548e-3p, miR-2284h-5p, miR-383-5p, miR-487a-3p, miR-219b-3p, miR-151-3p, miR-30a-3p, miR-338-5p, miR-29b-2-5p, let-7f, miR-208b-5p, miR-708-5p, miR-129-5p, novel_74, miR-6516-3p, miR-425-5p, miR-3074-2-3p, let-7b, let-7b-5p, miR-346, miR-2285c, novel_99, miR-2312, miR-345-5p, miR-3065-3p, miR-532-5p, miR-296-3p, miR-548o-3p, miR-22-3p, let-7i-5p, miR-6516, miR-193b-3p, miR-330-3p, miR-452-5p, miR-193a-3p, miR-378a-5p, miR-483-3p, miR-196b-5p, miR-2483-5p, let-7f-2-3p, let-7f-2-3p, miR-6240, miR-345-5p, let-7a-2-3p, miR-2285r, miR-654-3p, miR-432-3p, miR-22-3p, miR-145a-5p, miR-708-3p, miR-27b-3p, miR-30b-3p, miR-380-3p, miR-16-1-3p, miR-31-5p, miR-187-3p, miR-6238, miR-194-3p, miR-411-5p, miR-2355-3p, miR-2285g, miR-185-5p, let-7g-5p, miR-3431, let-7i, miR-502-5p, miR-22-5p, miR-625-3p, miR-193b-3p, miR-135a-2-3p, miR-326-3p, miR-2284w, miR-211, miR-1306, miR-150-5p, miR-376b-5p, miR-2285ab, miR-135b-5p, let-7e-5p, miR-26b-5p, miR-3120-3p, miR-216b-3p, miR-144-3p, miR-669, miR-374c-3p, miR-3071-3p, let-7c-5p, miR-664-3p, miR-219-

GHITM	ENSOARG0(	growth h	miR-194-3p,let-7g-5p,miR-2285g,miR-377-5p,miR-187-3p,miR-340-3p,miR-188-5p,miR-497-5p,miR-16-1-3p,miR-16b,miR-374b-3p,miR-145a-5p,miR-7b-5p,miR-1434-3p,miR-345-5p,miR-539-3p,miR-493-5p,miR-196b-5p,miR-146a,miR-7-5p,miR-320c,miR-374c-3p,miR-146b-3p,miR-2404,miR-7-1-3p,miR-26b-5p,miR-329-5p,miR-3074-5p,miR-195a-5p,let-7e-5p,miR-146b-5p,miR-2285k,miR-18a-3p,miR-3065-5p,miR-2285ab,miR-199b-5p,miR-24-3p,miR-211,miR-495-3p,miR-17-3p,miR-3957-3p,miR-1839-3p,novel_1,miR-6119-3p,novel_87,let-7i,miR-2284o,miR-16a,miR-15a,miR-486-5p,miR-758-5p,miR-505-5p,miR-322-5p,miR-431,miR-1224-5p,miR-196a-2-3p,miR-2285ad,miR-496,miR-582-3p,let-7d,miR-196a-5p,miR-615-3p,miR-2285b,miR-1271-3p,miR-1957a,let-7g,miR-199a-5p,miR-148b-5p,miR-4286,miR-2284f,miR-2385-3p,novel_127,let-7f-5p,miR-17-3p,let-7c-5p,miR-30f,miR-125a-3p,miR-2285af,miR-199a-5p,miR-365a-3p,miR-374c-3p,miR-300-3p,miR-215-5p,miR-874-3p,miR-503-3p,miR-487a-3p,miR-1248,miR-1814c,miR-7144-5p,miR-345-5p,miR-876-3p,miR-2474,miR-320a,miR-503-3p,miR-16-5p,miR-204-5p,miR-592,miR-28-5p,novel_9,miR-26c,miR-100-3p,miR-2285e,miR-148b-5p,miR-455-3p,miR-147a,miR-145b,miR-409b,miR-2426,miR-541,miR-484,miR-2310,miR-652-5p,miR-33a-5p,miR-424-5p,miR-1839-3p,miR-494-3p,let-7a-5p,miR-214-3p,let-7e,miR-146b,miR-199b-5p,miR-487b-5p,novel_124,miR-16b,miR-2957,miR-431-5p,miR-320b,let-7d-5p,miR-3082-5p,miR-211-5p,miR-7689-3p,miR-2285u,miR-544-5p,miR-493-5p,miR-668-5p,miR-136-5p,miR-129b-5p,miR-199b-5p,miR-154a,miR-1285-5p,novel_111,miR-2285f,miR-541-3p,miR-885-5p,miR-154-3p,miR-499a-5p,miR-3968,miR-615,miR-218-5p,miR-2285n,miR-15a-5p,miR-222-5p,miR-487a-3p,miR-485-3p,miR-18a-3p,miR-133a-3p,miR-16-1-3p,miR-218-1-3p,miR-

WWC3	ENSOARGO(	WWC fami	miR-2411,miR-486-3p,miR-377-5p,miR-29b-1-5p,miR-216c-5p,miR-329a-5p,miR-299-3p,miR-760-3p,miR-92a-1-5p,miR-500a-5p,miR-2285p,miR-210-5p,miR-196a-3p,miR-1247-5p,novel_83,miR-340-5p,miR-148b-3p,miR-1b-3p,miR-378d,miR-2284u,miR-218-1-3p,miR-16-1-3p,miR-5703,miR-217-5p,miR-188-3p,miR-124-3p,miR-362-3p,miR-224-5p,miR-6525,miR-145a-3p,miR-3074-5p,miR-669a-3p,miR-454-3p,miR-1468-5p,miR-6516-3p,miR-450b-3p,miR-338-5p,miR-665,miR-204-3p,miR-139-5p,miR-301a-3p,miR-582-5p,miR-2284s,miR-182-5p,miR-548o-3p,miR-504-5p,miR-582-5p,novel_99,miR-186-5p,miR-450a-1-3p,miR-21a-3p,miR-299,miR-1b-5p,miR-758-3p,miR-8485,miR-124a,miR-1468,miR-103a-2-5p,miR-487b-5p,miR-130b-3p,miR-3955-3p,miR-182-5p,miR-29b-1-5p,miR-362-3p,novel_103,miR-1185-3p,miR-2459,miR-455-3p,miR-1298-3p,miR-539-5p,miR-105-1,novel_78,miR-409b,miR-101c,miR-362-3p,miR-758-3p,miR-2284r,miR-329-3p,miR-484,miR-7705,miR-9-5p,miR-212-3p,miR-2447,miR-2284q,miR-2285t,miR-499a-5p,miR-107-5p,miR-382-3p,miR-1185-2-3p,miR-3082-5p,miR-2483-3p,miR-2319b,miR-10b,miR-139-5p,miR-2284g,miR-493-5p,miR-504,miR-361-3p,miR-140-5p,miR-2284a,miR-2285b,miR-2284y,miR-28b,miR-495-5p,miR-3529-3p,miR-152-3p,miR-200a-3p,miR-217,miR-214-5p,miR-450a-1-3p,miR-2411-5p,miR-9-5p,miR-299a-3p,miR-2285ad,miR-105-2,miR-128-3p,miR-7134-5p,miR-1290,miR-486b-3p,miR-331-3p,miR-2332,miR-133c,miR-374c-3p,miR-141-3p,miR-2355-5p,miR-370-3p,miR-671-5p,miR-329a,miR-3154,miR-488-3p,miR-503-3p,miR-432-3p,miR-2285r,miR-493-5p,miR-105-5p,miR-224-5p,miR-3431,miR-377-5p,miR-130a-3p,miR-31-5p,miR-188-5p,miR-2284x,miR-219a-1-3p,miR-16-1-3p,miR-450c-3p,miR-1434-3p,miR-542-3p,miR-301b-3p,miR-24-3p,miR-2285x,novel_101,miR-135a-2-3p,miR-656-5p,miR-22-
ATF6	ENSOARGO(	activati	novel_68,miR-489,miR-3959-5p,miR-2483-3p,miR-199a-5p,miR-154b-5p,miR-21a-3p,miR-199b-5p,miR-137-3p,miR-23b,miR-412-3p,miR-146b-5p,miR-544-5p,miR-1827,miR-1983,miR-539-5p,miR-3059-5p,miR-193a-5p,miR-214,miR-194b-5p,miR-9-5p,miR-877-3p,novel_27,miR-326-3p,miR-145-3p,miR-199b-5p,miR-2440,miR-133a-5p,miR-331-3p,miR-147-5p,miR-6517,miR-3600,miR-23b-3p,miR-9-5p,miR-503-5p,miR-23c,miR-330-5p,miR-3071-5p,miR-1193,miR-194-5p,miR-218-1-3p,miR-2284x,miR-503-5p,miR-23a-3p,miR-628-5p,miR-146a-5p,miR-199a-5p,miR-1271-3p,miR-503-5p,miR-618,miR-125a-3p,miR-326,miR-146a,miR-6119-5p,miR-185-3p,miR-194a,miR-450a-2-3p,miR-760-3p,miR-199b-5p,miR-146b,miR-452-5p,miR-214-3p,miR-21-3p

CCDC22	ENSOARG0(	coiled-c	miR-505-5p,miR-9-5p,miR-129-1-3p,miR-486-5p,miR-450a-1-3p,miR-299a-3p,miR-2285ad,novel_79,miR-412,miR-1224-5p,miR-155-5p,miR-23b-5p,miR-330-5p,miR-200a,novel_39,miR-3064-5p,miR-152-3p,miR-200a-3p,miR-125a-3p,miR-205-5p,miR-331-5p,miR-874-3p,miR-3956,miR-125a,miR-2355-5p,miR-329a,miR-2474,miR-1248,miR-20b-5p,miR-93-5p,miR-1197-3p,miR-1260b,novel_42,novel_63,miR-2433,miR-125b-5p,miR-6740-5p,miR-3431,miR-134-3p,miR-412-5p,miR-194-3p,miR-340-3p,miR-339-5p,miR-188-5p,miR-27b-3p,miR-7b-5p,miR-1843b-5p,miR-2113,miR-223-3p,miR-432,miR-450c-3p,miR-432-3p,miR-6240,miR-483-3p,miR-378a-5p,miR-377-3p,miR-7-5p,miR-2411-3p,novel_51,miR-299b-3p,novel_69,miR-181b-3p,miR-677,miR-3074-5p,miR-3120-3p,miR-412-3p,miR-378f,miR-148a-3p,miR-1306,miR-199b-5p,miR-335-5p,miR-6119-3p,miR-6123,miR-576-3p,miR-4324,miR-7859,miR-328-3p,miR-106a,miR-493-3p,miR-34a-5p,miR-218-1-3p,miR-2366,miR-378i,miR-217-5p,miR-378a-3p,miR-181b-1-3p,miR-377-5p,miR-486-3p,miR-503-5p,miR-487a,miR-345-3p,miR-202-5p,miR-618,miR-210-5p,miR-422a,miR-122-3p,miR-345-3p,miR-92a-1-5p,miR-324-3p,miR-216b-5p,miR-377-3p,miR-10a,miR-2284s,miR-1277-3p,miR-30c-1-3p,miR-504-5p,miR-2331-3p,miR-552-3p,miR-154a-3p,miR-339b,miR-1827,miR-450a-1-3p,miR-2285c,miR-299,miR-433-3p,miR-598-3p,miR-769-5p,miR-3184-5p,miR-129-2-3p,miR-3074-5p,miR-214,miR-4492,miR-874-5p,miR-665,miR-147a,miR-2459,miR-421,miR-455-3p,miR-1343-3p,novel_78,miR-330-3p,miR-181b-2-3p,miR-362-3p,miR-541,miR-301b-5p,miR-2284r,miR-329-3p,miR-378d,miR-8485,miR-214-3p,miR-199b-5p,miR-106b-5p,miR-505,miR-29b-1-5p,miR-378g,miR-3596,miR-4443,miR-2319b,miR-4792,miR-211-5p,miR-504,miR-1983,miR-140-5p,novel_111,miR-877-3p,miR-361-3p,miR-423-
SUCLG2	ENSOARG0(	succinat	miR-216a-3p,miR-105-1,miR-151a-3p,miR-7641,miR-27a-3p,miR-105-2,miR-128-3p,miR-188-5p,miR-378d,miR-27b-3p,miR-3120-5p,miR-8485,miR-200a,miR-345-5p,miR-466f-3p,miR-2285p,miR-105-5p,miR-182-5p,miR-27a-3p,miR-4792,miR-370-5p,miR-182-5p,miR-345-5p,miR-345-5p,miR-136-5p,miR-668-5p,miR-216a-3p,miR-323c,miR-1193,miR-370-5p,miR-151-3p,miR-382-5p

LEMD2	ENSOARG0(	LEM doma	miR-4443,miR-323a-5p,miR-378g,miR-2285u,miR-7689-3p,miR-3064-5p,miR-6395,miR-1306,miR-361-3p,miR-3141,miR-326-3p,miR-423-5p,miR-2428,miR-24-3p,miR-193b-3p,miR-1193,miR-342-3p,miR-541-3p,miR-7134-3p,miR-147-5p,miR-4726-5p,miR-769-5p,miR-107-5p,miR-502-5p,novel_116,miR-149-5p,miR-2459,miR-134-3p,miR-340-3p,miR-187-3p,novel_4,miR-1343-3p,miR-744-5p,miR-7862,miR-2889,miR-3432b,miR-22-3p,miR-873a-5p,miR-484,miR-541,miR-4532,miR-432-3p,miR-2483-5p,miR-574-3p,miR-124a,miR-130b-5p,miR-125a-5p,miR-103a-2-5p,miR-193a-3p,miR-3085-3p,miR-483-3p,miR-505,miR-197-5p,miR-3141,novel_51,miR-342,miR-326,miR-1306-5p,miR-22-3p,novel_32,miR-323-5p,miR-193b-3p,miR-769,miR-6130,miR-3065-3p,miR-216b-3p,miR-2355-5p,miR-370-5p,miR-532-5p,miR-1271,miR-141-3p,miR-3956,miR-125a,miR-140-5p,miR-3068-3p,miR-503-3p,miR-23a-5p,miR-885-3p,miR-7144-5p,miR-1827,miR-1248,miR-2474,miR-137-3p,miR-503-3p,miR-3184-5p,miR-769-5p,miR-3074-2-3p,miR-2330-3p,miR-1306-5p,miR-129-2-3p,miR-4510,miR-874-5p,miR-486b-3p,miR-130b-5p,miR-331-3p,miR-125b-5p,miR-6740-5p,miR-3604,miR-2285j,miR-2433,miR-370-5p,miR-328-3p,miR-129-1-3p,miR-6516-5p,miR-483-3p,miR-340-5p,miR-3600,miR-1224-5p,miR-3960,miR-378d,miR-885-3p,miR-493-3p,novel_73,miR-124-3p,miR-2904,miR-330-5p,miR-23b-5p,miR-3065-3p,miR-486-3p,miR-3064-5p,miR-200a-3p,miR-191-3p,miR-324-3p,miR-125b

PSMD11	ENSOARG0(	proteaso	miR-2284q,miR-7134-3p,miR-4726-5p,miR-2285y,miR-769-5p,miR-1973,miR-2285n,miR-502-3p,miR-362-5p,miR-452-5p,miR-2285f,miR-541-3p,miR-212-3p,novel_60,novel_94,miR-877-3p,miR-129b-5p,miR-361-3p,miR-378e,miR-7857-3p,miR-7977,miR-452-3p,miR-3064-5p,miR-3085-3p,miR-362-3p,miR-5126,miR-320b,miR-758-3p,miR-4532,miR-3187-3p,miR-652-5p,miR-494-3p,miR-424-5p,miR-3120-5p,miR-2284aa,miR-2426,miR-378c,miR-30b-3p,miR-412-3p,miR-378b,miR-484,miR-296-5p,miR-2285e,miR-500-5p,miR-221-3p,miR-216a-3p,miR-142-3p,miR-2300a-5p,miR-424-5p,miR-664a,miR-320e,miR-324-3p,miR-222-3p,miR-3074-2-3p,miR-145a-3p,miR-4510,miR-378c,novel_99,miR-2312,miR-22-3p,novel_32,miR-204-3p,miR-6130,miR-323-5p,miR-193b-3p,miR-21-3p,miR-378a-3p,miR-21-3p,miR-30b-3p,miR-760-3p,miR-320d,miR-125b,miR-216a-5p,miR-146a-5p,miR-2432,miR-29b-1-5p,miR-216c-5p,miR-941,miR-4429,miR-188-3p,miR-1197-5p,miR-1193,miR-2408,miR-362-3p,novel_133,miR-532-3p,miR-15a-5p,miR-200b,miR-383-5p,miR-3600,miR-3960,miR-548e-3p,miR-502b,miR-147-5p,miR-33a-3p,miR-16a,miR-133a-5p,miR-502-5p,miR-15a,miR-2428,novel_27,miR-24-3p,novel_101,miR-3957-3p,miR-6395,miR-195a-5p,miR-107,miR-1291,miR-21c,miR-18a-3p,miR-378b,miR-2387,miR-2285ab,miR-323a-5p,miR-2404,miR-378b,miR-125a-5p,miR-330-3p,miR-382,miR-7862,miR-1260b,miR-138,miR-30b-3p,miR-21-5p,miR-3071-5p,miR-145a-5p,miR-103,miR-185-5p,miR-2285g,miR-377-5p,miR-486b-3p,miR-138-5p,miR-331-3p,miR-1306-3p,miR-378c,miR-500b-5p,miR-6128,miR-23a-5p,miR-7144-5p,novel_48,miR-10b-3p,miR-320a,miR-362-5p,miR-154b-5p,miR-3965,miR-365a-3p,miR-769,miR-374c-3p,miR-141-3p,miR-107,miR-200c-3p,miR-450a-2-3p,miR-2898,miR-6119-5p,miR-200c,miR-1260a,miR-490-3p,miR-28b,miR-2478,miR-

CTR9	ENSOARG0(	CTR9 hom	miR-144,miR-331-3p,miR-29b-3p,miR-486b-3p,miR-204-5p,miR-410-3p,miR-767-5p,miR-3607-3p,miR-93,miR-17-5p,miR-3154,miR-503-3p,miR-3074-1-3p,miR-7144-5p,miR-10b-3p,miR-370-3p,miR-365a-3p,miR-34c-5p,miR-339a,miR-200c,miR-543-5p,miR-200c-3p,miR-450a-2-3p,miR-17-3p,miR-4286,miR-181a-2-3p,miR-1271-3p,miR-2284a,miR-30a-5p,miR-29d-3p,miR-134,miR-10a-5p,miR-200b-3p,miR-582-3p,miR-7641,miR-222,miR-214-5p,miR-1271-5p,miR-33a-3p,miR-34c,miR-16a,miR-20a-5p,miR-15a,miR-199c,miR-147-5p,miR-656-5p,miR-128-1-5p,miR-17-3p,novel_101,miR-219b-3p,miR-342-3p,miR-449a,miR-211,miR-18a-3p,miR-378b,miR-2285ab,miR-195a-5p,miR-505-3p,miR-19a-3p,miR-1291,miR-10a-5p,miR-670-3p,miR-29a-3p,miR-34b,miR-17-5p,miR-106a-5p,miR-330-3p,miR-1343-5p,miR-125a-5p,miR-2483-5p,miR-130b-5p,miR-345-5p,miR-1285,miR-653,miR-6239,miR-29b,miR-26b-3p,miR-665,miR-122-3p,miR-20b,miR-2285g,miR-338-5p,miR-320e,miR-132-5p,miR-424-5p,miR-17-3p,miR-6516-3p,miR-155-5p,miR-425-5p,miR-3074-2-3p,miR-1271,miR-145a-3p,miR-4510,miR-338-3p,miR-10b-5p,miR-27b-5p,let-7f-1-3p,miR-30f,miR-2312,let-7g-3p,miR-532-5p,miR-21-3p,novel_25,miR-6130,miR-193b-3p,miR-125b,miR-185-3p,miR-216a-5p,miR-299-3p,miR-21-3p,miR-30d,miR-34b-5p,miR-1277-5p,miR-466i-5p,miR-106a,miR-20a,miR-2432,miR-1197-5p,miR-2904,miR-376a-5p,miR-378d,let-7a-3p,miR-19b-3p,miR-505-3p,miR-3600,miR-548e-3p,miR-199a-3p,miR-200b,miR-15a-5p,miR-107-5p,miR-2285y,miR-30b-5p,miR-2284q,miR-335,miR-3958-3p,miR-452-5p,miR-2285f,miR-212-3p,miR-154a,miR-219a-2-3p,miR-129b-5p,miR-361-3p,miR-7977,miR-499b-5p,miR-10b,miR-664b,miR-7857-3p,miR-1306-5p,miR-342,miR-494-3p,miR-424-5p,miR-106a-5p,miR-758-3p,miR-412-3p,miR-10b,miR-484,miR-296-5p,miR-3120-5p,novel_4,miR-2285o,miR-

CD164	ENSOARG0(	CD164 mo	miR-2284y,miR-29a-5p,miR-2284a,miR-2285b,miR-1,miR-30a-3p,miR-29b-2-5p,miR-205-5p,miR-2285af,miR-6119-5p,miR-30c-2-3p,miR-322-5p,miR-1271-5p,miR-545-5p,miR-134-5p,miR-582-3p,novel_121,miR-222,miR-2285ad,miR-299a-3p,miR-423-3p,miR-18b-5p,miR-345-3p,miR-134,miR-3578,miR-2284v,miR-6128,miR-16-5p,miR-767-5p,miR-592,miR-134-5p,miR-34c-3p,miR-2284k,miR-1-5p,miR-99b-3p,miR-331-5p,miR-223-5p,miR-2284n,miR-99a-3p,miR-215-5p,miR-767,miR-18a,miR-6240,miR-2411-3p,miR-2285g,miR-2284d,miR-133b-5p,miR-377-5p,miR-26b-3p,miR-2284ab,miR-380-3p,miR-16b,miR-16-1-3p,miR-497-5p,miR-7862,miR-1434-3p,miR-3071-5p,novel_27,miR-1a-1-5p,miR-495-3p,miR-6119-3p,miR-206,miR-502b,miR-502-5p,miR-15a,miR-16a,miR-33a-3p,miR-299b-3p,miR-7-1-3p,miR-410-5p,miR-9851-3p,miR-195a-5p,miR-18b,miR-3970,miR-377-5p,miR-183-5p,miR-2285w,miR-345-3p,miR-2284m,miR-99a-3p,miR-3653-3p,miR-21-3p,miR-299-3p,miR-2285p,miR-345-3p,miR-23b-3p,miR-15a-5p,miR-2284h-5p,miR-30d-3p,miR-16-1-3p,miR-2284u,miR-215-5p,miR-124-3p,miR-188-3p,miR-142a-5p,miR-206-3p,miR-590-3p,miR-3958-5p,miR-1271,miR-3074-2-3p,miR-664a,miR-424-5p,miR-2285j,miR-222-3p,miR-30e-3p,miR-30a-3p,miR-29b-2-5p,miR-582-5p,miR-192-5p,miR-15b-5p,miR-30c-1-3p,miR-18a-5p,miR-2285c,miR-30c-1-3p,miR-124a,miR-424-5p,miR-23a-3p,miR-2284b,novel_103,miR-16b,miR-147a,miR-142-5p,miR-9788-3p,miR-221-3p,miR-2285e,miR-421,miR-299a-5p,miR-101c,miR-1a-3p,novel_111,miR-2284j,miR-2284q,miR-2285y,miR-2483-3p,miR-203-3p,miR-2284g,miR-2319b,miR-96-5p,miR-335-3p,novel_94,miR-221,miR-378j
ATF3	ENSOARG0(	activati	let-7g-3p,let-7g-3p,miR-1827,miR-548o-3p,miR-32-5p,miR-378a-3p,miR-1271,miR-215-5p,miR-2355-5p,miR-370-3p,miR-25-3p,miR-6130,miR-192-5p,miR-544-3p,miR-374c-3p,miR-154b-5p,miR-5100,miR-135a-5p,miR-320e,miR-324-3p,miR-874-5p,miR-4510,miR-129-2-3p,miR-378c,miR-1271,miR-1260b,miR-224-5p,miR-6134,miR-378c,miR-215-5p,miR-378i,miR-378a-3p,miR-218-2-3p,miR-142-3p,let-7a-2-3p,miR-548e-3p,miR-1224-5p,miR-3600,miR-1271-5p,miR-222-5p,miR-378h,miR-7859,miR-486-5p,miR-129-1-3p,miR-2284z,miR-532-3p,miR-8117,miR-1260a,miR-422a,miR-2898,miR-92a-3p,miR-105-3p,miR-503-5p,miR-181b-1-3p,miR-450b-5p,miR-92b-3p,miR-96-5p,miR-135b-5p,miR-378f,miR-3120-3p,miR-374c-3p,miR-378e,miR-181b-3p,miR-4443,miR-677,novel_82,miR-4324,miR-92a-3p,miR-6123,miR-490-5p,miR-3956-3p,miR-363-3p,novel_60,miR-361-3p,miR-542-3p,miR-296-5p,miR-378b,miR-450b-5p,miR-544a,miR-1260b,miR-2426,miR-378c,miR-2284aa,novel_78,miR-3955-5p,miR-181b-2-3p,miR-503-5p,miR-544b,miR-2459,novel_116,miR-216a-3p,miR-503-5p,miR-224-5p,miR-32,miR-378a-5p,miR-452-5p,miR-153-3p,miR-432-3p,miR-378d,let-7a-2-3p,miR-3187-3p
FAM160B2	ENSOARG0(	family w	miR-877-5p,miR-9788-3p,miR-216a-3p,miR-29a-3p,miR-505-5p,miR-552-3p,miR-3065-3p,miR-29b,miR-29d-3p,miR-2387,miR-3065-3p,miR-486-3p,miR-29c-3p,miR-486b-3p,miR-29a,miR-378a-5p,miR-29b-3p

C2CD2	ENSOARG0(	C2 calci	miR-450a-1-3p,miR-328-3p,miR-450c-3p,miR-3141,miR-450a-1-3p,miR-6395,miR-3963,miR-1291,miR-450b-5p,miR-2426,miR-3957-3p,miR-122-5p,miR-2285r,miR-432-3p,miR-3141,miR-331-3p,miR-92a-1-5p,miR-2285n,miR-543-5p,miR-2898,miR-450b-3p,miR-124-5p
SIAE	ENSOARG0(	sialic a	miR-331-5p,miR-376c-3p,miR-542-5p,miR-2355-5p,miR-30e-5p,miR-301,miR-23b,miR-1197-3p,let-7e-3p,miR-2284v,novel_9,miR-505-5p,miR-2284z,miR-429-3p,miR-30c,miR-196a-5p,miR-1343-5p,miR-450b-5p,miR-148b-5p,novel_39,miR-2284y,miR-200a,miR-3064-5p,miR-200a-3p,miR-152-3p,novel_127,miR-664-3p,miR-144-3p,miR-677,miR-181b-3p,miR-3074-5p,miR-410-5p,miR-146b-5p,miR-378f,miR-148a-3p,miR-150-5p,miR-1306,miR-216a-3p,miR-3591-3p,miR-301b-3p,miR-335-5p,miR-6119-3p,miR-206,miR-379-3p,miR-1247-3p,miR-208a-3p,miR-412-5p,miR-3535,miR-3431,novel_116,miR-16-1-3p,miR-27b-3p,miR-542-3p,novel_23,let-7a-2-3p,miR-503-3p,miR-6240,miR-668-3p,miR-196b-5p,miR-106b-3p,miR-146a,miR-365b-5p,miR-105-5p,miR-194a,miR-301a-3p,miR-376e-3p,miR-216b-5p,miR-2331-3p,miR-216b-3p,miR-625-5p,miR-1827,miR-299,miR-129b-3p,miR-411,miR-30d-5p,miR-6525,miR-224-5p,miR-3074-5p,miR-3184-3p,miR-3958-5p,novel_74,miR-190a-5p,miR-454-3p,miR-130b-5p,miR-30e-3p,miR-30a-3p,miR-151-3p,miR-3604,miR-664a-5p,miR-23b-3p,miR-7859,miR-23c,miR-18a-3p,miR-30d-3p,miR-493-3p,miR-33b-3p,miR-16-1-3p,miR-361-5p,miR-378i,miR-30c-5p,miR-1434-5p,miR-378a-3p,miR-664b-3p,miR-376a-5p,miR-376c-3p,miR-486-3p,miR-2285p,miR-208b-3p,miR-500a-5p,miR-422a,miR-25,miR-139-5p,miR-2284g,miR-335-3p,miR-140-5p,miR-378j,novel_111,miR-2447,miR-499a-5p,miR-3964,miR-218-5p,miR-153,miR-147a,miR-455-3p,miR-421,miR-2459,miR-199b-3p,miR-330-3p,miR-1343-3p,novel_78,miR-1388-5p,miR-1839-5p,miR-1a-3p,miR-378d,miR-2310,miR-1388-3p,miR-23a-3p,miR-487b-5p,miR-130b-3p,miR-146b,miR-153-3p,miR-103a-2-5p,miR-199a-3p,miR-365a-3p,miR-3965,miR-370-3p,miR-141-3p,let-7g-3p,novel_48,miR-7144-5p,miR-1814c,miR-488-3p,miR-362-

HIF3A	ENSOARG0(	hypoxia	miR-6525,miR-206-3p,let-7b-5p,miR-6134,miR-433-5p,let-7b,miR-129-2-3p,miR-3184-3p,miR-3074-5p,miR-4510,miR-214,miR-665-5p,miR-3074-2-3p,miR-450b-3p,miR-6516-3p,miR-17-3p,miR-664a,miR-190a-5p,miR-130b-5p,miR-2285j,let-7f,miR-151-3p,miR-320e,miR-3604,miR-655-3p,miR-377-3p,miR-376e-3p,miR-216b-5p,miR-323-5p,miR-6130,let-7i-5p,miR-140-5p,miR-30c-1-3p,miR-1277-3p,miR-370-5p,miR-21-3p,miR-296-3p,miR-1827,miR-450a-1-3p,miR-129b-3p,miR-655,miR-376a-5p,miR-377-5p,miR-146a-5p,miR-2411,miR-2432,miR-2424,miR-143-3p,miR-345-3p,miR-320d,miR-98-5p,let-7k,miR-26a-5p,miR-125b,miR-92a-1-5p,miR-185-3p,miR-210-3p,miR-383-5p,miR-148b-3p,novel_83,miR-380-5p,miR-505-3p,miR-212-5p,miR-4429,miR-33b-3p,miR-664-5p,miR-148a-5p,miR-2366,miR-1961,miR-2904,miR-188-3p,miR-5703,miR-140-3p,miR-361-3p,miR-154a,miR-9-5p,miR-190b-5p,miR-4726-5p,miR-3968,miR-101-3p,miR-3596,miR-378g,miR-489,let-7d-5p,miR-4443,miR-147-3p,miR-361-3p,miR-140-5p,miR-378j,miR-1306-3p,miR-30c-1-3p,miR-8485,let-7a-5p,miR-1839-3p,miR-494-3p,miR-652-5p,miR-491-5p,miR-487b-5p,miR-146b,let-7e,miR-214-3p,miR-342,miR-320b,novel_103,miR-5126,miR-2957,miR-148b-5p,miR-9788-3p,novel_78,novel_4,miR-1343-3p,miR-151a-3p,miR-101c,miR-6529a,miR-2284r,miR-4791,miR-143-5p,miR-1a-3p,miR-30b-3p,miR-125b-2-3p,miR-6128,novel_9,miR-767-5p,miR-2433,miR-551b-3p,miR-29b-3p,miR-1306-3p,miR-370-5p,miR-5100,miR-125b-5p,miR-100-3p,miR-409-3p,miR-300-3p,miR-376c-3p,miR-154b-5p,miR-574-5p,miR-3068-3p,miR-2355-5p,miR-370-3p,miR-125a,miR-767,miR-876-3p,miR-10b-3p,miR-329b,miR-23a-5p,miR-671-5p,miR-485-5p,miR-424-3p,miR-2448-3p,miR-320a,miR-380-5p,let-7g,miR-376c-5p,miR-1271-3p,miR-3529-3p,miR-2385-3p,miR-1,miR-17-3p,miR-450a-2-

MTMR4	ENSOARG0(	myotubul	miR-181b-1-3p,miR-376a-5p,novel_17,miR-210-5p,miR-125b,miR-185-3p,miR-324-3p,miR-2284m,miR-299-3p,miR-21-3p,miR-3653-3p,miR-3960,miR-548e-3p,miR-30d-3p,miR-3600,miR-15a-5p,miR-95-3p,miR-7859,miR-2300b-3p,miR-215-5p,miR-191,miR-2904,miR-33b-3p,miR-2284u,miR-148a-5p,miR-218-1-3p,miR-4510,miR-3074-2-3p,miR-346,miR-30e-3p,miR-2285j,miR-29b-2-5p,miR-665,miR-151-3p,miR-30a-3p,miR-487b-5p,novel_74,miR-454-3p,miR-424-5p,miR-548o-3p,miR-496-3p,miR-140-5p,miR-30c-1-3p,miR-6130,miR-301a-3p,miR-499b-3p,miR-192-5p,miR-15b-5p,miR-22-3p,novel_25,miR-487b-5p,miR-133b-3p,let-7g-3p,miR-2312,miR-1827,miR-885-3p,miR-424-5p,miR-1388-3p,miR-2284b,miR-30c-1-3p,miR-758-3p,miR-4532,miR-16b,miR-130b-3p,miR-330-3p,miR-151a-3p,miR-181b-2-3p,miR-27a-3p,miR-9788-3p,miR-148b-5p,miR-6529a,miR-758-3p,miR-2426,miR-3120-5p,miR-2284aa,miR-885-5p,miR-3956-3p,miR-3059-5p,novel_60,miR-2284j,miR-2285f,novel_111,miR-361-3p,miR-615,miR-335,miR-452-3p,miR-2284g,miR-2285u,miR-499a-3p,miR-27a-3p,miR-140-5p,miR-361-3p,miR-668-5p,miR-136-5p,miR-493-5p,miR-335-3p,miR-29b-2-5p,miR-30a-3p,miR-148b-5p,miR-2284a,miR-1271-3p,miR-192-3p,novel_39,miR-2284y,miR-6119-5p,miR-205-5p,miR-30c-2-3p,miR-2898,miR-107,miR-6517,miR-299a-3p,miR-322-5p,miR-214-5p,miR-545-5p,miR-2284z,miR-1290,miR-615-3p,miR-29d-3p,miR-885-3p,miR-496,miR-128-3p,miR-2284v,novel_42,miR-133a-3p,miR-16-5p,miR-494-5p,miR-2284k,miR-29b-3p,miR-125b-5p,miR-6740-5p,miR-331-5p,miR-138-5p,miR-191-5p,miR-503-3p,miR-215-5p,miR-125a,miR-2285aa,miR-331-5p,miR-133c,miR-424-3p,miR-331-5p,miR-488-3p,miR-137-3p,miR-10b-3p,miR-23a-5p,miR-6240,miR-493-5p,miR-668-3p,miR-654-3p,novel_51,miR-330-3p,miR-124-5p,miR-125a-5p,miR-103a-3p,miR-3955-5p,miR-

SHOC2	ENSOARG0(	SHOC2 le	miR-491-3p,let-7c-3p,miR-376b,miR-106a-5p,miR-424-5p,miR-494-3p,miR-1185-5p,miR-491-5p,miR-3955-3p,miR-505,miR-106b-5p,miR-16b,miR-182-5p,miR-539-5p,miR-147a,miR-199b-3p,miR-1839-5p,miR-6529a,miR-301b-5p,miR-1285-5p,miR-219a-2-3p,miR-181d-5p,miR-2284j,miR-4454,miR-3059-5p,miR-374a-3p,miR-301a-5p,miR-218-5p,miR-3596,miR-25,miR-489,miR-2285u,miR-499b-5p,miR-381-3p,miR-129b-5p,miR-664b-3p,miR-376a-5p,miR-125b-2-3p,miR-130a-5p,miR-20a,miR-1277-5p,miR-106a,miR-2284m,miR-466i-5p,miR-216a-5p,miR-500a-5p,miR-345-3p,miR-2285p,miR-328-3p,miR-7859,miR-200b,miR-15a-5p,miR-548e-3p,miR-106a,miR-664-5p,miR-2284u,miR-98-3p,miR-1193,miR-181c-5p,miR-6525,miR-98-3p,miR-2330-3p,miR-433-5p,miR-590-3p,miR-539-5p,miR-487a-5p,miR-208b-5p,miR-424-5p,miR-450b-3p,miR-6516-3p,miR-17-3p,miR-219b-3p,miR-2285j,miR-15b-5p,miR-656-3p,miR-216b-5p,miR-216b-3p,miR-182-5p,miR-660-5p,miR-543-3p,novel_99,miR-450a-1-3p,miR-296-3p,miR-668-3p,miR-382,miR-660,miR-466f-3p,miR-378a-5p,miR-17-5p,miR-106a-5p,miR-2411-3p,miR-296-3p,miR-20b,miR-2284d,miR-497-5p,miR-2284x,miR-188-5p,miR-16b,miR-450c-3p,miR-374b-3p,miR-542-3p,miR-181a-5p,miR-323c,miR-371b-3p,miR-17-3p,miR-2285x,miR-1a-1-5p,miR-199c,miR-6119-3p,miR-379-3p,miR-16a,miR-15a,miR-20a-5p,miR-142b,miR-133a-5p,novel_69,miR-2319a,miR-195a-5p,miR-6395,miR-21c,miR-3065-5p,miR-216a-3p,miR-200a,miR-29a-5p,miR-2284y,miR-192-3p,miR-181a-2-3p,novel_96,miR-17-3p,miR-181b-5p,miR-1a-2-5p,miR-200c-3p,miR-2285af,miR-200c,miR-219-3p,miR-582-3p,miR-545-5p,miR-450a-1-3p,miR-322-5p,miR-6516-5p,miR-429-3p,miR-128-3p,miR-200b-3p,miR-345-3p,miR-3578,miR-145-3p,miR-93,miR-374a-3p,miR-16-5p,miR-3607-3p,miR-374b-3p,miR-100-3p,miR-2284k,miR-1-5p,miR-365a-
MAPKAPK5	ENSOARG0(	mitogen-	miR-502b,miR-3969,miR-101-3p,miR-877-3p,miR-1271,novel_101,novel_48,miR-9851-3p,miR-186-5p,miR-96-5p,miR-10b-3p,miR-329a,miR-148a-3p,miR-146b-3p,miR-452-5p,miR-152-3p,miR-127-5p,miR-30f,miR-362-3p,miR-127-3p,miR-101c,miR-362-3p,miR-2426,miR-5703,miR-329-3p,miR-126-3p,miR-362-3p,miR-542-3p,miR-3578,novel_83,miR-24-2-5p,miR-1271-5p,miR-148b-3p

VSIG2	ENSOARG0(	V-set an	miR-217,miR-2285af,novel_96,miR-200a-3p,miR-152-3p,miR-107,miR-191-3p,miR-181b-5p,miR-4286,miR-3064-5p,miR-1,miR-2285b,miR-330-5p,miR-134,miR-18b-5p,miR-423-3p,miR-299a-3p,miR-486-5p,miR-450a-1-3p,miR-134-5p,miR-378h,miR-505-5p,miR-331-3p,miR-100-3p,miR-125b-5p,miR-6740-5p,miR-135a-1-3p,miR-2433,miR-134-5p,miR-126b-3p,novel_42,miR-378c,miR-18a,miR-3154,miR-23b,miR-1248,miR-485-5p,miR-23a-5p,miR-329a,miR-1271,miR-141-3p,miR-125a,miR-2355-5p,miR-542-5p,miR-370-3p,miR-324-5p,miR-365a-3p,novel_51,miR-197-5p,miR-296-3p,miR-106b-3p,miR-125a-5p,miR-124-5p,miR-296-3p,miR-432-3p,miR-3071-5p,miR-450c-3p,miR-1843b-5p,miR-181a-5p,miR-1434-3p,miR-873a-5p,miR-188-5p,miR-7862,miR-103a-3p,miR-187-3p,miR-103,miR-194-3p,novel_116,miR-185-5p,miR-134-3p,miR-3431,miR-6529b,miR-147-5p,novel_1,miR-206,miR-542-5p,miR-326-3p,miR-18b,miR-148a-3p,miR-2387,miR-378f,miR-6395,miR-412-3p,miR-107,miR-450b-3p,miR-1291,miR-320b,miR-299b-3p,miR-411b-3p,miR-125b,miR-185-3p,miR-422a,miR-500a-5p,miR-324-3p,novel_17,miR-542-5p,miR-210-5p,miR-299-3p,miR-466i-5p,miR-345-3p,miR-1197-5p,miR-378a-3p,miR-5703,miR-217-5p,miR-362-3p,miR-2366,miR-378i,novel_133,miR-133a-3p,miR-184-3p,miR-212-5p,miR-3960,miR-328-3p,novel_83,miR-151-5p,miR-23c,miR-148b-3p,miR-23b-3p,miR-383-5p,miR-665,miR-132-5p,miR-324-3p,miR-450b-3p,miR-214,miR-3184-3p,miR-4510,miR-378c,miR-433-5p,miR-338-3p,miR-6134,miR-181c-5p,miR-206-3p,miR-346,miR-433-3p,miR-450a-1-3p,miR-299,miR-1895,miR-625-5p,miR-18a-5p,miR-296-3p,miR-140-5p,miR-378a-3p,miR-504-5p,miR-877-5p,miR-151b,miR-204-3p,novel_32,miR-6130,miR-2284s,miR-362-3p,miR-505,miR-326,miR-5126,miR-103a-2-5p,miR-542-5p,miR-214-3p,miR-25-5p,miR-3085-3p,miR-491-
ARCN1	ENSOARG0(	archain	miR-204-5p,miR-592,miR-424-5p,miR-31-3p,miR-664a,miR-16-5p,miR-3074-5p,miR-1248,miR-7144-5p,let-7g-3p,miR-320a,miR-301,miR-488-3p,miR-15b-5p,miR-182-5p,miR-141-3p,miR-370-3p,miR-200a-3p,miR-320d,miR-210-5p,miR-2285af,miR-377-5p,miR-142a-3p,let-7a-2-3p,miR-582-3p,miR-4429,miR-23b-5p,miR-3578,miR-545-5p,miR-616-3p,miR-15a-5p,miR-322-5p,miR-107-5p,miR-16a,miR-6402,miR-15a,miR-502-5p,miR-211,miR-1983,miR-195a-5p,miR-9851-3p,miR-412-3p,miR-381-3p,miR-378b,miR-320c,miR-2404,miR-211-5p,miR-541-5p,miR-320b,miR-3074-5p,miR-16b,miR-455-5p,miR-182-5p,miR-320b,let-7a-2-3p,miR-424-5p,miR-2330-5p,miR-455-5p,miR-497-5p,miR-219a-1-3p,miR-16b,miR-1434-3p,miR-455-3p,miR-185-5p,miR-142-3p,miR-377-5p
CPD	ENSOARG0(	carboxyp	miR-548o-3p,miR-452-3p,miR-548e-3p,miR-30c-1-3p,miR-185-5p,miR-7859,miR-300-3p,miR-143-5p,miR-150-5p,miR-19a-3p,miR-19b-3p,miR-30c-1-3p,novel_17,miR-2285af,miR-30c-2-3p,miR-3964
CLDN34	ENSOARG0(	claudin	miR-299a-5p,miR-2284q,miR-2285af,miR-299,miR-2285p,miR-188-3p

YIPF4	ENSOARG0(	Yip1 dom	miR-6128, miR-194b-5p, miR-452-5p, miR-181d-5p, miR-142a-5p, miR-3959-3p, miR-7705, miR-181c-5p, miR-142b, miR-29b-2-5p, miR-203b-5p, miR-24-1-5p, miR-3120-3p, miR-877-5p, miR-140-5p, miR-2285w, miR-494-3p, miR-29b-2-5p, miR-24-2-5p, miR-421-5p, miR-2432, miR-618, miR-194a, miR-181b-5p, miR-548w, miR-3600, miR-142-5p, miR-9788-3p, miR-148b-5p, miR-2284z, miR-2284h-5p, miR-181a-5p, miR-194-5p, novel_23, miR-2426, miR-142-3p, miR-2284aa, miR-148a-5p
NBEA	ENSOARG0(	neurobea	miR-543-5p, miR-339a, miR-2285af, miR-6119-5p, miR-376c-5p, miR-1271-3p, miR-380-5p, miR-1, miR-3529-3p, miR-105-2, miR-10a-5p, let-7a-2-3p, miR-300, miR-3578, miR-545-5p, miR-582-3p, miR-214-5p, miR-24-2-5p, miR-302a-5p, miR-7641, miR-204-5p, miR-138-5p, miR-331-3p, miR-135a-5p, miR-34c-3p, miR-370-5p, miR-99b-3p, miR-135a-1-3p, miR-5100, miR-145-3p, miR-323-3p, miR-93, miR-103b, miR-1306-5p, miR-23a-5p, miR-485-5p, let-7g-3p, miR-17-5p, miR-18a, miR-381-3p, miR-488-3p, miR-2448-3p, miR-2284n, miR-365a-3p, miR-370-3p, miR-660, miR-125a-5p, miR-124-5p, miR-190a, miR-106a-5p, miR-17-5p, miR-539-3p, miR-138, miR-2284ab, miR-194-5p, miR-20b, miR-2284d, miR-130a-3p, miR-665, miR-22-5p, novel_1, miR-382-5p, miR-20a-5p, miR-133a-5p, miR-326-3p, miR-2428, miR-211, miR-1193, novel_101, novel_120, miR-342-3p, miR-505-3p, miR-135b-5p, miR-1291, miR-19a-3p, miR-2387, miR-18b, miR-670-3p, miR-320b, miR-10a-5p, miR-30b-3p, miR-21-3p, miR-106a, miR-2284m, miR-216a-5p, miR-125b, miR-374a-5p, miR-20a, miR-216c-5p, miR-19b-3p, miR-2904, miR-188-3p, miR-140-3p, miR-5703, miR-2300b-3p, novel_83, miR-532-3p, miR-151-5p, miR-487a-3p, miR-505-3p, miR-485-3p, miR-450b-3p, miR-664a, miR-324-3p, miR-338-5p, miR-338-3p, miR-10b-5p, miR-206-3p, miR-3074-2-3p, miR-4510, novel_99, miR-18a-5p, miR-6130, miR-139-5p, miR-370-5p, miR-660-5p, miR-342, novel_103, miR-326, miR-758-3p, miR-106a-5p, miR-2284aa, miR-450b-5p, miR-101a-5p, miR-10b, miR-9788-3p, miR-2320-3p, miR-105-1, novel_4, miR-151a-3p, miR-2284q, miR-3958-3p, miR-2285y, miR-1973, miR-107-5p, miR-219a-2-3p, miR-194b-5p, miR-541-3p, miR-7857-3p, miR-219-3p, novel_39, miR-192-3p, miR-450b-5p, miR-148b-5p, miR-330-5p, miR-23b-5p, miR-450a-1-3p, miR-2284z, miR-129-1-3p, novel_79, miR-18b-5p, miR-2285ad, miR-125b-5p, miR-2284k, miR-2284y, miR-20b-5p, miR-
DOK2	ENSOARG0(	docking	miR-16b, miR-885-3p, miR-4508, miR-497-5p, miR-484, miR-217-5p, miR-1343-5p, miR-3431, miR-15a-5p, miR-322-5p, miR-487a-3p, miR-328-3p, miR-485-3p, miR-339-5p, novel_79, miR-6536, miR-3085-3p, miR-543-5p, miR-1343-5p, miR-210-5p, miR-339a, miR-16b, miR-217, miR-539-3p, miR-4532, miR-424-5p, miR-3064-5p, miR-339b, miR-195a-5p, miR-6395, miR-885-3p, miR-361-3p, miR-15b-5p, miR-4443, miR-669, miR-3064-5p, miR-2355-5p, miR-424-5p, miR-15a, miR-485-3p, miR-16a, miR-2428, miR-665-5p, miR-16-5p

ABCA7	ENSOARG0(	ATP-bind	miR-199a-5p,miR-3065-3p,novel_39,miR-30b-3p,novel_127,miR-324-3p,miR-9-5p,miR-2285ad,miR-18a-3p,miR-3600,miR-133a-3p,miR-362-3p,miR-2300b-3p,miR-140-3p,miR-133a-3p,miR-138-5p,miR-4492,miR-204-5p,miR-5100,miR-100-3p,miR-664a-5p,miR-665,miR-22-3p,miR-199a-5p,miR-660-5p,miR-3065-3p,miR-2355-5p,miR-133b-3p,let-7g-3p,miR-329a,miR-2285c,miR-129b-3p,miR-363-5p,miR-199b-5p,miR-660,miR-362-3p,miR-2285g,miR-134-3p,miR-2285e,novel_78,miR-138,miR-30b-3p,miR-362-3p,miR-329-3p,miR-484,miR-541,miR-22-3p,miR-30b-3p,miR-361-3p,miR-211,novel_111,miR-199b-5p,novel_60,miR-3956-3p,miR-541-3p,miR-9-5p,miR-6123,miR-378g,miR-3596,miR-4443,miR-2319b,miR-3120-3p,miR-211-5p,miR-199b-5p,miR-18a-3p
CALM2	ENSOARG0(	calmodul	miR-324-3p,miR-326,miR-30b-3p,miR-214-3p,miR-760-3p,miR-345-3p,miR-4532,miR-503-3p,miR-2285i,miR-376a-5p,miR-22-3p,miR-330-5p,miR-484,miR-6529a,miR-653,miR-361-5p,miR-582-3p,miR-455-3p,miR-129-1-3p,miR-185-5p,miR-3431,miR-576-3p,miR-374a-3p,miR-4492,miR-374b-3p,miR-664a,miR-495-3p,miR-214,novel_42,miR-129-2-3p,miR-122-5p,miR-326-3p,miR-2428,miR-374a-3p,miR-21a-3p,miR-2387,miR-485-5p,miR-10b-3p,miR-2285k,miR-2355-5p,miR-3120-3p,miR-22-3p
ZNF207	ENSOARG0(	zinc fin	miR-2284j,miR-194b-5p,miR-181d-5p,miR-877-3p,miR-502-3p,miR-8095,miR-101-3p,miR-2284q,miR-452-3p,miR-10b,miR-664b,miR-2284g,miR-541-5p,miR-7689-3p,miR-499b-5p,miR-1185-2-3p,miR-500-3p,miR-203-3p,miR-221,miR-381-3p,miR-668-5p,miR-544-5p,miR-1983,miR-493-5p,miR-494-3p,miR-101b-3p,miR-424-5p,miR-33a-5p,miR-676-3p,let-7c-3p,miR-30c-1-3p,miR-2310,miR-199a-3p,miR-320b,miR-16b,miR-505,miR-29b-1-5p,miR-214-3p,miR-199b-3p,miR-2300a-5p,novel_78,miR-151a-3p,miR-145b,miR-1185-3p,miR-148b-5p,miR-221-3p,miR-10b,miR-501-3p,miR-3120-5p,miR-101c,miR-145a-3p,miR-3074-5p,miR-3184-3p,miR-590-3p,miR-539-5p,miR-214,miR-2330-3p,miR-224-5p,miR-10b-5p,miR-181c-5p,miR-222-3p,miR-338-5p,miR-320e,miR-151-3p,miR-17-3p,miR-450b-3p,miR-500,miR-424-5p,miR-548o-3p,miR-660-5p,miR-30c-1-3p,miR-545-3p,miR-2331-3p,miR-10a,miR-192-5p,miR-15b-5p,miR-450a-1-3p,miR-21a-3p,miR-500a-3p,let-7g-3p,miR-186-5p,miR-183-5p,miR-125b-2-3p,miR-29b-1-5p,miR-376a-5p,miR-421-5p,miR-143-3p,miR-380-3p,miR-2432,miR-2285p,miR-196a-3p,miR-92a-1-5p,miR-345-3p,miR-324-3p,miR-216a-5p,miR-2284m,miR-320d,miR-2484,miR-3653-3p,miR-34b-5p,miR-485-3p,miR-505-3p,miR-15a-5p,miR-487a-3p,miR-199a-3p,miR-2300b-3p,miR-215-5p,miR-376a-5p,miR-217-5p,miR-1434-5p,miR-4429,novel_73,miR-2284u,miR-378d,miR-34a-5p,miR-2285x,miR-17-3p,novel_27,miR-449a,miR-6402,miR-502-5p,miR-15a,miR-382-5p,miR-34c,miR-485-3p,miR-16a,miR-379-3p,miR-199c,miR-421-5p,miR-203b-5p,miR-3074-5p,miR-10a-5p,miR-3120-3p,miR-7-1-3p,miR-320b,miR-34b,miR-320c,miR-21c,miR-412-3p,miR-195a-5p,miR-505-3p,miR-6516-3p,miR-2319a,miR-493-5p,miR-628-5p,miR-382,miR-2284l,miR-224-5p,miR-194a,miR-2411-3p,miR-455-5p,miR-197-5p,miR-660,miR-432-5p,miR-2284d,miR-1434-

CTNND1	ENSOARG0(	catenin	miR-107-5p,miR-410-5p,miR-15b-3p,novel_63,miR-6402,miR-374a-3p,miR-190a-5p,miR-374b-3p,miR-134-5p,miR-379-3p,miR-6516-3p,miR-1296-5p,miR-1271,miR-665-5p,miR-190b-5p,miR-2285x,miR-342-3p,miR-212-3p,miR-1285-5p,miR-374a-3p,miR-625-5p,miR-1248,miR-1827,miR-96-5p,miR-216b-3p,miR-221-5p,miR-216b-3p,miR-190a,miR-455-5p,novel_51,miR-342,novel_17,miR-221-5p,miR-30b-3p,miR-103a-2-5p,miR-450a-2-3p,miR-4286,miR-216c-5p,miR-2411,miR-2285r,miR-197-3p,miR-296-5p,miR-134,miR-21b,miR-361-5p,miR-455-5p,miR-411-3p,miR-671-3p,miR-134-5p,miR-455-3p,miR-185-5p,miR-2411-5p,miR-1271-5p
PRKD2	ENSOARG0(	protein	miR-1a-1-5p,miR-576-3p,miR-142b,miR-3074-5p,miR-3120-3p,miR-320c,miR-6535,miR-3141,miR-216a-3p,miR-146b-5p,miR-378f,miR-668-3p,miR-432-3p,miR-7-5p,miR-296-3p,miR-146a,miR-3141,miR-194a,novel_51,miR-31-5p,miR-3535,miR-134-3p,miR-194-3p,miR-149-5p,miR-432,miR-19b-2-5p,miR-2113,miR-7b-5p,miR-197-3p,miR-450c-3p,novel_23,miR-381-5p,miR-4508,miR-193a-5p,miR-2433,miR-125b-5p,miR-331-5p,novel_9,miR-592,miR-574-5p,miR-2355-5p,miR-125a,miR-376c-3p,miR-301,miR-331-5p,miR-345-5p,miR-450b-5p,miR-148b-5p,miR-664-3p,miR-3071-3p,miR-1a-2-5p,novel_127,novel_96,miR-2285ad,miR-429-3p,miR-412,miR-450a-1-3p,novel_121,miR-1290,miR-2427,miR-1343-5p,miR-23b-5p,miR-330-5p,miR-2447,miR-877-3p,novel_111,miR-1285-5p,miR-1843a-3p,miR-490-5p,miR-139-5p,miR-4792,miR-211-5p,miR-3596,miR-25,miR-378g,miR-4443,miR-140-5p,miR-409-5p,miR-378j,miR-504,miR-335-3p,miR-9-3p,miR-1388-3p,miR-378d,miR-29b-1-5p,miR-491-5p,miR-146b,miR-381-5p,miR-1343-3p,miR-145b,miR-425-3p,miR-147a,miR-455-3p,miR-2459,miR-2284r,miR-758-3p,miR-3074-5p,miR-665-5p,miR-6525,miR-224-5p,miR-181c-5p,miR-2285j,miR-4492,miR-130b-5p,miR-2331-3p,miR-504-5p,miR-548o-3p,miR-2284s,miR-376e-3p,miR-582-5p,miR-450a-1-3p,miR-433-3p,miR-345-5p,miR-543-3p,miR-1827,miR-186-5p,miR-10a-3p,miR-2285w,miR-345-3p,miR-376c-3p,miR-143-3p,miR-210-5p,novel_17,miR-500a-5p,miR-324-3p,miR-422a,miR-18a-3p,miR-210-3p,miR-2284h-5p,miR-378i,miR-378a-3p,miR-493-3p,miR-34a-5p,miR-3957-3p,miR-542-5p,miR-211,miR-24-3p,miR-371b-3p,miR-449a,miR-326-3p,miR-133a-5p,miR-34c,miR-656-5p,miR-378b,miR-34b,miR-18a-3p,miR-130b-5p,miR-382,miR-654-3p,miR-296-3p,miR-345-5p,miR-224-5p,miR-660,miR-452-5p,miR-125a-5p,miR-1343-5p,miR-187-

MPG	ENSOARG0(	N-methyl	miR-30b-3p,miR-296-5p,miR-2284r,miR-542-3p,miR-4508,miR-2889,miR-744-5p,miR-30b-3p,miR-187-3p,miR-2300a-5p,novel_4,miR-1343-3p,miR-9788-3p,miR-149-5p,miR-185-5p,miR-134-3p,miR-1298-3p,let-7g-5p,miR-3431,miR-2957,miR-342,miR-1306-5p,miR-125a-5p,let-7e,miR-491-5p,miR-3085-3p,let-7a-5p,miR-493-5p,miR-4532,miR-2285r,miR-1306,miR-216a-3p,miR-3591-3p,miR-2285ab,miR-18b,miR-9851-3p,miR-493-5p,miR-6395,miR-6516-3p,let-7e-5p,miR-1291,miR-499b-5p,miR-3064-5p,miR-4792,let-7d-5p,miR-669,miR-4443,miR-3596,miR-7857-3p,miR-378g,let-7i,miR-4324,miR-6123,miR-128-1-5p,miR-2285f,miR-17-3p,miR-342-3p,miR-423-5p,miR-2428,miR-361-3p,miR-24-3p,miR-188-3p,novel_133,miR-134,miR-1961,miR-193b-5p,miR-133a-3p,miR-128-3p,miR-378d,miR-885-3p,let-7d,miR-1224-5p,miR-18b-5p,miR-3960,miR-532-3p,miR-134-5p,miR-328-3p,miR-219b-5p,miR-125b,let-7c-5p,miR-185-3p,let-7k,miR-543-5p,novel_96,miR-17-3p,miR-450a-2-3p,miR-760-3p,miR-98-5p,let-7f-5p,miR-3064-5p,miR-4286,miR-486-3p,let-7g,miR-130a-5p,miR-18a,miR-885-3p,miR-1895,miR-485-5p,let-7g-3p,miR-2312,miR-18a-5p,miR-2355-5p,miR-21-3p,miR-296-3p,miR-125a,miR-324-5p,let-7i-5p,miR-154b-5p,miR-324-3p,miR-132-5p,miR-125b-5p,miR-6740-5p,let-7f,miR-4492,miR-486b-3p,miR-134-5p,miR-17-3p,let-7b,miR-3184-5p,let-7b-5p,miR-1197-3p,miR-346
SESN1	ENSOARG0(	sestrin	miR-1843a-3p,miR-133b-3p,miR-185-3p,miR-133a-3p,miR-1343-3p

RFC3	ENSOARG0(	replicat	miR-664a,miR-487a-5p,miR-500,miR-4492,miR-15b-3p,miR-30e-3p,miR-30a-3p,miR-338-5p,miR-320e,miR-6525,miR-346,miR-214,miR-30f,miR-582-5p,miR-186-5p,miR-21a-3p,miR-2285c,miR-500a-3p,miR-30d-5p,miR-377-3p,miR-582-5p,miR-216b-5p,miR-139-5p,miR-3065-3p,miR-532-5p,miR-320d,miR-2284m,miR-466i-5p,miR-30b,miR-26a-5p,miR-2411,miR-329a-5p,miR-195a-3p,miR-4429,miR-142-3p,miR-16-1-3p,miR-2284u,miR-30c-5p,miR-217-5p,miR-23b-3p,miR-23c,miR-30d-3p,miR-2284q,miR-30b-5p,miR-101-3p,miR-502-3p,miR-3059-5p,miR-2284j,novel_94,miR-140-5p,miR-378j,miR-489,miR-500-3p,miR-139-5p,miR-2284g,miR-2319b,miR-499b-5p,miR-211-5p,miR-487b-5p,miR-214-3p,miR-320b,miR-326,miR-505,miR-1b-5p,miR-23a-3p,miR-2284b,miR-376b,miR-101a-5p,miR-21b,miR-16-2-3p,miR-101c,miR-501-3p,miR-30b-3p,miR-539-5p,miR-216a-3p,miR-421,miR-2285e,miR-548w,miR-145b,miR-767-5p,miR-410-3p,miR-204-5p,miR-2284k,miR-29b-3p,miR-1-5p,miR-2284v,miR-767,miR-329b,miR-376c-5p,miR-7144-5p,miR-190a-3p,miR-485-5p,miR-30e-5p,miR-2448-3p,miR-376c-5p,miR-320a,miR-23b,miR-2284n,miR-574-5p,miR-370-3p,miR-141-3p,miR-1a-2-5p,miR-543-5p,miR-200a-3p,miR-2285af,miR-6119-5p,miR-217,miR-380-5p,miR-2284y,miR-1957a,miR-29a-5p,miR-3065-3p,miR-2284a,miR-2285b,miR-192-3p,miR-3529-3p,miR-2385-3p,miR-30a-3p,miR-30c,miR-29d-3p,miR-2427,miR-30a-5p,miR-330-5p,miR-501-3p,miR-2411-5p,miR-423-3p,miR-7641,miR-29a,miR-147-5p,miR-6123,miR-4324,miR-6402,miR-576-3p,miR-142b,miR-211,miR-326-3p,miR-2428,miR-1a-1-5p,miR-29c-3p,miR-495-3p,miR-2319a,miR-376b-5p,miR-150-5p,miR-3065-5p,miR-29a-3p,miR-374c-3p,miR-320c,miR-329-5p,miR-26b-5p,miR-7-5p,miR-377-3p,novel_51,miR-432-3p,miR-380-5p,miR-101-5p,miR-29b,miR-30b-3p,miR-16-1-3p,miR-219a-1-3p,miR-2284x,miR-7b-5p,miR-

SLC18A1	ENSOARG0(	solute c	miR-195a-5p,miR-21c,miR-216a-3p,miR-181b-3p,miR-677,miR-374c-3p,miR-299b-3p,miR-10a-5p,miR-3074-5p,miR-203b-5p,miR-421-5p,miR-16a,miR-576-3p,miR-15a,miR-502-5p,miR-2428,miR-371b-3p,miR-24-3p,miR-1193,miR-495-3p,miR-1839-3p,miR-27b-3p,miR-497-5p,miR-219a-1-3p,miR-16-1-3p,miR-1260b,miR-30b-3p,miR-16b,miR-21-5p,miR-2284ab,miR-145a-5p,miR-3071-5p,miR-450c-3p,miR-7b-5p,miR-1434-3p,miR-873a-5p,miR-19b-2-5p,miR-2355-3p,miR-503-5p,miR-3431,miR-185-5p,miR-660,miR-378a-5p,miR-483-3p,miR-296-3p,miR-7-5p,miR-503-5p,miR-296-3p,miR-654-3p,miR-668-3p,miR-329b,miR-324-5p,miR-769,miR-374c-3p,miR-574-5p,miR-486b-3p,novel_9,miR-331-3p,miR-370-5p,miR-2284k,miR-1197-3p,miR-1260b,miR-16-5p,miR-6128,miR-193a-5p,miR-10a-5p,miR-128-3p,miR-214-5p,miR-450a-1-3p,miR-6516-5p,miR-483-3p,miR-322-5p,miR-1224-5p,miR-2285ad,miR-299a-3p,miR-543-5p,miR-127-5p,miR-2898,miR-1260a,miR-1271-3p,miR-490-3p,miR-2285b,miR-192-3p,miR-1957a,miR-3065-3p,miR-29b-2-5p,miR-381-3p,miR-27a-3p,miR-203-3p,miR-3082-5p,miR-25,miR-452-3p,miR-20a-3p,miR-2319b,miR-139-5p,miR-3964,miR-2285t,miR-3968,miR-2285y,miR-769-5p,miR-382-3p,miR-452-5p,miR-212-3p,miR-541-3p,miR-450b-5p,miR-30b-3p,miR-10b,miR-541,miR-9788-3p,miR-216a-3p,miR-455-3p,miR-142-3p,miR-27a-3p,miR-145b,miR-181b-2-3p,novel_78,miR-151a-3p,miR-3955-3p,miR-16b,miR-29b-1-5p,novel_103,novel_115,miR-1839-3p,miR-424-5p,miR-2330-5p,miR-411,miR-129b-3p,miR-450a-1-3p,miR-21a-3p,miR-15b-5p,miR-151b,miR-139-5p,miR-10a,miR-6516,miR-552-3p,miR-3065-3p,miR-370-5p,miR-660-5p,miR-4492,miR-424-5p,novel_74,miR-450b-3p,miR-320e,miR-29b-2-5p,miR-151-3p,miR-324-3p,miR-19b-1-5p,miR-346,miR-10b-5p,miR-769-5p,miR-3074-2-3p,miR-3074-5p,miR-16-1-3p,miR-140-3p,miR-

CAMTA1	ENSOARG0(	calmodul	miR-8485,miR-23a-3p,miR-124a,miR-1388-3p,miR-101b-3p,miR-146b,miR-214-3p,miR-103a-2-5p,miR-491-5p,miR-130b-3p,miR-106b-5p,miR-182-5p,miR-431-5p,miR-421,miR-147a,miR-181b-2-3p,miR-548w,miR-199b-3p,miR-1388-5p,miR-758-3p,miR-877-3p,novel_111,miR-2284j,miR-2447,miR-363-3p,miR-154-3p,miR-499a-5p,miR-101-3p,miR-615,miR-2440,miR-144-5p,miR-203-3p,miR-3596,miR-139-5p,miR-2319b,miR-335-3p,miR-544-5p,miR-1983,miR-96-5p,miR-668-5p,miR-378j,miR-221,miR-2424,miR-380-3p,miR-377-5p,miR-181b-1-3p,miR-142a-3p,miR-329a-5p,miR-487a,miR-92a-1-5p,miR-30b,miR-2285p,miR-210-3p,miR-7859,miR-23b-3p,miR-23c,miR-30d-3p,miR-106a,miR-34a-5p,miR-217-5p,miR-30c-5p,miR-6525,miR-214,miR-129-2-3p,miR-3184-3p,miR-129-5p,miR-454-3p,miR-130b-5p,miR-30a-3p,miR-219b-3p,miR-30e-3p,miR-377-3p,miR-301a-3p,miR-582-5p,miR-552-3p,miR-30c-1-3p,miR-32-5p,miR-548o-3p,miR-625-5p,miR-543-3p,miR-433-3p,miR-30d-5p,let-7a-2-3p,miR-503-3p,miR-432-3p,miR-2284l,miR-628-5p,miR-193a-3p,miR-32,miR-105-5p,miR-7-5p,miR-146a,miR-377-3p,novel_116,miR-194-3p,miR-188-5p,miR-27b-3p,miR-223-3p,miR-7b-5p,miR-542-3p,miR-301b-3p,miR-2285m,miR-193b-3p,miR-29c-3p,miR-335-5p,miR-421-5p,miR-29a,miR-4324,miR-320c,miR-677,miR-181b-3p,novel_68,miR-216b-3p,miR-329-5p,miR-146b-5p,miR-2319a,miR-412-3p,miR-3591-3p,miR-216a-3p,miR-3065-5p,miR-29a-5p,miR-2285b,miR-92b-3p,miR-3064-5p,miR-200a-3p,novel_96,miR-6536,miR-30f,miR-219-3p,miR-129-1-3p,miR-18b-5p,miR-431,miR-2285ad,miR-30c,miR-2427,miR-23b-5p,miR-3591-5p,miR-144-5p,miR-2433,miR-300-3p,miR-25-3p,miR-2285aa,miR-3068-3p,miR-20b-5p,miR-767,miR-2318,miR-93-5p,miR-23b,miR-30e-5p,miR-424-3p,miR-4532,miR-758-3p,miR-30c-1-3p,miR-106a-5p,miR-382-3p,miR-3085-3p,miR-3955-3p,miR-320b,novel_115,miR-221-
CALN1	ENSOARG0(	calneuro	miR-30c-1-3p,miR-296-3p,miR-204-3p,miR-376c-5p,miR-320a,miR-10b-3p,miR-7144-5p,miR-376c-5p,miR-671-5p,miR-1827,miR-129-2-3p,miR-346,miR-365a-5p,miR-6134,miR-2433,miR-551b-3p,miR-331-3p,miR-320e,miR-664a-5p,miR-135a-5p,miR-138-5p,novel_9,miR-487a-5p,miR-134-5p,miR-412,miR-196a-2-3p,miR-758-5p,miR-1247-5p,miR-134-5p,miR-545-5p,miR-129-1-3p,miR-134,miR-1193,miR-142-3p,miR-4429,miR-130a-5p,miR-376c-5p,miR-376b-5p,miR-490-3p,miR-1271-3p,miR-2285b,miR-143-3p,novel_17,miR-30c-2-3p,miR-320d,miR-2898,miR-30b-3p,miR-4792,miR-2404,miR-25,miR-411b-3p,miR-2483-3p,miR-320c,miR-376b-5p,miR-3065-5p,miR-378b,miR-135b-5p,novel_94,miR-2447,miR-342-3p,miR-495-3p,miR-154a,novel_1,miR-147-5p,novel_4,miR-330-3p,miR-5010-3p,miR-145b,miR-3535,miR-1298-3p,miR-147a,miR-411-5p,miR-1843b-5p,miR-4791,miR-223-3p,miR-197-3p,miR-412-3p,miR-145a-5p,miR-138,miR-3120-5p,miR-744-5p,miR-2284x,miR-6240,miR-363-5p,miR-376b,miR-30c-1-3p,miR-432-3p,miR-432-3p,miR-320b,miR-342,miR-330-3p,miR-365b-5p,miR-124-5p,miR-25-5p

TSPAN9	ENSOARG0(	tetraspa	novel_60,miR-885-5p,miR-3956-3p,miR-2284j,miR-194b-5p,miR-361-3p,miR-877-3p,miR-2285n,miR-2440,miR-301a-5p,miR-3968,miR-3964,miR-2284g,miR-3064-5p,miR-452-3p,miR-7977,miR-378g,miR-4443,miR-454-5p,miR-361-3p,miR-877-3p,miR-494-3p,miR-424-5p,miR-2284b,miR-652-5p,miR-33a-5p,miR-342,miR-326,novel_103,miR-182-5p,miR-16b,miR-491-5p,miR-3085-3p,miR-214-3p,miR-330-3p,miR-105-1,miR-1343-3p,miR-181b-2-3p,miR-9788-3p,miR-148b-5p,miR-296-5p,miR-301b-5p,miR-10b,miR-412-3p,miR-1a-3p,miR-30b-3p,miR-544a,miR-450b-5p,miR-2284aa,miR-3120-5p,miR-4510,miR-145a-3p,miR-214,miR-6525,miR-10b-5p,miR-206-3p,miR-6134,miR-2285j,miR-665,miR-874-5p,miR-424-5p,miR-708-5p,miR-4492,miR-2331-3p,miR-221-5p,miR-532-5p,miR-182-5p,miR-544-3p,miR-10a,miR-323-5p,miR-193b-3p,miR-6130,miR-22-3p,miR-15b-5p,miR-339b,miR-345-5p,miR-1895,miR-1827,miR-376a-5p,miR-181b-1-3p,miR-486-3p,miR-210-5p,miR-324-3p,miR-345-3p,miR-125b,miR-99a-3p,miR-760-3p,miR-34b-5p,miR-221-5p,miR-21-3p,miR-18a-3p,miR-212-5p,miR-148b-3p,miR-15a-5p,miR-532-3p,miR-191,novel_133,miR-2300b-3p,miR-2904,miR-188-3p,miR-1197-5p,miR-133a-3p,miR-2284u,miR-34a-5p,miR-148a-5p,miR-342-3p,miR-3957-3p,miR-193b-3p,miR-495-3p,miR-449a,miR-326-3p,miR-2428,miR-2284w,miR-15a,miR-16a,miR-34c,miR-128-1-5p,miR-206,miR-6123,miR-502b,miR-320b,miR-10a-5p,miR-146b-3p,miR-323a-5p,miR-2404,miR-34b,miR-677,miR-181b-3p,miR-148a-3p,miR-3141,miR-2387,miR-150-5p,miR-216a-3p,miR-18a-3p,miR-107,miR-195a-5p,miR-6240,miR-363-5p,miR-668-3p,miR-654-3p,let-7j,miR-345-5p,miR-3141,miR-330-3p,miR-365b-5p,miR-105-5p,miR-2411-3p,miR-194a,novel_51,miR-483-3p,miR-452-5p,miR-1343-5p,miR-125a-5p,miR-193a-3p,miR-31-5p,miR-103a-3p,miR-339-5p,miR-

KLHL25	ENSOARG0(	kelch-li	miR-363-5p,miR-668-3p,miR-628-5p,miR-6240,miR-660,miR-193a-3p,miR-124-5p,miR-378a-5p,miR-2285l,miR-330-3p,novel_51,miR-2411-3p,miR-377-3p,miR-149-5p,miR-134-3p,miR-665,miR-339-5p,miR-26b-3p,miR-31-5p,miR-187-3p,miR-4508,miR-7862,miR-138,miR-30b-3p,miR-29b,miR-1260b,miR-16-1-3p,miR-450c-3p,miR-873a-5p,miR-2113,miR-326-3p,miR-199b-5p,miR-2428,miR-24-3p,miR-193b-3p,miR-495-3p,miR-29c-3p,miR-135a-2-3p,miR-6123,miR-128-1-5p,miR-29a,novel_1,miR-677,miR-181b-3p,miR-299b-3p,miR-29a-3p,miR-10a-5p,miR-3074-5p,miR-7975,miR-2319a,miR-1291,miR-18a-3p,miR-2387,novel_39,miR-199a-5p,miR-490-3p,miR-1271-3p,miR-2285b,miR-380-5p,miR-148b-5p,miR-24-2-5p,miR-3064-5p,miR-2403,miR-4286,novel_127,miR-543-5p,novel_96,miR-200a-3p,miR-2898,miR-127-5p,miR-191-3p,miR-30c-2-3p,miR-339a,miR-1260a,miR-545-5p,miR-450a-1-3p,miR-486-5p,miR-129-1-3p,novel_79,miR-1224-5p,miR-412,miR-299a-3p,miR-10a-5p,miR-29d-3p,miR-501-3p,miR-330-5p,miR-345-3p,miR-3591-5p,miR-3578,miR-145-3p,miR-2903,miR-500b-5p,miR-1260b,miR-592,miR-486b-3p,miR-138-5p,novel_9,miR-331-3p,miR-409-3p,miR-3969,miR-29b-3p,miR-99b-3p,miR-199a-5p,miR-324-5p,miR-133c,miR-374c-3p,miR-769,miR-3959-5p,miR-2355-5p,miR-99a-3p,miR-141-3p,miR-1271,miR-615-5p,miR-7144-5p,miR-671-5p,miR-485-5p,miR-1248,miR-329b,miR-362-5p,miR-2448-3p,miR-4532,miR-193a,miR-30c-1-3p,miR-23a-3p,miR-744-3p,miR-199b-5p,miR-214-3p,miR-2285o,miR-103a-2-5p,miR-3085-3p,miR-491-5p,miR-326,miR-455-3p,miR-500-5p,miR-148b-5p,miR-425-3p,miR-147a,miR-222-5p,miR-330-3p,miR-2300a-5p,miR-1343-3p,miR-151a-3p,miR-3120-5p,miR-1388-5p,miR-2426,miR-412-3p,miR-501-3p,miR-30b-3p,miR-6529a,miR-541,miR-10b,miR-502-3p,miR-362-5p,miR-361-3p,miR-2447,novel_60,miR-3956-

WDR19	ENSOARG0(	WD repea	miR-29b-2-5p,miR-199a-5p,miR-200a,novel_39,miR-29a-5p,miR-148b-5p,miR-24-2-5p,miR-30c-2-3p,miR-205-5p,miR-107,miR-200a-3p,miR-450a-2-3p,miR-222,miR-545-5p,miR-450a-1-3p,miR-24-2-5p,miR-9-5p,miR-505-5p,miR-6516-5p,miR-1271-5p,miR-155-5p,let-7a-2-3p,miR-10a-5p,miR-411-3p,miR-6128,miR-144-5p,miR-1306-5p,miR-500b-5p,miR-6740-5p,miR-331-3p,miR-2332,miR-204-5p,miR-486b-3p,miR-138-5p,miR-331-5p,miR-598-3p,miR-370-3p,miR-141-3p,miR-503-3p,miR-199a-5p,miR-133c,miR-154b-5p,miR-300-3p,miR-320a,miR-362-5p,miR-503-3p,miR-331-5p,miR-424-3p,miR-190a-3p,miR-485-5p,miR-1814c,miR-10b-3p,miR-329a,let-7g-3p,miR-2483-5p,miR-668-3p,let-7j,let-7a-2-3p,miR-503-3p,miR-432-3p,miR-432-3p,miR-22841,miR-103a-3p,miR-149-5p,miR-194-3p,miR-103,miR-3535,miR-450c-3p,miR-197-3p,miR-181a-5p,miR-1843b-5p,miR-2889,miR-30b-3p,miR-138,miR-2285x,novel_27,miR-199b-5p,miR-2284w,miR-211,miR-24-3p,miR-382-5p,miR-33a-3p,miR-502-5p,miR-576-3p,miR-147-5p,miR-10a-5p,miR-3120-3p,miR-320c,miR-146b-3p,miR-3970,miR-376a-2-5p,miR-107,miR-412-3p,miR-369-3p,miR-486-3p,miR-29b-1-5p,miR-500a-5p,novel_17,miR-320d,miR-3600,miR-616-3p,miR-2904,miR-1434-5p,miR-362-3p,miR-4429,miR-142-3p,miR-1271,miR-214,miR-3074-2-3p,miR-10b-5p,miR-6525,miR-655-3p,miR-29b-2-5p,miR-222-3p,miR-487a-5p,miR-450b-3p,miR-664a,miR-155-5p,miR-216b-3p,miR-30c-1-3p,miR-155-5p,miR-6516,miR-10a,miR-598-3p,miR-655,miR-450a-1-3p,novel_99,miR-376b,miR-1388-3p,miR-8485,miR-2310,miR-30c-1-3p,miR-29b-1-5p,miR-362-3p,miR-320b,miR-199b-5p,miR-214-3p,miR-3955-3p,miR-5010-3p,miR-500-5p,miR-221-3p,miR-7857,miR-30b-3p,miR-329-3p,miR-484,miR-10b,miR-4791,miR-21b,miR-362-3p,miR-2426,miR-450b-5p,miR-9-5p,miR-219a-5p,miR-181d-5p,miR-3059-5p,miR-8095,miR-
WBSCR17	ENSOARG0(	Williams	miR-576-3p,miR-6740-5p,miR-320e,novel_42,miR-3607-3p,miR-3184-5p,miR-423-5p,miR-2428,miR-301,miR-3154,miR-18a-3p,miR-320a,miR-7144-5p,miR-23a-5p,miR-625-5p,miR-1248,miR-874-3p,miR-378g,miR-320c,miR-320b,miR-378a-5p,miR-320d,novel_127,miR-4286,miR-490-3p,miR-2284r,miR-432,miR-5703,miR-4429,miR-18a-3p,miR-432-5p,miR-1298-3p,miR-134-3p,miR-9788-3p,miR-1247-5p

MAGI1	ENSOARG0(	membrane	miR-183-5p,miR-329a-5p,miR-216c-5p,miR-503-5p,miR-487a,miR-146a-5p,miR-664b-3p,miR-376a-5p,miR-29b-1-5p,miR-181b-1-3p,miR-618,novel_17,miR-2484,miR-485-3p,miR-18a-3p,novel_83,miR-2284h-5p,miR-199a-3p,miR-487a-3p,miR-7859,miR-23b-3p,miR-210-3p,miR-200b,miR-23c,miR-2904,miR-124-3p,miR-140-3p,miR-1246,miR-362-3p,miR-16-1-3p,miR-361-5p,miR-493-3p,miR-33b-3p,miR-214,miR-433-5p,miR-10b-5p,miR-6525,miR-206-3p,miR-324-3p,miR-665,miR-208b-5p,miR-500,miR-130b-5p,miR-450b-3p,novel_74,miR-6516-3p,miR-664a,miR-487b-5p,miR-21-3p,miR-154a-3p,miR-3065-3p,miR-182-5p,miR-504-5p,miR-140-5p,miR-548o-3p,miR-204-3p,miR-10a,miR-582-5p,miR-139-5p,miR-193b-3p,miR-129b-3p,miR-500a-3p,miR-433-3p,miR-543-3p,miR-625-5p,miR-186-5p,miR-582-5p,let-7g-3p,miR-339b,miR-345-5p,miR-23a-3p,miR-33a-5p,miR-124a,miR-101b-3p,miR-2330-5p,miR-4532,miR-1b-5p,miR-362-3p,miR-505,miR-29b-1-5p,miR-182-5p,miR-342,miR-199a-3p,miR-146b,miR-153-3p,miR-214-3p,miR-491-5p,miR-181b-2-3p,miR-199b-3p,miR-105-1,miR-330-3p,miR-2300a-5p,miR-421,miR-153,miR-7857,miR-30b-3p,miR-329-3p,miR-2284r,miR-484,miR-10b,miR-301b-5p,miR-101c,miR-362-3p,miR-3059-5p,miR-2447,miR-212-3p,miR-9-3p,miR-8095,miR-877-3p,novel_111,miR-1973,miR-371a-5p,miR-301a-5p,novel_82,miR-218-5p,miR-101-3p,miR-490-5p,miR-154-3p,miR-2284q,miR-499b-5p,miR-541-5p,miR-211-5p,miR-664b,miR-139-5p,miR-10b,miR-452-3p,miR-2319b,miR-144-5p,miR-7857-3p,miR-3082-5p,miR-489,miR-2483-3p,miR-378j,miR-381-3p,miR-129b-5p,miR-493-5p,miR-9-3p,miR-136-5p,miR-668-5p,miR-504,miR-3529-3p,miR-200a,miR-29a-5p,miR-3065-3p,miR-450b-5p,miR-2478,miR-3071-3p,miR-339a,miR-2285af,miR-205-5p,miR-2397-5p,miR-664-3p,miR-200c,miR-200a-3p,novel_96,novel_127,miR-200c-

FOXO3	ENSOARG0(	forkhead	miR-412-3p,miR-7857,miR-758-3p,miR-30b-3p,miR-6529a,miR-296-5p,miR-1388-5p,miR-544a,miR-27a-3p,miR-181b-2-3p,novel_4,miR-548w,miR-1343-3p,miR-2300a-5p,miR-409b,miR-330-3p,miR-455-3p,miR-1185-3p,novel_19,miR-505,miR-182-5p,miR-342,miR-431-5p,miR-146b,miR-3085-3p,miR-491-5p,miR-1185-5p,miR-3955-3p,miR-1388-3p,miR-382-3p,miR-8485,miR-4532,miR-193a,miR-1b-5p,miR-758-3p,miR-2310,miR-378j,miR-129b-5p,miR-493-5p,miR-9-3p,miR-299b-5p,miR-668-5p,miR-3064-5p,miR-2319b,miR-452-3p,miR-20a-3p,miR-27a-3p,miR-1185-2-3p,miR-769-5p,miR-1973,miR-2285y,novel_82,miR-101-3p,miR-9-5p,miR-3059-5p,novel_60,miR-3956-3p,miR-212-3p,miR-361-3p,miR-1434-5p,miR-217-5p,miR-140-3p,miR-5703,miR-1193,miR-376a-5p,miR-218-1-3p,miR-378d,miR-19b-3p,miR-212-5p,miR-485-3p,miR-18a-3p,novel_83,miR-487a-3p,miR-95-3p,miR-345-3p,miR-125b,miR-210-5p,novel_17,miR-2285p,miR-1277-5p,miR-21-3p,miR-760-3p,miR-345-3p,miR-2432,miR-146a-5p,miR-376a-5p,miR-181b-1-3p,miR-130a-5p,miR-125b-2-3p,miR-21a-3p,miR-450a-1-3p,miR-625-5p,miR-1827,let-7g-3p,miR-339b,miR-345-5p,miR-3065-3p,miR-21-3p,miR-216b-3p,miR-182-5p,miR-496-3p,miR-22-3p,novel_25,miR-155-5p,miR-544-3p,miR-377-3p,miR-323-5p,miR-6130,miR-19b-1-5p,miR-665,miR-708-5p,miR-450b-3p,miR-155-5p,miR-664a,miR-129-2-3p,miR-145a-3p,miR-3184-3p,miR-4510,miR-338-3p,miR-6525,miR-769-5p,miR-22-3p,miR-450c-3p,miR-197-3p,miR-432,miR-19b-2-5p,miR-1434-3p,miR-223-3p,miR-2284x,miR-27b-3p,miR-455-5p,miR-138,miR-29b,miR-30b-3p,miR-219a-1-3p,miR-339-5p,miR-3955-5p,miR-103a-3p,novel_116,miR-544b,miR-103,miR-432-5p,miR-185-5p,miR-330-3p,miR-365b-5p,miR-197-5p,novel_51,miR-2411-3p,miR-455-5p,miR-377-3p,miR-296-3p,miR-146a,miR-106b-3p,miR-
UHMK1	ENSOARG0(	U2AF hom	miR-199b-5p,miR-665,miR-381-3p,miR-374b-3p,miR-324-3p,let-7f-1-3p,miR-199b-5p,miR-199b-5p,miR-9788-3p,miR-199a-5p,miR-199a-5p,miR-20a-3p,miR-212-3p

TMPRSS3	ENSOARG0(	transmem	miR-29b,miR-380-3p,miR-26a-2-3p,miR-744-5p,miR-873a-5p,miR-653,miR-1843b-5p,miR-1434-3p,miR-3535,miR-2285g,let-7g-5p,miR-185-5p,miR-6238,miR-187-3p,miR-31-5p,miR-483-3p,miR-378a-5p,miR-660,miR-452-5p,miR-224-5p,miR-296-3p,miR-330-3p,miR-365b-5p,miR-432-3p,miR-296-3p,let-7a-2-3p,miR-130b-5p,miR-6240,miR-2483-5p,miR-19a-3p,miR-412-3p,miR-2319a,miR-6395,miR-376a-2-5p,miR-135b-5p,let-7e-5p,miR-18a-3p,miR-3065-5p,miR-323a-5p,miR-670-3p,miR-299b-3p,miR-29a-3p,miR-3074-5p,miR-3120-3p,miR-206,miR-29a,miR-502b,miR-6402,let-7i,miR-3959-3p,miR-219b-3p,miR-29c-3p,miR-17-3p,miR-1193,miR-2285x,let-7d,miR-582-3p,let-7a-2-3p,miR-3591-5p,miR-29d-3p,miR-483-3p,miR-582-3p,miR-545-5p,miR-129-1-3p,miR-299a-3p,miR-7641,miR-1224-5p,miR-412,miR-376a-5p,miR-17-3p,let-7f-5p,miR-543-5p,miR-200a-3p,novel_127,miR-205-5p,miR-664-3p,miR-219-3p,miR-181c-3p,let-7c-5p,miR-450b-5p,let-7g,miR-3956-5p,miR-1,miR-3064-5p,miR-4286,miR-10b-3p,miR-7144-5p,miR-3074-1-3p,miR-1814c,miR-671-5p,miR-365a-3p,miR-223-5p,miR-133c,miR-2355-5p,miR-141-3p,miR-29b-3p,miR-5100,miR-370-5p,miR-135a-5p,miR-145-3p,miR-365a-5p,miR-1388-5p,miR-1839-5p,miR-101c,miR-6529a,miR-4791,miR-1298-5p,miR-412-3p,miR-1a-3p,miR-3962,miR-142-3p,miR-9788-3p,miR-2285e,novel_4,miR-330-3p,miR-1343-3p,miR-3085-3p,miR-103a-2-5p,let-7e,miR-29b-1-5p,miR-2310,let-7a-5p,miR-652-5p,miR-376b,miR-744-3p,miR-1983,novel_94,miR-877-3p,miR-361-3p,miR-129b-5p,miR-7857-3p,miR-2483-3p,let-7d-5p,miR-4443,miR-664b,miR-3064-5p,miR-541-5p,miR-4726-5p,miR-490-5p,miR-2440,miR-107-5p,miR-423-5p,miR-8095,miR-219a-2-3p,miR-2447,miR-3059-5p,miR-452-5p,miR-19b-3p,novel_133,miR-2300b-3p,miR-2366,miR-1961,miR-1193,miR-1197-5p,miR-222-5p,miR-328-

MRVI1	ENSOARG0(	murine r	miR-1197-3p,miR-1260b,miR-93,miR-1306-5p,miR-133a-3p,miR-103b,miR-193a-5p,miR-3607-3p,miR-138-5p,miR-134-5p,novel_63,miR-331-3p,miR-6740-5p,miR-34c-5p,miR-199a-5p,miR-574-5p,miR-141-3p,miR-215-5p,miR-2285aa,miR-2355-5p,let-7g-3p,miR-345-5p,miR-329a,miR-671-5p,miR-485-5p,miR-1248,miR-7144-5p,miR-20b-5p,miR-424-3p,miR-17-5p,miR-362-5p,miR-320a,miR-3154,miR-93-5p,miR-1271-3p,miR-2285b,novel_39,miR-29a-5p,miR-199a-5p,miR-181a-2-3p,miR-3064-5p,miR-200c-3p,miR-200a-3p,miR-200c,miR-1260a,novel_121,miR-129-1-3p,miR-545-5p,miR-134-5p,miR-450a-1-3p,miR-429-3p,miR-1224-5p,miR-582-3p,miR-885-3p,miR-10a-5p,miR-200b-3p,miR-1290,miR-134,miR-330-5p,miR-218-2-3p,miR-1343-5p,miR-199b-5p,miR-326-3p,miR-449a,novel_1,miR-656-5p,miR-20a-5p,miR-142b,miR-576-3p,miR-33a-3p,miR-34c,miR-34b,miR-181b-3p,miR-677,miR-320c,miR-6535,miR-216b-3p,miR-363,miR-10a-5p,miR-320b,miR-412-3p,miR-450b-3p,miR-379-5p,miR-3065-5p,miR-150-5p,miR-2285r,miR-345-5p,miR-1343-5p,miR-106a-5p,miR-17-5p,miR-197-5p,miR-432-5p,miR-20b,miR-31-5p,miR-1260b,miR-138,miR-2113,miR-1843b-5p,miR-432,miR-873a-5p,miR-450c-3p,miR-197-3p,miR-10b-5p,miR-129-2-3p,miR-4510,miR-425-5p,miR-665-5p,miR-450b-3p,miR-208b-5p,miR-487a-5p,miR-129-5p,miR-2285j,miR-6130,miR-192-5p,miR-10a,miR-204-3p,miR-32-3p,miR-548o-3p,miR-182-5p,miR-296-3p,miR-345-5p,miR-133b-3p,miR-1827,miR-885-3p,miR-450a-1-3p,miR-125b-2-3p,miR-181b-1-3p,miR-29b-1-5p,miR-20a,miR-466i-5p,miR-106a,miR-320d,miR-34b-5p,miR-374a-5p,miR-185-3p,miR-122-3p,miR-500a-5p,miR-324-3p,miR-200b,miR-7859,miR-106a,miR-548e-3p,miR-212-5p,miR-4429,novel_73,miR-142-3p,miR-33b-3p,miR-34a-5p,miR-215-5p,miR-362-3p,miR-2408,novel_133,miR-2904,miR-362-5p,miR-

MAP2K2	ENSOARG0(	mitogen-	miR-455-3p,miR-181b-2-3p,miR-330-3p,novel_78,miR-1343-3p,miR-1388-5p,miR-362-3p,miR-329-3p,miR-8485,miR-378d,miR-744-3p,miR-1388-3p,miR-214-3p,miR-381-5p,miR-103a-2-5p,miR-146b,miR-199b-5p,miR-130b-3p,miR-491-5p,miR-16b,miR-29b-1-5p,miR-431-5p,miR-4443,miR-144-5p,miR-378g,miR-3596,miR-2285u,miR-2319b,miR-1983,miR-378j,miR-423-5p,miR-1285-5p,novel_111,miR-361-3p,miR-3956-3p,miR-2447,novel_82,miR-210-3p,miR-212-5p,miR-18a-3p,miR-16-1-3p,miR-218-1-3p,miR-378a-3p,miR-193b-5p,miR-378i,miR-486-3p,miR-181b-1-3p,miR-376c-3p,miR-376a-5p,miR-345-3p,miR-92a-1-5p,miR-345-3p,miR-422a,miR-500a-5p,miR-324-3p,novel_17,miR-15b-5p,miR-301a-3p,miR-376e-3p,miR-377-3p,miR-552-3p,miR-296-3p,miR-30c-1-3p,miR-1827,miR-625-5p,miR-345-5p,miR-500a-3p,miR-2285c,miR-3184-5p,miR-224-5p,miR-769-5p,miR-214,miR-3074-5p,miR-874-5p,miR-500,miR-4492,miR-454-3p,miR-194-3p,novel_116,miR-134-3p,miR-412-5p,miR-103a-3p,miR-3955-5p,miR-31-5p,miR-27b-3p,miR-497-5p,miR-381-5p,miR-16-1-3p,miR-16b,miR-432,miR-2285r,miR-6240,miR-193a-3p,miR-378a-5p,miR-2411-3p,novel_51,miR-296-3p,miR-377-3p,miR-146a,miR-181b-3p,miR-677,miR-6535,novel_69,miR-329-5p,miR-216b-3p,miR-3074-5p,novel_68,miR-378f,miR-146b-5p,miR-412-3p,miR-216a-3p,miR-3970,miR-1306,miR-148a-3p,miR-199b-5p,miR-2284w,miR-301b-3p,miR-193b-3p,miR-335-5p,miR-28c,miR-4324,miR-134-5p,miR-322-5p,miR-431,miR-412,miR-2285ad,miR-330-5p,miR-1343-5p,miR-192-3p,miR-450c-5p,miR-29a-5p,novel_39,miR-3064-5p,novel_96,novel_127,miR-152-3p,miR-127-5p,miR-376c-3p,miR-1271,miR-542-5p,miR-2355-5p,miR-874-3p,miR-329a,miR-345-5p,miR-1260b,miR-1197-3p,miR-144-5p,miR-16-5p,miR-592,miR-134-5p,miR-2433,miR-2285o,miR-216a-3p,miR-

WDR78	ENSOARG0(	WD repea	miR-495-3p,miR-301b-3p,miR-625-3p,miR-22-5p,miR-655-5p,miR-3120-3p,miR-26b-5p,miR-2404,miR-320c,miR-677,miR-181b-3p,miR-376b-5p,miR-2285ab,miR-148a-3p,miR-3591-3p,miR-18a-3p,miR-1291,miR-135b-5p,miR-432-3p,let-7j,miR-539-3p,miR-2411-3p,miR-483-3p,miR-31-5p,miR-665,miR-432-5p,miR-2284d,miR-2285g,miR-432,miR-1434-3p,miR-1843b-5p,miR-181a-5p,novel_23,miR-30b-3p,miR-219a-1-3p,miR-2284v,novel_63,miR-2284k,miR-34c-3p,miR-3969,miR-135a-5p,miR-331-5p,miR-486b-3p,miR-28-5p,miR-574-5p,miR-874-3p,miR-141-3p,miR-331-5p,miR-365a-3p,miR-376c-3p,miR-331-5p,miR-376c-5p,miR-320a,miR-3154,miR-10b-3p,miR-329a,miR-2318,miR-376c-5p,miR-30a-3p,miR-181a-2-3p,miR-24-2-5p,miR-376b-5p,miR-200a,novel_39,miR-376c-5p,miR-2284y,miR-2284a,miR-127-5p,miR-181b-5p,miR-200a-3p,novel_127,miR-152-3p,miR-2285ad,miR-302a-5p,miR-483-3p,miR-345-3p,miR-582-3p,miR-155-5p,miR-2285f,miR-181d-5p,miR-2284j,miR-9-3p,miR-154a,miR-2285n,miR-2285y,miR-490-5p,miR-2284g,miR-24-1-5p,miR-7977,miR-25,miR-203-3p,miR-335-3p,miR-9-3p,let-7c-3p,miR-652-5p,miR-2284b,miR-2310,miR-320b,novel_115,miR-323b-3p,miR-362-3p,miR-130b-3p,miR-3955-3p,miR-181b-2-3p,miR-142-3p,miR-1298-3p,miR-455-3p,miR-9788-3p,miR-148b-5p,miR-216a-3p,miR-2285e,miR-126a-5p,miR-329-3p,miR-323b,miR-125b-2-3p,miR-30b-3p,miR-362-3p,miR-101c,miR-122-5p,miR-181c-5p,miR-30e-3p,miR-30a-3p,miR-155-5p,miR-454-3p,miR-708-5p,miR-548o-3p,miR-21-3p,miR-301a-3p,miR-376e-3p,miR-582-5p,miR-656-3p,miR-155-5p,miR-21a-3p,miR-2285c,miR-2312,miR-582-5p,miR-183-5p,miR-376a-5p,miR-181b-1-3p,miR-376c-3p,miR-142a-3p,miR-486-3p,miR-618,miR-345-3p,miR-320d,miR-18a-3p,miR-3600,miR-485-3p,miR-30d-3p,miR-487a-3p,miR-148b-3p,miR-23c,miR-340-5p,miR-532-3p,miR-362-
MIF4GD	ENSOARG0(	MIF4G do	miR-1388-3p,miR-541-3p,miR-769-5p,miR-769,novel_39,miR-4443,miR-541,miR-769-5p,miR-671-5p

FBXL20	ENSOARG0(	F-box an	miR-30f,novel_99,miR-582-5p,miR-625-5p,miR-450a-1-3p,miR-2285c,miR-21a-3p,miR-30d-5p,miR-129b-3p,miR-216b-5p,miR-6130,miR-582-5p,miR-377-3p,miR-192-5p,miR-15b-5p,novel_32,miR-548o-3p,miR-504-5p,miR-532-5p,miR-552-3p,miR-664a,miR-6516-3p,miR-17-3p,miR-450b-3p,miR-130b-5p,miR-424-5p,miR-2285j,miR-665,miR-224-5p,miR-181c-5p,miR-4510,miR-3074-5p,miR-3074-2-3p,miR-34a-5p,miR-148a-5p,miR-133a-3p,miR-362-3p,miR-215-5p,miR-376a-5p,miR-30c-5p,miR-140-3p,miR-124-3p,miR-15a-5p,miR-200b,miR-23c,miR-23b-3p,miR-2284h-5p,miR-466i-5p,miR-760-3p,miR-299-3p,miR-21-3p,miR-34b-5p,miR-30d,miR-210-5p,novel_17,miR-618,miR-30b,miR-216a-5p,miR-130a-5p,miR-421-5p,miR-143-3p,miR-2285w,miR-503-5p,miR-329a-5p,miR-504,novel_94,miR-203-3p,miR-2319b,miR-30b-5p,miR-1285-5p,miR-541-3p,miR-3059-5p,miR-181d-5p,miR-9-5p,miR-362-3p,miR-541,miR-329-3p,miR-30b-3p,miR-7857,miR-147a,miR-2285e,miR-148b-5p,novel_4,miR-199b-3p,miR-222-5p,miR-3955-3p,miR-1185-5p,miR-326,miR-1306-5p,miR-16b,miR-362-3p,miR-8485,miR-424-5p,miR-124a,miR-744-3p,let-7g-3p,miR-329a,miR-1248,miR-485-5p,miR-30e-5p,miR-365a-3p,miR-34c-5p,miR-324-5p,miR-574-5p,miR-141-3p,miR-215-5p,miR-409-3p,miR-100-3p,miR-331-3p,miR-145-3p,miR-6128,miR-16-5p,miR-3607-3p,let-7a-2-3p,miR-200b-3p,miR-30c,miR-128-3p,miR-3578,miR-30a-5p,miR-330-5p,miR-2427,miR-322-5p,miR-9-5p,miR-6517,miR-429-3p,miR-299a-3p,miR-376a-5p,miR-181b-5p,miR-200c-3p,miR-17-3p,miR-200a-3p,miR-107,miR-200c,miR-3071-3p,miR-28b,miR-148b-5p,miR-376b-5p,miR-376c-5p,miR-3529-3p,miR-181a-2-3p,miR-107,miR-450b-3p,miR-6516-3p,miR-195a-5p,miR-376a-2-5p,miR-2319a,miR-3065-5p,miR-216a-3p,miR-378b,miR-1306,miR-299b-3p,miR-34b,miR-329-5p,miR-3074-5p,novel_68,miR-199c,miR-
NBN	ENSOARG0(	nibrin [	miR-1434-5p,miR-107-5p,miR-103a-2-5p
SLC25A27	ENSOARG0(	solute c	miR-20b-5p,miR-133b-3p,miR-93-5p,miR-17-5p,let-7i-5p,miR-323-5p,miR-141-3p,miR-3068-3p,miR-30a-3p,miR-3969,let-7f,miR-30e-3p,let-7b-5p,miR-93,miR-374a-3p,miR-665-5p,miR-133a-3p,let-7b,miR-133a-3p,miR-200b-3p,miR-361-5p,let-7d,miR-1197-5p,miR-1961,miR-200b,miR-30d-3p,miR-429-3p,miR-106a,let-7k,miR-106a,let-7f-5p,miR-200c-3p,miR-466i-5p,miR-98-5p,miR-324-3p,let-7c-5p,miR-200c,miR-2432,miR-200a,miR-146a-5p,let-7g,miR-30a-3p,miR-20a,miR-105-3p,miR-146b-5p,let-7e-5p,miR-1306,let-7d-5p,miR-27a-3p,miR-323a-5p,miR-452-3p,miR-374a-3p,miR-203b-5p,novel_1,miR-33a-3p,let-7i,miR-20a-5p,novel_27,miR-1839-5p,miR-27b-3p,miR-2284x,miR-484,miR-20b,miR-412-5p,let-7g-5p,miR-27a-3p,miR-146b,let-7e,miR-505,novel_51,miR-17-5p,miR-106b-5p,miR-106a-5p,miR-146a,miR-1306-5p,let-7a-5p,miR-382-3p,miR-106a-5p

DVL2	ENSOARG0(	dishevel	miR-23a-3p,miR-424-5p,miR-127-3p,miR-214-3p,miR-125a-5p,miR-16b,miR-455-5p,novel_51,miR-323b-3p,novel_19,miR-224-5p,miR-1185-3p,miR-2459,miR-216a-3p,miR-27a-3p,miR-122-3p,miR-744-5p,miR-455-5p,miR-27b-3p,miR-497-5p,miR-1260b,miR-29b,miR-138,miR-16b,miR-323b,miR-542-3p,miR-2284r,miR-2428,miR-9-3p,miR-8095,miR-361-3p,miR-17-3p,miR-29c-3p,miR-1193,miR-1296-5p,miR-4454,miR-29a,novel_1,miR-16a,miR-769-5p,miR-576-3p,miR-28c,miR-15a,miR-27a-3p,miR-1185-2-3p,miR-29a-3p,miR-541-5p,miR-216b-3p,miR-3074-5p,miR-9-3p,novel_94,miR-195a-5p,miR-1291,miR-216a-3p,miR-150-5p,miR-361-3p,miR-1271-3p,miR-2411,miR-125b-2-3p,miR-28b,miR-29b-2-5p,miR-2385-3p,miR-200a-3p,miR-21-3p,miR-221-5p,miR-17-3p,miR-2898,miR-125b,miR-185-3p,miR-125a-3p,miR-196a-3p,miR-1260a,miR-2284h-5p,miR-532-3p,miR-2411-5p,miR-15a-5p,miR-23b-3p,miR-322-5p,miR-505-5p,miR-218-1-3p,miR-128-3p,miR-29d-3p,miR-2300b-3p,miR-2366,miR-3591-5p,miR-338-3p,miR-1260b,miR-224-5p,miR-769-5p,miR-214,miR-16-5p,miR-3074-5p,miR-4510,miR-28-5p,miR-424-5p,miR-874-5p,miR-708-5p,miR-4492,miR-17-3p,miR-138-5p,miR-29b-2-5p,miR-665,miR-125b-5p,miR-370-5p,miR-29b-3p,miR-15b-5p,miR-769,miR-6130,miR-2284s,miR-1271,miR-125a,miR-141-3p,miR-552-3p,miR-2355-5p,miR-370-5p,miR-221-5p,miR-1827,miR-7144-5p,miR-876-3p,miR-23b,miR-129b-3p,miR-301

ARHGAP35	ENSOARG0(	Rho GTPa	miR-125b-5p,miR-331-3p,miR-6740-5p,miR-29b-3p,miR-28-5p,miR-486b-3p,miR-138-5p,miR-93,miR-93-5p,miR-17-5p,miR-20b-5p,miR-485-5p,miR-671-5p,miR-370-3p,miR-615-5p,miR-125a,miR-874-3p,miR-199a-5p,miR-154b-5p,miR-34c-5p,novel_127,novel_96,miR-4286,miR-199a-5p,miR-3065-3p,miR-1271-3p,miR-29d-3p,miR-1343-5p,miR-330-5p,miR-501-3p,miR-885-3p,miR-196a-2-3p,miR-431,miR-6517,miR-485-3p,miR-34c,miR-20a-5p,miR-656-5p,miR-128-1-5p,novel_1,miR-29a,miR-29c-3p,miR-326-3p,miR-449a,miR-301b-3p,miR-2428,miR-199b-5p,miR-24-3p,miR-1306,miR-18a-3p,miR-21c,miR-3591-3p,miR-2387,miR-3141,miR-6395,miR-1291,miR-19a-3p,miR-329-5p,miR-6535,novel_69,miR-374c-3p,miR-34b,miR-29a-3p,miR-3141,novel_51,miR-106a-5p,miR-17-5p,miR-1343-5p,miR-125a-5p,miR-378a-5p,miR-363-5p,miR-628-5p,miR-130b-5p,miR-374b-3p,miR-708-3p,miR-542-3p,miR-138,miR-29b,miR-21-5p,miR-130a-3p,miR-26b-3p,miR-194-3p,miR-20b,miR-134-3p,miR-665,miR-3604,miR-454-3p,miR-708-5p,miR-4492,miR-130b-5p,miR-664a,miR-214,miR-3184-5p,miR-6134,miR-129b-3p,miR-885-3p,miR-625-5p,let-7g-3p,miR-21-3p,miR-3065-3p,miR-552-3p,miR-2331-3p,miR-301a-3p,miR-3601,miR-125b,miR-185-3p,miR-34b-5p,miR-106a,miR-760-3p,miR-20a,miR-329a-5p,miR-486-3p,miR-5703,miR-140-3p,novel_133,miR-34a-5p,miR-378d,miR-19b-3p,miR-18a-3p,miR-3960,miR-106a,miR-1247-5p,miR-328-3p,miR-107-5p,novel_82,miR-2440,novel_60,miR-3956-3p,miR-502-3p,miR-423-5p,miR-361-3p,miR-361-3p,miR-199b-5p,miR-136-5p,miR-4792,miR-500-3p,miR-378g,novel_19,miR-106b-5p,miR-1306-5p,miR-431-5p,miR-5126,miR-326,miR-199b-5p,miR-103a-2-5p,miR-214-3p,miR-491-5p,miR-130b-3p,let-7c-3p,miR-1388-3p,miR-106a-5p,miR-501-3p,miR-484,miR-2284r,miR-296-5p,miR-1388-5p,miR-9788-3p

TPMT	ENSOARG0(	thiopuri	miR-6535,miR-320c,miR-677,miR-181b-3p,miR-2404,miR-29a-3p,miR-299b-3p,miR-655-5p,miR-320b,miR-10a-5p,miR-135b-5p,miR-195a-5p,miR-6516-3p,miR-21c,miR-3065-5p,miR-2387,miR-326-3p,miR-374c-5p,miR-199b-5p,miR-2428,miR-211,miR-371b-3p,miR-193b-3p,miR-17-3p,miR-29c-3p,miR-2285x,miR-29a,miR-206,miR-16a,miR-15a,miR-142b,miR-28c,miR-576-3p,miR-20a-5p,miR-544b,miR-20b,miR-3535,miR-665,miR-377-5p,miR-31-5p,miR-4508,miR-497-5p,miR-29b,miR-21-5p,miR-16b,miR-219a-1-3p,miR-3071-5p,miR-653,miR-345-5p,miR-2285r,miR-654-3p,miR-432-3p,miR-432-3p,miR-382,miR-452-5p,miR-1343-5p,miR-193a-3p,miR-2411-3p,miR-106a-5p,miR-17-5p,miR-223-5p,miR-133c,miR-199a-5p,miR-376c-3p,miR-769,miR-3965,miR-3956,miR-574-5p,miR-3068-3p,miR-376c-5p,miR-20b-5p,miR-671-5p,miR-2318,miR-345-5p,miR-93-5p,miR-17-5p,miR-320a,miR-3154,miR-181c-3p,miR-93,miR-374a-3p,miR-1197-3p,miR-16-5p,miR-193a-5p,miR-134-5p,miR-374b-3p,miR-204-5p,miR-592,miR-331-3p,miR-3969,miR-135a-5p,miR-29b-3p,miR-5100,miR-494-5p,miR-134-5p,miR-9-5p,miR-322-5p,miR-758-5p,miR-1224-5p,miR-299a-3p,miR-2285ad,miR-6517,miR-10a-5p,miR-29d-3p,miR-1343-5p,miR-330-5p,miR-345-3p,miR-1290,miR-3578,miR-134,miR-29a-5p,miR-199a-5p,miR-2284y,miR-2284a,miR-28b,miR-2478,miR-450b-5p,miR-1,miR-2403,miR-3529-3p,miR-17-3p,miR-127-5p,miR-217,miR-6119-5p,miR-125a-3p,miR-203-3p,miR-25,miR-499a-3p,miR-3596,miR-489,miR-374b-5p,miR-211-5p,miR-2284g,miR-20a-3p,miR-335-3p,miR-181a-3p,miR-504,miR-140-5p,miR-199b-5p,miR-154a,miR-9-5p,miR-2447,miR-212-3p,miR-885-5p,miR-541-3p,miR-374a-3p,miR-490-5p,miR-769-5p,miR-2285y,miR-2285n,miR-382-3p,miR-455-3p,miR-9788-3p,miR-216a-3p,miR-148b-5p,miR-425-3p,miR-153,miR-539-5p,miR-5010-3p,miR-181b-2-
UBTD1	ENSOARG0(	ubiquiti	miR-378e,miR-2404,miR-193b-3p,miR-615-5p,miR-182-5p,miR-378a-3p,miR-2331-3p,miR-885-3p,miR-378f,miR-877-3p,miR-6395,miR-1291,miR-378c,miR-193b-3p,miR-378c,miR-145a-3p,miR-138-5p,miR-664a,miR-301a-5p,miR-328-3p,miR-378h,miR-212-5p,miR-6517,miR-1343-3p,miR-2889,miR-378d,miR-30b-3p,miR-138,miR-885-3p,miR-378c,miR-23b-5p,miR-378a-3p,miR-30b-3p,miR-378i,miR-296-5p,miR-378b,miR-301b-5p,miR-2300b-3p,novel_39,miR-378d,miR-345-3p,miR-2330-5p,miR-193a-3p,miR-422a,miR-182-5p
DDR2	ENSOARG0(	discoidi	miR-484,miR-1246,miR-301b-5p,miR-497-5p,miR-195a-5p,miR-16b,miR-222-5p,miR-340-3p,miR-31-5p,miR-133b-5p,miR-15b-5p,miR-322-5p,miR-15a-5p,miR-16a,miR-485-3p,miR-100-3p,miR-16b,miR-301a-5p,miR-15a,miR-424-5p,miR-490-5p,miR-760-3p,miR-16-5p,miR-744-3p,miR-424-5p

WIBG	ENSOARG0(	within b	novel_9,miR-374b-3p,miR-134-5p,miR-135a-1-3p,miR-135a-5p,miR-1260b,miR-500b-5p,miR-145-3p,miR-10b-3p,miR-365a-5p,miR-3607-3p,miR-16-5p,miR-381-3p,miR-362-5p,miR-503-3p,miR-141-3p,miR-2355-5p,miR-17-3p,miR-2898,miR-200a-3p,miR-664-3p,miR-2285af,miR-1260a,miR-3071-3p,miR-450b-5p,miR-200a,miR-181a-2-3p,miR-4286,miR-29b-2-5p,miR-300,miR-411-3p,miR-496,miR-155-5p,miR-105-2,miR-134,miR-330-5p,miR-23b-5p,miR-483-3p,miR-322-5p,miR-134-5p,miR-545-5p,miR-450a-1-3p,miR-6119-3p,miR-147-5p,miR-656-5p,miR-421-5p,miR-6402,miR-142b,miR-15a,miR-16a,miR-24-3p,novel_27,miR-374c-5p,miR-326-3p,novel_120,novel_101,miR-542-5p,miR-17-3p,miR-195a-5p,miR-135b-5p,miR-2319a,miR-378b,miR-3065-5p,miR-3591-3p,miR-1306,miR-323a-5p,miR-181b-3p,miR-677,miR-378b,miR-483-3p,miR-660,miR-452-5p,miR-330-3p,miR-365b-5p,miR-105-5p,miR-6240,miR-101-5p,miR-363-5p,miR-1260b,miR-16b,miR-380-3p,miR-497-5p,miR-542-3p,miR-1434-3p,miR-450c-3p,miR-185-5p,miR-412-5p,miR-544b,miR-133b-5p,miR-31-5p,miR-155-5p,miR-17-3p,novel_74,miR-450b-3p,miR-424-5p,miR-208b-5p,miR-129-5p,miR-655-3p,miR-665,miR-29b-2-5p,miR-98-3p,miR-6134,miR-122-5p,miR-4510,miR-145a-3p,miR-539-5p,novel_99,miR-186-5p,miR-450a-1-3p,miR-655,miR-411,miR-323-5p,miR-6130,miR-2284s,miR-155-5p,miR-15b-5p,miR-877-5p,miR-660-5p,miR-496-3p,miR-548o-3p,miR-182-5p,miR-21-3p,miR-760-3p,miR-30b-3p,miR-1277-5p,novel_17,miR-210-5p,miR-542-5p,miR-500a-5p,miR-181b-1-3p,miR-664b-3p,miR-421-5p,miR-380-3p,miR-369-3p,miR-184-3p,let-7a-3p,miR-148a-5p,miR-2284u,miR-218-1-3p,miR-664-5p,miR-376a-5p,miR-5703,miR-98-3p,miR-124-3p,miR-15a-5p,miR-383-5p,miR-532-3p,miR-3600,miR-499a-5p,miR-2284q,miR-3964,miR-107-5p,miR-362-5p,miR-877-3p,miR-212-3p,miR-

TACR3	ENSOARG0(	tachykin	miR-6516-3p,miR-155-5p,novel_74,miR-130b-5p,miR-129-5p,miR-208b-5p,miR-424-5p,miR-1468-5p,miR-29b-2-5p,miR-338-5p,miR-3184-5p,miR-425-5p,miR-1271,miR-30f,miR-186-5p,miR-582-5p,miR-625-5p,miR-299,miR-30d-5p,miR-129b-3p,miR-139-5p,miR-216b-5p,miR-376e-3p,miR-544-3p,miR-582-5p,miR-2284s,miR-15b-5p,miR-155-5p,miR-496-3p,miR-32-3p,miR-532-5p,miR-182-5p,miR-216b-3p,miR-320d,miR-21-3p,miR-30d,miR-30b,miR-216a-5p,miR-29b-1-5p,miR-376a-5p,miR-380-3p,miR-146a-5p,miR-2432,miR-369-3p,miR-4429,miR-148a-5p,miR-133a-3p,miR-16-1-3p,miR-30c-5p,miR-124-3p,miR-15a-5p,miR-23c,miR-23b-3p,miR-7859,miR-210-3p,miR-505-3p,miR-212-5p,miR-3600,miR-3968,miR-4726-5p,miR-30b-5p,miR-107-5p,miR-371a-5p,miR-361-3p,miR-362-5p,miR-7705,miR-1285-5p,miR-423-5p,miR-32-3p,miR-2284j,miR-194b-5p,miR-96-5p,miR-299b-5p,miR-335-3p,miR-3082-5p,miR-489,miR-3596,miR-1185-2-3p,miR-27a-3p,miR-144-5p,miR-2319b,miR-139-5p,miR-499b-5p,miR-103a-2-5p,miR-1468,miR-146b,miR-320b,miR-182-5p,miR-16b,miR-29b-1-5p,miR-424-5p,miR-124a,miR-2284b,miR-33a-5p,miR-23a-3p,miR-544a,miR-299a-5p,miR-3120-5p,miR-2284r,miR-2459,miR-1185-3p,miR-421,miR-500-5p,miR-148b-5p,miR-105-1,miR-330-3p,novel_78,miR-27a-3p,miR-331-5p,miR-592,miR-2284k,miR-34c-3p,miR-135a-5p,miR-100-3p,miR-144-5p,miR-16-5p,miR-329b,miR-10b-3p,miR-671-5p,miR-1248,miR-7144-5p,miR-3074-1-3p,miR-331-5p,miR-381-3p,miR-30e-5p,miR-320a,miR-362-5p,miR-200a-5p,miR-376c-3p,miR-154b-5p,miR-2284n,miR-324-5p,miR-503-3p,miR-574-5p,miR-2285aa,miR-127-5p,miR-107,miR-28b,miR-2284a,miR-490-3p,miR-200a,miR-1957a,miR-29b-2-5p,miR-300,miR-496,miR-30c,miR-105-2,miR-128-3p,miR-155-5p,miR-3591-5p,miR-30a-5p,miR-218-2-3p,miR-1271-5p,miR-322-5p,miR-302a-5p,miR-545-5p,miR-582-
SPTLC3	ENSOARG0(	serine p	miR-877-3p,miR-1197-3p,novel_27,miR-181c-5p,miR-2285m,miR-2428,miR-539-5p,miR-145a-3p,miR-885-5p,miR-6128,miR-3607-3p,miR-9-5p,miR-181d-5p,miR-199c,miR-371a-5p,miR-324-3p,miR-135a-5p,miR-582-5p,miR-139-5p,miR-411b-3p,miR-154b-5p,miR-139-5p,miR-548o-3p,miR-542-5p,miR-9851-3p,miR-543-3p,novel_94,miR-485-5p,miR-582-5p,miR-135b-5p,miR-301,miR-18a-3p,miR-664b-3p,miR-3529-3p,miR-668-3p,miR-466i-5p,miR-181b-5p,novel_127,miR-196a-3p,novel_17,miR-431-5p,miR-618,miR-7859,miR-9-5p,miR-222-5p,miR-147a,miR-2284z,miR-9788-3p,miR-2355-3p,miR-18a-3p,miR-199b-3p,novel_78,miR-222-5p,miR-431,miR-2284aa,miR-873a-5p,miR-181a-5p,miR-3071-5p

LCN2	ENSOARGO(	lipocali	miR-323a-5p,novel_69,miR-320c,miR-677,miR-669,miR-181b-3p,miR-150-5p,miR-1291,miR-7975,miR-6516-3p,miR-195a-5p,miR-326-3p,novel_27,miR-199b-5p,miR-15a,miR-20a-5p,miR-6529b,miR-16a,miR-128-1-5p,miR-31-5p,miR-340-3p,miR-665,miR-412-5p,miR-134-3p,miR-20b,miR-873a-5p,miR-542-3p,miR-1843b-5p,miR-22-3p,miR-450c-3p,miR-374b-3p,miR-138,miR-16b,miR-4508,miR-497-5p,miR-2889,miR-744-5p,miR-363-5p,miR-432-3p,miR-432-3p,novel_51,miR-106a-5p,miR-17-5p,miR-483-3p,miR-370-3p,miR-2355-5p,miR-615-5p,miR-1271,miR-199a-5p,miR-93-5p,miR-320a,miR-17-5p,miR-3154,miR-329b,miR-20b-5p,miR-23a-5p,miR-16-5p,miR-93,miR-2433,miR-331-3p,miR-3969,miR-138-5p,miR-486b-3p,miR-1224-5p,miR-322-5p,miR-505-5p,miR-302a-5p,miR-483-3p,miR-758-5p,miR-450a-1-3p,miR-214-5p,miR-129-1-3p,miR-2427,miR-330-5p,miR-23b-5p,miR-3064-5p,miR-199a-5p,miR-3065-3p,miR-490-3p,miR-30c-2-3p,miR-2898,novel_96,miR-543-5p,miR-3064-5p,miR-10b,miR-378g,miR-361-3p,miR-199b-5p,miR-129b-5p,miR-504,miR-9-3p,miR-361-3p,miR-423-5p,miR-4726-5p,miR-2285t,miR-2300a-5p,miR-181b-2-3p,miR-539-5p,miR-2284r,miR-323b,miR-450b-5p,miR-2426,miR-2330-5p,miR-106a-5p,miR-424-5p,miR-30c-1-3p,miR-3187-3p,miR-320b,miR-326,miR-323b-3p,miR-16b,miR-106b-5p,miR-3085-3p,miR-491-5p,miR-199b-5p,miR-2331-3p,miR-504-5p,miR-221-5p,miR-30c-1-3p,miR-3065-3p,miR-27a-5p,miR-323-5p,novel_32,miR-22-3p,miR-15b-5p,miR-21a-3p,miR-450a-1-3p,miR-1827,miR-145a-3p,miR-129-2-3p,miR-3184-5p,miR-338-3p,miR-6134,miR-320e,miR-665,miR-450b-3p,miR-664a,miR-424-5p,miR-487a-5p,miR-129-5p,miR-106a,miR-383-5p,miR-15a-5p,miR-2284h-5p,miR-2300b-3p,miR-2366,miR-5703,miR-140-3p,miR-142-3p,miR-4429,miR-361-5p,miR-378d,miR-2285w,miR-20a,miR-345-3p,miR-181b-1-3p,miR-486-3p,miR-
NPAS1	ENSOARGO(	neuronal	miR-1247-5p,miR-615-5p,miR-6527,novel_79,miR-339-5p,miR-339b,miR-128-1-5p,miR-339a,miR-324-3p
DDX43	ENSOARGO(	DEAD (As	miR-664a,miR-338-3p,miR-101-3p,miR-2285x,miR-323b,miR-323b-3p
PDPN	ENSOARGO(	podoplan	miR-3187-3p,miR-8485,miR-130a-5p,miR-181b-1-3p,miR-4286,miR-382-3p,miR-130b-5p,miR-181b-5p,miR-377-3p,miR-383-5p,miR-181b-2-3p,miR-122-3p,miR-1290,miR-181a-5p,miR-484,miR-7705,miR-181c-5p,miR-181d-5p,miR-130b-5p,miR-142b,novel_63,miR-181b-3p,miR-677,miR-132-5p,miR-377-3p,miR-3956,miR-2285u,miR-329-5p,miR-574-5p,miR-136-5p,miR-129b-5p,miR-200a-5p,miR-150-5p
MRPS11	ENSOARGO(	mitochon	miR-2426,miR-16b,miR-493-3p,miR-2284x,miR-27b-3p,miR-497-5p,miR-873a-5p,miR-22-3p,miR-15a-5p,miR-425-3p,miR-2284d,miR-322-5p,miR-103,miR-103a-3p,miR-6517,miR-18a-3p,miR-27a-3p,miR-2284m,novel_127,miR-107,miR-2285af,miR-16b,miR-324-3p,miR-2284l,miR-2285r,miR-424-5p,miR-2403,miR-107,miR-485-5p,miR-195a-5p,miR-1827,miR-625-5p,miR-361-3p,miR-18a-3p,miR-200a-5p,miR-15b-5p,miR-27a-3p,miR-4443,miR-22-3p,miR-664a,miR-424-5p,miR-15a,miR-2284k,miR-382-5p,miR-16a,miR-2428,miR-2284v,miR-145a-3p,miR-2284j,miR-16-5p,miR-193a-5p

MIER1	ENSOARG0(	mesoderm	miR-216c-5p,miR-494-3p,miR-6240,let-7c-3p,miR-345-3p,miR-376c-5p,miR-376b-5p,miR-2432,miR-320b,miR-330-3p,miR-500a-5p,miR-320d,miR-330-3p,miR-548w,miR-5010-3p,miR-2285g,miR-185-5p,miR-3431,novel_116,miR-2285e,miR-4429,let-7a-3p,novel_120,miR-3059-5p,miR-590-3p,miR-2285f,miR-6527,miR-3607-3p,miR-2285x,let-7b-3p,novel_63,miR-100-3p,miR-2285t,miR-2285u,miR-3956,miR-7-1-3p,miR-374c-3p,miR-320c,miR-2285ab,miR-2285c,miR-129b-3p,miR-320a,let-7g-3p,miR-299b-5p,miR-186-5p
SEC63	ENSOARG0(	SEC63 ho	miR-23c,miR-148b-3p,miR-383-5p,miR-23b-3p,miR-3600,novel_73,miR-133a-3p,miR-362-3p,miR-217-5p,miR-140-3p,miR-188-3p,miR-124-3p,miR-181b-1-3p,miR-664b-3p,miR-369-3p,miR-2484,miR-1277-5p,miR-2285p,miR-139-5p,miR-544-3p,miR-377-3p,miR-301a-3p,miR-877-5p,novel_25,miR-660-5p,miR-552-3p,miR-532-5p,miR-216b-3p,miR-370-5p,novel_99,miR-186-5p,miR-543-3p,miR-625-5p,miR-21a-3p,miR-655,miR-27b-5p,miR-206-3p,miR-181c-5p,miR-2330-3p,miR-17-3p,miR-6516-3p,miR-454-3p,miR-2285j,miR-320e,miR-655-3p,miR-19b-1-5p,miR-151-3p,miR-376b-3p,miR-1185-3p,miR-126a-5p,miR-500-5p,miR-216a-3p,miR-151a-3p,miR-1343-3p,miR-330-3p,miR-181b-2-3p,miR-450b-5p,miR-544a,miR-101a-5p,miR-362-3p,miR-1388-5p,miR-1839-5p,miR-101c,miR-329-3p,miR-484,miR-30b-3p,miR-1a-3p,miR-501-3p,miR-1b-5p,miR-8485,miR-2330-5p,miR-124a,miR-2284b,miR-33a-5p,let-7c-3p,miR-23a-3p,miR-130b-3p,miR-491-5p,miR-3085-3p,miR-326,miR-505,miR-362-3p,miR-7857-3p,miR-500-3p,miR-4443,miR-1185-2-3p,miR-376d,miR-20a-3p,miR-139-5p,miR-3064-5p,miR-664b,miR-126b-5p,miR-7977,miR-1983,miR-9-3p,miR-493-5p,miR-376a-3p,miR-362-5p,miR-7705,miR-502-3p,miR-9-3p,miR-885-5p,miR-181d-5p,miR-6527,miR-628-3p,novel_82,miR-2285n,miR-382-3p,miR-302a-5p,miR-24-2-5p,miR-545-5p,miR-376a-5p,miR-501-3p,miR-330-5p,miR-1343-5p,miR-7134-5p,miR-148b-5p,miR-450b-5p,miR-2285b,miR-192-3p,miR-1957a,miR-3064-5p,miR-1,miR-2385-3p,miR-1a-2-5p,miR-181b-5p,miR-17-3p,miR-152-3p,novel_96,miR-200a-3p,miR-107,miR-664-3p,miR-2397-5p,miR-2285af,miR-205-5p,miR-376b-3p,miR-217,miR-574-5p,miR-141-3p,miR-1271,miR-2285aa,miR-329a,miR-767,miR-7144-5p,miR-190a-3p,novel_48,miR-424-3p,miR-301,miR-488-3p,miR-362-5p,miR-3154,miR-500b-5p,miR-103b,miR-3607-

HBM	ENSOARG0(	hemoglob	miR-139-5p,miR-378e,miR-3596,miR-4443,miR-181b-3p,miR-677,miR-148a-3p,miR-361-3p,miR-378f,miR-379-5p,miR-342-3p,novel_101,miR-24-3p,miR-423-5p,miR-206,miR-4726-5p,miR-2284q,miR-1843a-3p,miR-1343-3p,miR-181b-2-3p,miR-185-5p,miR-432-5p,miR-153,miR-194-3p,miR-9788-3p,miR-143-5p,miR-432,miR-378b,miR-1a-3p,miR-1388-5p,miR-378c,miR-744-5p,miR-3120-5p,miR-101c,miR-1388-3p,miR-363-5p,miR-30c-1-3p,miR-378d,miR-296-3p,miR-296-3p,miR-377-3p,miR-342,miR-197-5p,miR-3955-3p,miR-1468,miR-153-3p,miR-2331-3p,miR-30c-1-3p,miR-378a-3p,miR-615-5p,miR-296-3p,miR-216b-3p,miR-139-5p,miR-377-3p,miR-6516,miR-151b,miR-324-5p,novel_32,miR-876-3p,miR-485-5p,miR-378c,miR-206-3p,miR-338-3p,miR-6134,miR-3184-5p,miR-10b-3p,miR-378c,miR-665,miR-331-3p,miR-1468-5p,miR-31-3p,miR-1224-5p,miR-378h,miR-6516-5p,miR-148b-3p,miR-2284h-5p,miR-151-5p,miR-1290,miR-378i,miR-1197-5p,miR-140-3p,miR-378a-3p,miR-2285w,miR-181b-1-3p,miR-450b-5p,novel_39,miR-92a-1-5p,miR-422a,miR-324-3p,miR-30c-2-3p,miR-760-3p,miR-466i-5p,miR-450a-2-3p,miR-2898,miR-152-3p
GPKOW	ENSOARG0(	G patch	miR-23b,let-7g-3p,let-7a-2-3p,miR-147-5p,miR-23a-3p,miR-23b-3p,miR-23c,miR-4532,let-7a-2-3p
CDCP1	ENSOARG0(	CUB doma	miR-17-3p,miR-29c-3p,novel_27,miR-2285m,miR-2428,miR-24-3p,miR-371b-3p,miR-425-5p,miR-576-3p,miR-133a-5p,miR-421-5p,miR-29a,miR-206,miR-320b,miR-3120-3p,miR-329-5p,miR-677,miR-181b-3p,miR-2404,miR-411b-3p,miR-374c-3p,miR-299b-3p,miR-29a-3p,miR-146b-5p,miR-1291,miR-450b-3p,miR-668-3p,miR-2483-5p,miR-363-5p,miR-628-5p,miR-130b-5p,miR-6240,let-7j,miR-330-3p,miR-2411-3p,miR-224-5p,miR-146a,miR-377-3p,miR-22851,miR-339-5p,miR-187-3p,miR-149-5p,miR-2285g,miR-134-3p,miR-185-5p,miR-22-3p,novel_23,miR-19b-2-5p,miR-2889,miR-29b,miR-30b-3p,miR-1260b,miR-193a-5p,miR-374b,miR-1260b,miR-331-3p,miR-3969,miR-409-3p,miR-29b-3p,miR-135a-1-3p,miR-28-5p,miR-370-3p,miR-2355-5p,miR-3956,miR-3957-5p,miR-874-3p,miR-154b-5p,miR-3957,miR-300-3p,miR-769,miR-488-3p,miR-30a-3p,miR-1,miR-181a-2-3p,novel_39,miR-200a,miR-30c-2-3p,miR-339a,miR-1260a,miR-125a-3p,miR-543-5p,miR-17-3p,miR-2898,novel_79,miR-7641,miR-2285ad,miR-299a-3p,miR-486-5p,miR-505-5p,miR-2411-5p,miR-6516-5p,miR-29d-3p,miR-30a-5p,miR-345-3p,miR-1290,miR-30c,miR-582-3p,miR-6527,novel_60,miR-3956-3p,miR-423-5p,miR-2285y,miR-769-5p,miR-371a-5p,miR-30b-5p,novel_82,miR-374b-5p,miR-7977,miR-4443,miR-3596,miR-25,miR-378g,miR-129b-5p,miR-409-5p,miR-335-3p,miR-504,miR-652-5p,miR-1388-3p,miR-124a,miR-2330-5p,miR-8485,miR-30c-1-3p,miR-214-3p,miR-25-5p,miR-222-5p,miR-181b-2-3p,miR-2300a-5p,miR-1343-3p,miR-330-3p,miR-455-3p,miR-2285e,miR-147a,miR-1a-3p,miR-30b-3p,miR-6529a,miR-484,miR-143-5p,miR-1388-5p,miR-450b-5p,miR-214,miR-3184-3p,miR-145a-3p,miR-3184-5p,miR-338-3p,miR-769-5p,miR-206-3p,miR-224-5p,miR-30a-3p,miR-19b-1-5p,miR-320e,miR-30e-3p,miR-500,miR-129-5p,miR-708-5p,miR-4492,miR-130b-5p,novel_74,miR-17-3p,miR-664a,miR-2331-

GOSR1	ENSOARG0(	golgi SN	miR-7641,miR-2284z,novel_83,miR-199a-3p,miR-328-3p,novel_121,miR-302a-5p,miR-6516-5p,miR-222-5p,miR-2411-5p,miR-188-3p,miR-217-5p,miR-1193,novel_133,miR-2300b-3p,miR-128-3p,miR-582-3p,miR-329a-5p,miR-105-3p,miR-2411,miR-29b-1-5p,miR-181b-1-3p,miR-219b-5p,miR-217,miR-181c-3p,miR-2285af,miR-2285p,miR-618,miR-543-5p,miR-21-3p,miR-450a-2-3p,miR-370-3p,miR-2285aa,miR-21-3p,miR-615-5p,miR-140-5p,miR-574-5p,miR-204-3p,novel_25,miR-324-5p,miR-582-5p,miR-6516,miR-769,miR-376c-5p,miR-488-3p,miR-2285c,miR-376c-5p,miR-582-5p,miR-1814c,miR-876-3p,miR-10b-3p,miR-2312,miR-6128,miR-3958-5p,miR-3074-2-3p,miR-145a-3p,miR-2284v,miR-181c-3p,miR-769-5p,miR-26c,miR-665,miR-34c-3p,miR-2284k,miR-2332,miR-129-5p,miR-181b-2-3p,miR-27a-3p,miR-3955-5p,miR-548w,miR-133b-5p,miR-216a-3p,miR-194-3p,miR-1185-3p,miR-2285e,miR-2285g,miR-147a,miR-185-5p,miR-374b-3p,miR-653,miR-2284aa,miR-27b-3p,miR-2284x,miR-299a-5p,miR-450b-5p,miR-668-3p,let-7f-2-3p,let-7f-2-3p,miR-493-5p,miR-2310,miR-432-3p,miR-2284l,miR-29b-1-5p,miR-505,miR-323b-3p,miR-2957,miR-199a-3p,miR-103a-2-5p,miR-2285o,miR-655-5p,miR-329-5p,miR-677,miR-669,miR-181b-3p,miR-1185-2-3p,miR-27a-3p,miR-7857-3p,miR-670-3p,miR-411b-3p,miR-216a-3p,miR-129b-5p,miR-2285ab,miR-376b-5p,miR-493-5p,miR-6395,miR-1983,miR-181a-3p,miR-410-5p,miR-412-3p,miR-2285f,miR-2447,miR-323c,novel_111,miR-769-5p,miR-107-5p,novel_82
HSD17B7	ENSOARG0(	hydroxys	miR-3578,miR-124-3p,miR-2285y,miR-105-5p,miR-105-2,miR-2285ad,miR-124a,miR-105-1,miR-3074-2-3p
MMP19	ENSOARG0(	matrix m	miR-2285f,miR-193b-3p,miR-190b-5p,miR-3059-5p,miR-2447,novel_27,miR-326-3p,miR-877-3p,miR-361-3p,miR-211,novel_82,miR-656-5p,novel_1,miR-655-5p,miR-211-5p,miR-7977,miR-4792,miR-20a-3p,miR-677,miR-144-3p,miR-4443,miR-181b-3p,miR-670-3p,miR-299b-3p,miR-2285ab,miR-18b,novel_94,miR-877-3p,miR-1983,miR-3963,miR-450b-3p,miR-412-3p,miR-33a-5p,miR-1388-3p,miR-8485,miR-3187-3p,miR-2285r,miR-30c-1-3p,miR-190a,miR-455-5p,miR-7-5p,miR-377-3p,miR-326,miR-660,miR-103a-2-5p,miR-193a-3p,miR-491-5p,miR-3955-3p,miR-181b-2-3p,miR-2300a-5p,novel_78,miR-2285e,miR-2285g,miR-412-5p,miR-147a,miR-3431,miR-3071-5p,miR-30b-3p,miR-2284r,miR-7b-5p,miR-455-5p,miR-30b-3p,miR-1388-5p,miR-425-5p,miR-539-5p,novel_42,miR-145a-3p,miR-6134,miR-6525,miR-144,miR-331-3p,miR-665,miR-190a-5p,miR-134-5p,miR-204-5p,miR-370-3p,miR-3065-3p,miR-532-5p,miR-1271,miR-141-3p,miR-30c-1-3p,miR-2331-3p,miR-221-5p,miR-660-5p,miR-204-3p,miR-154b-5p,miR-2284s,miR-6516,miR-377-3p,miR-193b-3p,miR-18a,miR-488-3p,miR-2285c,miR-671-5p,miR-485-5p,miR-18a-5p,miR-3065-3p,miR-2432,miR-490-3p,miR-181b-1-3p,miR-130a-5p,miR-30c-2-3p,miR-221-5p,miR-200a-3p,miR-299-3p,novel_96,miR-466i-5p,miR-18b-5p,miR-299a-3p,miR-134-5p,novel_121,miR-6516-5p,miR-218-2-3p,miR-2904,miR-2427,miR-330-5p,miR-23b-5p,miR-140-3p,novel_133,miR-134,miR-2300b-3p,miR-218-1-3p,miR-33b-3p,miR-184-3p

NSF	ENSOARG0(	N-ethylm	miR-6535,miR-144-3p,miR-2483-3p,miR-25,miR-499a-3p,miR-211-5p,miR-216b-3p,miR-4792,miR-195a-5p,miR-140-5p,miR-2284w,miR-423-5p,miR-211,miR-363-3p,miR-135a-2-3p,novel_1,miR-92a-3p,miR-16a,miR-15a,miR-9788-3p,miR-142-3p,miR-26b-3p,miR-497-5p,miR-219a-1-3p,miR-16b,miR-19b-2-5p,miR-653,miR-296-3p,miR-432-3p,miR-30c-1-3p,miR-424-5p,miR-101b-3p,miR-214-3p,miR-466f-3p,miR-124-5p,miR-16b,miR-32,miR-296-3p,miR-15b-5p,miR-6130,miR-25-3p,miR-499b-3p,miR-532-5p,miR-552-3p,miR-32-5p,miR-140-5p,miR-30c-1-3p,miR-625-5p,miR-27b-5p,miR-21a-3p,miR-3184-5p,miR-214,miR-103b,miR-16-5p,miR-4510,miR-204-5p,miR-424-5p,miR-19b-1-5p,miR-144,novel_83,miR-15a-5p,miR-302a-5p,miR-322-5p,miR-33b-3p,miR-142a-3p,miR-92b-3p,miR-450b-5p,miR-181a-2-3p,miR-183-5p,miR-92a-3p,miR-127-5p,miR-30c-2-3p
NAIF1	ENSOARG0(	nuclear	miR-2447,miR-3956-3p,miR-6527,miR-361-3p,miR-1285-5p,miR-7705,miR-218-5p,miR-101-3p,miR-2440,miR-499a-5p,miR-2285u,miR-3596,miR-378g,miR-2483-3p,miR-203-3p,miR-4443,miR-500-3p,miR-140-5p,miR-668-5p,miR-376a-3p,miR-1983,miR-124a,miR-744-3p,miR-8485,miR-199a-3p,miR-16b,miR-491-5p,miR-1185-5p,miR-487b-5p,miR-146b,miR-199b-5p,miR-548w,miR-1343-3p,miR-376b-3p,miR-147a,miR-1298-3p,miR-455-3p,miR-2459,miR-501-3p,miR-758-3p,miR-1388-5p,miR-101c,miR-6525,miR-224-5p,miR-122-5p,miR-2285j,miR-30e-3p,miR-30a-3p,miR-151-3p,miR-665,miR-3604,miR-190a-5p,miR-874-5p,miR-30c-1-3p,miR-2331-3p,miR-15b-5p,miR-2285c,miR-129b-3p,miR-411,miR-345-5p,miR-339b,miR-625-5p,miR-186-5p,miR-329a-5p,miR-195a-3p,miR-377-5p,miR-142a-3p,miR-486-3p,miR-210-5p,miR-2285p,miR-324-3p,miR-92a-1-5p,miR-18a-3p,miR-30d-3p,miR-193b-5p,miR-2366,miR-218-1-3p,miR-133a-3p,miR-361-5p,miR-29c-3p,miR-199b-5p,miR-2284w,miR-4324,miR-1247-3p,miR-142b,novel_87,miR-6119-3p,miR-379-3p,miR-29a,novel_68,miR-329-5p,miR-216b-3p,miR-411b-3p,miR-374c-3p,miR-677,miR-320c,miR-3141,miR-1306,miR-150-5p,miR-216a-3p,miR-3065-5p,miR-412-3p,miR-146b-5p,miR-668-3p,miR-503-3p,miR-7-5p,miR-146a,miR-3141,miR-365b-5p,miR-378a-5p,miR-31-5p,miR-339-5p,miR-412-5p,miR-134-3p,miR-149-5p,miR-708-3p,miR-223-3p,miR-7b-5p,miR-22-3p,miR-16b,miR-4508,miR-497-5p,novel_42,miR-16-5p,miR-1260b,miR-2433,miR-125b-5p,miR-574-5p,miR-2355-5p,miR-125a,miR-3959-5p,miR-301,miR-767,miR-2318,miR-345-5p,miR-1248,miR-450b-5p,novel_39,miR-29a-5p,novel_96,novel_127,miR-6517,miR-429-3p,miR-412,novel_79,miR-18b-5p,miR-322-5p,miR-1290,miR-155-5p,novel_60,miR-3059-5p,miR-2285f,miR-502-3p,miR-371a-5p,miR-4726-5p,miR-452-3p,miR-20a-3p,miR-499b-5p,miR-454-

RHNO1	ENSOARG0(	RAD9-HUS	miR-30b-3p,miR-16-1-3p,miR-101c,miR-27b-3p,miR-2284x,miR-412-3p,miR-30b-3p,miR-1298-3p,miR-1343-3p,miR-548w,miR-27a-3p,miR-3085-3p,miR-2285l,miR-224-5p,miR-296-3p,miR-431-5p,miR-654-3p,miR-296-3p,miR-127-3p,miR-652-5p,novel_94,miR-18a-3p,miR-378b,miR-146b-3p,miR-6535,miR-27a-3p,miR-3064-5p,miR-20a-3p,miR-499b-5p,miR-320b,miR-211-5p,miR-128-1-5p,novel_1,miR-147-5p,miR-218-5p,miR-371a-5p,miR-211,miR-154a,miR-2428,miR-2285m,miR-2447,miR-452-5p,miR-16-1-3p,miR-3591-5p,miR-140-3p,miR-671-3p,miR-545-5p,miR-18a-3p,miR-3960,miR-412,miR-431,novel_127,miR-21-3p,novel_17,miR-3064-5p,miR-2385-3p,miR-1827,miR-503-3p,miR-331-5p,miR-204-3p,miR-154b-5p,miR-2331-3p,miR-874-3p,miR-548o-3p,miR-2355-5p,miR-331-5p,miR-204-5p,miR-224-5p,miR-145a-3p,miR-425-5p
SLC35D1	ENSOARG0(	solute c	miR-16-1-3p,miR-199b-5p,miR-143-5p,miR-148a-3p,miR-199a-5p,miR-301a-3p,miR-148b-3p,miR-130a-3p,miR-199b-5p,miR-454-3p,miR-152-3p,miR-4726-5p,miR-130b-3p,miR-2285p,miR-199a-5p,miR-301b-3p,miR-199b-5p,miR-219b-3p
OOEP	ENSOARG0(	oocyte e	miR-450a-5p,miR-3958-3p,miR-2285y,miR-382-3p,miR-1285-5p,miR-362-5p,miR-194b-5p,miR-9-5p,miR-212-3p,miR-3059-5p,miR-1983,miR-544-5p,miR-493-5p,miR-504,miR-4443,miR-1185-2-3p,miR-203-3p,miR-3082-5p,miR-499a-3p,miR-2285u,miR-20a-3p,miR-3064-5p,miR-3085-3p,miR-491-5p,miR-106b-5p,miR-16b,miR-182-5p,miR-29b-1-5p,miR-2957,miR-320b,miR-199a-3p,miR-758-3p,miR-2284b,miR-23a-3p,miR-106a-5p,miR-424-5p,miR-16-2-3p,miR-101c,miR-2284aa,miR-2426,miR-1388-5p,miR-758-3p,miR-484,miR-2284r,miR-1185-3p,miR-421,miR-500-5p,miR-148b-5p,miR-5010-3p,miR-199b-3p,miR-151a-3p,miR-2300a-5p,miR-208b-5p,miR-424-5p,miR-17-3p,novel_74,miR-29b-2-5p,miR-338-5p,miR-664a-5p,miR-151-3p,miR-19b-1-5p,miR-2285j,miR-122-5p,miR-345-5p,let-7g-3p,miR-129b-3p,miR-15b-5p,miR-22-3p,miR-193b-3p,miR-216b-5p,miR-499b-3p,miR-6516,miR-296-3p,miR-182-5p,miR-532-5p,miR-496-3p,miR-2331-3p,miR-504-5p,miR-466i-5p,miR-202-5p,miR-106a,miR-320d,miR-185-3p,miR-92a-1-5p,miR-500a-5p,miR-196a-3p,miR-2424,miR-125b-2-3p,miR-376a-5p,miR-29b-1-5p,miR-20a,miR-195a-3p,miR-2285w,miR-216c-5p,miR-183-5p,miR-2284u,miR-378d,miR-148a-5p,miR-16-1-3p,miR-218-1-3p,novel_73,miR-4429,miR-140-3p,miR-5703,miR-1434-5p,miR-1246,miR-2300b-3p,miR-2284h-5p,miR-199a-3p,miR-15a-5p,miR-487a-3p,miR-23b-3p,miR-212-5p,miR-485-3p,miR-505-3p,miR-3600,miR-106a,miR-18a-3p,miR-199c,miR-22-5p,miR-29a,miR-16a,miR-485-3p,miR-20a-5p,miR-28c,miR-142b,miR-576-3p,miR-502-5p,miR-15a,miR-2428,novel_27,miR-3959-3p,miR-17-3p,miR-29c-3p,miR-193b-3p,miR-505-3p,miR-135b-5p,miR-195a-5p,miR-412-3p,miR-107,miR-18a-3p,miR-21c,miR-216a-3p,miR-376b-5p,miR-320c,novel_69,miR-29a-3p,miR-2404,miR-466f-3p,miR-193a-3p,miR-196b-5p,miR-194a,miR-106a-5p,miR-17-5p,miR-296-

RGS4	ENSOARG00	regulato	miR-452-5p,miR-541-3p,miR-8095,miR-129b-5p,miR-199b-5p,miR-541-5p,miR-664b,miR-3064-5p,let-7d-5p,miR-7857-3p,miR-489,miR-3082-5p,miR-362-3p,miR-342,miR-1306-5p,miR-326,let-7e,miR-1468,miR-3085-3p,miR-376b,let-7a-5p,miR-106a-5p,miR-424-5p,miR-3187-3p,miR-491-3p,miR-7857,miR-30b-3p,miR-484,miR-143-5p,miR-3120-5p,miR-450b-5p,miR-5010-3p,miR-105-1,miR-151a-3p,miR-2300a-5p,miR-29d-5p,miR-424-5p,miR-487a-5p,miR-450b-3p,miR-6516-3p,miR-17-3p,miR-2330-3p,miR-3074-2-3p,miR-4510,miR-145a-3p,let-7b-5p,miR-338-3p,miR-29c-5p,miR-3613-5p,let-7f-1-3p,novel_99,miR-582-5p,miR-370-5p,miR-21-3p,miR-182-5p,miR-496-3p,miR-6516,miR-139-5p,miR-6130,miR-30b-3p,miR-1277-5p,miR-299-3p,miR-21-3p,miR-106a,miR-466i-5p,miR-760-3p,miR-20a,miR-216c-5p,miR-183-5p,miR-146a-5p,miR-29b-1-5p,miR-130a-5p,miR-2904,novel_133,miR-362-3p,let-7a-3p,miR-1b-3p,miR-3600,novel_83,miR-199a-3p,miR-383-5p,miR-148b-3p,miR-15a-5p,miR-16a,let-7i,miR-382-5p,miR-15a,miR-20a-5p,miR-656-5p,miR-199c,novel_1,miR-17-3p,miR-1193,miR-342-3p,novel_120,miR-219b-3p,miR-136-3p,novel_27,miR-326-3p,miR-211,miR-323c,miR-3959-3p,miR-21c,miR-18a-3p,miR-378b,miR-195a-5p,miR-107,miR-146b-3p,miR-670-3p,miR-17-5p,miR-106a-5p,miR-455-5p,miR-452-5p,miR-466f-3p,miR-124-5p,miR-363-5p,miR-296-3p,miR-345-5p,miR-3071-5p,miR-145a-5p,miR-873a-5p,miR-1434-3p,miR-455-5p,miR-21-5p,miR-30b-3p,miR-380-3p,miR-219a-1-3p,miR-103,miR-20b,miR-185-5p,let-7g-5p,miR-409-3p,miR-370-5p,miR-5100,miR-410-3p,miR-374b-3p,miR-204-5p,miR-103b,miR-1306-5p,miR-323-3p,miR-93,miR-374a-3p,miR-3154,miR-17-5p,miR-488-3p,miR-7144-5p,miR-485-5p,miR-1814c,miR-10b-3p,let-7g-3p,miR-199a-5p,miR-324-5p,miR-154b-5p,miR-374c-3p,miR-365a-3p,let-7c-5p,miR-107,miR-
HEPACAM	ENSOARG00	hepatic	miR-147-3p,miR-27b-3p,miR-2889,miR-484,miR-665,miR-331-3p,miR-27a-3p,miR-2285m,novel_25,miR-181a-2-3p,miR-27a-3p

PDSS2	ENSOARG0(	prenyl (	miR-320b,miR-3120-3p,miR-26b-5p,miR-378b,miR-6535,miR-146b-3p,miR-2404,miR-411b-3p,miR-29a-3p,miR-374c-3p,miR-216a-3p,miR-18a-3p,miR-2387,miR-146b-5p,miR-9851-3p,miR-195a-5p,miR-1291,novel_101,miR-29c-3p,miR-219b-3p,miR-2284w,miR-16a,miR-33a-3p,miR-15a,miR-502-5p,miR-147-5p,miR-6123,miR-502b,miR-6119-3p,miR-29a,novel_1,miR-26b-3p,miR-31-5p,miR-411-5p,novel_116,miR-194-3p,miR-544b,miR-185-5p,miR-450c-3p,miR-22-3p,miR-197-3p,miR-873a-5p,miR-19b-2-5p,miR-653,miR-1434-3p,miR-542-3p,miR-497-5p,miR-455-5p,miR-27b-3p,miR-16b,miR-138,miR-29b,miR-363-5p,miR-668-3p,miR-6240,miR-432-3p,miR-330-3p,miR-197-5p,miR-365b-5p,miR-455-5p,miR-224-5p,miR-146a,miR-1343-5p,miR-466f-3p,miR-483-3p,miR-370-3p,miR-300-3p,miR-331-5p,miR-488-3p,miR-3074-1-3p,miR-1814c,miR-671-5p,miR-193a-5p,miR-16-5p,miR-1306-5p,miR-365a-5p,miR-1197-3p,miR-29b-3p,miR-135a-1-3p,miR-5100,miR-134-5p,miR-138-5p,miR-412,miR-1224-5p,miR-431,miR-2285ad,miR-6517,miR-134-5p,miR-582-3p,miR-450a-1-3p,miR-302a-5p,miR-322-5p,miR-505-5p,miR-483-3p,miR-2411-5p,miR-6516-5p,miR-218-2-3p,miR-1343-5p,miR-29d-3p,miR-134,miR-1290,miR-128-3p,miR-3064-5p,miR-181a-2-3p,miR-3065-3p,miR-29a-5p,miR-2285b,miR-450c-5p,miR-30c-2-3p,miR-2285af,miR-200c,miR-125a-3p,miR-543-5p,novel_96,novel_127,miR-2898,miR-450a-2-3p,miR-200c-3p,miR-499b-5p,miR-139-5p,miR-3064-5p,miR-2319b,miR-20a-3p,miR-203-3p,miR-27a-3p,miR-499a-3p,miR-2483-3p,miR-3082-5p,miR-378j,miR-129b-5p,miR-140-5p,miR-452-5p,let-7i-3p,miR-3059-5p,miR-541-3p,miR-1285-5p,miR-2285y,miR-218-5p,miR-2440,miR-101-3p,miR-2284q,miR-628-3p,miR-450a-5p,miR-27a-3p,novel_4,novel_78,miR-330-3p,miR-409b,miR-216a-3p,miR-455-3p,miR-147a,miR-412-3p,miR-484,miR-6529a,miR-541,miR-101c,miR-1388-5p,miR-

TASP1	ENSOARG0(	taspase,	miR-3074-5p,miR-655-5p,miR-10a-5p,miR-3120-3p,miR-2404,miR-323a-5p,miR-670-3p,miR-677,miR-144-3p,miR-181b-3p,miR-2387,miR-148a-3p,miR-150-5p,miR-3591-3p,miR-376b-3p,miR-21c,miR-410-5p,miR-450b-3p,miR-412-3p,miR-9851-3p,miR-2319a,miR-219b-3p,miR-342-3p,miR-1839-3p,miR-17-3p,miR-502-5p,miR-6119-3p,miR-421-5p,miR-6123,miR-31-5p,miR-412-5p,miR-3431,miR-6238,miR-708-3p,miR-1285,miR-2113,miR-542-3p,miR-1434-3p,miR-450c-3p,miR-22-3p,miR-197-3p,novel_23,miR-145a-5p,miR-21-5p,miR-138,miR-380-3p,miR-6240,let-7f-2-3p,let-7f-2-3p,miR-432-3p,miR-539-3p,let-7j,miR-296-3p,miR-224-5p,miR-377-3p,miR-296-3p,miR-330-3p,miR-105-5p,miR-365b-5p,novel_51,miR-487a-3p,miR-3068-3p,miR-2285aa,miR-300-3p,miR-365a-3p,miR-324-5p,miR-376c-3p,miR-381-3p,miR-10b-3p,miR-329b,novel_48,miR-671-5p,miR-485-5p,miR-1248,miR-3607-3p,miR-103b,miR-323-3p,miR-1197-3p,miR-374a-3p,miR-145-3p,miR-365a-5p,miR-34c-3p,miR-144,miR-138-5p,miR-410-3p,miR-374b-3p,miR-302a-5p,miR-450a-1-3p,miR-486-5p,miR-129-1-3p,miR-885-3p,miR-300,miR-582-3p,miR-105-2,miR-30c,miR-10a-5p,miR-29b-2-5p,miR-450c-5p,miR-376b-3p,miR-205-5p,miR-30f,miR-30c-2-3p,miR-3071-3p,miR-217,miR-17-3p,miR-152-3p,miR-10b,miR-2319b,miR-20a-3p,miR-2285u,miR-7977,miR-499a-3p,miR-3082-5p,miR-1185-2-3p,miR-376d,miR-378j,miR-129b-5p,miR-136-5p,miR-376a-3p,novel_94,miR-1983,novel_60,miR-3956-3p,miR-212-3p,miR-885-5p,miR-452-5p,miR-194b-5p,miR-423-5p,miR-2285n,miR-30b-5p,miR-382-3p,miR-101-3p,miR-371a-5p,miR-107-5p,miR-450a-5p,miR-3964,miR-374a-3p,miR-154-3p,miR-409b,miR-105-1,novel_78,novel_4,miR-2300a-5p,miR-548w,miR-330-3p,miR-145b,miR-1298-3p,miR-142-3p,miR-376b-3p,miR-9788-3p,miR-148b-5p,miR-216a-3p,miR-1185-3p,miR-10b,miR-2426,miR-
ZC3HAV1	ENSOARG0(	zinc fin	miR-17-3p,novel_60,miR-3956-3p,miR-361-3p,miR-877-3p,miR-3959-3p,novel_87,miR-218-5p,miR-101-3p,miR-374a-3p,miR-3964,miR-6119-3p,miR-206,miR-4792,miR-20a-3p,miR-452-3p,miR-203-3p,miR-146b-3p,miR-3596,miR-489,miR-374c-3p,miR-1306,miR-6516-3p,miR-6395,miR-450b-3p,miR-107,miR-2483-5p,miR-101b-3p,miR-654-3p,novel_115,miR-431-5p,miR-1306-5p,miR-452-5p,miR-106b-3p,miR-145b,miR-103a-3p,miR-2300a-5p,miR-216a-3p,miR-6238,miR-194-3p,miR-103,miR-185-5p,miR-147a,miR-450c-3p,miR-1a-3p,miR-197-3p,miR-145a-5p,miR-374b-3p,miR-1434-3p,miR-101c,miR-2284aa,miR-450b-5p,miR-101a-5p,miR-3607-3p,miR-425-5p,miR-133a-3p,miR-374a-3p,miR-206-3p,miR-6740-5p,miR-409-3p,miR-450b-3p,miR-6516-3p,miR-17-3p,miR-487b-5p,miR-155-5p,miR-331-5p,miR-1277-3p,novel_25,miR-155-5p,miR-374c-3p,miR-21a-3p,miR-331-5p,miR-450a-1-3p,novel_99,miR-485-5p,miR-133b-3p,miR-1,miR-130a-5p,miR-107,miR-202-5p,miR-17-3p,miR-191-3p,miR-431,miR-548e-3p,miR-450a-1-3p,miR-2284z,miR-328-3p,miR-7859,miR-140-3p,miR-155-5p,novel_73

ABR	ENSOARG0(active B	miR-2433,miR-29b-3p,miR-125b-5p,miR-331-3p,miR-6740-5p,miR-767-5p,miR-191-5p,miR-1260b,miR-18a,miR-767,miR-345-5p,miR-7144-5p,miR-1248,miR-615-5p,miR-1271,miR-125a,miR-34c-5p,miR-1260a,miR-125a-3p,miR-107,miR-3064-5p,miR-148b-5p,novel_39,miR-3065-3p,miR-2285b,miR-1343-5p,miR-29d-3p,miR-885-3p,miR-2285ad,miR-6517,miR-18b-5p,miR-758-5p,miR-6516-5p,miR-129-1-3p,miR-502-5p,miR-34c,miR-128-1-5p,miR-29a,miR-656-5p,novel_101,miR-29c-3p,miR-449a,miR-2428,miR-18b,miR-1306,miR-1291,miR-107,miR-9851-3p,miR-320b,miR-34b,miR-29a-3p,miR-503-5p,miR-330-3p,miR-194a,miR-378a-5p,miR-125a-5p,miR-1343-5p,miR-6240,miR-345-5p,miR-432,miR-145a-5p,novel_23,miR-194-5p,miR-29b,miR-1260b,miR-4508,miR-103a-3p,miR-432-5p,miR-2285g,miR-503-5p,miR-185-5p,miR-149-5p,miR-103,miR-544b,miR-29d-5p,miR-320e,miR-665,miR-874-5p,miR-4492,miR-129-2-3p,miR-214,miR-29c-5p,miR-122-5p,miR-21a-3p,miR-2285c,miR-18a-5p,miR-345-5p,miR-885-3p,miR-1895,miR-1827,miR-2331-3p,miR-504-5p,miR-221-5p,miR-3065-3p,miR-552-3p,miR-296-3p,miR-182-5p,miR-544-3p,miR-6516,miR-877-5p,novel_17,miR-324-3p,miR-185-3p,miR-92a-1-5p,miR-125b,miR-221-5p,miR-34b-5p,miR-2484,miR-21-3p,miR-503-5p,miR-191,miR-2300b-3p,miR-2904,miR-5703,miR-184-3p,miR-218-1-3p,miR-34a-5p,miR-3600,miR-212-5p,miR-383-5p,novel_83,miR-532-3p,miR-2285n,miR-2285y,miR-194b-5p,miR-361-3p,miR-361-3p,miR-504,miR-3064-5p,miR-7977,miR-4443,miR-1306-5p,miR-182-5p,miR-3085-3p,miR-214-3p,miR-1388-3p,miR-2330-5p,miR-296-5p,miR-544a,miR-101c,miR-330-3p,miR-145b,miR-1298-3p,miR-2285e

FAM71B	ENSOARG0(	family w	miR-210-5p,miR-30b,miR-422a,miR-760-3p,miR-320d,miR-30d,miR-3653-3p,miR-376c-3p,miR-380-3p,miR-2432,miR-2411,miR-146a-5p,miR-2408,miR-2300b-3p,novel_133,miR-378i,miR-30c-5p,miR-217-5p,miR-378a-3p,miR-188-3p,miR-5703,miR-4429,novel_73,miR-2284u,miR-133a-3p,miR-18a-3p,miR-485-3p,miR-3600,miR-380-5p,miR-200b,miR-23c,miR-487a-3p,miR-23b-3p,miR-2285j,miR-320e,miR-338-5p,miR-29b-2-5p,miR-655-3p,miR-151-3p,miR-6516-3p,miR-155-5p,miR-190a-5p,miR-129-2-3p,miR-3074-5p,miR-145a-3p,miR-378c,miR-224-5p,miR-6134,miR-30d-5p,miR-655,miR-30f,let-7g-3p,miR-133b-3p,novel_99,miR-140-5p,miR-378a-3p,miR-182-5p,miR-216b-3p,miR-370-5p,miR-499b-3p,miR-155-5p,miR-204-3p,novel_103,miR-1306-5p,miR-320b,miR-182-5p,miR-3955-3p,miR-3085-3p,miR-146b,miR-494-3p,miR-1388-3p,miR-676-3p,miR-23a-3p,miR-378d,miR-1b-5p,miR-541,miR-484,miR-378b,miR-450b-5p,miR-1388-5p,miR-378c,miR-151a-3p,miR-1298-3p,miR-9788-3p,miR-30b-5p,miR-3964,miR-541-3p,miR-2447,miR-3059-5p,miR-190b-5p,miR-452-5p,miR-361-3p,miR-136-5p,miR-1983,miR-2319b,miR-2284g,miR-3064-5p,miR-7689-3p,miR-2483-3p,miR-378e,miR-499a-3p,miR-454-5p,miR-200c,miR-205-5p,miR-217,miR-30f,miR-127-5p,miR-200c-3p,novel_127,miR-3529-3p,miR-4286,miR-495-5p,miR-29b-2-5p,miR-3064-5p,miR-192-3p,miR-2284y,miR-29a-5p,miR-1290,miR-23b-5p,miR-30a-5p,let-7a-2-3p,miR-30c,miR-200b-3p,miR-155-5p,miR-429-3p,miR-2285ad,miR-483-3p,miR-378h,miR-2411-5p,miR-129-1-3p,miR-370-5p,miR-5100,novel_9,miR-133a-3p,miR-6128,miR-3607-3p,miR-1197-3p,miR-378c,miR-30e-5p,miR-23b,miR-320a,miR-3154,let-7g-3p,miR-23a-5p,miR-3068-3p,miR-874-3p,miR-1271,miR-370-3p,miR-2355-5p,miR-3965,miR-376c-3p,miR-146a,miR-224-5p,miR-2331-5p,novel_51,miR-190a,miR-483-3p,miR-126a-

EEF2	ENSOARG0(	eukaryot	miR-378d,miR-8485,miR-23a-3p,miR-744-3p,miR-2284b,miR-199b-5p,miR-153-3p,miR-103a-2-5p,miR-323b-3p,miR-16b,miR-425-3p,miR-153,miR-148b-5p,miR-455-3p,novel_78,miR-548w,miR-1343-3p,miR-330-3p,miR-1388-5p,miR-21b,miR-2284r,miR-301b-5p,miR-323b,miR-758-3p,miR-877-3p,miR-2447,miR-3956-3p,miR-2284j,miR-9-5p,miR-3968,miR-490-5p,miR-218-5p,miR-301a-5p,miR-378g,miR-2483-3p,miR-2284g,miR-2285u,miR-211-5p,miR-504,miR-221,miR-421-5p,miR-377-5p,miR-486-3p,miR-2285w,miR-195a-3p,miR-345-3p,novel_17,miR-324-3p,miR-3601,miR-345-3p,miR-422a,miR-23b-3p,miR-23c,miR-2284h-5p,miR-328-3p,miR-18a-3p,miR-16-1-3p,miR-148a-5p,miR-2284u,miR-191,miR-378i,miR-193b-5p,miR-378a-3p,miR-217-5p,miR-769-5p,miR-6525,miR-224-5p,miR-346,miR-3184-3p,miR-3074-5p,miR-3958-5p,miR-874-5p,miR-130b-5p,miR-151-3p,miR-665,miR-377-3p,miR-10a,miR-15b-5p,miR-504-5p,miR-216b-3p,miR-339b,miR-1895,miR-450a-1-3p,miR-129b-3p,miR-411,miR-655,miR-433-3p,miR-432-3p,miR-432-3p,miR-380-5p,miR-6240,miR-668-3p,miR-193a-3p,miR-7-5p,miR-582,miR-377-3p,miR-296-3p,miR-365b-5p,miR-2285v,miR-2411-3p,miR-3431,miR-134-3p,miR-31-5p,miR-339-5p,miR-16b,miR-16-1-3p,miR-497-5p,miR-542-3p,miR-7b-5p,miR-1843b-5p,miR-450c-3p,novel_23,miR-374c-5p,miR-199b-5p,miR-193b-3p,miR-29c-3p,miR-29a,miR-374c-3p,miR-320c,miR-3074-5p,miR-410-5p,miR-2319a,miR-378f,miR-150-5p,miR-3065-5p,miR-450b-5p,miR-200a,novel_39,miR-2284y,miR-127-5p,novel_127,miR-9-5p,miR-505-5p,miR-322-5p,miR-2284z,miR-450a-1-3p,miR-486-5p,miR-2285ad,miR-429-3p,novel_79,miR-1224-5p,miR-23b-5p,miR-1260b,miR-2284v,miR-16-5p,miR-191-5p,miR-2284k,miR-100-3p,miR-1271,miR-1248,miR-200a-5p,miR-23b,miR-758-3p,miR-424-5p,miR-33a-5p,miR-652-5p,miR-
MEIS3	ENSOARG0(	Meis hom	miR-503-3p,let-7a-5p,miR-130b-5p,miR-363-5p,miR-483-3p,miR-378a-5p,miR-214-3p,let-7e,miR-362-3p,novel_51,miR-2284d,miR-134-3p,miR-185-5p,miR-1298-3p,let-7g-5p,miR-216a-3p,miR-149-5p,miR-194-3p,miR-455-3p,miR-1343-3p,miR-27a-3p,miR-26b-3p,miR-2284ab,miR-362-3p,miR-30b-3p,miR-329-3p,miR-541,miR-30b-3p,miR-541-3p,miR-9-5p,miR-495-3p,miR-4726-5p,miR-499a-5p,miR-2285t,miR-4324,let-7i,miR-378g,miR-411b-3p,let-7d-5p,miR-6535,miR-27a-3p,novel_68,let-7e-5p,miR-216a-3p,let-7g,novel_39,miR-183-5p,miR-4286,miR-2898,miR-2284m,miR-127-5p,let-7f-5p,miR-98-5p,let-7k,miR-21-3p,miR-205-5p,miR-125a-3p,let-7c-5p,miR-24-2-5p,miR-9-5p,miR-483-3p,miR-6517,miR-196a-2-3p,let-7d,miR-128-3p,miR-362-3p,miR-1961,miR-2904,miR-1197-5p,miR-1197-3p,let-7b-5p,miR-6134,novel_42,let-7b,miR-4510,miR-214,novel_9,miR-874-5p,miR-4492,miR-130b-5p,miR-15b-3p,let-7f,miR-2433,miR-2284k,miR-5100,miR-324-3p,miR-6130,miR-2284n,let-7i-5p,miR-370-3p,miR-2355-5p,miR-1271,miR-329a

VGLL1	ENSOARG0(	vestigia	miR-320b,miR-677,miR-320c,miR-181b-3p,miR-299b-3p,miR-411b-3p,miR-34b,miR-146b-5p,miR-6516-3p,miR-378f,miR-376a-2-5p,miR-450b-3p,miR-219b-3p,novel_27,miR-449a,miR-374c-5p,miR-34c,miR-4324,miR-28c,miR-656-5p,miR-147-5p,miR-502b,miR-128-1-5p,novel_1,miR-379-3p,miR-339-5p,miR-3955-5p,miR-31-5p,miR-133b-5p,miR-185-5p,miR-134-3p,miR-3431,miR-450c-3p,miR-145a-5p,novel_23,miR-873a-5p,miR-223-3p,miR-1285,miR-668-3p,miR-345-5p,miR-654-3p,miR-365b-5p,miR-105-5p,let-7d-3p,miR-224-5p,miR-146a,miR-452-5p,miR-125a-5p,miR-2285aa,miR-542-5p,miR-125a,miR-141-3p,miR-34c-5p,miR-137-3p,miR-320a,miR-7144-5p,miR-485-5p,miR-345-5p,miR-6128,miR-193a-5p,miR-365a-5p,miR-378c,miR-125b-5p,miR-6740-5p,novel_63,miR-5100,miR-486b-3p,novel_79,miR-376a-5p,miR-431,miR-299a-3p,miR-582-3p,miR-450a-1-3p,miR-9-5p,miR-378h,miR-218-2-3p,miR-2427,miR-1290,miR-105-2,miR-411-3p,miR-495-5p,miR-2284y,novel_39,miR-2284a,miR-450b-5p,miR-28b,miR-24-2-5p,miR-30c-2-3p,miR-3071-3p,miR-339a,miR-376b-3p,novel_96,miR-200a-3p,miR-2898,miR-191-3p,miR-7977,miR-24-1-5p,miR-139-5p,miR-2319b,miR-378g,miR-378e,miR-378j,miR-129b-5p,miR-140-5p,miR-452-5p,miR-9-5p,novel_111,miR-2440,miR-181b-2-3p,miR-145b,miR-105-1,miR-216a-3p,miR-455-3p,miR-376b-3p,miR-1298-3p,miR-147a,miR-125b-2-3p,miR-378b,miR-484,miR-143-5p,miR-1388-5p,miR-378c,miR-2284b,miR-30c-1-3p,miR-378d,miR-320b,miR-431-5p,miR-146b,miR-491-5p,miR-3955-3p,miR-30c-1-3p,miR-378a-3p,miR-548o-3p,novel_25,miR-139-5p,miR-216b-5p,miR-6130,miR-411,miR-655,miR-21a-3p,miR-450a-1-3p,miR-625-5p,miR-1827,novel_99,miR-345-5p,miR-339b,miR-665-5p,miR-433-5p,miR-378c,miR-3184-3p,miR-4510,miR-122-5p,miR-224-5p,miR-320[illegible],miR-655-3p,miR-2285[illegible],miR-15b-3p,miR-129-

KIF13A	ENSOARG0(	kinesin	miR-671-5p,miR-485-5p,miR-20b-5p,miR-329b,miR-10b-3p,miR-17-5p,miR-200a-5p,miR-137-3p,miR-93-5p,miR-2448-3p,miR-488-3p,miR-324-5p,miR-34c-5p,miR-141-3p,miR-138-5p,miR-409-3p,miR-29b-3p,miR-410-5p,miR-365a-5p,miR-1260b,miR-93,miR-200b-3p,miR-885-3p,miR-29d-3p,miR-1290,miR-615-3p,miR-1271-5p,miR-6516-5p,miR-9-5p,miR-505-5p,novel_79,miR-429-3p,miR-200a-3p,miR-107,miR-181b-5p,miR-200c-3p,miR-339a,miR-200c,miR-1260a,miR-200a,miR-450b-5p,miR-4286,miR-3064-5p,miR-30a-3p,miR-3529-3p,miR-19a-3p,miR-107,miR-3065-5p,miR-29a-3p,miR-411b-3p,miR-34b,miR-3120-3p,miR-378b,miR-147-5p,miR-625-3p,miR-29a,miR-1307-5p,miR-34c,miR-20a-5p,miR-502-5p,miR-301b-3p,miR-2428,miR-449a,miR-29c-3p,miR-495-3p,miR-2285x,miR-7862,miR-188-5p,miR-1260b,miR-138,miR-29b,miR-3071-5p,miR-1843b-5p,miR-7b-5p,miR-181a-5p,miR-432,miR-544b,miR-103,miR-20b,miR-3431,miR-134-3p,miR-432-5p,miR-130a-3p,miR-339-5p,miR-122-3p,miR-103a-3p,miR-31-5p,miR-17-5p,novel_51,miR-106a-5p,miR-365b-5p,miR-7-5p,miR-2284l,miR-668-3p,miR-130b-5p,miR-186-5p,miR-885-3p,miR-543-3p,miR-339b,let-7g-3p,miR-129b-3p,miR-21a-3p,miR-139-5p,miR-6516,miR-544-3p,miR-301a-3p,miR-132-5p,miR-130b-5p,miR-4492,miR-454-3p,miR-665,miR-132-5p,miR-30a-3p,miR-30e-3p,miR-2285j,miR-6134,miR-181c-5p,novel_44,miR-142a-5p,miR-214,miR-1271,miR-34a-5p,miR-19b-3p,miR-33b-3p,miR-532-3p,miR-200b,miR-383-5p,miR-95-3p,miR-30d-3p,miR-106a,miR-548e-3p,miR-21-3p,miR-34b-5p,miR-106a,novel_17,miR-2432,miR-376a-5p,miR-20a,miR-96-5p,miR-361-3p,miR-4443,miR-3596,miR-378g,miR-2285u,miR-139-5p,miR-3064-5p,miR-2285t,miR-615,novel_82,miR-218-5p,miR-181d-5p,miR-9-5p,miR-16-2-3p,miR-450b-5p,miR-2426,miR-544a,miR-143-5p,miR-6529a,miR-148b-5p,miR-455-3p,miR-142-
SERF2	ENSOARG0(	small ED	miR-200a-3p,miR-204-3p,novel_4,miR-2447,miR-4792,miR-141-3p,miR-2355-5p
ZDHHC7	ENSOARG0(	zinc fin	miR-148a-5p,miR-1343-5p,miR-378a-3p,miR-378i,novel_133,miR-2366,miR-328-3p,miR-378h,miR-2411-5p,novel_79,miR-200a-3p,novel_96,miR-181b-5p,miR-422a,miR-339a,miR-196a-3p,miR-210-5p,miR-199a-5p,miR-2411,miR-148b-5p,miR-29b-1-5p,miR-29b-2-5p,miR-345-3p,miR-181a-2-3p,miR-339b,miR-199a-5p,miR-499b-3p,miR-132-5p,miR-6130,miR-370-3p,miR-141-3p,miR-378a-3p,miR-132-5p,miR-665,miR-29b-2-5p,miR-378c,miR-181c-5p,miR-6525,miR-425-5p,miR-378c,miR-4510,miR-378c,miR-450b-5p,miR-873a-5p,miR-378b,miR-296-5p,miR-181a-5p,miR-149-5p,miR-148b-5p,miR-185-5p,miR-339-5p,miR-199b-5p,miR-452-5p,miR-1343-5p,miR-491-5p,miR-378a-5p,miR-29b-1-5p,miR-2957,miR-8485,miR-378d,miR-378f,miR-6395,miR-1291,miR-136-5p,miR-361-3p,miR-199b-5p,miR-499a-3p,miR-378e,miR-3596,miR-489,miR-425-5p,miR-199b-5p,miR-877-3p,miR-3957-3p,miR-495-3p,miR-181d-5p,miR-885-5p

AIM1	ENSOARG0(	absent i	miR-143-5p,miR-450b-5p,miR-2300a-5p,novel_4,miR-27a-3p,miR-142-3p,miR-500-5p,miR-326,miR-320b,miR-342,miR-2957,miR-362-3p,miR-3085-3p,miR-494-3p,miR-424-5p,miR-2330-5p,miR-30c-1-3p,miR-361-3p,miR-129b-5p,miR-877-3p,novel_94,miR-20a-3p,miR-3064-5p,miR-27a-3p,miR-454-5p,miR-769-5p,miR-1973,miR-371a-5p,miR-335,miR-541-3p,novel_60,miR-3059-5p,miR-452-5p,miR-181d-5p,miR-362-5p,miR-362-3p,miR-2408,miR-2300b-3p,miR-1197-5p,miR-5703,miR-1193,miR-2904,miR-4429,miR-3600,miR-148b-3p,miR-200b,miR-15a-5p,miR-222-5p,miR-532-3p,miR-196a-3p,miR-185-3p,miR-466i-5p,miR-760-3p,miR-320d,miR-21-3p,miR-221-5p,miR-216c-5p,miR-21a-3p,miR-27b-5p,miR-582-5p,miR-221-5p,miR-532-5p,miR-323-5p,miR-324-3p,miR-664a,miR-208b-5p,miR-424-5p,miR-145a-3p,miR-3074-2-3p,miR-6134,miR-181a-5p,miR-873a-5p,miR-145a-5p,miR-219a-1-3p,miR-1260b,miR-138,miR-744-5p,miR-133b-5p,miR-26b-3p,miR-377-5p,miR-665,miR-185-5p,miR-2284d,miR-103,miR-2355-3p,miR-2483-5p,miR-2387,miR-18a-3p,miR-21c,miR-107,miR-1291,miR-379-5p,miR-195a-5p,miR-135b-5p,miR-320b,miR-323a-5p,miR-2404,miR-15a,miR-16a,novel_1,miR-203b-5p,miR-22-5p,miR-342-3p,miR-24-3p,miR-371b-3p,miR-211,miR-2428,miR-326-3p,miR-218-2-3p,miR-582-3p,let-7a-2-3p,miR-200b-3p,miR-128-3p,miR-214-5p,miR-582-3p,miR-200c,miR-1260a,miR-30c-2-3p,miR-200c-3p,miR-181b-5p,miR-107,miR-4286,miR-30a-3p,miR-2385-3p,miR-1271-3p,miR-2284a,miR-376c-5p,miR-488-3p,miR-362-5p,miR-3154,miR-320a,novel_91,let-7g-3p,miR-10b-3p,miR-485-5p,miR-7144-5p,miR-23a-5p,miR-370-3p,miR-769,miR-410-5p,miR-1-5p,miR-138-5p,miR-204-5p,miR-3607-3p,miR-500b-5p,miR-145-3p,miR-541,miR-329-3p,miR-2284r,miR-362-3p,miR-1839-5p,miR-101c,novel_78,miR-145b,miR-181b-2-3p,miR-2459,miR-421,miR-455-3p,miR-

TEX36	ENSOARG0(	testis e	miR-196b-5p,miR-125a-5p,miR-7-5p,miR-194a,miR-345-5p,miR-493-5p,miR-101-5p,miR-29b,miR-7b-5p,miR-181a-5p,miR-194-5p,novel_23,miR-145a-5p,miR-185-5p,miR-412-5p,miR-2355-3p,miR-133b-5p,miR-130a-3p,miR-379-3p,miR-29a,miR-301b-3p,miR-1a-1-5p,novel_120,miR-495-3p,miR-29c-3p,miR-19a-3p,miR-6516-3p,miR-148a-3p,miR-216a-3p,miR-29a-3p,miR-323a-5p,miR-6535,miR-7-1-3p,miR-655-5p,miR-1a-2-5p,miR-181b-5p,miR-152-3p,novel_96,miR-2285af,miR-30f,miR-2285b,miR-1957a,miR-29b-2-5p,miR-411-3p,miR-30c,miR-128-3p,miR-196a-5p,miR-30a-5p,miR-29d-3p,miR-1271-5p,miR-302a-5p,miR-545-5p,miR-494-5p,miR-29b-3p,novel_63,miR-1-5p,miR-125b-5p,miR-133a-3p,let-7g-3p,miR-345-5p,miR-424-3p,miR-30e-5p,miR-376c-3p,miR-125a,miR-130b-3p,miR-491-5p,miR-153-3p,miR-2957,miR-29b-1-5p,miR-1b-5p,miR-193a,miR-8485,miR-124a,miR-744-3p,miR-21b,miR-143-5p,miR-153,miR-145b,miR-499a-5p,miR-30b-5p,miR-154a,miR-7705,miR-885-5p,miR-3059-5p,miR-181d-5p,miR-194b-5p,miR-96-5p,miR-493-5p,miR-2483-3p,miR-652-3p,miR-4792,miR-541-5p,miR-3653-3p,miR-30d,miR-30b,miR-125b,miR-376c-3p,miR-29b-1-5p,miR-2285w,miR-216c-5p,miR-345-3p,miR-195a-3p,miR-19b-3p,miR-30c-5p,miR-188-3p,miR-98-3p,miR-124-3p,miR-148b-3p,miR-2284h-5p,miR-451a,miR-454-3p,miR-29b-2-5p,miR-98-3p,miR-6525,miR-181c-5p,miR-145a-3p,miR-433-5p,miR-1271,miR-30f,miR-345-5p,let-7g-3p,miR-133b-3p,miR-487b-5p,miR-411,miR-30d-5p,miR-323-5p,miR-301a-3p,miR-376e-3p

C9orf156	ENSOARG0(	chromoso	miR-200b,miR-148b-3p,miR-199a-3p,miR-532-3p,miR-3600,miR-184-3p,miR-4429,miR-142-3p,miR-361-5p,miR-2284u,miR-16-1-3p,miR-2366,miR-191,novel_133,miR-1197-5p,miR-217-5p,miR-188-3p,miR-124-3p,miR-181b-1-3p,miR-125b-2-3p,miR-142a-3p,miR-130a-5p,miR-664b-3p,miR-143-3p,miR-380-3p,miR-2424,miR-146a-5p,miR-183-5p,miR-216c-5p,miR-2887,miR-345-3p,miR-202-5p,miR-2284m,miR-1277-5p,miR-196a-3p,miR-125b,miR-216a-5p,miR-139-5p,miR-377-3p,miR-27a-5p,miR-204-3p,miR-504-5p,miR-552-3p,novel_99,miR-1827,miR-186-5p,miR-625-5p,miR-433-3p,miR-224-5p,miR-6525,miR-142a-5p,miR-338-3p,miR-3184-5p,miR-3184-3p,miR-214,miR-17-3p,miR-451a,miR-130b-5p,miR-129-5p,miR-2285j,miR-320e,miR-142-3p,miR-142-5p,miR-1298-3p,miR-153,miR-1185-3p,miR-455-3p,miR-500-5p,novel_78,miR-199b-3p,novel_4,miR-181b-2-3p,miR-145b,miR-27a-3p,miR-5010-3p,miR-299a-5p,miR-2426,miR-2284aa,miR-541,miR-6529a,miR-484,miR-1b-5p,miR-491-3p,miR-8485,miR-1388-3p,miR-124a,miR-491-5p,miR-1185-5p,miR-103a-2-5p,miR-214-3p,miR-153-3p,miR-146b,miR-326,miR-320b,miR-199a-3p,miR-342,miR-27a-3p,miR-1185-2-3p,miR-144-5p,miR-20a-3p,miR-452-3p,miR-139-5p,miR-2284g,miR-499b-5p,miR-541-5p,miR-504,novel_94,miR-544-5p,miR-877-3p,miR-9-3p,miR-1983,miR-362-5p,miR-877-3p,miR-423-5p,miR-1285-5p,miR-9-3p,miR-212-3p,miR-3956-3p,miR-541-3p,novel_60,miR-2447,miR-3059-5p,miR-2284j,miR-452-5p,miR-2440,miR-218-5p,miR-107-5p,miR-758-5p,miR-2284z,miR-429-3p,miR-7641,let-7a-2-3p,miR-200b-3p,miR-3578,miR-330-5p,miR-450b-5p,miR-380-5p,miR-148b-5p,miR-490-3p,miR-2284a,miR-2284y,miR-376c-5p,miR-3529-3p,miR-4286,miR-181a-2-3p,miR-495-5p,miR-200c-3p,miR-17-3p,miR-200a-3p,novel_96,miR-152-3p,miR-200c,miR-2285af,miR-217,miR-374c-3p,miR-2284p,miR-

ATG5	ENSOARG0(	autophag	miR-15b-5p,miR-6516,miR-34c-5p,miR-3959-5p,miR-769,miR-216b-3p,miR-221-5p,miR-625-5p,miR-20b-5p,miR-2312,miR-2318,miR-329b,miR-129b-3p,miR-30d-5p,miR-17-5p,miR-3154,miR-21a-3p,miR-30e-5p,miR-2285c,miR-2448-3p,miR-181c-5p,miR-769-5p,miR-3607-3p,miR-16-5p,miR-424-5p,novel_74,miR-17-3p,miR-3604,miR-1-5p,miR-2332,miR-322-5p,miR-483-3p,miR-6516-5p,miR-758-5p,miR-15a-5p,miR-380-5p,miR-2285ad,miR-106a,miR-128-3p,miR-2284u,miR-34a-5p,miR-300,miR-142-3p,miR-30a-5p,miR-140-3p,miR-30c-5p,miR-3591-5p,miR-2424,miR-2284y,miR-192-3p,miR-2284a,miR-380-5p,miR-125b-2-3p,miR-181b-1-3p,miR-20a,miR-329a-5p,miR-183-5p,miR-34b-5p,miR-30d,miR-221-5p,miR-107,miR-17-3p,miR-106a,miR-202-5p,miR-1a-2-5p,miR-181b-5p,miR-500a-5p,miR-6535,miR-677,miR-181b-3p,miR-1185-2-3p,miR-34b,miR-374c-3p,miR-3120-3p,miR-2284g,miR-329-5p,miR-195a-5p,miR-107,miR-216a-3p,miR-129b-5p,miR-2285ab,miR-1285-5p,novel_27,miR-449a,miR-371b-3p,miR-2285f,miR-758-5p,miR-17-3p,miR-181d-5p,miR-1a-1-5p,miR-203b-5p,miR-769-5p,miR-16a,miR-34c,miR-33a-3p,miR-15a,miR-4324,miR-133a-5p,miR-20a-5p,miR-2440,miR-194-3p,miR-1185-3p,miR-103,miR-2285e,miR-2285g,miR-181b-2-3p,miR-548w,novel_78,miR-133b-5p,miR-103a-3p,miR-497-5p,miR-101c,miR-16b,novel_23,miR-1434-3p,miR-181a-5p,miR-654-3p,miR-101-5p,miR-2284b,miR-380-5p,miR-106a-5p,miR-424-5p,miR-483-3p,miR-106a-5p,miR-16b,miR-106b-5p,miR-17-5p

GRIPAP1	ENSOARG0(	GRIP1 as	miR-16b,miR-199a-3p,miR-320b,miR-1306-5p,miR-146b,miR-1468,miR-3085-3p,let-7c-3p,miR-33a-5p,miR-424-5p,miR-8485,miR-758-3p,miR-30c-1-3p,miR-7857,miR-758-3p,miR-296-5p,miR-143-5p,miR-541,miR-3120-5p,miR-5010-3p,miR-181b-2-3p,novel_78,miR-409b,miR-1343-3p,miR-2300a-5p,miR-455-3p,miR-421,miR-2459,miR-142-3p,miR-2285n,miR-3958-3p,miR-541-3p,miR-423-5p,miR-7705,miR-219a-2-3p,miR-877-3p,miR-361-3p,miR-378j,miR-361-3p,miR-504,miR-136-5p,miR-3064-5p,miR-452-3p,miR-3596,miR-324-3p,miR-92a-1-5p,miR-185-3p,miR-210-5p,novel_17,miR-299-3p,miR-320d,miR-760-3p,miR-183-5p,miR-216c-5p,miR-2432,miR-146a-5p,miR-380-3p,miR-143-3p,miR-376a-5p,miR-181b-1-3p,miR-142a-3p,miR-2904,miR-1197-5p,miR-140-3p,miR-2300b-3p,miR-2366,miR-218-1-3p,miR-4429,miR-33b-3p,miR-505-3p,miR-212-5p,miR-616-3p,novel_83,miR-199a-3p,miR-222-5p,miR-15a-5p,miR-200b,miR-324-3p,miR-219b-3p,miR-665,miR-3604,miR-320e,miR-4492,miR-874-5p,miR-208b-5p,miR-424-5p,miR-450b-3p,miR-664a,miR-3074-5p,miR-4510,miR-145a-3p,miR-3184-5p,miR-6525,miR-129b-3p,miR-450a-1-3p,miR-625-5p,miR-1827,miR-18a-5p,miR-504-5p,miR-30c-1-3p,miR-660-5p,miR-15b-5p,miR-6130,novel_51,miR-146a,miR-660,miR-1343-5p,miR-2285l,miR-503-3p,miR-654-3p,miR-450c-3p,miR-374b-3p,miR-497-5p,miR-188-5p,miR-16b,miR-380-3p,miR-1260b,miR-149-5p,miR-3431,miR-16a,novel_87,miR-4324,miR-15a,miR-576-3p,miR-28c,miR-502b,miR-6119-3p,novel_1,miR-136-3p,miR-2428,miR-1306,miR-150-5p,miR-18b,miR-146b-5p,miR-195a-5p,miR-505-3p,novel_68,miR-378b,miR-3074-5p,miR-677,miR-320c,miR-669,miR-181b-3p,miR-146b-3p,miR-374c-3p,miR-670-3p,miR-299b-3p,miR-30c-2-3p,miR-1260a,miR-219-3p,miR-200c,miR-200c-3p,miR-3064-5p,miR-181a-2-3p,miR-29a-5p,miR-490-3p,miR-192-3p,miR-1271-
FIGLA	ENSOARG0(	follicul	miR-217,miR-4726-5p,miR-668-3p,miR-2285f,miR-219a-5p,miR-346,miR-423-5p,miR-3184-5p,miR-2285ab,miR-433-3p,miR-217-5p,miR-5703,miR-2312,miR-668-5p,miR-625-5p,miR-20a-3p

MEP1A	ENSOARG0(	meprin A	miR-143-5p,miR-484,miR-296-5p,miR-378b,miR-30b-3p,miR-7857,miR-2426,miR-544a,miR-378c,miR-2300a-5p,miR-500-5p,miR-1306-5p,miR-326,miR-342,miR-362-3p,miR-3085-3p,let-7e,miR-424-5p,miR-382-3p,miR-2330-5p,let-7a-5p,miR-129b-5p,novel_94,miR-452-3p,miR-20a-3p,miR-3064-5p,miR-3082-5p,miR-378e,let-7d-5p,miR-30b-5p,miR-4726-5p,miR-3958-3p,novel_60,miR-194b-5p,miR-362-5p,miR-502-3p,miR-362-3p,miR-215-5p,miR-2300b-3p,novel_133,miR-5703,miR-1193,miR-2904,miR-941,miR-19b-3p,miR-3960,miR-3600,miR-1b-3p,miR-15a-5p,miR-383-5p,novel_83,miR-199a-3p,miR-532-3p,miR-299-3p,miR-34b-5p,miR-30d,miR-216c-5p,miR-130a-5p,miR-27b-5p,miR-30f,miR-18a-5p,miR-133b-3p,miR-378a-3p,miR-6130,miR-323-5p,miR-139-5p,miR-544-3p,miR-192-5p,miR-27a-5p,miR-877-5p,miR-22-3p,novel_32,miR-204-3p,miR-664a,miR-17-3p,miR-450b-3p,miR-708-5p,miR-424-5p,miR-4510,miR-378c,miR-2330-3p,miR-425-5p,miR-206-3p,let-7b-5p,miR-3071-5p,miR-194-5p,miR-30b-3p,miR-138,miR-133b-5p,miR-185-5p,miR-503-5p,let-7g-5p,miR-432-5p,miR-544b,miR-503-5p,miR-363-5p,miR-2483-5p,miR-345-5p,miR-18b,miR-2387,miR-2285k,miR-19a-3p,miR-450b-3p,miR-195a-5p,miR-379-5p,miR-655-5p,miR-34b,miR-323a-5p,miR-6402,miR-133a-5p,miR-502-5p,miR-15a,let-7i,miR-34c,miR-16a,miR-92a-3p,miR-502b,miR-342-3p,miR-495-3p,novel_101,miR-17-3p,miR-3959-3p,miR-2428,miR-326-3p,miR-449a,miR-3578,miR-30a-5p,miR-501-3p,let-7d,miR-411-3p,miR-105-2,miR-7641,miR-378h,let-7c-5p,miR-17-3p,miR-2898,miR-92a-3p,miR-3529-3p,miR-181a-2-3p,miR-2403,miR-1,miR-1271-3p,miR-2284a,miR-488-3p,miR-18a,miR-3154,miR-362-5p,miR-10a-3p,miR-485-5p,miR-7144-5p,miR-215-5p,miR-34c-5p,miR-3969,miR-331-3p,miR-138-5p,miR-28-5p,miR-486b-3p,miR-103b,miR-133a-3p,miR-500b-
KIAA0513	ENSOARG0(	KIAA0513	miR-542-3p,miR-3141,miR-1343-5p,miR-1306,miR-10b-3p,miR-96-5p,miR-1343-3p,miR-1271-5p,miR-6130,miR-1306-5p,miR-3141,miR-491-5p,miR-17-3p,miR-17-3p,miR-1343-5p,novel_96,miR-543-5p,miR-4510,miR-1271,miR-17-3p,miR-376a-5p

NCBP1	ENSOARGO(	nuclear	miR-6134,miR-224-5p,miR-206-3p,miR-323-3p,miR-425-5p,miR-6128,miR-190a-5p,miR-767-5p,miR-6516-3p,miR-487b-5p,miR-151-3p,miR-144,miR-494-5p,miR-2284k,miR-34c-3p,miR-133c,miR-216b-5p,miR-139-5p,miR-374c-3p,miR-21-3p,miR-216b-3p,miR-548o-3p,miR-1814c,miR-3074-1-3p,novel_48,let-7g-3p,miR-767,miR-487b-5p,miR-23b,miR-137-3p,miR-490-3p,miR-143-3p,miR-181b-1-3p,miR-148b-5p,miR-29b-1-5p,miR-450b-5p,miR-1,miR-152-3p,novel_96,miR-2484,miR-2284m,miR-202-5p,novel_17,miR-125a-3p,miR-2285p,miR-26a-5p,miR-376b-3p,miR-205-5p,miR-23c,miR-148b-3p,miR-23b-3p,miR-383-5p,miR-505-5p,miR-376a-5p,miR-1224-5p,miR-3600,miR-6517,miR-148a-5p,miR-361-5p,miR-142-3p,miR-501-3p,miR-2904,miR-2366,miR-502-3p,miR-8095,miR-2284j,miR-190b-5p,miR-2285f,miR-885-5p,miR-541-3p,miR-219b-3p,miR-206,miR-499a-5p,miR-628-3p,miR-107-5p,miR-425-5p,miR-382-5p,miR-6402,miR-500-3p,miR-181b-3p,miR-376d,miR-144-3p,miR-677,miR-26b-5p,miR-541-5p,miR-139-5p,miR-877-3p,miR-544-5p,miR-376a-3p,miR-376b-3p,miR-3591-3p,miR-409-5p,miR-148a-3p,miR-2285ab,miR-2310,let-7a-2-3p,miR-654-3p,miR-2285r,miR-101-5p,miR-23a-3p,miR-6240,miR-1388-3p,miR-103a-2-5p,miR-491-5p,miR-190a,miR-2957,miR-330-3p,miR-29b-1-5p,miR-224-5p,miR-411-5p,miR-148b-5p,miR-376b-3p,miR-181b-2-3p,miR-26b-3p,miR-151a-3p,miR-330-3p,miR-1a-3p,miR-501-3p,miR-143-5p,miR-542-3p,miR-1843b-5p,miR-541,miR-2113,miR-1434-3p
KIAA0232	ENSOARGO(	KIAA0232	miR-7144-5p,miR-6529b,miR-2433

RDH5	ENSOARG0(	retinol	let-7b-5p,miR-6134,miR-338-3p,miR-145a-3p,miR-3074-2-3p,miR-664a,miR-6516-3p,miR-155-5p,miR-17-3p,miR-487a-5p,miR-324-3p,miR-19b-1-5p,miR-320e,novel_25,miR-204-3p,miR-155-5p,miR-2312,miR-125b-2-3p,miR-146a-5p,miR-320d,miR-760-3p,miR-34b-5p,miR-30b-3p,miR-299-3p,miR-185-3p,miR-125b,miR-383-5p,miR-671-3p,miR-532-3p,miR-505-3p,miR-4429,miR-941,miR-378d,miR-2300b-3p,miR-2904,miR-124-3p,miR-5703,miR-1197-5p,miR-188-3p,miR-8095,miR-3059-5p,novel_60,miR-2285f,miR-452-5p,miR-4726-5p,miR-450a-5p,miR-2285t,miR-107-5p,let-7d-5p,miR-3064-5p,miR-20a-3p,miR-452-3p,miR-541-5p,miR-369-5p,miR-30c-1-3p,let-7a-5p,miR-33a-5p,miR-376b,miR-3085-3p,let-7e,miR-320b,miR-326,miR-2957,miR-9788-3p,miR-2285e,miR-409b,miR-5010-3p,miR-450b-5p,miR-3120-5p,miR-484,miR-143-5p,miR-412-3p,miR-30b-3p,miR-323-3p,miR-365a-5p,miR-1306-5p,miR-138-5p,miR-34c-3p,miR-551b-3p,miR-3969,miR-34c-5p,miR-370-3p,miR-7144-5p,miR-671-5p,miR-488-3p,miR-320a,miR-3154,miR-28b,miR-1957a,miR-1271-3p,miR-495-5p,miR-2385-3p,miR-4286,miR-450a-2-3p,miR-2898,miR-17-3p,miR-543-5p,miR-1260a,miR-30c-2-3p,let-7c-5p,miR-24-2-5p,let-7d,miR-128-3p,miR-615-3p,miR-345-3p,miR-134,miR-24-3p,miR-449a,miR-326-3p,miR-2428,miR-17-3p,miR-203b-5p,miR-22-5p,miR-502-5p,miR-133a-5p,miR-34c,let-7i,miR-34b,miR-450b-3p,miR-9851-3p,miR-505-3p,miR-6516-3p,miR-2285ab,miR-18a-3p,miR-378b,miR-345-5p,miR-363-5p,miR-124-5p,miR-125a-5p,miR-1343-5p,miR-2285g,let-7g-5p,miR-377-5p,miR-26b-3p,miR-138,miR-30b-3p,miR-1260b,miR-7862,miR-744-5p,miR-3071-5p,miR-6525,miR-433-5p,let-7b,miR-3074-5p,miR-3958-5p,miR-665-5p,novel_74,miR-4492,miR-500,let-7f,miR-664a-5p,miR-499b-3p,let-7i-5p,miR-30c-1-3p,miR-504-5p,miR-345-5p,miR-2285c,miR-500a-3p,miR-411,miR-376a-
VAX2	ENSOARG0(	ventral	miR-218-5p,novel_82,miR-34c,novel_111,miR-449a,miR-326-3p,miR-423-5p,miR-342-3p,miR-17-3p,novel_101,miR-299b-5p,miR-136-5p,miR-7975,miR-877-3p,miR-378f,miR-505-3p,miR-3065-5p,miR-378e,miR-323a-5p,miR-34b,miR-299b-3p,miR-669,miR-3064-5p,miR-10b,miR-216b-3p,miR-499b-5p,miR-491-5p,miR-3085-3p,miR-342,miR-326,miR-377-3p,miR-505,miR-197-5p,miR-30c-1-3p,miR-432-3p,miR-378d,miR-8485,miR-363-5p,miR-33a-5p,miR-138,miR-378c,miR-219a-1-3p,miR-299a-5p,miR-4508,miR-2889,miR-378b,miR-223-3p,novel_23,miR-147a,miR-421,miR-31-5p,novel_4,miR-665,miR-222-5p,miR-138-5p,miR-17-3p,miR-4492,miR-874-5p,miR-486b-3p,miR-30e-3p,miR-331-3p,miR-30a-3p,miR-665,miR-3604,miR-3184-5p,miR-378c,miR-6134,miR-378c,miR-10b-3p,miR-885-3p,miR-1827,miR-1248,miR-21a-3p,miR-200a-5p,miR-377-3p,miR-376e-3p,miR-34c-5p,miR-323-5p,novel_32,miR-376c-3p,miR-30c-1-3p,miR-378a-3p,miR-2285aa,miR-296-3p,miR-450a-2-3p,miR-17-3p,miR-466i-5p,miR-34b-5p,novel_96,miR-299-3p,miR-196a-3p,novel_17,miR-30c-2-3p,miR-92a-1-5p,miR-422a,miR-185-3p,miR-376c-3p,miR-486-3p,miR-3064-5p,miR-30a-3p,miR-885-3p,miR-133a-3p,miR-378d,miR-34a-5p,novel_133,miR-378i,miR-378a-3p,miR-330-5p,miR-378h,miR-299a-3p,miR-1b-3p,miR-30d-3p,miR-505-3p

NDUFAF5	ENSOARG0(	NADH deh	miR-148a-5p,miR-218-1-3p,miR-378a-3p,miR-193b-5p,miR-378i,miR-23c,miR-23b-3p,miR-7859,miR-422a,miR-324-3p,miR-26a-5p,novel_17,miR-376c-3p,miR-376a-5p,miR-345-3p,miR-195a-3p,miR-503-5p,miR-186-5p,miR-339b,miR-411,miR-487b-5p,miR-500a-3p,miR-129b-3p,miR-15b-5p,miR-216b-5p,miR-376e-3p,miR-582-5p,miR-10a,miR-301a-3p,miR-552-3p,miR-548o-3p,miR-2331-3p,miR-130b-5p,miR-500,miR-874-5p,miR-454-3p,miR-487b-5p,miR-665,miR-3604,miR-151-3p,miR-2285j,miR-590-3p,miR-1839-5p,miR-362-3p,miR-1388-5p,miR-501-3p,miR-1a-3p,miR-329-3p,miR-148b-5p,miR-496-5p,miR-181b-2-3p,miR-103a-2-5p,miR-130b-3p,miR-16b,miR-431-5p,miR-378d,miR-23a-3p,miR-3963,miR-96-5p,miR-140-5p,miR-500-3p,miR-4443,miR-2483-3p,miR-2285u,miR-139-5p,miR-3964,miR-7705,novel_111,miR-877-3p,miR-23b-5p,miR-330-5p,miR-1343-5p,miR-1290,novel_121,miR-2284z,miR-758-5p,miR-322-5p,miR-431,novel_79,miR-429-3p,miR-6517,miR-152-3p,miR-200a-3p,miR-28-3p,miR-30f,miR-2285b,miR-200a,novel_39,miR-148b-5p,miR-450b-5p,miR-1248,miR-329a,miR-876-3p,miR-23b,miR-200a-5p,miR-137-3p,miR-331-5p,miR-301,miR-376c-3p,miR-331-5p,miR-300-3p,miR-2285aa,miR-503-3p,miR-574-5p,miR-331-5p,miR-494-5p,novel_63,miR-2433,miR-1260b,miR-16-5p,miR-2284x,miR-497-5p,miR-16b,miR-197-3p,miR-1843b-5p,miR-7b-5p,miR-194-3p,miR-149-5p,miR-3431,miR-3535,miR-339-5p,miR-31-5p,novel_51,miR-194a,miR-7-5p,miR-2284l,miR-2285r,miR-668-3p,miR-378f,miR-3065-5p,miR-1306,miR-148a-3p,miR-677,miR-144-3p,miR-374c-3p,miR-26b-5p,miR-3120-3p,miR-6123,miR-379-3p,miR-206,miR-576-3p,miR-4324,miR-301b-3p,miR-1197-5p,miR-188-3p,miR-5703,miR-2904,miR-362-3p,novel_133,novel_83,miR-148b-3p,miR-340-5p,miR-15a-5p,miR-200b,miR-383-5p,miR-548o-3p,miR-130a-5p,miR-216c-

TSTD2	ENSOARG0(	thiosulf	miR-3071-5p,miR-145a-5p,miR-197-3p,miR-188-5p,miR-497-5p,miR-369-5p,miR-380-3p,miR-16b,miR-544b,miR-2284d,miR-2411-3p,miR-190a,miR-32,miR-224-5p,miR-363-5p,miR-380-5p,miR-432-3p,miR-2284l,miR-432-3p,miR-2285r,miR-18a-3p,miR-216a-3p,miR-135b-5p,miR-195a-5p,miR-450b-3p,miR-10a-5p,miR-329-5p,novel_68,novel_69,miR-34b,novel_87,miR-34c,miR-16a,miR-6529b,miR-142b,miR-133a-5p,miR-15a,miR-6123,miR-22-5p,novel_1,miR-92a-3p,miR-128-1-5p,novel_101,miR-1193,miR-17-3p,miR-495-3p,miR-449a,miR-323c,miR-211,miR-30a-5p,miR-345-3p,miR-10a-5p,miR-128-3p,miR-411-3p,miR-431,miR-7641,miR-214-5p,miR-2284z,miR-758-5p,miR-322-5p,miR-505-5p,miR-219-3p,miR-92a-3p,novel_127,miR-200a-3p,miR-17-3p,miR-450a-2-3p,miR-4286,miR-30a-3p,miR-490-3p,miR-3956-5p,miR-2284a,miR-2285b,miR-1271-3p,miR-200a,miR-3065-3p,miR-2284y,miR-92b-3p,miR-380-5p,miR-148b-5p,miR-3154,miR-200a-5p,miR-2448-3p,miR-301,miR-30e-5p,miR-1814c,miR-485-5p,miR-876-3p,miR-2474,miR-141-3p,miR-3068-3p,miR-376c-3p,miR-25-3p,miR-34c-5p,miR-135a-5p,miR-370-5p,miR-2284k,miR-34c-3p,miR-486b-3p,miR-204-5p,miR-3607-3p,miR-16-5p,miR-193a-5p,miR-2284v,miR-758-3p,miR-323b,miR-10b,miR-541,miR-101c,miR-2284aa,miR-450b-5p,miR-544a,miR-145b,miR-1343-3p,miR-2459,miR-455-3p,miR-1298-3p,miR-16b,miR-323b-3p,miR-431-5p,novel_103,miR-199a-3p,miR-3955-3p,novel_124,miR-2284b,miR-424-5p,miR-101b-3p,miR-2310,miR-758-3p,miR-3187-3p,miR-129b-5p,miR-409-5p,miR-140-5p,miR-211-5p,miR-499b-5p,miR-2284g,miR-2483-3p,miR-499a-3p,miR-2440,miR-101-3p,miR-382-3p,miR-30b-5p,miR-2285t,miR-190b-5p,miR-2284j,miR-541-3p,miR-363-3p,miR-2447,miR-219a-2-3p,miR-423-5p,novel_111,miR-877-3p,miR-140-3p,miR-188-3p,miR-1193,miR-30c-5p,miR-34a-5p,miR-
SDHB	ENSOARG0(	succinat	miR-184-3p,miR-4429,miR-29d-3p,miR-217-5p,miR-362-3p,miR-2300b-3p,miR-532-3p,miR-210-3p,miR-107,miR-92a-3p,miR-320d,miR-6536,miR-760-3p,miR-127-5p,miR-217,miR-185-3p,miR-92a-1-5p,miR-486-3p,miR-146a-5p,miR-92b-3p,miR-28b,miR-148b-5p,miR-181a-2-3p,novel_48,miR-767,miR-329a,miR-320a,miR-488-3p,miR-424-3p,miR-22-3p,miR-377-3p,miR-25-3p,miR-323-5p,miR-6130,miR-32-5p,miR-708-5p,miR-31-3p,miR-4492,miR-486b-3p,miR-28-5p,miR-767-5p,miR-320e,miR-29b-3p,miR-410-5p,miR-3184-5p,miR-122-5p,miR-6134,miR-214,miR-4510,miR-1839-5p,miR-6239,miR-3120-5p,miR-27b-3p,miR-455-5p,miR-29b,miR-362-3p,miR-30b-3p,miR-22-3p,miR-30b-3p,miR-3071-5p,miR-329-3p,miR-143-5p,miR-301b-5p,miR-103,miR-421,miR-222-5p,miR-27a-3p,miR-103a-3p,miR-199b-3p,miR-146b,miR-214-3p,miR-124-5p,novel_124,miR-3141,miR-32,miR-362-3p,miR-455-5p,miR-320b,miR-342,miR-377-3p,miR-146a,miR-4532,miR-146b-5p,miR-107,miR-3141,miR-320c,miR-27a-3p,miR-323a-5p,miR-29a-3p,miR-20a-3p,miR-22-5p,miR-3964,miR-656-5p,miR-199c,miR-92a-3p,miR-4726-5p,miR-29a,miR-301a-5p,miR-4324,miR-28c,miR-423-5p,miR-2428,miR-24-3p,miR-6527,novel_101,miR-29c-3p,novel_60,miR-342-3p,miR-363-3p,miR-3956-3p

SERBP1	ENSOARG0(	SERPINE1	miR-545-5p,miR-582-3p,miR-322-5p,miR-9-5p,miR-7859,miR-15a-5p,miR-548e-3p,miR-1193,miR-5703,miR-29a-5p,miR-2424,miR-376c-3p,miR-195a-3p,miR-29b-2-5p,miR-487a,miR-26a-5p,miR-6119-5p,novel_32,miR-133c,miR-15b-5p,miR-376c-3p,miR-376e-3p,miR-377-3p,miR-300-3p,miR-323-5p,miR-216b-5p,miR-154a-3p,miR-2285aa,miR-542-5p,miR-2355-5p,miR-487a-3p,miR-548o-3p,miR-625-5p,miR-1814c,miR-186-5p,miR-154b-3p,miR-142a-5p,miR-16-5p,miR-424-5p,miR-6516-3p,miR-151-3p,miR-6740-5p,miR-29b-2-5p,miR-409-3p,miR-3604,miR-410-5p,miR-2285j,miR-126a-5p,miR-142-5p,miR-539-5p,miR-151a-3p,miR-330-3p,miR-497-5p,miR-16b,miR-323b,miR-194-5p,miR-1285,miR-296-3p,miR-2310,miR-432-3p,miR-6240,miR-424-5p,miR-330-3p,miR-323b-3p,miR-16b,miR-194a,miR-377-3p,miR-296-3p,miR-144-3p,miR-6535,miR-323a-5p,miR-2483-3p,miR-26b-5p,miR-216b-3p,miR-544-5p,miR-195a-5p,miR-2285ab,miR-371b-3p,miR-2285f,miR-194b-5p,miR-9-5p,miR-2285x,miR-490-5p,miR-154-3p,miR-6119-3p,novel_1,miR-16a,miR-15a,miR-142b,miR-6402
RPL13	ENSOARG0(	ribosoma	miR-3120-3p,miR-139-5p,miR-10b,miR-320c,miR-7857-3p,miR-216a-3p,miR-3065-5p,miR-18a-3p,miR-219a-5p,miR-452-5p,miR-181d-5p,miR-9-5p,miR-2428,miR-769-5p,novel_87,miR-502-5p,miR-218-5p,miR-1343-3p,miR-455-3p,miR-9788-3p,miR-2355-3p,miR-432-5p,miR-185-5p,miR-22-3p,miR-30b-3p,miR-432,miR-653,miR-181a-5p,miR-1285,miR-2284x,miR-30b-3p,miR-3187-3p,miR-2310,miR-2411-3p,miR-320b,miR-552-3p,miR-2331-3p,miR-221-5p,miR-22-3p,miR-139-5p,miR-769,miR-656-3p,miR-3154,miR-433-3p,miR-320a,miR-23a-5p,miR-543-3p,miR-186-5p,miR-1814c,miR-193a-5p,miR-28a-3p,miR-145a-3p,miR-145-3p,miR-338-3p,miR-181c-5p,miR-769-5p,miR-2332,miR-708-5p,miR-28-5p,miR-134-5p,miR-3600,miR-7641,miR-18a-3p,miR-134-5p,miR-532-3p,miR-9-5p,miR-5703,miR-188-3p,miR-140-3p,miR-345-3p,miR-134,miR-193b-5p,miR-128-3p,miR-4429,miR-2385-3p,miR-181a-2-3p,miR-345-3p,miR-216c-5p,novel_39,miR-192-3p,miR-490-3p,miR-345-3p,miR-6119-5p,miR-1277-5p,miR-221-5p,miR-2484,novel_127,miR-320d,miR-28-3p,miR-181b-5p

EXOSC7	ENSOARG0(	exosome	miR-3591-3p,miR-3065-5p,miR-2387,miR-2285ab,miR-6395,miR-378f,miR-6516-3p,miR-412-3p,miR-10a-5p,miR-378b,miR-3074-5p,miR-216b-3p,miR-181b-3p,miR-320c,miR-677,miR-6535,miR-299b-3p,miR-146b-3p,miR-28c,miR-6402,miR-17-3p,miR-199b-5p,miR-2285m,miR-326-3p,miR-371b-3p,miR-24-3p,miR-3071-5p,miR-1434-3p,miR-653,miR-2889,miR-369-5p,miR-219a-1-3p,miR-30b-3p,miR-26b-3p,miR-665,miR-149-5p,miR-134-3p,miR-185-5p,miR-2411-3p,miR-105-5p,miR-1343-5p,miR-124-5p,miR-483-3p,miR-2483-5p,miR-130b-5p,miR-345-5p,miR-6412,miR-432-3p,miR-432-3p,miR-3154,miR-320a,miR-200a-5p,miR-485-5p,miR-671-5p,miR-1814c,miR-1248,miR-345-5p,miR-10a-3p,miR-141-3p,miR-2355-5p,miR-199a-5p,miR-3969,miR-409-3p,miR-331-3p,miR-6740-5p,miR-486b-3p,miR-144-5p,miR-3607-3p,miR-378c,miR-323-3p,miR-330-5p,miR-1343-5p,miR-345-3p,miR-3591-5p,miR-10a-5p,miR-105-2,miR-1224-5p,miR-299a-3p,miR-758-5p,miR-483-3p,miR-378h,miR-505-5p,miR-30c-2-3p,miR-2397-5p,miR-205-5p,miR-200a-3p,miR-127-5p,miR-17-3p,miR-4286,miR-2403,miR-30a-3p,miR-490-3p,miR-200a,novel_39,miR-199a-5p,miR-29a-5p,miR-28b,miR-381-3p,miR-199b-5p,miR-140-5p,miR-361-3p,miR-1983,miR-504,miR-136-5p,miR-452-3p,miR-4443,miR-144-5p,miR-7857-3p,miR-378e,miR-378g,miR-3596,miR-107-5p,novel_82,miR-4726-5p,miR-2285f,miR-2447,miR-30b-3p,miR-323b,miR-10b,miR-378b,miR-3120-5p,miR-378c,miR-1388-5p,miR-181b-2-3p,miR-222-5p,novel_4,miR-105-1,miR-455-3p,miR-9788-3p,miR-425-3p,miR-505,miR-323b-3p,novel_115,miR-326,miR-320b,miR-214-3p,miR-103a-2-5p,miR-199b-5p,miR-487b-5p,miR-25-5p,miR-744-3p,miR-23a-3p,miR-2310,miR-378d,miR-30c-1-3p,let-7f-1-3p,miR-186-5p,miR-543-3p,miR-345-5p,miR-2312,miR-532-5p,miR-21-3p,miR-548o-3p,miR-221-5p,miR-30c-1-3p,miR-504-5p,miR-
ST6GALNA(	ENSOARG0(	ST6 (alp	miR-1843b-5p,miR-30b-3p,miR-2426,miR-1839-5p,miR-1343-3p,miR-18a-3p,miR-503-5p,miR-500-5p,novel_17,miR-2285p,miR-503-5p,miR-3601,miR-500a-5p,miR-2898,novel_127,miR-503-5p,miR-4286,miR-363-5p,miR-376c-3p,miR-432-3p,miR-432-3p,miR-362-5p,miR-18a-3p,miR-129b-3p,miR-504,miR-876-3p,miR-1291,miR-485-5p,miR-504-5p,miR-320b,novel_69,miR-374c-3p,miR-376e-3p,miR-4443,miR-376c-3p,miR-410-5p,miR-3969,miR-665,miR-335,miR-335-5p,miR-145a-3p,miR-361-3p,miR-362-5p,miR-500b-5p,miR-2428,miR-6134,miR-145-3p,miR-423-5p,miR-3184-5p

CCDC180	ENSOARG0(	coiled-c	miR-411b-3p,miR-299b-3p,novel_69,miR-669,miR-378b,miR-10a-5p,miR-505-3p,miR-135b-5p,miR-378f,miR-148a-3p,miR-3591-3p,miR-378b,miR-150-5p,miR-371b-3p,miR-2428,miR-17-3p,miR-379-3p,miR-199c,miR-502b,miR-28c,miR-502-5p,miR-134-3p,miR-412-5p,miR-544b,miR-149-5p,miR-194-3p,miR-133b-5p,miR-26b-3p,miR-339-5p,miR-30b-3p,miR-455-5p,miR-27b-3p,miR-873a-5p,miR-432-3p,miR-296-3p,miR-6240,miR-130b-5p,miR-628-5p,miR-363-5p,miR-1343-5p,miR-452-5p,miR-296-3p,miR-455-5p,miR-330-3p,miR-197-5p,miR-223-5p,miR-141-3p,miR-2285aa,miR-1248,miR-485-5p,miR-23a-5p,miR-3074-1-3p,miR-301,miR-3154,miR-378c,miR-145-3p,miR-133a-3p,miR-103b,miR-3607-3p,miR-134-5p,miR-28-5p,miR-494-5p,miR-135a-5p,miR-100-3p,miR-378h,miR-6516-5p,miR-9-5p,miR-486-5p,miR-134-5p,miR-299a-3p,novel_79,miR-412,miR-1224-5p,miR-411-3p,miR-10a-5p,miR-134,miR-1343-5p,miR-28b,miR-200a,novel_39,miR-4286,miR-17-3p,miR-2898,novel_96,miR-200a-3p,miR-152-3p,miR-664-3p,miR-339a,miR-3082-5p,miR-378g,miR-378e,miR-27a-3p,miR-664b,miR-4792,miR-129b-5p,miR-154a,miR-423-5p,miR-541-3p,miR-9-5p,miR-452-5p,miR-2284q,miR-2440,miR-107-5p,miR-2459,miR-455-3p,miR-9788-3p,miR-199b-3p,novel_4,miR-330-3p,miR-2300a-5p,miR-27a-3p,miR-544a,miR-450b-5p,miR-2426,miR-378c,miR-3120-5p,miR-143-5p,miR-10b,miR-541,miR-378b,miR-2284r,miR-484,miR-30b-3p,miR-323b,miR-412-3p,miR-378d,miR-491-5p,miR-103a-2-5p,miR-214-3p,miR-323b-3p,miR-6130,miR-10a,miR-544-3p,miR-6516,miR-378a-3p,miR-532-5p,miR-296-3p,miR-21-3p,miR-339b,miR-133b-3p,miR-186-5p,miR-21a-3p,miR-487b-5p,miR-346,miR-6525,miR-10b-5p,miR-6134,miR-3184-5p,miR-4510,miR-378c,miR-3074-2-3p,miR-214,miR-664a,miR-17-3p,miR-130b-5p,miR-708-5p,miR-4492,miR-129-5p,miR-320e,miR-148b-3p,novel_83,miR-2284b-

PPP1CA	ENSOARG0(	protein	miR-10b-5p,miR-206-3p,miR-29c-5p,miR-338-3p,miR-378c,miR-145a-3p,miR-664a,miR-708-5p,miR-324-3p,miR-29d-5p,miR-132-5p,miR-193b-3p,miR-139-5p,miR-22-3p,miR-204-3p,miR-378a-3p,miR-370-5p,let-7g-3p,miR-27b-5p,miR-2432,miR-216c-5p,miR-2887,miR-760-3p,miR-34b-5p,miR-30b-3p,miR-299-3p,miR-412-5p,miR-125b,miR-185-3p,novel_83,miR-532-3p,miR-3600,miR-378d,novel_133,miR-2300b-3p,miR-3059-5p,miR-541-3p,miR-4726-5p,miR-335,miR-2284q,miR-769-5p,miR-378e,miR-3064-5p,miR-452-3p,miR-10b,miR-877-3p,miR-361-3p,miR-199b-5p,miR-30c-1-3p,miR-3187-3p,miR-2330-5p,miR-382-3p,let-7c-3p,miR-3085-3p,miR-25-5p,miR-326,miR-1306-5p,novel_4,miR-409b,miR-2300a-5p,miR-378c,miR-450b-5p,miR-2426,miR-6529a,miR-296-5p,miR-378b,miR-484,miR-10b,miR-378c,miR-1306-5p,miR-204-5p,miR-486b-3p,miR-28-5p,miR-410-5p,miR-370-5p,miR-135a-1-3p,miR-331-3p,miR-34c-5p,miR-769,miR-199a-5p,miR-3957,miR-3957-5p,miR-370-3p,miR-7144-5p,miR-485-5p,miR-488-3p,miR-3154,miR-28b,miR-199a-5p,miR-1271-3p,miR-1,miR-4286,miR-450a-2-3p,miR-2898,miR-1260a,miR-30c-2-3p,miR-378h,miR-214-5p,miR-128-3p,miR-10a-5p,miR-615-3p,miR-345-3p,miR-3578,miR-134,miR-211,miR-326-3p,miR-449a,miR-2428,miR-1193,miR-656-5p,miR-502b,miR-6402,miR-133a-5p,miR-34c,miR-2404,miR-34b,miR-378b,miR-10a-5p,miR-450b-3p,miR-1291,miR-6395,miR-18a-3p,miR-378b,miR-654-3p,miR-124-5p,miR-125a-5p,miR-1343-5p,miR-197-5p,miR-2355-3p,miR-187-3p,miR-26b-3p,miR-1260b,miR-653,miR-1434-3p,miR-145a-5p,miR-6525,miR-769-5p,miR-346,miR-3074-5p,miR-500,miR-874-5p,miR-4492,miR-2285j,miR-10a,miR-2331-3p,miR-504-5p,miR-30c-1-3p,miR-1277-3p,miR-296-3p,miR-552-3p,miR-186-5p,miR-1827,miR-500a-3p,miR-129b-3p,miR-486-3p,miR-143-3p,miR-2285w,miR-345-3p,miR-3653-
ATP6V1B1	ENSOARG0(	ATPase,	miR-3591-3p,miR-150-5p,miR-409-5p,miR-2387,miR-9-3p,miR-378f,miR-504,miR-7977,miR-3120-3p,miR-20a-3p,miR-320c,miR-3082-5p,miR-374c-3p,miR-378e,miR-33a-3p,miR-382-5p,miR-218-5p,miR-490-5p,miR-3964,miR-499a-5p,miR-3958-3p,miR-2284j,miR-9-5p,novel_120,miR-2428,miR-30b-3p,miR-412-3p,miR-2284r,miR-378b,miR-2284x,miR-101c,miR-1839-5p,miR-219a-1-3p,miR-30b-3p,miR-378c,miR-2284ab,novel_116,miR-6238,miR-147a,miR-2411-3p,miR-29b-1-5p,miR-197-5p,novel_115,miR-5126,miR-320b,miR-660,miR-487b-5p,miR-33a-5p,miR-376b,miR-1388-3p,miR-2310,miR-1b-5p,miR-432-3p,miR-22841,miR-378d,miR-432-3p,miR-320a,miR-1814c,miR-671-5p,miR-7144-5p,miR-2318,miR-2355-5p,miR-542-5p,miR-21-3p,miR-874-3p,miR-660-5p,miR-2331-3p,miR-504-5p,miR-378a-3p,miR-2284n,novel_25,miR-324-5p,miR-6130,miR-132-5p,miR-374c-3p,miR-132-5p,miR-410-5p,miR-2284k,miR-669a-3p,miR-487a-5p,miR-6516-3p,miR-103b,miR-4510,miR-378c,miR-539-5p,miR-1306-5p,miR-338-3p,miR-378c,miR-378a-3p,miR-1434-5p,miR-3578,miR-378i,miR-345-3p,miR-361-5p,miR-184-3p,miR-4429,miR-412,miR-2284h-5p,miR-2411-5p,miR-378h,miR-9-5p,miR-345-3p,miR-422a,miR-3071-3p,miR-324-3p,miR-2285p,novel_17,miR-543-5p,miR-3653-3p,miR-30b-3p,miR-320d,miR-450a-2-3p,miR-2284m,miR-5100,miR-2285w,novel_39,miR-2411,miR-29b-1-5p,miR-450b-5p

TMEM59L	ENSOARG0(	transmem	miR-874-3p,miR-370-3p,miR-125a,miR-34c-5p,miR-301,miR-93-5p,miR-17-5p,miR-320a,miR-345-5p,miR-3074-1-3p,miR-20b-5p,miR-671-5p,miR-193a-5p,miR-93,miR-1260b,miR-365a-5p,miR-410-5p,miR-2433,miR-125b-5p,miR-331-3p,miR-138-5p,miR-28-5p,miR-6517,novel_79,miR-129-1-3p,miR-345-3p,miR-615-3p,miR-1343-5p,miR-330-5p,miR-582-3p,miR-3064-5p,miR-3065-3p,novel_39,miR-1260a,miR-339a,miR-2898,miR-450a-2-3p,novel_96,miR-543-5p,novel_127,miR-34b,novel_69,miR-320c,miR-6535,miR-2387,miR-1306,miR-18a-3p,miR-1291,miR-135a-2-3p,novel_101,novel_27,miR-326-3p,miR-449a,miR-2428,miR-4324,miR-20a-5p,miR-576-3p,miR-34c,miR-625-3p,miR-656-5p,miR-22-5p,miR-31-5p,miR-339-5p,miR-432-5p,miR-412-5p,miR-185-5p,miR-3431,miR-20b,miR-432,miR-138,miR-1260b,miR-4508,miR-455-5p,miR-2284x,miR-130b-5p,miR-345-5p,miR-365b-5p,miR-17-5p,miR-106a-5p,miR-455-5p,miR-1343-5p,miR-125a-5p,miR-3065-3p,miR-6130,miR-216b-5p,miR-299,miR-433-3p,miR-339b,miR-345-5p,miR-625-5p,miR-1827,miR-539-5p,miR-4510,miR-3184-3p,miR-129-2-3p,miR-145a-3p,miR-214,miR-6525,miR-3184-5p,miR-6134,miR-338-3p,miR-665,miR-320e,miR-708-5p,miR-487a-5p,miR-4492,miR-208b-5p,miR-130b-5p,miR-18a-3p,miR-106a,miR-2284h-5p,miR-2366,miR-2904,miR-1193,miR-188-3p,miR-4429,miR-133a-3p,miR-34a-5p,miR-20a,miR-345-3p,miR-196a-3p,miR-618,miR-324-3p,miR-345-3p,miR-125b,miR-106a,miR-320d,miR-34b-5p,miR-3064-5p,miR-7977,miR-3082-5p,miR-4443,miR-361-3p,miR-136-5p,miR-877-3p,miR-541-3p,miR-1296-5p,miR-361-3p,miR-423-5p,miR-615,miR-3968,miR-1343-3p,novel_78,miR-409b,miR-2300a-5p,miR-455-3p,miR-296-5p,miR-541,miR-758-3p,miR-1388-3p,miR-106a-5p,miR-744-3p,miR-376b,miR-4532,miR-758-3p,miR-320b,miR-326,miR-1306-5p,miR-106b-5p,miR-491-5p,miR-3085-3p,miR-3955-

MMP16	ENSOARG0(	matrix m	miR-200b-3p,miR-345-3p,miR-134,miR-1290,miR-330-5p,miR-9-5p,miR-134-5p,miR-545-5p,novel_121,miR-129-1-3p,miR-429-3p,novel_79,miR-450a-2-3p,miR-200c-3p,miR-1260a,miR-200c,miR-30f,miR-339a,miR-2478,miR-3065-3p,miR-29a-5p,miR-2284y,miR-2284a,miR-450c-5p,miR-1271-3p,miR-192-3p,miR-3529-3p,miR-30a-3p,miR-1,miR-345-5p,miR-329b,miR-20b-5p,miR-1814c,miR-1248,miR-488-3p,miR-93-5p,miR-320a,miR-17-5p,miR-223-5p,miR-376c-3p,miR-154b-5p,miR-574-5p,miR-1271,miR-134-5p,novel_63,miR-2284k,miR-370-5p,miR-409-3p,miR-93,miR-1260b,miR-193a-5p,miR-3607-3p,miR-2284ab,miR-1260b,miR-19b-2-5p,miR-653,miR-1434-3p,miR-1285,miR-223-3p,miR-197-3p,novel_23,miR-145a-5p,miR-3071-5p,miR-2284d,miR-2285g,miR-6238,miR-20b,miR-3955-5p,miR-339-5p,miR-26b-3p,miR-378a-5p,miR-2285l,miR-452-5p,miR-466f-3p,miR-193a-3p,miR-224-5p,miR-146a,miR-377-3p,miR-330-3p,miR-106a-5p,miR-17-5p,miR-2411-3p,miR-432-3p,miR-654-3p,miR-2285r,miR-432-3p,miR-345-5p,miR-6240,miR-382,miR-628-5p,miR-146b-5p,miR-505-3p,miR-2285ab,miR-2387,miR-3970,miR-3591-3p,miR-3065-5p,miR-146b-3p,miR-323a-5p,miR-670-3p,novel_69,miR-320c,novel_68,miR-3074-5p,miR-329-5p,miR-320b,miR-26b-5p,miR-6119-3p,novel_1,miR-206,miR-421-5p,miR-147-5p,miR-20a-5p,miR-382-5p,miR-33a-3p,miR-24-3p,miR-326-3p,novel_27,novel_120,miR-136-3p,miR-193b-3p,miR-1193,miR-33b-3p,novel_73,miR-4429,miR-148a-5p,miR-2284u,miR-1246,miR-1197-5p,miR-5703,miR-23b-3p,miR-7859,miR-200b,miR-532-3p,novel_83,miR-548e-3p,miR-106a,miR-30d-3p,miR-505-3p,miR-212-5p,miR-106a,miR-320d,miR-760-3p,miR-1277-5p,miR-21-3p,miR-26a-5p,miR-216a-5p,miR-345-3p,miR-421-5p,miR-376c-3p,miR-146a-5p,miR-329a-5p,miR-216c-5p,miR-20a,miR-2312,miR-345-
ARRB1	ENSOARG0(	arrestin	miR-3141,miR-331-5p,miR-1306,miR-150-5p,miR-136-5p,miR-23a-5p,miR-2319b,miR-370-5p,miR-2285aa,miR-552-3p,miR-376e-3p,miR-6130,miR-6535,miR-22-3p,miR-376c-3p,miR-370-5p,miR-331-3p,miR-331-5p,miR-486b-3p,miR-6123,miR-145a-3p,miR-129-2-3p,miR-4510,miR-425-5p,miR-665-5p,miR-1193,miR-1197-3p,miR-2284r,miR-7b-5p,miR-412-3p,miR-1343-5p,miR-22-3p,miR-5703,miR-378d,miR-187-3p,miR-31-5p,miR-1343-3p,miR-412,miR-505-5p,miR-147a,miR-216a-3p,miR-129-1-3p,miR-421,miR-214-5p,miR-7-5p,miR-1306-5p,miR-3141,novel_51,miR-1343-5p,miR-543-5p,miR-744-3p,miR-125b-2-3p,miR-376c-3p,miR-486-3p
NAGK	ENSOARG0(	N-acetyl	miR-3120-5p,miR-4508,miR-142-3p,miR-138,miR-330-5p,miR-194-5p,miR-140-3p,miR-1246,miR-1290,miR-455-3p,miR-147a,miR-134-3p,miR-1271-5p,novel_4,miR-487b-5p,miR-185-3p,miR-194a,miR-197-5p,miR-219b-5p,miR-26a-5p,miR-326,miR-6119-5p,let-7j,miR-486-3p,miR-3065-3p,miR-376a-5p,miR-345-3p,miR-10b-3p,miR-96-5p,miR-140-5p,miR-769,miR-27a-5p,miR-378g,miR-3120-3p,miR-26b-5p,miR-370-3p,miR-7689-3p,miR-3065-3p,miR-541-5p,miR-486b-3p,miR-4492,miR-138-5p,miR-769-5p,miR-2285j,miR-218-5p,miR-154a,miR-326-3p,miR-3959-3p,miR-769-5p,miR-194b-5p,miR-1271,miR-212-3p,miR-145a-3p

FANCI	ENSOARG0(	Fanconi	miR-3968,miR-486b-3p,miR-208b-5p,miR-4492,miR-129-5p,miR-30a-3p,miR-378c,miR-129-2-3p,miR-378c,miR-539-5p,miR-495-3p,miR-18a-5p,miR-378f,miR-885-3p,miR-18b,miR-376b-5p,miR-18a,miR-129b-5p,miR-376c-5p,miR-378e,miR-4443,miR-378a-3p,miR-182-5p,miR-760-3p,miR-125a-3p,miR-182-5p,miR-345-3p,miR-422a,miR-2411-3p,miR-500a-5p,miR-378d,miR-450b-5p,miR-486-3p,miR-30a-3p,miR-885-3p,miR-378c,miR-143-5p,miR-345-3p,miR-378b,miR-378i,miR-378a-3p,miR-378h,miR-340-5p,miR-222-5p,miR-129-1-3p,miR-222-5p,miR-30d-3p,miR-18b-5p
LPGAT1	ENSOARG0(	lysophos	miR-7641,miR-1224-5p,miR-2285ad,novel_121,miR-505-5p,miR-30a-5p,miR-2427,let-7a-2-3p,miR-128-3p,miR-1,miR-3064-5p,miR-1271-3p,miR-200a,miR-2478,miR-24-2-5p,miR-217,miR-30c-2-3p,miR-125a-3p,miR-205-5p,miR-543-5p,miR-152-3p,miR-200a-3p,miR-107,miR-127-5p,miR-141-3p,miR-2285aa,miR-324-5p,miR-133c,miR-374c-3p,miR-17-5p,miR-93-5p,miR-200a-5p,miR-30e-5p,miR-671-5p,miR-20b-5p,miR-7144-5p,miR-345-5p,let-7g-3p,miR-329b,miR-3607-3p,miR-93,miR-323-3p,miR-3969,miR-370-5p,miR-135a-1-3p,miR-592,miR-486b-3p,miR-130a-3p,miR-665,miR-103a-3p,miR-31-5p,miR-103,miR-544b,miR-20b,miR-149-5p,miR-412-5p,miR-374b-3p,novel_23,miR-145a-5p,miR-1285,miR-27b-3p,miR-219a-1-3p,miR-668-3p,miR-345-5p,let-7a-2-3p,let-7j,miR-296-3p,miR-432-3p,miR-106a-5p,miR-17-5p,miR-105-5p,miR-296-3p,miR-146a,miR-193a-3p,miR-452-5p,miR-7-1-3p,novel_68,miR-6535,novel_69,miR-374c-3p,miR-323a-5p,miR-3591-3p,miR-216a-3p,miR-378b,miR-148a-3p,miR-6395,miR-146b-5p,miR-107,miR-1291,miR-2285x,miR-1193,miR-193b-3p,miR-3957-3p,miR-342-3p,miR-301b-3p,miR-2284w,miR-323c,miR-133a-5p,miR-142b,miR-20a-5p,miR-199c,miR-6123,miR-656-5p,miR-206,miR-106a,miR-548e-3p,miR-328-3p,miR-199a-3p,novel_83,miR-148b-3p,miR-7859,miR-217-5p,miR-140-3p,miR-188-3p,miR-376a-5p,miR-378d,miR-33b-3p,miR-142-3p,miR-20a,miR-195a-3p,miR-216c-5p,miR-146a-5p,miR-486-3p,miR-2424,miR-142a-3p,miR-376a-5p,miR-500a-5p,novel_17,miR-196a-3p,miR-30d,miR-760-3p,miR-106a,miR-296-3p,miR-532-5p,miR-21-3p,miR-370-5p,miR-548o-3p,miR-140-5p,miR-545-3p,miR-30c-1-3p,novel_25,miR-204-3p,miR-323-5p,miR-193b-3p,miR-216b-5p,miR-377-3p,miR-544-3p,miR-301a-3p,miR-30d-5p,miR-500a-3p,novel_99,miR-186-5p,miR-345-5p,miR-30f,let-7g-3p,miR-214,miR-122-5p,miR-206-
ATXN7	ENSOARG0(	ataxin 7	miR-205-5p,miR-2285y,novel_124,miR-219a-1-3p,miR-378d,miR-2285ad,miR-181a-2-3p,miR-3957-3p,miR-3082-5p,miR-3959-5p

MIDN	ENSOARG0(	midnolin	miR-331-3p,miR-2433,miR-191-5p,miR-134-5p,miR-486b-3p,miR-3607-3p,miR-6128,miR-193a-5p,miR-365a-5p,miR-378c,miR-1197-3p,miR-500b-5p,miR-93,miR-18a,miR-17-5p,miR-362-5p,miR-3154,miR-93-5p,miR-488-3p,miR-7144-5p,miR-20b-5p,miR-615-5p,miR-2355-5p,miR-874-3p,miR-574-5p,miR-154b-5p,miR-199a-5p,miR-769,miR-300-3p,miR-339a,miR-30c-2-3p,miR-125a-3p,novel_96,novel_127,miR-152-3p,miR-2898,miR-2385-3p,miR-490-3p,miR-199a-5p,miR-3065-3p,miR-330-5p,miR-1343-5p,miR-134,miR-128-3p,miR-155-5p,miR-411-3p,miR-18b-5p,novel_79,miR-1224-5p,miR-222,miR-134-5p,miR-545-5p,miR-483-3p,miR-378h,miR-505-5p,miR-302a-5p,miR-425-5p,miR-6529b,miR-20a-5p,miR-379-3p,miR-6119-3p,miR-495-3p,miR-758-5p,miR-3957-3p,miR-342-3p,miR-2284w,miR-199b-5p,miR-2285m,miR-326-3p,miR-24-3p,miR-216a-3p,miR-18a-3p,miR-378b,miR-150-5p,miR-148a-3p,miR-3141,miR-18b,miR-2285ab,miR-6395,miR-378f,miR-450b-3p,miR-1291,miR-320b,miR-3074-5p,novel_69,miR-374c-3p,novel_51,miR-106a-5p,miR-17-5p,miR-365b-5p,miR-2285v,miR-3141,miR-296-3p,miR-224-5p,miR-1343-5p,miR-124-5p,miR-483-3p,miR-363-5p,miR-296-3p,miR-873a-5p,miR-4508,miR-30b-3p,miR-339-5p,miR-20b,miR-3431,miR-134-3p,miR-2285g,miR-132-5p,miR-2285j,miR-222-3p,miR-500,miR-4492,miR-874-5p,miR-155-5p,miR-664a,miR-3074-5p,miR-145a-3p,miR-378c,miR-338-3p,miR-3184-5p,miR-122-5p,miR-346,miR-224-5p,miR-6525,miR-769-5p,miR-500a-3p,miR-2285c,miR-1827,miR-18a-5p,miR-339b,miR-2312,miR-296-3p,miR-552-3p,miR-3065-3p,miR-30c-1-3p,miR-221-5p,miR-378a-3p,miR-2331-3p,miR-155-5p,miR-204-3p,miR-2284s,miR-27a-5p,miR-92a-1-5p,miR-422a,miR-500a-5p,miR-324-3p,novel_17,miR-221-5p,miR-760-3p,miR-466i-5p,miR-106a,miR-20a,miR-105-3p,miR-486-3p,miR-188-3p,miR-378a-

ENG	ENSOARG0(	endoglin	miR-330-5p,miR-23b-5p,miR-2284z,miR-450a-1-3p,miR-129-1-3p,miR-9-5p,novel_79,miR-1224-5p,miR-431,miR-6517,novel_127,miR-127-5p,miR-3071-3p,miR-205-5p,miR-125a-3p,novel_39,miR-29a-5p,miR-148b-5p,miR-3064-5p,miR-20b-5p,miR-345-5p,miR-93-5p,miR-30e-5p,miR-376c-3p,miR-3959-5p,miR-2285aa,miR-125a,miR-1271,miR-874-3p,miR-125b-5p,miR-193a-5p,miR-144-5p,novel_42,miR-28a-3p,miR-2284v,miR-27b-3p,miR-16-1-3p,miR-450c-3p,miR-432,miR-1843b-5p,miR-542-3p,miR-2113,miR-194-3p,miR-149-5p,miR-3431,miR-339-5p,miR-3955-5p,miR-365b-5p,novel_51,miR-2411-3p,miR-377-3p,miR-378f,miR-3970,miR-216a-3p,miR-6535,miR-669,miR-677,miR-181b-3p,novel_69,miR-3120-3p,novel_68,miR-6119-3p,miR-142b,miR-335-5p,miR-16-1-3p,miR-133a-3p,miR-34a-5p,miR-184-3p,miR-378a-3p,miR-378i,miR-2366,miR-7859,miR-18a-3p,miR-106a,miR-324-3p,miR-92a-1-5p,miR-422a,miR-345-3p,miR-210-5p,miR-376c-3p,miR-181b-1-3p,miR-503-5p,miR-1827,miR-186-5p,miR-345-5p,miR-339b,miR-500a-3p,miR-299,miR-450a-1-3p,miR-376e-3p,miR-377-3p,miR-10a,miR-2331-3p,miR-504-5p,miR-500,miR-874-5p,miR-4492,miR-1468-5p,novel_74,miR-151-3p,miR-665,miR-664a-5p,miR-3184-5p,miR-122-5p,miR-769-5p,miR-6525,miR-346,miR-214,miR-129-2-3p,miR-758-3p,miR-2284r,miR-301b-5p,miR-541,miR-455-3p,miR-421,miR-153,miR-147a,miR-376b-3p,miR-145b,miR-181b-2-3p,miR-1343-3p,miR-153-3p,miR-214-3p,miR-29b-1-5p,novel_19,miR-106b-5p,miR-431-5p,miR-2310,miR-378d,miR-1388-3p,miR-376a-3p,miR-504,miR-378j,miR-140-5p,miR-409-5p,miR-203-3p,miR-144-5p,miR-4443,miR-7689-3p,miR-211-5p,miR-139-5p,miR-490-5p,miR-3968,miR-499a-5p,miR-301a-5p,miR-2440,miR-423-5p,miR-1285-5p,miR-877-3p,miR-361-3p,novel_111,miR-9-5p,miR-2447,miR-128-3p,miR-10a-5p,miR-218-2-3p,miR-345-

ASNA1	ENSOARG0(	arsA ars	miR-450a-1-3p,miR-129-1-3p,novel_79,miR-330-5p,miR-148b-5p,let-7g,let-7f-5p,miR-200a-3p,novel_96,novel_127,miR-205-5p,miR-125a-3p,miR-3068-3p,miR-2355-5p,miR-125a,miR-329a,miR-345-5p,miR-331-5p,miR-1197-3p,miR-1260b,miR-331-5p,miR-125b-5p,miR-100-3p,miR-412-5p,miR-149-5p,miR-340-3p,miR-3955-5p,miR-339-5p,miR-1843b-5p,miR-2113,miR-7b-5p,miR-542-3p,miR-450c-3p,let-7f-2-3p,miR-628-5p,let-7f-2-3p,miR-378a-5p,miR-483-3p,miR-7-5p,miR-377-3p,miR-105-5p,miR-365b-5p,miR-194a,miR-2411-3p,novel_51,novel_69,miR-6535,miR-669,novel_68,miR-329-5p,miR-412-3p,let-7e-5p,miR-216a-3p,miR-3591-3p,miR-199b-5p,miR-142b,miR-328-3p,miR-18a-3p,miR-212-5p,miR-33b-3p,miR-664-5p,miR-133a-3p,miR-34a-5p,miR-1961,miR-2366,miR-1434-5p,miR-486-3p,miR-329a-5p,miR-345-3p,miR-98-5p,let-7k,miR-2285p,miR-210-5p,miR-324-3p,miR-345-3p,miR-92a-1-5p,miR-10a,miR-582-5p,miR-377-3p,miR-216b-5p,let-7i-5p,miR-548o-3p,miR-216b-3p,miR-296-3p,miR-345-5p,miR-339b,miR-625-5p,miR-186-5p,miR-1827,miR-450a-1-3p,miR-129b-3p,miR-411,miR-769-5p,miR-346,miR-224-5p,miR-3184-5p,let-7b,miR-3184-3p,miR-129-2-3p,miR-214,miR-665-5p,miR-129-5p,miR-1468-5p,miR-4492,miR-130b-5p,let-7f,miR-665,miR-153,miR-455-3p,miR-1185-3p,miR-2459,miR-1343-3p,miR-1388-5p,miR-362-3p,miR-329-3p,miR-758-3p,miR-8485,miR-491-5p,miR-153-3p,miR-199b-5p,miR-214-3p,miR-505,miR-3596,miR-203-3p,miR-1185-2-3p,miR-4443,miR-139-5p,miR-96-5p,miR-3963,miR-493-5p,miR-1983,miR-140-5p,miR-361-3p,novel_111,miR-423-5p,miR-7705,miR-3956-3p,miR-3968,novel_82,miR-483-3p,miR-1271-5p,miR-6516-5p,miR-423-3p,miR-7641,let-7d,miR-105-2,miR-128-3p,miR-10a-5p,miR-345-3p,miR-3578,miR-199a-5p,miR-3065-3p,miR-490-3p,miR-181a-2-3p,miR-17-3p,miR-2898,miR-191-3p,miR-1260a,miR-

ATP5H	ENSOARG0(	ATP synt	miR-17-3p,novel_74,miR-424-5p,miR-129-5p,miR-145a-3p,miR-4510,miR-378c,novel_99,miR-487b-5p,miR-6130,miR-216b-5p,miR-544-3p,miR-15b-5p,miR-204-3p,miR-378a-3p,miR-221-5p,miR-545-3p,miR-2331-3p,miR-504-5p,miR-532-5p,miR-370-5p,miR-3065-3p,miR-2284m,miR-106a,miR-21-3p,miR-221-5p,novel_17,miR-422a,miR-181b-1-3p,miR-376a-5p,miR-380-3p,miR-2432,miR-105-3p,miR-2285w,miR-183-5p,miR-20a,miR-184-3p,miR-493-3p,miR-362-3p,miR-378i,miR-1197-5p,miR-378a-3p,miR-5703,miR-217-5p,miR-2904,miR-222-5p,miR-15a-5p,miR-199a-3p,miR-2284h-5p,miR-106a,miR-18a-3p,miR-4726-5p,miR-3968,miR-2440,miR-301a-5p,miR-361-3p,miR-541-3p,miR-2447,miR-2284j,miR-504,novel_94,miR-199b-5p,miR-378j,miR-3596,miR-378e,miR-203-3p,miR-20a-3p,miR-374b-5p,miR-491-5p,miR-199b-5p,miR-326,novel_115,miR-431-5p,miR-199a-3p,miR-106b-5p,miR-16b,miR-362-3p,miR-378d,miR-8485,miR-424-5p,miR-127-3p,miR-106a-5p,miR-744-3p,miR-33a-5p,miR-544a,miR-2426,miR-362-3p,miR-378c,miR-101c,miR-541,miR-301b-5p,miR-329-3p,miR-378b,miR-126a-5p,miR-455-3p,miR-409b,miR-105-1,miR-2300a-5p,miR-145b,miR-181b-2-3p,miR-767-5p,miR-374b-3p,miR-370-5p,miR-494-5p,miR-2284k,miR-29b-3p,miR-93,miR-145-3p,miR-378c,miR-374b,miR-16-5p,miR-193a-5p,miR-329a,miR-10b-3p,miR-767,miR-7144-5p,miR-20b-5p,miR-381-3p,miR-488-3p,miR-3154,miR-17-5p,miR-93-5p,miR-200a-5p,miR-3959-5p,miR-374c-3p,miR-2284n,miR-324-5p,miR-199a-5p,miR-127-5p,miR-17-3p,miR-2898,novel_127,miR-543-5p,novel_96,miR-217,miR-3071-3p,miR-192-3p,miR-3065-3p,miR-199a-5p,miR-3529-3p,miR-181a-2-3p,miR-2385-3p,miR-300,miR-105-2,miR-1290,miR-330-5p,miR-29d-3p,miR-1343-5p,miR-378h,miR-322-5p,miR-6517,miR-431,miR-7641,miR-29a,miR-20a-5p,miR-142b,miR-15a,miR-16a,miR-371b-3p,miR-199b-5p,miR-
USP38	ENSOARG0(	ubiquiti	let-7d,miR-1839-5p,miR-1961,miR-412-3p,miR-758-3p,miR-412-5p,miR-7859,miR-148b-3p,let-7g-5p,novel_121,miR-548e-3p,miR-412,miR-376a-5p,miR-98-5p,let-7f-5p,let-7e,miR-152-3p,let-7k,novel_17,miR-1306-5p,let-7c-5p,let-7g,miR-758-3p,let-7a-5p,miR-195a-3p,let-7e-5p,miR-376a-2-5p,miR-148a-3p,miR-1306,let-7d-5p,let-7i-5p,miR-574-5p,let-7f,miR-142b,let-7i,miR-338-5p,let-7b-5p,let-7b,miR-3059-5p,miR-212-3p

PRR14L	ENSOARG0(	proline	miR-17-5p,miR-106a-5p,miR-455-5p,miR-1343-5p,miR-130b-5p,miR-6412,miR-345-5p,miR-654-3p,miR-455-5p,miR-2889,miR-29b,miR-138,miR-219a-1-3p,miR-130a-3p,miR-26b-3p,miR-20b,miR-103,miR-2285g,miR-185-5p,miR-20a-5p,miR-3957-3p,miR-17-3p,miR-342-3p,miR-326-3p,miR-18a-3p,miR-2285ab,miR-2387,miR-9851-3p,miR-6395,miR-1291,miR-107,miR-19a-3p,miR-320b,miR-10a-5p,miR-2404,miR-29a-3p,miR-670-3p,miR-30c-2-3p,miR-339a,miR-217,miR-2285af,miR-6119-5p,miR-200c,miR-107,miR-17-3p,miR-450a-2-3p,miR-200c-3p,miR-181a-2-3p,miR-199a-5p,miR-3065-3p,miR-29d-3p,miR-615-3p,miR-3578,miR-105-2,miR-200b-3p,miR-10a-5p,miR-222,miR-214-5p,miR-483-3p,miR-378h,miR-331-3p,miR-34c-3p,miR-29b-3p,miR-135a-1-3p,miR-2332,miR-138-5p,miR-378c,miR-365a-5p,miR-93,miR-323-3p,miR-17-5p,miR-488-3p,miR-7144-5p,miR-485-5p,miR-10b-3p,miR-370-3p,miR-199a-5p,miR-324-5p,miR-769,miR-342,miR-326,miR-3085-3p,miR-3955-3p, let-7c-3p,miR-376b,miR-2330-5p,miR-106a-5p,miR-3187-3p,miR-193a,miR-30c-1-3p,miR-484,miR-296-5p,miR-378b,miR-10b,miR-378c,miR-2426,miR-5010-3p,miR-409b,miR-105-1,miR-221-3p,miR-216a-3p,miR-2285e,miR-769-5p,miR-1973,miR-4726-5p,miR-335,miR-2285f,miR-212-3p,miR-541-3p,miR-361-3p,miR-199b-5p,novel_94,miR-541-5p,miR-499b-5p,miR-7977,miR-3064-5p,miR-20a-3p,miR-378e,miR-185-3p,miR-221-5p,miR-299-3p,miR-106a,miR-760-3p,miR-466i-5p,miR-20a,miR-5100,miR-124-3p,miR-378d,miR-19b-3p,miR-548e-3p,miR-200b,miR-324-3p,miR-222-3p,miR-487a-5p,miR-17-3p,miR-664a,miR-378c,miR-145a-3p,miR-10b-5p,miR-21a-3p,miR-2312,miR-3065-3p,miR-21-3p,miR-221-5p,miR-378a-3p,novel_32,novel_25,miR-204-3p,miR-193b-3p,miR-105-5p,miR-365b-5p,miR-193a-3p,miR-483-3p,miR-628-5p,miR-2285r,miR-432-3p,miR-197-3p,novel_23,miR-2284x,miR-339-
TMEM163	ENSOARG0(	transmem	miR-486-3p,miR-767-5p,miR-378a-5p,miR-767,miR-379-5p,miR-486b-3p,miR-218-1-3p,miR-9851-3p,novel_82,miR-100-3p,miR-197-5p,miR-30f
ITGA7	ENSOARG0(	integrin	miR-486b-3p,miR-4726-5p,miR-665,miR-331-3p,miR-324-3p,miR-326-3p,miR-1285-5p,novel_111,miR-1197-3p,miR-6525,miR-18a-3p,miR-361-3p,miR-1185-2-3p,miR-323-5p,novel_69,miR-323a-5p,miR-27a-5p,miR-300-3p,miR-378g,novel_127,miR-2898,miR-326,miR-2285b,miR-486-3p,miR-29a-5p,miR-432-3p,let-7f-2-3p,let-7f-2-3p,miR-4286,miR-363-5p,miR-124a,miR-330-5p,miR-5703,miR-124-3p,miR-2300b-3p,miR-6529a,miR-1185-3p,miR-134-3p,miR-505-5p,miR-18a-3p

GINS2	ENSOARG0(GINS com	miR-149-5p,miR-3431,miR-26b-3p,miR-133b-5p,miR-340-3p,miR-744-5p,miR-497-5p,miR-4508,miR-138,miR-16b,novel_23,miR-374b-3p,miR-22-3p,miR-432-3p,miR-660,miR-2411-3p,miR-2331-5p,miR-377-3p,miR-374c-3p,miR-299b-3p,miR-216b-3p,miR-6395,miR-195a-5p,miR-376a-2-5p,miR-412-3p,miR-3591-3p,miR-18a-3p,miR-2285ab,miR-326-3p,miR-2285x,miR-6123,miR-203b-5p,miR-16a,miR-142b,miR-15a,miR-214-5p,miR-129-1-3p,miR-6516-5p,miR-2411-5p,miR-322-5p,miR-376a-5p,miR-299a-3p,miR-2285ad,miR-582-3p,miR-330-5p,miR-345-3p,novel_39,miR-181a-2-3p,novel_127,miR-200a-3p,miR-331-5p,miR-374c-3p,miR-1271,miR-141-3p,miR-2355-5p,miR-874-3p,miR-7144-5p,miR-3154,miR-181c-3p,miR-103b,miR-16-5p,miR-486b-3p,miR-28-5p,miR-138-5p,miR-331-3p,miR-2332,miR-410-5p,miR-2459,miR-455-3p,miR-1298-3p,miR-147a,miR-222-5p,miR-2300a-5p,miR-2426,miR-296-5p,miR-8485,miR-424-5p,miR-494-3p,miR-214-3p,miR-16b,miR-326,miR-3082-5p,miR-499a-3p,miR-2285u,novel_94,miR-1983,miR-181a-3p,miR-147-3p,miR-2285f,miR-628-3p,miR-2285y,miR-1973,miR-2285n,miR-328-3p,miR-1247-5p,miR-15a-5p,miR-18a-3p,miR-378d,miR-5703,miR-1197-5p,miR-2904,miR-376a-5p,novel_133,miR-486-3p,miR-2411,miR-2887,miR-299-3p,miR-221-5p,miR-30b-3p,miR-466i-5p,miR-345-3p,miR-210-5p,miR-196a-3p,miR-15b-5p,novel_32,miR-22-3p,miR-377-3p,miR-499b-3p,miR-27a-5p,miR-6516,miR-21-3p,miR-660-5p,miR-221-5p,miR-1895,miR-214,miR-129-2-3p,miR-708-5p,miR-424-5p,miR-874-5p,novel_74,miR-665,miR-132-5p

MGAT5	ENSOARG0(	mannosyl	miR-3065-3p,miR-490-3p,miR-2284a,miR-28b,miR-1,miR-4286,miR-181a-2-3p,miR-543-5p,miR-2898,miR-30c-2-3p,miR-217,miR-2285af,miR-1260a,miR-582-3p,miR-214-5p,miR-302a-5p,miR-483-3p,miR-378h,miR-1271-5p,miR-128-3p,miR-582-3p,miR-29d-3p,miR-378c,miR-365a-5p,miR-145-3p,miR-133a-3p,miR-1306-5p,miR-410-3p,miR-486b-3p,miR-28-5p,miR-204-5p,miR-331-3p,miR-26c,miR-29b-3p,miR-135a-1-3p,miR-2284n,miR-34c-5p,miR-769,miR-370-3p,miR-141-3p,miR-7144-5p,miR-1814c,miR-485-5p,miR-10b-3p,miR-3154,miR-488-3p,miR-296-3p,miR-654-3p,miR-2483-5p,miR-363-5p,miR-130b-5p,miR-125a-5p,miR-1343-5p,miR-2285l,miR-330-3p,miR-544b,miR-432-5p,miR-2284d,miR-2285g,miR-130a-3p,miR-377-5p,miR-133b-5p,miR-2889,miR-30b-3p,miR-29b,miR-2284ab,miR-1260b,miR-873a-5p,miR-326-3p,miR-449a,miR-2428,miR-211,miR-24-3p,miR-3957-3p,novel_101,novel_120,miR-342-3p,miR-135a-2-3p,miR-656-5p,miR-147-5p,miR-199c,miR-34c,miR-16a,miR-382-5p,miR-15a,miR-2404,miR-34b,miR-29a-3p,miR-320b,miR-378b,miR-195a-5p,miR-450b-3p,miR-1291,miR-19a-3p,miR-18a-3p,miR-2285ab,miR-2387,miR-146a-5p,miR-2432,miR-29b-1-5p,miR-130a-5p,miR-216c-5p,miR-30b-3p,miR-34b-5p,miR-221-5p,miR-21-3p,miR-2284m,miR-466i-5p,miR-760-3p,miR-185-3p,miR-125b,miR-671-3p,miR-151-5p,miR-532-3p,miR-148b-3p,miR-15a-5p,miR-3600,miR-378d,miR-19b-3p,miR-124-3p,miR-1197-5p,miR-140-3p,miR-5703,miR-362-3p,miR-206-3p,miR-1271,miR-378c,miR-424-5p,miR-708-5p,miR-664a,miR-320e,miR-22-3p,novel_32,miR-204-3p,miR-544-3p,miR-193b-3p,miR-139-5p,miR-3065-3p,miR-221-5p,miR-545-3p,miR-378a-3p,miR-133b-3p,miR-27b-5p,miR-21a-3p,miR-193a,miR-30c-1-3p,let-7c-3p,miR-424-5p,miR-494-3p,miR-2285o,miR-3085-3p,novel_124,miR-362-3p,miR-342,miR-326,miR-1306-5p,miR-
ATP13A2	ENSOARG0(	ATPase t	miR-7977

ADM	ENSOARG0(	adrenome	miR-16-5p,novel_42,miR-378c,miR-2903,miR-1260b,miR-2433,miR-29b-3p,miR-28-5p,miR-486b-3p,miR-141-3p,miR-331-5p,miR-34c-5p,miR-365a-3p,miR-25-3p,miR-769,miR-3154,miR-23b,miR-301,miR-671-5p,miR-345-5p,miR-3064-5p,miR-4286,miR-200a,miR-2284a,miR-490-3p,miR-2285b,miR-92b-3p,miR-28b,miR-1260a,miR-6119-5p,miR-107,novel_127,miR-92a-3p,miR-200a-3p,miR-181b-5p,miR-412,miR-2284z,miR-322-5p,miR-378h,miR-29d-3p,miR-7134-5p,miR-3591-5p,miR-193b-3p,miR-29c-3p,miR-449a,miR-24-3p,miR-34c,miR-16a,miR-15a,miR-28c,miR-142b,miR-92a-3p,miR-29a,miR-3120-3p,miR-26b-5p,miR-3074-5p,miR-6535,miR-34b,miR-29a-3p,miR-150-5p,miR-1306,miR-18a-3p,miR-195a-5p,miR-378f,miR-450b-3p,miR-107,miR-2483-5p,miR-628-5p,let-7f-2-3p,let-7f-2-3p,miR-130b-5p,miR-345-5p,miR-32,miR-126a-3p,miR-193a-3p,miR-26b-3p,miR-103a-3p,miR-103,miR-412-5p,miR-185-5p,miR-497-5p,miR-4508,miR-16b,miR-29b,miR-1260b,miR-378c,miR-4510,miR-3074-5p,miR-3184-5p,miR-769-5p,novel_44,miR-98-3p,let-7b-3p,miR-708-5p,miR-874-5p,miR-424-5p,miR-129-5p,miR-130b-5p,miR-32-5p,miR-221-5p,miR-378a-3p,miR-204-3p,miR-15b-5p,miR-193b-3p,miR-6130,miR-27b-5p,let-7f-1-3p,miR-1895,miR-625-5p,miR-345-5p,miR-486-3p,miR-421-5p,miR-324-3p,miR-3601,miR-185-3p,miR-422a,miR-26a-5p,miR-221-5p,miR-34b-5p,miR-1277-5p,miR-18a-3p,miR-532-3p,miR-23b-3p,miR-23c,miR-15a-5p,miR-98-3p,miR-378a-3p,miR-378i,miR-34a-5p,let-7a-3p,miR-493-3p,miR-941,miR-452-5p,miR-181d-5p,let-7i-3p,miR-3059-5p,miR-2447,miR-363-3p,miR-423-5p,miR-361-3p,miR-877-3p,miR-769-5p,miR-3064-5p,miR-2319b,miR-1185-2-3p,miR-378e,miR-25,miR-2483-3p,miR-129b-5p,miR-381-3p,miR-361-3p,miR-23a-3p,miR-676-3p,miR-1388-3p,miR-424-5p,miR-378d,miR-505,miR-2957,miR-16b,novel_115,miR-1306-

GLYAT	ENSOARG0(	glycine-	miR-329a,miR-329b,miR-767,miR-2474,miR-424-3p,miR-30e-5p,miR-2355-5p,miR-1197-3p,miR-1260b,miR-16-5p,miR-193a-5p,miR-196a-5p,miR-330-5p,miR-23b-5p,miR-1343-5p,miR-758-5p,miR-322-5p,novel_121,miR-2284z,miR-429-3p,miR-6517,miR-1224-5p,novel_79,miR-412,let-7f-5p,miR-200a-3p,novel_96,miR-219-3p,miR-205-5p,miR-3956-5p,miR-450c-5p,let-7g,miR-200a,novel_39,let-7e-5p,miR-378f,miR-3065-5p,miR-216a-3p,miR-3970,miR-150-5p,miR-1306,miR-669,miR-216b-3p,miR-206,miR-6123,miR-625-3p,miR-28c,miR-4324,miR-335-5p,miR-16b,miR-497-5p,miR-7b-5p,miR-2113,miR-432,novel_23,miR-197-3p,miR-3431,miR-3535,miR-31-5p,miR-339-5p,miR-196b-5p,miR-378a-5p,miR-377-3p,miR-7-5p,novel_51,miR-432-3p,let-7j,miR-6240,miR-493-5p,miR-380-5p,let-7f-2-3p,let-7f-2-3p,miR-628-5p,miR-339b,miR-1827,miR-625-5p,miR-299,miR-30d-5p,miR-10a,miR-377-3p,let-7i-5p,miR-15b-5p,miR-548o-3p,miR-2331-3p,miR-296-3p,miR-154a-3p,novel_74,miR-669a-3p,miR-4492,let-7f,miR-219b-3p,miR-769-5p,let-7b,miR-433-5p,miR-214,miR-665-5p,miR-142-3p,miR-218-1-3p,miR-193b-5p,miR-1961,miR-378i,miR-378a-3p,miR-2284h-5p,miR-98-5p,miR-202-5p,let-7k,miR-3653-3p,miR-122-3p,miR-422a,miR-500a-5p,miR-2424,miR-487a,miR-2285w,miR-493-5p,miR-1185-2-3p,miR-4443,miR-203-3p,miR-139-5p,miR-499a-5p,miR-154-3p,miR-218-5p,miR-1285-5p,miR-3956-3p,miR-2447,miR-6527,miR-362-3p,miR-1388-5p,miR-329-3p,miR-1a-3p,miR-147a,miR-425-3p,miR-1185-3p,miR-2459,miR-548w,miR-330-3p,miR-491-5p,miR-214-3p,miR-103a-2-5p,miR-16b,miR-505,miR-29b-1-5p,miR-378d,miR-2310,miR-671-5p,miR-1814c,miR-485-5p,miR-23a-5p,novel_48,miR-503-3p,miR-2448-3p,miR-381-3p,miR-362-5p,miR-769,miR-487a-3p,miR-141-3p,miR-615-5p,miR-370-3p,miR-767-5p,miR-138-5p,miR-135a-1-3p,miR-410-5p,miR-
SCYL3	ENSOARG0(	SCY1-lik	miR-410-5p,miR-210-5p,miR-500a-5p,miR-324-3p,miR-17-3p,miR-378a-5p,miR-17-3p,miR-193a-3p,miR-3059-5p,miR-193b-3p,miR-3957-3p,miR-17-3p,let-7j,miR-1271-3p,miR-484,miR-1434-3p,miR-3154,miR-33b-3p,miR-2284x,miR-378d,miR-2285aa,miR-1271,miR-2404,miR-193b-3p

DDX6	ENSOARG0(	DEAD (As	miR-206-3p,miR-98-3p,miR-3184-5p,miR-122-5p,miR-3074-5p,miR-2330-3p,miR-6516-3p,miR-17-3p,miR-664a,miR-130b-5p,miR-487a-5p,miR-454-3p,miR-708-5p,miR-208b-5p,miR-2285j,let-7b-3p,miR-665,miR-3604,miR-664a-5p,miR-320e,miR-324-3p,miR-151-3p,miR-139-5p,miR-192-5p,miR-301a-3p,miR-22-3p,novel_32,miR-204-3p,miR-548o-3p,miR-532-5p,miR-216b-3p,novel_99,miR-625-5p,miR-411,miR-130a-5p,miR-2432,miR-760-3p,miR-2284m,miR-320d,miR-1277-5p,novel_17,miR-196a-3p,miR-500a-5p,miR-148b-3p,miR-7859,miR-383-5p,miR-532-3p,novel_83,miR-548e-3p,miR-4429,novel_73,miR-142-3p,miR-19b-3p,miR-2284u,miR-218-1-3p,miR-2300b-3p,miR-362-3p,miR-215-5p,miR-5703,miR-98-3p,miR-124-3p,miR-1285-5p,miR-423-5p,miR-541-3p,miR-885-5p,miR-212-3p,miR-2447,miR-2284j,miR-194b-5p,miR-499a-5p,miR-2284q,miR-7134-3p,miR-374a-3p,miR-2440,miR-218-5p,miR-2285n,miR-301a-5p,miR-2285y,miR-371a-5p,miR-2483-3p,miR-378g,miR-4443,miR-203-3p,miR-2284g,miR-139-5p,miR-24-1-5p,miR-7977,miR-211-5p,miR-2285u,miR-668-5p,miR-136-5p,miR-381-3p,miR-2330-5p,miR-101b-3p,miR-124a,miR-1388-3p,miR-376b,miR-2284b,miR-130b-3p,miR-320b,miR-323b-3p,miR-505,miR-362-3p,miR-2320-3p,miR-2459,miR-455-3p,miR-548w,miR-330-3p,miR-151a-3p,miR-450b-5p,miR-362-3p,miR-3120-5p,miR-21b,miR-2284aa,miR-301b-5p,miR-541,miR-484,miR-329-3p,miR-30b-3p,miR-323b,miR-1a-3p,miR-412-3p,miR-374a-3p,miR-1260b,miR-323-3p,miR-145-3p,miR-2284v,miR-3607-3p,miR-6128,miR-204-5p,miR-374b-3p,miR-592,miR-28-5p,miR-29b-3p,miR-2284k,miR-135a-5p,miR-144,miR-100-3p,miR-154b-5p,miR-2284n,miR-133c,miR-574-5p,miR-215-5p,miR-2285aa,miR-2355-5p,miR-329a,miR-485-5p,miR-1248,miR-381-3p,miR-320a,miR-24-2-5p,miR-2284a,miR-2284y,miR-1,miR-191-3p,miR-127-5p,miR-17-
CNBD1	ENSOARG0(	cyclic n	miR-103a-2-5p,miR-3074-1-3p,novel_1,miR-107-5p,miR-2355-5p,miR-885-5p

STARD3	ENSOARG0(	StAR-rel	miR-330-5p,miR-23b-5p,miR-18b-5p,miR-423-3p,miR-6517,novel_121,miR-322-5p,miR-205-5p,novel_127,miR-2898,miR-4286,miR-3064-5p,miR-490-3p,novel_39,miR-28b,miR-24-2-5p,miR-18a,miR-362-5p,miR-200a-5p,miR-671-5p,miR-485-5p,miR-23a-5p,miR-125a,miR-2355-5p,miR-769,miR-300-3p,miR-125b-5p,miR-370-5p,miR-410-5p,miR-2433,miR-486b-3p,miR-16-5p,miR-500b-5p,miR-22-3p,miR-744-5p,miR-497-5p,miR-30b-3p,miR-16b,miR-130a-3p,miR-149-5p,miR-194-3p,miR-134-3p,miR-503-5p,miR-330-3p,miR-3141,miR-146a,miR-503-5p,miR-660,miR-125a-5p,miR-668-3p,miR-363-5p,miR-6240,miR-3141,miR-18b,miR-2387,miR-195a-5p,miR-9851-3p,miR-2319a,miR-146b-5p,novel_68,miR-181b-3p,miR-669,miR-677,miR-323a-5p,miR-146b-3p,miR-16a,miR-28c,miR-15a,miR-1193,miR-301b-3p,miR-2428,miR-326-3p,miR-371b-3p,miR-24-3p,miR-188-3p,miR-1197-5p,miR-5703,miR-2366,miR-133a-3p,miR-3960,miR-532-3p,miR-15a-5p,miR-125b,miR-185-3p,miR-324-3p,miR-196a-3p,miR-760-3p,miR-466i-5p,miR-503-5p,miR-486-3p,miR-146a-5p,miR-181b-1-3p,miR-129b-3p,miR-1827,miR-18a-5p,miR-370-5p,miR-660-5p,miR-504-5p,miR-15b-5p,miR-22-3p,miR-323-5p,miR-2284s,miR-301a-3p,miR-665,miR-132-5p,miR-324-3p,miR-2285j,miR-4492,miR-424-5p,miR-454-3p,miR-664a,miR-214,miR-145a-3p,miR-769-5p,miR-30b-3p,miR-484,novel_4,miR-330-3p,miR-2300a-5p,miR-1343-3p,miR-500-5p,miR-216a-3p,miR-16b,miR-326,miR-214-3p,miR-146b,miR-130b-3p,miR-3085-3p,miR-424-5p,miR-4532,miR-361-3p,miR-877-3p,miR-504,miR-668-5p,miR-7977,miR-24-1-5p,miR-4792,miR-3064-5p,miR-4443,miR-378g,miR-769-5p,miR-2440,miR-4726-5p,miR-361-3p,miR-362-5p

GRHPR	ENSOARG0(	glyoxyla	miR-21b,miR-101c,miR-758-3p,miR-323b,miR-147a,miR-1185-3p,miR-455-3p,miR-148b-5p,miR-1343-3p,miR-181b-2-3p,miR-487b-5p,miR-491-5p,miR-103a-2-5p,miR-199b-5p,miR-16b,miR-323b-3p,miR-378d,miR-744-3p,miR-504,miR-1983,miR-493-5p,miR-378j,miR-378g,miR-3596,miR-1185-2-3p,miR-4443,miR-2319b,miR-2284g,miR-211-5p,miR-2285u,miR-499a-5p,novel_82,novel_111,miR-1285-5p,miR-3956-3p,miR-2447,miR-34a-5p,miR-2284u,miR-148a-5p,miR-133a-3p,miR-193b-5p,miR-2366,miR-378i,miR-378a-3p,miR-2284h-5p,miR-212-5p,miR-202-5p,miR-345-3p,miR-422a,miR-500a-5p,miR-181b-1-3p,miR-486-3p,miR-2424,miR-2285w,miR-345-3p,miR-1827,miR-186-5p,miR-625-5p,miR-129b-3p,miR-216b-5p,miR-10a,miR-582-5p,miR-377-3p,miR-301a-3p,miR-15b-5p,miR-504-5p,miR-296-3p,miR-552-3p,miR-216b-3p,miR-4492,miR-454-3p,miR-2285j,miR-665,miR-769-5p,miR-142a-5p,miR-6525,miR-16b,miR-4508,miR-497-5p,miR-2113,miR-223-3p,miR-7b-5p,miR-432,novel_23,miR-197-3p,miR-134-3p,miR-3431,miR-412-5p,miR-149-5p,miR-103a-3p,miR-3955-5p,miR-31-5p,miR-340-3p,miR-377-3p,miR-296-3p,miR-7-5p,miR-2411-3p,miR-105-5p,miR-432-3p,miR-2285r,miR-432-3p,miR-493-5p,miR-628-5p,miR-412-3p,miR-378f,miR-2319a,miR-148a-3p,miR-3065-5p,miR-374c-3p,miR-181b-3p,miR-677,miR-216b-3p,novel_68,miR-301b-3p,miR-199b-5p,miR-155-5p,miR-330-5p,miR-23b-5p,miR-2427,miR-322-5p,miR-486-5p,novel_121,miR-2284z,miR-134-5p,miR-429-3p,miR-2285ad,miR-1224-5p,miR-412,miR-152-3p,novel_96,miR-125a-3p,miR-2284y,novel_39,miR-29a-5p,miR-876-3p,miR-331-5p,miR-223-5p,miR-503-3p,miR-125a,miR-542-5p,miR-2355-5p,miR-331-5p,miR-134-5p,miR-6740-5p,miR-125b-5p,miR-1197-3p,miR-1260b,miR-16-5p,miR-450b-5p,miR-378c,miR-3120-5p,miR-2284aa,miR-10b,miR-143-5p,miR-378b,miR-30b-

FLOT1	ENSOARG0(flotilli	let-7c-5p,miR-339a,miR-217,miR-125a-3p,miR-1260a,miR-205-5p,miR-2285af,miR-200a-3p,let-7f-5p,miR-2898,miR-30a-3p,miR-490-3p,miR-450c-5p,miR-1271-3p,miR-200a,let-7g,novel_39,miR-380-5p,miR-450b-5p,miR-28b,miR-134,miR-3591-5p,miR-345-3p,miR-10a-5p,let-7d,novel_79,miR-299a-3p,miR-134-5p,miR-450a-1-3p,miR-483-3p,miR-409-3p,miR-125b-5p,miR-5100,miR-374b-3p,miR-134-5p,miR-28-5p,miR-204-5p,miR-138-5p,miR-3607-3p,miR-1306-5p,miR-374a-3p,miR-1260b,miR-2448-3p,miR-671-5p,miR-1248,miR-485-5p,miR-7144-5p,miR-141-3p,miR-125a,miR-2355-5p,miR-769,miR-2411-3p,miR-197-5p,miR-296-3p,miR-224-5p,miR-503-5p,miR-125a-5p,miR-483-3p,miR-130b-5p,miR-380-5p,miR-503-3p,miR-296-3p,miR-22841,miR-2285r,miR-145a-5p,novel_23,miR-3071-5p,miR-197-3p,miR-450c-3p,miR-223-3p,miR-653,miR-188-5p,miR-26a-2-3p,miR-1260b,miR-138,miR-339-5p,miR-2355-3p,miR-544b,miR-503-5p,let-7g-5p,let-7i,novel_87,miR-6402,miR-28c,miR-147-5p,miR-335-5p,novel_27,miR-24-3p,miR-211,miR-2387,miR-2285ab,let-7e-5p,miR-505-3p,miR-2319a,miR-412-3p,miR-10a-5p,miR-320b,novel_69,miR-670-3p,miR-299b-3p,miR-146b-3p,miR-125b,miR-345-3p,miR-92a-1-5p,miR-216a-5p,miR-219b-5p,miR-2285p,miR-196a-3p,miR-21-3p,miR-299-3p,let-7k,miR-98-5p,miR-503-5p,miR-105-3p,miR-216c-5p,miR-217-5p,miR-1197-5p,miR-124-3p,miR-1961,miR-2408,novel_133,miR-218-1-3p,miR-33b-3p,miR-493-3p,miR-505-3p,miR-30d-3p,miR-532-3p,novel_83,miR-320e,miR-665,miR-132-5p,miR-30a-3p,miR-30e-3p,let-7f,miR-130b-5p,miR-708-5p,miR-664a,miR-450b-3p,miR-214,miR-2330-3p,let-7b,let-7b-5p,miR-224-5p,miR-769-5p,miR-10b-5p,miR-450a-1-3p,miR-21a-3p,miR-339b,miR-552-3p,miR-296-3p,miR-182-5p,let-7i-5p,novel_32,miR-544-3p,miR-10a,miR-132-5p,miR-182-5p,let-7e,miR-214-3p,miR-

MGST3	ENSOARG0(	microsom	miR-2285ad,miR-7641,miR-30d-3p,miR-199a-3p,miR-376a-5p,miR-1246,miR-23b-5p,miR-330-5p,miR-217-5p,miR-142-3p,miR-4429,miR-16-1-3p,miR-133a-3p,miR-34a-5p,miR-329a-5p,miR-195a-3p,miR-181a-2-3p,miR-2478,miR-181b-1-3p,miR-29a-5p,miR-486-3p,miR-490-3p,miR-2285af,miR-205-5p,miR-2397-5p,miR-618,miR-30c-2-3p,miR-219b-5p,miR-92a-1-5p,miR-217,miR-320d,miR-202-5p,miR-6536,miR-181b-5p,miR-34b-5p,miR-543-5p,miR-30c-1-3p,miR-548o-3p,miR-182-5p,miR-34c-5p,miR-139-5p,miR-769,novel_25,miR-137-3p,miR-500a-3p,miR-320a,miR-433-3p,miR-3154,miR-7144-5p,miR-543-3p,miR-1814c,novel_99,miR-186-5p,miR-3607-3p,miR-769-5p,miR-224-5p,miR-122-5p,miR-145-3p,miR-181c-5p,miR-5100,miR-30e-3p,miR-324-3p,miR-129-5p,miR-500,miR-486b-3p,miR-199b-3p,miR-409b,miR-665,miR-222-5p,miR-181b-2-3p,miR-185-5p,miR-194-3p,miR-2355-3p,miR-6529a,miR-181a-5p,novel_23,miR-194-5p,miR-3071-5p,miR-16-1-3p,miR-188-5p,miR-382-3p,miR-668-3p,miR-628-5p,miR-652-5p,miR-432-3p,miR-30c-1-3p,miR-2310,miR-199a-3p,miR-224-5p,miR-320b,miR-326,miR-197-5p,miR-182-5p,miR-194a,miR-124-5p,miR-466f-3p,miR-103a-2-5p,miR-139-5p,miR-20a-3p,miR-329-5p,miR-374c-3p,miR-34b,miR-677,miR-320c,miR-181b-3p,miR-378b,miR-136-5p,miR-2319a,miR-194b-5p,miR-2285x,miR-181d-5p,miR-24-3p,miR-449a,miR-326-3p,miR-2284w,miR-2285n,miR-769-5p,miR-34c,miR-107-5p,miR-490-5p,miR-199c

SPATA17	ENSOARG0(	spermato	miR-424-5p,let-7a-5p,miR-33a-5p,miR-30c-1-3p,miR-16b,miR-2957,miR-130b-3p,let-7e,miR-199b-5p,miR-330-3p,miR-2300a-5p,miR-222-5p,miR-376b-3p,miR-539-5p,miR-425-3p,miR-421,miR-455-3p,miR-148b-5p,miR-541,miR-125b-2-3p,miR-101a-5p,miR-1388-5p,miR-101c,miR-1839-5p,miR-541-3p,miR-3059-5p,miR-181d-5p,miR-154a,miR-423-5p,miR-218-5p,novel_82,miR-382-3p,miR-2285y,miR-450a-5p,miR-499a-5p,miR-3968,miR-2284q,miR-490-5p,miR-7977,miR-499b-5p,miR-3082-5p,miR-499a-3p,miR-376d,let-7d-5p,miR-199b-5p,miR-668-5p,miR-376a-3p,miR-329a-5p,miR-2411,novel_17,miR-2285p,miR-92a-1-5p,miR-185-3p,miR-98-5p,miR-466i-5p,miR-202-5p,let-7k,miR-1277-5p,miR-548e-3p,miR-18a-3p,miR-212-5p,miR-222-5p,miR-15a-5p,miR-7859,miR-383-5p,miR-2300b-3p,miR-1961,miR-2366,miR-191,miR-5703,miR-1197-5p,miR-148a-5p,miR-3074-5p,let-7b,miR-539-5p,miR-2330-3p,miR-6525,miR-181c-5p,let-7b-5p,miR-3184-5p,let-7f,miR-338-5p,miR-1843b-3p,miR-6516-3p,miR-17-3p,miR-664a,miR-129-5p,miR-708-5p,miR-424-5p,miR-454-3p,miR-496-3p,miR-660-5p,miR-548o-3p,miR-30c-1-3p,miR-296-3p,miR-21-3p,miR-499b-3p,miR-301a-3p,let-7i-5p,miR-15b-5p,miR-129b-3p,miR-345-5p,miR-133b-3p,novel_99,miR-186-5p,miR-6240,miR-432-3p,miR-345-5p,novel_51,miR-330-3p,miR-660,miR-31-5p,miR-3955-5p,miR-130a-3p,miR-3431,let-7g-5p,miR-432-5p,miR-2355-3p,novel_116,miR-6238,miR-1434-3p,miR-1843b-5p,miR-181a-5p,miR-432,miR-374b-3p,novel_23,miR-219a-1-3p,miR-16b,miR-6239,miR-497-5p,novel_120,miR-135a-2-3p,miR-2285x,miR-17-3p,miR-3957-3p,miR-301b-3p,miR-199b-5p,miR-502-5p,miR-15a,let-7i,miR-382-5p,miR-16a,miR-22-5p,miR-3074-5p,miR-329-5p,miR-374c-3p,miR-411b-3p,miR-3591-3p,miR-376b-3p,miR-18a-3p,miR-378b,miR-195a-5p,miR-379-

TMCO1	ENSOARG0(	transmem	miR-199b-5p,miR-378j,miR-299b-5p,miR-493-5p,miR-1983,miR-20a-3p,miR-499b-5p,miR-2285u,miR-211-5p,miR-378g,miR-499a-3p,miR-3596,miR-3082-5p,miR-371a-5p,miR-3968,miR-3964,miR-3059-5p,miR-877-3p,novel_111,miR-6529a,miR-412-3p,miR-758-3p,miR-299a-5p,miR-3120-5p,miR-409b,miR-539-5p,miR-9788-3p,miR-320b,novel_103,miR-16b,miR-199b-5p,miR-146b,miR-214-3p,miR-1388-3p,miR-2330-5p,miR-424-5p,miR-1b-5p,miR-299,miR-21a-3p,miR-18a-5p,miR-140-5p,miR-216b-3p,miR-27a-5p,miR-499b-3p,miR-377-3p,novel_32,miR-22-3p,miR-15b-5p,miR-324-3p,miR-19b-1-5p,miR-29b-2-5p,miR-6516-3p,miR-424-5p,miR-3074-5p,miR-214,miR-6525,novel_133,miR-2366,miR-2904,miR-5703,miR-188-3p,miR-4429,miR-340-5p,miR-15a-5p,miR-2284h-5p,novel_17,miR-320d,miR-21-3p,miR-183-5p,miR-2285w,miR-146a-5p,miR-2432,miR-18b,miR-412-3p,miR-146b-5p,miR-135b-5p,miR-195a-5p,novel_68,miR-3074-5p,miR-374c-3p,miR-320c,miR-144-3p,miR-6535,miR-15a,miR-28c,miR-16a,miR-33a-3p,novel_87,miR-6119-3p,miR-22-5p,miR-656-5p,miR-1a-1-5p,miR-211,novel_27,miR-2284w,miR-199b-5p,miR-708-3p,miR-873a-5p,miR-19b-2-5p,miR-7b-5p,miR-22-3p,miR-197-3p,miR-374b-3p,miR-16b,miR-497-5p,miR-7862,miR-2284x,miR-31-5p,miR-3955-5p,miR-134-3p,miR-7-5p,miR-377-3p,miR-146a,miR-1343-5p,miR-493-5p,miR-18a,miR-320a,miR-7144-5p,miR-671-5p,miR-574-5p,miR-141-3p,miR-374c-3p,miR-199a-5p,miR-1-5p,miR-144,miR-331-3p,miR-135a-5p,novel_9,miR-204-5p,miR-374b-3p,miR-16-5p,miR-3607-3p,miR-1197-3p,miR-374a-3p,miR-1343-5p,miR-412,miR-18b-5p,miR-322-5p,miR-205-5p,miR-30f,miR-28-3p,miR-6536,miR-1a-2-5p,miR-200a-3p,miR-29b-2-5p,miR-4286,miR-181a-2-3p,miR-28b,miR-450b-5p,miR-2478,miR-199a-5p
HID1	ENSOARG0(	HID1 dom	novel_32,novel_127,miR-324-3p,miR-18a-3p,miR-7977,miR-18a-3p

BRIX1	ENSOARG0(	BRX1, bi	miR-379-3p,miR-206,miR-4324,miR-374c-5p,miR-758-5p,miR-2319a,let-7e-5p,miR-3065-5p,miR-411b-3p,miR-144-3p,novel_68,miR-329-5p,miR-3120-3p,miR-378a-5p,miR-2411-3p,miR-194a,novel_51,miR-2285r,let-7j,let-7a-2-3p,let-7f-2-3p,let-7f-2-3p,miR-16-1-3p,miR-2284x,miR-19b-2-5p,miR-450c-3p,miR-134-3p,miR-3431,miR-194-3p,miR-3955-5p,miR-339-5p,novel_63,miR-2284k,miR-125b-5p,miR-2284v,miR-193a-5p,miR-20b-5p,miR-30e-5p,miR-301,miR-93-5p,miR-376c-3p,miR-874-3p,miR-2355-5p,miR-125a,let-7f-5p,miR-6536,novel_127,miR-200a-3p,miR-148b-5p,miR-450b-5p,let-7g,miR-200a,miR-2284y,miR-2285b,miR-450c-5p,miR-23b-5p,miR-450a-1-3p,miR-2285ad,miR-429-3p,miR-6517,miR-412,novel_79,miR-218-5p,miR-101-3p,miR-2440,miR-423-5p,miR-3956-3p,miR-2284j,miR-96-5p,miR-299b-5p,miR-409-5p,miR-378j,miR-381-3p,miR-3596,miR-378g,miR-2483-3p,miR-1185-2-3p,miR-2284g,miR-139-5p,miR-2285u,miR-130b-3p,miR-153-3p,miR-214-3p,miR-29b-1-5p,miR-323b-3p,miR-106b-5p,miR-182-5p,miR-1b-5p,miR-101b-3p,miR-2284b,miR-1839-5p,miR-101c,miR-21b,miR-323b,miR-1a-3p,miR-758-3p,miR-153,miR-496-5p,miR-539-5p,miR-148b-5p,miR-455-3p,miR-1185-3p,miR-199b-3p,novel_78,miR-1343-3p,miR-145b,miR-454-3p,miR-1468-5p,miR-130b-5p,let-7f,miR-2285j,miR-151-3p,miR-3604,miR-224-5p,miR-3184-5p,let-7b,miR-214,miR-339b,miR-450a-1-3p,miR-129b-3p,miR-433-3p,miR-30d-5p,miR-10a,miR-376e-3p,miR-216b-5p,let-7i-5p,miR-98-5p,let-7k,miR-210-5p,miR-345-3p,miR-2285i,miR-376a-5p,miR-376c-3p,miR-33b-3p,novel_73,miR-184-3p,miR-16-1-3p,miR-2284u,miR-1961,miR-1434-5p,miR-7859,miR-328-3p,miR-18a-3p,miR-106a,miR-380-5p,miR-128-1-5p,novel_1,miR-656-5p,miR-22-5p,miR-502b,miR-147-5p,miR-199c,miR-20a-5p,let-7i,miR-33a-3p,novel_27,miR-17-3p,miR-2285k,miR-6395,miR-

MELK	ENSOARG0(	maternal	miR-483-3p,miR-1343-5p,miR-193a-3p,miR-466f-3p,miR-146a,miR-365b-5p,novel_51,miR-2411-3p,miR-2285r,miR-22841,miR-130b-5p,miR-380-5p,miR-493-5p,miR-2483-5p,miR-138,miR-16b,miR-1260b,miR-497-5p,miR-1285,miR-181a-5p,miR-223-3p,miR-374b-3p,miR-103,miR-103a-3p,miR-26b-3p,miR-22-5p,miR-656-5p,miR-15a,miR-133a-5p,miR-485-3p,miR-16a,miR-326-3p,miR-3957-3p,miR-193b-3p,miR-107,miR-146b-5p,miR-195a-5p,miR-6395,miR-505-3p,miR-376a-2-5p,miR-6516-3p,miR-2285ab,miR-1306,miR-216a-3p,miR-18a-3p,miR-378b,miR-670-3p,miR-374c-3p,miR-6535,miR-216b-3p,miR-378b,miR-329-5p,miR-26b-5p,miR-181b-5p,miR-107,novel_127,miR-200a-3p,miR-1260a,miR-380-5p,miR-1957a,miR-2385-3p,miR-30a-3p,miR-181a-2-3p,miR-4286,miR-128-3p,miR-345-3p,miR-3591-5p,miR-1343-5p,miR-330-5p,miR-322-5p,miR-758-5p,miR-483-3p,miR-545-5p,miR-129-1-3p,novel_121,miR-486-5p,miR-376a-5p,miR-138-5p,miR-331-5p,miR-410-3p,miR-34c-3p,miR-331-3p,miR-1260b,miR-365a-5p,miR-16-5p,miR-10a-3p,miR-329b,miR-331-5p,miR-2448-3p,miR-331-5p,miR-374c-3p,miR-3959-5p,miR-3068-3p,miR-141-3p,miR-1271,miR-146b,miR-326,miR-1306-5p,miR-2957,miR-505,miR-16b,miR-8485,miR-491-3p,miR-1b-5p,miR-2330-5p,miR-424-5p,miR-33a-5p,miR-21b,miR-101c,miR-484,miR-541,miR-9788-3p,miR-409b,miR-151a-3p,miR-548w,miR-3958-3p,miR-3968,miR-541-3p,miR-2285f,miR-181d-5p,miR-493-5p,miR-140-5p,miR-2483-3p,miR-3082-5p,miR-7689-3p,miR-1277-5p,miR-26a-5p,miR-345-3p,miR-92a-1-5p,miR-146a-5p,miR-143-3p,miR-10a-3p,miR-183-5p,miR-329a-5p,miR-664-5p,miR-2366,miR-2904,miR-140-3p,miR-5703,miR-487a-3p,miR-340-5p,miR-15a-5p,miR-532-3p,miR-328-3p,miR-18a-3p,miR-30d-3p,miR-485-3p,miR-505-3p,miR-6516-3p,miR-424-5p,miR-130b-5p,miR-30e-3p,miR-151-3p,miR-30a-3p,miR-

ADGRF1	ENSOARG0(	adhesion	miR-361-3p,miR-3082-5p,miR-452-3p,miR-20a-3p,miR-7977,miR-2284q,miR-30b-5p,miR-371a-5p,miR-362-5p,miR-212-3p,miR-181d-5p,miR-2285f,miR-3120-5p,miR-6529a,miR-484,miR-30b-3p,miR-125b-2-3p,miR-412-3p,miR-2285e,miR-9788-3p,miR-500-5p,miR-221-3p,miR-151a-3p,novel_4,miR-3955-3p,miR-326,miR-320b,miR-382-3p,miR-33a-5p,miR-30f,miR-2312,miR-323-5p,miR-27a-5p,miR-155-5p,miR-532-5p,miR-155-5p,miR-6516-3p,miR-222-3p,miR-29b-2-5p,miR-320e,miR-539-5p,miR-3074-2-3p,miR-4429,miR-376a-5p,miR-5703,miR-140-3p,miR-200b,miR-148b-3p,miR-760-3p,miR-466i-5p,miR-320d,miR-21-3p,miR-34b-5p,miR-30d,miR-1277-5p,miR-30b-3p,miR-125b,miR-185-3p,miR-125b-2-3p,miR-29b-1-5p,miR-183-5p,miR-2285k,miR-1291,miR-376a-2-5p,miR-2285ab,miR-29a-3p,miR-34b,miR-323a-5p,miR-378b,miR-320b,novel_1,miR-92a-3p,miR-22-5p,miR-656-5p,miR-33a-3p,miR-34c,miR-371b-3p,miR-211,miR-2428,novel_27,miR-326-3p,miR-449a,miR-135a-2-3p,miR-219a-1-3p,miR-30b-3p,miR-29b,miR-1434-3p,miR-181a-5p,miR-653,miR-2285g,miR-125a-5p,miR-224-5p,miR-330-3p,miR-345-5p,miR-671-5p,miR-7144-5p,miR-3074-1-3p,miR-488-3p,miR-362-5p,miR-3154,miR-320a,miR-34c-5p,miR-141-3p,miR-370-3p,miR-486b-3p,miR-204-5p,miR-29b-3p,miR-1306-5p,miR-6128,miR-200b-3p,miR-134,miR-345-3p,miR-30a-5p,miR-218-2-3p,miR-29d-3p,miR-483-3p,miR-545-5p,miR-222,miR-376a-5p,miR-7641,miR-200c-3p,miR-181b-5p,miR-450a-2-3p,miR-92a-3p,miR-200c,miR-217,miR-2478,miR-490-3p,miR-2284a,miR-1271-3p,miR-376c-5p,miR-376b-5p,miR-30a-3p,miR-29b-2-5p,miR-136-5p,miR-504,miR-544-5p,miR-9-3p,miR-1983,miR-221,miR-2483-3p,miR-3596,miR-378g,miR-25,miR-203-3p,miR-2319b,miR-4792,miR-211-5p,miR-7689-3p,miR-2440,novel_82,miR-301a-5p,miR-1285-5p,miR-363-3p,miR-1839-5p,miR-101c,miR-301b-5p,miR-

MUM1	ENSOARG0(	melanoma	miR-206,miR-4324,miR-485-3p,miR-199b-5p,miR-2285m,miR-376a-3p,miR-335-5p,miR-193b-3p,miR-1839-3p,miR-412-3p,miR-378f,miR-148a-3p,miR-216a-3p,miR-299b-3p,miR-669,miR-3120-3p,miR-483-3p,miR-106b-3p,miR-193a-3p,miR-432-3p,miR-2284l,miR-432-3p,miR-628-5p,miR-16b,miR-497-5p,miR-27b-3p,miR-432,miR-197-3p,miR-450c-3p,novel_23,miR-134-3p,miR-149-5p,miR-194-3p,miR-31-5p,miR-103a-3p,miR-1260b,miR-1197-3p,miR-16-5p,miR-345-5p,miR-137-3p,miR-574-5p,miR-874-3p,miR-2355-5p,miR-1271,miR-6536,novel_127,novel_96,miR-200a-3p,miR-152-3p,miR-125a-3p,novel_39,miR-29a-5p,miR-3064-5p,miR-1290,miR-322-5p,miR-505-5p,miR-450a-1-3p,novel_121,miR-129-1-3p,miR-299a-3p,miR-2285ad,miR-6517,miR-1224-5p,miR-412,miR-18b-5p,novel_82,miR-2440,miR-361-3p,miR-877-3p,miR-423-5p,miR-7705,miR-2447,miR-299b-5p,miR-504,miR-1983,miR-140-5p,miR-499a-3p,miR-378g,miR-3596,miR-4792,miR-7689-3p,miR-1185-5p,miR-491-5p,miR-199b-5p,miR-214-3p,miR-199a-3p,novel_19,miR-29b-1-5p,miR-16b,miR-378d,miR-8485,miR-2284r,miR-541,miR-758-3p,miR-1298-3p,miR-421,miR-199b-3p,miR-1343-3p,miR-4492,miR-500,miR-130b-5p,miR-2285j,miR-151-3p,miR-3604,miR-665,miR-769-5p,miR-224-5p,miR-346,miR-3184-5p,miR-129-2-3p,miR-665-5p,miR-214,miR-345-5p,miR-1895,miR-625-5p,miR-299,miR-450a-1-3p,miR-500a-3p,miR-499b-3p,miR-151b,miR-15b-5p,miR-30c-1-3p,miR-504-5p,miR-202-5p,miR-500a-5p,miR-324-3p,miR-422a,miR-376a-5p,miR-486-3p,miR-2285w,miR-142-3p,miR-33b-3p,miR-133a-3p,miR-218-1-3p,miR-378i,miR-378a-3p,miR-2284h-5p,miR-1247-5p,miR-18a-3p,miR-212-5p,miR-128-1-5p,miR-147-5p,miR-199c,miR-15a,miR-133a-5p,miR-6402,miR-16a,miR-382-5p,miR-24-3p,miR-3959-3p,miR-371b-3p,miR-2428,miR-3957-3p,novel_101,miR-1193,miR-17-3p,miR-
MFAP2	ENSOARG0(	microfib	miR-10a-5p,miR-34b,miR-670-3p,miR-2404,miR-323a-5p,miR-

GRPEL1	ENSOARG0(	GrpE-lik	miR-145b,miR-1343-3p,miR-330-3p,miR-148b-5p,miR-147a,miR-758-3p,miR-301b-5p,miR-1388-5p,miR-23a-3p,miR-127-3p,miR-1388-3p,miR-574-3p,miR-2310,miR-16b,novel_19,miR-103a-2-5p,miR-146b,miR-2319b,miR-139-5p,miR-4443,miR-378g,miR-378j,miR-544-5p,miR-1983,miR-504,miR-299b-5p,miR-6527,miR-2447,novel_111,miR-301a-5p,miR-218-5p,miR-3968,miR-212-5p,miR-18a-3p,miR-328-3p,miR-23c,miR-23b-3p,miR-210-3p,miR-7859,miR-361-5p,miR-148a-5p,miR-16-1-3p,miR-33b-3p,miR-345-3p,miR-503-5p,miR-329a-5p,miR-486-3p,miR-324-3p,miR-26a-5p,novel_17,miR-216b-3p,miR-548o-3p,miR-504-5p,miR-2331-3p,miR-15b-5p,miR-30d-5p,miR-450a-1-3p,miR-625-5p,miR-885-3p,miR-3958-5p,miR-129-2-3p,miR-3074-5p,miR-224-5p,miR-6525,miR-769-5p,miR-665,miR-103a-3p,miR-31-5p,miR-149-5p,miR-134-3p,novel_23,miR-450c-3p,miR-22-3p,miR-2113,miR-7b-5p,miR-432,miR-497-5p,miR-4508,miR-16-1-3p,miR-16b,miR-6240,miR-432-3p,miR-432-3p,miR-2285v,miR-3141,miR-365b-5p,miR-146a,miR-7-5p,miR-196b-5p,miR-26b-5p,miR-3074-5p,miR-329-5p,novel_68,miR-6535,miR-299b-3p,miR-3591-3p,miR-216a-3p,miR-3970,miR-3141,miR-148a-3p,miR-146b-5p,miR-2319a,miR-412-3p,miR-410-5p,miR-335-5p,novel_87,miR-28c,miR-6123,miR-379-3p,miR-6119-3p,miR-1224-5p,miR-6517,miR-299a-3p,miR-2285ad,miR-129-1-3p,miR-134-5p,miR-450a-1-3p,miR-322-5p,miR-505-5p,miR-330-5p,miR-23b-5p,miR-1343-5p,miR-3591-5p,miR-196a-5p,miR-885-3p,novel_39,miR-148b-5p,miR-205-5p,novel_127,miR-152-3p,miR-6536,miR-874-3p,miR-23b,miR-200a-5p,miR-30e-5p,miR-329b,miR-16-5p,novel_42,miR-134-5p,novel_4,miR-2300a-5p,miR-2285e,miR-30b-3p,miR-3120-5p,miR-424-5p,miR-2330-5p,miR-758-3p,miR-4532,miR-193a,miR-3187-3p,miR-326,novel_103,miR-25-5p,miR-499b-5p,miR-452-3p,miR-10b,novel_94,miR-877-3p,miR-452-5p,miR-
RNF38	ENSOARG0(	ring fin	miR-376a-2-5p,miR-671-5p,miR-148a-3p,miR-216a-3p,miR-377-3p,miR-301a-3p,miR-27a-3p,miR-206,miR-3964,miR-4492,miR-454-3p,miR-2285j,miR-485-3p,miR-206-3p,miR-224-5p,miR-1285-5p,miR-301b-3p,miR-2428,miR-342-3p,miR-212-3p,miR-19b-3p,miR-1839-5p,miR-128-3p,miR-2284aa,miR-27b-3p,miR-2113,miR-1a-3p,miR-3071-5p,miR-125b-2-3p,miR-148b-3p,miR-2284z,miR-421,miR-130a-3p,miR-27a-3p,miR-376a-5p,miR-130b-3p,miR-152-3p,miR-342,miR-224-5p,miR-377-3p,novel_17,miR-505,miR-8485,miR-1

MTHFSD	ENSOARG0(	methenyl	novel_94,miR-147-3p,miR-199b-5p,miR-361-3p,miR-144-5p,miR-203-3p,miR-2483-3p,miR-378e,miR-378g,miR-452-3p,miR-20a-3p,miR-4792,miR-2284q,miR-301a-5p,miR-1973,miR-2285y,miR-371a-5p,miR-2440,miR-7705,miR-361-3p,miR-2285f,miR-3956-3p,miR-3059-5p,novel_60,miR-2447,miR-544a,miR-378c,miR-362-3p,miR-125b-2-3p,miR-301b-5p,miR-329-3p,miR-6529a,miR-2284r,miR-484,miR-378b,miR-421,miR-9788-3p,miR-216a-3p,miR-455-3p,miR-147a,miR-1298-3p,miR-153,miR-181b-2-3p,miR-105-1,miR-151a-3p,novel_78,novel_4,miR-214-3p,miR-146b,miR-153-3p,miR-199b-5p,miR-25-5p,miR-491-5p,miR-362-3p,miR-2957,miR-1306-5p,miR-2310,miR-378d,miR-33a-5p,let-7c-3p,miR-382-3p,miR-1827,miR-582-5p,miR-543-3p,miR-625-5p,miR-1895,miR-133b-3p,miR-500a-3p,miR-21a-3p,miR-877-5p,miR-155-5p,novel_32,miR-204-3p,miR-193b-3p,miR-6130,miR-582-5p,miR-532-5p,miR-221-5p,miR-545-3p,miR-378a-3p,miR-500,miR-708-5p,miR-874-5p,miR-664a,miR-155-5p,miR-17-3p,miR-665,miR-664a-5p,miR-151-3p,miR-338-3p,miR-214,miR-425-5p,miR-2330-3p,miR-3074-5p,miR-4510,miR-539-5p,miR-378c,miR-34a-5p,miR-664-5p,miR-133a-3p,miR-184-3p,miR-378a-3p,miR-5703,miR-2408,miR-362-3p,miR-2300b-3p,miR-2366,miR-1246,novel_133,miR-378i,novel_83,miR-340-5p,miR-95-3p,miR-380-5p,miR-3600,miR-21-3p,miR-221-5p,miR-34b-5p,miR-760-3p,miR-422a,miR-324-3p,miR-143-3p,miR-146a-5p,miR-2411,miR-2424,miR-486-3p,miR-181b-1-3p,miR-2887,miR-378f,miR-2319a,miR-146b-5p,miR-450b-3p,miR-3065-5p,miR-378b,miR-1306,miR-2387,miR-2285ab,miR-181b-3p,miR-677,miR-6535,miR-34b,miR-3074-5p,novel_68,miR-22-5p,novel_1,miR-34c,miR-2284o,miR-502-5p,miR-199b-5p,miR-449a,miR-24-3p,miR-323c,novel_101,miR-758-5p,miR-17-3p,miR-2285x,miR-3957-3p,miR-193b-3p,miR-188-5p,miR-26a-

CYP26B1	ENSOARG0(	cytochro	miR-105-1,miR-1343-3p,novel_4,miR-2300a-5p,miR-1185-3p,miR-9788-3p,miR-216a-3p,miR-1298-3p,miR-125b-2-3p,miR-30b-3p,miR-541,miR-2284r,miR-296-5p,miR-484,miR-3120-5p,miR-21b,miR-450b-5p,miR-106a-5p,miR-424-5p,miR-2330-5p,miR-494-3p,miR-4532,miR-30c-1-3p,miR-16b,miR-106b-5p,miR-505,miR-1306-5p,miR-431-5p,miR-342,miR-103a-2-5p,miR-2319b,miR-10b,miR-20a-3p,miR-1185-2-3p,miR-3082-5p,miR-378g,miR-499a-3p,miR-378j,miR-129b-5p,miR-99b-5p,miR-3956-3p,miR-541-3p,miR-2447,novel_60,miR-423-5p,miR-107-5p,miR-30b-5p,novel_82,miR-2284q,miR-2285t,miR-335,miR-4726-5p,miR-30d-3p,miR-3600,miR-106a,miR-18a-3p,miR-2284h-5p,miR-15a-5p,miR-1197-5p,miR-188-3p,miR-2366,miR-193b-5p,miR-30c-5p,novel_133,miR-133a-3p,miR-33b-3p,miR-493-3p,miR-20a,miR-195a-3p,miR-486-3p,miR-130a-5p,miR-377-5p,miR-125b,miR-185-3p,miR-324-3p,miR-500a-5p,miR-26a-5p,miR-210-5p,miR-299-3p,miR-30d,miR-30b-3p,miR-760-3p,miR-106a,miR-21-3p,miR-496-3p,miR-30c-1-3p,miR-155-5p,miR-15b-5p,miR-877-5p,miR-204-3p,novel_32,miR-6130,miR-656-3p,miR-499b-3p,miR-2284s,miR-30d-5p,miR-129b-3p,miR-21a-3p,miR-1827,miR-625-5p,miR-885-3p,miR-1895,miR-345-5p,miR-30f,miR-133b-3p,miR-3074-2-3p,miR-129-2-3p,miR-4510,miR-3074-5p,miR-6134,miR-3184-5p,miR-346,miR-665,miR-29b-2-5p,miR-320e,miR-30a-3p,miR-324-3p,miR-19b-1-5p,miR-30e-3p,miR-874-5p,miR-708-5p,miR-424-5p,miR-4492,miR-664a,miR-155-5p,miR-377-5p,miR-20b,miR-149-5p,miR-194-3p,miR-134-3p,miR-185-5p,miR-3071-5p,miR-1285,miR-19b-2-5p,miR-2889,miR-744-5p,miR-455-5p,miR-497-5p,miR-30b-3p,miR-138,miR-16b,miR-2483-5p,miR-345-5p,miR-654-3p,novel_51,miR-455-5p,miR-106a-5p,miR-99a-5p,miR-17-5p,miR-365b-5p,miR-105-5p,miR-125a-5p,miR-1343-5p,miR-
HACD1	ENSOARG0(	3-hydrox	miR-192-3p,miR-2285w,miR-4286,miR-101-5p,miR-369-3p,miR-127-5p,miR-491-5p,miR-320d,novel_96,miR-26a-5p,miR-377-3p,miR-320b,miR-182-5p,miR-323b-3p,novel_121,miR-2284h-5p,miR-409b,miR-4429,miR-1434-3p,miR-374c-5p,miR-539-5p,novel_101,miR-487b-5p,miR-208b-5p,miR-665,miR-377-3p,miR-320c,miR-504-5p,miR-182-5p,miR-26b-5p,miR-504,miR-544-5p,miR-320a

NDUFS7	ENSOARG0(	NADH deh	miR-1827,novel_99,miR-18a-5p,miR-339b,miR-30f,miR-30d-5p,miR-129b-3p,miR-155-5p,miR-151b,miR-204-3p,novel_25,miR-22-3p,miR-193b-3p,miR-192-5p,miR-301a-3p,miR-499b-3p,miR-532-5p,miR-2331-3p,miR-221-5p,miR-4492,miR-487a-5p,miR-454-3p,miR-155-5p,miR-664a-5p,miR-665,miR-29b-2-5p,miR-324-3p,miR-6134,miR-3184-5p,miR-206-3p,miR-346,miR-769-5p,miR-2330-3p,miR-3958-5p,miR-3074-5p,miR-378d,miR-34a-5p,miR-133a-3p,miR-184-3p,miR-4429,miR-142-3p,miR-33b-3p,miR-188-3p,miR-1197-5p,miR-2904,miR-2366,miR-215-5p,miR-2300b-3p,miR-30c-5p,novel_133,miR-328-3p,miR-151-5p,miR-532-3p,miR-210-3p,miR-212-5p,miR-18a-3p,miR-3960,miR-299-3p,miR-221-5p,miR-30d,miR-34b-5p,miR-760-3p,miR-320d,miR-324-3p,miR-542-5p,miR-210-5p,miR-30b,miR-2432,miR-29b-1-5p,miR-183-5p,miR-493-5p,miR-181a-3p,miR-361-3p,miR-500-3p,miR-454-5p,miR-4443,miR-489,miR-499a-3p,miR-378g,miR-3596,miR-452-3p,miR-7134-3p,miR-4726-5p,miR-301a-5p,miR-769-5p,miR-2440,miR-30b-5p,miR-7705,miR-502-3p,miR-423-5p,miR-361-3p,miR-877-3p,miR-194b-5p,miR-1296-5p,miR-3956-3p,miR-3059-5p,novel_60,miR-2447,miR-3120-5p,miR-2426,miR-758-3p,miR-125b-2-3p,miR-30b-3p,miR-1a-3p,miR-501-3p,miR-1298-5p,miR-301b-5p,miR-2284r,miR-296-5p,miR-9788-3p,miR-1298-3p,novel_4,novel_78,miR-1343-3p,miR-2300a-5p,miR-542-5p,miR-130b-3p,miR-25-5p,miR-491-5p,miR-29b-1-5p,miR-5126,miR-431-5p,miR-326,miR-1306-5p,miR-320b,miR-758-3p,miR-3187-3p,miR-376b,miR-1388-3p,miR-485-5p,miR-876-3p,miR-320a,miR-18a,miR-488-3p,miR-30e-5p,miR-769,miR-34c-5p,miR-1271,miR-542-5p,miR-370-3p,miR-3432a,novel_9,miR-331-3p,miR-2433,miR-365a-5p,miR-181c-3p,miR-1260b,miR-181a-2-3p,novel_42,miR-155-5p,miR-30c,miR-501-3p,miR-330-5p,miR-30a-5p,miR-1343-5p,miR-486-
GPM6B	ENSOARG0(	glycopro	miR-7705,miR-491-3p,miR-7859,miR-2330-3p,miR-548o-3p,miR-548e-3p,miR-6516-3p,miR-760-3p,miR-219a-1-3p
TUBB	ENSOARG0(	tubulin,	miR-200b,miR-421,miR-2285ad,miR-429-3p,miR-3600,miR-505-3p,miR-26b-3p,miR-30b-3p,miR-200b-3p,miR-181a-5p,miR-30b-3p,miR-194-5p,miR-432-3p,miR-329a-5p,miR-181b-5p,miR-200c-3p,miR-221-5p,miR-2285af,miR-200c,miR-365b-5p,miR-194a,miR-6130,novel_32,miR-221-5p,miR-329-5p,miR-7-1-3p,miR-9851-3p,miR-505-3p,miR-1827,miR-409-5p,miR-3065-5p,miR-877-3p,miR-423-5p,miR-3184-5p,miR-365a-5p,miR-181c-5p,miR-135a-2-3p,miR-335-5p,miR-4510,miR-181d-5p,miR-194b-5p,miR-4726-5p,miR-3968,miR-335,novel_1,miR-135a-1-3p,miR-331-3p,miR-2285y,miR-371a-5p
AXIN1	ENSOARG0(	axin 1 [	miR-328-3p,novel_32,miR-204-3p,miR-125a,miR-21-3p,miR-184-3p,miR-450b-3p,miR-6395,miR-541,miR-3154,miR-3591-3p,miR-877-3p,miR-4532,miR-541-3p,miR-2447,novel_124,miR-4492,miR-125a-5p,miR-185-3p,miR-125b,miR-125b-5p
SPR	ENSOARG0(	sepiapte	miR-210-5p,miR-296-3p,miR-483-3p,miR-107,miR-135a-2-3p,miR-296-3p,miR-1271-3p,miR-296-5p,miR-150-5p,miR-3591-3p,miR-876-3p,miR-885-3p,miR-107,miR-7975,miR-885-3p,miR-625-5p,miR-548e-3p,miR-103a-3p,miR-1343-3p,miR-2285aa,miR-21-3p,miR-296-3p,miR-552-3p,miR-7859,miR-483-3p,miR-3965,miR-6535,miR-103

TNFRSF21	ENSOARG0(	tumor ne	miR-106a-5p,miR-3187-3p,miR-8485,miR-758-3p,miR-362-3p,miR-2957,miR-106b-5p,miR-106a-5p,miR-17-5p,miR-320b,miR-1343-5p,miR-199b-5p,miR-193a-3p,miR-20b,miR-2459,miR-758-3p,miR-125b-2-3p,miR-329-3p,miR-1285,miR-143-5p,miR-362-3p,miR-16-1-3p,miR-193b-3p,miR-9-5p,miR-212-3p,miR-199b-5p,miR-211,miR-2285y,miR-2285n,miR-20a-5p,miR-211-5p,miR-20a-3p,miR-320c,miR-144-5p,miR-18b,miR-199b-5p,miR-135b-5p,miR-410-5p,miR-20a,miR-199a-5p,miR-122-3p,miR-217,miR-2285af,miR-8117,miR-2285p,miR-125a-3p,miR-106a,miR-320d,miR-466i-5p,miR-1224-5p,miR-18b-5p,miR-2285ad,miR-106a,miR-9-5p,miR-1343-5p,miR-217-5p,miR-1290,miR-362-3p,miR-16-1-3p,miR-4429,miR-3958-5p,miR-193a-5p,miR-144-5p,miR-665-5p,miR-3074-2-3p,miR-1306-5p,miR-93,miR-320e,miR-135a-5p,miR-410-5p,miR-135a-1-3p,miR-204-5p,miR-3068-3p,miR-199a-5p,miR-193b-3p,miR-93-5p,miR-320a,miR-18a,miR-17-5p,miR-20b-5p,miR-3074-1-3p,miR-329a,miR-18a-5p
STAM	ENSOARG0(	signal t	miR-665,miR-210-3p,miR-6516-5p,miR-199a-3p,miR-197-3p,miR-218-1-3p,miR-493-5p,miR-494-3p,miR-2310,miR-503-3p,miR-205-5p,miR-199a-3p,miR-125a-3p,miR-3601,miR-1277-5p,miR-139-5p,miR-3120-3p,miR-6516,miR-582-5p,miR-670-3p,miR-139-5p,miR-488-3p,miR-150-5p,miR-411,miR-493-5p,miR-6516-3p,miR-1814c,miR-1248,miR-582-5p,miR-145-3p,miR-6402,miR-3604,miR-669a-3p

SH2D3C	ENSOARG0(	SH2 doma	miR-455-3p,miR-9788-3p,miR-1298-3p,miR-27a-3p,miR-145b,miR-1343-3p,miR-2300a-5p,miR-378c,miR-1388-5p,miR-323b,miR-378b,miR-296-5p,miR-484,miR-8485,miR-378d,miR-1388-3p,miR-214-3p,miR-130b-3p,miR-487b-5p,miR-323b-3p,miR-342,miR-326,miR-27a-3p,miR-4443,miR-378e,miR-2483-3p,miR-493-5p,miR-1983,miR-504,miR-361-3p,miR-219a-2-3p,miR-6527,miR-2447,miR-499a-5p,miR-2285y,novel_82,miR-2285n,miR-3600,miR-18a-3p,miR-218-1-3p,miR-378d,miR-142-3p,miR-184-3p,miR-5703,miR-1197-5p,miR-378a-3p,miR-378i,miR-664b-3p,miR-181b-1-3p,miR-30b-3p,miR-3653-3p,miR-324-3p,miR-422a,miR-125b,miR-345-3p,miR-204-3p,miR-377-3p,miR-301a-3p,miR-323-5p,miR-6130,miR-216b-3p,miR-378a-3p,miR-504-5p,miR-660-5p,miR-1827,let-7g-3p,miR-345-5p,miR-122-5p,miR-6525,miR-214,miR-378c,miR-129-2-3p,miR-3074-5p,miR-4510,miR-874-5p,miR-454-3p,miR-17-3p,miR-664a,miR-324-3p,miR-29b-2-5p,miR-320e,miR-665,miR-149-5p,miR-412-5p,miR-26b-3p,miR-133b-5p,miR-7862,miR-27b-3p,miR-2284ab,miR-219a-1-3p,miR-145a-5p,miR-3071-5p,miR-1843b-5p,let-7j,miR-345-5p,miR-432-3p,miR-432-3p,miR-493-5p,miR-660,miR-125a-5p,miR-124-5p,miR-197-5p,miR-2411-3p,miR-377-3p,miR-677,miR-181b-3p,miR-323a-5p,miR-670-3p,miR-3074-5p,miR-378f,miR-379-5p,miR-412-3p,miR-216a-3p,miR-18a-3p,miR-326-3p,miR-301b-3p,novel_101,miR-17-3p,miR-342-3p,miR-22-5p,miR-4324,miR-134-5p,miR-129-1-3p,miR-378h,miR-2285ad,miR-128-3p,miR-330-5p,miR-345-3p,miR-134,novel_39,miR-490-3p,miR-148b-5p,miR-29b-2-5p,miR-543-5p,novel_127,miR-17-3p,miR-127-5p,miR-2285aa,miR-2355-5p,miR-125a,miR-574-5p,miR-503-3p,miR-23a-5p,miR-671-5p,miR-876-3p,miR-345-5p,miR-3154,miR-378c,miR-145-3p,miR-6128,miR-134-5p,miR-125b-5p,miR-6740-5p,miR-3969,miR-2433

RXFP2	ENSOARG0(	relaxin/	miR-299a-3p,miR-376a-5p,miR-196a-2-3p,miR-505-5p,miR-129-1-3p,novel_121,miR-545-5p,miR-196a-5p,miR-23b-5p,miR-501-3p,miR-218-2-3p,miR-300,miR-99a-5p,miR-2284f,miR-3064-5p,miR-30a-3p,miR-192-3p,miR-490-3p,miR-2284a,miR-2285b,miR-3065-3p,novel_39,miR-2284y,miR-125a-3p,miR-99a,miR-2285af,miR-3071-3p,miR-30c-2-3p,miR-127-5p,miR-543-5p,miR-152-3p,miR-3068-3p,miR-1271,miR-598-3p,miR-370-3p,miR-365a-3p,miR-769,miR-381-3p,miR-23b,miR-376c-5p,miR-320a,miR-100-5p,miR-10a-3p,miR-767,miR-376c-5p,miR-2284v,miR-6128,miR-193a-5p,miR-1197-3p,miR-145-3p,miR-370-5p,miR-2284k,miR-331-3p,miR-767-5p,miR-138-5p,miR-3432a,miR-133b-5p,miR-187-3p,miR-130a-3p,miR-2284d,miR-3535,miR-1285,miR-542-3p,miR-2113,miR-1434-3p,miR-19b-2-5p,novel_23,miR-16-1-3p,miR-138,miR-2284ab,miR-2284x,miR-369-5p,miR-6240,miR-224-5p,miR-99a-5p,miR-330-3p,miR-196b-5p,miR-193a-3p,miR-3120-3p,miR-655-5p,miR-670-3p,miR-299b-3p,miR-323a-5p,miR-669,miR-320c,miR-148a-3p,miR-376b-5p,miR-2285ab,miR-3591-3p,miR-412-3p,miR-19a-3p,miR-6395,novel_120,miR-135a-2-3p,miR-1193,miR-193b-3p,miR-24-3p,miR-301b-3p,miR-2428,miR-2284o,miR-379-3p,miR-199c,miR-502b,miR-203b-5p,miR-30d-3p,miR-148b-3p,miR-23c,miR-210-3p,miR-23b-3p,miR-328-3p,miR-616-3p,novel_83,miR-376a-5p,miR-1246,miR-2300b-3p,miR-5703,miR-1197-5p,miR-2904,miR-4429,miR-19b-3p,miR-378d,miR-2284u,miR-16-1-3p,miR-10a-3p,miR-369-3p,miR-219b-5p,miR-216a-5p,miR-2284m,miR-320d,miR-299-3p,miR-221-5p,miR-1277-3p,miR-30c-1-3p,miR-221-5p,miR-552-3p,miR-3065-3p,miR-21-3p,miR-370-5p,miR-323-5p,miR-139-5p,miR-193b-3p,miR-301a-3p,miR-499b-3p,miR-377-3p,novel_25,miR-204-3p,miR-655,miR-433-3p,miR-500a-3p,miR-129b-3p,miR-598-3p,miR-186-5p,miR-1827,miR-625-

CADPS	ENSOARG0(	Ca++-dep	miR-1285,miR-3071-5p,miR-30b-3p,miR-29b,miR-455-5p,miR-185-5p,miR-544b,miR-455-5p,miR-197-5p,miR-124-5p,miR-1343-5p,miR-130b-5p,miR-539-3p,miR-376b-3p,miR-7975,miR-655-5p,miR-34b,miR-29a-3p,miR-6402,miR-133a-5p,miR-34c,novel_1,miR-147-5p,miR-3959-3p,miR-211,miR-326-3p,miR-449a,miR-615-3p,miR-345-3p,miR-501-3p,miR-29d-3p,miR-105-2,miR-222,miR-378h,miR-1271-5p,miR-2411-5p,miR-376b-3p,miR-339a,miR-217,miR-30c-2-3p,miR-450a-2-3p,miR-29b-2-5p,miR-2385-3p,miR-199a-5p,miR-1957a,miR-488-3p,miR-10b-3p,miR-671-5p,miR-7144-5p,miR-487a-3p,miR-141-3p,miR-365a-3p,miR-34c-5p,miR-199a-5p,miR-29b-3p,miR-144,miR-767-5p,miR-28-5p,miR-204-5p,miR-323-3p,miR-365a-5p,miR-378c,miR-484,miR-378b,miR-30b-3p,miR-125b-2-3p,miR-450b-5p,miR-544a,miR-2426,miR-378c,miR-16-2-3p,miR-105-1,miR-409b,miR-221-3p,miR-216a-3p,miR-326,miR-1306-5p,miR-362-3p,miR-494-3p,miR-33a-5p,miR-30c-1-3p,miR-758-3p,miR-193a,miR-199b-5p,miR-361-3p,novel_94,miR-877-3p,miR-20a-3p,miR-664b,miR-499b-5p,miR-378e,miR-376d,miR-2285n,miR-2285y,miR-371a-5p,miR-7134-3p,miR-3059-5p,miR-452-5p,miR-219a-2-3p,miR-502-3p,miR-362-3p,miR-2300b-3p,miR-1246,miR-1197-5p,miR-5703,miR-1193,miR-485-3p,miR-3600,miR-1b-3p,miR-487a-3p,miR-760-3p,miR-34b-5p,miR-30b-3p,miR-1277-5p,miR-105-3p,miR-216c-5p,miR-125b-2-3p,miR-2411,miR-21a-3p,miR-154b-3p, let-7g-3p,miR-582-5p,miR-378a-3p,miR-544-3p,miR-204-3p,miR-222-3p,miR-29b-2-5p,miR-19b-1-5p,miR-6516-3p,miR-708-5p,miR-145a-3p,miR-378c,miR-3074-2-3p,miR-2113,miR-19b-2-5p,miR-16-1-3p,miR-31-5p,miR-339-5p,miR-194-3p,miR-2411-3p,novel_51,miR-365b-5p,miR-105-5p,miR-493-5p,miR-6240,let-7f-2-3p,let-7f-2-3p,miR-2285r,let-7i,miR-1306,miR-410-5p,miR-378f,miR-216b-3p,novel_68,miR-

CD2AP	ENSOARG0(	CD2-asso	miR-300,miR-2284z,novel_121,miR-222,miR-429-3p,miR-181b-5p,miR-200c-3p,miR-200a-3p,miR-205-5p,miR-6119-5p,miR-200c,miR-3071-3p,miR-200a,miR-2285b,miR-30a-3p,miR-10b-3p,miR-190a-3p,miR-381-3p,miR-137-3p,miR-23b,miR-2284n,miR-3068-3p,miR-141-3p,miR-138-5p,miR-204-5p,miR-6740-5p,miR-28a-3p,miR-138,miR-188-5p,miR-27b-3p,miR-181a-5p,miR-1434-3p,miR-412-5p,miR-2285g,miR-544b,miR-133b-5p,miR-452-5p,miR-466f-3p,miR-2284l,miR-2285ab,miR-3065-5p,miR-3120-3p,miR-26b-5p,novel_1,miR-203b-5p,miR-147-5p,miR-6123,miR-6402,miR-142b,miR-485-3p,miR-382-5p,novel_87,miR-33a-3p,miR-211,miR-374c-5p,novel_27,miR-219b-3p,miR-335-5p,novel_101,miR-495-3p,miR-2285x,miR-193b-5p,miR-23b-3p,miR-487a-3p,miR-23c,miR-222-5p,miR-340-5p,miR-199a-3p,miR-30d-3p,miR-485-3p,miR-2284m,miR-3653-3p,miR-1277-5p,miR-26a-5p,miR-125b-2-3p,miR-130a-5p,miR-216c-5p,let-7g-3p,miR-2312,miR-1827,novel_99,miR-21a-3p,miR-2285c,let-7f-1-3p,miR-544-3p,miR-301a-3p,miR-545-3p,miR-182-5p,miR-664a,miR-454-3p,miR-129-5p,miR-222-3p,miR-30e-3p,miR-30a-3p,miR-181c-5p,miR-544a,miR-2284aa,miR-101c,miR-541,miR-221-3p,miR-1185-3p,miR-2285e,miR-548w,novel_4,miR-1343-3p,miR-27a-3p,miR-199a-3p,miR-323b-3p,miR-182-5p,miR-193a,miR-491-3p,miR-2310,miR-382-3p,miR-23a-3p,miR-299b-5p,miR-9-3p,miR-221,miR-381-3p,miR-2483-3p,miR-1185-2-3p,miR-27a-3p,miR-20a-3p,miR-499b-5p,miR-126b-5p,miR-7977,miR-211-5p,miR-3968,miR-499a-5p,miR-335,miR-2285t,miR-382-3p,miR-9-3p,miR-7705,miR-541-3p,miR-2285f,miR-2284j,miR-181d-5p,miR-219a-5p

DEF6	ENSOARG0(	DEF6 gua	miR-378d,miR-652-5p,miR-744-3p,miR-33a-5p,miR-1839-3p,miR-101b-3p,miR-2330-5p,miR-124a,miR-214-3p,miR-25-5p,miR-3955-3p,miR-323b-3p,miR-1306-5p,miR-342,miR-199a-3p,miR-2285e,miR-9788-3p,miR-148b-5p,miR-455-3p,miR-376b-3p,miR-142-5p,miR-27a-3p,miR-5010-3p,miR-2300a-5p,novel_4,miR-1343-3p,miR-21b,miR-378c,miR-125b-2-3p,miR-323b,miR-378b,miR-296-5p,miR-2284r,miR-1285-5p,miR-877-3p,miR-219a-5p,miR-181d-5p,miR-452-5p,miR-1296-5p,miR-2285f,miR-3956-3p,novel_60,miR-2447,miR-2285t,miR-4726-5p,miR-27a-3p,miR-376d,miR-203-3p,miR-378g,miR-378e,miR-7977,miR-2319b,miR-20a-3p,miR-139-5p,miR-376a-3p,miR-361-3p,miR-143-3p,miR-486-3p,miR-130a-5p,miR-125b-2-3p,miR-345-3p,miR-195a-3p,miR-2285w,miR-329a-5p,miR-216c-5p,miR-21-3p,miR-1277-5p,miR-466i-5p,miR-422a,miR-185-3p,miR-324-3p,miR-210-5p,novel_17,miR-199a-3p,miR-2284h-5p,miR-222-5p,miR-210-3p,miR-212-5p,miR-148a-5p,miR-378d,miR-664-5p,miR-16-1-3p,miR-217-5p,miR-1197-5p,miR-378a-3p,miR-1434-5p,miR-188-3p,miR-124-3p,miR-1246,novel_133,miR-378i,miR-181c-5p,miR-142a-5p,miR-214,miR-2330-3p,miR-145a-3p,miR-129-2-3p,miR-3074-5p,miR-3184-3p,miR-378c,miR-539-5p,miR-130b-5p,miR-129-5p,miR-4492,miR-664a,miR-17-3p,miR-338-5p,miR-29b-2-5p,miR-204-3p,novel_32,novel_25,miR-139-5p,miR-323-5p,miR-216b-5p,miR-377-3p,miR-21-3p,miR-378a-3p,miR-2331-3p,miR-543-3p,miR-345-5p,let-7g-3p,miR-2312,miR-2285c,miR-21a-3p,miR-345-5p,miR-296-3p,miR-2284l,miR-2285r,miR-101-5p,miR-668-3p,miR-2483-5p,miR-130b-5p,miR-452-5p,miR-196b-5p,miR-378a-5p,novel_51,miR-455-5p,miR-377-3p,miR-296-3p,miR-544b,miR-2355-3p,miR-134-3p,miR-185-5p,miR-412-5p,miR-432-5p,miR-31-5p,miR-27b-3p,miR-455-5p,miR-16-1-3p,miR-138,miR-3071-5p,miR-1434-3p,miR-

DDB1	ENSOARG0(	damage-s	miR-654-3p,miR-432-3p,miR-345-5p,miR-380-5p,miR-6240,miR-668-3p,let-7f-2-3p,let-7f-2-3p,miR-124-5p,miR-125a-5p,miR-330-3p,miR-197-5p,let-7d-3p,miR-455-5p,novel_51,miR-432-5p,miR-134-3p,miR-185-5p,miR-3431,miR-149-5p,miR-340-3p,miR-31-5p,miR-339-5p,miR-665,miR-1260b,miR-455-5p,miR-27b-3p,miR-873a-5p,miR-432,miR-19b-2-5p,miR-1434-3p,miR-1843b-5p,miR-22-3p,miR-3071-5p,miR-323c,miR-326-3p,miR-2284w,miR-342-3p,miR-135a-2-3p,miR-542-5p,miR-758-5p,miR-17-3p,miR-656-5p,miR-199c,miR-4324,miR-142b,miR-2404,miR-670-3p,miR-677,miR-6535,miR-329-5p,miR-655-5p,miR-1291,miR-412-3p,miR-19a-3p,miR-6395,miR-135b-5p,miR-2285ab,miR-376b-5p,miR-2387,miR-150-5p,miR-376b-3p,miR-380-5p,miR-200a,miR-29a-5p,miR-1271-3p,miR-29b-2-5p,miR-450a-2-3p,miR-17-3p,miR-200c-3p,miR-1260a,miR-376b-3p,miR-200c,miR-339a,miR-758-5p,miR-6516-5p,miR-2411-5p,miR-214-5p,novel_121,miR-6517,miR-429-3p,novel_79,miR-582-3p,miR-200b-3p,miR-1290,miR-23b-5p,miR-330-5p,miR-374a-3p,miR-1260b,miR-1306-5p,miR-31-3p,miR-374b-3p,miR-592,miR-410-5p,miR-2332,miR-331-3p,miR-6740-5p,miR-125b-5p,miR-135a-5p,miR-300-3p,miR-769,miR-133c,miR-2285aa,miR-615-5p,miR-125a,miR-10b-3p,miR-345-5p,miR-488-3p,miR-2448-3p,miR-3154,miR-193a,miR-3187-3p,miR-758-3p,miR-1388-3p,miR-382-3p,miR-101b-3p,miR-3955-3p,miR-542-5p,miR-103a-2-5p,miR-214-3p,miR-342,miR-326,miR-29b-1-5p,miR-1298-3p,miR-142-3p,miR-376b-3p,miR-147a,miR-9788-3p,miR-455-3p,miR-2459,miR-1185-3p,miR-330-3p,miR-199b-3p,novel_78,novel_4,miR-1343-3p,miR-181b-2-3p,miR-27a-3p,miR-544a,miR-21b,miR-484,miR-125b-2-3p,miR-758-3p,miR-361-3p,novel_111,miR-8095,miR-2447,miR-212-3p,miR-6527,miR-2285f,miR-4726-5p,miR-374a-3p,novel_82,miR-218-

AADACL3	ENSOARGO(	arylacet	novel_9,miR-134-5p,miR-204-5p,miR-2284k,miR-331-3p,miR-100-3p,miR-323-3p,miR-2284v,miR-6128,miR-2318,miR-7144-5p,miR-137-3p,miR-23b,miR-25-3p,miR-2284n,miR-2355-5p,miR-17-3p,miR-181b-5p,miR-92a-3p,novel_127,miR-205-5p,miR-92b-3p,miR-28b,miR-450b-5p,novel_39,miR-2284y,miR-2284a,miR-3064-5p,miR-582-3p,let-7a-2-3p,miR-345-3p,miR-134,miR-2411-5p,miR-483-3p,miR-1271-5p,miR-134-5p,miR-129-1-3p,miR-92a-3p,novel_1,miR-28c,miR-211,miR-2428,miR-335-5p,miR-17-3p,miR-412-3p,miR-9851-3p,miR-2285ab,miR-21c,miR-18a-3p,miR-411b-3p,miR-3074-5p,miR-3120-3p,miR-483-3p,miR-452-5p,miR-32,miR-2411-3p,miR-432-3p,let-7a-2-3p,miR-2483-5p,miR-2284ab,miR-21-5p,miR-181a-5p,miR-2285g,miR-2284d,miR-411-5p,miR-26b-3p,miR-17-3p,miR-6516-3p,miR-3604,miR-338-5p,miR-142a-5p,miR-181c-5p,miR-338-3p,miR-3074-5p,miR-145a-3p,miR-129-2-3p,miR-1271,miR-2330-3p,miR-425-5p,novel_99,miR-2285c,miR-216b-5p,miR-32-5p,miR-548o-3p,miR-2284m,miR-324-3p,miR-345-3p,miR-2411,miR-105-3p,miR-2285w,miR-195a-3p,miR-2284u,miR-1434-5p,miR-5703,miR-23b-3p,miR-7859,miR-222-5p,miR-23c,miR-2284h-5p,miR-18a-3p,miR-548e-3p,miR-212-5p,miR-3958-3p,miR-335,miR-490-5p,miR-2285t,miR-301a-5p,novel_111,miR-363-3p,miR-2285f,let-7i-3p,miR-2284j,miR-181d-5p,miR-96-5p,miR-1983,miR-129b-5p,miR-7857-3p,miR-2483-3p,miR-203-3p,miR-2284g,miR-3064-5p,miR-452-3p,miR-211-5p,miR-7977,miR-3085-3p,novel_103,miR-8485,miR-4532,miR-758-3p,miR-23a-3p,miR-2284b,miR-450b-5p,miR-299a-5p,miR-101c,miR-301b-5p,miR-758-3p,miR-142-5p,miR-455-3p,miR-2459,miR-2285e

TOR2A	ENSOARG0(	torsin f	novel_51,miR-146a,miR-1343-5p,miR-125a-5p,miR-2285l,miR-483-3p,miR-345-5p,miR-22-3p,miR-1285,miR-542-3p,miR-432,miR-27b-3p,miR-138,miR-30b-3p,novel_116,miR-194-3p,miR-185-5p,miR-134-3p,miR-432-5p,miR-28c,miR-4324,miR-502-5p,miR-542-5p,novel_101,miR-17-3p,miR-136-3p,miR-219b-3p,miR-2428,miR-326-3p,miR-24-3p,miR-211,miR-18a-3p,miR-2387,miR-2285ab,miR-6516-3p,miR-146b-5p,miR-3074-5p,miR-320c,miR-144-3p,miR-6535,novel_69,miR-146b-3p,miR-30c-2-3p,miR-205-5p,novel_127,novel_96,miR-200a-3p,miR-17-3p,miR-4286,miR-490-3p,novel_39,miR-28b,miR-330-5p,miR-1343-5p,miR-582-3p,miR-1224-5p,miR-222,miR-214-5p,miR-129-1-3p,miR-483-3p,miR-505-5p,miR-144,miR-125b-5p,miR-331-3p,miR-6740-5p,miR-204-5p,miR-28-5p,miR-138-5p,miR-133a-3p,miR-6128,miR-1197-3p,miR-500b-5p,miR-2903,miR-362-5p,miR-3154,miR-320a,miR-301,miR-488-3p,miR-671-5p,miR-485-5p,miR-7144-5p,miR-329a,miR-345-5p,miR-10b-3p,miR-1271,miR-141-3p,miR-615-5p,miR-125a,miR-542-5p,miR-3957-5p,miR-3957,miR-505,miR-362-3p,miR-29b-1-5p,miR-326,miR-320b,miR-542-5p,miR-214-3p,miR-146b,miR-25-5p,miR-491-5p,miR-2330-5p,miR-758-3p,miR-3187-3p,miR-30c-1-3p,miR-758-3p,miR-30b-3p,miR-301b-5p,miR-2284r,miR-6529a,miR-484,miR-329-3p,miR-296-5p,miR-3120-5p,miR-1839-5p,miR-450b-5p,miR-1388-5p,miR-362-3p,miR-27a-3p,miR-1343-3p,miR-455-3p,miR-500-5p,miR-216a-3p,miR-221-3p,miR-1298-3p,miR-301a-5p,miR-2285n,miR-2285t,miR-4726-5p,miR-2285f,miR-362-5p,miR-361-3p,miR-877-3p,miR-378j,miR-221,miR-361-3p,novel_94,miR-211-5p,miR-7977,miR-2285u,miR-2319b,miR-452-3p,miR-27a-3p,miR-4443,miR-378g,miR-125b,miR-324-3p,miR-210-5p,miR-542-5p,miR-2285p,miR-21-3p,miR-30b-3p,miR-202-5p,miR-320d,miR-345-3p,miR-143-3p,miR-146a-5p,miR-29b-1-5p,miR-188-3p,miR-
DAZAP1	ENSOARG0(	DAZ asso	miR-2285w,miR-219a-5p,miR-341-3p,miR-3431,miR-455-3p,miR-423-5p,miR-3184-5p,miR-2284h-5p,miR-125b-2-3p,miR-185-3p,miR-2284q
TTC16	ENSOARG0(	tetratri	novel_133,miR-760-3p,miR-1185-5p,miR-153-3p,miR-335-3p,miR-1343-3p,miR-20a-3p,miR-153

RFC2	ENSOARGO(	replicat	novel_32,miR-204-3p,miR-155-5p,miR-877-5p,miR-15b-5p,miR-10a,miR-6516,miR-323-5p,miR-6130,miR-552-3p,miR-221-5p,miR-2331-3p,miR-30c-1-3p,miR-548o-3p,novel_99,miR-1827,miR-338-3p,miR-10b-5p,miR-6525,miR-129-2-3p,miR-4510,miR-3184-3p,miR-874-5p,miR-424-5p,miR-4492,miR-130b-5p,miR-155-5p,miR-664a,miR-324-3p,miR-320e,miR-2285j,miR-532-3p,miR-7859,miR-487a-3p,miR-15a-5p,miR-380-5p,miR-485-3p,miR-548e-3p,miR-3960,miR-218-1-3p,miR-2284u,miR-493-3p,miR-941,miR-2904,novel_133,miR-2300b-3p,miR-486-3p,miR-143-3p,miR-221-5p,miR-324-3p,miR-219b-5p,miR-125b,novel_17,miR-2285u,miR-211-5p,miR-3064-5p,miR-2319b,miR-1983,miR-361-3p,miR-361-3p,miR-219a-5p,miR-2447,miR-7134-3p,miR-2284q,miR-499a-5p,miR-2285n,novel_82,miR-2440,miR-2459,miR-1298-3p,miR-145b,novel_4,miR-330-3p,miR-409b,miR-548w,miR-2284aa,miR-3120-5p,miR-296-5p,miR-10b,miR-30c-1-3p,miR-2284b,miR-652-5p,miR-424-5p,miR-3085-3p,miR-16b,novel_103,miR-326,miR-324-5p,miR-125a,miR-874-3p,miR-23a-5p,miR-7144-5p,miR-485-5p,miR-876-3p,miR-301,miR-1260b,miR-16-5p,miR-1306-5p,novel_42,miR-181a-2-3p,miR-486b-3p,miR-204-5p,miR-125b-5p,miR-135a-5p,miR-2284z,miR-129-1-3p,miR-214-5p,miR-322-5p,miR-6516-5p,miR-1224-5p,miR-2285ad,miR-155-5p,miR-10a-5p,miR-23b-5p,miR-330-5p,miR-2284y,miR-192-3p,miR-2285b,miR-3064-5p,miR-543-5p,miR-30f,miR-30c-2-3p,miR-2285af,miR-1260a,miR-323a-5p,novel_69,miR-10a-5p,miR-3120-3p,miR-135b-5p,miR-195a-5p,miR-1291,miR-412-3p,miR-326-3p,miR-211,miR-371b-3p,miR-24-3p,miR-495-3p,miR-758-5p,miR-203b-5p,miR-6123,miR-128-1-5p,miR-16a,novel_87,miR-15a,miR-1247-3p,miR-133a-5p,miR-576-3p,miR-6238,miR-2355-3p,miR-185-5p,miR-26a-2-3p,miR-497-5p,miR-188-5p,miR-16b,miR-1260b,miR-145a-5p,miR-873a-
LRRC43	ENSOARGO(	leucine	novel_111,miR-338-3p,miR-2428,miR-3065-3p,miR-1306-5p,miR-214,miR-214-3p,miR-210-5p,novel_51,miR-149-5p,novel_68,miR-3960,miR-1271,miR-3065-3p,miR-2889,miR-1895,miR-7857

ADGRF4	ENSOARG0(	adhesion	miR-3074-5p,miR-6134,miR-224-5p,miR-142a-5p,miR-10b-5p,miR-665,miR-4492,miR-208b-5p,miR-129-5p,miR-17-3p,miR-552-3p,miR-21-3p,miR-370-5p,miR-2331-3p,miR-140-5p,miR-221-5p,miR-204-3p,miR-139-5p,miR-2284s,miR-10a,miR-582-5p,miR-129b-3p,miR-299,miR-582-5p,novel_99,miR-885-3p,miR-339b,miR-2312,miR-5100,miR-20a,miR-2285w,miR-143-3p,miR-146a-5p,miR-486-3p,miR-377-5p,miR-181b-1-3p,miR-125b-2-3p,miR-664b-3p,miR-125b,miR-324-3p,miR-2484,miR-221-5p,miR-30b-3p,miR-760-3p,miR-106a,miR-3600,miR-106a,miR-2284h-5p,miR-217-5p,miR-188-3p,miR-5703,miR-191,miR-181d-5p,miR-2285f,miR-2447,miR-1285-5p,miR-877-3p,miR-107-5p,miR-1973,miR-2440,novel_82,miR-3964,miR-211-5p,miR-7977,miR-7689-3p,miR-20a-3p,miR-139-5p,miR-3064-5p,miR-4443,miR-376d,miR-1185-2-3p,miR-378g,miR-129b-5p,miR-140-5p,miR-361-3p,miR-877-3p,miR-544-5p,novel_94,miR-376a-3p,miR-744-3p,miR-106a-5p,miR-1388-3p,miR-8485,miR-106b-5p,miR-431-5p,miR-1306-5p,miR-103a-2-5p,miR-146b,miR-487b-5p,miR-3085-3p,miR-145b,miR-181b-2-3p,novel_78,miR-105-1,miR-330-3p,miR-1185-3p,miR-216a-3p,miR-9788-3p,miR-376b-3p,miR-1298-3p,miR-142-5p,miR-30b-3p,miR-412-3p,miR-10b,miR-4791,miR-2284r,miR-296-5p,miR-544a,miR-450b-5p,miR-193a-5p,miR-1306-5p,novel_42,miR-93,miR-125b-5p,miR-370-5p,miR-2332,miR-204-5p,miR-486b-3p,miR-191-5p,miR-125a,miR-1271,miR-370-3p,miR-2355-5p,miR-874-3p,miR-17-5p,miR-3154,miR-93-5p,miR-200a-5p,miR-488-3p,miR-1248,miR-485-5p,miR-7144-5p,miR-20b-5p,novel_48,miR-4286,miR-3064-5p,miR-490-3p,miR-192-3p,miR-148b-5p,miR-217,miR-339a,miR-376b-3p,miR-543-5p,miR-181b-5p,miR-17-3p,miR-450a-2-3p,miR-431,novel_79,miR-412,miR-483-3p,miR-3578,miR-10a-5p,miR-105-2,miR-885-3p,miR-1193,novel_101,miR-17-3p,miR-24-

PGAP3	ENSOARG0(	post-GPI	novel_39,miR-1271-3p,miR-490-3p,miR-28b,miR-3064-5p,miR-4286,novel_127,miR-543-5p,miR-450a-2-3p,miR-2898,miR-127-5p,miR-339a,miR-205-5p,miR-125a-3p,miR-214-5p,miR-505-5p,novel_79,miR-1224-5p,miR-412,miR-299a-3p,miR-1343-5p,miR-330-5p,miR-145-3p,miR-3432a,miR-28-5p,miR-138-5p,novel_9,miR-125b-5p,miR-2284k,miR-324-5p,miR-154b-5p,miR-34c-5p,miR-2355-5p,miR-1271,miR-125a,miR-615-5p,miR-874-3p,miR-7144-5p,miR-485-5p,miR-329a,miR-329b,miR-320a,miR-3154,miR-488-3p,miR-654-3p,miR-432-3p,miR-106b-3p,miR-125a-5p,miR-1343-5p,miR-124-5p,miR-2285v,miR-194a,novel_51,miR-377-3p,miR-149-5p,miR-411-5p,miR-412-5p,miR-185-5p,miR-134-3p,miR-130a-3p,miR-339-5p,miR-377-5p,miR-2889,miR-138,miR-30b-3p,miR-197-3p,miR-194-5p,miR-145a-5p,miR-873a-5p,miR-223-3p,miR-1285,miR-1434-3p,miR-326-3p,miR-449a,novel_120,miR-342-3p,miR-656-5p,miR-199c,miR-6119-3p,miR-206,miR-34c,miR-576-3p,miR-28c,miR-320c,novel_69,miR-670-3p,miR-299b-3p,miR-34b,novel_68,miR-3074-5p,miR-9851-3p,miR-6395,miR-450b-3p,miR-1291,miR-412-3p,miR-1306,miR-150-5p,miR-18a-3p,miR-2387,miR-2432,miR-377-5p,miR-183-5p,miR-30b-3p,miR-34b-5p,miR-299-3p,miR-2284m,miR-324-3p,miR-185-3p,miR-125b,novel_83,miR-199a-3p,miR-151-5p,miR-328-3p,miR-383-5p,miR-18a-3p,miR-378d,miR-34a-5p,miR-4429,miR-188-3p,miR-1197-5p,novel_133,miR-376a-5p,miR-362-3p,miR-2366,miR-6134,miR-6525,miR-206-3p,miR-665-5p,miR-214,miR-3074-5p,miR-4510,miR-708-5p,miR-454-3p,miR-664a,miR-324-3p,miR-3604,miR-204-3p,miR-151b,miR-499b-3p,miR-377-3p,miR-6130,miR-216b-3p,let-7g-3p,miR-339b,miR-27b-5p,miR-299,miR-4532,miR-8485,miR-193a,miR-744-3p,miR-214-3p,miR-3085-3p,miR-130b-3p,miR-2957,miR-362-3p,miR-199a-3p,miR-320b,miR-342,miR-1306-5p,miR-
HSPH1	ENSOARG0(	heat sho	miR-2310,miR-6240,miR-103a-2-5p,miR-106b-3p,miR-200c-3p,miR-330-3p,miR-200c,miR-129-1-3p,miR-544b,miR-200b,miR-383-5p,miR-7859,miR-212-5p,miR-429-3p,miR-200b-3p,miR-142-3p,miR-544a,miR-140-3p,miR-1290,miR-1246,miR-3959-3p,miR-211,miR-129-2-3p,miR-204-5p,miR-2285t,miR-208b-5p,miR-107-5p,miR-7857-3p,miR-300-3p,miR-544-3p,miR-211-5p,miR-598-3p,miR-499b-5p,miR-374b-5p,miR-1814c,miR-6516-3p,miR-3074-1-3p,miR-598-3p
SRI	ENSOARG0(	sorcin [	miR-335,miR-206,miR-129-2-3p,miR-335-5p,miR-1271,miR-206-3p,miR-142a-5p,miR-154a,miR-378b,miR-96-5p,miR-146b-5p,miR-329-5p,miR-6516,miR-146a,miR-2285p,miR-455-5p,miR-330-3p,miR-378a-5p,miR-146b,miR-6240,miR-329a-5p,miR-1,miR-146a-5p,miR-2300b-3p,miR-1a-3p,miR-101a-5p,miR-2426,miR-148a-5p,miR-455-5p,miR-330-3p,miR-485-3p,miR-142-5p,miR-6516-5p,miR-1271-5p,miR-487a-3p,miR-302a-5p,miR-129-1-3p,miR-148b-5p,miR-216a-3p

GDPD5	ENSOARG0(	glycerop	miR-3141,miR-135b-5p,miR-6395,miR-9851-3p,miR-1291,miR-450b-3p,miR-655-5p,miR-329-5p,miR-3074-5p,novel_68,miR-320c,miR-669,miR-6535,novel_69,miR-299b-3p,miR-29a-3p,miR-670-3p,miR-34b,miR-34c,miR-485-3p,miR-576-3p,miR-28c,miR-22-5p,miR-656-5p,miR-29a,miR-29c-3p,miR-193b-3p,miR-342-3p,miR-199b-5p,miR-2428,miR-326-3p,miR-449a,miR-24-3p,novel_23,miR-542-3p,miR-432,miR-455-5p,miR-30b-3p,miR-29b,miR-377-5p,miR-339-5p,miR-194-3p,miR-149-5p,miR-134-3p,miR-185-5p,miR-3431,miR-432-5p,miR-455-5p,miR-365b-5p,miR-3141,miR-296-3p,miR-193a-3p,miR-125a-5p,miR-1343-5p,miR-378a-5p,miR-628-5p,miR-493-5p,miR-296-3p,miR-432-3p,miR-3154,miR-320a,miR-485-5p,miR-1814c,miR-329a,miR-767,miR-125a,miR-1271,miR-615-5p,miR-3956,miR-2285aa,miR-874-3p,miR-154b-5p,miR-199a-5p,miR-34c-5p,miR-135a-5p,miR-125b-5p,miR-6740-5p,miR-29b-3p,miR-28-5p,miR-134-5p,miR-486b-3p,miR-767-5p,miR-103b,miR-193a-5p,novel_42,miR-365a-5p,miR-10b-3p,miR-1197-3p,miR-501-3p,miR-330-5p,miR-29d-3p,miR-1343-5p,miR-134,miR-3591-5p,novel_79,miR-299a-3p,miR-2285ad,miR-129-1-3p,miR-134-5p,miR-505-5p,miR-339a,miR-219-3p,miR-205-5p,miR-4286,miR-181a-2-3p,miR-3064-5p,miR-29b-2-5p,miR-490-3p,miR-3956-5p,miR-199a-5p,miR-28b,miR-199b-5p,miR-361-3p,novel_94,miR-877-3p,miR-493-5p,miR-7977,miR-541-5p,miR-3064-5p,miR-139-5p,miR-500-3p,miR-378g,miR-7857-3p,miR-107-5p,miR-2285y,novel_82,miR-4726-5p,miR-3968,miR-6527,miR-1296-5p,miR-3956-3p,miR-541-3p,miR-2447,novel_60,miR-219a-2-3p,miR-8095,miR-423-5p,miR-502-3p,miR-361-3p,miR-30b-3p,miR-501-3p,miR-541,miR-143-5p,miR-329-3p,miR-296-5p,miR-484,miR-362-3p,miR-1388-5p,miR-5010-3p,novel_78,miR-1343-3p,novel_4,miR-409b,miR-421,miR-216a-3p,miR-455-3p,miR-147a,miR-29b-1-5p,miR-362-
NAALADL1	ENSOARG0(	N-acetyl	novel_32,miR-6535,miR-7859,miR-2285aa,miR-668-3p,miR-216b-3p,miR-543-5p,miR-1197-5p,miR-3141,miR-135a-1-3p,miR-3141

NPR2	ENSOARG0(	natriure	novel_96,miR-200a-3p,novel_127,miR-2898,miR-17-3p,miR-376b-3p,miR-3065-3p,novel_39,miR-490-3p,miR-450c-5p,miR-1271-3p,miR-148b-5p,miR-29b-2-5p,miR-3064-5p,let-7a-2-3p,miR-1343-5p,miR-214-5p,miR-322-5p,miR-758-5p,miR-2285ad,miR-222,miR-6517,miR-486b-3p,miR-28-5p,miR-204-5p,miR-138-5p,miR-409-3p,miR-2332,miR-1197-3p,miR-16-5p,miR-103b,miR-144-5p,miR-133a-3p,miR-1306-5p,miR-3074-1-3p,miR-7144-5p,miR-671-5p,miR-485-5p,miR-1814c,miR-329a,miR-345-5p,let-7g-3p,miR-488-3p,miR-769,miR-365a-3p,miR-370-3p,miR-2355-5p,miR-141-3p,miR-3068-3p,miR-1343-5p,miR-378a-5p,miR-146a,let-7j,miR-345-5p,let-7a-2-3p,miR-2285r,miR-432-3p,miR-668-3p,miR-628-5p,miR-497-5p,miR-26a-2-3p,miR-188-5p,miR-30b-3p,miR-138,miR-16b,miR-16-1-3p,miR-145a-5p,novel_23,miR-873a-5p,miR-542-3p,miR-1285,novel_116,miR-149-5p,miR-3431,miR-185-5p,miR-665,miR-26b-3p,miR-340-3p,miR-31-5p,miR-133b-5p,miR-16a,miR-33a-3p,miR-4324,miR-15a,miR-142b,miR-576-3p,miR-2284w,miR-211,miR-24-3p,miR-17-3p,miR-135a-2-3p,miR-146b-5p,miR-195a-5p,miR-6395,miR-1291,miR-410-5p,miR-376b-3p,miR-3065-5p,miR-18a-3p,miR-3591-3p,miR-2387,miR-6535,miR-669,miR-146b-3p,miR-411b-3p,miR-320b,novel_68,miR-30b-3p,miR-221-5p,miR-760-3p,miR-466i-5p,miR-92a-1-5p,miR-210-5p,novel_17,miR-2424,miR-146a-5p,miR-486-3p,miR-143-3p,miR-29b-1-5p,miR-16-1-3p,miR-33b-3p,miR-184-3p,miR-124-3p,miR-2904,miR-140-3p,miR-362-3p,miR-193b-5p,miR-328-3p,miR-15a-5p,miR-3600,miR-18a-3p,miR-424-5p,miR-874-5p,miR-708-5p,novel_74,miR-664a,miR-17-3p,miR-324-3p,miR-29b-2-5p,miR-664a-5p,miR-222-3p,miR-3184-5p,miR-6134,miR-769-5p,miR-3958-5p,miR-3184-3p,miR-145a-3p,miR-186-5p,novel_99,miR-133b-3p,miR-345-5p,novel_25,novel_32,miR-15b-5p,miR-21-3p,miR-3065-

SLC17A9	ENSOARG0(	solute c	miR-452-5p,miR-1296-5p,miR-6527,miR-423-5p,miR-361-3p,miR-2285y,miR-2440,novel_82,miR-30b-5p,miR-382-3p,miR-4726-5p,miR-211-5p,miR-7977,miR-499b-5p,miR-2285u,miR-20a-3p,miR-3064-5p,miR-376d,let-7d-5p,miR-378g,miR-361-3p,miR-1983,miR-877-3p,miR-376a-3p,miR-504,miR-424-5p,miR-2330-5p,miR-106a-5p,miR-124a,let-7a-5p,miR-8485,miR-30c-1-3p,miR-16b,miR-106b-5p,miR-362-3p,miR-326,miR-214-3p,let-7e,miR-153-3p,miR-25-5p,miR-1185-5p,miR-3085-3p,miR-145b,novel_78,miR-105-1,miR-1343-3p,miR-2285e,miR-421,miR-9788-3p,miR-216a-3p,miR-147a,miR-376b-3p,miR-30b-3p,miR-125b-2-3p,miR-412-3p,miR-296-5p,miR-2284r,miR-329-3p,miR-3120-5p,miR-1839-5p,miR-362-3p,miR-214,miR-665-5p,miR-129-2-3p,let-7b,miR-6134,let-7b-5p,miR-3184-5p,miR-665,let-7f,miR-4492,miR-424-5p,miR-450b-3p,miR-3065-3p,miR-504-5p,miR-30c-1-3p,miR-2331-3p,miR-877-5p,miR-15b-5p,miR-151b,let-7i-5p,miR-216b-5p,miR-132-5p,miR-582-5p,miR-2284s,miR-30d-5p,miR-411,miR-129b-3p,miR-2285c,miR-21a-3p,miR-1827,miR-625-5p,miR-30f,miR-339b,miR-20a,miR-329a-5p,miR-486-3p,miR-2411,miR-185-3p,miR-125b,miR-324-3p,miR-30b,miR-21-3p,let-7k,miR-30d,miR-760-3p,miR-466i-5p,miR-98-5p,miR-3600,miR-151-5p,miR-15a-5p,miR-1197-5p,miR-188-3p,miR-1434-5p,miR-5703,miR-124-3p,miR-2408,miR-362-3p,miR-1961,miR-2300b-3p,miR-193b-5p,miR-30c-5p,novel_133,miR-16-1-3p,miR-3957-3p,miR-326-3p,novel_27,miR-371b-3p,miR-211,let-7i,miR-16a,miR-20a-5p,miR-15a,miR-1247-3p,miR-502b,miR-6123,miR-378b,miR-329-5p,miR-669,novel_69,miR-2404,miR-376b-3p,miR-216a-3p,miR-150-5p,miR-2387,miR-3141,let-7e-5p,miR-195a-5p,miR-9851-3p,miR-412-3p,miR-1291,miR-432-3p,miR-432-3p,miR-17-5p,novel_51,miR-106a-5p,miR-105-5p,miR-

MESP2	ENSOARGO(	mesoderm	novel_4,miR-2300a-5p,miR-216a-3p,miR-9788-3p,miR-500-5p,miR-484,miR-296-5p,miR-378b,miR-30b-3p,miR-125b-2-3p,miR-378c,miR-450b-5p,miR-2426,let-7a-5p,miR-424-5p,miR-1839-3p,miR-2330-5p,miR-33a-5p,miR-30c-1-3p,miR-758-3p,miR-342,novel_115,miR-326,miR-5126,miR-1306-5p,miR-3085-3p,let-7e,miR-3064-5p,miR-10b,miR-452-3p,miR-20a-3p,miR-7977,miR-378e,miR-3082-5p,let-7d-5p,miR-454-5p,miR-361-3p,miR-877-3p,novel_60,miR-3059-5p,miR-181d-5p,miR-362-5p,miR-502-3p,miR-2285n,miR-769-5p,miR-1973,miR-4726-5p,miR-335,miR-2285t,miR-222-5p,miR-200b,miR-15a-5p,miR-151-5p,miR-616-3p,novel_133,miR-215-5p,miR-2300b-3p,miR-124-3p,miR-1193,miR-2411,miR-196a-3p,miR-125b,miR-185-3p,miR-760-3p,miR-34b-5p,miR-30b-3p,miR-221-5p,miR-21-3p,miR-299-3p,miR-140-5p,miR-221-5p,miR-378a-3p,miR-182-5p,miR-192-5p,miR-323-5p,miR-204-3p,miR-21a-3p,miR-378c,miR-145a-3p,miR-3074-2-3p,miR-2330-3p,let-7b-5p,miR-6134,miR-29b-2-5p,miR-655-3p,miR-450b-3p,miR-424-5p,miR-133b-5p,let-7g-5p,miR-103,miR-181a-5p,miR-30b-3p,miR-138,miR-1260b,miR-455-5p,miR-130b-5p,miR-654-3p,miR-296-3p,miR-197-5p,miR-455-5p,miR-124-5p,miR-452-5p,miR-125a-5p,miR-146b-3p,miR-323a-5p,miR-2404,miR-34b,miR-2387,miR-18a-3p,miR-1291,miR-450b-3p,miR-107,miR-195a-5p,miR-135b-5p,miR-379-5p,miR-342-3p,miR-24-3p,miR-326-3p,miR-449a,miR-2428,miR-502-5p,miR-15a,miR-34c,miR-16a,let-7i,miR-656-5p,miR-147-5p,miR-502b,miR-7641,miR-378h,miR-2411-5p,miR-214-5p,miR-134,miR-501-3p,let-7d,miR-582-3p,miR-200b-3p,miR-128-3p,miR-29b-2-5p,miR-30a-3p,miR-181a-2-3p,miR-4286,miR-28b,miR-1271-3p,miR-490-3p,miR-1260a,miR-200c,miR-30c-2-3p,let-7c-5p,miR-450a-2-3p,miR-2898,miR-200c-3p,miR-191-3p,miR-181b-5p,miR-107,miR-543-5p,miR-598-3p,miR-141-

GPS2	ENSOARG0(	G protei	miR-22-3p,miR-197-3p,miR-2113,miR-432,miR-2284x,miR-339-5p,miR-31-5p,miR-194-3p,miR-149-5p,miR-412-5p,miR-194a,novel_51,miR-32,miR-105-5p,miR-193a-3p,miR-378a-5p,miR-493-5p,let-7a-2-3p,miR-503-3p,miR-1306,miR-150-5p,miR-148a-3p,miR-378f,miR-3074-5p,miR-181b-3p,miR-677,miR-320c,miR-669,miR-4324,miR-29a,miR-29c-3p,miR-193b-3p,miR-376a-3p,miR-23b-5p,miR-1343-5p,miR-3591-5p,novel_79,miR-412,miR-129-1-3p,miR-486-5p,miR-125a-3p,miR-205-5p,novel_127,novel_96,miR-152-3p,miR-127-5p,miR-3064-5p,miR-3956-5p,miR-2285b,novel_39,miR-2284y,miR-148b-5p,miR-92b-3p,miR-329a,miR-767,miR-876-3p,miR-1271,miR-542-5p,miR-574-5p,miR-133c,miR-25-3p,miR-300-3p,miR-494-5p,miR-2284k,novel_42,miR-501-3p,miR-329-3p,miR-2284r,miR-1839-5p,miR-101c,miR-362-3p,miR-181b-2-3p,miR-1343-3p,miR-199b-3p,novel_78,miR-455-3p,miR-148b-5p,miR-147a,miR-106b-5p,miR-182-5p,miR-29b-1-5p,miR-103a-2-5p,miR-491-5p,miR-2284b,miR-127-3p,miR-1388-3p,miR-8485,miR-378d,miR-409-5p,miR-544-5p,miR-493-5p,miR-504,miR-299b-5p,miR-211-5p,miR-7689-3p,miR-2284g,miR-500-3p,miR-25,miR-2440,miR-218-5p,novel_82,miR-490-5p,miR-3968,miR-499a-5p,miR-2284j,miR-363-3p,miR-2447,miR-361-3p,miR-877-3p,miR-378a-3p,miR-378i,miR-148a-5p,miR-2284u,miR-34a-5p,miR-361-5p,miR-33b-3p,miR-30d-3p,miR-212-5p,miR-106a,miR-18a-3p,miR-328-3p,miR-92a-1-5p,miR-422a,miR-324-3p,miR-219b-5p,miR-210-5p,novel_17,miR-486-3p,miR-2424,miR-181b-1-3p,miR-664b-3p,miR-487b-5p,miR-500a-3p,miR-2285c,miR-339b,miR-296-3p,miR-548o-3p,miR-32-5p,miR-504-5p,miR-30c-1-3p,miR-10a,miR-665,miR-30a-3p,miR-30e-3p,miR-500,miR-129-5p,novel_74,miR-129-2-3p,miR-3074-5p,miR-346,miR-6525,miR-769-5p,miR-194-5p,miR-1434-3p,miR-1285,miR-7862,miR-219a-1-3p,miR-30b-
RGP1	ENSOARG0(	RGP1 hom	miR-574-3p,miR-1388-3p,miR-2330-5p,miR-628-5p,miR-378a-5p,miR-3085-3p,miR-3955-5p,miR-31-5p,novel_4,miR-339-5p,miR-26b-3p,miR-2285g,miR-185-5p,miR-149-5p,miR-9788-3p,miR-2285e,miR-296-5p,miR-873a-5p,miR-301b-5p,miR-22-3p,miR-30b-3p,miR-138,miR-30b-3p,miR-3120-5p,miR-2285x,let-7i-3p,miR-24-3p,novel_27,miR-2285m,miR-33a-3p,miR-301a-5p,miR-128-1-5p,miR-421-5p,miR-22-5p,miR-3064-5p,novel_68,miR-3074-5p,miR-7689-3p,miR-7977,miR-3596,miR-3082-5p,miR-6535,miR-203-3p,miR-361-3p,miR-18a-3p,miR-19a-3p,miR-3064-5p,miR-4286,novel_39,miR-486-3p,miR-3065-3p,miR-2285b,miR-1271-3p,miR-200c,miR-125a-3p,miR-210-5p,miR-339a,miR-185-3p,miR-28-3p,miR-200c-3p,novel_127,miR-18a-3p,miR-429-3p,miR-3600,novel_79,miR-200b,novel_83,miR-2300b-3p,miR-885-3p,miR-19b-3p,miR-133a-3p,miR-200b-3p,miR-539-5p,miR-28a-3p,miR-3074-5p,miR-3074-2-3p,miR-6134,miR-338-3p,miR-410-5p,miR-551b-3p,miR-132-5p,miR-665,miR-138-5p,miR-664a,miR-487b-5p,miR-500,miR-486b-3p,miR-2331-3p,miR-874-3p,miR-3065-3p,miR-2355-5p,miR-1271,miR-22-3p,miR-2285c,miR-500a-3p,miR-339b,miR-885-3p,miR-1827,miR-485-5p

STEAP4	ENSOARG0(	STEAP fa	miR-124a,miR-101b-3p,miR-199b-5p,miR-103a-2-5p,miR-342,miR-320b,novel_115,miR-1306-5p,miR-326,miR-29b-1-5p,miR-455-3p,miR-9788-3p,miR-221-3p,miR-2285e,miR-1185-3p,miR-330-3p,miR-2284aa,miR-21b,miR-1839-5p,miR-2284r,miR-1a-3p,miR-877-3p,novel_111,miR-2447,miR-2285f,miR-181d-5p,miR-3968,miR-499a-5p,miR-490-5p,miR-2284q,miR-2285n,miR-769-5p,miR-107-5p,miR-2483-3p,miR-1185-2-3p,miR-664b,miR-499b-5p,miR-668-5p,miR-1983,miR-140-5p,miR-221,miR-199b-5p,miR-378j,miR-129b-5p,miR-29b-1-5p,miR-2424,miR-380-3p,miR-183-5p,miR-195a-3p,miR-320d,miR-3653-3p,novel_17,miR-210-5p,miR-2285p,miR-216a-5p,miR-345-3p,miR-122-3p,miR-487a-3p,miR-7859,miR-200b,miR-151-5p,miR-328-3p,miR-548e-3p,miR-212-5p,miR-485-3p,miR-493-3p,miR-19b-3p,miR-142-3p,miR-378d,miR-2366,miR-124-3p,miR-5703,miR-769-5p,miR-206-3p,miR-181c-5p,miR-3184-3p,miR-129-2-3p,miR-425-5p,miR-155-5p,miR-6516-3p,miR-500,miR-129-5p,miR-708-5p,miR-130b-5p,miR-2285j,miR-29b-2-5p,miR-2284s,miR-204-3p,miR-22-3p,miR-151b,miR-155-5p,miR-660-5p,miR-1277-3p,miR-216b-3p,miR-296-3p,miR-2312,novel_99,miR-1827,miR-2285c,miR-500a-3p,miR-27b-5p,miR-432-3p,miR-432-3p,miR-539-3p,miR-130b-5p,miR-6240,miR-378a-5p,miR-660,miR-466f-3p,miR-330-3p,miR-105-5p,miR-2331-5p,miR-2411-3p,miR-2285g,novel_116,miR-149-5p,miR-411-5p,miR-187-3p,miR-3955-5p,miR-122-3p,miR-380-3p,miR-1260b,miR-181a-5p,miR-2113,miR-1434-3p,miR-22-3p,novel_23,miR-326-3p,novel_27,miR-199b-5p,miR-2428,miR-342-3p,novel_120,miR-495-3p,miR-2285x,miR-206,miR-502b,miR-4324,miR-485-3p,novel_87,miR-320c,miR-144-3p,miR-669,miR-3120-3p,miR-412-3p,miR-19a-3p,miR-9851-3p,miR-135b-5p,miR-6395,miR-2285ab,miR-376b-5p,miR-2387,miR-1306,miR-
ELL	ENSOARG0(	elongati	miR-331-3p,miR-301a-5p,miR-665,miR-29b-2-5p,miR-138-5p,miR-664a,miR-874-5p,miR-145a-3p,miR-371b-3p,miR-502-3p,miR-361-3p,miR-625-5p,miR-485-5p,miR-3064-5p,miR-2331-3p,miR-370-3p,miR-296-3p,miR-300-3p,miR-6535,miR-4443,miR-500-3p,miR-29b-1-5p,miR-92a-1-5p,miR-185-3p,miR-2898,miR-3085-3p,miR-491-5p,novel_96,miR-29b-2-5p,miR-3064-5p,miR-29b-1-5p,miR-432-3p,miR-296-5p,miR-542-3p,miR-301b-5p,miR-501-3p,miR-2904,miR-501-3p,miR-138
LRRC40	ENSOARG0(	leucine	miR-380-3p,miR-3653-3p,miR-380-3p
PTPRG	ENSOARG0(	protein	miR-19a-3p,miR-136-5p,miR-1291,miR-9-3p,miR-6395,miR-411b-3p,miR-2284g,miR-541-5p,miR-206,miR-374a-3p,miR-301b-3p,miR-8095,miR-9-3p,miR-2426,miR-2284aa,miR-412-3p,miR-1a-3p,miR-3431,miR-539-5p,miR-148b-5p,novel_116,miR-548w,miR-105-1,miR-130a-3p,miR-130b-3p,miR-105-5p,miR-8485,let-7j,miR-130b-5p,miR-2284b,miR-1248,miR-331-5p,miR-655,miR-487b-5p,miR-301a-3p,miR-548o-3p,miR-141-3p,miR-1271,miR-331-5p,miR-130b-5p,miR-669a-3p,miR-486b-3p,miR-410-3p,miR-454-3p,miR-494-5p,miR-655-3p,miR-206-3p,miR-374a-3p,miR-19b-3p,miR-33b-3p,miR-2284u,miR-361-5p,miR-148a-5p,miR-105-2,miR-222-5p,miR-383-5p,miR-328-3p,miR-2284z,miR-412,miR-200a-3p,novel_17,miR-486-3p,miR-2284y,miR-29a-5p,miR-200a,miR-1

SERPINH1	ENSOARG0(	serpin p	miR-24-3p,miR-30c-1-3p,miR-361-3p,miR-2428,miR-6134,miR-1285-5p,miR-3065-3p,miR-2330-5p,miR-1388-3p,miR-665-5p,miR-664a,miR-200a-3p,miR-185-3p,miR-30c-2-3p,miR-323-5p,miR-134-3p,miR-378g,miR-323a-5p,novel_4,miR-30c-1-3p,miR-141-3p,miR-3065-3p,miR-30b-3p,miR-744-5p,miR-1827,miR-4508,miR-296-5p,miR-5703,miR-30b-3p
UPK2	ENSOARG0(	uroplaki	miR-502b,miR-625-3p,miR-22-5p,miR-6529b,miR-1247-3p,miR-326-3p,miR-24-3p,miR-371b-3p,novel_101,miR-17-3p,miR-335-5p,miR-135a-2-3p,miR-6395,miR-505-3p,miR-9851-3p,miR-410-5p,miR-669,miR-670-3p,novel_69,miR-374c-3p,miR-299b-3p,miR-146b-3p,miR-323a-5p,miR-3120-3p,miR-320b,miR-216b-3p,miR-378b,miR-3074-5p,miR-378a-5p,novel_51,miR-2411-3p,miR-2331-5p,miR-197-5p,miR-377-3p,miR-224-5p,miR-7-5p,miR-345-5p,miR-432-3p,miR-654-3p,let-7f-2-3p,let-7f-2-3p,miR-363-5p,miR-493-5p,miR-130b-5p,miR-2889,miR-27b-3p,miR-4508,miR-16-1-3p,miR-1260b,miR-1843b-5p,miR-7b-5p,miR-544b,miR-3431,miR-185-5p,miR-26b-3p,miR-339-5p,miR-31-5p,miR-592,miR-486b-3p,miR-767-5p,miR-331-3p,miR-135a-1-3p,miR-1260b,novel_42,miR-671-5p,miR-23a-5p,miR-7144-5p,miR-345-5p,miR-767,miR-10b-3p,miR-3154,miR-381-3p,miR-488-3p,miR-374c-3p,miR-300-3p,miR-1271,miR-2355-5p,miR-543-5p,miR-191-3p,miR-450a-2-3p,miR-17-3p,miR-2898,miR-339a,miR-30c-2-3p,miR-1260a,miR-1271-3p,miR-490-3p,miR-3065-3p,miR-181a-2-3p,miR-300,miR-330-5p,miR-1290,miR-345-3p,miR-3591-5p,miR-2284z,novel_79,miR-429-3p,miR-6517,miR-299a-3p,miR-2285ad,miR-490-5p,miR-2285t,miR-335,miR-4726-5p,miR-3968,miR-101-3p,miR-2285n,miR-361-3p,miR-877-3p,miR-541-3p,miR-2447,novel_94,miR-493-5p,miR-378j,miR-1185-2-3p,miR-27a-3p,miR-4443,miR-203-3p,miR-7857-3p,miR-20a-3p,miR-3955-3p,miR-326,miR-199a-3p,miR-4532,miR-30c-1-3p,miR-33a-5p,let-7c-3p,miR-2330-5p,miR-1388-3p,miR-3120-5p,miR-2284aa,miR-21b,miR-544a,miR-541,miR-484,miR-421,miR-1185-3p,miR-455-3p,miR-147a,miR-27a-3p,miR-1343-3p,miR-2300a-5p,miR-130b-5p,miR-4492,miR-17-3p,miR-338-5p,miR-665,miR-3604,miR-320e,miR-122-5p,miR-224-5p,miR-6525,miR-425-5p,miR-3958-5p,miR-145a-3p,miR-4510,miR-
OSBP	ENSOARG0(	oxystero	miR-3064-5p,miR-503-5p,miR-503-5p,miR-5126,miR-326,miR-3085-3p,miR-25-5p,miR-6517,miR-1343-3p,miR-194-3p,miR-134-5p,miR-503-5p,miR-197-3p,miR-330-5p,miR-134,miR-7862,miR-885-3p,miR-142-3p,novel_101,miR-326-3p,miR-338-3p,miR-1307-3p,miR-1973,miR-665,miR-2433,miR-6529b,miR-134-5p,miR-4726-5p,miR-615-5p,miR-3064-5p,miR-361-3p,miR-2387,miR-885-3p
RAPGEF5	ENSOARG0(	Rap guan	miR-425-5p,miR-324-3p,miR-34c,miR-669a-3p,miR-2330-3p,miR-877-3p,miR-449a,novel_27,miR-9-3p,miR-200a-5p,miR-339b,miR-147-3p,miR-1827,miR-9-3p,miR-7975,miR-3064-5p,miR-499b-5p,miR-34b,miR-489,miR-34c-5p,miR-499b-3p,miR-499a-3p,miR-22-3p,miR-339a,miR-219b-5p,miR-124-5p,miR-3085-3p,miR-34b-5p,miR-3064-5p,miR-1246,miR-873a-5p,miR-125b-2-3p,miR-3071-5p,miR-22-3p,miR-34a-5p,miR-3120-5p,miR-133b-5p,miR-340-3p,novel_79,miR-339-5p,miR-455-3p

METTL11B	ENSOARG0(	methyltr	miR-199a-5p,miR-130a-5p,miR-181b-1-3p,miR-2478,miR-29b-1-5p,miR-29b-2-5p,miR-2484,miR-152-3p,miR-181b-5p,miR-500a-5p,miR-30f,miR-219-3p,miR-374a-5p,miR-1260a,miR-376b-3p,miR-148b-3p,miR-23c,miR-23b-3p,miR-18b-5p,miR-3600,miR-1b-3p,miR-142-3p,miR-2300b-3p,miR-1260b,miR-224-5p,miR-3074-5p,miR-130b-5p,miR-374b-3p,miR-592,miR-6516-3p,miR-29b-2-5p,miR-409-3p,miR-219b-3p,novel_63,miR-877-5p,miR-199a-5p,miR-323-5p,miR-582-5p,miR-3068-3p,miR-32-3p,novel_99,miR-1827,miR-582-5p,miR-543-3p,miR-18a-5p,miR-433-3p,miR-18a,miR-23b,miR-488-3p,miR-491-3p,miR-1b-5p,let-7f-2-3p,let-7f-2-3p,miR-23a-3p,miR-494-3p,miR-6240,miR-382-3p,miR-130b-5p,miR-199b-5p,miR-378a-5p,miR-29b-1-5p,miR-330-3p,miR-2285v,miR-224-5p,miR-2459,miR-1185-3p,miR-455-3p,miR-376b-3p,miR-27a-3p,miR-145b,miR-181b-2-3p,miR-330-3p,miR-2284x,miR-27b-3p,miR-299a-5p,miR-1260b,miR-101a-5p,miR-145a-5p,miR-143-5p,miR-873a-5p,miR-219a-2-3p,miR-154a,miR-199b-5p,miR-877-3p,miR-181d-5p,miR-452-5p,miR-3956-3p,miR-3059-5p,novel_60,miR-32-3p,miR-147-5p,miR-490-5p,novel_1,miR-371a-5p,miR-1185-2-3p,miR-376d,miR-27a-3p,miR-181b-3p,miR-203-3p,miR-677,miR-6535,miR-489,miR-411b-3p,miR-323a-5p,miR-499b-5p,miR-655-5p,miR-3074-5p,novel_94,miR-376a-3p,miR-2319a,miR-299b-5p,miR-376b-3p,miR-148a-3p,miR-199b-5p,miR-18b
PFKL	ENSOARG0(	phosphof	miR-669,miR-27a-3p,miR-323a-5p,miR-2404,miR-34b,miR-2285u,miR-4792,novel_68,miR-195a-5p,miR-216a-3p,miR-361-3p,miR-148a-3p,miR-449a,miR-423-5p,miR-361-3p,miR-371b-3p,miR-1296-5p,miR-1193,novel_60,miR-335-5p,miR-3956-3p,miR-34c,miR-16a,miR-15a,miR-4324,miR-1247-3p,miR-2440,miR-9788-3p,miR-2355-3p,miR-185-5p,miR-27a-3p,miR-145b,miR-31-5p,miR-2300a-5p,miR-497-5p,miR-744-5p,miR-27b-3p,miR-16b,miR-1260b,miR-2426,miR-22-3p,miR-197-3p,miR-125b-2-3p,miR-145a-5p,miR-873a-5p,miR-2284r,miR-542-3p,miR-4532,let-7a-2-3p,miR-424-5p,miR-125a-5p,miR-214-3p,miR-378a-5p,miR-365b-5p,miR-16b,miR-22-3p,miR-15b-5p,miR-151b,miR-34c-5p,miR-323-5p,miR-370-5p,miR-2355-5p,miR-125a,miR-1271,miR-2331-3p,miR-1827,let-7g-3p,miR-21a-3p,miR-365a-5p,miR-3184-5p,miR-6525,miR-1260b,miR-16-5p,miR-214,miR-1306-5p,miR-424-5p,miR-874-5p,miR-486b-3p,miR-132-5p,miR-125b-5p,miR-370-5p,miR-151-5p,miR-532-3p,miR-505-5p,miR-322-5p,miR-15a-5p,miR-148b-3p,miR-3600,miR-212-5p,miR-6517,miR-128-3p,let-7a-2-3p,miR-34a-5p,miR-2904,miR-188-3p,miR-2366,miR-486-3p,miR-125b-2-3p,miR-34b-5p,miR-152-3p,miR-21-3p,miR-760-3p,miR-125b,miR-185-3p,miR-2285af,miR-1260a,miR-125a-3p

PNISR	ENSOARG0(	PNN-inte	miR-134,miR-3591-5p,miR-10a-5p,let-7a-2-3p,miR-105-2,miR-128-3p,miR-582-3p,miR-411-3p,miR-18b-5p,miR-6517,miR-2285ad,miR-129-1-3p,novel_121,miR-582-3p,miR-134-5p,miR-450a-1-3p,miR-2411-5p,miR-322-5p,miR-205-5p,miR-200a-3p,miR-543-5p,miR-92a-3p,miR-107,miR-1a-2-5p,miR-29b-2-5p,miR-30a-3p,miR-3529-3p,miR-490-3p,miR-1271-3p,miR-376b-5p,miR-1957a,miR-376c-5p,miR-148b-5p,miR-92b-3p,miR-376c-5p,miR-18a,miR-200a-5p,miR-137-3p,miR-671-5p,miR-3074-1-3p,miR-376c-5p,miR-329b,novel_91,let-7g-3p,miR-10a-3p,miR-141-3p,miR-2284n,miR-34c-5p,miR-135a-5p,miR-144,novel_63,miR-1-5p,miR-2284k,miR-134-5p,miR-28-5p,miR-103b,miR-6128,miR-16-5p,miR-3607-3p,miR-2284v,miR-1306-5p,miR-323-3p,miR-3071-5p,miR-22-3p,miR-450c-3p,miR-181a-5p,miR-1285,miR-1434-3p,miR-432,miR-2284x,miR-27b-3p,miR-188-5p,miR-497-5p,miR-16-1-3p,miR-380-3p,miR-2284ab,miR-103a-3p,miR-31-5p,miR-2355-3p,miR-103,miR-432-5p,miR-2284d,miR-412-5p,miR-32,miR-105-5p,miR-330-3p,miR-377-3p,miR-224-5p,miR-124-5p,miR-6240,let-7a-2-3p,miR-2284l,miR-216a-3p,miR-3065-5p,miR-1306,miR-150-5p,miR-18b,miR-2285ab,miR-376b-5p,miR-195a-5p,miR-6395,miR-135b-5p,miR-107,miR-410-5p,miR-10a-5p,novel_68,miR-181b-3p,miR-669,miR-144-3p,miR-677,novel_69,miR-34b,miR-2404,miR-382-5p,miR-34c,miR-16a,miR-576-3p,miR-133a-5p,miR-147-5p,miR-625-3p,novel_1,miR-379-3p,miR-92a-3p,miR-6119-3p,novel_101,miR-495-3p,miR-2285x,miR-1a-1-5p,miR-335-5p,miR-219b-3p,novel_120,miR-2284w,miR-449a,miR-24-3p,miR-5703,miR-124-3p,miR-98-3p,miR-2366,miR-30c-5p,let-7a-3p,miR-361-5p,miR-34a-5p,miR-16-1-3p,miR-664-5p,miR-33b-3p,miR-30d-3p,miR-1b-3p,miR-548e-3p,miR-328-3p,miR-15a-5p,miR-7859,miR-92a-1-5p,miR-618,miR-2484,miR-34b-5p,miR-760-3p,miR-2284m,miR-10a-
DPP10	ENSOARG0(	dipeptid	miR-544a,miR-22-3p,miR-18a-3p,miR-411-5p,miR-22-3p,miR-544b,miR-544-3p,miR-25,miR-2404,miR-212-5p,miR-182-5p,miR-574-5p,miR-18a-3p,miR-199b-3p,miR-378b,miR-452-3p,novel_127,miR-199c,miR-182-5p,miR-196a-3p,miR-2330-3p

MAEL	ENSOARG0(	maelstro	miR-24-1-5p, miR-2285u, miR-25, miR-3082-5p, miR-203-3p, miR-4443, miR-140-5p, miR-221, miR-381-3p, miR-2447, miR-363-3p, miR-2285f, miR-2284j, miR-101-3p, miR-2285y, miR-7134-3p, novel_78, miR-1343-3p, miR-330-3p, miR-2300a-5p, miR-147a, miR-221-3p, miR-455-3p, miR-421, miR-2285e, miR-484, miR-6529a, miR-323b, miR-125b-2-3p, miR-758-3p, miR-1388-5p, miR-2284aa, miR-106a-5p, miR-23a-3p, miR-30c-1-3p, miR-758-3p, miR-320b, miR-199a-3p, miR-326, novel_103, miR-323b-3p, miR-29b-1-5p, miR-106b-5p, novel_124, miR-1185-5p, miR-487b-5p, miR-30c-1-3p, miR-32-5p, miR-2331-3p, miR-548o-3p, miR-216b-5p, miR-193b-3p, novel_25, novel_32, miR-204-3p, miR-877-5p, let-7f-1-3p, miR-2285c, miR-433-3p, miR-2312, miR-345-5p, miR-18a-5p, miR-543-3p, novel_99, miR-186-5p, miR-1827, miR-539-5p, miR-425-5p, miR-3074-2-3p, miR-98-3p, miR-224-5p, miR-181c-5p, let-7b-3p, miR-222-3p, miR-324-3p, miR-3604, miR-29b-2-5p, miR-320e, miR-664a, miR-6516-3p, miR-17-3p, miR-487b-5p, miR-155-5p, miR-208b-5p, miR-106a, miR-485-3p, miR-23b-3p, miR-487a-3p, miR-340-5p, miR-200b, miR-222-5p, novel_83, miR-199a-3p, miR-191, miR-2366, miR-1246, miR-98-3p, miR-1434-5p, miR-5703, miR-33b-3p, novel_73, miR-4429, miR-133a-3p, let-7a-3p, miR-361-5p, miR-503-5p, miR-20a, miR-345-3p, miR-376a-5p, miR-29b-1-5p, miR-130a-5p, miR-377-5p, miR-125b-2-3p, miR-26a-5p, miR-210-5p, miR-219b-5p, miR-106a, miR-2284m, miR-320d, miR-466i-5p, miR-2484, miR-2404, miR-374c-3p, miR-320c, miR-2285ab, miR-18b, miR-3970, miR-378b, miR-3065-5p, miR-6516-3p, miR-193b-3p, miR-2285x, miR-17-3p, miR-495-3p, miR-3959-3p, miR-326-3p, novel_27, miR-2428, miR-2284w, miR-4324, miR-142b, miR-133a-5p, miR-20a-5p, miR-576-3p, miR-485-3p, novel_87, miR-92a-3p, miR-656-5p, miR-625-3p, miR-203b-5p, miR-31-5p, miR-187-3p, miR-133b-5p, miR-665, miR-377-5p, miR-2284d, miR-

PLEKHG5	ENSOARG0(	pleckstr	miR-18b-5p,miR-106a,miR-18a-3p,miR-299a-3p,miR-2284h-5p,miR-134-5p,miR-222-5p,miR-1193,miR-140-3p,miR-1343-5p,miR-134,miR-2300b-3p,miR-19b-3p,miR-20a,miR-2285w,miR-1271-3p,miR-1957a,novel_39,miR-185-3p,miR-92a-1-5p,miR-324-3p,miR-542-5p,miR-299-3p,novel_96,novel_127,miR-106a,miR-2898,miR-296-3p,miR-874-3p,miR-504-5p,miR-574-5p,novel_25,miR-204-3p,miR-301a-3p,miR-17-5p,miR-18a,miR-93-5p,miR-301,miR-1827,miR-20b-5p,miR-18a-5p,novel_42,miR-3184-5p,miR-93,miR-665,miR-135a-1-3p,miR-134-5p,miR-130b-5p,miR-454-3p,miR-874-5p,miR-664a,novel_9,miR-138-5p,miR-222-5p,miR-130a-3p,novel_4,miR-2300a-5p,miR-133b-5p,miR-2459,miR-20b,miR-9788-3p,miR-216a-3p,miR-455-3p,miR-134-3p,miR-147a,miR-30b-3p,miR-1285,miR-541,miR-542-3p,miR-1843b-5p,miR-296-5p,miR-2284r,miR-3120-5p,miR-744-5p,miR-4508,miR-138,miR-30b-3p,miR-106a-5p,miR-2330-5p,miR-130b-5p,miR-4532,miR-106b-5p,novel_51,miR-17-5p,miR-106a-5p,miR-3141,miR-542-5p,miR-1343-5p,miR-130b-3p,miR-491-5p,miR-452-3p,miR-6535,novel_69,miR-299b-3p,miR-378g,miR-381-3p,miR-18a-3p,miR-2387,miR-3141,miR-18b,miR-361-3p,miR-379-5p,novel_94,miR-1983,miR-19a-3p,miR-412-3p,miR-504,miR-542-5p,miR-1296-5p,miR-541-3p,miR-3956-3p,miR-2447,novel_60,miR-301b-3p,miR-423-5p,miR-877-3p,miR-361-3p,miR-20a-5p,miR-4324,miR-128-1-5p,miR-4726-5p
MOSPD2	ENSOARG0(	motile s	miR-190a-3p,miR-493-5p,miR-876-3p,miR-378b,miR-144-3p,miR-216b-5p,miR-377-3p,miR-7857-3p,miR-532-5p,miR-2319b,miR-140-5p,miR-31-3p,novel_74,miR-33a-3p,miR-144,miR-100-3p,miR-576-3p,miR-24-3p,miR-224-5p,miR-452-5p,miR-532-3p,miR-2284h-5p,miR-330-3p,miR-2484,miR-330-3p,miR-3071-3p,miR-324-3p,miR-377-3p,miR-224-5p,novel_39,miR-6240,miR-493-5p,miR-2285w
NECAP2	ENSOARG0(	NECAP en	miR-10a-3p,miR-124a,miR-6240,miR-2330-5p,miR-495-5p,miR-29b-2-5p,miR-5100,miR-377-5p,miR-486-3p,miR-143-3p,miR-320b,miR-1306-5p,novel_17,miR-542-5p,miR-6119-5p,miR-505,miR-330-3p,miR-320d,miR-378a-5p,miR-127-5p,miR-543-5p,miR-21-3p,miR-542-5p,miR-2300a-5p,miR-330-3p,miR-1224-5p,miR-377-5p,miR-185-5p,novel_83,miR-9788-3p,miR-129-1-3p,miR-2459,miR-2284r,miR-873a-5p,miR-22-3p,miR-124-3p,miR-1197-5p,miR-4429,miR-3120-5p,miR-129-2-3p,miR-542-5p,miR-2440,miR-29b-2-5p,miR-320e,miR-425-5p,miR-3968,miR-664a,miR-1843a-3p,miR-147-5p,miR-486b-3p,miR-2355-5p,miR-320c,miR-22-3p,miR-1306,miR-320a,miR-10a-3p,miR-1248,miR-485-5p

EFCAB3	ENSOARG0(	EF-hand	novel_9,miR-138-5p,miR-204-5p,miR-486b-3p,miR-28-5p,miR-378c,miR-345-5p,let-7g-3p,miR-876-3p,miR-1814c,miR-7144-5p,miR-488-3p,miR-137-3p,miR-769,miR-199a-5p,miR-487a-3p,miR-141-3p,let-7f-5p,miR-200c-3p,miR-200a-3p,novel_127,miR-107,miR-200c,miR-6119-5p,miR-2285af,miR-205-5p,let-7c-5p,miR-24-2-5p,miR-450b-5p,miR-2478,miR-200a,let-7g,miR-2284y,miR-199a-5p,miR-495-5p,miR-30a-3p,miR-3064-5p,let-7d,miR-200b-3p,miR-1290,miR-196a-5p,miR-2427,miR-2411-5p,miR-378h,miR-9-5p,miR-302a-5p,miR-24-2-5p,miR-2284z,miR-429-3p,miR-2285ad,miR-147-5p,miR-656-5p,miR-133a-5p,miR-142b,miR-576-3p,let-7i,miR-33a-3p,miR-211,miR-199b-5p,novel_27,miR-342-3p,novel_101,miR-495-3p,miR-2285x,miR-107,miR-505-3p,miR-378f,let-7e-5p,miR-21c,miR-3591-3p,miR-18a-3p,miR-677,miR-669,miR-3074-5p,miR-329-5p,miR-2285l,miR-196b-5p,miR-224-5p,novel_51,miR-330-3p,miR-432-3p,miR-654-3p,let-7a-2-3p,miR-345-5p,miR-6240,miR-130b-5p,miR-382,miR-21-5p,miR-138,miR-2113,novel_23,let-7g-5p,miR-3431,miR-2285g,miR-103,miR-6238,miR-194-3p,miR-103a-3p,miR-133b-5p,miR-187-3p,miR-31-5p,miR-26b-3p,miR-130b-5p,miR-708-5p,miR-30e-3p,let-7f,miR-30a-3p,miR-224-5p,miR-346,miR-769-5p,let-7b-5p,miR-3074-5p,miR-145a-3p,miR-539-5p,let-7b,miR-378c,miR-590-3p,miR-3074-2-3p,miR-154b-3p,miR-345-5p,miR-2285c,let-7f-1-3p,miR-216b-5p,let-7i-5p,miR-221-5p,miR-140-5p,miR-378a-3p,miR-504-5p,miR-21-3p,miR-154a-3p,miR-466i-5p,miR-98-5p,let-7k,miR-221-5p,miR-2285p,novel_17,miR-210-5p,miR-422a,miR-500a-5p,miR-130a-5p,miR-2432,miR-2411,miR-486-3p,miR-487a,miR-329a-5p,miR-345-3p,miR-142-3p,miR-378d,miR-148a-5p,miR-2284u,miR-361-5p,miR-1961,miR-378i,miR-1197-5p,miR-378a-3p,miR-2904,miR-200b,miR-340-5p,miR-1247-
RHOXF1	ENSOARG0(	Rhox hom	miR-193b-3p,miR-377-3p,miR-3082-5p,miR-193b-3p,let-7j,miR-377-3p,miR-137-3p,novel_74,miR-300,miR-874-5p,miR-193a-3p

TPRKB	ENSOARG0(	TP53RK b	novel_74,miR-204-5p,miR-454-3p,miR-665,miR-664a-5p,miR-1197-3p,miR-181c-5p,miR-338-3p,miR-122-5p,miR-1306-5p,miR-1271,miR-3607-3p,let-7g-3p,miR-1248,miR-320a,miR-376c-5p,miR-25-3p,miR-499b-3p,miR-301a-3p,miR-2284s,miR-324-5p,miR-140-5p,miR-32-5p,miR-2355-5p,miR-216b-3p,miR-2285aa,miR-760-3p,miR-320d,miR-92a-3p,miR-2285p,miR-26a-5p,miR-376b-3p,miR-217,miR-450b-5p,miR-92b-3p,miR-3529-3p,miR-4429,miR-2366,miR-5703,miR-217-5p,miR-124-3p,miR-2904,miR-1271-5p,miR-483-3p,miR-505-5p,miR-7859,miR-199a-3p,miR-582-3p,miR-548e-3p,miR-412,novel_1,miR-6119-3p,miR-92a-3p,miR-576-3p,novel_87,miR-371b-3p,miR-211,miR-877-3p,miR-301b-3p,miR-363-3p,novel_101,miR-2285x,miR-181d-5p,miR-6527,miR-668-5p,miR-96-5p,miR-877-3p,miR-376a-3p,miR-140-5p,miR-376b-5p,miR-376b-3p,miR-3065-5p,miR-378b,miR-2404,miR-25,miR-146b-3p,miR-499a-3p,miR-376d,miR-320c,miR-211-5p,miR-26b-5p,miR-130b-3p,miR-483-3p,miR-452-5p,miR-153-3p,novel_103,miR-199a-3p,miR-320b,miR-455-5p,miR-32,miR-491-3p,miR-124a,let-7c-3p,miR-455-5p,miR-188-5p,miR-181a-5p,miR-2284r,miR-412-3p,miR-7857,miR-376b-3p,miR-1298-3p,miR-147a,miR-153,miR-126a-5p,miR-2459,miR-665,miR-130a-3p
C3orf67	ENSOARG0(	chromoso	miR-224-5p,miR-500b-5p,miR-323-3p,miR-6525,miR-142a-5p,miR-6516-3p,miR-134-5p,miR-486b-3p,miR-2284k,miR-665,novel_25,miR-140-5p,miR-182-5p,miR-141-3p,miR-216b-3p,miR-345-5p,miR-345-5p,let-7g-3p,miR-543-3p,miR-30e-5p,miR-3154,miR-362-5p,miR-30d-5p,miR-320a,miR-148b-5p,miR-192-3p,novel_39,miR-2424,miR-486-3p,miR-495-5p,miR-181b-5p,miR-320d,miR-2284m,miR-450a-2-3p,miR-200a-3p,miR-374a-5p,miR-345-3p,miR-500a-5p,miR-328-3p,miR-134-5p,miR-212-5p,miR-3600,miR-4429,miR-664-5p,miR-133a-3p,miR-16-1-3p,miR-2366,miR-134,miR-345-3p,novel_133,miR-5703,miR-30a-5p,miR-362-5p,miR-2428,miR-374c-5p,novel_101,miR-181d-5p,miR-2284j,miR-4726-5p,miR-147-5p,miR-203b-5p,miR-6402,miR-107-5p,miR-485-3p,miR-1185-2-3p,miR-27a-3p,miR-203-3p,miR-320c,miR-216b-3p,miR-499b-5p,miR-136-5p,miR-1291,miR-9-3p,miR-6516-3p,miR-6395,miR-335-3p,miR-3065-5p,miR-432-3p,miR-432-3p,miR-345-5p,miR-758-3p,miR-127-3p,let-7c-3p,miR-3955-3p,miR-124-5p,miR-103a-2-5p,miR-582,novel_115,miR-224-5p,miR-7-5p,miR-320b,miR-182-5p,miR-2411-3p,miR-1185-3p,miR-216a-3p,miR-9788-3p,miR-500-5p,miR-548w,miR-3955-5p,miR-27a-3p,miR-2426,miR-16-1-3p,miR-1388-5p,miR-27b-3p,miR-2284x,miR-7b-5p,miR-181a-5p,miR-758-3p,novel_23

FANK1	ENSOARG0(	fibronec	miR-320d,miR-466i-5p,miR-199a-5p,miR-3065-3p,miR-4429,novel_133,miR-2300b-3p,miR-7134-5p,miR-29d-3p,miR-322-5p,miR-15a-5p,miR-1224-5p,miR-505-3p,miR-212-5p,miR-664a,miR-487a-5p,miR-31-3p,miR-424-5p,miR-29b-3p,miR-665,miR-224-5p,miR-1306-5p,miR-4510,miR-3074-5p,miR-16-5p,miR-2474,miR-1248,miR-320a,miR-6130,miR-199a-5p,miR-324-5p,miR-15b-5p,miR-21-3p,miR-3065-3p,miR-182-5p,miR-199b-5p,miR-224-5p,miR-320b,miR-3141,miR-2957,miR-16b,miR-182-5p,miR-424-5p,miR-376b,miR-30b-3p,miR-29b,miR-16b,miR-497-5p,miR-744-5p,miR-30b-3p,miR-145a-5p,miR-134-3p,miR-147a,miR-421,novel_4,miR-1343-3p,miR-5010-3p,miR-145b,miR-26b-3p,miR-29a,miR-15a,miR-16a,miR-199b-5p,miR-29c-3p,miR-450b-3p,novel_94,miR-505-3p,miR-195a-5p,miR-3141,miR-199b-5p,miR-3591-3p,miR-378g,miR-29a-3p,miR-320c,miR-3074-5p
FDXR	ENSOARG0(	ferredox	miR-6130,miR-3957,miR-204-3p,miR-504-5p,miR-3957-5p,miR-2355-5p,miR-485-5p,miR-671-5p,miR-1827,miR-20b-5p,miR-488-3p,miR-17-5p,miR-487b-5p,miR-93-5p,miR-346,miR-93,miR-338-3p,miR-3184-5p,miR-4510,novel_42,miR-214,miR-664a,miR-486b-3p,miR-4492,miR-494-5p,miR-2433,miR-331-3p,miR-532-3p,novel_83,miR-106a,miR-431,miR-33b-3p,miR-2300b-3p,miR-2366,novel_133,miR-5703,miR-1271-3p,miR-486-3p,miR-20a,miR-495-5p,miR-466i-5p,miR-760-3p,miR-106a,miR-185-3p,miR-3082-5p,miR-25,miR-146b-3p,miR-378g,novel_68,miR-504,miR-361-3p,miR-129b-5p,miR-877-3p,miR-154a,miR-423-5p,miR-3956-3p,miR-2447,novel_60,miR-4726-5p,miR-2284q,miR-20a-5p,miR-133a-5p,miR-2285n,miR-502-5p,miR-185-5p,miR-134-3p,miR-1298-3p,miR-20b,miR-149-5p,miR-455-3p,novel_78,miR-744-5p,miR-484,miR-4532,miR-106a-5p,miR-378a-5p,miR-214-3p,novel_115,miR-431-5p,miR-17-5p,miR-106b-5p,miR-106a-5p

CD72	ENSOARG0(	CD72 mol	miR-361-3p,miR-147-3p,novel_94,miR-877-3p,miR-664b,miR-3064-5p,miR-10b,miR-452-3p,miR-499b-5p,miR-7977,miR-454-5p,miR-2285n,miR-2285y,miR-769-5p,miR-107-5p,miR-4726-5p,novel_60,miR-4454,miR-212-3p,miR-219a-5p,miR-362-5p,miR-484,miR-296-5p,miR-143-5p,miR-4791,miR-30b-3p,miR-125b-2-3p,miR-544a,miR-3120-5p,miR-2300a-5p,miR-9788-3p,miR-500-5p,miR-2285e,miR-1306-5p,novel_115,miR-2957,novel_124,miR-3085-3p,miR-3955-3p,miR-424-5p,miR-2330-5p,miR-33a-5p,miR-30c-1-3p,miR-4532,miR-193a,miR-3187-3p,miR-758-3p,let-7g-3p,novel_99,miR-221-5p,miR-660-5p,miR-496-3p,miR-3065-3p,miR-192-5p,miR-6130,novel_32,miR-22-3p,miR-204-3p,miR-324-3p,miR-132-5p,miR-450b-3p,miR-6516-3p,miR-664a,miR-17-3p,miR-424-5p,miR-4510,miR-6134,novel_133,miR-2300b-3p,miR-215-5p,miR-140-3p,miR-19b-3p,miR-378d,miR-548e-3p,miR-1b-3p,miR-383-5p,miR-15a-5p,miR-148b-3p,miR-196a-3p,miR-125b,miR-221-5p,miR-30b-3p,miR-299-3p,miR-21-3p,miR-130a-5p,miR-146a-5p,miR-2387,miR-378b,miR-18a-3p,miR-1291,miR-450b-3p,miR-19a-3p,miR-107,miR-9851-3p,miR-195a-5p,miR-6395,miR-670-3p,miR-15a,miR-16a,miR-128-1-5p,miR-22-5p,miR-17-3p,miR-211,miR-2428,miR-873a-5p,miR-374b-3p,miR-145a-5p,miR-30b-3p,miR-1260b,miR-455-5p,miR-26b-3p,miR-2285g,miR-544b,miR-103,miR-2355-3p,miR-455-5p,miR-660,miR-125a-5p,miR-1343-5p,miR-130b-5p,miR-2483-5p,miR-363-5p,miR-345-5p,miR-488-3p,miR-503-3p,miR-362-5p,miR-3154,let-7g-3p,miR-7144-5p,miR-23a-5p,miR-485-5p,miR-370-3p,miR-615-5p,miR-215-5p,miR-769,miR-365a-3p,miR-324-5p,miR-154b-5p,miR-2332,miR-331-3p,miR-204-5p,miR-486b-3p,miR-6128,miR-103b,miR-500b-5p,miR-145-3p,miR-345-3p,miR-615-3p,miR-7134-5p,miR-496,let-7a-2-3p,miR-483-3p,miR-214-5p,miR-1260a,miR-30c-2-3p,miR-339a,miR-

ASB9	ENSOARG0(	ankyrin	novel_63, miR-125b-5p, miR-6740-5p, novel_9, miR-134-5p, miR-125a, miR-331-5p, miR-300-3p, miR-200a-5p, miR-376c-5p, miR-329a, miR-3064-5p, miR-450b-5p, miR-148b-5p, let-7g, miR-200a, miR-29a-5p, miR-205-5p, miR-30f, let-7f-5p, miR-1a-2-5p, novel_96, novel_127, miR-152-3p, miR-412, novel_79, miR-431, miR-505-5p, miR-450a-1-3p, miR-134-5p, miR-486-5p, novel_121, miR-1290, miR-1343-5p, miR-330-5p, miR-1a-1-5p, miR-1839-3p, miR-193b-3p, miR-374c-5p, miR-2285m, miR-2284w, miR-4324, miR-28c, miR-206, miR-3074-5p, miR-374c-3p, miR-411b-3p, novel_69, miR-677, miR-669, miR-320c, miR-181b-3p, miR-376b-5p, miR-148a-3p, miR-1306, miR-150-5p, miR-3591-3p, miR-412-3p, miR-146b-5p, let-7e-5p, miR-380-5p, miR-6240, miR-668-3p, miR-2285r, let-7a-2-3p, miR-377-3p, miR-146a, miR-2285v, miR-2411-3p, novel_51, miR-193a-3p, miR-3955-5p, miR-339-5p, miR-412-5p, miR-3431, miR-134-3p, novel_116, miR-194-3p, miR-149-5p, miR-19b-2-5p, miR-1843b-5p, miR-22-3p, miR-450c-3p, let-7b, miR-3074-5p, miR-214, miR-6525, miR-142a-5p, miR-346, let-7f, miR-2285j, miR-151-3p, miR-129-5p, miR-1468-5p, miR-216b-3p, miR-296-3p, miR-552-3p, miR-377-3p, miR-10a, let-7i-5p, miR-2285c, miR-450a-1-3p, miR-129b-3p, miR-339b, miR-1827, miR-186-5p, miR-503-5p, miR-195a-3p, miR-664b-3p, miR-376a-5p, miR-181b-1-3p, miR-143-3p, miR-2285p, novel_17, miR-618, miR-210-5p, miR-219b-5p, miR-324-3p, miR-92a-1-5p, miR-345-3p, miR-98-5p, let-7k, miR-18a-3p, miR-212-5p, miR-1961, miR-33b-3p, miR-133a-3p, miR-2447, miR-3956-3p, miR-877-3p, miR-1285-5p, novel_82, miR-2440, miR-301a-5p, miR-499a-5p, miR-628-3p, miR-490-5p, miR-139-5p, miR-2285u, miR-378g, miR-2483-3p, miR-378j, miR-96-5p, miR-668-5p, miR-335-3p, miR-1983, miR-124a, miR-1388-3p, miR-101b-3p, miR-744-3p, miR-8485, miR-1b-5p, miR-199a-3p, miR-431-5p, miR-323b-3p, miR-505, miR-182-
GPA33	ENSOARG0(	glycopro	novel_116, miR-6535, miR-324-5p, miR-532-3p, miR-4443, miR-6525, miR-193b-3p, miR-193b-3p, miR-214, miR-218-1-3p, miR-874-5p, miR-193a-3p, miR-1827, miR-2284x, miR-214-3p

MOB1B	ENSOARG0(	MOB kina	miR-202-5p,miR-2284m,novel_17,miR-122-3p,miR-422a,miR-181b-1-3p,miR-130a-5p,miR-216c-5p,miR-142-3p,miR-148a-5p,let-7a-3p,miR-378i,miR-1246,miR-362-3p,miR-124-3p,miR-188-3p,miR-378a-3p,miR-148b-3p,miR-340-5p,miR-30d-3p,novel_74,miR-487b-5p,miR-17-3p,miR-155-5p,miR-500,miR-15b-3p,miR-2285j,miR-30e-3p,miR-30a-3p,miR-324-3p,miR-29b-2-5p,miR-378c,miR-3184-3p,miR-543-3p,miR-625-5p,miR-186-5p,novel_99,let-7f-1-3p,miR-2285c,miR-500a-3p,miR-544-3p,miR-139-5p,miR-155-5p,miR-378a-3p,miR-30c-1-3p,miR-32-3p,miR-182-5p,miR-552-3p,miR-1185-5p,miR-487b-5p,miR-153-3p,miR-323b-3p,miR-362-3p,miR-182-5p,miR-30c-1-3p,miR-378d,miR-193a,miR-1b-5p,miR-124a,miR-101b-3p,miR-382-3p,miR-2284b,miR-378c,miR-1388-5p,miR-362-3p,miR-2426,miR-450b-5p,miR-544a,miR-101a-5p,miR-16-2-3p,miR-101c,miR-378b,miR-329-3p,miR-143-5p,miR-323b,miR-153,miR-216a-3p,miR-148b-5p,miR-2285e,miR-105-1,miR-330-3p,miR-222-5p,miR-27a-3p,miR-181b-2-3p,miR-3958-3p,miR-499a-5p,miR-374a-3p,miR-3964,miR-2284q,miR-218-5p,miR-101-3p,miR-877-3p,novel_111,miR-32-3p,novel_60,miR-3059-5p,miR-885-5p,miR-3956-3p,miR-6527,miR-452-5p,miR-194b-5p,miR-2284j,miR-668-5p,miR-136-5p,miR-140-5p,miR-381-3p,miR-378e,miR-3596,miR-3082-5p,miR-2483-3p,miR-144-5p,miR-203-3p,miR-27a-3p,miR-454-5p,miR-139-5p,miR-2319b,miR-499b-5p,miR-450a-2-3p,miR-17-3p,miR-1a-2-5p,miR-152-3p,miR-30c-2-3p,miR-450b-5p,miR-29a-5p,miR-2284y,miR-2284a,miR-3956-5p,miR-30a-3p,miR-2385-3p,miR-29b-2-5p,miR-105-2,miR-155-5p,miR-128-3p,let-7a-2-3p,miR-1290,miR-29d-3p,miR-378h,novel_121,miR-374b-3p,miR-29b-3p,miR-1-5p,miR-2284k,miR-5100,miR-135a-5p,miR-374a-3p,miR-365a-5p,miR-378c,miR-145-3p,miR-1306-5p,miR-2284v,miR-144-5p,miR-329a,let-7g-3p,miR-200a-5p,miR-874-
GORAB	ENSOARG0(	golgin,	novel_99,miR-3154,miR-411,miR-3957,miR-15b-5p,miR-374c-3p,miR-377-3p,miR-3957-5p,miR-221-5p,miR-424-5p,miR-6516-3p,miR-324-3p,miR-181c-5p,miR-16-5p,miR-411-3p,miR-1343-5p,miR-2904,miR-5703,novel_133,miR-2366,miR-2300b-3p,miR-1246,miR-322-5p,miR-15a-5p,miR-107,miR-221-5p,miR-181b-5p,miR-130a-5p,miR-2385-3p,miR-4286,miR-183-5p,miR-1983,miR-195a-5p,miR-412-3p,miR-107,miR-129b-5p,miR-378j,miR-3082-5p,novel_69,miR-374c-3p,miR-203b-5p,miR-3968,miR-379-3p,miR-16a,miR-382-5p,miR-15a,novel_27,miR-495-3p,miR-181d-5p,miR-101c,miR-26a-2-3p,miR-497-5p,miR-16b,miR-3071-5p,miR-374b-3p,miR-7b-5p,miR-181a-5p,novel_116,miR-2355-3p,miR-103,miR-412-5p,miR-5010-3p,miR-103a-3p,miR-1343-5p,miR-16b,miR-7-5p,miR-377-3p,miR-8485,miR-382-3p,miR-424-5p

UBA7	ENSOARG0(	ubiquiti	miR-148a-3p,miR-2387,miR-150-5p,miR-18a-3p,miR-450b-3p,miR-19a-3p,miR-2319a,miR-378f,miR-320b,miR-146b-3p,miR-670-3p,miR-4324,miR-142b,miR-128-1-5p,miR-206,miR-147-5p,miR-24-3p,miR-301b-3p,miR-199b-5p,miR-7b-5p,miR-2113,miR-1434-3p,miR-450c-3p,novel_23,miR-380-3p,miR-26a-2-3p,miR-27b-3p,miR-7862,miR-31-5p,miR-130a-3p,miR-3535,miR-2285g,miR-3431,miR-149-5p,miR-2355-3p,miR-7-5p,miR-377-3p,miR-125a-5p,miR-654-3p,miR-345-5p,miR-331-5p,miR-345-5p,miR-190a-3p,miR-7144-5p,miR-671-5p,miR-1814c,miR-874-3p,miR-370-3p,miR-2355-5p,miR-125a,miR-374c-3p,miR-300-3p,miR-199a-5p,miR-551b-3p,miR-410-5p,miR-2284k,miR-2433,miR-5100,miR-331-3p,miR-26c,miR-125b-5p,miR-331-5p,miR-134-5p,novel_42,miR-378c,miR-134,miR-1290,miR-885-3p,miR-429-3p,miR-423-3p,miR-2411-5p,miR-378h,miR-582-3p,miR-134-5p,miR-450a-1-3p,miR-214-5p,miR-129-1-3p,miR-205-5p,miR-200c,miR-30c-2-3p,miR-200c-3p,novel_127,miR-152-3p,miR-3529-3p,miR-495-5p,miR-1,miR-450b-5p,novel_39,miR-2284y,miR-199a-5p,miR-450c-5p,miR-490-3p,miR-361-3p,miR-199b-5p,miR-409-5p,miR-668-5p,miR-877-3p,miR-378e,miR-203-3p,miR-27a-3p,miR-4443,miR-450a-5p,miR-3059-5p,miR-541-3p,miR-2284j,miR-361-3p,miR-296-5p,miR-484,miR-378b,miR-6529a,miR-541,miR-4791,miR-1a-3p,miR-758-3p,miR-125b-2-3p,miR-378c,miR-1388-5p,miR-544a,miR-3120-5p,miR-2300a-5p,miR-27a-3p,miR-2459,miR-2285e,miR-421,novel_103,novel_19,miR-1185-5p,miR-3955-3p,miR-130b-3p,miR-199b-5p,miR-214-3p,miR-2330-5p,let-7c-3p,miR-378d,miR-30c-1-3p,miR-758-3p,miR-2310,miR-21a-3p,miR-450a-1-3p,miR-2285c,let-7g-3p,miR-345-5p,miR-885-3p,miR-582-5p,miR-30c-1-3p,miR-545-3p,miR-221-5p,miR-378a-3p,miR-21-3p,novel_107,miR-216b-3p,miR-532-5p,miR-377-3p,miR-544-3p,miR-301a-3p,miR-582-5p,miR-
CA11	ENSOARG0(	carbonic	miR-6240,miR-1839-3p,miR-2330-5p,miR-424-5p,miR-130b-5p,miR-30c-1-3p,miR-16b,miR-330-3p,miR-326,miR-503-5p,miR-214-3p,miR-542-5p,miR-3955-3p,miR-25-5p,miR-491-5p,miR-378a-5p,miR-330-3p,miR-149-5p,miR-503-5p,miR-496-5p,miR-30b-3p,miR-450c-3p,miR-541,miR-484,miR-497-5p,miR-2426,miR-450b-5p,miR-16b,miR-30b-3p,miR-542-5p,miR-3957-3p,miR-1839-3p,miR-3956-3p,miR-541-3p,novel_60,miR-423-5p,miR-326-3p,miR-24-3p,miR-211,miR-33a-3p,miR-16a,miR-6529b,miR-615,miR-15a,miR-2284q,miR-128-1-5p,miR-211-5p,miR-2285u,miR-10b,novel_68,miR-6535,miR-378j,miR-195a-5p,miR-504,miR-181a-2-3p,miR-2887,miR-29b-2-5p,miR-503-5p,miR-2285b,miR-450b-5p,miR-30c-2-3p,miR-542-5p,miR-200c,miR-2285af,novel_96,miR-760-3p,miR-200c-3p,miR-450a-1-3p,novel_83,miR-6516-5p,miR-200b,miR-15a-5p,miR-322-5p,miR-330-5p,miR-5703,miR-218-2-3p,miR-2904,miR-215-5p,miR-193b-5p,miR-615-3p,miR-133a-3p,miR-200b-3p,miR-214,miR-16-5p,miR-4510,miR-3184-5p,miR-346,miR-665,miR-29b-2-5p,miR-331-3p,miR-2285j,miR-2433,miR-204-5p,miR-28-5p,miR-130b-5p,miR-708-5p,miR-424-5p,miR-450b-3p,miR-215-5p,miR-615-5p,miR-504-5p,miR-30c-1-3p,miR-15b-5p,miR-6130,miR-6516,miR-192-5p,miR-450a-1-3p,miR-1827,miR-671-5p,miR-625-5p

MMP24	ENSOARG0(	matrix m	miR-10a-5p,miR-128-3p,miR-330-5p,miR-501-3p,miR-18b-5p,novel_79,miR-6517,miR-2285ad,miR-152-3p,miR-543-5p,novel_127,miR-200a-3p,miR-339a,miR-125a-3p,miR-1260a,miR-3065-3p,miR-450b-5p,miR-345-5p,miR-18a,miR-34c-5p,miR-615-5p,miR-141-3p,miR-370-3p,miR-874-3p,miR-3969,miR-331-3p,miR-365a-5p,miR-1197-3p,miR-1260b,miR-1260b,miR-374b-3p,miR-22-3p,miR-542-3p,miR-2355-3p,miR-134-3p,miR-412-5p,miR-339-5p,miR-378a-5p,miR-365b-5p,miR-3141,miR-345-5p,miR-1291,miR-18a-3p,miR-216a-3p,miR-3141,miR-18b,miR-148a-3p,miR-6535,miR-34b,miR-146b-3p,miR-10a-5p,miR-320b,miR-3074-5p,miR-6123,miR-128-1-5p,miR-34c,miR-6402,miR-2428,miR-326-3p,miR-449a,miR-371b-3p,miR-135a-2-3p,miR-378d,miR-34a-5p,miR-188-3p,miR-1193,miR-140-3p,miR-2408,miR-1246,miR-532-3p,miR-148b-3p,miR-18a-3p,miR-34b-5p,miR-221-5p,miR-760-3p,miR-185-3p,miR-324-3p,miR-345-5p,miR-339b,miR-18a-5p,miR-21a-3p,miR-877-5p,novel_32,miR-22-3p,miR-10a,miR-552-3p,miR-182-5p,miR-3065-3p,miR-221-5p,miR-504-5p,miR-4492,miR-664a,miR-3184-5p,miR-346,miR-10b-5p,miR-214,miR-2330-3p,miR-665-5p,miR-3074-5p,miR-145a-3p,miR-539-5p,miR-3120-5p,miR-101a-5p,miR-501-3p,miR-10b,miR-541,miR-301b-5p,miR-296-5p,miR-2459,miR-9788-3p,miR-216a-3p,miR-1343-3p,miR-214-3p,miR-182-5p,miR-326,miR-193a,miR-1388-3p,miR-504,miR-361-3p,miR-500-3p,miR-489,miR-378g,miR-10b,miR-490-5p,miR-301a-5p,miR-2285y,miR-2440,miR-502-3p,miR-423-5p,miR-361-3p,miR-877-3p,miR-1296-5p,miR-541-3p
IDH2	ENSOARG0(	isocitra	miR-187-3p,miR-3064-5p,miR-3064-5p,miR-6535,miR-486-3p,miR-331-3p,novel_19,miR-3085-3p,miR-486b-3p,miR-744-5p
CPNE3	ENSOARG0(	copine I	miR-10b-3p,miR-30b-3p,miR-8485,miR-186-5p,miR-3154
SSBP4	ENSOARG0(	single s	miR-4492,miR-324-5p,miR-30b-3p,miR-361-3p,miR-30b-3p,novel_51

PIGA	ENSOARG0(	phosphat	miR-193a-3p,miR-377-3p,miR-2285r,miR-539-3p,miR-628-5p,miR-2284ab,miR-27b-3p,miR-1285,miR-223-3p,miR-145a-5p,let-7g-5p,miR-2284d,miR-2285g,miR-544b,miR-194-3p,novel_116,miR-130a-3p,let-7i,miR-323c,miR-301b-3p,miR-326-3p,miR-219b-3p,miR-2285x,novel_101,miR-193b-3p,miR-6395,let-7e-5p,miR-2285ab,miR-216a-3p,miR-376b-3p,miR-150-5p,miR-670-3p,miR-2404,miR-216b-3p,novel_68,let-7f-5p,miR-200c-3p,miR-200c,miR-376b-3p,miR-2285af,let-7c-5p,miR-30f,miR-148b-5p,miR-2284a,let-7g,miR-29a-5p,miR-4286,miR-30a-3p,miR-582-3p,let-7d,miR-200b-3p,miR-30c,miR-128-3p,miR-30a-5p,miR-330-5p,miR-23b-5p,miR-429-3p,miR-2285ad,novel_9,miR-374b-3p,miR-410-3p,miR-2332,miR-2284k,miR-409-3p,miR-3969,miR-6740-5p,miR-331-3p,miR-374a-3p,miR-2284v,miR-3607-3p,miR-329a,miR-30e-5p,miR-200a-5p,miR-365a-3p,miR-300-3p,miR-324-5p,miR-215-5p,miR-2285aa,miR-130b-3p,miR-3955-3p,let-7e,miR-326,miR-199a-3p,miR-505,miR-362-3p,miR-758-3p,let-7a-5p,miR-652-5p,miR-376b,miR-544a,miR-362-3p,miR-329-3p,miR-125b-2-3p,miR-758-3p,miR-376b-3p,miR-2285e,miR-421,miR-148b-5p,miR-216a-3p,novel_78,miR-145b,miR-27a-3p,miR-2284q,miR-374a-3p,miR-30b-5p,miR-2285n,miR-2285y,miR-154a,miR-1285-5p,miR-2284j,miR-6527,miR-2285f,miR-668-5p,miR-376a-3p,miR-335-3p,miR-2483-3p,miR-376d,miR-27a-3p,let-7d-5p,miR-2285u,miR-760-3p,miR-98-5p,let-7k,miR-30d,miR-221-5p,novel_17,miR-196a-3p,miR-30b,miR-216a-5p,miR-216c-5p,miR-183-5p,miR-33b-3p,miR-148a-5p,miR-193b-5p,miR-362-3p,miR-1961,miR-215-5p,miR-30c-5p,miR-200b,miR-383-5p,miR-487a-3p,miR-7859,miR-328-3p,miR-199a-3p,miR-548e-3p,miR-30d-3p,miR-485-3p,miR-664a,novel_74,miR-454-3p,miR-487a-5p,let-7f,miR-2285[illegible],miR-3604,miR-664a-5p,miR-30a-3p,let-7b-5p,miR-145a-
TCFL5	ENSOARG0(	transcri	miR-582-5p,miR-3963,miR-411,miR-199b-5p,miR-199a-5p,miR-154b-5p,miR-582-5p,miR-25,miR-193b-3p,miR-25-3p,miR-7-1-3p,miR-32-5p,miR-20a-3p,miR-708-5p,miR-28-5p,miR-134-5p,miR-499a-5p,novel_74,miR-92a-3p,novel_1,miR-107-5p,miR-199b-5p,miR-193b-3p,miR-425-5p,miR-212-3p,miR-363-3p,miR-21b,miR-361-5p,miR-188-5p,miR-16-1-3p,miR-3071-5p,miR-217-5p,miR-873a-5p,miR-126-3p,miR-1434-3p,miR-134,miR-134-5p,miR-2284h-5p,miR-539-5p,miR-6517,miR-199b-5p,miR-193a-3p,miR-92a-3p,miR-103a-2-5p,miR-124-5p,miR-32,miR-217,miR-199a-5p,miR-8485,miR-29a-5p,miR-2310,miR-92b-3p,miR-450b-5p,miR-664b-3p,miR-382

UNC13B	ENSOARG0(	unc-13 h	novel_127,miR-21-3p,let-7k,miR-34b-5p,let-7f-5p,miR-98-5p,let-7c-5p,novel_17,miR-490-3p,miR-380-3p,miR-2411,let-7g,miR-29a-5p,miR-183-5p,miR-34a-5p,let-7a-2-3p,let-7d,miR-140-3p,miR-1197-5p,miR-1193,miR-2904,miR-29d-3p,miR-215-5p,miR-1961,miR-486-5p,miR-2284h-5p,miR-582-3p,miR-2411-5p,miR-1271-5p,miR-95-3p,miR-1224-5p,miR-18a-3p,miR-190a-5p,miR-664a,miR-767-5p,miR-3969,miR-331-3p,miR-151-3p,let-7f,miR-29b-3p,let-7b-5p,miR-122-5p,miR-1271,miR-3074-5p,let-7b,miR-23a-5p,miR-625-5p,let-7g-3p,let-7g-3p,miR-767,miR-301,miR-21a-3p,miR-488-3p,let-7i-5p,miR-204-3p,miR-365a-3p,miR-27a-5p,miR-34c-5p,miR-192-5p,miR-215-5p,miR-370-3p,miR-216b-3p,miR-874-3p,let-7e,miR-1185-5p,miR-190a,miR-758-3p,let-7a-2-3p,miR-3187-3p,let-7a-5p,miR-27b-3p,miR-2889,miR-3120-5p,miR-26a-2-3p,miR-219a-1-3p,miR-380-3p,miR-29b,miR-758-3p,miR-145a-5p,miR-1285,miR-1434-3p,miR-147a,miR-1298-3p,let-7g-5p,miR-27a-3p,miR-145b,miR-133b-5p,miR-151a-3p,miR-1343-3p,miR-29a,let-7i,miR-34c,miR-7705,miR-2428,miR-449a,miR-24-3p,miR-29c-3p,miR-3956-3p,novel_60,miR-3059-5p,miR-135a-2-3p,miR-2447,miR-6516-3p,let-7e-5p,miR-96-5p,miR-378j,miR-378b,miR-18a-3p,miR-27a-3p,let-7d-5p,miR-6535,miR-411b-3p,miR-34b,miR-29a-3p,miR-3120-3p,miR-320b,miR-452-3p,miR-10b,miR-3074-5p
REEP6	ENSOARG0(	receptor	miR-326,miR-3141,novel_19,miR-3085-3p,miR-25-5p,miR-1343-5p,miR-2330-5p,miR-378d,miR-30c-1-3p,miR-4532,miR-503-3p,miR-378b,miR-296-5p,miR-2284r,miR-1843b-5p,miR-378c,miR-138,miR-3432b,miR-2426,miR-4508,miR-744-5p,miR-340-3p,novel_4,miR-377-5p,miR-147a,miR-1247-3p,miR-28c,miR-2447,miR-423-5p,miR-326-3p,miR-2428,miR-3141,miR-1291,miR-2319a,miR-378f,miR-3064-5p,miR-378b,miR-378e,miR-210-5p,miR-30c-2-3p,miR-185-3p,miR-422a,miR-92a-1-5p,miR-2898,miR-760-3p,miR-3064-5p,miR-28b,miR-377-5p,miR-378i,miR-3578,miR-1343-5p,miR-378a-3p,miR-330-5p,miR-378h,miR-2433,miR-331-3p,miR-664a-5p,miR-138-5p,miR-664a,miR-4492,miR-378c,miR-3184-5p,miR-378c,miR-6134,miR-378a-3p,miR-30c-1-3p,miR-27a-5p,miR-204-3p,miR-877-5p
C2orf78	ENSOARG0(	chromoso	miR-361-3p,miR-539-5p,miR-361-5p,miR-9851-3p,miR-582-3p,miR-3074-1-3p

MSI1	ENSOARG0(	musashi	miR-6402,miR-33a-3p,miR-361-3p,novel_111,miR-2428,miR-32-3p,miR-3059-5p,miR-212-3p,miR-2285f,miR-2285x,miR-412-3p,miR-379-5p,miR-1983,miR-376b-5p,miR-2285ab,miR-148a-3p,miR-1306,miR-378j,miR-129b-5p,miR-18a-3p,miR-2404,miR-411b-3p,miR-203-3p,miR-144-3p,miR-3074-5p,miR-2285u,miR-503-5p,miR-1306-5p,miR-105-5p,miR-190a,novel_19,miR-432-3p,miR-432-3p,miR-1388-3p,miR-130b-5p,miR-382,miR-376b,miR-628-5p,miR-2426,miR-101a-5p,miR-1839-5p,miR-188-5p,miR-3120-5p,miR-143-5p,miR-2285g,miR-412-5p,miR-503-5p,miR-2285e,miR-105-1,miR-339-5p,miR-190a-5p,miR-487a-5p,miR-130b-5p,miR-144,miR-100-3p,miR-3074-5p,miR-193a-5p,miR-2312,let-7g-3p,miR-339b,miR-7144-5p,miR-23a-5p,miR-376c-5p,miR-2285c,miR-376c-5p,miR-32-3p,miR-296-3p,miR-466i-5p,miR-127-5p,miR-191-3p,novel_127,miR-152-3p,miR-339a,miR-503-5p,miR-4286,miR-345-3p,miR-181a-2-3p,miR-105-2,miR-23b-5p,miR-1197-5p,miR-148b-3p,miR-18a-3p,miR-7641,novel_79
PHF6	ENSOARG0(	PHD fing	miR-654-3p,miR-296-3p,miR-6240,miR-380-5p,miR-466f-3p,miR-193a-3p,miR-452-5p,miR-296-3p,miR-224-5p,miR-106a-5p,miR-17-5p,miR-330-3p,miR-2284d,miR-412-5p,miR-2355-3p,miR-20b,miR-133b-5p,miR-1260b,miR-21-5p,miR-380-3p,miR-16b,miR-2284x,miR-7862,miR-497-5p,miR-873a-5p,miR-3071-5p,miR-450c-3p,miR-323c,miR-199b-5p,miR-1a-1-5p,miR-135a-2-3p,miR-495-3p,miR-758-5p,miR-1193,miR-2285x,miR-193b-3p,miR-206,miR-199c,miR-421-5p,miR-625-3p,miR-203b-5p,miR-133a-5p,miR-6402,miR-28c,miR-142b,miR-20a-5p,miR-15a,novel_87,miR-33a-3p,miR-382-5p,miR-485-3p,miR-16a,miR-2404,miR-320c,miR-26b-5p,miR-320b,miR-450b-3p,miR-195a-5p,miR-18b,miR-21c,miR-18a-3p,miR-216a-3p,miR-380-5p,miR-28b,miR-2285b,miR-199a-5p,miR-200a,miR-1957a,miR-127-5p,miR-1a-2-5p,miR-200c-3p,novel_127,miR-664-3p,miR-200c,miR-1260a,miR-2285af,miR-6516-5p,miR-1271-5p,miR-322-5p,miR-450a-1-3p,miR-545-5p,miR-429-3p,miR-18b-5p,miR-200b-3p,miR-128-3p,miR-3578,miR-30a-5p,miR-1260b,miR-93,miR-16-5p,miR-370-5p,miR-410-5p,miR-34c-3p,miR-2284k,miR-1-5p,miR-6740-5p,miR-769,miR-331-5p,miR-300-3p,miR-2284n,miR-376c-3p,miR-199a-5p,miR-3068-3p,miR-487a-3p,miR-329b,miR-20b-5p,miR-7144-5p,novel_48,miR-2448-3p,miR-30e-5p,miR-488-3p,miR-17-5p,miR-18a,miR-320a,miR-362-5p,miR-93-5p,miR-491-3p,miR-1b-5p,miR-8485,miR-3187-3p,miR-424-5p,miR-106a-5p,miR-124a,miR-744-3p,let-7c-3p,miR-214-3p,miR-199b-5p,miR-320b,miR-199a-3p,miR-16b,miR-106b-5p,miR-539-5p,miR-126a-5p,miR-421,miR-455-3p,miR-500-5p,miR-2300a-5p,miR-199b-3p,miR-330-3p,miR-548w,miR-299a-5p,miR-1839-5p,miR-16-2-3p,miR-7857,miR-361-3p,miR-362-5p,miR-9-3p,miR-3059-5p,miR-2284j,miR-452-5p,miR-3968,miR-490-5p,miR-769-5p,miR-371a-5p,miR-2285y,miR-2483-3p,miR-25,miR-

ZDHHC6	ENSOARG0(	zinc fin	miR-21c,miR-135b-5p,miR-195a-5p,miR-6516-3p,miR-655-5p,miR-146b-3p,miR-29a-3p,miR-670-3p,miR-15a,miR-6402,miR-20a-5p,miR-16a,novel_1,miR-502b,miR-342-3p,miR-371b-3p,miR-873a-5p,miR-181a-5p,miR-29b,miR-138,miR-30b-3p,miR-21-5p,miR-1260b,miR-133b-5p,miR-2285g,miR-432-5p,miR-185-5p,miR-20b,miR-106a-5p,miR-17-5p,miR-124-5p,miR-125a-5p,miR-466f-3p,miR-130b-5p,miR-2483-5p,miR-539-3p,miR-345-5p,miR-381-3p,miR-3154,miR-17-5p,miR-10a-3p,miR-3074-1-3p,miR-23a-5p,miR-370-3p,miR-141-3p,miR-3965,miR-324-5p,miR-29b-3p,miR-135a-1-3p,miR-3969,miR-135a-5p,miR-138-5p,miR-31-3p,miR-28-5p,miR-1306-5p,miR-6128,miR-93,miR-145-3p,miR-3578,miR-29d-3p,miR-501-3p,miR-300,miR-128-3p,miR-222,miR-7641,miR-483-3p,miR-1271-5p,miR-582-3p,miR-545-5p,miR-2285af,miR-1260a,miR-200c,miR-30c-2-3p,miR-181b-5p,miR-200c-3p,miR-1,miR-2478,miR-24-2-5p,miR-1957a,miR-3065-3p,miR-490-3p,miR-664b,miR-3064-5p,miR-7857-3p,miR-3082-5p,miR-371a-5p,miR-107-5p,miR-2285t,miR-181d-5p,miR-219a-5p,miR-452-5p,miR-502-3p,miR-154a,miR-296-5p,miR-484,miR-30b-3p,miR-125b-2-3p,miR-16-2-3p,miR-2284aa,novel_4,miR-9788-3p,miR-216a-3p,miR-221-3p,miR-2285e,miR-342,miR-1306-5p,novel_103,miR-362-3p,miR-2957,miR-3085-3p,miR-3955-3p,miR-424-5p,miR-106a-5p,miR-376b,miR-30c-1-3p,miR-27b-5p,miR-582-5p,miR-3065-3p,miR-139-5p,miR-204-3p,miR-222-3p,miR-324-3p,miR-655-3p,miR-487a-5p,miR-708-5p,miR-424-5p,miR-145a-3p,miR-1271,miR-206-3p,miR-6134,miR-1246,miR-362-3p,miR-2300b-3p,miR-2904,miR-188-3p,miR-1197-5p,miR-140-3p,miR-5703,miR-548e-3p,miR-485-3p,miR-487a-3p,miR-383-5p,miR-15a-5p,novel_83,miR-532-3p,miR-374a-5p,miR-216a-5p,miR-125b,miR-106a,miR-30b-3p,miR-21-3p,miR-183-5p,miR-216c-5p,miR-20a,miR-2432,miR-1306,miR-216a-

RAB11FIP3	ENSOARG0(	RAB11 fa	miR-543-5p,miR-181b-5p,miR-450a-2-3p,miR-339a,miR-1271-3p,miR-199a-5p,miR-3065-3p,miR-28b,miR-4286,miR-128-3p,miR-29d-3p,miR-134,miR-345-3p,miR-214-5p,miR-1271-5p,miR-302a-5p,miR-28-5p,miR-486b-3p,miR-204-5p,miR-767-5p,miR-331-3p,miR-26c,miR-29b-3p,miR-3607-3p,miR-6128,miR-1306-5p,miR-7144-5p,miR-320a,miR-154b-5p,miR-199a-5p,miR-769,miR-34c-5p,miR-660,miR-125a-5p,miR-1343-5p,miR-197-5p,miR-330-3p,miR-503-5p,miR-345-5p,miR-363-5p,miR-744-5p,miR-7862,miR-29b,miR-30b-3p,miR-374b-3p,miR-145a-5p,miR-181a-5p,miR-185-5p,miR-503-5p,miR-665,miR-187-3p,miR-16a,miR-34c,miR-15a,miR-502-5p,miR-449a,miR-326-3p,miR-24-3p,miR-323c,miR-371b-3p,miR-211,miR-2285x,miR-542-5p,miR-135a-2-3p,miR-342-3p,miR-6395,miR-195a-5p,miR-7975,miR-9851-3p,miR-1291,miR-18a-3p,miR-2387,miR-670-3p,miR-29a-3p,miR-34b,miR-323a-5p,miR-320b,miR-21-3p,miR-34b-5p,miR-221-5p,miR-760-3p,miR-320d,miR-125b,miR-185-3p,miR-542-5p,miR-196a-3p,miR-125b-2-3p,miR-183-5p,miR-378d,miR-4429,miR-1197-5p,miR-5703,miR-124-3p,miR-2904,miR-362-3p,novel_133,miR-222-5p,miR-148b-3p,miR-15a-5p,miR-3960,miR-708-5p,miR-424-5p,miR-664a,miR-320e,miR-324-3p,miR-1271,miR-204-3p,novel_32,miR-323-5p,miR-3065-3p,miR-660-5p,miR-221-5p,miR-542-5p,miR-3085-3p,miR-362-3p,miR-326,miR-320b,miR-342,miR-4532,miR-33a-5p,miR-424-5p,miR-125b-2-3p,miR-30b-3p,miR-143-5p,miR-484,miR-296-5p,novel_4,miR-2300a-5p,miR-409b,miR-4726-5p,miR-371a-5p,miR-769-5p,miR-181d-5p,miR-1296-5p,miR-541-3p,miR-199b-5p,miR-361-3p,miR-3082-5p,miR-7977,miR-499b-5p,miR-3064-5p,miR-152-3p,novel_127,miR-29a-5p,miR-3064-5p,miR-885-3p,miR-23b-5p,miR-330-5p,miR-1343-5p,miR-3591-5p,miR-129-1-3p,miR-134-5p,miR-505-5p,miR-322-5p,novel_79,miR-1224-5p,miR-

CISD2	ENSOARG0(	CDGSH ir	miR-2284d,miR-3955-5p,miR-339-5p,miR-665,miR-16-1-3p,miR-380-3p,miR-2284ab,miR-16b,miR-7862,miR-4508,miR-497-5p,miR-7b-5p,miR-542-3p,miR-653,miR-345-5p,miR-493-5p,miR-382,miR-466f-3p,miR-7-5p,miR-2411-3p,miR-190a,miR-299b-3p,miR-6535,miR-10a-5p,miR-3120-3p,miR-195a-5p,miR-2387,miR-216a-3p,miR-3959-3p,miR-2284w,miR-135a-2-3p,novel_120,miR-17-3p,novel_1,miR-199c,miR-133a-5p,miR-15a,miR-33a-3p,miR-16a,miR-322-5p,miR-582-3p,miR-299a-3p,novel_79,miR-10a-5p,miR-30c,miR-128-3p,miR-345-3p,miR-30a-5p,miR-1957a,miR-6536,miR-17-3p,miR-200a-3p,novel_96,miR-125a-3p,miR-6119-5p,miR-339a,miR-30c-2-3p,miR-2284n,miR-503-3p,miR-141-3p,miR-370-3p,miR-345-5p,miR-1814c,novel_48,miR-23b,miR-3154,miR-6128,miR-16-5p,miR-28-5p,miR-34c-3p,novel_63,miR-331-3p,miR-216a-3p,miR-455-3p,miR-199b-3p,miR-409b,miR-222-5p,miR-299a-5p,miR-10b,miR-301b-5p,miR-143-5p,miR-541,miR-30c-1-3p,miR-2310,miR-8485,miR-101b-3p,miR-424-5p,miR-1388-3p,miR-652-5p,miR-23a-3p,let-7c-3p,miR-491-5p,novel_103,miR-199a-3p,miR-16b,miR-29b-1-5p,miR-3082-5p,miR-499a-3p,miR-25,miR-139-5p,miR-541-5p,miR-668-5p,miR-136-5p,miR-504,miR-493-5p,miR-129b-5p,miR-381-3p,miR-8095,miR-3956-3p,miR-541-3p,novel_60,miR-499a-5p,miR-3968,miR-2284q,miR-2440,miR-30b-5p,miR-218-5p,miR-301a-5p,miR-23c,miR-15a-5p,miR-23b-3p,miR-2284h-5p,miR-199a-3p,miR-548e-3p,miR-16-1-3p,miR-30c-5p,miR-140-3p,miR-142a-3p,miR-130a-5p,miR-376a-5p,miR-29b-1-5p,miR-421-5p,miR-380-3p,miR-143-3p,miR-2432,miR-2285w,miR-760-3p,miR-21-3p,miR-299-3p,miR-1277-5p,miR-30b,miR-345-3p,miR-3601,miR-139-5p,miR-6130,miR-10a,miR-499b-3p,miR-15b-5p,miR-548o-3p,miR-504-5p,miR-30c-1-3p,miR-30f,miR-339b,miR-345-5p,miR-186-5p,miR-625-5p,miR-21a-3p,miR-[illegible]
CIB1	ENSOARG0(	calcium	miR-134-5p,miR-421,miR-3535,miR-483-3p,miR-412,miR-31-5p,miR-128-3p,miR-412-3p,miR-140-3p,miR-296-5p,miR-484,miR-134,miR-4791,miR-490-3p,miR-450c-5p,miR-376a-5p,miR-483-3p,miR-378a-5p,miR-377-3p,miR-2285p,miR-377-3p,miR-2355-5p,miR-541-5p,miR-370-3p,miR-548o-3p,miR-3074-5p,miR-20a-3p,miR-6516-3p,miR-10b-3p,miR-1291,miR-216a-3p,miR-3154,miR-361-3p,miR-8095,miR-877-3p,novel_42,miR-3074-5p,miR-656-5p,miR-134-5p,miR-4726-5p,miR-450a-5p,miR-664a

B4GALNT3	ENSOARG0(	beta-1,4	miR-345-3p,miR-146a-5p,miR-486-3p,miR-210-5p,miR-324-3p,miR-185-3p,miR-125b,miR-466i-5p,miR-34b-5p,miR-221-5p,miR-18a-3p,miR-30d-3p,miR-15a-5p,novel_133,miR-193b-5p,miR-124-3p,miR-1197-5p,miR-133a-3p,miR-34a-5p,miR-4510,miR-30e-3p,miR-30a-3p,miR-665,miR-17-3p,miR-664a,miR-4492,miR-874-5p,miR-454-3p,miR-424-5p,miR-140-5p,miR-221-5p,miR-552-3p,miR-301a-3p,miR-193b-3p,miR-6130,miR-15b-5p,miR-133b-3p,miR-885-3p,miR-124a,miR-424-5p,miR-494-3p,miR-1306-5p,miR-326,miR-16b,miR-130b-3p,miR-146b,novel_4,novel_78,miR-27a-3p,miR-147a,miR-455-3p,miR-296-5p,miR-30b-3p,miR-361-3p,miR-877-3p,miR-2285n,miR-218-5p,miR-4726-5p,miR-450a-5p,miR-1843a-3p,miR-2319b,miR-378g,miR-27a-3p,miR-4443,miR-361-3p,novel_94,miR-30a-3p,novel_39,miR-450c-5p,miR-17-3p,novel_127,miR-322-5p,miR-505-5p,miR-7134-5p,miR-1343-5p,miR-330-5p,miR-885-3p,miR-16-5p,miR-193a-5p,miR-133a-3p,miR-331-3p,miR-125b-5p,miR-486b-3p,miR-874-3p,miR-370-3p,miR-2355-5p,miR-125a,miR-34c-5p,miR-365a-3p,miR-3154,miR-7144-5p,miR-485-5p,miR-432-3p,let-7j,miR-146a,novel_51,miR-1343-5p,miR-125a-5p,miR-193a-3p,miR-133b-5p,miR-130a-3p,miR-134-3p,miR-194-3p,miR-542-3p,miR-30b-3p,miR-16b,miR-497-5p,miR-27b-3p,miR-744-5p,miR-193b-3p,miR-17-3p,miR-371b-3p,miR-326-3p,miR-449a,miR-2428,miR-4324,miR-15a,miR-34c,miR-16a,miR-320b,miR-655-5p,miR-34b,miR-670-3p,miR-2387,miR-1306,miR-18a-3p,miR-1291,miR-146b-5p,miR-9851-3p,miR-195a-5p

STOML2	ENSOARG0(	stomatin	miR-18a-3p,miR-548e-3p,miR-212-5p,miR-3600,miR-15a-5p,miR-23c,miR-7859,miR-23b-3p,miR-328-3p,miR-199a-3p,miR-2284h-5p,miR-2408,miR-1193,miR-1197-5p,miR-188-3p,miR-361-5p,miR-664-5p,miR-2285w,miR-181b-1-3p,miR-130a-5p,miR-143-3p,miR-2424,miR-210-5p,miR-542-5p,miR-2285p,miR-125b,miR-500a-5p,miR-3601,miR-2284m,miR-221-5p,miR-140-5p,miR-30c-1-3p,miR-221-5p,miR-2331-3p,miR-182-5p,miR-216b-3p,miR-6130,miR-323-5p,miR-6516,miR-301a-3p,miR-582-5p,miR-15b-5p,miR-22-3p,novel_32,miR-339b,let-7g-3p,miR-1827,miR-582-5p,miR-4510,miR-145a-3p,miR-3074-5p,miR-2330-3p,miR-3958-5p,miR-222-3p,miR-29b-2-5p,miR-219b-3p,miR-664a,novel_74,miR-130b-5p,miR-424-5p,miR-129-5p,miR-454-3p,miR-199b-3p,novel_78,miR-2300a-5p,miR-181b-2-3p,miR-222-5p,miR-27a-3p,miR-1298-3p,miR-221-3p,miR-143-5p,miR-301b-5p,miR-6529a,miR-296-5p,miR-758-3p,miR-501-3p,miR-101a-5p,miR-21b,miR-424-5p,miR-1388-3p,miR-23a-3p,let-7c-3p,miR-30c-1-3p,miR-758-3p,miR-1306-5p,miR-199a-3p,miR-16b,miR-182-5p,miR-505,miR-2957,miR-3955-3p,miR-130b-3p,miR-542-5p,miR-2319b,miR-211-5p,miR-7689-3p,miR-2285u,miR-3082-5p,miR-2483-3p,miR-25,miR-454-5p,miR-500-3p,miR-27a-3p,miR-203-3p,miR-221,miR-140-5p,novel_94,miR-877-3p,miR-9-3p,miR-3956-3p,novel_60,miR-3059-5p,miR-9-5p,miR-2284j,miR-1296-5p,miR-219a-2-3p,miR-502-3p,miR-9-3p,miR-2440,novel_82,miR-2285n,miR-301a-5p,miR-2284q,miR-222,novel_79,miR-1224-5p,miR-7641,miR-6516-5p,miR-9-5p,miR-505-5p,miR-322-5p,miR-486-5p,miR-23b-5p,miR-501-3p,let-7a-2-3p,miR-128-3p,miR-29b-2-5p,miR-2478,miR-2285b,miR-125a-3p,miR-219-3p,miR-205-5p,miR-339a,miR-30c-2-3p,miR-450a-2-3p,miR-543-5p,miR-200a-3p,novel_127,miR-107,miR-3068-3p,miR-125a,miR-141-3p,miR-2355-5p,miR-331-5p,miR-374c-3p,miR-223-5p,miR-
SUB1	ENSOARG0(	SUB1 hom	miR-381-3p,miR-411,miR-200a-5p,miR-7144-5p,miR-7975,miR-329-5p,novel_68,miR-3064-5p,miR-1271,miR-7857-3p,miR-544-3p,miR-144-5p,miR-677,miR-101-3p,miR-33a-3p,miR-338-5p,miR-379-3p,miR-6119-3p,miR-199c,miR-2284q,miR-191-5p,miR-130b-5p,miR-2330-3p,miR-144-5p,miR-154a,miR-708-3p,miR-191,miR-125b-2-3p,miR-33b-3p,miR-544a,miR-411-3p,miR-1839-5p,miR-199b-3p,novel_78,miR-330-3p,miR-548e-3p,miR-665,miR-7859,miR-302a-5p,miR-544b,miR-2285af,miR-330-3p,miR-760-3p,miR-3085-3p,miR-2484,miR-6240,miR-130b-5p,miR-329a-5p,miR-3064-5p,miR-1271-3p,miR-29a-5p

PIGO	ENSOARG0(	phosphat	miR-152-3p,novel_96,miR-30c-2-3p,miR-380-5p,miR-2478,miR-28b,miR-450b-5p,miR-1271-3p,miR-3065-3p,novel_39,miR-4286,miR-181a-2-3p,miR-29d-3p,miR-378h,miR-9-5p,miR-302a-5p,miR-505-5p,miR-214-5p,novel_121,miR-450a-1-3p,miR-18b-5p,miR-29b-3p,miR-181c-3p,miR-145-3p,miR-378c,miR-193a-5p,miR-10b-3p,miR-485-5p,miR-23a-5p,miR-2448-3p,miR-18a,miR-3154,miR-769,miR-34c-5p,miR-376c-3p,miR-3068-3p,miR-2285aa,miR-378a-5p,miR-224-5p,miR-330-3p,miR-6240,miR-380-5p,miR-29b,miR-2284x,miR-188-5p,miR-1434-3p,miR-1285,miR-708-3p,miR-873a-5p,miR-450c-3p,miR-431-3p,miR-185-5p,miR-194-3p,miR-133b-5p,miR-377-5p,miR-26b-3p,miR-29a,miR-6119-3p,miR-199c,miR-656-5p,miR-203b-5p,miR-28c,miR-34c,miR-449a,miR-542-5p,miR-29c-3p,novel_101,miR-495-3p,miR-378f,miR-18b,miR-148a-3p,miR-1306,miR-34b,miR-670-3p,miR-29a-3p,miR-323a-5p,miR-2404,miR-760-3p,miR-221-5p,miR-34b-5p,miR-542-5p,miR-618,miR-422a,miR-185-3p,miR-500a-5p,miR-324-3p,miR-377-5p,miR-376c-3p,miR-125b-2-3p,miR-34a-5p,miR-664-5p,miR-2300b-3p,miR-2408,miR-378i,miR-5703,miR-378a-3p,miR-188-3p,miR-124-3p,miR-148b-3p,miR-199a-3p,miR-151-5p,miR-3960,miR-664a,miR-450b-3p,miR-874-5p,miR-665,miR-224-5p,miR-769-5p,miR-3184-5p,miR-3184-3p,miR-4510,miR-145a-3p,miR-539-5p,miR-378c,miR-2330-3p,miR-18a-5p,let-7g-3p,miR-450a-1-3p,miR-129b-3p,miR-6130,miR-323-5p,miR-376e-3p,miR-151b,miR-378a-3p,miR-2331-3p,miR-221-5p,miR-30c-1-3p,miR-182-5p,miR-3065-3p,miR-25-5p,miR-491-5p,miR-542-5p,miR-1306-5p,miR-199a-3p,miR-182-5p,miR-505,miR-378d,miR-30c-1-3p,miR-124a,miR-378c,miR-2284r,miR-378b,miR-126a-5p,miR-9788-3p,miR-1343-3p,miR-199b-3p,miR-4726-5p,miR-769-5p,miR-371a-5p,miR-361-3p,miR-877-3p,miR-423-5p,miR-9-5p,miR-181a-3p,miR-140-5p,miR-378e,miR-

ZNF330	ENSOARG0(	zinc fin	miR-301,miR-876-3p,miR-1248,miR-874-3p,miR-1271,miR-2355-5p,miR-25-3p,miR-133c,miR-134-5p,miR-16-5p,miR-193a-5p,miR-1197-3p,miR-1260b,miR-6517,miR-429-3p,miR-18b-5p,novel_79,miR-758-5p,miR-322-5p,miR-450a-1-3p,miR-134-5p,miR-8117,novel_127,miR-3064-5p,miR-92b-3p,miR-148b-5p,miR-450c-5p,miR-192-3p,novel_39,miR-200a,miR-376b-5p,miR-410-5p,miR-378f,miR-2319a,miR-3074-5p,miR-3120-3p,miR-181b-3p,miR-677,novel_87,miR-485-3p,miR-374c-5p,miR-19b-2-5p,miR-450c-3p,miR-16b,miR-497-5p,miR-103a-3p,miR-3955-5p,miR-339-5p,miR-3431,miR-134-3p,miR-377-3p,miR-2411-3p,miR-194a,miR-105-5p,miR-380-5p,miR-432-3p,miR-450a-1-3p,miR-411,miR-339b,miR-186-5p,miR-1827,miR-1895,miR-543-3p,miR-30c-1-3p,miR-32-5p,miR-552-3p,miR-377-3p,miR-582-5p,miR-15b-5p,miR-151-3p,miR-4492,miR-3074-5p,miR-3958-5p,miR-224-5p,miR-346,miR-6525,miR-181c-5p,miR-378i,miR-217-5p,miR-378a-3p,miR-361-5p,miR-34a-5p,miR-148a-5p,miR-18a-3p,novel_17,miR-210-5p,miR-618,miR-345-3p,miR-422a,miR-181b-1-3p,miR-376a-5p,miR-143-3p,miR-136-5p,miR-2319b,miR-2483-3p,miR-378g,miR-3596,miR-4443,miR-101-3p,miR-3968,miR-490-5p,miR-885-5p,miR-363-3p,miR-2447,miR-361-3p,miR-1285-5p,miR-323b,miR-21b,novel_78,miR-1343-3p,miR-145b,miR-181b-2-3p,miR-1298-3p,miR-148b-5p,miR-455-3p,miR-182-5p,miR-16b,miR-323b-3p,miR-29b-1-5p,miR-378d,miR-8485,miR-2448-3p,miR-503-3p,miR-18a,miR-3154,miR-10b-3p,miR-7144-5p,miR-370-3p,miR-34c-5p,miR-2332,miR-370-5p,miR-138-5p,miR-374b-3p,miR-31-3p,miR-133a-3p,miR-3607-3p,miR-374a-3p,miR-378c,miR-134,miR-345-3p,miR-200b-3p,miR-105-2,miR-378h,miR-2411-5p,miR-545-5p,miR-200c,miR-1260a,miR-217,miR-339a,miR-30c-2-3p,miR-200c-3p,miR-181b-5p,miR-2898,miR-92a-3p,miR-107,miR-4286,miR-
MRC1	ENSOARG0(	mannose	miR-155-5p,miR-27b-3p,miR-544a,miR-22-3p,miR-544b,miR-412-5p,miR-302a-5p,miR-23b-3p,miR-23c,miR-5010-3p,miR-3600,miR-27a-3p,miR-199b-5p,miR-199a-5p,miR-148b-5p,miR-376a-5p,miR-23a-3p,miR-1983,miR-412-3p,miR-27b-5p,miR-378b,miR-23b,miR-488-3p,miR-199b-5p,miR-199a-5p,novel_25,miR-22-3p,miR-27a-3p,miR-544-3p,miR-655-5p,miR-216b-3p,miR-592,miR-155-5p,novel_87,miR-425-5p,miR-199b-5p,miR-154a,miR-6128
HPSE2	ENSOARG0(	heparana	miR-30b-3p,miR-129b-3p,miR-134,miR-2113,miR-744-5p,miR-6239,miR-4508,miR-30b-3p,miR-125a,miR-3600,miR-378b,miR-134-5p,miR-185-5p,miR-2404,miR-185-3p,miR-125b,miR-2331-5p,novel_19,miR-500a-5p,miR-125b-5p,miR-2433,miR-34c-3p,miR-4324,miR-134-5p,miR-125a-5p,miR-4492,miR-128-1-5p,miR-193a,miR-148b-5p
TLK2	ENSOARG0(	tousled-	miR-2411,miR-2411-5p,miR-2319b,miR-212-3p,miR-122-3p,miR-466i-5p,miR-2285l,miR-122-3p

STX3	ENSOARG0(	syntaxin	miR-495-3p,miR-2285m,miR-425-5p,let-7i,miR-28c,miR-4324,miR-1247-3p,miR-502b,miR-147-5p,miR-3120-3p,miR-655-5p,miR-320b,miR-411b-3p,miR-323a-5p,miR-2387,miR-148a-3p,miR-379-5p,miR-6395,let-7e-5p,miR-1291,miR-2331-5p,miR-377-3p,miR-224-5p,miR-125a-5p,miR-1343-5p,miR-187-3p,novel_116,let-7g-5p,miR-134-3p,miR-145a-5p,miR-22-3p,miR-197-3p,miR-542-3p,miR-744-5p,miR-27b-3p,miR-188-5p,miR-4508,miR-30b-3p,novel_42,miR-145-3p,miR-1197-3p,miR-125b-5p,miR-2433,miR-486b-3p,miR-125a,miR-2285aa,miR-2355-5p,miR-154b-5p,miR-671-5p,miR-876-3p,miR-4286,miR-181a-2-3p,miR-3064-5p,miR-1271-3p,miR-29a-5p,let-7g,miR-3065-3p,miR-2478,miR-28b,let-7c-5p,miR-30f,miR-8117,miR-152-3p,novel_96,let-7f-5p,miR-222,miR-505-5p,miR-1343-5p,let-7d,miR-3956-3p,miR-885-5p,miR-541-3p,novel_60,miR-2440,miR-101-3p,miR-2285n,miR-7134-3p,miR-490-5p,miR-3968,miR-499a-5p,miR-2285u,miR-3064-5p,miR-4792,miR-27a-3p,let-7d-5p,miR-499a-3p,miR-378g,miR-221,miR-361-3p,novel_94,miR-744-3p,miR-23a-3p,miR-382-3p,let-7a-5p,miR-1388-3p,miR-124a,miR-8485,let-7e,miR-491-5p,miR-3085-3p,miR-27a-3p,miR-145b,novel_4,miR-221-3p,miR-1298-3p,miR-30b-3p,miR-541,miR-484,miR-1839-5p,miR-3074-2-3p,miR-4510,miR-433-5p,let-7b,miR-6134,let-7b-5p,miR-224-5p,miR-320e,miR-222-3p,let-7f,miR-2285j,miR-664a,miR-487b-5p,miR-3065-3p,miR-221-5p,let-7i-5p,miR-22-3p,miR-6130,miR-323-5p,miR-499b-3p,miR-377-3p,miR-433-3p,miR-129b-3p,miR-21a-3p,miR-1827,miR-625-5p,miR-216c-5p,miR-486-3p,miR-2424,miR-125b-2-3p,miR-125b,miR-324-3p,novel_17,miR-21-3p,let-7k,miR-221-5p,miR-98-5p,miR-212-5p,miR-328-3p,novel_83,miR-148b-3p,miR-23c,miR-23b-3p,miR-188-3p,miR-5703,miR-140-3p,miR-124-3p,miR-1961,novel_133,miR-142-3p

FAM126A	ENSOARG0(	family w	miR-142a-5p,miR-181c-5p,miR-2330-3p,miR-214,miR-3958-5p,miR-155-5p,miR-450b-3p,miR-874-5p,miR-487a-5p,miR-4492,miR-424-5p,miR-30e-3p,miR-2285j,miR-222-3p,miR-664a-5p,miR-139-5p,miR-192-5p,miR-155-5p,miR-15b-5p,miR-204-3p,miR-548o-3p,miR-504-5p,miR-21-3p,miR-370-5p,miR-2312,miR-1827,novel_99,miR-450a-1-3p,miR-2285c,miR-3613-5p,miR-21a-3p,miR-129b-3p,miR-377-5p,miR-130a-5p,miR-376a-5p,miR-2432,miR-2424,miR-216c-5p,miR-20a,miR-760-3p,miR-106a,miR-299-3p,miR-21-3p,miR-2484,novel_17,miR-196a-3p,miR-92a-1-5p,miR-500a-5p,miR-15a-5p,miR-7859,miR-2284h-5p,miR-106a,miR-18a-3p,miR-548e-3p,miR-1b-3p,miR-380-5p,miR-3600,miR-33b-3p,miR-19b-3p,miR-16-1-3p,miR-1246,miR-215-5p,miR-1434-5p,miR-217-5p,miR-877-3p,miR-8095,miR-3956-3p,miR-885-5p,novel_60,miR-2447,miR-181d-5p,miR-2285f,miR-499a-5p,miR-490-5p,miR-371a-5p,miR-3082-5p,miR-25,miR-1185-2-3p,miR-27a-3p,miR-10b,miR-20a-3p,miR-139-5p,miR-3064-5p,miR-24-1-5p,miR-211-5p,miR-7977,miR-541-5p,miR-499b-5p,miR-504,miR-1983,miR-544-5p,miR-221,miR-2310,miR-424-5p,miR-1839-3p,miR-106a-5p,miR-376b,miR-3085-3p,miR-2285o,miR-214-3p,miR-106b-5p,miR-16b,miR-505,miR-2957,miR-147a,miR-142-5p,miR-1185-3p,miR-2285e,miR-221-3p,miR-27a-3p,miR-374a-3p,miR-365a-5p,miR-1306-5p,miR-3607-3p,miR-6128,miR-16-5p,miR-331-5p,miR-204-5p,miR-374b-3p,miR-31-3p,miR-2332,miR-370-5p,miR-135a-5p,miR-154b-5p,miR-141-3p,miR-215-5p,miR-2355-5p,miR-329b,miR-10b-3p,miR-23a-5p,miR-3074-1-3p,miR-20b-5p,miR-331-5p,miR-137-3p,miR-450b-5p,miR-24-2-5p,miR-490-3p,miR-192-3p,miR-29a-5p,miR-181a-2-3p,miR-3064-5p,miR-181b-5p,miR-200a-3p,novel_127,miR-125a-3p,miR-205-5p,miR-217,miR-24-2-5p,miR-322-5p,miR-450a-1-3p,miR-582-3p,miR-545-5p,miR-6517,miR-299a-3p,miR-222,miR-7641,miR-

GLYATL3	ENSOARG0(	glycine-	miR-3591-3p,miR-18a-3p,miR-150-5p,miR-2387,miR-135b-5p,miR-378f,miR-146b-5p,miR-335-3p,miR-136-5p,miR-7977,miR-3074-5p,miR-320c,miR-34b,miR-378g,miR-378e,miR-34c,miR-371a-5p,miR-4324,miR-450a-5p,miR-499a-5p,miR-17-3p,miR-885-5p,miR-3956-3p,novel_60,miR-3059-5p,miR-2428,miR-449a,miR-24-3p,miR-145a-5p,miR-378b,miR-6529a,miR-3120-5p,miR-2284x,miR-380-3p,miR-378c,miR-145b,miR-5010-3p,miR-105-1,miR-2300a-5p,miR-31-5p,miR-149-5p,miR-9788-3p,miR-539-5p,miR-3431,miR-134-3p,miR-185-5p,miR-147a,miR-197-5p,miR-105-5p,miR-146a,novel_103,miR-320b,miR-466f-3p,miR-146b,miR-3955-3p,miR-668-3p,miR-345-5p,miR-378d,miR-320a,miR-487b-5p,miR-129b-3p,miR-200a-5p,miR-331-5p,miR-543-3p,miR-625-5p,miR-345-5p,miR-345-5p,miR-552-3p,miR-215-5p,miR-21-3p,miR-574-5p,miR-378a-3p,miR-877-5p,miR-34c-5p,miR-135a-5p,miR-494-5p,miR-669a-3p,miR-874-5p,miR-208b-5p,miR-4492,miR-17-3p,miR-487b-5p,miR-331-5p,miR-6128,miR-3074-5p,miR-3184-3p,miR-378c,miR-539-5p,let-7e-3p,miR-378c,miR-122-5p,miR-1197-3p,miR-378a-3p,miR-188-3p,miR-215-5p,miR-1290,miR-2300b-3p,miR-378i,miR-34a-5p,miR-664-5p,miR-105-2,miR-4429,miR-18a-3p,novel_121,miR-378h,miR-758-5p,miR-422a,miR-185-3p,miR-30f,miR-125a-3p,miR-200c,novel_127,miR-1277-5p,miR-34b-5p,miR-466i-5p,miR-200c-3p,miR-127-5p,miR-320d,miR-450a-2-3p,miR-17-3p,miR-495-5p,miR-450c-5p,miR-146a-5p,miR-664b-3p
CCDC93	ENSOARG0(	coiled-c	miR-331-3p,miR-324-3p,miR-135a-5p,miR-4492,miR-134-5p,miR-486b-3p,miR-592,miR-539-5p,miR-3074-5p,miR-214,miR-145-3p,miR-3184-5p,miR-338-3p,let-7g-3p,miR-329a,miR-1895,miR-7144-5p,miR-671-5p,miR-582-5p,miR-574-5p,miR-2331-3p,miR-370-3p,miR-182-5p,miR-141-3p,miR-532-5p,miR-2284s,miR-582-5p,miR-3959-5p,miR-216b-5p,miR-193b-3p,miR-204-3p,miR-154b-5p,miR-877-5p,miR-205-5p,miR-324-3p,miR-21-3p,miR-200a-3p,miR-195a-3p,miR-3064-5p,miR-28b,miR-2478,miR-142a-3p,miR-181b-1-3p,miR-2432,miR-486-3p,miR-143-3p,novel_133,miR-134,miR-362-3p,miR-5703,miR-330-5p,miR-128-3p,miR-378d,miR-6517,miR-3600,miR-302a-5p,miR-134-5p,miR-2284z,novel_82,miR-4324,miR-2285n,miR-28c,miR-382-5p,miR-107-5p,miR-2447,novel_60,miR-3956-3p,miR-2285f,miR-193b-3p,miR-495-3p,miR-24-3p,miR-423-5p,miR-326-3p,miR-2285ab,miR-150-5p,miR-216a-3p,miR-135b-5p,miR-3064-5p,miR-3074-5p,miR-320b,miR-677,miR-203-3p,miR-6535,miR-181b-3p,miR-7-5p,miR-326,miR-362-3p,miR-182-5p,miR-3085-3p,miR-25-5p,miR-193a-3p,miR-214-3p,miR-103a-2-5p,miR-382,miR-628-5p,miR-432-3p,miR-654-3p,miR-873a-5p,miR-329-3p,miR-7b-5p,miR-1843b-5p,miR-125b-2-3p,miR-362-3p,miR-16-2-3p,miR-2284aa,miR-31-5p,novel_4,miR-2300a-5p,miR-409b,miR-181b-2-3p,miR-147a,miR-142-3p

VPS33B	ENSOARGO(	vacuolar	miR-2404,miR-320c,miR-144-3p,miR-216b-3p,miR-19a-3p,miR-107,miR-9851-3p,miR-195a-5p,miR-379-5p,miR-148a-3p,miR-18b,miR-3970,miR-3065-5p,miR-216a-3p,miR-3591-3p,miR-323c,novel_27,miR-2284w,miR-135a-2-3p,miR-92a-3p,novel_1,miR-656-5p,miR-199c,miR-147-5p,miR-4324,miR-15a,miR-6529b,miR-20a-5p,miR-16a,miR-425-5p,miR-149-5p,miR-103,miR-20b,miR-2355-3p,miR-31-5p,miR-103a-3p,miR-16b,miR-16-1-3p,miR-497-5p,miR-188-5p,miR-2284x,miR-19b-2-5p,miR-542-3p,miR-1843b-5p,miR-1434-3p,miR-197-3p,miR-3071-5p,miR-374b-3p,miR-2284l,let-7j,miR-130b-5p,miR-125a-5p,miR-32,miR-17-5p,miR-106a-5p,miR-25-3p,miR-3959-5p,miR-324-5p,miR-574-5p,miR-3068-3p,miR-2355-5p,miR-141-3p,miR-125a,miR-215-5p,miR-876-3p,miR-10a-3p,miR-20b-5p,miR-671-5p,miR-30e-5p,miR-503-3p,miR-331-5p,miR-93-5p,miR-320a,miR-18a,miR-17-5p,miR-93,miR-1306-5p,novel_42,miR-16-5p,miR-193a-5p,miR-331-5p,miR-486b-3p,miR-144,miR-6740-5p,miR-125b-5p,miR-100-3p,miR-322-5p,miR-9-5p,miR-450a-1-3p,miR-214-5p,miR-429-3p,miR-7641,miR-18b-5p,miR-376a-5p,miR-128-3p,miR-30c,miR-200b-3p,miR-155-5p,miR-3591-5p,miR-30a-5p,miR-92b-3p,miR-148b-5p,miR-450b-5p,miR-200a,miR-376c-5p,miR-1271-3p,miR-3529-3p,miR-30a-3p,miR-181a-2-3p,miR-450a-2-3p,miR-127-5p,miR-200c-3p,miR-107,miR-543-5p,miR-92a-3p,miR-152-3p,miR-200a-3p,miR-205-5p,miR-200c,miR-217,miR-20a-3p,miR-7977,miR-3963,miR-668-5p,miR-376a-3p,novel_94,miR-544-5p,miR-2447,miR-363-3p,miR-6527,miR-9-5p,miR-2284q,miR-30b-5p,novel_82,miR-142-3p,miR-199b-3p,miR-1343-3p,miR-2300a-5p,miR-548w,miR-1388-5p,miR-2426,miR-21b,miR-3120-5p,miR-484,miR-143-5p,miR-758-3p,miR-3187-3p,miR-758-3p,miR-127-3p,miR-106a-5p,miR-101b-3p,miR-494-3p,miR-424-5p,miR-33a-5p,miR-487b-5p,miR-199a-3p,miR-320b,miR-
CA5A	ENSOARGO(	carbonic	miR-320c,miR-412-5p,miR-3082-5p,miR-26b-3p,miR-1983,miR-9-3p,miR-1248,miR-412-3p,miR-4429,miR-1a-3p,miR-320a,miR-206-3p,miR-1,miR-425-5p,miR-22-5p,miR-124-5p,miR-320d,miR-206,miR-425-5p,miR-320e,miR-320b,miR-4324

C9orf131	ENSOARG0(	chromoso	miR-2447,miR-363-3p,miR-3956-3p,miR-361-3p,novel_82,miR-218-5p,miR-101-3p,miR-628-3p,miR-3968,miR-2319b,miR-25,miR-378g,miR-140-5p,miR-221,miR-381-3p,miR-299b-5p,miR-96-5p,miR-504,miR-376a-3p,miR-493-5p,miR-9-3p,miR-101b-3p,miR-23a-3p,miR-29b-1-5p,miR-16b,miR-1185-5p,miR-487b-5p,miR-199b-5p,miR-153-3p,miR-214-3p,miR-153,miR-376b-3p,miR-2284r,miR-758-3p,miR-101c,miR-21b,let-7b,miR-3184-3p,miR-3074-5p,miR-214,miR-6525,miR-122-5p,let-7f,let-7b-3p,miR-2285j,miR-151-3p,miR-664a-5p,miR-665,miR-4492,miR-129-5p,miR-32-5p,miR-30c-1-3p,miR-504-5p,miR-2284s,let-7i-5p,miR-15b-5p,miR-2285c,miR-129b-3p,miR-345-5p,miR-625-5p,miR-1827,miR-329a-5p,miR-376a-5p,miR-421-5p,miR-143-3p,miR-618,miR-2285p,miR-324-3p,miR-500a-5p,miR-98-5p,let-7k,miR-380-5p,miR-212-5p,miR-23b-3p,miR-23c,miR-1247-5p,miR-328-3p,miR-193b-5p,miR-1961,miR-218-1-3p,miR-16-1-3p,miR-361-5p,miR-758-5p,miR-2284w,miR-199b-5p,miR-4324,miR-421-5p,miR-3074-5p,miR-144-3p,miR-376b-5p,let-7e-5p,miR-493-5p,miR-432-3p,miR-105-5p,miR-378a-5p,miR-196b-5p,miR-483-3p,miR-31-5p,miR-103a-3p,miR-134-3p,miR-411-5p,novel_116,miR-19b-2-5p,miR-1843b-5p,miR-22-3p,novel_23,miR-16b,miR-16-1-3p,miR-497-5p,miR-16-5p,miR-1260b,miR-6740-5p,miR-125b-5p,miR-574-5p,miR-3068-3p,miR-125a,miR-25-3p,miR-3959-5p,miR-301,miR-23b,miR-376c-5p,miR-345-5p,miR-3064-5p,miR-92b-3p,let-7g,let-7f-5p,miR-429-3p,miR-322-5p,novel_121,miR-196a-5p,miR-1290,miR-330-5p,miR-155-5p,novel_60,miR-212-3p,miR-2285f,miR-452-5p,miR-362-5p,miR-3064-5p,miR-452-3p,miR-499b-5p,miR-7977,miR-3082-5p,let-7d-5p,miR-376d,miR-199b-5p,miR-129b-5p,novel_94,miR-877-3p,let-7a-5p,miR-424-5p,miR-30c-1-3p,miR-193a,miR-758-3p,miR-326,novel_103,miR-3085-3p,miR-

SNRPB2	ENSOARG0(	small nu	miR-151-3p,miR-19b-1-5p,miR-3604,miR-338-5p,let-7b-3p,miR-2285j,miR-874-5p,miR-424-5p,miR-454-3p,miR-425-5p,miR-214,miR-3074-2-3p,miR-590-3p,miR-539-5p,miR-129-2-3p,miR-338-3p,miR-6525,miR-98-3p,miR-129b-3p,miR-21a-3p,let-7f-1-3p,miR-543-3p,miR-582-5p,miR-1827,let-7g-3p,miR-133b-3p,miR-21-3p,miR-216b-3p,miR-296-3p,miR-496-3p,miR-548o-3p,miR-15b-5p,miR-301a-3p,miR-582-5p,miR-500a-5p,miR-92a-1-5p,miR-345-3p,miR-26a-5p,miR-34b-5p,miR-1277-5p,miR-30b-3p,miR-3653-3p,miR-2284m,miR-195a-3p,miR-2424,miR-2285i,miR-181b-1-3p,miR-98-3p,miR-188-3p,miR-1434-5p,miR-133a-3p,let-7a-3p,miR-34a-5p,miR-2284u,miR-23b-3p,miR-148b-3p,miR-15a-5p,miR-23c,miR-2440,miR-490-5p,miR-7134-3p,miR-2284q,miR-499a-5p,miR-335,miR-1296-5p,miR-2284j,miR-194b-5p,miR-3059-5p,miR-885-5p,miR-877-3p,miR-362-5p,miR-381-3p,novel_94,miR-147-3p,miR-499b-5p,miR-2285u,miR-211-5p,miR-7977,miR-2284g,miR-24-1-5p,miR-20a-3p,miR-144-5p,miR-203-3p,miR-27a-3p,miR-454-5p,miR-2483-3p,miR-16b,miR-214-3p,miR-491-5p,miR-130b-3p,let-7c-3p,miR-23a-3p,miR-1388-3p,miR-424-5p,miR-30b-3p,miR-6529a,miR-2284aa,miR-5010-3p,miR-27a-3p,miR-181b-2-3p,novel_78,miR-409b,miR-151a-3p,miR-548w,miR-500-5p,miR-331-3p,miR-2284k,miR-29b-3p,miR-1-5p,miR-5100,miR-204-5p,miR-592,miR-331-5p,miR-16-5p,miR-133a-3p,miR-144-5p,miR-28a-3p,miR-2284v,miR-365a-5p,miR-500b-5p,miR-362-5p,miR-23b,miR-381-3p,miR-331-5p,miR-503-3p,miR-7144-5p,miR-671-5p,miR-876-3p,miR-2355-5p,miR-141-3p,miR-503-3p,miR-223-5p,miR-324-5p,miR-2284n,miR-34c-5p,miR-365a-3p,miR-181c-3p,miR-125a-3p,miR-200a-3p,novel_96,miR-152-3p,miR-28-3p,miR-1a-2-5p,miR-200a,miR-2284y,miR-2284a,miR-24-2-5p,miR-148b-5p,miR-2427,miR-29d-3p,miR-345-3p,miR-3578,miR-1290,miR-496,miR-
HHAT	ENSOARG0(	hedgehog	miR-6130,miR-499b-3p,miR-377-3p,miR-598-3p,miR-21-3p,miR-3065-3p,miR-1277-3p,miR-30c-1-3p,miR-504-5p,novel_99,miR-2318,miR-655,miR-598-3p,miR-301,miR-6134,miR-214,miR-4510,miR-433-5p,miR-655-3p,miR-29b-2-5p,miR-29b-3p,miR-2285j,miR-214-5p,miR-483-3p,miR-376a-5p,miR-3960,miR-105-2,miR-218-1-3p,miR-140-3p,miR-5703,miR-29d-3p,miR-196a-5p,novel_133,miR-2424,miR-3065-3p,miR-2478,miR-369-3p,miR-29b-2-5p,miR-30b-3p,miR-127-5p,miR-500a-5p,miR-324-3p,miR-30c-2-3p,miR-29a-3p,miR-3596,miR-499a-3p,novel_94,miR-6516-3p,miR-376a-2-5p,miR-504,miR-3591-3p,miR-378b,novel_27,miR-371b-3p,miR-361-3p,miR-29c-3p,miR-3957-3p,miR-335-5p,miR-342-3p,miR-6123,miR-335,miR-29a,miR-4726-5p,miR-4324,miR-665,miR-1343-3p,miR-105-1,miR-31-5p,miR-7862,miR-29b,miR-3071-5p,miR-1434-3p,miR-1843b-5p,miR-2284r,miR-296-5p,miR-503-3p,miR-30c-1-3p,miR-432-3p,miR-214-3p,miR-483-3p,miR-196b-5p,miR-105-5p,miR-377-3p,miR-342

DNAJB5	ENSOARG0(	DnaJ (Hs	miR-181c-5p,miR-6525,miR-214,miR-4510,miR-129-2-3p,miR-145a-3p,miR-4492,miR-129-5p,miR-655-3p,miR-2285j,miR-22-3p,miR-204-3p,miR-877-5p,miR-27a-5p,miR-377-3p,miR-582-5p,miR-216b-5p,miR-6130,miR-296-3p,miR-552-3p,miR-504-5p,miR-543-3p,miR-582-5p,miR-2312,miR-655,miR-345-3p,miR-30b-3p,miR-21-3p,miR-320d,miR-466i-5p,miR-324-3p,miR-125b,miR-185-3p,miR-210-5p,miR-26a-5p,miR-671-3p,miR-378d,miR-4429,miR-1197-5p,miR-2366,miR-361-3p,miR-877-3p,miR-2285f,miR-181d-5p,miR-3059-5p,miR-2447,miR-2285t,miR-2285n,novel_82,miR-1185-2-3p,miR-25,miR-378g,miR-2285u,miR-4792,miR-24-1-5p,miR-3064-5p,miR-335-3p,miR-504,miR-378j,miR-361-3p,miR-199b-5p,miR-8485,miR-758-3p,miR-382-3p,miR-2330-5p,miR-199b-5p,miR-214-3p,miR-491-5p,miR-3085-3p,miR-3955-3p,miR-320b,miR-326,novel_103,miR-1306-5p,miR-1185-3p,miR-1298-3p,miR-1343-3p,novel_78,miR-758-3p,miR-331-3p,miR-125b-5p,miR-199a-5p,miR-125a,miR-574-5p,miR-7144-5p,miR-320a,miR-488-3p,miR-424-3p,novel_39,miR-200a,miR-199a-5p,miR-490-3p,miR-450b-5p,miR-24-2-5p,miR-3064-5p,novel_96,miR-200c-3p,miR-181b-5p,miR-200c,miR-129-1-3p,miR-429-3p,miR-6517,miR-582-3p,miR-2427,miR-330-5p,miR-326-3p,miR-199b-5p,miR-24-3p,novel_87,miR-320c,miR-26b-5p,miR-378b,miR-379-5p,miR-450b-3p,miR-1291,miR-3970,miR-1306,miR-2285ab,let-7j,miR-503-3p,miR-2285r,miR-432-3p,miR-125a-5p,novel_51,miR-7-5p,miR-377-3p,miR-134-3p,miR-26b-3p,miR-2284x,miR-22-3p,novel_23,miR-181a-5p,miR-7b-5p

RNF150	ENSOARG0(	ring fin	miR-1895,miR-18a-5p,miR-345-5p,miR-21-3p,miR-216b-3p,miR-221-5p,miR-140-5p,miR-204-3p,novel_25,miR-15b-5p,miR-30a-3p,miR-324-3p,miR-132-5p,miR-151-3p,miR-3604,miR-320e,miR-30e-3p,miR-424-5p,miR-130b-5p,miR-214,miR-665-5p,miR-3074-2-3p,miR-6525,miR-224-5p,miR-2904,miR-188-3p,miR-1197-5p,novel_133,miR-2366,miR-376a-5p,miR-378d,miR-4429,miR-30d-3p,miR-212-5p,miR-548e-3p,miR-532-3p,miR-15a-5p,miR-216a-5p,miR-500a-5p,miR-185-3p,miR-345-3p,miR-26a-5p,miR-221-5p,miR-3653-3p,miR-320d,miR-466i-5p,miR-2424,miR-2411,miR-143-3p,miR-376a-5p,miR-29b-1-5p,miR-125b-2-3p,miR-377-5p,miR-378j,miR-1983,miR-544-5p,novel_94,miR-541-5p,miR-7977,miR-211-5p,miR-664b,miR-24-1-5p,miR-452-3p,miR-2319b,miR-652-3p,miR-203-3p,miR-378g,miR-489,miR-1973,miR-3958-3p,miR-194b-5p,miR-9-5p,miR-452-5p,miR-3059-5p,miR-2447,miR-885-5p,miR-8095,miR-877-3p,novel_111,miR-1839-5p,miR-299a-5p,miR-2426,miR-151a-3p,miR-330-3p,miR-147a,miR-29b-1-5p,miR-16b,miR-320b,miR-1306-5p,novel_115,novel_103,miR-326,miR-214-3p,miR-676-3p,miR-424-5p,miR-2330-5p,miR-193a,miR-137-3p,miR-376c-5p,miR-18a,miR-3154,miR-320a,miR-376c-5p,miR-7144-5p,miR-23a-5p,miR-671-5p,novel_91,miR-345-5p,miR-370-3p,miR-2285aa,miR-1271,miR-3956,miR-574-5p,miR-154b-5p,miR-365a-3p,miR-331-3p,miR-3969,miR-135a-1-3p,miR-204-5p,miR-592,miR-16-5p,miR-6128,miR-365a-5p,miR-1260b,miR-1343-5p,miR-330-5p,miR-345-3p,miR-1290,miR-18b-5p,miR-582-3p,miR-24-2-5p,miR-9-5p,miR-322-5p,miR-483-3p,miR-2411-5p,miR-1260a,miR-664-3p,miR-6119-5p,miR-107,miR-450a-2-3p,miR-30a-3p,miR-4286,miR-181a-2-3p,miR-200a,miR-2285b,miR-24-2-5p,miR-1306,miR-376b-5p,miR-18b,miR-195a-5p,miR-107,miR-412-3p,miR-26b-5p,miR-320c,miR-16a,miR-15a,miR-6402,miR-576-3p,miR-421-5p,miR-

ADAM12	ENSOARG0(	ADAM met	novel_68,miR-2404,miR-29a-3p,novel_69,miR-144-3p,miR-<br>3591-3p,miR-450b-3p,miR-19a-3p,miR-146b-5p,miR-135b-<br>5p,miR-29c-3p,miR-17-3p,miR-211,miR-24-3p,miR-301b-<br>3p,miR-142b,novel_87,miR-206,novel_1,miR-29a,miR-<br>2284d,miR-149-5p,miR-544b,miR-653,miR-7b-5p,miR-<br>1285,novel_23,miR-138,miR-29b,miR-219a-1-3p,let-7f-2-<br>3p,let-7f-2-3p,miR-7-5p,miR-146a,miR-105-5p,miR-106b-<br>3p,miR-452-5p,miR-2285aa,miR-2355-5p,miR-1271,miR-365a-<br>3p,miR-2284n,miR-331-5p,miR-137-3p,miR-3154,miR-767,miR-<br>329b,miR-7144-5p,miR-28a-3p,miR-2284v,miR-3607-3p,miR-<br>145-3p,miR-29b-3p,miR-2284k,miR-1306-3p,miR-2332,miR-331-<br>3p,miR-144,miR-135a-5p,miR-138-5p,miR-767-5p,miR-331-<br>5p,miR-204-5p,miR-429-3p,miR-1224-5p,miR-1271-5p,miR-29d-<br>3p,miR-23b-5p,miR-501-3p,miR-582-3p,miR-105-2,miR-200b-<br>3p,miR-155-5p,miR-3529-3p,miR-1,miR-4286,miR-450b-5p,miR-<br>2284y,novel_39,miR-3065-3p,miR-2284a,miR-200c,miR-<br>217,miR-17-3p,miR-28-3p,miR-200c-3p,miR-127-5p,miR-<br>2284g,miR-139-5p,miR-4792,miR-20a-3p,miR-541-5p,miR-499b-<br>5p,miR-211-5p,miR-2483-3p,miR-3082-5p,miR-489,miR-1185-2-<br>3p,miR-500-3p,miR-4443,miR-1306-3p,miR-96-5p,miR-3059-<br>5p,miR-541-3p,miR-2284j,miR-877-3p,miR-502-3p,miR-<br>8095,miR-628-3p,miR-4726-5p,miR-490-5p,miR-409b,miR-105-<br>1,miR-222-5p,miR-216a-3p,miR-148b-5p,miR-421,miR-1185-<br>3p,miR-2459,miR-484,miR-541,miR-1a-3p,miR-501-3p,miR-<br>450b-5p,miR-544a,miR-21b,miR-3120-5p,miR-574-3p,miR-101b-<br>3p,miR-2284b,miR-193a,miR-2957,miR-146b,miR-2331-3p,miR-<br>3065-3p,miR-21-3p,miR-544-3p,miR-301a-3p,miR-139-<br>5p,novel_25,miR-155-5p,miR-21a-3p,miR-129b-3p,miR-543-<br>3p,miR-145a-3p,miR-1271,miR-665-5p,miR-206-3p,miR-<br>346,miR-2285j,miR-324-3p,miR-664a-5p,miR-<br>320e,novel_74,miR-155-5p,miR-17-3p,miR-129-5p,miR-454-
FMO3	ENSOARG0(	flavin c	miR-181a-5p,miR-4791,miR-301b-5p,miR-758-3p,novel_23,miR-<br>187-3p,novel_78,miR-330-3p,miR-339-5p,miR-3535,miR-<br>147a,miR-3431,novel_116,miR-221-3p,miR-2355-3p,miR-330-<br>3p,miR-487b-5p,miR-130b-5p,miR-6240,miR-494-3p,let-7c-<br>3p,miR-22841,miR-193a,miR-758-3p,miR-221,miR-129b-5p,miR-<br>2319a,novel_94,miR-329-5p,miR-452-3p,miR-30b-5p,miR-133a-<br>5p,miR-382-5p,miR-301a-5p,miR-335,miR-22-5p,miR-335-<br>5p,miR-181d-5p,miR-2284w,miR-30c-5p,novel_133,miR-30a-<br>5p,miR-155-5p,miR-30c,miR-222,miR-6517,novel_79,miR-582-<br>3p,miR-30b,miR-205-5p,miR-8117,miR-30f,miR-339a,miR-181b-<br>5p,miR-30d,miR-21-3p,miR-369-3p,miR-181a-2-3p,miR-21a-<br>3p,miR-30e-5p,miR-424-3p,miR-30d-5p,miR-339b,miR-<br>30f,novel_99,miR-582-5p,miR-27a-5p,miR-365a-3p,miR-155-<br>5p,miR-222-3p,miR-338-5p,miR-487b-5p,novel_9,miR-155-<br>5p,miR-410-3p,miR-130b-5p,miR-1306-5p,miR-145a-3p,miR-<br>193a-5p,miR-1197-3p,miR-181c-5p

LTA4H	ENSOARG0(	leukotri	miR-2284r,miR-541,miR-1388-5p,miR-199b-3p,miR-330-3p,novel_78,miR-181b-2-3p,miR-539-5p,miR-148b-5p,miR-455-3p,miR-1185-3p,miR-199a-3p,miR-16b,miR-182-5p,miR-103a-2-5p,miR-214-3p,miR-1388-3p,miR-378d,miR-140-5p,miR-221,miR-381-3p,miR-136-5p,miR-376a-3p,miR-9-3p,miR-2319b,miR-378g,miR-1185-2-3p,miR-500-3p,miR-3968,miR-490-5p,miR-6527,miR-361-3p,novel_111,miR-378i,miR-2366,miR-378a-3p,miR-217-5p,miR-142-3p,miR-33b-3p,miR-16-1-3p,miR-148a-5p,miR-380-5p,miR-328-3p,miR-618,novel_17,miR-210-5p,miR-500a-5p,miR-345-3p,miR-92a-1-5p,miR-422a,miR-202-5p,miR-487a,miR-376a-5p,miR-181b-1-3p,miR-2424,miR-486-3p,miR-143-3p,miR-450a-1-3p,miR-433-3p,miR-345-5p,miR-543-3p,miR-186-5p,miR-1827,miR-30c-1-3p,miR-548o-3p,miR-154a-3p,miR-296-3p,miR-2284s,miR-10a,miR-216b-5p,miR-15b-5p,let-7b-3p,miR-2285j,miR-665,novel_74,miR-451a,miR-874-5p,miR-129-5p,miR-3074-5p,miR-3184-3p,miR-214,miR-224-5p,miR-181c-5p,miR-432,miR-7b-5p,miR-22-3p,miR-450c-3p,miR-16b,miR-16-1-3p,miR-497-5p,miR-26a-2-3p,miR-27b-3p,miR-31-5p,miR-412-5p,miR-134-3p,miR-194-3p,miR-149-5p,miR-7-5p,miR-296-3p,miR-2411-3p,miR-194a,miR-378a-5p,miR-6240,let-7f-2-3p,let-7f-2-3p,miR-432-3p,miR-432-3p,miR-503-3p,miR-376b-5p,miR-148a-3p,miR-1306,miR-216a-3p,miR-2319a,miR-378f,miR-216b-3p,miR-3074-5p,miR-3120-3p,miR-677,miR-6535,miR-320c,miR-144-3p,miR-181b-3p,miR-425-5p,novel_87,miR-29a,miR-379-3p,miR-625-3p,miR-1a-1-5p,miR-29c-3p,miR-758-5p,miR-2284w,miR-23b-5p,miR-330-5p,miR-2285ad,miR-429-3p,miR-412,miR-322-5p,miR-450a-1-3p,miR-1a-2-5p,miR-152-3p,miR-200a-3p,miR-148b-5p,novel_39,miR-29a-5p,miR-450c-5p,miR-301,miR-137-3p,miR-376c-5p,miR-767,miR-2318,miR-345-5p,miR-1248,miR-874-3p,miR-2355-5p,miR-3956,miR-3959-5p,miR-223-5p,miR-
OTOR	ENSOARG0(	otorapli	miR-101-5p,miR-345-3p,miR-125b-2-3p,miR-2285af,miR-7-5p,miR-200c,miR-17-3p,miR-196b-5p,miR-200c-3p,miR-1b-3p,miR-412,miR-3600,miR-145b,miR-429-3p,miR-200b,miR-412-3p,miR-7857,miR-145a-5p,miR-196a-5p,miR-7b-5p,miR-200b-3p,miR-188-5p,miR-17-3p,miR-2447,miR-211,miR-665,miR-135a-1-3p,miR-204-5p,miR-17-3p,miR-21-3p,miR-211-5p,miR-204-3p,miR-2284s,miR-3591-3p,miR-301,miR-1248

CRK	ENSOARG0(	v-crk av	miR-2285af,miR-6119-5p,miR-450a-2-3p,miR-181b-5p,miR-200c-3p,miR-29b-2-5p,miR-181a-2-3p,miR-345-3p,miR-3591-5p,miR-128-3p,miR-105-2,miR-200b-3p,miR-300,miR-429-3p,miR-2284z,miR-6740-5p,miR-2284k,miR-31-3p,miR-6128,miR-144-5p,miR-133a-3p,miR-1306-5p,miR-2284v,miR-93,miR-93-5p,miR-17-5p,miR-381-3p,miR-20b-5p,miR-2284n,miR-3965,miR-330-3p,miR-105-5p,miR-2411-3p,miR-106a-5p,miR-17-5p,miR-7-5p,miR-377-3p,miR-363-5p,miR-6240,miR-493-5p,miR-432-3p,miR-145a-5p,miR-708-3p,miR-181a-5p,miR-7b-5p,miR-2284ab,miR-380-3p,miR-31-5p,novel_116,miR-544b,miR-20b,miR-2284d,miR-382-5p,miR-4324,miR-142b,miR-20a-5p,miR-421-5p,miR-542-5p,novel_101,miR-216a-3p,miR-450b-3p,miR-320b,miR-2404,miR-345-3p,miR-196a-3p,miR-542-5p,miR-2484,miR-106a,miR-2284m,miR-20a,miR-345-3p,miR-216c-5p,miR-183-5p,miR-380-3p,miR-142a-3p,miR-140-3p,miR-142-3p,miR-33b-3p,miR-106a,miR-1247-5p,miR-200b,miR-151-3p,miR-29b-2-5p,miR-665,miR-2285j,miR-181c-5p,miR-6525,miR-433-3p,miR-133b-3p,miR-204-3p,miR-377-3p,miR-2284s,miR-544-3p,miR-499b-3p,miR-323b-3p,miR-505,miR-106b-5p,miR-542-5p,miR-652-5p,miR-1388-3p,miR-106a-5p,miR-8485,miR-3187-3p,miR-7857,miR-323b,miR-2284aa,miR-544a,miR-145b,novel_4,miR-151a-3p,miR-330-3p,novel_78,miR-105-1,miR-142-3p,miR-2285n,miR-2440,miR-3968,miR-181d-5p,miR-2284j,miR-452-5p,miR-2447,miR-212-3p,miR-493-5p,miR-9-3p,miR-668-5p,miR-144-5p,miR-454-5p,miR-499a-3p,miR-489

ELMOD2	ENSOARG0(	ELMO/CED	miR-767,miR-2318,miR-345-5p,miR-30e-5p,miR-331-5p,miR-376c-3p,miR-574-5p,miR-874-3p,miR-3068-3p,miR-1271,miR-3432a,miR-592,miR-100-3p,miR-30c,miR-3591-5p,miR-1290,miR-2427,miR-505-5p,miR-129-1-3p,miR-429-3p,novel_79,miR-127-5p,miR-200a-3p,miR-152-3p,novel_127,miR-125a-3p,miR-664-3p,miR-450b-5p,miR-148b-5p,miR-29a-5p,novel_39,miR-148a-3p,miR-1306,miR-677,miR-669,miR-181b-3p,miR-29a,miR-421-5p,miR-6123,novel_87,miR-374c-5p,miR-1839-3p,miR-29c-3p,miR-16-1-3p,miR-26a-2-3p,miR-432,miR-19b-2-5p,miR-542-3p,miR-197-3p,miR-412-5p,miR-3535,miR-411-5p,novel_116,miR-194-3p,miR-31-5p,miR-339-5p,miR-296-3p,miR-377-3p,miR-2285v,miR-380-5p,miR-493-5p,miR-668-3p,miR-628-5p,miR-345-5p,miR-339b,miR-186-5p,miR-129b-3p,miR-500a-3p,miR-655,miR-30d-5p,miR-377-3p,miR-376e-3p,miR-582-5p,miR-499b-3p,miR-30c-1-3p,miR-2331-3p,miR-451a,miR-1468-5p,miR-500,miR-129-5p,miR-130b-5p,miR-15b-3p,miR-151-3p,miR-122-5p,miR-129-2-3p,miR-214,miR-16-1-3p,miR-148a-5p,miR-30c-5p,miR-210-3p,miR-2284h-5p,miR-1247-5p,miR-328-3p,miR-18a-3p,miR-212-5p,miR-30b,miR-210-5p,novel_17,miR-345-3p,miR-421-5p,miR-376a-5p,miR-181b-1-3p,miR-376c-3p,miR-143-3p,miR-2285w,miR-369-3p,miR-195a-3p,miR-335-3p,miR-493-5p,miR-409-5p,miR-1306-3p,miR-378j,miR-499a-3p,miR-203-3p,miR-500-3p,miR-126b-5p,miR-3968,novel_82,miR-218-5p,miR-877-3p,miR-2447,miR-3956-3p,miR-501-3p,miR-758-3p,miR-153,miR-147a,miR-455-3p,miR-148b-5p,miR-2459,miR-126a-5p,novel_78,miR-1343-3p,miR-181b-2-3p,miR-1185-5p,miR-153-3p,miR-214-3p,miR-505,miR-124a,miR-10b-3p,miR-7144-5p,miR-671-5p,miR-488-3p,miR-381-3p,miR-2448-3p,miR-362-5p,miR-3154,miR-141-3p,miR-615-5p,miR-138-5p,miR-767-5p,miR-28-5p,miR-410-5p,miR-29b-3p,miR-370-5p,miR-1306-

RHAG	ENSOARG0(	Rh-assoc	miR-30a-3p,miR-1,miR-29b-2-5p,miR-3529-3p,miR-490-3p,miR-30f,miR-152-3p,miR-2898,miR-18b-5p,miR-299a-3p,novel_121,miR-30a-5p,miR-10a-5p,miR-300,miR-6128,miR-3607-3p,miR-93,miR-144,miR-2285aa,miR-574-5p,miR-376c-3p,miR-365a-3p,miR-93-5p,miR-3154,miR-18a,miR-17-5p,miR-20b-5p,miR-1814c,miR-1248,miR-382,miR-493-5p,miR-432-3p,miR-190a,miR-106a-5p,miR-17-5p,miR-377-3p,miR-466f-3p,miR-31-5p,miR-3535,miR-1285,miR-1434-3p,miR-16-1-3p,miR-495-3p,miR-24-3p,miR-382-5p,miR-33a-3p,miR-20a-5p,miR-576-3p,miR-203b-5p,miR-147-5p,miR-206,miR-10a-5p,miR-144-3p,miR-677,miR-181b-3p,miR-299b-3p,miR-1306,miR-148a-3p,miR-18b,miR-412-3p,miR-19a-3p,miR-2285w,miR-29b-1-5p,miR-376c-3p,miR-181b-1-3p,miR-299-3p,miR-106a,miR-202-5p,miR-466i-5p,miR-30d-3p,miR-106a,miR-2284h-5p,miR-532-3p,miR-148b-3p,miR-124-3p,miR-5703,miR-140-3p,novel_133,miR-16-1-3p,miR-361-5p,miR-19b-3p,miR-539-5p,miR-10b-5p,miR-346,miR-206-3p,miR-30a-3p,miR-29b-2-5p,miR-30e-3p,miR-190a-5p,miR-500,novel_74,miR-548o-3p,novel_32,miR-377-3p,miR-10a,miR-376e-3p,miR-500a-3p,miR-30d-5p,miR-18a-5p,miR-124a,miR-106a-5p,miR-2310,miR-29b-1-5p,miR-106b-5p,miR-1306-5p,miR-181b-2-3p,novel_78,miR-2300a-5p,miR-2459,miR-421,miR-147a,miR-1a-3p,miR-301b-5p,miR-4791,miR-10b,miR-101c,miR-101a-5p,miR-450b-5p,miR-1296-5p,miR-452-5p,miR-190b-5p,miR-212-3p,miR-361-3p,miR-301a-5p,miR-101-3p,miR-2284q,miR-499b-5p,miR-4792,miR-10b,miR-493-5p,miR-1983,miR-544-5p
PLEKHA4	ENSOARG0(	pleckstr	miR-2284n,miR-323a-5p,miR-425-3p,miR-2284d,miR-323-5p,miR-370-5p,novel_78,miR-133a-3p,miR-485-5p,miR-2284u,miR-301,miR-3578,miR-2284y,miR-450c-5p,miR-2284a,miR-2284j,miR-1193,miR-2403,miR-2284b,miR-4286,miR-2284v,miR-450a-5p,miR-371a-5p,miR-2284k,miR-551b-3p,miR-370-5p
ARHGEF19	ENSOARG0(	Rho guan	miR-224-5p,miR-539-5p,miR-4510,miR-4492,miR-331-3p,miR-324-3p,miR-26c,miR-29b-3p,miR-204-3p,miR-3957,miR-499b-3p,miR-2284s,miR-216b-5p,miR-6130,miR-3065-3p,miR-2355-5p,miR-2331-3p,miR-3957-5p,miR-30c-1-3p,miR-3154,miR-100-5p,miR-3065-3p,miR-345-3p,miR-2898,miR-30c-2-3p,miR-345-3p,miR-185-3p,miR-210-5p,miR-99a,miR-328-3p,miR-1224-5p,miR-99a-5p,miR-29d-3p,miR-5703,miR-345-3p,novel_133,miR-361-3p,miR-24-3p,miR-29c-3p,miR-342-3p,miR-2447,miR-335-5p,miR-4726-5p,miR-29a,miR-335,miR-425-5p,miR-6402,miR-6535,miR-499a-3p,miR-29a-3p,miR-7-1-3p,miR-7977,miR-3120-3p,miR-4792,miR-20a-3p,miR-6395,miR-3963,miR-99b-5p,miR-378j,miR-30c-1-3p,miR-363-5p,miR-33a-5p,miR-2411-3p,miR-99a-5p,miR-342,miR-224-5p,miR-149-5p,miR-9788-3p,novel_116,miR-1298-3p,miR-133b-5p,miR-4508,miR-7862,miR-744-5p,miR-29b,miR-296-5p,miR-484

GOLPH3	ENSOARG0(	golgi ph	miR-378i,miR-378a-3p,miR-128-3p,miR-200b-3p,miR-2285ad,miR-429-3p,miR-505-3p,miR-9-5p,miR-378h,miR-200b,miR-545-5p,miR-151-5p,miR-2284h-5p,miR-196a-3p,miR-2285af,miR-618,miR-200c,miR-6119-5p,miR-422a,miR-2284m,miR-200c-3p,miR-1277-5p,miR-543-5p,miR-21-3p,miR-200a-3p,miR-183-5p,miR-2285w,miR-369-3p,miR-3064-5p,miR-195a-3p,miR-4286,miR-3065-3p,miR-2285b,miR-192-3p,miR-21a-3p,novel_99,miR-378a-3p,miR-660-5p,miR-548o-3p,miR-3065-3p,miR-2285aa,miR-141-3p,miR-22-3p,miR-151b,miR-154b-5p,miR-2284n,miR-2284k,miR-135a-1-3p,miR-3604,miR-409-3p,miR-378c,miR-6128,miR-3184-5p,miR-378c,miR-378b,miR-1434-3p,miR-1843b-5p,miR-197-3p,miR-22-3p,novel_23,miR-30b-3p,miR-758-3p,miR-30b-3p,miR-378c,miR-219a-1-3p,miR-16-2-3p,miR-3120-5p,miR-330-3p,miR-133b-5p,miR-2284d,miR-412-5p,miR-9788-3p,miR-421,miR-1185-3p,miR-330-3p,miR-3085-3p,miR-2285l,miR-660,miR-466f-3p,miR-6240,let-7c-3p,miR-33a-5p,let-7f-2-3p,let-7f-2-3p,miR-378d,miR-432-3p,miR-758-3p,miR-2387,miR-216a-3p,miR-2319a,miR-9851-3p,novel_94,miR-378f,miR-505-3p,miR-3064-5p,miR-655-5p,miR-2285u,miR-126b-5p,miR-3120-3p,miR-378e,miR-2483-3p,novel_69,miR-3082-5p,miR-6535,miR-1185-2-3p,miR-576-3p,miR-371a-5p,miR-3968,miR-3958-3p,miR-628-3p,miR-147-5p,miR-219b-3p,miR-495-3p,miR-2284j,miR-9-5p,miR-2285x,miR-877-3p,miR-423-5p,miR-7705
DDX27	ENSOARG0(	DEAD (As	let-7a-3p,miR-34a-5p,miR-5703,miR-345-3p,miR-1290,miR-450a-1-3p,miR-1b-3p,miR-212-5p,miR-34b-5p,miR-6536,miR-216a-5p,miR-345-3p,novel_39,miR-199a-5p,miR-486-3p,miR-1248,miR-2312,miR-3154,miR-424-3p,miR-450a-1-3p,miR-2285c,miR-22-3p,miR-199a-5p,miR-34c-5p,miR-323-5p,miR-182-5p,miR-208b-5p,miR-486b-3p,miR-450b-3p,miR-151-3p,miR-331-3p,let-7b-3p,miR-122-5p,miR-665-5p,miR-433-5p,miR-145a-3p,miR-30b-3p,miR-3432b,miR-219a-1-3p,miR-197-3p,miR-450c-3p,miR-22-3p,miR-30b-3p,miR-6529a,miR-9788-3p,miR-2285e,miR-496-5p,miR-2285g,miR-147a,miR-2300a-5p,miR-151a-3p,miR-409b,miR-199b-5p,miR-3955-3p,miR-182-5p,miR-2411-3p,novel_103,miR-432-3p,miR-432-3p,miR-101b-3p,miR-136-5p,miR-381-3p,miR-2285ab,miR-199b-5p,miR-323a-5p,miR-3082-5p,miR-34b,miR-670-3p,miR-6123,miR-628-3p,miR-499a-5p,miR-34c,miR-101-3p,miR-449a,miR-199b-5p,miR-2284w,miR-2428,miR-361-3p,miR-2285f

MBD3	ENSOARG0(	methyl-C	miR-501-3p,miR-345-3p,miR-378h,miR-2411-5p,miR-1271-5p,miR-339a,miR-1260a,miR-6119-5p,miR-543-5p,miR-450a-2-3p,miR-2898,miR-191-3p,miR-181a-2-3p,miR-3065-3p,miR-1957a,miR-2284a,miR-490-3p,miR-1271-3p,miR-28b,miR-671-5p,miR-615-5p,miR-3957-5p,miR-154b-5p,miR-3957,miR-34c-5p,miR-331-3p,miR-28-5p,miR-486b-3p,miR-138-5p,miR-1306-5p,miR-378c,miR-873a-5p,miR-1434-3p,miR-7862,miR-455-5p,miR-30b-3p,miR-138,miR-1260b,miR-219a-1-3p,miR-377-5p,miR-133b-5p,miR-2355-3p,miR-503-5p,miR-455-5p,miR-224-5p,miR-503-5p,miR-1343-5p,miR-125a-5p,miR-124-5p,miR-22851,miR-363-5p,miR-654-3p,miR-7975,miR-9851-3p,miR-505-3p,miR-6395,miR-1291,miR-2404,miR-323a-5p,miR-146b-3p,miR-34b,miR-34c,miR-382-5p,miR-33a-3p,miR-6402,miR-22-5p,miR-502b,miR-199c,novel_101,miR-342-3p,novel_120,miR-326-3p,miR-449a,miR-2428,miR-371b-3p,miR-24-3p,miR-2904,miR-188-3p,miR-1197-5p,miR-1193,miR-140-3p,miR-362-3p,miR-2408,miR-2300b-3p,miR-941,miR-3600,miR-505-3p,miR-532-3p,novel_83,miR-383-5p,miR-185-3p,miR-125b,miR-196a-3p,miR-221-5p,miR-34b-5p,miR-299-3p,miR-466i-5p,miR-216c-5p,miR-105-3p,miR-2411,miR-125b-2-3p,miR-21a-3p,miR-3065-3p,miR-221-5p,miR-378a-3p,miR-204-3p,miR-22-3p,miR-877-5p,miR-323-5p,miR-193b-3p,miR-6130,miR-139-5p,miR-320e,miR-708-5p,miR-487a-5p,miR-664a,miR-1271,miR-3074-2-3p,miR-539-5p,miR-378c,miR-145a-3p,miR-4510,miR-338-3p,miR-6134,miR-30b-3p,miR-125b-2-3p,miR-378b,miR-296-5p,miR-4791,miR-378c,miR-222-5p,miR-409b,miR-2300a-5p,miR-9788-3p,miR-362-3p,miR-2957,miR-342,miR-326,miR-3085-3p,miR-376b,miR-2330-5p,miR-494-3p,miR-4532,miR-3187-3p,miR-129b-5p,miR-147-3p,miR-7977,miR-3064-5p,miR-10b,miR-7857-3p,miR-378e,miR-3082-5p,miR-489,miR-2285p,miR-1296-5p,miR-3059-5p,miR-541-3p,miR-502-3p,miR-
CEP78	ENSOARG0(	centroso	miR-1260a,miR-200c,miR-210-5p,miR-202-5p,miR-200c-3p,novel_127,miR-195a-3p,miR-30a-3p,miR-181a-2-3p,miR-1957a,miR-29a-5p,miR-3065-3p,miR-490-3p,miR-23b-5p,miR-582-3p,miR-19b-3p,miR-105-2,miR-200b-3p,miR-18a-3p,miR-429-3p,miR-30d-3p,miR-200b,miR-222-5p,miR-532-3p,miR-486-5p,miR-30e-3p,miR-30a-3p,miR-135a-5p,miR-129-5p,miR-874-5p,miR-130b-5p,miR-2330-3p,miR-1260b,miR-346,miR-21a-3p,miR-137-3p,miR-376c-5p,let-7g-3p,miR-376c-5p,miR-140-5p,miR-21-3p,miR-3065-3p,miR-296-3p,miR-105-5p,miR-378a-5p,miR-130b-5p,miR-493-5p,miR-3187-3p,miR-296-3p,miR-1285,novel_23,miR-1260b,miR-2284x,miR-31-5p,miR-199b-3p,miR-105-1,miR-665,miR-5010-3p,miR-222-5p,miR-411-5p,miR-6402,miR-199c,miR-3059-5p,novel_60,miR-3956-3p,miR-6527,miR-376b-5p,miR-140-5p,miR-3591-3p,miR-18a-3p,miR-19a-3p,miR-335-3p,miR-493-5p,miR-135b-5p,miR-670-3p

ADAM19	ENSOARG0(	ADAM met	miR-3959-5p,miR-300-3p,miR-133c,miR-125a,miR-370-3p,miR-329b,miR-345-5p,miR-10b-3p,miR-20b-5p,miR-23a-5p,miR-30e-5p,miR-17-5p,miR-376c-5p,miR-3154,miR-320a,miR-93-5p,miR-93,miR-16-5p,miR-28-5p,miR-370-5p,miR-29b-3p,miR-3969,miR-135a-5p,miR-6740-5p,miR-331-3p,miR-125b-5p,miR-483-3p,miR-6516-5p,miR-322-5p,novel_121,miR-545-5p,miR-2285ad,miR-431,miR-1224-5p,let-7d,miR-330-5p,miR-29d-3p,miR-92b-3p,miR-28b,miR-1271-3p,novel_39,miR-200a,let-7g,miR-3065-3p,miR-181a-2-3p,let-7f-5p,miR-450a-2-3p,miR-92a-3p,miR-543-5p,novel_96,let-7c-5p,miR-30c-2-3p,miR-29a-3p,miR-320c,miR-329-5p,miR-3120-3p,miR-1291,miR-195a-5p,let-7e-5p,miR-135b-5p,miR-146b-5p,miR-2387,miR-1306,miR-371b-3p,miR-24-3p,miR-2284w,miR-326-3p,miR-335-5p,miR-342-3p,miR-29c-3p,novel_101,miR-495-3p,miR-1193,miR-3957-3p,miR-29a,miR-92a-3p,miR-199c,miR-147-5p,miR-656-5p,miR-28c,miR-20a-5p,miR-15a,miR-3613,let-7i,miR-16a,miR-185-5p,miR-134-3p,let-7g-5p,miR-20b,miR-3955-5p,miR-16b,miR-29b,miR-30b-3p,miR-188-5p,miR-27b-3p,miR-497-5p,miR-1843b-5p,miR-7b-5p,miR-145a-5p,miR-432-3p,miR-432-3p,miR-345-5p,miR-296-3p,miR-6412,miR-6240,miR-493-5p,miR-130b-5p,miR-363-5p,miR-483-3p,miR-125a-5p,miR-660,miR-296-3p,miR-146a,miR-7-5p,miR-224-5p,miR-106a-5p,novel_51,miR-17-5p,miR-197-5p,miR-32,miR-330-3p,miR-216b-5p,miR-499b-3p,miR-6516,miR-27a-5p,miR-15b-5p,let-7i-5p,miR-204-3p,miR-660-5p,miR-30c-1-3p,miR-504-5p,miR-32-5p,miR-296-3p,miR-370-5p,miR-3065-3p,miR-345-5p,miR-625-5p,miR-3613-5p,miR-224-5p,miR-6525,miR-6134,miR-338-3p,let-7b-5p,miR-539-5p,let-7b,miR-130b-5p,miR-708-5p,miR-424-5p,miR-2285j,let-7f,miR-320e,miR-665,miR-15a-5p,novel_83,miR-532-3p,miR-2284h-5p,miR-106a,miR-33b-3p,miR-4429,miR-493-3p,miR-378d,miR-148a-

GSDMA	ENSOARG0(	gasdermi	miR-450c-3p,miR-19b-2-5p,miR-432,miR-7b-5p,miR-497-5p,miR-188-5p,miR-16b,miR-16-1-3p,miR-340-3p,miR-31-5p,miR-149-5p,miR-412-5p,miR-134-3p,miR-3431,miR-7-5p,miR-377-3p,miR-146a,miR-378a-5p,let-7f-2-3p,miR-628-5p,let-7f-2-3p,miR-432-3p,miR-432-3p,miR-1306,miR-3591-3p,miR-146b-5p,let-7e-5p,miR-2284e,novel_68,miR-677,miR-181b-3p,miR-374c-3p,miR-485-3p,miR-142b,miR-421-5p,miR-1a-1-5p,miR-199b-5p,miR-1343-5p,miR-330-5p,miR-412,miR-2285ad,miR-450a-1-3p,novel_121,miR-486-5p,miR-322-5p,miR-3071-3p,miR-205-5p,miR-1a-2-5p,let-7f-5p,miR-29a-5p,let-7g,miR-200a,miR-23b,miR-876-3p,miR-345-5p,miR-2285aa,miR-542-5p,miR-223-5p,miR-2433,novel_63,miR-193a-5p,miR-16-5p,miR-541,miR-1839-5p,miR-1388-5p,miR-181b-2-3p,miR-145b,miR-1343-3p,miR-1185-3p,miR-126a-5p,miR-539-5p,miR-376b-3p,miR-1298-3p,miR-182-5p,miR-16b,miR-199b-5p,miR-146b,miR-103a-2-5p,miR-1185-5p,miR-23a-3p,miR-335-3p,miR-9-3p,miR-3963,miR-504,miR-139-5p,miR-203-3p,miR-1185-2-3p,miR-499a-3p,miR-378g,miR-218-5p,miR-3968,miR-2447,miR-7705,miR-361-3p,miR-877-3p,novel_111,miR-217-5p,miR-1961,miR-193b-5p,miR-16-1-3p,miR-361-5p,miR-493-3p,miR-30d-3p,miR-1247-5p,miR-2284h-5p,miR-23b-3p,miR-23c,miR-219b-5p,miR-92a-1-5p,miR-618,miR-3653-3p,miR-2484,let-7k,miR-98-5p,miR-2285w,miR-143-3p,miR-421-5p,miR-376a-5p,miR-450a-1-3p,miR-186-5p,miR-345-5p,miR-296-3p,miR-504-5p,miR-2331-3p,miR-30c-1-3p,miR-548o-3p,let-7i-5p,miR-15b-5p,miR-499b-3p,miR-377-3p,miR-582-5p,miR-30a-3p,let-7f,miR-15b-3p,miR-30e-3p,miR-129-5p,miR-4492,miR-874-5p,novel_74,let-7b,miR-6525,miR-374b-3p,miR-145a-5p,miR-30b-3p,miR-219a-1-3p,miR-133b-5p,miR-2355-3p,miR-544b,miR-432-5p,let-7g-5p,miR-1343-5p,miR-124-5p,miR-22851,miR-363-5p,miR-345-5p,miR-376b-3p,miR-378b,miR-

SLC4A4	ENSOARG0(	solute c	miR-885-5p,miR-194b-5p,miR-2284j,miR-181d-5p,novel_111,miR-301a-5p,miR-628-3p,miR-374a-3p,miR-2284q,miR-664b,miR-20a-3p,miR-374b-5p,miR-3082-5p,let-7d-5p,miR-140-5p,miR-378j,miR-96-5p,miR-493-5p,let-7a-5p,miR-1388-3p,miR-424-5p,miR-23a-3p,miR-33a-5p,miR-8485,miR-2957,miR-16b,miR-146b,miR-153-3p,let-7e,miR-548w,miR-153,miR-142-3p,miR-147a,miR-376b-3p,miR-1298-3p,miR-148b-5p,miR-421,miR-484,miR-301b-5p,miR-1388-5p,miR-101a-5p,miR-1839-5p,miR-2284aa,miR-3120-5p,let-7b,miR-3074-5p,miR-3958-5p,miR-1271,let-7b-5p,let-7f,miR-2285j,miR-655-3p,miR-29b-2-5p,miR-3604,novel_74,miR-874-5p,miR-424-5p,miR-130b-5p,miR-140-5p,miR-221-5p,miR-548o-3p,miR-582-5p,miR-216b-5p,novel_25,let-7i-5p,miR-15b-5p,miR-433-3p,miR-655,miR-345-5p,miR-18a-5p,miR-582-5p,miR-186-5p,miR-2285w,miR-369-3p,miR-376a-5p,miR-664b-3p,miR-142a-3p,miR-146a-5p,miR-374a-5p,miR-216a-5p,miR-2284m,miR-466i-5p,miR-98-5p,miR-221-5p,let-7k,miR-548e-3p,miR-7859,miR-487a-3p,miR-23b-3p,miR-383-5p,miR-23c,miR-200b,miR-15a-5p,miR-376a-5p,miR-1961,miR-217-5p,miR-218-1-3p,miR-361-5p,miR-148a-5p,novel_101,miR-323c,miR-15a,miR-142b,miR-16a,let-7i,novel_1,miR-3074-5p,miR-7-1-3p,miR-18b,miR-3065-5p,miR-410-5p,miR-146b-5p,miR-376a-2-5p,miR-195a-5p,let-7e-5p,miR-130b-5p,miR-345-5p,miR-146a,miR-194a,miR-196b-5p,miR-452-5p,miR-466f-3p,miR-2284d,let-7g-5p,novel_116,miR-1843b-5p,miR-542-3p,miR-223-3p,miR-194-5p,miR-3071-5p,miR-16b,miR-2284ab,miR-497-5p,miR-374b,miR-2284v,miR-16-5p,miR-374a-3p,miR-1197-3p,miR-2332,miR-409-3p,miR-134-5p,miR-503-3p,miR-370-3p,miR-300-3p,miR-365a-3p,miR-381-3p,miR-488-3p,miR-503-3p,miR-18a,miR-3154,miR-23b,miR-10b-3p,miR-345-5p,miR-23a-5p,miR-1248,miR-3529-3p,miR-29b-2-5p,let-

FLT3	ENSOARG0(	fms-rela	miR-33a-5p,miR-424-5p,miR-8485,miR-491-3p,miR-30c-1-3p,miR-29b-1-5p,miR-16b,miR-1306-5p,miR-214-3p,miR-330-3p,miR-9788-3p,miR-1185-3p,miR-539-5p,miR-1839-5p,miR-2426,miR-544a,miR-2447,miR-1285-5p,miR-877-3p,miR-30b-5p,miR-3964,miR-499a-5p,miR-7689-3p,miR-211-5p,miR-139-5p,miR-20a-3p,miR-203-3p,miR-1185-2-3p,miR-499a-3p,miR-3082-5p,miR-378j,miR-381-3p,miR-493-5p,miR-329a-5p,miR-2285w,miR-486-3p,miR-29b-1-5p,miR-216a-5p,miR-219b-5p,miR-30b,miR-2285p,miR-21-3p,miR-2484,miR-760-3p,miR-485-3p,miR-548e-3p,miR-2284h-5p,miR-7859,miR-383-5p,miR-15a-5p,miR-98-3p,miR-2904,miR-217-5p,miR-1197-5p,miR-30c-5p,miR-1246,let-7a-3p,miR-361-5p,miR-19b-3p,miR-214,miR-2330-3p,miR-3184-3p,miR-6134,miR-98-3p,miR-15b-3p,let-7b-3p,miR-208b-5p,miR-424-5p,miR-450b-3p,miR-21-3p,miR-30c-1-3p,miR-496-3p,miR-1277-3p,miR-204-3p,miR-877-5p,miR-544-3p,miR-499b-3p,miR-139-5p,miR-216b-5p,miR-129b-3p,miR-30d-5p,let-7f-1-3p,miR-450a-1-3p,novel_99,miR-30f,miR-2483-5p,let-7f-2-3p,let-7f-2-3p,miR-493-5p,miR-6240,miR-539-3p,miR-330-3p,novel_51,miR-31-5p,miR-133b-5p,miR-103a-3p,novel_116,miR-544b,miR-103,miR-450c-3p,miR-374b-3p,miR-653,miR-708-3p,miR-497-5p,miR-2284x,miR-16b,miR-3432b,miR-2285x,miR-211,miR-485-3p,miR-16a,miR-15a,miR-147-5p,miR-502b,miR-6119-3p,miR-379-3p,novel_1,miR-655-5p,miR-329-5p,miR-670-3p,miR-374c-3p,miR-1306,miR-3065-5p,miR-3591-3p,miR-2387,miR-195a-5p,miR-379-5p,miR-107,miR-19a-3p,miR-200a,miR-2285b,miR-3071-3p,miR-30c-2-3p,miR-217,miR-107,miR-200a-3p,miR-28-3p,miR-582-3p,miR-450a-1-3p,novel_121,miR-505-5p,miR-322-5p,miR-30a-5p,miR-3578,miR-1290,miR-30c,miR-496,miR-411-3p,miR-300,miR-16-5p,miR-28a-3p,miR-6740-5p,miR-409-3p,miR-486b-3p,miR-374b-3p,miR-204-5p,miR-2285aa,miR-141-

			miR-30f,miR-154b-3p,let-7g-3p,novel_99,let-7f-1-3p,miR-27b-5p,miR-6130,miR-6516,miR-544-3p,novel_25,miR-664a,miR-17-3p,miR-487a-5p,miR-708-5p,miR-338-5p,miR-320e,miR-324-3p,miR-10b-5p,miR-6134,let-7b-5p,miR-145a-3p,miR-4510,miR-539-5p,miR-4429,miR-378d,miR-376a-5p,miR-2300b-3p,miR-1246,miR-126-3p,miR-5703,miR-1197-5p,miR-188-3p,miR-2904,miR-532-3p,miR-548e-3p,miR-3600,miR-760-3p,miR-2284m,miR-106a,miR-320d,miR-21-3p,miR-34b-5p,miR-125b,miR-216c-5p,miR-20a,let-7d-5p,miR-10b,miR-20a-3p,miR-3064-5p,miR-7977,miR-541-5p,miR-3958-3p,miR-4726-5p,miR-30b-5p,miR-382-3p,miR-362-5p,miR-8095,miR-541-3p,miR-3059-5p,miR-452-5p,miR-181d-5p,miR-2426,miR-101a-5p,miR-544a,miR-16-2-3p,miR-10b,miR-4791,miR-6529a,miR-30b-3p,miR-2285e,miR-500-5p,miR-409b,miR-2300a-5p,miR-222-5p,miR-25-5p,miR-3085-3p,let-7e,miR-2285o,novel_103,miR-5126,miR-1306-5p,miR-326,miR-320b,miR-30c-1-3p,miR-758-3p,miR-193a,miR-106a-5p,let-7a-5p,miR-33a-5p,let-7c-3p,miR-485-5p,miR-376c-5p,miR-7144-5p,miR-2448-3p,miR-17-5p,miR-320a,miR-362-5p,miR-3154,miR-365a-3p,miR-34c-5p,miR-370-3p,miR-138-5p,miR-28-5p,miR-1306-3p,miR-409-3p,miR-93,miR-500b-5p,miR-365a-5p,miR-6128,let-7d,miR-10a-5p,miR-3578,miR-483-3p,miR-6516-5p,miR-376a-5p,miR-196a-2-3p,miR-191-3p,miR-181b-5p,miR-17-3p,miR-92a-3p,miR-107,miR-1260a,miR-217,miR-339a,let-7c-5p,miR-30c-2-3p,miR-380-5p,miR-490-3p,miR-181a-2-3p,miR-107,miR-1291,miR-376a-2-5p,miR-7975,miR-2387,miR-34b,miR-670-3p,miR-2404,miR-10a-5p,miR-7-1-3p,miR-320b,novel_1,miR-92a-3p,miR-128-1-5p,miR-502b,miR-133a-5p,miR-6402,miR-20a-5p,let-7i,miR-382-5p,miR-34c,miR-1307-3p,miR-2428,miR-449a,miR-326-3p,miR-17-3p,miR-1260b,miR-30b-3p,miR-138,miR-2284ab,miR-7862,miR-
SLC37A4	ENSOARG0(	solute c	
			miR-487b-5p,miR-331-5p,miR-134-5p,miR-374b-3p,miR-204-5p,miR-190a-5p,miR-2332,novel_63,miR-665,miR-219b-3p,miR-374a-3p,miR-145a-3p,miR-425-5p,miR-1271,miR-329a,let-7g-3p,miR-1827,miR-625-5p,miR-2285c,miR-331-5p,miR-374c-3p,miR-192-5p,miR-548o-3p,miR-3068-3p,miR-487a-3p,miR-532-5p,miR-215-5p,miR-1a-2-5p,miR-210-5p,miR-219-3p,miR-2285p,miR-2411,miR-487a,miR-300,miR-411-3p,miR-378d,miR-148a-5p,miR-362-3p,miR-134,miR-215-5p,miR-5703,miR-758-5p,miR-1271-5p,miR-2411-5p,miR-505-5p,novel_121,miR-134-5p,miR-505-3p,novel_1,miR-628-3p,miR-154-3p,miR-22-5p,miR-4324,miR-211,miR-219a-2-3p,miR-190b-5p,miR-96-5p,miR-544-5p,miR-505-3p,miR-493-5p,miR-3065-5p,miR-150-5p,miR-374c-3p,miR-3082-5p,miR-7857-3p,miR-2319b,miR-4792,miR-211-5p,miR-7-1-3p,miR-190a,miR-362-3p,miR-330-3p,miR-758-3p,miR-8485,let-7j,miR-3187-3p,miR-6240,miR-493-5p,miR-219a-1-3p,miR-362-3p,miR-1434-3p,miR-542-3p,miR-329-3p,miR-758-3p,novel_23,miR-374b-3p,miR-125b-2-3p,miR-539-5p,miR-2285g,miR-2285e,novel_116,miR-148b-5p,miR-330-3p,novel_78,miR-3955-5p
KLHL7	ENSOARG0(	kelch-li	

SRPR	ENSOARG0(	signal r	miR-320a,miR-411,miR-487b-5p,miR-378j,miR-625-5p,novel_94,miR-6395,miR-485-5p,miR-181a-3p,miR-1291,miR-2331-3p,miR-6535,miR-320c,miR-4443,miR-34c-5p,miR-3596,miR-378g,miR-34b,miR-34c,miR-502-5p,miR-135a-1-3p,miR-494-5p,miR-2440,miR-874-5p,miR-134-5p,miR-374b-3p,miR-486b-3p,miR-664a,miR-335,miR-487b-5p,miR-335-5p,miR-181c-3p,miR-3184-5p,miR-449a,miR-365a-5p,miR-423-5p,miR-877-3p,miR-374a-3p,miR-134,miR-34a-5p,miR-4429,miR-31-5p,miR-134-5p,miR-328-3p,miR-134-3p,miR-365b-5p,miR-185-3p,miR-320b,miR-34b-5p,miR-2898,miR-320d,miR-486-3p,miR-193a,miR-1b-5p
MBNL3	ENSOARG0(	musclebl	miR-185-5p,miR-664b,miR-664-3p,miR-2319b,miR-1843b-5p,miR-133a-5p,miR-5010-3p
CASP8AP2	ENSOARG0(	caspase	miR-4792,miR-18a-3p,miR-548e-3p,miR-7859,novel_25,miR-873a-5p,miR-18a-3p,miR-582-3p,miR-136-5p,miR-17-3p,miR-219a-2-3p,miR-219-3p,miR-133a-5p,miR-100-3p,miR-219b-3p,miR-105-5p,miR-17-3p,miR-17-3p,miR-499a-5p,novel_127
CD300E	ENSOARG0(	CD300e m	miR-493-3p,miR-450b-3p,miR-18a-3p,miR-204-3p,miR-18a-3p,miR-378b,miR-874-3p,miR-6517,miR-1343-3p,miR-2285t,novel_127,novel_51,miR-1247-3p,miR-361-3p,miR-371b-3p,miR-2447,miR-145a-3p
HUS1	ENSOARG0(	HUS1 che	miR-421-5p,miR-20a,miR-2284m,miR-106a,miR-320d,miR-26a-5p,miR-196a-3p,miR-15a-5p,miR-23c,miR-7859,miR-23b-3p,miR-106a,miR-548e-3p,miR-18a-3p,miR-4429,let-7a-3p,miR-218-1-3p,miR-362-3p,miR-217-5p,miR-188-3p,miR-98-3p,novel_44,miR-98-3p,miR-10b-5p,miR-6134,miR-4510,miR-130b-5p,miR-424-5p,let-7b-3p,miR-338-5p,miR-6130,miR-10a,miR-15b-5p,miR-32-5p,miR-504-5p,miR-532-5p,miR-182-5p,miR-18a-5p,let-7g-3p,miR-1827,let-7f-1-3p,miR-3187-3p,miR-494-3p,miR-106a-5p,miR-424-5p,miR-652-5p,miR-23a-3p,let-7c-3p,miR-199b-5p,miR-320b,miR-106b-5p,miR-16b,miR-182-5p,miR-362-3p,miR-505,miR-147a,miR-216a-3p,miR-1343-3p,miR-330-3p,miR-27a-3p,miR-362-3p,miR-10b,miR-301b-5p,miR-2284r,miR-329-3p,miR-30b-3p,miR-7705,miR-363-3p,miR-3956-3p,novel_60,miR-2284j,miR-4726-5p,miR-615,miR-301a-5p,miR-371a-5p,miR-25,miR-27a-3p,miR-20a-3p,miR-10b,miR-504,miR-147-3p,novel_94,miR-493-5p,miR-199b-5p,miR-378j,miR-92b-3p,miR-2285b,miR-1271-3p,miR-200a,miR-199a-5p,miR-92a-3p,novel_127,miR-217,miR-758-5p,miR-322-5p,miR-486-5p,miR-18b-5p,miR-411-3p,let-7a-2-3p,miR-10a-5p,miR-128-3p,miR-615-3p,miR-93,miR-145-3p,miR-2284v,miR-16-5p,miR-2284k,miR-25-3p,miR-374c-3p,miR-300-3p,miR-199a-5p,miR-874-3p,let-7g-3p,miR-329a,miR-10b-3p,miR-1248,miR-20b-5p,miR-320a,miR-18a,miR-23b,miR-17-5p,miR-93-5p,miR-432-3p,miR-2285r,miR-654-3p,let-7a-2-3p,miR-493-5p,miR-6240,miR-130b-5p,miR-378a-5p,miR-124-5p,miR-7-5p,miR-106a-5p,miR-17-5p,miR-330-3p,miR-20b,miR-149-5p,miR-31-5p,miR-2284ab,miR-30b-3p,miR-16b,miR-27b-3p,miR-2284x,miR-497-5p,miR-1843b-5p,miR-7b-5p,miR-1434-3p,miR-873a-5p,novel_23,miR-199b-5p,miR-2285x,novel_1,miR-379-3p,miR-92a-3p,miR-147-5p,miR-421-5p,miR-576-3p,miR-20a-5p,miR-15a,miR-33a-

VPS13A	ENSOARG0(	vacuolar	miR-3071-3p,miR-205-5p,miR-3064-5p,miR-192-3p,miR-2284y,miR-200a,miR-29a-5p,novel_39,miR-450b-5p,miR-148b-5p,miR-330-5p,miR-2427,miR-1343-5p,miR-3591-5p,miR-155-5p,miR-30c,miR-431,miR-412,miR-1224-5p,miR-429-3p,novel_121,miR-9-5p,miR-505-5p,miR-6740-5p,novel_63,miR-191-5p,miR-193a-5p,miR-137-3p,miR-301,miR-424-3p,miR-30e-5p,miR-1248,miR-345-5p,miR-767,miR-1271,miR-542-5p,miR-2285aa,miR-133c,miR-223-5p,miR-331-5p,miR-2411-3p,miR-194a,miR-365b-5p,let-7f-2-3p,let-7f-2-3p,miR-668-3p,miR-6240,miR-493-5p,miR-503-3p,let-7a-2-3p,miR-432-3p,miR-432-3p,miR-450c-3p,miR-22-3p,miR-1843b-5p,miR-2284x,miR-26a-2-3p,miR-3955-5p,novel_116,miR-28c,miR-421-5p,miR-379-3p,miR-29a,miR-6119-3p,miR-29c-3p,miR-3065-5p,miR-150-5p,miR-412-3p,miR-3120-3p,miR-216b-3p,novel_68,miR-677,miR-411b-3p,miR-345-3p,novel_17,miR-210-5p,miR-30b,miR-345-3p,miR-487a,miR-143-3p,miR-376a-5p,miR-421-5p,miR-217-5p,miR-1434-5p,miR-191,miR-30c-5p,miR-148a-5p,miR-34a-5p,novel_73,miR-30d-3p,miR-7859,miR-665,miR-30a-3p,miR-30e-3p,miR-2285j,miR-130b-5p,miR-1468-5p,miR-3184-3p,miR-433-5p,miR-3184-5p,miR-411,miR-30d-5p,miR-299,miR-186-5p,miR-1827,miR-345-5p,miR-548o-3p,miR-504-5p,miR-505,miR-323b-3p,miR-431-5p,miR-103a-2-5p,miR-1185-5p,miR-2310,miR-323b,miR-301b-5p,miR-1839-5p,miR-145b,miR-330-3p,miR-548w,miR-126a-5p,miR-148b-5p,miR-301a-5p,miR-218-5p,miR-3964,miR-9-5p,miR-3956-3p,miR-2447,miR-423-5p,miR-1983,miR-493-5p,miR-335-3p,miR-504,miR-136-5p,miR-126b-5p,miR-7689-3p,miR-2285u,miR-2319b,miR-4443,miR-203-3p,miR-2483-3p,miR-25,miR-217,miR-200c,miR-200c-3p,miR-450a-2-3p,miR-30a-3p,miR-2284a,miR-3065-3p,miR-28b,miR-30a-5p,miR-29d-3p,miR-345-3p,let-7a-2-3p,miR-200b-3p,miR-582-3p,miR-411-
CDX2	ENSOARG0(	caudal t	miR-30b,miR-30b-5p,miR-133a-5p,miR-30f,miR-29b-2-5p,miR-4726-5p,miR-30d,miR-29b-2-5p,miR-193a-5p,miR-665-5p,miR-500b-5p,miR-362-5p,miR-24-3p,miR-361-3p,miR-30c-5p,miR-30e-5p,miR-30a-5p,miR-362-5p,miR-140-3p,miR-30d-5p,miR-30c,miR-7144-5p,miR-2355-5p,miR-2483-3p,miR-669,miR-500-5p
TCN1	ENSOARG0(	transcob	miR-345-3p,let-7e-3p,miR-345-3p,miR-455-5p,miR-2411-3p,miR-148b-5p,miR-455-5p

GYS1	ENSOARG0(	glycogen	miR-487a-5p,miR-208b-5p,miR-708-5p,miR-664a,miR-29b-2-5p,miR-320e,miR-338-3p,miR-378c,miR-145a-3p,miR-2312,miR-30f,novel_32,miR-6516,miR-193b-3p,miR-532-5p,miR-140-5p,miR-378a-3p,miR-30d,miR-299-3p,miR-320d,miR-106a,miR-466i-5p,miR-196a-3p,miR-29b-1-5p,miR-20a,miR-378d,miR-4429,miR-19b-3p,miR-124-3p,miR-1197-5p,miR-5703,miR-199a-3p,novel_83,miR-4726-5p,miR-335,miR-30b-5p,miR-2285n,miR-2285f,miR-219a-5p,novel_60,miR-369-5p,miR-129b-5p,miR-361-3p,miR-378e,miR-3082-5p,miR-3064-5p,miR-3085-3p,miR-320b,novel_103,miR-326,miR-30c-1-3p,miR-376b,miR-106a-5p,miR-3120-5p,miR-378c,miR-2426,miR-450b-5p,miR-412-3p,miR-30b-3p,miR-378b,miR-216a-3p,miR-2285e,miR-2300a-5p,miR-28-5p,miR-486b-3p,miR-138-5p,miR-767-5p,miR-29b-3p,miR-410-5p,miR-5100,miR-378c,miR-365a-5p,miR-93,miR-6128,miR-1306-5p,miR-3074-1-3p,miR-671-5p,miR-10b-3p,miR-320a,miR-17-5p,miR-3154,miR-488-3p,miR-370-3p,miR-107,miR-543-5p,miR-2898,miR-450a-2-3p,miR-191-3p,miR-30c-2-3p,miR-217,miR-2285af,miR-376b-5p,miR-376c-5p,miR-490-3p,miR-28b,miR-29b-2-5p,miR-4286,miR-29d-3p,miR-30a-5p,miR-345-3p,miR-582-3p,miR-378h,miR-6516-5p,miR-203b-5p,miR-147-5p,miR-20a-5p,novel_27,miR-326-3p,miR-2428,miR-24-3p,novel_101,miR-219b-3p,miR-9851-3p,miR-379-5p,miR-6395,miR-1291,miR-19a-3p,miR-107,miR-18a-3p,miR-378b,miR-2285ab,miR-2387,miR-2404,miR-29a-3p,miR-320b,miR-378b,miR-197-5p,miR-17-5p,miR-106a-5p,miR-296-3p,miR-654-3p,miR-363-5p,miR-130b-5p,miR-744-5p,miR-138,miR-30b-3p,miR-29b,miR-219a-1-3p,miR-374b-3p,miR-20b,miR-103,miR-2285g,miR-130a-3p,miR-665,miR-454-3p,miR-129-5p,miR-130b-5p,miR-451a,miR-664a-5p,miR-2285j,miR-3184-5p,miR-6525,miR-346,miR-665-5p,miR-3074-5p,miR-885-3p,miR-625-5p,miR-30d-5p,miR-2285c,miR-301a-3p,miR-216b-
RAD52	ENSOARG0(	RAD52 ho	miR-125a-3p,miR-664-3p,miR-542-5p,miR-2285af,miR-125b,miR-422a,miR-450a-2-3p,novel_96,miR-200a-3p,miR-2285w,miR-29b-2-5p,miR-30a-3p,miR-380-5p,miR-421-5p,miR-200a,miR-378i,miR-378a-3p,miR-23b-5p,miR-140-3p,miR-124-3p,miR-885-3p,miR-105-2,miR-30d-3p,miR-378h,miR-483-3p,miR-1271-5p,miR-222-5p,miR-9-5p,miR-2284h-5p,miR-30e-3p,novel_63,miR-29b-2-5p,miR-125b-5p,miR-30a-3p,miR-1468-5p,miR-3074-5p,novel_42,miR-378c,miR-1271,miR-3607-3p,miR-1197-3p,miR-378c,miR-2448-3p,miR-876-3p,miR-1827,miR-1814c,miR-23a-5p,miR-885-3p,miR-874-3p,miR-378a-3p,miR-125a,miR-141-3p,miR-542-5p,miR-370-3p,miR-365a-3p,miR-499b-3p,miR-22-3p,miR-105-5p,miR-483-3p,miR-491-5p,miR-1185-5p,miR-1468,miR-542-5p,miR-125a-5p,miR-124a,miR-380-5p,miR-378d,miR-2310,miR-143-5p,miR-873a-5p,miR-378b,miR-22-3p,miR-378c,miR-1388-5p,miR-1839-5p,novel_78,miR-105-1,miR-134-3p,miR-2284q,miR-3956-3p,novel_60,miR-542-5p,miR-9-5p,miR-361-3p,miR-2284w,miR-96-5p,miR-378f,miR-9851-3p,miR-3074-5p,miR-664b,miR-3120-3p,miR-7689-3p,miR-499a-3p,miR-25,miR-378e,miR-378g

LNX2	ENSOARG0(	ligand o	miR-598-3p,miR-320a,miR-655,miR-503-3p,miR-424-3p,miR-625-5p,miR-543-3p,miR-10b-3p,miR-876-3p,miR-598-3p,miR-370-3p,miR-1271,miR-140-5p,miR-574-5p,miR-374c-3p,miR-544-3p,miR-193b-3p,miR-6740-5p,miR-144,miR-151-3p,miR-26c,miR-29b-2-5p,miR-409-3p,miR-135a-1-3p,miR-4492,miR-669a-3p,miR-17-3p,miR-1271,miR-3074-2-3p,miR-214,miR-374b,miR-1343-5p,miR-345-3p,miR-193b-5p,miR-4429,miR-328-3p,miR-23c,miR-345-3p,miR-26a-5p,miR-374a-5p,miR-2397-5p,miR-17-3p,miR-320d,miR-2898,miR-29b-2-5p,miR-143-3p,miR-490-3p,miR-2285b,miR-376a-5p,miR-150-5p,miR-378j,miR-6395,miR-96-5p,miR-1291,miR-374b-5p,miR-499b-5p,miR-126b-5p,miR-26b-5p,miR-7977,miR-203-3p,miR-144-3p,miR-320c,miR-6535,miR-27a-3p,miR-22-5p,miR-203b-5p,novel_1,miR-193b-3p,miR-2285x,miR-17-3p,miR-374c-5p,miR-374b-3p,miR-3071-5p,miR-223-3p,miR-7b-5p,miR-542-3p,miR-27b-3p,miR-16-1-3p,miR-544a,miR-27a-3p,miR-340-3p,miR-151a-3p,miR-133b-5p,miR-149-5p,miR-544b,miR-2411-3p,miR-320b,miR-7-5p,novel_103,miR-1343-5p,miR-193a-3p,miR-214-3p,miR-628-5p,miR-494-3p
ZNFX1	ENSOARG0(	zinc fin	miR-18a,miR-23b,miR-17-5p,miR-320a,miR-93-5p,miR-10b-3p,miR-671-5p,miR-20b-5p,miR-574-5p,miR-125a,miR-1271,miR-370-3p,miR-3959-5p,miR-34c-5p,miR-154b-5p,miR-135a-1-3p,miR-1306-3p,miR-2332,miR-100-3p,miR-125b-5p,miR-374b-3p,miR-204-5p,miR-133a-3p,miR-193a-5p,miR-6128,miR-374a-3p,miR-93,miR-378c,miR-1290,miR-330-5p,miR-582-3p,let-7a-2-3p,miR-105-2,miR-299a-3p,miR-18b-5p,miR-1224-5p,miR-7641,miR-378h,miR-483-3p,miR-505-5p,novel_121,miR-6119-5p,miR-2285af,miR-127-5p,miR-17-3p,miR-152-3p,miR-543-5p,miR-3529-3p,miR-4286,miR-3956-5p,miR-2285b,novel_39,miR-18b,miR-148a-3p,miR-2387,miR-2285ab,miR-3065-5p,miR-378b,miR-1306,miR-1291,miR-378f,miR-505-3p,miR-7975,novel_68,miR-3120-3p,miR-34b,miR-299b-3p,miR-670-3p,miR-181b-3p,miR-669,miR-320c,miR-677,miR-6535,miR-20a-5p,miR-4324,miR-1247-3p,miR-34c,miR-22-5p,miR-342-3p,miR-17-3p,miR-211,miR-301b-3p,miR-449a,miR-326-3p,miR-1285,miR-7b-5p,miR-1843b-5p,miR-432,miR-374b-3p,miR-22-3p,miR-219a-1-3p,miR-380-3p,miR-27b-3p,miR-188-5p,miR-7862,miR-340-3p,miR-377-5p,miR-130a-3p,miR-185-5p,miR-2285g,miR-432-5p,miR-20b,miR-149-5p,novel_116,miR-411-5p,miR-194-3p,miR-224-5p,miR-7-5p,miR-106a-5p,novel_51,miR-17-5p,miR-105-5p,miR-483-3p,miR-125a-5p,miR-2483-5p,miR-363-5p,miR-432-3p,miR-432-3p,let-7a-2-3p,miR-2285c,miR-21a-3p,miR-433-3p,miR-500a-3p,miR-129b-3p,miR-18a-5p,miR-2312,miR-133b-3p,miR-625-5p,miR-32-3p,miR-548o-3p,miR-2331-3p,miR-221-5p,miR-378a-3p,miR-552-3p,miR-182-5p,miR-532-5p,miR-216b-3p,miR-6130,miR-301a-3p,miR-204-3p,miR-22-3p,miR-2285j,miR-665,miR-324-3p,miR-17-3p,novel_74,miR-4492,miR-500,miR-208b-5p,miR-454-3p,miR-4510,miR-3184-3p,miR-378c,miR-2330-3p,miR-214,miR-665-5p,miR-224-5p,miR-

N4BP2	ENSOARG0(	NEDD4 bi	miR-6402,miR-133a-5p,miR-15a,novel_63,miR-16a,miR-34c,novel_74,miR-499a-5p,miR-424-5p,miR-28a-3p,miR-590-3p,miR-16-5p,miR-1839-3p,miR-346,miR-7705,miR-449a,novel_27,miR-488-3p,miR-411,miR-329a,miR-1248,miR-195a-5p,miR-574-5p,miR-215-5p,miR-216b-3p,miR-34b,miR-216b-5p,miR-34c-5p,miR-499b-3p,miR-499a-3p,miR-192-5p,miR-15b-5p,novel_25,miR-2285p,miR-16b,miR-216a-5p,miR-330-3p,miR-362-3p,miR-28-3p,miR-483-3p,miR-21-3p,miR-34b-5p,miR-1839-3p,miR-424-5p,miR-6240,miR-362-3p,miR-1290,miR-215-5p,miR-329-3p,miR-582-3p,miR-16b,miR-362-3p,miR-34a-5p,miR-361-5p,miR-3120-5p,miR-497-5p,miR-330-3p,miR-3600,miR-483-3p,miR-15a-5p,miR-322-5p,miR-545-5p
SLC38A5	ENSOARG0(	solute c	miR-323b,miR-6529a,miR-378b,miR-1388-5p,miR-378c,miR-450b-5p,miR-145b,miR-548w,novel_4,miR-221-3p,miR-9788-3p,miR-216a-3p,miR-455-3p,miR-2459,miR-1185-3p,miR-142-5p,miR-1298-3p,miR-147a,miR-323b-3p,miR-106b-5p,miR-320b,miR-199a-3p,miR-326,novel_115,miR-1468,miR-1185-5p,miR-3085-3p,miR-3955-3p,miR-124a,miR-2330-5p,miR-106a-5p,miR-3187-3p,miR-2310,miR-30c-1-3p,miR-378d,miR-378j,miR-381-3p,miR-140-5p,miR-221,miR-493-5p,miR-9-3p,miR-504,miR-499b-5p,miR-3064-5p,miR-1185-2-3p,miR-378e,miR-25,miR-378g,miR-2483-3p,miR-615,miR-4726-5p,miR-628-3p,miR-335,miR-1296-5p,miR-9-5p,miR-181d-5p,miR-2447,novel_60,miR-32-3p,miR-3059-5p,miR-363-3p,miR-3956-3p,miR-1285-5p,miR-124-3p,miR-5703,miR-1197-5p,miR-378a-3p,miR-1434-5p,miR-378i,novel_133,miR-133a-3p,let-7a-3p,miR-34a-5p,novel_73,miR-4429,miR-106a,miR-199a-3p,miR-616-3p,miR-383-5p,miR-95-3p,miR-200b,miR-324-3p,miR-92a-1-5p,miR-125b,miR-422a,miR-618,miR-210-5p,miR-34b-5p,miR-3653-3p,miR-106a,miR-320d,miR-760-3p,miR-20a,miR-2411,miR-664b-3p,miR-27b-5p,miR-433-3p,miR-411,miR-450a-1-3p,let-7f-1-3p,novel_99,miR-1827,miR-186-5p,let-7g-3p,miR-345-5p,miR-3065-3p,miR-552-3p,miR-296-3p,miR-32-5p,miR-140-5p,miR-30c-1-3p,miR-378a-3p,miR-504-5p,miR-32-3p,novel_25,miR-204-3p,miR-320e,let-7b-3p,miR-222-3p,miR-1468-5p,miR-450b-3p,miR-6516-3p,miR-17-3p,miR-425-5p,miR-433-5p,miR-539-5p,miR-378c,miR-122-5p,miR-6134,miR-181c-5p,miR-142a-5p,miR-6525,miR-450c-3p,miR-145a-5p,miR-3071-5p,miR-542-3p,miR-1843b-5p,miR-1434-3p,miR-181a-5p,miR-665,miR-31-5p,miR-6238,miR-194-3p,miR-20b,miR-134-3p,miR-185-5p,miR-32,miR-2285v,miR-17-5p,miR-106a-5p,miR-125a-5p,miR-124-5p,miR-378a-5p,let-7f-2-3p,miR-493-5p,miR-345-5p,let-7a-2-3p,miR-432-3p,miR-432-3p,miR-378b,miR-3065-

DROSHA	ENSOARG0(	drosha,	miR-361-3p,miR-3591-3p,miR-3065-5p,miR-2284e,miR-412-3p,miR-9851-3p,miR-9-3p,miR-1983,miR-6516-3p,miR-452-3p,miR-499b-5p,miR-320c,miR-27a-3p,miR-4324,miR-133a-5p,miR-2284o,novel_1,miR-421-5p,miR-147-5p,miR-181d-5p,miR-2284j,miR-432,miR-181a-5p,miR-7857,novel_23,miR-30b-3p,miR-30b-3p,miR-2284ab,miR-1388-5p,miR-219a-1-3p,miR-450b-5p,miR-101c,miR-2284aa,miR-27b-3p,miR-2284x,miR-188-5p,miR-27a-3p,miR-432-5p,miR-2284d,miR-9788-3p,miR-216a-3p,miR-320b,novel_115,miR-2411-3p,miR-1343-5p,miR-452-5p,miR-660,miR-466f-3p,miR-33a-5p,miR-2284l,miR-8485,miR-3187-3p,miR-433-3p,miR-320a,miR-3154,miR-767,miR-7144-5p,miR-3074-1-3p,miR-660-5p,miR-21-3p,miR-3965,novel_25,miR-154b-5p,miR-2284k,miR-15b-3p,miR-6516-3p,miR-767-5p,miR-708-5p,miR-28-5p,miR-486b-3p,miR-539-5p,miR-1306-5p,miR-129-2-3p,miR-2284v,miR-425-5p,miR-6525,miR-323-3p,miR-145-3p,miR-181c-5p,miR-345-3p,miR-2366,miR-2904,miR-1343-5p,miR-5703,miR-1197-5p,miR-4429,miR-133a-3p,miR-664-5p,miR-1224-5p,miR-2284z,miR-545-5p,miR-129-1-3p,novel_121,miR-205-5p,miR-345-3p,miR-2284m,miR-450a-2-3p,miR-320d,miR-181b-5p,miR-3653-3p,miR-2484,miR-543-5p,miR-2284f,miR-181a-2-3p,miR-421-5p,miR-486-3p
HYOU1	ENSOARG0(	hypoxia	miR-452-3p,miR-2319b,miR-139-5p,miR-24-1-5p,miR-7977,miR-378e,miR-376d,miR-27a-3p,miR-361-3p,miR-378j,miR-504,miR-376a-3p,miR-541-3p,miR-181d-5p,miR-9-5p,miR-1296-5p,miR-2285f,miR-361-3p,miR-877-3p,miR-1285-5p,miR-4726-5p,miR-199b-3p,miR-27a-3p,miR-376b-3p,miR-147a,miR-2285e,miR-2459,miR-216a-3p,miR-455-3p,miR-143-5p,miR-541,miR-2284r,miR-378b,miR-329-3p,miR-296-5p,miR-484,miR-30b-3p,miR-378c,miR-362-3p,miR-1388-5p,miR-2284aa,miR-494-3p,miR-101b-3p,miR-378d,miR-1b-5p,miR-3187-3p,miR-1306-5p,miR-326,miR-342,miR-505,miR-362-3p,miR-491-5p,miR-548o-3p,miR-378a-3p,miR-504-5p,miR-296-3p,miR-139-5p,miR-193b-3p,miR-6130,miR-377-3p,miR-2285c,miR-2312,miR-186-5p,miR-1827,miR-145a-3p,miR-4510,miR-378c,miR-6525,miR-6134,miR-181c-5p,miR-3604,miR-665,miR-664a,miR-6516-3p,miR-487b-5p,miR-4492,miR-18a-3p,miR-3600,miR-2408,miR-362-3p,miR-378i,miR-1197-5p,miR-140-3p,miR-378a-3p,miR-1193,miR-378d,miR-218-1-3p,miR-2285i,miR-486-3p,miR-2285p,novel_17,miR-185-3p,miR-422a,miR-219b-5p,miR-324-3p,miR-216a-5p,miR-760-3p,miR-30b-3p,miR-1277-5p,novel_68,novel_69,miR-669,miR-2387,miR-2285ab,miR-376b-3p,miR-216a-3p,miR-18a-3p,miR-1306,miR-2285k,miR-450b-3p,miR-378f,miR-135b-5p,miR-2319a,miR-135a-2-3p,miR-342-3p,novel_101,miR-495-3p,miR-193b-3p,miR-2428,miR-326-3p,novel_87,miR-6119-3p,miR-199c,miR-147-5p,miR-22-5p,miR-656-5p,miR-185-5p,miR-2285g,miR-432-5p,miR-194-3p,miR-149-5p,miR-181a-5p,miR-432,miR-873a-5p,miR-30b-3p,miR-27b-3p,miR-432-3p,miR-377-3p,miR-193a-3p,miR-1343-5p,miR-874-3p,miR-301,miR-3154,miR-329a,miR-10b-3p,miR-1306-5p,miR-193a-5p,miR-1197-3p,miR-378c,miR-145-3p,novel_63,miR-135a-5p,miR-486b-3p,miR-6517,miR-378b,miR-24-2-5p,miR-9-5p,miR-214-5p,miR-486-5p,miR-

LSM4	ENSOARG0(	LSM4 hom	miR-28a-3p,miR-145a-3p,miR-4510,miR-16-5p,miR-3074-2-3p,miR-214,miR-1197-3p,miR-1260b,miR-338-3p,miR-181c-5p,miR-6134,miR-2285j,miR-370-5p,miR-664a-5p,miR-424-5p,miR-454-3p,miR-370-5p,miR-21-3p,miR-301a-3p,miR-34c-5p,miR-6130,miR-199a-5p,miR-15b-5p,miR-21a-3p,miR-3154,miR-23b,miR-339b,miR-23a-5p,miR-485-5p,miR-1827,miR-28b,miR-199a-5p,miR-1260a,miR-6119-5p,miR-2285p,miR-216a-5p,miR-185-3p,miR-339a,miR-28-3p,miR-181b-5p,miR-34b-5p,novel_127,miR-152-3p,miR-18a-3p,miR-6517,novel_79,miR-1224-5p,miR-23b-3p,miR-322-5p,miR-148b-3p,miR-15a-5p,miR-23c,miR-532-3p,novel_121,miR-376a-5p,miR-1246,miR-1197-5p,miR-184-3p,miR-582-3p,miR-142-3p,miR-19b-3p,miR-218-1-3p,miR-34a-5p,miR-2285f,miR-1296-5p,miR-181d-5p,miR-1193,miR-361-3p,miR-24-3p,miR-371b-3p,miR-1307-3p,miR-449a,miR-301b-3p,miR-199b-5p,miR-15a,miR-28c,miR-34c,miR-16a,miR-2284q,miR-2285u,miR-3596,novel_69,miR-670-3p,miR-34b,miR-203-3p,miR-669,miR-4443,miR-2285ab,miR-140-5p,miR-148a-3p,miR-199b-5p,miR-3591-3p,miR-18a-3p,miR-299b-5p,miR-450b-3p,miR-19a-3p,miR-9851-3p,miR-195a-5p,miR-379-5p,miR-1388-3p,miR-424-5p,miR-23a-3p,miR-582,miR-2285v,miR-16b,miR-130b-3p,miR-199b-5p,miR-214-3p,miR-1343-3p,miR-130a-3p,miR-339-5p,miR-145b,miR-185-5p,miR-411-5p,miR-149-5p,miR-296-5p,miR-708-3p,miR-181a-5p,miR-125b-2-3p,miR-145a-5p,miR-16b,miR-2426,miR-1260b,miR-101c,miR-1839-5p,miR-497-5p,miR-7862
UMOD	ENSOARG0(	uromodul	miR-10b,miR-219a-1-3p,miR-544a,miR-1388-5p,miR-29b,miR-16b,miR-7862,miR-2284x,miR-497-5p,novel_4,miR-103a-3p,miR-130a-3p,miR-376b-3p,miR-103,miR-544b,miR-20b,miR-326,miR-320b,miR-17-5p,miR-16b,miR-106b-5p,miR-106a-5p,miR-29b-1-5p,miR-130b-3p,miR-424-5p,miR-106a-5p,miR-2284b,miR-432-3p,miR-432-3p,miR-2387,miR-376b-3p,miR-107,miR-136-5p,miR-3963,miR-195a-5p,miR-376a-3p,miR-2284g,miR-7977,miR-10a-5p,miR-499b-5p,miR-29a-3p,miR-3082-5p,miR-2404,miR-499a-3p,miR-376d,miR-320c,miR-20a-5p,novel_82,miR-15a,miR-16a,miR-29a,miR-2447,miR-29c-3p,miR-301b-3p,miR-326-3p,miR-134,miR-330-5p,miR-29d-3p,miR-4429,miR-33b-3p,miR-10a-5p,miR-2284u,miR-106a,miR-15a-5p,miR-24-2-5p,miR-322-5p,miR-134-5p,miR-210-5p,miR-376b-3p,miR-3601,miR-106a,miR-320d,miR-221-5p,miR-107,miR-181a-2-3p,miR-20a,miR-29b-1-5p,miR-450b-5p,miR-1271-3p,miR-2284a,miR-3065-3p,miR-2432,miR-2284y,miR-488-3p,miR-433-3p,miR-17-5p,miR-320a,miR-93-5p,miR-20b-5p,miR-221-5p,miR-2355-5p,miR-3065-3p,miR-499b-3p,miR-10a,miR-15b-5p,miR-204-3p,miR-5100,miR-2285j,miR-29b-3p,miR-134-5p,miR-454-3p,miR-424-5p,miR-16-5p,miR-93,miR-10b-5p

ERC1	ENSOARG0(	ELKS/RAB	miR-541,miR-2284r,novel_78,miR-1343-3p,miR-1298-3p,miR-376b-3p,miR-2459,miR-431-5p,miR-16b,novel_19,miR-29b-1-5p,miR-130b-3p,miR-214-3p,miR-1388-3p,miR-23a-3p,miR-2310,miR-1306-3p,miR-504,miR-96-5p,miR-376a-3p,miR-2319b,miR-4792,miR-2285u,miR-3596,miR-378g,miR-2440,miR-628-3p,miR-3968,miR-499a-5p,miR-2447,miR-877-3p,miR-361-3p,miR-423-5p,miR-2366,miR-217-5p,miR-361-5p,miR-34a-5p,miR-218-1-3p,miR-664-5p,miR-133a-3p,miR-18a-3p,miR-30d-3p,miR-212-5p,miR-23b-3p,miR-208b-3p,miR-92a-1-5p,miR-3601,miR-324-3p,miR-142a-3p,miR-143-3p,miR-486-3p,miR-129b-3p,miR-1827,miR-625-5p,miR-1895,miR-504-5p,miR-2331-3p,miR-30c-1-3p,miR-552-3p,miR-296-3p,miR-582-5p,miR-301a-3p,miR-15b-5p,miR-30e-3p,miR-2285j,miR-665,miR-3604,miR-30a-3p,miR-130b-5p,miR-874-5p,miR-1468-5p,miR-4492,miR-454-3p,miR-129-2-3p,miR-214,miR-665-5p,miR-6525,miR-769-5p,miR-181c-5p,miR-3184-5p,miR-7b-5p,miR-542-3p,miR-22-3p,miR-16b,miR-497-5p,miR-103a-3p,miR-31-5p,miR-3955-5p,miR-134-3p,miR-149-5p,novel_116,miR-7-5p,novel_51,miR-628-5p,miR-668-3p,miR-432-3p,miR-432-3p,miR-148a-3p,miR-3591-3p,miR-216a-3p,miR-1306,miR-2319a,miR-216b-3p,miR-320c,miR-576-3p,miR-208a-3p,miR-142b,miR-28c,miR-29a,miR-335-5p,miR-29c-3p,miR-1839-3p,miR-301b-3p,miR-3591-5p,miR-330-5p,miR-23b-5p,miR-1343-5p,miR-6517,miR-2285ad,miR-431,miR-1224-5p,miR-758-5p,miR-322-5p,miR-129-1-3p,miR-2284z,miR-8117,miR-125a-3p,miR-205-5p,novel_127,miR-152-3p,miR-200a-3p,miR-3064-5p,miR-450b-5p,miR-29a-5p,novel_39,miR-23b,miR-2474,miR-1248,miR-874-3p,miR-3956,miR-2355-5p,miR-542-5p,miR-2284k,novel_63,miR-2284v,novel_42,miR-16-5p,miR-296-5p,miR-484,miR-6529a,miR-3120-5p,miR-2284aa,miR-409b,miR-222-5p,miR-142-

ACOT7	ENSOARG0(	acyl-CoA	miR-885-3p,miR-30c,miR-200b-3p,miR-345-3p,miR-134,miR-30a-5p,miR-330-5p,miR-9-5p,miR-322-5p,miR-505-5p,miR-483-3p,miR-134-5p,miR-486-5p,novel_121,miR-2285ad,miR-429-3p,miR-7641,miR-1224-5p,miR-431,miR-17-3p,miR-200c-3p,miR-127-5p,miR-107,miR-543-5p,novel_96,miR-200a-3p,novel_127,miR-1260a,miR-200c,miR-30c-2-3p,miR-28b,novel_39,miR-3956-5p,miR-29b-2-5p,miR-3064-5p,miR-181a-2-3p,miR-329b,miR-671-5p,miR-485-5p,miR-30e-5p,miR-320a,miR-300-3p,miR-374c-3p,miR-874-3p,miR-542-5p,miR-141-3p,miR-125a,miR-615-5p,miR-3432a,miR-134-5p,miR-486b-3p,miR-28-5p,miR-2433,miR-370-5p,miR-125b-5p,miR-331-3p,miR-1260b,miR-181c-3p,miR-365a-5p,miR-16-5p,miR-103b,miR-30b-3p,miR-16b,miR-1260b,miR-497-5p,miR-744-5p,miR-22-3p,miR-197-3p,miR-3071-5p,miR-145a-5p,miR-6238,novel_116,miR-149-5p,miR-103,miR-2355-3p,miR-544b,miR-103a-3p,miR-130a-3p,miR-665,miR-483-3p,miR-125a-5p,miR-224-5p,miR-377-3p,miR-365b-5p,novel_51,miR-2411-3p,miR-668-3p,miR-2483-5p,miR-1291,miR-107,miR-195a-5p,miR-6395,miR-505-3p,miR-18a-3p,miR-670-3p,miR-320c,miR-669,novel_68,miR-329-5p,miR-320b,miR-655-5p,miR-7-1-3p,miR-128-1-5p,miR-147-5p,miR-199c,miR-15a,miR-28c,miR-16a,miR-24-3p,miR-371b-3p,miR-326-3p,novel_27,miR-301b-3p,miR-2285m,miR-342-3p,miR-17-3p,miR-542-5p,miR-1193,miR-493-3p,miR-4429,miR-664-5p,miR-378d,novel_133,miR-30c-5p,miR-2300b-3p,miR-193b-5p,miR-124-3p,miR-15a-5p,miR-200b,novel_83,miR-199a-3p,miR-532-3p,miR-328-3p,miR-18a-3p,miR-505-3p,miR-212-5p,miR-320d,miR-202-5p,miR-760-3p,miR-466i-5p,miR-30d,miR-221-5p,miR-30b,novel_17,miR-618,miR-210-5p,miR-542-5p,miR-324-3p,miR-125b,miR-185-3p,miR-92a-1-5p,miR-345-3p,miR-130a-5p,miR-125b-2-3p,miR-486-3p,miR-143-

PRAME	ENSOARG0(	preferen	miR-218-2-3p,miR-29d-3p,miR-30a-5p,miR-128-3p,let-7a-2-3p,miR-10a-5p,miR-222,miR-7641,miR-376a-5p,miR-24-2-5p,miR-1271-5p,miR-483-3p,miR-2411-5p,miR-214-5p,miR-2285af,miR-1260a,miR-339a,miR-450a-2-3p,miR-17-3p,miR-107,miR-29b-2-5p,miR-4286,miR-380-5p,miR-3065-3p,miR-1271-3p,miR-488-3p,miR-2448-3p,miR-362-5p,miR-10b-3p,let-7g-3p,miR-485-5p,miR-370-3p,miR-141-3p,miR-215-5p,miR-615-5p,miR-34c-5p,miR-365a-3p,miR-3965,miR-154b-5p,miR-2284n,miR-551b-3p,miR-29b-3p,miR-1306-3p,miR-2332,miR-138-5p,miR-204-5p,miR-6128,miR-103b,miR-500b-5p,miR-365a-5p,miR-873a-5p,miR-1434-3p,miR-138,miR-21-5p,miR-29b,miR-1260b,miR-7862,miR-187-3p,miR-133b-5p,miR-432-5p,miR-185-5p,miR-6238,miR-544b,miR-103,miR-330-3p,miR-2331-5p,miR-125a-5p,miR-130b-5p,miR-2483-5p,miR-296-3p,miR-2387,miR-21c,miR-378b,miR-18a-3p,miR-1291,miR-450b-3p,miR-107,miR-19a-3p,miR-376a-2-5p,miR-195a-5p,miR-10a-5p,miR-2404,miR-323a-5p,miR-29a-3p,miR-34b,miR-502-5p,miR-15a,miR-6529b,miR-34c,miR-16a,miR-342-3p,miR-219b-3p,novel_101,miR-17-3p,miR-2285x,miR-211,miR-24-3p,miR-371b-3p,miR-326-3p,novel_27,miR-449a,miR-376a-5p,miR-215-5p,miR-2300b-3p,miR-1246,miR-124-3p,miR-2904,miR-140-3p,miR-188-3p,miR-1193,miR-19b-3p,miR-3600,miR-15a-5p,miR-532-3p,miR-185-3p,miR-125b,miR-2284m,miR-760-3p,miR-466i-5p,miR-30d,miR-34b-5p,miR-2887,miR-29b-1-5p,miR-2432,miR-2411,miR-27b-5p,miR-30f,novel_99,miR-140-5p,miR-3065-3p,miR-192-5p,miR-544-3p,miR-323-5p,miR-204-3p,novel_32,miR-877-5p,miR-222-3p,miR-324-3p,miR-29b-2-5p,miR-320e,miR-17-3p,miR-664a,miR-208b-5p,miR-424-5p,miR-539-5p,miR-1271,miR-10b-5p,miR-206-3p,miR-338-3p,miR-484,miR-296-5p,miR-6529a,miR-10b,miR-7857,miR-412-3p,miR-544a,miR-450b-5p,miR-2426,miR-16-2-3p,miR-

LHX8	ENSOARG0(	LIM home	miR-382-5p,miR-33a-3p,miR-412-5p,miR-4324,miR-133a-5p,miR-20a-5p,miR-576-3p,miR-6123,novel_101,miR-1193,miR-342-3p,novel_120,miR-335-5p,novel_27,miR-199b-5p,miR-301b-3p,miR-1306,miR-216a-3p,miR-146b-5p,miR-379-5p,miR-450b-3p,miR-107,miR-26b-5p,miR-216b-3p,miR-144-3p,miR-320c,miR-374c-3p,miR-106a-5p,miR-194a,miR-17-5p,miR-2411-3p,miR-146a,miR-125a-5p,miR-452-5p,miR-466f-3p,miR-378a-5p,miR-2285l,miR-382,miR-130b-5p,miR-380-5p,miR-539-3p,let-7a-2-3p,miR-197-3p,novel_23,miR-194-5p,miR-3071-5p,miR-708-3p,miR-1434-3p,miR-27b-3p,miR-7862,miR-2284ab,miR-138,miR-130a-3p,miR-339-5p,miR-377-5p,miR-31-5p,miR-103a-3p,miR-20b,miR-103,miR-2355-3p,miR-3431,miR-144,miR-100-3p,miR-125b-5p,miR-3969,miR-5100,miR-370-5p,miR-138-5p,miR-6128,miR-3607-3p,miR-93,miR-93-5p,miR-137-3p,miR-3154,miR-17-5p,miR-320a,miR-30e-5p,miR-2448-3p,miR-301,miR-503-3p,miR-20b-5p,novel_48,miR-10b-3p,miR-329b,let-7g-3p,miR-141-3p,miR-125a,miR-223-5p,miR-199a-5p,miR-133c,miR-374c-3p,miR-3071-3p,miR-339a,miR-2285af,miR-200c,miR-107,miR-200a-3p,miR-200c-3p,miR-127-5p,miR-199a-5p,miR-1271-3p,miR-192-3p,miR-380-5p,miR-2478,miR-30a-5p,miR-345-3p,miR-3591-5p,miR-128-3p,miR-30c,miR-200b-3p,let-7a-2-3p,novel_79,miR-412,miR-431,miR-222,miR-429-3p,miR-2284z,miR-24-2-5p,miR-302a-5p,miR-371a-5p,miR-30b-5p,miR-2284q,miR-3958-3p,miR-335,miR-194b-5p,novel_60,miR-2447,miR-3956-3p,miR-541-3p,miR-8095,miR-361-3p,miR-129b-5p,miR-361-3p,miR-199b-5p,miR-221,novel_94,miR-299b-5p,miR-668-5p,miR-541-5p,miR-27a-3p,miR-2483-3p,miR-3082-5p,miR-29b-1-5p,miR-182-5p,miR-106b-5p,miR-342,miR-320b,miR-431-5p,miR-1306-5p,miR-146b,miR-199b-5p,miR-130b-3p,miR-376b,miR-106a-5p,miR-412-3p,miR-2284r,miR-484,miR-541,miR-16-2-3p,miR-21b,miR-
PDILT	ENSOARG0(	protein	miR-21-3p,miR-454-5p,miR-216b-5p,miR-670-3p,miR-30d-5p,miR-30e-5p,miR-146b-5p,miR-485-5p,miR-767,miR-30f,miR-3607-3p,miR-181d-5p,miR-145a-3p,miR-326-3p,novel_87,miR-30b-5p,miR-576-3p,miR-130b-5p,novel_1,miR-767-5p,novel_116,miR-2411-5p,miR-185-5p,miR-3962,miR-330-5p,miR-30a-5p,miR-3071-5p,miR-30c-5p,miR-181a-5p,miR-30c,miR-130b-5p,miR-146a-5p,miR-2411,miR-2285r,miR-30b,miR-196a-3p,miR-326,miR-146a,miR-146b,miR-30d,miR-181b-5p

			miR-320d,miR-202-5p,miR-30b-3p,miR-299-3p,miR-26a-5p,miR-324-3p,miR-345-3p,miR-125b,miR-664b-3p,miR-376a-5p,miR-181b-1-3p,miR-125b-2-3p,miR-486-3p,miR-2411,miR-143-3p,miR-329a-5p,miR-183-5p,miR-142-3p,miR-4429,miR-133a-3p,miR-148a-5p,novel_133,miR-362-3p,miR-2366,miR-98-3p,miR-5703,miR-217-5p,miR-188-3p,miR-23b-3p,miR-7859,miR-23c,miR-199a-3p,novel_74,miR-487b-5p,miR-6516-3p,miR-708-5p,miR-130b-5p,miR-222-3p,miR-2285j,miR-324-3p,miR-338-5p,miR-665,miR-6525,miR-98-3p,miR-206-3p,miR-346,miR-181c-5p,miR-3074-5p,miR-214,miR-2312,miR-133b-3p,miR-345-5p,miR-885-3p,miR-299,miR-129b-3p,miR-377-3p,miR-499b-3p,miR-139-5p,miR-204-3p,miR-30c-1-3p,miR-548o-3p,miR-370-5p,miR-3085-3p,miR-487b-5p,miR-214-3p,miR-320b,miR-199a-3p,miR-326,miR-505,miR-362-3p,miR-30c-1-3p,miR-8485,miR-2310,miR-23a-3p,let-7c-3p,miR-33a-5p,miR-1388-5p,miR-362-3p,miR-2426,miR-101c,miR-1839-5p,miR-2284aa,miR-3120-5p,miR-329-3p,miR-1a-3p,miR-30b-3p,miR-9788-3p,miR-148b-5p,miR-221-3p,miR-455-3p,miR-421,miR-1185-3p,miR-1343-3p,novel_4,miR-330-3p,miR-181b-2-3p,miR-3968,miR-335,miR-450a-5p,miR-374a-3p,miR-218-5p,novel_82,miR-101-3p,miR-2285y,miR-1285-5p,miR-9-3p,miR-2447,miR-1296-5p,miR-2285f,miR-2284j,miR-452-5p,miR-181d-5p,miR-299b-5p,miR-147-3p,miR-9-3p,miR-361-3p,miR-409-5p,miR-221,miR-378j,miR-378g,miR-3596,miR-499a-3p,miR-3082-5p,miR-1185-2-3p,miR-3064-5p,miR-139-5p,miR-20a-3p,miR-7689-3p,miR-450a-2-3p,miR-181b-5p,miR-127-5p,miR-191-3p,miR-543-5p,miR-200a-3p,miR-30c-2-3p,miR-217,novel_39,miR-450c-5p,miR-490-3p,miR-192-3p,miR-3064-5p,miR-1,miR-4286,miR-411-3p,miR-885-3p,miR-345-3p,miR-134,miR-330-5p,miR-2411-5p,miR-483-3p,miR-134-5p,miR-2284z,miR-545-5p,miR-299a-3p,miR-222,miR-
THG1L	ENSOARG0(	tRNA-his	
MVB12B	ENSOARG0(	multives	miR-128-1-5p,miR-34c,miR-769-5p,miR-107-5p,novel_82,miR-326-3p,miR-449a,miR-199b-5p,miR-9851-3p,miR-96-5p,miR-199b-5p,miR-378g,miR-34b,miR-655-5p,miR-1343-5p,miR-199b-5p,miR-103a-2-5p,miR-491-5p,miR-199a-3p,miR-326,miR-377-3p,miR-5126,miR-296-3p,miR-4532,miR-8485,miR-296-3p,miR-432-3p,miR-432-3p,miR-2889,miR-744-5p,miR-138,miR-30b-3p,miR-22-3p,miR-30b-3p,miR-296-5p,miR-1285,miR-1843b-5p,miR-148b-5p,miR-134-3p,novel_4,miR-133b-5p,miR-4492,miR-3432a,miR-138-5p,miR-664a,miR-331-3p,miR-665,miR-2433,miR-410-5p,miR-769-5p,miR-1271,miR-193a-5p,miR-129-2-3p,miR-1827,miR-485-5p,miR-199a-5p,miR-22-3p,miR-324-5p,miR-34c-5p,miR-377-3p,miR-769,miR-365a-3p,miR-542-5p,miR-296-3p,miR-874-3p,miR-34b-5p,novel_96,miR-466i-5p,miR-196a-3p,miR-199a-5p,miR-143-3p,miR-34a-5p,miR-148a-5p,miR-142-3p,miR-1343-5p,miR-330-5p,miR-199a-3p,miR-1247-5p,miR-129-1-3p,miR-505-5p,miR-222-5p,miR-1271-5p,miR-212-5p,miR-3960,miR-6517

MRPS18B	ENSOARG0(	mitochon	miR-16-5p,miR-1306-5p,miR-424-5p,miR-204-5p,miR-324-3p,novel_63,miR-204-3p,miR-15b-5p,miR-374c-3p,miR-216b-5p,miR-193b-3p,miR-3065-3p,miR-296-3p,miR-503-3p,miR-496-3p,miR-660-5p,miR-885-3p,miR-2312,miR-329b,miR-21a-3p,miR-2285c,miR-503-3p,miR-3065-3p,miR-2285b,miR-664b-3p,miR-107,miR-21-3p,miR-664-3p,miR-532-3p,miR-322-5p,miR-15a-5p,miR-133a-3p,miR-496,miR-885-3p,novel_27,miR-211,miR-877-3p,miR-2285f,miR-193b-3p,miR-2447,miR-335-5p,miR-128-1-5p,novel_1,miR-335,miR-16a,miR-371a-5p,miR-15a,miR-218-5p,miR-374c-3p,miR-211-5p,miR-664b,miR-195a-5p,miR-107,miR-1306,miR-2285ab,miR-4532,miR-296-3p,miR-668-3p,miR-424-5p,miR-660,miR-193a-3p,miR-16b,miR-2331-5p,miR-296-3p,miR-1306-5p,miR-2285e,miR-103,miR-2285g,miR-103a-3p,novel_78,miR-2300a-5p,miR-497-5p,miR-188-5p,miR-16b,miR-3071-5p,miR-125b-2-3p
RAB3GAP2	ENSOARG0(	RAB3 GTP	miR-34c,miR-382-5p,let-7i,miR-133a-5p,miR-147-5p,novel_1,miR-495-3p,miR-135a-2-3p,miR-326-3p,novel_27,miR-449a,miR-2428,miR-24-3p,miR-371b-3p,miR-9851-3p,miR-505-3p,miR-6516-3p,miR-450b-3p,miR-320b,miR-7-1-3p,miR-378b,miR-34b,miR-224-5p,miR-660,miR-452-5p,miR-466f-3p,miR-124-5p,miR-539-3p,miR-145a-5p,miR-3071-5p,miR-374b-3p,miR-873a-5p,miR-21-5p,miR-130a-3p,miR-26b-3p,miR-185-5p,let-7g-5p,miR-3969,miR-1-5p,miR-2332,miR-410-3p,miR-486b-3p,miR-145-3p,miR-323-3p,miR-320a,miR-2448-3p,miR-503-3p,miR-1814c,miR-10b-3p,let-7g-3p,miR-598-3p,miR-141-3p,miR-154b-5p,miR-34c-5p,miR-365a-3p,miR-30c-2-3p,let-7c-5p,miR-217,miR-543-5p,miR-450a-2-3p,miR-1,miR-376c-5p,miR-376b-5p,miR-2284a,miR-380-5p,miR-128-3p,let-7a-2-3p,miR-411-3p,miR-582-3p,let-7d,miR-222,miR-545-5p,miR-214-5p,miR-24-2-5p,miR-2285t,miR-335,miR-452-5p,miR-3059-5p,miR-32-3p,miR-361-3p,miR-499b-5p,miR-7977,let-7d-5p,miR-3082-5p,miR-320b,miR-326,novel_103,let-7e,miR-3955-3p,miR-652-5p,let-7a-5p,miR-494-3p,miR-30c-1-3p,miR-412-3p,miR-125b-2-3p,miR-3120-5p,miR-2426,miR-5010-3p,novel_4,miR-151a-3p,miR-9788-3p,miR-221-3p,miR-216a-3p,miR-655-3p,miR-222-3p,miR-208b-5p,miR-450b-3p,miR-6516-3p,miR-3074-2-3p,miR-539-5p,miR-4510,let-7b-5p,miR-6134,miR-338-3p,miR-206-3p,miR-27b-5p,miR-21a-3p,novel_99,miR-182-5p,miR-545-3p,miR-221-5p,miR-32-3p,miR-660-5p,miR-204-3p,miR-6130,miR-139-5p,miR-185-3p,miR-34b-5p,miR-30d,miR-221-5p,miR-299-3p,miR-320d,miR-466i-5p,miR-183-5p,miR-146a-5p,miR-125b-2-3p,miR-124-3p,miR-140-3p,miR-188-3p,miR-1193,novel_133,miR-1246,miR-376a-5p,miR-4429,miR-3600,miR-1b-3p,miR-505-3p,miR-616-3p,miR-487a-3p,miR-222-5p,miR-148b-3p,miR-206,miR-379-3p,miR-335-5p,miR-1a-1-

PBX3	ENSOARG0(	pre-B-ce	miR-93-5p,miR-137-3p,miR-30e-5p,miR-20b-5p,miR-1248,miR-329b,miR-345-5p,miR-2285aa,miR-2355-5p,miR-1271,miR-574-5p,miR-874-3p,miR-503-3p,miR-300-3p,miR-3959-5p,novel_63,miR-2284k,miR-592,novel_9,miR-16-5p,miR-2284v,miR-1197-3p,miR-330-5p,miR-196a-5p,miR-1224-5p,miR-2285ad,miR-429-3p,miR-2284z,miR-129-1-3p,miR-322-5p,novel_127,miR-6536,let-7f-5p,miR-127-5p,let-7g,miR-29a-5p,miR-2285b,miR-1306,miR-216a-3p,miR-146b-5p,miR-378f,let-7e-5p,miR-412-3p,miR-3074-5p,miR-329-5p,miR-320c,miR-677,miR-181b-3p,miR-374c-3p,miR-28c,miR-1839-3p,novel_23,miR-19b-2-5p,miR-7b-5p,miR-497-5p,miR-2284x,miR-27b-3p,miR-16b,miR-16-1-3p,miR-149-5p,miR-412-5p,miR-3535,miR-365b-5p,novel_51,miR-2411-3p,miR-7-5p,miR-377-3p,miR-146a,miR-196b-5p,let-7f-2-3p,let-7f-2-3p,let-7j,let-7a-2-3p,miR-2284l,miR-30d-5p,miR-655,miR-543-3p,miR-345-5p,miR-216b-3p,miR-2331-3p,let-7i-5p,miR-15b-5p,miR-10a,miR-377-3p,miR-2284s,let-7f,let-7b-3p,miR-129-5p,miR-214,let-7b,miR-129-2-3p,miR-3074-5p,miR-181c-5p,miR-6525,miR-769-5p,novel_44,miR-224-5p,miR-378a-3p,miR-378i,miR-2366,miR-1961,miR-16-1-3p,miR-361-5p,miR-148a-5p,miR-142-3p,miR-33b-3p,miR-212-5p,miR-18a-3p,miR-106a,miR-328-3p,miR-422a,miR-345-3p,let-7k,miR-98-5p,miR-329a-5p,miR-181b-1-3p,miR-378j,miR-376a-3p,miR-335-3p,miR-1983,miR-136-5p,miR-7689-3p,miR-1185-2-3p,miR-301a-5p,novel_82,miR-101-3p,miR-490-5p,miR-3968,miR-2284j,miR-2447,miR-1285-5p,miR-361-3p,novel_111,miR-2284r,miR-301b-5p,miR-541,miR-101c,miR-1388-5p,miR-181b-2-3p,miR-145b,miR-1343-3p,miR-148b-5p,miR-1185-3p,miR-539-5p,miR-376b-3p,miR-147a,miR-505,miR-29b-1-5p,miR-106b-5p,miR-16b,miR-182-5p,miR-146b,miR-214-3p,miR-1388-3p,miR-101b-3p,miR-8485,miR-378d,miR-17-5p,miR-320a,miR-
ANXA1	ENSOARG0(	annexin	miR-3071-3p,miR-2284aa,miR-2284z

ZNF70	ENSOARG0(	zinc fin	miR-29b-1-5p,miR-20a,miR-105-3p,miR-21-3p,miR-30b-3p,miR-1277-5p,miR-106a,miR-185-3p,miR-542-5p,novel_83,miR-532-3p,miR-15a-5p,miR-200b,miR-505-3p,miR-1b-3p,miR-3960,miR-548e-3p,miR-378d,miR-19b-3p,miR-140-3p,miR-188-3p,miR-124-3p,miR-2904,miR-362-3p,miR-2408,miR-6134,let-7b-5p,miR-206-3p,miR-10b-5p,miR-145a-3p,miR-424-5p,miR-17-3p,miR-320e,miR-877-5p,miR-22-3p,miR-204-3p,miR-193b-3p,miR-27a-5p,miR-140-5p,miR-30f,miR-21a-3p,miR-4532,miR-30c-1-3p,miR-2330-5p,miR-424-5p,miR-106a-5p,let-7a-5p,miR-542-5p,let-7e,miR-25-5p,miR-3085-3p,miR-362-3p,miR-1306-5p,miR-5126,miR-342,miR-9788-3p,novel_4,miR-2300a-5p,miR-3120-5p,miR-2284aa,miR-299a-5p,miR-30b-3p,miR-125b-2-3p,miR-10b,miR-296-5p,miR-484,miR-154a,miR-1296-5p,miR-212-3p,miR-7134-3p,miR-335,miR-4726-5p,miR-107-5p,miR-2285y,miR-2285n,miR-454-5p,let-7d-5p,miR-499b-5p,miR-20a-3p,miR-3064-5p,miR-361-3p,miR-1271-3p,miR-28b,miR-4286,miR-181a-2-3p,miR-30a-3p,miR-3529-3p,miR-200c-3p,miR-17-3p,miR-2898,let-7c-5p,miR-339a,miR-30c-2-3p,miR-200c,miR-2285af,miR-214-5p,miR-423-3p,miR-10a-5p,miR-200b-3p,let-7d,miR-30a-5p,miR-29d-3p,miR-345-3p,miR-93,miR-486b-3p,miR-138-5p,miR-99b-3p,miR-29b-3p,miR-324-5p,miR-365a-3p,miR-370-3p,miR-7144-5p,miR-3154,miR-17-5p,miR-2483-5p,miR-1343-5p,miR-124-5p,miR-106a-5p,miR-17-5p,miR-330-3p,miR-197-5p,miR-20b,let-7g-5p,miR-377-5p,miR-7862,miR-744-5p,miR-2889,miR-138,miR-30b-3p,miR-29b,miR-653,miR-1307-3p,miR-371b-3p,miR-24-3p,novel_101,miR-542-5p,miR-17-3p,miR-495-3p,miR-3957-3p,miR-342-3p,miR-147-5p,miR-203b-5p,miR-128-1-5p,let-7i,miR-33a-3p,miR-16a,miR-20a-5p,miR-15a,miR-29a-3p,miR-10a-5p,miR-655-5p,miR-505-3p,miR-195a-5p,miR-7975,miR-9851-3p,miR-19a-3p,miR-2285k,miR-1291,miR-18a-3p,miR-
ACSM5	ENSOARG0(	acyl-CoA	miR-3059-5p,miR-296-3p,miR-1260b,miR-345-3p,miR-2411-3p,miR-296-3p,miR-1260a,novel_96,miR-760-3p,miR-487b-5p,miR-491-5p,miR-345-3p,miR-488-3p,miR-885-3p,miR-1260b,miR-2426,miR-885-3p,miR-876-3p
MOGS	ENSOARG0(	mannosyl	miR-320b,miR-1260a,miR-185-3p,miR-217,miR-422a,miR-32,miR-181b-5p,miR-320d,miR-299-3p,miR-92a-3p,miR-30b-3p,miR-33a-5p,miR-23a-3p,miR-378d,miR-92b-3p,miR-143-3p,miR-181a-5p,miR-2300b-3p,miR-223-3p,miR-378i,miR-378b,miR-378a-3p,miR-217-5p,miR-4429,miR-1260b,miR-378c,miR-299a-3p,miR-378h,miR-23c,miR-23b-3p,novel_116,miR-532-3p,miR-410-5p,miR-4324,miR-320e,miR-769-5p,miR-92a-3p,miR-204-5p,miR-4492,miR-363-3p,miR-378c,miR-181d-5p,miR-1260b,miR-211,miR-769-5p,miR-181c-5p,miR-378c,miR-361-3p,miR-23b,miR-320a,miR-378f,miR-20a-3p,miR-378a-3p,miR-32-5p,miR-211-5p,miR-769,miR-299b-3p,miR-25-3p,miR-378e,miR-25,miR-320c

GPR153	ENSOARGO(G protei	miR-98-5p,miR-320d,let-7k,novel_17,miR-412-5p,miR-216a-5p,miR-376c-3p,miR-29b-1-5p,miR-183-5p,miR-4429,miR-378d,miR-1961,novel_133,miR-1197-5p,miR-140-3p,miR-487a-3p,miR-616-3p,miR-151-5p,miR-485-3p,miR-3600,miR-487b-5p,novel_74,let-7f,miR-224-5p,let-7b-5p,miR-145a-3p,let-7b,miR-665-5p,miR-345-5p,miR-133b-3p,miR-1827,miR-2285c,miR-377-3p,miR-376e-3p,miR-151b,let-7i-5p,novel_25,novel_32,miR-660-5p,miR-532-5p,miR-370-5p,miR-491-5p,novel_124,miR-103a-2-5p,let-7e,miR-320b,miR-342,miR-29b-1-5p,miR-2957,miR-8485,let-7a-5p,miR-3120-5p,miR-21b,miR-101c,miR-301b-5p,miR-296-5p,miR-411b-5p,miR-2285e,miR-2300a-5p,miR-145b,miR-335,miR-101-3p,novel_82,miR-107-5p,miR-301a-5p,miR-371a-5p,miR-361-3p,miR-3059-5p,miR-9-5p,miR-99b-5p,miR-335-3p,miR-361-3p,miR-378g,let-7d-5p,miR-203-3p,let-7f-5p,miR-543-5p,novel_96,miR-99a,let-7c-5p,miR-148b-5p,novel_39,let-7g,let-7d,miR-99a-5p,miR-23b-5p,miR-2427,miR-302a-5p,miR-9-5p,miR-214-5p,miR-370-5p,miR-2433,miR-133a-3p,miR-6128,miR-193a-5p,miR-345-5p,miR-485-5p,miR-23a-5p,miR-100-5p,miR-320a,miR-154b-5p,miR-376c-3p,miR-2355-5p,miR-660,miR-377-3p,miR-7-5p,miR-224-5p,miR-99a-5p,novel_51,miR-190a,miR-654-3p,miR-345-5p,miR-503-3p,miR-668-3p,miR-7b-5p,miR-145a-5p,let-7g-5p,miR-134-3p,miR-2285g,miR-31-5p,novel_1,miR-203b-5p,miR-22-5p,miR-412-5p,let-7i,miR-335-5p,miR-342-3p,miR-1193,let-7e-5p,miR-146b-3p,miR-320c,miR-655-5p,miR-320b

CFAP221	ENSOARG0(	cilia an	miR-34c,miR-6402,miR-28c,miR-625-3p,miR-199c,miR-502b,miR-17-3p,miR-342-3p,novel_27,miR-449a,miR-2428,miR-150-5p,miR-18a-3p,miR-376b-3p,miR-216a-3p,miR-2387,miR-505-3p,miR-450b-3p,miR-1291,miR-2285k,miR-3120-3p,miR-3074-5p,miR-6535,miR-320c,miR-669,miR-2404,miR-34b,miR-374c-3p,miR-194a,miR-582,miR-1343-5p,miR-125a-5p,miR-466f-3p,miR-378a-5p,miR-2483-5p,miR-363-5p,miR-130b-5p,miR-493-5p,miR-2284l,miR-22-3p,miR-3071-5p,miR-194-5p,miR-145a-5p,miR-19b-2-5p,miR-432,miR-2113,miR-27b-3p,miR-2284x,miR-7862,miR-138,miR-665,miR-133b-5p,miR-194-3p,novel_116,miR-432-5p,miR-2285g,miR-2284d,miR-125b-5p,miR-331-3p,miR-6740-5p,miR-3969,miR-2284k,miR-2433,miR-2332,miR-486b-3p,miR-28-5p,miR-138-5p,miR-331-5p,miR-193a-5p,miR-320a,miR-362-5p,miR-3154,miR-331-5p,miR-7144-5p,miR-671-5p,miR-876-3p,miR-141-3p,miR-1271,miR-125a,miR-487a-3p,miR-874-3p,miR-374c-3p,miR-34c-5p,miR-217,miR-376b-3p,miR-200c,miR-6119-5p,miR-125a-3p,miR-200a-3p,novel_96,novel_127,miR-2898,miR-17-3p,miR-127-5p,miR-200c-3p,miR-29b-2-5p,miR-30a-3p,miR-3064-5p,miR-4286,novel_39,miR-29a-5p,miR-1957a,miR-3065-3p,miR-1271-3p,miR-28b,miR-450b-5p,miR-148b-5p,miR-1343-5p,miR-128-3p,miR-200b-3p,miR-431,miR-429-3p,miR-214-5p,miR-486-5p,miR-505-5p,miR-9-5p,miR-6516-5p,novel_82,miR-2440,miR-499a-5p,miR-194b-5p,miR-9-5p,miR-452-5p,miR-3059-5p,novel_60,miR-2447,miR-3956-3p,miR-154a,miR-7705,miR-362-5p,miR-361-3p,miR-140-5p,miR-409-5p,miR-493-5p,miR-376a-3p,miR-877-3p,miR-504,miR-136-5p,miR-7977,miR-126b-5p,miR-3064-5p,miR-452-3p,miR-10b,miR-27a-3p,miR-376d,miR-4443,miR-489,miR-29b-1-5p,miR-2957,miR-342,miR-320b,miR-431-5p,miR-491-5p,miR-3085-3p,miR-2330-5p,miR-125b-2-3p,miR-3962,miR-484,miR-

TFAP2D	ENSOARGO(	transcri	miR-106a,miR-450a-2-3p,miR-181b-5p,miR-2484,miR-125a-3p,miR-30f,miR-345-3p,miR-422a,miR-92a-1-5p,miR-376a-5p,miR-130a-5p,miR-486-3p,miR-487a,miR-20a,miR-181a-2-3p,miR-345-3p,miR-378i,miR-376a-5p,miR-215-5p,miR-2300b-3p,miR-124-3p,miR-378a-3p,miR-140-3p,miR-7859,miR-487a-3p,miR-322-5p,miR-302a-5p,miR-378h,miR-15a-5p,miR-106a,miR-7641,miR-30d-3p,miR-138-5p,miR-487b-5p,miR-424-5p,miR-4492,miR-130b-5p,miR-374b-3p,miR-486b-3p,miR-331-3p,miR-93,miR-374a-3p,miR-3184-5p,miR-378c,miR-378c,miR-145a-3p,miR-16-5p,miR-154b-3p,miR-3074-1-3p,miR-543-3p,miR-20b-5p,miR-23a-5p,miR-93-5p,miR-17-5p,miR-377-3p,miR-365a-3p,miR-15b-5p,miR-378a-3p,miR-487a-3p,miR-874-3p,miR-154a-3p,miR-215-5p,miR-377-3p,miR-505,miR-330-3p,miR-106b-5p,miR-16b,miR-106a-5p,miR-17-5p,miR-2411-3p,miR-378d,miR-539-3p,miR-193a,miR-130b-5p,miR-124a,miR-1839-3p,miR-424-5p,miR-6240,miR-106a-5p,miR-378c,miR-138,miR-16b,miR-450b-5p,miR-21b,miR-497-5p,miR-378b,miR-873a-5p,miR-143-5p,miR-145a-5p,miR-9788-3p,miR-20b,miR-2355-3p,miR-3955-5p,miR-330-3p,miR-199b-3p,miR-409b,miR-145b,miR-1843a-3p,miR-154-3p,miR-199c,miR-15a,miR-576-3p,miR-20a-5p,miR-16a,miR-323c,miR-423-5p,miR-2284w,novel_120,novel_60,miR-3059-5p,miR-3956-3p,miR-1839-3p,miR-181d-5p,miR-335-3p,miR-378f,miR-195a-5p,miR-378e,miR-3082-5p,miR-329-5p,miR-378b

ZNF791	ENSOARG0(	zinc fin	miR-27b-3p,miR-188-5p,miR-223-3p,novel_23,miR-3431,miR-194-3p,miR-31-5p,miR-378a-5p,miR-146a,let-7a-2-3p,miR-668-3p,let-7f-2-3p,let-7f-2-3p,miR-2319a,miR-378f,miR-148a-3p,miR-411b-3p,miR-299b-3p,miR-677,miR-320c,miR-181b-3p,miR-216b-3p,miR-199b-5p,miR-301b-3p,miR-1a-1-5p,miR-335-5p,miR-1839-3p,miR-23b-5p,novel_121,miR-299a-3p,miR-2285ad,miR-429-3p,miR-18b-5p,miR-6536,miR-1a-2-5p,miR-127-5p,miR-152-3p,miR-125a-3p,miR-219-3p,miR-30f,miR-148b-5p,miR-2285b,miR-3064-5p,miR-1248,miR-301,miR-137-3p,miR-331-5p,miR-300-3p,miR-3068-3p,novel_9,miR-6740-5p,miR-193a-5p,miR-144-5p,miR-1388-5p,miR-21b,miR-758-3p,miR-147a,miR-2459,miR-1185-3p,miR-421,miR-181b-2-3p,miR-145b,miR-1185-5p,miR-130b-3p,miR-199b-5p,miR-214-3p,miR-199a-3p,miR-182-5p,miR-378d,miR-8485,miR-124a,miR-744-3p,miR-96-5p,miR-335-3p,miR-544-5p,miR-203-3p,miR-144-5p,miR-1185-2-3p,miR-139-5p,miR-628-3p,miR-490-5p,novel_82,miR-361-3p,miR-877-3p,miR-3956-3p,miR-885-5p,miR-33b-3p,miR-664-5p,miR-218-1-3p,miR-34a-5p,miR-361-5p,miR-378i,miR-2366,miR-98-3p,miR-217-5p,miR-378a-3p,miR-7859,miR-30d-3p,miR-202-5p,miR-3653-3p,miR-210-5p,miR-345-3p,miR-422a,miR-664b-3p,miR-376a-5p,miR-181b-1-3p,miR-486-3p,miR-2424,miR-143-3p,miR-195a-3p,miR-625-5p,miR-301a-3p,miR-548o-3p,novel_74,miR-4492,miR-454-3p,miR-874-5p,miR-130b-5p,let-7b-3p,miR-30e-3p,miR-30a-3p,miR-219b-3p,miR-98-3p,miR-122-5p,miR-214,miR-2284ab,miR-219a-1-3p,miR-653,miR-1434-3p,miR-145a-5p,miR-2285g,miR-187-3p,miR-665,miR-130a-3p,miR-654-3p,miR-130b-5p,miR-382,miR-9851-3p,miR-18b,miR-378b,miR-323a-5p,miR-670-3p,miR-34b,miR-320b,novel_1,miR-34c,miR-382-5p,miR-449a,miR-2428,miR-17-3p,miR-1193,miR-2285x,miR-300,miR-200b-3p,let-7a-2-3p,miR-345-3p,miR-
ALDH1A1	ENSOARG0(	aldehyde	miR-2355-3p,miR-155-5p,miR-877-3p,miR-376b,miR-2330-5p,miR-216c-5p,miR-877-3p,miR-487a-5p,miR-155-5p,miR-155-5p,miR-382-5p,miR-210-5p
RBM47	ENSOARG0(	RNA bind	novel_83,miR-2355-3p,miR-2411-5p,miR-2285ad,miR-3960,let-7a-3p,miR-380-3p,miR-98-3p,miR-124-3p,novel_23,miR-542-3p,miR-199a-5p,miR-2411,miR-1b-5p,miR-491-3p,miR-380-3p,miR-92b-3p,miR-2483-5p,miR-195a-3p,miR-652-5p,miR-124a,miR-199b-5p,miR-92a-3p,miR-2484,miR-214-3p,miR-466i-5p,miR-32,miR-181c-3p,miR-205-5p,miR-125a-3p,miR-199a-5p,miR-25,novel_69,miR-216b-5p,miR-25-3p,miR-2285u,miR-532-5p,miR-32-5p,miR-2319a,miR-543-3p,novel_94,miR-329b,miR-2318,miR-378b,let-7f-1-3p,miR-199b-5p,miR-199b-5p,miR-7705,miR-6525,miR-98-3p,miR-214,miR-452-5p,miR-363-3p,miR-212-3p,miR-31-3p,miR-92a-3p,miR-2285y,let-7b-3p,miR-2285n,miR-218-5p

DDX10	ENSOARG0(	DEAD (As	miR-103a-2-5p,miR-136-3p,miR-210-5p,miR-205-5p,miR-2285af,miR-345-5p,miR-29a-5p,miR-30a-3p,miR-133a-3p,miR-544a,miR-411-3p,miR-2355-3p,miR-544b,miR-500-5p,miR-30d-3p,miR-3600,miR-2300a-5p,miR-204-5p,miR-147-5p,miR-22-5p,miR-129-5p,miR-379-3p,miR-107-5p,miR-30a-3p,miR-30e-3p,miR-218-5p,miR-338-3p,novel_111,miR-211,miR-362-5p,miR-877-3p,miR-3059-5p,miR-877-3p,miR-345-5p,miR-345-5p,miR-411,miR-3591-3p,miR-362-5p,miR-378j,miR-677,miR-670-3p,miR-544-3p,miR-211-5p,miR-21-3p
TMC1	ENSOARG0(	transmem	miR-223-5p,miR-15b-5p,miR-34c-5p,miR-323-5p,miR-125a,miR-140-5p,novel_99,miR-671-5p,miR-10b-3p,miR-488-3p,miR-450a-1-3p,miR-365a-5p,miR-145-3p,miR-6134,miR-6525,miR-224-5p,miR-16-5p,miR-425-5p,miR-214,miR-590-3p,miR-487a-5p,miR-424-5p,miR-129-5p,miR-4492,miR-486b-3p,miR-450b-3p,miR-664a,miR-125b-5p,miR-29b-2-5p,miR-409-3p,miR-2285j,miR-494-5p,miR-2332,miR-450a-1-3p,miR-302a-5p,miR-322-5p,miR-15a-5p,miR-361-5p,miR-34a-5p,miR-1197-5p,miR-486-3p,miR-1271-3p,miR-2285b,miR-181b-1-3p,miR-29b-2-5p,miR-345-3p,miR-3529-3p,miR-105-3p,miR-107,miR-34b-5p,miR-466i-5p,miR-122-3p,miR-125b,miR-185-3p,miR-677,miR-181b-3p,miR-323a-5p,miR-34b,miR-499b-5p,miR-2285u,miR-7-1-3p,miR-195a-5p,miR-877-3p,miR-450b-3p,miR-107,miR-21c,miR-378j,miR-140-5p,miR-449a,miR-154a,miR-2428,miR-877-3p,miR-1296-5p,novel_101,miR-3059-5p,miR-335-5p,miR-147-5p,miR-335,miR-34c,miR-16a,miR-301a-5p,miR-4324,miR-15a,miR-218-5p,miR-6402,miR-103,miR-432-5p,miR-1298-3p,miR-181b-2-3p,miR-222-5p,miR-3955-5p,novel_4,miR-103a-3p,miR-497-5p,miR-21-5p,miR-16b,miR-101a-5p,miR-450b-5p,miR-197-3p,miR-450c-3p,miR-758-3p,miR-296-5p,miR-432,miR-2284r,miR-1285,miR-301b-5p,miR-758-3p,miR-376b,miR-494-3p,miR-424-5p,miR-125a-5p,miR-214-3p,miR-124-5p,miR-365b-5p,miR-16b,miR-224-5p

WNT5B	ENSOARG0(	wingless	miR-224-5p,miR-6525,miR-10b-5p,miR-338-3p,miR-6134,miR-122-5p,miR-3184-5p,miR-4510,miR-214,miR-665-5p,miR-6516-3p,miR-17-3p,miR-130b-5p,miR-708-5p,miR-874-5p,miR-222-3p,miR-320e,miR-3604,miR-665,miR-216b-5p,miR-6130,miR-10a,miR-544-3p,miR-548o-3p,miR-504-5p,miR-21-3p,miR-345-5p,miR-543-3p,miR-2285c,miR-21a-3p,miR-299,miR-129b-3p,miR-376a-5p,miR-2285i,miR-664b-3p,miR-486-3p,miR-466i-5p,miR-760-3p,miR-320d,miR-299-3p,miR-2484,miR-21-3p,miR-185-3p,miR-125b,miR-345-3p,miR-95-3p,miR-328-3p,miR-1247-5p,miR-199a-3p,miR-18a-3p,miR-4429,miR-19b-3p,miR-361-5p,miR-378d,miR-2366,novel_133,miR-191,miR-5703,miR-1197-5p,miR-140-3p,miR-124-3p,miR-423-5p,miR-3059-5p,miR-335,miR-2285t,miR-615,miR-1973,miR-489,miR-2483-3p,miR-3596,miR-2319b,miR-211-5p,miR-7977,miR-504,miR-221,miR-378j,miR-8485,miR-124a,miR-214-3p,miR-1306-5p,novel_103,miR-320b,miR-199a-3p,miR-323b-3p,miR-1298-3p,miR-496-5p,miR-2459,miR-2285e,miR-9788-3p,miR-221-3p,miR-548w,novel_4,miR-1343-3p,miR-544a,miR-101c,miR-10b,miR-484,miR-30b-3p,miR-323b,miR-1260b,miR-1306-5p,miR-331-5p,novel_9,miR-134-5p,miR-486b-3p,miR-28-5p,miR-204-5p,miR-191-5p,miR-2332,miR-5100,miR-2433,miR-3969,miR-125b-5p,miR-324-5p,miR-874-3p,miR-574-5p,miR-615-5p,miR-125a,miR-542-5p,miR-370-3p,miR-329b,miR-345-5p,miR-2474,miR-485-5p,miR-671-5p,miR-331-5p,miR-3154,miR-320a,miR-2478,novel_39,miR-3529-3p,miR-495-5p,miR-127-5p,miR-17-3p,miR-543-5p,novel_127,miR-107,miR-1260a,miR-758-5p,miR-505-5p,miR-134-5p,miR-222,miR-299a-3p,miR-10a-5p,miR-134,miR-615-3p,miR-345-3p,miR-24-3p,miR-211,miR-2428,miR-335-5p,miR-17-3p,miR-656-5p,miR-22-5p,novel_69,miR-299b-3p,miR-374c-3p,miR-320c,miR-10a-5p,miR-19a-3p,miR-107,miR-2285k,miR-1291,miR-6516-3p,miR-

ZFAND5	ENSOARG0(	zinc fin	let-7a-2-3p,miR-10a-5p,miR-218-2-3p,miR-322-5p,miR-302a-5p,miR-1271-5p,miR-2285ad,miR-200a-3p,novel_96,miR-1a-2-5p,miR-205-5p,miR-8117,miR-200a,miR-2284y,miR-29b-2-5p,miR-20b-5p,novel_48,miR-1814c,let-7g-3p,miR-93-5p,miR-17-5p,miR-3154,miR-301,miR-223-5p,miR-141-3p,miR-3969,miR-135a-5p,miR-1-5p,miR-181c-3p,miR-323-3p,miR-93,miR-3607-3p,miR-16-5p,miR-497-5p,miR-188-5p,miR-16b,miR-3071-5p,miR-542-3p,miR-181a-5p,miR-411-5p,miR-2355-3p,miR-20b,miR-2285g,miR-378a-5p,miR-17-5p,miR-106a-5p,miR-539-3p,let-7a-2-3p,miR-654-3p,miR-493-5p,miR-9851-3p,miR-7975,miR-505-3p,miR-135b-5p,miR-195a-5p,miR-412-3p,miR-3065-5p,miR-3591-3p,miR-2285ab,miR-7-1-3p,miR-10a-5p,miR-147-5p,novel_1,miR-16a,miR-33a-3p,miR-4324,miR-15a,miR-20a-5p,miR-374c-5p,miR-495-3p,novel_101,miR-1a-1-5p,miR-218-1-3p,miR-2284u,miR-33b-3p,novel_83,miR-487a-3p,miR-7859,miR-340-5p,miR-15a-5p,miR-1b-3p,miR-485-3p,miR-505-3p,miR-548e-3p,miR-106a,miR-3653-3p,miR-106a,miR-216a-5p,miR-29b-1-5p,miR-125b-2-3p,miR-20a,miR-183-5p,miR-543-3p,miR-2312,let-7g-3p,miR-129b-3p,miR-411,miR-2285c,miR-15b-5p,miR-499b-3p,miR-10a,miR-21-3p,miR-216b-3p,miR-552-3p,miR-504-5p,miR-548o-3p,miR-424-5p,miR-487b-5p,miR-151-3p,miR-29b-2-5p,miR-338-5p,miR-2285j,miR-15b-3p,miR-338-3p,miR-181c-5p,miR-10b-5p,miR-1271,miR-539-5p,miR-3184-3p,miR-3120-5p,miR-125b-2-3p,miR-10b,miR-143-5p,miR-2285e,miR-539-5p,miR-151a-3p,miR-491-5p,miR-29b-1-5p,miR-106b-5p,miR-16b,miR-3187-3p,miR-1b-5p,miR-2310,miR-33a-5p,miR-424-5p,miR-494-3p,miR-106a-5p,miR-493-5p,miR-1983,miR-181a-3p,miR-96-5p,miR-504,miR-129b-5p,miR-203-3p,miR-499a-3p,miR-2285u,miR-2284g,miR-452-3p,miR-499a-5p,miR-2285y,miR-2285n,miR-1285-5p,miR-7705,miR-154a,miR-877-3p,miR-
IGSF1	ENSOARG0(	immunogl	miR-199c,miR-379-3p,miR-4324,miR-502-3p,miR-423-5p,miR-199b-5p,miR-24-3p,miR-495-3p,miR-17-3p,miR-135a-2-3p,miR-335-3p,miR-504,miR-216a-3p,miR-199b-5p,miR-454-5p,miR-500-3p,miR-320b,miR-664b,miR-125a-5p,miR-199b-5p,miR-2957,miR-330-3p,miR-323b-3p,novel_51,miR-654-3p,miR-2285r,miR-1388-3p,miR-6240,miR-30b-3p,miR-450b-5p,miR-544a,miR-501-3p,miR-323b,miR-30b-3p,miR-432,miR-9788-3p,miR-455-3p,miR-148b-5p,miR-432-5p,miR-185-5p,miR-199b-3p,miR-330-3p,miR-1343-3p,miR-129-5p,miR-17-3p,miR-125b-5p,miR-331-3p,miR-3604,miR-2332,miR-3184-5p,miR-6525,miR-590-3p,miR-7144-5p,miR-671-5p,novel_99,miR-301,miR-199a-5p,novel_32,miR-154b-5p,miR-6516,miR-544-3p,miR-216b-5p,miR-2285aa,miR-3065-3p,miR-125a,miR-574-5p,miR-504-5p,miR-496-3p,miR-503-3p,miR-17-3p,miR-500a-5p,miR-125b,miR-6119-5p,miR-664-3p,miR-125a-3p,miR-3065-3p,miR-199a-5p,miR-148b-5p,miR-2385-3p,miR-128-3p,miR-148a-5p,miR-496,miR-411-3p,miR-501-3p,miR-2408,miR-545-5p,miR-6516-5p

AKR1E2	ENSOARG0(	aldo-ket	miR-134-5p,miR-125b-5p,miR-100-3p,miR-193a-5p,miR-20b-5p,miR-93-5p,miR-25-3p,miR-874-3p,miR-125a,miR-2285aa,miR-127-5p,miR-152-3p,novel_127,novel_96,miR-8117,miR-148b-5p,miR-2285b,miR-1290,miR-23b-5p,miR-134-5p,miR-450a-1-3p,miR-6517,miR-429-3p,miR-299a-3p,novel_79,miR-6119-3p,miR-4324,novel_87,miR-425-5p,miR-148a-3p,miR-374c-3p,miR-299b-3p,miR-320c,novel_68,novel_51,miR-2411-3p,miR-32,miR-105-5p,miR-503-3p,miR-6240,miR-16-1-3p,miR-1843b-5p,miR-19b-2-5p,miR-450c-3p,miR-134-3p,miR-194-3p,miR-103a-3p,miR-3955-5p,miR-129-5p,miR-30e-3p,miR-2285j,miR-665,miR-30a-3p,miR-769-5p,miR-181c-5p,miR-186-5p,miR-543-3p,miR-625-5p,miR-2285c,miR-450a-1-3p,miR-129b-3p,miR-548o-3p,miR-30c-1-3p,miR-32-5p,miR-2331-3p,miR-504-5p,miR-296-3p,miR-210-5p,miR-2285p,miR-345-3p,miR-92a-1-5p,miR-377-5p,miR-143-3p,miR-380-3p,miR-486-3p,miR-369-3p,miR-33b-3p,miR-148a-5p,miR-16-1-3p,miR-218-1-3p,miR-2366,miR-217-5p,miR-7859,miR-106a,miR-18a-3p,miR-3968,miR-499a-5p,miR-490-5p,novel_82,miR-218-5p,novel_111,miR-885-5p,miR-363-3p,miR-2447,miR-504,miR-96-5p,miR-140-5p,miR-3596,miR-378g,miR-139-5p,miR-2284g,miR-374b-5p,miR-2285u,miR-487b-5p,miR-491-5p,miR-153-3p,miR-106b-5p,miR-29b-1-5p,miR-323b-3p,miR-2310,miR-8485,miR-1388-3p,miR-1388-5p,miR-1298-5p,miR-541,miR-758-3p,miR-323b,miR-1298-3p,miR-153,miR-2459,miR-455-3p,miR-148b-5p,novel_78,miR-330-3p,miR-1343-3p,miR-486b-3p,miR-370-5p,miR-2332,miR-3969,miR-409-3p,miR-500b-5p,miR-93,miR-10b-3p,miR-145-3p,miR-374b,miR-1814c,miR-485-5p,miR-23a-5p,miR-488-3p,miR-381-3p,miR-17-5p,miR-362-5p,miR-3154,miR-320a,miR-769,miR-200c-3p,miR-181b-5p,miR-450a-2-3p,miR-2898,miR-92a-3p,miR-107,miR-200c,miR-2285af,miR-217,miR-30c-2-

GPNMB	ENSOARG0(	glycopro	novel_27,miR-2428,miR-24-3p,miR-1193,miR-17-3p,miR-376a-3p,miR-135a-2-3p,miR-342-3p,novel_1,miR-16a,miR-33a-3p,miR-15a,miR-20a-5p,miR-28c,miR-576-3p,miR-320c,miR-299b-3p,miR-374c-3p,miR-26b-5p,novel_68,miR-2319a,miR-7975,miR-146b-5p,miR-6395,miR-195a-5p,miR-505-3p,miR-450b-3p,miR-1306,miR-150-5p,miR-18a-3p,miR-2387,miR-432-3p,miR-654-3p,miR-432-3p,miR-101-5p,miR-380-5p,miR-130b-5p,miR-452-5p,miR-1343-5p,miR-125a-5p,miR-106a-5p,miR-455-5p,miR-17-5p,miR-2411-3p,miR-146a,miR-377-3p,miR-20b,miR-412-5p,miR-31-5p,miR-26a-2-3p,miR-497-5p,miR-455-5p,miR-16b,miR-16-1-3p,miR-219a-1-3p,miR-450c-3p,miR-145a-5p,miR-3071-5p,miR-374b-3p,miR-1285,miR-145-3p,miR-93,miR-1197-3p,miR-374a-3p,miR-16-5p,miR-374b-3p,miR-331-3p,miR-125b-5p,miR-370-5p,miR-376c-3p,miR-374c-3p,miR-769,miR-215-5p,miR-125a,novel_48,miR-20b-5p,miR-1814c,miR-329b,miR-93-5p,miR-320a,miR-17-5p,miR-3154,miR-488-3p,miR-301,miR-503-3p,miR-2448-3p,novel_39,miR-2285b,miR-1271-3p,miR-28b,miR-380-5p,miR-2385-3p,miR-30a-3p,miR-29b-2-5p,miR-4286,novel_127,miR-17-3p,miR-127-5p,miR-30c-2-3p,miR-450a-1-3p,miR-214-5p,miR-322-5p,miR-758-5p,miR-6516-5p,miR-412,miR-299a-3p,miR-218-2-3p,miR-1343-5p,miR-345-3p,miR-3591-5p,miR-219a-5p,novel_60,miR-3956-3p,miR-541-3p,miR-374a-3p,miR-3968,miR-769-5p,novel_82,miR-2285n,miR-203-3p,miR-1185-2-3p,miR-499a-3p,miR-3082-5p,miR-126b-5p,miR-139-5p,miR-452-3p,miR-2310,miR-30c-1-3p,miR-744-3p,miR-33a-5p,miR-106a-5p,miR-424-5p,miR-494-3p,miR-146b,miR-29b-1-5p,miR-16b,miR-106b-5p,miR-342,miR-199a-3p,miR-320b,novel_115,miR-1306-5p,miR-148b-5p,miR-455-3p,miR-1185-3p,miR-539-5p,miR-145b,miR-2300a-5p,miR-409b,miR-548w,miR-151a-3p,miR-101c,miR-101a-5p,miR-412-3p,miR-125b-2-3p,miR-2284r,miR-
CELF5	ENSOARG0(	CUGBP, E	miR-361-3p,miR-200a-5p,miR-1291,miR-668-5p,miR-504,miR-671-5p,miR-31-5p,miR-504-5p,miR-199b-3p,miR-133b-5p,miR-1b-3p,miR-320b,miR-370-3p,miR-125a,miR-3120-3p,miR-3596,novel_69,miR-2440,miR-125b-5p,miR-324-3p,miR-125b,miR-22851,miR-125a-5p,miR-199c,novel_42,miR-361-3p

NEDD1	ENSOARG0(neural p	novel_79,miR-429-3p,miR-2285ad,miR-214-5p,miR-486-5p,miR-30a-5p,miR-2427,miR-1290,miR-345-3p,miR-10a-5p,let-7a-2-3p,miR-30c,miR-105-2,miR-582-3p,miR-181a-2-3p,miR-4286,miR-495-5p,miR-2284a,miR-2285b,novel_39,miR-29a-5p,miR-199a-5p,miR-376c-5p,miR-376b-5p,miR-2284y,miR-148b-5p,miR-217,miR-339a,miR-3071-3p,miR-200c,miR-125a-3p,miR-1260a,miR-200a-3p,miR-200c-3p,miR-127-5p,miR-125a,miR-141-3p,miR-542-5p,miR-2355-5p,miR-874-3p,miR-574-5p,miR-199a-5p,miR-223-5p,miR-17-5p,miR-376c-5p,miR-3154,miR-93-5p,miR-30e-5p,miR-488-3p,miR-1248,miR-671-5p,miR-7144-5p,novel_48,miR-376c-5p,miR-20b-5p,let-7g-3p,miR-329b,miR-10b-3p,miR-2284v,miR-1306-5p,let-7e-3p,miR-145-3p,miR-1260b,miR-93,miR-409-3p,miR-135a-5p,miR-125b-5p,miR-6740-5p,miR-5100,miR-2284k,miR-592,miR-31-3p,miR-26b-3p,miR-339-5p,miR-122-3p,miR-31-5p,miR-544b,miR-20b,miR-6238,miR-3431,miR-432-5p,miR-2284d,miR-145a-5p,miR-223-3p,miR-1434-3p,miR-708-3p,miR-432,miR-188-5p,miR-2284x,miR-26a-2-3p,miR-1260b,miR-668-3p,miR-493-5p,miR-130b-5p,let-7a-2-3p,miR-2411-3p,miR-106a-5p,miR-17-5p,miR-197-5p,miR-105-5p,miR-660,miR-125a-5p,miR-7-1-3p,miR-10a-5p,miR-378b,miR-329-5p,miR-670-3p,miR-411b-3p,miR-2404,miR-378b,miR-1306,miR-2387,miR-376b-5p,miR-135b-5p,miR-450b-3p,miR-495-3p,miR-542-5p,miR-2284w,miR-199b-5p,novel_27,miR-323c,miR-485-3p,miR-6402,miR-20a-5p,miR-142b,miR-502-5p,miR-421-5p,miR-22-5p,miR-6119-3p,miR-3600,miR-106a,miR-548e-3p,miR-199a-3p,miR-532-3p,miR-7859,miR-188-3p,miR-217-5p,miR-140-3p,miR-1193,miR-30c-5p,miR-148a-5p,miR-2284u,miR-361-5p,miR-142-3p,miR-33b-3p,miR-20a,miR-329a-5p,miR-2424,miR-130a-5p,miR-376a-5p,miR-125b,miR-92a-1-5p,miR-345-3p,miR-324-3p,miR-500a-5p,miR-219b-5p,miR-219-

POP5	ENSOARG0(	POP5 hom	miR-106b-3p,miR-487b-5p,miR-491-5p,miR-194a,novel_51,miR-106b-5p,miR-182-5p,miR-17-5p,miR-106a-5p,miR-365b-5p,miR-29b-1-5p,miR-431-5p,miR-326,miR-193a,miR-33a-5p,miR-363-5p,miR-106a-5p,miR-382-3p,miR-2284x,miR-30b-3p,novel_23,miR-194-5p,miR-30b-3p,miR-145a-5p,miR-450c-3p,miR-181a-5p,miR-542-3p,miR-484,miR-296-5p,miR-126a-5p,miR-20b,miR-2459,miR-194-3p,miR-216a-3p,miR-1298-3p,miR-134-3p,miR-185-5p,miR-145b,miR-339-5p,miR-133b-5p,miR-3964,miR-2285t,miR-656-5p,miR-499a-5p,miR-20a-5p,novel_82,miR-326-3p,miR-361-3p,miR-194b-5p,miR-181d-5p,novel_101,miR-1296-5p,miR-1983,miR-2319a,miR-412-3p,miR-450b-3p,miR-378j,miR-409-5p,miR-140-5p,miR-669,miR-489,miR-3596,miR-146b-3p,miR-378g,miR-320b,miR-2319b,novel_96,miR-200a-3p,miR-6536,miR-181b-5p,miR-106a,miR-339a,miR-324-3p,miR-219b-5p,miR-125a-3p,miR-29b-1-5p,miR-4286,miR-20a,miR-29b-2-5p,miR-30a-3p,miR-216c-5p,miR-378d,miR-330-5p,miR-2427,novel_133,miR-450a-1-3p,miR-616-3p,miR-222-5p,miR-23c,miR-383-5p,miR-30d-3p,miR-431,novel_79,miR-106a,miR-28-5p,miR-708-5p,miR-331-5p,novel_9,miR-450b-3p,miR-29b-2-5p,miR-30a-3p,miR-6740-5p,miR-30e-3p,miR-181c-5p,miR-365a-5p,miR-93,miR-186-5p,miR-1827,miR-20b-5p,miR-339b,miR-3154,miR-17-5p,miR-93-5p,miR-331-5p,miR-450a-1-3p,miR-331-5p,miR-182-5p,miR-141-3p,miR-370-3p,miR-548o-3p
UBE2J1	ENSOARG0(	ubiquiti	miR-221-5p,miR-376e-3p,miR-877-5p,miR-450a-1-3p,miR-145a-3p,miR-142a-5p,miR-181c-5p,miR-338-5p,miR-320e,miR-664a,miR-190a-5p,miR-129-5p,miR-7859,miR-193b-5p,miR-1246,miR-4429,miR-378d,miR-329a-5p,miR-195a-3p,miR-376c-3p,miR-143-3p,miR-374a-5p,miR-618,miR-216a-5p,miR-125b,miR-320d,miR-466i-5p,miR-221-5p,miR-10b,miR-374b-5p,miR-211-5p,miR-378g,miR-25,miR-3082-5p,miR-376d,miR-1185-2-3p,miR-140-5p,miR-376a-3p,miR-9-3p,miR-541-3p,miR-190b-5p,miR-181d-5p,miR-9-3p,novel_82,miR-628-3p,miR-490-5p,miR-142-5p,miR-376b-3p,miR-2320-3p,miR-216a-3p,miR-1185-3p,miR-541,miR-1839-5p,miR-1388-3p,miR-193a,miR-491-3p,miR-320b,miR-431-5p,miR-487b-5p,miR-125a,miR-141-3p,miR-331-5p,miR-154b-5p,miR-376c-3p,miR-30e-5p,miR-488-3p,miR-381-3p,miR-331-5p,miR-301,miR-200a-5p,miR-320a,miR-3154,miR-329b,miR-2318,miR-1248,miR-485-5p,miR-374b,miR-1306-5p,miR-3607-3p,miR-1197-3p,miR-410-5p,miR-5100,miR-125b-5p,miR-331-5p,miR-204-5p,miR-7641,miR-431,miR-302a-5p,miR-450a-1-3p,miR-1290,miR-30a-5p,miR-300,miR-3529-3p,miR-2385-3p,miR-181a-2-3p,miR-450b-5p,miR-376b-3p,miR-2285af,miR-125a-3p,miR-450a-2-3p,miR-6536,miR-181b-5p,miR-200a-3p,miR-329-5p,miR-7-1-3p,miR-2404,miR-670-3p,miR-320c,miR-376b-3p,miR-1291,miR-2285x,miR-211,miR-371b-3p,miR-24-3p,novel_27,miR-374c-5p,novel_87,miR-33a-3p,miR-382-5p,novel_1,miR-22-5p,miR-134-3p,miR-185-5p,miR-149-5p,miR-194-3p,miR-1285,miR-181a-5p,miR-3071-5p,miR-380-3p,miR-2284x,miR-382,let-7f-2-3p,let-7f-2-3p,miR-22841,miR-190a,miR-105-5p,miR-125a-5p

			miR-3064-5p,miR-664b,miR-10b,miR-452-3p,miR-20a-3p,miR-499b-5p,miR-211-5p,miR-499a-3p,miR-203-3p,miR-4443,miR-376d,miR-140-5p,miR-199b-5p,miR-129b-5p,miR-493-5p,miR-376a-3p,novel_94,miR-544-5p,novel_60,miR-2447,miR-3956-3p,miR-362-5p,miR-877-3p,novel_82,miR-2285y,miR-4726-5p,miR-1343-3p,miR-145b,miR-376b-3p,miR-455-3p,miR-500-5p,miR-2459,miR-484,miR-1388-5p,miR-101a-5p,miR-299a-5p,miR-1388-3p,miR-424-5p,miR-193a,miR-2310,miR-320b,miR-1306-5p,miR-29b-1-5p,miR-16b,miR-3085-3p,miR-3955-3p,miR-199b-5p,miR-146b,miR-214-3p,miR-140-5p,miR-21-3p,miR-499b-3p,miR-193b-3p,miR-204-3p,miR-877-5p,miR-15b-5p,miR-299,miR-450a-1-3p,miR-433-3p,miR-133b-3p,miR-345-5p,miR-186-5p,miR-3074-5p,miR-214,miR-665-5p,miR-224-5p,miR-664a-5p,miR-338-5p,miR-665,miR-450b-3p,miR-424-5p,miR-129-5p,miR-485-3p,miR-15a-5p,miR-148b-3p,miR-200b,novel_133,miR-2904,miR-5703,miR-142-3p,miR-4429,miR-19b-3p,miR-378d,miR-329a-5p,miR-195a-3p,miR-29b-1-5p,miR-377-5p,miR-125b-2-3p,miR-146a-5p,novel_17,miR-324-3p,miR-500a-5p,miR-125b,miR-320d,miR-299-3p,miR-21-3p,miR-329-5p,miR-3074-5p,miR-299b-3p,miR-670-3p,miR-320c,miR-3141,miR-148a-3p,miR-3970,miR-150-5p,miR-1306,miR-3591-3p,miR-376b-3p,miR-450b-3p,miR-19a-3p,miR-146b-5p,miR-195a-5p,miR-193b-3p,miR-495-3p,miR-211,miR-24-3p,miR-199b-5p,miR-15a,miR-133a-5p,miR-485-3p,miR-16a,novel_87,miR-656-5p,miR-31-5p,miR-3955-5p,miR-377-5p,miR-432-5p,miR-3431,miR-185-5p,miR-149-5p,miR-432,miR-1285,miR-450c-3p,novel_23,miR-374b-3p,miR-145a-5p,miR-16b,miR-138,miR-497-5p,miR-188-5p,miR-455-5p,miR-7862,miR-493-5p,miR-101-5p,miR-2285r,miR-539-3p,miR-345-5p,miR-224-5p,miR-146a,miR-365b-5p,miR-3141,miR-455-5p,miR-125a-5p,miR-193a-3p,miR-125a,miR-3959-5p,miR-199a-
IL15RA	ENSOARG0(	interleu	
SLC37A3	ENSOARG0(	solute c	miR-2285w,miR-382-3p,miR-143-3p,miR-142a-3p,miR-2285i,miR-2331-5p,miR-185-3p,miR-2957,miR-181b-5p,miR-2898,miR-548w,novel_78,miR-133b-5p,miR-2285ad,miR-9788-3p,miR-582-3p,miR-2284h-5p,miR-142-3p,miR-181a-5p,miR-369-5p,miR-219a-1-3p,miR-2426,miR-181d-5p,novel_101,miR-3607-3p,miR-181c-5p,miR-2284w,miR-369-5p,miR-2284q,miR-1843a-3p,miR-655-5p,miR-3068-3p,miR-3082-5p,miR-671-5p,miR-9851-3p,miR-2285k,miR-2474,miR-10b-3p
CLINT1	ENSOARG0(	clathrin	miR-3187-3p,miR-1285-5p,miR-371b-3p,miR-3074-2-3p,miR-22-5p,miR-3071-3p,miR-500a-5p,miR-33a-3p,miR-499a-3p,miR-499b-3p,miR-340-5p,novel_68,miR-26a-2-3p,novel_99,miR-219a-1-3p,miR-129b-5p,miR-188-3p

GAPVD1	ENSOARG0(	GTPase a	miR-2478,miR-380-5p,miR-148b-5p,miR-200a,miR-30a-3p,miR-3064-5p,miR-4286,miR-200c-3p,miR-127-5p,miR-181b-5p,miR-6536,miR-543-5p,novel_96,miR-200a-3p,miR-152-3p,miR-205-5p,miR-664-3p,miR-200c,miR-219-3p,miR-3071-3p,miR-30f,miR-9-5p,miR-302a-5p,miR-758-5p,miR-6516-5p,miR-486-5p,miR-299a-3p,miR-2285ad,miR-429-3p,miR-411-3p,miR-128-3p,miR-200b-3p,miR-10a-5p,miR-345-3p,miR-3578,miR-1343-5p,miR-1197-3p,miR-1306-5p,miR-181a-2-3p,novel_9,miR-410-3p,miR-204-5p,miR-34c-3p,miR-3969,miR-409-3p,miR-135a-5p,miR-3959-5p,miR-223-5p,miR-574-5p,miR-487a-3p,miR-215-5p,miR-141-3p,miR-345-5p,miR-2318,miR-7144-5p,miR-671-5p,miR-1248,miR-2448-3p,miR-2285r,miR-539-3p,miR-345-5p,miR-503-3p,miR-380-5p,miR-493-5p,miR-6240,miR-382,miR-668-3p,miR-378a-5p,miR-1343-5p,miR-330-3p,miR-2411-3p,miR-2285g,miR-134-3p,miR-185-5p,miR-31-5p,miR-130a-3p,miR-380-3p,miR-30b-3p,miR-873a-5p,miR-708-3p,miR-542-3p,miR-181a-5p,miR-197-3p,miR-211,miR-323c,miR-301b-3p,miR-1839-3p,miR-495-3p,miR-421-5p,miR-625-3p,miR-147-5p,miR-142b,miR-1307-5p,miR-299b-3p,miR-677,miR-181b-3p,miR-10a-5p,miR-410-5p,miR-135b-5p,miR-2285ab,miR-148a-3p,miR-1306,miR-216a-3p,miR-3591-3p,miR-421-5p,miR-181b-1-3p,miR-2424,miR-380-3p,miR-487a,miR-105-3p,miR-1277-5p,miR-221-5p,miR-299-3p,miR-196a-3p,miR-219b-5p,miR-324-3p,miR-500a-5p,miR-345-3p,miR-487a-3p,miR-7859,miR-148b-3p,miR-200b,miR-548e-3p,miR-1b-3p,miR-485-3p,miR-212-5p,miR-133a-3p,miR-148a-5p,let-7a-3p,novel_133,miR-215-5p,miR-2408,miR-124-3p,miR-1434-5p,miR-10b-5p,miR-181c-5p,miR-539-5p,miR-145a-3p,miR-3958-5p,novel_74,miR-6516-3p,miR-454-3p,miR-129-5p,miR-669a-3p,miR-2285j,miR-15b-3p,miR-30e-3p,miR-219b-3p,miR-30a-3p,miR-324-3p,miR-10a,miR-6516,miR-301a-3p,miR-192-5p,miR-582-5p,miR-656-
ARHGAP36	ENSOARG0(	rho GTPa	miR-326,miR-193a-3p,miR-660,miR-491-5p,miR-124-5p,miR-101b-3p,miR-4791,miR-188-5p,miR-27b-3p,miR-1839-5p,miR-16-1-3p,miR-1260b,miR-29b,miR-27a-3p,miR-339-5p,miR-216a-3p,miR-3535,miR-33a-3p,miR-34c,miR-485-3p,miR-101-3p,novel_82,miR-29a,miR-450a-5p,miR-17-3p,miR-29c-3p,miR-495-3p,miR-452-5p,miR-193b-3p,miR-3059-5p,miR-326-3p,novel_27,miR-8095,miR-1285-5p,miR-449a,miR-877-3p,miR-211,miR-361-3p,miR-216a-3p,miR-361-3p,miR-9851-3p,miR-211-5p,miR-541-5p,miR-27a-3p,miR-34b,miR-411b-3p,novel_69,miR-29a-3p,miR-489,miR-2483-3p,miR-499a-3p,miR-339a,miR-185-3p,miR-122-3p,miR-1260a,novel_96,miR-34b-5p,miR-17-3p,miR-181a-2-3p,miR-450c-5p,miR-1271-3p,miR-2411,miR-2424,miR-2478,miR-330-5p,miR-29d-3p,miR-134,miR-126-3p,miR-34a-5p,miR-378d,miR-128-3p,miR-212-5p,novel_79,novel_121,miR-134-5p,miR-2411-5p,miR-5100,miR-29b-3p,miR-204-5p,miR-134-5p,miR-4492,miR-767-5p,miR-17-3p,miR-425-5p,miR-2330-3p,miR-338-3p,miR-1260b,miR-485-5p,miR-1248,miR-7144-5p,miR-625-5p,miR-339b,miR-767,miR-660-5p,miR-877-5p,miR-324-5p,miR-193b-3p,miR-34c-5p,miR-499b-3p

MS4A7	ENSOARG0(	membrane	miR-3068-3p,miR-30c-1-3p,miR-141-3p,miR-7689-3p,miR-216b-5p,miR-320c,miR-320a,miR-129b-3p,miR-19a-3p,miR-1291,miR-147-3p,miR-671-5p,miR-543-3p,miR-136-3p,miR-342-3p,miR-9-5p,novel_27,miR-5100,miR-147-5p,miR-2300a-5p,novel_78,miR-548w,miR-145b,miR-431,miR-3600,miR-9-5p,miR-302a-5p,miR-149-5p,miR-484,miR-873a-5p,miR-145a-5p,miR-758-3p,miR-101a-5p,miR-4429,miR-19b-3p,miR-1388-5p,miR-26a-2-3p,miR-101b-3p,miR-345-3p,miR-30c-1-3p,miR-490-3p,miR-758-3p,miR-8485,miR-2424,miR-431-5p,miR-320b,miR-342,miR-185-3p,miR-136-3p,miR-30c-2-3p,miR-466i-5p,miR-124-5p,miR-378a-5p,miR-320d,miR-1185-5p,miR-2898,miR-200a-3p
LOXL3	ENSOARG0(	lysyl ox	miR-148a-3p,miR-34b,miR-365a-3p,miR-323-5p,miR-323a-5p,miR-34c-5p,miR-1271,miR-370-3p,miR-4792,miR-3064-5p,novel_9,miR-17-3p,miR-4726-5p,miR-34c,miR-2440,miR-218-5p,miR-145-3p,miR-449a,miR-17-3p,miR-378d,miR-34a-5p,miR-218-2-3p,miR-223-3p,miR-196a-5p,miR-2355-3p,miR-148b-3p,miR-1343-3p,miR-3955-5p,miR-152-3p,miR-34b-5p,miR-3085-3p,miR-196b-5p,miR-17-3p,miR-6119-5p,miR-1271-3p,miR-628-5p,miR-3064-5p
GRPR	ENSOARG0(	gastrin-	miR-301,miR-18a-5p,miR-21a-3p,novel_25,miR-204-3p,miR-139-5p,miR-323-5p,miR-182-5p,miR-660-5p,miR-140-5p,miR-17-3p,miR-450b-3p,miR-338-5p,miR-19b-1-5p,miR-338-3p,miR-6134,miR-10b-5p,miR-425-5p,miR-145a-3p,miR-539-5p,miR-1197-5p,miR-5703,miR-1193,miR-2904,miR-124-3p,novel_133,miR-3600,miR-299-3p,miR-21-3p,miR-30b-3p,miR-30d,miR-760-3p,miR-125b,miR-185-3p,miR-216a-5p,miR-2411,miR-125b-2-3p,miR-2887,miR-183-5p,miR-216c-5p,novel_94,miR-361-3p,miR-27a-3p,miR-3082-5p,miR-541-5p,miR-20a-3p,miR-3064-5p,miR-2284q,miR-4726-5p,miR-371a-5p,miR-2285y,miR-30b-5p,miR-2285n,miR-502-3p,miR-181d-5p,miR-541-3p,miR-101a-5p,miR-2426,miR-125b-2-3p,miR-4791,miR-10b,miR-484,miR-6529a,miR-216a-3p,miR-27a-3p,novel_4,miR-2300a-5p,miR-3955-3p,miR-3085-3p,miR-326,novel_103,miR-1306-5p,miR-33a-5p,miR-382-3p,miR-485-5p,miR-7144-5p,miR-23a-5p,novel_91,miR-18a,miR-3154,miR-2448-3p,miR-154b-5p,miR-3957,miR-374c-3p,miR-598-3p,miR-3957-5p,miR-31-3p,miR-331-3p,miR-5100,miR-1-5p,miR-10a-5p,miR-128-3p,miR-501-3p,miR-30a-5p,miR-218-2-3p,miR-3578,miR-345-3p,miR-483-3p,miR-2411-5p,miR-543-5p,miR-107,miR-200c-3p,miR-181b-5p,miR-450a-2-3p,miR-2898,miR-17-3p,miR-217,miR-200c,miR-1957a,miR-380-5p,miR-2478,miR-4286,miR-181a-2-3p,miR-107,miR-450b-3p,miR-18a-3p,miR-18b,miR-670-3p,miR-323a-5p,miR-10a-5p,miR-656-5p,miR-22-5p,miR-6402,miR-133a-5p,miR-502-5p,miR-326-3p,miR-17-3p,miR-495-3p,miR-455-5p,miR-219a-1-3p,miR-380-3p,miR-181a-5p,miR-1285,miR-873a-5p,miR-103,miR-185-5p,miR-26b-3p,miR-665,miR-125a-5p,miR-660,miR-455-5p,miR-345-5p,miR-363-5p,miR-130b-5p,miR-345-5p,miR-411,miR-30d-5p,miR-598-3p,miR-129b-3p,miR-450a-1-3p,miR-499b-3p,miR-377-3p,miR-10a,miR-552-3p,miR-296-3p,miR-2331-3p,miR-504-5p,miR-

ATP6V0D2	ENSOARG0(	ATPase,	miR-488-3p,miR-485-5p,miR-7144-5p,miR-345-5p,miR-10b-3p,miR-2285aa,miR-2355-5p,miR-223-5p,miR-769,miR-3969,miR-6740-5p,miR-331-3p,miR-100-3p,miR-135a-1-3p,miR-204-5p,miR-374b-3p,miR-486b-3p,miR-410-3p,novel_42,miR-378c,miR-10b-3p,miR-374a-3p,miR-323-3p,miR-23b-5p,miR-3578,miR-1290,miR-128-3p,miR-200b-3p,miR-429-3p,miR-2285ad,novel_121,miR-378h,miR-24-2-5p,miR-30c-2-3p,miR-200c,miR-6119-5p,miR-543-5p,miR-200c-3p,miR-29b-2-5p,miR-2285b,miR-29a-5p,miR-450b-5p,miR-216a-3p,miR-3591-3p,miR-378f,miR-146b-5p,miR-7-1-3p,miR-378b,miR-3074-5p,miR-181b-3p,miR-669,novel_69,novel_87,miR-485-3p,miR-133a-5p,miR-502-5p,miR-4324,miR-6123,miR-128-1-5p,miR-3957-3p,novel_120,miR-301b-3p,miR-2428,miR-24-3p,miR-211,miR-2113,miR-542-3p,miR-432,miR-455-5p,miR-2284ab,miR-130a-3p,miR-544b,miR-134-3p,miR-432-5p,miR-455-5p,miR-296-3p,miR-146a,miR-345-5p,let-7j,miR-296-3p,miR-432-3p,miR-2285r,miR-500a-3p,miR-1895,miR-345-5p,miR-532-5p,miR-21-3p,miR-140-5p,miR-30c-1-3p,miR-378a-3p,miR-2331-3p,novel_32,miR-139-5p,miR-301a-3p,miR-544-3p,miR-29b-2-5p,miR-664a-5p,miR-500,miR-487a-5p,miR-454-3p,miR-129-5p,miR-4492,miR-3074-5p,miR-145a-3p,miR-539-5p,miR-378c,miR-6134,miR-338-3p,miR-346,miR-769-5p,miR-378a-3p,miR-140-3p,miR-2904,miR-2408,miR-1246,miR-378i,miR-378d,miR-133a-3p,miR-142-3p,miR-493-3p,miR-1b-3p,miR-200b,miR-422a,miR-185-3p,miR-324-3p,miR-210-5p,miR-195a-3p,miR-146a-5p,miR-486-3p,miR-181b-1-3p,miR-29b-1-5p,miR-664b-3p,miR-378j,miR-211-5p,miR-2285u,miR-452-3p,miR-20a-3p,miR-139-5p,miR-378e,miR-378g,miR-769-5p,miR-2285n,novel_82,miR-374a-3p,miR-490-5p,miR-499a-5p,miR-3968,miR-3059-5p,novel_111,miR-378b,miR-296-5p,miR-21b,miR-16-2-3p,miR-450b-5p,miR-2426,miR-544a,miR-

CDK2AP2	ENSOARG0(	cyclin-d	miR-147a,miR-27a-3p,miR-145b,miR-1343-3p,miR-409b,novel_4,novel_78,miR-105-1,miR-125b-2-3p,miR-301b-5p,miR-541,miR-2284r,miR-484,miR-1388-3p,miR-199b-5p,miR-130b-3p,miR-487b-5p,miR-491-5p,miR-326,miR-4443,miR-27a-3p,miR-489,miR-378g,miR-499a-3p,miR-211-5p,miR-668-5p,miR-199b-5p,miR-361-3p,miR-140-5p,miR-452-5p,miR-541-3p,miR-2447,miR-3059-5p,miR-4726-5p,miR-301a-5p,miR-615,miR-532-3p,miR-148b-3p,miR-3600,miR-378d,miR-1197-5p,miR-5703,miR-193b-5p,miR-2300b-3p,miR-345-3p,miR-2887,miR-185-3p,miR-324-3p,novel_17,miR-204-3p,miR-193b-3p,miR-27a-5p,miR-499b-3p,miR-3065-3p,miR-1827,novel_99,miR-885-3p,miR-625-5p,miR-129b-3p,miR-6134,miR-6525,miR-665-5p,miR-145a-3p,miR-130b-5p,miR-454-3p,miR-874-5p,miR-4492,miR-664a,miR-17-3p,novel_74,miR-665,miR-134-3p,miR-130a-3p,miR-27b-3p,miR-7862,miR-145a-5p,miR-374b-3p,miR-1285,miR-2113,miR-382,miR-130b-5p,miR-193a-3p,novel_51,miR-105-5p,miR-374c-3p,miR-9851-3p,miR-1291,miR-216a-3p,miR-148a-3p,miR-2428,miR-199b-5p,miR-326-3p,miR-24-3p,miR-211,miR-17-3p,miR-193b-3p,miR-3957-3p,miR-6517,miR-105-2,miR-128-3p,miR-885-3p,miR-330-5p,miR-23b-5p,miR-2427,miR-615-3p,novel_39,miR-3065-3p,miR-199a-5p,miR-376c-5p,miR-376b-5p,miR-152-3p,novel_96,miR-450a-2-3p,miR-2898,miR-17-3p,miR-199a-5p,miR-223-5p,miR-3959-5p,miR-2355-5p,miR-671-5p,miR-1248,miR-7144-5p,miR-23a-5p,miR-3154,miR-1197-3p,novel_42,miR-204-5p,miR-100-3p,miR-331-3p
CASP7	ENSOARG0(	caspase	miR-34a-5p,miR-501-3p,miR-2427,miR-218-2-3p,miR-29d-3p,miR-2284z,miR-23c,miR-23b-3p,miR-376a-5p,miR-34b-5p,miR-760-3p,miR-2284m,miR-3071-3p,miR-324-3p,miR-6119-5p,miR-2284a,miR-3065-3p,miR-2284y,miR-486-3p,miR-195a-3p,miR-29b-2-5p,miR-216c-5p,miR-186-5p,miR-7144-5p,novel_91,miR-345-5p,miR-345-5p,miR-23b,miR-2284n,novel_25,miR-204-3p,miR-323-5p,miR-301a-3p,miR-544-3p,miR-34c-5p,miR-3065-3p,miR-545-3p,miR-140-5p,miR-204-5p,miR-486b-3p,miR-454-3p,miR-1468-5p,miR-664a-5p,miR-29b-2-5p,miR-331-3p,miR-34c-3p,miR-15b-3p,miR-29b-3p,miR-2284k,miR-6134,miR-224-5p,miR-6128,miR-3184-3p,miR-2284v,miR-1306-5p,miR-2284x,miR-188-5p,miR-1839-5p,miR-2284aa,miR-125b-1-3p,miR-544a,miR-2284ab,miR-29b,miR-1388-5p,miR-197-3p,miR-501-3p,miR-301b-5p,miR-7b-5p,miR-484,miR-653,miR-2459,miR-9788-3p,miR-1298-3p,miR-2284d,miR-130a-3p,miR-1468,miR-130b-3p,miR-378a-5p,miR-1306-5p,miR-224-5p,miR-7-5p,miR-345-5p,miR-652-5p,miR-2284b,miR-23a-3p,miR-376a-2-5p,miR-668-5p,miR-136-5p,miR-378b,miR-3065-5p,miR-150-5p,miR-1306,miR-361-3p,miR-140-5p,miR-500-3p,miR-29a-3p,miR-34b,miR-3596,miR-323a-5p,miR-211-5p,miR-7-1-3p,miR-2319b,miR-22-5p,miR-29a,miR-301a-5p,miR-371a-5p,miR-34c,miR-301b-3p,miR-2284w,miR-449a,miR-502-3p,miR-3959-3p,novel_111,miR-361-3p,miR-211,miR-219a-5p,miR-29c-3p,miR-2284j,miR-2447

PIGK	ENSOARG0(	phosphat	miR-654-3p,miR-8485,miR-106a-5p,miR-382-3p,miR-2330-5p,miR-2483-5p,miR-23a-3p,miR-3955-3p,miR-224-5p,miR-377-3p,miR-106b-5p,miR-17-5p,miR-106a-5p,miR-3535,miR-134-3p,miR-1298-3p,miR-194-3p,miR-20b,miR-31-5p,miR-145b,miR-299a-5p,miR-2284aa,miR-4791,miR-197-3p,novel_23,miR-145a-5p,novel_111,miR-17-3p,miR-625-3p,novel_82,miR-20a-5p,miR-33a-3p,miR-7857-3p,miR-499a-3p,miR-378g,miR-3082-5p,miR-374c-3p,miR-3074-5p,miR-7689-3p,miR-3963,miR-668-5p,miR-2284e,miR-376a-5p,miR-2478,miR-125b-2-3p,miR-2432,miR-2411,miR-2424,miR-143-3p,miR-490-3p,miR-20a,miR-30a-3p,miR-195a-3p,miR-2887,miR-17-3p,miR-106a,miR-216a-5p,miR-500a-5p,miR-487a-3p,miR-2411-5p,novel_83,miR-2284z,miR-545-5p,miR-548e-3p,miR-106a,miR-7641,miR-30d-3p,miR-485-3p,miR-140-3p,miR-93,miR-224-5p,miR-3074-5p,miR-425-5p,miR-17-3p,miR-551b-3p,miR-30e-3p,miR-30a-3p,miR-338-5p,miR-665,miR-3604,miR-499b-3p,miR-377-3p,miR-582-5p,miR-877-5p,miR-154b-5p,let-7g-3p,miR-10b-3p,miR-329b,miR-20b-5p,miR-186-5p,miR-1248,miR-488-3p,miR-93-5p,miR-17-5p
FKBP6	ENSOARG0(	FK506 bi	miR-431-3p,miR-22-3p,miR-497-5p,miR-16b,miR-18a-3p,miR-103a-3p,miR-151-5p,miR-9788-3p,miR-103,miR-322-5p,miR-412-5p,miR-15a-5p,miR-324-3p,miR-16b,miR-146a,miR-1306-5p,miR-146b,miR-107,novel_127,miR-424-5p,miR-146a-5p,miR-1306,miR-18a-3p,miR-1895,miR-146b-5p,miR-195a-5p,miR-107,miR-22-3p,miR-151b,miR-15b-5p,miR-132-5p,miR-16a,miR-15a,miR-6529b,miR-424-5p,miR-16-5p,miR-877-3p,miR-346
BLM	ENSOARG0(	Bloom sy	miR-22-5p,miR-2284q,miR-20a-5p,miR-107-5p,miR-877-3p,miR-423-5p,miR-2428,miR-2447,miR-3059-5p,miR-17-3p,miR-136-5p,miR-504,miR-493-5p,miR-146b-5p,novel_94,miR-148a-3p,miR-2387,miR-18a-3p,miR-21c,miR-7857-3p,novel_69,miR-669,miR-677,miR-4443,miR-181b-3p,miR-3064-5p,miR-452-3p,miR-499b-5p,miR-2285u,miR-10a-5p,miR-7977,miR-378a-5p,miR-3085-3p,miR-146b,miR-452-5p,miR-125a-5p,miR-103a-2-5p,miR-7-5p,miR-146a,miR-296-3p,miR-365b-5p,miR-17-5p,miR-106b-5p,miR-106a-5p,miR-296-3p,miR-106a-5p,miR-628-5p,miR-21-5p,miR-16-1-3p,miR-873a-5p,miR-2284r,miR-1434-3p,miR-10b,miR-7b-5p,miR-496-5p,miR-147a,miR-455-3p,miR-20b,miR-2300a-5p,miR-181b-2-3p,miR-26b-3p,novel_9,miR-17-3p,miR-4492,miR-2285j,miR-125b-5p,miR-324-3p,miR-664a-5p,miR-655-3p,miR-10b-5p,miR-93,miR-3184-5p,miR-365a-5p,miR-193a-5p,miR-20b-5p,miR-7144-5p,miR-485-5p,miR-1248,miR-93-5p,miR-655,miR-17-5p,miR-2284s,miR-10a,miR-6516,miR-204-3p,miR-3957,miR-3957-5p,miR-2331-3p,miR-504-5p,miR-874-3p,miR-2355-5p,miR-552-3p,miR-296-3p,miR-125a,miR-106a,miR-17-3p,miR-152-3p,novel_127,miR-324-3p,miR-125b,miR-185-3p,miR-181b-1-3p,novel_39,miR-1957a,miR-146a-5p,miR-143-3p,miR-3064-5p,miR-20a,miR-345-3p,miR-16-1-3p,miR-10a-5p,miR-1434-5p,miR-148b-3p,miR-6516-5p,miR-18a-3p,miR-106a,miR-196a-2-3p

CFP	ENSOARG0(	compleme	miR-216a-3p,miR-204-3p,miR-483-3p,miR-429-3p,miR-1343-3p,miR-485-5p,miR-128-3p,miR-140-3p,miR-216a-3p,miR-29a-5p,miR-181a-2-3p,miR-4286,miR-452-5p,miR-2447,miR-874-5p,miR-200c-3p,miR-191-3p,miR-483-3p,miR-210-5p
HK2	ENSOARG0(	hexokina	miR-15b-5p,miR-322-5p,miR-15a-5p,miR-3074-5p,miR-497-5p,miR-101c,miR-195a-5p,miR-16b,let-7j,miR-2285b,miR-16-5p,novel_120,miR-3074-5p,miR-424-5p,miR-424-5p,miR-628-3p,miR-16a,miR-16b,miR-15a
SMC5	ENSOARG0(	structur	miR-200c-3p,miR-491-5p,novel_127,novel_96,miR-200a-3p,miR-660,miR-3071-3p,miR-30c-2-3p,miR-450b-5p,miR-30c-1-3p,miR-2284y,miR-2284b,miR-195a-3p,miR-2284u,miR-188-5p,miR-2284x,miR-1434-3p,miR-2459,novel_4,miR-548w,miR-429-3p,miR-18a-3p,miR-3964,miR-1197-3p,miR-3074-5p,miR-3607-3p,miR-9-3p,miR-7975,miR-23a-5p,miR-18a-3p,miR-3074-5p,miR-216b-3p,miR-660-5p,miR-30c-1-3p,miR-2284g,miR-141-3p,miR-126b-5p,miR-216b-3p
CDH18	ENSOARG0(	cadherin	miR-877-5p,miR-6516,miR-132-5p,miR-139-5p,miR-21-3p,miR-140-5p,miR-545-3p,miR-2312,miR-338-3p,miR-206-3p,miR-208b-5p,miR-424-5p,miR-450b-3p,miR-17-3p,miR-19b-1-5p,miR-324-3p,miR-29b-2-5p,miR-222-3p,miR-383-5p,miR-148b-3p,miR-15a-5p,miR-19b-3p,miR-4429,miR-2904,miR-215-5p,miR-1246,miR-2300b-3p,miR-29b-1-5p,miR-130a-5p,miR-20a,miR-216c-5p,miR-30b-3p,miR-320d,miR-106a,miR-466i-5p,miR-125b,miR-185-3p,miR-7857-3p,miR-541-5p,miR-499b-5p,miR-664b,miR-877-3p,miR-362-5p,miR-2285f,miR-181d-5p,miR-452-5p,miR-3059-5p,miR-374a-3p,miR-2285t,miR-3958-3p,miR-2285y,miR-107-5p,miR-2285n,miR-500-5p,miR-221-3p,miR-222-5p,miR-2284aa,miR-16-2-3p,miR-3120-5p,miR-450b-5p,miR-412-3p,miR-125b-2-3p,miR-4791,miR-382-3p,miR-106a-5p,miR-424-5p,miR-3955-3p,miR-320b,miR-1306-5p,miR-374c-3p,miR-141-3p,miR-215-5p,miR-3074-1-3p,miR-7144-5p,miR-376c-5p,miR-671-5p,let-7g-3p,miR-17-5p,miR-320a,miR-362-5p,miR-503-3p,miR-500b-5p,miR-93,miR-374a-3p,miR-3607-3p,miR-6128,miR-374b-3p,miR-204-5p,miR-331-3p,miR-3969,miR-582-3p,miR-545-5p,miR-214-5p,miR-6516-5p,miR-222,miR-128-3p,miR-411-3p,miR-490-3p,miR-29b-2-5p,miR-1,miR-181a-2-3p,miR-3529-3p,miR-107,miR-450a-2-3p,miR-17-3p,miR-181b-5p,miR-2285af,miR-670-3p,miR-655-5p,miR-320b,miR-195a-5p,miR-19a-3p,miR-107,miR-378b,miR-2285ab,miR-211,miR-17-3p,miR-2285x,miR-495-3p,miR-203b-5p,miR-199c,novel_1,miR-16a,miR-33a-3p,miR-502-5p,miR-15a,miR-20a-5p,miR-103,miR-20b,miR-432-5p,miR-130a-3p,miR-26b-3p,miR-377-5p,miR-219a-1-3p,miR-3071-5p,miR-873a-5p,miR-1434-3p,miR-181a-5p,miR-654-3p,miR-382,miR-130b-5p,miR-125a-5p,miR-452-5p,miR-466f-3p,miR-330-3p,miR-17-5p,miR-106a-5p,miR-224-5p,miR-15b-5p,miR-301a-3p,miR-625-5p,miR-543-3p,miR-186-5p,miR-433-3p,miR-450a-

RECQL4	ENSOARG0(	RecQ pro	miR-345-5p,miR-628-5p,miR-130b-5p,miR-1343-5p,miR-125a-5p,miR-378a-5p,miR-103,miR-544b,miR-194-3p,miR-149-5p,miR-134-3p,miR-339-5p,miR-103a-3p,miR-340-3p,miR-138,miR-22-3p,miR-326-3p,miR-371b-3p,miR-17-3p,miR-1839-3p,miR-576-3p,miR-28c,miR-4324,miR-323a-5p,miR-10a-5p,miR-320b,miR-378b,miR-107,miR-1291,miR-216a-3p,miR-18a-3p,miR-1306,miR-1271-3p,miR-3065-3p,miR-28b,miR-4286,miR-3064-5p,novel_127,miR-543-5p,novel_96,miR-107,miR-127-5p,miR-17-3p,miR-217,miR-339a,miR-125a-3p,miR-134-5p,miR-582-3p,miR-9-5p,miR-431,miR-412,novel_79,miR-1224-5p,miR-10a-5p,miR-128-3p,miR-330-5p,miR-1343-5p,miR-134,miR-1306-5p,miR-486b-3p,miR-134-5p,miR-28-5p,miR-138-5p,miR-125b-5p,miR-2433,miR-324-5p,miR-133c,miR-125a,miR-615-5p,miR-215-5p,miR-2355-5p,miR-370-3p,miR-874-3p,miR-574-5p,miR-23a-5p,miR-345-5p,miR-301,miR-2330-5p,miR-1839-3p,miR-214-3p,miR-3085-3p,miR-491-5p,miR-326,miR-1306-5p,miR-431-5p,miR-216a-3p,miR-9788-3p,miR-222-5p,miR-2300a-5p,miR-1343-3p,miR-3120-5p,miR-544a,miR-412-3p,miR-10b,miR-541,miR-301b-5p,miR-877-3p,miR-9-5p,miR-541-3p,miR-3956-3p,novel_60,miR-4726-5p,miR-301a-5p,miR-378g,miR-7977,miR-452-3p,miR-139-5p,miR-4792,miR-3064-5p,miR-877-3p,miR-136-5p,miR-504,miR-486-3p,miR-130a-5p,miR-345-3p,miR-21-3p,miR-221-5p,miR-185-3p,miR-125b,miR-324-3p,novel_17,miR-210-5p,novel_83,miR-1247-5p,miR-212-5p,miR-18a-3p,miR-378d,miR-218-1-3p,miR-1197-5p,miR-5703,miR-217-5p,miR-2904,miR-215-5p,novel_133,miR-122-5p,miR-6525,miR-10b-5p,miR-214,miR-130b-5p,miR-4492,miR-874-5p,miR-708-5p,miR-17-3p,miR-320e,miR-22-3p,miR-323-5p,miR-139-5p,miR-192-5p,miR-544-3p,miR-10a,miR-552-3p,miR-3065-3p,miR-2331-3p,miR-504-5p,miR-221-5p,miR-

DKKL1	ENSOARG0(	dickkopf	miR-365a-5p,miR-3969,miR-135a-5p,miR-26c,miR-2332,miR-486b-3p,miR-28-5p,novel_9,miR-141-3p,miR-370-3p,miR-223-5p,miR-324-5p,miR-769,miR-34c-5p,miR-17-5p,miR-93-5p,miR-381-3p,miR-671-5p,miR-20b-5p,miR-7144-5p,miR-10b-3p,miR-490-3p,miR-200a,miR-200c,miR-2285af,miR-200a-3p,miR-200c-3p,miR-7641,miR-429-3p,miR-345-3p,miR-200b-3p,miR-300,miR-582-3p,miR-135a-2-3p,miR-2428,miR-449a,miR-34c,miR-576-3p,miR-20a-5p,miR-147-5p,miR-655-5p,miR-216b-3p,miR-669,miR-34b,miR-323a-5p,miR-2404,miR-2387,miR-135b-5p,miR-450b-3p,miR-410-5p,miR-455-5p,miR-106a-5p,miR-17-5p,miR-2411-3p,miR-197-5p,miR-365b-5p,miR-7-5p,miR-224-5p,miR-31-5p,miR-194-3p,miR-185-5p,miR-134-3p,miR-3431,miR-2285g,miR-194-5p,miR-1285,miR-7b-5p,miR-542-3p,miR-1843b-5p,miR-708-3p,miR-455-5p,miR-188-5p,miR-2889,miR-665-5p,miR-3958-5p,miR-145a-3p,miR-4510,miR-224-5p,miR-769-5p,miR-664a-5p,miR-665,miR-130b-5p,miR-4492,miR-708-5p,miR-296-3p,miR-552-3p,miR-204-3p,miR-6130,miR-323-5p,miR-2284s,miR-129b-3p,miR-2285c,miR-1827,miR-20a,miR-2285w,miR-216c-5p,miR-329a-5p,miR-143-3p,miR-486-3p,miR-130a-5p,miR-376a-5p,miR-185-3p,miR-345-3p,miR-92a-1-5p,novel_17,miR-34b-5p,miR-106a,miR-1b-3p,miR-106a,miR-2284h-5p,miR-200b,miR-5703,miR-188-3p,miR-2904,miR-2300b-3p,miR-34a-5p,miR-452-5p,miR-194b-5p,miR-541-3p,miR-2447,miR-769-5p,miR-2440,novel_82,miR-218-5p,miR-499a-5p,miR-3082-5p,miR-378g,miR-378j,miR-744-3p,miR-106a-5p,miR-1388-3p,miR-4532,miR-8485,miR-505,novel_103,miR-153-3p,miR-1185-5p,miR-2459,miR-2285e,miR-216a-3p,miR-9788-3p,miR-148b-5p,miR-147a,miR-153,miR-541,miR-2284r,miR-484,miR-2426,miR-1388-5p
NSUN5	ENSOARG0(	NOP2/Sun	miR-128-1-5p,miR-668-5p,miR-330-3p,novel_133,miR-4324,novel_17,miR-193b-5p,miR-6529b,miR-122-5p,miR-1271,miR-340-3p,miR-6240,miR-330-3p
AK5	ENSOARG0(	adenylat	miR-329a,miR-1827,miR-544-5p,miR-21a-3p,miR-21c,miR-3965,miR-216b-5p,miR-2404,miR-500-3p,miR-204-3p,miR-3120-3p,miR-141-3p,miR-7-1-3p,miR-552-3p,miR-2355-5p,miR-664a,miR-6119-3p,miR-147-5p,miR-2332,miR-576-3p,miR-320e,miR-1197-3p,miR-877-3p,miR-2428,miR-502-3p,miR-2447,novel_120,miR-539-5p,miR-3607-3p,miR-16-1-3p,miR-21-5p,miR-362-3p,miR-3120-5p,miR-16-1-3p,miR-223-3p,miR-362-3p,miR-1290,miR-329-3p,miR-145a-5p,miR-501-3p,miR-124-3p,miR-501-3p,miR-7857,miR-147a,miR-2459,miR-9788-3p,novel_83,miR-616-3p,miR-532-3p,miR-145b,miR-124-5p,miR-200a-3p,miR-452-5p,miR-125a-3p,novel_51,miR-362-3p,miR-2310,miR-1b-5p,miR-2432,miR-200a,miR-494-3p,miR-124a,miR-1388-3p

RABEPK	ENSOARG0(	Rab9 eff	miR-124-5p,miR-190a,miR-330-3p,miR-2411-3p,miR-224-5p,miR-146a,let-7j,miR-2285r,miR-628-5p,miR-130b-5p,miR-6240,miR-497-5p,miR-30b-3p,miR-138,miR-380-3p,miR-16b,miR-219a-1-3p,miR-450c-3p,miR-145a-5p,miR-374b-3p,miR-432,miR-873a-5p,miR-708-3p,miR-1434-3p,miR-542-3p,miR-1843b-5p,miR-411-5p,miR-412-5p,miR-432-5p,miR-185-5p,miR-339-5p,miR-26b-3p,miR-31-5p,miR-147-5p,miR-199c,novel_1,miR-16a,miR-502-5p,miR-15a,miR-576-3p,miR-6402,miR-2428,miR-2284w,miR-3959-3p,miR-335-5p,miR-2319a,miR-378f,miR-195a-5p,miR-379-5p,miR-450b-3p,miR-3591-3p,miR-216a-3p,miR-2285ab,miR-320c,miR-677,miR-181b-3p,miR-670-3p,miR-320b,novel_68,miR-378b,miR-3074-5p,miR-329-5p,miR-200a-3p,novel_96,miR-28-3p,miR-200c-3p,miR-6536,miR-3071-3p,miR-339a,miR-6119-5p,miR-200c,miR-125a-3p,miR-29a-5p,miR-200a,miR-2284y,miR-2284a,miR-2285b,miR-24-2-5p,miR-2385-3p,miR-128-3p,miR-3591-5p,miR-345-3p,miR-450a-1-3p,miR-322-5p,miR-24-2-5p,miR-505-5p,miR-378h,miR-6516-5p,miR-412,novel_79,miR-2285ad,miR-429-3p,miR-31-3p,miR-28-5p,miR-138-5p,miR-5100,miR-378c,miR-500b-5p,miR-16-5p,miR-6128,miR-28a-3p,miR-7144-5p,miR-485-5p,miR-1814c,miR-876-3p,miR-329b,miR-362-5p,miR-320a,miR-3154,miR-301,miR-133c,miR-2355-5p,miR-2285aa,miR-615-5p,miR-141-3p,miR-874-3p,miR-503-3p,miR-153-3p,miR-491-5p,miR-16b,miR-320b,novel_103,miR-2310,miR-378d,miR-2284b,miR-744-3p,miR-424-5p,miR-101c,miR-378c,miR-450b-5p,miR-412-3p,miR-30b-3p,miR-378b,miR-6529a,miR-301b-5p,miR-500-5p,miR-216a-3p,miR-455-3p,miR-2459,miR-421,miR-153,miR-142-5p,miR-222-5p,miR-145b,miR-181b-2-3p,miR-1343-3p,miR-199b-3p,miR-330-3p,miR-3958-3p,miR-3968,miR-628-3p,miR-499a-5p,miR-335,miR-301a-5p,miR-2285p,miR-2440,miR-101-3p,miR-362-
CA2	ENSOARG0(	carbonic	miR-125a-3p,miR-664-3p,miR-181c-3p,miR-339a,novel_39,miR-143-3p,miR-450c-5p,miR-2285b,miR-329a-5p,miR-503-5p,miR-30a-3p,miR-369-3p,miR-4286,miR-2403,miR-300,miR-1290,miR-1246,miR-29d-3p,miR-1197-5p,miR-23c,novel_121,novel_79,miR-412,miR-30d-3p,miR-29b-3p,miR-34c-3p,miR-30e-3p,miR-144,miR-324-3p,miR-30a-3p,miR-655-3p,miR-135a-5p,miR-323-3p,miR-145-3p,miR-338-3p,miR-3184-3p,miR-339b,miR-381-3p,miR-655,miR-2284s,miR-370-3p,miR-503-5p,miR-330-3p,novel_51,miR-4532,miR-1388-3p,miR-493-5p,miR-6240,miR-101b-3p,miR-2330-5p,miR-29b,miR-1839-5p,miR-873a-5p,miR-296-5p,miR-541,miR-412-3p,novel_23,miR-425-3p,miR-503-5p,miR-3431,miR-2459,novel_4,miR-330-3p,miR-339-5p,miR-26b-3p,miR-628-3p,miR-128-1-5p,miR-335,miR-450a-5p,miR-29a,miR-421-5p,miR-142b,miR-24-3p,miR-3059-5p,miR-541-3p,miR-335-5p,miR-29c-3p,miR-299b-5p,miR-493-5p,miR-2319a,miR-135b-5p,miR-25,miR-29a-3p,miR-144-3p,miR-664b,miR-2319b,miR-329-5p,miR-20a-3p,miR-3120-3p
SPACA1	ENSOARG0(	sperm ac	miR-200c-3p,miR-2285t,miR-200b-3p,miR-200c,miR-374a-5p,miR-2404,miR-9-5p,miR-200b,miR-216b-5p,novel_32,miR-429-3p,miR-7641,miR-9-5p

RNF145	ENSOARG0(	ring fin	miR-1434-5p,miR-664-5p,miR-361-5p,miR-2284u,miR-30d-3p,miR-18a-3p,miR-2284h-5p,miR-2285p,miR-3653-3p,miR-2484,miR-329a-5p,miR-2285w,miR-421-5p,miR-377-5p,miR-129b-3p,miR-411,miR-186-5p,miR-339b,miR-30c-1-3p,miR-504-5p,miR-15b-5p,miR-377-3p,miR-301a-3p,miR-30a-3p,miR-3604,miR-665,miR-15b-3p,miR-30e-3p,miR-454-3p,miR-4492,novel_74,miR-665-5p,miR-129-2-3p,miR-3074-5p,miR-181c-5p,miR-142a-5p,miR-224-5p,miR-501-3p,miR-1a-3p,miR-323b,miR-301b-5p,miR-1388-5p,miR-145b,miR-330-3p,miR-548w,novel_78,miR-455-3p,miR-421,miR-2459,miR-142-5p,miR-539-5p,miR-323b-3p,miR-16b,miR-182-5p,miR-199a-3p,miR-199b-5p,miR-146b,miR-103a-2-5p,miR-2284b,miR-124a,miR-378j,miR-1983,miR-504,miR-2284g,miR-2319b,miR-144-5p,miR-500-3p,miR-301a-5p,miR-2440,miR-499a-5p,miR-6527,miR-9-5p,miR-2447,miR-361-3p,miR-877-3p,novel_79,miR-18b-5p,miR-429-3p,miR-134-5p,miR-129-1-3p,miR-9-5p,miR-322-5p,miR-505-5p,miR-758-5p,miR-205-5p,novel_127,miR-200a-3p,miR-2284y,miR-200a,miR-29a-5p,miR-2285b,miR-450b-5p,miR-1248,miR-1271,miR-574-5p,miR-223-5p,miR-3959-5p,miR-2433,miR-3432a,miR-134-5p,miR-16-5p,miR-144-5p,miR-197-3p,miR-542-3p,miR-497-5p,miR-188-5p,miR-16b,miR-339-5p,miR-31-5p,miR-365b-5p,novel_51,miR-296-3p,miR-146a,miR-377-3p,let-7f-2-3p,let-7f-2-3p,miR-380-5p,miR-6240,let-7a-2-3p,miR-2284l,miR-3970,miR-146b-5p,miR-2319a,miR-412-3p,miR-3074-5p,miR-329-5p,miR-320c,miR-144-3p,novel_87,miR-4324,miR-142b,miR-421-5p,miR-6123,miR-29a,miR-206,miR-29c-3p,miR-301b-3p,miR-199b-5p,miR-2284w,miR-2904,miR-124-3p,miR-1197-5p,miR-5703,miR-188-3p,miR-140-3p,novel_133,miR-19b-3p,miR-4429,miR-3600,miR-485-3p,miR-505-3p,miR-199a-3p,miR-487a-3p,miR-222-5p,miR-340-5p,miR-15a-5p,miR-200b,miR-185-

APBA1	ENSOARG0(	amyloid	miR-1260b, miR-16-5p, miR-193a-5p, miR-2284v, miR-134-5p, miR-191-5p, miR-6740-5p, miR-125b-5p, miR-2433, miR-2284k, miR-300-3p, miR-125a, miR-2285aa, miR-20b-5p, miR-345-5p, miR-2318, miR-23b, miR-93-5p, miR-301, miR-30e-5p, let-7g, miR-3064-5p, let-7f-5p, miR-30f, miR-450a-1-3p, miR-134-5p, miR-322-5p, miR-505-5p, miR-412, novel_79, miR-6517, miR-2285ad, miR-299a-3p, miR-30c, miR-330-5p, miR-23b-5p, miR-1343-5p, miR-196a-5p, miR-199b-5p, miR-1839-3p, miR-379-3p, miR-485-3p, miR-576-3p, miR-4324, miR-181b-3p, miR-677, miR-320c, miR-669, miR-411b-3p, novel_69, miR-374c-3p, miR-299b-3p, miR-3074-5p, novel_68, let-7e-5p, miR-2319a, miR-412-3p, miR-3591-3p, miR-150-5p, miR-3141, let-7j, let-7f-2-3p, let-7f-2-3p, miR-493-5p, miR-6240, miR-196b-5p, miR-378a-5p, novel_51, miR-2411-3p, miR-3141, miR-296-3p, novel_116, miR-194-3p, miR-149-5p, miR-134-3p, miR-339-5p, miR-103a-3p, miR-4508, miR-497-5p, miR-369-5p, miR-16b, miR-450c-3p, miR-1843b-5p, miR-432, miR-181c-5p, miR-122-5p, miR-3184-5p, miR-3074-5p, miR-3184-3p, let-7b, miR-500, miR-874-5p, miR-129-5p, miR-4492, miR-487b-5p, novel_74, miR-664a-5p, miR-665, miR-30a-3p, miR-30e-3p, let-7f, miR-2285j, miR-15b-5p, let-7i-5p, miR-499b-3p, miR-10a, miR-582-5p, miR-30c-1-3p, miR-1827, miR-625-5p, miR-543-3p, miR-1895, miR-339b, miR-345-5p, miR-30d-5p, miR-500a-3p, miR-450a-1-3p, miR-486-3p, miR-181b-1-3p, miR-376a-5p, miR-345-3p, let-7k, miR-98-5p, miR-345-3p, miR-3601, novel_17, miR-210-5p, miR-618, miR-30b, miR-328-3p, miR-7859, miR-23b-3p, miR-30d-3p, miR-106a, miR-34a-5p, miR-133a-3p, miR-493-3p, miR-217-5p, miR-1961, miR-30c-5p, miR-191, miR-423-5p, miR-2284j, miR-3956-3p, miR-885-5p, miR-2447, miR-218-5p, novel_82, miR-1185-2-3p, miR-652-3p, miR-2483-3p, miR-378g, miR-499a-3p, miR-139-5p, miR-1983, miR-493-5p, miR-299b-

ORC3	ENSOARG0(origin r	miR-134,miR-345-3p,miR-214-5p,miR-582-3p,miR-6516-5p,miR-339a,miR-30c-2-3p,miR-2285af,miR-376b-3p,miR-543-5p,miR-181b-5p,miR-2284a,miR-17-5p,miR-488-3p,miR-141-3p,miR-215-5p,miR-487a-3p,miR-154b-5p,miR-3969,miR-135a-5p,miR-204-5p,miR-486b-3p,miR-103b,miR-323-3p,miR-93,miR-3071-5p,miR-1434-3p,miR-181a-5p,miR-219a-1-3p,miR-30b-3p,miR-2284ab,miR-130a-3p,miR-20b,miR-2355-3p,miR-2285g,miR-2284d,miR-17-5p,miR-106a-5p,miR-660,miR-1343-5p,miR-2483-5p,miR-382,miR-101-5p,miR-539-3p,miR-296-3p,miR-376b-3p,miR-135b-5p,miR-6516-3p,miR-7975,miR-7-1-3p,miR-378b,miR-6402,miR-133a-5p,miR-6529b,miR-20a-5p,miR-502-5p,miR-147-5p,miR-203b-5p,novel_1,miR-2285x,novel_120,miR-219b-3p,miR-2428,miR-326-3p,novel_27,miR-211,miR-188-3p,miR-5703,miR-1193,miR-215-5p,miR-378d,miR-485-3p,miR-1b-3p,miR-3600,miR-532-3p,miR-148b-3p,miR-487a-3p,miR-185-3p,miR-216a-5p,miR-196a-3p,miR-221-5p,miR-760-3p,miR-2284m,miR-106a,miR-20a,novel_99,miR-154b-3p,miR-182-5p,miR-21-3p,miR-660-5p,miR-221-5p,novel_32,miR-193b-3p,miR-6516,miR-192-5p,miR-338-5p,miR-19b-1-5p,miR-208b-5p,miR-6516-3p,miR-425-5p,miR-539-5p,miR-30b-3p,miR-125b-2-3p,miR-7857,miR-484,miR-2284aa,miR-450b-5p,miR-101a-5p,miR-299a-5p,miR-5010-3p,novel_4,miR-2285e,miR-216a-3p,miR-9788-3p,novel_103,miR-326,miR-3955-3p,miR-33a-5p,miR-106a-5p,miR-1839-3p,miR-193a,miR-30c-1-3p,miR-129b-5p,novel_94,miR-877-3p,miR-499b-5p,miR-541-5p,miR-664b,miR-376d,miR-2285t,miR-181d-5p,miR-1296-5p,miR-212-3p,miR-8095,miR-9-3p,miR-330-5p,miR-1343-5p,novel_79,miR-129-1-3p,miR-134-5p,miR-2284z,miR-9-5p,miR-664-3p,miR-205-5p,miR-152-3p,miR-200a-3p,novel_96,miR-28-3p,miR-29a-5p,miR-2284y,novel_39,miR-200a,miR-148b-5p,miR-93-5p,miR-

MS4A5	ENSOARG0(	membrane	miR-155-5p,miR-664a,novel_74,miR-450b-3p,miR-130b-5p,miR-4492,miR-129-5p,miR-2285j,miR-665,miR-29b-2-5p,miR-664a-5p,miR-324-3p,miR-19b-1-5p,miR-206-3p,miR-346,miR-6134,miR-129-2-3p,miR-3074-5p,miR-214,miR-154b-3p,miR-2312,miR-1827,miR-625-5p,miR-885-3p,miR-450a-1-3p,miR-299,miR-21a-3p,miR-30d-5p,miR-323-5p,miR-139-5p,miR-192-5p,miR-155-5p,novel_32,miR-204-3p,miR-1277-3p,miR-30c-1-3p,miR-140-5p,miR-221-5p,miR-532-5p,miR-154a-3p,miR-3065-3p,miR-760-3p,miR-466i-5p,miR-106a,miR-299-3p,miR-221-5p,miR-542-5p,novel_17,miR-196a-3p,miR-185-3p,miR-130a-5p,miR-181b-1-3p,miR-377-5p,miR-143-3p,miR-2432,miR-486-3p,miR-487a,miR-216c-5p,miR-2887,miR-345-3p,miR-20a,novel_73,miR-493-3p,miR-215-5p,miR-362-3p,miR-193b-5p,miR-2300b-3p,miR-1197-5p,miR-188-3p,miR-1434-5p,miR-5703,miR-2904,miR-23c,miR-23b-3p,miR-106a,miR-212-5p,miR-335,miR-154-3p,miR-2284q,miR-2285t,miR-2285n,miR-2285y,miR-877-3p,miR-361-3p,miR-9-3p,miR-541-3p,miR-2447,miR-2285f,miR-877-3p,miR-9-3p,miR-376a-3p,miR-335-3p,miR-140-5p,miR-1306-3p,miR-378j,miR-378g,miR-27a-3p,miR-376d,miR-139-5p,miR-24-1-5p,miR-211-5p,miR-3955-3p,miR-1185-5p,miR-542-5p,miR-214-3p,miR-1468,novel_115,miR-326,miR-431-5p,miR-342,miR-106b-5p,novel_19,miR-362-3p,miR-30c-1-3p,miR-758-3p,miR-4532,miR-106a-5p,miR-382-3p,miR-652-5p,miR-33a-5p,miR-23a-3p,miR-362-3p,miR-3120-5p,miR-541,miR-143-5p,miR-329-3p,miR-2284r,miR-3962,miR-758-3p,miR-1a-3p,miR-1298-3p,miR-376b-3p,miR-2285e,miR-9788-3p,miR-455-3p,miR-330-3p,novel_4,miR-27a-3p,miR-181b-2-3p,miR-138-5p,miR-486b-3p,miR-134-5p,miR-204-5p,miR-1306-3p,miR-3969,miR-6740-5p,miR-1260b,miR-93,miR-145-3p,novel_42,miR-6128,miR-329a,miR-1248,miR-20b-5p,miR-17-5p,miR-23b,miR-93-5p,miR-
CRYZL1	ENSOARG0(	crystall	miR-301b-5p,miR-3591-5p,miR-23b-5p,miR-450c-3p,miR-22-3p,miR-2411-5p,miR-383-5p,miR-411b-5p,miR-582-3p,miR-450a-1-3p,miR-2285p,miR-2331-5p,miR-412-5p,novel_124,miR-200a-3p,miR-214-3p,miR-2330-5p,miR-382-3p,miR-28b,miR-2411,miR-200a,miR-503-3p,miR-450a-1-3p,miR-378b,miR-150-5p,miR-671-5p,miR-2319a,miR-369-5p,miR-211-5p,miR-3120-3p,miR-141-3p,miR-216b-3p,miR-370-5p,miR-489,miR-154b-5p,miR-22-3p,miR-28c,miR-370-5p,miR-412-5p,miR-301a-5p,miR-6740-5p,miR-132-5p,miR-450b-3p,miR-204-5p,miR-874-5p,miR-1193,miR-214,miR-211,miR-361-3p,miR-423-5p,miR-3184-5p

RARS2	ENSOARG0(	arginyl-	miR-2285w,miR-143-3p,miR-486-3p,miR-181b-1-3p,miR-142a-3p,miR-376a-5p,miR-422a,miR-92a-1-5p,miR-345-3p,miR-618,miR-2484,miR-212-5p,miR-380-5p,miR-106a,miR-2284h-5p,miR-7859,miR-378a-3p,miR-378i,miR-664-5p,miR-16-1-3p,miR-142-3p,miR-122-5p,miR-665,miR-151-3p,miR-500,miR-4492,novel_74,miR-296-3p,miR-504-5p,miR-15b-5p,miR-500a-3p,miR-129b-3p,miR-543-3p,miR-625-5p,miR-345-5p,miR-101b-3p,miR-1b-5p,miR-8485,miR-378d,miR-106b-5p,miR-16b,miR-182-5p,miR-199a-3p,miR-491-5p,miR-1185-5p,miR-181b-2-3p,miR-1343-3p,miR-199b-3p,miR-1298-3p,miR-147a,miR-1a-3p,miR-1839-5p,miR-101c,miR-1388-5p,miR-2284j,let-7i-3p,miR-3956-3p,miR-2447,novel_111,miR-361-3p,miR-628-3p,miR-499a-5p,miR-3968,miR-7689-3p,miR-378g,miR-409-5p,miR-9-3p,miR-544-5p,miR-493-5p,miR-136-5p,miR-504,miR-668-5p,miR-200a-3p,novel_96,miR-486-5p,miR-2284z,miR-134-5p,miR-322-5p,miR-330-5p,miR-23b-5p,miR-16-5p,miR-2284v,miR-6740-5p,miR-2284k,miR-592,miR-134-5p,miR-2285aa,miR-574-5p,miR-93-5p,miR-20b-5p,miR-345-5p,miR-329b,miR-668-3p,miR-493-5p,let-7a-2-3p,miR-432-3p,miR-2284l,miR-432-3p,miR-2285r,miR-2411-3p,miR-296-3p,miR-103a-3p,miR-411-5p,miR-134-3p,miR-1843b-5p,miR-2113,miR-432,miR-708-3p,miR-497-5p,miR-16-1-3p,miR-16b,miR-758-5p,miR-2284w,miR-142b,miR-576-3p,miR-379-3p,miR-206,miR-6119-3p,miR-3120-3p,novel_68,miR-181b-3p,miR-677,miR-320c,miR-411b-3p,miR-3591-3p,miR-3065-5p,miR-3970,miR-378f,miR-20a,miR-2411,miR-185-3p,miR-21-3p,miR-466i-5p,miR-320d,miR-106a,miR-199a-3p,miR-616-3p,miR-222-5p,miR-340-5p,miR-15a-5p,miR-383-5p,miR-5703,miR-376a-5p,miR-4429,miR-2330-3p,miR-378c,miR-206-3p,miR-338-5p,miR-320e,miR-708-5p,miR-424-5p,miR-664a,miR-21-3p,miR-370-5p,miR-378a-3p,miR-877-5p,miR-204-3p,miR-656-3p,miR-

ZZZ3	ENSOARG0(	zinc fin	miR-1434-5p,miR-378a-3p,miR-378i,miR-362-3p,let-7a-3p,miR-148a-5p,miR-142-3p,miR-3600,miR-30d-3p,miR-548e-3p,miR-18a-3p,novel_83,miR-7859,miR-422a,miR-374a-5p,novel_17,miR-760-3p,miR-216c-5p,miR-421-5p,miR-130a-5p,miR-129b-3p,miR-543-3p,miR-186-5p,miR-1827,miR-21-3p,miR-378a-3p,miR-548o-3p,miR-22-3p,miR-877-5p,miR-6516,miR-499b-3p,miR-377-3p,miR-30a-3p,let-7b-3p,miR-30e-3p,miR-130b-5p,miR-3074-2-3p,miR-378c,miR-3074-5p,miR-129-2-3p,miR-181c-5p,miR-142a-5p,miR-224-5p,miR-378b,miR-329-3p,miR-1839-5p,miR-21b,miR-378c,miR-362-3p,miR-2426,miR-27a-3p,miR-105-1,novel_4,miR-330-3p,novel_78,miR-148b-5p,miR-539-5p,miR-142-5p,miR-2957,miR-362-3p,miR-1185-5p,miR-3955-3p,miR-494-3p,miR-382-3p,miR-1b-5p,miR-378d,miR-381-3p,miR-7689-3p,miR-211-5p,miR-126b-5p,miR-27a-3p,miR-499a-3p,miR-378e,miR-25,miR-3082-5p,miR-181d-5p,miR-452-5p,miR-218-2-3p,miR-3591-5p,miR-1290,miR-105-2,miR-1224-5p,miR-129-1-3p,miR-6516-5p,miR-378h,miR-3071-3p,novel_127,miR-181b-5p,miR-30a-3p,miR-3529-3p,miR-200a-5p,miR-671-5p,miR-329a,miR-2318,miR-2285aa,miR-3068-3p,miR-365a-3p,novel_63,miR-204-5p,miR-592,miR-6128,miR-1306-5p,miR-378c,miR-22-3p,miR-197-3p,novel_23,miR-653,miR-181a-5p,miR-223-3p,miR-27b-3p,miR-105-5p,miR-330-3p,miR-224-5p,miR-377-3p,miR-466f-3p,miR-378a-5p,miR-668-3p,miR-130b-5p,miR-6240,miR-2285r,miR-18a-3p,miR-3065-5p,miR-3591-3p,miR-378f,novel_68,miR-378b,miR-3074-5p,miR-2404,miR-133a-5p,miR-421-5p,novel_27,miR-2284w,miR-211,miR-323c

TRH	ENSOARG0(	thyrotro	miR-4286,miR-30a-3p,miR-29b-2-5p,miR-3529-3p,miR-1271-3p,miR-3065-3p,miR-199a-5p,miR-28b,miR-339a,miR-3071-3p,miR-30c-2-3p,miR-6119-5p,miR-205-5p,miR-200a-3p,miR-152-3p,miR-2898,miR-450a-2-3p,miR-18b-5p,miR-1224-5p,novel_79,miR-6517,miR-214-5p,miR-129-1-3p,miR-486-5p,miR-322-5p,miR-30a-5p,miR-330-5p,miR-1343-5p,miR-29d-3p,miR-3578,miR-1290,miR-10a-5p,let-7a-2-3p,miR-103b,miR-16-5p,miR-1306-5p,miR-1197-3p,miR-93,miR-125b-5p,miR-331-3p,miR-370-5p,miR-29b-3p,miR-551b-3p,miR-410-3p,miR-1271,miR-615-5p,miR-141-3p,miR-125a,miR-2355-5p,miR-370-3p,miR-2285aa,miR-874-3p,miR-574-5p,miR-199a-5p,miR-3154,miR-18a,miR-17-5p,miR-93-5p,miR-30e-5p,miR-488-3p,miR-1248,miR-671-5p,miR-20b-5p,miR-7144-5p,miR-329a,let-7g-3p,miR-668-3p,let-7a-2-3p,miR-654-3p,novel_51,miR-106a-5p,miR-17-5p,miR-197-5p,miR-125a-5p,miR-1343-5p,miR-124-5p,miR-339-5p,miR-133b-5p,miR-544b,miR-20b,miR-194-3p,novel_116,miR-3431,miR-22-3p,miR-1285,miR-708-3p,miR-873a-5p,miR-27b-3p,miR-497-5p,miR-29b,miR-16b,miR-1193,novel_101,miR-29c-3p,miR-2285x,miR-3957-3p,miR-342-3p,miR-2428,miR-199b-5p,miR-2284w,miR-326-3p,miR-24-3p,miR-371b-3p,miR-33a-3p,miR-16a,miR-20a-5p,miR-28c,miR-15a,miR-502b,miR-29a,miR-6119-3p,miR-10a-5p,miR-320b,miR-3074-5p,miR-378b,novel_68,novel_69,miR-411b-3p,miR-29a-3p,miR-670-3p,miR-146b-3p,miR-3065-5p,miR-1306,miR-150-5p,miR-148a-3p,miR-18b,miR-2387,miR-6395,miR-195a-5p,miR-345-3p,miR-20a,miR-216c-5p,miR-143-3p,miR-125b-2-3p,miR-29b-1-5p,miR-125b,miR-185-3p,miR-219b-5p,miR-324-3p,novel_17,miR-210-5p,miR-21-3p,miR-30d,miR-221-5p,miR-3653-3p,miR-466i-5p,miR-760-3p,miR-106a,miR-30d-3p,miR-106a,miR-548e-3p,miR-328-3p,miR-1247-5p,novel_83,miR-15a-5p,miR-148b-3p,miR-7859,miR-383-
HSD11B1	ENSOARG0(	hydroxys	miR-30b-3p,miR-204-5p,novel_74,miR-664a,miR-331-3p,miR-205-5p,miR-664-3p,miR-326,miR-28c,miR-326-3p,miR-145-3p,miR-2428,miR-28b,miR-211,novel_73,miR-2426,miR-433-3p,miR-330-5p,miR-125b-2-3p,miR-378j,miR-671-3p,miR-211-5p,miR-664b
KIRREL3	ENSOARG0(	kin of I	miR-3184-3p,miR-2284w,miR-2433,miR-122-3p,miR-185-3p,miR-2331-5p,miR-2898,miR-4726-5p,miR-4492,miR-615-5p,miR-7977,miR-2355-5p,miR-1b-3p,miR-134-3p,miR-582-5p,miR-378g,miR-328-3p,miR-4443,miR-671-3p,miR-22-3p,miR-2113,miR-361-3p,miR-30b-3p,miR-22-3p,miR-147-3p,miR-30b-3p,miR-582-5p,miR-6395,miR-4508

USP33	ENSOARG0(	ubiquiti	miR-106a,miR-299-3p,miR-21-3p,miR-20a,miR-130a-5p,miR-146a-5p,miR-1246,miR-2300b-3p,miR-376a-5p,miR-188-3p,miR-5703,miR-19b-3p,let-7a-3p,miR-3600,miR-340-5p,miR-148b-3p,miR-200b,miR-324-3p,miR-655-3p,miR-29b-2-5p,miR-6516-3p,miR-155-5p,miR-17-3p,miR-487a-5p,miR-539-5p,miR-145a-3p,miR-2330-3p,miR-3074-2-3p,miR-206-3p,miR-21a-3p,let-7f-1-3p,let-7g-3p,novel_99,miR-140-5p,miR-21-3p,miR-532-5p,miR-182-5p,miR-6516,miR-656-3p,miR-22-3p,novel_32,miR-204-3p,miR-155-5p,miR-326,miR-106a-5p,miR-652-5p,miR-376b,miR-3187-3p,miR-412-3p,miR-450b-5p,miR-299a-5p,miR-101a-5p,miR-409b,miR-216a-3p,miR-500-5p,miR-2285e,miR-1973,miR-4726-5p,miR-452-5p,miR-194b-5p,miR-181d-5p,miR-362-5p,miR-361-3p,miR-199b-5p,miR-20a-3p,miR-489,miR-200c,miR-17-3p,miR-181b-5p,miR-200c-3p,miR-92a-3p,miR-1,miR-30a-3p,miR-29b-2-5p,miR-2478,miR-199a-5p,miR-1957a,miR-1271-3p,miR-345-3p,miR-582-3p,miR-200b-3p,miR-222,miR-6516-5p,miR-483-3p,miR-135a-1-3p,miR-3969,miR-409-3p,miR-135a-5p,miR-138-5p,miR-410-3p,miR-204-5p,miR-486b-3p,miR-93,miR-500b-5p,miR-17-5p,miR-3154,miR-362-5p,miR-376c-5p,miR-23a-5p,miR-190a-3p,miR-1814c,miR-370-3p,miR-374c-3p,miR-365a-3p,miR-199a-5p,miR-190a,miR-330-3p,miR-17-5p,miR-106a-5p,miR-130b-5p,miR-101-5p,miR-296-3p,miR-1285,miR-1434-3p,miR-194-5p,miR-138,miR-122-3p,miR-130a-3p,miR-432-5p,miR-2285g,miR-2284d,miR-20b,miR-20a-5p,miR-92a-3p,novel_1,miR-22-5p,miR-17-3p,miR-2285x,miR-211,miR-371b-3p,miR-326-3p,miR-2428,miR-18a-3p,miR-450b-3p,miR-19a-3p,miR-135b-5p,miR-655-5p,miR-7-1-3p,miR-618,miR-26a-5p,novel_17,miR-210-5p,miR-324-3p,miR-500a-5p,miR-345-3p,miR-329a-5p,miR-369-3p,miR-181b-1-3p,miR-486-3p,miR-2366,miR-33b-3p,novel_73,miR-16-1-3p,miR-18a-3p,miR-106a,miR-30d-3p,miR-30a-3p,miR-

TJP2	ENSOARG0(	tight ju	miR-539-5p,miR-425-5p,miR-10b-5p,miR-206-3p,miR-6134,miR-320e,miR-450b-3p,miR-6516-3p,miR-3065-3p,miR-532-5p,miR-6516,miR-656-3p,miR-139-5p,novel_25,miR-204-3p,miR-877-5p,miR-133b-3p,miR-2312,let-7g-3p,miR-18a-5p,novel_99,miR-20a,miR-29b-1-5p,miR-125b,miR-320d,miR-106a,miR-2284m,miR-548e-3p,miR-485-3p,miR-487a-3p,miR-383-5p,miR-148b-3p,miR-200b,miR-199a-3p,novel_83,novel_133,miR-1246,miR-124-3p,miR-1197-5p,miR-1193,miR-140-3p,miR-5703,miR-19b-3p,miR-4429,novel_60,miR-3059-5p,miR-212-3p,miR-2285f,miR-194b-5p,miR-8095,miR-219a-2-3p,miR-154a,miR-371a-5p,miR-769-5p,miR-335,miR-3064-5p,miR-499b-5p,miR-541-5p,miR-3082-5p,miR-361-3p,novel_94,miR-106a-5p,let-7c-3p,miR-30c-1-3p,miR-758-3p,miR-342,miR-320b,novel_103,miR-3085-3p,novel_4,miR-105-1,miR-9788-3p,miR-216a-3p,miR-484,miR-6529a,miR-10b,miR-2426,miR-1306-5p,miR-6128,miR-133a-3p,miR-323-3p,miR-93,miR-34c-3p,miR-29b-3p,miR-331-3p,miR-144,miR-135a-5p,miR-3969,miR-769,miR-488-3p,miR-17-5p,miR-18a,miR-320a,miR-10b-3p,miR-671-5p,miR-30a-3p,miR-1,miR-3065-3p,miR-490-3p,miR-200c,miR-30c-2-3p,miR-181c-3p,miR-450a-2-3p,miR-200c-3p,miR-423-3p,miR-6516-5p,miR-545-5p,miR-345-3p,miR-134,miR-29d-3p,miR-200b-3p,miR-10a-5p,miR-135a-2-3p,miR-342-3p,novel_101,miR-20a-5p,miR-656-5p,miR-147-5p,miR-10a-5p,miR-2404,miR-670-3p,miR-29a-3p,miR-2285ab,miR-18b,miR-18a-3p,miR-1291,miR-19a-3p,miR-2285k,miR-6516-3p,miR-6395,miR-135b-5p,miR-130b-5p,miR-363-5p,miR-539-3p,miR-345-5p,miR-224-5p,miR-106a-5p,miR-17-5p,miR-452-5p,miR-125a-5p,miR-665,miR-185-5p,miR-2355-3p,miR-20b,miR-1434-3p,miR-145a-5p,miR-3071-5p,miR-194-5p,miR-29b,miR-769-5p,miR-6525,miR-224-5p,miR-30e-3p,miR-30a-3p,miR-219b-
IQGAP1	ENSOARG0(	IQ motif	miR-124-5p,miR-191-3p,miR-660,miR-146b,miR-130b-5p,miR-146a,miR-135a-1-3p,miR-338-5p,miR-146a-5p,miR-380-3p,miR-130b-5p,miR-124a,miR-181a-2-3p,miR-4286,miR-380-3p,miR-146b-5p,miR-1434-3p,miR-124-3p,miR-660-5p

PIH1D1	ENSOARG0(	PIH1 dom	miR-125b,miR-422a,miR-185-3p,miR-221-5p,miR-30b-3p,miR-3653-3p,miR-195a-3p,miR-486-3p,miR-2300b-3p,novel_133,miR-378i,miR-217-5p,miR-378a-3p,miR-1193,miR-5703,miR-140-3p,miR-2904,miR-124-3p,miR-19b-3p,miR-3960,miR-30d-3p,miR-15a-5p,miR-532-3p,miR-2285j,miR-664a-5p,miR-19b-1-5p,miR-155-5p,miR-664a,miR-4492,miR-708-5p,miR-424-5p,miR-4510,miR-378c,miR-769-5p,miR-3184-5p,miR-122-5p,miR-2285c,miR-433-3p,miR-2312,miR-496-3p,miR-378a-3p,miR-221-5p,miR-2331-3p,miR-504-5p,miR-182-5p,miR-6130,miR-193b-3p,miR-155-5p,miR-15b-5p,miR-204-3p,miR-5126,miR-182-5p,miR-25-5p,miR-199b-5p,miR-424-5p,miR-2330-5p,miR-124a,miR-376b,miR-378d,miR-758-3p,miR-378b,miR-30b-3p,miR-758-3p,miR-412-3p,miR-7857,miR-378c,miR-16-2-3p,miR-1343-3p,miR-27a-3p,miR-145b,miR-2285e,miR-421,miR-9788-3p,miR-216a-3p,miR-615,novel_82,miR-382-3p,miR-769-5p,miR-2284q,miR-2447,miR-2285f,miR-423-5p,miR-199b-5p,miR-504,miR-664b,miR-7977,miR-499b-5p,miR-378e,miR-27a-3p,miR-664-3p,miR-2285af,miR-217,miR-127-5p,miR-450a-2-3p,miR-200a-3p,miR-181a-2-3p,miR-28b,miR-199a-5p,miR-134,miR-615-3p,miR-23b-5p,miR-1343-5p,miR-496,miR-155-5p,miR-412,miR-1224-5p,miR-378h,miR-483-3p,miR-322-5p,novel_121,miR-134-5p,miR-125b-5p,miR-486b-3p,miR-134-5p,miR-28-5p,miR-193a-5p,miR-16-5p,miR-378c,miR-503-3p,miR-488-3p,miR-3154,miR-485-5p,miR-23a-5p,miR-503-3p,miR-574-5p,miR-141-3p,miR-125a,miR-769,miR-374c-3p,miR-199a-5p,miR-7-5p,miR-2285v,miR-483-3p,miR-193a-3p,miR-125a-5p,miR-1343-5p,miR-1285,miR-7b-5p,miR-19b-2-5p,miR-145a-5p,miR-30b-3p,miR-16b,miR-27b-3p,miR-497-5p,miR-2285g,miR-28c,miR-15a,miR-4324,miR-16a,miR-128-1-5p,miR-22-5p,miR-136-3p,miR-193b-3p,miR-1307-3p,miR-199b-5p,miR-2387,miR-2285ah,miR-19a-

IKBIP	ENSOARG0(	IKBKB in	miR-345-3p,let-7a-2-3p,miR-582-3p,miR-7641,miR-2285af,miR-181b-5p,miR-450a-2-3p,miR-17-3p,miR-181a-2-3p,miR-2403,miR-2385-3p,miR-2284a,miR-380-5p,miR-320a,miR-17-5p,miR-3154,miR-2448-3p,miR-1814c,miR-485-5p,let-7g-3p,miR-769,miR-374c-3p,miR-144,miR-331-3p,miR-370-5p,miR-410-5p,miR-34c-3p,miR-1-5p,miR-31-3p,miR-410-3p,miR-3607-3p,miR-6128,miR-1306-5p,miR-145-3p,miR-365a-5p,miR-93,miR-323-3p,miR-194-5p,miR-3071-5p,miR-145a-5p,miR-374b-3p,miR-181a-5p,miR-30b-3p,miR-133b-5p,miR-2284d,miR-2285g,miR-17-5p,miR-330-3p,miR-224-5p,miR-1343-5p,miR-130b-5p,miR-345-5p,miR-539-3p,miR-18a-3p,miR-2285ab,miR-6516-3p,miR-195a-5p,miR-320b,miR-323a-5p,miR-33a-3p,miR-16a,miR-20a-5p,miR-15a,miR-199c,miR-147-5p,novel_1,miR-2285x,miR-17-3p,miR-1193,miR-3957-3p,novel_27,miR-326-3p,miR-371b-3p,miR-24-3p,miR-5703,miR-2904,miR-4429,miR-485-3p,miR-548e-3p,miR-616-3p,miR-340-5p,miR-148b-3p,miR-15a-5p,miR-222-5p,miR-487a-3p,miR-216a-5p,miR-196a-3p,miR-21-3p,miR-1277-5p,miR-221-5p,miR-106a,miR-320d,miR-20a,miR-183-5p,miR-216c-5p,miR-21a-3p,novel_99,let-7g-3p,miR-370-5p,miR-21-3p,miR-32-3p,miR-140-5p,miR-221-5p,miR-545-3p,miR-155-5p,miR-877-5p,novel_25,miR-139-5p,miR-323-5p,miR-320e,miR-324-3p,miR-424-5p,miR-208b-5p,miR-17-3p,miR-664a,miR-155-5p,miR-450b-3p,miR-2330-3p,miR-30b-3p,miR-484,miR-6529a,miR-450b-5p,miR-222-5p,miR-27a-3p,miR-409b,miR-2285e,novel_103,miR-326,miR-320b,miR-25-5p,miR-3085-3p,miR-33a-5p,miR-1839-3p,miR-424-5p,miR-106a-5p,miR-758-3p,miR-129b-5p,miR-361-3p,miR-7977,miR-541-5p,miR-10b,miR-664b,miR-3064-5p,miR-27a-3p,miR-3082-5p,miR-769-5p,miR-2284q,miR-2285t,miR-181d-5p,miR-194b-5p,miR-2285f,miR-541-3p,miR-32-3p,miR-3059-5p,miR-330-5p,miR-
RIN3	ENSOARG0(	Ras and	miR-466i-5p,miR-128-1-5p,miR-2408,miR-324-3p,miR-222-5p,miR-346,miR-122-5p,miR-324-5p,miR-669,miR-423-5p,miR-3184-5p,miR-574-5p,novel_42,miR-363-5p
FAM134B	ENSOARG0(	family w	miR-148b-5p,miR-30a-3p,miR-3064-5p,miR-2887,miR-466i-5p,miR-1277-5p,miR-3653-3p,novel_96,miR-2285af,miR-376b-3p,miR-125a-3p,miR-505-5p,miR-758-5p,miR-545-5p,miR-3600,novel_73,miR-133a-3p,miR-218-1-3p,miR-3578,miR-1197-5p,miR-1434-5p,miR-6525,miR-3184-5p,miR-10b-3p,miR-6134,miR-539-5p,miR-592,miR-2285j,miR-2332,miR-30e-3p,miR-30a-3p,miR-26c,miR-338-5p,miR-3969,miR-1277-3p,miR-3068-3p,miR-660-5p,miR-671-5p,miR-424-3p,miR-3154,miR-628-5p,miR-3085-3p,miR-124-5p,miR-491-5p,miR-660,miR-376b-3p,novel_116,miR-548w,miR-27a-3p,miR-1388-5p,miR-30b-3p,miR-27b-3p,miR-6529a,miR-873a-5p,miR-2113,miR-30b-3p,miR-24-3p,miR-423-5p,miR-2285f,novel_1,miR-2284q,miR-6123,miR-502b,miR-669,miR-27a-3p,miR-376d,miR-3064-5p,miR-329-5p,miR-376a-3p,miR-2285ab,miR-376b-3p

DENND5A	ENSOARG0(	DENN/MAD	miR-380-5p,miR-2478,novel_39,miR-199a-5p,miR-1957a,miR-376b-5p,miR-376c-5p,miR-192-3p,miR-29b-2-5p,miR-3064-5p,miR-200c-3p,novel_96,miR-200c,miR-6119-5p,miR-134-5p,miR-214-5p,miR-429-3p,miR-582-3p,miR-300,miR-128-3p,miR-345-3p,miR-134,miR-330-5p,miR-1197-3p,miR-145-3p,miR-138-5p,miR-134-5p,miR-486b-3p,miR-204-5p,miR-5100,miR-34c-5p,miR-199a-5p,miR-574-5p,miR-370-3p,miR-1271,miR-345-5p,miR-1814c,miR-381-3p,miR-2448-3p,miR-3154,miR-432-3p,miR-432-3p,miR-345-5p,miR-380-5p,miR-130b-5p,miR-6240,miR-7-5p,miR-330-3p,miR-2411-3p,novel_51,miR-2285g,miR-3431,miR-30b-3p,miR-138,miR-16-1-3p,miR-188-5p,miR-7b-5p,miR-223-3p,miR-1434-3p,novel_23,miR-211,miR-24-3p,miR-326-3p,miR-449a,miR-199b-5p,miR-6119-3p,miR-421-5p,miR-34c,novel_69,miR-411b-3p,miR-34b,miR-677,miR-181b-3p,miR-3074-5p,miR-7-1-3p,miR-450b-3p,miR-412-3p,miR-9851-3p,miR-2285ab,miR-1306,miR-216a-3p,miR-29b-1-5p,miR-421-5p,miR-181b-1-3p,miR-125b-2-3p,miR-486-3p,miR-143-3p,miR-345-3p,miR-3653-3p,miR-34b-5p,miR-221-5p,miR-324-3p,miR-500a-5p,miR-345-3p,miR-185-3p,miR-222-5p,miR-151-5p,novel_73,miR-16-1-3p,miR-34a-5p,miR-2366,miR-2300b-3p,miR-2904,miR-1197-5p,miR-5703,miR-140-3p,miR-3184-5p,miR-122-5p,miR-338-3p,miR-3074-5p,miR-4510,miR-3074-2-3p,miR-130b-5p,miR-151-3p,miR-29b-2-5p,miR-320e,miR-2284s,miR-6130,novel_25,novel_32,miR-204-3p,miR-151b,miR-221-5p,miR-2312,miR-345-5p,miR-1827,miR-2285c,miR-8485,miR-2310,miR-3085-3p,miR-491-5p,miR-199b-5p,miR-326,miR-1306-5p,miR-29b-1-5p,miR-2320-3p,miR-147a,miR-216a-3p,miR-9788-3p,miR-455-3p,miR-2285e,novel_4,miR-330-3p,miR-151a-3p,miR-181b-2-3p,miR-2426,miR-484,miR-541,miR-30b-3p,miR-423-5p,miR-2447,miR-3059-5p,miR-541-3p,miR-2285f,miR-

MCM3AP	ENSOARG0(	minichro	miR-493-5p,let-7a-2-3p,miR-22841,novel_51,miR-296-3p,miR-7-5p,miR-31-5p,miR-412-5p,miR-22-3p,miR-1843b-5p,miR-7b-5p,miR-542-3p,miR-188-5p,miR-497-5p,miR-16-1-3p,miR-16b,miR-335-5p,miR-301b-3p,miR-374c-5p,miR-1247-3p,miR-4324,miR-6123,miR-3120-3p,miR-329-5p,miR-181b-3p,miR-669,miR-320c,miR-677,miR-3065-5p,miR-1306,miR-150-5p,miR-2319a,miR-412-3p,miR-192-3p,miR-29a-5p,miR-3071-3p,miR-219-3p,miR-127-5p,miR-429-3p,miR-2285ad,novel_121,miR-486-5p,miR-758-5p,miR-322-5p,miR-23b-5p,miR-2427,miR-16-5p,miR-1197-3p,miR-6740-5p,miR-125b-5p,novel_63,novel_9,miR-1271,miR-125a,miR-2355-5p,miR-2285aa,miR-503-3p,miR-3068-3p,miR-376c-3p,miR-300-3p,miR-23b,miR-137-3p,miR-329a,miR-329b,miR-345-5p,miR-23a-3p,miR-101b-3p,miR-16b,miR-182-5p,miR-130b-3p,miR-1185-5p,miR-181b-2-3p,miR-199b-3p,miR-148b-5p,miR-539-5p,miR-376b-3p,miR-147a,miR-329-3p,miR-101c,miR-362-3p,miR-190b-5p,miR-3956-3p,miR-2447,miR-1285-5p,novel_111,miR-101-3p,miR-218-5p,miR-154-3p,miR-3968,miR-2319b,miR-2483-3p,miR-499a-3p,miR-25,miR-378j,miR-140-5p,miR-1983,miR-493-5p,miR-96-5p,miR-487a,miR-329a-5p,miR-181b-1-3p,miR-376c-3p,miR-664b-3p,miR-92a-1-5p,novel_17,miR-2285p,miR-202-5p,miR-30d-3p,miR-23c,miR-7859,miR-23b-3p,miR-217-5p,miR-2366,miR-193b-5p,miR-148a-5p,miR-16-1-3p,miR-3184-3p,miR-122-5p,miR-224-5p,miR-769-5p,miR-3604,miR-30a-3p,miR-219b-3p,miR-30e-3p,miR-15b-3p,miR-454-3p,miR-500,miR-129-5p,miR-190a-5p,novel_74,miR-552-3p,miR-154a-3p,miR-30c-1-3p,miR-2331-3p,miR-15b-5p,miR-376e-3p,miR-499b-3p,miR-301a-3p,miR-500a-3p,miR-186-5p,miR-543-3p,miR-345-5p,miR-363-5p,miR-345-5p,miR-296-3p,miR-197-5p,miR-190a,miR-224-5p,miR-660,miR-125a-5p,miR-130a-3p,miR-665,miR-6238,miR-3071-5p,miR-181a-5p,miR-1434-

IL18R1	ENSOARG0(	interleu	novel_94,miR-877-3p,let-7d-5p,miR-376d,miR-7977,miR-664b,miR-20a-3p,miR-10b,miR-2285t,miR-374a-3p,miR-3958-3p,miR-335,miR-769-5p,miR-371a-5p,miR-382-3p,miR-219a-2-3p,miR-2285f,miR-452-5p,miR-181d-5p,miR-3059-5p,miR-450b-5p,miR-125b-2-3p,miR-30b-3p,miR-484,miR-4791,miR-9788-3p,miR-216a-3p,miR-2285e,miR-2320-3p,miR-5010-3p,novel_4,miR-409b,let-7e,miR-3955-3p,miR-2957,miR-342,miR-1306-5p,novel_115,miR-326,miR-4532,miR-758-3p,miR-30c-1-3p,let-7c-3p,miR-33a-5p,let-7a-5p,miR-494-3p,miR-1839-3p,miR-133b-3p,miR-2312,let-7g-3p,novel_25,miR-6516,miR-139-5p,miR-221-5p,miR-450b-3p,miR-664a,miR-6516-3p,miR-17-3p,miR-19b-1-5p,miR-338-5p,let-7b-5p,miR-206-3p,miR-145a-3p,miR-19b-3p,miR-2904,miR-140-3p,miR-188-3p,miR-5703,miR-2300b-3p,miR-532-3p,miR-199a-3p,miR-222-5p,miR-148b-3p,miR-200b,miR-3600,miR-221-5p,miR-1277-5p,miR-299-3p,miR-466i-5p,miR-125b,miR-125b-2-3p,miR-183-5p,miR-216c-5p,miR-6516-3p,miR-19a-3p,miR-21c,miR-376b-3p,miR-18a-3p,miR-2285ab,miR-2387,miR-656-5p,miR-199c,novel_1,miR-382-5p,let-7i,miR-33a-3p,miR-502-5p,miR-326-3p,miR-323c,miR-24-3p,novel_101,miR-17-3p,miR-342-3p,miR-2889,miR-30b-3p,miR-21-5p,miR-1260b,miR-145a-5p,miR-181a-5p,miR-1434-3p,miR-2285g,miR-432-5p,miR-185-5p,let-7g-5p,miR-377-5p,miR-187-3p,miR-452-5p,miR-125a-5p,miR-190a,miR-99a-5p,miR-224-5p,miR-345-5p,miR-654-3p,miR-130b-5p,miR-376c-5p,miR-671-5p,miR-10b-3p,miR-3154,miR-100-5p,miR-374c-3p,miR-3965,miR-365a-3p,miR-769,miR-370-3p,miR-615-5p,miR-374b-3p,miR-767-5p,miR-144,miR-331-3p,miR-409-3p,miR-145-3p,miR-374a-3p,miR-133a-3p,miR-200b-3p,miR-128-3p,miR-99a-5p,let-7a-2-3p,let-7d,miR-134,miR-6516-5p,miR-450a-2-3p,miR-17-3p,miR-200c-3p,miR-181b-5p,miR-

ETS1	ENSOARG0(	v-ets av	miR-429-3p,miR-299a-3p,miR-2285ad,miR-376a-5p,miR-200b,miR-95-3p,miR-532-3p,miR-450a-1-3p,miR-3578,miR-1246,miR-615-3p,miR-5703,miR-1197-5p,miR-361-5p,miR-200b-3p,miR-133a-3p,miR-155-5p,miR-345-3p,miR-181a-2-3p,miR-4286,miR-130a-5p,miR-125b-2-3p,miR-376a-5p,miR-450b-5p,miR-208b-3p,miR-200c,novel_17,miR-1260a,miR-216a-5p,miR-200c-3p,miR-202-5p,miR-299-3p,novel_96,miR-30b-3p,miR-3653-3p,miR-182-5p,miR-532-5p,miR-365a-3p,miR-139-5p,miR-377-3p,miR-374c-3p,miR-155-5p,miR-450a-1-3p,miR-137-3p,miR-27b-5p,miR-500a-3p,miR-10b-3p,miR-133b-3p,let-7g-3p,novel_99,miR-181a-2-3p,miR-1306-5p,miR-539-5p,miR-214,miR-665-5p,miR-425-5p,miR-133a-3p,miR-3074-2-3p,miR-1260b,miR-374a-3p,miR-6525,miR-181c-5p,miR-2285j,miR-135a-5p,miR-100-3p,miR-6516-3p,miR-450b-3p,miR-130b-5p,miR-374b-3p,miR-500,miR-129-5p,miR-330-3p,miR-2300a-5p,miR-665,novel_116,miR-194-3p,miR-149-5p,miR-450c-3p,miR-1260b,miR-2889,miR-6240,miR-493-5p,miR-130b-5p,miR-377-3p,miR-182-5p,novel_51,miR-330-3p,miR-491-5p,miR-103a-2-5p,miR-214-3p,miR-452-3p,miR-139-5p,novel_68,miR-2285u,miR-3082-5p,miR-299b-3p,miR-7857-3p,miR-25,miR-203-3p,miR-652-3p,miR-136-5p,miR-412-3p,miR-135b-5p,miR-1983,miR-6516-3p,miR-376a-2-5p,miR-493-5p,miR-615,miR-208a-3p,miR-2285n,miR-425-5p,miR-107-5p,miR-1973,miR-2285y,miR-499a-5p,miR-374a-3p
RILP	ENSOARG0(	Rab inte	miR-6535,miR-6130,miR-377-3p,miR-370-5p,miR-655-5p,miR-541-5p,miR-221-5p,miR-625-5p,miR-8095,miR-326-3p,miR-214,miR-17-3p,miR-1193,miR-4510,miR-486b-3p,miR-664a,miR-17-3p,miR-370-5p,miR-455-3p,miR-2411-5p,miR-361-5p,miR-30b-3p,miR-330-5p,miR-30b-3p,miR-2904,miR-197-3p,miR-345-3p,miR-2411,miR-486-3p,miR-2432,miR-181a-2-3p,miR-214-3p,miR-21-3p,miR-221-5p,miR-17-3p,miR-345-3p,miR-2411-3p,miR-326,miR-377-3p,novel_115
PGM5	ENSOARG0(	phosphog	miR-34b-5p,miR-30b-3p,miR-221-5p,miR-21-3p,miR-664-3p,novel_17,miR-30c-2-3p,miR-500a-5p,miR-490-3p,miR-181a-2-3p,miR-10a-5p,miR-34a-5p,novel_133,miR-193b-5p,miR-1290,miR-140-3p,novel_83,miR-328-3p,miR-2285ad,novel_74,miR-6516-3p,miR-190a-5p,miR-410-3p,miR-551b-3p,miR-5100,miR-369-5p,miR-10b-5p,miR-346,miR-338-3p,miR-6134,miR-10b-3p,miR-1814c,miR-3613-5p,miR-34c-5p,miR-10a,miR-504-5p,miR-140-5p,miR-30c-1-3p,miR-221-5p,miR-874-3p,novel_103,miR-190a,miR-30c-1-3p,miR-3187-3p,miR-758-3p,miR-2310,miR-668-3p,let-7c-3p,miR-33a-5p,miR-369-5p,miR-653,miR-541,miR-10b,novel_23,miR-758-3p,miR-9788-3p,miR-194-3p,miR-340-3p,novel_4,miR-6119-3p,novel_1,miR-147-5p,miR-3613,miR-2285n,miR-142b,miR-576-3p,miR-34c,miR-2285y,miR-449a,miR-541-3p,miR-190b-5p,miR-1291,miR-504,miR-412-3p,miR-9851-3p,miR-1983,miR-6395,miR-140-5p,miR-34b,miR-411b-3p,miR-664b,miR-10a-5p
ANKH	ENSOARG0(	ANKH ino	miR-3960,miR-3074-5p,miR-154b-5p,novel_133,miR-335-3p,miR-1983,miR-2426,miR-412-3p,miR-1388-3p,miR-3074-5p,miR-4532,miR-486-3p,let-7j,miR-1307-3p,miR-3959-3p,miR-219b-5p,miR-185-3p,miR-2285af,miR-2285j,miR-2433,miR-4492,miR-486b-3p

CTU2	ENSOARG0(	cytosoli	miR-22-3p,miR-616-3p,miR-378g,miR-134-3p,miR-574-5p,miR-3074-5p,miR-2889,miR-2427,miR-22-3p,miR-1197-5p,miR-486-3p,miR-665-5p,miR-3074-5p,miR-145a-3p,miR-31-3p,miR-708-5p,miR-2484,miR-28-5p,miR-486b-3p
SNTG1	ENSOARG0(	syntroph	miR-2432,miR-486-3p,miR-181b-1-3p,miR-125b-2-3p,miR-376a-5p,miR-503-5p,miR-329a-5p,miR-21-3p,miR-1277-5p,miR-466i-5p,miR-320d,miR-125b,miR-324-3p,miR-216a-5p,miR-210-5p,miR-618,miR-26a-5p,miR-23c,miR-222-5p,miR-23b-3p,miR-487a-3p,miR-30d-3p,miR-485-3p,miR-3600,miR-1b-3p,let-7a-3p,miR-218-1-3p,miR-4429,miR-1193,miR-124-3p,miR-193b-5p,miR-2300b-3p,miR-2366,miR-338-3p,miR-122-5p,miR-425-5p,miR-3074-2-3p,miR-145a-3p,miR-4510,miR-3184-3p,miR-590-3p,miR-130b-5p,miR-4492,miR-129-5p,miR-6516-3p,novel_74,miR-29b-2-5p,miR-324-3p,miR-30a-3p,miR-30e-3p,miR-877-5p,miR-6130,miR-139-5p,miR-544-3p,miR-296-3p,miR-32-3p,miR-32-5p,miR-1827,miR-1895,miR-625-5p,miR-27b-5p,let-7f-1-3p,miR-21a-3p,miR-2310,miR-8485,miR-3187-3p,miR-23a-3p,miR-124a,miR-3085-3p,miR-491-5p,novel_19,miR-326,miR-320b,miR-126a-5p,miR-376b-3p,miR-1298-3p,miR-181b-2-3p,miR-145b,miR-27a-3p,novel_78,miR-105-1,miR-1343-3p,miR-3120-5p,miR-1839-5p,miR-2284aa,miR-101a-5p,miR-544a,miR-2426,miR-501-3p,miR-143-5p,miR-301b-5p,miR-541,miR-6529a,miR-502-3p,miR-452-5p,miR-9-5p,miR-1296-5p,miR-363-3p,miR-541-3p,miR-32-3p,miR-490-5p,miR-3964,miR-4726-5p,miR-301a-5p,miR-371a-5p,miR-101-3p,miR-218-5p,miR-500-3p,miR-27a-3p,miR-4443,miR-376d,miR-2483-3p,miR-3082-5p,miR-378g,miR-211-5p,miR-7977,miR-139-5p,miR-4792,miR-3064-5p,miR-544-5p,miR-376a-3p,miR-136-5p,miR-129b-5p,miR-361-3p,novel_39,miR-29a-5p,miR-450b-5p,miR-29b-2-5p,miR-30a-3p,miR-3064-5p,miR-543-5p,miR-200a-3p,novel_96,miR-1a-2-5p,miR-127-5p,miR-450a-2-3p,miR-125a-3p,miR-205-5p,miR-376b-3p,novel_121,miR-2284z,miR-134-5p,miR-9-5p,miR-1224-5p,miR-7641,miR-429-3p,miR-105-2,miR-128-3p,miR-300,miR-330-5p,miR-501-3p,miR-23b-5p,miR-1290,miR-134,miR-1197-3p,miR-323-
NUPL1	ENSOARG0(	nucleopo	miR-2284m,miR-17-3p,miR-17-3p,miR-30b-3p,miR-669a-3p,miR-2284k,miR-2285p,miR-217,miR-382-5p,miR-376a-5p,miR-2284l,miR-2284v,miR-382,miR-17-3p,miR-2284j,miR-10b-3p,miR-496,miR-2284x,miR-3065-5p,miR-381-3p,miR-217-5p,miR-3082-5p,miR-545-3p,miR-496-3p,miR-503-3p,miR-1343-3p

YBEY	ENSOARG0(	ybeY met	miR-874-3p,miR-2355-5p,miR-125a,miR-141-3p,miR-215-5p,miR-34c-5p,miR-154b-5p,miR-10b-3p,miR-329a,miR-2318,miR-345-5p,miR-23a-5p,miR-1814c,miR-3607-3p,miR-1197-3p,miR-2433,miR-125b-5p,miR-100-3p,miR-138-5p,miR-31-3p,miR-486b-3p,miR-7641,miR-23b-5p,miR-330-5p,miR-10a-5p,miR-3064-5p,miR-4286,miR-148b-5p,miR-28b,miR-2285b,miR-125a-3p,miR-30c-2-3p,miR-217,miR-107,miR-200a-3p,miR-543-5p,novel_68,miR-320b,miR-10a-5p,miR-34b,miR-2387,miR-1306,miR-450b-3p,miR-1291,miR-412-3p,miR-107,miR-146b-5p,miR-1839-3p,novel_101,miR-24-3p,miR-371b-3p,miR-326-3p,miR-449a,miR-4324,miR-6402,miR-28c,miR-34c,novel_1,miR-147-5p,miR-103a-3p,miR-665,miR-185-5p,miR-149-5p,miR-194-3p,miR-103,miR-1285,miR-223-3p,miR-22-3p,miR-138,miR-628-5p,miR-432-3p,miR-345-5p,miR-146a,miR-125a-5p,miR-30c-1-3p,miR-296-3p,miR-10a,miR-192-5p,miR-27a-5p,miR-22-3p,miR-21a-3p,miR-345-5p,miR-1827,miR-214,miR-10b-5p,miR-6525,miR-3184-5p,miR-15b-3p,miR-2285j,miR-3604,miR-4492,miR-532-3p,novel_133,miR-362-3p,miR-215-5p,miR-124-3p,miR-217-5p,miR-378d,miR-34a-5p,miR-2424,miR-486-3p,miR-146a-5p,miR-210-5p,miR-618,miR-324-3p,miR-125b,miR-92a-1-5p,miR-185-3p,miR-202-5p,miR-760-3p,miR-34b-5p,miR-3064-5p,miR-10b,miR-499b-5p,miR-1983,miR-541-3p,miR-423-5p,miR-329-3p,miR-541,miR-10b,miR-362-3p,miR-124a,miR-1388-3p,miR-744-3p,miR-30c-1-3p,miR-4532,miR-2310,miR-1b-5p,miR-1306-5p,miR-326,miR-362-3p,miR-505,miR-3085-3p,miR-146b,miR-214-3p
LGMN	ENSOARG0(	legumain	miR-324-5p,miR-545-5p,miR-23b-3p,miR-34c-5p,miR-34b,miR-23c,miR-30c-1-3p,miR-34a-5p,miR-1827,miR-30b-3p,miR-2904,miR-30b-3p,miR-23b,miR-181a-5p,miR-449a,miR-181c-5p,miR-30c-1-3p,miR-23a-3p,miR-181d-5p,miR-433-5p,miR-4492,miR-34b-5p,miR-181b-5p,miR-34c,miR-30c-2-3p,novel_51,miR-185-3p
GSTP1	ENSOARG0(	glutathi	miR-133b-3p,miR-6525,miR-1343-3p,miR-133a-3p

IFNGR2	ENSOARG0(	interfer	miR-452-3p,miR-2319b,miR-664b,miR-211-5p,miR-499b-5p,miR-378g,miR-25,miR-499a-3p,miR-378e,miR-361-3p,miR-129b-5p,miR-504,miR-136-5p,novel_94,miR-877-3p,miR-212-3p,miR-3956-3p,novel_60,miR-3059-5p,miR-2447,miR-452-5p,miR-1296-5p,miR-423-5p,miR-107-5p,miR-769-5p,miR-4726-5p,miR-7134-3p,miR-2285t,novel_78,novel_4,miR-409b,miR-181b-2-3p,miR-142-5p,miR-2285e,miR-455-3p,miR-10b,miR-378b,miR-296-5p,miR-484,miR-758-3p,miR-125b-2-3p,miR-450b-5p,miR-544a,miR-378c,miR-1388-5p,miR-424-5p,miR-1839-3p,miR-30c-1-3p,miR-378d,miR-758-3p,miR-3187-3p,miR-8485,miR-431-5p,miR-326,miR-1306-5p,miR-320b,miR-16b,miR-29b-1-5p,novel_124,miR-103a-2-5p,miR-214-3p,miR-496-3p,miR-30c-1-3p,miR-504-5p,miR-545-3p,miR-378a-3p,miR-2331-3p,miR-532-5p,miR-296-3p,miR-3065-3p,miR-544-3p,miR-377-3p,miR-10a,miR-582-5p,miR-499b-3p,miR-15b-5p,novel_25,novel_32,miR-22-3p,miR-204-3p,let-7f-1-3p,miR-2285c,miR-433-3p,miR-411,miR-129b-3p,miR-27b-5p,miR-339b,let-7g-3p,novel_99,miR-3074-5p,miR-3184-3p,miR-378c,miR-214,miR-665-5p,miR-98-3p,miR-346,miR-142a-5p,miR-769-5p,miR-10b-5p,miR-6134,miR-3184-5p,let-7b-3p,miR-665,miR-664a,miR-17-3p,miR-130b-5p,miR-4492,miR-424-5p,miR-3600,miR-200b,miR-15a-5p,miR-383-5p,miR-1246,miR-2366,miR-378i,miR-1197-5p,miR-5703,miR-378a-3p,miR-98-3p,miR-4429,miR-19b-3p,miR-493-3p,let-7a-3p,miR-133a-3p,miR-216c-5p,miR-345-3p,miR-181b-1-3p,miR-125b-2-3p,miR-376a-5p,miR-29b-1-5p,miR-380-3p,miR-2432,novel_17,miR-210-5p,miR-196a-3p,miR-125b,miR-422a,miR-324-3p,miR-320d,miR-21-3p,miR-3074-5p,novel_68,miR-10a-5p,miR-411b-3p,miR-29a-3p,miR-181b-3p,miR-320c,miR-677,miR-1306,miR-19a-3p,miR-1291,miR-378f,miR-195a-5p,miR-7975,miR-135a-2-3p,miR-29c-3p,miR-

CCDC126	ENSOARG0(	coiled-c	miR-7857,miR-412-3p,miR-30b-3p,miR-329-3p,miR-362-3p,miR-101a-5p,miR-216a-3p,miR-9788-3p,miR-1185-3p,miR-421,miR-505,miR-362-3p,miR-16b,miR-326,miR-3955-3p,miR-130b-3p,let-7c-3p,miR-1839-3p,miR-424-5p,miR-494-3p,miR-8485,miR-2310,miR-381-3p,novel_94,miR-1983,miR-3963,miR-499b-5p,miR-211-5p,miR-2284g,miR-452-3p,miR-1185-2-3p,miR-371a-5p,miR-2285n,miR-218-5p,miR-490-5p,miR-154-3p,miR-3958-3p,miR-181d-5p,miR-98-3p,miR-5703,novel_133,miR-362-3p,miR-2366,miR-16-1-3p,miR-218-1-3p,let-7a-3p,miR-2284u,miR-1b-3p,miR-30d-3p,miR-485-3p,miR-487a-3p,miR-15a-5p,miR-200b,miR-92a-1-5p,miR-196a-3p,miR-345-3p,miR-487a,miR-486-3p,miR-130a-5p,miR-655,let-7f-1-3p,let-7g-3p,miR-154b-3p,miR-3065-3p,miR-154a-3p,miR-296-3p,miR-545-3p,miR-660-5p,miR-15b-5p,miR-6130,miR-30a-3p,miR-338-5p,miR-655-3p,let-7b-3p,miR-30e-3p,miR-424-5p,miR-454-3p,miR-669a-3p,miR-4510,miR-338-3p,miR-181c-5p,miR-98-3p,miR-181a-5p,miR-497-5p,miR-455-5p,miR-29b,miR-30b-3p,miR-16b,miR-16-1-3p,miR-130a-3p,miR-3955-5p,miR-6238,miR-412-5p,miR-197-5p,novel_51,miR-455-5p,miR-452-5p,miR-660,miR-466f-3p,miR-539-3p,miR-432-3p,miR-432-3p,miR-21c,miR-9851-3p,miR-195a-5p,miR-412-3p,miR-19a-3p,miR-7-1-3p,miR-216b-3p,miR-6535,miR-29a-3p,miR-670-3p,miR-16a,miR-15a,miR-576-3p,miR-203b-5p,novel_1,miR-29a,miR-1839-3p,miR-29c-3p,miR-2285x,miR-1a-1-5p,miR-326-3p,miR-2428,miR-301b-3p,miR-2284w,miR-211,miR-29d-3p,miR-330-5p,miR-134,miR-200b-3p,miR-412,miR-429-3p,miR-545-5p,miR-134-5p,miR-322-5p,miR-125a-3p,miR-200c,miR-200a-3p,miR-1a-2-5p,miR-181b-5p,miR-200c-3p,miR-30a-3p,novel_39,miR-200a,miR-3065-3p,miR-2284y,miR-1271-3p,miR-450b-5p,miR-137-3p,miR-3154,miR-301,miR-1814c,miR-767,miR-329a,miR-141-3p,miR-
LAMP2	ENSOARG0(	lysosoma	miR-499a-5p,miR-2284ab,miR-6516-3p,miR-544-5p,miR-186-5p,miR-19b-2-5p,miR-19b-1-5p,miR-3065-5p,miR-582-5p,miR-29a-5p,miR-7641,miR-26b-3p,miR-7-1-3p
SYT7	ENSOARG0(	synaptot	miR-212-5p,miR-331-3p,miR-132-5p,miR-2300a-5p,miR-874-5p,miR-941,miR-4726-5p
IFNAR1	ENSOARG0(	interfer	miR-877-5p,miR-155-5p,miR-29a-3p,miR-6130,miR-378e,miR-3596,miR-3120-3p,miR-3065-3p,miR-378b,miR-378a-3p,miR-378f,miR-1827,miR-345-5p,miR-345-5p,miR-129b-3p,miR-378c,miR-3074-2-3p,miR-29c-3p,miR-4510,miR-145a-3p,miR-378c,miR-134-5p,miR-22-5p,miR-29a,miR-155-5p,miR-499a-5p,miR-3968,miR-29b-3p,miR-134-5p,miR-582-3p,miR-378h,miR-155-5p,miR-378c,miR-29b,miR-378a-3p,miR-29d-3p,miR-2427,miR-1285,miR-3578,miR-134,miR-484,miR-378b,miR-378i,miR-345-5p,miR-3065-3p,miR-8485,miR-378d,miR-345-3p,miR-466f-3p,miR-422a
MAP4K4	ENSOARG0(	mitogen-	novel_42,miR-29c-3p,miR-2903,miR-3065-3p,miR-133a-5p,miR-2433,miR-29b-3p,novel_19,miR-29a,miR-452-3p,miR-145b,miR-3065-3p,miR-29a-3p,miR-421,miR-9788-3p,miR-21a-3p,miR-145a-5p,miR-29d-3p,miR-29b

RNF166	ENSOARG0(	ring fin	miR-1895,miR-744-5p,miR-19b-3p,miR-19a-3p,miR-2285k,miR-1306,miR-2366,miR-148a-3p,miR-669,miR-9788-3p,miR-301a-3p,miR-148b-3p,miR-130a-3p,miR-3065-3p,miR-454-3p,miR-152-3p,miR-4726-5p,miR-2285l,miR-130b-3p,novel_82,miR-1306-5p,miR-423-5p,miR-3065-3p,miR-3184-5p,miR-301b-3p,miR-2285i,miR-361-3p,novel_42,miR-183-5p
MTMR6	ENSOARG0(	myotubul	miR-34c,miR-301a-5p,miR-107-5p,miR-34c-3p,novel_63,miR-502-5p,miR-370-5p,miR-101-3p,miR-449a,miR-181c-5p,miR-877-3p,miR-1296-5p,miR-181d-5p,miR-1193,miR-495-3p,miR-543-3p,miR-1983,miR-329b,miR-412-3p,miR-203-3p,miR-1185-2-3p,miR-34c-5p,miR-34b,miR-370-5p,miR-664b,miR-329-5p,miR-34b-5p,miR-103a-2-5p,miR-181b-5p,miR-105-5p,miR-196a-3p,miR-2285af,novel_17,miR-664-3p,miR-377-5p,miR-668-3p,let-7f-2-3p,let-7f-2-3p,miR-329a-5p,miR-216c-5p,miR-101b-3p,miR-382-3p,miR-105-2,miR-16-2-3p,miR-34a-5p,miR-181a-5p,miR-301b-5p,miR-143-5p,miR-126a-5p,miR-1185-3p,miR-377-5p,miR-105-1
HIP1	ENSOARG0(	huntingt	miR-744-5p,miR-29b,miR-19b-3p,miR-29d-3p,miR-124-3p,miR-1343-5p,miR-1197-5p,miR-125b-2-3p,miR-191,miR-541,miR-301b-5p,miR-194-3p,miR-2284h-5p,miR-134-3p,miR-212-5p,miR-6517,miR-133b-5p,miR-409b,miR-125a-5p,miR-1343-5p,miR-214-3p,miR-487b-5p,miR-125b,miR-185-3p,novel_17,miR-3065-3p,miR-4532,miR-2284l,miR-124a,miR-329a-5p,miR-2285w,novel_94,miR-19a-3p,miR-378g,miR-29a-3p,miR-3065-3p,miR-320b,miR-125a,miR-329-5p,miR-4492,miR-487a-5p,miR-191-5p,miR-2284q,novel_74,miR-29a,miR-125b-5p,miR-301a-5p,miR-425-5p,novel_82,miR-29b-3p,miR-4324,miR-6529b,miR-1197-3p,miR-214,miR-29c-3p,miR-3059-5p,miR-541-3p
DUSP10	ENSOARG0(	dual spe	miR-92a-3p,miR-502-5p,miR-2332,miR-371a-5p,miR-320e,miR-3969,miR-877-3p,novel_44,novel_120,miR-3059-5p,miR-342-3p,miR-363-3p,miR-212-3p,miR-2285x,miR-214,miR-186-5p,miR-433-3p,miR-18a-3p,miR-3591-3p,miR-25,miR-25-3p,miR-22-3p,miR-144-3p,miR-32-5p,miR-363,miR-874-3p,miR-329-5p,miR-21-3p,novel_127,miR-214-3p,miR-92a-3p,miR-21-3p,miR-342,miR-210-5p,miR-618,miR-216a-5p,miR-32,miR-664b-3p,miR-92b-3p,miR-8485,miR-216c-5p,miR-2330-5p,miR-382-3p,miR-181a-2-3p,miR-2426,miR-2284x,miR-223-3p,miR-22-3p,novel_23,miR-1197-5p,miR-3071-5p,miR-95-3p,miR-421,miR-18a-3p,miR-548w

[illegible]
3p,miR-1260b,miR-23b,miR-876-3p,miR-2318,miR-345-5p,miR-
1248,miR-874-3p,miR-2355-5p,miR-223-5p,miR-133c,miR-127-
5p,novel_127,miR-450b-5p,miR-200a,miR-23b-5p,miR-330-
5p,miR-2285ad,miR-6517,novel_79,miR-322-5p,miR-9-5p,miR-
134-5p,miR-486-5p,miR-129-1-3p,miR-485-3p,miR-379-3p,miR-
301b-3p,miR-1306,miR-411b-3p,miR-677,miR-669,miR-144-
3p,miR-320c,miR-181b-3p,miR-7-5p,miR-377-3p,miR-2411-
3p,miR-194a,novel_51,miR-6240,miR-628-5p,miR-432-3p,miR-
432-3p,let-7a-2-3p,miR-19b-2-5p,miR-432,miR-7b-5p,miR-
16b,miR-26a-2-3p,miR-497-5p,miR-27b-3p,miR-188-5p,miR-
2284x,miR-339-5p,miR-3535,miR-134-3p,miR-15b-
3p,novel_74,miR-451a,miR-500,miR-129-5p,miR-454-3p,miR-
3184-3p,miR-129-2-3p,miR-129b-3p,miR-500a-3p,miR-411,miR-
345-5p,miR-339b,miR-543-3p,miR-625-5p,miR-186-5p,miR-
1827,miR-2331-3p,miR-30c-1-3p,miR-548o-3p,miR-301a-
3p,miR-582-5p,miR-377-3p,miR-216b-5p,miR-15b-5p,miR-210-
5p,miR-618,miR-219b-5p,miR-345-3p,miR-486-3p,miR-133a-
3p,miR-148a-5p,miR-34a-5p,miR-361-5p,miR-18a-3p,miR-212-
5p,miR-7859,miR-95-3p,miR-210-3p,miR-23b-3p,miR-
23c,novel_82,miR-2440,miR-301a-5p,miR-499a-5p,miR-490-
5p,miR-3956-3p,miR-9-5p,miR-877-3p,miR-7705,miR-140-
5p,miR-299b-5p,miR-376a-3p,miR-544-5p,miR-9-3p,miR-139-
5p,miR-7689-3p,miR-211-5p,miR-378g,miR-25,miR-3596,miR-
2483-3p,miR-203-3p,miR-500-3p,miR-182-5p,miR-16b,miR-
130b-3p,miR-23a-3p,miR-8485,miR-2310,miR-301b-5p,miR-501-
3p,miR-101c,novel_78,miR-330-3p,miR-1343-3p,miR-181b-2-
3p,miR-1298-3p,miR-147a,miR-148b-5p,miR-455-3p,miR-
2459,miR-5100,miR-2332,miR-331-3p,miR-144,miR-31-3p,miR-
486b-3p,miR-204-5p,miR-6128,miR-488-3p,miR-320a,miR-
NOSIP ENSOARG0(nitric o 3154,let-7g-3p,miR-7144-5p,miR-671-5p,miR-1814c,miR-370-
miR-496-3p,miR-574-5p,miR-378a-3p,miR-6516,miR-544-
3p,miR-877-5p,miR-2448-3p,miR-450a-1-3p,miR-2285c,miR-
23b,miR-27b-5p,miR-329b,miR-2312,let-7g-3p,miR-186-
5p,miR-485-5p,miR-2284v,miR-145a-3p,miR-378c,miR-
1271,miR-6134,miR-378c,miR-2332,miR-2284k,miR-3969,miR-
144,miR-664a,miR-450b-3p,miR-204-5p,miR-3432a,miR-874-
5p,miR-3600,miR-380-5p,miR-1271-5p,miR-6516-5p,miR-
23c,miR-378h,miR-23b-3p,miR-450a-1-3p,miR-199a-3p,miR-
378i,miR-3591-5p,miR-1197-5p,miR-1434-5p,miR-378a-3p,miR-
218-2-3p,miR-142-3p,miR-496,miR-378d,miR-105-2,miR-128-
3p,miR-183-5p,miR-369-3p,miR-380-5p,miR-2285p,miR-
RABGAP1L ENSOARG0(RAB GTPa 422a,miR-466i-5p,miR-127-5p,miR-2284m,miR-20a-3p,miR-
378b,miR-2319b,novel_68,miR-7-1-3p,miR-211-5p,miR-541-
5p,novel_69,miR-378e,miR-144-3p,miR-2285ab,miR-216a-
3p,miR-136-5p,miR-450b-3p,miR-96-5p,novel_94,miR-6516-
3p,miR-505-3p,miR-378f,miR-2319a,miR-3956-
3p,novel_60,novel_120,miR-758-5p,miR-2284j,miR-2285f,miR-
211,miR-2428,miR-8095,miR-133a-5p,miR-218-5p,novel_1,miR-
199c,miR-502b,miR-1343-3p,miR-199b-3p,miR-105-1,miR-26b-
3p,miR-2285g,miR-544b,miR-421,miR-2459,miR-2285e,miR-
9788-3p,novel_116,miR-378b,miR-450c-3p,miR-544a,miR-
2284ab,miR-378c,miR-188-5p,miR-2284x,miR-101b-3p,miR-380-
5p,miR-23a-3p,miR-378d,miR-2285r,miR-199a-3p,miR-105-5p

VSTM2A	ENSOARG0(	V-set an	miR-877-3p,novel_1
SPPL3	ENSOARG0(	signal p	miR-6128,miR-1306-5p,miR-145-3p,miR-323-3p,miR-331-3p,miR-134-5p,miR-28-5p,miR-486b-3p,miR-31-3p,miR-410-3p,miR-370-3p,miR-2355-5p,miR-2285aa,miR-223-5p,miR-300-3p,miR-381-3p,miR-485-5p,miR-671-5p,miR-7144-5p,miR-329a,miR-4286,miR-3064-5p,miR-29b-2-5p,miR-2285b,miR-192-3p,miR-2284a,novel_39,miR-200a,miR-1957a,miR-2284y,miR-3065-3p,miR-28b,miR-217,miR-8117,miR-2285af,novel_96,miR-181b-5p,miR-6517,miR-299a-3p,novel_121,miR-134-5p,miR-582-3p,miR-2411-5p,miR-330-5p,miR-7134-5p,miR-2427,miR-1343-5p,miR-134,miR-3591-5p,miR-300,novel_101,miR-219b-3p,miR-2428,miR-326-3p,miR-371b-3p,miR-24-3p,miR-142b,miR-28c,miR-502-5p,miR-1247-3p,miR-147-5p,miR-421-5p,novel_1,miR-3120-3p,miR-3074-5p,miR-363,miR-669,miR-677,miR-299b-3p,miR-3065-5p,miR-1306,miR-505-3p,miR-2285k,miR-450b-3p,miR-363-5p,miR-668-3p,miR-503-3p,miR-654-3p,miR-194a,miR-377-3p,miR-224-5p,miR-1343-5p,miR-124-5p,miR-187-3p,miR-340-3p,miR-194-3p,miR-6238,miR-185-5p,miR-134-3p,miR-194-5p,miR-181a-5p,miR-188-5p,miR-2889,miR-3074-5p,miR-4510,miR-181c-5p,miR-3184-5p,miR-224-5p,miR-655-3p,miR-665,miR-29b-2-5p,miR-324-3p,miR-2285j,miR-708-5p,miR-664a,miR-552-3p,miR-3065-3p,miR-216b-3p,miR-221-5p,miR-204-3p,miR-6130,miR-377-3p,miR-27a-5p,miR-655,miR-27b-5p,miR-21a-3p,miR-186-5p,miR-2312,miR-369-3p,miR-2411,miR-486-3p,miR-29b-1-5p,miR-2285i,miR-299-3p,miR-21-3p,miR-3653-3p,miR-221-5p,miR-505-3p,miR-3600,novel_83,miR-616-3p,miR-1247-5p,miR-383-5p,miR-140-3p,miR-217-5p,miR-362-3p,miR-2408,miR-148a-5p,miR-2284u,miR-664-5p,miR-133a-3p,miR-941,miR-184-3p,miR-181d-5p,miR-194b-5p,miR-212-3p,miR-2447,miR-423-5p,miR-361-3p,novel_82,miR-2285n,miR-2284q,miR-3968,miR-3064-5p,miR-378g,miR-3596,miR-381-3p,miR-361-
CLTC	ENSOARG0(	clathrin	miR-22-5p,miR-7134-3p,miR-421-5p,miR-206,miR-6119-3p,miR-382-5p,let-7i,miR-6402,miR-28c,miR-218-5p,miR-323c,novel_101,miR-2285f,miR-3956-3p,novel_60,miR-4454,let-7e-5p,miR-19a-3p,miR-3591-3p,miR-2285ab,miR-203-3p,let-7d-5p,miR-3082-5p,miR-7977,miR-3120-3p,miR-329-5p,miR-10b,miR-466f-3p,let-7e,miR-153-3p,miR-3955-3p,miR-130b-3p,miR-491-5p,miR-1185-5p,miR-455-5p,miR-29b-1-5p,miR-197-5p,miR-431-5p,miR-2310,miR-1b-5p,miR-8485,miR-2285r,let-7a-5p,miR-455-5p,miR-7862,miR-16-1-3p,miR-3071-5p,miR-1a-3p,miR-1843b-5p,miR-455-3p,let-7g-5p,miR-3535,miR-153,miR-130a-3p,novel_78,miR-454-3p,novel_9,novel_74,miR-338-5p,miR-2332,let-7f,miR-15b-3p,miR-6134,let-7b-5p,miR-206-3p,miR-1306-5p,let-7b,miR-186-5p,miR-1814c,miR-7144-5p,miR-329b,let-7g-3p,miR-129b-3p,miR-21a-3p,let-7i-5p,miR-301a-3p,miR-141-3p,miR-21-3p,miR-548o-3p,miR-496-3p,miR-503-3p,miR-140-5p,let-7k,novel_96,miR-6536,miR-98-5p,let-7f-5p,let-7c-5p,miR-2285p,miR-2285af,miR-205-5p,miR-1271-3p,let-7g,miR-28b,miR-1,miR-329a-5p,miR-496,miR-133a-3p,miR-16-1-3p,miR-19b-3p,let-7d,miR-188-3p,miR-5703,miR-218-2-3p,miR-1961,miR-3591-5p,miR-486-5p,miR-7859,miR-431,miR-7641,miR-3600,miR-548e-3p

SMARCB1	ENSOARG0(	SWI/SNF	novel_69,miR-134-3p,miR-378g,miR-326-3p,miR-1271,novel_79,miR-2355-5p,miR-339-5p,miR-339b,miR-1895,miR-326,miR-361-3p,miR-330-5p,miR-339a,miR-324-3p
DCAF7	ENSOARG0(	DDB1 and	miR-30f,miR-21a-3p,miR-193b-3p,miR-6130,miR-27a-5p,miR-877-5p,miR-204-3p,novel_25,miR-3065-3p,miR-6516-3p,miR-664a,miR-708-5p,miR-424-5p,miR-320e,miR-29b-2-5p,miR-10b-5p,miR-338-3p,miR-6134,miR-145a-3p,miR-4510,miR-539-5p,miR-4429,miR-19b-3p,miR-2300b-3p,novel_133,miR-1197-5p,miR-188-3p,miR-2904,miR-200b,miR-148b-3p,miR-15a-5p,miR-222-5p,novel_83,miR-532-3p,miR-151-5p,miR-505-3p,miR-466i-5p,miR-760-3p,miR-2284m,miR-106a,miR-320d,miR-21-3p,miR-34b-5p,miR-30d,miR-30b-3p,miR-196a-3p,miR-185-3p,miR-125b,miR-216a-5p,miR-2432,miR-20a,novel_94,miR-27a-3p,miR-3064-5p,miR-7977,miR-335,miR-4726-5p,miR-2285n,miR-30b-5p,miR-2285y,miR-362-5p,miR-541-3p,novel_60,miR-181d-5p,miR-2426,miR-10b,miR-296-5p,miR-30b-3p,miR-216a-3p,miR-9788-3p,miR-500-5p,miR-2300a-5p,miR-27a-3p,miR-3085-3p,novel_124,novel_115,novel_103,miR-326,miR-320b,miR-193a,miR-3187-3p,miR-424-5p,miR-106a-5p,miR-2330-5p,miR-320a,miR-17-5p,miR-362-5p,miR-3154,miR-365a-3p,miR-3965,miR-374c-3p,miR-34c-5p,miR-324-5p,miR-141-3p,miR-615-5p,miR-28-5p,miR-486b-3p,miR-31-3p,miR-331-3p,miR-26c,miR-93,miR-500b-5p,miR-2903,miR-365a-5p,miR-10a-5p,miR-200b-3p,miR-128-3p,miR-345-3p,miR-30a-5p,miR-302a-5p,miR-24-2-5p,miR-582-3p,miR-200c-3p,miR-181b-5p,miR-191-3p,miR-450a-2-3p,miR-543-5p,miR-200c,miR-1260a,miR-2285af,miR-339a,miR-28b,miR-3065-3p,miR-181a-2-3p,miR-2385-3p,miR-29b-2-5p,miR-19a-3p,miR-1291,miR-505-3p,miR-6395,miR-195a-5p,miR-7975,miR-34b,miR-670-3p,miR-10a-5p,miR-147-5p,miR-6402,miR-20a-5p,miR-502-5p,miR-15a,miR-382-5p,miR-34c,miR-16a,miR-3959-3p,miR-24-3p,miR-2428,miR-449a,miR-326-3p,miR-136-3p,miR-1260b,miR-30b-3p,miR-2284ab,miR-744-5p,miR-181a-5p,miR-185-5p,miR-

CA1	ENSOARG0(	carbonic	miR-16a,miR-218-5p,miR-15a,miR-2285t,miR-495-3p,miR-1296-5p,miR-3956-3p,novel_60,miR-3059-5p,miR-301b-3p,miR-199b-5p,miR-374c-5p,miR-148a-3p,miR-199b-5p,miR-195a-5p,miR-6516-3p,miR-107,miR-96-5p,miR-374b-5p,miR-4792,miR-374c-3p,miR-3082-5p,miR-299b-3p,miR-378g,miR-16b,miR-29b-1-5p,miR-431-5p,miR-452-5p,miR-199b-5p,miR-130b-3p,miR-628-5p,miR-363-5p,miR-424-5p,miR-2310,miR-3187-3p,miR-432-3p,miR-374b-3p,miR-145a-5p,miR-125b-2-3p,miR-1434-3p,miR-223-3p,miR-296-5p,miR-497-5p,miR-16-1-3p,miR-544a,miR-2426,miR-16b,miR-145b,miR-130a-3p,miR-103a-3p,miR-3955-5p,miR-103,miR-134-3p,miR-29b-2-5p,miR-409-3p,miR-424-5p,miR-454-3p,miR-665-5p,miR-133a-3p,miR-3074-2-3p,miR-16-5p,miR-1271,miR-145-3p,miR-186-5p,miR-1814c,miR-2318,let-7g-3p,miR-133b-3p,miR-10a-3p,miR-10b-3p,miR-552-3p,miR-532-5p,miR-548o-3p,miR-15b-5p,novel_25,miR-199a-5p,miR-544-3p,miR-301a-3p,miR-374c-3p,miR-185-3p,miR-125a-3p,miR-26a-5p,miR-2397-5p,miR-374a-5p,miR-205-5p,miR-152-3p,miR-299-3p,miR-107,miR-6536,miR-2898,miR-29b-2-5p,miR-10a-3p,miR-216c-5p,miR-2424,miR-199a-5p,miR-376a-5p,miR-29b-1-5p,miR-2904,miR-2427,miR-1290,miR-16-1-3p,miR-212-5p,miR-431,miR-3600,miR-299a-3p,miR-548e-3p,novel_121,miR-15a-5p,miR-148b-3p,miR-1271-5p,miR-7859,miR-322-5p
RDX	ENSOARG0(	radixin	novel_120,miR-211,let-7i,miR-133a-5p,miR-20a-5p,miR-22-5p,miR-421-5p,miR-6119-3p,novel_1,miR-379-3p,miR-3120-3p,miR-216b-3p,miR-670-3p,miR-18a-3p,miR-21c,miR-3591-3p,let-7e-5p,miR-2285k,miR-382,miR-539-3p,miR-654-3p,miR-17-5p,miR-106a-5p,miR-224-5p,miR-377-3p,miR-466f-3p,miR-196b-5p,miR-26b-3p,miR-194-3p,miR-2355-3p,miR-20b,miR-412-5p,let-7g-5p,miR-1285,miR-1434-3p,miR-2284x,miR-2284ab,miR-21-5p,miR-380-3p,miR-144-5p,miR-103b,miR-93,miR-2284k,miR-2332,miR-204-5p,miR-2285aa,miR-370-3p,miR-1271,miR-141-3p,miR-3957-5p,miR-487a-3p,miR-3957,miR-2284n,miR-93-5p,miR-17-5p,miR-20b-5p,miR-3074-1-3p,miR-876-3p,let-7g,miR-192-3p,miR-3071-3p,let-7c-5p,novel_127,miR-200a-3p,let-7f-5p,miR-2285ad,miR-582-3p,miR-6516-5p,miR-196a-5p,miR-155-5p,let-7d,miR-3059-5p,miR-212-3p,miR-2285y,miR-2285n,miR-218-5p,miR-3964,miR-2285t,miR-154-3p,miR-2285u,miR-211-5p,miR-144-5p,let-7d-5p,miR-3596,miR-2483-3p,miR-335-3p,miR-668-5p,let-7c-3p,let-7a-5p,miR-494-3p,miR-382-3p,miR-106a-5p,miR-8485,miR-505,miR-106b-5p,miR-182-5p,miR-199a-3p,let-7e,miR-2459,miR-142-3p,miR-3962,miR-2284r,miR-450b-5p,miR-590-3p,let-7b,let-7b-5p,miR-224-5p,miR-346,miR-324-3p,miR-3604,let-7f,miR-208b-5p,miR-6516-3p,miR-155-5p,miR-21-3p,miR-154a-3p,miR-216b-3p,miR-182-5p,let-7i-5p,miR-155-5p,miR-2284s,miR-6516,miR-377-3p,miR-27b-5p,miR-433-3p,miR-299,miR-21a-3p,miR-543-3p,let-7g-3p,miR-154b-3p,miR-20a,miR-487a,miR-380-3p,miR-143-3p,miR-421-5p,miR-2285i,miR-142a-3p,miR-500a-5p,novel_17,miR-30b-3p,miR-1277-5p,miR-3653-3p,let-7k,miR-2284m,miR-106a,miR-98-5p,miR-485-3p,miR-18a-3p,miR-106a,miR-199a-3p,miR-2284h-5p,miR-487a-3p,miR-340-5p,miR-140-3p,miR-188-3p,miR-1961,miR-2300b-3p,miR-142-

IFI30	ENSOARG0(	interfer	miR-137-3p,miR-767,miR-1248,miR-874-3p,miR-3956,miR-125a,miR-99a-3p,miR-2355-5p,miR-2285aa,miR-376c-3p,miR-125b-5p,miR-193a-5p,miR-16-5p,miR-1197-3p,miR-1260b,miR-885-3p,miR-429-3p,miR-322-5p,miR-505-5p,miR-125a-3p,miR-664-3p,miR-205-5p,miR-127-5p,let-7f-5p,miR-200a-3p,miR-2285b,miR-3956-5p,let-7g,miR-3065-5p,miR-1306,miR-412-3p,miR-410-5p,miR-378f,let-7e-5p,miR-146b-5p,novel_68,novel_69,miR-320c,novel_87,miR-29a,miR-29c-3p,miR-199b-5p,miR-223-3p,miR-708-3p,miR-432,miR-19b-2-5p,miR-16b,miR-188-5p,miR-27b-3p,miR-497-5p,miR-31-5p,miR-134-3p,miR-3431,miR-149-5p,miR-411-5p,miR-146a,miR-296-3p,miR-194a,novel_51,miR-2411-3p,miR-2284l,miR-411,miR-885-3p,miR-625-5p,miR-548o-3p,miR-30c-1-3p,miR-296-3p,miR-216b-3p,miR-376e-3p,let-7i-5p,miR-15b-5p,miR-30e-3p,let-7f,miR-2285j,miR-664a-5p,miR-151-3p,miR-30a-3p,miR-669a-3p,let-7b,miR-214,miR-3958-5p,miR-346,miR-6525,miR-769-5p,miR-1961,miR-378i,miR-378a-3p,miR-217-5p,miR-142-3p,miR-493-3p,miR-30d-3p,miR-210-5p,miR-345-3p,miR-422a,miR-92a-1-5p,miR-324-3p,miR-98-5p,miR-99a-3p,miR-202-5p,let-7k,miR-2285w,miR-376c-3p,miR-376a-5p,miR-486-3p,miR-221,miR-136-5p,miR-1983,miR-335-3p,miR-2285u,miR-378g,novel_82,miR-3964,miR-6527,miR-2284r,miR-1388-5p,miR-1839-5p,miR-1343-3p,miR-145b,miR-1298-3p,miR-16b,miR-29b-1-5p,miR-487b-5p,miR-214-3p,miR-199b-5p,miR-146b,miR-127-3p,miR-1388-3p,miR-378d,miR-2310,miR-320a,miR-362-5p,novel_91,miR-10b-3p,miR-1814c,miR-3074-1-3p,miR-141-3p,miR-370-3p,miR-769,miR-199a-5p,miR-99b-3p,miR-370-5p,miR-29b-3p,miR-767-5p,miR-374b-3p,miR-486b-3p,miR-1306-5p,miR-374a-3p,miR-378c,miR-145-3p,miR-345-3p,miR-29d-3p,let-7d,miR-582-3p,miR-200b-3p,miR-222,miR-7641,miR-196a-2-3p,miR-

ANKS1B	ENSOARG0(	ankyrin	miR-181c-3p,miR-145-3p,miR-1306-5p,miR-193a-5p,miR-204-5p,miR-28-5p,miR-592,miR-2332,miR-135a-1-3p,miR-34c-5p,miR-769,miR-199a-5p,miR-376c-3p,miR-2355-5p,miR-370-3p,miR-876-3p,miR-671-5p,miR-1248,miR-488-3p,miR-3154,let-7g,miR-199a-5p,miR-29a-5p,let-7f-5p,miR-125a-3p,let-7c-5p,miR-9-5p,let-7d,miR-155-5p,miR-10a-5p,miR-1290,miR-211,miR-449a,novel_27,miR-2428,miR-199b-5p,miR-2284w,miR-22-5p,miR-203b-5p,miR-576-3p,miR-142b,miR-34c,let-7i,novel_87,miR-34b,novel_69,miR-7-1-3p,miR-10a-5p,let-7e-5p,miR-3065-5p,miR-378b,miR-296-3p,miR-124-5p,miR-296-3p,miR-432-5p,let-7g-5p,miR-26b-3p,miR-27b-3p,miR-432,miR-1843b-5p,miR-3071-5p,miR-145a-5p,miR-10b-5p,miR-769-5p,let-7b-5p,let-7b,miR-145a-3p,miR-214,miR-664a,miR-155-5p,miR-708-5p,let-7f,miR-338-5p,miR-10a,miR-376e-3p,miR-204-3p,let-7i-5p,miR-155-5p,miR-296-3p,miR-186-5p,novel_99,miR-1827,miR-376c-3p,miR-130a-5p,miR-143-3p,miR-195a-3p,miR-98-5p,miR-30b-3p,miR-34b-5p,let-7k,novel_17,miR-500a-5p,miR-383-5p,miR-3600,miR-1b-3p,miR-133a-3p,miR-34a-5p,miR-148a-5p,miR-1961,miR-1246,miR-5703,miR-140-3p,miR-1193,miR-361-3p,miR-877-3p,miR-9-3p,miR-8095,miR-2447,miR-3059-5p,miR-452-5p,miR-9-5p,miR-3958-3p,miR-769-5p,miR-107-5p,miR-7857-3p,miR-2483-3p,miR-203-3p,let-7d-5p,miR-27a-3p,miR-4792,miR-10b,miR-541-5p,miR-211-5p,miR-181a-3p,miR-9-3p,miR-361-3p,miR-199b-5p,miR-378j,miR-574-3p,let-7a-5p,miR-199b-5p,miR-103a-2-5p,let-7e,miR-214-3p,miR-2957,miR-1298-3p,miR-147a,miR-148b-5p,miR-9788-3p,miR-421,novel_78,miR-145b,miR-27a-3p,miR-1388-5p,miR-6529a,miR-296-5p,miR-1298-5p,miR-10b,miR-125b-2-3p
SCAF1	ENSOARG0(	SR-relat	miR-484,miR-330-5p,miR-885-3p,miR-885-3p,miR-7144-5p,miR-744-5p,miR-1827,miR-505-5p,miR-6130,novel_63,miR-326,miR-760-3p,miR-130b-5p,miR-130b-5p,miR-4510,miR-181a-2-3p,miR-2285r,miR-3184-5p,miR-326-3p,miR-423-5p

RBBP7	ENSOARG0(	retinobl	novel_87,miR-4324,miR-20a-5p,miR-142b,miR-22-5p,miR-421-5p,miR-6123,miR-6119-3p,miR-128-1-5p,miR-495-3p,miR-2285x,miR-219b-3p,novel_27,miR-2284w,miR-9851-3p,miR-6516-3p,miR-378f,miR-450b-3p,miR-3120-3p,miR-10a-5p,miR-320c,miR-2404,miR-374c-3p,miR-17-5p,novel_51,miR-106a-5p,miR-125a-5p,miR-1343-5p,miR-466f-3p,miR-196b-5p,miR-130b-5p,miR-380-5p,miR-493-5p,let-7j,miR-345-5p,let-7a-2-3p,miR-2284l,miR-22-3p,miR-450c-3p,miR-3071-5p,miR-145a-5p,miR-181a-5p,miR-1843b-5p,miR-2113,miR-223-3p,miR-16-1-3p,miR-339-5p,miR-377-5p,miR-3955-5p,miR-122-3p,miR-133b-5p,novel_116,miR-20b,miR-544b,miR-2355-3p,miR-412-5p,miR-125b-5p,miR-100-3p,miR-99b-3p,miR-494-5p,miR-135a-1-3p,miR-767-5p,novel_9,miR-193a-5p,miR-3607-3p,miR-1306-5p,miR-378c,miR-93,miR-93-5p,miR-17-5p,miR-3154,miR-320a,miR-2448-3p,miR-301,miR-20b-5p,miR-190a-3p,miR-1814c,miR-876-3p,miR-767,miR-345-5p,miR-2318,let-7g-3p,miR-370-3p,miR-2285aa,miR-99a-3p,miR-1271,miR-125a,miR-574-5p,miR-223-5p,miR-376c-3p,miR-217,miR-339a,novel_96,miR-181b-5p,miR-3064-5p,miR-30a-3p,miR-181a-2-3p,miR-4286,miR-29a-5p,miR-200a,miR-3065-3p,miR-450c-5p,miR-380-5p,miR-1343-5p,miR-3591-5p,miR-196a-5p,let-7a-2-3p,miR-10a-5p,novel_79,miR-2285ad,miR-450a-1-3p,novel_121,miR-2411-5p,miR-378h,miR-2285y,novel_82,miR-101-3p,miR-2285t,miR-490-5p,miR-4726-5p,miR-499a-5p,miR-450a-5p,miR-181d-5p,miR-452-5p,miR-3059-5p,novel_60,miR-3956-3p,miR-8095,miR-7705,miR-219a-2-3p,miR-361-3p,miR-129b-5p,miR-140-5p,miR-493-5p,miR-9-3p,miR-136-5p,miR-499b-5p,miR-541-5p,miR-126b-5p,miR-3064-5p,miR-139-5p,miR-4792,miR-20a-3p,miR-499a-3p,miR-7857-3p,miR-378e,miR-2483-3p,miR-106b-5p,miR-320b,miR-1185-5p,miR-3085-3p,miR-491-5p,miR-487b-5p,miR-3955-3p,miR-33a-

CAMK1G	ENSOARG0(	calcium/	miR-3064-5p,miR-499b-5p,miR-378g,miR-27a-3p,miR-4443,miR-376d,miR-361-3p,miR-221,miR-136-5p,miR-376a-3p,novel_60,miR-3956-3p,miR-541-3p,miR-212-3p,miR-361-3p,miR-7705,novel_82,miR-101-3p,miR-371a-5p,miR-2285y,miR-4726-5p,miR-499a-5p,miR-490-5p,miR-1343-3p,novel_4,miR-5010-3p,miR-27a-3p,miR-145b,miR-147a,miR-376b-3p,miR-221-3p,miR-2284r,miR-296-5p,miR-541,miR-412-3p,miR-30b-3p,miR-2426,miR-101c,miR-3120-5p,miR-106a-5p,miR-424-5p,miR-101b-3p,miR-4532,miR-5126,miR-106b-5p,miR-16b,miR-3085-3p,miR-491-5p,miR-3955-3p,miR-381-5p,miR-296-3p,miR-132-5p,miR-377-3p,miR-15b-5p,miR-450a-1-3p,miR-129b-3p,miR-433-3p,miR-1827,miR-3074-5p,miR-6134,miR-222-3p,miR-665,miR-320e,miR-450b-3p,miR-664a,miR-708-5p,miR-208b-5p,miR-4492,miR-424-5p,miR-18a-3p,miR-106a,miR-3600,miR-212-5p,miR-210-3p,miR-383-5p,miR-95-3p,miR-200b,miR-15a-5p,miR-2284h-5p,miR-328-3p,miR-2300b-3p,miR-2904,miR-1197-5p,miR-34a-5p,miR-503-5p,miR-2285w,miR-20a,miR-345-3p,miR-486-3p,miR-196a-3p,miR-125b,miR-185-3p,miR-92a-1-5p,miR-106a,miR-466i-5p,miR-760-3p,miR-34b-5p,miR-3074-5p,miR-320b,miR-34b,novel_69,miR-18a-3p,miR-376b-3p,miR-107,miR-9851-3p,miR-6395,miR-195a-5p,miR-135b-5p,miR-2285x,miR-1307-3p,miR-449a,miR-2428,miR-502-5p,miR-15a,miR-6402,miR-20a-5p,miR-576-3p,miR-16a,miR-34c,miR-382-5p,miR-103a-3p,miR-432-5p,miR-503-5p,miR-134-3p,miR-185-5p,miR-20b,miR-103,miR-432,miR-873a-5p,miR-1843b-5p,miR-450c-3p,miR-145a-5p,miR-3071-5p,miR-30b-3p,miR-138,miR-16b,miR-381-5p,miR-219a-1-3p,miR-497-5p,miR-4508,miR-27b-3p,miR-455-5p,miR-2284x,miR-2889,miR-382,miR-363-5p,miR-668-3p,miR-432-3p,miR-503-5p,miR-377-3p,miR-106a-5p,miR-17-5p,miR-455-5p,miR-125a-5p,miR-1343-5p,miR-574-5p,miR-3068-

ZNF157	ENSOARG0(	zinc fin	miR-3120-3p,miR-655-5p,miR-329-5p,novel_68,miR-6535,miR-299b-3p,miR-374c-3p,miR-216a-3p,miR-378b,miR-376a-2-5p,miR-505-3p,miR-2319a,miR-19a-3p,miR-410-5p,miR-1193,miR-1a-1-5p,miR-301b-3p,miR-199b-5p,novel_27,miR-24-3p,miR-142b,miR-576-3p,miR-147-5p,miR-203b-5p,miR-206,novel_1,miR-130a-3p,miR-665,miR-31-5p,miR-194-3p,miR-149-5p,miR-3431,miR-134-3p,miR-185-5p,miR-374b-3p,novel_23,miR-1434-3p,miR-181a-5p,miR-653,miR-4508,miR-380-3p,miR-345-5p,miR-2285r,miR-654-3p,miR-17-5p,miR-106a-5p,miR-2411-3p,miR-32,miR-197-5p,miR-105-5p,miR-466f-3p,miR-2285aa,miR-223-5p,miR-199a-5p,miR-365a-3p,miR-3965,miR-25-3p,miR-3154,miR-23b,miR-17-5p,miR-1248,miR-671-5p,miR-7144-5p,miR-345-5p,miR-10b-3p,miR-193a-5p,miR-6128,miR-409-3p,miR-494-5p,miR-370-5p,miR-1-5p,miR-134-5p,miR-410-3p,miR-376a-5p,miR-429-3p,miR-299a-3p,miR-2284z,miR-134-5p,miR-2411-5p,miR-505-5p,miR-2427,miR-134,miR-345-3p,miR-105-2,miR-200b-3p,miR-155-5p,miR-128-3p,miR-181a-2-3p,miR-1,miR-3529-3p,miR-490-3p,miR-1957a,miR-200a,miR-199a-5p,miR-200c,miR-1a-2-5p,miR-181b-5p,miR-200c-3p,miR-541-5p,miR-20a-3p,miR-2319b,miR-1185-2-3p,miR-203-3p,miR-378g,miR-381-3p,miR-378j,miR-199b-5p,miR-181d-5p,miR-212-3p,miR-363-3p,miR-2447,miR-8095,miR-371a-5p,miR-101-3p,miR-218-5p,miR-490-5p,miR-105-1,miR-409b,novel_78,miR-1185-3p,miR-2459,miR-421,miR-9788-3p,miR-1a-3p,miR-7857,miR-143-5p,miR-484,miR-2284r,miR-101c,miR-2284aa,miR-2426,miR-33a-5p,miR-23a-3p,miR-494-3p,miR-106a-5p,miR-2330-5p,miR-1b-5p,miR-8485,miR-106b-5p,miR-182-5p,miR-199b-5p,miR-130b-3p,miR-182-5p,miR-532-5p,miR-370-5p,miR-548o-3p,miR-32-5p,miR-155-5p,miR-204-3p,miR-656-3p,miR-301a-3p,miR-2284s,miR-27a-5p,miR-433-3p,miR-21a-3p,miR-186-5p,miR-
PRPF8	ENSOARG0(	pre-mRNA	let-7c-5p,let-7f-5p,miR-760-3p,miR-98-5p,let-7k,miR-543-5p,miR-28b,miR-181b-1-3p,miR-125b-2-3p,miR-199a-5p,let-7g,miR-134,miR-1961,let-7d,miR-10a-5p,miR-7859,miR-322-5p,miR-505-5p,miR-15a-5p,miR-134-5p,miR-2433,let-7f,miR-370-5p,miR-665,miR-320e,miR-664a,miR-874-5p,miR-708-5p,miR-424-5p,miR-28-5p,miR-134-5p,let-7b,miR-16-5p,miR-214,miR-10b-5p,miR-6525,miR-224-5p,let-7b-5p,miR-129b-3p,miR-27b-5p,miR-3154,miR-1827,miR-2331-3p,miR-545-3p,miR-2355-5p,miR-2285aa,miR-370-3p,miR-370-5p,miR-1271,miR-499b-3p,miR-10a,miR-199a-5p,miR-204-3p,miR-15b-5p,miR-877-5p,let-7i-5p,miR-224-5p,miR-5126,miR-3141,miR-197-5p,miR-16b,miR-1185-5p,novel_124,miR-199b-5p,let-7e,miR-214-3p,let-7a-5p,miR-1388-3p,miR-424-5p,miR-668-3p,miR-654-3p,miR-2284r,miR-484,miR-296-5p,miR-10b,miR-16b,miR-2426,miR-497-5p,miR-187-3p,miR-2300a-5p,miR-199b-3p,miR-181b-2-3p,let-7g-5p,miR-134-3p,miR-3431,miR-9788-3p,miR-455-3p,miR-15a,miR-6529b,miR-28c,miR-16a,let-7i,miR-203b-5p,miR-2285t,miR-199c,miR-6123,miR-2447,novel_60,miR-3956-3p,miR-1193,miR-24-3p,miR-199b-5p,miR-2428,miR-361-3p,miR-199b-5p,miR-2387,miR-3141,miR-1291,miR-147-3p,let-7e-5p,miR-195a-5p,miR-4792,miR-10a-5p,miR-499a-3p,miR-378g,miR-411b-3p,novel_69,let-7d-5p,miR-6535,miR-677,miR-181b-3p

PUS1	ENSOARG0(	pseudour	miR-18a-3p,miR-505-3p,miR-758-5p,miR-2366,miR-345-3p,miR-615-3p,miR-3591-5p,miR-1197-5p,miR-188-3p,miR-124-3p,miR-4429,miR-10a-5p,miR-216c-5p,miR-219-3p,novel_17,miR-125b,miR-345-3p,miR-466i-5p,miR-127-5p,miR-450a-2-3p,miR-17-3p,miR-2898,miR-320d,miR-2484,novel_127,miR-21-3p,miR-140-5p,miR-504-5p,miR-574-5p,miR-125a,miR-370-5p,miR-216b-3p,miR-499b-3p,miR-544-3p,miR-10a,miR-377-3p,miR-204-3p,novel_32,miR-21a-3p,miR-320a,miR-186-5p,miR-671-5p,miR-214,miR-665-5p,miR-425-5p,miR-6525,miR-323-3p,miR-10b-5p,miR-370-5p,miR-410-5p,miR-2285j,miR-665,miR-664a-5p,miR-125b-5p,miR-219b-3p,miR-664a,miR-6516-3p,miR-17-3p,miR-874-5p,miR-340-3p,miR-26b-3p,miR-2459,miR-149-5p,miR-455-3p,miR-10b,miR-323b,miR-544a,miR-450b-5p,miR-188-5p,miR-744-5p,miR-455-5p,miR-124a,miR-628-5p,miR-363-5p,miR-8485,miR-1306-5p,miR-377-3p,novel_115,miR-320b,novel_51,miR-455-5p,miR-2411-3p,miR-323b-3p,miR-124-5p,miR-214-3p,miR-103a-2-5p,miR-125a-5p,miR-106b-3p,miR-10a-5p,miR-499a-3p,miR-4443,miR-320c,miR-18a-3p,miR-3065-5p,miR-1306,miR-504,miR-147-3p,miR-6516-3p,novel_94,miR-505-3p,miR-2447,miR-1193,miR-17-3p,miR-877-3p,miR-219a-2-3p,miR-615,miR-142b,miR-502-5p,novel_82,miR-107-5p,miR-4726-5p,miR-22-5p,miR-1843a-3p
TBX18	ENSOARG0(	T-box 18	miR-1b-5p,miR-8485,miR-193a,miR-124a,miR-23a-3p,miR-487b-5p,miR-326,miR-320b,miR-342,miR-505,miR-323b-3p,miR-147a,miR-142-3p,miR-539-5p,miR-2285e,miR-148b-5p,miR-500-5p,miR-409b,miR-105-1,miR-2300a-5p,miR-330-3p,miR-145b,miR-5010-3p,miR-2426,miR-450b-5p,miR-101a-5p,miR-101c,miR-30b-3p,miR-323b,miR-1a-3p,miR-412-3p,miR-362-5p,miR-877-3p,miR-1285-5p,miR-3059-5p,miR-190b-5p,miR-2284j,miR-9-5p,miR-2285f,miR-499a-5p,miR-628-3p,miR-374a-3p,miR-3082-5p,miR-7857-3p,miR-25,miR-2319b,miR-2284g,miR-2285u,miR-499b-5p,miR-668-5p,miR-1983,miR-493-5p,miR-140-5p,miR-378j,miR-142a-3p,miR-143-3p,miR-2411,miR-2285w,miR-345-3p,miR-195a-3p,miR-320d,miR-2284m,miR-221-5p,miR-1277-5p,miR-618,miR-26a-5p,miR-185-3p,miR-500a-5p,miR-216a-5p,miR-23c,miR-7859,miR-23b-3p,miR-2284h-5p,miR-548e-3p,miR-18a-3p,novel_73,miR-4429,miR-33b-3p,miR-2284u,miR-664-5p,novel_133,miR-1197-5p,miR-1434-5p,miR-1193,miR-124-3p,miR-346,miR-224-5p,miR-206-3p,miR-6134,miR-145a-3p,miR-3184-3p,miR-4510,miR-2330-3p,miR-3958-5p,miR-6516-3p,miR-487b-5p,miR-664a,miR-155-5p,miR-450b-3p,miR-190a-5p,miR-500,miR-4492,miR-129-5p,miR-2285j,miR-3604,miR-338-5p,miR-6130,miR-877-5p,miR-548o-3p,miR-140-5p,miR-221-5p,miR-532-5p,miR-216b-3p,miR-21-3p,miR-2312,miR-625-5p,miR-2285c,miR-450a-1-3p,miR-433-3p,miR-487b-5p,miR-500a-3p,miR-2285r,miR-6240,miR-493-5p,miR-101-5p,miR-124-5p,miR-483-3p,miR-378a-5p,miR-466f-3p,miR-224-5p,miR-190a,miR-105-5p,miR-330-3p,miR-412-5p,miR-2285g,miR-2284d,miR-133b-5p,miR-138,miR-30b-3p,miR-2284ab,miR-26a-2-3p,miR-1434-3p,miR-1285,miR-2113,miR-873a-5p,miR-145a-5p,miR-450c-3p,miR-3959-3p,miR-326-3p,novel_27,miR-342-3p,novel_120,miR-206,miR-6119-3p,miR-576-3p,miR-142b,miR-

DNAJB4	ENSOARG0(	DnaJ (Hs	miR-545-5p,miR-322-5p,miR-24-2-5p,miR-758-5p,miR-6517,miR-10a-5p,miR-300,miR-345-3p,miR-3591-5p,miR-1957a,novel_39,miR-490-3p,miR-29b-2-5p,miR-4286,miR-107,miR-450a-2-3p,miR-181b-5p,miR-30c-2-3p,miR-2285af,miR-205-5p,miR-664-3p,miR-133c,miR-2284n,miR-376c-3p,miR-331-5p,miR-34c-5p,miR-365a-3p,novel_48,miR-3074-1-3p,miR-1814c,miR-345-5p,miR-320a,miR-3154,miR-381-3p,miR-503-3p,miR-301,miR-16-5p,miR-204-5p,novel_63,miR-494-5p,miR-2332,miR-103,miR-340-3p,miR-103a-3p,miR-497-5p,miR-188-5p,miR-380-3p,miR-30b-3p,miR-16b,miR-145a-5p,miR-1434-3p,miR-542-3p,miR-345-5p,miR-432-3p,miR-2285r,miR-432-3p,let-7f-2-3p,let-7f-2-3p,miR-2411-3p,miR-320c,miR-677,miR-181b-3p,miR-34b,miR-10a-5p,miR-216b-3p,miR-505-3p,miR-195a-5p,miR-376a-2-5p,miR-19a-3p,miR-107,miR-3591-3p,miR-449a,novel_27,miR-2428,miR-211,miR-323c,miR-3957-3p,miR-495-3p,novel_101,miR-22-5p,miR-6119-3p,novel_1,miR-34c,miR-16a,miR-502-5p,miR-15a,miR-6402,miR-142b,miR-2284h-5p,miR-7859,miR-15a-5p,miR-212-5p,miR-505-3p,miR-361-5p,miR-34a-5p,miR-19b-3p,miR-33b-3p,miR-4429,miR-5703,miR-193b-5p,miR-143-3p,miR-380-3p,miR-29b-1-5p,miR-376c-3p,miR-181b-1-3p,miR-125b-2-3p,miR-130a-5p,miR-2285w,miR-221-5p,miR-34b-5p,miR-1277-5p,miR-320d,miR-219b-5p,miR-216a-5p,miR-185-3p,miR-345-3p,miR-2285p,miR-618,miR-15b-5p,miR-10a,miR-376e-3p,miR-21-3p,miR-216b-3p,miR-552-3p,miR-140-5p,miR-30c-1-3p,miR-221-5p,miR-548o-3p,miR-345-5p,miR-655,miR-487b-5p,miR-299,miR-10b-5p,miR-539-5p,miR-4492,miR-487a-5p,miR-424-5p,novel_74,miR-487b-5p,miR-6516-3p,miR-664a,miR-338-5p,miR-665,miR-29b-2-5p,miR-655-3p,miR-455-3p,miR-1185-3p,miR-153,miR-539-5p,miR-145b,miR-181b-2-3p,novel_78,miR-299a-5p,miR-30b-3p,miR-10b,miR-301b-

NOL7	ENSOARG0(	nucleola	miR-4661-5p,miR-299-3p,miR-2484,miR-26a-5p,miR-2285p,miR-125b,miR-345-3p,miR-324-3p,miR-216c-5p,miR-195a-3p,novel_73,miR-184-3p,miR-33b-3p,miR-148a-5p,miR-378d,miR-16-1-3p,miR-218-1-3p,miR-1246,miR-1193,miR-148b-3p,miR-23c,miR-222-5p,miR-23b-3p,miR-1247-5p,miR-199a-3p,miR-616-3p,miR-505-3p,miR-212-5p,miR-487b-5p,novel_74,miR-454-3p,miR-708-5p,miR-665,miR-219b-3p,miR-181c-5p,miR-145a-3p,miR-3074-5p,let-7g-3p,miR-625-5p,miR-2285c,miR-411,miR-433-3p,miR-193b-3p,miR-301a-3p,miR-496-3p,miR-32-3p,miR-296-3p,miR-182-5p,miR-216b-3p,miR-130b-3p,miR-487b-5p,miR-199b-5p,miR-431-5p,novel_103,miR-342,miR-199a-3p,miR-182-5p,miR-2310,miR-3187-3p,miR-382-3p,miR-127-3p,miR-574-3p,miR-2284b,miR-23a-3p,miR-101a-5p,miR-2284aa,miR-143-5p,miR-484,miR-30b-3p,miR-125b-2-3p,miR-7857,miR-147a,miR-1298-3p,miR-421,miR-2285e,miR-500-5p,miR-216a-3p,miR-148b-5p,miR-330-3p,miR-199b-3p,miR-548w,miR-27a-3p,miR-222-5p,miR-2285n,miR-218-5p,miR-2285y,miR-1973,miR-362-5p,miR-219a-2-3p,miR-212-3p,miR-32-3p,miR-181d-5p,miR-9-5p,miR-2285f,miR-668-5p,miR-3963,miR-544-5p,miR-199b-5p,miR-140-5p,miR-129b-5p,miR-2483-3p,miR-27a-3p,miR-203-3p,miR-2319b,miR-452-3p,miR-499b-5p,miR-181b-5p,miR-152-3p,miR-200a-3p,miR-219-3p,miR-2285af,miR-30f,miR-28b,miR-148b-5p,miR-2285b,miR-2284a,miR-2284y,miR-199a-5p,miR-2385-3p,miR-495-5p,miR-582-3p,miR-300,miR-496,miR-128-3p,miR-1290,miR-3591-5p,miR-345-3p,miR-2427,miR-1343-5p,miR-505-5p,miR-9-5p,miR-302a-5p,novel_121,miR-2284z,miR-582-3p,miR-545-5p,miR-2285ad,miR-299a-3p,miR-431,miR-331-5p,miR-191-5p,miR-28-5p,miR-31-3p,miR-5100,miR-144,miR-6740-5p,miR-125b-5p,miR-500b-5p,miR-365a-5p,miR-1306-5p,miR-3607-3p,miR-10b-3p,miR-1814c,miR-1248,miR-331-
TSN	ENSOARG0(	translin	miR-652-5p,miR-668-3p,miR-2330-5p,miR-1839-3p,miR-432-3p,miR-432-3p,miR-182-5p,miR-582,miR-214-3p,miR-1468,miR-1343-5p,miR-483-3p,miR-181b-2-3p,novel_78,miR-3955-5p,miR-2459,miR-148b-5p,miR-134-3p,miR-3431,miR-3535,miR-30b-3p,novel_23,miR-4791,miR-541,miR-6529a,miR-26a-2-3p,miR-544a,miR-450b-5p,miR-30b-3p,miR-6527,miR-1839-3p,miR-2285f,miR-335-5p,miR-541-3p,miR-449a,miR-34c,miR-2440,miR-133a-5p,miR-147-5p,novel_1,miR-335,miR-181b-3p,miR-677,miR-203-3p,miR-34b,miR-146b-3p,miR-3596,miR-378g,miR-3591-3p,miR-150-5p,miR-376b-5p,miR-2285ab,miR-9851-3p,miR-3529-3p,miR-216c-5p,miR-200a,miR-29a-5p,miR-181b-1-3p,miR-500a-5p,miR-196a-3p,miR-30d,miR-34b-5p,miR-2284z,miR-6516-5p,miR-483-3p,miR-30a-5p,miR-5703,miR-1343-5p,novel_133,miR-34a-5p,miR-148a-5p,miR-142-3p,miR-582-3p,miR-214,miR-664a-5p,miR-1468-5p,miR-4492,miR-182-5p,miR-370-3p,miR-21-3p,miR-544-3p,miR-6516,miR-34c-5p,miR-30d-5p,miR-30e-5p,miR-329b,miR-2312

BCL2L12	ENSOARG0(	BCL2-lik	novel_115,miR-1306-5p,miR-342,miR-320b,miR-542-5p,miR-424-5p,miR-33a-5p,miR-4532,miR-10b,miR-125b-2-3p,novel_4,miR-5010-3p,miR-9788-3p,miR-216a-3p,miR-2285n,miR-769-5p,miR-2285y,miR-371a-5p,miR-2285t,miR-3059-5p,miR-219a-5p,miR-452-5p,miR-219a-2-3p,novel_94,miR-20a-3p,miR-7977,miR-3082-5p,miR-7857-3p,miR-542-5p,miR-125b,miR-320d,miR-299-3p,miR-221-5p,miR-130a-5p,miR-125b-2-3p,miR-146a-5p,miR-2411,miR-2300b-3p,novel_133,miR-1197-5p,miR-140-3p,miR-124-3p,miR-2904,miR-19b-3p,miR-4429,miR-378d,miR-3600,miR-1b-3p,miR-15a-5p,miR-532-3p,miR-29b-2-5p,miR-424-5p,miR-145a-3p,miR-4510,miR-3074-2-3p,miR-10b-5p,miR-18a-5p,miR-140-5p,miR-221-5p,miR-182-5p,miR-3065-3p,miR-323-5p,miR-6130,miR-139-5p,miR-877-5p,novel_32,miR-204-3p,miR-1343-5p,miR-125a-5p,miR-382,miR-653,miR-1260b,miR-2284ab,miR-7862,miR-130a-3p,miR-103,miR-15a,miR-33a-3p,miR-16a,miR-656-5p,novel_120,miR-342-3p,miR-135a-2-3p,miR-542-5p,novel_101,miR-495-3p,miR-24-3p,miR-371b-3p,miR-2428,novel_27,miR-18b,miR-2387,miR-378b,miR-19a-3p,miR-107,miR-379-5p,miR-195a-5p,miR-9851-3p,miR-10a-5p,miR-323a-5p,miR-146b-3p,miR-2397-5p,miR-1260a,miR-181c-3p,miR-217,miR-191-3p,miR-450a-2-3p,miR-107,miR-4286,miR-30a-3p,miR-1,miR-29b-2-5p,miR-2385-3p,miR-2478,miR-24-2-5p,miR-2284a,miR-1271-3p,miR-3065-3p,miR-10a-5p,miR-128-3p,miR-2411-5p,miR-214-5p,miR-135a-1-3p,miR-409-3p,miR-31-3p,miR-1306-5p,miR-103b,miR-488-3p,miR-18a,miR-320a,miR-3154,miR-10b-3p,miR-7144-5p,miR-598-3p,miR-769,miR-3965,miR-154b-5p,miR-431-5p,miR-16b,miR-182-5p,miR-505,novel_19,miR-487b-5p,miR-130b-3p,miR-214-3p,miR-146b,miR-101b-3p,miR-124a,miR-2284b,miR-676-3p,miR-23a-3p,miR-2284r,miR-758-3p,miR-1a-3p,miR-

TLE6	ENSOARG0(	transduc	miR-497-5p,miR-2284x,miR-29b,miR-138,miR-16b,miR-145a-5p,miR-542-3p,miR-181a-5p,miR-149-5p,miR-20b,miR-103,miR-134-3p,miR-26b-3p,miR-3955-5p,miR-103a-3p,miR-133b-5p,miR-1343-5p,miR-466f-3p,miR-124-5p,miR-106a-5p,miR-17-5p,miR-377-3p,miR-296-3p,miR-296-3p,miR-345-5p,miR-654-3p,miR-432-3p,miR-130b-5p,miR-2319a,miR-6516-3p,miR-195a-5p,miR-378f,miR-410-5p,miR-412-3p,miR-107,miR-677,miR-181b-3p,miR-146b-3p,miR-323a-5p,miR-299b-3p,miR-29a-3p,novel_68,miR-378b,miR-216b-3p,miR-3074-5p,miR-656-5p,miR-379-3p,miR-29a,miR-16a,miR-502-5p,miR-15a,miR-20a-5p,miR-326-3p,miR-2428,miR-24-3p,miR-1839-3p,miR-29c-3p,miR-219b-3p,miR-335-5p,miR-136-3p,miR-411-3p,miR-2427,miR-29d-3p,miR-1343-5p,miR-330-5p,miR-322-5p,miR-378h,miR-1271-5p,miR-1224-5p,miR-299a-3p,miR-429-3p,miR-107,miR-200a-3p,miR-543-5p,miR-450a-2-3p,miR-181b-5p,miR-6536,miR-200c-3p,miR-8117,miR-200c,miR-3065-3p,miR-29a-5p,miR-490-3p,miR-450b-5p,miR-3064-5p,miR-30a-3p,miR-7144-5p,miR-20b-5p,miR-23a-5p,miR-1248,miR-876-3p,miR-345-5p,miR-2318,miR-329a,miR-93-5p,miR-3154,miR-17-5p,miR-301,miR-324-5p,miR-154b-5p,miR-769,miR-370-3p,miR-141-3p,miR-3068-3p,miR-138-5p,miR-331-3p,miR-29b-3p,miR-378c,miR-93,miR-1197-3p,miR-16-5p,miR-3607-3p,miR-133a-3p,miR-362-3p,miR-1388-5p,miR-378c,miR-758-3p,miR-378b,miR-329-3p,miR-484,miR-301b-5p,miR-455-3p,miR-2459,miR-147a,miR-181b-2-3p,miR-145b,miR-1343-3p,novel_78,novel_4,miR-3085-3p,miR-362-3p,miR-505,miR-106b-5p,miR-16b,miR-199a-3p,miR-326,novel_103,miR-758-3p,miR-378d,miR-33a-5p,miR-1388-3p,miR-1839-3p,miR-424-5p,miR-106a-5p,miR-335-3p,miR-1983,miR-96-5p,miR-129b-5p,miR-378e,miR-25,miR-378g,miR-489,miR-126b-5p,miR-7977,miR-3064-5p,miR-4792,miR-452-3p,miR-10b,miR-20a-

LRPAP1	ENSOARG0(	low dens	miR-758-3p,miR-541,miR-301b-5p,miR-329-3p,miR-2459,miR-147a,miR-142-5p,miR-145b,miR-181b-2-3p,novel_78,miR-1343-3p,miR-491-5p,miR-106b-5p,miR-1388-3p,miR-124a,miR-1983,miR-504,miR-378g,miR-2285u,miR-4792,miR-301a-5p,miR-2440,miR-615,miR-218-5p,novel_82,miR-423-5p,miR-877-3p,miR-361-3p,miR-3956-3p,miR-2447,miR-34a-5p,miR-493-3p,miR-212-5p,miR-106a,miR-18a-3p,miR-345-3p,miR-500a-5p,miR-324-3p,miR-210-5p,novel_17,miR-486-3p,miR-181b-1-3p,miR-345-3p,miR-1895,miR-345-5p,miR-339b,miR-129b-3p,miR-450a-1-3p,miR-10a,miR-2331-3p,miR-504-5p,miR-130b-5p,miR-4492,miR-1468-5p,miR-874-5p,miR-664a-5p,miR-665,miR-2285j,miR-3184-5p,novel_44,miR-142a-5p,miR-6525,miR-665-5p,miR-3074-5p,miR-433-5p,miR-4508,miR-450c-3p,miR-7b-5p,miR-149-5p,miR-194-3p,miR-134-3p,miR-3431,miR-339-5p,miR-103a-3p,miR-31-5p,miR-340-3p,miR-378a-5p,novel_51,miR-2411-3p,miR-365b-5p,miR-7-5p,let-7a-2-3p,miR-432-3p,miR-628-5p,miR-412-3p,miR-2284e,miR-216a-3p,miR-1306,miR-181b-3p,miR-669,miR-677,miR-3074-5p,novel_68,miR-6123,miR-485-3p,miR-28c,miR-330-5p,miR-2427,miR-1343-5p,miR-1290,miR-486-5p,miR-450a-1-3p,novel_79,miR-429-3p,novel_96,novel_127,miR-205-5p,novel_39,miR-29a-5p,miR-450b-5p,miR-3064-5p,miR-20b-5p,miR-329b,miR-345-5p,miR-329a,miR-2474,miR-767,miR-93-5p,miR-125a,miR-1271,miR-2355-5p,miR-874-3p,miR-574-5p,miR-6740-5p,miR-125b-5p,miR-2433,miR-1260b,miR-193a-5p,novel_42,miR-30b-3p,miR-10b,miR-484,miR-296-5p,miR-9788-3p,miR-2300a-5p,miR-2285o,miR-1468,miR-3955-3p,novel_124,miR-3085-3p,miR-1306-5p,miR-326,miR-342,miR-758-3p,miR-4532,miR-2330-5p,miR-106a-5p,miR-877-3p,miR-361-3p,miR-10b,miR-3064-5p,miR-7134-3p,miR-4726-5p,miR-2285n,miR-452-5p,miR-212-3p,miR-541-3p,novel_60,miR-3059-

HHIPL2	ENSOARG0(	HHIP-lik	miR-129b-5p,miR-1306-3p,miR-199b-5p,miR-361-3p,miR-1983,miR-504,miR-181a-3p,miR-499b-5p,miR-452-3p,miR-139-5p,miR-2284g,miR-4792,miR-664b,miR-4443,miR-203-3p,miR-3082-5p,miR-769-5p,miR-1973,miR-2285y,novel_82,miR-2285t,miR-4726-5p,miR-181d-5p,miR-9-5p,miR-1296-5p,miR-2285f,miR-212-3p,miR-2447,miR-361-3p,miR-362-5p,miR-30b-3p,miR-323b,miR-143-5p,miR-484,miR-101c,miR-450b-5p,miR-544a,miR-1388-5p,miR-199b-3p,novel_78,novel_4,miR-548w,miR-409b,miR-330-3p,miR-126a-5p,miR-455-3p,miR-500-5p,miR-147a,miR-539-5p,miR-16b,miR-323b-3p,miR-326,miR-199a-3p,miR-199b-5p,miR-3955-3p,miR-491-5p,miR-2284b,miR-744-3p,miR-33a-5p,miR-424-5p,miR-124a,miR-2310,miR-8485,miR-3187-3p,miR-30c-1-3p,miR-500a-3p,miR-450a-1-3p,miR-186-5p,miR-1827,miR-339b,miR-296-3p,miR-370-5p,miR-21-3p,miR-660-5p,miR-30c-1-3p,miR-221-5p,miR-504-5p,miR-15b-5p,miR-204-3p,miR-6130,miR-139-5p,miR-544-3p,miR-132-5p,miR-376e-3p,miR-30a-3p,miR-132-5p,miR-30e-3p,miR-130b-5p,miR-500,miR-708-5p,miR-190a-5p,miR-424-5p,miR-487b-5p,miR-450b-3p,novel_74,miR-4510,miR-539-5p,miR-338-3p,miR-769-5p,miR-124-3p,miR-2904,miR-2366,miR-2300b-3p,miR-2284u,miR-941,miR-30d-3p,miR-18a-3p,novel_83,miR-199a-3p,miR-15a-5p,miR-125b,miR-345-3p,miR-216a-5p,miR-219b-5p,miR-21-3p,miR-221-5p,miR-345-3p,miR-216c-5p,miR-143-3p,miR-2411,miR-2424,miR-376c-3p,miR-130a-5p,miR-18a-3p,miR-3591-3p,miR-2387,miR-135b-5p,miR-195a-5p,miR-107,miR-412-3p,miR-450b-3p,miR-216b-3p,novel_68,miR-6535,miR-670-3p,miR-411b-3p,miR-374c-3p,miR-16a,miR-28c,miR-4324,miR-15a,miR-199c,novel_1,miR-1193,miR-199b-5p,novel_27,miR-326-3p,miR-3071-5p,novel_23,miR-450c-3p,miR-432,miR-653,miR-188-5p,miR-497-5p,miR-138,miR-30b-3p,miR-16b,miR-26b-3p,miR-339-
USP11	ENSOARG0(	ubiquiti	novel_42,miR-296-5p

REV1	ENSOARG0(	REV1, po	miR-450b-5p,miR-148b-5p,miR-2285b,miR-3956-5p,novel_39,miR-3064-5p,miR-127-5p,miR-6536,miR-28-3p,novel_127,miR-200a-3p,miR-8117,miR-125a-3p,miR-322-5p,miR-9-5p,miR-486-5p,miR-129-1-3p,miR-134-5p,miR-429-3p,miR-299a-3p,miR-412,miR-30c,miR-1290,miR-181c-3p,novel_42,miR-16-5p,miR-592,miR-134-5p,miR-100-3p,miR-125b-5p,miR-503-3p,miR-125a,miR-1271,miR-345-5p,miR-876-3p,miR-424-3p,miR-30e-5p,miR-432-3p,miR-432-3p,miR-377-3p,novel_51,miR-365b-5p,miR-3141,miR-134-3p,miR-3431,miR-149-5p,novel_116,miR-194-3p,miR-16-1-3p,miR-16b,miR-2284x,miR-497-5p,miR-1843b-5p,miR-542-3p,miR-432,miR-576-3p,miR-142b,miR-4324,miR-299b-3p,miR-374c-3p,miR-181b-3p,miR-677,miR-320c,miR-669,miR-329-5p,miR-216b-3p,miR-378f,miR-3141,miR-3591-3p,miR-216a-3p,miR-150-5p,miR-181b-1-3p,miR-2424,miR-486-3p,miR-329a-5p,miR-2285p,miR-30b,miR-92a-1-5p,miR-422a,miR-500a-5p,miR-324-3p,miR-210-3p,miR-328-3p,miR-18a-3p,miR-212-5p,miR-33b-3p,miR-16-1-3p,miR-218-1-3p,miR-193b-5p,miR-30c-5p,miR-378i,miR-378a-3p,novel_44,miR-224-5p,miR-3184-5p,miR-129-2-3p,miR-214,miR-487b-5p,novel_74,miR-130b-5p,miR-665,miR-582-5p,miR-377-3p,miR-15b-5p,miR-30c-1-3p,miR-2331-3p,miR-504-5p,miR-552-3p,miR-296-3p,miR-345-5p,miR-30d-5p,miR-378d,miR-2310,miR-1b-5p,miR-1388-3p,miR-103a-2-5p,miR-214-3p,miR-16b,miR-29b-1-5p,miR-147a,miR-425-3p,miR-2459,miR-126a-5p,miR-455-3p,miR-548w,novel_78,miR-181b-2-3p,miR-101c,miR-541,novel_111,miR-361-3p,miR-423-5p,miR-2447,miR-9-5p,miR-1843a-3p,miR-3964,novel_82,miR-378g,miR-139-5p,miR-504,miR-181a-3p,miR-96-5p,miR-378j,miR-1271-3p,miR-3065-3p,miR-2403,miR-29b-2-5p,miR-200c-3p,miR-17-3p,miR-2898,miR-200c,miR-6119-5p,miR-30c-2-3p,miR-378h,miR-2411-5p,miR-1271-5p,miR-24-2-5p,miR-

CPN1	ENSOARG0(	carboxyp	miR-1271-5p,miR-378h,novel_121,miR-450a-1-3p,miR-6517,miR-431,novel_79,miR-496,miR-105-2,miR-345-3p,miR-218-2-3p,miR-450b-5p,miR-380-5p,miR-376c-5p,miR-376b-5p,miR-29a-5p,miR-4286,miR-181a-2-3p,miR-17-3p,miR-2898,novel_127,novel_96,miR-219-3p,miR-125a-3p,miR-2285af,miR-205-5p,miR-339a,miR-217,miR-30c-2-3p,miR-34c-5p,miR-503-3p,miR-874-3p,miR-3068-3p,miR-125a,miR-215-5p,miR-2355-5p,miR-598-3p,miR-2318,miR-671-5p,miR-485-5p,miR-1248,miR-3074-1-3p,miR-7144-5p,miR-301,miR-2448-3p,miR-3154,miR-1197-3p,miR-378c,miR-103b,miR-370-5p,novel_63,miR-125b-5p,miR-3431,miR-185-5p,novel_116,miR-149-5p,miR-377-5p,miR-339-5p,miR-6239,miR-27b-3p,miR-2113,miR-873a-5p,miR-653,miR-708-3p,miR-3071-5p,miR-22-3p,miR-450c-3p,miR-197-3p,miR-432-3p,miR-2285r,miR-380-5p,let-7f-2-3p,let-7f-2-3p,miR-363-5p,miR-124-5p,miR-125a-5p,miR-105-5p,miR-34b,miR-323a-5p,novel_68,miR-26b-5p,miR-3120-3p,miR-412-3p,miR-1291,miR-378f,miR-2285ab,miR-378b,miR-18a-3p,miR-1306,miR-24-3p,miR-449a,miR-1193,miR-17-3p,miR-3957-3p,miR-22-5p,miR-4324,miR-485-3p,miR-34c,miR-487a-3p,miR-383-5p,miR-18a-3p,miR-485-3p,miR-142-3p,miR-34a-5p,miR-133a-3p,miR-215-5p,miR-378i,novel_133,miR-217-5p,miR-378a-3p,miR-140-3p,miR-2904,miR-377-5p,miR-143-3p,miR-216c-5p,miR-345-3p,miR-34b-5p,miR-3653-3p,miR-26a-5p,novel_17,miR-125b,miR-185-3p,miR-422a,miR-345-3p,miR-324-3p,miR-323-5p,miR-6130,miR-216b-5p,miR-192-5p,novel_32,miR-204-3p,miR-22-3p,miR-496-3p,miR-378a-3p,miR-140-5p,miR-30c-1-3p,miR-2331-3p,miR-370-5p,miR-339b,miR-2312,miR-186-5p,miR-625-5p,miR-450a-1-3p,miR-299,miR-598-3p,miR-500a-3p,miR-6525,miR-6134,miR-4510,miR-590-3p,miR-378[illegible],miR-1271,miR-17-3p,miR-6516-

JMJD8	ENSOARG0(	jumonji	miR-324-5p,miR-223-5p,miR-370-3p,miR-2355-5p,miR-874-3p,miR-574-5p,miR-485-5p,miR-7144-5p,miR-3074-1-3p,miR-345-5p,miR-2474,miR-2448-3p,miR-133a-3p,miR-16-5p,novel_42,miR-1306-5p,miR-486b-3p,novel_9,miR-138-5p,miR-331-3p,miR-410-5p,miR-322-5p,miR-412,miR-10a-5p,miR-105-2,miR-582-3p,miR-615-3p,miR-1271-3p,miR-490-3p,miR-380-5p,miR-148b-5p,miR-3064-5p,miR-543-5p,novel_127,miR-450a-2-3p,miR-125a-3p,miR-205-5p,miR-669,novel_69,miR-10a-5p,miR-320b,miR-379-5p,miR-195a-5p,miR-146b-5p,miR-450b-3p,miR-18a-3p,miR-2285ab,miR-342-3p,miR-6123,miR-502b,novel_1,miR-128-1-5p,miR-16a,miR-6529b,miR-15a,miR-149-5p,miR-503-5p,miR-134-3p,miR-31-5p,miR-497-5p,miR-381-5p,miR-138,miR-16b,miR-22-3p,miR-197-3p,miR-1285,miR-873a-5p,miR-345-5p,miR-668-3p,miR-363-5p,miR-2483-5p,miR-106b-3p,novel_51,miR-105-5p,miR-197-5p,miR-146a,miR-503-5p,miR-877-5p,miR-151b,miR-15b-5p,novel_32,miR-22-3p,miR-6130,miR-10a,miR-27a-5p,miR-532-5p,miR-216b-3p,miR-504-5p,miR-2331-3p,miR-345-5p,miR-2312,miR-133b-3p,miR-21a-3p,miR-6134,miR-3184-5p,miR-10b-5p,miR-214,miR-665-5p,miR-4510,miR-145a-3p,miR-424-5p,miR-4492,miR-664a,miR-151-5p,miR-15a-5p,miR-210-3p,miR-3600,miR-18a-3p,miR-133a-3p,miR-664-5p,miR-184-3p,miR-124-3p,miR-486-3p,miR-146a-5p,miR-503-5p,miR-2484,miR-466i-5p,miR-324-3p,miR-196a-3p,miR-489,miR-25,miR-378g,miR-7977,miR-499b-5p,miR-20a-3p,miR-10b,miR-4792,miR-3064-5p,novel_94,miR-504,miR-147-3p,miR-361-3p,miR-423-5p,miR-877-3p,miR-361-3p,miR-2285f,miR-541-3p,miR-3956-3p,novel_60,miR-615,novel_82,miR-5010-3p,miR-1343-3p,miR-105-1,novel_4,miR-1839-5p,miR-125b-2-3p,miR-412-3p,miR-10b,miR-541,miR-296-5p,miR-3187-3p,miR-33a-5p,miR-424-5p,miR-2330-5p,miR-124a,miR-381-5p,miR-214-

CAPN5	ENSOARG0(	calpain	miR-625-5p,miR-339b,miR-345-5p,miR-30d-5p,miR-129b-3p,let-7i-5p,miR-301a-3p,miR-2284s,miR-377-3p,miR-499b-3p,miR-548o-3p,miR-504-5p,miR-2331-3p,miR-454-3p,miR-874-5p,miR-664a-5p,miR-665,miR-30a-3p,miR-30e-3p,let-7f,miR-122-5p,miR-224-5p,miR-769-5p,miR-214,let-7b,miR-148a-5p,miR-2284u,miR-34a-5p,miR-664-5p,miR-142-3p,miR-493-3p,miR-378a-3p,miR-1961,miR-2366,miR-378i,miR-191,miR-30c-5p,miR-328-3p,miR-7859,miR-30d-3p,miR-18a-3p,let-7k,miR-98-5p,miR-202-5p,miR-422a,miR-92a-1-5p,miR-324-3p,novel_17,miR-30b,miR-486-3p,miR-664b-3p,miR-345-3p,miR-329a-5p,miR-1983,miR-544-5p,miR-335-3p,miR-504,miR-96-5p,miR-1306-3p,miR-499a-3p,miR-25,miR-211-5p,miR-2284g,miR-2440,miR-877-3p,miR-361-3p,miR-6527,miR-2447,miR-1a-3p,miR-541,miR-2284r,miR-148b-5p,miR-147a,miR-181b-2-3p,miR-199b-3p,miR-214-3p,miR-199b-5p,miR-130b-3p,miR-491-5p,miR-182-5p,miR-505,miR-431-5p,miR-8485,miR-378d,miR-1388-3p,miR-124a,miR-329b,miR-345-5p,miR-30e-5p,miR-1271,miR-125a,miR-2285aa,miR-874-3p,miR-191-5p,miR-134-5p,miR-125b-5p,miR-100-3p,miR-2433,miR-1260b,miR-1197-3p,miR-30c,miR-23b-5p,miR-330-5p,miR-1343-5p,miR-134-5p,miR-2284z,miR-505-5p,miR-431,miR-412,novel_79,miR-1224-5p,miR-200a-3p,novel_96,novel_127,miR-152-3p,let-7f-5p,miR-127-5p,miR-125a-3p,miR-2284y,let-7g,miR-29a-5p,miR-148b-5p,miR-3064-5p,miR-378f,let-7e-5p,miR-2284e,miR-412-3p,miR-3591-3p,miR-1306,miR-150-5p,miR-148a-3p,miR-669,miR-6535,miR-677,miR-411b-3p,miR-3120-3p,miR-329-5p,miR-6123,miR-206,miR-4324,miR-199b-5p,miR-301b-3p,miR-193b-3p,miR-335-5p,miR-4508,miR-197-3p,miR-22-3p,miR-542-3p,miR-7b-5p,miR-19b-2-5p,miR-149-5p,miR-194-3p,miR-3431,miR-339-5p,miR-103a-3p,miR-31-5p,miR-193a-3p,miR-
MCTS1	ENSOARG0(	malignan	miR-338-5p,miR-140-3p,miR-127-5p,let-7j

KCTD20	ENSOARG0(	potassiu	miR-499b-5p,miR-211-5p,miR-139-5p,miR-3064-5p,miR-329-5p,miR-320c,miR-144-3p,miR-4443,miR-3082-5p,miR-29a-3p,miR-378j,miR-2387,miR-335-3p,miR-758-5p,novel_101,miR-29c-3p,miR-2285x,miR-17-3p,miR-2447,miR-211,miR-107-5p,miR-2285n,miR-22-5p,miR-502b,miR-29a,miR-26b-3p,novel_4,novel_78,miR-122-3p,miR-2459,miR-484,miR-329-3p,miR-29b,miR-362-3p,miR-1388-5p,miR-101a-5p,miR-544a,miR-101b-3p,miR-491-3p,miR-30c-1-3p,miR-362-3p,miR-320b,miR-103a-2-5p,miR-483-3p,miR-3085-3p,miR-378a-5p,miR-3065-3p,miR-370-3p,miR-141-3p,miR-2331-3p,miR-30c-1-3p,miR-221-5p,miR-548o-3p,miR-3068-3p,miR-324-5p,miR-204-3p,miR-154b-5p,miR-544-3p,miR-139-5p,miR-320a,miR-21a-3p,miR-7144-5p,miR-1248,miR-186-5p,miR-10b-3p,miR-133b-3p,miR-329a,miR-329b,miR-133a-3p,miR-3184-3p,miR-144,miR-665,miR-320e,miR-29b-3p,miR-2285j,miR-5100,miR-134-5p,miR-204-5p,miR-17-3p,miR-380-5p,miR-548e-3p,miR-423-3p,miR-134-5p,miR-7859,miR-2411-5p,miR-483-3p,miR-29d-3p,miR-1197-5p,miR-1193,miR-188-3p,miR-5703,miR-126-3p,miR-362-3p,miR-134,miR-133a-3p,miR-218-1-3p,miR-4429,miR-3064-5p,miR-4286,miR-329a-5p,miR-216c-5p,miR-2411,miR-3065-3p,miR-200a,miR-3071-3p,miR-30c-2-3p,miR-196a-3p,miR-8117,miR-125a-3p,miR-221-5p,miR-200a-3p,miR-320d,miR-17-3p,miR-127-5p
ST18	ENSOARG0(	suppress	miR-664a,miR-190a-5p,miR-4492,miR-454-3p,miR-134-5p,miR-99b-3p,miR-30e-3p,miR-5100,miR-324-3p,miR-30a-3p,miR-409-3p,let-7g-3p,miR-2312,miR-625-5p,miR-485-5p,miR-2285c,miR-320a,miR-301a-3p,novel_25,miR-22-3p,miR-199a-5p,miR-223-5p,miR-574-5p,miR-2355-5p,miR-216b-3p,miR-99a-3p,miR-296-3p,miR-320d,miR-466i-5p,miR-99a-3p,miR-152-3p,miR-205-5p,miR-8117,miR-210-5p,miR-3601,miR-185-3p,miR-92a-1-5p,miR-2285i,miR-29b-1-5p,miR-199a-5p,miR-30a-3p,miR-4429,miR-128-3p,miR-134,miR-140-3p,miR-148b-3p,miR-483-3p,miR-2284h-5p,miR-134-5p,miR-2285ad,miR-30d-3p,miR-218-5p,novel_82,miR-2285n,miR-2285y,miR-199b-5p,miR-301b-3p,miR-2285f,miR-190b-5p,miR-2285k,miR-361-3p,miR-2285ab,miR-148a-3p,miR-199b-5p,miR-216a-3p,miR-411b-3p,miR-320c,miR-669,miR-4792,miR-20a-3p,miR-499b-5p,miR-2285u,miR-7-1-3p,miR-483-3p,miR-130b-3p,miR-199b-5p,miR-320b,miR-197-5p,miR-190a,miR-29b-1-5p,miR-2483-5p,let-7c-3p,miR-33a-5p,miR-744-3p,miR-450b-5p,miR-3120-5p,miR-143-5p,miR-4791,miR-22-3p,miR-3535,miR-2285g,novel_116,miR-2285e,miR-187-3p,miR-130a-3p
SPATA6L	ENSOARG0(	spermato	miR-1b-3p,miR-433-3p,miR-141-3p,miR-452-3p,miR-200a-3p,miR-758-5p

ZNF77	ENSOARG0(	zinc fin	miR-125b-2-3p,miR-484,miR-143-5p,miR-3120-5p,miR-5010-3p,novel_78,novel_4,miR-409b,miR-500-5p,miR-216a-3p,miR-455-3p,miR-2285e,miR-2459,miR-421,miR-153,miR-539-5p,miR-2320-3p,miR-106b-5p,miR-326,miR-153-3p,miR-214-3p,miR-494-3p,miR-101b-3p,miR-106a-5p,miR-8485,miR-1b-5p,miR-2310,miR-361-3p,novel_94,miR-2285u,miR-499b-5p,miR-7977,miR-452-3p,miR-2483-3p,miR-2440,miR-194b-5p,miR-3059-5p,miR-423-5p,miR-154a,miR-362-5p,miR-361-3p,miR-877-3p,novel_111,miR-5703,miR-140-3p,miR-188-3p,miR-2366,miR-133a-3p,miR-218-1-3p,miR-493-3p,miR-1b-3p,miR-30d-3p,miR-18a-3p,miR-106a,miR-222-5p,miR-324-3p,miR-500a-5p,miR-125b,miR-26a-5p,miR-21-3p,miR-106a,miR-20a,miR-329a-5p,miR-2411,miR-486-3p,miR-2424,miR-380-3p,miR-376c-3p,miR-125b-2-3p,miR-433-3p,miR-450a-1-3p,miR-2285c,novel_99,miR-345-5p,miR-21-3p,miR-216b-3p,miR-552-3p,miR-2331-3p,miR-545-3p,miR-877-5p,miR-6516,miR-376e-3p,miR-216b-5p,miR-30a-3p,miR-2285j,miR-15b-3p,miR-30e-3p,miR-874-5p,miR-130b-5p,miR-450b-3p,miR-17-3p,miR-214,miR-425-5p,miR-3184-5p,miR-6134,miR-338-3p,miR-450c-3p,miR-194-5p,miR-432,miR-873a-5p,miR-223-3p,miR-1434-3p,miR-455-5p,miR-380-3p,miR-2284ab,miR-1260b,miR-31-5p,miR-20b,miR-412-5p,miR-432-5p,miR-2284d,miR-2285g,miR-106a-5p,miR-194a,miR-17-5p,miR-455-5p,miR-125a-5p,miR-124-5p,miR-628-5p,miR-130b-5p,miR-345-5p,miR-18a-3p,miR-3591-3p,miR-378b,miR-376b-5p,miR-2319a,miR-135b-5p,miR-450b-3p,miR-655-5p,miR-320b,miR-26b-5p,miR-7-1-3p,miR-329-5p,miR-144-3p,miR-669,miR-670-3p,novel_69,miR-374c-3p,miR-502-5p,miR-28c,miR-20a-5p,miR-147-5p,miR-17-3p,miR-326-3p,miR-330-5p,miR-7641,miR-429-3p,miR-450a-1-3p,miR-6516-5p,miR-2411-5p,miR-1260a,miR-200c,miR-543-5p,novel_127,miR-2898,miR-

[truncated: 1,175,064 more chars]
